# Highly Distinct Microbial Communities in Elevated Strings and Submerged Flarks in the Boreal Aapa-Type Mire

**DOI:** 10.3390/microorganisms10010170

**Published:** 2022-01-13

**Authors:** Andrey L. Rakitin, Shahjahon Begmatov, Alexey V. Beletsky, Dmitriy A. Philippov, Vitaly V. Kadnikov, Andrey V. Mardanov, Svetlana N. Dedysh, Nikolai V. Ravin

**Affiliations:** 1 Institute of Bioengineering, Research Center of Biotechnology of the Russian Academy of Sciences, 119071 Moscow, Russia; rakitin@biengi.ac.ru (A.L.R.); shabegmatov@gmail.com (S.B.); mortu@yandex.ru (A.V.B.); vkadnikov@bk.ru (V.V.K.); mardanov@biengi.ac.ru (A.V.M.); 2 Papanin Institute for Biology of Inland Waters, Russian Academy of Sciences, 152742 Borok, Russia; philippov_d@mail.ru; 3 Winogradsky Institute of Microbiology, Research Center of Biotechnology of the Russian Academy of Sciences, 119071 Moscow, Russia; dedysh@mail.ru

**Keywords:** aapa mire, microbial diversity, methanogens, *Acidobacteriota*, *Chloroflexi*, *Planctomycetota*

## Abstract

Large areas in the northern hemisphere are covered by extensive wetlands, which represent a complex mosaic of raised bogs, eutrophic fens, and aapa mires all in proximity to each other. Aapa mires differ from other types of wetlands by their concave surface, heavily watered by the central part, as well as by the presence of large-patterned string-flark complexes. In this paper, we characterized microbial diversity patterns in the surface peat layers of the neighboring string and flark structures located within the mire site in the Vologda region of European North Russia, using 16S rRNA gene sequencing. The microbial communities in raised strings were clearly distinct from those in submerged flarks. Strings were dominated by the *Alpha-* and *Gammaproteobacteria*. Other abundant groups were the *Acidobacteriota, Bacteroidota, Verrucomicrobiota, Actinobacteriota,* and *Planctomycetota*. Archaea accounted for only 0.4% of 16S rRNA gene sequences retrieved from strings. By contrast, they comprised about 22% of all sequences in submerged flarks and mostly belonged to methanogenic lineages. Methanotrophs were nearly absent. Other flark-specific microorganisms included the phyla *Chloroflexi, Spirochaetota, Desulfobacterota*, *Beijerinckiaceae*- and *Rhodomicrobiaceae*-affiliated *Alphaproteobacteria*, and uncultivated groups env.OPS_17 and vadinHA17 of the *Bacteroidota*. Such pattern probably reflects local anaerobic conditions in the submerged peat layers in flarks.

## 1. Introduction

Wetlands are among the most productive ecosystems on Earth, performing various ecosystem functions important for human life and sustainable development [1]. Wetlands play an important role in the global water balance, ensuring the accumulation, long-term conservation and releasing of water, thereby maintaining the resilience of water flows to short-term fluctuations in the level of precipitation [1,2]. Wetlands are also important for their high nutrient recycling capacities and significant contributions to both carbon accumulation and storage and greenhouse gas emissions [3,4]. The total area of wetlands worldwide is about 5.5 million km^2^ [4,5], of which the peat accumulating wetlands (peatlands) account for about 4 million km^2^. About 80% of peatlands are located in zones with a temperate and cold climate in the northern hemisphere, mainly in Russia, Canada, the United States and Scandinavia [5,6].

Peatlands differ in several types depending on the water source and the type of vegetation. The two most contrasting types of peatlands are raised bogs, fed by rainwater, and eutrophic fens, which are primarily filled with groundwater and runoff [3,7]. Raised bogs are peat bogs dominated by *Sphagnum* mosses. Generally, raised bogs are highly acidic (pH around 4) and nutrient-poor. Microbial communities of raised bogs were studied using both cultivation-based and molecular methods, including fluorescence in situ hybridization, high-throughput sequencing of 16S rRNA gene fragments, metagenomics and metatrancriptomics [8,9,10,11,12,13,14,15,16,17]. The microbial communities of this type of peatlands are usually dominated by bacteria of the phyla *Acidobacteriota* and *Proteobacteria* (classes *Alpha-* and *Gammaproteobacteria*); *Verrucomicrobiota* and *Planctomycetota* also make up a significant share of the communities [8,9,11,12,13,14,15,16,17].

Eutrophic fens are fed primarily by groundwater. They are generally less acidic and more nutrient-rich than bogs, with sedges and grasses being the main vegetation type. Microbial communities of fens are characterized to a lesser extent; most studies were focused on microorganisms participating in the methane cycle [8,18,19]. Microbial communities of eutrophic fens are more diverse and differ greatly in composition from raised bogs [11]. In these communities, the dominant groups are *Chloroflexi* of the class *Anaerolineae*, some lineages of *Acidobacteriota* and *Betaproteobacteria*, and *Planctomycetota* of the uncultivated OM190 group [20].

Aapa mires is a term used for large patterned or ribbed fens [21]. Mires of this type differ from other types of wetlands by their concave surface, heavily watered by the central part, as well as by the presence of string-flark complexes [22,23,24,25]. The latter means that strings and flarks have significant differences in origin, trophic status, and the type of vegetation. So, the strings (elevated forms of microrelief) pass in their development sequentially eutrophic, mesotrophic, and oligotrophic stages and currently have dwarf shrub-*Sphagnum* and, as a rule, forested communities, underlain by raised peats. At the same time, flarks throughout their development are in the eutrophic stage; therefore, they have herbal, herbal-*Hypnum* or herbal-*Hypnum-Sphagnum* communities on fen peats. The regular recurrence of such strings and flarks (provided that the strings are located perpendicular to the water flow direction) creates aapa complexes that provide the characteristic landscape to this type of wetlands [25,26,27,28,29].

Aapa mires have a pan-boreal distribution range [30]. In northern Europe, the prevalent territory for their distribution is the northern part of Fennoscandia, where aapa is the dominant zonal type of mire massifs and occupies tens of thousands of square kilometres [26]. In the European part of Russia, aapa mire massifs belong to one of three groups based on their morphology and composition of their vegetation: (1) northern European forest-tundra (Lapland) aapa; (2) Karelian ring (boreal Fennoscandian) aapa; and (3) Onega-Pechora (north-eastern) aapa. The southern boundary of the sporadic distribution of aapa mires in the north of European Russia lies between 60° N and 61° N, near the southern boundary of the middle taiga subzone [31].

Russia accounts for a significant share of all the wetlands in the world. Large areas of wetlands are located in the region of Western Siberia, but in the European part of the country their area is about 15 million hectares [29]. Many large mire regions in North European Russia include closely located peatlands of various types, mainly raised bogs and eutrophic fens, and less often aapa type wetlands. While microbial communities of raised bogs and eutrophic fens located in this region were characterized in several studies [13,15,20], there is no such information on the aapa-mires. To fill this gap, in this study, we are comparing the structure of microbial communities in the surface peat layers of the adjacent strings and flarks of the aapa-type mire in the Vologda region, in the north of the European part of Russia. Our analysis showed that microbial communities of strings clearly differ from those in flarks and resemble the microbial communities of nearby raised bogs. At the same time, microbial community of the flark sites differs from that in both raised bogs and eutrophic fens.

## 2. Materials and Methods

### 2.1. Study Site

**Figure 1:**
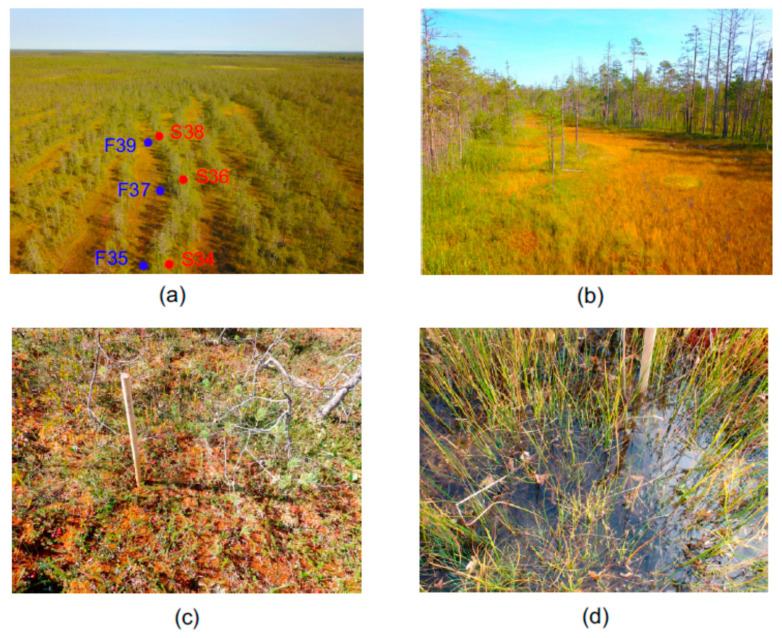
Aapa-type mire in Piyavochnoe mire system. (**a**) Aerial view, approximate positions of sampling sites are marked; (**b**) surface view showing a flark in center, and forest-covered strings on left and right sides; (**c**) sampling site in string; (**d**) sampling site in flark.

The object of this study was Piyavochnoe mire located in the north-west of Vologda region of North European Russia in the southern part of the middle taiga subzone. This is a large (80 km^2^) mire system composed of several raised bogs, aapa-mires and fen massifs, and a number of intramire primary lakes [31]. In the Piyavochnoe mire, the mire massif of the aapa type (coordinates 60.475 N, 36.504 E) is located in a separate depression and has a pronounced string-flark microrelief (alternation of forested *Sphagnum* ridges, grassed depression and low *Sphagnum* carpets located between them) in the central part (Figure 1). This aapa mire belongs to the Onega–Pechora aapa group, but has some features characteristic of the Karelian ring aapa mires [31].

The strings are relatively young in origin [31], have a height of 0.20–0.35 m, a width of 5–20 m, and are stretched over the entire width of the massif (400–600 m). They are occupied by pine-grass-shrub-*Sphagnum* oligotrophic plant communities.

The flarks are completely closed, watered (depth 0.1–0.3 m, width varies from 1–3 to 25–30 m, and even 30–55 m with a length of 150–400 m), and occupied by hydrophilic herb-*Hypnum* vegetation. Among the herbs, *Carex lasiocarpa* and *Menyanthes trifoliata* dominate; *Rhynchospora alba, Trichophorum alpinum*, *Scheuchzeria palustris* are also present; *Utricularia intermedia* and *U. minor* are abundant under water. Unlike strings, mosses do not form a continuous cover here; the dominant moss is *Scorpidium scorpioides* submerged in water. The plant species were identified using the guide to vascular plants of the North-West of Russia [32].

### 2.2. Sampling Procedure

The sampling was performed on 3 September 2020. Samples were taken in aapa complex (string-flark pattern) in string and flark sites (Figure 1). Three individual plots, approximately 50–60 m from each other, were chosen within one flark for sampling purposes. Three sampling points on a neighboring string were chosen near the sampling points in the flark so that the distance between the adjacent string and flark sampling sites was from 5 to 10 m.

The peat cores (30 × 30 × 20 cm; each sample of approximately 5 kg) were collected from the surface layer of the sampling plots without breaking the integrity and structure of the peat into 10 L plastic flasks and were transported to the laboratory in boxes containing ice packs. Each of the six collected peat cores was processed separately. The samples used for molecular analysis were taken from the upper peat layer at a depth of 0–10 cm. The samples were homogenized by cutting the peat material with sterile scissors into small fragments (about 0.5 cm) that were crushed and mixed thoroughly to prepare one composite sample for each of the cores. Three replicate samples were taken from each core and frozen at −20 °C for DNA extraction.

### 2.3. Chemical Analyses

Field measurements of pH and temperatures were performed using Combo HI 98129 analyzer (Hanna Instruments, Vöhringen, Germany). The total organic carbon and ammonium were measured for the average sample from each plot according to standard protocols for characterization of soils (GOST 26213-91 Soils. Methods for determination of organic matter and GOST 26489-85, Soils. Determination of exchangeable ammonium by CINAO method). Concentrations of Fe, Ca, Mg and P were determined by inductively coupled plasma mass spectrometry (ICP-MS Agilent 7500a, Agilent, Santa Clara, CA, USA). The concentration of nitrate and sulfate ions was determined by ion chromatography using the Dionex ICS-1100 (Dionex, Sunnyvale, CA, USA).

### 2.4. DNA Isolation, 16S rRNA Gene Fragment Amplification and Sequencing

Total genomic DNA from 0.6 g of each of 18 peat samples was extracted using a Power Soil DNA isolation kit (MO BIO Laboratories, Carlsbad, CA, USA), and stored at −20 °C. From 1.5 to 5.7 μg of DNA per sample was isolated (Supplemental Table S1).

PCR amplification of 16S rRNA gene fragments comprising the V3–V4 variable regions was performed using the universal prokaryotic primers 341F (5′-CCTAYG GGDBGCWSCAG) and 806R (5′-GGA CTA CNVGGG THTCTAAT) [33]. The obtained PCR fragments were bar-coded using the Nextera XT Index Kit v. 2 (Illumina, San Diego, CA, USA) and purified using Agencourt AMPure beads (Beckman Coulter, Brea, CA, USA). The concentrations of PCR products were measured using the Qubit dsDNA HS Assay Kit (Invitrogen, Carlsbad, CA, USA). All PCR fragments were then mixed and sequenced on Illumina MiSeq (2 × 300 nt reads). Pairwise overlapping reads were merged using FLASH v.1.2.11 [34]. The final dataset consisted of 655,536 16S rRNA gene reads (Supplemental Table S1).

### 2.5. Bioinformatics Analysis of Microbial Community Composition and Diversity

All sequences were clustered into operational taxonomic units (OTUs) at 97% identity using the USEARCH v. 11 program [35]. Low quality reads were removed prior to clustering, chimeric sequences were removed during clustering by the USEARCH algorithms (Supplemental Table S1). To calculate OTU abundances, all reads obtained for a given sample (including low-quality reads) were mapped to OTU sequences at a 97% global identity threshold by USEARCH. OTUs containing only one read in the entire dataset and likely resulting from sequencing errors were discarded using Usearch commands.

The taxonomic assignment of OTUs was performed by searching against the SILVA v.138 rRNA sequence database using the VSEARCH v. 2.14.1 algorithm [36]. OTUs assigned to chloroplasts, mitochondria, and eukaryotes were excluded from the analysis. OTU sequences are provided in the Supplemental File S1.

The diversity indices at a 97% OTU cut-off level were calculated using USEARCH v.11 [35]. To avoid sequencing depth bias, the number of reads generated for each sample were randomly subsampled to the size of the smallest dataset (50,023 reads) using the «single_rarefaction.py» script of QIIME v.1.9.1 [37]. The rarefaction curve was generated using QIIME v.1.9.1.

Calculation of weighted Unifrac distance metrics was performed applying “beta_div” command in USEARCH. Principle Coordinate Analysis (PCoA) was carried out in R programming language, applying the cmdscale function. The percent variation values were calculated using eig_perc function of metagMisc R package. The significance of difference between two groups of samples was calculated with the PERMANOVA test [38] using QIIME v.1.9.1 [37], weighted Unifrac distances were used as the input matrix.

One-way ANOVA test was performed using online ANOVA Calculator (https://goodcalculators.com/one-way-anova-calculator/, accessed on 15 October 2021). The difference was considered significant if the *p* value was less than 0.05.

### 2.6. Data Availability

The raw data generated from 16S rRNA gene sequencing were deposited in the NCBI Sequence Read Archive (SRA) and are available via the BioProject PRJNA776823.

## 3. Results and Discussion

### 3.1. Main Characteristics of Peat in Strings and Flarks

The analyzed peat samples obtained from string and flark sites showed a number of important differences regarding their chemical composition (Table 1). First, at the string sites, the groundwater level was at a depth of 10 to 20 cm, and at the flark sites the peat samples were completely submerged in water. The water in string samples had lower pH (4.6–5.2) than in flarks (5.5–5.9). While the total organic carbon contents in peat collected from string and flark sites were similar (97–98%), the flark water contained more ammonium as the strings, although this difference was not statistically significant (*p* = 0.10).

The concentrations of nitrate were an order of magnitude lower than of ammonium. The content of sulfate in string peat by far exceeded that in peat from flarks. The concentrations of phosphorous and magnesium in peat from strings were higher than those in flarks, while the content of iron and calcium did not differ significantly (Table 1).

### 3.2. Diversity of Microbial Communities

To characterize the compositions of microbial communities, between 12,421 and 59,099 sequences of 16S rRNA gene fragments (655,536 in total) were determined for 18 analyzed peat samples. As a result of clustering the obtained sequences, 5264 bacterial and 234 archaeal OTUs were identified at the level of 97% sequence identity. The rarefaction curve of the observed OTUs approached saturation indicating that most of microbial diversity was covered (Supplemental File S1).

**Figure 2:**
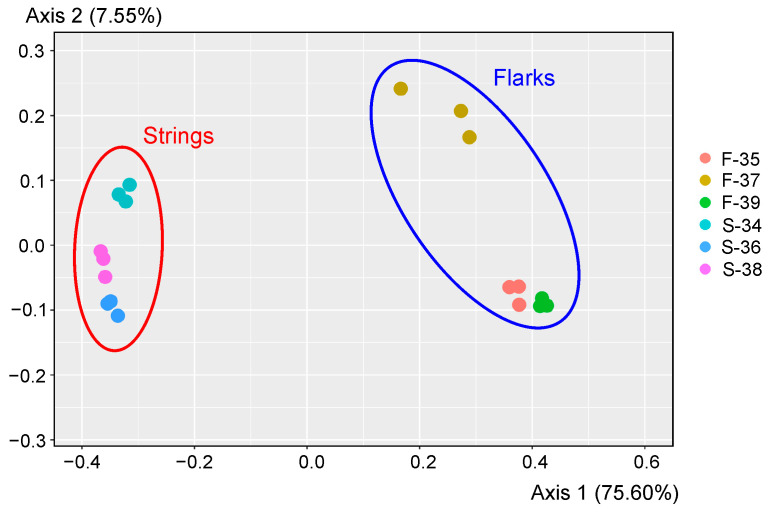
Comparison of microbial community composition in analyzed peat samples by principle coordinate analyses (PCoA). PCoA plot is based on weighted UniFrac distance of 16S rRNA sequencing dataset.

As revealed by the UniFrac analysis, replicate samples clustered together (Figure 2). Moreover, the microbial communities in different string peat samples were highly similar to each other and were significantly different from those in samples collected from flarks (*p* < 0.0001 as revealed by PERMANOVA test) (Figure 2). Therefore, for subsequent analysis, for each of the peat samples, three replicates were combined into one dataset. Only 445 OTUs were shared between flark and string peat samples further emphasizing the differences between these microbial communities.

The number of species-level OTUs present in individual peat samples ranged between 583 and 1485, these values are typical for peatlands [39]. Alpha diversity indices (Table 2) indicate that the microbial community composition was more diverse and even in the peat from strings then in the flarks according to the Shannon and Peilous indices.

The effective number of species in string samples (Jost index) was nearly twice as high as that in flarks. The differences in Peilous evenness, Jost and Shannon indices between strings and flarks were statistically significant (*p* < 0.05).

### 3.3. Microbial Community Composition at the Phylum Level

Taxonomic assignment of OTUs revealed the presence of 50 phylum-level bacterial and archaeal lineages defined in the genome-based taxonomy system [40], according to the SILVA v.138 database. However, OTUs representing top 12 phyla of Bacteria and top 4 phyla of Archaea, comprising on average more than 1% of all the 16S rRNA gene sequences in strings and/or flarks, together accumulated more than 92% of the microbiomes (Figure 2 and Supplemental Table S2).

The composition of microbial communities of peat from strings and flarks was strikingly different already at the level of domains and phyla (Figure 2). Archaea accounted for 21.9 ± 2.0% (mean ± standard error, of all 16S rRNA gene sequences) in flarks, but only 0.4 ± 0.1% in string samples. Archaeal populations in flarks were represented by members of the *Halobacterota* (13.6 ± 1.1%), *Crenarchaeota* (3.2 ± 0.5%), *Nanoarchaeota* (2.6 ± 0.5%) and *Thermoplasmatota* (1.9 ± 0.2%).

Bacterial communities in strings were dominated by the *Proteobacteria* (27.8 ± 1.0%), mostly of classes *Alpha-* (14.3 ± 0.7%) and *Gammaproteobacteria* (9.6 ± 0.4%). Other abundant groups were the *Acidobacteriota* (19.2 ± 0.6%), *Bacteroidota* (12.2 ± 0.7%), *Verrucomicrobiota* (11.0 ± 0.4%), *Actinobacteriota* (8.0 ± 0.5%), *Planctomycetota* (6.8 ± 0.3%), and *Patescibacteria* (6.8 ± 0.7%). All these lineages were also found in flark samples, but in most cases their relative abundancies were several times lower than in string peats. The exceptions are the *Bacteroidota*, the dominant phylum in flarks accounting for 14.5 ± 0.4% of the communities, and the *Gammaproteobacteria* which relative abundance in flarks was about 10%. Three phyla abundant in flarks, *Chloroflexi* (13.1 ± 0.5%), *Spirochaetota* (5.2 ± 0.4%) and *Desulfobacterota* (3.3 ± 0.5%) accounted on average for less than 1% of 16S rRNA gene sequences in the peat string samples. The differences in the relative abundance of all above mentioned phyla between the strings and flarks were statistically significant (*p* < 0.05).

### 3.4. Microbial Lineages Characteristic for Strings and Flarks

**Figure 3:**
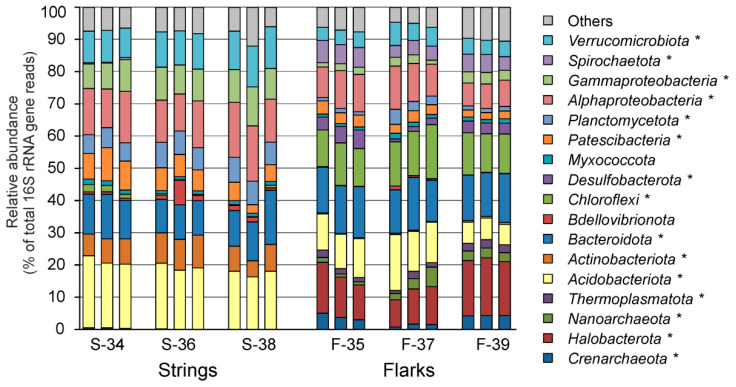
Prokaryotic community composition in string and flark peat samples, according to results of 16S rRNA gene profiling. Community composition is shown at the phylum level, with exception of *Proteobacteria*, for which classes *Alpha-* and *Gammaproteobacteria* are shown. All replicate samples (three per sampling site) are presented. Lineages with statistically significant differences (*p* < 0.05) in relative abundance in strings and flarks are marked with an asterisk.

The high relative abundance of archaea is the most striking specific characteristic of flarks (Figure 3). Most of Archaea represented known methanogenic lineages, including the genera *Methanoregula* (10.8 ± 1.1%), *Methanocella* (1.4 ± 0.2%), *Methanosaeta* (0.6 ± 0.2%), *Methanosarcina* (0.3 ± 0.05%), and the family *Methanomassiliicoccaceae* (1.8 ± 0.2%) of the *Thermoplasmatota*. Therefore, all three types of methanogens, hydrogenotrophic, acetoclastic and methyl-reducing, were present in the flark peat. Hydrogenotrophic methanogens (*Methanoregula, Methanocella* and *Methanosarcina*), were by far the most abundant. Members of the family *Methanomassiliicoccaceae* that produce methane by reducing methanol and other methylated compounds with hydrogen as an electron donor [41], ranked second in abundance, and the share of acetoclastic methanogens (*Methanosaeta*) was the lowest (Supplemental Table S2). Anaerobic methane oxidizing archaea were not detected.

Besides methanogens the archaeal populations in flark samples comprised members of the phyla *Nanoarchaeota*, known to be partner-dependent parasites or symbionts with small genome size and limited metabolic capacities [42], and the *Crenarchaeota*. The latter were mostly represented by the candidate class *Bathyarchaeia*, an anaerobic organotrophic fermentative archaea utilizing various proteinaceous substrates and polysaccharides of plant origin, as predicted by analysis of their genomes [43].

The phylum *Chloroflexi* was the second most abundant group of bacteria in flarks after the *Bacteroidota* (Figure 2). The majority of *Chloroflexi* were assigned to the class *Anaerolinea* (12.0 ± 0.6%), members of which were identified in diverse environments, including marine and freshwater sediments and soils [44]. They are metabolically versatile heterotrophs capable of growing on various polysaccharides by fermentation, and some species are capable of aerobic and/or anaerobic respiration [44,45].

The phylum *Spirochaetota* was found mainly in flarks, and almost all of its representatives belonged to the *Spirochaetaceae* family but were not classified at the genus level (Supplemental Table S2). A GenBank search revealed 16S rRNA sequences closely related (>99% identity) to most numerous *Spirochaetacea* OTU5 in lake sediments and rice paddy soil (GenBank KY691040 and AB660818). The phylum *Desulfobacterota*, found almost exclusively in flarks, was mostly represented by classes *Desulfuromonadia* (1.7 ± 0.3%), *Syntrophia* (0.9 ± 0.2%), *Syntrophobacteria* (0.4 ± 0.1%) and *Syntrophorhabdia* (0.3 ± 0.1%) (Supplemental Table S2). All OTUs from *Desulfuromonadia* belong to the family *Geobacteraceae*, the cultivated representatives of which are anaerobic iron-reducing organotrophs ubiquitous in soils and sediments [46]. Most of other *Desulfobacterota* belonged to uncultured genus-level lineages of the families *Smithellaceae, Syntrophaceae, Syntrophobacteraceae*, and *Syntrophorhabdaceae*, but some OTUs were classified at the genus level and assigned to the *Smithella, Syntrophus, Syntrophobacter,* and *Syntrophorhabdus*. Cultured members of these families are strictly anaerobic organisms that degrade intermediates of organic matter decomposition, such as short-chain fatty acids and aromatic compounds, in syntrophic associations with hydrogen-consuming methanogens [47,48,49,50,51]. Members of the *Syntrophobacteraceae* are major acetate- and propionate-degrading sulfate reducers in paddy soil [52,53].

*Actinobacteriota* were among the dominant groups of microorganisms in strings (8.0 ± 0.5%) but were much less abundant in peat samples from flarks (0.4 ± 0.05%). *Actinobacteriota* represented the classes *Acidimicrobiia* (5.3 ± 0.4%), *Thermoleophilia* (1.4 ± 0.2%), and *Actinobacteria* (1.3 ± 0.2%). The OTUs, which comprised the absolute majority of the 16S rRNA gene sequences of the *Actinobacteriota* were assigned to uncultivated lineages of the family level and higher (Supplemental Table S2).

The relative abundance of *Planctomycetota* in strings was three times higher than in flarks (6.8 ± 0.3% vs. 1.9 ± 0.4%). Most planctomycetes in strings belonged to the classes *Planctomycetia* (4.7 ± 0.2%) and *Phycisphaerae* (1.8 ± 0.2%) (Supplemental Table S2), which accommodate aerobic and anaerobic chemo-organotrophic bacteria utilizing a wide range of organic compounds [54,55]. Most of the corresponding OTUs were assigned to the families *Gemmataceae*, *Isosphaeraceae* and WD2101 soil group (*Tepidisphaeraceae*), which are typical inhabitants of the peatlands [56,57] and organic-rich soils [58,59]. The phylum *Verrucomicrobiota* accumulated 11.0 ± 0.4% of 16S rRNA gene reads in strings and twice less (5.1 ± 0.3%) in flarks. The families *Chthoniobacteraceae, Opitutaceae* and *Methylacidiphilaceae* were found mostly in strings, while *Pedosphaeraceae* accounted for about 4% of microbial communities in both types of peat (Supplemental Table S2).

**Figure 4:**
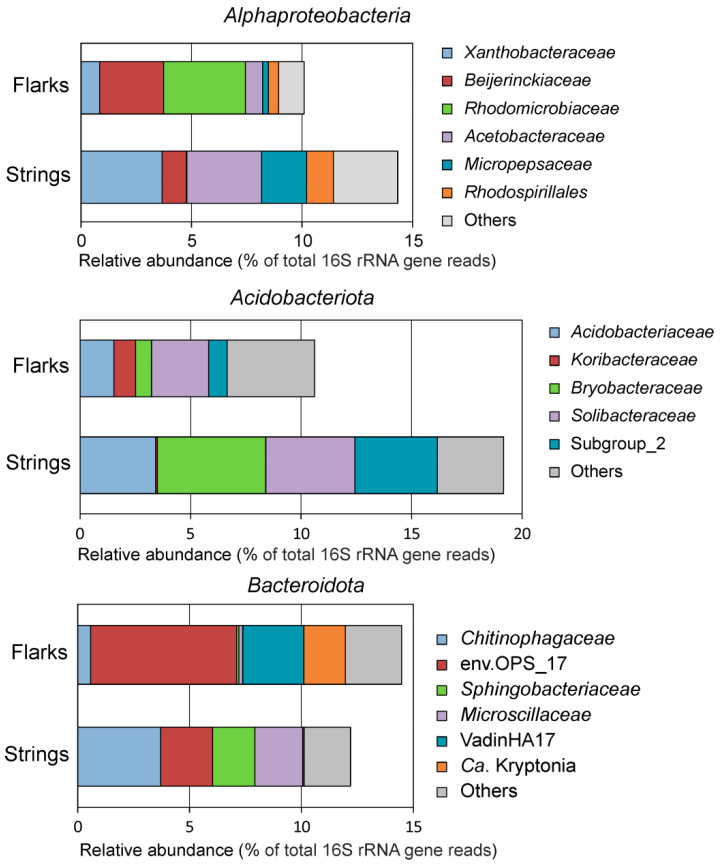
Composition of *Alphaproteobacteria*, *Acidobacteriota,* and *Bacteroidota* at family level. Mean values of relative abundances in strings and flarks are shown.

For other dominant phyla the difference between the microbial communities in strings and flarks became evident when the analysis was performed at lower taxonomic levels (Figure 4). Thus, a high relative abundance of the *Alphaproteobacteria* was detected both in strings (14.3 ± 0.7%) and flarks (10.1 ± 0.7%). In the strings this class was mostly represented by members of the *Xanthobacteraceae*, *Acetobacteraceae, Micropepsaceae,* and *Rhodospirillales*. In the flarks, however, *Beijerinckiaceae* (the genera *Roseiarcus* and *Rhodoblastus*) and *Rhodomicrobiaceae* (the genus *Rhodomicrobium*) were more numerous than in strings. The genus *Rhodomicrobium* was one of the most numerous in strings (3.7 ± 0.8%) but nearly absent in flark peat (0.02 ± 0.005%). Cultured species of these genera are photoheterotrophic bacteria found in acidic *Sphagnum* peat bogs [60,61,62].

Habitat-specific diversity patterns were also observed for the *Acidobacteriota* (Figure 4). Thus, members of the *Bryobacteraceae* (*Bryobacter* sp.), Subgroup 2 lineage and the genus *Granulicella* were more abundant in strings, while Ca. Koribacter was present almost exclusively in flarks (1.0 ± 0.30% vs. 0.07 ± 0.02% in strings). Ca. Solibacter and *Occallatibacter* were found ubiquitously present in strings as well as in flarks (4.0 ± 0.2% vs. 2.6 ± 0.2% and 1.3 ± 0.1% vs. 1.3 ± 0.3%, respectively).

The phylum *Bacteroidota* was one of the dominant lineages in both types of peats, accounting on average for 12.2 ± 0.7% and 14.5 ± 0.4% of 16S rRNA gene reads in strings and flarks, respectively. However, at the family level the difference between flarks and strings is clear (Figure 4). The *Chitinophagaceae*, *Sphingobacteriaceae*, and *Microscillaceae* were more abundant in strings, while uncultured family-level lineages env.OPS_17 and vadinHA17 were prevalent in flarks, and the later was detected exclusively in flarks. Members of *Bacteroidota*-related Ca. Kryptonia lineage were also specific for flarks.

### 3.5. Most Abundant OTUs in Two Types of Peat

The list of most abundant OTUs (on average >1% of all reads retrieved from the corresponding peat type), which were characteristics for either flarks or strings, is given in Table 3. These OTUs accumulated on average 18% of 16S rRNA gene reads in strings and 34% in flark samples. These data are consistent with a lower evenness and effective number of species (Jost index) in microbial communities of flark peats (Table 2).

Four of twelve string-specific OTUs were represented by members of the class *Acidobacteriia*, orders *Acidobacteriales, Bryobacterales* and Subgroup 2. Only two of them could be identified at the genus level, both representing the *Bryobacter*, one of the dominant phylotypes identified in raised bogs [20]. The most abundant string-specific OTU (3.0%) was affiliated with the *Gammaproteobacteria* and belonged to uncultured WD260 group. Two OTUs were assigned to *Actinobacteriota* (uncultured *Acidimicrobiia*), one represented the family *Microscillaceae* (*Bacteroidota*), and two belonged to the *Xanthobacteraceae* (*Alphaproteobacteria*). Two *Verrucomicrobiota*-affiliated OTUs in strings were represented by members of the family *Opitutaceae* and the genus *Chthoniobacter*. None of these OTUs accounted for more than 1% of 16S rRNA gene reads in flarks.

Sixteen OTUs with and average relative abundance above 1% were identified in flarks, and five of them represented Archaea (Table 3). Among archaeal OTUs three belonged to the genus *Methanoregula*, one—to the candidate class *Bathyarchaeia*, and one to the family *Methanomassiliicoccaceae*. Bacterial OTUs included vadinHA17- and env.OPS_17-related *Bacteroidota*, Ca. Solibacter of the *Acidobacteriota, Anaerolineaceae*-related *Chloroflexi*, *Alphaproteobacteria* of the genera *Rhodoblastus, Roseiarcus* and *Rhodomicrobium*, and unclassified members of the families *Geobacteraceae* (*Desulfobacterota*) and *Spirochaetaceae* (*Spirochaetota*).

### 3.6. Comparison of Microbial Communities of the Aapa Mire with Nearly Located Raised Bog and Eutrophic Fen

The presented data show that the microbial communities of peat samples from strings were clearly distinct from those in flarks, which are located in a close proximity, at a distance of only several meters. The trophic status and geochemical characteristics were probably the major factors that determined the microbial community composition of these ecosystems. Although microbial communities of string and flark peats were similar in overall richness, flark communities were characterized by lower evenness (Table 2). This may be due to the predominantly anoxic conditions in flarks, which promotes the development of several groups of anaerobic microorganisms, in particular, methanogens. For example, *Methanoregula*, accounted for about 10.8 ± 1.1% of flarks communities (Supplemental Table S2).

The composition of microbial communities from acidic and nutrient-poor *Sphagnum*-dominated peat bogs described in various studies is rather conservative [8,9,10,11,12,13,14,16,18]. These communities are usually dominated by *Acidobacteriota, Alphaproteobacteria*, *Planctomycetota* and *Verrucomicrobiota*. It is precisely such a community that we previously revealed in peat samples from raised bog in the Piyavochnoe mire massif [20]. This community was mostly composed of members of the phyla *Acidobacteriota* (33.5 ± 1.4% of 16S rRNA gene reads), *Proteobacteria* (15.6 ± 1.5%), *Verrucomicrobiota* (14.6 ± 0.6%), and *Planctomycetota* (10.9 ± 1.6%) [20]. All these phyla were among the dominant groups detected in this study in string samples in the aapa-type mire within the same Piyavochnoe mire massif, although the share of the *Acidobacteriota* was lower (19.2 ± 0.6%). Moreover, a clear similarity between the microbial communities of peat from strings and raised bog was observed at lower taxonomic levels. Thus, almost all of the detected *Acidobacteriota* belonged to the same class, the *Acidobacteriae* [63]. Its cultivated members are aerobic or facultatively anaerobic acidophilic chemohetrotrophs, which can utilize various sugars and polysaccharides, including cellulose and chitin [64,65]. Two of the most abundant string-specific OTUs detected in this study belonged to the genus *Bryobacter* [66]. OTUs assigned to this genus were found among the most abundant phylotypes in Piyavochnoe raised bog [20]. Representatives of the genus *Bryobacter* were isolated from boreal peat bogs and are capable of utilizing galacturonic and glucuronic acids, which are released during decomposition of *Sphagnum* moss [67]. Another acidobacterial lineage abundant both in strings and raised bogs in Piyavochnoe mire massif was yet uncultured Subdivision 2 of this phylum, which is often detected in *Sphagnum*-dominated wetlands [13,68]. Among the most numerous OTUs detected in both strings and raised bogs, there was also OTU assigned to uncultured lineage WD260 of the *Gammaproteobacteria* [20]. Significant similarities between strings and the raised bog were also found for the phyla *Verrucomicrobiota* (e.g., the prevalence of *Pedosphaeraceae, Opitutaceae,* and *Methylacidiphilaceae*) and *Planctomycetota* (WD2101 soil group) [56,57].

However, the relative abundance of the two bacterial phyla in strings and raised bogs was very different. The phylum *Actinobacteriota* accounted for 8.0 ± 0.5% of the community in the strings but only 2.2 ± 0.6% in Piyavochnoe raised bog [20]. Nearly all OTUs assigned to this phylum represented uncultured lineages within the classes *Acidimicrobiia, Thermoleophilia* and *Actinobacteria*. Even more pronounced differences were observed in the relative abundance of *Bacteroidota*, amounting to 2.1 ± 0.3% in the Piyavochnoe raised bog microbiome and 12.0 ± 0.7% in the strings. Most *Bacteroidota* in strings belonged to the families *Chitinophagaceae, Sphingobacteriaceae*, and *Microscillaceae*, comprising chemoheterotrophic species occurring in soils [69,70,71]. An important difference between the Piyavochnoe raised bog and strings is the pH value, which in these peat samples was 3.7 and 4.62–5.19, respectively. Probably more acidic conditions in the raised bog are favourable for the *Acidobacteriota* and negatively impacted the growth of the members of the *Actinobacteriota* and the *Bacteroidota*.

Contrary to the raised bog, the microbial community of Piyavochnoe fen was dominated by members of the *Proteobacteria* (20.9 ± 1.4%), *Chloroflexi* (17.9 ± 1.9%), *Acidobacteriota* (9.6 ± 0.5%) and *Planctomycetota* (7.0 ± 0.8%) [20]. The high relative abundance of *Chloroflexi*, mostly assigned to the class *Anaerolineae*, is a common trait of flarks and the fen. However, the high abundance of Archaea (21.9 ± 2.0%), mostly representing known methanogenic lineages (e.g., *Methanoregula* sp.) is specific for flark peats distinguishing them from Piyavochnoe bog and fen samples, the share of methanogenic archaea in which was several times lower [20]. An important feature of flarks was their complete immersion in water to a depth of 7–10 cm (Table 1), which was not observed in cases of strings and the previously studied Piyavochnoe bog and fen samples [20]. Apparently, the analyzed layer of peat from flarks was under fully or partially anoxic conditions necessary for the growth of strictly anaerobic methanogenic archaea. Anaerobes are also members of the *Spirochaetaceae*, as well as *Desulfobacterota*, which were found in flarks but missing in string, bog, and fen samples. *Chloroflexi* of the class *Anaerolineae*, metabolically versatile heterotrophs frequently reported as dominant microbial lineages in organic-rich aquatic anoxic habitats such as marine and freshwater sediments are also obligate or facultative anaerobes [45]. Two uncultured lineages of the *Bacteroidota*, env.OPS_17 and vadinHA17, were numerous in flarks comprising 6.5 ± 0.7% and 2.7 ± 0.5% of 16S rRNA gene reads, respectively. The env.OPS_17 group is poorly characterized, while VadinHA17 clade was found to be dominant and active in full-scale anaerobic digesters where these bacteria played a key role in the degradation of proteinaceous substances [72].

Unlike numerous methanogens, known methanotrophs were found in flarks only in small quantities and were represented exclusively by bacteria. Among the known aerobic methanotrophs, members of the order *Methylococcales* were found, but their relative abundance was less than 0.1%. Bacteria of the phylum *Methylomirabilota* (NC10), capable of nitrite-driven oxidation of methane under anoxic conditions [73], accounted for about 0.08% of the community. Considering the detected low relative abundance of methanotrophs and the high content of methanogens, flark areas of aapa-type mires may be sources of methane emissions into the atmosphere.

## 4. Conclusions

Overall, our results revealed that the surface peat layers of elevated strings in the aapa mire harbored microbial communities characteristic of the neighboring raised bog and dominated by the *Proteobacteria* (mostly by classes *Alpha* and *Gamma*), *Acidobacteriota, Bacteroidota, Verrucomicrobiota, Actinobacteriota*, and *Planctomycetota*. The flark structures are submerged in water and harbored unique microbial communities distinct not only from raised bog and eutrophic fen, but also from closely located strings. The hallmark of microbial communities of flarks is the high relative abundance of methanogenic archaea and bacterial phyla *Chloroflexi, Spirochaetota,* and *Desulfobacterota*. Such a pattern probably reflects local anaerobic conditions in the submerged peat layers.

## Figures and Tables

**Table 1:** Physical and chemical characteristics of sampling sites.

Sample ID	S-34	S-36	S-38	F-35	F-37	F-39
Sample type	string	string	string	flark	flark	flark
Water level (cm) *	−17…−19	−10…−12	−11…−13	+9…+10	+8…+10	+7…+9
pH **	4.62	5.19	5.04	5.5	5.94	5.74
T, °C	18.9	17.4	16.5	13.4	17.7	15.1
**Peat characteristics**						
Total organic carbon (%)	97.2	98.1	97.3	97.2	97.2	97.4
N-NH_4_ (mg kg ^−1^)	171.9	141.1	155.9	182.5	329.1	237.8
N-NO_3_ (mg kg ^−1^)	19.7	10.1	20.6	8.4	11.3	9.9
SO_4_ (mg L ^−1^) **	259	185	317	52	61	37,5
Fe (mg kg ^−1^)	380	450	460	500	750	830
Ca (mg kg ^−1^)	8100	6500	8000	3700	6000	7200
Mg (mg kg ^−1^) **	740	980	1040	430	570	670
P (mg kg ^−1^) **	1210	1340	1190	1070	1130	1110
Plant community (dominant species)	*Pinus sylvestris–Empetrum hermaphroditum–Sphagnum fuscum*			*Carex lasiocarpa–Scorpidium scorpioides*		
Vegetation coverage	97–99%			50–60%		

* minus sign means the depth of groundwater; plus sign—depth of water covering surface; ranges of values are shown; ** chemical parameters for which differences between strings and flarks were statistically significant according to ANOVA test (*p* < 0.05).

**Table 2:** Alpha-diversity metrics.

Peat Type	Sample ID	Richness	Peilous Evenness	Jost	Shannon
String	S-34	1937	0.803	249.7	6.08
	S-36	1352	0.805	199.8	5.81
	S-38	1657	0.797	218.0	5.90
Flark	F-35	1230	0.706	80.8	5.03
	F-37	1705	0.759	152.4	5.65
	F-39	1816	0.729	107.5	5.47

**Table 3:** Most abundant OTUs in microbial communities of string and flark sites.

OTU ID	Share in Strings (%)	Share in Flarks (%)	Taxonomy (Silva)
**Strings**			
Otu16	1.4 ± 0.2%	0.04 ± 0.01%	d:Bacteria, p:Acidobacteriota, c:Acidobacteriae, o:Acidobacteriales
Otu45	1.4 ± 0.2%	0.00 ± 0.00%	d:Bacteria, p:Acidobacteriota, c:Acidobacteriae, o:Bryobacterales, f:Bryobacteraceae, g:Bryobacter
Otu227	1.5 ± 0.2%	0.05 ± 0.02%	d:Bacteria, p:Acidobacteriota, c:Acidobacteriae, o:Bryobacterales, f:Bryobacteraceae, g:Bryobacter
Otu42	1.3 ± 0.3%	0.00 ± 0.00%	d:Bacteria, p:Acidobacteriota, c:Acidobacteriae, o:Subgroup_2
Otu29	2.0 ± 0.2%	0.00 ± 0.00%	d:Bacteria, p:Actinobacteriota, c:Acidimicrobiia
Otu27	1.1 ± 0.1%	0.01 ± 0.00%	d:Bacteria, p:Actinobacteriota, c:Acidimicrobiia
Otu13	1.7 ± 0.6%	0.04 ± 0.03%	d:Bacteria, p:Bacteroidota, c:Bacteroidia, o:Cytophagales, f:Microscillaceae
Otu2	1.6± 0.3%	0.57 ± 0.06%	d:Bacteria, p:Proteobacteria, c:Alphaproteobacteria, o:Rhizobiales, f:Xanthobacteraceae
Otu85	1.2 ± 0.2%	0.16± 0.03%	d:Bacteria, p:Proteobacteria, c:Alphaproteobacteria, o:Rhizobiales, f:Xanthobacteraceae
Otu24	3.0 ± 0.2%	0.02 ± 0.01%	d:Bacteria, p:Proteobacteria, c:Gammaproteobacteria, o:WD260
Otu104	1.0 ± 0.2%	0.01 ± 0.00%	d:Bacteria, p:Verrucomicrobiota, c:Verrucomicrobiae, o:Chthoniobacterales, f:Chthoniobacteraceae, g:Chthoniobacter
Otu126	1.0 ± 0.1%	0.02 ± 0.01%	d:Bacteria, p:Verrucomicrobiota, c:Verrucomicrobiae, o:Opitutales, f:Opitutaceae
**Flarks**			
Otu6	0.01 ± 0.00%	2.7 ± 0.4%	d:Archaea, p:Crenarchaeota, c:Bathyarchaeia
Otu4	0.01 ± 0.00%	5.2 ± 0.9%	d:Archaea, p:Halobacterota, c:Methanomicrobia, o:Methanomicrobiales, f:Methanoregulaceae, g:Methanoregula
Otu11	0.00 ± 0.00%	2.1 ± 0.5%	d:Archaea, p:Halobacterota, c:Methanomicrobia, o:Methanomicrobiales, f:Methanoregulaceae, g:Methanoregula
Otu21	0.01 ± 0.01%	1.7 ± 0.3%	d:Archaea, p:Halobacterota, c:Methanomicrobia, o:Methanomicrobiales, f:Methanoregulaceae, g:Methanoregula
Otu8	0.00 ± 0.00%	1.6 ± 0.1%	d:Archaea, p:Thermoplasmatota, c:Thermoplasmata, o:Methanomassiliicoccales, f:Methanomassiliicoccaceae
Otu28	0.01 ± 0.01%	1.1 ± 0.2%	d:Bacteria, p:Acidobacteriota, c:Acidobacteriae, o:Solibacterales, f:Solibacteraceae, g:Candidatus Solibacter
Otu72	0.00 ± 0.00%	1.2 ± 0.3%	d:Bacteria, p:Bacteroidota, c:Bacteroidia, o:Bacteroidales, f:Bacteroidetes_vadinHA17
Otu1	0.93 ± 0.34%	3.0 ± 0.6%	d:Bacteria, p:Bacteroidota, c:Bacteroidia, o:Sphingobacteriales, f:env.OPS_17
Otu77	0.39 ± 0.05%	1.2 ± 0.2%	d:Bacteria, p:Bacteroidota, c:Bacteroidia, o:Sphingobacteriales, f:env.OPS_17
Otu10	0.01 ± 0.00%	2.3 ± 0.2%	d:Bacteria, p:Chloroflexi, c:Anaerolineae, o:Anaerolineales, f:Anaerolineaceae
Otu9	0.01 ± 0.00%	2.2 ± 0.4%	d:Bacteria, p:Chloroflexi, c:Anaerolineae, o:Anaerolineales, f:Anaerolineaceae
Otu36	0.00 ± 0.00%	1.3 ± 0.3%	d:Bacteria, p:Desulfobacterota, c:Desulfuromonadia, o:Geobacterales, f:Geobacteraceae
Otu30	0.04 ± 0.02%	1.4 ± 0.3%	d:Bacteria, p:Proteobacteria, c:Alphaproteobacteria, o:Rhizobiales, f:Beijerinckiaceae, g:Rhodoblastus
Otu25	0.55 ± 0.03%	1.2 ± 0.1%	d:Bacteria, p:Proteobacteria, c:Alphaproteobacteria, o:Rhizobiales, f:Beijerinckiaceae, g:Roseiarcus
Otu7	0.02 ± 0.00%	3.6 ± 0.8%	d:Bacteria, p:Proteobacteria, c:Alphaproteobacteria, o:Rhizobiales, f:Rhodomicrobiaceae, g:Rhodomicrobium
Otu5	0.00 ± 0.00%	2.3 ± 0.3%	d:Bacteria, p:Spirochaetota, c:Spirochaetia, o:Spirochaetales, f:Spirochaetaceae

d, domain; p, phylum; c, class; o, order; f, family; g, genus.

## Data Availability

The obtained 16S rRNA gene sequences were deposited in the NCBI Sequence Read Archive (SRA) and are available via the BioProject PRJNA776823. OTU sequences are presented in the Supplemental File S1.

## Supplementary Materials

The following supporting information can be downloaded at: www.mdpi.com/article/10.3390/microorganisms10010170/s1, Supplemental File S1: Rarefaction curve of the observed OTUs and OTU sequences; Table S1: DNA isolation and sequencing statistics; Table S2: Relative abundance and taxonomic classification of OTUs.
