# Supplementary material for: Highly Distinct Microbial Communities in Elevated Strings and Submerged Flarks in the Boreal Aapa-Type Mire
Source: Microorganisms. 2022 Jan 13;10(1):170. doi: 10.3390/microorganisms10010170 (PMC8778904; doi:10.3390/microorganisms10010170)
Supplement: Supplementary file 1 [file microorganisms-10-00170-s001.zip › Supplemental File S1.pdf]

Rarefaction curve of the observed OTUs

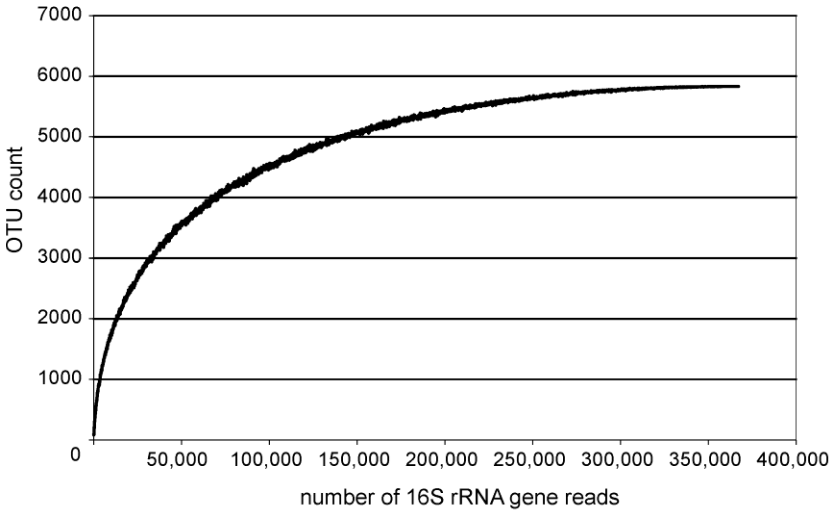

OTU sequences

>Otu1
CCAGCCTATGGGGGGCAGCAGTAAGGAATATTGGACAATGGTGGCAACACTGATCCAGCC
ATGCCGCGTGCAGGATGAAGGCGCTACGCGTTGTAAACTGCTTTTGTACCAGAGAAAACC
TATCTACGTGTAGATAGCTGATAGTATGGTAAGAATAAGCATCGGCTAACTTCGTGCCAG
CAGCCGCGGTAATACGAAGGATGCAAGCGTTATCCGGATTTATTGGGTTTAAAGGGTGCG
TAGGCGGACTTATAAGTCAGTGGTGAAATCTCGTCGCTTAACGACGAACGTGCCATTGAT
ACTGTAGGTCTTGAGTACAGATGCCGTTGGCGGAATGTGTCATGTAGCGGTGAAATGCAT
AGATATGACACAGAACACCGATTGCGAAGGCAGCTGACGAAACTGTAACTGACGCTGAGG
CACGAAAGCGTGGGGATCAAACAGGATTAGATACCCCAGTAGTCC
>Otu2
CCAGCCTATGGGGGGCAGCAGTGGGGAATATTGGACAATGGGCGCAAGCCTGATCCAGCC
ATGCCGCGTGAGTGATGAAGGCCCTAGGGTTGTAAAGCTCTTTTGTGCGGGAAGATAATG
ACGGTACCGCAAGAATAAGCCCCGGCTAACTTCGTGCCAGCAGCCGCGGTAATACGAAGG
GGGCTAGCGTTGCTCGGAATCACTGGGCGTAAAGGGTGCGTAGGCGGGTCTTTAAGTCAG
GGGTGAAATCCTGGAGCTCAACTCCAGAACTGCCTTTGATACTGAGGATCTTGAGTTCGG
GAGAGGTGAGTGGAACTGCGAGTGTAGAGGTGAAATTCGTAGATATTCGCAAGAACACCA
GTGGCGAAGGCGGCTCACTGGCCCGATACTGACGCTGAGGCACGAAAGCGTGGGGAGCAA
ACAGGATTAGAAACCCTAGTAGTCC
>Otu3
CCAGCCTATGGGGGGCAGCAGCTAAGAATATTCCGCAATGGGAGAAATCCTGACGGAGCG
ACGCCGCGTGGAAGATGAAGGCCGAAAGGTTGTAAATTCCTTTTATACGGGAAGAATAAG
TGTATTGAGAGAAACGGTGCATGATGACGGTACCGTATGAATAAGCCCCGGCTAACTACG
TGCCAGCAGCCGCGGTAATACGTAGGGGGCAAGCGTTGTTCGGATTCATTGGGCGTAAAG
GGCATGTAGGCTGTTATGTAAGTGTGGATTTAAAGGCGGAGGCTTAACCTCTAGTTTGGT
CTGCAAACTGCGTAGCTTGAGTGTAGAAGGGGAAACTGGAATTCCGCGTGTAGGGGTGAA
ATCTGTAGATATGCGGAAGAATACCTGTGGCGAAGGCGAGTTTCTGGTCTATAACTGACG
CTGAAATGCGAAAGTGTGGGTAGCAAACAGGATTAGAAACCCCAGTAGTCC
>Otu4
CCAGCCTATGGGGGGCACCAGGCGCGAAAACTTTACAATGCGGGAAACCGTGATAGGGGA
ACTCTGAGTGCCCGTTAAATCGGGCTGTCCATCAGTTTAAATAACTGGTGAAGAAAGGGC
CGGGCAAGACCGGTGCCAGCCGCCGCGGTAATACCGGCGGCTCGAGTGGTGGCCACTATT
ACTGGGCTTAAAGCGTTCGTAGCTGGTTTGTTAAGTCTCTGGGGAAATCTTCCGGCTTAA
CCGGAAGGCGTCTCAGGGATACTGGCAGACTAGGGACCGGGAGAGGTGAGGGGTACTCCA
GGGGTAGGAGTGAAATCCTGTAATCCTTGGGGGACCACCTGTGGCGAAGGCGCCTCACCA
GAACGGCTCCGACAGTGAGGGACGAAAGCTGGGGGAGCAAACCGGATTAGATACCCCCGT
AGTCC

>Otu5
CCAGCCTATGGGGGGCAGCAGCTAAGAATATTCCGCAATGGACGAAAGTCTGACGGAGCG
ACGCCGCGTGGATGACGAAGGCCGAAAGGTTGTAAAGTCCTTTTGTTGGGGAAGAATAAC
CATGGGAGGGAATGCCCGTGGGATGACATGAACCGACGAATAAGCCCCGGCTAACTACGT
GCCAGCAGCCGCGGTAACACGTAGGGGGCGAGCGTTGTTCGGAATTACTGGGCGTAAAGG
GCATGTAGGCGGCTTTGTAAGCCCGGCGTGGAAGCCCACGGCTCAACCGTGGGATTGCGT
TGGGAACTGCGAGGCTTGAATCATGGAGAGGGAGCTAGAATTCCTGGTGTAGGGGTGAAA
TCTGTAGAGATCAGGAAGAATACCAGTGGCGAAGGCGAGCTCCTGGCCAATGATTGACGC
TGAGGTGCGAAAGTGTGGGGATCAAACAGGATTAGATACCCCCGTAGTCC
>Otu6
CCAGCCTATGGGGGGCACCAGTCGCGAAAACTTTGCAATAAGCGAAAGCTTGACAGGGCT
ATCCCGAGTGCCATCCGCTGAGGAAGGCTTTTACCCAGTCTAGAACGCTGGGGGAATAAG
GAGAGGGCAAGTCTGGTGTCAGCCGCCGCGGTAATACCAGCTCTCCGAGTGGTGTGGATG
TTTATTGGGCCTAAAGCATCCGTAGCTGGCTAGGTTAGTCCCCTGTTAAATCCACCGAAT
TAATCGTTGGATTGCGGGGGATACTGCTTGGCTAGGGGACGAGAGAGGCAGACGGTATTT
CCGGGGTAGGGGTGAAATCCTATAATCCCGGGAAGACCACCAGTGGCGAAGGCTGTCTGC
TAGAACGCGCCCGACGGTGAGGGATGAAAGCTGGGGGAGCGAACCGGATTAGATACCCTC
GTAGTCC
>Otu7
CCAGCCTATGGGGGGCAGCAGTGGGGAATATTGGACAATGGGCGCAAGCCTGATCCAGCC
ATGCCGCGTGAGTGAAGAAGGCCTTAGGGTTGTAAAGCTCTTTTGGCGGGGACGATAATG
ACGGTACCCGCAGAATAAGCCCCGGCTAACTTCGTGCCAGCAGCCGCGGTAATACGAAGG
GGGCTAGCGTTGTTCGGAATCACTGGGCGTAAAGCGTACGCAGGCGGATTGATAAGTCAG
GGGTGAAATCCCGGAGCTCAACTTCGGAATTGCCTTTGATACTGTCTATCTTCGAGTTCG
GGAGAGGTTGGCGGAATTCCTAGTGTAGAGGTGAAATTCGTAGATATTAGGAAGAACACC
AGTGGCGAAGGCGGCCAACTGGCCCGATACTGACGCTCATGTACGAAAGCGTGGGGAGCA
AACAGGATTAGAAACCCCCGTAGTCC
>Otu8
CCAGCCTATGGGGGGCAGCAGTCGCGAAAACTTCACAATGGGGGCAACCCCGATGAGGGA
ATTCCAAGTGCTAGCACTTTTTGTGTTAGCTGTTCTTTTGTCTAAAAAACAAGAGAAGTA
AGGGCCGGGTAAGACGGGTGCCAGCCGCCGCGGTAATACCCGCGGCCCGAGTGGTGGTCG
ATATTATTGAGCCTAAAACGTTCGTAGCCGGTCTTGTAAATCCTTGGGTAAATCGGCCAG
CTTAACTGTCCGAAGTCCGGGGAGACTGCAAGACTTGGGATCGGGAGAGGTCAGAGGTAC
TTCTGGGGTAGGGGTAAAATCCTGTAATCCTAGAAGGACCACCGGTGGCGAAGGCGTCTG
ACTAGAACGAATTCGACGGTGAGGAACGAAGCCCTGGGGCGCAAACGGGATTAGATACCC
CAGTAGTCC
>Otu9
CCAGCCTATGGGGGGCTGCAGTAGGGAATATTGGTAATCTGCGAAAGCGGGAACCAGCAA
CGCCGCGTGTGCGATGAAGGCCTTCGGGTCGTAAAGCACTTTTTGAGGGGACGAGGAAGG
ACGGTACCCTCAGAATAAGTCTCGGCTAACTACGTGCCAGCAGCCGCGGTAACACGTAGG
AGACGAGCGTTATCCGGATTTACTGGGCGTAAAGCGCGTGCAGGCGGTTTGGTAAGTTGG
ATGTGAAAGCTCCCGGCTTAACTGGGAGAGGTCGTTCAATACTGCCAGACTAGAGGATGG
TAGAGGGAGGTGGAATTCTGGGTGTAGTGGTGAAATGCGTAGATATCCAGAGGAACACCA
GTGGCGAAAGCGGCCTCCTGGACCATTTCTGACGCTCATACGCGAAAGCTAGGGTAGCAA
ACGGGATTAGATACCCCAGTAGTCC
>Otu10
CCAGCCTATGGGGGGCAGCAGTAGGGAATATTGGGCAATGGGCGAAAGCCTGACCCAGCA
ACGCCGCGTGCACGATGAAGGTCTTCGGATCGTAAAGTGCTTTTCTGAGAGATGAGAAAG
GACAGTATCTCAGGAATAAGTCTCGGCTAACTACGTGCCAGCAGCCGCGGTAACACGTAG
GAGGCAAGCGTTATCCGGATTTACTGGGCGTAAAGCGCATGCAGGTGGTTCGGTAAGTTG
GATGTGAAAGCTCCTGGCTCAACTGGGAGAGGTCGTTCAATACTACCGGACTTGAGAGCA
GTAGAGGAAGGTGGAATTCCCGGTGTAGTGGTGAAATGCGTAGATATCGGGAGGAACACC
AGTGGCGAAAGCGGCCTTCTGGACTGTTTCTGACACTCATATGCGAAAGCTAGGGTAGCA
AACGGGATTAGAAACCCGAGTAGTCC
>Otu11
CCAGCCTACGGGTGGCAGCAGGCGCGAAAACTTTGCAATGCGGGAAACCGTGACAAGGGA
ACTCTGAGTGCCCGTAAAATCGGGCTGTCCATCGGTTTAAAAAACCGGTGAAGAAAGGGC
CGGGCAAGACCGGTGCCAGCCGCCGCGGTAATACCGGCGGCTCGAGTGGTGGCCACTATT

ACTGGGCTTAAAGCGTTCGTAGCTGGTTTGTTAAGTCTCTGGGGAAATCTTCCGGCTTAA
CCGGAAGGCGTCTCAGGGATACTGGCAGACTAGGGACCGGGAGAGGTGAGAGGTACTCCA
GGGGTAGGAGTGAAATCCTGTAATCCTTGGGGGACCACCTGTGGCGAAGGCGTCTCACCA
GAACGGCTCCGACAGTGAGGGACGAAAGCTGGGGGAGCAAACCGGATTAGATACCCGAGT
AGTCC
>Otu13
CCAGCCTACGGGGTGCAGCAGTAGGGAATATTGGACAATGGGCGAGAGCCTGATCCAGCC
ATGCCGCGTGCAGGAAGAAGGCCTTCTGGGTTGTAAACTGCTTTTGCCAGGGGATAAAAG
ACCCGTGCGCGGGGAATTGAAGGTACCTGGTGAATAAGCCACGGCTAACTACGTGCCAGC
AGCCGCGGTAATACGTAGGTGGCAAGCGTTGTCCGGATTTATTGGGTTTAAAGGGTGCGT
AGGCGGCCCTGTAAGTCAGTGGTGAAATACGGCAGCTCAACTGTCGAGGTGCCATTGATA
CTGCAGGGCTTGAGTACAGACGAGGTAGGCGGAATTGACGGTGTAGCGGTGAAATGCTTA
GATATCGTCAAGAACACCGATAGCGAAGGCAGCTTACTAGGCTGTAACTGACGCTGAGGC
ACGAAAGTGTGGGGATCAAACAGGATTAGATACCCCCGTAGTCC
>Otu15
CCAGCCTATGGGGGGCAGCAGTGGGGAATATTGCGCAATGGGGGAAACCCTGACGCAGCA
ACGCCGCGTGAGTGAAGAAGGCCTTCGGGTTGTAAAGCTCTTTCGGTTGGGAAGAAGGGG
AGCACAGTTAATAAGTGTGTTCTTTGATGGTACCAAAAGAAGAAGCACCGGCAAACTTCG
TGCCAGCAGCCGCGGTAATACGAAGGGTGCGAGCGTTGTTCGGAATTACTGGGCGTAAAG
GGTTCGTAGGCGGGAATGCAAGTCAAGTGTGAAATCCCCAGGCTCAACCTGGGACGTGCA
TTTGAGACTGTGTTTCTTGAGTTTCGGAGAGGGTGGTGGAATTGCTGGTGTAGGAGTGAC
ATCCGTAGAGATCAGCAGGAACACCGGAGGCGAAGGCGACCACCTGGCCGAATACTGACG
CTGAGGAACGAAAGCGTGGGGAGCAAACAGGATTAGATACCCCCGTAGTCC
>Otu16
CCAGCCTATGGGGGGCAGCAGTGGGGAATTTTGCGCAATGGGGGAAACCCTGACGCAGCA
ACGCCGCGTGGAGGATGAAGCCCCTTGGGGCGTAAACTCCTTTCGACCAAGACGATAATG
ACGGTACTGGTGGAAGAAGCACCGGCTAACTCTGTGCCAGCAGCCGCGGTAATACAGAGG
GTGCGAGCGTTGTTCGGAATTATTGGGCGTAAAGGGTGCGTAGGCGGTGCCACAAGTCAC
TTGTGAAATCCCCAGGCTTAACCTGGGGCCTGCAGGCGAAACTGTGGTGCTGGAGTATGG
GAGAGGTGCGTGGAATTCCCGGTGTAGCGGTGAAATGCGTAGATATCGGGAGGAACACCT
GTGGCGAAAGCGGCGCACTGGACCATAACTGACGCTGATGCACGAAAGCTAGGGGAGCAA
ACAGGATTAGAAACCCCCGTAGTCC
>Otu17
CCAGCCTACGGGGGGCAGCAGTAGGGAATATTGGTAATGTGCGAAAGCGCGAACCAGCAA
CGCCGCGTGCACGATGAAGGCCTTCGGGTTGTAAAGTGCTTTTAGAGAGGATGAGGAAGG
ACAGTACTCTCTGAATAAGCCTCGGCTAACTACGTGCCAGCAGCCGCGGTAAAACGTAGG
AGGCAAGCGTTATCCGGATTTACTGGGCGTAAAGCGCGTGTAGGTGGTTTTGTAAGTTGG
ATGTGAAAGCTCCCGGCTTAACTGGGAGAGGTCGTTCAAAACTACAAGACTTGAGAGTGG
TAGAGGAAGGTGGAATTCCGGGTGTAGTGGTGAAATGCGTAGATATCCGGAGGAACACCA
GTGGCGAAAGCGGCCTTCTGGACCATTTCTGACACTGATACGCGAAAGCTAAGGTAGCAA
ACGGGATTAGATACCCCAGTAGTCC
>Otu18
CCAGCCTATGGGTGGCAGCAGGCGCGAAAACTCTACAATGCAGGCAACTGCGATAGGGGA
ACATCGAGTGGCATCCACCATGGATGTCTGTCCAATCGTCTAAAAAACGATTGTCAGCAA
GGGCCGGGCAAGACCGGTGCCAGCCGCCGCGGTAATACCGGCGGCTCGAGTGGTGGCCGA
TATTATTGAGTCTAAAGGGTCCGTAGCCGGCTTTGCAAGTCCTATGGGAAATCCAGCGGC
TTAACCGTTGGGCTTTCATGGGATACTACATTGCTTGGGACTGGGAGAGGTAGGAGGTAC
TCAGGGGGTAGGGGTGAAATCCTGTAATCCTTTGGGGACCACCGGTGGCGAAGGCGTCCT
ACCAGAACAGGTCCGACGGTGAGGGACGAAAGCTAGGGGCACGAACCGGATTAGAAACCC
GAGTAGTCC
>Otu19
CCAGCCTATGGGGGGCAGCAGGCGCGAAAACTTTACAATGCGAGCAATCGTGATAAGGGA
ACTCCGAGTGCCTGTAAAATCAGGCTGTCCGCCAGTGTAAATAACTGGTGAAGAAAGGGC
CGGGCAAGACCGGTGCCAGCCGCCGCGGTAATACCGGCGGCTCGAGTGGTGGCCACTATT
ACTGGGCTTAAAGCGTTCGTAGCTGGTCTGTTAAGTCTCTGGGGAAATCTTCCGGCTTAA
CCGGAAGGCGTCTCAGGGATACTGGCAGACTAGGGACCGGAAGAGGTGAGAGGTACTCCA
GGGGTAGGAGTGAAATCCTGTAATCCTTGGGGGACCACCTGTGGCGAAGGCGTCTCACCA
GGACGGCTCCGACAGTGAGGAACGAAAGCTGGGGGAGCAAACCGGATTAGATACCCCCGT

AGTCC
>Otu20
CCAGCCTACGGGGGGCAGCAGTGGGGAATTTTGCGCAATGGGGGAAACCCTGACGCAGCA
ACGCCGCGTGGAGGATGAAGCTCCTTGGAGTGTAAACTCCTTTCGACCGGGAAAATTATG
ATGGTACCGGTGGAAGAAGCACCGGCTAACTCTGTGCCAGCAGCCGCGGTAATACAGAGG
GTGCGAGCGTTGTTCGGAATTATTGGGCGTAAAGGGCGCGTAGGCGGTGTTGTAAGTCAC
CTGTGAAACCTCTGGGCTTAACTCAGAGCCTGCAGGCGAAACTGCAATGCTGGAGGGTGG
GAGAGGTGCGTGGAATTCCCGGTGTAGCGGTGAAATGCGTAGATATCGGGAGGAACACCT
GTGGCGAAAGCGGCGCACTGGACCACTACTGACGCTGAGGCGCGAAAGCTAGGGGAGCAA
ACAGGATTAGATACCCGAGTAGTCC
>Otu21
CCAGCCTACGGGGGGCACCAGGCGCGAAAACTTTACAATGCGAGAAATCGTGATAAGGGA
ACTCTGAGTGCCCGTAAAATCGGGCTGTCCATCTGTTTAAATAACAGGTGAAGAAAGGGC
CGGGCAAGACCGGTGCCAGCCGCCGCGGTAATACCGGCGGCTCGAGTGGTGGCCACTATT
ACTGGGCTTAAAGCGTCCGTAGCTGGTCTGTTAAGTCTCTGGGGAAATCTTCCGGCTCAA
CCGGAAGGCGTCTCAGGGATACTGGCAGACTAGGGACCGGGAGAGGTGAGAGGTACTCCA
GGGGTAGGAGTGAAATCCTGTAATCCTTGGGGGACCACCTGTGGCGAAGGCGTCTCACCA
GAACGGCTCCGACAGTGAGGGACGAAAGCTGGGGGAGCAAACCGGATTAGATACCCGCGT
AGTCC
>Otu22
CCAGCCTATGGGGGGCAGCAGTGAGGAATATTGCGCAATGGACGAAAGTCTGACGCAGCG
ACGCCGCGTGAAGGATGAAGGCTCTTTGAGTCGTAAACTTCTGTAGAGAGGGAAGAATGT
CCCGTTCTTCGGGATTGACGGTACCTCTAAAGTAAGGATCGGCCAACTACGTGCCAGCAG
CCGCGGTAATACGTAGGATCCGAGCGTTGTCCGGAATCACTGGGTGTAAAGGGAGCGCAG
GCGGGCTTTCAAGTCGGTGGTGAAATCTTACAGCTTAACTGTAAAACTGCCTCCGATACT
GCAAGTCTTGAGTGTGGAAGAGGGCGATGGAATTCATGGTGTAGCGGTGAAATGCGTAGA
TATCATGAAGAACACCGGTCGCGAAGGCGGTCGCCTGGTCCATTACTGACGCTCATGCTC
GAAAGTGTGGGGAGCAAACAGGATTAGAGACCCGAGTAGTCC
>Otu23
CCAGCCTATGGGGGGCAGCAGCAAGGAATAGTAGGCAATGGGCGAAAGCCTGACCTCGCG
ACGCCGCGTGAAGGATGAAGGCCTTCGGGTCGTAAACTTCTTTTCTCGGGGATGAATAAT
GACAGTACTCGGGGAATAAGTCACGGCTAACTACGTGCCAGCAGCCGCGGTAAGACGTAG
GTGGCTAGCGTTATCCGGATTTACTGGGCGTAAAGAGTGCGCAGGCGGCCTTTCAAGTCA
GATGTAAAATCTCCCGGCTCAACTGGGAGGGACCATTTGATACTGTTTGGCTAGAGTACA
GCAGAGGGAAGTGGAATTCCCGGTGTAGTGGTGGAATGCGTAGATATCGGGAGGAACACC
AGTGGCGAAAGCGGCTTTCTGGGCTGTTACTGACGCTGTTGCACGAAAGCGTGGGGAGCA
AACAGGATTAGATACCCCTGTAGTCC
>Otu24
CCAGCCTATGGGAGGCAGCAGTGGGGAATATTGGACAATGGGGGAAACCCTGATCCAGCG
ACGCCGCGTGTGTGAAGAAGGCCTGCGGGTTGTAAAGCACTTTTAGTGGGGACAAAAAGC
TACGGATTAATACTTCGTGGTCTTGATTTAACCCAAAGAAAAAGCACCGGCTAACTCTGT
GCCAGCAGCCGCGGTAATACAGAGGGTGCGAGCGTTAATCGGAATTACTGGGCGTAAAGC
GCGCGTAGACGGTTTTGTAAGTCAGATGTGAAATCCCCGGGCTCAACCTGGGAACTGCAT
TTGAGACTGCATGGCTAGAGTATCGAAGAGGGAAGTGGAATTTCCGGTGTAGCGGTGAAA
TGCGTAGATATCGGAAGGAACACCAGTGGCGAAAGCGACTTCCTGGTCGAATACTGACGT
TCATGTGCGAAAGCGTGGGGAGCAAACAGGATTAGATACCCCAGTAGTCC
>Otu25
CCAGCCTACGGGGGGCAGCAGTGGGGAATATTGGACAATGGGCGCAAGCCTGATCCAGCC
ATGCCGCGTGAGTGATGACGGCCTTAGGGTTGTAAAGCTCTTTCGCCAGGGACGATAATG
ACGGTACCTGGATAAGAAGCCCCGGCTAACTTCGTGCCAGCAGCCGCGGTAATACGAAGG
GGGCTAGCGTTGTTCGGAATTACTGGGCGTAAAGCGCGTGTAGGCGGGTCTTTAAGTCAG
GGGTGAAATGCCAAGGCTCAACCTTGGAACTGCCTTTGATACTGGAGATCTTGAGTCCGG
GAGAGGTGAGTGGAACTGCGAGTGTAGAGGTGAAATTCGTAGATATTCGCAAGAACACCA
GTGGCGAAGGCGGCTCACTGGCCCGGAACTGACGCTGAGACGCGAAAGCGTGGGGAGCAA
ACAGGATTAGAAACCCGTGTAGTCC
>Otu26
CCAGCCTATGGGGGGCAGCAGTAAGGAATATTGGACAATGGTGGCAACACTGATCCAGCC
ATGCCGCGTGCAGGATGAAGGCGCTACGCGTTGTAAACTGCTTTTGTACGGGAGAAAACC

CTGCTACGTGTAGCAGGCTGATAGTACCGTAAGAATAAGCATCGGCTAACTTCGTGCCAG
CAGCCGCGGTAATACGAAGGATGCAAGCGTTATCCGGATTTATTGGGTTTAAAGGGAGCG
TAGGCGGACTTATAAGTCAGTGGTGAAATCTCGTTGCTTAACAACGAACGTGCCATTGAT
ACTGTAGGTCTTGAGTACAGATGATGTTGGCGGAATGTGTCATGTAGCGGTGAAATGCTT
AGATATGACACAGAACACCGATTGCGAAGGCAGCTGACAAAACTGTAACTGACGCTGAGG
CTCGAAAGCGTGGGGATCAAACAGGATTAGAAACCCTTGTAGTCC
>Otu27
CCAGCCTATGGGGGGCAGCAGTGGGGAATCTTGCGCAATGGGCGAAAGCCTGACGCAGCA
ACGCCGCGTGGAGGACGAAGGCTTTCTGAGTTGTAAACTCCTTTCAGCAGGGACGATTGT
GACGGTACCTGCAGAAGAAGCACCGGCCAACTACGTGCCAGCAGCCGCGGTGATACGTAG
GGTGCAAGCGTTGTCCGGATTTATTGGGCGTAAAGAGCTCGTAGGCGGTTTGATTAGTCG
GGTGTGAAACCTCCAGGCTTAACCTGGAGCCGCCACTCGATACTGTCATGACTAGAATTC
GGTAGGGGACCACGGAATTCCTGGTGTAGCGGTGAAATGCGCAGATATCAGGAGGAACAC
CAGCAGCGAAGGCGGTGGTCTGGGCCGACATTGACGCTGAGGAGCGAAAGCGTGGGGAGC
GAACAGGATTAGAAACCCCCGTAGTCC
>Otu28
CCAGCCTATGGGGGGCTGCAGTGGGGAATCTTGCACAATGGGGGAAACCCTGATGCAGCG
ACGCCGCGTGAGCGATGAAGCCCTTCGGGGTGTAAAGCTCTTTCGTCAGGGAAGATAGTG
ACGGTACCTGGAGAAGCAGCTGCGGCTAACTACGTGCCAGCAGCCGCGGTAATACGTAGG
CAGCAAGCGTTGTTCGGAGTTACTGGGCGTAAAGGGTGTGTAGGCGGTTCTTTAAGTTCG
CTGTGAAATCTCCCGGCTTAACCGGGAGGGTGCGGTGAATACTGAGGGACTAGAGTACGG
GAGAGGAAAGTGGAATTCCTGGTGTAGCGGTGAAATGCGTAGATATCAGGAGGAACACCG
GTGGTGTAGACGGCTTTCTGGACCGTAACTGACGCTGAGACACGAAAGCGTGGGTAGCAA
ACAGGATTAGAAACCCCCGTAGTCC
>Otu29
CCAGCCTACGGGTGGCACCAGTGGGGAATCTTGCGCAATGGACGAAAGTCTGACGCAGCA
ACGCCGCGTGAGGGACGAAGGCTTTCTGAGTTGTAAACCTCTTTCGACAGGAACGATTGT
GACGGTACCTGTAGAAGAAGCACCGGCCAACTATGTGCCAGCAGCCGCGGTGATACATAG
GGTGCGAGCGTTATTCGGATTTATTGGGCGTAAAGAGCTCGTAGGCGGTTCGACAAGTCG
GGTGTTAAACCCCCAGGCTCAACCTGGGGCCGCCACCCGAAACTGTTGTGACTAGAGTTT
GGTAGGGGATCACGGAATTCCTGGTGTAGCGGTGGAATGCGCAGATATCAGGAGGAACAC
CAGTAGCGAAGGCGGTGATCTGGGCCAATACTGACGCTGAGGAGCGAAAGCGTGGGGAGC
GAACAGGATTAGAGACCCCAGTAGTCC
>Otu30
CCAGCCTACGGGGGGCAGCAGTGGGGAATATTGGACAATGGGCGCAAGCCTGATCCAGCC
ATGCCGCGTGAGTGATGACGGCCCTAGGGTTGTAAAGCTCTTTCACCCACGACGATAATG
ACGGTAGTGGGAGAAGAAGCCCCGGCTAACTTCGTGCCAGCAGCCGCGGTAATACGAAGG
GGGCTAGCGTTGTTCGGATTTACTGGGCGTAAAGCGCACGTAGGCGGATCTTTAAGTCAG
GGGTGAAATGCCGAGGCTCAACTTCGGAACTGCCTTTGATACTGGAGATCTTGAGTTCGG
GAGAGGTGAGTGGAACTGCGAGTGTAGAGGTGAAATTCGTAGATATTCGCAAGAACACCA
GTGGCGAAGGCGGCTCACTGGCCCGATACTGACGCTGAGGTGCGAAAGCGTGGGGAGCAA
ACAGGATTAGATACCCGCGTAGTCC
>Otu31
CCAGCCTATGGGGGGCAGCAGTAAGGAATATTGGACAATGGTGGCAACACTGATCCAGCC
ATGCCGCGTGCAGGATGAAGGCGCTACGCGTTGTAAACTGCTTTTGTACGGGAGAAAACC
CTCGCTCGTGAGCGAGGCTGATAGTACCGTAAGAATAAGCATCGGCTAACTTCGTGCCAG
CAGCCGCGGTAATACGAAGGATGCAAGCGTTATCCGGATTTATTGGGTTTAAAGGGAGCG
TAGGCGGTTTTATAAGTCAGTGGTGAAATCTCGTTGCTTAACAACGAACGTGCCATTGAT
ACTGTAGAACTTGAGTACAGATGATGTTGGCGGAATGTGTCATGTAGCGGTGAAATGCTT
AGATATGACACAGAACACCGATTGCGAAGGCAGCTGACAAAACTGTAACTGACGCTGAGG
CTCGAAAGCGTGGGGATCAAACAGGATTAGATACCCGAGTAGTCC
>Otu32
CCAGCCTATGGGTGGCAGCAGTGGGGAATCTTGCACAATGGACGAAAGTCTGATGCAGCA
ACGCCGCGTGGGGGATGAAGCTTTTCGGAGTGTAAACCCCTTTCGACCCGGAAGAATGCC
CGCAAGGGCTTGACGGTACGGGTATAAGAAGCCCCGGCTAACTACGTGCCAGCAGCCGCG
GTAATACGTAGGGGGCCAGCGTTGCTCGGAATTACTGGGCGTAAAGGGTCTGTAGGCGGT
GTAGCAAGTCGGTAGTGAAATCCCTGGGCTTAACCCAGGGGCTGCTAACGAAACTGCTAT
GCTAGAGTGTGAGAGAGGCAAGTGGAATTACGGGTGTAGCGGTGAAATGCGTAGATATCC

GTAGGAACATCCGAGGCGAAGGCGGCTTGCTGGATCACAACTGACGCTGAGAGACGAAAG
CTAGGGGAGCAAACAGGATTAGAAACCCCCGTAGTCC
>Otu33
CCAGCCTATGGGGCGCACCAGTAGGGGATATTGGTAATCTACGCAAGTGGGAACCAGCAA
CGCCGCGTGGACGATGACGGCCTTCGGGTTGTAAAGTCCTTTTCAGTGGGAAGAGGAAGG
ACGGTACCACTGGAATAAGCCTCGGCTAACTACGTGCCAGCAGCCGCGGTAAAACGTAGG
AGGCGAGCGTTATCCGGATTTACTGGGTGTAAAGCGCATGCAGGCGGCTCGTTAAGTTGG
GTGTGAAAGCTTCCGGCTCAACTGGGAGAGGTTGCTCAAGACTGGCGAGCTAGAGAGTGG
TAGGGGAAGGCGGAATTCCGGGTGTAGTGGTGAAATGCGTAGAGATCCGGAGGAACACCA
GTGGCGAAGGCGGCCTTCTGGGCCACGACTGACGCTCAGATGCGACAGCTAGGGGAGCAA
ACGGGATTAGAAACCCGAGTAGTCC
>Otu34
CCAGCCTACGGGGGGCAGCAGTGGGGAATATTGGACAATGGGCGAAAGCCTGATCCAGCA
ATGCCGCGTGAGTGATGAAGGCCTTAGGGTTGTAAAGCTCTTTCGGGTGGGACGATGATG
ACGGTACCACCAGAAGAAGCCCCGGCTAACTTCGTGCCAGCAGCCGCGGTAATACGAAGG
GGGCTAGCGTTGTTCGGAATTACTGGGCGTAAAGCGCACGCAGGCGGCTCGATAAATTAG
AAGTGAAAGCCTTGGGCTTAACCTGAGAATTGCTTTTAAGACTGTCGAGCTAGAATCCAG
AAGAGGGTAGTGGAATTCCGAGTGTAGAGGTGAAATTCGTAGATATTCGGAAGAACACCA
GTGGCGAAGGCGACTACCTGGTCTGGCATTGACGCTCATGTGCGAAAGCGTGGGGAGCAA
ACAGGATTAGATACCCGAGTAGTCC
>Otu35
CCAGCCTACGGGGTGCTGCAGTGGGGAATTTTGCGCAATGGGGGAAACCCTGACGCAGCA
ACGCCGCGTGGAGGATGAAGCCCCTTGGGGTGTAAACTCCTTTCGATCGGGAAGATAATG
ACGGTACCGATATAAGAAGCCCCGGCTAACTTCGTGCCAGCAGCCGCGGTAATACGAGGG
GGGCAAGCGTTGTTCGGAATTATTGGGCGTAAAGGGTGCGTAGGCGGTTTGACAAGTCTG
ATGTGAAATCTATGGGCTCAACCCATAGTCTGCATCGGAAACTGTCGGGCTTGAGTATGG
GAGAGGTGAGTGGAATTTCCGGTGTAGCGGTGAAATGCGTAGATATCGGAAGGAACACCT
GTGGCGAAAGCGGCTCACTGGACCATTACTGACGCTGAGGCACGAAAGCTAGGGGAGCAA
ACAGGATTAGAAACCCTCGTAGTCC
>Otu36
CCAGCCTATGGGAGGCAGCAGTGGGGAATTTTGCGCAATGGGGGAAACCCTGACGCAGCA
ACGCCGCGTGAGTGATGAAGGCCTTCGGGTCGTAAAGCTCTGTCAGAGGGAAAGAAGTGT
AGGAGGGTTAATACCCCTTTTATTTGACGGTACCCTCAAAGGAAGCACCGGCTAACTCCG
TGCCAGCAGCCGCGGTAATACGGAGGGTGCGAGCGTTGTTCGGAATTATTGGGCGTAAAG
CGCGTGTAGGCGGTTTTTTAAGTCTGATGTGAAAGCCCTGGGCTCAACCCGGGAAGTGCA
TTGGAAACTGGGAGACTTGAATACGGGAGAGGGTAGTGGAATTCCTGGTGTAGGAGTGAA
ATCCGTAGATATCAGGAGGAACACCGGTGGCGAAGGCGGCTACCTGGACCGATATTGACG
CTGAGACGCGAAAGCGTGGGGAGCAAACAGGATTAGATACCCCTGTAGTCC
>Otu37
CCAGCCTACGGGGGGCAGCAGTCGGGAATTTTGCTCAATGGGCGCAAGCCTGAAGCAGCA
ACGCCGCGTGGGGGATGACGGCCTTCGGGTTGTAAACCTCTTTTCCCGGGGACGATGATG
ACGGTACTCGGGGAATAAGTCACGGCTAACTACGTGCCAGCAGCCGCGGTAATACGTAGG
TGACGAGCGTTGTCCGGATTTACTGGGCGTAAAGAGCGCGCAGGCGGTCGTACAAGTCGA
GTGTGAAATCCCCCGGCTCAACTGGGGAACGTCGCTCGATACTGTTCGACTTGAAGGCGG
GAGAGGGAAGCGGAATTCCCGGTGTAGTGGTGAAATGCGTAGATATCGGGAGGAACACCA
GTGGCGAAGGCGGCTTTCTGGCCCGTTCTTGACGCTGAGGCGCGAAAGCTAGGGTAGCAA
ACGGGATTAGAAACCCCAGTAGTCC
>Otu38
CCAGCCTATGGGGCGCTGCAGTGGGGAATCTTGCACAATGGGGGAAACCCTGATGCAGCG
ACGCCGCGTGAGCGATGAAGCCCTTCGGGGTGTAAAGCTCTTTCGGCAGGGACGATAATG
ACGGTACCTGCAGAAGAAGCTGCGGCTAACTACGTGCCAGCAGCCGCGGTAATACGTAGG
CAGCAAGCGTTGTTCGGAATTACTGGGCGTAAAGAGTGCGTAGGCGGTTTGGTAAGTTTG
GTGTGAAATCTCCCGGCTTAACTGGGAGGGTGCGCCGAAAACTGCCGGGCTAGAGTGTGG
GATAGGGAAGTGGAATTCCTGGTGTAGCGGTGAAATGCGTAGATATCAGGAGGAACACCT
GCGGTGTAGACGGCTTTCTGGACCACTACTGACGCTGAGGCACGAAAGCGTGGGGAGCAA
ACAGGATTAGAAACCCCAGTAGTCC
>Otu40
CCAGCCTATGGGGTGCTGCAGTCGGGAAAACTTCGCAATGCACGTAAGTGTGACGAGGGA

ATTCTCAGTGCTTAGGTTATACCTAGGCTTTTGCCAAGAGCAAGAATCTTGGCGAATAAG
TGGTGGGCAAGACCGGTGCCAGCCGCCGCGGTAACCCCGGCGCCACAAGTGGGAATCGCT
ATTATTGGGTCTAAAGCATCCGTAGCCGGTCTGTTAAATGCTCTGTGAAATCGTGGGGCT
CAACTTCACGGTGTGCAGAGTACACTGACAGACTAGGGACCAGGAAAGGCGTTCGGTATT
CCAGGGGGAGCGGTAAAATGTGATAATCCTTGGAGGACCACCAATGGCGAAGGCAGAACG
CTAGAATGGATCCGACGGTGAGGGATGAAAGCTAGGGGAGCGATCCGGATTAGATACCCG
TGTAGTCC
>Otu41
CCAGCCTATGGGGGGCAGCAGTAAGGAATATTGGTCAATGGACGCAAGTCTGAACCAGCC
ATGCCGCGTGGAGGATGAAGGCCCTCTGGGTTGTAAACTTCTTTTATTGGGGAAGAAACC
TGTTTTTTCTAAAGCAGTTGACGGTACCCGATGAATAAGCACCGGCTAACTCCGTGCCAG
CAGCCGCGGTAATACGGAGGGTGCAAGCGTTATCCGGATTCACTGGGTTTAAAGGGTGCG
TAGGTGGGCAGGTAAGTCAGTGGTGAAATCTCTGGGCTTAACCCAGAAACTGCCGTTGAT
ACTATCTGTCTTGAATGTAGTGGAGGTGAGCGGAATATGTCATGTAGCGGTGAAATGCTT
AGATATGACATAGAACATCTATTGCGAAGGCAGCTCGCTACACTATTATTGACACTGAGG
CACGAAAGCGTGGGGATCAAACAGGATTAGATACCCGAGTAGTCC
>Otu42
CCAGCCTATGGGGGGCAGCAGTGGGGAATCTTGCACAATGGACGAAAGTCTGATGCAGCA
ACGCCGCGTGGGGGATGAAGCTTTTCGGAGTGTAAACCCCTTTCGACCCGGAAGAATGCC
CGCAAGGGTTTGACGGTACGGGTATAAGAAGCCCCGGCTAACTACGTGCCAGCAGCCGCG
GTAATACGTAGGGGGCCAGCGTTGCTCGGAATTACTGGGCGTAAAGGGTCCGTAGGCGGT
GTGGCAAGTCGGGAGTGAAATCTCTGGGCTTAACCCAGAGGCTGCTTCCGAAACTGCCAT
GCTAGAGTGTGAGAGAGGCGAGTGGAATTACGGGTGTAGCGGTGAAATGCGTAGATATCC
GTAGGAACATCCGAGGCGAAGGCGGCTCGCTGGATCACAACTGACGCTGAGGGACGAAAG
CTAGGGGAGCAAACAGGATTAGAAACCCCTGTAGTCC
>Otu43
CCAGCCTATGGGGGGCAGCAGTAAGGAATATTGGACAATGCTCGCAAGAGTGATCCAGCC
ATGCCGCGTGCAGGAAGACGGCCCTATGGGTTGTAAACTGCTTTTGAACTAGAGAAAACC
CTCCTACGTGTAGGAGGCTGATAGTATAGTTAGAATAAGCATCGGCTAACTTCGTGCCAG
CAGCCGCGGTAAGACGAAGGATGCAAGCGTTATCCGGATTCATTGGGTTTAAAGGGAGCG
TAGGTGGACTTATAAGTCAGTGGTGAAATCTTTGGGCTTAACCCAAAAATTGCCATTGAT
ACTGTAGGTCTTGAGTACAGTTGCTGTGGGCGGAATATGACATGTAGTGGTGAAATACAT
AGAGATGTCATAGAACACCGATTGCGAAGGCAGCTCACAAAGCTGTAACTGACACTGAGG
CTCGAAAGTGCGGGGATCAAACAGGATTAGATACCCTTGTAGTCC
>Otu44
CCAGCCTACGGGTGGCAGCAGTCGAGAATTTTTCTCAATGGGCGAAAGCCTGAAGGAGCG
ACGCCGCGTGGGGGATGAATGGCTTCGGCCCGTAAACCCCTGTCATTTGCGAACAAATTA
TTTTACCTAACACGTAAAGTATTGATTGTAGCGGAAGAGGAAGGGACGGCTAACTCTGTG
CCAGCAGCCGCGGTAATACAGAGGTCCCAAGCGTTGTTCGGATTCACTGGGCGTAAAGGG
TGCGTAGGCGGTAGGGTAAGTCTGTTGTGAAATCTCCGAGCTTAACTCGGAAACTGCAAT
GGAAACTATCCGACTAGAGGAATGGAGGGGAGACTGGAATACTTGGTGTAGCAGTGAAAT
GCGTAGATATCAAGTGGAACACCAGTGGCGAAGGCGAGTCTCTGGACATTTCCTGACGCT
GAGGCACGAAAGCCAGGGGAGCAAACGGGATTAGATACCCCAGTAGTCC
>Otu45
CCAGCCTATGGGGGGCACCAGTGGGGAATCTTGCACAATGGAGGAAACTCTGATGCAGCG
ACGCCGCGTGAGCGATGAAGCCCTTCGGGGTGTAAAGCTCTTTCGACGGGAACGATAATG
ACGGTACCCGGAGAAGAAGCTGCGGCTAACTACGTGCCAGCAGCCGCGGTAATACGTAGG
CAGCAAGCGTTGTTCGGAATTACTGGGCGTAAAGAGTGTGTAGGCGGTTCTCTATGTTCG
GTGTGAAATCTCCCGGCTTAACTGGGAGGGTGCGCCGAAAACTGGAGGGCTTGAGTATGG
GAGAGGAAAGCGGAATTCCTGGTGTAGCGGTGAAATGCGTAGATATCAGGAGGAACACCT
GCGGTGTAGACGGCTTTCTGGACCATCACTGACGCTGAGACACGAAAGCGTGGGGAGCAA
ACAGGATTAGAAACCCCCGTAGTCC
>Otu46
CCAGCCTATGGGGGGCAGCAGTGGGGAATTTTGCGCAATGGGGGAAACCCTGACGCAGCA
ACGCCGCGTGGAGGATGAAGTCCCTTGGGACGTAAACTCCTTTCGACTGGGAAGATAATG
ACGGTACCAGTGGAAGAAGCCCCGGCTAACTTCGTGCCAGCAGCCGCGGTAATACGAGGG
GGGCAAGCGTTGTTCGGAATTATTGGGCGTAAAGGGCGCGTAGGCGGTGCGGTAAGTCAC
CTGTGAAACCTCCGGGCTCAACCCGGAGCCTGCAGGCGAAACTGCCGTGCTTGAGGGTGG

GAGAGGTGCGTGGAATTCCCGGTGTAGCGGTGAAATGCGTAGATATCGGGAGGAACACCT
GTGGCGAAAGCGGCGCACTGGACCACATCTGACGCTGAGGCGCGAAAGCTAGGGGAGCAA
ACAGGATTAGATACCCTAGTAGTCC
>Otu47
CCAGCCTATGGGGGGCAGCAGTGAGGAATTTTCGTCAATGGGGGCAACCCTGAACGAGCA
ACGCCGCGTGCAGGATGAAGGTTTTCGGATCGTAAACTGCTTTTCTCTGGGACGAGAACG
GACGGTACCAGAGGAATCAGCCCCGGCTAACTACGTGCCAGCAGCCGCGGTAATACGTAG
GGGGCAAGCGTTGTCCGGATTTATTGGGCGTAAAGCGCACGCAGGCGGTCAGATCAGTCT
GGGGTGACAGTCGTCGGCTCAACCGACGAAGTACTTCGGATACTGTCTGACTTGAGGGCT
TCAGAGGGACACGGAATTCCGGGTGGAGTGGTGAAATGCGTAGATATCCGGAGGAACACC
GATGGCGAAGGCAGTGTCCTGGGAAGTACCTGACGCTCAGGTGCGAAAGCTAGGGGAGCG
AACAGGATTAGATACCCCAGTAGTCC
>Otu48
CCAGCCTATGGGGTGCACCAGAAACTAAGGCAGGCTGTTTTAAACCCCTTATTCTTTCAA
ATATAATGACTGTACGTAGAACTCTTATCTTCTTATAAGCACTGGACTTGAAGAAAATCA
TCAAATGTTTGACAGATAAGATCTTAAGTATTTTTGCATGTTGCTTTTTTACTTAAAAGA
ACCAAGCCCGACTAACTTCGTGCAGCAGTCGCGGTAAAACGGGGGGGGTAGTGTTATTCG
TCATGACTGGGCGTAGGGAACGTAGGTGGATAGTTCAATATATCGTCAAAGACTGAGGAA
GGCCCTCTTTAATCGATATTTGCTGATTATCTTGAGTTTTAACGGAGATCTATTGAACTG
CAGCAGTAAAGGTTAAATTTGTTGATAATGTAGGATATTCGAAGGCGAAAGATTTATCTA
GTTAAATCTGACACTAAGGTTCGAATGATGGGGTTCAAAGCGGATTAGAAACCCGAGTAG
TCC
>Otu49
CCAGCCTACGGGGGGCAGCAGTGGGGAATCTTGGACAATGGGGGCAACCCTGATCCAGCC
ATGCCGCGTGAGTGATGAAGGCCTTCGGGTTGTAAAACTCTTTCGACGGGGACGATAATG
ACGGTACCCGTAGAAGAAGCTCCGGCTAACTTCGTGCCAGCAGCCGCGGTAATACGAAGG
GGGCTAGCGTTGTTCGGAATTACTGGGCGTAAAGCGCGCGCAGGCGGCCTTTCAAGTCAG
GGGTGAAAGCCCGGAGCTCAACTCCGGAATTGCCTTTGAAACTGTAAGGCTTGAGTACGG
GAGAGGTGAGTGGAATTCCCAGTGTAGAGGTGAAATTCGTAGATATTGGGAAGAACACCG
GTGGCGAAGGCGGCTCACTGGCCCGTTACTGACGCTCAGGCGCGACAGCGTGGGGATCAA
ACAGGATTAGATACCCCCGTAGTCC
>Otu50
CCAGCCTATGGGTTGCAGCAGTGGGGAATATTGCGCAATGGGCGAAAGCCTGACGCAGCA
ACGCCGCGTGGGGGATGAAGGTTCTCGGATTGTAAACCCCTTTCGATCGGAACGAATCCC
CCCCCGGTAAATAATCGGGGGGATCGACGGTACCGAGAGAAGAAGCCACGGCTAACTCTG
TGCCAGCAGCCGCGGTAAGACAGAGGTGGCGAGCGTTGTTCGGATTTATTGGGCGTAAAG
CGAACGCAGGCGGTTTGGTAAGTCAGAGGTGAAAGTCCACAGCTTAACTGTGGCACTGCC
TTTGATACTGCCTTTCTTGAGTGCATGAGGGGGAAGCGGAATTCCGAGTGTAGAGGTGAA
ATTCGTAGATATTCGGAGGAACACCGGTGGCGAAGGCGGCTTCCTGGCATGTAACTGACG
CTCATGTTCGAAAGCGTGGGTAGCAAACAGGATTAGAAACCCCCGTAGTCC
>Otu51
CCAGCCTATGGGGGGCAGCAGTGGGGAATCTTGCACAATGGAGGAAACTCTGATGCAGCG
ACGCCGCGTGAGCGATGAAGCCCCTCGGGGTGTAAAGCTCTTTCGGCAGGAACGATAATG
ACGGTACCTGAAGAAGAAGCTGCGGCTAACTACGTGCCAGCAGCCGCGGTAATACGTAGG
CAGCGAGCGTTGTTCGGAATTACTGGGCGTAAAGAGTGTGTAGGCGGTGCTCTAAGTTTG
GTGTGAAATCTCCCGGCTCAACTGGGAGGGTGCGCCGAAAACTGGGGTGCTCGAGCGTGG
GAGAGGAAAGCGGAATTCCTGGTGTAGCGGTGAAATGCGTAGATATCAGGAGGAACACCT
GTGGTGTAGACGGCTTTCTGGACCATTGCTGACGCTGAGACACGAAAGCGTGGGTAGCAA
ACAGGATTAGATACCCCCGTAGTCC
>Otu52
CCAGCCTATGGGGGGCAGCAGTGGGGAATTTTGCGCAATGGGGGAAACCCTGACGCAGCA
ACGCCGCGTGGAGGATGAAGTCCTTCGGGACGTAAACTCCTTTCGATCGGGACGATAATG
ACGGTACCGGAAGAAGAAGCCCCGGCTAACTTCGTGCCAGCAGCCGCGGTAATACGAGGG
GGGCGAGCGTTGTTCGGAATTATTGGGCGTAAAGGGTGCGTAGGCGGTTTTGTAAGTCTT
ATGTGAAATCTATGGGCTCAACTCATAGACTGCATGAGAAACTGCAGGGCTTGAGTGTGG
GAGAGGTGAGTGGAATTTCCGGTGTAGCGGTGAAATGCGTAGATATCGGAAGGAACACCT
GTGGCGAAAGCGGCTCACTGGACCACAACTGACGCTGATGCACGAAAGCTAGGGGAGCAA
ACAGGATTAGATACCCGCGTAGTCC

>Otu53
CCAGCCTATGGGGGGCTGCAGTCGAGAATTTTTCTCAATGGGGGAAACCCTGAAGGAGCG
ACGCCGCGTGGGGGATGAATGGCTTCGGCCCGTAAACCCCTGTCATTTGTGAACAAATTA
ATTCACCTAACACGTGGATTATTGATTGTAACGAAAGAGGAAGGGACGGCTAACTCTGTG
CCAGCAGCCGCGGTAATACAGAGGTCCCAAGCGTTGTTCGGATTCACTGGGCGTAAAGGG
TGCGTAGGCGGTTGGGTAAGTTTGACGTGAAATCTCCGGGCCTAACCCGGAAACTGCGTT
GAATACTATCCGACTAGAGGAATGGAGGGGAGACTGGAATACTTGGTGTAGCAGTGAAAT
GCGTAGATATCAAGTGGAACACCAGTGGCGAAGGCGAGTCTCTGGACATTTCCTGACGCT
GAGGCACGAAAGCCAGGGGAGCAAACGGGATTAGATACCCCCGTAGTCC
>Otu54
CCAGCCTATGGGGGGCAGCAGTGAGGAATTTTCGTCAATGGGGGAAACCCTGAACGAGCA
ACGCCGCGTGCAGGAAGAAGGTTTTCGGATCGTAAACTGCAAATCTCTGGGAAGAGAGAG
GACGGTACCAGAGGAGAAGCCCCGGCTAACTACGTGCCAGCAGCCGCGGTAATACGTAGG
GGGCGAGCGTTGTCCGGAGTTACTGGGCGTAAAGGGCACGCAGGCGGTCTGCTACGTTCG
GAGTGACAGTTGTCAGCTTAACTGACAAAGGTCTTCGAAAACGGGTAGACTTGAGGCCTT
GAGAGGGAGACGGAATTCCGGGTGGAGCGGTGAAATGCGTAGAGATCCGGAGGAACACCG
AAGGCGAAGGCAGTCTCCTGGCAAGGTACTGACGCTCAGGTGCGAAAGCTAGGGGAGCGA
ACAGGATTAGAAACCCCAGTAGTCC
>Otu55
CCAGCCTATGGGGGGCAGCAGTGAGGAATATTGGTCAATGGACGGAAGTCTGAACCAGCC
ATCCCGCGTGCAGGAAGAAGGCGCTATGCGTTGTAAACTGCTTTTCCGAGGGAAGAAAAC
CCCCGACGCGTCGGGGCTTGGCGGTACCTTGGGAATAAGCATCGGCTAACTCCGTGCCAG
CAGCCGCGGTAATACGGAGGATGCGAGCGTTATCCGGATTCATTGGGTTTAAAGGGTGCG
TAGGCGGACTGATAAGTCAGTGGTGAAAACCTGCAGCTTAACTGTAGAACTGCCATTGAT
ACTGTCGGACTTGAGTATGGTCAAGGTAGGCGGAATGTGTAATGTAGCGGTGAAATGCTT
AGATATTACACAGAACACCGATTGCGAAGGCAGCTTGCTGGGCCATTACTGACGCTGATG
CACGAAAGCGTGGGGAGCAAACAGGATTAGAAACCCGCGTAGTCC
>Otu56
CCAGCCTACGGGATGCAGCAGTGGGGAATCTTGCACAATGCGCGAAAGCGTGATGCAGCA
ACGCCGCGTGGGAGAAGAAGGCCTTCGGGTTGTAAATCCCTTTCAGTTGGGACGAAGCTT
CGTCGGTTAATAGCCGGTTGGAGTGACGGTACCTTCAGAAGAAGCCCCGGCTAACTACGT
GCCAGCAGCCGCGGTAATACGTAGGGGGCTAGCGTTGTCCGGAATCATTGGGCGTAAAGC
GCGTGTAGGCGGCTCAACAAGTCTGCTGTGAAAGTCCAGGGCTCAACCCTGGAATGCCGG
TGGAAACTGTTGGGCTAGAGTCCGGAAGGGGCGAGTGGAATTCCTGGTGTAGCGGTGAAA
TGCGCAGATATCAGGAGGAACACCAATGGCGAAGGCAGCTCGCTGGGACGGTACTGACGC
TGAGACGCGAAAGCGTGGGGAGCAAACAGGATTAGATACCCTAGTAGTCC
>Otu57
CCAGCCTATGGGAGGCAGCAGTTGGGAATCTTGGACAATGGGGGAAACCCTGATCCAGCC
ATGCCGCGTGAGTGATGAAGGCCTTCGGGTTGTAAAACTCTTTCGACGGGGACGATAATG
ACGGTACCCGTAGAAGAAGCTCCGGCTAACTTCGTGCCAGCAGCCGCGGTAATACGAAGG
GGGCTAGCGTTGTTCGGAATTACTGGGCGTAAAGCGCGCGCAGGCGGTCTTCCAAGTCAG
TGGTGAAAGCCCGGAGCTCAACTCCGGAACTGCCATTGAAACTGTGAGACTTGAGTACGA
GAGAGGTGAGTGGAATTCCCAGTGTAGAGGTGAAATTCGTAGATATTGGGAAGAACACCG
GTGGCGAAGGCGGCTCACTGGCTCGTAACTGACGCTCAGGCGCGACAGCGTGGGGATCAA
ACAGGATTAGAAACCCCCGTAGTCC
>Otu58
CCAGCCTATGGGGGGCTGCAGTAAGGAATATTGGACAATGGAGGCAACTCTGATCCAGCC
ATGCCGCGTGCAGGAAGAAGGCGCTACGCGTTGTAAACTGCTTTTGTACCAGAGAAAACC
TATGTACGTGTACGTAGTTGATAGTATGGTAAGAATAAGCATCGGCTAACTTCGTGCCAG
CAGCCGCGGTAATACGAAGGATGCAAGCGTTATCCGGATTTATTGGGTTTAAAGGGAGCG
TAGGCGGTCTTATAAGTCAGTGGTGAAATCTTGTCGCTTAACGACAAACGTGCCATTGAT
ACTGTAGGACTTGAGTACAGATGCCGTTGGCGGAATGTGTCATGTAGCGGTGAAATGCAT
AGATATGACACAGAACACCGATTGCGAAGGCAGCTGACGAAACTGTAACTGACGCTGAGG
CTCGAAAGCGTGGGGATCAAACAGGATTAGATACCCGTGTAGTCC
>Otu59
CCAGCCTACGGGAGGCAGCAGTGGGGAATATTGGACAATGGGCGCAAGCCTGATCCAGCC
ATGCCGCGTGAGTGATGAAGGCCTTAGGGTTGTAAAGCTCTTTTAGCAGGGAAGATAATG
ACGGTACCTGCAGAAAAAGCCCCGGCTAACTTCGTGCCAGCAGCCGCGGTAATACGAAGG

GGGCTAGCGTTGCTCGGAATTACTGGGCGTAAAGCGCACGTAGGCGGCTTCTTAAGTCGG
TGGTGAAATCCTGGAGCTCAACTCCAGAACTGCCTTCGATACTGGGAAGCTCGAGTCCGG
GAGAGGTGAGTGGAACTGCGAGTGTAGAGGTGAAATTCGTAGATATTCGCAAGAACACCA
GTGGCGAAGGCGGCTCACTGGCCCGGTACTGACGCTGAGGTGCGAAAGCGTGGGGAGCAA
ACAGGATTAGATACCCCAGTAGTCC
>Otu60
CCAGCCTATGGGGGGCAGCAGTCGAGAATTTTTCTCAATGGGCGAAAGCCTGAAGGAGCG
ACGCCGCGTGGGGGATGAATGGCTTCGGCCCGTAAACCCCTGTCATTTGCGAACAAATTG
ACTCACCTAACACGTGTGTCATTGATTGTAGCGGAAGAGGAAGGGACGGCTAACTCTGTG
CCAGCAGCCGCGGTAATACAGAGGTCCCAAGCGTTGTTCGGATTCACTGGGCGTAAAGGG
TGCGTAGGCGGTTGGGTAAGTCTGACGTGAAATCTCCGGGCCTAACCCGGAAACTGCGTT
GGATACTATCCGGCTAGAGGAATGGAGGGGAGACTGGAATACTTGGTGTAGCAGTGAAAT
GCGTAGATATCAAGTGGAACACCAGTGGCGAAGGCGAGTCTCTGGACATTTCCTGACGCT
GAGGCACGAAAGCCAGGGGAGCAAACGGGATTAGAAACCCTTGTAGTCC
>Otu61
CCAGCCTATGGGGGGCAGCAGTGGGGAATCTTGCACAATGGGGGAAACCCTGATGCAGCG
ACGCCGCGTGAGCGATGAAGCCCCTCGGGGTGTAAAGCTCTTTCGGCAGGGAAGATTATG
ACGGTACCTGCAGAAGCAGCTGCGGCTAACTACGTGCCAGCAGCCGCGGTAATACGTAGG
CAGCAAGCGTTGTTCGGAGTTACTGGGCGTAAAGGGTGTGTAGGCGGTTGCCTAAGTTTG
GTGTGAAATCTCCCGGCTTAACCGGGAGGGTGCGCCGAAAACTGGGTGACTAGAGTATGG
GATGGGTAAGTGGAATTCCTGGTGTAGCGGTGAAATGCGTAGATATCAGGAGGAACACCG
GTGGTGTAGACGGCTTACTGGACCATAACTGACGCTGAGACACGAAAGCGTGGGTAGCAA
ACAGGATTAGATACCCTAGTAGTCC
>Otu62
CCAGCCTATGGGTGGCTGCAGTTGGGAATCTTGGACAATGGGGGAAACCCTGATCCAGCC
ATGCCGCGTGAGTGATGAAGGCCTTCGGGTTGTAAAACTCTTTCGCGCACGACGATAATG
ACGGTAGTGCGAGAAGAAGCTCCGGCTAACTTCGTGCCAGCAGCCGCGGTAATACGAAGG
GGGCTAGCGTTGTTCGGAATTACTGGGCGTAAAGCGCGCGCAGGCGGCTATCCAAGTCAG
TGGTGAAAGCCCGGAGCTCAACTCCGGAACTGCCATTGAAACTGTTTAGCTTGAGGACGA
GAGAGGTGAGTGGAATTCCCAGTGTAGAGGTGAAATTCGTAGATATTGGGAAGAACACCG
GTGGCGAAGGCGGCTCACTGGCTCGTTTCTGACGCTCAGGCGCGACAGCGTGGGGATCAA
ACAGGATTAGAAACCCTCGTAGTCC
>Otu63
CCAGCCTATGGGGGGCAGCAGTCGAGAATTTTTCTCAATGGGGGAAACCCTGAAGGAGCG
ACGCCGCGTGGAGGATGAAGGCTTTCGGGTTGTAAACTCCTGTCATTTGGGAACAAGGCC
TTACGTTAACTGCGTGGAGGTTTGATAGTACCGAAAGAGGAAGAGACGGCTAACTCTGTG
CCAGCAGCCGCGGTAATACAGAGGTCTCAAGCGTTGTTCGGATTTATTGGGCGTAAAGGG
CGCGTAGGCGGCGGGGTAAGTCGGGTGTGAAATCTCAGGGCTCAACCCTGAAAATGCACT
TGATACTGCTCTGCTTGAGGACTGGAGAGGAGACTGGAATTCACGGTGTAGCAGTGAAAT
GCGTAGAGATCGTGAGGAAGACCAGTGGCGAAGGCGGGTCTCTGGACAGTTCCTGACGCT
GAGGCGCGAAGGCCAGGGGAGCAAACGGGATTAGAAACCCCCGTAGTCC
>Otu64
CCAGCCTATGGGGGGCAGCAGTGGGGAATCTTGCACAATGGGCGAAAGCCTGATGCAGCA
ACGCCGCGTGAGGGACGAAGGCTTTCTGAGTTGTAAACCTCTTTCAGCAGGGACGATTGT
GACGGTACCTGCAGAAGAAGCCCCGGCCAACTACGTGCCAGCAGCCGCGGTAATACGTAG
GGGGCAAGCGTTGTCCGGATTTATTGGGCGTAAAGAGCTCGTAGGCGGCTTGGCAAGTCG
GATGTGAAACCTCCAGGCTCAACCTGGAGTCGCCATTCGATACTGCCATGGCTAGAGTTT
GGTAGGGGACCACGGAATTCCTGGTGTAGCGGTGAAATGCGCAGATATCAGGAGGAACAC
CAGCGGCGAAGGCGGTGGTCTGGGCCAATACTGACGCTGAGGAGCGAAAGCGTGGGGAGC
GAACAGGATTAGAAACCCCAGTAGTCC
>Otu65
CCAGCCTACGGGGGGCAGCAGTAAGGGATATTGCGCAATGGGCGAAAGCCTGACGCAGCA
ACGCCGCGTGGAGGATGACGGCCTTCGGGTTGTAAACTTCTTTGGCGAGGGACGAGGAAG
GACGGTACCTCGCGAACAAGTCACGGCTAACTACGTGCCAGCAGCCGCGGTAAAACGTAG
GTGGCGAGCGTTATCCGGATTTACTGGGTGTAAAGCGTGTGCAGGCGGATGGGTAAGTGG
TGTATGAAATCGCCCGGCTCAACCGGGCGGGGTTATGCCAGACTGCCGGTCTAGAGGACG
AGAGAGGGGCGTGGAATTCCGGGTGTAGTGGTGAAATGCGTAGAGATCCGGAGGAACCCC
AGAGGCGAAGGCGGCGCCCTGGCTCGTACCTGACGCTCAGACACGACAGCATGGGGAGCG

AACGGGATTAGAAACCCCAGTAGTCC
>Otu66
CCAGCCTATGGGGGGCAGCAGTCGAGAATCTTCCGCAATGGACGAAAGTCTGACGGAGCG
ACGCCGCGTGATTGATGAAGTCCCTCTGGGACGTAAAGATCTTTTATGAGGGAAGAAGTT
TATTGACTGTACCTCATGAATAAGAGGCTCCTAATCTCGTGCCAGCAGGAGCGGTAATAC
GAGAGCCTCGAGCGTTATCCGGAATTATTGGGCGTAAAGGGTGCGTAGGTTGTTCTGTTA
GTCTTTTGTTAAAACCCCTGGCTTAACCAGGGATCCGCGAAAGAAACGGCAGAACTTGAA
AGTGTGAGAGGCGTACGGAACTCATGGTGTAGGGGTGAAATCCGTTGATATCATGGGGAA
CACCAAATGCGAAGGCAGTACGCTGGCGCATATTTGACACTGAAGCACGAAAGCGTGGGT
AGCGAATGGGATTAGAGACCCCCGTAGTCC
>Otu67
CCAGCCTACGGGGGGCAGCAGTGGGGAATCTTGCACAATGGACGAAAGTCTGATGCAGCA
ACGCCGCGTGGGGGATGAAGCTTCTCGGAGTGTAAACCCCTTTCGACCCGGAAGAAAGCC
CGCAAGGGTTTGACGGTACGGGTATAAGAAGCCCCGGCTAACTACGTGCCAGCAGCCGCG
GTAAAACGTAGGGGGCCAGCGTTGCTCGGAATTACTGGGCGTAAAGGGTCCGTAGGCGGT
GTGGCAAGTCGGTAGTGAAATCTCTGGGCTTAACTCAGAGGCTGCTGCCGAAACTGCTGT
GCTAGAGTGCGAGAGAGGCGAGTGGAATTGCGGGTGTAGCGGTGAAATGCGTAGATATCC
GCAGGAACATCCGAGGCGAAAGCGGCTCGCTGGATCGCAACTGACGCTGAGGGACGAAAG
CTAGGGGAGCAAACAGGATTAGATACCCTTGTAGTCC
>Otu68
CCAGCCTATGGGGGGCAGCAGTGGGGAATATTGGACAATGGGCGAAAGCCTGATCCAGCA
ATGCCGCGTGAATGATGAAGGCCTTAGGGTTGTAAAGTTCTTTCGGGTGGGACGATGATG
ACGGTACCACCAGAAGAAGCCCCGGCTAACTTCGTGCCAGCAGCCGCGGTAATACGAAGG
GGGCTAGCGTTGTTCGGAATTACTGGGCGTAAAGCGCACGCAGGCGGCTCGTTAAATTAG
AAGTGAAAGCCCTGGGCTCAACCCGGGAATTGCTTTTAAGACTGGCGAGCTTGAATCCGG
AAGAGGGTAGTGGAATTCCCAGTGTAGAGGTGAAATTCGTAGATATTGGGAAGAACACCA
GTGGCGAAGGCGACTACCTGGTCCGGCATTGACGCTCATGTGCGAAAGCGTGGGGAGCAA
ACAGGATTAGATACCCCCGTAGTCC
>Otu69
CCAGCCTATGGGGGGCAGCAGTGGGGAATTTTGCGCAATGGGGGAAACCCTGACGCAGCA
ACGCCGCGTGGAGGATGAAGTACTTCGGTACGTAAACTCCTTTCGATCGGGACGATAATG
ACGGTACCGGAAGAAGAAGCCCCGGCTAACTTCGTGCCAGCAGCCGCGGTAATACGAGGG
GGGCGAGCGTTGTTCGGAATTATTGGGCGTAAAGGGTGCGTAGGCGGTTTGGTAAGTCTT
TTGTGAAATCTACAGGCTCAACTTGTAGACTGCAAGGGAAACTGCCGGGCTTGAGTGTGG
GAGAGGTGAGTGGAATTCCCGGTGTAGCGGTGAAATGCGTAGATATCGGGAGGAACACCT
GTGGCGAAAGCGGCTCACTGGACCACAACTGACGCTGATGCACGAAAGCTAGGGGAGCAA
ACAGGATTAGATACCCCCGTAGTCC
>Otu70
CCAGCCTACGGGGGGCAGCAGTGGGGAATTTTGCACAATGGGGGAAACCCTGATGCAGCA
ACGCCGCGTGGAGGATGAAGTCCCTTGGGATGTAAACTCCTTTCGACCGGGACGATAATG
ACGGTACCGGTGGAAGAAGCCCCGGCTAACTCTGTGCCAGCAGCCGCGGTAATACAGAGG
GGGCAAGCGTTGTTCGGAATTATTGGGCGTAAAGGGTGCGTAGGCGGTGCGGTAAGTCTT
CTGTGAAATCTCCGGGCTCAACTCGGAGTCTGCAGGAGAAACTGCCGTGCTTGAGTATGG
GAGAGGTGAGTGGAATTCCTGGTGTAGCGGTGAAATGCGTAGATATCAGGAGGAACACCT
GTGGCGAAAGCGGCTCACTGGACCATAACTGACGCTGATGCACGAAAGCTAGGGGAGCAA
ACAGGATTAGAAACCCTAGTAGTCC
>Otu71
CCAGCCTATGGGTGGCAGCAGTCGAGAATATTCGACAATGGGCGAAAGCCTGATCGAGCG
ACACCGCGTGCAGGATGAAGGCCTTCGGGTCGTAAACTGCGGTAGTAAGATAACAATGTA
AATGAGTGTCTTACGGAAAGAGGTGGGTAACTACGTGCCAGCACCAGCGGTAATACGTAG
ACCTCAAGCGTTATCCGGATTTATTGGGCGTAAAGCGCGTGTAGGTGGCTTCGCGCGTCT
TCTGTTAAAGCCCAGGGCCTAACCCTGGAAGTGCAGGAGATACGGCGGAGCTCGAGGAGG
TTAGAGGTGCATGGAACGCACGGTGTAGGGGTGAAATCCGTTGATATCGTGCGGAATACC
AAAGGCGAAGGCATTGCACTGGGACCTTCCTGACACTGAGACGCGAAAGCGTGGGGAGCA
AAAAGGATTAGAAACCCTCGTAGTCC
>Otu72
CCAGCCTATGGGGGGCAGCAGTGAGGAATATTGGTCAATGGGCGCAAGCCTGAACCAGCC
ATCCCGCGTGCAGGAAGAAGGCGCTATGCGTCGTAAACTGCTTTTCCAGGGGAAGAAAAC

CCGTTACGAGTAACGGCTTGCCGGTACCCTGGGAATAAGCATCGGCTAACTCCGTGCCAG
CAGCCGCGGTAATACGGAGGATGCGAGCGTTATCCGGATTTATTGGGTTTAAAGGGTGCG
TAGGCGGATTAATAAGTCAGTGGTGAAAACCTTCAGCTTAACTGGAGACTTGCCATTGAT
ACTGTTAGTCTTGAGTACGGTCAAGGTAGGCGGAATGTGTAATGTAGCGGTGAAATGCTT
AGATATTACACAGAACACCGATTGCGAAGGCAGCTTACTGGGCCATTACTGACGCTGATG
CACGAAAGCGTGGGGAGCGAACAGGATTAGATACCCTAGTAGTCC
>Otu74
CCAGCCTATGGGGGGCAGCAGTGGGGAATCTTGCACAATGGGCGAAAGCCTGATGCAGCG
ACGCCGCGTGGGGGATGAAGCTTTTCGGAGTGTAAACCCCTTTCGACTGGGACGAAAACC
CGCAAGGGATTGACGGTACCAGTATAAGAAGCCCCGGCTAACTACGTGCCAGCAGCCGCG
GTAAGACGTAGGGGGCCAGCGTTGTTCGGAATTACTGGGTGTAAAGGGTTCGTAGGCGGT
GCGGCAAGTTGGGAGTGAAATCTCTGGGCTTAACCCAGAGGCTGCTTCCAAAACTGCTGT
GCTTGAGTGTGAGAGAGGCTCGTGGAATTGCAGGTGTAGCGGTGAAATGCGTAGATATCT
GCAGGAACACCCGTGGCGAAAGCGGCGAGCTGGATCACTACTGACGCTGAGGAACGAAAG
CTAGGGGAGCAAACAGGATTAGAAACCCCCGTAGTCC
>Otu75
CCAGCCTATGGGGGGCAGCAGTGGGGAATATTGGACAATGGGCGAAAGCCTGATCCAGCA
ATGCCGCGTGTGTGAAGAAGGTCTTCGGATTGTAAAGCACTTTCGACGGGGACGATGATG
ACGGTACCCGTAGAAGAAGCCCCGGCTAACTTCGTGCCAGCAGCCGCGGTAATACGAAGG
GGGCTAGCGTTGCTCGGAATGACTGGGCGTAAAGGGCGCGTAGGCGGATGGCACAGTCAG
ATGTGAAATTCCCGGGCTTAACCTGGGGGCTGCATTTGATACGTGTTGTCTAGAGTGAGG
AAGAGGGTCGTGGAATTCCCAGTGTAGAGGTGAAATTCGTAGATATTGGGAAGAACACCG
GTGGCGAAGGCGGCGACCTGGTCCTTGACTGACGCTGAGGCGCGAAAGCGTGGGGAGCAA
ACAGGATTAGAAACCCCCGTAGTCC
>Otu76
CCAGCCTATGGGGGGCAGCAGTAGGGAATTTTGCGCAATGGACGAAAGTCTGACGCAGCA
ACGCCGCGTGAGTGATGAAGGTCTTCGGATTGTAAAGCTCTGTTGAGTGGGAAGAAAAGT
TCAGGGAATAATACCTCTGAACGTGACGGTACCATTCGAGGAAGCCCCGGCTAACTACGT
GCCAGCAGCCGCGGTAATACGTAGGGGGCAAGCGTTGTTCGGAATCACTGGGCGTAAAGG
GAGAGTAGGCGGGAAAATAAGTTAGAAGTTTAATGTCCGGGCTTAACCCGGAATCTGCTT
CTAATACTGTTTTTCTTGAGTATTGGAGAGGGAGATGGAATTCCAGGTGTAGCGGTGGAA
TGCGTAGATATCTGGAAGAACACCAGCTGCGAAGGCGGTCTCCTGGCCAAATACTGACGC
TGATGCTCGAAAGCTAGGGGAGCAAACAGGATTAGAAACCCCAGTAGTCC
>Otu77
CCAGCCTACGGGTGGCTGCAGTAAGGAATATTGGACAATGGTGGCAACACTGATCCAGCC
ATGCCGCGTGCAGGATGAAGGCGCTACGCGTTGTAAACTGCTTTTGTACGGGAGAAAACC
TATCTACGTGTAGATAGCTGATAGTACCGTAAGAATAAGCATCGGCTAACTTCGTGCCAG
CAGCCGCGGTAATACGAAGGATGCAAGCGTTATCCGGATTTATTGGGTTTAAAGGGTGCG
TAGGCGGACTTATAAGTCAGTGGTGAAATCTCGATGCTTAACATCGAACGTGCCATTGAT
ACTGTAGGTCTTGAGTACAGATGCCGTTGGCGGAATGTGTCATGTAGCGGTGAAATGCAT
AGATATGACACAGAACACCGATTGCGAAGGCAGCTGACGAAACTGTAACTGACGCTGAGG
CACGAAAGCGTGGGGATCAAACAGGATTAGAAACCCTTGTAGTCC
>Otu78
CCAGCCTACGGGGGGCAGCAGTGGGGAATTTTGCGCAATGGGGGAAACCCTGACGCAGCA
ACGCCGCGTGGAGGATGAAGCCCCTTGGGGTGTAAACTCCTTTCGACTGGGAAGATTATG
ACGGTACCAGTGGAAGAAGCACCGGCTAACTCTGTGCCAGCAGCCGCGGTAATACAGAGG
GTGCGAGCGTTGTTCGGAATTATTGGGCGTAAAGGGCGCGTAGGCGGTGCGGTAAGTCAC
CTGTGAAATCCCCGGGCTTAACTCGGGGCCTGCAGGCGAAACTGCCGTGCTGGAGTATGG
GAGAGGTGCGTGGAATTCCCGGTGTAGCGGTGAAATGCGTAGATATCGGGAGGAACACCT
GTGGCGAAAGCGGCGCACTGGACCATTACTGACGCTGAGGCGCGAAAGCTAGGGGAGCAA
ACAGGATTAGATACCCGCGTAGTCC
>Otu79
CCAGCCTACGGGGGGCAGCAGTGGGGAATATTGGACAATGGGCGCAAGCCTGATCCAGCA
ATGCCGCGTGTGTGAAGAAGGCCTTCGGGTTGTAAAGCACTTTTATCAGGAGCGAAATAC
CATCGGCTAATACCCGGTGGGGCTGACGGTACCTGAGGAATAAGCACCGGCTAACTTCGT
GCCAGCAGCCGCGGTAATACGAAGGGTGCAAGCGTTAATCGGAATTACTGGGCGTAAAGC
GTGCGTAGGCGGTGAGTTAAGTCTGCTGTGAAATCCCCGGGCTCAACCTGGGAATGGCAG
TGGATACTGGCTCGCTAGAGTGTGATAGAGGATGGTGGAATTCCCGGTGTAGCGGTGAAA

TGCGTAGAGATCGGGAGGAACATCAGTGGCGAAGGCGGCCATCTGGATCAACACTGACGC
TGAGGCACGAAAGCGTGGGGAGCAAACAGGATTAGAAACCCCAGTAGTCC
>Otu80
CCAGCCTATGGGTGGCAGCAGTGGGGAATATTGGACAATGGGCGCAAGCCTGATCCAGCA
ATGCCGCGTGAGTGAAGAAGGTCTTCGGATTGTAAAGCTCTTTCGACGGGGACGATGATG
ACGGTACCCGTAGAAGAAGCCCCGGCTAACTTCGTGCCAGCAGCCGCGGTAATACGAAGG
GGGCTAGCGTTGCTCGGAATGACTGGGCGTAAAGGGCGCGTAGGCGGTTCGTACAGTCAG
ACGTGAAATTCCCGGGCTCAACCTGGGGGCTGCGTTTGATACGTGCGGGCTTGAGTTCGG
AAGAGGGTCGTGGAATTCCCAGTGTAGAGGTGAAATTCGTAGATATTGGGAAGAACACCG
GTGGCGAAGGCGGCGACCTGGTCCGATACTGACGCTGAGGCGCGAAAGCGTGGGGAGCAA
ACAGGATTAGATACCCCAGTAGTCC
>Otu81
CCAGCCTATGGGGGGCAGCAGTAAGGAATATTGGTCAATGGGCGGAAGCCTGAACCAGCC
ATGCCGCGTGCAGGAAGACGGCCCTACGGGTTGTAAACTGCTTTTATACCGGAATAAACC
CTTTTACGTGTAAGAGGCTGAATGTACGGTAAGAATAAGGATCGGCTAACTCCGTGCCAG
CAGCCGCGGTAATACGGAGGATCCAAGCGTTATCCGGATTTATTGGGTTTAAAGGGTGCG
TAGGCGGCTTTTTAAGTCAGGGGTGAAAGACGGTAGCTTAACTATCGCAGTGCCCTTGAT
ACTGAAGAGCTTGAATATACTAGAGGTAGGCGGAATGTGACAAGTAGCGGTGAAATGCAT
AGATATGTCACAGAACACCAATTGCGAAGGCAGCTTACTATGGTATGATTGACGCTGAGG
CACGAAAGCGTGGGGATCAAACAGGATTAGATACCCCCGTAGTCC
>Otu82
CCAGCCTACGGGTGGCAGCAGTAAGGAATATTGGTCAATGGGCGGAAGCCTGAACCAGCC
ATGCCGCGTGCAGGAAGACGGCCCTACGGGTTGTAAACTGCTTTTGCAGGGGAATAAACC
CCGTTACGTGTAACGGGTTGAATGTACTCTGAGAATAAGGATCGGCTAACTCCGTGCCAG
CAGCCGCGGTAATACGGAGGATCCGAGCGTTATCCGGATTTATTGGGTTTAAAGGGTGCG
TAGGCGGCTTATTAAGTCAGGGGTGAAAGACGGTAGCTTAACTATCGCAGTGCCCTTGAT
ACTGATGAGCTTGAATGTAGTTGAGGTAGGCGGAATGTGACAAGTAGCGGTGAAATGCAT
AGATATGTCACAGAACACCAATTGCGAAGGCAGCTTACTAAAGTATGATTGACGCTGAGG
CACGAAAGCGTGGGGATCAAACAGGATTAGAAACCCCAGTAGTCC
>Otu83
CCAGCCTATGGGGGGCTGCAGTGGGGAATTTTGCGCAATGGGGGAAACCCTGACGCAGCA
ACGCCGCGTGGAGGATGAAGGCCCTTGGGTCGTAAACTCCTTTCGACCGGGACGATTATG
ACGGTACCGGTGGAAGAAGCACCGGCTAACTCTGTGCCAGCAGCCGCGGTAATACAGAGG
GTGCGAGCGTTGTTCGGAATTATTGGGCGTAAAGGGCGCGTAGGCTGTGCGGTAAGTCAC
CTGTGAAACCTCCAGGCTTAACTTGGAGCCTGCAGGCGAAACTGCCGTGCTGGAGGGTGG
GAGAGGTGCGTGGAATTCCCGGTGTAGCGGTGAAATGCGTAGATATCGGGAGGAACACCT
GTGGCGAAAGCGGCGCACTGGACCACTTCTGACGCTGATGCGCGAAAGCTAGGGGAGCAA
ACAGGATTAGAAACCCCAGTAGTCC
>Otu84
CCAGCCTATGGGAGGCAGCAGTGGGGAATCTTGCGCAATGGGCGAAAGCCTGACGCAGCG
ACGCCGCGTGGGGGATGAAGCTTCTCGGAGTGTAAACCCCTTTCGACTGGGACGAATGCC
TCGCAAGAGGAGTGACGGTACCAGTAGAAGAAGCCCCGGCTAACTACGTGCCAGCAGCCG
CGGTAAGACGTAGGGGGCCAGCGTTGTTCGGAATTACTGGGTGTAAAGGGTTCGTAGGCG
GTGCGGCAAGTTGGGAGTGAAATCTCTGGGCTTAACCCAGAGGCTGCTTCCAAAACTGCC
GTGCTTGAGTGTGGGAGAGGCGCGTGGAATTGCAGGTGTAGCGGTGAAATGCGTAGATAT
CTGCAGGAACACCCGTGGCGAAAGCGGCGCGCTGGACCACAACTGACGCTGAGGAACGAA
AGCTAGGGGAGCAAACAGGATTAGAAACCCCCGTAGTCC
>Otu85
CCAGCCTATGGGGCGCTGCAGTGGGGAATATTGGACAATGGGCGCAAGCCTGATCCAGCC
ATGCCGCGTGGGTGATGAAGGCCCTAGGGTTGTAAAGCCCTTTCGGCGGGGAAGATAATG
ACGGTACCCGCAGAAGAAGCCCCGGCTAACTTCGTGCCAGCAGCCGCGGTAATACGAAGG
GGGCTAGCGTTGCTCGGAATCACTGGGCGTAAAGCGCACGTAGGCGGCTTTTTAAGTCAG
GGGTGAAATCCTGGAGCTCAACTCCAGAACTGCCTTTGATACTGAGGAGCTTGAGTCCGG
GAGAGGTGAGTGGAACTGCGAGTGTAGAGGTGAAATTCGTAGATATTCGCAAGAACACCA
GTGGCGAAGGCGGCTCACTGGCCCGGTACTGACGCTGAGGTGCGAAAGCGTGGGGAGCAA
ACAGGATTAGATACCCGCGTAGTCC
>Otu86
CCAGCCTATGGGTTGCAGCAGGCGGGTAACTTTTGCAATGCGCGCAAGCGTGACAAAGCA

AGCCAGAGTGATTTTCTATAATTGAAAATCTTTTGCGAGATGCAAAAAGTCTCGTGAATA
AGGACTGGGCAAGACTGGTGCCAGCCGCCGCGGTAATCCCAGCAGTCCGAGTCGCAGCCA
CATTTGTTGGGTCTAAAACATCCGTAGCTCGCCATCTAAGTCTCTTGTCAAATCGGGCCT
CTTAAGGGTCCGGCGAGCAAGAGATACTGTTTGGCTAGAGACCGGGAGACGTAAGGAGTA
CTATGGGAGTAGCGGTAAAATGCGTTGATCCTCATAGGACTAACAATAGCGAAGGCACCT
TACGATAACGGTTCTGACAGTGAGGGATGAAGGCTAGGGGCGCAAAGTGGATTAGAAACC
CGCGTAGTCC
>Otu87
CCAGCCTACGGGTTGCTCCAGTGGGGAATCTTGCGCAATGGGCGAAAGCCTGACGCAGCA
ACGCCGCGTGGGGGATGAAGCTTTTCGGAGTGTAAACCCCTTTCGACCCGGAAGAAGGCC
CGCAAGGGTCCGACGGTACGGGTATAAGAAGCCCCGGCTAACTACGTGCCAGCAGCCGCG
GTAAAACGTAGGGGGCCAGCGTTGCTCGGAATTACTGGGCGTAAAGGGTCTGTAGGCGGT
GTGGCAAGTTGGAAGTGAAATTTCAGGGCTTAACCCTGAAACTGCTTCCAAAACTGCCGT
GCTCGAGTGCGAGAGAGGCGAGTGGAATTGCAGGTGTAGCGGTGAAATGCGTAGATATCT
GCAGGAACATCCGAGGCGAAAGCGGCTCGCTGGATCGCAACTGACGCTGAGAGACGAAAG
CTAGGGGAGCAAACAGGATTAGATACCCCTGTAGTCC
>Otu88
CCAGCCTATGGGTTGCTGCAGTAAGGGATATTGCGCAATGGGCGAAAGCCTGACGCAGCA
ACGCCGCGTGCCCGAGGAAGGCCTTCGGGTCGTAAAGGGCTTTTAGGGCTGAAGAGCAAG
GACGGTAAGCCCGGAAGAAGTGTCGGCTAACTACGTGCCAGCAGCCGCGGTAAAACGTAG
GACGCAAGCGTTATCCGGAGTTACTGGGCGTAAAGGGCAGGCAGGCGGGCGGACAAGTCG
GGTGTGAAATCTCTCTGGCTTAACCGGGAGGGTGCATTCGAAACTGTCGGCCTGGAGGGC
AGCAGAGGAGCGTGGAATTCCGGGTGGAGTGGTGAAATGCGTAGAGATCCGGAGGAACAC
CAGAGGCGAAAGCGGCGCTCTGGGCTGCACCTGACGCTGAACTGCGAAAGCCAGGGGAGC
GAACGGGATTAGAAACCCCTGTAGTCC
>Otu89
CCAGCCTACGGGGGGCTGCAGTGGGGAATATTGGACAATGGGGGAAACCCTGATCCAGCG
ACGCCGCGTGTGTGAAGAAGGCCTGCGGGTTGTAAAGCACTTTTAGTGGGGACGAAAAGC
CTAGGGCTAATACCCTTGGGTCTTGACTTAACCCAAAGAAAAAGCACCGGCTAACTCTGT
GCCAGCAGCCGCGGTAATACAGAGGGTGCAAGCGTTAATCGGAATTACTGGGCGTAAAGC
GTGCGTAGACGGTTTTTTAAGTCGGATGTGAAATCCCCGGGCTCAACCTGGGAATTGCAT
TCGAGACTGGAAGGCTAGGGTGCGGAAGAGGGAAGCGGAATTTCTGGTGTAGCGGTGAAA
TGCGTAGATATCAGAAGGAACATCAGTGGCGAAAGCGGCTTCCTGGTCCAGCACCGACGT
TCAGGCACGAAAGCGTGGGGAGCAAACAGGATTAGATACCCCCGTAGTCC
>Otu90
CCAGCCTACGGGTGGCAGCAGTGAGGAATATTGGTCAATGGGCGGAAGCCTGAACCAGCC
ATCCCGCGTGCAGGAAGACGGCGCTATGCGTTGTAAACTGCTTTTCCAGGGGAAGAAAAC
CAGCTACGGGTAGCGGCTTGCCGGTACCCTGGGAATAAGCATCGGCTAACTCCGTGCCAG
CAGCCGCGGTAATACGGAGGATGCGAGCGTTATCCGGATTTATTGGGTTTAAAGGGTGCG
TAGGCGGACAAATAAGTCAGTGGTGAAAACCTGCAGCTTAACTGTAGACTTGCCATTGAT
ACTGTTAGTCTTGAGGGTGGTCAAGGTAGGCGGAATGTGTAATGTAGCGGTGAAATGCTT
AGATATTACACAGAACACCGATTGCGAAGGCAGCTTACTGGGCCATTACTGACGCTGATG
CACGAAAGCGTGGGGAGCGAACAGGATTAGAAACCCGAGTAGTCC
>Otu91
CCAGCCTACGGGTGGCAGCAGTAGGGAATATTGGACAATGGGTGAGAGCCTGATCCAGCC
ATGCCGCGTGCAGGAAGAAGGCCTTCTGGGTTGTAAACTGCTTTTGCCAGGGGATAAAAG
ACCCTTGCGAGGGAAATTGAAGGTACCTGGTGAATAAGCCACGGCTAACTACGTGCCAGC
AGCCGCGGTAATACGTAGGTGGCAAGCGTTGTCCGGATTTATTGGGTTTAAAGGGTGCGT
AGGCGGTTCTTTAAGTCAGTGGTGAAATACTTCGGCTCAACCGGAGATGTGCCAATGATA
CTGGGGAACTTGAGTACAGACGAGGTAGGCGGAATTGACGGTGTAGCGGTGAAATGCATA
GATATCGTCAAGAACACCGATAGCGAAGGCAGCTTACTAGACTGTAACTGACGCTGATGC
ACGAAAGTGTGGGGATCAAACAGGATTAGATACCCGAGTAGTCC
>Otu92
CCAGCCTATGGGAGGCAGCAGTAGGGAATATTGCGCAATGGACGAAAGTCTGACGCAGCA
ACGCCGCGTGTGGGATGAAGCTCCTTGGAGCGTAAACCACTGTCGGGGGGGACGAACGAT
TATGACTCTAACATAGTCATAGTTTGACTGTACCCCTAAAGGAAGCTCCGGCTAACTCCG
TGCCAGCAGCCGCGGTAATACGGGGGGAGCAAGCGTTGTTCGGAATCACTGGGCGTAAAG
GGTGCGCAGGCGGGAGATTAAGTCGGATGTGAAAACCTCGGGCTCAACCCGAGGCCTGCA

TTTGAAACTGATCTTCTTGAGTATCGGAGAGGAAAGCGGAACTCCAGGTGTAGCGGTGAA
ATGCGTAGATATCTGGAGGAACACCTGTGGCGAAGGCGGCTTTCTGGCCGAATACTGACG
CTCATGCACGAAGGCTAGGGGAGCAAACAGGATTAGATACCCTCGTAGTCC
>Otu93
CCAGCCTATGGGTTGCAGCAGTGGGGAATATTGGACAATGGGCGAAAGCCTGATCCAGCC
ATGCCGCGTGTGTGAAGAAGGTCTTCGGATTGTAAAGCACTTTAAGTTGGGAGGAAGGGC
ATGCACCTAATACGTGTGTGTCTTGACGTTACCGACAGAATAAGCACCGGCTAACTCTGT
GCCAGCAGCCGCGGTAATACAGAGGGTGCAAGCGTTAATCGGAATTACTGGGCGTAAAGC
GCGCGTAGGTGGTTCGTTAAGTTGAATGTGAAATCCCCGGGCTCAACCTGGGAACTGCAT
CCAAAACTGGCGAGCTAGAGTATGGTAGAGGGTGGTGGAATTTCCTGTGTAGCGGTGAAA
TGCGTAGATATAGGAAGGAACACCAGTGGCGAAGGCGACCACCTGGACTGATACTGACAC
TGAGGTGCGAAAGCGTGGGGAGCAAACAGGATTAGATACCCCAGTAGTCC
>Otu94
CCAGCCTATGGGGGGCTGCAGTGGGGAATCTTGCACAATGGGGGAAACCCTGATGCAGCG
ACGCCGCGTGAGCGATGAAGCCCTTCGGGGTGTAAAGCTCTTTCGGCAGGGAAGATTATG
ACGGTACCTGCAGAAGCAGCTGCGGCTAACTACGTGCCAGCAGCCGCGGTAATACGTAGG
CAGCAAGCGTTGTTCGGAGTTACTGGGCGTAAAGGGTGCGTAGGCGGCTTTTTAAGTTTG
GTGTGAAATCTCCCGGCTCAACTGGGAGGGTGCGCCGAATACTGAGAGGCTAGAGTGCGG
GAGAGGAAAGTGGAATTCCTGGTGTAGCGGTGAAATGCGTAGATATCAGGAGGAACACCG
GTGGTGTAAACGGCTTTCTGGACCGTAACTGACGCTGAGGCACGAAAGCGTGGGTAGCAA
ACAGGATTAGAAACCCCCGTAGTCC
>Otu95
CCAGCCTACGGGGGGCAGCAGGCGCGAAAACTTTACAATGCGGGAAACCGTGATAAGGGG
ACACCGAGTGCCAGCATCCTATGCTGGCTGTCCGGGTGTGTAAAATACACCTGTTAGCAA
GGGCCGGGCAAGACCGGTGCCAGCCGCCGCGGTAACACCGGCGGCCCGAGTGGTGATCGT
GATTATTGGGTCTAAAGGGTCCGTAGCCGGTTTGGTCAGTCCTCCGGGAAATCTGACAGC
TTAACTGTTAGGCTATCGGAGGATACTGCCAGACTTGGAACCGGGAGAGGTAAGAGGTAC
TACAGGGGTAGGAGTGAAATCTTGTAATCCCTGTGGGACCACCTGTGGCGAAGGCGTCTT
ACCAGAACGGGTTCGACGGTGAGGGACGAAAGCTGGGGGCACGAACCGGATTAGAAACCC
CCGTAGTCC
>Otu96
CCAGCCTATGGGGTGCAGCAGTGGGGAATCTTGCGCAATGGACGAAAGTCTGACGCAGCA
ACGCCGCGTGAGGGACGAAGGCTTTCTGAGTTGTAAACCTCTTTCGACAGGAACGATTGT
GACGGTACCTGTAGAAGAAGCACCGGCCAACTATGTGCCAGCAGCCGCGGTGATACATAG
GGTGCAAGCGTTATTCGGATTTATTGGGCGTAAAGAGCTCGTAGGCGGTTCAATAAGTGA
CGTGTTAATCCCCCAGGCTCAACCTGGGGCCGCCACGTCAAACTGTTGTGACTAGAGTTT
GGTAGGGGATCACGGAATTCCTGGTGTAGCGGTGGAATGCGCAGATATCAGGAGGAACAC
CAGTAGCGAAGGCGGTGATCTGGGCCAATACTGACGCTGAGGAGCGAAAGCGTGGGGAGC
GAACAGGATTAGAAACCCTAGTAGTCC
>Otu97
CCAGCCTACGGGTTGCTCCAGGCGCGAAAACTTCGCAATGTGGGTAACCACGACGAGGGA
ATTCCAAGTACTAGTACTTTGTATTAGTGGTTTTCCTGTCCAAAGAACAGGGAGGGTAAG
GGCTGGGTAAGACTGGTGCCAGCCGCCGCGGTAATACCAGCAGCCCAAGTGGTGATCATT
ATTATTGGGTCTAAAACGTCCGTAGTCGGCTTAGTAAATTCTTGGGTAAATTGTGAAGCT
CAACTTCACGAATTCCGAGGAGACTGCTAGGCTTGGGACCGGGGGAGGTTGGAGGTACTT
CGGGGGTAGGGGTGAAATCCTGTAATCCTCGAGGGACCACCAGTGGCGAAGGCGTCTGAC
CAAAACGGGTCCGACGACAAGGGACGAAGCCTAGGGGCGCAAACGGGATTAGATACCCTA
GTAGTCC
>Otu99
CCAGCCTATGGGGCGCTGCAGTGGGGAATATTGGACAATGGGCGCAAGCCTGATCCAGCC
ATGCCGCGTGTGTGAAGAAGGCCTTCGGGTTGTAAAGCACTTTAAGCGGGGAGGAAGGGC
TTATGGCTAATAACCGTGAGCATTGACGTTACCCGCAGAATAAGCACCGGCTAACTCTGT
GCCAGCAGCCGCGGTAATACAGAGGGTGCAAGCGTTAATCGGAATTACTGGGCGTAAAGC
GCGCGTAGGCGGTTGTGCAAGTTGGAGGTGAAATCCCCGGGCTTAACCTGGGCACTGCCT
TCAAAACTGCACGGCTAGAGTATGGGAGAGGAAGGTAGAATTCCAGGTGTAGCGGTGAAA
TGCGTAGAGATCTGGAGGAATACCGATGGCGAAGGCAGCCTTCTGGCCTAATACTGACGC
TGAGGTGCGAAAGCATGGGGAGCAAACAGGATTAGAAACCCCCGTAGTCC
>Otu100

CCAGCCTACGGGTGGCAGCAGTCGCGAAAACTTCACAATGGGAGAAATCCCGATGAGGGA
ATTCCAAGTGCTAGCACTATGTGTTAGCTGTTCTTTTGTCTAAAAAACAGAAGAAGTAAG
GGCCGGGTAAGACGGGTGCCAGCCGCCGCGGTAATACCCGCGGCCCGAGTGGTGGTCGAT
ATTATTGAGCCTAAAACGTCCGTAGCCGGTCTTGTAAATCCTTGGGTAAATCGGCCAGCT
TAACTGTCCGAAGTCCGGGGAGACTGCAAGACTTGGGATCGGGAGAGGTCAGAGGTACTT
CTGGGGTAGGGGTAAAATCCTGTAATCCTGGAAGGACCACCGGTGGCGAAGGCGTCTGAC
TAGAACGAATTCGACGGTGAGGGACGAAGCCCTGGGGCGCAAACGGGATTAGATACCCCT
GTAGTCC
>Otu101
CCAGCCTATGGGTGGCACCAGTCGAGAATATTCGACAATGGACGAAAGTCTGATCGAGCG
ACACCGCGTGTAGGATGAAGGCCCTCGGGTCGTAAACTACGGTAGTATGATAAGAATGCA
AATGACTGTCATACGGAAAGAGGTGGGTAACTACGTGCCAGCACCAGCGGTAATACGTAG
ACCTCAAGCGTTATCCGGATTTATTGGGCGTAAAGAGCATGTAGGAGGTTTCGCGCGTCT
TTTGTTAAAGCCCAGGGCCTAACCCTGGAAGTGCAGGAGATACGGCGGAACTAGAGGAGG
TTAGAGGTGCATGGAACTCACGGTGTAGGGGTGAAATCCGTTGATATCGTGGGGAACACC
AAAGGCGAAGGCAGTGCACTGGGACCTTCCTGACTCTGAGATGCGAAAGCGTGGGGAGCA
AAAAGGATTAGAAACCCGAGTAGTCC
>Otu102
CCAGCCTACGGGGGGCTGCAGTGAGGAATATTGCGCAATGGGGGCAACCCTGACGCAGCA
ACGCCGCGTGAGTGAGGAAGGTTTTCGGATTGTAAAGCTCTGTCGAGCGGAAAGAAATGC
ATGGTGGCTAATATCCATCATGCTTGACGGTACCGCTAAAGGAAGCACCGGCTAACTCCG
TGCCAGCAGCCGCGGTAATACGGGGGGTGCAAGCGTTGTTCGGAATTATTGGGCGTAAAG
AGCGTGTAGGCGGCTGGACAAGTCAGATGTGAAAGCCCTGGGCTTAACCTAGGAAGTGCA
TTTGAAACTGTCCGGCTTGAGTAAGGGAGAGGAAAGTGGAATTCCTGGTGTAGAGGTGAA
ATTCGTAGATATCAGGAGGAACACCGGTGGCGAAGGCGACTTTCTGGCCCTATACTGACG
CTGAGACGCGAAAGCGTGGGTAGCAAACAGGATTAGATACCCCCGTAGTCC
>Otu103
CCAGCCTATGGGTGGCAGCAGTAAGGAATATTGGTCAATGGACGAAAGTCTGAACCAGCC
ATGCCGCGTGGAGGATGAAGGCCCTCTGGGTTGTAAACTTCTTTTATTTGGGAAGAAACC
CATTTTTTCTAAGATGGTTGACGGTACCAGATGAATAAGCACCGGCTAACTCCGTGCCAG
CAGCCGCGGTAATACGGAGGGTGCAAGCGTTATCCGGATTCACTGGGTTTAAAGGGTGCG
TAGGCGGGCAGGTAAGTCAGTGGTGAAATCTCCGGGCTTAACCCGGAAACTGCCGTTGAT
ACTATCTGTCTTGAATGTCGTGGAGGTTAGCGGAATATGTCATGTAGCGGTGAAATGCTT
AGATATGACATAGAACACCAATTGCGAAGGCAGCTAACTACACGGTTATTGACGCTGAGG
CACGAAAGCGTGGGGATCAAACAGGATTAGAAACCCTCGTAGTCC
>Otu104
CCAGCCTATGGGTGGCTGCAGTCGAGAATTTTTCTCAATGGGGGAAACCCTGAAGGAGCG
ACGCCGCGTGGAGGATGAAGGTCTTCGGATTGTAAACTCCTGTCATGAGGGAACAAACCT
GCATGTTTAACTGATGTGCAGCTGATAGTACCTCAAGAGGAAGAGACGGCTAACTCTGTG
CCAGCAGCCGCGGTAATACAGAGGTCTCAAGCGTTGTTCGGATTCATTGGGCGTAAAGGG
TGCGTAGGCGGCGAAGCAAGTCAGGTGTGAAATCCCGGGGCTCAACCCCGGAACTGCACT
TGATACTGCTTTGCTTGAGGACTGGAGAGGAGATCGGAATTCACGGTGTAGCAGTGAAAT
GCGTAGATATCGTGAGGAAGGCCAGTGGCGAAGGCGGATCTCTGGACAGTTCCTGACGCT
GAGGCACGAAGGCCAGGGGAGCAAACGGGATTAGAAACCCTTGTAGTCC
>Otu105
CCAGCCTATGGGGCGCAGCAGTCGAGGATCTTCGGCAATGGGCGCAAGCCTGACCGAGCG
ACGCCGCGTGTGCGATGAAGGCCTTCGGGTTGTAAAGCACTGTCGAGGGGGAGGAAAGCC
CGCAAGGGCCTGACCTATCCCTGGAGGAAGCACGGGCTAAGTTCGTGCCAGCAGCCGCGG
TAAGACGAACCGTGCGAACGTTGTTCGGAATCACTGGGCTTAAAGGGCGCGTAGGCGGGT
GGTCAAGTCAGGGGTGAAATCCTCCAGCTCAACTGGAGAACAGCTTTTGATACTGGCCAT
CTTGAGGGAGGTAGGGGCATGTGGAACTTCCGGTGGAGCGGTGAAATGCGTAGATATCGG
AAGGAACGCCGGTGGCGAAAGCGACGTGCTGGACCTTTTCTGACGCTGAGGCGCGAAAGC
CAGGGGAGCAAACGGGATTAGAAACCCCCGTAGTCC
>Otu106
CCAGCCTATGGGGGGCAGCAGTGGGGAATATTGGACAATGGGCGCAAGCCTGATCCAGCC
ATGCCGCGTGAGTGATGACGGCCTTAGGGTTGTAAAGCTCTTTCGCACGCGACGATAATG
ACGGTAGCGTGAGAAGAAGCCCCGGCTAACTTCGTGCCAGCAGCCGCGGTAATACGAAGG
GGGCTAGCGTTGTTCGGAATTACTGGGCGTAAAGCGCGTGTAGGCGGGCTTTTAAGTCAG

GGGTGAAATGCCGAGGCTCAACCTCGGAACTGCCTTTGATACTGGAAGTCTTGAGTCCGG
GAGAGGTGAGTGGAACTGCGAGTGTAGAGGTGAAATTCGTAGATATTCGCAAGAACACCA
GTGGCGAAGGCGGCTCACTGGCCCGGAACTGACGCTGAGACGCGAAAGCGTGGGGAGCAA
ACAGGATTAGATACCCCAGTAGTCC
>Otu107
CCAGCCTACGGGGGGCTGCAGTCGAGAATTTTTCACAATGGGCGCAAGCCTGATGGAGCG
ACGCCGCGTGGGGGATGAATGGCTTCGGCCCGTAAACCCCTGTCATTTGCGATCAACCGT
TGTTGTTTAAGAGATGACAACCTGATAGTAGCGAAAGAGGAAGGGACGGCTAACTCTGTG
CCAGCAGCCGCGGTAATACAGAGGTCCCAAGCGTTGTTCGGATTCACTGGGCGTAAAGGG
TGCGTAGGTGGCGGGGTAAGTCGGATGTGAAATCTCCGAGCTCAACTCGGAAATGGCATT
GGAAACTGCTCTGCTCGAGGGTTGGAGGGGGGACTGGAATACTTGGTGTAGCAGTGAAAT
GCGTAGATATCAAGTGGAACACCAGTGGCGAAGGCGAGTCCCTGGACAACTCCTGACACT
GAGGCACGAAAGCTAGGGGAGCAAACAGGATTAGAGACCCTCGTAGTCC
>Otu108
CCAGCCTATGGGGGGCAGCAGTGGGGAATATTGGACAATGGGGGAAACCCTGATCCAGCA
ATGCCGCGTGTGTGAAGAAGGTCTTCGGATTGTAAAGCACTTTTGGCAGGGACGATGATG
ACGGTACCTGCAGAATAAGCCCCGGCTAACTTCGTGCCAGCAGCCGCGGTAATACGAAGG
GGGCTAGCGTTGCTCGGAATGACTGGGCGTAAAGGGCGCGTAGGCGGCACATACAGTCAG
AAGTGAAATTCCTGGGCTCAACCTGGGGACTGCTTTTGATACGTGTGAGCTAGAGTGGAG
AAGAGGGTTGTGGAATTTCCAGTGTAGAGGTGAAATTCGTAGATATTGGAAAGAACACCG
GTGGCGAAGGCGGCAACCTGGTCTTCTACTGACGCTGAGGCGCGAAAGCGTGGGGAGCAA
ACAGGATTAGAAACCCCCGTAGTCC
>Otu109
CCAGCCTACGGGGGGCAGCAGTGGGGAATCTTGCACAATGGGGGAAACCCTGATGCAGCG
ACGCCGCGTGAGCGATGAAGCCCTTCGGGGTGTAAAGCTCTTTCGGCAGGGAAGATTATG
ACGGTACCTGCAGAAGCAGCTGCGGCTAACTACGTGCCAGCAGCCGCGGTAATACGTAGG
CAGCGAGCGTTGTTCGGAGTTACTGGGCGTAAAGGGTGTGTAGGCGGTTGTTTAAGTTTG
GTGTGAAATCTCCCGGCTTAACCGGGAGGGTGCGCCGAATACTGAGCGACTAGAGTATGG
GAGAGGAAAGTGGAATTCCTGGTGTAGCGGTGAAATGCGTAGATATCAGGAGGAACACCG
GCGGTGTAGACGGCTTTCTGGACCATAACTGACGCTGAAACACGAAAGCGTGGGTAGCAA
ACAGGATTAGATACCCTAGTAGTCC
>Otu110
CCAGCCTATGGGACGCTGCAGGCGCGAAACCTTTACAATGCACGTAAGTGTGACGAGGGA
ATTCTCAGTGCTTAGGTTATACCTAGGCTTTTGCCAAGACCAAGACTCTTGGCGAATAAG
TGGTGGGCAAGACCGGTGCCAGCCGCCGCGGTAACCCCGGCGCCACGAGTGGGAATCGCT
TTTATTGGGTCTAAAGCATCCGTAGCCGGTTTGTTAAATGTTCTGTGAAATCGTGAAGCT
CAACTTCACGGTGTGCAGAGCACACTGACAAACTTGGGACCAGGAAAGGTGTTCGGTATT
CCAGGGGGAGCGGTAAAATGTGATAATCCTTGGAGGACCACCAATGGCGAAGGCAGAACG
CTAGAATGGATCCGACGGTGAGGGATGAAAGCTAGGGGAGCGATCCGGATTAGAAACCCC
TGTAGTCC
>Otu111
CCAGCCTACGGGGGGCAGCAGTGGGGAATCTTGCACAATGGAGGAAACTCTGATGCAGCG
ACGCCGCGTGAGCGATGAAGCCCTTCGGGGTGTAAAGCTCTTTCGACGGGAACGATAATG
ACGGTACCCGAAGAAGAAGCTGCGGCTAACTACGTGCCAGCAGCCGCGGTAATACGTAGG
CAGCAAGCGTTGTTCGGAATTACTGGGCGTAAAGAGTGTGTAGGCGGTGTTCTAAGTCTG
TTGTGAAATCTCCCGGCTTAACTGGGAGGGTGCGGCGGAGACTGGAATGCTAGAGTATGG
GAGAGGTAAGCGGAATTCCTGGTGTAGCGGTGAAATGCGTAGATATCAGGAGGAACACCT
GTGGTGTAGACAGCTTACTGGACCATGACTGACGCTGAGACACGAAAGCGTGGGTAGCAA
ACAGGATTAGATACCCTCGTAGTCC
>Otu112
CCAGCCTATGGGGCGCAGCAGGCGCGAAAACTTTACACTACACGAAAGTGTGATAAGGGG
ATTCCAAGTGGTTATACACATGTATAATCTTTTTTTGAGAGCAAAGATCTCGAAGAATAA
GTGGTGGGTAAGACCGGTGCCAGCAGCCGCGGTAATCCCGGCGCCACAAGTAGGGACCAG
GGTTATTGGGCCTAAAGCGTTCGTAGCAGGTCGGGTAAATCTCTTGTGAAATCGTTGGGC
TTAACCTAACGGCGTGCAGGAGAGACTGCTCGACTTGAGACCGGGAGGGGTTGAGAGTAT
TCCTTGGGGAGCGGTAAAATGCTATAATCCTTGGAAGACTACCTGTGGCGAAGGCGCTCA
ACCAGAACGGGTCTGACTGTGAGGGACGAAAGCCAGGGGAGCAATCCGGATTAGATACCC
TAGTAGTCC

>Otu113
CCAGCCTACGGGGTGCTGCAGTGGGGAATCTTGCACAATGGGCGAAAGCCTGATGCAGCG
ACGCCGCGTGGGGGATGAAGCATTTCGGTGTGTAAACCCCTTTCGACCGGGAAGAATACC
TAGCAATAGGGGTGACGGTACCGGTAGAAGAAGCCCCGGCTAACTACGTGCCAGCAGCCG
CGGTAAGACGTAGGGGGCCAGCGTTGTTCGGAATTACTGGGTGTAAAGGGTTCGTAGGCG
GTGTGGCAAGTTGGGAGTGAAATCTCTGGGCTTAACCCAGAGGCTGCTTCCAAAACTGCT
GTGCTTGAGTGTGGGAGAGGTGCGTGGAATTGCAGGTGTAGCGGTGAAATGCGTAGATAT
CTGCAGGAACACCCGTGGCGAAAGCGGCGCACTGGACCACAACTGACGCTGAGGAACGAA
AGCTAGGGGAGCAAACAGGATTAGATACCCCTGTAGTCC
>Otu114
CCAGCCTATGGGGGGCAGCAGTCGAGAATATTCGACAATGGGCGAAAGCCTGATCGAGCG
ACACCGCGTGCAGGATGAAGGCCTTCGGGTCGTAAACTGCGGTAGATAAGTAACAACGTA
AGTGAGTGCTTATCGGAAAGAGGTGGGTAACTACGTGCCAGCACCAGCGGTAAAACGTAG
ACCTCAAGCGTTATCCGGATTTATTGGGCGTAAAGCGCGTGTAGGTGGCTTCGCACGTCT
TCTGTTAAAGCCCAGGGCCTAACCCTGGAAGTGCAGGAGATACGGCGGAGCTAGAGGAGG
TTAGAGGTGCATGGAACGCACGGTGTAGGGGTGAAATCCGTTGATATCGTGCGGAATACC
AAAGGCGAAGGCATTGCACTGGGACCTTCCTGACACTGAGCCGCGAAAGCGTGGGGAGCA
AAAAGGATTAGAGACCCGTGTAGTCC
>Otu115
CCAGCCTATGGGAGGCAGCAGTGGGGAATCTTGCACAATGGGGGAAACCCTGATGCAGCG
ACGCCGCGTGAGCGATGAAGCCCTTCGGGGTGTAAAGCTCTTTCGACGGGAACGATAATG
ACGGTACCCGGAGAAGAAGCTGCGGCTAACTACGTGCCAGCAGCCGCGGTAATACGTAGG
CAGCAAGCGTTGTTCGGAGTTACTGGGCGTAAAGAGTGCGTAGGCGGTTCCGCAAGTTTG
GTGTGAAATCTCCCGGCTTAACTGGGAGGGTGCGCCGAAAACTGCGGGGCTGGAGTGTGG
GAGAGGTAAGCGGAATTCCTGGTGTAGCGGTGAAATGCGTAGATATCAGGAGGAACACCG
GCGGTGTAGACGGCTTACTGGACCATGACTGACGCTGAGGCACGAAAGCGTGGGGAGCAA
ACAGGATTAGAAACCCCTGTAGTCC
>Otu116
CCAGCCTATGGGGGGCTGCAGTCGCGAAAACTTTGCAATACACGAAAGTGTGACAGGGTC
ATCCCGAGTGCCGACCGCTGAGGTTGGCTTTTACCCAGTCTAGAAAGCTGGGGGAATAAG
GAGAGGGCAAGTCTGGTGTCAGCCGCCGCGGTAATACCAGCTCTCCGAGTGGTGTGGACG
TTTATTGGGCCTAAAGCATCCGTAGCTGGCTGGACAAGTCCCCTGTTAAACCCACCGATT
TAATCGTTGGCTTGCGGGGGATACTGCTCGGCTAGGGGACGAGAGAGGCAGACGGTATTC
CCGGGGTAGGGGTGAAATCCTATAATCCCGAGAAGACCACCAGTGGCGAAGGCTGTCTGC
TAGAACGCGCCCGACGGTGAGGGATGAAAGCTGGGGGAGCGAACCGGATTAGAGACCCCA
GTAGTCC
>Otu117
CCAGCCTACGGGTTGCTGCAGTCGAGAATATTCCACAATGGGGGAAACCCTGATGGAGCG
ACACCGCGTGGGGGATGAAGGCCTTCGGGTCGTAAACCCCTTTTATATGGGACGAAGCGC
AAGCTGACGGTACCATACGAATAAGAGGTTGCTAACTCTGTGCCAGCAGCAGCGGTAATA
CAGAGACCTCAAGCGTTATCCGGATTCACTGGGCGTAAAGCGTCCGCAGGTGGTTTGTTA
AGTCGTTTGTCAAATCCCGGTGCTCAACATCGGAACTGCGAACGATACTGGCAAACTTGA
GGATGGAAGAGGTAAGAGGAACTGCTGGTGTAGTAGTAAAATGCGTTAATATCAGCAGGA
ACACCAAAGGCGAAGGCATCTTACTGGGACATTCCTGACACTCATGGACGAAAGCGTGGG
GAGCAAAACGGATTAGAAACCCTTGTAGTCC
>Otu118
CCAGCCTATGGGGGGCACCAGTCGAGGATCTTCGTCAATGGGCGCAAGCCTGAACGAGCG
ACGCCGCGTGCGCGATGAAGGCCTTCGGGTTGTAAAGCGCGAAAGTGGGGAGAAAGGGAA
ACTTGATCAATCCACAGTAAGGACGGGCTAAGTTCGTGCCAGCAGCCGCGGTAAGACGAA
CTGTCCTAACGTTGTTCGGAATCACTGGGCTTAAAGGGCGCGTAGGCGGTCCAACAAGTC
GGGGGTGAAATCCTGCAGCTTAACTGCAGAAGTGCCTTCGATACTGTTGGTCTCGAGGGA
GATAGGGGTGTGCGGAACTTCCAGTGGAGCGGTGAAATGCGTTGATATTGGAAGGAACGC
CGGAGGCGAAAGCGGCGCACTGGATCTCTTCTGACGCTGAGGCGCGAAAGCTAGGGGAGC
AAACGGGATTAGATACCCCCGTAGTCC
>Otu119
CCAGCCTATGGGTCGCAGCAGTGGGGAATCTTGCACAATGGGGGAAACCCTGATGCAGCG
ACGCCGCGTGAGCGATGAAGCCCTTCGGGGTGTAAAGCTCTTTCGGCAGGGAAGATAGTG
ACGGTACCTGCAGAAGCAGCTGCGGCTAACTACGTGCCAGCAGCCGCGGTAATACGTAGG

CAGCGAGCGTTGTTCGGAGTTACTGGGCGTAAAGGGTGTGTAGGCGGTTGTTTAAGTTTG
GTGTGAAATCTCCCGGCTCAACTGGGAGGGTGCGCCGAATACTGAATGACTTCGAGTGCG
GGAGAGGAAAGTGGAATTCCTGGTGTAGCGGTGAAATGCGTAGATATCAGGAGGAACACC
GGTGGTGTAGACGGCTTTCTGGACCGTAACTGACGCTGAGACACGAAAGCGTGGGTAGCA
AACAGGATTAGAAACCCTTGTAGTCC
>Otu120
CCAGCCTATGGGTCGCTGCAGTGGGGAATTTTGCGCAATGGGGGAAACCCTGACGCAGCA
ACGCCGCGTGGAGGATGAAGTCCCTTGGGACGTAAACTCCTTTCGACCAAGACGATAATG
ACGGTACTGGTGGAAGAAGCACCGGCTAACTCTGTGCCAGCAGCCGCGGTAATACAGAGG
GTGCAAGCGTTGTTCGGAATTATTGGGCGTAAAGGGCGCGTAGGCGGCGCGACAAGTCAC
CTGTGAAATCTCCGGGCTTAACTCGGAGCCTGCAGGCGAAACTGTCGTGCTGGAGTATGG
GAGAGGTGCGTGGAATTCCCGGTGTAGCGGTGAAATGCGTAGATATCGGGAGGAACACCT
GTGGCGAAAGCGGCGCACTGGACCATAACTGACGCTGAGGCGCGAAAGCTAGGGGAGCAA
ACAGGATTAGATACCCTTGTAGTCC
>Otu121
CCAGCCTATGGGTGGCAGCAGTGGGGAATCTTGCACAATGGACGAAAGTCTGATGCAGCA
ACGCCGCGTGGGGGATGAAGCTTCTCGGAGTGTAAACCCCTTTCGACCCGGAAGAATGCC
CGCAAGGGTTTGACGGTACGGGTATAAGAAGCCCCGGCTAACTACGTGCCAGCAGCCGCG
GTAATACGTAGGGGGCCAGCGTTGCTCGGAATTACTGGGCGTAAAGGGTCCGTAGGCGGT
GCGGCAAGTCGATAGTGAAATCCCTAGGCTTAACCTAGGGGCTGCTAGCGAAACTGCCGT
GCTAGAGTGTGAGAGAGGCGAGTGGAATTACGGGTGTAGCGGTGAAATGCGTAGATATCC
GTAGGAACATCCGAGGCGAAAGCGGCTCGCTGGATCACAACTGACGCTGAGGGACGAAAG
CTAGGGGAGCAAACAGGATTAGATACCCTAGTAGTCC
>Otu122
CCAGCCTATGGGGGGCAGCAGTAGGGAATATTGGTAATGGGCGAAAGCCTGAACCAGCAA
CGCCGCGTGAACGATGAAGGCCTTCGGGTTGTAAAGTTCTTTTTGGAGGGATGAGGAAGG
ACAGTACCTCCAGAATAAGTCTCGGCTAACTACGTGCCAGCAGCCGCGGTAACACGTAGG
AGACTAGCGTTATCCGGATTTACTGGGCGTAAAGCGTGTGCAGGCGGCTCGGATAGTTGG
ATGTGAAAGCTCCTGGCTTAACTGGGAGAGGTCGTTCAATACTTCCGAGCTAGAGGATGA
GAGAGGGAGGTGGAATTCCGGGTGTAGTGGTGAAATGCGTAGATATCCGGAGGAACACCA
GTGGCGAAAGCGGCCTCCTGGCTCATTTCTGACGCTCATACACGAAAGCTAGGGTAGCAA
ACGGGATTAGAAACCCCCGTAGTCC
>Otu123
CCAGCCTATGGGGTGCAGCAGTAGGGAATATTGGACAATGGGCGCAAGCCTGATCCAGCC
ATCCCGCGTGAAGGATTAAGGCCCTATGGGTTGTAAACTTCTTTTCTCTGGGAATAAAAA
GTGGGATTTATCCTGCCTTGAAGGTACCAGAGGAATAAGCACCGGCTAACTCCGTGCCAG
CAGCCGCGGTAATACGGAGGGTGCAAGCGTTATCCGGATTCACTGGGTTTAAAGGGTGCG
TAGGTGGCTTTGTAAGTCAGTGGTGAAAGCCCGGAGCTCAACTCCGGAACTGCCATTGAT
ACTGCTTAGCTTGAATCAGATTGAGGTGGATGGAATATTACATGTAGCGGTGAAATGCTT
AGATATGTAATAGAACACCGATTGCGAAGGCAGTTCACTAAGTCTGTATTGACACTGAGG
CACGAAAGCGTGGGGATCAAACAGGATTAGATACCCTAGTAGTCC
>Otu124
CCAGCCTACGGGGGGCTGCAGTTGGGAATTTTGGACAATGGGGGAAACCCTGATCCAGCC
ATGCCGCGTGAGTGATGAAGGCCTTAGGGTTGTAAAGCTCTTTTACCCGGGAAGATAATG
ACTGTACCGGGAGAATAAGCTCCGGCTAACTTCGTGCCAGCAGCCGCGGTAATACGAAGG
GGGCTAGCGTTGTTCGGAATTACTGGGCGTAAAGCGCGCGCAGGCGGCCAATCAAGTCAG
GGGTGAAAGCCCAGAGCTCAACTCTGGAACTGCCTTTGAAACTGTTTGGCTAGAGGACGG
GAGAGGTGAGTGGAATTCCCAGTGTAGAGGTGAAATTCGTAGATATTGGGAAGAACACCG
GTGGCGAAGGCGGCTCACTGGCCCGTTTCTGACGCTCAGGCGCGACAGCGTGGGGATCAA
ACAGGATTAGAGACCCGTGTAGTCC
>Otu125
CCAGCCTATGGGGGGCAGCAGTGGGGAATTTTGCGCAATGGGGGAAACCCTGACGCAGCA
ACGCCGCGTGGAGGATGAAGTACTTCGGTACGTAAACTCCTTTCGATCGGGACGATAATG
ACGGTACCGGAAGAAGAAGCCCCGGCTAACTTCGTGCCAGCAGCCGCGGTAATACGAGGG
GGGCAAGCGTTGTTCGGAATTATTGGGCGTAAAGGGTGCGTAGGCGGTTTGGTAAGTCTT
GTGTGAAATCTCCGGGCTCAACTCGGAGTCTGCATAGGAAACTGCCGGGCTTGAGTGTGG
GAGAGGTGAGTGGAATTTCCGGTGTAGCGGTGAAATGCGTAGATATCGGAAGGAACACCT
GTGGCGAAAGCGGCTCACTGGACCACAACTGACGCTGATGCACGAAAGCTAGGGGAGCAA

ACAGGATTAGAGACCCGTGTAGTCC
>Otu126
CCAGCCTATGGGTGGCTGCAGTTTCGAATCATTCACAATGGGCGAAAGCCTGATGGTGCG
ACGCCGCGTGAGGGATGAAGGTCTTCGGATTGTAAACCTCTGTCACTGGGGAAGAAACGC
TTCAGGTTAATAGCCTGAAGCCTGACTTAACCCGGAGAGGAAGCAGTGGCTAACTCTGTG
CCAGCAGCCGCGGTAATACAGAGACTGCAAGCGTTATTCGGATTCACTGGGCGTAAAGGG
TGCGCAGGTGGCCAAGTGTGTGAGGCGTGAAAGCCCGTTGCTCAACAGCGGAATTGCACC
TCAAACTACATGGCTAGAGCATTGGAGAGGGGAGCAGAATTCACGGTGTAGCAGTGAAAT
GCGTAGATATCGTGAGGAATACCAGAGGCGAAGGCGGCTCCCTGGACAATTGCTGACACT
CAGGCACGAAAGCGTGGGGAGCAAAAGGGATTAGAAACCCCCGTAGTCC
>Otu127
CCAGCCTATGGGAGGCAGCAGTCGAGAATTTTTCTCAATGGGCGCAAGCCTGAAGGAGCG
ACGCCGCGTGGAGGATGAATGGCTTCGGCCCGTAAACTCCTGTCATTTGCGAACAAATTA
ATCCGCCCAACACGCGGATTATTGATTGTAGCGGAAGAGGAAGGGACGGCTAACTCTGTG
CCAGCAGCCGCGGTAATACAGAGGTCCCGAGCGTTGTTCGGATTTACTGGGCGTAAAGGG
TGCGTAGGCGGCGGGGTGAGTCTGACGTGAAATCTCCGGGCCCAACCCGGAAACGGCGTT
GGATACTGCCCCGCTCGAGGATTGGAAGGGAGACTGGAATACTTGGTGTAGCAGTGAAAT
GCGTAGATATCAAGTGGAACACCAGTGGCGAAGGCGAGTCTCTGGACAATTCCTGACGCT
GAGGCACGAAAGCCAGGGGAGCAAACGGGATTAGAGACCCCCGTAGTCC
>Otu128
CCAGCCTACGGGGGGCAGCAGGCGCGAAAACTTTACAATGCCGGCAACGGCGATAAGGGG
ACCTCGAGTGCCAGGATACAATCCTGGCTGTCGTAATGCCTAAAAAGCATTGCATAGCAA
GGGCCGGGCAAGACCGGTGCCAGCCGCCGCGGTAACACCGGCGGCTCGAGTGGTAACCGC
TATTATTGGGTCTAAAGGGTCTGTAGCCGGCTGACTAAGTCTCTTGGGAAATCTGGCAGC
TTAACTGTCAGGCTTTCAGGGGATACTGATCGGCTCGAGACCGGGAGAGGTGAGAGGTAC
CTCAGGGGTAGGGGTGAAATCTTGTAATCCTTGAGGGACCACCAGTGGCGAAGGCGTCTC
ACCAGAACGGATCTGACGGCAAGGGACGAAAGCTAGGGGCACGAACCGGATTAGAAACCC
CGGTAGTCC
>Otu130
CCAGCCTACGGGTTGCTGCAGTCGAGAATTTTTCTCAATGGGCGAAAGCCTGAAGGAGCG
ACGCCGCGTGGGGGATGAATGGCTTCGGCCCGTAAACCCCTGTCATTTGCGAACAAATTG
GTTCACCTAACACGTGAACCATTGATAGTAGCGGAAGAGGAAGGGACGGCTAACTCTGTG
CCAGCAGCCGCGGTAATACAGAGGTCCCAAGCGTTGTTCGGATTCACTGGGCGTAAAGGG
TGCGTAGGTGGTCGGGTAAGTCTGTTGTGAAATCTCCGAGCTTAACTCGGAAATGGCAGT
GGATACTATCCGGCTAGAGGAATGGAGGGGAGACTGGAATACTTGGTGTAGCAGTGAAAT
GCGTAGATATCAAGTGGAACACCAGTGGCGAAGGCGAGTCTCTGGACATTTCCTGACGCT
GAGGCACGAAAGCCAGGGGAGCAAACGGGATTAGAAACCCCCGTAGTCC
>Otu131
CCAGCCTATGGGTCGCACCAGGGAGGAATATTGGGCAATGGACGAAAGTCTGACCCAGCA
ACGCCGCGTGAAGGATGAAGGCCTTCGGGTCGTAAACTTCTTTTAAGGGAGACGAGAAAG
GACGGTATCCCTTGAATAAGCCACGGCTAACTACGTGCCAGCAGCCGCGGTAAAACGTAG
GTGGCGAGCGTTATCCGGATTCATTGGGCGTAAAGAGCTTGTAGGTGGCTAGATAAGTTG
GACGTGAAAACTCCGGGCTTAACTTGGAGAGGCCGTTCAATACTGTTTGGCTAGAGGATG
TCAGAGGGAAGTAGAATTCCTAGTGTAGCAGTGAAATGCGTAGATATTAGGAGGAATACC
CGTGGCGAAGGCGGCTTCCTGGGGCAAACCTGACACTGAGAAGCGAAAGCTAGGGGAGCA
AACGGGATTAGAAACCCCAGTAGTCC
>Otu132
CCAGCCTATGGGGTGCAGCAGTAGGGAATATTGCACAATGGGCGAAAGCCTGATGCAGCA
ACGCCGCGTGCACGAAGAAGGTCTTCGGATCGTAAAGTGCTTTTCTGAGAGATGAGAAAG
GACAGTATCTCAGGAATAAGTCTCGGCTAACTACGTGCCAGCAGCCGCGGTAAAACGTAG
GAGGCAAGCGTTATCCGGATTCACTGGGCGTAAAGCGCATGCAGGCGGTTCGGTAAGTTG
GGCGTGAAATCTCCCGGCTCAACTGGGAGAGGTCGTTCAATACTACCGGGCTAGAGAGCA
GTAGAGGAAGATGGAATTCCCGGTGTAGTGGTGAAATGCGTAGATATCGGGAGGAACACC
AGTGGCGAAGGCGATCTTCTGGACTGTTTCTGACGCTCATATGCGAAAGCTAGGGTAGTA
AACGGGATTAGATACCCTAGTAGTCC
>Otu133
CCAGCCTACGGGGGGCAGCAGTAAGGAATATTGGTCAATGGACGCAAGTCTGAACCAGCC
ATGCCGCGTGCAGGATGACAGCCCTACGGGTTGTAAACTGCTTTTGTACGGGAAAAAACC

TTCGGTCGTGAACCGGAGCTGATGGTACCGTAAGAATAAGCATCGGCTAACTCCGTGCCA
GCAGCCGCGGTAATACGGAGGATGCAAGCGTTATCCGGATTCATTGGGTTTAAAGGGTGC
GCAGGCGGAATGATAAGTCAGTGGTGAAATCCTACAGCTTAACTGTAGAACTGCCATTGA
TACTGTCGTTCTTGAGTACAGTTGGAGTGGGCGGAATGTGTCATGTAGCGGTGAAATGCT
TAGATATGACACAGAACACCAGTCGCGAAGGCAGCTCGCTAAACTGTAACTGACGCTCAT
GCACGAAAGCGTGGGGATCAAACAGGATTAGAAACCCTCGTAGTCC
>Otu134
CCAGCCTACGGGTGGCACCAGTCGCGAAAACTTCACAATGGGGGAAACCCCGATGAGGGA
ATTCCAAGTGCTAGCACTATGTGTTAGCTGTTCCCCTGTCTAAAAAACAGGAGAAGTAAG
GGCCGGGTAAGACGGGTGCCAGCCGCCGCGGTAATACCCGCGGCCCGAGTGGTGGTCGAT
ATTATTGAGCCTAAAACGTCCGTAGCCGGTCTTGTAAATCCTTGAGTAAATCGGCCAGCT
CAACTGTCCGAAGTTCGGGGAGACTGCAAGACTTGGGATCGGGAGAGGTCAGAGGTACTT
CTGGGGTAGGGGTAAAATCCTGTAATCCTGGAAGGACCACCGGTGGCGAAGGCGTCTGAC
TAGAACGAATTCGACGGTGAGGGACGAAGCCCTGGGGCGCAAACGGGATTAGAAACCCTC
GTAGTCC
>Otu135
CCAGCCTACGGGTGGCACCAGTAGGGAATATTGCACAATGGGCGAAAGCCTGATGCAGCA
ACGCCGCGTGCACGATGAAGGCCTTCGGGTCGTAAAGTGCTTTTCGGGGAGATGAGGAAG
GACAGTATCCCCGGAATAAGGCTCGGCTAACTACGTGCCAGCAGCCGCGGTAAAACGTAG
GAGCCTAGCGTTATCCGAATTCACTGGGCGTAAAGCGCGTGCAGGCGGTCCGGTAAGTTG
GATGTGAAATCTCCTGGCTCAACTGGGAGAGGACGTTCAATACTGTCGGACTAGAGGACG
GTAGAGGGAGGTGGAATTCCCGGTGTAGTGGTGAAATGCGTAGATATCGGGAGGAACACC
AGTGGCGAAAGCGGCCTTCTGGGCCGTTCCTGACGCTCAGACGCGAAAGCTAGGGGAGCA
AACGGGATTAGAAACCCTTGTAGTCC
>Otu136
CCAGCCTACGGGTCGCTGCAGCAGGGAATATTGGTAATCTGCGAAAGCGGGAACCAGCAA
CGCCGCGTGTGCGATGAAGGCCTTCGGGTCGTAAAGCACTTTTCGAGGGGATGAGGAAGG
ACAGTACCCTCGGAATAAGTCTCGGCTAACTACGTGCCAGCAGCCGCGGTAAAACGTAGG
AGGCGAGCGTTATCCGGATTTACTGGGCGTAAAGCGTGTGCAGGCGGTTCGGTAAGTTGG
ATGTGAAAGCTCCCGGCTCAACTGGGAGAGGTCGTTCAATACTGCTGGACTTGAGGATGG
TAGAGGGAGGTGGAATTCCGGGTGTAGTGGTGAAATGCGTAGATATCCGGAGGAACACCA
GTGGCGAAAGCGGCCTCCTGGACCATTTCTGACGCTCAGACACGAAAGCTAGGGGAGCAA
ACGGGATTAGATACCCCGGTAGTCC
>Otu137
CCAGCCTATGGGGGGCACCAGGCGCGAAACCTTTACAATGCGCTAACGCGTGATAAGGGT
ACTCCAAGTGCTCATGTTTTACATGGGCTTTTGCCAAGGGCGAATACCTTGGCGAATAAG
TGGTGGGTAAGACTGGTGCCAGCAGCCGCGGTAACCCCAGCGCCACAAGTGGTGATCGGT
TTTATTGGGCCTAAAGCATCCGTAGCTGGCCAAAAAAGTCTCTTGTGAAATCGGGACGCT
TAACGTACCGGACTGCAGGAGATACTATTTGGCTTGGAACTGGGAGGAGCAAGAGGAATT
TTGGGGGAAGCGGTAAAATGCTATAATCCCCAAAAGACCACCGATGGCGAAGGCATCTTG
CTAGAACAGATTCGACAGTCAGGGATGAAAGCTAGGGGAGCAAACCGGATTAGATACCCT
CGTAGTCC
>Otu138
CCAGCCTATGGGTGGCTGCAGTTGGGAATCTTGCACAATGGGGGAAACCCTGATGCAGCG
ACGCCGCGTGGAGGATGACGGGTCTAGGCCTGTAAACTCCTTTTAACGGGAAAGACTTAG
GACGGTACCCGTTGAATAAGGACCGGCTAACTACGTGCCAGCAGCCGCGGTAAGACGTAG
GGTCCAAGCGTTGTCCGGATTTACTGGGCGTAAAGAGCGCGTAGGCGGTCTGTTAAGTGT
GAAGTGAAATCTCCAGGGCTCAACCCGGAAACTGCTTCGCATACTGGCAGACTGGAGGGA
TGCAGAGGTTTGTGGAATTCCTGGTGTAGCGGTGAAATGCATAGATATCAGGAGGAACAC
CCATGGCGAAGGCAGCAAACTGGGCATCATCTGACGCTGAGGCGCGAAAGCGTGGGTAGC
AAACAGGATTAGAAACCCCTGTAGTCC
>Otu139
CCAGCCTATGGGATGCAGCAGTGGGGAATCTTGGACAATGGAGGAAACTCTGATCCAGCC
ATGCCGCGTGAGTGATGAAGGCCTTAGGGTTGTAAAACTCTTTCGGCGGGGACGATAATG
ACGGTACCCGCAGAAGAAGCTCCGGCTAACTTCGTGCCAGCAGCCGCGGTAATACGAAGG
GGGCTAGCGTTGTTCGGAATTACTGGGCGTAAAGCGCGCGCAGGCGGCCTTTCAAGTCAG
GGGTGAAAGCCCGGAGCTCAACTCCGGAATTGCCTTTGAAACTGCAAGGCTTGAGTACGG
GAGAGGTGAGTGGAATTCCCAGTGTAGAGGTGAAATTCGTAGATATTGGGAAGAACACCG

GTGGCGAAGGCGGCTCACTGGCCCGTAACTGACGCTCAAGCGCGACAGCGTGGGGATCAA
ACAGGATTAGAGACCCGAGTAGTCC
>Otu141
CCAGCCTATGGGTTGCTGCAGTAAGGGATATTGCGCAATGGGCGAAAGCCTGACGCAGCA
ACGCCGCGTGAAGGATGAAGGCCTTCGGGTTGTAAACTTCTTTTACCGGGGACGAGGAAG
GACGGTACCCGGCGAACAAGTCACGGCTAACTACGTGCCAGCAGCCGCGGTAAAACGTAG
GTGGCGAGCGTTATCCGGATTTACTGGGTGTAAAGCGTGTGCAGGCGGAAGGGTAAGTGG
TGTATGAAATCGCCCGGCTCAACCGGGCGGGGTTATGCCAGACTGCTCATCTAGAGGACG
AGAGAGGGGCGTGGAATTCCGGGTGTAGTGGTGAAATGCGTAGAGATCCGGAGGAACCCC
CGAGGCGAAGGCGGCGCCCTGGCTCGTACCTGACGCTCAGACACGACAGCATGGGGAGCG
AACGGGATTAGAAACCCGTGTAGTCC
>Otu142
CCAGCCTATGGGGGGCAGCAGTGGGGAATATTGGACAATGGGCGAAAGCCTGATCCAGCC
ATGCCGCGTGAGTGATGAAGGCCTTAGGGTTGTAAAGCTCTTTTACCCGGGAAGATAATG
ACGGTACCGGGAGAATAAGCCCCGGCTAACTTCGTGCCAGCAGCCGCGGTAATACGAAGG
GGGCTAGCGTTGTTCGGAATTACTGGGCGTAAAGCGCACGTAGGCGGATTTTTAAGTCAG
GGGTGAAATCCCGAGGCTCAACCTCGGAACTGCCTTTGATACTGGGAATCTTGAGTCCGG
GAGAGGTGAGTGGAACTGCGAGTGTAGAGGTGAAATTCGTAGATATTCGCAAGAACACCA
GTGGCGAAGGCGGCTCACTGGCCCGGTACTGACGCTGAGGTGCGAAAGCGTGGGGAGCAA
ACAGGATTAGAAACCCTAGTAGTCC
>Otu143
CCAGCCTACGGGGGGCAGCAGTAAGGAATATTGGTCAATGGACGCAAGTCTGAACCAGCC
ACGCCGCGTGCAGGATGAAGGCGCTCTGCGTCGTAAACTGCTTTTTACAGGGAAGAAAAG
ATTTGTTTCTACAGATTTTGACGGTACCTGAGGAATAAGCACCGGCTAACTCCGTGCCAG
CAGCCGCGGTAATACGGAGGGTGCAAGCGTTATCCGGATTTACTGGGTTTAAAGGGTGCG
TAGGCTGATTGATAAGTCAGTGGTGAAATCCTCGGGCTCAACCCGGGAATTGCCGTTGAT
ACTATTAGTCTTGAATATAGTTGAGGTAAGCAGAATGCGTGGTGTAGCGGTGAAATGCTT
AGATATCACGCAGAATACCGATTGCGAAGGCAGCTTGCTAAACTATTATTGACGCTGAGG
CACGAAAGCGTGGGGATCAAACAGGATTAGAGACCCTAGTAGTCC
>Otu144
CCAGCCTATGGGGGGCAGCAGTAGGGAATATTGCACAATGGGCGAAAGCCTGATGCAGCA
ACGCCGCGTGCATGATGAAGGCCTTCGGGTCGTAAAGTGCTTTTCTGAGAGATGAGAAAG
GACAGTATCTCAGGAATAAGTCTCGGCTAACTACGTGCCAGCAGCCGCGGTAAAACGTAG
GAGGCGAGCGTTATCCGGATTCACTGGGCGTAAAGCGTGTGCAGGCGGTTCGGTAAGTTG
GGCGTGAAATCTCCTGGCTTAACTAGGAGAGGTCGTTCAATACTACCAGACTTGAGAGCA
GTAGAGGAAGATGGAATTCCCGGTGTAGTGGTGAAATGCGTAGATATCGGGAGGAACACC
AGTGGCGAAGGCGATCTTCTGGACTGTTTCTGACGCTCATACACGAAAGCTAGGGTAGTA
AACGGGATTAGAGACCCTTGTAGTCC
>Otu145
CCAGCCTATGGGGGGCACCAGGCGCGAAAACTCTACAATGCAGGCAACTGCGATAGGGGG
ACATCGAGTGGCACCCACCTTGGGTGTCTGTCCAACCGTCTAAAAAACGGTTGTTAGCAA
GGGCCGGGTAAGACCGGTGCCAGCCGCCGCGGTAATACCGGCGGCTCGAGTGGTGGCCGA
TATTATTGAGTCTAAAGGGTCCGTAGCCGGCTTTGCAAGTCCTCTGGGAAATCCAGCGGC
TTAACCGTTGGGCTTCCATGGGAAACTACATTGCTTGGGACTGGGAGAGGTAGGAGGTAC
TCGAGGGGTAGGGGTGAAATCCTGTAATCCTTCGGGGACCACCGGTGGCGAAGGCGTCCT
ACCAGAACAGGTCCGACGGTGAGGGACGAAAGCTAGGGGCACGAACCGGATTAGAAACCC
TAGTAGTCC
>Otu146
CAGCCTACGGGGGGCTGCAGTGGGGAATATTGGACAATGGGCGAAAGCCTGATCCAGCAA
TGCCGCGTGAGTGATGAAGGCCTTAGGGTTGTAAAGCTCTTTCGCACGCGACGATGATGA
CGGTAGCGTGAGAAGAAGCCCCGGCTAACTTCGTGCCAGCAGCCGCGGTAATACGAAGGG
GGCTAGCGTTGTTCGGAATTACTGGGCGTAAAGGGCGCGTAGGCGGTTTATCTAGTCAGG
CGTGAAAGCCCCGGGCTCAACCTGGGAATTGCGTTTGATACTGGTAGACTTGAGTTCGGG
AGAGGTGAGTGGAATTCCCAGTGTAGAGGTGAAATTCGTAGATATTGGGAAGAACACCGG
TGGCGAAGGCGGCTCACTGGACCGATACTGACGCTGAGGCGCGAAAGCGTGGGGAGCAAA
CAGGATTAGATACCCTAGTAGTCC
>Otu148
CCAGCCTACGGGGGGCAGCAGTCGAGAATATTCGACAATGGGCGAAAGCCTGATCGAGCG

ACACCGCGTGCAGGATGAAGGCCTTCGGGTCGTAAACTGCGGTAGTATGGTAACAATGCA
AATGAGTGCCATACGGAAAGAGGTGGGTAACTACGTGCCAGCACCAGCGGTAATACGTAG
ACCTCAAGCGTTATCCGGATTTATTGGGCGTAAAGAGCATGTAGGAGGTTTTGTGCGTCT
TCTGTTAAAGCCCGGAGCCTAACTTCGGAAATGCAGGAGATACGACAGAACTAGAGGAGG
TTAGAGGTGCATGGAACTCACGGTGTAGGGGTGAAATCCGTTGATATCGTGGGGAACACC
AAAGGCGAAGGCAGTGCACTGGGACCTTCCTGACTCTGAGATGCGAAAGCGTGGGGAGCA
AAAAGGATTAGATACCCTCGTAGTCC
>Otu149
CCAGCCTATGGGGGGCACCAGTAAGGAATATTGGTCAATGGGCGGAAGCCTGAACCAGCC
ATGCCGCGTGCAGGAAGACGGCCCTACGGGTTGTAAACTGCTTTTGCAGGGGAATAAACC
TCCCGACGAGTCGGGAGCTGAATGTACCTTGAGAATAAGGATCGGCTAACTCCGTGCCAG
CAGCCGCGGTAATACGGAGGATCCGAGCGTTATCCGGATTTATTGGGTTTAAAGGGTGCG
TAGGCGGCCTATTAAGTCAGGGGTGAAAGACGGTAGCTCAACTATCGCAGTGCCTTTGAT
ACTGATGGGCTTGAATGTACTTGAGGTAGGCGGAATGTGACAAGTAGCGGTGAAATGCAT
AGATATGTCACAGAACACCAATTGCGAAGGCAGCTTACTAAAGTATGATTGACGCTGAGG
CACGAAAGCGTGGGGATCAAACAGGATTAGAAACCCCAGTAGTCC
>Otu150
CCAGCCTATGGGGGGCTGCAGTTTCGAATCATTCACAATGGGCGAAAGCCTGATGGTGCG
ACGCCGCGTGAGGGATGAAGGTCTTCGGATTGTAAACCTCTGTCACCGGGGAAGAAACGC
TTTGAGCTAATAGTTCAAAGCCTGACTTAACCCGGAGAGGAAGCAGTGGCTAACTCTGTG
CCAGCAGCCGCGGTAATACAGAGACTGCGAGCGTTATTCGGATTCACTGGGCGTAAAGGG
TGCGCAGGCGGCCATGTGTGTGAGGCGTGAAAGCCCGGAGCTTAACTCCGGAATTGCACC
TCAAACTACATGGCTAGAGCATTGGAGAGGGTAGCAGAATTCACGGTGTAGCAGTGAAAT
GCGTAGATATCGTGAGGAATACCAGAGGCGAAGGCGGCTACCTGGACAATTGCTGACGCT
CAGGCACGAAAGCGTGGGGAGCAAAAGGGATTAGAGACCCCAGTAGTCC
>Otu151
CCAGCCTATGGGGGGCACCAGTAAGGAATATTGGTCAATGGGCGGAAGCCTGAACCAGCC
ATGCCGCGTGCAGGAAGACGGCCCTACGGGTTGTAAACTGCTTTTGCAGGGGAATAAACC
CCCCAACGAGTTGGGGGCTGAAAGTACTCTGAGAATAAGGATCGGCTAACTCCGTGCCAG
CAGCCGCGGTAATACGGAGGATCCGAGCGTTATCCGGATTTATTGGGTTTAAAGGGTGCG
TAGGCGGCCTTGTAAGTCAGGGGTGAAAGACGGTAGCTTAACTATCGCAGTGCCTTTGAT
ACTGCAGGGCTTGAATGTACTTGAGGCAGGCGGAATGTGACAAGTAGCGGTGAAATGCAT
AGATATGTCACAGAACACCAATTGCGAAGGCAGCTTGCTAAAGTATGATTGACGCTGAGG
CACGAAAGCGTGGGGATCAAACAGGATTAGAAACCCTTGTAGTCC
>Otu153
CCAGCCTACGGGGGGCTCCAGTGGGGAATTTTGCGCAATGGGGGAAACCCTGACGCAGCA
ACGCCGCGTGGAGGATGAAGTCCCTTGGGACGTAAACTCCTTTCGACCAAGACGATGATG
ACGGTACTGGTGGAAGAAGCACCGGCTAACTCTGTGCCAGCAGCCGCGGTAATACAGAGG
GTGCGAGCGTTGTTCGGAATTATTGGGCGTAAAGGGCGCGTAGGCGGCGCCACAAGTCAC
TTGTGAAAACTCTGGGCTCAACTCAGAGCCTGCAAGCGAAACTGTGGTGCTGGAGTATGG
GAGAGGTGCGTGGAATTCCCGGTGTAGCGGTGAAATGCGTAGATATCGGGAGGAACACCT
GTGGCGAAAGCGGCGCACTGGACCATAACTGACGCTGAGGCGCGAAAGCTAGGGGAGCAA
ACAGGATTAGATACCCTTGTAGTCC
>Otu154
CCAGTTCGCAATGCCCGAAAGGGTGACGAAGCGACGCCGCGTGCGGGAAGAAGGCCTTCG
GGTTGTAAACCGCTGTCGGGAGTTAAGAAATGCAAGGATGTTAATAGCATCTTTGTTTGA
CTAAGGCTCCGGAGGAAGCCACGGCTAACTCTGTGCCAGCAGCCGCGGTAATACAGAGGC
GGCAAGCGTTGTTCGGAATTATTGGGCGTAAAGAGCACGTAGGCGGCTGTGTAAGTCGGT
TGTGAAAGCCTTCCGCTCAACGGAAGAACGGCATCCGATACTGCATGGCTTGAGTGTGGG
AGGGGAGAGTGGAACTTCTGGTGGAGCGGTGAAATGCGTAGATATCAGAAGGAACACCGG
CGGCGAAGGCGACTCTCTGGTCCATAACTGACGCTGAGTGTGCGAAAGCTAGGGGAGCAA
ACGGGATTAGAAACCCCCGTAGTC
>Otu155
CCAGCCTACGGGGGGCACCAGTCGAGAATTTTTCTCAATGGGCGAAAGCCTGAAGGAGCG
ACGCCGCGTGGGGGATGAATGGCTTCGGCCCGTAAACCCCTGTCATTTGTGAACAAATTA
ATCTACCCAACACGTGGATTATTGATTGTAACGGAAGAGGAAGGGACGGCTAACTCTGTG
CCAGCAGCCGCGGTAATACAGAGGTCCCAAGCGTTGTTCGGATTCACTGGGCGTAAAGGG
TGCGTAGGCGGTTTGACAAGTCTGATGTGAAATCCCGCAGCTTAACTGCGGAACTGCATT

GGAAACTGTCTGACTAGAGGAATGGAGGGGAGACTGGAATGCTTGGTGTAGCAGTGAAAT
GCGTAGATATCAAGCGGAACACCAGTGGCGAAGGCGAGTCTCTGGACATTTCCTGACGCT
GAGGCACGAAAGCCAGGGGAGCAAACGGGATTAGATACCCCAGTAGTCC
>Otu156
CCAGCCTACGGGGGGCAGCAGGCGCGAAAACTTTACAATGCGGGAAACCGTGATAAGGGA
ACCCCGAGTGCCCGTAAAATCGGGCTGTTAACCTGTTTAAAAAGCAGGTGGAGAAAGGGC
CGGGCAAGACCGGTGCCAGCCGCCGCGGTAATACCGGCGGCTCGAGTGGTGGCCACTATT
ACTGGGCTTAAAGCGTCCGTAGCTGGATTGTTAAGTCTCTTGGGAAATCTACCGGCTTAA
CCGATAGGCGTTCAAGGGATACTGGCAACCTAGGGACCGGAAGAGGTGAGAGGTACTCCA
GGGGTAGGAGTGAAATCCTGTAATCCTTGGGGGACCACCTGTGGCGAAGGCGTCTCACCA
GAACGGCTCCGACAGTGAGGGACGAAAGCTGGGGGAGCAAACCGGATTAGATACCCCAGT
AGTCC
>Otu157
CCAGCCTATGGGTGGCACCAGTGGGGAATATTGGACAATGGGCGAAAGCCTGATCCAGCC
ATGCCGCGTGAGTGATGAAGGCCTTAGGGTTGTAAAGCTCTTTCGCTGGGGAAGATAATG
ACGGTACCCAGAGAAGAAGCCCCGGCCAACTTCGTGCCAGCAGCCGCGGTAATACGAAGG
GGGCTAGCGTTGCTCGGAATCACTGGGCGTAAAGCGCACGTAGGCGGATTTCTAAGTCAG
GGGTGAAATCCCGGAGCTCAACTCCGGAACTGCCTTTGATACTGGAGATCTTGAGTCCGG
GAGAGGTGAGTGGAACTCCGAGTGTAGAGGTGAAATTCGTAGATATTCGGAAGAACACCA
GTGGCGAAGGCGGCTCACTGGCCCGGTACTGACGCTGAGGTGCGAAAGCGTGGGGAGCAA
ACAGGATTAGAAACCCCAGTAGTCC
>Otu158
CCAGCCTACGGGGTGCTGCAGTGGGGAATCTTGCGCAATGGGGGAAACCCTGACGCAGCA
ACGCCGCGTGGGTGATGAAGGCCTTCGGGTCGTAAAGCCCTGTCAGGTGGGAAGAAACAT
GTTGATGCCAATACCATCAACACTTGACGGTACCACCGAAGGAAGCACCGGCTAACTCCG
TGCCAGCAGCCGCGGTAATACGGAGGGTGCAAGCGTTGTTCGGAATTATTGGGCGTAAAG
CGCGTGTAGGCGGTGATGTAAGTCAGATGTGAAAGCCTTCCGCTTAACGGGAGAAGTGCA
TTTGAAACTGCATGACTAGAGTACGGGAGAGGAGAGTGGAATTCCCGGTGTAGAGGTGAA
ATTCGTAGATATCGGGAGGAACACCAGTGGCGAAGGCGGCTCTCTGGATCGATACTGACG
CTGAGACGCGAAAGCGTGGGTAGCAAACAGGATTAGATACCCCCGTAGTCC
>Otu159
CCAGCCTACGGGGTGCTGCAGTGGGGAATTTTGCGCAATGGGGGAAACCCTGACGCAGCA
ACGCCGCGTGGAGGATGAAGTCCTTCGGGACGTAAACTCCTTTCGATCGGGACGATTATG
ACGGTACCGGAAGAAGAAGCCCCGGCTAACTTCGTGCCAGCAGCCGCGGTAATACGAGGG
GGGCGAGCGTTGTTCGGAATTATTGGGCGTAAAGGGTGCGTAGGCGGTTTTGTAAGTCTT
GTGTGAAATCTTCAGGCTCAACTTGAAGACTGCACGAGAAACTGCAGGGCTTGAGTGTGG
GAGAGGTGAGTGGAATTCCTGGTGTAGCGGTGAAATGCGTAGATATCAGGAGGAACACCT
GTGGCGAAAGCGGCTCACTGGACCACAACTGACGCTGAAGCACGAAAGCTAGGGGAGCAA
ACAGGATTAGATACCCCAGTAGTCC
>Otu160
CCAGCCTATGGGGGGCAGCAGTGAGGAATATTGGTCAATGGGCGCAAGCCTGAACCAGCC
ATGCCGCGTGAAGGATGAAGGCCTTCTGGGTTGTAAACTTCTTTTATGAGGGAAGAAAAC
TATGGTTTCTACTATAGCCGACGGTACCTTAGGAATAAGCACCGGCTAACTCCGTGCCAG
CAGCCGCGGTAATACGGAGGGTGCAAGCGTTATCCGGATTTACTGGGTTTAAAGGGTGTG
TAGGCGGGCTTTTAAGTCAGTGGTGAAATCTCCGGGCTCAACCCGGAAACTGCCATTGAT
ACTATTAGTCTTGAATTTTGTTGAGGTAGGCGGAATAAGTCATGTAGCGGTGAAATGCAT
AGATATGACTTAGAACACCAATTGCGAAGGCAGCTTACTAAGCAAATATTGACGCTGAGG
CACGAAAGCGTGGGGATCAAACAGGATTAGATACCCTAGTAGTCC
>Otu161
CCAGCCTATGGGGTGCAGCAGTGGGGAATTTTGCGCAATGGGGGAAACCCTGACGCAGCA
ACGCCGCGTGGAGGATGAAGCCCCTTGGGGTGTAAACTCCTTTCGATCGGGACGATGATG
ACGGTACCGGATGAAGAAGCACCGGCTAACTCTGTGCCAGCAGCCGCGGTAATACAGAGG
GTGCAAGCGTTGTTCGGAATTATTGGGCGTAAAGGGTGCGTAGGCGGTGCGGTAAGTCTT
CTGTGAAATCTCCGGGCTTAACTCGGAGCCTGCAGGGGAAACTGCCGTGCTGGAGTATGG
GAGAGGTGAGTGGAATTCCCGGTGTAGCGGTGAAATGCGTAGATATCGGGAGGAACACCT
GTGGCGAAAGCGGCTCACTGGACCATTACTGACGCTGATGCACGAAAGCTAGGGGAGCAA
ACAGGATTAGAAACCCCCGTAGTCC
>Otu162

CCAGCCTATGGGGTGCAGCAGTGGGGAATATTGGACAATGGGCGCAAGCCTGATCCAGCA
ATGCCGCGTGGGTGAAGAAGGCCTGCGGGTTGTAAAGCCCTTTAAGTGGGGAGGAAAGGT
GGCGTGCGAACAGTGCGCGGCTCTGACGTTACCCACAGAATAAGCACCGGCTAACTCCGT
GCCAGCAGCCGCGGTAATACGGGGGGTGCAAGCGTTAATCGGAATTACTGGGCGTAAAGC
GTGCGTAGGCGGTTCGGACAGTCAGCCGTGAAAGCCCTGGGCTTAACCTGGGAACGGCGG
TTGAGACGGTCGGACTAGAGTGGGCTAGAGGGTCGTGGAATTCCCGGTGTAGCGGTGAAA
TGCGTAGAGATCGGGAGGAACACCGATGGCGAAGGCAGCGGTCTGGGGCCACACTGACGC
TAAGGCACGAAAGCGTGGGGAGCAAACAGGATTAGATACCCGAGTAGTCC
>Otu164
CCAGCCTACGGGTGGCAGCAGTAAGGAATATTGGTCAATGGGCGCAAGCCTGAACCAGCC
ATGCCGCGTGCAGGATGAAGGTCCTACGGATTGTAAACTGCTTTTGTACGGGAAAAAACC
CCTTGTTTCTACGAGGGTTGATGGTACTGTAAGAATAAGGATCGGCTAACTCCGTGCCAG
CAGCCGCGGTAATACGGAGGATCCAAGCGTTATCCGGATTCACTGGGTTTAAAGGGTGCG
TAGGCGGAATGACAAGTCAGTGGTGAAAACCTATAGCTTAACTATAGAACTGCCATTGAT
ACTGTTATTCTTGAGTGTATTGGATGTGAATGGAATGTGTCGTGTAGCGGTGAAATGCTT
AGATATGACACAGAACACCCATTGCGAAGGCAGTTCACAACACTACAACTGACGCTGAGG
CACGAAAGCGTGGGGATCAAACAGGATTAGAAACCCTAGTAGTCC
>Otu165
CCAGCCTATGGGTTGCAGCAGTGGGGAATTTTGCGCAATGGGGGAAACCCTGACGCAGCA
ACGCCGCGTGGAGGATGAAGTCCCTTGGGACGTAAACTCCTTTCGATCGGGACGATAATG
ACGGTACCGGAAGAAGAAGCCCCGGCTAACTTCGTGCCAGCAGCCGCGGTAATACGAGGG
GGGCAAGCGTTGTTCGGAATTATTGGGCGTAAAGGGAGCGTAGGCGGTTTGGTAAGTCTT
GTGTGAAATCTTCGGGCTCAACCCGAAGTCTGCACGAGAAACTGCCGGGCTTGAGTATGG
GAGAGGTGAGTGGAATTCCTGGTGTAGCGGTGAAATGCGTAGATATCAGGAGGAACACCT
GTGGCGAAAGCGGCTCACTGGACCATAACTGACGCTGAGGCTCGAAAGCTAGGGGAGCAA
ACAGGATTAGAAACCCGAGTAGTCC
>Otu166
CCAGCCTATGGGTGGCAGCAGTGGGGAATATTGGACAATGGGCGCAAGCCTGATCCAGCC
ATGCCGCGTGAGTGATGAAGGCCTTAGGGTTGTAAAGCTCTTTCGCTCGCGAGGATAATG
ACGGTAGCGAGAGAAGAAGCCCCGGCTAACTTCGTGCCAGCAGCCGCGGTAATACGAAGG
GGGCTAGCGTTGTTCGGAATCACTGGGCGTAAAGCGCTCGTAGGCGGATATTTAAGTCAG
AGGTGAAAGCCTGGGGCTCAACTCCAGAACTGCCTTTGATACTGGGTATCTTGAGTCCGG
TAGAGGTGAGTGGAACTGCTAGTGTAGAGGTGAAATTCGTAGATATTAGCAAGAACACCA
GTGGCGAAGGCGGCTCACTGGACCGGTACTGACGCTGAGGAGCGAAAGCGTGGGGAGCAA
ACAGGATTAGATACCCCCGTAGTCC
>Otu167
CCAGCCTATGGGGGGCAGCAGTAAGGAATATTGGTCAATGGGGGCAACCCTGAACCAGCC
ATGCCGCGTGCAGGATGACGGCCCTATGGGTTGTAAACTGCTTTTATACAAGAGAAAACC
CGAGTACGTGTACTCGGTTGATAGTATTGTAAGAATAAGCATCGGCTAACTTCGTGCCAG
CAGCCGCGGTAAGACGAAGGATGCAAGCGTTATCCGGATTCATTGGGTTTAAAGGGTGCG
TAGGCGGACTTGTAAGTCAGTGGTGAAATCTCTTTGCTTAACAAAGAAACTGCCATTGAT
ACTGCAGGTCTAGAGTATAGATGACGTTGGCGGAATATGACATGTAGTGGTGAAATACTT
AGATATGTCATAGAACACCGATTGCGAAGGCAGCTAACGAAACTATAACTGACGCTGAGG
CACGAAAGTGCGGGGATCAAACAGGATTAGAAACCCGTGTAGTCC
>Otu168
CCAGCCTATGGGGGGCAGCAGTGGGGGATATTGGACAATGGGGGGAACCCTGATCCAGCG
ATGCCGCGTGGGTGAAGAAGGCCTTTGGGTTGTAAAGCCCTTTAAGTTGGGAAGAAGGAC
TGATGTTTAAGAGATATTAGTTTTGACGGTACCGACGAAATAAGCACCGGCAAACTCTGT
GCCAGCAGCCGCGGTAATACAGAGGGTGCGAGCGTTAATCGGAATTACTGGGCGTAAAGG
GCGCGTAGGCGGTGAGTTTAGTGGGATGTGAAAGTCCAGGGCTTAACCTTGGGATTGCAT
ACTATACGGACTGACTGGAGTACGGGAGAGGGTGGCGGAATTTCCGGTGTAGCGGTGAAA
TGCGTAGAGATCGGAAGGAACGTCGATGGCGAAGGCAGCCACCTGGCCTGATACTGACGC
TGAGGCGCGAAAGCGTGGGGAGCGAACAGGATTAGATACCCTAGTAGTCC
>Otu169
CCAGCCTATGGGTTGCTCCAGGCGCGAAAACTTTACAATGCGGGAAACCGTGATAAGGGA
ACTCCGAGTGCCCGTTAAATCGGGCTGTCCACCAGTTTAAATAACTGGTGAAGAAAGGGC
CGGGCAAGACCGGTGCCAGCCGCCGCGGTAATACCGGCGGCTCGAGTGGTGGCCACTATT
ACTGGGCTTAAAGCGTTCGTAGCTGGTTTGTTAAGTCTCTGGGGAAATCTACCGGCTTAA

CCGGTAGGCGTCTCAGGGATACTGGCAGACTAGGGACCGGGAGAGGTGAGAGGTACTCCA
GGGGTAGGAGTGAAATCCTGTAATCCTTGGGGGACCACCTGTGGCGAAGGCGTCTCACCA
GAACGGCTCCGACAGTGAGGGACGAAAGCTGGGGGAGCAAACCGGATTAGAAACCCTTGT
AGTCC
>Otu170
CCAGCCTACGGGGCGCTGCAGTCGAGAATTTTTCTCAATGGGCGAAAGCTTGAAGGAGCG
ACGCCGCGTGGGGGATGAAGGGCTTCGGCCTGTAAACCCCTGTCATTTGCGAACAAACCC
ACTCATCGAATACATGGGTGGCTGATAGTAGTGAAAGAGGAAGGGACGGCTAACTCTGTG
CCAGCAGCCGCGGTAATACAGAGGTCCCAAGCGTTGTTCGGATTCACTGGGCGTAAAGGG
TGCGTAGGTGGCGGGAAAAGTTTGATGTGAAATCTCCGGGCTCAACCCGGAAATGGCATT
GAATACTGTCTCGCTCGAGGGTCGGAGGGGAGACTGGAATTCTCGGTGTAGCAGTGAAAT
GCGTAGATATCGAGAGGAACACCAGTGGCGAAGGCGAGTCTCTGGACGACTCCTGACACT
GAGGCACGAAAGCTAGGGGATCAAACAGGATTAGATACCCCTGTAGTCC
>Otu171
CCAGCCTATGGGGGGCAGCAGCTAAGAATCTTCCGCAATGGGGGAAACCCTGACGGAGCG
ACGCCGCGTGGATGATGAAGGCCGTAAGGTTGTAAAATCCTTTTGTCGGTGAAGAATAAG
CCAGGGAGTGGAAAGCCCTGGTGATGACGTTAACCGGCGAATAAGCCCCGGCTAATTACG
TGCCAGCAGCCGCGGTAATACGTATGGGGCAAGCGTTGTTCGGATTTATTGGGCGTAAAG
GGCGTGTAGGCGGCTCATCAAGTCTGGTGTGAAATCGCAGGGCTTAACCCTGCATATGCG
CTGGAAACTGGTGGGCTTGAGTTCTTGAGGGGAAGCTGGAATTCCAGGTGTAGGGGTGAA
ATCTGTAGATATCTGGAAGAACACCGGTGGCGAAGGCGAGCTTCTATCAGAGGACTGACG
CTGAGGCGCGAAAGCGTGGGGAGCAAACAGGATTAGAAACCCTAGTAGTCC
>Otu172
CCAGCCTACGGGGGGCAGCAGTAAGGAATATTGGTCAATGGACGAAAGTCTGAACCAGCC
ATGCCGCGTGAAGGATGAAGGCCCTCTGGGTTGTAAACTTCTTTTATCTGGGACGAAAAA
AGGGAATTCTTTCCCACTTGACGGTACCAGAGGAATAAGCACCGGCTAACTCCGTGCCAG
CAGCCGCGGTAATACGGAGGGTGCAAGCGTTATCCGGATTTACTGGGTTTAAAGGGTGCG
TAGGTGGGTCTGTAAGTCAGTGGTGAAATCTTCGAGCTTAACTCGGAAACTGCCATTGAT
ACTATAGATCTTGAATTATCTGGAGGTAAGCGGAATATGTCATGTAGCGGTGAAATGCTT
AGATATGACATAGAACACCAATTGCGAAGGCAGCTTACTACGGATCGATTGACACTGAGG
CACGAAAGCGTGGGGATCAAACAGGATTAGAGACCCGTGTAGTCC
>Otu173
CCAGCCTACGGGAGGCTGCAGTGGGGAATTTTGGACAATGGGGGCAACCCTGATCCAGCC
ATGCCGCGTGAGTGAAGAAGGCCTTCGGGTTGTAAAGCTCTTTCGGCGGGGACAAAACCG
CCACTTTTAATAAAGGTGGTGAATGATGGTACCCGAAGAAGAAGCACCGGCTAACTACGT
GCCAGCAGCCGCGGTAATACGTAGGGTGCGAGCGTTAATCGGAATTACTGGGCGTAAAGC
GTGCGCAGGTGGTTTCGTAAGTCAGATGTGAAATCCCCGGGCTTAACCTGGGAATGGCAT
TTGAGACTGCGAAGCTAGAGTACGGCAGAGGGAGGTGGAATTCCACGTGTAGCAGTGAAA
TGCGTAGATATGTGGAGGAACACCGATGGCGAAGGCAGCCTCCTGGGCCAGTACTGACGC
TCATGCACGAAAGCGTGGGGAGCAAACAGGATTAGATACCCTCGTAGTCC
>Otu174
CCAGCCTACGGGGGGCAGCAGTGAGGAATATTGGTCAATGGACGCAAGTCTGAACCAGCC
ATGCCGCGTGAAGGATGAAGGCCTTCTGGGTTGTAAACTTCTTTTACCTGGGAAGAAACC
CCGATTTTCTAATCGGGTTGACGGTACCAGAGGAATAAGCACCGGCTAACTCCGTGCCAG
CAGCCGCGGTAATACGGAGGGTGCAAGCGTTATCCGGATTTACTGGGTTTAAAGGGTGTG
TAGGCGGGCTTTTAAGTCAGTGGTGAAATCTCCGGGCTCAACCCGGAAACTGCCATTGAT
ACTATTAGTCTTGAATTCTGTTGAGGTAGGCGGAATAAGTCATGTAGCGGTGAAATGCAT
AGATATGACTTAGAACACCAATTGCGAAGGCAGCTTACTAAGCAGATATTGACGCTGAGG
CACGAAAGCGTGGGGATCAAACAGGATTAGAAACCCCAGTAGTCC
>Otu175
CCAGCCTATGGGGGGCTCCAGGGACGAATCCTTCGCAATGCGCGCAAGCGTGACGAGGGG
ACTCCGAGTGGTGGACTTAGTCCACCTTTTTATCAGAATAAACACCTGGTAGAATAAGGG
CCGGGCAATATCGGTGCCAGCCGCCGCGGTAACACCGAAGGCTCAAGTGGTATCCACGAA
TATTGGGCTTAAAGAGTCCGTAGCCGGCTTGTTAAGTTCTCTGTGAAATCTTCTGGCTTA
ACCAGAAGGCGTGCAGGGGGTACTGGCAGGCATGGGAGTGGGGGAAGTCAGGAGTACTCA
CGGGGTAGAGGTAAAATTCTGTAATCCTGTGAGGACTACCAGTGGCGAAGGCGCCTGACT
AAAACATGTCCGACGGTGAGGGACGAAGGCTAGGAGAACGAATCGGATTAGAAACCCCCG
TAGTCC

>Otu176
CCAGCCTATGGGTGGCAGCAGTAGGGAATATTGGTAATGGGCGAAAGCCTGAACCAGCAA
CGCCGCGTGAGCGATGAAGGCCTTCGGGTTGTAAAGCTCTTTTTATGGGGACGAGGAAGG
ACGGTACCCATAGAATAAGCCTCGGCTAACTACGTGCCAGCAGCCGCGGTAAAACGTAGG
AGGCAAGCGTTATCCGGATTTACTGGGCGTAAAGCGCATGCAGGCGGTTTGGAAAGTTGG
ATGTGAAAGCTCCTGGCTTAACTGGGAGAGGTCGTTCAATACTTCCAGACTAGAGGATGT
GAGAGGAGAGTGGAATTCCGAGTGTAGTGGTGAAATGCGTAGATATTCGGAGGAACACCA
GTGGCGAAAGCGGCTCTCTGGCACATTTCTGACGCTCATATGCGAAAGCTAGGGTAGCAA
ACGGGATTAGAAACCCCCGTAGTCC
>Otu177
CCAGCCTATGGGGGGCAGCAGTGGGGAATCTTGCACAATGGGGGAAACCCTGATGCAGCG
ACGCCGCGTGAGCGATGAAGCCCTTCGGGGTGTAAAGCTCTTTCGACAGGAACGATAATG
ACGGTACCTGTGGAAGAAGCTGCGGCTAACTACGTGCCAGCAGCCGCGGTAATACGTAGG
CAGCAAGCGTTGTTCGGAGTTACTGGGCGTAAAGGGTGTGTAGGCGGTTCACTAAGTTCG
GTGTGAAATCTCCCGGCTTAACTGGGAGGGTGCATTGAAAACTGGTGGGCTTGAGTATGG
GAGAGGTAAGCGGAATTCCTGGTGTAGCGGTGAAATGCGTAGATATCAGGAGGAACACCT
GTGGTGTAGACAGCTTACTGGACCATCACTGACGCTGAGACACGAAAGCGTGGGTAGCAA
ACAGGATTAGAAACCCGAGTAGTCC
>Otu178
CCAGCCTATGGGAGGCAGCAGTCAAGAATATTGGTCAATGGACGAAAGTCTGAACCAGCG
ATGTTGCCGCGTGAAGAAGGCTTATTGTCGTAAAACGCCGATGCGTGGATCCAGAATGAG
ATCTCCACGCTACGCAGCCCTGACGAATCTCGGTGCCAGTAGTCACGGTCACGCCGAAAG
GGCGAGGCATTCATAAAGGAAATGGGTGTAAAGCGTCATTAGGTGGATTCAATCAACGCA
TCTTGAAAAATCGGGGCCTAACCCCTAGAAAGAGAGGCGCATTGTGAATCTTGAGTGATG
CCGGGGATCGTCGAATGTCAGGGGTACCGGTGAAATGGGTCAATCCCTGATGGAAGTCCA
AAGGCGAAGGCAACGATCTAGGCATTCACTGACGCTGACTGACGAAAGTGCAGGGAGCAA
AGAGGATTAGAAACCCGCGTAGTCC
>Otu179
CCAGCCTATGGGTTGCACCAGTCGAGAATCTTCCACAATGGACGAAAGTCTGATGGAGCG
ACGCCGCGTGATTGATGAAGTCCCTCTGGGACGTAAAGATCTTTTATGAGGGAAGAAGTT
TATTGACTGTACCTCATGAATAAGAGGCTCCTAATCTCGTGCCAGCAGGAGCGGTAATAC
GAGAGCCTCGAGCGTTATCCGGAATTATTGGGCGTAAAGGGTGCGTAGGTTGTTCTGTTA
GTCTTTTGTCAAAGCCCCGAGCTTAACTTGGGATATGCGAAAGAAACGGCAGAACTTGAA
AGTGCGAGAGGTGTACGGAACTCATGGTGTAGGGGTGAAATCCGTTGATATCATGGGGAA
CACCAAATGCGAAGGCAGTACACTGGCGCATATTTGACACTGAAGCACGAAAGCGTGGGT
AGCGAATGGGATTAGAAACCCGCGTAGTCC
>Otu180
CCAGCCTATGGGGGGCAGCAGTGGGGAATATTGCGCAATGGGCGAAAGCCTGACGCAGCG
ACGCCGCGTGGGGGATGAAGGCCTTCGGGTTGTAAACCCCTTTCAGCAGGGACGAAGCGA
AAGTGACGGTACCTGCAGAAGAAGCGCCGGCTAACTACGTGCCAGCAGCCGCGGTAACAC
GTAGGGCGCAAGCGTTGTCCGGAATTATTGGGCGTAAAGAGCTCGTAGGCGGTCTGTTGC
GTCCGCTGTGAAAACTCGGGGCTTAACCCCGAGCCTGCAGTGGATACGGGCAGACTAGAG
GTAGGTAGGGGAGAATGGAATTCCCGGTGTAGCGGTGAAATGCGCAGATATCGGGAGGAA
CACCGGTGGCGAAGGCGGTTCTCTGGGCCTTACCTGACGCTGAGGAGCGAAAGCGTGGGG
AGCAAACAGGATTAGAAACCCTAGTAGTCC
>Otu181
CCAGCCTATGGGGGGCAGCAGTGGGGAATTTTCCGCAATGGGCGAAAGCCTGACGGAGCA
ATACCGCGTGAGGGAGGAAGGCTCTTGGGTTGTAAACCTCTTTTCTTAGGGAATAAAAAA
ATGAAGGTACCTAAGGAATAAGCATCGGCTAACTCCGTGCCAGCAGCCGCGGTAATACGG
AGGATGCAAGCGTTATCCGGAATGATTGGGCGTAAAGCGTCCGCAGGTGGCGATGTAAGT
CTGCTGTTAAAGAGTTTGGCTTAACCAAATAAAGGCAGTGGAAACTACATGGCTAGAGTG
CGTTCGGGGCAGAGGGAATTCCTGGTGTAGCGGTGAAATGCGTAGAGATCAGGAAGAACA
CCGGTGGCGAAAGCGCTCTGCTAGGCCGCAACTGACACTGAGGGACGAAAGCTAGGGGAG
CGAATGGGATTAGATACCCCAGTAGTCC
>Otu182
CCAGCCTACGGGGTGCACCAGTCAAGAAACTTCCACAATGGACGAAAGTCTGATGGAGCG
ACGCCGCGTGGTTGATGAAGTCCTTCGGGACGTAAAAACCTTTTATGAGGGAGAAAGTAA
TTGATGTTACCTCATGAATAAGGGGCTCCTAACTCTGTGCCAGCAGGAGCGGTAATACAG

AGGCCCCAAGCATTATCCGGAATCACTGGGCGTAAAGGGTGTGTAGGCGGTCGTGTTAGT
CTTTCGTGAAAGATCCTGGGCTCAACCCAAGAGACGCGGAGGAAACGGCACGACTCAGAG
GACGTAAGAGGTAAAGGGAACTCATGGTGTAGGGGTGAAATCCGTTGATATCATGGGGAA
CACCAAATGCGAAGGCACTTTACTGGTACGCTCCTGACGCTGAAACACGAAAGCGTGGGA
ATCGAACGGGATTAGAGACCCGAGTAGTCC
>Otu183
CCAGCCTACGGGGGGCTCCAGTGGGGAATCTTGGACAATGGGGGCAACCCTGATCCAGCC
ATGCCGCGTGAGTGATGAAGGCCTTCGGGTTGTAAAACTCTTTCGACGGGGACGATAATG
ACGGTACCCGTAGAAGAAGCTCCGGCTAACTTCGTGCCAGCAGCCGCGGTAATACGAAGG
GGGCTAGCGTTGTTCGGAATTACTGGGCGTAAAGCGCGCGCAGGCGGCTTCTCAAGTCAG
GGGTGAAAGCCCAGAGCTCAACTCTGGAACTGCCCTTGAAACTGTGGAGCTTGAGTACGG
TAGAGGTGAGTGGAATTCCCAGTGTAGAGGTGAAATTCGTAGATATTGGGAAGAACACCG
GTGGCGAAGGCGGCTCACTGGACCGTTTCTGACGCTCAGGCGCGACAGCGTGGGGATCAA
ACAGGATTAGAAACCCCCGTAGTCC
>Otu184
CCAGCCTACGGGGTGCAGCAGTCGAGAATTTTTCACAATGGACGAAAGTCTGATGGAGCG
ACGCCGCGTGGAGGATGAAGGTTTTCGGATTGTAAACTCCTGTCACTGCAGAACAAGGAT
GCGTTTATGAATAGTAGATGCATTTGATGGTATGCGGAGAGGAAGGGACGGCTAACTCTG
TGCCAGCAGCCGCGGTAAGACAGAGGTCCCGAGCGTTGTTCGGATTCATTGGGCGTAAAG
GGTGTGTAGGAGGTCGGGTAAGTCAGGTGTGAAATCTCACAGCTTAACTGTGAAACTGCG
CTTGATACTGCCCGGCTAGAGGATCGGAGGGGGTAACGGAATTTATGGTGTAGCAGTGAA
ATGCGTAGATATCATAAGGAACACCGGTGGCGAAGGCGGTTACCTGGAAGATTCCTGACT
CTGAAACACGAAAGCCAGGGGAGCAAACGGGATTAGATACCCCCGTAGTCC
>Otu185
CCAGCCTATGGGGGGCAGCAGTAGGGAATATTGGTTAATCTGCGAAAGCGGGAACCAGCA
ACGCCGCGTGTGCGATGAAGGCCTTCGGGTTGTAAAGCACTTTTTGGAGGGATGAGGAAG
GACAGTACCTCCAGAATAAGCCTCGGCTAACTACGTGCCAGCAGCCGCGGTAAAACGTAG
GAGGCAAGCGTTATTCGGATTTACTGGGCGTAAAGCGTGTGCAGGCGGTTCAGAAAGTTG
GATGTGAAAGCTCCTGGCTTAACTGGGAGAGGTCGTTCAATACTTCTGGACTAGAGGATG
AGAGAGGGAGGTGGAATTCCGGGTGTAGTGGTGAAATGCGTAGATATCCGGAGGAACACC
AGTGGCGAAAGCGGCCTCCTGGCTCATTTCTGACGCTCATACACGAAAGCTAGGGTAGCA
AACGGGATTAGAAACCCCAGTAGTCC
>Otu186
CCAGCCTATGGGGGGCAGCAGTGGGGAATATTGCGCAATGGGCGAAAGCCTGACGCAGCG
ACGCCGCGTGGGTGATGAAGGCCTTCGGGTCGTAAAACCCTGTCGGGTGGAACGAATCTC
CATCTCGCTAATACCGAAATGGATTGACGGTACCACCGAAGGAAGCCCCGGCTAACTACG
TGCCAGCAGCCGCGGTAATACGTAGGGGGCGAGCGTTGTTCGGATTTACTGGGCGTAAAG
AGCGCGTAGGTGGTTCGATCAGTCAGAGGTGAAATCCCTCGGCTCAACCGAGGACCCGCG
TCTGATACTGTCGAACTTGAGTGCAGGAGAGGAGAGCGGAATTCCGAGTGTAGCGGTGAA
ATGCGTAGATATTCGGAGGAACACCGGTGGCGAAGGCGGCTCTCTGGACTGTCACTGACA
CTGAGGCGCGAAAGCTAGGGGAGCAAACGGGATTAGATACCCGCGTAGTCC
>Otu187
CCAGCCTATGGGGGGCAGCAGTCGAGAATATTCGACAATGGGCGAAAGCCTGATCGAGCG
ACACCGCGTGCAGGATGAAGGCCCTCGGGTCGTAAACTGCGGTAGTATGATAAGAATGCA
AATGACTGTCATACGGAAAGAGGTGGGTAACTACGTGCCAGCACCAGCGGTAATACGTAG
ACCTCAAGCATTATCCGGATTTATTGGGCGTAAAGCGCACGTAGGAGGTTTTGCGCGTCT
TTTGTTAAAGCCCACCGCCTAACGGTGGAAGTGCAGGAGATACGGCAGAACTAGAGGGGG
TTAGAGGTGCATGGAACTCACGGTGTAGGGGTGAAATCCGTTGATATCGTGGGGAACACC
AAAGGCGAAGGCAGTGCACTGGGACCTTCCTGACTCTGAGTTGCGAAAGCGTGGGGAGCA
AAAAGGATTAGATACCCTCGTAGTCC
>Otu188
CCAGCCTACGGGATGCAGCAGTGGGGAATCTTGCACAATGGGGGCAACCCTGATGCAGCG
ACGCCGCGTGAACGATGAAGCCCTTCGGGGTGTAAAGTTCTTTCGGCAGGGAAGAATCAT
GACGGTACCTGCAGAAGCAGCTGCGGCTAACTACGTGCCAGCAGCCGCGGTAATACGTAG
GCAGCGAGCGTTGTTCGGAGTTACTGGGCGTAAAGGGTGTGTAGGCGGTTCTCTAAGTTT
GGTGTGAAATCTCCCGGCTTAACCGGGAGGGTGCGCCGAAAACTGGGGAACTAGAGTGCG
GGAGGGGAGAGTGGAATTCCTGGTGTAGCGGTGAAATGCGTAGATATCAGGAGGAACACC
GGTGGTGTAGACGGCTCTCTGGACCGTAACTGACGCTGAGACACGAAAGCGTGGGTAGCA

AACAGGATTAGAAACCCTCGTAGTCC
>Otu189
CCAGCCTACGGGGGGCAGCAGTGAGGAATATTGCGCAATGGCCGAAAGGCTGACGCAGCG
ACGCCGCGTGAAGGATGAAGTCCGTTAGGATGTAAACTTCTTTTGCAGGGGATGAATGTG
GTGCCTTCGGGTGCCATTGACCGTACCCTGCGAATAAGGATCGGCTAACTACGTGCCAGC
AGCCGCGGTAATACGTAGGATCCGAGCGTTGTCCGGAATTACTGGGTGTAAAGGGCGTGT
AGGTGGAACTTTAAGTCGGTGGTGAAAGCCTGCAGCTTAACTGCAGAACTGCCCTCGATA
CTGAAGTTCTTGAGTACGGCAGAGGGAGATGGAATTCATGGTGTAGCGGTGAAATGTGTA
GATATCATGAAGAACACCGGTAGCGAAGGCGGTCTCCTGGTCCGTAACTGACACTGAGGC
GCGAAAGCGTGGGGAGCAAACAGGATTAGAAACCCCCGTAGTCC
>Otu190
CCAGCCTACGGGGGGCAGCAGTGGGGAATTTTGGACAATGGGGGCAACCCTGATCCAGCC
ATGCCGCGTGTGTGAAGAAGGCCTTCGGGTTGTAAAGCACTTTTGTCCGGAACGAAACGG
CGGATGCTAATAACATCCGTTAATGACGGTACCGGAAGAATAAGCACCGGCTAACTACGT
GCCAGCAGCCGCGGTAATACGTAGGGTGCAAGCGTTAATCGGAATTACTGGGCGTAAAGC
GTGCGCAGGCGGTTTCGCAAGTCAGATGTGAAATCCCCGGGCTTAACCTGGGAACTGCAT
TTGAAACTACGAGGCTAGAGTGTGTCAGAGGGGGGTAGAATTCCACGTGTAGCAGTGAAA
TGCGTAGAGATGTGGAGGAATACCGATGGCGAAGGCAGCCCCCTGGGATAACACTGACGC
TCATGCACGAAAGCGTGGGGAGCAAACAGGATTAGATACCCTAGTAGTCC
>Otu191
CCAGCCTACGGGTGGCAGCAGCTAAGAATCTTCCGCAATGGGGGAAACCCTGACGGAGCG
ACGCCGCGTGGATGATGAAGGCCGTAAGGTTGTAAAATCCTTTTGTCGGTGAAGAATAAG
GGTGGGAGTGGAAAGCCCATCTGATGACGTTAGCCGGCGAATAAGCCCCGGCCAATTACG
TGCCAGCAGCCGCGGTAATACGTATGGGGCAAGCGTTGTTCGGAGTTATTGGGCGTAAAG
GGCGTGTAGGCGGCTCATCAAGTCTGGTGTGAAATCGTCAGGCTCAACCTGACATATGCG
CTGGAAACTGGTGGGCTTGAGTTCTTGAGGGGAAGCTGGAATTCCAGGTGTAGGGGTGAA
ATCTGTAGATATCTGGAAGAACACCGGTGGCGAAGGCGAGCTTCTATCAGAGGACTGACG
CTGAGGCGCGAAAGCGTGGGGAGCAAACAGGATTAGAAACCCTAGTAGTCC
>Otu192
CCAGCCTATGGGTGGCAGCAGTGGGGAATATTGGACAATGGGGGAAACCCTGATCCAGCG
ACGCCGCGTGTGTGAAGAAGGCCTGCGGGTTGTAAAGCACTTTTAGTGGGGATGAAAAGT
CCAGGGCTAATACCCTTGGATCTTGACCTAACCCAAAGAAAAAGCACCGGCTAACTCTGT
GCCAGCAGCCGCGGTAATACAGAGGGTGCGAGCGTTAATCGGAATTACTGGGCGTAAAGC
GTGCGTAGACGGTTTCGTAAGTCGGGTGTGAAATCCCCGGGCTCAACCTGGGAATTGCAT
TCGAGACTGCGAGGCTAGGGTGCGGAAGAGGGAAGCGGAATTTCCGGTGTAGCGGTGAAA
TGCGTAGATATCGGAAGGAACATCAGTGGCGAAGGCGGCTTCCTGGTCCAGCACCGACGT
TCAGGCACGAAAGCGTGGGGAGCAAACAGGATTAGATACCCTAGTAGTCC
>Otu193
CCAGCCTACGGGACGCAGCAGTGGGGAATCTTGGACAATGGGCGAAAGCTTGATCCGGTG
ATATTAAATGGAGGAATTAAGACAGCCAAAATGCTGTAAACTTCTATGCGTTAGATTGAA
ATAATTATGAAAGAGGAGTCTTAAACGAAGCCCTGGCTAAACTCGTGCCAGCAGCCGCGG
TAACACGAGTGGGGCGAGCGTTATTCAGTATTATTAGGCGTAAAAGGTTCGTAGATGGTC
TGATTATGTTGAAAAAGAAATCCTAAAGCCTAACTTTAGAAAAATTTTTCAATTTAAGTT
TGACTAAGAGTCTAAACGGGATAATAGAATTTCTTAACGAGGAGTAAAGTCCGAAGATCT
AGGAAGGAACACCATAAGTGAAAACATTTATCTAGTTTAGTCTGACATTGAGGAACGAAA
GCATGGGGATCAAAAAGGATTAGAAACCCCCGTAGTCC
>Otu194
CCAGCCTACGGGGGGCACCAGTTTCGAATCATTCACAATGGGCGAAAGCCTGATGGTGCG
ACGCCGCGTGGGGGATGAAGGTCTTCGGATTGTAAACCCCTGTCACCTGGGACTAAACTC
CGGCGAAGAGCCGGACTGAATTAACCAGGAGAGGAAGCAGTGGCAAACTCTGTGCCAGCA
GCCGCGGTAATACAGAGACTGCAAGCGTTACTCGGATTCACTGGGCGTAAAGGGAGCGCA
GGTGGGCTGGTGTGTCGGGCGTGAAATCCCGGGGCTTAACTCCGGAACGGCGCCCGAAAC
TACCGGCCTAGAGGATTGGAGAGGCGGGTGGAATTCCAGGTGTAGCGGTGAAATGCGTAG
ATATCTGGAGGAACACCGACGGCGAAGGCAGCCCGCTGGACAAATCCTGACACTCAGGCT
CGAAAGTATGGGGAGCAAAAGGGATTAGATACCCGTGTAGTCC
>Otu195
CCAGCCTACGGGGGGCAGCAGTGGGGAATATTGGACAATGGGCGCAAGCCTGATCCAGCA
ATGCCGCGTGTGTGAAGAAGGTCTTCGGATTGTAAAGCACTTTCGGCGGGGACGATGATG

ACGGTACCCGCAGAAGAAGCCCCGGCTAACTTCGTGCCAGCAGCCGCGGTAATACGAAGG
GGGCTAGCGTTGCTCGGAATGACTGGGCGTAAAGGGCGCGTAGGCGGTTTGTTTAGTCAG
GCGTGAAATTCCTGGGCTTAACCTGGGGGCTGCGCTTGATACAGGCAGACTTGAGTGTGG
AAGAGGGTCGTGGAATTCCCAGTGTAGAGGTGAAATTCGTAGATATTGGGAAGAACACCG
GTGGCGAAGGCGGCGACCTGGTCCATTACTGACGCTGAGGCGCGAAAGCGTGGGGAGCAA
ACAGGATTAGAAACCCTCGTAGTCC
>Otu196
CCAGCCTACGGGGGGCAGCAGTCAGGAATATTGCGCAATGGAGGAAACTCTGACGCAGCG
ACGCCGCGTGGGTGATGAAGGCCTTCGGGTCGTAAAGCCCTGTCGGAGGGGAAGAAAAGC
TTGGTGGCTAATATCCACCAGGCCCTTGACGGTACCCTCAAAGGAAGCACCGGCTAACTC
CGTGCCAGCAGCCGCGGTAAGACGGAGGGTGCAAGCGTTGTTCGGATTGACTGGGCGTAA
AGGGCGTGTAGGCGGTTGCCCAAGTCCGATGTGAAAGCCCGAGGCCTACCCTCGGAAGTG
CATCGGAAACTAGGCAGCTGGAGCGCGATAGAGGAGGGTAGAATTCCTGGTGTAGCGGTG
AAATGCGTAGATATCAGGAGGAATACCGGCGGCGAAGGCGGCCCTCTGGATCGTTGCTGA
CGCTGAGACGCGAAAGCGTGGGGAGCAAACAGGATTAGAAACCCCCGTAGTCC
>Otu197
CCAGCCTACGGGGTGCAGCAGTCGAGAATTTTTCACAATGGGGGAAACCCTGATGGAGCG
ACGCCGCGTGGGGGATGAATGGCTTCGGCCCGTAAACCCCTGTCATTTGCGAACAAATCC
GCACACCCAACACGTGTGTGGTTGATAGTAGCGGAAGAGGAAGGGACGGCTAACTCTGTG
CCAGCAGCCGCGGTAATACAGAGGTCCCAAGCGTTGTTCGGATTCACTGGGCGTAAAGGG
TGCGTAGGTGGCCGGGTAAGTCTGATGTGAAATCTCGGAGCTCAACTCCGAAACGGCATT
GGATACTCTCCGGCTGGAGGGTCGGAGGGGGGACTGGAATTCTCGGTGTAGCAGTGAAAT
GCGTAGATATCGAGAGGAACACCAGTGGCGAAGGCGAGTCCCTGGACGACTCCTGACACT
GAGGCACGAAAGCTAGGGGAGCAAACAGGATTAGAAACCCGAGTAGTCC
>Otu198
CCAGCCTATGGGGGGCAGCAGTAGGGAATCTTGCGCAATGCGCGAAAGCGTGACGCAGCA
ACGCCGCGTGGGGGAAGAAGGCCTTCGGGTTGTAAACCCCTTTCAGTTGGGACGAAGCTT
CGTCGGTTAATAGCCGACTGGAGTGACGGTACCTTCACAAGAAGCACCGGCTAACTACGT
GCCAGCAGCCGCGGTAATACGTAGGGTGCAAGCGTTGTCCGGAATTATTGGGCGTAAAGA
GCGTGTAGGCGGTCCGATCAGTCCGCTGTGAAAGTCCAGGGCTCAACCCTGGGATGCCGG
TGGATACTGTCGGACTAGAGTCCGGAAGAGGCGAGTGGAATTCCTGGTGTAGCGGTGAAA
TGCGCAGATATCAGGAGGAACACCAATGGCGAAGGCAGCTCGCTGGGACGGTACTGACGC
TGAGACGCGAAAGCGTGGGGAGCAAACAGGATTAGAAACCCTTGTAGTCC
>Otu199
CCAGCCTACGGGGGGCTGCAGTCGAGAATATTCGACAATGGGCGAAAGCCTGATCGAGCG
ACACCGCGTGCAGGATGAAGGCCCTCGGGTCGTAAACTGCGGTAGTATGGGAAGAATGTA
AATGACCGTACCATACGGAAAGAGGTGGGTAACTACGTGCCAGCACCAGCGGTAATACGT
AGACCTCAAGCGTTATCCGGATTTATTGGGCGTAAAGCGCATGTAGGAGGTTTTGCGCGT
CTTTTGTTAAAGCCCACCGCCCAACGGTGGAGATGCAGGAGATACGGCAGGACTAGAGGA
GGTTAGAGGTGCATGGAACTCACGGTGTAGGGGTGAAATCCGTTGATATCGTGGGGAACA
CCAAAGGCGAAGGCAGTGCACTGGGACCTTCCTGACTCTGAGATGCGAAAGCGTGGGGAG
CAAAAAGGATTAGATACCCTAGTAGTCC
>Otu200
CCAGCCTACGGGTTGCTGCAGTAAGGGATATTGCGCAATGGGCGAAAGCCTGACGCAGCA
ACGCCGCGTGGAGGATGAAGGCCTTCGGGTCGTAAACTCGTTTTGCCTGTGACGAGCAAG
GACGGTAGCAGGCGAATAAGTCACGGCTAACTACGTGCCAGCAGCCGCGGTAAAACGTAG
GTGGCGAGCGTTATCCGGATTTACTGGGCGTAAAGGGTGTGCAGGCGGGCGACCAAGTGG
TGTATGAAATCTCCGGGCCTAAGTCGGAGGGGTTATGCCAGACTGGTCGTCTTGAGCGTG
AGAGAGGGACATGGAATTCCGGGTGTAGTGGTGAAATGCGTAGAGATCCGGAGGAACCCC
AGAGGCGAAGGCGGTGTCCTGGCTCACGGCTGACGCTCAGCCACGACAGCATGGGGAGCG
AACGGGATTAGATACCCTAGTAGTCC
>Otu201
CCAGCCTATGGGTTGCACCAGGCGCGAAAACTTTGCAATGCGAGAAATCGTGACAAGGGA
ACTCCGAGTGCCCGTAAATTCGGGCTGTCCATCTGTGTAAAAAACAGGTGAAGAAAGGGC
CGGGCAAGACCGGTGCCAGCCGCCGCGGTAATACCGGCGGCTCGAGTGGTGGCCACTATT
ACTGGGCTTAAAGCGTCCGTAGCTGGTCTGTTAAGTCTCTGGGGAAATCTTCCGGCTTAA
CCGGAAGGCGTCCCAGGGATACTGGCAGACTAGGGACCGGGAGAGGTGAGAGGTACTCCA
GGGGTAGGAGTGAAATCCTGTAATCCTTGGGGGACCACCTGTGGCGAAGGCGTCTCACCA

GAACGGCTCCGACAGTGAGGGACGAAAGCTGGGGGAGCAAACCGGATTAGAAACCCCCGT
AGTCC
>Otu202
CCAGCCTATGGGGGGCTGCAGTGGGGAATCTTCCGCAATGGACGAAAGTCTGACGGAGCA
ACGCCGCGTGAGTGAAGAAGGTTTTCGGATCGTAAAACTCTGTTTTCAGGGACGAATGTG
CGTCGTGTAAATAATGCGATGTAATGACGGTACCTGAGTAGAAAGCCACGGCTAACTACG
TGCCAGCAGCCGCGGTAATACGTAGGTGGCAAGCGTTGTCCGGAATTATTGGGCGTAAAG
CGCGCGCAGGTGGGATATTAAGTCTGGTGTTAAAGTTCGGGGCTCAACCCCGTGAAGCAT
TTGGAAACTGATATTCTTGAGTGCAGGAGAGGAAAGTGGAATTCCTAGTGTAGCGGTGAA
ATGCGTAGATATTAGGAGGAACACCAGTGGCGAAGGCGACTTTCTGGACTGTGTCTGACA
CTGAGGCGCGAAAGCCAGGGTAGTGAACGGGATTAGATACCCCAGTAGTCC
>Otu203
CCAGCCTATGGGGGGCAGCAGTGGGGAATTTTGCGCAATGGGCGAAAGCCTGACGCAGCA
ACGCCGCGTGGGTGATGAAGGTCTTCGGACTGTAAAACCCTGTCGTCAGGGACGAAGGTG
CGCATGTTAATAACGTGTGTACTTGACGGTACCTGGAGAGGAAGCCCCGGCTAACTCTGT
GCCAGCAGCCGCGGTAATACAGAGGGGGCAAGCGTTATTCGGAATTATTGGGCGTAAAGG
GCGCGTAGGCGGTTTTTTAAGTCAGATGTGTAATCCCCGAGCTTAACTTGGGAACTGCAT
CTGAGACTGGAAGGCTAGAGTACTGGAGAGGGTGGTGGAATTCCTCGTGTAGCGGTGAAA
TGCGTAGAGATGAGGAGGAACACCAGTGGCGAAGGCGGCCACCTGGACAGTAACTGACGC
TGAGGCGCGAAAGTGTGGGTAGCAAACAGGATTAGAAACCCCAGTAGTCC
>Otu204
CCAGCCTATGGGGGGCAGCAGTGGGGAATCTTGCGCAATGGACGAAAGTCTGACGCAGCA
ACGCCGCGTGAGGGACGAAGGTTTTCTGAATTGTAAACCTCTTTCGACAGGGACGATAAT
GACGGTACCTGTAGAAGAAGCACCGGCCAACTATGTGCCAGCAGCCGCGGTGATACATAG
GGTGCGAGCGTTATTCGGATTTATTGGGCGTAAAGAGCTCGTAGGCGGTTCGATAAGTCG
GGTGTTAAATCCACAGGCTCAACCTGTGGCAGCCATCCGAAACTGTCGTGACTAGAGTTT
GGTAGGGGATCACGGAATTCCTGGTGTAGCGGTGGAATGCGCAGATATCAGGAGGAACAC
CAGCAGCGAAGGCGGTGATCTGGGCCAATACTGACGCTGAGGAGCGAAAGCGTGGGGAGC
GAACAGGATTAGAAACCCGCGTAGTCC
>Otu205
CCAGCCTATGGGTTGCAGCAGTTTCGAATCATTCACAATGGGGGAAACCCTGATGGTGCG
ACGCCGCGTGGGGGATGAAGGTCTTCGGATTGTAAACCCCTGTCACCTGGGACTAAACCT
CGGCGAAGAGCCGAGCTGAATTAACCAGGAGAGGAAGCAGTGGCTAACTCTGTGCCAGCA
GCCGCGGTGATACAGAGACTGCAAGCGTTACTCGGATTCACTGGGCGTAAAGGGAGCGCA
GGTGGGCAGGTGTGTCGGGTGTGAAATCCCGGGGCTTAACCCCGGAATGGCGCCCGAAAC
TATCTGTCTAGAGGATTGGAGAGGCGGGTGGAATTCCAGGTGTAGCGGTGAAATGCGTAG
ATATCTGGAGGAACACCGACGGCGAAGGCAGCCCGCTGGACAAATCCTGACACTCAGGCT
CGAAAGTATGGGGAGCAAAAGGGATTAGAAACCCTAGTAGTCC
>Otu206
CCAGCCTACGGGGCGCAGCAGTGGGGAATCTTGCACAATGGGCGAAAGCCTGATGCAGCG
ACGCCGCGTGAGCGATGAAGCCCTTCGGGGTGTAAAGCTCTTTCGACGGGAACGATAATG
ACGGTACCCGGAGAAGAAGCTGCGGCTAACTACGTGCCAGCAGCCGCGGTAATACGTAGG
CAGCGAGCGTTGTTCGGAATTACTGGGCGTAAAGAGTGTGTAGGCGGTGTTCCCAGTCTG
TTGTGAAATCTCCCGGCTTAACTGGGAGGGTGCAGCGGAAACTAGAATGCTAGAGGGTGG
GAGAGGTAAGCGGAATTCCTGGTGTAGCGGTGAAATGCGTAGATATCAGGAGGAACACCT
GTGGTGTAGACAGCTTACTGGACCACTTCTGACGCTGAGACACGAAAGCGTGGGTAGCAA
ACAGGATTAGAAACCCTTGTAGTCC
>Otu207
CCAGCCTACGGGAGGCAGCAGTGAGGAATATTGGGCAATGGGCGCAAGCCTGACCCAGCC
ATGCCGCGTGAAGGATGAATGCCCTATGGGTTGTAAACTTCTTTTGTACGGGACGAAACT
CCTGGTCGTGACCGGGATTGACTGTACCGTGCGAATAAGCATCGGCTAACTCCGTGCCAG
CAGCCGCGGTAATACGGAGGATGCAAGCGTTATCCGGATTCATTGGGTTTAAAGGGTGCG
TAGGCGGAATAATAAGTCAGTGGTGAAAGCCTGCGGCTTAACCGTAGAAATGCCATTGAT
ACTGTTATTCTTGAGTATAGTTGAGGTGGGCGGAATGTGTAATGTAGCGGTGAAATGCTT
AGATATTACACAGAACACCAATTGCGAAGGCAGCTCACTAAGCTGTTACTGACGCTGAGG
CACGAAAGCGTGGGGAGCAAACAGGATTAGATACCCCAGTAGTCC
>Otu208
CCAGCCTACGGGGGGCAGCAGTGGGGAATATTGGACAATGGGCGCAAGCCTGATCCAGCA

ATGCCGCGTGGGTGAAGAAGGTTTTCGGATTGTAAAGCCCTTTTGGCGGGGACGATGATG
ACGGTACCCGCAGAATAAGCCCCGGCTAACTTCGTGCCAGCAGCCGCGGTAATACGAAGG
GGGCTAGCGTTGCTCGGAATGACTGGGCGTAAAGGGCGCGTAGGTGGCGATCAAAGTCAG
GCGTGAAATTCCTGGGCTCAACCTGGGGGCTGCGTTTGATACTTGGTTGCTAGAGTGAGG
AAGAGGGTCGTGGAATTCCCAGTGTAGAGGTGAAATTCGTAGATATTGGGAAGAACACCG
GTGGCGAAGGCGGCGACCTGGTCCTTGACTGACGCTGAGGCGCGAAAGCGTGGGGAGCAA
ACAGGATTAGATACCCCCGTAGTCC
>Otu209
CCAGCCTATGGGAGGCAGCAGTCGAGAATTTTTCTCAATGGGCGAAAGCCTGAAGGAGCG
ACGCCGCGTGGGGGATGAAGGGCTTCGGCCCGTAAACCCCTGTCATTTGCGAACAAACCT
TATGGTATAATACATCATGAGCTGATTGTAGCGAAAGAGGAAGGGACGGCTAACTCTGTG
CCAGCAGCCGCGGTAATACAGAGGTCCCAAGCGTTGTTCGGATTCACTGGGCGTAAAGGG
TGCGTAGGTGGCGGGGTAAGTTTGATGTGAAATCTCCGGGCTTAACCCGGAAACTGCATT
GAATACTATTCTGCTCGAGGGTTGGAAGGGGGACTGGAATACTTGGTGTAGCAGTGAAAT
GCGTAGATATCAAGTGGAACACCAGTGGCGAAGGCGAGTCCCTGGACAACTCCTGACACT
GAGGCACGAAAGCTAGGGGAGCAAACAGGATTAGATACCCCCGTAGTCC
>Otu210
CCAGCCTATGGGGGGCTGCAGTGGGGAATCTTGCGCAATGGGCGAAAGCCTGACGCAGCC
ATGCCGCGTGGATGATGAAGGTCTTAGGATTGTAAAATCCTTTCAGCGGGGACGATAATG
ACGGTACCCGCAGAAGAAGCCCCGGCTAACTTCGTGCCAGCAGCCGCGGTAATACGAAGG
GGGCTAGCGTTGCTCGGAATTACTGGGCGTAAAGGGCGCGTAGGCGGACAGTTAAGTCGG
GGGTGAAAGCCCGGGGCTCAACCTCGGAATTGCCTTCGATACTGGCTGTCTTGAGTACGG
GAGAGGTGTGTGGAACTCCGAGTGTAGAGGTGAAATTCGTAGATATTCGGAAGAACACCA
GTGGCGAAGGCGACACACTGGCCCGTTACTGACGCTGAGGCGCGAAAGCGTGGGGAGCAA
ACAGGATTAGAAACCCGCGTAGTCC
>Otu211
CCAGCCTATGGGGGGCACCAGTAAGGAATATTGGACAATGGAGGCAACTCTGATCCAGCC
ATGCCGCGTGCAGGAAGAAGGCGCTACGCGTTGTAAACTGCTTTTGTACCAGAGAAAATC
ATTGTACGTGTACGATGTTGATAGTATGGTAAGAATAAGCATCGGCTAACTTCGTGCCAG
CAGCCGCGGTAATACGAAGGATGCAAGCGTTATCCGGATTTATTGGGTTTAAAGGGAGCG
TAGGCGGTCTTATAAGTCAGTGGTGAAATCTCGTTGCTTAACAACGAACGTGCCATTGAT
ACTGTAGGACTTGAGTACAGTTGCCGTTGGCGGAATGTGTCATGTAGCGGTGAAATGCAT
AGATATGACACAGAACACCGATTGCGAAGGCAGCTGACGAAACTGTAACTGACGCTGAGG
CTCGAAAGCGTGGGGATCAAACAGGATTAGAGACCCCCGTAGTCC
>Otu212
CCAGCCTATGGGGGGCAGCAGCTAAGAATCTTCCGCAATGGGGGAAACCCTGACGGAGCG
ACGCCGCGTGGATGATGAAGGCCGTAAGGTTGTAAAATCCTTTTGTTGGTGAAGAATAAG
TGGAGGAGTGGAAAGCCTCCATGATGACGTTAACCGACGAATAAGCCCCGGCTAATTACG
TGCCAGCAGCCGCGGTAATACGTATGGGGCAAGCGTTGTTCGGAGTTATTGGGCGTAAAG
GGCGTGTAGGCGGCTCATGAAGTCTGGTGTGAAATCGACAGGCTCAACCTGTCATATGCG
CTGGAAACTGGTGGGCTTGAGTTCTTGAGGGGAAGCTGGAATTCCAGGTGTAGGGGTGAA
ATCTGTAGATATCTGGAAGAACACCGGTGGCGAAGGCGAGCTTCTATCAGAGGACTGACG
CTGAGGCGCGAAAGCGTGGGGAGCAAACAGGATTAGATACCCGAGTAGTCC
>Otu213
CCAGCCTACGGGAGGCAGCAGTAGGGAATATTGGGCAATGGGCGAAAGCCTGACCCAGCA
ACGCCGCGTGCACGATGAAGGTCTTCGGATCGTAAAGTGCTTTTCTGAGAGATGAGAAAG
GACAGTATCTCAGGAATAAGTCTCGGCTAACTACGTGCCAGCAGCCGCGGTAAAACGTAG
GAGGCGAGCGTTATCCGGATTTACTGGGCGTAAAGCGCGTGCAGGTGGTTTGGTAAGTTG
GATGTGAAAGCTCCCGGCTCAACTGGGAGAGGTCGTTCAATACTACCAGACTTGAGAGCA
GTAGAGGGAGGTGGAATTCCCGGTGTAGTGGTGAAATGCGTAGATATCGGGAGGAACACC
AGTGGCGAAAGCGGCCTCCTGGACTGTTTCTGACACTCAGACGCGAAAGCTAGGGTAGCA
AACGGGATTAGATACCCCCGTAGTCC
>Otu214
CCAGCCTATGGGGGGCACCAGTGGGGAATTTTGCGCAATGGGCGAAAGCCTGACGCAGCA
ACGCCGCGTGAGTGATGAAGGCTTTCGGGTCGTAAAGCTCTGTCAAGGGGAAAGAAATGC
TTAGCGGTTAATACCCGTTATGCTTGACGGTACCCCTAAAGGAAGCACCGGCTAACTCCG
TGCCAGCAGCCGCGGTAATACGGAGGGTGCGAGCGTTGTTCGGAATTATTGGGCGTAAAG
CGCGTGTAGGCGGTTTGGTAAGTCTGATGTGAAAGCCCTGGGCTCAACCCGGGAAGTGCA

TTGGAAACTGTCAGACTTGAATACGGGAGAGGGTAGTGGAATTCCTGGTGTAGGAGTGAA
ATCCGTAGATATCAGGAGGAACACCGGTGGCGAAGGCGGCTACCTGGACCGATATTGACG
CTGAGACGCGAAAGCGTGGGTAGCAAACAGGATTAGATACCCCAGTAGTCC
>Otu215
CCAGCCTATGGGTGGCAGCAGTGGGGAATATTGGGCAATGGACGCAAGTCTGACCCAGCG
ACGCCGCGTGCGGGATGAAGGCCTTCGGGTTGTAAACCGCTTTCAGCAGGGAAGAAGCGA
AAGTGACGGTACCTGCAAAAGAAGCACCGGCTAACTATGTGCCAGCAGCCGCGGTAATAC
ATAGGGTGCAAGCGTTGTCCGGAATTATTGGGCGTAAAGAGCTCGTAGGTGGTTAGTTAC
GTCGGGTGTGAAAATCTGGGGCTCAACCCCAGACCTGCACTCGATACGGGCTAGCTAGAG
TTTGGTAGGGGAGACTGGAATTCCTGGTGTAGCGGTGGAATGCGCAGATATCAGGAGGAA
CACCAATGGCGAAGGCAGGTCTCTGGGCCAATACTGACACTGAGGAGCGAAAGCGCGGGG
AGCGAACAGGATTAGAAACCCCCGTAGTCC
>Otu216
CCAGCCTATGGGTTGCAGCAGTAGGGAATATTGCGCAATGGACGAAAGTCTGACGCAGCA
ACGCCGCGTGGATGATGAAGCATTTCGGTGTGTAAAATCCTGTCAGTGGGGAAGAAACGC
ACGGTGAGTAACTGCTCCGTGCTTGACGGTACCCGCAGAGGAAGTCCCGGCTAACTCCGT
GCCAGCAGCCGCGGTAATACGGGGGGGACAAGCGTTGTTCGGATTTACTGGGCGTAAAGG
GCGCGTAGGCGGACCTTCAAGTCGGATGTGAAACCTGGCGGCTTAACCACCAGCCTGCAT
TCGATACTGTTGGCCTTGAGTACGGGAGAGGAGAATGGAATTCCAGGTGTAGCGGTGAAA
TGCGTAGATATCTGGAAGAACACCAGTAGCGAAGGCGGTTCTCTGGACCGATACTGACGC
TAAAGCGCGAAGGCTTGGGGAGCAAACAGGATTAGATACCCTAGTAGTCC
>Otu217
CCAGCCTATGGGGGGCAGCAGTGGGGAATATTGGGCAATGGGCGAAAGCCTGACCCAGCC
ACGCCGCGTGAGTGATGAAGGCCTTCGGGTCGTAAAGCTCTGTGGGGAGGGACGAACAAG
TGCGCATCCAATACATGCGTGCCCTGACGGTACCTCCTTAGCAAGCACCGGCTAACCATG
TGCCAGCAGCCGCGGTAATACATGGGGTGCAAACGTTGCTCGGAATTATTGGGCGTAAAG
CGCGCGTAGGCGGTTACTTAAGTCGGATGTGAAAGCCCTTGGCTTAACTGAGGAAGTGCA
TCCGAGACTGAATAGCTAGAGTACGAAAGAGGGTCGCGGAATTCCCGGTGTAGAGGTGAA
ATTCGTAGATATCGGGAGGAACACCGGCGGCGAAGGCGGCGACCTGGTTCGAGACTGACG
CTGAGGCGCGAAAGCGTGGGGAGCAAACAGGATTAGAAACCCTCGTAGTCC
>Otu218
CCAGCCTACGGGGGGCAGCAGTCGCGAAACCTTGACAATGCGCGAAAGCGTGATCAGGGA
ACTCTGAGTGTCAACCTAACGGTTGACTTTTGATCAGCTTGAAAAGCTGGTAGAATAAGG
GCTGGGTAAATCTCGTGCCAGCCGCCGCGGTAACACGAGTAGCCCGAGTGGTGCCCACGA
ATATTGAGTCTAAAACGCATGTAGCCGGCTTGGAAAGTTCCCCGTGAAATCTCTCGGCTC
AACCGAGAGGCGCGCGGGGAATACTAACAGGCTCGGGAATGGAAGAGGCCAAGGGTACTG
TAAGGGGAGGAGTAAAATCCTGTAATCCTTGCAGGACCATCGGTGGCGAAAGCGCTTGGC
CAAGACATGTCCGACGGTGAGATGCGAAGGCTAGGGGAGCAAATCGGATTAGAGACCCCG
GTAGTCC
>Otu219
CCAGCCTATGGGGGGCAGCAGCTGGGAATCTTCTGCAATACACGAAAGTGTGACAGAGCG
AACCAGAGTGTTCCTCATTGAGGAACTTTTGCCAAATCTAAAAAGTTTGGCGAATAAGGA
CTGGGCAAGACTAGTGCCAGCCGCCGCGGTAATACTAGCTGTCCAAGTCGCAACCATCTT
TATTGGGTCTAAAACATCCGTAGCTTGCTTAGTAAGTTCTTTGTGAAATCCTGTCTCTCA
AGGGCAGGGCGTGCAAGGAATACTGCTTCGCTAGAGACTGGAAGACGTAAGAAGTACGTT
TGAAGTAGCGGTTAAATGCGTTAATCTTAGACGGACTCACAACAGCGAAGGCATCTTACG
AGGACAGATCTGACAGTGAGGGATGAAGGCCAGGGGCGCAAAACGGATTAGAAACCCTAG
TAGTCC
>Otu220
CCAGCCTACGGGTGGCAGCAGGTAGGAATCTTTTGCAATGCGCGCAAGCGTGACAAAGCG
AGCCAGAGTGCTTTTCAATTGAAAAGCTTTTGCCAAATCCAAAAAGTTTGGCGAATAAGG
ACTGGGCAAGACTAGTGCCAGCCGCCGCGGTAATACTAGCGGTCCAAGTCGCAGCCATCA
TTATTGGGTCTAAAATATCCGTAGCTTGCTTAATAAGTTCCCTGTGAAATCTTATATCTT
AAGTATAAGGCGTGCAGGGAATACTGCTAAGCTAGAGACTGGAAGACGTAGAAAGTACGT
TCAAAGTAGCGGTAAAATGTGTTAATCTTGAACGGACTCACAATAGCGAAGGCATTCTAC
GAGGACAGTTCTGACAGTAAAGGATGAAGGCTAGGGGCGCAAAATGGATTAGATACCCCC
GTAGTCC
>Otu221

CCAGCCTACGGGAGGCTGCAGTGGGGAATATTGCACAATGGGCGCAAGCCTGATGCAGCG
ACGCCGCGTGGGGGATGACGGCCTTCGGGTTGTAAACCTCTTTCAGCAGGGACGAAGCGC
AAGTGACGGTACCTGCAGAAGAAGCACCGGCCAACTACGTGCCAGCAGCCGCGGTAATAC
GTAGGGTGCGAGCGTTGTCCGGAATTACTGGGCGTAAAGAGCTCGTAGGTGGTTTGTCGC
GTTGTTCGTGAAAACCGAGGGCTTAACTCTCGGCGTGCGGGCGATACGGGCAGACTAGAG
TACTGCAGGGGAGACTGGAATTCCTGGTGTAGCGGTGGAATGCGCAGATATCAGGAGGAA
CACCGGTGGCGAAGGCGGGTCTCTGGGCAGTAACTGACGCTGAGGAGCGAAAGCGTGGGG
AGCGAACAGGATTAGAAACCCCAGTAGTCC
>Otu222
CCAGCCTATGGGAGGCTCCAGGCGCGAAACCTTCGCAATGCGCGAAAGCGTGACGAGGTT
AATCCGAGTGGTTCCCGCTGAGGGAATCTTTTGTTGGCTCTAAAATGGCCAGTGAATAAG
GGGAGGGCAAGTCTGGTGTCAGCCGCCGCGGTAATACCAGCTCCTCGAGTGGTCGGGGCG
ATTATTGGGCCTAAAGCATCCGTAGCCTGTTCCGTAGGTCTTCTGTTAAATCCAACGGCT
TAACCGTTGGCCTGCAGAAGATACCGCGGGACTAGGAGGCGGGAGAGGTGGACGGTACTC
CACGTGTAGGGGTAAAATCCTTTGATCCGTGGAAGACCACCAGTGGCGAAGGCGGTCCAC
CAGAACGCGCTCGACGGTGAGGGATGAAAGCTGGGGGAGCGAACCGGATTAGAAACCCGT
GTAGTCC
>Otu223
CCAGCCTATGGGGGGCAGCAGTAGGGAATTTTACGCAATGGGCGAAAGCCTGACGTAGCG
ACACCGCGTGAACGAAGAAGCCTTTTGGGGTGTAAAGTTCTGTCGGCTGGGAAGAAAAAA
ATGACGGTACCAGCAAAGGAAGCATCGGCTAACTACGTGCCAGCAGCCGCGGTAAGACGT
AGGATGCAAGCATTGTCCGGATTTATTGGGCGTAAAGAGTTCGTAGGCGGTTTGTTAAGT
CTAATGTTAAAGATCAGGGCTCAACCCTGGGACTGCATTGGATACTGGCAGACTGGAGTG
TGGTAGAGGTAAGTGGAATTTCTGGTGTAGCGGTGAAATGCGTAGATATCAGAAAGAACA
CCAGTGGCGTAGGCGACTTACTGGGCCATAACTGACGCTGAGGAACGAAAGCCAGGGGAG
CAAATGGGATTAGAAACCCGCGTAGTCC
>Otu224
CCAGCCTATGGGAGGCAGCAGGAGGGAATATTGGTAATGGGCGAAAGCCCGAACCAGCAA
CGCCGCGTGTGCGATGAAGGCCTTCGGGTCGTAAAGCACTTTTCGAGGGGATGAGGAAGG
ACAGTACCCTCGGAATAAGTCTCGGCTAACTACGTGCCAGCAGCCGCGGTAACACGTAGG
AGGCGAGCGTTATCCGGATTTACTGGGCGTAAAGCGTGTGCAGGCGGTCTAGCAAGTTGG
ATGTGAAAGCTCCTGGCTCAACTAGGAGAGGTCGTTCAATACTGCCAGACTCGAGGATGG
TAGAGGGAGGTGGAATTCCGAGTGTAGTGGTGAAATGCGTAGATATTCGGAGGAACACCA
GTGGCGAAAGCGGCCTCCTGGACCATTTCTGACGCTCATACACGAAAGCTAGGGGAGCAA
ACGGGATTAGAGACCCCCGTAGTCC
>Otu225
CCAGCCTACGGGTGGCAGCAGTGGGGAATATTGGACAATGGGGGCAACCCTGATCCAGCG
ACGCCGCGTGTGTGAAGAAGGCCTGCGGGTTGTAAAGCACTTTTAGTGGGGATGAAAAGC
TCAGGGTTAACACCTCTGGGTCTTGACCTAACCCACAGAAAAAGCACCGGCTAACTCTGT
GCCAGCAGCCGCGGTAATACAGAGGGTGCAAGCGTTAATCGGAATTACTGGGCGTAAAGC
GTGCGTAGACGGTTACATAAGTCGGGTGTGAAAGCCCCGGGCTCAACCTGGGAATTGCAT
TCGAGACTGCGTAGCTAGGGTGCGGAAGAGGGAAGCGGAATTTCCGGTGTAGCGGTGAAA
TGCGTAGATATCGGAAGGAACACCAGTGGCGAAGGCGGCTTCCTGGTCCAGCACCGACGT
TCAGGCACGAAAGCGTGGGGAGCAAACAGGATTAGAAACCCGCGTAGTCC
>Otu226
CCAGCCTATGGGTGGCAGCAGTAAGGAATATTGGACAATGGGCGCAAGCCTGATCCAGCC
ATGCCGCGTGAAGGATGAAGGTCCTCTGGATTGTAAACTTCTTTTATATGGGACGAAAAA
AGGGGAATCCTCCCCTCTTGACGGTACCGTATGAATAAGCACCGGCTAACTCCGTGCCAG
CAGCCGCGGTAATACGGAGGGTGCGAGCGTTATCCGGATTCACTGGGTTTAAAGGGTGCG
TAGGCGGGCAGGTAAGTCAGTGGTGAAATCCCGGAGCTTAACTCTGGAACTGCCATTGAT
ACTATCTGCCTTGAATATTGTGGAGGTTTGCGGAATATGTCATGTAGCGGTGAAATGCAT
AGATATGACATAGAACACCTATTGCGAAGGCAGCAGGCTACGCATATATTGACGCTGAGG
CACGAAAGCGTGGGGATCAAACAGGATTAGATACCCGAGTAGTCC
>Otu227
CCAGCCTACGGGGTGCAGCAGTGGGGAATCTTGCACAATGGGGGCAACCCTGATGCAGCG
ACGCCGCGTGAGCGATGAAGCCCCTCGGGGTGTAAAGCTCTTTCGGCAGGGACGATAATG
ACGGTACCTGAAGAAGAAGCTGCGGCTAACTACGTGCCAGCAGCCGCGGTAATACGTAGG
CAGCGAGCGTTGTTCGGAATTACTGGGCGTAAAGAGTGTGTAGGCGGTGCTCTAAGTTCG

GTGTGAAATCTCCTGGCTTAACTGGGAGGGTGCGCCGGAAACTGGAGTGCTCGAGCGTGG
GAGAGGAAAGCGGAATTCCTGGTGTAGCGGTGAAATGCGTAGATATCAGGAGGAACACCT
GCGGTGTAGACGGCTTTCTGGACCATTGCTGACGCTGAGACACGAAAGCGTGGGTAGCAA
ACAGGATTAGAAACCCTAGTAGTCC
>Otu228
CCAGCCTATGGGGCGCTCCAGGCGCGAAACCTCTCCAATGCACGCAAGTGTGAGAGGGGA
ATCCCAAGTGCTCAGGCAAAGCCTGGGCTTTTGCTATGTGTAAATAGCATGGCGAATAAG
TGGTGGGTAAGACTGGTGCCAGCCGCCGCGGTAACCCCAGCGCCACAAGTGGCACTCGCG
TTTATTGGGCCTAAAGCGTCCGTAGCAGGTCCTATACATCTCTTGTGAAATTGCCAGGCT
CAACTTGGCAGAGTGCAGGAGGCACTGTAGGACTTGAGACCGGGAAGGGTTAGAGGTATT
CCTGAGGGAGCGGTAAAATGCTATAATCTCAGGAGGACCACCTGTGGCGAAGGCGTCTAA
CTAGAACGGATCTGACTGTGAGGGACGAAAGCTAGGGGAGCAAAACGGATTAGAGACCCG
CGTAGTCC
>Otu229
CCAGCCTATGGGTGGCAGCAGTGGGGAATTTTGCGCAATGGGGGAAACCCTGACGCAGCA
ACGCCGCGTGGAGGATGAAATCCCTTGGGATGTAAACTCCTTTCGATAGGGAAGATTATG
ACGGTACCTATAGAAGAAGCCCCGGCTAACTTCGTGCCAGCAGCCGCGGTAATACGAGGG
GGGCAAGCGTTGTTCGGAATTATTGGGCGTAAAGGGTGCGTAGGCGGTTTGGCAAGTCTT
ATGTGAAATCTATGGGCTCAACCCATAGTCTGCATGAGAAACTGCCGGGCTTGAGTATGG
GAGAGGTGAGTGGAATTTCCGGTGTAGCGGTGAAATGCGTAGATATCGGAAGGAACACCT
GTGGCGAAAGCGGCTCACTGGACCATAACTGACGCTGAGGCACGAAAGCTAGGGGAGCAA
ACAGGATTAGATACCCCCGTAGTCC
>Otu230
CCAGCCTATGGGAGGCAGCAGTGGGGAATTTTGCGCAATGGGGGAAACCCTGACGCAGCA
ACGCCGCGTGGAGGATGAAGTATCTCGGTACGTAAACTCCTTTCGATGGGGAAGATAATG
ACGGTACCCATAGAAGAAGCCCCGGCTAACTTCGTGCCAGCAGCCGCGGTAATACGAGGG
GGGCAAGCGTTGTTCGGATTTATTGGGCGTAAAGGGTGCGTAGGCGGTTTGGCAAGTCTT
GTGTGAAATCTATGGGCTCAACCCATAGCCTGCACGAGAAACTACCGGGCTTGAGTGATG
GAGAGGTGAGTGGAATTTCCGGTGTAGCGGTGAAATGCGTAGATATCGGAAGGAACACCT
GTGGCGAAAGCGGCTCACTGGACATTAACTGACGCTGATGCACGAAAGCTAGGGGAGCAA
ACAGGATTAGAAACCCGCGTAGTCC
>Otu231
CCAGCCTATGGGGGGCAGCAGTGAGGAATATTGGTCAATGGGCGGAAGCCTGAACCAGCC
ATCCCGCGTGCAGGAAGACGGCGCTATGCGTTGTAAACTGCTTTTCCAGGGGAAGAAAAC
CCACGACGAGTCGTGGCTTGCCTGTACCCTGGGAATAAGCATCGGCTAACTCCGTGCCAG
CAGCCGCGGTAATACGGAGGATGCGAGCGTTATCCGGATTTATTGGGTTTAAAGGGTGCG
TAGGCGGAATAATAAGTCAGTGGTGAAAACCTTCAGCTTAACTGGAGACTTGCCATTGAT
ACTGTTAATCTTGAGTACGGTCAAGGTAGGCGGAATGTGTAATGTAGCGGTGAAATGCTT
AGATATTACACAGAACACCGATTGCGAAGGCAGCTTACTGGGCCAATACTGACGCTGATG
CACGAAAGCGTGGGGAGCGAACAGGATTAGAAACCCCAGTAGTCC
>Otu232
CCAGCCTATGGGGGGCTGCAGTGGGGAATATTGGACAATGGGCGCAAGCCTGATCCAGCA
ATGCCGCGTGAGTGATGAAGGCCTTAGGGTTGTAAAGCTCTTTCGCACGCGACGATGATG
ACGGTAGCGTGAGAAGAAGCCCCGGCTAACTTCGTGCCAGCAGCCGCGGTAATACGAAGG
GGGCTAGCGTTGTTCGGAATTACTGGGCGTAAAGGGCGCGTAGGCGGCGTTACAAGTTAG
GCGTGAAAGCCCCGGGCTCAACCTGGGAATTGCGCTTGATACTGTAGTGCTAGAGTTCGG
GAGAGGTGAGTGGAATTCCCAGTGTAGAGGTGAAATTCGTAGATATTGGGAAGAACACCG
GTGGCGAAGGCGGCTCACTGGACCGATACTGACGCTGAGGCGCGAAAGCGTGGGGAGCAA
ACAGGATTAGATACCCCAGTAGTCC
>Otu233
CCAGCCTATGGGGCGCAGCAGTGGAGAATATTTGACAATGAGCGAAAGCTTGATCGAGTA
ATGTTACGTGGATGATAAGAAAGCTTAATTGCTGTAAAGTTCGTTTTGTCAAGGATTATA
ATGTAATTGTACTTGAAGGAGAAGCCCTGACTAACTTCGTGCCAGCAGTCGCGGTAATAC
GGAGAGGGCGAGTGTTATTCGTATTGACTGGGTGTAAAGGGTAAGTAGGTTGTTTTTCAA
GTTTTAAATTAAATACCAAGTATTATTTTGGACTATTTTCAATACTGATAAACTAGAGTT
TAAGCGGAGGAGGTTGAACATTAGGAGTAGGGGTAAAATCCTTCAATATTTAAAGGGCAG
CCAATAAGCGAAGGCTACTTTCTAGCTTAAAACTGACACTGAGGTACAAAAGCGTAGGGA
TCGAACGGGATTAGAAACCCTCGTAGTCC

>Otu234
CCAGCCTATGGGAGGCAGCAGTGGGGAATCTTGCACAATGGAGGAAACTCTGATGCAGCG
ACGCCGCGTGAGCGATGAAGCCCTTCGGGGTGTAAAGCTCTTTCGACGGGAACGATAATG
ACGGTACCCGGAGAAGAAGCTGCGGCTAACTACGTGCCAGCAGCCGCGGTAATACGTAGG
CAGCAAGCGTTGTTCGGAATTACTGGGCGTAAAGAGTGTGTAGGTGGTGCTCCAAGTCTG
TTGTGAAATCTCCCGGCTTAACTGGGAGGGTGCAGCGGAAACTGGAATGCTTGAGGATGG
GAGAGGAAAGCGGAATTCCTGGTGTAGCGGTGAAATGCGTAGATATCAGGAGGAACACCC
GTGGTGTAGACAGCTTTCTGGACCATATCTGACACTGAGACACGAAAGCGTGGGTAGCAA
ACAGGATTAGATACCCCCGTAGTCC
>Otu235
CCAGCCTATGGGAGGCACCAGTCGAGAATATTCGACAATGGGCGAAAGCCTGATCGAGCG
ACGCCGCGTGCAGGATGAAGTGCTTCGGTATGTAAACTGCTTTTGTAGACCAGAAAGTTT
ATTGATCGGTCTAAGAATAAGAGGTTGCTAAACTCGTGCCAGCAGCAGCGGTAATACGAG
TGCCTCGAGCGTTATCCGGAATCATTGGGCGTAAAGGGTGTGCAGGCGGTCTGATTAGTC
TTCTGTTAAATTCTTCGGCTTAACCGGGGACATGCAGGGGAAACGGTCAGACTTGAGGAT
GCGAGAGGTTAACGGAACTCATAGTGTAGCGGTGAAATGCGTTGATATTATGGGGAACAC
CAAATGCGAAGGCAGTTAACTGGAGCACTCCTGACGCTGAAACACGAAAGCGTGGGTAGC
GAATGGGATTAGATACCCTCGTAGTCC
>Otu237
CCAGCCTATGGGGGGCACCAGTGAGGAATATTGGGCAATAGGCGCAAGCCTGACCCAGCC
ATGCCGCGTGAAGGATGACTGCCCTATGGGTTGTAAACTTCTTTTATACGGGAAGAAACT
TTCGAACGTGTTCGGAATTGACGGTACTGTATGAATAAGCATCGGCTAACTCCGTGCCAG
CAGCCGCGGTAATACGGAGGATGCAAGCGTTATCCGGATTCATTGGGTTTAAAGGGTGCG
TAGGCGGGTTGATAAGTCAGTGGTGAAATCCTGCAGCTTAACTGTAGAACTGCCATTGAT
ACTGTTAACCTTGAATGCAGTTGAGGTAGGCGGAATGTGTAATGTAGCGGTGAAATGCGT
AGATATTACACAGAACACCGATTGCGAAGGCAGCTTGCTAAACTGTGATTGACGCTGAGG
CACGAAAGCGTGGGGATCAAACAGGATTAGATACCCGCGTAGTCC
>Otu238
CCAGCCTATGGGGGGCAGCAGTAAGGGATATTGGGCAATGGGCGAAAGCCTGACCCAGCA
ACGCCGCGTGGAGGAAGACAGTCTTCGGATTGTAAACTCCTTTTGCAGGGGAAGAGGAAG
GACGGTACCCTGCGAATCAGTCTCGGCTAACTACGTGCCAGCAGCCGCGGTAAAACGTAG
GAGGCGAGCGTTATCCGGATTTACTGGGCGTAAAGGGTGTGCAGGCGGGCCGTTAAGTGG
TGCATGAAAGCGAAGGGCTCAACCCTTCGAGGCTGTGCCAGACTGGCGGGCTAGAGTGCG
GGAGAGGGCTGTGGAATTCCGGGTGTAGTGGTGAAATGCGTAGAGATCCGGAGGAACACC
AGAGGCGAAGGCGGCAGCCTGGTCCGCAACTGACGCTGAGACACGACAGCATGGGGAGCG
AACGGGATTAGAAACCCTCGTAGTCC
>Otu239
CCAGCCTATGGGGGGCTGCAGTGGGGAATATTGGACAATGGGCGCAAGCCTGATCCAGCA
ATGCCGCGTGAGTGAAGAAGGTCTTCGGATTGTAAAGCTCTTTCGGCGGGGACGATGATG
ACGGTACCCGCAGAAGAAGCCCCGGCTAACTTCGTGCCAGCAGCCGCGGTAATACGAAGG
GGGCTAGCGTTGCTCGGAATGACTGGGCGTAAAGGGCGCGTAGGCGGCTGTCATAGTCAG
ATGTGAAATTCCTGGGCTTAACCTGGGGGCTGCATTTGATACGTGGCGGCTAGAGTGCGG
AAGAGGGTCGTGGAATTCCCAGTGTAGAGGTGAAATTCGTAGATATTGGGAAGAACACCG
GTGGCGAAGGCGGCGACCTGGTCCGTAACTGACGCTGAGGCGCGAAAGCGTGGGGAGCAA
ACAGGATTAGATACCCTTGTAGTCC
>Otu240
CCAGCCTATGGGGTGCTGCAGTGGGGAATATTGCGCAATGGGCGAAAGCCTGACGCAGCG
ACGCCGCGTGAGGGATGAAGGCCTTCGGGTTGTAAACCTCTTTCAGCAGGGACGAAGCGA
AAGTGACGGTACCTGCAGAAGAAGCACCGGCTAACTACGTGCCAGCAGCCGCGGTAATAC
GTAGGGTGCAAGCGTTGTCCGGATTTATTGGGCGTAAAGAGCTCGTAGGCGGTTTGTCGC
GTCGGATGTGAAAACCCAGGGCTCAACCCTGGGCCTGCATTCGATACGGGCAGACTAGAG
TATGGCAGGGGAGACTGGAATTCCTGGTGTAGCGGTGAAATGCGCAGATATCAGGAGGAA
CACCGGTGGCGAAGGCGGGTCTCTGGGCCAAAACTGACGCTGAGGAGCGAAAGCGTGGGG
AGCGAACAGGATTAGATACCCTAGTAGTCC
>Otu241
CCAGCCTATGGGGGGCTCCAGTCGAGGATCTTCGGCAATGGGCGCAAGCCTGACCGAGCG
ACGCCGCGTGTGCGATGAAGGCCTTCGGGTTGTAAAGCACTGTCGAGGGAGAGGAAGCCG
CAAGGTTGACCGACCCCTGGAGGAAGCACGGGCTAAGTTCGTGCCAGCAGCCGCGGTAAG

ACGAACCGTGCGAACGTTGTTCGGAATCACTGGGCTTAAAGGGCGCGTAGGCGGGCTGTC
AGGTCCGGGGTGAAAGCACACGGCTTAACCGTGTAATTGCCTGGGATACCGGCAGTCTGG
AGGAGGGTAGGGGCATGTGGAACTTCCGGTGGAGCGGTGAAATGCGTTGATATCGGAAGG
AACGCCGGTGGCGAAAGCGACGTGCTGGGCCCTTCCTGACGCTGAGGCGCGAAAGCCAGG
GGAGCAAACGGGATTAGATACCCCAGTAGTCC
>Otu242
CCAGCCTACGGGGTGCAGCAGTGGGGAATTTTGCGCAATGGGGGAAACCCTGACGCAGCA
ATGCCGCGTGGAGGATGAAGTCCTTTGGGATGTAAACTCCTTTCGATCGGGAAGATAATG
ACGGTACCGGAAGAAGAAGCCCCGGCTAACTCTGTGCCAGCAGCCGCGGTAATACAGAGG
GGGCAAGCGTTGTTCGGAATTATTGGGCGTAAAGGGCGCGTAGGCGGTGCGGTAAGTCAC
CTGTGAAATCTCTGGGCTCAACTCAGAGTCTGCAGGCGAAACTGCCGTGCTTGAGTGTGG
GAGAGGTGAGTGGAATTCCCGGTGTAGCGGTGAAATGCGTAGATATCGGGAGGAACACCT
GTGGCGAAAGCGGCTCACTGGACCACAACTGACGCTGATGCGCGAAAGCTAGGGGAGCAA
ACAGGATTAGATACCCTTGTAGTCC
>Otu243
CCAGCCTACGGGGGGCTCCAGTGGGGAATTTTGCGCAATGGGGGAAACCCTGACGCAGCA
ACGCCGCGTGGAGGATGAAATCCCTTGGGATGTAAACTCCTTTCGATCGGGACGATTATG
ACGGTACCGGAAGAAGAAGCCCCGGCTAACTTCGTGCCAGCAGCCGCGGTAATACGAGGG
GGGCAAGCGTTGTTCGGAATTATTGGGCGTAAAGGGAGTGTAGGCGGTTTGGTAAGTTGT
GTGTGAAATCTTCGGGCTCAACCCGAAGTCTGCACACAAAACTGCCGGGCTTGAGGGTGG
GAGAGGTGAGTGGAATTTCCGGTGTAGCGGTGAAATGCGTAGATATCGGAAGGAACACCT
GTGGCGAAAGCGGCTCACTGGACCATTTCTGACGCTGAAACTCGAAAGCTAGGGGAGCAA
ACAGGATTAGAGACCCGAGTAGTCC
>Otu244
CCAGCCTATGGGGGGCAGCAGTAAGGAATATTGGTCAATGGAGGCAACTCTGAACCAGCC
ATGCCGCGTGCAGGATGACGGCCCTATGGGTTGTAAACTGCTTTTGTACGGGAGAAAACC
CTTTCACGTGTGAGAGGTTGATAGTACCGTAAGAATAAGCATCGGCTAACTTCGTGCCAG
CAGCCGCGGTAAGACGAAGGATGCAAGCGTTATCCGGATTCATTGGGTTTAAAGGGAGCG
TAGGTGGACTTGTAAGTCAGTGGTGAAATCTCTTCGCTTAACGAAGAAACTGCCATTGAT
ACTGCAGGTCTTGAGTACAGATGATGTGGGCGGAATATGACATGTAGTGGTGAAATACTT
AGATATGTCATAGAACACCGATTGCGAAGGCAGCTCACAAAACTGTAACTGACACTGAGG
CTCGAAAGTGCGGGGATCAAACAGGATTAGAAACCCGAGTAGTCC
>Otu245
CCAGCCTATGGGGGGCAGCAGTGGGGAATATTGGACAATGGGCGCAAGCCTGATCCAGCA
ATGCCGCGTGAGTGAAGAAGGTCTTCGGATTGTAAAGCTCTTTCGACGGGGACGATGATG
ACGGTACCCGTAGAAGAAGCCCCGGCTAACTTCGTGCCAGCAGCCGCGGTAATACGAAGG
GGGCTAGCGTTGCTCGGAATGACTGGGCGTAAAGGGCGCGTAGGCGGATGACACAGTCAG
GCGTGAAATTCCCGGGCTCAACCTGGGGACTGCGCTTGATACATGTTGTCTTGAGTTCGG
AAGAGGGTCGTGGAATTCCCAGTGTAGAGGTGAAATTCGTAGATATTGGGAAGAACACCG
GTGGCGAAGGCGGCGACCTGGTCCGACACTGACGCTGAGGCGCGAAAGCGTGGGGAGCAA
ACAGGATTAGAAACCCCCGTAGTCC
>Otu246
CCAGCCTATGGGAGGCTCCAGTGGGGAATATTGGACAATGGGCGCAAGCCTGATCCAGCC
ATGCCGCGTGGGTGATGAAGGCCTTAGGGTTGTAAAGCCCTTTCGGCGGGGAAGATAATG
ACGGTACCCGCAGAAGAAGCCCCGGCTAACTTCGTGCCAGCAGCCGCGGTAATACGAAGG
GGGCTAGCGTTGCTCGGAATCACTGGGCGTAAAGCGCACGTAGGCGGATCGTTAAGTCAG
GGGTGAAATCCTGGAGCTCAACTCCAGAACTGCCTTTGATACTGGCGATCTCGAGTTCGG
GAGAGGTGAGTGGAACTGCGAGTGTAGAGGTGAAATTCGTAGATATTCGCAAGAACACCA
GTGGCGAAGGCGGCTCACTGGCCCGATACTGACGCTGAGGTGCGAAAGCGTGGGGAGCAA
ACAGGATTAGAAACCCCAGTAGTCC
>Otu247
CCAGCCTATGGGGTGCACCAGTGGGGAATTTTGGACAATGGGCGCAAGCCTGATCCAGCC
ATGCCGCGTGAGTGAAGAAGGCCTTCGGGTTGTAAAGCTCTTTCGGTGGGGAAGAAATTG
CTTCGGTTAATACCTGGAGTAGATGACGGTACCTGCATAAGAAGCACCGGCTAACTACGT
GCCAGCAGCCGCGGTAATACGTAGGGTGCGAGCGTTAATCGGAATTACTGGGCGTAAAGC
GTGCGCAGGCGGTTGTATAAGTCAGATGTGAAATCCCCGGGCTCAACCTGGGAACTGCAT
TTGAGACTGTACGGCTAGAGTTTGGCAGAGGGGGGTGGAATTCCACGTGTAGCAGTGAAA
TGCGTAGAGATGTGGAGGAACACCGATGGCGAAGGCAGCCCCCTGGGCCAATACTGACGC

TCATGCACGAAAGCGTGGGGAGCAAACAGGATTAGATACCCTAGTAGTCC
>Otu248
CCAGCCTATGGGTGGCAGCAGTAGGGAATATTGCGCAATGGACGAAAGTCTGACGCAGCA
ACGCCGCGTGTGGGATGAAGCTCCTTGGAGTGTAAACCACTGTCGGGAGGGACGAACGAT
TACGACTCTAACATAGTCGTAGTTTGACTGTACCTTCAGAGGAAGCTCCGGCTAACTCCG
TGCCAGCAGCCGCGGTAATACGGGGGGAGCGAGCGTTGTTCGGAATCACTGGGCGTAAAG
GGTGCGCAGGCGGGAGACTAAGTCGGATGTGAAAACCTCGGGCTCAACCCGAGGCCTGCA
TTCGAAACTGATCTTCTTGAGTATCGGAGAGGAAAGTAGAACTCCAGGTGTAGCGGTGAA
ATGCGTAGATATCTGGAGGAATACCTGTGGCGAAGGCGGCTTTCTGGCCGAATACTGACG
CTCATGCACGAAGGCTAGGGGAGCAAACAGGATTAGAAACCCCCGTAGTCC
>Otu249
CCAGCCTACGGGGGGCTGCAGTGAGGAATTTTGCGCAATGGCCGCAAGGCTGACGCAGCA
ACGCCGCGTGGGTGAAGAAGGCCTTCGGGTCGTAAAGCCCTGTCAGGTGGGAAGAATGGC
TGGGGGACTAATAATCTCTCAGATTGACGGTACCACCAGAGGAAGCACCGGCTAACTCCG
TGCCAGCAGCCGCGGTAATACGGAGGGTGCAAGCGTTATTCGGAATTACTGGGCGTAAAG
CGCGTGCAGGCGGGCGGACAAGTCTGATGTGAAAGCCCCGGGCTCAACCTGGGAAGTGCA
TTGGAAACTGTTCGTCTTGAGTTCTGGAGAGGAAGGGGGAATTCCCGGTGTAGAGGTGAA
ATTCGTAGAGATCGGGAGGAATACCAGTGGCGAAGGCGCCCTTCTGGACGGCAACTGACG
CTGAGACGCGAAAGCGTGGGGAGCAAACAGGATTAGAGACCCGGGTAGTCC
>Otu250
CCAGCCTATGGGGGGCAGCAGTCGAGAATATTCGACAATGGGCGAAAGCCTGATCGAGCG
ACACCGCGTGCAGGATGAAGGCCTTCGGGTTGTAAACTGCGGTAGTATGGTAACAATGCA
AATGAGTGCCATACGGAAAGAGGTGGGTAACTACGTGCCAGCACCAGCGGTAATACGTAG
ACCTCAAGCGTTATCCGGATTTATTGGGCGTAAAGAGCATGTAGGAGGTTTTGCGCGTCT
TTTGTTAAATCCCATCGCCCAACGATGGAGCCGCAAGAGATACGGCAGAACTAGAGGGGG
TTAGAGGTGCATGGAGCGCACGGTGTAGGGGTGAAATCCGTTGATATCGTGCGAAACACC
AAAGGCGAAGGCAGTGCACTGGGACCTTCCTGACTCTGAGATGCGAAAGCGTGGGGAGCA
AAAAGGATTAGATACCCGAGTAGTCC
>Otu251
CCAGCCTACGGGAGGCAGCAGTGAGGAATATTGCGCAATGGCCGAAAGGCTGACGCAGCG
ACGCCGCGTGAAGGATGAAGTCCGTTAGGATGTAAACTTCTTTTGCAGGGGATGAATGTT
CCCGCTTCGGCGGGACTTGACCGTACCCTGCGAATAAGGATCGGCTAACTACGTGCCAGC
AGCCGCGGTAATACGTAGGATCCGAGCGTTGTCCGGAATTACTGGGTGTAAAGGGCGTGT
AGGTGGGGCTTTAAGTCGGTGGTGAAATCCTGCAGCTTAACTGCAGAATTGCCCTCGATA
CTGAGGCTCTTGAGTACGGCAGAGGGAGATGGAATTCATGGTGTAGCGGTGAAATGTGTA
GATATCATGAAGAACACCAGTAGCGAAGGCGGTCTCCTGGTCCGTAACTGACACTGAGGC
GCGAAAGCGTGGGGAGCAAACAGGATTAGATACCCCAGTAGTCC
>Otu252
CCAGCCTACGGGGGGCAGCAGTGGGGAATATTGGACAATGGGGGCAACCCTGATCCAGCA
ATGCCGCGTGGGTGAAGACGGCCTGCGGGTTGTAAAGCCCTTTAGGCGGGGAGGAACGGC
GGTACGCGAACAGCGCGCCGCTCTGACGTTACCCGCAGAATAAGCACCGGCTAACTCCGT
GCCAGCAGCCGCGGTAATACGGAGGGTGCGAGCGTTAATCGGAATTACTGGGCGTAAAGC
GTGCGTAGGCGGTTCGGTCAGTCAGCCGTGAAAGCCCCGGGCTCAACCTGGGAACGGCGG
TTGAGACGGCCGGACTGGAGTGGGCTAGAGGATCGTGGAACTCCCGGTGTAGCGGTGAAA
TGCGTAGAGATCGGGAAGAACACCGATGGCGAAGGCAGCGATCTGGGGCCACACTGACGC
TGAGGCACGACAGCGTGGGGAGCAAACAGGATTAGATACCCCAGTAGTCC
>Otu253
CCAGCCTATGGGGCGCACCAGCCAAGAATCTTCCGCAATGGGGGAAACCCTGACGGAGCG
ACGCCGCGTGAGGGATGAAGGTCTTCGGATTGTAAACCTCTTTTGTTCGGGAAGAATAAG
TCTGTCAGGAAATGGGCAGATGATGACGGTACCGAATGAATAAGCCCCGGCTAATTACGT
GCCAGCAGCCGCGGTAATACGTAAGGGGCTAGCGTTGCTCGGAATTACTGGGCGTAAAGG
GCGTGTAGGCGGCCGGATAAGTCGAATGTAAAAAACCAGGGCTTAACCCTGGGGCTGCGT
TCGAAACTGCTCGGCTTGAGTCTGGTGGAGGGTAATGGAATTCCCGGTGTAGCGGTGAAA
TGCGTAGATATCGGGAAGAACACCGAAGGCGAAGGCAGTTACCTATGCCGAGACTGACGC
TGAGGCGCGAAAGCATGGGGATCAAACAGGATTAGATACCCCGGTAGTCC
>Otu254
CCAGCCTACGGGAGGCAGCAGTAAGGAATATTGGTCAATGGACGCAAGTCTGAACCAGCC
ATGCCGCGTGGAGGATTAAGGCCCTCTGGGTTGTAAACTCCTTTTATCTGGGAAGAAATC

CATATATTCTTATGTGGTTGACGGTACCAGAGGAATAAGCACCGGCTAACTCCGTGCCAG
CAGCCGCGGTAATACGGAGGGTGCAAGCGTTATCCGGATTCACTGGGTTTAAAGGGTGCG
TAGGCGGGTAAGTAAGTCCGTGGTGAAATCTCTGAGCTTAACTCAGAAACTGCCGTGGAT
ACTATTTGCCTTGAATATTGTGGAGGTGAGCGGAATATGTCATGTAGCGGTGAAATGCTT
AGATATGACATAGAACACCAATTGCGAAGGCAGCTCACTACACAATCATTGACGCTGAGG
CACGAAAGCGTGGGGATCAAACAGGATTAGATACCCCCGTAGTCC
>Otu255
CCAGCCTACGGGATGCTGCAGTAAGGAATATTGGTCAATGGGCGCAAGCCTGAACCAGCC
ATGCCGCGTGCAGGAAGACGGCCCTACGGGTTGTAAACTGCTTTTGCAGGGGAATAAACC
CCCGTACGTGTACGGGGCTGAATGTACTCTGAGAATAAGGATCGGCTAACTCCGTGCCAG
CAGCCGCGGTAATACGGAGGATCCAAGCGTTATCCGGATTTATTGGGTTTAAAGGGTGCG
TAGGCGGCCTATTAAGTCAGGGGTGAAAGACGGTAGCTTAACTATCGCAGTGCCTTTGAT
ACTGATGGGCTTGAATGTACTTGAGGTAGGCGGAATGTGACAAGTAGCGGTGAAATGCAT
AGATATGTCACAGAACACCAATTGCGAAGGCAGCTTACTAAAGTATGATTGACGCTGAGG
CACGAAAGCGTGGGGATCAAACAGGATTAGATACCCGAGTAGTCC
>Otu256
CCAGCCTACGGGGCGCAGCAGTGGGGAATTTTGCGCAATGGGGGAAACCCTGACGCAGCA
ACGCCGCGTGGAGGATGAAGGCCCTTGGGTCGTAAACTCCTTTCGACCGGGAAAATTATG
ATGGTACCGGTGGAAGAAGCACCGGCTAACTCTGTGCCAGCAGCCGCGGTAATACAGAGG
GTGCGAGCGTTGTTCGGAATTATTGGGCGTAAAGGGCGCGTAGGCGGTACGGTAAGTCAC
CTGTGAAACCTCCGGGCTTAACTCGGAGCCTGCAGGCGAAACTGCCGTGCTGGAGTATGG
GAGAGGTGCGTGGAATTCCCGGTGTAGCGGTGAAATGCGTAGATATCGGGAGGAACACCT
GTGGCGAAAGCGGCGCACTGGACCATAACTGACGCTGAGGCGCGAAAGCTAGGGGAGCAA
ACAGGATTAGATACCCCCGTAGTCC
>Otu257
CCAGCCTATGGGTGGCTGCAGTGGGGAATCTTGCGCAATGGGCGAAAGCCTGACGCAGCC
ATGCCGCGTGGATGATGAAGGTCTTAGGATTGTAAAATCCTTTCACCGGGGACGATAATG
ACGGTACCCGGAGAAGAAGCCCCGGCTAACTTCGTGCCAGCAGCCGCGGTAATACGAAGG
GGGCTAGCGTTGCTCGGAATTACTGGGCGTAAAGGGAGCGTAGGCGGGTCGTTAAGTTGG
GGGTGAAAGCCCGGGGCTCAACCTCGGAAATGCCTTCAATACTGGCGACCTTGAGTGTGG
GAGAGGTGAGTGGAACTCCGAGTGTAGAGGTGAAATTCGTAGATATTCGGAAGAACACCA
GTGGCGAAGGCGACTCACTGGCCCATTACTGACGCTGAGGCTCGAAAGCGTGGGGAGCAA
ACAGGATTAGATACCCCCGTAGTCC
>Otu258
CCAGCCTACGGGGTGCAGCAGTTAGGAATCTTCTGCAATGCGCGCAAGCGTGACAGATTG
AGCCAGAGTGCTTTTCATTTGAAAAGCTTTTGCCAAATGCAAAAAGTTTGGCGAATAAGG
ACTGGGTAAGACGGGTGCCAGCCGCCGCGGTAATACCCGCGGTCCAAGTCGCAGCCATCA
TTATTGGGTCTAAAACATCCGTAGCTTGCTTAATAAGTTTCTTGTGAAATTCTATATCTC
AAGTATAGATCGGGCAAGAAGTACTGTTAAGCTAGAGACTGGAAGACGTAAGAAGTACGT
TTGGAGTAGCGGTAAAATGTGTTAATCCCAGACGGACTAACAACAGCGAAGGCATCTTAC
GAGGACAGTTCTGACAGTAAGGGATGAAGGCTAGGGGCGCAAAACGGATTAGAGACCCGT
GTAGTCC
>Otu259
CCAGCCTATGGGAGGCAGCAGTAAGGGATATTGCGCAATGGGCGAAAGCCTGACGCAGCA
ACGCCGCGTGGAGGATGAAGGCCTTCGGGTCGTAAACTCGTTTTGCGTCTGACGAGGAAG
GACGGTAGGACGCGAAGAAGTCACGGCTAACTACGTGCCAGCAGCCGCGGTAAAACGTAG
GTGGCGAGCGTTATCCGGATTTACTGGGCGTAAAGGGTGTGCAGGCGGACCGTCAAGTGG
TGTATGAAAACGCCGGGCTTAACTCGGCGATGGTAGGCCAGACTGACGGTCTAGAGGACG
AGAGAGGGGCATGGAATTCCGGGTGTAGTGGTGAAATGCGTAGAGATCCGGAAGAACCCC
AGAGGCGAAGGCGGTGCCCTGGCTCGTATCTGACGCTCAGCCACGACAGCATGGGGAGCG
AACGGGATTAGAAACCCCAGTAGTCC
>Otu260
CCAGCCTATGGGGGGCAGCAGTAGGGAATTTTCCACAATGGGCGAGAGCCTGATGGAGCA
ACACCGCGTGCAGGATGAAGGCCTTAGGGTCGTAAACTGCTTTTATGTATGAAGATTATG
ACGGTAGTACATGAATAAGGATCGGCTAACTCCGTGCCAGCAGCCGCGGTCATACGGAGG
ATCCAAGCGTTATCCGGAATTACTGGGCGTAAAGAGTTGCGTAGGTGGCAGAGTAAGCAG
GGCATGAAAGCGTGTGGCTCAACCATACATACATGTTCTGAACTGCTCAGCTTGAGGACG
AGAGAGGTAATTGGAATTCCTAGTGTAGGAGTGAAATCCGTAGATATTAGGAGGAACACC

GATGGCGTAAGCAGATTACTGGCTCGTTTCTGACACTAAGGCACGAAAGCGTGGGGAGCA
AACGGGATTAGAGACCCCTGTAGTCC
>Otu261
CCAGCCTATGGGGCGCAGCAGTCGGGAATTTTGGGCAATGGGCGAAAGCCTGACCCAGCA
ACGCCGCGTGAGGGATGAAGGCCTTCGGGTCGTAAACCTCTTTTCTCAGGGACGATAATG
ACGGTACCTGAGGAAGAAGCCACGGCTAACTACGTGCCAGCAGCCGCGGTAATACGTAGG
TGGCGAGCGTTGTCCGGATTTACTGGGCGTAAAGCGCACGCAGGCGGTCGTTCAAGTCGA
ATGTGAAAGCCCCCGGCTCAACTGGGGAGGGTCATTCGATACTGATCGACTTGAAGGCAG
GAGAGGGAAGCGGAATTCCCGGTGTAGTGGTGAAATGCGTAGATATCGGGAGGAACACCA
GTGGCGAAGGCGGCTTCCTGGCCTGTTCTTGACGCTGATGTGCGAAAGCTAGGGTAGCAA
ACGGGATTAGAAACCCCAGTAGTCC
>Otu262
CCAGCCTATGGGTCGCTGCAGTAAGGGATATTGCACAATGGGCGAAAGCCTGATGCAGCA
ACGCCGCGTGGAGGATGAAGGCCTTCGGGTCGTAAACTCGTTTTGACGCTGACGAGCAAG
GACGGTAGGCGTCGAATAAGTCACGGCTAACTACGTGCCAGCAGCCGCGGTAAAACGTAG
GTGGCGAGCGTTATCCGGATTTACTGGGCGTAAAGGGTGTGCAGGCGGGTCATCAAGTGG
TGTATGAAAGCCCCCAGCTAAACTGGGAAGGGTTATGCCAGACTGATGGTCTAGAGGACG
AGAGAGGGGCGTGGAATTCCGGGTGTAGTGGTGAAATGCGTAGAGATCCGGAGGAACCCC
AGAGGCGAAGGCGGCGCCCTGGCTCGTACCTGACGCTGATACACGAAAGCATGGGTAGCG
AACGGGATTAGAAACCCCCGTAGTCC
>Otu263
CCAGCCTATGGGGGGCAGCAGACGAGAATATTCCGCAATGGACGAAAGTCTGACGGAGCG
ACGCCGCGTGATGGATGAAGTGCCTTGGTACGTAAACATCTTTTATCGGGGACGAAGTTT
ATTGACGGTACCCGATGAATAAGGGGCTCCTAACTCTGTGCCAGCAGGAGCGGTAATACA
GAGGCCCCAAGCATTACCCGGAATCACTGGGCGTAAAGGGTGTCTAGGCGGTCATATTAG
TCTCTCGTTAAATGTCCGGGCTTAACCTGGTCACCGCGAGGGAAACGGTATGACTCGAGG
GCGTAAGAGGTGCACGGAACTCATGGTGGAGGGGTGAAATCCGTTGATATCATGGGGAAC
ACCAAAGGCGAAGGCAGTGCACTGGTACGTTCCTGACGCTCAAACACGAAAGCCAGGGTA
GCGAACGGGATTAGATACCCCCGTAGTCC
>Otu264
CCAGCCTACGGGTCGCAGCAGTCGAGGATCTTCGGCAATGGGCGCAAGCCTGACCGAGCG
ACGCCGCGTGCGCGATGAAGGCCTTCGGGTTGTAAAGCGCTGTCGAGGGGGAGGAAAGGG
CAACCCTGACCTATCCCTGGAGGAAGCACGGGCTAAGTTCGTGCCAGCAGCCGCGGTAAG
ACGAACCGTGCGAACGTTGTTCGGAATCACTGGGCTTAAAGGGCGCGTAGGCGGATCATC
AAGTCCGGGGTGAAAGCCCCCAGCTTAACTGGGGAAGTGCCTTGGATACTGGTGGTCTGG
AGGGAGATAGGGGCAGTTGGAACTTCCGGTGGAGCGGTGAAATGCGTTGATATCGGAAGG
AACGCCGGTGGCGAAAGCGAACTGCTGGATCTCTTCTGACGCTGAGGCGCGAAAGCCAGG
GGAGCAAACGGGATTAGATACCCCTGTAGTCC
>Otu265
CCAGCCTATGGGGGGCTCCAGTCGAGAATTTTTCACAATGGGCGAAAGCCTGATGGAGCG
ACGCCGCGTGGGGGATGAATGGCTTCGGCCCGTAAACCCCTGTCATTCGGGATCAATGCG
TCTGGGTGAACATCCCAGACGTTGATAGTACCGGAAGAGGAAGGGACGGCTAACTCTGTG
CCAGCAGCCGCGGTAATACAGAGGTCCCAAGCGTTGTTCGGATTCACTGGGCGTAAAGGG
TGCGTAGGTGGTTGGGTAAGTCTGATGTGAAATCTCGGAGCTTAACTCCGAAACTGCATT
GGATACTATCTAGCTTGAGAGTTGGAGGGGGGACTGGAATTCTCGGTGTAGCAGTGAAAT
GCGTAGATATCGAGAGGAACACCAGTGGCGAAGGCGAGTCCCTGGACAACTTCTGACACT
GAGGCACGAAAGCCAGGGGAGCAAACGGGATTAGATACCCGCGTAGTCC
>Otu266
CCAGCCTATGGGTTGCAGCAGTGGGGAATCTTGGACAATGGGCGCAAGCCTGATCCAGCA
ATGCCGCGTGGGTGAAGACGGCCTGCGGGTTGTAAAGCCCTTTTGGCGGGGAAGAAAGCC
CTGGGTTGAACAGACTCAGGGGGTGACGTTACCTGCTGAAGAAGCACCGGCCAACTCCGT
GCCAGCAGCCGCGGTAATACGGAGGGTGCGAGCGTTAATCGGAATTACTGGGCGTAAAGC
GCGCGTAGGCGGTGGGTCAAGTCAGTTGTGAAAGCCCCGGGCTCAACCTGGGAATGGCAA
TTGAGACTGACCGACTGGAGTGTAACAGAGGACCGTGGAATTCCCGGTGTAGCGGTGAAA
TGCGTAGAGATCGGGAAGAACACCGATGGCGAAGGCAGCGGTCTGGGTTAACACTGACGC
TGAGGTGCGAAAGCGTGGGGAGCAAACAGGATTAGAAACCCGAGTAGTCC
>Otu267
CCAGCCTATGGGTGGCAGCAGTGGGGAATATTGGGCAATGGGCGAAAGCCTGACCCAGCC

ACGCCGCGTGAGTGATGAAGGCCTTCGGGTCGTAAAGCTCTGTGGGGAGGGACGAATAAG
CGCGTATTAAATAAATACGTGCCCTGACGGTACCTCCTTAGCAAGCACCGGCTAACCATG
TGCCAGCAGCCGCGGTAATACATGGGGTGCAAACGTTGCTCGGAATCATTGGGCGTAAAG
CGCGCGTAGGCGGTCGCTTAAGTCGGATGTGAAATCCCTCGGCTTAACTGAGGAAGTGCA
TCCGAGACTGAGCAGCTAGAGTACGAAAGAGGGTCGCGGAATTCCCGGTGTAGAGGTGAA
ATTCGTAGATATCGGGAGGAACACCGGCGGCGAAGGCGGCGACCTGGTTCGAGACTGACG
CTGAGGCGCGAAAGCGTGGGGAGCAAACAGGATTAGAGACCCCCGTAGTCC
>Otu268
CCAGCCTATGGGATGCAGCAGTGGGGAATATTGGACAATGGGCGCAAGCCTGATCCAGCC
ATGCCGCGTGGATGATGAAGGCCTTAGGGTTGTAAAATCCTTTCGACGGGGAAGATAATG
ACGGTACCCGTAGAAGAAGCCCCGGCTAACTTCGTGCCAGCAGCCGCGGTAATACGAAGG
GGGCTAGCGTTGCTCGGAATCACTGGGCGTAAAGCGCACGTAGGCGGCTTTTTAAGTCAG
AGGTGAAATCCTGGAGCTCAACTCCAGAACTGCCTTTGATACTGAAGAGCTTGAGTCCGG
GAGAGGTGAGTGGAACTGCGAGTGTAGAGGTGAAATTCGTAGATATTCGCAAGAACACCA
GTGGCGAAGGCGGCTCACTGGCCCGGTACTGACGCTGAGGTGCGAAAGCGTGGGGAGCAA
ACAGGATTAGAAACCCCAGTAGTCC
>Otu269
CCAGCCTATGGGTTGCAGCAGTCGAGAATATTCGACAATGGAGGAAACTCTGATCGAGCG
ACGCCGCGTGCAGGAAGAAGTGCTTCGGTATGTAAACTGCTTTTGTAGACTAGTAATTTT
TGAACAGTCTAAGAATAAGAGGTTGCTAAACTCGTGCCAGCAGCAGCGGTAATACGAGTG
CCTCAAGCGTTATCCGGAATTATTGGGCGTAAAGGGTGTGTAGGTGGTTTTGTTAGTCTT
CTGTTAAATCTCTCGGCTTAACCGAGAGCGTGCAGGGGAAACGGCAAGACTTAGAGTGTG
CGAAGGGTCTGTGGAACTCATAGTGTAGCGGTGAAATGCGTTGATATTATGGGGAACACC
AAAAGCGAAGGCAGCAGACTGGAGCATTACTGACACTGAAACACGAAAGCGTGGGTAGCG
AATGGGATTAGAAACCCTCGTAGTCC
>Otu270
CCAGCCTATGGGGGGCTCCAGTAGGGAATATTGGGCAATGGGCGAAAGCCTGACCCAGCA
ACGCCGCGTGTGCGATGAAGGTCTTCGGATCGTAAAGCACTTTTCGAGGAGATGAGAAAG
GACAGTATCCTCGGAATAAGTCTCGGCTAACTACGTGCCAGCAGCCGCGGTAAAACGTAG
GAGGCAAGCGTTATCCGGATTTACTGGGCGTAAAGCGTGTGCAGGCGGTTTGGTAAGTTG
GATGTGAAAGCTCCTGGCTCAACTGGGAGAGGTCGTTCAATACTACCAGACTTGAGAGTG
GTAGAGGGAGGTGGAATTCCCGGTGTAGTGGTGAAATGCGTAGATATCGGGAGGAACACC
AGTGGCGAAAGCGGCCTCCTGGACCATTTCTGACGCTCAGACACGAAAGCTAGGGGAGCA
AACGGGATTAGAGACCCCAGTAGTCC
>Otu271
CCAGCCTACGGGGGGCAGCAGTGGGGAATTTTGCGCAATGGGGGAAACCCTGACGCAGCA
ACGCCGCGTGGAGGATGAAGTCCCTTGGGACGTAAACTCCTTTCGACCGGGACGATAATG
ACGGTACCGGTGGAAGAAGCCCCGGCTAACTCCGTGCCAGCAGCCGCGGTAATACGGGGG
GGGCAAGCGTTGTTCGGAATTATTGGGCGTAAAGGGCGCGTAGGCGGTGCGGTAAGTCAT
CTGTGAAATCTTCCGGCTCAACCGGGAGACTGCAGGCGAAACTGCCGTGCTGGAGTGTGG
GAGAGGTGAGTGGAATTCCCGGTGTAGCGGTGAAATGCGTAGATATCGGGAGGAACACCT
GTGGCGAAAGCGGCTCACTGGACCACAACTGACGCTGAGGCGCGAAAGCTAGGGGAGCAA
ACAGGATTAGAAACCCCAGTAGTCC
>Otu272
CCAGCCTACGGGGGGCAGCAGTAAGGAATATTGGTCAATGGGCGGAAGCCTGAACCAGCC
ATGCCGCGTGCAGGAAGACGGCCCTATGGGTTGTAAACTGCTTTTGCAGGGGAATAAACC
TACCAATGTATTGGTAGCTGAATGTACTCTGAGAATAAGGATCGGCTAACTCCGTGCCAG
CAGCCGCGGTAATACGGAGGATCCGAGCGTTATCCGGATTTATTGGGTTTAAAGGGTGCG
TAGGCGGCCTATTAAGTCAGGGGTGAAAGACGGTAGCTTAACTATCGCAGTGCCTTTGAT
ACTGATGGGCTTGAATGTACTTGAGGTAGGCGGAATGTGACAAGTAGCGGTGAAATGCAT
AGATATGTCACAGAACACCAATTGCGAAGGCAGCTTACTAAAGTATGATTGACGCTGAGG
CACGAAAGCGTGGGGATCAAACAGGATTAGAAACCCGAGTAGTCC
>Otu273
CCAGCCTATGGGACGCTGCAGTCGAGAATATTCGACAATGGGCGAAAGCCTGATCGAGCG
ACACCGCGTGCAGGATGAAGGCCTTCGGGTCGTAAACTGCGGTAGCTAGGGAGGAATGCA
AATGACTGTACCTAGCGGAAAGAGGTGGGTAACTACGTGCCAGCACCAGCGGTAAAACGT
AGACCTCAAGCGTTATCCGGATTTATTGGGCGTAAAGCGCGTGTAGGTGGTTCGGTACGT
TTCTGGTTAAATCCCATTGCCTAACAATGGACCCGCCAGGAATACGGCCGGACTAGAGGA

GGTTAGAGGTGCATAGAACGCACGGTGTAGGGGTGAAATCCGTTGATATCGTGCGGAATA
CCAAAGGCGAAGGCATTGCACTGGGACCTTCCTGACACTGAGACGCGAAAGCGTGGGGAG
CAAAAAGGATTAGAAACCCTAGTAGTCC
>Otu274
CCAGCCTACGGGAGGCACCAGTAGGGAATTTTCCACAATGGGGGCAACCCTGATGGAGCA
ACGCCGCGTGCAGGATGAAGGTCTTCGGATTGTAAACTGCTTTTATCTGTGACGAATATG
ACGGTAGCAGATGAATAAGGATCGGCTAACTCCGTGCCAGCAGCCGCGGTCATACGGAGG
ATCCAAGCGTTATCCGGAATTACTGGGCGTAAAGAGTTGCGTAGGTGGCATTGTAAGTTG
GTAGTGAAATCCTGGGGCTCAACCCCTTACCCATTACCAAAACTGCAAAGCTAGAGGATG
AGAGAGGTTATTGGAATTCCTAGTGTAGGAGTGAAATCCGTAGATATTAGGAGGAACACC
GATGGCGTAGGCAGATAACTGGCTCATTCCTGACACTAAGGCACGAAAGCGTGGGTAGCA
AACGGGATTAGATACCCCTGTAGTCC
>Otu275
CCAGCCTATGGGTCGCTGCAGTAAGGAATATTGGACAATGGGCGAGAGCCTGATCCAGCC
ATGCCGCGTGCAGGAAGAAGGACCTATGGTTTGTAAACTGCTTTTGAACAAGAGAAAACC
CATCTACGTGTAGATGGCTGATAGTATTGTTAGAATAAGCATCGGCTAACTTCGTGCCAG
CAGCCGCGGTAAGACGAAGGATGCAAGCGTTATCCGGATTCATTGGGTTTAAAGGGAGCG
TAGGCGGATAAATAAGTCAGTGGTGAAATCTCCGGGCTTAACCCGGAAACTGCCATTGAT
ACTGTTTATCTAGAGTACATTTGCTGTGGGCGGAATATGACATGTAGTGGTGAAATACTT
AGATATGTCATAGAACACCGATTGCGAAGGCAGCTCACAAAAATGTAACTGACGCTGAGG
CTCGAAAGTGCGGGGATCAAACAGGATTAGATACCCGTGTAGTCC
>Otu276
CCAGCCTACGGGAGGCTCCAGTAACGAATCTTCCGCAATGCACGAAAGTGTGACGGAGCG
ACGCCGCGTGTGGGACGAAGCCCTTCGGGGTGTAAACCACTGTCAGGGGTTAGAAAGTTC
TGATCAACCCCAGAGGAAGGCACGGCTAACTCTGTGCCAGCAGCCGCGGTAAGACAGAGG
TGCCAAGCGTTAGGCGGAATCACTGGGCTTAAAGCGTGTGTAGGCGGACTTCTAAGTACC
TTGTGAAATCCCACGGCTCAACCGTGGAACTGCTGGGTATACTGGGAGTCTTGAGCCATC
TAGGGGCTGCCGGAACAAATGGTGGAGCGGTGAAATGCGTAGATATCATTTGGAACGCCA
AAGGTGAAAACAGGCAGCTGGGGATGTTCTGACGCTGAGACACGAAAGCCAGGGGAGCGA
ACGGGATTAGAGACCCTCGTAGTCC
>Otu277
CCAGCCTACGGGTTGCAGCAGTGGGGAATATTGGACAATGGGCGCAAGCCTGATCCAGCA
ATGCCGCGTGAGTGAAGAAGGTCTTCGGATTGTAAAGCTCTTTCGACGGGGACGATGATG
ACGGTACCCGTAGAAGAAGCCCCGGCTAACTTCGTGCCAGCAGCCGCGGTAATACGAAGG
GGGCTAGCGTTGCTCGGAATGACTGGGCGTAAAGGGCGCGTAGGCGGTGAACACAGTTAG
GCGTGAAATTCCCGGGCTCAACCTGGGGACTGCGCTTAATACATGTTCGCTTGAGTTCGG
AAGAGGGTCGTGGAATTCCCAGTGTAGAGGTGAAATTCGTAGATATTGGGAAGAACACCG
GTGGCGAAGGCGGCGACCTGGTCCGATACTGACGCTGAGGCGCGAAAGCGTGGGGAGCAA
ACAGGATTAGATACCCTTGTAGTCC
>Otu278
CCAGCCTACGGGGGGCTGCAGTCGAGAATATTCGACAATGGGCGAAAGCCTGATCGAGCG
ACACCGCGTGCAGGATGAAGGCCTTCGGGTCGTAAACTGCGGTAGTAAGATAACAATGCA
AATGAGTGTCTTACGGAAAGAGGTGGGTAACTACGTGCCAGCACCAGCGGTAAAACGTAG
ACCTCAAGCGTTATCCGGATTTATTGGGCGTAAAGCGCGTGTAGGTGGCTTCGCGCGTCT
TCTGTTAAAGATCAGGGCCCAACCCTGAAAGTGCAGGAGATACGGCGGAGCTCGAGGAGG
TTAGAGGTGCATAGAACGCACGGTGTAGGGGTGAAATCCGTTGATATCGTGCGGAATACC
AAAGGCGAAGGCAGTGCACTGGGACCTTCCTGACACTGAGACGCGAAAGCGTGGGGAGCA
AAAAGGATTAGATACCCCTGTAGTCC
>Otu279
CCAGCCTATGGGTGGCTGCAGTGGGGAATCTTGCACAATGGGGGCAACCCTGATGCAGCG
ACGCCGCGTGAGCGATGAAGCCCTTCGGGGTGTAAAGCTCTTTCGGTAGGGAAGATAATG
ACGGTACCTACAGAAGCAGCTGCGGCTAACTACGTGCCAGCAGCCGCGGTAATACGTAGG
CAGCAAGCGTTGTTCGGAGTTACTGGGCGTAAAGGGTGTGTAGGCGGCTTTCTAAGTTTG
TTGTGAAATCTCCCGGCTTAACCGGGAGGGTGCGGCGAAGACTGGAAGGCTAGAGTATGG
GATGGGAGAGTGGAATTCCTGGTGTAGCGGTGAAATGCGTAGATATCAGGAGGAACACCT
GCGGTGTAGACGGCTCTCTGGACCATTACTGACGCTGAGACACGAAAGCGTGGGTAGCAA
ACAGGATTAGAAACCCGCGTAGTCC
>Otu280

CCAGCCTATGGGAGGCAGCAGTGGGGAATATTGGACAATGGGCGAAAGCCTGATCCAGCC
ATGCCGCGTGAGTGAAGAAGGCCTTCGGGTTGTAAAGCTCTTTCGGCGGGGACGATGATG
ACGGTACCCGCATAAGAAGCCCCGGCAAACTTCGTGCCAGCAGCCGCGGTAATACGAAGG
GGGCTAGCGTTGTTCGGAATTACTGGGCGTAAAGCGCGCGTAGGCGGTGATCTTTGTCAG
AGGTGAAAGCCCGGGGCTCAACTCCGGAATTGCCTTTGAAACGGGATTGCTTGAGTCCGA
GAGAGGATGGCGGAATTCCTAGTGTAGAGGTGAAATTCGTAGATATTAGGAAGAACACCG
GTGGCGAAGGCGGCCATCTGGCTCGGTACTGACGCTCAGGCGCGAAAGCGTGGGGAGCAA
ACAGGATTAGATACCCCCGTAGTCC
>Otu281
CCAGCCTATGGGGTGCAGCAGTGGGGAATATTGCACAATGGGCGCAAGCCTGATGCAGCA
ACGCCGCGTGAGGGACGACGGCCTTCGGGTTGTAAACCTCTTTTAGTAGGGAAGAAGCGA
AAGTGACGGTACCTGCAGAAAAAGCACCGGCTAACTACGTGCCAGCAGCCGCGGTAATAC
GTAGGGTGCAAGCGTTATCCGGAATTATTGGGCGTAAAGAGCTCGTAGGCGGTTTGTCGC
GTCTGCTGTGAAAACTGGAGGCTCAACCTCCAGCCTGCAGTGGGTACGGGCAGACTAGAG
TGCGGTAGGGGAGATTGGAATTCCTGGTGTAGCGGTGGAATGCGCAGATATCAGGAGGAA
CACCGATGGCGAAGGCAGATCTCTGGGCCGTAACTGACGCTGAGGAGCGAAAGCATGGGG
AGCGAACAGGATTAGATACCCTTGTAGTCC
>Otu282
CCAGCCTACGGGGGGCAGCAGTGGGGAATATTGGACAATGGGCGCAAGCCTGATCCAGCC
ATGCCGCGTGTGTGAAGAAGGCCTTCGGGTTGTAAAGCACTTTAAGCGGGGAGGAAGTGA
TCTGGATTAATAATCTGGATTATTGACGTTACCCGCAGAATAAGCACCGGCTAACTCTGT
GCCAGCAGCCGCGGTAATACAGAGGGTGCAAGCGTTAATCGGAATTACTGGGCGTAAAGC
GCGCGTAGGCGGTTGTGCAAGTTGGAGGTGAAATCCCCGGGCTTAACCTGGGCACTGCCT
TCAAAACTGCACGGCTAGAGTATGGGAGAGGAAGGTAGAATTCCAGGTGTAGCGGTGAAA
TGCGTAGAGATCTGGAGGAATACCGATGGCGAAGGCAGCCTTCTGGCCTAATACTGACGC
TGAGGTGCGAAAGCATGGGGAGCAAACAGGATTAGAGACCCCCGTAGTCC
>Otu283
CCAGCCTATGGGGCGCTCCAGTCGAGAATCTTCCACAATGGGCGAAAGCCTGATGGAGCG
ACGTTTCGTGAATGAAGAAGCCCTTCGGGGTGTAAAGTTCTTTTTTGGGGGAGGAACCCG
CAAGGGTGACAGTACTCCAAGAATAAGGAGGTCCTAACTCTGTGCCAGCAGGAGCGGTAA
TACAGAGCCTCCAAGCGTTATCCGGATTTACTGGGCGTAAAGGATTCGTAGGTGATTGAG
TAAGTTAAAAGTTAAAACTTATCGGCTCAACCTTTAAGCTGCTTTTAATACTGCTCGAAT
AGAGGATGTTAGAGGCTAACGGAACCGACGGTGTAGGGGTGAAATCCGTTGATATCGTCG
GGAACACCAAAAGCGAAGGCAGTTAGCTGGGACAATCCTGACACTGAGGAATGAAAGCGT
GGGGATCAAAAAGGATTAGATACCCCTGTAGTCC
>Otu284
CCAGCCTACGGGATGCTGCAGTCGAGAATCTTCCACAATGGACGAAAGTCTGATGGAGCG
ACGCCGCGTGATTGATGAAGTCCTTCGGGATGTAAAGATCTTTTATAGGGGACGAAGTTT
ATTGACGGTACCCTATGAATAAGGAGTTGCTAAACTCGTGCCAGCAGCAGCGGTAATACG
AGTGCTCCAAGCGTTATCCGGAATTATTGGGCGTAAAGGGTGTGTAGGCGGTCGCGTTAG
TCTTCTGTTAAATTCTTCGGCTTAACCGGGGGCATGCGGAGGAAACGGCGCGACTAGAGG
ATGCGAGAGGTGAATGGAACTCATGGAGTAGGGGTGAAATCCGTTGATATCATGGGGAAC
ACCAAAAGCGAAGGCAATTCACTGGCGCACTCCTGACGCTGAAACACGAAAGCGTAGGTA
GCGAATGGGATTAGAAACCCCTGTAGTCC
>Otu285
CCAGCCTACGGGGTGCTGCAGCGGGGAATATTGCACAATGGGCGCAAGCCTGATGCAGCG
ACGCCGCGTGAGGGATGACGGCCTTCGGGTTGTAAACCACTGTCGAAGGGGACGATAATG
ACGGTACCCTTGGAGGAAGCCCCGGCTAACTACGTGCCAGCAGCCGCGGTAAGACGTAGG
GGGCGAGCGTTATTCGGAATTATTGGGCGTAAAGCGCTCGTAGGCGGGATCGATAGTCCG
TGAAGAAAGACCTGGGCTCAACTCAGGGAACGGCACGGATACTTCGATTCTTGAGGCAAT
CAGAGGGTGATGGAATTCCCGGTGTAGCGGTGAAATGCGTAGATATCGGGAGGAACACCA
GTGGCGAAGGCGATCACCTGGGGTTGTTCTGACGCTGAGGAGCGAAAGCTAGGGGAGCAA
ACGGGATTAGATACCCCTGTAGTCC
>Otu286
CCAGCCTACGGGTGGCTCCAGGCGCGAAACCTCCGCAATGCGAGCAATCGCGACGGGGGG
ACCCCAAGTGCCACTCTTAACGGGGTGGCTTTTCTGAAGTGTAAAAAGCTTTAGGAATAA
GGGCTGGGCAAGACCGGTGCCAGCCGCCGCGGTAACACCGGCAGCTCAAGTGGTGGCCAT
TATTATTGGGCCTAAAGCGTTCGTAGCCGGTTTGATAAGTCTCTGGTGAAATCCCGCAGC

TTAACTGTGGGACTTGCTGGAGATACTATCAGACTTGAGGTCGGGAGAGGTTAGGGGTAC
TCCCAGGGTAGGGGTGAAATCCTATAATCCTGGGAGGACCACCTGTGGCGAAGGCGCCTA
ACTGGAACGAACCTGACGGTGAGTAACGAAAGCCAGGGGCGCGAACCGGATTAGAAACCC
TCGTAGTCC
>Otu287
CCAGCCTATGGGGGGCACCAGTGGGGAATTTTGGACAATGGGGGCAACCCTGATCCAGCA
ATGCCGCGTGTGTGAAGAAGGCCTTCGGGTTGTAAAGCACTTTTGTCAGGAACGAAACGG
ACTGAGTTAATACCTTGGTCTAATGACGGTACCTGAAGAATAAGCACCGGCTAACTACGT
GCCAGCAGCCGCGGTAATACGTAGGGTGCAAGCGTTAATCGGAATTACTGGGCGTAAAGC
GTGCGCAGGCGGTTATGTAAGACAGATGTGAAATGCCCGGGCTTAACCTGGGAACTGCAT
TTGTGACTGCATGGCTAGAATCTGGCAGAGGGGGGTAGAATTCCACGTGTAGCAGTGAAA
TGCGTAGATATGTGGAGGAATACCGATGGCGAAGGCAGCCCCCTGGGCTAAGATTGACGC
TCATGCACGAAAGCGTGGGGAGCAAACAGGATTAGAAACCCGTGTAGTCC
>Otu288
CCAGCCTATGGGGGGCAGCAGTAAGGAATATTGGTCAATGGACGCAAGTCTGAACCAGCC
ATGCCGCGTGAAGGATTAAGGTCCTCTGGATTGTAAACTTCTTTTATCTGGGACGAAAAA
AGGCGATTCTTCGTCACTTGACGGTACCAGATGAATAAGCACCGGCTAACTCCGTGCCAG
CAGCCGCGGTAATACGGAGGGTGCAAGCGTTATCCGGATTCACTGGGTTTAAAGGGTGCG
TAGGCGGGCAGGTAAGTCAGTGGTGAAATCCTTGAGCTTAACTCGAGAACTGCCATTGAT
ACTATTTGTCTTGAATATTGTGGAGGTAAGCGGAATATGTCATGTAGCGGTGAAATGCTT
AGATATGACATAGAACACCTATTGCGAAGGCAGCTTACTACGCATATATTGACGCTGAGG
CACGAAAGCGTGGGGATCAAACAGGATTAGAAACCCCTGTAGTCC
>Otu289
CCAGCCTACGGGTCGCAGCAGTAGGGAATATTGGACAATGGGCGAGAGCCTGATCCAGCC
ATGCCGCGTGCAGGAAGAAGGCCTTCTGGGTTGTAAACTGCTTTTGCCAGGGGATAAAAA
GTCCCTGCGGGGAAAATTGAAGGTACCTGGTGAATAAGCCACGGCTAACTACGTGCCAGC
AGCCGCGGTAATACGTAGGTGGCAAGCGTTGTCCGGATTTATTGGGTTTAAAGGGTGCGT
AGGCGGCCCTGTAAGTCAGTGGTGAAATACGGCAGCTCAACTGTCGAGGTGCCATTGATA
CTGCAGGGCTTGAGTACAGACGAGGTAGGCGGAATTGACGGTGTAGCGGTGAAATGCTTA
GATATCGTCAAGAACACCGATAGCGAAGGCAGCTTACTAGACTGTAACTGACGCTGAGGC
ACGAAAGTGCGGGGATCAAACAGGATTAGAAACCCGTGTAGTCC
>Otu290
CCAGCCTATGGGATGCTGCAGTGAGGAATATTGCGCAATGCCCGCAAGGGTGACGCAGCG
ACGCCGCGTGAAGGATGAAGGCCCTCTGGGTCGTAAACTTCTTTTGTGAGGGAATAATCT
GCCGCTACAGGCGGCATTGAATGTACCTCAGGAAAAAGCATCGGCTAACTACGTGCCAGC
AGCCGCGGTAATACGTAGGATGCGAGCGTTGTCCGGAATCACTGGGTGTAAAGGGAGCGC
AGGCTGGTCGGTAAGTCACTGGTGAAATTCCCGGGCTCAACCCGGGGACTGCCAGTGATA
CTGCTGATCTTGAGTGCAGAAGAGGTTGATGGAATTCTTGGTGTAGCGGTGAAATGCGTA
GATATCAAGAAGAACACCGATGGCGAAGGCAGTCAACTGGTCTGTAACTGACGCTCATGC
TCGAAAGCGTGGGGATCAAACAGGATTAGATACCCGTGTAGTCC
>Otu291
CCAGCCTACGGGGGGCAGCAGTAAGGAATATTGGTCAATGGACGCAAGTCTGAACCAGCC
ATGCCGCGTGGAGGATGAAGGCCCTCTGGGTTGTAAACTTCTTTTATCTGGGAAGAAACC
ACTCTTTTCTAAGGGTGTTGACGGTACCAGTTGAATAAGCACCGGCTAACTCCGTGCCAG
CAGCCGCGGTAATACGGAGGGTGCAAGCGTTATCCGGATTCACTGGGTTTAAAGGGTGCG
TAGGTGGGCAGGTAAGTCAGTGGTGAAATCTCCGGGCTTAACCCGGAAACTGCCGTTGAT
ACTATCTGTCTTGAATGTAGTGGAGGTGAGCGGAATATGTCATGTAGCGGTGAAATGCTT
AGATATGACATAGAACACCCATTGCGAAGGCAGCTCGCTACACTATTATTGACACTGAGG
CACGAAAGCGTGGGGATCAAACAGGATTAGAAACCCGAGTAGTCC
>Otu292
CCAGCCTATGGGCGCTGCAGTCGAGAATCTTCCGCAATGGACGAAAGTCTGACGGAGCG
ACGCCGCGTGGAGGATGAAGTGCTTCGGCATGTAAACTCCTTTTGCCAGGGAAAAAGTCT
ATTGATTGTACCTGGAGAATAAGAGGTTGCTAAACTCGTGCCAGCAGCAGCGGTAATACG
AGTGCCTCGAGCGTTATCCGGAATCATTGGGCGTAAAGGGTGCGTAGGCGGCATTGTTAG
TCTCCCGTAAAATCTTCCGGCTCAACCGGGAGTCCGCGGGAGAAACGGCATTGCTCGAGA
TTGGAAGGGGCCTCTGGAACTCATGGTGTAGCGGTGAAATGCGTTGATATCATGGGGAAC
ACCGAAAGCGAAGGCAGGAGGCTGGTCCATTTCTGACGCTGAAGCACGAAAGCGTGGGTC
GCGAATGGGATTAGAAACCCGAGTAGTCC

>Otu293
CCAGCCTATGGGTGGCTGCAGTCGAGGATCTTCGTCAATGGGCGAAAGCCTGAACGAGCG
ACGCCGCGTGCGCGATGAAGGCCTTCGGGTTGTAAAGCGCGAAAGATGGGACGAAAGCCG
AAAGGTTTGACCGATCATCAGTAAGCTCGGGCTAAGTTCGTGCCAGCAGCCGCGGTAAGA
CGAACCGAGCGAACGTTATTCGGAATCACTGGGCTTAAAGGGCGCGTAGGCGGGCATCAA
AGTCTGTGGTGAAATCCGGCAGCTTAACTGTCGAACTGCCGTGGATACTAGGTGTCTCGA
GGGAGGTAGGGGCGTGCGGAACTATTGGTGGAGCGGTGAAATGCGTTGATATCAATAGGA
ACTCCGGTGGCGAAGGCGGCACGCTGGACCTCATCTGACGCTGAGGCGCGAAAGCTAGGG
GAGCAAACGGGATTAGAAACCCCAGTAGTCC
>Otu294
CCAGCCTATGGGTGGCAGCAGTGAGGAATATTGGGCAATGGGCGAAAGCCTGACCCAGCA
ACGCCGCGTGGGCGATGAAGATCTGAGGATCGTAAAGCCCTGTTGAGAGGGACGAAACCC
CGACTTGTCGGGATTGACGGTACCTTTATAGAAAGCTCCGGCTAACTCCGTGCCAGCAGC
CGCGGTAATACGGGGGGAGCAAGCGTTGTTCGGATTTATTGGGCGTAAAGCGCGTGTAGG
CGGGACAATAAGTCAGAGGTGAAATCTTTAGGCTTAACCTAAAAACTGCCTCTGATACTG
TTGTTCTTGAGTGCGGAAGAGGGAAGTGGAATTCCAGGTGTAGCGGTGAAATGCGTAGAT
ATCTGGAAGAACACCAGTAGCGAAGGCGGCTTCCTGGTCCGCAACTGACGCTGAGACGCG
AAAGCGTGGGGAGCAAACAGGATTAGATACCCCCGTAGTCC
>Otu295
CCAGCCTATGGGTGGCACCAGTAGGGAATATTGGTTAATGGGCGAAAGCCTGACCCAGCA
ACGCCGCGTGTGCGATGAAGGCCTTCGGGTTGTAAAGCACTTTTGAAGGGGATGAGGAAG
GACAGTACCCTTCGAATAAGCCTCGGCTAACTACGTGCCAGCAGCCGCGGTAACACGTAG
GAGGCGAGCGTTATCCGGATTTACTGGGCGTAAAGCGTGTGCAGGCGGTTTGGTAAGTTG
GATGTGAAAGCTCCTGGCTTAACTGGGAGAGGTCGTTCAATACTACCAGGCTAGAGGATG
GGAGAGGGAGGTGGAATTCCGGGTGTAGTGGTGAAATGCGTAGATATCCGGAGGAACACC
AGTGGCGAAAGCGGCCTCCTGGCCCATTTCTGACGCTCATACACGAAAGCTAGGGTAGCA
AACGGGATTAGAAACCCCAGTAGTCC
>Otu296
CCAGCCTACGGGTGGCTGCAGTGGGGAATTTTGGACAATGGGCGCAAGCCTGATCCAGCC
ATGCCGCGTGTGGGAAGAAGGCCTTCGGGTTGTAAACCACTTTTGTCAGGGAAGAAACGG
CTCTTACTAATATTAGGGGCTAATGACGGTACCTGAAGAATAAGCACCGGCTAACTACGT
GCCAGCAGCCGCGGTAATACGTAGGGTGCAAGCGTTAATCGGAATTACTGGGCGTAAAGC
GTGCGCAGGCGGCTTTGCAAGACAGATGTGAAATCCCCGGGCTCAACCTGGGAACTGCAT
TTGTGACTGCATGGCTAGAGTACGGTAGAGGGGGATGGAATTCCGCGTGTAGCAGTGAAA
TGCGTGGATATCGGAAGGAACACCTGTGGCGAAAGCGGCTCACTGGACCATAACTGACGC
TGAGGCACGAAAGCTAGGGGAGCAAACAGGATTAGAGACCCGTGTAGTCC
>Otu297
CCAGCCTATGGGGCGCACCAGTCGAGAATAATTCGCAATGGGCGAAAGCCTGACGATGCG
ACGCCGTGTGAACGATGAAGGCCTTCGGGTTGTAAAGTTCTGTCGCCTATGAACAAGGTT
GCTTCGTGAATAACGAGGTAATTTGAGTGTAATAGGAAAGGAAGCACCGGCTAACTCCGT
GCCAGCAGCTGCGGTAATACGGAGGGTGCAAGCGTTAGTCGGAATTACTGGGCGTAAAGG
GTGCGTAGGCGGGGATATAAGTCAGATGTGAAAAACTGAAGCTCAACTTCAGCAGTGCAT
CTGAAACTGTATTTCTAGAGGGATAGCGGAGAAAACGGAATTCCACGTGTAGCGGTGAAA
TGCGTAGATATGTGGAAGAACACCTGTGGCGAAAGCGGTTTTCTAGCTTAAACCTGACGC
TGAGGCACGAAAGCAAGGGGAGCAAACAGGATTAGATACCCGAGTAGTCC
>Otu298
CCAGCCTACGGGGTGCTGCAGTAGGGAATCTTGGACAATAGGCGAAAGCCTGATCCAGCA
ATGCCGCGTGCACGATGAAGGTCTTCGGATTGTAAAGTGCTTTTAGAAGGGATGAGTAAG
GACAGTACCTTCTGAATAAGCCTCGGCGAACTACGTGCCAGCAGCCGCGGTAAGACGTAG
GAGGCAAGCGTTATCCGGATTTATTGGGCGTAAAGCGCGTGCAGGCGGTTTGGTAAGTTG
GATGTGAAATCTCTCGGCTTAACCGGGAGAGGTCGTTCAAAACTACCAGACTAGAGAATG
GTAGAGGAAGGTGGAATTCCGGGTGTAGTGGTGAAATGCGTAGATATCCGGAGGAACACC
AGTGGCGAAAGCGGCCTTCTGGACCATTTCTGACGCTAAGATGCGAAAGCTAGGGCAGTA
AACGGGATTAGAGACCCGAGTAGTCC
>Otu299
CCAGCCTATGGGGGGCAGCAGTTTGGGATATTCCACAATGGGCGAAAGCCTGATGGAGCG
ACACCGCGTGAAGGATGAAGGTCTTCGGATTGTAAACTTCTTTAGACGCAGATGAAGGTT
TCAGGGTGAATAATCCTGAAACTTGACAGTATGCGTAGAATAAGCCACGGCTAACTCTGT

GCCAGCAGCCGCGGTAATACAGAGGTGGCAAACGTTGTCCGGATTTATTGGGTGTAAAGG
GCATGTAGGTGGTTTTGTAAGTCAAAGGTGAAATGGCCCGGCTCAACCAGGTCATTGCCT
TTGAAACTGCAGAGCTTGAGTACGGAAGAGGAGAGCGGAATTCCCAGTGTAGCGGTGAAA
TGCGTAGATATTGGGAGGAACACCGGTGGCGAAAGCGGCTCTCTGGTCCGAAACTGACAC
TGAGATGCGAAAGCCAGGGGAGCAAACGGGATTAGAAACCCTCGTAGTCC
>Otu300
CCAGCCTATGGGAGGCTGCAGTGGGGAATCTTGCACAATGGAGGAAACTCTGATGCAGCG
ACGCCGCGTGAGCGATGAAGCCCTTCGGGGTGTAAAGCTCTTTCGACGGGAACGATAATG
ACGGTACCCGAAGAAGAAGCTGCGGCTAACTACGTGCCAGCAGCCGCGGTAATACGTAGG
CAGCGAGCGTTGTTCGGAATTACTGGGCGTAAAGAGTGTGTAGGTGGTGCTCTAAGTTTG
GTGTGAAATCTCCCGGCTTAACTGGGAGGGTGCGCCGAAAACTGGAGTGCTTGAGTATGG
GAGAGGTAAGCGGAATTCCTGGTGTAGCGGTGAAATGCGTAGATATCAGGAGGAACACCT
GTGGTGTAGACAGCTTACTGGACCATCACTGACACTGAGACACGAAAGCGTGGGTAGCAA
ACAGGATTAGAAACCCGCGTAGTCC
>Otu301
CCAGCCTACGGGGGGCTGCAGTGGGGAATATTGCGCAATGGGCGAAAGCCTGACGCAGCG
ACGCCGCGTGGGGGATGAAGCTTTTCGGAGTGTAAACCCCTTTCGACCCGGACGAATGAC
CCGCAAGGGTTTGACGGTACGGGTATAAGAAGCCCCGGCTAACTACGTGCCAGCAGCCGC
GGTAATACGTAGGGGGCCAGCGTTGCTCGGAATTACTGGGCGTAAAGGGTTCGTAGGTGG
TGCGGCAAGTTGGGAGTGAAATCTCTGGGCTTAACCCAGAGGCTGCTTTCAAAACTGCCG
TGCTCGAGTGCGAGAGAGGTCAGTGGAATTGCAGGTGTAGCGGTGAAATGCGTAGATATC
TGCAGGAACACCCGTGGCGAAGGCGGCTGACTGGATCGCAACTGACACTGAGGAACGAAA
GCTAGGGGAGCAAACAGGATTAGATACCCTCGTAGTCC
>Otu302
CCAGCCTACGGGTCGCTGCAGTCGAGGATATTTAGCAATGGGGGAAACCCTGACTATGTG
ACGCCGCGTGAGGGATGAAGGTTTTCGGATCGTAAACCTCTTTCGATGGAGAAGAAGCTC
AGCCTACTAATATTGGGTTGGGTTGACGGTACCCAGAGAAGAAGCCACGGCCAACTCCGT
GCCAGCAGCCGCGGTAATACGGGGGTGGCGAGCGTTACTCGGATTTATTGGGTGTAAAGG
ACAGGTAGGCGACCTGCCAAGTCAGAAGTGAAATCCTAAGGCTCAACCTTAGAACTGCTT
CTGAAACTGGCGGGATTGAGGCTAGGAGAGGAAAGCGGAATTCCCGGTGTAAGGGTGAAA
TCTGTAGATATCGGGAGGAACACCAGTGGCGAAAGCGGCTTTCTGGCCTAGATCTGACGC
TGAACTGTGAAAGCTAGGGGAGCAAACAGGATTAGAAACCCTAGTAGTCC
>Otu303
CCAGCCTATGGGGTGCAGCAGACGAGAATATTCGACAATGGGCGAAAGCCTGATCGAGCG
ACGCCGCGTGGTTGATGAAGTCCTTCGGGACGTAAAAACCTTTTATGAGGGAGGAAGTAA
TTGACGTTACCTCATGAATAAGGGGCTCCTAACTCTGTGCCAGCAGGAGCGGTAATACAG
AGGCCCCAAGCATTATCCGGAATCACTGGGCGTAAAGGGTGTGTAGGCGGTCGTATTAGT
CTTTCGTGAAAGATCTCGGGCTTAACCCGGGAGACGCGGAGGAAACGGTACGACTTCGAG
GATGCGAGAGGTATAGGGAACTCATGGTGTAGGGGTGAAATCCGTTGATATCATGGGGAA
CACCAAATGCGAAGGCACTATACTGGCGCACTCCTGACGCTGAAACACGAAAGCGTGGGA
ATCGAAAAGGATTAGAAACCCTCGTAGTCC
>Otu304
CCAGCCTACGGGGGGCAGCAGTGGGGAATTTTGCGCAATGGGGGAAACCCTGACGCAGCA
ACGCCGCGTGGAGGATGAAATCCCTTGGGATGTAAACTCCTTTCGATCGGGAAGATAATG
ACGGTACCGAGAGAAGAAGCCCCGGCTAACTTCGTGCCAGCAGCCGCGGTAATACGAGGG
GGGCAAGCGTTGTTCGGAATTATTGGGCGTAAAGGGTGCGTAGGCGGTTTGACAAGTCAT
CTGTGAAATCTCTGGGCTCAACCCAGAGTCTGCAGGCGAAACTGTCGGGCTTGAGTATGG
GAGAGGTGAGTGGAATTTCCGGTGTAGCGGTGAAATGCGTAGATATCGGAAGGAACACCT
GTGGCGAAAGCGGCTCACTGGACCATAACTGACGCTGATGCACGAAAGCTAGGGGAGCAA
ACAGGATTAGAAACCCCAGTAGTCC
>Otu305
CCAGCCTATGGGACGCACCAGCTAAGAATCTTCTGCAATGGACGAAAGTCTGACAGAGCG
ACGCCGCGTGAACGATGAAGGCCGCAAGGTTGTAAAGTTCTTTTCTTTGTGAGGAATAAG
GGGCAGAGGAAATGCTGTCCTGATGACGTAAGCGAAGGAATAAGCCCCGGCCAATTACGT
GCCAGCAGCCGCGGTAATACGTATGGGGCGAGCGTTGTTCGGAATCATTGGGCGTAAAGG
GCGTGTAGGCGGCTTGGCAAGCTTGGTGTGAAAGGTCACGGCTTAACCGTGAATGTGCGC
TGAGAACTGCCAGGCTGGAGTTTTGGGGGGGAAGCTGGAATTCCTGGTGTAGGGGTGAAA
TCTGTAGATATCAGGAAGAACACCGATGGCGAAGGCAAGCTTCTATCCAAAGACTGACGC

TGAGGCGCGAAAGTGTGGGGAGCAAACAGGATTAGATACCCCAGTAGTCC
>Otu306
CCAGCCTACGGGTCGCAGCAGTGGGGAATTTTGGACAATGGGCGCAAGCCTGATCCAGCC
ATGCCGCGTGTCTGAAGAAGGCCTTCGGGTTGTAAAGGACTTTTGTCAGGGAGGAAATCC
CTAAGGTTAATACCCTTGGGGGATGACAGTACCTGAAGAATAAGCACCGGCTAACTACGT
GCCAGCAGCCGCGGTAATACGTAGGGTGCAAGCGTTAATCGGAATTACTGGGCGTAAAGC
GTGCGCAGGCGGTTGTGTAAGTCTGATGTGAAAGCCCCGGGCTCAACCTGGGAACTGCAT
TGGAGACTGCACGGCTAGAGTGCGTCAGAGGGGGGTAGAATTCCGCGTGTAGCAGTGAAA
TGCGTAGAGATGCGGAGGAATACCGATGGCGAAGGCAGCCCCCTGGGATGACACTGACGC
TCATGCACGAAAGCGTGGGGAGCAAACAGGATTAGAAACCCTAGTAGTCC
>Otu307
CCAGCCTACGGGGGGCAGCAGTCGAGGATCTTCGTCAATGGGCGCAAGCCTGAACGAGCG
ACGCCGCGTGCGCGATGAAGGCCTTCGGGTTGTAAAGCGCGAAAGTGGGGACAAAACCGC
AAGGTTGATCGATCCACAGTAAGGACGGGCTAAGTTCGTGCCAGCAGCCGCGGTAAGACG
AACTGTCCTAACGTTGTTCGGAATCACTGGGCTTAAAGGGCGCGTAGGCGGTCGGACAAG
TCGGGGGTGAAATCCTGCAGCTTAACTGTAGAAGTGCCTTCGATACTGTCCGTCTGGAGG
GAGATAGGGGTGTGCGGAACTTCCAGTGGAGCGGTGAAATGCGTTGATATTGGAAGGAAC
GCCGGAGGCGAAAGCGGCGCACTGGATCTCTTCTGACGCTGAGGCGCGAAAGCTAGGGGA
GCAAACGGGATTAGAGACCCGCGTAGTCC
>Otu308
CCAGCCTATGGGTGGCAGCAGTGGGGAATCTTGCGCAATGGGGGAAACCCTGACGCAGCG
ACGCCGCGTGAGTGATGAAGGCCTTCGGGTTGTAAAGCTCTGTGGGGAGAGAAGAATTAG
TGGAGGCTAATCACTCCATGATGACGGTATCTCCTTAGCAAGCACCGGCTAACTCTGTGC
CAGCAGCCGCGGTAAGACAGAGGGTGCAAACGTTGTTCGGAATTACTGGGCGTAAAGCGT
GTGTAGGTGGTCGCGTAAGTCGGATGTGAAAGCCCTGGGCTCAACCCAGGAAGTGCACTC
GATACTGCGTGACTTGAGTACCGGAGAGGTTGGTAGAATTCTCGGTGTAGAGGTGAAATT
CGTAGATATCGAGAGGAATACCTGTGGCGAAGGCGGCCAACTGGACGAGTACTGACACTG
AGACACGAAAGCGTGGGGAGCAAACAGGATTAGATACCCCAGTAGTCC
>Otu309
CCAGCCTATGGGGGGCAGCAGTAGGGAATCTTCCACAATGGGCGAAAGCCTGATGGAGCA
ACGCCGCGTGCAGGATGAAGGCCTTCGGGTCGTAAACTGCTTTTATCTGTGACGATTATG
ACGGTAGCAGATGAATAAGGATCGGCTAACTCCGTGCCAGCAGCCGCGGTCATACGGAGG
ATCCAAGCGTTATCCGGAATTACTGGGCGTAAAGAGTTGCGTAGGTGGCATAGTAAGCAA
ATAGTGAAAGCCTGGGGCTCAACCCCTTACCCATTATTTGAACTGCTAAGCTCGAGGATG
AGAGAGGTTATTGGAATTCCTAGTGTAGGAGTGAAATCCGTAGATATTAGGAGGAACACC
GATGGCGTAGGCAGATAACTGGCTCATTCCTGACACTAAGGCACGAAAGCATGGGGAGCA
AACGGGATTAGAAACCCTCGTAGTCC
>Otu310
CCAGCCTATGGGGGGCTGCAGTGGGGAATATTGGACAATGGGCGCAAGCCTGATCCAGCA
ATGCCGCGTGAGTGATGAAGGCCTTAGGGTTGTAAAGCTCTTTCGGCGGGGACGATGATG
ACGGTACCCGCAGAAGAAGCCCCGGCTAACTTCGTGCCAGCAGCCGCGGTAATACGAAGG
GGGCTAGCGTTGTTCGGAATTACTGGGCGTAAAGGGCGCGTAGGCGGCGAAGCAAGTTAG
GCGTGAAAGCCCCGGGCTCAACCTGGGAATTGCGCTTGATACTGCTTTGCTTGAGTTCGG
GAGAGGTGAGTGGAATTCCCAGTGTAGAGGTGAAATTCGTAGATATTGGGAAGAACACCG
GTGGCGAAGGCGGCTCACTGGACCGATACTGACGCTGAGGCGCGAAAGCGTGGGGAGCAA
ACAGGATTAGAAACCCCAGTAGTCC
>Otu311
CCAGCCTACGGGGGGCAGCAGTGGGGAATCTTGCGCAATGCGCGAAAGCGTGACGCAGCA
ACGCCGCGTGGGGGAAGAAGGCTTTCGGGTTGTAAACCCCTTTCAGTTGGGACGAAGCTT
CGCCGGTGAACAGCCGGCTGGAGTGACGGTACCTTCACAAGAAGCACCGGCTAACTACGT
GCCAGCAGCCGCGGTAATACGTAGGGTGCAAGCGTTGTCCGGAATTATTGGGCGTAAAGA
GCGTGTAGGCGGCCCGATTAGTCCGCTGTGAAAGTCCAGGGCTCAACCCTGGAATGCCGG
TGGATACTGTCGGGCTAGAGTCCGGAAGGGGCGAGTGGAATTCCTGGTGTAGCGGTGAAA
TGCGCAGATATCAGGAGGAACACCAATGGCGAAGGCAGCTCGCTGGGACGTGACTGACGC
TGAGACGCGAAAGCGTGGGGAGCAAACAGGATTAGATACCCTCGTAGTCC
>Otu312
CCAGCCTATGGGGGGCAGCAGTCGAGAATCTTCGGCAATGGGCGCAAGCCTGACCGAGCG
ACGCCGCGTGGAGGATGAAGGCCTTCGGGTTGTAAACTCCTGTCGAGGGGGAGGAAGGGG

TAACCTTGACCTATCCCTGGAGGAAGCACGGCTAAGTTCGTGCCAGCAGCCGCGGTAAG
ACGAACCGTGCGAACGTTATTCGGAATCACTGGGCTTAAAGCGCGTGTAGGCGGATGGGG
ACGTCGGTAGCTGAAATCCCCCGGCTCAACCGGGGAACTGGCGCCGAAACGCCCCGTCTG
GAGGGACGTAGGGGGACCTGGAACTTCCGGTGGAGCGGTGAAATGCGTTGAGATCGGAAG
GAACGCCCGTGGCGAAAGCGAGGTCCTGGACGTTTTCTGACGCTGAGACGCGAAAGCTAG
GGTAGCGAACGGGATTAGAAACCCGAGTAGTCC
>Otu313
CCAGCCTATGGGGGGCAGCAGTGGGGAATATTGGACAATGGGCGAAAGCCTGATCCAGCA
ATGCCGCGTGAGTGATGAAGGCCTTAGGGTTGTAAAGCTCTTTCGGGTGGGACGATGATG
ACGGTACCACCAGAAGAAGCCCCGGCTAACTTCGTGCCAGCAGCCGCGGTAATACGAAGG
GGGCTAGCGTTGTTCGGAATTACTGGGCGTAAAGCGCACGCAGGCGGTCCGTTAAATTAG
AAGTGAAAGCCCTGGGCTCAACCTGGGAATTGCTTTTAAGACTGGCGGACTTGAATCCAG
AAGAGGGCAGTGGAATTCCAAGTGTAGAGGTGAAATTCGTAGATATTTGGAAGAACACCA
GTGGCGAAGGCGACTGCCTGGTCTGGCATTGACGCTCATGTGCGAAAGCGTGGGGAGCAA
ACAGGATTAGATACCCCAGTAGTCC
>Otu314
CCAGCCTATGGGGTGCACCAGTGGGGAATTTTGGACAATGGGCGCAAGCCTGATCCAGCA
ATGCCGCGTGTGTGAAGAAGGCCTTCGGGTTGTAAAGCACTTTTGTCCGGAAAGAAATCC
TTTGGGCTAATACCCTGAGGGGATGACGGTACCGGAAGAATAAGCACCGGCTAACTACGT
GCCAGCAGCCGCGGTAATACGTAGGGTGCAAGCGTTAATCGGAATTACTGGGCGTAAAGC
GTGCGCAGGCGGTCCGCTAAGACAGATGTGAAATCCCCGGGCTTAACCTGGGAACTGCAT
TTGTGACTGGCGGGCTAGAGTATGGCAGAGGGGGGTAGAATTCCACGTGTAGCAGTGAAA
TGCGTAGAGATGTGGAGGAATACCGATGGCGAAGGCAGCCCCCTGGGCCAATACTGACGC
TCATGCACGAAAGCGTGGGGAGCAAACAGGATTAGATACCCTCGTAGTCC
>Otu315
CCAGCCTACGGGGGGCAGCAGTGGGGAATCTTGCACAATGGAGGAAACTCTGATGCAGCG
ACGCCGCGTGAGTGATGAAGCCCTTCGGGGTGTAAAGCTCTTTCGGCAGGAACGATAATG
ACGGTACCTGCAGAAGAAGCTGCGGCTAACTACGTGCCAGCAGCCGCGGTAATACGTAGG
CAGCAAGCGTTGTTCGGAATTACTGGGCGTAAAGAGTGTGTAGGCGGTTCTCTAAGTTCC
GTGTGAAATCTCCCGGCTCAACTGGGAGGGTGCGCGGGAAACTGGAGGGCTCGAGTATGG
GAGAGGAAAGCGGAATTCCTGGTGTAGCGGTGAAATGCGTAGATATCAGGAGGAACACCT
GCGGTGTAGACAGCTTTCTGGACCATCACTGACGCTGAGACACGAAAGCGTGGGTAGCAA
ACAGGATTAGATACCCCCGTAGTCC
>Otu316
CCAGCCTATGGGGGGCAGCAGTAAGGAATATTGGTCAATGGGCGGAAGCCTGAACCAGCC
ATGCCGCGTGCAGGAAGACGGCCCTACGGGTTGTAAACTGCTTTTATACGGGAATAAACC
CCCCAACGTGTTGGGGGCTGAATGTACTGTAAGAATAAGGATCGGCTAACTCCGTGCCAG
CAGCCGCGGTAATACGGAGGATCCAAGCGTTATCCGGATTTATTGGGTTTAAAGGGTGCG
TAGGCGGCCTGTTAAGTCAGGGGTGAAAGACGGTAGCTCAACTATCGCAGTGCCCTTGAT
ACTGACGGGCTTGAATACACTAGAGGTAGGCGGAATGTGACAAGTAGCGGTGAAATGCAT
AGATATGTCACAGAACACCAATTGCGAAGGCAGCTTACTATGGTGTCATTGACGCTGAGG
CACGAAAGCGTGGGGATCAAACAGGATTAGATACCCCCGTAGTCC
>Otu318
CCAGCCTATGGGAGGCAGCAGTAGGGAATCTTGCGCAATGGGCGAAAGCCTGACGCAGCA
ATGCCGCGTGGGGGATGAAGGCCTTCGGGTTGTAAACTCCTTTTAGTGGGAACGAAATTG
ACGGTACCCACAGAAAAAGCCCCGGCTAACTACGTGCCAGCAGCCGCGGTGATACGTAGG
GGGCAAGCGTTGTCCGGATTCACTGGGCGTAAAGAGCTCGTAGGTGGTTTGGTAAGTCGG
GTGTGAAACCTCCAGGCTCAACCTGGAGACGCCACTCGATACTGCCATGGCTAGAGTCCG
GTAGGGGACCATGGAATTCCAGGTGTAGCGGTGAAATGCGCAGATATCTGGAGGAACACC
AGTAGCGAAGGCGGTGGTCTGGGCCGGTACTGACACTGAGGAGCGAAAGCGTGGGGAGCG
AACAGGATTAGAAACCCCCGTAGTCC
>Otu320
CCAGCCTATGGGGGGCAGCAGTAACGAATCTTCCGCAATGCACGAAAGTGTGACGGAGCG
ACGCCGCGTGTGGGACAAAGTTCTTCGGAATGTAAACCACTGTCAGGGGTTAGAAAGTTC
TGATCAACCCCAAAGGAAGGCACGGCTAACTCTGTGCCAGCAGCCGCGGTAATACAGAGG
TGCCAAGCGTTAGGCGGAATCACTGGGCTTAAAGCGTGTGTAGGCGGACTTCTAAGTGCC
TTGTGAAATCCCACGGCTCAACCGTGGAACTGCTGGGTATACTGGGAGTCTTGAGCGACC
TAGGGGCGAGTGGAACAAATGGTGGAGCGGTGAAATGCGTAGATATCATTTGGAACGCCA

ATGGTGAAAACAACTCGCTGGGGGTTTGCTGACGCTGAGACACGAAAGCTAGGGGAGCAA
ACGGGATTAGATACCCTTGTAGTCC
>Otu321
CCAGCCTACGGGGGGCTCCAGTCAAGAAACTTCCACAATGGACGAAAGTCTGATGGAGCG
ACGCCGCGTGGTTGATGAAGTCCTTCGGGACGTAAAAACCTTTTATGAGGGAGGAAGTAA
TTGACGTTACCTCATGAATAAGGGGCTCCTAACTCTGTGCCAGCAGGAGCGGTAATACAG
AGGCCCCAAGCATTATCCGGAATCACTGGGCGTAAAGGGTGTGTAGGCGGTCGTGTTAGT
CTTTCGTGAAAGCTTTTGGGCTTAACCCAGAAAACGCGGGGGAAACGGCACGACTTAGAG
GATGCGAGAGGTATATGGAACTCATGGTGTAGGGGTGAAATCCGTTGATATCATGGGGAA
CACCAAAAGCGAAGGCAATATACTGGCGCACTCCTGACGCTGAGACACGAAAGCGTGGGA
ATCGAACGGGATTAGAAACCCCAGTAGTCC
>Otu322
CCAGCCTATGGGGGGCACCAGTGGGGAATCTTGCACAATGGGCGAAAGCCTGATGCAGCG
ACGCCGCGTGGGGGATGAAGCTTTTCGGAGTGTAAACCCCTTTCGACCCGGACGAATGCC
CCGCAAGGGGACTGACGGTACGGGTATAAGAAGCCCCGGCTAACTACGTGCCAGCAGCCG
CGGTAAGACGTGGGGGGCCAGCGTTGCTCGGAATTACTGGGTGTAAAGGGTTCGTAGGCG
GTGTGGCAAGTCGGGAGTGAAATCTCTGGGCTTAACCCAGAGACTGCTTCCGAAACTGCT
GTGCTTGAGTGTGGGAGAGGCGCGTGGAATTGCAGGTGTAGCGGTGAAATGCGTAGATAT
CTGCAGGAACACCCGTGGCGAAAGCGGCGCGCTGGACCACTACTGACGCTGAGGAACGAA
AGCTAGGGGAGCAAACAGGATTAGAAACCCCTGTAGTCC
>Otu323
CCAGCCTATGGGTCGCAGCAGTGGGGAATCTTGCACAATGGGGGAAACCCTGATGCAGCG
ACGCCGCGTGAGCGATGAAGCCCTTCGGGGTGTAAAGCTCTTTCGGTAGGGAAGATCATG
ACGGTACCTGCAGAAGCAGCTGCGGCTAACTACGTGCCAGCAGCCGCGGTAATACGTAGG
CAGCAAGCGTTGTTCGGAGTTACTGGGCGTAAAGGGTGTGTAGGCGGTTGGATAAGTGTG
GTGTGAAATCTCCCGGCTTAACTGGGAGGGTGCGCTACAAACTGTTTGACTAGAGTGTGG
GATGGGAGAGTGGAATTCCTGGTGTAGCGGTGAAATGCGTAGATATCAGGAGGAACACCG
GTGGTGTAGACGGCTCTCTGGACCATAACTGACGCTGAGACACGAAAGCGTGGGTAGCAA
ACAGGATTAGAAACCCTAGTAGTCC
>Otu324
CCAGCCTACGGGGGGCAGCAGTCGAGAATTTTTCTCAATGGGCGAAAGCCTGAAGGAGCG
ACGCCGCGTGGGGGATGAATGGCTTCGGCCCGTAAACCCCTGTCATTTGTGAACAAATTG
TTTCACCTAACACGTGAAATATTGATTGTAACGGAAGAGGAAGGGACGGCTAACTCTGTG
CCAGCAGCCGCGGTAATACAGAGGTCCCAAGCGTTGTTCGGATTCACTGGGCGTAAAGGG
TGCGTAGGTGGCCAGGTAAGTTTGATGTGAAATCCCGCAGCTTAACTGCGGAACTGCATT
GAATACTATCTGGCTAGAGGAATGGAGGGGAGACTGGAATTCTCGGTGTAGCAGTGAAAT
GCGTAGATATCGAGAGGAACACCAGTGGCGAAGGCGAGTCTCTAGACATTTCCTGACGCT
GAGGCACGAAAGCCAGGGGAGCAAACGGGATTAGATACCCTGGTAGTCC
>Otu325
CCAGCCTATGGGGTGCACCAGTCGAGAATTTTTCACAATGGACGAAAGTCTGATGGAGCG
ACGCCGCGTGGAGGATGAAGGTTTTCGGATTGTAAACTCCTGTCACTGCAGAACAAGGCA
GTGCGTATGAATAGTACGTGCTGTTGATGGTATGCGGAGAGGAAGGGACGGCTAACTCTG
TGCCAGCAGCCGCGGTAAGACAGAGGTCCCGAGCGTTGTTCGGATTCATTGGGCGTAAAG
GGTGTGTAGGAGGTCGGGTAAGTCAGGTGTGAAATCTCAGAGCTTAACTCTGAAACTGCG
CTTGATACTGCCCGGCTAGAGGATCGGAGGGGGTATCGGAATTTATGGTGTAGCAGTGAA
ATGCGTAGATATCATAAGGAACACCGGTGGCGAAGGCGGATACCTGGAAGATTCCTGACT
CTGAAACACGAAAGCCAGGGGAGCAAACGGGATTAGATACCCTTGTAGTCC
>Otu326
CCAGCCTATGGGTTGCAGCAGTAAGGAATATTGGTCAATGGGCGAAAGCCTGAACCAGCC
ATGCCGCGTGGAGGATGAAGGCCCTCTGGGTTGTAAACTTCTTTTATCTGGGAAGAAACC
CGCGATTTCTATTGCGGTTGACGGTACCAGTTGAATAAGCACCGGCTAACTCCGTGCCAG
CAGCCGCGGTAATACGGAGGGTGCAAGCGTTATCCGGATTCACTGGGTTTAAAGGGTGCG
TAGGTGGGCAGGTAAGTCAGTGGTGAAATCTCCGAGCTTAACTCGGAAACTGCCGTTGAT
ACTATCTGTCTTGAATGTCGTGGAGGCAGGCGGAATATGTCATGTAGCGGTGAAATGCTT
AGATATGACATAGAACACCAATTGCGAAGGCAGCTTGCTACACGATGATTGACGCTGAGG
CACGAAAGCGTGGGGATCAAACAGGATTAGAAACCCGAGTAGTCC
>Otu327
CCAGCCTATGGGGCGCACCAGTGGGGAATATTGGACAATGGGGGCAACCCTGATCCAGCG

ACGCCGCGTGTGTGAAGAAGGCCTGCGGGTTGTAAAGCACTTTTAGTGGGGATGAAAAGC
TCAGGGCTAATACCCCCGGGTCTTGACCTAACCTACAGAAAAAGCACCGGCTAACTCTGT
GCCAGCAGCCGCGGTAATACAGAGGGTGCGAGCGTTAATCGGAATTACTGGGCGTAAAGC
GTGCGTAGACGGTTTCGTAAGTCGGATGTGAAATCCCCGGGCTCAACCTGGGAATTGCAT
TCGAAACTGCAAAGCTAGGGTGCGGAAGAGGGAAGCGGAATTTCCGGTGTAGCGGTGAAA
TGCGTAGATATCGGAAGGAACATCAGTGGCGAAAGCGGCTTCCTGGTCCAGCACCGACGT
TCAGGCACGAAAGCGTGGGGAGCAAACAGGATTAGAAACCCGAGTAGTCC
>Otu328
CCAGCCTATGGGGGGCACCAGTAGGGAATCTTGGACAATGGGGGAAACCCTGATCCAGCC
ATGCCGCGTGAGTGATGAAGGCCTTCGGGTTGTAAAACTCTTTCGACGGGGACGATAATG
ACGGTACCCGTAGAAGAAGCTCCGGCTAACTTCGTGCCAGCAGCCGCGGTAATACGAAGG
GGGCTAGCGTTGTTCGGAATTACTGGGCGTAAAGCGCGCGCAGGCGGCCATTCAAGTCAG
GGGTGAAAGCCCAGAGCTCAACTCTGGAACTGCCCTTGAAACTAGACGGCTAGAGTACGG
GAGAGGTGAGTGGAATTCCCAGTGTAGAGGTGAAATTCGTAGATATTGGGAAGAACACCG
GTGGCGAAGGCGGCTCACTGGCCCGTTACTGACGCTCAGGCGCGACAGCGTGGGGATCAA
ACAGGATTAGAAACCCTAGTAGTCC
>Otu329
CCAGCCTACGGGGGGCACCAGTAAGGAATATTGGTCAATGGGCGCAAGCCTGAACCAGCC
ATGCCGCGTGCAGGAAGACGGCCCTACGGGTTGTAAACTGCTTTTGCAGGGGAATAAACC
CCCATTCGTGAATGGGGTTGAATGTACTCTGAGAATAAGGATCGGCTAACTCCGTGCCAG
CAGCCGCGGTAATACGGAGGATCCGAGCGTTATCCGGATTTATTGGGTTTAAAGGGTGCG
TAGGCGGCCTTGTAAGTCAGGGGTGAAAGACGGTAGCTTAACTATCGCAGTGCCTTTGAT
ACTGCAGGGCTTGAATGTACTTGAGGCAGGCGGAATGTGACAAGTAGCGGTGAAATGCAT
AGATATGTCACAGAACACCAATTGCGAAGGCAGCTTGCTAAAGTATGATTGACGCTGAGG
CACGAAAGCGTGGGGATCAAACAGGATTAGAGACCCGAGTAGTCC
>Otu330
CCAGCCTATGGGTGGCAGCAGTGGGGAATTTTGCGCAATGGGGGAAACCCTGACGCAGCA
ACGCCGCGTGGAGGATGAAGCCCCTTGGGGCGTAAACTCCTTTCGATCGGGACGATTATG
ACGGTACCGGAAGAAGAAGCCCCGGCTAACTTCGTGCCAGCAGCCGCGGTAATACGAGGG
GGGCGAGCGTTGTTCGGAATTATTGGGCGTAAAGGGTGCGTAGGCGGTTCAGTAAGTCTT
GTGTGAAATCTTCGGGCTCAACTCGAAGTCTGCATGAGAAACTGCTGGGCTTGAGTATGG
GAGAGGTGAGTGGAATTCCCGGTGTAGCGGTGAAATGCGTAGATATCGGGAGGAACACCT
GTGGCGAAAGCGGCTCACTGGACCATAACTGACGCTGAGGCACGAAAGCTAGGGGAGCAA
ACAGGATTAGAGACCCCAGTAGTCC
>Otu331
CCAGCCTATGGGGGGCAGCAGTGGGGAATTTTACGCAATGGGCGAAAGCCTGACGTAGCG
ACACCGCGTGAGCGAAGAAGCCTTTTGGGGTGTAAAGCTCTGTCAGCTGGGAAGAAAAAA
ATGACGGTACCAGCAGAGGAAGCATCGGCTAACTACGTGCCAGCAGCCGCGGTAAGACGT
AGGATGCAAGCGTTGTCCGGATTTATTGGGCGTAAAGAGTTCGTAGGCGGTTTGTTAAGT
CTGATGTTAAATATCGGGGCTCAACCCCGGGACTGCATTGGATACTGGCAGACTTGAGTG
CGGTAGAGGTAAGTGGAATTTCTGGTGTAGCGGTGAAATGCGTAGATATCAGAAAGAACA
CCAGTGGCGTAGGCGACTTACTGGGCCGTAACTGACGCTGAGGAACGAAAGCCAGGGGAG
CGAATGGGATTAGATACCCGCGTAGTCC
>Otu332
CCAGCCTATGGGGGGCAGCAGTGAGGAATATTGGTCAATGGGCGAAAGCCTGAACCAGCC
ATCCCGCGTGAAGGAAGAAGGCGCTATGCGTCGTAAACTTCTTTTCCAGGGGAAGAAAAC
TTCCTATGTATAGGGACTTGCCGGTACCTTGGGAATAAGCATCGGCTAACTCCGTGCCAG
CAGCCGCGGTAATACGGAGGATGCAAGCGTTATCCGGATTCATTGGGTTTAAAGGGTGCG
TAGGCGGATTAATAAGTCAGTGGTGAAAACCTGCAGCTTAACTGTAGAGGTGCCATTGAT
ACTGTTAGTCTTGAGTGTGGTCAAGGTAGGCGGAATGTGTAATGTAGCGGTGAAATGCTT
AGATATTACACAGAACACCAATTGCGAAGGCAGCTTACTGGGCCATTACTGACGCTGATG
CACGAAAGCGTGGGGAGCAAACAGGATTAGATACCCCCGTAGTCC
>Otu333
CCAGCCTATGGGAGGCTCCAGTAGGGAATCTTGCGCAATGGACGAAAGTCTGACGCAGCA
ACGCCGCGTGGGGGATGAAGGCCTTCGGGTTGTAAACTCCTTTCAGTGGGAACGAAATTG
ACGGTACCCACAGAAGAAGCCCCGGCTAACTACGTGCCAGCAGCCGCGGTGATACGTAGG
GGGCGAGCGTTGTCCGGATTTACTGGGCGTAAAGAGCTCGTAGGCGGCTAAATAAGTCGG
GTGTGAAACCCCCAGGCTCAACCTGGGAACGCCACTCGATACTGTTTTGGCTAGAGTCCG

GTAGGGGACCATGGAATTCCTGGTGTAGCGGTGAAATGCGCAGATATCAGGAGGAACACC
AGTAGCGAAGGCGGTGGTCTGGGCCGGTACTGACGCTGAGGAGCGAAAGCGTGGGGAGCG
AACAGGATTAGATACCCTAGTAGTCC
>Otu334
CCAGCCTATGGGTCGCTGCAGTGGGGAATCTTGCACAATGGGGGCAACCCTGATGCAGCG
ACGCCGCGTGAGCGATGAAGCCCTTCGGGGTGTAAAGCTCTTTCGGCAGGGAAGATTATG
ACGGTACCTGCAGAAGCAGCTGCGGCTAACTACGTGCCAGCAGCCGCGGTAATACGTAGG
CAGCAAGCGTTGTTCGGAGTTACTGGGCGTAAAGGGTGCGTAGGCGGCTCGATAAGTATG
GTGTGAAATCTCCCGGCTTAACTGGGAGGGTGCGCTATAGACTGCCGGGCTAGAGTGTGG
GAGAGGAAAGTGGAATTCCTGGTGTAGCGGTGAAATGCGTAGATATCAGGAGGAACACCG
GTGGTGTAAACGGCTTTCTGGACCATAACTGACGCTGAGGCACGAAAGCGTGGGTAGCAA
ACAGGATTAGAAACCCGAGTAGTCC
>Otu335
CCAGCCTATGGGGCGCTGCAGTGGGGAATATTGGACAATGGGCGCAAGCCTGATCCAGCA
ATGCCGCGTGAATGATGAAGGCCTTAGGGTTGTAAAGTTCTTTCGTCGGGGACGATGATG
ACGGTACCCGAAGAAGAAGCCCCGGCTAACTTCGTGCCAGCAGCCGCGGTAATACGAAGG
GGGCTAGCGTTGTTCGGAATTACTGGGCGTAAAGGGCGCGTAGGCGGCCATTCAAGTTAG
GCGTGAAAGCCCTGGGCTCAACCTAGGAATTGCGCTTGATACTGTTTGGCTTGAGTTCGG
GAGAGGTGAGTGGAATTCCCAGTGTAGAGGTGAAATTCGTAGATATTGGGAAGAACACCA
GTGGCGAAGGCGGCTCACTGGACCGACACTGACGCTGAGGCGCGAAAGCGTGGGGAGCAA
ACAGGATTAGATACCCGCGTAGTCC
>Otu336
CCAGCCTATGGGGGGCACCAGTGGGGAATTTTGCGCAATGGGGGAAACCCTGACGCAGCA
ACGCCGCGTGGAGGATGAAGTCTCTTGGGACGTAAACTCCTTTCGATTGGGAAGATAATG
ACGGTACCAATAGAAGAAGCCCCGGCTAACTTCGTGCCAGCAGCCGCGGTAATACGAGGG
GGGCAAGCGTTGTTCGGAATTATTGGGCGTAAAGGGTGCGTAGGCGGTTTGGTAAGTCTT
GTGTGAAATCTTCGGGCTCAACTCGAAGTCTGCACGAGAAACTGCCGGGCTTGAGTGTGG
GAGAGGTGAGTGGAATTTCCGGTGTAGCGGTGAAATGCGTAGATATCGGAAGGAACACCT
GTGGCGAAAGCGGCTCACTGGACCATAACTGACGCTGATGCACGAAAGCTAGGGGAGCAA
ACAGGATTAGATACCCTCGTAGTCC
>Otu337
CCAGCCTATGGGGGGCAGCAGTAAGGGATATTGGGCAATGGGCGAAAGCCTGACCCAGCA
ACGCCGCGTGGAGGATGACGGTCTTCGGATTGTAAACTCCTTTTGCCAGGGACGAGGAAG
GACGGTACCTGGTGAATCAGTCTCGGCTAACTACGTGCCAGCAGCCGCGGTAAAACGTAG
GAGGCGAGCGTTATCCGGATTTACTGGGCGTAAAGGGTGTGCAGGCGGGTCCCTAAGTGG
CGCATGAAAGCTCCGCGCTTAACGTGGAGAGGCTGTGTCAGACTGGGGGTCTGGAGTGCG
CGAGAGGGCTGTGGAATTCCGGGTGTAGTGGTGAAATGCGTAGAGATCCGGAGGAACACC
AGAGGCGAAGGCGGCAGCCTGGCGCGCAACTGACGCTGAGACACGACAGCATGGGGAGCG
AACGGGATTAGAAACCCCAGTAGTCC
>Otu338
CCAGCCTATGGGTCGCAGCAGTAACGAATCTTCCGCAATGCACGAAAGTGTGACGGAGCG
ACGCCGCGTGTGGGACGAAGTCCTTCGGGATGTAAACCACTGTCAGGGGTTACCAAGTTC
TGAGGAGCCCCAGAGGAAGGGACGGCTAACTCTGTGCCAGCAGCCGCGGTAATACAGAGG
TCCCGAGCGTTAGGCGGAATCACTGGGCTTAAAGCGTGTGTAGGCGGATGCCTAAGTACC
TTGTGAAATCCCACGGCTTAACCGTGGAACTGCTTGGTATACTGGGTGTCTTGAGCCACT
TAGGGGCTACCGGAACAAATGGTGGAGCGGTGAAATGCGTAGATATCATTTGGAACGCCA
ATGGTGAAAACAGGTAGCTGGGAGTGTGCTGACGCTGAGACACGAAAGCCAGGGGAGCGA
ACGGGATTAGAAACCCCAGTAGTCC
>Otu339
CCAGCCTATGGGTGGCAGCAGTCGAGGATCTTCGGCAATGGGCGCAAGCCTGACCGAGCG
ACGCCGCGTGGGCGATGAAGGCCTTCGGGTTGTAAAGCCCTGTCGAGGGGGAGAAAAGAG
CAATCTTGATCTATCCCTGGAGGAAGCACGGGCTAAGTTCGTGCCAGCAGCCGCGGTAAG
ACGAACCGTGCGAACGTTATTCGGAATCACTGGGCTTAAAGGGTGCGTAGGCGGGTTGCC
AAGTCTGTGGTGAAATCCTCCGGCTTAACCGGAGAACTGCCGTGGATACTGGCGACCTCG
AGGGGGATAGGGGCTTGCGGAACTGTGCGTGGAGCGGTGAAATGCGTTGATATGCACAGG
AACTCCGGTGGCGAAGGCGGCGAGCTGGATCCTTTCTGACGCTGAGGCACGAAAGCCAGG
GGAGCGAACGGGATTAGATACCCCCGTAGTCC
>Otu340

CCAGCCTATGGGTGGCAGCAGTGGGGAATATTGGACAATGGGCGCAAGCCTGATCCAGCC
ATGCCGCGTGAGTGACGAAGGCCTTAGGGTTGTAAAGCTCTTTCGCAAGCGACGATAATG
ACGGTAGCTTGAGAAGAAGCCCCGGCTAACTCCGTGCCAGCAGCCGCGGTAAGACGGAGG
GGGCTAGCGTTGTTCGGAATTACTGGGCGTAAAGCGCACGTAGGCGGCGTTGCAAGTTGG
GGGTGAAATCCCGGGGCTCAACCCCGGAATTGCCTCCAAGACTGTGGCGCTGGAGTCCGG
AAGAGGAGAGCGGAATTCCCAGTGTAGAGGTGAAATTCGTAGATATTGGGAAGAACACCA
GTGGCGAAGGCGGCTCTCTGGTCCGGTACTGACGCTGAGGTGCGAAAGCGTGGGGAGCAA
ACAGGATTAGAAACCCGAGTAGTCC
>Otu341
CCAGCCTACGGGTGGCAGCAGTGGGGAATATTGGACAATGGGCGAAAGCCTGATCCAGCA
ATGCCGCGTGAGTGATGAAGGCCTTAGGGTTGTAAAGCTCTTTTGGCAGGGACGATGATG
ACGGTACCTGCAGAATAAGCCCCGGCTAACTTCGTGCCAGCAGCCGCGGTAATACGAAGG
GGGCTAGCGTTGTTCGGAGTTACTGGGCGTAAAGGGCGCGTAGGCGGCCTTGTCAGTCAG
ATGTGAAAGCCCTGGGCTCAACCCGGGATGTGCATTTGATACTGCAAGGCTAGAGGACGG
GAGAGGATAGTGGAATTCCCAGTGTAGAGGTGAAATTCGTAGATATTGGGAAGAACACCA
GTGGCGAAGGCGGCTATCTGGACCGTAACTGACGCTGAGGCGCGAAAGCGTGGGGAGCAA
ACAGGATTAGAGACCCCCGTAGTCC
>Otu342
CCAGCCTACGGGGGGCAGCAGTCGAGAATTTTTCTCAATGGGCGAAAGCCTGAAGGAGCG
ACGCCGCGTGGGGGATGAAGGGCTTCGGCTCGTAAACCCCTGTCATTTGCGAACAAACCT
TACGATTGAATAGATCGTGAGCTGATTGTAGCGAAAGAGGAAGGGACGGCTAACTCTGTG
CCAGCAGCCGCGGTAATACAGAGGTCCCAAGCGTTGTTCGGATTCACTGGGCGTAAAGGG
TGCGTAGGTGGCAAGGTAAGTTTGATGTGAAATCTCCGGGCTTAACCCGGAAACTGCATT
GAATACTATCTCGCTCGAGAGTTGGAGGGGAGACTGGAATACTTGGTGTAGCAGTGAAAT
GCGTAGATATCAAGTGGAACACCAGTGGCGAAGGCGAGTCTCTGGACAACTTCTGACACT
GAGGCACGAAAGCTAGGGGAGCAAACAGGATTAGAAACCCTAGTAGTCC
>Otu343
CCAGCCTACGGGGCGCTCCAGGCGCGCAAACTCCACAATGCACATACGTGTGATGGGGGG
AATCTGAGTGGTCGACTTCGGTTGACCTTTTGCCAAGTTTAGCAAGCTTGGAGAATAAGT
GCTGGGTAAGACCGGTGGCAGCCGCCACGGTAATACCGGCGGCACGAGTGGTATTCACAA
TTATTGGGCTTAAAGCGTTAGTAGCCGGCTGAAACAGTCTTGTGTGAAATATTGGCGCAT
GACGTCAATACGTGCATAAGATACCTTTCGGCTAGAGAGTGGGAGACGTTAGGAGTATTC
ATGGGGTAAGGGTAAAATCTCGTAATCCTATGAAGACTACCGGTGGCGAAGGCGCCTAAC
GAGAACACATCTGACGGTGAGTGACGAAGGCTAGGAGAACGAATCGGATTAGATACCCGC
GTAGTCC
>Otu344
CCAGCCTACGGGGGGCAGCAGTGGGGAATCTTGCGCAATGGGCGAAAGCCTGACGCAGCA
ACGCCGCGTGGGGGATGAAGCTTTTCGGAGTGTAAACCCCTTTCGACCCGGAAGAATGCC
CGCAAGGGTTTGACGGTACGGGTATAAGAAGCCCCGGCTAACTACGTGCCAGCAGCCGCG
GTAAAACGTAGGGGGCCAGCGTTGCTCGGAATTACTGGGCGTAAAGGGTCCGTAGGCGGT
GTGGCAAGTCGGAAGTGAAATCTCTGGGCTTAACCCAGAGGCTGCTCCCGAAACTGCTAT
GCTAGAGTGTGAGAGAGGCGAGTGGAATTGCAGGTGTAGCGGTGAAATGCGTAGATATCT
GCAGGAACATCCGAGGCGAAAGCGGCTCGCTGGATCACAACTGACGCTGAGGGACGAAAG
CTAGGGGAGCAAACAGGATTAGATACCCTCGTAGTCC
>Otu345
CCAGCCTATGGGGGGCAGCAGTGGGGAATATTGGACAATGGGGGAAACCCTGATCCAGCG
ACGCCGCGTGTGTGAAGAAGGCCTGCGGGTTGTAAAGCACTTTTAGTGGGGACAAAAAGT
CACGGGCTAACACCTTGTGATCTTGATTTAACTCACAGAAAAAGCACCGGCTAACTCTGT
GCCAGCAGCCGCGGTAATACAGAGGGTGCGAGCGTTAATCGGAATTACTGGGCGTAAAGC
GCGCGTAGACGGTTTTGTAAGTCAGGTGTGAAATCCCCGGGCTCAACCTGGGAACTGCAT
TTGATACTGCATGGCTAGAGTATCGAAGAGGGAAGTGGAATTTCCGGTGTAGCGGTGAAA
TGCGTAGATATCGGAAGGAACACCAGTGGCGAAAGCGACTTCCTGGTCGAATACTGACGT
TCATGTGCGAAAGCGTGGGGAGCAAACAGGATTAGAAACCCCCGTAGTCC
>Otu346
CCAGCCTATGGGGGGCACCAGTGGGGAATTTTGGACAATGGGCGCAAGCCTGATCCAGCA
ATGCCGCGTGCAGGACGAAGGCCTTCGGGTTGTAAACTGCTTTTGTACGGAACGAAACGG
TCCTTTCTAATACAAGGGGCTAATGACGGTACCGTAAGAATAAGCACCGGCTAACTACGT
GCCAGCAGCCGCGGTAATACGTAGGGTGCGAGCGTTAATCGGAATTACTGGGCGTAAAGC

GTGCGCAGGCGGTGATGTAAGACAGATGTGAAATCCCCGGGCTCAACCTGGGACCTGCAT
TTGTGACTGCATCGCTAGAGTACGGTAGAGGGGGATGGAATTCCGCGTGTAGCAGTGAAA
TGCGTAGATATGCGGAGGAACACCGATGGCGAAGGCAATCCCCTGGACCTGTACTGACGC
TCATGCACGAAAGCGTGGGGAGCAAACAGGATTAGAAACCCCCGTAGTCC
>Otu347
CCAGCCTATGGGGTGCAGCAGTCGAGGATCTTCGGCAATGGGCGCAAGCCTGACCGAGCG
ACGCCGCGTGCGCGATGAAGGCCTTCGGGTTGTAAAGCGCTGTCGAGGGGGAGGAAGCCG
AAAGGTTGACCTATCCCTGGAGGAAGCACGGGCTAAGTTCGTGCCAGCAGCCGCGGTAAG
ACGAACCGTGCGAACGTTGTTCGGAATCACTGGGCTTAAAGGGCGCGTAGGCGGTTGATC
AAGTCCGGGGTGAAATCTTTCGGCTTAACCGGAAAAGTGCCTTGGATACTGATCAGCTGG
AGGGAGGTAGGGGCAGCTGGAACTTCCAGTGGAGCGGTGAAATGCGTTGATATTGGAAGG
AACGCCGGTGGCGAAAGCGAGCTGCTGGACCTCTTCTGACGCTGAGGCGCGAAAGCTAGG
GGAGCAAACGGGATTAGATACCCTAGTAGTCC
>Otu348
CCAGCCTATGGGGTGCAGCAGTCGAGAATTTTTCACAATGGGCGAAAGCCTGATGGAGCG
ACGCCGCGTGGGGGATGAATGGCTTCGGCCCGTAAACCCCTGTCATTCGGGATCAATGTC
CCGGCAATAACATTGTCGGGATTGATAGTACCGGAAGAGGAAGGGACGGCTAACTCTGTG
CCAGCAGCCGCGGTAATACAGAGGTCCCAAGCGTTGTTCGGATTCACTGGGCGTAAAGGG
TGCGTAGGTGGTCGGGTAAGTTTGATGTGAAATCTCCGGGCTTAACCCGGAAATGGCATT
GAATACTATTCGGCTGGAGGGTTGGAGGGGGGACTGGAATTCTCGGTGTAGCAGTGAAAT
GCGTAGATATCGAGAGGAACACCAGTGGCGAAGGCGAGTCCCTGGACAACTCCTGACACT
AAGGCACGAAAGCTAGGGGAGCAAACAGGATTAGATACCCCTGTAGTCC
>Otu349
CCAGCCTACGGGGGGCTGCAGTGGGGAATATTGGACAATGGGCGCAAGCCTGATCCAGCC
ATGCCGCGTGAGTGAAGAAGGCCTTAGGGTTGTAAAGCTCTTTCGGCGGGGAAGATAATG
ACGGTACCCGCAGAAGAAGCCCCGGCTAACTTCGTGCCAGCAGCCGCGGTAATACGAAGG
GGGCTAGCGTTGTTCGGAATCACTGGGCGTAAAGCGCACGCAGGCGGACTGATAAGTCGG
GGGTGAAAGCCCGGGGCTCAACCTCGGAACTGCCTTCGATACTGTTAGTCTTGAGTCCGG
GAGAGGTGAGTGGAATTCCTAGTGTAGAGGTGAAATTCGTAGATATTAGGAAGAACACCA
GTGGCGAAGGCGACTCACTGGCCCGGTACTGACGCTCAGGTGCGAAAGCGTGGGGAGCAA
ACAGGATTAGAGACCCCAGTAGTCC
>Otu350
CCAGCCTACGGGAGGCAGCAGTGGGGAATCTTGCACAATGGGGGAAACCCTGATGCAGCG
ACGCCGCGTGAGCGATGAAGCCCTTCGGGGTGTAAAGCTCTTTCGGCAGGGAAGATTATG
ACGGTACCTGCATAAGCAGCTGCGGCTAACTACGTGCCAGCAGCCGCGGTAATACGTAGG
CAGCGAGCGTTGTTCGGAGTTACTGGGCGTAAAGGGTGTGTAGGCGGCTTTTTAAGTTTG
GTGTGAAATCTCCCGGCTCAACTGGGAGGGTGCGCCGAATACTGAAGGGCTAGAGTGCGG
GAGAGGAGAGTGGAATTCCTGGTGTAGCGGTGAAATGCGTAGATATCAGGAGGAACACCG
GTGGTGTAGACGGCTCTCTGGACCGTAACTGACGCTGAGACACGAAAGCGTGGGTAGCAA
ACAGGATTAGATACCCTCGTAGTCC
>Otu351
CCAGCCTACGGGGGGCAGCAGTAAGGAATATTGGTCAATGGACGCAAGTCTGAACCAGCC
ATGCCGCGTGAAGGATGAAGGCCCTCTGGGTTGTAAACTTCTTTTATCTGGGACGAAAAA
CAGGAATTCTTTTCTGCTCGACGGTACCAGGGGAATAAGCACCGGCTAACTCCGTGCCAG
CAGCCGCGGTAATACGGAGGGTGCAAGCGTTATCCGGATTTACTGGGTTTAAAGGGTGCG
TAGGTGGGTTGGTAAGTCAGTGGTGAAATCTTCGAGCTTAACTCGGAAACTGCCATTGAT
ACTATCAATCTTGAATTATCTGGAGGTGAGCGGAATATGTCATGTAGCGGTGAAATGCTT
AGATATGACATAGAACACCCATTGCGAAGGCAGCTCACTACGGATATATTGACACTGAGG
CACGAAAGCGTGGGGATCAAACAGGATTAGAAACCCCTGTAGTCC
>Otu352
CCAGCCTATGGGGGGCAGCAGTTTCGAATCATTCACAATGGGCGAAAGCCTGATGGTGCG
ACGCCGCGTGAGGGATGAAGGCCTTCGGGTTGTAAACCTCTGTCACCGGGGAAGAAACGC
TTCAAGTTAACAGCTTGAAGCCTGACTTAACCCGGAGAGGAAGCAGTGGCTAACTCTGTG
CCAGCAGCCGCGGTAATACAGAGACTGCGAGCGTTATTCGGATTCACTGGGCGTAAAGGG
TGCGCAGGTGGCCAAGTGTGTTAGGCGTGAAAGCCCGGGGCTTAACCCCGGAATTGCACC
TAAAACTACATGGCTAGAGCATTGGAGAGGGGAGCAGAATTCACGGTGTAGCAGTGAAAT
GCGTAGATATCGTGAGGAATACCAGAGGCGAAGGCGGCTCCCTGGACAATTGCTGACACT
CAGGCACGAAAGCGTGGGGAGCAAAAGGGATTAGAAACCCCTGTAGTCC

>Otu353
CCAGCCTATGGGGGGCAGCAGTGGGGAATCTTGGACAATGGGGGAAACCCTGATCCAGCC
ATGCCGCGTGAGTGAAGAAGGCCTTCGGGTTGTAAAGCTCTTTTGTCAGGGACGATAATG
ACGGTACCTGAAGAATAAGCCCCGGCAAACTTCGTGCCAGCAGCCGCGGTAATACGAAGG
GGGCTAGCGTTGTTCGGATTTACTGGGCGTAAAGCGCACGTAGGTGGATTGTTAAGTGAG
AGGTGAAATCCCAGGGCTCAACCCTGGAACTGCCTTTCATACTGGCAATCTAGAGTTCGG
AAGAGGTGAGTGGAATTCCAAGTGTAGAGGTGAAATTCGTAGATATTTGGAAGAACACCG
GTGGCGAAGGCGGCTCACTGGTCCGATACTGACACTGAGGTGCGAAAGCGTGGGGAGCAA
ACAGGATTAGAAACCCTCGTAGTCC
>Otu354
CCAGCCTATGGGGGGCAGCAGCTAAGAATATTCCGCAATGGACGAAAGTCTGACGGAGCG
ACGCCGCGTGGATGACGAAGGCCGAAAGGTTGTAAAATCCTTTTGTTGGGGAAGAATAAC
CATGGGAGGGAATGCCCGTGGGATGACATGAACCGACGAATAAGTCCCGGCTAACTACGT
GCCAGCAGCCGCGGTAACACGTAGGGGACAAGCGTTATTCGGAATTACTGGGCGTAAAGG
GCATGTAGGCGGCTTTGTAAGCCTGGCGTGAAAGTCACCGGCTCAACCGGTGGATTGCGT
CGGGAACTGCAGAGCTTGAGTCATGGAGAGGAAGGCAGAATTCCTGGTGTAGGGGTGAAA
TCTGTAGAGATCAGGAAGAATACCAGTGGCGAAGGCGGCCTTCTGGCCAATGACTGACGC
TGAGGTGCGAAAGTGTGGGGATCAAACAGGATTAGATACCCCAGTAGTCC
>Otu355
CCAGCCTACGGGGTGCAGCAGTAGGGAATATTGGGCAATGGGCGAAAGCCTGACCCAGCA
ACGCCGCGTGTGTGATGAAGGCCTTCGGGTCGTAAAACACTTTTCTGAGGGATGAGCAAG
GACAGTACCTCAGGAATAAGTCTCGGCTAACTACGTGCCAGCAGCCGCGGTAAAACGTAG
GAGACGAGCGTTATCCGGATTTACTGGGCGTAAAGCGTGTGCAGGCGGTTCGGCAAGTTG
GATGTGAAATCTCCCGGCTCAACTGGGAGGGGTCGTTCAAAACTACCGAACTTGAGGGCA
TCAGAGGAAGGTGGAATTCCGGGTGTAGTGGTGAAATGCGTAGATATCCGGAGGAACACC
AGTGGCGAAGGCGGCCTTCTGGGATGCTCCTGACGCTCATACACGAAAGCTAGGGGAGCG
AACGGGATTAGAAACCCCAGTAGTCC
>Otu356
CCAGCCTACGGGGGGCAGCAGTAGGGAATATTGCACAATGGGCGAAAGCCTGATGCAGCA
ACGCCGCGTGCGCGAAGAAGGCCTTCGGGTCGTAAAGCGCTTTTCGGGGAGATGAGGAAG
GACAGTATCCCCGGAATAAGGCTCGGCTAACTACGTGCCAGCAGCCGCGGTAACACGTAG
GAGCCAAGCGTTATCCGAATTCACTGGGCGTAAAGCGCGTGTAGGCGGCTTGATAAGTTG
GATGTGAAAGCTCCCGGCTCAACTGGGAGAGGACGTTCAATACTGTCAGGCTAGAGGACG
GCAGAGGGAGGTGGAATTCCCGGTGTAGTGGTGAAATGCGTAGATATCGGGAGGAACACC
AGTGGCGAAGGCGGCCTTCTGGGCCGTTTCTGACGCTCAGACGCGAAAGCTAGGGGAGCA
AACGGGATTAGAAACCCGCGTAGTCC
>Otu357
CCAGCCTACGGGTCGCTGCAGTAACGAATCTTCCGCAATGCACGCAAGTGTGACGGAGCG
ACGCCGCGTGTGGGACGAAGCCCTTCGGGGTGTAAACCACTGTCAGAGGATAGAAAGTTC
TGATCGTCCTCAGAGGAAGGCACGGCTAACTCTGTGCCAGCAGCCGCGGTAAGACAGAGG
TGCCGAGCGTTAGGCGGAATCACTGGGCTTAAAGCGTGTGTAGGCGGATCGTTAAGTGTC
TTGTGAAATCCCATGGCTTAACCATGGAACTGCTGGACATACTGGCGATCTTGGGTCACC
TAGGGGCAGCCGGAACAAATGGTGGAGCGGTAAAATGCGTTGATATCATTTGGAACGCCG
ATGGCGAAAGCAAGCTGCTGGGGGTGCACCGACGCTGAGACACGAAAGCCAGGGGAGCAA
ACGGGATTAGATACCCGCGTAGTCC
>Otu358
CCAGCCTACGGGGGGCAGCAGTGAGGAATTTTGCGCAATGGCCGCAAGGCTGACGCAGCA
ACGCCGCGTGGGTGAAGAAGGCCTTCGGGTCGTAAAGCCCTGTCAGGTGGGAAGAACGAC
CGGGGGACTAATAGTTTCCCGGATTGACGGTACCACCAAAGGAAGCACCGGCTAACTCCG
TGCCAGCAGCCGCGGTAATACGGAGGGTGCGAGCGTTATTCGGAATTACTGGGCGTAAAG
CGCGTGCAGGCGGGCCGGCAAGTCTGATGTGAAAGCCCCGGGCTTAACCTGGGAAGTGCA
TTGGAAACTGCTGGTCTTGAGTTCTGGAGAGGAAGGGGGAATTCCCGGTGTAGAGGTGAA
ATTCGTAGAGATCGGGAGGAATACCAGTGGCGAAGGCGCCCTTCTGGACGGCAACTGACG
CTGAGACGCGAAAGCGTGGGGAGCAAACAGGATTAGAAACCCCGGTAGTCC
>Otu359
CCAGCCTACGGGGGGCAGCAGTAAGGAATATTGGTCAATGGAGGCAACTCTGAACCAGCC
ATGCCGCGTGCAGGAAGACGGCCCTATGGGTTGTAAACTGCTTTTGTACCAGAGAAAACC
CGAGTACGTGTACTCGGTTGATAGTACGGTAAGAATAAGCATCGGCTAACTTCGTGCCAG

CAGCCGCGGTAAGACGAAGGATGCAAGCGTTATCCGGATTCATTGGGTTTAAAGGGAGCG
TAGGTGGATTTGTAAGTCAGTGGTGAAATCTCTCAGCTTAACTGAGAAACTGCCATTGAT
ACTGCAGATCTTGAGTACAGATGATGTGGGCGGAATATGACATGTAGTGGTGAAATACTT
AGATATGTCATAGAACACCGATTGCGAAGGCAGCTCACAAAACTGTAACTGACACTGAGG
CTCGAAAGTGCGGGGATCAAACAGGATTAGATACCCCAGTAGTCC
>Otu360
CCAGCCTACGGGGGGCAGCAGCTAAGAATATTCCGCAATGGGCGAAAGCCTGACGGAGCG
ACGCCGCGTGGACGAAGAAGGCCGAAAGGTTGTAAAGTCCTTTTGCTGGGGAAGAATAAG
GGCGGGAGGGAATGCCCGTCTGATGACGTGAACCGGTGAATAAGCCCCGGCTAACTACGT
GCCAGCAGCCGCGGTAACACGTAGGGGGCGAGCGTTGTTCGGAATTATTGGGCGTAAAGG
GCATGTAGGCGGTTACGTAAGCCCGGCGTAAAATACCTCAGCTTAACTGAGGGGCTGCGT
TGGGAACCGCGTGACTTGAGTTACCGAGAGGAAACCAGAATTCCAGGTGTAGGGGTGAAA
TCTGTAGATATCTGGAAGAATACCAGTGGCGAAGGCGGGTTTCTAGCGGATGACTGACGC
TGAGGTGCGAAAGCGTGGGGAGCAAACAGGATTAGAAACCCCTGTAGTCC
>Otu362
CCAGCCTATGGGGGGCACCAGGCGCGAAAACTTTGCAATGCGAGAAATCGTGACAAGGGA
ACTCCGAGTGCCTGTAAAATCAGGCTGTCCGCCAGTTTAAACAACTGGTGAAGAAAGGGC
CGGGCAAGACCGGTGCCAGCCGCCGCGGTAATACCGGCGGCTCGAGTGGTGGCCACTATT
ACTGGGCTTAAAGCGTTCGTAGCTGGCTTGTTAAGTCTCTGGGGAAATCTTCCGGCTTAA
CCGGAAGGCGTCTCAGGGATACTGGCAGGCTAGGGACCGGAAGAGGTGAGAGGTACTTCA
GGGGTAGGAGTGAAATCCTGTAATCCTTGAGGGACCACCTGTGGCGAAGGCGTCTCACCA
GGACGGCTCCGACAGTGAGGAACGAAAGCTGGGGGAGCAAACCGGATTAGAAACCCTTGT
AGTCC
>Otu364
CCAGCCTATGGGTGGCAGCAGCTAAGAATCTTCCGCAATGGGGGAAACCCTGACGGAGCG
ACGCCGCGTGGATGATGAAGGCCGTAAGGTTGTAAAATCCTTTTGTCGGTGAAGAATAAT
CGAAGGAGTGGAAAGCCTTTGAGATGACGTTAGCCGACGAATAAGCCCCGGCTAATTACG
TGCCAGCAGCCGCGGTAATACGTATGGGGCAAGCGTTGTTCGGATTTATTGGGCGTAAAG
GGCGTGTAGGCGGCTCATCAAGTCTGGTGTGAAAATGCAGGGCCTAACTCTGCAAATGCG
CTGGAAACTGGTGGGCTTGAGTTCTTGAGGGGAAGCTGGAATTCCAGGTGTAGGGGTGAA
ATCTGTAGATATCTGGAAGAACACCGGTGGCGAAGGCGAGCTTCTATCAGAGGACTGACG
CTGAGGCGCGAAAGCGTGGGGAGCAAACAGGATTAGATACCCTCGTAGTCC
>Otu365
CCAGCCTACGGGTGGCAGCAGTAACGAATCTTCCGCAATGCACGAAAGTGTGACGGAGCG
ACGCCGCGTGTGGGATGAAGTTCTTCGGAATGTAAACCACTGTCAGGGGTTAGAAAGTTC
TGATCAACCCCAGAGGAAGGCACGGCTAACTCTGTGCCAGCAGCCGCGGTAAGACAGAGG
TGCCAAGCGTTAGGCGGAATCACTGGGCTTAAAGCGTGTGTAGGTGGACTGGTAAGTACT
TTGTGAAATCCCGCGGCTTAACCGTGGAACTGCTTGGTATACTGCTGGTCTTGAGCCTTC
TAGGGGTCAGTGGAACAAATGGTGGAGCGGTGAAATGCGTAGATATCATTTGGAACGCCA
ATGGTGAAAACAACTGACTGGGGAAGTGCTGACACTGAGACACGAAAGCCAGGGGAGCAA
ACGGGATTAGAAACCCTAGTAGTCC
>Otu366
CCAGCCTACGGGTCGCACCAGTGGGGAATATTGGACAATGGGGGAAACCCTGATCCAGCA
ATGCCGCGTGAGTGATGAAGGCCTTCGGGTTGTAAAGCTCTTTTGGCAGGGACGATGATG
ACGGTACCTGCAGAATAAGCCCCGGCTAACTTCGTGCCAGCAGCCGCGGTAATACGAAGG
GGGCTAGCGTTGTTCGGAATTACTGGGCGTAAAGCGCACGCAGGCGGCTCGCTAAATTAG
AAGTGAAAGCCCTGGGCTCAACCCGGGAATTGCTTTTAAGACTGGCGAGCTAGAATCCAG
AAGAGGGTAGCGGAATTCCCAGTGTAGAGGTGAAATTCGTAGATATTGGGAAGAACACCG
GTGGCGAAGGCGGCTACCTGGTCTGGTATTGACGCTCAGGTGCGAAAGCGTGGGGAGCAA
ACAGGATTAGATACCCTTGTAGTCC
>Otu367
CCAGCCTACGGGAGGCAGCAGTAGGGAATCTTGCGCAATGGGCGAAAGCCTGACGCAGCA
ACGCCGCGTGGGGGATGAAGGCCTTCGGGTTGTAAACTCCTTTTAGTGGGAACGAAATTG
ACGGTACCCACAGAAAAAGCCCCGGCTAACTACGTGCCAGCAGCCGCGGTGATACGTAGG
GGGCGAGCGTTGTCCGGATTTACTGGGCGTAAAGAGCTCGTAGGCGGTTTAGTAAGTCGG
GTGTGAAACCTCTAGGCTTAACCTAGAGACGCCACTCGAAACTACTATGACTAGAGTCTG
GTAGGGGACCATGGAATTCCTGGTGTAGCGGTGAAATGCGCAGATATCAGGAGGAACACC
AGTAGCGAAGGCGGTGGTCTGGGCCAGTACTGACGCTGAGGAGCGAAAGCGTGGGGAGCG

AACAGGATTAGAAACCCCCGTAGTCC
>Otu368
CCAGCCTACGGGTGGCAGCAGTAAGGAATATTGGTCAATGGAGGCAACTCTGAACCAGCC
ATGCCGCGTGAACGATGAAGGCGCTATGCGTCGTAAAGTTCTTTTGTACGAGAAAAAACC
TATCTACGTGTAGATAGCTGATGGTATCGTAAGAATAAGGATCGGCTAACTCCGTGCCAG
CAGCCGCGGTAATACGGAGGATCCAAGCGTTATCCGGATTCATTGGGTTTAAAGGGTGCG
TAGGCGGCTTATTAAGTCAGCGGTGAAATCCTGGGGCTTAACCCCAGAACTGCCATTGAT
ACTGATGAGCTTGAGTACACTTGAAGTGGGCGGAATGTGTCGTGTAGCGGTGAAATGCTT
AGATATGACACAGAACACCGATTGCGAAGGCAGCTCACTAAATTGTCACTGACGCTGAGG
CACGAAAGCGTGGGGATCAAACAGGATTAGATACCCCAGTAGTCC
>Otu369
CCAGCCTACGGGGGGCTCCAGTGGGGAATTTTGGACAATGGGGGCAACCCTGATCCAGCA
ATGCCGCGTGTGTGAAGAAGGCCTTCGGGTTGTAAAGCACTTTTGTCCGGAAAGAAATCA
TCCTGGTTAATACCTGGGGTGGATGACGGTACCGGAAGAATAAGCACCGGCTAACTACGT
GCCAGCAGCCGCGGTAATACGTAGGGTGCAAGCGTTAATCGGAATTACTGGGCGTAAAGC
GTGCGCAGGCGGTTCGCTAAGACAGATGTGAAATCCCCGGGCTTAACCTGGGAACTGCAT
TTGTGACTGGCGGGCTAGAGTATGGCAGAGGGGGGTAGAATTCCACGTGTAGCAGTGAAA
TGCGTAGAGATGTGGAGGAATACCGATGGCGAAGGCAGCCCCCTGGGCCAATACTGACGC
TCATGCACGAAAGCGTGGGGAGCAAACAGGATTAGAAACCCCAGTAGTCC
>Otu370
CCAGCCTACGGGGCGCAGCAGTAAGGGATATTGCGCAATGGGCGAAAGCCTGACGCAGCA
ACGCCGCGTGGAGGATGACGGCCTTCGGGTTGTAAACTCCTTTTGGCCCGGACGAGGAAG
GACGGTACGGGTTGAAGAAGTCACGGCTAACTACGTGCCAGCAGCCGCGGTAAAACGTAG
GTGGCAAGCGTTATCCGGATTTACTGGGCGTAAAGCGTGTGCAGGCGGTGGTTCAAGTGG
TGTATGAAATCGCCCGGCTTAACCGGGCGGGGTTATGCCAGACTGGGCCACTCGAGTGCG
GGAGAGGGGCGTGGAATTCCGGGTGTAGTGGTGAAATGCGTAGAGATCCGGAGGAACCCC
AGAGGCGAAGGCGGCGCCCTGGCCCGACACTGACGCTCAGACACGACAGCATGGGGAGCG
AACGGGATTAGAAACCCTCGTAGTCC
>Otu371
CCAGCCTACGGGTTGCAGCAGTGGGGAATATTGCACAATGGGCGAAAGCCTGATGCAGCA
ACGCCGCGTGAGTGATGAAGGCCTTCGGGTTGTAAAGCTCTGTCTTTGGGGACGATAATG
ACGGTACCCAAGGAGGAAGCCACGGCTAACTACGTGCCAGCAGCCGCGGTAATACGTAGG
TGGCGAGCGTTATCCGGAATTATTGGGCGTAAAGAATGCGTAGGTGGACTTTTAAGTCAG
ATGTGAAATCCCCGGGCTTAACCCGGGAGCTGCATTTGAAACTGGAAGCCTGGAGTGCAG
GAGAGGTAAGTGGAATTCCAAGTGTAGCGGTGAAATGCGTAGATATTTGGAAGAACATCG
GTGGCGAAGGCGACTTACTGGACTGTTACTGACACTGAGGCATGAAAGCGTGGGGAGCAA
ACAGGATTAGAAACCCCGGTAGTCC
>Otu372
CCAGCCTATGGGGGGCAGCAGTAGGGAATCTTGCACAATGGACGAAAGTCTGATGCAGCG
ACGCCGCGTGAGTGATGAAGGCCTTCGGGTTGTAAAGCTCTGTTCTCAGGGAATAAAAAA
ATGAAGGTACCTGAGGAGAAAGGACCGGCTAACTTCGTGCCAGCAGCCGCGGTAATACGA
GGGGTCCAAGCGTTGCTCGGAATCATTGGGCGTAAAGCGGGTGTAGGTGGCTTTGTAAGT
CGGGAGTGAAAGCCCTGGGCTTAACCCAGGAAGTGCTTTCGATACTGCGAAGCTTGAGTG
TGGGAGAGGATCGTGGAATTCCAGGTGTAGTGGTGAAATACGTAGATATCTGGAGGAACA
CCGGTGGCGAAGGCGGCGATCTGGCCCAACACTGACACTCAGACCCGAAAGTGCGGGGAT
CAAACAGGATTAGAAACCCCCGTAGTCC
>Otu373
CCAGCCTATGGGGCGCTGCAGTCGAGAATATTCCACAATGGACGAAAGTCTGATGGAGCG
ACGCCGCGTGCAGGATGAAGGTATTCGTATCGTAAACTGCTTTTTTCAGGGATGAATTTC
GATGACAGTACCTGGAGAATAAGAGGTTACTAACTCTGTGCCAGCAGTAGCGGTAATACA
GAGACCTCAAGCGTTATCCGGATTTATTGGGCGTAAAGAGCTGGTAGGTGGTCATATTAG
TCAGATGTCAAATCTTCGAGCTCAACTCGAAAACTGCATTTGAAACGGTATGACTAGAGG
GTGTGAGAGATCACTGGAACTCATGGTGTAGCAGTGAAATGCGTTGATATCATGAGGAAC
ACCAAAGGCGAAGGCATGTGATTGGCACACTCCTGACACTGAGCAGCGAAAGCGTGGGTA
GCGAATGGGATTAGATACCCCAGTAGTCC
>Otu374
CCAGCCTATGGGATGCACCAGTGAGGAATATTGGTCAATGGACGCAAGTCTGAACCAGCC
ATGCCGCGTGAAGGATGAAGGCCTTCTGGGTTGTAAACTTCTTTTACCTGGGAAGAAACC

ACTGTTTTCTAACAGTGTTGACGGTACCAGAGGAATAAGCACCGGCTAACTCCGTGCCAG
CAGCCGCGGTAATACGGAGGGTGCAAGCGTTATCCGGATTTACTGGGTTTAAAGGGTGTG
TAGGCGGGCTTTTAAGTCAGTGGTGAAATCTCCGGGCTCAACCTGGAAACTGCCATTGAT
ACTATTAGTCTTGAATTTTGTTGAGGTAGGCGGAATAAGTCATGTAGCGGTGAAATGCAT
AGATATGACTTAGAACACCAATTGCGAAGGCAGCTTGCTAAACAAATATTGACGCTGAGG
CACGAAAGCGTGGGGATCAAACAGGATTAGATACCCCAGTAGTCC
>Otu375
CCAGCCTATGGGGGGCAGCAGTGGGGAATTTTGGACAATGGGGGCAACCCTGATCCAGCA
ATGCCGCGTGAGTGAAGAAGGCCTTCGGGTTGTAAAGCTCTTTTGTCAGGGAAGAAACGG
TGGTCTCTAATATAGGCTGCTAATGACGGTACCTGAAGAATAAGCACCGGCTAACTACGT
GCCAGCAGCCGCGGTAATACGTAGGGTGCAAGCGTTAATCGGAATTACTGGGCGTAAAGC
GTGCGCAGGCGGTTATATAAGTCAGATGTGAAATCCCCGGGCTCAACCTGGGAACTGCAT
TTGAGACTGTATGGCTAGAGTGTGTCAGAGGGGGGTAGAATTCCACGTGTAGCAGTGAAA
TGCGTAGATATGTGGAGGAATACCGATGGCGAAGGCAGCCCCCTGGGATAACACTGACGC
TCATGCACGAAAGCGTGGGGAGCAAACAGGATTAGAAACCCGAGTAGTCC
>Otu376
CCAGCCTATGGGGCGCTGCAGTGGGGAATTTTGGACAATGGGGGCAACCCTGATCCAGCC
ATGCCGCGTGAGTGAAGAAGGCCTTCGGGTTGTAAAGCTCTTTCAGCCGGGAAGAAATCG
CATCAGTTAATACCTGGTGTGGATGACGGTACCGGAATAAGAAGCACCGGCTAACTACGT
GCCAGCAGCCGCGGTAATACGTAGGGTGCGAGCGTTAATCGGAATTACTGGGCGTAAAGC
GTGCGCAGGCGGTTTGTTAAGATAGGCGTGAAATCCCCGGGCTCAACCTGGGAACTGCGT
TTATGACTGGCAGGCTAGAGTATGGCAGAGGGGGGTGGAATTCCACGTGTAGCAGTGAAA
TGCGTAGAGATGTGGAGGAACACCGATGGCGAAGGCAGCCCCCTGGGCCAATACTGACGC
TCATGCACGAAAGCGTGGGTAGCAAACAGGATTAGATACCCCTGTAGTCC
>Otu377
CCAGCCTACGGGGGGCTGCAGTCGAGAATCTTCGGCAATGGGCGCAAGCCTGACCGAGCG
ACGCCGCGTGGGGGATGAAGGATTTCGGTTCGTAAACCTCTTTTGGCAGGGAAGAAAAAG
TTGCATCTAACAGATGCAAAACTGACGGTACCTGCAGAATAAGCCACGGCTAACTCCGTG
CCAGCAGCCGCGGTAATACGGAGGCCCCGAGCGTTGTTCGGAATTACTGGGCTTAAAGCG
CGTGTAGGCGGGCTGGTAAGTGTCTTGTGAAATCCCCGGGCTCGACCCGGGAATTGCTCG
GCATACTGCCGGTCTTGAGGCAGGTATGGGTCACTGGAACCTTAGGTGTAGCGGTGAAAT
GCGTAGATATCTAAGGGAACGCCGGTGGCGAAAGCGGGTGACTGGGCCTGTCCTGACGCT
GAGACGCGAAAGCGTAGGGAGCAAACGGGATTAGATACCCCCGTAGTCC
>Otu378
CCAGCCTACGGGGTGCTGCAGTCGAGGATCTTTGGCAATGAGCGCAAGCTTGACCAAGCG
ACGCCGCGTGTGCGATGAAGGCCTTCGGGTTGTAAAGCACTGTCGAGGGGGACGAAAGCC
GAAAGGTTTGACCGATCCCTGGAGGAAGCACGGGCTAAGTTCGTGCCAGCAGCCGCGGTA
AGACGAACCGTGCGAACGTTGTTCGGAATCACTGGGCTTAAAGGGCGCGTAGGCGGCCCA
TCAAGTCAGGGGTGAAATCCTCCAGCTTAACTGGAGAAGTGCCTTTGATACTGGTGGGCT
CGAGGGAGGTAGGGGCATGTGGAACTTCTGGTGGAGCGGTGAAATGCGTTGATATCAGAA
GGAACGCCGGTGGCGAAAGCGACGTGCTGGACCTCTTCTGACGCTGAGGCGCGAAAGCTA
GGGGAGCAAACGGGATTAGAAACCCCAGTAGTCC
>Otu379
CCAGCCTATGGGGGGCAGCAGTGGGGAATTTTGGACAATGGGCGCAAGCCTGATCCAGCA
ATGCCGCGTGCAGGATGAAGGCCTTCGGGTTGTAAACTGCTTTTGTACGGAACGAAACGG
CGAGCTCTAATACAGTTTGCTAATGACGGTACCGTAAGAATAAGCACCGGCTAACTACGT
GCCAGCAGCCGCGGTAATACGTAGGGTGCAAGCGTTAATCGGAATTACTGGGCGTAAAGC
GTGCGCAGGCGGTTATATAAGACAGATGTGAAATCCCCGGGCTCAACCTGGGACCTGCAT
TTGTGACTGTATAGCTAGAGTACGGCAGAGGGGGATGGAATTCCGCGTGTAGCAGTGAAA
TGCGTAGATATGCGGAGGAACACCGATGGCGAAGGCAATCCCCTGGGCCTGTACTGACGC
TCATGCACGAAAGCGTGGGGAGCAAACAGGATTAGATACCCCCGTAGTCC
>Otu380
CCAGCCTACGGGGTGCTGCAGGCGCGAAACCTTTACAATGCACGAAAGTGTGATAGGGGG
ATACTCAGTGCTTACGATTATGTCGTAGGCTTTTGTCGATCGTAAATAGATTGGTGAATA
AGTGGTGGGTAAGACCGGTGCCAGCCGCCGCGGTAACCCCGGCGCCACGAGTGGTCATCA
CGATTATTGGGCCTAAAGCGTTCGTAGCCGGTTTGGTAAATCTCTTGTGAAATTGTTCGG
CTTAACCGGACAGCGTGCAGGAGACACTACCAGACTCGAGACCGGGAGGCGCAAGAAGTA
TGGCATGGGGACTGGTAAAATGGGATAATCCATGCTAGACTACCGATGGCGAAGGCATCT

TGCGAGAACGGATCTGACGGTGAGGAACGAAAGCTAGGGGAGCGAACCGGATTAGAAACC
CTAGTAGTCC
>Otu381
CCAGCCTATGGGTTGCTGCAGTCGAGAATTTTTCACAATGGGCGCAAGCCTGATGGAGCG
ACGCCGCGTGGGGGATGAATGGCTTCGGCCCGTAAACCCCTGTCATTTGCGATCAACCGT
TATTGTTTAAGAGATGATAACCTGATAGTAGCGAAAGAGGAAGGGACGGCTAACTCTGTG
CCAGCAGCCGCGGTAATACAGAGGTCCCAAGCGTTGTTCGGATTCACTGGGCGTAAAGGG
TGCGTAGGTGGCGAGGTAAGTCGGATGTGAAAGCTCGGAGCTCAACTCCGAAATGGCATT
GGAAACTACCTTGCTCGAGGATTGGAGGGGGGACTGGAATACTTGGTGTAGCAGTGAAAT
GCGTAGATATCAAGTGGAACACCAGTGGCGAAGGCGAGTCCCTGGACAACTCCTGACACT
GAGGCACGAAAGCTAGGGGAGCAAACAGGATTAGATACCCGAGTAGTCC
>Otu382
CCAGCCTATGGGTGGCAGCAGTGGGGAATCTTGCGCAATGGGCGAAAGCCTGACGCAGCG
ACGCCGCGTGAGCGATGAAGGCCTTCGGGTTGTAAAGCTCTGTGGGGAGGGAAGAATAAG
GTTTGGCTAATATCCAAACTGATGACGGTACCTCCTTAGCAAGCACCGGCTAACTCTGTG
CCAGCAGCCGCGGTAAGACAGAGGGTGCAAACGTTGTTCGGAATTACTGGGCGTAAAGCG
CGTGTAGGCGGCTAAGTAAGTCGGGCGTGAAATCCCACGGCTCAACCGTGGAAGTGCACT
CGAAACTGCATAGCTAGAGTCCTGGAGAGGAAGGTGGAATGCTTGGTGTAGAGGTGAAAT
TCGTAGATATCAAGCGGAACACCGGTGGCGAAGGCGGCCTTCTGGACAGTGACTGACGCT
GAGACGCGAAAGCGTGGGGAGCAAACAGGATTAGAAACCCCCGTAGTCC
>Otu383
CCAGCCTATGGGAGGCAGCAGTCGAGAATTTTTCACAATGGGGGAAACCCTGATGGAGCG
ACGCCGCGTGGGGGATGAATGGCTTCGGCCCGTAAACCCCTGTCATTTGTGACTAAACCT
TGCCATTTAACAGACGGCAAGCTGATTGTAGCAGAAGAGGAAGGGACGGCTAACTCTGTG
CCAGCAGCCGCGGTAATACAGAGGTCCCAAGCGTTGTTCGGATTCACTGGGCGTAAAGGG
TGCGTAGGTGGTTGGATAAGTTTGATGTGAAATCTCGGAGCTTAACTCCGAAACTGCATT
GAATACTATCCGGCTTGAGGGTCGGAGGGGGGACTGGAATTCTCGGTGTAGCAGTGAAAT
GCGTAGATATCGAGAGGAACACCAGTGGCGAAGGCGAGTCCCTGGACGACTCCTGACACT
GAGGCACGAAAGCTAGGGGAGCAAACAGGATTAGATACCCGAGTAGTCC
>Otu384
CCAGCCTATGGGGGGCTGCAGTGGGGAATATTGGACAATGGGGGCAACCCTGATCCAGCA
ATGCCGCGTGTGTGAAGAAGGTCTTCGGATTGTAAAGCACTTTTGGCAGGGACGATGATG
ACGGTACCTGCAGAATAAGCCCCGGCTAACTTCGTGCCAGCAGCCGCGGTAATACGAAGG
GGGCTAGCGTTGCTCGGAATGACTGGGCGTAAAGGGCGCGTAGGCGGTTTATACAGTCAG
ATGTGAAATTCCTGGGCTCAACCTGGGGACTGCATTTGAGACGTGTAGACTTGAGTGGAG
AAGAGGGTTGTGGAATTTCCAGTGTAGAGGTGAAATTCGTAGATATTGGAAAGAACACCG
GTGGCGAAGGCGGCAACCTGGTCTTCAACTGACGCTGAGGCGCGAAAGCGTGGGGAGCAA
ACAGGATTAGATACCCCTGTAGTCC
>Otu385
CCAGCCTACGGGTGGCTCCAGTAACGAATCTTCCGCAATGCACGAAAGTGTGACGGAGCG
ACGCCGCGTGGAGGACGAAGTTCTTCGGAATGTAAACTTCTGTCAGGGGATACCAAGTTC
TGAGGATCCCCAGAGGAAGGGGCGACTAACTCTGTGCCAGCAGTCGCGGTAAGACAGAGG
CCCCGAGCGTTAGGCGGAATCACTGGGCTTAAAGCGCGTGTAGGCGGAGGGCTAAGTACC
TTGTGAAATCCCCGGGCTCAACCCGGGAACTGCTGGGTATACTGGTCTTCTTGAGGCATC
TAGGGGCGAGCGGAACAAACGGTGGAGCGGTGAAATGCGTAGATATCGTTTGGAACGCCA
ATGGTGAAAACAGCTCGCTGGGGATGTCCTGACGCTGAGACGCGAAAGCCAGGGGAGCAA
ACGGGATTAGATACCCCTGTAGTCC
>Otu386
CCAGCCTATGGGTTGCTCCAGTCGAGAATTTTTCTCAATGGGGGCAACCCTGAAGGAGCG
ACGCCGCGTGGAGGATGAAGGTCTTCGGATTGTAAACTCCTGTCATTGGTGAACAAAGCC
GCGCGTTAACTGCGATGCGGTTTGATAGTAGCCGAAGAGGAAGAGACGGCTAACTCTGTG
CCAGCAGCCGCGGTAATACAGAGGTCTCAAGCGTTGTTCGGATTCATTGGGCGTAAAGGG
TGCGTAGGCGGCGGGGCAAGTCTGATGTGAAATCCCGGGGCTCAACCCTGGAACTGCATT
GGATACTGCCTTGCTAGAGTACTGGAGAGGAAACTGGAATTTACGGTGTAGCAGTGAAAT
GCGTAGAGATCGTAAGGAAGGCCAGTGGCGAAGGCGAGTTTCTGGACAGTTACTGACGCT
GAGGCACGAAGGCCAGGGGAGCAAACGGGATTAGATACCCGAGTAGTCC
>Otu387
CCAGCCTATGGGGGGCAGCAGTGAGGAATATTGCGCAATGCCCGAAAGGGTGACGCAGCG

ACGCCGCGTGGAGGATGAAGGCCCTACGGGTCGTAAACTCCTTTTATGAGGGAATAATTT
CTCGCTATAGGCGAGATTGAATGTACCTCAAGAAAAAGCATCGGCTAACTACGTGCCAGC
AGCCGCGGTAATACGTAGGATGCTAGCGTTGTCCGGATTTACTGGGTGTAAAGGGAGCGC
AGGCGGGTTGGTAAGTCGGTGGTGAAATTTTCGGGCTTAACCCGAAAACTGCCATCGATA
CTGCTAATCTTGAGTGCAGAAGAGGTTGATGGAATTCTAGGTGTAGCGGTGAAATGCGTA
GATATCTAGAAGAACACCGATGGCGAAGGCAGTCAACTGGTCTGCTACTGACGCTCATGC
TCGAAAGCGTGGGGATCAAACAGGATTAGAAACCCGCGTAGTCC
>Otu388
CCAGCCTACGGGTGGCTGCAGTGAGGAATATTGCTCAATGGGCGAAAGCCTGAAGCAGCG
ACGCCGCGTGAAGGATGAAATCCGTTAGGATGTAAACTTCTTTTGCGGGGGATGAATGTT
CCGGTTTCCGGAATTGACCGTACCCCGTGAATAAGCACCGGCCAACTACGTGCCAGCAGC
CGCGGTAATACGTAGGGTGCAAGCGTTGTCCGGAATTACTGGGTGTAAAGGGTCCGCAGG
CGGGCGAACAAGTCGGTGGTGAAATCTTGTGGCTCAACCACAAAACTGCCTCCGATACTG
TTTGTCTTGAGTTCGAGAGAGGATGATGGAATTCATGGTGTAGCGGTGAAATGCGTAGAG
ATCATGAAGAACACCGGTAGCGAAGGCGGTCATCTGGCTCGATACTGACGCTCAGGGACG
AAAGCGTGGGTAGCAAACAGGATTAGATACCCCAGTAGTCC
>Otu389
CCAGCCTACGGGAGGCAGCAGTAACGAATCTTCCACAATGCACGAAAGTGTGATGGAGCG
ACGCCGCGTGTAGGATGAAGTCCTTCGGGATGTAAACTACTGTCAGGGGAAAGAAAGTTC
TGATCTACCCCAGAGGAAGGCACGGCTAACTCTGTGCCAGCAGCCGCGGTAAGACAGAGG
TGCCAAGCGTTAGGCGGAATCACTGGGCTTAAAGCGTGTGTAGGCGGATTGACAAGTACC
TTGTGAAATCCCACGGCTCAACCGTGGAACTGCTTGGTATACTGTCGATCTTGAGGCATC
TAGGGGCAACTGGAACAAACGGTGGAGCGGTGAAATGCGTAGATATCGTTTGGAACGCCA
ATGGTGAAGACAGGTTGCTGGGGATGTACTGACGCTGAGACACGAAAGCCAGGGGAGCAA
ACGGGATTAGAGACCCCAGTAGTCC
>Otu390
CCAGCCTATGGGGGGCACCAGTCGAGAATATTCCACAATGGACGAAAGTCTGATGGAGCG
ACACCGCGTGCAGGATGAAGGCCTTCGGGTCGTAAACTGCGGTAGTATGGTAAGAATGCA
AATGACTGTCATACGGAAAGAGGTGGGTAACTACGTGCCAGCACCAGCGGTAATACGTAG
ACCTCAAGCGTTATCCGGATTTATTGGGCGTAAAGCGCACGTAGGAGGTTTTGCGCGTCT
TTTGTTAAAGCCCAGGGCCTAACCCTGGAGATGCAGGAGATACGGCAGAACTAGAGGAGG
TTAGAGGTGCATGGAACTCACGGTGTAGGGGTGAAATCCGTTGATATCGTGGGGAACACC
AAAGGCGAAGGCAGTGCACTGGGACCTTCCTGACTCTGAGTTGCGAAAGCGTGGGGAGCA
AAAAGGATTAGAAACCCGTGTAGTCC
>Otu391
CCAGCCTATGGGGGGCAGCAGTGAGGAATATTGGGCAATGCCCGCAAGGGTGACCCAGCA
ACGCCGCGTGAAGGATGAAAGCCGTAAGGTTGTAAACTTCTGTTAGAGGGGACGAATAGC
TTCGATCAAATCGAAGTTTGACGGTACCCTCAGAGAAAGCCCCGGCTAACTACGTGCCAG
CAGCCGCGGTAATACGTAGGGGGCAAGCGTTGTCCGGATTTACTGGGTGTAAAGGGCGCG
TAGGCGGCTATATAAGTCAGTGGTGAAATCCTGCGGCTTAACTGCAGAACTGCCATTGAT
ACTGTATAGCTTGAGTGCGGAAGAGAGAGACGGAATTCCAGGTGTAGTGGTGAAATACGT
AGATATCTGGAAGAACACCAGTTGCGAAGGCGGTCTCTTGGTCCGTTACTGACGCTGAGG
CGCGAAAGCGTGGGTAGCAAACAGGATTAGATACCCCAGTAGTCC
>Otu392
CCAGCCTACGGGGTGCAGCAGGCGCGAAACCTTTACAATGCGCGAAAGCGTGATAAGGGA
ACTCTGAGTGGCAGCCGGCTTTTGCCGACTGTCTTTTGGCAACCCCAAACCGGTTGCAGA
ATAAGTTCTGGGTAAGACCAGTGCCAGCCGCCATGTGCTCACGCACAATGCGCACGGTAC
GCGGTAATACTGGCAGAACGAGTGGTGTCCACGAATATTGGGTCTAAAGAGACCGTAGCT
GGCCTGTTAAGTCCACTGTGAAATCTTGGGGCTCAACCTCAAGGCGTGCAGTGGATACTG
GTAGGCTTGAGAGCGGGGGAGGTCAGGAGTACTTACGGGGTAAGGGTAAAATCTGTTGAT
CCTGTAAGGACTACCAGTGGCGAAGGCGCCTGACCAAAACGCGTCTGACAGTGAGGGTCG
AAGGCTAGGAGAACGAATCGGATTAGATACCCTAGTAGTCC
>Otu393
CCAGCCTACGGGGGGCAGCAGTGGGGAATATTGGACAATGGGCGAAAGCTTGATCCAGCG
ATATCACATGAAAGAAGAAGGCTTTATGTCGTAACTTTCTTACGTTAAGGAGGATAATGA
CGTGTACTTAAAAGGGAGTCCCGGCTAACCTCGTGCCAGCAGCCGCGGTAATACGGGGGG
GACGAGTGTTACTCATAATGACTGGGCGTAAAGGGTATGTAGATGGTTTAATTAGTTAAG
TGCTAAATAATAAATTAAAAGTTTATATAATGTACGTAAAACGTTTAAACTAGAGTATAT

TAGAGGAATATGGAATTTTTAATGTAAGGGTAAAATTTGTCAATAATAAAAAGACGACCG
AAGGCGAAGGCAATATTCTGGGATATTACTGACATTGAGGTACTGAAGCATGGGGATCAA
ATCGGATTAGAAACCCTCGTAGTCC
>Otu394
CCAGCCTATGGGGGGCAGCAGTAGGGAATATTGCATAATGGGCGAAAGCCTGATGCAGCA
ACGCCGCGTGTGCGATGAAGGCCTTCGGGTTGTAAAGCACTTTTCGAGGGGATGAGGAAG
GACAGTACCCTCGGAATAAGCCTCGGCTAACTACGTGCCAGCAGCCGCGGTAAAACGTAG
GAGGCGAGCGTTATCCGGATTTACTGGGCGTAAAGCGTGTGCAGGCGGTTCGGCAAGTTG
GATGTGAAAGCTCCCGGCTTAACTGGGAGAGGTCGTTCAAAACTACCGGACTTGAGGATG
GGAGAGGGAGGTGGAATTCCGGGTGTAGTGGTGAAATGCGTAGATATCCGGAGGAACACC
AGTGGCGAAGGCGGCCTCCTGGCCCATTTCTGACGCTCATACACGAAAGCTAGGGGAGCG
AACGGGATTAGAAACCCGAGTAGTCC
>Otu395
CCAGCCTATGGGTGGCAGCAGTGGGGAATTTTGCGCAATGGGCGAAAGCCTGACGCAGCA
ACGCCGCGTGGGTGATGAAGGTCTTCGGATCGTAAAACCCTGTCGTCAGGGACGAAGGTT
GTATGTCTAATAGATGTGCAGCTTGACGGTACCTGGAGAGGAAGCCCCGGCTAACTCTGT
GCCAGCAGCCGCGGTAATACAGAGGGGGCAAGCGTTATTCGGAATTATTGGGCGTAAAGG
GCGCGTAGGCGGTGATGTAAGTGAGATGTGTAATCCCCGAGCTTAACTTGGGAACTGCAT
CTCAGACTGTGTCGCTAGAGTGCTGGAGAGGGTGGTGGAATTCCACGTGTAGCGGTGAAA
TGCGTAGAGATGTGGAGGAACACCAGTGGCGAAGGCGGCCACCTGGACAGTAACTGACGC
TGAGGCGCGAAAGTGTGGGGAGCAAACAGGATTAGATACCCTCGTAGTCC
>Otu396
CCAGCCTACGGGTGGCTGCAGTAACGAATCTTCCGCAATGCACGCAAGTGTGACGGAGCG
ACGCCGCGTGTGGGATGAAGTTCTTCGGAATGTAAACCACTGTCAGGGGTTAGAAAGTTC
TGATCAACCCCAGAGGAAGGCACGGCTAACTCTGTGCCAGCAGCCGCGGTAAGACAGAGG
TGCCAAGCGTTAGGCGGAATCACTGGGCTTAAAGCGTGTGTAGGCGGATTTCTAAGTACC
TTGTGAAATCCCACGGCTCAACCGTGGAACTGCTGGGTATACTGGTTATCTTGAGCCACT
TAGGGGTCAGCGGAACAAATGGTGGAGCGGTGAAATGCGTAGATATCATTTGGAACGCCA
ATGGTGAAAACAGCTGACTGGGAGTGTGCTGACGCTGAGACACGAAAGCTAGGGGAGCAA
ACGGGATTAGAAACCCTCGTAGTCC
>Otu397
CCAGCCTACGGGGGGCTGCAGTGAGGAATATTGGTCAATAGGGGGAACCCTGAACCAGCC
ATGCCGCGTGAAGGAAGAATGCCCTAAGGGTTGTAAACTTCTTTTGTATGGGAGTAAAGA
CAGGTACGTGTACCTGGAAGAAAGTACTGTACGAATAAGCATCGGCTAACTCCGTGCCAG
CAGCCGCGGTAATACGGAGGATGCGAGCGTTATCCGGATTTATTGGGTTTAAAGGGTGCG
TAGGCGGGTTTGTAAGTCAGCGGTGAAAGACTGTCGCTTAACGATAGCATTGCCATTGAT
ACTGCAGATCTTGAGTACAGTTGAGGTAGGCGGAATGTGTAGTGTAGCGGTGAAATGCTT
AGATATTACACAGAACACCAATTGCGAAGGCAGCTTACTAAAGTGTAACTGACGCTGAGG
CACGAAAGCGTGGGGATCAAACAGGATTAGAAACCCGCGTAGTCC
>Otu398
CCAGCCTATGGGTGGCACCAGTGGGGAATATTGGACAATGGGGGCAACCCTGATCCAGCA
ATGCCGCGTGGGTGATGAAGGCCTTCGGGTTGTAAAGCCCTTTCGGCGGGGAAGATGATG
ACGGTACCCGCAGAAGAAGCCCCGGCTAACTTCGTGCCAGCAGCCGCGGTAATACGAAGG
GGGCTAGCGTTGTTCGGAATTACTGGGCGTAAAGGGCGCGTAGGCTGTGCTGTAAGTCAG
GCGTGAAAGGCCCGGGCTCAACCTGGGAACTGCGCTTGATACTGCGGCGCTGGAAGGCGG
GAGAGGATGGCGGAATTCCCAGTGTAGAGGTGAAATTCGTAGATATTGGGAAGAACACCG
GTGGCGAAGGCGGCCATCTGGCCCGCAATTGACGCTGAGGCGCGAAAGCGTGGGGAGCAA
ACAGGATTAGATACCCCAGTAGTCC
>Otu399
CCAGCCTACGGGTGGCAGCAGTCGAGAATATTCGACAATGGACGAAAGTCTGATCGAGCG
ACGCCGCGTGCAGGATGAAGGCCTTCGGGTCGTAAACTGCGGTAATATAGTAACAATGTA
AATGAGTGCTATATGGAAAGAGGTGGGTAACTACGTGCCAGCACCAGCGGTAAAACGTAG
ACCTCAAGCGTTATCCGGATTTATTGGGCGTAAAGCGCATGTAGGAGGTTTTGTGCGTCT
TCTGTTAAATCCCACCGCCTAACGGTGGACCTGCAGGAGATACGACATGACTAGAGGAGG
TTAGAGGTGCATGGAACTCACGGTGTAGGGGTGAAATCCGTTGATATCGTGGGGAACACC
AAAGGCGAAGGCAGTGCACTGGGACCTTCCTGACTCTGAGATGCGAAAGCGTGGGGAGCA
AAAAGGATTAGAGACCCTGGTAGTCC
>Otu400

CCAGCCTATGGGGCGCTGCAGTAACGAATCTTCCGCAATGCACGAAAGTGTGACGGAGCG
ACGCCGCGTGTGGGACGAAGTCCTTCGGGATGTAAACCACTGTCAGGGGTTAGAAAGTTC
TGATCAACCCCAGAGGAAGGCACGGCTAACTCTGTGCCAGCAGCCGCGGTAAGACAGAGG
TGCCAAGCGTTAGGCGGAATCACTGGGCTTAAAGCGTGTGTAGGTGGACTGGTAAGTACC
TTGTGAAATCCCACGGCTCAACCGTGGAACTGCTCGGTATACTGCTGGTCTTGAGCAACT
TAGGGGCGAGCGGAACAAATGGTGGAGCGGTGAAATGCGTAGATATCATTTGGAACGCCA
ATGGTGAAAACAGCTCGCTGGGAGTTTGCTGACACTGAGACACGAAAGCTAGGGGAGCAA
ACGGGATTAGATACCCCAGTAGTCC
>Otu401
CCAGCCTATGGGGCGCAGCAGTTAAGGAACTTTTACAATGCTCGAAAGAGTGATAAAATC
AACCAGAGTGTTTTCTTTTAGAAAACTTTTGTCTGCTGTAAAAAGGCAGATGAATAAGGA
CTGGGCAAGACCGGTGCCAGCCGCCGCGGTAATCCCGGCGGTCCGAGTCGCATCCACATT
TATTGGGTCTAAAACATCCGTAGCTTGTTTTGTAAGTCTCTTGTGAAATCCCATCTCTCA
AATTTGGGGCGTGCAAGAGATACTACATTGCTAGAGATCGGAAGACGTAAAGAGAACGAT
TGAGGTAGCGGTAAAATGTGTTAATCTTGATCGGACTAACAATGGCGAAGGCACTTTACG
AGTACGAATCTGACAGTGAGGGATGAAGGCTAGGGGCGCAAAAGGGATTAGATACCCCAG
TAGTCC
>Otu402
CCAGCCTATGGGGGGCACCAGCTAAGAATCTTCCGCAATGGGGGAAACCCTGACGGAGCG
ACGCCGCGTGGATGATGAAGGCCGTAAGGTTGTAAAATCCTTTTGTCGGTGAAGAATAAG
CGGGGGAGTGGAAAGCCCTCGTGATGACGTTAGCCGACGAATAAGCCCCGGCTAATTACG
TGCCAGCAGCCGCGGTAATACGTATGGGGCAAGCGTTGTTCGGATTTATTGGGCGTAAAG
GGCGTGTAGGCGGCTTATCAAGTCCGGTGTGAAATAGCAGAGCCCAACTCTGCGTATGCG
CTGGAAACTGGTAGGCTTGAGTTCTTGAGGGGAAGCTGGAATTCCAGGTGTAGGAGTGAA
ATCTGTAGATATCTGGAAGAACACCGGTGGCGAAGGCGAGCTTCTATCAGAGGACTGACG
CTGAGGCGCGAAAGCGTGGGGAGCAAACAGGATTAGATACCCTAGTAGTCC
>Otu403
CCAGCCTACGGGGGGCACCAGGCGCGAAAACTTTACAATGCGGGAAACCGTGATAAGGGA
ACTCCGAGTGCCCGTAAAATCGGGCTGTCCATCTGTTTAAATAACAGGTGAAGAAAGGGC
CGGGCAAGACCGGTGCCAGCCGCCGCGGTAATACCGGCGGCTCGAGTGGTGGCCACTATT
ACTGGGCTTAAAGCGTTCGTAGCTGGTCTGTTAAGTCTCTGGGGAAATCTACTGGCTTAA
CCAATAGGCGTTTCAGGGATACTGGCAGACTAGGGACCGGGAGAGGTGAGGGGTACTCCA
GGGGTAGGAGTGAAATCCTGTAATCCTTGGGGGACCACCTGTGGCGAAGGCGCCTCACCA
GAACGGCTCCGACAGTGAGGGACGAAAGCTGGGGGAGCAAACCGGATTAGATACCCCAGT
AGTCC
>Otu404
CCAGCCTATGGGGGGCTCCAGTCGAGAATCTTCGGCAATGGGCGCAAGCCTGACCGAGCG
ACGCCGCGTGGAGGATGAAGGCCTTCGGGTTGTAAACTCCTGTCGAGGGGGAGGAAGGGG
CAACCTTGACCGATCCCTGGAGGAAGCACGGGCTAAGTTCGTGCCAGCAGCCGCGGTAAG
ACGAACCGTGCGAACGTTATTCGGAATCACTGGGCTTAAAGCGCGTGTAGGCGGGCCGCC
ACGTCGGCTGCTGAAATCCCCCGGCTCAACCGGGGAACGGGCACCGATACGAGCGGCCTC
GAGGGGGGTAGGGGGCACTGGAACTTCCGGTGGAGCGGTGAAATGCGTTGAGATCGGAAG
GAACGCCCGTGGCGAAAGCGAGTGCCTGGACCCTTTCTGACGCTGAGACGCGAAAGCCAG
GGGAGCGAACGGGATTAGAAACCCTTGTAGTCC
>Otu405
CCAGCCTATGGGTGGCACCAGCCAAGAATCTTCCGCAATGGGGGAAACCCTGACGGAGCG
ACGCCGCGTGAAGGATGAAGGTCTTCGGATTGTAAACTTCTTTTGTTCGGGAAGAATAAG
GGTGATAGGAAATGATCATCTGATGACGGTACCGAATGAATAAGCCCCGGCTAATTACGT
GCCAGCAGCCGCGGTAATACGTAAGGGGCGAGCGTTGCTCGGAATTACTGGGCGTAAAGG
GCTTGTAGGCGGTCTTGTAAGTCGAATGTAAAAAACCACGGCTCAACCGTGGGGCTGCGT
TCGAAACTGCAAGGCTAGAGTTTGGTGGAGGGCGATGGAATTCCCGGTGTAGCGGTGAAA
TGCGTAGATATCGGGAAGAACACCGAAGGCTAAAGCAGTCGTCTATGCCGAAACTGACGC
TGAGGAGCGAAAGCATGGGGATCAAACAGGATTAGATACCCCCGTAGTCC
>Otu406
CCAGCCTATGGGTTGCACCAGTAGGGAATCTTGCGCAATGGGCGAAAGCCTGACGCAGCC
ATGCCGCGTGAATGATGAAGGTCTTAGGATTGTAAAATTCTTTCAGCGGGGACGATAATG
ACGGTACCCGCAGAAGAAGCCCCGGCTAACTTCGTGCCAGCAGCCGCGGTAATACGAAGG
GGGCTAGCGTTGCTCGGAATTACTGGGCGTAAAGGGCGCGTAGGCGGATGCTTAAGTCAG

AGGTGAAAGCCCAGGGCTCAACCTTGGAACTGCCTTTGATACTGGGCATCTTGAGTATGG
GAGAGGTGAGTGGAACTCCGAGTGTAGAGGTGAAATTCGTAGATATTCGGAAGAACACCA
GTGGCGAAGGCGACTCACTGGCCCATTACTGACGCTGAGGCGCGAAAGCGTGGGGAGCAA
ACAGGATTAGATACCCGAGTAGTCC
>Otu407
CCAGCCTATGGGGGGCTGCAGGCGCGAAACCTTCGCAATGCGCGCAAGCGCGACGAGGTT
ATTCTGAGTGATTCCCGCTGAGGGAATCTTTTCTTAGATCTAAAAAGTCTGGGGAATAAG
GGGAGGGCAAGTCTGGTGTCAGCCGCCGCGGTAATACCAGCTCCTCGAGTGGTCAGGGCG
TTTATTGGGCCTAAAGCATCCGTAGCCTGCTCAGTAGGTCTCCTGTTAAATCCAACGGCT
CAACCGTTGGTCTGCAGGAGATACCGCTGGGCTAGGAGGCGGGAGAGGCGGACGGTACTC
CGAGGGTAGGGGTAAAATCCTTTGATCCCCGGAAGACCACCAGTGGCGAAGGCGGTCCGC
CAGAACGCGCTCGACGGTGAGGGATGAAAGCTGGGGGAGCGAACCGGATTAGAAACCCTC
GTAGTCC
>Otu408
CCAGCCTACGGGAGGCAGCAGTAAGGAATATTGGTCAATGGACGCAAGTCTGAACCAGCC
ATGCCGCGTGAAGGATGAAGGCCCTCTGGGTTGTAAACTTCTTTTATCTGGGACGAAAAA
CAGGGATTCTTCCTTGCTCGACGGTACCAGAGGAATAAGCACCGGCTAACTCCGTGCCAG
CAGCCGCGGTAATACGGAGGGTGCAAGCGTTATCCGGATTTACTGGGTTTAAAGGGTGCG
TAGGTGGGCAGGTAAGTCAGTGGTGAAATCTCCGGGCTTAACTCGGAAACTGCCATTGAT
ACTATCTGTCTTGAATTATCTGGAGGTAAGCGGAATATGTCATGTAGCGGTGAAATGCTT
AGATATGACATAGAACACCCATTGCGAAGGCAGCTTACTACGGATCGATTGACACTGAGG
CACGAAAGCGTGGGGATCAAACAGGATTAGAAACCCCCGTAGTCC
>Otu409
CCAGCCTACGGGTCGCTGCAGTAGGGAATATTGGTAATCTGCGAAAGCGGGAACCAGCAA
CGCCGCGTGTGCGATGAAGGCCTTCGGGTCGTAAAGCACTTTTTAGAGGGATGAGGAAGG
ACAGTACCTTTAGAATAAGCCTCGGCTAACTACGTGCCAGCAGCCGCGGTAAAACGTAGG
AGGCAAGCGTTATCCGGATTTACTGGGCGTAAAGCGTGTGCAGGCGGTTCGGTAAGTTGG
ATGTGAAAGCTCCCGGCTTAACTGGGAGAGGTCGTTCAATACTGCCGGGCTAGAGAGTGG
TAGAGGGAGGTGGAATTCCGGGTGTAGTGGTGAAATGCGTAGATATCCGGAGGAACACCA
GTGGCGAAAGCGGCCTCCTGGACCATCTCTGACGCTCATACACGAAAGCTAGGGGAGCAA
ACGGGATTAGATACCCGAGTAGTCC
>Otu410
CCAGCCTATGGGGGGCTGCAGTAGGGAATATTGCATAATGGGCGAAAGCCTGATGCAGCA
ACGCCGCGTGTGCGATGAAGGCCTTCGGGTCGTAAAGCACTTTTAGAGGGGATGAGGAAG
GACAGTACCCTCTGAATAAGCCTCGGCTAACTACGTGCCAGCAGCCGCGGTAAAACGTAG
GAGGCGAGCGTTATCCGGATTTACTGGGCGTAAAGCGCGCGCAGGTGGTTTGGTAAGTTG
GATGTGAAAGCTCCTGGCTTAACTGGGAGAGGTCGTTCAAGACTGCTAAACTTGAGTGTG
GGAAAGGAAGGCGGAATTCCGGGTGTAGTGGTGAAATGCGTAGATATCCGGAGGAACACC
AGTGGCGAAAGCGGCCTTCTGGCCCACAACTGACACTCATGAGCGAAAGCTAGGGGAGCG
AACGGGATTAGAAACCCTCGTAGTCC
>Otu411
CCAGCCTATGGGGGGCTGCAGTCGAGGATCTTCGTCAATGGGCGAAAGCCTGAACGAGCG
ACGCCGCGTGTGCGATGAAGGCCTTCGGGTTGTAAAGCACAAAAGTGGGGATAAAAGCCG
AAAGGTCTGATAGATCCACAGTAAGCACGGGCTAAGTTCGTGCCAGCAGCCGCGGTAAGA
CGAACCGTGCGAACGTTGTTCGGAATCACTGGGCTTAAAGGGCGCGTAGGCGGGCGGTCA
AGTCTATGGTGAAATCCTCCAGCTCAACTGGAGAACTGCCGTGGATACTGATTGTCTCGA
GGGAGGTAGGGGCGTGCGGAACGGTCGGTGGAGCGGTGAAATGCGTTGATATCGATCGGA
ACTCCGGTGGCGAAGGCGGCACGCTGGACCTCTTCTGACGCTGAGGCGCGAAAGCCAGGG
GAGCAAACGGGATTAGAAACCCCTGTAGTCC
>Otu412
CCAGCCTATGGGGGGCACCAGTGGGGAATATTGGACAATGGGGGGAACCCTGATCCAGCA
ATGCCGCGTGTGTGAAGAAGGCCTGAGGGTTGTAAAGCACTTTCAGTGGGGAGGAGGTTA
GTTTGGTTAAGAGCTGGATTAATGGACGTTACCCACAGAAGAAGCACCGGCTAACTCCGT
GCCAGCAGCCGCGGTAATACGGAGGGTGCGAGCGTTAATCGGAATTACTGGGCGTAAAGG
GTGCGTAGGTGGTTAATTAAGTTATGTGTAAAATCCCCGGGCTTAACCTGGGCAGGTCAT
ATAATACTGATTGACTCGAGTATGGGAGAGGGTAGTGGAATTTCCGGTGTAGCGGTGAAA
TGCGTAGAGATCGGAAGGAACACCAGTGGCGAAGGCGGCTACCTGGCCTAATACTGACAC
TGAGGCACGAAAGCGTGGGGAGCAAACAGGATTAGATACCCCCGTAGTCC

>Otu413
CCAGCCTATGGGTGGCAGCAGTCGAGGATTTTTCTCAATGGGGGAAACCCTGAAGGAGCG
ACGCCGCGTGGGGGATGAAGGTCTTCGGATTGTAAACCCCTGTCATCCGGGAACAATGTA
ATCTACCTAACACGTGGATTATTGATAGTACTGGAAGAGGAAGCCGTGGCTAACTCTGTG
CCAGCAGCCGCGGTAATACAGAGACGGCAAGCGTTGCTCGGATTCATTGGGCGTAAAGGG
TCCGCAGGCGGTCGGTTAAGTCGGATGTGAAATCTCACAGCCTAACTGTGATCTGTCATT
CGATACTAGCCGGCTCGAGGACTGGAGAGGAGACTGGAATTGTCGGTGTAGCGGTGAAAT
GCGTAGAGATCGACAAGAACACCGGTGGCGAAGGCGAGTCTCTGGACAGTTCCTGACGCT
CAGGGACGAAAGCTAGGGGAGCAAACGGGATTAGATACCCCAGTAGTCC
>Otu414
CCAGCCTACGGGTCGCAGCAGTCGAGAATATTTGGCAATGGGCGAAAGCCTGACCATGCG
ACGCCGCGTGCTGGACGAAGTCCTTCGGGACGTAAACAGCTTTTATGAGGGAGGAAGTAA
TTGACGTTACCTCATGAATAAGGGGCTCCTAACTCTGTGCCAGCAGGAGCGGTAATACAG
AGGCCCCGAGCATTATCCGGAATCACTGGGCGTAAAGGGTGTGTAGGCGGTCGTGTTAGT
CCTTCGTCAAAGATCTTGGGCTTAACCCAGGAGATGCGCGGGAAACGGCACGACTTCGAG
GACGCGAGAGGTTGAGGGAACTCATGGTGTAGGGGTGAAATCCGTTGATATCATGGGGAA
CACCAAATGCGAAGGCACTCAACTGGCGCGCTCCTGACGCTGAAACACGAAAGCGTGGGT
AGCGAATGGGATTAGAAACCCTAGTAGTCC
>Otu415
CCAGCCTATGGGTTGCAGCAGTGAGGAATATTGCGCAATGGGGGAAACCCTGACGCAGCG
ACGCCGCGTGGGTGAAGAAGGCCTTCGGGTTGTAAAGCCCTGTCAGGTGGGAAGAAACCT
TTAAAGGTGAATAGCCTTTGAAACTGACGGTACCACCAAAGGAAGCACCGGCTAACTCCG
TGCCAGCAGCCGCGGTAAGACGGAGGGTGCAAGCGTTGCTCGGAATTACTGGGCGTAAAG
CGCGTGTAGGCGGTCCGATAAGTCGAGTGTGAAAGCCCGGGGCTCAACCTCGGAAGTGCA
TTCGAAACTGTCGGACTTGAGTACGGGAGAGGGAAGTGGAATTCCTGGTGTAGAGGTGAA
ATTCGTAGATATCAGGAGGAACACCGGTGGCGAAGGCGGCTTCCTGGACCGATACTGACG
CTGAGACGCGAAAGCGTGGGGAGCAAACAGGATTAGAAACCCCCGTAGTCC
>Otu416
CCAGCCTATGGGTGGCAGCAGTGGGGAATATTGCGCAATGGGCGAAAGCCTGACGCAGCG
ACGCCGCGTGAGGGATGAAGGCCTTCGGGTTGTAAACCTCTTTCAGCAGGGACGAAGCGA
AAGTGACGGTACCTGCAGAAGAAGCACCGGCCAACTACGTGCCAGCAGCCGCGGTAATAC
GTAGGGTGCAAGCGTTGTCCGGAATTATTGGGCGTAAAGAGCTCGTAGGCGGTTTGTCGC
GTCGGCTGTGAAAACTCGGGGCTCAACCCCGAGCCTGCAGTCGATACGGGCAAACTAGAG
TGTGGTAGGGGAGACTGGAATTCCTGGTGTAGCGGTGAAATGCGCAGATATCAGGAGGAA
CACCGGCGGCGAAGGCGGGTCTCTGGGCCATAACTGACGCTGAGGAGCGAAAGCGTGGGG
AGCGAACAGGATTAGATACCCGTGTAGTCC
>Otu417
CCAGCCTACGGGACGCTCCAGGCGCGAAAACTTTACACTGCGCGAAAGCGCGATAAGGGG
ACCTCGAGTGCGTGCGCAATGCGTACGCTTTTCAGATGCCTAAAAAGCATCTGGAATAAG
GGCCGGGCAAGACCGGTGCCAGCCGCCGCGGTAACACCGGCGGCTCAAGTGGTGGCCGCT
ATTATTGGGCTTAAAGGGTCCGTAGCCGGACCAGTTAGTCCCTTGGGAAATCTTACGGCT
TAACCGTAAGGCTGCCAATGGATACTGCTGGTCTTGGGACCGGGAGAGGCAAGAGGTACC
TCAGGGGTAGGAGTGAAATCCTGTAATCCTTGAGGGACCGCCAGTGGCGAAGGCGTCTTG
CTAGAACGGGTCCGACGGTGAGGGACGAAAGCTAGGGGCACGAACCGGATTAGAAACCCT
CGTAGTCC
>Otu418
CCAGCCTATGGGTCGCAGCAGTCGAGAATTTTTCTCAATGGGGGAAACCCTGAAGGAGCG
ACGCCGCGTGGAGGATGAAGGTCTTCGGATTGTAAACTCCTGTCATTAGGGAACAATTAC
CAGCGATTAACTGTCGCGGGTTTGATAGTACCTGAAGAGGAAGAGACGGCTAACTCTGTG
CCAGCAGCCGCGGTAATACAGAGGTCTCAAGCGTTGTTCGGATTCATTGGGCGTAAAGGG
TGCGTAGGCGGCGAGGCAAGTCAGGTGTGAAATCTCGGGGCTCAACTCCGAAACTGCACT
TGATACTGCCTTGCTAGAGTACTGGAGAGGAGATTGGAATTTACGGTGTAGCAGTGAAAT
GCGTAGATATCGTAAGGAAGACCAGTGGCGAAGGCGAATCTCTGGACAGTTACTGACGCT
GAGGCACGAAGGCCAGGGGAGCAAACGGGATTAGAAACCCTCGTAGTCC
>Otu419
CCAGCCTACGGGGGGCTGCAGCAACGAATATTCCGCAATGGGCGCAAGCCTGACGGAGCG
ACGCCGCGTGGAGGATGAAGTCCTTCGGGATGTAAACTCCAATAGTTTGTGAGGAAGTGA
CCCGGCCTAATACGCCGGGAAGTTGACCAGCAGATGAAAGGGGCGGCTAACTCCGTGCCA

GCAGCCGCGGTAATACGGAGGCCCCGAGCGTTGTTCGGAATTACTGGGCTTAAAGCGCGT
GTAGGCGGGCTGGTAAGTGTCTTGTGAAATCCCCGGGCTCAACCCGGGAATTGCTCGGCA
TACTGCCGGTCTTGAGGCAGGTATGGGTCACTGGAACCTTAGGTGTAGCGGTGAAATGCG
TAGATATCTAAGGGAACGCCGGTGGCGAAAGCGGGTGACTGGGCCTGTCCTGACGCTGAG
ACGCGAAAGCGTAGGGAGCAAACGGGATTAGAAACCCGTGTAGTCC
>Otu420
CCAGCCTATGGGGGGCAGCAGTGAGGAATATTGCACAATGGGCGAAAGCCTGATGCAGCG
ACACCGCGTGAAGGATGAAGGTCTTAGGATTGTAAACTTCTGTTAAGTGGGAAAAAAGAC
TCATTTCAAATAAAAATGAGGGATGATGGTACCACTAGAGAAAGCACCGGCTAACTTCGT
GCCAGCAGCCGCGGTAATACGAGGGGTGCAAACGTTATTCGGAATTACTGGGTGTAAAGG
GTACGTAGACGGCATATTAAGTCAGTTGTTAAATTCCCCAGCTTAACTGGGGGTAGGCGA
TTGAAACTGATAAGCTAGAGGATGAGAGAGAGAAGTGGAATTCTCGGAGTAGCGGTAAAA
TGCGTAGATCTCGAGAGGAACACCGATGGCGAAGGCAGCTTCTTGGCTCATTTCTGACGT
TGAGGTACGAAAGCGTGGGGAGCAAACAGGATTAGAAACCCGAGTAGTCC
>Otu421
CCAGCCTATGGGTCGCTGCAGTGGGGAATCTTGCACAATGGGGGCAACCCTGATGCAGCG
ACGCCGCGTGAGCGATGAAGCCCTTCGGGGTGTAAAGCTCTTTCGGCAGGGACGATAATG
ACGGTACCTGAAGAAGAAGCTGCGGCTAACTACGTGCCAGCAGCCGCGGTAATACGTAGG
CAGCGAGCGTTGTTCGGAATTACTGGGCGTAAAGCGTGCGCAGGCGGTCCGCTAAGACAG
ATGTGAAATCCCCGGGCTTAACCTGGGAACTGCATTTGTGACTGGCGGGCTAGAGTATGG
CAGAGGGGGGTAGAATTCCACGTGTAGCAGTGAAATGCGTAGAGATGTGGAGGAATACCG
ATGGCGAAGGCAGCCCCCTGGGCCAATACTGACGCTCATGCACGAAAGCGTGGGTAGCAA
ACAGGATTAGAGACCCCTGTAGTCC
>Otu422
CCAGCCTATGGGAGGCAGCAGTGGGGAATATTGCACAATGGGCGCAAGCCTGATGCAGCC
ATGCCGCGTGTGTGAAGAAGGCCTTCGGGTTGTAAAGCACTTTCAGCGAGGAGGAAGGGT
ATCGTGTTAATAGCACGGTACATTGACGTTACTCGCAGAAGAAGCACCGGCTAACTCCGT
GCCAGCAGCCGCGGTAATACGGAGGGTGCAAGCGTTAATCGGAATTACTGGGCGTAAAGC
GCACGCAGGCGGTTTGTTAAGTCAGATGTGAAATCCCCGAGCTTAACTTGGGAACTGCAT
TTGAAACTGGCAAGCTAGAGTCTTGTAGAGGGGGGTAGAATTCCAGGTGTAGCGGTGAAA
TGCGTAGAGATCTGGAGGAATACCGGTGGCGAAGGCGGCCCCCTGGACAAAGACTGACGC
TCAGGTGCGAAAGCGTGGGGAGCAAACAGGATTAGAAACCCTTGTAGTCC
>Otu423
CCAGCCTACGGGAGGCTCCAGTAGGGAATATTGCACAATGGAGGAAACTCTGATGCAGCG
ACGCCGCGTGAGTGATGAAGGACTTCGGTTTGTAAAGCTCTGTTCTTAGGGAATAAAAAA
ATGAATGTACCTGAGGAGAAAGCACCGGCTAACTTCGTGCCAGCAGCCGCGGTAATACGA
AGGGTGCAAGCGTTGCTCGGAATCATTGGGCGTAAAGCGAGCGCAGGTGGTCTTGTAAGT
CAGGAGTGAAATCCCAGGGCTCAACCCTGGAAGTGCTTTTGATACTGCGAGACTTGAATG
TGGGAGAGGGTACTAGAATACATAGTGTAGTGGTGAAATACGTAGATATTATGTGGAACA
CCGGTGGCGAAGGCGGGTGCCTGGCCCAACATTGACACTTAGGCTCGAAAGCGTGGGGAT
CAAACAGGATTAGAAACCCCTGTAGTCC
>Otu424
CCAGCCTACGGGGGGCACCAGTGGGGAATTTTGCGCAATGGGCGAAAGCCTGACGCAGCA
ACGCCGCGTGCGGGATGAAGGCCTTCGGGTTGTAAACCGCTTTCAGCAGGGATGAAATTG
ACAGTACCTGCAGAAGAAGCCCCGGCTAACTACGTGCCAGCAGCCGCGGTAATACGTAGG
GGGCAAGCGTTATCCGGATTCATTGGGCGTAAAGAGCGCGTAGGCGGATTGTTAAGTCAG
ATGTGAAAACTGGGGGCTCAACCCGCAGCCTGCATCTGAAACTGGCAGTCTTGAGTCTGG
TAGAGGAAAGTGGAATTCCCAGTGTAGCGGTGAAATGCGCAGATATTGGGAGGAACACCA
GTGGCGAAGGCGGCTTTCTGGGCCACGACTGACGCTGAGGCGCGAAAGCTAGGGGAGCAA
ACAGGATTAGAAACCCCTGTAGTCC
>Otu425
CCAGCCTACGGGGTGCAGCAGTGGGGAATATTGGACAATGGGGGAAACCCTGATCCAGCG
ACGCCGCGTGTGTGAAGAAGGCCTGCGGGTTGTAAAGCACTTTTAGTGGGGACAAAAAGC
CACGAACTAATACTACGTGGTCTTGATTTAACCTACAGAAAAAGCACCGGCTAACTCTGT
GCCAGCAGCCGCGGTAATACAGAGGGTGCGAGCGTTAATCGGAATTACTGGGCGTAAAGC
GCGCGTAGACGGTTTGGTAAGTCAGATGTGAAATCCCTGGGCTCAACCTGGGAACTGCAT
TTGATACTGCCTGGCTAGAGTATGGAAGAGGGAAGTGGAATTTCCGGTGTAGCGGTGAAA
TGCGTAGATATCGGAAGGAACACCAGTGGCGAAAGCGACTTCCTGGTCCAATACTGACGT

TCATGTGCGAAAGCGTGGGGAGCAAACAGGATTAGAAACCCCCGTAGTCC
>Otu426
CCAGCCTATGGGGGGCTCCAGTGGGGAATTTTGCGCAATGGGGGAAACCCTGACGCAGCA
ACGCCGCGTGGAGGATGAAGCCCCTTGGGGTGTAAACTCCTTTCGACCGGGAAAATAATG
ATGGTACCGGTGGAAGAAGCACCGGCTAACTCTGTGCCAGCAGCCGCGGTAATACAGAGG
GTGCGAGCGTTGTTCGGAATTATTGGGCGTAAAGGGCGCGTAGGCGGTATTGTAAGTCAC
CTGTGAAACCTCTAGGCTTAACCTAGAGCCTGCAGGCGAAACTGCAATGCTGGAGGGTGG
GAGAGGTGCGTGGAATTCCCGGTGTAGCGGTGAAATGCGTAGATATCGGGAGGAACACCT
GTGGCGAAAGCGGCGCACTGGACCACTACTGACGCTGATGCGCGAAAGCTAGGGGAGCAA
ACAGGATTAGAAACCCCCGTAGTCC
>Otu427
CCAGCCTACGGGGTGCAGCAGTCGAGAATTTTTCACAATGGGGGAAACCCTGATGGAGCG
ACGCCGCGTGGGGGATGACTGGCTTCGGCCCGTAAACCCCTGTCATTTGCGAACAAATTG
ATTCACCTAACACGTGAAGCATTGATAGTAGCGGAAGAGGAAGGGACGGCTAACTCTGTG
CCAGCAGCCGCGGTAATACAGAGGTCCCAAGCGTTGTTCGGATTCACTGGGCGTAAAGGG
TGCGTAGGTGGCCGGGTAAGTCTGATGTGAAATCTCGCAGCTTAACTGCGAAACTGCATT
GGATACTATCCGGCTGGAGGGTCGGAGGGGGGACTGGAATTCTCGGTGTAGCAGTGAAAT
GCGTAGATATCGAGAGGAACACCAGTGGCGAAGGCGAGTCCCTGGACGACTCCTGACACT
GAGGCACGAAAGCTAGGGGAGCAAACAGGATTAGATACCCCCGTAGTCC
>Otu428
CCAGCCTACGGGGCGCTGCAGTGGGGAATATTGGACAATGGGCGCAAGCCTGATCCAGCC
ATGCCGCGTGAGTGATGAAGGCCCTAGGGTTGTAAAGCTCTTTCGGCGGGGACGATGATG
ACGGTACCCGCAGAAGAAGCCCCGGCTAACTTCGTGCCAGCAGCCGCGGTAATACGAAGG
GGGCTAGCGTTGTTCGGAATTACTGGGCGTAAAGCGCACGCAGGCGGTTCGTTTAGTTAG
AAGTGAAAGCCCAGGGCTCAACCTTGGAACTGCTTTTAATACTGGCGAACTAGAATCCGT
GAGAGGGCAGTGGAATTCCGAGTGTAGAGGTGAAATTCGTAGATATTCGGAAGAACACCA
GTGGCGAAGGCGACTGCCTGGCACGGTATTGACGCTCATGTGCGAAAGCGTGGGGAGCAA
ACAGGATTAGAAACCCTCGTAGTCC
>Otu429
CCAGCCTATGGGGGGCTGCAGTAAGGAATATTGGTCAATGGACGCAAGTCTGAACCAGCC
ATGCCGCGTGCAGGATGAAGGTCCTACGGATCGTAAACTGCTTTTGTACGGGAAAAAATC
CCGGGTTTCTACCCGGGTTGATGGTACCGTAAGAATAAGGATCGGCTAACTCCGTGCCAG
CAGCCGCGGTAATACGGAGGATCCGAGCGTTATCCGGATTTACTGGGTTTAAAGGGTGCG
TAGGTGGAATGTCAAGTCAGTGGTGAAAACCTGCAGCTTAACTGTAGAATTGCCATTGAT
ACTGTCATTCTTGAGTGTATTTGATGTAAGTGGAATGTGTCGTGTAGCGGTGAAATGCTT
AGATATGACACAGAACACCCATTGCGAAGGCAGCTTACAAAACTACTACTGACACTGAGG
CACGAAAGCGTGGGGATCAAACAGGATTAGAAACCCTGGTAGTCC
>Otu430
CCAGCCTATGGGGGGCAGCAGTCGAGAATCTTCCACAATGGACGAAAGTCTGATGGAGCG
ACGCCGCGTGGTTGATGAAGTCCTTCGGGACGTAAAAACCTTTTATGGGGGAGGAAGTAA
TTGACGTTACCCCATGAATAAGGGGCTCCTAACTCTGTGCCAGCAGGAGCGGTAATACAG
AGGCCCCAAGCGTTATCCGGAATCACTGGGCGTAAAGGGTGTGTAGGCGGTCGTGTTAGT
CTTTTGTCAAAGCTTTTCGGCTTAACCGAGAAAACGCAGGAGAAACGGCACGGCTTTGAG
GATGCGAGAGGTAGAGGGAACTCATAGTGTAGGGGTGAAATCCGTTGATATTATGGGGAA
CACCAAATGCGAAGGCACTCTACTGGCGCATTCCTGACGCTGAGACACGAAAGCGTGGGA
ATCGAACGGGATTAGAGACCCGTGTAGTCC
>Otu431
CCAGCCTACGGGATGCAGCAGTCGAGGATCTTCGGCAATGGGCGCAAGCCTGACCGAGCG
ACGCCGCGTGTGCGATGAAGGCCTTCGGGTTGTAAAGCACTGTCGAGGGGGAGAAAGATC
CGCAAGGATTTGATCTATCCCTGGAGGAAGCACGGGCTAAGTTCGTGCCAGCAGCCGCGG
TAAGACGAACCGTGCGAACGTTATTCGGAATCACTGGGCTTAAAGGGCGCGTAGGCGGGT
CATCAAGTCAGGGGTGAAATACTCCGGCCTAACCGGAGAACGGCTTCTGATACTGGTGAC
CTCGAGGGATGTAGGGGCATGTGGAACTTCCGGTGGAGCGGTGAAATGCGTAGATATCGG
AAGGAACGCCGGTGGCGAAAGCGACGTGCTGGACATCTTCTGACGCTGAGGCGCGAAAGC
CAGGGGAGCAAACGGGATTAGAAACCCCTGTAGTCC
>Otu432
CCAGCCTATGGGATGCTGCAGTGGGGAATCTTGCGCAATGGGGGCAACCCTGACGCAGCC
ATGCCGCGTGAATGATGAAGGTCTTAGGATTGTAAAATTCTTTCAGCAGGGACGATAATG

ACGGTACCTGCAGAAGAAGCCCCGGCTAACTTCGTGCCAGCAGCCGCGGTAATACGAAGG
GGGCTAGCGTTGCTCGGAATTACTGGGCGTAAAGGGCGCGTAGGCGGGTCGTTTAGTTAG
AGGTGAAAGCCCAGGGCTCAACCCTGGAATTGCCTTTAATACTGGCGGCCTTGAGTATGA
GAGAGGTGTGTGGAACTCCGAGTGTAGAGGTGAAATTCGTAGATATTCGGAAGAACACCA
GTGGCGAAGGCGACACACTGGCTCATTACTGACGCTGAGGCGCGAAAGCGTGGGGAGCAA
ACAGGATTAGATACCCTCGTAGTCC
>Otu433
CCAGCCTATGGGGTGCAGCAGTAAGGAATATTGGTCAATGGAGGCAACTCTGAACCAGCC
ATGCCGCGTGCAGGAAGACGGCCCTACGGGTTGTAAACTGCTTTTGTACCGGAATAAACC
TTGATACGAGTATCGAGCTGAATGTACGGTAAGAATAAGGATCGGCTAACTCCGTGCCAG
CAGCCGCGGTAATACGGAGGATCCAAGCGTTATCCGGATTTATTGGGTTTAAAGGGTGCG
TAGGCGGCTTGTTAAGTCAGGGGTGAAAGGCGGTAGCTTAACTATCGGAGTGCCCTTGAT
ACTGACGAGCTTGAATGCAGCTGAGGTAGGCGGAATGTGACAAGTAGCGGTGAAATGCAT
AGATATGTCACAGAACACCGATTGCGAAGGCAGCTTACTAAAGTGCGATTGACGCTGAGG
CACGAAAGCGTGGGGATCAAACAGGATTAGATACCCTCGTAGTCC
>Otu434
CCAGCCTACGGGGCGCAGCAGTAGGGAATATTGGACAATGGGCGAAAGCCTGATCCAGCC
ATCCCGCGTGAAGGATTAAGGCCCTATGGGTTGTAAACTTCTTTTCTCTGGGAATAAAAA
CCGGTATTTATACTGGCTTGAAGGTACCAGAGGAATAAGCACCGGCTAACTCCGTGCCAG
CAGCCGCGGTAATACGGAGGGTGCAAGCGTTATCCGGATTCACTGGGTTTAAAGGGTGCG
TAGGTGGCTTTGTAAGTCAGTGGTGAAAGCCCGGAGCTCAACTCCGGAACTGCCATTGAT
ACTGCTTAGCTTGAATCACGTCGAGGTGGATGGAATATTACATGTAGCGGTGAAATGCTT
AGATATGTAATAGAACACCGATTGCGAAGGCAGTTCACTAGGCGTGTATTGACACTGAGG
CACGAAAGCGTGGGGATCAAACAGGATTAGATACCCTGGTAGTCC
>Otu435
CCAGCCTACGGGTTGCTGCAGTAGGGAATATTGGACAATGGACGCAAGTCTGATCCAGCC
ATCCCGCGTGAAGGATTAAGGCCCTCTGGGTTGTAAACTTCTTTTCTCTGGGAATAAAAA
GCGGTATTCTTATCGCCTTGAAGGTACCAGAGGAATAAGCACCGGCTAACTCCGTGCCAG
CAGCCGCGGTAATACGGAGGGTGCAAGCGTTATCCGGATTCACTGGGTTTAAAGGGTGCG
TAGGTGGTCTGTTAAGTCAGCGGTGAAAGCCTGGAGCTCAACTCCAGAATTGCCATTGAT
ACTGACGGACTTGAATCAGGTTGAGGTGGATGGAATATTACATGTAGCGGTGAAATGCTT
AGATATGTAATAGAACACCGATTGCGAAGGCAGTTCACTAAGCCTGCATTGACACTGAGG
CACGAAAGCGTGGGGATCAAACAGGATTAGAAACCCTTGTAGTCC
>Otu436
CCAGCCTACGGGGGGCAGCAGTTAGGAATCTTGCGCAATGGGCGAAAGCCTGACGCAGCC
ATGCCGCGTGAATGATGAAGGTCTTAGGATTGTAAAATTCTTTCAGCGGGGACGATAATG
ACGGTACCCGCAGAAGAAGCTCCGGCTAACTTCGTGCCAGCAGCCGCGGTAATACGAAGG
GGGCTAGCGTTGCTCGGAATTACTGGGCGTAAAGGGCGCGTAGGCGGACAGTTTAGTCAG
AGGTGAAAGCCCAGGGCTCAACCTTGGAAATGCCTTTGATACTGGCTGTCTTGAGTTCGG
GAGAGGTGTGTGGAACTCCGAGTGTAGAGGTGAAATTCGTAGATATTCGGAAGAACACCA
GTGGCGAAGGCGACACACTGGCCCGATACTGACGCTGAGGCGCGAAAGCGTGGGGAGCAA
ACAGGATTAGATACCCCAGTAGTCC
>Otu437
CCAGCCTACGGGGGGCACCAGTGGGGAATCTTGCACAATGGAGGAAACTCTGATGCAGCG
ACGCCGCGTGAGCGATGAAGCCCTTCGGGGTGTAAAGCTCTTTCGGCAGGGAAGATTATG
ACGGTACCTGCAGAAGCAGCTGCGGCTAACTACGTGCCAGCAGCCGCGGTAATACGTAGG
CAGCAAGCGTTGTTCGGAGTTACTGGGCGTAAAGGGTGCGTAGGCGGTCTTTTAAGTTTG
GTGTGAAATCTCCCGGCTCAACCGGGAGGGTGCGCCGAATACTGAGAGGCTAGAGTGTCG
GAGAGGTAAGTGGAATTCCTGGTGTAGCGGTGAAATGCGTAGATATCAGGAGGAACACCG
GTGGTGTAAACGGCTTACTGGACGATAACTGACGCTGAAGCACGAAAGCGTGGGTAGCAA
ACAGGATTAGATACCCTAGTAGTCC
>Otu438
CCAGCCTATGGGGTGCTGCAGTGGGGAATATTGGGCAATGGGCGAAAGCCTGACCCAGCC
ACGCCGCGTGAGTGATGAAGGCCTTCGGGTCGTAAAGCTCTGTGGGGAGGGACGAATAAG
CACGGACCTAATACGTCCGGGCCCTGACGGTACCTCCTTAGCAAGCACCGGCTAACCATG
TGCCAGCAGCCGCGGTAATACATGGGGTGCAAACGTTGCTCGGAATCATTGGGCGTAAAG
CGCACGTAGGCGGCCACTTAAGTCGGATGTGAAATCCCTCGGCTTAACTGAGGAAGTGCA
TCCGAAACTGAATGGCTTGAGTACGAAAGAGGGTCGCGGAATTCCCGGTGTAGAGGTGAA

ATTCGTAGATATCGGGAGGAACACCGGCGGCGAAGGCGGCGACCTGGTTCGAGACTGACG
CTGAGGTGCGAAAGCGTGGGGAGCAAACAGGATTAGATACCCCCGTAGTCC
>Otu439
CCAGCCTACGGGAGGCAGCAGCGACGAATATTCCGCAATGCACGAAAGTGTGACGGAGCA
ATGCCGCGTGCAGGATGAAGCGGCTTCGCCGTGTAAACTGCTGTCAGGGGTCAGAAACAC
TGATCGACCCCAAAGGAAGAGCCGGCTAACCCTGTGCCAGCAGCCGCGGTAATACAGGGG
GCTCAAGCGTTAATCGGAATCACTGGGCTTAAAGGGTGCGTAGGCGGACGAGAAAGTGTT
CTGTGAAATCCCCCGGCTCAACCGGGGAATGGCGGGGCAAACTTCTCGTCTTGAGGCAAG
TAGAGGCTGTCGGAACGCTAGGTGGAGCGGTGAAATGCGTAGAGATCTAGCGGAACGCCG
AAGGTGAAGACAGGCAGCTGGGCTTGTCCTGACGCTCAGGCACGAAAGCGTGGGGAGCAA
ACGGGATTAGAGACCCGCGTAGTCC
>Otu440
CCAGCCTACGGGGGGCAGCAGTGGGGAATCTTGCGCAATGGGCGAAAGCCTGACGCAGCA
ACGCCGCGTGAGGGACGAAGGCCTTCTGGGTTGTAAACCTCTTTCAGCAGGGACGATGAT
GACGGTACCTGCAGAAGAAGCCCCGGCCAACTACGTGCCAGCAGCCGCGGTAATACGTAG
GGGGCGAGCGTTGTCCGGATTTATTGGGCGTAAAGAGCTCGTAGGCGGCTTAGCAAGTCG
GGTGTGAAACCTCCAGGCTCAACCTGGAGCCGCCACTCGATACTGCTATGGCTAGAGTCC
GGTAGGGGACCACGGAATTCCTGGTGTAGCGGTGAAATGCGCAGATATCAGGAGGAACAC
CAGTGGCGAAGGCGGTGGTCTGGGCCGGTACTGACGCTGAGGAGCGAAAGCGTGGGGAGC
GAACAGGATTAGATACCCCAGTAGTCC
>Otu441
CCAGCCTACGGGGTGCTGCAGTGGGGAATATTGCACAATGGGCGAAAGCCTGATGCAGCG
ACGCCGCGTGAGGGATGACGGCCTTCGGGTTGTAAACCTCTTTCAGCAGGGACGAAGCGA
AAGTGACGGTACCTGCAGAAGAAGCACCGGCCAACTACGTGCCAGCAGCCGCGGTAATAC
GTAGGGTGCAAGCGTTGTCCGGAATTATTGGGCGTAAAGAGCTCGTAGGCGGTTTGTCGC
GTCGGCTGTGAAAACCTGGGGCTCAACCCCGGGCCTGCAGCCGATACGGGCAAGCTAGAA
TTCGGTAGGGGAGACTGGAATTCCTGGTGTAGCGGTGAAATGCGCAGATATCAGGAGGAA
CACCGGTGGCGAAGGCGGGTCTCTGGGCCGATATTGACGCTGAGGAGCGAAAGCGTGGGG
AGCAAACAGGATTAGAGACCCTCGTAGTCC
>Otu442
CCAGCCTATGGGGGGCACCAGGCGCGAAAACTCCACAATGTACGGAAGTACGATGGGGTT
ATCTCGAGTGCAGTCCGATAAGGGCTGCTTTTCCATAGAGTAATATCCTGTGGGAATAAG
AGGGGGGCAAGACTGGTGTCAGCCGCCGCGGTAACACCAGCCCCTCGAGTGGTGGGGACT
TTTATTTGGCCTAAAGCGTCCGTAGCTGGCTGTATCCGTCCCCCGTTAAATCCAGCGTCC
TAAGCGTTGGACCGCGGGTGATACCATACAGCTAGAGGGTGGGATAGGTGAGCGGTATTT
CTGGGGTAGGGGCGAAATCCTTTGATCCCAGGAGGACCACCAGTGGCGAAGGCTGCTCAC
TAGAACACGCCTGACGGTCAGGGACGAAAGCTGGGGCAGCGAACCGGATTAGATACCCGA
GTAGTCC
>Otu443
CCAGCCTATGGGGTGCAGCAGTGGGGAATATTGGACAATGGGGGCAACCCTGATCCAGCA
ATGCCGCGTGTGTGAAGAAGGCCTTCGGGTTGTAAAGCACTTTTATCCGGAACGAAACGC
GCAGGGTGAATATCCTTGCGAACTGACGGTACCGGAGGAATAAGCACCGGCTAACTTCGT
GCCAGCAGCCGCGGTAATACGAAGGGTGCAAGCGTTACTCGGAATTACTGGGCGTAAAGG
GTGCGTAGGTGGTTGTTTAAGTTTGCTGTGAAAGCCCCGGGCTCAACCTGGGAATGGCAG
TGAATACTGGACAGCTAGAGTGCGGTAGAGGGTAGTGGAATTCCCGGTGTAGCAGTGAAA
TGCGTAGAGATCGGGAGGAACACCAGTGGCGAAGGCGGCTACCTGGACCAGCACTGACAC
TGAAGCACGAAAGCGTGGGGAGCAAACAGGATTAGAAACCCCAGTAGTCC
>Otu444
CCAGCCTACGGGTGGCTGCAGTCGAGAATTTTTCACAATGGACGAAAGTCTGATGGAGCG
ACGCCGCGTGGAGGATGAAGGTTCTTGGATTGTAAACTCCTGTCACTGCAGAACAAGGCT
ACGCATATGAATAGTACGCGTAGTTGATGGTATGCGGAGAGGAAGGGACGGCTAACTCTG
TGCCAGCAGCCGCGGTGAGACAGAGGTCCCGAGCGTTGTTCGGATTCATTGGGCGTAAAG
GGTGTGTAGGAGGTCAGGTAAGTCGTGTGTGAAATCTCAGAGCTTAACTCTGAAACTGCG
CTCGATACTGCTTGGCTAGAGGATCGGAGGGGGTAACGGAATTTATGGTGTAGCAGTGAA
ATGCGTAGATATCATAAGGAACACCGGTGGCGAAGGCGGTTACCTGGAAGATTCCTGACT
CTGAAACACGAAAGCCAGGGGAGCAAACGGGATTAGAAACCCTAGTAGTCC
>Otu445
CCAGCCTATGGGGGGCACCAGGTCATGGGAACGAGCACCTCATTATCCCGCATCTGGCGC

CGCAGACTCGCGATCGCCTGCTTGCCATGTTCGATGCCCGAACGCGTGCCGCCCTGCGCC
TGGATATAAAACCATTCACTCGGGCGGCCTGAGTTCGCAACAACTTCAGCAATCTTCGCC
GGCTGCGTGTATTCGCATCCCAGCCCCACGGCGAGACAGGCTCCGACATTGGGATGGGTC
CCGAGGGCGAGCATGAGCCTGATCGCGTAGGCGTTATCGTAACAACCCGGAAAACCTATA
ACGTGCACGCCGTCTTCGCCGCGTGCGATCTCATGCGCGACATAGCTCGCGCACTCCACG
GTATAGATCACGAGGATGAGGTTCCTGATTCCCTTTCTGCCGTCCTTGCGCAGAAAGCCT
TGCCAGACGCGATTTGTCAATTCCAGCTCATTCATAGGCAATATCATTAGATACCCGAGT
AGTCC
>Otu446
CCAGCCTATGGGGCGCTGCAGTAGGGAATCTTCCGCAATGGGCGAAAGCCTGACGGAGCA
ATGCCGCGTGAGTGATGAAGGTTTTCGGATCGTAAAGCTCTGTTGCCAGGGAAGAACGCT
TGGGAGAGTAACTGCTCTCAAGGTGACGGTACCTGAGAAGAAAGCCCCGGCTAACTACGT
GCCAGCAGCCGCGGTAATACGTAGGGGGCAAGCGTTGTCCGGAATTATTGGGCGTAAAGC
GCGCGCAGGCGGTCATTTAAGTCTGGTGTTTAATCCCGGGGCTCAACCCCGGATCGCACT
GGAAACTGGGTGACTTGAGTGCAGAAGAGGAGAGTGGAATTCCACGTGTAGCGGTGAAAT
GCGTAGATATGTGGAGGAACACCAGTGGCGAAGGCGACTCTCTGGGCTGTAACTGACGCT
GAGGCGCGAAAGCGTGGGGAGCAAACAGGATTAGAAACCCTTGTAGTCC
>Otu447
CCAGCCTATGGGGGGCAGCAGTCGAGGATCTTCGTCAATGGGCGCAAGCCTGAACGAGCG
ACGCCGCGTGCGCGATGAAGGCCTTCGGGTTGTAAAGCGCGAAAGTGGAGAGAAAGGGAA
ACTTGATCAATCCACAGTAAGGACGGGCTAAGTTCGTGCCAGCAGCCGCGGTAAGACGAA
CTGTCCTAACGTTGTTCGGAATCACTGGGCTTAAAGGGCGCGTAGGCGGTCCGACAAGTC
AGGGGTGAAATCCTGCAGCTTAACTGCAGAAGTGCCTTTGATACTGTCGGTCTGGAGGGA
GGTAGGGGTGTGTGGAACTTCCAGTGGAGCGGTGAAATGCGTTGATATTGGAAGGAACGC
CGGAGGCGAAAGCGACGCACTGGACCTTTTCTGACGCTGAGGCGCGAAAGCTAGGGGAGC
AAACGGGATTAGATACCCGAGTAGTCC
>Otu448
CCAGCCTACGGGGGGCAGCAGGTAGGAATCTTTTGCAATGCGCGCAAGCGTGACAAAGCG
AGCCAGAGTGCTTTTCAATTGAAAAGCTTTTGCCAAATCTAAAAATTTTGGCGAATAAGG
ACTGGGTAAGACTAGTGCCAGCCGCCGCGGTAATACTAGCGGTCCAAGTCGCAGCCATCA
TTATTGGGTCTAAAACATCCGTAGCTTGTTTAATAAGTTCCCTGTGAAATCTCATCTCTT
AAGGATGAGGCGTGCAGGGAATACTGCTAAGCTAGAGACTGGAAGACGTAGAAAGTATGT
ATAAAGTAACGGTAAAATGTGTTAATCTTATGCAGACTCACAATAGCGAAGGCATTCTAC
GAGGACAGTTCTGACAGTAAAGGATGAAGGCTAGGGGCGCAAAATGGATTAGAGACCCTC
GTAGTCC
>Otu449
CCAGCCTATGGGGCGCTGCAGTCGAGGATATTTAGCAATGGGGGAAACCCTGACTATGTG
ACGCCGCGTGAGGGATGAAGGTTTTCGGATCGTAAACCTCTTTCGATGGAGAAGAAGTCT
TTAGCTCTAACATAGCTGAAGATTGACGGTACCCAGAGAAGAAGCCACGGCCAACTCCGT
GCCAGCAGCCGCGGTAATACGGGGGTGGCAAGCGTTACTCGGATTTATTGGGTGTAAAGG
ACAGGTAGGCGTTCTGCCAAGTTAGAAGTGAAATCCTAAGGCTTAACCTTAGAACTGCTT
CTAAAACTGGCGGAATTGAGGCTAGAAGAGAAGAGCGGAATTCCCGGTGTAAGGGTGAAA
TCTGTAGATATCGGGAGGAACACCAGTGGCGAAAGCGGCTCTTTGGTCTAGATCTGACGC
TAAACTGTGAAAGCTAGGGGAGCAAACAGGATTAGAAACCCCAGTAGTCC
>Otu450
CCAGCCTACGGGGGGCAGCAGTGGGGAATATTGGGCAATGGGCGAAAGCCTGACCCAGCC
ACGCCGCGTGAGTGATGAAGGCCTTCGGGTCGTAAAGCTCTGTGGGGAGGGACGAATAAG
CTCGGTATTAATACCACCGGGCCCTGACGGTACCTCCTTAGCAAGCACCGGCTAACCCTG
TGCCAGCAGCCGCGGTAATACAGGGGGTGCAAACGTTGCTCGGAATTATTGGGCGTAAAG
CGCACGTAGGCGGTTCGTTATGTCAGATGTGAAAGCCCTTGGCTCAACTGAGGAAGTGCA
TCTGAAACTGGCGAGCTAGAGTATGTAAGAGGGTCGCGGAATTCCCGGTGTAGAGGTGAA
ATTCGTAGATATCGGGAGGAACACCGGTGGCGAAGGCGGCGACCTGGGACAATACTGACG
CTGAGGTGCGAAAGCGTGGGGAGCAAACAGGATTAGATACCCTCGTAGTCC
>Otu451
CCAGCCTATGGGTGGCAGCAGTAAGGAATATTGGTCAATGGAGGCAACTCTGAACCAGCC
ATGCCGCGTGCAGGAAGAAGGCCCTATGGGTCGTAAACTGCTTTTGTACCAGAGAAAACC
CGAGTACGTGTACTCGGTTGATAGTATGGTAAGAATAAGCATCGGCTAACTTCGTGCCAG
CAGCCGCGGTAAGACGAAGGATGCAAGCGTTATCCGGATTCATTGGGTTTAAAGGGTGCG

TAGGCGGACTGATAAGTCAGTGGTGAAATCTCTCAGCTTAACTGAGAAACTGCCATTGAT
ACTGTTAGTCTAGAGTATAGATGACGTTGGCGGAATATGACATGTAGTGGTGAAATACTT
AGATATGTCATAGAACACCGATTGCGAAGGCAGCTAACGAAACTATAACTGACGCTGAGG
CACGAAAGTGCGGGGATCAAACAGGATTAGATACCCCCGTAGTCC
>Otu452
CCAGCCTACGGGAGGCAGCAGTCGAGGATCTTCGTCAATGGGCGCAAGCCTGAACGAGCG
ACGCCGCGTGCGCGATGAAGGCCTTCGGGTTGTAAAGCGCGAAAGTGGAGAGAAAGCGAA
AGTTGATCAATCCACAGTAAGGACGGGCTAAGTTCGTGCCAGCAGCCGCGGTAAGACGAA
CTGTCCTAACGTTGTTCGGAATCACTGGGCTTAAAGGGCGCGTAGGCGGTCGAACAAGTC
AGGGGTGAAATCCTGCAGCTTAACTGTAGAAGTGCCTCTGATACTGTTCGTCTGGAGGGA
GATAGGGGTGTGCGGAACTTCCAGTGGAGCGGTGAAATGCGTTGATATTGGAAGGAACGC
CGGAGGCGAAAGCGGCGCACTGGATCTCTACTGACGCTGAGGCGCGAAAGCCAGGGGAGC
AAACGGGATTAGATACCCTCGTAGTCC
>Otu453
CCAGCCTATGGGGCGCAGCAGTCAAGAATCTTGGTCAATGGGCGAAAGCCTGAACCAGCG
ATGTCGCAGCGTGATGAAGGCTTATAGTCGTAAAGCGCCAAAGGATGGATACAGAATGAG
ATGTCCATCCAGAGTAGCCCTGTGTAATCTCGGTGCCAGCACACGCGGTCATACCGAAAG
GGCGAGGCATTCCTATTCGAAATGGGTGTAAAGAGTCATTAGGCGGACTTATCCAATCCT
ATCTGAAAGAACGAGGCAAAACCTTAAGGGAGGATGGGTTGATTAGAAGTCTAGAGTATA
GCAGGGGGTCATGGAATGCTTGGCGTACCGATGGAATGGATTGATGCTAAGTGGAACTCC
AATGGCGAAGGCAATGACCTGGGCTATTACTGACGCTGACTGACGAAAGTGTGGGGAGCA
AAGTGGATTAGAAACCCGCGTAGTCC
>Otu455
CCAGCCTACGGGAGGCTGCAGTAGGGAATATTGCATAATGGGCGCAAGCCTGATGCAGCA
ACGCCGCGTGTGCGATGAAGGCCTTCGGGTCGTAAAGCACTTTTAGAGCGGATGAGGAAG
GACAGTACGCTCTGAATAAGCCTCGGCTAACTACGTGCCAGCAGCCGCGGTAAAACGTAG
GAGGCGAGCGTTATCCGGATTTACTGGGCGTAAAGCGTGTGCAGGCGGTTGAGAAAGTTG
GATGTGAAAGCTCCTGGCCTAACTGGGAGAGGTCGTTCAATACTACTTGACTAGAGAGTG
GGAGAGGGAGGTGGAATTCCGGGTGTAGTGGTGAAATGCGTAGATATCCGGAGGAACACC
AGTGGCGAAAGCGGCCTCCTGGCCCATTTCTGACGCTCATACACGAAAGCTAGGGGAGCG
AACGGGATTAGAAACCCTCGTAGTCC
>Otu456
CCAGCCTACGGGGGGCAGCAGTGGGGAATTTTGCGCAATGGGCGAAAGCCTGACGCAGCA
ACGCCGCGTGAGTGATGAAGGCTTTCGGGTCGTAAAGCTCTGTCAAGGGGAAAGAAGTGT
ATTGTGGTTAATACCCATGATACTTGACGGTACCCCTAAAGGAAGCACCGGCTAACTCCG
TGCCAGCAGCCGCGGTAATACGGGGGGTGCAAGCGTTGTTCGGAATTATTGGGCGTAAAG
CGCGTGTAGGCGGTTTGTTAAGTCTGATGTGAAAGCCCTGGGCTCAACCCAGGAAGTGCA
TTGGAAACTGGCAGACTTGAATACGGGAGAGGGTAGTGGAATTCCTGGTGTAGGAGTGAA
ATCCGTAGATATCAGGAGGAACACCGGTGGCGAAGGCGGCTGCCTGGACCGATATTGACG
CTGAGACGCGAAAGCGTGGGTAGCAAACAGGATTAGAAACCCCCGTAGTCC
>Otu457
CCAGCCTATGGGAGGCTCCAGTGGGGAATTTTGCGCAATGGGCGAAAGCCTGACGCAGCA
ACGCCGCGTGGGTGATGAAGGTCTTCGGATCGTAAAACCCTGTCGTCGGGGACGAAGGTC
GCATGTCTAATAGATGTGTGGCTTGACGGTACCCGGAGAGGAAGCCCCGGCTAACTCTGT
GCCAGCAGCCGCGGTAATACAGAGGGGGCAAGCGTTATTCGGAATTATTGGGCGTAAAGG
GCGCGTAGGCGGTTCGTTAAGTCAGATGTGTAATCCCCGAGCTCAACTTGGGAACTGCAT
CTGAGACTGGCGGGCTAGAGTACTGGAGAGGGTGGTAGAATTCCACGTGTAGCGGTGAAA
TGCGTAGAGATGTGGAGGAATACCAGTGGCGAAGGCGGCCACCTGGACAGTAACTGACGC
TGAGGCGCGAAAGTGTGGGTAGCAAACAGGATTAGATACCCTCGTAGTCC
>Otu458
CCAGCCTATGGGTCGCAGCAGTGGGGAATTTTGGACAATGGGGGCAACCCTGATCCAGCC
ATGCCGCGTGAGTGAAGAAGGCCTTCGGGTTGTAAAGCTCTTTCGGCGGGGACAAAACGG
CGACTGCGAATAACGGTCGTTAATGATGGTACCCGAAGAAGAAGCACCGGCTAACTACGT
GCCAGCAGCCGCGGTAATACGTAGGGTGCGAGCGTTAATCGGAATTACTGGGCGTAAAGC
GTGCGCAGGTGGTTTCGTAAGTCAGATGTGAAAGCCCCGGGCTTAACCTGGGAATGGCAT
TTGAGACTGCGAAGCTAGAGTACGGCAGAGGGAGGTGGAATTCCACGTGTAGCAGTGAAA
TGCGTAGATATGTGGAGGAACACCGATGGCGAAGGCAGCCTCCTGGGCCAGTACTGACGC
TCATGCACGAAAGCGTGGGGAGCAAACAGGATTAGATACCCGAGTAGTCC

>Otu459
CCAGCCTATGGGGCGCAGCAGTGAGGAATATTGCACAATGGGAGAAATCCTGATGCAGCA
ACGCCGCGTGAATGATGAAGGTCTTCGGATCGTAAAATTCTTTTGTAAGGGACGAATGTG
CCGCTCTGTGGCATTGACGGTACCTTGCGAATAAGACACGGCTAACTCTGTGCCAGCAGC
CGCGGTAATACAGAGGTGTCAAGCGTTGTCCGGATTTACTGGGTGTAAAGGGTGCGTAGG
CGGTTATATAAGTCAGAGGTTAAAGCTCCTTGCTTAACAAGGAAATCGCTTTTGATACTG
TATAACTTGAGTACTGAAAAGGAAGATGGAATTTCCGGTGTAGCGGTGGAATGCGTAGAT
ATCGGAAAGAACACCAGCAGCGAAGGCAGTCTTCTGGTCAGTAACTGACGCTCATGCACG
AAAGCGTGGGGATCAAACAGGATTAGATACCCCCGTAGTCC
>Otu460
CCAGCCTATGGGGGGCTGCAGTAAGGAATATTGGTCAATGGACGCAAGTCTGAACCAGCC
ATGCCGCGTGCAGGATGACAGCCCTATGGGTTGTAAACTGCTTTTGTACGGAAAAAAACC
TCCGGTCGTGAACCGGAGCTGATGGTACCGTAAGAATAAGCATCGGCTAACTCCGTGCCA
GCAGCCGCGGTAATACGGAGGATGCAAGCGTTATCCGGATTCATTGGGTTTAAAGGGTGC
GCAGGCGGAATGATAAGTCAGTGGTGAAATCCTACAGCTTAACTGTAGAACTGCCATTGA
TACTGTCGTTCTTGAGTACACTTGAAGTGGGCGGAATGTGTCATGTAGCGGTGAAATGCT
TAGATATGACACAGAACACCGATCGCGAAGGCAGCTCACTAAAGTGTTACTGACGCTCAT
GCACGAAAGCGTGGGGATCAAACAGGATTAGAAACCCGAGTAGTCC
>Otu461
CCAGCCTATGGGAGGCAGCAGTAACGAATCTTCCGCAATGCACGAAAGTGTGACGGAGCG
ACGCCGCGTGTGGGACGAAGCCCTTCGGGGTGTAAACCACTGTCAAGAGATAGAAAGTTC
TGATCATCTCTAGAGGAAGGCACGGCTAACTCTGTGCCAGCAGCCGCGGTAAGACAGAGG
TGCCAAGCGTTAGGCGGAATCACTGGGCTTAAAGCGTGTGTAGGTGGACCTGTAAGTACC
TTGTGAAATCCCACGGCTTAACCGTGGAACTGCTCGGTATACTGCGGGTCTTGAGGTACC
TAGGGGCGAGTGGAACAAATGGTGGAGCGGTGAAATGCGTAGATATCATTTGGAACGCCA
ATGGAGAAATCAACTCGCTGGGGGTATCCTGACACTGAGACACGAAAGCCAGGGGAGCAA
ACGGGATTAGAGACCCTAGTAGTCC
>Otu462
CCAGCCTATGGGTTGCTCCAGTGGGGAATCTTGCGCAATGGGCGAAAGCCTGACGCAGCG
ACGCCGTGTGTGCGATGAAGGTTTTCGGATCGTAAAGCACTGTCGCCCGGGAAAAAGAAA
TGATGGTACCGGGAGAGGAAGCACCGGCTAACTCTGTGCCAGCAGCCGCGGTAATACAGA
GGGTGCGAGCGTTGTTCGGAATTATTGGGCGTAAAGCGCTGGTAGGTGGCTTGTTAAGTC
GCGTGTGAAATCTCCAGGCTCAACCTGGAAAGTGCGCGCGAAACTGGCGAGCTTGAGTGC
CGGAGAGGGTCGCGGAATTCCCAGTGTAGAGGTGAAATTCGTAGATATTGGGAGGAACAC
CAGAGGCGAAGGCGGCGACCTGGAAGGTAACTGACACTGAGCAGCGAAAGCGTGGGGAGC
AAACAGGATTAGAGACCCTCGTAGTCC
>Otu463
CCAGCCTATGGGTGGCAGCAGTGAGGAATCTTGCGCAATGGGGGCAACCCTGACGCAGCA
ACGCCGCGTGAGTGATGAAGGCCTTCGGGTCGTAAAGCTCTGTCAGATGGGAAGAAAGTT
TCGGGCGTGAATAATTCCCGGAATTGACGGTACCGTCAGAGGAAGCACCGGCTAACTCCG
TGCCAGCAGCCGCGGTAATACGGAGGGTGCAAGCGTTGTTCGGAATTATTGGGCGTAAAG
AGCGTGTAGGCGGCCGGGTAAGTCAGATGTGAAAGCCCTTGGCTTAACCAAGGAAGTGCA
TTTGAAACTGCCTGGCTTGAGTACGGGAGGGGGAAGTGGAATTCCCGGTGTAGAGGTGAA
ATTCGTAGATATCGGGAGGAACACCGGTGGCGAAGGCGGCTTCCTGGACCGATACTGACG
CTGAGACGCGAAAGCGTGGGGAGCAAACAGGATTAGATACCCCCGTAGTCC
>Otu464
CCGCCTATGGGGGGCAGCAGTGAGGAATATTGCACAATGGGCGAAAGCCTGATGCAGCGA
CGCCGCGTGGAGGATGACGGTCTTCGGATTGTAAACTCCTGTTAAGTGTGAAAAAAGACT
TGTTTTTAATAGAAGCAAGGGATGATAGTAACACTAGAGAAAGCACCGGCTAACTTCGTG
CCAGCAGCCGCGGTAATACGAGGGGTGCAAACGTTATTCGGAATCACTGGGCGTAAAGGG
TGCGTAGACGGCAAAATAAGTCAATTATTAAATCTCCTAGCTTAACTAGGAAACTGTGAT
TGAAACTGATTTGCTTGAGGATGGAAGAGAGAAGTGGAATTCTCGGAGTAGCGGTAAAAT
GCGTAGATCTCGAGAGGAACACCGATGGCGAAGGCAGCTTCTTGGTCCATTTCTGACGTT
GAGGCACGAAAGCGTGGGGAGCAAACAGGATTAGAGACCCGAGTAGTCC
>Otu468
CCAGCCTACGGGAGGCAGCAGTCGAGAATATTCGACAATGGGCGAAAGCCTGATCGAGCG
ACGCCGCGTGCAGGATGAAGGCCTTCGGGTCGTAAACTGCGGTAGTATGGTAACAATGCA
AATGAGTGCCATACGGAAAGAGGTGGGTAACTACGTGCCAGCACCAGCGGTAATACGTAG

ACCTCAAGCGTTATCCGGATTTATTGGGCGTAAAGCGCATGTAGGTGGTTTCGCGCGTCT
TTTGTTAAAGCCCACCGCCCAACGGTGGAGATGCAGGAGATACGGCGAGACTAGAGGGGG
TTAGAGGTGCATAGAACGCACGGTGTAGGGGTGAAATCCGTTGATATCGTGCGGAATACC
AAAGGCGAAGGCATTGCACTGGGACCTTCCTGACATTGAGATGCGAAAGCGTGGGGAGCA
AAAAGGATTAGAAACCCTGGTAGTCC
>Otu470
CCAGCCTATGGGAGGCAGCAGTAAGGAATATTGGTCAATGGACGCAAGTCTGAACCAGCC
ATGCCGCGTGGAGGATGAAGGTCCTCTGGATTGTAAACTTCTTTTATTTGGGAAGAAACC
ACCGATTTCTATTGGTGTTGACGGTACCAGATGAATAAGCACCGGCTAACTCCGTGCCAG
CAGCCGCGGTAATACGGAGGGTGCAAGCGTTATCCGGATTCACTGGGTTTAAAGGGTGCG
TAGGCGGGTTGGTAAGTCCGTGGTGAAATCTCCAAGCTTAACTTGGAAACTGCCGTGGAT
ACTATCAATCTTGAATATCGTGGAGGTGAGCGGAATATGTCATGTAGCGGTGAAATGCTT
AGATATGACATAGAACACCATTGCGAAGGCAGCTCGCTACACGGTTATTGACGCTGAGGC
ACGAAAGCGTGGGGATCAAACAGGATTAGAGACCCCCGTAGTCC
>Otu471
CCAGCCTATGGGATGCAGCAGTGGGGAATCTTGGACAATGAGTGAAAACTTGATCCAGCG
ATATCACATGAGTGAAGAAGGCCATTTGGTTGTAAAGCTCTTTCGTTAACGAAGATAATG
ACAGTAGTTAAAGTAAGAAGTCCTGGCAAATCCTGTGCCAGCAGCCGCGGTAATACAGGA
GGGGCAAGCGTTATTCGGATTTAATGGGCGTAAAGGGTGCGTAGGCTATAGATTAGATTA
AATATGAAATACCAAGGCTTAACCTTGGAACTGTATTTGAAATCGATTTATTTGAGTTAG
ATAGAGGTTTGTAGAATTCTTAAAGTAAAGGTAATATTTTTCGATATTAGGAGGAATACC
TGAGGCGAAGGCGACATTCTGGATCTATACTGACGCTGAGGCACGAAAGCGTGGGGAGCA
AACAGGATTAGATACCCTGGTAGTCC
>Otu473
CCAGCCTACGGGAGGCAGCAGTGGGGAATATTGGACAATGGGCGCAAGCCTGATCCAGCC
ATGCCGCGTGAGTGATGAAGGCCTTAGGGTTGTAAAGCTCTTTTGTCAGGGAAGATAATG
ACGGTACCTGAAGAATAAGCACCGGCTAACTTCGTGCCAGCAGCCGCGGTAATACGAAGG
GTGCTTGCGTTGTTCGGAATTACTGGGCGTAAAGCGCGCGTAGGCGGCTATCCAAGTCAG
AGGTGAAAGCCCTGGGCTCAACCCAGGAATTGCCTTTGAAACTGTATAGCTAGAGTGTCG
GAGGGGATAGCGGAATTCCTAATGTAGAGGTGAAATTCGTAGATATTAGGAGGAACACCG
GTGGCGAAGGCGGCTATCTGGACGACAACTGACGCTGAGGCGCGAAAGCACGGGGATCAA
ACAGGATTAGAAACCCGAGTAGTCC
>Otu477
CCAGCCTACGGGTTGCTGCAGTAGGGAATTTTCCACAGTGGGCGAAAGCCTGATGGAGCA
ACGCCGCGTGCAGGATGAAGGCCTTTGGGTTGTAAACTGCTTTTCTCCGTGAGGAATATG
ACAGTAGCAGAGGAATAAGGATCGGCTAACTCCGTGCCAGCAGCCGCGGTCATACGGAGG
ATCCAAGCGTTATCCGGAATTACTGGGCGTAAAGAGTTGCGTAGGCGGCAGAGTAAGCAT
GGCATGAAAGCGTATGGCTCAACCATATATACATGTTGTGAACTGCTCAGCTAGAGGATG
AGAGAGGTGGCTGGAATTCCCAGTGTAGGAGTGAAATCCGTAGATATTGGGAGGAACACC
GATGGCGTAGGCAGGCCACTGGCTCATTCCTGACGCTCAGGCACGAAAGCGTGGGGAGCG
ACCGGGATTAGAAACCCCGGTAGTCC
>Otu479
CCAGCCTACGGGTGGCTGCAGCCGAGAATATTCGACAATGGGCGAAAGCCTGATCGAGCG
ACGCCGCGTGATGGATGAAGTGCTTCGGCATGTAAACATCTTTTATAGGCGAGAAAGTTT
ATTGATCAGCCTAAGAATAAGGGGTTGCTAAACTCGTGCCAGCAGCAGCGGTAATACGAG
TGCCCCAAGCGTTATCCGGAATCATTGGGCGTAAAGGGTGTGTAGGCGGTCTGGTTAGTC
TTCTGTTAAATTCTTCGGCTTAACCGGGGGCATGCAGGGGAAACGGCCAAACTAGAGGAT
GCGAGAGGTGAATGGAACTCATGGAGTAGGGGTGAAATCCGTTGATATCATGGGGAACAC
CAAATGCGAAGGCAATTCACTGGCGCACTCCTGACGCTGAAACACGAAAGCGTAGGTAGC
GAATGGGATTAGATACCCCAGTAGTCC
>Otu480
CCAGCCTATGGGGGGCTGCAGTAGGGAATATTGCACAATGGAGGAAACTCTGATGCAGCG
ACGCCGCGTGAGTGACGAAGGACTTCGGTTCGTAAAGCTCTGTTGCAGGGGAATAACAAA
ATGAAGGTACTCTGCGAGAAAGGGTCGGCTAACTTCGTGCCAGCAGCCGCGGTAAGACGA
GGGACCCGAGCGTTGTTCGGAATCATTGGGCGTAAAGCGGGTGTAGGTGGCTTTGTAAGT
CAGGTGTGAAAGCCCAGGGCTTAACCTTGGAAGTGCATTTGATACTGCGAAGCTTGAGTG
TGGGAGAGGTAAGTAGAATTCCAGGTATAGTGGTGAAATACGTAGATATCTGGAGGAATA
CCGGAGGCGAAGGCGGCTTACTGGCCCAACACTGACACTCAGACCCGAAAGCGTGGGGAT

CAAACAGGATTAGAAGCCCGCGTAGTCC
>Otu481
CCAGCCTATGGGTTGCAGCAGTCGAGGATCTTCGGCAATGGGCGCAAGCCTGACCGAGCG
ACGCCGCGTGTGCGATGAAGGCCTTCGGGTTGTAAAGCACTGTCGAGGGGGATAAAGCCG
CAAGGTTGATTGATCCCTGGAGGAAGCACGGGCTAAGTTCGTGCCAGCAGCCGCGGTAAG
ACGAACCGTGGGGACGTTGTTCGGAATCACTGGGCTTAAAGGGCGCGTAGGCGGCAAACC
AAGTCCGGGGTGAAAGCCCCCGGCTCAACCGGGGAAGTGCCTTGGATACTGGTTTGCTCG
AGGGGGGTAGGGGCAGAGGGAACTTCTGGTGGAGCGGTGAAATGCGTTGATATCAGAAGG
AACGCCGGTGGCGAAAGCGCTCTGCTGGATCTCTACTGATGCTGAGGCGCGAAAGCTAGG
GGAGCAAACGGGATTAGAAACCCTTGTAGTCC
>Otu482
CCAGCCTATGGGGGGCAGCAGTGGGGAATTTTGGACAATGGGCGCAAGCCTGATCCAGCC
ATGCCGCGTGTGGGAAGAAGGCCTTCGGGTTGTAAACCACTTTTGTCAGGGAAGAAACGG
TCTGATCTAATACATTGGACTAATGACGGTACCTGAAGAATAAGCACCGGCTAACTACGT
GCCAGCAGCCGCGGTAATACGTAGGGTGCGAGCGTTAATCGGAATTACTGGGCGTAAAGC
GTGCGCAGGCGGCTTTGCAAGACAGATGTGAAATCCCCGGGCTCAACCTGGGAACTGCAT
TTGTGACTGCATGGCTAGAGTACGGTAGAGGGGGATGGAATTCCGCGTGTAGCAGTGAAA
TGCGCAGATATGCGGAGGAACACCGATGGCGAAGGCAATCCCCTGGACCTGTACTGACGC
TCATGCACGAAAGCGTGGGGAGCAAACAGGATTAGATACCCGTGTAGTCC
>Otu486
CCAGCCTACGGGGCGCTCCAGTGGGGAATTTTGCGCAATGGGCGAAAGCCTGACGCAGCA
ACGCCGCGTGGGTGATGAAGGTCTTCGGATCGTAAAACCCTGTCGTCAGGGACGAAGGTT
ACGGGTTGAATATTCCCGTGACTTGACGGTACCTGGAGAGGAAGCCCCGGCTAACTCTGT
GCCAGCAGCCGCGGTAATACAGAGGGGGCAAGCGTTATTCGGAATTATTGGGCGTAAAGG
GCGCGTAGGCGGTGTTTTAAGTGAGATGTGCAATCCCCGAGCTTAACTTGGGAACTGCAT
CTCAGACTGGAACGCTAGAGTACTGGAGAGGGTGGTAGAATTCCACGTGTAGCGGTGAAA
TGCGTAGAGATGTGGAGGAATACCAGTGGCGAAGGCGGCCACCTGGACAGTAACTGACGC
TGAGGCGCGAAAGTGTGGGTAGCAAACAGGATTAGAAACCCGGGTAGTCC
>Otu487
CCAGCCTATGGGGGGCACCAGGCGCGAAAACTTTACAATGCGGGCAACCGTGATAAGGAA
ACCCCGAGTGCCAGCACAGGCTGGCTGTCCACCAGTGTAAAAAACTGGTGAAGAAAGGGC
CGGGCAAGACCGGTGCCAGCCGCCGCGGTAATACCGGCGGCTCGAGTGGTGGCCGCTTTT
ACTGGGCTTAAAGGGTCCGTAGCTGGATTGACAAGTCCCTTGAGAAATCTATCGGCTTAA
CTGATAGGCGTTCAGGGGATACTGTTATTCTAGGGACCGGGAGAGGTGAGAGGTACTGCC
GGGGTAGGAGTGAAATCCTGTAATCCCGGTGGGACGACCTATGGCGAAGGCATCTCACCA
GAACGGCTCCGACAGTGAGGGACGAAAGCTGGGGGAGCAAACCGGATTAGAAACCCCTGT
AGTCC
>Otu488
CCAGCCTATGGGTGGCTGCAGTTCGGAATATTCGGCAATGGACGAAAGTCTGACCGAGCG
ACGCCGCGTGCGGGATGAAGGCCCTTTGGGTTGTAAACCGCTGTCACAGGTGAGCAAATC
ATGTCCGGTGAACAATCGGATGTGTTGAGTTAAGCTTGAGAGGAAGCCCCCGCTAATCAC
GTGCCAGCAGCGGCGGTAATACGTGAGGGGCGAACGTTGTTCGGTGTCACTGGGCCTAAA
GGGCGCGTAGGTGGCTGCGTAAGTAAGGTGTGAAAGCCCTCGGCTTACCCGGGGAATTGC
GCCTTATACTGCGTGGCTTGAGGATTTCAGGGGAAGGGGGAACTCCGAGTGTAGCGGTGA
AATGCGCAGATATTCGGGGGAAGGCCGGCGGCGAAGGCGCCCTTCTGGGAAATTCCTGAC
ACTGAGGCGCGAAAGCGTGGGGAGCAAACAGGATTAGATACCCTCGTAGTCC
>Otu489
CCAGCCTACGGGTCGCAGCAGTAAGGAATATTGGACAATGGTGGCAACACTGATCCAGCC
ATGCCGCGTGCAGGATGAAGGCGCTACGCGTTGTAAACTGCTTTTGTACGGGAGAAAACC
TATCTACGTGTAGATAGCTGATAGTACCGTAAGAATAAGCATCGGCTAACTACGTGCCAG
CAGCCGCGGTAATACGTAGGGTGCGAGCGTTAATCGGAATTACTGGGCGTAAAGCGTGCG
CAGGCGGTTGTGTAAGACAGGTGTGAAATCCCCGGGCTTAACCTGGGAACTGCGCTTGTG
ACTGCACGACTGGAGTACGGCAGAGGGGGGTGGAATTCCACGTGTAGCAGTGAAATGCGT
AGAGATGTGGAGGAACACCGATGGCGAAGGCAGCCCCCTGGGCTAGTACTGACGCTCATG
CACGAAAGCGTGGGTAGCAAACAGGATTAGATACCCCAGTAGTCC
>Otu492
CCAGCCTACGGGAGGCTCCAGTAGGGAATCTTCCACAATGGGCGAAAGCCTGATGGAGCA
ACGCCGCGTGCAGGATGAATGCCTTAGGGTTGTAAACTGCTTTTATCTGTGACGAATATT

ACGGTAGCAGATGAATAAGGATCGGCTAACTCCGTGCCAGCAGCCGCGGTCATACGGAGG
ATCCAAGCGTTATCCGGAATTACTGGGCGTAAAGAGTTGCGTAGGTGGCAGAGTAAGTTG
GTAGTGAAAGAGTTCGGCTCAACCGAATAGACATTATCAAAACTGCTTAGCTAGAGGATG
AGAGAGGTTATTGGAATTCCCAGTGTAGGAGTGAAATCCGTAGATATTGGGAGGAACACC
GATGGCGTAGGCAGATAACTGGCTCATTCCTGACACTAAGGCACGAAAGCGTGGGGAGCA
AACGGGATTAGAGACCCGCGTAGTCC
>Otu493
CCAGCCTACGGGTCGCACCAGTGGGGAATATTGGACAATGGGCGCAAGCCTGATCCAGCC
ATGCCGCGTGGATGATGAAGGCCCTAGGGTTGTAAAGTCCTTTCGGCGGGGAAGATAATG
ACGGTACCCGCAGAAGAAGCCCCGGCTAACTTCGTGCCAGCAGCCGCGGTAATACGAAGG
GGGCTAGCGTTGCTCGGAATGACTGGGCGTAAAGGGCGCGTAGGCGGATTGTACAGTCAG
ATGTGAAATTCCCGGGCTTAACCTGGGGGCTGCATTTGATACGTATAGTCTTGAGTGAGG
AAGAGGGTTGTGGAATTCCCAGTGTAGAGGTGAAATTCGTAGATATTGGGAAGAACACCG
GTGGCGAAGGCGGCAACCTGGTCCTTTACTGACGCTGAGGCGCGAAAGCGTGGGGAGCAA
ACAGGATTAGAAACCCGCGTAGTCC
>Otu494
CCAGCCTACGGGGGGCAGCAGTAACGAATCTTCCGCAATGCACGAAAGTGTGACGGAGCG
ACGCCGCGTGTAGGACGAAGCCCTTCGGGGTGTAAACTACTGTCAGGGGTTAGAAAGTTC
TGATCAACCCCAGAGGAAGGCACGGCTAACTCTGTGCCAGCAGCCGCGGTAAGACAGAGG
TGCCAAGCGTTAGGCGGAATCACTGGGCTTAAAGCGTGTGTAGGCGGATTCCTAAGTACC
TTGTGAAATCCCACGGCTCAACCGTGGAACTGCTTGGTATACTGGGTGTCTTGAGCTACT
TAGGGGCGACTGGAACAAATGGTGGAGCGGTGAAATGCGTAGATATCATTTGGAATGCCA
ATGGTGAAAACAGGTCGCTGGGAGTATGCTGACGCTGAGACACGAAAGCCAGGGGAGCGA
ACGGGATTAGATACCCGTGTAGTCC
>Otu495
CCAGCCTACGGGAGGCAGCAGTCGAGAATCTTCGGCAATGGGCGCAAGCCTGACCGAGCG
ACGCCGCGTGGAGGACGAAGGCCTTCGGGTTGTAAACTCCTGTCGAGGGGGAGGAAGCCG
CAAGGTTGACCGATCCCTGGAGGAAGCACGGGCTAAGTTCGTGCCAGCAGCCGCGGTAAG
ACGAACCGTGCGAACGTTATTCGGAATCACTGGGCTTAAAGCGCGTGTAGGCGGGAAGGC
ACGTCGGTTGCTGAAATCCCCCGGCTCAACCGGGGAACGGGCACCGATACGACCTTCCTA
GAGGGGGGTAGGGGGAGCTGGAACTTCCGGTGGAGCGGTGAAATGCGTTGAGATCGGAAG
GAACGCCCGTGGCGAAAGCGAGCTCCTGGACCCTTACTGACGCTGAGACGCGAAAGCCAG
GGGAGCGAACGGGATTAGATACCCCTGTAGTCC
>Otu500
CCAGCCTACGGGATGCTCCAGTGGGGAATCTTGCACAATGGGGGCAACCCTGATGCAGCG
ACGCCGCGTGAGCGATGAAGCCCTTCGGGGTGTAAAGCTCTTTCGTCAGGGAAGAAACAG
ACGGTACCTGGAGAAGCAGCTGCGGCTAACTACGTGCCAGCAGCCGCGGTAATACGTAGG
CAGCGAGCGTTGTTCGGAGTTACTGGGCGTAAAGGGTGTGCAGGCGGTTCTTTAAGTTTG
TTGTGAAATCTCCCGGCTTAACCGGGAGGGTGCGGCGAATACTGAGGGACTAGAGTATGG
GAGAGGAAAGTGGAATTCCTGGTGTAGCGGTGAAATGCGTAGATATCAGGAGGAACACCG
GTGGTGTAGACGGCTTTCTGGACCATAACTGACGCTGATGCACGAAAGCTAGGGGAGCAA
ACAGGATTAGATACCCTGTAGTCC
>Otu501
CCAGCCTATGGGGCGCTGCAGTCGAGGATTTTTCTCAATGGGGGAAACCCTGAAGGAGCG
ACGCCGCGTGAGGGATGAAGGTCTTCGGATTGTAAACCTCTGTCATCTGGGAACAATGTA
ATCTACCTAACACGTGGATTATTGATAGTACCGGAAGAGGAAGCCGTGGCTAACTCTGTG
CCAGCAGCCGCGGTAATACAGAGACGGCGAGCGTTGTTCGGATTCATTGGGCGTAAAGGG
TGCGCAGGCGGTTCGTTAAGTCGGATGTGAAAGCTCACAGCCTAACTGTGATAGGTCATT
CGAAACTGGCGGACTCGAGGGCTGGAGAGGAGACTGGAATAGTCGGTGTAGCGGTGAAAT
GCGTAGAGATCGACTAGAACACCGGTGGCGAAGGCGGGTCTCTGGACAGTTCCTGACGCT
CATGCACGAAAGCCAGGGGAGCAAACGGGATTAGAGACCCTTGTAGTCC
>Otu504
CCAGCCTACGGGAGGCTCCAGGAGGCCGACGAATCCCAGCTTTGGCTGGAACTGCTCCGC
GAAGAGTGTGCTATCGAACCGAAGTTTACCCAATCTTTGGAGCAGGAGTCTTCGGAGTTT
ATCGCCATCTTCACCACGATGATCAACAAAACCAAAGGAAAAGGCTGAAAACCAAAGCAG
AAAGCTGAAACATGGAAAGTTGAATAAATTTCTGTTTTCTGCTTTCCGAATTTCTTTTTC
TACCCTTGGTTAATGTCCTTGGCCCGTGAAGCCCTCGGTTTGCTACTTCACAGGCCGAGA
CCCGTGAAGCCTGCATTTGATGCTTCACCGGGCAGCGCATCAGCTCTATAAAGACCCGG

TACATAAAATGCTATTTTATATTCTTGAACGAGTTGAAGGAGCGCAGCACTATTACGGCT
GTTGCAACCAAGTAGCGGACACGATTATGAACGCGAACGGAGCGATTAGATACCCTAGTA
GTCC
>Otu505
CCAGCCTATGGGGGGCTGCAGTCGAGGATCTTCGGCAATGAGCGCAAGCTTGACCGAGCG
ACGCCGCGTGTGCGATGAAGGCCTTCGGGTTGTAAAGCACTGTCGAGGGGGAGAAAAGGG
TAAAACCTTGATCTATCCCTGGAGGAAGCACGGGCTAAGTTCGTGCCAGCAGCCGCGGTA
AGACGAACCGTGCGAACGTTGTTCGGAATCACTGGGCTTAAAGGGCGCGTAGGCGGGCTG
TCAAGTCTGGGGTGAAATCCCGCGGCTCAACCGTGGAACTGCCTTAGATACTGACGGCCT
CGAGGGAGGTAGGGGCGAGCGGAACTGTGGGTGGAGCGGTGAAATGCGTTGATATTCACA
GGAACTCCGGTGGCGAAGGCGGCTCGCTGGCCCTCTTCTGACGCTGATGCGCGAAAGCTA
GGGGAGCAAACGGGATTAGAAACCCCCGTAGTCC
>Otu507
CCAGCCTATGGGTTGCTGCAGTGGGGAATCTTGCGCAATGGGCGAAAGCCTGACGCAGCA
ACGCCGCGTGGAGGACGAAGGCTTTCTGAGTTGTAAACTCCTTTCAGCAGGGACGATTGT
GACGGTACCTCCAGAAGAAGCCCCGGCTAACTACGTGCCAGCAGCCGCGGTAATACGTAG
GGGGCAAGCGTTGTCCGGATTTATTGGGCGTAAAGAGCGTGTAGGCGGCCAGATAGGTCT
GTTGTGAAAACTCGAGGCTCAACCTCGAGACGCCGATGGAAACCATTTGGCTAGAGTCCG
GAAGAGGAGAGTGGAATTCCTGGTGTAGCGGTGAAATGCGCAGATATCAGGAAGAACACC
CGTGGCTAAGGCGGCTCTCTAGTACGGTACTGACGCTGAGACGCGAAAGCGTGGGGAGCG
AACAGGATTAGAAACCCCCGTAGTCC
>Otu508
CCAGCCTACGGGTTGCTGCAGTGGGGAATCTTGCGCAATGCGCGAAAGCGTGACGCAGCA
ACGCCGCGTGGGGGAAGACGGCCTTCGGGTTGTAAACCCCTTTCAGTTGGGACGAAGCCC
AGCCGGTTAATAGCCGGTATGGGTGACGGTACCTTCACAAGAAGCACCGGCTAACTACGT
GCCAGCAGCCGCGGTAATACGTAGGGTGCAAGCGTTGTCCGGAATTATTGGGCGTAAAGA
GCGTGTAGGTGGTCTGATCAGTCCGCTGTGAAAGCCCAGGGCTCAACCCTGGAATGCCGG
TGGATACTGTCAGACTTGAGTCCGGAAGGGGCGAGTGGAATTCCTGGTGTAGCGGTGAAA
TGCGCAGATATCAGGAGGAACACCTATGGCGAAGGCAGCTCGCTGGGACGGTACTGACGC
TGAGACGCGAAAGCGTGGGGGTCAAACAGGATTAGATACCCGAGTAGTCC
>Otu509
CCAGCCTACGGGTGGCTCCAGTGGGGAATCTTGCACAATGGGGGAAACCCTGATGCAGCG
ACGCCGCGTGAGTGATGAAGCCCTTCGGGGTGTAAAGCTCTTTCGACGGGAACGATAATG
ACGGTACCCGGAGAAGAAGCTGCGGCTAACTACGTGCCAGCAGCCGCGGTAATACGTAGG
CAGCAAGCGTTGTTCGGAATTACTGGGCGTAAAGCGAGTGTAGGTGGTGCTATAAGTTTG
GTGTGAAATCTCCCGGCTCAACTGGGAGGGTGCGCCGAAGACTGTAGTGCTAGAGTGTGG
GAGAGGTAGGCGGAATTCCTGGTGTAGCGGTGAAATGCGTAGATATCAGGAGGAACACCT
GCGGTGTAGACGGCTTACTGGACCACGACTGACACTGAAACTCGAAAGTGTGGGGAGCAA
ACAGGATTAGATACCCTAGTAGTCC
>Otu510
CCAGCCTATGGGGGGCAGCAGTGGAGAATCTTGCGCAATGGGCGAAAGCCTGACGCAGCA
ACGCCGCGTGTGTGATGAAGGTCTTCGGATCGTAAAGCACTGTCGCGAGGGAAGATACAC
TTCACCATAATACGGTGAGGATAAGACGGTACCTCGAGAGGAAGCACCGGCTAACTCTGT
GCCAGCAGCCGCGGTAATACAGAGGGTGCGAGCGTTGTTCGGAATTATTGGGCGTAAAGC
GCTTGTAGGCGGCTTGTCAAGTCTCGTGTGAAATCCCCAGGCTTAGCCTGGGAAGTGCGC
GGGAAACTGGCGAGCTTGAGTGCCGGAGAGGGTCGCGGAATTCCCGGTGTAGAGGTGAAA
TTCGTAGATATCGGGAGGAACACCAGTGGCGAAGGCGGCGACCTGGACGGACACTGACGC
TGAGACGCGAAAGCGTGGGGAGCAAACAGGATTAGAGACCCTCGTAGTCC
>Otu511
CCAGCCTATGGGGGGCTGCAGTCGAGGATCTTCGGCAATGGGCGCAAGCCTGACCGAGCG
ACGCCGCGTGTGCGATGAAGGCCTTCGGGTTGTAAAGCACTGTCGAGGGGGAGGAAGCCC
GCAAGGGTTGACTAATCCCTGGAGGAAGCACGGGCTAAGTTCGTGCCAGCAGCCGCGGTA
AGACGAACCGTGCGAACGTTGTTCGGAATCACTGGGCATAAAGGGCGCGTAGGCGGGTCA
TAAAGTCCGTGGTGAAAGCCCCCAGCTTAACTGGGGAAGTGCTTCGGATACTGATGGTCT
CGAGTGGGGTAGGGGCATGCGGAACTTCCGGTGGAGCGGTGAAATGCGTAGATATCGGAA
GGAACGCCGGTGGCGAAAGCGGCGTGCTGGACCCCAACTGACGCTGAGGCGCGAAAGCCA
GGGGAGCAAACGGGATTAGAAACCCGAGTAGTCC
>Otu513

CCAGCCTATGGGGGGCAGCAGTGGGGAATATTGGACAATGGGGGAAACCCTGATCCAGCC
ATGCCGCGTGTGTGATGAAGGCCTTCGGGTTGTAAAGCACTTTCGCACGTGACGATGATG
ACGGTAACGTGAGAAGAAGCCCCGGCTAACTTCGTGCCAGCAGCCGCGGTAATACGAAGG
GGGCAAGCGTTGTTCGGAATTACTGGGCGTAAAGGGCGCGTAGGCGGTGTACTAAGTTAG
GCGTGAAAGCCCTGGGCTCAACCCGGGAATTGCGCTTGATACTGGTACGCTAGAATCCAG
GAGAGGTTCGTGGAATTCCCGGTGTAGAGGTGAAATTCGTAGAGATTGGGAAGAACACCG
GTGGCGAAGGCGGCCAACTGGACTGGTATTGACGCTGAGGCGCGAAAGCGTGGGGAGCAA
ACAGGATTAGATACCCGCGTAGTCC
>Otu517
CCAGCCTACGGGAGGCAGCAGTGGGGAACCTTGCGCAATGGACGAAAGTCTGACGCAGCC
ACGCCGCGTGAGTGAAGAAGGCCTTCGGGTTGTAAAGCTCTGTCGGGCGGGACGAAAATA
CTTAGGGTGAATAGCCCTAGGGACTGACGGTACCGCCAAAGGAAGCACCGGCTAACTTCG
TGCCAGCAGCCGCGGTAATACGAGGGGTGCAAGCGTTGCTCGGAATTATTGGGCGTAAAG
GGTAGGTAGGTGGTCTCGTTTGTCTGGGGTGAAATCCTTGAGCTTAACTCAAGAAGTGCC
CTAGAAACGGCGAGACTAGAGTTCTGGAGAGGGTCGTGGAATTCCCGGTGTAGCGGTGAA
ATGCGTAGAGATCGGGAGGAACACCAGAGGCGAAGGCGGCGACCTGGACAGATACTGACA
CTCAACTACGAAAGCGTGGGGAGCAAACAGGATTAGAAACCCCGGTAGTCC
>Otu518
CCAGCCTATGGGTGGCAGCAGTGGGGAATCTTGCGCAATGGGCGAAAGCCTGACGCAGCG
ACGCCGCGTGTGCGATGAAGGTCTTCGGATCGTAAAGCACTGTCGCGAGGGAAGAAACCT
GACGGTACCTCGAGAGGAAGCACCGGCTAACTCTGTGCCAGCAGCCGCGGTAATACAGAG
GGTGCGAGCGTTGTTCGGAATTATTGGGCGTAAAGCGCTGGTAGGTGGCCTGTCAAGTCG
CGTGTGAAAGCTCCCGGCTCAACCGGGAAAGTGCGCGCGAAACTGGCTGGGCTTGAGTGC
CGGAGAGGGTTGCGGAATTCCCGGTGTAGAGGTGAAATTCGTAGATATCGGGAGGAACAC
CAGAGGCGAAGGCGGCAACCTGGAAGGTGACTGACACTGAGCAGCGAAAGCGTGGGTAGC
AAACAGGATTAGATACCCGAGTAGTCC
>Otu520
CCAGCCTATGGGGTGCACCAGTCGAGAATATTCGACAATGGGCGAAAGCCTGATCGAGCG
ACACCGCGTGCAGGATGAAGGCCCTCGGGTCGTAAACTGCGGTAGATAATTAACAATGTA
AATGAGTGATTATCGGAAAGAGGTGGGTAACTACGCGCCAGCACCAGCGGTAATACGTAG
ACCTCAAGCGTTATCCGGACTTATTGGGCGTAAAGAGCATGTAGGAGGTTTCGCGCGTCT
TTTGTTAAAGCCCACCGCCCAACGGTGGAAGTGCAGGAGATACGGCGGGACTAGAGGAGG
TTAGAGGTGCATGGAACTCACGGTGTAGGGGTGAAATCCGTTGATATCGTGGGGAACACC
AAAGGCGAAGGCAGTGCACTGGGACCTTCCTGACTCTGAGATGCGAAAGCGTGGGGAGCA
AAAAGGATTAGAGACCCTAGTAGTCC
>Otu521
CCAGCCTATGGGTCGCTCCAGTGGGGAATTTTACGCAATGGGCGAAAGCCTGACGTTGCG
ACACCGCGTGAGCGAAGAAGCCCTTTGGGGTGTAAAGCTCTGTCGGCTGGGACGAAAAAA
ATGACGGTACCAGCAAAGGAAGCATCGGCTAACTACGTGCCAGCAGCCGCGGTAAGACGT
AGGATGCAAGCGTTGTCCGGATTTATTGGGCGTAAAGAGTTCGTAGGTGGTTTGTTAAGT
CTGATGTTAAAGATCGGGGCTCAACCCCGGGAGTGCATTGGATACTGGCAGACTTGAGTG
CAGTAGAGGTAAGTGGAATTCCCAGTGTAGCGGTGAAATGCGTAGATATTGGGAAGAACA
CCAGTGGCGTAGGCGACTTACTGGGCTGTAACTGACACTGAGGAACGAAAGCCAGGGGAG
CAAATGGGATTAGATACCCTCGTAGTCC
>Otu524
CCAGCCTATGGGATGCAGCAGTTTCGAATCATTCACAATGGGCGAAAGCCTGATGGTGCG
ACACCGCGTGGGGGATGAAGGTCTTCGGAATGTAAACCCCTGTCACCGGGGAAGAAACGC
TTCAATTTAACAGACTGAAGCCTGACTTAACCCGGAGAGGAAGCAGTGGCTAACTCTGTG
CCAGCAGCCGCGGTAATACAGAGACTGCAAGCGTTATTCGGATTCACTGGGCGTAAAGGG
TGCGCAGGCGGCCAAGTGTGTTAGATGTGAAATCCCGGGGCTCAACCCCGGAACTGCGTC
TAAAACTACTTGGCTAGAGCATTGGAGAGGGTAGCGGAATTCACGGTGTAGCAGTGAAAT
GCGTAGATATCGTGAGGAACACCAGAGGCGAAGGCGGCTACCTGGACAATTGCTGACGCT
CAGGCACGAAAGCGTGGGGAGCAAAAGGGATTAGATACCCCTGTAGTCC
>Otu525
CCAGCCTACGGGTGGCACCAGTCGAGAATTTTTCTCAATGGGGGAAACCCTGGAGGAGCG
ACGCCGCGTGAAGGATGAAGGTCTTCGGATTGTAAACTTCTGTCATTAGAGAACAAGTGC
CGCCGAGTAACTACCGGCGGCTTGATAGTACCTGAAGAGGAAGAGACGGCTAACTCTGTG
CCAGCAGCCGCGGTAATACAGAGGTCTCAAGCGTTGTTCGGATTCATTGGGCGTAAAGGG

TGCGTAGGTGGCGAGGTAAGTTGGATGTGAAATCCCGGGGCTTAACCCCGGAACTGCATT
CAATACTGCCTTGCTCGAGTACTGGAGAGGAGGTTGGAATTTACGGTGTAGCAGTGAAAT
GCGTAGATATCGTAAGGAAGACCGGTGGCGAAGGCGGATCTCTGGACAGTAACTGACACT
GAGGCACGAAGGCCAGGGGAGCAAACGGGATTAGAGACCCGCGTAGTCC
>Otu526
CCAGCCTACGGGGTGCACCAGTGGGGAATCTTGCGCAATGCGCGAAAGCGTGACGCAGCA
ACGCCGCGTGAGTGAAGACGGCCTTCGGGTTGTAAAGCTCTTTCAGTTGGGACGAAGCTT
TGCCGGTTAATAGCCGGTCGGAGTGACGGTACCTTCAGAAGAAGCCCCGGCTAACTACGT
GCCAGCAGCCGCGGTAATACGTAGGGGGCGAGCGTTGTCCGGAATCATAGGGCGTAAAGC
GCGTGTAGGCGGCTTGGAAAGTCTGCTGTGAAAGTCCAGGGCTCAACCCTGGAATGCCGG
TGGAAACTATCAAGCTAGAGTCCGGAAGGGGAGTGTGGAATTCCTGGTGTAGCGGTGAAA
TGCGCAGATATCAGGAGGAACACCAATGGCGAAGGCAGCACTCTGGGACGTGACTGACGC
TGAGACGCGAAAGCGTGGGGAGCAAACAGGATTAGAACCCCAGTAGTCC
>Otu527
CCAGCCTATGGGGGGCAGCAGTGGGGAATCTTGCGCAATGGGCGAAAGCCTGACGCAGCA
ACGCCGCGTGAGCGAAGAAGGCCTTCGGGTTGTAAAGCTCTGTCAGGGGGAAAGAAGGCA
CCTGGGTTAATAGCCCAAGGGCTGACGGTACCTCCAAAGGAAGCCACGGCTAACTACGTG
CCAGCAGCCGCGGTAATACGTAGGTGGCGAGCGTTGTCCGGAATCATTGGGCGTAAAGAG
CGCGTAGGTGGCTTGTTAGGTCTGGTGTGAAATCTTGGGGCTCAACCCCAAGCGTGCACT
GGAAACCGGCTTGCTAGAGTCTGGGAGAGGAGAGTGGAATTCCTGGTGTAGCGGTGAAAT
GCGTAGATATCAGGAGGAACACCGGTAGCGAAGGCGGCTCTCTGGAACAGTACTGACGCT
GAGGCGCGAAAGCTAGGGGAGCGAACAGGATTAGAGACCCGTGTAGTCC
>Otu528
CCAGCCTACGGGGGGCTCCAGTCGAGAATCATTTGCAATGGACGAAAGTCTGACAATGCG
ACGCCGCGTGCAGGAGGAAGGCCTTCGGGTTGTAAACTGCTGTCAGGGGTTATGAACGTC
ACGGGACTAACACTTCTGTGATCTGACAAAGGCCCCAGAGGAAGCTCCGGCTAAATCCGT
GCCAGCAGCCGCGGTAATACGGATGGAGCAAGCGTTGTTCGGAATCACTGGGCATAAAGC
GCATGTAGGCGGCTCTGAAAGTAGGGTGTGAAATCCCCTGGCCCACCCAGGGAACTGCAT
TCTAAACTTCAGGGCTTGAGTGAAGGAGGGGAGAGAGGAACTCCTGGTGGAGCGGTAAAA
TGCGTAGATATCAGGAGGAACGCCGGTGGAGAAGTCGTCTCTCTGGCCTTTTACTGACGC
TGAGATGCGAAAGCTAGGGGAGCAAACGGGATTAGAAACCCGAGTAGTCCC
>Otu530
CCAGCCTATGGGGGGCACCAGACGAGAATATTCCGCAATGGACGAAAGTCTGACGGAGCG
ACATCGCGTGCTGGATGAAGTGCTTAGGTACGTAAACAGCTTTTATCGGGGACGAAGTAA
TTGACGGTACCCGATGAATAAGGGGCTCCTAACTCTGTGCCAGCAGGAGCGGTAATACAG
AGGCCCCAAGCATTACCCGGAATCACTGGGCGTAAAGGGTGTCCAGGCGGCCCTATTAGT
CTCTTGTAAAATCCGTGGGCTCAACCTACGGCGCGCAAGGGAAACGGTAGGGCTCGAGGG
CGCGAGAGGTACAGGGAACTCATGGTGGAGGGGTGAAATCCGTTGATATCATGGGGAACA
CCAAAGGCGAAGGCACTGTACTGGCGCGTTCCTGACGCTCACACACGAAAGCCAGGGTAG
CGAACGGGATTAGATACCCCAGTAGTCC
>Otu533
CCAGCCTATGGGAGGCACCAGTGGGGAATATTGCACAATGGGCGCAAGCCTGATGCAGCG
ACGCCGCGTGCGGGATGAAGGCCTTCGGGTTGTAAACCACTTTCACCAGGGACGAAGCGA
GAGTGACGGTACCTGGGGAAGAAGCGCCGGCTAACTACGTGCCAGCAGCCGCGGTAATAC
GTAGGGCGCAAGCGTTGTCCGGAATTATTGGGCGTAAAGAGCTCGTAGGCGGCTTGTCAC
GTCTGCTGTGAAATCCCGGGGCTCAACCCCGGGCGTGCAGTGGATACGGGCTTGCTAGAG
GCAGGCAGGGGAGAACGGAATTCCCGGTGTAGCGGTGAAATGCGCAGATATCGGGAGGAA
CACCGGTGGCGAAGGCGGTTCTCTGGGCCTGTTCTGACGCTGAGGAGCGAAAGCGTGGGG
AGCAAACAGGATTAGAAACCCCCGTAGTCC
>Otu534
CCAGCCTATGGGATGCAGCAGTGGGGAATATTGGACAATGGGGGAAACCCTGATCCAGCG
ACGCCGCGTGGGTGAAGAAGGCCTGCGGGTTGTAAAGCCCTTTCAGTGGAGAAGAAAAGC
CTCGACCTAATACGTCGGGGTCTTGACGTAACTCACAAAAGAAGCACCGGCTAACTCTGT
GCCAGCAGCCGCGGTAATACAGAGGGTGCGAGCGTTAATCGGAATTACTGGGCGTAAAGC
GCGCGTAGGCGGCTTTGCAAGTCGAGTGTGAAATCCCCGGGCTCAACCTGGGAACTGCAT
TCGAGACTGCATTGCTAGAGTATGGGAGAGGGAGGTGGAATTTCAGGTGTAGCGGTGAAA
TGCGTAGATATCTGAAGGAACATCAGTGGCGAAAGCGACCTCCTGGACCAATACTGACGC
TCATGTGCGAAAGCGTGGGGAGCAAACAGGATTAGAAACCCTTGTAGTCC

>Otu535
CCAGCCTACGGGTGGCACCAGGCGCGAAACCTCTACAATGCACGCAAGTGTGATAGGGGA
ATCCCAAGTGCTCAAGCACAGCTTGGGCTTTTGCTATGTGTAAATAGCATGGCGAATAAG
TGGTGGGCAAGACTGGTGCCAGCCGCCGCGGTAACACCAGCGCCACGAGTGGCAATCGCG
TTTATTGGGCCTAAAGCGTCCGTAGCAGGTTTTGTAAATTTCTTGTGAAACTGCCAGGCT
CAACTTGGCAATGTGCAGGAAACACTGCAGAACTAGAGACCGGGAAGGGTCAGAGGTATT
CCTGAGGGAGCGGTAAAATGCTATAATCTCAGGAGGACCACCGGTGGCGAAGGCGTCTGA
CTAGAACGGATCTGACTGTGAGGGACGAAAGCTAGGGGAGCAAACCGGATTAGATACCCT
CGTAGTCC
>Otu536
CCAGCCTACGGGGGGCACCAGTCGAGAATCTTCGGCAATGAGCGCAAGCTTGACCGAGCG
ACGCCGCGTGCGGGATGAAGGCCTTCGGGTTGTAAACCGCTTTCAGTGGGGAGGAAATCG
CTAGGGGTTCTCCCCTAGTGTTGACCTATCTTCAGAAGAAGGACGGGCTAAGTTCGTGCC
AGCAGCCGCGGTAATACGAACCGTCCAAACGTTATTCGGATTCATTGGGCTTAAAGGGTA
CGTAGGCGGCGCCGTCAGTCGGGTGTGAAAGCCCTCGGCTCAACCGAGGAACTGCGCCCG
ATACTGCGGTGCTTGAGGGAGACAGAGGTAAGCGGAACTGATGGTGGAGCGGTGAAATGC
GTTGATATCATCAGGAACACCGGTGGCGAAAGCGGCTTACTGGGTCTCTTCTGACGCTGA
GGTACGAAAGCTAGGGGAGCGAACGGGATTAGAAACCCTAGTAGTCC
>Otu537
CCAGCCTATGGGTTGCACCAGTGGGGAATATTGCACAATGGGCGCAAGCCTGATGCAGCG
ACGCCGCGTGGGGGATGACGGCCTTCGGGTTGTAAACCTCTTTCACCATCGGCGAAGCCG
GATGCTGTTGTGTCTGGTGACGGTAGGTGGAGAAGAAGCACCGGCCAACTACGTGCCAGC
AGCCGCGGTAATACGTAGGGTGCGAGCGTTGTCCGGAATTACTGGGCGTAAAGAGCTCGT
AGGTGGTTTGTCGCGTTGTCCGTGAAATTCCCTGGCTTAACTGGGGGCGTGCGGGCGATA
CGGGCAGACTGGAGTACTGCAGGGGAGACTGGAATTCCTGGTGTAGCGGTGGAATGCGCA
GATATCAGGAGGAACACCGGTGGCGAAGGCGGGTCTCTGGGCAGTAACTGACGCTGAGGA
GCGAAAGCGTGGGGAGCGAACAGGATTAGAAACCCCCGTAGTCC
>Otu539
CCAGCCTATGGGTGGCACCAGTAGGGAATATTGCACAATGGAGGAAACTCTGATGCAGCG
ACGCCGCGTGAGTGATGAAGGACTTCGGTTCGTAAAGCTCTGTTGCAGGGGAATAAGACA
GTGAATGTACTCTGCGAGAAAGGGTCGGCTAACTTCGTGCCAGCAGCCGCGGTAAGACGA
GGGACCCGAGCGTTGTTCGGAATCATTGGGCGTAAAGCGGGTGTAGGTGGCTCTGTAAGT
CAGGCGTGAAAGCCCGGGGCTTAACCTCGGAAGTGCGCTTGATACTGCGAAGCTTGAGTG
CCGGAGAGGCTACTAGAATTCCTGGTGTAGTGGTGAAATACGTAGATATCAGGAGGAATA
CCGGTGGCGAAGGCGGGTAGCTGGCCAGACACTGACACTCAGACCCGAAAGCGTGGGGAT
CAAACAGGATTAGATACCCGGGTAGTCC
>Otu542
CCAGCCTATGGGAGGCTGCAGTGGGGAATTTTACACAATGGGCGAAAGCCTGATGTAGCG
ACACCGCGTGAGCGAAGAAGCCCTTTGGGGTGTAAAGCTCTGTCAACAGGGACGAAAAAA
ATGACGGTACCTGTGGAGGAAGCATCGGCTAACTACGTGCCAGCAGCCGCGGTAAGACGT
AGGATGCGAGCGTTGTCCGGATTTATTGGGCGTAAAGAGTTCGTAGGCGGTTTGTTAAGT
CTGATGATAAAGACTGGGGCTCAACCCTGGGAGTGCATTGGATACTGGCAGACTAGAGTG
TGGTAGAGGCAAGCGGAATTCCCAGTGTAGCGGTGAAATGCGTAGATATTGGGAAGAACA
CCGGTGGCGCAAGCGGCTTGCTGGGCCATAACTGACGCTGAGGAACGAAAGCTAGGGTAG
CGAATGGGATTAGAAACCCGTGTAGTCC
>Otu546
CCAGCCTACGGGTGGCTGCAGTCGAGAATCATTCGCAATGGGCGAAAGCCTGACGATGCG
ACGCCGTGTGAGCGATGAAGGCCTTAGGGTTGTAAAGCTCTTTCGCTTGGGAACAAGAGA
CGCTGGCTAATATCCAGCAGATTTGAGGGTACCAGGTAAAGAAGCACCGGCTAACTCCGT
GCCAGCAGCTGCGGTAATACGGAGGGTGCAAGCATTAATCGGATTTATTGGGCGTAAAGG
GCGCGTAGGCGGAAAGAAAAGTCAGATGTGAAATCCTGGGGCTCAACCTCAGAACAGCAT
TTGAAACTCTCTTTCTAGAGGGTAGGCGGAGAAAACGGAATTCCACAAGTAGCGGTGAAA
TGCGTAGATATGTGGAAGAACACCGGTGGCGAAGGCGGTTTTCTAGCTTATTCCTGACGC
TGAGGCGCGAAAGCAAGGGGATCAAACAGGATTAGATACCCTTGTAGTCC
>Otu549
CCAGCCTACGGGAGGCTCCAGTCGAGAATTTTTCACAATGGGGGAAACCCTGATGAAGCG
ACGCCGCGTGGGGGATGAATGGCTTCGGCCCGTAAACCCCTGTCATTTGTGAACAAACCT
TTCCGCTGAACAAGCGGGAAGCTGATTGTAACGGAAGAGGAAGGGACGGCTAACTCTGTG

CCAGCAGCCGCGGTAATACAGAGGTCCCAAGCGTTGTTCGGATTCACTGGGCGTAAAGGG
TGCGTAGGTGGCCGGGTAAGTCTGATGTGAAATCTCGGAGCTTAACTCCGAAACGGCATT
GGATACTATCCGGCTCGAGGGTCGGAAGGGGGACTGGAATTCTCGGTGTAGCAGTGAAAT
GCGTAGATATCGAGAGGAACACCAGTGGCGAAGGCGAGTCCCTGGACGACTCCTGACACT
GAGGCACGAAAGCTAGGGGAGCAAACAGGATTAGATACCCCCGTAGTCC
>Otu550
CCAGCCTACGGGATGCAGCAGTAGGGAATTTTCCACAATGGGCGAAAGCCTGATGGAGCA
ACACCGCGTGCAGGATGAAACCTTTAGGGGCGTAAACTGCTTTTATATGTGACGAATATG
ACGGTAGCATATGAATAAGGATCGGCTAACTCCGTGCCAGCAGCCGCGGTCATACGGAGG
ATCCAAGCGTTATCCGGAATTACTGGGCGTAAAGAGTTGCGTAGGTGGCAAAGTAAGCAG
AGCATGAAAGCGTGAGGCTCAACCTCATATCCATGTTTTGAACTGCTTAGCTAGAGGATG
AGAGAGGTTATTGGAATTCCTAGTGTAGGAGTGAAATCCGTAGATATTAGGAGGAACACC
GATGGCGTAGGCAGATAACTGGCTCATTCCTGACACTAAGGCACGAAAGCGTGGGGAGCA
AACGGGATTAGAAACCCGGGTAGTCC
>Otu551
CCAGCCTATGGGTGGCAGCAGTGGGGAATCTTGCGCAATGGACGAAAGTCTGACGCAGCC
ACGCCGCGTGAGTGAAGAAGGCCTTCGGGTTGTAAAGCTCTGTCGGAGGGGACGAAAATG
CGGTCGGTGAATAGCCGATCGTACTGACGGTACCCTCAAAGGAAGCACCGGCTAACTTCG
TGCCAGCAGCCGCGGTAATACGAGGGGTGCGAGCGTTGCTCGGAATTATTGGGCGTAAAG
GGTAGGTAGGTGGTCTGATTTGTCCGGGGTGAAAGCCTTGAGCTTAACTCAAGAAGTGCC
TCGGAAACGGTCAGACTCGAGTCCTGGAGAGGGTCGTGGAATTCCCGGTGTAGCGGTGAA
ATGCGTAGAGATCGGGAGGAACACCAGCGGCGAAGGCGGCGACCTGGACAGGTACTGACA
CTCAACTACGAAAGCGTGGGGATCAAACAGGATTAGATACCCGCGTAGTCC
>Otu553
CCAGCCTATGGGTCGCTGCAGTGGGGAATATTGGACAATGGGCGCAAGCCTGATCCAGCC
ATCCCGCGTGCAGGAAGAAGGCCCTATGGGTTGTAAACTGCTTTTGTGCAGGAAGAACAG
TATCCTCGAGAGGGTATTTGACGGTACTGTGCGAATAAGCACCGGCTAACTCCGTGCCAG
CAGCCGCGGTAATACGGAGGGTGCAAGCGTTATCCGGATTTATTGGGTTTAAAGGGTGCG
TAGGCGGTAAACTAAGTCAGTGGTGAAATCCTGCAGCTTAACTGTAGCATTGCCATTGAA
ACTGATTTACTTGAGTTATCTTGAAGTAGGCGGAATGTGTAGTGTAGCGGTGAAATGCTT
AGATATTACACAGAACACCGATTGCGAAGGCAGCTTACTAAGGGTGAACTGACGCTGAGG
CACGAAAGCGTGGGGATCGAACAGGATTAGATACCCTCGTAGTCC
>Otu556
CCAGCCTATGGGATGCTGCAGTGGGGAATTTTGGACAATGGGCGCAAGCCTGATCCAGCA
ATGCCGCGTGAGTGAAGAAGGCCTTCGGGTTGTAAAGCTCTTTTGTCCGGGAAGAAACGG
CACTTACTAAAAATGAGCGTTATTGACGGTACCGGAAGAATAAGCACCGGCTAACTACGT
GCCAGCAGCCGCGGTAATACGTAGGGTGCGAGCGTTAATCGGAATTACTGGGCGTAAAGC
GTGCGCAGGCGGTTATGTAAGTCAGATGTGAAATCCCCGGGCTCAACCTGGGCATGGCAT
TTGAGACTGCAAGGCTAGAGTGTGTCAGAGGGGGGTGGAATTCCACGTGTAGCAGTGAAA
TGCGTAGAGATGTGGAGGAACACCGATGGCGAAGGCAGCCCCCTGGGATAACACTGACGC
TCATGCACGAAAGCGTGGGGAGCAAACAGGATTAGAAACCCTCGTAGTCC
>Otu558
CCAGCCTACGGGGCGCTGCAGTGGGGAATATTGGACAATGGGCGCAAGCCTGATCCAGCC
ATGCCGCGTGAGTGATGAAGGCCCTAGGGTTGTAAAGCTCTTTTGTGCGGGAAGATAATG
ACTGTACCGGGAGAATAAGCTCCGGCTAACTTCGTGCCAGCAGCCGCGGTAATACGAAGG
GGGCTAGCGTTGTTCGGAATTACTGGGCGTAAAGCGTGCGCAGGCGGCTTCCCAAGTCAG
TGGTGAAAGTCCGGAGCTCAACTCCGGAACTGCCATTGAAACTGTGAAGCTCGAGGACGA
GAGAGGTGAGTGGAATTCCCAGTGTAGAGGTGAAATTCGCAGATATTGGGAAGAACACCG
GTGGCGAAGGCGGCTCACTGGCTCGTTTCTGACGCTCATGCACGACAGCGTGGGGATCAA
ACAGGATTAGAAACCCTTGTAGTCC
>Otu559
CCAGCCTACGGGGGGCAGCAGGTAGGAATCTTTTGCAATGTACGCAAGTATGACAAAGCA
AGCCAAAGTGAATTTCCTTTTTGGAAAATCTTTTGCCAGCTGTAAAAAGGCTGGCGAATA
AGGAGTGGGCAAGACCGGTGCCAGCCGCCGCGGTAATCCCGGCAGTCCAAGTCGCAGCCA
ACATTATTGGGTCTAAAACATCCGTAGCTTGCCTCGTAAGTCTCTTGTGAAATTCTGTCT
CTTAAGGGCAGATCGTGCAAGGGATACTGCGAGGCTAGAGACCGGTGGACGTAAGGAGTA
CATTCGAGGTAGTGGTAAAATACGTTAATCTCGAGTGGACTAACACTAGCGAAGGCACCT
TACGATGACGGTTCTGACAGTGAGGGATGAAGGCTAGGGGCGCAAAATGGATTAGAAACC

CTTGTAGTCC
>Otu561
CCAGCCTATGGGGTGCAGCAGTGGGGAATCTTGCACAATGGGGGAAACCCTGATGCAGCG
ACGCCGCGTGGGTGATGAAGCTCTTCGGAGTGTAAAGCCCTTTCGGCAGGGAAGAGAAAA
GACGGTACCTGCAAAAGAAGCCCCGGCTAACTACGTGCCAGCAGCCGCGGTAATACGTAG
GGGGCTAGCGTTGTTCGGAATTACTGGGCGTAAAGAGAGCGTAGGTGGTGCGGTGTGTTT
GGTGTGAAATCTCCCGGCTCAACCGGGAGGGGCCATCGAAAACTGCCGTGCTTGAATATG
GGAGAGGGTAGTGGAATTCCCGGTGTAGCGGTGAAATGCGTAGAGATCGGGAGGAACACC
AGCGACGAAGGTGGCTACCTGGACCATTATTGACACTGAGGCTCGAAAGCTAGGGGAGCA
AACAGGATTAGATACCCCTGTAGTCC
>Otu562
CCGCCTACGGGTCGCAGCAGTCGAGGATCTTCGTCAATGGGCGCAAGCCTGAACGAGCGA
CGCCGCGTGCGCGATGAAGGCCTTCGGGTTGTAAAGCGCGAAAGTGGAGAGAAAGGGAAA
CTTGATCAATCCACAGTAAGGACGGGCTAAGTTCGTGCCAGCAGCCGCGGTAAGACGAAC
TGTCCTAACGTTGTTCGGAATCACTGGGCTTAAAGGGCGCGTAGGCGGTCGGACAAGTCG
GGGGTGAAATCCTGCAGCTTAACTGTAGAAGTGCCTTCGATACTGTCCGTCTGGAGGGAG
ATAGGGGTGTGCGGAACTTCCAGTGGAGCGGTGAAATGCGTAGATATCGGAAGGAACACC
TGTGGCGAAAGCGGCTCACTGGACCATAACTGACGCTGATGCACGAAAGCTAGGGGAGCA
AACAGGATTAGATACCCTAGTAGTCC
>Otu564
CCAGCCTACGGGTCGCAGCAGTAGGGAATCTTCCGCAATGGGCGAAAGCCTGACGGAGCA
ATGCCGCGTGAGTGATGAAGGCCTTCGGGTCGTAAAGCTCTGTTGAGGGGGAAGAATGAT
TCATCCGATAATACCGGATGAGTTTTGACGGTACTCCTCGAGGAAGCCCCGGCTAATTAC
GTGCCAGCAGCCGCGGTAATACGTAAGGGGCAAGCGTTGTCCGGAATCACTGGGCGTAAA
GCGCACGTAGGCGGCTTGGCAAGTCAGGGGTGCAAGCTCTAGGCTTAACCTAGAAATTGC
CTCTGAAACTACCTCGCTTGAGGGTCAGAGGGGAAACTGGAATTCACGGTGTAGCGGTGA
AATGCGTAGATATCGTGGAGAACACCCGTGGCGAAGGCGGGTTTCTGGCTGACAACTGAC
GCTGAGGTGCGAAAGCTAGGGGAGCGAACGGGATTAGATACCCCCGTAGTCC
>Otu565
CCAGCCTACGGGTCGCAGCAGTGGGGAATATTGGACAATGGGGGCAACCCTGATCCAGCG
ACGCCGCGTGGGTGAAGAAGGCCTGCGGGTTGTAAAGCCCTTTCGTTGGGGAAGAAAAGT
TCCGACCTAATACGTCGGGACCGTGACGTTACCCAAAGAAGAAGCACCGGCTAACTCTGT
GCCAGCAGCCGCGGTAATACAGAGGGTGCGAGCGTTAATCGGAATTACTGGGCGTAAAGC
GCACGTAGATGGCTTTGTAAGTCAGATGTGAAATCCCCGGGCTCAACCTGGGAACTGCAT
TTGAAACTGCATTGCTCGAATGTGGAAGAGGGAAGCGGAATTCCAGGTGTAGCGGTGAAA
TGCGTAGATATCTGGAGGAACATCAGTGGCGAAGGCGGCTTCCTGGTCCAACATTGACAT
TCAGGTGCGAAAGCGTGGGGAGCAAACAGGATTAGAAACCCTTGTAGTCC
>Otu566
CCAGCCTACGGGTGGCAGCAGTCGAGGATCATTCGCAATGGGCGAAAGCCTGACGATGCG
ACGCTGCGTGAACGATGAAGGCCTTCGGGTCGTAAAGTTCTTTCGCGCAAGAACAAGGAA
GGTATGTGAATAACATACCGACTTGAGGGTACTGCGTAAAGAAGCACCGGCTAACTCCGT
GCCAGCAGCTGCGGTAATACGGAGAGTGCAAGCATTAATCGGAATTATTGGGCGTAAAGG
GCGCGTAGGCGGAATGATAAGTCAGATGTGAAATACCGAAGCTCAACTTCGGTGCTGCAT
TTGAAACTATCAATCTAGAGGGTTGACGGAGAAAACGGAATTCCACGTGTAGCGGTGAAA
TGCGTAGATATGTGGAAGAACACCAGTGGCGAAAGCGGTTTTCTAGTTAATACCTGACGC
TGAGGCGCGAGAGCAAGGGGAGCAAACAGGATTAGATACCCGTGTAGTCC
>Otu568
CCAGCCTACGGGTGGCAGCAGCAAGGAATTTTGCGCAATGGGCGAAAGCCTGACGCAGCA
ACGCCGCGTGAGGGATGAAGGCCTTCGGGTTGTAAACCTCTTTTGTCAGGGACGAATAAT
GACGGTACCTGGCGAATAAGCTTCGGCTAACTACGTGCCAGCAGCCGCGGTAATACGTAG
GAAGCAAGCGTTATCCGGATTCACTGGGCGTAAAGTGAGCGTAGGTGGCCTTTCAAGTCG
GATGTGAAATCTCCTGGCTTAACTGGGAGGGGTCATTCGATACTGTTGGGCTAGAGGACA
TCAAGGGAAGGTGGAATTCCGGGTGTAGTGGTGAAATGCGTAGATATCCGGAGGAACACC
AGTGGCGAAGGCGGCCTTCTGGGATGTTTCTGACACTGAGGCTCGAAAGTGTGGGGAGCG
AACAGGATTAGAGACCCTCGTAGTCC
>Otu573
CCAGCCTACGGGATGCTCCAGTAAGGAATATTGGTCAATGGGCGGAAGCCTGAACCAGCC
ATGCCGCGTGCAGGAAGACGGCCCTACGGGTTGTAAACTGCTTTTGTGCCAGAATAAACC

TTCGTTCGTGAACGAAGCTGAATGTATGGTAAGAATAAGGATCGGCTAACTCCGTGCCAG
CAGCCGCGGTAATACGGAGGATCCGAGCGTTATCCGGATTTATTGGGTTTAAAGGGTGCG
TAGGCGGCCTGTTAAGTCAGTGGTGAGAGACGGTAGCTCAACTATCGCAGTGCCCTTGAT
ACTGATGGGCTTGAATGTACTAGAGGCAGGCGGAATGTGACAAGTAGCGGTGAAATGCAT
AGATATGTCACAGAACACCAATTGCGAAGGCAGCTTACTATGGTATGATTGACGCTGAGG
CACGAAAGCGTGGGGATCAAACAGGATTAGAGACCCGCGTAGTCC
>Otu576
CCAGCCTACGGGGTGCTGCAGTAGGGAATCTTGCGCAATGGGCGAAAGCCTGACGCAGCA
ACGCCGCGTGGGGGATGAAGGCCTTCGGGTTGTAAAGCGCGAAAGTGGGGACAAAACCGC
AAGGTTGATCGATCCACAGTAAGGACGGGCTAAGTTCGTGCCAGCAGCCGCGGTAAGACG
AACTGTCCAAACGTTGTTCGGAATCACTGGGCTTAAAGGGCGCGTAGGCGGTCCATCAAG
TCGGGGGTGAAATCCTGCAGCTTAACTGTAGAAGTGCCTTCGATACTGATGGTCTGGAGG
GAGGTAGGGGTGTGTGGAACTTCCAGTGGAGTGGTGAAATGCGTTGATATTGGAAGGAAC
GCCGGTGGCGAAAGCGACGCACTGGACCTCTTCTGACGCTGAGGCGCGAAAGCTAGGGGA
GCAAACGGGATTAGAAACCCCGGTAGTCC
>Otu577
CCAGCCTACGGGGTGCAGCAGTCGAGAATTTTTCTCAATGGGGGCAACCCTGAAGGAGCG
ACGCCGCGTGGAGGATGAAGGTCTTCGGATTGTAAACTCCTGTCATTGGGGAACAAGTGC
CGGTAATTAACTACTACCGGCTTGATAGTACCTGAAGAGGAAGAGACGGCTAACTCTGTG
CCAGCAGCCGCGGTAATACAGAGGTCTCAAGCGTTGTTCGGATTCATTGGGCGTAAAGGG
TGCGTAGGTGGCGCCGTAAGTCAGGTGTGAAATCTCGGGGCTCAACCCCGAAACTGCACT
TGATACTACGGTGCTCGAGTACTGGAGAGGAGATTGGAATTTACGGTGTAGCAGTGAAAT
GCGTAGATATCGTAAGGAAGACCAGTGGCGAAGGCGAATCTCTGGACAGTTGCTGACACT
GAGGCACGAAGGCCAGGGGAGCAAACGGGATTAGATACCCCAGTAGTCC
>Otu579
CCAGCCTATGGGTTGCTGCAGTTAGGAATCTTGCGCAATGGGCGAAAGCCTGACGCAGCC
ATGCCGCGTGAATGATGAAGGTCTTAGGATTGTAAAATTCTTTCAGTGGGGAAGATAATG
ACGGTACCCACAGAAGAAGCTCCGGCTAACTTCGTGCCAGCAGCCGCGGTAATACGAAGG
GGGCTAGCGTTGCTCGGAATTACTGGGCGTAAAGGGCGCGTAGGCGGATGACTAAGTTGG
GGGTGAAAGCCCGGGGCTCAACCTCGGAACTGCCTTCAATACTGGTCATCTTGAGTTCGG
GAGAGGTGTGTGGAACTCCGAGTGTAGAGGTGAAATTCGTAGATATTCGGAAGAACACCA
GTGGCGAAGGCGACACACTGGCCCGATACTGACGCTGAGGCGCGAAAGCGTGGGGAGCAA
ACAGGATTAGAAACCCCTGTAGTCC
>Otu580
CCAGCCTATGGGGGCTGCAGTCGAGGATCTTCGGCAATGGGGGCAACCCTGACCGAGCGA
TGCCGCGTGCGCGATGAAGGCCTTCGGGTTGTAAAGCGCGAAAGTGGGGAGAAAAGCGAA
AGTTTGATCTATCCACAGTAAGCTCGGGCTAAGTTCGTGCCAGCAGCCGCGGTAAGACGA
ACCGAGCGAACGTTGTTCGGAATCACTGGGCTTAAAGGGCGCGTAGGCGGGCTGTCAAGT
TTGGGGTGAAATCCCGCGGCTCAACCGTGGAACTGCCTCAAATACTGACGGTCTCGAGGG
AGGTAGGGGCGAGCGGAACTAATGGTGGAGCGGTGAAATGCGTTGATATCATCAGGAACT
CCGGTGGCGAAGGCGGCTCGCTGGACCTTTTCTGACGCTGAGGCGCGAAAGCTAGGGGAG
CAAACGGGATTAGAAACCCTGGTAGTCC
>Otu581
CCAGCCTACGGGGTGCTGCAGTGAGGAATTTTCCGCAATGGGCGAAAGCCTGACGGAGCG
ACGCCGTGTGTGCGATGAAGTCCTTCGGGATGTAAAGCACTGTCAGTGGGGAAGATATTG
ACGGTACCCACAGAGGAAGGCCCGGCTAACTACGTGCCAGCAGCCGCGGTAATACGTAGG
GGCCAAGCGTTGTCCGGAATTATTGGGCGTAAAGGGCGTGTAGGCGGCCTGATAAGTCAG
ATGTTAAATCTACAGGCTCAACCTGTATCCGCATTTGATACTGTCGGGCTAGAGAGTGGT
AGAGGAAAGTGGAACTTACGGTGTAGCGGTGAAATGCGTAGATATCGTAAGGAACACCAG
TGGCGAAGGCGACTTTCTGGGCCACTTCTGACGCTGAGACGCGAAAGCCAGGGGAGCAAA
CGGGATTAGATACCCCAGTAGTCC
>Otu582
CCAGCCTACGGGGGGCTCCAGTGGGGAATATTGGACAATGGGCGCAAGCCTGATCCAGCC
ACGCCGCGTGAGTGATGAAGGCCTTCGGGTCGTAAAGCTCTGTGGGGAGGGACGAACCGC
TGGGTATTAATACCATCCAGCATGACGGTACCTCCTTAGCAAGCACCGGCTAACTTCGTG
CCAGCAGCCGCGGTAATACGAAGGGTGCAAACGTTGCTCGGAATTATTGGGCGTAAAGCG
CACGTAGGCGGCTTATCAAGTCGGATGTGAAATCCCTCGGCTCAACCAAGGAAGTGCATC
CGAAACTGACAAGCTTGAGTTCGAAAGAGGATCGCGGAATTCCCGGTGTAGAGGTGAAAT

TCGTAGATATCGGGAGGAACACCAGTGGCGAAGGCGGCGATCTGGGTCGATACTGACGCT
GAGGTGCGAAAGCGTGGGGAGCAAACAGGATTAGAGACCCTCGTAGTCC
>Otu583
CCAGCCTACGGGGCGCTGCAGTCGAGAATCTTCCGCAATGGACGAAAGTCTGACGGAGCG
ACGCCGCGTGCAGGATGAAGTGCTTCGGTATGTAAACTGCTTTTGCCAGGGAGAAAGTTT
ATTGATAGTACTTGGCGAATAAGAGGTTGCTAAACTCGTGCCAGCAGCAGCGGTAATACG
AGTGCCTCAAGCGTTATCCGGAATTATTGGGCGTAAAGGATGTGTAGGTGGTCGTGTTAG
TCTCTTGTTAAATTCTTCGGCTTAACCGGGGGCATGCAAGGGAAACGGCACGACTCGAGG
ATGCGAGGGGTCTGCGGAACTCATAGTGTAGCGGTGAAATGCGTTGATATTATGGGGAAC
ACCAAAAGCGAAGGCAGCAGACTGGAGCACTCCTGACACTGAAACATGAAAGCGTGGGTA
GCGAATGGGATTAGAAACCCTCGTAGTCC
>Otu585
CCAGCCTATGGGTCGCAGCAGTGAAGAATCTTGGGCAATGGAGGAAACTCTGACCCAGTG
AGAATACGTGTATGACGAAGGCGTCCAGTTTTCTGTAAAGTACTTTCGGGGATGAAGATG
TAGGCATCAGTCCCAGTAAGAAGCACTGGCCAACTCCGTGCCAGCAGCCGCGGTAATACG
GAGAGTGCGGGCGTTATTCGTTTTGATTGGGTGTAAAGGGTACGTAGGTTGCTGTTGCTA
ATTCTTGTCCGAAATCGTGGAGTATTCCTACACGGCGGCCTTTTAAAGATTCAGCTAGTG
TCAAGGATAGGTAGGGGCAATTTCCTGTGTAGGGGTAGAATCCAGCCATATAGGAACGAA
CGCCAGCTGGCGAAGGCGCCTTTCTAGTTTTGACAGACGCTAAGGTACGGAAGCGTGGGG
AGCAAACAGGATTAGAAACCCGAGTAGTCC
>Otu588
CCAGCCTACGGGATGCTCCAGTCAAGAACTTTCCACAATGGGCGAAAGCCTGATGGAGCG
ACGCCGCGTGACTGATGAAGTCCTTCGGGACGTAAAGGTCTTTTATGGGGGAGGAAGTAA
TTGACGTTACCCCATGAATAGGGGGCTCCTAACTCTGTGCCAGCAGGAGCGGTAATACAG
AGGCCCCAAGCGTTATCCGGAATCACTGGGCGTAAAGGATGTGTAGGCGGTCGTGTTAGT
CGTCTGTGAAAGGCCGTGGGCTTAACCCATGGACCGCAAACGAAACGGCACGACTTCGAG
GATGCGAGAGGTATATGGAACTCATGGTGTAGGGGTGAAATCCGTTGATATCATGGGGAA
CACCAAATGCGAAGGCAATATACTGGCGCACTCCTGACGCTGAGACATGAAAGCGTGGGA
ATCGAACGGGATTAGAGACCCTAGTAGTCC
>Otu589
CCAGCCTATGGGGGGCTGCAGTGGGGAATATTGGACAATGGGCGAAAGCCTGATCCAGCA
ACTCTGCGTGAGGGACGAAGGTCTTCGGATCGTAAACCTCTTTTACATGGGACGAAATCC
CGCAAGGGTCTGACGGTACCATGCGAATAAGCAACGGCTAACTATGTGCCAGCAGCCGCG
GTAAGACATAGGTTGCGAGCGTTATTCGGAATTACTAGGCGTAAAGCGAGTGTAGGCGGA
CCTTTAAGTCTGACGTTAAATCTCCTGGCTTAACTAGGAGCTGTCGTCGGATACTGAAGG
CCTCGAATGGGGTAGGGGGCAGCGGAATTCCCGGTGTAGCGGTGAAATGCGTAGATATCG
GGAGGAACACCTATGGCGAAAGCAGCTGCCTGGGCCTCTATTGACGCTAAGACTCGAAAG
CTGGGGGAGCAAACAGGATTAGATACCCTCGTAGTCC
>Otu590
CCAGCCTATGGGTTGCTGCAGTAAGGAATCTTCCACAATGGGCGAAAGCCTGATGGAGCA
ATGCCGCGTGCAGGATGACGGCCTTCGGGTTGTAAACTGCTTTTATATGGGAAGATTATG
ACGGTACCATATGAATAAGGACCGGCTAACTACGTGCCAGCAGCCGCGGTAATACGTAGG
GTCCAAGCGTTATCCGGAATCACTGGGCGTAAAGCGTGCGTAGGCGGACGTTTAAGTGCA
ATGCGAAATCTCGTGGCTCAACCATGTAGACTGTATTGCATACTGGATGTCTAGAGGATG
GCAGAGGCAAGTGGAATTACTGGTGTAGCAGTGAAATGCGTAGATATCAGTAGGAACACC
AATGGCGAAGGCAGCTTGCTGGGCCATTTCTGACGCTAAGGCACGAAAGCGTGGGGGGCG
AACAGGATTAGAGACCCGCGTAGTCC
>Otu591
CCAGCCTATGGGGCGCACCAGTAGGGAATATTGGACAATGGGGGCAACCCTGATCCAGCC
ATGCCGCGTGAATGATGAAGGCCTTCGGGTCGTAAAGTTCTTTTGGCGGGGACGATGATG
ACGGTACCCGCAGAATAAGCTCCGGCTAACTTCGTGCCAGCAGCCGCGGTAATACGAAGG
GAGCTAGCGTTGTTCGGAATTACTGGGCGTAAAGGGCGCGTAGGCGGCTTGGCAAGTTGG
GTGTGAAAGCCCTGGGCTCAACCCGGGAACTGCACTCAAGACTGCCTTGCTTGAATTCGG
TAGAGGGTGGTAGAATTCCCAGTGTAGAGGTGAAATTCGTAGAGATTGGGAAGAATACCC
GTGGCGAAGGCGGCCAACTGGACCGACATTGACGCTGAGGCGCGAAAGCGTGGGGAGCAA
ACAGGATTAGAAACCCCCGTAGTCC
>Otu593
CCCTACGGGTGGCTGCAGTCGAGGATCTTCGGCAATGTGCGCAAGCACGACCGAGCGACG

CCGCGTGTGCGACGAAGGCCTTCGGGTTGTAAAGCACTGTCGAGGGGGAGAAAAGCCTGC
AAGGGTGTGATCTATCCCTGGAGGAAGCTCGGGCTAAGTTCGTGCCAGCAGCCGCGGTAA
GACGAACCGAGCGAACGTTGTTCGGAATCACTGGGCTTAAAGGGCGCGTAGGCGGGCTGC
CAAGTTTGTGGTGAAATCCTCCGGCTCAACCGGGGAAGTGCCTCGAATACTGGCGGTCTG
GAGGGAGGTAGGGGCGTGCGGAACTGTAGGTGGAGCGGTGAAATGCGTTGATATCTACAG
GAACTCCGGTGGCGAAGGCGGCACGCTGGACCTTTTCTGACGCTGAGGCGCGAAAGCCAG
GGGAGCGAACGGGATTAGAAACCCTCGTAGTCC
>Otu595
CAGCCTACGGGGGGCTGCAGTCGAGGATCTTTGGCAATGAGCGCAAGCTTGACCAAGCGA
CGCCGCGTGTGCGATGAAGGCCTTCGGGTTGTAAACCACTGTCAGAGGGGATGAAATGCA
GGTGGGTTCTCCCATCTGTTTGACATAGCCTCAGAGGAAGGACGGGCTAAGTTCGTGCCA
GCAGCCGCGGTAAGACGAACCGTCCAAACGTTATTCGAAATCACTGGGCTTAAAGGGTGC
GTAGGCGGCGCGGCAAGTTGGGTGTGAAATCCCTCGGCTCAACCGAGGAACTGCGCCCAA
AACTGCCGTGCTCGAGGGAGATAGAGGTGAGCGGAACTTAGGGTGGAGCGGTGAAATGCG
TTGATATCCTAAGGAACACCGGTGGCGAAAGCGGCTCACTGGATCTCTTCTGACGCTGAG
GCACGAAAGCTAGGGTAGCGAACGGGATTAGATACCCCCGTAGTCC
>Otu600
CCAGCCTATGGGTGGCACCAGTCGAGAATTTTTCTCAATGGGCGAAAGCCTGAAGGAGCG
ACGCCGCGTGGGGGATGAAGGGCTTCGGCTCGTAAACCCCTGTCATTTGCGAACAAACCT
TACGATTGAACAGATCGTGAGCTGATTGTAGCGAAAGAGGAAGGGACGGCTAACCCTGTG
CCAGCAGCCGCGGTAATACAGAGGTCCCAAGCGTTGTTCGGATTCACTGGGCGTAAAGGG
TGCGTAGGCGGTTGAATAAGTCTGATGTGAAATCTCCGGGCTTAACCCGGAAATTGCATC
GGATACTATCCGACTAGAGGAATGGAGGGGAGACTGGAATACTTGGTGTAGCAGTGAAAT
GCGTAGATATCAAGTGGAACACCAGTGGCGAAGGCGAGTCTCTGGACATTTCCTGACGCT
GAGGCACGAAAGCCAGGGGAGCAAACGGGATTAGATACCCCTGTAGTCC
>Otu601
CCAGCCTATGGGTCGCTGCAGCAACGAATCTTCCCCAATGCCGGAAACGGTGAGGGAGCG
ACGCCGCGTGAAGGACGAAGTTCTTCGGAATGTAAACTTCTGCAAGGGTCATGAAAGCCT
TAGGGCCAATATCCCAAAAGGTTGATCTGACCCCTAAAAGGGGCGGCTAACTCTGTGCCA
GCAGCCGCGGTAATACAGAGGCCCCAAGCGTTACTGAGAATCACTGGGTTTAAAGGGTGC
GTAGGTGGTCCGTTAAGTTCGTTGTGAAAGCCCTGGGCTCAACCCAGGAACTGCTTCGAA
TACTGGCGGACTTGAGGCCGGTAGGGGTCACTGGAACTGACGGTGGAGCGGTGAAATGCG
TAGATATCGTCAGGAACGCCAATGGTGAAGACAGGTGACTGGGCCGGATCTGACACTGAG
GCACGAAAGCGTGGGGAGCGAACGGGATTAGATACCCCCGTAGTCC
>Otu604
CCAGCCTATGGGGGGCACCAGTGGGGAATTTTGGACAATGGGCGCAAGCCTGATCCAGCA
ATGCCGCGTGGATGAAGAAGGTTTTCGGATTGTAAAGTCCTTTCGGCGGGGACGATGATG
ACGGTACCCGCAGAAGAAGCCCCGGCTAACTTCGTGCCAGCAGCCGCGGTAATACGAAGG
GGGCTAGCGTTGCTCGGAATGACTGGGCGTAAAGGGCGCGTAGGCGGTTTGGACAGTTAG
GCGTGAAATTCCTGGGCTCAACCTGGGGACTGCGCTTAATACATCCAGACTTGAGTGCGG
AAGAGGGTTGTGGAATTCCCAGTGTAGAGGTGAAATTCGTAGATATTGGGAAGAACACCG
GTGGCGAAGGCGGCAACCTGGTCCGTGACTGACGCTGAGGCGCGAAAGCGTGGGGAGCAA
ACAGGATTAGAGACCCCAGTAGTCC
>Otu607
CCAGCCTACGGGATGCTGCAGTGGGGAATCTTGCGCAATGGACGAAAGTCTGACGCAGCG
ACGCCGCGTGGGTGATGAAGGCCTTCGGGTTGTAAAGCCCTGTGGGGAGAGACGAATAAG
TCGGATCCAATAACTCCGATGATGACGGTATCTCCTTAGCAAGCACCGGCTAACTCTGTG
CCAGCAGCCGCGGTAAGACAGAGGGTGCAAACGTTGTTCGGAATTACTGGGCGTAAAGCG
TGTGTAGGCGGCTACGCAAGTCGGATGTGAAATCCCCGGGCTCAACCCGGGAAGTGCACC
CGATACTGCGCGGCTTGAGTACCAGAGAGGTTGGTGGAATTCTCGGTGTAGAGGTGAAAT
TCGTAGATATCGAGAGGAACACCGGTGGCGAAGGCGGCCAACTGGACGAGTACTGACGCT
GAGACACGAAAGCGTGGGGAGCAAACAGGATTAGAAACCCGGGTAGTCC
>Otu613
CCAGCCTACGGGTCGCAGCAGTGGGGAATCTTGCGCAATGGGCGAAAGCCTGACGCAGCG
ACGCCGCGTGAGGGAAGAAGGCCTTCGGGTTGTAAACCTCTTTCAGGAGGGACGAAGCCA
CTCGGGTTAATAGCCCAGAGGGTGACGGTACCCCCAGAAGAAGCCCCGGCTAACTACGTG
CCAGCAGCCGCGGTAATACGTAGGGGGCAAGCGTTGTCCGGATTTATTGGGCGTAAAGAG
CGTGTAGGCGGTTACACAGGTCCGCTGTGAAAACTCGAGGCTCAACCTCGAGCTGTCGGT

GGAAACCGTGTGGCTAGAGTCCGGAAGAGGAGAGTGGAATTCCCGGTGTAGCGGTGAAAT
GCGCAGATATCGGGAAGAACACCCGTGGCTAAGGCGGCTCTCTAGTACGGTACTGACGCT
GAGACGCGAAAGCGTGGGGAGCGAACAGGATTAGAAACCCCAGTAGTCC
>Otu616
CCAGCCTATGGGTGGCAGCAGTCGAGAATCTTCGGCAATGGACGAAAGTCTGACCGAGCG
ACGCCGCGTGCGGGATGAAGGCCTTCGGGTTGTAAACCGCTGTCAGAGGGGATGAAATTC
ATTTGGGTGCTCCCAGATGATTGACAGAGCCTCAGAGGAAGCACGGGCTAAGTACGTGCC
AGCAGCCGCGGTAATACGTACTGTGCGAACGTTATTCGGAATCACTGGGCTTAAAGGGTG
TGTAGGCGGTTGATTAAGTAGGGTGTGAAAGGCCTTGGCCTAACCAAGGCATTGCGCTCT
AAACTGGTCGACTTGAGTAAGATAGGGGTGTGCGGAACTTCCAGTGGAGCGGTGAAATGT
GTTGATATTGGAAGGAACGCCGGTGGCGAAAGCGGCACACTGGGTCTTAACTGACGCTGA
GACACGAAAGCCAGGGGAGCGAACGGGATTAGAACCCCCGTAGTCC
>Otu621
CCAGCCTATGGGGTGCAGCAGTCGAGAATCTTTCACAATGGGGGCAACCCTGATGGAGCG
ACGCCGCGTGGAGGATGAAGGGCTTCGTCTTGTAAACTCCTGTCACCACAGAACAAGGTC
TGTGTATTAATAACATGCAGATTTGATGGTATGTGGAGAGGAAGGGACGGCTAACTCTGT
GCCAGCAGCCGCGGTAATACAGAGGTCCCAAGCGTTGTTCGGATTCACTGGGCGTAAAGG
GTGCGTAGGAGGCCGGGAAAGTCTGATGTGAAATCCCACCGCTTAACGGTGGAATGGCAT
TGGATACTGCTCGGCTCGAGGACTGGAGGGGAAAGCGGAATTCCTGGTGTAGCGGTGAAA
TGCGTAGATATCAGGAGGAACACCAACGGCGAAGGCAGCTTTCTAGACAGTTCCTGACTC
TGAGGCACGAAGGCCAGGGGAGCAAACGGGATTAGAGACCCTAGTAGTCC
>Otu622
CCAGCCTATGGGTCGCTGCAGTTAAGAATCTTGCTCAATGGGCGAAAGCCTGAAGCAGCG
ACGCCGCGTGGACGATGAAGGTCTTCGGATTGTAAAGTCCACTAAGCAGGGATGAATAAG
CAACGGGTAATATTCGTTGTGATGACTGTACCTGCCTAAAGCCTCGGCTAACTACGTGCC
AGCAGCCGCGGTAAGACGTATGAGGCAAGCGTTGTTCGGAATTATTGGGCGTAAAGGGCG
TGTAGGCGGGGGATTAAGTTAGGTGTGAAATCCATGGGCTTAACCCATGAACTGCATTTA
ATACTGGTTCTCTTGAGTTCGGGAGAGGTGAGCGGAATTCCCGGTGTAGCGGTGAAATGC
GTAGATATCGGGAAGAACACCAGTGGCGAAGGCGGCTCACTGGCCTAGAACTGACGCTAA
GGCGCGAAAGCGTGGGGAGCGAACGGGATTAGAAACCCTTGTAGTCC
>Otu623
CCAGCCTACGGGATGCAGCAGTAGGGAATATTGCACAATGGGCGAAAGCCTGATGCAGCG
ATGCCACGTGAGCGATGAAGGCCTTCGGGTCGTAAAGCTCTTTTGTCTAGGAAAAAGAGG
CGTGATTGAAAGGTTGCGTTTTTTGATGGTACTAGAAGAAAAAGCACCTGCTAACTACGT
GCCAGCAGCAGCGGTAATACGTAGGGTGCAAGCGTTAATCGGAATTATTGGGCGTAAAGG
GCGCGTAGGCGGATTTTTAAGTCAGTGGTTTAATCTCGGAGCTCAACTCCGAAAGTGCCA
TTGATACTGAAAGTCTAGAGTATCGGCGGAGAAAATGGAATTCCTGGTGTAGCGGTGAAA
TGCGTAGATATCAGGAGGAACACCAAGAGCGAAGGCAGTTTTCTAGCCGATAACTGACGC
TGAGGCGCGAAAGCATGGGGAGCAAACAGGATTAGATACCCGCGTAGTCC
>Otu626
CCAGCCTACGGGTTGCACCAGTGGGGAATCTTGCGCAATGGACGAAAGTCTGACGCAGCC
ACGCCGCGTGAGTGAAGAAGGCCTTCGGGTTGTAAAGCTCTGTCGGGAGGGACGAAAAGC
CAATAGGTGAATAGCCTGTTGGTGTGACGGTACCTTCAAAGGAAGCACCGGCTAACTCTG
TGCCAGCAGCCGCGGTAATACAGAGGGTGCAAGCGTTGCTCGGAATTATTGGGCGTAAAG
GGCAGGTAGGTGGTCTCATTTGTCTGGGGTGAAAGCCTTGAGCTTAACTCAAGAAGTGCC
CCAGAAACGGTGAGACTGGAGTCTCGGAGAGGGTCGTGGAATTCCCGGTGTAGCGGTGAA
ATGCGTAGAGATCGGGAGGAACACCAGAGGCGAAGGCGGCGACCTGGACGAGCACTGACA
CTCAACTGCGAAAGCGTGGGGAGCAAACAGGATTAGATACCCCTGTAGTCC
>Otu627
CCAGCCTATGGGTGGCAGCAGTGGGGAATCTTGCGCAATGGGCGAAAGCCTGACGCAGCG
ACGCCGCGTGAGTGATGAAGGCCTTCGGGTTGTAAAGCTCTGTGGAGGGGGACGAATAAG
TCGTGGCCAACATCCACGATGATGACGGTACCTCTTTAGCAAGCACCGGCTAACTCTGTG
CCAGCAGCCGCGGTAAGACAGAGGGTGCAAACGTTGTTCGGAATTACTGGGCGTAAAGCG
CGTGTAGGCTGCTCTGCAAGTCGGATGTGAAAGCCCTGGGCTCAACCTAGGAAGTGCATT
CGAAACTGCAAAGCTTGAGTCCTGGAGAGGAAGGCGGAATTCTCGGTGTAGAGGTGAAAT
TCGTAGATATCGAGAGGAACACCGGTGGCGAAGGCGGCCTTCTGGACAGTGACTGACGCT
GAGACGCGAAAGCGTGGGGAGCAAACAGGATTAGATACCCGTGTAGTCC
>Otu628

CCAGCCTATGGGGGGCTCCAGTGAGGAATTTTGCGCAATGGCCGCAAGGCTGACGCAGCA
ACGCCGCGTGGGTGAAGAAGGCCTTCGGGTCGTAAAGCCCTGTCAGGTGGGAAGAATGGC
CCGGGGATTAACAGTCTCCGGGAGTGACGGTACCACCAGAGGAAGCACCGGCTAACTCCG
TGCCAGCAGCCGCGGTAATACGGAGGGTGCAAGCGTTATTCGGAATTACTGGGCGTAAAG
CGCGTGCAGGCGGGCTAGCAAGTCTGATGTGAAAGCCCTGGGCTTAACCTGGGAAGTGCA
TTGGAAACTGCTGGTCTTGAGTGCTGGAGAGGAAGGGGGAATTCCCGGTGTAGAGGTGAA
ATGCGTAGATATCGGGAAGAACACTAGTGGCGAAGGCGGTCACCTGGGCTGTAACTGACA
CTGAGACCCGAAGGCGTGGGGAGCAAACAGGATTAGATACCCGGGTAGTCC
>Otu629
CCAGCCTACGGGTGGCAGCAGTTTGGAATATTCCACAATGGGCGCAAGCCTGATGGAGCG
ACACCGCGTGAAGGATGAAGGTCTTCGGATCGTAAACTTCTTTAGACCTAGATGAAGATT
GCAAGGTAAATAATCTTGCAATTTGACAGTATAGGTAGAATAAGCCACGGCTAACTCTGT
GCCAGCAGCCGCGGTAATACAGAGGTGGCAAACGTTGTCCGGATTTATTGGGTGTAAAGG
GCATGTAGGTGGTCTTATAAGTCAAAGGTGAAATGGCCCGGCTCAACCAGGTCATTGCCT
TTGAAACTGCAAGACTTGAGTTCGGAAGAGGAAAGCGGAATTCCCAGTGTAGCGGTGAAA
TGCGTAGATATTGGGAGGAACACCGGTGGCGAAGGCGGCTTTCTGGTCCGAAACTGACAC
TGAGATGCGAAAGCCAGGGGAGCAAACGGGATTAGAGACCCTCGTAGTCC
>Otu630
CCAGCCTATGGGTTGCTCCAGTGGGGAATTTTGCGCAATGGGCGAAAGCCTGACGCAGCA
ACGCCGCGTGAGTGATGAAGGCTTTCGGGTCGTAAAGCTCTGTCGAGGGGAAAGAAGTGT
AAGGAGGTTAATACCCTCTTTATTTGACGGTACCCCTAAAGGAAGCACCGGCTAACTCCG
TGCCAGCAGCCGCGGTAATACGGGGGGTGCAAGCGTTGTTCGGATTTATTGGGCGTAAAG
AGCGTGTAGGCGGTTTGTTAAGTCTGATGTGAAAGCCCTGGGCTCAACCCAGGAAGTGCA
TTGGAAACTGTCAGACTTGAATACGGGAGAGGGCAGTGGAATTCCTAGTGTAGAGGTGAA
ATTCGTAGATATTAGGAAGAACACCAGTGGCGAAGGCGGCCAACTGGCCCGATACTGACG
CTCATGTGCGAAAGCGTGGGGAGCAAACAGGATTAGAAACCCCAGTAGTCC
>Otu632
CCAGCCTATGGGAGGCACCAGTGGGGAATTTTGGACAATGGGGGCAACCCTGATCCAGCA
ATGCCGCGTGAGTGAAGAAGGCCTTCGGGTTGTAAAGCTCTTTTGTCAGGGAAGAAAAGG
GACTGGCTAATATCTGGTCCTCATGACGGTACCTGAAGAATAAGCACCGGCTAACTACGT
GCCAGCAGCCGCGGTAATACGTAGGGTGCAAGCGTTAATCGGAATTACTGGGCGTAAGGC
GTGCGCAGGCGGTTTTGTAAGACTGTCGTGAAATCCCCGGGCTCAACCTGGGAATGGCGA
TGGTGACTGCAAGGCTAGAGTTTGGCAGAGGGGGGTAGAATTCCACGTGTAGCAGTGAAA
TGCGTAGATATGTGGAGGAACACCGATGGCGAAGGCAGCCCCCTGGGTCAAAACTGACGC
TCATGCACGAAAGCGTGGGGAGCAAACAGGATTAGAACCCCAGTAGTCC
>Otu633
CCAGCCTATGGGTTGCAGCAGTGGAGAATATTGGTCAATAAACGAAAGTTTGAACTAGCG
TTTTGCGGAGATGAAAGATTCATAGGATCGAGAAGGCTACGATAGCTGTAAAGTCTGTGC
GTGGGTTAGAAAAATGATTCTACGCCTAAAGAGAACCCCGGGCCAACAACGTGCCAGCAG
CTGCGGTAAGACGTTGACGGGGAAGCGTTACTGGCCGTTACTAGGTGTAAAGAGTGGCTA
GACTGTTTAAGAAATTGTATCTAAAATCATAGGATTGACGGAGGCATATACAATACCTTA
ATACTGGAGATTTGGGGAGGTGGGCGATAAGTATAATCTAGAGATAAAATTCCGAGAGAT
TATAAGGATATCCTGTAGCCGCGGCGGCTTTCCATCCATTATCTGACGTCGAATCACTAA
AGTACGAGCATCAATCAGGATTAGATACCCGGGTAGTCC
>Otu634
CCTACGGGGGGCTGCAGTGGGGATTTTGGGCAATGGGCAAAAGCCTGACCCAGCGACGCC
GTGTGGGCGATGAAGGCCTTCGGGTTGTAAAGCCCTGTTGGGTGGAAAGAAGGGTTCGGA
GACGAATAATCTCTGAACCTTGACGGTACCACCAGAGAAAGCACCGGCTAACTCCGTGCC
AGCAGCCGCGGTAATACGGAGGGTGCAAGCGTTGTTCGGAATTACTGGGCGTAAAGGGCG
CGTAGGTGGTTGCGTAAGTCGGATGTGAAATCCCCGGGCTTAACCCGGGAACTGCACCCG
ATACTGCGTGACTCGAGTGCAGCAGAGGGAAACGGAATTCCTGGTGTAGAGGTGAAATTC
GTAGATATCAGGAGGAACACCGGTGGCGAAGGCGGTTTCCTGGGCTGACACTGACACTGA
GGCGCGAAAGCGTGGGTAGCAAACAGGATTAGAAACCCTAGTAGTCC
>Otu636
CCAGCCTACGGGTTGCAGCAGTAAGGAATATTGGTCAATGGACGCAAGTCTGAACCAGCC
ATGCCGCGTGAAGGATGAAGGCCCTAGTGGTCGTAAACTTCTTTTATACGGGAAAAATAG
CATCTACGTGATAGGTGTTTGATGGTACTGTATGAATAAGCATCGGCTAACTCCGTGCCA
GCAGCCGCGGTAATACGGAGGATGCAAGCGTTATCCGGATTTATTGGGTTTAAAGGGTGC

GCAGGCGGGGCTATAAGTCAGTGGTGAAAGCCTACAGCTTAACTGTAGAATTGCCATTGA
TACTGTAGTCCTTGAGTGTAGGTGAAGAAGGCGGAATGTGTTGTGTAGCGGTGAAATGCT
TAGATATAACACAGAACACCGATTGCGAAGGCAGCTTTCTAAACTACAACTGACGCTCAT
GCACGAAAGCGTGGGGAGCAAACAGGATTAGATACCCGAGTAGTCC
>Otu638
CCAGCCTACGGGGCGCTGCAGTCGAGAATTTTTCTCAATGGGGGAAACCCTGAAGGAGCG
ACGCCGCGTGGAGGATGAAGGTCTTCGGATTGTAAACTCCTGTCATTAGAGAACAAAACC
CACTTAGTAACTGATGTTGGGTTTGATAGTATCTGAAGAGGAAGAGACGGCTAACTCTGT
GCCAGCAGCCGCGGTAATACAGAGGTCTCAAGCGTTGTTCGGATTCATTGGGCGTAAAGG
GTGCGCAGGCGGCGGGGCAAGTCGGATGTGAAATCTCGGGGCTTAACCCCGAAACTGCAT
TCGATACTGCCTTGCTTGAGGACTGGAGAGGAGACTGGAATTCCTGGTGTAGCAGTGAAA
TGCGTAGATATCAGGAGGAACGCCAATGGCGAAGGCAAGTCTCTGGACAGTTCCTGACGC
TCATGCACGAAGGCCAGGGGAGCAAACGGGATTAGAAACCCTGGTAGTCC
>Otu639
CCAGCCTATGGGGGTGCAGCAGTCGAGAATTTTTCACAATGGGCGAAAGCCTGATGGAGC
GACGCCGCGTGGGGGATGAATGGCTTCGGCCCGTAAACCCCTGTCATTCGCGAACAAACC
TTTCCATTTAATACGTGGAAAGCTGATAGTAGCGGAAGAGGAAGGGACGGCTAACTCTGT
GCCAGCAGCCGCGGTAATACAGAGGTCCCAAGCGTTGTTCGGATTCACTGGGCGTAAAGG
GTGCGTAGGCGGCCAGGTAAGTCTGATGTGAAATCTCGGAGCCTAACTCCGAAACTGCAT
TGGATACTATCTGGCTTGAGGAATGGAGGGGAGACTGGAATGCTCGGTGTAGCAGTGAAA
TGCGTAGATATCGAGCGGAACACCAGTGGCGAAGGCGAGTCTCTGGACATTTCCTGACGC
TGAGGCACGAAAGCCAGGGGAGCAAACGGGATTAGAAACCCCAGTAGTCC
>Otu640
CCAGCCTATGGGTGGCTGCAGTGAGGAATATTGGTCAATGGGCGCAAGCCTGAACCAGCC
ATCCCGCGTGCAGGAAGAAGGCGCTATGCGTCGTAAACTGCTTTTCCAGAGGAAGAAAAC
CCCCGACGTGTCGGGGCTTGCCGGTACTCTGGGAATAAGCATCGGCTAACTCCGTGCCAG
CAGCCGCGGTAATACGGAGGATGCGAGCGTTATCCGGATTTATTGGGTTTAAAGGGTGCG
TAGGCGGACTTATAAGTCAGTGGTGAAATCTCGTTGCTTAACAACGAACGTGCCATTGAT
ACTGTAGGTCTTGAGTACAGATGCCGTTGGCGGAATGTGTCATGTAGCGGTGAAATGCAT
AGATATGACACAGAACACCGATTGCGAAGGCAGCTGACGAAACTGTAACTGACGCTGAGG
CACGAAAGCGTGGGGATCAAACAGGATTAGATACCCGCGTAGTCC
>Otu644
CCAGCCTACGGGTCGCAGCAGCCGAGAATATTCGACAATGGGCGAAAGCCTGATCGAGCG
ATACCGCGTGGTGGATGAAGTGCTTCGGCACGTAAACACCTTTTATGGAGGAGGAAGTAA
TTGACGTTACTCCATGAATAAGGGGCTCCTAACTCTGTGCCAGCAGGAGCGGTAATACAG
AGGCCCCGAGCATTACCCGGAATCATTGGGCGTAAAGAGTGCGTAGGTGGTCATGTTAGT
CGGGCGTTAAACCCCGGGGCTCAACCCCGGAACCGCGCCCGAAACGGCATGACTCGAGGG
CGTGAGAGGTACATGGAACTCATGGTGTAGGGGTGAAATCCGTTGATATCATGGGGAACA
CCGAAAGCGAAGGCAATGTACTGGCGCGTTCCTGACACTCAAGCACGAAAGCCAGGGGAG
CAAACGGGATTAGAAACCCGCGTAGTCC
>Otu649
CCAGCCTACGGGTGGCTCCAGTCGAGGATCTTCGGCAATGGGCGCAAGCCCGACCGAGCG
ATACCGCGTGTGCGACGAAGGCCTTCGGGTTGTAAAGCACTGTCGAGGGGGAGAAAACCG
CAAGGTTGATCTATCCCTGGAGGAAGCACGGGCTAAGTTCGTGCCAGCAGCCGCGGTAAG
ACGAACCGTGCGAACGTTGTTCGGAATCACTGGGCTTAAAGGGCGCGTAGGCGGGCTGCC
AAGTTTGGGGTGAAATCCTCCGGCTCAACCGGGGAAGTGCCTCGAATACTGGCGGCCTCG
AGGGGGGTAGGGGCGTGCGGAACTGTGGGTGGAGCGGTGAAATGCGTTGATATCCACAGG
AACTCCGGTGGCGAAGGCGGCACGCTGGACCTTTTCTGACGCTGAGGCGCGAAAGCCAGG
GGAGCGAACGGGATTAGATACCCGTGTAGTCC
>Otu651
CCAGCCTACGGGGTGCACCAGTGGGGAATTTTGGACAATGGGCGCAAGCCTGATCCGGCC
ATGCCGCGTGCGGGAAGAAGGCCTTCGGGTTGTAAACCGCTTTTGTCAGGGAAGAAACGG
CTTTCTCTAATACAGAGAGTTAATGACGGTACCTGAAGAATAAGCACCGGCTAACTACGT
GCCAGCAGCCGCGGTAATACGTAGGGTGCAAGCGTTAATCGGAATTACTGGGCGTAAAGC
GTGCGCAGGCGGTTATGCAAGACAGAGGTGAAATCCCCGGGCTCAACCTGGGAACTGCCT
TTGTGACTGCATAGCTAGAGTACGGTAGAGGGGGATGGAATTCCGCGTGTAGCAGTGAAA
TGCGTAGATATGCGGAGGAACACCGATGGCGAAGGCAATCCCCTGGACCTGTACTGACGC
TCATGCACGAAAGCGTGGGGAGCAAACAGGATTAGAAACCCTCGTAGTCC

>Otu654
CCAGCCTACGGGTGGCAGCAGTTTCGAATCATTCACAATGGGCGAAAGCCTGATGGTGCG
ACGCCGCGTGAGGGATGAAGGTCTTCGGATTGTAAACCTCTGTCACCGGGGAAGAAACGC
TTTGCGTTAATACTGCAAAGCCTGACTTAACCCGGAGAGGAAGCAGTGGCTAACTCTGTG
CCAGCAGCCGCGGTAATACAGAGACTGCGAGCGTTATACGGATTCACTGGGCGTAAAGGG
TGCGCAGGCGGCCAAGTGTGTTAGGCGTGAAAGCCCGGGGCTTAACCCCGGAATTGCACC
TAAAACTACATGGCTAGAGCATTGGAGAGGGTAGCAGAATTCACGGTGTAGCAGTGAAAT
GCGTAGATATCAAGCGGAACACCAGTGGCGAAGGCGAGTCTCTGGACATTTCCTGACGCT
GAGGCACGAAAGCCAGGGGAGCAAACGGGATTAGATACCCCTGTAGTCC
>Otu657
CCAGCCTACGGGGCGCAGCAGTAAGGGATATTGCACAATGGGCGAAAGCCTGATGCAGCA
ACGCCGCGTGGAGGATGAAGGCTTTCGGGTCGTAAACTCCTTTTGGACCTGACGAGGAAG
GACGGTAGGGTCCGAATAAGTCACGGCTAACTACGTGCCAGCAGCCGCGGTAAAACGTAG
GTGGCGAGCGTTATCCGGATTTACTGGGCGTAAAGCGTGTGCAGGCGGGGCACCAAGTGG
TGTATGAAGGGTCTCGGCTCAACCGGGATAGGTTATGCCAGACTGGTGCTCTAGAGTACG
AGAGAGGGGCGTGGAATTCCGGGTGTAGTGGTGAAATGCGTAGAGATCCGGAGGAACCCC
AGAGGCGAAGGCGGCGCCCTGGCTCGATACTGACGCTCAGACACGACAGCATGGGGAGCG
AACGGGATTAGAGACCCTAGTAGTCC
>Otu658
CCAGCCTATGGGCGCTCCAGTGGGGAATCTTGCGCAATGGGCGAAAGCCTGACGCAGCAA
CGCCGCGTGAGGGATGAAGGCCTTCGGGTTGTAAACCTCTTTCAAGCAGGGACGATAATG
ACGGTACCTGCAGAAGAAGCTCCGGCCAACTACGTGCCAGCAGCCGCGGTAATACGTAGG
GAGCGAGCGTTGTCCGGATTCATTGGGCGTAAAGAGCTCGTAGGCGGTTTGATAAGTCGG
ATGTGAAACCTCCAGGCTCAACCTGGAGTCGCCATCTGATACTGTCATGACTAGAGTCCG
GTAGGGGCCCACGGAACTCCTGGTGTAGCGGTGAAATGCGCAGATATCAGGAAGAACACC
GGTGGCGAAGGCGGTGGGCTGGGCCGGCACTGACGCTGAGGAGCGAAAGCGTGGGGAGCG
AACAGGATTAGAGACCCGAGTAGTCC
>Otu662
CCAGCCTACGGGGGGCTGCAGTGGGGAATATTGGACAATGGGCGCAAGCCTGATCCAGCA
ATGCCGCGTGAGTGATGACGGCCTTAGGGTTGTAAAGCTCTTTCGGCGGGGACGATGATG
ACGGTACCCGCAGAAGAAGCCCCGGCTAACTTCGTGCCAGCAGCCGCGATAATACGAAGG
GGGCAAGCGTTGTTCGGAATTACTGGGCGTAAAGGGCGCGCAGGCGGCCTATCAAGTCAG
ATGTGAAAGCCCCGGGCTCAACCTGGGAATAGCATTTGGGACTGTTAGGCTAGAGATCGG
GAGAGGAGAGTGGAATTCCCAGTGTAGAGGTGAAATTCGTAGATATTGGGAAGAACACCG
GTGGCGAAGGCGGCTCTCTGGACCGATACTGACGCTGAGGCGCGAAAGCGTGGGGAGCAA
ACAGGATTAGAAACCCCCGTAGTCC
>Otu665
CCAGCCTACGGGTTGCTCCAGCAACGAATCTTCCCCAATGCCGGAAACGGTGAGGGAGCG
ACGCCGCGTGAAGGACGAAGTACTTCGGTATGTAAACTTCTAAAAGGGTTTTGAAAGACG
AGCGCCTAATACGCGCAAAATTTGATCTGATCCTGATAAGGGGCGGCTAACTCTGTGCCA
GCAGCCGCGGTAATACAGAGGCCCCAAGCGTTACTGAGAATCACTGGGTTTAAAGGGTGC
GTAGGTGGTCCGTTAAGTCAGTTGTGAAATCCCCGGGCTCAACCCGGGAACTGCTTCTGA
TACTGGCGGACTTGAGGCCAGTAGGGGTCACTAGAACAGACGGTGGAGCGGTGAAATGCG
TAGATATCGTCTGGAATGCCTGTGGTGAAGACGGGTGACTGGGCTGGTTCTGACACTGAG
GCACGAAAGCGTGGGGAGCGAACGGGATTAGAAACCCTTGTAGTCC
>Otu666
CCAGCCTACGGGGGGCACCAGTCGAGAATATTTGGCAATGGGCGAAAGCCTGACCATGCG
ACACCGCGTGCTGGATGAAGTCCTTCGGGACGTAAACAGCTTTTATGGGGGAAGAAGTAA
TTGACATTACCCCATGAATAAGGGGCTCCTAACTCTGTGCCAGCAGGAGCGGTAATACAG
AGGCCCCGAGCATTATCCGGAATCACTGGGCGTAAAGGGTGTGTAGGCGGTCGTGTTAGT
CCTTTGTAAAAGGTCTCTGGCTTAACCAGGGAACTGCAGGGGAAACGGCACGACTTCGAG
GACGCGAGAGGTCTGGGGAACTCATGGTGTAGGGGTGAAATCCGTTGATATCATGGGGAA
CACCAAATGCGAAGGCACCAGACTGGCGCGCTCCTGACGCTGAGACACGAAAGCGTGGGT
AGCGAATGGGATTAGAAACCCGCGTAGTCC
>Otu670
CCAGCCTATGGGGGGCAGCAGTCGAGGATCATTCGCAATGGGCGAAAGCCTGACGATGCG
ACGCTGTGTGAGCGATGAAGGCCTTCGGGTCGTAAAGCTCTTTCGCCTGGGAACAAGACA
AAGGGGCGAATAGTCTCATAGTTTGAGGGTACCAGGTAAAGAAGCACCGGCTAACTCCGT

GCCAGCAGCTGCGGTAATACGGAGGGTGCAAGCATTAATCGGATTTATTGGGCGTAAAGG
GCGCGTAGGCGGTTTCTTAAGTCAGATGTGAAATTCCGGGGCTCAAACTCGGAGCTGCAT
TTGAAACTGGGAGACTGGAGTGTTGACGAAGAAAACGGAATTCCACATGTAGCGGTGAAA
TGCGTAGATATGTGGAAGAACACCTGTGGCGAAAGCGGTTTTCTAGTTAACTACTGACGC
TGAGGCGCGAGAGCAAGGGGAGCAAACAGGATTAGATACCCCAGTAGTCC
>Otu671
CCAGCCTACGGGAGGCACCAGTGGGGAAAATTGGACAATGGGGGAAACCCTGATCCAGCG
ACGCCGCGTGTGTGAAGAAGGCCTGCGGGTTGTAAAGCACTTTTAGTGGGGATGAAAAGT
TCAGGGCTAATACCCTTGGATCTTGACCTAACCTACAGAAAAAGCACCGGCTAACTCTGT
GCCAGCAGCCGCGGTAATACAGAGGGTGCGAGCGTTAATCGGAATTACTGGGCGTAAAGC
GTGCGTAGACGGTTCTGTAAGTCGGATGTGAAATCCCCGGGCTCAACCTGGGAATTGCAT
TCGAGACTGCAAAGCTAGGGTGCGGAAGAGGGAAGCGGAATTTCCGGTGTAGCGGTGAAA
TGCGTAGATATTCGCAAGAACACCAGTGGCGAAGGCGGCTCACTGGCCCGATACTGACGC
TGAGGCACGAAAGCGTGGGGAGCAAACAGGATTAGAGACCCGAGTAGTCC
>Otu672
CCAGCCTACGGGGGGCAGCAGTCAAGAATATTCCTCAATGGCCGAAAGGCTGAAGGAGCG
ACGCCGCGTGTAGGATGAAGGTCTTCGGATCGTAAACTACTTTTACATGGGAAGAATTTG
TGACGGTACCATGCGAATAAGAGGTTGCTAACTCTGTGCCAGCAGCAGCGGTAATACAGA
GACCTCAAGCGTTATCCGGATTTATTGGGCGTAAAGCGTCCGCAGGTGGCTCAGCGGGTC
AGGGGTTAAAACTTACTGCTCAACGGTAAGACTGCTCCTGAAACTACTAAGCTAGAGTAT
GGAAGAGGTAAGCGGAATTCTCGGTGTAGTCGTAATAAGCGTTGATATCGAGAAGAACAC
CAAATGCGAAGGCAGCTTACTGGTACATTACTGACACTCAGGGACGAAAGCGTGGGGAGC
AAACAGGATTAGATACCCCTGTAGTCC
>Otu673
CCAGCCCACGGGTGGCAGCAGGGAATATTGGACAATGGGCGCAAGCCTGATCCAGCCATG
CCGCGTGAGTGATGAAGGCCTTAGGGTTGTAAAGCTCTTTTGGCGGGGAAGATAATGACG
GTACCCGCAGAATAAGCCCCGGCTAACTTCGTGCCAGCAGCCGCGGTAATACGAAGGGGG
CAAGCATTGTTCGGAATTACTGGGCGTAAAGGGTGCGTAGGCGGATTGGATAGTCAGATG
TGAAAGCCCAGGGCTCAACCCTGGAATTGCATTTGATACTGCCAGTCTGGAATATTGGAG
AGGAACGTGGAATTCTCAGTGTAGAGGTGAAATTCGTAGATATTGAGAGGAACACCAGTG
GCGTAGGCGACGTTCTGGCCAAATATTGACGCTGAGGCACGAAAGTGTGGGGAGCAAACA
GGATTAGAAACCCTCGTAGTCC
>Otu674
CCAGCCTACGGGAGGCTCCAGTCGAGAATTTTTCTCAATGGGGGAAACCCTGAAGGAGCG
ACGCCGCGTGGAGGATGAAGGTCTTCGGATTGTAAACTCCTGTCATTGGTGAACAAGATT
GCCGGTTAACTGCCGGTGGATTTGATAGTAGCCGAAGAGGAAGAGACGGCTAACTCTGTG
CCAGCAGCCGCGGTAATACAGAGGTCTCGAGCGTTGTTCGGATTCATTGGGCGTAAAGGG
TGCGTAGGCGGCGTGGCAAGTCTGATGTGAAATCCCGGGGCTCAACCCCGGAACTGCATT
GGATACTGCCATGCTAGAGTACTGGAGAGGAGATTGGAATTTACGGTGTAGCAGTGAAAT
GCGTATATATCGTAAGGAAGACCAGTGGCGAAGGCGAATCTCTGGGCAGTTACTGACGCT
GAGGCACGAAGGCCAGGGGAGCAAACGGGATTAGATACCCGTGTAGTCC
>Otu675
CCAGCCTACGGGAGGCACCAGTAGGGAATTTTCCACAATGGACGAAAGTCTGATGGAGCA
ACGCCGCGTGCAGGATGAACCCCTTCGGGGCGTAAACTGCTTTTATCTGTGACGATTATG
ACGGTAGCAGATGAATAAGGATCGGCTAACTCCGTGCCAGCAGCCGCGGTCATACGGAGG
ATCCAAGCGTTATCCGGAATTACTGGGCGTAAAGAGTTGCGTAGGTGGCATGGTAAGTTG
GTAGTGAAAGCGTTCGGCTCAACCGAATACACATTACCAAAACTGCCAAGCTAGAGGATG
AGAGAGGTTATTGAAATTCCCAGTGTAGGAGTGAAATCCGTAGATATTGGGAGGAACACC
GATGGCGTAGGCAGATAACTGGCTCATTCCTGACACTAAGGCACGAAAGCGTAGGTAGCA
AACGGGATTAGAAACCCTAGTAGTCC
>Otu676
CCAGCCTATGGGTGGCACCAGTAGGGAATCTTGCGCAATGGGCGAAAGCCTGACGCAGCA
ACGCCGCGTGGGGGATGAAGCTTCTCGGAGTGTAAACCCCTTTCGACCCGGACGAATGCC
CGCAAGGGTTTGACGGTACGGGTATAAGAAGCCCCGGCTAACTACGTGCCAGCAGCCGCG
GTAATACGTAGGGGGCCAGCGTTGCTCGGAATTACTGGGCGTAAAGGGTCTGTAGGCGGT
GCGGCAAGTCGGATGTGAAATCTCTGGGCTTAACCCAGAGGCTGCTTCCGAAACTGCCGC
GCTAGAGGATGAGAGAGGCGAGTGGAATTGCGGGTGTAGCGGTGAAATGCGTAGATATCC
GCAGGAACATCCGAGGCGAAGGCGGCTCGCTGGATCATTTCTGACGCTGAGAGACGAAAG

CTAGGGGAGCAAACAGGATTAGATACCCGTGTAGTCC
>Otu677
CCAGCCTATGGGGGGCAGCAGTAGGGAATTTTCCACAATGGGCGAAAGCCTGATGGAGCA
ACGCCGCGTGCAGGATGAAGGCCTTAGGGTCGTAAACTGCTTTTATATGTGAAGAATATG
ACGGTAGCATATGAATAAGGATCGGCTAACTCCGTGCCAGCAGCCGCGGTCATACGGAGG
ATCCAAGCGTTATCCGGAATTACTGGGCGTAAAGAGTTGCGTAGGTGGCAGAGTAAGTAG
ATAGTGAAAGCGTACGGCTCAACCGTATATCCATTATCTAAACTGCTCAGCTTGAGGACG
AGAGAGGTAACTGGAATTTCTAGTGTAGGAGTGAAATCCGTGGATATTAGAAGGAACACC
GATGGCGTAGGCAGGTTACTGGCTCGTTTCTGACACTAAGGCACGAAAGCGTGGGGAGCA
AACGGGATTAGAAACCCCTGTAGTCC
>Otu680
CCAGCCTATGGGGGGCAGCAGTGGGGAATATTGGACAATGGGGGAAACCCTGATCCAGCA
ATGCCGCGTGAGTGATGAAGGCCTTAGGGTTGTAAAGCTCTTTCGGCGGGGACGATGATG
ACGGTACCCGCAGAAGAAGCCCCGGCTAACTTCGTGCCAGCAGCCGCGGTAATACGAAGG
GGGCGAGCGTTGTTCGGAATTACTGGGCGTAAAGGGCGCGCAGGCGGCTCAGACAGTCAG
ATGTGAAAGCCCCGGGCTTAACCTGGGAACTGCATTTGATACTACTGAGCTTGAGTACGG
GAGAGGATAGTGGAATTCCCAGTGTGGAGGCGAAATTCGTAGATATTGGGAAGAACACCG
GTGGCGAAGGCGGCTATCTGGACCGTAACTGACGCTGAGGCGCGAAAGCGTGGGGAGCAA
ACAGGATTAGAAACCCCTGTAGTCC
>Otu681
CCAGCCTACGGGAGGCAGCAGTGGGGAATTTTGTGCAATGGGCGAAAGCCTGACACAGCG
ACACCGCGTGAGCGAAGAAGCCCTTTGGGGTGTAAAGCTCTGTCAGCTGGAACGAAAAAA
ATGACGGTACCAGCAGAGGAAGCATCGGCTAACTACGTGCCAGCAGCCGCGGTAAGACGT
AGGATGCGAGCGTTGTCCGGATTTATTGGGCGTAAAGAGTTCGTAGGTGGTTTGTTAAGT
CTGATGTTAAAGATTGGGGCTCAACCCTGAACATGCATTGGATACTGGCAGACTTGAGTG
TGGTAGAGGCTAGTGGAATTCCCAGTGTAGCGGTGAAATGCGTAGATATTGGGAAGAACA
CCGGTGGCGTAGGCGACTAGCTGGGCCATAACTGACACTGAGGAACGAAAGCCAGGGTAG
CGAATGGGATTAGATACCCTTGTAGTC
>Otu682
CCAGCCTATGGGGGGCACCAGTCGAGAATCTTTCGCAATGGGGGAAACCCTGACGACGCG
ACGCCGCGTGGAGGATGAAGGCCTTCGGGTTGTAAACTCCTGTCAGCAGGGGAGAACAAG
CTTTTGATGAATAATCGAGAGCTTTGACAGTACCTGCAGAGGAAGCCACGGCTAACTCTG
TGCCAGCAGCCGCGGTAATACAGAGGTGGCAAGCGTTGTTCGGATTTATTGGGCGTAAAG
GGTCCGTAGGCGGCCTGGAAAGTTGGATGTGAAATCCCACAGCTTAACTGTGGAACTGCA
TTCAAAACTTCTAGGCTCGAGTGTGATAGGGGTAAAGGGAATTCCCGGTGTAAGGGTGAA
ATCTGCAGAGATCGGGAGGAACACCAGTCGCGAAGGCGCTTTACTGGATCACTACTGACG
CTGAGGGACGAAAGCCAGGGGAGCAAACGGGATTAGATACCCGAGTAGTCC
>Otu683
CCAGCCTATGGGGCGCAGCAGTCGAGAATTTTTCACAATGGGCGAAAGCCTGATGGAGCG
ACGCCGCGTGGGGGATGAATGGCTTCGGCCCGTAAACCCCTGTCATTCGCGAACAATGCC
TGCGGCCTAACACGTCGCAGGTTGATAGTAGCGGAAGAGGAAGGGACGGCTAACTCTGTG
CCAGCAGCCGCGGTAATACAGAGGTCCCGAGCGTTGTTCGGATTCACTGGGCGTAAAGGG
TGCGTAGGTGGCCAGGTAAGTCTGATGTGAAATCTCGGAGCTTAACTCCGAAACTGCATT
GGATACTATCTGGCTGGAGGGTTGGAGGGGGGACTGGAATTCTCGGTGTAGCAGTGAAAT
GCGTAGATATCGAGAGGAACACCAGTGGCGAAGGCGAGTCCCTGGACAACTCCTGACACT
AAGGCACGAAAGCTAGGGGAGCAAACAGGATTAGAAACCCGCGTAGTCC
>Otu686
CCAGCCTATGGGGTGCAGCAGACGAGAATATTCCGCAATGGACGAAAGTCTGACGGAGCG
AGGCCGCGTGATGGATGAAGTGCTTAGGTACGTAAACATCTTTTATCGGGGAAGAAATTA
TTGACGGTACCCGATGAATAAGGGGCTCCTAACTCTGTGCCAGCAGGAGCGGTAATACAG
AGGCCCCGAGCGTTACCCGGAATCACTGGGCGTAAAGGGTGTCCAGGCGGGCATATTAGT
CTCTCGTAAAATCTATGGGCTTAACCTATAGCGCGCGAGGGAAACGGTATGTCTAGAGGG
CGCGAGAGATAGAGGGAACTCATGGTGGAGGGGTGAAATCCGTTGATATCATGGGGAACA
CCGAAGGCGAAGGCACTCTATTGGCGCGTTCCTGACGCTCACACACGAAAGCCAGGGGAG
CGAACGGGATTAGATACCCCCGTAGTCC
>Otu687
CCAGCCTATGGGGTGCTCCAGTGGGGAATATTGGACAATGGGCGCAAGCCTGATCCAGCA
ATGCCGCGTGAGTGAAGAAGGTCTTCGGATTGTAAAGCTCTTTCGACGGGGACGATGATG

ACGGTACCTAGACAAGAAGCCCCGGCTAACTTCGTGCCAGCAGCCGCGGTAATACGAAGG
GGGCTAGCGTTGTTCGGAATTACTGGGCGTAAAGCGCGTGTAGGCGGGCGAATAAGTCAG
GGGTGAAATGCCAAGGCTCAACCTTGGAACTGCCTTTGATACTGTTCGTCTTGAGTCCGG
GAGAGGTGAGTGGAACTGCGAGTGTAGAGGTGAAATTCGTAGATATTCGCAAGAACACCA
GTGGCGAAGGCGGCTCACTGGCCCGGAACTGACGCTGAGACGCGAAAGCGTGGGGAGCAA
ACAGGATTAGATACCCCCGTAGTCC
>Otu688
CCAGCCTATGGGTCGCACCAGTGGGGAATATTGGACAATGGGGGAAACCCTGATCCAGCA
ATGCCGCGTGAGTGATGAAGGCCTTAGGGTTGTAAAGCTCTTTCGCCTGCGACGATGATG
GCGGTAGCAGGAGAAGAAGCCCCGGCTAATTCCGTGCCAGCAGCCGCGGTAATACGGAAG
GGGCTAGCGTTGTTCGGAATTACTGGGCGTAAAGCGCGCGTAGGCGGCTCGTCAAGTGAG
GCGTGAAAGTCCTGGGCTCAACCTGGGGACGGCGCTTCAGACTGTCGGGCTAGAGTCCGG
AAGAGGGTGGTGGAATTCCGAGTGTAGAGGTGAAATTCGTAGATATTCGGAGGAACACCG
GTGGCGAAGGCGGCCACCTGGTCCGGTACTGACGCTCAAGTGCGAAAGCGTGGGGAGCAA
ACAGGATTAGAGACCCTCGTAGTCC
>Otu691
CCAGCCTATGGGTCGCAGCAGTGGGGAATCTTGCACAATGGAGGAAACTCTGATGCAGCG
ACGCCGCGTGAGCGATGAAGCCCTTCGGGGTGTAAAGCTCTTTCGACGGGAACGATAATG
ACGGTACCCGGAGAAGAAGCTGCGGCTAACTACGTGCCAGCAGCCGCGGTAATACGTAGG
GGGCCAGCGTTGCTCGGAATTACTGGGCGTAAAGGGTCCGTAGGCGGTGTGGCAAGTCGG
GAGTGAAATCTCTAGGCTTAACCTAGAGGCTGCTTTCGAAACTGCCGTGCTAGAGTGTGA
GAGAGGCGAGTGGAATTACGGGTGTAGCGGTGAAATGCGTAGATATCCGTAGGAACATCC
GAGGCGAAAGCGGCTCGCTGGATCACAACTGACGCTGAGGGACGAAAGCTAGGGGAGCAA
ACAGGATTAGATACCCTGGTAGTCC
>Otu696
CCAGCCTACGGGGTGCTCCAGTAGGGAATTTTCCACAATGGGCGAAAGCCTGATGGAGCA
ACGCCGCGTGCAGGACGAAGGCCTTCGGGTTGTAAACTGCTTTTATATGTGACGATTATG
ACGGTAGCATATGAATAAGGATCGGCTAACTCCGTGCCAGCAGCCGCGGTCATACGGAGG
ATCCAAGCGTTATCCGGAATTACTGGGCGTAAAGAGTTGCGTAGGTGGCGGTACAAGTTG
GTAGTGAAAGCGTTCGGCTCAACCGAATATACATTATCAAAACTGTACAGCTAGAGGATG
AGAGAGGTTATTGGAATTCCTAGTGTAGGAGTGAAATCCGTAGATATTAGGAGGAACACC
GATGGCGTAGGCAGATAACTGGCTCATTCCTGACACTAAGGCACGAAAGCGTGGGGAGCG
AACTGGATTAGATACCCGCGTAGTCC
>Otu699
CCAGCCTACGGGGGGCAGCAGTCGAGGATCTTCGGCAATGGACGCAAGTCTGACCGAGCG
ACGCCGCGTGCGGGATGAAGGCCTTCGGGTTGTAAACCGCTGTCAGTGGGGAGAAAGTCT
ATAAGGGTACTCTCTTATAGTTGATCTATCCGCAGAGGAAGTGTGGGCTAAGTTCGTGCC
AGCAGCCGCGGTAAGACGAACCACACAAACGTTATTCGGAATTACTGGGCTTAAAGGGTG
CGTAGGCTGCGCAGAAAGTTGGGTGTGAAATACCTCGGCTCAACCGAGGAACTGCGCCCA
AGACTACTGTGCTTGAGGGAGACAGAGGTGAGCGGAACTGAGGGTGGAGCGGTGAAATGC
GTTGATATCCTCAGGAACACCCGTGGCGAAAGCGGCTCACTGGGTCTCTTCTGACGCTGA
GGCACGAAAGCTAGGGTAGCGAACGGGATTAGAAACCCCCGTAGTCC
>Otu700
CCAGCCTACGGGTGGCTGCAGTCGAGAATTTTTCTCAATGAGCGCAAGCTTGACGGAGCG
ACACTGTGTGAGTGATGAAGGCCTTCGGGTTGTAAAGCTCTTTTGTTTGGGAACAAGAAA
GATACGTGAATAACGTATCAATTTGAGGGTACTAGAAGAAAAAGTACCGGCTAACTACGT
GCCAGCAGCTGCGGTAATACGTAGGGTACAAGCATTAATCGGAATTATTGGGCGTAAAGG
GCGCGTAGGCGGGGACGTAAGTCAGATGTGAAATACCAAAGCTCAACTTTGGTGCTGCAT
TTGAAACTGCGTTTCTTGAGGACTGACGGAGAAAGCGGAATTCCATGTGTAGCGGTGAAA
TGCGTAGATACATGGAGGAACACCGGTGGCGAAAGCGGCTTTCTAGTTTTTACCTGACGC
TGAGGCGCGAGAGCAAGGGGAGCAAACAGGATTAGAGACCCCAGTAGTCC
>Otu701
CCAGCCTACGGGGGGCTCCAGTCGAGAATCTTCCGCAATGGGCGAAAGCCTGACGGAGCG
ACGTTACGTGAATGATGAAGCTCTTCGGAGTGTAAAGTTCTTTTCTATGGGAGGAAGTTA
TTGACAGTACCATAGGAATAAGGGGATCCTAATTCTGTGCCAGCAGGAGCGGTGAGACAG
AATCCCCAAGCGTTACCCGGATTTATTGGGCGTAAAGGGTCCGTAGGTGGTTTGGTAAGT
TGAAAGTTAAATCTCATCGGCTTAACCTTTGAGGTGCTTTCAATACTGCCAGACTTGAGG
GCGTTAGGGGTTAGCGGAACCGACGGAGTAGGGGTGAAATCCGTTGATATCGTCGGGAAC

ACCAAAAGCGAAGGCAGCTAACTAGGACGACCCTGACACTGAGGGACGAAAGCGTGGGGA
GCAAAAAGGATTAGAAACCCCCGTAGTCC
>Otu705
CCAGCCTACGGGGTGCAGCAGTGGGGAATCTTGCACAATGGGCGAAAGCCTGATGCAGCG
ACGCCGCGTGAGCGATGAAGGCCTTCGGGTTGTAAAGCTCTGTGGGGAGGGACGAATAAG
AATAGGCCAATATCCTATTCGATGACGGTACCTCCTTAGCAAGCACCGGCTAACTCTGTG
CCAGCAGCCGCGGTAAGACAGAGGGTGCAAACGTTGTTCGGAATCACTGGGCGTAAAGCG
CGTGTAGGCGGCTATGTAAGTCGGACGTGAAAGCCCACGGCTCAACTGTGGAAGTGCGCT
CGAAACTGCGTAGCTTGAGTTTTGGAGAGGAAGGCGGAATACTTGGTGTAGAGGTGAAAT
TCGTAGATATCAGGTAGAACACCAGTGGCGAAGGCGGCCTTCTGGACAATGACTGACGCT
GAGACGCGAAAGCGTGGGGAGCAAACAGGATTAGAAACCCCTGTAGTCC
>Otu706
CCAGCCTATGGGTGGCTGCAGTCGAGGATCTTTGGCAATGGGCGCAAGCCTGACCAAGCG
ACGCCGCGTGTGCGATGAAGGCCTTCGGGTTGTAAAGCACTGTCGAGGGGGAGTAAGACC
CGAAAGGGTTTGAACTATCCCTGGAGGAAGCTCGGGCTAAGTTCGTGCCAGCAGCCGCGG
TAAGACGAACCGAGCGAACGTTGTTCGGAATCACTGGGCTTAAAGGGCGCGTAGGCGGGT
ATGAAAGTCTGCGGTGAAATCCTCCAGCTTAACTGGAGAAGTGCCGTGGATACTGCACGT
CTTGAGGAGGGTAGGGGCATCTGGAACGGTCGGTGGAGCGGTGAAATGCGTTGATATCGA
CCGGAACTCCGGTGGCGAAGGCGAGGTGCTGGACCCTTTCTGACGCTGAGGCGCGAAAGC
CAGGGGAGCAAACGGGATTAGAAACCCCCGTAGTCC
>Otu707
CCAGCCTACGGGATGCAGCAGTAGGGAATATTGCACAATGGACGAAAGTCTGATGCAGCA
ACGCCGCGTGCGCGATGAAGGCCTTCGGGTCGTAAAGCGCTTTTCGGGAGGATGAGGAAG
GACGGTACTCCCGGAATAAGTCACGGCTAACTACGTGCCAGCAGCCGCGGTAAAACGTAG
GTGGCGAGCGTTATCCGGATTTACTGGGCGTAAAGCGTGTGCAGGCGGTCCGGTCAGTCG
GATGTGAAATCTCTCGGCTCAACCGAGAGGGGTCATTCGATACTTCCGGACTTGAGGGCA
GTAGAGGAAGGTGGAATTCCCGGTGTAGTGGTAAAATGCGTAGAGATCGGGAGGAACACC
AGTGGCGAAGGCGGCCTTCTGGGCTGCCCCTGACGCTCAGACACGAAAGCTAGGGGAGCG
AACGGGATTAGATACCCCAGTAGTCC
>Otu709
CCAGCCTATGGGAGGCAGCAGTAACGAATCTTCCGCAATGCACGCAAGTGTGACGGAGCG
ACGCCGCGTGTGGGACGAAGCCCCTCGGGGTGTAAACCACTGTCAGGGGATAGAAAGTTC
TGATCATCCCCAGAGGAAGGCACGGCTAACTCTGTGCCAGCAGCCGCGGTAAGACAGAGG
TGCCAAGCGTTAGGCGGAATCACTGGGCTTAAAGCGTGTGTAGGCGGACCGTTAAGTACC
TTGTGAAATCCCCCGGCTCAACCGGGGAACTGCTGGGTATACTGGCGGTCTTGGGCCACT
CAGGGGCAGATGGAACAGGCGGTGGAGCGGTGAAATGCGTAGATATCGCCTGGAACGCCA
AAGGAGAAATCAGTCTGCTGGGGGTGTGCCGACGCTGAGATACGAAAGCCAGGGGAGCGA
ACGGGATTAGAGACCCCTGTAGTCC
>Otu710
CCAGCCTACGGGATGCTGCAGTAGGGAATTTTGCGCAATGGGCGGAAGCCTGACGCAGCA
ACGCCGCGTGAGTGATGAAGGCCTTCGGGTTGTAAAGCTCTGTTGAGTGGGAAGAAACTT
CTCATGGATAATACCCATGGGGACTGACGGTACCACTTGAGGAAGCCCCGGCTAACTACG
TGCCAGCAGCCGCGGTAATACGTAGGGGGCAAGCGTTGTTCGGAATCACTGGGCGTAAAG
GGAGCGTAGGCGGAGATGTAAGTTGGGAGTGAAATTCACAGGCTCAACCTGTGACTTGCT
TCCAATACTGCGTCTCTTGAGTATAGGAGAGGGCGATGGAATTCCAGGTGTAGCGGTGGA
ATGCGTAGATATCTGGAAGAACACCAGTGGCGAAGGCGCCCATCTGGCCTAATACTGACG
CTCAGGCACGAAAGCTAGGGGAGCAAACAGGATTAGAGACCCCCGTAGTCC
>Otu712
CCAGCCTATGGGGGGCTGCAGTCGGGAAAATTTTGCAATGCGCGAAAGCGTGACAGAGCA
AGCCAGAGTGATTTTCCGTATTGGAAAATCTTTTGTGAGATGTAAACATTCTTACGAATA
AGGAGTGGGAGAGACTGGTGCCAGCCGCCGCGGTAATCCCAGCACTCCAAGTCGCAGCCA
CAATTATTGGGTCTAAAACATCCGTAGCTTGCTTTGTAAGTCTTTTGTCAAATCGGGCCT
CTTAAGGGTCCGGCGAGCAAAAGATACTGCTTAGCTAGGGACCGGGAGATGTAAGGAGTA
CGAATGGAGTAGCGGTTAAATGCGTTGATCCTATTTGGACTAACAATAGCGAAGGCACCT
TACAAGAACGGATCCGACAGTGAGGGATGAAGGCTAGGGGCGCAAATTGGATTAGAAACC
CCTGTAGTCC
>Otu713
CCAGCCTATGGGAGGCACCAGTCGAGAATCATTCGCAATGGGCGAAAGCCTGACGATGCG

ACGCCGTGTGTATGATGAAGGCCTTCGGGTTGTAAAGTACTTTCGCCTGGGAACAAGGGA
AGCCGGCTAATATCCGGCGGACTTGAGGGTACCAGGTAAAGAAGCACCGGCTAACTACGT
GCCAGCAGCTGCGGTAATACGTAGGGTGCAAGCATTAATCGGATTTATTGGGCGTAAAGG
GCGCGTAGGCGGAAGCGTAAGTCAGATGTGAAAGCCCGGGGCTCAACCCCGGAACAGCAT
TTGAAACTGCGTTTCTAGAGGGTAGGCGGAGAAAATGGAATTCCACAAGTAGCGGTGAAA
TGCGTAGATATGTGGAAGAACACCAGTGGCGAAGGCGGTTTTCTAGCTTACACCTGACGC
TGATGCGCGAAAGCAAGGGGATCAAACAGGATTAGAAACCCTCGTAGTCC
>Otu714
CCAGCCTACGGGAGGCAGCAGTCGAGAATCTTCGGCAATGGGCGCAAGCCTGACCGAGCG
ACGCCGCGTGCGGGATGAAGGCCTTCGGGTTGTAAACCGCTGTCAGTGCGGAGGAAATAT
AGTGGGGTTCTCCCCATTATTTGACCTATGCGCAGAGGAAGGACGGGCTAAGTTCGTGCC
AGCAGCCGCGGTAAGACGAACCGTCCAAACGTTATTCGGAATTACTGGGCTTAAAGGGTG
CGTAGGCGGTCCTGTAAGTTGGGTGTGAAAGACCTCGGCCTAACCGAGGAATTGCGCCCA
AAACTGCCGGACTTGAGCAAGGCAGAGGTGAACGGAACTTAGGGTGGAGCGGTGAAATGC
GTTGATATCCTAAGGAACACCAGTAGCGAAAGCGGTTCACTGGGCCTTTTCTGACGCTGA
TGCACGAAAGCCAGGGGAGCGAACGGGATTAGATACCCCTGTAGTCC
>Otu716
CCAGCCTACGGGTCGCTGCAGTGGGGAATATTGGACAATGGGCGAAAGCCTGATCCAGCA
ATGCCGCGTGAGTGATGAAGGCCTTAGGGTTGTAAAGCTCTTTTACCAGGGATGATAATG
ACAGTACCTGGAGAATAAGCTCCGGCTAACTCCGTGCCAGCAGCCGCGGTAATACGGAGG
GAGCTAGCGTTGTTCGGAATTACTGGGCGTAAAGCGCACGTAGGCGGCGACTCAAGTCAG
AGGTGAAAGCCCGGGGCTCAACCCCGGAACTGCCTTTGAAACTAGGTTGCTAGAATTCTG
GAGAGGTCAGTGGAATTCCGAGTGTAGAGGTGAAATTCGTAGATATTCGGAAGAACACCA
GTGGCGAAGGCGACTGACTGGACAGATATTGACGCTGAGGTGCGAAAGCGTGGGGAGCAA
ACAGGATTAGATACCCTAGTAGTCC
>Otu719
CCAGCCTATGGGGTGCACCAGTAAGGAATATTGGACAATGGTGGCAACACTGATCCAGCC
ATGCCGCGTGCAGGATGAAGGCGCTACGCGTTGTAAACTGCTTTTGTACGGGAGAAAACC
TATCTACGTGTAGATAGCTGATAGTACCGTAAGAATAAGCATCGGCTAACTTCGTGCCAG
CAGCCGCGGTAATACGGAGGGTGCAAGCGTTATCCGGATTCACTGGGTTTAAAGGGTGCG
TAGGTGGTCTGTTAAGTCAGCGGTGAAAGCCTGGAGCTTAACTCCAGAATTGCCATTGAT
ACTGACGGACTTGAATCAAGTTGAGGTGGATGGAATATTACATGTAGCGGTGAAATGCTT
AGATATGTAATAGAACACCGATTGCGAAGGCAGTTCACTAAGCTTGCATTGACACTGAGG
CACGAAAGCGTGGGGATCAAACAGGATTAGAGACCCCCGTAGTCC
>Otu720
CCAGCCTATGGGTTGCTCCAGTGGGGAATATTGGACAATGGGGGCAACCCTGATCCAGCC
ATGCCGCGTGAGTGATGAAGGCCTTCGGGTTGTAAAGCTCTTTTGGCGGGGACGATGATG
ACGGTACCCGCAGAATAAGCCCCGGCTAACTTCGTGCCAGCAGCCGCGGTAAGACGAAGG
GGGCAAGCGTTGTTCGGAATTACTGGGCGTAAAGCGAGTGTAGGTGGTTGTCGTAGTCAG
GCGTGAAAGCCTTGAGCTCAACTCAAGAAATGCGCTTGATACTGGGCAGCTAGAGGACTG
GAGAGGATAGTGGAATTCCCAGTGTAGTGGTGAAATACGTAGAGATTGGGAAGAACACCA
GTGGCGAAGGCGGCTATCTGGACGGTTTCTGACACTAAGACTCGAAAGCGTGGGGAGCAA
ACAGGATTAGATACCCCCGTAGTCC
>Otu726
CCAGCCTATGGGGTGCTGCAGTGGAGAATATTGGTCAATAAACGAAAGTTTGAACCAGCG
TTTTGCGGGGATGATAGATTCATAGAATCATGAAGGCTATGACAGCTGTAAAGTCCGTCC
GCAGGTTAGAAAAATGATTTTACACCTGAAGAGAACCCCGGGCCAACAACGTGCCAGCAG
CTGCGGTAAGACGTTGACGGGGAAGCGTTACTGGCCATTACTAGGCGTAAAGAGTGGCTA
GATGGTTTAAGAACTTGAAAAAAAATCATAGAATAGTCGAAGACTTTTTCAAAACCTTAA
TACTTGAGGATTAAAGAGGTAAGTGATATATACAATTTAGAGATGAAATTCTAATAGATT
GTAAAGATCTTCAGCGGCCTCGGCGACTTTCCATTTAATTTCCTAACATTGAACCACTAA
AGCACGAGTATCAATCAGGATTAGATACCCGAGTAGTCC
>Otu727
CCAGCCTACGGGATGCTGCAGTGGGGAATCTTGCACAATGGGGGAAACCCTGATGCAGCG
ACGCCGCGTGAGCGATGAAGCCCTTCGGGGTGTAAAGCTCTTTCGGCAGGAACGATTATG
ACGGTACCTGCAGAAGAAGCTGCGGCTAACTACGTGCCAGCAGCCGCGGTAATACGTAGG
CAGCGAGCGTTGTTCGGAGTTACTGGGCGTAAAGAGTATGTAGGCGGTTTCGTAAGTTTG
GTGTGAAATCTCCCGGCTTAACTGGGAGGGTGCGCCGAAAACTGCGAGGCTAGAGTGCGG

GAGAGGAGAGCGGAATTCCTGGTGTAGCGGTGAAATGCGTAGATATCAGGAGGAACACCG
GCGGTGTAGACGGCTCTCTGGACCGTTACTGACGCTGAGATACGAAAGCGTGGGTAGCAA
ACAGGATTAGAAACCAGAGTAGTCC
>Otu731
CCAGCCTACGGGGTGCTCCAGTAAGGAATATTGGACAATGGGGGCAACCCTGATCCAGCC
ATGCCGCGTGAAGGATGAAGGCGCTCTGCGTCGTAAACTTCTTTTAGTTGGGAAAAATTG
TTGGGTTTCTACCCGACTTGATTGTACCAAAAGAATAAGGACCGGCTAACTTCGTGCCAG
CAGCCGCGGTAATACGAAGGGTCCAAGCGTTATCCGGATTTATTGGGTTTAAAGGGTGCG
TAGGCGGGTTGTTAAGTCAGTGGTGAAATCTTGCAGCTTAACTGTAAAATTGCCATTGAT
ACTGACGATCTTGAGTACACTTGAAGTGGGCGGAATATGTCATGTAGAGGTGAAATTCTT
AGAGATGGCATAGAACACCGATTGCGAAGGCAGCTCACTAAATTGTAACTGACGCTGAGG
CACGAAAGCGTGGGGATCAAACAGGATTAGATACCCTGGTAGTCC
>Otu732
CCAGCCTATGGGAGGCAGCAGTGGGGAATCTTGCGCAATGGACGAAAGTCTGACGCAGCC
ACGCCGCGTGAGTGAAGAAGGCCTTCGGGTCGTAAAGCTCTGTCGGAAGGGACGAAAAGT
CTATGAGTTAATAGCTCATAGATCTGACGGTACCCTCAAAGGAAGCACTGGCTAACTTCG
TGCCAGCAGCCGCGGTAATACGAGGGGTGCAAGCGTTGCTCGGAATTATCGGGCGTAAAG
GGTAGGTAGGTGGTCTCGTTTGTCTGGGGTGAAAGCCTTGAGCTTAACTCAAGAAGTGCC
CCAGAAACGGCGAGACTCGAGTCTTGGAGAGGGTCGTGGAATTCCCGGTGTAGCGGTGAA
ATGCGTAGAGATCGGGAGGAACACCAGAGGCGAAGGCGGCGACCTGGACAAGTACTGACA
CTCAACTACGAAAGCGTGGGTAGCAAACAGGATTAGAGACCCGCGTAGTCC
>Otu733
CCAGCCTATGGGTGGCTCCAGAACAGTCAAGGGCCGCTTGCCGGAGGGCCGTGGAAGGGC
CAGTGGTCCACCGCCCGCAGTGCCTTCACCCTTAGCGGTCCTTGCGGCGCCAGCTATAAT
TCCGCGAAGCTCTTGCGTCTCGTTTTGCCATGATGCTGTAATGCCGTAGCTGCTTGCGAA
CAAACGTCATTTTTGCGGAAGAGGCGATCCGCCTGGCTATTCGTAGCGATAATCTTGCCG
CTTTCATCGAGCAAAATGACACCGACCGCCAGCCGGTCAAGGGCTTCAAGGGATGCTCGC
TGGCGGCCTTCTAATTCCTCAAACCTTCGGTGAAGCTGTATTGCCCGTTGGAGATGGGGG
AACAAAAGCCGCAGCAAGTTTACTTCGTCGGGTCCAAAGGGCTGCTTTCTGGAAGATCTG
TGACAAGAAAGATTAGAAACCCGTGTAGTCC
>Otu734
CCAGCCTACGGGACGCACCAGTGGGGAATATTGGACAATGGGCGAAAGCCTGATCCAGCA
ATGCCGCGTGTGTGAAGAAGGTCTTCGGATTGTAAAGCACTTTCGACAGGGACGATAATG
ACGGTACCCGTAGAAGAAGCCCCGGCTAACTTCGTGCCAGCAGCCGCGGTAATACGAAGG
GGGCTAGCGTTGCTCGGAATGACTGGGCGTAAAGGGCGCGTAGGCGGCGATTATAGTCAG
ATGTGAAATTCCTGGGCTCAACCTGGGGACTGCATTTGATACGTGATTGCTTGAGTGTGG
AAGAGGGTCGTGGAATTCCCAGTGTAGAGGTGAAATTCGTAGATATTGGGAAGAACACCG
GTGGCGAAGGCGGCGACCTGGTCCATAACTGACGCTGAGGCGCGAAAGCGTGGGGAGCAA
ACAGGATTAGATACCCGCGTAGTCC
>Otu735
CCAGCCTACGGGGCGCAGCAGTGGGGAATATTGGACAATGGGGGAAACCCTGATCCAGCA
ATGCCGCGTGAATGATGAAGGCCTTAGGGTTGTAAAGTTCTTTCACCTGTGAAGATAATG
ACGGTAGCAGGAGAAGAAGCCCCGGCTAACTCCGTGCCAGCAGCCGCGGTAAGACGGAGG
GGGCTAGCGTTGTTCGGAATGACTGGGCGTAAAGGGCGCGTAGGCGGTTTTTTAAGTGAG
GCGTGAAAGCCCTGGGCTTAACCCAGGAGGTGCGTTTCATACTGGAGAACTTGAGTACGA
GAGAGGAAAGTGGAATTCCTAGTGTAGAGGTGAAATTCGTAGATATTAGGAAGAACACCA
GAGGCGAAGGCGGCTTTCTGGCTCGTAACTGACGCTGAGGCGCGAAAGCGTGGGGAGCAA
ACAGGATTAGAGACCCGTGTAGTCC
>Otu742
CCAGCCTATGGGGATGCTCCAGTCAAGAACATTTGGCAATGGGCGAAAGCCTGACCATGC
GACGCCGCGTGCTGGACGAAGTCCTTCGGGACGTAAACAGCTTTTATGCGTGAAGAAGTA
ATTGACATTAGCGCATGAATAAGGGGCTCCTAACTCTGTGCCAGCAGGAGCGGTAATACA
GAGGCCCCAAGCATTATCCGGAATCACTGGGCGTAAAGGGTGTGTAGGCGGCGATATTAG
TCTTCTGTTAAAGTTTCAGGGCTTAACCCAGAAAATGCGGGAGAAACGGTATTGCTTGAG
GTTGCGAGAGGTAGAGGGAACTCATGGTGTAGGGGTGAAATCCGTTGATATCATGGGGAA
CACCAAATGCGAAGGCACTCTACTGGCGCACACCTGACGCTGAGACACGAAAGCGTGGGG
ATCGAACGGGATTAGAGACCCTTGTAGTCC
>Otu746

CCAGCCTACGGGGTGCACCAGTAGGGAATCTTCCGCAATGGACGCAAGTCTGACGGAGCA
ACGCCGCGTGAGTGATGAAGGTTTTCGGATCGTAAAGCTCTGTTGCCCTAGACGAACAGC
AAGGAGAGTAACTGCTCTTTGTGTGACGGTATAGGAGAAGAAAGCCCCGGCTAACTACGT
GCCAGCAGCCGCGGTAATACGTAGGGGGCAAGCGTTGTCCGGAATTATTGGGCGTAAAGC
GCGCGCAGGCGGTCATTTAAGTTTGGTGTTTAAGCCCGGGGCTCAACCCCGGTTCGCATC
GAAAACTGGGTGACTGGAGTGTAGGAGAGGAAAGTGGAATTCCACGTGTAGCGGTGAAAT
GCGTAGAGATGTGGAGGAACACCAGTGGCGAAGGCGACTTTCTGGCCTATAACTGACGCT
GAGGCGCGAAAGCGTGGGGAGCAAACAGGATTAGAAACCCGAGTAGTCC
>Otu751
CCAGCCTATGGGGGGCAGCAGTGTAGAATTTTAGACAATGCACGAAAGTGTGATCTAGTG
ATATTTCGTAAGTGATGAATGACAGTATTACCACTGTAAAACTTTTTGGTTAAAGATAAT
AATGATAGTATTTAATTAAAAACACCGGCTAATTCCGTGCCAGCAGCCGCGGTAATACAG
AAGGTGTAAACATTATCCATCTTAATTGGATGTAAAGGATATGTAGACGATTTGTTAAAA
TTTTTCTGAAAGTTTTAATAGGTATTAAAGATGAGATTTTTAACTAACAATTTATGAGTT
TAAAATAGGTCGGAATAATTTTATATGTAAGGGTAAAATCTCTATATATATGAAGGAATA
TCAAAATAATTGTGAAAACATCCCTCTAATTTAAACTGACGTCGAGATATGAAAGTGTAG
GTATCAAATTGGATTAGAAACCCCGGTAGTCC
>Otu755
CCAGCCTATGGGGTGCTCCAGTCGAGAATCTTCGGCAATGGGCGCAAGCCTGACCGAGCG
ACGCCGCGTGGAGGACGAAGGCCTTCGGGTTGTAAACTCCTGTCGAGGGGGAAGAAGGGC
CTTAAAACCTTGACTGATCCCTGGAGGAAGCACGGGCTAAGTTCGTGCCAGCAGCCGCGG
TAAGACGAACCGTGCGAACGTTATTCGGAATCACTGGGCTTAAAGCGCGTGTAGGTGGGA
GGGTACGTCCGTCGTTGAAAGCCCCCGGCTCAACCGGGGAACTGGCGCGGATACGGCCTT
CCTGGAGGGACGTAGGGGGACCTGGAACTTCCGGTGGAGCGGTGAAATGCGTTGAGATCG
GAAGGAACGCCCGTGGCGAAAGCGAGGTCCTGGACGTTTTCTGACACTGAGACGCGAAAG
CCAGGGGAGCGAACGGGATTAGAAACCCCAGTAGTCC
>Otu756
CCAGCCTTGGGGTGCACCAGTCGAGAATTTTTCTCAATGGGGGAGACCCTGAAGGAGCGA
CGCCGCGTGGAGGATGAAGGTCTTCGGATTGTAAACTCCTGTCATTTGGGAACAAGTTGC
ATCGGTTAACTGCCGATGCATTGATAGTACCAGAAGAGGAAGAGACGGCTAACTCTGTGC
CAGCAGCCGCGGTAATACAGAGGTCTCAAGCGTTGTTCGGATTCATTGGGCGTAAAGGGT
GCGTAGGTGGCGGGGCAAGTCAGGTGTGAAATCTCGGGGCTCAACCCCGAAACTGCACTT
GATACTGCCTTGCTTGAGTACTGGAGAGGAGATTGGAATTTACGGTGTAGCAGTGGAATG
CGTAGATATCGTAAGGAAGACCAGTGGCGAAGGCGAATCTCTGGACAGTTACTGACACTG
AGGCACGAAGGCCAGGGGAGCAAACGGGATTAGATACCCCAGTAGTCC
>Otu759
CCAGCCTATGGGGGGCACCAGCTAAGAATCTTCCGCAATGGGCGAAAGCCTGACGGAGCG
ATGCCGCGTGATCGATGAAGGTCGAGAGATTGTAAAGATCTTTTCCACGTGAGGAATAAC
CATCGGAGGGAATGCCGGTGGGATGACGTTAGCGTGGGAATAAGCCCCGGCTAATTACGT
GCCAGCAGCCGCGGTAACACGTAAGGGGCGAGCGTTGTTCGGAATTATTGGGCGTAAAGG
GCACGCAGGCGGTTATACAAGTCCGATGTTAAAGACCTGGGCTCAACTCAGGGGCTGCGT
TGGAAACTGTATGACTAGAGTATTAGAGGGGGAGTCGGAATTCCTAGTGTAGGGGTGAAA
TCTGTTGATATTAGGAAGAACACCGGTGGCGAAGGCGGACTCCTGGCTGGATACTGACGC
TGAGGTGCGAAAGCATAGGGAGCAAACAGGATTAGATACCCCGGTAGTCC
>Otu761
CCAGCCTACGGGGCGCAGCAGTAGGGAATATTGCTTAATGGGCGAAAGCCTGAAGCAGCA
ACGCCGCGTGTACGATGAAGGCCTTCGGGTTGTAAAGTACTTTTTGAGGGGATGAGGAAG
GACAGTACCCTCAGAATAAGCCTCGGCTAACTACGTGCCAGCAGCCGCGGTAACACGTAG
GAGGCGAGCGTTATCCGGATTTACTGGGTGTAAAGCGCGTGTAGGCGGTCTGGATAGTTG
GATGTGAAAGCTCCCGGCTTAACTGGGAGAGGTCGTTCAATACTTCCAGACTAGAGTGTG
AGAGAGGGAGGTGGAATTCCGGGTGTAGTGGTGAAATGCGTAGATATCCGGAGGAACACC
AGTGGCGAAAGCGGCCTCCTGGCTCACAACTGACGCTCAGACGCGAAAGCTAGGGTAGCA
AACGGGATTAGAGACCCGTGTAGTCC
>Otu762
CCAGCCTACGGGTCGCACCAGTCGAGGATCTTCGTCAATGGGGGAAACCCTGAACGAGCG
ACGCCGCGTGCGCGATGAAGGCCTTCGGGTTGTAAAGCGCGAAAGTGGGGACGAAAGCCG
AAAGGTTTGACCGATCCACAGTAAGCTCGGGCTAAGTTCGTGCCAGCAGCCGCGGTAAGA
CGAACCGAGCGAACGTTGTTCGGAATCACTGGGCTTAAAGGGCGCGTAGGCGGGCATTCA

AGTCTATGGTGAAATCCTCCAGCTCAACTGGAGAACTGCCGTGGATACTGGGTGCCTCGA
GGGAGGTAGGGGCGTGCGGAACTAATGGTGGAGCGGTGAAATGCGTTGATATCATTAGGA
ACTCCGGTGGCGAAGGCGGCACGCTGGACCTCTTCTGACGCTGAGGCGCGAAAGCCAGGG
GAGCAAACGGGATTAGAAACCCCTGTAGTCC
>Otu763
CCAGCCTACGGGTGGCAGCAGTCGAGAATCTTTCGCAATGGGCGCAAGCCTGACGAAGCG
ACGCCGTGTGAGCGAAGAAGGCCTTCGGGTTGTAAAGCTCTTTCGCTAGGGAACAAGAGA
GACGCAGTAATATTGCGTCAATTTGATGGTACTTGGTAAAGAAGCACCGGCTAACTCCGT
GCCAGCAGCTGCGGTAATACGGAGGGTGCAAGCATTGATCGGAATTACTGGGCGTAAAGG
GCGCGCAGGCGGTTAGGAAAGTCAGATGTGAAATTCCGGGGCTCAACCCCGGAGCTGCAT
TTGAAACTTCCTTTCTAGAGTTTAGTTAGGGAAAACGGAATTCCACGTGTAGCGGTGAAA
TGCGTAGATATGTGGAAGAACACCAGTGGTGAAGACGGATTTCTGGGCTATAACTGACGC
TGAGGCGCGAAAGCATGGGGAGCAAACAGGATTAGAGACCCCCGTAGTCC
>Otu764
CCAGCCTATGGGGCGCACCAGTGAGGAATTTTGGACAATGGGCGCAAGCCTGATCCAGCC
ATTCCGCGTGAGTGAAGAAGGCCTTCGGGTTGTAAAGCTCTTTCGGCAGGAACGAAACGG
TGGAGGCTAATACCTTTCATCAATGACGGTACCTGAAGAAGAAGCACCGGCTAACTACGT
GCCAGCAGCCGCGGTAATACGTAGGGTGCGAGCGTTAATCGGAATTACTGGGCGTAAAGC
GTGCGCAGGCGGTTTTGTAAGACAGATGTGAAATCCCCGGGCTTAACCTGGGAACTGCGT
TTGTGACTGCAAGACTTGAGTGCGGCAGAGGGGGGTGGAATTCCACGTGTAGCAGTGAAA
TGCGTAGAGATGTGGAGGAACACCGATGGCGAAGGCAGCCCCCTGGGTCGACACTGACGC
TCATGCACGAAAGCGTGGGTAGCAAACAGGATTAGAAACCCGCGTAGTCC
>Otu765
CCAGCCTATGGGTTGCTGCAGTAGGGAATTTTCCACAATGGGCGAAAGCCTGATGGAGCA
ACGCCGCGTGCAGGATGAAGGCTCTCGGGTCGTAAACTGCTTTTATATGTGACGATTATG
ACGGTAACATATGAATAAGGATCGGCTAACTCCGTGCCAGCAGCCGCGGTCATACGGAGG
ATCCAAGCGTTATCCGGAATTACTGGGCGTAAAGAGTTGCGTAGGTGGCATTGTAAGTTG
GTAGTGAAAGCGTGGGGCTCAACCCCATAAACATTATCAAAACTACAAAGCTAGAGGATG
AGAGAGGTTATTGGAATTCCCAGTGTAGGAGTGAAATCCGTAGATATTGGGAGGAACACC
GATGGCGTAGGCAGATAACTGGCTCATTCCTGACACTAAGGCACGAAAGCGTGGGTAGCA
AACGGGATTAGATACCCGAGTAGTCC
>Otu766
CCAGCCTACGGGGTGCAGCAGTGGGGAATATTGGACAATGGGCGCAAGCCTGATCCAGCC
ATGCCGCGTGGGTGATGAAGGCCCTAGGGTTGTAAAGCTCTTTCGCTGATGACGATGATG
ACGGTAATCAGAGAAGAAGCCCCGGCTAACTTCGTGCCAGCAGCCGCGGTAATACGAAGG
GGGCTAGCGTTGTTCGGAATTACTGGGCGTAAAGGGCGCGTAGGCGGCATAGCAAGTTAG
AAGTGAAAGCCCCGGGCTCAACCTGGGAATTGCTTTTGATACTGCTAAGCTTGAGTTCGG
GAGAGGTGAGTGGAATTCCCAGTGTAGAGGTGAAATTCGTAGATATTGGGAAGAACACCG
GTGGCGAAGGCGGCTCACTGGACCGACACTGACGCTGAGGCGCGAAAGCGTGGGGAGCAA
ACAGGATTAGATACCCTAGTAGTCC
>Otu772
CCAGCCTATGGGTTGCACCAGTGGGGAATATTGGACAATGGGCGCAAGCCTGATCCAGCC
ATGCCGCGTGAGTGATGAAGGCCTTAGGGTTGTAAAGCTCTTTTGTCCGGGAAGATAATG
ACTGTACCGGAAGAATAAGCCCCGGCTAACTTCGTGCCAGCAGCCGCGGTAATACGAAGG
GGGCTAGCGTTGTTCGGAATCACTGGGCGTAAAGCGCACGTAGGTGGACATTTAAGTCGG
AGGTGAAATCCCAAGGCTCAACCTTGGAACTGCCTTCGATACTGGGTGTCTTGAGTATGG
TAGAGGTTAGTGGAACTGCAAGTGTAGAGGTGAAATTCGTAGATATTTGCAAGAACACCA
GTGGCGAAGGCGGCTAACTGGACCATTACTGACACTGAGGTGCGAAAGCGTGGGGAGCAA
ACAGGATTAGAGACCCCAGTAGTCC
>Otu774
CCAGCCTATGGGTTGCACCAGTGGGGAATATTGGACAATGGGCGCAAGCCTGATCCAGCC
ATGCCGCGTGAGTGATGAAGGCCTTAGGGTTGTAAAGCTCTTTCGTCAGGGACGATAATG
ACGGTACCTGAAGAAGAAGCCCCGGCTAACTTCGTGCCAGCAGCCGCGGTAATACGAAGG
GGGCTAGCGTTGTTCGGAATTACTGGGCGTAAAGCGCACGTAGGCGGGCTTTTAAGTCAG
GGGTGAAATCCCGGGGCTCAACCTCGGAACTGCCTCTGATACTGGAAGTCTTGAGTTCGA
GAGAGGTGAGCGGAATTGCGAGTGTAGAGGTGAAATTCGTAGATATTCGCAGGAACACCA
GTGGCGAAGGCGGCTCACTGGCTCGATACTGACGCTGAGGTGCGAGAGCGTGGGGAGCAA
ACAGGATTAGAAACCCGCGTAGTCC

>Otu777
CCAGCCTATGGGGTGCAGCAGTGGGGAATTTTGGACAATGGACGCAAGTCTGATCCAGCC
ATGCCGCGTGCGGGAAGAAGGCCTTCGGGTTGTAAACCGCTTTTGTCAGGGAAGAAAAGT
CTCGGGTTAATACCTTGGGATGATGACGGTACCTGAAGAATAAGCACCGGCTAACTACGT
GCCAGCAGCCGCGGTAATACGTAGGGTGCAAGCGTTAATCGGAATTACTGGGCGTAAAGC
GTGCGCAGGCGGTTCTATAAGACAGATGTGAAATCCCCGGGCTCAACCTGGGAACTGCAT
TTGTGACTGTAGAGCTGGAGTACGGCAGAGGGGGATGGAATTCCGCGTGTAGCAGTGAAA
TGCGTAGATATGCGGAGGAACACCGATGGCGAAGGCAATCCCCTGGGCCTGTACTGACGC
TCATGCACGAAAGCGTGGGGAGCAAACAGGATTAGAAACCCCAGTAGTCC
>Otu779
CCAGCCTACGGGGTGCTGCAGTCGAGAATTTTTCACAATGGGCGCAAGCCTGATGGAGCG
ACGCCGCGTGGGGGATGAATGGCTTCGGCCCGTAAACCCCTGTCATTTGCGATCAAGGCG
CATTATTTAAAAGATGATGTGTTGATAGTAGCGAAAGAGGAAGGGACGGCTAACTCTGTG
CCAGCAGCCGCGGTAATACAGAGGTCCCGAGCGTTGTTCGGATTCACTGGGCGTAAAGGG
TGCGTAGGTGGTGAGGTAAGTCGGATGTGAAATCTCGGAGCTCAACTCCGAAACGGCATT
GGAAACTACCTTGCTGGAGGGTTGGAAGGGGGACTGGAATGCTTGGTGTAGCAGTGAAAT
GCGTAGATATCAAGCGGAACACCAGTGGCGAAGGCGAGTCCCTGGACAACACCTGACACT
GAGGCACGAAAGCTAGGGGAGCAAACAGGATTAGATACCCCAGTAGTCC
>Otu780
CCCAGCCTATGGGTGGCACCAGTGGGGAATATTGGACAATGGGGGCAACCCTGATCCAGC
AATGCCGCGTGAGTGATGAAGGCCTTCGGGTTGTAAAGCTCTTTCGCCCACGGCGATGGT
GACGGTAGTGGGAGAAGAAGCCCCGGCTAACTTCGTGCCAGCAGCCGCGGTAATACGAAG
GGGGCTAGCGTTGTTCGGAGTTACTGGGCGTAAAGGGCGCGTAGGCGGCTGTCCAAGTCG
GGTGTGAAAGCCCAGGGCTCAACCCTGGATGTGCACTCGAGACTGGCCGGCCAGAGGACG
GGAGAGGATGGTGGAATTCCCAGTGTAGAGGTGAAATTCGTAGATATTGGGAAGAACACC
GGTGGCGAAGGCGGCCATCTGGACCGTTTCTGACGCTGAGGCGCGAAAGCGTGGGGAGCA
AACAGGATTAGATACCCTGGTAGTCC
>Otu785
CCAGCCTACGGGGGGCTCCAGTCGAGAATCTTCCGCAATGGACGAAAGTCTGACGGAGCG
ACGCCGCGTGGAGGATGAAGTGCTTCGGCATGTAAACTCCTTTTGCCAGGGAAAAAGTTA
TTGATTGTACCTGGAGAATAAGAGGTTGCTAAACTCGTGCCAGCAGCAGCGGTAATACGA
GTGCCTCGAGCGTTATCCGGAATCATTGGGCGTAAAGGGTGTGTAGGCGGCGATGTTAGT
CTCGCGTAAAATCTTTCGGCTCAACCGAGAGTCCGCGCGGGAAACGGCATCGCTTAGAGG
ATGGTAGGGGTCTCTGGAACTCTAGGTGTAGCGGTGAAATGCGTTGATATCTAGGGGAAC
ACCGAAAGCGAAGGCAAGAGACTGGACCACTCCTGACGCTGAAACACGAAAGTGTGGGTC
GCGAATGGGATTAGAAACCCCTGTAGTCC
>Otu786
CCAGCCTACGGGGCGCACCAGCAACGAATCTTCCCCAATGGGCGAAAGCCTGAGGGAGCG
ACGCCGCGTGTGGGATGAAGTCTTTCGGGATGTAAACCACTGTTAGGATTACGAAAGGTT
AGGGTCTAATACGCCCAAAACTTGATCTAGTCCAGAGAAAGGGACGGCTAACTCTGTGCC
AGCAGCCGCGGTAATACAGAGGTCCCAAGCGTTACTGAGAATCACTGGGTTTAAAGGGTG
CGTAGGTGGTCTGTTAAGTTTGTTGTGAAATCCCCGGGCTCAACCCGGGAATTGCTTCGA
ATACTGGCAGACTTGAGTCCTGTAGGGGTCACTGGAACAGACGGTGGAGCGGTGAAATGC
GTAGATATCGTCTGGAACGCCTGTGGTGAAGACGGGTGACTGGGCAGGTTCTGACACTGA
GGCACGAAAGCGTGGGGAGCGAACGGGATTAGATACCCCTGTAGTCC
>Otu787
CCAGCCTACGGGAGGCTGCAGTGGGGAATATTGCACAATGGGCGAAAGCCTGATGCAGCA
ACGCCGCGTGAGTGATGAAGGTCTTCGGATTGTAAAACTCTGTCTTTGGGGACGATAATG
ACGGTACCCAAGGAGGAAGCCACGGCTAACTACGTGCCAGCAGCCGCGGTAATACGTAGG
TGGCAAGCGTTGTCCGGATTTACTGGGCGTAAAGGATGTGTAGGTGGATGCTTAAGTCAG
ATGTGAAAGCCCCGAGCTTAACTTGGGGACTGCATTTGAAACTGGGCATCTAGAGTGCAG
GAGAGGAAAGTGGAATTCCTAGTGTAGCGGTGAAATGCGTAGAGATTAGGAAGAACATCA
GTGGCGAAGGCGACTTTCTGGACTGTAACTGACACTGAGGCATGAAAGCGTGGGGAGCAA
ACAGGATTAGATACCCCTGTAGTCC
>Otu788
CCAGCCTATGGGGGGCAGCAGTCGAGAATTTTTCACAATGGACGAAAGTCTGATGGAGCG
ACGCCGCGTGGAGGATGAAGGTTTTCGGATTGTAAACTCCTGTCACTGCAGAACAAGGAT
ATGCTCGTTAACAGCGAATGTATTTGATGGTATGCGGAGAGGAAGGGACGGCTAACTCTG

TGCCAGCAGCCGCGGTAAGACAGAGGTCCCGAGCGTTGTTCGGATTCATTGGGCGTAAAG
GGTGTGTAGGAGGTTGAGTAAGTCAGGTGTGAAATCTCAGGGCTTAACCCTGAAACTGCG
CTTGATACTGCTCGGCTAGAGGATCGGAGGGGGTATCGGAATTTATGGTGTAGCAGTGAA
ATGCGTAGATATCATAAGGAACACCGGTGGCGAAGGCGGATACCTGGAAGATTCCTGACT
CTGGAACACGAAAGCCAGGGGAGCAAACGGGATTAGAAACCCCCGTAGTCC
>Otu789
CCAGCCTACGGGTGGCTGCAGTGGGGAATATTGGACAATGGGCGAAAGCCTGATCCAGCC
ATGCCGCGTGAGTGATGAAGGCCTTAGGGTTGTAAAGCTCTTTTGCCAGGGAAGATAATG
ACGGTACCTGGAGAATAAGCACCGGCTAACTTCGTGCCAGCAGCCGCGGTAATACGAAGG
GTGCTAGCGTTGTTCGGAATTACTGGGCGTAAAGCGCGCGTAGGCGGTCATGCAAGTCAG
GGGTGAAATCCCGGAGCCTAACTCCGGAACTGCCTTTGAAACTGCATGGCTGGAGTGTGT
GAGGGGATAGCGGAATTGCTAATGTAGAGGTGAAATTCGTAGATATTAGCAGGAACACCG
GTGGCGAAGGCGGCTATCTGGCACACAACTGACGCTGAGGCGCGAAAGCGTGGGGATCAA
ACAGGATTAGATACCCCGGTAGTCC
>Otu790
CCAGCCTACGGGAGGCACCAGTCGAGAATCTTCCGCAATGGACGAAAGTCTGACGGAGCG
ACGCCGCGTGGAGGATGAAGTTCTTCGGAATGTAAACTCCTTTTGCCAGGGAAAAAGTTA
TTGATTGTACCTGGAGAATAAGAGGTTGCTAAACTCGTGCCAGCAGCAGCGGTAATACGA
GTGCCTCAAGCGTTATCCGAAATCATTGGGCGTAAAGGGTGTGTAGGTGGCTGTGTTAGT
CTTGTGTTAAATTTACCGGCTCAACCGGTAAGCTGCATGGGAAACGGCACGGCTTCGAGG
ACGGTAGAGGTCTCTGGAACTCTAGGTGTAGCGGTGAAATGCGTTGATATCTAGGGGAAC
ACCAAAAGCGAAGGCAAGAGACTGGACCGCTCCTGACACTGAAACACGAAAGCGTGGGTC
GCGAATGGGATTAGATACCCTCGTAGTCC
>Otu791
CCAGCCTATGGGAGGCACCAGTGGGGAATATTGGACAATGGGCGAAAGCCTGATCCAGCA
ATACCGCGTGAATGATGAAGGCCTTAGGGTTGTAAAGTTCTTTTAGTTGGGAAGATAATG
ACGGTACCAACAGAAAAAGCCCCGGCTAACTCCGTGCCAGCAGCCGCGGTAAGACGGAGG
GGGCTAGCGTTGTTCGGAATTACTGGGCGTAAAGGGCGCGTAGGCGGATTAGTAAGTTGG
GAGTGAAAGCCCGGGGCTTAACCTCGGAACTGCTTTCAAAACTGCTAGTCTAGAGTGAAG
TAGGGGGTGATGGAATTCCTAGTGTAGAGGTGAAATTCTTAGATATTAGGAGGAACACCG
GTGGCGAAGGCGGTCACCTGGACTTCAACTGACGCTGAGGCGCGAAAGCGTGGGGAGCAA
ACAGGATTAGAAACCCCAGTAGTCC
>Otu792
CCAGCCTATGGGTCGCTCCAGTAGGGAATCTTGCGCAATGGGCGAAAGCCTGACGCAGCA
ACGCCGCGTGGGGGATGAATGCCTTCGGGTTGTAAACTCCTTTCAGCGGGAACGAAATTG
ACGGTACCCGCAGAAGAAGCCCCGGCCAACTACGTGCCAGCAGCCGCGGTGATACGTAGG
GGGCGAGCGTTGTCCGGATTTATTGGGCGTAAAGAGCTCGTAGGCGGTTCGACAAGTCGG
GTGTGAAACCCCCAGGCTTAACCTGGGGACGCCACTCGAAACTGTCGTGACTAGAGTCCG
GTAGGGGATCACGGAATTCCTGGTGTAGCGGTGAAATGCGCAGATATCAGGAGGAACACC
AGTGGCGAAGGCGGTGATCTGGGCCGGTACTGACGCTGAGGAGCGAAAGCGTGGGGAGCG
AACAGGATTAGAGACCCCAGTAGTCC
>Otu793
CCAGCCTATGGGTTGCACCAGTCGAGGATCTTCGGCAATGGGCGAAAGCCTGACCGAGCG
ACGCCGCGTGCGGGATGAAGGCCTTCGGGTTGTAAACCGCTGTCAGTGGGGAGGAAGTCT
GGGAGGGATCACCCTTCCAGTTGACCTATCCGCAGAGGAAGGACGGCTAAGTTCGTGCC
AGCAGCCGCGGTAATACGAACCGTCCTAACGTTATTCGGAATCACTGGGCTTAAAGGGTG
CGTAGGCGGTTCGGTAAGTTGGGTGTGAAATCCCTCGGCTCAACCGAGGAATTGCGCCCA
AAACTGTCGAACTTGAGGGAGACAGAGGTGAGTGGAACTTAGGGTGGAGCGGTGAAATGC
GTTGATATCCTAAGGAACACCGGTGGCGAAAGCGACTCACTGGGTCTTTTCTGACGCTGA
GGCACGAAAGCTAGGGTAGCGAACGGGATTAGATACCCTCGTAGTCC
>Otu794
CCAGCCTATGGGTTGCTCCAGGCGAGAATATTCCACAATGGACGCAAGTCTGATGGAGCG
ACGCCGCGTGATGGATGAAGTGCTTCGGTACGTAAACATCTTTTATGGGGGACGAAGTTA
TTGACGGTACCCCATGAATAAGGACCTCCTAACTCTGTGCCAGCAGGAGCGGTAATACAG
AGGGTCCAAGCGTTACCCGGAATCACTGGGCGTAAAGGGTGTCTAGGCGGCCTGGTTAGT
CGTTCGTTAAATCCGTGGGCCTAACCTACGGTCTGCGAGCGAAACGGCCAGGCTCGAGGG
CGCGAGAGGTGCACGGAACTCATGGTGGAGGGGTGAAATCCGTTGATATCATGGGGAACA
CCAAAGGCGAAGGCAGTGCACTGGTGCGTTCCTGACGCTCAAACACGAAAGCTAGGGTAG

CGAACGGGATTAGAAACCCCCGTAGTCC
>Otu796
CCAGCCTATGGGACGCAGCAGTGGGGAATTTTCCGCAATGGGCGAAAGCCTGACGGAGCA
ATACCGCGTGAGGGAGGAAGGCTCTTGGGTTGTAAACCTCTTTTCTCAGGGAATAAGAAA
GTGAAGGTACCTGAGGAATAAGCATCGGCTAACTCCGTGTCAGCAGCCGCGGTAATACGG
AGGATGCAAGCGTTATCCGGAATGATTGGGCGTAAAGCGTCCGCAGGTGGTGATGTAAGT
CTGCTGTTAAAAAGTTCAGCTTAACTGGATAAGGGCGGTGGAAACTACATGACTAGAGTA
CAAACGGGGCAAGAGGAATTCCTGGTGTAGCGGTGAAATGCGTAGATATCAGGAAGAACA
TCGGTGGCGAAGGCGTCTTGCTAGAATGAAACTGACACTGAGGGACGAAAGCTAGGGGAG
CGAATGGGATTAGAAACCCCTGTAGTCC
>Otu797
CCAGCCTATGGGGCGCAGCAGTCAAGAACATTCGACAATGGGCGAAAGCCTGATCGAGCG
ACACCGCGTGCAGGATGAAGGCCCTCGGGTCGTAAACTGCGGTAGTAACATAACAATGTA
AATGAGTGTGTTACGGAAAGAGGTGGGTAACTACGTGCCAGCACCAGCGGTAATACGTAG
ACCTCAAGCGTTATCCGGATTTATTGGGCGTAAAGAGTATGTAGGAGGTTTTGCGCGTCT
TTTGTTAAAGCCCAGGGCCTAACCCTGGAAGTGCAGGAGATACGGCAGAACTAGAGGAGG
TTAGAGGTGCATGGAACTCACGGTGTAGGGGTGAAATCCGTTGATATCGTGGGGAACACC
AAAGGCGAAGGCAGTGCACTGGGACCTTCCTGACTCTGATATACGAAAGCGTGGGGAGCA
AAAAGGATTAGAGACCCCCGTAGTCC
>Otu798
CCAGCCTATGGGTGGCAGCAGTGGGGAATTTTGGACAATGGGGGCAACCCTGATCCAGCC
ATTCCGCGTGAGTGAAGAAGGCCTTCGGGTTGTAAAGCTCTTTCAGCAGGAACGAAACGG
TCATTTTTAATAAAGATGGCTAATGACGGTACCTGAAGAAGAAGCACCGGCTAACTACGT
GCCAGCAGCCGCGGTAATACGTAGGGTGCGAGCGTTAATCGGAATTACTGGGCGTAAAGC
GTGCGCAGGCGGTTCCATAAGACAGATGTGAAATCCCCGGGCTTAACCTGGGAACTGCGT
TTGTGACTGTGGGACTGGAGTGTGGCAGAGGGGGGTGGAATTCCACGTGTAGCAGTGAAA
TGCGTAGAGATGTGGAGGAACACCGATGGCGAAGGCAGCCCCCTGGGTCAACACTGACGC
TCATGCACGAAGGCGTGGGGAGCAAACAGGATTAGATACCCGAGTAGTCC
>Otu800
CCAGCCTACGGGTTGCTGCAGTCGAGGATCTTCGTCAATGGGCGCAAGCCTGAACGAGCG
ACGCCGCGTGCGCGATGAAGGCCTTCGGGTTGTAAAGCGCGAAAGAGAAGATAAAACCGC
AAGGTTGATAGACTCTCAGTAAGGACGGGCTAAGTTCGTGCCAGCAGCCGCGGTAAGACG
AACTGTCCTAACGTTGTTCGGAATCACTGGGCTTAAAGGGCGCGTAGGCAGTCGACCAAG
TCAGGGGTGAAATCCTGCAGCTTAACTGTAGAAGTGCCTTTGATACTGGTCGTCTCGAGG
GAGGTAGGGGTGTGTGGAACTTCCAGTGGAGCGGTGAAATGCGTTGATATTGGAAGGAAC
GCCGGAGGCGAAAGCGACGCACTGGACCTCTTCTGACGCTGAGGCGCGAAAGCTAGGGGA
GCAAACGGGATTAGATACCCGTGTAGTCC
>Otu801
CCAGCCTATGGGAGGCAGCAGCCGAGAATCTTTAGCAATGGACGAAAGTCTGACTATGCG
ACGCCGCGTGAGGGATGAAGGATTTCGGTTCGTAAACCTCTTTTGGCAGGGAAGAAAAAG
TTGCAGTTAATAGCTGCAAAACTGACGGTACCTGCAGAATAAGCCACGGCTAACTCCGTG
CCAGCAGCCGCGGTAATACGGAGGTGGCTAGCGTTACTCGGAATTATTGGGTGTAAAGGG
CAAGTAGGCGGCTTAGTAAGTAGGGGGTGAAATACTCTGGCTCAACCGGAGAACTGCCTT
CTAAACTACTAGGCTTGAGTATGACAGAGGAATATGGAATTCTCGGTGTAAGGGTGAAAT
CTGTAGATATCGAGAAGAACACCAGCGGCGAAGGCGATATTCTGGGTCATTACTGACGCT
AAATTGCGAAAGCTAGGGGAGCAAACAGAATTAGAGACCCCCGTAGTCC
>Otu804
CCAGCCTACGGGGGGCTGCAGGCGAGAATATTCCGCAATGGACGCAAGTCTGACGGAGCG
ACGCCGCGTGAAGGATGAAGTGCTTAGGTACGTAAACTTCTTTTATCGAGGAAGAACTAA
TTGACGGTACTCGATGAATAAGGGGCTCCTAACTCTGTGCCAGCAGGAGCGGTAATACAG
AGGCCCCGAGCATTACCCGGAATCACTGGGCGTAAAGGGTGTCCAGGCGGCCTTGTTAGT
CTCGCGTAAAATCCATGAGCTCAACTTATGGCGCGCGCGGGAAACGGCAAGGCTCGAGGG
CGCGAGAGATAGAGGGAACTCATGGTGGAGGGGTGAAATCCGTTGATATCATGGGGAACA
CCAAAGGCGAAGGCACTCTATTAGCGCGTTCCTGACGCTCACACACGAAAGCCAGGGGAG
CGAACGGGATTAGATACCCTGGTAGTCC
>Otu807
CCAGCCTATGGGAGGCAGCAGTCGAGAATCTTTCGCAATGGGCGCAAGCCTGACGAAGCG
ACGCCGTGTGAGCGAAGAAGGCCTTCGGGTTGTAAAGCTCTTTCGCCAGGGAACAAGAAA

GCAGTGATAATAACGCTGCGATTTGATGGTACTTGGTAAAGAAGCACCGGCTAACTCCGT
GCCAGCAGCTGCGGTAATACGGAGGGTGCAAGCATTGATCGGAATTACTGGGCGTAAAGG
GCGCGTAGGCGGATTGGCAAGTCAGATGTGAAATTCCGCGGCTCAACTGCGGAGCTGCAT
TTGAAACTGCCAGACTTGAGCATAGTTAGGGAAAACGGAATTCCACGTGTAGCGGTGAAA
TGCGTAGATATGTGGAAGAACACCAGTGGTGAAGACGGTTTTCTGGGCTATTGCTGACGC
TGAGGCGCGAAAGCTAGGGGAGCAAACAGGATTAGAAACCCTCGTAGTCC
>Otu808
CCAGCCTACGGGTGGCAGCAGTCGAGGATCTTCGGCAATGGGCGCAAGCCTGACCGAGCG
ACGCCGCGTGAAGGATGAAGGCCTTCGGGTTGTAAACTTCTGTCGAGGGGGATCAAGGGC
AACTTGAGTGATCCCTGGAGGAAGCACGGGCTAAGTTCGTGCCAGCAGCCGCGGTAAGAC
GAACCGTGCGAACGTTGTTCGGAATCACTGGGCATAAAGGGCGCGTAGGCGGCTTATCAA
GTCAGGGGTGAAATCCGGCAGCTCAACTGTCGAAGTGCCTTTGATACTGATGAGCTCGAG
GGAGGTAGGGGTATGTGGAACTTCCGGTGGAGCGGTGAAATGCGTTGATATCGGAAGGAA
CGCCGGTGGCGAAAGCGACGTACTGGACCTCTTCTGACGCTGAGGCGCGAAAGCTAGGGG
AGCAAACGGGATTAGAAACCCCCGTAGTCC
>Otu810
CCAGCCTATGGGGCGCAGCAGCCAAGAATCTTCCGCAATGGGCGAAAGCCTGACGGAGCG
ACGCCGCGTGAAGGAAGAAGGTCTTCGGATTGTAAACTTCTTTTGTCAGGGAAGAATAAG
GAGCGAGGAAATGCGTCTCCGATGACGGTACCTGATGAATAAGCCCCGGCTAATTACGTG
CCAGCAGCCGCGGTAATACGTAAGGGGCGAGCGTTGCTCGGAATTACTGGGCGTAAAGGG
CGTGTAGGCGGTCTCACAAGTCGAATGTAAAAAACCAGGGCTTAACCCTGGGGCTGCGTT
TGAAACTGTGAGACTTGAGTCTGGTGGAGGGCAATGGAATTCCCGGTGTAGCGGTGAAAT
GCGTAGATATCGGGAAGAACACCGAAGGCGAAAGCAGTTGTCTATGCCAAGACTGACGCT
GAGGCGCGAAAGCATGGGGATCAAACAGGATTAGATACCCCCGTAGTCC
>Otu811
CCAGCCTATGGGGGGCTCCAGTGGGGAATTTTGGACAATGGGCGCAAGCCTGATCCAGCC
ATGCCGCGTGAGTGAAGAAGGCCTTCGGGTTGTAAAGCTCTTTCAGCAGGAACGAAATGG
TTCGTGCTAATACCACGGACTGATGACGGTACCTGAAGAAGAAGCACCGGCTAACTACGT
GCCAGCAGCCGCGGTAATACGTAGGGTGCAGGCGTTAATCGGAATTACTGGGCGTAAAGC
GTGCGCAGGCGGTTTGTTAAGACAGGTGTGAAATCCCCGGGCTCAACCTGGGAACTGCGC
TTGTTACTGGCAGACTGGAGTGTGGCAGAGGGGGGTGGAATTCCACGTGTAGCAGTGAAA
TGCGTAGAGATGTGGAGGAACACCGATGGCGAAGGCAACCCCCTGGGCTAACACTGACGC
TCATGCACGAAAGCGTGGGGAGCAAACAGGATTAGAGACCCTTGTAGTCC
>Otu812
CCAGCCTACGGGAGGCACCAGTAAGGAATATTGGTCAATGGGCGGAAGCCTGAACCAGCC
ATGCCGCGTGCAGGAAGACGGCCCTACGGGTTGTAAACTGCTTTTGCAGGGGAATAAACC
TCGATATGTATATCGAGCTGAATGTACTCTGAGAATAAGGATCGGTTAACTCCGTGCCAG
CAGCCGCGGTAATACGGAGGATCCAAGCGTTATCCGGATTTATTGGGTTTAAAGGGTGCG
TAGGTGGCTTTTTAAGTCAGGGGTGAAAGACGGTAGCTTAACTATCGCAGTGCCTTTGAT
ACTGAAGAGCTTGAATGAACTGGAGGTAAGCGGAATGTGACAAGTAGCGGTGAAATGCAT
AGATATGTCACAGAACACCAATTGCGAAGGCAGCTTACTACGGTTTTATTGACACTGAGG
CACGAAAGCGTGGGGAGCAAACGGGATTAGAGACCCCGGTAGTCC
>Otu814
CCAGCCTACGGGGGGCACCAGTCAAGAACATTCGACAATGGGCGAAAGCCTGATCGAGCG
ACACCGCGTGCAGGATGAAGGCCTTCGGGTTGTAAACTGCGGTAGATAGCTAACAATGCA
AATGAGTGACTATTGGAAAGAGGTGGGTAACTACGTGCCAGCACCAGCGGTAATACGTAG
ACCTCAAGCGTTATCCGGATTTATTGGGCGTAAAGCGCACGTAGGAGGTTTTGCGCGTCT
TTTGTTAAAGCCCAGGGCCTAACCCTGGAAGTGCAGGAGATACGGCAGAACTAGAGGAGG
TTAGAGGTGCATGGAACTCACGGTGTAGGGGTGAAATCCGTTGATATCGTGGGGAACACC
GAAGGCGAAGGCAGTGCACTGGGACCTTCCTGACTCTGAGTTGCGAAAGCGTGGGGAGCA
AAAAGGATTAGATACCCCTGTAGTCC
>Otu815
CCAGCCTATGGGGGGCTGCAGTGGGGAATCTTGCGCAATGGGGGAAACCCTGACGCAGCG
ACGCCGCGTGCGGGATGAAGGCCTTCGGGTTGTAAACCGCTTTCAGCAGGGACGAAATTG
ACGGTACCTGCAGAAGAAGGCCCGGCCAACTACGTGCCAGCAGCCGCGGTAATACGTAGG
GGCCTAGCGTTGTCCGGAATTATTGGGCGTAAAGAGCTCGTAGGCGGTTCGGCAAGTCGG
ATGTGAAATCTCCACGCTTAACGTGGAGGGGCCATTCGAAACTGCTGTGACTCGAGTCCG
GTAGGGGAGTGTGGAATTCCCGGTGTAGCGGTGAAATGCGCAGATATCGGGAGGAACACC

AGTAGCGAAGGCGGCACTCTGGGCCGGTACTGACGCTGAGGAGCGAAAGCGTGGGGAGCG
AACAGGATTAGAGACCCGAGTAGTCC
>Otu816
CCAGCCTACGGGTTGCTGCAGTCGAGAATCTTCGGCAATGGGCGCAAGCCTGACCGAGCG
ACGCCGCGTGTGCGATGAAGGCCTTCGGGTTGTAAAGCACTGTCGAGGGGGAGGAAGCCG
CAAGGTTGACCTACCCCTGGAGGAAGCACGGGCTAAGTTCGTGCCAGCAGCCGCGGTAAG
ACGAACCGTGCGAACGTTGTTCGGAATCACTGGGCTTAAAGGGCGCGTAGGCGGGTCACC
AAGTCTGGGGTGAAATACTTCGGCTTAACCGGAGAACAGCTTCAGATACTGGTGGCCTAG
AGGGAGGTAGGGGCCTGTGGAACTTCCGGTGGAGCGGTGAAATGCGTTGATATCGGAAGG
AACGCCGGTGGCGAAAGCGACGGGCTGGACCTCTTCTGACGCTGAGGCGCGAAAGCTAGG
GGAGCAAACGGGATTAGAAACCCGAGTAGTCC
>Otu817
CCAGCCTATGGGAGGCTGCAGTGGGGAATCTTGCACAATGGGCGAAAGCCTGATGCAGCG
ACGCCGCGTGAGGGAAGAGGGCTTTCGGGTTGTAAACCTCTTTCAGGTGGGACGAAGCTC
CTCGGGTTAATAGCCCAGAGGGTGACGGTACCGCCAGAAGAAGCCCCGGCTAACTACGTG
CCAGCAGCCGCGGTAATACGTAGGGGGCAAGCGTTGTCCGGATTTATTGGGCGTAAAGAG
CGTGTAGGCGGTTAGGTAGGTCCGTTGTGAAAACTCGAGGCTCAACCTCGAGACGCCGAT
GGAAACCATCTAACTAGAGTCCGGAAGAGGAGAGTGGAATTCCTGGTGTAGCGGTGAAAT
GCGCAGATATCAGGAAGAACACCCGTGGCTAAGGCGGCTCTCTAGTACGGTACTGACGCT
GAGACGCGAAAGCGTGGGGAGCGAACAGGATTAGAAACCCCGGTAGTCC
>Otu819
CCAGCCTATGGGTGGCACCAGTGGGGAATCTTGCGCAATGCGCGAAAGCGTGACGCAGCA
ACGCCGCGTGGGGGAAGACGGCCTTCGGGTTGTAAACCCCTTTCAGTTGGGACGAAGGTT
CACCGGTGAATAGCCGGTTGGATTGATGGTACCTTCAGAAGAAGCCCCGGCTAACTACGT
GCCAGCAGCCGCGGTAATACGTAGGGGGCAAGCGTTGTCCGGAATCATTGGGCGTAAAGA
GCGTGTAGGCGGCTCGGTAAGTCCGCTGTGAAAGTCCAGGGCTCAACCCTGGAATGCCGG
TGGAAACTGTCGAGCTAGAGTCCGGAAGAGGCGAGTGGAATTCCTGGTGTAGCGGTGAAA
TGCGCAGATATCAGGAGGAACACCAATGGCGAAGGCAGCTCGCTGGGACGGTACTGACGC
TGAGACGCGAAAGTGTGGGGAGCAAACAGGATTAGATACCCGCGTAGTCC
>Otu821
CCAGCCTACGGGTGGCAGCAGTGGGGAATTTTACGCAATGGGGGAAACCCTGACGTAGCG
ACACCGCGTGAGCGAAGAAGCCCTTTGGGGTGTAAAGCTCTGTCGGCTGGGACGAATAAA
TGACGGTACCAGCAGAGGAAGCATCGGCTAACTACGTGCCAGCAGCCGCGGTAAGACGTA
GGATGCGAGCGTTGTCCGGATTTATTGGGCGTAAAGAGTTCGTAGGCGGTTTGTTAAGTC
TGATGTTAAAGATCAGGGCCCAACCCTGGGAGTGCATTGGATACTGGCAGACTGGAGTGC
AGTAGAGGCGAGTGGAATTCCCAGTGTAGCGGTGAAATGCGTAGATATTGGGAAGAACAC
CGGTGGCGCAGGCGACTCGCTGGGCTGTAACTGACGCTGAGGAACGAAAGCTAGGGGAGC
AAATGGGATTAGATACCCCCGTAGTCC
>Otu824
CCAGCCTACGGGACGCACCAGTAGGGAATATTGGACAATGGGCGCAAGCCTGATCCAGCA
ATGCCGCGTGAGTGATGAAGGCCTTAGGGTTGTAAAGCTCTTTTGGCGGGGACGATGATG
ACGGTACCCGCAGAATAAGCTCCGGCTAACTTCGTGCCAGCAGCCGCGGTAATACGAAGG
GAGCTAGCGTTGTTCGGAATTACTGGGCGTAAAGGGTGCGTAGGCGGCTTTTTAAGTTTG
GTGTGAAATCTCCCGGCTCAACTGGGAGGGTGCGCCGAATACTGAGAGGCTAGAGTGTGG
GAGAGGAAAGTGGAATTCCTGGTGTAGCGGTGAGATGCGTAGATATCAGGAGGAACACCG
GTGGTGTAAACGGCTTTCTGGACCATAACTGACGCTGAGGCACGAAAGCGTGGGTAGCAA
ACAGGATTAGAAACCCGAGTAGTCC
>Otu828
CCAGCCTATGGGGGGCTCCAGTAGGGAATATTGCGCAATGGGCGAAAGCCTGACGCAGCA
ATTCCGCGTGGAGGACGAAGGATCTTGGTCTGTAAACTCCTTTCGGTGGTGACGATAATG
ACGGTAGCCACAGAAGAAGCACCGGCTAACTACGTGCCAGCAGCCGCGGTAAGACGTAGG
GTGCAAGCGTTGTCCGGATTTATTGGGCGTAAAGCGTTCGTAGGCGGTTTGCCAAGTCTG
GCGTTAAAGGCGGAGCTCAACTCCCGTATGGCGTTGGAAACTGGCAAGCTAGAGTGTGGT
AGAGGTAAGGGGAATTCCCGGTGTAGCGGTGAAATGCGTAGATATCGGGAGGAACACCAG
TGGCGAAAGCGCCTTACTGGACCATTACTGACGCTGAGGAACGAAAGCCAGGGTAGCGAA
AGGGATTAGAAACCCGCGTAGTCC
>Otu829
CCAGCCTATGGGTCGCAGCAGTGGGGAATCTTGCACAATGGGGGCAACCCTGATGCAGCG

ACGCCGCGTGAGCGATGAAGCCCTTCGGGGTGTAAAGCTCTTTCGGCAGGAACGATAATG
ACGGTACCTGAAGAAGAAGCTGCGGCTAACTACGTGCCAGCAGCCGCGGTAATACGTAGG
CAGCAAGCGTTGTTCGGAGTTACTGGGCGTAAAGAGAGCGTAGGCGGTCCTCTAAGTTTG
GTGTGAAATCTCCCGGCTCAACCGGGAGGGTGCGCCGAAGACTGGAGGGCTCGAGTATGG
GAGAGGTAAGCGGAATTCCTGGTGTAGCGGTGAAATGCGTAGATATCAGGAGGAACACCT
GTGGTGTAGACGGCTTACTGGACCATCACTGACGCTGAGGCACGAAAGCGTGGGTAGCAA
ACAGGATTAGAAACCCGCGTAGTCC
>Otu830
CCAGCCTATGGGGTGCAGCAGTGAGGAATATTGGTCAATGGGCGCAAGCCTGAACCAGCC
ATCCCGCGTGAAGGAAGAAGGCGCTATGCGTTGTAAACTTCTTTTCCAGGGGACGAAAAT
TTTGCACGTGTGCAGAACTGACGGTACCCTGGGAATAAGCATCGGCTAACTCCGTGCCAG
CAGCCGCGGTAATACGGAGGATGCAAGCGTTATCCGGATTTATTGGGTTTAAAGGGTGCG
TAGGCGGAAAAATAAGTCAGTGGTGAAAACCTGCAGCTTAACTGTAGAATTGCCATTGAT
ACTGTTATTCTTGAGTGTGGTCAAGGTAGGCGGAATGTATAATGTAGCGGTGAAATGCTT
AGATATTACACAGAACACCAATTGCGAAGGCAGCTTGCTGGCCATTACTGACGCTGATG
CACGAAAGCGTGGGGAGCGAACAGGATTAGATACCCTCGTAGTCC
>Otu831
CCAGCCTATGGGGGGCACCAGTGGGGAATCTTGCGCAATGGACGAAAGTCTGACGCAGCG
ACGCCGCGTGGGTGATGAAGGCCTTCGGGTTGTAAAGCTCTGTGGGGGGAGACGAATAAG
TGCAGCCTAATACGCTGCATGATGACGGTATCTCCTTAGCAAGCACCGGCTAACTCTGTG
CCAGCAGCCGCGGTAAGACAGAGGGTGCAAACGTTGTTCGGAATTACTGGGCGTAAAGCG
TGTGTAGGCGGCCATGTAAGTCAGATGTGAAAGCCCCAGGCTCAACCTGGGAAGTGCACC
TGATACTGCATGGCTTGAGTATCGGAGAGGTTGGTGGAATTCTCGGTGTAGAGGTGAAAT
TCGTAGATATCGAGAGGAACGCCGGCGGCGAAGGCGGCCAACTGGACGAATACTGACGCT
GAGACACGAAAGCGTGGGGAGCAAACAGGATTAGATACCCTTGTAGTCC
>Otu833
CCAGCCTACGGGTGGCAGCAGTCAAGAATATTCCTCAATGGCCGAAAGGCTGAAGGAGCG
ACGCCGCGTGTAGGATGAAGGTCTTCGGATTGTAAACTACTTTTACTAGGGAAGAATTTG
TGACGGTACCTAGTGAATAAGAGGTTGCTAACTCTGTGCCAGCAGCAGCGGTAATACAGA
GACCTCAAGCGTTATCCGGATTTATTGGGCGTAAAGCGTCCGTCGGTGGTTTAGTAAGTC
AGGGGTCAAAACCTGCCGCTTAACGGCAGGATCGCCTTTGATACTGCTAGACTCGAGTAT
GGAAGAGGCAAGCGGAATTCTAGGTGTAGTCGTAATAAGCGTTGATATCTAGAAGAACAC
CAAATGCGAAGGCAGCTTGCTGGTACATTACTGACACTCAGGGACGAAAGCGTGGGGAGC
AAACAGGATTAGAGACCCGAGTAGTCC
>Otu834
CCAGCCTATGGGGGGCACCAGTCGAGAATTTTTCTCAATGGGGGAAACCCTGAAGGAGCG
ACGCCGCGTGGGGGATGAATGGCTTCGGCCCGTAAACCCCTGTCATTCGGGAGCAATGCG
TCTGGGTGAACATCTCAGACGTTGATAGTACCGGAAGAGGAAGGGACGGCTAACTCTGTG
CCAGCAGCCGCGGTAATACAGAGGTCCCAAGCGTTGTTCGGATTTACTGGGCGTAAAGGG
TGCGTAGGTGGTTGGGTAAGTTTGATGTGAAATCTCCGGGCTTAACCCGGAAAATGCATT
GAATACTATTCAGCTTGAGGATTGGAGGGGGGACTGGAATGCTCGGTGTAGCAGTGAAAT
GCGTAGATATCGAGCGGAACACCAGTGGCGAAGGCGAGTCCCTAGACAATTCCTGACACT
GAGGCACGAAAGCCAGGGGAGCAAACGGGATTAGATACCCGCGTAGTCC
>Otu835
CCAGCCTATGGGGCGCAGCAGTGGGGAATCTTGCGCAATGGGCGGAAGCCTGACGCAGCA
ACGCCGCGTGAGCGAAGAAGGCCTTCGGGTTGTAAAGCTCTGTCAGGAGGGACGAAGCCA
CTCGGGTTAATAGCCCAGAGGGTGACGGTACCTCCAGAGGAAGGTCCGGCTAACTACGTG
CCAGCAGCCGCGGTAATACGTAGGGACCTAGCGTTGTCCGGAATTATTGGGCGTAAAGAG
CGTGTAGGCGGCTTGCCAAGTCCGATGTGAAAACTCGGGGCTTAACCCCGAGCCTGCATT
GGAAACTGTCTTGCTAGAGTCCGGAAGAGGGGACTGGAATTCCTGGTGTAGCGGTGAAAT
GCGCAGATATCAGGAAGAACACCCGTGGCGAAGGCGGGTCCCTGGGACGGTACTGACGCT
GAGACGCGAAAGCGTGGGGAGCGAACAGGATTAGAAACCCTCGTAGTCC
>Otu836
CCAGCCTATGGGGGGCAGCAGCAACGAATCTTCCCCAATGGGCGAAAGCCTGAGGGAGCG
ACGCCGCGTGAAGGACGAAGTTCTTCGGAATGTAAACTTCTGTTAGGGTTATGAAAGTGA
AGGGACCTAATACGTCCACAATTGATCTAGCCCAAAGAAAGGGACGGCTAACTCTGTGCC
AGCAGCCGCGGTAATACAGAGGTCCCAAGCGTTACTGAGAATCACTGGGTTTAAAGGGTG
CGTAGGTGGTCCGTTAAGTTCGTTGTGAAATCCCTGGGCTCAACCCAGGAACTGCTTCGA

ATACTGGCGGACTTGAGGCCGGTAGGGGTCACTGGAACTGACGGTGGAGCGGTGAAATGC
GTAGATATCGTCAGGAACGCCGGTGGTGAAGACGGGTGACTTGGCCGGTTCTGACACTGA
GGCACGAAAGCGTGGGGAGCGAACGGGATTAGAGACCCGAGTAGTCC
>Otu837
CCAGCCTATGGGGCGCAGCAGTTGGGAATCTTGCACAATGGGGGAAACCCTGATGCAGCG
ACGCCGCGTGGAGGACGAAGGGTCTAGGCTCGTAAACTCCTTTTATCGGGAAAGACTTAG
GACGGTACCCGATGAATAAGCACCGGCTAACTACGTGCCAGCAGCCGCGGTAAGACGTAG
GGTGCAAGCGTTGTCCGGATTTACTGGGCATAAAGAGCGCGTAGGCGGTCTGTTAAGTGT
AGAGTGAAATCTCCAGGGCTCAACCCGGAAACTGCTCTGCATACTGACAGACTAGAGGGA
CGGAGAGGTATGTGGAATTCCTGGTGTAGCGGTGAAATGCGTTGATATCAGGAGGAACAC
CCATGGCGAAGGCAGCATACTGGTCGTCTCCTGACGCTGAGGCGCGAAAGCGTGGGTAGC
AAACAGGATTAGATACCCTAGTAGTCC
>Otu839
CCAGCCTATGGGGGGCTGCAGTAGGGAATATTGCGCAATGGAGGAAACTCTGACGCAGCG
ACGCCGCGTGGGTGATGAAGGCTTTCGGGTCGTAAAGCCCTGTCGGAAGGAAAGAAAATC
ATTATGGCTAACATCCATAGTGTTTGACGGTACCTTTAAAGGAAGCACCGGCTAACTACG
CGCCAGCAGCCGCGGTAATACGTAGGGTGCTAGCGTTGTTCGGAATCATTGGGCGTAAAG
CGCGTGTAGGTGGTTATGTAAGTCGAGTGTGAAATCCCTGGGCTCAACCGAGGAAGTGCA
TCCGAAACTACATAGCTAGAGGACAGTAGAGGAAGGTGGAATTCCTAGTGTAGAGGTGAA
ATTCGTAGATATTAGGAGGAATACCGGTGGCGAAGGCGGCCTTCTGGGCTGGACCTGACA
CTGAGACGCGAAAGCGTGGGTAGCAAACAGGATTAGAGACCCCTGTAGTCC
>Otu840
CCAGCCTACGGGACGCTGCAGTAGGGAATTTTCCACAATGGACGAAAGTCTGATGGAGCA
ACGCCGCGTGCAGGAGGAAGGCCCTCGGGTCGTAAACTGCTTTTATTGGTGACAATTTTG
ACGGTAGCCAATGAATAAGGACCTGCTAACTACGTGCCAGCAGCCGCGGTCATACGTAGG
GTCCAAGCGTTATCCGGAATTACTGGGCGTAAAGAGTTGCGTAGGTGGCATAGTAAGCAA
ATAGTGAAAGCGTGAGGCTCAACCTCATAACCATTATTTGAACTACTAAGCTTGAGTATG
AGAGAGGTAGATGGAATTTCTAGTGTAGGAGTGAAATCCGTAGATATTAGAAGGAACACC
GATGGCGTAAGCAGTCTACTAGCTCATTACTGACACTAAGGCACGAAAGCGTGGGGAGCG
AACGGGATTAGATACCCTTGTAGTCC
>Otu842
CCAGCCTATGGGTGGCTGCAGTAACGAATCTTCCGCAATGCACGCAAGTGTGACGGAGCG
ACGCCGCGTGTGGGACGAAGTCCTTCGGGATGTAAACCACTGTCAGGGGTTACCAAGTTC
TGAGGAGCCCCAGAGGAAGGCACGGCTAACTCTGTGCCAGCAGCCGCGGTAAGACAGAGG
TGCCGAGCGTTAGGCGGAATCACTGGGCTTAAAGCGTGTGTAGGCGGGCCGTTAAGTATC
TTGTGAAATCCCCCGGCTCAACCGGGGAATGGCTGGATATACTGGCGGTCTTGAGCAATC
TAGGGGCAGATGGAACAAGTGGTGGAGCGGTGAAATGCGTAGATATCACTTGGAACGCCA
AAGGTGAAGACAGTCTGCTGGGGATTTGCTGACGCTGAGACACGAAAGCCAGGGGAGCGA
ACGGGATTAGAAACCCTCGTAGTCC
>Otu843
CCAGCCTATGGGGGGCAGCAGTCGAGAGGCTTCGGCAATGGGGGAAACCCTGACCGAGCG
ACGCCGCGTGGGCGATGAAGGCCGCTAGGTTGTAAAGCCCTGTCGAGTAGGAAGAAACGC
AGGTGGGCGAACAGTCCATCTGCTTGACGGTACTGCTAAAGGAAGCTCCGGCCAACTCCG
TGCCAGCAGCCGCGGTAATACGGGGGGAGCGAACGTTGTTCGGAATTACTGGGCGTAAAG
GGCGTGCAGGCGGCCTTGTAAGTGGGATGTGAAATGCCCTGGCTCAACCAGGGAAATGCA
TCTCAGACTGCAAGGCTTGAGTATAGGAGAGGATGGGGGAATTCCCGGTGTAGCGGTGAA
ATGCATTGATATCGGGAGGAACACCAGTGGCGAAGGCGCCCATCTGGCCTAATACTGACG
CTCAGGCGCGAAAGCTAGGGGAGCAAACAGGATTAGAAACCCGGGTAGTCC
>Otu845
CCAGCCTACGGGTCGCTGCAGTTTCGAATCATCCACAATGGGCGAAAGCCTGATGGTGCG
ACGCCGCGTGAGGGATGAAGGCCTTCGGGTCGTAAACCTCTGTCACCGGGGAGCAAATGT
AAGACTTATACTCTTACTTGAGTTAACCCGGAGAGGAAGCAGTGGCAAACTCTGTGCCAG
CAGCCGCGGTAATACAGAGACTGCGAGCGTTACTCGGATTCACTGGGCGTAAAGGGTGCG
TAGGCGGCTAAATGTGTCAGATGTGAAATCCCGGGGCTCAACTCCGGAACTGCGTCTGAA
ACTATTTAGCTAGAGACTCGGCGAGGTAAGCGGAATTCCAAGTGTAGCAGTGAAATGCGT
AGATATTTGGAGGAACACCGAAGGCGAAGGCAGCTTACTGGAAGAGATCTGACGCTGAGG
CACGAAAGCGTGGGGAGCAAAAGGGATTAGATACCCGCGTAGTCC
>Otu847

CCAGCCTATGGGGGGCAGCAGTCGAGGATCTTTCGCAATGGGCGAAAGCCTGACGGAGCG
ACGCTGTGTGAGTGATGAAGGCCTTCGGGTCGTAAAACTCTTTTGCCTGAGAACAAGAGC
GATTCGTGAATAACGAGTCAATTTGAGGGTATCAGGAGAAAAAGCACCGGCTAACTACGT
GCCAGCAGCTGCGGTAATACGTAGGGTGCAAGCATTAATCGGAATTATTGGGCGTAAAGG
GCGCGTAGGCGGATGGATAAGTCAGATGTGAAATACCAAAGCTCAACTTTGGTGCTGCAT
TTGAAACTGTTTATCTAGAGGATTGACGGAGAAAGCGGAATTCCACGAGTAGCGGTGAAA
TGCGTAGATACGTGGAGGAACACCGGTGGCGAAAGCGGCTTTCTAGTTTTTACCTGACGC
TGAGGCGCGAAAGCGTGGGGAGCAAACAGGATTAGATACCCGCGTAGTCC
>Otu849
CCAGCCTATGGGGGGCAGCAGTAGGGAATATTGCACAATGGAGGAAACTCTGATGCAGCG
ACGCCGCGTGAGTGATGAAGGACTTCGGTTCGTAAAGCTCTGTTGCAGGGGAATAATAAA
GTGAAGGTACTCTGCAAGAAAGGGTCGGCTAACTTCGTGCCAGCAGCCGCGGTAAGACGA
GGGACCCTAGCGTTGTTCGGAATCATTGGGCGTAAAGCGGGTGTAGGTGGCTTTGTAAGT
CGGATGTGAAAGCCCAGGGCTCAACCTTGGAAGTGCATCTGATACTGCGAAGCTTGAGTG
CCGGAGAGGTTACTAGAATTCCTGGTGTAGTGGTGAAATACGTAGATATCAGGAGGAATA
CCGGAGGCGAAGGCGGGTAACTGGCCGGACACTGACACTCAGACCCGAAAGCGTGGGATC
AAACAGGATTAGAAACCCCAGTAGTCC
>Otu851
CCAGCCTATGGGTGGCACCAGTCGAGAATCTTCGGCAATGGACGCAAGTCTGACCGAGCG
ACGCCGCGTGTGGGATGAAGGCCTTCGGGTTGTAAACCACTGTCAGAGGGGATGAAATGC
CGGTGGGTACTCCCATCGGTTTGACATAGCCTCAGAGGAAGGACGGGCTAAGTTCGTGCC
AGCAGCCGCGGTAAGACGAACCGTCCGAACGTTATTCGGAATCACTGGGCTTAAAGGGTG
CGTAGGCGGTGCGGAAAGTTGGGTGTGAAATCCCTCGGCTTAACCGAGGAACTGCGCTCA
AAACTACCGTGCTCGAGGGAGATAGAGGTGAGCGGAACTTAGGGTGGAGCGGTGAAATGC
GTTGATATCCTAAGGAACACCGGTGGCGAAAGCGGCTCACTGGATCTCTTCTGACGCTGA
GGCACGAAAGCTAGGGTAGCGAACGGGATTAGAAACCCTAGTAGTCC
>Otu852
CCAGCCTACGGGATGCTGCAGTGGGGAATCTTGGGCAATGGGCGACAGCCTGACCCAGTG
AGAACACATGCATGACGACGGCGTCTAGTTTTCTGTAAAGTGCTTTTCGCGGTTTGACGA
TAATGACTAAAAACCGAGAAGAAGTGCCGGCCAACTCCGTGCCAGCAGCCGCGGTAAGAC
GGAGGGCGCGGGCGTTATTCGTTTTGATTGGGTGTAAAGGGTACGTAGGCGGTTCCGATT
TTTTAGGCTAAAAGAACAGAGTGATCCTTTGTGATGGCCTTTTATAAACAGGAGCTTGTG
TTGGAGATAGGTTGGGTCAAATTCTTCTTCAGGGGTAATATCCATAGAAATTGGGATGAA
AGTCAGCATGCGGAGGCGCCTTTCTTGCTCCAACAGACGCTAAGGTACGGAAGCTTAGGT
AGCAAACAGGATTAGAAACCCTCGTAGTCC
>Otu853
CCAGCCTACGGGAGGCAGCAGTTAGGAATCGTCTACAATGCGCGCAAGCGTGATAGCGCG
AGTCAGAGTGTTTCTCAACGAGAAACTTTTGCCAAATCTAAAACGTTTGGCGAATAAGGA
CTGGGTAAGACTAGTGCCAGCCGCCGCGGTAATCCTAGCGGTCCAAGTCGCAGCCAACAT
TATTGGGTCTAAAACATCCGTAGCTTGCTTAATAAGTTCTTTGTGAAATCCTGCGTCTCA
AATGCAGGGCGTGCAAAGAGTACTGTAAAGCTAGAGACTGGAAGACGTAGAGAGTACGTT
CGAAGTAGTGGTAAAATACGTTAATCTCGGACGGACTTACAATAGCGAAGGCACTCTACG
AGGACAGTTCTGACAGTGAGGGATGAAGGCTAGGGTCGCGAAAGGGATTAGAAACCCCCG
TAGTCC
>Otu856
CCAGCCTACGGGTGGCAGCAGTGGGGAATATTGGACAATGGGCGCAAGCCTGATCCAGCA
ATTCCGCGTGGGTGAAGAAGGTCTTCGGATTGTAAAGCCCTTTCGGCGGGGACGATGATG
ACGGTACCCGCAGAAGAAGCCCCGGCTAACTTCGTGCCAGCAGCCGCGGTAATACGAAGG
GGGCTAGCGTTGCTCGGAATTACTGGGCGTAAAGGGCGCGTAGGCGGCGCAAGTAGTCAG
GCGTGAAATTCCTGGGCTCAACCTGGGGACTGCGCTTGATACGCTTGTGCTAGAGGACGG
AAGAGGCTCGCGGAATTCCCAGTGTAGAGGTGAAATTCGTAGATATTGGGAAGAACACCG
GTGGCGAAGGCGGCGAGCTGGTCCGTTACTGACGCTGAGGCGCGACAGCGTGGGGAGCAA
ACAGGATTAGAAACCCGTGTAGTCC
>Otu857
CCAGCCTATGGGGTGCTGCAGTGGGGAATATTGGACAATGGGCGCAAGCCTGATCCAGCC
ATGCCGCGTGAGTGATGAAGGCCTTAGGGTTGTAAAGCTCTTTTGCCGGGGACGATAATG
ACTGTACCCGGAGAATAAGTCCCGGCTAACTTCGTGCCAGCAGCCGCGGTAATACGAAGG
GGACTAGCGTTGTTCGGAATCACTGGGCGTAAAGCGCACGTAGGTGGATTTGTAAGTCAG

GGGTGAAATCCCGGGGCTCAACCTCGGAACTGCCTTTGATACTGCAAGTCTTGAGTCCGA
TAGAGGTGGGTGGAATTCCTAGTGTAGAGGTGAAATTCGTAGATATTAGGAAGAACACCA
GTGGCGAAGGCGGCCCACTGGATCGGTACTGACACTGAGGTGCGAAAGCGTGGGGAGCAA
ACAGGATTAGAGACCCCAGTAGTCC
>Otu859
CCAGCCTATGGGGCGCTGCAGTCGAGAATCTTTCGCAATGGGCGCAAGCCTGACGAAGCG
ACGCCGTGTGAGCGAAGAAGGCCTTCGGGTTGTAAAGCTCTTTCGCTAGGGAACAAGAGA
GGCGCGATAACACCGCGCTGATTTGAGGGTACTTGGTAAAGAAGCACCGGCTAACTCCGT
GCCAGCAGCTGCGGTAATACGGAGGGTGCAAGCATTGATCGGAATTACTGGGCGTAAAGG
GTGCGTAGGCGGCTCGATAAGTCAGATGTGAAATCCCACGGCTCAACCGTGGAACAGCAT
TTGAAACTGTCGGACTTGAGCATAGTTAGGGAAAACGGAATTCCACGTGTAGCGGTGAAA
TGCGTAGATATGTGGAAGAACACCAGTGGTGAAGACGGTTTTCTGGGCTATAGCTGACGC
TGAGGCACGAAAGCTAGGGGAGCAAACAGGATTAGAGACCCTCGTAGTCC
>Otu860
CCAGCCTACGGGGTGCTGCAGCCGAGAATATTCGACAATGGGCGCAAGCCTGATCGAGCG
ACGCCGCGTGATGGATGAAGTGCTTCGGCATGTAAACATCTTTTATAGACGAGAAAGTAA
TTGATCAGTCTAAGAATAAGGAGTTGCTAAACTCGTGCCAGCAGCAGTGGTAATACGAGT
GCTCCAAGCGTTATCCGGAATCATTGGGCGTAAAGGGTGTGTAGGTGGTTATGTTAGTCT
TCTGTTAAAGCTCTCGGCTTAACCGGGAAAATGCAGGGGAAACGGCACAACTAGAGGATG
CGAGAGGTATATGGAACTCATGGAGTAGGGGTGAAATCCGTTGATATCATGGGGAACACC
AAATGCGAAGGCAGTATACTGGCGCACTCCTGACACTGAGACACGAAAGCGTAGGTAGCG
AATGGGATCAGATACCCCAGTAGTCC
>Otu861
CCAGCCTACGGGGGGCAGCAGTGGGGAATTTTGCGCAATGGGGGAAACCCTGACGCAGCA
ACGCCGCGTGGAGGATGAAGTATCTTGGTACGTAAACTCCTTTCGATGGGGAAGATAATG
ACGGTACCCATAGAAGAAGCCCCGGCTAACTTCGTGCCAGCAGCCGCGGTAATACGAGGG
GGGCAAGCGTTGTTCGGAATTATTGGGCGTAAAGGGTGCGTAGGCGGTTTGATAAGTCTG
GTGTGAAATCTATGGGCTCAACCCATAGTCTGCACTAGAAACTGTCGGGCTTGAGTATGG
GAGAGGTGAGTGGAATTTCCGGTGTAGCGGTGAAATGCGTAGATATCGGAAGGAACACCT
GTGGCGAAAGCGGCTCACTGGACCATAACTGACGCTGAGGCACAAAAGCGTGGGTAGCAA
ACAGGATTAGATACCCGTGTAGTCC
>Otu862
CCAGCCTATGGGGTGCTGCAGCGGGGAATATTGGACAATGGGGGAAACCCTGATCCAGCC
ATGCCGCGTGAGTGATGAAGGCCTTAGGGTTGTAAAGCTCTTTTACCAGGGAAGATGATG
ACGGTACCTGGAGAATAAGCACCGGCTAACTTCGTGCCAGCAGCCGCGGTAATACGAAGG
GTGCTAGCGTTGTTCGGAATCACTGGGCGTAAAGCGTGCGTAGGCGGCTTTCCAAGTCAG
GGGTGAAATCCTGGGGCTCAACCCCAGAATTGCCTTTGAAACTGGAAAGCTAGAGTATTG
GAGGGGAGTGCGGAATTCCTAATGTAGAGGTGAAATTCGTAGATATTAGGAGGAACACCG
GTGGCGAAGGCGGCGCTCTGGACAATAACTGACGCTCTGGCACGAAAGTGCGGGGATCAA
ACAGGATTAGATACCCTTGTAGTCC
>Otu863
CAGCCTATGGGGGGCAGCAGTGGGGAATATTGGACAATGGGCGCAAGCCTGATCCAGCAA
TGCCGCGTGGATGAAGAAGGTCTTCGGATTGTAAAGTCCTTTCGGCGGAGACGATGATGA
CGGTACCCGCAGAAGAAGCCCCGGCTAACTTCGTGCCAGCAGCCGCGGTAATACGAAGGG
GGCTAGCGTTGCTCGGAATGACTGGGCGTAAAGGGCGCGTAGGCGGCTGTCACAGTCGGG
TGTGAAATTCCTGGGCTCAACCTGGGGGCTGCACCCGAGACGTGATGGCTAGAGTTTGAA
AGAGGGTCGTGGAATTCCCAGTGTAGAGGTGAAATTCGTAGATATTGGGAAGAACACCGG
TGGCGAAGGCGGCGACCTGGTTCATTACTGACGCTGAGGCGCGAAAGCGTGGGGAGCAAA
CAGGATTAGAAACCCCAGTAGTCC
>Otu864
CCAGCCTACGGGAGGCAGCAGTAGGGAATCTTCCGCAATGGGCGAAAGCCTGACGGAGCA
ATGCCGCGTGAGGGATGAAGGCCTTCGGGTTGTAAACCTCTGTTGTTAGGGAAGAAGGTC
GCCGTGAAAACGGTTGGCTTGACGGTACCTGACGAGGAAGCCCCGGCTAATTACGTGCCA
GCAGCCGCGGTAATACGTAAGGGGCAAGCGTTGTCCGGAATCACTGGGCGTAGAGGGCGC
GTAGGCGGTTTGATAAGTAGAAGGTGAAAGATTCGGGCTCAACCCGAAAATTGCCTTCTA
AACTGTCTTACTTGAGGGTCTGAGGGGAAACTGGAATTCACGGTGTAGCGGTGAAATGCG
TAGATATCGTGGAGAACACCCGTGGCGAAAGCGGGTTTCTGGCAGACTCCTGACGCTGAG
GCGCGAAGGCTAGGGGAGCAAACGGGATTAGATACCCGCGTAGTCC

>Otu865
CCAGCCTATGGGGTGCTGCAGTGGGGAATATTGGACAATGGGGGCAACCCTGATCCAGCA
ATGCCGCGTGTGTGAAGAAGGCCTGAGGGTTGTAAAGCACTTTCAGTGGGGAGGAGGGTA
TGTAGGTTAAGAGCTGACATACTGGACGTTACCCACAGAAGAAGCACCGGCTAACTCCGT
GCCAGCAGCCGCGGTAATACGGAGGGTGCGAGCGTTAATCGGAATTACTGGGCGTAAAGG
GTGCGTAGGTGGTTGATTAAGTTATCTGTGAAATTCCTGGGCTCAACCTGGGCAGGTCAG
ATAAGACTGGTTAACTGGAGTATGGGAGAGGGTAGTGGAATTTCCGGTGTAGCGGTGGAA
TGCGTAGAGATCGGAAGGAACACCAGTGGCGAAGGCGGCTACCTGGCCTAATACTGACAC
TGAGGCACGAAAGCGTGGGGAGCAAACAGGATTAGAAACCCCCGTAGTCC
>Otu866
CCAGCCTACGGGAGGCTGCAGTCGAGAATTTTTCACAATGGGGGAAACCCTGATGGAGCG
ACGCCGCGTGGAGGACGAAGGGCTTCGTGCTTGTAAACTCCTGTCAAGCGGGAACAAGAA
AGTGATAGTACCGCTAGAGGAAGAGACGGCTAACTCTGTGCCAGCAGCCGCGGTAATACA
GAGGTCCCAAGCGTTGTTCGGATTCATTGGGCGTAAAGGGTGCGTAGGCGGCGTGGTAAG
TCGAATGTGAAATCTCGGGGCTCAACCCCGAAACTGCACTCGATACTACCATGCTAGAGG
ATTGTAGAGGAGATCGGAATTCACGGTGTAGCAGTGAAATGCGTAGATATCGTGAGGAAG
ACCAGTTGCGAAGGCGGATCTCTGGGCAATTCCTGACGCTGAGGCACGAAGGCTAGGGGA
GCAAACGGGATTAGATACCCTCGTAGTCC
>Otu876
CCAGCCTACGGGGGGCAGCAGTCGAGAATATTCCCAATGGCCGAAAGGCTGAGGGAGCGA
CGCCGCGTGCAGGACGAAGGGCTTCGGCTCGTAAACTGCTTTTTTCAGGGAAGAACACTG
ACGGTACCTGAAGGATAAGAGGTTGCTAACTCTGTGCCAGCAGCAGCGGTAATACAGAGA
CCTCAAGCGTTATCCGGATTTACTGGGCGTAAAGGGTCCGCAGGTGGTCATGTGCGTCGA
TGGTTAAATCCTCGGGCTCAACCCGGAAACCGCCGTCGATACGGCATGACTAGAGGCCGG
AAGAGGTAAGCGGAATTGCCGGTGTAGTAGTAATATGCGTTAATATCGGCAAGAACACCA
AATGCGAAGGCAGCTTACTGGTACGCGCCTGACACTCAGGGACGAAAGCGTGGGGGAGCG
AAAGGGATTAGAAACCCGTGTAGTCC
>Otu878
CCAGCCTACGGGAGGCTCCAGGCGCGAAACCTTTGCCATGCGCGAAAGCGTGACAGGGGA
ATTCCGAGTGGTAGGGGGGTTTACCTCCCTATCTTTTGGCCAATCCAAACAATTGGCAGA
ATAAGATCTGGGTAAGACCAGTGCCAGCCGCCATGCGCGTGATCATTCGCACGCACGGCA
CGGTACGCGGTAATACTGGCAGAGTAAGTGGTACCCACGAATATTGGGTCTAAAGAGTCC
GTAGCGGGCCTGTTAAGTCCACTGTGAAATCTGGCGGCTCAACCGTCAGGCGTGCAGTGG
ATACTGGCAGGCTTGGGAGTGGGGGAGGTCGGGAGTACTCACGGGGTAACGGTAAAATGT
TGTAATCCTGTGAGGACTACCAGTGGCGAAGGCGCCCGACCAAAACACGTCCGACCGTGA
GGGACGAAGGCTAGGAGAACGAATCGGATTAGAAACCCGAGTAGTCC
>Otu880
CCAGCCTACGGGTCGCAGCAGTCGAGAATCTTTCGCAATGGGCGAAAGCCTGACGAAGCG
ACGCCGTGTGAGCGAAGAAGGCCTTCGGGTTGTAAAGCTCTTTCGCTAGGGAACAAGAAA
GTCCGACTAATAATTGGATGATTTGATGGTACTTGGTAAAGAAGCACCGGCTAACTCCGT
GCCAGCAGCTGCGGTAATACGGAGGGTGCAAGCATTGATCGGAATTACTGGGTGTAAAGG
GCGCGTAGGCGGCAGAATAAGTCGGATGTGAAATTCCGGGGCTCAACCCCGGAGCTGCAT
TTGAAACTATTCAGCTAGAGGTTAGTAAGGGAAAACGGAATTCCACGTGTAGCGGTGAAA
TGCGTAGATATGTGGAAGAACACCGGTGGTGAAGACGGTTTTCTGTGCTAAACCTGACGC
TGAGGCGCGAAAGCTAGGGGAGCAAACAGGATTAGATACCCCCGTAGTCC
>Otu881
CCAGCCTATGGGGGGCAGCAGGTCGATAAACTGACGGGCGTTGCCCGGGCTGTCCAGCGG
CAGATAGAAGATATGGTCTGCTCCGGTATAGTCCTTTTTGGCCTCATATCCGGATGGAGA
AAAGAAAGTCAGCACGATCCGGGCCGCAGGCTCCTGTCTGCGCAGCCCCTCGATCACCGG
CCGGCCCTGCTCGAATTCTCCGAGCGAAGAACAATGCATCCAGATCACCGGGTGTTTCGC
GTCCGCCGCACTCGCCGCGCCGGCATCTCCGGCACCTCGCTCATCTGCGCCGGCCCTGAT
AGCTGTCATTTCTACCCTTATCCGGTCAAAAATTCCTTTCCTGCCCTCCAGCCATGCCTT
CGCTTTCGGATTCCAGGGCGCGACAAGGCGAATGCCGACTTTATACAACCACAAAAAAAC
ATGATAAAAAATGATACTCAACTTCCGGCATTTATTTGATTAGAAACCCTTGTAGTCC
>Otu883
CCAGCCTATGGGAGGCTCCAGTGGGGAATTTTGCGCAATGGGCGAAAGCCTGACGCAGCA
ACGCCGCGTGGAGGATGAAGGCCTTCGGGTCGTAAACTCCTGTCAGTAGGGACGAATGCT
TCGCAAGAGGTTTGACTGTACCTGCTGAGGAAGCCACGGCTAACTCCGTGCCAGCAGCCG

CGGTAATACGGAGGTGGCAAGCGTTGTTCGGAATTACTGGGCGTAAAGGGCGCGTAGGCG
GCTTTGCAAGTCAGATGTGAAAGCCCCGAGCTTAACTCGGGAACTGCATCTGAAACTGCT
TAGCTTGAGTCCTGGAGGGGGTAGTGGAATTCCCAGTGTAGCGGTGAAATGCGTAGATAT
TGGGAGGAACACCTGCGGCGAAGGCGGCTACCTGGACAGTGACTGACGCTGAGGCGCGAA
AGCTAGGGGAGCGAACGGGATTAGAAACCCCCGTAGTCC
>Otu884
CCAGCCTATGGGGGGCAGCAGTGGGGAATATTGGACAATGGGGGAAACCCTGATCCAGCA
ATGCCGCGTGAGTGATGAAGGCCTTCGGGTTGTAAAGCTCTTTTATCAGGGACGATAATG
ACGGTACCTGAGGAATAAGCACCGGCTAACTTCGTGCCAGCAGCCGCGGTAATACGAAGG
GTGCTAGCGTTGTTCGGAATAACTGGGCGTAAAGGGAGCGTAGGCGGCTAAGTACGTTCG
ATGTGAAAGTCCTGGGCTTAACCTGGGGACTGCATTGGATACGGCTTAGCTTGAGGCATA
GAGGGGAAGATGGAATTGCGTGTGTAGAGGTGAAATTCGTAGATATACGCAAGAACACCA
GTGGCGAAGGCGATCTTCTGGCTATGACCTGACGCTAAGGCTCGAAAGCGTGGGGAGCAA
ACAGGATTAGAGACCCTAGTAGTCC
>Otu885
CCAGCCTACGGGGGGCTGCAGTGAGGAATATTGGTCAATGGGCGCGAGCCTGAACCAGCC
ATCCCGCGTGCAGGAAGACGGCCCTATGGGTTGTAAACTGCTTTTGTGCAGGAAGAACGC
TCTCTACGTGTAGGGAGGTGACGGTACTGCATGAATAAGCACCGGCTAACTCCGTGCCAG
CAGCCGCGGTAATACGGAGGGTGCAAGCGCTATCCGGATTTATTGGGTTTAAAGGGTGCG
TAGGCGGTTAAATAAGTCAGTGGTGAAAATCTGCAGCTCAACTGTAGGGGTGCCATTGAA
ACTGTTTTACTTGAGTAACCATGAGGTAGGCGGAATGTGTAGTGTAGCGGTGAAATGCTT
AGATATTACACAGAACACCGATTGCGTAGGCAGCTTACTAGCGGTTTACTGACGCTGATG
CACGAAAGCGTGGGGATCAAACAGGATTAGATACGTAGTCC
>Otu886
CCAGCCTATGGGGCGCACCAGTGGGGAATCTTGCGCAATGGACGAAAGTCTGACGCAGCC
ACGCCGCGTGAGTGAAGAAGGCCTTCGGGTCGTAAAGCTCTGTCGGAGGGGACGAAAAGT
CGTACGGTTAATAGCCGTGCGATGTGACGGTACCCTCAAAGGAAGCACCGGCTAACTTCG
TGCCAGCAGCCGCAGTAATACGAGGGGTGCGAGCGTTGCTCGGAATTATTGGGCGTAAAG
GGTAGGTAGGTGGTCTCGTTTGTCTGGGGTGAAAGCCTTGAGCTTAACTCAAGAAGTGCC
CTAGAAACGGCGAGACTAGAATCTGTGAGAGGGTCGTGGAATTCCCGGTGTAGCGGTGAA
ATGCGTAGAGATCGGGAGGAACACCAGAGGCGAAGGCGGCGACCTGGAACAGTATTGACA
CTCAACTACGAAAGCGTGGGTAGCAAACAGGATTAGAAACCCCAGTAGTCC
>Otu887
CCAGCCTATGGGGGGCAGCAGTCGAGAATTTTTCTCAATGGGGGCAACCCTGAAGGAGCG
ACGCCGCGTGAAGGATGAAGGTCTTCGGATTGTAAACTTCTGTCATTAGAGAACAAGTGC
CGCCGAGTAACTACCGGCGGCTTGATAGTATCTGAAGAGGAAGGGACGGCTAACTCTGTG
CCAGCAGCCGCGGTAATACAGAGGTCCCAAGCGTTGTTCGGATTCATTGGGCGTAAAGGG
TGCGTAGGTGGTATCGTAAGTTGGATGTGAAATCCCGGAGCTCAACTCCGGAACTGCATT
CAATACTGCGGTGCTTGAGTACTGGAGAGGAGATTGGAATTCACGGTGTAGCAGTGAAAT
GCGTAGATATCGTGAGGAAGACCAGTGGCGAAGGCGGATCTCTGGACAGTAACTGACACT
GAGGCACGAAGGCCAGGGGAGCAAACGGGATTAGATACCCGAGTAGTCC
>Otu890
CCAGCCTACGGGGCGCAGCAGGGAATATTGCGCAATGAACGAAAGTCTGACGCAGCAACG
CCGCGTGGATGATGAAGTATTTCGGTATGTAAAATCCTGTCGGTGGGGAAGAAACGTGTC
GGTAGTAACTGATCGGCACCTGACGGTACCCACAGAGGAAGTCCCGGCTAACTCCGTGCC
AGCAGCCGCGGTAATACGGGGGGGACAAGCGTTGTTCGGATTTACTGGGCGTAAAGGGCG
CGTAGGCGGGCCTTCAAGTCAGATGTGAAAACTTCGAGCTTAACTCGGAGCCTGCATTTG
ATACTGTTGGTCTTGAGTACAGGAGAGGAGAGTGGAATTCCAGGTGTAGCGGTGAAATGC
GTAGATATCTGGAAGAACACCAGTAGCGAAGGCGGCTCTCTGGACTGATACTGACGCTCA
AGCGCGAAGGCTTGGGGAGCAAACAGGATTAGAAACCCTCGTAGTCC
>Otu892
CCAGCCTATGGGGGGCTGCAGTGGGGAATTTTGGACAATGGGGGCAACCCTGATCCAGCC
ATGCCGCGTGTCTGAAGAAGGCCTTCGGGTTGTAAAGGACTTTTGCCGGGGAGCAAAACT
TACGGCTGAATAAGCCGTGGGGATGAGAGTACCTGGAGAATAAGCACCGGCTAACTACGT
GCCAGCAGCCGCGGTAATACGTAGGGTGCGAGCGTTAATCGGAATTACTGGGCGTAAAGC
GTGCGCAGGCGGTTTTGCAAGTCTGATGTGAAAGCCCCGGGCTTAACCTGGGAACGGCAT
TGGAGACTGCAAGACTAGAGTGCGTCAGAGGGGGGTAGAATTCCACGTGTAGCAGTGAAA
TGCGTAGAGATGTGGAGGAATACCGATGGCGAAGGCAGCCCCCTGGGATGACACTGACGC

TCATGCACGAAAGCGTGGGGAGCAAACAGGATTAGATACCCGCGTAGTCC
>Otu893
CCAGCCTACGGGGCGCACCAGTGGGGAATTTTGGACAATGGGCGCAAGCCTGATCCAGCC
ATGCCGCGTGTGTGAAGAAGGCCTTCGGGTTGTAAAGCACTTTCGGCCGGAACGAAATCG
CGCGGATTAATAACCCGCGTGGATGACGGTACCGGAAGAAGAAGCACCGGCTAACTACGT
GCCAGCAGCCGCGGTAATACGTAGGGTGCGAGCGTTAATCGGAATTACTGGGCGTAAAGG
GTGCGCAGGCGGTTCCGCAAGTCAGGCGTGAAATCCCCGGGCTTAACCTGGGAATGGCGC
TTGAAACTACGGGACTCGAGTATGGCAGAGGGAGGTGGAATTCCACGTGTAGCGGTGAAA
TGCGTAGAGATGTGGAGGAACACCGATGGCGAAGGCAGCCTCCTGGGCCAATACTGGCGC
TCATGCACGAAAGCGTGGGGAGCAAACAGGATTAGATACCCCTGTAGTCC
>Otu894
CCAGCCTACGGGGCGCAGCAGACGAGAATATTCCGCAATGGACGAAAGTCTGACGGAGCG
ACGCCGCGTGATGGATGAAGTGCTTTGGTATGTAAACATCTTTTATCGGGGACGAAGTTT
ATTGACGGTACCCGATGAATAAGGGGCTCCTAACTCTGTGCCAGCAGGAGCGGTAATACA
GAGGCCCCAAGCATTACCCGGAATAACTGGGCGTAAAGGGTGTGTAGGCGGTCATGGTAG
TCGTTCGTTAAATCCGCGGGCCTAACCTGCGGTCTGCGAACGAAACGCTATGACTCGAGG
GCGCGAGAGGTGCACGGAACTCATGGTGGAGGGGTGAAATCCGTTGATATCATGGGGAAC
ACCAAAGGCGAAGGCAGTGCACTGGTGCGTTCCTGACGCTGAAACACGAAAGCCAGGGTA
GCGAACGGGATTAGATACCCGTGTAGTCC
>Otu895
CCAGCCTACGGGGGGCTCCAGTCGAGGATCTTCGGCAATGGGCGCAAGCCTGACCGAGCG
ACGCCGCGTGTGCGATGAAGGCCTTCGGGTTGTAAAGCACTGTCGAGGGGGAGGAAGACC
CGCAAGGGTTTGACCTATCCCTGGAGGAAGCACGGGCTAAGTTCGTGCCAGCAGCCGCGG
TAAGACGAACCGTGCGAACGTTGTTCGGATTCACTGGGCTTAAAGGGCGCGTAGGCGGCT
TGCCAAGTCAGGGGTGAAATCTTTCGGCTTAACCGGAAAAGTGCTTCTGATACTGGCAGG
CTGGAGGGAGGTAGGGGCAGATGGAACTTCCGGTGGAGCGGTGAAATGCGTAGATATCGG
AAGGAACGCCGGTGGCGAAAGCGATCTGCTGGACCTCTTCTGACGCTGAGGCGCGAAAGC
CAGGGGAGCAAACGGGATTAGAAACCCCGGTAGTCC
>Otu896
CCAGCCTATGGGGGGCAGCAGTTGGGAATATTGCGCAATGGGCGAAAGCCTGACGCAGCA
ACGCCGCGTGAGCGATGAAGGTCTTCGGATCGTAAAGCTCTTTCGGCCGGGACGAAAACT
CACCGTTTAATAAGCGGTGACTTGACGGTACCGGAAGAAGAAGCACCGGCTAACTCTGTG
CCAGCAGCCGCGGTAATACAGAGGGTGCAAGCGTTGTTCGGAGTTATTGGGCGTAAAGCG
CGTGTAGGCGGCTTTGTGTGTCGGGTGTGAAAGCCCTCGGCTTAACCGAGGAAGTGCGCC
CGAAACTGCAGAGCTTGAGTGCCGGAGAGGAGAGCGGAACACCCGGTGTAGAGGTGAAAT
TCGTAGATATCGGGTAGAACACCAGTGGCGAAGGCGGCTCTCTGGACGGCAACTGACGCT
GAGACGCGAAAGCGTGGGGAGCAAACAGGATTAGAAACCCGCGTAGTCC
>Otu897
CCAGCCTACGGGTGGCACCAGTAGGGAATATTGGGCAATGGAGGCAACTCTGACCCAGCC
ATGCCGCGTGCAGGACGAAGGCGCACAGCGTTGTAAACTGCTTTTGTCAGGGAAGAATGG
TGGTTGTGCGCAATCATGTGACGGTACCTGATGAATAAGCACCGGCTAACTCCGTGCCAG
CAGCCGCGGTAATACGGAGGGTGCAAGCGTTGTCCGGATTTATTGGGTTTAAAGGGTGCG
TAGGCGGGCTGATAAGTCTGGTTTGAAAGCTGGTCGCTTAACGATCAGATGTGGCTGGAT
ACTGTTAGTCTTGAAATTGTTGGAGGCAGCCGGAACGGGTCGCGTAGCGGTGAAATGCAT
AGATATGACCCAGAACACCAATTGCGTAGGCAGGCTGCTACGACTGATTTGACGCTGAGG
CACGAGAGCATGGGGAGCAAACAGGATTAGAGACCCTTGTAGTCC
>Otu902
CCAGCCTACGGGGTGCTGCAGTCAAGAATATTCCTCAATGGACGAAAGTCTGAAGGAGCG
ACGCCGCGTGACCGATGAAGTCCCTCGGGATGTAAAGGTCTTTTCTTAGGGAAGAACTAA
TGACGGTACCCAAGGAATAAGAGGTTGCTAACTCTGTGCCAGCAGCAGCGGTAATACAGA
GACCTCAAGCGTTATCCGGATTGATTGGGTGTAAAGCGTGCGCAGGCGGCTGATTAAGCC
GACGGTTAAATCTTGCGGCTCAACTGCAAGATTGCCGCCGGAACTGGTCAACTCGAGCCT
GGAAGAGGTGAGCGGAATTGCCGGTGTAACGGTAAAATGTGTTAATATCGGCAAGAACAC
CAGAGGCGAAAGCGGCTCACTGGTACATGGCTGACGCTGTGGCACGAAAGCGCGGGGAGC
GAATGGGATTAGAAACCCCAGTAGTCC
>Otu903
CCAGCCTATGGGGGGCAGCAGTGGGGAATCTTGCACAATGGGGGAAACCCTGATGCAGCG
ACGCCGCGTGAGTGATGAAGCTCTTCGGAGTGTAAAGCTCTTTCGGCAGGGAAGAGATAA

GACGGTACCTGCAAAAGAAGCCCCGGCTAACTACGTGCCAGCAGCCGCGGTAATACGTAG
GGGGCCAGCGTTGTTCGGAATTACTGGGCGTAAAGAGCGCGTAGGCGGCGCAGTGTGTTT
GGTGTGAAATCTCCCGGCTTAACCGGGAGGGGCCATCGAAAACTGCTGTGCTTCGGGTTT
GGGAGAGGGGAGTGGAATTCCCGGTGTAGCGGTGAAATGCGTAGAGATCGGGAGGAACAC
CAGCGACGAAGGTGGCTCCCTGGACCAATACCGACGCTGAGGCACGAAAGCTAGGGGAGC
AAACAGGATTAGAAACCCTGGTAGTCC
>Otu908
CCAGCCTATGGGTTGCTGCAGTGGGGAATATTGGACAATGGAGGAAACTCTGATCCAGCG
ATGTTTTGTGAGTGATTGACGGCATTCGCTGTAAAGCTCTTTCGGCAACAGGGATAACGA
CGCTGTTGAGGTAAGAAGTCCCGGCTAATTCCGTGCCAGCAGCCGCGGTAAAACGGGGGG
GACCGGCGTTATTCGTAAGAATTTGGCGTAAAGGGTACGTAGACGCGCCCGAGAACACTT
CGGTCGAAAATCCATGGTCTGGATCGTGGGATAGACGCTGTAACCTCGGGGATAGAGTGG
TGGCCACGTTACCGGAATTCTCGGTGTAGCAGGGGTTAAATCCAACCATACCGGGAGGAA
TGCTTTTGGTGAAGACAGGTTTCCGGGCTGCCACTGACGTTCAGGTACGGAGGTGTGAGT
ATCAAACGGGATTAGATACCCGAGTAGTCC
>Otu910
CCAGCCTATGGGTTGCTGCAGTGGGGAATATTGGACAATGGGCGAAAGCCTGATCCAGCA
ATGCCGCGTGTGTGAAGAAGGCCTTCGGGTTGTAAAGCACTTTCAGTAGGAAAGAAAAGC
ACAGGGTTAATACCCCTGTGCCATGACGTTACCTAAAGAAGAAGCACCGGCTAACTCTGT
GCCAGCAGCCGCGGTAATACAGAGGGTGCGAGCGTTAATCGGAATTACTGGGCGTAAAGC
GTGCGTAGGCGGATATTTAAGTCAAATGTGAAATCCCCGGGCTTAACCTGGGAACTGCAT
CCGATACTGGATATCTTAGAGTATGGTAGAGGAAAGTGGAATTCCCGGTGTAGCGGTGAA
ATGCGTAGATATCGGGAGGAACATCAGTGGCGAAGGCGGCTTTCTGGACCAATACTGACG
CTGAGGTACGAAAGCGTGGGGAGCAAACAGGATTAGAAACCCCAGTAGTCC
>Otu911
CCAGCCTACGGGTGGCTCCAGGCGCGAAAACTTCGCAATGCGCGAAAGCGTGACGAGGCT
ACCCGGAGTGATTTTTGAAGAAAAAATCTGTGGAGAGTGTCTAACAAGCCTCTCAAGAAA
GGAGAGGGCAAGGCTGGTGCCAGCCGCCGCGGTAAAACCAGCTCTTCAAGTTGTCGGGAC
GATTACTGGGCTTAAAGAGTCCGTAGCCGGTTTAGCAAGTCCCTGGTAAAATCTGGCAGC
TTAACTGTCAGTACGCTAGGGAAACTACTTTACTAGAGGGCGGGAAAGGTCTGGGGTACT
TCAGGGGTAGGGGTGGAATCCTATAATCCTTGGGGGACCACCAGTGGCGAAAGCGCCAGA
CTGGAACGCGCCTGACGGTGAGGGACGAAAGCCAGGGGAGCAAACCGGATTAGATACCCT
GGTAGTCC
>Otu912
CCAGCCTACGGGTTGCTCCAGTAGGGAATCTTCCACAATGGGCGAAAGCCTGATGGAGCA
ACGCCGCGTGCAAGATGAAGGCCTTCGGGTTGTAAACTGCTTTTATATGTGACGATTATG
ACGGTAGCATATGAATAAGGATCGGCTAACTCCGTGCCAGCAGCCGCGGTCATACGGAGG
ATCCAAGCGTTATCCGGAATTACTGGGCGTAAAGAGTTGCGTAGGTGGCATAGTAAGCGG
ACAGTGAAATCGTGTGGCTCAACCACATAAACATTGCCTGAACTACTAAGCTAGAGGACG
AGAGAGGTACCTGGAATTACCAGTGTAGGAGTGAAATCCGTAGATATTGGTAGGAACACC
GATGGCGTAGGCAGGGTACTGGCTCGTTTCTGACACTAAGGCACGAAAGCGTGGGTAGCA
AACTGGATTAGATACCCCAGTAGTCC
>Otu914
CCAGCCTACGGGGGGCACCAGGAGGGAACATTGGTAATGGGCGAAAGCCCGAACCAGCAA
CGCCGCGTGTGCGATGAAGGCCTTCGGGTTGTAAAGCACTTTTCACTCGGATGAGGAAGG
ACAGTACGGGTGGAATAAGCCTCGGCTAACTACGTGCCAGCAGCCGCGGTAAAACGTAGG
AGGCGAGCGTTATCCGGATTTACTGGGCGTAAAGCGCGTGTAGGCGGCTTGGAAAGTTGG
ATGTGAAAGCTCCCGGCTAAACTGGGAGAGGTCGTTCAAAACTTCCAGGCTAGAGGATGG
GAGAGGGAGGTGGAATTCCGGGTGTAGTGGTGAAATGCGTAGATATCCGGAGGAACACCA
GTGGCGAAAGCGGCCTCCTGGCCCACCCCTGACGCTCAGACGCGAAAGCTAGGGTAGCAA
ACGGGATTAGAAACCCTAGTAGTCC
>Otu916
CCAGCCTATGGGTGGCAGCAGGCTAGGACAATCAGGCGTGCGCATTGATTCCGATTTGTC
GATTAACAGGGCTATGTCGTGGCGACCGAGTTGTTCCAGTTCTGAAGCCGTCAGTTTGAC
TGTACTTACTGTTTTTTCAACCCGAATTTGTATGGGTTTAATGCTGGTTTGACCCGAGCT
AAAATTGGAAACATCGTTAGTGGGTGCCGTTGTTGGCGATGAAGTTGGAACGGGCGCCCC
GCTATTGGTGGCATCACCGGGGCGCTGAAGCCTGTATTTATAGATATACGGGGCCGCGCT
GTCGGCGGCGGACGAGCGCGTGTTGAAAGATGATCCTGCGGAAATCACCGTTAGAAACAG

ACTTGCACCTAGCCGTAAAAACTTTGAAAAGGATGGTCGATTGAGACCGGGAAAAAGTTG
CAATTAGAAACCCCCGTAGTCC
>Otu919
CCAGCCTATTGGGGGCACCAGTAGGGAATTTTCCACAATGGACGAAAGTCTGATGGAGCA
ACGCCGCGTGCAGGATGAAGGCCTTTGGGTCGTAAACTGCTTTTCTCTGTGAGGAATATG
ACAGTAGCAGAGGAATAAGGATCGGCTAACTCCGTGCCAGCAGCCGCGGTCATACGGAGG
ATCCAAGCGTTATCCGGAATTACTGGGCGTAAAGAGTTGCGTAGGCGGCAGAGTAAGCAG
AATGTGAAATCGTGTGGCTCAACCATACACCCATATTTTGAACTGCTCAGCTAGAGGATG
AGAGAGGTGGCTGGAATTCCCAGTGTAGGAGTGAAATCCGTAGATATTGGGAGGAACACC
GATGGCGTAGGCAGGCCACTGGCTCATTCCTGACGCTCAGGCACGAAAGCGTGGGGAGCG
ACCGGGATTAGAAACCCCCGTAGTCC
>Otu920
CCAGCCTACGGGTCGCAGCAGTGAGGAATATTGGACAATGGGCGCAAGCCTGATCCAGCC
ATGCCGCGTGAGTGATGAAGGCCTTAGGGTTGTAAAACTCTTTTAGTGGGGAAAATAATG
ATGGTACCCACAGAAAAAGCTCCGGCTAACTTCGTGCCAGCAGCCGCGGTAATACGAAGG
GAGCAAGCGTTACTCGGAATTACTGGGCGTAAAGCGCATGTAGGCGGAATTATAAGTTAG
AGGTGAAAGCCCGGGGCTCAACCCTGGAATTGCCTTTAAAACTGTAATTCTTGAGTATGA
CAGAGGATGGTGGAATTTCCAGTGTAGAGGTGAAATTCGTAGATATTGGAAGGAACACCG
GTGGCGAAGGCGACCATCTGGGTCATAACTGACGCTGAGATGCGAAAGCGTGGGGAGCAA
ACAGGATTAGAAACCCTGGTAGTCC
>Otu921
CCAGCCTACGGGAGGCTGCAGTGGGGAATATTGGACAATGGGCGCAAGCCTGATCCAGCC
ATGCCGCGTGAGTGATGAAGGCCCTAGGGTTGTAAAGCTCTTTTGGCAGGGAGGAAGGTA
TGTATTTTAAGAGATAACATATTTGACTGTACCTGCAGAATAAGCACCGGCTAACTCTGT
GCCAGCAGCCGCGGTAATACAGAGGGTGCAAGCGTTAATCGGAGTTACTGGGCGTAAAGG
GCGCGTAGGCGGTCGTATAAGTGAGATGTGAAATCCCTGGGCTTAACCTAGGAACTGCAT
CTCATACTGTGCGACTAGAGTATTGTAGAGGGTAGTGGAATTTCCGGTGTAGCGGTGAAA
TGCGTAGAGATCGGAAGGAACACCAGTGGCGAAGGCGGCTACCTGGACAAATACTGACGC
TGAGGCGCGAAAGCGTGGGGAGCAAACAGGATTAGATACCCCAGTAGTCC
>Otu922
CCAGCCTACGGGGGGCAGCAGTGGGGAATATTGGACAATGGACGAAAGTCTGATCCAGCA
ATACCGCATGAGTGATGAAGGTCCATTTTGATTGTAAAGCTCTTTCGTTAAGGAAGATTA
TGACATTACTTAAAAAAGAAGCCCCGGCTAACTTCGTGCCAGCAGTCGCGGTAAAACGGG
GGGGGCGAGTGTTACTCTACATAACTGGGCGTAAAGGGCATGTAGGTTGTTTTTTATGTT
TTTAGTAAAAGTCTAAAGCTCAACTTTAGAATCGCTATTAATACTAATAGACTTGAGTAT
TTAAGAAGATTATTGAATTCTTAATGGAGAGGTGAAATTCTAAGAGATTAAGAAGGCACT
CAAAGGTGAAGACAATAATCTATTATATACTGACGCTGATATGCAAAAGCATAGGGATCA
AACAGGATTAGAAACCCCGGTAGTCC
>Otu924
CAGCCTACGGGGTGCACCAGTTGGGAATCTTGCACAATGGGGGAAACCCTGATGCAGCGA
CGCCGCGTGGAGGATGACGGGTCTAGGCCTGTAAACTCCTTTTACCAGGAAAGACTTAGG
ACGGTACCTGGTGAATAAGCACCGGCTAACTACGTGCCAGCAGCCGCGGTAAGACGTAGG
GTGCAAGCGTTGTCCGGATTTACTGGGCGTAAAGGGCGCGTAGGCGGCTCGTTAAGTGTG
AAGTGTAATCCCTAGGGCTCAACCCAGGAACTGCTTTACATACTGGCGGGCTTGAGGATT
GGAGAGGCTTGTGGAATTCCTGGTGTAGCGGTGAAATGCGTTGATATCAGGAGGAACACC
CATGGCGAAGGCAGCAAGCTGGCCAATTTCTGACGCTGAGGCGCGAAAGCGTGGGTAGCA
AACAGGATTAGATACCCTTGTAGTCC
>Otu925
CCAGCCTACGGGATGCTCCAGTGGGGAATTTTGGACAATGGGCGAAAGCCTGATCCAGCA
ACGCCGCGTGAAGGATGAAGGTCTTCGGATTGTAAACTTCTTTTAGAGGGAACGAATAAA
TTGACGGTACTCTCAGAAAAAGCCACGGCTAACTACGTGCCAGCAGCCGCGGTAATACGT
AGGTGGCAAGCGTTACTCGGATTTACTAGGCGTAAAGCGTGTGTAGGTGGTTGAATAAGT
CTGTAGTGAAATCTCTCGGCTTAACCGGGAGGGGTCTACAGAAACTATTCGGCTTGAGTG
TGGTAGGGGAGAACGGAATTCCTGGTGTAGCGGTGAAATGCGTAGATATCAGGAGGAACA
CCGATGGCGAAAGCGGTTCTCTGGACCAATACTGACACTGATATACGAAAGCTAGGGGAG
CAAACAGGATTAGAAACCCTCGTAGTCC
>Otu926
CCAGCCTATGGGTGGCAGCAGTGGGGAATATTGGACAATGGGGGAAACCCTGATCCAGCC

ATGCCGCGTGAATGATGAAGGCCTTCGGGTTGTAAAATTCTTTTGGTGGGGACGATGATG
ACGGTACCCACAGAATAAGCTCCGGCTAACTTCGTGCCAGCAGCCGCGGTAATACGAAGG
GGGCTAGCGTTGTTCGGAATTACTGGGCGTAAAGGGCGCGTAGGCGGCTTGATAAGTTGG
ATGTGAAAGCCCAGGGCTCAACCCTGGAATAGCATTCAAGACTATCTCGCTTGAATTCGG
TAGAGGTTGGTGGAATTCCCAGTGTAGAGGTGAAATTCGTAGAGATTGGGAAGAATATCC
GTGGCGAAGGCGGCCAACTGGACCGACATTGACGCTGAGGCGCGAAAGCGTGGGGAGCAA
ACAGGATTAGAGACCCTCGTAGTCC
>Otu928
CCAGCCTACGGGGGGCTGCAGTGGGGAATCTTGCGCAATGGGCGAAAGCCTGACGCAGCA
ACGCCGCGTGTGTGATGAAGGTCTTCGGATCGTAAAGCACTGTCGCGAGGGAAGAGAAGC
CCTGGGTGAACAATCCAGGGATAGGACGGTACCTCGAGAGGAAGCACCGGCTAACTCTGT
GCCAGCAGCCGCGGTAATACAGAGGGTGCGAGCGTTGTTCGGAAGTATTGGGCGTAAAGC
GCTTGTAGGCGGCAGTTTAAGTCTCATGTGAAAGCCCTAGGCTTAACCTAGGAAGTGCAT
GAGATACTGGACGGCTTGGATGCCGGAGAGGGTCGCGGAATTCCCGGTGTAGAGGTGAAA
TTCGTAGATATCGGGAGGAACACCAGAGGCGAAGGCGGCGACCTGGACGGACATTGACGC
TGAGACGCGAAAGCGTGGGTAGCAAACAGGATTAGAAACCCCAGTAGTCC
>Otu933
CCAGCCTACGGGTGGCTCCAGTGAGGAATATTGCGCAATGGGGGAAACCCTGACGCAGCG
ACGCCGCGTGAGTGATGAAGGCCTTCGGGTTGTAAAGCTCTGTCAGAGGGAAAGAAAGGG
GTGTTGGAATAATACCCAACACCATTGACGGTACCCTCGGAGGAAGCACCGGCTAACTCC
GTGCCAGCAGCCGCGGTAATACGGAGGGTGCAAGTGTTATTCGGAATCACTGGGCGTAAA
GAGCACGTAGGCGGATGGGTAAGTCAGATGTGAAATCCCGGGGCTTAACTTCGGAACTGC
ATTTGATACTGCTCGTCTTGAGTATGGTAGAGGGGGATGGAATTCCCGGTGTAGAGGTGA
AATTCGTAGATATCGGGAGGAACACCAGAGGCGAAGGCGATCCCCTGGGCCATTACTGAC
GCTGAGGTGCGAAAGCGTGGGGAGCAAACAGGATTAGAGACCCTTGTAGTCC
>Otu937
CCAGCCTATGGGGGGCTGCAGTAAGGAATATTGGTCAATGGGCGCAAGCCTGAACCAGCC
ATGCCGCGTGCAGGATGAAGGCTCTATGGGTCGTAAACTGCTTTTATACGGGAAGAAACC
CCTCTACGTGTAGAGGGTTGACGGTACTGTAAGAATAAGGATCGGCTAACTCCGTGCCAG
CAGCCGCGGTAATACGGAGGATCCAAGCGTTATCCGGATTTATTGGGTTTAAAGGGTGCG
TAGGCGGACTTGTAAGTCAGTGGTGAAAGCCCGCAGCTTAACTGCGGAACTGCCATTGAT
ACTGCTAGTCTTGAGTATAACTGAGGTGGGCGGAATGTGTCATGTAGCGGTGAAATGCTT
AGATATGACACAGAACACCGATTGCGAAGGCAGCTCGCTAAACTATAACTGACGCTGAGG
CACGAAAGTGTGGGTAGCAAACAGGATTAGAAACCCGTGTAGTCC
>Otu938
CCACGGGGTTGCAGCAGTGGGGAATATTGGACAATGGGCGCAAGCCTGATCCAGCAATGC
CGCGTGTGTGAAGAAGGCCTTCGGGTTGTAAAGCACTTTTATCAGGAGCGAAACGCTGTC
GATTAATACTCGGCGGAACTGACGGTACCTGAGGAATAAGCACCGGCTAACTTCGTGCCA
GCAGCCGCGGTAATACGAAGGGTGCAAGCGTTAATCGGAATTACTGGGCGTAAAGCGTGC
GTAGGCGGTTTGATAAGTCTGTTGTGAAATCCCCGGGCTCAACCTGGGAATGGCAATGGA
TACTGGCAAGCTAGAGTGTGATAGAGGATGGTGGAATTCCCGGTGTAGCGGTGAAATGCG
TAGAGATCGGGAGGAACATCAGTGGCGAAGGCGGCCATCTGGATCAACACTGACGCTGAG
GCACGAAAGCGTGGGGAGCAAACAGGATTAGATACCCGGGTAGTCC
>Otu939
CCAGCCTATGGGTGCAGCAGTTTGGAATTTTGGTCAATGGGGGAAACCCTGAACCAGTGA
CGCCGCGTGGACGAAGACGCCCTTCGGGTTGTAAAGTCCTTTTGGCAGGGAAAAATTTTG
ATTGTACCTGCAGAATAAGAGGTTGCTAACTCTGTGCCAGCAGCAGCGGTAATACAGAGA
CCTCAAGCGTTATCCGGAATTATTGGGCGTAAAGCGTACCGATCGGTTGTTTTGATAGTC
AGAGGTGAAATCACGAAGCTCAACTTCGTGCTCGTCCTTTGAAACTTCAAGACTAGAAGG
GCAAAGAGGAAGCTGGAACAAACGGTGTAGTAGTGAAATGCGTTGATATCGTTTGGAACA
CCAATAGCGAAGGCAGGCTTCTGGGTGCCACTTGACACTGCTAGGACGAAAGCGTGGGTA
GCGAATGGGATTAGAAACCCCTGTAGTCC
>Otu940
CAGCCTATGGGTGGCTGCAGTCGAGAATCTTCCGCAATGGGCGAAAGCCTGACGGAGCGA
CGCCGCGTGATTGATGAAGTCCTTCGGGACGTAAAGATCTTTTATGCGCGAGAAAGTAAT
TGATAGTAGCGCATGAATAAGGGGCTCCTAACTCTGTGCCAGCAGGAGCGGTAATACAGA
GGCCCCAAGCATTATCCGGAATCACTGGGCGTAAAGGGTGTGTAGGCGGCTATGTTAGTC
TTTTGTGAAAGATCCCCGGCTTAACCGGGGAGACGCAGGGGAAACGGCATGGCTTCGAGG

GTATGAGAGGTAAAGGGAACTCATGGTGTAGGGGTGAAATCCGTTGATATCATGGGGAAC
ACCAAATGCGAAGGCACTTTACTGGCATACTCCTGACGCTGAAACACGAAAGCGTGGGAA
TCGAATGGGATTAGATACCCGTGTAGTCC
>Otu941
CCAGCCTACGGGAGGCAGCAGTTTCGAATCATTCACAATGGGCGCAAGCCTGATGGTGCG
ACGCCGCGTGGGGGACGAAGGTCTTCGGACTGTAAACCCCTGTCACCGGGGAAGAAACGC
TTTGAGCTAACAGTTCAAAGCCTGACTTAACCCGGAGAGGAAGCAGTGGCTAACTCTGTG
CCAGCAGCCGCGGTAATACAGAGACTGCAAGCGTTATTCGGATTCACTGGGCGTAAAGGG
TGCGCAGGCGGCTGAGTGTGTCAGGTGTGAAAGCCCGAGGCTTAACCTCGGAATTGCGCC
TGAAACTGCTCGGCTAGAGGACTGGAGAGGGTAGCGGAATTCACGGTGTAGCAGTGAAAT
GCGTAGATATCGTGAGGAACACCAGAGGCGAAGGCGGCTACCTGGACAGTACCTGACGCT
CAGGCACGAAAGCGGGGGGAGCAAAAGGGATTAGATACCCTCGTAGTCC
>Otu943
CCAGCCTACGGGGTGCTCCAGTGGGGAATCTTGCGCAATGGACGAAAGTCTGACGCAGCC
ACGCCGCGTGAGTGAAGAAGGCCTTCGGGTTGTAAAGCTCTGTCGGGCGGGACGAAAATT
TTTAGGGTTAACAGCCCTAGAAACTGACGGTACCGCCAAAGGAAGCACCGGCTAACTTCG
TGCCAGCAGCCGCGGTAATACGAAGGGTGCAAGCGTTGCTCGGAATTATTGGGCGTAAAG
GGTAGGTAGGTTGTCTCATTTGTCTGGGGTGAAAGCCTTGAGCTTAACTCAAGAAGTGCC
CCAGAAACGGTGAGACTAGAATCTGTGAGAGGGTCGTGGAATTCCCGGTGTAGCGGTGAA
ATGCGTAGAGATCGGGAGGAACACCAGAGGCGAAGGCGGCGACCTGGAACAGTATTGACA
CTCAACTACGAAAGCGTGGGTAGCAAACAGGATTAGAAACCCCTGTAGTCC
>Otu948
CCAGCCTATGGGGTGCTGCAGTAGGGAATATTGCGCAATGGGGGAAACCCTGACGCAGCG
ACGCCGCGTGAGTGATGAAGGCCTTAGGGTCGTAAAGCTCTGTTGAATGGGAAGAAAAAA
ATGACGGTACCATTCGAGAAAGGACCGGCTAACTTCGTGCCAGCAGCCGCGGTAAGACGA
GGGGTCCTAGCGTTGTTCGGAATCATTGGGCGTAAAGGGTGTGTAGGTTGCCATTCAAGT
CAGTTGTGAAAGCCCCGGGCTCAACCCGGGAAGTGCTTCTGATACTGTTTGGCTTGAGTA
CTGGAGAGGGTAGTGGAATTCCTGGTGTAGTGGTGAAATACGTAGATATCAGGAGGAATA
CCGGTGGCGAAGGCGGCTACCTGGCCATGTACTGACACTGAAGCACGAAAGCGTGGGTAG
CAAACAGGATTAGATACCCCCGTAGTCC
>Otu949
CCAGCCTATGGGATGCTGCAGTGGGGAATATTGGTCAATGGGCGCAAGCCTGAACCAGCC
ATCCCGCGTGCAGGATGAAAGCCCTATGGGTTGTAAACTGCTTTTATACGGGAAAAAACT
CTTGATTTCGTATCGAGACTGATGGTACCGTAAGAATAAGGATCGGCTAACTCCGTGCCA
GCAGCCGCGGTAATACGGAGGATCCAAGCGTTATCCGGATTTATTGGGTTTAAAGGGTGC
GCAGGCGGAGTAATAAGTCAGTGGTGAAATCCTACAGCTTAACTGTAGAACTGCCATTGA
TACTGTTATTCTTGAGTATATTTGAAGTGGGCGGAATGTGTCATGTAGCGGTGAAATGCT
TAGATATGACACAGAACACCAATCGCGAAGGCAGCTCACTAAACTATAACTGACGCTCAG
GCACGAAAGCGTGGGGATCAAACAGGATTAGAAACCCTGGTAGTCC
>Otu953
CCAGCCTACGGGTGGCTGCAGTGGGGAATTTTGCGCAATGGGCGAAAGCCTGACGCAGCA
ACGCCGCGTGGAGGATGAAGGTCTTCGGATCGTAAACTCCTGTCGATGGGGACGAAAAAA
ATGACTGTACCCGTTGAGGAAGCCACGGCTAACTCCGTGCCAGCAGCCGCGGTAATACGG
AGGTGGCAAGCGTTGTTCGGAATTACTGGGCGTAAAGGGCGCGTAGGCGGTTGGGCAAGT
CAGATGTGAAAGCCCCGGGCTTAACTCGGGAACTGCATCTGAAACTGCCTTACTTGAGTT
CTGGATGGGGTAGTGGAATTCCCAGTGTAGCGGTGAAATGCGTAGATATTGGGAGGAACA
CCTGCGGCGAAGGCGGCTACCTGGACAGTGACTGACGCTGAGGCGCGAAAGCTAGGGGAG
CGAACGGGATTAGAAACCCCGGTAGTCC
>Otu955
CCAGCCTCGGGGGGCAGCAGTAGGGAATTTTGCGCAATGGACGAAAGTCTGACGCAGCGA
CGCCGCGTGAGCGATGAAGGTCTTCGGATCGTAAAGCTCTGTTGGATGGAAGAACGGTTT
TGCATCTAATACATGCGGAACATGACCGTACCATTCGAGTAAGCACCGGCCAACTACGTG
CCAGCAGCCGCGGTAGTACGTAGGGTGCAAGCGTTGTTCGGAATTACTGGGTGTAAAGGG
AGAGTAGGCGGTTTGGTAAGTTAGGAGTGAAATGCCCACGCTCAACGTGGGACCTGCTTT
TAATACTGCCGGACTTGAGTATGGGAGAGGAAGATAGAATTCCAGGTGTAGCGGTGGAAT
GCGTAGATATCTGGAAGAATACCAGTTGCGTAGGCGGTCTTCTGGCCCAATACTGACGCT
GAGGCTCGAAAGCTAGGGGAGCAAACAGGATTAGATACCCTGGTAGTCC
>Otu956

CCAGCCTATGGGGGGCAGCAGTGGGGAATATTGGTCAATGGGCGCAAGCCTGAACCAGCC
ATCCCGCGTGAAGGATGAAGGCTCTAGTAGTTGTAAACTTCTTTTATACAGGAAGAAACC
TGAGTACGTGTACCCAGTTGACGGTACTGTATGAATAAGCATCGGCTAACTCCGTGCCAG
CAGCCGCGGTAATACGGAGGATGCAAGCGTTATCCGGATTTATTGGGTTTAAAGGGTCCG
CAGGCGGAATTTCAAGTCAGTGGTGAAATCCTACAGCTTAACTGTAGAACTGCCATTGAT
ACTGTTATTCTTGAGTGCGGTCGACGTGGGCGGAATATGACATGTAGCGGTGAAATGCAT
AGATATGTCATAGAACACCAATTGCGAAGGCAGCTCACGAGACCGCAACTGACGCTCAGG
GACGAAAGCATGGGTAGCGAACAGGATTAGATACCCCTGTAGTCC
>Otu957
CCAGCCTATGGGTGGCTGCAGTGGGGAATATTGGACAATGGGCGCAAGCCTGATCCAGCA
ATGCCGCGTGAGTGATGAAGGCCTTAGGGTTGTAAAGCTCTTTCGACGGGAACGATAATG
ACGGTACCCGAAGAAGAAGCTGCGGCTAACTACGTGCCAGCAGCCGCGGTAATACGTAGG
GTGCAAGCGTTAATCGGAATTACTGGGCGTAAAGCGTGCGCAGGCGGTTATGTAAGACAG
ATGTGAAATGCCCGGGCTTAACCTGGGAACTGCATTTGTGACTGCATGGCTAGAATCTGG
CAGAGGGGGGTAGAATTCCACGTGTAGCAGTGAAATGCGTAGATATGTGGAGGAATACCG
ATGGCGAAGGCAGCCCCCTGGGCTAAGATTGACGCTCATGCACGAAAGCGTGGGGAGCAA
ACAGGATTAGAAACCCCAGTAGTCC
>Otu959
CCAGCCTACGGGGCGCTGCAGTCGAGAATTTTTCACAATGGGCGAAAGCCTGATGGAGCG
ACGCCGCGTGGGGGATGAATGGCTTCGGCCCGTAAACCCCTGTCATTTGCGAGCAAAGCT
GCCGGCCTAACACGCTGGTAGTTGATCGTAGCGAAAGAGGAAGGGACGGCTAACTCTGTG
CCAGCAGCCGCGGTAATACAGAGGTCCCGAGCGTTGTTCGGATTTACTGGGCGTAAAGGG
TGCGTAGGTGGCTGGGTAAGTTTGATGTGAAATCTCCGGGCTCAACCCGGAAATGGCATT
GAATACTGCTTGGATAGAGGGTTGGAGAGGGGACTGGAATTCTCGGTGTAGCAGTGAAAT
GCGTAGATATCGAGAGGAACACCAGTGGCGAAGGCGAGTCCCTGGACAACTCCTGACACT
GAGGCACGAAAGCTAGGGGAGCAAACAGGATTAGAGACCCGAGTAGTCC
>Otu962
CCAGCCTATGGGACGCAGCAGTCGAGAATCTTCCGCAATGGACGAAAGTCTGACGGAGCG
ACGTTACGTGAATGATGAAGCCCTTCGGGGTGTAAAGTTCTTTTATATGGGAAGAAGCGC
AAGCAGACTGTACCATATGAATAAGGGGATCCTAATTCTGTGCCAGCAGGAGCGGTAATA
CAGAATCCCCGAGCGTTACCCGGATTTATTGGGCGTAAAGGGTCCGTAGGTGGTAAAGTA
AGTTGAAAGTTAAAACTCATCGGCTTAACCTTTGAGGTGCTTTCAATACTGCTATACTAG
AGGATGTTAGGGGTGAACGGAACCGACGGAGTAGGGGTGAAATCCGTTGATATCGTCGGG
AACACCAAAAGCGAAGGCAGTTAACTAGGACGACCCTGACACTGAGGAACGAAAGCGTGG
GGAGCAAAAAGGATTAGAGACCCTAGTAGTCC
>Otu963
CCAGCCTATGGGTGGCACCAGTGGGGAATCTTGCGCAATGGACGAAAGTCTGACGCAGCC
ACGCCGCGTGAGTGAAGAAGGCCTTCGGGTTGTAAAGCTCTGTCGAGCGGGACGAAAAAT
CTTTAGGTGAATAGCCTAGAGATCTGACGGTACCGCTAAAGGAAGCACCGGCTAACTCCG
TGCCAGCAGCCGCGGTAATACGGAGGGTGCAAGCGTTGCTCGGAATTATTGGGCGTAAAG
GGTAGGTAGGTGGTCTCATTTGTCTGGGGTGAAAGCCTTGAGCTTAACTCAAGAAGTGCC
CCAGAAACGGTGAGACTAGAGTCCTGGAGAGGGTCGTGGAATTCCCGGTGTAGCGGTGAA
ATGCGTAGAGATCGGGAGGAACACCAGAGGCGAAGGCGGCGACCTGGACAGGTACTGACA
CTCAACTACGAAAGCGTGGGGAGCAAACAGGATTAGAAACCCGTGTAGTCC
>Otu964
CCAGCCTACGGGTGGCAGCAGTAAGGGATATTGCGCAATGGGCGAAAGCCTGACGCAGCA
ACGCCGCGTGCCCGAGGAAGGCCTTCGGGTCGTAAAGGGCTTTTAGGGCTGAAGAGCAAG
GACGGTAAGCCCGGAAGAAGTGTCGGCTAACTACGTGCCAGCAGCCGCGGTAACACGTAG
GAGGCAAGCGTTATCCGGATTTACTGGGCGTAAAGCGCATGCAGGTGGTTCGGTAAGTTG
GATGTGAAAGCTCCTGGCTCAACTGGGAGAGGTCGTTCAATACTACCGGACTTGAGAGCA
GTAGAGGAAAGTGGAATTCCCGGTGTAGTGGTGAAATGCGTAGATATCGGGAGGAACACC
AGTGGCGAAAGCGGCTTTCTGGACTGTTTCTGACACTCATATGCGAAAGCTAGGGTAGCA
AACGGGATTAGATACCCCCGTAGTCC
>Otu965
CCAGCCTATGGGGGGCAGCAGTCGAGGATCTTCGGCAATGGGCGCAAGCCTGACCGAGCG
ACGCCGCGTGTGCGATGAAGGCCTTCGGGTTGTAAAGCACTGTCGAGGGGGAGGAAAGCC
GCAAGGTTTGACCTATCCCTGGAGGAAGCACGGGCTAAGTTCGTGCCAGCAGCCACGGTA
AGACGAACCGTGCGAACGTTGTTCGGAATCACTGGGCTTAAAGGGCGCGTAGGCGGGTCT

TCAAGTCCGGGGTGAAAGCCCCCAGCTCAACTGGGGAAGTGCCTTGGAGACTGAAGGTCT
CGAGGGAGGTAGGGGTCCGTGGAACTTCGGGTGGAGCGGTGAAATGCGTTGATATCCGAA
GGAACGCCGGTGGCGAAAGCGACGGACTGGATCTCTTCTGACGCTGAGGCGCGAAAGCCA
GGGGAGCAAACGGGATTAGATACCCTGGTAGTCC
>Otu966
CCAGCCTATGGGGGGCTGCAGTAAGGAATATTGGACAATGCCCGCAAGGGTGATCCAGCC
ATGCCGCGTGCAGGATGAAGGCCCTATGGGTCGTAAACTGCTTTTGAACAAGAGAAAACC
CTCCTACGTGTAGGAGGCTGATAGTATTGTTAGAATAAGCATCGGCTAACTTCGTGCCAG
CAGCCGCGGTAAGACGAAGGATGCAAGCGTTATCCGGATTCATTGGGTTTAAAGGGAGCG
TAGGCGGTCTTATAAGTCAGTGGTGAAAGCTCCTCGCTTAACGAGGAAATTGCCATTGAT
ACTGTAGGACTTGAGTACAGTTGCTGTGGGCGGAATATGACATGTAGTGGTGAAATACAT
AGAGATGTCATAGAACACCGATTGCGAAGGCAGCTCACAAAACTGTAACTGACGCTGAGG
CTCGAAAGTGCGGGGATCAAACAGGATTAGAAACCCTGGTAGTCC
>Otu967
CCAGCCTATGGGGTGCTGCAGTAGGGAATATTGGGCAATGGAGGCAACTCTGACCCAGCC
ATGCCGCGTGCAGGAAGAAGGCCCTCCGGGTTGTAAACTGCTTTTGAACGGGAAGAAAAG
ACCCTGCGGGGGAAAGTGACGGTACCGTTAGAATAAGCCACGGCTAACTACGTGCCAGCA
GCCGCGGTAATACGTAGGTGGCAAGCGTTGTCCGGATTTATTGGGTTTAAAGGGTGCGTG
GGCGGCCGCGCAAGTCAGTAGTGAAATACCGCAGCTCAACTGTGGGGGTGCCATTGATAC
TTCGCGGCTTGAGTGGGGTCGGGGTGGCCGGAACTTACGGTGTAGCGGTGAAATGCATAG
ATACCGTAAAGAACACCGATAGCGAAGGCAGGCCACTGGGCCCCAACTGACGCTGAGGCA
CGAAAGCGTGGGGATCGAACAGGATTAGATACCCTCGTAGTCC
>Otu968
CCAGCCTACGGGGGGCAGCAGCAAGGAATCTTGGGCAATGGGCGAAAGCCTGACCCAGCG
ACGCCGCGTGAGGGATGAAGGCCTTCGGGTTGTAAACCTCTTTTCTCAGGGAAGAATAAT
GACGGTACCTGAGGAATAAGTCTCGGCTAACTACGTGCCAGCAGCCGCGGTAATACGTAG
GAGGCGAGCGTTATCCGGATTTATTGGGCGTAAAGTGGGCGTAGGTGGTCTTTCAAGTCA
GATGTGAAATCTCCCGGCTTAACTGGGAGGGGTCATCTGATACTGTTGGACTTGAGTACA
GCAGGGGAAAATGGAATTCCCGGTGTAGTGGTGAAATGCGTAGATATCGGGAGGAACACC
AGAGGCGAAGGCGATTTTCCAGGCTGGAACTGACACTGAGGCCCGAAAGCGTGGGGAGCG
AACAGGATTAGATACCCGAGTAGTCC
>Otu970
CCAGCCTATGGGTGGCTCCAGATCGAACATGACTTCAAACTGATCCAAGAGACGGAGGCA
GGAGGAATCTGATGGGTACCGCAGAACGTCGTCAATTTACCGCAGAGCAGAAGGTCGCAC
ATGGGACCGAAAACCATGAAATCAATGCGATCCGCACCGGCAGCCCGCAGAGCCTACATG
AGGCACGTGGCCACGACCGTAGACGATGTCGGCGACCGACCCTTCTTGTGCCTTAACCGG
CAAGAGATGTTGCACTTGCTCTGGGCGCTCAATCTCCGAGCTCGGGAATACTCCGTCAGC
GTCAATCCTCGAGACTGCGCTTGACGGGACTGCGCTTGACCAAAATGGACCAGGTATCGC
CGCGCTCCTGAAACAATTCAGCCTGTTTCGGTGGAAACCTAGATATGAGCAATCTCAGAA
GTACCTCGCTGATCTAATAAAGCTCAGGTAGATTGATTAGATACCCCCGTAGTCC
>Otu971
CCAGCCTACGGGTGGCAGCAGTGAGGAATATTGGACAATGGGCGGAAGCCTGATCCAGCA
ATACCGCGTGTGTGAAGAAGGCCTGAGGGTTGTAAAGCACTTTCAATGGGAAGGAATACC
TGGTGGTTAATACCTGCCAGACTGACATTACCATACAAGAAGCACCGGCTAACTCCGTGC
CAGCAGCCGCGGTCATACGGAGGGTGCAAGCGTTAATCGGAATTACTGGGCGTAAAGCGT
GCGTAGGCGGTTCGTTAAGTCAGATGTGAAAGCCCTGGGCTTAACCTGGGAACTGCATTT
GATACTGGCGGACTTGAGTTGAGTAGAGGAGAGTGGAATTTCAGGTGTAGCGGTGAAATG
CGTAGAGATCTGAAGGAACACCAGTGGCGAAGGCGGCTCTCTGGACTCAAACTGACGCTG
AGGTACGAAAGCGTGGGTAGCAAACAGGATTAGAAACCCCCGTAGTCC
>Otu973
CCAGCCTACGGGGGGCTGCAGTGGGGAATCTTGCACAATGGGCGAAAGCCTGATGCAGCA
ACGCCGCGTGGAGGACGAATGCTTTCTGAGTTGTAAACTCCTTTCAGCAGGGACGATTGT
GACGGTACCTGCAGAAGAAGCACCGGCCAACTACGTGCCAGCAGCCGCGGTGATACGTAG
GGTGCAAGCGTTGTCCCGATTTATTGGGCGTAAAGAGCTCGTAGGCGGTTTGATAAGTCG
GATGTGAAATCTCCAGGCTCAACCTGGAGCTGCCATTCGATACTGTCATGACTTGAGGCC
GGTAGGGGACCATGGAATTCCTAGTGTAGCGGTGAAATGCGCAGATATTAGGAGGAACAC
CAGCAGCGAAGGCGGTGGTCTGGGCCGGTACTGACGCTGAGGAGCGAAAGCGTGGGGAGC
GAACAGGATTAGATACCCTCGTAGTCC

>Otu977
CCAGCCTATGGGTGGCAGCAGTGGGGAATATTGGACAATGGGCGCAAGCCTGGTCCAGCA
ATGCCGCGTGTGTGAAGAAGGTCTTCGGATTGTAAAGCTCTTTCGGCAGGGAAGATGATG
ACGGTACCTGCAGAAGCAGCTGCGGCTAACTACGTGCCAGCAGCCGCGGTAATACGTAGG
CAGCAAGCGTTGTTCGGAGTTACTGGGCGTAAAGGGTGCGTAGGCGGCCTTGTAAGTATG
GTGTGAAATCTCCCGGCTTAACTGGGAGGGTGCGCTATTAACTGCAGGGCTAGAGTGCGG
GAGAGGAAAGTGGAATTCCTGGTGTAGCGGTGAAATGCGTAGATATCAGGAGGAACACCG
GTGGTGTAAACGGCTTTCTGGACCGTAACTGACGCTGAGGCACGAAAGCGTGGGTAGCAA
ACAGGATTAGAGACCCCAGTAGTCC
>Otu978
CCAGCCTATGGGTCGCTGCAGTCAAGAATATTCCTCAATGGACGAAAGTCTGAAGGAGCG
ACGCCGCGTGACTGAAGAAGTCCTTCGGGATGTAAAGGTCTTTTCTCAGGGAAGAAATCT
GACGGTACCTGAGGAATAAGAGGTTGCTAACTCTGTGCCAGCAGCAGCGGTAATACAGAG
ACCTCGAGCGTTATCCGGATTGATTGGGCGTAAAGCGTGCGCAGACGGCTTTCTAAGTCT
GCGGTCAAATTCTCTAGCTCAACTGGGGAACTGCCGCGGAAACTGGAAGGCTAGAGTGCG
GAAGAGGTCAGCGGAATTGCCGGTGTAACGGTAAAATGTGTTAATATCGGCAAGAACACC
AGAGGCGAAAGCGGCTGACTGGGACGCAACTGACGTTGTTGCACGAAAGCGTGGGGAGCG
AATGGGATTAGATACCCTAGTAGTCC
>Otu979
CCAGCCTATGGGAGGCTCCAGCGGGGAATCTTGCGCAATGGGCGAAAGCCTGACGCAGCA
ACGCCGCGTGTGTGATGAAGGTCTTCGGATCGTAAAGCACTGTCGCGAGGGACGAGAAGC
ATTGGGTGAACAATCCAGTGATAGGACGGTACCTCGAGAGGAAGCACCGGCTAACTCTGT
GCCAGCAGCCGCGGTAATACAGAGGGTGCGAGCGTTGTTCGGAATTATTGGGCGTAAAGC
GCTTGTAGGCGGTCTACTAAGTCCATTGTGAAAGCCCTTGGCTCAACCAAGGAAGTGCAA
TGGATACTGGAAGACTTGAATGCCGGAGAGGGTCGCGGAATTCCCGGTGTAGAGGTGAAA
TTCGTAGATATCGGGAGGAACACCAGTGGCGAAGGCGGCGACCTGGACGGACATTGACGC
TGAGACGCGAAAGCGTGGGGAGCAAACAGGATTAGAAACCCGAGTAGTCC
>Otu983
CCAGCCTATGGGGGGCAGCAGTGGGGAATTTTGGACAATGGGGGCAACCCTGATCCAGCC
ATGCCGCGTGCGGGAAGAAGGCCTTCGGGTTGTAAACCGCTTTTGGCAGGAACGAAACGG
CGCGCGCCAATACCGCGCGTTAATGACGGTACCTGCAGAATAAGCACCGGCTAACTACGT
GCCAGCAGCCGCGGTAATACGTAGGGTGCGAGCGTTAATCGGAATTACTGGGCGTAAAGC
GTACGCAGGCGGCTTGGTAAGACAGGTGTGAAATCCCCGGGCTCAACCTGGGAACTGCAT
TTGTGACTGCCAGACTCGAGTTTGTCAGAGGGGGGTGGAATTCCACGTGTAGCAGTGAAA
TGCGTAGAGATGTGGAGGAACACCGATGGCGAAGGCAGCCCCCTGGGATGAGACTGACGC
TCATGTACGAAAGCGTGGGGAGCAAACAGGATTAGAGACCCGAGTAGTCC
>Otu984
CCAGCCTATGGGGGGCACCAGTAGGGAATATTGGTTAATGGGCGAAAGCCTGACCCAGCA
ACGCCGCGTGTGCGATGAAGGCCTTCGGGTCGTAAAGCACTTTTTAGTGGGATGAGGAAG
GACAGTACCACTAGAATAAGTCTCGGCTAACTACGTGCCAGCAGCCGCGGTAAAACGTAG
GAGGCAAGCGTTATCCGGATTTACTGGGCGTAAAGCGCGTGTAGGTGGTTTTGTAAGTTG
GATGTGAAAGCTCCCGGCTTAACTGGGAGAGGTCGTTCAAAACTACAAGACTTGAGAGTG
GTAGAGGAAGGTGGAATTCCGGGTGTAGTGGTGAAATGCGTAGATATCCGGAGGAACACC
AGTGGCGAAAGCGGCCTTCTGGACCATTTCTGACACTGATACGCGAAAGCTAAGGTAGCA
AACGGGATTAGATACCCGCGTAGTCC
>Otu988
CCAGCCTATGGGGCGCACCAGTCGAGAATCTTCCGCAATGGGCGAAAGCCTGACGGAGCG
ACGTCGCGTGGTTGATGAAGTCTTTCGGGATGTAAAAACCTTTTATGAGCTACTAAGTTA
TTGAAGGGCTCATGAATAAGGGGTTGCTAAACTCGTGCCAGCAGCAGCGGTAATACGAGT
GCCCCGAGCGTTATCCGGAATTATTGGGCGTAAAGGATGTGTAGGCGGTTCTGTTAGTCT
CATGTTAAAGCTCCCGGCTTAACCGGGAAAGTGCGTGGGAAACGGCAGAACTAGAGGATG
TGAGAGGTATGCGGAACTCATAGTGTAGGGGTGAAATCCGTTGATATTATGGGGAACACC
AAAAGCGAAGGCAGCATACTGGCACACTCCTGACGTTGAGACATGAAAGCGTGGGTAGCG
AATGGGATTAGAGACCCCCGTAGTCC
>Otu991
CCAGCCTACGGGGCGCACCAGTCGAGAATCTTCCGCAATGGGCGAAAGCCTGACGGAGCG
ACGCCGCGTGACTGATGAAGTCCTTCGGGACGTAAAGGTCTTTTGTGAGGTAGAAAGTTA
TTGATCGCCTCAAGAATAAGGGGTTGCTAAACTCGTGCCAGCAGCAGCGGTAATACGAGT

GCTCCAAGCGTTATCCGGAATTATTGGGCGTAAAGGGTGTGTAGGCGGTCTTATTAGTCT
TTTGTTAAAACTCCCGGCTCAACCGGGAATCTGCAAAAGAAACGGTAAGACTTGAGGGTG
CGAGGGGTGAATGGAACTCATAGTGTAGGGGTGAAATCCGTTGATATTATGGGGAACACC
AAAAGCGAAGGCAATTCACTGGCGCACTTCTGACGCTGAAACACGAAAGCGTGGGTAGCG
AATGGGATTAGAAACCCGCGAGTCC
>Otu993
CCAGCCTACGGGAGGCTGCAGTCGAGAATTTTTCACAATGGGGGAAACCCTGATGGAGCG
ACGCCGCGTGGGGGATGAATGGCTTCGGCCCGTAAACCCCTGTCATTTGCGAATAAACCT
TGTTGTTTAAGAGATGACGAGTTGACAGTAGCGAAAGAGGAAGGGACGGCTAACTCTGTG
CCAGCAGCCGCGGTAATACAGAGGTCCCAAGCGTTGTTCGGATTCACTGGGCGTAAAGGG
TGCGTAGGTGGTCGGGTAAGTCTGATGTGAAATCTCGGAGCTCAACTCCGAAACTGCATT
GGATACTATCCGGCTAGAGGATCGGAGGGGGGACTGGAATTCTCGGTGTAGCAGTGAAAT
GCGTAGATATCGAGAGGAACACCAGTGGCGAAGGCGAGTCCCTGGACGATTCCTGACACT
GAGGCACGAAAGCTAGGGGAGCAAACAGGATTAGAGACCCGCGTAGTCC
>Otu994
CCAGCCTACGGGGGGCTGCAGTAACGAATCTTCCGCAATGCACGCAAGTGTGACGGAGCG
ACGCCGCGTGTGGGACGAAGTCCTTCGGGATGTAAACCACTGTCAGGGGTAAGAAAGTTC
TGATCTACCCCAGAGGAAGGCACGGCTAACTCTGTGCCAGCAGCCGCGGTAAGACAGAGG
TGCCGAGCGTTAGGCGGAATCACTGGGCTTAAAGCGTGTGTAGGCGGGTCGTTAAGTATC
TTGTGAAATCCCCCGGCTCAACCGGGGAACTGCTGGGTATACTGGCGATCTTGAGCAATT
CAGGGGCAGATGGAACAAGTGGTGGAGCGGTGAAATGCGTAGATATCACTTGGAACGCCA
ATGGTGAAAACAACTCGCTGGGGGTTTGCTGACGCTGAGACACGAAAGCTAGGGGAGCAA
ACGGGATTAGATACCCCTGTAGTCC
>Otu995
CCAGCCTATGGGTGGCAGCAGTCGAGGATCTTCGGCAATGAGCGCAAGCTTGACCGAGCG
ACGCCGCGTGAGCGATGAAGGCCTTCGGGTTGTAAAGCTCTTTAGTGGGGGAGAAAAGCC
GAAAGGTGTGATCTATCCCAAAAATAAGCACGGGCTAAGTTCGTGCCAGCAGCCGCGGTA
AGACGAACCGTGCGAACGTTATTCGGAATCACTGGGCTTAAAGGGCGCGTAGGCGGGCTG
TCAAGTCTGGGGTGAAATCCCACGGCTTAACCGTGGAACTGCCTCAGATACTGACGGCCT
CGAGGAAGGTAGGGGCATGCGGAACTGTAGGTGGAGCGGTGAAATGCGTTGATATCTACA
GGAACTCCGGTGGCGAAAGCGGCGTGCTGGACCTTTTCTGACGCTGAGGCGCGAAAGCTA
GGGGAGCAAACGGGATTAGAAACCCCGGTAGTCC
>Otu996
CCAGCCTACGGGTCGCAGCAGTGGGGAATATTGGACAATGGGGGCAACCCTGATCCAGCA
ATGCCGCGTGTGTGAAGAAGGCCTTCGGGTTGTAAAGCACTTTTGTCAGGGAAGAAAAGG
TTTGCCCTAATACGGTGGACTGATGACGGTACCTGAAGAATAAGCACCGGCTAACTACGT
GCCAGCAGCCGCGGTAATACGTAGGGTGCAAGCGTTAATCGGAATTACTGGGCGTAAAGC
GTGCGCAGGCGGTTTGTTAAGACGGATGTGAAATCCCCGGGCTCAACCTGGGAACTGCAT
TCGTGACTGGCAGGCTAGAGTATGGCAGAGGGGGGTGGAATTCCACGTGTAGCAGTGAAA
TGCGTAGAGATGTGGAGGAACACCGATGGCGAAGGCAGCCCCCTGGGCCAATACTGACGC
TCATGCACGAAAGCGTGGGGAGCAAACAGGATTAGATACCCCTGTAGTCC
>Otu1004
CCAGCCTATGGGTGGCACCAGTGGGGAATATTGGACAATGGGGGAAACCCTGATCCAGCC
ATGCCGCGTGAGTGATGAAGGCCTTAGGGTTGTAAAGCTCTTTTAGCAGGGAAGATGATG
ACGGTACCTGCAGAATAAGCACCGGCTAACTTCGTGCCAGCAGCCGCGGTAATACGAAGG
GTGCTAGCGTTGTCCGGAATCACTGGGCGTAAAGCGCGCGTAGGCGGCTTTTCAAGTCAG
GGGTGAAATGCCAAGGCTTAACCTTGGAACTGCCTTTGAAACTGTAAAGCTGGAGTGTCG
GAGGGGATAGCGGAATTCCTAATGTAGTGGTGAAATACGTAGATATTAGGAGGAACACCG
GTGGCGAAGGCGGCTATCTGGACGACAACTGACGCTGAGGCGCGAAAGCGTGGGGATCAA
ACAGGATTAGAACCCTCGTAGTCC
>Otu1005
CCAGCCTACGGGGGGCAGCAGTGGGGAATTTTGGACAATGGGCGCAAGCCTGATCCAGCC
ATGCCGCGTGAGTGAAGAAGGCCTTCGGGTTGTAAAGCTCTTTCAGCCGGAAAGAAATCG
CATCGGATAATACCTGATGTGGATGACGGTACCGGAAGAAGAAGCACCGGCTAACTACGT
GCCAGCAGCCGCGGTAATACGTAGGGTGCGAGCGTTAATCGGAATTACTGGGCGTAAAGC
GTGCGCAGGCGGTTGTGTAAGACAGATGTGAAATCCCCGGGCTCAACCTGGGAACTGCGT
TTGTGACTGCACGACTAGAGTACGGCAGAGGGAGGTGGAATTCCACGTGTAGCAGTGAAA
TGCGTAGAGATGTGGAGGAACACCGATGGCGAAGGCAGCCTCCTGGGCCAGTACTGACGC

TCATGCACGAAAGCGTGGGGAGCAAACAGGATTAGATACCCTAGTAGTCC
>Otu1007
CCAGCCTACGGGTCGCAGCAGTGGGGAATTTTGCGCAATGGGGGAAACCCTGACGCAGCA
ACGCCGCGTGGAGGATGAAGTACTTCGGTACGTAAACTCCTTTCGATCGGGACGATAATG
ACGGTACCGGAAGAAGAAGCCCCGGCTAACTTCGTGCCAGCAGCCGCGGTAATACAGAGG
GTGCGAGCGTTAATCGGAATTACTGGGCGTAAAGCGCACGCAGGCGGCTCGATAAATTAG
AAGTGAAAGCCTTGGGCTTAACCTGAGAATTGCTTTTAAGACTGTCGAGCTAGAATCCAG
AAGAGGGTAGTGGAATTCCGAGTGTAGAGGTGAAATTCGTAGATATTCGGAAGAACACCA
GTGGCGAAGGCGACTACCTGGTCTGGCATTGACGCTCATGTGCGAAAGCGTGGGGAGCAA
ACAGGATTAGATACCCGAGTAGTCC
>Otu1008
CCAGCCTATGGGGTGCAGCAGTCGAGGATCTTCGGCAATGGGCGCAAGCCTGACCGAGCG
ACGCCGCGTGTGGGATGAAGGCCTTCGGGTTGTAAACCACTGTCAGTGGGGAGAAAATGC
TGGTGGGTACTCCCATCAGTTTGACCTATCCGCAGAGGAAGTGTGGGCTAAGTTCGTGCC
AGCAGCCGCGGTAAGACGAACCACACAAACGTTATTCGGAATCACTGGGCTTAAAGGGTG
CGTAGGCTGTGCAGAAAGTTGGGTGTGAAATACCTCGGCTCAACCGAGGAACTGCGCCCA
AAACTACTGTGCTCGAGGAAGACAGAGGTAAGCGGAACTGAGGGTGGAGCGGTGAAATGC
GTTGATATCCTCAGGAACACCCGTGGCGAAAGCGGCTTACTGGGTCTTTTCTGACGCTGA
TGCACGAAAGCTAGGGGAGCAAACGGGATTAGAAACCCCAGTAGTCC
>Otu1009
CCAGCCTACGGGGCGCTGCAGCAGTGGGGAATATTGCACAATGGGCGCAAGCCTGATGCA
GCGACGCCGCGTGAGGGATGACGGCCTTCGGGTTGTAAACCTCTTTCAGTACCGACGAAG
CGTGAGTGACGGTAGGTACAGAAGAAGCACCGGCCAACTACGTGCCAGCAGCCGCGGTAA
TACGTAGGGTGCGAGCGTTGTCCGGAATTACTGGGCGTAAAGAGCTCGTAGGTGGTTTGT
CGCGTTGTTCGTGAAAACTCACAGCTCAACTGTGGGCGTGCGGGCGATACGGGCAGACTG
GAGTACTGCAGGGGAGACTGGAATTCCTGGTGTAGCGGTGGAATGCGCAGATATCAGGAG
GAACACCGGTGGCGAAGGCGGGTCTCTGGGCAGTAACTGACGCTGAGGAGCGAAAGCGTG
GGGAGCGAACAGGATTAGAAACCCGAGTAGTCC
>Otu1010
CCAGCCTATGGGTGGCACCAGCAAGGAATATTGCGCAATGGGCGAAAGCCTGACGCAGCG
ACGCCGCGTGAGGGAAGAAGGCCTTCGGGTCGTAAACCTCTTTTCTTGGGGAAGAATAAT
GACGGTACCCAGGGAATAAGTCACGGCTAACTACGTGCCAGCAGCCGCGGTAATACGTAG
GCGGCGAGCATTATCCGGATTTATTGGGCGTAAAGCGGGTGTAGGTGGTTTTTCAAGTCA
GATGTGAAATTTCTCGGCTTAACCGGGAAGAGTCATTTGAGACTGTTAGACTTGAGGGCA
GGAGAAGGAGATGGAATTCCCGGTGTAGTGGTGAAATGCGTAGATATCGGGAGGAACACC
AGTGGCGAAAGCGGTCTCCCGGCCTGTTCCTGACACTGAGGCCCTAAAGCGTGGGGAGCA
AACAGGATTAGATACCCTTGTAGTCC
>Otu1011
CCAGCCTATGGGTCGCAGCAGTGGGGAATATTGGACAATGGGGGCAACCCTGATCCAGCC
ATGCCGCGTGAATGATGAAGGCCTTCGGGTTGTAAAGTTCTTTTGACAGGGACGATGATG
ACGGTACCTGTAGAATAAGCTCCGGCTAACTTCGTGCCAGCAGCCGCGGTAATACGAAGG
GAGCTAGCGTTGTTCGGAATTACTGGGCGTAAAGGGCGCGTAGGCGGCTTGGCAAGTTGG
GTGTGAGAGCCCAGGGCTCAACCCTGGAATTGCACTCAAGACTGCCTTGCTCGAATTAGG
TAGAGGTTGGCGGAATTCCCAGTGTAGAGGTGAAATTCGTAGATATTGGGAAGAACACCC
GTGGCGAAGGCGGCCAACTGGACCTATATTGACGCTGAGGCGCGAAAGCGTGGGGAGCAA
ACAGGATTAGATACCCGTGTAGTCC
>Otu1012
CCAGCCTATGGGTGGCAGCAGTGGGGAATATTGGACAATGGGCGAAAGCCTGATCCAGCA
ATGCCGCGTGGATGAAGAAGGTCTTCGGATTGTAAAGTCCTTTCGGCGGGGACGATGATG
ACGGTACCCGCAGAAGAAGCCCCGGCTAACTTCGTGCCAGCAGCCGCGGTAATACGAAGG
GGGCTAGCGTTGCTCGGAATGACTGGGCGTAAAGGGCGCGTAGGCGGTGCGGATAGTCAG
ATGTGAAATTCCTGGGCTTAACCTGGGGACTGCATTTGATACGTCCGCGCTTGAGTGTGG
AAGAGGGTCGTGGAATTCCCAGTGTAGAGGTGAAATTCGTAGATATTGGGAAGAACACCG
GTGGCGAAGGCGGCGACCTGGTCCATAACTGACGCTGAGGCGCGAAAGCGTGGGGAGCAA
ACAGGATTAGAAACCCCCGTTAGTCC
>Otu1015
CCAGCCTACGGGGGGCTCCAGTGGGGAATATTGGACAATGGGCGAAAGCCTGATCCAGCC
ATGCCGCGTGTGTGAAGAAGGTCTTCGGATTGTAAAGCACTTTAAGTTGGGAGGAAGGGT

TGTAGATTAATACTCTGCAATTTTGACGTTACCGACAGAATAAGCACCGGCTAACTCTGT
GCCAGCAGCCGCGGTAATACAGAGGGTGCAAGCGTTAATCGGAATTACTGGGCGTAAAGC
GCGCGTAGGTGGTTTGTTAAGTTGGATGTGAAATCCCCGGGCTCAACCTGGGAACTGCAT
CCAAAACTGGCAAGCTAGAGTATGGTAGAGGGTGGTGGAATTTCCTGTGTAGCGGTGAAA
TGCGTAGATATAGGAAGGAACACCAGTGGCGAAGGCGACCACCTGGATTGATACTGACAC
TGAGGTGCGAAAGCGTGGGGAGCAAACAGGATTAGATACCCTCGTAGTCC
>Otu1016
CCAGCCTACGGGAGGCAGCAGTAGGGAATATTGGACAATGAGCGAAAGCTTGATCCAGCA
ATATTAAATTAGTGAAGAAGACTGATTTTGTTGTAAAGCTTTTTCGTTAAAAAGGATAAT
GACGTTATTTAAAGGAAGAAGTCCTGGCCAATGCCGTGCCAGCAGCCGCGGAAAAACGGT
AGGGGCAAGTGTTATCCATATTGAATGGGTGTAAAGGGTGCGTAGATCGTTAAATTGGGT
ATTAAAAGTTTAATAGGCATATTAATATTATGGGTAATTTTTAATAAGATAACTTGAGTT
AATTTTAGGTAAAGAGAATTCTTAGTGTAAAAATAAAATTTTACAATATTAAGAGGAATA
TCGGGAGCGAAGGCGCTTTTCTGGAATTAAACTGACGTTGAGGCACGAAAGCATAGGGAT
CAAATGGGATTAGATACCCCCGTAGTCC
>Otu1017
CCAGCCTATGGGGGGCTGCAGTGGGGAATATTGGACAATGGGCGCAAGCCTGATCCAGCC
ATGCCGCGTGAGTGATGAAGGCCCTAGGGTTGTAAAGCTCTTTTGTGCGGGAAGATAATG
ACGGTACCGCAAGAATAAGCCCCGGCTAACTTCGTGCCAGCAGCCGCGGTAATACGAAGG
GGGCTAGCGTTGCTCGGAATCACTGGGCGTAAAGGGTGCGTAGGCGGTTTGGTAAGTCTT
TTGTGAAATCTATGGGCTCAACTCATAGACTGCAAGGGAAACTGCCGGGCTTGAGTGTGG
GAGAGGTGAGTGGAATTCCCGGTGTAGCGGTGAAATGCGTAGATATCGGGAGGAACACCT
GTGGCGAAAGCGGCTCACTGGACCACAACTGACGCTGATGCACGAAAGCGTGGGGAGCAA
ACAGGATTAGAAACCCTAGTAGTCC
>Otu1018
CCAGCCTATGGGGCGCTGCAGTAGGGAATATTGGGCAATGGAGGCAACTCTGACCCAGCC
ATACCGCGTGCAGGATGAAGGCCCTCTGGGTTGTAAACTGCTTTTATCAGGGAAGAAACG
CTAGGATTTATCTTAGCCTGACGGTACCTGAGGAATAAGCACCGGCTAACTCCGTGCCAG
CAGCCGCGGTAATACGGAGGGTGCAAGCGTTATCCGGATTTACTGGGTTTAAAGGGTGCG
TAGGCGGATTGTTAAGTCAGTGGTGAAAGATTCGAGCTCAACTCGGAAACTGCCATTGAT
ACTGATGATCTCGAATACAGTTGAGGTAGGCGGAATATTACATGTAGCGGTGAAATGCTT
AGATATGTAATAGAACACCGATTGCGAAGGCAGCTTGCTAAACTGTTATTGACGCTGAGG
CACGAAAGCGTGGGGATCAAACAGGATTAGAAACCCGTGTAGTCC
>Otu1020
CCAGCCTATGGGGTGCAGCAGTAAGGAATATTGGTCAATGGACGCAAGTCTGAACCAGCC
ATGCCGCGTGAAGGATGAAGGCCCTCTGGGTTGTAAACTTCTTTTATCTGGGACGAAACA
CTGTTTTTCTAAACAGCTTGACGGTACCAGAGGAATAAGCACCGGCTAACTCCGTGCCAG
CAGCCGCGGTAATACGGAGGGTGCAAGCGTTATCCGGATTCACTGGGTTTAAAGGGTGCG
TAGGCGGGTATGTAAGTCCGTGGTGAAATCCTGCAGCTTAACTGCAGAACTGCCGTGGAT
ACTATATATCTTGAATGTTGTTGAGGTGAGTGGAATATGTCATGTAGCGGTGAAATGCTT
AGAGATGACATAGAACACCGATTGCGAAGGCAGCTCGCTAAACAAATATTGACGCTGAGG
CACGAAAGCGTGGGTAGCAAACAGGATTAGATACCCTCGTAGTCC
>Otu1024
CCAGCCTATGGGAGGCTGCAGTGGGGAATTTTGCGCAATGGGGGAAACCCTGACGCAGCA
ACGCCGCGTGGAGGATGAAGTACTTCGGTACGTAAACTCCTTTCGATCGGGACGATAATG
ACGGTACCGGAAGAAGAAGCCCCGGCTAACTTCGTGCCAGCAGCCGCGGTAATACGAAGG
GGGCTAGCGTTGTTCGGAATTACTGGGCGTAAAGGGTGCGTAGGCGGGTCTTTAAGTCAG
GGGTGAAATCCTGGAGCTCAACTCCAGAACTGCCTTTGATACTGAGGATCTTGAGTACGG
GAGAGGTGAGTGGAACTGCGAGTGTAGAGGTGAAATTCGTAGATATTCGCAAGAACACCA
GTGGCGAAGGCGGCTCACTGGCCCGTTACTGACGCTGAGGCACGAAAGCGTGGGGAGCAA
ACAGGATTAGATACCCGCGTAGTCA
>Otu1025
CCAGCCTACGGGTTGCTCCAGGCGAGCGAACTCCACAATGCGCGCAAGCGTGATGGGGCA
ATTCGAAGTGCTATCTCTGATAAAGGTAGCTGTGGCGCGGCCTAAGGAGTCGCGCAAGAA
AGGTGAGGGCAAGGCTGGTGCCAGCAGCCGCGGTAAAACCAGCTCATCAAGCGCTCAGGA
CGATTATTGAGCTTAAAGCATTCGTAGCGGGCCCTCCAAGTCCCCGGTTAAATGCCAGTG
CCTAACATTGGACCTGCCGGGGATACTATTGGGCTTGGGAGTGGGAGACGCTGCGAGTAT
GGTCGGGGTACCGGTGAAATGGGATAATCCCGACTAGACTACCGGTGGCGAAGGCGAGCA

GCGAGAACACGTCCGACCGTGATGGATGAAAGCGTGGGGAGCGAACCGGATTAGAGACCC
CAGTAGTCC
>Otu1026
CCAGCCTACGGGTGGCAGCAGTAAGGAATATTGGTCAATGGGCGCAAGCCTGAACCAGCC
ATGCCGCGTGAGGGATGAAGGTCTTCGGATTGTAAACCTCTGTCACTGGGGAAGAAACGC
TTCAGGTTAATAGCCTGAAGCCTGACTTAACCCGGAGAGGAAGCAGTGGCTAACTCTGTG
CCAGCAGCCGCGGTAATACAGAGACTGCAAGCGTTATTCGGATTCACTGGGCGTAAAGGG
TGCGCAGGTGGCCAAGTGTGTGAGGCGTGAAAGCCCGGAGCTCAACTCCGGAATTGCGCC
TCAAACTACATGGCTAGAGCATTGGAGAGGGGAGCAGAATTCACGGTGTAGCAGTGAAAT
GCGTAGATATCGTGAGGAATACCAGAGGCGAAGGCGGCTCCCTGGACAATAGCTGACACT
CAGGCACGAAAGCGTGGGGAGCAAAAGGGATTAGATACCCCCGTAGTCC
>Otu1031
CCAGCCTACGGGTTGCTGCAGCCAAGAACATTTGGCAATGGGCGAAAGCCTGACCATGCG
ACGCCGCGTGGTTGATGAAGTCCTTCGGGACGTAAAAACCTTTTGCGCGTGAGGAAGTAA
TTGACGTTAGCGCGCGAATAAGGGGCTCCTAACTCTGTGCCAGCAGGAGCGGTAATACAG
AGGCCCCGAGCATTATCCGGAATCACTGGGCGTAAAGGGTGTGTAGGCGGCGATATTAGT
CTTTTGTTAAAGTCTTTGGGCTTAACCCAGAGAATGCAGGAGAAACGGTATCGCTTTGAG
GACGCGAGAGGTAAGGGGAACTCATGGTGTAGGGGTGAAATCCGTTGATATCATGGGGAA
CACCAAATGCGAAGGCACCTTACTGGCGCGCTCCTGACGCTGAAACACGAAAGCGTGGGT
AGCGAACGGGATTAGATACCCTGGTAGTCC
>Otu1034
CCAGCCTACGGGATGCTCCAGTAACGAATCTTCCGCAATGCACGAAAGTGTGACGGAGCG
ACGCCGCGTGTGGGACGAAGCCCTTCGGGGTGTAAACCACTGTCAGAGGATAGAAAGTTC
TGATCATCCTCAGAGGAAGGCACGGCTAACTCTGTGCCAGCAGCCGCGGTAAGACAGAGG
TGCCAAGCGTTAGGCGGAATCACTGGGCTTAAAGCGTGTGTAGGCGGAATCGTAAGTATC
TTGTGAAATCCCACGGCTCAACCGTGGAACTGCTTGATATACTGCGATTCTTGAGCTGCC
TAGGGGCGAGTGGAACAAATGGTGGAGCGGTGAAATGCGTAGATATCATTTGGAACGCCA
ATGGTGAAAACAACTCGCTGGGGGCATGCTGACGCTGAGACACGAAAGCCAGGGGAGCAA
ACGGGATTAGAAACCCGTGTAGTCC
>Otu1037
CCAGCCTATGGGAGGCAGCAGTAAGGAATTTTGGGCAATGAGGTAACCCTTGACCCGGCG
AGAATGCGTGCATGATGAATGCGGTCAGTTTTCTGTAAAGTGCTTTACGGAACGAAGATA
CTGACTGTAGTTCTTAGAAGAAGCACCGGCTAACTCCGTGCCAGCAGCTGCGGTAAGACG
GAGGGTGCGGGCCTTATTCATTATGATTGGGTGTAAAGGGTGCGTAGGTGGCTACTAACG
ACACTGATAGAAATTGCAGAGAGTTCCTATGCAGTTATCAGTGAACTCGAACGTACGCTA
GAGTTGGGTAATGGATCATGGCAAAATCTCACGTAAAGGTTGAATTTTCAGAGATGAGAT
TGAACACCGGCAGGCGAAGGCGCGTCTTCTGTCCCAACTGACACTCAGGCACGGAAGCTA
CGGGAGCAAACCGGATTAGAAACCCGCGTAGTCC
>Otu1038
CCAGCCTATGGGGGGCTCCAGTAAGGGATATTGCGCAATGGGCGAAAGCCTGATGCAGCA
ACGCCGCGTGGAGGAAGAAGGCCTTCGGGTCGTAAACTCCTTTTCTCACTGACGAGGAAG
GACGGTAGGTGAGGAATAAGTCACGGCTAACTACGTGCCAGCAGCCGCGGTAAAACGTAG
GTGGCAAGCGTTATCCGGATTTACTGGGCGTAAAGGGTGTGCAGGCGGGCGGTCAAGTGG
TGTATGAAATATCCCGGCTCAACCGGGAAGGGTTATGCCAGACTGACCGTCTTGAGTGCG
AGAGAGGGGCGTGGAATTCCGGGTGTAGTGGTGAAATGCGTAGAGATCCGGAGGAACCCC
AGAGGCGAAGGCGGCGCCCTGGCTCGCAACTGACGCCCATACACGACAGCATGGGGAGCG
AACGGGATTAGAAACCCTAGTAGTCC
>Otu1040
CCAGCCTACGGGGTGCTGCAGTGAGGAATTTTCCACAATGGACGCAAGTCTGATGGAGCA
ATGCTACATGAAGGAAGAAGGTCCTTTGGATTGTAAACTTCTGTTCATCAGGAAGAAAAA
AGTAAGCGAAAGTTTGCTCTTGACGGTACTGATGTAGAAAGCCACGACTAACTATGTGCC
AGCAGTCGCGGTAAGACATAGGTGGCGAACGTTATCCGGATTTATTGGGCGTAAAGGATG
CGTAGATGTTATTCATAAGTTATTAGTAGAAAATAGAAGCTCAACTTCTGGCGGCTAATA
ATACTATAATAATTGAGGGCAGAGGAGGTCGACGGAATTCTGTGTGGAGCGGTGAAATGC
TTAGATATACAGAGGAACACCAAAATAGGCGAAGGCAGTTAACTATTCTGTTCCTGACAT
TGAGGCATGAAAGCGTGGGGAGCAAAACGGATTAGAAACCCGAGTAGTCC
>Otu1042
CCAGCCTATGGGGGGCTCCAGTCGAGGATCTTTCGCAATGGGCGCAAGCCTGACGAAGCG

ACGCTGCGTGAGCGATGAAGGCCTTCGGGTCGTAAAGCTCTTTCGCGCAAGAACAAGGAA
AGGATGTGAACAACATTCAGACTTGAGGGTACTGCGTAAAGAAGCACCGGCTAACTCCGT
GCCAGCAGCTGCGGTAATACGGAGAGTGCAAGCATTAATCGGAATTATTGGGCGTAAAGG
GCGCGTAGGCTGAAGGATAAGTCAGATGTGAAATTCCGAAGCTCAACTTCGGAGCTGCAT
TTGAAACTATCTTTCTAGAGGATTGACGGAGAAAACGGAATTCCACGTGTAGCGGTGAAA
TGCGTAGATATGTGGAAGAACACCTGTGGCGAAAGCGGTTTTCTAGTTTATACCTGACGC
TGAGGCGCGAGAGCAAGGGGAGCAAACAGGATTAGATACCCGGGTAGTCC
>Otu1043
CCAGCCTACGGGGCGCACCAGTCAAGAATCTTCCACAATGGACGAAAGTCTGATGGAGCG
ACGCCGCGTGATTGATGAAGTGGTTCGCCACGTAAAGATCTTTTATGAGGGAAGAAGTAT
ATTGACGGTACCTCATGAATAAGGGGCTCCTAACTCTGTGCCAGCAGGAGCGGTAATACA
GAGGCCCCGAGCGTTATCCGGAATTATTGGGCGTAAAGGGTGTGTAGGTGGCATTGTTAG
TCGTTTGTGAAATCCCGGGGCTTAACCTCGGAAACGCGAACGAAACGGCAAAGCTTGAGT
ATGTGAGAGGTAAGCGGAACTCATGGTGTAGGGGTGAAATCCGTTGATATCATGGGGAAC
ACCAAAAGCGAAGGCAGCTTACTGGCACATTACTGACACTGAAACACGAAAGCGTGGGGA
TCAAACAGGATTAGATACCCTCGTAGTCC
>Otu1044
CCAGCCTACGGGGTGCTGCAGTCGAGAATCTTTCGCAATGGGCGAAAGCCTGACGAAGCG
ACGCTGTGTGAGCGAAGAAGGCCTTCGGGTTGTAAAGCTCTTTCGCTAGGGAACAAGAAA
GTCGGATTAATAATCCGATAATTTGATGGTACTTGGTAAAGAAGCACCGGCTAACTCCGT
GCCAGCAGCTGCGGTAATACGGAGGGTGCAAGCATTGATCGGAATTACTGGGCGTAAAGG
GCGCGTAGGCGGTTAGGAAAGTCAGATGTGAAATTCCGGGGCTCAACCCCGGAGCTGCAT
TTGAAACTTCCAGACTAGAGTTCAGTTAGGGAAAACAGAATTCCACGTGTAGCGGTGAAA
TGCGTAGATATGTGGAAGAATACCGGTGGTGAAGACGGTTCTCTGGGCTGATACTGACGC
TGAGGCGCGAAAGCAAGGGGAGCAAACAGGATTAGAAACCCCCGTAGTCC
>Otu1045
CCAGCCTATGGGGCGCACCAGTGGGGAATATTCCGCAATGGGCGAAAGCCTGACGGAGCG
ACGCCGCGTGGATGATGAAGGCCTTCGGGTTGTAAAATCCTGTAGAGGGGGAAGAAGGGT
GGCGTAAGCCATTTGACAGTACTCCTAAAGTAAGCTACGGCAAACTCTGTGCCAGCAGCC
GCGGTAATACAGAGGTAGCTAGCGTTGTTCGGAATGACTGGGTGTAAAGCGCGTGTAGGC
GGTTTTGTAAGTTGAATGTGAAATCCCTTGGCTCAACCAAGGAACGGCATTCAAAACTGC
ATTACTTGAGTGCGGAAGAGGAAAGTGGAATTTCTGGTGTAGCGGTGAAATGCGTAGATA
TCAGAAGGAACACCGGTGGCGAAGGCGACTTTCTGGTCCGCAACTGACGCTGAGACGCGA
AAGCTAGGGGAGCAAACAGGATTAGATACCCGTGTAGTCC
>Otu1047
CCAGCCTATGGGTCGCTGCAGTGAGGAATCTTGCGCAATGGGCGAAAGCCTGACGCAGCA
ACGCCGCGTGTGGGACGAAGGCCTTCTGGGTTGTAAACCACTTTCAGCAGGGACGATTGT
GACGGTACCTGCAGAAGAAGCCCCGGCCAACTACGTGCCAGCAGCCGCGGTAATACGTAG
GGGGCGAGCGTTGTCCGGAATTATTGGGCGTAAAGAGCTCGTGGGCGGCTTGGCAAGTCA
CGTGTGAAATCTCCAGGCTCAACCTGGTGATGCCACGTGATACTGCCATGGCTTGAGTCC
GGTAGGGGACCATGGAATTCCTGGTGTAGCGGTGAAATGCGCAGATATCAGGAGGAACAC
CAGTGGCGAAGGCGGTGGTCTGGGCCGGTACTGACGCTGAGGAGCGAAAGCGTGGGGAGC
GAACAGGATTAGAGACCCTCGTAGTCC
>Otu1048
CCAGCCTACGGGATGCTGCAGTGGGGAATTTTGCGCAATGGGGGAAACCCTGACGCAGCA
ACGCCGCGTGAGTGATGAAGGCTTTCGGGTCGTAAAGCTCTGTCAGTAGGGAAGAAATGG
GAACGGTTAATACCCGTTTTCCTTGACGGTACCTACAAAGGAAGCACCGGCTAACTCCGT
GCCAGCAGCCGCGGTAATACGGAGGGTGCAAGCGTTGTTCGGATTTATTGGGCGTAAAGC
GCGTGTAGGCGGTTTTTTAAGTCTGATGTGAAAGCCCCGGGCTCAACCCGGGAAGTGCAT
TGGATACTGGAAGACTTGAATACGGGAGAGGGTAGTGGAATTCCTAGTGTAGGAGTGAAA
TCCGTAGATATCAGGAGGAACACCGGTGGCGAAGGCGGCTGCCTGGACCGATATTGACGC
TGAGACGCGAAAGCGTGGGGAGCAAACAGGATTAGAAACCCCTGTAGTCC
>Otu1051
CCAGCCTATGGGGTGCTGCAGTAAGGAATATTGGTCAATGGGCGAAAGCCTGAACCAGCC
ATGCCGCGTGGAGGATGAAGGTCCTCTGGATTGTAAACTTCTTTTATCTGGGACGAAAAA
AGGGAATTCTTTCTCACTTGACGGTACCAGATGAATAAGCACCGGCTAACTCCGTGCCAG
CAGCCGCGGTAATACGGAGGGTGCAAGCGTTATCCGGATTCACTGGGTTTAAAGGGTGCG
TAGGTGGGCAGGTAAGTCAGTGGTGAAATCTCTGGGCTTAACCCAGAAACTGCCGTTGAT

ACTATCTGTCTTGAATGTAGTGGAGGTGAGCGGAATATGTCATGTAGCGGTGAAATGCTT
AGATATGACATAGAACATCTATTGCGAAGGCAGCTCGCTACACTATTATTGACACTGAGG
CACGAAAGCGTGGGGATCAAACAGGATTAGATACCCTTGTAGTCC
>Otu1053
CCAGCCTACGGGGGGCAGCAGTGGGGAATATTGGGCAATGGGCGAAAGCCTGACCCAGCA
ACGCCGCGTGAAGGAAGAAGGCTTTCGGGTTGTAAACTTCTTTGACGAGGGACGAAAAAA
ATGACGGTACCTCGAAAACAAGCCACGGCTAACTACGTGCCAGCAGCCGCGGTAATACGT
AGGTGGCAAGCGTTATCCGGATTTACTGGGTGTAAAGGGCGCGTAGGCGGGATTGCAAGT
CAGATGTGAAATGCGGGGACTTAACCTCCGAACTGCATTTGAAACTGTGATTCTTGAGTA
TCGGAGAGGCAGGCGGAATTCCTAGTGTAGCGGTGAAATGCGTAGATATTAGGAGGAACA
CCAGTGGCGAAGGCGGCCTGCTGGACGACAACTGACGCTGAGGCACGAAAGCTGGGGGAG
CAAACCGGATTAGATACCCCAGTAGTCC
>Otu1054
CCAGCCTACGGGTCGCACCAGTGGGGAATTTTACACAATGGGCGAAAGCCTGATGTAGCG
ACACCGCGTGAGCGAAGAAGCCCTTTGGGGTGTAAAGCTCTGTCAACAGGGACGAAAAAA
ATGACGGTACCTGTGGAGGAAGCATCGGCTAACTACGTGCCAGCAGCCGCGGTAAGACGT
AGGATGCGAGCGTTGTCCGGATTTATTGGGCGTAAAGAGTTCGTAGGCGGTTCGTTAAGT
CTGATGTAAAAGGCACGGGCTCAACCCGTGTATCGCATTAGATACTGGCGGACTAGAGTG
CAGTAGAGGCAAGCGGAATTCCCAGTGTAGCGGTGAAATGCGTAGATATTGGGAAGAACA
CCGGTGGCGCAAGCGGCTTGCTGGGCTGTAACTGACGCTGAGGAACGAAAGCTAGGGTAG
CGAATGGGATTAGATACCCGCGTAGTCG
>Otu1055
CCAGCCTACGGGGGGCAGCAGTGGGGAATCTTGCGCAATGGGCGAAAGCCTGACGCAGCA
ACGCCGCGTGTGTGATGAAGGTCTTCGGATCGTAAAGCACTGTCGCGAGGGACGAGAAGC
CGCTTACAAATAGTGAGTGGATAGGACGGTACCTCGAGAGGAAGCACCGGCTAACTCTGT
GCCAGCAGCCGCGGTAATACAGAGGGTGCGAGCGTTGTTCGGAATTATTGGGCGTAAAGC
GCTTGTAGGCGGCACTATAAGTCCACTGTGAAAGCCCTTGGCTCAACCAAGGAAGAGCAG
TGGATACTGTAGAGCTTGAATGCCGGAGAGGGTCGCGGAATTCCCGGTGTAGAGGTGAAA
TTCGTAGATATCGGGAGGAACACCAGTGGCGAAGGCGGCGACCTGGACGGACATTGACGC
TGAGACGCGAAAGCGTGGGGAGCAAACAGGATTAGATACCCGCGTAGTCC
>Otu1057
CCAGCCTATGGGTCGCTGCAGTGGGGAATATTGGACAATGGGGGCAACCCTGATCCAGCG
ACGCCGCGTGTGTGAAGAAGGCCTGCGGGTTGTAAAGCACTTTTAGTGGGGACAAAAGGC
TACGGACTAATACTCTGTGGTCTTGATTTAACCCAAAGAAAAAGCACCGGCTAACTCTGT
GCCAGCAGCCGCGGTAATACAGAGGGTGCGAGCGTTAATCGGAATTACTGGGCGTAAAGC
GTGCGTAGACGGTTCTGTAAGTCGGATGTGAAATCCCCGGGCTCAACCTGGGAATTGCAT
TCGAAACTGCAAAGCTTGGGTGCGGAAGAGGGAAGCGGAATTTCCGGTGTAGCGGTGAAA
TGCGTAGATATCGGAAGGAACACCAGTGGCGAAAGCGACTTCCTGGTCGAATACTGACGT
TCATGTGCGAAAGCGTGGGGAGCAAACAGGATTAGATACCCTTGTAGTCC
>Otu1058
CCAGCCTATGGGTGGCTCCAGTGAGGGATTTTGCACAATGGAGGAAACTCTGATGCAGCG
ACGCCGCGTGCGGGACGAAGCCTTTCGGGGTGTAAACCGCTGTCAGTGGAGAAGAGTAGG
GACGGTACCCACTGAGGAAGCATCGGCTAACTACGTGCCAGCAGCCGCGGTAAGACGTAG
GGTGCAAGCGTTGTCCGGAATCACTGGGCGTAAAGAGCTCGTAGGCGGGGTCTTGCGTCG
TGGAGTAAATTTTTCTGCTTAACGGAGAAATCTTCCGCGATACGGGGACTCTTGAGGACA
GGAGAGGAAAGCGGAATTCCGAGTGTAGCGGTGAAATGCGTAGATATTCGGAGGAACACC
GGTGGCGAAGGCGGCTTTCTGGCCTGATTCTGACGCTGAGGAGCGAAAGCCAGGGTAGCG
AACGGGATTAGAAACCCCAGTAGTCC
>Otu1060
CCAGCCTATGGGTGGCAGCAGTCAAGAAACTTCCACAATGGACGAAAGTCTGATGGAGCG
ACGCCGCGTGGTTGATGAAGTCCTTCGGGACGTAAAGACCTTTTATGAGGGAGAAAGTAA
TTGATGTTACCTCATGGATAAGGGGCTCCTAACTCTGTGCCAGCAGGAGCGGTAATACAG
AGGCCCCAAGCATTATCCGGAATCACTGGGCGTAAAGGGTGTGTAGGCGGTCGTATTAGT
CTTTTGTTAAAGTTCTGGGGCTTACCCTCGGAAGTGCAAGGGAAACGGTACGACTAAGAG
GATGCGAGAGGTAAAGGGAACTCATGGTGTAGGGGTGAGATCCGTTGATATCATGGGGAA
CACCAAATGCGAAGGCACTTTACTGGCGCACTCCTGACGCTGAAACACGAAAGCGTGGGA
ATCGAACGGGATTAGAGACCCTAGTAGTCC
>Otu1062

CCAGCCTACGGGGGGCAGCAGTGGGGAATATTGCGCAATGGGCGAAAGCCTGACGCAGCG
ACGCCGCGTGGGGGATGAAGGCCTTCGGGTTGTAAACCTCTTTCAGCGCCGAAGAAGCGA
AAGTGACGGTAGGCGCAGAAGAAGCACCGGCTAACTACGTGCCAGCAGCCGCGGTAATAC
GTAGGGTGCAAGCGTTGTCCGGAATTATTGGGCGTAAAGAGCTCGTAGGCGGTTTGTCGC
GTCTGCTGTGAAAACTCGGGGCTTAACTCCGGGCCTGCAGTGGGTACGGGCAGACTAGAG
TGCGGTAGGGGAGACTGGAATTCCTGGTGCAGCGGTGAAATGCGCAGATATCAGGAGGAA
CACCGGTGGCGAAGGCGGGTCTCTGGGCCGTAACTGACGCTGAGGAGCGAAAGCGTGGGG
AGCGAACAGGATTAGAAACCCGAGTAGTCC
>Otu1064
CCAGCCTACGGGGTGCACCAGTGGGGAATCTTGCGCAATGGACGAAAGTCTGACGCAGCG
ACGCCGCGTGGGTGATGAAGGCCTTCGGGTTGTAAAGCCCTGTGGGGCGGGACGAATTAG
TAGAAGCCAACATCTTCTATGATGACGGTACCGCCTTAGCAAGCACCGGCTAACTCTGTG
CCAGCAGCCGCGGTAAGACAGAGGGTGCAAACGTTGTTCGGAATTACTGGGCGTAAAGCG
TGTGTAGGCGGTCATGTAGGTCGGATGTGAAAGCCCCAGGCTCAACCTGGGAAGTGCACC
CGAAACCGCACGACTTGAGTATCGGAGAGGTTGGTGGAATTCTCGGTGTAGAGGTGAAAT
TCGTAGATATCGAGAGGAACACCGGCGGCGAAGGCGGCCAACTGGACGAATACTGACGCC
GAGACACGAAAGCGTGGGGAGCAAACAGGATTAGATACCCGTGTAGTCC
>Otu1068
CCAGCCTATGGGGTGCTCCAGTCGAGAATCTTTTGCAATGCTCGAAAGAGTGACAATGCG
ACGCCGCGTGTGCGATGAAGGTCTTCGGATCGTAAGGCACTGTCAGCCGGGAGGAAGGTC
TTTCCCTTTAATAAAGGGGAAGGTTTGACAGTACCGGCAGAGGAAGCCACGGCTAACTCC
ATGCCAGCAGCCGCGGTAATACGGAGGTGGCAAGCGTTACTCGGATTGATTGGGTGTAAA
GGGCGTGTAGGTGGTGAATTAAGTCGGGTGTGAAATCCCTTGGCTCAACCAAGGAACTGC
ACCCGATACTGATTTGCTTGAGCGACAGAGAGGTAAGTGGAATTCCCGGTGTAACAGTGA
AATGTGTAGATATCGGGAGGAACACCAGTGGCGAAGGCGACTTACTGGATGTCTGCTGAC
ACTGAAACGCGAGAGCTAGGGGAGCAAACAGGATTAGATACCCTAGTAGTCC
>Otu1074
CCAGCCTATGGGTGGCACCAGTCGAGAATCTTCGGCAATGGGCGCAAGCCTGACCGAGCG
ACGCCGCGTGTGGGACGAAGTTCTTCGGAATGTAAACCACTGTCAGGGGTTAGAAAGTTC
TGATCAACCCCAGAGGAAGGCACGGCTAACTCTGTGCCAGCAGCCGCGGTAATACAGAGG
TGCCAAGCGTTAGGCGGAATCACTGGGCTTAAAGCGTGTGTAGGCGGAATTCTAAGTGTC
TTGTGAAATCCCACGGCTCAACCGTGGAACTGCTCGATATACTGGAATTCTTGAGCCACT
TAGGGGCGACCGGAACAAATGGTGGAGCGGTGAAATGCGTAGATATCATTTGGAACGCCA
ATGGTGAAAACAGGTCGCTGGGAGTGTGCTGACGCTGAGACACGAAAGCTAGGGGAGCAA
ACGGGATTAGATACCCCAGTAGTCC
>Otu1075
CCAGCCTATGGGGTGCTGCAGTCGAGGATCTTCGGCAATGGGCGCAAGCCTGACCGAGCG
ACGCCGCGTGCGGGATGAAGGCCTTCGGGTTGTAAACCGCCGTCGGAGTGGAGGAAATTT
CAAGGGGTACTCCCTTTGAATTGACTAATACTCAGAGGAAGGACGGGCTAAGTTCGTGCC
AGCAGCTGCGGTAAGACGAACCGTCCAAACGTTATTCGGAATCACTGGGCTTAAAGGGTG
CGTAGGCGGCTCAGAAAGTAGGGTGTGAAAGCCCTCGGCTCAACCGAGGAATTGCGCCCT
AAACTACTGGGCTCGAGGAAGACAGAGGTAAGCGGAACTTACGGTGGAGCGGTGAAATGC
GTCGATATCGTAAGGAACACCAGGAGCGAAAGCGGCTTACTGGGTCTTTTCTGACGCTGA
GGCACGAAAGCTAGGGGAGCGAACGGGATTAGATACCCCAGTAGTCC
>Otu1077
CCAGCCTATGGGGGGCACCAGTCGAGAATTTTTCACAATGGGCGCAAGCCTGATGGAGCG
ACGCCGCGTGGGGGATGAATGGCTTCGGCCCGTAAACCCCTGTCATTCGCGAACAATGCC
TGCGGCTTAATACGTTGCAGGTTGATAGTAGCGGAAGAGGAAGGGACGGCTAACTCTGTG
CCAGCAGCCGCGGTAATACAGAGGTCCCAAGCGTTGTTCGGATTCACTGGGCGTAAAGGG
TGCGTAGGTGGTCGGGTAAGTCTGACGTGAAATCTCCGAGCTTAACTCGGAAACGGCGTC
GGATACTATTCGGCTCGAGGGTTGGAGGGGGGACTGGGATACTTGGTGTAGCAGTGAAAT
GCGTAGATATCAAGTGGAACACCAGTGGCGAAGGTGAGTCCCTGGACAACTCCTGACGCT
GAGGCACGAAAGCTAGGGGAGCAAACAGGATTAGATACCCCAGTAGTCC
>Otu1078
CCAGCCTATGGGTCGCTCCAGTGGGGAATTTTGCGCAATGGGGGAAACCCTGACGCAGCA
ACGCCGCGTGGAGGATGAAATCCCTTGGGATGTAAACTCCTTTCGATCGGGAAGATAATG
ACGGTACCGAGAGAAGAAGCCCCGGCTAACTTCGTGCCAGCAGCCGCGGTAATACGAGGG
GGGCAAGCGTTGTTCGGAATTATTGGGCGTAAAGGGTGCGTAGGCGGTTTGATAAGTCAC

ATGTGAAATCTTCGGGCTCAACCCGAAGTCTGCATGCGAAACTGTCGGGCTTGAGTATGG
GAGAGGTGAATGGAATTCCTGGTGTAGCGGTGAAATGCGTAGATATCAGGAGGAACACCT
GTGGCGAAAGCGGCTCACTGGACCATCACTGACGCTGAAACACGAAAGCTAGGGGAGCAA
ACAGGATTAGATACCCTCGTAGTCC
>Otu1082
CCAGCCTATGGGGGGCAGCAGTAAGGAATCTTCCACAATGGACGAAAGTCTGATGGAGCA
ATGCCGCGTGCAGGATGACGGCCTTCGGGTTGTAAACTGCTTTTATATGGGAAGATTATG
ACGGTACCATATGAATAAGGACCGGCTAACTACGTGCCAGCAGCCGCGGTAATACGTAGG
GTCCAAGCGTTATCCGGAATCACTGGGCGTAAAGAGTGTGTAGGCGGGAGTTCGCGTGCA
ATGCGAAATCGTGTGGCTCAACCATATCGACTGTATTGCATACGGGACACCTAGAGGATA
TGAGAGGCAAGTGGAATTGGTAGTGTAGCAGTGAAATGCGTAGATATTACCAGGAACACC
AATGGCGAAGGCAGCTTGCTGGCATATTTCTGACGCTGAGACACGAAAGCGTGGGGAGCA
AACAGGATTAGAAACCCTAGTAGTCC
>Otu1083
CCAGCCTATGGGTTGCTGCAGTGAGGAATATTGCGCAATGGCCGAAAGGCTGACGCAGCG
ACGCCGCGTGGAGGATGAAGTCCGTTAGGATGTAAACTCCTTTTGCAGGGGATGAATGTG
CCCTTTCAGGGCATTGACCGTACCTTGCGAATAGGGATCGGCTAACTACGTGCCAGCAGC
CGCGGTAATACGTAGGATCCGAGCGTTGTCCGGAATTACTGGGTGTAAAGGGCGTGTAGG
TGGGCTTTTAAGTCGGTAGTGAAATCCCGCAGCTCAACTGCGGAATTGCTTCCGATACTG
GAAGTCTTGAGTACGGCAGAGGGAGATGGAATTCATGGTGTAGCGGTGAAATGTGTAGAT
ATCATGAAGAACACCAGTAGCGAAGGCGGTCTCCTGGTCCGTTACTGACACTGGGGCGCG
AAAGCGTGGGGAGCAAACAGGATTAGATACCCGTGTAGTCC
>Otu1084
CCAGCCTATGGGTGGCTCCAGTCGAGAATTTTTCACAATGGGGGAAACCCTGATCCAGCG
ACGCCGCGTGTGTGAAGAAGGCCTGCGGGTTGTAAAGCACTTTTAGTGGGGATGAAATGT
GCAGGGCTAATACCTCTGCATTTTGACCTAACCCACAGAAAAAGCACCGGCTAACTCTGT
GCCAGCAGTCGCGGTAATACAGAGGGTGCAAGCGTTAATCGGAATTACTGGGCGTAAAGC
GTGCGCAGACGGTTACATAAGTCGGGTGTGAAAGCCCCGGGCTCAACCTGGGAATTGCAT
TCGAGACTGTGTAGCTAGGGTACGGAAGAGGGAAGCGGAATTTCAGGTGTAGCGGTGAAA
TGCGTAGATATCTGAAGGAACACCAGTGGCGAAAGCGGCTTCCTGGTCCAGTACCGACGT
TCAGGCACGAAAGCGTGGGGAGCAAACAGGATTAGAAACCCGCGTAGTCC
>Otu1085
CCAGCCTATGGGTGGCAGCAGTGGGGAATATTGGACAATGGGCGCAAGCCTGATCCAGCC
ATGCCGCGTGAGTGATGAAGGCCTTAGGGTTGTAAAGCTCTTTCGACGGGGACGATAATG
ACGGTACCCGTAGAAGAAGCCCCGGCTAACTTCGTGCCAGCAGCCGCAGTAATACGAAGG
GGGCTAGCGTTGTTCGGAATCACTGGGCGTAAAGCGCACGCAGGCGGATCGTTAAGTCAG
GGGTGAAATCCCGAGGCTCAACCTCGGAACTGCCTCTGATACTGGCGATCTTCGAGTCCG
GGAGAGGTGAGTGGAACCCCGAGTGTAGAGGTGAAATTCGTAGATATTCGGAAGAACACC
AGTGGCGAAGGCGGCTCACTGGCCCGGTACTGACGCTCAGGTGCGAAAGCGTGGGGAGCA
AACAGGATTAGAAACCCCTGTAGTCC
>Otu1086
CCAGCCTATGGGTTGCACCAGTGGGGAATCTTGCGCAATGGACGAAAGTCTGACGCAGCC
ACGCCGCGTGAGTGAAGAAGGCCTTCGGGTTGTAAAGCTCTGTCGGAGGGGACAAAAACG
TTTAGGGTTAATAGCCCTAGATCTTGATGGTACCCTTAAAGGAAGCACCGGCTAACTTCG
TGCCAGCAGCCGCGGTAATACGAAGGGTGCAAGCGTTGCTCGGAAGTATTGGGCGTAAAG
GGTAGGTAGGTGGTTACGTATGTCTGGGGTGAAATCCCTGAGCTCAACTCAGGAAGTGCC
TTGGAAACGGCGTAACTAGAGTACTGGAGAGGTTCGTAGAATTCCTGGTGTAGCGGTGAA
ATGCGTAGAGATCAGGAGGAATACCAGAGGCGAAGGCGGCGAACTGGACAGTTACTGACA
CTCAACTACGAAAGCGTGGGGAGCAAACAGGATTAGATACCCCCGTAGTCC
>Otu1090
CCAGCCTATGGGAGGCAGCAGTCGAGAATTTTTCACAATGGACGAAAGTCTGATGGAGCG
ACGCCGCGTGGAGGATGAAGGTTTTCGGATTGTAAACTCCTGTCACTGCAGAACAAGGAT
GCATCAGTTAACAGCCGATGCATTTGATGGTATGCGGAGAGGAAGGGACGGCTAACTCTG
TGCCAGCAGCCGCGGTAAGACAGAGGTCCCGAGCGTTGTTCGGATTCATTGGGCGTAAAG
GGTGTGTAGGAGGTCGGGTAAGTCAGGTGTGAAATCTCAGAGCTTAACTCTGAAACTGCG
CTTGATACTGCTCGGCTAGAGGATCGGAGGGGGTAACGGAATTTACGGTGTAGCAGTGAA
ATGCGTAGATATCGTAAGGAAGACCAGTGGCGAAGGCGAATCTCTGGACAGTTACTGACA
CTGAGGCACGAAGGCCAGGGGAGCAAACAGGATTAGAAACCCCCGTAGTCC

>Otu1095
CCAGCCTACGGGTTGCAGCAGCTACATGTGCGCGCGCGCCGCTGATGTCGCCCACCTCTT
CAGCGGCGCACAATGAGCGCGCCGCCGGAGGATGGTCCTGTGAAACGAACTGAGGGCCGA
GTTGTATGACATCGGGTTAGGCGCGTTATTGGCGTTGGTCGGAATACTATTCCTTCGGTT
TCGGCGCGACATCGGGGATGCGCTCAGCGGGAGGAGCTCTACGCCATTGGGCCCGCAGCC
CGCGAGACGACGAAATACAACTTACAACCCGATTTTGGGCGGCATTTTGTTTCTCTCCGC
TGGCATTTTTTGCGCCATTTGGGGTGTCGTAGCTCTCGTCACCAGTCACTGAATGTGCCA
GGCAACTGCACAACAGGCTTCTGCCACGACGATTTCGAAGCGATTCGCGTTAGCCCGCAC
CCTCGCGTCTGCGGATTAGATACCCTAGTAGTCC
>Otu1096
CCAGCCTACGGGATGCACCAGTGGGGAATATTGGACAATGGGCGCAAGCCTGATCCAGCA
ATGCCGCGTGAGTGATGAAGGCCTTAGGGTTGTAAAGCTCTTTCGCACGCGACGATGATG
ACGGTAGCGTGAGAAGAAGCCCCGGCTAACTTCGTGCCAGCAGCCGCGGTAATACGAAGG
GGGCGAGCGTTGTTCGGAATTACTGGGCGTAAAGGGCGCGCAGGCGGCCCTTCAAGTCAG
GCGTGAAAGCCCCGGGCTTAACCTGGGAAATGCGCTTGAGACTGGAAGGCTTGAGTTCGG
GAGAGGATAGTGGAATTCCCAGTGTAGAGGTGAAATTCGTAGATATTGGGAAGAACACCG
GTGGCGAAGGCGGCTATCTGGACCGATACTGACGCTGAGGCGCGAAAGCGTGGGGAGCAA
ACAGGATTAGAAACCCTAGTAGTCC
>Otu1097
CCAGCCTATGGGTTGCACCAGTAAGGAATATTGGACAATGGAGGCAACTCTGATCCAGCC
ATGCCGCGTGAAGGATGAAGGCGCTACGCGTTGTAAACTTCTTTTCCCTTAGAAAAAACT
CTTGGTTTCGTACCGAGACTGATTGTACAAGAGGAATAAGCATCGGCTAACTTCGTGCCA
GCAGCCGCGGTAATACGAAGGATGCAAGCGTTATCCGGATTCACTGGGTTTAAAGGGTGT
GCAGGTGGGGTAATAAGTCAGTGGTGATAGCCCCCGACTCAATCGGGGAACTGCCATAGA
TACTGTTATTCTTGAGTGTAGTTGAGGCGGATGGAATATGACATGTAGCGGTGAAATGCT
TAGATATGTCATAGAACACCGATCGCGAAGGCAGTCCGCCAAACTATTACTGACACTGAA
ACACGAAAGTGCGGGGATCAAACAGGATTAGAACCCGCGTAGTCC
>Otu1099
CCAGCCTATGGGGTGCACCAGTGGGGAATATTGGACAATGGGCGCAAGCCTGATCCAGCC
ATGCCGCGTGGATGATGAAGGCCCTAGGGTTGTAAAGCTCTTTTGGCGGGGACGATGATG
ACGGTACCCGCAGAATAAGCCCCGGCTAACTTCGTGCCAGCAGCCGCGGTAAGACGAAGG
GGGCTAGCGTTGTTCGGAATTACTGGGCGTAAAGCGTGTGTAGGTGGTTGTCCAAGTTGG
ATGTGAAAGCCTTGAGCTCAACTCAAGAAATGCATTCAGGACTGGATGGCTAGAGGACCG
GAGAGGATAGTGGAATTCCCAGTGTAGTGGTGAAATACGTAGAGATTGGGAAGAACACCA
GTGGCGAAGGCGGCTATCTGGACGGTTTCTGACACTAAGACACGAAAGCGTGGGGAGCAA
ACAGGATTAGAGACCCTAGTAGTCC
>Otu1100
CCAGCCTACGGGTGGCAGCAGTGGGGAATATTGGACAATGGGCGCAAGCCTGATCCAGCA
ATGCCGCGTGTGTGAAGAAGGCCTGCGGGTTGTAAAGTACTTTCGGTGGGGAGAAAGAGG
CTATCACTAATACTGATAGCGGATGATGTTACCCACAGAAGAAGCACCGGCTAACTCTGT
GCCAGCAGCCGCGGTAATACAGAGGGTGCGAGCGTTAATCGGAATTACTGGGCGTAAAGG
GTGCGTAGGTGGTTGCGTAAGTTAGGTGTGAAAGCCCCGGGCTTAACCTGGGAATTGCGC
TTGATACTGCGTGGCTAGAGTACTGTAGAGGAAGGTGGAATTTCCAGTGTAGCGGTGAAA
TGCGTAGATATTGGAAGGAACATCGGTGGCGAAGGCGACCTTCTGGACAGATACTGACAC
TGAGGCACGAAAGCATGGGGAGCAAACAGGATTAGATACCCCCGTAGTCC
>Otu1102
CCAGCCTATGGGTGGCAGCAGTGGGGAATTTTGTGCAATGGGGGAAACCCTGACACAGCG
ACACCGCGTGAGCGAAGAAGCCCTTTGGGGTGTAAAGCTCTGTCGGCTGGAACGAAAAAA
ATGACGGTACCAGCAGAGGAAGCATCGGCTAACTACGTGCCAGCAGCCGCGGTAAGACGT
AGGATGCGAGCGTTGTCCGGATTTATTGGGCGTAAAGAGTTCGTAGGTGGTTTGTTAAGT
CTGATGTTAAAGACTGAGGCTCAACCTCGGAAATGCATTGGATACTGGCAGACTGGAGTG
CAGTAGAGGCAAGTGGAATTCCCAGTGTAGCGGTGAAATGCGTAGATATTGGGAAGAACA
CCAGTGGCGTAAGCGACTTGCTGGGCTGTAACTGACACTGAGGAACGAAAGCCAGGGGAG
CGAATGGGATTAGAAACCCTCGTAGTCC
>Otu1105
CCAGCCTATGGGTGGCAGCAGTGAGGAATCTTCCACAATGGACGAAAGTCTGATGGAGCA
ACGCCGCGTGCAGGATGACGGCTTTCGGGTTGTAAACTGCTTTTACTTGGGAAGATTATG
ACGGTACCAAGTGAATAAGGACCGGCTAACTACGTGCCAGCAGCCGCGGTAATACGTAGG

GTCCAAGCGTTATCCGGAATTACTGGGCGTAAAGCGTGCGCAGGTGGCTCGTTAAGTGAG
ACATTAAATCCAGCGGCTCAACCGTTTGGCTGTGTTTCATACTGGCGGGCTAGAGGATGG
CAGAGGCAAGTGGAATTTGTAGTGTAGCAGTGAAATGCGTAGATATTACAAGGAACACCA
ATGGCGAAGGCAGCTTGCTGGGCCACACCTGACACTCAGGCACGAAAGCGTGGGGAGCGA
ACAGGATTAGATACCCCGGTAGTCG
>Otu1109
CCAGCCTACGGGTCGCTCCAGTAGGGAATCTTCCGCAATGGACGAAAGTCTGGCGGAGCA
ACGCCGCGTGAGTGATGAAGGTTTTCGGATCGTAAAGCTCCGTTGCCAGGGAAGAACGCT
TGGGATAGTAACTGCTCCCAAGGTGACGGTACCTGAGAAGAAAGCCCCGGCTAACTACGT
GCCAGCAGCCGCGGTAATACGTAGGGGGCAAGCGTTGTCCGGAATTATTGGGCGTAAAGC
GCGCGCAGGCGGCCTTGTAAGTCTGTTGTTTAAACTCGGGGCTCAACCCCGAGTCGCAAT
GGAAACTGCAAAGCTTGAGTACAGAAGAGGAAAGTGGAATTCCACGTGTAGCGGTGAAAT
GCGTAGAGATGTGGAGGAACACCAGTGGCGAAGGCGACTTTCTGGGCTGTAACTGACGCT
GAGGCGCGAAAGCGTGGGGAGCAAACAGGATTAGAAACCCGCGTAGTCC
>Otu1111
CCAGCCTATGGGTTGCTCCAGTCGAGAATTTTTCTCAATGGGGGAAACCCTGAAGGAGCG
ACGCCGCGTGGAGGATGAAGGTCTTCGGATTGTAAACTCCTGTCATGAGGGAACAAACCC
GCACGTTTAACTGATGTGCGGCTGATAGTACCTCAAGAGGAAGAGACGGCTAACTCTGTG
CCAGCAGCCGCGGTAATACAGAGGTCCCAAGCGTTGTTCGGATTTACTGGGCGTAAAGGG
TGCGTAGGCGGCGGGGCCAGTCTGACGTGAAATCTCCGGGCCCAACCCGGAAACTGCGTT
GGATACTATCCGGCTAGAGGATTGGAAGGGAGACTGGAATACTTGGTGTAGCAGTGAAAT
GCGCAGATATCAAGTGGAACACCAGTGGCGAAGGCGAGTCTCTGGACATTTCCTGACGCT
GAGGCACGAAAGCCAGGGGAGCAAACGGGATTAGATACCCGAGTAGTCC
>Otu1112
CCAGCCTACGGGGTGCACCAGTAGGGAATCGTCGGCAATGCCCGAAAGGGTGACCGCGCG
ACGCCGCGTGCAGGAAGAAGGCCTTCGGGTTGTAAACTGCTTTTGTAGGGGATAATTATG
ATAGTACCCTAAGAATAAGGGCCTGCTAACTACGTGCCAGCAGCAGCGGTAATACGTAGG
GCCCGAGCGTTATCCGGAATTATTGGGCGTAAAGAGCGTGTAGGTGTTCTACCAAGTTGG
TCGTTAAATCCGAGAGCTTAACTCTCGGGCGGCGAGCAAAACTGGTGGAATTGAGGTTTG
CAGGGGTGAGCAGAATTTCTGGAGTAGGGGTGAAATCCGTTGATACCAGAAGGAATACCA
ATGGCGAAGGCAGCTCACTGGGCAATACCTGACACTGAGACGCGAAAGCGTGGGGAGCGA
ACGGGATTAGAAACCCTAGTAGTCC
>Otu1116
CCAGCCTATGGGTCGCAGCAGTAGGGAATTTTCCACAATGGGCGAAAGCCTGATGGAGCA
ACGCCGCGTGCAGGATGAATGCCTTCGGGTTGTAAACTGCTTTTATCTGTGACGAATATG
ACGGTAGCAGATGAATAAGGATCGGCTAACTCCGTGCCAGCAGCCGCGGTCATACGGAGG
ATCCAAGCGTTATCCGGAATTACTGGGCGTAAAGAGTTGCGTAGGTGGCAGTGTAAGCAA
GTAGTGAAAGCCTGGGGCTCAACCCCTTACCCATTACTTGAACTGCACAGCTAGAGGACG
AGAGAGGTAATTGGAATTCCTAATGTAGGGGTAAAATCCGTTGATCTTAGGAGGAACACC
GATGGCGTAGGCAGATTACTGGCTCGTTTCTGACACTAAGGCACGAAAGCGTGGGGAGCA
AACAGGATTAGAAACCCGTGTAGTCC
>Otu1117
CCAGCCTATGGGGTGCACCAGTGGGGAATATTGCGCAATGGGCGAAAGCCTGACGCAGCG
ACGCCGCGTGTGGGATGACGGCCTTCGGGTTGTAAACCACTGTCGGGAGGGACGAAACAA
ATGACGGTGCCTCCAAAGGAAGCACCGGCTAACTCTGTGCCAGCAGCCGCGGTAATACAG
AGGGTGCGAGCGTTGTCCGGAATTACTGGGCGTAAAGGGCGCGTAGGCGGCCCGATAAGT
AGGGGGTGAAATTCCGTGGCTTAACCACGGAACTGCCTTCTAAACTGTCAGGCTTGAGCA
CGGTAGAGGCAGATGGAATTCCCGGTGTAGCGGTGGAATGCGTAGAGATCGGGAAGAACA
CCAGTGGCGAAGGCGGTCTGCTGGGCCGTTGCTGACGCTGAGGCGCGACAGCGTGGGGAG
CAAACAGGATTAGAAACCCGCGTAGTCC
>Otu1118
CCAGCCTATGGGATGCTGCAGGCGCGAAACCTTTACAATGCACGAAAGTGTGATAGGGGG
ATACTCAGTGCTTACGACTCTGTTGTAGGCTTTTGTCGATCGTAAACAGATCGGCGAATA
AGTGGTGGGTAAGACTGGTGCCAGCCGCCGCGGTAACCCCAGCGCCACAAGTGGTCATCG
CGATTATTGGGCCTAAAGCGTTCGTAGCCGGTTTGGTGCGTCTCTTGTGAAATTGTTCGG
CTTAACCGAACAGCGTGCAGGAGACACGGCCAGACTCGAGACCGGGAGGCGTGCAAAGTA
TTCCATGGGGACTGGTAAAATGGGATAATCCATGGAAGACTACCGATGGCGAAGGCATTG
CACGAGAACGGATCTGACGGTGAGGAACGAAAGCTAGGGGAGCGAACCGGATTAGAAACC

CTGGTAGTCC
>Otu1121
CCAGCCTACGGGGTGCAGCAGTGGGGAATTTTACGCAATGGGCGAAAGCCTGACGTAGCG
ACACCGCGTGAGCGAAGAAGCCCTTTGGGGTGTAAAGCTCTGTCAGCTGGAACGAAACAA
ATGACGGTACCAGCAGAGGAAGCATCGGCTAACTACGTGCCAGCAGCCGCGGTAAGACGT
AGGATGCAAGCGTTGTCCGGATTTATTGGGCGTAAAGAGTTCGTAGGCGGTTTGGTAAGT
CTGATGTTAAAGACCGAGGCTCAACTTCGGGACCGCATTGGATACTGCCAGACTGGAGTG
TGGTAGAGGCTAGTGGAATTCCTAGTGTAGCGGTGAAATGCGTAGATATTAGGAAGAACA
CCAGTGGCGTAGGCGACTAGCTGGGCCATTACTGACGCTGAGGAACGAAAGCCAGGGGAG
CAAATGGGATTAGATACCCGCGTAGTCC
>Otu1122
CCAGCCTATGGGTTGCAGCAGTCGCGAAACCTTCGCAATACGCGAAAGCGTGACGAGGGA
ACCCCAAGTGTGCATGTACAGCATGCACTTTTGCTAAGTTTAGAACACTTGGAGAATAAG
GGCTGGGTAAGACTGGTGCCAGCCTCCGCGGTAACACCAGCAGCTCAAGTGGCGATCACG
TTTACTGGGTTTAAAGGGTGCGTAGTCGGTTTGGCAAGTTTTCTGTGAAATCCTGGCGCT
TAACGTCAGGGATTGCAGAAAATACTACCAAGCTAGAGACCGGGGTAGGAGTGAGGTATT
CTGGGGGTAGCGGTGAAATGCTATAATCCCCGGAGGACCACCAGTAGCGAAGGCGTCACT
CTGGAACGGGTCTGACGATGAGGTACGAGAGCTAGGGGAGCAAACAGGATTAGAAACCCG
AGTAGTCC
>Otu1125
CCAGCCTACGGGATGCAGCAGTCGAGGATCTTCGGCAATGGGCGCAAGCCTGACCGAGCG
ACGCCGCGTGTGCGATGAAGGCCTTCGGGTTGTAAAGCACTGTCGAGAGAAAGAAAGCCG
CAAGGTTGATCGATCTCTGGAGGAAGCACGGGCTAAGTTCGTGCCAGCAGCCGCGGTAAG
ACGAACCGTGCGAACGTTGTTCGGATTCACTGGGCTTAAAGGGCGCGTAGGCGGGTGATC
AAGTCAGGGGTGAAATCCTCCAGCTTAACTGGAGAATAGCCTTTGATACTGGTCGTCTTG
AGGAAGGTAGGGGCATGTGGAACTTCCGGTGGAGCGGTGAAATGCGTAGATATCGGAAGG
AACGCCGGTGGCGAAAGCGACGTGCTGGACCTTTTCTGACGCTGAGGCGCGAAAGCCAGG
GGAGCAAACGGGATTAGATACCCCAGTAGTCC
>Otu1128
CCAGCCTATGGGACGCACCAGTAAGGAATATTGGACAATGGGGGCAACCCTGATCCAGCC
ATGCCGCGTGAAGGATGAAGGCGCTCTGCGTCGTAAACTTCTTTTACTCGGGAAAAATTG
TTGGGTTTCTACCCGACTTGATTGTACCGAGAGAATAAGGACCGGCTAACTTCGTGCCAG
CAGCCGCGGTAATACGAAGGGTCCAAGCGTTATCCGGATTTATTGGGTTTAAAGGGTGCG
TAGGCGGATTATTAAGTCAGTGGTGAAAGCTCCATGCTCAACATGGAAATTGCCATTGAT
ACTGATGATCTTGAGTACACTTGAAGTGGGCGGAATATGTCATGTAGAGGTGAAATTCTT
AGAGATGACATAGAACACCGATTGCGAAGGCAGCTCACTAAATTGTAACTGACGCTGAGG
CACGAAAGCGTGGGGATCAAACAGGATTAGATACCCGTGTAGTCC
>Otu1130
CCAGCCTACGGGGCGCTCCAGTGGGGAATATTGGACAATGGGGGAAACCCTGATCCAGCC
ATGCCGCGTGAGTGAAGAAGGCCTTAGGGTTGTAAAGCTCTTTTGGCGGGGACGATAATG
ACGGTACCCGCAGAATAAGCCCCGGCTAACTTCGTGCCAGCAGCCGCGGTAATACGAAGG
ATGCAAGCGTTATCCGGATTTATTGGGTTTAAAGGGTGCGTAGGCGGACTTATAAGTCAG
TGGTGAAATCTCGTTGCTTAACAACGAACGTGCCATTGATACTGTAGGTCTTGAGTACAG
ATGCCGTTGGCGGAATGTGTCATGTAGCGGTGAAATGCATAGATATGACACAGAACACCG
ATTGCGAAGGCAGCTGACGAAACTGTAACTGACGCTGAGGCACGAAAGCGTGGGGATCAA
ACAGGATTAGAAACCCTGGTAGTCC
>Otu1132
CCAGCCTATGGGGGGCACCAGTGGGGAATCTTGCGCAATGGGCGAAAGCCTGACGCAGCA
ACGCCGCGTGAGGGATGAAGGTCTTCGGGTTGTAAACCTCTTTCAAGCAGGGACGATAGT
GACGGTACCTGCAGAAGAAGCTCCGGCCAACTACGTGCCAGCAGCCGCGGTGATACGTAG
GGAGCGAGCGTTGTCCGGATTCATTGGGCGTAAAGAGCTCGTAGGCGGCTCAGTAAGTCA
GGTGTGAAATCCTCAGGCTCAACCTGAGGTCGCCATCTGATACTGCTGTGGCTAGAGCCC
GGTAGGGGTCCACGGAATTCCTGGTGTAGCGGTGAAATGCGCAGATATCAGGAGGAACAC
CAGTGGCGAAGGCGGTGGACTGGGCCGGTACTGACGCTGAGGAGCGAAAGCGTGGGGAGC
GAACAGGATTAGAAACCCCAGTAGTCC
>Otu1135
CCAGCCTACGGGGTGCTGCAGCAACGAATCTTCCCCAATGCCGGAAACGGTGAGGGAGCG
ACGCCGCGTGAAGGATGAAGTACTTCGGTATGTAAACTTCTAAAGGGGTTATGAAAGCTG

AGGACCTAATACGTCCGGAGGTTGATCAGACCCTGATAAGGGGCGGCTAACTCTGTGCCA
GCAGCCGCGGTAATACAGAGGCCCCAAGCGTTACTGAGAATCACTGGGTTTAAAGGGTGC
GTAGGTGGCGCGTTAAGTCCGTTGTGAAATCCCTTGGCTCAACCAAGGAACTGCTTCGGA
TACTGGCGTGCTTGAGGCCGGTAGGGGTCACTGGAACTGACGGTGGAGCGGTGAAATGCG
TAGATATCGTCAGGAACGCCGGTGGTGAAGACGGGTGACTGGGCCGGTTCTGACACTGAG
GCACGAAAGCGTGGGTAGCAAACAGGATTAGAAACCCCTGTAGTCC
>Otu1136
CCAGCCTATGGGTCGCAGCAGTGGGGAATTTTGGACAATGGTGGCAACACTGATCCAGCC
ATGCCGCGTGCAGGATGAAGGCGCTACGCGTTGTAAACTGCTTTTGTACGGGAGAAAACC
TATCTACGTGTAGATAGCTGATAGTACCGTAAGAATAAGCATCGGCTAACTTCGTGCCAG
CAGCCGCGGTAATACGAAGGATGCAAGCGTTATCCGGATTTATTGGGTTTAAAGGGTGCG
TAGGCGGACTTATAAGTCAGTGGTGAAATCTCGTCGCTTAACGACGAACGTGCCATTGAT
ACTGTAGGTCTTGAGTACAGATGCCGTTGGCGGAATGTGTCATGTAGCGGTGAAATGCTT
AGATATTACACAGAACACCGATTGCGAAGGCAGCTTGCTGGGCCATTACTGACGCTGATG
CACGAAAGCGTGGGGAGCAAACAGGATTAGAGACCCCTGTAGTCC
>Otu1137
CCAGCCTACGGGTGGCAGCAGTGGGGAATACTGGACAATGAGCTAACGCTTGATCCGGCA
ATGTTAAACGAATGAAATGAAAGTAATGAGCTGTAAAATTCACAAAGTTGGTTTATATAT
ATAATGAATGAATTGATAAAGCCCCGACTAACTTCGTGCCAGCAGTCGCGGTAATACGGA
GGGGGCGAGTGTTACTCATCATGACTAGGCGTAAAGGGTGCTAAGACGGCTATGGTTTGT
TCTGGTATAAAACTCAATGGAAATATTGAATTTAGCCAAATAAACAATTTAGCTAGGGTA
GGTTGGAGAATAGGGTTACTGATTTTTGAGGGGTAGAATCCGACGAGAAGATTAGGAGAT
ACAAAGGCGAAGGCAACTATTTATGACAAACTAACGTTGAGACACAAAAGCGTGGGTAGC
AAACAGGATTAGAAACCCGTGTAGTCC
>Otu1138
CCAGCCTATGGGAGGCTGCAGTGGGGAATCTTGCGCAATGCGCGAAAGCGTGACGCAGCA
ACGCCGCGTGGGTGAAGAAGGCCCTCGGGTTGTAAAGCCCTTTCAGTTGGGACGAAGCTT
CGTCGGTGAATAGCCGACCGGAGTGACGGTACCTTCAGAAGAAGCCCCGGCTAACTACGT
GCCAGCAGCCGCGGTAATACGTAGGGGGCAAGCGTTGTCCGGAATCATTGGGCGTAAAGC
GCGTGTAGGCGGCTCGATAAGTCTGCTGTGAAAGTCCAGGGCTCAACCCTGGAATGCCGG
TGGATACTGTCGGGCTAGAGTACGGAAGAGGTGAGTGGAATTCCTGGTGTAGCGGTGAAA
TGCGCAGATATCAGGAGGAACACCAATGGCGAAGGCAGCTCACTGGGACGTTACTGACGC
TGAGACGCGAAAGCGTGGGGAGCAAACAGGATTAGATACCCGCGTAGTCC
>Otu1140
CCAGCCTATGGGGTGCAGCAGTCGAGGATCTTCGGCAATGGGCGCAAGCCTGACCGAGCG
ACGCCGCGTGCGCGACGAAGGCCTTCGGGTTGTAAAGCGCTGTCGAGGGGGAGGAAGCCG
CAAGGTTGACCTATCCCGGGAGGAAGCACGGGCTAAGTTCGTGCCAGCAGCCGCGGTAAG
ACGAACCGTGCGAACGTTGTTCGGAATCACTGGGCTTAAAGGGCGCGTAGGCGGGCCGTC
TGGTCCGGGGTGAAATCCCACCGCTTAACGGTGGGACTGCCTGGGATATCGGCGGTCTGG
AGGGGGGTAGGGGCACGTGGAACTTCCGGTGGAGCGGTGAAATGCGTTGATATCGGAAGG
AACGCCGGTGGCGAAAGCGACGTGCTGGGCCTTTCCTGACGCTGAGGCGCGAAAGCCAGG
GGAGCGAACGGGATTAGAGACCCCTGTAGTCC
>Otu1141
CCAGCCTATGGGTGGCTGCAGTGGAACTGAAACAAGGTCCACACTCTTGAAGAGGCAGCA
GTGAAGAATCATGGTCAATGCGCGCAAGCGTGAACCTGCGATCCGTCCAGTGGGATGAAG
GAGGTTTCTGTAAACCACCGTCCGTTCTCTGAAGCGCGATGGGAGAACGGGGCAGTCGTG
ATGAATCCTTGTGCCAGCAGTCGCGGTCACACAAGAGCGGCAAATGTTACTCGTGCGGAA
TGGGTCTAAAGCGTGCGTAGGTGGAGGGATGGGAGGGCGTTTGGAGTGAGCATGGGATCC
ATGTGCAAGGACGCCCGTCCTCCCTCTGGAGGCACTGAGAGGGGAGAAGAATGTCCAGGG
GACCGATGAAATGGGAGGATCCTGGATGGAACACCAAAGGCGCAGGCATCTCTCTGGCAG
TGCCTGACACTGAGAGCACGAAGGCGTGGGGAGCAAACGGGATTAGATACCCTTCC
>Otu1142
CCAGCCTACGGGAGGCAGCAGTTGGGAATCTTGGACAATGGTGGCAACACTGATCCAGCC
ATGCCGCGTGAAGGATGAAGGCGCTATGCGTCGTAAACTTCTTTTGAACGGGAGAAACCC
CTCGTACGTGTACGGGGCTGATAGTACCGTTAGAATAAGGGTCGGCTAACTTCGTGCCAG
CAGCCGCGGTAATACGAAGGACCCGAGCGTTATCCGGATTCATTGGGTTTAAAGGGTGCG
TAGGCGGAATGATAAGTCAGTGGTGAAATCCTGCAGCTTAACTGCAGAACTGCCATTGAT
ACTGTCAATCTTGAATACAACTGATGTGGGCGGAATGTGCCATGTAGTGGTGAAATACTT

AGATATGGCACAGAACACCGATTGCGAAGGCAGCTCACAAAATTGTTATTGACGCTGATG
CACGAAAGCGTGGGGATCAAACAGGATTAGATACCCCTGTAGTCG
>Otu1145
CCAGCCTACGGGATGCAGCAGTGGGGAATATTGGACAATGGGGGAAACCCTGATCCAGCG
ACGCCGCGTGTGTGAAGAAGGCCTGCGGGTTGTAAAGCACTTTTGTCCGGAAAGAAATCC
TCTTCGATAATACCGAGGGGGGATGACGGTACCGGAAGAATAAGCACCGGCTAACTACGT
GCCAGCAGCCGCGGTAATACGTAGGGTGCAAGCGTTAATCGGAATTACTGGGCGTAAAGC
GCGCGTAGACGGTTTTGTAAGTCAGATGTGAAATCCCTGGGCTCAACCTGGGAACTGCAT
TTGAGACTGCATGGCTAGAGTATGGAAGAGGGAAGTGGAATTTCCGGTGTAGCGGTGAAA
TGCGTAGATATCGGAAGGAACACCAGTGGCGAAAGCGACTTCCTGGTCCAATACTGACGT
TCATGTGCGAAAGCGTGGGGAGCAAACAGGATTAGATACCCCCGTAGTCC
>Otu1146
CCAGCCTATGGGGGGCACCAGTGGGGAATCTTGGACAATGGGGGAAACCCTGATCCAGCC
ATGCCGCGTGAGTGATGAAGGCCTTCGGGTTGTAAAACTCTTTTACCTGGGAAGATAATG
ACTGTACCAGGAGAATAAGCCCCGGCTAACTTCGTGCCAGCAGCCGCGGTAATACGAAGG
GGGCTAGCGTTGTTCGGAATTACTGGGCGTAAAGCGTGCGCAGGCGGCTTCTCAAGTCAG
GGGTGAAAGCCCAGAGCTCATCTCTGGAATTGCCTTTGAAACTGTGAAGCTTGAGTACGA
GAGAGGTGAGTGGAATTCCCAGTGTAGAGGTGAAATTCGTAGATATTGGGAAGAACACCG
GTGGCGAAGGCGGCTCACTGGCTCGTAACTGACGCTCAGGCACGACAGCGTGGGGATCAA
ACAGGATTAGATACCCGAGTAGTCC
>Otu1147
CCAGCCTATGGGGGGCACCAGTGGGGAATTTTCCGCAATGGGCGAAAGCCTGACGGAGCA
ACGCCGCGTGGATGATGAAGTCTTTCGGGACGTAAAATCCTTTCAGCAGGGACGAATAAA
GTGACGGTACCTGCAGAAGAAGCATCGGCTAACTACGTGCCAGCAGCCGCGGTAATACGT
AGGATGCGAGCGTTGTCCGGAATTATTGGGCGTAAAGCGAACGTAGGCGGCGAGTTAAGT
TTGGTCTTAAAGACTGGGGCTCAACCCCAGGAGTGGACTGAAAACTGATTTGCTAGAGGA
TGTCAGAGGAAAGTGGAATTCCGAGTGTAGCGGTGAAATGCGTAGATATTCGGAGGAACA
CCAGTGGCGAAAGCGACTTTCTGGGGCATTCCTGACGCTGAGGTTCGAAAGCTCGGGGGG
CAAACAGGATTAGAGACCCCCGTAGTCC
>Otu1148
CTAGCCTATGGGGGGCTGCAGTCGAGGATCTTCGGCAATGGGCGCAAGCCTGACCGAGCG
ACGCCGCGTGCGCGATGAAGGCCTTCGGGTTGTAAAGCGCTGTCGAGGGGGAGGAAAGCC
GTAAGGTCTGACCTATCCCTGGAGGAAGGACGGGCTAAGTTCGTGCCAGCAGCCGCGGTA
AGACGAACCGTCCAAACGTTGTTCGGAATCACTGGGCTTAAAGGGCGCGTAGGCGGGCCA
GCAAGTCCGGGGTGAAATCTTTCGGCTTAACCGGAAAAGTGCCTTGGAAACTGCTGGTCT
AGAGGGAGGTAGGGGCATGTGGAACTTCCGGTGGAGCGGTGAAATGCGTTGATATCGGAA
GGAACGCCGGTGGCGAAAGCGACGTGCTGGATCTCTTCTGACGCTGAGGCGCGAAAGCCA
GGGGAGCAAACGGGATTAGAAACCCGCGTAGTCC
>Otu1149
CCAGCCTACGGGTCGCTGCAGTGGGGAATCTTGCACAATGGAGGCAACTCTGATGCAGCG
ACGCCGCGTGAGCGATGAAGCCCCTCGGGGTGTAAAGCTCTTTCGGCAGGGAAGATTATG
ACGGTACCCATAGAAGAAGCCCCGGCTAACTTCGTGCCAGCAGCCGCGGTAATACGAGGG
GGGCAAGCGTTGTTCGGATTTATTGGGCGTAAAGGGTGCGTAGGCGGTTTGGCAAGTCTT
GTGTGAAATCTTCGGGCTCAACCCGAAGCCTGCATAAGAAACTGCCGGGCTTGAGTATGG
GAGAGGTGAGTGGAATTTCCGGTGTAGCGGTGAAATGCGTAGATATCGGAAGGAACACCT
GTGGCGAAAGCGGCTCACTGGACCATAACTGACGCTGATGCACGAAAGCTAGGGGAGCAA
ACAGGATTAGAAACCCCCGTAGTCC
>Otu1151
CCAGCCTACGGGTCGCACCAGTGGGGAATTTTGGACAATGGGGGCAACCCTGATCCAGCG
ACGCCGCGTGAAGGATGAAGGTCTTCGGATTGTAAACTTCTTTTAGAGGAGACGAATGCC
TGCGTGAAAAGCGCAGGGTGACGGTATTCTCTGAATAAGCCACGGCTAACTACGTGCCAG
CAGCCGCGGTAATACGTAGGTGGCAAGCGTTATTCGGAATCACTAGGCGTAAAGCGCAGG
TAGGCGGTTGGGTAAGTCTCTTGTGAAAGCTCCCGGCTTAACTGGGAGAGGCCAAGGGAA
ACTACCCGGCTAGAGTGTGGTAGAGGAAACTGGAATTCCCGGTGTAGCGGTGAAATGCGT
AGATATCGGGAGGAACACCTATGGCGAAAGCAGGTTTCTGGGCCATCACTGACGCTGAGC
TGCGAAAGCTAGGGGAGCAAACAGGATTAGATACCCTAGTAGTCC
>Otu1152
CCAGCCTATGGGGCGCTGCAGTGGGGAATTTTGTGCAATGGGCGAAAGCCTGACACAGCG

ACACCGCGTGAACGAAGAAGCTCTTTGGAGTGTAAAGTTCTGTCGGCTGGAACGAAAACA
ATGACGGTACCAGCAAAGGAAGCATCGGCTGACTACGTGCCAGCAGCCGCGGTAAGACGT
AGGATGCAAGCGTTGTCCGGATTTATTGGGCGTAAAGAGTTCGTAGGCGGTTTACTAAGT
CTGATGTTAAAGATCGGGGCTCAACCCTGAGAGTGCATTGGATACTGGTAGGCTTGAGTG
TGGTAGAGGCTAGTGGAATTCCCAGTGTAGCGGTGAAATGCGTAGATATTGGGAAGAACA
CCAGTGGCGTAGGCGACTAGCTGGGCCATAACTGACGCTGAGGAACGGAAGCCAGGGGAG
CGAATGGGATTAGAACCCCCGTAGTCC
>Otu1153
CCAGCCTATGGGTGGCAGCAGTGGGGAATCTTGGACAATGGGCGAAAGCCTGATCCAGCA
ATGTCGCGTGTGTGAAGAAGGCCTTAGGGTTGTAAAACACTTTCAGCAATGAAGAAGTAA
AACTTGCTAATATCTAGTTTTATTGACGTTAATTGAAGAAGAAGTACTGGCTAACTCTGT
GCCAGCAGCCGCGGTAATACAGAGAGTGCAAGCGTTAATCGGAATTATTGGGTGTAAAGG
GTGTGTAGGTGGATAAAAAAGTTACATGTGAAATCCCTGAGCTTAACTTAGGAACTGCAT
GTAATACTTTTTATCTAGAGTATTGTAGAGGAAAGTAGAATTTCCGGTGTAGCGGTGAAA
TGCGTAGATATCGGAAGGAATACCAGAGGCGAAGGCGACTCTCTGGCCAATTACTGACAC
TGAGGCACGAAAGCATGGGGAGCAAACAGGATTAGATACCCCAGTAGTCC
>Otu1155
CCAGCCTACGGGATGCTGCAGTTTGGAATTTTGGACAATGGGCGAAAGCCTGATCCAGCG
ACGCCGCGTGGACGATGAAGGCCTTCGGGTCGTAAAGTCCTTTTGCTCGGGAACAATATT
TTGAGCGTACCGAGAGAATAAGAGGTTGCTAACTCTGTGCCAGCAGCAGCGGTAATACAG
AGACCTCAAGCGTTATCCGGAATCATTGGGCGTAAAGCGTACCGATAGGTGGTTTTACAA
GTCAGAAGTGAAATCTCTCAGCTTAACTGGGAGACTGTCTTTTGAAACTGTAAAACTAGA
GGGGCAAAGAGGAAGCTGGAACAAGCGGTGTAGTAGTGAAATGCGTTGATATCGCTTGGA
ACACCAATAGCGTAGGCAGGCTTCTGGGTGCCACCTGACACTGCTAGGACGAAAGCGTGG
GGAGCGAATGGGATTAGATACCCCCGTAGTCC
>Otu1156
CCAGCCTATGGGAGGCTCCAGTGGGGAATCTTGCGCAATGGGCGAAAGCCTGACGCAGCG
ACGCCGCGTGAGTGATGAAGGCCTTCGGGTTGTAAAGCTCTGTGGGGAGAGACGAATAAG
TGCCGCCTAATACGTTGCATGATGACGGTATCTCCTTAGCAAGCACCGGCTAACTCTGTG
CCAGCAGCCGCGGTAAGACAGAGGGTGCAAACGTTGTTCGGAATTACTGGGCGTAAAGCG
AGTGTAGGCGGTCTGAAAAGTCGGGTGTGAAAGCCCTGGGCTCAACCCAGGAAGTGCACT
CGAAACTGCCAGGCTAGAGTGTCGGAGAGGTTGGTGGAATTCTCGGTGTAGAGGTGAAAT
TCGTAGATATCGAGAGGAACACCGGTGGCGAAGGCGGCCAACTGGACGAACACTGACGCT
GAGACTCGAAAGCGTGGGGAGCAAACAGGATTAGAAACCCTAGTAGTCC
>Otu1157
CCAGCCTACGGGGTGCTGCAGTCGAGAATATTTAGCAATGGACGAAAGTCTGACTATGCG
ACGCCGCGTGAGGGATGAAGTATTTCGGTACGTAAACCTCTTTCGATAGGGAAGATACTA
CCTGTAGTAATACAGGAACTGACGGTACCTAGAGAAGAAGCCATGGCCAACTCCGTGCCA
GCAGCCGCGGTAATACGGGGATGGCGAGCGTTACTCGGATTTACTGGGTGTAAAGGGCAG
GTAGGCGGCTCCGCAAGTTGAAGGTGAAATGCTCTGACTCAATCAGAGAACTGCCTTCAA
AACTACGGGGCTTGAGGATTTGAGAGGAGAGCGGAATTCCCGGTGTAAGGGTGAAATCTG
TAGATATCGGGAGGAACACCCGTGGCGAAAGCGGCTCTCTGGCAAATCCCTGACGCTGAG
CTGCGAAAGCTAGGGGAGCAAACAGGATTAGAAACCCCAGTAGTCC
>Otu1158
CCAGCCTATGGGGGGCTCCAGTAGGGAATATTGCACAATGGAGGAAACTCTGATGCAGCG
ACGCCGCGTGAGTGACGAAGGCCTTCGGGTTGTAAAGCTCTGTTCTCAGGGAAAAAGAAA
GTGATTGCACTTGAGAAGAAAGGACCGGCTAACTTCGTGCCAGCAGCCGCGGTAAGACGA
GGGGTCCAAGCGTTGTTCGGAATCATTGGGCGTAAAGCGGGTGTAGGCGGCTCTATAAGT
CAGGAGTGAAAGCCCCGAGCTTAACTCGGGAAGTGCTTTTGATACTGCAGAGCTTGAATG
TGGGAGAGGGTAGTGGAATTCCAGGTGTAGTGGTGAAATACGTAGATATCTGGAGGAACA
CCGGTGGCGAAGGCGGCTACCTGGCCCAACATTGACGCTGAGACCCGAAAGCATGGGGAT
CAAACAGGATTAGATACCCTCGTAGTCC
>Otu1159
CCAGCCTATGGGTCGCACCAGTCGAGAATTTTTCACAATGGGGGAAACCCTGATGGAGCG
ACGCCGCGTGGAGGATGAAGGTTTTCGGATTGTAAACTCCTGTCATGAGAGAACAAAACC
CGCAAGGGCTTGATAGTATCTCAAGAGGAAGGGACGGCTAACTCTGTGCCAGCAGCCGCG
GTAATACAGAGGTCCCAAGCGTTGTTCGGACTCACTGGGCGTAAAGGGTGCGTAGGTGGC
GGGGCAAGTTGCGTGTTAAATCTCAAGGCTCAACCTTGAAACCGCTCGCAATACTGCTCT

GCTGGAGGACTGTAGAGGAGACTGGAATTCACGGTGTAGCAGTGAAATGCGTAGATATCG
TGAGGAAGACCAGTGGCGAAGGCGAGTCTCTGGGCAGTTCCTGACACTGAGGCACGAAGG
CCAGGGGAGCAAACGGGATTAGAGACCCCCGTAGTCC
>Otu1161
CCAGCCTATGGGAGGCAGCAGTCGAGAATTTTTCTCAATGGGGGAAACCCTGAAGGAGCG
ACGCCGCGTGGAGGATGAAGGTTTTCGGATTGTAAACTCCTGTCATTGGGGAACAAGTGT
TCGGGGAGTAACTGCCCTGGATTTGATAGTACCCGAAGAGGAAGAGACGGCTAACTCTGT
GCCAGCAGCCGCGGTAATACAGAGGTCTCAAGCGTTGTTCGGATTCATTGGGCGTAAAGG
GTGCGCAGGCTGTTGTGTAAGTCGGATGTGAAATCCCGGGGCTCAACCCCGGAACTGCAT
TCGATACTGCATGACTAGAGGACTGGAGAGGAGATTGGAATTCACGGTGTAGCAGTGAAA
TGCGTAGATATCGTGAGGAAGACCAGTGGCGAAGGCGAATCTCTGGACAGTTCCTGACGC
TCATGCACGAAGGCTAGGGGAGCAAACGGGATTAGAAACCCGAGTAGTCC
>Otu1162
CCAGCCTATGGGTTGCTGCAGTCGAGAATCTTCCGCAATGGGCGAAAGCCTGACGGAGCG
ACGCCGCGTGACTGATGAAGTTCTTCGGAACGTAAAGGTCTTTTGTGAGGTAGAAAGTAA
TTGATCGCCTCAAGAATAAGGGGTTGCTAAACTCGTGCCAGCAGCAGCGGTAATACGAGT
GCCCCGAGCGTTATCCGGAATTATTGGGCGTAAAGGGTGTGTAGGCGGCTCTGTTAGTCT
TTTGTTAAAACTCCCGGCTCAACCGGGAAGGTGCAAAAGAAACGGCAGAACTTGAGGGTG
CGAGAGGTGAATGGAACTCATAGTGTAGGGGTGAAATCCGTTGATATTATGGGGAACACC
AAAAGCGAAGGCAATTCACTGGCGCATTCCTGACGTTGAAACACGAAAGCGTGGGTAGCG
AATGGGATTAGAAACCCTCGTAGTCC
>Otu1163
CCAGCCTACGGGTCGCACCAGTCGAGAATCTTCCGCAATGGACGAAAGTCTGACGGAGCG
ACGCCGCGTGGTTGATGAAGCGCTTCGGCGCGTAAAAACCTTTTATGAGCGACTAAGTTT
ATTGAAGAGCTCATGAATAAGAGGTTGCTAAACTCGTGCCAGCAGCAGCGGTAATACGAG
TGCCTCAAGCGTTATCCGGAATTATTGGGCGTAAAGGGTGTGTAGGCGGCTATGTTAGTC
TCGCGTTAAATTCTTCGGCTCAACCGGGGGTCTGCGCGGGAAACGGCATGGCTTGAGGAT
GCGAGGGGTCTCTGGAACTCATGGTGTAGCGGTGAAATGCGTTGACATCATGGGGAACAC
CAAGAGCGAAGGCAGGAGACTGGAGCATTTCTGACGCTGAAACACGAAAGCGTGGGTAGC
GAATGGGATTAGAAACCCCCGTAGTCC
>Otu1164
CCAGCCTATGGGGTGCTGCAGTGGGGAATTTTGGACAATGGGGGAAACCCTGATCCAGCA
ACGCCGCGTGAATGAAGAAGGCCTTAGGGTTGTAAAGTTCTTTTATCTGGGAGCAAAGTG
TATGTGTTAATAGCGCATATATATGAGAGTACCAGAGGAATAAGCACCGGCTAACTACGT
GCCAGCAGCCGCGGTAATACGTAGGGTGCGAGCGTTAATCGGAATTACTGGGCGTAAAGG
GTGTGTAGGCGGTTGTGTAAGTTAGATGTGAAATACCTGGGCTTAACCTGGGAACTGCAT
TTAAGACTGCATGGCTAGAGTTTGTCAGAGGGGGGTAGAATTCCAAGTGTAGCAGTGAAA
TGCGTAGAGATTTGGAGGAATACCGATGGCGAAGGCAGCCCCCTGGGATAGAACTGACGC
TGAAACACGAAAGCGTGGGTAGCAAACAGGATTAGAAACCCCCGTAGTCC
>Otu1165
CCAGCCTACGGGGTGCAGCAGTAAGGAATCTTCCACAATGGACGAAAGTCTGATGGAGCA
ACACCGCGTGCAGGATGAAGGCTTTCGGGTCGTAAACTGCTTTTATAGAGGAAGATTATG
ACGGTACTCTATGAATAAGGACCGGCTAACTACGTGCCAGCAGCCGCGGTAATACGTAGG
GTCCAAGCGTTATCCGGAATCACTGGGCGTAAAGCGTGCGTAGGTGGTCTGTTAAGTGAG
ATATTAAAGCGTGTGGCTCAACCATATCGTTGTGTCTCATACTGGCAGACTAGAGGACGG
CAGAGGCAAGTGGAATTACTAGTGTAGCAGTGAAATGCGTAGATATTAGTAGGAACACCA
ATGGCGAAGGCAGCTTGCTGGGCCGCCCCTGACACTGAGGTACGAAAGCGTGGGGAGCGA
ACAGGATTAGAGACCCCAGTAGTCC
>Otu1169
CCAGCCTACGGGAGGCTGCAGTGGGGAATATTGGACAATGGGCGCAAGCCTGATCCAGCA
ATGCCGCGTGAGTGATGAAGGCCTTAGGGTTGTAAAGCTCTTTCGGGTGGGACGATGATG
ACGGTACCACCAGAAGAAGCACCGGCTAACTCCGTGCCAGCAGCCGCGGTAATACGGAGG
GTGCGAGCGTTGTTCGGAATTATTGGGCGTAAAGCGCGTGTAGGCGGTTTTTTAAGTCTG
ATGTGAAAGCCCTGGGCTCAACCCGGGAAGTGCATTGGAAACTGGGAGACTTGAATACGG
GAGAGGGTAGTGGAATTCCTGGTGTAGGAGTGAAATTCGTAGATATCAGGAGGAACACCG
GTGGCGAAGGCGGCTTCCTGGACCGATACTGACGCTGAGACGCGAAAGCGTGGGGAGCAA
ACAGGATTAGAAACCCCGGTAGTCC
>Otu1173

CCAGCCTACGGGAGGCTGCAGTAGGGAATTTTCCACAATGGACGAAAGTCTGATGGAGCA
ACGCCGCGTGCAGGATGAAGGCCTTAGGGTTGTAAACTGCTTTTATTTGTGACGAATATG
ACGGTAGCAAATGAATAAGGATCGGCTAACTCCGTGCCAGCAGCCGCGGTCATACGGAGG
ATCCAAGCGTTATCCGGAATTACTGGGCGTAAAGAGTTGCGTAGGTGGCATTGTAAGCGA
GTAGTGAAAGCGTTCGGCTCAACCGAATACACATTACCCGAACTACAAAGCTAGAGGACG
AGAGAGGTTATTGGAATTCCTAGTGTAGGAGTGAAATCCGTAGATATTAGGAGGAACACC
GATGGCGTAGGCAGATAACTGGCTCGTTCCTGACACTAAGGCACGAAAGCGTGGGTAGCA
AACGGGATTAGATACCCCAGTAGTCC
>Otu1175
CCAGCCTACGGGAGCGCAGCAGTAGGGAATTTTGCGCAATGGACGAAAGTCTGACGCAGC
AACGCCGCGTGAGTGATGAAGGTCTTCGGATTGTAAAGCTCTGTTGAGTGGGAAGAACGA
TTCTGCATCTAATACATGCGGGATATGACGGTACCATTCGAGGAAGCTCCGGCCAACTAC
GTGCCAGCAGCCGCGGTAATACGTAGGGAGCAAGCGTTGTTCGGAATTACTGGGCGTAAA
GGGAGAGTAGGCGGAAAGGTAAGTTAGGAGTGAAATGTACGGGCTTAACCCGTAATCTGC
TTTTAATACTGCCATTCTTGAGTATGGGAGAGGATGATAGAATTCCAGGTGTAGCGGTGG
AATGCGTAGATATCTGGAAGAATACCAGTTGCGAAGGCGGTCATCTGGCCCAATACTGGC
GCTGAGGCTCGAAAGCTAGGGGAGCAAACAGGATTAGAGACCCGAGTAGTCC
>Otu1176
CCAGCCTATGGGTGCTGCAGTGGGGAATCTTGCGCAATGGGCGAAAGCCTGACGCAGCGA
CGCCGCGTGGGTGATGAAGGCCTTCGGGTTGTAAAGCCCTGTGGGGAGAGACGAATAAGG
TCGCGCTAATACCTCGACCGATGACGGTATCTCCTTAGCAAGCACCGGCTAACTCTGTGC
CAGCAGCCGCGGTAAGACAGAGGGTGCAAACGTTGTTCGGAATTACTGGGCGTAAAGCGT
GTGTAGGCTGCCGCGCAAGTCGGATGTGAAAGCCCCGGGCTCAACCCGGGAAGTGCACCC
GAAACTGCGTGGCTCGAGTACCGGAGAGGTTGGCGGAATTCTCGGTGTAGAGGTGAAATT
CGTAGATATCGAGAGGAACACCGGTGGCGAAGGCGGCCAACTGGACGAGTACTGACGCTG
AGACACGAAAGCGTGGGGAGCAAACAGGATTAGATACCCCCGTAGTCC
>Otu1177
CCAGCCTACGGGGGGCTCCAGTGGGGAATTTTGCGCAATGGACGGAAGTCTGACGCAGCA
ACGCCGCGTGGGTGATGAAGGTCTTCGGATCGTAAAACCCTGTCGTCAGGGACGAAGGTT
ACGAGTTTAAGAGATGCGTAGCTTGACGGTACCTGGAGAGGAAGCCCCGGCTAACTCTGT
GCCAGCAGCCGCGGTAATACAGAGGGGGCAAGCGTTATTCGGAATTATTGGGCGTAAAGG
GCGCGTAGGCGGTTTTGTAAGTGAGATGTGTAATCCCCGGGCTCAACTTGGGAACTGCAT
CTGGGACTGCGAGACTAGAGTGCTGGAGAGGGTGGTAGAATTCCATGTGTAGCGGTGAAA
TGCGTAGAGATGTGGAGGAATACCAGTGGCGAAGGCGGCCACCTAGACAGTAACTGACGC
TGAGGCGCGAAAGTGTGGGTAGCAAACAGGATTAGATACCCCAGTAGTCC
>Otu1178
CCAGCCTATGGGGGGCTGCAGTGGGGAATATTGCGCAATGGGGGAAACCCTGACGCAGCA
ACGCCGCGTGAATGATGAAGGCCTTCGGGTTGTAAAGTTCTGTCTTCTGGGACGATAATG
ACGGTACCAGAGGAGGAAGCCACGGCTAACTACGTGCCAGCAGCCGCGGTAATACGTAGG
TGGCAAGCGTTGTCCGGATTTACTGGGCGTAAAGGATGCGTAGGCGGACATTTAAGTCAG
ATGTGAAATACCCGAGCTTAACTTGGGTGCTGCATTTGAAACTGGGTGTCTAGAGTGCAG
GAGAGGTAAGTGGAATTCCTAGTGTAGCGGTGAAATGCGTAGAGATTAGGAAGAACACCA
GTGGCGAAGGCGACTTACTGGACTGTAACTGACGCTGAGGCATGAAAGCGTGGGGAGCAA
ACAGGATTAGAAACCCCAGTAGTCC
>Otu1179
CCAGCCTACGGGGTGCACCAGTAGGGAATATTGGACAATGGGCGCAAGCCTGATCCAGCC
ATGCCGCGTGAGTGATGAAGGCCTTCGGGTTGTAAAGCTCTTTTCTGAGGGAAGAACAAC
CGGAACAGGAAATGGTTCCGGTCTGACGGTACCTCAGGAATAAGGGACGGCTAACTCCGT
GCCAGCAGCCGCGGTAATACGGAGGTCCCGAGCGTTGTTCGGAATTACTGGGCGTAAAGG
GAGCGCAGGCGGACAGGTCAGTCTGAAGTGGAAATCCGGGGCTCAACCTCGGACTTGCTT
CGGATACTGCCTGTCTTGAGTTCGTGAGAGGAAGATGGAATTCAGGGTGTAGCGGTGAAA
TGCTCAGATATCCTGAGGAACACCGGTGGCGAAAGCGGTCTTCTGGCGCGAAACTGACGC
TGAGGCTCGAAAGCGTGGGGAGCAAACGGGATTAGATACCCTAGTAGTCC
>Otu1180
CCAGCCTACGGGGTGCTGCAGTGAGGAATATTGGACAATGGGGGAAACCCTGATCCAGCC
ATGCCGCGTGAGTGATGAAGGCCTTCGGGTTGTAAAACTCTTTTAGATGTGACGATGATG
ACGGTAACATCAGAAAAAGCTCCGGCTAACTTCGTGCCAGCAGCCGCGGTAATACGAAGG
GAGCGAGCGTTGTTCGGAATCACTGGGCTTAAAGCGTGCGTAGGCGGATTGGTAAGTAAG

AAGTGAAATCCCAGGGCTTAACCCTGGAACTGCTTTTAGACTGCCTGTCTGGAATTCGG
TAGAGGTTGGTGGAACTCCTAGTGTAGAGGTGAAATTCGCAGATGTTAGGAAGAACACCG
GTGGCGAAGGCGACCAACTGGACCGATATTGACGCTGAGGCACGAAAGCGTGGGTAGCAA
ACAGGATTAGATACCCCCGTAGTCC
>Otu1182
CCAGCCTATGGGGGGCAGCAGTAAGGAATATTGGACAATGGACGCAAGTCTGATCCAGCC
ATGCCGCGTGAAGGATTAAGGCCCTCTGGGTTGTAAACTTCTTTTATAGGGGACGAAAAA
GGGATTTTCTAATCCAACTGACGGTACCCTATGAATAAGCACCGGCTAACTCCGTGCCAG
CAGCCGCGGTAATACGGAGGGTGCAAGCGTTATCCGGATTCACTGGGTTTAAAGGGTGCG
TAGGCGGGCAATTAAGTCAGTGGTGAAATCTTGGGGCTCAACCCCGGAACTGCCATTGAT
ACTATTTGTCTTGAATACTGTGGAGGTTAGCGGAATATGTCATGTAGCGGTGAAATGCTT
AGATATGACATAGAACACCGATTGTGAAGGCAGCTGGCTACACAAGCATTGACGCTGAGG
CACGAAAGCGTGGGGATCAAACAGGATTAGATACCCCGTAGTCC
>Otu1183
CCAGCCTACGGGAGGCAGCAGTGGGGAATCTTGCGCAATGGGCGAAAGCCTGACGCAGCC
ACGCCGCGTGAGTGAAGAAGGCCTTCGGGTTGTAAAGCTCTGTCGGGCAGGACGAAAACT
TATGAGGTTAACAGCCTCATGAATTGACGGTACCGCCAAAGGAAGCACCGGCTAACTTCG
TGCCAGCAGCCGCGGTAATACGAGGGGTGCAAGCGTTGCTCGGAATTATTGGGCGTAAAG
GGTAGGTAGGTGGTCTGATTTGTCTGGGGTGAAAGCCTTGAGCTTAACTCAAGAAGTGCC
TCAGAAACGGTCAGACTGGAATCTTGGAGAGGGTCGTGGAATTCCCGGTGTAGCGGTGAA
ATGCGTAGAGATCGGGAGGAACACCAGAGGCGAAGGCGGCGACCTGGACAAGTATTGACA
CTCAACTACGAAAGCGTGGGTAGCAAACAGGATTAGATACCCTGGTAGTCC
>Otu1185
CCAGCCTACGGGTGGCACCAGTGAGGAATCTTGCGCAATGGGGGCAACCCTGACGCAGCA
ACGCCGCGTGAGTGAAGAAGGCCTTCGGGTCGTAAAGCTCTGTCGGGTGGGAAGAAATGT
ATGCAAGCTAATACCTTGTATACTTGACGGTACCGCCAAAGGAAGCACCGGCTAACTCCG
TGCCAGCAGCCGCGGTAATACGGGGGGTGCAAGCGTTGTTCGGAATTATTGGGCGTAAAG
AGCGTGTAGGCGGCGGGATAAGTCAGATGTGAAAGCCCTGGGCTCAACTCAGGAAGTGCA
TTTGATACTGTTTCGCTTGAGTACTGGAGAGGGAAGAGGAATTCCTGGTGTAGAGGTGAA
ATTCGTAGATATCAGGAGGAACACCGGTGGCGAAGGCGACTTCCTGGCCAGATACTGACG
CTGAGACGCGAGAGCGTGGGGAGCAAACAGGATTAGATACCCGAGTAGTCC
>Otu1186
CCAGCCTACGGGAGGCACCAGTAACGAATCTTCCCCAATGCACGAAAGTGTGAGGGAGCG
ACGCTGCGTGGAGGATGAAGTCCTTCGGGATGTAAACTCCTGTCGGGAGCTACCAAGTTC
TGAGGAGCTCCAAAGGAAGAGGCGGCTAACTCTGTGCCAGCAGCCGCGGTAAGACAGAGG
CCTCGAGCGTTAGGCGGAATCACTGGGCTTAAAGCGTGTGTAGGTGGATTCTTAAGTGCT
TTGTGAAATCCCACGGCTTAACCGTGGAACTGCTTGGCATACTGGGAATCTTGAGCTATC
TAGGGGCAACTGGAACAGGCGGTGGAGCGGTGAAATGCGTAGATATCGTCTGGAACGCCA
ATGGTGAAGACAGGTTGCTGGGGATATGCTGACACTGAGACACGAAAGCCAGGGGAGCAA
ACGGGATTAGATACCCGCGTAGTCC
>Otu1187
CCAGCCTACGGGGGGCTGCAGTCGAGAATCTTCCGCAATGGGCGAAAGCCTGACGGAGCG
ACGTCGCGTGATGGATGAAGTTCTTCGGAACGTAAACATCTTTTATTAGGGAGAAATCCG
CCGCAAGGCGGAGTGATAGTACTTAATGAATAAGGGGTTGCTAAACTCGTGCCAGCAGCA
GCGGTAATACGAGTGCCCCAAGCGTTATCCGGAATTACTGGGCGTAAAGCGTGCGTAGGC
GGTGTGATTAGTCGTCCGTTAAAGCTCCCGGCTTAACCGGGAAAAGGCGAACGAAACGGT
CACGCTGGAGGAGGTGAGGGGTCTATGGAACTCATGGTGTAGGGGTGAAATCCGTTGATA
TCATGGGGAACACCAAAAGCGAAGGCAGTAGACTGGCACCTTACTGACGCTGTAACACGA
AAGCGTGGGGATCAAACGGGATTAGATACCCTCGTAGTCC
>Otu1188
CCAGCCTACGGGATGCTGCAGTGAGGAATATTGCGCAATGTCCGAAAGGGTGACGCAGCG
ACGCCGCGTGGAGGATGAAGGCCCTATGGGTCGTAAACTCCTTTTGAGGGGGAAGAATAG
CCTCGTATAGCGAGGTTTGACGGTACCTCTAGAAAAAGCACCGGCCAACTACGTGCCAGC
AGCCGCGGTAATACGTAGGGTGCAAGCGTTGTCCGGATTTACTGGGTGTAAAGGGAGCGC
AGGCGGGTTAGCAAGTCGTTGGTGAAATTCTGAGGCTCAACCTCAGAACTGCCAATGATA
CTGCTGATCTTGAGTACAGAAGAGGAAGACGGAATTCCTGGTGTAGCGGTGAAATGCGTA
GATATCAGGAAGAACACCGGTGGCGAAGGCGGTCTTCTGGTCTGTAACTGACGCTCATGC
GCGAAAGCGTGGGGAGCAAACAGGATTAGATACCCCTGTAGTCC

>Otu1190
CCAGCCTATGGGGTGCAGCAGTGGGGAATTTTCCGCAATGGGCGAAAGCCTGACGGAGCA
AGACCGCGTGAGGGAGGAAGGCTCTTGGGTTGTAAACCTCTTTTCTCAAGGAAGAAGAAA
GTGACGGTACTTGAGGAATCAGCATCGGCTAACTCCGTGCCGGCAGCCGCGGTAATACGG
AGGATGCAAGCGTTATCCGGAATTATTGGGCGTAAAGCGTCCGTAGGTGGCCATTCAAGT
CTGCCGTTAAAACCAGTGGCTTAACCACTGACAGGCGGTGGAAACTGAAGAGCTAGAGTG
TGGTAGGGGTAGAGGGAATTCCCAGTGTAGCGGTGAAATGCGTAGAGATTGGGAAGAACA
CCGGTGGCGAAAGCGCTCTGCTGGACCACAACTGACACTCACAGGACGAAAGCTAGGGGA
GCGAAAGGGATTAGAAACCCGCGTAGTCC
>Otu1191
CCAGCCTACGGGGGGCAGCAGTAAGGGATATTGCGCAATGGGCGAAAGCCTGACGCAGCA
ACGCCGCGTGGAGGATGAAGGCTTTCGGGTTGTAAACTCCTTTTGACGCTGACGAGGAAG
GACGGTAGGCGTCGAAGAAGTCACGGCTAACTACGTGCCAGCAGCCGCGGTAAAACGTAG
GTGGCAAGCGTTATCCGGATTTACTGGGCGTAAAGCGTGTGCAGAGGGATGACTAAGTGG
TGTATGAAAGCGCCAGGCTCAACCTGGCAGGGTTATGCCAGACTGGTCGTCTAGAAACGG
AGAGAGGGACGTGGAATGCCGGGTGTAGTGGTGAAATGCGTAGAGATCCGGCAGAACAAC
AGAGGCGAAGGCGGCGTCCTGGCTCCGATTTGACCTTCAGACACGACAGCATGGGGAGCG
AACGGGATTAGAAACCCTAGTAGTCC
>Otu1192
CCAGCCTATGGGGGGCAGCAGTTTCGAATTATTCCCAATGGGCGCAAGCCTGAGGGTGCA
ATACCGCGTGGAGGATGAAGGTCTTCGGATCGTAAACTCCTGTCGTAAGGGACAAAACCA
CCTATTTTAATAGAATAGGTGCTGATTTAACTTTAAGAGGAAGAGGTGGCTAACTCTGTG
CCAGCAGCCGCGGTGATACAGAGACCTCAAGCGTTACTCGGATTCACTGGGCGTAAAGGG
TACGTAGGTGGCATGGTGTGTTGGATGTGAAATTCCGGAGCTTAACTCCGGCGCTGCGTC
CAAAACTATCGTGCTAGAGTACTGTAGAGATGAGCGGAATTTCTAGTGTAGCGGTAAAAT
GCGTAGATATTAGAAAGAACACCAACGGCGAAGGCAGCTCATTGGGCAGTTACTGACACT
GAGGTACGAAAGCGTGGGGAGCAAAAAGGATTAGATACCCCAGTAGTCC
>Otu1195
CCAGCCTACGGGGTGCTGCAGTCGAGGATCATTCGCAATGGGCGAAAGCCTGACGATGCG
ACGCTGCGTGGAGGATGAAGGCCTTCGGGTTGTAAACTCCTGTCATGAGGGGAAAAGGTG
CCGGGCGTGAATAACGTCTGGCCTCGATGGTACCTCAAGAGGAAGCCACGGCCAACTCTG
TGCCAGCAGCCGCGGTAATACAGAGGTGGCGAGCGTTGTTCGGATTTACTGGGCGTAAAG
GGCGCGTAGGCGGTTATGTACGTCGAGTGTGAAATCTCTGGGCTCAACCCGGAAAGTGCG
CTCGAAACTGCATGACTTGAGCATGGAATGGGAGAGCGGAATACACGGTGTAGCGGTGAA
ATGCGTTGATATCGTGTAGAACACCGGTGGCGAAGGCGGCTCTCTGGAACATTGCTGACG
CTGAGGCGCGAAAGTTGGGGGAGCAAACAGGATTAGATACCCTCGTAGTCC
>Otu1197
CCAGCCTATGGGGTGCACCAGTGGGGAATCTTGCGCAATGGACGAAAGTCTGACGCAGCG
ACGCCGCGTGGGTGAAGAAGGCCTTCGGGTTGTAAAGCCCTGTCGAGTGGGAAAAACGAG
GGATGGAATAATACGTCATCTCAGTGATTGTACCACTAGAGGAAGCACCGGCTAACTCCG
TGCCAGCAGCCGCGGTAATACGGAGGGTGCAAGCGTTGTTCGGAATTATTGGGCGTAAAG
GGCAGGTAGGTGGTCTAGTAAGTCTACTGTGAAATCCCTGAGCTTAACTCAGGAAGTGCG
GTAGATACTGCAAGACTAGAGTATCGGAGGGGTGCGTGGAATTCCCGGTGTAGCGGTGAA
ATGCGTAGATATCGGGAGGAACACCAGGGGCGAAGGCGGCGCACTTGACGAAAACTGACA
CTCAACTGCGAAAGCGTGGGGAGCAAACAGGATTAGATACCCCCGTAGTCC
>Otu1200
CCAGCCTATGGGGTGCAGCAGTCGAGAATTTTTCACAATGGGCGCAAGCCTGATGGAGCG
ACGCCGCGTGGGGGATGAATGGCTTCGGCCCGTAAACCCCTGTCATTTGCGACCAATGCT
GCCGGGTGAATATCCCGGCAGTTGATAGTAGCGAAAGAGGAAGGGACGGCTAACTCTGTG
CCAGCAGCCGCGGTAATACAGAGGTCCCGAGCGTTGTTCGGATTTACTGGGCGTAAAGGG
TGCGTAGGCGGCAGGGTAAGTTTGATGTGAAATCTCCGGGCCCAACCCGGAAACGGCATT
GAATACTGCCTTGCTTGAGGGTTGAAGAGGAGACTGGAATTCTCGGTGTAGCAGTGAAAT
GCGTAGATATCGAGAGGAACACCAGCGGCGAAGGCGAGTCTCTGGGCAACCACTGACGCT
GAGGCACGAAAGCTAGGGGAGCAAACAGGATTAGAGACCCTTGTAGTCC
>Otu1201
CCAGCCTACGGGTGGCTGCAGTGAGGAATATTGGTCAATGGGCGGAAGCCTGAACCAGCC
ATCCCGCGTGCAGGAAGAAGGCCCTATGGGTTGTAAACTGTTTTTCTGAGGGAATAAAAC
CCCCGACGTGTCGGGGCTTGTAGGTACTTCAGGAATAAGCATCGGCTAACTCCGTGCCAG

CAGCCGCGGTAATACGGAGGATGCGAGCGTTATCCGGATTCATTGGGTTTAAAGGGTGCG
TAGGCGGACTAATAAGTCAGTGGTGAAATTCTGCAGCTTAACTGTAGACGTGCCATTGAT
ACTGTTAGACTTGAGTATGGTCAAGGTAGGCGGAATGTGTAATGTAGCGGTGAAATGCTT
AGATATTACACAGAACACCGATTGCGAAGGCAGCTTGCTGGCCATTACTGACGCTGATG
CACGAAAGCGTGGGGAGCAAACAGGATTAGAGACCCCGGTAGTCC
>Otu1202
CCAGCCTACGGGGGGCAGCAGTGGGGAATATTGGACAATGGACGCAAGTCTGATCCAGCG
ACACCGCGCGGAGGATGAAGGTCTTCGGATCGTAAACTCCTCTCGGAGATGATAAAGTCT
TGGTGAACCCAAGATTGACGGTAATCTCAGAAGAAGTCACGGCTAACTACGTGCCAGCAG
CCGCGGTAATACGTAGGTGGCGAGCGTTACTCGGAATTACTAGGTGTAAAGCGCGAGTAG
GTGGTTCGGTAAGTTGGGAGTTAAAGCTCCAATAGAATTGGAGAGGTCTTCTAATACTGC
CGAACTTGAGTGTAGCATGGGGAACTGGAATTCCCGGTGTAGCGGTGAAATGCGTAAATA
TCGGGAGGAATACCTATGGCGAAGGCGAGTTCCTGGGCTACTACTGACACTGAGCAGCGA
AGACGTGGGGAGCAAACAGGATTAGAGACCCCCGTAGTCC
>Otu1204
CCAGCCTACGGGGTGCTGCAGTAGAGCTCGATCAGTTCAGTCGCGTATTGGCCGCTGTCG
AGTTTGGGCGGCGTGGGAACGTCCATATCGCCGAACTGGCTGCCGTCGACGCCTACGAAG
GCGTAGGCATAAGAGCCCATTGGGCTGTTGAGCGTGCGTGGGCCGCCCATGGTCAGAGCT
TGAAAGTCGGCGAAGCTGCCGGTGTCGATCGCGTTACGAAAGGCGTTGAAAGCGGGGATG
TTTACCTTGCCCGGCGAATCTTGCAGGATGCACTTCGAGTAGGTGCCGCATTTGTCGGCG
AGGTTTTCGTCGCCGTTGGGCGTGTGAGTCACCGGCGGGATGAGAGCGTCGCGGGTGGCG
GCGTTGAATCGGCGGGCGAAGCAGTTCAGCACGCGAGGATCGGTCACGGTGGACGGCACG
CATGTTTGCGGGGGCTGGGTTGAGCATGGGCTTGGCATTAGAAACCCTTGTAGTCC
>Otu1205
CCAGCCTACGGGACGCTGCAGTCGAGAATATTCCACAATGGACGAAAGTCTGATGGAGCG
ACGCCGCGTGCAGGATGAAGGCCTTCGGGTTGTAAACTGCGGTAGTATGGTAACAATGCA
AATGAGTGCCATACGGAAAGAGGTGGGTAACTACGTGCCAGCACCAGCGGTAAAACGTAG
ACCTCGAGCGTTATTCGGATTTATTGGGCGTAAAGCGCGTGTAGGGGGTTTTGTACGTCT
TTTGTTAAAGCCCACCGCTCAACGGTGGAAGTGCAGGAGATACGGCAAGACTAGAGGATG
TTAGAGGTGCCAGGAACACACGGTGTAGGGGTGAAATCCGTTGATATCGTGTGGAACACC
AAAGGCGAAGGCAAGGCACTGGGACACTCCTGACCCTGAGACGCGAAAGCGTGGGGAGCA
AACAGGATTAGATACCCCAGTAGTCC
>Otu1206
CCAGCCTATGGGGTGCACCAGTGGGGAATTTTCCGCAATGGGCGAAAGCCTGACGGAGCG
ACGCCGCGTGGAGGATGAAGGATTTCGGTCTGTAAACTCCTGTCAGGGGGGACGATAATG
ACGGTACCCCCAAAGGAAGCATCGGCTAACTACGTGCCAGCAGCCGCGGTAAGACGTAGG
ATGCGAGCGTTGTTCGGAATTATTGGGCGTAAAGCGTGCGTAGGCGGCTTGTTAAGTCTG
GTGTTAAAGCTAGGGGCTCAACCCCTGTGTTGCACTGGAAACTGGCAGGCTTGAGTGCGG
TAGGGGAAGGTGGAATTCCGAGTGTAGCGGTGAAATGCGTAGATATTCGGAAGAACACCG
GTGGCGAAGGCGACCTTCTGGGCCGCCACTGACGCTGAGGCACGAAAGCGTGGGGAGCAA
ACGGGATTAGAAACCCTTGTAGTCC
>Otu1207
CCAGCCTATGGGGGGCAGCAGTGGGGAATTTTACGCAATGGGCGAAAGCCTGACGTAGCG
ACACCGCGTGAGCGAAGAAGCCCTTTGGGGTGTAAAGCTCTGTCAGCAGGGACGAAACAA
ATGACGGTACCTGCAGAGGAAGCATCGGCTAACTACGTGCCAACAGCCGCGGTAAGACGT
AGGATGCAAGCGTTGTCCGGATTTATTGGGCGTAAAGAGTTCGTAGGTGGTTTGTTAAGT
CCGGTGTTAAAGATCAGGGCTCAACCCTGGGACCGCACTGGATACTGGCAGACTGGAGTG
CAGTAGAGGCGAGTGGAATTCCCAGTGTAGCGGTGAAATGCGTAGATATTGGGAAGAACA
CCGGTGGCGTAAGCGACTCGCTGGGCTGTAACTGACACTGAGGAACGAAAGCCAGGGGAG
CGAATGGGATTAGAAACCCCCGTAGTCC
>Otu1208
TCAGCCTATGGGGGGCAGCAGTAGGGAATATTGGTAATCTGCGAAAGCGGGAACCAGCAA
CGCCGCGTGTGCGATGAAGGCCTTCGGGTTGTAAAGCACTTTTTGGAGGGATGAGGTAGG
ACAGTACCTTCAGAATAAGTCTCGGCTAACTACGTGCCAGCAGCCGCGGTAACACGTAGG
AGACAAGCGTTATCCGGATTTACTGGGCGTAAAGCGCATGCAGGCGGTTGAATAAGTTGG
ATGTGAAAGCTCCCGGCTTAACTGGGAGAGGTCGTTCAATACTGTTTAACTAGAGGATGG
AAGAGGAAGGTGGAATTCCGGGTGTAGTGGTGAAATGCGTAGATATCCGGAGGAACACCA
GTGGCGAAAGCGACCTTCTGGTCCATAACTGACGCTCATATGCGAAAGCTAGGGTAGCAA

ACGGGATTAGAAACCCTTGTAGTCC
>Otu1210
CCAGCCTATGGGGGGCAGCAGTGGGGAATCTTGCGCAATGGGCGAAAGCCTGACGCAGCC
ATGCCGCGTGAATGATGAAGGTCTTAGGATTGTAAAATTCTTTTAGCAGGGACGATAATG
ACGGTACCTGCAGAAAAAGCCCCGGCTAACTTCGTGCCAGCAGCCGCGGTAATACGAAGG
GGGCTAGCGTTGCTCGGAATTACTGGGCGTAAAGGGAGCGTAGGCGGGTTATCAAGTTGG
AGGTGAAAGCCCAGGGCTCAACCTTGGAATTGCCTTCAAAACTGATAGCCTAGAGTATGG
AAGAGGTAAGTGGAACTCCGAGTGTAGAGGTGAAATTCGTAGATATTCGGAAGAACACCA
GTGGCGAAGGCGACTTACTGGTCCATGACTGACGCTGAGGCTTGAAAGCTAGGGTAGCGA
ACGGGATTAGAGACCCGAGTAGTCC
>Otu1211
CCAGCCTATGGGTGGCAGCAGTTAGGGAACTTTTCCAATGCGCGCAAGCGTGAGAGAGCA
AGTCAGAGTGATTTTGATATATTTTCAAGATCTTTTGCCGAATGTAAAAAGTTCGGAGAA
TAAGGACTGGGCAAGACTGGTGCCAGCCGCCGCGGTAATCCCAGCGGTCCGAGTCGCATC
CACAATTATTGGGTCTAAAACATTCGTAGCTTGTTTGTTAAGTTCCTTGTGAAATCTTAA
ATCTTAAATTTAAGGCGTGCAGGGAATACTGGCAAACTTGAGACCGGAAGAAGTAAAGAG
TATAGCCGAGGTAGTGGTAAAATACGTTAATCTCGGTTAGACTAACAACAGCGAAGGCAC
TTTACTAGTACGGATCTGACAGTGAGGAATGAAGGCTAGGGTCGCGAAAGGGATTAGAAA
CCCTAGTAGTCC
>Otu1212
CCAGCCTACGGGAGGCTCCAGTGGGGAATCTTGCGCAATGGGCGAAAGCCTGACGCAGCG
ACGCCGCGTGTGCGATGAAGGTCTTCGGATCGTAAAGCACTGTCGCGGGGGAAAAAGCAA
TGATGGTACCCCGAGAGGAAGCACCGGCTAACTCTGTGCCAGCAGCCGCGGTAATACAGA
GGGTGCGAGCGTTGTTCGGAATTATTGGGCGTAAAGCGCTGGTAGGTGGCTTGTTAAGTC
GCGTGTGAAAGCTTCCGGCTCAACCGGAAAAGTGCGCGCGAAACTGGTGAGCTTGAGTAT
GGGAGAGGGTCGCGGAATTCCCGGTGTAGAGGTGAAATTCGTAGATATCGGGAGGAACAC
CAGAGGCGAAGGCGGCGACCTGGAACATCACTGACACTGAGCAGCGAAAGCGTGGGGAGC
AAACAGGATTAGAAACCCCCGTAGTCC
>Otu1218
CCAGCCTACGGGTGGCTGCAGTGGGGAATCTTGGACAATGGGCGCAAGCCTGATCCAGCC
ATGCCGCGTGAGTGAAGAAGGCCTTAGGGTTGTAAAACTCTTTCGCCGGTGAAGATAATG
ACGGTAACCGGAGAAGAAGCCCCGGCTAACTTCGTGCCAGCAGCCGCGGTAATACGAAGG
GGGCTAGCGTTGTTCGGAATTACTGGGCGTAAAGCGCACGTAGGCGGATTGGTAAGTAGG
GGGTGAAATCCCCGGGCTCAACCCGGGAACTGCCTTCTAGACTGCCAGTCTTGAGGTCAG
GAGAGGCGAGTGGAATTCCGAGTGTAGAGGTGAAATTCGTAGATATTCGGAGGAACACCA
GTGGCGAAGGCGACTCGCTGGACTGATACTGACGCTGAGGTGCGAAAGCGTGGGGAGCAA
ACAGGATTAGAAACCCCAGTAGTCC
>Otu1220
CCAGCCTACGGGGGGCAGCAGTCGAGAATCTTCGGCAATGGGCGAAAGCCTGACCGAGCG
ACGCCGCGTGCGGGATGAAGGCCTTCGGGTTGTAAACCGCTGTCGGTGGGGAGGAAATAC
TGGGGGGTTCTCCCTTCAGTTTGACCTATCCGCAGAGGAAGTACGGGCTAAGTTCGTGCC
AGCAGCCGCGGTAACACGAACCGTACGAACGTTATTCGGAATCACTGGGCTTAAAGGGTG
CGTAGGCGGCCTGGTAAGTTGGGTGTGAAATCCCTCGGCTTAACCGAGGAATTGCGCTCA
AGACTGCCAGGCTCGAGGGAGTCAGAGGTGAGCGGAACTTAGGGTGGAGCGGTGAAATGC
GTTGATATCCTAAGGAACACCAGGGGCGAAAGCGGCTCACTGGGACTCTTCTGACGCTGA
GGCACGAAAGCTAGGGGAGCGAACGGGATTAGAAACCCCCGTAGTCC
>Otu1221
CCAGCCTATGGGTTGCAGCAGTAGGGAATATTGGACAATGGGTGAGAGCCTGATCCAGCC
ATGCCGCGTGCAGGAAGACGGCCTTCTGGGTTGTAAACTGCTTTAGCCTGGGGATAAAAC
GACCATGCGTGGTTAATTGAAGGTACCAGGATAATAAGCCACGGCTAACTACGTGCCAGC
AGCCGCGGTAATACGTAGGTGGCAAGCGTTGTCCGGATTTATTGGGTTTAAAGGGTGCGT
AGGCGGTTTTATAAGTCAGTGGTGAAATACGGCAGCTTAACTGTCGAGGTGCCATTGATA
CTGTAGAACTTGAGTACAGACGAGGTAGGCGGAATTGACGGTGTAGCGGTGAAATGCTTA
GATATCGTCAAGAACACCGATAGCGAAGGCAGCTTACTAGACTGTAACTGACGCTGAGGC
ACGAAAGTGTGGGGATCAAACAGGATTAGAAACCCCAGTAGTCC
>Otu1222
CCAGCCTACGGGATGCTGCAGTGAGGAATATTGCGCAATGTCCGAAAGGGTGACGCAGCG
ACGCCGCGTGGAGGATGAAGGCCCTATGGGTCGTAAACTCCTTTTGAGGGGGAAGAATAG

TCCCATATACTGGGATTTGACGGTACCTCTAGAAAAAGCACCGGCTAACTACGTGCCAGC
AGCCGCGGTAATACGTAGGGTGCGAGCGTTGTCCGGAATCACTGGGTGTAAAGGGAGCGC
AGGCGGGTCGGCAAGTCATTGGTGAAATCCGGAGGCTTAACCTCAGGACTGCCAATGATA
CTTCCGATCTTGAGTGCAGAAGAGGAAGACGGAATTCCTGGTGTAGCGGTGAAATGCGTA
GATATCAGGAAGAACACCGGTGGCGAAGGCGGTCTTCTGGTCTGTAACTGACGCTCATGC
TCGAAAGCGTGGGGAGCAAACAGGATTAGAAACCCTAGTAGTCC
>Otu1223
CCAGCCTATGGGTGGCTGCAGTAGGGAATATTGCGCAATGGAGGCAACTCTGACGCAGCG
ACGCCGCGTGGGTGATGAAGGCTTTCGGGTCGTAAAGCCCTGTCGGAAGGGAAGAAAAAT
AGAGTAGCTAATATCTACTTTACTTGACGGTACCTTAAGAGGAAGCACCGGCTAACTACG
TGCCAGCAGCCGCGGTAATACGTAGGGTGCAAGCGTTGTTCGGAATTATTGGGCGTAAAG
CGCGTGTAGGCGGTTTTCTAAGTCGGATGTGAAATCCCTCGGCTCAACCGGGGAAGTGCA
TTCGAAACTAGTAAGCTAGAAGATGGTAGAGGAAGGTGGAATTCCTAGTGTAGAGGTGAA
ATTCGTAGATATTAGGAGGAATACCGGAGGCGAAGGCGGCCTTCTAGACCATTCTTGACG
CTGAGACGCGAAAGCGTGGGGAGCAAACAGGATTAGAAACCCCCGTAGTCC
>Otu1224
CCAGCCTACGGGTGGCTCCAGTGGGGAATATTGGACAATGGGGGCAACCCTGATCCAGCG
ACGCCGCGTGTGTGAAGAAGGCCTGCGGGTTGTAAAGCACTTTTAGTGGGGATGAAATGT
GCAGGGTTAATACCTCTGCATTTTGACCTAACCCACAGAAAAAGCACCGGCTAACTCTGT
GCCAGCAGCCGCGGTAATACAGAGGGTGCAAGCGTTAATCGGAATTACTGGGCGTAAAGC
GTGCGCAGGCGGTCCGCTAAGACAGATGTGAAATCCCCGGGCTTAACCTGGGAACTGCAT
TTGTGACTGGCGGGCTAGAGTATGGCAGAGGGGGGTAGAATTCCACGTGTAGCAGTGAAA
TGCGTAGAGATGTGGAGGAATACCGATGGCGAAGGCAGCCCCCTGGGCCAATACTGACGC
TCATGCACGAAAGCGTGGGGAGCAAACAGGATTAGATACCCGCGTAGTCC
>Otu1233
CCAGCCTACGGGACGCAGCAGTGGGGAATTTTGCGCAATGGGGGAAACCCTGACGCAGCA
ACGCCGCGTGGAGGATGAAGTCCCTTGGGACGTAAACTCCTTTCGACTGGGAAGATAATG
ACGGTACCAGTGGAAGAAGCCCCGGCTAACTTCGTGCCAGCAGCCGCGGTAATACGAAGG
GGGCTAGCGTTGCTCGGAATCACTGGGCGTAAAGGGTGCGTAGGCGGGTCTTTAAGTCAG
GGGTGAAATCCTGGAGCTCAACTCCAGAACTGCCTTTGATACTGAGGATCTTGAGTTCGG
GAGAGGTGAGTGGAACTGCGAGTGTAGAGGTGAAATTCGTAGATATTCGCAAGAACACCA
GTGGCGAAGGCGGCCAACTGGTCCGATACTGACGCTGAGGTGCGAAAGCGTGGGGAGCAA
ACAGGATTAGATACTCCGGTAGTCC
>Otu1234
CCAGCCTATGGGTCGCTGCAGTCGGGAAAATTTTGCAATACACGAAAGTGTGACAGAGCA
AGCCAGAGTGCTTTTCTTTTTGAAAAGCTTTTGTGAGATGTAAAAAGTCTTACGAATAAG
GACTGGGCAAGACTGGTGCCAGCCGCCGCGGTAATCCCAGCAGTCCAAGTCGCAGCCACA
TTTGTTGGGTCTAAAATATCCGTAGCTTGCTATTCAAGTCTTTTGTGAAATCGGGACTCT
TAAGGTTCCGGCGGGCAAAAGATACTGTTTAGCTAGAGACCGGGAGGTGCAGGAAGTACG
TTTAGGGTAGCGGTAAAATGCTCTGATCCTAAATGGACTAACAGTCGCGAAGGCGTCCTG
TAAGAACGGTTCTGACAGTGAGGGATGAAGGCTAGGGGCGCAAAGTGGATTAGAAACCCC
AGTAGTCC
>Otu1236
CCAGCCTACGGGACGCTGCAGTGAGGAATTTTCCGCAATGGGGGAAACCCTGACGGAGCG
ACGCCGCGTGTGGGAAGAAGGCCTTCGGGTCGTAAACCACTGTCGAGGGGGACGATATTG
ACGGTACCCCTGGAGGAAGCCCCGGCTAACTACGTGCCAGCAGCCGCGGTAAGACGTAGG
GGGCAAGCGTTGTCCGGAATTATTGGGCGTAAAGCGCTCGTAGGTGGATCGATAAGTCCG
CAGAGAAAGACCCGGGCTCAACTCGGGGAACGGTGTGGATACTATTGATCTTGAGGCAAT
CAGAGGATGATGGAATTCCCGGTGTAGCGGTGAAATGCGTAGATATCGGGAGGAACACCA
GTGGCGAAGGCGATCATCTGGGGTTGTTCTGACACTGAGGAGCGAAAGCTAGGGGAGCAA
ACGGGATTAGAAACCCGGGTAGTCC
>Otu1238
CCAGCCTACGGGTCGCTGCAGTCGAGAATCTTTCACAATGGGGGCAACCCTGATGGAGCG
ACGCCGCGTGGAGGATAAGGTCTTCGGATTGTAAACTCCTGTCATGCGAGAGCAAGGCAT
AGCAATATGTTTGATAGTATCGCAAGAGGAAGAGACGGCTAACTCTGTGCCAGCAGCCGC
GGTAATACAGAGGTCTCAAGCGTTGTTCGGAATCACTGGGCGTAAAGGGTGCGTAGGCTG
CGGAGTAAGTCGGGTGTGAAATTTGAGGGCTCAACCCTCAACTAGCACCTGATACTGCTT
CGCTAGAGACCTGGAGAGGAGTTTGGAATTCTCGGTGTAGCAGTGAAATGCGTAGATTTC

GAGAAGAACGCTCGCAGCGAAAGCGAAACTCTGGACAGCGTCTGACGCTGAGGCACGAAG
GCTAGGGGAGCAAACGGGATTAGATACCCTCGTAGTCC
>Otu1240
CCAGCCTATGGGGGGCTGCAGTAGGGAATATTGCACAATGGAGGAAACTCTGATGCAGCG
ACGCCGCGTGAGTGATGAAGGCCTTCGGGTCGTAAAGCTCTGTCGCAAGGGAATAACACA
ATGAATGTACCTTGCAAGAAAGGATCGGCTAACTTCGTGCCAGCAGCCGCGGTAAGACGA
GGGATCCTAGCGTTGTTCGGAATTATTGGGCGTAAAGCGGGTGTAGGTGGCCATGTAAGT
CAGATGTGAAAGCCCAGGGCTTAACCCTGGAAGTGCATTTGATACTGCGTGGCTTGAGTG
TAAGAGAGGATAGTAGAATTCCTGGTGTAGTGGTGAAATACGTAGATATCAGGAGGAATA
CCGGCGGCGAAGGCGGCTATCTGGCTTAACACTGACACTCAGACCCGAAAGCGTGGGGAT
CAAACAGGATTAGAAACCCTCGTAGTCC
>Otu1243
CCAGCCTATGGGGGGCTCCAGTCGAGAATTTTTCACAATGGGGGCAACCCTGATGGAGCG
ACGCCGCGTGGAGGATGAAGGTCTTCGGATTGTAAACTCCTGTCAAACGGGAACAAGAAA
GTGATAGTACCGTTAGAGGAAGAGACGGCTAACTCTGTGCCAGCAGCCGCGGTAATACAG
AGGTCTCAAGCGTTGTTCGGATTCATTGGGCGTAAAGGGTGCGCAGGCTGTGAGGTAAGT
CGGGTGTGAAATTTCGGGGCTCAACCCCTAAACTGCATTCGATACTGCTTCGCTAGAGGA
CTGTAGAGGAGATTGGAATTCACGGTGTAGCAGTGAAATGCGTAGATATCGTGAGGAAGA
CCAGTGGCGAAGGCGAATCTCTGGGCAGTTCCTGACGCTCATGCACGAAGGCCAGGGGAG
CAAACGGGATTAGAAACCCCGGTAGTCC
>Otu1244
CCAGCCTATGGGAGGCTCCAGTGGGGAATTTTGGACAATGGGGGCAACCCTGATCCAGCG
ACGCCGCGTGAAGGATGAAGGTCTTCGGATCGTAAACTTCTTTTAGAGGGGATGAATGAG
TTAGTGAATAGCTAACTGTGACAGTACTCTTTGAATAAGCCACGGCTAACTGCGTGCCAG
CAGCCGCGGTAATACGTAGGTGGCAAGCGTTACTCGGAATTACTAGGCGTAAAGCGCAGG
TAGGCGGTTGGATAAGTTTCTTGTGAAAGCTCCCGGCTTAACTGGGAGAGGCCAAGGAAT
ACTATCCGACTTGAGTATGGTAGGGGTAACTGGAATTCCCGGTGTAGCGGTGAAATGCGT
AGATATCGGGAGGAATACCTATGGCGAAGGCAGGTTACTGGGCCATTACTGACGCTGAGC
TGCGAAAGCTAGGGGAGCAAAAAGGATTAGATACCCGCGTAGTCC
>Otu1246
CCAGCCTACGGGTTGCAGCAGTGGGGAATATTGCGCAATGGGCGAAAGCCTGACGCAGCA
ACGCCGCGTGAGTGATGAAGGTCTTCGGATCGTAAAGCTCTGTCGGCAGGAACGATGGGC
TCGTTGGCTAACATCCAACGGGTTTGACGGTACCTGCAAAGGAAGCACCGGCTAACTCTG
TGCCAGCAGCCGCGGTAAGACAGAGGGTGCAAGCGTTGTTCGGAACTACTGGGCGTAAAG
CGCGCGTAGGCGGCTTTTCAAGTCGGGTGTGAAATCCCACGGCCTAACCGTGGAAGTGCA
TTCGATACTGAGAGGCTTGAGTCCTGGAGAGGGTGGTGGAATTCCCAGTGTAGAGGTGAA
ATTCGTAGATATTGGGAGGAACACCGGTGGCGAAGGCGACCACCTGGACAGAGACTGACG
CTGAGGTGCGAAAGCGTGGGGAGCAAACAGGATTAGAAACCCCAGTAGTCC
>Otu1248
CCAGCCTACGGGTCGCAGCAGTGGGGAATATTGGACAATGGGCGCAAGCCTGATCCAGCC
ATGCCGCGTGAGTGATGAAGGCCTTAGGGTTGTAAAGCTCTTTTGCCAGGGAAGATGATG
ACGGTACCTGGAGAATAAGCACCGGCTAACTTCGTGCCAGCAGCCGCGGTAATACGAAGG
GTGCTAGCGTTGTTCGGAATTACTGGGCGTAAAGCGCGCGTAGGCGGCTTGTCAAGTCAG
GGGTGAAATCCCACAGCTTAACTGTGGAACTGCCTTTGAAACTGTCAAGCTTGAGTGTCG
GAGGGGATAGCGGAATTGCTAATGTAGAGGTGAAATTCGTAGATATTAGCAGGAACACCG
GTGGCGAAGGCGGCTATCTGGACGACAACTGACGCTGAGGCGCGAAAGCGTGGGGATCAA
ACAGGATTAGATACCCTTGTAGTCC
>Otu1250
CCAGCCTATGGGTGGCAGCAGTGGGGAATATTGCGCAATGGGGGAAACCCTGACGCAGCG
ACGCCGCGTGAAGGATGAAGGTCTTCGGATTGTAAACTTCTGTTGAGAGGGAAGAAAAGT
CTATATTTAAAAGATGTAGATGTTGACGGTACCTCTATAGAAAGCCCCGGCTAACTTCGT
GCCAGCAGCCGCGGTAATACGAAGGGGGCAAGCGTTATTCGGAATAACTGGGCGTAAAGA
GCGTGTAGGTGGTTCTATAAGTTGATTGTTAAATTTCACGGCCTAACCGTGAGCATGCAG
TCAAAACTGTGGGACTAGAGGACAGGAGGGAGGAGTGGAATTCTCGGAGTAGCGGTAAAA
TGCGTAGATCTCGAGAGGAACACCGATGGCGAAGGCAGCTCCTTGGCCTGTTCCTGACAC
TGAAATGCGAAAGCGTGGGGAGCAAACAGGATTAGAAACCCTTGTAGTCC
>Otu1253
CCAGCCTATGGGTTGCAGCAGTAAGGAATATTGGACAATGGGGGCAACCCTGATCCAGCG

ATGCCGCGTGAGTGAAGAAGGCCCTCGGGTCGTAAAGCTCTTTAGGCTGGGAAGAAGGGT
ATGTGGTGAATAGCCATGTATCTTGACGGTACCGGCAGAATAAGCACCGGCAAACTCTGT
GCCAGCAGCCGCGGTAATACAGAGGGTGCGAGCGTTAATCGGAATTACTGGGTGTAAAGG
GCGCGTAGGCGGCGCGATGTGTGTGATGTGAAAGCCCCGGGCTTAACCTGGGAAGTGCAT
TGCAAACTGTCGTGCTGGAGTATATGAGAGGGTGGCGGAATTTCCGGTGTAGCGGTGAAA
TGCGTAGAGATCGGAAGGAACGTCGATGGCGAAGGCAGCCACCTGGCATAATACTGACGC
TGAGGCGCGAAAGCGTGGGGAGCGAACAGGATTAGAAACCCGCGTAGTCC
>Otu1255
CCAGCCTACGGGGGGCTGCAGTCGAGAATTTTTCACAATGGGCGAAAGCCTGATGGAGCG
ACGCCGCGTGGGGGATGAATGGCTTCGGCCCGTAAACCCCTGTCATTTGCGAACAAACCT
ATTCACCTAACACGTGAAGAGCTGATTGTAGCGGAAGAGGAAGGGACGGCTAACTCTGTG
CCAGCAGCCGCGGTAAGACAGAGGTCCCGAGCGTTGTTCGGATTCATTGGGCGTAAAGGG
TGTGTAGGAGGTCGGGTAAGTCAGGTGTGAAATCTCAGAGCTTAACTCTGAAACTGCGCT
TGATACTGCTCGGCTAGAGGATCGGAGGGGGTATCGGAATTTATGGTGTAGCAGTGAAAT
GCGTAGATATCATAAGGAACACCGGTGGCGAAGGCGGATACCTGGAAGATTCCTGACTCT
GAAACACGAAAGCCAGGGGAGCAAACGGGATTAGATACCCGCGTAGTCC
>Otu1257
CCAGCCTATGGGGTGCAGCAGTGAGGAATTTTGCGCAATGGACGAAGGTCTGACGCAGCA
ACGCCGCGTGAGTGATGAAGGTCTTCGGATTGTAAAACTCTGTTGAGTGGGAAGAAAAGT
TCCGGGAATAACACCTCGGGACCTGACGGTACCACTTGAGGAAGCCCCGGCTAACTACGT
GCCAGCAGCCGCGGTAATACGTAGGGGGCAAGCGTTGTTCGGAATCACTGGGCGTAAAGG
GAGCGTAGGCGGAGGAATAAGTTAGGAGTTTAATGTACGGGCTTAACCCGTAATCTGCTC
CTAATACCGTTCTTCTTGAGTATGGGAGAGGGAGATGGAATTCCAGGTGTAGCGGTGGAA
TGCGTAGATATCTGGAAGAACACCAGCTGCGAAGGCGGTCTCCTGGCCCAATACTGACGC
TGAGGCTCGAAAGCTAGGGGAGCAAACAGGATTAGAAACCCCGTAGTCC
>Otu1259
CCAGCCTACGGGTCGCAGCAGTGGGGAATTTTACACAATGGGGGAAACCCTGATGTAGCG
ACACCGCGTGAGCGAAGAAGCCCTTTGGGGTGTAAAGCTCTGTCAGCAGGGACGAAAAAA
ATGACGGTACCTGCAGAGGAAGCATCGGCTAACTACGTGCCAGCAGCCGCGGTAAGACGT
AGGATGCGAGCGTTGTCCGGATTTATTGGGCGTAAAGAGTTCGTAGGTGGTTCATTAAGT
CTGGTGTAAAAGACTGGGGCTCAACCCTGGGATGGCACTGGATACTGGTGGACTGGAGTG
AGGTAGAGGCAAGCGGAATTCCCAGTGTAGCGGTGAAATACGTAGATATTGGGAAGAACA
CCGGTGGCGCAAGCGGCTTGCTGGGCCTTAACTGACGCTGAGGAACGAAAGCTAGGGGAG
CAAATGGGATTAGAAACCCGCGTAGTCC
>Otu1260
CCAGCCTACGGGTCGCAGCAGTGGGGAATTTTCCGCAATGGGCGAAAGCCTGACGGAGCA
ACGCCGCGTGAGTGATGAAGACCTTCGGGTTGTAAAGCTCTGTCGGAGGGGAAGAACACA
ATGACGGTACCCTCCAAGAAAGCCCCGGCTAACTACGTGCCAGCAGCCGCGGTAATACGT
AGGGGGCGAGCGTTATCCGGATTTACTGGGCGTAAAGCGCGTTAAGGCGGCCGACAAAGT
TGGTGGTGAAATTTCAGGGCTCAACTCTGAAACTGCCTCCAATACTGGTTGGCTTGAGTG
CGAGAGAGGAAGGTGGAATTCCCGGTGTAGTAGTGAAATGCGTAGATATCGGGAGGAACA
CCAGTGGCGAAGGCGGCCTTCTGGCTCGCAACTGACGCTGTAGCGCGAAAGCGTGGGGAG
CGAACGGGATTAGAAACCCCCGTAGTCC
>Otu1261
CCAGCCTACGGGTTGCTCCAGTGGGGAATATTGGACAATGGGGGAAACCCTGATCCAGCG
ACGCCGCGTGTGTGAAGAAGGCCTGCGGGTTGTAAAGCACTTTTAGTGGGGACAAAAAGC
TACGGATTAATACTCTGTGGTCTTGATTTAACCCAAAGAATAAGCACCGGCTAACTACGT
GCCAGCAGCCGCGGTAATACGTAGGGTGCAAGCGTTAATCGGAATTACTGGGCGTAAAGC
GTACGCAGGCGGTTCGCTAAGACAGATGTGAAATCCCCGGGCTTAACCTGGGAACTGCAT
TTGTGACTGGCGGGCTAGAGTATGGCAGAGGGGGGTAGAATTCCACGTGTAGCAGTGAAA
TGCGTAGAGATGTGGAGGAATACCGATGGCGAAGGCAGCCCCCTGGGCCAATACTGACGC
TCATGCACGAAAGCGTGGGGAGCAAACAGGATTAGAAACCCGCGTAGTCC
>Otu1264
CCAGCCTACGGGTTGCAGCAGTGAGGAATCTTCCGCAATGGGCGAAAGCCTGACGGAGCG
ACACCGCGTGGAGGACGAAGGCCTTTTGGTTGTAAACTCCTTTTTTGAGGGAATAAATGA
TGAAGTCACCTCAAGAATAAGCACCGGCTAACTACGTGCCAGCAGCTGCGGTAATACGTA
GGGTGCAAGCGTTATCCGGAATTATTGGGCGTAAAGCGTTCGCAGGCGGTTTTGTAAGTC
TGTGGTTAAATCTGGTCGCTCAACGATCAACATGCCACAAATACTGCAAAGCTAGAGGAT

GGCAGAGGCAACTGGAATTCTGCGTGTAGGGGTAAAATCCGTTGATACGCAGAGGAACAC
CAAAAGCGAAGGCAGGTTGCTGGGCCATTTCTGACGCTGTTGAACGAAAGCGTGGGTAGC
AAACAGGATTAGAAACCCCAGTCC
>Otu1266
CCAGCCTATGGGTTGCAGCAGTGGGGAATTTTGTGCAATGGGCGAAAGCCTGACACAGCG
ACACCGCGTGAGCGACGAAGCCTTTTGGGGTGTAAAGCTCTGTCGGCAGGGAAGAAAAAA
ATGACGGTACCTGCAGAGGAAGCATCGGCTAACTACGTGCCAGCAGCCGCGGTAAGACGT
AGGATGCAAGCGTTGTCCGGATTTATTGGGCGTAAAGAGTTCGTAGGCGGTTCGTTAAGT
CTGATGTTAAAGACTGGGGCTCAACCTCGGGAGTGCATTGGATACTGGCGGACTGGAGTA
CAGTAGAGGCAAGTGGAATTCCCAGTGTAGCGGTGAAATGCGTAGATATTGGGAAGAACA
CCGGTGGCGCAAGCGACTTGCTGGGCTGTAACTGACGCTGAGGAACGAAAGCTAGGGTAG
CGAATGGGATTAGAGACCCGCGTAGTCC
>Otu1268
CCAGCCTATGGGGCGCTCCAGTGGGGAATATTGGACAATGGGCGCAAGCCTGATCCAGCA
ATGCCGCGTGTGTGAAGAAGGCCTGCGGGTTGTAAAGCACTTTCAGTTGGGAGGAAGGCC
TTAGCGTGAATATCGCTGAGGATTGACGTTACCAACAGAAGAAGCACCGGCTAACTCTGT
GCCAGCAGCCGCGGTAATACAGAGGGTGCAAGCGTTAATCGGAATTACCGGGCGTAAAGG
GCGCGTAGGTGGTTATTTAAGTTAGAGGTGAAATCCCCGGGCTTAACCTGGGAATTGCCT
ATAAGACTGGATAGCTAGAGTTCGGGAGAGGGAAGTGGAATTTCCGGTGTAGCGGTGAAA
TGCGTAGATATCGGAAGGAACACCAGTGGCGAAGGCGACTTCCTGGCCCGATACTGACAC
TGAGGCGCGAAAGCGTGGGGAGCGAACAGGATTAGAAACCCCCGTAGTCC
>Otu1269
CCAGCCTATGGGGTGCAGCAGTCGAGGATCTTCGGCAATGGGCGCAAGCCTGACCGAGCG
ATGCCGCGTGCGGGATGAAGGCCTTCGGGTTGTAAACCGCTGTCAGTGGGGAGGAAATGT
ACGTGGGTACTCCCACGTATTTGACCTATCCGCAGAGGAAGTGTGGGCTAAGTTCGTGCC
AGCAGCCGCGGTAAGACGAACCACACAAACGTTATTCGGAATCACTGGGCTTAAAGGGTG
CGTAGGCTGCGCAGCAAGTTGGGTGTGAAATCCCTCGGCTCAACCGAGGAACTGCGCCCA
AAACTGCTGTGCTCGAGGGAGATAGAGGTGAGCGGAACTTAGGGTGGAGCGGTGAAATGC
GTTGATATCCTAAGGAACACCCGTGGAGAAAGCGGCTCACTGGATCTCTTCTGACGCTGA
GGCACGAAAGCTAGGGTAGCGAACGGGATTAGATACCCCAGTAGTCC
>Otu1272
CCAGCCTACGGGTCGCTCCAGGCGCGAAAACTTTACAATGCTGGAAACAGCGATAAGGGG
ACCTCGAGTGCCAGGTTACAAATCTGGCTGTCGTGATGCCTAAAAAGCATTGCATAGCAA
GGGCCGGGCAAGACCGGTGCCAGCCGCCGCGGTAACACCGGCGGCTCGAGTGGTAACCGT
TATTATTGGGTCTAAAGGGTCTGTAGCCGGCCGGATAAGTCTCTTGGTAAATCTGGCAGC
TTAACTGTCAGGCTTTCAGGAGATACTGTCTGGCTCGAGGCCGGGAGAGGTGAGAGGTAC
TTCAGGGGTAGGGGTGAAATCTTGTAATCCTTGAAGGACCACCAGTGGCGAAGGCGTCTC
ACCAGAACGGGCCTGACGGCAAGGGACGAAAGCTAGGGGCACGAACCGGATTAGAAACCC
GAGTAGTCC
>Otu1274
CCAGCCTATGGGACGCACCAGTGGGGAATATTGCGCAATGGGCGAAAGCCTGACGCAGCG
ACGCCGCGTGGGTGATGAAGGCCTTCGGGTTGTAAAGCCCTGTGGGGGGAGAAGAATAAG
TCGGTGTAATATCCCGATGATGACGGTATCCCCTTAGCAAGCACCGGCTAACTCTGTGCC
AGCAGCCGCGGTAAGACAGAGGGTGCAAACGTTGTTCGGAATTACTGAGCGTAAAGCGTG
TGTAGGCGGCTACGTAAGTCGGATGTGAAAGCCCTGGGCTCAACCCAGGAAGTGCACCCG
ATACTGCATAGCTTGAGACTCGGAGAGGTTGGTGGAATTCTCGGTGTAGAGGTGAAATTC
GTAGATATCGAGAGGAACATCGGTGGCGAAGGCGGCCAACTGGACGAAGTCTGACGCTGA
GACACGAAAGCGTGGGGAGCAAACAGGATTAGATACCCCCGTAGTCC
>Otu1278
CCAGCCTATGGGTCGCAGCAGTAAGGAATATTGGGCAATGGACGCAAGTCTGACCCAGCC
ATGCCGCGTGCAGGATGAATGCCCTATGGGTTGTAAACTGCTTTTATACGGGAAAAACCC
CTTCGACGTGTCGAAGGCTGATGGTACCGTAAGAATAAGGATCGGCTAACTCCGTGCCAG
CAGCCGCGGTAATACGGAGGATCCAAGCGTTATCCGGATTTATTGGGTTTAAAGGGTGCG
TAGGCGGAATTGTAAGTCAGTGGTGAAATCCTACAGCTTAACTGTAGAATGGCCATTGAT
ACTGCAGATCTTGAGTATATTTGAAGTGGGCGGAATGTGTCATGTAGCGGTGAAATGCTT
AGATATGACACAGAACACCGATTGCGAAGGCAGCTCACTAAACTACAACTGACGCTGAGG
CACGAAAGCGTGGGGAGCAAACAGGATTAGAGACCCCGGTAGTCC
>Otu1282

CCAGCCTATGGGTGGCAGCAGTGGGGAATATTAGACAATGGGCGACAGCCTGATCTAGCG
ATACTTCGTGGAGGACTAAAGCCTCAGTTGTAAACTCCTATTTCGATAATAACAATAATG
ATTATAATTATAAAAGAAGCCCCGGTAAATGTCTGTGCCAGCAGCCGCGGTAATACAGGG
GGGGCAAGTATTATTCTACATGACTGGGCGTAAAGGATATGTAGGCGGTTTTTTAATAC
TAAAAAAAATTCCATATTTAAGTTATGGGTTATTTTTTTATATTGTAAAACTTCGAGTTT
ATAAAAGATATCAGAACCCACTAGAGTAACAGTGAAATGTGGATAAACTAAGGGGAATTC
CAAAAGGCGAAAGCAATTATCTTGTTAAAACTGACGCTGAAATATGAAGGTTTGGGGAGC
AAACAGGATTAGAAACCCTCGTAGTCC
>Otu1283
CCAGCCTACGGGACGCTCCAGTGGGGAATATTGGACAATGGGCGAAAGCCTGATCCAGCA
ATGCCGCGTGAGTGATGAAGGCCTTAGGGTTGTAAAGCTCTTTTACCCGGGAAGATAATG
ACTGTACCGGGAGAATAAGCCCCGGCTAACTCCGTGCCAGCAGCCGCGGTAATACGGAGG
GGGCTAGCGTTGTTCGGAATTACTGGGCGTAAAGCGCACGTAGGCGGCTTTGTAAGTTAG
AGGTGAAAGCCTGGGGCTCAACTCCAGAATTGCCTTTAAGACTGCATCGCTTGAACGTCG
GAGAGGTGAGTGGAATTCCGAGTGTAGAGGTGAAATTCGTAGATATTCGGAAGAACACCA
GTGGCGAAGGCGGCTCACTGGACGACTGTTGACGCTGAGGTGCGAAAGCGTGGGGAGCAA
ACGGGATTAGATACCCCTGTAGTCC
>Otu1284
CCAGCCCATGGGGGGCTGCAGTCGAGAATTTTTCTCAATGGGGGAAACCCTGAAGGAGCG
ACGCCGCGTGGAGGATGAAGGTCTTCGGATCGTAAACTCCTGTCATTTGCGAACAAACCT
ATTCACCTAACACGTGAAGAGCTGATTGTAGCGGAAGAGGAAGGGACGGCTAACTCTGTG
CCAGCAGCCGCGGTAATACAGAGGTCCCAAGCGTTGTTCGGATTCACTGGGCGTAAAGGG
TGCGTAGGCGGTCAGGTAAGTCTGATGTGAAATCCCGAAGCCTAACTTCGGAACTGCATT
GGATACTATCTGGCTAGAGGAATGGAGGGGAGACTGGAATGCTCGGTGTAGCAGTGAAAT
GCGTAGATATCGAGCGGAACACCAGTGGCGAAGGCGAGTCTCTGGACATTTCCTGACGCT
GAGGCACGAAAGCCAGGGGAGCAAACGGGATTAGAGACCCTCGTAGTCC
>Otu1286
CAGCTACGGGAGGCAGCAGTCGAGGATCTTCGTCAATGGGCGCAAGCCTGAACGAGCGAC
GCCGCGTGTGCGATGAAGGCCTTCGGGTTGTAAAGCACTGTCGAGGGGGATCAAGGCGTA
AGCTTGATTGATCCCTGGAGGAAGCGCGGGCTAAGTTCGTGCCAGCAGCCGCGGTAAGAC
GAACCGTGCGAACGTTGTTCGGAATCACTGGGCTTAAAGGGCGCGTAGGCGGCAGGCGAG
GTCGGGGGTGAAATCCCACAGCTTAACTGTGGAAGAGCCTCCGATACCGGCTTGCTGGAG
GAGGGTAGGGGTGCGCGGAACTTCTGGTGGAGCGGTGAAATGCGTTGATATCAGAAGGAA
CGCCGGCGGCGAAAGCGGCGCACTGGACCCTTTCTGACGCTGAGGCGCGAAAGCCAGGGG
AGCAAACGGGATTAGATACCCCAGTAGTCC
>Otu1290
CCAGCCTACGGGAGGCTGCAGTGGGGAATATTGCGCAATGGGCGAAAGCCTGACGCAGCG
ACGCCGCGTGGGGGATGAAGCTTCTCGGAGTGTAAACCCCTTTCGACCCGGAAGAATGTC
CCGCAAGGGATTGACGGTACGGGTATAAGAAGCCCCGGCTAACTACGTGCCAGCAGCCGC
GGTAATACGTAGGGGGCCAGCGTTGCTCGGAATTACTGGGCGTAAAGGGTTCGTAGGCGG
TGCGGCAAGTCGGGAGTGAAATCTCTGGGCTCAACTCAGAGGCTGCTTCCGAAACTGCCG
TGCTTGAGTGCGGGAGAGGCGAGTGGAATTACAGGTGTAGCGGTGAAATGCGTAGATATC
TGTAGGAACACCCGTGGCGAAGGCGGCTCGCTGGACCGCAACTGACGCTGAGGAACGAAA
GCTAGGGGAGCAAACAGGATTAGAAACCCGTGTAGTCC
>Otu1291
CCAGCCTACGGGTGGCAGCAGCAAGGAATTTTGCGCAATGGGCGAAAGCCTGACGCAGCG
ACGCCGCGTGGGGGATGACGGCCTTCGGGTTGTAAACCCCTTTTCACGAGGAAGAAATAT
GACGGTACTCGTGGAATAAGTCTTGGCTAACTACGTGCCAGCAGCCGCGGTAATACGTAG
AAGGCGAACGTTATCCGGATTTACTGGGCGTAAAGCGGGTGTAGACGGTTTTTTAAGTCA
GTTGTGAAATCTCCCGACTCAATCTGGAGTCTGCAATTGATACTGGAGAGCTCGAGGGCA
TCAGAAGAAAGCGGAATTCACGGTGTAGCGGTGAAATGCGTAGATATCGTGAGGAACACC
AGTGGCGAAGGCGGCTTTCTAGGATGCTCCTGACGTTGAGGCCCGAAAGTGTGGGGAGCG
AAACGGATTAGATACCCGCGTAGTCC
>Otu1293
CCAGCCTATGGGTCGCTGCAGCAAGGAATTTTTCTCAATGGGCGAAAGCCTGAAGGAGCG
ACGCCGCGTGGGGGATGAATGGCTTCGGCCCGTAAACCCCTGTCATTCGGGATCAATGCC
TGTGGCCTAATACGCTGCAGGTTGATAGTACCGGAAGAGGAAGGGACGGCTAACTCTGTG
CCAGCAGCCGCGGTAATACAGAGGTCCCAAGCGTTGTTCGGATTCACTGGGCGTAAAGGG

TGCGTAGGCGGTCGGGTAAGTCTGATGTGAAATCCCGCAGCTTAACTGCGGAACTGCATT
GGATACTATTCGGCTAGAGGAGTGGAGGGGAGACTGGAATTCTCGGTGTAGCAGTGAAAT
GCGTAGATATCGAGAGGAACACCTGTGGCGAAGGCGAGTCTCTGGACACTTCCTGACGCT
GAGGCACGAAAGCCAGGGGAGCAAACGGGATTAGAAACCCCAGTAGTCC
>Otu1296
CCAGCCTATGGGAGGCAGCAGTGGGGAATATTGCGCAATGGACGAAAGTCTGACGCAGCA
ACGCCGCGTGAGTGAAGAAGGCCTTTGGGTTGTAAAGCTCTTTCGGCCGGGAAGAAGGGC
GTGGGTGTTAATAGTATCCACATTTGATGGTACCGGAAGAAGAAGCACCGGCTAACTTCG
TGCCAGCAGCCGCGGTAATACGAAGGGTGCGAGCGTTGTTCGGAATTACTGGGCGTAAAG
GGTTTGTAGGCTGAAATGTAAGTCAAGTGTGAAATCCCCGAGCTAAACTTGGGACGTGCA
TTTGAAACTGTGTTTCTTGAGTATCGTAGAGGGTGGTGGAATTGTAGGTGTAGGAGTGAC
ATCCGTAGAGATCTGCAGGAACATCAGAGGCGAAGGCGACCACCTGGCCGATTACTGACG
CTGAGGAACGAAAGCGTGGGGAGCAAACAGGATTAGAGACCCGCGTAGTCC
>Otu1297
CCAGCCTACGGGTGGCTCCAGTCGAGGATCTTCGGCAATGGGCGCAAGCCTGACCGAGCG
ACGCCGCGTGTGCGATGAAGGCCTTCGGGTTGTAAAGCACTGTCGAGGGGGAGGAAGCCG
CAAGGTTGACCTATCCCTGGAGGAAGCACGGGCTAAGTTCGTGCCAGCAGCCGCGGTAAG
ACGAACCGTGCGAACGTTGTTCGGAATCACTGGGCTTAAAGGGCGCGTAGGCGGATCATC
AAGTCCGGGGTGAAATCTTTCGGCTTAACCGGAAAACAGCCTTGGATACTGGTGGTCTCG
AGTGAGGTAGGGGCCGTTGGAACTTCCGGTGGAGCGGTGAAATGCGTTGATATCGGAAGG
AACGCCGGTGGCGAAAGCGAACGGCTGGACCTCTTCTGACGCTGAGGCGCGAAAGCCAGG
GGAGCAAACGGGATTAGAGACCCTTGTAGTCC
>Otu1298
CAGCCTATGGGTCGCAGCAGTCGAGGATCTTCGGCAATGGGCGCAAGCCTGACCGAGCGA
CGCCGCGTGCGGGATGAAGGCCTTCGGGTTGTAAACCGCTGTCAGTGGGGAGAAAATGCT
GGTGGGTACTCTCATCAGTTTGATCTATCCGCAGAGGAAGTCAGGGCTAAGTTCGTGCCA
GCAGCCGCGGTAAGACGAACCTGACGAACGTTATTCGGAATTACTGGGCTTAAAGGGTGC
GTAGGCTGTGCAGAAAGATGGGTGTGAAATACCTCGGCTTAACCGAGGAACTGCGCCCAA
AACTACTGTGCTTGAGGGAGACAGAGGTGAGCGGAACTTAGGGTGGAGCGGTGAAATGCG
TTGATATCCTAAGGAACACCCGTGACGAAAGCGGCTCACCGGGTCCCTTCTGACGCTGAG
GCACGAAAGCTAGGGGAGCAAACGGGATTAGATACCCCGTAGCC
>Otu1301
CCAGCCTATGGGTCGCTGCAGTGAGGAATATTGGACAATGGACGCAAGTTTGATCCAGCA
TTAAGGCGTGAATCAGTGGAAATTTTTCCACCCTTATTACTTGTGAATAAGGGAAGAATT
TATTTTGAAAGATTCTAAAAAAAAGGACTAGTAAAAATGAATGTACTTTTTGAATAAGTT
CCGGCTAACTTCGTGCCAGCAGCCGCGGTAACACGGGGGGAACGAGTGTTATTCGTCTTG
ACTGGGCTTAAAGGGTACGTAGGTGGTCGTATTGTGTGTGTAATTATTAACGAAAATATG
ACTGAGGGTATATAGGAGATTAATGGTACTCTTGATGTAGGGGTAAAATCTTATGATATC
AAAGGGGACCATCGATGGGCGAAAACGATTAATCATTATATTACTAACATTGAGGTACTA
AAGCATAGGGAGCAAACAGGATTAGATACCCCCGTAGTCC
>Otu1302
CCAGCCTATGGGGGGCACCAGTGGGGAATCTTGCACAATGGGCGAAAGCCTGACGCAGCG
ACGCCGCGTGAGTGATGAAGGCCCTCGGGTTGTAAAGCTCTGTGGAGAGGGACGAATAAG
TGTTGGCCAATATCCAACATGATGACGGTACCTTTTTAGCAAGCACCGGCTAACTCTGTG
CCAGCAGCCGCGGTAAGACAGAGGGTGCAAACGTTGTTCGGAATTACTGGGCGTAAAGCG
CGTGTAGGCTGCTTGGAAAGTCGGATGTGAAAGCCCTGGGCTTAACCCAGGAAGTGCACT
CGAAACTTCCAAGCTCGAGTCCTGGAGAGGGTGGCGGAATTCTCGGTGTAGAGGTGAAAT
TCGTAGATATCGAGAGGAACACCGGTGGCGAAGGCGGCTACCTGGACAGTGACTGACGCT
GAGACGCGAAAGCGTGGGGAGCAAACAGGATTAGAAACCCGAGTAGTCC
>Otu1303
CCAGCCTATGGGGGGCTGCAGTGGGGAATATTGGACAATGGGGGCAACCCTGATCCAGCC
ATGCCGCGTGAATGAAGAAGGCCTTCGGGTTGTAAAGTTCTTTTGGCGGGGACGATGATG
ACGGTACCCGCAGAATAAGCTCCGGCTAACTTCGTGCCAGCAGCCGCGGTAATACGAAGG
GAGCAAGCGTTATCCGGATTCACTGGGTTTAAAGGGTGCGTAGGTGGGCAGGTAAGTCAG
TGGTGAAATCTCTGGGCTTAACCCAGAAACTGCCGTTGATACTATCTGTCTTGAATGTAG
TGGAGGTGAGCGGAATATGTCATGTAGCGGTGAAATGCTTAGATATGACATAGAACATCT
ATTGCGAAGGCAGCTCGCTACACTATTATTGACACTGAGGCACGAAAGCGTGGGGATCAA
ACAGGATTAGATACCCCAGTAGTCC

>Otu1305
CCAGCCTACGGGAGGCAGCAGTGGGGAATATTGGACAATGGGGGCAACCCTGATCCAGCC
ATGCCGCGTGAATGATGAAGGCCTTCGGGTTGTAAAGTTCTTTTGACGGGGACGATGATG
ACGGTACCCGTAGAATAAGCTCCGGCTAACTTCGTGCCAGCAGCCGCGGTAATACGAAGG
GAGCTAGCGTTGTTCGGAATTACTGGGCGTAAAGGGCGCGTAGGCGGTTTGACAAGTTGG
ATGTGAAAGCCCAGGGCTCAACCCTGGAATTGCATTCAAGACTGTTTTACTTGGATTCGG
TAGAGGTGAGTGGAATTCCCAGTGTAGAGGTGAAATTCGTAGATATTGGGAAGAACACCA
GTGGCGAAGGCGGCTCACTGGACCGACATTGACGCCGAGGCGCGAAAGCGTGGGGATCAA
ACAGGATTAGATACCCCCGTAGTCC
>Otu1307
CCAGCCTATGGGGGGCAGCAGTGGGGAATCTTGCGCAATGGGCGAAAGCCTGACGCAGCA
ACGCCGCGTGAGTGATGAAGGCCTTCGGGTCGTAAAGCTCTTTCGGAAGGGACGAAACCG
GTCCGGGATAATAAACCGGGCTCTTGACGGTACCTTAAGAAGAAGCACCGGCTAACTCTG
TGCCAGCAGCCGCGGTAATACAGAGGGTGCAAGCGTTGTTCGGAATTATTGGGCGTAAAG
CGCGTGTAGGCGGCAAGGCATGTCGGGTGTGAAAGCCCTCGGCTTAACCGAGGAAGTGCG
CCCGAAACTACTTTGCTTGAGTACTGGAGAGGGTGGCGGAACTCCCGGTGTAGAGGTGAA
ATTCGTAGGTATCGGGAAGAACACCAGTGGCGAAGGCGGCCACCTGGACAGATACTGACG
CTGAGACGCGAAAGCGTGGGGAGCAAACAGGATTAGAAACCCTGGTAGTCC
>Otu1309
CCAGCCTATGGGGGGCAGCAGTGGGGAATCTTGCGCAATGGGCGAAAGCTTGACGCAGCG
ACGCCGCGTGGGTGATGAAGGCCTTCGGGTTGTAAAGCCCTGTGGGGAGAGAAGAATAAG
GCATGCCGAATACGCATGTTGATGACGGTATCTCCTTAGCAAGCACCGGCTAACTCTGTG
CCAGCAGCCGCGGTAAGACAGAGGGTGCAAACGTTGTTCGGAATTACTGGGCGTAAAGCG
CGTGTAGGCGGCAACGCAAGTCGGATGTGAAAGCCCTGGGCTCAACCCAGGAAGCGCATT
CGATACTGCGGAGCTCGAGTCTCGGAGAGGAGGGTGGAACTCTCGGTGTAGGGGTGAAAT
TCGTAGATATCGAGAAGAACACCGGTGGCGAAGGCGGCCCTCTGGACGATGACTGACGCT
GAGACGCGAAAGCGTGGGGAGCAAACAGGATTAGAGACCCCCGTAGTCC
>Otu1311
CCAGCCTACGGGGTGCTGCAGTCGAGAATTCTTCTCAATGGGGGAAACCCTGAAGGAGCG
ACGCCGCGTGAAGGATGAAGGTCTTCGGATTGTAAACTTCTGTCATTGGAGAACAAACCG
CCGGGTAGTAACTGCCCCGGCGTTGATAGTACCTGAAGAGGAAGAGACGGCTAACTCTGT
GCCAGCAGCCGCGGTAATACAGAGGTCTCAAGCGTTGTTCGGATTCATTGGGCGTAAAGG
GTGCGTAGGTGGTGCCGTAAGTCGGATGTGAAATCCCGGAGCTTAACTCCGGAACTGCAT
TCGATACTGCGGTGCTTGAGGACTGGAGAGGAGATCGGAATTCACGGTGTAGCAGTGAAA
TGCGTAGATATCGTGAGGAAGACCAGTGGCGAAGGCGGATCTCTGGACAGTTCCTGACAC
TGAGGCACGAAGGCCAGGGTAGCAAACAGGATTAGAAACCCTAGTAGTCC
>Otu1313
CCAGCCTACGGGTTGCAGCAGTCGAGAATCTTCGGCAATGGGCGCAAGCCTGACCGAGCG
ACGCCGCGTGCGGGATGAAGGCCTTCGGGTTGTAAACCGCTGTCGGAGGGGAGGAAATGC
CAAGGGGTTCTCTCCTTGGTTTGACCTATCCTCAGAGGAAGTACGGGCTAAGTTCGTGCC
AGCAGCCGCGGTAAGACGAACCGTACAAACGTTATTCGGAATTACTGGGCTTAAAGGGTG
CGTAGGCGGCCCGACAAGTTGGGTGTGAAAGCCCTCGGCTTAACCGAGGAATGGCACTCA
AAACTGCCGGGCTTGAGGGAGATAGAGGTGAGCGGAACTTAGGGTGGAGCGGTGAAATGC
GTTGATATCCTAAGGAACACCGGTAGCGAAAGCGGCTCACTGGGTCTTCTCTGACGCTGA
GGTGCGAAAGCGTGGGGAGCAAACAGGATTAGAGACCCTAGTAGTCC
>Otu1314
CCAGCCTACGGGGTGCTGCAGTGGGGAATTTTCCACAATGGGCGAAAGCCTGATGGAGCG
ACGCCGCGTGAGGGATGAAGGTCTTCGGATTGTAAACCTCTTTTGACAGGGAATAAGTGT
AACAAAGCTAATATCTTTGTTGCTTGACGGTACCTGTAGAATAAGCTACGGCTAACTCTG
TGCCAGCAGCCGCGGTAATACAGAGGTAGCGAACGTTGTCCGGATTCACTGGGCGTAAAG
GGCTTGTAGGTTGCCGAATAAGTCAGGCGTGAAATCGTATAGCTTAACTATACAACCGCG
TTTGATACTGTTTGGCTTGAGTGCAGGAGGGGTAATTGGAATTTCCGGTGTAGCGGTGAA
ATGCGTAGATATCGGAAAGAACACCTGTGGCGAAGGCGGATTACTGGCCTGCAACTGACA
CTGAGGAGCGAAAGCTAGGGGAGCAAACAGGATTAGATACCCCCGTAGTCC
>Otu1315
CCAGCCTATGGGGGGCTGCAGGCGAGAAACTTCCGCAATGCGCGAAAGCGTGACGGGGGT
AGTCCGAGTGCTAGCCTAACGGCTAGCTTTTCGCGAGCTTGATTCGCTCGTGGAATAAGG
GTCTGGGCAAATCCTGTGCCAGCCGCCGCGGTAATACAGGTGGCACGAGTGGTGCCCGCG

AATATTGAGCTTAAAGCGCCCGTAGCCGGCTTCGCAAGTTTCCCGTCAAATCCCTCAGCT
TAACTGAGGGGCGCGCGGGAAATACTACGAGGCTCGAGAGTGGGGGAAGCCAAGAGTACG
TTAGGGGGAGCGGTAAAATGCTTTAATCCTTAACGGACTACCAGTGGCGAAAGCGCCTGG
CTAAAACACGTCTGACGGTGAGGGACGAAGGCTAGGGGAGCAAACGGGATTAGAAACCCC
CGTAGTCC
>Otu1316
CCAGCCTATGGGGGGCAGCAGTAGGGAATTTTGCGCAATGGACGGAAGTCTGACGCAGCA
ACGCCGCGTGAGTGATGAAGGTCTTCGGATTGTAAAGCTCTGTTGAGTGGGAAGAATAAC
CCTGTAATTAATACGTACAGGGGATGACGGTACCATTCGAGGAAGCCCCGGCTAACTACG
TGCCAGCAGCCGCGGTAATACGTAGGGGGCAAGCGTTGTTCGGAATCACTGGGCGTAAAG
GGAGCGTAGGCGGTTCGGTAAGTTAGGAGTGAAATCTACAGGCTTAACCTGTAGATTGCT
TTTAATACTGCCGATCTTGAGTATTGGAGAGGATGATGGAATTCCAGGTGTAGCGGTGGA
ATGCGTAGATATCTGGAAGAACACCAGTTGCGTAGGCGGTCATCTGGCCAAATACTGACG
CTGAGGCTCGAAAGCTAGGGGAGCAAACAGGATTAGAAACCCGTGTAGTCA
>Otu1317
CCAGCCTATGGGGGGCTGCAGTCGAGAATTTTTCACAATGGGGGAAACCCTGATGGAGCG
ACGCCGCGTGGAGGACGAAGGTCTTCGGATTGTAAACTCCTGTCATGCGGGAACAAGAAA
GTGATAGTACCGCAAGAGGAAGAGACGGCTAACTCTGTGCCAGCAGCCGCGGTAATACAG
AGGTCTCAAGCGTTGTTCGGATTCATTGGGCGTAAAGGGTGCGCAGGCGGCGTGGAAAGT
TGAGTGTGAAATCCTGAGGCTTAACCTCAGAACTGCACTCAATACTCCCATGCTAGAGGA
ATGTAGAGGAGAGTGGAATTCACGGTGTAGCAGTGAAATGCGTAGATATCGTGAGGAAGA
CCAGTTGCGAAGGCGACTCTCTGGGCATTTCCTGACGCTCATGCACGAAGGCCAGGGGAG
CAAACGGGATTAGAAACCCTTGTAGTCC
>Otu1318
CCAGCCTACGGGAGGCACCAGTCGAGGATCTTCGGCAATGCGCGCAAGCGCGACCGAGCG
ACGCCGCGTGGGCGACGAAGGCCTTCGGGTTGTAAAGCCCTGTCGAGGGGGAGAAAGCCG
CAAGGCGGATCCATCCCTGGAGGAAGCTCGGGCTAAGTTCGTGCCAGCAGCCGCGGTAAG
ACGAACCGAGCGAACGTTGTTCGGAATCACTGGGCTTAAAGGGCGCGTAGGCGGGCTGCC
GCGTCCGGGGTGAAATCCTCCGGCTCAACCGGAGAACGGCCCCGGGTACGGGCGGCCTCG
AGGGGGGTAGGGGCGTGCGGAACTGTGGGTGGAGCGGTGAAATGCGTTGATATCCACAGG
AACTCCGGTGGCGAAGGCGGCACGCTGGACCCCATCTGACGCTGAGGCGCGAAAGCCAGG
GGAGCGAACGGGATTAGATACCCCGGTAGTCC
>Otu1321
CCAGCCTATGGGTGGCAGCAGTGGGGAATTTTCCGCAATGGGCGAAAGCCTGACGGAGCA
ATACCGCGTGAGGGAGGAAGGCTTTTGGGTCGTAAACCTCTTTTGTCAGGGAAGAAGATC
TGACGGTACCTGACGAATCAGCATCGGCTAACTCCGTGCCAGCAGCCGCGGTAATACGGA
GGATGCAAGCGTTATCCGGAATTATTGGGCGTAAAGCGTCCGCAGGTGGTTTTTCAAGTC
TGCTGTTAAAGCGTGCGGCTTAACCGCATATCGGCAGTGGAAACTGGGAGACTAGAGTTT
GGTAGGGGTCACGGGAATTCCCAGTGTAGCGGTGAAATGCGTAGATATTGGGAAGAACAC
CAGCGGCGAAAGCGCGTGACTGGGCCTGAACTGACACTCATGGACGAAAGCTAGGGGAGC
GAAAGGGATTAGAAACCCTCGTAGTCC
>Otu1322
CCAGCCTACGGGTTGCAGCAGTGGGGAATTTTGGACAATGGGCGAAAGCCTGATCCAGCA
ACTCTGCGTGAGGGACGAAGTCCTTCGGGATGTAAACCTCTTTTGTCGGGGACGAACGCC
AGCAATGGCTTGACGGTACCCGGCGAATAAGCCACGGCTAACTACGTGCCAGCAGCCGCG
GTAAGACGTAGGTGGCGAGCGTTATTCGGAATTACTAGGCGTAAAGCGAGTGTAGGCGGG
TGCTTAAGTCCGTCGTGAAATCTCCCGGCTCAACTGGGATAGGTCGATGGATACTGGGCG
CCTAGAGTGGGGTAGGGGGCAGTGGAATTCCCGGTGTAGCGGTGAAATGCGTAGATATCG
GGAGGAACACCTATGGCGAAAGCAGCTGCCTGGGCCCTTACTGACGCTGAGGTGCGAAAG
CGTGGGGAGCAAACAGGATTAGAAACCCTCGTAGTCC
>Otu1323
CCAGCCTACGGGTTGCTGCAGTCGAGGATCTTCGGCAATGGGCGCAAGCCTGACCGAGCG
ACGCCGCGTGTGCGAAGAAGGCCTTCGGGTTGTAAAGCACTTTGGCAGGGGAGGAAAGCC
GTAAGGTTTGACCTATCCTGAGAACAAGCACGGGCTAAGTTCGTGCCAGCAGCCGCGGTA
AGACGAACCGTGCGAACGTTGTTCGGATTCACTGGGCTTAAAGGGCGCGTAGGCGGACTG
TCAAGTCAGGGGTGAAATCTTTCGGCTTAACCGGAAAACAGCTTTTGATACTGACAGTCT
AGAGGGAGGTAGGGGCATGCGGAACTTCCGGTGGAGCGGTGAAATGCGTTGATATCGGAA
GGAACTCCGGTGGCGAAAGCGGCGTGCTGGATCTCTTCTGACGCTGAGGCGCGAAAGCTA

GGGGAGCAAACGGGATTAGAAACCCTAGTAGTCC
>Otu1324
CCAGCCTATGGGAGGCTGCAGTGGGGAATCTTGCGCAATGGGCGAAAGCCTGACGCAGCG
ACGCCGCGTGGGTGATGAAGGCCTTCGGGTTGTAAAGCCCTGTGGGGAAGAACGAATAAG
GGCGGGTTAATAGCCCGTTTGATGACGGTACTTCCTTAGCAAGCACCGGCTAACTCTGTG
CCAGCAGCCGCGGTAAGACAGAGGGTGCGAACGTTGTTCGGAATTACTGGGCGTAAAGCG
CGTGTAGGCGGTCCAGCAAGTCGGGTGTGAAACCCCTAGGCTCAACCTGGGAAGTGCATT
CGATACTGTTGGACTGGAGTCCCGGAGAGGATGGTGGAACTCTCGGTGTAGAGGTGAAAT
TCGTAGATATCGGGAAGAACATCGGTGGCGAAGGCGGCCATCTGGACGGTGACTGACGCT
GAGACGCGAAAGCGTGGGGAGCAAACAGGATTAGATACCCGCGTAGTCC
>Otu1325
CCAGCCTATGGGGTGCAGCAGTGGGGAATATTGGACAATGGGCGCAAGCCTGATCCAGCC
ATGCCGCGTGAGTGACGAAGGTCTTCGGATTGTAAAGCTCTTTTGGCGGGGACGATAATG
ACGGTACCCGCAGAATAAGCCCCGGCTAACTTCGTGCCAGCAGCCGCGGTAATACGAAGG
GGGCTAGCGTTGTTCGGAATCACTGGGCGTAAAGCGCACGTAGGCGGACTTATAAGTCAG
GGGTGAAATCCCGGGGCTCAACCTCGGAACTGCCTTTGATACTGTGAGTCTCGAGACCGG
AAGAGGTGAGTGGAATTCCTAGCGTAGAGGTGAAATTCGTAGATATTAGGAAGAACACCA
GTGGCGAAGGCGGCTCACTGGTCCGGTTCTGACGCTGAGGTGCGAAAGCGTGGGGATCAA
ACAGGATTAGAAACCCTGGTAGTCC
>Otu1327
CCAGCCTACGGGGTGCAGCAGTAACGAATATTGGGCAATGGGCGAAAGCCTGACCCAGCG
ACGCCGCGTGTGGGATGAAGTCCCTCGGGATGTAAACCACTGTCAGGGGTTACCAAGTGA
CCGCCGCTAATATCGGCGGAAGTTGAGGAGCCCCAGAGGAAGCCACGGCTAACTTCGTGC
CAGCAGCCGCGGTAAGACGAAGGTGGCGAGCGTTGTTCGGAATCACTGGGCTTAAAGAGC
GCGTAGGCGGGCTGTTTTGCGCCTTGTGAAATCCCTCGGCTTAACCGAGGAATGGCTTGG
CGAACGGACAGCCTTGAGGTAGGTAGAGGTGTGGAGAACTCTTGGTGGAGCGGTGGAATG
CGTAGATATCAAGAGGAATGCCGGAGGCGAAGGCGCTGCACTGGGCCTATCCTGACGCTG
AGGCGCGAAAGCCAGGGGAGCAAACGGGATTAGATACCCTTGTAGTCC
>Otu1328
CCAGCCTACGGGTTGCAGCAGTGAGGAATATTGGTCAATGGGGGCAACCCTGAACCAGCC
ATGCCGCGTGAAGGATGAAGGCCTTATGGGTTGTAAATTTCTTTTGTATGGGGCGAACCG
TTTCTACGCGTTAGAGACCTGACAGTACCATACGAATAAGCATCGGCTAACTCCGTGCCA
GCAGCCGCGGTAATACGGAGGATGCGAGCGTTATCCGGATTCACTGGGTTTAAAGGGTGA
GTAGGCGGGTTAGTAAGTCAGTGGTGAAAGTCTGTCGCTTAACGATAGGATTGCCATTGA
TACTGCTAGTCTTGAATTCAGTTGAGGCAGGCGGAATGTGCAGTGTAGCGGTGAAATGCT
TAGATATTGCACAGAACACCGATTGCGAAGGCAGCTTGCTAAACTGATATTGACGCTGAG
TCACGAAAGCGTGGGGATCAAACAGGATTAGAAACCCTTGTAGTCC
>Otu1329
CCAGCCTATGGGTGGCAGCAGTGGGGAATATTGCGCAATGGGGGAAACCCCGACGCAGCA
ACACCGCGTGGAGGAAGAAGGCCTTCGGGTTGTAAACTCCTGTCAGTGGGGAAAAAGGTT
CCGGGGGTGAATAATCTCCGGAGTTTGATTGTACCCGCAGAGGAAGCCCCGGCTAACTAC
GTGCCAGCAGCCGCGGTAAAACGTAGGGGGCCAGCGTTACTCGGAATTATTGGGCGTAAA
GGGCGCGTAGGCGGCATGACAAGTCTTTGGTGAAATCCCTTGGCTTAACCAAGGAATTGC
CGGAGATACTGTTTTGCTTGAGTCCGGTAGAGGGAAGTGGAATTCCCGGTGTAGCGGTGA
AATGCGTAGATATCGGGAGGAACACCCATGGCGAAGGCAGCTTCCTGGACCGGCACTGAC
GCTGAGGCGCGAAAGCGTGGGTAGCAAACAGGATTAGAAACCCTCGTAGTCC
>Otu1330
CCAGCCTATGGGAGGCTGCAGTAACGAATATTCCACAATGGGCGCAAGCCTGATGGAGCG
ATGCCGCGTGCAGGATGAAGCCCTTCGGGGTGTAAACTGCTGTCAGGGGTAAGCAACTCA
ATGAGCTATCCCAGAGGAAGAGGCGACTAACTCTGTGCCAGCAGTCGCGGTAATACAGAG
GCCTCGAGCGTTAATCGGAATCACTGGGCTTAAAGGGTGCGTAGGCTGATTCGTAAGTGC
CTTGTGAAATCCCATGGCTCAACCATGGAACTGCTCGGCAGACTGCGAGTCTTGAGGTCG
GTAGAGGTTGCTGGAACTGTAGGTGGAGCGGTGAAATGCGTAGATATCTACAGGAACGCC
AAAGGTGAAGACAGGCAACTGGGCCGATTCTGACGCTGATGCACGAAAGCGTGGGGAGCA
AACAGGATTAGAAACCCGCGTAGTCA
>Otu1331
CCAGCCTATGGGGGGCTGCAGTAGGGAATATTGGTTAATGGGCGAAAGCCTGACCCAGCA
ACGCCGCGTGTGCGATGAAGGCCTTCGGGTCGTAAAGCACTTTTAGTGGGATGAGGAAG

GACAGTACCACTAGAATAAGTCACGGCTAACTACGTGCCAGCAGCCGCGGTAACACGTAG
GAGACAAGCGTTATCCGGATTTACTGGGTGTAAAGCGCGTGTAGGCGGCTCTGTAAGTTG
GATGTGAAAGCTCCTGGCTTAACTGGGAGAGGTTGTTCAAGACTGCAGGGCTTGAGGGTA
ACAGAGGGGAGTGGAATTCCGAGTGTAGCGGTAAAATGCATAGATATTCGGAAGAACACC
AGTGGCGAAAGCGGCTCTCTGGGTTACATCTGACGCTCAGACGCGAAAGCTAGGGGAGCA
AACGGGATTAGATACCCCTGTAGTCC
>Otu1332
CCAGCCTATGGGAGGCTGCAGCAAAGAATCTTCCGCAATGGACGAAAGTCTGACGGAGCG
ACGCCGCGTGTATGACGAAGGCCGAAAGGTTGTAAAGTACTTTTCTTGAGGAAGAATAAG
GCAGGGAGGGAATGCCCTGCTGATGACATGAATTGAGGAATAAGCCCCGGCTAACTACGT
GCCAGCAGCCGCGGTAACACGTAGGGGGCAAGCGTTGTTCGAAATTATTGGGCGTAAAGG
GCATGTAGGCGGTCATGCAAGCCTGGTGTGAAAAATCTCAGCTTAACTGAGAAGACGCGC
TGGGAACTGCATAACTTGAGTTCGTAAGGGGAAGCCGGAATTCCAGGTGTAGGGGTGAAA
TCTGTAGAGATCTGGAAGAACACCAATGGCGTAGGCAGGCTTCTAGTACTGAACTGACGC
TGAGATGCGAAAGTGCGGGGAGCAAACAGGATTAGATACCCGTGTAGTCC
>Otu1335
CCAGCCTATGGGGGGCTGCAGGAGGGAATATTGGTAATGGGCGAAAGCCCGAACCAGCAA
CGCCGCGTGTGCGATGAAGGCCTTCGGGTCGTAAAGCACTTTTCGAGGGGACGAGGAAGG
ACGGTACCCTCAGAATAAGTCTCGGCTAACTACGTGCCAGCAGCCGCGGTAACACGTAGG
AGACGAGCGTTATCCGGATTTACTGGGCGTAAAGCGCGTGCAGGCGGTTTGGTAAGTTGG
ATGTGAAATCTCCCGGCTCAACTGGGAGGGGTCGTTCAAAACTACCGAACTTGAGGGCAT
CAGAGGAAGGTGGAATTCCGGGTGTAGTGGTGAAATGCGTAGATATCCGGAGGAACACCA
GTGGCGAAGGCGGCCTTCTGGGATGCTCCTGACGCTCATACACGAAAGCTAGGGGAGCGA
ACGGGATTAGAAACCCTCGTAGTCC
>Otu1338
CCTACGGGGGGCAGCAGTGGGGAATATTGGACAATGGGCGAAAGCCTGATCCAGCAATGC
CGTGTGTGTGAAGAAGGCCTTCGGGTTGTAAAGCACTTTCAGTAGGGAAGAAAAGTTCAA
GGGTAATATCCTTGAATCTTGACGTTACCTACAGAAGAAGCACTGGCTAACTCTGTGCCA
GCAGCCGCGGTAATACAGAGAGTGCAAGCGTTAATCGGAATTACTGGGCGTAAAGCGCAC
GTAGGTGGATGCTTAAGTCGGATGTGAAAGCCCTGGGCTTAACCTAGGAATTGCATTCGA
TACTGGGCGTCTAGAGTATAGTAGAGGGAAGTGGAATTTCCGGTGTAGCGGTGAAATGCG
TAGATATCGGAAGGAACATCAGTGGCGAAAGCGGCTTCCTGGACTAATACTGACACTTAG
GTGCGAAAGCGTGGGGAGCAAACAGGATTAGATACCCTCGTAGTCC
>Otu1349
CCAGCCTACGGGGGGCTGCAGTGGGGAATCTTGGACAATGGGCGCAAGCCTGATCCAGCA
ATGCCGCGTGAGTGAAGAAGGCCTTCGGGTTGTAAAGCTCTTTTGTCAGGGAAGAAACGG
TAGAGGCTAATATCCTTTGCTAATGACGGTACCTGAAGAATAAGCACCGGCTAACTACGT
GCCAGCAGCCGCGGTAATACGTAGGGTGCAAGCGTTAATCGGAATTACTGGGCGTAAAGC
GTGCGCAGGCGGTTGTGCAAGACAGATGTGAAATCCCCGGGCTCAACCTGGGAATTGCAT
TTGTGACTGCGCAGCTAGAGTGTGTCAGAGGGGGGTAGAATTCCACGTGTAGCAGTGAAA
TGCGTAGATATGTGGAGGAATACCGATGGCGAAGGCAGCCCCCTGGGATAACACTGACGC
TCATGCACGAAAGCGTGGGGAGCAAACAGGATTAGAGACCCCCGTAGTCC
>Otu1350
CCAGCCTACGGGGGGCAGCAGTGAGGAATCTTGCGCAATGGGGGAAACCCTGACGCAGCA
ACGCCGCGTGAGTGAGGAAGGTCTTCGGGTCGTAAAGCTCTGTCAGGTGGGAAGAAATGT
ATGGGGTGAATAGCCACCATACTTGACGGTACCACCAGAGGAAGCACCGGCTAACTCCGT
GCCAGCAGCCGCGGTAATACGGGGGGTGCAAGCGTTGTTCGGAATCATTGGGCGTAAAGA
GCGTGTAGGCGGCTTGATCAGTCAGATGTGAAAGCCCTGGGCTTAACCCAGGAAGTGCAT
TTGAAACTGTCAGGCTAGAGTAGGGGAGAGGGAAGTGGAATTCCTGGTGTAGAGGTGAAA
TTCGTAGATATCAGGAGGAACACCGGTGGCGAAGGCGACTTCCTGGCCCTATACTGACGC
TGAGACGCGAGAGCGTGGGTAGCAAACAGGATTAGATACCCCTGTAGTCC
>Otu1351
CCAGCCTACGGGGGGCAGCAGTGGGGAATTTTGGACAATGGGCGCAAGCCTGATCCAGCA
ATGCCGCGTGAGTGAAGAAGGCCTTCGGGTTGTAAAGCTCTTTTGTCAGGGAAGAAACGG
CCTGTTCTAATACAGTGAGCTAATGACGGTACCTGAAGAATAAGCACCGGCTAACTACGT
GCCAGCAGCCGCGGTAATACGTAGGGTGCAAGCGTTAATCGGAATTACTGGGCGTAAAGC
GTGCGCAGGCGGTTTTGTAAGTCTGTCGTGAAATCCCCGGGCTCAACCTGGGAATGGCGA
TGGAGACTGCAAGGCTAGAGTTTGGCAGAGGGGGGTAGAATTCCACGTGTAGCAGTGAAA

TGCGTAGATATGTGGAGGAACACCGATGGCGAAGGCAGCCCCCTGGGTCAAAACTGACGC
TCATGCACGAAAGCGTGGGGAGCAAACAGGATTAGATACCCTAGTAGTCC
>Otu1355
CCAGCCTATGGGATGCAGCAGTGGGGAATTTTGGACAATGGGCGCAAGCCTGATCCAGCC
ATGCCGCGTGCGGGAAGAAGGCCTTCGGGTTGTAAACCGCTTTTGTCAGGGAAGAAATCC
TTTGGGCTAATACCCCGGAGGGATGACGGTACCTGAAGAATAAGCACCGGCTAACTACGT
GCCAGCAGCCGCGGTAATACGTAGGGTGCAAGCGTTAATCGGAATTACTGGGCGTAAAGC
GTGCGCAGGCGGTTGTGTAAGACAGATGTGAAATCCCCGGGCTTAACCTGGGAACTGCAT
TTGTGACTGCACGGCTGGAGTGCGGCAGAGGGGGATGGAATTCCGCGTGTAGCAGTGAAA
TGCGTAGATATGCGGAGGAACACCGATGGCGAAGGCAATCCCCTGGGCCTGCACTGACGC
TCATGCACGAAAGCGTGGGGAGCAAACAGGATTAGAGACCCGCGTAGTCC
>Otu1358
CCAGCCTATGGGTTGCAGCAGTCGAGGATCTTTTGCAATGCCCGCAAGGGTGACAATGCG
ACGCCGCGTGTGTGATGAAGGTCTTCGGATCGTAAAACACTGTCGGCCGGGATGAATCTC
TGGAGGATGAATAATCCTGCAGGGTGACAGTACCGGCAAAGGAAGCCACGGCTAACTCCG
TGCCAGCAGCCGCGGTAATACGGAGGTGGCAGGCGTTACTCGGATTGATTGGGTGTAAAG
GGTGTGTAGGTGGCAGTTTAAGTCGAATGTGAAATCCCTTGGCTTAACCAAGGAATTGCA
TTCGATACTGATCTGCTTGAGCGTCAGAGAGGAAAATGGAATTCACGGTGTAACAGTGAA
ATGTGTAGATATCGTGAGGAACACCAGTGGCGAAGGCGATTTTCTGGCTGACTGCTGACA
CTGAAACACGAGAGCTAGGGGAGCAAACAGGATTAGAAACCCTAGTAGTCC
>Otu1360
CCAGCCTACGGGGCGCTGCAGTCGAGAGTATTTAGCAATGGGCGAAAGCCTGACTATGCG
ACGCCGCGTGAAGGATGAAGGTTTTCGGATTGTAAACTTCTTTTGTCAGGGAAGAAACTC
CTGTCGTTAATAGCGCCAGGATTGACGGTACCTGATGAATAAGCCACGGCTAACTCCGTG
CCAGCAGCCGCGGTAATACGGAGGTGGCAAGCGTTACTCGGAATCATTGGGTGTAAAGGG
CAAGTAGGCGGTTTAATAAGTGAGGGGTGAAATCTTTCGACTCAATCGGGAGACTGCCTT
TCAAACTGTTAAACTTGGAGCAGGAAGGGGGAAATGGAATTCACGGTGTAAGGGTGAAAT
CTGTAGATATCGTGAAGAACACCGGCGGCGAAGGCGATTTCCTGGTCCTGTCTTGACGCT
GAATTGCGAAAGCTAGGGGAGCAAACAGAATTAGAAACCCTCGTAGTCC
>Otu1361
CCAGCCTACGGGGTGCTGCAGTGAGGAATCTTGCGCAATGGGGGAAACCCTGACGCAGCA
ACGCCGCGTGAGTGAGGAAGGCCTTCGGGTCGTAAAGCTCTGTCAGGTGGGAAGAAGTGT
ATGGGAGTTAATAGTTTTCATACTTGACGGTACCACCAGAGGAAGCACCGGCAAACTCCG
TGCCAGCAGCCGCGGTAATACGGGGGGTGCAAGCGTTGTTCGGAATCATTGGGCGTAAAG
AGCGTGTAGGCGGCTGGATAAGTCGGATGTGAAAGCCCTGGGCTCAACCCGGGAAGTGCA
TTTGAAACTGTTCAGCTTGAGTAAGGGAGAGGGAAGTGGAATTCCTGGTGTAGAGGTGAA
ATTCGTAGATATCAGGAGGAACACCGGCGGCGAAGGCGACTTCCTGGCCCTATACTGACG
CTGAGACGCGAGAGCGTGGGTAGCAAACAGGATTAGATACCCGAGTAGTCC
>Otu1362
CCAGCCTACGGGATGCACCAGTCGAGAATTTTTCACAATGGGCGCAAGCCTGATGGAGCG
ACGCCGCGTGGGGGATGAATGGCTTCGGCCCGTAAACCCCTGTCATTCGGGAGCAATGCA
ACGGATTGAATATTTTCGTTGTTGATAGTACCGGAAGAGGAAGGGACGGCTAACTCTGTG
CCAGCAGCCGCGGTAATACAGAGGTCCCAAGCGTTGTTCGGATTCACTGGGCGTAAAGGG
TGCGTAGGTGGCCGGGAAAGTCTGATGTGAAAGCTCGGAGCTTAACTCCGAAATGGCATT
GGAAACTATTCGGCTAGAGGTTGGGAGGGGGAACTGGAATTCTCGGTGTAGCAGTGAAAT
GCGTAGATATCGAGAGGAACACCAGTGGCGAAGGCGAGTTCCTGGACCAATCCTGACACT
GAGGCACGAAAGCCAGGGGAGCAAACGGGATTAGATACCCGAGTAGTCC
>Otu1364
CAGCCTACGGGTCGCACCAGTGGGGAATATTGCACAATGGGCGAAAGCCTGATGCAGCAA
CGCCGCGTGCACGAAGAAGGTCTTCGGATCGTAAAGTGCTTTTCTGAGAGATGAGAAAGG
ACAGTATCTCAGGAATAAGTCTCGGCTAACTACGTGCCAGCAGCCGCGGTAATACGTAGG
GGGCAAGCGTTGTCCGGATTTACTGGGCGTAAAGCGCACGCAGGCGGTCGTGCCAGTTTC
CTCTGACAGGTCGGGGCTTAACCCTGGCAGGCGAGGAAAGACGACACGACTTGAGGACTT
CAGAGGGACGTGGAATTCCAGGTGGAGCGGTGAAATGCGTAGAGATCTGGAAGAACACCA
AAGGCGAAGGCAGCGTCCTGGGAAGTAACTGACGCTCATGTGCGAAAGCTAGGGTAGCGA
ACGGGATTAGAGACCCGAGTAGTCC
>Otu1365
CCAGCCTATGGGGGGCTCCAGTCGAGAATTTTTCACAATGGGCGAAAGCCTGATGGAGCG

ACGCCGCGTGGGGGATGAATGGCTTCGGCCCGTAAACCCCTGTCATTCGCGATCAAACCT
TGCCATTTAAAAGATGGCAAGCTGATAGTAGCGGAAGAGGAAGGGACGGCTAACTCTGTG
CCAGCAGCCGCGGTAATACAGAGGTCCCAAGCGTTGTTCGGATTCACTGGGCGTAAAGGG
TGCGTAGGTGGTAGGGTAAGTCGGATGTGAAAGCTCGGAGCTCAACTCCGAAATGGCATT
GGATACTACCCTGCTAGAGGGTCGGAGGGGGGACTGGAATTCTCGGTGTAGCAGTGAAAT
GCGTAGATATCGAGCGGAACACCAGTGGCGAAGGCGGCTCACTGGACCACAACTGACGCT
GATGCACGAAAGCGTGGGGAGCAAACAGGATTAGAAACCCCTGTAGTCC
>Otu1368
CCAGCCTACGGGACGCAGCAGTCGAGAATTTTTCACAATGGGCGCAAGCCTGATGGAGCG
ACGCCGCGTGGGGGATGAATGGCTTCGGCCCGTAAACCCCTGTCATTCGCGATCAAACCT
TGTTATTTAAAAGATGGCGAGCTGATAGTAGCGGAAGAGGAAGGGACGGCTAACTCTGTG
CCAGCAGCCGCGGTAATACAGAGGTCCCGAGCGTTGTTCGGATTCACTGGGCGTAAAGGG
TGCGTAGGTGGCGAGGTAAGTCGGATGTGAAAGCTCGGAGCTCAACTCCGAAATGGCATT
GGAAACTACCTTGCTCGAGGAATGGAGGGGGGACTGGAATTCTCGGTGTAGCAGTGAAAT
GCGTAGATATCGAGAGGAACACCAGTGGCGAAGGCGAGTCCCTGGACATTTCCTGACACT
GAGGCACGAAAGCTAGGGGAGCAAACAGGATTAGATACCCCTGTAGTCC
>Otu1369
CCAGCCTACGGGGGGCTGCAGTCGAGAATCTTCCGCAATGGGCGAAAGCCTGACGGAGCG
ACGCCGCGTGCGGGATGAAGGCCTTCGGGTTGTAAACCGCTGTCAGTGGGGAGGAAATGC
ACGGGGGTTCTCCCTTGTGTTTGACCGATCCGCAGAGGAAGCACAGGCTAAGTTCGTGCC
AGCAGCCGCGGTAACACGAACTGTGCGAACGTTATTCGGAATTACTGGGCTTAAAGAGTT
CGTAGGCGGCCTGTTAAGTCGGGTGTGAAATCCCTCGTCTCAACCGAGGAATTGCGTTCG
AAACTGGCGGGCTTGAGTGAGATAGAGGTGAGCGGAACTGATGGTGGAGCGGTGAAATGC
GTTGATATCATCAGGAACACCGGTTGCGAAGGCGGCTCACTGGGTCTTTACTGACGCTGA
GGAACGAAAGCTAGGGGAGCGAACGGGATTAGATACCCTCGTAGTCC
>Otu1370
CCAGCCTATGGGTTGCAGCAGTTCGGAATATTCGGCAATGGGCGAAAGCCTGACCGAGCG
ACGCCGCGTGCGGGATGAAGGCCCTCTGGGTTGTAAACCGCTGTCACAGGTGAGCAAATC
ACGCGTAATTAACAACTACGTGTGTTGAGTTAAGCTTGAGAGGAAGCCCCCGCTAATCAC
GTGCCAGCAGCGGCGGTAATACGTGAGGGGCGAACGTTGTTCGGTGTCACTGGGCTTAAA
GGGCGCGTAGGTGGCTGCGTAAGTAACGTGTGAAAGCCCTCGGCTTACCCGGGGAATTGC
GCGTTATACTGCGTGGCTTGAGGATCTCAGGGGAAGGGGGAACTCCAGGTGTAGCGGTGA
AATGCGCAGATATCTGGGGGAAGGCCGGCGGCGAAGGCGCCCTTCTGGGAGACACCCGAC
ACTGAGGCGCGAAAGCGTGGGGAGCAAACAGGATTAGAAACCCGTGTAGTCC
>Otu1371
CCAGCCTATGGGGGGCTGCAGTGGGGAATATTGGACAATGGGCGAAAGCCTGATCCAGCA
ATACCGCGTGAGTGATGAAGGCCTTAGGGTTGTAAAGCTCTTTTAGTAGGGAAGATAATA
ACGGTACCTACAGAAAAAGCCCCGGCTAACTCCGTGCCAGCAGCCGCGGTAAGACGGAGG
GGGCTAGCGTTGTTCGGAATTACTGGGCGTAAAGAGCGCGTAGGCGGTATAGTAAGTTGG
AAGTGAAAGCCCGAGGCTTAACCTCGGAATTGCTTTCAAAACTGCTAAACTAGAGTGTGG
TAGGGGATGGTAGAATTCCTAGTGTAGAGGTGGAATTCTTAGATATTAGGAGGAATACCG
GTGGCGAAGGCGACCATCTGGGCCATTACTGACGCTGAGGTGCGAAAGCGTGGGGAGCAA
ACAGGATTAGAAACCCTCGTAGTCC
>Otu1372
CCAGCCTATGGGTGGCTGCAGTGGGGAATATTGCGCAATGGGGGAAACCCTGACGCAGCA
ACGCCGCGTGAGTGAGGAAGGACTTCGGTCTGTAAAGCTCTGTTAATGTGGAAAAATGGT
TAGTGGTCTAATAGGCCATTGATTTGATGGTACACATAGAGGAAGCACCGGCTAACTTCG
TGCCAGCAGCCGCGGTAATACGAAGGGTGCAAGCGTTGTTCGGATTTATTGGGCGTAAAG
CGCTCGTAGGCGGACCTATAAGTCAGATGTGAAATCTCGGGGCTCAACCTCGAAACTGCG
TCTGAAACTGTGGGTCTAGAATCTTGGAGGGGGAAGGGGAATTTCGCATGTAGGGGTAAA
ATCCGTAGATATGCGAAGGAACACCAGAGGCGAAGGCGCCTTCCTGGACAAGTATTGACG
CTAAGGAGCGAAAGCGTGGGGATCAAACAGGATTAGAAACCCGAGTAGTCC
>Otu1378
CCAGCCTACGGGGGGCACCAGCAAGGAATTTTGTGCAATGGGCGAAAGCCTGACACAGCG
ACGCCGCGTGAAGGATGAAGATCTTCGGATTGTAAACTTCTTTTTCAGGGAAGAATAAT
GACGGTACCTGAAGAATAAGCTTCGGCTAACTACGTGCCAGCAGCCGCGGTAATACGTAG
GAAGCGAGCGTTATCCGGATTTATTGGGCGTAAAGAGAGCGCAGGCGGTTCTATAAGTCA
GATGTTAAAACTCCCGGCTTAACTGGGAGAGGTCATCTGATACTGTTGGACTCGAGAGCA

GTAGGGGGAGATGGAATTCCCGGTGTAGCGGTGAAATGCGTAGATATCGGGGGGAACACC
AGTGGCGAAAGCGGTCTCCTTGGCTGTTTCTGACGCTTATGCTCGAAAGCGTGGGGAGTA
AACAGGATTAGAAACCCCTGTAGTCC
>Otu1381
CCAGCCTATGGGTGGCAGCAGCCGAGAATATTCGACAATGGGCGAAAGCCTGATCGAGCG
ACGCCGCGTGATGGATGAAGACCCTCTGGGTCGTAAACATCTTTTGTGAGGGAGAAAGTT
TATTGATAGTACCTCAAGAATAAGGGGTTGCTAAACTCGTGCCAGCAGCAGCGGTAATAC
GAGTGCCCCGAGCGTTATCCGGAATTATTGGGCGTAAAGGGTGTGTAGGTGGTTGCGTTA
GTCGCTTGTTAAAACCCGGGGCTTAACCCCGGAACCGCGAGCGAAACGGCGCGACTAGAG
GATGGCAGGGATGTGTGGAACTCATGGAGTAGGGGTGAAATCCGTTGATATCATGGGGAA
CACCAAAAGCGAAGGCAACACATTGGGCCACTCCTGACACTGAAACACGAAAGCGTGGGT
AGCGAATGGGATTAGATACCCCCGTAGTCC
>Otu1382
CCAGCCTATGGGTGGCTCCAGTGAGGAATATTGCACAATGGGCGCAAGCCTGATGCAGCG
ACGCCGCGTGGGTGAAGAAGGTTTTCGGATCGTAAAACCCTTTTCTGTGTGACGAGCAAG
GACGGTAGCACAGGAATAAGTCTCGGCTAACTACGTGCCAGCAGCCGCGGTAAAACGTAG
GAGACAAACGTTATCCGGAGTTACTGGGCGTAAAGGGCGTGTAGGTGGTTTCGTAAGTCT
GGCGTGAAATTTTTCGGCTTAACCGGGAAAGGTCGTCGGATACTGCGGGACTAGAGGACG
GTAGAGGCATGTGGAATTCCGGGTGTAGTGGTGAAATGCGTAGAGATCCGGAGGAACACC
AGTGGCGAAGGCGACATGCTGGGCCGTACCTGACACTGATGCGCGACAGCATGGGGAGCA
AACAGGATTAGATACCCTAGTAGTCC
>Otu1384
CCAGCCTACGGGGTGCACCAGTGGGGAATATTGGGCAATGGGCGCAAGCCTGACCCAGCG
ACGCCGCGTGGGGGATGAAGGTCTTCGGATCGTAAACCCCTGTCAGGAGAGACGAACGCC
TTGCGATTAATAGTCGCAAGGTCTGACGGTACCTCCAAAGGAAGCCCCGGCTAACTCCGT
GCCAGCAGCCGCGGTAATGCGGAGGGGGCAAGCGTTATTCGGAATTACTGGGCGTAAAGC
GCGCGCAGGTGGCGTGGTAAGTGGCAGGTGAAAGCCCTCGGCTCAACCGAGGAATTGCCT
GCCAAACTGTCATGCTTGAGGCCGGGAGGGGAAAGCGGAATTCCTGGTGTAGCGGTGAAA
TGCGTAGATACCAGGAGGAACACCGGTGGCGAAGGCGGCTTTCTGGACCGGTTCTGACAC
TCATGCGCGAAAGCGTGGGGAGCAAACAGGATTAGAAACCCGTGTAGTCC
>Otu1385
CCAGCCTACGGGGGGCTCCAGTCGAGAATTTTTCACAATGGGGGAAACCCTGATGGAGCG
ACGCCGCGTGGGGGATGAATGGCTTCGGCCCGTAAACCCCTGTCATTCGGGATCAATGCC
TGCGATTTAAAAGATAGCAGGTTGATAGTACCGGAAGAGGAAGGGACGGCTAACTCTGTG
CCAGCAGCCGCGGTAATACAGAGGTCCCAAGCGTTGTTCGGATTTACTGGGCGTAAAGGG
TGCGTAGGTGGTTGGGTAAGTCTGATGTGAAATCTCGGAGCTTAACTCCGAAACGGCATT
GGATACTATTCAGCTTGAGAGTTGGAGGGGGGACTGGAATTCTCGGTGTAGCAGTGAAAT
GCGTAGATATCGAGAGGAACACCAGTGGCGAAGGCGAGTCCCTGGACAACTTCTGACACT
GAGGCACGAAAGCCAGGGGAGCAAACGGGATTAGAGACCCCGGTAGTCC
>Otu1386
CTAGCCTATGGGTGGCTGCAGTCGAGAATAATTCGCAATGGACGAAAGTCTGACGATGCG
ACGCCGCGTGAACGATGAAGGCCTTCGGGTTGTAACGTTCTGTTGTCCTGGAACAAGAGT
AGTTGACGAATAGTTGACTACAATGAGGGTACAGGAAGAGGAAGCACCGGCTAATTCCGT
GCCAGCAGCTGCGGTAATACGGAAGGTGCAAGCGTTAGTCGGAATTACTGGGCGTAAAGG
GTGCGTAGGCGGTGATGTAAGTCGGACGTGAAATTCCGGGGCTCAACCTCGGAACTGCGT
ACGAAACTGCATCACTTGAGGAATGGCGGAGAAAATGGAATTCCATATGTAGCGGTGAAA
TGCGTAGATATATGGAAGAACACCTGTGGCGAAAGCGGTTTTCTAGCCTTCTCCTGACGC
TGAGGCACGAAAGCAAGGGGAGCAAACAGGATTAGAAACCCTCGTAGTCC
>Otu1387
CCAGCCTACGGGTGGCTGCAGTGGGGAATATTGGACAATGGGGGAAACCCTGATCCAGCA
ACGCCGCGTGTGGGATGACGGCCTTCGGGTTGTAAACCACTTTTTAGGGGGACGATGATG
ACGGTACCCCTGGAATAAGCCCCGGCTAACTCTGTGCCAGCAGCCGCGGTAAGACAGAGG
GGGCAAGCGTTGTCCGGAATTACTGGGCGTACAGCGCACGCAGGTGGCATGGCAAGTGTG
GTGTGAAAGCCCCGCGCTTAACGCGGGGAGGCCAGCACAGACTGCCACGCTAGAGGGATG
CAGGGGGTGGTGGAATTGCCGGTGTAGTGGTGAAATGCGTAGAGATCGGCAGGAACACCA
AGGACGAAGGTAGCCACCTGGGCATCACCTGACACTCATGTGCGACAGCGTGGGGAGCCA
ACCGGATTAGATACCCGAGTAGTCC
>Otu1388

CCAGCCTACGGGGGGCAGCAGTAGGGAATTTTGCGCAATGGGCGAAAGCCTGACGCAGCA
ACGCCGCGTGAGTGATGAAGGTCTTCGGATTGTAAAGCTCTGTCAGGAGGGAAGAACCTG
CCGATTTTATACCTCGGCACTGACGGTACCTCCAGAGGAAACTCCGGCTAACTCCGTGCC
AGCAGCCGCGGTAATACGGAGGGAGTGAGTGTTGTTCGGATTTATTGGGTGTAAAGGGAG
TGTAGGCGGAAATTTAAGTCAGGGGTGAAAAGTTAGGGCTTAACCCTAAACCTGCCTTTG
AGACTGAATTTCTTGAGTATGGAAGAGGGCGATAGAATTCCTGGTGTAGCGGTGGAATGC
GTAGATATCAGGAGGAATACCGATAGCGAAGGCAGTCGCCTGGGACAATACTGACGCTGA
AACTCGAAAGCATGGGGAGCAAACAGGATTAGAAACCCGAGTAGTCC
>Otu1389
CCAGCTACGGGTTGCACCAGTCGAGAATCTTCCACAATGGACGAAAGTCTGATGGAGCGA
CGCCGCGTGGTTGACGAAGTCCTTCGGGACGTAAAGATCTTTTATGAGGGAAGAAGTATA
CTGACTGTACCTCATGAATAAGGGGCTCCTAATCTCGTGCCAGCAGGAGCGGTAATACGA
GAGCCCCGAGCGTTATCCGGAATTATTGGGCGCAAAGGGTGCGTAGGCGGTTATGTTAGT
CGTAGGTCAAAGGCCCGAGCTTAACTCGGGAAAGGCACACGAAACGGCATTGACTTAGAG
AGGAGAAGAGGTGTGCGGAACTCATGGTGTAGGGGTGAAATCCGTTGGTATCATGGGGAA
CACCGAAAGCGAAAGCAGCACACTGGTCTCTTTCTGACGCTGAGGCACGAAAGCGTGGGT
AGCGAATGGGATTAGAAACCCTAGTAGTCC
>Otu1391
CCAGCCTACGGGAGGCAGCAGGCATTGCTTGCTGTCTTTTGGCAACCTTAAAACGGTTGC
AGAATAAGTTCTGGGTAAGACCAGTGCCAGCCGCCATGGCGCGCAAATTCGCACGCGTGG
CACGGTACGCGGTAATACTGGCAGAACGAGTGGTGCCCACGAATATTGGGTTTAAAGAGA
CCGTAGCTGGTCTGTTAAGTTCGCTGTGAAATCTTGGGGCTTAACCTCAAGGAGTGCAGC
AAATACTGGCGGACTTGAGGGCGGGGGAGGCCGGGAGTACTTACGGGGTAAGGGTAAAAT
CTCGTAATCCTGTAAGGACTACCAGTGGCGAAGGCGCCCGGCCAAAACGCGTCTGACAGT
GAGGGTCGAAGGCTAGGAGAACGAATCGGATTAGAGACCCCAGTAGTCC
>Otu1393
CCGCCTACGGGGCGCAGCAGTGGGGAATATTGCACAATGGACGAAAGTCTGATGCAGCAA
CGCCGCGTGAGTGATGAAGGTCTTCGGATTGTAAAGCTCTGTCTTCTGGGACGATAATGA
CGGTACCAGAGGAGGAAGCCACGGCTAACTACGTGCCAGCAGCCGCGGTAATACGTAGGT
GGCGAGCGTTGTCCGGAATTACTGGGCGTAAAGGATGCGTAGGCGGATATTTAAGTCAGA
TGTGAAACCCCCAGGCTTAACTTGGGGACTGCATTTGAAACTGGGTATCTAGAGTGCAGG
AGAGGTAAGTGGAATTCCTAGTGTAGCGGTGAAATGCGTAGAGATTAGGAAGAACATCAG
TGGCGAAGGCGACTTACTGGACTGTAACTGACGCTGAGGCATGAAAGCGTGGGTAGCAAA
CAGGATTAGATACCCCCGTAGTCC
>Otu1394
CCAGCCTATGGGTCGCACCAGTGGGGAATATTGGGCAATGGGCGCAAGCCTGACCCAGCC
ATGCCGCGTGAGTGATGAAGGCCTTCGGGTCGTAAAGCTCTGTGGGGAGGGACGAACAAG
CCTTTGGCTAATATCCAAGGGCCCTGACGGTACCTCCTTAGCAAGCACCGGCTAACTCCG
TGCCAGCAGCCGCGGTAATACGGAGGGTGCAAACGTTGCTCGGAATCATTGGGCGTAAAG
CGCGCGTAGGCGGTTCGTTAAGTCGGATGTGAAATCCCTGGGCTCAACCCAGGAAGTGCA
TACGAAACTGGCGAGCTTGAATATGGAAGAGGGTCGCGGAATTCCCGGTGTAGAGGTGAA
ATTCGTAGATATCGGGAGGAACACCAGTGGCGAAGGCGGCGACCTGGGCCAATATTGACG
CTGAGGTGCGAAAGCGTGGGGAGCAAACAGGATTAGATACCCGTGTAGTCC
>Otu1395
CCAGCCTTACGGGGGGCTGCAGTAGGGAATATTGGTAATCTGCGAAAGCGGGAACCAGCA
ACGCCGCGTGTGCGATGAAGGCCTTCGGGTCGTAAAGCACTTTTTGAGGGGACGAGGAAG
GACGGTACCCTCAGAATAAGTCTCGGCTAACTACGTGCCAGCAGCCGCGGTAACACGTAG
GGGGCAAGCGTTGTTCGGAATTACTGGGCGTAAAGGGCATGTAGGCGGCTCTGTAAGTCC
GGCGTGGAAGCCCACGGCTCAACCGTGGGATTGCGCTGGAAACTGCGAGGCTTGAATCAT
GGAGAGGGAGCTAGAATTCCTGGTGTAGGGGTGAAATCTGTAGAGATCAGGAAGAATACC
AATGGCGAAGGCAAGCTCCTGGCCAATGATTGACGCTGAGGTGCGAAAGTGTGGGGATCA
AACAGGATTAGAAACCCCCGTAGTCC
>Otu1397
CCAGCCTATGGGAGGCAGCAGTGGGGAATCTTCCGCAATGGACGAAAGTCTGACGGAGCG
ACGCCGCGTGAGGGATGAAGGTCTTCGGATTGTAAACCTCTTTCGAATGGGAATAAGTGC
AGCGGGCGAATAGTACCGCTGTTTGACTGTACCATATGAAGAAGCTACGGCTAACTCTGT
GCCAGCAGCCGCGGTAATACAGAGGTAGCAAGCGTTGTCCGGATTCACTGGGCGTAAAGG
GCGTGTAGGTGGCCTTATAAGTCGGGTGTGAAATCGTTCCGCTTAACGGAACAATTGCAC

CCGATACTGTTTGGCTTGAGTGCAGTAGGGGAAATTGGAATTTCCGGTGTAGCGGTGAAA
TGCGTAGATATCGGAAAGAACACCCGTGGCGAAGACGGATTTCTGGACTGCAACTGACAC
TGAGGCGCGAAAGCCAGGGGAGCAAACGGGATTAGAAACCCCCGTAGTCC
>Otu1399
CCAGCCTATGGGGTGCTCCAGTAGGGAATATTGGTAATGTGCGAAAGCGCGAACCAGCAA
CGCCGCGTGCACGATGAAGGCCTTCGGGTTGTAAAGTGCTTTTAGAGAGGATGAGGAAGG
ACAGTACTCTCTGAATAAGCCTCGGCTAACTACGTGCCAGCAGCCGCGGTAGAACGTAGG
AGGCGAGCGTTATCCGGATTTACTGGGTGTAAAGCGCATGCAGGCGGATGACTAAGTTGG
GTGTGAAAGCTCCTGGCCTAACTGGGAGAGGTCGCTCAAGACTGGTCGTCTCGAGAGTGG
TAAGGGAAGGTGGAATTCCGGGTGTAGTGGTGAAATGCGTAGATATCCGGAGGAACACCA
GTGGCGAAGGCGGCCTTCTGGGCCACATCTGACGCTCAGATGCGAAAGCTAGGGTAGCAA
ACGGGATTAGAGACCCTAGTAGTCC
>Otu1400
CCAGCCTATGGGGTGCTGCAGTCGAGAATTTTTCACAATGGGCGAAAGCCTGATGGAGCG
ACGCCGCGTGGGGGATGAATGGCTTCGGCCCGTAAACCCCTGTCATTCGCGAACAATTGT
TGCGGTTTAATACATCGCAGCTTGATAGTAGCGGGAGGGGAAGGGACGGCTAACTCTGTG
CCAGCAGCCGCGGTAATACAGAGGTCCCGAGCGTTGTTCGGATTCACTGGGCGTAAAGGG
TGCGTAGGTGGTTGGGTAAGTTTGATGTGAAATCTCCGGGCTTAACCCGGAAATGGCATT
GAATACTATTCAGCTCGAGGATTGGAGGGGGGACTGGAATTCTCGGTGTAGCAGTGAAAT
GCGTAGATATCGAGAGGAACACCAGTGGCAAAGGCGAGTCCCTGGACAACTCCTGACACT
AAGGCACGAAAGCTAGGGGAGCAAACAGGATTAGATACCCCCGTAGTCC
>Otu1405
CCAGCCTACGGGTGGCAGCAGTCGAGAATCTTCCGCAATGGACGAAAGTCTGACGGAGCG
ACGCCGCGTGATGGATGAAGTCCTTCGGGACGTAAACATCTTTTATGAGGGATGACTATT
CGACAGTACCTCATGAATAAGGGGCTCCTAACTCTGTGCCAGCAGGAGCGGTAATACAGA
GGCCCCAAGCATTACCCGGAATCACTGGGCGTAAAGGGTGCGTAGGTGGCGCCATTAGTC
CTCTTTCAAATCCTCCGAGCTCAACTCGGAGGCTCGAGAGGAAACGGTGGCGCTTGAGGG
TGTACGAGGTACGTGGAACTCTAGGTGTAGGGGTGAAATCCGTTGATATCTAGGGGAACA
CCGAATGCGAAGGCAGCGTACTGGGACATTCCTGACACTGAGGCACGAAAGCGTGGGTAG
CGAAAAGGATTAGAAACCCCAGTAGTCC
>Otu1409
CCAGCCTACGGGGGGCTGCAGTGGGGAATTTTCCGCAATGGGCGAAAGCCTGACGGAGCA
AGACCGCGTGGGGGAAGAAGGTTTGTGGATTGTAAACCCCTTTTGATTGGGAAGAACACA
ATGACGGTACCAATCGAATCAGCATCGGCTAACTCCGTGCCAGCAGCCGCGGTAATACGG
AGGATGCAAGCGTTATCCGGAATTATTGGGCGTAAAGCGTCCGTAGGTGGCATTGCAAGT
CAGTTGTTAAAGCGCGGGGCTTAACCCCGTAAAGGCAATTGAAACTGCGAAGCTAGAGTG
CGATAGGGGCAAGGGGAATTCCCAGTGTAGCGGTGAAATGCGTAGATATTGGGAAGAACA
CCGGTGGCGAAAGCGCCTTGCTGGGTCTGCACTGACACTGAGGGACGAAAGCTAGGGGAG
CGAAAGGGATTAGAGACCCCCGTAGTCC
>Otu1410
CCAGCCTATGGGGGGCAGCAGTCGAGGATCTTTGGCAATGGGCGCAAGCCTGACCAAGCG
ACGCCGCGTGTGCGATGAAGGCCTTCGGGTTGTAAAGCACTGTCGAGGGGGATAAAGCCG
CAAGGTTGATTGATCCCTGGAGGAAGCACGGGCTAAGTTCGTGCCAGCAGCCGCGGTAAG
ACGAACCGTGCGAACGTTGTTCGGAATCACTGGGCTTAAAGGGCGCGTAGGCGGCAGATC
AAGTCCGGGGTGAAATCCTCCAGCTCAACTGGAGAAGTGCCTTGGATACTGATTTGCTCG
AGGGAGGTAGGGGTCGAGGGAACTTCTGGTGGAGCGGTGAAATGCGTTGATATCAGAAGG
AACGCCGGTGGCGAAAGCGCTCGACTGGACCTCTTCTGACGCTGAGGCGCGAAAGCTAGG
GGAGCAAACGGGATTAGAAACCCCAGTAGTCC
>Otu1411
CCAGCCTACGGGGGGCAGCAGTAGGGAATATTGGTCAATGGATGCAAGTCTGAACCAGCC
ATGCCGCGTGCAGGAAGAAGGCCTTCTGGGTTGTAAACTGCTTTTGCCAGGGGATAAAAC
GGATCTGCGGATCTAATTGAAGGTACCTGGTGAATAAGCCACGGCTAACTACGTGCCAGC
AGCCGCGGTAATACGTAGGTGGCAAGCGTTGTCCGGATTTATTGGGTTTAAAGGGTGCGT
AGGCGGTCCTATAAGTCAGTGCTGAAATATCCCGGCTTAACCGGGAGGGTGGCATCGATA
CTGCAGGACTTGAGTAGAGTCGAAGTGGGCGGAATTGACGGTGTAGCGGTGAAATGCTTA
GATATCGTCAAGAACACCGATAGTGAAGACAGCTCACTAGGCTCAAACTGACGCTGAGGC
ACGAAAGTGTGGGGATCAAACAGGATTAGAAACCCGAGTAGTCC
>Otu1412

CCAGCCTATGGGGGGCACCAGTGGGGAATTTTGCACAATGGGGGAAACCCTGATGCAGCA
ACGCCGCGTGGAGGATGAAGTCCCTTGGGATGTAAACTCCTTTCGATCGGGACGATTATG
ACGGTACCGGAAGAAGAAGCACCGGCTAACTCTGTGCCAGCAGCCGCGGTAATACAGAGG
GTGCAAGCGTTGTTCGGAATTATTGGGCGTAAAGGGTGCGTAGGCGGTTGGGTAAGTCTG
ACGTGAAATCTCCGGGCCTAACCCGGAAACTGCGTTGGATACTATCCGGCTAGAGGAATG
GAGGGGAGACTGGAATACTTGGTGTAGCAGTGAAATGCGTAGATATCAAGTGGAACACCA
GTGGCGAAGGCGAGTCTCTGGACATTTCCTGACGCTGAGGCACGAAAGCCAGGGGAGCAA
ACGGGATTAGATACCCGGGTAGTCC
>Otu1417
CCAGCCTACGGGGTGCACCAGTGGGGAATCTTGCACAATGGGGGCAACCCTGATGCAGCG
ACGCCGCGTGAACGATGAAGCCCTTCGGGGTGTAAAGTTCTTTCGACGGGAACGATAATG
ACGGTACCCGGAGAAGAAGCTGCGGCTAACTACGTGCCAGCAGCCGCGGTAATACGTAGG
CAGCAAGCGTTGTTCGGAGTTACTGGGCGTAAAGAGTGCGTAGGCGGTCAGCCAAGTTTG
GTGTGAAATCTCCCGGCTCAACTGGGAGGGTGCGCCGAAAACTGGTTGGCTAGAGTATGG
GAGAGGAAAGCGGAATTCCTGGTGTAGCGGTGAAATGCGTAGATATCAGGAGGAACACCT
GTGGTGTAGACAGCTTTCTGGACCATAACTGACGCTGAGGCACGAAAGCGTGGGTAGCAA
ACAGGATTAGATACCCTTGTAGTCC
>Otu1419
CCAGCCTATGGGGGGCAGCAGTGGGGAATCTTGGACAATGGGGGCAACCCTGATCCAGCC
ATGCCGCGTGAGTGAAGAAGGCCTTAGGGTTGTAAAGCTCTTTCACACGTGACGATGATG
ACGGTAACGTGAGAAGAAGCCCCGGCTAACTTCGTGCCAGCAGCCGCGGTAATACGAAGG
GGGCTAGCGTTGTTCGGAATTACTGGGCGTAAAGCGCGCGTAGGCGGTTATGTAAGTCAG
GGGTGAAATCCCGGGGCTCAACCCTGGAACTGCCTTTGAGACTGCGTAGCTAGAGAACGG
GAGAGGTGAGTGGAATTCCCAGTGTAGAGGTGAAATTCGTAGATATTGGGAAGAACACCG
GTGGCGAAGGCGGCTCACTGGCCCGTTTCTGACGCTGAGGCGCGAAAGCGTGGGGAGCAA
ACAGGATTAGATACCCCCGTAGTCC
>Otu1421
CCAGCCTACGGGGGGCTCCAGTGAGGAATCTTGCGCAATGGGGGAAACCCTGACGCAGCA
ACGCCGTGTGAGTGATGAAGGTTTTCGGATCGTAAAGCTCTGTTTGAAGGGACGAAACGT
ATTTGATTAATACTCAAATAACTTGACGGTACCTTCAGAGGAAGCACCGGCTAACTCCGT
GCCAGCAGCCGCGGTAATACGGAGGGTGCAAGCGTTGTTCGGAATCACTGGGCGTAAAGG
GCGTGTAGGTGGCTAATTAAGTCAGGTGTGAAATCCTTCCGCTTAACGGGAGAAGTGCAC
TTGAAACTGCTTAGCTAGAGTACGGGAGAGGAAGGTGGAATTCCTGGTGTAGAGGTGAAA
TTCATAGATATCGGGAAGAACACCGGTGGCGAAGGCGGCCTTCTGGCCCGATACTGACGC
TGAGACGCGAAAGCGTGGGGAGCAAACAGGATTAGATACCCTCGTAGTCC
>Otu1422
CCAGCCTATGGGACGCTCCAGTGGGGAATCTTGCGCAATGGACGAAAGTCTGACGCAGCC
ACGCCGCGTGAGTGAAGAAGGCCTTCGGGTTGTAAAGCTCTGTCGGAGGGGACAAAAAAT
CTTAGGGTTAATAGCCCTAGGACTTGATGGTACCCTCAAAGGAAGCACCGGCTAACTTCG
TGCCAGCAGCCGCGGTAATACGAAGGGTGCAAGCGTTGCTCGGAATTATTGGGCGTAAAG
GGTTGGTAGGTGGTTACGTATGTCTGAGGTGAAATCCCTGAGCTCAACTCAGGACGTGCC
TTGGAAACGGCGTAACTAGAGTGCTAGAGAGGTTCGTGGAATTCCTGGTGTAGCGGTGAA
ATGCGTAGAGATCAGGAGGAACATCAGCGGCGAAGGCGGCGAACTGGATAGTAACTGACA
CTGAACAACGAAAGCGTGGGGAGCAAACAGGATTAGATACCCGAGTAGTCC
>Otu1424
CCAGCCTATGGGGGGCAGCAGTTTAGAAATTTGCGCAATGGGCGAAAGCCTGACGCAGCG
ACGCCGCGTGAGGGATGAAGGTCTTCGGATTGTAAACCTCTGTCGAACAAGACGAAAGCT
TGCCGGTAAATAATCGGCAAGTTTGACGGTACTGTTGGAGGAAGCTATGGCTAACTCTGT
GCCAGCAGCCGCGGTAATACAGAGGTAGCGAGCGTTGTCCGGAATTACTGGGCGTAAAGG
GCGTGTAGGCGGCCGGGTAAGTCTAGTGTTAAAACTTTCAGCTCAACTGAGAGATGTCAC
TGGATACTGCTTGGCTTGAGGACAGTAGAGGAGAGTGGAATTCCCGGTGTAGCGGTGAAA
TGCGTAGATATCGGGAGGAACACCTGTGGCGAAGGCGGCTCTCTGGTCTGTCTCTGACGC
TGAGGCGCGAAAGCTAGGGGAGCAAACGGGATTAGATACCCTCGTAGTCC
>Otu1426
CCAGCCTACGGGTTGCTCCAGTCGAGGATCTTTGGCAATGAGCGCAAGCTTGACCAAGCG
ACGCCGCGTGTGCGATGAAGGCCTTCGGGTTGTAAAGCACTGTCGAGGGGAACGAAGCCG
AAAGGTTGACCGATCCCTGGAGGAAGCACGGGCTAAGTTCGTGCCAGCAGCCGCGGTAAG
ACGAACCGTGCGAACGTTGTTCGGAATCACTGGGCTTAAAGGGCGCGTAGGCGGCTTGTC

AAGTCAGGGGTGAAATCTTTCGGCTCAACCGGAAAAGTGCCCTTGATACTGACAAGCTCG
AGGGAGGTAGGGGTGTGTGGAACTTCTGGTGGAGCGGTGAAATGCGTTGATATCAGAAGG
AACGCCGGTGGCGAAAGCGACGCACTGGACCTTTTCTGACGCTGAGGCGCGAAAGCTAGG
GGAGCAAACGGGATTAGAGACCCTTGTAGTCC
>Otu1427
CCAGCCTACGGGTCGCTGCAGTTCGGAATATTCGGCAATGGACGAAAGTCTGACCGAGCG
ACGCCGCGTGCGGGATGAAGGCCCTATGGGTTGTAAACCGCTTTCGCGAGTGAGCAAATC
AAGACTGGCGAACATCCAGTTCTGTTGAGTTAAGCTCGTAAAGAAGCCCCCGCTAATCAC
GTGCCAGCAGCGGCGGTAATACGTGAGGGGCGAACGTTGTTCGGTGTCACTGGGCTTAAA
GGGTGCGTAGGTGGCTTTGTAAGTAAGGAGTGAAATCCCTCGGCTTACCCGGGGAATTGC
TTTTATACTGCAAGGCTTGAGGATCAGAGGGGAAAGAGGAACTCCGAGTGTAGCGGTGAA
ATGCGCAGATATTCGGGGGAAGGCCGGCGGCGAAGGCGTCTTTCTGGCTGATTCCTGACA
CTGAGGCACGAAAGCGTGGGGAGCAAACAGGATTAGAAACCCCTGTAGTCC
>Otu1432
CCAGCCTATGGGAGGCTGCAGTGAGGAATATTGCACAATGGGCGAAAGCCTGATGCAGCA
ACGCCGCGTGAGCGATGAAGGCCTTCGGGTCGTAAAGCTCTTTTGAGAGGGACGAGAACG
GACGGTACCTCTTGAATAAGTCACGGCTAACTACGTGCCAGCAGCCGCGGTAAAACGTAG
GTGGCGAGCGTTATCCGGATTCACTGGGCGTAAAGCGAGTGCAGGTGGCCTCGTAAGTTG
GATGTGAAATTTCCCGGCTTAACCGGGAGCGGTCATTCAATACTGCGGGGCTTGAGGGTA
GAAGAGGGGGGTGGAATTGCCGGTGTAGTGGTGAAATGCGTAGATATCGGCAGGAACACC
AGTGGCGAAAGCGGCCCCCTGGTTTACTCCTGACACTCAGACTCGAAAGCTAGGGTAGCG
AACGGGATTAGATACCCCAGTAGTCC
>Otu1433
CCAGCCTATGGGTTGCAGCAGCAACGAATCTTCCCCAATGGGCGAAAGCCTGAGGGAGCG
ACGCCGCGTGAAGGATGAAGTACTTCGGTATGTAAACTTCTTTAGGGGCTACGAAAGCGT
AAGGACCTAATACGTCCTGAGTTGATCTAGTCCAAAATAAGGGACGGCTAACTCTGTGCC
AGCAGCCGCGGTAATACAGAGGTCCCGAGCGTTACTGAGAATCACTGGGTTTAAAGGGTG
CGTAGGTGGTCCGTTAAGTCCGTTGTGAAATCCCTGGGCTCAACCCAGGAGCTGCTTCGG
ATACTGGCGGACTTGAGGCCGGTAGAGGTCACTGGAACTGACGGTGGAGCGGTGAAATGC
GTAGATATCGTCAGGAACGCCGGTGGTGAAGACGGGTGACTGGGCCGGTTCTGACACTGA
GGCACGAAAGCGTGGGGAGCGAACGGGATTAGAGACCCCAGTAGTCC
>Otu1434
CCAGCCTACGGGTCGCACCAGCTAGGAATTTTCCGCAATGGGCGAAAGCCTGACGGAGCG
ACACTGCGTTGAGGATTGAAGGCCCTACGGGGTTGTAAACTCATTTTAAGCTAGAAGAGA
GAGGACTGTATAGCTGGAATAAGCCCCGGCAAACTACGTGCCAGCAGCCGCGGTAATACG
TAGGGGGCGAGTATTGTTCGGATTTACTGGGCATAAAGGGTGCGTAGGCGGGATATCAAG
CAGGATGTGAAATCTAATGGCTCAACCATTAAATTGCATTCTGAACTGGTATTCTTGAGT
GCAGCAGGGGAGTGCAGAATTCCCGGTGTAGGGGTGAAATCTATAGATATCGGGAAGAAT
ACCGGTCGCGAAGGCGGCACTCTGGGCTGTAACTGACGCTGAGGCACGAAAGCGTGGGGA
TCGAACCGGATTAGAAACCCTTGTAGTCC
>Otu1435
CCAGCCTACGGGGGGCAGCAGTTCGGAATATTCGGCAATGGGCGAAAGCCTGACCGAGCG
ACGCCGCGTGCGGGATGAAGGCCCTTGGGTTGTAAACCGCTGTCACAGGTGAGCAAATCA
TGCGCGAGGAACATTCGCGTATGTTGAGTTAAGCTTGAGAGGAAGCCCCCGCTAATCACG
TGCCAGCAGCGGCGGTAATACGTGAGGGGCGAACGTTGTTCGGTGTCACTGGGCTTAAAG
GGCGCGTAGGTGGTTGCGTAAGTAAGGTGTGAAAGCCCTCGGCTTACCCGGGGAATTGCG
CCTTATACTGCGTGACTTGAGGGTCATAGGGGAAGGGGGAACTCCAGGTGTAGCGGTGAA
ATGCGCAGATATCTGGGGGAAGGCCGGCGGCGAAGGCGCCCTTCTGGGTGATTCCTGACA
CTGAGGCGCGAAAGCGTGGGGAGCAAACAGGATTAGAGACCCTCGTAGTCC
>Otu1437
CCAGCCTACGGGGGGCACCAGTGGGGAATTTTGCACAATGCCCGAAAGGGTGATGCAGCA
ACGCCGCGTGAGGGATGAAGGCCTTCGGGTCGTAAACCTCTTTCGACCGGAAAAAATGGT
CCGCTGAAGAGGCGGGCCTGATGGTACCGGTGGAAGAAGCCCCGGCTAACTACGTGCCAG
CAGCCGCGGTAATACGTAGGGGGCGAGCGTTGTTCGGAATTACTGGGCGTAAAGGGCGTG
TAGGCGGTGCGGTAAGTCGGGCGTGAAATCTCTAGGCTTAACCTGGAGGGGCCGTTCGAA
ACTGCCGTGCTAGAGTGCGGGAGGGGGAAGTGGAATTCCCGGTGTAGCGGTGAAATGCGT
AGATATCGGGAGGAACACCTGTGGCGAAGGCGGCTTCCTGGACCGCAACTGACGCTGAGG
CGCGAAAGCTAGGGGAGCAAACAGGATTAGAGACCCTCGTAGTCC

>Otu1438
CCAGCCTATGGGAGGCAGCAGTGGGGAATTTTGCGCAATGGGGGAAACCCTGACGCAGCA
ACGCCGCGTGGAGGATGAAGTCCTTCGGGACGTAAACTCCTTTCGATCGGGACGATTATG
ACGGTACCGGAAGAAGAAGCCCCGGCTAACTACGTGCCAGCAGCCGCGGTAACACGTAGG
GGGCGAGCGTTGTTCGGAATTACTGGGCGTAAAGCGCGTGTAGGCGGGTCTTTAAGTCAG
GGGTGAAATGCCAAGGCTCAACCTTGGAACTGCCTTTGATACTGGAGATCTTGAGTCCGG
GAGAGGTGAGTGGAACTGCGAGTGTAGAGGTGAAATTCGTAGATATTCGCAAGAACACCA
GTGGCGAAGGCGGCTCACTGGCCCGGAACTGACGCTGAGACGCGAAAGCGTGGGGAGCAA
ACAGGATTAGAACCCTAGTAGTCC
>Otu1440
CCAGCCTACGGGCCGCACCAGCAACGAATCTTCCCCAATGCCGGAAACGGTGAGGGAGCG
ACGCCGCGTGAAGGATGAAGTCTTTCGGGATGTAAACTTCTGTTAGGGTTATGAAAGCGT
AAGGGGCTAATATCCCCTGAGTTGATCTGATCCAGAGAAAGGGACGGCTAACTCTGTGCC
AGCAGCCGCGGTAATACAGAGGTCCCAAGCGTTACTGAGAATCACTGGGTTTAAAGGGTG
CGTAGGTGGCGCGTTAAGTCCGTTGTGAAATCCCCGAGCTCAACTCGGGAACTGCTTCGG
ATACTGGCGTGCTTGAGGCCGGTAAGGGTCACTGGAACTGACGGTGGAGCGGTGAAATGC
GTAGATATCGTCAGGAACGCCGGTGGTGAAGACGGGTGACTAGGCCGGTTCTGACACTGA
GGCACGAAAGCGTGGGGAGCGAACGGGATTAGAAACCCGCGTAGTCC
>Otu1443
CCAGCCTATGGGGTGCACCAGTCACGAATCTTCCTCAATGGCCGAAAGGCTGAAGGAGCG
ACGCCGCGTGTATGAAGACGCCCTTCGGGGTGTAAAATACTTTTATCTGGGAAGAAACAA
TGACGGTACCAGATGAATAAGGAGCTGCTAACCTCGTGCCAGCAGCAGCGGTAATACGAG
GGCTCCAAGCGTTATCCGGATTTATTGGGCGTAAAGGGTCTGTAGGCGGTTGGTCGCATC
TCCTGTTAAAGCCCGAGGCTCAACCTCGGAACAGCGGGAGAGATGGACAAACTAGAGGCC
GGGAGAGGCAAATGGAATTGCTGGTGTAGGGGTTAAATCCGTTAATATCAGCAAGAACAC
CAAACGCGAAGGCATTTTGCTGGAACGGTCCTGACGCTAAGAGACGAAAGCGTGGGTAGC
GAATGGGATTAGAAACCCTTGTAGTCC
>Otu1444
CCAGCCTATGGGGTGCAGCAGGCGCGAAACCTTTACAATGCACGAAAGTGTGATAGGGGG
ATACTCAGTGCTTACGATTATGTCGTAGGCTTTTGTCAATCGTAAATAGATTGGCGAATA
AGTGGTGGGCAAGACCGGTGCCAGCCGCCGCGGTAACCCCGGCGCCACAAGTGGTCATCG
CGATTATTGGGCCTAAAGCGTTCGTAGCCGGTTTGGTAAATCTCTTGTGAAATTGTTCGG
CTTAACCGAACAGCGTGCAGGAGACACTGCCGAACTCGAGACCGGGAGGCGTAAGAAGTA
TTCCATGGGGACTGGTAAAATGGGATAATCCATGGAAGACTACCGATGGCGAAGGCATCT
GACCAGAACGGATCTGACGGTGAGGAACGAAAGCCAGGGGAGCGAACCGGATTAGAAACC
CCCGTAGTCC
>Otu1448
CCAGCCTACGGGAGGCAGCAGTAGGGAATATTGGTTAATGGGCGAAAGCCTGACCCAGCA
ACGCCGCGTGTGCGATGAAGTCCTTCGGGATGTAAACCGCTGTCAGGGGTAAGAAAGTTC
TGATCTACCCCAGAGGAAGAGACGGCTAACTCTGTGCCAGCAGCCGCGGTAATACAGAGG
TCTCAAGCGTTAGGCGGAATCACTGGGCTTAAAGCGTGTGTAGGCGGATCGTTAAGTGCC
TTGTGAAATCCCACGGCTCAACCGTGGAACTGCTCGGCATACTGGCGATCTTGAGCCATC
TAGGGGTTGCTGGAACAAACGGTGGAGCGGTGAAATGCGTAGATATCGTTTGGAACGCCA
AAGGTGAAAACAGGCAACTGGGGATGTGCTGACGCTGAGACACGAAAGCTAGGGGAGCAA
ACGGGATTAGAAACCCCTGTAGTTCC
>Otu1452
CCAGCCTATGGGGGGCACCAGTGAGGAATATTGCGCAATGGGGGCAACCCTGACGCAGCA
ACGCCGCGTGAGTGAGGAAGGTTTTCGGATCGTAAAGCACTGTCGGGAGGGACGAATAAG
GCGGCGGCTAACATCCGCAGCCGATGACGGTACCTCCAAAGGAAGCACCGGCTAACTCTG
TGCCAGCAGCCGCGGTAATACAGAGGGTGCTAGCGTTGTTCGGAATTATTGGGCGTAAAG
CGCGTGTAGGCGGCCTTGCAAGTTGGGTGTGAAAGCCCTCGGCTTAACCGAGGAAGTGCG
CCCAAAACTACAGGGCTTGAGTGCCGGAGAGGGTGGCGGAATTCCCGGTGTAGAGGTGAA
ATTCGTAGATATCGGGAGGAACACCAGCGGCGAAGGCGGCCACCTGGACGGTAACTGACG
CTGAGACGCGAAAGCGTGGGTAGCAAACAGGATTAGAAACCCGTGTAGTCC
>Otu1453
CCAGCCTATGGGGCGCTCCAGTGGGGAATCTTGCGCAATGGGCGAAAGCCTGACGCAGCA
ACGCCGCGTGAGTGATGAAGGTCTTCGGATCGTAAAGCTCTGTCGGAGGGAACGATGCCG
TCGTTGGCCAATATCCAACGGTGGTGACGGTACTCTCAAAGGAAGCACCGGCTAACTCTG

TGCCAGCAGCCGCGGTAAGACAGAGGGTGCAAGCGTTGTTCGGAATTATTGGGCGTAAAG
GGCGCGTAGGCGGCAGCGTAAGTCAGGCGTGAAAGCCCGGGGCTTAACCCCGGAAGTGCG
CCTGATACTGCGGTGCTTGAGTCCCGGAGAGGGTGGTGGAATTCCCAGTGTAGAGGTGAA
ATTCGTAGATATTGGGAGGAATACCGGTGGCGAAGGCGACCACCTGGACGGAGACTGACG
CTGAGGCGCGAAAGCGTGGGGAGCAAACAGGATTAGAAACCCCAGTAGTCC
>Otu1455
CCAGCCTACGGGTCGCTGCAGTCGAGAATCTTCCGCAATGGACGAAAGTCTGACGGAGCG
ATGTCGCGTGATGGATGAAGTTCTTAGGAACGTAAACATCTTTTATCGGGGAGAAAATTT
TGATAGTACCCGATGAATAAGGGGTTGCTAAACTCGTGCCAGCAGCAGCGGTAATACGAG
TGCCCCAAGCGTTATCCGGAATTACTGGGCGTAAAGCGTGTGTAGGCGGCGTGATTAGTC
TTTGGTCAAAGCTCCCGGCTTAACCGGGAAAAGGCCGGAGAAACGGTCACACTAGAGGGT
GCGAAAGGTCTATGGAACTCATGGTGTAGGAGTGAAATCCGTTGATATCATGGGGAACAC
CGAAAGCGAAGGCAATAGACTGGCGCATTACTGACGCTGCAACACGAAAGCGTGGGGATC
AAACGGGATTAGATACCCTTGTAGTCC
>Otu1459
CCAGCCTATGGGATGCTCCAGTCGAGAATCATTCGCAATGGGCGCAAGCCTGACGATGCG
ACGCCGCGTGCAGGATGAAGGTCTTCGGATTGTAAACTGCTGTGATGGGGGAGCAATGCA
GAACGTGCCAACACCACGTACTGTTGATAGTACCCCAAGAGGAAGCCACGGCTAACTCTG
TGCCAGCAGCCGCGGTAATACAGAGGTGGCAAGCGTTGTTCGGATTTACTGGGCGTAAAG
GGTGCGTAGGCGGTCTTGTGTGTCGGATGTGAAATCCCACGGATCATCCGTGGAACTGCA
TTCGAAACTGCAGGGCTGGAGTGCAGGAGAGGAAAGTGGAATTCTCGGTGTAGCGGTGAA
ATGCGTTGATATCGAGAGGAACGCCGGAGGCGAAGGCGACTTTCTGGACTGCAACTGACG
CTGAGGCACGAAAGCTAGGGGAGCAAACAGGATTAGAAACCCCCGTAGTCC
>Otu1464
CCAGCCTATGGGTCGCTGCAGTCGAGGATCTTTCGCAATGGGCGCAAGCCTGACGAAGCG
ACGCCGTGTGAGTGAAGAAGGCCTTTGGGTTGTAAAGCTCTTTCGCCTGGGAACAAGAGG
ATTTGGTGAATAACTGAATACTTTGAGGGTACTAGGTAAAGAAGCACCGGCTAACTCCGT
GCCAGCAGCTGCGGTAATACGGAGGGTGCAAGCATTAATCGGATTTACTGGGCGTAAAGG
GCGCGTAGGCGGTATGCCAAGTCATTTGTGAAATTTCGCGGCTCAACCGCGAAGCTGCAA
GTGAGACTGGTGAACTAGAGGATAAGCGGAGAAAATGGAATTCCACATGTAGCGGTGAAA
TGCGTAGATATGTGGAGGAACACCGGAGGCGAAAGCGGTTTTCTAGCTTACCCCTGACGC
TGAAGCGCGAAAGCAAGGGGATCAAACAGGATTAGATACCCGTGTAGTCC
>Otu1469
CCAGCCTATGGGGGCACCAGTGGGGAATATTGCGCAATGGGCGAAAGCCTGACGCAGCAA
CGCCGCGTGAGTGATGAAGGTCTTCGGATTGTAAAGCTCTGTTGAATGGGAAGAATAACT
CCGTAAATAATACGTACGGAGGATGACGGTACCATTCGAGGAAGCCCCGGCTAACTACGT
GCCAGCAGCCGCGGTAATACGTAGGGGGCAAGCGTTGTTCGGAATCACTGGGCGTAAGGG
AGCGTAGGCGGATTGGTAAGTTAGGAGTGAAATCTACAGGCTTAACCTGTACGATTGCTT
TTAATACTGCCTTTCTTGAGTATTGGAGAGGATGATGGAATTCCAGGTGTAGCGGTGGAA
TGCGTAGATATCTGGAAGAACACCAGTTGCGAAGGCGGTCATCTGGCCAAATACTGACGC
TGAGGCTCGAAAGCTAGGGGAGCAAACAGGATTAGAGACCCCCGTAGTCC
>Otu1474
CCAGCCTATGGGGGGCACCAGTGAGGAATATTGGGCAATGCCCGGAAGGGTGACCCAGCA
ACGCCGCGTGGAGGATGGCGGCCGTAAGGTTGTAAACTCCTCTTGAAGGGGACGAAAGAC
CAAATTTCGTAAGGGATTTGGGTTGACTGTACCCTTAGAAGAAGCCCCGGCTAACTACGT
GCCAGCAGCCGCGGTAATACGTAGGGGGCAAGCGTTGTCCGGATTTACTGGGTGTAAAGG
GCGCGTAGGCGGGTTTGTAAGTCAGTGGTGAAATCCTGCAGCTTAACTGCAGAACTGCCA
TTGATACTGCAAATCTTGAGTTCGGAAGAGAGAAGCGGAATTCCAGGTGTGGTGGTGAAA
TACGTAGATATCTGGAAGAACACCAGTGGCGAAGGCGGCTTCTTGGTCCGTAACTGACGC
TGAGGCGCGAAAGCGTGGGGATCAAACAGGATTAGATACCCGAGTAGTCC
>Otu1476
CCAGCCTATGGGGGGCACCAGTGGGGAATTTTGGACAATGGGCGAAAGCCTGATCCAGCG
ACGCCGCGTGGAGGATGAAGGCCTTCGGGTTGTAAACTCCTTTTAGAGGGAACGAATAAA
TTGACGGTACCCTCAGAAAAAGCCACGGCTAACTACGTGCCAGCAGCCGCGGTAATACGT
AGGTGGCGAGCGTTACTCGGAATTACTAGGCGTAAAGCGCTTGTAGGTGGTTGGATAAGT
CTGTTGTGAAATTTCACGGCTTAACTGTGAAAGTGCAACGGAAACTATCCGGCTTGAGTG
TGGTAGGGGTTGACGGAATTCCCGGTGTAGCGGTGAAATGCGTAGATATCGGGAGGAACA
CCAATGGCGAAAGCAGTCAACTGGGCCAATACTGACACTGAGGAGCGAAAGCTAGGGGAG

CAAACAGGATTAGAAACCCCAGTAGTCC
>Otu1478
CCAGCCTACGGGGGGCTCCAGTGAGGAATATTGCGCAATGGGCGAAAGCCTGACGCAGCG
ACGCCGCGTGGGTGACGAAGGCCTTCGGGTCGTAAAACCCTTTTGGTGGGGACGAGTGAG
GACGGTACCCATCGAATAAGTCTCGGCTAACTACGTGCCAGCAGCCGCGGTAAAACGTAG
GAGGCGAGCGTTATCCGGATTCACTGGGCGTAAAGCGCGCGTAGGCGGTTAGGTAAGTCG
GACGTTAAAGCCCCTGGCTCAACTGGGGGAGGTCGTTCGATACTGCTTGGCTTGAGGATG
AGAGAGGAAAGCGGAATTCCCGGTGTCGCGGTGGAATGCGTAGAGATCGGGAGGAACACC
AGTGGCGAAGGCGGCTTTCTGGTTCATTTCTGACGCTGATAGCGCGAAAGCGTGGGGAGC
AAACGGGATTAGATACCCCTGTAGTCC
>Otu1479
CCAGCCTATGGGTTGCAGCAGTTAGGAATCATCTGCAATGCGCGAAAGCGTGACAGTTTG
AACCAGAGTGCTTTTCATTTGAAAAGCTTTTGCCAAATGTAAAAAGTTTGGCGAATAAGG
ACTGGGTAAGACGAGTGCCAGCCGCCGCGGTAATACTCGCGGTCCAAGTCGCAGCCATCT
TTATTGGGTCTAAAACATCCGTAGCTTGCCTGGTAAGTCCCTTGTGAAATCTTGGCTCTT
AAGGTCAAGGCGTGCAAGGGGTACTGTCAGGCTAGAGACCGGCAGACGTAAGAAGTATGT
TCGAAGTAGCGGTAAAATGCATTAATCTCGAACAGACTCACAACAGCGAAGGCATCTTAC
GAGGACGGTTCTGACTGTCAGGGATGAAGGCTAGGGGCGCAAAACGGATTAGATACCCTA
GTAGTCC
>Otu1482
CCAGCCTACGGGGGGCTGCAGTGGGGAATCTTCCGCAATGGACGCAAGTCTGACGGAGCG
ACGCCGCGTGCAGGATGAAGGCCTTCGGGTTGTAAACTGCTTTCGAAAGGGAATAAGTGC
AGCAGGCGAATAGTACTGCTGTTTGACTGTACCTTAGGAAGAAGCTACGGCTAACTCTGT
GCCAGCAGCCGCGGTAATACAGAGGTAGCAAGCGTTGTCCGGATTCACTGGGCGTAAAGG
ACGTGTAGGCGGCCCTATAAGTCGGGTGTGAAATCGTTCTGCTTAACAGAACAATTGCTC
CCGATACTGTTTGGCTTGAGTGCAGGAGGGGAAAATGGAATTTCCGGTGTAGCGGTGGAA
TGCGTAGATATCGGAAAGAACACCCGTGGCGAAGGCGGTTTTCTGGCCTGCAACTGACGC
TGAGGCGTAAAAGCTAGGGGAGCAAACGGGATTAGAAACCCGCGTAGTCC
>Otu1483
CCAGCCTATGGGTTGCTGCAGCAACGAATCTTCCCCAATGGGCGAAAGCCTGAGGGAGCG
ACGCCGCGTGTAGGATGAAGTTCTTCGGAATGTAAACTACTGTTAGGGTTTTGAAAGCGA
TGCGCCATAACATGGCGCAAAGTTGATCTAGCCCAGAGAAAGGGACGGCTAACTCTGTGC
CAGCAGCCGCGGTAATACAGAGGTCCCGAGCGTTACTGAGAATCACTGGGTTTAAAGGGT
GCGTAGGTGGTCCGTTAAGTCCGTTGTGAAATCCCTTGGCTCAACCAAGGAACTGCTTCG
GATACTGGCGGACTTGAGGCCGGTATGGGTCACTGGAACTGACGGTGGAGCGGTGAAATG
CGTAGATATCGTCAGGAACGCCGGTGGTGAAGACGGGTGACTGGGCCGGTTCTGACACTG
AGGCACGAAAGCGTGGGGAGCGAACGGGATTAGATACCCCCGTAGTCC
>Otu1488
CCAGCCTATGGGACGCAGCAGTCGAGAATTTTCCGCAATGGGCGAAAGCCTGACGGAGCG
ACGCCGCGTGGAGGACGAAGTTCTTCGGAATGTAAACTCCTTTTGCCAGGGAAAAAGTTA
TTGATTGTACCTGGAGAATAAGAAGTTGCTAAACTCGTGCCAGCAGCAGCGGTAATACGA
GTGCTTCAAGCGTTATCCGGAATCATTGGGCGTAAAGGGTGTGTAGGCGGTAATGTTAGT
CTCCTGTCAAATCTTTCGGCTCAACCGAGAGTCCGCAGAGGAAACGGCATAACTAAGAGG
ACGGAAGAGGTCTCTGGAACTCATGGTGTAGCGGTGAAATGCGTTGATATCATGGGGAAC
ACCAAAAGCGAAGGCAAGAGACTGGTCCGCTCCCGACGCTGAAACACGAAAGCGTGGGTC
GCGAATGGGATTAGAGACCCTTGTAGTCC
>Otu1489
CCAGCCTATGGGGGGCTGCAGTGGGGAATTTTGCGCAATGGGCGAAAGCCTGACGCAGCG
ACGCCGCGTGGGTGACGAAGGCCTTCGGGTCGTAAAGCCCTGTGGGGAGGAACGAACCGG
CATCGGGCTAATATCCTGGTGCCCTGACGGTACCTTCTTAGCAAGCACCGGCTAACTCTG
TGCCAGCAGCCGCGGTAAGACAGAGGGTGCAAACGTTGCTCGGAATTACTGGGCGTAAAG
CGCGTGTAGGTGGTCTGGTAAGTCGGGTGTGAAAGCCCAGGGCTTAACCCTGGAAGTGCA
TTCGAAACTGCCAGGCTAGAGTACTGAAGAGGACAGCGGAATTCCCGGTGTAGAGGTGAA
ATTCGTAGATATCGGGAGGAACACCAGTGGCGAAGGCGGCTGTCTGGGCAGATACTGACA
CTGAGACGCGAAAGCGTGGGAAGCAAACAGGATTAGATACCCCAGTAGTCC
>Otu1491
CCAGCCTATGGGAGGCAGCAGTCGAGAATCTTCCGCAATGGACGAAAGTCTGACGGAGCG
ACGCCGCGTGACTGATGAAGACCCTACGGGTCGTAAAGGTCTTTTATGAGGGAAGAAGTT

TATTGACGGTACCTCATGAATAAGAGGCTCCTAATCTCGTGCCAGCAGGAGCGGTAATAC
GAGAGCCTCGAGCGTTATCCGGAATTATTGGGCGTAAAGGGTGAGTAGGCGGCGCTGTTA
GTCGAGTGTTAAATCTCCGGGCTTAACCTGGAAACCGCATTCGAAACGGCAGTGCTTGAG
ATAAGAAGAGGTGTGCAGAACTCAAGGTGTAGGGGTGAAATCCGTTGATATCTTGGGGAA
TACCAAAAGCGAAGGCAGCACACTGGTCTTTATCTGACGCTGAATCACGAAAGCGTGGGT
AGCGAATGGGATTAGAAACCCCAGTAGTCC
>Otu1492
CCAGCCTATGGGACGCTCCAGTGGGGAATTTTGCGCAATGGGCGAAAGCCTGACGCAGCA
ACGCCGCGTGGAGGATGAAGGCCTTCGGGTCGTAAACTCCTGTCAGGGGGGACGAAAAAA
ATGACTGTACCCCTTGAGGAAGCCACGGCTAACTCCGTGCCAGCAGCCGCGGTAATACGG
AGGTGGCAAGCGTTGTTCGGAATTACTGGGCGTAAAGGGCGCGTAGGCGGCTTTACAAGT
CAGATGTGAAAGCCCTGGGCTTAACCCAGGAATTGCATCTGAAACTGTTTTGCTTGAGTC
CTGGAGGGGGTAGTGGAATTCCCAGTGTAGCGGTGAAATGCGTAGATATTGGGAGGAACA
CCTGTGGCGAAGGCGGCTACCTGGACAGTGACTGACGCTGATGCGCGAAAGCTAGGGGAG
CAAACGGGATTAGAAACCCTTGTAGTCC
>Otu1493
CCAGCCTACGGGGGGCAGCAGTAGGGAATATTGCTCAATGGGGGAAACCCTGAAGCAGCA
ACGCCGCGTGAGTGATGAAGGCCTTCGGGTCGTAAAGCTCTGTTGTACGGGAAGAACGCG
GGTATGGCTAACATCCATATCCAGTGACGGTACCGTATAAGAAAGGACCGGCTAACTTCG
TGCCAGCAGCCGCGGTAAGACGGGGGGTCCTAGCGTTGTTCGGAATTATTGGGCGTAAAG
CGTGTGCAGGTGGTTTGGTAAGTTGGATGTGAAAGCCCCGGGCTTAACTCGGGAAGTGCA
TCCAAAACTCCCAAACTTGAGTATGGAAGAGGGTCGTGGAATTCCTGGTGTAGTGGTGAA
ATACGTAGATATCAGGAGGAACACCGGCGGCGAAGGCGGCGACCTGGTCCTATACTGACA
CTCATACACGAAAGCGTGGGTAGCAAACAGGATTAGAAACCCTTGTAGTCC
>Otu1496
CCAGCCTACGGGGTGCAGCAGTCGAGGAAATTTTTCAATGCCCGCAAGGGTGAACGAGTA
AGCCAGAGTGTTTTCTTTACGAAAACTTTTGCCTGATGCAAAAAGTCAGGCGAATAAGGA
CTGGGCAAGACCGGTGCCAGCCGCCGCGGTAATCCCGGCGGTCCAAGTCGCATCCACAAT
TATTGGGTCTAAAACATTCGTAGCTTGTTTTTTAAGTCTCTTGTGAAATTCCATATCTCA
AGTATGGAGCGTGCAAGAGATACTATTTAACTAGAGACCGGAAGACGCAAAGAGTACGAC
GGAGGTAGCGGTAAAATGTGTTAATCTTTGTCGGACTAACAATGGCGAAGGCACTTTGCG
AGTACGGATCTGACAGTGAGGAATGAAGGCTAGGGGCGCAAAAGGGATTAGATACCCGGG
TAGTCC
>Otu1497
CCAGCCTACGGGTTGCTGCAGTGGGGAATATTGGACAATGGGCGAAAGCCTGATCCAGCA
ATGCCGCGTGTGTGAAGAAGGCCTTCGGGTTGTAAAGCACTTTAGGTCGGAAAGAAAAAG
CTTCACCTAATACGTGAAGCCTTGACGGTACCGACAGAATAAGCACCGGCTAACTCTGTG
CCAGCAGCCGCGGTAATACAGAGGGTGCAAGCGTTAATCGGATTTACTGGGCGTAAAGCG
TGCGTAGGCGGTTTGTTAAGTCGGTTGTGAAAGCCCTGGGCTCAACCTGGGAATTGCAGT
CGATACTGGCAGACTAGAGAACGGTAGAGGGAGGCGGAACTCCAGGTGTAGCAGTGAAAT
GCGTAGATATCTGGAAGAACACCGATGGCGAAGGCAACCTCCTGGGCCTGTTCTGACGCT
GAGGCACGAAAGCGTGGGGAGCAAACAGGATTAGAGACCCGAGTAGTCC
>Otu1499
CCAGCCTACGGGTTGCAGCAGTGGGGAATATTGGACAATGGGCGCAAGCCTGATCCAGCA
ATGTCGCGTGAATGATGAAGGCCTTAGGGTTGTAAAGTTCTTTCGGCGGGGAAGAAATTG
ACGGTACCCGCAGAAGAAGCTCCGGCTAACTCCGTGCCAGCAGCCGCGGTGATACGGGGG
GAGCTAGCGTTTTTCGGAATTACTGGGCGTAAAGGGCGCGTAGGCGGCCAATAAAGTTGA
AAGTGAAAGCCCCGGGCTTAACCCGGGAACTGCTTTCAAAACTCATTGGCTAGAGGATGA
CAGAGGACAGTAGAATTCCTAGTGTAGAGGTGAAATTCTTAGATATTAGGAAGAATACCA
GAGGCGAAGGCGACTGTCTGGGTCATATCTGACGCTGAGGCGCGAAAGCGTGGGGAGCAA
ACAGGATTAGATACCCGCGTAGTCC
>Otu1500
CCAGCCTATGGGTTGCTGCAGTGGGGAATCTTGCGCAATGGACGAAAGTCTGACGCAGCC
ACGCCGCGTGAGTGAAGAAGGCCTTCGGGTTGTAGAGCTCTGTCGGGAGGGACGAAAACT
TTTGCGGTTAATAGCCGCAGAAATTGACGGTACCTTCAAAGGAAGCACCGGCTAACTCCG
TGCCAGCAGCCGCGGTAATACGGAGGGTGCAAGCGTTGCTCGGAATTATTGGGCGTAAAG
GGTAGGTAGGTGGTCTCATTTGTCAGGGGTGAAAGCCTTGGGCTTAACCCAAGAAGTGCC
CCTGAAACGGTGAGACTGGAGTACTGGAGAGGGTCGTGGAATTCCCGGTGTAGCGGTGAA

ATGCGTAGAGATCGGGAGGAACACCAGAGGCGAAGGCGGCGACCTGGACAGTTACTGACA
CTCAACTACGAAAGCGTGGGGAGCAAACAGGATTAGAAACCCGAGTAGTCC
>Otu1501
CCAGCCTATGGGTGGCAGCAGTCAAGAACATTCCGCAATGGACGAAAGTCTGACGGAGCG
ACGCCGCGTGCAGGAAGAAGGCCTTCGGGTTGTAAACTGCTTTTGCGTGGGAAGAAATTT
GACGGTACCACGAGAATAAGGGGTTGCTAACTCTGTGCCAGCAGCAGCGGTAATACAGAG
ACCCCGAGCGGTATCCGGAATTACTGGGCGTAAAGCGCAAGTAGTCGGTTTTTATAGTCT
GATGTTAAATCTCACGGCTCAACTGTGAGACTGCATCGGAAACGTAAAGACTAGAGCAAA
TCAGAGGCAAGCGGAACGTGTGGTGTAGCGGTGAAATGCGTTGATATCACACGGAACACC
AAAAGCGAAGGCAGCTTGCTAGGGTTTTGCTGACGATCAGTTGCGAAAGCGTGGGGATCA
AAAAGGATTAGATACCCTAGTAGTCC
>Otu1502
CCAGCCTATGGGGGGCTGCAGTGGGGAATTTTGCGCAATGGGGGAAACCCTGACGCAGCC
ATGCCGCGTGAACGATGAAGGCCTTCGGGTTGTAAAGTTCTTTTGACGGGGAAGATGATG
ACTGTACCCGTAGAATAAGCACCGGCTAACTTCGTGCCAGCAGCCGCGGTAATACGAAGG
GTGCTAGCGTTGTTCGGAATTACTGGGCGTAAAGGGCGCGTAGGCGGCTTGGCAAGTTGG
ATGTGAAAGCCCAGGGCTCAACCCTGGAATTGCATTCAAGACTGCTTTGCTTGAATTCGG
TAGAGGTTGGTGGAATTCCCAGTGTAGAGGTGAAATTCGTAGAGATTGGGAAGAACGCCC
GTGGCGAAGGCGGCCAACTGGACCGACATTGACGCTGAGGCGCGAAAGCGTGGGGATCAA
ACAGGATTAGAAACCCTCGTAGTCC
>Otu1504
CCAGCCTACGGGTGGCAGCAGTCGAGAATCTTCCACAATGGACGAAAGTCTGATGGAGCG
ACGCCGCGTGGTTGATGAAGTCCTTCGGGATGTAAAAACCTTTTATGAGGGAGAAAGTTA
TTGATAGTACCTCATGAATAAGGGGTTGCTAAACTCGTGCCAGCAGCAGCGGTAATACGA
GTGCCCCAAGCATTATCCGGAATTATTGGGCGTAAAGGGTTTGTAGGTGGTTCTATTAGT
CTTGTGTTAAAGCTCCTGGCTCAACTGGGAACGTGCATGAGAAACGGTAGAACTAGAGGA
TGCGAGAGGTGTGTAGAACTCATAGTGTAGGGGTGAAATCCGTTGATATTATGGGGAATA
CCAAAAGCGAAGGCAGCACACTGGCGCACTCCTGACACTGAAAAACGAAAGCGTGGGTAG
CGAATGGGATTAGATACCCGAGTAGTCC
>Otu1509
CCAGCCTATGGGGGGCACCAGTGGGGAATATTGGGCAATGGGCGAAAGCCTGACCCAGCG
ACGCCATGTGGGTGATGAAGGCCTTCGGGTTGTAAAGCCCTGTTAGGTGGGAAGAAGGGT
CCAGAGGTAAATAATTTCTGGATTTTGACGGTACCACCGGAGAAAGCACCGGCTAACTCC
GTGCCAGCAGCCGCGGTAATACGGAGGGTGCAAGCGTTGTTCGGAATTACTGGGCGTAAA
GGGCGTGTAGGCGGTAATGTAAGTCAGACGTGAAATCCTTTGGCTCAACTGAAGAACTGC
GTCTGAAACTGCATGACTAGAGTGCAGAAGAGGGAAACGGAATTCCTGGTGTAGAGGTGA
AATTCGTAGATATCAGGAGGAACACCGGTGGCGAAGGCGGTTTCCTGGTCTGACACTGAC
GCTGAGGCGCGAAAGCGTGGGGAGCAAACAGGATTAGATACCCCTGTAGTCC
>Otu1511
CCAGCCTACGGGAGGCTGCAGTGTAGAATTTTGGACAATGTGTGAAAACATGATTCAGTA
ATATTTCATAAGTGAAAATAAGACAGTATTATCACTGTAAAACTTTTTTGTTAAGAAAAA
TTATGATTGTACTTAAAGAATAAGCACTGGCTAATTCTGTGCCAGCAGCCGCGGTAATAC
AGAAAGTGCAAGCATTGTTCATCTTAATTGGGTGTAAAGAATATGTAGGTTTAAGATATA
TAAAATTTTGAAATTTTCAAAAAATTTGAGAAAAAAAATTTTACATACTTATATGAGTTC
AAAAAAGGTTTAAAGAATTTTATATGTAAGGGTAGAATCTCTAAAAATATAAAGGAATAC
TAAAGTAGTTGTGAAAACATTTTTCTAAAATGAACTGACGCTGAGATATGAAAGTATAGG
GATCAAATAGGATTAGAAACCCCGGTAGTCC
>Otu1513
CCAGCCTATGGGTCGCAGCAGTGAGGAATATTGCGCAATGGCCGAAAGGCTGACGCAGCG
ACGCCGCGTGAGGGATGAAGTCCGTTAGGATGTAAACCTCTTTTGCAGGGGATGAATAGT
CCCTTTCAGGGATCTGACAGTACCCTGCGAATAAGGATCGGCTAACTACGTGCCAGCAGC
CGCGGTAATACGTAGGATCCGAGCGTTGTCCGGATTTACTGGGTGTAAAGGGCGTGTAGG
TGGACCTTTAAGTCGATGGTGAAATCCTGCGGCTCAACCGCAGAATTGCTTTCGAAACTG
GAGGTCTTGAGTGCGGCAGAGGGAGATGGAATTCATGGTGTAGCGGTGAAATGTGTAGAT
ATCATGAAGAACACCAGTAGCGAAGGCGGTCTCCTGGTCCGTAACTGACACTGAGGCGCG
AAAGCGTGGGGAGCAAACAGGATTAGAAACCCTTGTAGTCC
>Otu1519
CCAGCCTATGGGACGCTGCAGTAGGGAATATTGGGCAATGGGCGAGAGCCTGACCCAGCC

ATGCCGCGTGAAGGAAGAAGGCCTTATGGGTTGTAAACTTCTTTTGATCGGGAACAAAGA
GACCCTGCGGGGTAAGAAGAGTGTACCGATAGAATAAGCACCGGCTAACTCCGTGCCAGC
AGCCGCGGTAATACGGAGGGTGCAAGCGTTGTCCGGATTTATTGGGTTTAAAGGGTGCGT
AGGCGGCTTTTTAAGTCAGTGGTGAAATACAGCAGCTCAACTGTTGAGGTGCCATTGATA
CTGAAGAGCTTGAGTACAGATGAGGTAGGCGGAATTTATGGTGTAGCGGTGAAATGCATA
GATACCATAAAGAACACCGATTGCGAAGGCAGCTTACTAAGCTGTAACTGACGCTGAGGC
ACGAAAGCGTGGGGAGCGAACAGGATTAGAGACCCTCGTAGTCC
>Otu1520
CCAGCCTATGGGTGGCAGCAGTGAGGAATATTGCTCAATGGGCGAAAGCCTGAAGCAGCA
ACGCCGCGTGCGCGGTGAAGGCCTTCGGGTCGTAAAGCGCTTTTGAGAGGGACGAGATAG
GACGGTACCTCTGGAATAAGTCACGGCTAACTACGTGCCAGCAGCCGCGGTAAAACGTAG
GTGGCAAGCGTTATCCGGATTCACTGGGCGTAAAGCGCGTGCAGGTGGTTCTGTAAGTTG
GATGTTAAATCTTCCGGCCTAACCGGGAGCGGTCATTCAATACTGCAGGACTTGAGGGCA
GAAGAGGGGGGTGGAATTGCCGGTGTAGTGGTGAAATGCGTAGATATCGGCAGGAACACC
TGTGGCGAAAGCGGCCCCCTGGTCTGCTCCTGACACTCAGACGCGAAGGCTAGGGTAGTG
AACGGGATTAGATACCCCAGTAGTCC
>Otu1524
CCAGCCTATGGGGGGCAGCAGTCGAGGACCTTCGGCAATGGGCGCAAGCCTGACCGAGCG
ACGCCGCGTGTGCGATGAAGGCCTTCGGGTTGTAAAGCACTGTCGAGGGGGAGAAAAGGG
CAACCTTGATCGACCCCTGGAGGAAGCACGGGCTAAGTTCGTGCCAGCAGCCGCGGTAAG
ACGAACCGTGCGAACGTTGTTCGGAATCACTGGGCTTAAAGGGCGCGTAGGCGGGCGGTC
AAGTCAGAGGTGAAATCCCACGGCTCAACCGTGGAAGCGCCCCTGATACTGATTGTCTCG
AGGGAGGTAGGGGCGTGCGGAACTTCCGGTGGAGCGGTGAAATGCGTAGATATCGGAAGG
AACACCGGTGGCGAAAGCGGCGCGCTGGACCTTTACTGACGCTGAGGCGCGAAAGCCAGG
GGAGCAAACGGGATTAGATACCCGTGTAGTCC
>Otu1525
CCAGCCTACGGGTGGCAGCAGTCGAGAATTTTTCTCAATGGGCGAAAGCCTGAAGGAGCG
ACGCCGCGTGGGGGATGAAGGGCTTCGGCTCGTAAACCCCTGTCATTTGCGAACAAACCT
TTTCATTTAACAGATGAAAAGCTGATTGTAGCGAAAGAGGAAGGGACGGCTAACTCTGTG
CCAGCAGCCGCGGTAATACAGAGGTCCCAAGCGTTGTTCGGATTCACTGGGCGTAAAGGG
TGCGCAGGTGGCGAGGTGAGTTTGGCGTGAAATCTCCGGGCTCAACCCGGAAAATGCGTT
GAATACTCTCTCGCTAGAGGGTTGGAGGGGAGACTGGAATTCTCGGTGTAGCAGTGAAAT
GCGTAGATATCGAGAGGAACACCAGTGGCGAAGGCGAGTCTCTGGACAACTCCTGACACT
GAGGCACGAAAGCTAGGGGAGCAAACAGGATTAGATACCCCAGTAGTCC
>Otu1526
CCAGCCTACGGGTTGCAGCAGTGGGGAATATTGGACAATGGGGGCAACCCTGATCCAGCG
ACGCCGCGTGTGTGAAGAAGGCCTGCGGGTTGTAAAGCACTTTTGGCAGGGACGATGATG
GCGGTACCTGCAGAATAAGCCCCGGCTAACTTCGTGCCAGCAGCCGCGGTAATACGAAGG
GGGCTAGCGTTGCTCGGAATGACTGGGCGTAAAGGGCGCGTAGGCGGCTGTCACAGTCAG
AAGTGAAATTCCTGGGCTCAACCTGGGGACTGCTTTTGATACGTGGCGGCTTGAGTGAGG
AAGAGGGTTGCGGAATTTCCAGTGTAGAGGTGAAATTCGTAGATATTGGAAAGAACACCG
GTGGCGAAGGCGGCAACCTGGTCCTTTACTGACGCTGAGGCGCGAAAGCGTGGGGAGCAA
ACAGGATTAGATACCCTTGTAGTCC
>Otu1529
CCAGCCTACGGGTCGCTGCAGTGGGGAATATTGGACAATGGGCGCAAGCCTGATCCAGCA
ATGCCGCGTGAGTGAAGAAGGTCTTCGGATTGTAAAGCTCTTTCGGCGGGGACGATGATG
ACGGTACCCGCAGAAGAAGCCCCGGCTAACTTCGTGCCAGCAGCCGCGGTAATACGAAGG
GGGCTAGCGTTGCTCGGAATGACTGGGCGTAAAGGGCGCGTAGGCGGCTGTCATAGTCAG
ATGTGAAATTCCCGGGCTTAACCTGGGGGCTGCATTTGATACGTGGCGGCTAGAGTGCGG
AAGAGGGTCGTGGAATTCCCAGTGTAGAGGTGAAATTCGTAGATATCAGGAGGAACACCT
GTGGTGTAGACAGCTTACTGGACCATCACTGACACTGAGACACGAAAGCGTGGGTAGCAA
ACAGGATTAGATACCCTGGTAGTCC
>Otu1530
CCAGCCTACGGGGGGCAGCAGTAGGGAATTTTCCACAATGGGCGCAAGCCTGATGGAGCA
ACGCCGCGTGCAGGATGAATGTCTTCGGATTGTAAACTGCTTTTCTCTGTGACGAATATG
ACGGTAGCAGAGGAATAAGGACCTGCTAACTACGTGCCAGCAGCCGCGGTCATACGTAGG
GTCCAAGCGTTATCCGGAATTACTGGGCGTAAAGAGTTGCGTAGGTGGCATAGTAAGCGG
GTAGTGAAATTGTGTGGCTCAACCATACACCCATTACCTGAACTGCTAAGCTAGAGGACG

AGAGAGGTTATTGGAATTCCCTGTGTAGGAGTGAAATCCGTAGATATAGGGAGGAACACC
GATGGCGTAGGCAGATAACTGGCTCGTTTCTGACACTAAGGCACGAAAGCGTGGGGAGCA
AACGGGATTAGATACCCGTGTAGTCC
>Otu1531
CCAGCCTACGGGTCGCTGCAGTCGAGAATCTTCCGCAATGGACGAAAGTCTGACGGAGCG
ACGCCGCGCGGAGGATGAAGTGCTTCGGCATGTAAACTCCTTTTGCCAGGGAAAAAGTCT
ATTGATTGTACCTGGAGAATAAGAGGTTGCTAAACTCGTGCCAGCAGCAGCGGTAATACG
AGTGCCTCGAGCGTTATCCGGAATTATTGGGCGTAAAGGGTGCGTAGGTGGTCGTGTTAG
TCTTGTGTTAAATTTCTCGGCTCAACCGGGAAGCTGCACGGGAAACGGCACGACTCAGAG
GTTGGAAGGGGTTTGCGGAACTCATAGTGTAGCGGTGAAATGCGTTGATATTATGGGGAA
CACCAAAAGCGAAGGCAGCAAACTGGTCCACACCTGACACTGAAGCACGAAAGCGTGGGT
CGCGAATGGGATTAGATACCCTTGTAGTCC
>Otu1532
CCAGCCTACGGGACGCTGCAGTTAGGGAATTTTCACACTGGGCGCAAGCCTGATGGAGCA
ACGCCGCGTGGGCGATGAAGGCCTTCGGGTCGTAAAGCCCTTTTCTGGGGGAAGACAAAA
GGACGGTACCCCAGGAAGAAGCCTCGGCTAACTACGTGCCAGCAGCCGCGGTAATACGTA
GGAGGCGAGCGTTGTCCGGAATTACTGGGCGTAAAGAGCGTGTAGGCGGTCGCCTACGTC
GTGGATGAAAGCCCTCTGCTCAACGGGGGGAGTGTTCGCGAAACGGGGTGACTCTGTGGA
GTGTGGTAGGGGGGTGCGGAATGGCCGGTGGAGCGGTGAAATGCGTAGAGATCGGTCAGA
ACACCAGTGGCGAAGGCGACACCCTGGGCCAGTACTGACGCTGAGACGCGAAAGCGTGGG
GAGCGAACGGAATTAGAAACCCTTGTAGTCC
>Otu1534
CCAGCCTATGGGACGCAGCAGTTGGGAATTTTGGACAATGGGCGCAAGCCTGATCCAGCC
ATGCCGCGTGCAGGATGAAAGCCTTCGGGTTGTAAACTGCTTTTGTACGGAACGAAAAGG
TTCTTTCTAATAAAGAGAGCTCATGACGGTACCGTAAGAATAAGCACCGGCTAACTACGT
GCCAGCAGCCGCGGTAATACGTAGGGTGCAAGCGTTAATCGGAATTACTGGGCGTAAAGC
GTGCGCAGGCGGTGATGTAAGACAGTTGTGAAATCCCCGGGCTCAACCTGGGAACTGCAA
TTGTGACTGCATTGCTGGAGTGCGGCAGAGGGGGATGGAATTCCGCGTGTAGCAGTGAAA
TGCGTAGATATGCGGAGGAACACCGATGGCGAAGGCAATCCCCTGGGCCTGCACTGACGC
TCATGCACGAAAGCGTGGGGAGCAAACAGGATTAGAAACCCCGGTAGTCC
>Otu1537
CAGCCTATGGGGTGCAGCAGTGAGGAATATTGCGCAATGGGGGAAACCCTGACGCAGCGA
CGCCGCGTGGAGGATGAAGGCCTTCGGGTTGTAAACTCCTGTCAGTAGGGAAGAGAACGG
ACGGTACCTACTGAGGAAGCTTCGGCTAACTACGTGCCAGCAGCCGCGGTAAGACGTAGG
GAGCAAGCGTTGTCCGGAATTACTGGGCGTAAAGAGCTCGCAGGCGGGAGCGTAAGTCTG
GAAGGAAATCTCAAGGCTCAACTTTGGGGCTTTTTCAGATACTGCGTTTCTTGAGGGTAT
CAGAGGATAGTAGAATTCCCGGTGTAGCGGTGAAATGCGTAGATATCGGGAGGAATACCA
GTGGCGAAGGCGACTGTCTGGGATATTCCTGACGCTGAGGAGCGAAAGCGTGGGGAGCAA
ACGGGATTAGAGACCCCCGTAGTCC
>Otu1538
CCAGCCTATGGGGCGCAGCAGTAACGAATCTTCCGCAATGCACGAAAGTGTGACGGAGCG
ACGCCGCGTGTGGGATGAAGTTCTTCGGAATGTAAACCACTGTCAGGGGTAAGAAAGTTC
TGATCTACCCCAGAGGAAGGCACGGCTAACTCTGTGCCAGCAGCCGCGGTAAGACAGAGG
TGCCAAGCGTTAGGCGGAATCACTGGGCTTAAAGCGTGTGTAGGCGGATCGTTAAGTGCT
TTGTGAAATCCCATGGCTCAACCATGGAACTGCTTGGCATACTGGCGATCTTGAGCCAGC
TAGGGGCAACCGGAACAAATGGTGGAGCGGTGAAATGCGTAGATATCATTTGGAACGCCA
ATGGTGAAAACAGGTTGCTGGGGCTGTGCTGACGCTGAGACACGAAAGCCAGGGGAGCAA
ACGGGATTAGAAACCCGCGTAGTCC
>Otu1540
CCAGCCTACGGGTTGCAGCAGTGGGGAATCTTGCGCAATGGGCGAAAGCCTGACGCAGCG
ACGCCGCGTGTGTGATGAAGGCCTTCGGGTTGTAAAGCACTGTGGGGAGAGAAGAATAAG
TGGTGGCTAATAGCCACCATGATGACGGTATCTCCTTAGCAAGCACCGGCTAACTCTGTG
CCAGCAGCCGCGGTAAGACAGAGGGTGCAAACGTTGTTCGGAATTACTGGGCGTAAAGCG
TGTGTAGGCGGCTAGGTAAGTCGGATGTGAAAGCCCTGGGCTCAACCCAGGAAGTGCACT
CGATACTGCTTAGCTAGAGTACCGGAGAGGTTGGTGGAATTCTCGGTGTAGAGGTGAAAT
TCGTAGATATCGAGAGGAACACCGGTGGCGAAGGCGGCCAACTGGACGAGTACTGACGCT
GAGACACGAAAGCGTGGGGAGCAAACAGGATTAGAAACCCGTGTAGTCC
>Otu1544

CCAGCCTATGGGTGGCAGCAGTGGGGAATATTGGACAATGGGGGGAACCCTGATCCAGCA
ATGCCGCGTGTGTGAAGAAGGCCTGAGGGTTGTAAAGCACTTTCAGTGGGGAGGAGGGTT
GATTGGTTAAGAGCTGATTGATTGGACGTTACCCACAGAAGAAGCACCGGCTAACTCCGT
GCCAGCAGCCGCGGTAATACGGAGGGTGCGAGCGTTAATCGGAATTACTGGGCGTAAAGG
GTGCGTAGGTGGTTTAATAAGTTATCTGTGAAAGCCCTGGGCTTAACCTGGGAAGGCCAG
ATAAGACTGTAAGACTAGAGTATGGGAGAGGGTAGTGGAATTTCCGGTGTAGCGGTGAAA
TGCGTAGATATCGGAAGGAACACCGGTGGCGAAGGCGGCTACCTGGCCTGATACTGACAC
TGAGGCACGAAAGCGTGGGGAGCAAACAGGATTAGAAACCCCCGTAGTCC
>Otu1549
CCAGCCTACGGGGTGCAGCAGTGGGGAATATTGGGCAATGGGCGAAAGCCTGACCCAGCT
ATGCCGCGTGAGTGATGAAGGCCTTCGGGTCGTAAAGCTCTGTGGAGAGGGAAGAACAAG
CATGTGGCTAATATCCACGTGCCCTGACGGTACCTCCTTAGCAAGCACCGGCTAACTCCG
TACCAGCAGCCGCGGTAATACGGAGGGTGCAAACGTTGCTCGGAATCATTGGGCGTAAAG
CGCACGTAGGTGGTCTACTAAGTCGGGTGTGAAAGCCCTGGGCTCAACCCAGGAAGTGCA
CTCGAAACTGATAGGCTTGAGTATGGAAGAGGGTCGCGGAATTCCCGGTGTAGAGGTGAA
ATTCGTAGATATCGGGAGGAACACCAGTGGCGAAGGCGGCGACCTGGGCCAATACTGACA
CTGAGGTGCGAAAGCGTGGGGAGCAAACAGGATTAGAAACCCTGGTAGTCC
>Otu1550
CCAGCCTATGGGGGGCAGCAGTCGAGGATCTTCGGCAATGGGCGCAAGCCTGACCGAGCG
ACGCCGCGTGTGCGATGAAGGCCTTCGGGTTGTAAAGCACTGTCGAGGGGGAGAAAGGGC
AACTTGATCGGCCCCTGGAGGAAGCACGGGCTAAGTTCGTGCCAGCAGCCGCGGTAAGAC
GAACCGTGCGAACGTTATACGGAATCACTGGGCTTAAAGGGCGCGTAGGCGGCTGTCAA
GTCAGAGGTGAAATCCTGCGGCTCAACCGTAGAAGAGCCCCTGATACTGACGGTCTTGAG
GGAGGTAGGGGCGTGTGGAACTTCCGGTGGAGCGGTGGAATGCGTAGATATCGGAAGGAA
CGCCGGTGGCGAAAGCGACACGCTGGCCCTCTACTGACGCTGAGGCGCGAAAGCCAGGGG
AGCAAACGGGATTAGATACCCCCGTAGTCC
>Otu1553
CCAGCCTACGGGTCGCACCAGTCGGGAAACTTTTGCAATACACGCAAGTGTGACAAAGCA
AGCCAAAGTGCCATTCTATATTTGAATAGCTTTTGTGGGATGCAAAAAGTCTCACGAATA
AGGACTGGGCAAGACTGGTGCCAGCCGCCGCGGTAATCCCAGCAGTCCAAGTCGCAGCCA
CATTTGTTGGGTCTAAAATATCCGTAGCTTGCCTTTCAAGTCTTTTGTGAAATCGGGACT
CTTAAAGTTCCGGCGGGCAAAAGATACTGTTAGGCTAGAGACCGGGAGGCGTAAGAAGTA
CAGATAGGGTAACGGTAAAATGTGTTGATCCTATTTGGACTAACAGTCGCGAAGGCGTCT
TACGAGAACGGTTCTGACAGTGAGGGATGAAGGCTAGGGGCGCAAAGTGGATTAGATACC
CCAGTAGTCC
>Otu1554
CCAGCCTATGGGGTGCTGCAGTGGGGAATATTGGTCAATGGGCGAAAGCCTGAACCAGCA
ATGCCGCGTGGGTGATGAAGGCCTTCGGGTTGTAAAGCCCTGTCAGTGGGAAAGAAAAAG
CCGGTTAACACCCGGACTTGACGGTACCCACAAAGGAAGCACCGGCTAACTCTGTGCCAG
CAGCCGCGGTAATACAGAGGGTGCAAGCGTTGTTCGGAATTACTGGGCGTAAAGCGCGTG
TAGGCGGCTTTTTAAGTCGGACGTGAAATCCCTAGGCTTAACCTAGGAACTGCGTCCGAT
ACTACTAAGCTCGAGTGTCGGAGGGGGTGGCGGAATTCCCGGTGTAGAGGTGAAATTCGT
AGATATCGGGAGGAACACCTGTGGCGAAGGCGGCCACCTGGAAGACAACTGACGCTGAGA
CGCGAAAGCGTGGGGAGCAAACAGGATTAGAAACCCTCGTAGTCC
>Otu1555
CCAGCCTATGGGGTGCTGCAGTGGGGAATTTTGCGCAATGGGCGAAAGCCTGACGCAGCA
ACGCCGCGTGCGGGATGAAGGCCTTCGGGTCGTAAACCGCTTTCAGCAGGGATGAAAATG
ACAGTACCTGCAGAAGAAGCCCCGGCTAAATACGTGCCAGCAGCCGCGGTAATACGTATG
GGGCAAGCGTTATCCGGATTCATTGGGCGTAAAGAGCGCGTAGGCGGCTTGTTAAGTTAG
GTGTGAAATCCGGGGGCTTAACCCGCGGCTTGCACCTAAAACTGGCAGGCTTGAGTCTGG
TAGAGGAAAGTGGAATTCCCAGTGTAGCGGTGAAATGCGCAGATATTGGGAGGAACACCA
GTGGCGAAGGCGGCTTTCTGGGCCACGACTGACGCTGAGGCGCGAAAGCGTGGGGATCAA
ACAGGATTAGATACCCTGGTAGTCC
>Otu1557
CCAGCCTACTGGTCGCAGCAGTCGAGGATCTTCGGCAATGGGCGCAAGCCTGACCGAGCG
ACGCCGCGTGTGCGATGAAGGCCTTCGGGTTGTAAAGCACTGTCGAGGGGGAGGAAAGCC
GCAAGGTCTGACCTATCCCTGGAGGAAGCACGGGCTAAGTTCGTGCCAGCAGCCGCGGTA
AGACGAACCGTGCGAACGTTGTTCGGAATCACTGGGCTTAAAGGGCGCGTAGGCGGGCC

GCAAGTCCGGGGTGAAATCCCCCAGCTCAACTGGGGAAGTGCCTTGGAAACTGCGGTTCT
AGAGGGAGGTAGGGGCATGTGGAACTTCGGGTGGAGCGGTGAAATGCGTTGATATCCGAA
GGAACGCCGGTGGCGAAAGCGACGTGCTGGATCTCTTCTGACGCTGAGGCGCGAAAGCCA
GGGTAGCGAACAGGATTAGATACCCGCGTAGTCC
>Otu1558
CCAGCCTATGGGATGCAGCAGTGGGGAATATTGGACAATGGGCGAAAGCTTGATCCAGCA
ATGCCGCGTGAGTGATGAAGGCCTTAGGGTTGTAAAACTCTTTCGACGGGGACGATAATG
ACGGTACCCGTAGAAGAAGCTCCGGCTAACTTCGTGCCAGCAGCCGCGGTAATACGAAGG
GGGCTAGCGTTGTTCGGAATTACTGGGCGTAAAGCGCGCGCAGGCGGCTATCCAAGTCAG
TGGTGAAAGCCCGGAGCTCAACTCCGGAACTGCCATTGAAACTGTTTAGCTTGAGGACGA
GAGAGGTGAGTGGAATTCCCAGTGTAGAGGTGAAATTCGTAGATATTGGGAAGAACACCA
GTGGCGAAGGCGGCTCACTGGCTCGTTTCTGACGCTCAGGCGCGATAGCGTGGGGATCAA
ACAGGATTAGATACCCTGGTAGTCC
>Otu1559
CCAGCCTATGGGGGGCAGCAGTGGGGAATTTTGGACAATGGACGCAAGTCTGATCCAGCC
ATGCCGCGTGAGTGAAGAAGGCCTTCGGGTTGTAAAGCTCTTTTGTCCGGGAGCAAAACT
TAACGCCTAATACGTGTTGAGGCTGAGAGTACCGGAAGAATAAGCACCGGCTAACTACGT
GCCAGCAGCCGCGGTAATACGTAGGGTGCGAGCGTTAATCGGAATTACTGGGCGTAAAGC
GTGCGCAGGTGGTTATGTAAGCTTGATGTGAAATCCCCGGGCTTAACCTGGGAACTGCAT
TGAGGACTGCATGGCTAGAGTGTGGCAGAGGGGGGTAGAATTCCACGTGTAGCAGTGAAA
TGCGTAGAGATGTGGAGGAATACCAATGGCGAAGGCAGCCCCCTGGGTTAACACTGACAC
TCATGCACGAAAGCGTGGGGAGCAAACAGGATTAGATACCCCTGTAGTCC
>Otu1560
CCAGCCTATGGGTGGCACCAGTAACGAATATTCCGCAATGCGCGAAAGCGTGACGGAGCG
ATGCCGCGTGCAGGATGAAGCCCCTCGGGGTGTAAACTGCTGTCAGGGTTTAGCAACACA
ATGAGCATACCCAGAGGAAGAGACGGCTAACTCTGTGCCAGCAGCCGCGGTAATACAGAG
GTCTCGAGCGTTAATCGGAGTCACTGGGCTTAAAGGGTGCGTAGGCGGGTCCGCAAGTGC
TTTGTGAAATCCCATGGCTCAACCATGGAATTGCTTGGCAGACTGCGGATCTTGAGGTCG
GTAGAGGCTGCTGGAACTCTAGGTGGAGCGGTGAAATGCGTAGATATCTAGAGGAACGCC
GATGGTGAAGACAGGCAGCTGGGCCGATTCTGACGCTGAGGCACGAAAGCGTGGGGAGCG
AACGGGATTAGATACCCCCGTAGTCC
>Otu1563
CCAGCCTACGGGAGGCTCCAGTTAGGAATCTTGCGCAATGGGCGAAAGCCTGACGCAGCC
ATGCCGCGTGAATGATGAAGGTCTTAGGATTGTAAAATTCTTTCAGTGGGGACGATAATG
ACGGTACCCATAGAAGAAGCCCCGGCTAACTTCGTGCCAGCAGCCGCGGTAATACGAGGG
GGGCAAGCGTTGTTCGGATTTATTGGGCGTAAAGGGTGCGTAGGCGGTTTGATAAGTCTG
ATGTGAAATCTATGGGCTCAACCCATAGTCTGCATTAGAAACTGTCGGGCTTGAGTATGG
GAGAGGTGAGTGGAATTTCCGGTGTAGCGGTGAAATGCGTAGATATCGGAAGGAACACCT
GTGGCGAAAGCGGCTCACTGGACCATAACTGACGCTGATGCACGAAAGCTAGGGGAGCAA
ACAGGATTAGAAACCCGGGTAGTCC
>Otu1566
CCAGCCTATGGGGGGCTCCAGTGGGGAATCTTCCGCAATGGACGAAAGTCTGACGGAGCA
ACGCCGCGTGAGTGAAGAAGGTTTTCGGATCGTAAAGCTCTGTTGTTTGAGACGAACGTG
CAGTATACGAATAATGTGCTGTAATGACGGTATCAAACTAGAAAGCCACGGCTAACTACG
TGCCAGCAGCCGCGGTAATACGTAGGTGGCAAGCGTTGTCCGGAATTATTGGGCGTAAAG
GGAGCGCAGGTGGGAATGTAAGTCAGTCTTAAAAGTGCGGGGCTCAACCCCGTGATGGGA
CAGAAACTACATTTCTTGAGTGCAGGAGAGGAAAGTGGAATTCCTAGTGTAGCGGTGAAA
TGCGTAGATATTAGGAGGAACACCAGTGGCGAAGGCGACTTTCTGGACTGTAACTGACAC
TGAGGCTCGAAAGCTAGGGGAGCGAACGGGATTAGAAACCCTAGTAGTCC
>Otu1572
CCAGCCTACGGGGGGCACCAGAAGTGGGCATGGGGGCATTATAAAAGACGGAGCCAAAAT
GGTAAACGCCATGGCAAATAGTGTGGTGCCTAAGTTCACCGTTATTATGGGCAACTCCTT
TGGTGCGGCCAATTACGCTATGTGTGGCAAAGCCTACGACCCCCGTTTAATTGTGGGTTG
GCCTACCGCGCAAATTGCTGTAATGGGTGGTGCACAAGCCGCCAAAGTATTGGTGCAAAT
AGAAGTAGCTTCTTTAAAAGCTAAAGGAGAAGTTATTACTCCCGAAAATGAAGCCGAGTT
ATACAACAAAACCAAAGACCGCTACGATACACAAACTACGCCCTATTACGCTGCATCTCG
TTTGTGGTTAGATGCCATTATAGACCCATTAGAAACCCTTGTAGTCC
>Otu1575

CCAGCCTATGGGGGGCAGCAGGCGCGAAACCTCTGCAATGCGCGAAAGCGTGACAGGGGA
ATTCTGAGTGCTACACTACGCGTGTAGCTTTTCGCAATTTTAAGACAATTGCGGAATAAG
GGCTGGGCAAGACTAGTGCCAGCCGCCGCGGTAACACTAGCGGCCCGAGTGGTGTCCACG
AATATTGGGTCTAAAGAGTCCGTAGCTGGTCCGTTAAGTTTCCTGTGAAATTTCAGGGCT
CAACTCTGAAGCGTGCAGGAAATACTGGCGAGACTTGAGAGCGGGGGAGGTCTAGAGTAC
TCATGGGGTAACGGTAAAATGTTGTAATCCTATGAGGACTTCCAGTGGCGAAGGCGCTAG
ACCAAAACGCGTCTGACAGTGAGGGACGAAGGCTAGGAGAACGAATCGGATTAGATACCC
GTGTAGTCC
>Otu1576
CCAGCCTACGGGGCGCAGCAGTAGGGAATATTGGGCAATGGGGGCAACCCTGACCCAGCC
ATGCCGCGTGCAGGAGAAAGGCCCTCTGGGTTGTAAACTGCTTTTGGGAGGGGACAAAAG
GCCCATGCGTGGGAGATTGAGTGTACCTCCAGAATAAGCACCGGCTAACTCCGTGCCAGC
AGCCGCGGTAATACGGAGGGTGCGAGCGTTGTCCGGATTTATTGGGTTTAAAGGGTGCGT
AGGCGGCCGCTTAAGTCGGGGGTGAAACCCACGGGCTCAACCCGTGGAGTGCCCCCGATA
CTGTGCGGCTTGGGTCGTCTCTGGGCGGGCGGAATGGAAGGTGTAGCGGTGAAATGCATA
GATATCTACCAGAACGCCGATAGCGAAGGCAGCTCGCTGGGGACGTACCGACGCTGAGGC
ACGATAGCGTGGGGATCAAACAGGATTAGATACCCCGGTAGTCC
>Otu1577
CCAGCCTACGGGTGGCACCAGCAACGAATATTGGACAATGGACGCAAGTCTGATCCAGCG
ACGCCGCGTGCGGGATGAAGCCCTTCGGGGTGTAAACCGCTGTCAGGGGTAATCAATAAA
TTGAGATATCCCAGAGGAAGCCACGGCTAACTCCGTGCCAGCAGCCGCGGTAAGACGGAG
GTGGCGAGCGTTACTCGGATTTACTGGGCTTAGAGCGGGTGCAGGCGGCCATACAAGTCC
GGAGTGAAATCCCCCGGCTCAACCGGGGAACTGCTTTGGATACTGTATGGCTTGAGGCAA
GCAGGGGTCAGTGGAACGTTTGGTGTAGCGGTGAAATGCGTAGATATCAAACGGAACGCC
GGTGGTGAAGACGGCTGACTGGGCTTGTCCTGACGCTCAGACCCGAAAGCGTGGGGAGTG
AACCGGATTAGATACCCCCGTAGTCC
>Otu1578
CCAGCCTATGGGATGCTCCAGTCGAGAATCTTTTGCAATGGGCGAAAGCCTGACAAAGCG
ACGCCGTGTGAATGAAGAAGGCCTTCGGGTTGTAAAGTTCTTTTGCCAACGAGCAAGAAA
GAGTAGTGAATAACTGCTCAATTTGAGAGTAGTTGGAGAATAAGCACCGGCAAACTCCGT
GCCAGCAGCCGCGGTAATACGGAGGGTGCAAGCATTAATCGGAATAATTGGGCGTAAAGG
GCGCGTAGGTGGATTAGTAAGTCATTTGTGAAATTCCGGAGCTTAACTCCGGAGCTGCAA
GTGAAACTCCTAGTCTAGAGGTTGGTCGGAGGAAACGGAATTCCACGTGTAGCGGTGAAA
TGCGTAGATATGTGGAAGAACACCGGTGGCGAAAGCGGTTTCCTAGATTACACCTGACAC
TGATGCGCGAGAGCGTGGGGAGCAAACAGGATTAGATACCCGCGTAGTCC
>Otu1579
CCAGCCTACGGGGGGCTCCAGTCGGGAAAATTCTGCAATGTACGAAAGTATGACAGAGCA
AGCCAAAGTGCTTTTCTTTTAGAAAAGCTTTTGTGTGCTGTAAAAAGGCACACGAATAAG
GACCGGGAGAGACTGGTGCCAGCCGCCGCGGTAATCCCAGCGGTCCAAGTCGCAGCCACA
TTTGTTGGGTCTAAAACATCCGTAGCTTGCCTTACAAGTCTTTTGTGAAATCGGGACTCT
TAAGGTTCCGTCGGGCAAAAGATACTGTTTGGCTAGAGACCGGGAGATGTAAAGAGTACA
GATAGGGTAGTGGTTGAATACGTTGATCCTTTCTGGATTAACGATAGCGAAGGCACTTTA
CAAGCACGGATCTGACAGTGAGGGATGAAGGCTAGGGGCGCAAAGTGGATTAGAAACCCC
AGTAGTCC
>Otu1581
CCAGCCTATGGGAGGCTGCAGTGGGGAATCTTGCACAATGGGCGAAAGCCTGATGCAGCA
ACGCCGCGTGGGTGAAGAAGGTTTTCGGATTGTAAAGCCCTTTCGGCTGGGAAGAAGGGT
GTTACGGTAAATAATCGTAGCATTTGACGGTACCAGAAGAAGAAGCACCGGCTAATTTCG
TGCCAGCAGCCGCGGTAATACGAAAGGTGCAAGCGTTGTTCGGAATGACTGGGCGTAAAG
CGCACGTAGGCGGGTCTGTAAGTCAGATGTGAAATCCCCGGGCTCAACCCGGGAACAGCA
CCTGAAACTGCGGATCTTGAATGCTTGAGAGGTTGGTGGAATTCCAGGTGTAGGAGTGAA
ATCCGTAGATATCTGGAGGAACACCGGAGGCGAAGGCGGCCAACTGGCGATGCATTGACG
CTGAGGTGCGAAAGCGTGGGGAGCAAACAGGATTAGAGACCCGTGTAGTCC
>Otu1583
CCAGCCTACGGGAGGCAGCAGTGAGGAATATTGGGCAATGGACGAAAGTCTGACCCAGCA
ACGCCGCGTGGAGGATGAAGGCCGTAAGGTCGTAAACTCCTTTTGAGAGGGACGAATATT
TATATTTCGTAAGGGATATAAACTGACGGTACCTTTAGAAGAAGCCCCGGCTAACTACGT
GCCAGCAGCCGCGGTAATACGTAGGGGGCAAGCGTTGTCCGGATTTACTGGGTGTAAAGG

GCGCGTAGGCGGGTTTGTAAGTCAGTGGTGAAAGCCTGCAGCTTAACTGCAGAACTGCCA
TTGATACTGCAAATCTTGAGTTCAGAAGAGAGAAGCGGAATTCCAGGTGTAGTGGTGAAA
TACGTAGATATCTGGAAGAACACCAGTGGCGAAGGCGGCTTCTTGGTCTGTAACTGACGC
TGAGGCGCGAAAGCGTGGGGATCAAACAGGATTAGAAACCCCCGTAGTCC
>Otu1586
CCAGCCTATGGGTTGCTGCAGTGGGGAATCTTGGACAATGGGCGCAAGCCTGATCCAGCC
ATGCCGCGTGAGTGACGAAGGCCTTAGGGTTGTAAAGCTCTTTTACCAGGGACGATAATG
ACGGTACCTGGAGAATAAGTCCCGGCTAACTTCGTGCCAGCAGCCGCGGTAATACGAAGG
GGACTAGCGTTGTTCGGAATTACTGGGCGTAAAGCGCACGTAGGCGGGATGGCCAGTCAG
AGGTGAAATCCCAGAGCTCAACTCTGGAACCGCCTTTGATACAGCCATTCTTGAGTCCGA
TAGAGGTGAGTGGAATTCCTAGTGTAGAGGTGAAATTCGTAGATATTAGGAAGAACACCA
GTGGCGAAGGCGGCTCACTGGATCGGTACTGACGCTGAGGTGCGAAAGCGTGGGGAGCAA
ACAGGATTAGAAACCCTTGTAGTCC
>Otu1589
CCAGCCTACGGGACGCACCAGTTTCGAATCATTCACAATGGGCGAAAGCCTGATGGTGCG
ACGCCGCGTGAGGGATGAAGGTCTTCGGATTGTAAACCTCTGTCACTGGGGAAGAAACGC
TTCAGGTTAATAGCCTGAAGCCTGACTTAACCCGGAGAGGAAGCAGTGGCTAACTCTGTG
CCAGCAGCCGCGGTAATACAGAGACTGCAAGCGTTATTCGGATTCACTGGGCGTAAAGGG
TGCGCAGGTGGCCAAGTGTGTGAGGCGTGAAAGCCCGGAGCTCAACTCCGGAATTGCACC
TCAAACTACATGGCTAGAGCATTGGAGAGGGGAGCAGAATTCACGGTGTAGCAGTGAAAT
GCGTAGATATCGTGAGGAAGACCAGTGGCGAAGGCGGGTCTCTGGACAGTTCCTGACGCT
GAGGCGCGAAGGCCAGGGGAGCAAACGGGATTAGAGACCCTGGTAGTCCCC
>Otu1590
CCAGCCTACGGGTTGCAGCAGTGAGGAATATTGGACAATGGGCGCAAGCCTGATCCAGCC
ATGCCGCGTGCAGGAAGACGGTCCTATGGATTGTAAACTGCTTTTATACAGGAAGAAACA
CTCCCTCGTGAGGGAACTTGACGGTACTGTAAGAATAAGGATCGGCTAACTCCGTGCCAG
CAGCCGCGGTAATACGGAGGATCTAAGCGTTATCCGGAATCATTGGGTTTAAAGGGTCCG
TAGGCGGTTTGATAAGTCAGTGGTGAAAGCCCATCGCTCAACGATGGAACGGCCATTGAT
ACTGTCAGACTTGAATTACTAGGAAGTAACTAGAATATGTAGTGTAGCGGTGAAATGCTT
AGATATTACATGGAATACCAATTGCGAAGGCAGGTTACTACTAGTGGATTGACGCTGATG
GACGAAAGCGTGGGGAGCGAACAGGATTAGAGACCCGTGTAGTCC
>Otu1591
CCAGCCTACGGGGGGCTCCAGTGGGGAATCTTGCGCAATGGACGAAAGTCTGACGCAGCC
ACGCCGCGTGAGTGATGAAGGCCTTCGGGTTGTAAAGCTCTGTCGCCCGGGACGAACGGC
CGTAAGGTTAACAGCCTTATGGATTGACGGTACCGGGAGAGGAAGCACCGGCTAACTCTG
TGCCAGCAGCCGCGGTAATACAGAGGGTGCAAGCGTTGCTCGGAATTATTGGGCGTAAAG
GGCAGGTAGGTGGTCTTATTTGTCTAGGGTGAAATCCCTGAGCTTAACTCAGGAAGTGCC
CTAGAAACGGTAAGACTGGAGTACTGGAGAGGGTCGTGGAATTCCCGGTGTAGCGGTGAA
ATGCGTAGAGATCGGGAGGAACACCAGAGGCGAAGGCGGCGACCTGGACAGTGACTGACA
CTCAACTGCGAAAGCGTGGGGAGCAAACAGGATTAGAAACCCTAGTAGTCC
>Otu1592
CCAGCCTACGGGTCGCTGCAGTAGGGAATTTTCCACAATGGGCGAAAGCCTGATGGAGCA
ACGCCGCGTGCAGGATGAAGGCCTTCGGGTTGTAAACTGCTTTTATCTGTGAAGATTATG
ACGGTAGCAGATGAATAAGGATCGGCTAACTCCGTGCCAGCAGCCGCGGTCATACGGAGG
ATCCAAGCGTTATCCGGAATTACTGGGCGTAAAGAGTTGCGTAGGTGGCATTGTAAGTTG
CTAGTGAAAGCATACGGCTCAACCGTATATACATTAGCAAAACTGCAAAGCTTGAGGACG
AGAGAGGTACCTGGAATTCCTAGTGTAGGAGTGAAATCCGTAGATATTAGGAGGAACACC
GATGGCGTAGGCAGGGTACTGGCTTGTTCCTGACACTAAGGCACGAAAGCCAGGGGAGCA
AACGGGATTAGAAACCCGCGTAGTCC
>Otu1593
CCAGCCTATGGGTGGCTGCAGTGGGGAATCTTGGACAATGGGGGAAACCCTGATCCAGCC
ATGCCGCGTGAGTGATGAAGGCCTTCGGGTTGTAAAACTCTTTCGACGGGGACGATAATG
ACAGTACCCGTAGAAGAAGCTCCGGCTAACTTCGTGCCAGCAGCCGCGGTAATACGAGGG
GGGCAAGCGTCGTTCGGAATTATTGGGCGTAAAGGGTGCGTAGGCGGTTTGGTAAGTCTT
GTGTGAAATCTCCGGGCTCAACTCGGAGTCTGCACGGGAAACTGCCGGGCTTGAGTGTGG
GAGAGGTGAGTGGAATTTCCGGTGTAGCGGTGAAATGCGTAGATATCGGAAGGAACACCT
GTGGCGAAAGCGGCTCACTGGACCACAACTGACGCTGATGCACGAAAGCTAGGGGAGCAA
ACAGGATTAGATACCCTCGTAGTCC

>Otu1594
CAGCCTATGGGTGGCAGCAGTGGGGAATATTGGACAATGGGCGCAAGCCTGATCCAGCCA
CGCCGCGTGAGTGATGAAGGCCTTCGGGTCGTAAAGCTCTGTGGGGAGGGACGAACCGCT
GGATGTGCATAGCTTCCAGCATGACGGTACCTCCTTAGCAAGCACCGGCTAACTTCGTGC
CAGCAGCCGCGGTAATACGAAGGGTGCAAACGTTGCTCGGAATTATTGGGCGTAAAGCGC
ACGTAGGCGGCGCTCTAAGTCGGATGTGAAAGCCTTTGGCTCAACCAAAGAAGTGCATTC
GAAACTGGAGCGCTTGAGTACTTAAGAGGATCGCGGAATTCCCGGTGTAGAGGTGAAATT
CGTAGATATCGGGAGGAACACCAGTGGCGAAGGCGGCGATCTGGAAAGATACCGACGCTG
AGGTGCGAAAGCGTGGGGAGCAAACAGGATTAGAAACCCTAGTAGTCC
>Otu1596
CCAGCCTATGGGAGGCTCCAGTCGAGAATCTTCCACAATGGACGAAAGTCTGATGGAGCG
ACGCCGCGTGATTGATGAAGTCCTTCGGGACGTAAAGATCTTTTATGAGGGAAGAAGTTT
ATTGACGGTACCTCATGAATAAGGGGCTCCCAATCTCGTGCCAGCAGGAGCGGTAATACG
AGAGCCTCAAGCGTTATCCGGAATTACTGGGCGTAAAGGGTGCGTAGGTGGTAGTGCTAG
TCGTGTGTTAAATCCCGGGGCTCAACCTCGGAATCGCATGCGAAACGGCACAACTTAGAG
AGGAGGAGAGGTGTGCGGAACTCATGGTGTAGGGGTGAAATCCGTTGATATCATGGGGAA
CACCAAAAGCGAAAGCAGCACACTGGCCTTTTTCTGACACTGAGGCACGAAAGCGTGGGT
AGCGAATGGGATTAGAACCCTGGTAGTCC
>Otu1599
CCAGCCTACGGGTTGCACCAGTCGAGGATTTTTCTCAATGGGGGAAACCCTGAAGGAGCG
ACGCCGCGTGAGGGATGAAGGTCTTCGGATTGTAAACCTCTGTCATCTGGGAACAATGCG
CTCCACCTAATACGTGGATCGTTGATAGTACCGGAAGAGGAAGCAGTGGCTAACTCTGTG
CCAGCAGCCGCGGTAATACAGAGACTGCAAGCGTTGTTCGGATTCATTGGGCGTAAAGGG
TGCGCAGGCGGTGTGCTAAGTTGGATGTGAAATCTCACAGCCTAACTGTGATAGGTCATT
CAAAACTGGCATGCTAGAGGACTGGAGAGGAGACTGGAATAGTCGGTGTAGCGGTGAAAT
GCGTAGAGATCGACTAGAACACCGGTGGCGAAGGCGGGTCTCTGGACAGTTCCTGACGCT
CAGGCACGAAAGCCAGGGGAGCAAACGGGATTAGAAACCCTTGTAGTCC
>Otu1602
CCAGCCTATGGGGGGCAGCAGTGAGGAATCTTGCGCAATGGGGGAAACCCTGACGCAGCA
ACGCCGCGTGAGTGAGGAAGGCTTTCGGGTCGTAAAGCTCTGTCAGATGGGAAGAAATGG
GTGAAGATCAATACCCTTCATTCTTGACGGTACCATCAGAGGAAGCACCGGCTAACTCCG
TGCCAGCAGCCGCGGTAATACGGGGGGTGCAAGCGTTGTTCGGAATCATTGGGCGTAAAG
CGCGCGCAGGCGGCTTGGTAAGTCAGATGTGAAAGCCCTGGGCTTAACCCAGGAAGTGCA
TTTGATACTGCCTCGCTAGAGTACGGGAGAGGAAAGTGGAATTCCTGGTGTAGAGGTGAA
ATTCGTAGATATCAGGAGGAACACCGGTGGCGAAGGCGACTTTCTGGCCCGATACTGACG
CTCAGGCGCGAAAGCGTGGGGAGCAAACAGGATTAGATACCCCCGTAGTCC
>Otu1604
CCAGCCTACGGGTCGCAGCAGTGGGGAATATTGGACAATGGGGGCAACCCTGATCCAGCG
ATGCCGCGTGGGTGAAGAAGGCCTTCGGGTTGTAAAGCCCTTTAGGATGGGAAGAAGCTG
CGTAGGTGAATAGCCTATGTAGTTGACGGTACCGTCAGAATAGGCACCGGCAAACTCTGT
GCCAGCAGCCGCGGTAATACAGAGGGTGCGAGCGTTAATCGGATTAACTGGGCGTAAAGG
GCGCGTAGGCGGTTGTATTAGTGTGATGTGAAAGCCCTGGGCTTAACCTAGGAAGAGCAT
CGCAAACGGTGCAACTTGAGTATAAGAGAGGGTGGCGGAATTTCCGGTGTAGCGGTGAAA
TGCGTAGAGATCGGAAGGAACGTCAATGGCGAAGGCAGCCACCTGGCTTAATACTGACGC
TGAGGCGCGAAAGCGTGGGTAGCGAACAGGATTAGAGACCCGCGTAGTCC
>Otu1605
CCAGCCTACGGGGCGCTCCAGCAAGGAATATTGGGCAATGGGCGAAAGCCTGACCCAGCG
ACACCGCGTGGGGGAAGAAGACCTTCGGGTTGTAAACTCCTTTTATTGGGGAAGAATAAT
GACGGTACCCGATGAATAAGCCCCGGCTAACTACGTGCCAGCAGCCGCGGTAATACGTAG
GGGGCGAGCGTTATCCGGATTTACTGGGCGTAAAGCGGGTGCAGGCGGCCTGTCAAGTCG
GATGTGAAATCTCCCGGCCCAACCGGGAGGGTGCATTCGAAACTGATGGGCTAGAGTGCA
GCAGAGGGAGATGGAATTCCCGGTGTAGTGGTGAAATGCGTAGATATCGGGAAGAACACT
AGTGGCGAAGGCGGTCTCCTGGGCTGTAACTGACGCTGGGGCCCGAAGGCGTGGGGAGCA
AACAGGATTAGATACCCTTGTAGTCC
>Otu1606
CCAGCCTATGGGTGGCAGCAGTAGGGAATATTGGGCAATGGAGGCAACTCTGACCCAGCC
ATGCCGCGTGCAGGAAGAAGGCCCTCTGGGTTGTAAACTGCTTTTGAACGGGAAGAAAAG
TCCATGCGTGGGCCTATGACGGTACCGTTAGAATAAGCCACGGCTAACTACGTGCCAGCA

GCCGCGGTAATACGTAGGTGGCAAGCGTTGTCCGGATTTATTGGGTTTAAAGGGTGCGTA
GGCGGCCCTGTAAGTCAGTGGTGAAATACCGCAGCTCAACTGTGGGGGTGCCATTGATAC
TGCAGGGCTTGAGTGGAGTCGGGGTGGCCGGAATTTACGGTGTAGCGGTGAAATGCATAG
ATACCGTAAAGAACACCGATAGCGAAGGCAGGCCACTGGGCTCCAACTGACGCTGAGGCA
CGAAAGCGTGGGGATCAAACAGGATTAGATACCCTAGTAGTCC
>Otu1608
CCAGCCTACGGGTCGCACCAGCTAAGAATCTTCCGCAATGGACGAAAGTCTGACGGAGCG
ACGTCGCGTGGGTGATGAAGGCGGAAACGTTGTAAAGCCCTTTTATAGACGAGGAATAAG
CCTGGGAGGGAATGCCTGGGTGATGACGGTAGTCTGTGAATAAGCCCCGGCTAATTACGT
GCCAGCAGCCGCGGTAACACGTATGGGGCGAGCGTTGTTCGGAATTATTGGGCGTAAAGG
GTATGCAGGCGGTTTTGTAAGTCTGGTGTGCAAGACCACAGCTTAACTGTGGGAACGCGC
TGGAAACTGTGAGACTAGAGTACAGGAAGGGGAGTTGGAATTCCAGGTGTAGGGGTGAAA
TCTGTAGATATCTGGAAGAACATCAGTGGCGAAGGCGAACTCCTGGCCATGTACTGACGC
TGAGATACGAAAGCGTGGGGAGCAAACAGGATTAGATACCCGCGTAGTCC
>Otu1609
CCAGCCTACGGGGGGCAGCAGTCTAGAGGCTTCGGCAATGGACGAAAGTCTGACCGAGCG
ACGCCGCGTGCGGGATTGAAGGTCGTGAGATTGTAAACCGCTGTCGGCGGGGAAGAATGC
CCGGATGGTGAATCCGGGAGAGACGGTACCCGCAGAGGAAGCCCCGGCTAACTCCGTGCC
AGCAGCCGCGGTAATACGGAGGGGGCAAACGTTGTTCGGATTTACTGGGCGTAAAGGGTG
CGTAGGCGGTTCGGTTAGTCTGGTGTGAAAATTCGTCGCTTAACGACGAAAACGCATCGG
AAACTGCCGAGCTCGAGGGTGGAAGAGGCAGGTGGAATTCCCGGTGTAGCGGTGGAGTGC
GTAGAGATCGGGGAGAACACTCGTGGCGAAGGCGGCCTGCTGGTCCATTTCTGACGCTGA
GGCACGAAAGCCAGGGGAGCAAACGGGATTAGATACCCGTGTAGTCC
>Otu1612
CCAGCCTATGGGACGCACCAGTGGGGAATATTGGACAATGGGCGAAAGCCTGATCCAGCC
ATGCCGCGTGGGTGAAGAAGGCCTTAGGGTTGTAAACCCCTTTCAGCGGGGAAGATAATG
ACGGTACCCGCAGAAGAAGTCCCGGCTAACTCCGTGCCAGCAGCCGCGGTAATACGGAGG
GGACTAGCGTTGTTCGGAATTACTGGGCGTAAAGCGCACGTAGGCGGATTTGTAAGTCAG
GGGTGAAATCCCGGGGCTCAACCTCGGAACTGCCTTTGATACTGCAAATCTCGAGTCCGG
AAGAGGTGAGTGGAATTCCTAGTGTAGAGGTGAAATTCGTAGATATTAGGAAGAACACCA
GTGGCGAAGGCGGCTCACTGGTCCGGTACTGACGCTGAGGTGCGAAAGCGTGGGGAGCAA
ACAGGATTAGAAACCCTCGTAGTCC
>Otu1613
CAGCCTACGGGGCGCAGCAGTGGGGAATTTTGGACAATGGGGGCAACCCTGATCCAGCGA
CGCCGCGTGAAGGATGAAGGTCTTCGGATTGTAAACTTCTTTTAGAGGGGATGAATGCCG
CCGTGAATAACGGTGGGTGACAGTACTCTTTGAATAAGCCACGGCTAACTACGTGCCAGC
AGCCGCGGTAATACGTAGGTGGCAAGCGATACTCGGAATCACTAGGCGTAAAGCGCAGGT
AGGCGGTATGGTAAGTCTTTTGTGAAAGCTCCCGGCTTAACTGGGAGAGGTCAAGGGAAA
CTACCGGACTAGAGTGTGGTAGGGGATACTGGAATTCCTGGTGTAGCGGTGAAATGCGTA
GATATCAGGAGGAATACCTATGGCGAAAGCAGGTATCTGGGCCATCACTGACGCTGAGCT
GCGAAAGCTAGGGGAGCAAACAGGATTAGATACCCTCGTAGTCC
>Otu1614
CCAGCCTACGGGTGGCACCAGTGGGGAATATTGGGCAATGGGCGCAAGCCTGACCCAGCC
ATGCCGCGTGAGTGATGAAGGCCTTCGGGTCGTAAAGCTCTGTGGGGAGGGAAGAAACCT
CTCGTAGCTAATATCTACGGGATTTGACGGTACCTCCTTAGCAAGCACCGGCTAACTCCG
TGCCAGCAGCCGCGGTAATACGGAGGGTGCAAACGTTGCTCGGAATCATTGGGCGTAAAG
CGCACGTAGGCGGCCTAATAAGTCGGATGTGAAAGCCCTGGGCTCAACCCAGGAAGTGCA
TTCGAAACTGCTAGGCTTGAATATGGAAGAGGGTCGCGGAATTCCCGGTGTAGAGGTGAA
ATTCGTAGATATCGGGAGGAACACCAGTGGCGAAGGCGGCGACCTGGGCCAATATTGACG
CTGAGGTGCGAAAGCGTGGGGAGCAAACAGGATTAGAGACCCTAGTAGTCC
>Otu1620
CCAGCCTATGGGACGCAGCAGGCGCGAAATTTCCGCAATGCGCGAAAGCGTGACGGGGGT
AATCCGAGTGATAGTCTAACGACTATCTTTTGGCCAGTCTGAATAACTGGCAGAATAAGT
GGTGGGTAAATCCTGTGCCAGCCGCCGCGGTAATACAGGTAGCACAAGTGGTGTCCATGA
ATATTGAGTCTAAAGCGCCCGTAGCTGGCCTTGCCAGTCTTCTGTGAAATCTCGCGGCCT
AACCGCGAGGCGTGCAGAAGATACTACAAGGCTTGGGAGTGGGGGAGGTCAGAGGTACTA
TAGGGGGAAGAGTAAAATCCTGTAATCCTTGTAGGACCCTCGGTGGCGAAGGCGTCTGAC
CAAAACACATCCGACAGTGAGGGACGAAGGCTAGGGGAGCGAACCGAATTAGATACCCTA

GTAGTCC
>Otu1621
CCAGCCTACGGGTCGCTGCAGTAGGGAATTTTCCACAATGGGCGAAAGCCTGATGGAGCA
ACGCCGCGTGCAGGATGAATGCCTTCGGGTTGTAAACTGCTTTTATATGTGACGATTATG
ACAGTAGCATATGAATAAGGATCGGCTAACTCCGTGCCAGCAGCCGCGGTCATACGGAGG
ATCCAAGCGTTATCCGGAATTACTGGGCGTAAAGAGTTGCGTAGGTGGCAGAGTAAGTTA
GTAGTGAAAGCGTTCGGCTCAACCGAATATCCATTACTAAAACTGCTCAGCTAGAGGACG
AGAGAGGTTATTGGAATTCCTAGTGTAGGAGTGAAATCCGTAGATATTAGGAGGAACACC
GATGGCGTAGGCAGATAACTGGCTCGTTCCTGACACTAAGGCACGAAAGCGTGGGTAGCA
AACGGGATTAGAAACCCGGGTAGTCC
>Otu1622
CCAGCCTACGGGAGGCTCCAGTAAGGAATATTGGTCAATGGGCGGAAGCCTGAACCAGCC
ATGCCGCGTGCAGGAAGACGGCCCTACGGGTTGTAAACTGCTTTTATACCGGAATAAACC
TCGTTATGTATAACGAGCTGAATGTACGGTAAGAATAAGGATCGGCTAACTCCGTGCCAG
CAGCCGCGGTAATACGGAGGATCCAAGCGTTATCCGGATTTATTGGGTTTAAAGGGTGCG
TAGGTGGCCTGTTAAGTCAGGGGTGAAAGACGGTAGCTCAACTATCGCAGTGCCCTTGAT
ACTGACGGGCTTGAATGAACTAGAGGTAGGCGGAATGTGACAAGTAGCGGTGAAATGCAT
AGATATGTCACAGAACACCAATTGCGAAGGCAGCTTACTATGGTTTGATTGACACTGAGG
CACGAAAGCGTGGGGATCAAACAGGATTAGAAACCCTAGTAGTCC
>Otu1623
CCAGCCTACGGGGTGCTGCAGTAGGGAATATTGCGCAATGGACGAAAGTCTGACGCAGCG
ACGCCGCGTGGGCGACGAAGGCCTTCGGGTCGTAAAGCCCTTTTAGCAGGGACGAGTGAG
GACGGTACCTGCTGAATAAGTCTCGGCTAACTACGTGCCAGCAGCCGCGGTAAAACGTAG
GAGGCGAGCGTTATCCGGATTCACTGGGCGTAAAGCGCGCGTAGGCTGTTGAGTAAGTCG
GACGTGAAAGCCCCTGGCTCAACTGGGGGAGGCCGTTCGATACTGCTCGGCTTGAGGGTG
AGAGGGGAAAGTGGAATTCCCGGTGTAGCGGTGGAATGCGTAGAGATCGGGAGGAACACC
AGTGGCGAAGGCGGTTTTCTGGCTCACCCCTGACGCTGATAGCGCGAAAGCGTGGGGAGC
AAACGGGATTAGATACCCGCGTAGTCC
>Otu1624
CCAGCCTACGGGACGCACCAGCAGGGGTTTTTCTTTTTTGTCTGAAACCAACCCGAAAAG
AGGCTGGATGACCGACGAAGCCACATTCGACGCAATAAGGATAAATCCTGCCATGGTATA
CGAAAGGTCGAGTTTATCCTTGAGAAATGGCAATGCCGCTGTAAGCGCGCCCTGGTAGAT
ATCAGTCATACAATGACCCAGGGAGAGTAGCATAAGAACTTTCAAATTGAATTTTTTTAT
ATCTGTTTCTTGGTTATTCACGCTTCTCATTCCTCTCCGGTAGTTTTGCCAATGCCCTCA
AGCCATAGACCGTACCCATATGCACGACCACACCCAGGTAGGCGCCCGCGATGATGTCGC
TTAAAAAGTGATAATCCATCATAATCAAAGCCAGTGCTACTGCCATTAGATACCCCTGTA
GTCC
>Otu1626
CCAGCCTACGGGTTGCAGCAGTGAGGAATATTACTCAATGGACGAAAGTCTGAAGCAGCA
ACGCCGCGTGAGGGATGAAGGTGCTCTGCATCGTAAACCTCTGTAGAGGGAGACAAACTT
CGAGTTACTACTCGATTGATGGTACCCCTAAAGTAAGGATCGGCTAACTACGTGCCAGCA
GCCGCGGTAATACGTAGGATCCAAGCGTTGTCCGGATTTACTGGGTGTAAAGGGTGCGCA
GGCGGACCAATAAGTCAGGAGTGAAATCTCGGCGCTCAACGCCGAAACGTCTTTTGATAC
TGTTGGTCTTGAATAAGCGAGAGATTCGTGGAATTCCGAGTGTAGCAGTGAAATGTGTAG
ATATTCGGAAGAACACCAGAGGCGAAGGCAGCGAATTGGCGCTTTATTGACGCTCAGGCA
CGAAAGCATGGGGATCAAACAGGATTAGAGACCCTCGTAGTCC
>Otu1631
CAGCCTACGGGAGGCAGCAGTAGGGAATATTGCACAATGGGCGAAAGCCTGATGCAGCGA
CGCCGCGTGAGGGATGAAGGGCTTCGGCTCGTAAACCTCTGTCAGGTGGGACGATGGGAC
ATATGGCTAACATCCATGTGTTTTGACGGTACCGCCGGAGGAAGCACCGGCTAACTACGT
GCCAGCAGGCGCGGTAATACGTAGGGTGCAAGCGTTGCTCGGAATCACTGGGCGTAAAGC
GTGAGTAGGTGGACTTTTGGTAAGTCAGATGTGAAAGCCTTGGGCCTAACCCAAGAAGTG
CATTTGATACTGCCAGTCTAGAGTTCGGGAGAGGGAAGCAGAATTCCAGGTGTAGGGGTG
AAATCCGTAGATATCTGGAGGAATACCAGTGGCGCAAGCGGCTTCCTGGACCGATACTGA
CACTGAGTCACGAAAGCGTGGGGATCAAACAGGATTAGAAACCCCTGTAGTCC
>Otu1634
CCAGCCTACGGGACGCTGCAGTGGGGAATATTGGGCAATGGACGCAAGTCTGACCCAGCC
ATCCCGCGTGAAGGATGAAGGATCTATGGTTCGTAAACTTCTTTTATTGGGGAAGAAAAA

GCCTGATTTATCAGGAACTGACGGTACCCAATGAATAAGCACCGGCTAACTCCGTGCCAG
CAGCCGCGGTAATACGGAGGGTGCAAGCGTTATCCGGATTCACTGGGTTTAAAGGGTGCG
TAGGCGGCTTTATAAGTCATTGGTGAAAGCTCGCAGCTTAACTGCGAAACTGCCATTGAA
ACTGTTTAGCTTGAATGTGGTTGAAGTGGGCGGAATACAACATGTAGCGGTGAAATGCTT
AGATATGTTTTAGAACACCGATTGCGAAGGCAGCTCACTAAGCCAATATTGACGCTGAGG
CACGAAAGCGTGGGTAGCAAACAGGATTAGAAACCCTTGTAGTCC
>Otu1635
CCAGCCTATGGGGTGCTCCAGTCGAGAATATTCGACAATGGGCGAAAGCCTGATCGAGCG
ACGCCGCGTGTAGGATGAAGGCCTTCGGGTCGTAAACTACGGTAGTAACATAACAATGTA
AATGAGTGTGTTACGGAAAGAGGTTGGTAACTACGTGCCAGCACCCGCGGTAAAACGTAG
ACCTCAAGCGTTATCCGGATTTATTGGGCGTAAAGCGCGTGTAGGAGGTTTTGTGCGTCT
TCTGTTAAAGCCCACCGCCTAACGGTGGAAGTGCAGGAGATACGGCAGAACTAGAGGAGG
TTAGAGGTGCATGGAACTCACGGTGTAGGGGTGAAATCCGTTGATATCGTGGGGAACACC
AAAGGCGAAGGCATTGCACTGGGACCTTCCTGACTCTGAGACGCGAAAGCGTGGGGAGCA
AAAAGGATTAGAAACCCTCGTAGTCC
>Otu1637
CCAGCCTATGGGAGGCTGCAGTGAGGAATATTGCGCAATGGACGAAAGTCTGACGCAGCA
ACGCCGCGTGTGGGATGAAGGCCTTTTAGGTCGTAAACCACTGTAGCAGGGGAAAAACAT
CCCATTCGATGGGCTTGATGGTACCCTGAAAGTAAGCCTCGGCTAACTACGTGCCAGCAG
CCGCGGTAATACGTAGGAGGCGAGCGTTGTCCGGATTTACTGGGTGTAAAGGGTCCGCAG
GCGGGCATCTAAGTCGGTGGTGAAATCCTTCAGCTTAACTGAAGAACTGCCTCCGATACT
GGGTGCCTTGAATATAGGAGAGGGCAATGGAATTCCAGGTGTAGCGGTGAAATGCTTAGA
TATCTGGAAGAACACCGGTTGCGAAGGCTGTTGCCTGGCCTATTATTGACGCTCATGGAC
GAAAGCGTGGGGATCAAACAGGATTAGAAACCCGTGTAGTCC
>Otu1638
CCAGCCTATGGGGGGCACCAGGTAGGAATCTTTTGCAATGCGCGCAAGCGTGACAAAGCG
AGCCAGAGTGCTTTTCAATGAAAAGCCTTTGCCAAATCTAAAAAGTTTGGAGAATAAGGA
CTGGGTAAGACTGGTGCCAGCCGCCGCGGTAATCCCAGCGGTCCAAGTCGCAGCCATCAT
TATTGGGTCTAAAATATCCGTAGCTTGCTTGGTAAGTTCCTTGTGAAATCTTATATCTCA
AGTATAAGGCGTGCGAGGAATACTGCTGAGCTAGAGACTGGAAGACGTAAAAAGTATGTT
CAAAGTAACGGTAAAATGTGTTAATCTTGAGCAGACTCACAACAGCGAAGGCATTTTACG
AGGACAGTTCTGACAGTTAAGGATGAAGGCTAGGGGCGCAAAAAGGATTAGATACCCGGG
TAGTCC
>Otu1640
CCAGCCTATGGGTGGCAGCAGTTAGGAATTTTGGGCAATGGGCGCAAGCCTGACCCAGCA
ACGCCGCGTGGGTGACGAAGGCCTTCGGGTTGTAAAGCCCTTTTCTGAGGGAAGATGATG
ACGGTACCTCAGGAAGAAGCACCGGCTAACTACGTGCCAGCAGCCGCGGTAATACGTAGG
GTGCGAGCGTTGCCCGGATTTATTGGGCGTAAAGGGCGCGTAGGCGGCCACGCACGTCCG
TTGTGAACTCGCCCGGCTCAACTGGGCGGGGTCAGCGGATACGGTGTGGCTTGAGCAAGC
TAGGGGGCAATGGAATTCCCGGTGTAGTGGTGGAATGCGTAGATATCGGGAGGAACACCA
GTGGCGAAGGCGGTTGCCTGGAGCTTTGCTGACGCTGAGGCGCGAAAGCGTGGGGAGCGA
TCCGGATTAGAGACCCCCGTAGTCC
>Otu1641
CCAGCCTACGGGGTGCAGCAGTCGAGAATCTTCGGCAATGGACGAAAGTCTGACCGAGCG
ACGCCGCGTACGGGATGAAGGTCTTCGGATTGTAAACCGCTGTCAGAGGGGATGAAGTAT
ACGAGAGCTATCTCTTGTATTTGACAGAGCCTCAGAGGAAGCACGGGCTAAGTTCGTGCC
AGCAGCCGCGGTAATACGAACTGTGCGAACGTTATTCGGAATCACTGGGCTTAAAGGGTG
CGTAGGCGGTTTTCTAAGTCAGGTGTGAAATCTTCCGGCTCAACCGGAAAACTGCGCCTG
AGACTGGATAACTTGAGTGAGGTAGGGGTGTGTGGAACTTCCGGTGGAGCGGTGAAATGT
GTAGAGATCGGAAGGAACGCCAGCAGCGAAAGCGGCACACTGGGCCTTTTCTGACGCTGA
GGCACGAAAGCTAGGGTAGTGAACGGGATTAGAAACCCCAGTAGTCC
>Otu1642
CCAGCCTACGGGTCGCAGCAGTCGAGAATTTTTCTCAATGGGGGAAACCCTGAAGGAGCG
ACGCCGCGTGGGGGATGAATGGCGTCGGCCCGTAAACCCCTGTCATTTGCGAACAAGTTG
CATCGCCTAACACGTGATGCATTGATTGTAGCGAAAGAGGAAGGGACGGCTAACTCTGTG
CCAGCAGCCGCGGTAATACAGAGGTCCCAAGCGTTGTTCGGATTCACTGGGCGTAAAGGG
TGCGTAGGCGGTGAGGTAAGTCTGACGTGAAATCTCCGGGCTTAACCCGGAAACTGCGTT
GGATACTATCTCACTCGAGGAATGGAGGGGAGACTGGAATACTTGGTGTAGCAGTGAAAT

GCGTAGATATCAAGTGGGACACCAGTGGCGAAGGCGAGTCTCTGGACATTTCCTGACGCT
GAGGCACGAAAGCCAGGGGAGCAAACGGGATTAGATACCCGCGTAGTCC
>Otu1643
CCAGCCTATGGGGGGCTCCAGTAACGAATCTTCCGCAATGCACGAAAGTGTGACGGAGCG
ACGCCGCGTGGGGGACGAAGCCCTTCGGGGTGTAAACCACTGTCAGGGGATAGAAAGCTC
TGATCATCCCCAGAGGAAGGCACGGCTAACTCTGTGCCAGCAGCCGCGGTAAGACAGAGG
TGCCAAGCGTTAGGCGGAATCACTGGGCTTAAAGCGTGTGTAGGCGGGTTCGTAAGTACC
TTGTGAAATCCCACGGCTTAACCGTGAAACTGCTCGGTATACTGCGAGTCTTGAGCCAAC
TAGGGGCTACTGGAACAAATGGTGGAGCGGTGAAATGCGTAGATATCATTTGGAACGCCA
ATGGTGAAAACAGGTAGCTGGGGTTGTGCTGACGCTGAGACACGAAAGCCAGGGGAGCAA
ACGGGATTAGATACCCTCAGTCC
>Otu1644
CCAGCCTACGGGGTGCAGCAGTACGGAATATTGGACAATGCACGACAGTGTGATCTAGTG
ATATTCCTCAAGTGATGATAGCCGAGTAGGCCGTAAAGCTTTTTATAAAAACTGAATAAT
GACTAACGTTTTTAAGAAAAAGTCCTGGCCAACTCTGTGCCAGCAGCCGCGGTAAGACAG
GGGGGGCAAGCGTTATTCGTCTTGACTGGGCGTAAAGGGTTCGTAGGCGGTTAGTCTAAA
TTAACGGTAAATCCTAGGGTAAATCTTAGAGGCTGTTTTCAATTAGTTGAACTTGAGTTT
ACGTTGCGTCAGCGGAATTTTTTTGGAGTAGTAAAATGCGTAGATATTAAAAGGAATACC
AATGGTGAAGACAACTGACTAGCTATAACTGACGCTGAGGAACGAAGGCGTAGGTAGCAA
ACAGGATTAGAAACCCCGGTAGTCC
>Otu1648
CCAGCCTACGGGGTGCTGCAGTTAGGAATTTTGCGCAATGGACGAAAGTCTGACGCAGCG
ACATCGCGTGAAGGATGAAGGCCCTCGGGTCGTAAACTTCTTTTATTTTGCCAAGAAGGC
ATTTTGAATCAACAGCTACTAACTACGTGCCAGCAGTCGCGGTAATACGTAGGCTGTAAG
CGTTACCCGGTTTTATTGGGCGTAAAGAGTCTGTAGGTGGTTTGATAAGTCTTGTTTCAA
AGACCCCGGCTTAACCGGGGGCAGGGGCAGGATACTGTCAGACTAGAGTGATATCGGGGT
TACTGGAATTTGTAGTGTAGGGGTTAAATCCGTGGATACTACAAAGAACACCAAGCGCGA
AGGCGGGTAACCAGGTATTTACTGACACTCAGAGACGAAAGCTAGGGTAGCCAATCAGAT
TAGATACCCTAGTAGTCC
>Otu1649
CAGCCTATGGGGGGCAGCAGTCGAGGATCTTTCGCAATGGGCGCAAGCCTGACGAAGCGA
CGCTGTGTGGACGATGAAGGCCTTCGGGTTGTAAAGTCCTTTCGCGTAAGAACAAGAGAG
ACTGTCTAATAAGCAGTTAATTTGAGGGTTCTACGTAAAGAAGCACCGGCTAACTCCGTG
CCAGCAGCTGCGGTAATACGGAGGGTGCAAGCATTAATCGGATTTACTGGGCGTAAAGGG
CGCGTAGGCGGATCTACAAGTCAAATGTGAAATTCCGGAGCTCAACTTCGGAGCGGCATT
TGAAACTGTAGGTCTATAGAGGGATGGCGGAGAAAATGGAATTCCACATGTAGCGGTGAA
ATGCGTAGATATGTGGAAGAACACCAGTGGCGAAAGCGGCTTTCTAGCTATTACCTGACG
CTGAGGCGCGAGAGCATGGGGAGCAAACAGGATTAGATACCCCTGTAGTCC
>Otu1651
CCAGCCTACGGGTCGCTGCAGTGGGGAATATTGGACAATGGGCGCAAGCCTGATCCAGCA
ATGCCGCGTGGGTGAAGAAGGTCTTCGGATTGTAAAGCCCTTTCGACGGGGACGATGATG
ACGGTACCCGTAGAAGAAGCCCCGGCTAACTTCGTGCCAGCAGCCGCGGTAATACGAAGG
GGGCTAGCGTTGCTCGGAATGACTGGGCGTAAAGGGCGCGTAGGCGGTTTGAACAGTTGG
GCGTGAAATTCCAGGGCTTAACCTTGGGACTGCGTTCAATACGTTTAGACTTGAGTTCGG
AAGAGGGTCGTGGAATTCCCAGTGTAGAGGTGAAATTCGTAGATATTGGGAAGAACACCG
GTGGCGGAGGCGGCGACCTGGTCCGAAACTGACGCTGAGGCGCGAAAGCGTGGGGAGCAA
ACAGGATTAGAAACCCCTGTAGCC
>Otu1654
CCAGCCTATGGGGGGCTCCAGTGGGGAATATTGGACAATGGGCGAAAGCCTGATCCAGCC
ATGCCGCGTGAATGATGAAGGCCTTAGGGTTGTAAAGTTCTTTTGCCAGGGAAGATAATG
ACGGTACCTGGAGAATAAGCACCGGCTAACTTCGTGCCAGCAGCCGCGGTAATACGAAGG
GTGCTAGCGTTGTTCGGAATTACTGGGCGTAAAGAGCGCGTAGGCGGTTGTACTAGTCAG
AGGTGAAATCCCGGAGCTTAACTCCGGAACTGCCTTTGAAACTGTGCAGCTGGAGTGTGT
GAGGGGATAGCGGAATTCTTAATGTAGAGGTGAAATTCGTAGATATTAAGAGGAACACCG
GTGGCGAAGGCGGCTATCTGGCGCACAACTGACGCTGAGGCGCGAAAGCGTGGGGATCAA
ACAGGATTAGAAACCCGAGTAGTCC
>Otu1656
CCAGCCTACGGGAGGCTGCAGTCGAGAATTTTTCTCAATGGGCGAAAGCCTGAAGGAGCG

ACGCCGCGTGGGGGATGAATGGCTTCGGCCCGTAAACCCCTGTCATTTGCGAACAAACCT
TTTCATTTAATATGTGAAAAGCTGATTGTAGCGAAAGAGGAAGAGACGGCTAACTCTGTG
CCAGCAGCCGCGGTAATACAGAGGTCTCAAGCGTTGTTCGGATTCACTGGGCGTAAAGGG
TGCGTAGGTGGCGGGGCAAGTCTGACGTGAAATCTCCGGGCTTAACCCGGAAATGGCGTT
GGAAACTGCCTTGCTAGAGGATTGGAGGGGAGACTGGAATACTTGGTGTAGCAGTGAAAT
GCGTAGATATCAAGTGGAACACCAGTGGCGAAGGCGAGTCTCTGGACAGTTCCTGACGCT
CATGCACGAAGGCTAGGGGAGCAAACGGGATTAGAGACCCCAGTAGTCC
>Otu1663
CCAGCCTATGGGGTGCTCCAGTCGAGAATTTTTCTCAATGGGGGAAACCCTGAAGGAGCG
ACGCCGCGTGGAGGATGAAGGCTTTCGGGTTGTAAACTCCTGTCATTTGGGAACAAGGCC
TTACGTTAACTGCGTGGAGGATTGATAGTACCAGAAGAGGAAGAGACGGCTAACTCTGTG
CCAGCAGCCGCGGTAATACAGAGGTCTCAAGCGTTGTTCGGATTCATTGGCGTAAAGGGC
GCGTAGGCGGCGAGGTAAGTCTGGTGTGAAATCTAGGAGCTCAACTCCTAAACTGCACTG
GATACTGCTTTGCTTGAGGACTGGAGAGGAGATTGGAATTCACGGTGTAGCAGTGAAATG
CGTAGATATCGTGAGGAAGACCAGTGGCGAAGGCGGATCTCTGGACAGTTCCTGACGCTG
AGGCGCGAAGGCCAGGGGAGCAAACGGGATTAGAAACCCGTGTAGTCC
>Otu1668
CCAGCCTACGGGTCGCTGCAGTCGAGAATCATTCGCAATGGGCGAAAGCCTGACGATGCG
ACGCCGTGTGTATGATGAAGGCCTTCGGGTTGTAAAGTACTTTCGCCTGGGAACAAGAGA
TGGTGGCTAACATCCATCAAATTTGAGGGTACCAGGTAAAGAAGCACCGGCTAACTCCGT
GCCAGCAGCTGCGGTAATACGGAGGGTGCAAGCATTAATCGGATTTATTGGGCGTAAAGG
GCGCGTAGGCGGGCTCGTAAGTCAGATGTGAAAGCCCGGGGCTCAACCCCGGAACAGCAT
TTGAAACTGCAAGTCTAGAGGGTAGGCGGAGAAAACAGAATTCCACAAGTAGCGGTGAAA
TGCGTAGATATGTGGAAGAATACCGGTGGCGAAGGCGGTTTTCTAGCTTATACCTGACGC
TGAGGCGCGAAAGCAAGGGGATCAAACAGGATTAGATACCCCCGTAGTCC
>Otu1670
CAGCCTATGGGGGGCTGCAGTGGGGAATATTGGACAATGGGCGAAAGCCTGATCCAGCCA
TGCCGCGTGAGTGATGAAGGCCTTAGGGTTGTAAAGCTCTTTTATCAGGGAAGATGATGA
CGGTACCTGAAGAATAAGCACCGGCTAACTTCGTGCCAGCAGCCGCGGTAATACGAAGGG
TGCTAGCGTTGTTCGGAATCACTGGGCGTAAAGCGTGCGTAGGCGGCTTATCAAGTCAGG
GGTGAAATCCTGGGGCTCAACCCCAGAATTGCCTTTGAAACTGGTAAGCTTAAGTCTCGG
AGGGGATAGCGGAATTGCTAATGTAGAGGTGAAATTCGTAGATATTAGCAGGAACACCGG
TGGCGAAGGCGGCTATCTGGACGAGTACTGACGCTCTGGCACGAAAGCGTGGGGATCAAA
CAGGATTAGAGACCCTGGTAGTCC
>Otu1680
CAGCCTATGGGGGGCAGCAGTCGAGAATCTTCGGCAATGGGCGCAAGCCTGACCGAGCGA
CGCCGCGTGCGGGATGAAGGCCCTTGGGTTGTAAACCGCTGTCAGAGGGGATGAAGTGCG
GGTGGGCAATCCCATCCGTTTGACATAGCCTCAGAGGAAGCACGGGCTAAGTTCGTGCCA
GCAGCCGCGGTAATACGAACTGTGCAAACGTTATTCGGAATCACTGGGCTTAAAGGGTGC
GTAGGCGGCCATTTAAGTAGGGTGTGAAAGCCCCCGGCTCAACCGGGGAATTGCGTCCTA
AACTGGCTGGCTAGAGTGAGATAGAGGTGTGCGGAACTTCCGGTGGAGCGGTGAAATGTG
TTGATATCGGAAGGAACGCCGGTGGCGAAAGCGGCACACTGGGTCTCAACTGACGCTGAG
GCACGAAAGCCAGGGGAGCGAACGGGATTAGAAACCCTAGTAGTCC
>Otu1681
CCAGCCTATGGGGTGCAGCAGTCGAGGATCTTCGTCAATGGGCGCAAGCCTGAACGAGCG
ACGCCGCGTGGGCGATGAAGGCCTTCGGGTTGTAAAGCCCTTTAGTGGGGGAGGAAAGCG
CAAGCTTGACCTATCCCAAGAATAAGCACGGGCTAAGTTCGTGCCAGCAGCCGCGGTAAG
ACGAACCGTGCGAACGTTGTTCGGAATCACTGGGCTTAAAGGGCGCGTAGGCGGTCTGTC
AAGTCAGGGGTGAAAGCCTCCAGCTTAACTGGAGAAGTGCCTTTGATACTGGCGGACTCG
AGTGAGGTAGGGGTAGTGGGAACTTCCGGTGGAGCGGTGAAATGCGTTGATATCGGAAGG
AACGCCGGTGGCGAAAGCGCACTACTGGACCTCTTCTGACGCTGAGGCGCGAAAGCCAGG
GGAGCAAACGGGATTAGAAACCCTTGTAGTCC
>Otu1685
CCAGCCTATGGGACGCAGCAGTGGAGAATCTTGGACAATGGGCGAAAGCCTGATCCAGTC
ACGTTGCGTGAAATCTTTCTTTTTTCAATGAAAATCATGACTGTAATTGAAGAATAAGTC
CTGGCTAACCTCGTGCCAGCAGCCGCGGTAAGACGAGGAGGACAAGCGTTCTTCGGTATC
ACTAGGCGTAAAGTCCAGTAAGGTGGTTACTCAATCAAAGAACTTCAACTGAAAGAAAAG
TTCAGCAAGTTCCAAGAAAGTCACTAGAGTTATAAGAAAGAAAAAGGAATTTCCAAAGTA

GTAGTAAAATACGTTGAGATTGGAAGGAAGACCGAAGGCGAAGGCATTTTTCTCCTTTTA
ACTGACACTGATCTGGGAAAGTCTGGGGAGCAAATGGGATTAGATACCCGTGTAGTCC
>Otu1686
CCAGCCTATGGGAGGCAGCAGTGGGGAATATTGGACAATGGGCGAAAGCCTGATCCAGCA
ATGCCGCGTGTGTGAAGAAGGCCTTCGGGTTGTAAAGCACTTTCAGTGGGGAGGAAAGCT
TTAAGGTTAATACCCTTCAAAGATTGACGCTACCTGCAGAAGAAGCACTGGCTAACTCTG
TGCCAGCAGCCGCGGTAATACAGAGAGTGCAAGCGTTAATCGGAATCACTGGGCGTAAAG
CGCGCGTAGGTGGATATCTAAGTCGGATGTGAAAGCCCTGGGCTTAACCTAGGAACTGCA
TCCGATACTGGATGACTAGAGTATGGTAGAGGGAAGTGGAATTTCCGGTGTAGCGGTGAA
ATGCGTAGATACCGGAAGGAACACCAGTGGCGAAGGCGACTTCCTGGACCAATACTGACG
TTCATGTGCGAAAGCGTGGGGAGCAAACAGGATTAGATACCCCCGTAGTCC
>Otu1688
CCAGCCTATGGGTCGCAGCAGTGGGGAATTTTGGACAATGGGCGCAAGCCTGATCCAGCC
ATGCCGCGTGAGTGAAGAAGGCCTTCGGGTTGTAAAGCTCTTTTGTCCGGGAGCAAACCT
TAGTGGTTAATAACCGCTGAGCTGAGAGTACCGGAAGAATAAGCACCGGCTAACTACGTG
CCAGCAGCCGCGGTAATACGTAGGGTGCAAGCGTTAATCGGAATTACTGGGCGTAAAGCG
TGCGCAGGTGGTTGTGCAAGTCTGATGTGAAATCCCCGAGCTCAACTTGGGAACTGCATT
GGAGACTGCATGACTAGAGTACGTCAGAGGGGGGTGGAATTCCACGTGTAGCAGTGAAAT
GCGTAGAGATGGGGAGGAACACCGATGGCGAAGGCAGCCCCCTGGGTCGACACTGACGCT
CATGCACGAAAGCGTGGGGAGCAAACAGGATTAGAAACCCTGGTAGTCC
>Otu1689
CCGCCTACGGGATGCTGCAGTGGGGAATATTGGACAATGGGCGAAAGCCTGATCCAGCAA
TGCCGCGTGAGTGATGAAGGCCTTAGGGTTGTAAAGCTCTTTTACCCGGGATGATAATGA
CAGTACCGGGAGAATAAGCTCCGGCTAACTCCGTGCCAGCAGCCGCGGTAATACGGAGGG
AGCTAGCGTTATTCGGAATTACTGGGCGTAAAGCGCACGTAGGCGGCTTTGTAAGTAAGA
GGTGAAAGCCTGGTGCTCAACACCAGAACTGCCTTTTAGACTGCATCGCTTGAATCCAGG
AGAGGTGAGTGGAATTCCGAGTGTAGAGGTGAAATTCGTAGATATTCGGAAGAACACCAG
TGGCGAAGGCGGCTCACTGGACTGGTATTGACGCTGAGGTGCGAAAGCGTGGGGAGCAAA
CAGGATTAGAAACCCCTGTAGTCC
>Otu1690
CCAGCCTATGGGGGGCTCCAGTCGAGGATCTTCGGCAATGGGCGCAAGCCTGACCGAGCG
ACGCCGCGTGTGCGATGAAGGCCTTCGGGTTGTAAAGCACTGTCGAGGGGGAGAAAGCCG
CAAGGTTGATCAATCCCTGGAGGAAGCACGGGCTAAGTTTGTGCCAGCAGCCGCGGTAAG
ACGAACCGTGCGAACGTTATTCGGAATCACTGGGCTTAAAGGGCGTGTAGGCGGGTCAGC
AAGTCTGTGGTGAAAGCCTCCAGCTTAACTGGAGAAGTGCCGTGGATACTGTTGATCTTG
AGGGAGGTAGGGGCATGTGGAACTTCCGGTGGAGCGGTGAAATGCGTAGATATCGGAAGG
AACGCCGGTGGCGAAAGCGACGTGCTGGACCTCTTCTGACGCTGAGGCGCGAAAGCTAGG
GGAGCAAACGGGATTAGATACCCCAGTAGTCC
>Otu1692
CCAGCCTACGGGTTGCACCAGTTTCGAATCATTCACAATGGGGGCAACCCTGATGGTGCG
ACGCCGCGTGGGGGATGAAGGTCTTCGGATTGTAAACCCCTGTCACCTGGGACAAAACCT
CGGCGAAGAGCCGAGCTGATTCAACCAGGAGAGGAAGCAGTGGCTAACTCTGTGCCAGCA
GCCGCGGTAATACAGAGACTGCAAGCGTTACTCGGATTCACTGGGCGTAAAGGGAGCGCA
GGTGGGCAAGTGTGTCGGGCGTGAAATCCTGGGGCTTAACTCCAGAACGGCGCCCGAAAC
TACTTGTCTGGAGGATTGGAGAGGCAGACGGAATTCCAGGTGTAGCGGTGAAATGCGTAG
ATATCTGGAGGAACACCGACGGCGAAGGCACTCTGCTGGACAAATCCTGACACTCAGGCT
CGAAAGCGTGGGGAGCAAAAGGGATTAGAGACCCGTGTAGTCC
>Otu1693
CCAGCCTACGGGGTGCTCCAGTAGGGAATATTGCGCAATGGGCGAAAGCCTGACGCAGCG
ACGCCGCGTGAGTGATGAAGGCCTTAGGGTCGTAAAGCTCTGTTGTAAGGGAAAATTATG
ATGGTACCTTATAAGAAAGGACCGGCTAACTTCGTGCCAGCAGCCGCGGTAAGACGAGGG
GTCCTAGCGTTGTTCGGAATCATTGGGCGTAAAGCGCATGTAGGCGGCCTATCAAGTCAG
GTGTGAAATCCCTGGGCTCAACCCAGGACGTGCACTTGATACTGGTGGGCTTGAATATAG
GAGAGGATAGTGGAACTCCAGGTGTAGTGGTGAAATACGTAGATATCTGGAGGAACACCG
GCGGCGAAGGCGGCTATCTGGCCTAATATTGACGCTGAGATGCGAAAGTGCGGGGATCAA
ACAGGATTAGAAACCCGAGTAGTCC
>Otu1695
CCAGCCTACGGGGGGCAGCAGTCGAGAATCTTCCTCAATGGCCGAAAGGCTGAAGGAGCG

ACGCCGCGTGGACGATGAAGGCCTTCGGGTCGTAAAGTCCTTTTTTCGGGGACGAACAAA
TGACGGTACCCGAAGAATAAGAGGTTGCTAACTCTGTGCCAGCAGCAGCGGTAATACAGA
GACCTCGAGCGTTATCCGGAATTATTGGGCGTAAAGGGTCCGCAGGTGGCTTGGTAAGTC
ATGCGTCAAATATTTGGGCTCAACCCAGATGCTGCGTGTGATACTGCTCGGCTTGAGGCT
GGGAGAGGTGCATGGAATTACTGGTGTAGCGGTAAAATGCGTTAATATCAGTAAGAACAC
CAAAGGCGAAGGCAATGCACTGGAACAGTCCTGACACTCAGGGACGAAAGCGTGGGGAGC
GAAAGGGATTAGAAACCCTAGTAGTCC
>Otu1696
CCAGCCTACGGGGGGCAGCAGTTTCGAATCATTCACAATGGGGGCAACCCTGATCCAGCG
ACGCCGCGTGTGTGAAGAAGGCCTGCGGGTTGTAAAGCACTTTTAGTGGGGATGAAAAGT
TCAGGGCTAATATCCTTGGGCATTGACCTAACCCACAGAAAAAGCACCGGCTAACTCTGT
GCCAGCAGCCGCGGTAATACAGAGGGTGCAAGCGTTAATCGGAATTACTGGGCGTAAAGC
GTGCGTAGACGGTTCCGTAAGTCGGGTGTGAAAGCCCCGGGCTCAACCTGGGAATTGCAT
TCGAGACTGCGAAGCTAGGGTACGGAAGAGGGAAGCGGAATTTCTGGTGTAGCGGTGAAA
TGCGTAGATATCAGAAGGAACATCAGTGGCGAAAGCGGCTTCCTGGTCCAGTGCCGACGT
TCAAGCACGAAAGCGTGGGGAGCAAACAGGATTAGAAACCCTCGTAGTCC
>Otu1698
CCAGCCTATGGGTCGCTGCAGTGGGGAATATTGCACAATGGGCGCAAGCCTGATGCAGCG
ACGCCGCGTGGGGGATGACGGCCTTCGGGTTGTAAACCTCTTTCACCATTGACGAAGTCT
GTGGGTTTTCTTGCAGGTGACGGTAGGTGGAGAAGAAGCACCGGCCAACTACGTGCCAGC
AGCCGCGGTAATACGTAGGGTGCGAGCGTTGTCCGGAATTACTGGGCGTAAAGAGCTCGT
AGGTGGTTTGTCGCGTTGTTCGTGAAATCTCACGGCTTAACTGTGAGCGTGCGGGCGATA
CGGGCAGACTGGAGTACTGCAGGGGAGACTGGAATTCCTGGTGTGGCGGTGGAATGCGCA
GGTATCAGGAGGAACACCGGTGGCGAAGGCGGGTCTCTGGGCAGTAACTGACGCTGAGGA
GCGAAAGCGTGGGGAGCGAACAGGATTAGATACCCGTGTAGTCC
>Otu1700
CAGCCTATGGGGGGCAGCAGTGGGGAATTTTGCGCAATGGGGGAAACCCTGACGCAGCAA
CGCCGCGTGGAGGATGAAGTACTTCGGTACGTAAACTCCTTTCGATCGGGACGATTATGA
CGGTACCGGAAGAAGAAGCCCCGGCTAACTTCGTGCCAGCAGCCGCGGTAATACGAGGGG
GGCAAGCGTTGTTCGGAATTATTGGGCGTAAAAGGTGCGTAGGCGGTTTTGTAAGTCTGA
TGTGAAATCTATGGGCTCAACTCATAGACTGCATCGGAAACTGCAGGGCTTGAGTGTGGG
AGAGGTGAGTGGAATTCCTGGTGTAGCGGTGAAATGCGTAGATATTCGCAAGAACACCAG
TGGCGAAGGCGGCTCACTGGCCCGATACTGACGCTGAGGTGCGAAAGCGTGGGGAGCAAA
CAGGATTAGATACCCCAGTAGTCC
>Otu1703
CCAGCCTATGGGGGGCAGCAGTGGGGAATCTTGCACAATGGGCGAAAGCCTGATGCAGCG
ACGCCGCGTGGAGGATGACGGCCTTCGGGTTGTAAACTCCTTTTGCAGGGGAAGATAATG
ACGGTACCCTGCGAATAAGCCATGGCTAACTCTGTGCCAGCAGCCGCGGTAAGACAGAGG
TGGCGAGCGTTGTTCGGAATTACTGGGCTTAAAGGGCGCGTAGGCGGTGATGTAAGTCTG
GGGTGGAATTCCACAGCTTAACTGTGGAATTGCCCTGGAAACTGCATTGCTTGAGTCGGA
CAGGGGAAGGCGGAATTCCAGGTGTAGCGGTGAAATGCGTAGATATCTGGAGGAAGGCCT
GTGGTGAAGACGGCCTTCTGGGTCTTGACTGACGCTGAGGCGCGAAAGCGTGGGTAGCAA
ACAGGATTAGAGACCCGCGTAGTCC
>Otu1705
CCAGCCTATGGGTGGCAGCAGTCGAGAATCTTCGGCAATGGGCGCAAGCCTGACCGAGCG
ACGCCGCGTGTGGGATGAAGGCCTTCGGGTTGTAAACCACTGTCGGAGGGGAGGAAATAC
ATGTGGGTACTCTCACATGCTTGACCTAACCTCGGAGGAAGGACGGGCTAAGTTCGTGCC
AGCAGCCGCGGTAAGACGAACCGTCCGAACGTTATTCGGAATTACTGGGCTTAAAGGGTG
CGTAGGCGGCGGAGAAAGTAGGGTGTGAAATCCCTCGGCTTAACCGAGGAACTGCGCTCT
AAACTACTTTGCTCGAGGGAGATAGAGGTGAGCGGAACTTGGGGTGGAGCGGTGAAATGC
GTTGATATCCTAAGGAACACCGGTGGCGAAAGCGGCTCACTGGATCTCTTCTGACGCTGA
GGCACGAAAGCTAGGGTAGCGAACGGGATTAGAAACCCTTGTAGTCC
>Otu1710
CCAGCCTACGGGTGGCTCCAGTGAGGAATATTGCGCAATGCCCGAAAGGGTGACGCAGCG
ACGCCGCGTGGAGGATGAAGGCCCTATGGGTCGTAAACTCCTTTTGCGAGGGAAGAACAG
AGCGCTACAGGCGCTCGTGACGGTACCTCGAGAAAAAGCATCGGCTAACTACGTGCCAGC
AGCCGCGGTAATACGTAGGATGCGAGCGTTGTCCGGATTCACTGGGTGTAAAGGGAGCGC
AGGCGGGTCGGTAAGTCATTGGTGAAATTTTCGGGCTCAACCCGAAGACTGCCACTGATA

CTGCCGATCTTGAGTGTGGAAGAGGTTGGTGGAATTCATGGTGTAGCGGTGAAATGCGTA
GATATCATGAAGAACACCGATGGCGAAGGCAGCCAACTGGTCCATTACTGACGCTCATGC
TCGAAAGCGTGGGGATCAAACAGGATTAGAGACCCGCGTAGTCC
>Otu1711
CCAGCCTACGGGAGGCAGCAGTGGGGAATATTGGTCAATGGGCGGAAGCCTGAACCAGCC
ATGCCGCGTGCAGGAAGACGGCCCTACGGGTTGTAAACTGCTTTTGTGCCAGAATAAACC
CTCGTTCGTGAATGAGGTTGAATGTATGGTAAGAATAAGGATCGGCTAACTCCGTGCCAG
CAGCCGCGGTAATACGGAGGATCCGAGCGTTATCCGGATTTATTGGGTTTAAAGGGTGCG
TAGGCGGCTTTGTAAGTCAGGGGTGAAAGACGGTAGCTTAACTATCGCAGTGCCTTTGAT
ACTGCAGGGCTTGAATGTACTTGAGGTAGGCGGAATGTGACAAGTAGCGGTGAAATGCAT
AGATATGTCACAGAACACCAATTACGAAGGCAGCTTACTAAAGTATGATTGACGCTGAGG
CACGAAAGCGTGGGGATCAAACAGGATTAGATACCCCAGTAGTCC
>Otu1715
CCAGCCTATGGGTCGCTGCAGCCGAGAATATTCGACAATGGGCGAAAGCCTGATCGAGCG
ACGCCGCGTGACTGATGAAGTCCTTCGGGACGTAAAGGTCTTTTGTGAGGTAGAAAGTTA
TTGATCGCCTCAAGAATAAGGGGTTGCTAAACTCGTGCCAGCAGCAGCGGTAATACGAGT
GCCCCAAGCGTTATCCGGAATCATTGGGCGCAAAGGGTGTGTAGGCGGTTATATTAGTCT
CTTGTTAAAGCTCCCGGCTCAACCGGGAAAATGCAAGAGAAACGGTATGACTAGAGGATG
CGAGAGGTGAACGGAACTCATAGTGTAGGGGTGAAATCCGTTGATATTATGGGGAACACC
AAATGCGAAGGCAGTTCACTGGCGCACTCCTGACGTTGAAACACGAAAGCGTGGGTAGCG
AATGGGATTAGAAACCCTTGTAGTCC
>Otu1716
CCAGCCTATGGGTGGCAGCAGTAACGAATATTGGGCAATGGGCGAAAGCCTGACCCAGCG
ACGCCGCGTGTGGGAAGAAGTTCTTCGGAATGTAAACCACTGTCAGGGATTACCAAGCAA
GTGCGCCTAATATGCGCGCAAGTTGAGGCGTCCCAGAGGAAGCCACGGCTAACTTCGTGC
CAGCAGCCGCGGTAATACGAAGGTGGCAAGCGTTGTTCGGAATCACTGGGCTTAAAGCGC
ACGTAGGCGGCCCATCAAGTGCCTTGTGAAATCCCCCGGCTCAACCGAGGAATTGCTTGG
CATACTGTTGGGCTTGAGGCAGGTAGGGGCGAGTGGAACTCTTGGTGGAGCGGTGGAATG
CGTAGATATCAAGAGGAACGCCGGTGGCGAAAGCGACTCGCTGGGCCTGTCCTGACGCTG
AGGTGCGAAAGCCAGGGGAGCAAACGGGATTAGATACCCTCGTAGTCC
>Otu1719
CCAGCCTACGGGGGGCAGCAGCTAAGAATCTTCCGCAATGGACGAAAGTCTGACGGAGCG
ACGTCGCGTGGGTGATGAAGGCGGAAACGTTGTAAAGCCCTTTTATAGACGAGGAATAAG
GTGGGGAGGGAATGCCCTGCTGATGACGGTAGTCTGTGAATAAGCCCCGGCTAATTACGT
GCCAGCAGCCGCGGTAACACGTATGGGGCGAGCGTTGTTCGGAATTATTGGGCGTAAAGG
GCATGCAGGCGGTCTTATAAGTCTGGTGTGTAAACCCGCAGCTTAACTGCGGAGCCGCAC
TGGAAACTGTGGGACTAGAGTACAGGAAGGGGAGTTGGAATTCCAGGTGTAGGGGTGAAA
TCTGTAGATATCTGGAAGAACATCAGTGGCGAAGGCGAACTTCTGGCCATGTACTGACGC
TGAGATGCGAAAGCGTGGGGAGCAAACAGGATTAGATACCCTTGTAGTCC
>Otu1720
CCAGCCTACGGGGTGCAGCAGTGAGGAATTTTGCGCAATGGGGGAAACCCTGACGCAGCG
ACGCCGCGTGGAGGATGAAGGCCTTCGGGTCGTAAACTCCTGTCAGAGGTGAAGAACGCC
CGCAAGGGCTTGACGGTAACCTCAGAGGAAGCCCCGGCTAACTACGTGCCAGCAGCCGCG
GTAATACGTAGGGGGCAAACGTTGCTCGGAATTACTGGGCGTAAAGGGTGAGTAGGCGGC
TTGGCAAGTCATGGATGAAATCCCGCAGCTCAACTGCGGAACGGTCCTTGAAACTGCCTG
GCTTGAGGATAGTTGAAGAGAATGGAGTTCCCGGTGGAGCGGTGAAATGCGTAGATATCG
GGAGGAACACCAGAGGCGTAGGCGATTCTCTAAGCTACTCCTGACGCTGAATCACGAAAG
CTAGGGGAGCAAACGGGATTAGAGACCCTCGTAGTCC
>Otu1722
CCAGCCTATGGGATGCTGCAGTCGAGAATTTTTCTCAATGGGGGAAACCCTGAAGGAGCG
ACGCCGCGTGGAGGATGAAGGTCTTCGGATTGTAAACTCCTGTCATTTGGGAACAATTGT
CACCGGTTAACTGCCGGGGGCTTGATAGTACCAGAAGAGGAAGGGACGGCTAACTCTGTG
CCAGCAGCCGCGGTAATACAGAGGTCCCAAGCGTTGTTCGGATTCATTGGGCGTAAAGGG
TGCGTAGGTGGTGCCGTAAGTTGGATGTGAAATCCCGGAGCTCAACTCCGGAACTGCATT
CAATACTGCGGCGCTCGAGTACTGGAGAGGAGATCGGAATTCACGGTGTAGCAGTGAAAT
GCGTAGATATCGTGAGGAAGACCAGTGGCGAAGGCGGATCTCTGGACAGTTACTGACACT
GAGGCACGAAGGCCAGGGGAGCAAACGGGATTAGATACCCGCGTAGTCC
>Otu1724

CCAGCCTATGGGTTGCTGCAGTCGAGGATCTTCGGCAATGGGCGCAAGCCTGACCGAGCG
ACGCCGCGTGCGGGATGAAGGCCTTCGGGTTGTAAACCGCTGTCAGAGGGGATGAAATGT
ATGAGGGTACTCTCTCATACTTGACATAGCCTCAAAGGAAGTACGGGCTAAGTTCGTGCC
AGCAGCCGCGGTAAGACGAACCGTACATACGTTATTCGGAATTACTGGGCTTAAAGGGTG
CGTAGGCGGCGGTGCAAGTTGGGTGTGAAATCCCTCGGCTCAACCGAGGAACTGCGCTCA
AAACTGCATTGCTTGAGGGAGATAGAGGTGAGTGGAACTTAGGGTGGAGCGGTGAAATGC
GTTGATATCCTAAGGAACACCAGTGGCGAAAGCGACTCACTGGGTCTCTTCTGACGCTGA
GGCACGAAAGCTAGGGTAGCGAACGGGATTAGAAACCCGAGTAGTCC
>Otu1725
CCAGCCTACGGGTGGCACCAGTGGGGAATCTTGGACAATGGGGGCAACCCTGATCCAGCG
ATGCCGCGTGTGTGAAGAAGGCCTGCGGGTTGTAAAGCACTTTTGTTAGGGAAGAAAGGT
TGGTGGTGAATAGCCATTGACTGTGACTGTACCTAAAGAATAAGCACCGGCTAACTCTGT
GCCAGCAGCCGCGGTAATACAGAGGGTGCAAGCGTTAATCGGAATGACTGGGCGTAAAGG
GCGAGTAGGCGGTTATATAGGTGTGGTGTGAAAGACACGGGCTTAACCTGTGGGTGTCAC
CGCAAACGGTATGACTAGAGTGCAGTAGAGGGGAGTGGAATTTCCGGAGGAGCGGTGAAA
TGCGTAGATCTCGGAAGGAACACCGGTGGCGAAGGCGACTCTCTGGACTGCAACTGACGC
TGAGGCGCGAAGGCGTGGGGAGCAAACAGGATTAGAGACCCTTGTAGTCC
>Otu1727
CCAGCCTACGGGGTGCACCAGTGGGGAATATTGGACAATGGGCGCAAGCCTGATCCAGCA
ATGCCGCGTGGGTGAAGAAGGTCTTCGGATTGTAAAGCCCTTTCGGCGGGGACGATGATG
ACTGTACCCGCAGAAGAAGCTCCGGCTAACTTCGTGCCAGCAGCCGCGGTAATACGAAGG
GGGCTAGCGTTGCTCGGAATTACTGGGCGTAAAGGGCGCGTAGGCGGGTGTTTAAGTCAG
AGGTCAAATCCCAGGGCTCAACCCTGGAACTGCCTTTGATACTGGGCACCTAGAGTTCGG
GAGAGGTGTGTGGAACTCCGAGTGTAGAGGTGAAATTCGTAGATATTCGGAAGAACACCA
GTGGCGAAGGCGACACACTGGCCCGATACTGACGCTGAGGCGCGAAAGCGTGGGGAGCAA
ACAGGATTAGATACCCGAGTAGTCC
>Otu1729
CCAGCCTATGGGGGGCACCAGCAAGGAATATTGGACAATGGTGGCAACACTGATCCAGCC
ATGCCGCGTGCAGGATGAAGGCGCTACGCGTTGTAAACTGCTTTTGTACGGGAGAAAACC
TATCTACGTGTAGATAGCTGATAGTACCGTAAGAATAAGCATCGGCTAACTTCGTGCCAG
CAGCCGCGGTAATACGAAGGATGCAAGCGTTATCCGGATTTATTGGGTTTAAAGGGTGCG
TAGGCGGACTTATAAGTCAGTGGTGAAAACCTGCAGCTTAACTGTAGAATTGCCATTGAT
ACTGTTAGTCTTGAGTACGGTCAAGGTAGGCGGAGTGTGTAATGTAGCGGTGAAATGCTT
AGATATTACACAGAACACCGATTGCGAAGGCAGCTTACTGGGCCATTACTGACGCTGATG
CACGAAAGCGTGGGGAGCGAACAGGATTAGAAACCCTCGTAGTCC
>Otu1730
CCAGCCTATGGGGGGCTGCAGTAGGGAATATTGCGCAATGGAGGAAACTCTGACGCAGCG
ACGCCGCGTGGGTGATGAAGGCCTTCGGGTCGTAAAGCCCTGTCGGAAGAGAAGAAAAAC
GATTTGGCTAATATCCAAGTCGCTTGTCGGTACCTTTAAAGGAAGCACCGGCTAACTACG
TGCCAGCAGCCGCGGTAATACGTAGGGTGCTAGCGTTGTTCGGAATCATTGGGCGTAAAG
CGCGTGTAGGTGGTTAAGTAAGTCGGGTGTGAAATCCCTCGGCTCAACCGAGGAAGTGCA
TCCGAAACTGCTTAGCTAGAGGACGGTAGAGGAAGGTGGAATTCCAAGTGTAGAGGTGAA
ATTCGTAGATATTTGGAGGAATACCGGTGGCGAAGGCGGCCTTCTGGGCCGTTCCTGACA
CTGAGACGCGAAAGCGTGGGTAGCAAACAGGATTAGAGACCCTCGTAGTCC
>Otu1731
CCAGCCTATGGGTCGCTGCAGTCGAGAATCTTCCACAATGGACGAAGGTCTGATGGAGCG
ACGCCGCGTGATTGATGAAGTCCTTCGGGACGTAAAGATCTTTTATGAGGGAAGAAGTTT
ATTGACGGTACCTCATGAATAAGGGGCTCCTAATCTCGTGCCAGCAGGAGCGGTAATACG
AGAGCCCCAAGCGTTATCCGGAATTATTGGGCGTAAAGGGTGCGTAGGCGGTTGTGTTAG
TCATGTGTTAAAGGCCCGGGCTTAACTTGGGACATGCACGCGAAACGGCACGACTTAGAA
TGAAGGAGAGGTGTGCGGAGCTCATGGTGTAGGGGTGAAATCCGTTGATATCATGGGGAA
CACCAAATGCGAAAGCAGCACACTGGCCTTTTATTGACGCTGAGGCACGAAAGCGTGGGT
AGCGAATGGGATTAGAAACCCTGGTAGTCC
>Otu1732
CCAGCCTATGGGGCGCAGCAGTCGAGGATCTTCGGCAATGGGCGCAAGCCTGACCGAGCG
ACGCCGCGTGTGCGATGAAGGCCTTCGGGTTGTAAAGCACTGTCGAGGGGAAGAAAGCCG
CAAGGTTGATCGACCCCTGGAGGAAGCACGGGCTAAGTTCGTGCCAGCAGCCGCGGTAAG
ACGAACCGTGCGAACGTTGTTCGGAATCACTGGGCTTAAAGGGCGCGTAGGCGGGTGGCC

AAGTCGGTGGTGAAATCCTCCAGCTTAACTGGAGAGCTGCCTTCGATACTGGTCGTCTGG
AGGGGGTAGGGGCAGACGGAACTTCCGGTGGAGCGGTGAAATGCGTAGATATCGGAAGGA
ACGCCGGCGGCGAAAGCGGTCTGCTGGACCCCCACTGACGCTGAGGCGCGAAAGCCAGGG
GAGCAAACGGGATTAGATACCCCAGTAGTCC
>Otu1733
CCAGCCTATGGGGCGCTGCAGTCGAGAATCTTCCGCAATGGACGAAAGTCTGACGGAGCG
ACGTCGCGTGGTTGATGAAGTTCTTAGGGATGTAAAAACCTTTTATGAGGGAGAAAGTTA
TTGATAGTACCTCATGAATAAGGGGTTGCTAAACTCGTGCCAGCAGCAGCGGTAATACGA
GTGCCCCAAGCGTTATCCGGAATTATTGGGCGTAAAGGGTGTGTAGGTGGTTTTGTTAGT
CTTGTGTTAAAGCTCCCGGCTCAACTGGGAAAATGCATAAGAAACGGCAAGACTAGAGGA
TGCGAGAGGTGTGTAGAACTCATAGTGTAGGGGTGAAATCCGTTGATATTATGGGGAATA
CCAAAAGCGAAGGCAGCACACTGGCGCACTCCTGACACTGAAACACGAAAGCGTGGGTAG
CGAATGGGATTAGAAACCCTAGTAGTCC
>Otu1734
CCAGCCTACGGGGGGCAGCAGTCATGAATCTTCCCCAATGGCCGAAAGGCTGAGGGAGCG
ACGCCGCGTGTATGATGAAGCTCTTCGGAGTGTAAAATACTTTTCTCTGGGACGAATTTT
GACTTTACCAGAGGAATAAGGAGCCGCTAACCTCGTGCCAGCAGCGGCGGTAATACGAGG
GCTCCAAGCGTTATCCGGACTTATTGGGCGTAAAGGGTACGTAGGCGGTCTGACGCATCT
TTCGTTAAATCTTCGGGCTCAACCCGGAGGCTGCGGAAGAGATGGTCAGACTGGAGATCG
GAAGAGGCAAGCGGAATTGTTGGTGTAGGGGTTAAATCCGTTAATATCAACAAGAACACC
AAAAGCGAAGGCAGCTTGCTAGGACGATTCTGACGCTGAGGTACTAAAGCGTGGGTAGCG
AATAGGATTAGAAACCCGCGTAGTCC
>Otu1735
CCAGCCTATGGGTCGCACCAGTGGGGAATCTTGCGCAATGGGCGAAAGCCTGACGCAGCG
ACGCCGCGTGGGTGATGAAGGCCTTCGGGTTGTAAAGCCCTGTGGGGAGGGACGAGTAAG
TCGGAGCCAATACCTCCGGCGATGACGGTACCTCTTTAGCAAGCACCGGCTAACTCTGTG
CCGGCAGCCGCGGTAAGACAGAGGGTGCAAACGTTGTTCGGAATTACTGGGCGTAAAGCG
TGTGTAGGCGGCCGTGAAAGTCGGATGTGAAAGCCCCGGGCTCAACTCGGGAAGTGCACC
CGATACTGCATGGCTTGAGTACCGGAGAGGTTGGTGGAATTCTCGGTGTAGAGGTGAAAT
TCGTAGATATCGAGAGGAACACCGGTGGCGAAGGCGGCCAACTGGACGAGTACTGACGCT
GAGACACGAAAGCGTGGGGAGCAAACAGGATTAGAAACCCCAGTAGTCC
>Otu1736
CCAGCCTATGGGTTGCTCCAGTCGAGAATCTTTCGCAATGGGCGAAAGCCTGACGAAGCG
ACGCCGTGTGAGCGAAGAAGGCCTTCGGGTTGTAAAGCTCTTTCGCTAGGGAACAAGAAA
GGGACGCTAATATCGTCCTGATTTGAGGGTACTTGGTAAAGAAGCACCGGCTAACTCCGT
GCCAGCAGCTGCGGTAATACGGAGGGTGCAAGCATTGATCGGAATTACTGGGCGTAAAGG
GCGCGTAGGCGGCTAGAAAAGTTGAATGTGAAATTCCGCGGCTCAACTGCGGAGCGGCAT
TCAAAACTCTTTAGCTCGAGCATAGTTAGGGAAAACGGAATTCCACGTGTAGCGGTGAAA
TGCGTAGATATGTGGAAGAACACCTGTGGTGAAGACGGTTTTCTGGGCTATTGCCGACGC
TGAGGCGCGAAAGCTAGGGGAGCAAACAGGATTAGAAACCCTAGTAGTCC
>Otu1737
CCAGCCTACGGGGGGCAGCAGTCGAGGATTTTTCTCAATGGGGGAAACCCTGAAGGAGCG
ACGCCGCGTGGGGGATGAAGGTCTTCGGATTGTAAACCCCTGTCATCTGGGAACAATGTG
GTCAACCTAACACGTGGATCATTGATAGTACCGGAAGAGGAAGCAGTGGCTAACTCTGTG
CCAGCAGCCGCGGTAATACAGAGACTGCAAGCGTTGTTCGGATTCATTGGGCGTAAAGGG
TGCGCAGGCGGTCGGCTAAGTCGGATGTGAAATCTCACAGCCTAACTGTGATAGGTCATT
CGAAACTGGCCGGCTTGAGGACTGGAGAGGAGACTGGAATGGTTGGTGTAGCAGTGAAAT
GCGTAGAGATCAACCAGAACACCGGTGGCGAAGGCGGGTCTCTGGACAGTTCCTGACGCT
CAGGCACGAAAGCCAGGGGAGCAAACGGGATTAGATACCCGAGTAGTCC
>Otu1738
CCAGCCTATGGGAGGCTGCAGTGGGGAATATTGGACAATGGGCGCAAGCCTGATCCAGCC
ATGCCGCGTGAGTGATGACGGCCTTAGGGTTGTAAAGCTCTTTCGACGGGGACGATAATG
ACGGTACCCGTAGAAGAAGCCCCGGCTAACTTCGTGCCAGCAGCCGCGGTAATACGAAGG
GGGCTAGCGTTGTTCGGAATCACTGGGCGTAAAGCGCACGTAGGCGGATTGTTAAGTCGG
GGGTGAAATCCTGAGGCTCAACCTCAGAACTGCCTCCGATACTGGCCATCTCGAGTTCGG
AAGAGGTTGGTGGAACAGCTAGTGTAGAGGTGAAATTCGTAGATATTAGCTAGAACACCA
GTGGCGAAGGCGGCCAACTGGTCCGACACTGACGCTGAGGTGCGAAAGCGTGGGGAGCAA
ACAGGATTAGATACCCTCGTAGTCC

>Otu1739
CCAGCCTACGGGACGCTGCAGTGGGGAATATTGGACAATGGGCGAAAGCCTGATCCAGCC
ATGCCGCGTGAGTGAAGAAGGCCTTCGGGTTGTAAAGCTCTTTCGACGGGGACGATGATG
ACGGTACCCGTATAAGAAGCCCCGGCAAACTTCGTGCCAGCAGCCGCGGTAATACGAAGG
GGGCTAGCGTTGTTCGGAATTACTGGGCGAAAAGCGAACGTAGGCGGTGTTCTTTGTCAG
AGGTGAAAGCCTGGGGCTCAACCCCAGAATTGCCTTTGAAACGGGGACGCTTGAGTCCGA
GAGAGGGTGGCGGAATTCCTAGTGTAGAGGTGAAATTCGTAGATATTAGGAAGAACACCA
GTGGCGAAGGCGGCCACCTGGCTCGGTACTGACGCTCAGGTTCGAAAGCGTGGGGAGCAA
ACAGGATTAGAGACCCGTGTAGTCC
>Otu1740
CCAGCCTATGGGTCGCAGCAGTGAGGAATTTTCCGCAATGGGCGAAAGCCTGACGGAGCA
ATGCCGCGTGTGGGAAGAAGGCCTTCGGGTCGTAAACCACTGTCGAAGGGGACGATACTG
ACGGTACCCTTGGAGGAAGCCCCGGCTAACTACGTGCCAGCAGCCGCGGTAAGACGTAGG
GGGCGAGCGTTATTCGGAATTATTGGGCGTAAAGCGCTCGTAGGCGGGACAGGAAGTCCG
TGAAGAAAGACCTGGGCTCAACTCAGGGAACGGCACGGATACTCTTGTTCTTGAGGCAGT
CAGAGGGTGATGGAATTCCCGGTGTAGCGGTGAAATGCGTAGATATCGGGAGGAACACCA
GTGGCGAAGGCGATCACCTGGGGTTGTTCTGACGCTGAGGAGCGAAAGCTAGGGGAGCAA
ACGGGATTAGATACCCGTGTAGTCC
>Otu1741
CCAGCCTATGGGTGGCTCCAGTCGAGAATCTTCGGCAATGGACGCAAGTCTGACCGAGCG
ACGCCGCGTGCGGGATGAAGGCCTTCGGGTTGTAAACCGCTGTCGGAGGGGATGAAATCG
TGGTGGGTTCTCCCACCTCGTTGACATAGCCTCAAAGGAAGTGTGGGCTAAGTTCGTGCC
AGCAGCCGCGGTAAGACGAACCACACAAACGTTATTCGGAATTACTGGGCTTAAAGGGTG
CGTAGGCGGCGCGGAAAGTAGGGTGTGAAATCCCTCGGCTCAACCGAGGAATAGCGCCCT
AAACTACCGTGCTCGAGGGAGATAGAGGTGAGCGGAACTTAGGGTGGAGCGGTGAAATGC
GTTGATATCCTAAGGAACACCGGTGGCGAAAGCGGCTCACTGGATCTCTTCTGACGCTGA
GGCACGAAAGCTAGGGTAGCGAACGGGATTAGATACCCCCGTAGTCC
>Otu1746
CCAGCCTACGGGTCGCAGCAGTGGGGAATATTGGGCAATATGTGAAAACATGACCCAATG
ATAATTCGAAAGTGAGAAATGGAAAGCATTATCGTTGTAAAACTTATTTTTCAAAAATGA
TAATGACAGTATTTGGAAAAGTAGCACTGGCTAATTCTGTGCCAGCAGCCGCGGTAATAC
AGGGAGTGCAGACGTTATTCGGCATAATTGGGTGTAAAGGATACGTAGGTTGATTTGGTT
TGGAAATTTCATGAAATACTTAGGTTAAAAGCTAAGAAAATTGAAAAAAAGACATTAATT
TGTTTATTTACCCAAAATCTATGAGTTTAAGATAGGTTAAGAGAATTTCTTGTGTAAGAG
TAGAATCTCTAAATATTTGAAGGACTACCAAAATAGTTGCTAAAGCACTTTTCTAGCTTA
AAACTGACACTGAGGTATGAAAGATTAAGGTATCAAACAGGATTAGAAACCCTGGTAGTC
A
>Otu1751
CCAGCCTATGGGATGCAGCAGTGGGGAATATTGGATAATGGGCGAAAGCCTGATCCAGCC
ATGCCGCGTGAGTGATGAAGGCCTTAGGGTTGTAAAGCTCTTTCAGTGGGGAAGATAATG
ACGGTACCCACAGAAGAAGCCCCGGCTAACTTCGTGCCAGCAGCCGCGGTAATACGAAGG
GGGCTAGCGTTGTTCGGATTTACTGGGCGTAAAGCGCACGTAGGCGGATCGTTAAGTCGG
GGGTGAAATCCTGGAGCTCAACTCCAGAACTGCCTTCGATACTGGCGATCTCGAGTCCGG
AAGAGGTGAGTGGAACTCCTAGTGTAGAGGTGGAATTCGTAGATATTAGGAAGAACACCA
GTGGCGAAGGCGGCTCACTGGTCCGGTACTGACGCTGAGGTGCGAAAGCGTGGGGAGCAA
ACAGGATTAGATACCCCAGTAGTCC
>Otu1753
CCAGCCTATGGGGGGCAGCAGTGGGGAATATTGGACAATGGGCGCAAGCCTGATCCAGCC
ATGCCGCGTGAGTGATGAAGGCCTTAGGGTTGTAAGGCTCTTTCGCTAGGGACGATAATG
ACGGTACCTAGACAAGAAGCCCCGGCTAACTTCGTGCCAGCAGCCGCGGTAATACGAGGG
GGGCAAGCGTTGTTCGGAATTATTGGGCGTAAAGGGTGCGTAGGCGGTTTGACAAGTCTC
ATGTGAAATCTTCGGGCTCAACCCGAAGTCTGCATGGGAAACTGTCGGGCTTGAGTATTG
GAGAGGTGAGTGGAATTTCCGGTGTAGCGGTGAAATGCGTAGATATCGGAAGGAACACCT
GTGGCGAAAGCGGCTCACTGGACAATAACTGACGCTGATGCACGAAAGCTAGGGGAGCAA
ACAGGATTAGATACCCCCGTAGTCC
>Otu1754
CCAGCCTATGGGGGGCAGCAGTCTAGAAATTTGGGCAATGGGCGAAAGCCTGACCCAGCG
ACACCGCGTGGAGGATGAAGTCTTTCGGGATGTAAACTCCTGACAGGCGGAGCGATGCCG

TTTCGGAGTAACTGCCGAGACGGTGACGGTACTGCCAAAGGAAGCCCCGGCTAACTACGT
GCCAGCAGCCGCGGTAATACGTAGGGGGCAAGCGCTGTCCGGATTTACTGGGCGTAAAGG
GTGTGCAGGCGGACTGGCGTGTCGATGGTGAAAGCTCCCGGCTTAACCGGGAAAGTGCTG
TCGAAACTACCAGTCTTGAGGGCGGAAGAGGAAACTGGAACTTCCGGTGTAGCGGTAAAA
TGCGTAGATATCGGAAGGAACGCCGATAGCGAAGGCAGGTTTCTGGGACGTACCTGACGC
TGAAACACGAAAGCTAGGGGAGCAAACAGGATTAGATACCCCAGTAGTCC
>Otu1755
CCAGCCTACGGGGGGCACCAGTGGGGAATCTTGCACAATGGGGGAAACCCTGATGCAGCA
ACGCCGCGTGAGGGACGAAGGCTTTCTGAGTTGTAAACCTCTTTCAGCAGGGACGATTGT
GACGGTACCTGCAGAAGAAGCACCGGCCAACTACGTGCCAGCAGCCGCGGTGATACGTAG
GGTGCAAGCGTTGTCCGGATTTATTGGGCGTAAAGAGCTCGTAGGCGGTTTGGTAAGTCG
GGTGTGAAATCCCCAGGCTCAACCTGGGGCTGCCACCCGATACTGCCCTGACTCGAGTCC
GGTAGGGGACCATGGAATTCCTGGTGTAGCGGTGAAATGCGCAGATATCAGGAGGAACAC
CAGTAGCGAAGGCGGTGGTCTGGGCCGGTACTGACGCTGAGGAGCGAAAGCGTGGGTAGC
AAACAGGATTAGATACCCCAGAGTCC
>Otu1757
CCAGCCTACGGGGGGCTGCAGTGGGGAATATTGGACAATGGGGGAAACCCTGATCCAGCC
ATGCCGCGTGAACGATGAAGGCCTTCGGGTTGTAAAGTTCTTTTGACAGGGAAGATGATG
ACTGTACCTGTAGAATAAGCACCGGCTAACTTCGTGCCAGCAGCCGCGGTAATACGAAGG
GTGCTAGCGTTGTTCGGAATTACTGGGCGTAAAGGGCGCGTAGGCGGGTCGACAAGTTGG
ATGTGAAAGCCCAGGGCTCAACCCTGGAATTGCAATCAAGACTGTCTTTCTTGAATTCGG
TAGAGGTTGGTGGAATTCCCAGTGTAGAGGTGAAATTCGTAGAGATTGGGAAGAACACCG
GTGGCGAAGGCGGCTCACTGGCCCGTTACTGACGCTCAGGCGCGACAGCGTGGGGATCAA
ACAGGATTAGATACCCTAGTAGTCC
>Otu1759
CCAGCCTACGGGTGGCTGCAGTCGAGAATTTTTCACAATGGGGGAAACCCTGATGGAGCG
ACGCCGCGTGGGGGATGAATGGCTTCGGCCCGTAAACCCCTGTCATTCGGGATCAATGCC
TACGGTTTAATAAATCGTGGGTTGATAGTACCGGAAGAGGAAGGGACGGCTAACTCTGTG
CCAGCAGCCGCGGTAATACAGAGGTCCCAAGCGTTGTTCGGATTCACTGGGCGTAAAGGG
TGCGTAGGTGGTCGGGTAAGTCTGATGTGAAATCTCGGAGCTCAACTCCGAAACGGCATT
GGATACTATTCGGCTTGAGGATTGGAGGGGGGACTGGAATACTTGGTGTAGCAGTGAAAT
GCGTAGATATCAAGTGGAACACCAGTGGCGAAGGCGAGTCCCTGGACAAATCCTGACACT
AAGGCACGAAAGCTAGGGGAGCAAACAGGATTAGAGACCCCTGTAGTCC
>Otu1761
CCAGCCTACGGGGCGCTGCAGTAACGAATCTTCCGCAATGCACGAAAGTGTGACGGAGCG
ACGCCGCGTGGAGGACGAAGTTCTTCGGAATGTAAACTCCTGTCAGAGGAAAGAAAGTTC
TGATCTACCTCAGAGGAAGAGGCGGCTAACTCTGTGCCAGCAGCCGCGGTAATACAGAGG
CCTCGAGCGTTAGGCGGAATCACTGGGCTTAAAGCGTGTGTAGGCGGACTTCTAAGTACC
TTGTGAAATCCCACGGCTTACCCGTGGAACTGCTTGGTATACTGGTTGTCTTGAGCCACC
TAGGGGCGAGTGGAACAAATGGTGGAGCGGTGAAATGCGTAGATATCATTTGGAACGCCA
ATGGTGAAAACAACTCGCTGGGGGTGTGCTGACGCTGAGACACGAAAGCCAGGGGAGCGA
ACGGGATTAGAGACCCCAGTAGTCC
>Otu1763
CCAGCCTACGGGTGGCACCAGTCGAGAATTTTTCACAATGGGCGCAAGCCTGATGGAGCG
ACGCCGCGTGGGGGATGAAGGGTTTCGATCCGTAAACCCCTGTCATTTGCGAACAATGGT
TATTCAATAACACTGGATAGCTTGATAGTAGCGGAAGAGGAAGGGACGGCTAACTCTGTG
CCAGCAGCCGCGGTAATACAGAGGTCCCAAGCGTTGTTCGGATTCACTGGGCGTAAAGGG
TGCGTAGGTGGCCGGGTAAGTTCGGTGTGAAAGCTCGGAGCTCAACTCCGAAAAGTCATC
GAATACTATCCGGCTGGAGGGTCGGAGGGGAGACTGGAATTCTCGGTGTAGCAGTGAAAT
GCGTAGACATCGAGAGGAACACCAGTGGCGAAGGCGAGTCTCTGGACGACACCTGACACT
GAGGCACGAAAGCCAGGGGAGCAAACAGGATTAGATACCCCCGTAGTCC
>Otu1764
CCAGCCTATGGGTCGCTCCAGTGGGGAATTTTGCGCAATGGGGGAAACCCTGACGCAGCA
ACGCCGCGTGGAGGATGAAGCCCCTTGGGGTGTAAACTCCTTTCGATCGGGAAGATAATG
ACGGTACCGATATAAGAAGCCCCGGCTAACTACGTGCCAGCAGCCGCGGTAAAACGTAGG
GGGCCAGCGTTGCTCGGAATTACTGGGCGTAAAGGGTCCGTAGGCGGTGTGGCAAGTCGG
TAGTGAAATCTCTGGGCTTAACCCAGAGGCTGCTGCCGAAACTGCTATGCTAGAGTGTGA
GAGAGGCGAGTGGAATTACGGGTGTAGCGGTGAAATGCGTAGATATCCGTAGGAACATCC

GAGGCGAAGGCGGCTCGCTGGATCACAACTGACGCTGAGGGACGAAAGCTAGGGGAGCAA
ACAGGATTAGAGACCCGAGTAGTCC
>Otu1765
CCAGCCTACGGGATGCACCAGTAAGGAATATTGGTCAATGGAGGCAACTCTGAACCAGCC
ATGCCGCGTGCAGGATGAAGGTCCTCTGGATTGTAAACTGCTTTTATCAGGGAAGAAATC
ACCTATTTCTATGGGTGTTGCCGGTACCTGAGGAATAAGCACCGGCTAACTCCGTGCCAG
CAGCCGCGGTAATACGGAGGGTGCAAGCGTTATCCGGATTTACTGGGTTTAAAAGGTGCG
TAGGCGGATTTTTAAGTCAGTGGTGAAAACTCCGAGCTTAACTCGGAAACTGCCATTGAT
ACTATTAATCCTGAATTTAGTTGAGGTTGGCGGAATGTGTCGTGTAGCGGTGAAATGCTT
AGATATGACACAGAACACCGATTGCGAAGGCAGCTGGCTAAACTAAAATTGACGCTGAGG
CACGAAAGTGTGGGGATCAAACAGGATTAGATACCCGAGTAGTCC
>Otu1768
CCAGCCTATGGGTTGCTCCAGCTGGGAATCTTCTGCAATACACGAAAGTGTGACAGAGCG
AGCCAGAGTGCTTCTCATTGAGAAGCTTTTGCCAAATCTAAAAAGTTTGGCGAATAAGGA
CTGGGCAAGACTAGTGCCAGCCGCCGCGGTAATACTAGCTGTCCAAGTCGCAGCCATCTT
TATTGGGTCTAAAACATCCGTAGCTTGTCTAGCAAGTTCTTTGTGAAATCTTATCTCTTA
AGGATAGGGCGCGCATGGAGTACTACTAGGCTAGAGACTGGAAGACGTAAGAAGTACGTT
TGAAGTAGCGGTTAAATGCGTTAATCTTAGACGGACTCACAACAGCGAAGGCATCTTACG
AGGACAGTTCTGACAGTGAGGGATGAAGGCCGGGGGCGCAAAATGGATTAGAAACCCCCG
TAGTCC
>Otu1769
CCAGCCTATGGGAGGCAGCAGTAAGGAATATTGGACAATGGTGGCAACACTGGTCCAGCC
ATGCCGCGTGCAGGATGACAGCCCTATGGGTCGTAAACTGCTTTTGTACGGGAAAAAAAC
ACTCTTCGTGAAAGAGTACTGATTGTACCGTAAGAATAAGGATCGGCTAACTCCGTGCCA
GCAGCCGCGGTAATACGGAGGATCCAAGCGTTATCCGGATTCATTGGGTTTAAAGGGTGC
GCAGGCGGAATTGTAAGTCAGTGGTGAAAGCCTGTTGCTTAACAACAGAACTGCCATTGA
TACTGCAAATCTTGAGTACATTTGATGTGGGCGGAATGTGTCATGTAGCGGTGAAATGCT
TAGATATGACACAGAACACCGATTGCGAAGGCAGCTCACAAAACTGTAACTGACGCTGAG
GCACGAAAGCGTGGGTATCAAACAGGATTAGATACCCTCGTAGTCC
>Otu1770
CCAGCCTACGGGGCGCAGCAGCAACGAATATTCCGCAATGCGCGAAAGCGTGACGGAGCG
ATGCCGCGTGCAGGATGAAGCCCCTCGGGGTGTAAACTGCTGTCAGGGTATAGCAAACAA
ATGAGCAAACCCAAAGGAAGAGACGGCTAACTCTGTGCCAGCAGCCGCGGTAATACAGAG
GTCTCGAGCGTTAATCGGAGTCACTGGGCTTAAAGGGTGCGTAGGCGGGTCTGTAAGTGT
CTTGTGAAATCCCACGGCTCAACTGTGGAATTGCTCGATAGACTACGGATCTTGAGGTTG
GTAGGGGTCGCTGGAACTCTAGGTGGAGCGGTGAAATGCGTAGATATCTAGAGGAACGCC
GAAGGTGAAGACAAGCGACTGGGCCAATTCTGACGCTGAGGCACGAAAGCGTGGGGAGCA
AACGGGATTAGAGACCCTTGTAGTCC
>Otu1771
CCAGCCTATGGGAGGCACCAGTGGGGAATATTGGGCTATGGGCGAAAGCCTGACCCAGCG
ACGCCGTGTGGGCGATGAAGGCCTTCGGGTTGTAAAGCCCTGTCAGGTGGGAAGAAGTCC
CGGCAACGGGATTGACGGTACCACCGGAGGAAGCACCGGCTAACTCCGTGCCAGCAGCCG
CGGTAATACGGAGGGTGCGAGCGTTGTTCGGAATTACTGGGCGTAAAGGGCGCGTAGGTG
GTTCGGTAAGTCAGATGTGAAATCCCGAGGCTCAACTTCGGAATGGCATCTGAAACTGCC
GGACTCGAGTACAGAAGAGGGAAACGGAATTCCCGGTGTAGAGGTGAAATTCGTAGATAT
CGGGAGGAACACCGGTGGCGCAGGCGGTTTCCTGGTCTGATACTGACACTGAGGCGCGAA
AGCGTGGGGGGCAAACAGGATTAGATACCCTCGTAGTCC
>Otu1773
CCAGCCTATGGGGGGGCAGCAGTGGGGAATTTTGTGCAATGGGGGAAACCCTGTCACAGC
GACACCGCGTGAGCGAAGAAGCCCTTTGGGGTGTAAAGCTCTGTCGGCTGGAACGAAAAA
AATGACGGTACCAGCAAAGGAAGCATCGGCTAACTACGTGCCAGCAGCCGCGGTAAGACG
TAGGATGCAAGCGTTGTCCGGATTTATTGGGCGTAAAGAGTTCGTAGGCGGTTCGTTAAG
TCTGATGTTAAAGATCGGGGCTCAACCCCGGGAGTGCATTGGATACTGGCGAGCTGGAGT
GCGGTAGAGGTTAGTGGAATTCCCAGTGTAGCGGTGAAATGCGTAGATATTGGGAAGAAC
ACCAGTGGCGTAGGCGACTAACTGGGCCGTAACTGACGCTGAGGAACGAAAGCCAGGGGA
GCGAATGGGATTAGATACCCCCGTAGTCC
>Otu1776
CCAGCCTACGGGTCGCACCAGCACCATGGATTGTTGACGGTGGGTGGGGGCCTCGGGATT

GGTCTTACCCATGACGATCGCCACCTTGCACTCGGGGTCCATCGCTCCCGATGTCCACCA
CTTACGTCCATTGATGAGGTAATCGTCGCCCTCGGGGGTGATGGCGCAGCCGATGTTGGT
GGCGTCGGAGCTGGCCACGTCCGGCTCGGTCATGGAGAAGCATGAGCGGATCTCGCCCTC
CAAGAGAGGGACGAGCCAGCGGTCCTTGTGCTCCGGCGTGCCGAACAGGTTGAGGATCTC
CATGTTGCCGGTGTCAGGCGCCGAGCAGTTGCAGGCCCGCGCCGCGAGGTGCGAGCGGCC
CATGATCTCGGCCAGGGGTGCGTAATCCAGATTAGATACCCGCGTAGTCC
>Otu1779
CCAGCCTACGGGTCGCACCAGTCGAGAATCATTCGCAATGGGCGAAAGCCTGACGATGCG
ACGCCGTGTGAACGATGAAGGCCTTCGGGTTGTAAAGTTCTTTCGCCTGGGAACAAGAGA
AGTTGATGAATAATCAGCTAATTTGAGGGTACTAGGTAAAGAAGCACCGGCTAACTCCGT
GCCAGCAGCTGCGGTAATACGGAGGGTGCAAGCATTAATCGGATTTATTGGGCGTAAAGG
GCGCGTAGGCGGGAATGTAAGTCAGATGTGAAAGCCCAGGGCTCAACCCTGGAACAGCAT
TTGAAACTGCATTTCTAGAGGGTAGGCGGAGAAAACAGAATTCCACGTGTAGCGGTGAAA
TGCGTAGATATGTGGAGGAATACCGGTGGCGAAGGCGGTTTTCTAGCTTATTCCTGACGC
TAATGCGCGAAAGCAAGGGGAGCAAACAGGATTAGATACCCTCGTAGTCC
>Otu1780
CCAGCCTATGGGACGCTCCAGTGGGGAATATTGGACAATGGGCGCAAGCCTGATCCAGCC
ATGCCGCGTGAGTGATGAAGGCCTTAGGGTTGTAAAGCTCTTTTGTCAGGGAAGATAATG
ACGGTACCTGAAGAATAAGCACCGGCTAACTTCGTGCCAGCAGCCGCGGTAATACGAAGG
GTGCTAGCGTTGTTCGGAATTACTGGGCGTAAAGCGCGCGTAGGCGGTCTATCAAGTCAG
AGGTGAAAGCCCTGGGCTCAACCCAGGAATTGCCTTTGAAACTGATAGACTTGAGTGTCG
GAGGGGATAGCGGAATTGCTAATGTAGAGGTGAAATTCGTAGATATTAGCAGGAACACCG
GTGGCGAAGGCGGCTATCTGGACGACAACTGACGCTGAGGCGCGAAAGCGTGGGGATCAA
ACAGGATTAGATACCCCCGTAGTCC
>Otu1783
CCAGCCTATGGGTGGCTGCAGTCGAGAATCTTCCGCAATGGACGCAAGTCTGACGGAGCG
ACGCCGCGTGATGGATGAAGTCTTTCGGGATGTAAACATCTTTTATGAGCGAGAAAGTTT
ATTGATCAGCTCATGAATAAGGGGTTGCTAAACTCGTGCCAGCAGCAGCGGTAATACGAG
TGCCCCAAGCGTTATCCGGAATTATTGGGCGTAAAGGGTGTGTAGGCGGTTTCATTAGTC
TCCTGTTAAATCTCCCGACTCAATCGGGATCCTGCAGGAGAAACGGTGAACCTAGAAGAT
GCGAGAGGTTTGCGGAACTCATAGTGTAGGGGTGAAATCCGTTGATATTATGGGGAACAC
CAAAAGCGAAGGCAGCAAACTAGCGCATTCTTGACGCTGAAACACGAAAGCGTGGGTAGC
GAATGGGATTAGATACCCCTGTAGTCC
>Otu1786
CCAGCCTATGGGATGCTGCAGTCGAGAATCTTCCGCAATGGACGAAAGTCTGACGGAGCG
ACGTTACGTGAATGATGAAGCCCTTCGGGGTGTAAAGTTCTTTTCTCTGGGAGGAAGCCG
CAAGGCAGACAGTACCAGAGGAATAAGGGGATCCTAATTCTGTGCCAGCAGGAGCGGTAA
GACAGAATCCCCGAGCGTTACCCGGATTTATTGGGCGTAAAGGGTCCGTAGGTGGTCAAG
TAAGTTGAAAGTTAAAACTCATCGGCTTAACCTTTGAGGTGCTTTCAATACTGCTCGACT
AGAGGATGTTAGGGGTGAACGGAACCGACGGAGTAGGGGTGAAATCCGTTGATATCGTCG
GGAACACCAAAAGCGAAGGCAGTTCACTAGGACAATCCTGACACTGAGGGACGAAAGCTA
GGGGAGCAAACGGGATTAGATACCCGAGTAGTCC
>Otu1787
CCAGCCTACGGGTTGCTCCAGTCGAGAATCTTCGGCAATGGGCGCAAGCCTGACCGAGCG
ACGCCGCGTGGAGGATGAAGGCCTTCGGGTTGTAAACTCCTGTCGAGGGGGAAGAAGGGT
CGAAAGACCTTGACTGATCCCTGGAGGAAGCACGGGCTAAGTTCGTGCCAGCAGCCGCGG
TAAGACGAACCGTGCGAACGTTATTCGGAATCACTGGGCTTAAAGCGCGCGTAGGCGGGA
CGGTGCGTCGACTGCTGAAATCCCCCGGCTTAACCGGGGAACGGGCATCGATACGGCCAT
CCTGGAGGAACGTAGGGGGACCTGGAACTTCCGGTGGAGCGGTGAAATGCGTTGAGATCG
GAAGGAACGCCCGTGGCGAAAGCGAGGTCCTGGACGTTTTCTGACGCTGAGACGCGAAAG
CTAGGGGAGCGAACGGGATTAGAGACCCTAGTAGTCC
>Otu1788
CCAGCCTACGGGAGGCAGCAGTGAGGAATATTGCGCAATGGGGGAAACCCTGACGCAGCA
ACGCCGCGTGAGTGAGGAAGGCCTTCGGGTCGTAAAGCTCTGTCAGTTGGAAAGAAATGT
CTGATAGTTAATACCTTTCAGGCTTGACGGTACCAACAGAGGAAGCACCGGCTAACTCCG
TGCCAGCAGCCGCGGTAATACGGGGGGTGCAAGCGTTGTTCGGAATTATTGGGCGTAAAG
AGCGTGTAGGCGGCCGGATGTGTCAGATGTGAAAGCCCTGGGCTTAACCCAGGAAGTGCA
TTTGAAACTATTCGGCTTGAGTAGGGGAGAGGGAAGTGGAATTCCTGGTGTAGAGGTGAA

ATTCGTAGATATCAGGAGGAACACCGGTGGCGAAGGCGACTTCCTGGCCCTATACTGACG
CTGAGACGCGAGAGCGTGGGTAGCAAACAGGATTAGAAACCCTCGTAGTCC
>Otu1789
CCAGCCTATGGGTGGCTGCAGTAACGAATCTTCCGCAATGCACGAAAGTGTGACGGAGCG
ACGCCGCGTGTAGGATGAAGTCCTTCGGGATGTAAACTACTGTCAGGGGTAAGAAAGTTC
TGATCTACCCCAGAGGAAGGGACGGCTAACTCTGTGCCAGCAGCCGCGGTAATACAGAGG
TCCCGAGCGTTAGGCGGAATCACTGGGCTTAAAGCGTGTGTAGGCGGATCGGTAAGTACC
TTGTGAAATCCCACGGCTTAACCGTGGAACTGCTTGGTATACTGCCAGTCTTGAGCCAGC
TAGGGGCTACCGGAACAAATGGTGGAGCGGTGAAATGCGTAGATATCATTTGGAACGCCA
ATGGTGAAAACAGGTAGCTGGGGCTGTGCTGACGCTGAGACAGGAAAGCCAGGGGAGCGA
ACGGGATTAGAGACCCCCGTAGTCC
>Otu1790
CCAGCCTATGGGTCGCTGCAGTCAAGAAACTTCCACAATGGACGAAAGTCTGATGGAGCG
ACGCCGCGTGGTTGATGAAGTCCTTCGGGACGTAAAGACCTTTTATGAGGGAGGAGGTAA
TTGACGTTACCTCATGAATAAGGGGCTCCTAACTCTGTGCCAGCAGGAGCGGTAATACAG
AGGCCCCAAGCATTATCCGGAATCACTGGGCGTAAAGGGTGTGTAGGCGGTCGTATTAGT
CGTTTGTGAAAGATCTCGGGCTTACTCCGGGAGACGCAAGCGAAACGGTACGACTTCGAG
GATGCGAGAGGTATATGGAACTCATGGTGTAGGGGTGAAATCCGTTGATATCATGGGGAA
CACCAAATGCGAAGGCAATATACTGGCGCACTCCTGACGCTGAAACACGAAAGCGTGGGA
ATCGAACGGGATTAGATACCCCCGTAGTCC
>Otu1792
CCAGCCTACGGGGGGCACCAGTGGGGAATTTTGGACAATGGGGGAAACCCTGATCCAGCA
ACGCTGCGTGAAGGATGAAGGTCTTCGGATTGTAAACTTCTTTTGTTCGGGACGAATACC
CGCAAGGGCTTGACGGTACCGGACGAATAAGCCACGGCTAACTACGTGCCAGCAGCCGCG
GTAAGACGTAGGTGGCGAGCGTTACTCGGAATCACTAGGCGTAAAGCGCGTGTAGGCGGG
TGCTTAAGTCTGCTGTGAAATCTCCTGGCTCAACTGGGAGGGGTCAGCAGATACTGGGCG
TCTGGAGTGAGGCAGGGGGTACTGGAATTCCCGGTGTAGCGGTGAAATGCGTAGATATCG
GGAGGAACACCAATGGCGAAGGCAGGTACCTGGGCCTTTACTGACGCTAAGACGCGAAAG
CTAGGGGAGCAAACAGGATTAGATACCCCTGTAGTCC
>Otu1795
CCAGCCTACGGGGGGCAGCAGTAGGGAATTTTGCGCAATGGACGAAAGTCTGACGCAGCA
ACGCCGTGTGGGTGATGAAGGTCTTCGGATCGTAAAGCTCTGTCAGGAGGGAAGAAACTA
TGCCATTAATACCGGCATACTGACGGTACCTCCAGAGGAAGCTCCGGCTAACTTCGTGCC
AGCAGCCGCGGTAATACGAGGGGAGCTAGTGTTGTTCGGATTTACTGGGCGTAAAGGGAG
TGTAGGCGGGCATCTAAGTCAGAGGTGAAATTTCAGGGCTCAACCCTGAACTTGCCTTCG
ATACTGGGTGTCTTGAGTATGGAAGAGGTCGATGGAATTCCTGGTGTAGCGGTGGAATGC
GTAGAGATCAGGAGGAACGCCAATAGCGAAAGCAGTCGACTGGGACAATACTGACGCTGA
AACTCGAAAGCATGGGGAGCAAACAGGATTAGAAACCCTGGTAGTCC
>Otu1797
CCAGCCTATGGGATGCAGCAGTGGGGAATTTTGCACAATGCCCGAAAGGGTGATGCAGCA
ACGCCGCGTGAGGGATGAAGGCCTTCGGGTCGTAAACCTCTTTCGACCGGGAAAAACGGG
CTGGTGAAGAGCCAGTCCTGATGGTACTGGTGGAAGAAGCCCCGGCCAACTACGTGCCAG
CAGCCGCGGTAATACGTAGGGGGCAAGCGTTGTTCGGAATTACTGGGCGTAAAGGGCGTG
TAGGCGGTGCGGTAAGTTGGGCGTGAAATCCCCGGGCTTAACCCGGGGGGGTCGTTCAAG
ACTGCCGTGCTAGGGTGCGGGAGAGGGAAGTGGAATTCCCGGTGTAGCGGTGAAATGCGT
AGATATCGGGAGGAACACCTGCGGCGAAGGCGGCTTCCTGGACCGTAACCGACGCTGAGG
CGCGAAAGCTAGGGGGGCAAACAGGATTAGATACCCGAGTAGTCC
>Otu1799
CCAGCCTACGGGGTGCTGCAGTCGAGAATCTTCCACAATGGACGAAAGTCTGATGGAGCG
ACGCCGCATGATTGATGAAGTGCTTCGGCACGTAAAGATCTTTTATGGGGGAAGAAGTCT
ATTGACGGTACCTCATGAATAAGGGGCTCCTAACTCTGTGCCAGCAGGAGCGGTAATACA
GAGGCCCCGAGCGTTATCCGGAATTATTGGGCGTAAAGGGTGCGTAGGTGGCGATACTAG
TCGTTTGTGAAAGATCCGGGCTCAACCTGGAAAACGCGAACGAAACGGTATTGCTTGAGT
GAGGAAGGGGTAAGCGGAACTCAAGGTGTAGGGGTGAAATCCGTTGATATCTTGGGGAAC
ACCAAAAGCGAAGGCAGCTTACTGGTCCTTTACTGACACTGAAGCACGAAAGCGTAGGTA
GCGAATGGGATTAGAGACCCGAGTAGTCC
>Otu1801
CCAGCCTACGGGGGGCTGCAGTAACGAATCTTCGTCAATGCACGAAAGTGTGAACGAGCG

ACGCCGCGTGGAGGATGAAGTTCTTCGGAATGTAAACTCCTGTCAGGGGAAAGAAAGTTC
TGATCTACCCCAGAGGAAGGGACGGCTAACTCTGTGCCAGCAGCCGCGGTAAGACAGAGG
TCCCAAGCGTTAGGCGGAATCACTGGGCTTAAAGCGTGTGTAGGCGGATTAGCAAGTACC
TTGTGAAATCCCATGGCTCAACCATGGAACTGCTTGGTATACTGCTAGTCTTGAGGCATC
TAGAGGCAACTGGAACAAACGGTGGAGCGGTGAAATGCGTAGATATCGTTTGGAACGCCC
AAGGTGAAAACAGGTTGCTGGGGATGTACTGACGCTGAGACACGAAAGCCAGGGGAGCAA
ACGGGATTAGAAACCCCAGTAGTCC
>Otu1802
CCAGCCTATGGGGGGCAGCAGTGAGGAATCTTGGACAATGAGCGAAAGCTTGATCCAGCA
ATGTCGCGTGAGTGATGCAGAACTATATCTATAAAACTCTTTCGTCAAGGAAGATTATGA
CATTACTTGAAAAAGAAGCTCCGGCCAACCTCGTGCCAGCAGCCGCGGTAATACGGGGGG
AGCTAGCGTTATTCGGGATAACTGGGCGTAAAGCGTGTGCCTACGGTTTATCATGTTAAA
AGTTAAATATCGGAGCACAACTTTCTAAAAACTTTTAAGACTGATATAAACTTAGAGTAT
GATTAGGGATAGTAGAATTTTTAAAGTAGCAGTGAAATGTTCAGATATTAAAAGGAATGC
CACTAGGCGAAGGCAACTATCTGGATCAATACTGACGTGTCTACACGAAAGTATGGGGAG
CAAACAGGATTAGAAACCCCAGTAGTCC
>Otu1805
CCAGCCTACGGGTCGCTCCAGTGGGGAATATTGGGCAATGGGCGAAAGCCTGACCCAGCC
ACGCCGCGTGAGTGATGAAGGCCTTTGGGTCGTAAAGCTCTGTGGAGGGGGACGAACAAG
CGCGCATTTAATAGATGCGTGCCCTGACGGTACCCCTTTAGCAAGCACCGGCTAACCATG
TGCCAGCAGCCGCGGTAATACATGGGGTGCAAACGTTGCTCGGAATTATTGGGCGTAAAG
CGCGCGTAGGCGGTTGCTTAAGTCAGATGTGAAAGCCCTCGGCTTAACTGAGGAAGTGCA
CCTGAGACTGAGCAGCTAGAGTATGAAAGAGGGTCGCGGAATTCCCGGTGTAGAGGTGAA
ATTCGTAGATATCGGGAGGAACACCGGCGGCGAAGGCGGCGACCTGGTTCAAGACTGACG
CTGAGGCGCGAAAGCGTGGGGAGCAAACAGGATTAGATACCCCTGTAGTCC
>Otu1806
CCAGCCTATGGGGGGCACCAGTGGGGAATATTGGACAATGGGGGCAACCCTGATCCAGCA
ATGCCGCGTGAGTGATGAAGGCCTTAGGGTTGTAAAGCTCTTTTGGCGGGGACGATGATG
ACGGTACCCGCAGAATAAGCCCCGGCTAACTTCGTGCCAGCAGCCGCGGTAATACGAAGG
GGGCGAGCGTTGTTCGGAATTACTGGGCGTAAAGGGCGCGTAGGCGGCCGATCAAGTTAG
ATGTGAAAGTCCTGGGCTCAACCTGGGGACTGCATTTGATACTGATTGGCTTGAATCCGG
GAGAGGATAGCGGAATTCCCAGTGTAGAGGTGAAATTCGTAGATATTGGGAAGAACACCA
GTGGCGAAGGCGGCTATCTGGACCGGTATTGACGCTGAGGCGCGAAAGCGTGGGGAGCAA
ACAGGATTAGATACCCCTGTAGTCC
>Otu1807
CCAGCCTATGGGTCGCTGCAGGGAATATTGGACAATGGGCGAGAGCCTGATCCAGCCATG
CCGCGTGCAGGAAGAAGGCCTTCTGGGTTGTAAACTGCTTTTGCCAGGGGATAAAAGACC
CGTGCGCGGGGAATTGAAGGTACCTGGTGAATAAGCCACGGCTAACTACGTGCCAGCAGC
CGCGGTAATACGTAGGTGGCAAGCGTTATCCGGATTCACTGGGTTTAAAGGGTGCGTAGG
TGGGCAGGTAAGTCAGTGGTGAAATCTCCGGGCTTAACCCGGAAACTGCCGTTGATACTA
TCTGTCTTGAATGTCGTGGAGGTGGGCGGAATATGTCATGTAGCGGTGAAATGCTTAGAT
ATGACATAGAACACCAATTGCGAAGGCAGCTCACTACACGATGATTGACGCTGAGGCACG
ATAGCGTGGGGATCAAACAGGATTAGATACCCCTGTAGTCC
>Otu1810
CCAGCCTATGGGACGCTGCAGTCGAGAATTTTTCTCAATGGGCGAAAGCCTGAAGGAGCG
ACGCCGCGTGAAGGATGAAGGTCTTCGGATTGTAAACTTCTGTCATTAGAGAACAATGCA
TGGGGGAGTAACTGCCTCCATGTTGATAGTATCTGAAGAGGAAGAGACGGCTAACTCTGT
GCCAGCAGCCGCGGTAATACAGAGGTCTCAAGCGTTGTTCGGATTCATTGGGCGTAAAGG
GTGCGTAGGTGGTGGAATAAGTCGGATGTGAAATCTCGGGGCTTAACCCCGAAACTGCAT
TCGATACTGTTTTGCTGGAGGACTGGAGAGGAGATTGGAATTCGTGGTGTAGCAGTGAAA
TGCGTAGATATCATGAGGAAGACCAGTGGCGAAGGCGGATCTCTGGACAGTTCCTGACAC
TGAGGCACGAAGGCCAGGGGAGCAAACGGGATTAGATACCCTTGTAGTCC
>Otu1812
CAGCCTATGGGTGGCTGCAGTAGGGAATATTGGACAATGGGGGCAACCCTGATCCAGCGA
TGCCGCGTGAGTGAAGAAGGCCTTCGGGTCGTAAAGCTCTTTAGGTTGGGAAGAAGGGTA
TGTGGTTAATAACCACGTACTTTGACGGTACCGACAGAATAAGCACCGGCAAACTCTGTG
CCAGCAGCCGCGGTAATACAGAGGGTGCGAGCGTTAATCGGAATTACTGGGTGTAAAGGG
CGCGTAGGCGGTAAGGTGTGTGTGGTGTGAAAGCCCCGGGCTTAACCTGGGAAGTGCATC

GCAAACTATCTAACTAGAGTATATGAGAGGGTGGCGGAATTTCCGGTGTAGCGGTGAAAT
GCGTAGAGATCGGAAGGAACGTCAATGGCGAAGGCAGCCACCTGGCATAATACTGACGCT
GAGGCGCGAAAGCGTGGGGAGCGAACAGGATTAGAGACCCTCGTAGTCC
>Otu1813
CCAGCCTACGGGATGCTGCAGTGGGGAATTTTGCGCAATGGGCGAAAGCCTGACGCAGCA
ACGCCGCGTGGAGGATGAAGGTCTTCGGATCGTAAACTCCTGTCGATGGGGACGAAAGCC
GAAAGGTATGACTGTACCCGTTGAGGAAGCCACGGCTAACTCCGTGCCAGCAGCCGCGGT
AATACGGAGGTGGCAAGCGTTGTTCGGAATTACTGGGCGTAAAGGGCGCGTAGGCGGCTA
AACAAGTCAGATGTGAAAGCCCCGAGCTTAACTCGGGAATTGCATCTGAAACTGTATAGC
TTGAGTTCTGGAGGGGGTAGTGGAATTCCCAGTGTAGCGGTGAAATGCGTAGATATTGGG
AGGAACACCAGCGGCGAAGGCGGCTACCTGGACAGTAACTGACGCTGAGGCGCGAAAGCT
AGGGGAGCGAACGGGATTAGAAACCCCCGTAGTCC
>Otu1814
CCAGCCTACGGGACGCTGCAGTAACGAATATTCCGCAATGGGCGAAAGCCTGACGGAGCA
ATGCCGCGTGTGGGATGAAGCGGTTTCGCCGTGTAAACCACTGTCAGGGAATAGAAAAGA
TGATCATTTCCAGAGGAAGGGGCGACTAACTCTGTGCCAGCAGTCGCGGTAAGACAGAGG
CCCCGAGCGTTAGTCGGAATTACTGGGCTTAAAGGGTGCGTAGGCGGGCGTGTCAGTGTC
TCGTGAAAGCCCCGGGCTCAACCCGGGAACCGCGGGGCATACTGCACGTCTCGAAGGAGC
TAGAGGCCGGTGGAACGATCGGTGGAGCGGTGGAATGCGTAGAGATCGATCGGAACGCCA
AGGGTGAAGACAACCGGCTGGGGCTCTCTTGACGCTGAGGTACGAAAGCGTGGGGATCAA
ACAGGATTAGAAACCCCCGTAGTCC
>Otu1815
CCAGCCTACGGGGGGCTGCAGTAAGGAATATTGGTCAATGGACGCAAGTCTGAACCAGCC
ATGCCGCGTGAAGGATGAAGGCCCTCTGGGTTGTAAACTTCTTTTATCTGGGAAGAAATC
CACTGTTTCTACGGTGGTTGACGGTACCAGAGGAATAAGCACCGGCTAACTCCGTGCCAG
CAGCCGCGGTAATACGGAGGGTGCAAGCGTTATCCGGATTTACTGGGTTTAAAGGGTGCG
TAGGCGGGCTTTTAAGTCAGTGGTGAAATCTCCGGGCTCAACTCGGAAATTGCCATTGAT
ACTATTAGTCTTGAATTAAGTTGAGGTGGGCGGAATGTGTCATGTAGCGGTGAAATGCTT
AGATATGACACAGAACACCGATTGCGAAGGCAGCTCGCTAAACTTATATTGACGCTGAGG
CACGAAAGCGTGGGGATCAAACAGGATTAGAGACCCCAGTAGTCC
>Otu1816
CCAGCCTACGGGACGCTCCAGTCGAGGATCTTCGGCAATGGGCGCAAGCCTGACCGAGCG
ACGCCGCGTGCGCGATGAAGGCCTTCGGGTTGTGAAGCGCTGTCGAGGGGGAGGAACGCC
GCAAGGCCTGACCGACCCCTGGAGGAAGCACGGGCTAAGTTCGTGCCAGCAGCCGCGGTA
AGACGAACCGTGCGAACGTTGTTCGGATTCACTGGGCTTAAAGGGCGCGTAGGCGGGCAA
CCAAGTCAGGGGTGAAATCGTTCAGCTTAACTGGACAAGAGCCTTTGATACTGGTGGTCT
TGAGGGAGGTAGGGGCAGGTGGAACTTCCGGTGGAGCGGTGAAATGCGTAGATATCGGAA
GGAACGCCGGTGGCGAAAGCGGCCTGCTGGATCTCTACTGACGCTGAGGCGCGAAAGCCA
GGGGAGCAAACGGGATTAGATACCCGTGTAGTCC
>Otu1817
CCAGCCTATGGGAGGCAGCAGTGGGGAATTTTGCACAATGGGCGAAAGCCTGATGCAGCA
ACGCCGCGTGTGTGATGAAGGTCTTCGGATCGTAAAGCACTTTCGACTGGGACGAACAAG
GCTGGCCACACCAGCCCCTGACTGTACCAGGAGAAGAAGCACCGGCTAACTCTGTGCCAG
CAGCCGCGGTAATACAGAGGGTGCAAGCGTTGTTCGGAATTATTGGGCGTAAAGCGCGTG
TAGGCGGCTTAGCAAGTCGGGCGTGAAAGCCCTCGGCTCAACCGAGGAAGTGCACCCGAA
ACTGCTGAGCTTGAGTGCCGGAGAGGATGGCGGAATTCCCCAAGTAGAGGTGAAATTCGT
AGATATGGGGAGGAACACCGGTGGCGAAGGCGGCCATCTGGACGGTAACTGACGCTGAGT
CGCGAAAGCGTGGGTAGCAAACAGGATTAGATACCCGCGTAGTCC
>Otu1820
CCAGCCTATGGGACGCAGCAGTCGAGAATCATTCGCAATGGGCGAAAGCCTGACGATGCG
ACGCCGTGTGTGTGATGAAGGCCTTTGGGTCGTAAAGCACTTTCGCTTGGGAACAAGAGA
AGCAGGCTAATACCCTGCTGATTTGAGGGTACCAGGTAAAGAAGCACCGGCTAACTACGT
GCCAGCAGCTGCGGTAATACGTAGGGTGCAAGCATTAATCGGAATTATTGGGCGTATAGG
GCGCGTAGGCGGGTAAACAAGTCGGATGTGAAATCCCAGAGCTCAACTCTGGAACAGCCT
CCGAAACTGTAAATCTAGAGGGTAGGCGGAGAAAACGGAATTCCACAAGTAGCGGTGAAA
TGCGTAGATATGTGGAAGAACACCCGTGGCGAAGGCGGTTTTCTAGCTTATTCCTGACGC
TGAGGCGCGAAAGCAAGGGGATCAAACAGGATTAGAAACCCTAGTAGTCC
>Otu1822

CCAGCCTACGGGTGGCACCAGTAGGGAATATTGCACAATGGGCGAAAGCCTGATGCAGCA
ACGCCGCGTGCGCGATGAAGGCCTTCGGGTCGTAAAGCGCTTCTCTGGGAGAAGAGGAAG
GACACTATCCCAGGAATAAGGCTCGGCTAACTACGTGCCAGCAGCCGCGGTAAAACGTAG
GAGCCAAGCGTTATCCGAATTCACTGGGCGTAAAGCGCGTGCAGGCGGTTTGGTAAGTTG
GATGTGAAATCTCCCGGCTCAACTGGGAGAGGACGTTCAATACTGTCAGACTAGAGGGTG
ACAGAGGGAGGTGGAATTCCCGGTGTAGTGGTGAAATGCGTAGATATCGGGAGGAACACC
AGTGGCGAAAGCGGCCTCCTAGGTCGCACCTGACGCTCAGACGCGAAAGCTAGGGGAGCA
AACGGGATTAGAGACCCGTGTAGTCC
>Otu1823
CCAGCCTACGGGAGGCAGCAGTCAAGAACCTTCCACAATGGACGAAAGTCTGATGGAGCG
ACGCCGCGTGGTTGATGAAGTGCTTCGGCACGTAAAGACCTTTTATGAGGGAAGAAGTAT
ATTGACGGTACCTCATGAATAAGGGGCTCCTAACTCTGTGCCAGCAGGAGCGGTAATACA
GAGGCCCCGAGCGTTATCCGGAATTATTGGGCGTAAAGGGTGCGTAGGTGGCGATGTTAG
TCGTTTGTGAAAGCCCGGGGCTTAACCTCGGAAACGCGAACGAAACGGCATTGCTTGAGT
AGAGAAGAGGTGTGCAGAACTCATGGTGTAGGGGTGAAATCCGTTGATATCATGGGGAAT
ACCAAAAGCGAAGGCAGCACACTGGTCTCATACTGACATTGAAGCACGAAAGCGTGGGTA
GCGAATGGGATTAGATACCCTGGTAGTCC
>Otu1825
CCAGCCTATGGGGGGCTGCAGGCGCGAAAACTTCGCAATGTGGGAAACCACGACGAGGGA
ATCCCAAGTGCCAACGATTTTTCGTTGGCTGTTGCCCTGTCTAAAAAACAGGGAGAGTAA
GGGCTGGGTAAGACGGGTGCCAGCCGCCGCGGTAATACCCGCGGCCCAAGTGGTGCTCGT
TATTATTGGGTTTAAAACGTCCGTAGCCGGTTCGGTAAATTCTTGGGTAAATCGACTGGC
CTAACCAGTCGAATTCCGGGGAGACTGCCGGACTCGGGACCGGGGGAGGTCGGAGGTACT
TCCAGGGTAGGGGTGAAATCCTGTAATCCTGGGGGGACCACCAGTGGCGAAGGCGTCCGA
CCAGAACGGCTCCGACGGTAAGGGACGAAGCCCTGGGGCGCAAACGGGATTAGAAACCCT
TGTAGTCC
>Otu1826
CCAGCCTACGGGTCGCACCAGTCGAGAATAATTCACAATGGGGGCAACCCTGATGGTGCA
ACGCCGCGTGGAGGATGACAGTCTTCGGATTGTAAACTCCTGTCATCCGGGAGTAAGACC
TGGCGGTTAACAGCCGACAGGGTTGATAGTACCGGAAGAGGAAGGGACGGCTAACTTCGT
GCCAGCAGCCGCGGTAATACGAAGGTCCCGAGCGTTGTTCGGATTCACTGGGCGTAAAGG
GAGCGTAGGCTGCGCGGTAAGTCAGATGTGAAATCCCGGGGCTCAACCCCGGAACCGCAT
CCGATACTGCCGCGCTGGAGGATTGGAGAGGTAGCTGGAATTCTTGGTGTAGCAGTGAAA
TGCGTGGATATCAAGAGGAACACTCGTGGCGAAAGCGAGCTACTGGACAACTCCTGACGC
TGAGGCTCGAAGGCCAGGGTAGCGAAAGGGATTAGAGACCCTCGTAGTCC
>Otu1828
CCAGCCTATGGGACGCTCCAGTCGAGAATTTTTCACAATGGGGGAAACCCTGATGGAGCG
ACGCCGCGTGGGGGATGAATGGCTTCGGCCCGTAAACCCCTGACATTCGTGAACAAACGT
TTCTACTGAACAAGTGGAAATTTGATAGTAGCGGAAGAGGAAGGGACGGCTAACTCTGTG
CCAGCAGCCGCGGTAATACAGAGGTCCCAAGCGTTGTTCGGATTCACTGGGCGTAAAGGG
TGCGTAGGTGGTCGGGTAAGTCTGATGTGAAAGCTCGGAGCTTAACTCCGAAATGGCATT
GGATACTATCTGGCTGGAGGATCGGAGGGGGGACTGGAATTCTCGGTGTAGCAGTGAAAT
GCGTAGATATCGAGAGGAACACCAGTGGCGAAGGCGAGTCCCTGGACGATTCCTGACACT
GAGGCACGAAAGCTAGGGGAGCAAACAGGATTAGAAACCCGAGTAGTCC
>Otu1829
CCAGCCTATGGGACGCAGCAGTAGGGAATATTGCTCAATGGGCGCAAGCCTGAAGCAGCA
ATGCCGCGTGAAGGATGAAGGTCTTCGGATCGTAAACTTCTTTTGTTTGGGATGAAGAAA
TTTGGGTTAATAGCCCAGAGATTTGACAGTACCAAAAGAATAACCACCGGCTAACTCCGT
GCCAGCAGCTGCGGTAATACGGAGGGTGGAAGCGTTGATCGGATTGACTGGGCGTAAAGG
GAGCGTAGGCGGCTCGATAAGTCGATGGTGAAATTTCTGGGCTTAACCCAGAAGCTGCCG
TCGAAACTATCGAACTAGAGGACATTAAGGGAAAGCGGAATACCACATGTAGCGGTGAAA
TGCGTAGATATGTGGTAGAACACCGGTGGTGAAGACGGCTTTCTGTGATGTTACTGACGC
TGAGGCTCGAAAGCATGGGGATCAAACAGGATTAGAAACCCGGGTAGTCC
>Otu1831
CCAGCCTACGGGTCGCTGCAGTTGGGAATCTTGGACAATGGGGGAAACCCTGATCCAGCC
ATGCCGCGTGAGTGATGAAGGCCTTCGGGTTGTAAAGCTCTTTTACCCGGGAAGATAATG
ACTGTACCGGGAGAATAAGCTCCGGCTAACTTCGTGCCAGCAGCCGCGGTAATACGAAGG
GGGCTAGCGTTGTTCGGAATTACTGGGCGTAAAGCGTGCGCAGGTGGTTTCCCAAGTCAG

TGGTGAAAGCCCGGAGCTCAACTCCGGAATTGCCATTGAAACTGTGAGACTTGAGGACGA
GAGAGGTGAGTGGAATTCCCAGTGTAGAGGTGAAATTCGTAGATATTGGGAAGAACACCG
GTGGCGAAGGCGGCTCACTGGCTCATTACTGACGCTCAGGCGCGACAGCGTGGGGATCAA
ACAGGATTAGATACCCCTGTAGTCC
>Otu1833
CCAGCCTATGGGGTGCACCAGTCGAGGATTTTTCTCAATGGGGGAAACCCTGAAGGAGCG
ACGCCGCGTGAGGGATGAAGGTCTTCGGATTGTAAACCTCTGTCATGCGGGATCAATGCA
TCCGACCTAATACGTCGGGTGTTGATAGTACTGCAAGAGGAAGACGTGGCTGACTCTGTG
CCAGCAGCCGCGGTAATACAGAGACGTCGAGCGTTGTTCGGATTCATTGGGCGTAAAGGG
TCCGCAGGCGGTCGGGTAAGTCGGATGTGAAATTTCGAGGCTTAACCTCGAACAGTCATT
CGAAACTGCTCGGCTTGAGGAGTGGAGAGGAGACTGGAATTGTCGGTGTAGCGGTGAAAT
GCGTAGAGATCGACAAGAACACCGGTGGCGAAGGCGGGTCTCTGGACACTTCCTGACGCT
CAGGGACGAAAGCCAGGGGAGCAAACGGGATTAGAGACCCCCGTAGTCC
>Otu1836
CCAGCCTATGGGATGCAGCAGTGGGGAATATTGGACAATGGGCGCAAGCCTGATCCAGCA
ATGCCGCGTGTGTGAAGAAGGCCTGCGGGTTGTAAAGCACTTTCAGTGGGGAGGAAGGCC
TCGATGTTAATAGTATCGAGGATTGACGTTACCTACAGAAGAAGCACCGGCTAACTCTGT
GCCAGCAGCCGCGGTAATACAGAGGGTGCAAGCGTTAATCGGAATTACTGGGCGTAAAGG
GCGCGTAGGTGGTTATTTAAGTCAGATGTGAAATCCCTGGGCTTAACCTAGGAATTGCAT
TTGAAACTAGATGGCTAGAGTATGGTAGAGGGAAGTGGAATTTCCGGTGTAGCGGTGAAA
TGCGTAGATATCGGAAGGAACACCAGTGGCGAAGGCGACTTCCTGGACCATTACTGACAC
TGAGGCGCGAAAGCGTGGGGAGCGAACAGGATTAGAAACCCCAGTAGTCC
>Otu1838
CCAGCCTATGGGGTGCTGCAGTGGGGAATCTTGCACAATGGGGGCAACCCTGATGCAGCG
ACGCCGCGTGAGCGATGAAGGCCTTAGGGTTGTAAAGCTCTTTCATCAGGGACGATAATG
ACGGTACCTGAAGAAGAAGCCCCGGCTAACTTCGTGCCAGCAGCCGCGGTAATACGAAGG
GGGCTAGCGTTGTTCGGAATCACTGGGCGTAAAGCGCACGTAGGCGGATCTTTAAGTCAG
GGGTGAAATGCCAGGGCTCAACCTTGGAACTGCCTTTGATACTGGAGATCTCGAGTCCGG
GAGAGGTGAGTGGAACTGCGAGTGTAGAGGTGAAATTCGTAGATATTCGCAAGAACACCA
GTGGCGAAGGCGGCTCACTGGCCCGGTACTGACGCTGAGGTGCGAAAGCGTGGGGAGCAA
ACAGGATTAGAGACCCGAGTAGTCC
>Otu1839
CCAGCCTATGGGGGGCTGCAGTCGAGAATTTTTCACAATGGGGGAAACCCTGATGGAGCG
ACGCCGCGTGGAGGATGAAGGCCCTCGGGTCGTAAACTCCTGTCATGCGGGAACAAGAAA
GTGATAGTACCGCAAGAGGAAGAGACGGCTAACTCTGTGCCAGCAGCCGCGGTAATACAG
AGGTCTCAAGCGTTGTTCGGATTCATTGGGCGTAAAGGGTGCGTAGGTGGCGGTGTAAGT
CTAACGTGAAATCTCGGGGCTCAACCTCGAAATTGCGTCGGATACTGCATTGCTAGAGGA
TTGTAGAGGAGAGTGGAATTCATGGTGTAGCAGTGAAATGCGTAGATATCATGAGGAAGA
CCAGTTGCGAAGGCGACTCTCTGGGCAACTCTTGACACTGAGGCACGAAGGCTAGGGGAG
CAAACGGGATTAGAAACCCCAGTAGTCC
>Otu1840
CCAGCCTATGGGTGGCAGCAGTTAGGGAATTTTGACAATGGGCGCAAGCCTGATCGAGTG
ACGCCGCGTGTGGGACGACGCCTTTCGGGGTGTAAACCACTTTTCTCGGGGACGAGAACG
GACGGTACCCGAGGAATAAGCCTCGGCTAACTACGTGCCAGCAGCCGCGGTAATACGTAG
GAGGCGAGCGTTGTCCGGAATTACTGGGCGTAAAGCGCGCGTAGGCGGTTTCTTGCGTAG
GCGGTGAAAGCCCCCGGCTCACCTGGGGGAAGGCCGCCTAAACGGGGAGACTTTGAGGGC
GGTAGGGGCTGGCGGAACACCCGGAGTAGCGGTGAAATGCGTTGAGATCGGGTAGAACAC
CAGTGGCGAAGGCGGCCAGCTGGGCCGTCCCTGACGCTGAGGCGCGAAAGCCAGGGGAGC
GAACGGAATTAGATACCCCTGTAGTCC
>Otu1844
CCAGCCTATGGGGTGCACCAGTCGAGAATTTTTCACAATGGGGGAAACCCTGATGGAGCG
ACGCCGCGTGGAGGATGAAGGTTTTCGGATTGTAAACTCCTGTCACCACAGAACAAGCAA
TGCAGGCTAATAATCTGCAAAGTTGATGGTATGTGGGGAGGAAGGGACGGCTAACTCTGT
GCCAGCAGCCGCGGTAATACAGAGGTCCCAAGCGTTGTTCGGATTCACTGGGCGTAAAGG
GTGTGTAGGAGGTCATTTAAGTCTGATGTGAAATCCCGTCGCTCAACGACGGAACTGCAT
TGGATACTATTTGGCTAGAGGATCTGAGGGGGAAGCGGAATTCTTGGTGTAGCGGTGAAA
TGCGTAGATATCAAGAGGAACACCCGTGGCGAAAGCGGCTTCCTGGAAGATTCCTGACTC
TGAAACACGAAAGGCAGGGGAGCGAATGGGATTAGAAACCCGGGTAGTCC

>Otu1847
CCAGCCTATGGGGGGCACCAGTCGAGAATTTTTCTCAATGGGCGAAAGCCTGAAGGAGCG
ACGCCGCGTGGAGGATGAATGGCTTCGGCCCGTAAACTCCTGTCATTTGCGAACAAACCT
TACGATTTAACAGATTGTGAGCTGATTGTAACGGAAGAGGAAGGGACGGCTAACTCTGTG
CCAGCAGCCGCGGTAATACAGAGGTCCCAAGCGTTGTTCGGATTCACTGGGCGTAAAGGG
TGCGTAGGCGGTTTGACAAGTCTGATGTGAAATCCCGCAGCTTAACTGCGGAACTGCATT
GGAAACTGTCTGACTAGAGGAATGGAGGGGAGACTGGAATGCTTGGTGTAGCAGTGAAAT
GCGTAGATATCAAGCGGAACACCAGTGGCGAAGGCGAGTCTCTGGACATTTCCTGGCGCT
GAGGCACGAAGGCCAGGGGAGCAAACGGGATTAGAAACCCGGGTAGTCC
>Otu1848
CCAGCCTATGGGTGGCACCAGGCGCGAAACCTTTACAATGCACGAAAGTGTGATAGGGGA
ACTCAGAGTGCTTATGCTTTTTGCATAAGCTTTTGCCAACTCAAAATTGGTTGGAGAATA
AGTGGTGGGCAAGACTGGTGCCAGCCGCCGCGGTAACCCCAGCGCCACAAGTGGGAATCA
GTATTATTGGGCCTAAAGCGTCCGTAGCAGGTTTGTTAAATTATTTGTGAAATCGCATCG
CTCAACGTTGCGGATGGCAGATAAGACTGGCAAACTAGAGACTGGGAGACGTTGGAGGTA
TTCAAAGGGGAGCGGTAAAATGCGAGAATCCTTTGAAGACCACCTGTTGCGAAGGCGTCC
AACAATAACAGGTCTGACTGTGAGGGTCGAAAGCTAGGGGAGCGAACCGGATTAGAGACC
CTCGTAGTCC
>Otu1849
CCAGCCTATGGGGCGCTCCAGCAAAAGGACAAGTCGGTCGGGGCTCCATCACGCTATCAG
CGATATGCGGGTACGGTCGCGAAAATAGTGCCAAAAGCCGGAACGCTCACTGCGGTGGAT
GTTACTAGCGCTGCCGATACGATTGATGACGCTACCGAGCAGTATCTGAGGGACGAGAAT
TTGCGGCAATGTCCTCTCTGTGAGGGCACAGGCGAGCACAACCGATATCGCTGTCCTATC
TGCGAAGGCGACGGAAGCGTTCCAAGGGAAGCTGAATTCGACCTTCGGGCATTCAAACAG
GTGGAATGCCCTCTCTGCAAGGGGACGGGGACGCATAATCGATTTGAATGCCCGATTTGC
TCGGGTATTGGAACGATCGATGAAGCTGCATTAGAGACCCCAGTAGTCC
>Otu1851
CCAGCCATGGGTCGCTGCAGGTAGGAATCTTTTGCAATGCACGAAAGTGTGACAAAGCGA
ATCAGAGTGCTTTTCAATGAAAAGCTTTTGTGAAATGTAAAAAGTTTCACGAATAAGGAC
TGGGTAAGACGGGTGCCAGCCGCCGCGGTAATACCCGCGGTCCAAGTCGCAGTCATTTTT
ATTGGGTCTAAAACATCCGTAGCTTGCTTGGTAAGTTTCCTGTGAAATTCTGTCTCTCAA
GGGCAGAGCGCGCAGGGAGTACTGCCTTGCTGGAGACTGGAAGACGTAAGAAGTATGTTC
AAAGTAGCGGTAAAATGCGTTAATCTTGGACAGACTAACAACAGCGAAGGCATCTTACGA
GGACAGTTCTGACAGTAAGGGATGAAGGCTAGGGGCGCGAAACGGATTAGATACCCCAGT
AGTCC
>Otu1853
CCAGCCTATGGGGTGCACCAGTCGAGAATCATTCGCAATGGGCGAAAGCCTGACGATGCG
ACGCCGTGTGAGTGAAGAAGGCCTTCGGGTCGTAAAGCTCTTTCGCCTGGGAACAAGAGA
AGCTGGCTAACATCCAGCGGATTTGAGGGTACCAGGATAAGAAGCACCGGCTAACTCCGT
GCCAGCAGCTGCGGTAATACGGAGGGTGCAAGCATTAATCGGATTTATTGGGCGTAAAGG
GCGCGTAGGCGGGAGACTAAGTCAGATGTGAAATCCCGGGGCTCAACCCCGGAACGGCAT
TTGAAACTATTTTTCTTGAGGGTAGGCGGAGAAAACGGAATTCCACGAGTAGCGGTGAAA
TGCGTAGATATGTGGAAGAACACCGGTGGCGAAGGCGGTTTTCTAGCTTATTCCTGACGC
TGAAGCGCGAAAGCAAGGGGATCAAACAGGATTAGAAACCCTGGTAGTCC
>Otu1860
CCAGCCTACGGGAGGCAGCAGTAAGGAATATTGGTCAATGGACGCAAGTCTGAACCAGCC
ATGCCGCGTGGAGGATGAAGGTCCTCTGGATTGTAAACTTCTTTTCTCTGGGGCGAAAAA
AGGATATTCTTATCCTCCTGACAGTACCAGAGGAATAAGCACCGGCTAACTCCGTGCCAG
CAGCCGCGGTAATACGGAGGGTGCAAGCGTTATCCGGATTCACTGGGTTTAAAGGGTGCG
TAGGAGGGCAGGTAAGTCAGTGGTGAAATCCCCGAGCTTAACTTGGGAACTGCCGTTGAT
ACTATCTGTCTTGAATATTGTGGAGGCAAGCGGAATATGTCATGTAGCGGTGAAATGCTT
AGATATGACATAGAACACCAATTGCGAAGGCAGCTTGCTACACAACTATTGACTCTGATG
CACGAAAGCGTGGGGATCATACAGGATTAGATACCCCCGTAGTCC
>Otu1867
CCAGCCTACGGGTTGCTGCAGTAAGGAATATTGGTCAATGGGCGAAAGCCTGAACCAGCC
GTGCCGCGTGAAGGATGAAGGCCCTCTGGGTTGTAAACTTCTTTTATTTGGGACGAAAAA
AGGGAATTCTTTCCCGTCTGACGGTACCAGATGAATAAGCACCGGCTAACTCCGTGCCAG
CAGCCGCGGTAATACGGAGGGTGCAAGCGTTATCCGGATTCACTGGGTTTAAAGGGTGCG

TAGGCGGGCAGGTAAGTCAGTGGTGAAATCTTTGGGCTTAACCCGAAAACTGCCATTGAT
ACTATCTGTCTTGAATATTGTGGAGGTGAGCAGAATATGTCATGTAGCGGTGAAATGCTT
AGATATGACATAGAATACCCATTGCGAAGGCAGCTCACTACGCATATATTGACGCTGAGG
CACGAAAGCGTGGGGATCAAACAGGATTAGATACCCGCGTAGTCC
>Otu1871
CCAGCCTACGGGGGGCAGCAGTGGGGAATATTGGACAATGGGGGAAACCCTGATCCAGCG
ATGCCGCGTGAGTGATGAAGGCCTTCGGGTTGTAAAGCTCTTTTGTCAGGGACGATGATG
ACGGTACCTGACGAATAAGCTCCGGCTAACTTCGTGCCAGCAGCCGCGGTAATACGAAGG
GAGCTAGCGTTGTTCGGAATTACTGGGCGTAAAGGGCGTGTAGGCGGTACCATAAGTCAG
ATGTGAAAGCCCTAGGCTTAACCTAGGAGGTGCATTTGATACTATGGAACTAGAGATCGA
GAGAGGAAAGTGGAATTACGAGTGTAGAGGTGAAATTCGTAGATATTCGTAAGAACACCA
GTGGCGAAGGCGACTTTCTGGCTCGATACTGACGCTAAGGCGCGAAAGCGTGGGGAGCAA
ACAGGATTAGAAACCCCCGTAGTCC
>Otu1872
CCAGCCTACGGGAGGCACCAGTAGGGAATATTGCGCAATGGAGGAAACTCTGACGCAGCG
ACGCCGCGTGGGTGATGAAGGCTTTCGGGTCGTAAAGCCCTGTCGGAAGGGACGAAAAAC
GGTATGGCTAACATCCATACCGCTTGACGGTACCTTCAGAGGAAGCACCGGCTAACTACG
TGCCAGCAGCCGCGGTAATACGTAGGGTGCAAGCGTTGTTCGGAATCATTGGGCGTAAAG
CGCGTGTAGGCTGTTAATTAAGTCGGGTGTGAAATCCCTGGGCTCAGCCGAGGAAGTGCA
TCCGAAACTAATTAGCTAGAGAATGGTAGAGGAAGGTGGAATTCCTAGTGTAGAGGTGAA
ATTCGTAGATATTAGGAGGAATACCGGTGGCGAAGGCGGCCTTCTGGACCATTTCTGACG
CTGAGACGCGAAAGCGTGGGGAGCAAACAGGATTAGAAACCCTAGTAGTCC
>Otu1877
CCAGCCTATGGGGGGCAGCAGTCGAGAATTTTCCGCAATGGGCGAAAGCCTGACGGAGCG
ACGCCGCGTGTGGGAAGAAGGCCTTCGGGTCGTAAACCACTGTCGAAGGGGACGATAATG
ACGGTACCCTTGGAGGAAGCCCCGGCTAACTACGTGCCAGCAGCCGCGGTAAGACGTAGG
GGGCGAGCGTTATTCGGAATTATTGGGCGTAAAGCGCTCGTAGGCGGGATTGCAAGTCCG
TGTTGAAAGACCTCGGCTCAACTGAGGGACCGGCTCGGATACTGTGATTCTTGAGGCAAT
CAGAGGGTGATGGAATTCCCGGTGTAGCGGTGAAATGCGTAGATATCGGGAGGAACACCA
GTGGCGAAGGCGATCACCTGGGGTTGTTCTGACGCTGAGGAGCGAAAGCTAGGGGAGCAA
ACGGGATTAGAAACCCCCGTAGTCC
>Otu1880
TCAGCCTATGGGAGGCTGCAGTTAGGGAACTTTTCCAATGCGCGCAAGCGTGAGAAAGTG
AGCCGGAGTGATACAGATATAATTTCTGTATCTTTTGTTCATTGCAAAAAGATGGACGAA
TAAGGAGTGGGCAAGATCGGTGCCAGCCGCCGCGGTAATCCCGACGCTCCAAGTCGCAGC
CACAATTATCGGGTCTAAAACATTCGTAGCTCGTTTGTTAAGTTCTTTGTGAAATCTGAA
ATCTTAAGTTTCAGGCGTGCAAAGAATACTGGCAGACTTGAGACCGGAAGAAGCAAGAAG
TATGATCAAGGTAGTGGTAAAATATGTTAATCTCGAGCAGACTAACAACAGCGAAGGCAT
CTTGCTAGTACGGATCTGACAGTGAGGAATGAAGGCTAGGGGCGCAAAGTGGATTAGATA
CCCGCGTAGTCC
>Otu1881
CCAGCCTATGGGATGCACCAGTCGAGAATTTTTCTCAGTGGGCGAAAGCCTGACTATGCG
ACGCCGCGTGAGGGATGAAGGATTTCGGTTCGTAAACCTCTTTTGGCAGGGAAGAAAATG
TTGCAGTTAATAGCTGCAAAACTGACGGTACCTGCAGAATAAGCCACGGCTAACTCCGTG
CCAGCAGCCGCGGTAATACGGAGGTGGCTAGCGTTACTCGGAATTATTGGGTGTAAAGGG
CAAGTAGGCGGCTTGGTAAGTGGGGGGTGAAATACTCCGGCTCAATCGGAGAACTGCCTT
CCAAACTACCAGGCTTGAGCGTGACAGAGGAAAATGGAATTCTCGGTGTAAGGGTGAAAT
CTGTAGATATCGAGAAGAACACCAGCGGCGAAGGCGATTTTCTGGGTCACTACTGACGCT
AAATTGCGAAAGCTAGGGGAGCAAACAGGATTAGAAACCCCAGTAGTCC
>Otu1885
CAGCCTATGGGTTGCAGCAGTGGGGAATCTTGCACAATGGGGGAAACCCTGATGCAGCGA
CGCCGCGTGAGCGATGAAGCCCTTCGGGGTGTAAAGCTCTTTCGACGGGAACGATAATGA
CGGTACCCGAAGAAGAAGCTGCGGCTAACTACGTGCCAGCAGCCGCGGTAATACGTAGGC
AGCAAGCGTTGTTCGGAATTACTGGGCGTAAAGAGTGCGTAGGCGGTGTTCTAAGTCTGT
TGTGAAATCTCCTGGCTCAACTGGGAGGGTGCGCCGAATACTGAGAGGCTAGAGTGTGGG
AGAGGAAAGTGGAATTCCTGGTGTAGCGGTGAAATGCGTAGATATCAGGAGGAACACCGG
TGGTGTAAACGGCTTTCTGGACCATAACTGACGCTGAGGCACGAAAGCGTGGGTAGCAAA
CAGGATTAGAAACCCTTGTAGTCC

>Otu1887
CCAGCCTACGGGTGGCTGCAGTCGAGAATATTCGACAATGGGCGAAAGCCTGATCGAGCG
ACGCCGCGTGCAGGATGAAGGCCTTCGGGTCGTAAACTGCGGTAGCTAGGTAACAATTTA
TGAGTGCTTAGCGGAAAGAGGTGGGTAACTACGTGCCAGCACCAGCGGTAATACGTAGAC
CTCAAGCGTTATCCGGATTTATTGGGCGTAAAGCGCGTGTAGGTGGTTTTGTGCGTCCCT
GGTTAAAGCCCACCGCCTAACGGTGGAAGTGCCAGAGATACGACAAGACTAGAGGAGGTT
AGAGGCAAGCAGAACGCACGGTGTAGGGGTGAAATCCGTTGATATCGTGCGGAATACCAA
AGGCGAAGGCAGCTTGCTGGGACTTTCCTGACACTGAGACGCGAAAGCGTGGGGAGCAAA
AAAGATTAGATACCCCCGGTAGTCC
>Otu1889
CCAGCCTACGGGTGGCAGCAGTAGGGAATATTGCTTAATGTGCGAAAGCGCGAAGCAGCA
ACGCCGCGTGTGTGATGAAGGCCTTCGGGTCGTAAAACACTTTTTTGAAGGATGAGGAAG
GACAGTACTTCAAGAATAAGCCTCGGCTAACTACGTGCCAGCAGCCGCGGTAACACGTAG
GAGGCGAGCGTTATCCGGATTTACTGGGCGTAAAGCGCGTGTAGGCGGTTCGGTAAGTTG
GATGTGAAAGCTCCCGGCTTAACTGGGAGAGGTCGTTCAATACTACCGGACTTGAGAGTG
GTAGAGGAAGGTGGAATTCCGCGTGTAGTGGTGAAATGCGTAGATATGCGGAGGAACACC
AGTGGCGAAAGCGGCCTTCTGGCCCACTTCTGACGCTCAGACGCGAAAGCTAGGGGAGCA
AACGGGATTAGAAACCCCTGTAGTCC
>Otu1890
CCAGCCTATGGGTGGCTGCAGTGGGGAATCTTGCGCAATGGACGAAAGTCTGACGCAGCC
ACGCCGCGTGAGTGAAGACGGCCTTCGGGTTGTAAAGCTCTGTCGAGCGGGACGGAAAAG
CGGAGGGTTAACAGCCCTTCGAATTGACGGTACCGCTAAAGGAAGCACCGGCTAACTCCG
TGCCAGCAGCCGCGGTAATACGGAGGGTGCAAGCGTTGCTCGGAATTATTGGGCGTAAAG
GGTAGGTAGGTGGTCTCATTTGTCAGGGGTGAAAACCTTGGGCTTAACCCAAGAAGTGCC
CCTGAAACGGTGGGACTGGAGTCCTGGAGAGGGTCGTGGAATTCCCGGTGTAGCGGTGAA
ATGCGTAGAGATCGGGAGGAACACCAGCGGCGAAGGCGGCGACCTGGACAGGTACTGACA
CTCAACTACGAAAGCGTGGGGAGCAAACAGGATTAGAGACCCGCGTAGTCC
>Otu1891
CCAGCCTACGGGTGGCACCAGTGGGGAATATTGGGCAATGGGCGAAAGCCTGACCCAGCG
ACGCCGCGTGGGTGATGAAGGCCTTCGGGTTGTAAAGCCCTTTCGTGTGGAAAGAACAGT
CCTGCGGCTAATACTCGCAGGATTTGACGGTACCACAGGAAGAAGCACCGGCTAACTCCG
TGCCAGCAGCCGCGGTAATACGGAGGGTGCGAGCGTTGTTCGGAATTACTGGGCGTAAAG
GGCGCGTAGGCGGTTGTGATAGTCAGGTGTGAAATCCTTCGGCTTAACCGGAGAACTGCA
TCTGAAACTTCACAACTAGAGTACAGGAGAGGGAAACGGAATTCCCGGTGTAGAGGTGAA
ATTCGTAGATATCGGGAGGAACACCAGTGGCGAAGGCGGTTTCCTGGCCTGATACTGACG
CTGAGGCGCGAAAGCGTGGGGAGCAAACAGGATTAGAAACCCTCGTAGTCC
>Otu1893
CCAGCCTATGGGGCGCTGCAGAGGAGAATCATTCGCAATGGGCGAAAGCCTGACGGTGCG
ACGCCGCGTGAGGGATGAAGGCCTTCGGGTTGTAAACCTCTGTCAGGAGTTATGAACGGC
GAGGGCGCCAATATCGCCTTCGCGTGACAAAGGCTCCAAAGGAAGCTCCGGCTAACTACG
TGCCAGCAGCCGCGGTAATACGTAGGGAGCGAGCGTTACTCGGAATCACTGGGCATAAAG
GGCGTGTAGGCGGGTGCGCAAGTCCGTCGTGAAATCCCTCGGCTCAACTGGGGAATTGCG
GCGGATACTGCGTGTCTTGAGTGTGGGAGGGGAGAGTGGAACTCTTGGTGGAGCGGTGAA
ATGCGTAGATATCAAGAGGAACGCCGGTGGCGAAAGCGGCTCTCTGGCCCACTTCTGACG
CTGAGGCGCGAAAGCTAGGGGAGCAAACAGGATTAGAAACCCGCTAGTCC
>Otu1895
CCAGCCTATGGGTGGCTGCAGTAGGGAATATTGGTTAATGGGCGAAAGCCTGACCCAGCA
ACGCCGCGTGAGTGATGAAGGCCTTCGGGTTGTAAAACTCTTTTTATGGGGATGAGGAAG
GACAGTACCCATAGAATAAGTCTCGGCTAACTACGTGCCAGCAGCCGCGGTAACACGTAG
GAGACGAGCGTTATCCGGATTTACTGGGTGTAAAGCGCGTGCAGGCGGTCCGATAAGTTG
GATGTGAAAGCTCCTGGCTTAACTGGGAGAGGTCGTTCAATACTGTTGGACTAGAGGATG
TCAGAGGGGAGTGGAATTCCGAGTGTAGCGGTAAAATGCATAGATATTCGGAAGAACACC
AGTGGCGAAGGCGGCTCTCTGGGGCATATCTGACGCTCAGACGCGAAAGCTAGGGTAGCA
AACGGGATTAGAAACCCCGGTAGTCC
>Otu1896
CCAGCCTATGGGATGCAGCAGTAACGAATCTTCCGCAATGCACGAAAGTGTGACGGAGCG
ACGCCGCGTGTGGGATGAAGTCCTTCGGGATGTAAACCACTGTCAGGGGAAAGAAAGTTC
TGATCTACCCCAGAGGAAGAGACGGCTAACTCTGTGCCAGCAGCCGCGGTAATACAGAGG

TCTCGAGCGTTAGGCGGAATCACTGGGCTTAAAGCGTGTGTAGGCGGCGTGCTAAGTACC
TTGTGAAATCCCACAGCTCAACTGTGGAACTGCTTGGTATACTGGTTCGCTTGAGCAACA
TAGGGGCTACCGGAACAAATGGTGGAGCGGTGAAATGCGTAGATATCATTTGGAACGCCA
ATGGTGAAAACAGGTAGCTGGGTGTTTGCTGACGCTGAGACACGAAAGCCAGGGGAGCAA
ACGGGATTAGATACCCGCGTAGTCC
>Otu1897
CCAGCCTATGGGTTGCACCAGGCGCGAAAATTCCGCAATGCGCGAAAGTGTGACGGGGGA
ATTCTGAGTGTTCCTCTTACGAGGAACTTTTGCCAAGATTGAATCTCTTGGAGAATAAGG
GGTGGGTAAATCCTGTGCCAGCCGCCGCGGTAATACAGGTAGCCCGAGTGGTATCCACGA
ATATTGAGCCTAAAGCGCTCGTAGCCGGTTTTGCAAGTCTTCTGTGAAATCCTCTGGCTC
AACCAGAGGGCGTGCAGGAGATACTACAAGGCTTGGGAGTGGGGGAGGTAACTGGTACGT
TAGGGGGAGGGGTAAAATCCTGTAATCCTTAACGGACCTTCAGTAGCGAAAGCGAGTTAC
CAAAACACGTCCGACGGTGAGGGGCGAAGGCTAGGGGAGCAAATCGGATTAGATACCCCA
GTAGTCC
>Otu1898
CCAGCCTATGGGAGGCAGCAGCAAGGAATTTTCGTCAATGGGCGCAAGCCTGAACGAGCA
ACGCCGCGTGCGGGATGACGGCCTTCGGGTTGTAAACCGCTTTTGCGGGGGACGATAATG
ACGGTACCCCGCGAACAAGCCCCGGCTAACTCTGTGCCAGCAGCCGCGGTAAGACAGAGG
GGGCAAGCGTTGTCCGGAATTACTGGGCGTACAGCGCACGCAGGTGGCCAAGCAAGTGTT
GTGTGAAAGCCCCGCGCTTAACGCGGGGAGGCCAGAGCAGACTGCTTGGCTCGAGGGGTG
CAGAGGGTCGTGGAATTGCCGGTGTAGTGGTGAAATGCGTAGAGATCGGCAGGAACACCA
AGGACGAAGGTAGCGACCTGGGCACTCACTGACACTCATGTGCGACAGCGTGGGGAGCAA
ACCGGATTAGAAACCCCGGTAGTCC
>Otu1903
CCAGCCTATGGGTGGCACCAGTCGAGAATTTTTCACAATGGTCGCAAGACTGATGGAGCG
ACGCCGCGTGGAGGATGAAGGTTTTCGGATTGTAAACTCCTGTCACTACAGAACAAGGGT
ATTTAGGTTAACAACCTAGGTATTTGATGGTATGTGGAGAGGAAGGGACGGCTAACTCTG
AGCCAGCAGCCGCGGTAATACAGAGGTCCCGAGCGTTGTTCGGATTCATTGGGCGTAAAG
GGTGTGTAGGAGGTCGGGTTAGTCAGGTGTGAAATCTCGGAGCTTAACTCTGAAACTGCG
CTTGATACTGCTCGGCTAGAGGATCGGAGGGGGTACTGGAATATATGGTGTAGCAGTGAA
ATGCGTAGATATCATATGGAACACCGGTGGCGTAGGCGGGTACCTGGAAGATTCCTGACT
CTGAAACACGAAAGCTAGGGGAGCAAACGGGATTAGAAACCCCCGTAGTCC
>Otu1907
CCAGCTTATGGGGCGCTGCAGTAAGGAATATTGGACAATGGGGGCAACCCTGATCCAGCC
ATGCCGCGTGAGTGATGAAGGCCTTCGGGTTGTAAAACTCTTTTAGATGTGAAGATGATG
ACGGTAGCATCAGAAAAAGCTCCGGCTAACTTCGTGCCAGCAGCCGCGGTAATACGAAGG
GAGCGAGCGTTGTTCGGAATCACTGGGCTTAAAGCGTGCGTAGGCGGATTTGTAAGTAAG
AAGTGAAAGCCCAGGGCTCAACCCTGGAACTGCTTTTTAGACTGCAAGTCTTGAATTCGG
TAGAGGTTAGTGGAATATCCAGTGTAGAGGTGAAATTCGCAGATATTGGATGGAACATCA
GTGGCGAAGGCGACTAACTGGACCGATATTGACGCTGAGGTACGAAAGCGTGGGTAGCAA
ACAGGATTAGATACCCCCGTAGTCC
>Otu1908
CCAGCCTATGGGTTGCACCAGTAACGAATCTTCCGCAATGCACGAAAGTGTGACGGAGCG
ACGCCGCGTGGAGGAGAAGTTCTTCGGAATGTAAACTCCTGTCATGAGAAAGAAAGTTCT
GATCTACTCAAGAGGAAGGGACGGCTAACTCTGTGCCAGCAGCCGCGGTAAGACAGAGGT
CCCGAGCGTTAGGCGGAATCACTGGGCTTAAAGCGTGTGTAGGCGGATCTGTAAGTACCT
TGTGAAATCCCACGGCTTAACCGTGGAACTGCTTGGTATACTGCGGATCTTGAGGTACTT
AGGGGCAACTGGAACAAATGGTGGAGCGGTGAAATGCGTAGATATCATTTGGGACGCCAA
TGGTGAAAACAAGTTGCTGGGAGTATTCTGACGCTGAGACACGAAAGCCAGGGGAGCAAA
CGGGATTAGAAACCCTCGTAGTCC
>Otu1909
CCAGCCTACGGGGGGCAGCAGTGGGGAATCTTGCGCAATGGGCGAAAGCCTGATGGAGCA
ACACCGCGTGCAGGATGACGGCCTTCGGGTTGTAAACTGCTTTTATATGGGAAGATTATG
ACGGTACCATATGAATAAGGACCGGCTAACTACGTGCCAGCAGCCGCGGTAATACGTAGG
GTCCAAGCGTTATCCGGAATTACTGGGCGTAAAGCGTGCGCAGGCGGCTCGTTAAGTGAG
ATGTGAAATCTGATGGCTCAACCGTTAGACTGTATCTCATACTGGCGAGCTAGAGGATGG
CAGAGGCAAGTGGAATTCGTAGTGTAGCAGTGAAATGCGTAGATATTACGAGGAACACCA
ATGGCGAAGGCAGCTTGCTGGGCTATTTCTGACGCTCAGGCACGAAAGCGTGGGGAGCGA

ACAGGATTAGATACCCTCGTAGTCC
>Otu1912
CCAGCCTATGGGACGCAGCAGTGGGGAATATTGGACAATGGGCGCAAGCCTGATCCAGCA
ATACCGCGTGTGTGAAGAAGGCCTTAGGGTTGTAAAGCACTTTCAATAAGGAGGAAAACC
TGCCGGTCAATACCCGGCAGCTTGACATTACCTTTAGAAGAAGCACCGGCTAACTCCGTG
CCAGCAGCCGCGGTAATACGGAGGGTGCAAGCGTTAATCGGAATTACTGGGCGTAAAGCG
TGCGTAGGCGGTTCGTTAAGTCAGATGTGAAAGCCCCGGGCTTAACCTGGGAACGGCATT
TGAAACTGGCGAACTCGAGTTGAGTAGAGGGGAGTGGAATTTCAGGTGTAGCGGTGAAAT
GCGTAGATATCTGAAGGAACACCAGTGGCGAAGGCGACTCCCTGGACTCAAACTGACGCT
GAGGTACGAAAGCGTGGGTAGCAAACAGGATTAGATACCCCCGTAGTCC
>Otu1916
CCAGCCTATGGGGTGCAGCAGTCGAGAATTTTTCACAATGGACGAAAGTCTGATGGAGCG
ACGCCGCGTGGAGGATGAAGGTTTTCGGATTGTAAACTCCTGTCACTGCAGAACAAGGTC
AGGCGATTGAACATTTCGTCTGGTTGATGGTATGCGGAGAGGAAGGGACGGCTAACTCTG
TGCCAGCAGCCGCGGTAAGACAGAGGTCCCGAGCGTTGTTCGGATTCACTGGGCGTAAAG
GGTGCGTAGGCGGTCGGGTAAGTCTGACGTGAAATCTCCGGGCCTAACCCGGAAACTGCG
TCGGATACTATCCGGCTAGAGGATTGGAAGGGAGACTGGAATACTTGGTGTAGCAGTGAA
ATGCGTAGATATCAAGTGGAACACCAGTGGCGAAGGCGAGTCTCTGGACAATTCCTGACG
CTGAGGCACGAAAGCCAGGGGAGCAAACGGGATTAGATACCCCTGTAGTCC
>Otu1918
CTAGCCTATGGGTGGCAGCAGTGGGGAATATTGGGCAATGGGCGAAAGCCTGACCCAGCG
ACGCCGTGTGGGTGATGAAGGCCTTCGGGTTGTAAAGCCCTGTCAGGTGGAACGAAGGGC
TCAGAGACGAATAATCTTTGAGCTTTGACGGTACCACCGGAGGAAGCACCGGCTAACTCC
GTGCCAGCAGCCGCGGTAATACGGAGGGTGCAAGCGTTGTTCGGAATTACTGGGCGTAAA
GGGCGCGTAGGTGGTTGTGTAAGTCAGATGTGAAATCCTGAGGCTCAACCTCAGAACTGC
ATCTGATACTGCATTGACTAGAGTGCAGCAGAGGGAAACGGAATTCCCGGTGTAGAGGTG
AAATTCGTAGATATCGGGAGGAACACCGGTGGCGAAGGCGGTTTCCTGGGCTGACACTGA
CACTGAGGCGCGAAAGCGTGGGTAGCAAACAGGATTAGAACCCCCGTAGTCC
>Otu1921
CCAGCCTACGGGAGGCAGCAGTGGGGAATATTGGACAATGGGCGCAAGCCTGATCCAGCC
ATGCCGCGTGAGTGAAGAAGGCCTTAGGGTTGTAAAGCTCTTTTGGCGGGGACGATAATG
ACGGTACCCGCAGAAGAAGCCCCGGCTAACTTCGTGCCAGCAGCCGCGGTAATACGAAGG
GGGCTAGCGTTGCTCGGAATGACTGGGCGTAAAGGGCGCGTAGGCGGATGATACAGTCAG
ATGTGAAATTCCTGGGCTTAACATGGGGGCTGCATTTGAGACGTGTTGTCTAGAGTGAGG
AAGAGGGTTGTGGAATTCCCAGTGTAGAGGTGAAATTCGTAGATATTGGGAAGAACACCG
GTGGCGAAGGCGGCAACCTGGTCCTTGACTGACGCTGAGGCGCGAAAGCGTGGGGAGCGA
ACAGGATTAGAAACCCTTGTAGTCC
>Otu1924
CCAGCCTATGGGTGGCAGCAGCTAAGAATCTTCCGCAATGGGCGAAAGCCTGACGGAGCG
ATGCCGCGTGATCGATGAAGGTCGAGAGATTGTAAAGATCTTTTACAGCTGAGGAATAAG
GGTGGGAGGGAATGCCCGCCTGATGACGTTAAGCTGTGAATAAGCCCCGGCTAATTACGT
GCCAGCAGCCGCGGTAACACGTAAGGGGCGAGCGTTGTTCGGAATTATTGGGCGTAAAGG
GCACGCAGGCGGTTATGCAAGTCTGATGTGAAATCTCCGGGCTTAACTCGGAATCCGCGT
TGGAAACTGCATGACTAGAGTATTAGAGGGGGAGTCGGAATTCCTAGTGTAGGGGTGAAA
TCTGTTGATATTAGGAAGAACACCGGTGGCGAAGGCGGACTCCTGGCTGAATACTGACGC
TGAGGTGCGAAAGCATAGGGAGCAAACAGGATTAGATACCCGTGTAGTCC
>Otu1925
CCAGCCTATGGGATGCACCAGTGGGGAATTTTACGCAATGGGCGAAAGCCTGACGTAGCG
ACACCGCGTGAGTGAAGAAGCCCTTTGGGGTGTAAAGCTCTGTCGGCTGGAACGAACACA
ATGACGGTACCAGCAAAGGAAGCATCGGCTAACTACGTGCCAGCAGCCGCGGTAAGACGT
AGGATGCAAGCGTTGCCCGGATTTATTGGGCGTAAAGAGTTCGTAGGCGGTTTGTTAAGT
CTGGTGTTAAAGACCGGGGCTCAACCTCGGAAATGCATTGGATACTGGCAGACTGGAGTG
TGGTAGAGGCGAGTGGAATTCCCAGTGTAGCGGTGAAATGCGTAGATATTGGGAAGAACA
CCAGTGGCGTAGGCGACTCGCTGGGCCATAACTGACGCTGAGGAACGAAAGCCAGGGGAG
CGAATGGGATTAGAAACCCGAGTAGTCC
>Otu1927
CCAGCCTACGGGTGGCAGCAGTCGAGAATTTTTCACAATGGGCGAAAGCCTGATGGAGCG
ACGCCGCGTGGGGGATGAATGGCTTCGGCCCGTAAACCCCTGTCATTCGGGATCAATGCG

TACTGCCTAATAAGCGGCGCGTTGATAGTACCGGAAGAGGAAGGGACGGCTAACTCTGTG
CCAGCAGCCGCGGTAATACAGAGGTCCCAAGCGTTGTTCGGATTCACTGGGCGTAAAGGG
TGCGTAGGTGGTCGGGTAAGTTTGATGTGAAATCTCCGGGCCTAACCCGGAAATGGCATT
GAATACTACTCGGCTGGAGGGTTGGAGGGGGGACTGGAATTCTCGGTGTAGCAGTGAAAT
GCGTAGATATCGAGAGGAACACCAGTGGCGAAGGCGAGTCCCTGGACAACTCCTGACACT
AAGGCACGAAAGCTAGGGGAGCAAACAGGATTAGATACCCCCGTAGTCC
>Otu1929
CCAGCCTACGGGGTGCACCAGCAACGAATCTTCCCCAATGGGCGAAAGCCTGAGGGAGCG
ACGCCGCGTGAAGGACGAAGTACTTCGGTATGTAGACTTCTGTTAGGGTTATGAAAGTAA
AAGGGGCTAATACCCTCTTAATTGATCTAGCCCAAAGAAAGGGACGGCTAACTCTGTGCC
AGCAGCCGCGGTAATACAGAGGTCCCAAGCGTTACTGAGAATCACTGGGTTTAAAGGGTG
CGTAGGTGGTCCGTTAAGTCAGTTGTGAAATCCCCGGGCTCAACCCGGGAACTGCTTCTG
ATACTGGCGGACTTGAGGCCAGTAGGGGTCACTAGAACTGACGGTGGAGCGGTGAAATGC
GTAGATATCGTCAGGAATGCCGGTGGTGAAGACGGGTGACTGGGCTGGTTCTGACACTGA
GGCACGAAAGCGTGGGGAGCGAACGGGATTAGAGACCCCAGTAGTCC
>Otu1931
CCAGCCTATGGGGTGCACCAGTGGGGAATATTGGACAATGGGCGAAAGCCTGATCCAGCA
ATGCCGCGTGAGTGATGAAGGCCTTAGGGTTGTAAAGCTCTTTTACCAGGGATGATAATG
ACAGTACCTGGAGAATAAGCTCCGGCTAACTCCGTGCCAGCAGCCGCGGTAATACGGAGG
GAGCTAGCGTTGTTCGGAATTACTGGGCGTAAAGCGCACGTAGGCGGTACATCAAGTCAG
GGGTGAAAGCCCGGAGCTCAACTCCGGAACTGCCCTTGAAACTAGTGTGCTAGAATCTTG
GAGAGGCGGGTGGAATTCCGAGTGTAGAGGTGAAATTCGTAGATATTCGGAAGAACACCA
GTGGCGAAGGCGGCCCGCTGGACAAGTATTGACGCTGAGGTGCGAAAGCGTGGGGAGCAA
ACAGGATTAGATACCCCTGTAGTC
>Otu1932
CCAGCCTACGGGGGGCAGCAGTCGAGAATACTCCACAATGGACGAAAGTCTGATGGAGCG
ACGCCGCGTGCGGGAAGAAGGCCTTCGGGTCGTAAACCGCTTTTATAAGGGAAGAATGTA
ATGACGGTACCTTATGAATAAGAGGTTACTAACTCTGTGCCAGCAGTAGCGGTAATACAG
AGACCTCAAGCGTTATCCGGATTTATTGGGCGTAAAGGGCGCGTAGGCGGATATATTAGT
CAGACGTCAAATCTCCGAGCTTAACTCGGAAACTGCGTTTGAAACGGTATATCTTGAGGA
AGTGGGAGACCAGTGGAACATATGGTGTAGCAGTGAAATGCGTTGATATCATATGGAACA
CCAAAGGCGAAGGCATCTGGTTGGCACTTTCCTGACGCTGAGGCGCGAAAGCGTGGGTAG
CGAATGGGATTAGAAACCCCAGTAGTCC
>Otu1935
CCAGCCTACGGGTGGCACCAGTCGAGGATCTTCAGCAATGGGCGCAAGCCTGACCGAGCG
ACGCCGCGTGTGCGAAGAAGGCCTTCGGGTTGTAAAGCACTGTCGAGGGGGAGAAAAGCC
GTAAGGTCTGATCTATCCCTGAAGGAAGCACGGGCTAAGTTCGTGCCAGCAGCCGCGGTA
AGACGAACCGTGCGAACGTTGTTCGGAATCACTGGGCTTAAAGGGCGCGTAGGCGGGTGA
TCAAGTCAGGGGTGAAATCTTTCGGCTTAACCGGAAAACAGCCTCTGATACTGGTCATCT
CGAGGGAGGTAGGGGCATGTGGAACTTCCGGTGGAGCGGTGAAATGCGTAGATATCGGAA
GGAACGCCGGTGGCGAAAGCGACGTGCTGGACCTTTTCTGACGCTGAGGCGCGAAAGCTA
GGGGAGCAAACGGGATTAGATACCCGAGTAGTCC
>Otu1937
CCAGCCTACGGGTGGCTGCAGCCGAGAATATTCGACAATGGGCGAAAGCCTGATCGAGCG
ATACCGCGTGGTGGATGAAGCGCTTCGGCGCGTAAACACCTTTTATGAGGGAGGAAGTTA
TTGACGTTACCTCATGAATAAGGGGCTCCTAGCTCTGTGCCAGCAGGAGCGGTAATACAG
AGGCCCCAAGCGTTACCCGGAATTACTGGGCGTAAAGAGTGCGTAGGTGGTCGTGTTAGT
CGTCTGTTAAACCCCGGAGCTCAACTCCGGATCTGCAGACGAAACGGCACGACTCGAGGG
CGTGAGAGGTGCATAGAACTCTAGGTGTAGGGGTGAAATCCGTTGATATCTAGGGGAATA
CCGAAAGCGAAGGCAGTGCACTGGCGCGTTCCTGACACTCAAGCACGAAAGCGTGGGTAG
CGAACGGGATTAGAAACCCTCGTAGTCC
>Otu1944
CCAGCCTACGGGATGCAGCAGTGGGGAATTTTGGACAATGGGGGCAACCCTGATCCAGCC
ATTCCGCGTGTCTGAAGAAGGCCTTCGGGTTGTGAAGGACTTTTGTCCGGGAGCAAAGCC
TGCTGGTTAATAACCGGCGGGGATGAGAGTACCGGAAGAATAAGCACCGGCTAACTACGT
GCCAGCAGCCGCGGTAATACGTAGGGTGCGAGCGTTAATCGGAATTACTGGGCGTAAAGC
GTGCGCAGGCGGTATTGCAAGTCTGATGTGAAAGCCCCGGGCTTAACCTGGGAACGGCAT
TGGAAACTGCAAAACTAGAGTACGTCAGAGGGGGGTAGAATTCCACGTGTAGCAGTGAAA

TGCGTAGAGATGTGGAGGAATACCGATGGCGAAGGCAGCCCCCTGGGATGATACTGACGC
TCATGCACGAAAGCGTGGGGAGCAAACAGGATTAGATACCCCCGTAGTCC
>Otu1945
CCAGCCTATGGGTCGCTGCAGTCGAGGATCTTCGGCAATGGGCGCAAGCCTGACCGAGCG
ACGCCGCGTGAGCGATGAAGGCCTTCGGGTTGTAAAGCTCTTTAGTGGGGGAGAAAAGCG
CAAGCTTGATCTATCCCAAGAATAAGCACGGGCTAAGTTCGTGCCAGCAGCCGCGGTAAG
ACGAACCGTGCGAACGTTGTTCGGAATCACTGGGCTTAATGGGCGCGTAGGCGGCTTGTC
AAGTCCGGGGTGAAAGCCTCCAGCTTAACTGGAGAAGTGCCTTGGATACTGGCGAGCTCG
AGGGAGGTAGGGGCAGATGGAACTTCCGGTGGAGCGGTGAAATGCGTTGATATCGGAAGG
AACGCCGGTGGCGAAAGCGATCTGCTGGACCTCTTCTGACGCTGAGGCGCGAAAGCTAGG
GGAGAGAACGGGATTAGAAACCCTTGTAGTCC
>Otu1948
CCAGCCTACGGGACGCTGCAGTAGGGAATTTTGGACAATGGGGGAAACCCTGATCCAGCC
ATGCCGCGTGAGTGAAGAAGGCCTTCGGGTTGTAAAGCTCTTTTGGTCGGGAAGAAAGGA
CTCCTTCTAATACAGGGGGTTTTTGACGGTACCGGCAGAATAAGCACCGGCTAACTACGT
GCCAGCAGCCGCGGTAATACGTAGGGTGCGAGCGTTAATCGGAATTACTGGGCGTAAAGA
GTGCGTAGGCGGTTCGTTATGTCTGCTGTGAAAGCCCTGGGCTTAACCCGGGAACTGCAG
TGGAAACTGGCGGGCTTGAGTATGGCAGAGGGGGGTGGAATTCCGCGTGTAGCAGTGAAA
TGCGTAGAGATGCGGAGGAACACCGATGGCGAAGGCAGCCCCCTGGGCTACTACTGACGC
TGAGGCACGAAAGCGTGGGGAGCAAACAGGATTAGAAACCCTCGTAGTCC
>Otu1949
CCAGCCTATGGGAGGCTGCAGTAGGGAATATTGCGCAATGGGCGAAAGCCTGACGCAGCA
ATTCCGCGTGGAGGATGAAGTTTCTTGGAATGTAAACTCCTTTCGGTGGTGACGATAATG
ACGGTAGCCACAGAAGAAGCACCGGCTAACTACGTGCCAGCAGCCGCGGTAAGACGTAGG
GTGCAAGCGTTGTCCGGATTTATTGGGCGTAAAGAGTTCGTAGGCGGTTTGCCAAGTCTG
TTGTTAAAGGCCTGAGCTCAACTCGGGTACGGCATCGGAAACTGGCAGGCTAGAGTGTGG
TAGAGGCAAGGGGAATTCCCGGTGTAGCGGTGAAATGCGTAGATGTCGGGAGGAACACCA
GTGGCGAAAGCGCCTTGCTGGCCCACTACTGACGCTGAGGAACGAAAGCCAGGGTAGCGA
AAGGGATTAGAGACCCCCGTAGTCC
>Otu1950
CCAGCCTATGGGTGGCAGCAGTCGAGAATTCTTCACAATGGGCGAAAGCCTGATGGAGCG
ACGCCGCGTGGGGGATGAATGGCTTCGGCCCGTAAACCCCTGTCATTTGCGAACAAACGC
CATTATTTAAAAGATGATGGCTTGATTGTAGCGAAAGAGGAAGGGACGGCTAACTCTGTG
CCAGCAGCCGCGGTAATACAGAGGTCCCAAGCGTTGTTCGGATTCACTGGGCGTAAAGGG
TGCGTAGGTGGTGGGGTAAGTCTGATGTGAAATCTCCGAGCTCAACTCGGAAATGGCATT
GGAAACTACCCCGCTCGAGGATTGGAGGGGGGACTGGAATTCTCGGTGTAGCAGTGAAAT
GCGTAGATATCGAGAGGAACACCAGTGGCGAAGGCGAGTCCCTGGACAATACCTGACACT
GAGGCACGAAAGCTAGGGGAGCAAACAGGATTAGAAACCCCCGTAGTCC
>Otu1952
CCAGCCTACGGGGGGCAGCAGTCGCGAATCATTCACAATGGGCGCAAGCCTGATGATGCC
ACGCCGCGTGCGGGATGAAGGCCCTCTGGGTTGTAAACCGCTGTCAAGGGAGGGGAAACG
TATGGTGTTAACAGCGCCATGCTTGACTGCTCCCTAAAGGAAGCCCCGGCAAAACATGTG
CCAGCAGCCGCGGTAATACATGTGGGGCAAGCGTTGTTCGGAATTACTGGGCATAAAGGG
TGCGTAGGTGGCTTGTTAAGTCACGGGTGAAAGTTTTCGGCCCAACCGGAAAATGGCCTG
CGATACTGGCGAGCTTGAGTGCGTGAGGGGTAACCGGAACGAGCAGTGTAGCGGTGAAAT
GCGTAGATATTGCTCGGAAGGCCAGAGGCGAAGGCGGGTTACTGGTACGCAACTGACACT
GAGGCACGAAAGCGTGGGGAGCAAACAGGATTAGATACCCGTGTAGTCC
>Otu1953
CCAGCCTACGGGAGGCACCAGTAGGGAATCTTGCGCAATGGGCGAAAGCCTGACGCAGCA
ACGCCGCGTGAGGGACGAAGGCTTTCTGAGTTGTAAACCTCTTTCAGCAGGGACGATTGT
GACGGTACCTGCAGAAGAAGCACCGGCCAACTACGTGCCAGCAGCCGCGGTGATACGTAG
GGTGCAAGCGTTGTCCGGATTTATTGGGCGTAAAGAGCTCGTAGGCGGTTCGATAAGTCG
GGTGTGAAATCTCCAGGCTCAACCTGGAGTCGCCACTTGATACTGTCGTGACTAGAGTCC
GGTAGGGGACCATGGAATTCCTGGTGTAGCGGTGAAATGCGCAGATATCAGGAGGAATAC
CAGTAGCGAAGGCGGTGGTCTGGGCCGGAACTGACGCTGAGGAGCGAAAGCGTGGGGAGC
GAACAGGATTAGAAACCCCAGTAGTCC
>Otu1954
CCAGCCTATGGGGCGCTGCAGTGAGGAATATTGGTCAATGGGCGGAAGCCTGAACCAGCC

ATGCCGCGTGCAGGAAGACGGCCCTACGGGTTGTAAACTGCTTTTCCGGGGGAAGAATAA
GGGCGATGCGTCGTCCGATGCCGGTACCCCGGGAATAAGGATCGGCTAACTCCGTGCCAG
CAGCCGCGGTAATACGGAGGATCCGAGCGTTATCCGGATTTACTGGGTTTAAAGGGTGCG
TAGGCGGGCGCTTAAGTCAGCGGTGAAATACGGGGGATCAACCTTCGAACGGCCGTTGAT
ACTGGGCGCCTTGGATTCGGTCGAGGCGGGCGGAATGCGACATGTAGCGGTGAAATGCTT
AGATATGTCGCAGAACGCCGATTGCGAAGGCAGCTCGCCAGGCCGACATTGACGCTGAGG
CACGAAAGCGTGGGGATCGAACAGGATTAGATACCCGCGTAGTCC
>Otu1956
CCAGCCTATGGGAGGCACCAGTAGGGAATATTGCACAATGGGCGCAAGCCTGATGCAGCA
ACGCCGCGTGCGCGATGAAGGCCTTCGGGTTGTAAAGCGCTTTTCGGGGAGATGAGGAAG
GACAGTATCCCCGGAATAAGTCTCGGCTAACTACGTGCCAGCAGCCGCGGTAAAACGTAG
GAGGCGAGCGTTATCCGAATTTACTGGGCGTAAAGCGTGTGCAGGCGGTTCGGTAAGTTG
GATGTGAAATCTCCTGGCTCAACTGGGAGAGGTCGTTCAAGACTGTCGGACTAGAGGGCG
GTAGAGGGAGGTGGAATTCCCGGTGTAGTGGTGAAATGCGTAGATATCGGGAGGAACACC
TGTGGCGAAAGCGGCCTCCTGGACCGTACCTGACGCTCAGACACGAAAGCTAGGGTAGCA
AACGGGATTAGAAACCCGCGTAGTCC
>Otu1960
CCAGCCTATGGGTGGCAGCAGTCGAGAATTTTCCACAATGGGGGCAACCCTGATGGAGCA
ACGCCGCGTGCAGGATGAATGCCTTCGGGTTGTAAACTGCTTTTATCTGTGACGATTTTG
ACGGTAGCAGATGAATAAGGATCGGCTAACTCCGTGCCAGCAGCCGCGGTCATACGGAGG
ATCCAAGCGTTATCCGGAATTACTGGGCGTAAAGAGTTGCGTAGGTGGCATTGTAAGCGA
GTAGTGAAAGCGTCCGGCTCAACCGGATATACATTACTCGAACTGCAAAGCTAGAGGATG
AGAGAGGTTATTGGAATTCCCAGTGTAGGAGTGAAATCCGTAGATATTGGGAGGAACACC
GATGGCGTAGGCAGATAACTGGCTCATTCCTGACACTAAGGCACGAAAGCGTGGGTAGCA
AACGGGATTAGAGACCCGGGTAGTCC
>Otu1962
CCAGCCTATGGGGCGCAGCAGCGGGGAATATTGCGCAATGGGGGCAACCCTGACGCAGCA
ACGCCGCGTGAGTGATGAAGGTCCTCGGATCGTAAAGCTCTGTCGGTAGGAAAGATGGTT
CGCATGGCTAACATCCATGTGAGCTGACGGTACCTACAAAGGAAGCACCGGCTAACTCTG
TGCCAGCAGCCGCGGTAATACAGAGGGTGCAAGCGTTGTTCGGAATTATTGGGCGTAAAG
GGCGCGTAGGCGGTTGTGTAAGTCGAATGTGAAAGCCCAGGGCTCAACCTTGGAAGTGCA
TCCGAAACTACACAACTTGAATCTCGGAGAGGGTGGTGGAATTCCCAGTGTAGAGGTGAA
ATTCGTAGATATTGGGAGGAACACCGGTGGCGAAGGCGACCGCCTGGACGAAGATTGACG
CTGAGGCGCGAGAGCGTGGGGAGCAAACAGGATTAGAAACCCTCGTAGTCC
>Otu1963
CCAGCCTATGGGGGGCTGCAGTTTCGAATCATTCACAATGGGCGCAAGCCTGATGGTGCG
ACGCCGCGTGAGCGATGAAGCCCTTCGGGGTGTAAAGCTCTTTCGGCAGGGACGATTATG
ACGGTACCTGCAGAAGAAGCTGCGGCTAACTACGTGCCAGCAGCCGCGGTAATACGTAGG
CAGCAAGCGTTGTTCGGAATTACTGGGCGTAAAGAGTGCGTAGGCGGTTGGGCAAGTCTG
TTGTGAAATCTCCCGGCTTAACTGGGAGGGTGCGATGGAAACTGCCCGGCTAGAGTGTGG
GAGAGGGAAGTGGAATTCCTGGTGTAGCGGTGAAATGCGTAGATATCAGGAGGAACACCT
GCGGTGTAGACGGCTTCCTGGACCACTACTGACGCTGAGGCACGAAAGCGTGGGGAGCAA
ACAGGATTAGATACCCTTGTAGTCC
>Otu1965
CCAGCCTATGGGAGGCACCAGTGGGGAATATTGGACAATGGGGGCAACCCTGATCCAGCC
ATGCCGCGTGCAGGAAGACGGCCCTACGGGTTGTAAACTGCTTTTGCGGGGGAATAAACC
CCCGTATGTATACGGGGTTGAATGTACCTTGAGAATAAGGATCGGCTAACTCCGTGCCAG
CAGCCGCGGTAATACGGAGGATCCGAGCGTTATCCGGATTTATTGGGTTTAAAGGGTGCG
TAGGCGGCCTGGTAAGTCAGGGGTGAAAGACGGTAGCTTAACTATCGCAGTGCCTTTGAT
ACTGCCGGGCTTGAATGTACTTGAAGTGGGCGGAATGTGACAAGTAGCGGTGAAATGCAT
AGATATGTCACAGAACACCGATTGCGAAGGCAGCTCACTAAAGTATGATTGACGCTGAGG
CACGAAAGCGTGGGGATCAAACAGGATTAGATACCCGAGTAGTCC
>Otu1968
CCAGCCTACGGGTCGCAGCAGTCGAGAATCTTCGGCAATGGGGGAAACCCTGACCGAGCG
ACGCCGCGTGCGAGATGAAGGCCTTCGGGTTGTAAATCGCTGTCAGAGGGGAGGAAATGC
CGGTGGGCTCTCCCACCGGTTTGACCTATCCTCAGAGGAAGGACGGGCTAAGTTCGTGCC
AGCAGCCGCGGTAAGACGGACCGTCCAAACGTTATTCGGAATCACTGGGCTTAAAGGGTG
CGTAGGCGGCCCGGGAAGTTGCGTGTGAAAGCCCTCGGCTCAACCGAGGAATTGCGCGCA

AAACTGCCGGGCTTGAGGGAGACATAGGTGAGCGGAACTTAGGGTGGAGCGGTGAAATGC
GTTGATATCCTAAGGAACACCAGTAGCGAAAGCGGCTCACTGGGTCTCTTCTGACGCTGA
AGCACGAAAGCTAGGGGAGCGAAGAGGATTAGAAACCCTGGTGTCC
>Otu1971
CCAGCCTATGGGGGGCTGCAGTGGGGAATATTGGGCAATGGGGGAAACCCTGATCCAGCC
ACGCCGCGTGAGTGATGAAGGCCTTCGGGTCGTAAAGCTCTGTGGGGAGGGACGAACCGC
TGATGGTTAATATCCAGCAGCATGACGGTACCTCCTTAGCAAGCACCGGCTAACTTCGTG
CCAGCAGCCGCGGTAATACGAAGGGTGCAAACGTTGCTCGGAATTATTGGGCGTAAAGCG
CACGTAGGCGGCTCTACAAGTCGGATGTGAAATCCCTCGGCTTAACCAAGGAAGTGCATC
CGAAACTGCAGAGCTTGAGTACCAAAGAGGATCGCGGAATTCCCGGTGTAGAGGTGAAGT
TCGTAGATATCGGGAGGAACACCAGTGGCGAAGGCGGCGATCTGGGTGGATACTGACGCT
GAGGTGCGAAAGCGTGGGGAGCAAACAGGATTAGATACCCGAGTAGTCC
>Otu1972
CCAGCCTATGGGACGCTGCAGCGGGGAATATTGGACAATGGGCGAAAGCCTGATCCAGCA
ATGCCGCGTGGATGAAGAAGGTCTTCGGATTGTAAAGTTCTTTTGACGGGGACGATGATG
ACGGTACCCGTAGAATAAGCTCCGGCTAACTTCGTGCCAGCAGCCGCGGTAATACGAAGG
GAGCTAGCGTTGTTCGGAATTACTGGGCGTAAAGGGCGCGTAGGCGGCTTGGCAAGTTGG
GTGTGAAAGCCCAGGGCTCAACCCTGGAACTGCACTCAAGACTGCCTTGCTTGAATTCGG
TAGAGGTTGGTGGAATTCCCAGTGTAGAGGTGAAATTCGTAGAGATTGGGAAGAACACCC
GTGGCGAAGGCGGCCAACTGGACCGACATTGACGCTGAGGCGCGAAAGCGTGGGGATCAA
ACAGGATTAGAAACCCTCGTAGTCC
>Otu1973
CCAGCCTACGGGTTGCAGCAGTAGGGAATATTGCGCAATGGAGGAAACTCTGACGCAGCG
ACGCCGCGTGGGTGATGAAGGCCTTCGGGTCGTAAAGCCCTGTCGAAAGGGAAGAATGAC
GAAGTAGAGAATATCTACTTCGAGTGACGGTACCTTTAAAGGAAGCACCGGCTAACTACG
TGCCAGCAGCCGCGGTAATACGTAGGGTGCAAGCGTTGTTCGGAATTATTGGGCGTAAAG
CGCGTGTAGGCTGTTAGCTAAGTCGATTGTGAAATCCCTGGGCTCAACCGAGGAAGTGCA
GTCGAAACTAGCTAGCTTGAAGACGGTAGAGGAAGGTAGAATTCCAAGTGTAGAGGTGAA
ATTCGTAGATATTTGGAGGAATACCGGTGGCGAAGGCGGCCTTCTGGGCCGTTCTTGACG
CTGAGACGCGAAAGCGTGGGTAGCAAACAGGATTAGATACCCCAGTAGTCC
>Otu1974
CCAGCCTATGGGGCGCTGCAGTGGGGAATTTTGGACAATGGGCGAAAGCCTGATCCAGCA
ATGCCGCGTGTGTGAAGAAGGCCTTCGGGTTGTAAAGCACTTTTGTTCGGGAAGAAAAGG
ACTGGGCTAATATCCTGGTCTGATGACGGTACCGGAAGAATAAGCACCGGCTAACTACGT
GCCAGCAGCCGCGGTAATACGTAGGGTGCAAGCGTTAATCGGAATTACTGGGCGTAAAGC
GTGCGCAGGCGGTTTGTTAAGACAGATGTGAAATCCCCGGGCTTAACCTGGGAACTGCAT
TTGTGACTGGCAAGCTAGAGTATGGCAGAGGGGGGTGGAATTCCGCGTGTAGCAGTGAAA
TGCGTAGATATGCGGAGGAACACCGATGGCGAAGGCAGCCCCCTGGGCCAATACTGACGC
TCATGCACGAAAGCGTGGGGAGCAAACAGGATTAGAGACCCCAGTAGTCA
>Otu1976
CCAGCCTACGGGGCGCTGCAGTCGAGAATTTTTCACAATGGGGGAAACCCTGATGGAGCG
ACGCCGCGTGGAGGATGAAGGCCCTTGGGTCGTAAACTCCTGTCATAAGAGAACAAGAAA
GTGATAGTATCTTAAGAGGAAGAGACGGCTAACTCTGTGCCAGCAGCCGCGGTAATACAG
AGGTCTCAAGCGTTGTTCGGATTCATTGGGCGTAAAGGGTGCGCAGGCTGTGTGGTAAGT
CGGGTGTGAAATTTAGAGGCTCAACCTCTAAACTGCATCTGATACTGCCATGCTAGAGGA
CTGTAGAGGAGATTGGAATTCACGGTGTAGCAGTGAAATGCGTAGATATCGTGAGGAAGA
CCAGTGGCGAAGGCGAATCTCTGGGCAGTTCCTGACGCTCATGCACGAAGGCTAGGGGAG
CAAACGGGATTAGATACCCGAGTAGTCC
>Otu1977
CCAGCCTATGGGTGGCTCCAGTTGGGAATCTTGGACAATGGGCGAAAGCCTGATCCAGCC
ATGCCGCGTGAGTGATGAAGGCCTTAGGGTTGTAAAGCTCTTTTACCCGGGAAGATAATG
ACGGTACCGGGAGAATAAGCTCCGGCTAACTTCGTGCCAGCAGCCGCGGTAATACGAAGG
GGGCTAGCGTTGTTCGGAATTACTGGGCGTAAAGCGTGCGCAGGCGGTTTCTCAAGTCAG
GGGTGAAAGCCCAGAGCTCAACTCTGGAAATGCCTTTGAAACTGTGAAGCTCGAGTGCGG
GAGAGGTGAGTGGAATTCCCAGTGTAGAGGTGAAATTCGTAGATATTGGGAAGAACACCG
GTGGCGAAGGCGGCTCACTGGCCCGTTTCTGACGCTCATGCACGATAGCGTGGGGATCAA
ACAGGATTAGAGACCCGCGTAGTCC
>Otu1978

CCAGCCTATGGGTCGCAGCAGTCGAGGATCTTCGGCAATGGGCGCAAGCCTGACCGAGCG
ACGCCGCGTGTGCGATGAAGGCCCTTGGGTTGTAAAGCACTGTCGAGGGGGAGGAAAGCC
GCAAGGTCTGACCTATCCCTGGAGGAAGCACGGGCTAAGTTCGTGCCAGCAGCCGCGGTA
AGACGAACCGTGCAAACGTTGTTCGGAATCACTGGGCTTAAAGGGCGCGTAGGCGGCTTG
CCAAGTCAGGGGTGAAATCTTTCGGCTTAACCGGAAAATTGCTTTTGATACTGGTGAGCT
TGAGGGAGGTAGGGGCAGGTGGAACTTCCGGTGGAGCGGTGAAATGCGTAGATATCGGAA
GGAACGCCGGTGGCGAAAGCGACCTGCTGGACCTCTACTGACGCTGAGGCGCGAAAGCCA
GGGGAGCAAACGGATTAGAGACCCTAGTAGTCC
>Otu1980
CCAGCCTATGGGGGGCAGCAGTCGAGAATCTTTCGCAATGGGCGAAAGCCTGACGAAGCG
ACGCCGTGTGAGCGAAGAAGGCCTTCGGGTTGTAAAGCTCTTTCGCTAGGGAACAAGAGA
ATCAGATTAATAATCTGGTAATTTGATGGTACTTGGTAAAGAAGCACCGGCTAACTCCGT
GCCAGCAGCTGCGGTAATACGGAGGGTGCAAGCATTGATCGGAATTACTGGGCGTAAAGG
GCGCGTAGGCGGCAAGAAAAGTCAGATGTGAAATTCCGGGGCTCAACCCCGGAGCTGCAT
TTGAAACTTTCTAGCTAGAGGTTGGTAAGGGAAAACGGAATTCCACGTGTAGCGGTGAAA
TGCGTAGATATGTGGAAGAACACCGGTGGTGAAGACGGTTTTCTGTGCCAAACCTGACGC
TGAGGCGCGAAAGCTAGGGGAGCAAACAGGATTAGAAACCCGGGTAGTCC
>Otu1984
CCAGCCTATGGGTTGCACCAGCAACGAATCTTCCGCAATGGGCGCAAGCCTGACGGAGCG
ACGCCGCGTGTGGGACGAAGTTCTTCGGAATGTAAACCACTGTTAGGGTTATGAAAGCAA
TGCGCGCTAATACCGCGCAAAGTTGATCTAGCCCAGAGAAAGGGACGGCTAACTCTGTGC
CAGCAGCCGCGGTAATACAGAGGTCCCAAGCGTTACTGAGATTCACTGGGTTTAAAGGGT
GCGTAGGTGGCGCGTTAAGTTCGTTGTGAAATCCCCGGGCTCAACCCGGGAATTGCTTCG
AATACTGGCGCGCTTGAGGTCAGCATGGGTTACTGGAACAGACGGTGGAGCGGTGAAATG
CGTAGATATCGTCTGGAACGCCAGTGGTGAAGCCGGGTAACTGGACTGATTCTGACACTG
AGGCACGAAAGCGTGGGGAGCGAACGGGATTAGATACCCCCGTAGTCC
>Otu1992
CCAGCCTATGGGTCGCAGCAGTGGGGAATATTGGACAATGGGGGAAACCCTGATCCAGCC
ATGCCGCGTGAGTGATGAAGGCCTTCGGGTTGTAAAGCTCTTTCGCACGCGACGATGATG
ACGGTAGCGTGAGAAGAAGCCCCGGCTAACTTCGTGCCAGCAGCCGCGGTAATACGAAGG
GGGCAAGCGTTGTTCGGAATTACTGGGCGTAAAGGGCGCGTAGGCGGTGCGTTAAGTCAG
ATGTGAAAGCCCTGGGCTCAACCCGGGAATTGCATTTGATACTGGCGTGCTCGAATCCAG
GAGAGGTTGGTGGAATTCCCAGTGTAGAGGTGAAATTCGTAGAGATTGGGAAGAACACCG
GTGGCGAAGGCGGCCAACTGGACTGGTATTGACGCCGAGGCGCGAAAGCGTGGGGAGCAA
ACAGGATTAGATACCCCAGTAGTCC
>Otu1995
CCAGCCTATGGGGGGCTGCAGTGGGGAATCTTCCGCAATGGACGAAAGTCTGACGGAGCA
ACGCCGCGTGTATGATGAAGGTCTTCGGATTGTAAAGTACTGTCTTTGGGGAAGAATGAT
TGGTTTGAAAATATTGAGCCAATATGACGGTACCCAAGGAGGAAGCCCCGGCTAACTACG
TGCCAGCAGCCGCGGTAATACGTAGGGGGCAAGCGTTGTCCGGAATTATTGGGCGTAAAG
GGCGCGTAGGCGGATAGTTAAGTCCGGTGTGAAAGATCAGGGCTCAACCCTGAGAGTGCA
TCGGAAACTGGTTATCTTGAGGACAGGAGAGGAAAGTGGAATTCCACGTGTAGCGGTGAA
ATGCGTAGATATGTGGAGGAACACCAGTGGCGAAGGCGACTTTCTGGACTGTAACTGACG
CTGAGGCGCGAAAGCGTGGGGAGCAAACAGGATTAGAAACCCCAGTAGTCC
>Otu1997
CCAGCCTATGGGGGGCTGCAGTGGGGAATATTGGACAATGGGCGCAAGCCTGATCCAGCC
ATGCCGCGTGAGTGATGACGGCCTTAGGGTTGTAAAGCTCTTTTGGTGGGGACGATGATG
ACGGTACCCACAGAATAAGCACCGGCTAACTTCGTGCCAGCAGCCGCGGTAATACGAAGG
GTGCTAGCGTTGTTCGGAATTACTGGGCGTAAAGGGCGCGTAGGCTGTCTTACAAGTTGG
GTGTGAAAGCCCAGGGCTCAACCCTGGAACTGCACTCAAAACTGTTTGACTTGAATTCGA
TAGAGGTTAGTGGAATTCCCAGTGTAGAGGTGAAATTCGTAGATATTGGGAAGAATATCC
GTGGCGAAGGCGGCTAACTGGACCGACATTGACGCTGAGGCGCGAAAGCGTGGGGATCAA
ACAGGATTAGAAACCCGAGTAGTCC
>Otu2005
CCAGCCTATGGGGCGCTGCAGTCGAGAATCTTTCGCAATGGGCGCAAGCCTGACGAAGCG
ACGCCGTGTGAGCGAAGAAGGCCTTCGGGTTGTAAAGCTCTTTCGCTAGGGAACAAGAAA
GAAGCACTAATACTGCTTCGATTTGAGGGTACTTGGTAAAGAAGCACCGGCTAACTCCGT
GCCAGCAGCTGCGGTAATACGGAGGGTGCAAGCATTGATCGGAATTACTGGGCGTAAAGG

GCGCGTAGGCGGATGAGAAAGTCAAATGTGAAATACCGTGGCTCAACTGCGGTGCTGCAT
TCGAAACTCCTCATCTCGAGCCTAGTTAGGGAAAACGGAATTCCACGTGTAGCGGTGAAA
TGCGTAGATATGTGGAAGAACACCTGTGGTGAAGACGGTTTTCTGGGCTAGAGCTGACGC
TGAGGCGCGAAAGCTAGGGGAGCAAACAGGATTAGAAACCCCAGTAGTCC
>Otu2009
CCAGCCTACGGGTGGCTCCAGTGGGGAATATTCCGCAATGGGCGAAAGCCTGACCCAGCG
ACGCCGTGTGGGTGATGAAGGCCTTCGGGTTGTAAAGCCCTGTCGGGTGGGAAGAAGGGT
CCCGCGGTAAATAATCGCGGGATTTTGACGGTACCACCGGAGGAAGCACCGGCTAACTCC
GTGCCAGCAGCCGCGGTAATACGGAGGGTGCAAGCGTTGTTCGGAATTACTGGGCGTAAA
GGGCGCGTAGGTGGTTCTGTAAGTCAGATGTGAAATCCTTCGGCTCAACCGGAGAACTGC
ATCTGATACTGCAGTGACTGGAGTACAGGAGAGGGAAACGGAATTCCAGGTGTAGAGGTG
AAATTCGTAGATATCTGGAGGAACACCGGTGGCGAAGGCGGTTTCCTGGCCTGATACTGA
CACTGAGGCGCGAAAGCGTGGGGAGCAAACAGGATTAGATACCCCAGTAGTCC
>Otu2010
CCAGCCTATGGGGCGCACCAGTCGAGAATCTTGGTCAATGGGCGAAAGCCTGAACCAGCG
ACGCCAAAGACTGAAGAAAGCATCAGCTGTAAAGGTCGGAACTTCACGAGGAGAATGACG
GTAGTGGAGGAGAAGTCTCGTCTAATGATTGTGCCAGCAGACGCGGTCATACAACGGAGG
CAAGTGTTGTTCATCATGACTCGGTGTAAAGGGCAGGTAGGCGGTGATCGGCATGACTTT
CTCGAAAACGATCCATGCAGGGTCAAGAAGGGAAGAGTAACGCGGTCACTGGAGTCGGAC
AGAGGTATACGGAATGATTTCAGGACTAGTGAAATGGGTTGATCGAAGTCGGAACTCCAG
AGGTGAAGACGGTATACTGGGTTTGAAAGAGAGGTCCAGAAGTGGACCAGACTGACGCTG
AACTGCGAAAGCATAGGGATCAAAAGGGATTAGAAACCCCCGTAGTCC
>Otu2011
CCAGCCTACGGGGGGCTCCAGTAGGGAATATTGCACAATGGAGGAAACTCTGATGCAGCG
ACGCCGCGTGAGTGATGAAGGCCTTCGGGTTGTAAAGCTCTGTTCTCAGGGAAAAAGAAA
GTGATTGTACCTGAGGAGAAAGGACCGGCTAACTTCGTGCCAGCAGCCGCGATAAGACGA
GGGGTCCTAGCGTTGCTCGGAATTACTGGGCGTAAAGCGGGTGTAGGTGGCTTTGTAAGT
CAGAAGTGAAAGCCCTGGGCTTAACCCAGGAAGTGCTTTTGATACTGCTTAGCTCGAGTG
TGGGAGAGGATAGTAGAATTCCAGGTGTAGTGGTGAAATACGTAGATATCTGGAGGAATA
CCGGTGGCGAAGGCGGCTATCTGGCCCAACACTGACACTCAGACCCGAAAGCGTGGGGAT
CAAACAGGATTAGAAACCCCCGTAGTCC
>Otu2012
CCAGCCTATGGGGCGCTCCAGTAGGGAATCTTGCGCAATGGGCGAAAGCCTGACGCAGCG
ACGCCGCGTGGACGATGAAGTTCTTCGGAATGTAAAGTCCTGTCAGGAGGGAAGAAACCT
TTGCATGTCAAATGCAAAGCTGACGGTACCTCCAGAGGAAGCTACGGCTAACTTCGTGCC
AGCAGCCGCGGTAATACGGGGGTAGCGAGTGTTGTTCGGATTTACTGGGCGTAAAGGGAG
CGCAGGCGGTAATGTAAGTCAGGAGTGAAATGTTGGGGCTTAACCCCAAATCTGCTTTTG
ATACTGCTTTACTAGAGTATGCAAGAGGAGGATGGAATTCTAGGTGTAGCGGTGGAATGT
GTAGATATCTAGAAGAACACCGATTGCGAAGGCAGTCCTCTGGTGCAATACTGACGCTCA
GGCTCGAAGGCGTGGGGAGCAAACAGGATTAGAAACCCCAGTAGTCC
>Otu2013
CCAGCCTACGGGAGGCACCAGTAACGAATCTTCCCCAATGCACGAAAGTGTGAGGGAGCG
ACGCCGCGTGGAGGACGAAGTCCTTCGGGATGTAAACTCCTGTCAGGGGTTAGAAAGTTC
TGATCAACCCCAGAGGAAGTGGCGGCTAACTCTGTGCCAGCAGCCGCGGTAAGACAGAGG
CCACAAGCGTTAGGCGGAATCACTGGGCTTAAAGCGTGTGTAGGCGGATCGTTAAGTGCC
TTGTGAAATCCCCCGGCTCAATCGGGGAACGGCTGGGCATACTGGCGATCTTGAGCTGCC
TAGGGGCGACTGGAACAAGCGGTGGAGCGGTGAAATGCGTAGATATCGCTTGGAACGCCA
ATGGTGAAAACAGGTCGCTGGGGGCAAGCTGACGCTGAGACACGAAAGCCAGGGGAGCAA
ACGGGATTAGAAACCCCAGTAGTCC
>Otu2015
CCAGCCTACGGGGGGCAGCAGTAGGGAATTTTCCACAATGGGCGAAAGCCTGATGGAGCA
ACGCCGCGTGCAGGATGAAGGCTCTAGGGTCGTAAACTGCTTTTATATGTGACGATAATG
ACGGTAGCATATGAATAAGGATCGGCTAACTCCGTGCCAGCAGCCGCGGTCATACGGAGG
ATCCAAGCGTTATCCGGAATTACTGGGCGTAAAGAGTTGCGTAGGTGGCATAGTAAGCGA
GTAGTGAAAGCGTTCGGCTCAACCGGATATCCATTACTTGAACTGCTAAGCTAGAGGATG
AGAGAGGTTATTGGAATTCCTTGTGTAGGAGTGAAATCCGTAGATATAGGGAGGAACACC
GATGGCGTAGGCAGATAACTGGCTCATTCCTGACACTAAGGCACGAAAGCGTGGGGAGCA
AACGGGATTAGAAACCCGTGTAGTCC

>Otu2016
CAGCCTATGGGGCGCACCAGTAGGGAATTTTGGGCAATGGGCGAAAGCCTGACCCAGCAA
CGCCGCGTGAAGGATGAAGGCCTTCGGGTTGTAAACTTCTGTTGGATGGGAAAAAAAGGT
AGCAATACCCTGATGGTACCATTCGAGTAAGCACCGGCTAACTACGTGCCAGCAGCCGCG
GTAATACGTAGGGTGCAAGCGTTGTTCGGAATTACTGGGCGTAAAGGGAGCGTAGGCGGA
AATTTAAGTTAGGAGTTTAATTCTCGGGCTTAACCCGAGGCTGGCTCTTAATACTGAGTT
TCTTGAGTATGGAAGAGGATGACGGAATTCCAGGTGTAGCGGTGGAATGCGTAGATATCT
GGAAGAACACCAGTAGCGAAGGCGGTCATCTGGGCCAATACTGACGCTGAGGCTCGAAAG
CTAGGGGAGCAAACAGGATTAGATACCCTTGTAGTCC
>Otu2017
CCAGCCTATGGGATGCACCAGTGGGGAATTTTGCGCAATGGGGGAAACCCTGACGCAGCA
GCGCCGCGTGAGTGATGAAGGCTTTCGGGTCGTAAAGCTCTGTCAGTGGGGAAGAAATGG
GGCGTGCTAATACCGCGTCTTCTTGACGGTACCCACAAAGGAAGCACCGGCTAACTCCGT
GCCAGCAGCCGCGGTAATACGGGGGGTGCAAGCGTTGTTCGGATTTATTGGGCGTAAAGC
GCGTGTAGGCGGTTTTTTAAGTCTGATGTGAAAGCCCTGGGCTCAACCCAGGAAGTGCAT
TGGATACTGGAAGACTTGAATACGGGAGAGGGTAGTGGAATTCCTAGTGTAGGAGTGAAA
TCCGTAGATATTAGGAGGAACACCGGTGGCGAAGGCGGCTACCTGGACCGATATTGACGC
TGAGACGCGAGAGCGTGGGGAGCAAACAGGATTAGATACCCGAGTAGTCC
>Otu2019
AGCCTATGGGATGCACCAGTCGAGAATTTTTCGCAATGGACGAAAGTCTGATGGAGCGAC
GCCGCGTGGAGGATGAAGGTTTTCGGATTGTAAACTCCTGTCACTGCAGAACAAGGCTAT
GTTCGTTAACAGCGAATGTAGTTGATGGTATGCGGAGAGGAAGGGACGGCTAACTCTGTG
CCAGCAGCCGCAGTAAGACAGAGGTCCCGAGCGTTGTTCGGATTCATTGGGCGTAAAGGG
TGTGTAGGAGGTGGGGTAAGTCAGGTGTGAAATCTCCGAGCTTAACTCGGAAACTGCAAT
GGAAACTATCCGACTAGAGGAATGGAGGGGAGACTGGAATACTTGGTGTAGCAGTGAAAT
GCGTAGATATCAAGTGGAACACCAGTGGCGAAGGGGAGTCTCTGGACATTTCCTGACGCT
GAGGCACGAAAGCCAGGGGAGCAAACGGGATTAGAAACCCTCGTAGTCC
>Otu2020
CCAGCCTATGGGGTGCACCAGCCAAGAATCTTCCGCAATGGGCGAAAGCCTGACGGAGCG
ACGCCGCGTGAGGGAAGAAGGTCTTCGGATTGTAAACCTCTTTTGTAAGGGAAGAATAAG
TTGCGTAGGAAATGACGCGATGATGACGGTACTTTATGAATAAGCCCCGGCTAATTACGT
GCCAGCAGCCGCGGTAATACGTAAGGGGCGAGCGTTACTCGGAATTACTGGGCGTAAAGG
GCGTGTAGGCGGTTTCACAAGTCGAATGTAAAAAACCACAGCTCAACCGTGGGTCTGCGT
TCGAAACTGTGAGACTTGAGTCTGGTGGAGGGCAATGGAATTCCCGGTGTAGCGGTGAAA
TGCGTAGATATCGGGAAGAACACCGAAGGCGAAAGCAGTTGTCTATGCCAAGACTGACGC
TGAGGCGCGAAAGCATGGGGATCAAACAGGATTAGAAACCCCCGTAGTCC
>Otu2022
CCAGCCTACGGGGGGCAGCAGTGGAGAATATGTGGGCAATGAGCGAAAGCTTAACCCCGG
AAAGTTTTGTGGGGGACGACAAGCCTGCTTGTAAACCCCTTTCGGCAAATGAGGATTATG
ACATTAGTTTGAGGCAAGATAGTCCTGACTAATGTTCGTGCCAGCAGTTGCGGTAAGACG
GATGGGGCCAGCGTTATTGGGCTTTACTGGGTGTAAAGGGTACGTAGACAGTTTTAATTT
ATTAGTGTAAAAGGTCTGGGTTAAACTTTTTCACCTTGCATATAATATAATGAAACTTGA
GTTTAGTAGAAGTAACTAGAATTTTTAATGAAGGGATGATATCCTTCGAAATTAAAAGGA
ATGCTAAATGTGAAAACATGTTGCTATTTTAAACTGACGTTAAAGTACGAAAGCATAGGG
ATCAAACAGGATTAGATACCCTTGTAGTCC
>Otu2023
CCAGCCTATGGGATGCACCAGTGAGGAATATTGGGCAATGGACGGAAGTCTGACCCAGCC
ATGTCGCGTGAAGGATGAAGGCGTTCTGCGTTGTAAACTTCTTTTACCTGGGAAGAAACC
ACCCTTTTCTAAGGGTGTTGACGGTACCAGGGGAATAAGCACCGGCTAACTCCGTGCCAG
CAGCCGCGGTAATACGGAGGGTGCAAGCGTTATCCGGATTTACTGGGTTTAAAGGGTGTG
TAGGCGGGCTATTAAGTCTGGGGTGAAATCTCCGAGCTCAACTCGGAAACTGCCTTGGAT
ACTATTAGCCTTGAATTATGTTGAGGTTAGCGGAATATAACATGTAGCGGTGAAATGCAT
AGATATGTTATAGAACACCGATTGCGAAGGCAGCTAACTAAGCATATATTGACGCTGAGG
CACGAAAGCGTGGGGATCAAACAGGATTAGATACCCCTGTAGTCC
>Otu2026
CCAGCCTACAGGTGGCACCAGTGGGGAATCTTGCGCAATGGGCGAAAGCCTGACGCAGCC
ACGCCGCGTGAGTGAAGAAGGCCTTCGGGTTGTAAAGCTCTGTCGGGCGGGACGAAAATG
CCTGGTGTTAACAGCGCCAGGTACTGACGGTACCGCCAAAGGAAGCACCGGCTAACTTCG

TGCCAGCAGCCGCGGTAATACGAGGGGTGCAAGCGTTGCTCGGAATTATTGGGCGTAAAG
GGTAGGTAGGTGGTCTCATTTGTCTAGGGTGAAAGCCTTGAGCTTAACCCAAGAAGTGCC
CCAGAAACGGTGAGACTCGAGTCTTGGAGAGGGTTGTGGAATTCCCGGTGTAGCGGTGAA
ATGCGTAGAGATCGGGAGGAACACCAGAGGCGAAGGCGGCAACCTGGACAAGTACTGACA
CTCAACTACGAAAGCGTGGGGAGCAAACAGGATTAGAAACCCGAGTAGTCC
>Otu2028
CCAGCCTATGGGTGGCTGCAGTAGGGAATATTGGTAATGGGCGAAAGCCTGAACCAGCAA
CGCCGCGTGAGCGATGAAGGCCTTCGGGTTGTAAAGCTCTTTTGGAGGGGATGAGGAAGG
ACAGTACCCTTCGAATAAGCCTCGGCTAACTACGTGCCAGCAGCCGCGGTAAAACGTAGG
AGGCGAGCGTTATCCGGATTTACTGGGCGTAAAGCGCGCGTAGGCGGCACGGCAAGTTGG
ATGTGAAAGCTCCCGGCTTAACTGGGAGAGGTCGTTCAAAACTGCCGAGCTAGAGGATGT
GAGAGGGAGGTGGAATTCCGGGTGTAGTGGTGAAATGCGTAGATATCCGGAGGAACACCA
GTGGCGAAAGCGGCCTCCTGGCACATAACTGACGCTCATGTGCGAAAGCTAGGGTAGCAA
ACAGGATTAGATACCCGTGTAGTCC
>Otu2029
CCAGCCTATGGGACGCAGCAGTCGAGGATCTTCGGCAGTGGGCGCAAGCCTGACCGAGCG
ACGCCGCGTGCGCGATGAAGGCCTTCGGGTTGTAAAGCGCTGTCGAGGGGGAGGAAACCG
CAAGGTTGACCTATCCCTGGAGGAAGGACAGGCTAAGTTCGTGCCAGCAGCCGCGGTAAG
ACGAACCGTCCTAACGTTGTTCGGAATCACTGGGCTTAAAGGGCGCGTAGGCGGGCTTCC
AAGTCCGGGGTGAAATCTTTCGGCTTAACCGGAAAATAGCCTTGGATACTGGGAGTCTCG
AGTGAGGTAGGGGCGGGTGGAACTTCCGGTGGAGCGGTGAAATGCGTTGATATCGGAAGG
AACGCCGGTGGCGAAAGCGACCCGCTGGACCTCTTCTGACGCTGAGGCGCGAAAGCCAGG
GGAGCAAACGGGATTAGAAACCCGTTAGTCC
>Otu2031
CCAGCCTACGGGACGCTGCAGTAAGGGATATTGCGCAATGGGCGAAAGCCTGACGCAGCA
ACGCCGCGTGGAGGAAGAAGGCCTTCGGGTCGTAAACTCGTTTTGACGCCGACGAGCAAG
GACGGTAAGCGTCGAATAAGTCACGGCTAACTACGTGCCAGCAGCCGCGGTAAAACGTAG
GTGGCGAGCGTTATCCGGATTTACTGGGCGTAAAGGGTGTGCAGGCGGGTCGTCAAGTGG
TGTATGAAAGCGCCCGGCTTAACCGGGCGTAGTTATGCCAGACTGACGACCTAGAGGACG
AGAGAGGGGCGTGGAATTCCGGGTGTAGTGGTGAAATGCGTAGAGATCCGGAAGAACCCC
AGAGGCGAAGGCGGCGCCCTGGCTCGTCCCTGACGCTCAGACACGACAGCATGGGGAGCG
AACGGGATTAGAAACCCGAGTAGTCC
>Otu2033
CCAGCCTATGGGTGGCAGCAGTGGGGAATCTTCCACAATGGGCGAAAGCCTGATGGAGCA
ACGCCGCGTGAGTGATGAAGGCCTTCGGGTTGTAAAACTCTTTCGTGCGAGAATAACGCT
GCAGGGTGAACAATCCTGCAGCTTGACTGTATCGCAGGAAGAAGACACGGCTAACTCTGT
GCCAGCAGCCGCGGTAATACAGAGGTGTCGAGCGTTGTCCGGATTTACTGGGCGTAAAGG
GCGTGTAGGCGGGTGGGTAAGTCGGATGTGAAATCGCTGGGCTTAACTCAGCAACTGCAT
CCGAAACTGCTTGTCTTGAGTGCAGAAGAGGAAAGCGGAATTCCCGGTGTAGCGGTGAAA
TGCGTAGATATCGGGAGGAACACCAGTGGCGAAGGCGGCTTTCTGGTCTGCAACTGACGC
TGAAACGCGAAAGCTAGGGGAGCAAACGGGATTAGAAACCCTAGTAGTCC
>Otu2034
CCAGCCTACGGGATGCTGCAGTCGAGAATCTTCGGCAATGGACGCAAGTCTGACCGAGCG
ACGCCGCGTGCGGGATGAAGGTCTTCGGATTGTAAACCGCTGTCAGAGGGGATGAAGTGT
ACGGGAGTTCTCTCCTGTATTTGACAGAGCCTCAGAGGAAGCACGGGCTAAGTTCGTGCC
AGCAGCCGCGGTAATACGAACTGTGCGAACGTTATTCGGAATCACTGGGCTTAAAGGGTG
CGTAGGCGGTTACCTAAGTCAGGTGTGAAATCTCTCGGCTCAACCGGGAAACTGCGCTTG
AAACTGGGTGACTGGAGTGAGGCAGGGGTGTGTGGAACTTCCGGTGGAGCGGTGAAATGT
GTAGATATCGGAAGGAACGCCAGCAGCGAAAGCGGCACACTGGGCCTTTTCTGACGCTGA
GGCACGAAAGCTAGGGGAGTGAACGGGATTAGATACCCTCGTAGTCC
>Otu2036
CCAGCCTACGGGTTGCTGCAGCCGAGAATATTCGACAATGGGCGAAAGCCTGATCGAGCG
ATACCGCGTGGTGGATGAAGCGATTCGTCGCGTAAACATCTTTTATGGAGGAGGAAGTAA
TTGACGTTACTCCATGAATAAGGGGCTCCAAACTCTGTGCCAGCAGGAGCGGTAATACAG
AGGCCCCGAGCGTTACCCGGAATTACTGGGCGTAAAGAGTGCGTAGGTGGTTGTGTTAGT
CGGGCGTTAAATATCCAGGGCTCAACCTTGGAAACCCGTCCGAAACGGCACAACTTGAGG
GAGTGAGAGGTGAATGGAACTCTAAGTGTAGGCGTGAAATCCGTTGATATTTAGGGGAAC
ACCGAAAGCGAAGGCAATTCACTGGCACTTTCCTGACACTCAAGCACGAAAGCGTGGGTA

GCGAACGGGATTAGAAACCCTCGTAGTCC
>Otu2045
CCAGCCTATGGGAGGCTCCAGTAAGGAATATTGGTCAATGGACGCAAGTCTGAACCAGCC
ATGCCGCGTGGAGGATGAAGGCCCTCTGGGTTGTAAACTCCTGTCATTGGTGAACAAGGC
GGCCCGTTAACTGCGGAGCCGCTCGATAGTAGCCGAAGAGGAAGAGACGGCTAACTCTGT
GCCAGCAGCCGCGGTAATACAGAGGTCTCGAGCGTTGTTCGGATTCATTGGGCGTAAAGG
GTGCGTAGGCGGCGGGGTAAGTCTGATGTGAAATCCCGGGGCTCAACCCTGGAACTGCAT
TGGATACTGCCTTGCTAGAGAGCTGGAGAGGAAACTGGAATTTACGGTGTAGCAGTGAAA
TGCGTAGAGATCGTAAGGAAGACCAGTGGCGAAGGCGAGTTTCTGGACAGCTACTGACGC
TGAGGCACGAAGGCCAGGGGAGCAAACGGGATTAGATACCCTAGTAGTCC
>Otu2046
CCAGCCTACGGGGCGCACCAGTGGGGAATTTTACACAATGGGCGAAAGCCTGATGTAGCG
ACACCGCGTGAGTGAAGAAGCCCCTTGGGGTGTAAAACTCTGTCGGCAGGGACGAAAAAA
ATGACGGTACCTGCAAAGGAAGCATCGGCTAACTACGTGCCAGCAGCCGCGGTAAGACGT
AGGATGCAAGCGTTGTCCGGATTTATTGGGCGTAAAGAGTTCGTAGGTGGTTTGTTAAGT
CTGATGTTAAAGACTGGGGCTCAACCTCGGGAGTGCATTGGATACTGGCAGACTGGAGTG
CAGTAGAGGCAAGCGGAATTCCCAGTGTAGCGGTGAAATGCGTAGATATTGGGAAGAACA
CCGGTGGCGTAAGCGGCTTGCTGGGCTGTAACTGACACTGAGGAACGAAAGCTAGGGGAG
CAAATGGGATTAGAGACCCGCGTAGTCC
>Otu2047
CCAGCCTATGGGGGGCACCAGTCGAGAGGCTTCGGCAATGGGGGAAACCCTGACCGAGCG
ACGCCGCGTGGGGGATGAAGGCCCTTGGGTTGTAAACCCCTTTTGTTCGGGAAGAAATGC
GATCAGGTGAACAATCTGGTCGTTTGACGGTACCGGACGAATAAGCTCCGGCTAACTCCG
TGCCAGCAGCCGCGGTAACACGGGGGGAGCAAGCGTTGTTCGGAATCACTGGGCGTAAAG
GGCGTGTAGGCGGTCTGGTAAGTGAGATGTGAAATCCCTCGGCTCACCCGAGGATCTGCA
TCTCATACTGCTAGGCTTGAGTACGGGAGAGGACGAGGAAATTCCTGGTGTAGCGGTGAA
ATGCGTAGATATCAGGAGGAACACCGGTGGCGAAAGCGCTCGTCTGGCCCGATACTGACG
CTGATGCGCGAAAGCTAGGGGAGCAAACGGGATTAGAAACCCCGGTAGTCC
>Otu2053
CCAGCCTATGGGGCGCTGCAGTCGAGAATCTTCGGCAATGGGCGCAAGCCTGACCGAGCG
ACGCCGCGTGCGGGATGAAGGCCCTCGGGTTGTAAACCGCTGTCAGAGGGAAAGAAATGC
CAAGGGGTTCTCTTCTTGGTTTGACTGACCTTCAGAGGAAGTACGGGCTAAGTTCGTGCC
AGCAGCCGCGGTAAGACGAACCGTACGAACGTTACTCGGAATCACTGGGCTTAAAGGGTG
CGTAGGCTGTCCGGTAAGTCGGGTGTGAAATACCTCAGCTCAACTGAGGAATTGCGCCCG
AAACTGTCGGACTCGAGGGAGACAGAGGTGAGCGGAACTTAGGGTGGAGCGGTGAAATGC
GTTGATATCCTAAGGAACACCAGTGGCGAAAGCGGCTCACTGGGTCTCTTCTGACGCTGA
GGCACGAAAGCTAGGGGAGCGAACGGGATTAGATACCCTCGTAGTCC
>Otu2054
CCAGCCTACGGGGTGCTGCAGTGGGGAATTTTGGACAATGGGGGCAACCCTGATCCAGCC
ATGCCGCGTGCGGGAAGAAGGCCTTCGGGTTGTAAACCGCTTTTGGTCGGGAAGAAAAGG
CATTTCCTAATAGGGGGTGTTCTTGACGGTACCGGCAGAATAAGCACCGGCTAACTACGT
GCCAGCAGCCGCGGTAATACGTAGGGTGCGAGTGTTAATCGGAATTACTGGGCGTAAAGC
GTGCGCAGGCGGTTTGTTAAGTCTGATGTGAAAGCCCCGGGCTCAACCTGGGAATGGCAT
TGGAAACTGGCAAGCTCGAGTTTGGCAGAGGGGGGTGGAATTCCGCGTGTAGCAGTGAAA
TGCGTAGAGATGCGGAGGAACACCGATGGCGAAGGCAGCCCCCTGGGCTAAGACTGACGC
TCAGGCACGAAAGCGTGGGGAGCAAACAGGATTAGATACCCCAGTAGTCC
>Otu2055
CCAGCCTACGGGTGGCTCCAGGCGCGAAAACTCTACAATGCAGGCAACTGCGATAGGGGA
ACATCGAGTGGCATTCATATTGGGTGTCTGTCCTCCAGCCTAAAAAGCTGGGGTTAGCAA
GGGCCGGGCAAGACCGGTGCCAGCCGCCGCGGTAATACCGGCGGCTCGAGTGGTGGCCGA
TATTATTGAGTCTAAAGGGTCCGTAGCCGGCTTTGCAAGTCTTTCGGGAAATCCAGCGGC
TTAACCGTTGGTCGTCCGGGGGGTACTACATTGCTTGGGACTGGGAGAGGTAGGAGGTAC
TCAGGGGGTAGGAGTGAAATCCTGTAATCCTTTGGGGACCACCGGTGGCGAAGGCGTCCT
ACCAGAACAGGTCCGACGGTGAGGGACGAAAGCTAGGGGCACGAACCGGATTAGAAACCC
TCGTAGTCC
>Otu2056
CCAGCCTATGGGTTGCAGCAGCCAGGAATATTGGGCAATGGGCGAAAGCCTGACCCAGCG
ACACCGCGTGGGGGAAGAAGGCCCTCGGGTTGTAAACCCCTTTTATTAGGGACGAATAAT

GACGGTACCTGATGAATAAGCTTCGGCTAACTACGTGCCAGCAGCCGCGGTAATACGTAG
GAAGCGAGCGTTATCCGGATTTATTGGGCGTAAAGCGGGCGTAGGTGGCTTTTCAAGTCC
GATGTGAAATCTCCTGGCTTAACTGGGAGGGTGCATCGGATACTGTTGAGCTGGAGTGCA
GCAGAGGAAGATGGAATTCCCGGTGTAGTGGTGAAATGCGTAGATATCGGGAGGAACACT
AGTGGCGAAGGCGGTCTTCTGGGCTGTAACTGACACTGAGGTCCGAAGGCGTGGGGAGCA
AACAGGATTAGAAACCCGCGTATCC
>Otu2058
CCAGCCTACGGGGCGCAGCAGGCGCGAAACCTTTACAATGCTGGAAACAGCGATAGGGGG
ACCTCGAGTGCCACCGTACAACGGTGGCTGTCGTTGTGTCTAAAAAACACGATATAGCAA
GGGCCGGGCAAGACCGGTGCCAGCCGCCGCGGTAACACCGGCGGCTCGAGTGGTAACCGC
TATTATTGGGTTTAAAGGGTCTGTAGCCGGCCGGATAAGTCTCTTGGGAAATCTGGCAGC
TCAACTGTCAGGCTTTCAGGAGATACTGTTCGGCTCGAGACCGGGAGAGGTGAGAGATAC
TTCAGGGGTAGGGGTGAAATCTTGTAATCCTTGAAGGACCACCAGTGGCGAAGGCGTCTC
ACCAGAACGGATCTGACGGCAAGGGACGAAAGCTAGGGGCACGAACCGGATTAGAAACCC
GCGTAGTCC
>Otu2061
CCAGCCTTGGGGGGCAGCAGTCGAGAATTTTTCACAATGGGCGAAAGCCTGATGGAGCGA
CGCCGCGTGGGGGATGAAGGGCTTCGGCTCGTAAACCCCTGTCATTCGTGAACAATACCT
GGGCATGAACAGTGTTCAGGTTGATAGTAACGGAAGAGGAAGGGACGGCTAACTCTGTGC
CAGCAGCCGCGGTAATACAGAGGTCCCGAGCGTTGTTCGGATTTACTGGGCGTAAAGGGT
GCGTAGGCGGTCGGGTAAGTCTAACGTGAAATCTCCAAGCTTAACTTGGAAAGTGCGTTG
GATACTATCCGACTTGAGTTCCGGAGAGGGGACTGGAATTCTCGGTGTAGCAGTGAAATG
CGTAGATATCGAGAGGAACACCAGTGGCGAAGGCGGGTCTCTGGACGGATACTGACGCTG
AGGCACGAAAGCCAGGGGAGCAAACGGGATTAGATACCCCCGTAGTCC
>Otu2065
CCAGCCTATGGGTGGCAGCAGTCGAGGATTTTTCTCAATGGGGGAAACCCTGAAGGAGCG
ACGCCGCGTGGGGGATGAAGGACTTCGGTTCGTAAACCCCTGTCACTTGGGAACAAAAGT
TTCAACCCAACACGTTGGAGCCTGATAGTACCAGGAGAGGAAGGCACGGCTAACTCTGTG
CCAGCAGCCGCGGTAATACAGAGGTGCCAAGCGTTGTTCGGATTTATTGGGCGTAAAGGG
TGCGTAGGCGGCCATGCAAGTCGGGTGTGAAATCCCGGAGCTCAACTCCGGAACGGCATT
CGGAACTGCGTGGCTGGAGATCTGGAGAGGTGACTGGAATTCTCGGTGTAGCAGTGAAAT
GCGTAGATATCGAGAGGAACACCAGAGGCGAAAGCGAGTCACTGGACAGAATCTGACGCT
GAGGCACGAAAGCCAGGGGAGCAAACGGGATTAGAAACCCCCGTAGTCC
>Otu2066
CCAGCCTACGGGTGGCACCAGTAGGGAATATTGGGCAATGGGCGAAAGCCTGACCCAGCA
ACGCCGCGTGCACGATGAAGGCCTTCGGGTCGTAAAGTGCTTTTCTGAGGAATGAGAAAG
GACAGTACCTCAGGAATAAGCCTCGGCTAACTACGTGCCAGCAGCCGCGGTAAAACGTAG
GAGGCGAGCGTTATCCGGATTTACTGGGCGTAAAGCGTGTGCAGGCGGTACGGCAAGTTG
GATGTGAAAGCTCCTGGCTTAACTAGGAGAGGTCGTTCAATACTACCGAACTTGAGGTCA
TCAGAGGAAGGTGGAATTCCCGGTGTAGTGGTGAAATGCGTAGATATCGGGAGGAGCACC
AGTGGCGAAAGCGGCCTTCTGGGATGATTCTGACGCTCATACACGAAAGCTAGGGTAGCG
AACGGGATTAGAGACCCGAGTAGTCC
>Otu2068
CCAGCCTACGGGGGGCACCAGTGAGGAATTTTGGACAATGGGGGCAACCCTGATCCAGCA
ACGCCGCGTGAAGGACGACGGTCTTCGGATTGTAAACTTCTTTTATTCGGGACTAAGAAA
TCTGTAGAAATACGGAGAGTGAATGTACCGGATGAATAAGCCACGGCTAACTTCGTGCCA
GCAGCCGCGGTAATACGAAGGTGGCAAGCGTTACTCGGAATTACTAGGCGTAAAGGGCAG
GTAGGTGGTTTGGTTAGTCTGTTGTTAAAACTTTCGGCTTAACCGAAAGAGGTCAATAGA
TACTGCCAGACTTGAGTATAGGAGAGGATACTGGAATTCCCGGTGTAGCGGTGAAATGCG
CAGAGTTCGGGAGGAACACCAATGGCGAAAGCAGGTATCTGGACTATTACTGACACTCAG
CTGCGAAAGCTAGGGGAGCAAACAGGATTAGATACCCCCGTAGTCC
>Otu2071
CCAGCCTACGGGATGCAGCAGTAACGAATATTCCACAATGCGCGAAAGCGTGATGGAGCG
ATGCCGCGTGCAGGATGAAGCCCCTCGGGGTGTAAACTGCTGTCAGGGTTTACCAACAAA
ATGAGGAGACCCAAAGGAAGAGACGGCTAACTCTGTGCCAGCAGCCGCGGTAATACAGAG
GTCTCGAGCGTTAATCGGAGTTACTGGGCTTAAAGGGTGCGTAGGCGGTTCCGTAAGTGC
TTTGTGAAATCCCATGGCTTAACCATGGAATGGCTTGGCAGACTGCGGAACTTGAGGTCG
GTAGGGGTCGCTGGAACTCTAGGTGGAGCGGTGAAATGCGTAGATATCTAGAGGAACGCC

AAAGGTGTAGACAGGCGACTGGGCCGATTCTGACGCTGAGGCACGAAAGCGTGGGGAGCG
AACGGGATTAGAAACCCGCGTAGTCC
>Otu2072
CCAGCCTACGGGTCGCTCCAGTGGGGAATTTTGCGCAATGGGGGAAACCCTGACGCAGCA
ACGCCGCGTGGAGGAAGAAGTATTTCGGTACGTAAACTCCTTTCGATCGGGACGATAATG
ACGGTACCGAGAGAAGAAGCCCCGGCTAACTTCGTGCCAGCAGCCGCGGTAATACGAGGG
GGGCAAGCGTTGTTCGGAATTATTGGGCGTAAAGGGTGCGTAGGCGGTTTGGTAAGTTTT
GTGTGAAAACTATGGGCTCAACCCATAGCCTGCACGAAAAACTGCCGGGCTAGAGTATTG
GAGAGGTGAGTGGAATTCCTGGTGTAGCGGTGAAATGCGTAGATATCAGGAGGAACACCT
GTGGCGAAAGCGGCTCACTGGACAATAACTGACGCTGATGCACGAAAGCTAGGGGAGCAA
ACAGGATTAGATACCCTCGTAGTCC
>Otu2073
CCAGCCTATGGGGGGCAGCAGTTGGGAATATTGGACAATGGGGGAAACCCTGATCCAGCA
ATCCCGCGTGAGTGATGAAGGCCTTCGGGTTGTAAAGCTCTTTTGGTGGGGAAAAAGGGA
AACTTGATGGTACCCACAGAAAAAGCACCGGCTAACTCCGTGCCAGCAGCCGCGGTAATA
CGGAGGGTGCAAGCGTTGTTCGGAATCATTGGGCGTAAAGCGCGTGTAGGCGGTTACCTA
AGTCTGGCGTGAAAGCCCGGGGCTCAACTCCGGAACTGCACTGGATACTGGGTGACCCGA
GGATGAGAGAGGATGGCGGAATTCCTGGTGTAGGGGTGAAATCCGTAGATATCAGGAGGA
ATATCGGTGGCGAAGGCGGCCATCTGGCTCATTTCTGACGCTGAGACGCGAAAGCGTGGG
GAGCAAACAGGATTAGAGACCCTCGTAGTCC
>Otu2074
CCAGCCTATGGGGGGCTCCAGTGAGGAATATTGGTCAATGGACGCAAGTCTGAACCAGCC
ATGCCGCGTGAAGGATGAAGGCCTTCTGGGTTGTAAACTTCTTTTATGGGGGAAGAAAAT
TACGGTATCTACTGTAACCGACGGTACCCTAGGAATAAGCACCGGCTAACTCCGTGCCAG
CAGCCGCGGTAATACGGAGGGTGCAAGCGTTATCCGGATTTACTGGGTTTAAAGGGTGTG
TAGGCGGGCATTTAAGTCAGTGGTGAAATCTTTCCGCTTAACGGGAAAACTGCCATTGAT
ACTATTTGTCTTGAATTTTGTTGAGGAGGGCGGAATATGTCATGTAGCGGTGAAATGCAT
AGATATGACATAGAACACCGATAGCGAAGGCAGCTCTCTAAGCAGATATTGACGCTGAGG
CACGAAAGCGTGGGGATCAAACAGGATTAGATACCCGCGTAGTCC
>Otu2076
CCAGCCTACGGGACGCTGCAGTGAGGAATCTTGCGCAATGGGGGAAACCCTGACGCAGCA
ACGCCGCGTGAGTGAGGAAGGTTTTCGAACCGTAAAGCTCTGTCAGGTGGAAAGAAATGT
ATGGGTGTTAATAGCGTCCATACTTGACGGTACCACCAGAGGAAGCACCGGCTAACTCCG
TGCCAGCAGCCGCGGTAATACGGGGGGTGCAAGCGTTGTTCGGAATCATTGGGCGTAAAG
AGCGTGTAGGCGGCTTGACAAGTCAGATGTGAAAGCCCTGGGCTTAACCCAGGAAGTGCA
TTTGAAACTGTCTGGCTAGAGTAAGGAAGAGGAAAGCGGAATTCCTGGTGTAGAGGTGAA
ATTCGTAGATATCAGGAGGAACACCGGTGGCGAAGGCGGCTTTCTGGTCCTATACTGACG
CTGAGACGCGAGAGCGTGGGTAGCAAACAGGATTAGAAACCCCGGTAGTCC
>Otu2079
CCAGCCTACGGGTCGCAGCAGTTAGGAAAATTCTGCAATGCGCGCAAGCGTGACAGAGCA
AGCCAAAGTGTGTTTCTATAATTGAAACACTTTTATGCAATCTAAAAAGTTGCATGAATA
AGGACTGGGCAAGACTGGTGCCAGCCGCCGCGGTAATCCCAGCGGTCCGAGTCGCAGCCA
CATTTATTGGGTCTAAAACATCCGTAGCTCGTTGATTAAGTTTCATGTAAAATCCAGCAT
CTTAAGTGTTGGGCTGCATGAAATACTGTTCAACTAGAGACCGGGAGACGCAAGAAGTAC
ATTTCGGGTAGCGGTAAAATGCGTTGATCCTTGATGGACTAACAACAGCGAAGGCATCTT
GCGAGAACGGTTCTGACAGTGAGGGATGAAGGCTAGGGGCGCAAATTGGATTAGAGACCC
CTGTAGTCC
>Otu2080
CCAGCCTATGGGTTGCTGCAGTCGAGAATCTTCGGCAATGGGCGCAAGCCTGACCGAGCG
ACGCCGCGTGTGGGATGAAGGCCCTTGGGTTGTAAACCACTGTCGGAGGGGATGAAGTGC
ATAGGAGCAATCTCCTGTGTTTGACATAGCCTCAGAGGAAGCACGGGCTAAGTTCGTGCC
AGCAGCCGCGGTAATACGAACTGTGCGAACGTTATTCGGAATCACTGGGCTTACAGGGTG
CGTAGGCGGCTTGCTAAGTAGGGTGTGAAAGCCCCCGGCTCAACCGGGGAATTGCGCCCT
AAACTGGCGAGCTAGAGTGAGATAGGGGTATGTGGAACTTCCGGTGGAGCGGTGAAATGT
GTTGATATCGGAAGGAACGCCGGTGGCGAAAGCGACATACTGGATCTTGTCTGACGCTGA
GGCACGAAAGCCAGGGGAGCGAACGGGATTAGAAACCCCAGTAGTCC
>Otu2081
CCAGCCTACGGGTCGCTGCAGTAGGGAATATTGCTCAATGGGCGAAAGCCTGAAGCAGCA

ACGCCGCGTGCACGAAGAAGGTCTTCGGATCGTAAAGTGCTTTTCTGGGAGATGAGAAAG
GACAGTATCCCAGGAATAAGTCTCGGCTAACTACGTGCCAGCAGCCGCGGTAAAACGTAG
GAGGCAAGCGTTATCCGGATTCACTGGGCGTAAAGCGCATGCAGGCGGTTCGGTAAGTTG
GGCGTGAAATCTCCCGGCTCAACTGGGAGAGGTCGTTCAATACTACCGGGCTAGAGAGCA
GTAGAGGAAGATGGAATTCCCGGTGTAGTGGTGAAATGCGTAGATATCGGGAGGAACACC
TGTGGCGAAAGCGGCGCACTGGACCATAACTGACGCTGAGGCGCGAAAGCTAGGGGAGCA
AACAGGATTAGAAACCCGAGTAGTCC
>Otu2082
CCAGCCTATGGGGGGCTGCAGTCGAGAATCTTCCGCAATGGGGGAAACCCTGACGGAGCG
ACGCCGCGTGCGGGATGAAGGTCTTCGGATTGTAAACCGCTGTCACCCGGGATGAACGTT
GACCGTACCGGGAGAGGAAGCCACGGCTAACTACGTGCCAGCAGCCGCGGTAATACGTAG
GTGGCGAGCGTTATTCGGATTTATTGGGCGTAAAGGGTCCGTAGGGGGCTTGGTGTGGCC
GGTGTGAAATGTCAAGGCTCAACCTTGAAATGGCACTGGCGACTGCCGGGCTGGAGTGCC
GCAGAGGAAAGTGGAATTCCTGGTGTAGCGGTGGAATGCGTAGATATCAGGAGGAACACC
GAAGGCGAAGGCAGCTTTCTGGGCGGCAACTGACCCTGAGGGACGAAAGCTGGGGGAGCA
AACAGGATTAGAAACCCCCGTAGTCC
>Otu2084
CCAGCCTATGGGTTGCAGCAGTAAGGGATATTGCTCAATGGCCGAAAGGCTGAAGCAGCA
ACGCCGCGTGAAGGATGAAGTCCTCTCGGATGTAAACTTCTGTAGAGAAGGAGAAAACCC
CCACTCTGTGGGGATTGATAGTACTTCTAAAGTAAGCCCCGGCTAACTACGTGCCAGCAG
CCGCGGTAATACGTAGGGGGCAAGCGTTGTCCGGATTTACTGGGTGTAAAGGGTGCTCAG
GTGGTTTTATAAGTCAGAGGTGAAATCTAAAAGCTTAACTTTTGAACGGCCTTTGATACT
GTAGAACTTGTGTACGGAAGAGGGCGATGGAATTCCAGGTGTAGCAGTGAAATGCGTAGA
TATCTGGAGAAACACCAGTGGCGAAGGCGGTCGCCTGGTCCGGTACAGACACTAAAGCAC
GAAAGCGTGGGTAGCAAACAGGATTAGATACCCGAGTAGTCC
>Otu2086
CCAGCCTACGGGAGGCAGCAGTAGGGAATATTGGGCAATGGGCGAAAGCCTGACCCAGCC
ATGCCGCGTGCAGGATTGAATGTTCTATGAATTGTAAACTGCTTTTATCTGGGACGAAAA
AATTCCGTTTTCGGAAAACTGACGGTACCAGATGAATAAGGACCGGCTAACTCCGTGCCA
GCAGCCGCGGTAATACGGAGGGTCCAAGCGTTATCCGGATTCACTGGGTTTAAAGGGTGC
GTAGGTGGTCAGAAAAGTCAGTGGTGAAATCCTACAGCTTAACTGTAGAACTGCCATTGA
AACTTTCTGGCTTGAGTGTCGTTAAGGCGAGTGGAATGTAACATGTAGCGGTGAAATGCT
TAGATATGTTACAGAACACCAATTGTGAAGACAGCTCGCTGGACGACAACTGACATTGAG
GCACGAAAGCGTGGGTAGCGAACAGGATTAGAAACCCCAGTAGTCC
>Otu2087
CCAGCCTACGGGTGGCAGCAGCAACGAATCTTCCCCAATGGACGAAAGCCTGAGGGAGCG
ACGCCGCGTGGAGGACGAAGTACTTCGGTATGTAAACTCCTGTTAGGGTTATGAAAGCGA
CTGCGCCTAATACGCGCGGAAGTTGATCTGATCCAGAGAAAGGGACGGCTAACTCTGTGC
CAGCAGCCGCGGTAATACAGAGGTCCCAAGCGTTACTGAGAATCACTGGGTTTAAAGGGT
GCGTAGGTGGTCCGTTAAGTTCGTTGTGAAATCCCTGGGCTCAACCCAGGAACTGCTTCG
AATACTGGCGGACTTGAGGCCGGTAGGGGTCACTGGAACAGATGGTGGAGCGGTGAAATG
CGTAGATATCATCTGGAACGCCGGTGGTGAAGACGGGTGACTGGGCCGGTTCTGACACTG
AGGCACGAAAGCATGGGGAGCGAACGGGATTAGAGACCCGCGTAGTCC
>Otu2088
CCAGCCTACGGGACGCAGCAGCAACGAATCTTCCCCAATGCCGGAAACGGTGAGGGAGCG
ACGCCGCGTGGAGGACGAAGTACTTCGGTATGTAAACTCCTGTTAGGGTTACGAAAGCGA
CGGGGCCTAATACGCCCCTAGTTGATCTAGCCCAGAGAAAGGGACGGCTAACTCTGTGCC
AGCAGCCGCGGTAATACAGAGGTCCCAAGCGTTACTGAGAATCACTGGGTTTAAAGGGTG
CGTAGGTGGTCCGTTAAGTCGGTTGTGAAATCCCCGGGCTCAACCCGGGAACTGCTTCCG
ATACTGGCGGACTTGAGGCCGGTAGGGGTCACTGGAACAGACGGTGGAGCGGTGAAATGC
GTAGATATCGTCTGGAACGCCGGTGGTGAAGACGGGTGACTGGGCCGGTTCTGACACTGA
GGCACGAAAGCGTGGGGAGCGAACGGGATTAGATACCCCAGTAGTCC
>Otu2089
CCAGCCTACGGGGTGCTGCAGTAACGAATCTTCCACAATGCACGAAAGTGTGATGGAGCG
ACGCCGCGTGTGGGATGAAGTCCTTCGGGATGTAAACCACTGTCAGGGGTTACCAAGTTC
TGAGGAGCCCCAGAGGAAGGCACGGCTAACTCTGTGCCAGCAGCCGCGGTAAGACAGAGG
TGCCAAGCGTTAGGCGGAATCACTGGGCTTAAAGCGTGTGTAGGTGGATGTCTAAGTACC
TTGTGAAATCCCACGGCTTAACCGTGGAACTGCTCGGTATACTGGATGTCTTGAGCCACC

TAGGGGCGAGCGGAACAAATGGTGGAGCGGTGAAATGCGTAGATATCATTTGGAACGCCA
ATGGTGAAAACAGCTCGCTGGGGGTGTGCTGACACTGAGACACGAAAGCCAGGGGAGCAA
ACGGGATTAGATACCCCAGTAGTC
>Otu2090
CCAGCCTATGGGGGGCAGCAGTGAGGAATATTGGTCAATGGGGGCAACCCTGAACCAGCC
ATGCCGCGTGGAGGAAGAAGGCCCTAAGGGTTGTAAACTTCTTTTGTACGGGGGTAACCT
CGGATACGTGTATCCGACTGAAAGTACTGTACGAATAAGCAACGGCTAACTCCGTGCCAG
CAGCCGCGGTAATACGGAGGTTGCAAGCGTTATCCGGATTTATTGGGTTTAAAGGGTGCG
TAGGCGGGTTTGTAAGTCAGTGGTGAAAGACTGTCGCTTAACGATAGCATTGCCATTGAT
ACTGCAGGTCTTGAGTACACTTGAGGTAGGCGGAATGTGTAGTGTAGCGGTGAAATGCTT
AGATATTACACAGAACACCAACTGCGAAGGCAGCTTACTAAATTGTCACTGACGCTGAGG
CACGAAAGCGTGGGGATCAAACAGGATTAGATACCCCTGTAGTCC
>Otu2091
CCAGCCTATGGGTTGCAGCAGTGGGGAATTTTGGACAATGGGGGCAACCCTGATCCAGCC
ATTCCGCGTGAGTGAAGAAGGCCTTCGGGTTGTAAAGCTCTTTCGGCAGGAACGAAAAGG
CAGGCTCTAACATAGTCTGTTGATGACGGTACCTGAAGAAGAAGCACCGGCTAACTACGT
GCCAGCAGCCGCGGTAATACGTAGGGTGCGAGCGTTAATCGGAATTACTGGGCGTAAAGC
GTGCGCAGGCGGTTTTGTAAGACAGATGTGAAATCCCCGGGCTTAACCTGGGAACTGCGT
TTGTGACTGCAAGACTTGAGTGCGGCAGAGGGGGGTGGAATTCCACGTGTAGCAGTGAAA
TGCGTAGAGATGTGGAGGAACACCGATGGCGAAGGCAGCCCCCTGGGCTAGTACTGACGC
TCATGCACGAAAGCGTGGGTAGCAAACAGGATTAGATACCCCCGTAGTCC
>Otu2093
CCAGCCTACGGGTTGCAGCAGCAGGGAATATTGCGCAATGGGCGAAAGCCTGACGCAGCA
ACACCGCGTGAGGGAAGAAGGCCTTCGGGTCGTAAACCTCTTTTGTTAGGGAAGAAAAAT
GACGGTACCTAATGAATAAGTCACGGCTAACTACGTGCCAGCAGCTGCGGTAATACGTAG
GTGGCAAGCGTTATCCGGATTCACTGGGCGTAAAGAGCGCGTAGGTGGTCCTTCAAGTCA
GATGTTAAATATTCCGGCTTAACTGGAAGAGGTCATCTGATACTGCTGGACTTGAGGACG
GTAGAGGGAGGTGGAATTCCCGGTGTAGTGGTGAAATGCGTAGATATCGGGAGGAACACC
AGTGGCGAAAGCGGCCTCCTAGGCCGTTCCTGACACTGAGGCGCGAAAGCGTGGGGAGCG
AACAGGATTAGAGACCCGCGTAGTCC
>Otu2095
CCAGCCTATGGGTCGCAGCAGTAAGGAATATTGCGCAATGGGCGAAAGCCTGACGCAGCG
ACGCCGCGTGGGGGATGAAGGTCTTCGGATTGTAAACCCCTTTCGGGAGGGAAGATGAAA
TGGGGTAACCCATTCGGACGGTACCTCCAGAAGCAGCCACGGCTAACTTCGTGCCAGCAG
CCGCGGTAATACGAAGGTGGCAAGCGTTATTCGGATTTACTGGGCGTACTGGGAGCGTAG
GCGGTTCGGTAAGCCCTCTGTGAAATCTTCAGGCTTAACCTGGAAAAGTCGGAGGGGACT
GCTGGGCTAGAGGACGGGAGAGGAGCGCGGAATTCCCGGTGTAGCGGTGAAATGCGTAGA
GATCGGGAGGAAGGCCGGTGGCGAAGGCGGCGCTCTGGAACGTTTCTGACGCTGAGGCTC
GAAAGCGTGGGGAGCAAACAGGATTAGATACCCCCGTAGTCC
>Otu2096
CCAGCCTATGGGGTGCAGCAGTGAGGAATTTTGCTCAATGGGCGCAAGCCTGAAGCAGCG
ACGCCGCGTGAATGATGAAGTTCTTCGGAATGTAAAGTTCTTTTGGCAGGGAAAATTATG
ATTGTACCTGCAGAATAAGCACCGGCAAACTATGTGCCAGCAGCCGCGGTAATACATAGG
GTGCAAGCGTTGTCCGGAATTACTGGGCGTAAAGAGCGTGTAGGCGGCACTTTAAGTCGC
AACCTTAAATACAGGGGCTTTACCCCTGTCAGGGTTCGATACTGATGTGCTAGAGATTGG
GAGAGGAAAGCGGAACTCACAGTGTAGCGGTGAAATGCGTAGATATTGTGAGGAACACCC
GTGGCGAAGGCGGCTTTCTGGACCATTTCTGACGCTGAGACGCGAGAGCGTGGGGATCAA
ACAGGATTAGAGACCCGGGTAGTCC
>Otu2098
CTAGCCTACGGGATGCAGCAGTAGGGAATTTTGCGCAATGGGCGAAAGTCTGACGCAGCA
ACGCCGCGTGAGTGATGACGGTCTTCGGATTGTAAAGCTCTGTTGAGTGGGAAGAAAAGT
TCCGTTAATAGCGGAGCCTGACGGTACCGCTCGAGGAAGCCCCGGCTAACTACGTGCCAG
CAGCCGCGGTAATACGTTGGGGGCGAGCGTTGTTCGGAATCACTGGGCGTAAAGGGAGCG
TAGGCGGGTCTGTAAGTTAGGAGTTTAATTCCCGGGCTTAACCCGGGGCCGGCTTTTAAT
ACTGCAGATCTTGAGTAGGGAAGAGAGAGATGGAATTTCAGGTGTAGCGGTGGAATGCGT
AGATATCTGAAAGAACACCAGTAGCGAAGGCGGTCTCCTGGTACCATACTGACGCTGAGG
CTCGAAAGTTAGGGGAGCAAACAGGATTAGATACCCTGGTAGTCC
>Otu2099

CCAGCCTATGGGTGGCAGCAGTGAGGAATATTGGTCAATGGGGGCAACCCTGAACCAGCC
ATGCCGCGTGAAGGAAGACTGCCCTAAGGGTTGTAAACTTCCTTTGTACTAGGGTAAACT
TGTTCTTGCGAGGACAACTGAAAGTATAGTACGAATAAGCATCGGCTAACTCCGTGCCAG
CAGCCGCGGTAATACGGGGGATGCAAGCGTTATCCGGATTTATTGGGTTTAAAGGGTGCG
TAGGCGGATACATAAGTCAGTGGTGAAAGACTGTTGCTTAACGATAGCATTGCCATTGAT
ACTGTGTGTCTTGAGTGTAGATGAGGTAGGCGGAATGTGTAGTGTAGCGGTGAAATGCTT
AGATATTACACAGAACACCGATTGCGAAGGCAGCTTACTAATCTACCACTGACGCTGAGG
CACGAAAGCGTGGGGATCAAACAGGATTAGAAACCCTTGTAGTCC
>Otu2101
CCAGCCTATGGGGGGCTGCAGTGGGGAATTTTGGACAATGGGCGAAAGCCTGATCCAGCA
ACTCTGCGTGAGGGATGAAGTCCTTCGGGATGTAAACCTCTTTTGCCGGGGACGAATACC
CGCAAGGGTCTGACGGTACCCGGCGAATAAGCCACGGCTGACTACGTGCCAGCAGCCGCG
GTAAGACGTAGGTGGCGAGCGTTATTCGGAATTACTAGGCGTAAAGCGAGTGTAGGCGGG
CGCTTAAGTCCGTCGTGAAATCTCCCGGCTCAACTGGGAGGGGTCGATGGATACTGGGTG
CCTTGAGTGGGGTAGGGGGCAGTGGAATTCCCGGTGTAGCGGTGAAATGCGTAGATATCG
GGAGGAACACCTATGGTGAAAGCAGCTGCCTGGGCCCTTACTGACGCTGAGACTCGAAAG
CTAGGGGAGCAAACAGGATTAGATACCCTAGTAGTCC
>Otu2102
CCAGCCTATGGGGCGCACCAGTGAGGAATTTTCCGCAATGGACGAAAGTCTGACGGAGCG
ACGCCGTGTGTGTGATGAAGTCTTTCGGGATGTAAAACACTGTCGAGAGGGACGATATTG
ACGGTACCTCTGGAGGAAGCCCCGGCTAACTACGTGCCAGCAGCCGCGGTAATACGTAGG
GGGCGAGCGTTGTCCGGAATTATTGGGCGTAAAGGGCGTGTAGGCGGTTCCGTAAGTTGG
ACGTTAAAGGTATGGGCTCAACTCATACATCGCGTTCAATACTGCGGTTCTGGAGAATGG
TAGAGGAAAACGGAACTTCCGGTGTAGCGGTGAAATGCGTAGATATCGGAAGGAACACCA
GTGGCGAAGGCGGTTTTCTGGGCCATTTCTGACGCTGAGGCGCGAAAGCTAGGGGAGCAA
ACAGGATTAGATACCCCCGTAGTCC
>Otu2103
CCAGCCTACGGGGTGCTCCAGGCGCGAAACCTTTGCAATGCACGTAAGTGTGACAAGGGA
ATTCAGAGTGCTCATACAAAGTATGGGCTTTTGCCAAGAACAAATATCTTGGAGAATAAG
TGGTGGGAAAGACTGGTGCCAGCCGCCGCGGTAACCCCAGCGCCACTAGTGGGAATCGCG
TTTATTGGGCCTAAAGCATCCGTAGCTGGTTATGTAAATCTCTTGTGAAATTGTTAGACT
CAATCTAACAGCGCGCAGGAGACACTGCAAAACTAGGGACCGGGAGGAGTCAGAGGTATG
CTATGGGGAGCGGTAAAATGCTATAATCCATGGTAGACCACCTGTGGCGAAGGCGTCTGA
CTAGAACGGGTTCGACAGTGAGGGATGAAAGCCAGGGGAGCAAACCGGATTAGATACCCC
TGTAGTCC
>Otu2106
CCAGCCTATGGGGCGCTGCAGTCGAGGATCTTCGTCAATGGGCGAAAGCCTGAACGAGCG
ATGCCGCGTGAGCGATGAAGGCCTTCGGGTTGTAAAGCTCAAAAGAGATGAGAAAGGGAA
ACTTGATCGAATCTCAGTAAGCTCGGGCTAAGTTCGTGCCAGCAGCCGCGGTAAGACGAA
CCGAGCGAACGTTGTTCGGAATCACTGGGCTTAAAGGGCGCGTAGGCGGGTGCTCAAGTC
CGGGGTGAAATCCTCCAGCTCAACTGGAGAACTGCCTCGGATACTGGGTGCCTCGAGGGT
GGCAGGGGTATGCGGAACTATCGGTGGAGCGGTGAAATGCGTTGATATCGATAGGAACTC
CGGTGGCGAAGGCGGCATACTGGTCCACTTCTGACGCTGAGGCGCGAAAGCCAGGGGAGC
AAACGGGATTAGATACCCTGGTAGTCC
>Otu2110
CCAGCCTATGGGTGGCAGCAGTGGGGAATTTTGCACAATGCCCGAAAGGGTGATGCAGCA
ACGCCGCGTGAGGGATGAAGGCCTTCGGGTCGTAAACCTCTTTCGACCGGGAAAAACGGG
CGGGTGAATAATCCGCTCTGATGGTACCGGTGGAAGAAGCCCCGGCCAACTACGTGCCAG
CAGCCGCGGTAATACGTAGGGGGCGAGCGTTGTTCGGAATTACTGGGCGTAAAGGGCGTG
TAGGCGGTGCGGTAAGTCGGACGTTAAATCCCCGGGCTTAACCCGGGGCAGTCGTTCGAA
ACTGCCGTGCTCGAGTACGGGAGAGGGAAGTGGAATTCCAGGTGTAGCGGTGAAATGCGT
AGATATCTGGAGGAACACCGGCGGCGAAGGCGGCTTCCTGGACCGTAACTGACGCTGAGG
CGCGAAAGCTAGGGGAGCAAACAGGATTAGATACCCTAGTAGTCC
>Otu2111
CCAGCCTACGGGGGGCAGCAGCTAAGAATCTTCCGCAATGCCGGAAACGGTGAGGGAGCG
ACGCCGCGTGAAGGATGAAGCACTTCGGTATGTAAACTTCTTTTAGGGTTAAGAAAGCAG
AGGGACCTAATACGTCCTGAGTTGATCTAATCCAGAATAAGGGGCGGCTAACTCTGTGCC
AGCAGCCGCGGTAATACAGAGGCCCCAAGCGTTACTGAGAATCACTGGGTTTAAAGGGTG

CGTAGGTGGTCCGTTAAGTTCGTTGTGAAATCCCCGGGCTCAACCCGGGAACTGCTTCGA
ATACTGGCGGACTTGAGGCCGGTAGGGGTCACTGGAACAGACGGTGGAGCGGTGAAATGC
GTAGATATCGTCTGGAACGCCGGTGGTGAAGACGGGTGACTGGGCCGGTTCTGACACTGA
GGCACGAAAGCGTGGGGAGCGAACGGGATTAGAAACCCCTGTAGTCC
>Otu2113
CCAGCCTATGGGGGGCACCAGGCGCGAAACCTTTGCCATGCGCGAAAGCGTGACAGGGGA
ACTCCGAGTGGTAGAGGGGTTTACCTCTCTATCTTTTGGCCGATCCAAACAATCGGCAGA
ATAAGATCTGGGTAAGACCAGTGCCAGCCGCCATGCGCGTGAACATTCGCACGCACGGCA
CGGTACGCGGTAATACTGGCAGAGCAAGTGGTACCCACGAATATTGGGCCTAAAGAGTCC
GTAGCGGGCCGATTAAGTCCACTGTGAAATCGGGCGGCTCAACCGTCCGGCGTGCAGTGG
ATACTGACCGGCTTGGGAGCGGGGGAGGTCGGGAGTACTTGCGGGGTAACGGTAAAATGT
TGTAATCCTGCAAGGACTACCAGTGGCGAAGGCGCCCGACCAAAACGCGTCCGACCGTGA
GGGACGAAGGCTAGGAGAACGAATCGGATTAGAAACCCGTGTAGTCC
>Otu2114
CCAGCCTATGGGGGGCAGCAGTGAGGAATATTGCGCAATGCCCGAAAGGGTGACGCAGCG
ACGCCGCGTGGAGGATGAAGGCCCTATGGGTCGTAAACTTCTTTTGTGAGGGAATAATTT
CTTGCTATACGCAGGATTGAATGTACCTTAAGAAAAAGCATCGGCTAACTACGTGCCAGC
AGCCGCGGTAATACGTAGGATGCTAGCGTTGTCCGGATTTACTGGGTGTAAAGGGAGCGC
AGGCGGGTTGGTAAGTCGGTGGTGAAATACTCGAGCTTAACTCGGGAACTGCCATCGATA
CTGCTGATCTTGAGTGTGGAAGAGGTTGATGGAATTCTTGGTGTAGCGGTGAAATGCGTA
GATATCAAGAAGAACACCAATGGCGAAGGCAGTCAACTGGTCCATTACTGACGCTCATGC
TCGAAAGCGTGGGGATCAAACAGGATTAGATACCCTCGTAGTCC
>Otu2116
CAGCCTATGGGGCGCAGCAGTGAGGAATATTGCGCGATGGACGAAAGTCTGACGCAGCAA
CGCCGCGTGGGGGATGAAGGTGCTTTGCATTGTAAACCCCTGTAGGGGGGGACGAATAAG
CCGGTTTTGCCGGTGATGACGGTACCCCCAAAGTAAGCACCGGCTAACTCCGTGCCAGCA
GCCGCGGTAATACGGAGGGTGCGAGCGTTGTCCGGATTTACTGGGTGTAAAGGGCGTGTA
GGCGGGAGAATGTGTCGGAAGTGAAATCGTGCGGCTTAACCGTGTCAAATGCTTCCGAAA
CTGTTCTTCTTGAATGCGGGAGAGGCAGATGGAATTCCAGGTGTAGCGGTGGAATGCGTA
GATATCTGGAAGAACACCGGTGGCGAAGGCGGTCTGCTGGCCCGTGATTGACGCTGAGGC
GCGAAAGTGTGGGGAGCAAACAGGATTAGAAACCCGAGTAGTCC
>Otu2118
CCAGCCTACGGGGTGCAGCAGTGGGGAATTTTGGACAATGGGGGAAACCCTGATCCAGCC
ATGCCGCGTGAGTGAAGAAGGCCTTCGGGTTGTAAAGCTCTTTCGGACGGAAAGAAATCG
CACGGATAAATAGTCCGTGTGGATGACGGTACCGTAAGAAGAAGCACCGGCTAACTACGT
GCCAGCAGCCGCGGTAATACGTAGGGTGCGAGCGTTAATCGGAATTACTGGGCGTAAAGC
GTGCGCAGGCGGTTGCATAAGACAGATGTGAAATCCCCGGGCTCAACCTGGGAACTGCGT
TTGTGACTGTGCGACTGGAGTACGGTAGAGGGGGGTGGAATTCCTGGTGTAGCAGTGAAA
TGCGTAGATATCAGGAGGAACATCGATGGCGAAGGCAGCCCCCTGGACCTGTACTGACGC
TCATGCACGAAAGCGTGGGGAGCAAACAGGATTAGAAACCCCCGTAGTCC
>Otu2119
CAGCCTATGGGGGGCAGCAGTGAGGAATATTGGGCAATGGGCGCAAGCCTGACCCAGCCA
TGCCGCGTGAAGGATGAATGCCCTATGGGTTGTAAACTTCTTTTGTACGGGACGAAACTC
TTGGTCGTGACCAGGATTGACTGTACCGTGCGAATAAGCATCGGCTAACTCCGTGCCAGC
AGCCGCGGTAATACGGAGGATGCAAGCGTTATCCGGATTCATTGGGTTTAAAGGGTGCGT
AGGCGGAATAATAAGTCAGTGGTGAAAACCTTCAGCTTAACTGGAGACTTGCCATTGATA
CTGTTAGTCTCGAGTACGGTCAAGGTAGGCGGAATGTGTAATGTAGCGGTGAAATGCTTA
GATATTACACAGAACACCGATTGCGAAGGCAGCTTACTGGGCCATTACTGACGCTGATGC
ACGAAAGCGTGGGGAGCGAACAGGATTAGAAACCCGCGTAGTCC
>Otu2122
CCAGCCTATGGGATGCAGCAGGCGCGAAACCTTTACAATGCTGGCAACAGCGATAAGGGG
ACCTCGAGTGCTACGATACAATCGTGGCTGTCTAGTTGCCTAAAAAGCAATTGATAGCAA
GGGCCGGGCAAGACCGGTGCCAGCCGCCGCGGTAACACCGGCGGCTCGAGTGGTAACCGC
TATTATTGGGTCTAAAGGGTCTGTAGCCGGCTTAGTTAGTCTCTTGAGAAATCTGGCCGC
TCAACGGTCAGGCGTTCAGGGGATACTGCTAAAGCTCGAGACCGGGAGAGGTGAGAGGTA
CTTCAGGGGTAGGGGTGAAATCTTGTAATCCTTGAAGGACCACCAGTGGCGAAGGCGTCT
CACCAGAACGGATCTGACGGCAAGGGACGAAAGCTAGGGGCACGAACCGGATTAGATACC
CCTGTAGTCC

>Otu2123
CCAGCCTACGGGTTGCACCAGCTAAGAATCTTCCGCAATGGGCGAAAGCCTGACGGAGCG
ACGCCGCGTGGATGATGAAGGCCGAAAGGTTGTAAAATCCTTTTCTTGGGGAAGAATAAT
GATTGGAGTGGAGTGCCAGTCAGATGACATTAACTAGGGAATAAGCCCCGGCTAATTACG
TGCCAGCAGCCGCGGTAATACGTATGGGGCAAGCGTTGTTCGGATTTATTGGGCGTAAAG
GGCGTGTAGGCGGTTTCGTAAGCTCGGCGTGAAATACTCTGGCTTAACCAGAGAAACGCG
TTGGGAACTGCGAGACTAGAGTTTAGGAGGGGAAGCTGGAATTCCTGGTGTAGGGGTGAA
ATCTGTAGATATCAGGAAGAACACCAAAGGCGAAGGCAAGCTTCTGGACATAGACTGACG
CTGAGGCGCGAAAGCGTGGGGAGCAAACAGGATTAGAAACCCCCGTAGTCC
>Otu2124
CCAGCCTATGGGGTGCAGCAGTCGAGAATATTCCACAATGGACGAAAGTCTGATGGAGCG
ACGCCGCGTGCAGGATGAAGGCCCTTGGGTTGTAAACTGCTTTTGTAAGGGAAGAATTTC
GTTGACGGTACCTTACGAATAAGAGGTTACTAACTCTGTGCCAGCAGTAGCGGTAATACA
GAGACCTCAAGCGTTATCCGGATTTATTGGGCGTAAAGAGCTGGTAGGTTGTTATACTAG
TCAGATGTCAAATCTTCGAGCTCAACTCGAAAACTGCATTTGAAACGGTATAGCTAGAGG
GTGTGAGAGATCTATGGAACTCATGGTGTAGCAGTGAAATGCGTTGATATCATGAGGAAC
ACCAAAGGCGAAGGCATTAGATTGGCACACTCCTGACACTGAGCAGCGAAAGCGTGGGTA
GCGAATGGGATTAGATACCCGTGTAGTCC
>Otu2125
CCAGCCTATGGGAGGCACCAGGCGCGAAACCTCTGCAATGCGCGAAAGCGTGACAGGGGA
ACTCTGAGTGGCAGCCGGCTTTTAGCCGACTGTCTTTTGGCGAACCTAAACCGTTCGCAG
AATAAGTTCTGGGTAAGACCAGTGCCAGCCGCCATGGGCATATGCATATTGCCCACGGTA
CGCGGTAATACTGGCAGAACGAGTGGTGCCCACGAATATTGGGTCTAAAGAGACCGTAGC
TGGTCTGTTAAGTCCACTGTGAAATCTTGGGGCTCAACCTCAAGGCGTGCAGTGGATACT
GGCAGGCTTGAGAGCGGGGGAGGTCAGGGGTACTTACGGGGTATGAGTAAAATCAGTTGA
TCCTGTAAGGACCACCAGTGGCGAAGGCGCCTGACCAAAACGCGTCTGACAGTGAGGGTC
GAAGGCTAGGAGAACGAATCGGATTAGAAACCCGAGTAGTCC
>Otu2126
CCAGCCTATGGGGGGCAGCAGTTTAGAATATTGTGCAATGGGCGAAAGCCTGACACAGCG
ACGCCGCGTGGGTGATGAAGGCCTTCGGGTCGTAAAGCCCTGTTGGTAGGGATGAGAAGG
GACAGTACCTACCGAGGAAGCCCCGGCTAACTACGTGCCAGCAGCTGCGGTAATACGTAG
GGGGCGAGCGTTGTCCGAATTTACTGGGCGTAAAGCGTACGTAGGCGGCTCTACAAGTTA
CATCTTAAAGGCGTCAGCTCAACTGGCGAGTGTGGTGTAAGACTGCAGGGCTTGAGGCAA
TCAGAAGAATGTGGAACTCCGAGTGTAGCGGTGGAATGCGTAGAGATTCGGAAGAACACC
CATGGCGAAGGCAGCATTCTGGGATTGACCTGACGCTGAGGTACGAAAGCCAGGGGAGCG
AACGGGATTAGAAACCCGTGTAGTCC
>Otu2127
CCAGCCTATGGGATGCTGCAGTGGGGAATATTGGACAATGGGCGCAAGCCTGATCCAGCC
ATGCCGCGTGAGTGATGACGGCCTTAGGGTTGTAAAGCTCTTTCGCCAGGGACGATAATG
ACGGTACCTGGATAAGAAGCCCCGGCTAACTACGTGCCAGCAGCCGCGGTAATACGTAGG
GGGCAAGCGTTGTTCGGAATCACTGGGCGTAAAGGGAGAGTAGGCGGGAAAATAAGTTAG
AAGTTTAATGTCCGGGCTTAACCCGGAATCTGCTTCTAATACTGTTTTTCTTGAGTATTG
GAGAGGGAGATGGAATTCCAGGTGTAGCGGTGGAATGCGTAGATATCTGGAAGAACACCA
GCTGCGAAGGCGGTCTCCTGGCCAAATACTGACGCTGATGCTCGAAAGCTAGGGGAGCAA
ACAGGATTAGAGACCCCCGTAGTCC
>Otu2128
CCAGCCTACGGGATGCAGCAGTGGGGAATATTGGACAATGGGCGAAAGCCTGATCCAGCA
ATGCCGCGTGTGTGATGAAGGCCTGCGGGTTGTAAAGCACTTTCGTTGGGGAAGAAATGC
TCAAAGCTAATACCTTTGAGAGTTGACGTTACCCAAAGAAGAAGCACCGGCTAACTCTGT
GCCAGCAGCCGCGGTAATACAGAGGGTGCAAGCGTTAATCGGAGTTACTGGGCGTAAAGG
GCGCGTAGGTGGATATTTAAGTCGGATGTGAAATCCCCGGGCTTAACCTGGGAATTGCAT
TCGATACTGGATATCTAGAGTATGGTAGAGGAAAGCGGAATTCCCGGTGTAGCGGTGAAA
TGCGTAGATATCGGGAAGAACATCAGGGCGAAGGCGGCTTTCTGGACCAATACTGACACT
GAGGCGCGAAAGCGTGGGGAGCAAACAGGATTAGAAACCCTGTAGTCC
>Otu2129
CCAGCCTATGGGGGGCAGCAGTCGGGAATATTGCCCAATGGACGAAAGTCTGAGGCAGCA
ACGCCGCGTGCGCGATGAAGGCCTTCGGGTCGTAAAGCGCTTTTCGGGTGGATGAGAGAG
GACAGTACACCCGGAATAAGATACGGCTAACTACGTGCCAGCAGCCGCGGTAAAACGTAG

GTACCGAGCGTTATCCGGATTTACTGGGCGTAAAGCGCGTTCAGGCGGTTTGGCAAGTCG
GGCATGAAATCCTTCGGCTCAACCGGGGGAGGTTGTCCGATACTGCTGAACTGGAGGGCA
GTAGAGGGTGGTGGAATTCCGGGTGTAGTGGTGAAATGCGTAGAGATCCGGAGGAACACC
AGTGGCGAAAGCGGCCACCTGGACTGTACCTGACGCTGAAACGCGAAAGCTAGGGGAGCG
AACGGGATTAGAAACCCTAGTAGTCC
>Otu2130
CCAGCCTATGGGGGGCTGCAGTCGAGAATCTTCCGCAATGGGCGAAAGCCTGACGGAGCG
ACGCCGCGTGGTTGATGAAGTCCTTCGGGACGTAAAAACCTTTTATGATTAAGAAAGTTT
ATTGATCTGATCATGAATAAGGGGCTCCAAACTCTGTGCCAGCAGGAGCGGTAATACAGA
GGCCCCAAGCGTTGTCCGGAATCACTGGGCGTAAAGGGTGTGTAGGTGGTTTTGTTAGTC
TCTCGTTAAATCTCCCGACTCAATCGGGAACATGCGAGAGATACGGCAAAACTTAGAGAG
TGCGAGAGGTGAATGGAACTCATGGAGTAGGGGTGAAATCCGTTGATATCATGGGGAACA
CCAAAAGCGAAGGCAATTCACTGGCGCATTTCTGACACTGAAACACGAAAGCGTGGGTAG
CGAATGGGATTAGATACCCCAGTAGTCC
>Otu2134
CCAGCCTATGGGTGGCTCCAGCTAAGAATATTCCGCAATGGACGAAAGTCTGACGGAGCG
ACGCCGCGTGGATGAAGAAGGCCGAAAGGTTGTAAAATCCTTTTGTTGGGGAAGAATAAG
CGTGGGAGGGAATGCCCGCGTGATGACATGAACCGACGAATAAGTCCCGGCTAACTACGT
GCCAGCAGCCGCGGTAACACGTAGGGGACAAGCGTTATTCGGAATTACTGGGCGTAAAGG
GCATGTAGGCGGCTTTGTAAGCCTGGCGTGAAAGTCACCGGCTCAACCGGTGGATTGCGT
CGGGAACTGCAGGGCTTGAGTCACGGAGAGGGAGCTAGAATTCCTGGTGTAGGGGTGAAA
TCTGTAGAGATCAGGAAGAATACCAACGGCGAAGGCAAGCTCCTGGCCGATGACTGACGC
TGAGGTGCGAAAATGTGGGGATCAAACAGGATTAGAGACCCTCGTAGTCC
>Otu2135
CCAGCCTATGGGATGCTCCAGGGCGTATTTTTCTTTGGGCCAGTTGCTCAGTCCACAGAG
GCGATATCCCGCCTCTTTCAACTCTCGAAGAATCTCTACCACCGGCTGGACAGCGCCCCT
CAGAGACTCGAGATAGCGTTCGTCGTAAGCCCGGATCAGATCATGGTATTCCGGAAAACG
CGCGATCAGTTCCGCTGTCCCCTCGGCGAAAGAGCGTCCACGGTCTTGCTCCAGGTTCCA
TCCGGTAAAATCGACCTCCTTCAAAAAACGTTCCACCGCCTGCTGGTCGTCGCCCAATAT
CTTGCTGTACAGGTAATAGGGGTCCCAATCCATCAGGACACCTCCAAAATCAAATACGAT
TACCTGCGCTTGCTTTTCATTTTTGTCCATTAGAAACCCCAGTAGTCC
>Otu2139
CCAGCCTACGGGTTGCACCAGTAGGGAATATTGCACAATGGGCGAAAGCCTGATGCAGCA
ACGCCGCGTGCACGATGAAGGTCTTCGGATCGTAAAGTGCTTTTCTGGGAGATGAGAAAG
GACAGTATCCCAGGAATAAGTCTCGGCTAACTACGTGCCAGCAGCCGCGGTAACACGTAG
GAGACGAGCGTTATCCGGATTTACTGGGCGTAAAGCGCGTGCAGGCGGTTTGGTAAGTTG
GATGTGAAAGCTCCCGGCTTAACTGGGAGAGGTCGTTCAATACTGCCAGACTAGAGGATG
GTAGAGGGAGGTGGAATTCTGGGTGTAGTGGTGAAATGCGTAGATATCCAGAGGAACACC
AGTGGCGAAAGCGGCCTCCTGGACCATTTCTGACGCTCAGACGCGAAAGCTAGGGTAGCA
AACGGGATTAGAAACCCGTGTAGTCC
>Otu2140
CCAGCCTATGGGACGCAGCAGGGCCAAGAGCGGAATTATGGCCAAGGTAAGGACCAGCAT
CAGTTGGAAATCACCGAAATAGGCCGCCATCTCTGCCTGCTCGCCGATCCTGCCATTAAG
CAAGGCCGTTCCATGTGGGGTCGCCAGTTGCGCGGCAAGCTGTGGCTCTCGGGCAAGAAG
ATTGTATGGCACTATCTGCTCGATCAGAGCGCTGCGGGCAAGCTGGGTGTTGGAAATCAG
CAGCGCGTGAACCATGGCTATTCCTACACTGCTGCCTAGGTTGCGGATCAAACTGAACAT
CGCGGCGCCTTCATTGCGCAAGGTTGTCGGCAGCGTTCCGAACGTCAGGGCTGCCATCGC
AACCCAGGCTAGACCGACGCCTAAACCCTGGGTGATGCCGGACCAGAATACTGGCGCGAC
GCCCATCTGTAGGTCGAACCAAGTCATCTGAGACAGCGATAGCGCGGTAATTAGATACCC
CCGTAGTCC
>Otu2144
CCAGCCTATGGGGTGCAGCAGTGGGGAATCTTGCACAATGGGCGACAGCCTGATGCAGCA
ATGCCGCGTGAGGGAAGACGGCCTTCGGGTTGTAAACCTCTTTCAGTTGGGATGAAGGTT
GGACGGTGAATAGCCGCGCCAACTGACCGTACCTTCAGAAGAAGCTCCGGCTAACTACGT
GCCAGCAGCCGCGGTAATACGTAGGGAGCAAGCGTTGTCCGGAATCATTGGGCGTAAAGC
GCGTGTAGGCGGTTCCATAAGTCTGCTGTGAAAGTCGGAGGCTCAACCTTCGAATGCCAG
TGGATACTGTGGGACTTGAGTACGGAAGAGGAGTGTGGAATTCCTGGTGTAGCGGTGAAA
TGCGCAGATATCAGGAGGAACACCAACGGCGAAGGCAGCACTCTGGGACGTTACTGACGC

TGAGACGCGAAAGCGTGGGGAGCAAACAGGATTAGAAACCCTAGTAGTCC
>Otu2145
CCAGCCTATGGGATGCAGCAGTGGGGAATATTGGACAATGGGGGCAACCCTGATCCAGCA
ATGCCGCGTGAGTGATGAAGGCCTTAGGGTTGTAAAGCTCTTTCACCCACGACGATGATG
ACGGTAGTGGGAGAAGAAGCCCCGGCTAACTTCGTGCCAGCAGCCGCGGTAATACGAAGG
GGGCTAGCGTTGCTCGGAATTACTGGGCGTAAAGGGCGCGTAGGCGGACCTGTCAGTTGG
GTGTGAAAGCCCGGGGCTCAACCCCGGAATTGCACTCAATACTTCAGGTCTTGAGTACCG
GAGAGGATGGTGGAATTCCCAGTGTAGAGGTGAAATTCGTAGATATTGGGAAGAACACCG
GTGGCGAAGGCGGCCATCTGGACGGTAACTGACGCTGAGGCGCGAAAGCGTGGGGAGCAA
ACAGGATTAGAAACCCCGGTAGTCC
>Otu2146
CCAGCCTATGGGGGGCTGCAGTCAAGAAACTTCCACAATGGACGAAAGTCTGATGGAGCG
ACGCCGCGTGGTTGACGAAGTCCTTCGGGACGTAAAAACCTTTTATGGGGGAGGAAGTAA
TTGACGTTACCCCATGAATAAGAGGCTCCTAACTCTGTGCCAGCAGGAGCGGTAATACAG
AGGCCTCAAGCATTATCCGGAATCACTGGGCGTAAAGGGTGTGTAGGCGGCGATGTTAGT
CTCTCGTGAAAGCCCGTGGGCTCAACCCATGGAACGCGGGGGAAACGGCATCGCTTTGAG
GACGCGAGAGGTATATGGAACTCATGGTGTAGGGGTGAAATCCGTTGATATCATGGGGAA
CACCAAATGCGAAGGCAATATACTGGCGCGCACCTGACGCTGAAACACGAAAGCGTGGGA
ATCGAACGGGATTAGAAACCCCAGTAGTCC
>Otu2148
CCAGCCTACGGGATGCAGCAGTCGAGAATATTCCTCAATGGACGAAAGTCTGAAGGAGCG
ACGCCGCGTGACTGAAGAAGTCCTTCGGGATGTAAAGGTCTTTTTTCCGGGACGAAATCT
GACGGTACCGGAAGAATAAGAGGTTGCTAACTCTGTGCCAGCAGCAGCGGTAATACAGAG
ACCTCGAGCGTTATCCGGATTGATTGGGCGTAAAGCGTGCGCAGGCGGCTTTCTAAGCTC
ACGGTCAAATCCCGCGGCTCAACCGCGGGACTGCCGCGAAAACTGGAAGGCTAGAGTACG
GAAGAGGCTAGCGGAATTGCCGGTGTAACGGTAAAATGTGTTAATATCGGCAAGAACACC
AGAGGCGAAAGCGGCTAGCTGGTACGTTACTGACGCTGTTGCACGAAAGCGTGGGGAGCG
AATGGGATTAGAAACCCCTGTAGTCC
>Otu2149
CCAGCCTACGGGGGGCAGCAGTGAGGAATATTGCACAATGGGCGAAAGCCTGATGCAGCA
ACGCCGCGTGAGGGACGAAGGCCTTCGGGTCGTAAACCTCTTTTCTGAGGGACGAGATCA
GGACGGTACCTCAGGAATAAGTCTCGGCTAACTACGTGCCAGCAGCCGCGGTAAAACGTA
GGAGGCGAGCGTTATCCGGATTTACTGGGCGTAAAGCGCACGCAGGCGGCTCCGTTAGTC
GGACGTGAAAGCTCCTGGCTTAACTGGGAGAGGCCGTTCGATACTGCGGGGCTTGAGGTT
GGGAGAGGGGTGTGGAATTCCCGGTGTAGTGGTGGAATGCGTAGATATCGGGAGGAACAC
CAGTGGCGAAGGCGGCACCCTGGCCCACACCTGACGCTGAGGTGCGAAAGCGTGGGTAGC
AAACAGGATTAGATACCCTAGTAGTCC
>Otu2150
CCAGCCTATGGGTCGCAGCAGGCGCGAAACCTTTACAATGCACGAAAGTGTGATAGGGGG
ATACTCAGTGCTTACGACTCTGTTGTAGGCTTTTGTCAATCGTAAATAGATCGGCGAATA
AGTGGTGGGTAAGACTGGTGCCAGCCGCCGCGGTAACCCCAGCGCCACGAGTGGTCATCG
CGATTATTGGGCCTAAAGCGTTCGTAGCCGGTTTGGTGCATCTCTTGTGAAATTGTTCGG
CTTAACCGGACAGCGTGCAGGAGACACGGCCAAACTCGAGACCGGGAGGCGTCAGAAGTA
TGCTGTGGGGACTGGTAAAATGGGATAATCCACAGCAGACTACCGATGGCGAAGGCATCT
GACGAGAACGGATCTGACGGTGAGGAACGAAAGCCAGGGGAGCGAACCGGATTAGAAACC
CTAGTAGTCC
>Otu2151
CCAGCCTACGGGACGCTCCAGCCAAGAATCTTCCGCAATGGGGGAAACCCTGACGGAGCG
ACGCCGCGTGAGGGAAGAAGGTCTTCGGATTGTAAACCTCTCTTTTCAGGGAAGAATAAG
GTTGACAGGAAATGGTCGACTGATGACGGTACCTGATGAATAAGCCCCGGCTAATTACGT
GCCAGCAGCCGCGGTAATACGTAAGGGGCGAGCGTTGCTCGGAATTACTGGGCGTAAAGG
GCGTGCAGGCGGCCTTACAAGTCGAATGTAAAATACCGCAGCTTAACTGCGGAAATGCGT
TCGAAACTGTAAAGCTTGAGTCTGGTGGAGGGTGATGGAATTCCCGGTGTAGCGGTGAAA
TGCGTAGATATCGGGAAGAACACCGAAGGCGAAAGCAGTCATCTATGCCGAGACTGACGC
TGAGGCGCGAAAGCATGGGGATCAAACAGGATTAGAAACCCTTGTAGTCC
>Otu2153
CCAGCCTATGGGAGGCAGCAGTGGGGAATATTGCGCAATGGGCGAAAGCCTGACGCAGCG
ACGCCGCGTGGGTGATGAAGGCCTTCGGGTCGTAAAGCCCTGTCGGGAGGGACGAAACCT

CGGCGACCTAATATGTCGCCGACTTGACGGTACCTCCGAAGGAAGCACCGGCTAACTCCG
TGCCAGCAGCCGCGGTAATACGGAGGGTGCGAGCGTTGTTCGGGATCACTGGGCGTAAAG
CGCGTGTAGGCGGCCTTGTAAGTTTGGTGTGAAAGCCCGGGGCTTACCCCCGGAAGTGCA
CTGGAGACTGCAAGGCTGGAGTACCGGAGAGGAAAGTGGAATTCCTGGTGTAGGAGTGAA
ATCCGTAGATATCAGGAGGAACACCGGTGGCGAAGGCGGCTACCTGGACCGATATTGACG
CTGAGACGCGAAAGCGTGGGGAGCAAACAGGATTAGAAACCCCAGTAGTCC
>Otu2154
CCAGCCTATGGGGTGCAGCAGTTAGGAATTTTGCACAATGGGGGAAACCCTGATGCAGCA
ACGCCGCGTGGGCGAAGAAGGTCTTCGGATCGTAAAGCCCTTTTCTGCGGGAAGAGCAAG
GACGGTACCGCAGGAATAAGCCTCGGCTAACTACGTGCCAGCAGCCGCGGTAATACGTAG
GAGGCGAGCGTTGTCCGGATTTATTGGGCGTAAAGAGCGCGTAGGTGGCTCGACAAGTTG
GGTGTGAAATCCTCTGGCTTAACTGGAGGGGGTCATCCGATACTGTCGGGCTGGAGGCAG
GCAGAGGAAGGTAGAATTCCCGGTGTAGTGGTGAAATGCGTAGATATCGGGAGGAATACC
AGTGGCGAAGGCGGCCTTCTGGGCCTGTTCTGACGCTGAGGCGCGAAAGCGTGGGGAGCG
AACCGGATTAGAAACCCGGGTAGTCC
>Otu2159
CCAGCCTATGGGGGGCAGCAGTGGGGAATCTTGCACAATGGGGGAAACCCTGATGCAGCG
ACGCCGCGTGAGCGATGAAGCCCTTCGGGGTGTAAAGCTCTTTCGACGGGAACGATAATG
ACGGTACCTGCAGAAGAAGCTGCGGCTAACTACGTGCCAGCAGCCGCGGTAATACGTAGG
CAGCGAGCGTTGTTCGGAGTTACTGGGCGTAAAGAGTGCGTAGGCGGTTCTCTAAGTTCG
GTGTGAAATCTCCCGGCTCAACTGGGAGGGTGCGCCGAAAACTGGAGGGCTTGAGTGCGG
GAGAGGAGAGCGGAATTCCTGGTGTAGCGGTGAAATGCGTAGATATCAGGAGGAACACCG
GTGGTGCAGACGGCTCTCTGGACCGCAACTGACGCTGAGGCACGAAAGCGTGGGGAGCAA
ACAGGATTAGATACCCTGGTAGTCC
>Otu2163
CCAGCCTATGGGGGGCTGCAGCAAGGAATAGTAGGCAATGGGGGAAACCCTGACCTCGCG
ACGCCGCGTGAGGGATGAAGGCTTTCGGGTCGTAAACCTCTTTTCCCGGGGATGAATAAT
GACAGTACTCGGGGAATAAGTCACGGCTAACTACGTGCCAGCAGCCGCGGTAAAACGTAG
GTGGCGAACGTTATCCGGATTCACTGGGCGTAAAGCGTGCGTAGGCGGTTCGTTAAGTCG
GATGTAAAATCTCCTGGCTCAACTGGGAGGCACCATCCGATACTGGCGAGCTGGAGTGCA
TCAGAGGAGAATGGAATTCCCGGTGTAGTGGTGGAATGCGTAGATATCGGGAGGGACACC
AGTGGCGAAAGCGGTTCTCTGGGATGTTACTGACGCTGTTGCACGAAAGCGTGGGGAGCA
AACAGGATTAGAGACCCCCGTAGTCC
>Otu2165
CCAGCCTACGGGGTGCACCAGTGAGGAATATTGGTCAATGGGCGAAAGCCTGAACCAGCC
ATGCCGCGTGAAGGATGAAGGCGCTATGCGTTGTAAACTTCTTTTGTACAGGACGAACCG
CACGCTCGTGAGCGTGCCTGACAGTACTGTGCGAATAAGCAACGGCTAACTCCGTGCCAG
CAGCCGCGGTAATACGGAGGTTGCAAGCGTTATCCGGATTTATTGGGTTTAAAGGGTGCG
TAGGCGGAATGATAAGTCAGTGGTGAAAGCCTGTAGCTTAACTATAGAATTGCCATTGAT
ACTGTCGTTCTTGAGTGCAGTTGAGGTAGGCGGAATGTGTAATGTAGCGGTGAAATGCTC
AGATATTACACAGAACACCAATTGCGTAGGCAGATTACTAAACTGTAACTGACGCTGAGG
CACGAAAGCGTGGGTAGCAAACAGGATTAGAGACCCGCGTAGTCC
>Otu2175
CCAGCCTACGGGGTGCTGCAGTGAGGAATTTTGGGCAATGAACGCAAGTTTGACCCATTA
ATAATTAATGAGTGAGAAACGGACAGTACCAGCATCGTAAAACTCTTTTACCAAAAAAGA
TAATGACCGTATTTGGAGAATAAACACCGGCTAATTCTGTGCCAGCAGCCGCGGTAACAC
AGGTAGTGTTAACGTTATTCATCATAATTGGGTGTAAAGGATATGTAGGCGGTTTATGTA
AGATTTTTTGTAAAATTCATTGGTATGACCCAGATGGTTGACAAAAAATACCTAAAAACT
ATGAGTTTAATTTAAGTTAAAAGAATTTCTAAAGTAAAAAAGTCTAAAATCTCTAGAGAT
TAGAACGACTGCCAGATTAGTTACGGAAGTATTTTTCTTAATTAAACTGACGCTGAGATA
TGAAAGTGTAGGGATCAAACAGGATTAGAAACCCCTGTAGTCC
>Otu2177
CCAGCCTATGGGGGGCAGCAGTCGGGAAACTTTTGCAATACACGAAAGTGTGACAAAGCA
AGCCAGAGTGATTTTCTATAATTGAAAATCTTTTGTGGGATGTAAAAAGTCTCACGAATA
AGGACTGGGCAAGACTGGTGCCAGCCGCCGCGGTAATCCCAGCAGTCCAAGTCGCAGCCA
CATTTGTTGGGTCTAAAATATCCGTAGCTTGCTTTTCAAGTCTCTTGTGAAATCGGGACT
CTTAAGGTTCCGGCGGGCAAGAGATACTGTTAAGCTAGAGACCGGGAGGCGTAAGAAGTA
CATTTAGGGTAACGGTAAAATGTGTTGATCCCAAATGGACTAACAGTCGCGAAGGCGTCT

TACGAGAACGGTTCTGACAGTGAGGGATGAAGGCTAGGGGCGCAAAGTGGATTAGAAACC
CTTGTAGTCC
>Otu2183
CCAGCCTACGGGGGGCACCAGCGGGGAATCTTGCACAATGGGGGAAACCCTGATGCAGCG
ACGCCGCGTGAGCGATGAAGCCCTTCGGGGTGTAAAGCTCTTTCGACGGGGAAGATTATG
ACGGTACCCGGAGAAGAAGCTGCGGCTAACTACGTGCCAGCAGCCGCGGTAATACGTAGG
CAGCAAGCGTTGTTCGGAATTACTGGGCGTAAAGAGTGCGTAGGTGGTTGGCTAAGTTTG
GTGTGAAATCTCCTGGCTTAACTGGGAGGGTGCGCCGAAAACTGGTCGACTAGAGTGTGG
GAGAGGGACGTGGAATTCCTGGTGTAGCGGTGAAATGCGTAGATATCAGGAGGAACACCT
GCGGTGTAGACGGCGTCCTGGACCATGACTGACACTGAGGCACGAAAGCGTGGGGAGCAA
ACAGGATTAGAAACCCGAGTAGTCC
>Otu2184
CAGCCTATGGGAGGCACCAGTCGAGGAAATTTTTCAATGCCCGAAAGGGTGAACGAGTAA
GTCAGAGTGTTTTCTTTACGAAAACTTTTGTCTGTTGTAAAAAGACAGACGAATAAGGAC
TGGGCAAGACCGGTGCCAGCCGCCGCGGTAATCCCGGCGGTCCAAGTCGCATCCACAATT
ATTGGGTCTAAAACATCCGTAGCTTGTTTTGTAAGTCTCTTGTGAAATTTCATATCTCAA
ATATGAAGCGTGCAAGAGATACTACATTGCTAGAGACCGGAAGACGCGAAGAGTACGACG
GAGGTAGCGGTAAAATGTGTTAATCTCTGTCGGACTAACAATGGCGAAGGCACTTTGCGA
GTACGGATCTGACAGTGAGGGATGAAGGCTAGGGGCGCAAAAGGGATTAGAAACCCCCGT
AGTCC
>Otu2185
CCAGCCTATGGGAGGCAGCAGCTAAGAATCTTCGGCAATGGGCGCAAGCCTGACCGAGCG
ACGCCGCGTGGAGGATGAAGGCCTTCGGGTTGTAAACTCCTGTCGAGGGGGAGGAAGGGG
CAACCTTGACCGACCCCTGGAGGAAGCACGGGCTAAGTTCGTGCCAGCAGCCGCGGTAAG
ACGAACCGTGCGAACGTTATTCGGAATTACTGGGCTTAAAGCGCGTGTAGGCGGATGGGA
ACGTCGGTCTTTGAAAGCCCCCGGCTCAACCGGGGAAGCGGAGCCGAAACGACCGATCTG
GAGGGACGTAGGGGGACCTGGAACTTCCGGTGGAGCGGTGAAATGCGTTGAGATCGGAAG
GAACGCCCGTGGCGAAAGCGAGGTCCTGGACGTCTGCTGACGCTGAGACGCGAAAGCTAG
GGGAGCAAACGGGATTAGAAACCCCGGTAGTCC
>Otu2188
CCAGCCTATGGGGGGCTGCAGTCGAGAATCTTTCACAATGGGCGAAAGCCTGATGGAGCG
ACGCCGCGTGGGGGATGAATGGCTTCGGCCCGTAAACCCCTGTCATTCGGGAGCAAGGCG
TTTGACCTAACACGTCAAATGTTGATAGTACCGGAAGAGGAAGGGACGGCTAACTCTGTG
CCAGCAGCCGCGGTAATACAGAGGTCCCAAGCGTTGTTCGGATTCACTGGGCGTAAAGGG
TGCGTAGGCGGCCGGGCAAGTCCGGTGTGAAATCTCGGAGCTTAACTCCGAAACGGCACT
GGATACTTTCCGGCTCGAGGTTGGGAGGGGGGACTGGAATTCTCGGTGTAGCAGTGAAAT
GCGTAGATATCGAGAGGAACACCAGTGGCGAAGGCGAGTCCCTGGACCAATCCTGACGCT
GAGGCACGAAAGCCAGGGGAGCAAACGGGATTAGAAACCCTTGTAGTCC
>Otu2189
CCAGCCTACGGGTGGCTGCAGTAGGGAATATTGGGCAATGGAGGCAACTCTGACCCAGCC
ATGCCGCGTGCAGGATGAAGGCCCTCTGGGTTGTAAACTGCTTTTATCAGGGAAGAAACG
CTTGGATTCATCTGAGCCTGACGGTACCTGAGGAATAAGCACCGGCTAACTCCGTGCCAG
CAGCCGCGGTAATACGGAGGGTGCAAGCGTTATCCGGATTTACTGGGTTTAAAGGGTGCG
TAGGCGGGTTATTAAGTCAGTGGTGAAAGATTTGGGCTCAACCCAAAAATTGCCATTGAT
ACTGGTAGCCTCGAGTACAGTTGAGGTGGGCGGAATGTGTCATGTAGCGGTGAAATGCAT
AGATATGACACAGAACACCAATTGCGAAGGCAGCTCGCTAAACTGTAACTGACGCTGAGG
CACGAAAGCGTGGGTAGCAAACAGGATTAGAAACCCCAGTAGTCC
>Otu2192
CCAGCCTACGGGTGGCAGCAGTGGGGAATTTTACGCAATGGGCGAAAGCCTGACGTAGCG
ACACCGCGTGAACGAAGAAGCCTTTTGGGGTGTAAAGTTCTGTCGGCTGGGAAGAAAAAA
ATGACGGTACCAGCAAAGGAAGCATCGGCTAACTACGTGCCAGCAGCCGCGGTAAGACGT
AGGATGCAAGCGTTGTCCGGATTTATTGGGCGTAAAGAGTTCGTAGGCGGTCTGTTAAGT
CTGATGTTAAAGATCGGGGCTCAACCCCGGGAGTGCATTGGATACTGGCAGGCTGGAGTG
CGGTAGAGGCGAGTGGAATTCCCAGTGTAGCGGTGAAATGCGTAGATATTGGGAAGAACA
CCAGTGGCGTAGGCGACTCGCTGGGCCGTAACTGACGCTGAGGAACGAAAGCCAGGGGAG
CAAACAGGATTAGATACCCTAGTAGTCC
>Otu2193
CCAGCCTACGGGGTGCACCAGTCGAGGATCTTTAGCAATGCCCGAAAGGGTGACTATGCG

ACGCCGCGTGCGGGATGAAGGCCTTCGGGTCGTAAACCGCTGTCAGGGAGGAAGAAATCT
CATACGTGAATATCGTATGAGTTTGACATCACTCCCAAAGGAAGCCACGGCTAACTCTGT
GCCAGCAGCCGCGGTAAGACAGAGGCGGCAAGCGTTGTTCGGATTTATTGGGTGTAAAGG
GCAGGTAGGTGGCCTTGCAAGTCCGGTGTGAAATCCCACGGCTCAACCGTGGAACTGCAT
TGGATACTGCTTGGCTTGAGTATCGGAGAGGTGAGGGGAATTCCCGGTGTAAGGGTGAAA
TCTGTAGATATTGGGAGGAATACCAGTGGCGAAGGCGCCTCACTGGCCGTTTACTGACAC
TGAGCTGCGAAAGCTAGGGGAGCAAACAGGATTAGAAACCCTTGTAGTCC
>Otu2195
CCAGCCTATGGGGGGCAGCAGCAAGGAATTTTGCGCAATGGGGGAAACCCTGACGCAGCG
ATGCCGCGTGGAGGACGAAGGTCTTCGGATTGTAAACTCCTTTTGCGGGGGAAAAATGTC
GGCGTGAATAACGTCGAATGATGGTACTCCGCGAATAAGCCACGGCTAACTTCGTGCCAG
CAGCCGCGGTAAGACGAAGGTGGCGAGCGTTACTCGGAATTACTAGGCGTAAAGCGCGAG
CAGGCGGTTCGGTAAGTCCGTCGTGAAAGCCCCGGGCTCAACCCGGGGAGGCCGACGGAT
ACTGCCGGACTTGAATGTGGGAGAGGGGACTGGAATTCCTGGTGTAGCGGTGAAATGCGT
AGATATCAGGAGGAACACCAAAGGCGAAGGCAGGTCCCTGGACCATTATTGACGCTGAGT
CGCGAAAGCCGGGGGAGCAAACAGGATTAGATACCCGCGTAGTCC
>Otu2198
CCAGCCTACGGGGTGCACCAGTGGGGAATATTGGACAATGAGCGAAAGCTTGATCCAGTA
ATAATTCGTGAGTGAGAAAAGGAAAGCAGTAACGCTGTAAAACTCAAAAAATAAAAAAAT
AATGATATTTTTTATGAAAAGCACTGGCTAATTCTGTGCCAGCAGCCGCGGTAAGACAGG
TAGTGCGGACGTTATTCATTTTAATTAGGTGTAAAGGATATGTAGGTTGCTTTTTTGAAT
GTTTTTAAAAATTTCTGAGCGTGATCCTTAGAGTTAAAAAGCATATTTAAAAGCTCGAGT
ATTTAAAACGTTAAAAGAATTTTCTTTGTAAAGGTAGAATTTCTAAATAATTAAAAGACT
ACCAAAAAAATTGTGAAAACATTCTTCGATTAATAACTGACGCTGAGATATGAAAGTATA
AGTATCAAATAGGATTAGAAACCCCTGTAGTCC
>Otu2199
CCAGCCTATGGGAGGCACCAGTGGGGAATCTTGCGCAATGGGCGAAAGCCTGACGCAGCG
ACGCCGCGTGGGTGATGAAGGCCTTCGGGTCGTAAAGCCCTGTCAAGAGGGACGAATCCT
CGTCGACCTAACACGTCGGCGACCTGACGGTACCTCTGAAGGAAGCACCGGCTAACTCCG
TGCCAGCAGCCGCGGTAATACGGAGGGTGCGAGCGTTGTTCGGAATTACTGGGCGTAAAG
CGCGTGTAGGCGGCCTTCTAAGTCTGGTGTGAAAGCCCGGGGCTCACCCCCGGAAGTGCA
TTGGATACTGGGAGGCTGGAGTACCGGAGAGGAGGGTGGAATTCCTGGTGTAGCGGTGAA
ATGCGTAGATATCAGGAGGAACACCGGTGGCGAAGGCGGCCCTCTGGACGGATACTGACG
CTGAGACGCGAAAGCGTGGGGAGCAAACAGGATTAGAAACCCGAGTAGTCC
>Otu2201
CAGCCTATGGGTGGCTGCAGTCGAGAATCTTCGGCAATGGGCGCAAGCCTGACCGAGCGA
CGCCGCGTGTGGGATGAAGGCCTTCGGGTTGTAAACCACTGTCAGAGGGGAGGAAATATG
TGGGGGTACTCTCCCATGTTTGACCTAGCCTCAGAGGAAGGACGGGCTAAGTTCGTGCCA
GCAGCCGCGGTAAGACGAACCGTCCAAACGTTATTCGGAATTACTGGGCTTAAAGGGTGC
GTAGGCGGCGCGGAAAGTAGGGTGTGAAATCCCTCGGCTTAACCGAGGAACTGCGTCCTA
AACTACCGTGCTCGAGGGAGATAGAGGTGAGCGGAACTTAGGGTGGAGCGGTGAAATGCG
TTGATATCCTAAGGAACACCGGTGGCGAAAGCGGCTCACTGGCCCGATACTGACGCTGAG
GCACGAAAGCGTGGGGAGCAAACAGGATTAGAAACCCTCGTAGTCC
>Otu2202
CCAGCCTATGGGGGGCAGCAGTGGGGAATTTTGGACAATGGGGGCAACCCTGATCCAGCC
ATGCCGCGTGTGGGAAGAAGGCCTTCGGGTTGTAAACCACTTTTGTCAGGGAAGAAATTG
GCCAGGTTAACACCTTGGCTAGATGACGGTACCTGGAGAATAAGCACCGGCTAACTACGT
GCCAGCAGCCGCGGTAATACGTAGGGTGCAAGCGTTAATCGGAATTACTGGGCGTAAAGC
GTGCGCAGGCGGTTTCGCAAGTCAGATGTGAAAGCCCCGGGCTCAACCTGGGAATTGCAT
TTGAAACTACGAGGCTAGAGTGTGTCAGAGGGGGGTAGAATTCCACGTGTAGCAGTGAAA
TGCGTAGAGATGTGGAGGAATACCGATGGCGAAGGCAGCCCCCTGGGCTAAGATTGACGC
TCATGCACGAAAGCGTGGGGAGCAAACAGGATTAGAAACCCCAGTAGTCC
>Otu2203
CCAGCCTACGGGAGGCAGCAGTCGAGAATCTTTCGCAATGGGCGCAAGCCTGACGAAGCG
ACGCCGTGTGAGCGAAGAAGGCCTTCGGGTTGTAAAGCTCTTTCGCTAGGGAACAAGAGA
GATAGGATAACACCCTATTGATTTGATGGTACTTGGTAAAGAAGCACCGGCTAACTCCGT
GCCAGCAGCTGCGGTAATACGGAGGGTGCAAGCATTGATCGGAATTACTGGGCGTAAAGG
GCGCGTAGGCGGTGAAATAAGTCGGATGTGAAATTCCGGGGCTCAACCCCGGAGCTGCAT

TTGAAACTGTTTCGCTAGAGTTCAGTTAGGGTAAACGGAATTCCACGTGTAGCGGTGAAA
TGCGTAGATATGTGGAAGAACACCGGTGGTGAAGACGGTTTACTGGGCTGAAACTGACGC
TGAGGCGCGAAAGCATGGGGAGCAAACAGGATTAGAGACCCCAGTAGTCC
>Otu2207
CCAGCCTATGGGTGGCTGCAGACGAGAATATTCCGCAATGGGCGAAAGCCTGACGGAGCG
ACGCCGCGTGATGGATGAAGTGCTTTGGTACGTAAACATCTTTTATCGGGGACGAAGTTT
ATTGACGGTACCCGATGAATAAGGGGCTCCTAACTCTGTGCCAGCAGGAGCGGTAATACA
GAGGCCCCAAGCATTACCCGGAATTACTGGGCGTAAAGAGTGTCGAGGCGGCCATATTAG
TCGTTCGTTAAATCCGCGGGCTCAACCTGCGGTCTGCGAGCGAAACGGTATGGCTTCGAG
GGCGCGAGAGGTGCACGGAACTCATGGTGGAGGGGTGAAATCCGTTGATATCATGGGGAA
CACCAAAGGCGAAGGCAGTGCACTGGTGCGTTCCTGACGCTTAAACACGAAAGCCAGGGG
AGCGAACGGGATTAGATACCCGAGTAGTCC
>Otu2208
CCAGCCTATGGGTCGCTGCAGGCGCGAAAACTCCACAATGTACGAAAGTACGATGGGGGA
ATCCTAAGTGCTTACACACAGTGTAGGCTTTTTTCGAAAGCAAAAATTTCGGAGAATAAG
TGGTGGGCAAGACTGGTGCCAGCCGCCGCGGTAACACCAGCGCCACTAGTGGGAGCCGTA
GTTATTGGGCCTAAAGCGTCCGTAGCCGGTCAGGTAAATCTTTTGTGAAATCGTTGGGCT
TAACTTAACGACGTGCAGAAGACACTGCCAGACTTGGAATTGGGAAGAGTCAGAGGTATT
TCTGGGGGAGCGGTAAAATGTGGTAATCCCGGAAGGACCACCGGTGGCGAAGGCGTCTGA
CTAGAACAACTTCGACGGTGAGGGACGAAAGCCAGGGGAGCGATCCGGATTAGATACCCC
CGTAGTCC
>Otu2209
CCAGCCTATGGGTCGCTCCAGTGGGGAATCTTGCGCAATGGAGGAAACTCTGACGCAGCG
ACGCCGCGTGTGCGATGAAGGTCTTCGGATCGTAAAGCACTGTCGCGAGGGAAAACAAAC
GATGGTACCTCGAGAGGAAGCACCGGCTAACTCTGTGCCAGCAGCCGCGGTAATACAGAG
GGTGCGAGCGTTGTTCGGAATTATTGGGCGTAAAGCGCTGGTAGGCGGCCCGACAAGTCG
CGTGTGAAAGCTCCCGGCTCAACCGGGAAAGTGCGCGCGAAACTGGCGGGCTTGAGTGTC
GGAGAGGGTCGCGGAATTCCCGGTGTAGAGGTGAAATTCGTAGATATCGGGAGGAACACC
AGAGGCGAAGGCGGCGACCTGGAAGACGACCGACGCTGAGCAGCGAAAGCCAGGGGAGCA
AACGGGATTAGAAACCCCCGTAGTCC
>Otu2210
CCAGCCTATGGGGTGCAGCAGTAACGAATATTCCGCAATGGGCGAAAGCCTGACGGAGCG
ACGCCGCGTGGAGGACGAATTCCTTCGGGATGTAAACTCCTTTTAGGTGTAAGAAAACGA
AGCGCGCTAACATCGCGCCAGTTGATCTATGCCAGAAAAAGCATCGGCTAACCCCGTGCC
AGCAGCCGCGGTAATACGGGAGATGCAAGCGTTGTTCGGAATCACTGGGCTTAAAGCGCG
TGTAGGCGGCTTGGTAAGTACTTTGTGAAATCCCTCGGCTCAACCGAGGACCTGCAAGGT
ATACTGCCGAGCTTGAGGCAGGTAGGGGTCACTGGAACTCTAGGTGGAGCGGTGAAATGC
GTAGATATCTAGAGGAACGCCCGTGGCGAAAGCGGGTGACTGGGCCTGCCCTGACGCTGA
GACGCGAAAGCGTGGGTAGCAAACGGGATTAGAAACCCGGGTAGTCC
>Otu2212
CCAGCCTATGGGGCGCTCCAGTGAGGAATTTTGCGCAATGGCCGCAAGGCTGACGCAGCA
ACGCCGCGTGGGTGAAGAAGGCCTTCGGGTCGTAAAGCCCTGTCAGGTGGGAAGAAAGGC
TTGATTGCTAATATCGATTGAGTTTGACGGTACCACCAGAGGAAGCACCGGCTAACTCCG
TGCCAGCAGCCGCGGTAATACGGAGGGTGCGAGCGTTATTCGGAATTATTGGGCGTAAAG
CGCGTGTAGGCGGGGATACAAGTCTGATGTGAAAGCCCTGGGCTTAACCTGGGAAGTGCA
TTGGAAACTGTATTTCTTGAGTACTGGAGAGGAAGGGGGAATTCCCGGTGTAGAGGTGAA
ATTCGTAGAGATCGGGAGGAATACCAGTGGCGTAGGCGCCTTTCTGGACGGTTACTGACG
CTGAGACGCGAAAGCGTGGGGAGCAAACAGGATTAGAGACCCCCGTAGTCC
>Otu2217
CCAGCCTATGGGGTGCAGCAGTCGAGGATCTTCGGCAATGGGCGCAAGCCTGACCGAGCG
ACGCCGCGTGTGCGATGAAGGCCTTCGGGTTGTAAAGCACTGTCGAGGGGGAGGAAGGCG
AAAGCTTGACCTATCCCTGGAGGAAGCACGGGCTAAGTTCGTGCCAGCAGCCGCGGTAAG
ACGAACCGTGCGAACGTTATTCGGAATCACTGGGCTTAAAGGGCGCGTAGGCGGGCGATC
AAGTCCGAGGTGAAATCCTCCAGCTTAACTGGAGAAGTGCCCCGGATACTGGTCGTCTCG
AGGAGGGTAGGGGCATCTGGAACTAGCGGTGGAGCGGTGAAATGCGTTGATATCGCTAGG
AACTCCGATGGCGAAGGCAAGGTGCTGGACCCTTTCTGACGCTGAGGCGCGAAAGCCAGG
GGAGCGAACGGGATTAGATACCCCTGTAGTCC
>Otu2222

CCAGCCTATGGGTTGCAGCAGTGGGGAATTTTGCACAATGCCCGAAAGGGTGATGCAGCA
ACGCCGCGTGAGGGATGAAGGCCTTCGGGTCGTAAACCTCTTTCGATCGGGAAAAACGGC
CCTGCGAAGATCGGGGTCTGATGGTACCGGAAGAAGAAGCCCCGGCCAACTACGTGCCAG
CAGCCGCGGTAATACGTAGGGGGCGAGCGTTGTTCGGAATTACTGGGCGTAAAGGGCGTG
TAGGCGGTGTGGTAAGTTGGGCGTGAAATCCCCGGGCTTAACCCGGGGGGTCATTCAAGA
CTGCCACGCTAGGGTGCGGGAGAGGGCAGTGGAATTCCCGGTGTAGCGGTGAAATGCGTA
GATATCGGGAGGAACACCTGCGGCGAAGGCGGCTGCCTGGGCCGTAACCGACGCTGAGGC
GCGAAAGCTAGGGGAGCAAACAGGATTAGATACCCCGGTAGTCC
>Otu2225
CCAGCCTATGGGGGGCTGCAGTGGGGAATCTTGCGCAATGGACGAAAGTCTGACGCAGCC
ACGCCGCGTGAGTGAAGAAGGCCTTCGGGTTGTAAAGCTCTGTCGTGCGGGACGAATCTC
GTATGGGTGAATAGCCCATACGACTGACGGTACCGCAAAAGGAAGCACCGGCTAACTTCG
TGCCAGCAGCCGCGGTAATACGAGGGGTGCAAGCGTTGCTCGGAATTATTGGGCGTAAAG
GGTAGGTAGGTGGTCTCATTTGTCTGGGGTGAAAGCCTTGAGCTTAACTCAAGAAGTGCC
CCGGAAACGGTGAGACTGGAGTCTTGGAGAGGGTCGTGGAATTCCCGGTGTAGCGGTGAA
ATGCGTAGAGATCGGGAGGAACACCAGAGGCGAAGGCGGCGACCTGGACAAGTACTGACA
CTCAACTACGAAAGCGTGGGGAGCAAACAGGATTAGAAACCCTTGTAGTCC
>Otu2226
CCAGCCTACGGGACGCAGCAGTGGGGAATTTTGGACAATGGGGGAAACCCTGATCCAGCA
ACTCTGCGTGGAGGACGAAGTCTCTCGGGATGTAAACTCCTTTTCCAGGGGACGAATAGG
CGCAAGCCTTTGACGGTACTCTGGGAATAAGCAACGGCTAACTACGTGCCAGCAGCCGCG
GTAAGACGTAGGTTGCGAGCGTTATTCGGATTCACTAGGCGTAAAGCGTGTGTAGGTGGA
TGGCTAAGTCTGTTGTGAAATCTCCTGGCTCAACTGGGAGAGGTCAATAGATACTGGCTG
TCTTGAGTTGGGTAGGGGGAACCGGAATTCCAGGTGTAGCGGTGAAATGCGCAGATATCT
GGAGGAACACCGGCGGCGAAGGCGGGTTCCTGGGCCTTAACTGACACTGAGACACGAAAG
CTAGGGGAGCAAACAGGATTAGATACCCCTGTAGTCC
>Otu2232
CCAGCCTATGGGAGGCAGCAGTGGGGAATCTTGTGCAATGGGCGAAAGCCTGACGCAGCG
ACGCCGCGTGGGTGATGAAGGCCTTCGGGTTGTAAAGCCCTTTGGGTGGAGACGAATAAG
TCGTAGCTAACACCTACGATGATGACGGTATCCACATAACAAGCACCGGCTAACTCTGTG
CCAGCAGCCGCGGTAAGACAGAGGGTGCAAACGTTGTTCGGAATTACTGGGCGTAAAGCG
CATGTAGGCGGCCAAGCAGGTCGGATGTGAAATCCCTCGGCTCAACCAAGGAAGTGCATT
CGAAACCGCATGGCTAGAGTCCCGGAGAGGAAGGTGGAATTCTCGGTGTAGAGGTGAAAT
TCGTAGATATCGAGAGGAACACCTGTGGCGAAAGCGGCCTTCTGGACGAGGACTGACGCT
GAGATGCGAAAGCGTGGGGAGCAAACAGGATTAGATACCCTTGTAGTCC
>Otu2233
CCAGCCTATGGGGTGCTGCAGTGGGGAATTTTCCGCAACGGGCGAAAGCCTGACGGAGCA
ATACCGCGTGAGGGAGGAAGGCTCTTGGGTTGTAAACCTCTTTTCTCAGGGAATAAGAAA
GTGAAGGTACCTGAGGAAGAAGCATCGGCTAACTCCGTGCCAGCAGCCGCGGTAATACGG
AGGATGCAAGCGTTATCCGGAATGATTGGGCGTAAAGGGTCCGCAGGTGGAAATGAAAGT
CTGCTGTTAAAGAGTTCTGCTCAACAGAATAAAGGCGGTGGAAACTACATAACTAGAGTA
CGTTCGGGGCAGAGGGAATTCCTGGTGTAGCGGTGAAATGCGTAGATATCAGGAAGAACA
CCGGTGGCGAAGGCGCTCTGCTAGGCCGTGACTGACACTGAGGGACGAAAGCTAGGGGAG
CGAATGGGATTAGATACCCGGGTAGTCC
>Otu2236
CCAGCCTATGGGGGGCAGCAGTCGAGAATCTTCCGCAATGGGCGAAAGCCTGACGGAGCG
ACGCCGCGTGATTGATGAAGTCCTTCGGGACGTAAAGATCTTTTATGCGTGAGAAAGTTA
TTGATGTTAGCGCATGAATAAGGGGCTCCTAACTCTGTGCCAGCAGGAGCGGTAATACAG
AGGCCCCAAGCATTATCCGGAATCACTGGGCGTAAAGGGTGTGTAGGCGGCGTTGTTAGT
CTTTCGTGAAGGGTCTCTGGCTTAACTAGGGAACCGCGGAGGAAACGGCAATGCTTCGAG
GACGTGAGAGGTATATGGAACTCAAGGTGTAGGGGTGAAATCCGTTGATATCTTGGGGAA
CACCGAATGCGAAGGCAATATACTGGCGCGCTCCTGACGCTGAAACACGAAAGCGTGGGA
ATCGAACGGGATTAGATACCCTGGTAGTCC
>Otu2237
CCAGCCTATGGGTGGCAGCAGTTGGGGATTTTGCACAATGGGCGCAAGCCTGATGCAGCA
ACACTGCGTGAAGGATGAAGGTTTTCGGATCGTAAACTTCTTTTATGAGGGAATAATCTT
GAAGGTACCTCATGAATAAGCACCGGCTAACTACGTGCCTGCAGCCGCGGTAATACGTAG
GGTGCAAACGTTATCCGGATTTATTGGGCGTAAAGAGCTCGTAGGCGGAATTGTAAGTTG

GATATTAAAGACTCAGGCTCAACTTGGGGAAAGTATCCAAAACTGCAAATCTAGAGTGAT
GCAGGGGAGAACGGAACTTACAGTGTAGCGGTGGAATGCGTAGATATTGTAAGGAACGCC
AATGGTGAAGACAGTTCTCTGGGCATTTTCTGACGCTGAGGAGCGAAAGCGTGGGTAGCG
ATCGGGATTAGAAACCCGAGTAGTCC
>Otu2242
CCAGCCTACGGGGGGCAGCAGTGGGGAATATTCCGCAATGGGCGCAAGCCTGACGGAGCG
ACGCCGCGTGGATGATGACAGTCTTCGGATTGTAAAGTCCTGTAGAGGGGGAAGAAGGGT
GCGCAAGCACTTGACAGTACTCCTAAAGTAAGCTACGGCAAACTCTGTGCCAGCAGCCGC
GGTAATACAGAGGTAGCAAGCGTTGTTCGGAATGACTGGGTGTAAAGCGCGTGTAGGCGG
TCTTTTAAGTTGAATGTGAAATCTCCGGGCTTAACCCGGAAACGGCATTCAATACTGATT
GACTTGAGTGCGAAAGAGGAAAGTGGAATTTCGGGTGTAGCGGTGAAATGCGTAGATATC
CGAAGGAACACCGGTGGCGAAGGCGACTTTCTGGTTCGCAACTGACGCTGAGACGCGAAA
GCTAGGGGAGCAAACAGGATTAGAAACCCGCGTAGTCC
>Otu2247
CCAGCCTACGGGAGGCAGCAGTCGAGAATATTCGACAATGGGCGAAAGCCTGATCGAGCG
ACGCCGCGTGCAGGATGAAGCTCTTCGGAGTGTAAACTGCTTTTATGTTCTAGAAAGTTA
TTGATCGGAACATGAATAAGGAGCTGCAAACTTCGTGCCAGCAGCCGCGGTAATACGAAG
GCTCCAAGCGTTATCCGGATTTATTGGGCGTAAAGGGCGCGTAGGGGGTTCCATGTGTTT
CTGGTTAAAGACCGAAGCTTAACTTCGGAGTTGCCAGGAAAACTATGAAACTTGAGGGTG
TTAGAGGTGAGTAGAACGCACGGTGTAGGGGTGAAATCCGTTGATATCGTGCGGAATACC
AAAGGCGAAGGCAGCTCACTGGGACATTCCTGACCCTGAGGCGCGAAAGCGTGGGGAGCA
AAAAGGATTAGATACCCCTGTAGTCC
>Otu2248
CCAGCCTATGGGGGGCAGCAGTGGGGAATATTGGACAATGGGCGCAAGCCTGATCCAGCA
ATACCGCGTGTGTGAAGAAGGCCTTAGGGTTGTAAAGCACTTTCAGTAGCTAAGAAATAC
TCAAGATTAATATCCTTGAGAGTTGACGTAAACTACGGAAGAAGCACCGGCTAACTCTGT
GCCAGCAGCCGCGGTAATACAGAGGGTGCAAGCGTTAATCGGAATTACTGGGCGTAGAGC
GCACGTAGGTGGGTGCTTAAGTCGAATGTGAAACCCCCGAGCTCAACTTGGGGACTGCAT
GCGATACTGAACACCTAGAGTATAGTAGAGGGAAGTGGAATTTCCGGAGTAGCGGTGAAA
TGCGTAGATATCGGAAGGAACACCAGTGGCGAAGGCGGCTTCCTGGGCTAATACTGACAC
TGAGGTGCGAAAGCGCGGGGAGCAAACAGGATTAGATACCCTTGTAGTCC
>Otu2250
CCAGCCTATGGGAGGCTCCAGTAGGGAATCTTGCGCAATGGGCGAAAGCCTGACGCAGCA
ACGCCGCGTGGGGGATGAATGCCTTCGGGTTGTAAACCCCTTTCAGTGGGAACGAAATTG
ACGGTACCCACAAAAGAAGCCCCGGCCAACTACGTGCCAGCAGCCGCGGTGATACGTAGG
GGGCGAGCGTTGTCCGGATTTATTGGGCGTAAAGAGCTCGTAGGCGGCTTAGTAAGTCGG
CGTGTTAAACCTCCAGGCTCAACCTGGAGTCGCACCCGATACTGCTATGGCTAGAGTCCG
GTAGGGGGTCACGGAATTCCTGGTGTAGCGGTGAAATGCGCAGATATCAGGAGGAACACC
AGTGGCGAAGGCGGTGGCCTGGGCCGGTACTGACGCTGAGGAGCGAAAGCGTGGGGAGCG
AACAGGATTAGAAACCCCTGTAGTCC
>Otu2251
CCAGCCTACGGGGCGCAGCAGTGAGGGATATTCGACAATGGGGGAAACCCTGATCGAGCG
ACGCCGCGTGCAGGAAGAAGCTTTTCGGAGCGTAAACTGCTTTTCTGTGAGACGAGAGAG
GACGGTATCACAGGAACTAAGTCACGGCTAACTACGTGCCAGCAGCCGCGGTAATACGTA
GGTGGCAAGCGTTGTCCGGAGTTACTGGGCGTAAAGGGTGAGTAGGCGGGATGTTGCGTC
CCTAGTGAAATCTCCTGGCTTAACTGGGAGGGGTTTAGGGATACGGGCATTCTTGAGGTA
TGGAGAGGAAAGCAGAATTCCTGGTGTAACAGTGAAATGTGTAGATATCAGGAGGAATAC
CGGAGGGGAAGCCGGCTTTCTGGCCACGACCTGACGCTGATGCACGAAAGCTTGGGGAGC
AAACAGGATTAGAAACCCTCGTAGTCC
>Otu2253
CCAGCCTACGGGTGGCTGCAGCAAGGAATATTGTACAATGGGCGAAAGCCTGATACAGCG
ACGCCGCGTGAGGGATGAAGATTCTCGGATTGTAAACCTCTTTTCTCAGGGACGAACAAT
GACGGTACCTGGGGAATAAGTCACGGCTAACTACGTGCCAGCAGCCGCGGTAATACGTAG
GTGGCTAGCGTTATCCGGATTTATTGGGCGTAAAGAGAGCGTAGGTGGTTTGGCAAGTCG
GATGTAAAATCTCCCGGCTTAACTGGGAGGGATCATTCGATACTGTTAGACTGGAGTGCA
GCAGAGGGAGTTGGAATTCCCGGTGTAGTGGTGAAATGCGTAGATATCGGGAGGAACACC
AGTGGCGAAGGCGAGCTCCTGGGCTGCTACTGACACTGAGGCTCGAAAGCGTGGGGAGCG
AACAGGATTAGAAACCCTGGTAGTCC

>Otu2256
CCAGCCTACGGGGTGCTCCAGCAAGGAATTTTGCGCAATGGGCGAAAGCCTGACGCAGCA
ACACCGCGTGAGGGAAGAAGGCCCTCGGGTTGTAAACCTCTTTTGTCAGGGAAGAATAAT
GACGGTACCTGGCGAATAAGTCACGGCTAACTACGTGCCAGCAGCTGCGGTAATACGTAG
GTGGCAAGCGTTATCCGGATTCACTGGGCGTAAAGAGCGCGTAGGTGGTCCTTCAAGTCA
GATGTTAAATTTTCCGGCTCAACTGGAAAATGTCATTTGATACTGCTGGACTTGAGGGTG
GTAGAAGGAGGTGGAATTCCCGGTGTAGTGGTGAAATGCGTAGATATCGGGAGGAACACC
AGTGGCGAAAGCGGCCTCCTAGGCCATTCCTGACACTGAGGCGCGAAAGCGTGGGGAGCG
AACAGGATTAGAAACCCTAGTAGTCC
>Otu2257
CCAGCCTACGGGTGGCTGCAGTAGGGAATATTGGACAATGGGCGCAAGCCTGATCCAGCC
ATGCCGCGTGAGTGATGAAGGCCTTAGGGTTGTAAAGCTCTTTCACCCACGACGATAATG
ACGGTAGTGGGAGAAGAAGCCCCGGCTAACTTCGTGCCAGCAGCCGCGGTAATACGAAGG
GGGCTAGCGTTGTTCGGAATCACTGGGCGTAAAGCGCGCGTAGGCGGACCGTTAAGTCGG
AGGTGAAAGCCTGGAGCTCAACTCCAGAACTGCCTTCGATACTGGCGGTCTCGAGTCCGG
GAGAGGTGAGTGGAACTGCTAGTGTAGAGGTGAAATTCGTAGATATTAGCAAGAACACCA
GTGGCGAAGGCGGCTCACTGGCCCGGTACTGACGCTGAGGTGCGAAAGCGTGGGGAGCAA
ACAGGATTAGAAACCCCCGTAGTCCG
>Otu2259
CCAGCCTATGGGTTGCTGCAGTGAGGAATTATGCGCAATGGGGGAAACCCTGACGCTGCG
ACGCCGCGTGGGTGATGAAGGTCTTCGGATCGTAAAACCCTGTCGAGAGGGGCGAAAAGC
CCTGGTGCTAATACCACTGGGGCCTGACGGTACCTCTGGAGGAAGTCCCGGCTAACTCCG
TGCCAGCAGCCGCGGTAATACGGAGGGGGCGAGCGTTGTTCGGAATTACTGGGCGTAAAG
CGCGCGTAGGCGGTCGTGTAAGTCGGATGTGAAAGCCCTCAGCTCAACTGAGGAATTGCG
TCCGATACTGCTCGGCTTGAGTCCGGTAGGGGATGGCAGAATTCCCGGTGTAGCGGTGGA
ATGCGTAGATATCGGGAGGAATACCGATGGCGAAGGCGGCCATCTGGGCCGGAACTGACG
CTGATGCGCGAAAGCTAGGGGAGCAAACAGGATTAGATACCCTCGTAGTCC
>Otu2260
CCAGCCTACGGGGCGCTGCAGTGGGGAATTTTGCGCAATGGGGGAAACCCTGACGCAGCA
ACGCCGCGTGGAGGATGAAGTACTTCGGTACGTAAACTCCTTTCGATCGGGACGATAATG
ACGGTACCGGAAGAAGAAGCCCCGGCTAACTTCGTGCCAGCAGCCGCGGTAATACGAGGG
GGGCAAGCGTTGTTCAGAATTATTGGGCGTAAAGGGTGCGTAGGCGGTTTGGTAAGTCTT
TTGTGAAATCTATGGGCTCAACTCATAGACTGCAAGGGAAACTGCCGGGCTTGAGTGTGG
GAGAGGTGAGTGGAATTTCCGGTGTAGCGGTGAAATGCGTAGATATCGGAAGGAACACCA
AAGGCGAAGGCGGCTACCTGCACCGATATTGACGCTGAGACGCGAAAGCGTGGGGAGCAA
ACAGGATTAGATACCCGAGTAGTCC
>Otu2263
CCAGCCTACGGGAGGCAGCAGTCGAGAATATTCCACAATGGACGAAAGTCTGATGGAGCG
ACGCCGCGTGCAGGATGAAGGCCTTCGGGTCGTAAACTGCGGTAGTAAGTCAGCAATGCA
AATGAGCAACTTACGGAAAGAGGTTGGTAACTACGTGCCAGCACCCGCGGTAAAACGTAG
ACCTCAAGCGTTATCCGGATTTATTGGGCGTAAAGAGCATGTAGGAGGTTTCGCGCGTCT
TCTGTTAAAGCCCACCGCCCAACGGTGGAAGTGCAGGAGATACGGCGAGACTAGAGGATG
TTAGAGGTACCAGGAACACACGGTGTAGGGGTGAAATCCGTTGATATCGTGTGGAACACC
AAAGGCGAAGGCAAGGTACTGGGACACTCCTGACTCTGAGATGCGAAAGCGTGGGGAGCA
AAAAGGATTAGAGACCCCTGTAGTCC
>Otu2264
CCAGCCTACGGGATGCAGCAGTAGGGAATTTTCCGCAATGGGCGAAAGCCTGACGGAGCG
ACGCCGCGTGAGGGACGAAGGTCTTCGGATTGTAAACCTCTTTGGAGTGAGACGAACGAG
CTTACGGTGAATAATCGTAGGCAGTGACGGTATCACTAGAACAAGTCACGGCTAACTCTG
TGCCAGCAGCCGCGGTAATACAGAGGTGACGAACGTTGTCCGGAATTACTGGGCGTAAAG
GGCGTGTAGGTGGTCAGATAAGTCAGATGTGAAAGCTCTGGGCTTAACCCGGGAATTGCA
TTTGATACTGTTTGGCTTGAGTACGGTAGAGGAGGGTGGAATTCCCAGTGTAGCGGTGAA
ATGTGTAGATATTGGGAGGAACACCGGTGGCGAAGGCGGCCCTCTGGACCGAGACTGACG
CTGAAACGCGAAAGCTAGGGGAGCAAACGGGATTAGATACCCGCGTAGTCC
>Otu2265
CAGCCTATGGGTGGCTGCAGTAACGAATATTGGGCAATGGGCGAAAGCCTGACCCAGCGA
CGCCGCGTGTGGGAAGAAGTTCTTCGGAATGTAAACCACTGTCAGGGATTACCAAGCGAT
CCCGACTAATAGTCGGGAGAGTTGAGGAGTCCCAGAGGAAGCAACGGCTAACTTCGTGCC

AGCAGCCGCGGTAATACGAAGGTTGCGAGCGTTGTTCGGAATCACTGGGCTTAAAGAGCA
CGTAGGCGGCCCATCAAGTGCCTTGTGAAATCCCCCGGCTCAACCGGGGAATTGCTTGGT
AGACTGCTGGGCTTGAGGCAAGTAGAGGCGCGTGGAACTCTTGGTGGAGCGGTGGAATGC
GTAGATATCAAGAGGAACGCCGGTGGCGAAAGCGGCGCGCTGGGCTTGTCCTGACGCTGA
GGTGCGAAAGCCAGGGGAGCAAACGGGATTAGAAACCCCGGTAGTCC
>Otu2266
CCAGCCTATGGGACGCACCAGTCGAGGATCATTCGCAATGGGCGAAAGCCTGACGATGCG
ACGCTGTGTGAGTGATGAAGGCCTTCGGGTCGTAAAGCTCTTTCGCCTTGGAACAAGAGA
GTGTAGTGAATAACTATACAATTTGAGGGTACAAGGTAAAGAAGCACCGGCTAACTCCGT
GCCAGCAGCTGCGGTAATACGGAGGGTGCAAGCATTAATCGGAATTATTGGGCGTAAAGG
GCGCGTAGGCGGATTTGTAAGTCAGATGTGAAATTCCGAAGCTCAACTTCGGAGCTGCAT
TTGAAACTGTAAGTCTAGAGGAATGGCGGAGAAAATGGAATTCCACGTGTAGCGGTGAAA
TGCGTAGATATGTGGAAGAACACCGGTGGCGAAAGCGGTTTTCTAGCTTTTTCCTGACGC
TGAGGCGCGAGAGCAAGGGGAGCAAACAGGATTAGATACCCCTGTAGTCC
>Otu2268
CCAGCCTACGGGTGGCTGCAGGTACGAGGTCTCCGCAATGCACGAAAGTGTGACGGGGGA
ATTCCAAGTGCTTTGCCTTCGGGCAAAGCTGTGTTAAAGTCTAAATAGCTTTAAAAGAAA
GGGGTGGGAAAGACCGGTGCCAGCAGCCGCGGTAATCCCGGCACCTCAAGTAGTGACCGC
TATTATTGGGCCTAAAGTATCCGTAGCCGGATTCATAAGTTCTCTGTGAAATCGCGTGGC
TTAACCATGCGGCGTGCAGAGAATACTGTTAATCTAGAGACCGGGTGGGGTCAGGGGTAT
TCCTAGGGTAGCGGCTAAATGTTATAATCCTAGGAGGTCCAACAGTGGTGGAGACGCCTG
ACTAGAACGGGTCTGACGGTGAATGGATGAAAGCTGGGGGAGCAAAGCGGATTAGAGACC
CCCGTAGTCC
>Otu2271
CCAGCCTACGGGTCGCTGCAGTGGGGAATCTTGCACAATGGGGGAAACCCTGATGCAGCG
ACGCCGCGTGAGCGATGAAGCCCTTCGGGGTGTAAAGCTCTTTCGGCAGGAAAGATTATG
ACGGTACCTGCGGAAGAAGCTGCGGCTAACTACGTGCCAGCAGCCGCGGTAATACGTAGG
CAGCGAGCGTTGTTCGGAGTTACTGGGCGTAAAGAGTGCGTAGGTGGTTTTCTAAGTTTG
GTGTGAAATCTCCCGGCTCAACTGGGAGGGTGCGCCGAAAACTGGAAGGCTAGAGTGTGG
GAGAGGAGAGCGGAATTCCTGGTGTAGCGGTGAAATGCGTAGATATCAGGAGGAACACCT
GCGGTGCAGACGGCTCTCTGGACCATTACCGACACTGAGGCACGAAAGCGTGGGGAGCAA
ACAGGATTAGAAACCCCAGTAGTCC
>Otu2273
CCAGCCTACGGGGTGCTCCAGGTAGGAAACTTCTGCAATACACGCAAGTGTGACAGAGCA
AGCCAGAGTGTTTTTCATATAATTTGAAAATCTTTTGCGGGATGTAAAAAGTCCCGAGAA
TAAGGACCGGGCAAGACTGGTGCCAGCCGCCGCGGTAATCCCAGCGGTCCAAGTCGCAGC
CACAATTATTGGGTCTAAAACATCCGTAGCTTGCTTTGCAAGTCTTTTGTGAAATCGGGA
ATCTTAAGTTTCCGGCGAGCATAAGATACTGCTTAGCTTGAGACCGGGAGATGCAAGGAG
TACGTTAGGAGTAGCGGTAAAATGCGTTGATCCTTAACGGACTAACAACAGCGAAGGCAC
CTTGCAAGAACGGATCTGACAGTGAGGGATGAAGGCTAGGGGCGCAAAGTGGATTAGATA
CCCCTGTAGTCC
>Otu2274
CCAGCCTACGGGTCGCAGCAGTTTCGAATCTTTCGCAATGGGCGCAAGCCTGACGAAGCG
ACGCCGCGTGGGGGATGAAGGTCTTCGGATCGTAAACCCCTGTCAGCCGTGAACAATGTC
ACTCATTTAACAGACGGGTGATTTGATGGTAACGGCAGAGGAAGAGGTTGCTAACTCTGT
GCCAGCAGCAGCGGTAATACAGAGACCTCAAGCGTTGTTCGGATTCATTGGGCGTAAAGA
GTGCGCAGGCGGCTGGGTGTGTCGGACGTGAAATCCCGAGGCTTAACCTCGGAAGTGCAT
CCGAAACTGCCCGGCTGGAGGTTTGCAGAGGCGAGCGGAATTCACGGTGTAGCGGTGAAA
TGCGTAGATATCGTGAGGAAGACCAGTGGCGAAGGCGGCTCGCTGGGCAAATCCTGACGC
TGGTGCACGAAAGCGTGGGGAGCCAATGGGATTAGATACCCCAGTAGTCC
>Otu2275
CCAGCCTACGGGTGGCAGCAGTGAGGAATATTGGTCAATGGGCGCAAGCCTGAACCAGCC
ATGCCGCGTGAAGGATGAATGCCCTATGGGTTGTAAACTTCTTTTGTACGGGACGAAACC
CCTGAACGTGTTTGGGGCTGACGGTACCGTACGAATAAGCATCGGCTAACTCCGTGCCAG
CAGCCGCGGTAATACGGAGGATGCAAGCGTTATCCGGATTCATTGGGTTTAAAGGGTGCG
TAGGCGGGAAAATAAGTCAGTGGTGAAAGCCTACAGCTTAACTGTAGAATTGCCATTGAT
ACTGTTATTCTTGAGTACATTTGAAGTGGGTGGAATGTGTGGTGTAGCGGTGAAATGCTT
AGATATCACACAGAACGCCAATTGCGAAGGCAGCTCACTAAGCTGTTACTGACGCTGAGG

CACGAAAGCGTGGGGATCAAACAGGATTAGATACCCGAGTAGTCC
>Otu2276
CCAGCCTATGGGGGGCAGCAGTGGGGAATATTGGGCAATGGGCGAAAGCCTGACCCAGCC
ATGCCGCGTGCAGGAAGACGGCCCTACGGGTTGTAAACTGCTTTTGCAGGGGAATAAACC
TCATTACGTGTAATGAGCTGAATGTACTCTGAGAATAAGGATCGGCTAACTCCGTGCCAG
CAGCCGCGGTAATACGGAGGATCCAAGCGTTATCCGGATTTATTGGGTTTAAAGGGTGCG
TAGGCGGCCAATTAAGTCAGGGGTGAAAGACGGTAGCTTAACTATCGCAGTGCCTTTGAT
ACTGATTGGCTTGAATGGACTAGAGGTAGGCGGAATGTGACAAGTAGCGGTGAAATGCAT
AGATATGTCACAGAACACCAATTGCGAAGGCAGCTTACTATGGTTTTATTGACGCTGAGG
CACGAAAGCGTGGGGATCAAACAGGATTAGAAACCCTGGTAGTCC
>Otu2277
CCAGCCTATGGGGGGCAGCAGTGGGGAATATTGGGCAATGGGCGCAAGCCTGACCCAGCC
ACGCCGCGTGAGTGATGGAGGCCTTCGGGTCGTAAAGCTCTGTGGGGAGGGACGAACCTC
TGCAACCCAATACGTTGCAGACTGACGGTACCTCCTTAGCAAGCACCGGCCAACTCCGTG
CCAGCAGCCGCGGTAATACGGAGGGTGCAAACGTTGCTCGGAATCATTGGGCGTAAAGCG
CGCGTAGGCGGTCCAGTCAGTCGGGTGTGAAAGCCCTCGGCTTAACCGAGGAAGTGCATC
CGAAACTACTGGGCTAGAGTACCGGAGAGGACGACGGAATTCCCGGTGTAGAGGTGAAAT
TCGTAGATATCGGGAGGAACACCAGCGGCGAAGGCGGTCGTCTGGACGGATACTGACGCT
GAGGCGCGAAAGCGTGGGGAGCAAACAGGATTAGAAACCCGTGTAGTCC
>Otu2278
CCAGCCTATGGGGGGCAGCAGGCGCGAGAATTCTCCAATGCACGCAAGTGTGAGAGAGGA
ATCTTGAGTGCTTTTCTTAATGAAAAGCTTTTGCGTACTGTAAAAAGGTACGAGAATAAG
GGCTGGGCAAGATCGGTGCCAGCAGCCGCGGTAATCCCGACGGCCCAAGTGGGACTCGCA
ATTATTGGGTCTAAAACATTCGTAGCTGGTTTTATAAGTTTCTTGTTAAATTGCAAGGCT
CAACCTTGTAGCTGCAAGAAATACTATCAAGCTGGAGACTGGGAGACGTAATGAGTACAG
CAAGGGTAATGGTAAAATATTCTAATCCTAGCTGGACTAACAATGGCGAAGGCACGTTAC
GAGAACAGTTCTGACAGTGAGGAATGAAGGCCAGGGGCGCAAATTGGATTAGAAACCCCA
GTAGTCC
>Otu2280
CCAGCCTATGGGTGGCAGCAGTCGAGAATCTTTAGCAATGGGCGAAAGCCTGACTATGCG
ACGCCGCGTGAGGGATGAAGGTTTTCGGATCGTAAACCTCTTTCGAAAGGGAAGAAGTCC
GCCTTTGGCGGATTGACGGTACCTTGAGAAGAAGCCACGGCCAACTCCGTGCCAGCAGCC
GCGGTAATACGGGGGTGGCGAGCGTTACTCGGAATTACTGGGTGTAAAGGGCAAGTAGGT
GGCTGTGTAAGTTGGGGGTGAAATGCTTTGACTCAATCAAAGAACTGCCTTCAAAACTGC
ATAGCTTGAGGCTGGAATAGGAGAGCGGAATTCCCGGTGTAAGGGTGGAATCTGTAGATA
TCGGGAGGAACACCAGTTGCGAAGGCGGCTCTCTAGTCTAGATCTGACACTAATTTGCGA
AAGCTAGGGTAGCAAACAGGATTAGATACCCCTGTAGTCC
>Otu2281
CCAGCCTATGGGGGGCTCCAGTGAGGAATATTGCGCAATGCCCGCAAGGGTGACGCAGCG
ACGCCGCGTGGAGGATGAAGGCCCTATGGGTCGTAAACTCCTTTTGAGAGGGAAGAATGG
TCTGCTACAGGCAGGCTTGACTGTACCTTTAGAAGAAGCACCGGCTAACTACGTGCCAGC
AGCCGCGGTAATACGTAGGGTGCGAGCGTTGTCCGGAATTACTGGGTGTAAAGGGAGCGC
AAGCGGGTCAGTAAGTCATTGGTGAAAACGCGGAGCTCAACTCCGACGATGCCATTGATA
CTATTGATCTTGAGTGCAGAAGAGGAAGATGGAATTCCTGGTGTAGCGGTGAAATGCGTA
GATATCAGGAAGAACACCGGTGGCGAAGGCGGTCTTCTGGTCTGCAACTGACGCCCGGGC
TCGAAAGCGTGGGGAGCAAACAGGATTAGAGACCCCAGTAGTCC
>Otu2283
CCAGCCTACGGGGTGCACCAGTCGAGAATTTTTCTCAATGGGGGAAACCCTGAAGGAGCG
ACGCCGCGTGAAGGATGAAGGTCTTCGGATTGTAAACTTCTGTCATTAGAGAACAAGTGC
ACGGGCAGTAACTGGCCTGTGTTTGATAGTATCTGAAGAGGAAGGGACGGCTAACTCTGT
GCCAGCAGCCGCGGTAATACAGAGGTCCCAAGCGTTGTTCGGATTCATTGGGCGTAAAGG
GTGCGTAGGCGGTGCCGTAAGTCGGATGTGAAATCTCGGAGCTCAACTCCGAAACTGCAT
TCGATACTGCGGTGCTTGAGGACTGGAGAGGAGATCGGAATTCACGGTGTAGCAGTGAAA
TGCGTAGATATCGTGAGGAAGACCGGTGGCGAAGGCGGATCTCTGGACAGTTCCTGACGC
TGAGGCACGAAGGCCAGGGGAGCAAACGGGATTAGAAACCCCAGTAGTCC
>Otu2291
CCAGCCTACGGGTGGCACCAGTGGGGAATATTGCGCAATGGTCGAGAGACTGACGCAGCG
ACGCCGCGTGTGGGATGAAGCTCTTCGGGGTGTAAACCACTGTTGCCTGGGACGAACGGG

CGGATCAATCCGCCGTGACGGTACCAGGTGAGGAAGCACCGGCTAACTCCGTGCCAGCAG
CCGCGGTAATACGGAGGGTGCGAGCGTTGTCCGGAGTCACTGGGCGTAAAGGGCGCGTAG
GTGGCTTTGTAAGCGGGCGGTGAAAGTCCGGGGCTCAACCCCGGATCGGCCGTGCGGACT
GCAAAGCTCGAGCACTGTAGAGGCAGGTGGAATTCCGGGTGTAGCGGTGGAATGCGTAGA
GATCCGGAGGAACACCAGTGGCGAAGGCGGCCTGCTGGGCAGTGGCTGACACTGAGGCGC
GACAGCGTGGGGAGCAAACAGGATTAGATACCCGAGTAGTCC
>Otu2292
CCAGCCTATGGGACGCAGCAGGCGCGAAACCTCCACAATGCACGAAAGTGTGATGGGGGG
ATCCTACGTGCTCATGTCTAACATGGGCTTTTGCTGAGACCAGACATCTCGGCGAATAAG
GGGTGGGCAAGACCGGTGCCAGCCGCCGCGGTAACCCCGGCGCCCCGAGTGGCAATCACG
ATTATTGGGCCTAAAGCGTCCGTAGCCGGACGGGTAAATCCTCTGTGAAATCGCTGCGCT
TAACGTAGCGGCACGCAGGGGAAACTGCTCGTCTCGAGACCGGGAGGGGTCAGAGGTATG
CCGTGGGGACTGGTAAAATGGTATAATCCACGGTAGACCACCAATGGCGAAGGCATCTGA
CCGGAACGGATCTGACGGTGAGGGACGAAAGCTGGGGGAGCGAACCGGATTAGATACCCT
TGTAGTCC
>Otu2295
CCAGCCTACGGGTCGCTCCAGGCGCGAAAACTTTCCAATGCACGCAAGTGTGAGAAGGGA
ATCCTAAGTGCTCAAGCAAAGCTTGGGCTTTTGCTATGTGTAAAAAACATGGCGAATAAG
TGGTGGGCAAGACTGGTGCCAGCCGCCGCGGTAACCCCAGCGCCACAAGTGGCATTCGCG
TTTATTGGGCCTAAAGCGTCCGTAGCAGGTTCTGAAAATTCTTTGTGAAATTGCCAGGCT
CAACTTGGCAGAGTGCAGGGAACACTACAGAGCTTGGGACCGGGAAGGGTCAGAGGTATT
CCTGAGGGGGCGGTAAAATGCTATAATCTCAGGAGGACCACCGGTGGCGAAGGCGTCTGA
CTAGAACGGATCCGACTGTGAGGGACGAAAGCCAGGGGATCAGAACGGATTAGATACCCG
TGTAGTCC
>Otu2297
CCAGCCTACGGGGCGCTGCAGTGAGGAATCTTGCGCAATGGGGGAAACCCTGACGCAGCA
ACGCCGCGTGAGTGAGGAAGGTTTTCGGATCGTAAAGCTCTGTCAGGTGGGAAGAAATGT
ATAAGGGTTAACAGCCTTTATGCTTGACGGTACCACCAAAGGAAGCACCGGCTAACTCCG
TGCCAGCAGCTGCGGTAATACGGGGGGTGCAAGCGTTGTTCGGAATTATTGGGCGTAAAG
AGCGTGTAGGCGGCTTGATAAGTCAGATGTGAAAGCCCTGGGCTTAACCCAGGAAGTGCA
TTTGAAACTGTCTTGCTTGAGTAGGGGAGAGGAAAGTGGAATTCCTGGTGTAGAGGTGAA
ATTCGTAGATATCAGGAGGAACACCGGTGGCGAAGGCGACTTTCTGGCCCTATACTGACG
CTGAGACGCGAGAGCGTGGGGAGCAAACAGGATTAGAAACCCTGGTAGTCC
>Otu2298
CCAGCCTATGGGGGGCAGCAGTGAGGAATATTGGGCAATGGGGGAAACCCTGACCCAGCG
ACGCCGCGTGGGTGACGAAGGCCTTCGGGTCGTAAAACCCTTTTGGCAGGGAAGAGTGAG
GACGGTACCTGTCGAATAAGTCTCGGCTAACTACGTGCCAGCAGCCGCGGTAAAACGTAG
GAGGCGAGCGTTATCCGGATTTACTGGGCGTAAAGCGCGCGTAGGCGGGTGGGTAAGTCT
GACGTGAAAGCCCCTGGCTCAACTGGGGGAGGCCGTTGGATACTGCCCGGCTTGAGGGTG
AGAGAGGAGAGTGGAATTCCCGGTGTAGCGGTGGAATGCGTAGAGATCGGGAGGAACACC
AGTGGCGAAAGCGACTCTCTGGCTCACCCCTGACGCTGAGTGCGCGAAAGCGTGGGGAGC
AAACGGGATTAGAGACCCCTGTAGTCC
>Otu2301
CCAGCCTACGGGAGGCACCAGTCGAGAATTTTTCTCAATGGGGGAAACCCTGAAGGAGCG
ACGCCGCGTGAAGGATGAAGGTCTTCGGATTGTAAACTTCTGTCATTGGGGAACAATGTG
TGGTCGTTAACTGCGATCGCATTGATAGTACCCAAAGAGGAAGAGACGGCTAACTCTGTG
CCAGCAGCCGCGGTAATACAGAGGTCTCAAGCGTTGTTCGGATTCATTGGGCGTAAAGGG
TGCGTAGGTGGTGTCGTAAGTCGGGTGTGAAATCTCGGGGCTTAACCCCGAAACTGCATT
CGATACTGCGATGCTCGAGGACTGGAGAGGAGACTGGAATTCACGGTGTAGCAGTGAAAT
GCGTAGATATCGTGAGGAAGGCCAGTGGCGAAGGCGGGTCTCTGGACAGTTCCTGACACT
GAGGCACGAAGGCCAGGGGAGCAAACGGGATTAGAAACCCGCGTAGTCC
>Otu2303
CCAGCCTATGGGAGGCTGCAGTAGGGAATATTGGGCAATGGGCGAAAGCCTGACGCAGCA
ACGCCGCGTGGGTGATGAAGGTCTTCGGATTGTAAAACCCTGTCGTCGGGGACGAAGGTG
CGGGATCTAATAGGTCTTGTACTTGACGGTACCCGGAGAGGAAGCCCCGGCTAACTCTGT
GCCGGCAGCCGCGGTAATACAGAGGGGGCAAGCGTTATTCGGAGTTACTGGGCGTAAAGG
GCGCGTAGGCGGTTCTTTAAGTCAGATGTGCAATCCCCGAGCTCAACTTGGGAACTGCAT
CTGAGACTGGAGAGCTAGAGTACTGGAGAGGGTGGTGGAATTCCTCGTGTAGCGGTGAAA

TGCGTAGAGATGAGGAGGAACACCAGTGGCGAAGGCGGCCACCTGGACAGTGACTGACGC
TGAGGCGCGAAAGCGTGGGTAGCAAACAGGATTAGAAACCCTAGTAGTC
>Otu2304
CCAGCCTATGGGTCGCTCCAGTGAGGAATATTGCGCAATGGGGGAAACCCTGACGCAGCG
ACGCCGCGTGAGTGATGAAGGCCTTCGGGTTGTAAAGCTCTGTCAGAGGGGAAGAAAGGA
GAAGTAGCTAATATCTGCTTCTATTGACGGTACCCTCGGAGGAAGCACCGGCTAACTCCG
TGCCAGCAGCCGCGGTAATACGGAGGGTGCAAGTGTTATTCGGATTTACTGGGCGTAAAG
AGCACGTAGGCGGATAGGTCAGTCAGATGTGAAAGCCCGGAGCTTAACTCCGGAACTGCA
TTTGATACTACCTGTCTTGAGTATGGTAGAGGGGGGTGGAATTCCCGGTGTAGAGGTGAA
ATTCGTAGATATCGGGAGGAACACCAGAGGCGAAGGCGACCCCCTGGGCAATAACTGACG
CTGAGGTGCGAAAGCGTGGGGAGCAAACAGGATTAGATACCCCAGTAGTCC
>Otu2306
CCAGCCTACGGGTTGCAGCAGCCGAGAATATTCGACAATGGGCGGAAGCCTGATCGAGCG
GTACCGCGTGGTGGATGAAGCGTTTCGACGCGTAAACACCTTTTATGGAGGAGGAATTTT
TGACGTTACTCCATGAATAAGGGGCTCCTAACTCTGTGCCAGCAGGAGCGGTAATACAGA
GGCCCCGAGCGTTACCCGGAATTACTGGGCGTAAAGAGTGTGTAGGTGGTTGTGTTAGTC
GTTTGTTAAAGACCAGGGCTCAACCTTGGAATCGCAAGCGAAACGGCACAACTTGAGGGC
ATGAGAGGTCTGCAGAACTCTAGGTGTAGGGGTAAAATCCGTTGATATCTAGGGGAATAC
CGAAAGCGAAGGCAGCAGACTGGCGTGTTCCTGACACTCAAACACGAAAGCGTGGGTAGC
GAATGGGATTAGATACCCGCGTAGTCC
>Otu2312
CCAGCCTATGGGTGGCTGCAGTCGAGGATCTTCGGCAATGGGCGCAAGCCTGACCGAGCG
ACGCCGCGTGCGGGATGAAGGCCTTCGGGTTGTAAACCGCCGTCGGAGTGGAGGAAATTT
CAGGGGGTTCTCCCCTTGGATTGACTAATACTCAGAGGAAGGACGGGCTAAGTTCGTGCC
AGCAGCCGCGGTAAGACGAACCGTCCAAACGTTATTCGGAATCACTGGGCTTAAAGGGTG
CGTAGGCGGCCCGGAAAGTTGGGTGTGAAAGCCCTCGGCTCAACCGAGGAATTGCGCCCA
AAACTACCGGGCTCGAGGAAGACAGAGGTAAGCGGAACTTAGGGTGGAGCGGTGAAATGC
GTTGATATTCTAAGGAACACCAGGAGCGAAAGCGGCTTACTGGGTCTTTTCTGACGCTGA
GGCGCGAAAGTGTGGGTAGCAAACAGGATTAGAAACCCCAGTAGTCC
>Otu2314
CCAGCCTATGGGGGGCAGCAGTGAGGAATATTGGTCAATGGGCGAGAGCCTGAACCAGCC
AAGTCGCGTGAAGGAAGAAGGTTCTATGGATTGTAAACTTCTTTTGTAAGGGAATAAAAT
GCGCTACGTGTAGTGTTTTGTATGTACTTTACGAATAGGGATCGGCTAACTCCGTGCCAG
CAGCCGCGGTAATACGGAGGATCCGAGCGTTATCCGGATTTATTGGGTTTAAAGGGTGCG
TAGGCGGACCCGTAAGTCAGTGGTGAAAGTTTGCAGCTTAACTGTAAAATTGCCATTGAA
ACTACGGATCTTGAGTGTAAATGAGGTAGGCGGAATGTGTTGTGTAGCGGTGAAATGCTT
AGATATAACACAGAACACCAATTGCGAAGGCAGCTTACTAGGATACAACTGACGCTGAGG
CACGAAAGCGTGGGGATCAAACAGGATTAGATACCCTAGTAGTCC
>Otu2316
CCAGCCTATGGGAGGCAGCAGTGGGGAATATTGCTCAATGGGCGAAAGCCTGAAGCAGCG
ACGCCGCGTGAAGGATGAAATCCGTTAGGATGTAAACTTCTTTTGCAGGGGAAGAATATC
CGCCTTGGGCGGAGTGACGGTACTCTGCGAATAAGCACCGGCTAACTACGTGCCAGCAGC
CGCGGTAATACGTAGGGTGCAAGCGTTGTCCGGAATTACTGGGTGTAAAGGGTCCGCAGG
CGGGCAGACAAGTCGATGGTGAAATCCCGTGGCTCAACCACGGAACTGCCGTTGATACTG
TTTGTCTTGAATTCGAGAGAGGATGATGGAATTCATGGTGTAGCGGTGAAATGCGTAGAG
ATCATGAAGAACACCGGAAGCGAAGGCGGTCATCTGGCTCGATATTGACGCTCAGGGACG
AAAGCGTGGGGAGCAAACAGGATTAGAGACCCGCGTAGTCC
>Otu2318
CCAGCCTATGGGGTGCAGCAGTGGGGAATCTTGCGCAATGGGCGAATGCCTGACGCAGCG
ACGCCGCGTGTGGGATGACGGCCTTCGGGTTGTAAACCACTGTCGGGAGGGACGAAGGCC
CGGCGGCGAATAGCCGACCGGGATTGACGGTACCTCCAAAGGAAGCACCGGCTAACTCCG
TGCCAGCAGCCGCGGTAATACGGAGGGTGCGAGCGTTGTCCGGAATCACTGGGCGTAAAG
GGCGCGTAGGCGGCTGGGTAAGTCACGCGTGAAATTCCGGGGCTCAACCCCGGAACGGCG
TGTGGGTCTGCCCGGCTAGAGCACGGTAGAGGCGAGTGGAATTCCAGGTGTAGCGGTGGA
ATGCGTAGATATCTGGAAGAACACCGGTGGCGAAGGCGGCTCGCTGGGCCGTTGCTGACG
CTGAGGCGCGAAAGCGTGGGGAGCAAACAGGATTAGATACCCCCGTAGTCC
>Otu2322
CCAGCCTACGGGGCGCAGCAGGTAGGAATCTTTTGCAATGCGCGCAAGCGTGACAAAGCG

AGCCAGAGTGCTTTTCATTTGAAAAGCTTTTGCTAAATCTAAAACGTTTGGCGAATAAGG
ACTGGGTAAGACTAGTGCCAGCCGCCGCGGTAATCCTAGCGGTCCAAGTCGCAGCCATCA
TTATTGGGTCTAAAACATCCGTAGCTTGTTTAATAGGTTCCCTGTGAAATCTTGTCTCTT
AAAGGCAAGGCGTGCAAGGAATACTGCTAAACTAGAGACTGGAAGACGTAAGAAGTATGT
TCAAAGTAGTGGTAAAATATGTTAATCTTGGACAGACTAACAACAGCGAAGGCATCTTAC
GAGGACAGTTCTGACAGTAAAGGATGAAGGCTAGGGTCGCGAAAGGGATTAGATACCCTG
GTAGTCC
>Otu2323
CCAGCCTACGGGATGCTCCAGTGGGGAATATTGGACAATGGGCGCAAGCCTGATCCAGCC
ATGCCGCGTGAGTGATGACGGCCTTCGGGTTGTAAAGCACTTTTGTCCGGAAAGAAAACT
TCTGCCCTAATACGGTGGGAGGATGACGGTACCGGAAGAATAAGCACCGGCTAACTACGT
GCCAGCAGCCGCGGTAATACGTAGGGTGCGAGCGTTAATCGGAATTACTGGGCGTAAAGC
GTGCGCAGGCGGTTCGTTAAGACAGATGTGAAATCCCCGGGCTTAACCTGGGAACTGCAT
TTGTGACTGGCGAGCTAGAGTATGGCAGAGGGGGGTAGAATTCCACGTGTAGCAGTGAAA
TGCGTAGAGATGTGGAGGAATACCGATGGCGAAGGCAGCCCCCTGGGCCAATACTGACGC
TCATGCACGAAAGCGTGGGGAGCAAACAGGATTAGAAACCCCTGTAGTCC
>Otu2325
CCAGCCTATGGGTGGCAGCAGTGAGGAATATTGGACAATGGGCGAAAGCCTGATCCAGCA
ATACCGCATGAGTGAAGAAGGCCTTCGGGTTGTAAAGCTCTTTCAGCAGGGAAAATAATG
ATGGTACCTGCAAAAGAAGTCCCGGCTAACTCCGTGCCAGCAGCCGCGGTAATACGGAGG
GAACTAGCGTTGTTCGGATTTACTGGGCGTAAAGGGCGCGTAGGCGGTTTGTTAAGTTAG
AAGTGAAAGCCCAGGGCTTAACCCTGGAACTGCTTCTAAAACTGGCAGACTAGAGTGCGG
GAGAGGATAGTGGAATTCCTAGTGTAGAGGTGAAATTCTTAGATATTAGGAGGAACACCA
GTGGCGAAGGCGACTATCTGGAACGTTACTGACGCTGAGGCGCGAAAGCGTGGGGAGCAA
ACAGGATTAGATACCCCCGTAGTCC
>Otu2327
CCAGCCTATGGGGGGCACCAGTGGGGGATATTGGACAATGGGCGAAAGCCTGATCCAGCA
ATGCCGCGTGAGTGATGAAGGCCTTAGGGTTGTAAAGCTCTTTCGGGTGGGACGATGATG
ACGGTACCACCAGAAGAAGCCCCGGCTAACTTCGTGCCAGCAGCCGCGGTAATACGAAGG
GAGCTAGCGTTGTTCGGAATTACTGGGCGTAAAGGGCGCGTAGGCGGTTTAACAAGTTGG
GTGTGAAAGCCCAGGGCTCAACCCTGGAACGGCACCCAAGACTGTTTTACTTGAATTCGG
TAGAGGTTGGTGGAATTCCCAGTGTAGAGGTGAAATTCGTAGATATTGGGAAGAATATCC
GTGGCGAAGGCGGCCAACTGGACCGACATTGACGCTGAGGCGCGAAAGCGTGGGGAGCAA
ACAGGATTAGAAACCCCCGTAGTCC
>Otu2331
CCAGCCTATGGGGTGCAGCAGTGGGGAATTTTGGACAATGGGGGAAACCCTGATCCAGCA
ATGCCGCGCGTGTGAAGAAGGCCTTCGGGTTGTAAAGCACTTTTGTCCGGAAAGAAATCC
TTTGAGTTAATACCTCGGGGGGATGACGGTACCGGAAGAATAAGCACCGGCTAACTACGT
GCCAGCAGCCGCGGTAATACGTAGGGTGCAAGCGTTAATCGGAATTACTGGGCGTAAAGC
GTGCGCAGGCGGTCCGCTAAGACAGATGTGAAATCCCCGGGCTTAACCTGGGAACTGCAT
TTGTGACTGTAGAGCTGGAGTACGGCAGAGGGGGATGGAATTCCGCGTGTAGCAGTGAAA
TGCGTAGATATGCGGAGGAACACCGATGGCGAAGGCAATCCCCTGGGCCTGTACTGACGC
TCATGCACGAAAGCGTGGGGAGCAAACAGGATTAGATACCCTCGTAGTCC
>Otu2335
CCAGCCTATGGGTTGCTCCAGTCGAGAATCTTTCTCAATGGGCGCAAGCCTGAAGGAGCG
ACGCCGCGTGGAGGATGAAGGTCTTCGGATTGTAAACTCCTGTCATTAGGGAACAAGTGG
CGCGATAGTAACTGCTCGCGCCTTGATAGTACCTGAAGAGGAAGAGACGGCTAACTCTGT
GCCAGCAGCCGCGGTAATACAGAGGTCTCAAGCGTTGTTCGGATTCATTGGGCGTAAAGG
GTGCGTAGGTGGTGTCGTAAGTCGGATGTGAAATCTCGGGGCTTAACCCCGAAACTGCAT
TCGATACTGCGGTGCTCGAGGACTGGAGAGGAGATCGGAATTCACGGTGTAGCAGTGAAA
TGCGTAGATATCGTGAGGAAGGCCAGTGGCGAAGGCGGATCTCTGGACAGTTCCTGACAC
TGAGGCACGAAGGCCAGGGGAGCAAACGGGATTAGAGACCCTAGTAGTCC
>Otu2338
CCAGCCTATGGGACGCACCAGGAAGAGACAGGCGGATTTACGGCGTTCACTCCCTTTGCG
TTTCAACCAACGGCCGCGGCCGCAAGTCTTCCAGGCTGGGAAGAGGCCACCGGGGTGGAG
TACCTGAAGATACTGGCGATCTGCAGGATGTATCTGGACACCATCGAGAACGTGCAATCG
AACCTGGAGACGCAGGGACTCAAAGTCCTGCAGATGGGCCTGCGGTTCGGCGGAAACGAT
GTGGGCTCGGTACCGGAAGGCGGCGGAGTAAAGCCAATCGTCGAGGAAGATTTGCGGCGC

GTCATTCGGGACGCCGGATTCCGGCCAGTGCAGCGGGATATTTTGTACAGGACAATGTTC
CTCAACTAGCTTTGGTCTGCGGAATATCGTGAAGGCATTTCCCCGCATCGTATCGCGTAG
AATCGGTTGATATTAGAAACCCTAGTAGTCC
>Otu2341
CCAGCCTACGGGATGCTGCAGTGGGGAATCTTGCGCAACGGGCGAAAGCCTGACGCAGCA
ACGCCGCGTGTGTGATGAAGGTCTTCGGATCGTAAAGCACTGTCGCGAGGGACGAATAGG
GGCCGGGATAATACTCCGGTTCGATGACGGTACCTCGAGAGGAAGCACCGGCTAACTCTG
TGCCAGCAGCCGCGGTAATACAGAGGGTGCAAGCGTTGTTCGGAATTATTGGGCGTAAAG
CGCGTGTAGGCGGCTCAGCAAGTCAGGTGTGAAAGCCCTCGGCTTAACCGAGGAAGTGCG
CCTGAAACTATTGAGCTTGAGTGCCGGAGAGGGTGGCGGAATTCCCGGTGTAGAGGTGAA
ATTCGTAGATATCGGGAGGAACACCAGCGGCGAAGGCGGCCACCTGGACGGTTACTGACG
TTGAGACGCGAAAGCGTGGGGAGCAAACAGGATTAGATACCCTAGTAGTCC
>Otu2343
CCAGCCTATGGGATGCAGCAGTCGAGAATCTTCGGCAATGGGGGAAACCCTGACCGAGCG
ACGCCGCGTGCGAGATGAAGGCCTTCGGGTTGTAAATCGCTGTCGTAGGGGAGGAAATGC
TGGTGGGTTCTCCCATCAGTTTGACCTATCCTAGGAGGAAGGACGGGCTAAGTTCGTGCC
AGCAGCCGCGGTAATACGGACCGTCCAAACGTTATTCGGAATCACTGGGCTTAAAGGGTG
CGTAGGCGGTCCGGGAAGTTGGGTGTGAAATACCTCGGCTCAACCGAGGAACTGCGCCCA
AAACTGCCGGGCTGGAGGGAGACATAGGTGTGCGGAACCTAGGGTGGAGCGGTGAAATGC
GTTGAGATCCTAGGGAACACCAGTAGCGAAAGCGGCACACTGGGTCTCTTCTGACGCTGA
TGCACGAAAGCTAGGGGAGCGAAGAGGATTAGATACCCCCGTAGTCC
>Otu2344
CCAGCCTACGGGTTGCAGCAGTGGGGAATTTTGCACAATGCCCGAAAGGGTGATGCAGCA
ACGCCGCGTGAGGGATGAAGGCCTTCGGGTCGTAAACCTCTTTCGATCGGGAAAAATGGG
TGGGTGAATAATCCACCCTGATGGTACCGGAAGAAGAAGCCCCGGCCAACTACGTGCCAG
CAGCCGCGGTAATACGTAGGGGGCGAGCGTTGTTCGGAATTACTGGGCGTAAAGGGCGTG
TAGGCGGTGTGGTAAGTTGGGCGTGAAATCCCCGGGCTTAACCCGGGGGGGCCGTTCAAG
ACTGCCATGCTAGGGTGTGGGAGAGGGTAGTGGAATTCCCGGTGTAGCGGTGAAATGCGT
AGATATCGGGAGGAACACCTGCGGCGAAAGCGGCTACCTGGACCATAACTGACGCTGAGG
CGCGAAAGCTAGGAGAGCAAACAGGATTAGAGACCCCAGTAGTCC
>Otu2346
CCAGCCTATGGGGTGCTCCAGTGGGGAATTTTGGACAATGGGGGCAACCCTGATCCAGCA
ATGCCGCGTGAGTGAAGAAGGCCTTCGGGTTGTAAAGCTCTTTTGTCCGGGAAGAAACGG
TAATTTTTAATAAAGATTGCTAATGTCGGTACCGGAAGAATAAGCACCGGCTAACTACGT
GCCAGCAGCCGCGGTAATACGTAGGGTGCAAGCGTTAATCGGAATTACTGGGCGTAAAGC
GTGCGCAGGCGGTTTATTAAGTCAGCTGTGAAATCCCCGGGCTTAACCTGGGAATGGCAG
TTGAGACTGGTAAGCTGGAGTGTGTCAGAGGGGGGTAGAATTCCACGTGTAGCAGTGAAA
TGCGTAGAGATGTGGAGGAATACCGATGGCGAAGGCAGCCCCCTGGGATAACACTGACGC
TCATGCACGAAAGCGTGGGGAGCAAACAGGATTAGAAACCCCCGTAGTCC
>Otu2347
CCAGCCTATGGGGTGCTCCAGTGGGGAATATTGGACAATGGGCGCAAGCCTGATCCAGCC
ATGCCGCGTGAGTGATGAAGGCCCTAGGGTTGTAAAGCTCTTTCGCACGCGACGATGATG
ACGGTAGCGTGAGAAGAAGCCCCGGCTAACTTCGTGCCAGCAGCCGCGGTAATACGAAGG
GGGCTAGCGTTGTTCGGAATTACTGGGCGTAAAGCGCACGCAGGCGGTCTGCATAGTCAG
AAGTGAAAGCCCCGGGCTCAACCTGGGAACTGCTTTTGATACTGGCAGGCTTGACTTATG
GAGAGGGTAGTGGAATTCCGAGTGTAGAGGTGAAATTCGTAGATATTCGGAAGAACACCA
GTGGCGAAGGCGACTACCTGGCCATCAAGTGACGCTCATGTGCGAAAGCGTGGGGAGCAA
ACAGGATTAGAAACCCCAGTAGTCC
>Otu2355
CCAGCCTATGGGAGGCTGCAGTAAGGAATATTGGACAATGGGCGCAAGCCTGATCCAGCA
ATGCTGCGTGTGTGAAGAAGGCCTTCGGGTTGTAAAGCACTTTAAGTTGGGAAGAAAAAA
TTTCACCTAATACGTGGAATCTTGACGGTACCGACAGAATAAGCACCGGCTAACTCTGTG
CCAGCAGCCGCGGTAATACAGAGGGTGCAAGCGTTAATCGGATTTACTGGGCGTAAAGCG
TGCGTAGGCGGTTTGTTAAGTCGATTGTGAAAGCCCTGGGCTCAACCTGGGAATTGCAGT
CGATACTGATGGACTAGAGAACGGTAGAGGGAGGCGGAACTCCAGGTGTAGCGGTGAAAT
GCGTAGATATCTGGAAGAACACCGATGGCGAAGGCAACCTCCTGGGCCTGTTCTGACGCT
GAGGCACGAAAGCGTGGGTAGCAAACAGGATTAGATACCCCCGTAGTCC
>Otu2356

CCAGCCTATGGGGTGCTGCAGTCGAGGATCTTCGGCAATGGGCGCAAGCCTGACCGAGCG
ACGCCGCGTGTGGGATGAAGGCCCTCGGGTTGTAAACCACTGTCAGAGGGGATGAAATGC
CATGGGGTTCTCTCCATGGTTTGACATATCCCCAGAGGAAGGACGGGCTAAGTTCGTGCC
AGCAGCCGCGGTAATACGAACCGTCCTAACGTTATTCGGAATCACTGGGCTTAAAGGGCG
CGTAGGCGGCGCTACAAGTTGGGTGTGAAAGCCCTCGGCTCAACCGAGGAATTGCGCCCA
AAACTGTAGTGCTTGAGGGAGATAGAGGTGAGCGGAACTTAGGGTGGAGCGGTGAAATGC
GTTGATATCCTAAGGAACACCTGGGGCGAAAGCGGCTCACTGGATCTCTTCTGACGCTGA
TGCACGAAAGCTAGGGTAGCGAACGGGATTAGAGACCCGAGTAGTCC
>Otu2357
CCAGCCTATGGGATGCAGCAGTGAGGAATATTGGTCAATGGACGCAAGTCTGAACCAGCC
ATGCCGCGTGAAGGAAGAAGGCCCTAAGGGTTGTAAACTTCTTTTGTACGGGGGTAATAG
TTTCTACGCGTAGAGACATGAAAGTACTGTACGAATAAGCGTCGGCTAACTACGTGCCAG
CAGCCGCGGTAATACGTAGGACGCAAGCGTTATCCGGATTCATTGGGTTTAAAGGGTGCG
TAGGCGGGCCTTTAAGTCAGTGGTGAAAGTTTGCCGCTTAACGGTAAGATTGCCATTGAT
ACTGGAGGCCTTGAGTGCATATGAGGTAGGCGGAATGTGTAGTGTAGTGGTGAAATACTT
AGATATTACACAGAACACTGATTGCGAAGGCAGCTTACTAAGATGCAACTGACGCTGAGG
CACGAAAGCGTGGGGATCAAACAGGATTAGAAACCCGAGTAGTCC
>Otu2364
CCAGCCTATGGGGGGCAGCAGTGGGGAATATTGCACAATGGGCGAAAGCCTGATGCAGCG
ACGCCGCGTGAGGGATGACGGCCTTCGGGTTGTAAACCTCTTTCAGCAGGGAAGAAGCGG
CAGTGATTTTTCACGGCGTGACGGTACCTGCAGAAGAAGCACCGGCTAACTACGTGCCAG
CGGCCGCGGTAATACGTAGGGTGCGAGCGTTGTCCGGAATTATTGGGCGTAAAGAGCTCG
TAGGCGGCTTGTCGCGTCGGATGTGAAAGCCCGGGGCTTAACTCCGGGTCTGCATTCGAT
ACGGGCAGGCTAGAGTGTGGTAGGGGAGATCGGAGTTCCTGGTGTAGCGGTGAAATGCGC
AGATATCAGGAGGAACACCGGTGGCGAAGGCGGATCTCTGGGCCATTACTGACGCTGAGG
AGCGAAAGCGTGGGGAGCGAACAGGATTAGATACCCTCGTAGTCC
>Otu2368
CCAGCCTACGGGGCGCTCCAGTAAGGAATATTGGGCAATGGACGCAAGTCTGACCCAGCC
ATGCCGCGTGAAGGATGACGGCCCTACGGGTTGTAAACTTCTTTTGTACGGGAAAAAACC
CCTGATCGTGTATCAGGGTTGATGGTACCGTAAGAATAAGCATCGGCTAACTCCGTGCCA
GCAGCCGCGGTAATACGGAGGATGCAAGTGTTATCCGGATTCATTGGGTTTAAAGGGTGC
GTAGGCGGGATGGTAAGTCAGTGGTGAAATCCTACAGCTTAACTGTAGAACTGCCATTGA
TACTGCCAACCTTGAGTACATTTGAAGTGGGCGGAATGTGTGGTGTAGCGGTGAAATGCT
TAGATATCACACAGAACACCGATAGCGAAGGCAGCTCACTAAACTGTAACTGACGCTGAG
GCACGAAAGCGTGGGGATCAAACAGGATTAGAAACCCCTGTAGTCC
>Otu2370
CCAGCCTATGGGTTGCACCAGTGGGGAATTTTGCGCAATGGGGGAAACCCTGACGCAGCA
ACGCCGCGTGGAGGATGAAATATCTTGGTATGTGAACTCCTTTCGATGGGGAAGATTATG
ACGGTACCTGCAAAAGCAGCTGCGGCTAACTACGTGCCAGCAGCCGCGGTAATACGTAGG
CAGCAAGCGTTGTTCGGAGTTACTGGGCGTAAAGGGTGTGTAGGCGGCTTTTTAAGTTTG
GTGTGAAATCTCCCGGCTCAACTGGGAGGGTGCGCCGAATACTGAGAGGCTAGAGTGTGG
GAGAGGAAAGTGGAATTCCTGGTGTAGCGGTGAAATGCGTAGATATCAGGAGGAACACCG
GTGGTGTAAACGGCTTTCTGGACCATAACTGACGCTGAGACACGAAAGCGTGGGTAGCAA
ACAGGATTAGAAACCCTTGTAGTCC
>Otu2371
CCAGCCTATGGGGGGCAGCAGTCGAGAATCTTCCGCAATGGACGAAAGTCTGACGGAGCG
ACGCCGCGTGGAGGATGAAGTTCTTCGGAACGTAAACTCCTTTTGCCAGGGAAAAAGTTA
TTGATTGTACCTGGAGAATAAGAAGTTGCTAAACTCGTGCCAGCAGCAGCGGTAATACGA
GTGCTTCAAGCGTTATCCGGAATCATTGGGCGTAAAGGGTGTGTAGGCGGTTTTGTTAGT
CTCGCGTAAAATCTTTCGGCTCAACCGAGAGTCCGCGTGGGAAACGGCAAGACTAAGAGG
ATGGAAGGGGTCTTTGGAACTCATGGTGTAGCGGTGAAATGCGTTGATATCATGGGGAAC
ACCGAAAGCGAAGGCAAAAGACTGGTCCACTCCTGACGCTGAAACACGAAAGCGTGGGTC
GCGAATGGGATTAGAAACCCCCGTAGTCC
>Otu2374
CCAGCCTACGGGATGCAGCAGCTAAGAATATTCCGCAATGGACGAAAGTCTGACGGAGCG
ACGCCGCGTGGATGACGAAGGCCGAAAGGTTGTAAAATCCTTTTGCTGGGGAAGAATAAG
CGTGGAAGGAAATGTCCGCGTGATGACATGAACCGGTGAATAAGTCCCGGCTAACTACGT
GCCAGCAGCCGCGGTAACACGTAGGGGATAAGCGTTGTTCGGAATTATTGGGCGTAAAGG

GCATGTAGGCGGTTTTGTAAGCCTGGCGTGAAATCCTGCAGCTTAACTGCGGAACTGCGC
TGGGAACTGCGAGACTCGAATCACTGAGGGGGAGATAGAATTCCAGGTGTAGGGGTGAAA
TCTGTAGATATCTGGAAGAATACCGGTGGCGAAGGCGATCTCCTGGCAGATGATTGACGC
TGAGGTGCGAAAGCGTGGGGAGCAAACAGGATTAGAAACCCCCGTAGTCC
>Otu2375
CCAGCCTACGGGTCGCAGCAGTCGAGAATCATTCGCAATGGGCGAAAGCCTGACGATGCG
ACGCCGTGTGAGCGATGAAGGCCTTAGGGTCGTAAAGCTCTTTCGCTTGGGAACAAGAGG
ATCCGGCTAATATCCGGTCGATTTGAGTGTACCAGGTAAAGAAGCACCGGCTAACTCCGT
GCCAGCAGCTGCGGTAATACGGAGGGTGCAAGCATTAATCGGATTTATTGGGCGTAAAGG
GCGCGTAGGCTGGTTTGTAAGTCAGATGTGAAATCCCGGGGCCCAACCTCGGAACAGCAT
TTGAGACTGCATACCTAGAGGGTAGGCGGAGAAAACGGAATTCCACAAGTAGCGGTGAAA
TGCGTAGATATGTGGAAGAACACCGGTGGCGAAGGCGGTTTTCTAGCTTACTCCTGACGC
TGAGGCGCGAAAGCAAGGGGATCAAACAGGATTAGAAACCCGAGTAGTCC
>Otu2378
CCAGCCTACGGTGGGCAGCAGTGGGGAATATTGGACAATGGGGGAAACCCTGATCCAGCG
ATGCCGCGTGTGTGAAGAAAGCCTGAGGGTTGTAAAGCACTTTAGTGGGGGAGGAGTAGT
ATCTGGTTAATAACTGGATATGAGGACGTTACCCTAAGAATAAGCACCGGCAAACTCTGT
GCCAGCAGCCGCGGTAATACAGAGGGTGCAAGCGTTAATCGGAATGACTGGGCGTAAAGG
GTACGTAGGTGGAGGAATAAGTATCATGTGAAAGTCCTGGGCTCAACCTGGGGATGGCAT
GGTAGACTGTTTTTCTGGAGTGCAGTAGAGGTAAGGGGAATTTCCGGTGTAGCGGTGAAA
TGCGTAGAGATCGGGGGGAACACCTGTGGCGAAGGCGCCTTACTGGGCTGTAACTGACAC
TGAGGTACGAAAGCGTGGGGAGCAAACAGGATTAGAAACCCGCGTAGTCC
>Otu2379
CCAGCCTACGGGTCGCACCAGTAAGGAATATTGGTCAATGGGCGCAAGCCTGAACCAGCC
ATGCCGCGTGAAGGATAAAGGTCCTCTGGATTGTAAACTTCTTTTATTTGGGACGAAAAA
TGGGGATTCTTCCTCACCTGACGGTACCAGATGAATAAGCACCGGCTAACTCCGTGCCAG
CAGCCGCGGTAATACGGAGGGTGCAAGCGTTATCCGGATTCACTGGGTTTAAAGGGTGCG
TAGGCGGGCAGTTAAGTCAGTGGTGAAATCTTTGGGCTTAACCCGGAAACTGCCATTGAT
ACTATCAGTCTTGAATATTGTGGAGGCCTGCGGAATATGTCATGTAGCGGTGAAATGCTT
AGAGATGACATAGAACACCAATTGCGAAGGCAGCAGGCTACGCATATATTGACGCTGAGG
CACGAAAGCGTGGGGATCAAACAGGATTAGATACCCTTGTAGTCC
>Otu2381
CCAGCCTACGGGTCGCTGCAGTCGAGAATCTTCCGCAATGGACGAAAGTCTGACGGAGCG
ACGCCGCGTGGAGGATGAAGTGCTTCGGCATGTAAACTCCTTTTGCCAGGGAAAAAGTTA
TTGATGGTACCTGGAGAATAAGAGGTTGCTAAACTCGTGCCAGCAGCAGCGGTAATACGA
GTGCCTCGAGCGTTATCCGGAATTATTGGGCGTAAAGGGTGTGTAGGTGGTTTCGTTAGT
CTTCCGTAAAATCTCTCGGCTCAACCGGGAACTCGCGGAGGAAACGGCGAGACTAAGAGG
ACGGTAGAGGTTTCTGGAACTCATAGTGTAGCGGTGAAATGCGTTGATATTATGGGGAAC
ACCAAAAGCGAAGGCAAGAAACTGGACCGCTCCTGACACTGAAACACGAAAGCGTGGGTC
GCGAATGGGATTAGATACCCCAGTAGTCC
>Otu2383
CCAGCCTATGGGTCGCAGCAGTGGGGAATATTGCTCAATGGGGGAAACCCTGAAGCAGCA
ACGCCGCGTGAGTGATGAAGGCCTTCGGGTCGTAAAGCTCTGTTGTACGGGGCGAATGTG
CTTATGGCTAACATCCATAGGCAGTGACAGTACCGTATAAGAAAGGACCGGCTAACTTCG
TGCCAGCAGCCGCGGTAAGACGGGGGGTCCAAGCGTTGTTCGGAATCATTGGGCGTAAAG
CGTGTGCAGGTGGTCTTGTAAGTTGGATGTGAAAGCCCCGAGCTTAACTCGGGAAGTGCA
TTCAAAACTGCAAGACTTGAGTATGGAAGAGGGTTGTGGAATTCCAGGTGTAGTGGTGAA
ATACGTAGATATCTGGAGGAACACCGGTGGCGAAAGCGGCAACCTGGTCCTATACTGGCA
CTCATACACGAAAGCGTGGGGATCAAACAGGATTAGATACCCCCGTAGTCC
>Otu2386
CCAGCCTACGGGGGGCTGCAGTCGAGAATTTTTCTCAATGGGCGAAAGCCTGAAGGAGCG
ACGCCGCGTGGGGGATGAAGGGCTTCGGCTCGTAAACCCCTGTCATTTGCGAACAAACCT
TACGATTTAACAGATCGTGAGCTGATTGTAGCGAAAGAGGAAGGGACGGCTAACTCTGTG
CCAGCAGCCGCGGTAATACAGAGGTCCCAAGCGTTGTTCGGATTCACTGGGCGTAAAGGG
TGCGTAGGTGGCAAGGTAAGTTTGGTGTGAAATCTCGGGGCTCAACTCCGAAACTGCACT
TGATACTGCCTTGCTAGAGTACTGGAGAGGAGATTGGAATTTACGGTGTAGCAGTGAAAT
GCGTAGATATCGTAAGGAAGACCAGTGGCGAAGGCGAATCTCTGGACAGTTACTGACGCT
GAGGCACGAAGGCCAGGGGAGCAAACGGGATTAGATACCCCAGTAGTCC

>Otu2387
CCAGCCTATGGGTTGCTCCAGTAGGGAATATTGCGCAACGGGGGAAACCCTGACGCAGCG
ACGCCGCGTGAGTGATGAAGGCTTTAGGGTCGTAAAGCTCTGTTGTACGAGAAGAAACAA
ATGACGGTATCGTACAAGAAAGGACCGGCTAACTTCGTGCCAGCAGCCGCGGTAATACGA
GGGGTCCTAGCGTTGTTCGGAATTATTGGGCGTAAAGGGTGTGTAGGCTGCTAGGTAAGT
CAGTTGTGAAAGCCCCGGGCTCAACCCGGGAAGTGCTTCTGATACTGCTTAGCTTGAGTA
CTGGAGAGGATAGTGGAATTCCAGGTGTAGTGGTGAAATACGTAGATATCTGGAGGAACA
CCGGTGGCGAAGGCGGCTATCTGGCCATGTACTGACGCTGAGACACGAAAGCGTGGGGAG
CAAACAGGATTAGAAACCCTAGTAGTCC
>Otu2388
CCAGCCTACGGGTCGCAGCAGTGAGGAATTTTGCGCAATGGCCGCAAGGCTGACGCAGCA
ACGCCGCGTGGGTGAAGAAGGCCTTCGGGTCGTAAAGCCCTGTCAGGTGGGAAGAAAGGT
CTTGAGTGAACGCTTGGGATTTGACGGTACCACCAGAGGAAGCACCGGCTAACTCCGTGC
CAGCAGCCGCGGTAATACGGAGGGTGCAAGCGTTATTCGGAATTACTGGGCGTAAAGCGC
GTGTAGGCGGGGGTACAAGTCTGATGTGAAAGCCCCGGGCTTAACCTGGGAAGTGCATTG
GAGACGGTATCTCTTGAGTACTGGAGAGGAAGGGGGAATTCCCGGTGTAGAGGTGAAATT
CGTAGAGATCGGGAGGAATACCAGTGGCGTAGGCGCCTTTCTGGACGGTTACTGACGCTG
AGACGCGAAAGCGTGGGGAGCAAACAGGATTAGATACCCGCGTAGTCC
>Otu2390
CCAGCCTATGGGAGGCTGCAGTAGGGAATATTGGACAATGGGCGCAAGCCTGATCCAGCC
ATCCCGCGTGAAGGATTAAGGTCCTATGGATTGTAAACTTCTTTTCTCTGGGAATAAAAA
GTGGGATTCATTCCCGCCTTGAAGGTACCAGAGGAATAAGCACCGGCTAACTCCGTGCCA
GCAGCCGCGGTAATACGGAGGGTGCAAGCGTTATCCGGATTCACTGGGTTTAAAGGGTGC
GTAGGTGGCTTTGTAAGTCAGTGGTGAAATATTACGGCTCAACCGTAAAACTGCCATTGA
TACTGCTTAGCTTGAATCAGGTCGAGGTGGATGGAATATTACATGTAGCGGTGAAATGCT
TAGATATGTAATAGAACACCGATTGCGAAGGCAGTTCACTAGGCCTGAATTGACACTGAG
GCACGAAAGCGTGGGGATCAAACAGGATTAGAGACCCTCGTAGTCC
>Otu2392
CCAGCCTATGGGGTGCAGCAGTCGAGGATCTTCGGCAATGGGCGCAAGCCTGACCGAGCG
ACGCCGCGTGTGCGATGAAGGCCTTCGGGTTGTAAAGCACTGTCGAGGGGGAGGAAGCCG
CAAGGTTGACCTATCCCTGGAGGAAGCACGGGCTAAGTTCGTGCCAGCAGCCGCGGTAAG
ACGAACCGTGCGAACGTTGTTCGGAATCACTGGGCTTAAAGGGCGCGTAGGCGGGCCACC
AAGTCCGGGGTGAAATCCTCCGGCTTAACCGGAGAATTGCCTGGGATACTGGTGGTCTTG
AGGGAGGTAGGGGCACGCGGAACTTCCGGTGGAGCGGTGAAATGCGTTGATATCGGAAGG
AACGCCGGTGGCGAAAGCGGCGTGCTGGCCCTCTTCTGACGCTGAGGCGCGAAAGCCAGG
GGAGCGAACGGGATTAGAAACCCGCGTAGTCC
>Otu2394
CCAGCCTATGGGTGGCAGCAGTCGAGAATAGTCTACAATGGACGAAAGTCTGATAGTGCG
ACGCCGCGTGAACGAAGAAACCTTTCGGGGTGTAAAGTTCTTTTCTGTGCGAGCAGGGGT
GGATAGAGTAATATGCTGTTCATCTGGATATTAGTACAGGAATAAGCCACGGCTAACTCC
GTGCCAGCAGCCGCGGTAACACGGGGGTGGCAAGCGTTGTCCGGAATTACTAGGCGTAAA
GGGCAGGTAGGCGGGTCTATAAGTCGGACGTTAAAGCCTCAATCTTAAGTTGAGAAATGC
GTTTGATACTGTAGGTCTTGAATGCGGTGGGGGAAGACGGAATTCCAGGTGTAGCGGTGG
AATGCGCAGATATCTGGAGGAACACCGAAGGCGAAGGCAGTCTTCTATACCGTAATTGAC
GCTAAACTGCGAAGGTGGGGGATCAAACAGTATTAGAAACCCTAGTAGTCC
>Otu2396
CCAGCCTACGGGTGGCAGCAGTGTGGAATATTGGACAATGGACTAAAATGTCTGATCCAG
TTACATCTCTCGGGTGATTGACGGCGATTGCCGTAAAACTGAACGGCTGATAATCTTGAT
CTTGACTAAAGAAGATTATTGGTATTAAATAGTCCAGGGCAACCTCTGTGCCAGCAGCCG
CGGGAATACAGAGTGGACAAGTGTTACCCATCCTAACTGGGCATAAAGAGTGCGTAGATG
GATATTAAAGAAATGGCTAAAAGAAAATTGCTGTAATGGGTAGAGATTTTTAGCAGCGGA
TATCTTTGAGAAATGAATAGGTTACTAGTATACATGAAGGATTTGATTTAGGAAATTGTC
GATCTCATGGGGACTACCAATAGCGAAGGCGTTTAACCAATCATAATCTGACATTAAGGC
ACGAACTCGTGGGTAGCAAGCGGGATTAGATACCCTTGTAGTCC
>Otu2397
CAGCCTACGGGTTGCAGCAGTGAGGAATATTGGTCAATGGGTGCAAGCCTGAACCAGCCA
TGCCGCGTGCAGGATTAAGGCCCTATGAGTTGTAAACTGCTTTTATACAGGACGAAACTC
TCGAACGTGTTCGGGATTGACTGTACTGTAAGGATAAGGATCGGCTAACTCCGTGCCAGC

AGCCGCGGTAATACGGGGGATCCAAGCGTTATCCGGATTTACTAGGTTTAAAGGGTGCGT
AGGCGGGATAGTAAGTCAATGGTGAGATCCTACAGCTTAACTGTAGAATTGCCTTTGATA
CTGCTGTTCTTGAGTATAGATGAGGTGGGCGGAATGTGTCAAGTAGCGGTGAAATGCTTA
GATATGACACAGAACTCCAATTGCGTAGGCAGCTCACTAACTTAATACTGACGCTGAGGC
ACGAAAGCGTGGGGAGCAAACAGGATTAGATACCCTAGTAGTCC
>Otu2404
CCAGCCTATGGGAGGCAGCAGTAACGAATATTCCGCAATGCGCGAAAGCGTGACGGAGCA
ATGCCGCGTGCAGGATGAAGCGGTTTCGCCGTGTAAACTGCTGTCAGGGGCGAGAAATAC
TGATCAGCCCCAGAGGAAGGACCGGCTAAACTCGTGCCAGCAGCCGCGGTAAGACGAGTG
GTCCTAGCGTTAGTCGGAATCACTGGGCTTAAAGGGTGCGTAGGTGGGCTTGTAAGTGCC
TTGTGAAAGCCCTCGGCTCAACCGAGGAACTGCAGGGCATACTGCACGTCTTGAGGAAAC
TAGAGGCCGGTAGAACGATAGGTGGAGCGGTGGAATGCGTAGAGATCTATCGGAATGCCG
AAGGTGAAGACAGCCGGCTGGGGTTTTCCTGACACTGAGGCACGAAAACGTGGGGATCAA
ACAGGATTAGAGACCCCCGTAGTCC
>Otu2407
CCAGCCTATGGGGCGCTGCAGTCGAGAATTTTTCACAATGGGCGCAAGCCTGATGGAGCG
ACGCCGCGTGGGGGATGAAGGGTTTCGATCCGTAAACCCCTGTCATTTGCGAACAAACCT
CGTCCGATAACACCGGACGAGCTGATAGTAGCGGAAGAGGAAGGGACGGCTAACTCTGTG
CCAACAGCCGCGGTAATACAGAGGTCCCAAGCGTTGTTCGGATTCACTGGGCGTAAAGGG
TGCGTAGGTGGCCGGGTAAGTTCGGTGTGAAATCTCGGAGCTCAACTCCGAAATGGCATC
GAATACTATCCGGCTGGAGGGTCGGAGGGGAGACTGGAATTCTCGGTGTAGCAGTGAAAT
GCGTAGATATCGAGAGGAACACCAGTGGCGAAGGCGAGTCTCTGGACGACTCCTGACACT
GAGGCACGAAAGCCAGGGGAGCAAACAGGATTAGAGACCCGTGTAGTCC
>Otu2411
CCAGCTACGGGGCGCTCCAGTGAGGAATATTGGGCAATGAGCGAAAGCTTGACCTAGTAA
TGGTTCAAGAGTGAGAAATAGACGGCATTATCGCTGTAAAGCTCTTTCAAAAATGAAGAT
AATGACTGTAGTTTAAGAATAAGCACTGGCTAATTCTGTGCCAGCAGCCGCGGTAATACA
GGGAGTGCAGACGTTATTCGGATGAAATTGGTGTAAAGTATATGTAGGCGGCTTTTTGAA
CTTTTTTAAAATACTAGAATTAATCTTTTAGAAAAAAAAATGTAATCGAAAGCTAAGAGT
TTAAGAAAGGTTGAAAGAATTTCTTGTCTAAGGGTTGAATCTTTAAAAATAAGGACGACT
ACCAAAATAGTAGCGAAAGCATTCTTCTGGCTTAATACTGACGCTGAGATATGAAAGTTT
AGGGATCAAACAGGATTAGATACCCGTGTAGTCC
>Otu2416
CCAGCCTATGGGTTGCAGCAGTGAGGAATTTTGCGCAATGGGAGCAATCCTGACGCAGCA
ACGCCGCGTGGGTGACGAAGGCCTTCGGGTCGTAAAGCCCTGTCAGGTGGGAAGAATGGG
CATAAGGCGAACAGCCTTGTGTTTTGACGGTACCACCAGAGGAAGCACCGGCTAACTCCG
TGCCAGCAGCCGCGGTAATACGGAGGGTGCGAGCGTTATTCGGAATTACTGGGCGTAAAG
CGCGTGCAGGCGGGTATGCAGGTCTGATGTGAAAGCCCCGGGCTCAACCCGGGAAGAGCA
TTGGAAACGGCATATCTTGAGTGCTGGAGAGGAAGGGGGAATTCCCGGTGTAGAGGTGAA
ATTCGTAGAGATCGGGAGGAATACCAGTGGCGAAGGCGCCCTTCTGGACGGCAACTGACG
CTGAGACGCGAAAGCGTGGGGAGCAAACAGGATTAGATACCCCCGTAGTCC
>Otu2418
CCAGCCTATGGGGTGCTGCAGCCGAGAATTTTTCTCAATGGGCGAAAGCCTGAAGGAGCG
ACGCCGCGTGGGGGATGAATGGCTTCGGCCCGTAAACCCCTGTCATTCGGGATCAATGCT
TTTCACTTAACACGTGAACAGTTGATAGTACCGGAAGAGGAAGGGACGGCTAACTCTGTG
CCAGCAGCCGCGGTAATACAGAGGTCCCAAGCGTTGTTCGGATTCACTGGGCGTAAAGGG
TGCGTAGGTGGTAGGGCAAGTCCGGCGTGAAATCTCCGAGCCTAACTCGGAAATGGCGTC
GGAAACTGCCCAACTAGAGGATTGGAGGGGAGACTGGAATACTTGGTGTAGCAGTGAAAT
GCGTAGATATCAAGTGGAACACCAGTGGCGAAGGCGAGTCTCTGGACAATTCCTGACACT
GAGGCACGAAAGCTAGGGGAGCAAACGGGATTAGAGACCCGAGTAGTCC
>Otu2419
CCAGCCTACGGGGGGCAGCAGTGGGGAATATTCTGCAATGAGCGAAAGCTTGACAGAGCA
ATACCGCGTGAAGGAAAACGACCTAAGGGTTGTAAACTTCTTTTCTCAAAAAAGAAATTT
GACATTATTTGAGGAATAAGCATCGGCAAACCCTGTGCCAGCAGCCGCGGCAAGACAGGG
GATGCAAGCGTTATCCGGAATTATTGGGCGTAAAGCGTCTGTAGATCGAAACTTAAGTCT
TTTGTTAAATTTTAAGGCTTAACCTTAAACTCGCAAAAGAAACTAAATTTCTGGAGTCTG
GTAGAGGCAAAGGGAACTTTCAGTGGAGCGGTGAAATGCGGAGATATTGAAATGAAGGCC
AAAAGCGAAGGCACTTTGCTAGACCTGTACTAACATTGAGAGACGAAAGCTAGGGGAGCG

ATGCAGATTAGAAACCCTTGTAGTCA
>Otu2420
CCAGCCTATGGGGCGCTCCAGTCGAGAATCTTCGGCAATGGGCGCAAGCCTGACCGAGCG
ACGCCGCGTGGAGGACGAAGGCCTTCGGGTTGTAAACTCCTGTCGAGGGGAAAGAAGGCC
CGTAAGGGCTTGACTGATCCCTGGAGGAAGCACGGGCTAAGTTCGTGCCAGCAGCCGCGG
TAAGACGAACCGTGCGAACGTTATTCGGAATCACTGGGCTTAAAGCGCGTGTAGGCGGGA
CGGCACGTCGGTCGCTGAAATCCCCCGGCTCAACCGGGGAACGGGCACCGATACGACCGC
CCTCGAGGGGCGTAGGGGGACCTGGAACTTCCGGTGGAGCGGTGAAATGCGTTGAGATCG
GAAGGAACGCCCGTGGCGAAAGCGAGGTCCTGGACGCTTACTGACGCTGAGACGCGAAAG
CCAGGGGAGCGAACGGGATTAGATACCCGGGTAGTCC
>Otu2423
CCAGCCTATGGGGTGCTGCAGTCGAGGATCTTCGGCAATGGGCGCAAGCCTGACCGAGCG
ACGCCGCGTGCGCGATGAAGGCCTTCGGGTTGTAAAGCGCTGTCGAGGGGGAGAAAGCTT
CGCAAGGAGTTGATCGACCCCTGGAGGAAGCACGGGCTAAGTTCGTGCCAGCAGCCGCGG
TAAGACGAACCGTGCGAACGTTATTCGGATTCATTGGGCTTAAAGGGCGCGTAGGCGGGC
CAGCAAGTCAGGGGTGAAATCTTTCAGCTCAACTGGAAAAGTGCCTTTGATACTGCTGGC
CTCGAGGGAGATAGGGGCGTGCGGAACTTCCGGTGGAGCGGTGAAATGCGTAGATATCGG
AAGGAACGCCGGTGGCGAAAGCGGCGCGCTGGATCTCTTCTGATGCTGAGGCGCGAAAGC
CAGGGGAGCAAACGGGATTAGAAACCCTCGTAGTCC
>Otu2424
CCAGCCTATGGGGGGCTCCAGTAGGGAATATTGCGCAATGGGGGAAACCCTGACGCAGCG
ACGCCGCGTGGGTGATGAAGGCCTTAGGGTCGTAAAGCTCTGTTGTACGGGAAGAAAAAA
ATGACGGTACCGTATAAGAAAGGATCGGCTAACTTCGTGCCAGCAGCCGCGGTAATACGA
GGGATCCTAGCGTTGTTCAGAATCATTGGGCGTAAAGGGTGTGTAGGCGGCTAGGCAAGT
CAGTTGTGAAAGCCCCGGGCTCAACCCGGGAAGTGCTTCTGATACTGCTTAGCTTGAGTA
CTGGAGAGGTGAGTAGAATTCCAGGTGTAGTGGTGAAATACGTAGATATCTGGAGGAATA
CCGGTGGCGAAGGCGGCTCACTGGCCATGTACTGACGCTGAAACACGAGAGTGCGGGGAT
CAAACAGGATTAGATACCCTCGTAGTCC
>Otu2427
CCAGCCTACGGGTGGCAGCAGTGGGGAATATTGGACAATGGGCGCAAGCCTGATCCAGCC
ATGCCGCGTGAGTGATGACGGCCTTAGGGTTGTAAAGCTCTTTCGCACGCGACGATAATG
ACGGTAGCGTGAGAAGAAGCCCCGGCTAACTTCGTGCCAGCAGCCGCGGTAATACGTAGG
CAGCAAGCGTTGTTCGGAGTTACTGGGCGTAAAGGGTGCGTAGGCGGCTCTATAAGTATG
GTGTGAAATCTCCCGGCTCAACTGGGAGGGTGCGCCGTAGACTGTGGGGCTAGAGTGTGG
GAGAGGAAAGTGGAATTCCTGGTGTAGCGGTGAAATGCGTAGATATCAGGAGGAACACCG
GTGGTGTAAACGGCTTTCTGGACCATAACTGACGCTGAGGCACGAAAGCGTGGGTAGCAA
ACAGGATTAGATACCCTTGTAGTCC
>Otu2429
CCAGCCTATGGGGGGCACCAGGGGGGGATCTTGGTCAATGGGGGAAACCCTGAACCAGCG
ACGCCGCGTGAGGGAAGAAGGTCTTTGGATCGTAAACCTCTTTTGCCAGGGAAGATGATG
ACGGTACCTGGCGAATAAGCCCCGGCTAACTACGTGCCAGCAGCCGCGGTAATACGTAGG
GGGCGAGCGTTGTCCGGATTTACTGGGCGTAAAGAGCGCGTAGGCGGTCTGAAGAGTCGG
CTGTGAAATCTCTGGGCTCAACCCAGAGGGGTCGGCCGAGACCTTTGGACTTGAGGGTGG
TAGAGGAAGGTGGAATTCCGGGTGTAGTGGTGAAATGCGTAGAGATCCGGAGGAACACCA
GTGGCGAAGGCGGCCTTCTGGGCCAAACCTGACGCTGAGGCGCGAAAGCGTGGGGAGCGA
ACCGAATTAGATACCCCCGTAGTCC
>Otu2432
CCAGCCTATGGGGTGCTCCAGTGGGGAATATTGGACAATGGGGGAAACCCTGATCCAGCG
ACGCCGCGTGTGTGAAGAAGGCCTGCGGGTTGTAAAGCACTTTTAGTGGGGACAAAAAGT
CACGGGCTAACACCGCGTGATCTTGATTTAACCCACAGAAAAAGCACCGGCTAACTCTGT
GCCAGCAGCCGCGGTAATACAAAGGGTGCGAGCGTTAATCGGAATTACTGGGCGTAAAGC
GTGCGCAGGCGGTTATGTAAGACAGATGTGAAATGCCCGGGCTTAACCTGGGAACTGCAT
TTGTGACTGCATGGCTAGAATCTGGCAGAGGGGGGTAGAATTCCACGTGTAGCAGTGAAA
TGCGTAGATATGTGGAGGAATACCGATGGCGAAGGCAGCCCCCTGGGCTAAGATTGACGC
TCATGCACGAAAGCGTGGGGAGCAAACAGGATTAGATACCCCAGTAGTCC
>Otu2433
CCAGCCTATGGGTCGCTGCAGTCGCGAATCATTCACAATGGGCGCAAGCCTGATGATGCC
ACGCCGCGTGCGGGATGAAGGCCCTCTGGGTTGTAAACCGCTGTCAATGGAGGGAAACG

TATGGTGTTAACAGCGCCATGCTTGACTGCTCTGTAAAGGAAGCCCCGGCAAAACATGTG
CCAGCAGCCGCGGTAATACATGTGGGGCAAGCGTTGTTCGGAATTACTGGGCATAAAGGG
TGCGTAGGCGGCATGCCAAGTCAGGGGTGAAAGGTTTCGGCCCAACCGGGACAGTGCCTT
TGATACTGGCGTGCTTGAGTGCGTGAGGGGTGACTGGAACGAGTGGTGTAGCGGTGAAAT
GCGTAGATATCACTCGGAAGGCCAACGGCGAAGGCAGGTCACTGGTACGCAACTGACGCT
GAGGCACGAAAGCGTGGGGAGCAAACAGGATTAGAAACCCTAGTAGTCC
>Otu2434
CCAGCCTATGGGTCGCTGCAGTGAGGAATATTGCTCAATGGACGAAAGTCTGAAGCAGCG
ACGCCGCGTGAGGGATGAAATCCGTTAGGATGTAAACCTCTTTTCTGAGGGACGAACAAG
ATGCCTTGAGCATCTCTGACGGTACCTCAGGAATAAGGATCGGCCAACTACGTGCCAGCA
GCCGCGGTAATACGTAAGATCCGAGCGTTGTCCGGAATTACTGGGTGTAAAGGGTCTGCA
GGCGGGCAAATAAGTCGGTGGTGAAATCCTATAGCTCAACTATAGAACTGCCTCCGATAC
TATTTGTCTTGAGTTCGAGAGAGGGTAATGGAATTCATGGTGTAGCGGTGAAATGCGTAG
ATATCATGAAGAACACCGGTTGCGAAGGCGATTACCTGGCTCGACACTGACGCTCAGGGA
CGAAAGCGTGGGGATCAAACAGGATTAGAAACCCTAGTAGTCC
>Otu2437
CCAGCCTACGGGTGGCTCCAGTGGGGAATATTGGACAATGGGGGCAACCCTGATCCAGCA
ATGCCGCGTGAGTGATGAAGGCCTTCGGGTTGTAAAGCTCTTTCGCCCACGACGATGGTG
ACGGTAGTGGGAGAAGAAGCCCCGGCTAACTTCGTGCCAGCAGCCGCGGTAATACGAAGG
GGGCGAGCGTTGTTCGGAATTACTGGGCGTAAAGGGCGCGTAGGCGGCTCATGAAGTCAG
GCGTGAAAGCCCCGGGCTCAACCTGGGAATTGCGTTTGATACTCGAGAGCTAGAGTTCGG
GAGAGGAGAGTGGAATTCCCAGTGTAGAGGTGAAATTCGTAGATATTGGGAAGAACACCG
GTGGCGAAGGCGGCTCTCTGGACCGATACTGACGCTGAGGCGCGAAAGCGTGGGGAGCAA
ACAGGATTAGAAACCCTAGTAGTCC
>Otu2438
CCAGCCTATGGGGCGCAGCAGTCGAGAATTTTTCACAATGGGGGAAACCCTGATGGAGCG
ACGCCGCGTGGAGGATGAAGGTTTTCGGATTGTAAACTCCTGTCACCACAGAACAAGATT
ACCGGTTCCAATAAAGCCAGTGATTGATGGTATGTGGAGAGGAAGGGACGGCTAACTCTG
TGCCAGCAGCCGCGGTAATACAGAGGTCCCGAGCGTTGTTCGGATTCATTGGGCGTAAAG
GGTGTGTAGGAGGTCGTGTAAGTCAGGCGTGAAATCTCACCGCTTAACGGTGAAATTGCG
TTTGATACTGCTCGGCTAGAGGATTTGAGGGGGTAACGGAATTCTTGGTGTAGCAGTGAA
ATGCGTAGATATCAAGAGGAACACCAGTGGCGAAGGCGGTTACCTGGAAAATTCCTGACT
CTGAAACACGAAAGCTAGGGGAGCAAACGGGATTAGATACCCGAGTAGTCC
>Otu2439
CAGCCTATGGGTTGCTGCAGTAGGGAATATTGGACAATGGGCGAGAGCCTGATCCAGCCA
CGCCGCGTGAGTGATGAAGGCCTTCGGGTCGTAAAGCTCTGTTGTACGGGAAGAACGCGG
ATATGGCTAACATCCATATCCAGTGACGGTACCGTATAAGAAAGGACCGGCTAACTTCGT
GCCAGCAGCCGCGGTAAGACGGGGGGTCCTAGCGTTGTTCGGAATCATTGGGCGTAAAGC
GTGTGCAGGTGGTCTTGAAAGTTGGATGTGAAAGCCCCGGGCTTAACTCGGGAAGTGCAT
TCAAAACTACAAGACTTGAGTAAGGAAGAGGTTCGTGGAATTCCTGGTGTAGTGGTGAAA
TACGTAGATATCAGGAGGAACACCGGCGGCGAAGGCGGCGAACTGGTCCTATACTGACAC
TCATACACGAAAGCGTGGGGATCAAACAGGATTAGATACCCGTGTAGTCC
>Otu2440
CCAGCTATGGGTGGCTGCAGTAGGGAATATTGCACAATGGAGGAAACTCTGATGCAGCGA
CGCCGCGTGAGTGACGAAGGCCTTCGGGTTGTAAAGCTCTGCTCTCAGGGAAAAAGCAAG
TGATTGTACCTGAGGAGAAAGGACCGGCTAACTTCGTGCCAGCAGCCGCGGTAAGACGAG
GGGTCCAAGCGTTGTTCGGAATCATTGGGCGTAAAGCGAGTGTAGGTGGCTCTATAAGTC
AGAAGTGAAAGCCCTGGGCTCAACCCAGGAAGTGCTTTTGATACTGCAGAGCTTGAATGT
GGGAGAGGATCGTGGAATTCCAGGTGTAGTGGTGAAATACGTAGATATCTGGAGGAACAC
CGGTGGCGAAGGCGGCGATCTGGCCCAACATTGACACTGAGACCCGAAAGCGTGGGGATC
AAACAGGATTAGATACCCCAGTAGTCC
>Otu2442
CCAGCCTATGGGGCGCACCAGTGGGGAATATTGGACAATGGGCGCAAGCCTGATCCAGCA
ATGCCGCGTGTGTGAAGAAGGCCTGCGGGTTGTAAAGCACTTTCAGTGGGGAGGAAGGGC
TTGGTGCTAATAACACCGAGCATTGACGCTACCCACAGAAGAAGCACCGGCTAACTCTGT
GCCAGCAGCCGCGGTAATACAGAGGGTGCAAGCGTTAATCGGAATTACTGGGCGTAAAGG
GCGCGTAGGTGGTTTGCTAAGTCAGATGTGAAAACCCTGGGCTTAACTTAGGAATTGCAT
TTGAAACTGGTAGACTAGAGTTTGGGAGAGGGAAGTGGAATTTCCGGTGTAGCGGTGAAA

TGCGTAGATATCGGAAGGAACATCAGTGGCGAAGGCGACTTCCTGGCCCGATACTGACAC
TGAGGCGCGAAAGCGTGGGGAGCGAACAGGATTAGAGACCCGAGTAGTCC
>Otu2443
CCAGCCTACGGATGCTGCAGTCGAGAATCTTCCGCAATGGGCGAAAGCCTGACGGAGCGA
CGTTACGTGAATGAAGAAGCCCTTCGGGGTGTAAAGTTCTTTTCTCTGGGAGGAAGTTAT
TGACAGTACCAGAGGAATAAGGGGATCCTAATTCTGTGCCAGCAGGAGCGGTAAGACAGA
ATCCCCAAGCGTTACCCGGATTTATTGGGCGTAAAGGGTCCGTAGGTGGTAAAGTAAGTT
GAAAGTCAAAACTCATCGGCTTAACCTTTGAGATTCTTTCAAAACTGCTATACTAGAGGG
CGTTAGGGGTTAGCGGAACCGACGGGGTAGGGGTGAAATCCGTTGATATCGTCGGAACA
CCAAAAGCGAAGGCAGCTAACTAGGACGACCCTGACACTGAGGGACGAAAGCGTGGGGAG
CAAAAAGGATTAGAAACCCCAGTAGTCC
>Otu2444
CCAGCCTATGGGTTGCAGCAGTGGGGAATCTTGCGCAATGGGCGAAAGCCTGACGCAGCA
ACGCCGCGTGAGGGACGAAGGCTTTCTGAGTTGTAAACCCCTTTCGACAGGAACGATTGT
GACGGTACCTGTAGAAGAAGCACCGGCCAACTATGTGCCAGCAGCCGCGGTGATACATAG
GGTGCAAGCGTTATCCGGATTCACTGGGTTTAAAGGGTGCGTAGGCGGGCAGGTAAGTCA
GTGGTGAAATCTCCGGGCTCAACCCGGAAACTGCCGTTGATACTATTTGTCTTGAATGTC
GTGGAGGTTAGCGGAATATGTCATGTAGCGGTGAAATGCTTAGATATGACATAGAACACC
AATTGCGAAGGCAGCTAGCTACACGGTTATTGACGCTGAGGCACGAAAGCGTGGGGATCA
AACAGGATTAGATACCCCAGTAGTCC
>Otu2445
CCAGCCTATGGGTGGCTGCAGTCGAGAATCTTTCGCAATGGGCGAAAGCCTGACGAAGCG
ACGCCGTGTGAGTGATGAAGGCCTTCGGGTTGTAAAGCTCTTTCGCTTGGGAACAAGGGT
TTGTGGTGAACAGCCATAGATCTTGAGGGTACCAGGTAAAGAAGCACCGGCTAACTCCGT
GCCAGCAGCTGCGGTAATACGGAGGGTGCAAGCATTAATCGGATTTATTGGGCGTAAAGG
GCGCGTAGGCGGGCCCTCAAGTCTGTTGTGAAATTCTGGGGCTCAACCTCGGAGCTGCAA
CGGAGACTGTGGGTCTGGAGGCTTGGCGGGGAAAATGGAATTCCACGTGTAGCGGTGAAA
TGCGTAGATATGTGGAAGAACACCGGTGGCGAAGGCGATTTTCCAGCTTATGCCTGACGC
TGAAGCGCGAGAGCAAGGGGAGCAAACAGGATTAGATACCCGGGTAGTCC
>Otu2447
CCAGCTACGGGTGGCTGCAGTGGGGAATATTGGACAATGGGCGCAAGCCTGATCCAGCAA
TGCCGCGTGAATGATGAAGGCCTTAGGGTTGTAAAGTCCTTTCGGCGGGGACGATGATGA
CGGTACCCGCAGAAGAAGCCCCGGCTAACTTCGTGCCAGCAGCCGCGGTAATACGAAGGG
GGCTAGCGTTGCTCGGAATGACTGGGCGTAAAGGGCGCGTAGGCGGTTTGGACAGTTGGG
CGTGAAATTCCTGGGCTTAACCCGGGGGCTGCGCTCAATACATCCAGGCTTGAGTGAGGA
AGAGGGTCGTGGAATTCCCAGTGTAGAGGTGAAATTCGTAGATATTGGGAAGAACACCGG
TGGCGAAGGCGGCGACCTGGTCCTTTACTGACGCTGAGGCGCGAAAGCGTGGGGAGCAAA
CAGGATTAGATACCCATGTAGTCC
>Otu2448
CCAGCCTATGGGGGGCTGCAGTCGAGAATCTTCCGCAATGGGCGCAAGCCTGACGGAGCG
ATGTCGCGTGAAGGATGAAGGTCTTCGGATTGTAAACTTCTGTCACTGGGGACGAATAAA
TGACGGTACCCGGAGAGGAAGTCACGGCTAACTACGTGCCAGCAGCCGCGGTAATACGTA
GGTGGCTAGCGTTGTTCGGATTTATTGGGCGTAAAGGGTCCGCAGGGGGCCGGCTAAGTC
TGATGTGAAATACCAAGGCTCAACCTTGGAACTGCATTGGAAACTGGTTGGCTAGAGCAT
CGGAGAGGAGATTGGAATTCCTGGTGTAGCGGTGGAATGCGTAGATATCAGGAGGAACAC
CAAAGGCGAAGGCAGATCTCTGGACGATTGCTGACCCTCAGGGACGAAAGCATGGGGATC
AAACAGGATTAGAAACCCCCGTAGTCC
>Otu2449
CCAGCCTACGGGGGGCAGCAGTGAGGAATATTGGGCAATGAGCGAAAGCTTGACCCAGCG
AGAATTTGTGTGTGAAGAAGGCTTACGTTGTAAAGCACTTTCGGGTAGAACGATAATGAC
GGTACTATCAGTAAAAGAAGCACTGGCTAAGTCTGTGCCAGCAGCCGCGGTAATACAGAC
AGTGCAAGCGTTATTCGGATTTAATGGGCGTAAAGGGTATGTTGGTGGCTCAAAAAAACT
ATATTAAAAAGGCTAGAGTAATAACTTTAGACATAATATAAAGATTAAAGGGCTTGAGTA
GATAAGGAGTAGGAGGAATTTTTCATGTTAACAGTAAAATGTAATGATATGAAAGGGACT
ACCAGAGGCGAAGGCATCTATCTATTATTTACTGACGCTGAGGTACGAAAGCATGGGGAG
CAAATCAGATTAGAAACCCCTGTAGTCC
>Otu2452
CCAGCCTATGGGAGGCAGCAGTGGGGAATTTTTGGCAATGGGGGAAACCCTGACCAAGCA

ACGCCGCGTGGGTGATGAAGTCTCTTGGGACGTAAAACCCTTTCGACGGGGAAGAAAGGC
CGCAAGGCTTTGACGGTACCCGTAGAAGAAGCCCCGGCTAACTCCGTGCCAGCAGCCGCG
GTAATACGGGGGGGGCAAGCGTTGTTCGGAATTACTGGGCGTAAAGGGCGCGTAGGCGGT
CTGCTAAGTTGGATGTGAAAACTCTGGGCTTAACCCAGAGCCTGCATTCAAAACTGACAG
GCTAGAGTTCTGGAGGGGATAGCGGAATTCCTGGTGTAGCGGTGAAATGCGTAGATATCA
GGAGGAACACCGGTGGCGAAGGCGGCTATCTGGACAGAGTCTGACGCTGAGGCGCGAAAG
CTAGGGGAGCAAACAGGATTAGATACCCCAGTAGTCC
>Otu2453
CCAGCCTACGGGGGGCTGCAGTAGGGAATATTGCGCAATGGGCGAAAGCCTGACGCAGCG
ACACCGTGTGAAGGATGAAGATTTTCGGATCGTAAACTTCTGTCAATTGGGGACGAAGGT
AGTAGGGTTAATAGCTCTTACTATTGACGGTACCCAATAAGGAAGTCTCGGCTTACTCCG
TGCCAGCAGCCGCGGTAAGACGGGGGAGGCAAGCGTTGTCCGGAATTATTGGGCGTAAAG
AGCGCGTAGGTGGTTTGTAAAGTCATTTGTGAAAACTTTAGGCTTAACTTAAAGACTGCA
AGTGATACTGACAAACTTGAGTACAGTAGAGGAAAGTGGAATTCCTGGTGTAGCGGTGAA
ATGCGCAGATATCAGGAAGAACACCAGTGGCGAAGGCGGCTTTCTGGGCTGTTACTGACA
CTGAGGCGCGAAAGCTAGGGGAGCGAACAGGATTAGATACCCCCGTAGTCC
>Otu2456
CCAGCCTATGGGTCGCAGCAGTCAAGAACTTTCCACAATGGACGCAAGTCTGATGGAGCG
ACGCCGCGTGATTGATGAAGTCCTTCGGGACGTAAAGATCTTTTATGAGGGAAGAAGTTT
ATTGACGGTACCTCATGAATAAGGGGCTCCTAATCTCGTGCCAGCAGGAGCGGTAATACG
AGAGCCCCAAGCGTTATCCGGAATTATTGGGCGTAAAGGGTGCGTAGGCGGTTATGTTAG
TCGTGTGTTAAAACCCGGGGCTCAACCTCGGATCTGCGCGCGAAACGGCATGACTTAGAA
TGAAGAAGAGGTGTGCGGAACTCATGGTGTAGGGGTGAAATCCGTTGATATCATGGGGAA
CACCAAATGCGAAAGCAGCACACTGGTCTTTTATTGACGCTGAGGCACGAAAGCGTGGGT
AGCGAATGGGATTAGATACCCGAGTAGTCC
>Otu2462
CCAGCCTACGGGAGGCAGCAGTGGGGAATATTGGACAATGGGGGCAACCCTGATCCAGCA
ACGCCGCGTGAATGAAGAAGGTCTTAGGATTGTAAAGTTCTTTTGCCTGAGAGCAAATAT
CATTAGTTAAAAGCTGATGGTTATGAGAGTATCAGGAGAATAAGCACCGGCTAACTACGT
GCCAGCAGCCGCGGTAATACGTAGGGTGCGAGCGTTAATCGGAATTACTGGGCGTAAAGA
GTGCGTAGGCGGATATGTAAGTTAGATGTGAAATACCTGGGCTTAACCTGGGAGGTGCAT
TTAAGACTGCATATCTAGAGTTTGTCAGAGGGGGGTAGAATTCCAAGTGTAGCAGTGAAA
TGCGTAGAGATTTGGAGGAATACCGATGGCGTAGGCAGCCCCCTGGGATAAGACTGACGC
TGAGGCACGAAAGCGTGGGTAGCAAACAGGATTAGATACCCTTGTAGTCC
>Otu2466
CCAGCCTACGGGTGGCTCCAGTGAGGAATCTTCCGCAATGGACGAAAGTCTGACGGAGCA
ACGCCGCGTGCAGGATGACGGCCTTCGGGTTGTAAACTGCTTTTATTTGGGAAGATTATG
ACGGTACCAAATGAATAAGGACCGGCTAACTACGTGCCAGCAGCCGCGGTAATACGTAGG
GTCCAAGCGTTATCCGGAATTACTGGGCGTAAAGCGTGCGTAGGCGGCGCGTTAAGTGAG
ATGTGAAATCTGGCGGCTCAACCGCGAGACTGCATCTCATACTGGCGTGCTAGAGGATGG
CAGAGGCAAGTGGAATTCGTAGTGTAGCAGTGAAATGCGTAGATATTACAAGGAACACCA
ATGGCGAAGGCAGCTTGCTGGGCCACACCTGACGCTGAGGTACGAAAGCGTGGGGAGCGA
ACAGGATTAGATACCCGTGTAGTCC
>Otu2469
CCACCTATGGGACGCAGCAGTGGGGAATATTGGACAATGGGGGCAACCCTGATCCAGCGA
TGCCGCGTGGGTGAAGAAGGCCTTCGGGTTGTAAAGCCCTTTAGGTCGGGACGAAGTTAT
GCGGGGGATAATCCCGTGTAGTTGACGGTACCGACAGAATAAGCACCGGCAAACTCTGTG
CCAGCAGCCGCGGTAATACAGAGGGTGCGAGCGTTAATCGGAGTTACTGGGCGTAAAGGG
CGCGTAGGCGGTTATGTGGGTGTGATGTGAAAGCCCCGGGCTTAACCTGGGAAGTGCATC
GCAAACTATGTAGCTAGAGTATAAGAGAGGGTGGCGGAATTTCCGGTGTAGCGGTGAAAT
GCGTAGAGATCGGAAGGAACGTCAATGGCGAAGGCAGCCACCTGGCTTAATACTGACGCT
GAAGCGCGAAAGCGTGGGGAGCGAACAGGATTAGATACCCCAGTAGTCC
>Otu2471
CCAGCCTATGGGACGCTCCAGCCGAGAATATTCGACAATGGGCGCAAGCCTGATCGAGCG
ATACCGCGTGGTGGATGAAGCGCTTCGGCGCGTAAACATCTTTTATGGAGGAGGAAGTTA
TTGACGTTACTCCATGAATAAGGGGCTCCTAACTCTGTGCCAGCAGGAGCGGTAATACAG
AGGCCCCAAGCGTTACCCGGAATTACTGGGCGTAAAGAGTGCGTAGGTGGTCGTGTTAGT
CGTGCGTCAAAACCCGGAGCTCAACTCCGGATCCGCGCACGAAACGGCACGACTCGAGAG

CGTGAGAGGTCTGTGGAACTCTGGGTGTAGGGGTGAAATCCGTTGATATCCAGGGGAACA
CCGAAAGCGAAGGCAGCAGACTGGCGCGTTTCTGACACTCAAGCACGAAAGCGTGGGTAG
CGAACGGGATTAGAGACCCCAGTAGTCC
>Otu2475
CCAGCCTATGGGTGGCAGCAGTGAGGAATATTGGTCAATGGACGCAAGTCTGAACCAGCC
ATGCCGCGTGAAGGATGAAGGCCTTCTGGGTTGTAAACTTCTTTTATCTGGGAAGAAACC
ACTTTTTTCTAAGAGTGTTGACGGTACCAGAGGAATAAGCACCGGCTAACTCCGTGCCAG
CAGCCGCGGTAATACGGAGGGTGCAAGCGTTATCCGGATTTACTGGGTTTAAAGGGTGTG
TAGGCGGGCACTTAAGTCAGTGGTGAAATCTCTAGGCTCAACCTAGAAACTGCCATTGAT
ACTATATGTCTTGAATTCTGTTGAGGAGGGCGGAATATGTCATGTAGCGGTGAAATGCAT
AGATATGACATAGAACACCGATAGCGAAGGCAGCTCTCTAAGCAGACATTGACGCTGAGG
CACGAAAGCGTGGGGATCAAACAGGATTAGAGACCCGTGTAGTCC
>Otu2479
CCAGCCTATGGGGGGCAGCAGTGGGGAATATTGGACAATGGGCGCAAGCCTGATCCAGCA
ATGCCGCGTGGGTGAAGAAGGTCTTCGGATTGTAAAGCCCTTTCGGCGGGGACGATAATG
ACGGTACCCGTAGAAGAAGCTCCGGCTAACTTCGTGCCAGCAGCCGCGGTAATACGAAGG
GGGCTAGCGTTGTTCGGAATTACTGGGCGTAAAGCGCGCGCAGGCGGCTTTTCAAGTCGA
GGGTGAAAGCCCAGAGCTCAACTCTGGAACTGCCTTCGAAACTGTGGAGCTTGAGTACGG
GAGAGGTGAGTGGAATTCCCAGTGTAGAGGTGAAATTCGTAGATATTGGGAAGAACACCG
GTGGCGAAGGCGGCTCACTGGCCCGTTACTGACGCTCAGGCGCGACAGCGTGGGGATCAA
ACAGGATTAGATACCCGCGTAGTCC
>Otu2481
CCAGCCTACGGGAGGCTCCAGTAGGGAATATTGGGAATGGGCGCAAGCCTGACCCAGCAA
CGCCGCGTGAGTGATGAAGGCCTTCGGGTTGTAAAACTCTTTTCACCGGGAAGAGGAAGG
ACGGTACCGGTGGAATAAGTCTCGGCTAACTACGTGCCAGCAGCCGCGGTAACACGTAGG
AGACAAGCGTTATCCGGATTTACTGGGTGTAAAGCGCGTGCAGGCGGTTAGGTAAGTTGG
ATGTGAAAGCTCCTGGCTTAACTGGGAGAGGTCGTTCAAAACTACCGAACTAGAGGATGG
CAGAGGGGAGTGGAATTCCGAGTGTAGCGGTAAAATGCATAGATATTCGGAAGAACACCA
GTGGCGAAGGCGGCTCTCTGGGCCATATCTGACGCTCATACGCGAAAGCTAGGGTAGCAA
ACGGGATTAGATACCCTCGTAGTCC
>Otu2489
CCAGCCTATGGGGGGCACCAGTCGAGAATTTTTCTCAATGGGCGAAACCCTGATGCAGCG
ACGCCGCGTGGAGGATGAAGGGTCTAGGCCTGTAAACTCCTTTTATGGGGAAAGACTTAG
GACGGTACCCCATGAATAAGGACCGGCTAACTACGTGCCAGCAGCCGCGGTAAGACGTAG
GGTCCAAGCGTTGTCCGGATTTACTGGGCGTAAAGAGCGCGTAGGCGGCTCGTTAAGTGT
GAAGTGAAATCCCTAGGGCTCAACCCAGAAACTGCTTTACATACTGGCGAGCTAGAGGGA
TGCAGAGGTTGGTGGAATTCCTGGTGTAGCGGTGAAATGCGTTGATATCAGGAGGAACAC
CCATGGCGAAGGCAGCAAACTGGGCATCATCTGACGCTGAGGCGCGAAAGCGTGGGTAGC
AAACAGGATTAGAAACCCGCGTAGTCC
>Otu2490
CCAGCCTACGGGGTGCTCCAGTGAGGAATATTGGTCAATGGGCGCAAGCCTGAACCAGCC
ATGCCGCGTGAAGGATGAAGGCCCTATGGGTTGTAAACTTCTTTTGTACGGGATGAAACT
TTCTTACGTGTAAGGAACTGACGGTACTGTACGAATAAGCAACGGCTAACTCCGTGCCAG
CAGCCGCGGTAATACGGAGGTTGCAAGCGTTATCCGGATTTATTGGGTTTAAAGGGTGCG
TAGGCGGGATTGTAAGTCAGTGGTGAAAACCTGCAGCTTAACTGTAGAACTGCCATTGAT
ACTGCCATTCTTGAGTATAGTTGAGGTAGGCGGAATGTGTAATGTAGCGGTGAAATGCTT
AGATATTACACAGAACACCAATTGCGTAGGCAGCTTACTAAACTAAAACTGACGCTGAGG
CACGAAAGCGTGGGGAGCAAACAGGATTAGAAACCCCCGTAGTCC
>Otu2493
CCAGCCTATGGGATGCTGCAGCAAGGAATCTTCCACAATGGGCGAAAGCCTGATGGAGCA
ACGCCGCGTGCAGGATGAATGCCTTCGGGTTGTAAACTGCTTTTATACGTGACGAATATG
ACGGTAGCGTATGAATAAGGATCGGCTAACTCCGTGCCAGCAGCCGCGGTCATACGGAGG
ATCCAAGCGTTATCCGGATTTACTGGGCGTAAAGAGTTGCGTAGGTGGTAGTTTAAGCGA
ATAGTGAAATCTTGTGGCTCAACCATGAGACTGTTATTCGAACTGAGCTACTCGAGAACG
AGAGAGGTCACTGGAATTCCCTGTGTAGGAGTGAAATCCGTAGATATAGGGAGGAACACC
AATGGCGTAGGCAGGTGACTGGCTCGTTTCTGACACTGAGGCACGAAAGCGCGGGGAGCA
AACAGGATTAGAAACCCCAGTAGTCC
>Otu2495

CCAGCCTACGGGTTGCTCCAGTCGAGAATCTTCCGCAATGGGCGAAAGCCTGACGGAGCG
ACGTTACGTGAATGATGAAGCCCTTAGGGGTGTAAAGTTCTTTTATATGGGAAGAAGTTA
TTGACTGTACCATATGAATAAGGAGATCCTAATTCTGTGCCAGCAGGAGCGGTAAGACAG
AATCTCCAAGCGTTACCCGGATTTATTGGGCGTAAAGGGTCCGTAGGTGGCACAGTAAGT
TGAAAGTTAAATCTCATCGGCTTAACCTTTGAGGTGCTTTCAATACTGCTGCGCTTGAGG
GCGTTAGGGGTTAGCGGAACCGACGGAGTAGGGGTGAAATCCGTTGATATCGTCGGGAAC
ACCAAAAGCGAAGGCAGCTAACTAAGACAGCCCTGACACTGAGGAACGAAAGCGTGGGGA
GCAAAAAGGATTAGAGACCCCCGTAGTCC
>Otu2496
CCAGCCTACGGGTTGCAGCAGTCGAGAATTTTTCACAATGGGCGCAAGCCTGATGGAGCG
ACGCCGCGTGGGGGATGAATGGCTTCGGCCCGTAAACCCCTGTCATTTGCGAACAATGCG
TTCGACCTAACACGTCGAACGTTGATAGTAGCGAAAGAGGAAGGGACGGCTAACTCTGTG
CCAGCAGCCGCGGTAATACAGAGGTCCCAAGCGTTGTTCGGATTCACTGGGCGTAAAGGG
TGCGTAGGTGGCGAGGCAAGTCGGATGTGAAAGCTCGGAGCTCAACTCCGAAATGGCATT
GGAAACTGCCTTGCTCGAGGGTTGGAGGGGGGACTGGAATACTTGGTGTAGCAGCGAAAT
GCGTAGATATCAAGTGGAACACCAGTGGCGAAGGCGAGTCCCTGGACAACTCCTGACACT
GAGGCACGAAAGCTGGGGGAGCAAACAGGATTAGAAACCCGCGTAGTCC
>Otu2499
CCAGCCTATGGGACGCACCAGTTAAGAATATTCCACAATGCACGAAAGTGTGATGGAGCG
ACGCCGCGTGAATGATGAAGCCCTTAGGGGTGTAAAGTTCTTTTATTTAGGAAAAATTTT
TGATGTTACTAAATGAATAAGGGGCTGCTAACTTCGTGCCAGCAGCAGCGGTAATACGAA
GGCCCCGAGCGTTATCCGGATTTACTGGGTGTAAAATGTCTGTAGACGGTTTTATAAGTT
AAATGTAAAATCTTTTAGCTTAACTAAAAGTTTGCATTTAATTCTATAAGACTAGAGGGT
GGGGGAAGTAAGCGGAATTGGTGGTGTAGTTGTAATAAGCGTTAATATCACCAAGAACAC
CAAAAGCGTAGGCAGCTTACTAAAACATACCTGACGTTGAGAGACGAAAGCGTGGGGAGC
GAAACAGATTAGATACCCGCGTAGTCC
>Otu2506
CCAGCCTACGGGTGGCTGCAGTGAGGAATATTGCGCAATGGACGGGAGTCTGACGCAGCA
ACGCCGCGTGAGCGATGAAGGCCTTCGGGTCGTAAAGCTCTTTTCTGGGGGACGAGGAAG
GACGGTACCTCAGGAATAAGCCTCGGCTAACTACGTGCCAGCAGCCGCGGTAAAACGTAG
GAGGCAAGCGTTATCCGGATTTATTGGGCGTAAAGCGCATGCAGGTGGTTCCACAAGTCG
GATGTGAAAGCTCCTGGCTTAACTGGGAGAGGTCGTTCGAAACTGTGGGACTGGAGGTCG
GAAGAGGGAAGCGGAATTCCCGGTGTAGTGGTGGAATGCGTAGATATCGGGAGGAACACC
AGTGGCGGAAGCGGCTTCCTGGTCCGCACCTGACACTCAGATGCGAAAGCGTGGGGAGCG
AACAGGATTAGAAACCCCGGTAGTCC
>Otu2509
CCAGCCTACGGGTGGCAGCAGTGGAGAATATTGCGCAATGGGCGAAAGCCTGACGCAGCA
ACGCCGCGTGTGTGATGAAGGCCTTCGGGTTGTAAAGCACTTTCGGCCGGGAAGAAGGGT
GTGCGAACTAATAATGCGTACATTTGATGGTACCGGAAGAAGAAGCACCGGCTAACTTCG
TGCCAGCAGCCGCGGTAATACGAAGGGTGCGAGCGTTGTTCGGAATTACTGGGCGTAAAG
GGTTCGTAGGCGGGAGGGTAAGTTAAGTGTGAAATGTCCGGGCTCAACCTGGAACGTGCA
CTTAAAACTGCTCTTCTTGAGTACTGGAGAGGGCAGTGGAATTGCTGGTGTAGGAGTGAC
ATCCGTAGAGATCAGCAGGAACACCGGAGGCGAAGGCGACTGCCTGGCCAAGTACTGACG
CTGAGGAACGAAAGCGTGGGGAGCAAACAGGATTAGATACCCCTGTAGTCC
>Otu2512
CCAGCCTACGGGAGGCTGCAGTGAGGAATATTGGTCAATGGGCGCAAGCCTGAACCAGCC
ATGCCGCGTGAAGGATGAAGGCCTTCTGGGTTGTAAACTTCTTTTAAGTGGGAAGAAACC
CCTTGTATCTACGAGGGTTGACGGTACCATAGGAATAAGCACCGGCTAACTCCGTGCCAG
CAGCCGCGGTAATACGGAGGGTGCAAGCGTTATCCGGATTTACTGGGTTTAAAGGGTGTG
TAGGCGGGCCTTTAAGTCAGTGGTGAAATCTCTGGGCTTAACCTGGAAACTGCCATTGAT
ACTATTGGTCTTGAATTCTGTTGAGGAGGGCGGAATATGTCATGTAGCGGTGAAATGCAT
AGAGATGACATAGAACCCCGATTGCGAAGGCAGCTCTCTAAACAGCGATTGACGCTGAGG
CACGAAAGCGTGGGGATCAAACAGGATTAGAAACCCCCGTAGTCC
>Otu2514
CCGCCTACGGGGTGCAGCAGTGGGGAATATTGGACAATGGAGGCAACCCTGATCCAGCAA
TGCCGCGTGAGTGATGAAGGCCTTCGGGTTGTAAAGCTCTTTCGCCTACGACGATGGTGA
CGGTAGTAGGAGAAGAAGCCCCGGCTAACTTCGTGCCAGCAGCCGCGGCAATACGAAGGG
GGCTAGCGTTGTTCGGAATTACTGGGCGTAAAGGGCGCGTAGGCGGCCGGCTCAGTCAGA

TGTGAAAGCCCGGGGCTCAACCCCGGATGTGCATTTGATACTGGTCGGCTTGAGGACGGG
AGAGGATAGTGGAATTCCCAGTGTAGAGGTGAAATTCGTAGATATTGGGAAGAACACCGG
TGGCGAAGGCGGCTATCTGGACCGTATCTGACGCTGAGGCGCGAAAGCGTGGGGAGCAAA
CAGGATTAGAAACCCGTGTAGTCC
>Otu2517
CCAGCCTATGGGGTGCAGCAGTCGAGAATCATTCGCAATGGGCGAAAGCCTGACGATGCG
ACGCCGTGTGAGCGAAGAAGGCCTTCGGGTTGTAAAGCTCTTTCGCTTGGGAACAAGAGA
TGTCGTTTAATAAACGGCAAATTTGAGGGTACCAGGTAAAGAAGCACCGGCTAACTCCGT
GCCAGCAGCTGCGGTAATACGGAGGGTGCAAGCATTAATCGGATTTATTGGGCGTAAAGG
GCGCGTAGGCTGAGATGTAAGTCAGATGTGAAATCCCTGGGCTCAACCCAGGAACTGCAT
TTGAAACTACATTTCTAGAGGGTAGGCGGAGAAAATGGAATTCCACATGTAGCGGTGAAA
TGCGTAGATATGTGGAGGAACACCAGTGGCGAAGGCGGTTTTCTAGCTTACTCCTGACGC
TGAGGCGCGAAAGCAAGGGGATCAAACAGGATTAGAGACCCGTGTAGTCC
>Otu2522
CCAGCCTATGGGTCGCACCAGTCGAGGATCTTTCGCAATGGGCGCAAGCCTGACGAAGCG
ACACTGCGTGAGTGATGAAGGCCTTCGGGTTGTAAAACTCTTTCGCGCAAGAACAAGGAA
GGGATGTGAATAACATTCTGACTTGAGGGTACTGCGTAAAGAAGCACCGGCTAACTCCGT
GCCAGCAGCTGCGGTAATACGGAGGGTGCAAGCATTAATCGGAATTATTGGGCGTAAAGG
GCGCGTAGGCGGAAATGTAAGTCAGATGTGAAATTCCGAAGCTCAACTTCGGAGCTGCAT
TTGAAACTACAATTCTAGAGGGTTGACGGAGAAAACGGAATTCCACGTGTAGCGGTGAAA
TGCGTAGATATGTGGAAGAACACCGGTGGCGAAAGCGGTTTTCTAGTTAATACCTGACGC
TGAGGCGCGAGAGCAAGGGGAGCAAACAGGATTAGAAACCCGCGTGTCC
>Otu2523
CCAGCCTACGGGGGGCTCCAGTGGGGAATTTTGCGCAATGGGCGAAAGCCTGACGCAGCA
ACGCCGCGTGCGGGACGAAGGCCTTAGGGTCGTAAACCGCTTTCAGCAGGGACGAAACTG
ACTGTACCTGCAGAAGAAGCTCCGGCTAACTACGTGCCAGCAGCCGCGGTAATACGTAGG
GAGCAAGCGTTATCCGGATTTATTGGGCGTAAAGCGCGCGTAGGCGGCTTGTTAAGTCAG
GTGTGAAAGCCGGGGGCTCAACCCGCGGATTGCATCTGAAACTGGCAGGCTTGAGTGTGG
TAGAGGAAAGTGGAATTCCCGGTGTAGCGGTGAAATGCGCAGATATCGGGAGGAACACCA
GTGGCGAAGGCGGCTTTCTGGGCCACCACTGACGCTGAGGCGCGAAAGCTAGGGGAGCGA
ACAGGATTAGATACCCTAGTAGTCC
>Otu2525
CCAGCTTACGGGACGCAGCAGTGAGGAATATTGGACAGTGGACGAAAGTTTGATCCAGCC
ATGGGGCATTGGGGAAAGGAAGGTTCAATGAGACTGTAAACCCTTTTGTCAGCCACGATT
ATGACGGTAGCTGAAGAAAAAAGTCCCGGCTAATCTCGTGCCAGCAGCCGCGGTAATACG
AGAGGGACAAGTGTTATTCCCACTAACTGGGCGTAAAGGGCTTCTAGGCGGTTCTTGCAG
TTAAAGGTGAAAGCCCAGTCATATCTTTGGAAGGGCCTTTAAAACGAAGAACTTCGGGTT
TTTTTAGAGAATGCACTATGTTGGAAGTAAAGGTTAAATTTGTTGATATCCAACGGGGTT
CCGAAGGCGAAAGCAGCATTCTAACTAAAACCGACGCTGAAGAGCAAAAGCATGGGGATC
GATGCGGATTAGAGACCCTTGTAGTCC
>Otu2526
CCAGCCTACGGGAGGCTGCAGTGGGGAATTGTTCGCAATGGGCGCAAGCCTGACGACGCA
ACGCCGCGTGGAGGATGAAGAATTTCGGTTCGTAAACTCCTTTCGACCGAGATGAAAACC
TCCCGACTTAATACGTTGGGAGGCTGACAGTATCGAGGGAAGCAGCCCCGGCTAACTCCG
TGCCAGCAGCCGCGGTAATACGGGGGGGGCAAGCGTTGTTCGGAATTACTGGGCGTAAAG
GGTTTGTAGGTGGTCGACTCAGTCAGACGTGAAATCCCTCAGCTTAACTGGGGAACTACG
TCTGATACTGGTTGGCTTGAGTGCAGGAGAGGAACGCGGAATTCCAGGTGTAGCGGTGAA
ATGCGTAGATATCTGGAGGAACACCGGTGGCGAAGGCGGCGTTCTGGATTGCAACTGACA
CTGAGAAACGAAAGCCAGGGGAGCAAACGGGATTAGATACCCGCGTAGTCC
>Otu2531
CCAGCCTATGGGTCGCACCAGTTAGGCATAATCTACAATGTACGCAAGTATGATAGTTTG
AGCCAGAGTGCTTTCCAATTGGAAAGCTTTTGCTAAATCTAAATAGTTTGGCGAATAAGG
ACTGGGTAAGACGGGTGCCAGCCGCCGCGGTAATACCCGCGGTCCAAGTCGCAGCCATCA
TTATTGGGTCTAAAACATCCGTAGCTCGTTTGGTAAGTTCCTTGTGAAATCCGGCATCTC
AAGTGTCGGGCGCGCAGGGAGTACTGCCAGGCTAGAGACTGGAAGACGTGAGAAGTACGT
TCTAAGTAGTGGTTAAATACGTTAATCTAGGACGGACTCACAATAGCGAAGGCATCTCAC
GAGGACAGTTCTGACAGTAAGGGATGAAGGCTAGGGGCGCAAAACGGATTAGAAACCCCG
GTAGTCC

>Otu2533
CCAGCCTACGGGGTGCTCCAGTAAGGAATATTGGTCAATGGACGCAAGTCTGAACCAGCC
ATGCCGCGTGCAGGATGAAGGCCCTCTGGGTTGTAAACTGCTTTTATATGGAAATAAAAA
AACCCTTGCGAGGGTTATTGAAGGTACCATAAGAATAAGCACCGGCTAACTCCGTGCCAG
CAGCCGCGGTAATACGGAGGGTGCAAGCGTTATCCGGATTTACTGGGTTTAAAGGGTGCG
TAGGCGGCTCTTTAAGTCAGTGGTGAAATCCTAGTGCTCAACACTAGAACTGCCATTGAA
ACTGAGGAGCTCGAGTCAAGGAGAGGTAAGCAGAATTTATGGTGTAGCAGTGAAATGCTT
AGATACCATAAGGAATACCAATAGCGAAGGCAGCTTACTGGCCTTGTACTGACGCTGAGG
CACGAAAGCGTGGGGAGCAAACAGGATTAGATACCCGCGTAGTCC
>Otu2534
CCAGCCTATGGGAGGCAGCAGTCGAGAATCTTTGGCAATGCACGAAAGTGTGACCAAGCG
ACGCCGCGTGATTGACGAAGCCCTTCGGGGTGTAAAGATCTTTTATGCATGAAAAAATTA
TTGATATCAGTGCATGAATAAGGGGCTCCTAACTCTGTGCCAGCAGGAGCGGTAATACAG
AGGCCCCGAGCGTTATTCGGAATTATTGGGCGTAAAGGGTGCGTAGGTGGTTTGATTAGT
CGACGGTCAAAGATCAGGGCTGAACCCTGGGAAGGCCATCGAAACGGTCAGACTCGAGTA
GGTGAGAGGTGAATGGAACTCATGGAGGAGGGGTGAAATCCGTTGATATCATGGGGAACA
CCAAATGCGAAGGCAATTCACTGGCGCCTTACTGACACTCAAGCACGAAAGCGTAGGTAG
CGAATGGGATTAGAAACCCCTGTAGTCC
>Otu2538
CCAGCCTATGGGATGCACCAGTGGGGAATCTTGCACAATGGGCGAAAGCCTGATGCAGCG
ACGCCGCGTGAGCGATGAAGGCCTTCGGGTTGTAAAGCTCTGTGGGGAGGGACGAATAAG
TGCTGGCTAATATCCAGCATGATGACGGTACCTCCTTAGCAAGCACCGGCTAACTCTGTG
CCAGCAGCCGCGGTAAGACAGAGGGTGCAAACGTTGTTCGGAATCACTGGGCGTAAAGCG
CGTGTAGGCGGCTACGTAAGTCGGACGTGAAAGCCCACGGCTCAACTGTGGAAGTGCGCT
CGAAACTGTGTAGCTTGAGTCTTGGAGAGGAAGGCGGAATACTTGGTGTAGAGGTGAAAT
GCGTAGATATCAAGTGGAACACCAGTGGCGAAGGCGAGTCCCTGGACAACTCCTGACACT
GAGGCACGAAAGCTAGGGGAGCAAACAGGATTAGAAACCCCGTAGTCC
>Otu2544
CCAGCCTATGGGTCGCTGCAGTGGGGAATATTGGACAATGGGCGCAAGCCTGATCCAGCA
ATGCCGCGTGTGTGAAGAAGGCCTTCGGGTTGTAAAGCACTTTTATCAGGAGCGAAATAC
CATCGGCTAATACCCGGTGGGGCTGACGGTACCTGAGGAATAAGCACCGGCTAACTTCGT
GCCAGCAGCCGCGGTAATACGGGGGGTGCAAGCCTTAATCGGAATTACTGGGCGTAAAGC
GTGCGCAGGCGGTTATGTAAGACAGATGTGAAATCCCCGGGCTTAACCTGGGAACTGCAT
TTGTGACTGGCGGGCTAGAGTATGGCAGAGGGGGGTAGAATTCCACGTGTAGCAGTGAAA
TGCGTAGAGATGTGGAGGAATACCGATGGCGAAGGCAGCCCCCTGGGCCAATACTGACGC
TCATGCACGAAAGCGTGGGGAGCAAACAGGATTAGAGACCCCCGTAGTCC
>Otu2546
CCAGCCTACGGGGTGCTCCAGTAAGGAATATTGGTCAATGGAGGCAACTCTGAACCAGCC
ATGCCGCGTGCAGGAAGACGGACCTATGGTTTGTAAACTGCTTTTGTACGAGAGAAAACC
CTACTACGTGTAGTAGGCTGATAGTATCGTAAGAATAAGGATCGGCTAACTTCGTGCCAG
CAGCCGCGGTAATACGAAGGATCCAAGCGTTATCCGGATTCATTGGGTTTAAAGGGAGCG
TAGGCGGTTTAATAAGTCAGTGGTGAAATCCCTCAGCTCAACTGAGGACCTGCCATTGAT
ACTGTCGAACTAGAGTACAGATGACGTTGGCGGAATGTGTCATGTAGCGGTGAAATGCAT
AGATATGACACAGAACACCGATTGCGAAGGCAGCTGACGAAACTGAAACTGACGCTGAGG
CTCGAAAGTGCGGGGATCAAACAGGATTAGAGACCCGTGTAGTCC
>Otu2549
CCAGCCTACGGGGGGCTGCAGTCGAGAATTTTTCTCAATGGGCGAAAGCCTGAAGGAGCG
ACGCCGCGTGGAGGATGAAGGTCTTCGGATTGTAAACTCCTGTCATGAGAGAACAATTCT
ACAGGTAATAACTGACCTGTAGTTGATAGTATCTCAAGAGGAAGAGACGGCTAACTCTGT
GCCAGCAGCCGCGGTAATACAGAGGTCTCAAGCGTTGTTCGGATTCATTGGGCGTAAAGG
GTGCGTAGGCGGCGCCGTAAGTCAGGTGTGAAATCTCGGGGCTTAACCTCGAAACTGCAC
TTGATACTGCGGTGCTTGAGGACTGGAGAGGAGATCGGAATTCATGGTGTAGCAGTGAAA
TGCGTAGATATCATGAGGAAGACCAGTGGCGAAGGCGGATCTCTGGACAGTTCCTGACGC
TGAGGCACGAAGGCCAGGGGAGCAAACGGGATTAGAGACCCCTGTAGTCCC
>Otu2551
CCAGCCTATGGGTTGCACCAGTTAAGAATCTTGCACAATGGGCGAAAGCCTGATGCAGCG
ACGCCGCGTGGATGATGAAGGCCTTCGGGTTGTAAAATCCGGTAAGCAGGGAAGAATAAG
TGAGATATAACACGTCTCATGATGACGGTACCTGTCTAAAGCCTCGGCTAACTACGTGCC

AGCAGCCGCGGTAACACGTATGAGGCGAGCGTTGTTCGGAATTATTGGGCGTAAAGCGCG
TGTAGGCGGGAAATTAAGTTATGTGTGAAATCCAAGGGCCTAACCCTTGAACTGCACCTA
ATACTGATTTTCTTGAGTTCGGGAGAGGAGAGCGGAATTCCCGGTGTAGCGGTGAAATGC
GTAGATATCGGGAAGAACACCAGTGGCGAAGGCGGCTCTCTGGCCTAGAACTGACGCTGA
GACGCGAAAGCGTGGGGAGCGAACGGGATTAGAAACCCGTGTAGTCC
>Otu2553
CCAGCCTATGGGGGGCACCAGTCGAGGATCTTTCGCAATGGGCGCAAGCCTGACGAAGCG
ACGCTGTGTGGACGATGAAGGCCTTCGGGTTGTAAAGTCCTTTCGCGCAAGAACAAGAGA
GGTTCACTAATAGTGAGCCGATTTGAGGGTACTGCGTAAAGAAGCACCGGCTAACTCCGT
GCCAGCAGCTGCGGTAATACGGAGGGTGCAAGCATTAATCGGATTTACTGGGCGTAAAGG
GCGCGTAGGCGGGTTTAAAAGTCAAATGTGAAATTCCGGGGCTCAACCTCGGAGCGGCAT
TTGAGACTATAAGTCTATAGAGGGATGGCGGAGAAAGCGGAATTCCACGTGTAGCGGTGA
AATGCGTAGATATGTGGAAGAACACCGGTGGCGAAAGCGGCTTTCTAGTTATTTCCTGAC
GCTGAGGCGCGAGAGCATGGGGAGCAAACAGGATTAGAAACCCTCGTAGTCC
>Otu2557
CCAGCCTATGGGTTGCAGCAGTCGAGAATTTTTCACAATGGGCGCAAGCCTGATGGAGCG
ACGCCGCGTGGGGGATGAATGGCTTCGGCCCGTAAACCCCTGTCATTCGCGATCAACCCT
TGCTATTTAAAAGATGGCAAGCTGATAGTAGCGGAAGAGGAAGGGACGGCTAACTCTGTG
CCAGCAGCCGCGGCAATACAGAGGTCCCAAGCGTTGTTCGGATTCACTGGGCGTAAAGGG
TGCGCAGGTGGTGGGGTAAGTCGGATGTGAAATCTCGGAGCTTAACTCCGAAATGGCATT
GGAAACTGCCCTGCTTGAGGGTCGGAGGGGGGACTGGAATTCTCGGTGTAGCAGTGAAAT
GCGTAGATATCGAGAGGAACACCAGTGGCGAAGGCGAGTCCCTGGACGACTCCTGACACT
GAGGCACGAAAGCTAGGGGAGCAAACAGGATTAGAGACCCTAGTAGTCC
>Otu2561
CCAGCCTACGGGAGGCAGCAGTGAGGAATATTGGTCAATGGGGGCAACCCTGAACCAGCC
ATGCCGCGTGAAGGAAGACGGCCCTAAGGGTTGTAAACTTCTTTTGTACTAGGGTAATTT
CATCTACGAGTAGGTGATTGAAAGTACAGTACGAATAAGCATCGGCTAACTCCGTGCCAG
CAGCCGCGGTAATACGGAGGATGCAAGCGTTATCCGGATTTATTGGGTTTAAAGGGTGCG
TAGGCGGGTAATTAAGTCAGTGGTGAAAGACTGTTGCTTAACGATAGCATTGCCATTGAT
ACTGAATATCTTGAGTATAGGTGAGGTAGGCGGAATGTGTAGTGTAGCGGTGAAATGCTT
AGATATTACACAGAACACCGATTGCGAAGGCAGCTTACTAATCTAGTACTGACGCTGAGG
CACGAAAGCGTGGGGAGCAAACAGGATTAGATACCCTTGTAGTCC
>Otu2563
CCAGCCTATGGGTTGCAGCAGTAGGGAATTTTCCACAATGGGCGAAAGCCTGATGGAGCA
ACGCCGCGTGCAGGACGAAGGCCTTCGGGTTGTAAACTGCTTTTATCTGTGACGAATATG
ACGGTAGCAGATGAATAAGGATCGGCTAACTCCGTGCCAGCAGCCGCGGTCATACGGAGG
ATCCAAGCGTTATCCGGAATTACTGGGCGTAAAGAGTTGCGTAGGTGGCATTGTAAGTCA
GTAGTGAAAGCGTTCGGCTCAACCGAATATCCATTACTGAAACTGCAGAGCTAGAGGATG
AGAGAGGTTATTGGAATTCCTAGTGTAGGAGTGAAATCCGTAGATATTAGGAGGAACACC
GATGGCGTAGGCAGATAACTGGCTCATTCCTGACACTAAGGCACGAAAGCATGGGTAGCA
AACGGGATTAGAAACCCCCGTAGTCC
>Otu2567
CCAGCCTACGGGGTGCTGCAGCAGGGAATCGTACGCAATGGACGAAGGTCTGACGTCGCA
ATGCCGCGTGGAGGATGAAGGCCTTCGGGTTGTAAACTCCTTTTATAGCAATAGAGATGT
TATTAAGAATAAGCACCTGCTAACTCTGTGCCAGCAGCCGCGGTAATACAGAGGGTGCAA
GCGTTATCCGGATTTATTGGGCGTAAAGCGTTTCGTAGGCGGTTCGAGAAGTTACTTTTC
AAAGGCCACGGCTTAACCGGGGAAAGGGAGGTAATACTAGCGAACTTGATTTTTGGTGGG
GTTTCTAGAACTGATGGTGTAGTAGTGAAATACGTTGATATCATCAGGAATTCCAAGGGC
GAAGGCAGGAAACTAACCAAGTAATGACGCTGAGGAACGACAGCTAGGGGAGCGAAAGGG
ATTAGAGACCCTAGTAGTCC
>Otu2568
CCAGCCTACGGGGCGCAGCAGTGGGGAATATTGGACAATGGGGGCAACCCTGATCCAGCC
ATGCCGCGTGAATGATGAAGGCCTTCGGGTTGTAAAGTTCTTTTGGTGGGGACGATGATG
ACGGCACCCACAGAATAAGCTCCGGCTAACTTCGTGCCAGCAGCCGCGGTAATACGAAGG
GAGCTAGCGTTGTTCGGAATTACTGGGCGTAAAGGGCGCGTAGGCGGTTTGACAAGTCGG
ATGTGAAAGCCCTGGGCTTAACCCGGGAATTGCATTCGATACTGTTTTACTCGAATTCGG
TAGAGGTTGGTAGAATTCCCAGTGTAGAGGTGAAATTCGTAGAGATTGGGAAGAATACCC
GTGGCGAAGGCGGCCAACTGGACCGACATTGACGCTGAGGCGCGAAAGCGTGGGTAGCAA

ACAGGATTAGATACCCCAGTAGTCC
>Otu2569
CCAGCCTATGGGGGGCAGCAGTGAGGAATCTTGGCAATGAAGGAAACTTTGACCCAGCG
AGAATTCGTGCATGACGAATGCGGTTGGTTTTCTGTAAAGTGCTTTTCGGCGATGAGGAT
AATGACTGTAATCGTAAATAAGAAGCACCGGCTAACTCCGTGCCAGCAGCTGCGGTAAGA
CGGAGGGTGTGGGCCTTATTCATTATGATTGGGTGTAAAGGGTATGTAGGTAGCTGCTGC
TCTTTTTGACTAAAATGATGGAGAGTTCCTACATCAATTGTCTTTTGAAGGTATACAGCT
GGAGTTATGTGAGAGGTAGCGGCAAACCCGCATGTAAGGGTAATATTTTGCTATATGGTG
GGCGAACATCAATAAGCGAAGGCGCGTTTCCATCTTTAACTGACGCTCAGATACGGAAGC
TACGGGAGCAAACAGGATTAGAGACCCGTGTAGTCC
>Otu2570
CCAGCCTACGGGGGGCACCAGGCGCGAAAACTCCACAATGCACGAAAGTGTGATGGGGGG
ATCCTACGTGCTCATGTCTGACATGGGCTTTTGCCAAGGCTAGAGATCTTGGCGAATAAG
GGGTGGGCAAGACCGGTGCCAGCCGCCGCGGTAACACCGGCGCCCCGAGTGGCAATCGCG
ATTATTGGGCCTAAAGCGTCCGTAGCCGGACAGGTAAATCCTCTGTGAAATCGTTGCGCT
TAACGTAACGGCGCGCAGGGGACACTGCCTGTCTCGAGACCGGGAGGAGTCAGAGGTATG
CCGTGGGGACTGGTAAAATGGTATAATCCACGGTAGACCACCAATGGCGAAGGCATTTGA
CCAGAACGGATCTGACGGTGAGGGACGAAAGCCAGGGGAGCGAACCGGATTAGATACCCC
TGTAGTCC
>Otu2572
CCAGCCTACGGGGGGCAGCAGTGAGGAATATTGGTCAATGGACGCAAGTCTGAACCAGCC
ATGCCGCGTGCAGGATGAAGGCCTTACAGGTTGTAAACTGCTTTTATACGGGACGAAACC
CCTGAACGTGTTCAGGGCTGACGGTACTGTATGAATAAGCATCGGCTAACTCCGTGCCAG
CAGCCGCGGTAATACGGAGGATGCAAGCGTTATCCGGATTTATTAGGTTTAAAGGGTGCG
TAGGCGGAATATTAAGTCAGTGGTGAAATCCTACAGCTTAACTGTAGAGCTGCCATTGAA
ACTGATATTCTTGAGTCTAGTTGAGGTGGGCGGAATGTGTCATGTAGCGGTGAAATGCAT
AGATATGACACAGAACACCGATTGCGTAGGCAGCTCACTAAGCTATAACTGACGCTGAGG
CACGAAAGCGTGGGGATCAAACAGGATTAGAGACCCTCGTAGTCC
>Otu2575
CCAGCCTACGGGTGGCTGCAGTTTCGAATCATTCACAACGGGCGAAAGCCTGATGGTGCG
ACGCCGCGTGAGGGATGAAGGTCTTCGGATTGTAAACCTCTGTCACTGGGGAGAAAGCGC
TTTGAGTTAATAGTTCAAAGCCTGATTTAACCCGGAGAGGAAGCAGTGGCTAACTCTGTG
CCAGCAGCCGCGGTAATACAGAGACTGCAAGCGTTATTCGGATTCACTGGGCGTAAAGGG
TGCGCAGGCGGCCATGTGTGTCAGATGTGAAATCCCGGGGCTCAACCCCAGAACTGCGTC
TGAAACTACATGGCTAGAGTATTGGAGAGGGTAGTGGAATTCACGGTGTAGCAGTGAAAT
GCGTAGATATCGTGAGGAACACCAGAGGCGAAGGCGACTACCTGGACAATTACTGACGCT
CAGGCACGAAAGCGTGGGGAGCCAAAGGGATTAGAAACCCGCGTAGTCC
>Otu2576
CAGTCTACGGGTTGCACCAGTGGGGAATTTTGGACAATGGGGGCAACCCTGATCCAGCCA
TTCCGCGTGAGTGAAGAAGGCCTTCGGGTTGTAAAGCTCTTTCGGCAGGAACGAAACGGC
GCGGGCGAATACCCTGCGTTAATGACGGTACCTGAAGAAGAAGCACCGGCTAACTACGTG
CCAGCAGCCGCGGTAATACGTAGGGTGCGAGCGTTAATCGGAATTACTGGGCGTAAAGCG
TGCGCAGGCGGTTTTGTAAGACAGCTGTGAAATCCCCGGGCTTAACCTGGGAACTGCGGT
TGTGACTGCAAGACTGGAGTAAGGCAGAGGGGGGTGGAATTCCACGTGTAGCAGTGAAAT
GCGTAGAGATGTGGAGGAACACCGATGGCGAAGGCAGCCCCCTGGGCTATTACTGACGCT
CATGCACGAAAGCGTGGGGAGCAAACAGGATTAGATACCCTTGTAGTCC
>Otu2577
CCAGCCTACGGGTCGCAGCAGTCGAGAATTTTTCACAATGGGGGCAACCCTGATGGAGCG
ACGCCGCGTGGAGGATGAAGGGCTTCGGCTCGTAAACTCCTGTCACCCCAGACAAAGCCC
CTCCGGTGAACATCCGGAGGGTTTGATGGTATGGGGAGGGGAAGGGACGGCTAACTCTGT
GCCAGCAGCCGCGGTAATACAGAGGTCCCAAGCGTTGTTCGGATTTACTGGGCGTAAAGG
GTGCGTAGGAGGCTTGGAAAGTCAGGTGTGAAATCTCACCGCTTAACGGTGAAATGGCGC
TTGATACTCCCGAGCTAGAAGATCGGAGGGGAGAACGGAATTCCTGGTGTAGCGGTGAAA
TGCGTAGATATCAGGAGGAACACCAACGGCGAAGGCAGTTCTCTGGACGATTCTTGACTC
TGAGGCACGAAGGCCAGGGGAGCAAACGGGATTAGAAACCCTTGTAGTCC
>Otu2579
CCAGCCTATGGGTCGCTCCAGTAGGGAATATTGCTCAATGGGGGAAACCCTGAAGCAGCA
ACGCCGCGTGAGTGATGAAGGCCTTCGGGTCGTAAAGCTCTGTTGTACGGGGAGAATGAG

GGTGTGTGTAATATGCTCACCAAGTGACAGTACCGTATAAGAAAGGACCGGCTAACTTCG
TGCCAGCGGCCGCGGTAAGACGGGGGGTCCTAGCGTTGTTCGGAATCATTGGGCGTAAAG
CGTGTGCAGGTGGTTTGGAAAGTTGGATGTGAAAGCCTTGGGCTTAACCCAAGAAGTGCA
TTCAAAACTACCAAACTTGAGTAAGGAAGAGGGTCGTGGAATTCCAGGTGTAGTGGTGAA
ATACGTAGATATCTGGAGGAACACCGGTGGCGAAGGCGGCGACCTGGTCCTATACTGACA
CTCATACACGAAAGCGTGGGGATCAAACAGGATTAGAGACCCTCGTAGTCC
>Otu2589
CCAGCCTATGGGATGCTCCAGACTGGACGAACTCGAATACGAAGCCGTCGGGATCATTCA
TCATCACGGCACGGGTATTATTCCGGGGCCGCTGCGTATACACGGGTCCGGCGCTTGTGG
TTGGCACTTCGATACCCCGGTCTTTGACCTTATCGAATGCCTTGTCGATGTCCCGTACCT
GAATCGAAAGGCGGGTTATACCGGGGTCGTAAATATGCTGCCTGAATGGCTTCCGTTCGA
TCCCTGAGAACTCAATGAGCACAAGCTGGAAATTAGACCCGGGAATGGACATCGTGGCAG
ACCTTGCTCTGGCAGCCGGATTCGTCGCCACAGCCTTCTGCACGAGTGTGGAGTTGGCAG
GAGCCAGTGGTGCGGGCGGGGAAACCACTTGCAGATCGAGACCGTCATGATAGAACTCGA
TGGATTCCTCGAGATTTGGGACGACATGAATTAGAGACCCTTGTAGTCC
>Otu2590
CAGCCTATGGGGCGCTGCAGTCGAGAATAATGGTCAATGCGCGAAAGCGTGAACCTGCGA
TCTACCAGCGGTGATGAAGGAGTTTTCTGTAAAGCCGATAAGGAGAGACCCAGAAGGAGG
GTCTTTCTGCTTAGTCGTGATAAATCCTTGTGCCAGCAGTCGCGGTCATACAAGAGCGGC
AAGTGTTGTTCGATGGAATTCGGTGTAAAGGGTAGGTAGGCGGCAGTGCTATTCTGCGAT
GTCTGTGTCAAGCGATTGACAAAGGCAACGATGTGGATGTGGATAGTGGATGGAATTTAT
AAGACAAGACGATCTCTTTGAAGGGATTCCTGTCAAGCTGCTTGAGAATACGAGAGGGAA
ATGGAATGACGAGAGTACCAATGAAATAGGTCGATCCTCGTTGGAACACCAATGGCGAAG
GCAATTTCCTGGAGTTTATCTGACGCTGAGCTACGAAAGCGTGGGGAGCAAACGAGATTA
GATACCCCAGTAGTCC
>Otu2594
CCAGCCTATGGGAGGCTGCAGCCGAGAATATTCCGCAATGGGCGAAAGCCTGACGGAGCG
ACGCCGCGTGGTTGATGAAGTCCTTCGGGACGTAAAAACCTTTTATGGGGGAGGAAGTAA
TTGACGTTACCTCATGAATAAGGGGCTCCTAACTCTGTGCCAGCAGGAGCGGTAATACAG
AGGCCCCGAGCATTATCCGGAATCACTGGGCGTAAAGGGTGTGTAGGCGGTCGCGTTAGT
CTTTTGTTAAAGTTCTTCGGCTTAACCGAGGAAATGCAGAGGAAACGGCGCGACTTCGAG
GACGCGAGAGGTAGAGGGAACTCATGGTGTAGGGGTGAAATCCGTTGATATCATGGGGAA
CACCAAATGCGAAGGCACTCTACTGGCGCGCTCCTGACGCTGAAACACGAAAGCGTGGGT
AGCGAATGGGATTAGAAACCCCAGTAGTCC
>Otu2599
CCAGCCTACGGGACGCTCCAGTTGGGAATTTTGCACAATGGACGAAAGTCTGATGCAGCA
ACGCCGCGTGGGGGATGAAGATTTTCGGATTGTAAACCCCTTTTATCAGGGAGTAATTTT
TGAACGTACCTGATGAATAAGCACCGGCTAACTCCGTGCCAGCAGCCGCGGTAATACGGA
GGGTGCAAACGTTGTCGGATTTACTGGGCGTAAAGCGTATGTAGGTGGCCTTATAAGTGA
ATGATTAAAGACCAACGCTTAACGTTGGAACTGTTGTTCATACTGTTTGGCTGGAGGGTG
GCAGGGGAGCATGGAACTTTGAGTGTAGGAGTGATATCCGTAGATATTCAAAGGAACACC
AATGGCGAAGGCAGTGCTCTGGGCCATTTCTGACACTGAGATGCGAAAGTGTGGGGAGCA
AACGGGATTAGATACCCTCGTAGTCC
>Otu2602
CCAGCCTATGGGTTGCTGCAGCAGGGAATCGTACGCAATGGACGAAAGTCTGACGTCGCA
ATGCCGCGTGGAGGATGAAGGCCTTCGGGTTGTAAACTCCTTTTATAGTAATAGAGATGT
TATTAAGAATAAGCACCCGCTAACTCTGTGCCAGCAGCCGCGGTAATACAGAGGGTGCAA
GCGTTATCCGGATTTATTGGGCGTAAAGCGTTTCGTAGGTGACCAAGTAAGTTATCTTTC
AAAGACCACGGCTTAACCGGGGGAAGGGAGGTAATACTGCTAGGTTCGATTTTTGGTGGG
GCTTCTGGAACTGATGGTGTAGTAGTGAAATACGTTGATATCATCAGGAACTCCGAGGGC
GAAGGCAGGAAGCTAACCAATTAATGACACTGATGAACGACAGCTAGGGGAGCGAAAGGG
ATTAGATACCCCTGTAGTCC
>Otu2606
CCAGCCTATGGGTCGCACCAGTAAGGAATATTGGTCAATGGGCGGAAGCCTGAACCAGCC
ATGCCGCGTGCAGGAAGACGGCCCTATGGGTTGTAAACTGCTTTTGCAGGGGAATAAACC
CCGGTACGAGTACCGGGCTGAATGTACTCTGAGAATAAGGATCGGCTAACTCCGTGCCAG
CAGCCGCGGTAATACGGAGGATGCGAGCGTTATCCGGATTTATTGGGTTTAAAGGGTGCG
TAGGCGGATTAATAAGTCAGTGGTGAAAACCTTCAGCTTAACTGGAGACTTGCCATTGAT

ACTGTTAGTCTTGAGTACGGTCAAGGTAGGCGGAATGTGTAATGTAGCGGTGAAATGCTT
AGATATTACACAGAACACCGATTGCGAAGGCAGCTTACTGGGCCATTACTGACGCTGATG
CACGAAAGCGTGGGGAGCGAACAGGATTAGAAACCCGCGTAGTCC
>Otu2607
CCAGCCTACGGGGCGCAGCAGTGGGGAATATTGCACAATGGGCGCAAGCTTGATGCAGCG
ACGCCGCGTGGAGGAAGAAGGTCTTTGGATTGTAAACTCCTGTTAAGTGGGAAAAAAGAA
GCTTATTTAAAAGATGAGTTGGATGATGGTACCACTAGAGAAAGCACCGGCAAACTTCGT
GCCAGCAGCCGCGGTAATACGAAGGGTGTAAGCGTTATTCGGAATAACTGGGCGTAAAGA
GCGCATAGGCGGGCTATTAAGTTATTCGTTAAATCTCCTGGCTTAACTGGGAAAGGGCGG
GTGAAACTGGTAGTCTAGAGTGTAAGAGAGAGAAGTGGAATTCTCGGAGTAGCGGTGAAA
TGCGTAGATCTCGAGAGGAACACCGGAGGCGAAGGCGGCTTCTTGGCTTACAACTGACGC
TCAAGCGCGAAAGCGTGGGGAGCAAACAGGATTAGAGACCCTTGTAGTCC
>Otu2609
CAGCCTACGGGGGCAGCAGTGGGGAATCTTGCACAATGGACGAAAGTCTGATGCAGCAAC
GCCGCGTGGGGGATGAAGCTTTTCGGAGTGTAAACCCCTTTCGACCCGGAAGAAAGCCCG
CAAGGGTTTGACGGTACGGGTATAAGAAGCCCCGGCTAACTACGTGCCAGCAGCCGCGGT
AAAACGTAGGGGGCCAGCGTTGCTCGGAATTACTGGGCGTAAAGGGTCCGTAGGCGGTGT
GGCAAGTCGGTAGTGAAATCTCTGGGCTTAACCCAGAGGCTGCTGCCGAAACTGCTATGC
TAGAGTGTGAGAGAGGCGAGTGGAATTACGGGTGTAGCGGTGAAATGCGTAGATATCCGT
AGGAACATCAGTGGCGAAGGCGACTACCTGGTCTGGCATTGACGCTCATGTGCGAAAGCG
TGGGGAGCAAACAGGATTAGAAACCCCCGTAGTCC
>Otu2610
CCAGCCTACGGGTGGCAGCAGTGGGGAATATTGCGCAATGGGCGGAAGCCTGACGCAGCG
ACGCCGCGTGGGGGATGAAGGCCTTCGGGTTGTAAACCCCTTTCACCAGGAGCGAAAACA
GACGGTACCTGGGGAAGAAGCGCCGGCTAACTACGTGCCAGCAGCCGCGGTGATACGTAG
GGCGCAAGCGTTGTCCGGAATTATTGGGCGTGAAGAGCTCGTAGGCGGCCTGTCGCGTCC
GCTGTGAAATCCTGGGGCTTAACCCCGGGCGTGCAGTGGATACGGGCTGGCTAGAGGCAG
GCAGGGGAGAACGGAATTCCCGGTGTAGCGGTGAAATGCGCAGATATCGGGAGGAACACC
GGTGGCGAAGGCGGTTCTCTGGGCCTGTTCTGACGCTGAGGAGCGAAAGCGTGGGGAGCA
AACAGGATTAGAGACCCTGGTAGTCC
>Otu2614
CCAGCCTACGGGGGGCAGCAGTCAAGGATCTTCGGCAATGGGCGCAAGCCTGACCGAGCG
ACGCCGCGTGCGGGATGAAGGCCTTCGGGTTGTAAACCGCTGTCAGTAGGGAGGAAATTT
CATGGGGTACCCTTCATGAATTGACCTATCTGCAGAGGAAGGACGGGCTAAGTACGTGCC
AGCAGCCGCGGTAATACGTACCGTCCAAACGTTATTCGGAATTACTGGGCTTAAAGGGTG
CGTAGGCGGTCCGGAAAGTTGTGTGTGAAAGCCCTCGGCTCAACCGAGGAATTGCGCGCA
AAACTACCGGACTTGAGGGAGACAGAGGTAAGCGGAACTTAAGGTGGAGCGGTGAAATGC
GTTGATATCTTAAGGAACACCAGGAGCGAAAGCGGCTTACTGGGTCTCTTCTGACGCTGA
GGCACGAAAGCTAGGGGAGCGAACGGGATTAGAGACCCTTGTAGTCC
>Otu2616
CCGCCTACGGGTCGCTGCAGTAGGGAATTTTCCACAATGGGCGAAAGCCTGATGGAGCAA
CGCCGCGTGCAGGATGAAGGCCTTAGGGTTGTAAACTGCTTTTATATGTGAAGAATATGA
CGGTAGCATATGAATAAGGATCGGCTAACTCCGTGCCAGCAGCCGCGGTCATACGGAGGA
TCCAAGCGTTATCCGGAATTACTGGGCGTAAAGAGTTGCGTAGGTGGCGCTGTAAGTTGG
TAGTGAAAGCCTGGGGCTCAACCCCTTACCCATTACCAAAACTGCAGAGCTAGAGGACGA
GAGAGGTAGTTGGAATTCCTAGTGTAGGAGTGAAATCCGTAGATATTAGGAGGAACACCG
ATGGCGTAGGCAGACTACTGGCTCGTTTCTGACACTAAGGCACGAAAGCATGGGGAGCGA
CCGGGATTAGAGACCCCAGTAGTCC
>Otu2618
CCAGCCTATGGGTGGCACCAGTCGAGAATTTTTCTCAATGGGGGAAACCCTGAAGGAGCG
ACGCCGCGTGGAGGAAGAAGGCCTTCGGATTGTAAACTCCTGTCACTGCAGAACAAGGAT
ACGCATGTTAATAGCAAGCGTATTTGATGGTATGCGGAGAGGAAGGGACGGCTAACTCTG
TGCCAGCAGCCGCGGTAAGACAGAGGTCCCGAGCGTTGTTCGGATTCATTGGGCGTAAAG
GGTGTGTAGGAGGTAGAGTAAGTCAGGTGTGAAATCTCAGAGCTTAACTCTGAAACTGCG
CTTGATACTGCTCTGCTAGAGGATCGGAGGGGGTATCGGAATTTATGGTGTAGCAGTGAA
ATGCGTAGATATCATAAGGAACACCGGTGGCGAAGGCGGATACCTGGAAGATTCCTGACT
CTGAAACACGAAAGCCAGGGGAGCAAACGGGATTAGAAACCCTCGTAGTCC
>Otu2619

CCAGCCTATGGGGGGCAGCAGGCGCGAAAACTTTACAATGCACGAAAGTGTGATAGGGGA
ATCCAGAGTGCTTATACAATGTATAGGCTTTTGCCAAGAGTAAATATCTTGGAGAATAAG
TGGTGGGAAAGACTGGTGCCAGCCGCCGCGGTAACCCCAGCGCCACTAGTGGGAATCGCG
TTTATTGGGCCTAAAGCATCCGTAGCTGGTTTTGTAAATCCCTTGTGAAATTGTTAGGCT
CAACCTAACAGCGTGCAGGGGACACTGCAGAACTAGGGACCGGGAGGAGTCAGAGGTATG
TTGAGGGGAGCGGTAAAATGCTATAATCCTCGATAGACCACCTGTGGCGAAGGCGTCTGA
CTAGAACGGGTCCGACAGTGAGGGATGAAAGCCAGGGGAGCAAACCGGATTAGATACCCT
CGTAGTCC
>Otu2627
CCAGCCTACGGGGTGCAGCAGTGAGGAATATTGCGCAATGGGCACAAGCCTGACGCAGCG
ACACCGCGTGAAGGATGAAGGCTTTCGGGTCGTAAACTTCTGTTAAGTGGGAAGAAAAAG
CTGGTCCTAATACGACCAGAAAATGACGGTACTACTAGAGAAAGCACCGGCTAACTTCGT
GCCAGCAGCCGCGGTAAGACGAGGGGTGCAAACGTTATTCGGAATGATTGGGTGTAAAGG
GTGCGTAGGCGGTATGTTAAGTCAATCGTTAAATTTTTCAGCTTAACTGAAAGTCAGCGG
TAGAAACTGGCGTACTTGAGGATGGAAGAGAGAAGTAGAATTCTCGGAGTAGCGGTAAAA
TGCGTAGATCTCGAGAGGAATACCAATGGCGAAGGCAGCTTCTTGGTCCATATCTGACGC
TGAGGCACGAAAGCGTGGGGAGCAAACAGGATTAGATACCCCAGTAGTCC
>Otu2630
CCAGCCTATGCGACGCAGCAGTAGGGAATTTTCCACAATGGGCGAAAGCCTGATGGAGCA
ACGCCGCGTGCAGGATGAATGCCTTAGGGCTGTAAACTGCTTTTATATGTGACGATTATG
ACGGTAGCATATGAATAAGGATCGGCTAACTCCGTGCCAGCAGCCGCGGTCATACGGAGG
ATCCAAGCGTTATCCGGAATTACTGGGCGTAAAGAGTTGCGTAGGTGGCATAGTAAGCGA
ATAGTGAAAGCGTGTGGCTCAACCACATACACATTGTTTGAACTGCTAAGCTAGAGGATG
AGAGAGGTTATTGGAATTCCCAGTGTAGGAGTGAAATCCGTAGATATTGGGAGGAACACC
GATGGCGTAGGCAGATAACTGGCTCATTCCTGACACTGAGGCACGAAAGCGTGGGTAGCA
AACGGGATTAGATACCCTCGTAGTCC
>Otu2631
CCAGCCTACGGGGGGCAGCAGTGGGAAATTTTACGCAATGGGGGAAACCCTGACGTAGCG
ACACCGCGTGAGCGAAGAAGCCCTTTGGGGTGTAAAGCTCTGTCAGCTGGAACGAAAATA
CATATGACGGTACCAGCAGAGGAAGCATCGGCTAACTACGTGCCAGCAGCCGCGGTAAGA
CGTAGGATGCGAGCGTTGTCCGGATTTATTGGGCGTAAAGAGTTCGTAGGTGGTTTGTTA
AGTCTGATGTTAAAGATTGGGGCTCAACCCTGAAAATGCATTGATACTGGCAGACTTGAG
TGTGGTAGAGGCTAGTGGAATTCCCAGTGTAGCGGTGAAATGCGTAGATATTGGGAAGAA
CACCGGTGGCGAAGGCGGCGACCTGGTCCGACACTGACGCTGAGGCGCGAAAGCGTGGGG
AGCAAACAGGATTAGATACCCCCGTAGTCC
>Otu2632
CCAGCCTATGGGTTGCAGCAGTGAGGAATTTTGGTCAATGGGGGAAACCCTGAACCAGCG
ACGCCGCGTGTGGGACGAAGGGGCTTTGCCCTGTAAACCACTGTCGGATGGGAAGAAAAG
TCCCCTTGGGGGACTGGGACTGTACCATCAAAGGAAGGATCGGCTAACTACGTGCCAGCA
GCCGCGGTAATACGTAGGATCCAAGCGTTGTCCGGATTTACTGGGTGTAAAGGGTGCGCA
GGCGGACTTGTGCGTCAGAGATTAAATACATCGGCTCAACCGGTGAACCGCCTTTGATAC
GGCAAGTCTTGAGTGCGAGAGAGGATGGTGGAATTCCTGGTGTAGCGGTGAAATGCGTAG
ATATCAGGAGGAACACCAGTGGCGAAGGCGGCCATCTGGCTCGCAACTGACGCTCAGGCA
CGAAAGCGTGGGGAGCAAACAGGATTAGAGACCCTAGTAGTCC
>Otu2633
CCAGCCTATGGGGTGCACCAGTTTGGAATATTCCACAACGGGCGAAAGCCTGATGGAGCG
ACACCGCGTGAAGGATGAAGGTCCTCGGATTGTAAACTTCTTTAGACCTAGATGAACGTT
CCATGGTGAATAATCCTGGAATTTGACAGTATAGGTAAAATAAGCCACGGCTAACTCTGT
GCCAGCAGCCGCGGTAATACAGAGGTGGCAAACGTTGTCCGGATTTATTGGGTGTAAAGG
GCATGTAGGTGGTCTTGTAAGTCAAAGGTGAAATGGCCAGGCTCAACCAGGTCTTTGCCT
TTGAAACTGCTAGGCTTGAGTACGGAAGAGGAGAGCGGAATTCCCAGTGTAGCGGTGAAA
TGCGTAGATATTGGGAGGAACACCGGTGGCGAAGGCGGCTCTCTGGTCCGAAACTGACAC
TGAGATGCGAAAGCCAGGGGAGCAAACGGGATTAGATACCCCCGTAGTCC
>Otu2637
CCAGCCTATGGGAGGCTCCAGTGGGGAATCTTGGACAATGGGCGAAAGCCTGACGCAGCG
ACGCCGCGTGGGTGACGAAGGCCTTCGGGTTGTAAAGCCCTGTGGGGCGGAACGAACATG
GGACGGGATAACACCCTGTTTCACTGACGGTACCGCCTTAGCAAGCACCGGCTAACTCTG
TGCCAGCAGCCGCGGTAAGACAGAGGGTGCAAACGTTGCTCGGAATTACTGGGCGTAAAG

CGCGTGTAGGTGGTCTGATCAGTCGGATGTGAAAGCCCTGGGCTCAACCCAGGAAGTGCA
TTCGATACTGCCAGACTGGAGTCTTGAAGAGGAGAGCGGAATTCCCGGTGTAGAGGTGAA
ATTCGTAGATATCGGGAGGAACACCAGTGGCGAAGGCGGCTCTCTGGGCAATGACTGACA
CTGAGACGCGAAAGCGTGGGAAGCAAACAGGATTAGATACCCGTGTAGTCC
>Otu2642
CCAGCCTACGGGTGGCTCCAGTAGGGAATCTTGCACAATGGGCGAAAGCCTGATGCAGCG
ACGCCGCGTGAGTGACGAAGGCCTTCGGGTTGTAAAGCTCTGTTCTCAGGGAAAAAGAAA
GTGATTGTACCTGAGGAGAAAGGACCGGCTAACTTCGTGCCAGCAGCCGCGGTAAGACGA
GGGGTCCAAGCGTTGTTCGGAATCATTGGGCGTAAAGCGGGTGTAGGTGGCTCTATAAGT
CAGGAGTGAAAGCCCCAAGCTTAACTTGGGAAGTGCTTTTGATACTGCAGAGCTTGAGTG
TGGGAGAGGATCGTGGAATTCCTGGTGTAGTGGTGAAATACGTAGATATCAGGAGGAACA
CCGGTGGCGAAGGCGGCGATCTGGCCCAACACTGACACTGAGACCCAAAAGCGTGGGGAT
CAAACAGGATTAGAAACCCGAGTAGTCC
>Otu2643
CCAGCCTATGGGGTGCAGCAGTGGGGAATTTTGGACAATGGGCGAAAGCCTGATCCAGCC
ATGCCGCGTGCAGGATGAAGGCCTTCGGGTTGTAAACTGCTTTTGTACGGAACGAAAAGC
CTCTTTCTAATAAAGAGGGGTCATGACGGTACCGTAAGAATAAGCACCGGCTAACTACGT
GCCAGCAGCCGCGGTAATACGTAGGGTGCAAGCGTTAATCGGAATTACTGGGCGTAAAGC
GTGCGCAGGCGGTTTTGTAAGACAGAGGTGAAATCCCCGGGCTCAACCTGGGAACTGCCT
TTGTGACTGCAAGGCTGGAGTGCGGCAGAGGGGGATGGAATTCCGCGTGTAGCAGTGAAA
TGCGTAGAGATGTGGAGGAACACCGATGGCGAAGGCAGCCCCCTGGGCCAATACTGACGC
TCATGCACGAAAGCGTGGGGAGCAAACAGGATTAGAGACCCTCGTAGTCC
>Otu2655
CCAGCCTATGGGGCGCTGCAGTCGAGAATTTTTCACAATGGACGTAAGTCTGATGGAGCG
ACGCCGCGTGGAGGATGAAGGTTTTCGGATTGTAAACTCCTGTCACTGCAGAACAAGGAT
GTGTTGATGGATAGTCAATGCATTTGATGGTATGCGGAGAGGAAGAGACGGCTAACTCTG
TGCCAGCAGCCGCGGTAATACAGAGGTCTCAAGCGTTGTTCGGATTCATTGGGCGTAAAG
GGTGCGTAGGCGGCGAAGCAAGTCAGGTGTGAAATCCCGGGGCTCAACCCCGGAACTGCA
TTTGATACTGCTTTGCTTGAGGACTGGAGAGGAGATCGGAATTCACGGTGTAGCAGTGAA
ATGCGTAGATATCGTGAGGAAGGCCAGTGGCGAAGGCGGATCTCTGGACAGTTCCTGACG
CTGAGGCACGAAGGCCAGGGGAGCAAACGGGATTAGAGACCCCTGTAGTCC
>Otu2656
CCAGCCTACGGGAGGCTCCAGTGGGGAATATTGGACAATGGGGGCAACCCTGATCCAGCC
ATGCCGCGTGGGTGAAGAAGGCCTTCGGGTTGTAAAGCCCTTTTGTACGGGAAGAAAAGC
TTTCGGTGAATATCCGGAGGTCATGACGGTACCGTAAGAATAAGCACCGGCTAACTTCGT
GCCAGCAGCCGCGGTAATACGAAGGGTGCAAGCGTTACTCGGAATTACTGGGCGTAAAGC
GTGCGTAGGTGGTTTGTTAAGTCTGATGTGAAATCCCCGGGCTCAACCTGGGAATGGCAT
TGGAAACTGGCAGGCTAGAGTGCGGTAGAGGGTGGTGGAATTCCCGGTGTAGCAGTGAAA
TGCGTAGAGATCGGGAGGAACATCTGTGGCGAAGGCGGCCACCTGGACCAGCACTGACAC
TGAGGCACGAAAGCGTGGGGAGCAAACAGGATTAGATACCCCTGTAGTCC
>Otu2657
CCAGCCTATGGGATGCAGCAGTCGAGAATCTTCCACAATGGACGAAAGTCTGATGGAGCG
ACGCCGCGTGGTTGATGAAGTCCTTCGGGACGTAAAAACCTTTTATGAGCCACTAAGTTA
TTGAAGAGCTCATGAATAAGGGGTTGCTAAACTCGTGCCAGCAGCAGCGGTAATACGAGT
GCCCCAAGCGTTATCCGGAATCATTGGGCGTAAAGGGTGTGTAGGTGGTCGTATTAGTCT
TGTGTTAAATCCTTAGGCTTAACCTAAGGCATGCATAGGAAACGGTACGACTAGAAGATG
CGAGGGGTGAATGGAACTCATAGTGGAGGGGTGAAATCCGTTGATATTATAGGGAACACC
AAAAGCGAAGGCAATTCACTGGCGCATTCTTGACACTGAAACACGAAAGCGTGGGTAGCG
AATGGGATTAGAAACCCGCGTAGTCC
>Otu2660
CCAGCCTACGGGTTGCACCAGTGGGGAATCTTGGTCAATGGGCGCAAGCCTGAACCAGCG
ACGCCGCGTGAGTGATGAAGGCCTTCGGGTTGTAAAGCTCTGTGGGGAGGGACGAAAGAC
TGAGGTCTAATAGGCTTCAGGTTGACGGTACCTCCTTAGCAAGCACCGGCTAACTCTGTG
CCAGCAGCCGCGGTAAGACAGAGGGTGCAAACGTTGCTCGGAATCACTGGGCGTAAAGGG
CGTGTAGGCGGATCGGCAAGTCGGGCGTGAAATCCCTGGGCTCGACCCAGGAACTGCACT
CGAAACTGCCTTTCTTGAGTGCCGGAGAGGAAAGCGGAATACCTGGTGTAGAGGTGAAAT
TCGTAGATATCAGGTGGAACACCAGTGGCGAAGGCGGCTTTCTGGACGGTAACTGACGCT
GAGACGCGAAAGCGTGGGGAGCAAACAGGATTAGATACCCCCGTAGTCC

>Otu2661
CCAGCCTACGGGATGCTGCAGTCGAGGATCTTCGGCAATGGGCGCAAGCCTGACCGAGCG
ACGCCGCGTGTGCGAAGAAGGCCTTCGGGTTGTAAAGCACTGTCGAGGGGGAGGAAAGCC
CGCAAGGGTCTGACCTATCCCTGGAGGAAGCACGGGCTAAGTTCGTGCCAGCAGCCGCGG
TAAGACGAACCGTGCGAACGTTGTTCGGATTCACTGGGCTTAAAGGGCGCGTAGGCGGGC
CATCAAGTCTGTGGTGAAAGCCCCCGGCTTAACCGGGGAAGTGCCGTGGATACTGGTGGT
CTCGAGGGGTGCAGGGGCATGCGGAACTTCCGGTGGAGCGGTGAAATGCGTAGATATCGG
AAGGAACGCCGGTGGCGAAAGCGGCGTGCTGGACACCTACTGACGCTGAGGCGCGAAAGC
CAGGGGAGCAAACGGGATTAGATACCCCCGTAGTCC
>Otu2662
CCAGCCTATGGGTGGCTCCAGTAAGGAATATTGGACAATGGACGCAAGTCTGATCCAGCC
ATGCCGCGTGAAGGATGAAGGTCCTCTGGATTGTAAACTTCTTTTATGTGGGACGAAAAA
AGGGGATTCTTCCTCGTCTGACGGTACCATATGAATAAGCACCGGCTAACTCCGTGCCAG
CAGCCGCGGTAATACGGAGGGTGCAAGCGTTATCCGGATTCACTGGGTTTAAAGGGTGCG
TAGGCGGGTATGTAAGTCAGTGGTGAAATCTCAGAGCTTAACTCTGAAACTGCCATTGAT
ACTATATGTCTTGAATATTGTGGAGGTAAGCGGAATATGTCATGTAGCGGTGAAATGCTT
AGATATGACATAGAACACCCATTGCGAAGGCAGCTTACTACACATATATTGACGCTGAGG
CACGAAAGCGTGGGGAGCAAACCGGATTAGAAACCCTTGTAGTCC
>Otu2663
CCAGCCTATGGGGCGCTCCAGTCGAGGATCTTCGGCAATGGACGCAAGTCTGACCGAGCG
ACGCCGCGTGCGGGATGAAGGCTTTCGGGTTGTAAACCGCTGTCAGTGGGGAGGAAATCG
GTGAGGGTAATCTCTCATTGTTGACCTATCCGCAGAGGAAGCAGGGGCTAAGTTCGTGCC
AGCAGCCGCGGTAAGACGAACCTTGCTAACGTTATTCGGAATTACTGGGCTTAAAGGGTG
CGTAGGCGGTGCAGAAAGTCGGGTGTGAAATCCCTCAGCTCAACTGAGGAATTGCGTCCG
AAACTACTGTGCTTGAGGAAGATAGAGGTACGCGGAACTTAGGGTGGAGCGGTGAAATGC
GTTGATATCCTAAGGAACACCGGTGGCGAAAGCGGCGTACTGGGTCTTATCTGACGCTGA
GGCACGAAAGCTAGGGTAGCGAACGGGATTAGAAACCCTTGTAGTCC
>Otu2669
CCAGCCTATGGGTCGCTGCAGTGAGGAATATTGCGCAATGGGGGAAACCCTGACGCAGCG
ACGCCGCGTGGAGGAAGAAGGTTTTCGGATTGTAAACTCCTGTCAGTGGGGAAGAGAATG
GACGGTACCCACTGAGGAAGCTTCGGCTAACTACGTGCCAGCAGCCGCGGTAAGACGTAG
GGAGCGAGCGTTGTCCGGAATTACTGGGCGTAAAGAGCTCGTAGGCGGGACCGTAAGTCC
GAGAGGAAATCTCAAGGCTCAACTTTGGGGCTTTCTCGGATACTGCGGTTCTTGAGGATA
TCAGAGGAAAGTAGAATTCCCGGTGTAGCGGTGAAATGCGTAGATATCGGGAGGAATACC
AGTGGCGAAGGCGACTTTCTGGGATATTCCTAACGCTGAGGAGCGAAAGCGTGGGGAGCA
AACGGGATTAGATACCCTTGTAGTCC
>Otu2671
CCAGCCTATGGGTGGCACCAGTAAGGAATCTTCCGCAATGGGCGAAAGCCTGACGGAGCA
ACGCCGCGTGCAGGATGACGGCCTTCGGGTTGTAAACTGCTTTTATTTGGGAAGATTATG
ACGGTACCAAATGAATAAGGACCGGCTAACTACGTGCCAGCAGCCGCGGTAATACGTAGG
GTCCAAGCGTTATCCGGAATCACTGGGCGTAAAGAGTGCGTAGGCGGGAGTCTAAGTGCA
ATGCGAAATCCTGTGGCTCAACCATATGGACTGTATTGCATACTGGACACCTAGAGGATA
TGAGAGGCAAGTGGAATTACCGGTGTAGCAGTGAAATGCGTAGATATCGGTAGGAACACC
AATGGCGAAGGCAGCTTGCTGGCATATTTCTGACGCTAAGGCACGAAAGCGTGGGGAGCG
AACAGGATTAGATACCCGTGTAGTCC
>Otu2674
CCAGCCTACGGGAGGCAGCAGTCGAGAATCTTTCGCAATGGGCGAAAGCCTGACGAAGCG
ACGCCGTGTGTGTGATGAAGGCCCTCGGGTCGTAAAGCACTTTCGCCTGGGAACAAGAAA
TTCCGGCTAATATCCGGGAAATTTGAGAGTACTAGGTAAAGAAGCACCGGCTAACTACGT
GCCAGCAGCTGCGGTAATACGTAGGGTGCAAGCATTAATCGGATTTATTGGGCGTAAAGG
GCGCGTAGGCGGAAATGCCAGTCAGATGTGAAATCCCGGGGCTCAACCCCGGAACAGCAT
TTGAAACTACATTTCTAGAGGGTAGGCGGAGAAAACGGAATTCCACAAGTAGCGGTGAAA
TGCGTAGATATGTGGAAGAACACCAGTGGCGAAGGCGGTTTTCTAGCTTATACCTGACGC
TGAGGCGCGAAGGCAAGGGGAGCAAACAGGATTAGATACCCGTGTAGTCC
>Otu2675
CCAGCCCGGGGTGCAGCAGTCGGGAATTTTGCTCAATGGGCGAAAGCCTGAAGCAGCAAC
GCCGCGTGCGGGATGAAGGCCTTCGGGTTGTAAACCGCTTTTACCAGGGACGATGATGAC
GGTACCTGATGAATAAGTCACGGCTAACTACGTGCCAGCAGCCGCGGTAATACGTAGGTG

ACCAGCGTTGTCCGGATTTACTGGGCGTAAAGAGCGCGCAGGCGGTCGTCCAAGTCGAGT
GTGAAAGCCCCCGGCTCAACTGGGGAGGGTCACTCGATACTGATCGACTCGAAGGCAGGA
GAGGGAAGCGGAATTCCCGGTGTAGTGGTGAAATGCGTAGATATCGGGAGGAACACCAGT
GGCGAAGGCGGCTTCCTGGCCTGTTCTTGACGCTGAGGCGCGAAAGCTAGGGGAGCAAAC
GGGATTAGAAACCCTCGTAGTCC
>Otu2681
CCAGCCTATGGGTTGCACCAGTCGAGGATCTTCGGCAATGGGCGCAAGCCTGACCGAGCG
ACGCCGCGTGTGGGATGAAGGCCTTCGGGTTGTAAACCACTGTCAGAGGGGATGAAATTC
CTGGGGGTTCTCCCCCAGGATTGACATATCCTCAGAGGAAGTACGGGCTAAGTTCGTGCC
AGCAACCGCGGTAAGATGAACCGTACGAACGTTATTCGGAATCACTGGGCTTAAAGGGTG
CGTAGGCGGCGCGGAAAGTTGGGTGTGAAATCCCTCGGCTTAACCGAGGAATTGCGCTCA
AAACTGTCGTGCTCGAGGGAGATAGAGGTGAGCGGAACTTAGGGTGGAGCGGTGAAATGC
GTTGATATCCTAAGGAACACCGGTGGCGAAGGCGGCTCACTGGATCTCTTCTGACGCTGA
GGCACGAAAGCTAGGGGAGCGAACGGGATTAGAAACCCGGGTAGTCC
>Otu2683
CCAGCCTATGGGGGGCTGCAGTCGAGAATCTTCGGCAATGGGGGCAACCCTGACCGAGCG
ACGCCGCGTGCGAGATGAAGGCCTTCGGGTTGTAAATCGCTGTCAGTGGGGAGGAAATGC
CGGTGGGTTCTCTCATCGGTTTGACCTATCCGCAGAGGAAGGACGGGCTAAGTTCGTGCC
AGCAGCCGCGGTAAGACGAACCGTCCAAACGTTATTCGGAATCACTGGGCTTAAAGGGTG
CGTAGGCGGCCCGGGAAGTTGGGTGTGAAATCCCTCGGCTCAACCGAGGAATTGCGCCCA
AAACTGCCGGGCTCGAGGAAGACAGAGGTGAGCGGAACTTAGGGTGGAGCGGTGAAATGC
GTTGATATCCTAAGGAACACCAGTAGCGAAAGCGGCTCACTGGGTCTTTTCTGACGCTGA
TGCACGAAAGCTAGGGGAGCGAAGAGGATTAGATACCCCGGTAGTCC
>Otu2684
CCAGCCTACGGGGGGCAGCAGTGGGGAATATTGCTCAATGGGCGAAAGCCTGAAGCAGCG
ACGCCGCGTGAACGATGAAGTCCTTCGGGATGTAAAGTTCTTTCGTCAGGGACAATTATG
ATGGTACCTGAATAAGAAGGCCCGGCTAACTATGTGCCAGCAGCCGCGGTAATACATAGG
GGCCAAGCGTTATCCGGATTTATTGGGCGTAAAGCGCGCGTAGGTGGTCGATCAAGTCGG
GTGTCAAATTTGGAAGCTCAACTTCCATACGCGCCCGAAACTGTTCGGCTTTGAGGTCGG
TAGAGGGAAGTGGAACTCACAGTGTAGCGGTGAAATGCGTAGATATTGTGAGGAACACCA
GTGGCGAAGGCGACTTCCTGGCCCGTACCTGACGCTAAAGTGCGAAAGCATGGGGAGCAA
ACAGGATTAGAGACCCTCGTAGTCC
>Otu2685
CCAGCCTATGGGACGCACCAGTGAAGAATATTGGTCAATGGACGCAAGTCTGAACCAGCC
ATGCCGCGTGAAGGATGAAGGCCCTATGGGTTGTAAACTTCTTTTATATGGGACGAATCT
CCCGCTCGTGAGCGGGATTGACGGTACTGTACGAATAAGCAACGGCTAACTCCGTGCCAG
CAGCCGCGGTAATACGGAGGTTGCAAGCGTTATCCGGATTTATTGGGTTTAAAGGGTGCG
TAGGCGGGACAATAAGTCAGTGGTGAAAGCCTGCAGCTTAACTGTAGAACTGCCATTGAT
ACTGTTGTTCTTGAGTATAGTTGAGGTGGGCGGAATGTGTAATGTAGCGGTGAAATGCTT
AGATATTACACAGAACACCAATTGTGTAGGCAGCTCACTAAACTAAAACTGACGCTGAGG
CACGAAAGCGTGGGGAGCAAACAGGATTAGATACCCCTGTAGTCG
>Otu2686
CCAGCCTATGGGTGGCACCAGTGGGGAATATTGGACAATGGGCGAAAGCCTGATCCAGCA
ACGCCGCGTGAGGGATGAAGCTCTTCGGGGTGTAAACCTCTGTAGGAAGGAAAGAAACCA
TGACGGTACTTTCAAAGTAAGCCCCGGCTAACTCCGTGCCAGCAGCCGCGGTAATACGGA
GGGGGCTAGCGTTACTCGGAATCACTGGGCGTAAAGCGCACGTAGGCGGTTTTCTAAGTT
TTTCGTGTAATCTCCCGGCTTAACTGGGAACCCGCGGAAAATACTGGGAGATTGGAGTAT
GGTAGAGGAGAGCGGAATTCCTGGTGTAGCGGTGAAATGCGTAGATATCAGGAGGAACAC
CCGTGGCGAAAGCGGCTCTCTGGACCATTACTGACGCTGAGGTGCGAAAGCCAGGGGAGC
GAACGGGATTAGATACCCCGGTAGTCC
>Otu2688
CCAGCCTACGGGGCGCAGCAGTTGATTCAGGTGGGCGGCAAAGCCGAAAGCCGCTTGTGG
CGCGAACTCGTGGAACGCCATCACTATCTCGCATGCCGGGTGCCGCTTGGCGCCCACCTG
CGCTACTTTGTGCGAGATCGCGAACGGGAGTTGGCCTGTCTGCTCTGGATTCTCGCCAGC
TTGGAAGATGGAGCCCCGCGATGCCTGGATCGGATGGAGCGATGAGCAGCGGCGTCGCAA
CCTGTAGTCGATCGTGAACAATGGCCGCTTCTGGATCTTGCCATGGGTGCATGTCAAAGG
ACTGGCCAGCAAGGTCCTGGCACTGAGCGCTCGGCAAGTGCCGAGCGACTGGAAAATCCG
CTATGGACCTCGCCCTCTGCTATTAGAAACCCTTGTAGTCC

>Otu2689
CCAGCCTATGGGTTGCAGCAGTGGGGAATCTTGCGCAATGGGCGAAAGCCTGACGCAGCG
ACGCCGCGTGGGTGATGAAGGCCTTCGGGTTGTAAAGCCCTGTGGGGAGGGACGAATAAA
CCATGGCTAATATCCATGGCGATGACGGTACCTCTTTAGCAAGCACCGGCTAACTCTGTG
CCAGCAGCCGCGGTAAGACAGAGGGTGCGAACGTTGTTCGGAATTACTGGGCGTAAAGCG
CGTGTAGGTGGCATCGCAAGTCGGGTGTGAAAGCCCGGGGCTCAACCCCGGAAGTGCATT
CGATACTGCGGAGCTAGAGTCTCGGAGAGGATGGTGGAACTCTCGGTGTAGAGGTGAAAT
TCGTAGATATCGAGAAGAACACCGGCGGCGAAGGCGGCCATCTGGACGATGACTGACACT
GAGACGCGAAAGCGTGGGGAGCAAACAGGATTAGATACCCTTGTAGTCC
>Otu2690
CCAGCCTATGGGGGGCAGCAGTGGGGAATCTTGGACAATGGGGGAAACCCTGATCCAGCC
ATGCCGCGTGAGTGATGAAGGCCTTAGGGTTGTAAAACTCTTTCGACGGGGACGATAATG
ACGGTACCCGTAGAAGAAGCTCCGGCTAACTTCGTGCCAGCAGCCGCGGTAATACGAAGG
GGGCTAGCGTTGTTCGGAATTACTGGGCGTAAAGCGTGCGCAGGCGGCTCATCAAGTCAG
GGGTGAAAGCCCAGAGCTCAACTCTGGAATTGCCTTTGAAACTAGTGAGCTTGAGTACGG
GAGAGGTGAGTGGAATTCCGAGTGTAGAGGTGAAATTCGTAGATATTCGGAAGAACACCA
GTGGCGAAGGCGGCTCACTGGACCGTAACTGACGCTCATGCACGACAGCGTGGGGAGCAA
ACAGGATTAGAAACCCGGGTAGTCC
>Otu2693
CAGCCTATGGGTCGCTGCAGTAACGAATATTCCGCAATGGGCGCAAGCCTGACGGAGCGA
TGCCGCGTGTGGGACGAAGCCCTTCGGGGTGTAAACCACTGTCAGGGTTTAGCAAGAGCG
TGAGCAAACCCAAAGGAAGAGGCGACTAACTCTGTGCCAGCAGTCGCGGTAATACAGAGG
CCTCGAGCGTTAATCGGAATCACTGGGCTTAAAGGGTGCGTAGGCGGCTTCGTAAGTGCC
TTGTGAAATCCCATGGCTCAACCATGGAACTGCTCGGCAGACTGCGGAGCTTGAGGTTGG
TAGGGGCTGCTGGAACTCTAGGTGGAGCGGTGAAATGCGTAGATATCTAGAGGAACGCCG
ACGGTGAAGACAGGCAGCTGGGCCAAAACTGACGCTGAGGCACGAAAGCGTGGGGAGCGA
ACGGGATTAGATACCCTCGTAGTCC
>Otu2694
CCAGCCTATGGGGTGCTGCAGGTCGGATTACCGGGACCAGTGAAGGCGGTGTTCTTCCGC
AGAGTGGTCAGGGTTCCCGTCGAGGAGTTGATCACCGCACCGGTGATCGTGCTGTCGGCA
TAATTGGCGGTGTAGAGATACTGGTTCGAGGGGTCCTCAAGAATGCATTGCGGAGCCGAG
CCGGCGCCAAAGATAACGCTGGTCCCGCTAGCTCCACCGGACAGGGTCGTCAATCTGCCT
GTAGTCGGGTCGACGAAGAAGGCGCTGACCGAACTTGCTGGACCGCTTGCATTGAGATTG
GGTCCCTGGTTTGCTACATAAAGAAACTTGCCAGTATGGTCCACCAGCATAGGTCCAGGA
TTTGCGACAGTCCCGGTATTGTCTACAGCTCCGCCAACCAGCGATTAGAGACCCTTGTAG
TCC
>Otu2695
CCAGCCTACGGGGGGCACCAGTCGAGAATCTTCGGCAATGGGCGCAAGCCTGACCGAGCG
ACGCCGCGTGTGCGATGAAGGCCTTCGGGTTGTAAAGCACTGTCGAGGGGGAGGAAGGGC
AACTTGACCGACCCCTGGAGGAAGCACGGGCTAAGTTCGTGCCAGCAGCCGCGGTAAGAC
GAACCGTGCGAACGTTATTCGGAATCACTGGGCTTAAAGGGCGCGTAGGCGGGCGATCAA
GTCAGAGGTGAAAGCCTCCCGCTCAACGGGAGAAGTGCCCCTGATACTGGTTGTCTCGAG
GGAGGTAGGGGCATGTGGAACTTCCGGTGGAGCGGTGAAATGCGTAGATATCGGAAGGAA
CGCCGGTGGCGAAAGCGACGTGCTGGACCTCTTCTGACGCTGAGGCGCGAAAGCTAGGGG
AGCAAACGGGATTAGATACCCTCGTAGTCC
>Otu2701
CCAGCCTATGGGGGGCACCAGTCGAGGATCTTCGGCAATGGGCGCAAGCCTGACCGAGCG
ACGCCGCGTGTGCGATGAAGGCCTTCGGGTTGTAAAGCACTGTCGAGGGGGAGGAACGCC
CGCAAGGGTTTGACCTATCCCTGGAGGAAGCACGGGCTAAGTTCGTGCCAGCAGCCGCGG
TAAGACGAACCGTGCGAACGTTGTTCGGATTCACTGGGCTTAAAGGGCGCGTAGGCGGGT
TTTCAAGTCCGGGGTGAAATACTCTCGCTCAACGAGAGAACTGCCCCGGATACTGGAGAT
CTCGAGGGAGGTAGGGGCATGTGGAACTTCCGGTGGAGCGGTGAAATGCGTAGATATCGG
AAGGAACGCCGGTGGCGAAAGCGACGTGCTGGACCTCTTCTGACGCTGAGGCGCGAAAGC
CAGGGGAGCAAACGGGATTAGATACCCTCGTAGTCC
>Otu2706
CCAGCCTATGGGACGCTCCAGTGAGGAATATTGGACAATGGGCGCAAGCCTGATCCAGCC
ATGCCGCGTGAGTGATGAAGGCCCTAGGGTTGTAAAGCTCTTTTGTCCGGGAAGATAATG
ACTGTACCGGAAGAATAAGCCCCGGCTAACTTCGTGCCAGCAGCCGCGGTAATACGAAGG

GGGCTAGCGTTGTTCGGATTTACTGGGCGTAAAGCGCACGTAGGCGGATCTTTAAGTCAG
AGGTGAAATCCCAAGGCTCAACCCTGGAACTGCCTTTGATACTGGGGATCTTGAGTTCGG
GAGAGGTGAGTGGAACTGCGAGTGTAGAGGTGAAATTCGTAGATATTCGCAAGAACACCA
GTGGCGAAGGCGGCTCACTGGCCCGATACTGACGCTGAGGTGCGAAAGCGTGGGGAGCAA
ACAGGATTAGATACCCCAGTAGTCC
>Otu2707
CCAGCCTATGGGTGGCACCAGTGGGGAATTTTGCGCAATGGGGGAAACCCTGATCCAGCA
ATGCCGCGTGAGTGAAGAAGGTCTTAGGATTGTAAAGCTCTTTCGGTGGGGACGATGATG
ACGGTACCCACAGAAGAAGCCCCGGCTAACTTCGTGCCAGCAGCCGCGGTAATACGAAGG
GGGCTAGCGTTGCTCGGAATGACTGGGCGTGAAGGGCGCGTAGGCGGCTTGTATAGTCAG
ATGTGAAATTCCTGGGCTCAACCTGGGGACTGCATTTGATACGTGCAGGCTTGAGTGTGG
AAGAGGGTCGTGGAATTCCCAGTGTAGAGGTGAAATTCGTAGATATTGGGAAGAACACCG
GTGGCGAAGGCGGCGACCTGGTCCATAACTGACGCTGAGGCGCGAAAGCGTGGGGAGCAA
ACAGGATTAGATACCCCAGTAGTCC
>Otu2711
CCAGCCTACGGGGGGCTGCAGTCGAGAATCTTCGGCAATGGGCGCAAGCCTGACCGAGCG
ACGCCGCGTGGAGGACGAAGGCCTTCGGGTTGTAAACTCCTGTCGAGGAGGAGGAAGGGG
CCGTGCAGAACGGTTCTTGACCGATTCCTGGAGGAAGCACGGGCTAAGTTCGTGCCAGCA
GCCGCGGTAAGACGAACCGTGCGAACGTTATTCGGAATTACTGGGCTTAAAGCGCGTGTA
GGCGGGTCGGCACGTCCGCGGCTGAAATCCCCTGGCTCAACCAGGGAAGTGGCCCGGATA
CGACCGGTCTGGAGGGGGGTAGGGGAATCGGGAACTCACGGTGGAGCGGTGAAATGCGTT
GAGATCGTGAGGAACGCCCGTGGCGAAAGCGCGGTTCTGGACCCCACCTGACGCTGAGAC
GCGAAAGCCAGGGGAGCGAACGGGATTAGATACCCTAGTAGTCC
>Otu2713
CCAGCCTACGGGGTGCTCCAGTCGAGAATTTTTCTCAATGGGCGAAAGCCTGAAGGAGCG
ACGCCGCGTGGGGGATGAATGGCTTCGGCCCGTAAACCCCTGTCATTTGCGAACAAATGG
TTTTACCTAACACGTAAAGCCTTGATAGTAGCGGAAGAGGAAGGGACGGCTAACTCTGTG
CCAGCAGCCGCGGTAATACAGAGGTCCCAAGCGTTGTTCGGATTCACTGGGCGTAAAGGG
TGCGTAGGCGGCCGGGTAAGTCTGATGTGAAATCCCGCAGCTCAACTGCGGAACGGCATT
GGATACTATTCGGCTAGAGGAGTGGAGGGGAGACTGGAATACTTGGTGTAGCAGTGAAAT
GCGTAGATATCAAGTGGAACACCAGTGGCGAAGGCGAGTCTCTGGACACTTCCTGACGCT
GAGGCACGAAAGCCAGGGGAGCAAACGGGATTAGAAACCCGCGTAGTCC
>Otu2714
CCAGCCTACGGGGGGCAGCAGTGGGGAATCTTGCACAATGGGCGAAAGCCTGATGCAGCA
ACGCCGCGTGGGTGAAGAAGGTTCTCGGATTGTAAAGCCCTTTCGGCTGGGGAGAAGGGT
GTCATGGTGAATAATCACGGCATCTGACGGTACCAGAAGAAGAAGCACCGGCTAATTTCG
TGCCAGCAGCCGCGGTAATACGAAAGGTGCAAGCGTTGTTCGGAATGACTGGGCGTAAAG
CGCACGTAGGCGGGTTTGCAAGTAGGATGTGAAAGCCCCAGGCTTAACCTGGGAATGGCA
TCTTAAACTACAAACCTTGAATGCTTGAGAGGTTGGTGGAATGCCTGGTGTAGGAGTGAA
ATCCGTAGATATCAGGTGGAATATCGGAGGCGAAGGCGGCCAACTGGTGGTGCATTGACG
CTAAGGTGCGAAAGCGTGGGGAGCAAACAGGATTAGATACCCGTGTAGTCC
>Otu2717
CCAGCCTACGGGGTGCTGCAGTCGAGAATTTTTCTCAATGGGCGAAAGCCTGAAGGAGCG
ACGCCGCGTGGGGGATGAATGGCTTCGGCCCGTAAACCCCTGTCATTTGCGAACAAATTA
TTTTACCTAACACGTGAAGTATTGATTGTAGTAGAAGAGGAAGGGACGGCTAACTCTGTG
CCAGCAGCCGCGGTAATACAGAGGTCCCAAGCGTTGTTCGGATTCACTGGGCGTAAAGGG
TGCGTAGGCGGTCGGGTAAGTCTGATGTGAAATCTCCAAGCCTAACTTGGAAACTGCATT
AGATACTATACGGCTCGAGGAATGGAGGGGAGACTGGAATACTTGGTGTAGCAGTGAAAT
GCGTAGATATCGTGAGGAAGGCCAGTGGCGAAGGCGGATCTCTGGACAGTTCCTGACGCT
GAGGCACGAAGGCCAGGGGAGCAAACGGGATTAGATACCCGCGTAGTCC
>Otu2719
CAGCCTACGGGGGCACCAGTAACGAATCTTCCGCAATGCACGAAAGTGTGACGGAGCGAC
GCCGCGTGTAGGATGAAGTCCTTCGGGATGTAAACTACTGTCAGGGGAAAGAAAGTTCTG
ATCTACCCCAGAGGAAGAGGCGGCTAACTCTGTGCCAGCAGCCGCGGTAATACAGAGGCC
TCGAGCGTTAGGCGGAATCACTGGGCTTAAAGCGTGTGTAGGTGGATGCCTAAGTGTCTT
GTGAGATCCCATGGCTTAACCATGGAATTGCTTGACATACTGGGTGTCTTGAGCCACTCA
GGGGCTACTGGAACAAGCGGTGGAGCGGTGAAATGCGTAGATATCGCTTGGAACGCCAAT
GGTGAAAACAGGTAGCTGGGGGTGTGCTGACACTGAGACACGAAAGCCAGGGGAGCAAAC

GGGATTAGAAACCCTAGTAGTCC
>Otu2721
CCAGCCTACGGGGCGCACCAGCAGATGATCGACTCGATGCCGTGCGGCGTGCTGGTTGTG
GATACCGCCGATGCGATTGTGATGATCAATCCGGAGAGCCGGCGGCTGTTGGAATTGGGT
AGCAAGCGCGTCAAAAGCTTGCGCGATCTCGCGGCGATCAGCCAGGTAGACTTTGAGTCT
CTAATGGATGGCTCAGCGGACCAGCTTGACACGGAAGTGTGCGTTCCTGCCACGGAAGGT
CAGCGCTGGCTGGCGATTGGCAACCGAAAACTAATGAGTGCATCGGCCAGCCAAGGACCA
GGAAATGAGAGAGCCGCGCTGCAGAGTATCTGGATTCTCCGGGACATTACGGCAAGCAAA
CAGGCCGAGCGGGAACGCGAAGCCGCGCGCAGCGCGATGGCACTGGCAGAGGTTGCGACG
ATCCTGGCACACGAAATTAGAAACCCCAGTAGTCC
>Otu2723
CCAGCCTACGGGGTGCAGCAGTGGGGAATATTGGACAATGGGCGCAAGCCTGATCCAGCA
ATGCCGCGTGAGTGATGAAGGCCTTCGGGTTGTAAAGCTCTTTCGTCTGCGACGATGATG
ACGGTAGCAGAAGAAGAAGCCCCGGCTAACTTCGTGCCAGCAGCCGCGGTAATACGAAGG
GGGCTAGCGTTGTTCGGAATTACTGGGCGTAAAGGGCGCGTAGGCGGTTGATCAAGTCAG
GCGTGAAAGCCCCGGGCTTAACCTGGGAAATGCGCTTGAGACTGGCCGGCTTGAGTTCGG
GAGAGGCGAGTGGAATTCCCAGTGTAGAGGTGAAATTCGTAGATATTGGGAAGAACACCA
GTGGCGAAGGCGGCTCGCTGGACCGAAACTGACGCTGAGGCGCGAAAGCGTGGGGAGCAA
ACAGGATTAGAGACCCCAGTAGTCC
>Otu2728
CCAGCCTATGGGTTGCTGCAGTAAGGAATATTGGTCAATGGACGAAAGTCTGAACCAGCC
ATGCCGCGTGAAGGATGAAGGCCCTCTGGGTTGTAAACTTCTTTTATCTGGGACGAAAAA
AGGGAATTCTTTCCCACTTGACGGTACCAGAGGAATAAGCACCGGCTAACTCCGTGCCAG
CAGCCGCGGTAATACGGAGGGTGCAAGCGTTATCCGGATTTACTGGGTTTAAAGGGTGCG
TAGGTGGGTAGGTAAGTCAGTGGTGAAATCTTCGAGCTTAACTCGGAAACTGCCATTGAT
ACTATTTATCTTGAATTTTGTTGAGGTAGGCGGAATAAGTCATGTAGCGGTGAAATGCAT
AGATATGACTTAGAACACCAATTGCGAAGGCAGCTTGCTAAACAAATATTGACGCTGAGG
CACGAAAGCGTGGGGATCAAACAGGATTAGAAACCCTGGTAGTCC
>Otu2729
CCAGCCTACGGGAGGCAGCAGTCGAGAATCATTCGCAATGGGCGAAAGCCTGACGATGCG
ACGCCGTGTGAGCGATGAAGGCCTTAGGGTCGTAAAGCTCTTTCGCTTGGGAACAAGAGA
CGCAGGCTAATATCCTGCAAATTTGAGGGTACCAGGTAAAGAAGCACCGGCTAACTCCGT
GCCAGCAGCTGCGGTAATACGGAGGGTGCAAGCATTAATCGGATTTATTGGGCGTAAAGG
GCGCGTAGGCGGAGCGATAAGTCAGATGTGAAATCCCGGGGCTCAACCCCGGAACAGCAT
TTGAAGCTGTCATTCTAGAGGATAGATGGAGAAAACGGAATTCCACAAGTAGCGGTGAAA
TGCGTAGATATGTGGAAGAACACCGGTGGCGAAAGCGGTTTTCTAATTTAGACCTGACGC
TGAGGCGCGAAAGCAAGGGGATCAAACAGGATTAGATACCCTGGTAGTCC
>Otu2733
CCAGCCTATGGGTTGCACCAGGTACGAAGTCTCCGCAATGCACGAAAGTGTGACGGGGGA
ACTCCAAGTGAATTGCTTTCGAGCAATTCTGTACAGAAGTGTAAATAGCTTCTGGAGAAA
GGGGTGGGTAAGACCGGTGCCAGCAGCCGCGGTAATCCCGGCACCTCAAGTAGTGACCGC
TATTATTGGGCCTAAAGAATCCGTAGCCGGATTAATAAGTTCCCTGTGAAATCGCGCAGC
TTAACTGTGCGGCGTGCAGGGAATACTGTTCAATCTAGGAACCGGGTGGGGTCACGGGTA
CTCCTGGGGTAGCGGTTAAATGTTGTAATCCCAGGAAGACCAACAGTGGTGGAGACGAGT
GACTAGAACGGGTTCGACGGTGAATGGATGAAAGCTGGGGGAGCAAAGCGGATTAGATAC
CCGTGTAGTCC
>Otu2740
CCAGCCTATGGGGGGCTCCAGTTAGGAATCTTCTGCAATGCGCGAAAGCGTGACAGAGCG
AGTCAGAGTGCTTTTCAACTGAAAGGCTTTTGCTAGATGTAAAGAGTCTGGCGAATAAGG
ACTGGGTAAGACTGGTGCCAGCCGCCGCGGTAATACCAGCGGTCCAAGTCGCAGCCATCA
TTATTGGGTCTAAAACATCCGTAGCTTGCTTAATAAGTTCCTTGTGAAATCTAATATCTT
AAGTATCAGGCGCGCAAGGAGTACTGTTAAGCTAGAGACTGGAAGACGTAGGAAGTACGT
TCAAAGTAGTGGTAAAATACGTTAATCTTGGACGGACTCACAACAGCGAAGGCATCCTAC
GAGGACAGTTCTGACAGTGAGGGATGAAGGCTAGGGTCGCGAAACAGATTAGAAACCCTA
GTAGTCC
>Otu2753
CCAGCCTACGGGTCGCTGCAGTGGGGAATTTTGCGCAATGGGGGAAACCCTGACGCAGCA
ACGCCGCGTGGAGGATGAAGTCCTTCGGGACGTAAACTCCTTTCGATCGGGACGATTATG

ACGGTACCGGAAGAAGAAGCCCCGGCTAACTTCGTGCCAGCAGCCGCGGTAATACGAGGG
GGGCGAGCGTTGTTCGGAATTATTGGGCGTAAAGGGTGCGTAGGCGGTTTGGTAAGTCTT
GTGTGAAATCTCCGGGCTCAACTCGGAGCCTGCACGAGAAACTGCCGGGCTTGAGTGTGG
GAGAGGTGAGTGGAATTTCCGGTGTAGCGGTGAAATGCGTAGATATCGGAAGGAACACCT
GTGGTGTAAACGGCTTTCTGGACCATAACTGACGCTGAGGCACGAAAGCGTGGGTAGCAA
ACAGGATTAGAGACCCTAGTAGTCC
>Otu2754
CCAGCCTACGGGGGGCTGCAGTGGGGAATCCTGCGCAATGGGCGAAAGCCTGACGCAGCA
ACGCCGCGTGGGGGATGAAGGCCTTCGGGTTGTAAACCCCTTTCAGTGGGAACGAAATTG
ACGGTACCCACAGAAGACGCCCCGGCCAACTACGCGCCAGCAGCCGCGGTGATACGTAGG
GGGCGAGCGTTGTCCGGATTTATTGGGCGTAAAGAGCTCGTAGGCGGTTCGGTAAGTCGG
GTGTGAAACCTCCAGGCTCAACCTGGAGACGCCACTCGATACTGCCATGACTAGAGTCCG
GTAGGGGATCACGGAATTCCTGGTGTAGCGGTGAAATGCGCAGATATCAGGAGGAGCACC
AGTGGCGAAGGCGGTGATCTGGGCCGGAACTGACGCTGAGGAGCGAAAGCGTGGGGAGCG
AACAGGATTAGAAACCCTCGTAGTCC
>Otu2755
CCAGCCTACGGGGTGCACCAGTGGGGAATATTGCGCAATGGGCGGAAGCCTGACGCAGCG
ACGCCGCGTGCGGGATGAAGGCCTTCGGGTTGTAAACCGCTTTCAGCAGGGACGAATTCA
GACGGTACCTGCAGAAGAAGCGCCGGCTAACTACGTGCCAGCAGCCGCGGTAATACGTAG
GGCGCAAGCGTTGTCCGGAATTATTGGGCGTAAAGAGCTCGTAGGCGGCCTGTTGCGTCC
GCTGTGAAATCCCGGGGCTCAACCCCGGGCGTGCAGTGGATACGGGCAGGCTAGAGGCAG
GCAGGGGAGAACGGAATTCCTGGTGTAGCGGTGAAATGCGCAGATATCAGGAGGAACACC
GGTGGCGAAGGCGGTTCTCTGGGCCTGTTCTGACGCTGAGGAGCGAAAGCGTGGGGAGCA
AACAGGATTAGAAACCCGAGTAGTCC
>Otu2759
CCAGCCTATGGGTTGCACCAGTAGGGAATTTTCCACAATGGGCGAAAGCCTGATGGAGCA
ACGCCGCGTGCAGGATGAAGGCTTTCGGGTCGTAAACTGCTTTTATATGTGAAGATTATG
ACGGTAGCATATGAATAAGGATCGGCTAACTCCGTGCCAGCAGCCGCGGTCATACGGAGG
ATCCAAGCGTTATCCGGAATTACTGGGCGTAAAGAGTTGCGTAGGTGGCAAAGTAAGTTG
GTAGTGAAAGCGTACGGCTCAACCGTATATCCATTACCAAAACTGCTTAGCTAGAGAGCG
AGAGAGGTACCTGGAATTCCTAGTGTAGGAGTGAAATCCGTAGATATTAGGAGGAACACC
GGTGGCGTAGGCAGGGTACTGGCTCGTTTCTGACACTAAGGCACGAAAGCGTGGGGAGCA
ACCCGGATTAGAGACCCTTGTAGTCC
>Otu2760
CCAGCCTATGGGGGGCAGCAGTGGGGAATATTGGGCAATGCGCAACCGCGTGACCCAGTG
ATATTTTGCGAGTGAGAAACGGACAGCAATATCGCTGTAAAGCTCTTTTATCAAGGAAGA
TAATGACAGTACTTGAAGAATCAGCACTGGCTAATTCTGTGCCAGCAGCCGCGGTAACAC
AGGGAGTGCAGATGTTATTCATCATTATTGGGTGTAAAGGATATGTAGATTGTTTAGGTT
TGGATATTTTGTAAAATTCTCGGGTAAATTCCTTAGATCGACAAAAGTTACCCAAAAACT
TAGAGTTTAAAATAGGTTGATAGAATTTCTTGTGTAAGGGTAGAATCTCTATATATTAGA
ACGACTTCTTAAAAAGTTGCGAAGGCAATTTTCTTGTTTAAACTGACATTGAGATATGAA
AGTATAGGGATCAAATAGGATTAGAAACCCGCGTAGTCC
>Otu2763
CCAGCCTATGGGGGGCTGCAGTCGAGAATCTTCCGCAATGGGGGCAACCCTGACGGAGCG
ACGCCGCGTGGAGGATGACGTACTTCGGTATGTAAACTCCTTTTATTGGGGAGAAAGTTA
TTGATAGTACCCGATGAATAAGAAGTTGCTAAACTCGTGCCAGCAGCAGCGGTAATACGA
GTGCTTCAAGCGTTATCCGGAATTATTGGGCGTAAAGGGTGTGTAGGCGGTCGTGTTAGT
CTCTCGTTAAAGACTTCGGCTTAACCGGGGTTCTGTGAGGGAAACGGCACGACTAGAGGA
TGCGAGAGGTGAATGGAACTCATAGTGTAGGGGTGAAATCCGTTGATATTATGGGGAACA
CCAAAAGCGAAGGCAATTCACTGGCGCACTCCTGACGCTGAAACACGAAAGCGTGGGTAG
CGAATGGGATTAGAAACCCTCGTAGTCC
>Otu2765
CCAGCCTATGGGGCGCAGCAGTGGGGAATTTTGGACAATGGGCGAAAGCCTGATCCAGCA
ACTCTGCGTGAGGGACGAAGATCTTAGGATTGTAAACCTCTTTTGCACGGGACGAAACCC
CGCAAGGGACTGACGGTACCGTGCGAATAAGCCACGGCTAACTATGTGCCAGCAGCCGCG
GTAAGACATAGGTGGCGAGCGTTATTCGGAATTACTAGGCGTAAAGCGAGTGTAGGCGGG
CGCTTAAGTCCGACGTTAAATCTCCCGGCTTAACTGGGAAACGTCGTCGGATACTGGGCG
TCTCGAGTAGGGTAGGGGGCAGCGGAATTCCCGGTGTAGCGGTGAAATGCGTAGATATCG

GGAGGAACACCTATGGCGAAAGCAGCTGCCTGGGCCTTTACTGACGCTAAGACTCGAAAG
CTGGGGGAGCAAACAGGATTAGATACCCCTGTAGTCC
>Otu2768
CCAGCCTATGGGGGGCAGCAGTCGAGAATTTTTCTCAATGGGCGAAAGCCTGAAGGAGCG
ACGCCGCGTGGGGGATGAATGGCTTCGGCCCGTAAACCCCTGTCATTTGTGAACAAATTG
CTTCACCCAACACGTGAAGCATTGATAGTAACGGAAGAGGAAGGGACGGCTAACTCTGTG
CCAGCAGCCGCGGTAATACAGAGGTCCCAAGCGTTGTTCGGATTCACTGGGCGTAAAGGG
TGCGCAGGTGGCTGGGTAAGTTTGACGTGAAATCTCCGGGCTCAACCCGGAAAATGCGTT
GAATACTATCTGGCTGGAGGGTTGGAGGGGAGACTGGAATTCTCGGTGTAGCAGTGAAAT
GCGTAGATATCGAGAGGAACACCAGTGGCGAAGGCGAGTCTCTGGACAACTCCTGACACT
GAGGCACGAAAGCTAGGGGAGCAAACAGGATTAGAAACCCTTGTAGTCC
>Otu2772
CCAGCCTACGGGGGGCTGCAGTGGGGAATATTGCGCAATGGGGGAAACCCTGACGCAGCA
ACGCCGCGTGAGTGAGGAAGGCCTTCGGGTTGTAAAGCTCTTTCGGCTGGGAAGAAGTGA
ACTATTTTTAATAAAGATAGTTTGTGATGGTACCAGAAGAAGAAGCACCGGCAAACTTCG
TGCCAGCAGCCGCGGTAATACGAAGGGTGCAAGCGTTATTCGGAATTATTGGGCGTAAAG
GGTTCGTAGGCGGGAGAGCAAGTCAAGTGTGAAATTTCTAGGCTAAACCTAGAGAGTGCA
TTTGAAACTGCTTTTCTTGAGTGTTAGAGAGGGTGATGGAATTGCTGGTGTAGGAGTGAC
ATCCGTAGAGATCAGCAGGAACATCGGAGGCGAAGGCGGTCACCTGGCTAAATACTGACG
CTGAGGAACGAAAGCATGGGGAGCAAACAGGATTAGATACCCTCGTAGTCC
>Otu2776
CCAGCCTACGGGTCGCAGCAGTAGGGAATATTGGGCAATGGTCGATAGACTGACCCAGCC
ATGCCGCGTGCAGGAAGAAGTATTTATGATATGTAAACTGCTTTAGCAGGGGAAGAAAAA
GGCCATGCGTGGCTCATTGACGGTACCCTGAGAATAAGCACCGGCTAACTCCGTGCCAGC
AGCCGCGGTAATACGGGGGGTGCAAGCGTTGTCCGGATTTATTGGGTTTAAAGGGTGCGC
AGGCGGCTCTGTAAGTCAGTGTTAAAAGTCATCAGCTTAACTGGTGTTATGGCAATGATA
CTGCAGAGCTTGAGTACAGATGAGGCAGGCGGAATTTATGGTGTAGCGGTGAAATGCATA
GATACCATAAAGAACACCGATAGCGAAGGCAGCTTGCCGCGTTGTAACTGACGCTCATGC
ACGAAAGCGTGGGGATCAAACAGGATTAGAAACCCGTGTAGTCC
>Otu2778
CCAGCCTATGGGTGGCAGCAGTGGGGAATTTTGCGCAATGGGGGAAACCCTGACGCAGCA
ACGCCGCGTGGAGGATGAAGTACTTCGGTACGTAAACTCCTTTCGATCGGGACGATTATG
ACGGTACCGGAAGAAAAAGCACCGGCTAACTCTGTGCCAGCAGCCGCGGTAATACAGAGG
GTGCGAGCGTTAATCGGATTCACTGGGCGTAAAGGGTGCGTAGGCGGTCGGGTAAGTCTG
ACGTGAAATCTCCGGGCCTAACCCGGAAACTGCGTCGGATACTATCCGGCTAGAGGATTG
GAAGGGAGACTGGAATACTTGGTGTAGCAGTGAAATGCGTAGATATCAAGTGGAACACCA
GTGGCGAAGGCGAGTCTCTGGACAATTCCTGACGCTGAGGCACGAAAGCCAGGGGAGCAA
ACGGGATTAGAAACCCTCGTAGTCC
>Otu2779
CCAGCCTATGGGTTGCAGCAGTGAGGAATCTTGCGCAATGGGCGAAAGCCTGACGCAGCG
ACGCCGCGTGGAGGATGAAGGTCTTAGGATTGTAAACTTCTGTTAAGCGGGAAGAAATGA
TTCGTTATAATACAGCGAATTTCCGACGGTACCGCTAGAGAAAGCACCGGCTAAACTCGT
GCCAGCAGCCGCGGTAATACGAGTGGTGCAAGCGTTATTCGGATTTATTGGGCGTAAAGG
GTGTGTAGACGGCTTGTTAAGTCATTTGTTTAATCCTCCGGCCTAACCGGAGTACAGCAG
GTGAAACTGGCGAACTTGAGGGTGGAAGAGAGAAGTGGAATTCTCGGAGTAGCGGTAAAA
TGCGTAGATCTCGAGAGGAACACCAATGGCGAAGGCAGCTTCTTGGTCCATACCTGACGT
TGAAACACGAAAGCGTGGGGAGCAAACAGGATTAGAGACCCCCGTAGTCC
>Otu2782
CCAGCCTATGGGTTGCACCAGTGGGGAATATTGGACAATGGGGGAAACCCTGATCCAGCA
ATGCCGCGTGGGTGAAGAAGGCCTTCGGGTTGTAAAGCCCTTTAGGCGGGGAAGAAGGTT
TTAGGGTGATAAGCCCTAGAATTTGACGGTACCCGCAGAATAAGCACCGGCTAACTCTGT
GCCAGCAGCCGCGGTAATACAGAGGGTGCGAGCGTTAATCGGATTTACTGGGCGTAAAGG
GCGCGTAGGTGGTAAAGCAAGTAAGATGTGAAAGCCCCGGGTTTAACCTGGGAAGTGCGT
CTTAAACTGCTTAACTAGAGTATGGTAGAGGGTAGTGGAATTTCCGGTGTAGCGGTGAAA
TGCGTAGAGATCGGAAGGAACGCCAGTGGTGAAGACGACTACCTGGACCAATACTGACAC
TGAGGCGCGAAAGCGTGGGGATCAAACAGGATTAGATACCCCTGTAGTCC
>Otu2784
CCAGCCTATGGGTTGCTGCAGTCGAGAATTTTTCTCAATGGGTGAAAGCCTGAAGGAGCG

ACGCCGCGTGGGGGATGAATGGCTTCGGCCCGTAAACCCCTGTCATTTGCGAACAAATTG
ACTCACTTAACACGTGTGTCATTGATTGTAGTAGAAGAGGAAGGGACGGCTAACTCTGTG
CCAGCAGCCGCGGTAATACAGAGGTCCCAAGCGTTGTTCGGATTCACTGGGCGTAAAGGG
TGCGTAGGCGGTTGAATAAGTCTGATGTGAAATCTCCGGGCTTAACCCGGAAATTGCATC
GGATACTATCCGACTAGAGGAATGGAGGGGAGACTGGAATACTTGGTGTAGCAGTGAAAT
GCGTAGATATCGTGAGGAAGGCCAGTGGCGAAGGCGGATCTCTGGACAGTTCCTGACGCT
GAGGCACGAAGGCCAGGGGAGCAAACGGGATTAGAAACCCTCGTAGTCC
>Otu2785
CCAGCCTACGGGATGCAGCAGTAGGGAATATTGCTCAATGGGGGAAACCCTGAAGCAGCA
ACGCCGCGTGAGTGATGAAGGTTTTCGGATTGTAAAGCTCTGTTGTGCGGGGAAAACGAT
ATTATGGCTAACATCCATAGTAAGTGATTGTACCGCATAAGAAAGGACCGGCTAACTTCG
TGCCAGCAGCCGCGGTAAGACGGGGGGTCCTAGCGTTGTTCGGAATTATTGGGCGTAAAG
CGTGTGCAGGTTGTCTGAAAAGTTAGATGTGAAATCCTTGGGCTCAACCCAAGACGTGCA
TTTAAAACTGTCAGACTTGAGTATGGAAGAGGGTCGTGGAATTCCAGGTGTAGTGGTGAA
ATACGTAGATATCTGGAGGAACACCGGCGGCGAAGGCGGCGACCTGGTCCAATACTGACA
CTCATACGCGAAAGCGTGGGGATCAAACAGGATTAGAGACCCTCGTAGTCC
>Otu2786
CAGCCTATGGGTGGCAGCAGTGGGGAATATTGGACAATGGGCGAAAGCCTGATCCAGCAA
TGCCGCGTGGGTGATGAAGGCCTTCGGGTTGTAAAGCCCTGTCGGCGGGAACGAAAAAAT
CGATGTTGAATCGATCTTGACGGTACCCGCAAAGGAAGCACCGGCTAACTCTGTGCCAGC
AGCCGCGGTAATACAGAGGGTGCAAGCGTTGTTCGGAATTACTGGGCGTAAAGCGCATGT
AGGCGGTCTAGTTAGTCAGATGTGAAAGCCCTGGGCTTAACCCAGGAACCGCATCTGATA
CTGCTAAACTTGAGTGTCGGAGGGGATGGCGGAATTCCCGGTGTAGAGGTGAAATTCGTA
GATATCGGGAGGAACACCTGTGGCGAAGGCGGCCATCTGGAAGACAACTGACGCTGAGAT
GCGAAAGCGTGGGGAGCAGACAGGATTAGAAACCCTAGTAGTCC
>Otu2788
CCAGCCTACGGGATGCAGCAGTGGGGAATCTTGCGCAATGGACGAAAGTCTGACGCAGCG
ACGCCGCGTGGGTGATGAAGGCCTTCGGGTTGTAAAGCCCTGTGGGGAGAGACGAATAAG
TGTTGGCTAATACCCAGCATGATGACGGTATCTCCTTAGCAAGCACCGGCTAACTCTGTG
CCAGCAGCCGCGGTAAGACAGAGGGTGCAAACGTTGTTCGGAATTACTGGGCGTAAAGCG
TGTGTAGGCGGCTCCGTAAGTCAGGTGTGAAAGCCCCAGGCTTAACCTGGGAAGTGCACT
TGATACTGCGGAGCTTGAGTATCGGAGAGGTTGGTGGAATTCTCGGTGTAGAGGTGAAAT
TCGTAGATATCGAGAGGAACACCGGTGGCGAAGGCGGCCAACTGGACGAATACTGACGCT
GAGACACGAAAGCGTGGGGAGCAAACAGGATTAGAAACCCGTGTAGTCC
>Otu2789
CCAGCCTACGGGGGGCAGCAGTGGGGAATATTGGGCAATGCGTGAAAACGTGACCCAGTG
ATATTTTGCGAGTGAGAAACGGACAGCGCTATCGCTGTAAAGCTCTTTTATCAAAGAAGA
TAATGACAGTATTTGAAGAATCAGCACTGGCTAATTCTGTGCCAGCAGCCGCGGTAACAC
AGGGAGTGCAGATGTTATTCATCATTATTGGGTGTAAAGGATATGTAGGCTGTTTAGGTT
TGGATATTTTGTAAAAGCCTTGGGTAAATTCCTTAGATAGACAAAAGTTACCCAAAAACT
TAGAGTTTAAAGTAGGTTATTAGAATTTCTTGTGTAAGGGTAGAATCTCTATATATTAGA
ACGACTTCCTAAAAAGTTGCGAAAGCATTTTTCTTGTTTAAACTGACGCTGAGATATGAA
AGTATAGGGATCAAATAGGATTAGAAACCCCGTAGTCC
>Otu2793
CCAGCCTACGGGGGGCTCCAGTCGAGGATCTTCGTCAATGGGCGAAAGCCTGAACGAGCG
ATGCCGCGTGCGCGATGAAGGCCTTCGGGTTGTAAAGCGCGAAAGTGGGAATAAAGGGAA
ACTTGATTGAACCACAGTAAGCTCGGGCTAAGTTCGTGCCAGCAGCCGCGGTAAGACGAA
CCGAGCGAACGTTGTTCGGAATCACTGGGCATAAAGGGCGCGTAGGCGGGCTTCTAAGTC
CGTGGTGAAATACTCTGGCTTAACCAGAGAACTGCCTCGGATACTGGGAGTCTCGAGTAA
GGTAGGGGCACGTGGAACGGCTGGTGGAGCGGTGAAATGCGTTGATATCGGAAGGAACGC
CGGTGGCGAAAGCGACGTGCTGGACCCTTTCTGACGCTGAGGCGCGAAAGCCAGGGGAGC
AAACGGGATTAGAAACCCTAGTAGTCC
>Otu2796
CCAGCCTATGGGATGCACCAGCGGGGAATTTTGGACAATGGGGGCAACCCCGATCCAGCA
ATGCCGCGTGAGTGAAGAAGGCCTTCGGGTTGTAAAGCTCTTTTGTCAGGGAAGAAACGG
TGTCTCCTAATACGGGGCGCTAATGACGGTACCTGAAGAATAAGCACCGGCTAACTACGT
GCCAGCAGCCGCGGTAATACGTAGGGTGCAAGCGTTAATCGGAATTACTGGGCGTAAAGC
GTGCGCAGGCGGGTATGTAAGTCAGCTGTGAAATCCCCGGGCTTAACCTGGGAATGGCAG

TTGAGACTGCATGGCTAGAGTGTGTCAGAGGGGGGTAGAATTCCACGCGTAGCAGTGAAA
TGCGTAGAGATGTGGAGGAATACCGATGGCGAAGGCAGCCCCCTGGGATAACACTGACGC
TCATGCACGAAAGCGTGGGGAGCAAACAGGATTAGAAACCCCGGTAGTCC
>Otu2802
CCAGCCTACGGGAGGCACCAGTGAGGAATATTGGTCAATGGACGCATGTCTGAACCAGCC
ATGCCGCGTGAAGGATGAAGGCCTTCTGGGTTGTAAACTTCTTTTATGAGGGAAGAAACC
CCAATATTCTTATTGGGTTGACGGTACCTTAGGAATAAGCACCGGCTAACTCCGTGCCAG
CAGCCGCGGTAATACGGAGGGTGCAAGCGTTATCCGGATTCACTGGGTTTAAAGGGTGTG
TAGGCGGGCCTGTAAGTCAGTGGTGAAATCTCCGGGCTCAACCCGGAAACTGCCATTGAT
ACTATAGGTCTTGAATTTTGTTGAGGTTGGCGGAATAAGTCATGTAGCGGTGAAATGCAT
AGATATGACTTAGAACACCGATTGCGAAGGCAGCTAACTAAGCAAACATTGACGCTGAGG
CACGAAAGCGTGGGGATCAAACAGGATTAGAAACCCTCGTAGTCC
>Otu2803
CCAGCCTACGGGGGGCTGCAGTAGGGAATATTGCGCAATGGAGGAAACTCTGACGCAGCG
ACGCCGCGTGGGTGATGAAGGCCTTCGGGTCGTAAAGCCCTGTCGGAAGGGAAGAATGAC
AAAATGGCTAACATCCATTTTGGTTGACGGTACCTTTAAAGGAAGCACCGGCTAACTACG
TGCCAGCAGCCGCGGTAATACGTAGGGTGCAAGCGTTGTTCGGAATCATTGGGCGTAAAG
CGCATGTAGGTGGTTAGGTAAGTCGGGTGTGAAATCCCTGGGCTCAACCGAGGAAGTGCA
TTCGAAACTACCTAGCTAGAGGACGGTAGAGGAAGGTGGAATTCCTAGTGTAGAGGTGAA
ATTCGTAGATATTAGGAGGAATACCTGTGGCGAAGGCGGCCTTCTGGGCCGTTCCTGACA
CTGAGATGCGAAAGCGTGGGTAGCAAACAGGATTAGATACCCGAGTAGTCC
>Otu2810
CCAGCCTATGGGGGGCTGCAGTGGGGAATTTTACGCAATGGGCGAAAGCCTGACGTAGCG
ACACCGCGTGAACGAAGAAGCCCTTTGGGGTGTAAAGTTCTGTCGGCTGGAACGAAAATA
AATATGACGGTACCAGCAGAGGAAGCATCGGCTAACTACGTGCCAGCAGCCGCGGTAAGA
CGTAGGATGCAAGCGTTGTCCGGATTCATTGGGCGTAAAGGGTTCGTAGGCGGTTTGTTA
AGTCTGGTGTTAAAGGCCCGGGCCCAACCCGGGTATCGCACTGGATACTGGCAGACTGGA
GTGCAGTAGAGGCGAGTGGAATTCCCAGTGTAGCGGTGAAATGCGTAGATATTGGGAAGA
ACACCGGTGGCGCAGGCGACTCGCTGGGCTGTAACTGACGCTGAGGAACGAAAGCTAGGG
GAGCAAATGGGATTAGATACCCCGGTAGTCC
>Otu2811
CCAGCCTACGGGGGGCACCAGTAAGGAATATTGGTCAATGGGCGGAAGCCTGAACCAGCC
ATGCCGCGTGCAGGAAGACGGCCCTACGGGTTGTAAACTGCTTTTGCAGGGGAATAAACC
CCCGTATGTATACGGGGTTGAATGTACTCTGAGAATAAGGATCGGCTAACTCCGTGCCAG
CAGCCGCGGTAATACGGAGGATCCGAGCGTTATCCGGATTTATTGGGTTTAAAGGGTGCG
TAGGCGGACTTATAAGTCAGTGGTGAAATCTCGTCGCTTAACGACGAACGTGCCATTGAT
ACTGTAGGTCTTGAGTACAGATGCCGTTGGCGGAATGTGTCATGTAGCGGTGAAATGCAT
AGATATGACACAGAACACCGATTGCGAAGGCAGCTGACGAAACTGTAACTGACGCTGAGG
CACGAAAGCGTGGGGATCAAACAGGATTAGAAACCCTAGTAGTCC
>Otu2812
CCAGCCTACGGGAGGCAGCAGTGAGGAATATTGGTCAATGGGCGCAAGCCTGAACCAGCC
ATGCCGCGTGAAGGATGAAGGCCTTCTGGGTTGTAAACTTCTTTTACAGGGGAAGAAAAG
TATTGTTTCTACGATATCCGACGGTACCCTGGGAATAAGCACCGGCTAACTCCGTGCCAG
CAGCCGCGGTAATACGGAGGGTGCAAGCGTTATCCGGATTTACTGGGTTTAAAGGGTGTG
TAGGCGGCATTTAAGTCAGTGGTGAAATCTCCGAGCTCAACTTGGAAACTGCCATTGAT
ACTATTTGTCTTGAATTCTGTTGAGGTGGGCGGAATAAGTCATGTAGCGGTGAAATGCAT
AGATATGACTTAGAACCCCAATTGCGAAGGCAGCTCACTAAACAGACATTGACGCTGAGG
CACGAAAGCGTGGGGATCAAACAGGATTAGATACCCCCGTAGTCA
>Otu2813
CCAGCCTACGGGTTGCACCAGTAGGGAATTTTCCACAATGGGCGAAAGCCTGATGGAGCA
ACGCCGCGTGCAGGATGAAGGCCTTCGGGTTGTAAACTGCTTTTATCTGTGACGAATATG
ACGGTAGCAGATGAATAAGGATCGGCTAACTCCGTGCCAGCAGCCGCGGTCATACGGAGG
ATCCAAGCGTTATCCGGAATTACTGGGCGTAAAGAGTTGCGTAGGTGGCAAAGTAAGCGG
ACAGTGAAATAGTGTGGCTCAACCATACAACCATTGTCTGAACTGCTTAGCTTGAGGACG
AGAGAGGTAGATGGAATTCCCAGTGTAGGAGTGAAATCCGTAGATATTGGGAGGAACACC
GATGGCGTAGGCAGTCTACTGGCTCGTTCCTGACACTAAGGCACGAAAGCGTGGGGAGCA
AACGGGATTAGAAACCCGAGTAGTCC
>Otu2815

CCAGCCTACGGGACGCACCAGTCGAGAATTTTTCACAATGGGGGAAACCCTGATGGAGCG
ACGCCGCGTGGGGGATGAATGGCTTCGGCCCGTAAACCCCTGTCATTTGCGAACAAACGT
TTTCGTTGAACAGGCGAAAACTTGATTGTAGCGGAAGAGGAAGGGACGGCTAACTCTGTG
CCAGCAGCCGCGGTAATACAGAGGTCCCAAGCGTTGTTCGGATTCACTGGGCGTAAAGGG
TGCGTAGGTGGTCAGATAAGTCTGATGTGAAATCTCGGAGCTTAACTCCGAAACTGCATT
GGAAACTAGTTGGCTGGAGGGTCGGAGGGGGGACTGGAATTCTCGGTGTAGCAGTGAAAT
GCGTAGATATCGAGAGGAACACCAGTGGCGAAGGCGAGTCCCTGGACGACTCCTGACACT
GAGGCACGAAAGCTAGGGGAGCAAACAGGATTAGATACCCTAGTAGTCC
>Otu2816
CCAGCCTATGGGTTGCTCCAGTGGGGAATATTGGACAATGAGGGAAACCCTGATCCAGCA
ATGCCGCGTGTGTGAAGAAGGCCTTCGGGTTGTAAAGCACTTTTGTCGAGGAGGAAGGCG
TGAAGGAATTCACGATTGACTTAACTCGAAGAAACAGCACCGGCTAACTCTGTGCCAGCA
GCCGCGGTAATACAGAGGGTGCAAGCGTTAATCGGAATTATTGGGCGTAAAGCGCACGTA
GGCGGTTCGATAAGTTAGATGTGAAATACCTGGGCTTAACTTGGGGATGTCATCTAAAAC
TGTGGGACTAGAGTAGAGTAGAGGAAGGGGGAATTTCCGGTGTAGCGGTGAAATGCGTAG
ATATCGGAAGGAACATCAGTGGCGAAGGCGCCCTTCTGGACAAATACTGACGCTGAGGAG
CGAAAGCGTGGGGAGCAAACAGGATTAGATACTCTTGTAGTCC
>Otu2817
CCAGCCTATGGGTGGCTCCAGTCAAGAAACTTTGGCAATGCACGAAAGTGTGACCAAGCG
ACGCCGCGTGATTGATGAAGCCCTTCGGGGTGTAAAGATCTTTTATGCATGAGAAAATTA
TTGATGTTAGTGCATGAATAAGGGGCTCCTAACTCTGTGCCAGCAGGAGCGGTAATACAG
AGGCCCCGAGCGTTATCCGGAATTATTGGGCGTAAAGGGTGCGTAGGTGGTTTTGTTAGT
CGAATGTCAAAAATCTGGGCTCAACCCAGGGGAGGCATTCGAAACGGCAAGACTCGAGTA
TGTGAGAGGTGAACGGAACTCATGGAGTAGGGGTGAAATCCGCTGATATCATGGGGGACA
CCAAATGCGAAGGCAGTTCACTGGCACATTACTGACACTCAAGCACGAAAGCGTGGGTAG
CGAATGGGATTAGATACCCCGGTAGTCC
>Otu2818
CCAGCTATGGTTGCTGCAGTCGAGAATTTTTCACAATGGACGAAAGTCTGATGGAGCGAC
GCCGCGTGGAGGATGAAGGTTTTCGGATTGTAAACTCCTGTCACTGCAGAACAAGGCCAT
GCGATTGAACATTTCGCATGGTTGATGGTATGCGGAGAGGAAGGGACGGCTAACTCTGTG
CCAGCAGCCGCGGTAAGACAGAGGTCCCGAGCGTTGTTCGGATTCATTGGGCGTAAAGGG
TGTGTAGGAGGCTGGGTAAGTCAGGTGTGAAATCTCACAGCTTAACTGTGAAACTGCGCT
TGATACTGCTCGGCTAGAGGATCGGAGGGGGTAACGGAATTTATGGTGTAGCAGTGAAAT
GCGTAGATATCATAAGGAACACCGGTGGCGAAGGCGGTTACCTGGAAGATTCCTGACTCT
GAAACACGAAAGCCAGGGGAGCAAACGGGATTAGAAACCCCAGTAGTCC
>Otu2825
CCAGCCTATGGGAGGCAGCAGCCGAGGATCATTCGCAATGGGCGCAAGCCTGACGATGCG
ACGCCGTGTGAGCGAAGAAGGCCTTCGGGTTGTAAAGCTCTTTCGCTTGGGAACAAGAGA
CGTTGGTGAACAATCAGCGAATTTGAGGGTACCAGGTAAAGAAGCACCGGCTAACTCCGT
GCCAGCAGCTGCGGTAATACGGAGGGTGCAAGCATTAATCGGATTTATTGGGCGTATAGG
GCGCGTAGGCGGGAAGGTAAGTCAGATGTGAAATCCCGGGGCTCAACCCCGGAACAGCAT
TTGAAACTCCCTTTCTTGAGGATAGGCGGAGAAAACGGAATTCCACATGTAGCGGTGAAA
TGCGTAGATATGTGGAGGAACACCGGTGGCGAAGGCGGTTTTCTAGCTTATTCCTGACGC
TGAGGCGCGAAAGCAAGGGGATCAAACAGGATTAGAAACCCTCGTAGTCC
>Otu2826
CCAGCCTATGGGTGGCTCCAGCCGAGAATATTCGACAATGGGCGAAAGCCTGATCGAGCG
ATACCGCGTGGTGGATGAAGCGCTTCGGCGCGTAAACACCTTTTATGAGGGAGGAAGTTA
TTGACGTTACCTCATGAATAAGGGGCTCCTAACTCTGTGCCAGCAGGAGCGGTAATACAG
AGGCCCCAAGCGTTACCCGGAATTACTGGGCGTAAAGAGTGCGTAGGTGGTCGTATTAGT
CGCGCGTCAAAACCCAGGGCTCAACCCTGGATCCGCGCGCGAAACGGTACGACTCGAGGG
CGTGAGAGGTCTGTGGAACTCTAGGTGTAGGGGTGAAATCCGTTGATATCTAGGGGAACA
CCGAAAGCGAAGGCAGCAGACTGGCGCGTTCCTGACACTCAAGCACGAAAGCGTGGGTAG
CGAACGGGATTAGAAACCCTTGTAGTCC
>Otu2827
CCAGCCTACGGGGGGCAGCAGTCGAGAATTTTTCTCAATGGGCGAAAGCCTGAAGGAGCG
ACGCCGCGTGGGGGATGAAGGGCTTCGGCTCGTAAACCCCTGTCATTTGCGAACAAACCT
TTCCGCTTAACCGGCGGAAAACTGATGGTAGCGAAAGAGGAAGGGACGGCTAACTCTGTG
CCAGCAGCCGCGGTAGTACAGAGGTCCCAAGCGTTGTTCGGATTCACTGGGCGTAAAGGG

TGCGCGGGTGGCGGGGTAAGTTTGATGTGAAATCTCCGGGCTCAACCCGGAAACGACATT
GAATACTATCCGGCTCGAGGGTTGGAGGGGGGACTGGAATTCTCGGTGTAGCAGTGAAAT
GCGTAGATATCGAGAGGAACACCAGCGGCGAAGGCGAGTCCCTGGACAACTCCTGACACT
GAGGCACGAAAGCTAGGGGAGCAAACAGGATTAGAAACCCGTGTAGTCC
>Otu2834
CCAGCCTACGGGTGGCAGCAGTGGGGAATTTTGGACAATGGGGGCAACCCTGATCCAGCC
ATGCCGCGTGTGTGAAGAAGGCCTTCGGGTTGTAAAGCACTTTTGTCCGGAACGAAACGG
CGGATGCGAATAACATCCGTTAATGACGGTACCGGAAGAATAAGCACCGGCTAACTTCGT
GCCAGCAGCCGCGGTAATACGAAGGGTGCGAGCGTTGTTCGGATTTATTGGGCGTAAAGA
GCTCGTAGGCGGTTCAGCAAGTCGGGTGTTAAACCCCCAGGCTCAACCTGGGGCCGCCAC
CCGAAACTGTTGTGACTAGAGTTTGGTAGGGGATCACGGAATTCCCGGTGTAGCGGTGGA
ATGCGCAGATATCAGGAGGAACACCAGTAGCGAAGGCGGTGATCTGGGCCAATACTGACG
CTGAGGAGCGAAAGCGTGGGGAGCGAACAGGATTAGAAACCCGAGTAGTCC
>Otu2836
CCAGCCTATGGGTCGCACCAGTCAGGAATACTGCGCAATGGAGGAAACTCTGACGCAGCG
ACGCCGCGTGGGTGATGAAGGCCTTCGGGTCGTAAAGCCCTGTCGGAGGGGAAGAAAAGC
TCGCTGGCTAATATCCAGCGGGCCTTTGACGGTACCCTCAAAGGAAGCACCGGCTAACTC
CGTGCCAGCAGCCGCGGTAAGACGGAGGGTGCAAGCGTTGTTCGGATTGACTGGGCGTAA
AGGGCGTGTAGGCGGTTGCCCAAGTCCGATGTGAAAGCCCGGGGCTTAGCCTCGGAAGTG
CATCGGAAACTAGGCAGCTGGAGCGCGATAGAGGAGGGTAGAATTCCTGGTGTAGCGGTG
AAATGCGTAGATATCAGGAGGAACACCAATGGCGAAGGCAGCTCGCTGGGACGGTACTGA
CGCTGAGACGCGAAAGCGTGGGGAGCAAACAGGATTAGAGACCCTCGTAGTCC
>Otu2837
CCAGCCTACGGGAGGCAGCAGTGGGGGATATTGGACAATGGGCGCAAGCCTGATCCAGCA
ATGCCGCGTGAATGATGAAGGCCTTCGGGTTGTAAAGTTCTTTCGACGGGGACGATGATG
ACGGTACCCGTAGAAGAAGCCCCGGCTAACTTCGTGCCAGCAGCCGCGGTAATACGAAGG
GGGCTAGCGTTGTTCGGAATTACTGGGCGTAAAGCGCACGCAGGCGGCTCGTTAAATTAG
AAGTGAAAGCCCTGGGCTCAACCCGGGAATAGCTTTTGAAACTGGCGAGCTAGAATTCGG
GAGAGGGTAGTGGAATTCCAAGTGTAGAGGTGAAATTCGTAGATATTTGGAAGAACACCG
GTGGCGAAGGCGGCTACCTGGCCCGATATTGACGCTCATGTGCGAAAGCGTGGGGAGCAA
ACAGGATTAGATACCCTTGTAGTCC
>Otu2838
CCAGCCTATGGGTGGCACCAGTCGAGGATTTTTCACAATGGGGGCAACCCTGATGGAGCG
ACGCCGCGTGGAGGATGAAGGTTTTCGGATTGTAAACTCCTGTCACTGCAGAGCAAAGCT
TGGCCGTTAATACCGGTTCAAGTCTGATAGTATGCAGGGAGGAAGGGACGGCTAACTCTG
TGCCAGCAGCCGCGGTAATACAGAGGTCCCGAGCGTTGTTCGGATTCACTGGGCGTAAAG
GGTGTGTAGGAGGTTTGGTAAGTCAGGTGTGAAATCCCGCAGCTTAACTGCGGAACTGCA
TTCGATACTGCCGAACTAGAGGATCGGAGGGGGAAGCGGAACTCTTGGTGTAGCGGTGAA
ATGCGTAGATATCAAGAGGAACACCGGTGGCGAAGGCGGCTTCCTGGAAGATTCCTGACT
CTGAAACACGAAAGCCAGGGGAGCGAATGGGATTAGAAACCCCGGTAGTCC
>Otu2842
CCAGCCTACGGGAGGCACCAGTGAGGAATTTTCCGCAATGGGCGAAAGCCTGACGGAGCG
ACGCCGCGTGTGGGAAGAAGGCCTTCGGGTCGTAAACCACTGTCGAAGGGGAAGATATTG
ACGGTACCCTTGGAGGAAGCCCCGGCTAACTACGTGCCAGCAGCCGCGGTAAGACGTAGG
GGGCAAGCGTTGTCCGGAATTATTGGGCGTAAAGCGCTCGTAGGCGGATCTACAAGTCTG
TGAAGAAAGACCCGGGCTCAACTCGGGGAACGGCATAGATACTGTTGATCTTGAGACGAT
CAGAGGATGATGGAATTCCCGGTGTAGCGGTGAAATGCGTAGATATCGGGAGGAACACCA
GTGGCGAAGGCGATCATCTGGGGTTGTTCTGACGCTGAGGAGCGAAAGCTAGGGGAGCAA
ACGGGATTAGAGACCCGCGTAGTCC
>Otu2844
CCAGCCTATGGGTGGCTGCAGTGGGGAATCTTGCACAATGGGCGAAAGCCTGATGCAGCA
ACGCCGCGTGAGTGAAGAAGGCCTTTGGGTTGTAAAGCTCTTTCGGTTGGGAAGAAGGGA
ATATAGGCTAATACCTTATATTTTTGACGGTACCAGCATAAGAAGCACCGGCTAATTCCG
TGCCAGCAGCCGCGGTAATACGGGAGGTGCAAGCGTTGTTCGGAGTGACTGGGCGTAAAG
CGCACGTAGGTGGCTTGTAAGTCGGATGTGAAATCCCCGGGCTTAACCTGGGAACTGCA
TCCGAAACTACAAGTCTTGAATGCTTCAGAGGTTGGTAGAATTCCAGGTGTAGGAGTGAA
ATCCGTAGAGATCTGGAGGAATACCGGAGGCGAAGGCGGCCAACTGGGAATGCATTGACA
CTGAGGTGCGAAAGCGTGAGGAGCAAACAGGATTAGATACCCGAGTAGTCC

>Otu2845
CCGCTATGGGGTGCTCCAGTCGAGAATCTTTGGCAATGCACGAAAGTGTGACCAAGCGAC
GCCGCGTGATTGATGAAGCCCTTCGGGGTGTAAAGATCTTTTATGCATGAAAAAATTATT
GATTTTAGTGCATGAATAAGGGGCTCCTAACTCTGTGCCAGCAGGAGCGGTAATACAGAG
GCCCCGAGCGTTATTCGGAATTATTGGGCGTAAAGGGTGCGTAGGTGGTTTTGTTAGTCG
ACTGTCAAAAATCAGGGCTCAACCCTGGGGAGGCAATCGAAACGGCAAGACTCGAGTAGG
TGAGAGGTGTTCGGAACTCATGGAGGAGGGGTGAAATCCGTTGATATCATGGGGAACACC
AAATGCGAAGGCAGCACACTGGCGCCTTACTGACACTCAAGCACGAAAGCGTAGGTAGCG
AATGGGATTAGATACCCCTGTAGTCC
>Otu2847
CCAGCCTATGGGTTGCACCAGTTAAGAATAATGGCCAATGCGCGAAAGCGTGAGCCTGCG
ATCTGTCAGCGGTGATGAAGGAGATTTCTGTAAAACCGAAAAGGGAAGCTCCATATCGAG
GACTTCCTGATCAGTCGTGATAAATCCTTGTGCCAACAGTCGCGGTCATACAAGAGCGGC
GAATGTTGCATGATAGAATTCGGTGTAAAGGGCATGTAGGCGGCAGTCGTCACTCTCATC
ATCCAGAGTGACATAAGGACGGAATTTAAACGATGAGACGACCTCTCTAAAGGGATTCCT
ATCCGACTGCTTTGAGAGAATAAGAGGAGAATGGAATGGCCAGTGTACCAATGAAATCGG
TTGATCCTGGCTGGAACATCCATGGCGAAGGCAATTCTCTGGTATTCATCTGACGCTGAG
GTGCGAAAGTATGGGGAGCGATCGGGATTAGAAACCCCCGTAGTCC
>Otu2848
CCAGCCTATGGGTCGCTGCAGTAGGGAATATTGGACAATGGAGGCAACTCTGATCCAGCC
ATGCCGCGTGAATGATGAAGGCCTTAGGGTTGTAAAGTTCTTTTAGTGGGGAAGATAATG
ACGGTACCCACAGAAAAAGCCCCGGCTAACTCTGTGCCAGCAGCCGCGGTAATACAGAGG
GGGCTAGCGTTGTTCGGAATTACTGGGCGTAAAGGGCGCGTAGGCGGTCCGTTAAGTTAG
AAGTGAAATCCCGGGGCTTAACCTCGGAATTGCTTTTAATACTGGCAGACTTGAGATCGG
TAGGGGATAGTGGAATTCTTAGTGTAGGAGTGAAATCCGTAGATATTAAGAGGAACACCA
GTGGCGAAGGCGACTATCTGGACCGATTCTGACGCTGAGGCGCGAAAGTGTGGGGAGCAA
ACAGGATTAGAGACCCGAGTAGTCC
>Otu2851
CCAGCCTATGGGGGGCACCAGTGGGGAATATTGCGCAATGGGGGAAACCCTGACGCAGCA
ACGCCGCGTGTGTGAAGAAGGCCTGCGGGTTGTAAAGCACTTTTAGTGGGGACAAAAAGC
CACGGACTAATACTCTGTGGTCTTGATTTAACCCAAAGAAAAAGCACCGGCTAACTCTGT
GCCAGCAGCCGCGGTAATACAGAGGGTGCGAGCGTTAATCGGAATTACTGGGCGTAAAGC
GTGCGTAGACGGTTTTGTAAGTCGGATGTGAAATCCCCGGGCTCAACCTGGGAATTGCAT
TCGAAACTGCAAGGCTTGGGTGCGGAAGAGGGAAGCGGAATTTCCGGTGTAGCGGTGAAA
TGCGTAGATATCGGAAGGAACATCAGTGGCGAAGGCGGCTTCCTGGTCCAGCACCGACGT
TCAGGCACGAAAGCGTGGGGAGCAAACAGGATTAGATACCCCGGTAGTCC
>Otu2852
CCAGCCTATGGGTTGCTGCAGTCGAGAATTTTTCTCAATGGGGGAAACCCTGAAGGAGCG
ACGCCGCGTGGAGGATGAAGGTCTTCGGATTGTAAACTCCTGTCATTAGGGAACAAGTTG
CATGCGTTAACTGCGCATGCATTGATAGTACCTGAAGAGGAAGAGACGGCTAACTCTGTG
CCAGCAGCCGCGGTAATACAGAGGTCTCAAGCGTTGTTCGGATTCATTGGGCGTAAAGGG
TGCGTAGGTGGCGCCGTAAGTCAGGTGTGAAATCTCGGGGCTTAACCCCGAAACTGCATT
TGATACTGCGGTGCTCGAGTACTGGAGAGGAGATTGGAATTTACGGTGTAGCAGTGAAAT
GCGTAGATATCGTAAGGAAGACCAGTGGCGAAGGCGAATCTCTGGACAGTTACTGACACT
GAGGCACGAAGGCCAGGGGGGCAAACGGGATTAGAAACCCTCGTAGTCC
>Otu2854
CCAGCCTACGGGGGGCACCAGTAAGGAATATTGGACAATGGGCGCAAGCCTGATCCAGCC
ATGCCGCGTGAAGGATGAAGGCCTTCGGGTTGTAAAGGACTTTTGTTCGGGAGGAAATCC
CGCTGGTTAATACCTGGCGGGGATGACAGTACCGGAAGAATAAGCACCGGCTAACTACGT
GCCAGCAGCCGCGGTAATACGTAGGGTGCAAGCGTTAATCGGAATTACTGGGCGTAAAGC
GTGCGCAGGCGGTTTTGCAAGTCTGATGTGAAAGCCCCGGGCTCAACCTGGGAACGGCAT
TGGAGACTGCAAGACTAGAGTGCGTCAGAGGGGGGTAGAATTCCGCGTGTAGCAGTGAAA
TGCGTAGAGATGCGGAGGAATACCGATGGCGAAGGCAGCCCCCTGGGATGACACTGACGC
TCATGCACGAAAGCGTGGGGAGCAAACGGGATTAGAGACCCCTGTAGTCC
>Otu2860
CCAGCCTATGGGATGCTCCAGTGGGGAATATTGGACAATGGGGGCAACCCTGATCCAGCA
ATGCCGCGTGTGTGAAGAAGGCCTTAGGGTTGTAAAGCACTTTCAGTAGGGAGGAAGGGG
TTAATGCTAATATCATTAATCATTGACGTTACCTACAGAAGAAGCACCGGCTAACTCTGT

GCCAGCAGCCGCGGTAATACAGAGGGTGCGAGCGTTAATCGGAATTACTGGGCGTAAAGC
GTGTGTAGGTGGTTAGATAAGTTAGATGTGAAATCCCCGGGCTTAACCTGGGCATTGCGT
TTAAGACTGTTTAACTAGAGTACTGTAGAGGATAGTGGAATTTCCAGTGTAGCGGTGAAA
TGCGTAGATATTGGAAGGAACACCAGTGGCGAAGGCGACTATCTGGACAGATACTGACAC
TGAGACACGAAAGCGTGGGGAGCAAACAGGATTAGAGACCCGTGTAGTCC
>Otu2861
CCAGCCTACGGGAGGCAGCAGTAGGGAATATTGGACAATGGGGGAAACCCTGATCCAGCC
ATGCCGCGTGAACGATGAAGGCCTTCGGGTTGTAAAGTTCTTTTGGTGGGGACGATGATG
ACGGTACCCACAGAATAAGCACCGGCTAACTTCGTGCCAGCAGCCGCGGTAATACGAAGG
GTGCTAGCGTTGTTCGGAATTACTGGGCGTAAAGGGCGCGTAGGCGGTTTAACAAGTTGG
GTGTGAAAGCCCAGGGCTTAACCCTGGAACTGCACTCAAGACTGTTTTACTTGAATTCGG
TAGAGGTTAGTGGAATTCCCAGTGTAGAGGTGAAATTCGTAGATATTGGGAAGAATATCC
GTGGCGAAGGCGGCTAACTGGACTGACATTGACGCTGAGGCGCGAAAGCGTGGGGATCAA
ACAGGATTAGATACCCTAGTAGTCG
>Otu2865
CCAGCCTATGGGGGGCTCCAGTGGGGAATATTGGACAATGGGGGGAACCCTGATCCAGCA
ATGCCGCGTGTGTGAAGAAGGCCTGAGGGTTGTAAAGCACTTTCGTTAGGGAGGAGGGTG
ATTAGGTTAAGAGCTAGATTACTGGACGTTACCTAAAGAAGAAGCACCGGCTAACTCCGT
GCCAGCAGCCGCGGTAATACGGAGGGTGCAAGCGTTAATCGGAATTACTGGGCGTAAAGG
GTGCGTATGTGGTTGATTAAGTTATCTGTGAAATACCTGGGCTTAACCTGGGCAGGTCAG
ATGATACTGATTGACTCGAGTACGGGAGAGGGTAGTGGAATCTCCGGTGTAGCGGTGAAA
TGCGTAGAGATCGGAAGGAACACCAGTGGCGAAGGCGGCTACCTGGCCTAGTACTGACAC
TGAGGCACGAAAGCGTGGGGAGCAAACAGGATTAGAAACCCCCGTAGTCC
>Otu2866
CCAGCCTATGGGGTGCAGCAGTGGGGAATATTGGACAATGGGCGCAAGCCTGATCCAGCC
ATGCCGCGTGAGTGATGAAGGCCTTCGGGTCGTAAAGCTCTGTGGGGAGGGACGAAAGGC
CAAGGAGCTAATACCTCTTGGTTTGACGGTACTTCCTTAGCAAGCACCGGCTAACTCCGT
GCCAGCAGCCGCGGTAATACGGAGGGTGCAAACGTTGCTCGGAATCATTGGGCGTAAAGC
GCACGTAGGCGGCTTGTTAAGTCGGATGTGAAAGCCCCGGGCTCAACCCGGGAAGGGCAT
TCGAAACTGGCGAGCTTGAGTATGGAAGAGGGTCTCGGAATTCCCGGTGTAGAGGTGAAA
TTCGTAGATATCGGGAGGAACACCAGTGGCGAAGGCGGAGACCTGGGCCAATACTGACGC
TGAGGTGCGAAAGCGTGGGGAGCAAACAGGATTAGAAACCCGAGTAGTCC
>Otu2867
CAGCCTACGGGAGGCAGCAGTGGGGAATTTTGGACAATGGGCGAAAGCTTGATCCAGCAA
AATCGCGTGAGTGAGGAAGACTTTATGTCGTAAAGCTATTTCGTTAAGGAAAATTGTGAT
TTACTTAAAGAAGAAGTCCCGACTAACTTCGTGCCAGCAGTCGCGGTAAAACGGGGGGGG
CAAGCGTTATTCAGATTAATTGGGCGTAAAGGGTATGTAGGTGGTTTATGGTATTAAAAG
TTAAAGATAAGGGCTTAACCTTTGAAAAGCTTTTAGAATTAATAAGCTTGAGTATAAAGG
GTGATAGTGGTATTTTTGGTGGAGAGGTGAAATTCTTAGATATTAAAAAGACCAGCAGAG
GCGAAGGCGACTATCAATTTTATTTACTGACGTTGAGGTACGAAAGCATGGGGAGCAAAC
AGGATTAGAGACCCCAGTAGTCC
>Otu2873
CCAGCCTATGGGGGGCAGCAGTCGAGGATCTTCGGCAATGGGCGCAAGCCTGACCGAGCG
ACGCCGCGTGCGCGATGAAGGCCTTCGGGTTGTAAAGCGCTGCCGAGGGGGAGGAAGCCG
CAAGGTTGACCTATCCCTGGAGGAAGCACGGGCTAAGTTCGTGCCAGCAGCCGCGGTAAG
ACGAACCGTGCGAACGTTGTTCGGAATCACTGGGCTTAAAGGGCGCGTAGGCGGGTCATC
CAGTCCGGGGTGAAATCTTTCGGCTTAACCGGAAAATAGCCTTGGATACGGGTGGTCTAG
AGGGAGGCAGGGGCAGCGGGAACTTCCGGTGGAGCGGTGAAATGCGTTGATATCGGAAGG
AACGCCGGTGGCGAAAGCGCGCTGCTGGACCTTTTCTGACGCTGAGGCGCGAAAGCCAGG
GGAGCAAACGGGATTAGATACCCCCGTAGTCC
>Otu2874
CCAGCCTATGGGAGGCTCCAGTGGGGAATTTTGGACAATGGGCGCAAGCCTGATCCAGCA
ATGCCGCGTGAGTGAAGAAGGCCCTTGGGTTGTAAAGCTCTTTCGGTCGGGAAGAAGGAG
GAGTAGCTAATATCTACTCCGACAGACGGTACCGGCATAAGAAGCACCGGCTAACTACGT
GCCAGCAGCCACGGTAATACGTAGGGTGCAAGCGTTAATCGGAATGACTGAGCGTAAAGG
GTGTGTAGGCGGTTTTTTAAGTCTGATGTGAAAGCCCCGGGCTCAACCTGGGAACGGCAT
TGGAAACTGGGAAACTGGAGTACGTAAGAGGGGGGTGGAATTCCACGTGTAGCAGTGAAA
TGCGTAGAGATGTGGAGGAACACCGATGGCGAAGGCAACCCCCTGGGACGATACTGACGC

TGAGGCACGAAAGCGTGGGGAGCAAACAGGATTAGAAACCCCAGTAGTCC
>Otu2875
CCAGCCTACGGGACGCTCCAGTAAAACCTGCACCGACAATCACCCAGTTGTAACGTGCCA
TCGAAAGATATCCAGTACCGTTGGTGTCAGTGAGTATCAGTCGGCATCTTGTCTTCGGCT
AAAAATTCAGGCTGACAATCAAAGAAAGTTCCGGCCCAAAAGCCGGTAACAATGACCCTT
AGTCGAAGGAATAATTTGTCGGTTCTTCCTAAAGTCCTGCGGAGATCGTTTAGGACTCGA
AGGATGAACGAAATTGTTCGTTTGGAGAGTCAACCGAAATGTTTACGCTGAGTGTATAAG
GTATTTCGGTAAAAATAATAATACAGTGCAATTCGCGCCGCCGATTCTTCTCTGTCAATT
CGAAAGGAAACATTTCCGGTCTTCAGATGCGTAACGTGACTATTACCAATTAGAGACCCT
CGTAGTCC
>Otu2881
CCAGCCTATGGGAGGCAGCAGTCGAGAATTTCTCACAATGGCGAAAGCCTGAAGGAGCGA
CGCCGCGTGGGGGATGAATGGCTTCGGCCCGTAAACCCCTGTCATTTGTGAACAAATTGC
TTCACCCAACACGTGAAGCATTGATAGTAACGGAAGAGGAAGAGACGGCTAACTCTGTGC
CAGCAGCCGCGGTAATACAGAGGTCTCAAGCGTTGTTCGGATTCATTGGGCGTAAAGGGT
GCGTAGGCGGCGGGGTAAGTCAGGTGTGAAATCTCGGGGCACAACTCCGAAACTGCACTT
GATACTGCCCTGCTTGAGTACTGGAGAGGAGATTGGAATTTACGGTGTAGCAGTGAAATG
CGTAGATATCGTAAGGAAGACCAGTGGCGAAGGCGAATCTCTGGACAGTTACTGACGCTG
AGGCACGAAGGCCAGGGGAGCAAACGGGATTAGATACCCCAGTAGTCC
>Otu2884
CCAGCCTATGGGGGGCAGCAGTGGGGAATTTTGTGCAATGGGCGAAAGCCTGACACAGCG
ACACCGCGTGGATGAAGAAGCCCTTTGGGGTGTAAAATCCTGTCGGCTGGAACGAAAAAA
ATGACGGTACCAGCAGAGGAAGCATCGGCTAACTACGTGCCAGCAGCCGCGGTAAGACGT
AGGATGCGAGCGTTGTCCGGATTTATTGGGCGTAAAGAGTTCGTAGGCGGTTTGTTAAGT
CTGATGTTAAAGATCGGGGCTCAACCCTGGGAGTGCATTGGATACTGGCAGACTGGAGTG
TGGTAGAGGCTAGTGGAATTCCCAGTGTAGCGGTGAAATGCGTAGATATTGGGAAGAACA
CCAGTGGCGTAGGCGACTAGCTGGGCCATAACTGACGCTGAGGAACGAGAGCCAGGGGAG
CGAATGGGATTAGAAACCCGAGTAGTCC
>Otu2886
CCAGCCTACGGGGGGCTGCAGTCGAGAATTTTTCTCAATGGGGGAAACCCTGAAGGAGCG
ACGCCGCGTGGAGGATGAAGGTCTTCGGATTGTAAACTCCTGTCATGAGGGAACAAACCC
GCACGTTTAACTGATGTGCGGCTGATAGTACCTCAAGAGGAAGAGACGGCTAACTCTGTG
CCAGCAGCCGCGGTAATACAGAGGTCTCAAGCGTTGTTCGGATTCATTGGGCGTAAAGGG
TGCGTAGGCGGCGAAGCAAGTCAGATGTGAAATCCCGGGGCTTAACCCCGGAACTGCATT
TGATACTGCTTTGCTTGAGGACTGGAGAGGAGATCGGAATTCACGGTGTAGCAGTGAAAT
GCGTAGATATCGAGAGGAACACCAGTGGCGAAGGCGAGTCCCTGGACGACTCCTGACACT
GAGGCACGAAAGCTAGGGGAGCAAACAGGATTAGAGACCCGAGTAGTCC
>Otu2887
CCAGCCTATGGGGTGCTGCAGTGGGGAATATTGGACAATGGGCGAAAGCCTGATCCAGCA
ATGCCGCGTGTGTGAAGAAGGCCTTCGGGTTGTAAAGCACTTTTAGTGAGGAGGAAAGTT
TCAAGGCTAATACCCTTGAAAATTGACGTTACTCACAGAAAAAGCACCGGCAAACTCTGT
GCCAGCAGCCGCGGTAATACAGAGGGTGCGAGCGTTAATCGGAATTACTGGGCGTAAAGC
GCACGTAGGCGGATATCTAAGTCGATTGTGAAATCCCCGGGCTCAACCTGGGAACTGCAT
TCGATACTGGATATCTAGAGTATGGTAGAGGAAAGTGGAATTCCCGGTGTAGCGGTGAAA
TGCGTAGATATCGGGAGGAACACCAGTGGCGAAGGCGGCTTTCTGGATCAATACTGACGC
TAAGGTGCGAAAGCGTGGGGAGCAAACAGGATTAGAAACCCTAGTAGTCC
>Otu2891
CCAGCCTACGGGGTGCAGCAGTGAGGAATATTGGTCAATGGACGCAAGTCTGAACCAGCC
ATGCCGCGTGAAGGATGAAGGCCTTCTGGGTTGTAAACTTCTTTTATCTGGGAAGAAACC
ACCTTTTTCTAAGGGTGTTGACGGTACCAGAGGAGTAAGCACCGGCTAACTCCGTGCCAG
CAGCCGCGGTAATACGGAGGGTGCAAGCGTTATCCGGATTCACTGGGTTTAAAGGGTGTG
TAGGCGGGTATTTAAGTCAGTGGTGAAATCTCCGGGCTCAACCCGGAAACTGCCATTGAT
ACTATATATCTTGAATACTGTTGAGGTAGGCGGAATATATCATGTAGCGGTGAAATGCAT
AGATATGATATAGAACACCGATTGCGAAGGCAGCTTACTAAACAGTTATTGACGCTGAGG
CACGAAAGCGTGGGGATCAAACAGGATTAGAAACCCGCGTAGTCC
>Otu2897
CAGCCTATGGGTCGCTGCAGTGGGGAATCTTGGACAATGGGGGCAACCCTGATCCAGCGA
TGCCGCGTGGGTGAAGAAGGCCTTCGGGTTGTAAAGCCCTTTAGGCTGGGACGAAGTGTG

TGGGGGGATAATACTCATGCATTGACGGTACCAGCAGAATAAGCACCGGCAAACTCTGTG
CCAGCAGCCGCGGTAATACAGAGGGTGCAAGCGTTAATCGGAATTACTGGGCGTAAAGGG
CGCGTAGGCGGTTTATTGGGTGTGATGTGAAAGCCTTGGGCTTAACCTGAGAAGTGCATC
GCGAACTAATAGACTTGAGTAGATGAGAGGGTGGCGGAATTTCCGGTGTAGCGGTGAAAT
GCGTAGAGATCGGAAGGAACGTCAATGGCGAAGGCAGCCACCTGGCATCATACTGACGCT
GAGGCGCGAAAGCGTGGGGAGCGAACAGGATTAGATACCCCCGTAGTCC
>Otu2902
CCAGCCTATGGGTGGCACCAGTAGGGAATATTGCACAATGGAGGAAACTCTGATGCAGCG
ACGCCGCGTGAGCGATGAAGGCCTTCGGGTTGTAAAGCTCTGTCGTATGGGAATAAGCAA
GTGAATGTACCATGCAAGAAAGGATCGGCTAACTTCGTGCCAGCAGCCGCGGTAAGACGA
GGGATCCTAGCGTTGTTCGGAATCATTGGGCGTAAAGCGGGTGCATGTGGCTCTGTAAGT
CAGGTGTGAAAGCCCAGGGCTTAACCCTGGAAGTGCATTTGATACTGCAGAGCTTGAGTG
CTGGAGAGGCTATTAGAATTCCTGGTGTAGTGGTGAAATACGTAGATATCAGGAGGAATA
CCGGAGGCGAAGGCGGATAGCTGGCCAGACACTGACACTCAGACCCGAAAGCGTGGGGAT
CAAACAGGATTAGAGACCCTCGTAGTCC
>Otu2903
CCAGCCTATGGGACGCTGCAGTGGGGAATTTTGCACAATGGACGCAAGTCTGATGCAGCG
ACGCCGCGTGAGTGAAGAAGGCCTTCGGGTTGTAAAGCTCTGTCGAATGGGACAAAGAGA
TATCTGCAATAATACGGCAGGTAAGTGATGTTACCATTAGAGGAAGCACCGGCTAACTCC
GTGCCAGCAGCCGCGGTAATACGGAGGGTGCAAGCGTTGTTCGGAATTATTGGGCGTAAA
GGGCAGGTAGGTGGTCTCAAAAGTCTACTGTGAAATCCCCGAGCTTAACTTGGGAAGTGC
GGTGGATACTCTGAGACTCGAGTACTGGATGGGTGCGTGGAATTCCCGGTGTAGCGGTGA
AATGCGTAGAGATCGGGAGGAACACCAGAGGCGAAGGCGGCGACCTGGACAGGTACTGAC
ACTCAACTACGAAAGCGTGGGGAGCAAACAGGATTAGAAACCCCCGTAGTCC
>Otu2906
CCAGCTATGGGTTGCACCAGTCGAGAATCTTTCGCAATGGGCGAAAGCCTGACGAAGCAA
CGCTGTGTGTGTGATGAAGGCCTTCGGGTTGTAAAGCACTTTCGCCTAAGAACAAGAGAG
CGTTATTAATACCAACGCAATTTGAGAGTACTAGGTAAAGAAGCACCGGCTAACTACGTG
CCAGCAGCTGCGGTAATACGTAGGGTGCAAGCATTAATCGGATTTATTGGGCGTAAAGGG
CGCGTAGGCGGAGGGGTAAGTCAGATGTGAAAGCCCGCAGCTCAACTGCGGAACAGCATT
TGAAACTACCCCCCTAGAGGGTAGGCGGAGAAAACGGAATTCCACAAGTAGCGGTGAAAT
GCGTAGATATGTGGAAGAACACCAGTGGCGAAGGCGGTTTTCTAGCTTATACCTGACGCT
GAGGCGCGAAAGCAAGGGGAGCAAACAGGATTAGAGACCCCAGTAGTCC
>Otu2908
CCAGCCTACGGGACGCTGCAGTGAGGAATATTGCACAATGGGCGAAAGCCTGATGCAGCG
ACACCGCGTGAAGGATGAAGGTCTTAGGATTGTAAACTTCTGTTAAGCGGGAAGAAAAGT
TTGTTGTTAATACCAACAAGAAATGACGGTACCGCTAGAGAAAGCACCGGCTAACTTCGT
GCCAGCAGCCGCGGTAATACGAAGGGTGCAAGCGTTATTCGGATTGATTGGGCGTAAAGG
GTGCGCAGACGGTAGGCTAAGTCCGTTAGTCAAATCCCTCAGCTTAACTGAGGAACAGCT
GCGGATACTGGTGTGCTAGAGGGTGGGAGAGAGAAGTGGAATTCCCGGAGTAGCGGTAAA
ATGCGTAGATCTCGGGAGGAACACCAATGGCGAAGGCAGCTTCTTGGCCCATTCCTGACG
TTGAGGCACGAAAGCGTGGGGAGCAAACAGGATTAGAAACCCTCGTAGTCC
>Otu2916
CCAGCCTATGGGTGGCAGCAGTGGGGAATCTTGCGCAATGGGCGAAAGCCTGACGCAGCG
ACGCCGCGTGGGTGATGAAGGCCTTCGGGTTGTAAAGCCCTGTGGGGAGGGAAGAATGAA
CCAGGGTGAACAATCCTGGCGATGACGGTACCTCCTTAGCAAGCACCGGCTAACTCTGTG
CCAGCAGCCGCGGTAATACAGAGGGTGCAAACGTTGTTCGGATTTACTGGCGTAAAGCGC
GTGTAGGCGGTTTTCCAAGTCGGACGTGAAAGCCCGGGGCTCAACCTCGGAAGTGCGCTC
GATACTGGGAGACTGGAGTCTCGGAGAGGATGGTGGAACTCTCGGTGTAGAGGTGAAATT
CGTAGATATCGAGAAGAACACCGGCGGCGAAGGCGGCCATCTGGACGAAGACTGACGCTG
AGACGCGAAAGCGTGGGGAGCAAACAGGATTAGATACCCCTGTAGTCC
>Otu2920
CCAGCCTATGGGATGCAGCAGTGGGGAATATTGCACAATGGGCGAAAGCCTGATGCAGCG
ACGCCGCGTGAGGGATGAAGGCCTTCGGGTTGTAAACCTCTTTCAGCAGGGACCAAGCGC
AAGTGAGTGTACCTGCAGAAGAAGCACCGGCTAACTACGTGCCAGCAGCCGCGGTAATAC
GTAGGGTGCGAGCGTTGTCCGGATTTATTGGGCGTAAAGGGCTCGTAGGCGGCCTGTCGC
GTCGGAAGTGAAAGCCCACTGCTTAACGGTGGGTCTGCTTCCGGTACGGGCAGGCTGGAG
TATGGCAGGGGAGACTGGAATTCCTGGTGTAGCGGTGGAATGCGCAGATATCAGGAGGAA

CACCGGTGGCGAAGGCGGGTCTCTGGGCCATTACTGACGCTGAGGAGCGAAAGCGTGGGG
AGCGAGCAGGATTAGATACCCTGGTAGTCC
>Otu2921
CCAGCCTACGGGGGGCTCCAGCCGAGAATATTCGACAATGGGCGAAAGCCTGATCGAGCG
ACGCCGCGTGATTGATGAAGTCTTTCGGGATGTAAAGATCTTTTATGGGGGAGAAAGCCA
TGTTTGACATGGTTGATGGTACTCCATGAATAAGGGGTTGCTAAACTCGTGCCAGCAGCA
GCGGTAATACGAGTGCCCCGAGCGTTATCCGGAATTATTGGGCGTAAAGGGTGTGTAGGT
GGTTGTGTTAGTCTTTTGTTAAAACTCTTGGCTTAACCAAGAAGGTGCGAAAGAAACGGC
ACGACTAGAGAGTGCGAGAGGTGAGCGGAACTCATGGTGTAGGGGTGAAATCCGTTAATA
TCATGGGGAACACCAAAGGCGAAGGCAGCTCACTGGCGTACTTCTGACACTGAAACACGA
AAGCGTGGGTAGCGAATGGGATTAGAAACCCCGGTAGTCC
>Otu2923
CCAGCCTACGGGATGCACCAGTGGGGAATTTTGCGCAATGGGGGAAACCCTGACGCAGCA
ACGCCGCGTGGAGGATGAAGCCCCTTGGGGCGTAAACTCCTTTCGATCGGGAAGATAATG
ACGGTACCGGAAGAAGAAGCCCCGGCTAACTTCGTGCCAGCAGCCGCGGTAATACGAGGG
GGGCGAGCGTTGTTCGGAATTATTGGGCGTAAAGGGCGCGTAGGCGGTTGGACAAGTCTT
GTGTGAAATCTTCAGGCTCAACCTGAAGTCTGCATAGGAAACTGTCCGGATTGAGTGTGG
GAGAGGTGCGTGGAATTCCCGGTGTAGCGGTGAAATGCGTAGATATCGGGAGGAACACCT
GTGGCGAAAGCGGCGCACTGGACCACAACTGACGCTGAGGCGCGAAAGCTAGGGGAGCAA
ACAGGATTAGATACCCTTGTAGTCC
>Otu2924
CCAGCCTATGGGTGGCACCAGTCAAGAACTTTCCACAATGGACGCAAGTCTGATGGAGCG
ACGCCGCGTGATTGATGAAGTCCTTCGGGACGTAAAGATCTTTTATGAGGGAAGAAGTTT
ATTGACGGTACCTCATGAATAAGGGGCTCCTAATCTCGTGCCAGCAGGAGCGGTAATACG
AGAGCCCCGAGCGTTATCCGGAATTATTGGGCGTAAAGGGTGCGTAGGTGGTTTTGTTAG
TCGTTTGTCAAATCCCAGAGCTTAACTTTGGACCCGCAAACGAAACGGCAAGACTAGAGG
ATGTGAGAGGTATAGGGAACTCATGGTGTAGGGGTGAAATCCGTTGATATCATGGGGAAC
ACCGAAAGCGAAGGCACTATACTGGCACACTCCTGACACTCAAGCACGAAAGCGTGGGTA
GCGAGTGGGATTAGATACCCGCGTAGTCC
>Otu2931
CCAGCCTATGGGGGGCAGCAGTCGAGAATTTTTCTCAATGGGGGAAACCCTGAAGGAGCG
ACGCCGCGTGGAGGATGAAGGTCTTCGGATTGTAAACTCCTGTCATTTGAGAACAAGGGG
TTGGGGAGTCACTGCCCTGATCATTGATAGTATCAGAAGAGGAAGAGACGGCTAACTCTG
TGCCAGCAGCCGCGGTAATACAGAGGTCTCAAGCGTTGTTCGGATTCATTGGGCGTAAAG
GGTGCGTAGGCGGTTCCGTAAGTCGGATGTGAAATCTTGGGGCTCAACCCCAAAACGGCA
TTCGATACTGCGGAGCTAGAGGACTGGAGAGGAGACTGGAATTCACGGTGTAGCAGTGAA
ATGCGTAGATATCGTGAGGAAGACCAGCGGCGAAGGCGGGTCTCTGGACAGTTCCTGACG
CTGATGCACGAAGGCCAGGGGAGCGAACGGGATTAGAAACCCTAGTAGTCC
>Otu2934
CCAGCCTATGGGGAGCACCAGTCGAGGATCTTCGTCAATGGGCGCAAGCCTGAACGAGCG
ATGCCGCGTGCGCGATGAAGGCCTTCGGGTTGTAAAGCGCGAAAGAGTGAATAAAGGCGA
AAGCTTGATTGATACTCAGTAAGCTCGGGCTAAGTTCGTGCCAGCAGCCGCGGTAAGACG
AACCGAGCGAACGTTGTTCGGAATCACTGGGCTTAAAGGGCGCGTAGGCGGGTGGACAAG
TCTGTGGTGAAATACTTCAGCTCAACTGGAGAACTGCCGTGGATACTGTTCATCTCGAGG
AAGGTAGGGGCAAGTGGAACTGGTGGTGGAGCGGTGAAATGCGTTGATATCATCAGGAAC
TCCGGTGGCGAAGGCGACTTGCTGGACCTTTTCTGACGCTGAGGCGCGAAAGCCAGGGGA
GCAAACGGGATTAGATACCCGAGTAGTCC
>Otu2937
CCAGCCTATGGGACGCAGCAGTCGAGAATTTTTCTCAATGGGCGAAAGCCTGAAGGAGCG
ACGCCGCGTGGGGGATGAAGGGCTTCGGCCCGTAAACCCCTGTCATTTGCGAACAAACCT
TACCATTTAATAGGTGGTGAGCTGATTGTAGCGAAAGAGGAAGGGACGGCTAACTCTGTG
CCAGCAGCCGCGGTAATACAGAGGTCCCAAGCGTTGTTCGGATTCACTGGGCGTAAAGGG
TGCGTAGGTGGTGGGGTAAGTTTGATGTGAAATCTCCGGGCTTAACCCGGAAACTGCATT
GAACACTATCTCACTCGAGGCTTGGAGGGGGGACTGGAATTCTCGGTGTAGCAGTGAAAT
GCGTAGATATCGAGAGGAACACCAGTGGCGAAGGCGAGTCCCTGGACAACACCTGACACT
GAGGCACGAAAGCTAGGGGAGCAAACAGGATTAGATACCCGAGTAGTCC
>Otu2944
CCAGCCTATGGGGTGCTGCAGTGGGGAATATTGGACAATGGGCGCAAGCCTGATCCAGCG

ATGCCGCGTGTGTGAAGAAGGCCTGCGGGTTGTAAAGCACTTTCGGTGGGGAGGAAAGAC
TCTAGGCTAATACCCTGGAGAGTTGACGTTACCCACAGAAGAAGCACCGGCTAACTCTGT
GCCAGCAGCCGCGGTAATACAGAGGGTGCAAGCGTTAATCGGAATTACTGGGCGTAAAGG
GTGCGTAGGTGGTTTGATAAGTTAGGTGTGAAAGCCCTGGGCTTAACCTGGGAATTGCGC
CTAATACTGTCAAACTAGAGTACTGTAGAGGGAGGTGGAATTTCCGGTGTAGCGGTGAAA
TGCGTAGATATCGGAAGGAACATCAGTGGCGAAGGCGACTTCCTGGACAGATACTGACAC
TGAGGCACGAAAGCGTGGGGAGCGAACAGGATTAGAAACCCCTGTAGTCC
>Otu2945
CCAGCCTACGGGGGGCTGCAGTGGGGAGTTTTGCGCAATGGACGAAAGTCTGACGCAGCG
ACGCCGCGTGGGTGAAGAAGGCCTTCGGGTTGTAAAGCCCTGTCGAGTGGGACAAATGGC
ATCCGTAATAATACGACGGGTGCGTGATGGTACCACTAAAGGAAGCACCGGCTAACTCCG
TGCCAGCAGCCGCGGTAATACGGAGGGTGCAAGCGTTGTTCGGAATTATTGGGCGTAAAG
GGCAGGTAGGTGGTCTTAGAAGTCAACTGTGAAAGCCCCGAGCTTAACTTGGGAAGTGCG
GTTGATACTCTTAGACTAGAGTGCTGGATGGGTGCGTGGAATTCTCGGTGTAGCGGTGAA
ATGCGTAGATATCGAGAGGAACACCAGAGGCGAAAGCGGCGCACTGGACAGTAACTGACA
CTCAGCTGCGAAAGCGTGGGGAGCAAACAGGATTAGAACCCCGGTAGTCC
>Otu2947
CCAGCCTATGGGTTGCTGCAGTCAAGAACTTTCCACAATGGACGAAAGTCTGATGGAGCG
ACGCCGCGTGTGGGAAGAAGGCCCTCGGGTCGTAAACCGCTGTCGAAGGGGACGATTTTG
ACGGTACCCTTGGAGGAAGCCCCGGCTAACTACGTGCCAGCAGCCGCGGTAAGACGTAGG
GGGCGAGCGTTATTCGGAATCATTGGGCGTAAAGCGCTCGTAGGCGGCTCCGCAAGTCCG
TGAAGAAAGACCTGGGCTCAACCCAGGGAACGGCACGGATACTGCGGGGATTGAGGGAAT
CAGAGGGTGATGGAATTCCCGGTGTAGCGGTGAAATGCGTAGATATCGGGAGGAACACCA
GTGGCGAAGGCGATCACCTGGGGTTCAACTGACGCTGAGGAGCGAAAGCTAGGGGAGCAA
ACGGGATTAGAGACCCGAGTAGTCC
>Otu2951
CCAGCCTACGGGGGGCAGCAGTAGGGAATCTTGCGCAATGGGGGAAACCCTGACGCAGCC
ATGCCGCGTGAATGATGAAGGTCTTAGGATTGTAAAATTCTTTCAGCGGGGAAGATAATG
ACTGTACCCGCAGAAGAAGCTCCGGCTAACTTCGTGCCAGCAGCCGCGGTAATACGAAGG
GGGCTAGCGTTGCTCGGAATTACTGGGCGTAAAGGGCGCGTAGGCGGGTGTTTAAGTCAG
AGGTCAAATCCCAGGGCTCAACCCTGGAACTGCCTTTGATACTGGGCACCTAGAGTACGG
GAGAGGTGTGTGGAACTCCGAGTGTAGAGGTGAAATTCGTAGATATTCGGAAGAACACCA
GTGGCGAAGGCGACACACTGGCCCGTTACTGACGCTGAGGCGCGAAAGCGTGGGGAGCAA
ACAGGATTAGAAACCCCCGTAGTCC
>Otu2952
CCAGCCTATGGGTTGCAGCAGTCGAGAATCTTCCGCAATGGGCGCAAGCCTGACGGAGCG
ACGCCGCGTGGTTGATGAGGTCCTTAGGGACGTAAAAACCTTTTATGAGCAAAAAAGTAA
TTGATTAGCTCATGAATAAGGGGTTGCTAAACTCGTGCCAGCAGCAGCGGTAATACGAGT
GCCCCAAGCGTTATCCGGAATCATTGGGCGTAAAGGGTGTGTAGGTGGTTGTATTAGTCT
TCTGTTAAATTCTTCGGCTCAACCGGGGACCTGCGGAGGAAACGGTACGACTAGAGGATG
CGAGGGGAATATGGAACTCATAGTGGAGGGGTGAAATCCGTTGATATTATGGGGAACACC
GAAAGCGAAGGCAGTATTCTGGCGCATTCCTGACACTGAAACACGAAAGCGTGGGTAGCG
AATGGGATTAGATACCCCTGTAGTCC
>Otu2953
CCAGCCTACGGGTTGCTGCAGTCGAGGATCTTCGGCAATGGGCGCAAGCCTGACCGAGCG
ACGCCGCGTGTGCGATGAAGGCCTTCGGGTTGTAAAGCACTGTCGAGGGGGAGAGACGCC
CGCAAGGGCTTGATCTATCCCTGGAGGAAGCACGGGCTAAGTTCGTGCCAGCAGCCGCGG
TAAGACGAACCGTGCGAACGTTGTTCGGAATCACTGGGCTTAAAGGGCGCGTAGGCGGCC
CATCAAGTCAGGGGTGAAATCCTCCAGCTTAACTGGAGAAGTGCCTTTGATACTGGTGGG
CTCGAGGGGTGCAGGGGCATGTGGAACTTCCGGTGGAGCGGTGAAATGCGTAGATATCGG
AAGGAACGCCGGTGGCGAAAGCGACGTGCTGGACACCTTCTGACGCTGAGGCGCGAAAGC
CAGGGGAGCAAACGGGATTAGATACCCGCGTAGTCC
>Otu2961
CCAGCCTACGGGTGGCTGCAGTGGGGAATATTGGACAATGGGGGCAACCCTGATCCAGCA
ACGCCGCGTGAATGAAGAAGGCCCTCGGGTTGTAAAGTTCTTTTGCGTGGGAACAAAAGT
AATGTGCTAATACCACATTGCGTTGATAGTACCACGAGAATAAGCACCGGCTAACTACGT
GCCTGCAGCCGCGGTAATACGTAGGGTGCAAGCGTTAATCGGAATTACTGGGCGTAAAGG
GTGCGCAGGCGGCTATATAAGTCAGATGTGAAATACCTAAGCTTAACTTGGGGGGTGCAT

TTGATACTGTATGGCTAGAGTTAGTCAGAGGGGGGTAGAATTCCTGGTGTAGCGGTGAAA
TGCGTAGAGATCAGGAGGAATACCAATGGCGTAGGCAGCCCCCTGGGACAAAACTGACGC
TCATGTACGAAAGCGTGGGAAGCAAACAGGATTAGAGACCCGAGTAGTCC
>Otu2966
CCAGCCTACGGGTGGCTCCAGCGGGGAATATTGGACAATGGGGGAAACCCTGATCCAGCA
ATGCCGCGTGTGTGAAGAAGGCCTTCGGGTTGTAAAGCACTTTCAGTGGGGAGAAAGGGG
TTTGCGTTAATACCGCAAATCATTGATGTTACCCAAAGAAGAAGCACCGGCTAACTCTGT
GCCAGCAGCCGCGGTAATACAGAGGGTGCAAGCGTTAATCGGAATGACTGGGCGTAAAGC
GTGTGTAGGTGGTTGGATAAGTTAGATGTGAAATCCCCGGGCTTAACCTGGGAACTGCGT
TTAAAACTGTTCAGCTAGAGTACTGTAGAGGATAGTGGAATTTCCAGTGTAGCGGTGAAA
TGCGTAGATATTGGAAGGAACACCAGTGGCGAAGGCGACTGTCTGGACAGATACTGACAC
TGAGACACGAAAGCGTGGGGAGCAAACAGGATTAGATACCCGTGTAGTCC
>Otu2970
CCAGCCTACGGGGTGCTGCAGTTAGGAATTTTCCGCAATGGGCGAAAGCCTGACGGAGCA
ACGCCGCGTGAGTGATGAAGACCCTAGGGTTGTAAAGCTCTGTCGGAGGGGAAGAAATAT
GACGGTACCCTCCAAGAAAGCCCCGGCTAACTACGTGCCAGCAGCCGCGGTAATACGTAG
GGGGCGAGCGTTATCCGGATTTACTGGGCGTAAAGCGCGTTAAGGCGGTTGGCAAAGTTG
GTTGTGAAATCTCAGGGCTCAACCCTGAAATTGCGACCAATACTGGTCGACTAGAGTGCA
AGAGAGGAAGGTGGAATTCCCGGTGTAGTAGTGAAATGCGTAGATATCGGGAGGAACACC
AGTGGCGAAGGCGGCCTTCTGGCTTGTAACTGACGCTGTAGCGCGAAAGCGTGGGGAGCG
AACCGGATTAGAAACCCTCGTAGTCG
>Otu2971
CCAGCCTATGGGGGGCAGCAGTGGGGAATATTGCGCAATGGGCGAAAGCCTGACGCAGCG
ACGCCGCGTGGAGGATGAAGGTCTTCGGATTGTAAACTTCTGTTAAGTGGGAAAAAAGTT
CATTTCTTAATACGAAATGAAGATGATGGTACCACTAAAGAAAGCACCGGCTAATCTCGT
GCCAGCAGCCGCGGTAATACGAGAGGTGCTAGCGTTATTCGGATTTATTGGGTGTAAAGG
GCGCGTAGACGGCGTGTTAAGTTTATTGTTAAATCCTCCGGCTCAACTGGAGATAGGCGG
TTAATACTGGCATGCTAGAGGATGGAAGAGAGAAGTGGAATTCTCGGAGTAGCGGTAAAA
TGCGTAGATCTCGAGAGGAACACCGATGGCGAAGGCAGCTTCTTGGTCCACTCCTGACGT
TGAGGCGCGAAAGCGTGGGGAGCAAACAGGATTAGAAACCCCAGTAGTCC
>Otu2973
CCAGCCTACGGGGTGCTCCAGTGGGGAATATTGGACAATGGGCGCAAGCCTGATCCAGCC
ATGCCGCGTGAGTGAAGAAGGCCTTAGGGTTGTAAAGCCCTTTTGGCGGGGACGATAATG
ACGGTACCCGCAGAATAAGCCCCGGCTAACTTCGTGCCAGCAGCCGCGGTAATACGAAGG
GGGCTAGCGTTGTTCGGAATCACTGGGCGTAAAGCGCACGCAGGCGGATTGATAAGTCAG
GGGTGAAATCCCGGAGCTCAACTTCGGAATTGCCTTTGATACTGAGGATCTTGAGTTCGG
GAGAGGTGAGTGGAACTGCGAGTGTAGAGGTGAAATTCGTAGATATTCGCAAGAACACCA
GTGGCGAAGGCGGCTCACTGGCCCGATACTGACGCTGAGGCACGAAAGCGTGGGGAGCAA
ACAGGATTAGATACCCGAGTAGTCC
>Otu2975
CCAGCCTACGGGTCGCTCCAGGGGGGAATATTGCGCAATGGGCGAAAGCCTGACGCAGCG
ACGCCGCGTGGAGGATGAAGGTCTTAGGATTGTAAACTCCTGTCAGATGGAAAGAAAGGT
CTGCGTTTAATAGACGCGGAATATGACGGTACCATCAGAGGAAGCACCGGCTAACTTCGT
GCCAGCAGCCGCGGTAATACGAAGGGTGCAAGCGTTATTCGGAATCACTGGGCGTAAAGG
GTGTGTAGACGGTATGTTAAGTTCGTCGTTAAAGGCTCCGGCCTAACTGGAGTATTGCGA
TGAAAACTGGCGTGCTAGAGGTGAGAGAGAGAAGTGGAATTCCCGGAGTAGCGGTAAAAT
GCGTGGATCTCGGGAGGAACACCAATGGCGAAGGCAGCTTCTTGGCTCATTCCTGACGTT
GAGGCACGAAAGCGTGGGGAGCAAACAGGATTAGAAACCCTAGTAGTCC
>Otu2976
CCAGCCTATGGGGCGCAGCAGTGGGGAATATTGGACAATGGGCGCAAGCCTGATCCAGCC
ATGCCGCGTGAGTGATGACGGCCTTAGGGTTGTAAAGCTCTTTCGCTAGGGACGATAATG
ACGGTACCAGTGGAAGAAGCCCCGGCTAACTTCGTGCCAGCAGCCGCGGTAATACGAGGG
GGGCAAGCGTTGTTCGGAATTATTGGGCGTAAAGGGCGCGTAGGCGGTGCGGCAAGTCAC
CTGTGAAACCTCCGGGCTCAACCCGGAGCCTGCAGGCGAAACTGCCGTGCTGGAGTATGG
TAGAGGTGCGTGGAATTCCCGGTGTAGCGGTGAAATGCGTAGATATCGGGAGGAACACCT
GTGGCGAAAGCGGCGCACTGGACCATAACTGACGCTGAGGCGCGAAAGCTAGGGGAGCAA
ACAGGATTAGAAACCCCTGTAGTCC
>Otu2981

CCAGCCTACGGGGGGCTGCAGTGGGGAATTTTGGACAATGGGCGCAAGCCTGATCCAGCC
ATTCCGCGTGAGTGAAGAAGGCCTTCGGGTTGTAAAGCTCTTTCGGCGGGGAAGAAATCG
GTAAGGATAATACCTTTACTGGACGACGGTACCTGAAGAAGAAGCACCGGCTAACTACGT
GCCAGCAGCCGCGGTAATACGTAGGGTGCGAGCGTTAATCGGAATTACTGGGCGTAAAGG
GTGCGCAGGCGGCTCTATAAGACAGATGTGAAATCCCCGGGCTCAACCTGGGAACTGCGT
TTGTGACTGTAGGGCTAGAGTACGGCAGAGGGGGGTAGAATTCCACGTGTAGCAGTGAAA
TGCGTAGAGATGTGGAGGAATACCGATGGCGAAGGCAGCCCCCTGGGTCGATACTGACGC
TCATGCACGAAAGCGTGGGGAGCAAACAGGATTAGATACCCTCGTAGTCC
>Otu2983
CCAGCCTATGGGATGCTGCAGTGGGGAATATTGCGCAATGGGCGAAAGCCTGACGCAGCA
ACGCCGCGTGAGTGAGGAAGGCCTTCGGGTTGTAAAGCACTTTCGGTTGGGAAGAAGGGA
ATTAGTCCTAATAAGATTGGTTCTTGATGGTACCAAAAGAAGAAGCACCGGCTAACTTCG
TGCCAGCAGCCGCGGTAATACGAAGGGTGCGAGCGTTATTCGGATTTATTGGGCGTAAAG
GGTTCGTAGGCGGGAAATCAAGTCAAGTGTGAAATATCCAGGCTTAACCTGGAAAGTGCA
TTTGAAACTGTTTTTCTTGAGTGTTGAAGAGGGTGGTGGAATTGCTGGTGTAGGAGTGAC
ATCCGTAGAGATCAGCAGGAACATCGGAGGCGAAGGCGGCCACCTGGTCAATCACTGACG
CTGAGGAACGAAAGCATGGGGAGCAAACAGGATTAGATACCCTCGTAGTCC
>Otu2986
CCAGCCTACGGGATGCACCAGTCGAGAATCTTCCGCAATGGGCGAAAGCCTGACGGAGCG
ACGCCGCGTGACTGATGAAGTCCTTAGGGACGTAAAGGTCTTTTATGAGGGAAGAAGTTT
ATTGACGGTACCTCAGGAATAAGGGGCTCCTAATCTCGTGCCAGCAGGAGCGGTAATACG
AGAGCCCCGAGCGTTATCCGGAATTACTGGGCGTAAAGGGTGCGTAGGTTGCATTGTTAG
TCACACGTTAAAACCCCAAGCTTAACTTGGGAAGAGCGTTTGAAACGGCAATGCTAGAGA
GTGCGAGGGGCATGCGGAACTCATGGTGTAGGGGTGAAATCCGTTGATATCATGGGGAAC
ACCAAAAGCGAAGGCAGCATGCTGGCGCATTTCTGACGCTGAAGCACGAAAGCGTGGGTA
GCGAATGGGATTAGATACCCGAGTAGTCC
>Otu2988
CCAGCCTACGGGGGGCAGCAGTAAGGAATATTGGTCAATGGACGAAAGTCTGAACCAGCC
ATGCCGCGTGCAGGATGAGAGCCTTATGGGTTTTAAACTGCTTTTATCGGGGAGTAAGAA
TGATTACGAGTAATTATTTAGCAAGTACCTGAGGAATAAGGATCGGCTAACTCCGTGCCA
GCAGCCGCGGTAATACGGAGGATTCGAGCGTTATCCGGATTTATTGGGTTTAAAGGGTGC
GTAGGCGGTTAATTAAGTAAGTGGTGAAATGTTTGTGCTTAACAGGAACAGTGCCATTTA
AACTGATTGACTTGAGTACAGATGAAGTTGGCGGAATGTGTCAAGTAGCGGTGAAATGCA
TAGATATGACACAGAACACCGACAGCGAAGGCAGCTGACTAAGGTGTAACTGACGCTGAG
GCACGAAGGCGTGGGGATCAAACAGGATTAGAAACCCTCGTAGTCC
>Otu2990
CCAGCCTATGGGTGGCAGCAGTAAGGAATATTGGTCAATGGACGCAAGTCTGACCGAGCG
ACGCCGCGTGCGGGATGAAGGCCTTCGGGTTGTAAACCGCTGTCAGAGGGGATGAAATTC
ATTTGGGTTCTCCCAGATGATTGACAGAGCCTCAGAGGAAGCACGGGCTAAGTACGTGCC
AGCAGCCGCGGTAATACGTACTGTGCGAACGTTATTCGGAATCACTGGGCTTAAAGGGTG
TGTAGGCGGCCAGCTAAGTAGGGTGTGAAAGGCCTCGGCCTAACCGAGGCATTGCGCTCT
AAACTGACTGGCTTGAGTGAGATAGGGGTGTGCGGAACTTCCAGTGGAGCGGTGAAATGT
GTTGATATTGGAAGGAACGCCGGTGGCGAAAGCGGCACACTGGGTCTCGTCTGACGCTGA
GACACGAAAGCCAGGGGAGCGAACGGGATTAGATACCCTTGTAGTCC
>Otu2996
CCAGCCTATGGGGGGCTCCAGTGGGGAATCTTGCACAATGGGGGCAACCCTGATGCAGCG
ACGCCGCGTGAGCGATGAAGCCCTTCGGGGTGTAAAGCTCTTTCGGCAGGAACGATAATG
ACGGTACCTGAAGAAGAAGCTGCGGCTAACTACGTGCCAGCAGCCGCGGTAATACGTAGG
CAGCAAGCGTTGTTCGGAGTTACTGGGCGTAAAGAGTGTGTAGGCGGCGTTCTAAGTCTG
GTGTGAAATCTCCCGGCTCAACTGGGAGGGTGCGCCGGAGACTGGGATGCTTGAGTATGG
GAGAGGAAAGCGGAATTCCTGGTGTAGCGGTGAAATGCGTAGGTATCAGGAGGAACACCT
GCGGTGTAGACGGCTTTCTGGACCATCACTGACGCTGAGACACGAAAGCGTGGGTAGCAA
ACAGGATTAGATACCCTTGTAGTCC
>Otu2997
CCAGCCTACGGGGCGCTGCAGTGGGGAATTTTGCGCAATGGGGGAAACCCTGACGCAGCA
ACGCCGCGTGGAGGATGAAGTATCTTGGTACGTAAACTCCTTTCGATGGGGAAGATTATG
ACGGTACCCATAGAAGAAGCCCCGGCTAACTTCGTGCCAGCAGCCGCGGTAATACGTAGG
GTGCAAGCGTTAATCGGAATTACTGGGCGTAAAGCGTGCGCAGGCGGTTCGCTAAGACAG

ATGTGAAATCCCCGGGCTTAACCTGGGAACTGCATTTGTGACTGGCGGGCTAGAGTATGG
CAGAGGGGGGTAGAATTCCACGTGTAGCAGTGAAATGCGTAGAGATGTGGAGGAATACCG
ATGGCGAAGGCAGCCCCCTGGGCCAATACTGACGCTCATGCACGAAAGCGTGGGGAGCAA
ACAGGATTAGATACCCGTGTAGTCC
>Otu2998
CCAGCCTATGGGTTGCTCCAGTGGGGAATCTTGCACAATGGGGGAAACCCTGATGCAGTG
ACGCCGCGTGAGCGATGAAGCCCCTCGGGGTGTAAAGCTCTTTCGGCAGGGAAGATTATG
ACGGTACCTGCAGAAGCAGCTGCGGCTAACTACGTGCCAGCAGCCGCGGTAATACGTAGG
CAGCAAGCGTTGTTCGGAATTACTGGGCGTAAAGAGTGCGTAGGCGGTGTTCTAAGTCTG
TTGTGAAATCTCCTGGCTCAACTGGGAGGGTGCGGCGGAGACTGGAATGCTAGAGTATGG
GAGAGGTAAGCGGAATTCCTGGTGTAGCGGTGAAATGCGTAGATATCAGGAGGAACACCT
GTGGTGTAGACAGCTTACTGGACCATGACTGTCGCTGAGGCACGAAAGCGTGGGTAGCAA
ACAGGATTAGAAACCCCTGTAGTCC
>Otu3000
CCAGCCTACGGGAGGCAGCAGTGGGGAATCTTGCGCAATGGGCGAAAGCCTGACGCAGCA
ACGCCGCGTGGGGGATGAAGGCTCTCGGGTCGTAAACCCCTTTCAGTGGGAACGAAAATG
ACGGTACCCACAGAAGAAGGACCGGCCAACTACGTGCCAGCAGCCGCGGTAACACGTAGG
GTCCGAGCGTTGTCCGGATTTATTGGGCGTAAAGGGCCCGTAGGCGGCTTGATAAGTCGG
GTGTGAAAACTCCAGGCTCAACCTGGAGACGCCACCCGATACTGTCATGGCTTGAGTCTG
GTAGGGGAGTGCGGAATTCCTGGTGTAGCGGTGAAATGCATAGATATCAGGAGGAACACC
GGTGGCGAAGGCGGCACTCTGGGCCAGTACTGACGCTGATGGGCGAAAGCGTGGGGAGCA
AACAGGATTAGAAACCCTCGTAGTCC
>Otu3003
CAGCCTACGGGTGGCAGCAGTGGGGAATATTGGACAATGGGGGCAACCCTGATCCAGCGA
TGCCGCGTGGGTGAAGAAGGCCTTCGGGTTGTAAAGCCCTTTAGGTTGGGACGAAGGATG
TTGGGTTAATACCCCAGCATTTTGACGGTACCGACAGAATAAGCACCGGCAAACTCTGTG
CCAGCAGCCGCGGTAAAACAGAGGGTGCGAGCGTTAATCGGAATTACTGGGCGTAAAGGG
CGCGTAGGCGGTCGAGTGAGTGTGATGTGAAAGCCCTGGGCTTAACCTAGGAAGTGCATC
GCAAACGACTCGACTGGAGTATAAGAGAGGGTGGCGGAATTTCCGGTGTAGCGGTGAAAT
GCGTAGAGATCGGAAGGAACGTCAATGGCGAAGGCAGCCACCTGGCTTAATACTGACGCT
GAGGCGCGAAAGCGTGGGGATCGAACAGGATTAGAAACCCGAGTAGTCC
>Otu3005
CCAGCCTACGGGTTGCAGCAGTAAGGAATATTGGTCAATGGGCGCAAGTCTGAACCAGCC
ATGCCGCGTGCAGGATGAAGGCCCTCTGGGTCGTAAACTGCTTTTATCTATGAAGAAATC
ACCTGTTTCTACGGGTGTTGACGGTAATAGAGGAATAAGCACCGGCTAACTCCGTGCCAG
CAGCCGCGGTAATACGGAGGGTGCAAGCGTTATCCGGATTTACTGGGTTTAAAGGGTGCG
TAGGCGGGTTGATAAGTCAGTGGTGAAAGCCCCGAGCCTAACTCGGGAACTGCCATTGAT
ACTATCAATCTTGAATTCAGTTGAGGTGGGCGGAATGTGTCGTGTAGCGGTGAAATGCTT
AGATATGACACAGAACACCAATTGCGAAGGCAGCTCGCTAAACTGACATTGACGCTGAGG
CACGAAAGCGTGGGGATCAAACAGGATTAGAAACCCCCGTAGTCC
>Otu3013
CCAGCCTACGGGTCGCACCAGTGGGGAATATTGGACAATGGGGGCAACCCTGATCCAGCA
ATGCCGCGTGAGTGATGAAGGCCTTCGGGTTGTAAAGCTCTTTTGTCGGGGACGATGATG
ACGGTACCCGACGAATAAGCTCCGGCTAACTTCGTGCCAGCAGCCGCGGTGATACGAAGG
GAGCTAGCGTTGTTCGGAATGACTGGGCGTAAAGGGCGCGTAGGCGGTTTTATAAGTTTG
ATGTGAAAGCCCTGGGCTTAACCTAGGAATAGCATTGGATACTGTAAGACTAGAGTCCGA
GAGGGGGAAATGGAATTGCGAGTGTAGAGGTGATATTCGTAGATATTCGCAAGAACACCA
GTGGCGAAGGCGATTTCCTGGTTCGGTACTGACGCTAAGGCGCGAAAGCGTGGGGAGCAA
ACAGGATTAGATACCCGAGTAGTCC
>Otu3016
CCAGCCTACGGGAGGCACCAGTAGGGAATTTTCCACAATGGGCGAAAGCCTGATGGAGCA
ACGCCGCGTGCAGGATGAATGTCTTCGGATTGTAAACTGCTTTTATCTGTGACGATTATG
ACGGTAGCAGATGAATAAGGATCGGCTAACTCCGTGCCAGCAGCCGCGGTCATACGGAGG
ATCCAAGCGTTATCCGGAATTACTGGGCGTAAAGAGTTGCGTAGGCGGCAAGTTAAGCAG
AGCATGAAAGCGTGTGGCTCAACCATACAGCTATGTTCTGAACTGGCTAGCTAGAGGACG
AGAGAGGTAACTGGAATTCCTAGTGTAGGAGTGAAATCCGTAGATATTAGGAGGAACACC
GATGGCGTAGGCAGGTTACTGGCTCGTTCCTGACGCTCAGGCACGAAAGCGTGGGTAGCG
AACTGGATTAGAAACCCGAGTAGTCC

>Otu3020
CCAGCCTATGGGTTGCACCAGTGGGGAATATTGCGCAATGGGCGAAAGCCTGACGCAGCG
ACGCCGCGTGGAGGATGAAGGTCTTCGGATTGTAAACTTCTGTTAAGTGGGAAAAAAGTG
TCATTCCTAATACGAATGAAAGATGATGGTACCACTAGAGAAAGCACCGGCTAATCTCGT
GCCAGCAGCCGCGGTAATACGAGAGGTGCTAGCGTTATTCGGATTAATTGGGTGTAAAGG
GTGCGTAGACGGCGCGTTAAGTTTGTTGTTAAATCCTCCGGCTTAACCGGAGATATGCGG
CAAAAACTGGCGTGCTAGAGGATGGAAGAGAGAAGTGGAATTCTCGGAGTAGCGGTAAAA
TGCGTAGATCTCGAGAGGAACACCGATGGCGAAGGCAGCTTCTTGGTCCATTCCTGACGT
TGAGGCACGAAAGCGTGGGGAGCAAACAGGATTAGAGACCCCCGTAGTCC
>Otu3021
CCAGCCTACGGGGGGCAGCAGTAGGGAATATTGCGCAATGGAGGAAACTCTGACGCAGCG
ACGCCGCGTGGAGGATGAAGGCTTTCGGGTCGTAAACTCCTGTCGGAAGGGACGAAAGTT
TTAGGGCTAATATCCCTAGAATCTGACGGTACCTTTAAAGGAAGCACCGGCTAACTACGT
GCCAGCAGCCGCGGTAATACGTAGGGTGCAAGCGTTGTTCGGAATTATTGGGCGTAAAGC
GCGTGTAGGTGGCTATATAAGTCGAGTGTGAAATCCCTGGGCTCAACCGAGGAAGTGCAC
TCGAAACTGCATAGCTAGAGGATAGTAGAGGAAGGTGGAATTCCTAGTGTAGAGGTGAAA
TTCGTAGATATTAGGAGGAATACCGGTGGCGAAGGCGACCTTCTGGGCTATTCCTGACAC
TGAGACGCGAAAGCATGGGTAGCGAACAGGATTAGATACCCTAGTAGTCC
>Otu3022
CAGCCTACGGGGGGCTGCAGTGAGGAATATTGGACAATGGGCGTAAGCTTGATCCAGCAA
TACCTACTGAGTGATGAAGAGAATTTTGTAAAACTTAGAGGATAAGGAAGATTGTGACTT
TACTTAATCCTTAGTCCTGGCTAACTTCGTGCCAGCAGCCGCGGTAAAACGGAGGGGACA
AGTGTTACTCATAATGACTGGGCGTAAAGGGTACGTAGATTGTTAAAAAAGTAGTGAGTA
AAATAAAAGTTTAATCACTTTTCATGCTTACTAAACTATTTAGCTTGAGTTAATTAGAGG
GTAGTGGTACTTTTGGTGGAGAGGTGATATTCGACGATATCAAAAGGACAATCGAAGGCG
AAGGCAACTACCTGGAGTTAACTGACGTTGAGGTACTGAAGCGTGGGGATCAAATCGGAT
TAGAAACCCCTGTAGTCC
>Otu3031
CCAGCCTACGGGTGGCAGCAGTGGGGAATCTTGCACAATGGGCGAAAGCCTGATGCAGCA
ATGCCGCGTGAGTGATGAAGGCCTTCGGGTTGTAAATCTCTGTCGGGCGGGAAAAATGTG
CTTATGGATAATATTCATAGGCAGTGATGGTACCGCTAAAGGAAGAGGCGGCTAATTCTG
TGCCAGCAGCCGCGGTAATACAGAAGCCTCGAGCGTTGTTCGGAATTACTGGGCGTAAAG
GGAATGTAGGTGGTGATGTAAGTCAGATGTGAAAGCCCCGGGCTTAACCTGGGAAGTGCA
TTTGATACTGCGTCGCTAGAGCACGAGAGAGGAAAGTGGAATTCCAGGTGTAGGGGTGAA
ATCCGTAGATATCTGGAGGAATACCAATGGCGAAGGCAGCTTTCTGGCTCGTAACTGACA
CTGAGATTCGAAAGCATGGGTAGCAAACAGGATTAGATACCCTCGTAGTCC
>Otu3034
GGACTACGCGGGTATCTAATCCTGTTTGATCCCCACGCTTTCGTGCCTCAGTGTCAGTTG
TAGTTTTGTAAGCTGCCTTCGCAATGGGTGTTCTGTGTCATATCTAAGCATTTCACCGCT
ACATGACACATTCCACTTACATCAAATACACTCAAGAACGACAGTATCAATGGCAGGTCT
GCAGTTGAGCTGCAGGTTTTCACCACTGACTTGACGTTCCACCTACGCACCCTTTAAACC
CAGTAAATCCGGATAACGCTCGGATCCTCCGTATTACCGCGGCTGCTGGCACGGAGTTAG
CCGATCCTTATTCTTACGGTACCATCAACCCTCGTAGAAACAAGGGGTTTTTTCCCGTAC
AAAAGCAGTTTACGATCCGTAGGACCTTCATCCTGCACGCGGCATGGCTGGTTCAGACTT
GCGTCCATTGACCAATATTCCTTACTGCTGCAACCCATAGGCTGG
>Otu3036
CCAGCCTATGGGGGGCAGCAGTGGGGAATATTGGACAATGGGGGGAACCCTGATCCAGCA
ATGCCGCGTGTGTGAAGAAGGCCTGAGGGTTGTAAAGCACTTTCAGTAGGGAGGAGTATT
TTCTGGTTAAGAGCTGGGGGATTGGACGTTACCTACAGAAGAAGCACCGGCTAACTCCGT
GCCAGCAGCCGCGGTAATACGGAGGGTGCGAGCGTTAATCGGAATTACTGGGCGTAAAGC
GTGCGTAGGTGGTTGAATAAGTTATCTGTGAAGTCCCTGGGCTCAACCTGGGCAGGTCAG
ATGATACTGTTTAACTCGAGTATGGGAGAGGGTAGTGGAATTTCCGGTGTAGCGGTGAAA
TGCGTAGAGATCGGAAGGAACACCAGTGGCGAAGGCGGCTACCTGGCCTAATACTGACAC
TGAGGCACGAAAGCGTGGGGAGCAAACAGGATTAGAGACCCGCGTAGTCC
>Otu3042
CCAGCCTACGGGACGCTCCAGTGGGGAATATTGGACAATGGGCGAAAGCCTGATCCAGCA
ATGCCGCGTGTGTGAAGAAGGCCTTCGGGTTGTAAAGCACTTTCAGTGGGGAGGAGGGTT
GATTGGTTAAGAGCTGATTGATTGGACGTTACCCACAGAAGAAGCACCGGCTAACTCCGT

GCCAGCAGCCGCGGTAATACGGAGGGTGCGAGCGTTAATCGGAATTACTGGGCGTAAAGG
GTGCGTAGGTGGTTAGTTAAGTTATCTGTGAAATCCCTGGGCTTAACCTGGGCAGGTCAG
ATAAGACTGATTGACTCGAGTATTGGAGAGGGTAGTGGAATTTCCGGTGTAGCGGTGAAA
TGCGTAGAGATCGGAAGGAACACCAGTGGCGAAGGCGGCTACCTGGCCAAATACTGACAC
TGAGGCACGAAAGCGTGGGGAGCAAACAGGATTAGATACCCTAGTAGTCC
>Otu3044
CCAGCCTATGGGGCGCACCAGTGAGGAATCTCGCACAATGGACGAAAGTCTGATGCAGCG
ACGCCGCGTGAAGGATGAAGGTCTTCGGATTGTAAACTTCTGTTAAGCGGGACGAAATAC
TCATACCTAATACGTATGAGAGATGACGGTACCGCTAGAGAAAGCACCGGCTAACTTCGT
GCCAGCAGCCGCGGTAATACGAGGGGTGCAAGCGTTATCCGGAATTATTGGGCGTAAAGG
GCGCGTAGGTTGCTTATTAAGTCTGTTGTTAAATCTCCCGGCCTAACCGGGAATCTGCGA
TGGATACTGATGAGCTAGAGGATGGAAGAGAGAGGTGGAATTCTCGGAGTAGCGGTAAAA
TGCGTAGATCTCGAGAGGAACACCGATGGCGAAGGCAGCCTCTTGGTCCATTCCTGACAC
TGATGCGCGAAAGCGTGGGGAGCAAACAGGATTAGATACCCCTGTAGTCC
>Otu3047
CCAGCCTATGGGGTGCTGCAGTGGGGAATTTTGGACAATGGGCGCAAGCCTGATCCAGCA
ATGCCGCGTGAGTGAAGAAGGCCTTCGGGTTGTAAAGCTCTTTTGTCAGGGAAGAAAAGG
CACGCTCGAATAAAGTGTGTTCATGACGGTACCTGAAGAATAAGCACCGGCTAACTACGT
GCCAGCAGCCGCGGTAATACGTAGGGTGCAAGCGTTAATCGGAATTACTGGGCGTAAAGC
GTGCGCAGGCGGTTTTTTAAGTCAGATGTGAAATCCCCGGGCTCAACCTGGGAATGGCAT
TTGAGACTGGAAAGCTGGAGTGTGTCAGAGGGGGGTGGAATTCCACGTGTAGCAGTGAAA
TGCGTAGAGATGTGGAGGAACACCGATGGCGAAGGCAGCCCCCTGGGATAACACTGACGC
TCATGCACGAAAGCGTGGGGAGCAAACAGGATTAGATACCCCGGTAGTCC
>Otu3049
CCAGCCTATGGGTCGCACCAGTGGGGAATTTTGCGCAATGGGGGAAACCCTGACGCAGCA
ACGCCGCGTGGAGGATGAAATATCTTGGTATGTAAACTCCTTTCGATGGGGAAGATTATG
ACGGTACCCATAGAAGAAGCCCCGGCTAACTTCGTGCCAGCAGCCGCGGTAATACGAGGG
GGGCAAGCGTTGTTCGGAATTATTGGGCGTAAAGGGTGCGTAGGCGGTTTTGTAAGTCTG
ATGTGAAATCTATGGGCTCAACTCATAGACTGCATCGGAAACTGCAGGGCTTGAGTGTGG
GAGAGGTGAGTGGAATTCCTGGTGTAGCGGTGAAATGCGTAGATATCAGGAGGAACACCT
GTGGCGAAAGCGGCTCACTGGACCACAACTGACGCTGATGCACGAAAGCTAGGGGAGCAA
ACAGGATTAGATACCCTTGTAGTCC
>Otu3056
CGCCTACGGGGGGCAGCAGTAGGGAATATTGGACAATGGGCGAAAGCCTGATCCAGCAAT
GCCGCGTGAGTGAGGAAGGCCTTAGGGTTGTAAAGCTCTTTCGGCGGGGACGATGATGAC
GGTACCCGCAGAAGAAGCCCCGGCTAACTTCGTGCCAGCAGCCGCGGTAATACGAAGGGG
GCGAGCGTTGTTCGGAATTACTGGGCGTAAAGGGCGCGTAGGCGGCCATCTTTGTCAGGC
GTGAAAGCCCCGGGCTCAACCTGGGAATTGCGCTTGGGACGGGATGGCTTGAGTTCGGGA
GAGGAGAGTGGAATTCCCAGTGTAGAGGTGAAATTCGTAGATATTGGGAAGAACACCGGT
GGCGAAGGCGGCTCTCTGGACCGATACTGACGCTGAGGCGCGAAAGCGTGGGGAGCAAAC
AGGATTAGATACCCCTGTAGTCC
>Otu3057
CCAGCCTACGGGGCGCAGCAGTAAGGAATATTGGTCAATGGGCGCAAGCCTGAACCAGCC
ACGCCGCGTGCAGGATGAAGGCCCTCTGGGTTGTAAACTGCTTTTTCCAGGGACGAAATC
CCCGTTTCTACGGGAGTTGACGGTACCTGGTGAATAAGCACCGGCTAACACCGTGCCAGC
AGCCGCGGTAATACGGAGGGTGCAAGCGTTATCCGGATTTACTGGGTTTAAAGGGTGCGT
AGGCGGACGTTTAAGTCAGTGGTGAAAGCCTCCCGCTTAACGGGAGAACTGCCATTGATA
CTATTCGTCTCGAATTCGGTTGAGGTAGATGGAACGTGACATGTAGCGGTGAAATGCTTA
GATATGTCACAGAACACCAATTGCGAAGGCAGTCTGCTAAACCGATATTGACGCTGAGGC
ACGAAAGCGTGGGGAGCAAACAGGATTAGAAACCCCTGTAGTCC
>Otu3060
CCAGCCTATGGGGGGCTGCAGTCGAGGATCTTTTGCAATGGGCGCAAGCCTGACAAAGCG
ACGCCGCGTGGGGGAGGAAGGCCTTCGGGTTGTAAACCCCTGTTGTCGGTTACGAATGGT
AGGGTGCCAATAGCGCTACTACTTGACTAAGGCCGATGAGGAAGCCACGGCTAACTACGT
GCCAGCAGCCGCGGTAATACGTAGGTGGCAAGCGTTGTTCGGAATCACTGGGCATAAAGC
GCGCGTAGGCGGCTATGTAAGTCGGGTGTGAAATCCCTCGGCTCAACCGAGGAACTGCGC
TCGATACTGCATGGCTTGAGGTCTGGAGGGGAGAGTGGAATTCCCGGTGGAGCGGTGAAA
TGCGTAGAGATCGGGAAGAACGCCGGCGGCGAAAGCGACTCTCTGGTCAGACCCTGACGC

TGAGGCGCGAAAGCTAGGGGAGCAAACGGGATTAGATACCCCCGTAGTCC
>Otu3062
CCAGCCTACGGGTGGCTCCAGTGGGGAATATTGGACAATGGGGGCAACCCTGATCCAGCG
ACGCCGCGTGTGTGAAGAAGGCCTGCGGGTTGTAAAGCACTTTTAGTGGGGATGAAATGT
GCAGGGTTAATACCTCTGCATTTTGACCTAACCCACAGAAAAAGCACCGGCTAACTCTGT
GCCAGCAGCCGCGGTAATACAGAGGGTGCAAGCGTTAATCGGAATTACTGGGCGTAAAGC
GTGCGCAGACGGTTATATAAGTCGGGTGTGAAAGCCCCGGGCTCAACCTGGGAATTGCAT
TCGAGACTGTGTAGCTAGGGTACGGAAGAGGGAAGCGGAATTTCAGGTGTAGCGGTGAAA
CGCGTAGATATCTGAAGGAACACCTGTGGCGAAAGCGGCTCACTGGACCACAACTGACGC
TGATGCACGAAAGCTAGGGGAGCAAATGGGATTAGATACCCTTGTAGTCC
>Otu3069
CCAGCCTATGGGTTGCACCAGTGAGGAATTTTCCGCAATGGGCGAAAGCCTGACGGAGCG
ACGCCGCGTGTGGGAAGAAGGCCTTCGGGTCGTAAACCCCTTTCAGCAGGGACGAAGCGA
GAGTGACGGTACCTGCAGAAGAAGCGCCGGCTAACTACGTGCCAGCAGCCGCGGTAACAC
GTAGGGCGCGAGCGTTGTCCGGAATTATTGGGCGTAAAGAGCTCGTAGGTGGTCCGCTAC
GTCCGCTGTGAAAACCTAGGGCTTAACCCTGGGCTTGCAGTGGATACGGGCAGACTAGAG
GTAGGTAGGGGAGAATGGAATTCCCGGTGTAGCGGTGAAATGCGCAGATATCGGGAGGAA
CACCGGTGGCGAAGGCGGTTCTCTGGGCCTTACCTGACACTGAGGAGCGAAAGCGTGGGG
AGCGAACAGGATTAGAACCCTGGTAGTCC
>Otu3070
CCAGCCTATGGGGGGCAGCAGTGGGGAATCTTACACAATGGGGGAAACCCTGATGTAGCG
ACACCGCGTGAGCGAAGAAGCCCTTTGGGGTGTAAAGCTCTGTCAGCAGGGACGAAAAAA
ATGACGGTACCTGCAGAGGAAGCATCGGCTAACTACGTGCCAGCAGCCGCGGTAAGACGT
AGGATGCGAGCGTTGTCCGGATTTATTGGGCGTAAAGAGTTCGTAGGTGGTTTCATAAGT
CTGGTGTAAAAGGCGCGGGCCTAACCCGTGTATAGCACTGGATACTGTGAGACTGGAGTT
AGGTAGAGGCAAGCGGAATTCCCAGTGTAGCGGTGAAGTGCGTAGATATTGGGAAGAACA
CCGGTGGCGCAAGCGGCTTGCTGGGCCTAAACTGACGCTGAGGAACGAAAGCCAGGGGAG
CAGATGGGATTAGATACCCTGGTAGTCC
>Otu3075
CCAGCCTACGGGAGGCTGCAGTGGGGAATATTGGACAATGGGGGAAACCCTGATCCAGCG
ACGCCGCGTGTGTAAAGAAGGCCTGCGGGTTGTAAAGCACTTTTAGTGGGGACAAAAAGC
TACGGACTAATACTCTGTGGTCTTGATTTAACCCAAAGAAAAAGCACCGGCTAACTCTGT
GCCAGCAGCCGCGGTAATACAGAGGGTGCGAGCGTTAATCGGAATTACTGGGCGTAAAGC
GCGCGTAGACGGTTTTGTAAGTCAGATGTGAAATCCCTGGGCTCAACCTGGGAACTGCAT
TTGATACTGCATGGCTAGAGTATCGAAGAGGGAAGTGGAATTTCCGGTGTAGCGGTGAAA
TGCGTAGATATCGGAAGGAACACCAGTGGCGAAAGCGGATCTCTGGACAGTTCCTGACGC
TGAGGCACGAAGGCCAGGGGAGCAAACGGGATTAGATACCCGAGTAGTCC
>Otu3081
CCAGCCTATGGGTTGCACCAGTAGGGAATATTGGGCAATGGAGGCAACTCTGACCCAGCC
ATCCCGCGTGCAGGACGAAGGCCCTCTGGGTTGTAAACTGCTTTTATTGGGGAAGAAAAA
TTCTGATTCATCAGGACTTGACGGTACCCAATGAATAAGCACCGGCTAACTCCGTGCCAG
CAGCCGCGGTAATACGGAGGGTGCAAGCGTTATCCGGATTCACTGGGTTTAAAGGGTGCG
TAGGCGGCTTTGCAAGTCAGTGGTGAAAGCCCGCAGCTCAACTGCGGAACTGCCATTGAA
ACTGCTTGGCTGGAGTGTTATTGAAGTGGGCGGAATACATCATGTAGCGGTGAAATGCTT
AGATATGATGTAGAACTCCGATTGCGAAGGCAGCTCACTAAATGACAACTGACGCTGAGG
CACGAAAGCGTGGGTAGCAAACAGGATTAGAAACCCTCGTAGTCC
>Otu3083
CCAGCCTATGGGACGCACCAGTCGAGGATCTTCGGCAATGGGCGCAAGCCTGACCGAGCG
ACGCCGCGTGTGCGATGAAGGCCTTCGGGTTGTAAAGCACTGTCGGGGGGGAGAAAAGGG
CAACCTTGATCGACCCCGGAGGAAGCACGGGCTAAGTTCGTGCCAGCAGCCGCGGTAAGA
CGAACCGTGCGAACGTTGCTCGGAATCACTGGGCGTAAGGGGTTCGTAGGCGGGAATGCA
AGTCAAGTGTGAAATCCCCAGGCTCAACCTGGGACGTGCATTTGAGACTGTGTTTCTTGA
GTTTCGGAGAGGGTGGTGGAATTGCTGGTGTAGGAGTGACATCCGTAGAGATCAGCAGGA
ACACCGGAGGCGAAGGCGACCACCTGGCCGAATACTGACGCTGAGGAACGAAAGCGTGGG
GAGCAAACAGGATTAGAAACCCGAGTAGTCC
>Otu3089
CCGCCTATGGGTGGCTCCAGTGGGGAATATTGGACAATGGGCGGAAGCCTGATCCAGCAA
TGCCGCGTGTGTGAAGAAGGCCTGCGGGTTGTAAAGCACTTTCAGTGGGGAGGAGAGGCC

TTTGGCTAATATCCAAAGGAAGAGACGTTACCCACAGAAGAAGCACCGGCTAACTCTGTG
CCAGCAGCCGCGGTAATACAGAGGGTGCAAGCGTTAATCGGAATTACTGGGCGTAAAGGG
TGCGTAGGTGGTTTGATAAGTTGGGTGTGAAATTCCTGGGCTTAACCTAGGAACTGCGCT
CAATACTGTTGAGCTAGAGTACTGTAGAGGAAGGTGGAATTTCCAGTGTAGCGGTGAAAT
GCGTAGATATTGGAAGGAACATCAGTGGCGAAGGCGACTTTCTGGACAGATACTGACACT
GAGGCACGAAAGCGTGGGGAGCAAACAGGATTAGAGACCCCTGTAGTCC
>Otu3093
CCAGCCTATGGGGCGCTCCAGTCGAGGATCTTCGGCAATGGGCGCAAGCCTGACCGAGCG
ACGCCGCGTGCGGGATGAAGGCCTTCGGGTTGTAAACCGCTGTCGAGGGGGATGAAGGGC
ATCTGGGTTCACCCAGGTGTCACGCTTGACACATCCCTGGAGGAAGCACGGGCTAAGTTC
GTGCCAGCAGCCGCGGTAAGACGAACCGTGCGAACGTTGTTCGGATTCACTGGGCTTAAA
GGGCGCGTAGGCGGTTTGCCAAGTCTGTGGTGAAATCTTTCGGCTTAACCGGAAAATGGC
CATGGATACTGGTGAACTCGAGGGAGGTAGGGGCAGTGGGAACTTCCGGTGGAGCGGTGA
AATGCGTTGATATCGGAAGGAACGCCGGTGGCGAAAGCGCACTGCTGGATCTCTTCTGAC
GCTGAGGCGCGAAAGCTAGGGGAGCAAACAGGATTAGAAACCCTAGTAGTCC
>Otu3094
CCAGCCTATGGGAGGCAGCAGTGGGGAATATTGCGCAATGGGCGAAAGCCTGACGCAGCA
ACGCTGCGTGAGTGAAGAAGGCCTTCGGGTTGTAAAGCTCTTTCGGCTGGGAAGAAGGGT
AGAAAGGTGAATAATCTTTTTATTTGATGGTACCAGAAGAAGAAGCACCGGCAAACTTCG
TGCCAGCAGCCGCGGTAATACGAAGGATGCGAGCGTTGTTCGGAATTATTGGGCGTAAAG
GGTTCGTAGGCGGGATTGTAAGTCAAGTGTGAAATCCCCGAGCTTAACTCGGGACGTGCA
TTTGAAACTGTGATTCTTGAGTCATAGAGAGGGTGGTGGAATTGTTGGTGTAGGAGTGAC
ATCCGTAGAGATCAACAGGAACATCAGAGGCGAAAGCGGCCACCTGGCTATGTACTGACG
CTGAGGAACGAAAGCGTGGGGAGCAAACAGGATTAGAAACCCGGGTAGTCC
>Otu3095
CCAGCCTACGGGTCGCTCCAGTAGGGAATTTTCCACAATGGGCGAAAGCCTGATGGAGCA
ACGCCGCGTGCAGGATGAATGCCTTAGGGTTGTAAACTGCTTTTATATGTGACGATAATG
ACGGTAGCATATGAATAAGGATCGGCTAACTCCGTGCCAGCAGCCGCGGTCATACGGAGG
ATCCAAGCGTTATCCGGAATTACTGGGCGTAAAGAGTTGCGTAGGTGGCAAAGTAAGTAG
ACAGTGAAATCGTGTGGCTCAACCATACATCCATTGCCTAAACTGCTTAGCTAGAGGATG
AGAGAGGTTATTGGAATTCCTAGTGTAGGAGTGAAATCCGTAGATATTAGGAGGAACACC
GATGGCGTAGGCAGATAACTGGCTCATTCCTGACACTCAGGCACGAAAGCGTGGGGAGCA
AACGGGATTAGATACCCCAGTAGTCC
>Otu3097
CAGCCTATGGGTTGCTGCAGTGGGGAATATTGGACAATGGGCGCAAGCCTGATCCAGCAA
TGCCGCGTGAGTGATGAAGGCCTTCGGGTTGTAAAGCTCTTTCGCACGCGACGATGATGA
CGGTAGCGGAAGAAGAAGCCCCGGCTAACTTCGTGCCAGCAGCCGCGGTAATACGAGGGG
GGCAAGCGTTGTTCGGAATTATTGGGCGTAAAGGGTGCGTAGGCGGTTTGGTAAGTCTTT
TGTGAAATCTACGGGCTCAACTCGTAGACTGCAAGGGAAACTGCCGGGCTTGAGTATGGG
AGAGGTGAGTGGAATTTCCGGTGTAGCGGTGAAATGCGTAGATATCGGAAGGAACACCTG
TGGCGAAAGCGGCTCACTGGACCATAACTGACGCTGATGCACGAAAGCTAGGGGAGCAAA
CAGGATTAGAAACCCTCGTAGTCC
>Otu3099
CCAGCCTACGGGGTGCAGCAGTGGGGAATTTTACGCAATGGGGGAAACCCTGACGTAGCG
ACACCGCGTGAGCGAAGAAGCCCTTTGGGGTGTAAAGCTCTGTCGGCTGGGACGAAAAAA
ATGACGGTACCAGCAAAGGAAGCATCGGCTAACTACGTGCCAGCAGCCGCGGTAAGACGT
AGGATGCAAGCGTTGTCCGGATTTATTGGGCGTAAAGAGTTCGTAGGCGGTTTGTTAAGT
CTGAAGTTAAAGACTGAGGCTCAACCTCAGGACTGTTTTGGATACTGACAAACTAGAGTG
CAATAGAGGCGAGTGGAATTTCCAGTGTAGCGGTGAAATGCGTAGATATTGGGAAGAACA
CCAGTGGCGAAGGCGACTCGCTGGGTTGTAACTGACGCTGAGGAACGAAAGCCAGGGTAG
CGAATGGGATTAGAAACCCTAGTAGTCC
>Otu3103
CCAGCCTATGGGTTGCAGCAGTGAGGAATATTGCTCAATGGGCGAAAGCCTGAAGCAGCG
ACGCCGCGTGAGGGATGAAATCCGTTAGGATGTAAACCTCTTTTGGTAGGGAAGAATGTT
CCGTTTCACGGAATTGACGGTACCTACAGAATAAGCACCGGCAAACTACGTGCCAGCAGC
CGCGGTAATACGTAGGGTGCAAGCGTTGTCCGGAATTACTGGGTGTAAAGGGTCCGCAGG
CGGAAATGTAAGTCAATGGTGAAATTCTTCGGCTCAACCGAAGACATGCTGTCGATACTA
CATTTCTTGAGTCCGAGAGAGGATAACAGAATTCATGGTGTAGCAGTGAAATGCGTAGAG

ATCATGAAGAATACCGGTAGCGAAGGTGGTTATCTGGCTCGGTACTGACGCTCAGGGACG
AAAGCGTGGGTAGCAAACAGGATTAGAAACCCTTGTAGTCC
>Otu3104
CCAGCCTATGGGGCGCAGCAGTGGGGAATATTGGACAATGGGGGCAACCCTGATCCAGCG
ATGCCGCGTGAGTGATGAAGGCCTTCGGGTTGTAAAGCTCTTTTGTTGGGGACGATGATG
ACGGTACCCGACGAATAAGCCCCGGCTAACTTCGTGCCAGCAGCCGCGGTAATACGAAGG
GGGCTAGCGTTGTTCGGAATGACTGGGCGTAAAGGGCGCGTAGGCGGTTTGATAAGTTTG
GTGTGAAAGTCCTGGGCTTAACCTGGGGATTGCATTGGAAACTGTCAGGCTAGAGGACGA
GAGAGGAAAATGGAATTGCGAGTGTAGAGGTGAAATTCGTAGATATTCGCAAGAACACCG
GTGGCGAAGGCGGTCTTCTGGCTCGGTACTGACGCTAAGGCGCGAAAGCGTGGGGAGCAA
ACAGGATTAGAAACCCCAGTAGTCC
>Otu3107
CCAGCCTATGGGTCGCAGCAGTAGGGAATTTTCCACAATGGGCGAAAGCCTGATGGAGCA
ACGCCGCGTGCAGGATGAATGCCTTAGGGTTGTAAACTGCTTTTATCTGTGACGAATATG
ACGGTAGCAGATGAATAAGCCTCGGCTAACTCCGTGCCAGCAGCCGCGGTCATACGGAGG
AGGCAGGCGTTATCCGGAATTACTGGGCGTAAAGAGTTGCGTAGGTGGCAAAGTGTGTCG
ATAGTGAAAGCGTGGGGCTCAACCTCATATCCATTATCGAAACTGCTTAGCTAGAGGACG
AGAGAGGTTATTGGAATTCCCAGTGTAGGAGTGAAATCCGTAGATATTGGGAGGAACACC
GATGGCGTAGGCAGATAACTGGCTCGTTCCTGACACTAAGGCACGAAAGCGTGGGTAGCA
AACGGGATTAGATACCCCCGTAGTCC
>Otu3108
CCAGCCTATGGGGGGCACCAGTCGCGAATCTTTCACAATGGGCGCAAGCCTGATGAAGCC
ACGCCGCGTGCGGGATGAAGGCCCTCTGGGTTGTAAACCGCTGTCAAGTGGGGGGAAACG
CCGGGTGTTAACAGCGCCCGGCTTGACTGCCCGCTAAAGGAAGCCCCGGCAAAACATGTG
CCAGCAGCCGCGGTAATACATGTGGGGCAAGCGTTGTTCGGAATTACTGGGCATAAAGGG
TGCGTAGGTGGCTTGTTAAGTCAGGGGTGAAAGTTTTCGGCCCAACCGGAAAATGGCCTT
TGATACTAGCGAGCTTGAGTGCGTGAGGGGTGACTGGAACGAGTGGTGTAGCGGTGAAAT
GCGTAGATATCACTCGGAAGGCCAACGGCGAAGGCAAGTCACTGGTGCGCAACTGACACT
GAGGCACGAAAGCGTGGGGAGCAAACAGGATTAGAAACCCCCGTAGTCC
>Otu3109
CCAGCCTATGGGTGGCTGCAGTCGAGGATCTTCGGCAATGGGCGCAAGCCTGACCGAGCG
ACGCCGCGTGTGCGATGAAGGCCTTCGGGTTGTAAAGCACTGTCGAGGGGAAGAAAGCCG
CAAGGTTGATCGACCCCTGGAGGAAGCACGGGCTAAGTTCGTGCCAGCAGCCGCGGTAAG
ACGAACCGTGCGAACGTTGTTCGGAATCACTGGGCTTAAAGGGCGCGTAGGCGGGCTGCC
GCGTCCGGGGTGAAATCCCACGGCTCAACCGTGGAACGGCCTCGGATACGGGCGGCCTCG
AGGGGGGTAGGGGCATCTGGAACTGTGAGTGGAGCGGTGAAATGCGTTGATATTCACAGG
AACTCCGGTGGCGAAGGCGAGGTGCTGGACCCCATCTGACGCTGAGGCGCGAAAGCCAGG
GGAGCGAACGGGATTAGAAACCCCCGTAGTCC
>Otu3111
CCAGCCTATGGTTGCTCCAGTCGAGAATTTTTCTCAATGGGCGAAAGCCTGAAGGAGCGA
CGCCGCGTGGGGGATGAATGGCTTCGGCCCGTAAACCCCTGTCATTTGTGAACAAATTAA
TCTACCCAACACGTGGATTATTGATAGTAACGGAAGAGGAAGGGACGGCTAACTCTGTGC
CAGCAGCCGCGGTAATACAGAGGTCCCAAGCGTTGTTCGGATTCATTGGGCGTAAAGGGT
GTGTAGGAGGTAGGGTAAGTCAGGTGTGAAATCTCAGAGCTTAACTCTGAAACTGCGCTT
GATACTGCTCTGCTAGAGGATCGGAGGGGGTATCGGAATTTATGGTGTAGCAGTGAAATG
CGTAGATATCATAAGGAACACCGGTGGCGAAGGCGGATACCTGGAAGACTCCTGACTCTG
AAACACGAAAGCCAGGGGAGCAAACGGGATTAGATACCCCAGTAGTCC
>Otu3113
CCAGCCTATGGGGCGCTCCAGTGGGGAATATTGGACAATGGGCGCAAGCCTGATCCAGCC
ATGCCGCGTGAGTGTTGAAGGCCTAGGGTTGTAAAGCCCTTTCGGCGGGGAAGATAATGA
CGGTACCCGTAGAAGAAGCCCCGGCTAACTTCGTGCCAGCAGCCGCGGTAATACGAAGGG
GGCTAGCGTTGCTCGGAATGACTGGGCGTAAAGGGCGCGTAGGCGGCGTGCAAAGTCAGG
CGTGAAATTCCTGGGCTCAACCTGGGGACTGCGCTTGATACGTGCATGCTTGAGTTTGGA
AGAGGGTCGTGGAATTCCCAGTGTAGAGGTGAAATTCGTAGATATTGGGAAGAACACCGG
TGGCGAAGGCGGCGACCTGGTCCTGGACTGACGCTGAGGCGCGAAAGCGTGGGGAGCAAA
CAGGATTAGATACCCCAGTAGTCC
>Otu3122
CCAGCCTACGGGTCGCTCCAGTCAAGAACCTTCCACAATGGACGAAAGTCTGATGGAGCG

ACGTCGCGTGGTTGATGAAGTCCTTAGGGACGTAAAAACCTTTTGTCGGGGAGAAAGTTA
TTGATAGTACCCGAAGAATAAGGGGTTGCTAAACTCGTGCCAGCAGCAGCGGTAATACGA
GTGCCCCGAGCGTTATCCGGAATTATTGGGCGTAAAGGGTGTGTAGGTGGTTGTGTTAGT
CTCCTGTTAAATTCCCGGGCTTAACCTGGGGCATGCAGGGGAAACGGCACAACTAGAGGA
TGCGAGGGGTGAATGGAACTCATGGTGTAGGGGTGAAATCCGTTGATATCATGGGGAACA
CCAAAAGCGAAGGCAATTCACTGGCGCATTCCTGACACTGAAACACGAAAGCGTGGGTAG
CGAATGGGATTAGAAACCCCAGTAGTCC
>Otu3125
CCAGCCTACGGGTCGCTGCAGTGGGGAATATTGCGCAATGGGCGAAAGCCTGACGCAGCG
ACGCCGCGTGAAGGATGAAGGTCTTCGGATCGTAAACTTCTGTTAAGTGGGAAGAAAGAC
CGCTCTCTAATATGGAGCGGGGATGACGGTACCACTAGAGAAAGCACCGGCTAACTTCGT
GCCAGCAGCCGCGGTAATACGAAGGGTGCAAACGTTATTCGGAATCACTGGGCGTAAAGG
GTGTGTAGACGGCATACTAAGTTCGTTGTTTAATCTTCCGGCCTAACCGGAAGCTCGCGA
TGAAAACTAGTAAGCTTGAGGATGGAAGAGAGAAGTGGAATTCTCGGAGTAGCGGTAAAA
TGCGTAGATCTCGAGAGGAACACCGATGGCGAAGGCAGCTTCTTGGTCCACTCCTGACGT
TGAGACACGAAAGCGTGGGTAGCAAACAGGATTAGATACCCGCGTAGTCC
>Otu3128
CCAGCCTACGGGAGGCTGCAGTGGGGAATATTGGACAATGGGCGAAAGCCTGACGCAGCA
ACGCCGCGTGGGGGATGAAGGCCTTCGGGTTGTAAACTCCTTTTAGTGGGAACGAAATTG
ACGGTACCCACAGAATAAGCCCCGGCCAACTACGTGCCAGCAGCCGCGGTGATACGTAGG
GGGCGAGCGTTGTCCGGATTCATTGGGCGTAAAGAGCTCGTAGGCGGTTCGGTAAGTCGG
GTGTGAAACCTCCAGGCTCAACTTGGAGACGCCACTCGATACTGCTGTGACTCGAGTCCG
GTAGGGGACCACGGAATTCCTGGTGTAGCGGTGAAATGCGCAGATATCAGGAGGAACACC
AGTAGCGAAGGCGGTGGTCTGGGCCGGCACTGACGCTGAGGAGCGAAAGCGTGGGGAGCG
AGCAGGATTAGATACCCCTGTAGTCC
>Otu3129
CCAGCCTATGGGTGGCTGCAGTGAGGAATATTCCGCAATGGGCGCAAGCCTGACGGAGTG
ATGTCGCGTGAGTGATGAAGGCCTTCGGGTTGTAAAGCTCTTTGGATAGGGAGTAAGAAA
ACTCGGATAACATCCGGGTAATTTGACAGTACTTATACAACAAGCACCGGCTAACCCCGT
GCCAGCAGCTGCGGTAATACGGGGGGTGCAAGCGTTGATCGGATTGACTGGGCGTAAAGG
GAGCGTAGGCGGTCGAATAAGTCAGTTGTGAAATTCCGGGGCTCAACCCCGGAGCTGCGA
CTGAAACTATTTGACTAGAGGGTGGTAGGGGAAAACAGAATTCTGCATGTAGCGGTGAAA
TGCGTAGATATGCAGAGGAATACCGGTGGCGAAGGCGGTTTTCTGTACCATTCCTGACGC
TGAGGCTCGAAGGTGTGGGGAGCAAACAGGATTAGAGACCCCTGTAGTCC
>Otu3130
CCAGCCTATGGGACGCACCAGGTGTAAAAACCATGTGGACTCCGAGTTTCTTTAAAGAAA
TGATATCATCCTCCGGAATAATTCCGCCTACAATAAAGAGGACGTCAGTCAGTCCTTCTT
TGTGGACTCCTTCGATAATATCAGGCACGAGTGCGTTGTGCGCACCTGACAAAATACTGA
GCCCAATCACGTGAACGTCCTCTTGAATGGCCGCATTGATCACCATTTCTGGGGTTTGGT
GTAAGCCAGTATAAATGACCTCAAATCCAGCATCGCGTAGTGCGCGCGCTATGACCTTTG
CTCCTCGATCATGCCCATCCAGGCCAGGTTTTGCGACAAGGACACGTATCATGCCTTTAT
TATTTAGGGCTTTGTCGCTTGTACCAGGTGTTATAATAGAGGATTGAGTAACGGACATAA
TTAGAGACCCGCGTAGTCC
>Otu3132
CCAGCCTACGGGTTGCAGCAGACGAGAATATTCGACAATGGGCGAAAGCCTGATCGAGCG
ACGCCGCGTGCAGGATGAAGTTCTTCGGGGTGTAAACTGCTTTTATGAGCGAGAAAGTTT
ATTGATCAGCTCATGAATAAGAGGTTGCTAAACTCGTGCCAGCAGCAGCGGTAATACGAG
TGCCTCAAGCGTTATCCGGAATCATTGGGCGTAAAGGGTGTGTAGGTGGCCGCGTTAGTC
TTCCGTTAAATTCTCCGACTCAATCGGAGTACTGCGGGGGAAACGGCGCGGCTTGAGGAT
GCGAGAGGTCTGTGGAACTCATAGTGTAGCGGTGAAATGCGTTGATATTATGGGGAACAC
CAAAAGCGAAGGCAGCAGACTGGAGCACTCCTGACACTGAAACACGAAAGCGTGGGTAGC
GAACGGGATTAGAGACCCTCGTAGTCC
>Otu3134
CCAGCCTACGGGTGGCAGCAGTCGAGAATTTTTCACAATGGGGGAAACCCTGATGGAGCG
ACGCCGCGTGGAGGATGAAGGTTTTCGGATTGTAAACTCCTGTCACTGCAGAACAAGGGT
GCGTTTGTGAACAGCAAACGCATTTGATGGTATGCGGAGAGGAAGGGACGGCTAACTCTG
TGCCAGCAGCCGCGGTAAGACAGAGGTCCCGAGCGTTGTTCGGATTCATTGGGCGTAAAG
GGTGTGTAGGAGGTCGGGTAAGTCAGGTGTGAAATCTCAGAGCTTAACTCTGAAACTGCG

CTTGATACTGCTCGGCTAGAGGATCGGAGGGGGTATCGGAATTTATGGTGTAGCAGTGAA
ATGCGTAGATATCATAAGGAACACCGGTGGCGAAGGCGGTGATCTGGGCCAATACTGACG
CTGAGGAGCGAAAGCGTGGGGAGCGAACAGGATTAGAGACCCCTGTAGTCC
>Otu3135
CCAGCCTACGGGGCGCTGCAGTGGGGAATATTGGACAATGGGGGCAACCCTGATCCAGCC
ATGCCGCGTGGGTGAAGAAGGCCTTCGGGTTGTAAGGCCCTTTTGTACGGGAAGAAAAGC
TGCGGGTGAATACTCCGTGGTCATGACGGTACCGTAAGAATAGGCACCGGCTAACTTCGT
GCCAGCAGCCGCGGTAATACGAAGGGTGCAAGCGTTACTCGGAATTACTGGGCGTAAAGC
GTGCGTAGGCGGTGGTTTAAGTCTGATGTGAAATCCCCGGGCTCAACCTGGGAATGGCAT
TGGATACTGGACCGCTAGAGTACGGTAGAGGGTGGTGGAATTCCCGGTGTAGCAGTGAAA
TGCGTAGAGATCGGGAGGAACATCTGTGGTGAAGGCGGCCACCTGGACCAGTACTGACGC
TGAGGCACGAAAGCGTGGGGAGCAAACAGGATTAGATACCCCTGTAGTCC
>Otu3136
CCAGCCTATGGGTCGCACCAGTGAGGAATATTGCGCAATGGGCGAAAGCCTGACGCAGCG
ACGCCGCGTGGAGGATGAAGGTCTTAGGATTGTAAACTTCTGTTAAGTGGGAAGAAAGAC
CTGTCTCTAATACAGACGGGGGATGACGGTACCATTAAAGAAAGCACCGGCTAATCTCGT
GCCAGCAGCCGCGGTAATACGAGAGGTGCAAGCGTTATTCGGAATTATTGGGCGTAAAGG
GTGCGTAGGCGGTATATTAAGTCAACTGTTAAATTTTCCGGCTCAACCGGAAGCAGGCGG
TAGATACCGGTATGCTAGAGGGTGAAAGAGAGGAGTGGAATTCTCGGAGTAGCGGTAAAA
TGCGTAGATCTCGAGAGGAACACCAATGGCGAAGGCAGCTCCTTGGTTCACTTCTGACGC
TGATGCACGAAAGCGTGGGGAGCAAACAGGATTAGAGACCCGTGTAGTCC
>Otu3141
CCAGCCTATGGGGGGCACCAGTCGAGAATCTTTCGCAATGGGCGAAAGCCTGACGAAGCG
ACGCCGTGTGTGCGATGAAGGCCTTCGGGTTGTAAAGCACTTTCGCTTGGGAACAAGAGA
AGCCGGCTAATATCCGGCAAATTTGAGTGTACCAGGTAAAGAAGCACCGGCTAACTACGT
GCCAGCAGCTGCGGTAATACGTAGGGTGCAAGCATTAATCGGATTTATTGGGCGTAAAGG
GCGCGTAGGCGGGGTGATAAGTCAGATGTGAAAGCCCGGGGCTCAACCCCGGAACTGCAT
TTGAAACTGTCACTCTTGAGGGTAGGCGGAGAAAATGGAATTCCACAAGTAGCGGTGAAA
TGCGTAGATATGTGGAAGAACACCAGTGGCGAAGGCGGTTTTCTAGCTTATACCTGACGC
TGAGGCGCGAAAGCAAGGGGAGCAAACAGGATTAGATACCCCAGTAGTCC
>Otu3143
CCAGCCTATGGGATGCACCAGTGGGGAATATTGGACAATGGGCGAAAGCCTGATCCAGCA
ATACCGAGTGAGTGATGAAGGCCTTAGGGTTGTAAAGCTCTTTTCAGCAGGGAAGATAAT
GACGGTACCTGACCAAGAAAGCCCCGGCTAACTCCGTGCCAGCAGCCGCGGTAAGACGGA
GGGGGCTAGCGTTGTTCGGAATTACTGGGCGTAAAGAGTGCGTAGGCGGTTTAGTAAGTT
GGAAGTGAAAGCCCGAGGCTTAACCTCGGAACTGCTTTCAAAACTATTAATCTAGAGTGT
AGTAGGGGATGATGGAATTCCTAGTGTAGAGGTGAAATTCTTAGATATTAGGAGGAACAC
CGGTGGCGAAGGCGGTCATCTGGGCTACAACTGACGCTGATGCACGAAAGCGTGGGGAGC
AAACAGGATTAGAAACCCGAGTAGTCC
>Otu3145
CCAGCCTATGGGGGGCAGCAGTGGGGAGTATTGGACAATGGGGGCAACCCTGATCCAGCC
ATGCCGCGTGAGTGATGAAGGCCTTCGGGTTGTAAAGCTCTTTTGGTGGGGACGATGATG
ACGGTACCCACAGAATAAGCTCCGGCTAACTTCGTGCCAGCAGCCGCGGTAATACGAAGG
GAGCTAGCGTTGTTCGGAGTTACTGGGCGTAAAGGGCGCGTAGGTGGCTTAGTAAGTTAG
GTGTGAAAGCCCAGGGCTCAACCCTGGAATAGCACTTAAGACTGCTTCGCTTGAATTCGG
TAGAGGTGAGTGGAATTCCCAGTGTAGAGGTGAAATTCGTAGAGATTGGGAAGAACACCC
GTGGCGAAGGCGGCTCACTGGACCGACATTGACACTGAGGCGCGAAAGCGTGGGGATCAA
ACAGGATTAGAAACCCTAGTAGTCC
>Otu3147
CCAGCCTACGGGTCGCTGCAGTGGGGAATATTGGACAATGGGCGCAAGCCTGATCCAGCA
ATGCCGCGTGTGTGAAGAAGGCCTGCGGGTTGTAAAGCACTTTCGGTGGGGAGGAAGGCC
TTAGTGTGAATATCACTGAGGATTGACGTTACCCACAGAAGAAGCACCGGCTAACTCTGT
GCCAGCAGCCGCGGTAATACAGAGGGTGCGAGCGTTAATCGGAATTACTGGGCGTAAAGG
GTGCGTAGGTGGTTAATTAAGTCAGATGTGAAATCCCAAGGCTTAACCTTGGAATGGCAT
TTGATACTGGTTGGCTAGAGTACAGAAGAGGGAAGTGGAATTTCCGGTGTAGCGGTGAAA
TGCGTAGATATCGGAAGGAACATCAGCGGCGAAGGCGACTTCCTGGTCTGTTACTGACAC
TGAGGCACGAAAGCGTGGGGAGCGAACAGGATTAGAAACCCCAGTAGTCC
>Otu3148

CCAGCCTACGGGTTGCTCCAGTGGGGAATCTTGCGCAATGGGCGAAAGCCTGACGCAGCA
ACGCCGCGTGGGGGATGAAGGCTCTCGGGTTGTAAACCCCTTTCAGCAGGAACGAAAACA
GACGGTACCTGCGGAAGAAGCCCCGGCCAACTACGTGCCAGCAGCCGCGGTGATACGTAG
GGGGCGAGCGTTGTCCGGATTTATTGGGCGTAAAGAGCTCGTAGGCGGCTCGGTAAGTCG
GGTGTGAAACCTCCAGGCTCAACCTGGAGATGCCACTCGATACTGCCATGGCTAGAGTCC
GGTAGGGGACCACGGAATTCCTGGTGTAGCGGTGAAATGCGCAGATATCAGGAGGAACAC
CGGTGGCGAAGGCGGTGGTCTGGGCCGGAACTGACGCTGAGGAGCGAAAGCGTGGGGAGC
GAACAGGATTAGATACCCCAGTAGTCC
>Otu3149
CCAGCCTATGGGACGCACCAGTCGAGAATCATTCGCAATGGGCGAAAGCCTGACGAAGCG
ACGCCGTGTGTGCGATGAAGGCCTTCGGGTTGTAAAGCACTTTCGCCTGGGAACAAGGGA
AGCCGGCTAATATCCGGCAGACTTGAGGGTACCAGGTAAAGAAGCACCGGCTAACTACGT
GCCAGCAGCTGCGGTAATACGTAGGGTGCGAGCATTAATCGGATTTATTGGGCGTAAAGG
GCGCGTAGGCGGGGATAAAAGTCATATGTGAAATCCCGGGGCTCAACCTCGGAACGGCAT
ATGAAACTCTATCTCTAGAGGGTAGGCGGAGAAAATGGAATTCCACAAGTAGCGGTGAAA
TGCGTAGATATGTGGAAGAACACCAGTGGCGAAGGCGGTTTTCTAGCTTATACCTGACGC
TGATGCGCGAAAGCAAGGGGATCAAACAGGATTAGATACCCCGGTAGTCC
>Otu3150
CCAGCCTACGGGAGGCAGCAGTAAGGGATATTGCGCAATGGGCGAAAGCCTGACGCAGCA
ACGCCGCGTGGAGGAAGAAGGCCTTCGGGTTGTAAACTTCTTTTGGGGCTGACGAGGAAG
GACGGTAAGCCCCGAATAAGTCACGGCTAACTACGTGCCAGCAGCCGCGGTAAAACGTAG
GTGGCGAGCGTTATCCGGATTTACTGGGCGTAAAGCGTGTGCAGACGGGGTTACAAGTGG
TGTATGAAATTTCCCAGCTTAACTGGGAGGGGTTATGCCAGACTGTAGCTCTAGAGAACG
GGAGAGGGACGTGGGATTCCGGGTGTAGTGGTGAAATGCGTAGAGATCCGGAGGAACCCC
CGAGGCGAAGGCGGCGTCCTGGCCCGGTTCTGACGTTCAGACACGACAGCATGGGGAGCG
AACGGGATTAGAAACCCCAGTAGTCC
>Otu3151
CCAGCTATGGGTGGCACCAGTGGGGAATATTGGACAATGGGCGCAAGCCTGATCCAGCAA
TGCCGCGTGAGTGAAGAAGGTCTTCGGATTGTAAAGCTCTTTCGACGGGGACGATGATGA
CGGTACCCGTAGAAGAAGCCCCGGCTAACTTCGTGCCAGCAGCCGCGGTAATACGAAGGG
GGCTAGCGTTGCTCGGAATGACTGGGCGTAAAGGGCGCGTAGGCGGCGATGATAGTCAGG
CGTGAAATTCCTGGGCTCAACCTGGGGACTGCGCTTGATACGTTATTGCTAGAGTTAGGA
AGAGGGTCGTGGAATTCCCAGTGCAGAGGTGAAATTCGTAGATATTGGGAAGAACACCGG
TGGCGAAGGCGGCGACCTGGTCCTTGACTGGCGCTGAGGCGCGAAAGCGTGGGGAGCAAA
CAGGATTAGAAACCCTAGTAGTCC
>Otu3158
CCAGCCTATGGGTGGCTGCAGTCAGGAATATTGCGCAATGGAGGAAACTCTGACGCAGCG
ACGCCGCGTGGGTGATGAAGGCCTTCGGGTCGTAAAGCCCCGTCGGAGGGGAAGAAAAGT
CTGTTGGCTAATATCCAGCGGACCTTTGACGGTACCCTCAAAGGAAGCACCGGCTAACTC
CGTGCCAGCAGCCGCGGTAAGACGGAGGGTGCGAGCGTTGTTCGGATTGACTGGGCGTAA
AGGGCGTGTAGGCGGTTGGTTAAGTCGGGTGTGAAAGCCTGGGGCTCAGCCCCAGAAGTG
CATCCGAAACTAGCCAGCTAGAGCGCGATAGAGGAGGGTGGAATTCCTGGTGTAGCGGTG
AAATGCGTAGATATCAGGAGGAACACCGGCGGCGAAGGCGGCCCTCTGGATCGTTGCTGA
CGCTGAGACGCGAAAGCGTGGGGAGCGAACAGGATTAGAGACCCTTGTAGTCC
>Otu3160
CCAGCCTACGGGGTGCAGCAGTGAGGAATTTTCCGCAATGGGCGAAAGCCTGACGGAGCG
ACGCCGCGTGTGGGAAGAAGGCCTCCGGGTCGTAAACCACTGTCGAAGGGGACTATACTG
AAGGTACCCTTGGAGGAAGCCCCGGCTAACTACGTGCCAGCAGCCGCGGTAAGACGTAGG
GGGCAAGCGTTATTCGGATTTATTGGGCGTAAAGCGCTCGTAGGCGGGAGGTCAAGTCCG
TGAAGAAAGACCTGGGCTCAACTCAGGGAACGGCACGGATACTGGCTTTCTTGAGGCATT
CAGAGGGTGATGGAATTCCCGGTGTAGCGGTGAAATGCGTAGACATCGGGAGGAACACCA
GTGGCGAAGGCGATCACCTGGGGATGTTCTGACGCTGAGGAGCGAAAGCTAGGGGAGCAA
ACGGGATTAGAGACCCGGGTAGTCC
>Otu3168
CCAGCCTATGGGGGGCTGCAGTGGGGAATCTTGCACAATGGAGGAAACTCTGATGCAGCG
ACGCCGCGTGAGCGATGAAGCCCTTCGGGGTGTAAAGCTCTTTCGACGGGAACGATAATG
ACGGTACCCACAGAAGAAGCCCCGGCTAACTACGTGCCAGCAGCCGCGGTGATACGTAGG
GGGCGAGCGTTGTCCGGATTTACTGGGCGTAAAGAGCTCGTAGGCGGTTTGATAAGTCGG

GTGTGAAACCTCCAGGCTCAACCTGGAGTCGCCACTTGATACTGTCATGACTAGAGTCCG
GTAGGGGACCATGGAATTCCTGGTGTAGCGGTGAAATGCGCAGATATCAGGAGGAACACC
AGTAGCGAAGGCGGTGGTCTGGGCCGGTACTGACGCTGAGGAGCGAAAGCGTGGGGAGCG
AACAGGATTAGATACCCTCGTAGTCC
>Otu3170
CCAGCCTATGGGAGGCTCCAGTGGGGAATATTGCACAATGGGCGAAAGCCTGATGCAGCG
ACGCCGCGTGGAGGATGACGGTCTTCGGATTGTAAACTCCTGTTAAGTGGGAAGAAAGAT
TCATACCTAATACGTATGGAGGATGACGGTACCACTAGAGAAAGCACCGGCAAACTTCGT
GCCAGCAGCCGCGGTAATACGAAGGGTGCGAGCGTTATTCGGAATAACTGGGCGTAAAGA
GCGTGTCGGCGGTTTCTTAAGTCATCTGTTAAAGAACTCGGCTCAACCGAGTGCCTGCGG
GTGAAACTGGGAGACTAGAGTGCGAGAGAGAAGGGTAGAATTCTCGGAGTAGCGGTAAAA
TGCGTAGATCTCGAGAGGAATACCGGTTGCGAAGGCGGCCCTTTGGCTCGTAACTGACGC
TTAAGCGCGAAAGCGTGGGGAGCAAACAGGATTAGAAACCCGAGTAGTCC
>Otu3174
CCAGCCCACGGGTCGCAGCAGTGGGGAATATTGGACAATGGGGGCAACCCTGATCCAGCA
ATGCCGTGTGTGTGAAGAAGGCCTTCGGGTTGTAAAGCACTTTTAGTGGGAAAGAAAAGC
TTAAGGGTAATATCCTTAAGCCTTGACGTTACCCACAGAAAAAGCACTGGCTAACTCTGT
GCCAGCAGCCGCGGTAATACAGAGAGTGCAAGCGTTAATCGGAATTACTGGGCGTAAAGC
GCACGTAGGTGGATATTTAAGTCGGATGTGAAATCCCTGAGCTTAACTTAGGAATTGCAT
TCGATACTGGATGTCTAGAGTATAGTAGAGGGAAGTGGAATTTCCGGTGTAGCGGTGAAA
TGCGTAGATATCGGAAGGAACATCAGTGGCGAAAGCGGCTTCCTGGACTAATACTGACAC
TTAGGTGCGAAAGCGTGGGGAGCAAACAGGATTAGATACCCCAGTAGTCC
>Otu3175
CCAGCCTATGGGTTGCTGCAGTTAGGAATCTTGCACAATGGGGGAAACCCTGATGCAGCG
ACGCCACGTGGAGGACGAAGGGTCTAGGCTCGTAAACTCCTTTTATCGGGAAAGACTTAG
GACGGTACCCGATGAATAAGCACCGGCTAACTACGTGCCAGCAGCCGCGGTAAGACGTAG
GGTGCAAGCGTTGTCCGGATTTACTGGGCGTAAAGAGCGCGTAGGCGGTCTGTTAAGTGT
AAAGTGAAATCTCCAGGGCTCAACCCGGAAATTGCTTTGCATACTGGCAGGCTAGAGGGT
TGCAGAGGTTTGTGGAATTCCCGGTGTAGCGGTGAAATACGTAGATATCGGGAGGAACAC
CCATGGCGAAGGCAGCAAACTGGGCAACATCTGACGCTGAGGCGCGAAAGCGTGGGTAGC
AAACAGGATTAGAACCCTTGTAGTCC
>Otu3186
CCAGCCTATGGGTCGCAGCAGTCGAGGATCTTCCGCAATGGGCGCAAGCCTGACGGAGCG
ACGCCGCGTGTGGGATGAAGGCCTTCGGGTTGTAAACCACTGTCACCGGTTATGAAAGGA
TCTGGTGTTAACAGCATCAGGGACTGACAAAGGCCGGAGAGGAAGCCACGGCTAACTTCG
TACCAGCAGCCGCGGTAAGACGAAGGTGGCGAGCGTTGTTCAGAATCACTGGGCATAAAG
CGCACGTAGGCGGCGACGTAAGTTGGGTGTGAAATCCCTCGGCCTAACTGAGGAACTGCG
CCCAGTACTGCGTTGCTTGAGGATCAAAGGGGTGCATGGAATTCCCGGTGGAGCGGTGAA
ATGCGTTGATATCGGGAGGAACTCCGGCGGCGAAAGCGGTGCACTGGTTGATTTCTGACG
CTGAGGTGCGAAAGCCAGGGGAGCAAACGGGATTAGATACCCCCGTAGTCC
>Otu3188
CCAGCCTATGGGTCGCTGCAGTGGGGAATTTTGCGCAGTGGGGGAAACCCTGACGCAGCA
ACGCCGCGTGGAGGATGCAGTCCCTTGGGACGTAAACTCCTTTCGATCGGGACGATTATG
ACGGTACCGGAAGAAGAAGCCCCGGCTAACTTCGTGCCAGCAGCCGCGGTAATACGAGGG
GGGCGAGCGTTGTTCGGAATTACTGGGCGTAAAGGGTTCGTAGGCGGGAATGCAAGTCAA
GTGTGAAATCCCCAGGCTCAACCTGGGACGTGCATTTGAGACTGTGTTTCTTGAGTTTCG
GAGAGGGTGGTGGAATTGCTGGTGTAGGAGTGACATCCGTAGAGATCAGCAGGAACACCG
GAGGCGAAGGCGACCACCTGGCCGAATACTGACGCTGAGGAACGAAAGCGTGGGGAGCAA
ACAGGATTAGAAACCCTAGTAGTCC
>Otu3192
CCAGCCTATGGGGGGCAGCAGTAGGGAATCTTCCGCAATGGGCGCAAGCCTGACGGAGCG
ACGCCGCGTGAGTGAAGACGGCCCTTGGGTTGTAAAGCTCTGTTCATCGGGAAGAAGATC
TGACGGTACCGATGAAGAAAGCCCCGGCTAACTACGTGCCAGCAGCCGCGGTAATACGTA
GGGGGCAAGCGTTGTCCGGAATGATTGGGCGTAAAGGGCGCGTAGGCGGATACATAAGTC
GGGCGTGAAAGGCACGGGCTCAACCCGTGTAAGCGTTCGAAACGGTGTATCTTGAGTGCG
GTAGAGGAAAGCGGAATTTCTGGTGTAGCGGTGGAATGCGTAGAGATCAGAAGGAACACC
GGTGGCGAAGGCGGCTTCCTGGACTGTAACTGACGCTGAGGCGCGAAGGCGTGGGGAGCA
AACAGGATTAGAGACCCTAGTAGTCC

>Otu3195
CCAGCCTATGGGGGGCAGCAGTGGGGAATATTGGACAATGGGCGCAAGCCTGATCCAGCC
ATGCCGCGTGAGTGATGACGGCCTTAGGGTTGTAAAGTTCTTTTGACAGGGAAGATGATG
ACTGTACCTGTAGAATAAGCACCGGCTAACTTCGTGCCAGCAGCCGCGGTAATACGAAGG
GTGCTAGCGTTGTTCGGAATTACTGGGCGTAAAGGGCGCGTAGGCGGGTTGACAAGTTGG
ATGTGAAAGCCCAGGGCTCAACCCTGGAATTGCATTCAAGACTGTCTTTCTTGAATTCGG
TAGAGGTTGGTAGAATTCCCAGTGTAGAGGTGAAATTCGTAGAGATTGGGAAGAATACCC
GTGGCGAAGGCGACCAACTGGACCGACATTGACGCTGAGGCGCGAAAGCGTGGGGATCAA
ACAGGATTAGAGACCCCCGTAGTCC
>Otu3197
CCAGCCTATGGGGCGCTGCAGTGGGGAATCTTGCACAATGGGGGCAACCCTGATGCAGCG
ACGCCGCGTGGAGGATGAAGGATCTAGGTCTGTAAACTCCTTTTGTTGGGAAAGACTTAG
GACGGTACCCAACGAATAAGCACCGGCTAACTACGTGCCAGCAGCCGCGGTAAGACGTAG
GGTGCAAGCGTTGTCCGGATTTACTGGGCGTAAAGAGCGCGTAGGCGGCTTGTTAAGTGT
GAAGTTAAATCTCCAGGCTCAACCTGGAAACCATTTCGCATACTGGCGAGCTGGAGGGAT
GGAGAGGTTAGTGGAATTCCTGGTGTAGCGGTGAAATGCGTAGATATCAGGAGGAACACC
CATGGCGAAGGCAGCTAACTGGCCATTTTCTGACGCTGAGGCGCGAAAGCGTGGGTAGCA
AACAGGATTAGATACCCTCGTAGTCC
>Otu3199
CCAGCCTACGGGTGGCAGCAGTCGAGAATCTTCGGCAATGGGCGCAAGCCTGACCGAGCG
ACGCCGCGTGGAGGATGAAGGCCTTCGGGTTGTAAAGCACTGTCGAGGGGGAGGAAGCTC
CGCAAGGGGTTGACCTATCCCTGGAGGAAGCACGGGCTAAATTCGTGCCAGCAGCCGCGG
TAATACGAATCGTGCGAACGTTGTTCGGAATCACTGGGCTTAAAGGGCGCGTAGGCGGTC
AATCAAGTCCGGGGTGAAATCTTTCGGCTTAACCGGAAAAGTGCCTTGGATACTGATTGG
CTGGAGGGAGGCAGGGGCAGCTGGAACTTCCAGTGGAGCGGTGAAATGCGTTGATATTGG
AAGGAACTCCGGTGGCGAAAGCGAGCTGCTGGTCCTCTTCTGACGCTGATGCGCGGAAGC
TAGGGGAGCAAACGGGATTAGAGACCCGTGTAGTCC
>Otu3200
CCAGCCTACGGGATGCAGCAGTCGAGGATCTTCGGCAATGGGCGCAAGCCTGACCGAGCG
ACGCCGCGTGTGCGATGAAGGCCTTCGGGTTGTAAAGCACTGTCGAGGGGGAGAAAAGCC
CGAAAGGGTTTGATCTATCCCTGGAGGAAGCACGGGCTAAGTTCGTGCCAGCAGCCGCGG
TAAGACGAACCGTGCGAACGTTGTTCGGAATCACTGGGCTTAAAGGGCGCGTAGGCGGGC
TGCCAAGTCTGTGGTGAAATCCCGCGGCTCAACCGTGGAACTGCCTTAGATACTGGCGGC
CTCGAGGGAGGTAGGGGCGAGCGGAACTGTGAGTGGAGCGGTGAAATGTGTTGATATTCA
CAGGAACTCCGGTGGCGAAGGCGGCTCGCTGGCCCTCTTCTGACGCTGAGGCGCGAAAGC
CAGGGGAGCAAACGGGATTAGAAACCCTAGTAGTCC
>Otu3201
CCAGCCTATGGGTCGCAGCAGTTGGGAATTTTGCACAATGGACGAAAGTCTGATGCAGCG
ACGCCGCGTGGAGGATGAAGGTCTTCGGATTGTAAACTCCTTTTACTTGGGAATAATTTT
TTGAAGGTACCAAGTGAATAAGCACCGGCTAACTCCGTGCCAGCAGCCGCGGTAATACGG
AGGGTGCAAACGTTGTCCGGAATTACTGGGCGTAAAGCGCATGTAGGTGGCTTTGTAAGT
CAGCGATCAAAGACCGACGCTTAACGTCGGGACCGTTGCTGATACTGCAAGGCTAGAGGA
AAACAGGGGTACGCGGAACTTTAGGTGTAGGAGTGAAATCCGTAGATATCTAAAGGAACA
CCAATGGCGAAGGCAGCGTACTGGGTTTTATCTGACACTAAGATGCGAAAGCGTGGGGAG
CAAACGGGATTAGAGACCCTAGTAGTCC
>Otu3203
CCAGCCTATGGGTGGCAGCAGTGGGGAATATTGGACAATGGGGGAAACCCTGATCCAGCG
ACGCCGCGTGTGTGAAGAAGGCCTGCGGGTTGTAAAGCACTTTTAGTGGGGACAAAAAGC
CATGGACTAATACTCTGTGGTCTTGATTTAACCCAAAGAAAAAGCACCGGCTAACTCTGT
GCCAGCAGCCGCGGTAATACAGAGGGTGCGAGCGTTAATCGGAATTACTGGGCGTAAAGC
GCGCGTAGACGGTTTTGTAAGTCAGATGTGAAATCCCTGGGCTCAACCTAGGAACTGCAT
TTGAGACTGCATGGCTAGAGTATCGAAGAGGGAAGTGGAATTTCCGGTGTAGCGGTGAAA
TGCGCAGATATCAGGAGGAACACCGGTGGTGTAAACGGCTTACTGGACGATAACTGACGC
TGATGCACGAAAGCGTGGGTAGCAAACAGGATTAGAAACCCCAGTAGTCC
>Otu3204
CCAGCCTATGGGGCGCAGCAGTGGGGAATATTGGACAATGGGGGCAACCCTGATCCAGCA
ATGCCGCGTGTGTGAAGAAGGCCTTCGGGTTGTAAAGCACTTTAGGCGAGGAGGAAGGTA
TAGTGTTTAAGAGATGCTATGCTTGACTGTACTCGCAGAATAAGCACCGGCTAACTCTGT

GCCAGCAGCCGCGGTAATACAGAGGGTGCGAGCGTTAATCGGAATTACTGGGCGTAAAGG
GCGCGTAGGCGGTTATTTAAGTTAGATGTGAAATCCCTGGGCTTAACCTAGGAACTGCAT
CTAAGACTGGATGACTAGAGTATGGCAGAGGGTAGTGGAATTTCCGGTGTAGCGGTGAAA
TGCGTAGAGATCGGAAGGAACACCAGTGGCGAAGGCGGCTACCTGGGCTACTACTGACGC
TGAGGCGCGAAAGCGTGGGGAGCAAACAGGATTAGATACCCTCGTAGTCC
>Otu3205
CCAGCCTACGGGGTGCAGCAGTGGGGAATTTTGCGCAATGGGGGAAACCCTGACGCAGCA
ACGCCGCGTGGAGGATGAAATCCCTTGGGATGTAAACTCCTTTCGATCGGGACGATTATG
ACGGTACCGGAAGAAGAAGCCCCGGCTAACTTCGTGCCAGCAGCCGCGGTAATACGAGGG
GGGCAAGCGTTGTTCGGAATTATTGGGCGTAAAGGGTGCGTAGGCGGTGCCACAAGTCAC
TTGTGAAATCCCCAGGCTTAACCTGGGGCCTGCAGGCGAAACTGTGGTGCTGGAGTATGG
GAGAGGTGCGTGGAATTCCCGGTGTAGCGGTGAAATGCGTAGATATCGGGAGGAACACCT
GTGGCGAAAGCGGCGCACTGGACCATAACTGACGCTGAGGCGCGAAAGCTAGGGGAGCAA
ACAGGATTAGATACCCGAGTAGTCC
>Otu3208
CCAGCCTATGGGAGGCTGCAGTGGGGAATATTGGACAATGGGGGCAACCCTGATCCAGCC
ATGCCGCGTGCAGGAAGAAGGCGTTATGCGTTGTAAACTGCTTTTGATTGGGAATAAAAA
GACCCTGCGGGGTAAATTGAATGTACCAATAGGATAAGCCACGGCTAACTACGTGCCAGC
AGCCGCGGTAATACGTAGGTGGCAAGCGTTATCCGGATTTATTGGGTTTAAAGGGAGCGT
AGGCGGTTTTGTAAGTCAGTGGTGAAAGACGGTCGCTCAACGATCGCATTGCCATTGATA
CCGCAAAGCTAGAGTACAGTATAGGTGGGCGGAATTTATGGTGTAGCGGTGAAATGCTTA
GATACCATAAAGAACACCGATTGCGTAGGCAGCTCACTGGACTGTAACTGACGCTGAGGC
TCGAAAGTGTGGGTATCAAACAGGATTAGAGACCCCAGTAGTCC
>Otu3210
CCAGCCTACGGGGTGCTGCAGTAGGGAATATTGCTCAATGGGGGAAACCCTGAAGCAGCA
ACGCCGCGTGAGTGATGAAGGCCTTCGGGTCGTAAAGCTCTGTTGTACGGGACGAATGCG
GCTAGAGATAACATTTCTAGCCAGTGACAGTACTGTATAAGAAAGGACAGGCTAACTTCG
TGCCAGCAGCCGCGGTAAGACGGGGGGTCCAAGCGTTGTTCGGAATTATTGGGCGTAAAG
CGTGTGTAGGCGGTGTTGTAAGTTGGATGTGAAAGCCCCGAGCTTAACTTGGGAAGTGCA
TTCAAAACTACAAAACTTGAGTATGGAAGAGGGTCGTGGAATTCCTGGTGTAGTGGTGAA
ATACGTAGATATCAGGAGGAACACCGGCGGCGAAGGCGGCGACCTGGTCCTATACTGACA
CTCAAACACGAAAGCGTGGGGATCAAACGGGATTAGAGACCCGCGTAGTCC
>Otu3211
CCAGCCTACGGGGGGCAGCAGTGGGGAATATTGCACAATGGACGAAAGTCTGATGCAGCG
ACGCCGCGTGGAGGATGAAGGTTTTCGGATTGTAAACTTCTGTTAAGTGGGAAGAAAGAC
TTTTACCTAATACGTAAAGGGGATGACGGTACCACTAGAGAAAGCGCCGGCAAACTTCGT
GCCAGCAGCCGCGGTAATACGAAGGGTGCTAGCGTTATTCGGAATAACTGGGTGTAAAGA
GCGTGCAGGCGGCCTGCTAAGTCGATCGTCGAAGCGCCCAGCTTAACTGGGCAAAGGCGA
GCGAAACTGATAGGCTAGAGTATGGGAGAGAAGAGTGGAATTCTCGGAGTAGCGGTAAAA
TGCGTAGATCTCGAGAGGAACACCGGTTGCGAAGGCGGCTCTTTGGCCCATTACTGACGC
TTAAGCGCGAAAGCGTGGGGAGCAAACAGGATTAGAAACCCTCGTAGTCC
>Otu3214
CCAGCCTATGGGTGGCAGCAGTGAGGAATATTGGTCAATGGGCGCAAGCCTGAACCAGCC
ATGCCGCGTGAAGGATGAAGGCCTTCTGGGTTGTAAACTTCTTTTATGTGGGAAGAAACC
CCGCTTTTCTAAGCGGGTTGACGGTACCATAGGAATAAGCACCGGCTAACTCCGTGCCAG
CAGCCGCGGTAATACGGAGGGTGCAAGCGTTATCCGGATTTACTGGGTTTAAAGGGTGTG
TAGGCGGGCAATTAAGTCAGTGGTGAAATCTCCGAGCTTAACTTGGAAACTGCCATTGAT
ACTATTTGTCTTGAATTCTGTTGAGGTGGGCGGAATATGTCATGTAGCGGTGAAATGCAT
AGAGATGACATAGAACCCCAATTGCGAAGGCAGCTCACTAAACAGTTATTGACGCTGAGG
CACGAAAGCGTGGGGAGCAAACAGGATTAGATACCCGGGTAGTCC
>Otu3215
CCAGCCTACGGGATGCAGCAGTGGGGAATCTTGCGCAATGGGCGAAAGCCTGACGCAGCC
ACGCCGCGTGAGTGAAGAAGGCCTTCGGGTTGTAAAGCTCTGTCGAGCGGGACGAATGTC
CAGTCGGTTCATAGCCGATTGGGGTGACGGTACCGCTAAAGGAAGCACCGGCTAACTCTG
TGCCAGCAGCCGCGGTAATACAGAGGGTGCAAGCGTTGCTCGGAATTATTGGGCGTAAAG
GGTAGGTAGGTGGTCTCATTTGTCTGGGGTGAAATCCTTGGGCTTAACCCAAGAAGTGCC
CCAGAAACGGTGAGACTGGAGTCCTGGAGAGGGTCGTGGAATTCCCGGTGTAGCGGTGAA
ATGCGTAGAGATCGGGAGGAACACCAGAGGCGAAGGCGGCGACCTGGACAGGTACTGACA

CTCAACTACGAAAGCGTGGGGAGCAAACAGGATTAGAAACCCCCGTAGTCC
>Otu3217
CCAGCCTACGGGTTGCTGCAGTGGGGAATATTGCGCAATGGGGGAAACCCTGACGCAGCA
ACGCCGCGTGAGTGAAGAAGGCCTTAGGGTTGTAAAGCTCTGTTGTACGGGAAAATTATG
ATGGTACCGTATAAGAAAGGACCGGCTAACTTCGTGCCAGCAGCCGCGGTAAGACGAGGG
GTCCTAGCGTTGTTCGGAATCATTGGGCGTAAAGCGCACGTAGGTGGCTTTGTAAGTCAG
ATGTGAAAGCCCTGGGCTCAACCCAGGAAGTGCATTTGATACTGCAAAGCTTGAATATGG
GAGAGGGTAGTAGAATTCCTGGTGTAGTGGTGAAATACGTAGATATCAGGAGGAATACCG
GTGGCGAAGGCGGCTACCTGGCCCAATATTGACACTGAGGTGCGAAAGCGTGGGGATCAA
ACAGGATTAGAGACCCGAGTAGTCC
>Otu3222
CCAGCCTATGGGGCGCACCAGTAGGGAATCTTCCACAATGGGCGAAAGCCTGATGGAGCA
ACGCCGCGTGCAGGACGAAGGCCTTAGGGTTGTAAACTGCTTTTATCTGTGACGAATATG
ACGGTAGCAGATGAATAAGGATCGGCTAACTCCGTGCCAGCAGCTGCGGTCATACGGAGG
ATCCAAGCGTTATCCGGAATTACTGGGCGTAAAGAGTTGCGTAGGTGGCATAGTAAGTAG
ATAGTGAAAGCGTGTGGCTCAACCATATACACATTATCTAAACTGCTGAGCTAGAGGATG
AGAGAGGTTATTGGAATTCCCAGCGTAGGAGTGAAATCCGTAGATATTGGGAGGAACACC
GATGGCGTAGGCAGATAACTGGCTCATTCCTGACACTAAGGCACGAAAGCGTGGGTAGCA
AACGGGATTAGAGACCCTAGTAGTCC
>Otu3227
CCAGCCTATGGGAGGCTCCAGTCGAGGATCTTCGGCAATGGACGCAAGTCTGACCGAGCG
ACGCCGCGTGCGGGATGAAGGCTTTCGGGTTGTAAAGCGCTGTCGAGGGGGAGGAAGCCG
CAAGGTGGACCTATCCCTGGAGGAAGCACGGGCTAAGTTCGTGCCAGCAGCCGCGGTAAG
ACGAACCGTGCGAACGTTGTTCGGAATCACTGGGCTTAAAGGGCGCGTAGGCGGATCACC
AAGTCCGGGGTGAAAGCCCCCAGCTTAACTGGGGAAGTGCCTTGGATACTGGTGGTCTCG
AGGGAGGTAGGGGCAGTGGGAACTTCCGGTGGAGCGGTGAAATGCGTTGATATCGGAAGG
AACGCCGGTGGCGAAAGCGCACTGCTGGACCTCTACTGACGCTGAGGCGCGAAAGCCAGG
GGAGCAAACGGGATTAGAAACCCTAGTAGTCC
>Otu3231
CCAGCCTATCGGGTGCTCCAGCGAGGGATTTTGCGCAATGGAGGAAACTCTGACGCAGCG
ACGCCGCGTGCGGGACGAAGCCTTTCGGGGTGTAAACCGCTGTCAGTGGAGAAGAGAATG
GACGGTACCCACTGAGGAAGCCTCGGCTAACTACGTGCCAGCAGCCGCGGTAAGACGTAG
GGGGCGAGCGTTGTCCGGAATCACTGGGCGTAAAGAGCTCGTAGGCGGGATTGTTAGTCT
CGGAGTAAATCCATCTGCTCAACGGATGGTTCATCTGGGATACGGCAATTCTTGAGGACA
GGAGAGGAAAGCGGAATTCCGAGTGTAGCGGTGAAATGCGTAGAGATTCGGAGGAACACC
AGTGGCGAAGGCGGCTTTCTGGCCTGATTCTGACGCTGAGGAGCGAAAGCCAGGGGAGCG
AACGGGATTAGATACCCCAGTAGTCC
>Otu3235
CCAGCCTATGGGGCGCTGCAGTCGAGGATCTTCGTCAATGGGCGCAAGCCTGAACGAGCG
ACGCCGCGTGTGCGATGAAGGCCTTCGGGTTGTAAAGCACTGTCGAGGGGGATCAAGGCG
CAAGCTTGAGTGATCCCTGGAGGAAGCACGGGCTAAGTTCGTGCCAGCAGCCGCGGTAAG
ACGAACCGTGCGAACGTTGTTCGGAATCACTGGGCTTAAAGGGCGCGTAGGCGGCCTGCA
AGGTCGGGGGTGAAATCCCACGGCTTAACCGTGGAAGTGCCTTCGATACCGGCGGGCTCG
AGGAAGGTAGGGGTGCGCGGAACTTCTGGTGGAGCGGTGAAATGCGTTGATATCAGAAGG
AACGCCGGCGGCGAAAGCGGCGCACTGGACCTTTTCTGACGCTGAGGCGCGAAAGCCAGG
GGAGCAAACGGGATTAGAGACCCGGGTAGTCC
>Otu3238
CCAGCCTATGGGTCGCTGCAGTCGAGAATTTTTCTCAATGGGGGAAACCCTGAAGGAGCG
ACGCCGCGTGAAGGATGAAGGTCTTCGGATTGTAAACTTCTGTCATTGGGGAACAATGTG
CGAGGGAGTAACTGCCCACGTATTGATAGTACCCGAAGAGGAAGAGACGGCTAACTCTGT
GCCAGCAGCCGCGGTAATACAGAGGTCTCAAGCGTTGTTCGGATTCATTGGGCGTAAAGG
GTGCGTAGGTGGCGAAGTAAGTCGGGTGTGAAATCTCGGGGCTTAACCCCGAAACTGCAT
TCGATACTGCTTTGCTCGAGGACTGGAGAGGAGATCGGAATTCATGGTGTAGCAGTGAAA
TGCGTAGATATCATGAGGAAGACCAGTGGCGAAGGCGGATCTCTGGACAGTTCCTGACAC
TGAGGCACGAAGGCCAGGGGAGCAAACGGGATTAGAGACCCCCGTAGTCC
>Otu3240
CCAGCCTATGGGTGGCACCAGGCCGACATCGGTCATCAGCGTTGATCCGACATTTAGGGA
TTCGAGCGCGGTGAGTCCTCCGATGTGTTCGAGTGCGCTATCTCCGGCTTTGGTTCCGTG

CAGGTTGAGGCGCTTCAGTTGTTTCCAATCGTGAATGGCGGCGATGCCTTCGTCGGTGAT
GTACTCGGCGTAGTAGAGGCTGAAGTCGGTGATGCCATGGAGATTCTTGATCTCCTGCAA
GCCGGCGTCGGTGATGTGGGTGAGTGAAAGATCGAGGGTGGTCAGGGAAGGATAGTTGTT
GAGCTGGCGCAGATCTGTGTCGTTGACCCAGGTTCCGCGCAGGCTGATGCCGGTGACCGC
GCCTTTAGCGTTGCGTTGAACGGTGCCGCCCAGATCGGTTATCCAGTGAGTGTCTTCCTG
CGCAAAGCTGGTGACGGCGGAGATTAGAAACCCTAGTAGTCC
>Otu3243
CCAGCCTACGGGTTGCAGCAGTGGGGAATATTGGACAATGGGCGAAAGCCTGATCCAGCA
ATGCCGCGTGAGTGATGAAGGCCCTAGGGTTGTAAAGCTCTTTCGCACGCGACGATGATG
ACGGTAGCGTGAGAAGAAGCCCCGGCTAACTTCGTGCCAGCAGCCGCGGTAATACGAAGG
GGGCTAGCGTTGTTCGGAATTACTGGGCGTAAAGCGCACGCAGGCGGTCCGTTAAATTAG
AAGTGAAAGCCCTGGGCTCAACCCGGGAATTGCTTTTAAGACTGGCGGACTAGAATCCGG
AAGAGGGTAGCGGAATTCCCAGTGTAGAGGTGAAATTCGTAGATATTGGGAAGAACACCG
GTGGCGAAGGCGGCTACCTGGTCCGGAATTGACGCTCATGTGCGAAAGCGTGGGGAGCAA
ACAGGATTAGATACCCGCGTAGTCC
>Otu3244
CCAGCCTACGGGAGGCTCCAGTCGAGGATCTTCCGCAATGAGCGCAAGCTTGACGGAGCA
ACGCCGCGTGTGGGATGAAGGCCTTCGGGTTGTAAACCACTGTCACTCGTTAAGAAAGGG
TTGGGGGTTAACAGCCTCCAGCACTGACTAAGGCGAGAGAGGAAGCCACGGCTAACTTCG
TGCCAGCAGCCGCGGTAAGACGAAGGTGGCGAGCGTTGTTCGGAATCACTGGGCATAAAG
CGCACGTAGGCGGTGCCGTAAGTCGGGTGTGAAATCCCTCGGCCTAACCGAGGAACTGCG
CCCGATACTGCGGTGCTTGAGGACCAGAGGGGTGCATGGAATTTCCGGTGGAGCGGTGAA
ATGCGTTGATATCGGAAGGAACTCCGGCGGCGAAAGCGATGCACTGGCTGGTTTCTGACG
CTGAGGTGCGAAAGCCAGGGGAGCAAACGGGATTAGAAACCCCCGTAGTCC
>Otu3245
CCAGCCTATGGGTGGCTCCAGTGGGGAATATTGCTCAATGGGGGAAACCCTGAAGCAGCA
ACGCCGCGTGAGTGATGAAGGCCTTCGGGTCGTAAAGCTCTGTTGTACGGGACGAATGCG
CCTATGGCTAACATCCATGGGCAGTGACAGTACTGTATAAGTAAGGACCGGCTAACTTCG
TGCCAGCAGCCGCGGTAAGACGGGGGGTCCTAGCGTTGTTCGGAATCATTGGGCGTAAAG
CGTGTGCAGGTGGTTCTGTAAGTTAGATGTGAAAGCCTTGGGCTTAACCCAAGAAGTGCA
TTTAAAACTACAAAACTTGAATATGGGAGAGGGTCGTGGAATTCCTGGTGTAGTGGTGAA
ATACGTAGATATCAGGAGGAACACCGGTGGCGAAGGCGGCGACCTGGTCCAATATTAACA
CTCATACACGAAAGCGTGGGGATCAAACAGGATTAGAGACCCGAGTAGTCC
>Otu3246
CCAGCCTACGGGTGGCTGCAGTGGGGAATCTTGCGCAATGGACGAAAGTCTGACGCAGCC
ACGCCGCGTGAGTGAAGAAGGCCTTCGGGTTGTAAAGCTCTGTCGCCCGGGACGAACAGC
CGATTGGTGAATAGCCAGTCGGTCTGACGGTACCGGGAAAGGAAGCACCGGCTAACTTCG
TGCCAGCAGCCGCGGTAATACGAGGGGTGCAAGCGTTGCTCGGAATTATTGGGCGTAAAG
GGTAGGTAGGTGGTCTCATTTGTCTGGGGTGAAAGCCTCGAGCTTAACTCGAGAAGTGCC
CCAGAAACGGTGAGACTGGAGTCCTGGAGAGGGTCGTGGAATTCCCGGTGTAGCGGTGAA
ATGCGTAGAGATCGGGAGGAACACCAGAGGCGAAGGCGGCGACCTGGACAGGTACTGACA
CTCAACTACGAAAGCGTGGGGAGCAAACAGGATTAGAGACCCCGGTAGTCC
>Otu3249
CCAGCCTACGGGATGCAGCAGTGGGGAATTTTCCGCAATGGGCGCAAGCCTGACGGAGCA
ACGCCGCGTGGGTGACGAAGTCTTTCGGGATGTAAAGCCCTTTCAGCTGGGAAGAAACAA
ATGACGGTACCAGCAGAAGAAGCATCGGCTAACTACGTGCCAGCAGCCGCGGTAATACGT
AGGATGCAAGCGTTGTCCGGATTTATTGGGCGTAAAGCGTACGTAGGCGGCCTTGTAAGT
CGGTGGTTAAAGCCCTCAGCTCAACTGCGGGACTGCCATCGAAACTGTGAGGCTGGAGGT
TGGTAGAGGAAGTCGGAATTCCCAGTGTAGCGGTGAAATGCGTAGATATTGGGAGGAACA
CCAGTGGCGAAAGCGGACTTCTGGGCCACACCTGACGCTGAGGTACGAAAGCCAGGGTAG
CGAACGGGATTAGAAACCCCTGTAGTCC
>Otu3253
CCAGCCTACGGGGCGCACCAGTGGGGAATTTTACGCAATGGGGGAAACCCTGACGTAGCG
ACACCGCGTGAACGAAGAAGCCTTTTGGGGTGTAAAGTTCTGTCAGCTGGAACGAAAAAA
ATGACGGTACCAGCAGAGGAAGCATCGGCTAACTACGTGCCAGCAGCCGCGGTAAGACGT
AGGATGCAAGCGTTGTCCGGATTTATTGGGCGTAAAGAGTTCGTAGGTGGTTTGTTAAGT
TTGATGTTAAAGATCAGGGCCCAACCCTGGGAGTGCATTGAATACTGGCAGACTGGAGTG
TAGTAGGGGCAAGCGGAATTCCCAGTGTAGCGGTGAAATGCGTAGATATTGGGAAGAACA

CCAGTGGCGTAAGCGGCTTGCTGGGCTACAACTGACACTGAGGAACGAAAGCCAGGGGAG
CAAATGGGATTAGATACCCCTGTAGTCC
>Otu3254
CCAGCCTATGGGTGGCACCAGTAGGGAATTTTCCACAACGGGCGAAAGCCTGATGGAGCA
ACGCCGCGTGCAGGATGAAGGCCTTCGGGTTGTAAACTGCTTTTATATGTGAAGATTATG
ACGGTAGCATATGAATAAGGATCGGCTAACTCCGTGCCAGCAGCCGCGGTCATACGGAGG
ATCCAAGCGTTATCCGGAATTACTGGGCGTAAAGAGTTGCGTAGGTGGCATAGTAAGCGG
ACAGTGAAAGCGTGTGGCTCAACCATATACACATTGTCTGAACTGCTAAGCTAGAGGACG
AGAGAGGTACCTGGAATTCCTAGTGTAGGAGTGAAATCCGTAGATATTAGGAGGAACACC
GATGGCGTAGGCAGGGTACTGGCTTGTTCCTGACACTAAGGCACGAAAGCGTGGGGAGCG
AACGGGATTAGATACCCGCGTAGTCC
>Otu3257
CCAGCCTACGGGGGGCAGCAGTGGGGAATCTTGCACAATGGGCGAAAGCCTGATGCAGCA
GCGCCGCGTGAGGGACGAATGCTTTCTGAGTTGTAAACCTCTTTCAGCAGGGACGATAAT
GACGGTACCTGCAGAAGAAGCACCGGCCAACTACGTGCCAGCAGCCGCGGTGATACGTAG
GGTGCAAGCGTTGTCCGGATTTATTGGGCGTAAAGAGCTCGTAGGCGGTTTGATAAGTCG
GGTGTGAAACCCCCAGGCTCAACCTGGGGACGCCACTCGATACTGTTATGACTAGAGTCT
GGTAGGGGATCACGGAATTCCTGGTGTAGCGGTGGAATGCGCAGATATCAGGAGGAACAC
CAGTAGCGAAGGCGGTGATCTGGGCCAGTACTGACGCTGAGGAGCGAAAGCGTGGGGAGC
GAACAGGATTAGAGACCCCAGTAGTCC
>Otu3258
CCAGCCTACGGGGTGCTGCAGTGGGGAATTTTACACAATGGGGGAAACCCTGATGTAGCG
ACACCGCGTGAGCGAAGAAGCCCTTTGGGGTGTAAAGCTCTGTCGGCAGGGACGAAAAAA
ATGACGGTACCTGCAGAGGAAGCATCGGCTAACTACGTGCCAGCAGCCGCGGTAAGACGT
AGGATGCGAGCGTTGTCCGGATTTATTGGGCGTAAAGAGTTCGTAGGTGGTTTTATAAGT
CTGGTGTAAAAGGCGCGGGCTCAACCCGTGTAGAGCACTGGATACTGTAAGACTTGAGTT
AAGTAGAGGCAAGCGGAATTCCCAGTGTAGCGGTGAAATGCGTAGATATTGGGAAGAACA
CCGGTGGCGCAAGCGGCTTGCTGGGCTTAAACTGACGCTGAGGCACGAAAGCGTGGGGAG
CAAACAGGATTAGAAACCCCCGTAGTCC
>Otu3260
CCAGCCTACGGGTGGCAGCAGTGGGGAATCTTGCGCAATGGACGAAAGTCTGACGCAGCC
ACGCCGCGTGAGTGAAGAAGGCCTTCGGGTTGTAAAGCTCTGTCGTGAGGGACAAAAACC
GCTTAGGTGAATAGCCTAGGCGCTTGATGGTACCTTGAAAGGAAGCACCGGCTAACTTCG
TGCCAGCAGCCGCGGTAATACGAAGGGTGCAAGCGTTGCTCGGAATTATTGGGCGTAAAG
GGTAGGTAGGTGGTTACGTATGTCTGAGGTGAAATCCCTGAGCTTAACTCAGGACGTGCC
TTGGAAACGGCGTAACTAGAGTGCTGGAGAGGTTCGCAGAATTCCCGGTGTAGCGGTGAA
ATGCGTAGAGATCGGGAGGAATACCAGAGGCGAAGGCGGCGAACTGGACAGTTACTGACA
CTAAACTACGAAAGCGTGGGGAGCAAACAGGATTAGATACCCTTGTAGTCC
>Otu3261
CCAGCCTACGGGAGGCTCCAGTGGGGAATTTTGGACAATGGGCGAAAGCCTGATCCAGCG
ACGCCGCGTGAAGGATGAAGGCCTTCGGGTTGTAAACTTCTTTTAGAGGGGACGAAAAAA
ATGACGGTACCCTCGGAATAAGCCACGGCTAACTACGTGCCAGCAGCCGCGGTAATACGT
AGGTGGCGAGCGTTACTCGGAATTACTAGGCGTAAAGCGCATGTAGGCGGTCTGATAAGT
CCATGGTGAAATTTCCCGGCTCAACCGGGATAGGCCCATGGAAACTATCTGACTTGAGTG
TGGCAGGGGTAGACGGAATTCCCGGTGTAGCGGTGAAATGCGTAGATATCGGGAGGAACA
CCGATGGCGAAAGCAGTCTACTGGGCCAATACTGACGCTGATGTGCGAAAGCTAGGGGAG
CAAACAGGATTAGAAACCCTTGTAGTCC
>Otu3262
CCAGCCTATGGGACGCTGCAGTGGGGAATATTGGACAATGGGCGCAAGCCTGATCCAGCC
ATGCCGCGTGAGTGAAGAAGGCCTTAGGGTTGTAAAGCTCTTTTGGCGGGGACGATAATG
ACAGTACCCGCAGAATAAGCCCCGGCTAACTTCGTGCCAGCAGCCGCGGTAATACGTAGG
CAGCAAGCGTTGTTCGGAGTTACTGGGCGTAAAGGGTGTGTAGGCGGTTCCTTAAGTTTG
TTGTGAAATCTCCCGGCTTAACCGGGAGGGTGCGGCGAATACTGAGGGACTAGAGTACGG
GAGAGGAAAGTGGAATTCCTGGTGTAGCGGTGAAATGCGTAGATATCAGGAGGAACACCG
GTGGTGTAGACGGCTTTCTGGACCGACACTGACGCTGAGACACGAAAGCGTGGGTAGCAA
ACAGGATTAGATACCCCCGTTAGTCC
>Otu3265
CCAGCCTACGGGATGCAGCAGTGGGGAATATTGGACAATGGGCGCAAGCCTGATCCAGCC

ATGCCGCGCGAGTGAAGAAGGCCTTCGGGTTGTAAAGCTCTTTCAGGTGGGACGATGATG
ACGGTACCACCAGAAGAAGCTCCGGCTAACTTCGTGCCAGCAGCCGCGGTAATACGAAGG
GAGCTAGCGTTGTTCGGAATTACTGGGCGTAAAGCGCGCGTAGGCGGTTTACTCAGTCAG
AGGTGAAATCCTGGGGCTCAACCTCGGCATTGCCTTTGATACTGGTAAGCTTGAGTTCGG
AAGAGGATGGTGGAATACCCAGTGTAGAGGTGAAATTCGTAGATATTGGGTGGAACACCG
GTGGCGAAGGCGACCATCTGGTCCGATACTGACGCTGAGGTGCGAAAGCGTGGGGAGCAA
ACAGGATTAGAACCCTGGTAGTCC
>Otu3274
CCAGCCTACGGGTGGCTGCAGTGAGGAATATTGGTCAATGGACGCAAGTCTGAACCAGCC
ATGCCGCGTGAAGGATGAAGGCCTTCTGGGTTGTAAACTTCTTTTATGTGGGAAGAAACC
TCTGATTTCTATTAGAGTTGACGGTACCATAGGAATAAGCACCGGCTAACTCCGTGCCAG
CAGCCGCGGTAATACGGAGGGTGCAAGCGTTATCCGGATTTACTGGGTTTAAAGGGTGTG
TAGGCGGGTCTTTAAGTCAGTGGTGAAATCTCCGAGCTCAACTTGGAAACTGCCATTGAT
ACTATTGATCTTGAATTCTGTTGAGGTGGGCGGAATAAGTCATGTAGCGGTGAAATGCAT
AGATATGACTTAGAACCCCGATTGCGAAGGCAGCTCACTAAGCAGACATTGACGCTGAGG
CACGAAAGCGTGGGGATCAAACAGGATTAGATACCCCAGTAGTCC
>Otu3275
CCAGCCTATGGGGTGCTCCAGTGGGGAATTTTGGACAATGGGCGAAAGCCTGATCCAGCC
ATGCCGCGTGAGTGAAGAAGGCCTTCGGGTTGTAAAGCTCTTTCGCAAGGAAAGAAAACT
TAGCTACTAATACTAGCTGAGGATGACGGTACCTTGATAAGAAGCACCGGCTAACTACGT
GCCAGCAGCCGCGGTAATACGTAGGGTGCGAGTGTTAATCGGAATTACTGGGCGTAAAGC
GTGCGCAGGCGGTTCGATAAGTCTGATGTGAAATCCCCGGACTCAACCTGGGAACTGCGT
TGGAAACTGTCGAGCTAGAGTGTAGAAGAGGGGGGTAGAATTCCACGTGTAGCAGTGAAA
TGCGTAGAGATGTGGAGGAATACCGATGGCGAAGGCAGCCCCCTGGGCTAACACTGACGC
TCATGCACGAAAGCGTGGGGAGCAAACAGGATTAGAAACCCCGGTAGTCC
>Otu3283
CTAGCCTACGGGGGGCTGCAGTGGGGAATATTGGACAATGGGCGAAAGCCTGATCCAGCC
ATGCCGCGTGAGTGATGAAGGCTCTAGGGTTGTAAAGCTCTTTCGCCGATGAAGATAATG
ACGGTAGTCGGAGAAGAAGCCCCGGCTAACTTCGTGCCAGCAGCCGCGGTAATACGAAGG
GGGCTAGCGTTGTTCGGAATTACTGGGCGTAAAGCGCGCGTAGGCTGTGCGACAAGTCAG
TGGTGAAATCCCGGAGCTCAACTTCGGAACTGCCATTGAAACTGTCGTGCTGGAATCTCA
GAGGGGGTAGCGGAATTCCAAATGTAGAGGTGAAATTCGTAGATATTTGGAGGAACACCG
GTGGCGAAGGCGGCTACCTGGCTGAGTATTGACGCTGAGGCGCGAAAGCGTGGGGATCAA
ACAGGATTAGAAACCCGAGTAGTCC
>Otu3284
CCAGCCTACGGGGGGCAGCAGCAAGGAATTTTGTGCAATGGGCGAAAGCCTGACACAGCG
ACACCGCGTGGGGGATGAAGGCCTTCGGGTTGTAAACCCCTTTTCTCAGGGAGGAAAACT
GACAGTACCTGAGGAATAAGTGACGGCTAACTACGTGCCAGCAGCCGCGGTAATACGTAG
GCCACGAGCGTTATCCGGATTTACTGGGCGTAAAGAGTGCGCAGACGGTCTTTCAAGTCG
GATGTAAAATCTCCTGGCTTAACTGGGAGGGATCACCCGATACTGTGAGACTTGAGGACA
ACATAGGAAGGTAGAATTCCCGGTGTAGTGGTGAAATGCGTAGATATCGGGAGGAATACC
AGTGGCGAAGGCGGCCTTCTGGGTTGTTTCTGACGTTCAGGCACGAAAGTGTGGGGAGCA
AACAGGATTAGATACCCTCGTAGTCC
>Otu3285
CCAGCCTATGGGGCGCTCCAGTCGAGAATCTTCCGCAATGGGCGAAAGCCTGACGGAGCG
ACGCCGCGTGATCGATGAAGTCCCTCTGGGACGTAAAGATCTTTTGTGGGATAGAAAGTT
TATTGATCATCCCAAGAATAAGAGGTTGCTAAACTCGTGCCAGCAGCAGCGGTAATACGA
GTGCCTCAAGCGTTATCCGGAATCATTGGGCGTAAAGGGTGTGTAGGCGGTCGCGTTAGT
CTTCTGTCAAATTCTTCGGCTTAACCGGGGGCTCGCAGAGGAAACGGCGCGACTTGAGAG
TGCGAGGGGTGAATGGAACTCATGGAGTAGGGGTGAAATCCGTTGATATCATGGGGAACA
CCAAAAGCGAAGGCAATTCACTGGCGCATTTCTGACGCTGAAACACGAAAGCGTGGGTAG
CGAATGGGATTAGAGACCCGTGTAGTCC
>Otu3289
CCAGCCTATGGGTCGCTCCAGTGAGGAATATTGGTCAATAGGGGGAACCCTGAACCAGCC
ATGCCGCGTGAAGGAAGAATGCCCTAAGGGTTGTAAACTTCTTTTGTACGGGAGTAAAGA
CAGGTACGCGTACCTGAGTGAAAGTACCGTGCGAATAAGCATCGGCTAACTCCGTGCCAG
CAGCCGCGGTAATACGGAGGATGCGAGCGTTATCCGGATTTATTGGGTTTAAAGGGTGCG
TAGGCGGACTTATAAGTCAGTGGTGAAATCTCATCGCTTAACGATGAACGTGCCATTGAT

ACTGTAGGTCTTGAGTACAGATGCCGTTGGCGGAATGTGTCATGTAGCGGTGAAATGCAT
AGATATGACACAGAACACCGATTGCGAAGGCAGCTGACGAAACTGTAACTGACGCTGAGG
CACGAAAGCGTGGGGATCAAACAGGATTAGATACCCCTGTAGTCC
>Otu3293
CCAGCCTACGGGAGGCACCAGTCGAGAATCATTCGCAATGGGCGAAAGCCTGACGATGCG
ACGCCGTGTGTGCGATGAAGGCCTTCGGGTTGTAAAGCACTTTCGCCTGGGAACAAGAGA
AGCTGGCTAACATCCAGTGGATTTGAGGGTACTAGGTAAAGAAGCACCGGCTAACTACGT
GCCAGCAGCTGCGGTAATACGTAGGGTGCAAGCATTAATCGGATTTATTGGGCGTAAAGG
GCGCGTAGGCTGATGTGTAAGTCAGATGTGAAATCCCGGGCTCAACCCCGGAACAGCAT
TTGAAACTGCACGTCTAGAGGGTAGGCGGAGAAAACGGAATTCCACAAGTAGCGGTGAAA
TGCGTAGATATGTGGAAGAACACCTGTGGCGAAGGCGGTTTTCTAGCTTATACCTGACGC
TGAGGCGCGAAAGCAAGGGGATCAAACAGGATTAGAAACCCGTGTAGTCC
>Otu3294
CCAGCCTATGGGGGGCTCCAGTCGAGAATTTTTCTCAATGGGGGAAACCCTGAAGGAGCG
ACGCCGCGTGGGGGATGAATGGCTTCGGCCCGTAAACCCCTGTCATTTGCGAACAAATTG
TTTCAGCTAACAAATGAAGCATTGATTGTAGCAGAAGAGGAAGGGACGGCTAACTCTGTG
CCAGCAGCCGCGGTAATACAGAGGTCCCAAGCGTTGTTCGGATTCACTGGGCGTAAAGGG
TGCGTAGGCGGTAGGGTAAGTCTGATGTGAAATCTCCAAGCCTAACTTGGAAACTGCATC
GGAAACTATCCTGCTCGAGGAATGGAGGGGAGACTGGAATACTTGGTGTAGCAGTGAAAT
GCGTAGATATCAAGTGGAACACCAGTGGCGAAGGCGAGTCTCTAGACATTTCCTGACGCT
GAGGCACGAAAGCCAGGGGAGCAAACGGGATTAGATACCCTAGTAGTCC
>Otu3295
CCAGCCTATGGGGCGCTCCAGAGGAGGATCATCGTCAATGGGCGAAAGCCTGAACGTGCG
ACGCCGCGTGGGGGACGAAGGCCTTCGGGTCGTAAACCCCTGTCAGTTGCTGAGAACATA
CCGGCGGTTAATAGCCGCCGGTATTGACTAGTAGCAGAGGAAGGGACGGCTAACTTCGTG
CCAGCAGCAGCGGTAATACGGAGGTCCCTAGCGTTGTTCGGATTTACTGGGCGTAAAGGG
CGTCTAGGCGGGACGGCAAGTCGGGTATGAAATGCTCTGGCTCAACCAGAGAATGGTGCC
CGAAACTGCCGCCCTTGAGTATAGCAGAGGACAGTGGAACTCTTGGTGGAGCGGTGAAAT
GCGTAGATATCAAGAGGAACGCCGGAGGCGAAAGCGATTGTCTGGGCTATTACTGACGCT
GAAGCGCGAAAGCTAGGGGAGCAAACGGGATTAGATACCCTAGTAGTCC
>Otu3298
CCAGCCTACGGGATGCAGCAGTGGGGAATCTTGCACAATGGGGGAAACCCTGATGCAGCG
ACGCCGCGTGAGCGATGAAGCCCTTCGGGGTGTAAAGCTCTTTCGGCAGGGACGATAATG
ACGGTACCTGCAGAAGAAGCTGCGGCTAACTACGTGCCAGCAGCCGCGGTAATACGTAGG
CAGCAAGCGTTGTTCGGAATTACTGGGCGTAAAGAGTGCGTAGGCGGTTTGGCAAGTTCG
GTGTGAAATCTCCCGGCTTAACTGGGAGGGTGCGCCGAAAACTGCCAGACTAGAGTGTGG
GAGAGGGGAGTGGAATTTCCGGTGTAGCGGTGAAATGCGTAGATATCGGAAGGAACACCT
GTGGCGAAAGCGGCTCACTGGACCACAACTGACGCTGATGCACGAAAGCGTGGGGAGCAA
ACAGGATTAGATACCCCCGTAGTCC
>Otu3300
CCAGCCTATGGGTGGCTGCAGCTAAGAATCTTCCGCAATGGGCGAAAGCCTGACGGAGCG
ATGCCGCGTGATCGATGAAGGTCGAGAGATTGTAAAGATCTTTTCCGCATGAGGAATAAA
GCCAGGAGGGAATGCCTGGCAGATGACGTTAGTGCGGGAATAAGCCCCGGCTAATTACGT
GCCAGCAGCCGCGGTAACACGTAAGGGGCGAGCGTTGTTCGGAATTATTGGGCGTAAAGG
GCACGCAGGCGGTTGTGCAAGTCTGATGTGAAAGACCTGGGCTTAACTCAGGGGCCGCGT
TGGAAACTGCATAACTAGAGTATTGGAGGGGGAGTCGGAATTCCTAGTGTAGGGGTGAAA
TCTGTTGATATTAGGAAGAACACCGGTGGCGAAGGCGGACTCCTGGCCAATGATTGACGC
TGAGGTGCGAAAGTGTGGGGATCAAACAGGATTAGATACCCGCGTAGTCC
>Otu3301
CCAGCCTACGGGGGGCAGCAGTGGGGAATATTGGACAATGGGCACAAGCCTGATCCAGCC
ATGCCGCGTGAGTGAAGAAGGCCTTAGGGTTGTAAAGCTCTTTTGGCGGGGACGATAATG
ACGGTACCCGCAGAATAAGCCCCGGCTAACTTCGTGTCAGCAGCCGCGGTAATACGAAGG
GGGCTAGCGTTGTTCGGAATTACTGGGCGTAAAGGGCGCGTAGGCGGTTCGACAAGTCAG
ATGTGAAAGCCCTGGGCTCAACCCAGGAAATGCGTTTGATACTGTCGTGCTTGAATTCGG
GAGAGGTTGGTAGAATTGCCAGTGTAGAGGTGAAATTCGTAGATATTGGCAAGAATACCC
GTGGCGAAGGCGGCCAACTGGACCGACATTGACGCTGAGGCGCGAAAGCGTGGGGAGCAA
ACAGGATTAGAAACCCCCGTAGTC
>Otu3302

CCAGCCTATGGGGTGCTGCAGTGGGGAATCTTGCACAATGGGGGAAACCCTGATGCAGCG
ACGCCGCGTGAGCGATGAAGCCCCTCGGGGTGTAAAGCTCTTTCGGCGGGGACGATAATG
ACGGTACCGGTGGAAGAAGCCCCGGCTAACTCTGTGCCAGCAGCCGCGGTAATACAGAGG
GGGCGAGCGTTGTTCGGAATTATTGGGCGTAAAGGGCGCGTAGGCGGTGCGGTAAGTCTT
TTGTGAAATCTCCGGGCTCAACTCGGAGTCTGCAGGAGAAACTGCCGTGCTTGAGTGTGG
GAGAGGTGAGTGGAATTCCCGGTGTAGCGGTGAAATGCGTAGATATCGGGAGGAACACCC
GTGGCGAAAGCGGCTCACTGGACCACAACTGACGCTGAGGCGCGAAAGCTAGGGGAGCAA
ACAGGATTAGAAACCCGAGTAGTCC
>Otu3303
CCAGCCTATGGGTGGCACCAGTTTCGAATTATTCCCAATGGGCGCAAGCCTGAGGGTGCA
ATACCGCGTGGAGGATGAAGGCCTTAGGGTCGTAAACTCCTGTCGTCAGGGACAAAACCT
TTTATTTTAATAGAATAAAAGCTGATTTAACTTGAAGAGGAAGAGGTGGCTAACTCTGTG
CCAGCAGCCGCGGTGATACAGAGACCTCAAGCGTTACTCGGATTCACTGGGCGTAAAGGG
TACGTAGGTGGTGGGATATGTTGGATGTGAAATTCCGGAGCTTAACTCCGGCGCTGCGTC
CAAAACTATCTCACTAGAGTACTGTAGAGAAGAGCGGAATTTCAGGTGTAGCGGTAAAAT
GCGTAGATATCTGAAAGAACACCAACGGCGAAGGCAGCTCTTTGGGCAGTAACTGACACT
GAGGTACGAAAGCGTGGGGAGCAAAAAGGATTAGAGACCCCAGTAGTCC
>Otu3304
CCAGCCTACGGGATGCAGCAGTGGGGAATATTGCGCAATGGGCGAAAGCCTGACGCAGCA
ACGCCGCGTGGGTGATGAAGGCCTTCGGGTCGTAAAACCCTGTCGGGTGGAACGAATTCC
CTCTTCGCTAATATCGGAGAGGATTGACGGTACCACCGAAGGAAGCCCCGGCTAACTACG
TGCCAGCAGCCGCGGTAATACGTAGGGGGCGAGCGTTGTTCGGATTTACTGGGCGTAAAG
AGCGCGTAGGCGGTACGATAAGTCGGAGGTGAAATCCCTCGGCTCAACCGAGGACCCGCG
TCCGATACTGTCGAACTTGAGTGCAGGAGAGGAGAGCGGAATTCCCAGTGTAGCGGTGAA
ATGCGTAGATACTGGGAGGAACACCGGTGGCGAAGGCGGCTCTCTGGACTGTCACTGACG
CTGAGGCGCGAAAGCTAGGGGAGCAAACGGGATTAGAAACCCTTGTAGTCC
>Otu3305
CCAGCCTATGGGAGGCTCCAGTGGGGAATTTTGCACAATGCCCGCAAGGGTGATGCAGCA
ACGCCGCGTGAGGGATGAAGGCCTTCGGGTCGTAAACCTCTTTCGATCGGGAGAAACGGT
CCGCTGAAGAGGCGGATCTGATGGTACCGGATGAAGAAGCCCCGGCCAACTACGTGCCAG
CAGCCGCGGTAATACGTAGGGGGCGAGCGTTGTTCGGAATTACTGGGCGTAAAGGGCGTG
TAGGCGGTGCGGTATGTGGGGCGTGAAATCCCCGGGCTTAACCCGGGGGGGCCGTTCCGG
ACTGCCGTGCTAGGGTGCGGGAGAGGGCAGTGGAATTCCCGGTGTAGCGGTGAAATGCGT
AGATATCGGGAGGAACACCTGCGGCGAAGGCGGCTGCCTGGACCGTAACCGACGCTGAGG
CGCGAAAGCTAGGGGAGCAAACAGGATTAGATACCCGAGTAGTCC
>Otu3307
CCAGCCTATGGGTGGCAGCAGTGGGGAATTTTGCGCAATGGGGGAAACCCTGACGCAGCA
ACGCCGCGTGGAGGATGAAGTACCTTGGTACGTAAACCCCTTTCGATCGGGACGATAATG
ACGGTACCGAGAGAAGAAGCCCCGGCTAACTTCGTGCCAGCAGCCGCGGTAATACGAAGG
GGGCTAGCGTTGTTCGGAATCACTGGGCGTAAAGCGCACGCAGGCGGATTGATAAGTCAG
GGGTGAAATCCCGGGGCTCAACCTCGGAATTGCCTTTGATACTGTCTATCTTCGAGTTCG
GGAGAGGTTGGCGGAATTCCTAGTGTAGAGGTGAAATTCGTAGATATTAGGAAGAACACC
AGTGGCGAAGGCGGCCAACTGGCCCGATACTGACGCTCATGTGCGAAAGCGTGGGGAGCA
AACAGGATTAGAAACCCGAGTAGTCC
>Otu3308
CCAGCCTATGGGTTGCTGCAGTGAGGAATTTTGCGCAATGGCCGCAAGGCTGACGCAGCA
ACGCCGCGTGGGTGAAGAAGGCCTTCGGGTCGTAAAGCCCTGTCAGGTGGGAAGAACGAC
CGGGGGACTAATAGTTTCCCGGATTGACGGTACCACCAAAGGAAGCACCGGCTAACTTCG
TGCCAGCAGCCGCGGTAATACGAAGGGGGCTAGCGTTGTTCGGAATCACTGGGCGTAAAG
CGTACGCAGGCGGATTGATAAGTCAGGGGTGAAATCCCGGGGCTCAACCTCGGAATTGCC
TTTGATACTGTCTATCTTCGAGTTCGGGAGAGGTTGGCGGAATTCCTAGTGTAGAGGTGA
AATTCGTAGATATTAGGAAGAACACCAGTGGCGAAGGCGGCCAACTGGCCCGATACTGAC
GCTCATGTACGAAAGCGTGGGGAGCAAACCGGATTAGAGACCCGAGTAGTCC
>Otu3314
CCAGCCTACGGGGCGCTGCAGTAAAGAATCTTTCACAATGGGGGAAACCCTGATGAAGCA
ACACCGCGTGGAGGATGAAGGCGTTTTCGTTGTAAACTCCTTTTCTGAAGGAATAACTTT
GAAGGTACTTCAGGAATAAGCAACGGCTAACTACGTGCCAGCAGCCGCGGTAATACGTAG
GTTGCGAGCGTTATCCGGAATTATTGGGCGTAAAGCGTGCGCAGGCGGTTTGTTAAGTTG

GATGTCAAAGCCTTGAGCTCAACTCAAGTCAGGTATCCAATACTGGCAAGCTGGAGATAG
GCAGAGGTAAGTGGAATTCTGTATGTAGGGGTAATATCCGTAGATATACAGAGGAACACC
AAAAGCGAAGGCAGCTTACTGGGCCTTTTCTGACGCTCATGCACGAAAGCGTGGGGAGCA
AACGGGATTAGATACCCCAGTAGTCC
>Otu3315
CCAGCCTATGGGGCGCAGCAGTCGCGAAAACTTTACAATGCACGAAAGTGTGATAAGGGA
ATTCCAAGTGCTTTATCTTCGGATAAGGCTTTTGCCAAGTCCAAACAGCTTGGCGAATAA
GTGGTGGGCAAGACCGGTGCCAGCCGCCGCGGTAACACCGGCGCCACGAGTGGGGATCAT
ATTTATTGGGTCTAAAGCATCCGTAGCCGGTTTAGCAACTCTTTTGTGAAATCGTTTCGC
TTAACGTAACGACGTGCAAAAGAAACTACTAGACTAGGAACCGGGAGAGGTTAGAGGTAT
TCTGGGGGGAGCGGTAAAATGTGATAATCCTCAGAGGACCACCAATGGTGTAGGCATCTA
ACTAGAACGGGTTCGACGGTGAGGGATGAAGGCTAGGGGAGCGATCCGGATTAGAGACCC
TAGTAGTCC
>Otu3317
CCAGCCTATGGGATGCTGCAGTGAGGAATATTGCTCAATGGACGAAAGTCTGAAGCAGCA
ACGCCGCGTGCGGGATGAAGGCCCTATGGGTCGTAAACCGCTGTAGAATGGGACAAATAT
CCGCCTCGGGCGGATTCGATGGTACCGTTAAAGTAAGCCTCGGCAAACTACGTGCCAGCA
GCCGCGGTAATACGTAGGAGGCAAGCGTTGTCCGGAATCACTGGGTGTAAAGGGTGCGCA
GGCGGGAAGGTAAGTTAGAGGTGAAATCTTGCAGCTTAACTGCAAAACTGCCTCTGATAC
TGCTTTTCTTGAGTACGGAAGAGAATGATGGAATTCCTGGTGTAGCGGTGAAATGCGTAG
ATATCAGGAAGAACACCAGTGGCGAAGGCGGTCATTTGGTCCGTTACTGACGCTCAGGCA
CGAAAGCGTGGGGATCAAACAGGATTAGAAACCCGAGTAGTCC
>Otu3318
CCAGCCTACGGGTGGCACCAGTGGGGAATCTTCCACAATGGGCGAAAGCCTGATGGAGCA
ACGCCGCGTGGGTGATGAAGGCCTTCGGGTTGTAAAACCCTGTCTTGCGAGAATAAGGTT
GCAGGGTGAATAATCCTGCAACTTGACTGTATCGCAGGAGGAAGACACGGCTAACTCTGT
GCCAGCAGCCGCGGTAATGCAGAGGTGTCAAGCGTTGTCCGGAATTACTGGGCGTAAAGG
GCGTGTAGGTGGGTCGGTAAGTCGGATGTGAAATCACTGGGCTTAACCCAGTAACTGCAT
CCGAAACTGCCGGTCTTGAGTGCAGAAGAGGAGAGCGGAATTCCCAGTGTAGCGGTGAAA
TGCGTAGATATTGGGAGGAACACCAGTGGCGAAGGCGGCTCTCTGGTCTGCAACTGACAC
TGAAACGCGAAAGCTAGGGGAGCAAACGGGATTAGAAACCCTAGTAGTCC
>Otu3321
CCAGCCTATGGGGGGCTGCAGGCGCGCAAACTCCACAATGCGCGTAAGCGTGATGGGGGG
AATCTGAGCGATTCACTTCGGTGAATCTTTTGCCAAGTCCAGTCAGCTTGGCGAATAAGT
GCTGGGCAAGACCGGTGGCAGCCGCCACGGTAATACCGGCAGCACAAGTGGTGTCCACGA
TTATTGGGCTTAAAGCGCTCGTAGCTGGCAAATACCGTCTCGTGTGAAATATTGGTGCTC
AACATCAATGCGGGCACGAGATACCTATTTGCTAGGGAGCGGGTGAGGTCAGGAGTACTT
ATGGGGAAGGGGTAAAATCCTATAATCCTATAAGGACTAACGGTGGCGAAGGCGCCTGAC
TAGAACGCATCCGACAGTGAGGAGCGAAGGCTAGGAGAACGAATCGGATTAGAGACCCTT
GTAGTCC
>Otu3322
CCAGCCTACGGGTGGCTCCAGTGAGGAATATTGGTCAATGGGCGAGAGCCTGAACCAGCC
ATCCCGCGTGCAGGAAGAAGGCCCTATGGGTTGTAAACTGCTTTTACGCAAGAAGAATGG
TACCCTCGTGAGGGTATTTGACGGTACTGCGTGAATAAGCACCGGCTAACTCCGTGCCAG
CAGCCGCGGTAATACGGAGGGTGCAAGCGTTATCCGGATTTATTGGGTTTAAAGGGTGCG
TAGGCGGTATTATAAGTCAGTGGTGAAATCCTGCAGCTTAACTGTAGCATTGCCATTGAA
ACTGCAGTACTTGAGTTATCTTGAAGTAGGCGGAATGTGTAGTGTAGCGGTGAAATGCTT
AGATATTACACAGAACACCGATTGCGAAGGCAGCTTACTAAGGGTACACTGACGCTGAGG
CACGAAAGCGTGGGGATCGAACAGGATTAGATACCCGCGTAGTCC
>Otu3328
CCAGCCTACGGGTGGCACCAGTCGAGAATCTTCCGCAATGGGCGAAAGCCTGACGGAGCG
ACGTTACGTGAATGAAGAAGCCCTTCGGGGTGTAAAGTTCTTTTATGCGGGAAAAAGTTA
TTGATTGTACCGCATGAATAAGGGGATCCTAATTCTGTGCCAGCAGGAGCGGTAAGACAG
AATCCCCGAGCGTTACCCGGATTTATTGGGCGTAAAGGGTCTGTAGGTGGTTTGGTAAGT
TGAAAGTTAAAATTCATCGGCTCAACCTTTGAAGTGCTTTCAATACTGCTAAACTAAGAG
GGCATTAGGGGCTAACGGAACCGACGGAGTAGGGGTGAAATCCGTTGATATCGTCGGGAA
CACCAAAAGCGAAGGCAGTTAGCTGGGATGACCCTGACACTGAGAGACGAAAGCGTGGGG
AGCAAAAAGGATTAGATACCCCCGTAGTCC

>Otu3330
CCAGCCTACGGGGTGCAGCAGTGAGGAATATTGGTCAATGGGGGCAACCCTGAACCAGCC
ATGCCGCGTGAAGGATGAAGGCCTTACGGGTTGTAAACTTCTTTTATACGGGGCGAATTG
TTTTTATGCGTAAAGACTTGACAGTACCGTACGAATAAGCATCGGCTAACTCCGTGCCAG
CAGCCGCGGTAATACGGAGGATGCAAGCGTTATCCGGATTCACTGGGTTTAAAGGGTGAG
TAGGCGGACTGATAAGTCAGTGGTGAAAGACTGTAGCTTAACTATAGAAATGCCATTGAT
ACTATCGGCCTTGAGTGAAGTTGAGGCAGGCGGAATGTGCAGTGTAGCGGTGAAATGCTT
AGATATTGCACAGAACACCAATTGCGAAGGCAGCTTGCTAAACTTCTACTGACGCTGAGT
CACGAAAGCGTGGGGATCAAACAGGATTAGATACCCCAGTAGTCC
>Otu3332
CCAGCCTACGGGTGGCTGCAGTGGGGAATATTGCGCAATGGGCGAAAGCCTGACGCAGCA
ACGCCGCGTGAGTGAAGAAGGCCTTCGGGTTGTAAAGCTCTTTCGGTTGGGAAGAAGGGA
ATGAGTGTTAATAGCGCTTGTTTTTGATGGTACCAAAAGAAGAAGCACCGGCTAACTTCG
TGCCAGCAGCCGCGGTAATACGAAGGGTGCAAGCGTTGTTCGGAATTACTGGGCGTAAAG
GGTTCGTAGGCGGGACATTAAGTCACATGTGAAATCCCCGAGCTTAACTCGGGACGTGCA
TTTGAAACTGATGTTCTTGAGTATCGGAGAGGGTAGTGGAATTGCTGGTGTAGGAGTGAC
ATCCGTAGAGATCAGCAGGAACACCGGAGGCGAAGGCGACTGCCTGGCCGATTACTGACG
CTCATGTACGAAAGCGTGGGGAGCAAACAGGATTAGAAACCCCAGTAGTCC
>Otu3335
CCAGCCTATGGGGGGCAGCAGTAGGGAATATTGGTCAATGGGCGCGAGCCTGAACCAGCC
ATGCCGCGTGCAGGATGACGGCCTTATGGGTTGTAAACTGCTTTTGTACGGGAATAAATC
TGATTACGTGTAATTAGTTGAAGGTACCGTAAGAATAAGCATCGGCTAACTCCGTGCCAG
CAGCCGCGGTAATACGGAGGATGCAAGCGTTATCCGGATTCATTGGGTTTAAAGGGTGCG
CAGGCGGACCGATAAGTCAGTGGTGAAATTCTACGGCTCAACTGTAGAACTGCCATTGAT
ACTGTCGGACTAGAGTATATTTGGAGTGGGCGGAATGTGTCATGTAGCGGTGAAATGCAT
AGATATGACACAGAACACCGATCGCGAAGGCAGCTCACTAAGATACAACTGACGCTCATG
CACGAAAGCGTGGGGATCAAACAGGATTAGATACCCCAGTAGTCC
>Otu3338
CCAGCCTACGGGGGGCAGCAGTGGGGAATTTTGCGCAATGGGCGAAAGCCTGACGCAGCA
ACGCCGCGTGAGTGATGAAGGTCTTCGGATCGTAAAGCTCTTTCGGCCGGGACGAAAACG
GGTCGGTGAATAGCCGGCTCCTTGACGGTACCGGAAGAAGAAGCACCGGCTAACTCTGTG
CCAGCAGCCGCGGTAATACAGAGGGTGCAAGCGTTGTTCGGAATTATTGGGCGTAAAGCG
CGTGTAGGCGGCGAAGCATGTCGGGTGTGAAAGCCCTCGGCTTAACCGAGGAAGTGCGCC
CGAAACTGCTTTGCTCGAGTGCTGGAGAGGGTAGCGGAATTCCCGGTGTAGAGGTGAAAT
TCGTAGATATCGGGAGGAACACCAGTGGCGAAGGCGGCTACCTGGACGGACACTGACGCT
GAGACGCGAAAGCGTGGGGAGCAAACAGGATTAGAAACCCTTGTAGTCC
>Otu3341
CCAGCCTACGGGTTGCTCCAGTGGGGAATTTTGGACAATGGGGGCAACCCTGATCCAGCG
ACGCCGCGTGGAGGACGAAGGTCTTCGGATTGTAAACTCCTTTTAGAGGGGATGAACGAC
CTGGTGAATAGCCAGGCCTGACAGTACTCTTTGAATAAGCCACGGCTAACTACGTGCCAG
CAGCCGCGGTAATACGTAGGTGGCAAGCGTTACTCGGAATCACTAGGCGTAAAGCGCAGG
TAGGTGGTTTGGTAAGTCTTTTGTGAAAGCTCCTGGCTCAACTGGGAGAGGTCAAAGGAA
ACTACCAGGCTAGAGTGTGGTAGGGGATACTGGAATTCCTGGTGTAGCGGTGAAATGCGT
AGATATCAGGAGGAATACCTATGGCGAAAGCAGGTATCTGGGCCATCACTGACACTGAGC
TGCGAAAGCTAGGGGAGCAAACAGGATTAGAGACCCCAGTAGTCC
>Otu3344
CCAGCCTATGGGGGGCAGCAGTGGGGAATTTTGGACAATGGGGGCAACCCTGATCCAGCG
ACGCCGCGTGGAGGACGAAGGTCTTCGGATTGTAAACTCCTTTTGGAGGGGAAGAATGCC
TGCGTGAAAAACGCAGGGTGACGGTACCCTTCGAATAAGCCACGGCTAACTACGTGCCAG
CAGCCGCGGTAATACGTAGGTGGCAAGCGTTACTCGGAATCACTAGGCGTAAAGCGCAGG
TAGGCGGTTCGATAAGTCTCTTGTGAAAGCTCCCGGCTCAACTGGGAGAGGCCAAGGGAA
ACTGTCGAGCTAGAATGTGGCAGAGGAAGCTGGAATTCCCGGTGTAGCGGTGAAATGCGT
AGATATCGGGAGGAACACCTATGGCGAAAGCAGGCTTCTGGGCCATCATTGACGCTGAGC
TGCGAAAGCTAGGGGAGCAAACAGGATTAGAAACCCCAGTAGTCC
>Otu3346
CCAGCCTATGGGGTGCTCCAGTGGGGAATCTTGCACAATGGGGGAAACCCTGATGCAGCG
ACGCCGCGTGAGCGATGAAGCCCTTCGGGGTGTAAAGCTCTTTCGACGGGAACGATAATG
ACGGTACCCGGAGAAGAAGCTGCGGCTAACTACGTGCCAGCAGCCGCGGTAATACGTAGG

CAGCGAGCGTTGTTCGGAGTTACTGGGCGTAAAGAGTACGTAGGCGGTGCTCCAAGTCTG
GTGTGAAATCTCCCGGCTTAACCGGGAGGGTGCGCCGGATACTGGAGTGCTTGAGTGTGG
GAGAGGAAAGCGGAATTCCTGGTGTAGCGGTGAAATGCGTAGATATCAGGAGGAACACCG
GTGGTGTAGACGGCTTTCTGGACCACTACTGACGCTGAGGCACGAAAGCGTGGGGAGCAA
ACAGGATTAGATACCCCCGTAGTCC
>Otu3347
CCAGCCTACGGGGGGCAGCAGTGGGGAATCTTGCGCAATGGGCGAAAGCCTGACGCAGCA
ACGCCGCGTGAGTGATGAAGGTCTTCGGATCGTAAAGCTCTGTCGACAGGAAAGATGGGG
AGCATGGCCAATATCCATGTTTCTTGACGGTACCTGTAAAGGAAGCACCGGCTAACTCTG
TGCCAGCAGCCGCGGTAAGACAGAGGGTGCGAGCGTTGTTCGGAATTATTGGGCGTAAAG
GGCGCGTAGGGGCGCCGTAAGTCACGTGTGAAAGCCCAGGGCTTAACCCTGGAAGTGCGC
GTGATACTGCGGTGCTTGAGTCTCGGAGAGGGTGGTGGAATTCCCAGTGTAGAGGTGAAA
TTCGTAGATATTGGGAGGAATACCGGTGGCGAAGGCGACCACCTGGACGAAGACTGACGC
TGAGGCGCGAAAGCGTGGGGAGCAAACAGGATTAGATACCCTAGTAGTCC
>Otu3348
CCAGCCTATGGGGGGCAGCAGTGGGGAATTTTGCGCAATGGGGGAAACCCTGACGCAGCA
ACGCCGCGTGAGTGATGAAGGCCTTCGGGTCGTAAAGCTCTGTCAGAGGGAAAGAAGTGT
AGGAGGGTTAATACCCCTTTTATTTGACGGTACCCTCAAAGGAAGCACCGGCTAACTCCG
TGCCAGCAGCCGCGGTAATACGAGGGGGGCGAGCGTTGTTCGGAATTATTGGGCGTAAAG
GGTGCGTAGGCGGTTTGGTAAGTCTTCTGTGAAATCTATGGGCTCAACTCATAGACTGCA
TGAGAAACTGCCGGGCTCGAGTATGGGAGAGGTGAGTGGAATTTCCGGTGTAGCGGTGAA
ATGCGTAGATATCGGAAGGAACACCTGTGGCGAAAGCGGCTCACTGGACCATAACTGACG
CTGATGCACGAAAGCTAGGGGAGCAAACAGGATTAGAAACCCCCGTAGTCC
>Otu3353
CCAGCCTATGGGTCGCAGCAGTGAGGAATATTGGGCAATGCCCGAAAGGGTGACCCAGCA
ACGCCGCGTGGAGGATGACGGCCGTAAGGTTGTAAACTCCTTTTGAGAGAGACGAATATC
CATATTTCGTAAGGAATATGGTTTGACTGTATCTCTAGAAGAAGCCCCGGCTAACTACGT
GCCAGCAGCCGCGGTAATACGTAGGGGGCAAGCGTTGTCCGGATTTACTGGGTGTAAAGG
GCGCGTAGGCGGATTTGTAAGTCAGTGGTGAAAGCCTGTAGCTTAACTACAGAACTGCCT
CTGATACTGCAAACCTTGAGTTCGGAAGAGAGAAGCGGAATTCCAGGTGTAGTAGTGAAA
TACGTAGATATCTGGAAGAACACCAGTGGCGAAGGCGGCTTCTTGGTCCGTAACTGACGC
TGAGGCGCGAAAGCGTGGGGAGCAAACAGGATTAGAGACCCTAGTAGTCC
>Otu3356
CCAGCCTACGGGTTGCTGCAGTGGGGAATATTGGACAATGGGGGCAACCCTGATCCAGCA
ATGCCGCGTGAATGAAGAAGGCCTTAGGGTTGTAAAGTTCTTTTGTCAGGGAAGATTATG
ACGGTACCTGACGAATAAGTCCCGGCTAACTCCGTGCCAGCAGCCGCGGTAATACGGAGG
GGGCAAGCGTTGTTCGGAATTACTGGGCGTAAAGGGTGTGTAGGCGGTACATCAAGTCAG
ACGTGAAAGACCTAGGCTTAACCTAGGAGGTGCGTTTGAGACTGATGAACTTGAGATCGG
GAGAGGAGAGTAGAATTCCAAGTGTAGAGGTGAAATTCGTAGATATTTGGAGGAATACCA
GTGGCGAAGGCGGCTCTCTGGACCGAATCTGACGCTGAGGCACGAAAGCGTGGGGAGCAA
ACAGGATTAGATACCCGGGTAGTCC
>Otu3357
CCGCCTATGGGGGGCTCCAGCTGGGAATCTTCTGCAATATACGAAAGTATGACAGAGCGA
ACCAAAGTGTTTCTCATTGAGAAACTTTTGCCAAATCTAAAAAGTTTGGCGAATAAGGAC
TGGGCAAGACTAGTGCCAGCCGCCGCGGTAATACTAGCTGTCCAAGTCGCAACCATCTTT
ATTGGGTCTAAAACATCCGTAGCTTGTTTAGCAAGTTCCTTGTGAAATCCTGTCTCTTAA
GGTCAGGGCGCGCAAGGAGTACTACTAGGCTAGAGACTGGAAGACGTAAGAAGTACGTTT
GAAGTAGCGGTTAAATGCGTTAATCTTAGACGGACTCACAACAGCGAAGGCATCTTACGA
GGACAGTTCTGACAGTGAGGGATGAAGGCTCGGGGCGCAAAACGGATTAGATACCCGCGT
AGTCC
>Otu3361
CCAGCCTATGGGGTGCTCCAGCCAAGAATCTTGGGCAATGGACGAAAGTCTGACCCAGCA
ACGCCGCGTGAAGGATTAAGGTCTTCGGATTGTAAACTTCTGTCAGAGGGGACGAAGGTG
TGCAGGCAAACAGTCTGTACAGTTGACGGTACTCTCAAAGGAAGCCCCGGCTAATTACGT
GCCAGCAGCCGCGGTAAGACGTATGGGGCAAGCGTTGCTCGGATTAACTGGGCGTAGAGG
GTGCGTAGGCGGTATTGAAAGTCAAATGTGAAATACCACGGCTCAACTGTGGGTCCGCAT
TTGAAACTTCGATACTTGAGTTCAGGTGAGGAGAATGGAATTCCAGGTGTAGGGGTGAAA
TCTGTAGATATCTGGAAGAACACCAGTGGCGTAGGCGATTCTCTAACTTGATACTGACGC

TGAGGCACGAAAGCATGGGGAGCAAACAGGATTAGAAACCCTGGTAGTCC
>Otu3362
CCAGCCTATGGGGGGCTGCAGTCGAGGATCTTCGGCAATGGGCGCAAGCCTGACCGAGCG
ACGCCGCGTGCGGGATGAAGGCCTTCGGGTTGTAAACCGCCGTCGGAGGGGAGGAAATAC
TAGGGGGTACTCTTCCTAGTTTGACCTATCCTCAGAGGAAGTACGGGCTAAGTTCGTGCC
AGCAGCCGCGGTAATACGAACCGTACAAACGTTATTCGGAATCACTGGGCTTAAAGGGTG
CGTAGGCGGCCCGGAAAGTTGGGTGTGAAAGCCCTCGGCTCAACCGAGGAATTGCGCCCA
AAACTACCGGGCTTGAGGGAGACAGAGGTAAGCGGAACTTAGGGTGGAGCGGTGAAATGC
GTTGATATCCTAAGGAACACCAGGAGCGAAAGCGGCTTACTGGGTCTCTTCTGACGCTGA
GGCACGAAAGCTAGGGGAGCGAACGGGATTAGAGACCCTCGTAGTCC
>Otu3364
CCAGCCTATGGGGGGCAGCAGTTAAGAATATTCCTCAATGGCCGAAAGGCTGAAGGAGCG
ACGCCGCGTGCGGGATGAAGGGCTTCGGCTCGTAAACCGCTTTTATTTGGGAAGAATTCT
GACGGTACCAAATGAATAAGAGGTTGCTAACTCTGTGCCAGCAGCAGCGGTAATACAGAG
ACCTCAAACGTTGTCCGGATTTATTGGGCGTAAAGGGTCCGCAGGTGGCCGTGCGCGTCG
GGAGTTAAATGTCGGGGCTCAACTCCGACACCGCTCTCGATACGGCATGGCTAGAGGCCG
GAAGAGGCACACGGAATTGCCGGTGTAGTAGTAATATGCGTTAATATCGGCAAGAACACC
AAATGCGAAGGCAGTGTGCTAGGACGGTTCTGACACTCAGGGACGAAAGCGTGGGGAGCG
AAAGGGATTAGAAACCCTAGTAGTCC
>Otu3365
CCAGCCTACGGGTGGCAGCAGTGGGGAATATTGGACAATGGGCGCAAGCCTGATCCAGCC
ATGCCGCGTGAGTGATGAAGGCCTTAGGGTTGTAAAGCTCTTTCGCAGGTGAAGATAATG
ACGGTAACCTGAGAAGAAGCCCCGGCTAACTTCGTGCCAGCAGCCGCGGTAATACGAAGG
GGGCTAGCGTTGTTCGGAATTACTGGGCGTAAAGCGCACGTAGGCGGATATTCAAGTCAG
GGGTGAAATCCCGGGGCTCAACCCCGGAACTGCCCTTGATACTGGGTATCTTGAGTGTAG
GAGAGGTGAGTGGAATTCCGAGTGTAGAGGTGAAATTCGTAGATATTCGGAAGAACACCA
GTGGCGAAGGCGGCTCACTGGCCTACAACTGACGCTGAGGTGCGAAAGCGTGGGGAGCAA
ACAGGATTAGAAACCCTGGTAGTCC
>Otu3367
CCAGCCTATGGGAGGCACCAGTTTCAAATCATTCACAATGGGCGAAACCCTGATGCAGCA
ACGCCGCGTGAGTGATGACGGTCTTCGGATTGTAAAACTCTGTCTTCAGGGACGATAATG
ACGGTACCTGAGGAGGAAGCCACGGCTAACTACGTGCCAGCAGCCGCGGTAATACGTAGG
TGGCAAGCGTTGTCCGGATTTACTGGGCGTAAAGGGAGCGTAGGTGGATATTTAAGTGGG
ATGTGAAATACTCGGGCTTAACCTGAGTGCTGCATTCCAAACTGGATATCTAGAGTGCAG
GAGAGGAAAGTAGAATTCCTAGTGTAGCGGTGAAATGCGTAGAGATTAGGAAGAATACCA
GTGGCGAAGGCGACTTTCTGGACTGTAACTGACACTGAGGCTCGAAAGCGTGGGGAGCAA
ACAGGATTAGAAACCCTCGTAGTCC
>Otu3368
CCAGCCTACGGGGCGCACCAGTGGGGAATCTTGCACAATGGGGGAAACCCTGATGCAGCG
ACGCCGCGTGAGCGATGAAGCCCTTCGGGGTGTAAAGCTCTTTCGTCGGGGACGATAATG
ACGGTACCCGGAGAAGCAGCTGCGGCTAACTACGTGCCAGCAGCCGCGGTAACACGTAGG
AGACGAGCGTTATCCGGATTTACTGGGCGTAAAGCGCGTGCAGGCGGTTTGGTAAGTTGG
ATGTGAAAGCTCCCGGCTTAACTGGGAGAGGTCGTTCAATACTGCCAGACTAGAGGATGG
TAGAGGGAGGTGGAATTCTGGGTGTAGTGGTGAAATGCGTAGATATCCAGAGGAACACCA
GTGGCGAAAGCGGCCTCCTGGACCATTTCTGACGCTCATACGCGAAAGCTAGGGTAGCAA
ACGGGATTAGATACCCCGGTAGTCC
>Otu3370
CCAGCCTACGGGAGGCAGCAGTAGGGAATTTTGCGCAATGGGGGAAACCCTGACGCAGCA
ACGCCGCGTGGGTGATGAAGGTCTTCGGATTGTAAAGCCCTGTCTTTTGGGACGATAATG
ACGGTACCAAAGGAGGAAGCCACGGCTAACTACGTGCCAGCAGCCGCGGTAATACGTAGG
TGGCAAGCGTTGTCCGGATTTACTGGGCGTAAAGGGTGCGTAGGTGGATATTTAAGTCAG
ATGTGAAATCCCCGGGCTTAACCTGGGAAGTGCATTTGAAACTGGATATCTTGAGTGCAG
GAGAGGTAAGTGGAATTCCTAGTGTAGCGGTGAAATGCGTAGATATTAGGAAGAACACCG
GTGGCGAAGGCGACTTACTGGACTGTAACTGACACTGAGGCACGAAAGCGTGGGTAGCAA
ACAGGATTAGATACCCCAGTAGTCC
>Otu3372
CCAGCCTATGGGGTGCAGCAGTGGGGAATTCTGCGCAATGGACGAGAGTCTGACGCAGCA
ACGCCGCGTGGGTGATGAAGGTCTTCGGATCGTAAAACCCTGTCGTCAGGGACGAAGGTT

GCGAGTTTAAGAGATGCGCAGCTTGACGGTACCTGGAGAGGAAGCCCCGGCTAACTCTGT
GCCAGCAGCCGCGGTAATACAGAGGGGGCAAGCGTTATTCGGAATTATTGGGCGTAAAGG
GCGCGTAGGCGGTGTTATAAGTCAGATGTGTAATCCCCGAGCTCAACTTGGGAACTGCAT
CTGAGACTGTAGTACTAGAGTGCTGGAGAGGGTGGTAGAATTCCACGTGTAGCGGTGAAA
TGCGTAGAGATGTGGAGGAATACCAGTGGCGAAGGCGGCCACCTGGACAGTGACTGACGC
TGAGGCGCGAAAGTGTGGGTAGCAAACAGGATTAGAAACCCGGGTAGTCC
>Otu3373
CCAGCCTATGGGGCGCAGCAGTCGAGAATCTTCCGCAATGGGCGAAAGCCTGACGGAGCA
ATGCCGCGTGCAGGATGAAGGCCTTCGGGTCGTAAACTGCTGTCACCGGGGATGAATAAT
GACAGTACCCGGGGAGGAAGTCACGGCTAACTACGTGCCAGCAGCCGCGGTAATACGTAG
GTGTCGAGCGTTGTTCGGATTTATTGGGCGTAAAGGGTTCGTAGGATGTTTATTAAGATT
GGTGTGAAATCCCAAGGCTCAACCTTGGAACTGCATTGATTACTGATGAACTAGAGTATC
GTAGAGGAAAGCGGGATTCCAGGTGTAGCGGTGGAATGCGTAGATATCTGGAAGAACACC
GACAGCGAAGGCAGCTTTCTGGGCGATAACTGACTCTGAGGAACGAAAGCATGGGGAGCA
AACAGGATTAGAGACCCCCGTAGTCC
>Otu3378
CCAGCCTATGGGGTGCAGCAGTGGGGAATATTGGACAATGGGGGCAACCCTGATCCAGCA
ATGCCGCGTGGGTGAAGAAGGTCTTCGGATTGTAAAGCCCTTTCGGCGGGGACGATGATG
ACGGTACCCGCAGAAGAAGCCCCGGCTAACTTCGTGCCAGCAGCCGCGGTAATACGAAGG
GGGCTAGCGTTGCTCGGAATGACTGGGCGTAAAGGGCGCGTAGGCGGTTGTATCAGTCAG
ACGTGAAATTCCTGGGCTTAACCTGGGGGCTGCGTTTGAGACGGTATAACTTGAGTGAGG
AAGAGAGTCGTGGAATTCCCAGTGTAGAGGTGAAATTCGTAGATATTGGGAAGAACACCG
GTGGCGAAGGCGGCGACCTGGTCCTTTACTGACGCTGAGGCGCGAAAGCGTGGGGAGCAA
ACAGGATTAGAAACCCCTGTAGTCC
>Otu3379
CCAGCCTATGGGTCGCAGCAGGTAGGGAAATTTTCCAATGCGCGCAAGCGTGAGAAAGCG
AGCCAGAGTGTTGTTCCTAAATTTGGAATAACTTTTGTCTGTTACAAAAAGACAGGCGAA
TAAGGAGTGGGCAAGACCGGTGCCAGCCGCCGCGGTAATCCCGGCGCTCCGAGTCGCAGC
CACAATTATTGGGTCTAAGACATTCGTAGCTTGTTTTGTAAGTTTCTTGTGAAATTCTAA
ATCTTAAATTTAGAGCGTGCAAGAAATACTGCTTAACTTGGGACTGGTAGAAGTAAGGAG
TACAATCAAGGTAGTAGTAAAATACGTTAATCTTAATTGGACTAACAAGAGCGAAGGCAC
CTTACTAGCACAGATCTGACAGTGAGGAATGAAGGCTAGGGTCGCGAAACGGATTAGAAA
CCCTAGTAGTCC
>Otu3380
CCAGCCTACGGGTGGCAGCAGTAGGGAATTTTGCGCAATGGACGAAAGTCTGACGCAGCA
ACGCCGCGTGAGTGATGAAGGCCTTCGGGTCGTAAAGCCCTGTCAGGTGGGAAGAATGAT
TCGGGTATGAATAATGCTCGAATGTGACGGTACCACCAGAGGAAGCACCGGCTAACTCCG
TGCCAGCAGCCGCGGTAATACGGAGGGTGCAAGCGTTATTCGGAATTACTGGGCGTAAAG
CGCGTGCAGGCGGGCGAGCAAGTCTGATGTGAAAGCCCCGGGCTTAACCTGGGAAGTGCA
TTGGAAACTGCTCGTCTTGAGTTGCGGAGAGGAAGGGGGAATTCCCGGTGTAGAGGTGAA
ATTCGTAGAGATCGGGAGGAATACCAGTGGCGAAGGCGCCCTTCTGGACGACAACTGACG
CTGAGACGCGAAAGCGTGGGGAGCAAACAGGATTAGAAACCCTCGTAGTCC
>Otu3383
CCAGCCTATGGGTGGCAGCAGTAACGAATCATGGGCAATGGGCGAAAGCCTGACCCTGCG
ACGCCGCGTGCGGGATGAAGTTCTTCGGAATGTAAACCGCTGTCAGGGGTTACCAAGCAA
ACGGGCCTAACACGCTCGCGCGTTGAGGAGCCCCAGAGGAAGCCACGGCTAACTTCGTGC
CAGCAGCCGCGGTAAGACGAAGGTGGCAAGCGTTGTTCGGAATCACTGGGCTTAAAGAGC
ACGTAGGCGGGCCGTCTTGCGCTTTGTGAAATCCCCCGGCTCAACCGGGGAATTGCAGGG
CGAACGGGCGGTCTTGAGGCAGGTAGAGGTGTGGGGAACTCTTGGTGGAGCGGTGGAATG
CGTAGATATCAAGAGGAACGCCGGAGACGAAGGTGCCGCACTGGGCCTGTCCTGACGCTG
AGGTGCGAAAGCCAGGGGAGCAAACGGGATTAGAAACCCTAGTAGTCC
>Otu3384
CCAGCCTACGGGTCGCTGCAGTGGGGAATATTGGACAATGGGCGCAAGCCTGATCCAGCC
ATGCCGCGTGAGTGAAGAAGGCCTTAGGGTTGTAAAATTCTTTTGGCGGGGACGATAATG
ACGGTACCCGCAGAATAAGCCCCGGCTAACTTCGTGCCAGCAGCCGCGGTAATACGAGGG
GGGCGAGCGTTGTTCGGAATTATTGGGCGTAAAGGGTGCGTAGGCGGTTTTGTAAGTCTT
GTGTGAAATCTTCAGGCTCAACTTGAAGACTGCACGAGAAACTGCAGGGCTTGAGTGTGG
GAGAGGTGAGTGGAATTCCTGGTGTAGCGGTGAAATGCGTAGATATCAGGAGGAACACCT

GTGGCGAAAGCGGCTCACTGGACCACAACTGACGCTGATGCACGAAAGCTAGGGGAGCAA
ACAGGATTAGAAACCCGAGTAGTCC
>Otu3393
CCAGCCTATGGGGCGCAGCAGTGGGGAATATTGGGCAATGGGCGAAAGCCTGACCCAGCG
ACGCCGCGTGGGCGATGAAGGCCTTCGGGTTGTAAAGCCCTTTCGTGCGGAAAGAACAGT
CTCGTGGCTAATATCCACGGGATTTGACGGTACCGCAGGAAGAAGCACCGGCTAACTCCG
TGCCAGCAGCCGCGGTAATACGGAGGGTGCGAGCGTTGTTCGGAATTATTGGGCGTAAAG
GACGCGTAGGCGGTAATGAAAGTCAGATGTGAAATACTTCGGCTCAACTGAAGAACTGCA
TCTGAAACTTCATAACTAGAGTACAGAAGAGGGAAACGGAATTCCCGGTGTAGAGGTGAA
ATTCGTAGATATCGGGAGGAACACCAGTGGCGAAGGCGGTTTCCTGGTCTGATACTGACG
CTGAGGCGTGAAAGTGTGGGGAGCAAACAGGATTAGAACCCCAGTAGTCC
>Otu3394
CCAGCCTATGGGGGGCTCCAGTGGGGAATATTGGACAATGGGCGAAAGCCTGATCCAGCC
ATGCCGCGTGAATGGTGAAGGCCCTAGGGTCGTAAAGTTCTTTTGTCAGGGAAGATAATG
ACGGTACCTGAAGAATAAGCACCGGCTAACTTCGTGCCAGCAGCCGCGGTAATACGAAGG
GTGCTAGCGTTGTTCGGAATTACTGGGCGTAAAGCGCGCGTAGGCGGGCTAACAAGTCAG
AGGTGAAATCCCAGGGCTCAACCCTGGAACTGCCTTTGAAACTGTTAGCCTGGAGTGTGT
GAGGGGATAGCGGAATTCCTAATGTAGAGGTGAAATTCGTAGATATTAGGAGGAACACCG
GTGGCGAAGGCGGCTATCTGGCACACAACTGACGCTGAGGCGCGAAAGCGTGGGGATCAA
ACAGGATTAGAAACCCTGGTAGTCC
>Otu3395
CCAGCCTATGGGTTGCTGCAGTCGGGAATTTTGGACAATGGGGGAAACCCTGATCCAGCG
ACGCCGCGTGCAGGATGACGTTCTTCGGAATGTAAACTGCTGTCGGGCGGGACGAACGTC
TTTCGAGCTAATATCTCGAGAGAGTGACGGTACCGCCGAAGGAAGCACCGGCTAACTCTG
TGCCAGCAGCCGCGGTAATACAGAGGGTGCAAGCGTTGTTCGGATTGACTGGGCGTAAAG
GGCGCGTAGGTGGTTAGGTAAGTCCGTTGTGAAATCAACATCGCTTAACGAGTTGTCCGC
ATCGGAAACTGTCTAACTTGAGTATTGGAGAGGATAACGGAATTCCTGGTGTAGCGGTGG
AATGTGTAGAGATCAGGAGGAACACCTGTGGCGAAGGCGGTTATCTGGCCAAATACTGAC
ACTGAGGCGCGAAAGCTAGGGGAGCAAACGGGATTAGATACCCTTGTAGTCC
>Otu3398
CCAGCCTATGGGTTGCTCCAGTGGGGAATATTGGACAATGGGCGAAAGCCTGATCCAGCA
ATGCCGCGTGTGTGAAGAAGGTCTTAGGATTGTAAAGCACTTTCGACGGGGACGATGATG
ACGGTACCCGTAGAAGAAGCCCCGGCTAACTTCGTGCCAGCAGCCGCGGTAATACGAAGG
GGGCTAGCGTTGCTCGGAATGACTGGGCGTAAAGGGCGCGTAGGCGGAGTTATCAGTCAG
ACGTGAAATTCCTGGGCTTAACCTGGGGGCTGCGTTTGATACGGTAAGTCTAGAGTGAGG
AAGAGGGTTGTGGAATTCCCAGTGTAGAGGTGAAATTCGTAGATATTGGGAAGAACACCG
GTGGCGAAGGCGGCAACCTGGTCCTTTACTGACGCTGAGGCGCGAAAGCGTGGGGAGCAA
ACAGGATTAGATACCCGAGTAGTCC
>Otu3405
CCAGCCTATGGGGGGCTGCAGTCGAGAATCTTCCGCAATGGACGAAAGTCTGACGGAGCG
ACGTTACGTGAATGAAGACGCCCTTCGGGGTGTAAAGTTCTTTTCTCTGGGAGGAAGCCG
CAAGGCAGACAGTACCAGAGGAATAAGGGGATCCTAATTCTGTGCCAGCAGGAGCGGTAA
GACAGAATCCCCAAGCGTTACCCGGATTTATTGGGCGTAAAGGGTTCGTAGGTGGTACAG
TAAGTTGAAAGTTAAAACTCATCGGCTTAACCTTTGAGGTGCTTTCAAAACTGCTGAACT
TGAGGGCGTTAGGGGTTAGCGGAACCGACGGAGTAGGGGTGAAATCCGTTGATATCGTCG
GGAACACCAAAAGCGAAGGCAGCTAACTAGGACGACCCTGACACTGAGGAACGAAAGCGT
GGGGAGCAAAAAGGATTAGAACCCTCGTAGTCC
>Otu3413
CCTATGGGGGGCTGCAGAGGAGGATCATTCGCAATGGGCGCAAGCCTGACGGTGCGACGC
CGCGTGAGGGATGAAGGCCTTCGGGTTGTAAACCTCTGTCAGGAGTTATGAACGGCGGGG
GCGTTAACACCGCCTCCGTGTGACAAAGGCTCCAAAGGAAGCCACGGCTAACTACGTGCC
AGCAGCCGCGGTAATACGTAGGTGGCAAGCGTTACTCGGAATCACTGGGCATAAAGGGCG
TGTAGGCGGATGTGTAAGTCCGGTGTAAAAGCCCCCGGCTCAACCGGGGAAGGCCATCGG
ATACTGCGCGTCTAGAGTGCGGGAGGGGAGAGTGGAACTCTTGGTGGAGCGGTGAAATGC
GTAGATATCAAGAGGAACGCCGGTGGCGAAAGCGGCTCTCTGGCCCGTTACTGACGCTGA
GGCGCGAAAGCTAGGGGAGCAAACGGGATTAGAAACCCCAGTAGTCC
>Otu3415
CCAGCCTATGGGGGGCACCAGTCGAGAATATTCGTCAATGCGCGAAAGCGTGAACGAGCG

ATGCCGCGTGCGGGATGAAGGCCTTCGGGTTGTAAACCGCTTTTGTATAGGAAGAATTTA
GATGACGGTACTATGCGAATAAGAGGTTACTAACTCTGTGCCAGCAGTAGCGGTAATACA
GAGACCTCAAGCGTTATCCGGATTTATTGGGCGTAAAGAGCTGGTAGGTGGTCATATTAG
TCAGATGTCAAATCTCGGGGCTCAACTCCGAAACTGCATTTGAAACGGTATGACTAGAGG
GTGTGAGAGATCACTGGAACTCATGGTGTAGCAGTGAAATGCGTTGATATCATGGGGAAC
ACCAAAGGCGAAGGCATGTGATTGGCACACTCCTGACACTGAGCAGCGAAAGCGTGGGTA
GCGAATGGGATTAGAAACCCTAGTAGTCC
>Otu3420
CCAGCCTACGGGGGGCAGCAGTGGGGAATTTTCCGCAATGGGCGAAAGCCTGACGGAGCA
AGACCGCGTGGGGGAGGAAGGTATATTTATTGTAAACCCCTTTTCCAAGGAAAGAAAAAA
ATGACGGTACCTTGGGAATAAGCATCGGCTAACTCTGTGCCAGCAGCCGCGGTAATACAG
AGGATGCAAGCGTTATCCGGAATTATTGGGCGTAAAGAGTCCGTAGGGGGCTCATCAAGT
CTGTCGTTAAAGCCCACAGCTTAACTGTGGTCAAGCGAGGGAAACTGAAGAGCTAGAGAG
CAGTAGGGGCAGAGGGAATTCTCAGTGTAGCGGTGAAATGCGTAGAGATTGGGAAGAACA
TCGGTGGCGAAAGCGCTCTGCTGGGCTGAATCTGACCCTGAGGGACGAAAGCTAGGGGAG
CAAAAGGGATTAGATACCCGCGTAGTCC
>Otu3422
CCAGCCTATGGGGGGCTCCAGTAAGGAATATTGCGCAATGGGCGCAAGCCTGACCGAGCG
ACGCCGCGTGTGGGATGAAGGCCTTCGGGTTGTAAACCACTGTCAGAGGGGATGAAATGC
GTAGGGGTTCTCTCCTACGTTTGACATAGCCTCAGAGGAAGTACGGGCTAAGTTCGTGCC
AGCAGCCGCGGTAAGACGAACCGTACAAACGTTATTCGGAATCACTGGGCTTAAAGGGTG
CGTAGGCGGCGGCGCAAGTTGGGTGTGAAATCCCTCGGCTCAACCGAGGAACTGCGCTCA
AAACTGCGTTGCTTGAGGGAGATAGAGGTGAGTGGAACTTAGGGTGGAGCGGTGAAATGC
GTTGATATCCTAAGGAACACCAGTGGCGAAAGCGACTCACTGGGTCTCCACTGACGCTGA
GGCACGAAAGCTAGGGTAGCGAACGGGATTAGATACCCTGGTAGTCC
>Otu3427
CCAGCCTACGGGTCGCTGCAGTCGAGAATCTTCGGCAATGGGCGCAAGCCTGACCGAGCG
ACGCCGCGTGTGGGATGAAGGCCTTCGGGTTGTAAACCGCTGTCAGTGGGGAGGAAATGC
GGGAGGGAACTCCCTTCCGTTTGACCTATCCGCAGAGGAAGTACGGGCTAAGTTCGTGCC
AGCAGCCGCGGTAATACGAACCGTACGAACGTTATTCGGAATTACTGGGCTTAAAGGGTG
CGTAGGCGGCTCAGAAAGTTGGGTGTGAAAGCCCTCGGCTCAACCGAGGAACTGCGCCCA
AAACTACTGAGCTCGAGGGAGACAGAGGTGAGTGGAACTTAGGGTGGAGCGGTGAAATGC
GTTGATATCCTAAGGAACACCAGTGGCGAAAGCGACTCACTGGGTCTCTTCTGACGCTGA
GGCACGAAAGCTAGGGGAGCGAACGGGATTAGAGACCCCCGTAGTCC
>Otu3438
CCAGCCTACGGGGTGCTCCAGTGAGGAATATTGGTCAATGGGGGCAACCCTGAACCAGCC
ATGCCGCGTGAAGGAAGAAGGCCCTAAGGGTTGTAAACTTCTTTTGTACGGGGGTAAACT
CTTCTACGAGTAGGAGATTGAAAGTACTGTACGAATAAGCAACGGCTAACTCCGTGCCAG
CAGCCGCGGTAATACGGAGGTTGCAAGCGTTATCCGGATTTATTGGGTTTAAAGGGTGCG
TAGGCGGGCTTATAAGTCAGCGGTGAAAGACTGTCGCTTAACGATAGCATTGCCGTTGAT
ACTGTAGGCCTTGAGTACGTTTGAGGTGGGCGGAATGTGTAGTGTAGCGGTGAAATGCTT
AGATATTACACAGAACACCAATTGCGAAGGCAGCTCACTAAACTGTCACTGACGCTGAGG
CACGAAAGCGTGGGGATCAAACAGGATTAGAAACCCGCGTAGTCC
>Otu3441
CCAGCCTACGGGGGGCAGCAGTGAGGAATTTTGGACAATGCCCGAAAGGGTGATCCAGCG
ACACCGCGTGAGTGATGAAAGCTTTCGAGTTTTAAAACTCTGTCAAGCGGGAAGAAAAAA
CGATTATATCGGAATTGACGGTACCGCTGAAGGAAGCCCCGGCTAATCCCGTGCCAGCAG
CCGCGGTAATACGGGAGGGGCAAACGTTACTCGGATTTACTGGGTGTAAAGGGTTCGTAA
GTTGTTTATAAAGTCAAATCTTAAATATTTCAGCTTAACTGAAAAAGCGATTTGAAACTG
TTAGACTAGAGTACGAAGGAGGAAAGTGGAACTGCCGGTGTAGCGGTGAAATGCGTAGAT
ATCGGCAGGAACACCAATTGCGAAGGCTACTTTCTATTTCGATACTGACACTGAGGAACG
AAAGCATGGGGAGCGATCGGGATTAGATACCCTTGTAGTCC
>Otu3442
CCAGCCTATGGGAGGCACCAGTCGAGAATATTCCGCAATGGGCGAAAGCCTGACGGAGCG
ACGCCGCGTGGTTGATGAAGTCCTTCGGGATGTAAAAACCTTTTATGATCTACTAAGTTT
ATTGAAGAGATCATGAATAAGAGGTTGCTAAACTCGTGCCAGCAGCAGCGGTAATACGAG
TGCCTCGAGCGTTATCCGGAATCATTGGGCGTAAAGGGTGTGTAGGAGGTTGTGTTAGTC
TTCTGTTAAATTCTTCGGCTTAACCGGGGACTCGCGGGTGATACGGCGCAACTAGAGGAT

GCGAGAGGTGAGTGGAACTCATAGTGTAGGGGTGAAATCCGTTGATATTATGGGGAACAC
CAAATGCGAAGGCAGCTCACTGGCGCACTCCTGACTCTGAAACACGAAAGCGTGGGTAGC
GAATGGGATTAGATACCCGAGTAGTCC
>Otu3444
CCAGCCTATGGGTGGCAGCAGGCGCGAAACCTCCACAATGCGCGTAAGCGCGATGGGGGG
AGTCTGATTGGCGTACTTCGGTATGCCTTTTGCTAAGTCTAAATCGCTTGGCGAATAAGT
GCTGGGCAAGACCGGTGGCAGCCGCCACGGTAATACCGGCGGCACTAGTGGTGTCCGCGA
TTATTGGGCTTAAAGGGTCAGTAGCCTGCTGGAACCGTCCCACGTGAAATGTTAGCGCCT
AACGCTAACGCGGGCGTGGGATACCTTCCGGCTAGGGAGTGGAAGAGGTCAGGGGTACTT
GTGGGGTAGGGGTAAAATCCTGTAATCCTATAAGGACCACCGGTGGCGAAGGCGCCTGAC
CAGGACATATCCGACGGTGAGTGGCTAAGGCTAGGAGAACGAATCGGATTAGAGACCCGA
GTAGTCC
>Otu3447
CCAGCCTATGGGGTGCTGCAGTCGAGAATTTTTCACAATGGGCGAAAGCCTGATGGAGCG
ACGCCGCGTGGGGGATGAATGGCTTCGGCCCGTAAACCCCTGTCATTCGGGAGCAATGCG
TCTGGGTGAACATCCCAGACGTTGATAGTACCGGAAGAGGAAGGGACGGCTAACTCTGTG
CCAGCAGCCGCGGTAATACAGAGGTCCCAAGCGTTGTTCGGATTTACTGGGCGTAAAGGG
TGCGTAGGCGGTGAGATAAGTCTGATGTGAAATCTCCGGGCTTAACCCGGAAAATGCATT
GGAAACTATCTGACTCGAGAGTTGGAGGGGGGACTGGAATACTTGGTGTAGCAGTGAAAT
GCGTAGATATGAAGTGGAACACCAGTGGCGAAGGCGAGTCCCTGGACAACTTCTGACGCT
GAGGCACGAAAGCTAGGGGAGCAAACAGGATTAGAAACCCCGGTAGTCC
>Otu3452
CCAGCCTATGGGGCGCAGCAGTAACGAATCTTCCGCAATGCACGAAAGTATGACGGAGCG
ACGCCGCGTGTGGGACGAAGTTCTTCGGAATGTAAACCACTGTCAGGGGTTAGAAAGTTC
TGATCAACCCCAGAGGAAGGCACGGCTAACTCTGTGCCAGCAGCCGCGGTAAGACAGAGG
TGCCAAGCGTTAGGCGGAATCACTGGGCTTAAAGCGTGTGTAGGCGGATTGACAAGTACT
TTGTGAAATCCCACGGCTTAACCGTGGAACTGCTCGGTATACTGTCAGTCTTGAGCTATC
TAGAGGCAACTGGAACAAACGGTGGAGCGGTGAAATGCGTAGATATCGTTTGGAATGCCA
AAGGTGAAAACAGGTTGCTGGGGATATGCTGACGCTGAGACACGAAAGCCAGGGGAGCAA
ACGGGATTAGAAACCCCTGTAGTCC
>Otu3453
CCAGCCTACGGGGGGCACCAGTAAGGAATATTGGACAATGGGGGAAACCCTGATCCAGCA
ATGCCGCGTGAATGAAGAAGGCCTTCGGGTTGTAAAGTTCTTTCGCTCGCGAAGATAGTG
ACGGTAGCGAGATAAGAAGGTCCGGCTAACTCTGTGCCAGCAGCCGCGGTGAGACAGAGG
GACCTAGCGTTGTTCGGAATGACTGGGCGTAAAGGGTGCGTAGGTGGTTGTTTAAGTAGA
GTGTAAAAGCCCCAGGCTTAACCTGGGAACAGCATTCTATACTGGACGACTAGAGTATGG
GAGAGGAAAGTGGAATTCCTAGTGTAGAGGTGAAATTCGTAGATATTAGGAGGAACACCA
GAGGCGAAGGCAACTTTCTGGTCCATAACTGACACTGAGGCACGAAAGCGTGGGGAGCAA
ACAGGATTAGATACCCTTGTAGTCC
>Otu3457
CCAGCCTACGGGGCGCTGCAGCTAAGAATCTTCCGCAATGGGGGAAACCCTGACGGAGCG
ACGCCGCGTGGATGATGAAGGCCGTAAGGTTGTAAAATCCTTTTGTCGGTGAAGAATAAG
GGAGGGAGTGGAAAGCCCTCCTGATGACGTTAGCCGGCGAATAAGCCCCGGCTAATTACG
TGCCAGCAGCCGCGGTAATACGTATGGGGCAAGCGTTGTTCGGATTTATTGGGCGTAAAG
GGCGTGTAGGCGGCTCATCAAGTCTGGTGTGAAATCGCAGGGCTTAACCCTGCATATGCG
CTGGAAACTGGTGGGCTTGAGTTCTTGAGGGGAAGCTGGAATTCCAGGTGTAGGGGTGAA
ATCTGTAGAGATCAGGAAGAATACCAGTGGCGAAGGCGAGCTCCTGGCCAATGATTGACG
CTGAGGTGCGAAAGTGTGGGGATCAAACAGGATTAGAAACCCGGGTAGTCC
>Otu3462
CCAGCCTATGGGGGGCACCAGTAAGGGATATTGCGCAATGGGCGAAAGCCTGACGCAGCA
ACGCCGCGTGGAGGAAGAAGGTCTTCGGATTGTAAACTCCTTTTAGTTGGGACGAGCAAG
GACGGTACCAACAGAATAAGCCTCGGCTAACTACGTGCCAGCAGCCGCGGTAAAACGTAG
GAGGCGAGCGTTATCCGGATTTACTGGGCTTAAAGGGTGTGCAGGCGGATGATTAAGTCG
TGCATGAAAGCCGTGCGCTCAACGCGCGGAGGCTGTGCGATACTGGTCGTCTAGGGTATG
CGAGAGGGATGCGGAATTCCGGGTGTAGTGGTGAAATGCGTAGATATCCGGAAGAACACC
AGAGGCGAAGGCGGCATCCTGGCGCATTACTGACGCTGAGACACGACAGCATGGGGAGCG
AACGGGATTAGATACCCTCGTAGTCC
>Otu3471

CCAGCACGGGGGGCTGCAGTCGAGGATTTTTCTCAATGGGGGAAACCCTGAAGGAGCGAC
GCCGCGTGGGGGATGAAGGTCTTCGGATTGTAAACCCCTGTCATCTGGGATCAAAGCGCA
CGACCCAATACGTCGTGTGTTGATAGTACCGGAAGAGGAAGCCGTGGCTAACTCTGTGTC
AGCAGCCGCGGTAATTCAGAGACGGCGAGCGTTGTTCGGATTCATTGGGCGTAAAGGGTG
CGCAGGCGGCCCAGCAAGTCGGATGTGAAATCTCATAGCCTAACTATGAGCGGTCATTCG
AAGCTGCTGGGCTCGAGGACTGGAGAGGAGACTGGAATTGTCGGTGTAGCGGTGAAATGC
GTAGAGATCGACAAGAACACCGGTGGCGAAGGCGGGTCTCTGGACAGTTCCTGACGCTCA
GGCACGAAAGCCAGGGGAGCAAACGGGATTAGATACCCGCGTAGTCC
>Otu3472
CCAGCCTATGGGACGCTCCAGTCGAGAATATTTGACAATGGAGGAAACTCTGATCAAGCG
ACGCCGCGTGGAGGATGAAGTCCTTAGGGACGTGAACTCCTTTTGTAGACTAATAAGTTT
TGAATGGCCTAAGAATAAGAGGTTGCTAAACTCGTGCCAGCAGCAGCGGTAATACGAGTG
CCTCAAGCGTTATCCGGAATTATTGGGCGTAAAGGGTATGTAGGTGGTTTTGTTAGTCTT
CTGTTAAAACTCTGGGCTTAACCCAGAACATGCAGGAGAAACGGCAAGACTTTAGAGTAT
GCGAGGGGTCTGTGGAACTCTAGGTGTAGCAGTGAAATGCGTTGATATCTAGGGGAACAC
CAAAAGCGAAAGCAGCAGACTGGAGCATTACTGACACTGAAATACGAAAGCGTGGGTAGC
GAATGGGATTAGAGACCCCCGTAGTCC
>Otu3479
CCAGCCTATGGGGCGCTGCAGTGAGGAATATTCGTCAATGGGGGAAACCCTGAACGAGCA
ACGCCGCGTGCAGGAAGAAGGTTTTCGGATCGTAAACTGCTTTTCTCTGGGACGAGAACG
AACGGTACCAGAGGAAGAAGCCCCGGCTAACTACGTGCCAGCAGCCGCGGTAATACGTAG
GGGGCAAGCGTTGTCCGGATTTATTGGGCGTAAAGCGCACGCAGGCGGTCAACCAAGTTC
GGAGTGACAGTTTCCGGCTTAACTGGGAAAGGTCTTCGAAAACTGGTGGACTTGAGAACT
TGAGGGGGAGACGGAATTCCGGGTGGAGCGGTGAAATGCGTAGAGATCCGGAGGAACACC
GAAAGCGAAGGCAGTCTCCTGGCAAGTTTCTGACGCTCAGGTGCGAAAGCTAGGGGAGCG
AACAGGATTAGAAACCCGAGTAGTCC
>Otu3482
CCAGCCTATGGGTGGCAGCAGTAGGGAATATTGGGCAATGGAGGCAACTCTGACCCAGCC
ATACCGCGTGCAGGACGAAGGCCCTCTGGGTTGTAAACTGCTTTTATCAGGGAAGAAACG
CTTGGATTCATCTGAGCCTGACGGTACCTGAGGAATAAGCACCGGCTAACTCCGTGCCAG
CAGCCGCGGTAATACGGAGGGTGCAAGCGTTATCCGGATTTACTGGGTTTAAAGGGTGCG
TAGGCGGACTGTTAAGTCAGTGGTGAAAGATTCGGGCTCAACCCGAAAATTGCCATTGAT
ACTGGCAGTCTTGAGTACAGTTGAGGTAGGCGGAATGTGTCATGTAGCGGTGAGATGCAT
AGATATGACACAGAACACCGATTGCGAAGGCAGCTTGCTGGGCCATTACTGACGCTGATG
CACGAAAGCGTGGGGAGCAAACAGGATTAGAGACCCGTGTAGTCC
>Otu3485
CCAGCCTATGGGTGGCACCAGGCGCGAAACCTCCACAATGCGCGAAAGCGTGATGGGGGG
AGTCTGAGTGGCGGACTTCGGTCCGCCTTTTGCCAAGTCTAAATAGCTTGGAGAATAAGT
GCTGGGTAAGACCGGTGGCAGCCGCCACGGTAATACCGGCGGCACGAGCGGTGTCCGCGA
TTATTGGGCTTAAAGGGCCAGTAGCCGGCTAGAACAGTCCCACGTGAAATGTTGACGCTC
AACGTTAACCCGGGCGTGGGATACCTTCTGGCTAGGGAGTGGGAGAGATCTGGGGTACTT
ATGGGGTAGGGGTAAAATCCTGTAATCCTATAAGGACCACCGGTGGCGAAGGCGCCAGAT
CAGAACACATCCGACGGTGAGTGGCGAAGGCTAGGGGAACGAATCGGATTAGAAACCCGA
GTAGTCC
>Otu3486
CCAGCCTATGGGTGGCAGCAGTCGAGAATCTTCCGCAATGGGCGAAAGCCTGACGGAGCG
ACGTTACGTGAATGAAGAAGCCCTTCGGGGTGTAAAGTTCTTTTCTCATTGAAGAACGCA
AGTGACTGTAAATGAGGAATAAGGGGATCCTAATCCTGTGCCAGCAGGAGCGGTAAGACG
GAATCCCCAAGCGTTACCCGGATTTATTGGGCGTAAAGGGTTCGTAGGTGGCGTAGTAAG
TTGAAAGTTAAATCTCATCGGCTCAACCTTTGAGGTGCTTTCAATACTGCTACGCTTGAG
GGCGTTAGGGGTTAACGGAACCGACGGAGTAGGGGTGAAATCCGTTGATATCGTCGGGAA
CACCAAAAGCGAAGGCAGTTAACTAGGACGACCCTGACACTGAGGAACGAAAGCGTGGGG
AGCAAAAAGGATTAGAGACCCCCGTAGTCC
>Otu3489
CCAGCCTACGGGATGCACCAGCATATCCACCGGTTGTAATCAGCCCTCCTATAACATTAC
CAATAAAATTACCACTTGTATCAATAATCGCTTTATTAGTAGTCCACCCAATAGGTTGCC
CAACTGTCTGTCCCGTTGCCGACGAAAAATATAGCTGTCCAGCATACATAGCAAGTTTAG
CTGGGTTTGGGTCTGATGCTGTCCAGGTTCCATTCCAATATGCTCCTTGTGTCCATTCTG

CGTCATTTGTCCCCGTTGAGACAAAGCTGGCACCCGAACCTATGGCAACCGCTCCTGCTC
CCAATGTCCCGTTTACATCCAATTTATAAGCAGGACTCGTTGTTCCAATACCGACTTCTC
CGGTCTTCAGTATTGTCATCCTCTCAGTGTTATTCATTAGATACCCTGGTAGTCC
>Otu3496
CCAGCCTACGGGTGGCACCAGTGGGGAATCTTGCACAATGGGGGCAACCCTGATGCAGCG
ACGCCGCGTGAACGATGAAGCCCTTCGGGGTGTAAGGTTCTATCGGCAGGGACGATAATG
ACGGTACCGGAAGAATAAGCACCGGCTAACTACGTGCCAGCAGCCGCGGTAATACGTAGG
GTGCAAGCGTTAATCGGAATTACTGGGCGTAAAGCGTGCGCAGGCGGTTTCGCAAGTCAG
ATGTGAAATCCCCGGGCTTAACCTGGGAACTGCATTTGAAACTACGAGGCTAGAGTGTGT
CAGAGGGGGGTAGAATTCCACGTGTAGCAGTGAAATGCGTAGAGATGTGGAGGAATACCG
ATGGCGAAGGCAGCCCCCTGGGATAACACTGACGCTCATGCACGAAAGCGTGGGGAGCAA
ACAGGATTAGAAACCCCCGTAGTCC
>Otu3498
CCAGCCTACGGGTGGCAGCAGTCGAGGATCTTCGGCAATGGGCGCAAGCCTGACCGAGCG
ACGCCGCGTGCGGGATGAAGGCCTTCGGGTTGTAAACCGCTGTCAGTGGGGAGAAAATGT
ACGGAGGTTCTCCTCCGTATTTGATCTATCCGCAGAGGAAGTGTGGGCTAAGTTCGTGCC
AGCAGCCGCGGTAAGACGAACCACACAAACGTTATTCGGAATTACTGGGCTTAAAGGGTG
CGTAGGCTGCGCGGAAAGTTGGGTGTGAAATCCCTCGGCTCAACCGAGGAACTGCGCCCA
AAACTACCGTGCTCGAGGGAGACAGAGGTGAGCGGAACTTAGGGTGGAGCGGTGAAATGC
GTTGATATCCTAAGGAACACCCGTGGCGAAAGCGGCTCACTGGGTCTCTTCTGACGCTGA
GGCACGAAAGCTAGGGTAGCGAACGGGATTAGAAACCCGAGTAGTCC
>Otu3507
CCAGCCTACGGGGGGCAGCAGTGGGGAATTTTGGACAATGGGGGCAACCCTGATCCAGCA
ATGCCGCGTGAGTGAAGAAGGCCTTCGGGTTGTAAAGCTCTTTTGTCCGGGAAGAAACGG
TTCGCCTTAATAAGGTGAGCTAATGACGGTACCGGAAGAATAAGCACCGGCTAACTACGT
GCCAGCAGCCGCGGTAATACGTAGGGTGCAAGCGTTATCCGGATTCACTGGGTTTAAAGG
GTGCGTAGGTGGGCAGGTAAGTCAGTGGTGAAATCTCCGGGCTTAACCCGGAAACTGCCG
TTGATACTATCTGTCTTGAATGTAGTGGAGGTGAGCGGAATATGTCATGTAGCGGTGAAA
TGCTTAGATATGACATAGAACACCTATTGCGAAGGCAGCTCGCTACACTATTATTGACAC
TGAGGCACGAAAGCGTGGGGATCAAACAGGATTAGAGACCCTGGTAGTCC
>Otu3508
CCAGCCTATGGGGCGCTGCAGTAGGGAATCTTGCGCAATGGGCGAAAGCCTGACGCAGCA
ACGCCGCGTGGGGGATGAAGGCCTTCGGGTTGTAAACTCCTTTTAGTGGGAACGAAATTG
ACGGTACCCACAGAAAAAGCCCCGGCTAACTACGTGCCAGCAGCCGCGGTGATACGTAGG
GGGCAAGCGTTGTCCGGATTTACTGGGCGTAAAGAGCTCGTAGGCGGCTTGGTTAGTCGG
CGTGTCAAATCTCCAGGCTTAACCTGGAGTCGCACTCGATACTGCCATAGCTAGAATCCG
GTAGGGGACCATGGAATTCCTGGTGTAGCGGTGAAATGCGCAGATATCAGGAGGAACACC
AGTAGCGAAGGCGGTGGTCTGGGCCGGTATTGACGCTGAGGAGCGAAAGCGTGGGGAGCG
AACAGGATTAGAGACCCGGGTAGTCC
>Otu3515
CCAGCCTATGGGTCGCTGCAGTTAGGGAATTTTGACAATGGGCGCAAGCCTGATCGAGTG
ACGCCGCGTGCGGGACGAAGCCTCTCGGGGTGTAAACCGCTTTTCTGTGGGACGAGCAAG
GACGGTACCGCAGGAATAAGCCTCGGCTAACTACGTGCCAGCAGCCGCGGTAATACGTAG
GAGGCGAGCGTTGTCCGGAATTACTGGGTGTAAAGAGCGCGTAGGCGGCTCGTTGCGTCG
GGCGTGAAAGCTCCCGGCTTACCTGGGAGAGGGCGACCGAAACGGGCGAGCTTAGAGGGC
GGTAGGGGCTGGCGGAACGCCCGGAGTAGCGGTGAAATGCGTTGAGATCGGGCAGAACAC
CAGTGGCGTAGGCGGCCAGCTGGGCCGACCCTGACGCTGAGGCGCGAAAGCCAGGGGAGC
GAACGGAATTAGAAACCCGAGTAGTCC
>Otu3521
CCAGCCTATGGGGGGCAGCAGTGGGGAATCTTGGACAATGGGGGCAACCCTGATCCAGCG
ATGCCGCGTGAGTGAAGAAGGCCTTCGGGTCGTAAAGCTCTTTAGGTTGGGAAGAAGTGT
ATATGGGGATAATCCATATACATTGACGGTGCCGACAGAATAAGCACCGGCAAACTCTGT
GCCAGCAGCCGCGGTAATACAGAGGGTGCAAGCGTTAATCGGAGTTACTGGGCGTAAAGG
GCGCGTAGGCGGTTGTATGAGTGTGATGTGAAAGCCCCGGGCTTAACCTGGGAAGTGCAT
CGCAAACGATACAACTGGAGTATATGAGAGGGTGGCGGAATTTCCGGTGTAGCGGTGAAA
TGCGTAGAGATCGGAAGGAACGTCAATGGCGAAGGCAGCCACCTGGCATAATACTGACGC
TGAGGCGCGAAAGCGTGGGGAGCGAACAGGATTAGATACCCGTGTAGTCC
>Otu3522

CCAGCCTATGGGTGGCTGCAGTGGAGAATATTAGTCAATAAACGAAAGTTCGAACTAGCA
CTTTACGGAAATGAACGATTTAAAAAAAATCATGAAGGCTATGACAGCTGTAAAGTTTAA
CCGTAGATTAACATGATGCTTTTATATCTAAAAAGAACCCCGGGCTAACAACGTGCCAGC
AGCTGCGGTAAGACGTTGACGGGGGAGCGTTACTGGTCGTTACTGGGTGTAGAGAGAAGC
TAGACTGTTTAAGGACTTGCATTAAAAACAAAAGGATGAGCGAATCCCTAATGCAAAAGC
TTAATACTAGAGTTTTAAAGAAATTCGCGATAAGTACAATCTAGAGATGAAATTCTAAGA
GATTGTAAGGATCTTCGAAAGCCACGGCAGCGAATTATTTAATCCTGACGTCGAATTTCT
AAAGTGCGGTTCTCAATTAGGATTAGAGACCCCCGTAGTCC
>Otu3526
CCAGCCTACGGGTCGCAGCAGTGGGGAATTTTGCGCAATGGGGGAAACCCTGACGCAGCA
ACGCCGCGTGGAGGATGAAATCCCTTGGGATGTAAACTCCTTTCGATAGGGAAGATAATG
ACGGTACCTATAGAAGAAGCCCCGGCTAACTTCGTGCCAGCAGCCGCGGTAATACGTGGG
GGCAAGCGTTGTTCGGAATTATTGGGCGTAAAGGGTGTGTAGGCGGTGCTCTAAGTTCGG
TGTGAAATCTCCTGGCTTAACTGGGAGGGTGCGCCGGAAACTGGAGTGCTCGAGCGTGGG
AGAGGAAAGCGGAATTCCTGGTGTAGCGGTGAAATGCGTAGATATCAGGAGGAACACCTG
CGGTGTAGACGGCTTTCTGGACCATTGCTGACGCTGAGACACGAAAGCGTGGGTAGCAAA
CAGGATTAGATACCCCTGTAGTCC
>Otu3530
CCAGCCTATGGGTGGCTCCAGTCGAGAATAATTCGCAATGGGCGAAAGCCTGACGATGCG
ACGCCGTGTGAACGAAGAAGGCCTTCGGGTTGTAAAGTTCTGTCGTCTTGGAACAAGTGT
AACGTGTAAATAATGCGTTACATTGATGGTACAAGAAAAGGAAGCACCGGCTAACTCCGT
GCCAGCAGCTGCGGTAATACGGAGGGTGCAAGCGTTAGTCGGAATTACTGGGCGTAAAGG
GTGCGTAGGCGGGTGAGTAAGTCAGTGGTGAAATTCCGGAGCTTAACTTCGGAGCTGCCA
CTGAAACTGCACACCTAGAGGAATAGCGGGGAAAACGGAATTCCATGTGTAGCGGTGAAA
TGCGTAGATATATGGAAGAACACCTGTGGCGTAGGCGGTTTTCTAGCTACATCCTGACGC
TGAGGCACGAAAGCAAGGGGAGCAAACAGGATTAGAGACCCCTGTGTCC
>Otu3538
CCAGCCTATGGGGCGCTGCAGTAGGGAATTTTCCGCAATGGACGAGAGTCTGATGGAGCA
ACGCCGCGTGCAGGATGAATGTCTTCGGATTGTAAACTGCTTTTCTCTGTGACGATTATG
ACGGTAGCAGAGGAATAAGGACCTGCTAACTACGTGCCAGCAGCCGCGGTCATACGTAGG
GTCCAAGCGTTATCCGGAATTACTGGGCGTAAAGAGTTGCGTAGGTGGCATAGTAAGCGA
ATAGTAAAATGACGGGGCTCAACCTCGTGTCTATTATTTGAACTGCTAAGCTAGAGGACG
AGAGAGGTTATTGGAATTCCTAAAGTAGGGGTAATATCCGTAGATATTAGGAGGAACACC
GATGGCGTAGGCAGATAACTGGCTCGTTTCTGACACTAAGGCACGAAAGCGTGGGTAGCA
AACAGGATTAGATACCCTAGTAGTCC
>Otu3546
CCAGCCTATGGGGGGCTGCAGTCGAGAATCTTTCGCAATGGGCGCAAGCCTGACGAAGCG
ACGCCGTGTGAGCGAAGAAGGCCTCCGGGTTGTAAAGCTCTTTCGCTAGGGAACAAGAGA
GACGGGATGATACTCCGTTGATTTGATGGTACTTGGTAAAGAAGCACCGGCTAACTCCGT
GCCAGCAGCTGCGGTAATACGGAGGGTGCAAGCATTGATCGGAATTACTGGGCGTAAAGG
GCGCGTAGGCGGTCAGGAAAGTCAGATGTGAAATTCCGGGGCTCAACCCCGGAGCTGCAT
TTGAAACTTCCTATCTAGAGTTCAGTTAGGGAAAACGGAATTCCACGTGTAGCGGTGAAA
TGCGTAGATATGTGGAAGAACATCGGTGGTGAAGACGGTTTTCTGGGCTGATACTGACGC
TGAGGCGCGAAAGCAAGGGGAGCAAACAGGATTAGATACCCGAGTAGTCC
>Otu3548
CCAGCCTATGGGGGGCAGCAGTCGAGAATCTTCCACAATGGACGAAAGTCTGATGGAGCG
ACGCCGCGTGATTGATGAAGTCCTTCGGGACGTAAAGATCTTTTATGCGTGAGAAAGTAA
TTGATGTTAGCGCATGAATAAGGGGCTCCTAACTCTGTGCCAGCAGGAGCGGTAATACAG
AGGCCCCGAGCATTATCCGGAATCACTGGGCGTAAAGGGTGTGTAGGCGGCAGCGTTAGT
CGACTGTGAAAGATCCAGGGCTTAACCCAGGAGACGCAATCGAAACGGCGCAGCTACGAG
GGCGCGAGAGGTACAGGGAACTCACGGTGTAGGGGTGAAATCCGTTGATATCATGGGGAA
CACCAAATGCGAAGGCACTATACTGGCGCACTCCTGACGCTGAAACACGAAAGCGTGGGA
ATCGAAAAGGATTAGAGACCCGTGTAGTCC
>Otu3551
CCAGCCTATGGGATGCTGCAGTGAGGAATCTTGCGCAATGGGCGAAAGCCTGACGCAGCG
ACGCCGCGTGGAGGATGACGGTCTTCGGATTGTAAACCCCTGTTAAGCGGGGAGAAAGGT
CTACTTCTAATACAAGTAGAATATGACGGTACCGCTAGAGAAAGCACCGGCTAACTTCGT
GCCAGCAGCCGCGGTAATACGAGGGGTGCAAGCGTTATTCGGATTAATTGGGCGTAAAGG

GTGTGCAGACGGTTTATTAAGTCATTTGTTAAATTCTTCGGCCTAACTGAAGGTATGCAA
GTGATACTGATGAACTAGAGGGTGGAAGGGAGAAGTGGAATTCTCGGAGTAGCGGTAAAA
TGCGTAGATCTCGAGAGGAACACCAATGGCGAAGGCAGCTTCTTGGTCCATTTCTGACGT
TCAAACACGAAAGCGTGGGGAGCAAACAGGATTAGAGACCCGAGTAGTCC
>Otu3555
CCAGCCTATGGGTGGCTGCAGTGGGGAATCCTGGACAATGGAGGCAACTCTGATCCGGCT
ATACCGTGTGTGTGAAGAAGGCCTTAGGGTTGTAAAGCACTTTTAGTAGGGAAGAAGATT
TTGGTGTTAATAGCATCAAGATTTGACGGTACCTAAAGAAAAAGCACCGGCAAACTCTGT
GCCAGCAGCCGCGGTAATACAGAGGGTGCAAGCATTAATCGGAATTATTGGGCGTAAAGG
GCATGTAGGTTGTTCGGTAAGTTAGATGTGAAAGAATCGGGCTCAACCTGATAAAGCATC
TAATACTGCCAAACTAGAGTGCGGTAGGGGATGATGGAATTTTCTGTGTAGCGGTAAAAT
GCTTAGAGATTGAAAGGAACATCGGAGGCGAAGGCGATCGTCTGGGCCGCAACTGACACT
GAAATGCGAAAGCGTGGGTAGCAAACAGGATTAGAAACCCCAGTAGTCC
>Otu3556
CCAGCCTACGGGGGGCTGCAGTGGGGAATATTGGACAATGGGGGAAACCCTGATCCAGCG
ACGCCGCGTGTGTGAAGAAGGCCTGCGGGTTGTAAAGCCCTTTCGACGGGGACGATGATG
ACGGTACCCGTAGAAGAAGCCCCGGCTAACTTCGTGCCAGCAGCCGCGGTAATACGAAGG
GGGCTAGCGTTGCTCGGAATGACTGGGCGTAAAGGGCGCGTAGGCGGCGATGATAGTCAG
GCGTGAAATTCCTGGGCTCAACCTGGGGACTGCGCTTGATACGTTATTGCTTGAGTACGG
AAGAGGGTCGTGGAATTCCCAGTGTAGAGGTGAAATTCGTAGATATCGGGAAGAACACCG
GTGGCGAAGGCGGCGACCTGGTCCGGAACTGACGCTGAGGCGCGAAAGCGTGGGGAGCAA
ACAGGATTAGAAACCCCAGTAGTCC
>Otu3558
CCAGCCTATGGGGGGCTGCAGTCGAGAATCTTGCACAATGGGCGAAAGCCTGATGCAGCG
ACGCCGCGTGGGGGATGAAGCATTTCGGTGTGTAAACCCCTTTCGACTGGGAAGAATGCC
TCGCAAGAGGACTGACGGTACCAGTATAAGAAGCCCCGGCTAACTACGTGCCAGCAGCCG
CGGTAAGACGTAGGGGGCCAGCGCTGTTCGGAATTACTGGGTGTAAAGGGTTCGTAGGCG
GTGTGGCAAGTTGGGAGTGAAATCTCTGGGCTCAACCCAGAGGCTGCTTCCAAAACTGCT
GTGCTTGAGTGTGAGAGAGGCTCGTGGAATTGCAGGTGTAGCGGTGAAATGCGTAGATAT
CCGCAGGAACATCCGAGGCGAAAGCGGCTCGCTGGATCGCAACTGACGCTGAGGGACGAA
AGCTAGGGGAGCAAACAGGATTAGAAACCCGCGTAGTCC
>Otu3559
CCAGCCTATGGGTGGCTCCAGTGGGGAATCTTGCGCAATGGAGGAAACTCTGACGCAGCG
ACGCCGCGTGGGTGAAGAAGGCCTTCGGGTTGTAAAGCCCTGTCGAGTGGGAAAAACGAT
TCCATAAATAATACGTATGGGAAGTGATTGTACCACTAGAGGAAGCACCGGCTAACTCCG
TGCCAGCAGCCGCGGTAATACGGAGGGTGCAAGCGTTGTTCGGAATTATTGGGCGTAAAG
GGCAGGTAGGTGGTCTTAAAAGTCTACTGTGAAATCCCTGAGCTTAACTCAGGAAGTGCG
GTGGAAACTGTAAGACTAGAGTGCTGGAGGGGTGCGTGGAATTGCCGGTGTAGCGGTGAA
ATGCGTAGATATCGGCAGGAACACCAGAGGCGAAAGCGGCGCACTGGACAGCAACTGACA
CTCAACTGCGAAAGCGTGGGGAGCAAACAGGATTAGAGACCCCGGTAGTCC
>Otu3560
CCAGCCTATGGGTCGCAGCAGTCGAGAATCATTCGCAATGGGCGAAAGCCTGACGATGCG
ACGCCGTGTGAGCGAAGAAGGCCTTAGGGTTGTAAAGCTCTTTCGCCCGGGAACAAGAGA
AGTCGGCTAATATCCGGCGGATTTGAGCGTACCGGGTAAAGAAGCACCGGCTAACTCCGT
GCCAGCAGCTGCGGTAATACGGAGGGTGCAAGCATTAATCGGATTAATTGGGCGTAAAGG
GCGCGTAGGCGGGGATGTAAGTCAGATGTGAAATCCCGAGGCTCAACCTCGGAACAGCAT
TTGAAACTACATCTCTTGAGGGTAGGCGGAGAAAACGGAATTCCACAAGTAGCGGTGAAA
TGCGTAGATATGTGGGAGAACACCGGTGGCGAAGGCGGTTTTCTAGCTTATTCCTGACGC
TGAAACGCGAAAGCAAGGGGATCAAACAGGATTAGATACCCGCGTAGTCC
>Otu3561
CCAGCCTATGGGTGGCTCCAGCCGAGAATATTCGACAATGGGCGAAAGCCTGATCGAGCG
ACGCCGCGTGCAGGATGAAGTGCTTCGGTATGTAAACTGCTTTTGCGGACTAAGAGGTTT
ATTGACTAGTCCGAGAATAAGGGGTTGCTAAACTCGTGCCAGCAGCAGCGGTAATACGAG
TGCCCCAAGCGTTATCCGGAATCATTGGGCGTAAAGGGTGTGTAGGCGGTCGCGTTAGTC
TTCTGTTAAATTCTTCGGCTTAACCGGGGGCATGCAGGGGAAACGGCGCGACTTGAGGAT
GCGAGAGGTGAATGGAACTCATGGAGTAGGGGTGAAATCCGTTGATATCATGGGAACAC
CGAAAGCGAAGGCAATTCACTGGCGCACTCCTGACGCTGAAACACGAAAGCGTAGGTAGC
GAATGGGATTAGAAACCCGTGTAGTCC

>Otu3562
CCAGCCTATGGGTCGCAGCAGTGGGGAATTTTGGACAATGGGCGCAAGCCTGATCCAGCA
ATGCCGCGTGTGGGATGAAGGCCTTCGGGTTGTAAACCACTGTCAGAGAGGATGAAATAC
TGGTGGGTACTCCCATCAGCTTGACATATTCTTAGAGGAAGGACGGGCTAAGTTCGTGCC
AGCAGCCGCGGTAAGACGAACCGTCCAAACGTTATTCGGAATCACTGGGCTTAAAGGGTG
CGTAGGCGGCGCGGAAAGTAGGGTGTGAAATCCCTCGGCTCAACCGAGGAACTGCGCTCT
AAACTACCGTGCTCGAGGGAGATAGAGGTGAGCGGAACTTGGGGTGGAGCGGTGAAATGC
GTTGATATCCTAAGGAACACCGGTGGCGAAAGCGGCTCACTGGATCTCTTCTGACGCTGA
GGCACGAAAGCTAGGGTAGCGAACGGGATTAGAAACCCGCGTAGTCC
>Otu3564
CCAGCCTATGGGAGGCAGCAGGCGCGCAAACTCCACACTGCGCGGAAGCGTGATGGGGGG
AATCTGAGCGGTTCACTTCGGTGAACCTTTTGCCAAGTGTAGCAAGCTTGGCGAATAAGT
GCCGGGCAAGACCGGTGGCAGCCGCCACGGTAATACCGGCGGCACAAGTGGTGTCCACGA
TTATTGGGCTTAAAGCGCTCGTAGCTGGCCAGAACCGTCTGATGTGAAATATTGGTGCTC
AACACCAATGCGTGCATCAGATACCTTCTGGCTAGGGAGTGGGTGAGGTTAGGAGTACTT
ACGGGGAAGGGGTAAAATCCTATAATCCTGTAAGGACTAACGGTGGCGAAGGCGCCTAAC
TAGAACACATCCGACAGTGAGGAGCGAAGGCTAGGAGAACGAATCGGATTAGATACCCTG
GTAGTCC
>Otu3568
CCAGCCTATGGGTGGCTCCAGTGGGGAATATTGGACAATGGGCGCAAGCCTGATCCAGCA
ACGCCGCGTGAGGGATGAAGCATTTCGGTGTGTAAACCTCTGTAAGTGGGGACGAAGGGG
CAACCCTGACGGTACCCGCAAAGTAAGCCCCGGCTAACTCCGTGCCAGCAGCCGCGGTAA
TACGGAGGGGGCTAGCGTTATTCGGAATTACTGGGCGTAAAGAGCGCGTAGGCGGACTCC
TAAGTCTGTTGTCTAATCCCCCGGCTTAACCGGGGAAAGGCAAGAGATACTGGGAGACTA
GAGTGTCGGAGGGGGAACTAGAATTCCTGGTGTAGCGGTGAAATGCGTAGAGATCAGGAG
GAATACCGGTGGCGTAAGCGAGTTCCTAGACGACAACTGACGCTGAGGCGCGAAAGCCAG
GGGAGCAAACGGGATTAGAAACCCCAGTAGTCC
>Otu3571
CCAGCCTATGGGAGGCAGCAGTCGAGAATTTTTCACAATGGGCGAAAGCCTGATGGAGCG
ACGCCGCGTGGAGGATGAAGGTCTTCGGATTGTAAACTCCTGTCACTACAGAACAAAACT
ACCAGGTAAATAATCTGGTGGCTTGATAGTATGTGGAGAGGAAGGGACGGCTAACTCTGT
GCCAGCAGCCGCGGTGATACAGAGGTCCCGAGCGTTGTTCGGATTTACTGGGCGTAAAGG
GTGCGTAGGAGGTTAAGAAAGTAAGGCGTGAAATCCCACCGCTCAACGGTGGAACTGCTT
CTTATACTACTTAGCTAGAGGACTGGAGGGGAGATCGGAATTCCTGGTGTAGCGGTGAAA
TGCGTAGATATCAGGAGGAACGCCAATAGCGAAGGCAGATCTCTGGAAAGTTCCTGACTC
TGAGGCACGAAGGCTAGGGTAGCAAACGGGATTAGATACCCCAGTAGTCC
>Otu3573
CCAGCCTATGGGGTGCTGCAGTGAGGAATATTGCGCAATGGGGGCAACCCTGACGCAGCA
ACGCCGCGTGAGTGAGGAAGGTTTTCGGACCGTAAAGCTCTGTCGAGTGGGAAGAAATGT
ATCATGGCTAATATCCATGGTATTTGACGGTACCGCTAAAGGAAGCACCGGCTAACTCCG
TGCCAGCAGCCGCGGTAATACGGGGGGTGCAAGCGTTGTTCGGAATTATTGGGCGTAAAG
AGCGTGTAGGCGGCTGAATAAGTCAGATGTGAAATCCCTGGGCTTAACCCAGGAAGTGCA
TTTGAAACTATTCAGCTTGAGTAGGGGAGAGGAAAGTGGAATTCCTGGTGTAGAGGTGAA
ATTCGTAGATATCAGGAGGAACACCGGTGGCGAAGGCGACTTTCTGGCCCTATACTGACG
CTGAGACGCGAGAGCGTGGGTAGCAAACAGGATTAGATACCCGAGTAGTCC
>Otu3574
CCAGCCTATGGGGGCTCCAGGGAATATTGGACAATGGAGGAAACTCTGATGCAGCGACGC
CGCGTGAGCGATGAAGCCCCTCGGGGTGTAAAGCTCTTTCGGCAGGAACGATAATGACGG
TACCTGAAGAAGAAGCTGCGGCTAACTACGTGCCAGCAGCCGCGGTAATACGTAGGCAGC
GAGCGTTGTTCGGAACTACTGGGCGTAAAGAGTGTGTAGGCGGTGCTCTAAGTTTGGTGT
GAAATCTCCCGGCTTAACCGGGAGGGTGCGCCGAAAACTGGGTGACTAGAGTATGGGATG
GGTAAGTGGAATTCCTGGTGTAGCGGTGAAATGCGTAGATATCAGGAGGAACACCGGTGG
TGTAGACGGCTTACTGGACCATAACTGACGCTGAGACACGAAAGCGTGGGTAGCAAACAG
GATTAGAAACCCGTGTAGTCC
>Otu3575
CCAGCCTACGGGGTGCACCAGGCGAGAATATTCCACAATGGACGAAAGTCTGATGGAGCG
ACGCCGCGTGCTGGATGAAGTGCTTCGGTACGTAAACAGCTTTTATCGGGGACGAAATTA
TTGACGGTACCCGATGAATAAGGACCTCCTAACTCTGTGCCAGCAGGAGCGGTAATACAG

AGGGTCCAAGCGTTACCCGGAATCACTGGGCGCAAAGGGCGTCAAGGCGGCCATGTTAGT
CGTTTGTAAAATCCGTGGGCTCAACCTACGGCGCGCAAGCGAAATGGCATGGCTCGAGGG
CGCGAGAGGTAGAGGGAACTCACGGTGGAGGGGTGAAATCCGTTGATATCGTGGGGAACA
CCAAAGGCGAAGGCACTCTACTAGCGCGCTCCTGACGCTCACACACGAAAGCCAGGGGAG
CGAACGGGATTAGAAACCCCTGTAGTCC
>Otu3594
CCAGCCTACGGGGGGCACCAGTGAGGAATTTTGCGCAATGGGCGAAAGCCTGACGCAGCG
ACGCCGCGTGCGGGACGAAGGTCTTCGGATCGTAAACCGCTGTCAGTGGGGAAGAGCAAG
GACGGTACCCGCTGAGGAAGCTTCGGCTAACTACGTGCCAGCAGCCGCGGTAATACGTAG
GAAGCGAGCGTTGTCCGGATTTACTGGGCGTAAAGGGCGTGTAGGCGGAGCGGCGCATCC
CGGAGTAAATCCAACGGCTCAACCGTTGGTTCATCTGGGAGATGGCCGCCCTTGAGGACA
GGAGAGGAAAGTGGAATTCCCGGTGTAGCGGTGAAATGCGTAGATATCGGGAGGAACACC
AGTGGCGAAGGCGACTTTCTGGCCTGTTCCTGACGCTGAGGCGCGAAAGCCAGGGGAGCG
AACGGGATTAGAAACCCTTGTAGTCC
>Otu3603
CCAGCCTACGGGACGCTCCAGTTAAGGAACTTTCCCAATGCTCGAAAGAGTGAGAAAATC
AACCAGAGTGTTTTCTTTTAGAAAACTTTTGTCTGCTGTAAAAAAGCAGATGAATAAGGA
CTGGGCAAGACCGGTGCCAGCCGCCGCGGTAATCCCGGCGGTCCGAGTCGCATCCACATT
TATTGGGTCTAAAACATCCGTAGCTTGTTATGTAAGTCTCTTGTGAAATCTCATTTCTCA
AACTTGAGGCGTGCAAGAGATACTACATTGCTAGAGATCGGAAGACGTAAAGAGTACGAC
GGAGGTAGTGGTTAAACATGTTAATCTTCGTCGGACTAACAATGGCGAAGGCACTTTACG
AGTACGAATCTGACAGTGAGGGATGAAGGCTAGGGGCGCAAAAGGGATTAGATACCCTAG
TAGTCC
>Otu3607
CCAGCCTATGGGTGGCTGCAGTGAGGAATATTGCGCAATGGGGGCAACCCTGACGCAGCA
ACGCCGCGTGAGTGAGGAAGGTTTTCGGATTGTAAAGCTCTGTCGAGCGGAAAGAAATGC
ATGGTGGCTAATATCCATCATGCTTGACGGTACCGCTAAAGGAAGCCCCGGCTAACTTCG
TGCCAGCAGCCGCGGTAATACGAGGGGGGCGAGCGTTGTTCGGAATTATTGGGCGTAAAG
GGTGCGTAGGCGGTTTGGTAAGTCTTTTGTGAAATCTATGGGCTCAACTCATAGACTGCA
AGGGAAACTGCCGGGCTTGAGTGTGGGAGAGGTGAGTGGAATTCCCGGTGTAGCGGTGAA
ATGCGTAGATATCGGGAGGAACACCTGTGGCGAAAGCGGCTCACTGGACCACAACTGACG
CTGATGCACGAAAGCTAGGGGAGCAAACAGGATTAGATACCCCAGTAGTCC
>Otu3611
CCAGCCTACGGGGGGCAGCAGTGGGGAATATTGGACAATGGGCGAAAGCCTGATCCAGCA
ATGCCGCGTGAATGATGAAGGCCTTCGGGTCGTAAACCTCTTTCGATCGGGAAAAACGGC
TCTGTGAATAGCAGGGTCTGATGGTACCGGAAGAAGAAGCCCCGGCCAACTACGTGCCAG
CAGCCGCGGTAATACGTAGGGGGCGAGCGTTGTTCGGAATTACTGGGCGTAAAGGGCGTG
TAGGCGGTGCGGTAAGTTGGGCGTGAAATCCCCGGGCTTAACCCGGGGGGGCCGTTCAGG
ACTGCCGTGCTAGGGTGCGGGAGAGGGCAGTGGAATTCCCGGTGTAGCGGTGAAATGCGT
AGATATCGGGAGGAACACCTGCGGCGAAGGCGGCTGCCTGGACCGTAACCGACGCTGAGG
CGCGAAAGCTAGGGGAGCAAACAGGATTAGATACCCGCGTAGTCC
>Otu3624
CCAGCCTATGGGGGGCTGCAGTTCGGAATATTCGGCAATGGACGCAAGTCTGACCGAGCG
ACGCCGCGTGCGGGATGAAGGCCCTTGGGTTGTAAACCGCTGTCACAGGTGAGCAAGTCA
CAGCCGCATAACACGCGGTTGTGTTGAGTTAAGCTTGAGAGGAAGCCCCCGCTAATCACG
TGCCAGCAGCGGCGGTAATACGTGAGGGGCGAACGTTGTTCGGTGTCACTGGGCTTAAAG
GGCGCGTAGGTGGCTGCGTAAGTAAGGTGTGAAAGCCCTCGGCTTACCCGGGGAATTGCG
CCTTATACTGCGTGGCTTGAGGGTCATAGGGGAAGGAGGAACTCCAGGTGTAGCGGTGAA
ATGCGCAGATATCTGGGGGAAGGCCGGCGGCGAAGGCGTCCTTCTGGGTGATTCCTGTCA
CTGAGGCGCGAAAGCGTGGGGAGCAAACAGGATTAGATACCCGTGTAGTCC
>Otu3625
CCAGCCTATGGGAGGCTCCAGTCTAGAGGCTTCGGCAATGGGGGCAACCCTGACCGAGCG
ACGCCGCGTGTGGGATTGAAGGCCTTCGGGTCGTAAACCACTGTCAAGAGGGAAGAATAC
CTGGATGGCGAATTCGGGGGAGACGGTACCTCTGGAGGAAGCCCCGGCTAACTCCGTGCC
AGCAGCCGCGGTAATACGGAGGGGGCAAGCGTTGTTCGGATTTACTGGGCGTAAAGGGTG
TGTAGGCGGTTGCGCAAGTCTGGTGTGAAATCTTCACCGCTTAACGGGAAGAGGTCACTG
GAAACTGCGTGACTCGAGTATGGTAGAGGCAAGCGGAATTCCTGGTGTAGCGGTGGAATG
TGTAGATATCAGGAAGAACATCTGTGGCGAAGGCGGCTTGTTGGGCCATCACTGACGCTG

AGACACGAAAGCGTGGGGAGCAAACGGGATTAGATACCCCCGTAGTCC
>Otu3626
CCAGCCTATGGGGGGCACCAGCAGGGAATCGTACGCAATGGACGAAAGTCTGACGTCGCA
ATGCCGCGTGGGGGATGAAGGCCTTCGGGTCGTAAACCCCTTTTGCCATATATGAGAGTA
TATGGAGAATAAGCAACTGCTAACTCTGTGCCAGCAGCCGCGGTAATACAGAGGTTGCAA
GCGTTATCCGGATTTATTGGGCGTAAAGCGTTTCGTAGGCGGATTGGAAAGTTACTCTTC
AAAGACCACGGCTTAACCGGGGGAAGGGGAGTAATACTACTAGTCTTGAAATATGGTGGG
GTATCTGGAACTGATGGTGTAGTAGTGAAATACGTTGATATCATCAGGAACTCCAAGGGC
GAAGGCAGGATACTAACCATTTTTTGACGCTGAGGAACGACAGCTAGGGGAGCGAAAGGG
ATTAGATACCCCCGTAGTCC
>Otu3629
CCAGCCTATGGGGCGCACCAGTGAGGAATTTTCCGCAATGGGCGAAAGCCTGATGGAGCG
ACGCCGCGTGGGGGATGAATGGCTTCGGCCCGTAAACCCCTGTCATTCGCGAACAATGTC
CCGACAATAATACTGGCGGGATTGATAGTAGCGGAAGAGGAAGGGACGGCTAACTCTGTG
CCAGCAGCCGCGGTAATACAGAGGTCCCAAGCGTTGTTCGGATTCACTGGGCGTAAAGGG
TGCGTAGGTGGTCGGGTAAGTTCGATGTGAAATCTCGGAGCTCAACTCCGAAACGGCATT
GGATACTATTCGGCTTGAGGACTGGAGGGGGGACTGGAATTCTCGGTGTAGCAGTGAAAT
GCGTAGATATCGAGAGGAACACCAGTGGCGAAGGCGAGTCCCTGGACAATCCCTGACACT
AAGGCACGAAAGCTAGGGGAGCAAACAGGATTAGAGACCCTCGTAGTCC
>Otu3631
CCAGCCTATGGGTGGCTCCAGTGGGGAATATTGGTCAATGGACGCAAGTCTGAACCAGCC
ATGCCGCGTGCAGGATGACAGCCCTACGGGTTGTAAACTGCTTTTGTACGGGAAAAAACC
TTCGGTCGTGAACCGGAGCTGATAGTATGGTAAGAATAAGCATCGGCTAACTTCGTGCCA
GCAGCCGCGGTAATACGAAGGATGCAAGCGTTATCCGGATTTATTGGGTTTAAAGGGTGC
GTAGGCGGACTTATAAGTCAGTGGTGAAATCTCATCGCTTAACGATGAACGTGCCATTGA
TACTGTAGGTCTTGAGTACAGATGCCGTTGGCGGAATGTGTCATGTAGCGGTGAAATGCA
TAGATATGACACAGAACACCGATTGCGAAGGCAGCTGACGAAACTGTAACTGACGCTGAG
GCACGAAAGCGTGGGGATCAAACAGGATTAGAAACCCCAGTAGTCC
>Otu3633
CCAGCCTACGGGTTGCAGCAGGCGCGAAACCTTTGCAATGCGCGTAAGCGTGACAGGGGA
ACTCTGAGTGGTAGAGGACACAGTCCTCTATCTTTTGACAACCCTAAATCGGTTGTAGAA
TAAGGTCTGGGCAAGACTAGTGCCAGCCGCCATGGTAGATGCTAAATGCACCCTACCCAC
GGTAAGCGGTAATACTAGCAGAACGAGTGGTATCCACGAATATTGGGTCTAAAGGGTCCG
TAGCTGGCCTGTTAAGTTCGCTGTGAAATCTTGGAGCTCAACTTCAAGGCGTGCAGCAAA
TACTGGCGGGCTTGAGAGTGGGGGAGGTCAGGGGTACTCACGGGGTAAGAGTAAAATCTT
GTAATCCTGTGAGGACTTCCAGTGGCGTAGGCGCCTGACCAAAACACGTCTGACAGTGAG
GGACGAAGGCTAGGAGAACGAATCGGATTAGATACCCCAGTAGTCA
>Otu3634
CCAGCCTATGGGAGGCTCCAGTCGAGAATTTTTCACAATGGGGGAAACCCTGATGGAGCG
ACGCCGCGTGGGGGATGAATGGCTTCGGCCCGTAAACCCCTGTCATTTGCGACCAAGATT
GCCGGTTGAATATTCCGGCGGTTTGATGGTAGCGGAAGAGGAAGGGACGGCTAACTCTGT
GCCAGCAGCCGCGGTAATACAGAGGTCCCGAGCGTTGTTCGGATTCACTGGGCGTAAAGG
GTGCGTAGGTGGCCGGGTAAGTCTGATGTGAAATCTCTGAGCTCAACTCAGAAACTGCAC
TGGATACTATCCGGCTCGAGGGTTGAAAGGGGGACTGGAATTCTCGGTGTAGCAGTGAAA
TGCGTAGATATCGAGAGGAACACCAGTGGCGAAGGCGAGTCCCTGGACAACTCCTGACGC
TGAGGCACGAAAGCCAGGGGATCAAACGGGATTAGAAACCCTCGTAGTCC
>Otu3635
CCAGCCTATGGGTGGCACCAGTAAGGAATATTGGACAATGGACGCAAGTCTGATCCAGCC
ATGCCGCGTGAAGGATTAAGGTCCTCTGGATTGTAAACTTCTTTTATCTGGGACGAAAAA
AGGGTTTTCTAACTCGACTGACGGTACCAGATGAATAAGCACCGGCTAACTCCGTGCCAG
CAGCCGCGGTAATACGGAGGGTGCAAGCGTTATCCGGATTCACTGGGTTTAAAGGGTGCG
TAGGCGGGTAGGTAAGTCAGTGGTGAAATCTCTAAGCTTAACTTGGAAACTGCCATTGAT
ACTATTTATCTTGAATATTGTGGAGGTAAGCGGAATATGTCATGTAGCGGTGAAATGCTT
AGATATGACATAGAACACCTATTGCGAAGGCAGCTTGCTATACATATATTGACGCTGAGG
CACGAAAACGTGGGGATCAAACAGGATTAGATACCCCGGTAGTCG
>Otu3637
CCAGCCTACGGGTGGCAGCAGTAACGAATATTCCGCAATGGGCGCAAGCCTGACGGAGCG
ATGCCGCGTGCAGGACGAAGCCCTTCGGGGTGTAAACTGCTGTCAGGGTTTAGCAACATA

ATGAGCAGACCCAAAGGAAGAGGCGACTAACTCTGTGCCAGCAGTCGCGGTAATACAGAG
GCCTCGAGCGTTAATCGGAATCACTGGGCTTAAAGGGTGCGTAGGCGGTCTCGTAAGTGC
CTTGTGAAATCCCATGGCTCAACCATGGAACGGCTCGGCAGACTGCGGGACTTGAGGTTG
GTAGGGGCTACTGGAACTCTAGGTGGAGCGGTGAAATGCGTAGATATCTAGAGGAACGCC
AAAGGTGAAGACAGGTAGCTGGGCCAATTCTGACGCTGAGGCACGAAAGCGTGGGGAGCG
AACGGGATTAGAGACCCTTGTAGTCC
>Otu3638
CCAGCCTACGGGGGGCTGCAGTGAGGAATCTTCGTCAATGGGGGAAACCCTGAACGAGCA
ACGCCGCGTGCAGGAAGACGGTCTTCGGATTGTAAACTGCTTTTCTCTGGGAAGAGGAAG
GACGGTACCAGAGGAAGAAGCCTCGGCTAACTACGTGCCAGCAGCCGCGGTAATACGTAG
GAGGCAAGCGTTGTCCGGATTTATTGGGCGTAAAGCGTCCGCAGGCGGTGAACCAAGTTT
GGAGTGACAGTTGCCGGCTTAACCGGGAGAGTGCTTCGAAAACTGGTTGACTTGAGGGCT
TCAGAGGGACACGGAATTCCGGGTGTAGTGGTGAAATGCGTAGATATCCGGAGGAACATC
AATGGCGTAGGCAGTGTCCTGGGAAGTACCTGACGCTCAGGGACGAAAGCTAGGGGAGCG
AACGGGATTAGATACCCCTGTAGTCC
>Otu3639
CCAGCCTACGGGGCGCACCAGTGAGGAATATTGCGCAATGGCCGAAAGGCTGACGCAGCG
ACGCCGCGTGAAGGATGAAGTCCGTTAGGATGTAAACTTCTTTTGCAGGGGATGAATGTG
GTGCCTTCGGGTGCCATTGACCGTACCCTGCGAATAAGGATCGGCTAACTACGTGCCAGC
AGCCGCGGTAATACGTAGGATCCGAGCGTTGTCCGGAATTACTGGGTGTAAAGGGCGTGT
AGGTGGAACTTTAAGTCGGTGGTGAAAGCCTGCAGCTCAACTGCAGAACTGCCTTCGATA
CTGAAGTTCTTGAGTACGGCAGAGGGAGATGGAATTCATGGTGTAGCGGTGAAATGCGTA
GATATCAGGAGGAACACCTGTGGCGAAAGCGGCTCACTGGACCATTTCTGACGCTCATAC
GCGAAAGCTAGGGTAGCAAACGGGATTAGATACCCGAGTAGTCC
>Otu3642
CCAGCCTACGGGTGGCTCCAGTCAAGAATCTTGGTCAATGGGCGAAAGCCTGAACCAGCG
ATGTCGCAGCGCGAATTGAAGGCTTATAGTCGTAAAGCGCCGAAGTATGGATCCAACAAG
GAGATGTCCATATGGAGTAGCCCTGTGTAATCTCGGTGCCAGCACACGCGGTCATACCGA
AAGGGCGAGGCATTCCTATTCGAAATGGGTGTAAAGAGTCATTAGGCGGACTTATCAAAT
TCTACCTGAAAGATCGAGGCGAAACCTTAAGCGAGGGTGGAATCATTTAGAGTCTAGAGT
ATAGCAGAGGGTTGTGGAATGCTTGGCGTACCGATGGAATGGATTGATGCTAAGTGGAAC
TCCAATGGCGAAGGCAACAACCTGGGCTTTACTGACGCTGACTGACGAAAGTGTGGGGAG
CAAAGTGGATTAGAGACCCTAGTAGTCC
>Otu3644
CCAGCCTACGGGGTGCACCAGTGGGGAATTTTGCGCAATGGGCGAAAGCCTGACGCAGCA
ACGCCGCGTGGAGGATGAAGGCCCCCGGGTCGTAAACTCCTGTCAGTGGGGACGAATGCC
TCGCAAGAGGTTTGACTGTACCCATTGAGGAAGCCACGGCTAACTCCGTGCCAGCAGCCG
CGGTAATACGGAGGTGGCAAGCGTTGTTCGGAATTACTGGGCGTAAAGGGCGCGTAGGCG
GTTTGGCAAGTCAGATGTGAAAGCCCCGGGCTTAACCCGGGAATTGCATCTGAAACTGCT
TAACTTGAGCTCTGGATGGGGTAGTGGAATTCCCAGTGTAGCGGTGAAATGCGTAGATAT
TGGGAGGAACACCTGCGGCGAAGGCGGCTACCTGGACAGTGACTGACGCTGATGCACGAA
AGCTAGGGGAGCAAACAGGATTAGAAACCCGCGTAGTCC
>Otu3653
CCAGCCTATGGGATGCTGCAGTCGAGAATTTTTCTCAATGGGCGAAAGCCTGAAGGAGCG
ACGCCGCGTGGGGGATGAATGGCTTCGGCCCGTAAACCCCTGTCATTCGGGATCAATGCG
TCTGGGTGAACATCCCAGACGTTGATAGTAACGGAAGAGGAAGGGACGGCTAACTCTGTG
CCAGCAGCCGCGGTAATACAGAGGTCCCAAGCGTTGTTCGGATTCACTGGGCGTAAAGGG
TGCGTAGGTGGCCGGGTAAGTCTGATGTGAAATCTCGGAGCTCAACTCCGGAACGGCATT
GGATACTATCCGGCTGGAGGGTCGGAAGGGGGACTGGAATTCTCGGTGTAGCAGTGAAAT
GCGTAGATATCGAGAGGAACACCAGCGGCGAAGGCGAGTCCCTGGACGACTCCTGACACT
GAGGCACGAAAGCTAGGGGAGCAAACAGGATTAGAGACCCTGGTAGTCC
>Otu3654
CCAGCCTACGGGAGGCTGCAGTAAGGGATATTGCGCAATGGGCGAAAGCCTGACGCAGCA
ACGCCGCGTGGAGGACGACGGCCTTCGGGTTGTAAACTCCTTTTCACGAGGACGAGGAAG
GACGGTACTCGTGGAATAAGCTTCGGCTAACTACGTGCCAGCAGCCGCGGTAAGACGTAG
GAAGCGAGCGTTATCCGGATTTACTGGGCTTAAAGGGTGTGTAGGCGGGTCGTTAAGTTG
TGCATGAAAGCTGCCCGCTCAACGGGCAGAGGCTGTGCAAGACTGGCGGTCTAGAGGCTG
TGAGAGGGATGCGGAATTCCGGGTGTAGTGGTGAAATGCGTAGAGATCCGGAAGAACACC

AGAGGCGAAGGCGGCATCCTGGCACAGAACTGACGCTGAGACACGACAGCATGGGGAGCG
AACGGGATTAGATACCCGCGTAGTCC
>Otu3659
CCAGCCTACGGGGGGCAGCAGTGAAGAATCTTGGGCAATGGAGTAACCTCTGACCCAGTA
AGAATTCATGCATGATGGATGCGTACAGTTTTCTGTAAAGTGCTAGCGATAACGAGGAAG
CTGTCCGTAGTTATCCCATAAAGCGCCGGCCAACTTCGTGCCAGCAGCCGCGGTAATACG
AAGGGCGCGTGTGTTATTCGTTTTGATTGGGTGTAAAGGGTACGTAGGCTGCTGTTGAAA
TTCTTGACTAAAAGCGTGTAGCATTCCTATACGATTGTTTTTAAAGACTCAGCTAGTGA
TGGGGAGTGGTGGGGGTAACTTCTTGTGTAGGGGTAGAATCCGACCATATAAGAATGAAC
ACCAGCTAGCGAAGGCGCCTCTCCGGCCTCATCAGACGCTAAGGTACGGAAGTTTAGGGA
GCAAACAGGATTAGAAACCCGCGTAGTCC
>Otu3660
CCAGCCTACGGGGGGCACCAGTCGAGGACCTTCGTCAATGGGCGAAAGCCTGAACGAGCG
ACGCCGCGTGCGTGATGAAGGCCTTCGGGTTGTAAAACGCGAAAGTGGGGACGAAAGCCG
AAAGGTTTGACCGATCCACAGTAAGCTCGGGCTAAGTTCGTGCCAGCAGCCGCGGTAAGA
CGAACCGAGCGAACGTTATTCGGAATCACTGGGCTTAAAGGGCGCGTAGGCGGGCATCAA
AGTCTGTGTTGAAATCCGGCAGCTCAACTGTCGAACCGGCATGGATACTGGGTGTCTCGA
GGGAGGTAGGGGCGTGCGGAACTATTGGTGGAGCGGTGAAATGCGTTGATATCAATAGGA
ACTCCGGTGGCGAAGGCGGCACGCTGGACCTCTTCTGACGCTGAGGCGCGAAAGCCAGGG
GAGCAAACGGGATTAGATACCCGTGTAGTCC
>Otu3662
CCAGCCTACGGGACGCAGCAGTGGGGAATTTTGCGCAATGGGGGAAACCCTGACGCAGCA
ACGCCGCGTGGAGGATGAAGCCCCTTGGGGCGTAAACTCCTTTCGACCAAGACGATAATG
ACGGTACTGGTGGAAGAAGCACCGGCTAACTCTGTGCCAGCAGCCGCGGTAATACAGAGG
GTGCAAGCGTTGTTCGGAATTATTGGGCGTAAAGGGCGCGTAGGCGGCGCGACAAGTCAC
CTGTGAAATCCCCAGGCTTAACCTGGGGCCTGCAGGCGAAACTGTCGTGCTGGAGTATGG
GAGAGGTGCGTGGAATTCCCGGTGTAGCGGTGAAATGCGTAGATATCAGGAGGAACACCG
GTGGTGTAAACGGCTTTCTGGACCATAACTGACGGTGAGGCACGAAAGCGTGGGTAGCAA
ACAGGATTAGATACCCCCGTAGTCC
>Otu3664
CCAGCCTATGGGTCGCAGCAGTAGGGAATTTTCCGCAATGGGCGAAAGCCTGACGGAGCG
ACGCCGCGTGAGAGAAGACGGTCTTCGGATTGTAAATCTCTGTCTTTGGGGAAGAAAACA
ATGACGGTACCCAAAGAGGAAGCCCCGGCTAACTACGTGCCAGCAGCCGCGGTAATACGT
AGGGGGCGAGCGTTATCCGGATTTATTGGGCGTAAAGGGCGTGTAGGCGGTAGATTAAGT
TGGTTGTAAAAGGCAATGGCTCAACCATTGTAAGCGACCAAAACTGGTTTACTTGAGTGC
AGAAGAGGAGAGTAGAATTCCCGGTGTAGCGGTGGAATGCGTAGATATCGGGAGGAATAC
CAGTGGCGAAGGCGGCTCTCTGGTCTGTAACTGACGCTGAGGCGCGAAAGCGTGGGGAGC
AAACAGGATTAGATACCCGAGTAGTCC
>Otu3666
CCAGCCTATGGGGCGCTGCAGTAAGGAATATTGGTCAATGGGCGGAAGCCTGAACCAGCC
ATGCCGCGTGCAGGAAGACGGCCCTATGGGTTGTAAACTGCTTTTGCAGGGGAATAAACC
TCCCGACGTGTCGGGAGTTGAATGTACTCTGAGAATAAGGATCGGCTAACTCCGTGCCAG
CAGCCGCGGTAATACGGAGGATCCGAGCGTTATCCGGATTTATTGGGTTTAAAGGGTGCG
TAGGTGGCTTTTTAAGTCAGGGGTGAAAGACGGCAGCTTAACTGTCGCAGTGCCTTTGAT
ACTGAAGAGCTTGAATACACTTGAGGTAGGCGGAATGTGACAAGTAGCGGTGAAATGCAT
AGATATGTCACAGAACACCAATTGCGAAGGCAGCTTACTAAAGTGGGATTGACACTGATG
CACGAAAGCGTGGGGATCAAACAGGATTAGAAACCCTCGTAGTCC
>Otu3669
CCAGCCTATGGGTCGCTCCAGTCGAGGATCTTCGGCAATGGGCGCAAGCCTGACCGAGCG
ACGCCGCGTGTGCGAAGAAGGCCTTCGGGTTGTAAAGCACTGTCGAGGGGGAGAAAAGCC
GCAAGGCCTGATCTATCCCTGGAGGAAGCACGGGCTAAGTTCGTGCCAGCAGCCGCGGTA
AGACGAACCGTGCAAACGTTATTCGGAATCACTGGGCTTAAAGGGCGCGTAGGCGGGTGG
TCAAGTCAGGGGTGAAATCGTTCGGCTTAACCGGACAATAGCCTCTGATACTGGCCGTCT
CGAGGGAGGTAGGGGCATGTGGAACTTCCGGTGGAGCGGTGAAATGCGTTGAGATCGGAA
GGAACGCCCGTGGCGAAAGCGAGGTCCTGGACGTCTACTGACGCTGAGACGCGAAAGCTA
GGGGAGCGAACGGGATTAGAAACCCGAGTAGTCC
>Otu3671
CCAGCCTATGGGTGGCAGCAGTCGAGGATCTTCGTCAATGGGCGAAAGCCTGAACGAGCG

ATGCCGCGTGCGCGATGAAGGCCTTCGGGTTGTAAAGCGCGAAAGAGGGAATAAAGGGAA
ACTTGATTGAACCTCAGTAAGCTCGGGCTAAGTTCGTGCCAGCAGCCGCGGTAAGACGAA
CCGAGCGAACGTTGTTCGGAATCACTGGGTATAAAGGGCGCGTAGGCGGGTTTCTAAGTC
CGTGGTGAAATACTCCAGCTCAACTGGAGAACTGCCTCGGATACTGGAAGTCTCGAGTAA
GGTAGGGGCACGTGGAACGGCTGGTGGAGCGGTGAAATGCGTTGATATCAGTCGGAACTC
CGGTGGCGAAGGCGATGTGCTGGACCTTTACTGACGCTGAGGCGCGAAAGCCAGGGGAGC
AAACGGGATTAGAAACCCCAGTAGTCC
>Otu3672
CCAGCCTACGGGATGCTGCAGTAACGAATCTTCCGCAATGCACGCAAGTGTGACGGAGCG
ACGCCGCGTGGAGGACGAAGTCCTTCGGGATGTAAACTCCTGTCAGGGGTTAGAAAGTTC
TGATCAACCCCAGAGGAAGGCACGGCTAACTCTGTGCCAGCAGCCGCGGTAAGACAGAGG
TGCCAAGCGTTAGGCGGAATCACTGGGCTTAAAGCGTGTGTAGGCGGGCCGTTAAGTGCT
TTGTGAAATCCCCCGGCTCAACCGGGGAACTGCTGAGCAGACTGGCGGTCTTGAGCGGCC
TAGGGGCCAGCGGAACAAGCGGTGGAGCGGTGAAATGCGTAGATATCGCTTGGAACGCCT
GCGGTGAAAACGGCTGGCTGGGGGCCTGCTGACGCTGAGACACGAAAGCCAGGGGAGCAA
ACGGGATTAGATACCCTCGTAGTCC
>Otu3673
CCAGCCTATGGGAGGCAGCAGTAGGGAATCTTGCGCAATGGACGAAAGTCTGACGCAGCA
ACGCCGCGTGAGGGACGAAGGCTTTCTGAGTTGTAAACCTCTTTCGACAGGAACGATTGT
GACGGTACCTGTAGAAGAAGCACCGGCCAACTATGTGCCAGCAGCCGCGGTGATACATAG
GGTGCAAGCGTTATTCGGATTTATTGGGCGTAAAGAGCTCGTAGGCGGTTCGACAAGTCG
GGTGTTAAACCCCCAGGCTTAACCTGGGGCCGCCACCCGAAACTGTTGTGACTAGAGTTT
GGTAGGGGATCACGGAATTCCTGGTGTAGCGGTGGAATGCGCAGATACCAGGAGGAACAC
CAGTAGCGAAAGCGGCTCACTGGACCATAACTGACGCTGATGCACGAAAGCTAGGGGAGC
AAACAGGATTAGAAACCCGAGTAGTCC
>Otu3685
CCAGCCTACGGGAGGCAGCAGTAGGGAATATTGCGCAATGGACGAAAGTCTGACGCAGCG
ACGCCGCGTGAGTGATGAAGGCCTTAGGGTCGTAAAGCTCTGTTGTACGGGAAGAAAAAA
ATGACGGTACCGTATAAGAAAGGATCGGCTAACTTCGTGCCAGCAGCCGCGGTAAGACGA
GGGATCCTAGCGTTGTTCGGAATCATTGGGCGTAAAGAGTATGTAGGTTGCTTTACAAGT
CAGTTGTGAAATCCCCAGGCTTAACCCGGGAAGTGCTTCTGATACTGTTTAGCTTGAGTA
CTGTAGAGGTGAGTGGAATTCCTGGTGTAGTGGTGAAATACGTAGGTATCAGGAGGAACA
CCGGCGGCGAAGGCGGCTCACTGGACATGTACTGACACTGAGATACGAAAGCGTGGGGAG
CAAACAGGATTAGAAACCCCCGTAGTCC
>Otu3690
CCAGCCTATGGGGGGCTGCAGTCGGGAATTTTGCTCAATGGGCGCAAGCCTGAAGCAGCA
ACGCCGCGTGGGGGATGACGGCCTTCGGGTTGTAAACCTCTTTTCCCGGGGACGATGATG
ACGGTACTCGGGGAATAAGTCTCGGCTAACTACGTGCCAGCAGCCGCGGTAACACGTAGG
AGGCAAGCGTTATCCGGATTTACTGGGCGTAAAGCGCATGCAGGTGGTTCGGTAAGTTGG
ATGTGAAAGCTCCTGGCTCAACTGGGAGAGGTCGTTCAATACTACCGGACTTGAGAGCAG
TAGAGGGAGGTGGAATTCCCGGTGTAGTGGTGAAATGCGTAGATATCGGGAGGAACACCA
GTGGCGAAAGCGGCCTCCTGGACTGTTTCTGACACTCATATGCGAAAGCTAGGGTAGCAA
ACGGGATTAGATACCCCGGTAGTCC
>Otu3692
CCAGCCTATGGGATGCAGCAGTAAGGAATATTGGTCAATGGACGCAAGTCTGAACCAGCC
ATGCCGCGTGAAGGAAGACGGCCCTACGGGTTGTAAACTTCTTTTGTACAGGAAAAAACC
ACCGGTCGTGAATCGGTGCTGATGGTACTGTAAGAATAAGGATCGGCTAACTCCGTGCCA
GCAGCCGCGGTAATACGGAGGATCCAAGCGTTATCCGGATTCATTGGGTTTAAAGGGTGC
GCAGGCGGAATGATAAGTCAGTGGTGAAATCCTACGGCTCAACTGTAGAACTGCCATTGA
TACTGTCGTTCTTGAGTACACTTGGAGTGGGCGGAATGTGTCATGTAGCGGTGAAATGCT
TAGATATGACACAGAACGCCGATCGCGAAGGCAGCTCACTAAGTTGTAACTGACGCTCAG
GCACGAAAGCGTGGGGATCAAACAGGATTAGATACCCGCGTAGTCC
>Otu3696
CCAGCCTACGGGGGGCAGCAGTGGGGAATTTTGCACAATGCCCGAAAGGGTGATGCAGCA
ACGCCGCGTGAGGGATGAAGGCCTTCGGGTCGTAAACCTCTTTCGATCGGGAAAAACGGC
CCTGTGAAGAGCGGGGTCTGATGGTACCGGTGGAAGAAGCCCCGGCTAACTACGTGCCAG
CAGCCGCGGTAATACGTAGGGGGCGAGCGTTGTTCGGAATTACTGGGCGTAAAGGGCGTG
TAGGCGGTGCGGTAAGTTGGGCGTGAAATCCCCGGGCTTAACCCGGGGGGGCCGTCCAAA

ACTGCCGTGCTAGGGTATGGGAGAGGGTAGCGGAATTCCCGGTGTAGCGGTGAAATGCGT
AGATATCGGGAGGAACACCTGCGGCGAAGGCGGCTGCCTGGACCATTACCGACGCTGAGG
CGCGAAAGCTAGGGGAGCAAACAGGATTAGATACCCGAGTAGTCC
>Otu3699
CCAGCCTATGGGTTGCACCAGTCGAGAATCTTCCACAATGGGCGAAAGCCTGATGGAGCG
ACGTTTCGTGAATGAAGAAGCCCTTCGGGGTGTAAAGTTCTTTTTTGGGGGAGGAATCTT
GCCGCAAGGCGGGATGACAGTACTCCAAGAATAAGGAGGTCCTAACTCTGCGCCAGCAGG
AGCGGTAATACAGAGCCTCCGAGCGTTATCCGGATTTACTGGGCGTAAAGGATTCGTAGG
TGATTGAGTTAGTCTTTGGTTAAAACTCATCGGCTCAACCTTTGAGCTGCCAAAGATACT
GCTCGAATAGAGGATGTTAGAGGCTAACGGAACCGACGGTGTAGGGGTGAAATCCGTTGA
TATCGTCGGGAACACCAAAAGCGAAGGCAGTTAGCTGGGACAATCCTGACACTGAGGAAT
GAAAGCGTGGGGATCAAAAAGGATTAGAGACCCTCGTAGTCC
>Otu3703
CCAGCCTATGGGGGGCTCCAGTGGGGAATCTTCCGCAATGGACGAAAGTCTGACGGAGCG
ACGCCGCGTGGAGGATGAAGTCCTTCGGGACGTAAACTCCTTTTGCCAGGGAAAAAGTTA
TTGATTGTACCTGGAGAATAAGAAGTTGCTAAACTCGTGCCAGCAGCAGCGGTAATACGA
GTGCTTCAAGCGTTATCCGAAATCATTGGGCGTAAAGGGTGTGTAGGCGGCTTCGTTAGT
CCAGCGTTAAATCTTCCGGCTCAACCGGGAGGCTGCGCAGGAAACGGCGAAGCCTAGAGG
ACGGTAGGGGTCTCTGGAACTCATGGTGTAGCGGTGAAATGCGTTGATATCATGGGGAAC
ACCGAAAGCGAAGGCAAGAGACTGGACCGCTCCTGACGCTGAAACACGAAAGCGTGGGTC
GCGAATGGGATTAGAAACCCCCGTAGTCC
>Otu3704
CCAGCCTACGGGTTGCACCAGTAGGGAATATTGCGCAATGGAGGAAACTCTGACGCAGCG
ACGCCGCGTGGGTGATGAAGGCCTTCGGGTCGTAAAGCCCTGTCGGAGGGGAAGAAAAAC
ACGTCTGTTAATAATAGATGTGCTTGACGGTACCCTTAAAGGAAGCACCGGCTAACTACG
TGCCAGCAGCCGCGGTAATACGTAGGGTGCAAGCGTTGTTCGGAATCATTGGGCGTAAAG
CGCGCGTAGGCTGTCAATTAAGTCGGGTGTGAAATCCCTGGGCTCAACCGAGGAAGTGCA
TCCGAAACTGATTGGCTAGAAGACGGTAGAGGAAGGTGGAATTCCTAGTGTAGAGGTGAA
ATTCGTAGATATTAGGAGGAATACCGGTGGCGAAGGCGGCCTTCTGGGCCGTTCTTGACG
CTGAGGCGCGAAAGCGTGGGTAGCAAACAGGATTAGAAACCCTCGTAGTCC
>Otu3710
CCAGCCTACGGGTGGCAGCAGACGAGAATATTCCGCAATGGACGAAAGTCTGACGGAGCG
ACGCCGCGTGATGGATGAAGTGCTTCGGTACGTAAACATCTTTTATCGGGGACGAAGTTA
TTGACGGTACCCGGTGAATAAGGGGCTCCTAACTCTGTGCCAGCAGGAGCGGTAATACAG
AGGCCCCAAGCATTACCCGGAATCACTGGGCGTAAAGGGTGTCCAGGCGGTCGTATTAGT
CGTTCGTAAAATCCGTGAGCTCAACTTACGGTCCGCGAGCGAAACGGTACGACTAGAGAG
TGCGAGAGGTATACGGAACTCATGGTGGAGGGGTGAAATCCGTTGATATCATGGGGAACA
CCAAAGGCGAAGGCAGTATACTGGCGCATTTCTGACGCTCACACACGAAAGCCAGGGGAG
CGAACGGGATTAGAAACCCCAGTAGTCC
>Otu3711
CCAGCCTATGGGGGGCAGCAGTAACGAATCTTCCGCAGTGGGCGAAAGCCTGACGGAGCG
ACGCCGCGTGGAGGAATAAGGCCTTCGGGTTGTAAACTCCTTTTAGGGTGACGAAATCAA
GGCGACCTAACACGTCGTCAAGGTGATCGATCCCAGAATAAGCCACGGCTAACTCTGTGC
CAGCAGCCGCGGTAATACAGAGGTAGCAAGCGTTGTTCGGAATTACTGGGCTTAAAGCGC
GTGTAGGCGGTCGCGCAAGTACTTGGTGAAATCCCTCGGCCTAACCGAGGAAGTGCCAGG
TATACTGCGTGACTTGAGGCGAATAGGGGTGCCTGGAACTCTAGGTGGAGCGGTGAAATG
CGTAGATATCTAGAGGAACGCCCGTGGCGAAAGCGGGGCACTGGAATCGTTCTGACGCTG
AGACGCGAAAGCGTGGGGAGCAAACGGGATTAGATACCCCTGTAGTCC
>Otu3712
CCAGCCTACGGGAGGCAGCAGCAGGGAATCGTGCGCAATGGACGAAAGTCTGACGCCGCA
ATGCCGCGTGGAGGATGAAGGCCTTCGGGTCGTAAACTCCTTTTGCCATAGAGGAGATGC
TATGGAGAATAAGTGACTGCTAACTCTGTGCCAGCAGCCGCGGTAATACAGAGGTCACAA
GCGTTATCCGGATTTATTGGGCGTAAAGCGTTTCGTAGGTGATTCATTAAGTTGCTCTTC
AAATACCGAGGCTCAACCTCGGGAAGGGGAGTAAGACTGATGGAATTGAATTGTGGTGGG
GTTTCTGGAACTGGTGGTGTAGTAGTGAAATACGTTGATATCATCAGGAACTCCAAGGGC
GAAGGCAGGAAACTAACCATATATTGACACTGAGGAACGACAGCTAGGGTAGCGAAAGGG
ATTAGAGACCCCCGTAGTCC
>Otu3714

CCAGCCTACGGGGGGCAGCAGTCGAGAATTTTTCACAATGGGCGCAAGCCTGATGGAGCG
ACGCCGCGTGGGGGATGAATGGCTTCGGCCCGTAAACCCCTGTCATTCGGGATCAATGCG
CTTGGTTGAACATTCCAAGCGTTGATAGTACCGGAAGAGGAAGGGACGGCTAACTCTGTG
CCAGCAGCCGCGGTAATACAGAGGTCCCAAGCGTTGTTCGGATTTACTGGGCGTAAAGGG
TGCGTAGGCGGTCGGGTAAGTCTGGTGTGAAATTTCGGAGCTCAACTCCGAAACGGCATT
GGATACTATTCGGCTAGAGGTTGGGAAGGGAGACTGGAATTCTCGGTGTAGCAGTGAAAT
GCGTAGATATCGAGAGGAACACCAGTGGCGAAGGCGAGTCTCTGGACCAATCCTGACGCT
GAGGCACGAAAGCCAGGGGAGCAAACGGGATTAGAAACCCTAGTAGTCC
>Otu3716
CCAGCCTACGGGACGCTCCAGTCGAGGATCTTCGGCAATGGGCGCAAGCCTGACCGAGCG
ACGCCGCGTGCGCGATGAAGGCCTTCGGGTTGTAAAGCGCTGTCGAGGGGGAGGAAGGCC
TGCAAGGGCTTGACCAATCCCTGGAGGAAGCACGGGCTAAGTTCGTGCCAGCAGCCGCGG
TAAGACGAACCGTGCGAACGTTGTTCGGATTCACTGGGCTTAAAGGGCGCGTAGGCGGGC
TGTCAAGTCGGGGGTGAAATCTTTCAGCTTAACTGGAAAAGTGCCTTTGATACTGACAGT
CTCGAGGGAGATAGGGGCGTGCGGAACTTCCGGTGGAGCGGTGAAATGCGTAGATATCGG
AAGGAACGCCGGTGGCGAAAGCGGCGCGCTGGATCTCTTCTGACGCTGAGGCGCGAAAGC
CAGGGGAGCAAACGGGATTAGAAACCCTCGTAGTCC
>Otu3717
CCAGCCTATGGGAGGCAGCAGTCGAGAATTTTTCACAATGGGCGAAAGCCTGATGGAGCG
ACGCCGCGTGGGGGATGAATGGCTTCGGCCCGTAAACCCCTGTCATTCGGGATCAATGCG
TCTGGTTGAACATCCCAGACGTTGATAGTACCGGAAGAGGAAGGGACGGCTAACTCTGTG
CCAGCAGCCGCGGTAATACAGAGGTCCCAAGCGTTGTTCGGATTTACTGGGCGTAAAGGG
TGCGTAGGCGGTCGGCCAAGTCTGACGTGAAATCTCCGGGCCTAACCCGGAAACGGCGTT
GGATACTGGCTGGCTCGAGGAATGGAGGGGAGACTGGAATACTTGGTGTAGCAGTGAAAT
GCGTAGATATCAAGTGGAACACCAGTGGCGAAGGCGAGTCTCTGGACATTTCCTGTCGCT
GAGGCACGAAAGCCAGGGGAGCAAACGGGATTAGAAACCCCTGTAGTCC
>Otu3718
CCAGCCTATGGGGGGCTGCAGTGAGGAATATTGAGCAATGAACGAAAGTTTGACACAGTG
ATAGTTTATGAGTGAGCAATGGACAGTATTATCACTGTAAAACTCTTTTAACAAAAAAGA
TAATGACATTTTTTGGAGAATAAGCACTGGCTAATTCTGTGCCAGCAGCCGCGGTAACAC
AGGTAGTGCTAACGTTATTCATCATAATTGGGTGTAAAGGATATGTAGGTGGTTTTAGAA
TTGAATTTTTGTAAAAAGCACTAAAAAAAAATAGTGATAGGCAAATTTTACCTTAGAACT
AGAGAGTTTTGATCAAGTAGATAGAATTTCTTATGTAAGAGTAGAATCTATATATATTGG
AACGACTGCCAAACGAGTTACGAAAGTATTTTTCTAATCAAAACTGACACTGAGATATGA
AAGTATAGGGATCAAACAGGATTAGAAACCCCAGTAGTCC
>Otu3720
CCAGCCTACGGGACGCACCAGTCGAGGATCTTCGGCAATGGGCGCAAGCCTGACCGAGCG
ACGCCGCGTGGATGATGAAGGCCTTCGGGTTGTAAAATCCTGTCGAGGGGGATCAAGAGC
AATTTGAGTGATCCCTGGAGGAAGCACGGGCTAAGTTCGTGCCAGCAGCCGCGGTAAGAC
GAACCGTGCAAACGTTATTCGGAATCACTGGGCATAAAGGGCGCGTAGGCGGCTCCAAAA
GTCAGGGGTGAAATCCGGCAGCTTAACTGTCGCAGTGCCTTTGATACTGTGGAGCTAGAG
GGAGGTAGGGGTCTGTGGAACTTCCGGTGGAGCGGTGAAATGCGTTGATATCGGAAGGAA
CGCCGGTGGCGAAAGCGACGGACTGGATCTCTTCTGACGCTGAGGCGCGAAAGCCAGGGG
AGCAAACGGGATTAGAGACCCGCGTAGTCC
>Otu3721
CCAGCCTATGGGGGGCTCCAGTGGGGAAATTTGGACAATGGACGCAAGTCTGATCCAGCC
ATGCCGCGTGAGTGAAGAAGGCCTTCGGGTTGTAAAGCTCTTTTGTCCGGGAGCAAACCC
ACATGGATAATACCTGTGTGGGATGAGAGTACCGGAAGAATAAGCACCGGCTAACTACGT
GCCAGCAGCCGCGGTAATACGTAGGGTGCAAGCGTTAATCGGAATTACTGGGCGTAAAGC
GTGCGCAGGTGGTTGTGTAAGCTTGATGTGAAATCCCCGGGCTCAACCTGGGAACTGCAT
TGAGGACTACACGACTAGAGTGTGGCAGAGGGGGGTGGAATTCCACGTGTAGCAGTGAAA
TGCGTAGAGATGTGGAGGAACACCGATGGCGAAGGCAGCCCCCTGGGCTAACACTGACAC
TCATGCACGAAAGCGGGGGGAGCAAACAGGATTAGAAACCCGAGTAGTCC
>Otu3724
CCAGCCTATGGGGGGCTGCAGTAACGAATCTTCCGCAATGCACGCAAGTGTGACGGAGCG
ACGCCGCGTGTGGGATGAAGTCCTTCGGGATGTAAACCACTGTCAGAGGAAAGAAAGTTC
TGATCTACCTCAGAGGAAGGCACGGCTAACTCTGTGCCAGCAGCCGCGGTAAGACAGAGG
TGCCAAGCGTTAGGCGGAATCACTGGGCTTAAAGCGTGTGTAGGCGGGTCCCTAAGTACC

TTGTAAAATCCCACGGCTCAACCGTGGAACTGCTCGGTATACTGAGGATCTTGAGCCACC
TAGGGGCGAGCGGAACAAATGGTGGAGCGGTGAAATGCGTAGATATCATTTGGAACGCCA
ATGGTGAAAACAGCTCGCTGGGGGTGTGCTGACGCTGAGACACGAAAGCCAGGGGAGCAA
ACGGGATTAGAAACCCGAGTAGTCC
>Otu3728
CCAGCCTATGGGTCGCTGCAGTGGGGAATTTTGGACAATGGGCGCAAGCCTGATCCAGCC
ATGCCGCGTGAGTGAAGAAGGCCTTCGGGTTGTAAAGCTCTTTCAGCCGGAAAGAAATCG
CACTTGCTAATACTAAGTGTGGATGACGGTACCGGAAGAAGAAGCACCGGCTAACTACGT
GCCAGCAGCCGCGGTAATACGTAGGGTGCGAGCGTTAATCGGAATTACTGGGCGTAAAGC
GTGCGCAGGCGGTTTTGTAAGACAGACGTGAAATCCCCGGGCTTAACCTGGGAACTGCGT
TTGTGACTGCAAGGCTAGAGTATGGCAGAGGGGGGTAGAATTCCACGTGTAGCAGTGAAA
TGCGTAGATATGTGGAGGAATACCGATGGCGAAGGCAGCCCCCTGGGTCAATACTGACGC
TCATGCACGAAAGCGTGGGGAGCAAACAGGATTAGATACCCTCGTAGTCC
>Otu3729
CCAGCCTACGGGGTGCTGCAGTCGAGAATATTCCCCAATGGACGAAAGTCTGAGGGAGCG
ACGCCGCGTGTGGGATGAAGGCCTTCGGGTCGTAAACCACTTTTGTCAGGGACGAATTTA
TGACGGTACCTGAAGAATAAGAGGTTGCTAACTCTGTGCCAGCAGCAGCGGTAATACAGA
GACCTCAAGCATTATCCGGATTTATTGGGCGTAAAGGGTCCGCAGGTGGCATGGCACGTC
TTGCGTCAAATGTTGAAGCTCAACTTTAACACTGCGCGGGATACGGCCAAGCTAGAGGGT
GGGAGAGGCGAGCGGAATTACCGGTGTAGTAGTAAAATGCGTTAATATCGGTAAGAACAC
CAAATGCGAAGGCAGCTCGCTAGAACACTTCTGACACTCAGGGACGAAAGCGTGGGTAGC
GAATGGGATTAGAACCCGTGTAGTCC
>Otu3734
CCAGCCTATGGGGGGCACCAGTGGGGAATACTGGACAATGAGCAAACGCTTGATCCGGCA
ATGTTAAACGAATGAACTGAAAGTAATGAGCTGTAAAATTCATAAAGTTAGTATGCATAT
AGTGAACTTATTAATAAAGCCCCGACTAACTTCGTGCCAGCAGTCGCGGTAATACGGGGG
GGGCGAGTGTTACTCATCATGACTAGGCGTAAAGGGTGTTAAGACCGCTAGGGTTTGTCT
TAGTCAAAAGTTCAATTGAGCTATTGAAAAGTACTAATTAAACAACTTAGCTAAGGGTAG
ATTGGAGAATAGAATTACTAGTTTTTAAGGGGTAGAATCCAACGAGAGAATTAAGAGGAA
CAAAGGCGAAAGCAACTATTTATAGTCGACCAACGTTGATACACTAAAGCGTGGGGAGCA
AACAGGATTAGATACCCTAGTAGTCC
>Otu3735
CCAGCCTACGGGGGGCACCAGGCACGAAAACTTTACACTGCACGAAAGTGTGATAGGGGA
ACCCTCAGTGCATTACTATTTGTAATGCTTTTGCCAAATGTAAATAGTTTGGCGAATAAG
TGGTGGGCAAGACCGGTGCCAGCCGCCGCGGTAACACCGGCGCCACGAGTGGGGATCGCG
TTTATTGGGCCTAAAGCGTCCGTAGCAGGTTTTGTAAATTCTTTGTGAAATCATTTCGCT
TAACGAAATGGCGTGCAGAGAACACTGCAAAACTTGGAACCGGTGAGGAGTGAGAGGAAT
TCTTTGGGGAGCGGTAAAATGCTATAATCCTTAGAAGACCACCTGTGGCGAAGGCGTCTC
ACTAGAACGGATTCGACTGTGAGGGACGAAAGCTAGGGGAGCGATCCGGATTAGATACCC
CCGTAGTCC
>Otu3736
CCAGCCTACGGGTCGCAGCAGTGGAGAATATTAGGCAATGGAGTTGCTTCTGACCTAGTG
AGGGTGAATGCATGAGGAAGGCTAGGACTCTGTAAAGTGCTTTAGGCAAGGAAGATAATA
TCAGTAGCTGCTTTAATAAGCGCCGGCCAATCTCGTGCCAGCAGCAGCGGTAAAACGAGA
GGCGCGGGTGTTATTCGTTTTGATTGGGTGTAAAGGGTATGTAGACAGTTGTGGAGGTTC
TGATATAAAACGTGGAGTATTCCTATGCGAAGAGTCTTTGAGCTTATCAAAAACAACTAG
TGTTGATGTTGGGTTTGGGCAATTTTGTGAGTAGGAGTAGAATCCAAACATACACGAAGG
AACACCAAAAGCAAAGGCACCTTTCTAGTTTCAACAGACGTTGAGGTACGGAAGCTTAGG
GATCAAACAGGATTAGAAACCCGGGTAGTCC
>Otu3740
CCAGCCTACGGGAGGCAGCAGTGAGGAATATTGGTCAATGGGCGCAAGCCTGAACCAGCC
ATGCCGCGTGAAGGATGAAGGCCCTACGGGTTGTAAACTTCTTTTGTACGGGACGAAACT
CTTGCTCGTGAGCAGGACTGACTGTACTGTATGAATAAGCATCGGCTAACTCCGTGCCAG
CAGCCGCGGTAATACGGAGGATGCAAGCGTTATCCGGATTCATTGGGTTTAAAGGGTGCG
TAGGCGGGATGATAAGTCAGTGGTGAAAGCCTGCGGCTTAACCGTAGAATTGCCATTGAT
ACTGTCGTTCTTGAGTGCAGTTGAGGTGGGTGGAATGTATAATGTAGCGGTAAAATGCGT
AGATATTATACAGAACACCAATTGCGAAGGCAGCTCACTAAACTGTAACTGACGCTGAGG
CACGAAAGCGTGGGGAGCAAACAGGATTAGAAACCCCAGTAGTCC

>Otu3744
CCAGCCTATGGGAGGCTCCAGTAGGGAATATTGGGCAATGGGCGAAAGCCTGACCCAGCA
ACGCCGCGTGCACGATGAAGGTCTTCGGATCGTAAAGTGCTTTTCTGAGAGATGAGAAAG
GACAGTATCTCAGGAATAAGCACCGGCTAACTCCGTGCCAGCAGCCGCGGTAATACGGAG
GGTGCAAGCGTTATCCGGATTTACTGGGTTTAAAGGGTGTGTAGGCGGGCTTTTAAGTCA
GTGGTGAAATCTCCGGGCTCAACCTGGAAACTGCCATTGATACTATTAGTCTTGAATTTT
GTTGAGGTAGGCGGAATAAGTCATGTAGCGGTGAAATGCATAGATATGACTTAGAACACC
AATTGCGAAGGCAGCTTGCTAAACAAACATTGACGCTGAGGCACGAAAGCGTGGGGATCA
AACAGGATTAGAAACCCCTGTAGTCC
>Otu3745
CCAGCCTATGGGGCGCAGCAGTGGGGAATCTTGCGCAATGGGCGAAAGCCTGACGCAGCA
ACGCCGCGTGTGTGATGAAGGTCTTCGGATCGTAAAGCACTGTCGCGAGGGAAGATACAC
GACCAGATAACACCTGGGCGTTAAGACGGTACCTCGAGAGGAAGCACCGGCTAACTCTGT
GCCAGCAGCCGCGGTAATACAGAGGGTGCGAGCGTTGTTCGGAATTATTGGGCGTGAAGC
GCTTGTAGGCGGCCGTTTAAGTCTCATGTGAAAGCCCTCGGCTCAACCGAGGAAGTGCAT
GAGATACTGGACGGCTTGAATGCCGGAGAGGGTCGCGGAATTCCCGGTGTAGAGGTGAAA
TTCGTAGAGATCGGGAGGAACACCAGTGGCGAAGGCGGCGACCTGGACGGACATTGACGC
TGAGACGCGAAAGCGTGGGGAGCAAACAGGATTAGAAACCCCTGTAGTCC
>Otu3746
CCAGCCTACGGGGTGCTCCAGTAGGGAATATTGGACAATGGGCGGAAGCCTGATCCAGCC
ATGCCGCGTGCAGGAAGAAGGCCTTCTGGGTCGTAAACTGCTTTTCCCGGGGGATAAAAA
GGCCCTGCGGGGCAAATTGAAGGTACCCGGGGAATAAGCCACGGCTAACTACGTGCCAGC
AGCCGCGGTAATACGTAGGTGGCGAGCGTTGTCCGGATTTATTGGGTTTAAAGGGTGCGT
AGGCGGATTAATAAGTCAGTGGTGAAAACCTTCAGCTTAACTGGAGACTTGCCATTGATA
CTGTTAGTCTTGAGTACGGTCAAGGTAGGCGGAATGTGTAATGTAGCGGTGAAATGCTTA
GATATTACACAGAACACCGATTGCGAAGGCAGCTTACTGGGCCATTACTGACGCTGATGC
ACGAAAGCGTGGGGAGCGAACAGGATTAGAGACCCGCGTAGTCC
>Otu3748
CCAGCCTATGGGTCGCTCCAGTGGGGAATTTTGGACAATGGGGGAAACCCTGATCCAGCC
ATGCCGCGTGAGTGAAGAAGGCCTTCGGGTTGTAAAGCTCTTTTGGCCGGAAAGAAAAGA
TCTCGTCTAATAAACGAGGTTCTTGACGGTACCGGCAGAATAAGCACCGGCTAACTACGT
GCCAGCAGCCGCGGTAATACGTAGGGTGCGAGCGTTAATCGGAATTACTGGGCGTAAAGA
GTGCGTAGGCGGTTCTTTGTGTCCGCTGTGAAAGCCCCGGGCTTAACCTGGGAATGGCAG
TGGAAACTAGAGGACTTGAATCCGGCAGAGGGGGGTGGAATGCCACGTGTAGCAGTGAAA
TGCGTAGATATGTGGCGGAACACCGATGGCGAAGGCAGCTCCCTGGGCTGAGATTGACGC
TCAGGCACGAAAGCGTGGGGAGCAAACAGGATTAGATACCCTAGTAGTCC
>Otu3753
CCAGCCTACGGGTTGCTGCAGTAGGGAATATTGGGCAATGGGCGAAAGCCTGACCCAGCA
ACGCCGCGTGCACGATGAAGGTCTTCGGATCGTAAAGTGCTTTTCTGAGAGATGAGAAAG
GACAGTATCTCAGGAATAAGTCTCGGCTAACTACGTGCCAGCAGCCGCGGTAACACGTAG
GAGGCAAGCGTTATCCGGATTTACTGGGCGTAAAGCGCATGCAGGTGGTTCGGTAAGTTG
GATGTGAAAGCTCCTGGCTTAACTGGGAGAGGTCGTTCAATACTTCCAGACTAGAGGATG
TGAGAGGAGAGTGGAATTCCGAGTGTAGTGGTGAAATGCGTAGATATTCGGAGGAACACC
AGTGGCGAAAGCGGCTCTCTGGCACATTTCTGACGCTCATATGCGAAAGCTAGGGTAGCA
AACGGGATTAGAGACCCGTGTAGTCC
>Otu3755
CCAGCCTACGGGGCGCTGCAGTGGGGAATTTTGCGCAATGGGCGCAAGCCTGACGCAGCA
ACGCCGCGTGAGTGATGAAGGTCTTCGGATCGTAAAGCTCTGTCGGCGGGAACGATGCGT
TCGCTGGATAATACCCAGCGGACTCGACGGTACTCGCAAAGGAAGCACCGGCTAACTCTG
TGCCAGCAGCCGCGGTAAGACAGAGGGTGCGAGCGTTGTTCGGAATTATTGGGCGTAAAG
GGCGCGTAGGCGGCTTGGCAAGTTGGATGTGAAAGCCCCGGGCTTAACCCGGGAAGTGCA
TCCAAAACTGCTTTGCTTGAGTGTTGGAGAGGGTGGCGGAATTCCCAGTGTAGAGGTGAA
ATTCGTAGATATTGGGAGGAACACCCGTGGCGAAGGCGGCCACCTGGACAAACACTGACG
CTGAGGCGCGAAAGCGTGGGGAGCAAACAGGATTAGAAACCCCGGTAGTCC
>Otu3756
CCAGCCTATGGGGGGCACCAGTAACGAATATTCCGCAATGGGCGAAAGCCTGACGGAGCA
ATGCCGCGTGTGGGATGAAGCGGTTTCGCCGTGTAAACCACTGTCAGAAGATAGAAAAGA
TGATCATCTTCAGAGGAAGGGGCGACTAACTCTGTGCCAGCAGTCGCGGTAAGACAGAGG

CCCCGAGCGTTAGTCGGAATCACTGGGCTTAAAGGGTGCGTAGGCGGGCGCGTAAGTGCT
TTGTGAAAGCCCTGGGCTCAACCCAGGAATTGCAGGGCATACTGCGCGTCTCGAGGTAGC
TAGAGGCCGGTAGAACGATCGGTGGAGCGGTGGAATGCGTAGAGATCGATCGGAATGCCA
AGGGTGAAGACAACCGGCTGGGGCTTACCTGACGCTGAGGCACGAAAGCGTGGGGAGCAA
ACAGGATTAGAGACCCCTGTAGTCC
>Otu3758
CCAGCCTACGGGTGGCAGCAGTCGCGAATCTTTTGCAATGCGCGTAAGCGTGACAAAGCG
AACCAAAGTGTTTTCCGCACAGGAAAACTTTTGCCAGCTGTAAAAAGGCTGGCGAATAAG
GACTGGGCAAGACCGGTGCCAGCCGCCGCGGTAATCCCGGCAGTCCAAGTCGCAGCCAAC
ATTATTGGGTCTAAAACATCCGTAGCTTGTTTATTAAGTTCTTTGTGAAATCCGGCATCT
TAAGTGTCGGGCGAGCAGAGAATACTGATAAGCTTGGGATCGGAAGATGCAAGGAGTACA
CTAAAGGTAGTGGTAAAATACGTTAATCTTTGGTGGACTAACAATTGGCGAAGGCACCTT
GCAAGTACGAATCTGACAGTGAGGGATGAAGGCCAGGGGCGCAAAATGGATTAGATACCC
GCGTAGTCC
>Otu3759
CCAGCCTATGGGGGGCTGCAGTCGAGAATCTTCCACAATGGACGAAAGTCTGATGGAGCG
ACGCCGCGTGATTGATGAAGTCCTTCGGGACGTAAAGATCTTTTATGAGGGAAGAAGTTT
ATTGACGGTACCTCAGGAATAAGGGGCTCCTAATCTCGTGCCAGCAGGAGCGGTAATACG
AGAGCCCCGAGCGTTATCCGGAATTATTGGGCGTAAAGGGTGCGTAGGTGGTTTTGTTAG
TCGTTTGTCAAATCTTCGGGCTTAACCCGAAATCCGCAAACGAAACGGCATAACTTGAGA
GTGTGAGAGGTATAGGGAACTCATGGTGTAGGGGTGAAATCCGTTGATATCATGGGGAAC
ACCAAAAGCGAAGGCACTATACTGGCACACATCTGACACTTAAGCACGAAAGCGTGGGTA
GCGAATGGGATTAGAGACCCGTGTAGTCC
>Otu3766
CCGCCTATGGGAGGCTGCAGTCGAGAATATTCGACAATGGACGAAAGCCTGATCGAGCGA
CGCCGCGTGCAGGATGAAGGCCTTCGGGTCGTAAACTGCGGTAAACACATGACAATGCAA
ATGAGTGTGTGTTGGAAAGAGGTGGGTAACTACGTGCCAGCACCAGCGGTAATACGTAGA
CCTCAAGCGTTATCCGGATTTATTGGGCGTAAAGCGCATGTAGGTGGTTTCGCGCGTCTT
CTGTTAAAGCCCACCGCCCAACGGTGGAAGTGCAGGAGATACGGCGAGACTAGAGGGGGT
TAGAGATGCATAGAACGCACGGTGTAGGGGTGAAATCCGTTGATATCGTGCGGAATACCA
AAGGCGAAGGCATTGCACTGGGACCTCCCTGACATTGAGATGCGAAAGCGTGGGGAGCAA
AAAGGATTAGATACCCCAGTAGTCC
>Otu3769
CCAGCCTATGGGGGGCACCAGTAGGGAATATTGGACAATGGGCGAAAGCCTGATCCAGCC
ATGCCGCGTGAGTGATGAAGGCCTTCGGGTCGTAAAACTCTTTTCTGAGGGAAGAACATC
CCGGGTAGGAAATGGCCCGGGCTTGACGGTACCTCAGGAATAAGCAACGGCTAACTCCGT
GCCAGCAGCCGCGGTAATACGGAGGTTGCGAGCGTTGTTCGGAATAACTGGGCGTAAAGG
GAGCGCAGGCGGTTCGACCAGTCTAAAGTGAAAGCCCGGGGCTTAACCTCGGAAGTGCTT
TGGATACTATCGAACTCGAGTTCGTGAGAGGAAAGCGGAATTCCAGGTGTAGCGGTGAAA
TGCGTAGATATCTGGAGGAACACCGGTGGCGAAAGCGGCTTTCTGGCACGATACTGACGC
TGAGGCTCGAAAGCATGGGGAGCGAACAGGATTAGAAACCCCCGTAGTCC
>Otu3770
CCAGCCTATGGGTTGCTCCAGCTACGAAACCTCTACAATGCACGCAAGTGTGATAGGGTA
ACCCCTCGTGCTTACCGACAAGGTAGGCTTTTGCTTAGCCTAGTGCGCTAAGCGAATAAG
TGGTGGGCAAGACCGGTGCCAGCAGCCGCGGTAATCCCGGCGCCACGAGTGGAAGTCACT
TCTATTGGGTCTAAAGTATTCGTAGCCGTTCCCATAAGTTTCTTGTGAAAGCCCTGTAGC
TTAACTCAGGAATTGCAAGAAATACTGTGGGAATTGGGACTGGGAGAGGTATGGGGTATT
GTTTGGGTAGCGGTAAAATGTTATAATCCTTACAAGACCGACTAATGCGAAGGCACCATA
CTTGAACAGATCCGACGGTGAGGAATGAGAGCTGGGGGAGCAAACCGGATTAGAGACCCG
AGTAGTCC
>Otu3771
CCAGCCTACGGGTTGCAGCAGTAGGGAATATTGGACAATGGGTGCGAGCCTGATCCAGCC
ATGCCGCGTGCAGGAAGAAGGCCTTCTGGGTTGTAAACTGCTTTTGCCAGGGGATAAAAC
GACCTTGCGAGGTTAATTGAAGGTACCTGGTGAATAAGCCACGGCTAACTACGTGCCAGC
AGCCGCGGTAATACGTAGGTGGCAAGCGTTGTCCGGATTTATTGGGTTTAAAGGGTGCGT
AGGCGGTCCTATAAGTCAGTGGTGAAATACAGCCGCTTAACGGTTGAGGTGCCATTGATA
CTGTTGGACTTGAGTACAGACGAGGTAGGCGGAATTGACGGTGTAGCGGTGAAATGCATA
GATATCGTCAAGAACACCGATAGCGAAGGCAGCTTACTAGACTGTAACTGACGCTGAGGC

ACGAAAGTGTGGGGATCAAACAGGATTAGAGACCCCAGTAGTCC
>Otu3772
CCAGCCTATGGGTCGCTGCAGTCGCGAAACCTTGACAATGCGCGAAAGCGTGATCGGGGA
ACTCTGAGTGCCAACCTTACGGTTGGCTTTTGATCAGCTTGAATAGCTGGTAGAATAAGG
GCTGGGTAAGTCTCGTGCCAGCCGCCGCGGTAACACGAGCAGCCCGAGTGGTGCCCACGA
ATATTGAGTCTAAAACGCATGTAGCCGGCTTGTTAAGTTCCCCGTGAAATCTTCAGGCTC
AACCTGAAGGCGCGCGGGGAATACTAGCAGGCTAGGGAATGGAAGAGGCCAAGGGTACTG
TAAGGGGAGGAGTAAAATCCTGTAATCCTTGCAGGACCATCGGTGGCGAAAGCGCTTGGC
CAAGACATGTCCGACGGTGAGATGCGAAGGCTAGGGGAGCAAATCGGATTAGAAACCCCC
GTAGTCC
>Otu3774
CCAGCCTATGGGGCGCAGCAGTGGGGAATTTTCCGCAATGGGCGCAAGCCTGACGGAGCA
AGACCGCGTGGGGGACGAAGGTTTGTGGATTGTAAACCCCTTTTGATTGGGGAGAAGTTC
TGACGGTACCAATCGAATCAGCATCGGCTAACTCCGTGCCAGCAGCCGCGGTAATACGGA
GGATGCAAGCGTTATCCGGAATTATTGGGCGTAAAGCGTCCGTAGGTGGTTATTCAAGTC
AGTTGTTAAAGCGTGGGGCTTAACCCCATAAAGGCAAATGAAACTGAATGGCTAGAGTGC
GATAGGGGCAAGGGGAATTTCCAGTGTAGCGGTGAAATGCGTAGATATTGGAAAGAACAC
CGGTGGCGAAAGCGCCTTGCTGGGTCTGCACTGACACTGAGGGACGAAAGCTAGGGGAGC
GAAAGGGATTAGAGACCCTCGTAGTCC
>Otu3775
CCAGCCTATGGGGTGCACCAGTCGAGAATCTTCCACAATGGACGGAAGTCTGATGGAGCG
ACGCCGCGTGATTGATGAAGTCCTTCGGGACGTAAAGATCTTTTATGAGGGAAGAAGTAT
ATTGACTGTACCTCATGAATAAGGGGCTCCTAATCTCGTGCCAGCAGGAGCGGTAATACG
AGAGCCCCAAGCGTTATCCGGAATTATTGGGCGTAAAGGGTGCGTAGGCGGTTGTGTTAG
TCGAATGTTAAAGACCCGAGCTTAACTCGGGGAACGCGTTCGAAACGGCACGACTTAGAA
TGAAGGAGGGGTGTGTAGAACTCATGGTGTAGGGGTGAAATCCGTTGATATCATGGGGAA
TACCAAAAGCGAAAGCAGCACACTGGCCTTTTATTGACGCTGAGGCGCGAAAGCGTGGGT
AGCGAATGGGATTAGATACCCTCGTAGTCC
>Otu3776
CCAGCCTATGGGAGGCTGCAGTGGGGAATATTGCACAATGGGCGAAAGCCTGATGCAGCG
ACGCCGCGTGAGGATGACGGCCTTCGGGTTGTAAACCTCTTTCAGCAGGGACGAAGCGTA
AGTGACGGTACCTGCAGAAGAAGCACCGGCTAACTACGTGCCAGCAGCCGCGGTAATACG
TAGGGTGCAAGCGTTGTCCGGATTTATTGGGCGTAAAGAGCTCGTAGGCGGTTTGTCGCG
TCGGATGTGAGAACCAGCAGCTTAACTGCTGGCCTGCATTCGATACGGGCAGACTTGAGG
ACGGTAGGGGAGACTGGAACTCCTGGTGTAGCGGTGGAATGCGCAGATATCAGGAAGAAC
ACCGGTGGCGAAGGCGGGTCTCTGGGCCGTTCCTGACGCTGAGGAGCGAAAGCGTGGGTA
GCAAACAGGATTAGAGACCCCTGTAGTCC
>Otu3777
CCAGCCTATGGGGGGCAGCAGCAACGAATATTCCGCAATGGGCGCAAGCCTGACGGAGCG
ACGCCGCGTGAAGGATGAAGTCCCTCGGGATGTAAACTTCAATAGTCTGATAGGAAGTAA
CCCGGTCGAACAGGCCGGGAAGTTGACCAGCAGATGAAAGGGGCGGCTAACTCCGTGCCA
GCAGCCGCGGTAAGACGGAGGCCCCGAGCGTTGTTCGGAATTATTGGGCTTAAAGCACAT
GTAGGCGGACTGGTAAGTGCTTCGTGAAATCCCCGGGCTCAACCCGGGAATTGCTTGGCA
TACTGTCGGTCTTGAGGCAGGTATGGGTCACTGGAACCTTAGGTGTAGCGGTGAAATGCG
TAGATATCTAAGGGAACGCCGGTGGCGAAAGCGGGTGACTGGGCCTGTCCTGACGCTGAG
ACGCGAAGGCGTAGGGAGCAAACGGGATTAGATACCCTAGTAGTCC
>Otu3781
CCAGCCTACGGGTCGCTGCAGGCGCGAAAACTCTACACTGCACGAAAGTGTGATAGGGGG
ATCCAAAGTGCTTATACATTAGTATAGGCTTTTGCCAAAGACAAATCCTTTGGGGAATAA
GTGGTGGGCAAGACCGGTGCCAGCCGCCGCGGTAACACCGGCGCCACAAGTGGCAACCGT
ATTTATTGGGCCTAAAGCGTTCGTAGCCGGCTTAGTAAATCTTCTGTGAAATTGTTTGGC
TTAACCAAACAACGTGCAGGAGACACTGCTATGCTTGAGACCGGGAGGGGCTAGGGGTAT
TCTTGGGGGAGCGGTAAAATGTTGTAATCCCTTGAAGACCACCTGTGGCGAAGGCGCCTA
GCCAGAACGGATCTGACGGTGAGGGACGAAAGCTAGGGGAGCGATCCGGATTAGATACCC
TCGTAGTCC
>Otu3782
CCAGCCTACGGGAGGCAGCAGTGGGGAATTTTGGACAATGGGCGAAAGCCTGATCCAGCG
ACGCCGCGTGGAGGATGAAGGTCTTCGGATTGTAAACTCCTTTTAGAGGGGACGAATAAA

TTGACAGTACCCTCAGAAAAAGCCACGGCTAACTACGTGCCAGCAGCCGCGGTAATACGT
AGGTGGCGAGCGTTACTCGGAATTACTAGGCGTAAAGCGCATGTAGGCGGTTGGATAAGT
CTGCGGTTAAATCTCCCGGCTCAACTGGGAACTGTCCGCAGAAACTATCCGGCTTGAGTG
TGGCAGGGGGAGACGGAATTCCCGGTGTAGCGGTGAAATGCGTAGATATCGGGAGGAACA
CCAATGGCGAAAGCAGTCTCCTGGGCCAATACTGACGCTGAGATGCGAAAGCTAGGGGAG
CAAACAGGATTAGAAACCCTTGTAGTCC
>Otu3785
CCAGCCTATGGGACGCAGCAGTGGGGAATCTTGCGCAATGGACGAAAGTCTGACGCAGCG
ACGCCGCGTGGGTGAAGAAGGCCTTCGGGTTGTAAAGCCCTGTCGAGTGGGAAAAACGAC
ATCCGAAATAATACGTCGGGTGAGTGCTGGTACCACTAGAGGAAGCACCGGCTAACTCCG
TGCCAGCAGCCGCGGTAATACGGAGGGTGCAAGCGTTGTTCGGAATTATTGGGCGTAAAG
GGCAGGTAGGTGGTCTTATAAGTCTACTGTGAAATCCCTGGGCTTAACCCAGGAAGTGCG
GTGGATACTGTAAGACTAGAGTGCTGGAGGGGTGCGTGGAATTCCCGGTGTAGCGGTGAA
ATGCGTAGATATCGGGAGGAACACCAGAGGCGAAGGCGGCGCACTGGACAGCAACTGACA
CTCAACTGCGAAAGCGTGGGGAGCAAACAGGATTAGAGACCCGCGTAGTCC
>Otu3787
CCAGCCTACGGGTGGCACCAGTGGGGAATATTGGACAATGGGCGCAAGCCTGATCCAGCC
ATGCCGCGTGAGTGATGACGGCCTTAGGGTTGTAAAGCTCTTTCACCCACGACGATAATG
ACGGTAGTGGGAGAAGAAGCCCCGGCTAACTTCGTGCCAGCAGCCGCGGTAATACGAAGG
GGGCTAGCGTTGTTCGGAATCACTGGGCGTAAAGCGTACGTAGGCGGATTTTTAAGTCAG
AGGTGAAATCCTGGAGCTCAACTCCAGAACTGCCTTTGATACTGGAAGTCTTGAGTTCGG
GAGAGGTGAGTGGAACTGCCAGTGTAGAGGTGAAATTCGTAGATATTGGCAAGAACACCA
GTGGCGAAGGCGGCTCACTGGCCCGGAACTGACGCTGAGACGCGAAAGCGTGGGGAGCAA
ACAGGATTAGAAACCCTAGTAGTCC
>Otu3793
CCAGCCTACGGGGCGCTGCAGGTAGGTAACTTTTGCAATGCACGAAAGTGTGACAAAGCG
AGCCAGAGTGTTTTTCTCTATTATGTGAAAATCTTTTGCGGGACGTAAAAAGTCCCGTGA
ATAAGGACTGGGCAAGACTGGTGCCAGCCGCCGCGGTAATCCCAGCAGTCCAAGTCGCAG
CCACAATTATTGGGTCTAAAATATCCGTAGCTTGCTTTGCAAGTCTCTTGTGAAATCGGG
ACTCTTAAAGTTCCGCCGTGCAAGAGATACTGCTTAGCTAGAGACCGGGAGACGTAAGAA
GTACATCTAGAGTAGCGGTAAAATGCGTTGATCCTTGATGGACTAACAACAGCGAAGGCA
TCTTACGAGAACGGATCTGACAGTGAGGGATGAAGGCTAGGGGCGCAAAGTGGATTAGAG
ACCCCAGTAGTCC
>Otu3801
CCAGCCTATGGGGTGCTCCAGTGGGGAATTTTGCACAATGGGGGAAACCCTGATGCAGCA
ACGCCGCGTGGAGGATGAAGTCCCTTGGGATGTAAACTCCTTTCGACCGGGACGATAATG
ACGGTACCGGTGGAAGAAGCCCCGGCTAACTCTGTGCCAGCAGCCGCGGTAATACAGAGG
GGGCGAGCGTTGTTCGGAATTATTGGGCGTAAAGGGCGCGTAGGCGGTGCGGTAAGTCTT
TTGTGAAATCTCCGGGCTCAACTCGGAGTCTGCAGGAGAAACTGCCGTGCTTGAGTGTGG
GAGAGGTGAGTGGAATTCCCGGTGTAGCGGTGAAATGCGTAGATATCAGGAGGAACACCG
GTGGTGTAGACGGCTTTCTGGACCGACACTGACGCTGAGACACGAAAGCGTGGGTAGCAA
ACAGGATTAGAAACCCTTGTAGTCC
>Otu3802
CCATGGGTGGCTGCAGTAGGGAATATTGGTTAATGGGCGAAAGCCCGAACCAGCAACGCC
GCGTGTGCGATGAAGGCCTTCGGGTCGTAAAGCACTTTTTGGAGGGAAGAGGAAGGACGG
TACCTCCAGAATAAGCCTCGGCTAACTACGTGCCAGCAGCCGCGGTAACACGTAGGAGGC
GAGCGTTATTCGGATTTACTGGGCGTAAAGCGCGTGTAGGCGGTTCGGTAAGTTGGATGT
GAAAGCTCCTGGCTTAACTAGGAGAGGTCGTTCAATACTGTCGGACTTGAGAGTGGTAGA
GGGAGATGGAATTCCGGGTGTAGTGGTGAAATGCGTAGATATCCGGAGGAACACCAGTGG
CGAAAGCGATCTCCTGGACCATTTCTGACGCTCAGACGCGAAAGCTAGGGTAGCAAACGG
GATTAGATACCCGCGTAGTCC
>Otu3808
CCAGCCTATGGGTCGCTCCAGTTCGGAATATTCGGCAATGGGCGGAAGCCTGACCGAGCG
ACGCCGCGTGCGGGATGAAGGCCCTTTGGGTTGTAAACCGCTGTCACAGGTGAGCAAATC
AGGTTGGTTTAATACGCCAGCTTGTTGAGTTAAGCTTGAGAGGAAGCCCCCGCTAATCAC
GTGCCAGCAGCGGCGGTAATACGTGAGGGGCGAACGTTGTTCGGTGTCACTGGGCTTAAA
GGGCGCGTAGGTGGCTGCGTAAGTAAGGTGTGAAAGCCCACGGCTTACCCGTGGAATTGC
GCCTTATACTGCGTGGCTTGAGGATCTCAGGGGAAGGGGGAACTCCAGGTGTAGCGGTGA

AATGCGCAGATATCTGGGGGAAGGCCGGCGGCGAAGGCGCCCTTCTGGGAGATTCCTGAC
ACTGAGGCGCGAAAGCGTGGGGAGCAAACAGGATTAGAAACCCTAGTAGTCC
>Otu3810
CCAGCCTACGGGTGGCACCAGTAACGAATATTCCGCAATGGGCGCAAGCCTGACGGAGCG
ACGCCGCGTGCAGGATGAAGCCCTTCGGGGTGTAAACTGCTGTCAGGGGTAAGCAAGAAC
GTGAGCTATCCCAGAGGAAGGGGCGACTAACTCTGTGCCAGCAGTCGCGGTAATACAGAG
GCCCCGAGCGTTAATCGGAATCACTGGGCTTAAAGGGTGCGTAGGCGTTTCCGCAAGTGC
CTTGTGAAATCCCACGGCTCAACCGTGGAACTGCTTGGTAGACTGCGGAAATTGAGGTTG
GTAGAGGCGGCTGGAACGGATGGTGGAGCGGTGAAATGCGTAGATATCATCCGGAACGCC
AAAGGTGAAGACAGGTCGCTGGGCCAATTCTGACGCTGAGGCACGAAAGCGTGGGGAGCA
AACGGGATTAGAAACCCTTGTAGTCC
>Otu3811
CCAGCCTACGGGACGCTGCAGTCAAGAAACTTTGGCAATGCACGAAAGTGTGACCAAGCG
ACGCCGCGTGATTGATGAAGCCCTTCGGGGTGTAAAGATCTTTTATGCATGAGGAAATTA
TTGACGTTAGTGCATGAATAAGGGGCTCCTAACTCTGTGCCAGCAGGAGCGGTAATACAG
AGGCCCCGAGCGTTATCCGGAATTATTGGGCGTAAAGGGTGAGTAGGTGGTTTAGTTAGT
CGAATTTCAAAGATCTGGGCTCAACCCAGGGAAGGGATTCGAAACGGCTAGACTTGAGTA
TGTGAGAGGTGAACGGAACTCATGGAGTAGGGGTGAAATCCGTTGATATCATGGGGAACA
CCAAATGCGAAGGCAGTACGCTGGCGCATATTTGACACTGAAGCACGAAAGCGTGGGTAG
CGAATGGGATTAGATACCCGAGTAGAC
>Otu3816
CCAGCCTATGGGTCGCAGCAGTGGGGAATTTTGCGCAATGGGCGAAAGCCTGACGCAGCG
ACGCCGCGTGGGTGATGAAGGCCTTCGGGTTGTAAAGCCCTGTGGGGAGGGAAGAATAAA
GGACGGCGAATACCCGTCCCGATGACGGTACCTCCTTAGCAAGCACCGGCTAACTCTGTG
CCAGCAGCCGCGGTAAGACAGAGGGTGCAAACGTTGTTCGGAATTACTGGGCGTAAAGCG
CGTGTAGGCGGCTACGCAAGTCGGGTGTGAAAGCCCTTGGCTCAACCGAGGAAGTGCATT
CGAAACTGCGCAGCTTGAGTCCCGGAGAGGATGGTGGAATTCTCGGTGTAGAGGTGAAAT
TCGTAGATATCGAGAGGAACACCGGTGGCGAAGGCGGCCATCTGGACGGTGACTGACGCT
GAGACGCGAAAGCGTGGGGAGCAAACAGGATTAGATACCCGAGTAGTCC
>Otu3822
CCAGCCTACGGGTGGCAGCAGTTTCGAATCATTCACAATGGGCGAAAGCCTGATGGTGCG
ATGCCGCGTGAGGGATGAAGGTCTTCGGATTGTAAACCTCTGTCACTGGGGAAGAAACGC
TTCAAGTTAACAGCTTGAAGCCTGACTTAACCCGGAGAGGAAGCAGTGGCTAACTCTGTG
CCAGCAGCCGCGGTAATACAGAGACTGCAAGCGTTATTCGGATTCACTGGGCGTAAAGGG
TGCGCAGGCAGCCAAGTGTGTGAGGCGTGAAAGCCCGGGGCTTAACCCCGGAATTGCACC
TCAAACTACATGGCTAGAGCATTGGAGAGGGTAGCAGAATTCATGGTGTAGCAGTGAAAT
GCGTAGATATCATGAGGAATACCAGAGGCGAAGGTGGCTATCTGGACGGCAACTGACGCT
CAGGCACGAAAGCGTGGGGAGCAAAAGGGATTAGATACCCTAGTAGTCC
>Otu3823
CCAGCCTATGGGACGCAGCAGTGGGGAATCTTGCGCAATGGACGAAAGTCTGACGCAGCC
ACGCCGCGTGAGTGAAGAAGGCCTTCGGGTTGTAAAGCTCTGTCGAGCGGGACGAACATC
TTGGTGGTTAATAGCCACCAGGACTGACGGTACCGCTAAAGGAAGCACCGGCTAACTCTG
TGCCAGCAGCCGCGGTAATACAGAGGGTGCAAGCGTTGCTCGGAATCATTGGGCGTAAAG
GGTAGGTAGGTGGTCTCATTTGTCTGGGGTGAAAGCCTTGAGCTTAACTCGAGAAGTGCC
CCGGAAACGGTGAGACTAGAGTCTTGGAGAGGGTCGTGGAATTCCCGGTGTAGCGGTGAA
ATGCGTAGAGATCGGGAGGAACACCAGAGGCGAAGGCGGCGACCTGGACAAGTACTGACA
CTCAACTACGAAAGCGTGGGGAGCAAACAGGATTAGATACCCCTGTAGTCC
>Otu3827
CCAGCCTACGGGGTGCACCAGTCGAGGATCTTCCGCAATGGGCGCAAGCCTGACGGAGCG
ACGCCGCGTGTGGGATGAAGGCCTTCGGGTTGTAAACCACTGTCACTCGTTATGAAAGGG
TTCAACGTTAACAGCGTTGGAAACTGACAAAGGCGAGAGAGGAAGCCACGGCTAACTTCG
TGCCAGCAGCCGCGGTAAGACGAAGGTGGCGAGCGTTGTTCGGAGTCACTGGGCATAAAG
CGCACGTAGGCGACGACGTAAGTTGGGTGTGAAATCCCTCGGCCCAACCGAGGAACTGCG
CCCAGTACTGCGTTGCTTGAGGATCAAAGGGGTGCATGGAATTCCCGGTGGAGCGGTGAA
ATGCGTTGATATCGGGAGGAACTCCGGCGGCGAAAGCGGTGCACTGGTTGATTTCTGACG
CTGAGGTGCGAAAGCCAGGGGAGCAAACGGGATTAGATACCCGAGTAGTCC
>Otu3828
CCAGCCTATGGGTCGCTCCAGCTAAGAATCTTCCGCAATGGGGGAAACTCTGACGGAGCG

ACGCCGCGTGGAGGATGAAGTGCTTCGGCATGTAAACTCCTTTTGCCAGGGAAAAAGTTA
TTGATTGTACCTGGAGAATAAGAGGTTGCTAAACTCGTGCCAGCAGCAGCGGTAATACGA
GTGCCTCGAGCGTTATCCGGAATCATTGGGCGTAAAGGGTGTGTAGGCGGTCGTGTTAGT
CTCGCGTAAAATCTTTCGGCTCAACCGAGAGTCCGCGCGGGAAACGGCATGACTAAGAGG
ATGGCAGGGGTCTTCGGAACTCATGGTGTAGCGGTGAAATGCGTTGATATCATGGGGAAC
ACCAAAAGCGAAGGCAGAAGACTGGGCCACTCCTGACGCTGAAACACGAAAGCGTGGGTC
GCGAATGGGATTAGATACCCCCGTAGTCC
>Otu3831
CCAGCCTACGGGATGCAGCAGTGGGGAATCTTGCACAATGGGGGAAACCCTGATGCAGCG
ACGCCGCGTGAGCGATGAAGCCCTTCGGGATGTAAAGCTCTTTCGGCAGGGACGATAATG
ACGGTACCTGCAGAAGAAGCTGCGGCTAACTACGTGTCAGCAGCCGCGGTAATACGTAGG
CAGCAAGCGTTGTTCGGAGTTACTGGGCGTAAAGAGTGTGTAGGCGGTTCCCTAAGTTCG
GTGTGAAATCTCCCGGCTCAACTGGGAGGGTGCGCCGAAAACTGGGGGACTGGAGTATGG
GAGAGGAAAGCGGAATTCCTGGTGTAGCGGTGAAATGCGTAGATATCAGGAGGAACACCG
GTGGTGTAGACGGCTTTCTGGACCATTACTGACGCTGAGACACGAAAGCGTGGGGAGCAA
GCAGGATTAGATACCCCAGTAGTCC
>Otu3833
CCAGCCTATGGGGGGCACCAGTGAGGAATATTGGTCAATGGACGCAAGTCTGAACCAGCC
ATGCCGCGTGAAGGATGAAGGCCTTCTGGGTTGTAAACTTCTTTTATGGGGGAAGAAACC
CTCCCTTTCTATGGAGGTTGACGGTACCCTAGGAATAAGCACCGGCTAACTCCGTGCCAG
CAGCCGCGGTAATACGGAGGGTGCAAGCGTTATCCGGATTTACTGGGTTTAAAGGGTGTG
TAGGCGGGTCTTTAAGTCAGTGGTGAAATCTCCGGGCTCAACCCGGAAACTGCCATTGAT
ACTATTGGCCTTGAATTTTGTTGAGGGGGGCGGAATAAGTCATGTAGCGGTGAAATGCAT
AGATATGACTTAGAACACCGATAGCGAAGGCAGCTCCCTAAGCAAACATTGACGCTGAGG
CACGAAAGCGTGGGGATCAAACAGGATTAGATACCCCCGTAGTCC
>Otu3837
CCAGCCTATGGGTGGCAGCAGTGGGGAATTTTGCGCAATGGGGGAAACCCTGACGCAGCA
ACGCCGCGTGGAGGGTGAAGTACCTTGGTACGTAAACTCCTTTCGATCGGGACGATAATG
ACGGTACCCGGAGAAGAAGCTGCGGCTAACTACGTGCCAGCAGCCGCGGTAATACGTAGG
CAGCAAGCGTTGTTCGGAGTTACTGGGCGTAAAGAGTGCGTAGGCGGTTCCGTAAGTTTG
TTGTGAAATCTCCCGGCTTAACTGGGAGGGTGCGGCGAAAACTGCGGGGCTGGAGTGTGG
GAGAGGTAAGCGAAATTCCCGGTGTAGCGGTGAAATGCGTAGTTATCGGGAGGAACACCA
GCGGTGTAGACGGCTTACTGGACCATGACTGACGCTGAGGCACGAAAGCGTGGGGAGCAA
ACAGGATTAGAAACCCCCGTAGTCC
>Otu3844
CCAGCCTACGGGGGGCTCCAGTGGGGAATATTGGACAATGGGGGAAACCCTGATCCAGCA
ATGCCGCGTGAGTGATGAAGGCCTTAGGGTTGTAAAGCTCTTTCACCCGTGACGATGATG
ACGGTAACGGGAGAAGAAGCCCCGGCTAACTTCGTGCCAGCAGCCGCGGTAATACGAAGG
GGGCAAGCGTTGTTCGGAATGACTGGGCGTAAAGGGCGCGTAGGCGGTTTGCCAAGTTGG
AGGTGAAATCCCGGGGCTCAACCTCGGAATTGCCTTCAAAACTGGCTGGCTAGAGTATGG
AAGAGGGTCGTGGAATTCCCAGTGTAGAGGTGAAATTCGTAGATATTGGGAAGAACACCA
GTGGCGAAGGCGGCGACCTGGTCCATAACTGACGCTGAGGCGCGACAGCGTGGGGAGCAA
ACAGGATTAGAGACCCCTGTAGTCC
>Otu3845
CCAGCCTATGGGATGCTGCAGTCAAGAATATTCGTCAATGGGCGAAAGCCTGAACGAGCG
ACACCGCGTGCAGGATGAAGGCCTTCGGGTCGTAAACTGCGGTAGATAAGTGACAATGCA
AATGAGTGCTTATCGGAAAGAGGTGGGTAACTACGTGCCAGCACCAGCGGTAAAACGTAG
ACCTCAAGCGTTATCCGGATTTATTGGGCGTAAAGCGCGTGTAGGGGGTTTTGTGCGTCT
TCTGTTAAAGCCCACCGCCTAACGGTGGAAGTGCAGGAGATACGGCAAGACTAGAGGAGG
TTAGAGGTGCATGGAACGCACGGTGTAGGGGTGAAATCCGTTGATATCGTGCGGAACACC
AAAGGCGAAGGCAATGCACTGGGACCTTCCTGACCCTGAGACGCGGAAGCGTGGGTAGCA
AAAAGGATTAGATACCCTAGTAGTCC
>Otu3847
CCAGCCTACGGGAGGCAGCAGCCAAGAATCTTCCGCAATGGGGGAAACCCTGACGGAGCG
ACGCCGCGTGAAGGAAGAAGGTCTTCGGATCGTAAACTTCTTTTATCAGGAAAGAATAAG
GAGCGAGGAAATGCGGCTCCGATGACGGTACCTGATGAATAAGCCCCGGCTAATTACGTG
CCAGCAGCCGCGGTAATACGTAAGGGGCGAGCGTTGCTCGGAATTACTGGGTGTAAAGGG
CTTGTAGGCGGGCTTACAAGTCGAGTGTAAAAGACCACGGCTCAACTGTGGGAATGCATT

CGAAACTGTAAGCCTTGAGTCTGATGGAGGGTAATGGAATTCCCGGTGTAGCGGTGAAAT
GCGTAGAGATCGGGAAGAACACCGAAGGCTAAAGCAGTTACCTATGTCAAGACTGACGCT
GAGGAGCGAAAGCATGGGGATCAAACAGGATTAGATACCCCTGTAGTCC
>Otu3851
CCAGCCTATGGGAGGCTCCAGTGGGGAATCTTGCGCAATGGGCGAAAGCCTGACGCAGCG
ACGCCGCGTGTGCGATGAAGGTCTTCGGATCGTAAAGCACTGTCGCGAGGGACAAAGGAA
GTGATGGTACCTCGAGAGGAAGCACCGGCTAACTCTGTGCCAGCAGCCGCGGTAATACAG
AGGGTGCGAGCGTTGTTCGGAATTATTGGGCGTAAAGCGCTGGTAGGTGGTTCTTCAAGT
CGCTTGTGAAAGCTCCCGGCTCAACCGGGAAAGTGCGGGCGAAACTGGAGGACTTGAGTG
CCGGAGAGGGTCGCGGAATTCCCGGTGTAGAGGTGAAATTCGTAGATATCGGGAGGAACA
CCAGAGGCGAAGGCGGCGACCTGGAAGGTGACTGACACTGAGCAGCGAAAGCGTGGGGAG
CAAACAGGATTAGATACCCCGGTAGTCC
>Otu3852
CCAGCCTATGGGAGGCAGCAGTAAGGGATATTGCGCAATGGGCGAAAGCCTGACGCAGCA
ACGCCGCGTGGAGGATGAAGGCCTTCGGGTCGTAAACTCGTTTTGCGTCTGACGAGGAAG
GACGGTAGGACGCGAAGAAGTCACGGCTAACTACGTGCCAGCAGCCGCGGTAAAACGTAG
GTGGCGAGCGTTATCCGGATTTACTGGGCGTAAAGGGTGTGCAGGCGGGGACACAAGTGG
TGTATGAAAGCACCCAGCTAAACTGGGTAAGGTTATGCCAGACTGTGTTTCTAGAGGACG
AGAGAGGGGCGTGGAATTCCGGGTGTAGTGGTGAAATGCGTAGAGATCCGGAGGAACCCC
AGAGGCGAAGGCGGCGCCCTGGCTCGTACCTGACGCTGATACACGAAAGCATGGGTAGCG
AACGGGATTAGATACCCGAGTAGTCC
>Otu3854
CCAGCCTATGGGATGCACCAGTCGAGGATCTTCGGCAATGGGCGCAAGCCTGACCGAGCG
ACGCCGCGTGCGGGATGAAGGCCTTCGGGTTGTAAACCGCTGTCAGTGGGGAGGAAAGGC
AGGAGGGTTCTCCCTCTTGTTTGACCTATCCGCAGAGGAAGTACGGGCTAAGTTCGTGCC
AGCAGCCGCGGTAAGACGAACCGTACGAACGTTATTCGGAATCACTGGGCTTAAAGGGTG
CGTAGGCGGCCCGGAAAGTTGGGTGTGAAAGCCCTCGGCTCAACCGAGGAATTGCGCCCA
AAACTACCGGGCTCGAGGGAGACAGAGGTGAGCGGAACTTAGGGTGGAGCGGTGAAATGC
GTTGATATCCTAAGGAACACCAGGAGCGAAAGCGGCTCACTAGGTCTCTTCTGACGCTGA
TGCACGAAAGCTAGGGGAGCGAACGGGATTAGAAACCCTCGTAGTCC
>Otu3855
CCAGCCTATGGGGGGCAGCAGTGGGGAATATTGGACAATGGGGGAAACCCTGATCCAGCA
ATGCCGCGTGAGTGATGAAGGCCTTCGGGTTGTAAACTGCTTTTGTACGGAACGAAAAGG
TTTGGCCTAATAAGCTGAGCTCATGACGGTACCGTAAGAATAAGCACCGGCTAACTACGT
GCCAGCAGCCGCGGTAATACGTAGGGTGCGAGCGTTAATCGGAATTACTGGGCGTAAAGC
GTGCGCAGGCGGTTATGTAAGACAGATGTGAAATCCCCGGGCTCAACCTGGGAACTGCAT
TAGTGACTGCATAGCTGGAGTACGGCAGAGGGGGATGGAATTCCGCGTGTAGCAGTGAAA
TGCGTAGATATGCGGAGGAACACCGATGGCGAAGGCAATCCCCTGGGCCTGTACTGACGC
TCATGCACGAAAGCGTGGGGAGCAAACAGGATTAGAAACCCTTGTAGTCC
>Otu3856
CCAGCCTATGGGGCGCAGCAGTAACGAATCTTCCCCAATGCACGAAAGTGTGAGGGAGCG
ACGCCGCGTGGAGGACGAAGTCCTTCGGGATGTAAACTCCTGTCAGGGGTTACCAAGTTC
TGAGGAACCCCAGAGGAAGTGGCGGCTAACTCTGTGCCAGCAGCCGCGGTAAGACAGAGG
CCACAAGCGTTAGGCGGAATCACTGGGCTTAAAGCGTGTGTAGGCGGACTCTTAAGTGCC
TTGTGAAATCCCACGGCTTAACCGTGGAACTGCTGGGCATACTGGGAGTCTTGAGCTGTC
TAGGGGCGACTGGAACAAACGGTGGAGCGGTGAAATGCGTAGATATCGTTTGGAACGCCA
ATGGTGAAAACAGGTCGCTGGGGACATGCTGACGCTGAGACACGAAAGCCAGGGGAGCAA
ACGGGATTAGATACCCCCGTAGTCC
>Otu3857
CCAGCCTATGGGTCGCTGCAGTGAGGAATATTGGTCAATGGGCGCAAGCCTGAACCAGCC
ATCCCGCGTGCAGGAAGAAGGCGCTATGCGTTGTAAACTGCTTTTCCAGGGGAAGAAAAC
CCGCTACGTGTAGCGGCTTGCCGGTACCTTGGGAATAAGCATCGGCTAACTCCGTGCCAG
CAGCCGCGGTAATACGGAGGATGCGAGCGTTATCCGGATTTATTGGGTTTAAAGGGAGCG
TAGGTGGATTTGTAAGTCAGTGGTGAAATCTCTCAGCTTAACTGAGAAACTGCCATTGAT
ACTGCAGATCTTGAGTACAGATGATGTGGGCGGAATATGACATGTAGTGGTGAAATACTT
AGATATGTCATAGAACACCGATTGCGAAGGCAGCTCACAAAACTGTAACTGACACTGAGG
CTCGAAAGTGCGGGGATCAAACAGGATTAGATACCCCCGTAGTCC
>Otu3859

CCAGCCTATGGGGGGCAGCAGCTAAGAATATTCCACAATGGACGAAAGTCTGATGGAGCG
ACGCCGCGTGGACGATGAAGGCCTTCGGGTTGTAAAGTCCTTTCGTGCGGGAAGATCTTT
CTACTAATGAACAATTAGTAGGATCGACTGTACCGCAGGAAGAAGCCCCGGCTAATTACG
TGCCAGCAGCCGCGGTAATACGTATGGGGCGAGCGTTATTCGGATTTATTGGGCGTAAAG
GGTATGCAGGGGGCTTGTTAAGTCGGAAGTGAAATTTCCAGGCTCAACCTGGAAATTGCT
CTCGATACTGGCAGGCTAGAGTTTGGCAGAGGAGAATGGAATTCCAGGTGTAGGGGTGAA
ATCTGTAGATATCTGGAAGAACACCAGTGGTGAGGACGATTCTCTGGGCCAAAACTGACC
CTCAGATACGAAAGCCAGGGGAGCAAACGGGATTAGATACCCGTGTAGTCC
>Otu3867
CCAGCCTATGGGGTGCTGCAGGCGCGAAACCTCTGCAATGCGCGAAAGCGTGACAGGGGA
ACTCTGAGTGCTACACCACGTGTGTAGCTTTTTGCAAGCTTAAGACGCTTGTAGAATAAG
GGCTGGGTAAGACTAGTGCCAGCCGCCGCGGTAACACTAGCGGCCCAAGTGGTGTCCACG
AATATTGGGTCTAAAGAGCCCGTAGCTGGTCCGTTAAGTTTCCTGTGAAATCTCAGAGCT
CAACTTTGAGGCGTGCAGGAAATACTGGCGCGACTTGAGAGCGGGGGAGGTCAAGAGTAC
TCATGGGGTAGCGGTAAAATGTTGTAATCCTATGAGGACTTCCAGTGGCGAAGGCGCTTG
ACCAAAACGCGTCTGACAGTGAGGGACGAAGGCTAGGAGAACGAATCGGATTAGAGACCC
GTGTAGTCC
>Otu3870
CCCAGCCTATGGGGTGCTGCAGCAACGAATATTGGGCAATGGGGGAAACCCTGACCCAGC
GACGCCGCGTGCAGGAGGAGGCCCTTCGGGGTGTAAACTGCTGTCGGGGACCAACAAGTG
ATACGGGTGAATAATCCGTAAAGTTGAGTCGTCCCGAAGGAAGCCTCGACTAACTCTGTG
CCAGCAGTCGCGGTAATACAGAGGAGGCAAGCGTTGCTCGGAATCATTGGGCTTAAAGAG
CGTGTAGGCGGCTTGGTAAGTGTCGCGTGAAATCCCATGGCTCAACCATGGAACTGCGCG
GCAAACTGCTTGGCTTGAGGTCGGTAGAGGTGCCTGGAACTCTAGGTGGAGCGGTGAAAT
GCGTAGATATCTAGAGGAACGCCGGAGGCGAAAGCGGGGCACTGGACCGATTCTGACGCT
GAGACGCGAAAGCGTGGGGAGCAAACAGGATTAGAAACCCTAGTAGTCC
>Otu3879
CCAGCCTATGGGGTGCAGCAGTGGGGAATTTTGGACAATGGGGGAACCCTGATCCAGCAA
TGCCGCGTGTGTGAAGAAGGCCTTCGGGTCGTAAAGCACTTTTGTCAGGAACGAAACGGT
TTTGGCTAATACCTAGAACTAATGACGGTACCTGAAGAATAAGCACCGGCTAACTACGTG
CCAGCAGCCGCGGTAATACGTAGGGTGCAAGCGTTAATCGGAATTACTGGGCGTAAAGCG
TGCGCAGGCGGTTGTGTAAGTCTGATGTGAAATCCCCGGGCTCAACCTGGGAATTGCATT
GGAGACTGCATAGCTAGAATCTGGCAGAGGGGGGTAGAATTCCACGTGTAGCAGTGAAAT
GCGTAGAGATGTGGAGGAACACCGATGGCGAAGGCAGCCCCCTGGGCTAAGATTGACGCT
CATGCACGAAAGCGTGGGGAGCAAACAGGATTAGATACCCTCGTAGTCC
>Otu3882
CCAGCCTACGGGTCGCAGCAGGCGCGAAACCTTTACAATGCACGAAAGTGTGATAGGGGG
ATACTCAGTGCTTACGGCTCTGCTGTAGGCTTTTGTCGATCGTAAATAGATCGGCGAATA
AGTGGTGGGTAAGACTGGTGCCAGCCGCCGCGGTAACCCCAGCGCCACAAGTGGTCATCG
CGATTATTGGGCCTAAAGCGTTCGTAGCCGGAGTGGCAAATCTCTTGTGAAATTGTTCGG
CTTAACCGGACAGCGTGCAGGAGACACTACCACCCTCGAGACCGGGAGGCGTAAGAAGTA
TGGCATGGGGACTGGTAAAATGGGATAATCCATGCTAGACTACCGATGGCGAAGGCATCT
TACGAGAACGGATCTGACGGTGAGGAACGAAAGCCAGGGGAGCGAACCGGATTAGAAACC
CCTGTAGTCC
>Otu3884
CCAGCCTATGGGTGGCTGCAGTGGGGAATATTGGACAATGGGCGCAAGCCTGATCCAGCC
ATGCCGCGTGAGTGATGAAGGCCCTAGGGTTGTAAAGCTCTTTCACCCACGACGATAATG
ACGGTACCCGGAGAAGAAGCCCCGGCTAACTTCGTGCCAGCAGCCGCGGTAATACGAAGG
GGGCTAGCGTTGCTCGGAATTACTGGGCGTAAAGGGAGCGTAGGCGGATCGTTAAGTTGG
GGGTGAAAGCCCGGGGCTCAACCTCGGAAATGCCTTCAATACTGGCGATCTTGAGTATGG
GAGAGGTAAGTGGAACTCCGAGTGTAGAGGTGAAATTCGTAGATATTCGGAAGAACACCA
GTGGCGAAGGCGACTTACTGGCCCATTACTGACGCTGAGGCTCGAAAGCGTGGGGAACAA
ACAGGATTAGAGACCCCCGTAGTCC
>Otu3885
CCAGCCTATGGGATGCTCCAGTAAGGAATATTGGTCAATGGAGGCAACTCTGAACCAGCC
ATGCCGCGTGAAGGATGAAGGCCCTATGGGTCGTAAACTTCTTTTGTACGGGAAAAAACC
CTTGAACGTGTTCGAGGCTGATGGTACCGTAAGAATAAGGATCGGCTAACTCCGTGCCAG
CAGCCGCGGTAATACGGAGGATCCAAGCGTTATCCGGATTCATTGGGTTTAAAGGGTGCG

TAGGCGGCTTATTAAGTCAGTGGTGAAATCCCGAGGCTCAACTTCGGAACTGCCATTGAT
ACTGATAAGCTTGAGTGTAAATGAAGTGGGCGGAATGTGTCGTGTAGCGGTGAAATGCTT
AGATATGACACAGAACACCGATTGCGAAGGCAGCTCACTAAACTACAACTGACGCTGAGG
CACGAAAGCGTGGGGATCAAACAGGATTAGAAACCCCTGTAGTCC
>Otu3887
CCAGCCTACGGGGTGCTGCAGCGGGGAATTTTGGACAATGGGGGCAACCCTGATCCAGCC
ATGCCGCGTGCGTGAAGAAGGCCTTCGGGTTGTAAAGCGCTTTTGTTGGGGAAGAAAAGG
GGTTCTTGAATAAAGGATTCTGCTGACGGTACCCAAAGAAAAAGCACCGGCTAACTACGT
GCCAGCAGCCGCGGTAATACGTAGGGTGCAAGCGTTAATCGGAATGACTGGGCGTAAAGA
GTGCGTAGGCGGTGACTTAAGATGGGTGTGAAATCCCCGGGCTTAACCTGGGAAGTGCAT
TCATGACTGGGTCGCTGGAGTGCGGTAGAGGGGGGTGGAATTCCTGGTGTAGCAGTGAAA
TGCGTAGAGATCAGGAGGAACACCGATGGCGAAGGCAGCCCCCTGGCCCGTGACTGACGC
TGAGGCACGAAAGCGTGGGGAGCAACCAGGATTAGAAACCCGCGTAGTCA
>Otu3888
CCAGCCTATGGGAGGCATCAGGGACGAATATTGGGCAATGGGCGAAAGCCTGACCCAGCG
ACGCCGCGTGTGGGAGGAAGTCCTTCGGGATGTAAACCACTGTCAGGGGTTACCAAGTGA
AAGGGCCTAATACGCCCTCTAATTGAGGAGCTCCAAAGGAAGCCACGGCTAACTTCGTGC
CAGCAGCCGCGGTAAGACGAAGGTGGCTAGCGTTGTTCGGAATCACTGGGCTTAAAGAGC
ATGTAGGCGGCCTGTTAAGCACTTTGTGAAATCCCTCGGCTTAACCGGGGAATTGCTTGG
TGTACTGGCAGGCTTGAGGCAGGTAGGGGCGCGTGGAACTCTTGGTGGAGCGGTGAAATG
CGTAGATATCAAGAGGAACGCCGGAGGCGAAAGCGATGCGCTGGGCCTGTCCTGACGCTG
AGATGCGAAAGCTAGGGGAGCAAACAGGATTAGAAACCCTAGTAGTCC
>Otu3891
CCAGCCTATGGGTTGCTCCAGGTCGGTCGGAACAGTGTACTGAACCAGGCCGCCTGCTTT
CTGACTGCTCTTCTCTTCGGAAAAGAAGAAGAACAGTCGGTCGCGGTTCCGGTTGAACTT
GCCCGGGAAATAGATTGGTCCTCCGACGGTGACACCGAATGTGTTGTAACGGTAGATCGC
GCGCGGCACAGAATTGAGGTTGTTGAAGTAGGTGTTCGCGTTCAATGATTCGTTGCGAAT
GTATTCATAGGCGCTGCCGTGGAACGTGCGTGTCCCGGACTTCGTCACGCCAACGATAAC
GGCGCTGCCGTTTCTTCCGTACTCGGCCCGGAAATTGTTGACGAGCACCGTCACTTCGCT
GATCGCGTCAATGTTGGAAGTGAACGGGTACTGCGGATTAGAGACCCCAGTAGTCC
>Otu3893
CCAGCCTATGGGGTGCACCAGTTAAGGAAATTTTTCGATGCCCGCAAGGGTGAACGAGTA
AGCCAGAGTGTTTCCTTTACGGAAACTTTTGTCTGCTGTAGAAAGGCAGACGAATAAGGA
CTGGGCAAGACCGGTGCCAGCCGCCGCGGTAATCCCGGCGGTCCAAGTCGCATCCACAAT
TATTAGGTCTAAAACATCCGTAGCTTGTTTTGTAAGTCTCTTGTGAAATCTCAAGTCTTA
AGCTTGAGGCGTGCAAGAGATACTGCTGAACTCGAGATCGGAAGACGCAAAGAGTACGAT
TGAGGTAGTGGTAAAATATGTTAATCTTAATCGGACTAACAATGGCGAAGGCACTTTGCG
AGTACGAATCTGACAGTGAGGGATGAAGGCTAGGGGCGCAAAAGGGATTAGAAACCCCGG
TAGTCC
>Otu3894
CCAGCCTACGGGATGCTCCAGTGGGGAATCTTGCACAATGGGGGCAACCCTGATGCAGCG
ACGCCGCGTGAGCGATGAAGCCTTTCGGGGTGTAAAGCTCTTTCGACAGGAACGATTATG
ACGGTACCTGGAGAAGAAGCTGCGGCTAACTACGTGCCAGCAGCCGCGGTAATACGTAGG
CAGCAAGCGTTGTTCGGAGTTACTGGGCGTAAGGAGTGCGTAGGCGGTCCTATAAGTTCC
GTGTGAAATCTCCCGGCTCAACTGGGAGGGTGCGCGGAAGACTGTAGGGCTTGAGTGTGG
GAGAGGTAAGCGGAATTCCTGGTGTAGCGGTGAAATGCGTAGATATCAGGAGGAACACCT
GTGGTGTAGACAGCTTACTGGACCATAACTGACGCTGAGGCACGAAAGCGTGGGTAGCAA
ACAGGATTAGAAACCCCTGTAGTCC
>Otu3897
CCAGCCTACGGGAGGCAGCAGTGAGGAATTTTCCGCAATGGGCGAAAGCCTGACGGAGCG
ACGCCGCGTGTGGGATGAAGGCCCTCGGGTCGTAAACCACTGTCGAAGGGGACTATACTG
AAGGTACCCTTGGAGGAAGCCCCGGCTAACTACGTGCCAGCAGCCGCGGTAAGACGTAGG
GGGCGAGCGTTATTCGGAATTATTGGGCGTAAAGCGCCAGTAGGCGGATTGGAAAGTTCG
TGAAGAAAGACCCGGGCTCAACTCGGGGAACGGCACGAATACTTCCAGTCTTGAGGCAAT
CAGAGGGTGATGGAATTCCCGGTGTAGCGGTGAAATGCGTGGATATCGGGAGGAACACCA
GTGGCGAAGGCGATCACCTGGGGTTGATCTGACGCTGAGTGGCGAAAGCTAGGGGAGCAA
ACGGGATTAGAAACCCCCGTATCC
>Otu3898

CCAGCCTATGGGTCGCAGCAGTGAGGAATATTGGGCAATGGACGCAAGTCTGACCCAGCA
ACGCCGCGTGGAGGATGAAGGTCTTTGGATTGTAAACTCCTTTTGCAGGGGAAGAGTGAG
GACGGTACCCTGAGAATAAGTCACGGCTAACTACGTGCCAGCAGCCGCGGTAATACGTAG
GTGACGAGCGTTATCCGGATTTATTGGGCGTAAAGCGCACGCAGGCGGCGCAACAAGTCT
GACGTGAAATCTCCCGGCTTAACTGGGAGCGGTCGTTGGAAACTGTTGTGCTTGAGGCGA
TGAGAGGGGTGTGGAATTCCCGGTGTAGTGGTGGAATGCGTAGATATCGGGAGGAACATC
AGTGGCGAAAGCGGCACCCTGGCATTAGCCTGACGCTCATGTGCGAAAGCGAGGGGAGCG
AACGGGATTAGAAACCCCGGTAGTCC
>Otu3901
CCAGCCTATGGGGGGCACCAGTAAGGGATATTGCACAATGGGCGAAAGCCTGACGCAGCA
ACGCCGCGTGAAGGATGAAGGCCTTCGGGTTGTAAACTTTTTTATTCGGGACGAGCAAG
GACGGTACCGAGTGAATAAGTCACGGCTAACTACGTGCCAGCAGCCGCGGTAAAACGTAG
GTGGCGAGCGTTATCCGGATTTACTGGGCGTAAAGCGTGTGCAGGCGGGCGGTCAAGTGG
TGTATGAAAGCACTGCGCTCAACGCAGTGACCGTAGGCCAGACTGACTGTCTGGAGGGCG
AGAGAGGGGCGTGGAATTCCGGGTGTAGTGGTGAAATGCGTAGAGATCTGGAGGAACCCC
AGAGGCGAAGGCGGCGCCCTGGCTCGCACCTGACGCTCAGACACGACAGCATGGGGAGCG
AACGGGATTAGATACCCGCGTAGTCC
>Otu3902
CCAGCCTATGGGGTGCAGCAGGCGCGAAAACTCTCCAATGTGCGAAAGCGTGAGAGGGGA
ATTCGCAGTGCTTACGGTTTAGCCGTAGGCTTTTGCCATGTGTAAATAGCATGGCGAATA
AGTGGTGGGTAAGACTGGTGCCAGCCGCCGCGGTAACACCAGCGCCACGAGTGGCTATCG
CGATTATTGGGCCTAAAGCGTCCGTAGTCGGTTCTGTACATCTTCTGTGAAATCGCAACG
CTCAACGTTGCGGCATGCGGGAGACACGGCAGAACTTGAGACCGGGAGGCGTGTAGGGTA
TTCCACGGGGACCGGTAAAATGGTATAATCCGTGGGGGACCACCTGTGGCGAAGGCGCTA
CACGAGAACGGATCTGACGATGAGGGACGAGAGCCAGGGGAGCAAACCGGATTAGATACC
CCCGTAGTCC
>Otu3904
CCAGCCTATGGGTTGCTGCAGTGAGGAATATTGGGCAATGGACGCAAGTCTGACCCAGCA
ACGCCGCGTGGAGGATGAAGGTCTTTGGATTGTAAACTCCTTTTGCCGGGGAAGAGTGAG
GACGGTACCCGGAGAATAAGTCACGGCTAACTACGTGCCAGCAGCCGCGGTAATACGTAG
GTGACAAGCGTTATCCGGATTTATTGGGCGTAAAGCGCACGCAGGCGGCTGATTAAGTCT
GACGTGAAATCTCTCGGCTTAACCGGGAGCGGTCGTTGGAAACTAATCGGCTTGAGGCGA
TGAGAGGGGTGTGGAATTCCCGGTGTAGTGGTGGAATGCGTAGATATCGGGAGGAACACC
AGTGGCGAAAGCGGCCTTCTGGACTGTTTCTGACACTCATATGCGAAAGCTAGGGTAGCA
AACAGGATTAGAGACCCCTGTAGTCC
>Otu3905
CCAGCCTATGGGTGGCAGCAGTGGGGAATTTTGGACAATGGGGGCAACCCTGATCCAGCC
ATTCCGCGTGAGTGAAGAAGGCCTTCGGGTTGTAAAGCTCTTTTGTCCGGAGCGAAAAGG
TTACGGTGAATATCCGTGACTACTGACGGTACCGGAAGAATAAGCACCGGCTAACTACGT
GCCAGCAGCCGCGGTAATACGTAGGGTGCGAGCGTTAATCGGAATTACTGGGCGTAAAGC
GTGCGCAGGCGGTTTTGTAAGCCAGACGTGAAATCCCCGGGCTTAACCTGGGAATGGCGT
TTGGGACTGCAAGGCTCGAGTACGGCAGAGGGGACTAGAATTCCTGGTGTAGCAGTGAAA
TGCGTAGATATCAGGAGGAATACCGATGGCGAAGGCAGGTCCCTGGGTTAGTACTGACGC
TCATGCACGAAAGCGTGGGGAGCAAACAGGATTAGAAACCCGCGTAGTCC
>Otu3913
CCAGCCTATGGGACGCTGCAGTTAAGAATCTTGCGCAATGGGCGCAAGCCTGACGCAGCG
ACGCCGCGTGCGCGATGAAGGCCTTCGGGTTGTAAAGCGCAATAGACAGGGACGAAGAAA
GTGACGGTACCTGTTGAAAGCACCGGCTAACTACGTGCCAGCAGCCGCGGTAATACGTAT
GGTGCAAGCGTTGTTCGGATTTACTGGGCGTAAAGAGCGCGCGGGCGGATAATTAAGTCA
GGTGTGAAATCTTCGGGCTCAACCCGGAGACTGCACTTGAAACTAATGATCTCGAGTCCC
GGAGAGGCAGGCAGAATTCCTGGTGTAGCGGTGAAATGCGTAGATATCAGGAGGAATACC
AATGGCGAAGGCAGCCTGCTGGACGGTGACTGACGCTGAGGCGCGAAAGCGTCGGGAGCA
AACAGGATTAGATACCCTCGTAGTCC
>Otu3914
CCAGCCTACGGGAGGCTCCAGTGGGGAATATTGGACAATGGGCGCAAGCCTGATCCAGCC
ATGCCGCGTGAGTGATGAAGGCCTTAGGGTTGTAAAGCTCTTTTACCTGGGAAGATAATG
ACGGTACCAGGAGAATAAGCCCCGGCTAACTTCGTGCCAGCAGCCGCGGTAATACGAAGG
GGGCTAGCGTTGTTCGGATTTACTGGGCGTAAAGCGCACGTAGGCGGATCTTTAAGTCAG

GGGTGAAATGCCGGGGCTCAACCCCGGAACTGCCTTTGATACTGGGGATCTCGAGTCCGG
GAGAGGTGAGCGGAACTGCGAGTGTAGAGGTGAAATTCGTAGATATTCGGAAGAACACCA
GTGGCGAAGGCGACTCGCTGGACAAGTATTGACGCTGAGGTGCGAAAGCGTGGGGAGCAA
ACAGGATTAGATACCCCCGTAGTCC
>Otu3916
CCAGCCTACGGGAGGCAGCAGTGGGGAATATTGGACAATGGGGGCAACCCTGATCCAGCC
ATGCCGCGTGAGTGAAGAAGGCCTTAGGGTTGTAAAGCTCTTTCGCTAGGGACGATAATG
ACGGTACCTAGATAAGAAGCCCCGGCTAACTTCGTGCCAGCAGCCGCGGTAATACGAAGG
GGGTTAGCGTTGTTCGGAATCACTGGGCGTAAAGCGCACGCAGGCGGATTGATAAGTCAG
AGGTGAAATCCCGGAGCTCAACTTCGGAATTGCCTTTGATACTGTCTATCTTGAGGTCGA
GAGAGGTTGGCGGAATTCCTAGTGTAGAGGTGAAATTCGTAGATATTAGGAAGAACACCA
GTGGCGAAGGCGGCCAACTGGCTCGATACTGACGCTCATGTGCGAAAGCGTGGGGAGCAA
ACAGGATTAGATACCCTTGTAGTCC
>Otu3917
CCAGCCTATGGGGCGCAGCAGTGGGGAATTTTGCGCAATGGGGGAAACCCTGACGCAGCA
ACGCCGCGTGGAGGATGAAGTCCCTTGGGACGTAAACTCCTTTCGATCGGGACGATTATG
ACGGTACCGGAAGAAGAAGCCCCGGCTAACTTCGTGCCAGCAGCCGCGGTAATACGAGGG
GGGCGAGCGTTGTTCGGAATTACTGGGCGTAAAGGGCATGTAGGCGGCTTTGTAAGCCCG
GCGTGGAAGCCCACGGCTCAACCGTGGGATTGCGTTGGGAACTGCAAGGCTTGAATCATG
GAGAGGGAGCTAGAATTCCTGGTGTAGGGGTGAAATCTGTAGAGATCAGGAAGAATACCA
GTGGCGAAGGCGAGCTCCTGGCCAATGATTGACGCTGAGGTGCGAAAGTGTGGGGATCAA
ACAGGATTAGAAACCCTTGTAGTCC
>Otu3918
CCAGCCTACGGGTTGCTGCAGTGGGGAATCTTGCGCAATGGGCGAAAGCCTGACGCAGCG
ACGCCGCGTGGGTGATGAAGGCCTTCGGGTTGTAAAGCCCTGTGGGGAGGGACGAATAAG
TCATGGCTAATATCCATGGTGATGACGGTACCTCCTTAGCAAGCACCGGCTAACTCTGTG
CCAGCAGCCGCGGTAAGACAGAGGGTGCGAACGTTGTTCGGAATTACTGGGCGTAAAGCG
CGTGTAGGCGGTGGGGTAAGTCGGATGTGAAAGCCCGGGGCTCAACCTCGGAAGTGCACT
CAATACTACCTCGCTGGAGTCCCGGAGAGGATGGTGGAACTCTCGGTGTAGAGGTGAAAT
TCGTAGATATCGAGAAGAACACCGGTGGCGAAGGCGGCCATCTGGACGGTGACTGACGCT
GAGACGCGAAAGCGTGGGGAGCAGACAGGATTAGATACCCCAGTAGTCC
>Otu3921
CCAGCCTACGGGTCGCTCCAGTGGGGAATATTGGACAATGGGCGCAAGCCTGATCCAGCC
ATGCCGCGTGAGTGACGAAGGCCTTAGGGTTGTAAAGCTCTTTTGGCGGGGAAGATAATG
ACGGTACCCGCAGAATAAGCTCCGGCTAACTTCGTGCCAGCAGCCGCGGTAATACGAAGG
GTGCGAGCGTTGTTCGGAATTACTGGGCGTAAAGGGTTCGTAGGCGGGAATGCAAGTCAA
GTGTGAAATCCCCAGGCTTAACCTGGGACGTGCATTTGAGACTGTGTTTCTTGAGTTTCG
GAGAGGGTGGTGGAATTGCTGGTGTAGGAGTGACATCCGTAGAGATCAGCAGGAACACCG
GAGGCGAAGGCGACCACCTGGCCGAATACTGACGCTGAGGAACGAAAGCGTGGGGAGCAA
ACAGGATTAGATACCCGAGTAGTCC
>Otu3922
CCAGCCTACGGGTGGCTGCAGTGGGGAATCTTGCGCAATGGACGAAAGTCTGACGCAGCG
ACGCCGCGTGGGTGATGAAGGCCTTCGGGTTGTAAAGCCCTGTGGGGAGAGACGAATAAG
TGTAGCCTAATACGTTACATGATGACGGTATCTCCTTAGCAAGCACCGGCTAACTCTGTG
CCAGCAGCCGCGGTAAGACAGAGGGTGCAAACGTTGTTCGGAATTACTGGGCGTAAAGCG
TGTGTAGGCGGTCTTGTAAGTCGGATGTGAAAGCCCCGGGCTCAACCCGGGAAGTGCACT
CGATACTGCGAGACTTGAGTATCGGAGAGGTTGGTGGAATTCTCGGTGTAGAGGTGAAAT
TCGTAGATATCGAGAGGAACACCGGTGGCGAAAGCGGCCAACTGGACGAATACTGACGCT
GAGACACGAAAGCGTGGGGAGCAAACAGGATTAGAGACCCGCGTAGTCC
>Otu3929
CCAGCCTACGGGGCGCTGCAGTAACGAATCTTCCGCAATGTACGCAAGTATGACGGAGCG
ACGCCGCGTGTGGGACGAAGCCCTTCGGGGTGTAAACCACTGTCAGGGGTTAGAAAGTTC
TGATCGACCCCAGAGGAAGGCACGGCTAACTCTGTGCCAGCAGCCGCGGTAAGACAGAGG
TGCCAAGCGTTAGGCGGAATCACTGGGCTTAAAGCGTGTGTAGGCGGATTTCTAAGTACC
TTGTGAAATCCCACGGCTCAACCGTGGAACTGCTTGGTATACTGGTTATCTTGAGCTGTC
CAGGGGCGACCGGAACAAATGGTGGAGCGGTGAAATGCGTAGATATCATTTGGAACGCCA
ATGGTGAAAACAGGTCGCTGGGGACATGCTGACGCTGAGACACGAAAGCCAGGGGAGCAA
ACGGGATTAGAAACCCTCGTAGTCC

>Otu3931
CCAGCCTACGGGGGGCAGCAGTTTGGAATTTTGCACAATGGGCGAAAGCCTGATGCAGCG
ACGCCGCGTGGACGATGAAGGCCTTCGGGTTGTAAAGTCCTTTTGCTCGGGAACAATTTT
TGAGCGTACCGAGAGAATAAGAGGTTGCTAACTCTGTGCCAGCAGCAGCGGTAATACAGA
GACCTCAAGCGTTATCCGGAATCATTGGGCGTAAAGCGTACCGATAGGTGGCTTACAAAG
TCAGAAGTGAAATCTCTCAGCTTAACTGGGAGAATGTCTTTTGAAACTTGTAAGCTAGAG
GGGCAAAGAGGAAGCTGGAACGAACGGTGTAGTAGTGAAATGCGTTGATATCGTTCGGAA
CACCAATAGCGTAGGCAGGCTTCTGGGTGCCACCTGACACTGCTAGGACGAAAGCGTGGG
TAGCGATAAGGATTAGAAACCCTGGTAGTCC
>Otu3934
CCAGCCTATGGGGCGCTCCAGTGGGGAATATTGCACAATGGGCGGAAGCCTGATGCAGCG
ACGCCGCGTGAGGGATGACGGCCTTCGGGTTGTAAACCTCTTTCAGCAGGGACGAAGCGA
GAGTGACGGTACCTGCAGAAGAAGCGCCGGCCAACTACGTGCCAGCAGCCGCGGTAAGAC
GTAGGGCGCGAGCGTTGTCCGGATTTATTGGGCGTAAAGAGCTCGTAGGCGGCTTGTCGC
GTCGACTGTGAAAACTCAGGGCTCAACCCTGAGCTTGCAGTCGATACGGGCAGGCTCGAG
TTCGGTAGGGGAGACTGGAATTCCTGGTGTAGCGGTGAAATGCGCAGATATCAGGTGGAA
CACCGGTGGCGAAGGCGGGTCTCTGGGCCGATACTGACGCTGAGGAGCGAAAGCGTGGGG
AGCGAACAGGATTAGAGACCCTAGTAGTCC
>Otu3939
CCAGCCTACGGGTGGCAGCAGTGGGGAATATTGGACAATGGGCGCAAGCCTGATCCAGCC
ATGCCGCGTGAGTGATGACGGCCTTAGGGTTGTAAAGCTCTTTCGGCGGGGACAAAACGG
CGACCGCGAATAACGGTTGTTAATGATGGTACCACCAGAAGAAGCCCCGGCTAACTACGT
GCCAGCAGCCGCGGTAATACGTAGGGGGCAAGCGTTGTTCGGATTCATTGGGCGTAAAGG
GCATGTAGGCTGTTATGTAAGTGTGGATTTAAAGGCGGAGGCTTAACCTCTAGTTTGGTC
TGCAAACTGCGTAGCTTGAGTGTAGAAGGGGAAACTGGAATTCCGCGTGTAGGGGTGAAA
TCTGTAGATATGCGGAAGAATACCTGTGGCGAAGGCGAGTTTCTGGTCTATAACTGACGC
TGAAATGCGAAAGTGTGGGTAGCAAACAGGATTAGAAACCCTAGTAGTCC
>Otu3940
CCAGCCTCGGGTCGCAGCAGTCGAGGATCTTTGGCAATGGGCGCAAGCCTGACCAAGCGA
CGCCGCGTGCGCGATGAAGGCCTTCGGGTTGTAAAGCGCTGTCGAGGGGGAGGAAGCCGC
AAGGTTGACCTATCCCTGGAGGAAGGACGGGCTAAGTTCGTGCCAGCAGCCGCGGTAAGA
CGAACCGTCCTAACGTTGTTCGGAATCACTGGGCTTAAAGGGCGCGTAGGCGGGCTGCCA
AGTCTAGGGTGAAATCTTTCGGCTTAACCGGGAAATAGCCTTGGATACTGGTAGTCTCGA
GGGGGGTAGGGGCCGTTGGAACTTCCGGTGGAGCGGTGAAATGCGTTGATATCGGAAGGA
ACGCCGGTGGCGAAAGCGAACGGCTGGACCTCTTCTGACGCTGAGGCGCGAAAGCCAGGG
GAGCAAACGGGATTAGATACCCCAGTAGTCC
>Otu3942
CCAGCCTATGGGGGGCAGCAGTGGGGAATTTTGGACAATGGGCGCAAGCCTGATCCAGCC
ATGCCGCGTGAGTGAAGAAGGCCTTCGGGTTGTAAAGCTCTTTCGGCGGGGAAGAAATTA
CTTGGGCGAATACCCTGAGTAGATGACGGTACCCGAAGAAGAAGCACCGGCTAACTACGT
GCCAGCAGCCGCGGTAATACGTAGGGTGCGAGCGTTAATCGGAATTACTGGGCGTAAAGC
GTGCGCAGGCGGTTTTGTAAGACAGGTGTGAAATCCCCGGGCTTAACCTGGGAACTGCGC
TTGTGACTGCAAGACTGGAGTACGGCAGAGGGGGGTGGAATTCCTGGTGTAGCAGTGAAA
TGCGTAGAGATCAGGAGGAACACCGATGGCGAAGGCAGCCCCCTGGGTCCGTACTGACGC
TCATGCACGAAAGCGTGGGGAGCAAACAGGATTAGAGACCCCAGTAGTCC
>Otu3943
CCAGCCTATGGGAGGCAGCAGGCGCGAAAACTCTCCAATGCGCGAAAGCGTGAGAGGGGA
ATCCCAAGTGCTTAAGCATTACGCTTAGGCTTTTTTCGATTGCAAAAAAATCGGAGAATA
AGTGGTGGGCAAGACCGGTGCCAGCAGCCGCGGTAACCCCGGCGCCACAAGTGGCAACCG
TGTATTTGGCCTAAAGCGTCCGTAGCCGGTTCTGTGCATCCCTTGTGAAATTGACCCGCT
TAACGGGTCAGCGTGCAGGGGACACGGCAGAACTTGGAACTGGGAGGCGTCAGAGGTATT
GTTGGGGGAACGGTAAAATGTTATAATCCCAGCAAGACCACCGATGGCGAAGGCATCTGA
CGAGAACAGCTTCGACGGTGAGGGACGAAAGCTAGGGGAGCAAACCGGATTAGATACCCG
AGTAGTCC
>Otu3945
CCAGCCTATGGGAGGCAGCAGTGAGGAATATTGGTCAATGGGCGCAAGCCCGAACCAGCC
ATGCCGCGTGAAGGACGAAGGCCCTCGTGGTCGTAAACTTCTTTAGTATAGGGCGAAAAA
CCAAACGGGCTTTGGGCTTGACAGTACTATGCCAAAAAGCATCGGCTAACTCCGTGCCAG

CAGCCGCGGTAATACGGAGGATGCGAGCGTTATTCGGATTTATTGGGTTTAAAGGGTGCG
CAGGCGGCCTAATAAGTCAGTGGTGAAATACCGTTGCTCAACAACGGGGCTGCCATTGAT
ACTGTAAGGCTAGGGCCTGTTCGACGTACGCGGAATGCGGCAAGTAGTGGTGAAATACAT
AGATATGCCGCAGAACACCGATAGCGAAGGCAGCGTACGAGGATATGGCCGACGCTCAGG
CACGAAAGCGTGGGGATCAAACAGGATTAGATACCCGCGTAGTCC
>Otu3947
CCAGCCTATGGGTTGCAGCAGTGGGGAATCTTGCACAATGGGCGAAAGCCTGATGCAGCG
ACGCCGCGTGGGTGAAGAAGGTCTTCGGATCGTAAAGCCCTTTTCTGCGTGACGAGACAA
GGACGGTAGCGCAGGAAGAAGTCTCGGCTAACTACGTGCCAGCAGCCGCGGTAAAACGTA
GGAGGCAAGCGTTATCCGGAGTTACTGGGCGTAAAGCGCGTGCAGGTGGTGATACAAGTT
CGGTGTGAAAGCGCCCGGCTCAACTGGGCGAGGACATCGAAGACTGTGTTACTCGAGGCG
GGTAGAGGGGCGTAGAATTCCGGGTGTAGCGGTGAAATGTGTAGAGATCCGGAGGAATAC
CAGTGGTGAAGACGGCGCCCTGGGCCCGACCTGACACTGAAACGCGAAAGCGTGGGGAGC
GAACGGGATTAGAGACCCGGGTAGTCC
>Otu3949
CCAGCCTATGGGGTGCTGCAGCCGAGAATATTCGACAATGGGCGAAAGCCTGATCGAGCG
ATACCGCGTGGTGGATGAAGTGCTTCGGCACGTAAACATCTTTTATGAGGGAGGAAGTTA
TTGACGTTACCTCGTGAATAAGGGGCTCCTAACTCTGTGCCAGCAGGAGCGGTAATACAG
AGGCCCCGAGCGTTACCCGGAATTACTGGGCGCAAAGAGTGCGTAGGTGGTCATGTTAGT
CGGCTGTTAAATACCCGAGGCTCAACCTTGGGAATCCGGTCGAAACGGCATGACTTGAGG
GCGTGAGAGGTACATGGAACTCTAGGTGTAGGGGTGAAATCCGTTGATATCTAGGGGAAC
ACCGAAAGCGAAGGCAATGTACTGGCGCGTTCCTGACACTCAAGCACGAAAGCGTGGGTA
GCGAACGGGATTAGATACCCGCGTAGTCC
>Otu3950
CCAGCCTACGGGGTGCTCCAGTGAGGAATATTGGTCAATGGACGCAAGTCTGAACCAGCC
ATGCCGCGTGAAGGATGAAGGCCTTCTGGGTTGTAAACTTCTTTTATCTGGGGAGAAACC
CCGACTTTTTAGTTGGGTTGACGGTACCAGAGGAATAAGCACCGGCTAACTCCGTGCCAG
CAGCCGCGGTAATACGGAGGGTGCAAGCGTTATCCGGATTTACTGGGTTTAAAGGGTGTG
TAGGCGGGTCAGTAAGTCAGTGGTGAAATCTCCGGGCTCAACCCGGAAACTGCCATTGAT
ACTATTGGTCTTGAATACTGTTGAGGTGGGCGGAATATGTCATGTAGCGGTGAAATGCAT
AGATATGACATAGAACACCGATTGCGAAGGCAGCTCACTAAGCAGCGATTGACGCTGAGG
CACGAAAGCGTGGGGATCAAACAGGATTAGAAACCCGGGTAGTCC
>Otu3952
CCAGCCTATGGGTGGCTGCAGTAAGGAATATTGGTCAATGGACGGAAGTCTGAACCAGCC
ATGCCGCGTGCAGGAAGACGGCCCTACGGGTTGTAAACTGCTTTTGCAGGGGAATAAACC
TTTGGTCGTGACCAAAGTTGAATGTACTCTGAGAATAAGGATCGGCTAACTCCGTGCCAG
CAGCCGCGGTAATACGGAGGATCCAAGCGTTATCCGGATTTATTGGGTTTAAAGGGTGCG
TAGGTGGCCTGATAAGTCAGGGGTGAAAGACGGTAGCTTAACTATCGCAGTGCCCTTGAT
ACTGCCGGGCTTGAATGAACTAGAGGTAGGCGGAATGTGACAAGTAGCGGTGAAATGCAT
AGATATGTCACAGAACACCAATTGCGAAGGCAGCTTACTATGGTTAGATTGACACTGAGG
CACGAAAGCGTGGGGATCAAACAGGATTAGAAACCCCTGTAGTCC
>Otu3954
CCAGCCTATGGGTGGCTCCAGTAACGAATATTGGGCAATGGGCGCAAGCCTGACCCAGCG
ACGCCGCGTGTAGGATGAAGTCCTTCGGGATGTAAACTACTGTCAGGGGTAATAAAGTCG
TAAGATTGATATAACCCAAAGGAAGTCACGGCTAACTCTGTGCCAGCAGCCGCGGTAATA
CAGAGGTGGCAAGCGTTGTTCGGAATCACTGGGCTTAAAGCGTGTGTAGGCGGACCAGTA
AGTGTCTTGTGAAATCCCTCGTCTCAACCGAGGAACTGCTCGGCAAACTACTGGTCTTGA
GGTAGGCATGGGTGCTTGGAACTCTTGGTGGAGCGGTGAAATGCGTAGATATCAAGAGGA
ACGCCTGTGGTGAAAACGGAGCACTTGACCTATTCTGACGCTGAGACACGAAAGCGTGGG
GAGCGAACGGGATTAGATACCCTTGTAGTCC
>Otu3955
CCAGCCTACGGGGCGCTGCAGTAGGGAATTTTGCGCAATGGACGAAAGTCTGACGCAGCA
ATACCGTGTGAGGGATGAAGCATTTCGGTGTGTAAACCTCTGTCGTCGGTGAATAAAGGT
GTTGGGAGTGGAAAGCCCAGCATTTGAATGTAGCCGAGAAGGAAGCACCGGCTAACTCTG
TGCCAGCAGCCGCGGTAATACAGGGGGTGCAAGCGTTGTTCGGAATCACTGGGCGTAAAG
GGAGCGTAGGCGGATCCACAAGACAGAGGTTAAATGTTCGGGCTCAACCTGAAATCTGCC
TTCGTGACTGTGGATCTTGAGTATGGCAGAGGCTAGTGGAATTACCTGTGTAGCGGTGGA
ATGCGTAGATATAGGTAAGAACACCAGAGGCGAAGGCGACTAGCTGGGCCAATACTGACG

CTGAGGCGCGAAAGCCGGGGGAGCAAACAGGATTAGATACCCCAGTAGTCC
>Otu3958
CCAGCCTATGGGTGGCTGCAGTCGAGGATTTTTCTCAATGGGGGAAACCCTGAAGGAGCG
ACGCCGCGTGAGGGATGAAGGTCTTCGGATTGTAAACCTCTGTCATGCGGGATCAAAGCA
TACGACCTAACACGTCGTATGTTGATAGTACTGCAAGAGGAAGCAGTGGCTAACTCTGTG
CCAGCAGCCGCGGTAATACAGAGACTGCAAGCGTTGTTCGGATTCATTGGGCGTAAAGGG
TCCGCAGGTGGTCCAGCAAGTCGGATGTGAAATCCCATGGCCTAACCATGGAAAGTCATT
CGAAACTGCTGGGCTAGAGGACTGGAGAGGAGACTGGAATAGTTGGTGTAGCAGTGAAAT
GCGTAGATATCAACTAGAACACCAGTAGCGAAGGCGAGTCTCTGGACAGTTCCTGACACT
CATGGACGAAAGCCAGGGGAGCAAACGGGATTAGAAACCCCAGTAGTCC
>Otu3959
CCAGCCTATGGGTGGCAGCAGTAGGGAATCTTCCACAATGGGCGAAAGCCTGATGGAGCG
ACGCCGCGTAGAGGATGAAGGTCTTCGGATTGTAAACTTTTTTCGTTTGGGAACAAGTGT
ATCTGCGTGAATAATGCAGGTATTTGATGGTACCAAAGGAAGAAGCTACGGCTAACTCTG
TGCCAGCAGCCGCGGTAATACAGAGGTAGCGAACGTTGTCCGGATTTACTGGGCGTAAAG
GGATCGTAGGTGGTCATATAAGTCGGATGTGAAATCGTATTGCTTAACAATACCATTGCA
TTTGATACTGTATGGCTTGAGTGCAGGATGGGTAAATGGAATTTCCGGTGTAGCGGTGAA
ATGCGTAGATATCGGAAAGAACACCCGTGGCGAAGGCGGTTTACTGGCCTGCAACTGACA
CTGAGGATCGAAAGCCAGGGGAGCAAACGGGATTAGATACCCCGGTAGTCC
>Otu3961
CCAGCCTACGGGGGGCTCCAGCAACGAATATTCCGCAATGGGCGCAAGCCTGACGGAGCG
ATGCCGCGTGAAGGATGAAGCCCCTCGGGGTGTAAACTTCTGTCAGGGTTCACCAACACA
ATGAGGAGACCCAGAGGAAGAGACGGCTAACTCTGTGCCAGCAGCCGCGGTAATACAGAG
GTCTCGAGCGTTAATCGGAATCACTGGGCTTAAAGGGTGCGTAGGCGGAACGACAAGTGT
TTTGTGAAAGCCCATGGCTCAACCATGGAATTGCTCGACATACTGTCGTTCTTGAGGTCG
GTAGAGGTTGCCGGAACTCTAGGTGGAGCGGTGAAATGCGTAGATATCTAGAGGAACGCC
AAAGGCGAAGGCAGGCAACTGGGCCGATTCTGACGCTGAGGCACGAAAGCGTGGGGAGCG
AACGGGATTAGAAACCCTCGTAGTCC
>Otu3963
CCAGCCTATGGGTCGCAGCAGTGGGGAATCTTGCGCAATGGGCGAAAGCCTGACGCAGCG
ACGCCGCGTGGGTGAAGAAGGCCTTCGGGTTGTAAAGCCCTGTCGAGTGGGAAAAACAGC
CGTGTCAATAATAAGGGCATGGTTTGATGGTACCACTAAAGGAAGCACCGGCTAACTCTG
TGCCAGCAGCCGCGGTAATACAGAGGGTGCAAGCGTTGTTCGGAATTATTGGGCGTAAAG
GGCAGGTAGGTGGTCTTAAAAGTCTGCTGTGAAATCCCTGGGCTTAACCCAGGAAGTGCG
GCGGATACTCTAAGACTTGAGTTCCGGAGGGGTGCGTGGAATTCCGAGTGTAGCGGTGAA
ATGCGTAGATATTCGGAGGAACACCAGAGGCGAAAGCGGCGCACTAGACGGCAACTGACA
CTCAACTGCGAAAGCGTGGGGAGCAAACAGGATTAGATACCCGAGTAGTCC
>Otu3964
CAGCCTACGGGGGGCAGCAGTAACGAATCTTCCGCAATGCACGAAAGTGTGACGGAGCGA
CGCCGCGTGTAGGAGAAGCCCTTCGGGGTGTAAACTACTGTCAGGGGTCAGAAAGTTCTG
ATCGACCCCAGAGGAAGGCACGGCTAACTCTGTGCCAGCAGCCGCGGTAAGACAGAGGTG
CCAAGCGTTAGGCGGAATCACTGGGCTTAAAGCGTGTGTAGGCGGATCGTTAAGTGTCTT
GTGAAATCCCACGGCTCAACCGTGGAACTGCTTGACAGACTGGCGGTCTTGAGCCACCCA
GGGGCGAGCGGAACAGATGGTGGAGCGGTGAAATGCGTAGATATCATCTGGAACGCCAAA
GGTGAAAACAGCTCGCTGGGGGTGTGCTGACGCTGAGACACGAAAGCCAGGGGAGCAAAT
GGGATTAGATACCCGCGTAGTCC
>Otu3965
CCAGCTATGGGGTGCAGCAGGCGCGAAAACTCTCCAATGCACGAAAGTGTGAGAGGGGAA
TCCCAAGTGCTTACACACTATGTGTAGGCTTTTTTCGATCGCAAAAAGATCGGAGAATAA
GTGGTGGGCAAGACCGGTGCCAGCAGCCGCGGTAACCCCGGCGCCACAAGTGGCAACCGT
GTATTTGGCCTAAAGCGTCCGTAGCCGGCTCTGTGCATCCTTTGTGAAATTGTCCCGCCT
AACGGGGCAGCGTGCAGAGGACACGGCAGAACTTGGAACTGGGAGGCGTGAGCGGTATTC
CTGGGGGAACGGTAAAACGTTATAATCCCAGGAAGACCACCGATGGCGAAGGCAGCTCAC
GAGAACAGCTTCGACGGTGAGGGACGAAAGCTAGGGGAGCAAACCGGATTAGAAACCCTC
GTAGTCC
>Otu3967
CCAGCCTACGGGTGGCAGCAGTAAGGAATCTTCCACAATGGGGGCAACCCTGATGGAGCA
ATACCGCGTGCAGGAAGACGCGTTTCGGCGTGTAAACTGCTTTTGTCACCGATGATTATG

ACAGTAGGTGAAGAATAAGGACCGGCTAATTACGTGCCAGCAGCCGCGGTAATACGTAAG
GTCCAAGCGTTATCCGGAATTACTGGGCGTAAAGCGTCTGTAGGCGGCGCAGTAAGTTAG
GTGCGAAATCTCGCGGCTCAACCGTGTAGACTGTGCTTAAAACTACTGTGCTAGAGGTTG
GTAGAGGCAAGTGGAACTTGCGGTGTAACAGTGACATGTGTAGATATCGCAAAGAACACC
AATGGCGAAGGCAGCTTGCTGGGCCACACCTGACGCTCAGAGACGAAAGCGTGGGGAGCG
AACGGGATTAGAAACCCGTGTAGTCC
>Otu3968
CCAGCCTACGGGGGGCACCAGTGGGGAATCTTGCGCAATGGGCGAAAGCCTGACGCAGCC
ATGCCGCGTGAGTGAAGAAGGTCTTAGGATTGTAAAGCTCTTTCGCCCGTGACGATGATG
ACGGTAACGGGAGAAGAAGCCCCGGCTAACTTCGTGCCAGCAGCCGCGGTAATACGAAGG
GGGCTAGCGTTGTTCGGAATTACTGGGCGTAAAGCGCGCGTAGGCGGTTTTGCAAGCCAG
AGGTGAAATGCCTGAGCTCAACTCAGGAATGGCCTTTGGGACTGCGAGGCTTTGAGGACT
GGAGAGGTGAGTGGAATTCCCAGTGTAGAGGTGAAATTCGTAGATATTGGGAAGAACACC
GGTGGCGAAGGCGGCTCACTGGCCAGTTTCTGACGCTGTAGCGCGAAAGCGTGGGGATCA
AACAGGATTAGAGACCCGAGTAGTCC
>Otu3969
CCAGCCTATGGGGGGCAGCAGTAACGAATCTTCCCCAATGCACGCAAGTGTGAGGGAGCG
ACGCCGCGTGGAGGATGAAGTCCTTCGGGATGTAAACTCCTGTCAGAGGTTAGAAAGTTC
TGATCAACCTCAGAGGAAGGCACGGCTAACTCTGTGCCAGCAGCCGCGGTAAGACAGAGG
TGCCAAGCGTTAGGCGGAATCACTGGGCTTAAAGCGTGTGTAGGCGGACTTGTAAGTGCC
TTGTGAAATCCCAAGGCTCAACCTTGGAATTGCTTGGCATACTGCGAGTCTTGAGCTGCT
TAGGGGTTGCCGGAACAAACGGTGGAGCGGTGAAATGCATAGATATCGTTTGGAGCGCCA
AAGGTGAAAACAGGCAACTGGGAGCATGCTGACGCTGAGACACGAAAGCCAGGGGAGCAA
ACGGGATTAGAGACCCGTGTAGTCC
>Otu3970
CCAGCCTACGGGGGGCTGCAGTCGAGGATCTTCGGCAATGGGCGAAAGCCTGACCGAGCG
ACGCCGCGTGCGGGATGAAGGCCTTCGGGTTGTAAACCGCTGTCAGTGGGGAGGAAGTGT
GGGAGGGTTCTCCCTCTCATTTGACCTATCCGCAGAGGAAGTACGGGCTAAGTTCGTGCC
AGCAGCCGCGGCAAGACGAACCGTACGAACGTTATTCGGAATCACTGGGCTTAAAGGGTG
CGTAGGCTGTCCGGTAAGTTGGGTGTGAAATCCCTCGGCTTAACCGAGGAATTGCGCCCA
AAACTGCCGGACTTGAGGGAGACAGAGGTGAGCGGAACTTAGGGTGGAGCGGTGAAATGC
GTTGATATCCTAAGGAACACCAGGGGCGAAAGCGGCTCACTGGGTCTCTTCTGACGCTGA
GGCACGAAAGCTAGGGGAGCGAACGGGATTAGAGACCCCTGTAGTCC
>Otu3975
CCAGCCTATGGGGGGCTCCAGTAAGGAATATTGGTCAATGGACGCAAGTCTGAACCAGCC
ATGCCGCGTGAAGGATGACAGCCCTATGGGTTGTAAACTTCTTTTATACAGGAAAAAACC
CCCGGACGTGTTCGGGGCTGATGGTACTGTAAGAATAAGGATCGGCTAACTCCGTGCCAG
CAGCCGCGGTAATACGGAGGATCCAAGCGTTATCCGGATTTATTGGGTTTAAAGGGTGCG
TAGGCGGATTGGTAAGTCAGTGGTGAAATCCTACAGCTTAACTGTAGAACTGCCATTGAT
ACTATTAGTCTTGAGTACAATTGAAGTGGGCGGAATGTGTCATGTAGCGGTGAAATGCAT
AGAGATGACACAGAACACCGATAGCGAAGGCAGCTCACTAAACTGTAACTGACGCTGAGG
CACGAAAGCGTGGGGATCAAACAGGATTAGAGACCCCCGTAGTCC
>Otu3976
CCAGCCTATGGGGGGCACCAGTGGGGAATATTGGACAATGGGGGCAACCCTGATCCAGCC
ATGCCGCGTGGGTGAAGAAGGCCTTCGGGTTGTAAAGCCCTTTCAGGTGGGACGATGATG
ACGGTACCACCAGAAGAAGCTCCGGCTAACTTCGTGCCAGCAGCCGCGGTAATACGAAGG
GAGCTAGCGTTGTTCGGAATTACTGGGCGTAAGCGCGCGTAGGCGGTTTGTTTAGTCAGA
GGTGAAATCCCGGGGCTCAACCTCGGAATTGCCTTTGATACTGGCAAGCTAGAGTTCGGA
AGAGGATAGTGGAATACCCAGTGTAGAGGTGAAATTCGTAGATATTGGGTGGAACACCGA
TGGCGAAGGCAGCTATCTGGTCCGATACTGACGCTGAGGTGCGAAAGCGTGGGGAGCAAA
CAGGATTAGATACCCGCGTAGTCC
>Otu3980
CCAGCCTACGGGAGGCAGCAGTGAGGAATATTGGACAATGGTGGCAACACTGATCCAGCC
ATGCCGCGTGAAGGATGACTGCCCTATGGGTTGTAAACTTCTTTTGTTGGGGACGAAAAA
GGGCTACGTGTAGCCCCCTGACGGTACCCAACGAATAAGCATCGGCTAACTCCGTGCCAG
CAGCCGCGGTAATACGGAGGATGCAAGCGTTATCCGGATTTATTGGGTTTAAAGGGTGCG
TAGGCGGGCCCATAAGTCAGTGGTGAAATCCTGTAGCTTAACTACAGCACTGCCATTGAT
ACTGTGGGTCTTGAGTACAGTTGAGGTAGGCGGAATGTACAGTGTAGCGGTGAAATGCTT

AGATATTGTACAGAACACCGATTGCGTAGGCAGCTTACTAAACTGTAACTGACGCTGAGG
CACGAAAGCATGGGGAGCAAACAGGATTAGATACCCCAGTAGTCC
>Otu3982
CCAGCCTATGGGGTGCAGCAGCTAAGAATCTTCCGCAATGGGCGAAAGCCTGACGGAGCG
ATGCCGCGTGATCGATGAAGGTCGAGAGATTGTAAAGATCTTTTCCGCATGAGGAATAAA
GCCAGGAGGGAATGCCTGGCAGATGACGTTAGTGCGGGAATAAGCCCCGGCTAATTACGT
GCCAGCAGCCGCGGTAACACGTAAGGGGCGAGCGTTGTTCGGAATTATTGGGCGTAAAGG
GCACGCAGGCGGTTATGCAAGTCCGATGTGAAATCTCCGGGCTCAACTCGGGAAACGCGT
TGGAAACTGCATGACTAGAGTATTAGAGGGGGAGTCGGAATTCCTAGTGTAGGGGTGAAA
TCTGTTGATATTAGGAAGAACACCGGTGGCGAAGGCGGACTCCTGGCTGGATACTGACGC
TGAGGTGCGAAAGCATAGGGAGCAAACAGGATTAGATACCCCCGTAGTCC
>Otu3983
CCAGCCTACGGGAGGCAGCAGTAGGGAATATTGGACAATGGACGCAAGTCTGATCCAGCC
ACCCCGCGTGAAGGATTAAGGCCCTATGGGTTGTAAACTTCTTTTCTTTGGGAATAAAAA
GCGGTATTCATATCGCCTTGAAGGTACCAGGGGAATAAGCACCGGCTAACTCCGTGCCAG
CAGCCGCGGTAATACGGAGGGTGCAAGCGTTATCCGGATTCACTGGGTTTAAAGGGTGCG
TAGGTGGTCTGCTAAGTCAGCGGTGAAAGCCCGGAGCTTAACTCCGGAATTGCCATTGAT
ACTGACGGACTTGAATCAAGTTGAGGTGGATGGAATATTACATGTAGCGGTGAAATGCTT
AGATATGTAATAGAACACCGATTGCGAAGGCAGCTGACGAAACTGTAACTGACGCTGAGG
CACGAAAGCGTGGGGATCAAACAGGATTAGAAACCCCAGTAGTCC
>Otu3984
CCAGCCTACGGGGTGCTGCAGTGGGGAATATTGGACAATGGGCGGAAGCCTGATCCAGCG
ATACCGCGTGTGTGAAGAAGGCCTGAGGGTTGTAAAGCACTTTCAATGGGAAGGAACACC
TATCGGCGAATACCCGGTAGACTGACATTACCCATACAAGAAGCACCGGCTAACTCCGTG
CCAGCAGCCGCGGTAATACGGAGGGTGCAAGCGTTAATCGGAATTACTGGGCGTAAAGCG
TGCGTAGGCGGTGATCTAAGTCAGATGTGAAAGCCCTGGGCTTAACCTGGGAACTGCATT
TGATACTGGGTCACTCGAGTTGAGTAGAGGAGAGTGGAATTTCAGGTGTAGCGGTGAAAT
GCGTAGAGATCCGAAGGAACACCAGTGGCGAAGGCGGCTCTCTGGACTCAAACTGACGCT
GAGGTACGAAAGCGTGGGTAGCAAACAGGATTAGAGACCCGTGTAGTCC
>Otu3991
CCAGCCTACGGGTTGCTGCAGTCGAGAATCTTCCGCAATGGACGAAAGTCTGACGGAGCG
ACGCCGCGTGATTGATGAAGCTCTTCGGAGTGTAAAGATCTTTTATGAGGGAAGAAGTTT
ATTGACGGTACCTCATGAATAAGGGGCTCCTAATCTCGTGCCAGCAGGAGCGGTAATACG
AGAGCCCCGAGCGTTATCCGGAATTATTGGGCGTAAAGGGTGCGTAGGTGGTAGTGTTAG
TCGTTTGTCAAATCCTCGGGCTTAACCCGAGAACTGCGAACGAAACGGCACAACTAGAGA
GTGTGAGAGGTATAGGGAACTCATGGTGTAGGGGTGAAATCCGTTGATATCATGGGGAAC
ACCAAAAGCGAAGGCACTATACTAGCACATATCTGACACTCAAGCACGAAAGCGTGGGTA
GCGAATGGGATTAGATACCCTGGTAGTCC
>Otu3992
CCAGCCTACGGGTGGCACCAGTAAGGAATATTGGACAATGGTCGCAAGACTGATCCAGCC
ATGCCGCGTGAAGGATGAAGGCCCTATGGGTTGTAAACTTCTTTTGTACGAGAAAAAACC
TCTTTACGTGTAAAGAGCTGATTGTATCGTAAGAATAAGGATCGGCTAACTCCGTGCCAG
CAGCCGCGGTAATACGGAGGATCCAAGCGTTATCCGGATTTATTGGGTTTAAAGGGTGCG
TAGGCGGAATGATAAGTCAGTGGTGAAAGCCTGCAGCTTAACTGTAGAATTGCCGTTGAT
ACTGTCGTTCTTGAGTATATTTGATGTGGGCGGAATGTGTCATGTAGCGGTGAAATGCTT
AGATATGACACAGAACACCGATTGCGAAGGCAGCTCACAAAACTATAACTGACGCTGAGG
CACGAAAGCGTGGGGATCAAACAGGATTAGAAACCCGTGTAGTCC
>Otu3994
CCAGCCTACGGGTGGCAGCAGTGGGGAATCTTGCACAATGGGGGAAACCCTGATGCAGCG
ACGCCGCGTGAGCGATGAAGCCCTTCGGGGTGTAAAGCTCTTTCGTCAGGGAAGATAGTG
ACGGTACCTGGAGAAGCAGCTGCGGCTAACTACGTGCCAGCAGCCGCGGTAATACGTAGG
CAGCAAGCGTTGTTCGGAGTTACTGGGCGTAAAGAGTGGGTAGGCGGTGCTCTAAGTTTG
GTGTGAAATCTCCCGGCTCAACTGGGAGGGTGCGCCGAAGACTGGAGTGCTTGAGTATGG
GAGAGGAAAGCGGAATTCCTGGTGTAGCGGTGAAATGCGTAGATATCAGGAGGAACACCT
GCGGTGAAGGCGGCTTTCTGGACCATTACTGACGCTGAGACACGAAAGCGTGGGTAGCAA
ACAGGATTAGAGACCCTCGTAGTCC
>Otu4000
CCAGCCTACGGGTGGCAGCAGGTAGGTATTTTTGCAATGCACGAAAGTGTGACAGAGCG

AGCCAGAGTGTTTTTCCATTTGGAAAAACTTTTGCCAGCTGTAAATAGGCTGGCGAATAA
GGACTGGGCAAGACCGGTGCCAGCCGCCGCGGTAATCCCGGCGGTCCAAGTCGCAGCCAA
CTTTATTGGGTCTAAAACATCCGTAGCTTGTTATGAAAGTCTTTTGTGAAACTCTGTCTC
TTAAGGGCAGAAATGGCAATAGATACTACATGACTAGAGACCGGTGGACGTAAGGAGTAC
ATTTGAGGTAGTGGTAAAATACGTTAATCTTAAATGGACTAACAACAGCGAAGGCACCTT
ACGATGACGGTTCTGACAGTGAGGGATGAAGGCTAGGGGCGCAAAATGGATTAGAAACCC
CTGTAGTCC
>Otu4001
CCAGCCTATGGGTGGCTCCAGTCGAGAATCTTCCGCAATGGACGAAAGTCTGACGGAGCG
ACGCCGCGTGGAGGATGAAGTGCTTCGGCATGTAAACTCCTTCTGCCAGGGAAAAAGTTT
ATTGATTGTACCTGGAGAATAAGAGGTTGCTAAACTCGTGCCAGCAGCAGCGGTAATACG
AGTGCCTCGAGCGTTATCCGGAATCATTGGGCGTAAAGGGTGCGTAGGCGGCGATGTTAG
TCTCCCGTAAAATTCCTTGGCTTAACCAAGGGCGCGCGGGAGAAACGGCATTGCTTGAGA
GCGGAAGGGGCCTCTGGAACTCATGGTGTAGCGGTGAAATGCGTTGATATCATGGGGAAC
ACCAAAAGCGAAGGCAGGAGGCTGGTCCGTTTCTGACGCTGAAGCACGAAAGCGTGGGTC
GCGAATGGGATTAGAAACCCCGGTAGTCC
>Otu4005
CCAGCCTACGGGGGGCTCCAGTGGGGAATATTCCGCAATGGGCGAAAGCCTGACGGAGCG
ACGCCGCGTGGATGATGAAGGTCTTCGGATTGTAAAGTCCTGTAGAGGGGGAAGAAGGGT
GTAGTAATACATTTGACAGTACTCCTAAAGTAAGCTGCGGCAAACTCTGTGCCAGCAGCC
GCGGTAATACAGAGGTAGCAAGCGTTGTTCGGAATGACTGGGTGTAAAGCGCATGTAGAT
GGTTTTGTAAGTTGAATGTGAAATCCCTTGGCTCAACCAAGGAACGGCATTCAATACTGC
TTAACTTGAGTGCAGGAGAGGAAAGTGGAATTTCTGGTGTAGCGGTGAAATGCGTAGATA
TCAGAAGGAACACCGGTGGCGAAGGCGACTTTCTGGCCTGTAACTGACATTGAGATGCGA
AAGCTAGGGGAGCAAACAGGATTAGATACCCGCGTAGTCC
>Otu4008
CCAGCCTATGGGGGGCTCCAGTGAGGAATTTTGCGCAATGGGGGAAACCCTGACGCAGCG
ACGCCGCGTGGAGGATGAAGGCCTTCGGGTCGTAAACTCCTGTCAGAGGTGAAGAATGCC
CGCAAGGGTTTGACGGTAACCTCAAAGGAAGCCCCGGCTAACTACGTGCCAGCAGCCGCG
GTAATACGTAGGGGGCAAACGTTGCTCGGAATTACTGGGCGTAAAGGGTGTGTAGGCGGC
TGGGCAAGTCAAAGATGAAATCCCGAGGCTTAACTTCGGAACTGTCTTTGAAACTGCCTA
GCTTGAGGATAGTTGATGAAAAGGGAATTCCCGGTGGAGCGGTGAAATGCGTAGATATCG
GGAGGAACACCTGAGGCGAAGGCGCTTTTCAAAACTACTCCTGACGCTGAAACACGAAAG
CTAGGGGAGCAAACGGGATTAGAGACCCGCGTAGTCC
>Otu4009
CCAGCCTATGGGAGGCACCAGTCGAGAATCTTGGTCAATGGGCGAAGGCCTGAACCAGCG
ATGCCGCAGCGTGAAGAAGGCCTCTGGTTGTAAAACGCATATGCACGAGTGCAGAATGAG
GTGCTCGTGCGGAATAGTCCTGTGCAATCTCGGTGCCAGCACACGCGGTCAGACCGAAAG
GGCAAGGCATATCTTATTGAAATGGGCGTAAAAGGTCATTAGGCTGGCTTGAAGAAGGCT
ACTTGAAAGAAAGAGGCATAACCTTGAGTGAGGGTAGTCGATTCTGAGCCTTGAGTATCG
TGGGGGGTTATAGAATGCTCGACGGACCGATGGAATGGGTTGATGTCGAGTGGAACATCG
GTGGCGAAGGCGATAACCTGGACGATAACTGACGCTGACTGACTAAGGTGTGGAGATCAA
AGTGGATTAGATACCCTAGTAGTCC
>Otu4010
CCAGCCTACGGGAGGCAGCAGTCGAGAATCATTCGCAATGGGCGAAAGCCTGACGATGCG
ACGCCGTGTGAGCGATGAAGGCCTTAGGGTCGTAAAGCTCTTTCGCTTGGGAACAAGAGA
GGCTGGCTAACATCCAGCTAATTTGAGGGTACCAGGTAAAGAAGCACCGGCTAACTCCGT
GCCAGCAGCTGCGGTAATACGGAGGGTGCAAGCATTAATCGGATTTATTGGGCGTAAAGG
GCGCGTAGGCTGAAGAGAAAGTCTTGTGTGAAATCCCGGGGCTCAACCCCGGAACAGCAC
GGGAAACTCCTTTTCTAGAGGGTAGGCGGAGAAAACGGAATTCCACAAGTAGCGGTGAAA
TGCGTAGATATGTGGAAGAACACCGGTGGCGAAGGCGGTTTTCTAGCTTACTCCTGACGC
TGAGGCGCGAAAGCAAGGGGATCAAACAGGATTAGATACCCCAGTAGTCC
>Otu4011
CCAGCCTACGGGGCGCAGCAGTGAGGAATATTGGTCAATGGACGCAAGTCTGAACCAGCC
ATGCCGCGTGAAGGATGAATGCCCTACGGGTTGTAAACTTCTTTTGTACGAGGCGAATCT
CTTCTACGTGTAGAGGACTGACAGTATCGTACGAATAAGCATCGGCTAACTCCGTGCCAG
CAGCCGCGGTAATACGGAGGATGCAAGCGTTATCCGGATTTATTGGGTTTAAAGGGTGCG
TAGGTGGATTATAAGTCAGAGGTGAAATATTGCAGCTTAACTGTAAGATTGCCTTTGAT

GCTGTAGTTCTTGAGTTCAGTTGAGGTGGGCGGAATGTGTAATGTAGCGGTGAAATGCTT
AGATATTACACAGAACACCGATTGCGTAGGCAGCTCACTAAACTGACACTGACACTGAGG
CACGAAAGCGTGGGGATCAAACAGGATTAGATACCCCGGTAGTCC
>Otu4013
CCAGCCTATGGGGTGCAGCAGTAAGGAATATTGGTCAATGGAGGCAACTCTGAACCAGCC
ATGCCGCGTGAAGGATGAAGGCGCTATGCGTCGTAAACTTCTTTTATATGGGAAAAAACC
TCTGAACGTGTTCGGAGCTGATGGTACCATAAGAATAAGGATCGGCTAACTCCGTGCCAG
CAGCCGCGGTGATACGGAGGATCCAAGCGTTATCCGGATTCATTGGGTTTAAAGGGTGCG
TAGGCGGCTTGTTAAGTCAGCGGTGAAATCTCAGGGCTTAACCCTGAAACTGCCATTGAT
ACTGACGAGCTTGAGTACACTTGAAGTGGGCGGAATGTGTCGTGTAGCGGTGAAATGCTT
AGATATGACACAGAACACCGATTGCGAAGGCAGCTCACTAAATTGTCACTGACGCTGAGG
CACGAAAGCGTGGGGATCAAACAGGATTAGAGACCCGCGTAGTCA
>Otu4015
CCAGCCTATGGGTTGCAGCAGTCGAGGATCTTCGGCAATGGGCGCAAGCCTGACCGAGCG
ACGCCGCGTGCGGGATGAAGGCCTTCGGGTTGTAAACCGCTGTCAGAGGGGAGGAAATTC
CGGTGGGTACTCCCATCGGTTTGACCTATCTTCAGAGGAAGTACGGGCTAAGTTCGTGCC
AGCAGCCGCGGTAAGACGAACCGTACAAACGTTATTCGGAATCACTGGGCTTTAAGGGTG
CGTAGGCGGCCCGGAAAGTTGGGTGTGAAAGCCCTCGGCTCAACCGAGGAACTGCGCCCA
AAACTACCGGGCTCGAGGAAGATAGAGGTGAGCGGAACTTAGGGTGGAGCGGTGAGATGC
GTTGATATCCTAAGGAACACCAGTAGCGAAAGCGGCTCACTGGGTCTTTTCTGACGCTGA
GGCACGAAAGCTAGGGGAGCGAAGAGGATTAGAAACCCTCGTAGTCC
>Otu4017
CCAGCCTATGGGGCGCTGCAGCAACGAATCTTCCCCAATGGCCGAAAGGCTGAGGGAGCG
ACGCCGCGTGTGGGACGAAGTACTTCGGTATGTAAACCACTGTTAGGGTTACGAAAGCAA
TGCGCGCTAACATCGCGCAAAGTTGATCTAATCCAGAGAAAGGGACGGCTAACTCTGTGC
CAGCAGCCGCGGTAATACAGAGGTCCCGAGCGTTACTGAGATTCACTGGGTTTAAAGGGT
GCGTAGGTGGTCCGTTAAGTCAGTTGTGAAATCCCCGGGCTCAACCCGGGAACTGCTTCT
GATACTGGCGGACTTGAGGCCTGTAGGGGTTACTAGAACAGACGGTGGAGCGGTGAAATG
CGTAGATATCGTCTGGAATGCCTGTGGTGAAGACGGGTAACTGGGCAGGTTCTGACACTG
AGGCGCGAAAGCGTGGGGAGCGAACGGGATTAGAAACCCGTGTAGTCC
>Otu4018
CCAGCCTATGGGTTGCTGCAGTCTAGAGGCTTCGGCAATGGGGGAAACCCTGACCGAGCG
ACGCCGCGTGCGGGATTGAAGGCCTTCGGGTCGTAAACCGCTGTCAGGTGGGAAGAATGC
CCGTATGGCGAATACGGGAGCGACGGTACCACCAGAGGAAGCCCCGGCTAACTCCGTGCC
AGCAGCCGCGGTAATACGGAGGGGGCAAGCGTTATTCGGATTTACTGGGCGTAAAGGGTG
CGTAGGCGGCCGCGCAAGTCTGACGTGAAATCTTCACCGCTCAACGGGAAGAGGTCGTTG
GAAACTGCGTGGCTCGAGTGTGGTAGAGGCAAGCGGAATTCCAGGTGTAGCGGTGGAATG
TGTAGATATCTGGAAGAACACCCGTGGCGAAGGCGGCTTGCTGGGCCATCACTGACGCTG
AGGCACGAAAGCGTGGGGAGCAAACGGGATTAGATACCCCCGTAGTCC
>Otu4021
CCAGCCTATGGGGTGCACCAGTGGGGAATTTTCCGCAATGGGCGAAAGCCTGACGGAGCA
ATACCGCGTGAGGGAGGAAGGCTCTTGGGTTGTAAACCTCTTTTCTCAGGGAAGAAAGAT
GACGGTACCTGAGGAATAAGCATCGGCTAACTCCGTGCCAGCAGCCGCGGTAATACGGAG
GATGCAAGCGTTATCCGGAATGATTGGGCGTAAAGGGTCCGCAGGTGGCATTGAAAGTCT
GCTGTTAAAGAGTCTGGCTCAACCAGATAAAGGCAGTGGAAACTACAAAGCTAGAGTGCG
TTCGGGGTAGAGGGAATTCCTGGTGTAGCGGTGAAATGCGTAGAGATCAGGAAGAACACC
GGTGGCGAAGGCGCTCTACTAGGCCGCAACTGACACTGAGGGACGAAAGCTAGGGGAGCG
AATGGGATTAGAAACCCCTGTAGTCC
>Otu4022
CCAGCCTATGGGGGGCACCAGTGAGGAATATTGCCCAATGGACGAAAGTCTGAGGCAGCG
ACGCCGCGTGGGTGATGAAGGCCTTCGGGTCGTAAAGCCCTTTTCTGCGTGACGAGACAA
AGGACGGTAGCGCGGGAAAAAGTCTCGGCTAACTACGTGCCAGCAGCCGCGGTAAAACGT
AGGAGGCAAGCGTTATCCGGAGTTACTGGGCGTAAAGAGTGCGTAGGCGGATTGATAAGT
GGGTGGTGAAAGCGCCCGGCTAAACCGGGCGAGGTCCATTCAGACTGTCAGTCTAGAGGC
GGATAGAGGGGTGCGGAATTCCGGGTGTAGCGGTGAAATGTGTAGAGATCCGGAGGAACA
CCAGTGGTGCAAACGGCGCCCTGGGTCCGAACTGACGCTGAAGCACGAAAGCGTGGGGAG
CGAACGGGATTAGAAACCCTAGTAGTCC
>Otu4025

CCAGCCTACGGGGGGCTGCAGTCGAGAATTTTTCTCAATGGGCGAAAGCTTGAAGGAGCG
ACGCCGCGTGGGGGATGAAGGGCTTCGGCCTGTAAACCCCTGTCATTTGCGAACAAACCT
GCTTATTGAATAGATGAGCAGCTGATTGTAGCGAAAGAGGAAGGGACGGCTAACTCTGTG
CCAGCAGCCGCGGTAATACAGAGGTCCCAAGCGTTGTTCGGATTCACTGGGCGTAAAGGG
TGCGTAGGTGGCGGAATAAGTTTGATGTGAAATCTCCGGGCTTAACCCGGAAAATGCATT
GAATACTATTTTGCTCGAGGGTCGGAGGGGGGACTGGAATTCCCGGTGTAGCAGTGAAAT
GCGTAGATATCGAGAGGAACACCAGTGGCGAAAGCGAGTCCCTGGACGACTCCTGACACT
GAGGCACGAAAGCTAGGGGATCAAACAGGATTAGAAACCCCGGTAGTCC
>Otu4027
CCAGCCTATGGGGGGCTCCAGTGGGGAATATTGGGCAATGGCGGAAACGCTGACCCAGCC
ATGCCGCGTGCCGGAAGAAGGTCCATTGGACTGTAAACGGCTTTTCTTTGGGGCTAACGT
ATGATTAGATCATACTTGAAAGTACTAAGGGAATAAGCACGGGCTAACTACGTGCCAGCA
GCCGCGGTAATACGTAGGGTGCAAGCGTTGTCCGGATTTACTGGGTTTAAAGGGTGCGTA
GGCGGCGCGATAAGTCTGGGATGAAATGGCTAGGCTCAACCTAGTCGCTGTCCTGGATAC
TGTCATGCTTGAGATCGGTTGAGGTTACTGGAATGAGTGGTGTAGCGGTGAAATGCGTAG
AGATCACTCAGAACACCAATTGCGAAGGCAGGTAACTGGGCCGTATCTGACGCTGAGGCA
CGAAAGCGTGGGGATCAAACAGGATTAGAAACCCCCGTAGTCC
>Otu4028
CCAGCCTACGGGAGGCAGCAGCTAAGAATCTTCCGCAATGGGCGAAAGCCTGACGGAGCG
ATGCCGCGTGATCGATGAAGGTCGAGAGATTGTAAAGATCTTTTCCACATGAGGAATAAG
TCTGGGAGGGAATGCCTGGATGATGACGTTAGTGTGGGAATAAGCCCCGGCTAATTACGT
GCCAGCAGCCGCGGTAACACGTAAGGGGCGAGCGTTGTTCGGAATTATTGGGCGTAAAGG
GCACGCAGGCGGTTTTGCAAGTCTGATGTGAAAGACCTGGGCTTAACTCAGGGGCCGCGT
TGGAAACTGCAAGACTAGAGTATTAGAGGGGGAGTCGGAATTCCTAGTGTAGGGGTGAAA
TCTGTTGATATTAGGAAGAACACCGGTGGCGAAGGCGGACTCCTGGCTGGATACTGACGC
TGAGGTGCGAAAGCATAGGGAGCAAACAGGATTAGAAACCCGAGTAGTCC
>Otu4031
CCAGCCTATGGGGGGCTGCAGTAGGGAATTTTGCGCAATGGGCGAAAGCCTGACGCAGCA
ACGCCGCGTGAAGGATGAAGGTCTTCGGATTGTAAACTTCTGTTGGATGGGAAAAAAATC
CTAGCAATAGGACTGATGGTACCATTCGAGTAAGCACCGGCTAACTACGTGCCAGCAGCC
GCGGTAATACGTAGGGTGCAAGCGTTGTTCGGAATCACTGGGCGTAAAGGGAGCGTAGGC
GGGGTTGTAAGTTAGGAGTTTAAACCTTGGGCTTAACCCAAGATCTGCTCTTAATACTGC
GATTCTTGAATATTGGAGAGGGTGATGGAATTCCAGGTGTAGCGGTGGAATGCGTAGATA
TCTGGAAGAACACCAGTAGCGAAGGCGGTCACCTGGCCAAATATTGACGCTGAGGCTCGA
AAGCTAGGGGAGCAAACAGGATTAGATACCCCAGTAGTCC
>Otu4032
CCAGCCTACGGGATGCAGCAGTGGGGAATATTGGGCAATGGGCGAAAGCCTGACCCAGCG
ACGCCGTGTGGGCGATGAAGGCCTTCGGGTTGTAAAGCCCTGTTGGGTGGAAAGAAGGGT
CCATCGACGAATAGTCTTTGGATCTTGACGGTACCACCAGAGGAAGCACCGGCTAACTCC
GTGCCAGCAGCCGCGGTAATACGGAGGGTGCAAGCGTTGTTCGGAATTACTGGGCGTAAA
GGGCGCGTAGGTGGTTGTGTAAGTCGGATGTGAAATCCCCAGGCTCAACTTGGGAACCGC
ATCTGATACTGCATGACTAGAGTACAGCAGAGGGAAACGGAATTCCTGGTGTAGAGGTGA
AATTCGTAGATATCAGGAGGAACACCGGTGGCGAAGGCGGTTTCCTGGGCTGATACTGAC
ACTGAGGCGCGAAAGCGTGGGTAGCAAACAGGATTAGAAACCCTAGTAGTCC
>Otu4033
CCAGCCTATGGGTGGCAGCAGTAGGGAATATTGGTAATGTGCGAAAGCGCGAACCAGCAA
CGCCGCGTGCACGATGAAGGTCTTCGGATTGTAAACTTCTTTTAGAGGGAACGAATAAAT
TGACGGTACTCTCAGAAAAAGCCACGGCTAACTACGTGCCAGCAGCCGCGGTAATACGTA
GGTGGCAAGCGTTACTCGGATTTACTAGGCGTAAAGCGTGTGTAGGTGGTTGGATAAGTC
TGTGGTGAAATCTCTCGGCTCAACCGGGAGGGGTTCACAGAAACTATCCGGCTTGAGTGT
GGTAGGGGAAGACGGAATTCCTGGTGTAGCGGTGAAATGCGTAGATATCAGGAGGAACAC
CGATGGCGAAAGCGGTCTTCTGGGCCAATACTGACACTGAAACACGAAAGCTAGGGGAGC
AAACAGGATTAGAAACCCCTGTAGTCC
>Otu4037
CCAGCCTACGGGTGGCTGCAGTCGAGGATCTTCGGCAATGGGCGAAAGCCTGACCGAGCG
ACGCCGCGTGCGGGATGAAGGCCTTCGGGTTGTAAACCGCCGTCGTAGAGGAGGAAATGC
AGGTGGGTTCTCCCATCTGTTTGACCTATTCTAAGAGGAAGGACGGGCTAAGTTCGTGCC
AGCAGCCGCGGTAAGACGAACCGTCCAAACGTTATTCGGAATCACTGGGCTTAAAGGGTG

CGTAGGCGGCTCAGAAAGTAGGGTGTGAAAGCCCTCGGCTTAACCGAGGAATTGCGCCCT
AAACTACTGGGCTTGAGGGACGCAGAGGTAAGCGGAACTTAGGGTGGAGCGGTGAAATGC
GTTGATATCCTAAGGAACACCAGGAGCGAAAGCGGCTTACTGGGCGTTTTCTGACGCTGA
TGCACGAAAGCTAGGGGAGCGAACGGGATTAGAGACCCGTGTAGTCC
>Otu4038
CCAGCCTACGGGGTGCAGCAGTCGAGGATCTTCGGCAATGGGCGCAAGCCTGACCGAGCG
ACGCCGCGTGTGCGATGAAGGCCTTCGGGTTGTAAAGCACTGTCGAGGGGGAGAAAGCCG
AAAGGTTGATCTATCCCTGGAGGAAGCACGGGCTAAGTTCGTGCCAGCAGCCGCGGTAAG
ACGAACCGTGCGAACGTTGTTCGGAATCACTGGGCTTAAAGGGCGCGTAGGCGGGCTGCC
AAGTCTGGGGTGAAATCCCACGGCTCAACCGTGGAACTGCCTCGGATACTGGCGGTCTCG
AGGGAGGTAGGGGCATGCGGAACTGTGGGTGGAGCGGTGAAATGCGTTGATATCCACAGG
AACTCCGGTGGCGAAAGCGGCGTGCTGGACCTTTTCTGACGCTGAGGCGCGAAAGCTAGG
GGAGCAAACGGGATTAGAAACCCTTGTAGTCC
>Otu4044
CCAGCCTATGGGGCGCTGCAGTAGGGAATATTGGGCAATGGGCGGAAGCCTGACCCAGCC
ACGCCGCGTGGGGGAAGAAGGCCCACCGGGTTGTAAACCCCTTTGGCCAGGGAATAACTA
CCCGCACGGCGGGGGATGAAGGTACCTGGAGAACAAGCGCCGGCAAACTACGTGCCAGCA
GCCGCGGTAATACGTAGGGCGCGAGCGTTGTCCGGATTTACTGGGTTTAAAGGGTGCGCA
GGCGGGACCTTAAGTCGGCGGTGAAATGCGGGGGCTCAACCCCCGCGCCGTTGCCGATAC
TGGGGATCTTGAGTGCGCCCGGGGCGGCCGGAACGGGCGGTGTAGCGGTGAAATGCGTAG
AGATCGCTCAGAACGCCGATAGCGAAGGCAGGCCGCCAGGGCGCAACTGACGCTCAGGCA
CGAAAGCGCGGGGAGCAAACAGGATTAGATACCCTCGTAGTCC
>Otu4049
CCAGCCTACGGGGGGCTGCAGTGAGGAATATTGCACAATGGGCGCAAGCCTGATGCAGCG
ACGCCGCGTGTAGGATGAAGGGGCTTCGCCCTGTAAACTACTGTTAAGAGGGAAGAAAAG
CTCGTTACGCGAGCTGGGACTGTACCTCTAGAGGAAGGATCGGCTAACTACGTGCCAGCA
GCCGCGGTAATACGTAGGATCCGAGCGTTGTCCGGAATCACTGGGTGTAAAGGGTGCGCA
GGCGGGCTTGTGCGTCAGAGGTTAAATATCCCGGCTTAACTGGGAAAATGCCTTTGATAC
GGCAAGTCTTGAATATGAGAGAGGATGGTGGAATTCCTGGTGTAGCGGTGAAATGCGTAG
ATATCAGGAGGAACACCAGTGGCGAAGGCGGCCATCTGGCTCATTATTGACGCTCAGGCA
CGAAAGCGTGGGGAGCAAACAGGATTAGAAACCCCCGTAGTCC
>Otu4050
CCAGCCTATGGGGGGCACCAGTGAGGAATATTGGTCAATGGGGGAAACCCTGAACCAGCG
ACGCCGCGTGAAGGATGAAGGGGCTTTGCCTCGTAAACTTCTGTCGGGAGGGAAGAAAAG
CCGGGTTGCCCGGCTGGGACTGTACCTCTAAAGGAAGGGTCGGCTAACTACGTGCCAGCA
GCCGCGGTAATACGTAGGACCCAAGCGTTGTCCGGATTTACTGGGTATAAAGGGTGCGTA
GGCGGATTTGTGCGTCAGAGGTTAAATACCCCGGCTTAACCGGGGAAATGCCTTTGATAC
GGCAGGTCTTGAGTGCGAGCGAGGATGGTAGAATTCATGGTGTAGCGGTGAAATGCGTAG
ATATCATGAGGAATACCCGTGGCGAAGGCGGCCATCTGGCTCGATACTGACGCTCAGGCA
CGAAAGCGTGGGGAGCAAACAGGATTAGATACCCGGGTAGTCC
>Otu4051
CCAGCCTTGGGTGGCTGCAGTGGGGAATATTCCGCAATGGGCGAAAGCCTGACGGAGCGA
CGCCGCGTGGATGATGACGGTCTTCGGATTGTAAAGTCCTGTAGAGGGGGAAGAAGGGTG
CAGCAATGCATTTGACAGTACTCCTAAAGTAGGCTACGGCAAACTCTGTGCCAGCAGCCG
CGGTAATACAGAGGTAGCAAGCGTTGTTCGGAATGACTGGGTGTAAAGCGCGTGTAGGCG
GTTTTGTAAGTTGAATGTGAAATCCCTTGGCTCAACCAAGGAACGGCATTCAATACTGCA
TAACTTGAGTGCGGAAGAGGAAAGTGGAATTTCGGGTGTAGCGGTGAAATGCGTAGATAT
CCGAAGGAACACCGGTGGCGAAGGCGACTTTCTGGTCCGCAACTGACGTTGAGACGCGAA
AGCTAGGGGAGCAAACAGGATTAGAAACCCTAGTAGTCC
>Otu4052
CCAGCCTACGGGGGGCAGCAGTTTCGAATCATTCACAATGGGCGAAAGCCTGATGGTGCG
ACGCCGCGTGAGGGATGAAGGTCTTCGGATTGTAAACCTCTGTCACTAGGGAAGAAACGT
CAAAACCTAATACGTTTTGGCCTGACTTAACCTGGAGAGGAAGCAGTGGCTAACTCTGTG
CCAGCAGCCGCGGTAATACAGAGACTGCAAGCGTTATTCGGATTCACTGGGCGTAAAGGG
TGCGCAGGCGGCCGTGTGTGTCAGATGTGAAATCCCGGGGCTTAACCCCGGAACTGCGTC
TGAAACTACACGGCTAGAGCATTGGAGAGGGAAGTGGAATTCATGGTGTAGCAGTGAAAT
GCGTAGATATCATGAGGAACACCAGAGGCGAGGCGACTTCCTGGACAATTGCTGACGCTC
AGGCACGAAAGCGTGGGTAGCAAAAGGGATTAGATACCCTAGTAGTCC

>Otu4055
CCAGCCTACGGGGTGCTGCAGGGAATCTTGGTAATCTGCGAAAGCGGGAACCAGCAACGC
CGCGTGTGCGATGAAGGCCTTCGGGTCGTAAAGCACTTTTCGAGGGGATGAGGAAGGACA
GTACCCTCGGAATAAGTCTCGGCTAACTACGTGCCAGCAGCCGCGGTAAAACGTAGGAGG
CAAGCGTTATCCGGATTTACTGGGCGTAAAGCGCGTGCAGGCGGTTCGGTAAGTTGGATG
TGAAAGCTCCTGGCTCAACTGGGAGAGGTCGTTCAATACTACCGGACTTGAGAGCAGTAG
AGGAAGGTGGAATTCCCGGTGTAGTGGTGAAATGCGTAAATATCGGGAGGAACACCAGTG
GCGAAAGCGGCCTTCTGGACTGTTTCTGACACTCATATGCGAAAGCTAGGGTAGCAAACG
GGATTAGAAACCCCAGTAGTCC
>Otu4060
CCAGCCTACGGGGGGCTGCAGTGGGGAATCTTGCGCAATGGACGAAAGTCTGACGCAGCC
ACGCCGCGTGAGTGAAGAAGGCCTTCGGGTTGTAAAGCTCTGTCGGAGGGGACAGAAACG
TCTAAGGTTAATAGCCTTAGATCTTGATGGTACCCTTAAAGGAAGCACCGGCTAACTTCG
TGCCAGCAGCCGCGGTAATACGAAGGGTGCAAGCGTTGCTCGGAATTATTGGGCGTAAAG
GGTAGGTAGGTGGTTACGTATGTCTGGGGTGAAATCCCTGAGCTCAACTCAGGAAGTGCC
TTGGAAACGGCGTAACTAGAGTGCTAGAGAGGTTCGTGGAATTCCCAGTGTAGCGGTGAA
ATGCGTAGAGATTGGGAGGAACACCAGAGGCGAAGGCGGCGAACTGGATAGCAACTGACA
CTGAACTACGAAAGCGTGGGTAGCAAACAGGATTAGATACCCGAGTAGTCC
>Otu4061
CCAGCCTATGGGGGGCTGCAGTAACGAATCTTCCACAATGCACGAAAGTGTGATGGAGCG
ACGCCGCGTGTGGGACGAAGCCCTTCGGGGTGTAAACCACTGTCAGGGGATAGAAAGTTC
TGATCATCCCCAGAGGAAGGCACGGCTAACTCTGTGCCAGCAGCCGCGGTAAGACAGAGG
TGCCAAGCGTTAGGCGGAATCACTGGGCTTAAAGCATGTGTAGGCGGTCCCGTAAGTATC
TTGTGAAATCCCATGGCTTAACCATGGAACTGCTCGATATACTGCGGGACTTGAGCCAAC
TAGGGGCTACCGGAACAAGTGGTGGAGCGGTGAAATGCGTAGATATCACTTGGAACGCCA
ATGGTGAAAACAGGTAGCTGGGGTTGTGCTGACGCTGAGACACGAAAGCCAGGGGAGCAA
ACGGGATTAGAAACCCTTGTAGTCC
>Otu4063
CCAGCCTATGGGTCGCTGCAGTGGGGAATTTTGCACAATGGGGGAAACCCTGATGCAGCA
ACGCCGCGTGGAGGATGAAGTCCCTTCGGGGCGTAAAGCTCTTTCGGCAGGGAAGACTAT
GACGGTACCTGCAGAAGCAGCTGCGGCTAACTACGTGCCAGCAGCCGCGGTAATACGTAG
GCAGCGAGCGTTGTTCGGAGTTACTGGGCGTAAAGGGTGTGTAGGCGGTTATTTAAGTTT
GGTGTGAAATCTCCCGGCTTAACCGGGAGGGTGCGCCGAATACTGAGTGACTAGAGTGCG
GGAGAGGAGAGTGGAATTCCTGGTGTAGCGGTGAAATGCGTAGATATCAGGAGGAACACC
GGCGGTGTAGACGGCTCTCTGGACCGCGACTGACGCTGAGACACGAAAGCGTGGGTAGCA
AACAGGATTAGATACCCCTGTAGTCC
>Otu4071
CCAGCCTACGGGACGCAGCAGTGAGGAATTTTCGTCAATGGGGGCAACCCTGAACGAGCA
ACGCCGCGTGCAGGATGAAGGTTTTCGGATCGTAAACTGCTTTTCTTGGGGACGAGAACG
GACGGTACCCAAGGAAGAAGCCCCGGCTAACTACGTGCCAGCAGCCGCGGTAATACGTAG
GGGGCAAGCGTTGTCCGGATTTATTGGGCGTAAAGCGCACGCAGGCGGTCCTATAAGTTT
GTACTGACAGTTTCCGGCTCAACCGGAAAAGGTGTATGAAGACTGTAGGACTTGAGGGCT
TCAGAGGAATGCGGAATTCCGGGTGGAGTGGTGAAATACGTAGATATCCGGAGGAACACC
AATGGCGAAGGCAGCATTCTGGGAAGTACCTGACGCTCAGGTGCGAAAGCCAGGGGAGCG
AACGGGATTAGATACCCCAGTAGTCC
>Otu4073
CCAGCCTACGGGGTGCTCCAGTTTAGAAATTTGCGCAATGGGCGAAAGCCTGACGCAGCG
ACGCCGCGTGAGGGATGAAGATCTTCGGATTGTAAACCTCTGTCGAACAAGACGAAAGCT
TATCGGTGAACAATCGATAAGTTTGACGGTACTGTTAAAGGAAGCTATGGCTAACTCTGT
GCCGGCAGCCGCGGTAATACAGAGGTAGCGAGCGTTGTCCGGAATTACTGGGCGTAAAGG
GCGTGTAGGCGGCCGGGTAAGTCTAGAGTTAAAACTCTCAGCTCAACTGAGAGGCGTCTC
CGGAGACTGCTTGGCTTGAGGACAGTAGAGGAGAGTGGAATTCCCGGTGTAGCGGTGAAA
TGCGTAGATATCGGGAGGAACACCAGTGGCGAAGGCGGCTCTCTGGTCTGTATCTGACGC
TGAGGCGCGAAAGCTGGGGGAGCAAACGGGATTAGATACCCCCGTAGTCC
>Otu4075
CCAGCCTACGGGACGCTCCAGTGGGGAATCTTGCACAATGGGGGAAACCCTGATGCAGCG
ACGCCGCGTGAGTGATGAAGCCCTTCGGGGTGTAAAGCTCTTTCGGCAGGGACGATAATG
ACGGTACCTGCAGAAGAAGCTGCGGCTAACTACGTGCCAGCAGCCGCGGTAATACGTAGG

CAGCAAGCGTTGTTCGGAATTATTGGGCGTAAAGGGTGCGTAGGCGGTGCGGTAAGTCTT
CTGTGAAATCTCCGGGCTTAACTCGGAGCCTGCAGGGGAAACTGCCGTGCTGGAGTATGG
GAGAGGTGAGTGGAATTCCCGGTGTAGCGGTGAAATGCGTAGATATCGGGAGGAACACCT
GTGGCGAAAGCGGCTCACTGGACCATTACTGACGCTGATGCACGAAAGCTAGGGGAGCAA
ACAGGATTAGATACCCGTGTAGTCC
>Otu4076
CCAGCCTACGGGGGGCTGCAGTCGAGGATCTTCGGCAATGGGCGCAAGCCTGACCGAGCG
ACGCCGCGTGTGCGATGAAGGCCTTCGGGTTGTAAAGCACTGTCGAGGGGGAGGAAGGCG
CAAGCTTGACCTATCCCTGGAGGAAGCACGGGCTAAGTTCGTGCCAGCAGCCGCGGTAAG
ACGAACCGTGCGAACGTTGTTCGGAATCACTGGGCTTAAAGGGCGCGTAGGCGGTCGCAG
AAGTCTCGGGTGAAATCTTTCGGCTCAACCGGAAACGTGCCTGGGATACTGTGCGACTGG
AGGGAGATAGGGGCGGACGGAACTTCCGGTGGAGCGGTGAAATGCGTTGATATCGGAAGG
AACGCCGGTGGCGAAAGCGGTCCGCTGGGTCTCTACTGACGCTGAAACACGAAAGCTAGG
GGAGCAAACGGGATTAGAAACCCCAGTAGTCC
>Otu4077
CCAGCCTATGGGTTGCTCCAGTCGAGAATCTTGGGCAATGCGCGAAAGCGTGACCCAGCA
ATGCCGCGTGCAGGATGAAGGCCTTCGGGTTGTAAACTGCTGTCACCCGGGACGAAGAAT
TGACGGTACCGGGAGAGGAAGCCACGGCTAACTACGTGCCAGCAGCCGCGGTAATACGTA
GGTGGCGAGCGTTGTTCGGATTTATTGGGCGTAAAGGGTCTGTAGGGGGTTTGCTAAGTC
TGATGTGAAATCCCGTGGCTCAACCACGGAACTGCATTGGAGACTGGCGGACTAGAGTTC
TGGAGAGGTAAGCGGAATTCCAGGTGTAGCGGTGGAATGCGTAGATATCTGGAAGAACAC
CGATAGCGAAGGCAGCTTACTGGACAGCGACTGACCCTGAAAGACGAAAGCGTGGGAAGC
AAACAGGATTAGATACCCGCGTAGTCC
>Otu4078
CCAGCCTATGGGTGGCAGCAGTAGGGAATATTGCGCAATGGAGGAAACTCTGACGCAGCG
ACGCCGCGTGGGTGACGAAGGCCTTCGGGTCGTAAAGCCCTGTCGGAAGGGAAGAAAAAC
GGTATGGCTAACATCCATACCGCTTGACGGTACCTTTAAAGGAAGCACCGGCTAACTACG
TGCCAGCAGCCGCGGTAATACGTAGGGTGCAAGCGTTGTTCGGAATCATTGGGCGTAAAG
CGCGTGTAGGTGGTTAGTTAAGTCGATTGTGAAATCCCTGGGCTCAACCGAGGAAGTGCA
GTCGAAACTAACTAGCTAGAGGACGGCAGAGGAAGGTGGAATTCCAAGTGTAGAGGTGAA
ATTCGTAGATATTTGGAGGAATACCGGTGGCGAAGGCGGCCTTCTGGACCGTTCCTGACA
CTGAGACGCGAAAGCGTGGGGAGCAAACAGGATTAGATACCCCAGTAGTCC
>Otu4079
CCAGCCTACGGGTGGCTGCAGTCGGGAATTTTGGGCAATCCCCGCAAGGGTGATCCAGCG
ATACCACGTGAGTGATGAAGTTCTTTGGAATGTAAAGCTCTGTCAGGGGATGAGAAAAGA
ATACGGTGAATAGCCAGATTCGTTGACTATCCCCAGAGGAAGCTCCGGCCAACTCCGTGC
CAGCAGCCGCGGTAATACGGGGGGAGCAAGCGTTGCCCGGAATCACTGGGCGTAAAGGGT
GTGTAGGTGGATTTGCAAGTCAAACGTGAAATCTTACAGCTCAACTGTAAAATGGCGTTT
GAAACTGCATTTCTTGAGTACGGGAGAGGAGAATGGAATTTCAGGTGTAGCGGTGAAATG
CGTAGATATCTGAAAGAACACCGGTTGCGAAGGCTATTCTCTATCCTAATACTGACACTG
TGACACGAAAGCTAGGGGAGCAAACAGGATTAGATACCCTTGTAGTCC
>Otu4080
CCAGCCTATGGGTCGCACCAGTAGGGAATATTGGTAATGTGCGAAAGCGCGAACCAGCAA
CGCCGCGTGCACGATGAAGGCCTTCGGGTTGTAAAGTGCTTTTAGAGAGGATGAGGAAGG
ACAGTACTCTCTGAATAAGCCTCGGCTAACTTCGTGCCAGCAGCCGCGGTAACACGTAGG
AGGCAAGCGTTATCCGGATTTACTGGGCGTAAAGCGCATGCAGGTGGTTCGGTAAGTTGG
ATGTGAAAGCTCCTGGCTCAACTGAGAGAGGTCGTTCAATACTACCGGACTTGAGAGCAG
TAGAGGGAGGTGGAATTCCCGGTGTAGTGGTGAAATGCGTAGATATCGGGAGGAACACCA
GTGGCGAAAGCGGCCTCCTGGACTGTTTCTGACACTCATATGCGAAAGCGTGGGTAGCAA
ACAGGATTAGAGACCCTCGTAGTCC
>Otu4081
CCAGCCTATGGGTCGCAGCAGTGGGGAATATTGGACAATGGGCGCAAGCCTGATCCAGCC
ATGCCGCGTGAGTGATGAAGCCCTTCGGGGTGTAAAGCTCTTTCGGCAGGGAAGATTATG
ACGGTACCTGCAGAAGCAGCTGCGGCTAACTACGTGCCAGCAGCCGCGGTAATACGTAGG
CAGCAAGCGTTGTTCGGAGTTACTGGGCGTAAAGGGTGTGTAGGCGGTTGTTTAAGTTTG
GTGTGAAATCTCCCGGCTCAACCGGGGGGGTGCGCCGAATACTGAGCGACTAGAGTGCGG
GAGAGGAGAGTGGAATTCCTGGTGTAGCGGTGAAATGCGTAGATATCAGGAGGAACACCG
GTGGTGCAGACGGCTCTCTGGACCGCGACTGACGCTGAGACACGAAAGCGTGGGTAGCAA

ACAGGATTAGAAACCCGCGTAGTCC
>Otu4083
CCAGCCTATGGGGTGCTCCAGTGGGGAATCTTGCGCAATGGGCGAAAGCCTGACGCAGCA
ACGCCGCGTGAGCGAAGAAGGCCTTCGGGTTGTAAAGCTCTGTCAGGAGGGACGAAGCCA
CTCGGGTTAATAGCCCAGAGGGTGACGGTACCTCCAGAGGAAGGTCCGGCTAACTACGTG
CCAGCAGCCGCGGTAATACGTAGGGACCAAGCGTTGTCCGGAATTATTGGGCGTAAAGAG
CGTGTAGGCGGTTTGCTAAGTCCGGTGTGAAATGTCGGGGCTCAACCCCGGAACTGCATT
GGAAACTGGCTGACTTGAGTCCGGAAGAGGAGACTGGAATTCCTGGTGTAGCGGTGAAAT
GCGCAGATATCAGGAAGAACACCCGTGGCGAAGGCGGGTCTCTGGGACGGTACTGACGCT
GAGACGCGAAAGCGTGGGGAGCAAACAGGATTAGATACCCCTGTAGTCC
>Otu4085
CCAGCCTATGGGTTGCAGCAGTCGAGAATCATTCACAATGGGGGAAACCCTGATGATGCG
ACGCCGTGTGAGCGATGAAGGCCTTCGGGTTGTAAAGCTCTTTCGTAAGGGAACAAGAAA
AAGAGACGAATAATCTTTTGATTTGAGAGTACCTTATAAAGAAGCACCGGCTAACTCCGT
GCCAGCAGCTGCGGTAATACGGAGGGTGCAAGCATTAATCGGAATTATTGGGCGTAAAGG
GCGCGTAGGCTGATAGGAAAGTCAGATGTGAAATTTCGGAGCTCAACTTCGAAGCGGCAT
TTGAAACTCCCTGTCTAGAGGATAGGCGGAGAAAACGGAATTCCACGTGTAGCGGTGAAA
TGCGTAGAGATGTGGAAGAACACCTGTGGCGAAAGCGGTTTTCTAGCTTACACCAGACGC
TGATGCACGAAAGCTAGGGGAGCAAACAGGATTAGAAACCCGCGTAGTCC
>Otu4091
CCAGCCTATGGGTTGCTCCAGTAGGGAATATTGGTTAATCTGCGAAAGCGGGAACCAGCA
ACGCCGCGTGTGCGATGAAGGCCTTCGGGTCGTAAAGCACTTTTTGGAGGGATGAGGAAG
GACAGTACCTCCAGAATAAGCCTCGGCTAACTACGTGCCAGCAGCCGCGGTAAAACGTAG
GAGGCAAGCGTTATTCGGATTTACTGGGCGTAAAGCGTGTGCAGGCGGTTCGGAAAGTTG
GATGTGAAAGCTCCCGGCTTAACTGGGAGAGGTCGTTCAATACTACCGGACTTGAGAGCA
GTAGAGGAAGGTGGAATTCCCGGTGTAGTGGTGAAATGCGTAGATATCGGGAGGAACACC
AGTGGCGAAAGCGGCCTTCTGGACTGTTTCTGACACTCATATGCGAAAGCTAGGGTAGCA
AACGGGATTAGAGACCCTCGTAGTCC
>Otu4098
CCAGCCTATGGGAGGCACCAGTGAGGAATATTGGTCAATGGACGCAAGTCTGAACCAGCC
ATGCCGCGTGAAGGATGAAGGCCCTACGGGTTGTAAACTTCTTTTGTACGGGACGAAACT
ATGTTACGTGTAATGTACTGACGGTACCGTATGAATAAGCAACGGCTAACTCCGTGCCAG
CAGCCGCGGTAATACGGAGGTTGCAAGCGTTATCCGGATTTATTGGGTTTAAAGGGTGCG
TAGGCGGGACGATAAGTCAGTGGTGAAAGCCTGCAGCTTAACCGTAGAATTGCCATTGAT
ACTGTCGTTCTTGAGTGTAGTTGAGGTGGGCGGAATGTGTAATGTAGCGGTGAAATGCTT
AGAGATTACACAGAACACCGATTGCGTAGGCAGCTCACTAAACTATAACTGACGCTGAGG
CACGAAAGCGTGGGGAGCAAACAGGATTAGAAACCCCTGTAGTCC
>Otu4099
CCGCCTATGGGGCGCTCCAGGCACGAAACCTCTACAATATACGCAAGTGTGATAGGGGGA
CCCAGAGTGCTTATCTCTAAGATAAGCTTTTGTCAAGGGTAAATACCTTGAAGAATAAGT
GGTGGGCAAGACTGGTGCCAGCCGCCGCGGTAACACCAGCGCCACGAGTAGGAACCGCGT
TTATTTGGTCTAAAGCATCCGTAGCAGGATTGACAAGTTCTTTGTGAAATTGTTGGGCTT
AACCTAACAGCGTGCAAAGAAGACTATCTTTCTCGAGACTGGAGAGGAGTTAGAGGTATT
CTGGGGGGAGCGGTAAAATGCTATAATCCCCAGACGACCACCTGTGGCGAAGGCGTCTAA
CTAGAACAGTTCTGACTGTGAGGGATGAAAGCTGGGGGAGCGATCCGGATTAGAAACCCC
CGTAGTCC
>Otu4100
CCAGCCTATGGGATGCTGCAGACGAGAATATTCGACAATGGACGAAGGTCTGATCGAGCG
ACGCCGCGTGGAGGATGAAGTGCTTCGGCATGTAAACTCCTTTTGCCAGGGAAAAAGTCT
ATTGATTGTACCTGGAGAATAAGAGGTTGCTAAACTCGTGCCAGCAGCAGCGGTAATACG
AGTGCCTCGAGCGTTATCCGGAATTATTGGGCGTAAAGGGTGCGTAGGCTGTTCTGTTAG
TCTCATGTTAAATTTCCCGGCTCAACCGGGAAGCCGCATGGGAAACGGCAGGACTTAGAG
GTTGGAAGGGGTTTGCGGAACTCATGGTGTAGCGGTGAAATGCGTTGATATCATGGGGAA
CACCAAAAGCGAAGGCAGCAAACTGGTCCAAATCTGACGCTGAAGCACGAAAGCGTGGGT
CGCGAATGGGATTAGATACCCCAGTAGTCC
>Otu4105
CCAGCCTACGGGTGGCAGCAGCCGAGAATATTCGACAATGGGCGAAAGCCTGATCGAGCG
ACGCCGCGTGGAGGACGAAGTCCTTAGGGACGTAAACTCCTTTTATAGGTTAGAAAGTTA

TTGATCAACCTAGGAATAAGAGGTTGCTAAACTCGTGCCAGCAGCAGCGGTAATACGAGT
GCCTCAAGCGTTATCCGGAATTACTGGGCGTAAAGGGTGTGTAGGCGGTTTTGTTAGTCT
TCTGTCAAATTCTTCGGCTTAACCGGGGGCTCGCAGAGGAAACGGCAAAACTAGAGGATG
CGAGAGGCACGTAGAACTCATGGTGTAGGGGTGAAATCCGTTGATATCATGGGGAATACC
AAAAGCGAAGGCAGCGTGCTGGCGCATTTCTGACGTTGAAACACGAAAGCGTAGGTAGCG
AACGGGATTAGAAACCCCCGTAGTCC
>Otu4110
CCAGCCTATGGGGCGCAGCAGTAAGGGATATTGCTCAATGGGCGAAAGCCTGAAGCAGCA
ACGCCGCGTGGAGGATGAAGGTCTTCGGATCGTAAACTTCTTTTTACAGGGACGAGATAG
GACGGTACCTGTAGAATAAGCCTCGGCTAACTACGTGCCAGCAGCCGCGGTAAAACGTAG
GAGGCGAGCGTTATCCGGATTTACTGGGCTTAAAGGGTGTGCAGGCGGTTGACTAAGTCG
TGCATGAAAGCCATGTGCTTAACACGTGGAGGCTGTGCGAGACTGGTCGGCTAGAGTGTG
TGAGAGGGAAGCAGAATTCCGGGTGTAGTGGTGAAATGCGTAGATATCCGGAGGAATACC
AGAGGCGAAGGCGGCTTCCTGGCACACTACTGACGCTGAGACACGACAGCATGGGGAGCG
AACGGGATTAGAAACCCCCGTAGTCC
>Otu4111
CCAGCCTATGGGGTGCTGCAGCGATACCACGTGAGTGATGAAGTTCTTTGGAATGTAAAG
CTCAGTCAGGGGATGAGAAAAGGATTTGGTGAATAGCCAGATCCGTTGACTATCCCCAGA
GGAAGCTCCGGCCAACTCCGTGCCAGCAGCCGCGGTAATACGGGGGGAGCAAGCGTTGCC
CGGAATCACTGGGCGTAAAGGGTGTGTAGGTGGATCTGCAAGTCAAGTGTGAAATCTTAC
GGCTTAACCGTAAAATTGCGCTTGAAACTGCATTTCTTGAGTACGGGAGAGGAGAATGGA
ATTTCAGGTGTAGCGGTGAAATGCGTAGATATCTGAAAGAACACCGGTTGCGAAGGCTAT
TCTCTATCCTAATACTGACACTGTGACACGAAAGCTAGGGGAGCAAACAGGATTAGATAC
CCGAGTAGTCC
>Otu4113
CCAGCCTACGGGGCGCTGCAGGGAGGAATATTGGGCAATGGACGAAAGTCTGACCCAGCA
ACGCCGCGTGAAGGATGAAGGCCTTCGGGTCGTAAACTTCTTTTGAGGGAGAAGAGAAAG
GACGGTATCCCTCGAATAAGCCACGGCTAACTACGTGCCAGCAGCCGCGGTAAAACGTAG
GTGGCGAGCGTTATCCGGATTCATTGGGCGTAAAGAGCTTGTAGGTGGTTGCGTAAGTTG
GACGTGAAAACTCCGGGCTTAACTTGGAGAGGCCGTTCAATACTGTGCGACTAGAGGATG
TCAGAGGGAAGTAGAATTCCTGGTGTAGCAGTGAAATGCGTAGATATCAGGAGGAATACC
CGTGGCGAAGGCAGCTTCCTGGGACATAACTGACACTGAGAAGCGAAAGCTAGGGGAGCA
AACGGGATTAGATACCCTTGTAGTCC
>Otu4116
CCAGCCTACGGGGTGCTCCAGTAAGGGATATTGCGCAATGGGCGAAAGCCTGACGCAGCA
ACGCCGCGTGGAGGAAGAAGGCCTTCGGGTCGTAAACTCCTTTTGTGGGGGACGAGGAAG
GACGGTACCTCACGAAGAAGTCACGGCTAACTACGTGCCAGCAGCCGCGGTAAAACGTAG
GTGGCGAGCGTTATCCGGATTTACTGGGCGTAAAGCGTGTGCAGATGGGTCACCAAGTGG
TGTATGAAATCCCTGAGCTTAACTTAGAGAGGTTATGCCAGACTGGTGGTCTAGAGAACG
GGAGAGGGACGTGGAATTCCGGGTGTAGTGGTGAAATGCGTAGAGATCCGGAGGAACCCC
TGAGGCGAAGGCGGCGTCCTGGCCCGATTCTGACATTCAGACACGACAGCATGGGGAGCG
AACGGGATTAGAGACCCTCGTAGTCC
>Otu4117
CCAGCCTACGGGGGGCTGCAGTCGAGAATCATTCGCAATGGGCGCAAGCCTGACGATGCG
ACGCCGTGTGTGCGATGAAGGCCTTCGGGTTGTAAAGCACTTTCGCTTGGGAACAAGGGA
AACTGGCTAATATCCAGCGTACTTGAGGGTACCAGGTAAAGAAGCACCGGCTAACTCCGT
GCCAGCAGCTGCGGTAATACGGAGGGTGCGAGCATTAATCGGATTTATTGGGCGTAAAGG
GCGCGTAGGCGGGAACATAAGTCGGATGTGAAATCCCAGGGCTCAACCCTGGAACAGCAT
TTGATACTGTGTTTCTAGAGGGTAGGCGGAGAAAATGGAATTCCACAAGTAGCGGTGAAA
TGCGTAGATATGTGGAAGAACACCGGTGGCGAAGGCGATTTTCTAGCTTATACCTGACGC
TGAGGCGCGAAAGCAAGGGGATCAAACAGGATTAGAGACCCTAGTAGTCC
>Otu4118
CCAGCCTACGGGTGGCAGCAGTAACGAATCTTCCGCAATGCACGAAAGTGTGACGGAGCG
ACGCCGCGTGTGGGACGAAGCCCTTCGGGGTGTAAACCACTGTCAGGGGAAAGAAAGTTC
TGATCTACCCCAAAGGAAGGGACGGCTAACTCTGTGCCAGCAGCCGCGGTAATACAGAGG
TCCCGAGCGTTAGGCGGAATCACTGGGCTTAAAGCGTGTGTAGGCGGATCTGTAAGTACC
TTGTGAAATCCCATGGCCCAACCATGGAACTGCTTGGTATACTGCGGATCTTGAGCTAGC
TAGGGGCAACCGGAACAGACGGTGGAGCGGTGAAATGCGTAGATATCGTCTGGAACGCCA

ATGGTGAAAACAGGTTGCTGGGGCTAAGCTGACGCTGAGACACGAAAGCCAGGGGAGCAA
ACGGGATTAGAAACCCCAGTAGTCC
>Otu4120
CCAGCCTATGGGGTGCAGCAGTTTCGAATCATTTACAATGGGCGAAAGTCTGATAGTGCG
ACGCCGCGTGGGGGATGAAGGTCTTCGGATTGTAAACCCCTGTCACCGGGGAACAAACGC
GTACGGTTAATAGCCGTCCGCCTGAGCTAACCCGGAGAGGAAGCCGTGGCTAACTCTGTG
CCAGCAGCCGCGGTAATACAGAGACGGCGAGCGTTACTCGGATTTACTGGGCGTAAAGGG
TGCGTAGGTGGCCAAGTGTGTCGGGTGTGAAATCCCGGAGCTCAACTCCGGAACTGCGCC
CGAAACTACTTGGCTTGAGGCTTGGAGAGGGTGATAGAATTCCTGGTGTAGCGGTGAAAT
GCGTAGATATCAGGAGGAATACCAACGGCGAAGGCAGTCACCTGGACAAGTTCTGACACT
GAGGCACGAAAGCGTGGGGAGCAAAAGGGATTAGAAACCCTCGTAGTCC
>Otu4121
CCAGCCTATGGGAGGCAGCAGTCGAGAATTTTTCTCAATGGGCGAAAGCCTGAAGGAGCG
ACGCCGCGTGGGGGATGAATGGCTTCGGCCCGTAAACCCCTGTCATTCGGGATCAATGCG
TCTGGGTGAACATCCCAGACGTTGATAGTACCGGAAGAGGAAGGGACGGCTAACTCTGTG
CCAGCAGCCGCGGTAATACAGAGGTCCCAAGCGTTGTTCGGATTTACTGGGCGTAAAGCG
CGTGCAGGCGGTTCTGTAAGTTGGATGTGAAATCTCCGGGCCTAACCCGGAAACTGCGTT
GGATACTATCCGGCTAGAGGAATGGAGGGGAGACTGGAATACTTGGTGTAGCAGTGAAAT
GCGTAGATATCAAGTGGAACACCAGTGGCGAAGGCGAGTCTCTGGACATTTCCTGACGCT
GAGGCACGAAAGCCAGGGGAGCAAACGGGATTAGAGACCCTAGTAGTCC
>Otu4123
CCAGCCTACGGGGGGCACCAGGCGCGAAAACTTTACAATGCGCGAAAGCGTGATAAGGGA
ATTCCAAGTGCTTGGCCTTTGAGTCGAGCTTTTGCCGAGTGTAAAAATCTCGGAGAATAA
GTGGTGGGTAAGACGGGTGCCAGCCGCCGCGGTAACACCTGCGCCACGAGTGGGAGCCAC
TTTTATTGGGTCTAAAGCGTTCGTAGCTGGAGTATTAACTCCCTTGTGAAATTGTTGTGC
TTAACATAACATCGTGCAAGGGAAACTGGTACTCTTGAGACTGGGAAGAGCTTGGAGTAT
GCCAAGGGGAGCGGTAAAATGTTATAATCCTTGGTAGACTACCTGTGGCGAAGGCGCCAA
GCTAGAACAGGTCTGACAGTGAGGGACGAAGGCTAGGGGAGCAAAACGGATTAGATACCC
CCGTAGTCC
>Otu4125
CCAGCCTATGGGTGGCACCAGGCGCGAAACCTTTACAATGCACGAAAGTGTGATAGGGGA
ACTCAGAGTGCATATACTTTTAGTATATGCTTTTGCCAACTCGAAAATGGTTGGAGAATA
AGTGGTGGGCAAGACTGGTGCCAGCCGCCGCGGTAACCCCAGCGCCACAAGTGGGAATCG
GTATTATTGGGTCTAAAGCGTCCGTAGCAGGTTTTCTAAATTACTTGTGAAATCACATCG
CTCAACGGTGTGGACTGCAGGTAAGACTGGAAAACTAGAGACTGGGAGACGTTGGAGGTA
TTCAAAGGGGAGCGGTAAAATGCGAGAATCCTTTGAAGACCACCTGTTGCGAAGGCGTCC
AACAATAACAGGTCTGACTGTGAGGGGCGAAAGCTAGGGGAGCGAACCGGATTAGGTACC
CGAGTAGTCC
>Otu4131
CCAGCCTATGGGTGGCAGCAGTAAGGAATATTGGACAATGCCCGCAAGGGTGATCCAGCC
ATGCCGCGTGCAGGAAGAAGGCCCTATGGGTCGTAAACTGCTTTTGAACAAGAGAAAACC
CCTCTACGTGTAGAGGGCTGATAGTATTGTTAGAATAAGCATCGGCTAACTTCGTGCCAG
CAGCCGCGGTAAGACGAAGGATGCAAGCGTTATCCGGATTCATTGGGTTTAAAGGGTGCG
TAGGCGGCCCGGTAAGTCAGTGGTGAAATCTCCTTGCTTAACAAGGAAACTGCCATCGAT
ACTGCTGGGCTTGAGTACAGATGCTGTGGGCGGAATATGACATGTAGTGGTGAAATACAT
AGAGATGTCATAGAACACCGATTGCGAAGGCAGCTCACAAAACTGTAACTGACGCTGAGG
CACGAAAGTGCGGGGATCAAACAGGATTAGAAACCCCAGTAGTCC
>Otu4135
CCAGCCTATGGGTGGCAGCAGTCGAGAATTTTTCACAATGGGCGAAAGCCTGATGGAGCG
ACGCCGCGTGGGGGATGAATGGCTTCGGCCCGTAAACCCCTGTCATTTGCGAACAAACCT
TGTTATTTAAAAGATGACAAGCTGATAGTAGCGAAAGAGGAAGGGACGGCTAACTCTGTG
CCAGCAGCCGCGGTAATACAGAGGTCCCAAGCGTTGTTCGGATTCACTGGGCGTAAAGGG
TGCGTAGGTGGTCGGGTAAGTCTGACGTGAAATCTCCGAGCTTAACTCGGAAACGGCGTC
GGATACTATTCGGCTGGAGGGTTGGAGGGGGGACTGGAATACTTGGTGTAGCAGTGAAAT
GCGTAGATATCAAGTGGAACACCAGTGGCGAAGGCGAGTCCCTGGACAACTCCTGACACT
GAGGCACGAAAGCTAGGGGAGCAAACAGGATTAGATACCCTCGTAGTCC
>Otu4136
CAGCCTACGGGAGGCAGCAGTGGGGAATATTGGACAATGGGCGCAAGCCTGATCCAGCCA

TGCCGCGTGAGTGATGAAGGCCTTAGGGTTGTAAAGCTCTTTTGTCCGGGACGATAATGA
CGGTACCGGAAGAATAAGCCCCGGCTAACTTCGTGCCAGCAGCCGCGGTAATACGAAGGG
GGCTAGCGTTGCTCGGAATCACTGGGCGTAAAGGGCGCGTAGGCGGCCATTCAAGTCGGG
GGTGAAAGCCTGTGGCCCAACCACAGAATTGCCTTCGATACTGTTTGGCTTGAGTATGGC
AGAGGTCAGTGGAACTGCGAGTGTAGAGGTGAAATTCGTAGATATTCGCAAGAACACCAG
TGGCGAAGGCGGCTGACTGGGCCATTACTGACGCTGAGGCGCGAAAGCGTGGGGAGCAAA
CAGGATTAGAAACCCCAGTAGTCC
>Otu4140
CCAGCCTATGGGTTGCAGCAGTCGAGAATTTTTCACAATGGGCGCAAGCCTGATGGAGCG
ACGCCGCGTGGGGGATGAATGGCTTCGGCCCGTAAACCCCTGTCATTCGCGAACAATGTC
CATGCATTAACACCGTATGGATTGATAGTAGCGGAAGAGGAAGGGACGGCTAACTCTGTG
CCAGCAGCCGCGGTAATACAGAGGTCCCAAGCGTTGTTCGGATTTACTGGGCGTAAAGGG
TGCGTAGGTGGTCGGGTAAGTCTGATGTGAAATCTCGGAGCCTAACTCCGAAACGGCATT
GGATACTATTCGGCTCGAGGGTTGGAGGGGGGACTGGAATTCTCGGTGTAGCAGTGAAAT
GCGTAGATATCGAGAGGAACACCGGTGGCGAAGGCGAGTCCCTGGACAACTCCTGACACT
AAGGCACGAAAGCTAGGGGAGCAAACAGGATTAGATACCCCGGTAGTCC
>Otu4141
CCAGCCTATGGGTGGCTGCAGTAGGGAATTTTGGACAATGGGCGAAAGCCTGATCCAGCA
ACGCCGCGTGCGCGATGACAGCCTTCGGGTTGTAAAGCGCTTTTAGGGGAGATGAGAAAG
GACAGTATCCCCTGAATAAGCCTCGGCGAACTACGTGCCAGCAGCCGCGGTAAAACGTAG
GAGGCTAGCGTTATCCGGATTTATTGGGCGTAAAACGTGTGCAGGCGGTTTAGCAAGTTG
GATGTGAAAGCTCCCGGCTTAACTGGGAGAGGTCGTTCAAAACTGCTAGACTAGAGGATG
GGAGAGGGAGGTGGAATTCCGGGTGTAGTGGTGAAATGCGTAGATATCCGGAGGAACACC
AGTGGCGAAAGCGGCCTCCTAGACCATTTCTGACGCTTAGACACGAAAGCTAGGGGAGCA
AACGGGATTAGAAACCCCGGTAGTCC
>Otu4148
CCAGCCTACGGGTCGCAGCAGCTAAGAATATTCCGCAATGGGAGATATCCTGACGGAGCG
ACGCCGCGTGAACGAAGAAGGCAGAAATGTTGTAAAGTTCTTTTATACGGGAAGAATAAG
GGCAGGAGTGGAAAGTTTGCCTGGTGACGGTACCGTATAAATAAGCCACGGCTAACTACG
TGCCAGCAGCCGCGGTAATACGTAGGTGGCAAGCGTTGTTCGGAATTATTGGGCGTAAAG
GGCATGTAGGCGGTAAGATAAGTGTGAATTGAAAGACTGAGGCTTAACCTCACGTTTGGT
TTGCAAACTGTCTAACTAGAGTGCAGAAGGGGAAACTGGAATTCCGCGTGTAGGGGTGAA
ATCTGTAGATATGCGGAAGAATACCTGTGGCGAAGGCGAGTTTCTGGTCTGTAACTGACG
CTGAAATGCGAAAGTGTGGGGAGCAAACAGGATTAGAAACCCTGGTAGTCC
>Otu4149
CCAGCCTATGGGGGGCACCAGCTAAGAATATTCCGCAATGGGGGAAACCCTGACGGAGCA
ATCTCGCGTGAGTGATGAAGGCTTTCGGGTTGTAAAGCTCTTTTGGCGGGGAAGAGTAAG
GTCATCAGGAAATGGGTGATCGACGACGGTACCCGCAGAAGAAGGACCGACTAATTACGT
GCCAGCAGTCGCGGTAATACGTAGGGTTCAAACGTTGTTCGGAATCATTGGGCGTAAAGG
GTACGCAGGCGGTTTTCTAAGTCAGTGGTGAAAATCTGAGGCTTAACCTCAGAACTGCCA
TTGATACTGGGAGACTAGAGTTGTAATAGGGGTGGCGGGAATTCCAAGTGTAGGAGTGAA
ATCTGTAGATATTTGGAGGAACACCAATAGCGAAGGCAAGCCACTGGTTAACGACTGACG
CTCAGGTACGAGAGCTAGGGGAGCAAATGGGATTAGAGACCCCCGTAGTCC
>Otu4151
CCAGCCTATGGGATGCTGCAGTGGGGAATCTTGCACAATGGGGGCAACCCTGATGCAGCG
ACGCCGCGTGAACGATGAAGCCCTTCGGGGCGTAAAGTTCTTTCGGCAGGAACGATAATG
ACGGTACCTGAAGAAGAAGCTGCGGCTAACTACGTGCCAGCAGCCGCGGTAATACGTAGG
CAGCAAGCGTTGTTCGGAGTTACTGGGCGTAAAGAGTGTGTAGGCGGTTTTCTAAGTTTG
TTGTGAAATCTCCCAGCTCAACCGGGAGGGTGCGGCGAAGACTGGAAAGCTTGAGTATGG
GAGAGGAAAGCGGAATTTCCGGTGTAGCGGTGAAATGCGTAGATATCGGAAGGAACACCT
GCGGTGTAGACGGCTTTCTGGACCATCACTGACGCTGAGACACGAAAGCGTGGGTAGCAA
ACAGGATTAGAAACCCGCGTAGTCC
>Otu4155
CCAGCCTATGGGTCGCTGCAGTGGGGAATATTGCACAATGGGCGCAAGCCTGATGCAGCG
ACGCCGCGTGGAGGAAGAAGGCTTTCGAGTTGTAAACTCCTGTTAAGTGGGAAGAAAGAC
TGTTGACTAATAATCAACAGGGATGACGGTACCACTAGAGAAAGCACCGGCAAACTTCGT
GCCAGCAGCCGCGGTAATACGAAGGGTGCAAGCGTTATTCGGAGTTACTGGGCGTAAAGA
GTATGTAGGCGGTTTATTAAGTTAGTTGTTAAATACCATGGCTTAACTATGGAAAGGCAG

CTGATACTGATGAGCTAGAGCGCAGAAGAGAGGAGTGGAATTCCCGGAGTAGCGGTAAAA
TGCGTAGATCTCGGGAGGAACACCGGTTGCGAAGGCGGCTTCTTGGTCTGTGGCTGACGC
TAAAGTACGAAAGCGTGGGGAGCAAACAGGATTAGAAACCCGTGTAGTCC
>Otu4162
CCAGCCTACGGGGTGCAGCAGTCAAGAACCTTCCGCAATGGACGAAAGTCTGACGGAGCG
ACGCCGCGTGGAGGATGAAGTGCTTCGGCATGTAAACTCCTTTTGCCAGGGAAAAAGTTT
ATTGATTGTACCTGGAGAATAAGAGGTTGCTAAACTCGTGCCAGCAGCAGCGGTAATACG
AGTGCCTCGAGCGTTATCCGGAATCATTGGGCGTAAAGGGTGTGTAGGCGGCGATGTTAG
TCTCCCGTAAAATCTTCCGGCTCAACCGGGAGTCCGCGGGAGAAACGGCATTGCTCGAGA
TTGGAAGGGGTCTCTGGAACTCTAGGTGTAGCGGTGAAATGCGTTGATATCTAGGGGAAC
ACCAAAAGCGAAGGCAGGAGACTGGTCCATTTCTGACGCTGAAACACGAAAGCGTGGGTA
GCGAATGGGATTAGAGACCCCAGTAGTCC
>Otu4163
CCAGCCTATGGGGGGCAGCAGTAGGGAATATTGCACAATGGAGGAAACTCTGATGCAGCG
ACGCCGCGTGAGTGATGAAGGACTTCGGTTCGTAAAGCTCTGTCGTATGGGAATAAGAAA
GTGAATGTACCATGCAAGAAAGGATCGGCTAACTTCGTGCCAGCAGCCGCGGTAAGACGA
GGGATCCTAGCGTTGTTCGGAATCATTGGGCGTAAAGCGGATGTAGGTGGCTTTGTAAGT
CAGATGTGAAAGCCCAGGGCTCAACCCTGGAAGTGCATTTGATACTGCGAAGCTTGAGTG
CTAGAGAGGTTACTAGAATACCTGGTGTAGTGGTGAAATACGTAGATATCAGGTGGAATA
CCGGAGGCGAAGGCGGGTAACTAGCTAGACACTGACACTGAGATCCGAAAGTGTGGGGAT
CAAACAGGATTAGATACCCGTGTAGTCC
>Otu4165
CCAGCCTATGGGAGGCTGCAGTTAGGAATTTTGCGCAATGGACGAAAGTCTGACGCAGCG
ACATCGCGTGGAGGATGACGGCCTTCGGGTTGTAAACTCCTTTTGCCTTTTCATGAAGAA
AAGGCGAATAAGCAACTACTAACTACGTGCCAGCAGTCGCGGTAATACGTAGGTTGCAAG
CGTTACCCGGTTTTACTGGGCGTAAAGAGTCTGTAGGTGGTTTTCCAAGTCTTGTTTCAA
AGACCCCGGCCTAACCGGGGGAAGGGGCAGGATACTGGGAGACTAGAGTTATATCGGGGT
TACTGGAATTTGCAGTGTAGGGGTAAAATCCGTGGATACTGCAAAGAACACCAAGCGCAA
AGGCGGGTAACCAGGTATGTACTGACACTCAGAGACGAAAGCTAGGGTAGCAAATCAGAT
TAGAAACCCTGGTAGTCC
>Otu4167
CCAGCCTACGGGGGGCTGCAGCTAAGAATATTCCGCAATGGGAGATATCCTGACGGAGCG
ACGCCGCGTGTATGAAGAAGGCAGAAATGTTGTAAAGTACTTTTATACGGGAAGAATAAG
GTAGGTAATGGAAAGATCTATTGATGACGGTACCGTATGAATAAGCCACGGCTAACTACG
TGCCAGCAGCCGCGGTAATACGTAGGTGGCAAGCGTTGTTCGGATTTATTGGGCGTAAAG
GGCATGTAGGCGGTTAGATAAGTGTGAATTGAAAGGCGTAGGCTTAACCTATCGTTTGGT
TTGCAAACTATCTGACTAGAGTGCAGAAGGGGAAACTGGAATTCCGCGTGTAGGGGTGAA
ATCTGTAGATATGCGGAAGAATACTTGTGGCGAAGGCGAGTTTCTGGTCTGTAACTGACG
CTGAAATGCGAAAGTGTGGGGAGCAAACAGGATTAGAGACCCGCGTAGTCC
>Otu4168
CCAGCCTACGGGACGCACCAGTAGGGAATATTGCGCAATGGGCGAAAGCCTGACGCAGCG
ACGCCGCGTGGACGATGAAGTTCTTCGGAATGTAAAGTCCTGTCAGGAGGGAAGAAAACC
TTGGGTGTTAAATCCAAGGCTGACGGTACCTCCAGAGGAAGCTACGGCTAACTTCGTGCC
AGCAGCCGCGGTAATACGGGGGTAGCGAGTGTTGTTCGGATTTACTGGGCGTAAAGGGAG
CGCAGGCGGTAAGATAAGTCAGGAGTGAAAGGTTGGGGCTTAACCCCAAACCTGCTTTTG
ATACTGTCTTGCTAGAGTATGCGAGAGGAGGATGGAATTCTAGGTGTAGCGGTGGAATGT
GTAGATATCTAGAAGAACACCGATTGCGAAGGCAGTCCTCTGGTGCAATACTGACGCTCA
AGCTCGAAAGCTAGGGGAGCAAACAGGATTAGATACCCCAGTAGTCC
>Otu4170
CCAGCCTACGGGTCGCACCAGTTAGGAATTTTGCACAATGGACGAAAGTCTGATGCAGCG
ACATCGCGTGAAGGATGAAGGCCCTCGGGTCGTAAACTTCTTTTCTATCTTCATGAAGTC
GATAGGAATCAGTGACTACTAACTACGTGCCAGCAGTCGCGGTAATACGTAGGTCACAAG
CGTTACCCGGTTTTATTGGGCGTAAAGCGTCTGTAGGCGGTTTTTCAAGTCTTATTTTAA
AGACCCCGGCTCAACCGGGGGAAGGGGTAGGATACTGAAAGACTAGAGTTATATCGGGGT
TACTGGAATTTGCAGTGTAGGGGTAAAATCCGTGGATACTGCAAAGAACACCAAGCGCGA
AGGCGGGTAACCAGGTATGTACTGACGCTCAGAGACGAAAGCTAGGGTAGCCAATCAGAT
TAGAAACCCTCGTAGTCC
>Otu4173

CCAGCCTACGGGGCGCTCCAGGGACGAATATTCCGCAATGGACGAAAGTCTGACGGAGTG
ACGCCGCGTGTGGGAAGAAGTCCTTCGGGATGTAAACCACTGTCAGGGGTCACCAAGTAA
CCGCGTCTAACATGCGCGGAAGTTGAGGAGCCCCAGAGGAAGCCACGGCTAACTTCGTGC
CAGCAGCCGCGGTAAGACGAAGGTGGCGAGCGTTGTTCGGAATTACTGGGCTTAAAGCGC
ACGTAGGCGGCCTGTTAAGTGCTTTGTGAAAGCCCTCGGCTCAACCGAGGATCTGCTTGG
CATACTGGCAGGCTTGAGGCAGGTATGGGCGAGAGGAACTCTTGGTGGAGCGGTGAAATG
CGTAGATATCAAGAGGAACGCCGGTGGTGAAGACGTCTCGCTGGGCCTGTCCTGACGCTG
AGGTGCGAAAGCCAGGGGAGCAAACGGGATTAGAAACCCGCGTAGTCC
>Otu4174
CCAGCCTATGGGGGGCTGCAGTGGGGAATTTTGCGCAATGGGCGAAGGCCTGACGCAGCA
ACGCCGCGTGTGTGATGAAGGTCTTCGGATCGTAAAGCACTGTCGCGGGGGAAGAAACCT
GACGGTACCCCGAGAGGAAGCACCGGCTAACTCTGTGCCAGCAGCCGCGGTAATACAGAG
GGTGCGAGCGTTGTTCGGAATTATTGGGCGTAAAGCGCTGGTAGGTGGCTCTCCAAGTCG
CGTGTGAAATCTTCCGGCTCAACCGGAAAAGTGCGCGCGAAACTGTGGAGCTTGAGTGCC
GGAGAGGGTCGCGGAATTCCCGGTGTAGAGGTGAAATTCGTAGATATCGGGAGGAACACC
AGAGGCGAAGGCGGCGACCTGGAAGGTAACTGACACTGAGCAGCGAAAGCGTGGGGAGCA
AACAGGATTAGAAACCCTAGTAGTCC
>Otu4180
CCAGCCTATGGGATGCTGCAGTAACGAATATTGGGCAATGGGCGAAAGCCTGACCCAGCG
ACGCCGCGTGTAGGAGGAAGTCCCTCGGGATGTAAACTACTGTCAGGGTGAAGAAAGACC
GCAAGGTTTGATCTAACCCAGAGGAAGCAACGGCTAACTTCGTGCCAGCAGCCGCGGTAA
TACGAAGGTTGCGAGCGTTGTTCGGAATCACTGGGCTTAAAGCGCATGTAGGCGGATGAG
TAAGCGTTTTGTGAAATCCCTCGGCTTAACCGAGGAATTGCTTGGCGAACTGCTCATCTT
GAGGCAAGTAGGGGTGCATGGAACTCTTGGTGGAGCGGTGGAATGCGTAGATATCAAGAG
GAACGCCAATGGTGAAAACAGTGCACTGGGCTTGTTCTGACGCTGAGATGCGAAAGCGTG
GGGAGCGAACGGGATTAGAAACCCCAGTAGTCC
>Otu4181
CCAGCCTATGGGTTGCAGCAGTAAGGAATATTGGTCAATGGACGCAAGTCTGAACCAGCC
ATGCCGCGTGAAGGATGAAGGCCCTCTGGGTTGTAAACTTCTTTTATCTGGGACGAAAAA
AGGGCATTCTTGCTCACTTGACGGTACCAGAGGAATAAGCACCGGCTAACTCCGTGCCAG
CAGCCGCGGTAATACGGAGGGTGCAAGCGTTATCCGGATTTACTGGGTTTAAAGGGTGCG
TAGGTGGGTAATTAAGTCAGCGGTGAAATCTTCGAGCTTAACTCGGAAACTGCCATTGAT
ACTATTTATCTTGAATATCCTGGAGGTAAGCGGAATATGTCGTGTAGCGGTGAAATGCTT
AGATATGACATAGAACACCCATTGCGAAGGCAGCTCACTACGGGATTATTGACACTGAGG
CACGAAAGCGTGGGGATCAAACAGGATTAGAGACCCGCGTAGTCC
>Otu4182
CCAGCCTACGGGGGGCTGCAGTTTGGAATATTCCGCAATGGGCGAAAGCCTGACGGAGTG
ACGCCGCGTGAGGGATGAAGGCCTTCGGGTTGTAGACCTCTTTTGTCCGTTAGAAAGAGC
CTCGGAGATAATATCTCCGGGATCTGATAAAGGCGGGTGAATAAGCACCGGCTAACTCTG
TGCCAGCAGCCGCGGTAATACAGAGGGTGCAAACGTTGTTCGGTGTCATTGGGCTTAAAG
GGTGCGTAGGCGGTCGTGCAAGTTGGGTGTGAAAGCCCCTGGCTTAACCAGGGAATTGCG
CCCAATACTACACGACTCGAGGGTCAAAGGGGAGAGCGGAACTCCAGGTGTAGCGGTAAA
ATGCGTAGATATCTGGGGGAACGCCGATGGTGAAGACAGCTCTCTGGGTGACTCCTGACG
CTGAGTGCACGAAAGCTGGGGGAGCAAACAGGATTAGAGACCCGAGTAGTCC
>Otu4183
CCAGCCTATGGGTTGCACCAGCAACGAATCTTCCCCAATGCCGGAAACGGTGAGGGAGCG
ACGCCGCGTGAAGGACGAAGTTCTTCGGAATGTAAACTTCTGTAAGGATCATGAAAGCTG
AGGACCTAATACGTCTAGAGGTTGATCAGGTCCCTAAAAGGGGCGGCTAACTCTGTGCCA
GCAGCCGCGGTAATACAGAGGCCCCAAGCGTTACTGAGAATCACTGGGTTTAAAGGGTGC
GTAGGTGGCGCGTTAAGTCCGTTGTGAAATCCCTTGGCTCAACCAAGGAATGGCTTCGGA
TACTGGCGCGCTTGAGGCCGGTAAGGGTCACTGGAACTGACGGTGGAGCGGTGAAATGCG
TAGATATCGTCAGGAACGCCAATGGTGAAGACAGGTGACTGGGCCGGTTCTGACACTGAG
GCACGAAAGCGTGGGGAGCGAACGGGATTAGAAACCCCAGTAGTCC
>Otu4184
CCAGCCTATGGGTGGCACCAGTAAGGGATATTGCGCAATGGGCGAAAGCCTGACGCAGCA
ACGCCGCGTGAAGGATGAAGGCCTTCGGGTCGTAAACTTCTTTGGCCGGGGACGAGCAAG
GACGGTACCCGGACAACGAGTCACGGCTAACTACGTGCCAGCAGCCGCGGTAAAACGTAG
GTGGCGAGCGTTATCCGGATTTACTGGGTGTAAAGCGCATGCAGGCGGCTCGTTAAGTTG

GGTGTGAAAGCTTCCGGCTCAACTGGGAGAGGTTGCTCAAGACTGGCGAGCTAGAGAGTG
GTAGGGGAAGGCGGAATTCCGGGTGTAGTGGCGAAATGCGTAGAGATCCGGAGGAACACC
AGTGGCGAAGGCGGCCTTCTGGGCCGCGACTGACGCTCAGATGCGACAGCTAGGGGAGCA
AACGGGATTAGAAACCCTTGTAGTCC
>Otu4185
CCAGCCTATGGGGCGCAGCAGTGGGGAATATTGGGTAATGGGCGAAAGCCTGACCCAGCG
ACGCCGCGTGGGGGATGAAGGCCTTCGGGTTGTAAACCCCTGTCAGGGGAGACGAAAGTC
TCGCGGTGAATAGCCGCGAGATCTGACGGTACCCCCAAAGGAAGCCCCGGCTAACTCCGT
GCCAGCAGCCGCGGTAATGCGGAGGGGGCGAGCGTTATTCGGAATTACTGGGCGTAAAGC
GCGCGCAGGTGGCGCGGTAAGTGGCAGGTGAAAGCCCTCGGCTCAACCGAGGAACTGCCT
GCCAAACTGCTGCGCTTGAGGCCGGGAGGGGAGAGTGGAATTCCTGGTGTAGCGGTGAAA
TGCGTAGATACCAGGAGGAACACCGGTGGCGAAGGCGGCTCTCTGGACCGGTTCTGACGC
TCATGCGCGAAAGCGTGGGGAGCAAACAGGATTAGATACCCTCGTAGTCC
>Otu4194
CCAGCCTACGGGTCGCAGCAGTGGGGAATTTTGGACAATGGGGGCAACCCTGATCCAGCA
ATGCCGCGTGTGTGAAGAAGGCCTTCGGGTTGTAAAGCACTTTTGTCAGGAACGAAACGG
TCAAGGCTAATACCCTGGGCTAATGACGGTACCTGAAGAATAAGCACCGGCTAACTACGT
GCCAGCAGCCGCGGTAATACGTAGGGTGCAAGCGTTAATCGGAATTACTGGGCGTAAAGC
GTGCGCAGGCGGTTATACAAGACAGATGTGAAATCCCCGGGCTCAACCTGGGAATTGCAT
TTGTGACTGTATGGCTAGAGTGTGTCAGAGGGGGGTAGAATTCCACGTGTAGCAGTGAAA
TGCGTAGAGATGTGGAGGAATACCGATGGCGAAGGCAGCCCCCTGGGATAACACTGACGC
TGAGGCGCGAAAGTGTGGGTAGCAAACAGGATTAGATACCCTCGTAGTCC
>Otu4197
CCAGCCTATGGGTGGCTCCAGTGGGGAATCTTGCACAATGGGGGAAACCCTGATGCAGCG
ACGCCGCGTGAGCGATGAAGCCCTTAGGGTTGTAAAGCTCTTTTGGCGGGGACGATAATG
ACGGTACCCGCAGAATAAGCCCCGGCTAACTTCGTGCCAGCAGCCGCGGTAATACGAAGG
GGGCTAGCGTTGTTCGGAATCACTGGGCGTAAAGCGCACGCAGGCGGATTGATAAGTCAG
GGGTGAGATCCCGGGGCTCAACCTCGGAATTGCCTTTGATACTGTCTGTCTTGAGTTCGG
GAGAGGTTGGCGGAATTCCTAGTGTAGAGGTGAAATTCGTAGATATTAGGAAGAGCACCA
GTGGCGAAGGCGGCCAACTGGCCCGACACTGACGCTCATGTGCGAAAGCGTGGGGAGCAA
ACAGGATTAGATACCCCAGTAGTCC
>Otu4198
CCAGCCTACGGGTCGCTCCAGCAACGAATCTTCCCCAATGCCGGAAACGGTGAGGGAGCG
ACGCCGCGTGGAGGATGAAGTCTTTCGAGATGTAAACTCCCGTTAGGGTTACGAAAGCAA
TGCGCGTGAATAATGCGCAAAGTTGATCTAGCCCAAAGAAAGGGGCGGCTAACTCTGTGC
CAGCAGCCGCGGTAATACAGAGGCCCCAAGCGTTACTGAGAATCACTGGGTTTAAAGGGT
GCGTAGGTGGCGCGTTAAGTCCGTTGTGAAATCCCCTGGCTCAACCAGGGAACTGCTTCG
GATACTGGCGTGCTTGAGGCCGGTAGGGGTCACTGGAACAGACGGTGGAGCGGTGAAATG
CGTAGATATCGTCTGGAACGCCGGTGGTGAAGACGGGTGACTGGGCCGGTTCTGACACTG
AGGCACGAAAGCGTGGGGAGCGAACGGGATTAGAAACCCTCGTAGTCC
>Otu4199
CCAGCCTACGGGTTGCTGCAGTGGGGAATCTTGCGCAATGGGCGAAAGCCTGACGCAGCG
ACGCCGCGTGTGCGATGAAGGTCTTCGGATCGTAAAGCACTGTCGCGGGGGACGAAAACC
GACGGTACCCCGAGAGGAAGCACCGGCTAACTCTGTGCCAGCAGCCGCGGTAATACAGAG
GGTGCGAGCGTTGTTCGGAATTATTGGGCGTAAAGCGCTTGTAGGCGGCGTGTCAAGTCG
CGTGTGAAAGCCCCGGGCTCAACCCGGGAAGTGCGCGCGAAACTGGCAGGCTCGAGTCCC
GGAGAGGGTCGCGGAATTCCCGGTGTAGAGGTGAAATTCGTAGATATCGGGAGGAACACC
AGAGGCGAAGGCGGCGACCTGGAAGGAGACTGACGCTGAGAAGCGAAAGCGTGGGGAGCA
AACAGGATTAGATACCCCGGTAGTCC
>Otu4202
CCAGCCTATGGGTGGCAGCAGCAACGAATCTTCCCCAATGGGCGAAAGCCTGAGGGAGCG
ACGCCGCGTGAAGGACGAAGTTCTTCGGAATGTAAACTTCTGTTAGGGTTATGAAAGCGA
CCGCGCCTAATACGCGCGGAAGTTGATCTGACCCAGGGAAAGGGACGGCTAACTCTGTGC
CAGCAGCCGCGGTAATACAGAGGTCCCAAGCGTTACTGAGAATCACTGGGTTTAAAGGGT
GCGTAGGTGGTCCGTTAAGTCCGTTGTGAAATCCCTTGGCTCAACCAAGGAACTGCTTCG
GATACTGGCGGACTTGAGGCCAGTAGAGGTCACTGGAACTGACGGTGGAGCGGTGAAATG
CGTAGATATCGTCAGGAACGCCGGTGGTGAAGACGGGTGACTGGGCTGGTTCTGACACTG
AGGCACGAAAGCGTGGGGAGCGAACGGGATTAGAAACCCTTGTAGTCC

>Otu4204
CCAGCCTATGGGGTGCTGCAGTCGAGAATTTTTCTCAATGGGGGAAACCCTGAAGGAGCG
ACGCCGCGTGGAGGATGAAGGTCTTCGGATTGTAAACTCCTGTCATTGAAGAACAAAGTA
CGTAAGTTAACTGCTTATGTGCCGATAGTACTCGAAGAGGAAGGGACGGCTAACTCTGTG
CCAGCAGCCGCGGTAATACAGAGGTCCCAAGCGTTGTTCGGATTCATTGGGCGTAAAGGG
AGCGTAGGTGGTCGGGTAAGTCAGATGTCAAATATCGATGCTCAACGTCGAATCGCATTT
GATACTGCCTGGCTAGAGGAATTGAGAGGAGGCCGGAATTCACGGTGTAGCAGTGAAATG
CGTAGATATCGTGAGGAAGACCAACGGCGAAAGCAGGCCTCTGGAAATCTCCTGACACTG
AGGCTCGAAGGCCAGGGGAGCAAACGGGATTAGAGACCCGTGTAGTCC
>Otu4208
CCAGCCTATGGGGGGCTGCAGCTAAGAATATTCCACAATGGGAGCAATCCTGATGGAGCG
ACGCCGCGTGTATGAAGAAGACAGAAATGTTGTAAAGTACTTTTATACGGGAAGAATAAG
AGTAGTGAGAGAAACGTTACTTGATGACGGTACCGTATGAATAAGCCACGGCTAACTACG
TGCCAGCAGCCGCGGTAATACGTAGGTGGCAAGCGTTGTTCGGATTTATTGGGCGTAAAG
GGCATGTAGGCGGTAAGATAAGTGTGAATTGAAATACCGAGGCTTAACCTCGCGTTTGGT
TTGCAAACTGTCTTGCTAGAGTGCAGAATGGGAAACTGGAATTCCGCGTGTAGGGGTGAA
ATCTGTAGATATGCGGAAGAATACTTGTGGCGAAGGCGAGTTTCTGGTCTGTAACTGACG
CTGAAATGCGAAAGTGCGGGGAGCAAACAGGATTAGATACCCCAGTAGTCC
>Otu4210
CCAGCCTACGGGTCGCTGCAGTGAGGAATTTTGCGCAATGGCCGCAAGGCTGACGCAGCA
ACGCCGCGTGGGTGAAGAAAGCCTTCGGGTCGTAAAGCCCTGTCGGGTGGGAAGAACTGC
TCGGGGATGAATAATCTTCGGGCTTGACGGTACCACCAAAGGAAGCACCGGCTAACTCCG
TGCCAGCAGCCGCGGTAATACGGAGGGTGCAAGCGTTATTCGGAATTACTGGGCGTAAAG
CGCGTGCAGGCGGGCCTGCAAGTCTGATGTGAAAGCCCTGGGCTCAACCTGGGAAGTGCA
TTGGAAACTGTGGGTCTTGAGTTCTGGAGAGGAAGGGGGAATTCCCGGTGTAGAGGTGAA
ATTCGTAGATATCGGGAGGAATACCAGTGGCGAAGGCGCCCTTCTGGACGGCAACTCACG
CTGAGACGCGAAAGCGTGGGGAGCAAACAGGATTAGATACCCTAGTAGTCC
>Otu4211
CCAGCCTATGGGAGGCAGCAGTAAGGAATATTGGTCAATGGACGCAAGTCTGAACCAGCC
ATGCCGCGTGGAGGATGAAGGCCCTCTGGGTTGTAAACTTCTTTTATCAGCGAAGAAACC
CTTGGTTTATACTGAGGTTGACGGTACCTGATGAATAAGCACCGGCTAACTCCGTGCCAG
CAGCCGCGGTAATACGGAGGGTGCAAGCGTTATCCGGATTCACTGGGTTTAAAGGGTGCG
TAGGTGGGTTTGTAAGTCAGTGGTGAAATCTCCAAGCTTAACTTGGAAACTGCCGTTGAT
ACTATAGATCTTGAATCTCGTGGAGGTAAGCGGAATATGTCATGTAGCGGTGAAATGCTT
AGATATGACATAGAACACCGATAGCGAAGGCAGCTTACTACACGAGTATTGACACTGATG
CACGAAAGCGTGGGGATCAAACAGGATTAGAGACCCTTGTAGTCC
>Otu4212
CCAGCCTATGGGGGGCAGCAGTAAGGAATATTGGGCAATGGGCGCAAGCCTGACCCAGCC
ATGCCGCGTGCAGGATTGACGGCCCTATGGGTTGTAAACTGCTTTTGTACGGGAAAAAAC
CCCCGGTCGTGTACCGGGGCTGATGTTACCGTAAGAATAAGCATCGGCTAACTCCGTGCC
AGCAGCCGCGGTAATACGGAGGATGCAAGCGTTATCCGGATTCATTGGGTTTAAAGGGTG
CGCAGGCGGAGCAATAAGTCAGTGGTGAAAGCCTACAGCTTAACTGTAGAATTGCCATTG
ATACTGTTGTTCTTGAGTATAGTTGGAGTGGGCGGAATGTATCATGTAGCGGTGAAATGC
TTAGATATGATACAGAACACCAATCGCGAAGGCAGCTCACTAAACTATAACTGACGCTCA
TGCACGAAAGCGTGGGGAGCAAACAGGATTAGAAACCCCAGTAGTCC
>Otu4217
CCAGCCTACGGGGGGCAGCAGTTAAGAATCTTGCACAATGGGCGCAAGCCTGATGCAGCG
ACGCCGCGTGAATGATGAAGGCCTTCGGGTTGTAAAGTTCAGTAAGCAGGGAAGAATAAG
CAAGAGGTAATATTTCTTGTGATGACGGTACCTGCCTAAAGCCCCGGCTAACTACGTGCC
AGCAGCCGCGGTAATACGTATGGGGCGAGCGTTGTTCGGAATTATTGGGCGTAAAGGGCG
TCTAGGCGGGAAATTAAGTTAGGTGTGAAAGCTATGGGCTCAACCCATAAACTGCATCTA
AAACTGATTTTCTTGAGTTCGGGAGAGGAGAGCGGAATTCCAGGTGTAGCGGTGAAATGC
GTAGATATCTGGAAGAACACCAGTGGCGAAGGCGGCTCTCTGGCCTAGAACTGACGCTGA
AGCGCGAAAGCGTGGGGAGCGAACAGGATTAGATACCCCTGTAGTCC
>Otu4218
CCAGCCTACGGGGGGCAGCAGGGAGGAATATTGGGCAATGGGGGAAACCCTGACCCAGCA
ACGCCGCGTGAAGGAAGAAGGCCTTCGGGTTGTAAACTTCTGTGACGGGGGACGAAAGAA
ATGACGGTACCCCGAGAGGAAGCCACGGCAAACTACGTGCCAGCAGCCGCGGTAATACGT

AGGTGGCGAGCGTTGTCCGGAATCACTGGGCGTAAAGGGCGCGTAGGCGGCGCTTCAAGT
CGCATGTGAAAAACCAGGGCTTAACTCTGGGGGGTCATGCGAAACTGGAGTGCTTGAGTG
CGGTAGAGGAAAGCGGAATTCCTGGTGTAGCGGTAAAATGCGTAGAGATCAGGAGGAACA
CCGGTGGCGAAGGCGGCTTTCTGGGCCGCAACTGACGCTGAGGCGCGAAAGCGTGGGGAG
CAAACAGGATTAGATACCCCAGTAGTCC
>Otu4221
CCAGCCTACGGGTTGCACCAGTCGAGAATATTCCACAATGGACGAAAGTCTGATGGAGCG
ACGCCGCGTGCGGGATGAAGGCCCTTGGGTTGTAAACCGCTCTTGTAAGTGAAAAATTTC
GATGATGGTAGCTTACGAATAAGAGGTTACTAACTCTGTGCCAGCAGTAGCGGTAATACA
GAGACCTCAAGCGTTATCCGGATTTATTGGGCGTAAAGAGCTGGTAGGTGGTTATATTAG
TCAGATGTCAAATCTCGGAGCTTAACTCTGAAACTGCATTTGAAACGGTATAACTCGAGG
GTGTGAGAGATCTATGGAACTCATGGTGTAGCAGTGAAATGCGTTGATATCATGAGGAAC
ACCAAAGGCGAAGGCATTAGATTGGCTCACTCCTGACACTGAGCAGCGAAAGCGTGGGTA
GCGAATGGGATTAGATACCCCAGTAGTCC
>Otu4222
CCAGCCTATGGGGGGCAGCAGTAGGGAATTTTCCACAATGGGCGAAAGCCTGATGGAGCA
ACGCCGCGCGCAGGATGAAAGCCTTCGGGTTGTAAACTGCTTTTATGTGCGAAGAATTTG
ACGGTAACACATGAATAAGGGTCGGCTAACTACGTGCCAGCAGCCGCGGTCATACGTAGG
ACCCAAGCGTTATCCGGATTTACTGGGCGTAAAGAGTTGCGTAGGTGGTTTTTTAAGTGG
AGCGTGAAAGCGTGTGGCTCAACCATACAGCTGCGTTCCAAACTGATTAACTTGAGTGCG
AGAGAGGTAGATGGAATTTCTAGTGTAGGAGTGAAATCCGTAGATATTAGAAGGAACACC
GATGGCGTAGGCAGTCTACTGGCTCGTTACTGACACTAAGGCACGAAAGCGTGGGGAGCA
AACGGGATTAGAAACCCCTGTAGTC
>Otu4225
CCAGCCTATGGGGCGCTGCAGTGGGGAATTTTGCGCAATGGGGGAAACCCTGACGCAGCA
ACGCCGCGTGGAGGATGAAGTCCTTCGGGACGTAAACTCCTTTCGATCGGGACGATAATG
ACGGTACCGGAAGAAGAAGCCCCGGCTAACTTCGTGCCAGCAGCCGCGGTAATACGAGGG
GGGCGAGCGTTGTTCGGAATTATTGGGCGTAAAGGGCGCGTAGGCGGTGCGGTAAGTCAC
CTGTGAAATCCCCAGGCTTAACCTGGGGCCTGCAGGCGAAACTACCGTGCTGGAGGGTGG
GAGAGGTGCGTGGAATTCCCGGTGTAGCGGTGAAATGCGTAGATATCGGGAGGAACACCT
GTGGCGAAAGCGGCGCACTGGACCACTACTGACGCTGAGGCACGAAAGCTAGGGGAGCAA
ACAGGATTAGATACCCCAGTAGTCC
>Otu4227
CCAGCCTACGGGGGGCTGCAGTGAGGAATATTGGTCAATGGATGCAAGTCTGAACCAGCC
ATGCCGCGTGAAGGATGACTGCCCTATGGGTTGTAAACTTCTTTTGTAGAAGGCGAAACG
CATGTCTTGAACATGAACTGACGGTACTCTACGAATAAGCATCGGCTAACTCCGTGCCAG
CAGCCGCGGTAATACGGAGGATGCGAGCGTTATCCGGATTTATTGGGTTTAAAGGGTTCG
TAGGCGGCTTTATAAGTCAGCGGTGAAATACCAGAGCTTAACTCTGGGGCTGCCGTTGAT
ACTGTTAAGCTTGAGTAGGATCGACGATGGCGGAATGAGACAAGTAGCGGTGAAATGCAT
AGATATGTCTCAGAACACCGATAGCGTAGGCAGCTGTCGAGGTCATCTACTGACGCTGAG
GAACGAAAGCGTGGGGATCAAACAGGATTAGATACCCGAGTAGTCC
>Otu4228
CCAGCCTACGGGGGGCAGCAGCAGGGAATTTTGCGCAATGGACGAAAGTCTGACGCAGCA
ACGCCGCGTGAGTGATGAAGGCCTTCGGGTTGTAAAGCTCTGTCGTTCGGGACGAAAATC
CAGTCGGTGAATAGCCGATTGGACTGACGGTACCGAAAAAGGAAGCACCGGCTAACTCCG
TGCCAGCAGCCGCGGTAATACGGAGGGTGCAAGCGTTGCTCGGAATTATTGGGCGTAAAG
GGCAGGTAGGTGGTCTCGTTTGTCTGGGGTGAAAGCCTTGAGCTTAACTCAAGTAGTGCC
CCAGAAACGGCGAGACTAGAGTATTGGAGAGGGTCGTGGAATTCCCGGTGTAGCGGTGAA
ATGCGTAGAGATCGGGAGGAACACCAGTGGCGAAGGCGGCGACCTGGACAATTACTGACA
CTCAACTGCGAAAGCGTGGGTAGCAAACAGGATTAGAGACCCGCGTAGTCC
>Otu4230
CCAGCCTATGGGGCGCAGCAGTGAGGAATATTGGGCAATGGGCGCAAGCCTGATCCAGCC
ATGCCGCGTGAGTGATGACGGCCTTAGGGTTGTAAAGCTCTTTCACCCACGACGATAATG
ACGGTAGTGGGAGAAGAAGCCCCGGCTAACTACGTGCCAGCAGCCGCGGTAATACGTAGG
GGGCAAGCGTTGTTCGGATTCATTGGGCGTAAGGGGCATGTAGGCTGTTATGTAAGTGTG
GATTTAAAGGCGGAGGCTTAACCTCTAGTTTGGTCTGCAAACTGCGTAGCTTGAGTGTAG
AAGGGGAAACTGGAATTCCGCGTGTAGGGGTGAAATCTGTAGATATGCGGAAGAATACCT
GTGGCGAAGGCGAGTTTCTGGTCTATAACTGACGCTGAAATGCGAAAGTGTGGGTAGCAA

ACAGGATTAGAGACCCCCGTAGTCC
>Otu4237
CCAGCCTATGGGGGGCTGCAGTAGGGAATATTGCGCAATGGAGGAAACTCTGACGCAGCG
ACGCCGCGTGGGTGATGAAGGCCTTCGGGTCGTAAAGCCCTGTCGGAAGGGAAGAAAAAT
ATCTGGGATAACACCCCAGATACTTGACGGTACCTTCAGAGGAAGCACCGGCTAACTACG
TGCCAGCAGCCGCGGTAATACGTAGGGTGCTAGCGTTGTTCGGAATCACTGGGCGTAAAG
CGCGTGTAGGTCGTTAGTTAAGTCGGGTGTGAAATCCCTGGGCTCAACCGAGGAAGTGCA
TCCGAAACTAACTAGCTAGAGAATGGTAGAGGAAGGTGGAATTCCTAGTGTAGAGGTGAA
ATTCGTAGATATTAGGAGGAATACCGGTGGCGAAGGCGGCCTTCTGGACCATTTCTGACA
CTGAGACGCGAAAGCGTGGGGAGCAAACAGGATTAGAAACCCTAGTAGTCC
>Otu4239
CCAGCCTATGGGTGGCACCAGTGGGGAATATTGGACAATGGGCGCAAGCCTGATCCAGCA
ATGCCGCGTGTGTGAAGAAGGCCTGCGGGTCGTAAAGCACTTTAAGCAGGAAAGAAGGGT
GGGGTGTGTATAGCACTTTGCATTGACGTTACCTGCAGAATAAGCACCGGCTAACTCCGT
GCCAGCAGCCGCGGTAATACGGAGGGTGCGAGCGTTAATCGGAATTACTGGGCGTAAAGC
GCGCGTAGGCGGTTTGATCAGTCTGAGGTGAAAGCCCTGGGCTTAACCTGGGAATGGCCT
TGGAAACTGTCAAGCTGGAGTGTGGGAGAGGGGCGTGGAATTTCCGGTGTAGCAGTGAAA
TGCGTAGAGATCGGAAGGAACACCAGTGGCGAAGGCGGCGCTCTGGCCCAACACTGACGC
TGAGGTGCGAAAGCGTGGGGAGCAAACAGGATTAGAAACCCCAGTAGTCC
>Otu4241
CCAGCCTACGGGTCGCAGCAGTAACGAATATTGGGCAATGGGCGAAAGCCTGACCCAGCG
ACGCCGCGTGTGGGAAGAAGTTCTTCGGAATGTAAACCACTGTCAGGGGTTACCAAGCGA
GTACGTCGAATAGGCGTGCGAGTTGAGGCGCCCCAGAGGAAGCAACGGCTAACTTCGTGC
CAGCAGCCGCGGTAATACGAAGGTTGCGAGCGTTGTTCGGAATCACTGGGCTTAAAGAGC
ACGTAGGCGGCCCATCAAGTGCCTTGTGAAATCCCACGGCTCAACCGTGGAGCTGCTTGG
CATACTGTTCGGGCTTGAGGCAGGTAGAGGCGCGTGGAACTCTTGGTGGAGCGGTGGAAT
GCGTAGATATCAAGAAGAACGCCGGAGGCGAAAGCGATGCGCTGGGCCTGTCCTGACGCT
GAGGTGCGAAAGCCAGGGGAGCAAACGGGATTAGAAACCCCTGTAGTCC
>Otu4242
CCAGCCTACGGGTTGCAGCAGTAGGGAATATTGGTCAATGGGGGCAACCCTGAACCAGCC
ATGCCGCGTGCAGGAAGAAGGCGTTATGCGTTGTAAACTGCTTTTGACAGGGAATAAAAA
GGCCATGCGTGGCAAATTGAATGTACCTGTAGAATAAGCCACGGCTAACTACGTGCCAGC
AGCCGCGGTAATACGTAGGTGGCGAGCGTTATCCGGATTTATTGGGTTTAAAGGGAGCGT
AGGCGGTTTTGTAAGTCAGTGGTGAAAGTCGGCCGCTTAACGGTTGGAATGCCATTGATA
CTGCAGAGCTAGAGTATAGTATAGGTAGGGAGAATTGACGGTGTAGCGGTGAAATGCATA
GATATCGTCAAGAATACCGATTGCGAAGGCACCTTACTGGACTATAACTGACGCTGAGGC
TCGAAAGTATGGGAATCAAACAGGATTAGATACCCTCGTAGTCC
>Otu4244
CCAGCCTACGGGGTGCTCCAGTAACGAATCTTCCGCAATGCACGAAAGTGTGACGGAGCG
ACGCCGCGTGTGGGACGAAGTTCTTCGGAATGTAAACCACTGTCAGGGGAAAGAAAGTTC
TGATCTACCCCAGAGGAAGAGACGGCTAACTCTGTGCCAGCAGCCGCGGTAAGACAGAGG
TCTCGAGCGTTAGGCGGAATCACTGGGCTTAAAGCGTGTGTAGGCGGATGTCTAAGTACC
TTGTGAAATCCCACGGCTCAACCGTGGAACTGCTTGGTATACTGGATGTCTTGAGCCATC
TAGGGGCAACTGGGACAAACGGTGGAGCGGTGAAATGCGTAGATATCGTTTGGAACGCCA
AAGGTGAAAACAAGTTGCTGGGGATGTGCTGACGCTGAGACACGAAAGCCAGGGGAGCAA
ACGGGATTAGAAACCCCAGTAGTCC
>Otu4248
CCAGCCTACGGGGGGCACCAGTGAGGAATCTTCGTCAATGGGCGCAAGCCTGAACGAGCG
ACGCCGCGTGCGGGAAGACGCATTTCGGTGTGTAAACCGCTTTTCTTGGGGACGAGAAGG
GACGGTACCCAAGGAATAAGCATCGGCTAACTACGTGCCAGCAGCCGCGGTAATACGTAG
GATGCGAGCGTTGTCCGGAGTTACTGGGCGTAAAGCGTGTGCAGGCGGTTGTCTGCGTCT
GGGGTGAAATCTCCCGGCTTAACTGGGAGGGTGCCACGGATACGAGGCGACTTGAGGACT
TGAGAGGGAAGTAGAACTCCCGGTGGAACAGTGAAATGTGTAGAGATCGGGAAGAATACC
AGAGGGGAAGCCGGCTTCCTGGCAAGTATCTGACGCTCAGCCACGAAAGCTTGGGGAGTA
AACAGGATTAGATACCCGGGTAGTCC
>Otu4251
CCAGCCTACGGGAGGCACCAGCCAAGAATCTTCCGCAATGGGGGAAACCCTGACGGAGCG
ACGCCGCGTGAAGGAAGAAGGTCTTCGGATTGTAAACTTCTTTTGTAAGGGAAGAATAAG

TTTGCGAGGAAATGCGCAGATGATGACGGTACTTTATGAATAAGCCCCGGCTAATTACGT
GCCAGCAGCCGCGGTAATACGTAAGGGGCGAGCGTTACTCGGAATTACTGGGCGTAAAGG
GCGTGTAGGCGGTTTTACAAGTCTAATGTAAAAAACCACGGCTTAACCGTGGGGCTGCGT
TAGAAACTGTAAGACTTGAGTCTGGTGGAGGGCAATGGAATTCCCGGTGTAGCGGTGAAA
TGCGTAGATATCGGGAAGAACACCGAAGGCGAAAGCAGTTGTCTATGCCAAGACTGACGC
TGAGGCGCGAAAGCATGGGGATCAAACAGGATTAGAGACCCTAGTAGTCC
>Otu4253
CCAGCCTATGGGAGGCAGCAGTGGGGAATATTGGACAATGGGGGCAACCCTGATCCAGCC
ATGCCGCGTGTGTGAAGAAGGCCTTCGGGTTGTAAAGCACTTTCAGTGGGGAGAAAGGTT
TGTCACCTAATACGTGACGAAATTGATGCTACCCACAGAAGAAGCACCGGCTAACTCCGT
GCCAGCAGCCGCGGTAATACGGAGGGTGCAAGCGTTAATCGGAATTACTGGGCGTAAAGC
GCACGTAGGTGGTTTTTTAAGCCAGCTGTGAAATCCCCGGGCTCAACCTGGGACTTGCAG
TTGGTACTGGAAAGCTAGAGTAGGGTAGAGGGGTGTGGAATTCCAGGCGTAGCGGTGAAA
TGCGTAGATATCTGGAGGAACATCAGTGGCGAAGGCGACACCCTGGACTCATACTGACAC
TGAGGTGCGAAAGCGTGGGGAGCAAACAGGATTAGATACCCCTGTAGTCC
>Otu4254
CCAGCCTACGGGATGCTGCAGTAAGGAATATTGGACAATGGTGGCAACACTGATCCAGCC
ATGCCGCGTGCAGGATGAAGGCGCTACGCGTTGTAAACTGCTTTTGTACGGGAGAAAACC
CTCGCTCGTGAGCGAGGCTGATAGTACCGTAAGAATAAGCATCGGCTAACTTCGTGCCAG
CAGCCGCGGTAACACGTAGGAGGCAAGCGTTATCCGGATTTATTGGGTTTAAAGGGTGCG
TAGGCGGATTAATAAGTCAGTGGTGAAAACCTTCAGCTTAACTGGAGACTTGCCATTGAT
ACTGTTAGTCTTGAGTACGGTCAAGGTAGGCGGAATGTGTAATGTAGCGGTGAAATGCTT
AGATATTACACAGAACACCGATTGCGAAGGCAGCTTGCTGGGCCATTACTGACGCTGATG
CACGAAAGCGTGGGGAGCAAACAGGATTAGATACCCGCGTAGTCC
>Otu4259
CCAGCCTACGGGTTGCAGCAGTTTCGAATCATTCACAATGGGCGAAAGCCTGATGGTGCG
ACGCCGCGTGGAGGATGAAGGTTTTCGGATTGTAAACTCCTGTCACTGCAGAACAAGGCC
GGGCGATTGAATATTTCGTCCGGTTGATGGTATGCGGAGAGGAAGGGACGGCTAACTCTG
TGCCAGCAGCCGCGGTAAGACGGAGGTCCCGAGCGTTGTTCGGATTCATTGGGCGTAAAG
GGTGTGTAGGAGGTCGGGTAAGTCAGGTGTGAAATCTCACAGCTTAACTGTGAAACTGCG
CTTGATACTGCCCGGCTAGAGGATCGGAGGGTGTAACGGAATTTATGGTGTAGCAGTGAA
ATGCGTAGATATCATAAGGAACACCGGTGGCGAAGGCGGTTACCTGGAAGATTCCTGACT
CTGAAACACGAAAGCCAGGGGAGCAAACGGGATTAGAAACCCCAGTAGTCC
>Otu4261
CCAGCCTATGGGACGCTCCAGTCGAGAATCTTCCACAATGGACGAAAGTCTGATGGAGCG
ACGCCGCGTGATTGATGAAGTCCTTCGGGACGTAAAGATCTTTTATGAGGGAAGAAGTTT
ATTGACGGTACCTCATGAATAAGGGGCTCCTAATCTCGTGCCAGCAGGAGCGGTAATACG
AGAGCCCCGAGCGTTATCCGGAATTATTGGGCGTAAAGGGTGTGTAGGCGGTTGTATTAG
TCGTGTGTCAAAACTCCCAGCTTAACTGGGAATCGGCATACGAAACGGTACGACTTAGAA
TGAAGGAGAGGTGTGCGGAACTCATGGTGTAAGGGTGAAATCCGTTGATATCATGGGGAA
CACCAAATGCGAAAGCAGCACACTGGCCTTTTATTGACGCTGAGACACGAAAGCGTGGGT
AGCGAATGGGATTAGATACCCTCGTAGTCC
>Otu4263
CCAGCCTACGGGGGGCAGCAGTAGGGAATCTTCCACAATGGGCGAAAGCCTGATGGAGCA
ACGCCGCGTGTAGGATGAAGGCCCTCGGGTCGTAAACTGCTTTTATCTGTGACGAATATG
ACGGTAGCAGATGAATAAGGATCGGCTAACTCCGTGCCAGCAGCCGCGGTCATACGGAGG
ATCCAAGCGTTATCCGGAATCACTGGGCGTAAAGAGTTGCGTAGGTGGCATGATAAGTTG
GTAGTGCAATCCTGGGGCTCAACCCCTTACCCATTACCAAAACTGTCAAGCTAGAGGATG
AGAGAGGTTATTGGAATTCCTAGTGTAGGAGTGAAATCCGTAGATATTGGGAGGAACACC
GATGGCGTAGGCAGGCCACTGGCTCATTCCTGACGCTCAGGCACGAAAGCGTGGGGAGCG
ACCGGGATTAGATACCCCAGTAGTCC
>Otu4264
CCAGCCTATGGGAGGCAGCAGTGGGGAATATTGGACAATGGGCGCAAGCCTGATCCAGCC
ACGCCGCGTGGGTGATGAAGGCCTTCGGGTCGTAAAGCCCTGTCGGAGGGGAAGAAAAGC
TCGCTGGCTAATATCCAGCGGGCCTTTGACGGTACCCTCAAAGGAAGCACCGGCTAACTC
CGTGCCAGCAGCCGCGGTAAGACGGAGGGTGCAAGCGTTGCTCGGATTGACTGGGCGTAA
AGGGCGTGTAGGCGGTTGCCCAAGTCCGATGTGAAAGCCCGGGGCTTAGCCTCGGAAGTG
CATCGGAAACTAGGCAGCTGGAGCGCGATAGAGGAGGGTAGAATTCCTGGTGTAGCGGTG

AAATGCGTAGATATCAGGAGGAATACCGGCGGCGAAGGCGGCCCTCTGGATCGTTGCTGA
CGCTGAGACGCGAAAGCGTGGGGAGCAAACAGGATTAGATACCCCTGTAGTCC
>Otu4269
CCAGCCTATGGGGGGCTGCAGTGGAGAATATTGATCAATAAACGAGAGTTTGAATTAGTG
TTTTGCGGGAATGAATGATTCTTAGAATCTCGAAGGCTATGATAGCTGTAAAGTTCGTCC
GTCGGTTAGAATAATGACATTAAACTGAAAAAGAATCCCGGGCTAACAACGTGCCAGCAG
CTGCGGTAAGACGTTGACGGGGGAGCGTTACTGGTCATTACTAGGCGTAAAGAATGGCTA
AACGGTTTAAGAAATTACACTTTAAATCAAAGATATTGTCGAAGGTTTGTGTAAACTCTT
AAGACTTGAGGAGTAAGGAAATTTGCGATAAGTATAATCTAGAGATAAAGTTCTAAAAGA
TTATAAAGATCTTCAAATGCCATGGCAGCATTTTATTTATTATCCTAACGCTAAACCACT
AAAGTATAAAAATCAATCAGGATTAGAAACCCCAGTAGTCC
>Otu4270
CCGCCTACGGGGGGCTGCAGTAAGGAATATTGGACAATGGGCGCAAGCCTGATCCAGCCA
TGCCGCGTGAAGGATGAAGGTCCTCTGGATTGTAAACTTCTTTTATAGGGGACGAAAAAA
GGGTTTGCCAACCCGTCTGGCGGTACCCTATGAATAAGCACCGGCTAACTCCGTGCCAGC
AGCCGCGGTAATACGGAGGGTGCAAGCGTTATCCGGATTCACTGGGTTTAAAGGGTGCGT
AGGCGGGTATGTAAGTCAGTGGTGAAATCTTGGGGCTCAACCCCGAAACTGCCATTGATA
CTATGTATCTTGAATATTGTGGAGGTTAGCGGAATATGTCATGTAGCGGTGAAATGCTTA
GATATGACATAGAACACCGATTGCGAAGGCAGCTGGCTACACAAATATTGACGCTGAGGC
ACGAAAGCGTGGGGATCAAACAGGATTAGATACCCCAGTAGTCC
>Otu4274
CCAGCCTACGGGGCGCAGCAGCCGAGAATATTCGACAATGGGCGAAAGCCTGATCGAGCG
ACGCCGCGTGGAGGATGAAGTCCTTAGGGACGTAAACTCCTTTTATAGGTTAGAAAGTTA
TTGATCAACCTAGGAATAAGAGGTTGCTAAACTCGTGCCAGCAGCAGCGGTAATACGAGT
GCCTCAAGCGTTATCCGGAATTACTGGGCGTAAAGGGTGTGTAGGCGGTTTTGTTAGTCT
TGTGTAAAAACTTCTCGCTTAACGAGAAATCTGCATAGGAAACGGCAAAACTAGAAGATG
AGAGGGGAGCATAGAACTCATGGTGTAGGGGTGAAATCCGTTGATATCATGGGGAATACC
AAAAGCGAAGGCAGTGCTCTGGCTCATTCTTGACGCTGAAACACGAAAGCGTGGGTAGCG
AACGGGATTAGAGACCCTTGTAGTCC
>Otu4278
CCAGCCTACGGGATGCTGCAGTCGAGAATTTTTCACAATGGGGGAAACCCTGATGGAGCG
ACGCCGCGTGGGGGATGAATGGCTTCGGCCCGTAAACCCCTGTCATTTGCGAACAAATTG
TTTCATTTAACACCTGAAACATTGATAGTAGTGGAAGAGGAAGGGACGGCTAACTCTGTG
CCAGCAGCCGCGGTAATACAGAGGTCCCAAGCGTTGTTCGGATTCACTGGGCGTAAAGGG
TGCGTAGGTGGCCAGGTAAGTCTGATGTGAAATCTCGAAGCTCAACTTCGAAACTGCATT
GGATACTATCTGGCTGGAGGATCGGAGGGGAAACTGGAATTCTTGGTGTAGCAGTGTAAT
GCGTAGATATCAAGAGGAACACCAGTGGCGAAGGCGAGTCTCTGGACGATTCCTGACACT
GGGGCACGAAAGCTAGGGGAGCAAACAGGATTAGAGACCCTAGTAGTCC
>Otu4284
CCAGCCTACGGGGGGCAGCAGTGGGGAATATTGGACAATGGGGGCAACCCTGATCCAGCA
ATGCCGCGTGTGTGAAGAAGGCCTTCGGGTTGTAAAGCACTTTCAGTGGGGAGGAAGGGT
TGTTAATTAATACTTAACAACATTGACGTTACCCACAGAAGAAGCACCGGCTAACTCTGT
GCCAGCAGCCGCGGTAATACAGAGGGTGCAAGCGTTAATCGGAATGACTGGGCGTAAAGC
GTGTGTAGGTGGTTGGATAAGTTAGATGTGAAATCCCTGGGCTTAACCTGGGAACTGCGT
TTAAGACTGTCCAACTAGAGTACTGTAGAGGATAGTGGAATTTCCAGTGTAGCGGTGAAA
TGCGTAGATATTGGAAGGAACACCAGTGGCGAAGGCGACTGTCTGGATAGATACTGACAC
TGAGACACGAAAGCGTGGGGAGCAAACAGGATTAGATACCCGCGTAGTCC
>Otu4285
CCAGCCTATGGGGGGCTGCAGTCGAGGATCATTCGCAATGAGCGAAAGCTTGACGATGCG
ACGCTGTGTGAACGATGAAGGCCTTCGGGTTGTAAAGTTCTTTCGCTTGGGAACAAGAGA
ATTTAGTGAATAACTGAGTAATTTGAGTGTACCAGGTAAAGAAGCACCGGCTAACTCCGT
GCCAGCAGCTGCGGTAATACGGAGGGTGCAAGCATTAATCGGATTTATTGGGCGTAAAGG
GCGCGTAGGCGGACTATCAAGTCAGATGTGAAATACCAAAGCTCAACTTTGGTGCTGCAT
TTGAAACTGGTAGACTTGAGGGTTGACGGAGAAAATGGAATTCCACGTGTAGCGGTGAAA
TGCGTAGATATGTGGAAGAACACCTGTGGCGAAAGCGATTTTCTAGTTAATACCTGACGC
TGAGGCGCGAGAGCAAGGGGAGCAAGCAGGATTAGAGACCCGTGTAGTCC
>Otu4286
CCAGCCTACGGGGTGCACCAGTAACGAATCTTCCGCAATGTACGAAAGTATGACGGAGCG

ACGCCGCGTGTAGGACGAAGCCCTTCGGGGTGTAAACTACTGTCAGGGGTTAGAAAGCTC
TGATCAACCCCAGAGGAAGGCACGGCTAACTCTGTGCCAGCAGCCGCGGTAAGATAGAGG
TGCCAAGCGTTAGGCGGAATCACTGGGCTTAAAGCGTGTGTAGGCGGAATCGTAAGCACC
TTGTGAGATCCCACGGCTCAACCGTGGAACTGCCTGGTATACTGCGATTCTTGGGCCAAT
TAGGGGCAGCCGGAACAAATGGTGGAGCGGTGAAATGCGTAGATATCATTTGGAACGCCA
AGGGCGAAAGCAGGCTGCTGGGATTGTGCCGACGCTGAGACACGAAAGCCAGGGGAGCAA
ACAGGATTAGAAACCCCGGTAGTCG
>Otu4287
CCAGCCTACGGGATGCTGCAGTCGAGAATTTTTCTCAATGGGCGAAAGCCTGAAGGAGCG
ACGCCGCGTGGGGGATGAAGGGCTTCGGCCCGTAAACCCCTGTCATTTGCGAACAAACCC
CGCGACCCAACACGTCGCGGGCTGATGTAGCGAAAGAGGAAGGGACGGCTAACTCTGTGC
CAGCAGCCGCGGTAATACAGAGGTCCCAAGCGTTGTTCGGATTCACTGGGCGTAAAGGGT
GCGTAGGTGGCGGGGTAAGTTTGATGTGAAATCTCCGGGCTTAACCCGGAAACTGCATTG
AATACTATCTTGCTCGAGGTTTGGAGGGGGGACTGGAATTCTCGGTGTAGCAGTGAAATG
CGTAGATATCGAGAGGAACACCAGTGGCGAAGGCGAGTCCCTGGACAACACCTGACACTG
GGGCACGAAAGCTAGGGGAGCAAACAGGATTAGATACCCCCGTAGTCC
>Otu4289
CCAGCCTACGGGAGGCTGCAGTGAGGAATATTGCGCAATGGAGGAAACTCTGACGCAGCG
ACGCCGCGTGGGTGATGAAGGCTTTCGGGTCGTAAAGCCCTGTCGGAAGGGACGAAAAAC
GTTGTGGCTAACATCCACGACGATTGACGGTACCTTTAAAGGAAGCACCGGCTAACTACG
TGCCAGCAGCCGCGGTAATACATAGGGTGCAAGCGTTGTTCGGAATCATTGGGCGTAAAG
CGCGTGTAGGCGGTCAAATAAGTCGAGTGTGAAATCCCTGGGCTCAACCGAGGAAGTGCA
TCCGAAACTATTTGGCTGGAAGGAGGTAGAGGAAGGTGGAATTCCTAGTGTAGAGGTGAA
ATTCGTAGATATTAGGAGGAATACCGGCGGCGAAGGCGGCCTTCTGGACCTCTCTTGACG
CTGAGACGCGAAAGCGTGGGTAGCAAACAGGATTAGAGACCCCGGTAGTCC
>Otu4290
CCAGCCTATGGGGTGCTCCAGTGGGGAATATTGGACAATGGGGGAAACCCTGATCCAGCG
ACGCCGCGTGTGTGAAGAAGGCCTGCGGGTTGTAAAGCACTTTTAGTGGGGACGAAAAGC
CTGGGGCTAATACCCTTTGGTCTTGACTTAACCCAAAGAAAAAGCACCGGCTAACTCTGT
GCCAGCAGCCGCGGTAATACAGAGGGTGCAAGCGTTAATCGGAATTACTGGGCGTAAAGG
GTGCGTAGGTGGTTGTTTAAGTTTGCTGTGAAAGCCCCGGGCTCAACCTGGGAATGGCAG
TGAATACTGGACAGCTAGAGTGCGGTAGAGGGTAGTGGAATTCCCGGTGTAGCAGTGAAA
TGCGTAGAGATCGGGAGGAACACCAGTGGCGAAGGCGGCTACCTGGACCAGCACTGACAC
TGAAGCACGAAAGCGTGGGGAGCAAACAGGATTAGATACCCGTGTAGTCC
>Otu4294
CCAGCCTACGGGGGGTACCAGTGGGGAATATTGGACAATGGGCGAAAGCCTGATCCAGCA
ATACCGCGTGAGTGATGAAGGCCTTAGGGTTGTAAAGCTCTTTTAATAGGGAAGATAATG
ACGGTACCTATAGAAAAAGCCCCGGCTAACTCCGTGCCAGCAGCCGCGGTAAGACGGAGG
GGGCTAGCGTTGTTCGGAATTACTGGGCGTAAAGAGCGCGTAGGCGGCTATGCAAGTTGG
GAGTGAAATCCCTGGGCTTAACCCAGGAACTGCTTCCAAAACTGCGTAGCTAGAGTATGG
TAGGGGGTGATAGAATTCCTAGTGTAGAGGTGAAATTCTTAGATATTAGGAGGAATACCG
GTGGCGAAGGCGATCACCTGGGCCATTACTGACGCTGAGGCGCGAAAGCGTGGGGAGCAA
ACAGGATTAGAAACCCCAGTAGTCC
>Otu4295
CCAGCCTATGGGGTGCTGCAGTGGGGAATTTTGGACAATGAGCGAAAGCTTGATCCAGCA
ATACCGAATGGGTGATGAAGACTGATTTGGTTGTAAAACTCTTTCGACAAAAAGGATAAT
GACGTTGTTTGGGAGAAGAAGTCCTGGCCAATAGCCGTGCCAGCAGCCGCGGAAATACGG
TAGGGGCAAGCGTTATTCACATTTAATGGGCGTAAAGGGTGCGTAGGTGGTCTTTTTTTA
ATTTTTTGTTAAAAAACATAGCATAACTATGTAGAAGCAAAAAAAATTTTAAGACTTTGA
GTGGGAGAGAGGTTTAGGGAATTTTCAGTGAAAAAGTGGAATTTTACCATATTGAAAGGA
ACGTCTACAGCGAAGGCACTTTTCCAGATCTTAACTGACATTGAGGCACGAAGGCATAGG
GATCAAACGGGATTAGATACCCCCGTAGTCC
>Otu4300
CCAGCCTATGGGGTGCTCCAGTGGGGAATTTTGGACAATGGGGGCAACCCTGATCCAGCA
ATGCCGCGTGAGTGAAGAAGGCCTTCGGGTTGTAAAGCTCTTTTGTCAGGGAAGAAACGG
TTCATCCTAATACGGTGGGCTAATGACGGTACCTGAAGAATAAGCACCGGCTAACTACGT
GCCAGCAGCCGCGGTAATACGTAGGGTGCAAGCGTTAATCGGAATTACTGGGCGTAAAGC
GTGCGCAGGCGGTTTCGCAAGTCAGATGTGAAATCCCCGGGCTTAACCTGGGAACTGCAT

TTGAAACTACGAGGCTAGAGTGTGTCAGAGGGGGGTAGAATTCCACGTGTAGCAGTGAAA
TGCGTAGAGATGTGGAGGAATACCGATGGCGAAGGCAGCCCCCTGGGATAACACTGGCGC
TCATGCACGAAAGCGTGGGGAGCAAACAGGATTAGATACCCCGGTAGTCC
>Otu4301
CCAGCCTACGGGAGGCAGCAGTCAGGAATCTTGGTCAATGGACGAACGTCTGAACCAGCG
ATGCCACAGTGGGAGGAAGGTATACACTGTAAACCACAGACGATCGAACGAAGGATGATG
GGTTCGATCTGACAAGCTCTATGTAATTTCGGTGCCAGCACATGCGGTCAATCCGAAAGA
GCGAGGCATGCTCTATGGAATTGGGTCTAAAACGTCTGTAGATGGTCGACGCGAGGCATC
TTGTTGAAGTTCGACACACGGATGGATGCGAGGTGGACCCGTCGTCTTGAGTACGAGAGA
GGAAGGTGGAATGTCGAGCGGACCGATGAAATGGGTTGATTCTCGATGGAACTCCAGAGG
CGAAGGCGACCTTCTGGCTCGTACTGACATTGAGAGACGAAAGCCTGGGGAGCAAATCCG
ATTAGATACCCGCGTAGTCC
>Otu4302
CCAGCCTACGGGGGGCAGCAGTGGGGAATCTTGCACAATGGAGGAAACTCTGATGCAGCG
ACGCCGCGTGAGCGATGAAGCCCTTCGGGTGTAAAGCTCTTTCGGCAGGGAAGATGATGA
CGGTACCTGCAGAAGCAGCTGCGGCTAACTACGTGCCAGCAGCCGCGGTAATACGTAGGC
AGCAAGCGTTGTTCGGAGTTACTGGGCGTAAAGGGTGCGTAGGCGGCCTTGTAAGTATGG
TGTGAAATCTCCCGGCTTAACTGGGAGGGTGCGCTATTAACTGCAGGGCTAGAGTGCGGG
AGAGGAAAGTGGAATTCCTGGTGTAGCGGTGAAATGCGTAGATATCAGGAGGAACACCTG
CGGTGTAGACGGCTTTCTGGACCATCACTGACGCTGAGACACGAAAGCGTGGGTAGCAAA
CAGGATTAGATACCCTCGTAGTCC
>Otu4303
CCAGCCTATGGGGGGCACCAGTGGGGAATCTTGCGCAATGGGCGAAAGCCTGACGCAGCA
ACGCCGCGTGGAGGACGAAGGCTTTCTGAGTTGTAAACTCCTTTCAGCAGGGACGATTGT
GACGGTATCTGAGGAATAAGCACCGGCTAACTCTGTGCCAGCAGCCGCGGTAATACAGAG
GGTGCGAGCGTTAATCGGAATTACTGGGCGTAAAGCGCGCGTAGACGGTTTTGTAAGTCA
GATGTGAAATCCCTGGGCTCAACCTGGGAACTGCATTTGATACTGCATGGCTAGAGTATC
GAAGAGGGAAGTGGAATTTCCGGTGTAGCGGTGAAATGCGTAGATATCGGAAGGAACACC
AGTGGCGAAAGCGACTTCCTGGTCGAATACTGACGTTCATGTGCGAAAGCGTGGGGAGCA
AACAGGATTAGAAACCCTAGTAGTCC
>Otu4305
CCAGCCTATGGGATGCACCAGTGGGGAATTTTGCGCAATGGGGGAAACCCTGACGCAGCA
ACGCCGCGTGGAGGATGAAGCCCCTTGGGGCGTAAACTCCTTTCGACCAAGACGATAATG
ACGGTACTGGTGGAAGAAGCACCGGCTAACTCTGTGCCAGCAGCCGCGGTAATACGTAGG
GGGCGAGCGTTGTCCGGGGTTACTGGGCGTAAAGGGCACGCAGGCGGTCTGCTACGTTCG
GAGTGACAGTTGTCAGCTTAACTGACAAAGGTCTTCGAAAACGGGTAGACTTGAGGCCTT
GAGAGGGAGACGGAATTCCGGGTGGAGCGGTGAAATGCGTAGAGATCCGGAGGAACACCG
AAGGCGAAGGCAGTCTCCTGGCAAGGTACTGACGCTCAGGTGCGAAAGCTAGGGGAGCGA
ACAGGATTAGATACCCCTGTAGTCC
>Otu4307
CCAGCCTATGGGGTGCTGCAGCTAAGAATCTTCCGCAATGGGCGAAAGCCTGACGGAGCG
ATGCCGCGTGATCGATGAAGGTCGAGAGATTGTAAAGATCTTTTCCAGTTGAGGAATAAG
TCTAGGAGGGAATGCCTGGATGATGACGTTAAACTGGGAATAAGCCCCGGCTAATTACGT
GCCAGCAGCCGCGGTAATACGTAAGGGGCGAGCGTTGTTCGGAATTATTGGGCGTAAAGG
GCACGCAGGCGGTTATGCAAGTCTGGTGTGAAAGACCTGGGCTTAACTCAGGGAACGCGC
TGGAAACTGTATGACTAGAATCTTGGAGGGGGAGTTGGAATTCCTAGTGTAGGGGTGAAA
TCTGTTGATATTAGGAAGAACACCGGTGGCGAAGGCGAACTCCTGGCTGTAGATTGACGC
TGAGGTGCGAAAGCATAGGGAGCAAACAGGATTAGAAACCCTTGTAGTCC
>Otu4308
CCAGCCTATGGGGGGCAGCAGTCGAGAATCTTTCGCAATGGACGAAAGTCTGACGAAGCG
ACGCCTCGTGAGTGATGAAGGCCTACGGGTTGTAAAGCTCTTTTGTCAGGGAACAAGAAA
AGAGGGTTAATAGCCTTCCATTTGAGTGTACCTGAAGAATAAGCACCGGCTAACTCCGTG
CCAGCAGCCGCGGTAATACGGAGGGTGCAAGCATTACTCGGAATAACTGGGCGTAAAGGG
CGCGTAGGCGGCCCTGTAAGTCGGGAGTAAAATTCCAGAACTTAATTCTGGAGCTGCTCT
CGAAACTACATGGGCTTGAGGTTAGGATGTGAAAGTAGAATTCCACGTGTAGCGGTGAAA
TGCGTAGATATGTGGAAGAAGACCTGTAGCGAAAGCGACTTTCAGAACTACACCTGACGC
TGAGGCGCGAAAGCGTGGGGAGCAAACAGGATTAGAAACCCTAGTAGTCC
>Otu4309

CCAGCCTATGGGTGGCTGCAGTAGGGAATCTTCCACAATGGGCGAAAGCCTGATGGAGCA
ACGCCGCGTGCAGGATGAAGGCCTTCGGGTCGTAAACTGCTTTTATCTGTGACGATTATG
ACGGTAGCAGATGAATAAGGATCGGCTAACTCCGTGCCAGCCAGCCTATGGGACGCACCA
GCGGTAATACGTAGACCTCAAGCGTTATCCGGATTTATTGGGCGTAAAGCGCGTGTAGGT
GGCTTCGCGCGTCTTCTGTTAAAGCCCAGGGCCTAACCCTGGAAGTGCAGGAGATACGGC
GGAGCTCGAGGAGGTTAGAGGTGCATGGAACGCACGGTGTAGGGGTGAAATCCGTTGATA
TCGTGCGGAATACCAAAGGCGAAGGCATTGCACTGGGACCTTCCTGACACTGAGACGCGA
AAGCGTGGGGAGCAAAAAGGATTAGATACCCTAGTAGTCC
>Otu4311
CCAGCCTATGGGAGGCACCAGTGGGGAATCTTGCGCAATGGACGAAAGTCTGACGCAGCG
ACGCCGCGTGGGTGAAGAAGGCCTTCGGGTTGTAAAGCCCTGTCGAGTGAGACAAATGGT
ACGTGGAGTAACTGCCACGTATTTGATGGTATCACTAAAGGAAGCACCGGCTAACTCTGT
GCCAGCAGCCGCGGTAATACAGAGGGTGCAAGCGTTGTTCGGAATTATTGGGCGTAAAGG
GCAAGTAGGTGGTCTCAAAAGTTTACTGTGAAATCCCTGGGCTTAACCCAGGAACTGCGG
TGGATACTCTGAGACTTGAGTGCTGGAGGGGTGCGTGGAATTCCCGGTGTAGCGGTGAAA
TGCGTAGATATCGGGAGGAACACCAGAGGCGAAAGCGGCGCACTGGACAGCAACTGACAC
TCAATTGCGAAAGCGTGGGGAGCAAACAGGATTAGAGACCCTCGTAGTCC
>Otu4315
CCAGCCTACGGGATGCACCAGTTGGGAATCTTGCACAATGGGGGAAACCCTGATGCAGCG
ACGCCGCGTGGAGGATGACGGATCTAGGTCTGTAAACTCCTTTTGCACGGAAAGACTTAG
GACGGTACCGTGCGAATAAGCACCGGCTAACTACGTGCCAGCAGCCGCGGTAAGACGTAG
GGTGCAAGCGTTGTCCGGATTTACTGGGCGTAAAGAGCGCGTAGGCGGTCTGTTAAGTGA
GAAGTGAAATCTCCAGGGCTCAACCCGGAAACTGCTTTTCATACTGGCAGACTAGAGGGA
TGCAGAGGTTTGTGGAATTCCTGGTGTAGCGGTGAAATGCGTTGATATCAGGAGGAACAC
CCATGGCGAAGGCAGCAAACTGGGCATCATCTGACGCTGAGGCGCGAAAGCGTGGGTAGC
AAACAGGATTAGATACCCTAGTAGTCC
>Otu4317
CCAGCCTATGGGTCGCAGCAGTAGGGAATATTGCGCAATGGCCGAAAGGCTGACGCAGCG
ACGCCGCGTGGAGGATGAAGTCCGTTAGGATGTAAACTCCTTTTGCAGGGGATGAATGTT
CCGGTTCATTCGGAATTGACCGTACCTTGCGAATAAGGATCGGCTAACTACGTGCCAGCA
GCCGCGGTAATACGTAGGATCCGAGCGTTGTCCGGATTTACTGGGTGTAAAGGGTGTGTA
GGTGGATTTCTAAGTCGGTGGTGAAATCCCGCGGCTTAACCGCGGAATTGCTTCCGATAC
TGGAGATCTTGAGTTCGGCAGAGGGAGATGGAATTCATGGTGTAGCGGTGAAATGTGTAG
ATATCATGAAGAACACCGGTAGCGAAGGCGGTCTCCTGGTCCGATACTGACACTGAGGCG
CGAAAGCGTGGGGAGCAAACAGGATTAGAGACCCCGGTAGTCC
>Otu4318
CCAGCCTACGGGGGGCTCCAGTGGGGAATTTTGTGCAATGGGCGAAAGCCTGACACAGCG
ACACCGCGTGAGCGAGGAAGCCCTTTGGGGTGTAAAGCTCTGTCAGCAGGAACGAATAAA
TGACGGTACCTGCAGAGGAAGCATCGGCTAACTACGTGCCAGCAGCCGCGGTAAGACGTA
GGATGCGAGCGTTGTCCGGATTTATTGGGCGTAAAGAGTTCGTAGGCGGTTTGTTAAGTC
TGATGTTAAAGATCAGGGCTCAACCCTGGGAGTGCATTGGATACTGGCAGACTTGAGTAT
GGTAGAGGCGAGTGGAATTCCCAGTGTAGCGGTGAAATGCGTAGATATTGGGAAGAACAC
CGGTGGCGCAAGCGACTCGCTGGGCCATTACTGACGCTGAGGAACGAAAGCTAGGGGAGC
GAATGGGATTAGAAACCCGGGTAGTCC
>Otu4319
CCAGCCTACGGGTTGCACCAGTGAGGAATTTTGCTCAATGGGCGAAAGCCTGAAGCAGCG
ACGCCGCGTGAATGATGAAGTCCTTCGGGATGTAAAGTTCTGTCAGCAGGGACAATTATG
ATGGTACCTGCAGAGGAAGCACCGGCAAACTATGTGCCAGCAGCCGCGGTAATACATAGG
GTGCAAGCGTTGTCCGGATTTACTGGGCGTAAAGAGCGTGTAGGCGGTATGATAAGTCAA
ACTTTAAAATCTCTTGGCTCAACCAGAGATCGGGTTTGATACTGTCGTGCTAGAGAATGG
GAGAGGAAAGTAGAACTAACAGTGTAGCGGTGAAATGCGTAGATATTGTTAGGAATACCC
GTGGCGAAGGCGGCTTTCTGGACCATTTCTGACGCTGAGACGCGAAAGCGTGGGGATCAA
ACAGGATTAGAAACCCGCGTAGTCC
>Otu4320
CCAGCCTACGGGTGGCTCCAGCGACGAATCTTCTGCAATGGGCGAAAGCCTGACAGAGCA
ATGCCGCGTGTGGGATGAAGCGTCTATGACGTGTAAACCACTGTCAGGGGGCGGAACCAA
TGATTGCCCCCAGAGGAAGAGCCGGCTAACCCTGTGCCAGCAGCCGCGGTAATACAGGGG
GCTCGGGCGTTACGCGGAATCACTGGGCTTAAAGGGTGCGTAGGTGGTTCGCAAAGCATT

ACGTGAAAGCCCCGGGCTCAACCCGGGAACAGCGCGATGAACTGGCGAACTTGAGGCAAG
TAGGGGCCGACGGAACAGTGGGTGGAGCGGTGAAATGCTTAGAGATCCACTGGAACGCCG
ATGGTGAAGACGGTCGGCTGGGCTTGTCCTGACACTGAGGCACGAAAGCGTGGGGAGCAA
ACAGGATTAGAAACCCCGGTAGTCC
>Otu4322
CCAGCCTACGGGGTGCTGCAGTAACGAATCTTCCGCAATGCAGGAAACTGTGACGGAGCG
ACGCCGCGTGTGGGACGAAGTTCTTCGGAATGTAAACCACTGTCAGGGGAAAGAAAGTTC
TGATCTACCCCAGAGGAAGGCACGGCTAACTCTGTGCCAGCAGCCGCGGTAAGACAGAGG
TGCCGAGCGTTAGGCGGAATCACTGGGCTTAAAGCGTGTGTAGGCGGGTTCGTAAGTACC
TTGTGAAATCCCACGGCTTAACCGTGGAACTGCTTGGTATACTGCGAGTCTTGAGTCATT
CAGGGGCAACCGGAACAAATGGTGGAGCGGTGAAATGCGTAGATATCATTTGGAACGCCA
ATGGTGAAAACAGGTTGCTGGGGATGTACTGACGCTGAGACACGAAAGCCAGGGGAGCAA
ACGGGATTAGATACCCGAGTAGTCC
>Otu4326
CCAGCCTATGGGAGGCACCAGTGGGGAATATTGGACAATGGGGGCAACCCTGATCCAGCA
ATGCCGCGTGTGTGAAGAAGGCCTTCGGGTTGTAAAGCACTTTAGGCGGGGAAGAAGGTA
TGTATTTTAAGAGATAACATATTTGACGGTACCCGCAGAATAAGCACCGGCTAACTCTGT
GCCAGCAGCCGCGGTAATACAGAGGGTGCAAGCGTTAATCGGATTTACTGGGCGTAAAGG
GTGCGTAGGTGGTTGTGTAAGTGAGATGTGAAATCCCCGGGCTTAACCTGGGAACTGCAT
CTTATACTGCGCAGCTGGAGTACATGAGAGGGTAGTGGAATTTCCGGTGTAGCGGTGAAA
TGCGTAGATATCGGAAGGAACACCAGTGGCGAAGGCGGCTACCTGGCATGATACTGACAC
TGAGGCACGAGAGCGTGGGGATCAAACAGGATTAGAAACCCCAGTAGTCC
>Otu4336
CCAGCCTACGGGGTGCAGCAGTAGGGAATATTGCGCAATGGAGGAAACTCTGACGCAGCG
ACGCCGCGTGGGTGACGAAGGCTTTCGGGTCGTAAAGCCCTGTCGGAAGGGACGAAAAAC
GTAATGGCTAACATCCATGACGATTGACGGTACCTTCAAAGGAAGCACCGGCTAACTACG
TGCCAGCAGCCGCGGTAATACGTAGGGTGCTAGCGTTGTTCGGAATCATTGGGCGTAAAG
CGCGTGTAGGCGGTTAGGTAAGTCGAATGTGAAATCCCTGGGCTCAACCGAGGAAGTGCA
TACGAAACTGCCTGGCTAGAGGACGGTAGAGGAAGGTGGAATTCCTAGTGTAGAGGTGAA
ATTCGTAGATATTAGGAGGAATACCGGTGGCGAAGGCGGCCTTCTGGGCCGATCCTAACG
CTGAGACGCGAAAGCGTGGGGAGCAAACAGGATTAGATACCCTGGTAGTCC
>Otu4338
CCAGCCTACGGGGCGCTGCAGTGGGGAATATTGGACAATGGGGGCAACCCTGATCCAGCA
ATGCCGCGTGAGTGATGAAGGCCTTAGGGTTGTAAAGCTCTTTTGTCTGTGATGATAATG
ACAGTAGCAGACGAATAAGCCCCGGCTAACTTCGTGCCAGCAGCCGCGGTAATACGAAGG
GGGCTAGCGTTGTTCGGAATAACTGGGCGTAAAGGGCGCGTAGGCGGTTAGATACGTCTG
ATGTGAAAGTCCTGGGCTTAACCTAGGGACTGCATTGGATACGGTTTAGCTTGAGTGCGA
GAGGGGAAAATGGAATTGCGCGTGTAGAGGTGAAATTCGTAGAGATGCGCAAGAACACCG
GTGGCGAAGGCGATTTTCTGGCTCGCAACTGACGCCGAGGCGCGAAAGCGTGGGGAGCAA
ACAGGATTAGAAACCCGGGTAGTCC
>Otu4339
CCAGCCTATGGGGGGCTGCAGTGGGGAATATTGGACAATGGGGGGAACCCTGATCCAGCA
ATGCCGCGTGTGTGAAGAAGGCCTTAGGGTTGTAAAGCACTTTCAGTGGGGAGGAAGGAG
TGTGCGCTAATACCGTACATTATTGACGTTACCCACAGAAGAAGCACCGGCTAACTCTGT
GCCAGCAGCCGCGGTAATACAGAGGGTGCAAGCGTTAATCGGAATGACTGGGCGTAAAGC
GTGTGTAGGTGGTTAGATAAGTTAGATGTGAAATCCCCGGGCTTAACCTGGGAACTGCGT
TTAAGACTGGTTAGCTAGAGTACTGTAGTGGATAGTGGAATTTCCAGTGTAGCGGTTAAA
TGCGTAGATATTGGAAGGAACACCAGTGGCGAAGGCGACTATCTGGACAGATACTGACAC
TGAGACACGAAAGCGTGGGGAGCAAACAGGATTAGAGACCCCCGTAGTCC
>Otu4342
CCAGCCTATGGGGCGCTGCAGTCGAGAATCTTCCGCAATGGACGAAAGTCTGACGGAGCG
ACGCCGCGTGATTGATGAAGCTTTTCGGAGTGTAAAGATCTTTTATGAGGGAAGAAGTTT
ATTGACGGTACCTCATGAATAAGGGGCTCCTAATCTCGTGCCAGCAGGAGCGGTAATACG
AGAGCCCCGAGCGTTATCCGGAATTATTGGGCGTAAAGGGTGCGTAGGTGGTTCTGTTAG
TCGTTTGTCAAATCTCTGGGCTTAACCCAGAAACCGCGAACGAAACGGCAGAACTTGAGA
GTGTGAGGGGTGAATGGAACTCATGGTGTAGGGGTGAAATCCGTTGATATCATGGGGAAC
ACCAAAAGCGAAGGCAATTCACTGGCACATATCTGACACTCAAGCACGAAAGCGTGGGTA
GCGAATGGGATTAGAAACCCGCGTAGTCC

>Otu4344
CCAGCCTACGGGTCGCTGCAGTCGAGAATCTTCCACAATGGACGAAAGTCTGATGGAGCG
ACGCCGCGTGATTGATGAAGTCCTTCGGGACGTAAAGATCTTTTATGGGGGAGGAAGTAA
TTGACGTTACCCCATGAATAAGAGGCTCCTAACTCTGTGCCAGCAGGAGCGGTAATACTG
AGGCCTCAAGCATTATCCGGAATCACTGGGCGTAAAGGGTGTGTAGGCGGCGTTGTTAGT
CTTTCGTAAAAGCTCTGGGGCTTAACCTCGGAAATGCGGGAGAAACGGCAATGCTCGAGG
ACGCGAGAGGTATATGGAACTCATGGTGTAGGGGTGAAATCCGTTGATATCATGGGGAAC
ACCAAATGCGAAGGCAATATACTGGCGCGCTCCTGACGCTGAAACACGAAAGCGTGGGAA
TCGAACGGGATTAGATACCCCCGTAGTCC
>Otu4350
CCAGCCTATGGGAGGCTGCAGTCGAGAATATTCCCCAATGGACGAAAGTCTGAGGGAGCG
ACGCCGCGTGCAGGATGAAGGCCTTTGGGTCGTAAACTGCTTTTTTCAGGGAAGAATCAT
GACGGTACCTGAAGAATAAGAGGTTGCTAACTCTGTGCCAGCAGCAGCGGTAATACAGAG
ACCTCAAGCGTTATCCGGATTTATTGGGCGTAAAGGGTCCGCAGGTGGTCGTGCGCGTCG
ATTGTTAAATCCCCGAGCTCAACTCGGAAATCGCAGTCGATACGGCAAGACTAGAGGCCG
GAAGAGGTAAGCGGAATTGCCGGTGTAGTAGTAATATGCGTTCATATCGGCAAGAACACC
AAATGCGAAGGCAGCTTACTGGGACGCGCCTGACACTCAGGGACGAAAGCGTGGGGAGCG
AAAGGGATTAGAAACCCTAGTAGTCC
>Otu4355
CCAGCCTACGGGGGGCAGCAGTGGGGAATATTGCGCAATGGGCGAAAGCCTGACGCAGCG
ACGCCGCGTGTGCGATGAAGGTCTTCGGATCGTAAAGCACTGTCGCGGGGGAAAAAAGAA
ATGATGGTACCCCGAGAGGAAGCACCGGCTAACTCTGTGCCAGCAGCCGCGGTAATACAG
AGGGTGCGAGCGTTGTTCGGAATTATTGGGCGTAAAGCGCTGGTAGGTGGCCTGACAAGT
CGCGTGTGAAAGCTCCCGGCTCAACCGGGAAAGTGCGCGCGAAACTGGCGGGCTTGAGTG
CCGGAGAGGACCGCGGAATTCCCGGTGTAGAGGTGAAATTCGTAGATATCGGGAGGAACA
CCAGAGGCGAAGGCGGCGGTCTGGAAGGCGACTGACACTGAGCAGCGAAAGCGTGGGGAG
CAAACAGGATTAGAAACCCCTGTAGTCC
>Otu4358
CCAGCCTATGGGAGGCAGCAGTGGGGAATCTTGCGCAATGGGCGAAAGCCTGACGCAGCG
ACGCCGCGTGGGTGATGAAGGCCTTCGGGTTGTAAAGCCCTGTGGGGAGGGACGAATAAG
TCAGGGTGAACAATCCTGATGATGACGGTACCTTCTTAGCAAGCACCGGCTAACTCTGTG
CCAGCAGCCGCGGTAAGACAGAGGGTGCGAACGTTGTTCGGAATTACTGGGCGTAAAGCG
CGTGTAGGCGGCACGGTAAGTCGGGTGTGAAAGCCCGGGGCTCAACCCCGGAAGTGCATT
CGATACTACCAAGCTGGAGTCTCGGAGAGGCGGGTGGAACTCTCGGTGTAGAGGTGAAAT
TCGTAGATATCGAGAAGAACACCGGTGGCGAAGGCGGCCCGCTGGACGAAGACTGACGCT
GAGACGCGAAAGCGTGGGGAGCAAACAGGATTAGAGACCCCCGTAGTCC
>Otu4364
CCAGCCTACGGGTGGCTGCAGTGGGGAATATTGCGCAATGGGGGAAACCCTGACGCAGCA
ACGCCGCGTGAAGGATGAAGGTCTTCGGATTGTAAACTTCTTTGATTGGGGAAGAATAAT
GACGGTACCCAAAAAACAAGCCACGGCTAACTACGTGCCAGCAGCCGCGGTAATACGTAG
GTGGCGAGCGTTGTCCGGAATTACTGGGTGTAAAGGGCGTGTAGGCGGGGAGACAAGTCA
GATGTGAAATTCCAGAGCTCAACTCTGGCGCTGCATCTGAAACTGTATCTCTTGAGTGCT
GGAGAGGAAAGCGGAATTCCTAGTGTAGCGGTGAAATGCGTAGATATTAGGAGGAACACC
AGTGGCGAAGGCGGCTTTCTGGACAGTAACTGACGCTGAGGCGCGAAAGCGTGGGGAGCA
AACAGGATTAGATACCCGCGTAGTCC
>Otu4366
CAAGCCTATGGGGGGCTGCAGTGAGGAATCTTGGGCAATGAGGTAAACCTTGACCCAGCG
AGAATTCATGCACGATGAATGCGGTTAGTTTACTGTAAAGTGCTTTTGACGACGAAGATA
ATGACTGTAGTTGAGACGTAAGAAGCATCGGCTAACTCCGTGCCAGCAGCTGCGGTAAGA
CGGAGGATGCGGGCCTTATTCATTATGATTGGGTGTAAAGGGTGCGTAGATGGCTGTTGA
AATTTAAACAAAAAGCGTGGAGTATTCCTATGCGTGTGTTTTCTTATAGATTCAGCTAGT
GTTGAGGAAAGGTAGCGATAATTCCTTGTGTAAGGGTAAAATCTTAAGAGTCAAGGAAGA
ATACCAGCCGGCGGAGGCGCGTTTCCCGCCTCAACAGACGTTAAGGTACGGAAGCTATGG
AAGCAAACAGGATTAGATACCCCAGTAGTCC
>Otu4372
CCAGCCTACGGGGCGCTGCAGTCGAGGATCTTTCGCAATGGGCGCAAGCCTGACGAAGCG
ACGCTGCGTGAGCGATGAAGGCCTTCGGGTCGTAAAGCTCTTTCGCGCAAGATCAAGGGA
GATGTGTGAATAACACATTGCCTTGAGGGTACTGCGTAAAGAAGCACCGGCTAACTCCGT

GCCAGCAGCTGCGGTAATACGGAGAGTGCAAGCATTAATCGGAATTATTGGGCGTAAAGG
GCGCGTAGGCGGGAATGAAAGTCAGATGTGAAATTCCGAAGCTCAACTTCGGAGCTGCAT
TTGAAACTACAATTCTAGAGGATTGACGGAGAAAACGGAATTCCACGTGTAGCGGTGAAA
TGCGTAGATATGTGGAGGAACACCTGTGGCGAAAGCGGTTTTCTAGCTTATACCTGACGC
TGAGGCGCGAGAGCAAGGGGAGCAAACAGGATTAGAAACCCTAGTAGTCC
>Otu4373
CCAGCCTATGGGTCGCACCAGTAGGGAATATTGCGCAATGGAGGAAACTCTGACGCAGCG
ACGCCGCGTGGGTGATGAAGGTCTTCGGATCGTAAAGTCCTGTCGGATGGGAAGAAACAC
GGTATGGCTAATATCCATACCGCTTGACGGTACCATTAAAGGAAGCACCGGCTAACTACG
TGCCAGCAGCCGCGGTAATACGTAGGGTGCAAGCGTTGTTCGGAATCATTGGGCGTAAAG
CGCGTGTAGGTGGTTTGTTAAGTCGATTGTGAAATCCCTGGGCTCAACCGAGGAAGTGCA
GTCGAAACTAACTGACTAGAAGATGGTAGAGGAAGGTGGATTTCCAAGTGTAGAGGTGAA
ATTCGTAGATATTTGGAGGAATACCGGCGGCGAAGGCGGCCTTCTGGGCCATTCTTGACA
CTGAGACGCGAAAGCGTGGGTAGCAAACAGGATTAGAAACCCCCGTAGTCC
>Otu4375
CCAGCCTATGGGAGGCTGCAGTCGAGGATCTTCGGCAATGGGCGCAAGCCTGACCGAGCG
ACGCCGCGTGTGCGATGAAGGCCTTCGGGTTGTAAAGCACTGTCGAGGGGGAGAAAGTCC
GCAAGGATTGATCTATCCCTGGAGGAAGCACGGGCTAAGTTCGTGTCAGCAGCCGCGGTA
AGACGAACCGTGCGAACGTTGTTCGGATTCACTGGGCTTAAAGGGCGCGTAGGCGGGTGG
TCAAGTCAGGGGTGAAATCTTGCGGCTCAACCGCAAAAGTGCCTCTGATACTGGCCATCT
CGAGGGAGGTAGGGGCATGTGGAACTTCCGGTGGAGCGGTGAAATGCGTAGATATCAGAA
GGAACGCCGGTGGCGAAAGCGACGTGCTGGACCTCTTCTGACGCTGAGGCGCGAAAGCTA
GGGGAGCAAACGGGATTAGAGACCCCAGTAGTCC
>Otu4376
CCAGCCTATGGGGTGCTCCAGTGGGGAATATTGGACAATGGGCGAAAGCCTGATCCAGCC
ATGCCGCGTGAGTGATGAAGGCCTTAGGGTTGTAAAGCTCTTTTACCAGGGAAGATGATG
ACGGTACCTGGAGAATAAGCACCGGCTAACTTCGTGCCAGCAGCCGCGGTAATACGAAGG
GTGCTAGCGTTGTTCGGAATTACTGGGCGTAAAGCGCGTGTAGGCGGCTTACCAAGTCAG
AGGTGAAATGCCGGGGCTCAACCCCGGAACTGCCTTTGGAACTAGTAAGCTTCGAGTATT
GGAGGGGAGTGCGGAATTGCTAATGTAGAGGTGAAATTCGTAGATATTAGCAGGAACGCC
GGTGGCGAAGGCGGCACTCTGGACAATAACTGACGCTTAGGCGCGAAAGCGTGGGGATCA
AACAGGATTAGATACCCGAGTAGTCC
>Otu4377
CCAGCCTATGGGAGGCAGCAGTAGGGAATTTTGCGCAATGGACGAAAGTCTGACGCAGCG
ACGCCGCGTGAGGGATGAAGGTCTTCGGATTGTAAACCTCTGTTGGGTGGGAAGAAACTT
TTCCGGAATAATACGCCGGAAAATTGACGGTACCGCTTGAGGAAGCCCCGGCTAACTACG
TGCCAGCAGCCGCGGTAATACGTAGGGGGCAAGCGTTGTTCGGAATCACTGGGCGTAAAG
GGAGCGTAGGCGGAGAGGTAAGTTGGAAGTTTAATTCCCGGGCTCAACCCGGGACCAGCT
TTCAATACTGCCATTCTTGAGTATGGGAGAGGATGATGGAATTCCAGGTGTAGCGGTGGA
ATGCGTAGATATCTGGAAGAACACCAGTTGCGAAGGCGGTCATCTGGCCCAATACTGACG
CTGAGGCTCGAAAGCTAGGGGAGCAAACAGGATTAGAAACCCTAGTAGTCC
>Otu4386
CCAGCCTATGGGAGGCTCCAGTGGGGAATATTGGACAATGGGCGAAAGCCTGATCCAGCA
ATGCCGCGTGTGTGAAGAAGGTCTTCGGATTGTAAAGCACTTTCGACGGGGACGATGATG
ACGGTACCCGTAGAAGAAGCCCCGGCTAACTTCGTGCCAGCAGCCGCGGTAATACGAAGG
GGGCTAGCGTTGCTCGGAATCACTGGGCGTAAAGCGCACGTAGGCGGCTTTTTAAGTCAG
AGGTGAAATCCTGGAGCTCAACTCCAGAACTGCCTTTGATACTGAGGAGCTCGAGTCCGG
GAGAGGTGAGTGGAACTGCGAGTGTAGAGGTGAAATTCGTAGATATTGGGAAGAACACCG
GTGGCGAAGGCGGCTCACTGGCCCGTTACTGACGCTCAGGCGCGACAGCGTGGGGATCAA
ACAGGATTAGAAACCCCCGTAGTCC
>Otu4389
CCAGCCTATGGGTGGCTCCAGTGGGGAATCTTGCACAATGGGGGCAACCCTGATGCAGCG
ACGCCGCGTGAGCGATGAAGCCCTTCGGGGTGTAAAGCTCTTTCGGCAGGGAAGATAATG
ACGGTACCTGCAGAAGCAGCTGCGGCTAACTACGTGCCAGCAGCCGCGGTAATACGTAGG
CAGCAAGCGTTGTTCGGAGTTACTGGGCGTAAAGGGTGCGCAGGCGGCTATATAAGTCAG
ATGTGAAATACCTAAGCTTAACTTGGGGGGTGCATTTGATACTGTATGGCTAGAGTTAGT
CAGAGGGGGGTAGAATTCCTGGTGTAGCAGTGAAATGCGTAGAGATCAGGAGGAATACCA
ATGGCGTAGGCAGCCCCCTGGGACAAAACTGACGCTCATGTACGAAAGCGTGGGAAGCAA

ACAGGATTAGATACCCCCGTAGTCC
>Otu4391
CCAGCCTATGGGAGGCTCCAGTCGAGAATTTTTCTCAATGGGCGAAAGCCTGAAGGAGCG
ACGCCGCGTGGAGGATGAATGGCTTCGGCCCGTAAACTCCTGTCATTTGCGAACAAATTA
ATTCACCCAACACGTGGATTATTGATTGTAGCGGAAGAGGAAGGGACGGCTAACTCTGTG
CCAGCAGCCGCGGTAATACAGAGGTCCCAAGCGTTGTTCGGATTTACTGGGCGTAAAGGG
TGCGTAGGCGGTGAGGTAAGTCTGACGTGAAATCTCCGGGCCTAACCCGGAAACTGCGTT
GGATACTATCTCGCTAGAGGATTGGAAGGGAGACTGGAATACTTGGTGTAGCAGTGAAAT
GCGTAGATATCAAGCGGAACACCAGTGGCGAAGGCGAGTCTCTGGACAATTCCTGACGCT
GAGACACGAAAGCGTGGGTAGCAAACAGGATTAGAAACCCGGGTAGTCC
>Otu4393
CCAGCCTACGGGGTGCAGCAGCGGGGAATATTGGACAATGGGGGCAACCCTGATCCAGCG
ATGCCGCGTGGGTGAAGAAGGCCTTCGGGTTGTAAAGCCCTTTAGGTTGGGAAGAAGAGT
GTGTACTGAATAAGTGCATGCTTTGACGGTACCAACAGAATAAGCACCGGCAAACTCTGT
GCCAGCAGCCGCGGTAATACAGAGGGTGCGAGCGTTAATCGGATTTACTGGGCGTAAAGG
GCGCGTAGGCGGTTGTGTGAGTGTGATGTGAAAGCCCCGGGCTTAACCTGGGAAGTGCAT
CGCAAACGACACGACTGGAGTATAAGAGAGGGTGGCGGAATTTCCGGTGTAGCGGTGAAA
TGCGTAGAGATCGGAAGGAACGTCAATGGCGAAGGCAGCCACCTGGCTTAATACTGACGC
TGAGGCGCGAAAGCGTGGGGATCAAACAGGATTAGATACCCTAGTAGTCC
>Otu4394
CCAGCCTACGGGACGCTGCAGTCGAGAATTTTTCACAATGGGCGCAAGCCTGATGGAGCG
ACGCCGCGTGGAGGATGAAGGTTCTTGGATTGTAAACTCCTGTCACTGCAGAACAAGGAT
ACACATGTGAATAGCGCGTGTATTTGATGGTATGCGGAGAGGAAGGGACGGCTAACTCTG
TGCCAGCAGCCGCGGTAAGACAGAGGTCCCAAGCGTTGTTCGGATTCATTGGGCGTAAAG
GGTGTGTAGGAGGTCGGGTTAGTCAGGTGTGAAATCTCCCAGCTTAACTGGGAAACTGCG
CTTGATACTGCCCGGCTAGAGGATCGGAGGGGGTAACGGAATTCATGGTGTAGCAGTGAA
ATGCGTAGATATCATGAGGAACACCGGTGGCGAAGGCGGTTACCTGGAAGATTCCTGACT
CTGAAACACGAAAGCCAGGGGAGCAAACAGGATTAGATACCCTAGTAGTCC
>Otu4395
CCAGCCTACGGGAGGCTCCAGTGAGGAATCTTGGGCAATGAGCGAAAGCTTGACCCAGCG
AGAATTTGTGTGTGAGGAAGGCGTTGAGTTGTAAAGCACTTTCAGGTAGGAAGATAATGA
CTGTACTACAGCAGAAGAAGTACTGGCTAAGTCTGTGCCAGCAGCCGCGGTAATACAGAC
AGTGCAAGCGTTATTCAGATTTAATGGGCGTAAAGGGTATGTAGACGGTTCTAAAAACTA
GATAAAAAATGCTAGATTTATAACTTTAGCTAAATATCTAAAGATTAAAGAACTTTGAGT
TAATTAGAGGTTAGACGAATTTGTTGTGTTAACAGTAAAATGTTATTATATAATAAGGAC
GACCAAAGGTGAAGACATCTTTCTGCAATTAACTAACGTTGAGGTACGAAGGCATGGGGA
GCAAACAGGATTAGAAACCCGCGTAGTCC
>Otu4396
CCAGCCTACGGGTCGCTCCAGTGGGGAATATTGGACAATGGGGGAAACCCTGATCCAGCC
ATGCCGCGTGAATGATGAAGGCCTTCGGGTTGTAAAGTTCTTTTGCAGGGGACGATGATG
ACGGTACCCTGAGAATAAGCTCCGGCTAACTTCGTGCCAGCAGCCGCGGTAATACGAAGG
GAGCTAGCGTTGTTCGGATTACTGGGCGTAAAGGGCGCGTAGGCGGTTAGACAAGTTGGA
AGTGAAAGCCCTGGGCTCAACCCGGGAATTGCTTTCAAAACTGTCTTACTTGAATTCGGT
AGAGGTTGGTAGAATTCCCAGTGTAGAGGTGAAATTCGTAGAGATTGGGAAGAATACCCG
TGGCGAAGGCGGCCAACTGGACCGACATTGACGCTGAGGCGCGAAAGCGTGGGGAGCAAA
CAGGATTAGAAACCCGCGTAGTCC
>Otu4400
CCAGCCTACGGGTGCAGCAGTAAGGAATATTGGTCAATGGGCGAAAGCCTGAACCAGCCA
TGCCGCGTGCAGGAAGACGGCCCTACGGGTTGTAAACTGCTTTTGCAGGGGAATAAACCC
CCGGTCGTGACCGGGGTTGAATGTACTCTGAGAATAAGGATCGGCTAACTCCGTGCCAGC
AGCCGCGGTAATACGGAGGATCCGAGCGTTATCCGGATTTATTGGGTTTAAAGGGTGCGT
AGGCGGCTTTTTAAGTCAGGGGTGAAAGACGGTAGCTTAACTATCGCAGTGCCTTTGATA
CTGAAGAGCTCGAATACACTAGAGGTAGGCGGAATGTGACAAGTAGCGGTGAAATGCATA
GATATGTCACAGAACACCAATTGCGAAGGCAGCTTACTATGGTGCAATTGACGCTGAGGC
ACGAAAGCGTGGGGATCAAACAGGATTAGAGACCCTCGTAGTCC
>Otu4402
CAGCCTACGGGTGGCTGCAGTCGAGGATCTTCGGCAATGGGCGCAAGCCTGACCGAGCGA
CGCCGCGTGTGCGATGAAGGCCTTCGGGTTGTAAAGCACTGTCGAGGGGAAGAAAGCTCC

GCAAGGGGTTGATCGACCTCTGGAGGAAGCACGGGCTAAGTTCGTGCCAGCAGCCGCGGT
AAGACGAACCGTGCGAACGTTGTTCGGATTCACTGGGCTTAAAGGGCGCGTAAGCGGGCC
ATCAAGTCAGGGGTGAAATCTTTCGGCTCAACCGGAAAAGTGCCTTTGATACTGGTGGTC
TCGAGGGAGGTAGGGGCATGCGGAACTTCCGGTGGAGCGGTGAAATGCATAAATATCGGA
AGGAACGCCGGTGGCGAAAGCGGCGTGCTGGACCTCTTCTGACGCTGAGGCGCGAAAGCT
AGGGGAGCAAACGGGATTAGAAACCCGAGTAGTCC
>Otu4404
CCAGCCTACGGGACGCTGCAGTGGGGAATATTGGGCAATGGGCGCAAGCCTGATCCAGCA
ATGCCGCGTGAGTGAGGAAGGCCTTAGGGTTGTAAAGCTCTTTCGCACGTGACGATGATG
ACGGTAGCGTGAGAAGAAGCCCCGGCTAACTTCGTGCCAGCAGCCGCGGTAATACGAAGG
GGGCGAGCGTTGTTCGGATTTACTGGGCGTAAAGGGCGCGCAGGCGGCTATCTTTGTCAG
GCGTGAAAGCCCCGGGCTTAACCTGGGAACTGCGTTTGGGACGGGATGGCTAGAGATCGG
GAGAGGAGAGTGGAATTCCCAGTGTAGAGGTGAAATTCGTAGATATTGGGAAGAACACCG
GTGGCGAAGGCGGCTCTCTGGACCGATACTGACGCTGAGGCGCGAAAGCGTGGGGAGCAA
ACAGGATTAGATACCCCCGTAGTCC
>Otu4409
CCAGCCTACGGGTTGCTCCAGTGGGGAATATTGGACAATGGGCGCAAGCCTGATCCAGCC
ATGCCGCGTGAGTGATGACGGCCTTAGGGTTGTAAAGCTCTTTCGCACGCGACGATAATG
ACGGTAGCGTGAGAAGAAGCCCCGGCTAACTTCGTGCCAGCAGCCGCGGTAATACGAAGG
GGGCTAGCGTTGTTCGGAATTACTGGGCGTAAAGCGTGCGCAGGCGGTTATGTAAGACAG
ATGTGAAATGCCCGGGCTTAACCTGGGAACTGCATTTGTGACTGCATGGCTAGAATCTAG
CAGAGGGGGGTAGAATTCCACGTGTAGCAGTGAAATGCGTAGATATGTGGAGGAATACCG
ATGGCGAAGGCAGCCCCCTGGGCTAAGATTGACGCTCATGCACGAAAGCGTGGGGAGCAA
ACAGGATTAGAGACCCTAGTAGTTCC
>Otu4411
CCAGCCTATGGGATGCTGCAGTAGGGAATATTGCGCAATGGGGGAAACCCTGACGCAGCG
ACGCCGCGTGAGCGATGAAGGCCTTCGGGTCGTAAAGCTCTGTTGAACGGGACAAAAAAA
ATGATGGTACCGTTCGAGAAAGGATCGGCTAACTTCGTGCCAGCAGCCGCGGTAAGACGA
GGGATCCTAGCGTTGTTCGGAATCATTGGGCGTAAAGCGTGCGTAGGTGGCCTGGTAAGT
CAGGTGTGAAAGCCCCGAGCTCAACTCGGGAAGTGCATCTGATACTGCTGGGCTTGAGTA
CTGAAGAGGGTAGTAGAATTCCTGGTGTAGTGGTGAAATACGTAGATATCAGGAGGAATA
CCGGTGGCGAAGGCGGCTACCTGGTCATGTACTGACACTGAGGCACGAAAGCGTGGGGAT
CAAACAGGATTAGATACCCCAGTAGTCC
>Otu4415
CCAGCCTACGGGTCGCAGCAGTGGGGAATTTTGGACAATGGGCGAAAGCCTGATCCAGCG
ACGCCGCGTGAGTGAAGAAGGCCTTCGGGTTGTAAAGCTCTTTCGTTAGGGAAGATAATG
ACGGTACCTAAATAAGAAGCTCCGGCTAACTTCGTGCCAGCAGCCGCGGTAATACGAAGG
GAGCTAGCGTTACTCGGAATCACTGGGCGTAAAGCGTGCGTAGGCGGCTTTGTAAGTCAG
AAGTGAAATCCAGGGGCTCAACCCCAGAACTGCTTTTGAAACTGCAAGGCTAGAGTATCA
TAGGGGATGGCGGAATTTCTAGTGTAGAGGTGAAATTCGTAGATATTAGAAGGAACACCG
GTGGCGAAGGCGGCCATCTGGATGATAACTGACGCTGTTGCACGAAAGCGTGGGGAGCAA
ACAGGATTAGATACCCGCGTAGTCC
>Otu4422
CCAGCCTACGGGTGGCACCAGTGGGGAATTTTGCGTAATGGGCGAAAGCCTGACGCAGCA
ACGCCGCGTGTGTGATGAAGGTCTTCGGATCGTAAAGCACTTTCGACCGGGAAGAAACGC
TGTTGGCTAACATCCAGCAGTCTGACGGTACCGGGAGAAGAAGCACCGGCTAACTCTGTG
CCAGCAGCCGCGGTAATACAGAGGGTGCAAGCGTTGTTCGGAATTATTGGGCGTAAAGCG
CGTGTAGGCGGCTTAGCAAGTCGGGTGTGAAAGCCCTCGGCTCAACCGAGGAAGTGCGCC
CGAAACTGCAGAGCTTGAGTACCGGAGAGGATGGCGGAATTCCCCAAGTAGAGGTGAAAT
TCGTAGATATGGGGAGGAACACCGGTGGCGAAGGCGGCCATCTGGACGGATACTGACGCT
GAGACGCGAAAGCGTGGGGAGCAAACAGGATTAGAAACCCCGGTAGTCC
>Otu4423
CCAGCCTACGGGGGGCAGCAGTTAGGAATCTTGCGCAATGGGCGAAAGCCTGACGCAGCC
ATGCCGCGTGAATGATGAAGGTCTTAGGATTGTAAAATTCTTTCAGCGGGGACGATAATG
ACGGTACCTGTAGAAGAAGCTCCGGCTAACTTCGTGCCAGCAGCCGCGGTAATACGAAGG
GGGCTAGCGTTGTTCGGAATTACTGGGCGTAAAGCGCGCGCAGGCGGCTTCTCAAGTCAG
GGGTGAAAGCCCAGAGCTCAACTCTGGAACTGCCCTTGAAACTGTGGAGCTTGAGTACGG
GAGAGGTGAGTGGAATTCCCAGTGTAGAGGTGAAATTCGTAGATATTGGGAAGAACACCG

GTGGCGAAGGCGGCTCACTGGCCCGTTACTGACGCTCAGGCGCGACAGCGTGGGGATCAA
ACAGGATTAGAGACCCTCGTAGTCC
>Otu4441
CCAGCCTACGGGTGGCACCAGTGGGGAATTTTGCGCAATGGGGGAAACCCTGACGCAGCA
ACGCCGCGTGGAGGATGAAGTACTTCGGTACGTAAACTCCTTTCGATCGGGACGATTATG
ACGGTACCGGAAGAAGAAGCCCCGGCTAACTTCGTGCCAGCAGCCGCGGTAATACGAGGG
GGGCAAGCGTTGTTCGGAATTATTGGGCGTAAAGGGTGTGTAGGCGGTTTGGTAAGTCTC
TTGTGAAATCTTCAGGCTCAACTTGAAGACTGCAAGGGAAACTGCCGGGCTTGAGTATGG
GAGAGGTGAGTGGAATTCCTGGTGTAGCGGTGAAATGCGTAGATATCAGGAGGAACACCT
GTGGCGAAAGCGGCTCACTGGACCATAACTGACGCTGATGCACGAAAGCTAGGGGAGCAA
ACAGGATTAGAAACCCCCGTAGTCC
>Otu4442
CCAGCCTACGGGGTGCACCAGTAGGGAATATTGGGCAATGGAGGGAACTCTGACCCAGCC
ATGCCGCGTGCAGGACGAAGGAGCTATGCTCTGTAAACTGCTTTAGCAGGGGAAGAAAAG
GCGTCTGCGGACGAGATTGACGGTACCCTGAGAATAAGCACCGGCTAACTACGTGCCAGC
AGCCGCGGTAATACGTAGGGTGCAAGCGTTGTCCGGATTTATTAGGTTTAAAGGGTGCGT
AGGCGGCTTTATAAGTCAGTGTTTAAAGCACATAGCTCAACTATGGAACTGGCATTGATA
CTGTAGAGCTTGAGTAGAAACGGGGCAGGCGGAATTTATGGTGTAGCGGTGAAATGCATA
GATACCATAAAGAACACCGATAGCGAAGGCAGCTTGCCAGGATCTAACTGACGCTGAGGC
ACGAAAGCGTGGGGATCAAACAGGATTAGATACCCCAGTAGTCC
>Otu4443
CCAGCCTATGGGTGGCACCAGTAAGGGATATTGCGCAATGGGCGAAAGCCTGACGCAGCA
ACGCCGCGTGGAGGAAGAAGGCCTTCGGGTCGTAAACTCCTTTGGACGGAGACGAGGAAG
GACGGTATCCGTCGAACAAGTCACGGCTAACTACGTGCCAGCAGCCGCGGTAAAACGTAG
GTGGCAAGCGTTATCCGGATTTACTGGGCGTAAAGCGTGTGCAGGCTGTTCACCAAGTGG
TGTATGAAATCGCTCAGCTCAACTGGGCGCGGTTATGCCAGACTGGTGGACTGGAGTCTG
AAAGAGGGTGGTGGAATTCCGGGTGTAGTGGTGAAATGCGTAGAGATCCGGAGGAACCCC
AGAGGCGAAGGCGGCCACCTGGTTCAGCACTGACGCTCAGACACGACAGCATGGGGAGCG
AACGGGATTAGATACCCCTGTAGTCC
>Otu4449
CCAGCCTATGGGAGGCACCAGTGGGGAATATTGGGCAATGGACGAAAGTCTGACCCAGCC
ATGCCGCGTGAATGATGAAGGCTTTAGGGTTGTAAAGTTCTTTCGCCGATGAAGATAATG
ACGGTAGTCGGAGAAGAAGCCCCGGCTAACTTCGTGCCAGCAGCCGCGGTAATACGAAGG
GGGCTAGCGTTGTTCGGAATTACTGGGCGTAAAGCGCGCGTAGGCTGTGCTACAAGCCAG
AGGTGAAATCCCGGAGCTTAACTTCGGAACTGCCTTTGAAACTGTAGTGCTGGAATCTCA
GAGGGGGTAGCGGAATTCCAAATGTAGGGGTGAAATCCGTAGATATTTGGAGGAACACCG
GTGGCGAAGGCGGCTACCTGGCTGAGTATTGACGCTGAGGCGCGAAAGCGTGGGGAGCAA
ACAGGATTAGAGACCCCCGTAGTCC
>Otu4453
CCAGCCTATGGGTGGCACCAGTCGAGAATCTTCCACAATGGACGAAAGTCTGATGGAGCG
ACGCCGCGTGCAGGATGAAGTGCTTCGGTATGTAAACTGCTTTTACGGGGGACGAAGTTT
ATTGACGGTACCCCGAGAATAAGAGGTTGCTAAACTCGTGCCAGCAGCAGCGGTAATACG
AGTGCCTCAAGCGTTATCCGGAATTATTGGGCGTAAAGGGTGTGTAGGTGGTCATGTTAG
TCTTTTGTTAAATTCTTCGGCTCAACCGGGGGCATGCAAAGGAAACGGCACGACTAGAGG
ATGCGAGAGGCATATGGAACTCATGGTGTAGGGGTGAAATCCGTTGATATCATGGGGAAC
ACCGAAAGCGAAGGCAGTATGCTGGCGCATTCCTGACACTCAAACACGAAAGCGTGGGTA
GCGAATGGGATTAGATACCCCGGTAGTCC
>Otu4454
CCAGCCTATGGGGGGCTCCAGTTTCGAATCATTCACAATGGGCGCAAGCCTGATGGTGCG
ACGCCGCGTGAGGGATGAAGGCCTTCGGGTTGTAAACCTCTGTCACCGGGGAAGAAACGT
TTCAAGTTAACAGCTTGAAGCCTGACTTAACCCGGAGAGGAAGCAGTGGCTAACTCTGTG
CCAGCAGCCGCGGTAATACAGAGACTGCAAGCGTTATTCGGATTCACTGGGCGTAAAGGG
TGCGCAGGCGGTTGAGTGTGTCAGATGTGAAATCCCGAGGCTCAACCTCGGAACTGCGTC
TGAAACTACTCGACTAGAGTACTGGAGAGGGTAACGGAATTCACGGTGTAGCAGTGAAAT
GCGTAGATATCGTGAGGAACACCAGAGGCGAAGGCGGTTACCTGGACAGTTACTGACGCT
CAGGCACGAAAGCATGGGGAGCAAAAGGGATTAGAAACCCCAGTAGTCC
>Otu4456
CCAGCCTACGGGTGGCTGCAGTGGGGAATCTTGCGCAATGGGCGAAAGCCTGACGCAGCG

ACGCCGCGTGGGTGATGAAGGCCTTCGGGTTGTAAAGCCCTGTGGGGAGGAGTGAATAAG
GCTTGGTTAATAGCCGGGTCGATGACGGTACCTCCTTAGCAAGCTCCGGCTAACTCTGTG
CCAGCAGCCGCGGTAATACAGAGGGTGCGAACGTTGTTCGGAATTACTGGGCGTAAAGCG
CGTGTAGGCGGTCTGAAAAGTCGGATGTGAAAACCCTGGGCTCAACCCGGGAAGCGCATT
CGATACTGTCGGACTGGAGTCCCGGAGAGGATGGTGGAACTCTCGGTGTAGAGGTGAAAT
TCGTAGATATCGGGAAGAACATCGGTGGCGAAGGCGGCCATCTGGACGGTGACTGACGCT
GAGACGCGAAAGCGTGGGGAGCAAACAGGATTAGAAACCCTAGTAGTCC
>Otu4457
CCAGCCTATGGGGGGCAGCAGTGAGGGATATTGCACAATGGGGGAAACCCTGATGCAGCG
ACGCCGCGTGGGTGATGAAGGGCTTCGGCTCGTAAAACCCTTTTGCCACTGACGAGAACG
GACGGTAGGTGGCGAATAAGCCTCGGCTAACTACGTGCCAGCAGCCGCGGTAAAACGTAG
GAGGCAAGCGTTATCCGGAGTTACTGGGCGTAAAGCGTGCGTAGGCGGTGGTGTAAGTGG
GACGTGAAAGCTTCCGGCTAAACTGGAAGAGGACGACTCAGACTGCACGACTAGAACGGG
TCAGAGGTTCGCGGAATTCGGGGTGTAGCGGTGAAATGTGTAGAGATCCCGAGGAACACC
AGTGGTGCAAACGGCGGACTGGGACTTTGTTGACGCTGAGGCACGAAAGCGTGGGGAGCG
AACGGGATTAGATACCCCCGTAGTCC
>Otu4460
CCAGCCTATGGGTGGCTGCAGTCGAGAATTTTTCTCAATGGGCGAAAGCCTGAAGGAGTG
ACGCCGCGTGGGGGATGAAGGGCTTCGGCTCGTAAACCCCTGTCATTTGCGAACAAACCT
TGCAGTTTAACAGATTGCAAGCTGATTGTAGCAGAAGAGGAAGGGACGGCTAACTCTGTG
CCAGCAGCCGCGGTAATACAGAGGTCCCAAGCGTTGTTCGGATTCACTGGGCGTAAAGGG
TGCGTAGGTGGCAGGGTAAGTTTGATGTGAAATCTCCGGGCTTAACCCGGAAACTGCATT
GAATACTATCCTGCTAGAGGATTGGAGGGGGGACTGGAATACTTGGTGTAGCAGTGAAAT
GCGTAGATATCAAGTGGAACACCAGTGGCGAAGGCGAGTCCCTGGACAATACCTGACACT
GAGGCACGAAAGCTAGGGGAGCAAACAGGATTAGATACCCCTGTAGTCC
>Otu4461
CCAGCCTATGGGAGGCTGCAGTCGAGGATCATTCGCAATGGGCGAAAGCCTGACGATGCG
ACGCTGCGTGGAGGATGAAGGCCTTCGGGTTGTAAACTCCTGTCATGAGGGGAAAAGGTG
CCGGGTGTTAATAGCATCCGGCCTCGATAGTACCTCAAGAGGAAGCCACGGCTAACTCTG
TGCCAGCAGCCGCGGTAATACAGAGGTGGCGAGCGTTGTTCGGATTTACTGGGCGTAAAG
GGCGCGTAGGCGGTTTGGTATGTCGAGTGTGAAATCTCCAGGCTCAACCTGGAAAGTGCG
CTCGAAACTGCTGAACTCGAGCATGGGAGGGGAGAGCGGAATACACGGTGTAGCGGTGAA
ATGCGTTGATATCGTGTAGAACACCGGTGGCGAAGGCGGCTCTCTGGAACATTGCTGACG
CTGAGGCGCGAAAGTTGGGGGAGCAAACAGGATTAGAAACCCTAGTAGTCC
>Otu4465
CCAGCCTACGGGAGGCAGCAGTCGAGAATTTTTCTCAATGGGCGAAAGCCTGAAGGAGCG
ACGCCGCGTGGGGGATGAATGGCTTCGGCCCGTAAACCCCTGTCATTTGCGAACAAATTG
ACTCGCCTAACACGTGTGTCATTGATTGTAGCGGAAGAGGAAGGGACGGCTAACTCTGTG
CCAGCAGCCGCGGTAATACAGAGGTCCCAAGCGTTGTTCGGATTCACTGGGCGTAAAGGG
TGCGTAGGTGGTGAGGTAAGTCGGATGTGAAAGCTCGGAGCTCAACTCTGAAATGGCATT
GGAAACTACCTGACTGGAGGGTCGGAGGGGGGACTGGAATTCTCGGTGTAGCAGTGAAAT
GCGTAGATATCGAGAGGAACACCAGTGGCGAAGGCGAGTCCCTGGACGACTCCTGACACT
GAGGCACGAAAGCTAGGGGAGCAAACAGGATTAGATACCCGTGTAGTCC
>Otu4466
CTAGCCTATGGGGGGCAGCAGTGGGGAATTTTGGACAATGGGCGCAAGCCTGATCCAGCA
ATGCCGCGTGCAGGATGAAGGCCTTCGGGTTGTAAACTGCTTTTGTACGGAGCGAAAAGG
TTCTTGCTAATACCAGGGACACATGACGGTACCGTAAGAATAAGCACCGGCTAACTACGT
GCCAGCAGCCGCGGTAATACGTAGGGTGCAAGCGTTAATCGGAATTACTGGGCGTAAAGC
GTGCGCAGGCGGTTTTGTAAGACAGATGTGAAAACCCCGGGCTCAACCTGGGAACTGCAT
TTGTGACTGCAAAGCTAGAGTACGGCAGAGGGGGATGGAATTCCGCGTGTAGCAGTGAAA
TGCGTAGATATGCGGAGGAACGCCGATGGCGAAGGCAATCCCCCGGGCCTGTACTGACGC
TCATGCACGAAAGCGTGGGGAGCAAACAGGATTAGATACCCCCGTAGTCC
>Otu4473
CCAGCCTACGGGGCGCTGCAGTGGGGAATATTGGACAATGGGCGAAAGCCTGATCCAGCA
ATGCCGCGTGTGTGAAGAAGGTCTTAGGATTGTAAAGCACTTTCGGCGGGGACGATGATG
ACGGTACCCGCAGAAGAAGCCCCGGCTAACTTCGTGCCAGCAGCCGCGGTAATACGAAGG
GGGCTAGCGTTGCTCGGAATGACTGGGCGTAAAGGGCGCGTAGGCGGATGATTTAGTTAG
GTGTGAAATTCCTGGGCTTAACTTGGGGACTGCACTTAATACAGGTCGTCTAGAGTGGGA

TAGAGGGTTGTGGAATTCCCAGTGTAGAGGTGAAATTCGTAGATATTGGGAAGAACACCG
GTGGCGAAGGCGGCAACCTGGATCTTTACTGACGCTGAGGCGCGAAAGCGTGGGGAGCAA
ACAGGATTAGAGACCCCTGTAGTCC
>Otu4474
CCAGCCTATGGGTGGCTCCAGGCGCGAAACCTCCACAATGCGCGAAAGCGTGATGGGGGG
ATTCCGAGTGCTTACATTTTATGTAAGCTTTTGCCAAGAGTTCACATCTTGGAGAATAAG
TGGTGGGTAAGACCGGTGCCAGCAGCCGCGGTAACCCCGGCGCCACGAGTGGCAATCTTA
TATTGGGCCTAAAGCATTCGTAGCAGGTTTGAAAAATCCTTTGTGAAATTGTTGAACTCA
ATACAACAGCGTGCAGGGGACACTTTCAGACTAGAGACTGAGAGGAGTTGGGGGTATGCC
TTGGGGAGCGGTTAAATGTGATAATCCAAGGTAGACCACCTGTGGCGAAGGCGCCCAACT
AGAACAGTTCTGACTGTGAGGGATGAAAGCTAGGGGAGCGATCCGGATTAGAGACCCTGG
TAGTCC
>Otu4477
CCAGCCTATGGGAGGCAGCAGTCGAGAATCTTCCACAATGGACGAAAGTCTGATGGAGCG
ACGCCGCGTGGGGGACGAAGGTCTTCGGATTGTAAACCCCTGTCACTGGGGAAGAAACGC
TTTGAATTAATCGTTCAAAGCCTGACTTAACCCGGAGAGGAAGCAGTGGCTAACTCTGTG
CCAGCAGCCGCGGTAATGCAGAGACTGCAAGCGTTATTCGGATTCACTGGGCGTAAAGGG
TGCGCAGGCGGCCAGGTGTGTCAGGTGTGAAAGCCCGAGGCTTAACCTCGGAATTGCGCC
TGAAACTGCTTGGCTAGAGCATTGGAGAGGGTAGCGGAATTCACGGTGTAGCAGTGAAAT
GCGTAGATATCGTGAGGAACACCAGAGGCGAAGGCGGCTACCTGGACAATTGCTGACGCT
CAGGCACGAAAGCGTGGGGAGCAAAAGGGATTAGAAACCCGAGTAGTCC
>Otu4478
CCAGCGTGGCAGCAGCAAGGAATTTTCGGCAATGGGGGAAACCCTGACCGAGCAACGCCG
CGTGGGCGACGAAGTCCTTCGGGATGTAAAGCCCTTTTCTGTGGGAAGAAAAAGGACGGT
ACCACAGGAATAAGCATCGGCTAACTACGTGCCAGCAGCCGCGGTAATACGTAGGATGCA
AGCGTTGTCCGGATTTACTGGGCGTAAAGGGCGCGCAGGCGGTATAGCAAGTCTATCGTG
AAAGCCCCCGGCTAAACCGGGGAGGGTCGGTAGATACTGCTAAACTAGAGTGCAGCAGAG
GATAGTGGAATTCCCGGTGTAGTGGTGAAATGCGTAGATATCGGGAGGAACACCAGTGGC
GAAAGCGGCTATCTGGGCTGTAACTGACGCTGAGGCGCGAAAGCTAGGGGAGCAAACAGG
ATTAGATACCCTAGTAGTCC
>Otu4490
CCAGCCTACGGGGGGCACCAGTGGGGAATCTTGGACAATGGGGGCAACCCTGATTCAGCC
ATGCCGCGTGAGTGAAGAAGGCCTTAGGGTTGTAAAGCTCTTTCGCACGTGACGATAATG
ACGGTACCCGTAGAATAAGCTCCGGCTAACTTCGTGCCAGCAGCCGCGGTAATACGAAGG
GAGCTAGCGTTGTTCGGAATTACTGGGCGTAAAGGGCGCGTAGGCGGTTCGACAAGTTGG
ATGTGAAAGCCCTGGGCTCAACCCGGGAATTGCATTCAATACTGTCTTACTTGAATTCGG
TAGAGGTTGGTAGAATTCCCAGTGTAGAGGTGAAATTCGTAGATATTGGGAAGAATACCC
GTGGCGAAGGCGGCCAACTGGACCGACATTGACGCTGAGGCGCGAAAGCGTGGGGAGCAA
ACAGGATTAGATACCCTCGTAGTCC
>Otu4491
CCAGCCTATGGGGTGCTCCAGTCGAGAATTTTTCTCAATGGGCGAAAGCCTGAAGGAGCG
ACGCCGCGTGGGGGATGAATGGCTTCGGCCCGTAAACCCCTGTCATTTGTGAACAAATTG
CTTCACCCAACACGTGAAGCATTGATTGTAACGGAAGAGGAAGGGACGGCTAACTCTGTG
CCAGCAGCCGCGGTAATACAGAGGTCCCAAGCGTTGTTCGGAATCACTGGGCGTAAAGGG
TGCGTAGGCGGGTTTTTAAGTCAGGGGTGAAATCCTGGAGCTCAACTCCAGAACTGCCTT
TGATACTGAAGATCTTGAGTCCGGGAGAGGTGAGTGGAACTGCGAGTGTAGAGGTGAAAT
TCGTAGATATTCGCAAGAACACCAGTGGCGAAGGCGGCTCACTGGCCCGGTACTGACGCT
GAGGCACGAAAGCGTGGGGAGCAAACAGGATTAGATACCCTCGTAGTCC
>Otu4493
CCAGCCTATGGGTGGCAGCAGTGGGGAATTTTCCGCAATGGGCGAAAGCCTGACGGAGCA
AGACCGCGTGAGGGAGGAAGGCTCTTGGGTTGTAAACCTCTTTTCTCAGGGAAGAAGCTC
TGACGGTACCTGAGGAATCAGCATCGGCTAACTCCGTGCCAGCAGCCGCGGTAATACGGA
GGATGCAAGCGTTATCCGGAATCATTGGGCGTAAAGCGTTCGTAGGTGGCTAGTCAAGTC
TGCTGTCAAAGGCTGGGGCTTAACTCCAGAAAGGCAGTGGAAACTGAGTGGCTAGAGTGC
GGTAGGGGTAGAGGGAATTCCCGGTGTAGCGGTGAAATGCGTAGAGCTCGGGAAGAACAC
CGGTGGCGAAAGCGCTCTGCTGGGCCTGCACTGACACTGAGGGACGAAAGCTAGGGGAGC
GAATGGGATTAGAAACCCGTGTAGTCC
>Otu4494

CCAGCCTATGGGTCGCTGCAGTAGGGAATATTGCACAATGGAGGAAACTCTGATGCAGCG
ACGCCGCGTGAGTGACGAAGGACTTCGGTTCGTAAAGCTCTGTTGCATGGGAATAAGAAA
GTGAATGTACCCTGTGAGGAAGGGTCGGCTAACTTCGTGCCAGCAGCCGCGGTAAGACGA
GGGACCCTAGCGTTGTTCGGAATCATTGGGCGTAAAGCGGTGGCAGGTGGCTTGGTAAGT
CAGGTGTGAAAGCCCTGGGCTCAACCCAGGAAGTGCATTTGATACTGCTGAGCTTGAGTG
CTGGAGAGGCTACTAGAATTCCTGGTGTAGTGGTGAAATACGTAGATATCAGGAGGAATA
CCGGAGGCGAAGGCGGGTAGCTGGCCAGACACTGACACTCATCACCGAAAGCGTGGGGAT
CAAACAGGATTAGATACCCGCGTAGTCC
>Otu4496
CCAGCCTACGGGGTGCTGCAGTGGGGAGTCTTGCGCAATGGACGAAAGTCTGACGCAGCC
ACGCCGCGTGAGTGAAGAAGGCCTTCGGGTTGTAAAGCTCTGTCGGGAGGGACGAAAATG
CTTTTAGTGAATAGCTGAGAGTACTGACGGTACCTCCAAAGGAAGCACCGGCTAACTTCG
TGCCAGCAGCCGCGGTAATACGAAGGGTGCAAGCGTTGCTCGGAATTATTGGGCGTAAAG
GGTAGGTAGGTGGTTTCATTTGTCTGGGGTGAAAGCCTTTGGCTTAACCAAAGAAGTGCC
CCGGAAACGGTGGAACTAGAGTGCTAGAGAGGGTCGTGGAATTCCCGGTGTAGCGGTGAA
ATGCGTAGAGATCGGGAGGAACACCAGAGGCGAAGGCGACGACCTGGATAGCAACTGACA
CTCAACTACGAAAGCGTGGGGAGCAAACAGGATTAGATACCCCTGTAGTCC
>Otu4497
CCAGCCTATGGGATGCAGCAGTGAGGAATTATGCGCAATGGGGGAAACCCTGACGCAGCC
ACGCCGCGTGAGTGAAGAAGGCCTTCGGGTTGTAAAGCTCTGTCGGAGGGGACAAAAACC
TTTTGGGTTAATAGCCTAAGAGCTTGATGGTACCCTCAAAGGAAGCACCGGCTAACTTCG
TGCCAGCAGCCGCGGTAATACGAAGGGTGCAAGCGTTGCTCGGAATTATTGGGCGTAAAG
GGTAGGTAGGTGGTGGCAAAAGTCTGAGGTGAAATCCCCGAGCTTAACTCGGGACGTGCC
TTGGAAACTTTGTCACTAGAGTACTAGAGAGGTTCGTGGAATTCCCGGTGTAGCGGTGAA
ATGCGTAGAGATCGGGAGGAACATCAGTGGCGAAGGCGACGAACTGGATAGTAACTGACA
CTCAACTACGAAAGCGTGGGGAGCAAACAGGATTAGAAACCCGTGTAGTCC
>Otu4498
CCAGCCTATGGGACGCAGCAGTTCGGAATATTCGGCAATGGGCGAAAGCCTGACCGAGCG
ACGCCGCGTGCGGGATGAAGGTCCTTGGGTTGTAAACCGCTGTCACAGGTGAGCAAATTA
CGGAGAGTCAACAACTCGCCGTATTGAGTTAAGCTTGAGAGGAAGCCCCCGCTAATCACG
TGCCAGCAGCGGCGGTAATACGTGAGGGGCGAACGTTGTTCGGTGTCACTGGGCTTAAAG
GGCGCGTAGGTGGCTGCGTAAGTAAGGTGTGAAAGCCCTCGGCTTACCCGGGGAATTGCG
CCTTATACTGCGTGGCTTGAGGGTCATAGGGGAAGGGGGAACTCCAGGTGTAGCGGTGAA
ATGCGCAGATATCTGGGGGAAGGCCGGCGGCGAAGGCGCCCTTCTGGGTGATTCCTGACA
CTGAGGCGCGAAAGCGTGGGGAGCAAACAGGATTAGAAACCCTCGTAGTCC
>Otu4501
CCAGCCTACGGGATGCTGCAGTGGGGAATATTGGGCAATGGGCGCAAGCCTGACCCAGCC
ATGCCGCGTGAGTGATGAAGGCCTTCGGGTCGTAAAGCTCTGTGGGAAGGGACGAAAAAA
CGGGTGCTAATACCACTCGGCTTGACGGTACCTTCTTAGCAAGCACCGGCTAACTCCGTG
CCAGCGGCCGCGGTAATACGGAGGGTGCAAACGTTGCTCGGAATTATTGGGCGTAAAGCG
CACGTAGGCGGTTCGCTATGTCGGATGTGAAAGCCCTCGGCTTAACCGAGGAAGTGCACC
TGAAACTGGCGAGCTTGAGTACGGAAGAGGGTCTCGGAATTCCCGGTGTAGAGGTGAAAT
TCGTAGATATCGGGAGGAACACCAGTGGCGAAGACGGAGACCTGGGCCGATACTGACGCT
GAGGTGCGAAAGCGTGGGGAGCAAACAGGATTAGATACCCTAGTAGTCC
>Otu4506
CCAGCCTATGGGAGGCAGCAGTGGGGAATATTGGGCAATGGGCGCAAGCCTGACCCAGCC
ATGCCGCGTGAGTGATGAAGGCCTTCGGGTCGTAAAGCTCTGTGGGGAGGGACGAAAACG
ATGCGGAAGAAACGACGCATCCTTGACGGTACCTCCTTAGCAAGCACCGGTTAACTCTGT
GCCAGCAGCCGCGGTAATACAGAGGGTGCGAACGTTGCTCGGAATTACTGGGCGTAAAGC
GCGTGTAGGCGGATCCGTAAGTCAGGCGTGAAATCCCCAGGCTCAACCTGGGAACTGCGC
TTGAAACTGCGGGTCTTGAGTGTCGGAGAGGGTGGCGGAATTCCTGGTGTAGAGGTGAAA
TTCGTAGATATCAGGAGGAACACCGGTGGCGAAGGCGGCCACCTGGACGACAACTGACGC
TGAGACGCGAAAGCGTGGGGAGCAAACAGGATTAGAACCCGAGTAGTCC
>Otu4507
CCAGCCTATGGGTCGCTCCAGCGAGGAATTTTCCGCAATGGACGAAAGTCTGACGGAGCA
ACGCCGCGTGGGTGAAGAAGTTTTTCGGAATGTAAAACCCTTTTCTGTGGGAAGAAAGAA
AGACGGTGCCACAGGAATAAGCATCGGCTAACTACGTGCCAGCAGCCGCGGTAATACGTA
GGATGCAAGCGTTGTCCGGAATTACTGGGCGTAAAGGGCGAGTAGGCGGCCTGCCAAGTC

CGATGTGAAAGCTCCCGGCTTAACTGGGAGAGGCCATTGGAAACTGGCAAGCTGGAGTGC
AGGAGAGGCTAACGGAATTCCCGGTGTAGTGGTGAAATGCGTAGATATCGGGAGGAACAC
CAGTGGCGAAGGCGGTTAGCTGGCCTGTAACTGACGCTGAGGCGCGAAGGCTAGGGGAGC
AAACAGGATTAGAAACCCCAGTAGTCC
>Otu4509
CCAGCCTACGGGAGGCTGCAGTGGGGAATATTGGACAATGGGCGAAAGCCTGATGCAGCC
ATGCCGCGTGTGTGAAGAAGGCCTTCGGGTTGTAAAGCACTTTCAGCGAGGAGGAAGGCA
TCATACTTAATACGTGTGGTGATTGACGTTACTCGCAGAAGAAGCACCGGCTAACTCCGT
GCCAGCAGCCGCGGTAATACGGAGGGTGCAAGCGTTAATCGGAATTACTGGGCGTAAAGC
GCACGCAGGCGGTTTGTTAAGTCAGATGTGAAATCCCCGCGCTTAACGTGGGAACTGCAT
TTGAAACTGGCAAGCTAGAGTCTTGTAGAGGGGGGTAGAATTCCAGGTGTAGCGGTGAAA
TGCGTAGAGATCTGGAGGAATACCGGTGGCGAAGGCGGCCCCCTGGACAAAGACTGACGC
TCAGGTGCGAAAGCGTGGGGAGCAAACAGGATTAGAGACCCCCGTAGTCC
>Otu4512
GGACTACGAGGGTTTCTAATCCTGTTTGATCCCCACGCTTTCGGGTCTGAGTGTCAGTGT
CTGGCCAGTTACCCGCCTTCGCCTCCGGTATTCCTCCTGATATCTACGTATTTCACCACT
ACACCAGGAATTCTAGTAACCTCTCCAGCACTCAAGCTTCGCAGTATCAAACGCACTTCC
AAGGTTAAGCCCTGGGCTTTCACGTCTGACTTACAAAGCAACCTACACCCGCTTTACGCC
CAATAATTCCGAACAACGCTAGGGTCCCTCGTCTTACCGCGGCTGCTGGCACGAAGTTAG
CCGACCCTTTCTCGCAAAGTACCTTCATTTTTTTATTCCCTTGCAACAGAGCTTTACGAA
CCGAAGTCCTTCATCACTCACGCGGCGTCGCTGCATCAGAGTTTCCTCCATTGTGCAATA
TTCCCTACTGCTGCACCCCGTAGGCTG
>Otu4514
CCAGCCTATGGGGCGCACCAGTGAGGAATTTTGCACAATGGGGGAAACCCTGATGCAGCA
ACGCCGCGTGTATGATGAAGGGGCTATGCCTTGTAAAGTACTGTCGGACAGGAAAAAAAG
TTCCCTTTGGGGACTGGGATTGTACTGTCAAAGGAAGGGTCGGCTAACTACGTGCCAGCA
GCCGCGGTAATACGTAGGACCCAAGCGTTGTCCGGAATTACTGGGTGTAAAGGGTGCGTA
GGCGGGCTTGCGCGTCGAAGGTTAAAGACCACAGCTTAACTGCGGAAGTGCCTTCGATAC
GGTGAGTCTTGAATATGAGAGAGGTAAGTAGAATTCCTGGTGTAGCGGTGAAATGCGTAG
ATATCAGGAGGAATACCAGTGGCGAAGGCGGCTTACTGGCTCATTATTGACGCTGAGGCA
CGAAAGCGTGGGGATCAAACAGGATTAGAAACCCCAGTAGTCC
>Otu4515
CCAGCCTATGGGTCGCACCAGTCGAGGATTTTTCTCAATGGGCGAAAGCCTGAAGGAGCG
ACGCCGCGTGGGGGATGAAGGTCTTCGGATTGTAAACCCCTGTCATCTGGGAACAATGTG
ATTCACCTAACACGTGGATCATTGATAGTACCGGAAGAGGAAGCAGTGGCTAACTCTGTG
CCAGCAGCCGCGGTAATACAGAGACTGCAAGCGTTGTTCGGATTCATTGGGCGTAAAGGG
TCCGCAGGCGGTCTGTTAAGTCGGATGTGAAATCTCACAGCCTAACTGTGATAGGTCATT
CGAAACTGGCAGGCTCGAGGAGTGGAGAGGAGACTGGAATTGCTGGTGTAGCGGTGAAAT
GCGTAGAGATCAGCAAGAACACCGGTGGCGAAGGCGAGTCTCTGGACACTTCCTGACGCT
CAGGGACGAAAGCCAGGGGAGCAAACGGGATTAGAAACCCGTGTAGTCC
>Otu4518
CCAGCCTACGGGGGGCTGCAGTGGGGAATTTTGCACAATGGGGGAAACCCTGATGCAGCA
ACGCCGCGTGGAGGATGAAGTCCCTTGGGATGTAAACTCCTTTCGACCGGGACGATAATG
ACGGTACCGGTGGAAGAAGCCCCGGCTAACTCTGTGCCAGCAGCCGCGGTAATACGAGGG
GGGCGAGCGTTGTTCGGAATTATTGGGCGTAAAGGGTGCGTAGGCGGTTTGACAAGTCTG
ATGTGAAATCCCGCAGCTTAACTGCGGAACGGCATTGGAAACTGTTTGACTAGAGGAATG
GAGGGGAGACTGGAATACTTGGTGTAGCAGTGAAATGCGTAGATATCAAGTGGAACACCA
GTGGCGAAGGCGAGTCTCTGGACATTTCCTGACGCTGAGGCACGAAAGCCAGGGGAGCAA
ACGGGATTAGAAACCCTAGTAGTCC
>Otu4519
CCAGCCTACGGGGGGCAGCAGTTTCGAATCATTCACAATGGGCGCAAGCCTGATGGTGCG
ACGCCGCGTGGGGGATGAAGGTCCTTGGATTGTAAATCCCTGTCACCGGGGAAGAAACGT
ACACACTTAACACGTGTGTGCCTGACTTAACCCGGAGAGGAAGCAGTGGCTAACTCTGTG
CCAGCAGCCGCGGTAATACAGAGGCTGCAAGCGTTATTCGGATTCACTGGGCGTAAAGGG
TGCGCAGGCGGCCATGTGTGTTAGGCGTGAAAGCCCGAGGCTCAACCTCGGAATTGCACC
TAAAACTACATGGCTAGAGTACTGGAGAGGGTAGCAGAATTCATGGTGTAGCAGTGAAAT
GCGTAGATATCATGAGGAATACCAATGGCGAAGGCAGCTACCTGGACAGTTACTGACGCT
CAGGCACGAAAGCGTGGGGAGCAAAAGGGATTAGAGACCCGAGTAGTCC

>Otu4520
CCAGCCTACGGGATGCTGCAGTCGAGGATCTTCGGCAATGGGCGCAAGCCTGACCGAGCG
ACGCCGCGTGTGCGATGAAGGCCTTCGGGTTGTAAAGCACTGTCGGGGGGGAGAAAGCCG
CAAGGTTGATCGACCCCCGGAGGAAGCACGGGCTAAGTTCGTGCCAGCAGCCGCGGTAAG
ACGAACCGTGCGAACGTTGTTCGGAATCACTGGGCTTAAAGGGCGCGTAGGCGGGCGATC
CAGTCAAGGGTGAAATCCTGCGGCTTAACCGTAGAAGAGCCCCTGATACTGATTGTCTCG
AGGGAGGTAGGGGCGTGTGGAACTTCCGGTGGAGCGGTGAAATGCGTAGATATCGGAAGG
AACGCCGGTGGCGAAAGCGACGCGCTGGACCTCTTCTGACGCTGAGGCGCGAAAGCCAGG
GGAGCAAACGGGATTAGAAACCCCAGTAGTCC
>Otu4522
CCAGCCTACGGGAGGCTGCAGTGGGGAATTTTGCGCAATGGGCGAAAGCCTGACGCAACA
ACGCCGCGTGGAGGACGAAGGTTTTCGAATTGTAAACTCCTGTCCCGGGGGACGATAATG
ACGGTACCCCGGGAGGAAGCCCCGGCTAACCTCGTGCCAGCAGCCGCGGTAATACGAGGG
GGGCAAGCGTTGTTCGGAATCATTGGGCGTAAAGGGCGCGTAGGCGGCCTGGGAAGTTCT
GGGTGAAATCCCTCGGCTCAACCGAGGAAGTGCCTGGGAAACCACTGGGCTGGAGTGCTG
GAGAGGGAAGCGGAATTCCTGGTGTAGCGGTGAAATGCGTAGAGATCAGGAGGAACATCT
GAGGCGAAGGCGGCTTCCTGGACAGACACTGACGCTGAGGCGCGAAAGCCAGGGGAGCAA
ACAGGATTAGATACCCTAGTAGTCC
>Otu4527
CCAGCCTACGGGTGGCACCAGCAAGGAATTTTCGGCAATGGGGGAAACCCTGACCGAGCA
ACGCCGCGTGGGCGACGAAGTCCTTCGGGATGTAAAGCCCTTTTCTGTGGGAAGACTAAG
GACGGTACCACAGGAATAAGCACCGGCTAACTACGTGCCAGCAGCCGCGGTAATACGTAG
GGTGCAAGCGTTGTCCGGATTTACTGGGCGTAAAGGGCGCGCAGGCGGCCTGATAAGTCA
CTCGTGAAATTTCCCGGCTAAACTGGGAAGGGTCGAGTGATACTGTCAAGCTAGAGAGCA
GCAGAGGATAGTAGAATTCCCGGTGTAGTGGTGAAATGCGTAGATATCGGGAGGAATACC
AGTGGCGAAAGCGGCTATCTGGGCTGTGTCTGACGCTGAGGCGCGAAGGCTAGGGGAGCA
AACAGGATTAGAAACCCTCGTAGTCC
>Otu4531
CCAGCCTATGGGGCGCTGCAGTCGAGGATCTTCGGCAATGGGCGAAAGCCTGACCGAGCG
ACGCCGCGTGCGGGATGAAGGCCTTCGGGTTGTAAACCGCTGTCAGTGGGGAGGAGGAAA
TTCCAAGGGGTTCTCCCTTTGGATTGACCTATACTCAGAGGAAGGACGGGCTAAGTTCGT
GCCAGCAGCCGCGGTAAGACGAACCGTCCAAACGTTATTCGGATTCACTGGGCTTAAAGG
GTGCGTAGGCGGCTCAGAAAGTAGGGTGTGAAAGCCCTCGGCTCAACCGAGGAATTGCGC
CCTAAACTACTGGGCTTGAGGAACGCAGAGGTAAGCGGAACTTAGGGTGGAGCGGTGAAA
TGCGTTGATATCCTAAGGAACACCAGGAGCGAAAGCGGCTTACTGGGCGTTTTCTGACGC
TGAGGCACGAAAGCTAGGGGAGCGAACGGGATTAGATACCCCGGTAGTCC
>Otu4532
CCAGCCTACGGGGGGCAGCAGTCGAGAATTTTTCACAATGGGGGAAACCCTGATGGAGCG
ACGCCGCGTGGGGGATGAATGGCTTCGGCCCGTAAACCCCTGTCATTTGCGAACAAACCT
CATGCATGAACAATGCATGAATTGATTGTAGCGGAAGAGGAAGGGACGGCTAACTCTGTG
CCAGCAGCCGCGGTAATACAGAGGTCCCAAGCGTTGTTCGGATTCACTGGGCGTAAAGGG
TGCGTAGGTGGTCGGGTAAGTCTGATGTGAAATCTCGCAGCTTAACTGCGAAACTGCATT
GGATACTATCCGGCTGGAGGATTGGAGGGGGGACTGGAATTCTTGGTGTAGCAGTGAAAT
GCGTAGATATCAAGAGGAACACCAGTGGCGAAGGCGAGTCCCTGGACAATTCCTGACACT
GAGGCACGAAAGCTAGGGGAGCAAACAGGATTAGAAACCCCCGTAGTCC
>Otu4536
CCAGCCTACGGGGGGCTGCAGTCGAGGATCTTCGTCAATGGGCGCAAGCCTGAACGAGCG
ATGCCGCGTGCGCGATGAAGGCCTTCGGGTTGTAAAGCGCTGTCGAGGGGGAGAAAAGCC
GAAAGGTTTGATCTATCCCTGGAGGAAGCTCGGGCTAAGTTCGTGCCAGCAGCCGCGGCA
AGACGAACCGAGCGAACGTTGTTCGGAATCACTGGGCTTAAAGGGCGCGTAGGCGGGCTG
TCAAGTCTGGGGTGAAATCCCGCGGCTCAACCGTGGAACTGCCTCAGATACTGACGGCCT
CGAGGGAGGTAGGGGCGAGCGGAACGGGTGGTGGAGCGGTGAAATGCGTTGATATCACTC
GGAACTCCGGTGGCGAAAGCGGCTCGCTGGATCTCTTCTGACGCTGAGGCGCGAAAGCTA
GGGGAGCAAACGGGATTAGATACCCGTGTAGTCC
>Otu4537
CCAGCCTACGGGAGGCTGCAGTAGGGAATATTGCGCAATGGAGGAAACTCTGACGCAGCG
ACGCCACGTGGGTGATGAAGGTTTTCGGATCGTAAAGCCCTGTCGGATGGGACGAAAAAC
GAGGTGGCTAACATCCACTTCGCTTGACGGTACCATCAAAGGAAGCACCGGCTAACTACG

TGCCAGCAGCCGCGGTAATACGTAGGGTGCTAGCGTTGTTCGGAATCATTGGGCGTAAAG
CGCGTGTAGGCGGTTAGTAAAGTTGGGTGTGAAATCCCTGGGCTCAACCGAGGAAGTGCA
TCCAAAACTAACTAGCTAGAGAGTGGTAGAGGGAGGTGGAATTCCGTGTGTAGAGGTGAA
ATTCGTAGATATACGGAGGAATACCGGTGGCGAAGGCGGCCTCCTGGACCATTTCTGACG
CTGAGACGCGAAAGCGTGGGGAGCAAACAGGATTAGATACCCCTGTAGTCC
>Otu4540
CCAGCCTACGGGGGGCTGCAGTGGGGAATATTGGGCAATGGAGGAAACTCTGACCCAGCC
ATGCCGCGTGAATGATGAAGGCCTTAGGGTTGTAAAGTTCTTTCGCCGATGAAGATAATG
ACGGTAGTCGGAGAAGAAGCCCCGGCTAACTTCGTGCCAGCAGCCGCGGTAATACGAAGG
GGGCTAGCGTTGTTCGGAATTACTGGGCGTAAAGCGAGCGTAGGTTGCTCGACAAGTCAG
TGGTGAAAGCCCAAGGCTCAACCTTGGAACTGCCATTGAAACTGTCGAGCTGGAATCTTA
GAGGGGGTAGCGGAATTCCAAATGTAGGGGTGAAATCCGTAGATATTTGGAGGAACACCG
GTGGCGAAGGCGGCTACCTGGCTAAGTATTGACACTGAGGCTCGAAAGCGTGGGGATCAA
ACAGGATTAGAGACCCTTGTAGTCC
>Otu4543
CCAGCCTACGGGGGGCTGCAGTTCGGAATATTCGGCAATGGACGAAAGTCTGACCGAGCG
ACGCCGCGTGCGGGATGAAGGCCCTATGGGTTGTAAACCGCTTTCGCGATTAGGTAAATC
ACGGTTGATTAACAATTGACTGTGTTGAATAAGGATCGTAAAGAAGCCCCCGCTAATCAC
GTGCCAGCAGCGGCGGTAATACGTGAGGGGCGAACGTTGTTCGGTGTCACTGGGCTTAAA
GGGTGCGTAGGTGGTTGCGTAAGTAACGTGTGAAATCCCTCGGCTTACCCGGGGAATTGC
ACGTTATACTGCGTGACTTGAGGGCTAAAGGGGAAAAGGGAACTCCAGGTGTAGCGGTGA
AATGCGCAGATATCTGGGGGAAGGCCGGCGGCGAAGGCGCTTTTCTGGTTAGTACCTGAC
ACTGAGGCACGAAAGCGTGGGGAGCAAACAGGATTAGATACCCTAGTAGTCC
>Otu4544
CCAGCCTATGGGGTGCTCCAGTCGAGAATCTTCCGCAATGGACGAAAGTCTGACGGAGCG
ACGCCGCGTGGAGGATGAAGTGCTTCGGCATGTAAACTCCTTTTGCCAGGGAAAAAGTTT
ATTGATTGTACCTGGAGAATAAGAAGTTGCTAAACTCGTGCCAGCAGCAGCGGTAATACG
AGTGCTTCGAGCGTTATCCGGAATCATTGGGCGTAAAGGGTGTGTAGGCGGCGATGTTAG
TCTCATGTAAAATCCTTCGGCTCAACCGAGGGCTCGCACGAGAAACGGCATTGCTTAGAG
GACGGAAGGGGTCTCTGGAACTCATGGTGTAGCGGTGAAATGCGTTGATATCATGGGGAA
CACCAAAAGCGAAGGCAAGAGACTGGTCCGCTCCTGACGCTGAAACACGAAAGCGTGGGT
CGCGAATGGGATTAGATACCCTTGTAGTCC
>Otu4546
CCAGCCTACGGGGTGCTCCAGTGGGGAATATTGGACAATGGGGGCAACCCTGATCCAGCA
ATGCCGCGTGAGTGATGAAGGCCTTAGGGTTGTAAAGCTCTTTCGGGTGGGACGATGATG
ACGGTACCACCAGAAGAAGCCCCGGCTAACTTCGTGCCAGCAGCCGCGGTAATACGAAGG
GGGCTAGCGTTGTTCGGAATTACTGGGCGTAAAGCGCACGCAGGCGGCGCGTTAAATAAG
GAGTGAAAGCCCTGGGCTCAACCCGGGAATTGCTCTTTAGACTGGCGCGCTAGAATTCGG
AAGAGGGCAGTGGAATTCCAAGTGTAGAGGTGAAATTCGTAGATATTTGGAAGAACACCA
GTGGCGAAGGCGACTGCCTGGTCCGATATTGACGCTCATGTGCGAAAGCGTGGGGAGCAA
ACAGGATTAGAAACCCCTGTAGTCC
>Otu4547
CCAGCCTACGGGAGGCTGCAGCGACGAATCTTCCGCAATGGGCGAAAGCCTGACGGAGCG
ATGCCGCGTGTGGGATGAAGCGGCTTTGCCGTGTAAACCACTGTCAGGAGAAAGGAATAT
TGACCATCTCCAGAGGAAGGGGCGACTAACTCTGTGCCAGCAGTCGCGGTAATACAGAGG
CCCCGAGCGTTAGTCGGAATCACTGGGCTTAAAGGGTGCGTAGGCGGCTGGATAAGTGTC
GTGTGAAATCCCACTGCTCAACGGTGGAACTGCACGGCAAACTGTTCGGCTAGAAGAATG
TAGAGGTCGCTAGAACGCATGGTGGAGCGGTGGAATGCGTAGATATCATGCGGAATGCCA
AAGGTGAAGACAGGCGACTGGGCATTTCTTGACGCTGAGGCACGAAAGCGTGGGGAGCAA
ACAGGATTAGATACCCTTGTAGTCC
>Otu4552
CAGCCTACGGGGGGCTCCAGCTAGGAATTTTCCGCAATGGGCGAAAGCCTGACGGAGCGA
CGCCGCGTAAGGGATGAAGGCTCTACGGAGTTGTAAACCTTTTTTCTGCGAGACGAGAGA
GGACGGTATCGCAGGAATAAGCCCCGGCAAACTACGTGCCAGCAGCCGCGGTAATACGTA
GGGGGCGAGTGTTGTTCGGAATTACTGGGCATAAAGGGTGCGTAGGCGGGAAGCCAAGCA
AGGCGTGAAAGACCCTGGCTTAACTAGGGAACTGCGCTTTGAACTGGTTTTCTGGAGTGC
AGGATGGGTGAGCAGAATTCCCGGTGTAGGGGTGAAATCTATAGATATCGGGAGGAATAC
CGGTCGCGAAGGCGGCTCACTGGCCTGTAACTGACGCTGAAGCACGAAAGCGTGGGGATC

AAACCGGATTAGAGACCCCTGTAGTCC
>Otu4560
CCAGCCTACGGGATGCTGCAGCAACGAATCTTCCCCAATGGGCGAAAGCCTGAAGGAGCG
ACGCCGCGTGGGGGATGAATGGCTTCGGCCCGTAAACCCCTGTCATTTGGGAACAATGCG
TTTGGGTGAACATCCCAAACGTTGATAGTACCGGAAGAGGAAGGGACGGCTAACTCTGTG
CCAGCAGCCGCGGTAATACAGAGGTCCCAAGCGTTGTTCGGATTTACTGGGCGTAAAGGG
TGCGTAGGTGGTCGGGTAAGTCTGATGTGAAATCTCGGAGCTTAACTCCGAAACTGCATT
GGATACTATTCGGCTTGAGGGTTGGAGGGGAGACTGGAATTCTCGGTGTAGCAGTGAAAT
GCGTAGATATCGAGAGGAACACCAGTGGCGAAGGCGAGTCTCTGGACAACTCCTGACACT
GAGGCACGAAAGCCAGGGGAGCAAACGGGATTAGAAACCCTAGTAGTCC
>Otu4564
CCAGCCTACGGGTGGCTGCAGTGGGGAATCTTGCGCAATGGGCGAAAGCCTGACGCAGCG
ACGCCGCGTGGGTGATGAAGGCCTTCGGGTTGTAAAGCCCTGTGGGGAGAGACGAATAAG
TCGAGGCGAATACCCTCGATGATGACGGTATCTCCTTAGCAAGCACCGGCTAACTCTGTG
CCAGCAGCCGCGGTAAGACAGAGGGTGCAAACGTTGTTCGGAATTACTGGGCGTAAAGCG
CATGTAGGCGGCTCTGCAGGTCGGATGTGAAATCCCTCGGCTCAACCAAGGAAGTGCATT
CGAAACCGCAGAGCTAGAGTCCCGGAGAGGAAGGTGGAATTCTCGGTGTAGAGGTGAAAT
TCGTAGATATCGAGAGGAACACCGGTGGCGAAAGCGGCCTTCTGGACGAGGACTGACGCT
GAGATGCGAAAGCGTAGGGAGCAAACAGGATTAGAAACCCTTGTAGTCC
>Otu4565
CCAGCCTATGGGAGGCTCCAGTAAGGAATATTGGTCAATGGACGCAAGTCTGAACCAGCC
ATGCCGCGTGAAGGATGAAGGCCCTAGTGGTTGTAAACTTCTTTTATACGGGAAAAATTC
TTGGGTCGTGAATCCGAGTTGATGGTACTGTATGAATAAGCACCGGCTAACTCCGTGCTA
GCAGCCGCGGTAATACGGAGGGTGCAAGCGTTATCCGGATTTATTGGGTTTAAAGGGTGC
GCAGGCGGAACTATAAGTCAGCGGTGAAAGCCTACAGCTTAACTGTAGAATTGCCATTGA
TACTGTAGTTCTTGAGTGTAGGTGAAGAAGGCGGAATGTGTTGTGTAGCGGTGAAATGCT
TAGATATAACACAGAACACCGATTGCGAAGGCAGCTTTCTAAACTACAACTGACGCTCAT
GCACGAAAGCGTGGGAGCAAACAGGATTAGAAACCCTCGTAGTCC
>Otu4566
CCAGCCTATGGGTCGCAGCAGTCGAGAATTTTTCACAATGGGCGAAAGCCTGACGCAGCA
ACGCCGCGTGGAGGATGAAGGCCTTCGGGTCGTAAACTCCTTTTGGTCGAGACGAGGAAG
GACGGTATCGACCGAATAAGTCACGGCTAACTACGTGCCAGCAGCCGCGGTAAAACGTAG
GTGGTGAGCGTTATCCGGATTTACTGGGTGTAAAGCGTGTGCAGGCGGTGCAGTAAGTGG
TGTATGAAATCTCCGCGCTTAACGTGGAGGGGTTATGCCAGACTGCTGGACTAGAGGACG
TGAGAGGGGCATGGAATTCCGGGTGTAGTGGTGAAATGCGTAGAGATCCGGAGGAACCCC
AGAGGCGAAGGCGGTGCCCTAGCACGTCCCTGACGCTCAGACACGACAGCATGGGGAGCG
AACGGGATTAGATACCCCCGTAGTCC
>Otu4567
CCAGCCTATGGGGTGCTGCAGTCGAGGATCTTCGGCAATGGGCGCAAGCCTGACCGAGCG
ACGCCGCGTGTGCGATGAAGGCCTTCGGGTTGTAAAGCACTGTCGAGGGGGAGGAAGCCG
CAAGGTTGACCTATCCCTGGAGGAAGCACGGGCTAAGTTCGTGCCAGCAGCCGCGGTAAG
ACGAACCGTGCGAACGTTGTTCGGAATCACTGGGCTTAAAGGGCGCGTAGGCGGACGCTC
AAGTCCGGGGTGAAATCTTTCGGCTCAACCGGAAAATAGCCCTGGATACTGAGAGTCTCG
AGGCAGGTAGGGGCAGCTGGAACTTCCGGTGGAGCGGTGAAATGCGTTGATATCGGAAGG
AACGCCGGTGGCGAAGGCGAGTCTCTGGACATTTCCTGACGCTGAGGCACGAAAGCCAGG
GGAGCAAACGGGATTAGAAACCCGCGTAGTCC
>Otu4568
CCAGCCTACGGGTTGCTGCAGTCGAGAATATTCGACAATGGGCGAAAGCCTGATCGAGCG
ACACCGCGTGCAGGATGAAGGCCTTCGGGTTGTAAACTGCGGTAGTATAGTAACAATGTA
AATGAGTGCTATACGGAAAGAGGTGGGTAACTACGTGCCAGCACCAGCGGTAATACGTAG
ACCTCAAGCGTTATCCGGATTTATTGGGCGTAAAGCGCATGTAGGGGGGTTCGGTGCGTCT
TCTCTTAAAGCCCACGGCCTAACCGTGGAAGTGGAGGAGATACGACCGGACTAGAGGGGG
TTAGAGGTGCATGGAGCTCACGGTGTAGGGGTGAAATCCGTTGATATCGTGGGAAACACC
AAAGGCGAAGGCAGTGCACTGGGACCTTCCTGACCCTGAGATGCGAAAGCGTGGGGAGCA
AAAAGGATTAGAAACCCCCGTAGTCC
>Otu4575
CCAGCCTATGGGACGCAGCAGCAAGGAATTTTCGGCAATGGGGGGAACCCTGACCGAGCA
ACGCCGCGTGGAGGACGAAGTATTTCGGTATGTAAACTCCTTTTTGATGGGAAGACAGGA

AAGAGGACGGTACCATCAGAATAAGCTCCGGCTAACTACGTGCCAGCAGCCGCGGTAATA
CGTAGGGAGCGGGCGTTGTCCGGAATTACTGGGCGTAAAGGGCGAGTAGGCGGTATAGCA
AGTTCGATGTGAAAGCCTTCGGCTTAACCGGAGAGGGTCATCGGAGACTGCTAAACTAGA
GTGCAAAAGAGGCTAACAGAATTCCCGGTGTTGTGGTGAAATGCGTAGATATCGGGAGGA
ATACCAGTGGCGAAGGCGGTTAGCTGGGTTGTAACTGACGCTGAGGCGCGAAGGCTAGGG
TAGCAAACAGGATTAGAAACCCCCGTAGTCC
>Otu4579
CCAGCCTATGGGTCGCTCCAGTCGAGAATTTTTCACAATGGGCGCAAGCCTGATGGAGCG
ACGCCGCGTGGGGGATGAATGGCTTCGGCCCGTAAACCCCTGTCATTTGCGATCAAACCT
CGTTATTTAAAAGATGACGAACTGATAGTAGCGAAAGAGGAAGGGACGGCTAACTCTGTG
CCAGCAGCCGCGGTAATACAGAGGTCCCAAGCGTTGTTCGGATTCACTGGGCGTAAAGGG
TGCGTAGGTGGCGAGGTAAGTCGGATGTGAAAGCTCGGGGCTTAACCCCGAAATGGCATT
GGAAACTACCTTGCTTGAGGGTTGGAGGGGGGACTGGAATTCTCGGTGTAGCAGTGAAAT
GCGTAGATATCGAGAGGAACACCAGTGGCGAAGGCGAGTCCCTGGACAACACCTGACACT
GAGGCACGAAAGCGTGGGGAGCAAACAGGATTAGATACCCGTGTAGTCC
>Otu4586
CCAGCCTATGGGGTGCTCCAGTTGGGAATATTGGACAATGGGCGCAAGCCTGATCCAGCC
ATGCCGCGTGAGTGAAGAAGGCCTTAGGGTTGTAAAGCTCTTTCGCCGACGAAGATAATG
ACGGTAGTCGGAGAAGAAGCCCCGGCTAACTTCGTGCCAGCAGCCGCGGTGATACGAAGG
GGGCTAGCGTTGTTCGGAATTACTGGGCGTAAAGCGCACGTAGGCGGATTGTTAAGTTAG
AGGTGAAAGCCCAGGGCTCAACCCTGGAATTGCCTTTGATACTGGCAGTCTAGAGATCGG
AAGAGGTGAGTGGAATTCCTAGTGTAGAGGTGAAATTCGTAGATATTAGGAAGAACACCA
GTGGCGAAGGCGGCTCACTGGTCCGATTCTGACGCTGAGGTGCGAAAGCGTGGGGAGCAA
ACAGGATTAGATACCCTAGTAGTCC
>Otu4587
CCAGCCTACGGGATGCACCAGTGGGGAATATTGGACAATGGGCGCAAGCCTGATCCAGCG
ACGCCGCGTGAGTGATGAAGGCCTTCGGGTTGTAAAGCTCTGTTGAGGGAGAAGAAAACA
GTTGGCGCGAATAGCTCCAGCTCTTGACGGTATCCCTTTAGAAAGCACCGGCTAACTCTG
TGCCAGCAGCCGCGGTAAGACAGAGGGTGCAAACGTTGCTCGGAATTACTGGGCGTAAAG
CGCGTGTAGGCGGACCCACAAGTCGGACGTGAAAGCCCTGGGCTCAACCCAGGAACTGCA
TCCGAAACTGTGGGTCTGGAGTGGTGGAGAGGGAAGCAGAATTCCTGGTGTAGAGGTGAA
ATTCGTAGATATCAGGAGGAACACCGGTGGCGAAGGCGGCTTCCTGGACACTTACTGACG
CTGAGACGCGAAAGCGTGGGGAGCAAACAGGATTAGAGACCCTCGTAGTCC
>Otu4589
CCAGCCTATGGGTGGCAGCAGTTAGGAATCTTGGGCAATGGGCGCAAGCCTGACCCAGCC
ATGCCGCGTGTGTGAAGAAGGCCTTCGGGTTGTAAAGCACTTTCAGCGGTGAAGAAAAAC
TTAGGGCTAATACCCTTGAGTCTTGACGTTAACCACAGAAGAAGCACCGGCTAACTCCGT
GCCAGCAGCCGCGGTAATACGGAGGGTGCGAACGTTAATCGGAATCACTGGGCGTAAAGC
GCGCGTAGGCGGTTTGTTAAGTGGGATGTGAAATCCCCGGACTTAACCTGGGAATTGCAT
TTCAAACTGGCCGACTAGAGTACGGTAGAGGGAGGTAGAATTTCCTGTGTAGCGGTGAAA
TGCGTAGATATAGGAAGGAATACCAGTGGCGAAGGCGGCCTCCTGGATCGATACTGACGC
TGAGGCGCGAAAGCGTGGGGAGCAAACAGGATTAGAAACCCCTGTAGTCC
>Otu4591
CCAGCCTACGGGATGCAGCAGTAAGGAATATTGGTCAATGGAGGCAACTCTGAACCAGCC
ATGCCGCGTGCAGGATGACGGCCCTATGGGTTGTAAACTGCTTTTGTACGGGAGAAAACC
CTTTCACGTGTGAGAGGTTGATAGTACCGTAAGAATAAGCATCGGCTAACTTCGTGCCAG
CAGCCGCGGTAAGACGAAGGATGCAAGCGTTATCCGGATTTATTGGGTTTAAAGGGAGCG
TAGGCGGTCTTATAAGTCAGTGGTGAAATCTCGTTGCTTAACAACGAACGTGCCATTGAT
ACTGTAGGGCTTGAGTACAGTTGCCGTTGGCGGAATGTGTCATGTAGCGGTGAAATGCAT
AGATATGACACAGAACACCGATTGCGAAGGCAGCTGACGAAACTGTAACTGACGCTGAGG
CTCGAAAGCGTGGGGATCAAACAGGATTAGATACCCGAGTAGTCC
>Otu4592
CCAGCCTATGGGTCGCAGCAGTCGAGAATCTTCCGCAATGGACGAAAGTCTGACGGAGCG
ACGCCGCGTGGAGGATGAAGTGCTTCGGCATGTAAACTCCTTCTGCCAGGGAAAAAGTTT
ATTGATTGTACCTGGAGAATAAGAGGTTGCTAAACTCGTGCCAGCAGCAGCGGTAATACG
AGTGCCTCGAGCGTTATCCGGAATCATTGGGCGTAAAGGGTGCGTAGGCGGCGATGTTAG
TCTCCCGTAAAATCTTTCGGCTCAACCGAGAGTCTGCGGGGGAAACGGCATTGCTTGAGG
ACGGGAGAGGTCTCTGGAACTCTAGGTGTAGCGGTGAAATGCGTTGATATCTAGGGGAAC

ACCAAAAGCGAAGGCAAGAGACTGGCCCGCTCCTGACGCTGAAGCACGAAAGCGTGGGTC
GCGAATGGGATTAGATACCCGTGTAGTCC
>Otu4593
CCAGCCTACGGGGGGCTGCAGTCGAGAATCTTCCGCAATGGGCGAAAGCCTGACGGAGCG
ACGTTACGTGAATGATGAAGCCCTTAGGGGTGTAAAGTTCTTTTCTCTGGGAGGAAGCCG
CAAGGCAGACAGTACCAGAGGAATAAGAGGGTCCTAACTCTGTGCCAGCAGGAGCGGTAA
TACAGAGCCCTCGAGCGTTACCCGGATTTATTGGGCGTAAAGGGTCCGTAGGTGGTAAAG
TAAGTTGAAGGTTAAATCTCATCGGCTCAACCTTTGAGGTGCTTTCAATACTGCTATACT
AGAGGGCGTTAGGGGTTAGCGGAACCGACGGAGTAGGGGTGAAATCCGTTGATATCGTCG
GGAACACCAAAAGCGAAGGCAGCTAACTAGGACGACCCTGACACTGAGGGACGAAAGCGT
GGGGAGCAAAAAGGATTAGAAACCCTCGTAGTCC
>Otu4599
CCAGCCTACGGGTGGCAGCAGTGGGGAATCTTGCACAATGGGGGAAACCCTGATGCAGCG
ACGCCGCGTGAGCGATGAAGCCCTTCGGGGTGTAAAGCTCTTTCGGCAGGGACGATAATG
ACGGTACCTGCAGAAGAAGCTGCGGCTAACTACGTGCCAGCAGCCGCGGTAATACGTAGG
CAGCAAGCGTTGTTCGGAATTACTGGGCGTAAAGAGTGTGTAGGCGGTGCTCTAAGTTTG
GTGTGAAATCTCCCGGCTTAACTGGGAGGGTGCGCCGAAGACTGGAGTGCTCGAACGTGG
GAGAGGAAAGCGGAATTCCTGGTGTAGCGGTGAAATGCGTAGATATCAGGGGGAACACCA
ATGGCGAAGGCAGCTCGCTGGGACGTGACTGACGCTGAGACGCGAAAGCGTGGGGAGCAA
ACAGGATTAGAAACCCTAGTAGTCC
>Otu4601
CCTACGGGTTGCAGCAGTCGAGAATATTCGACAATGGGCGAAAGCCTGATCGAGCGACGC
CGCGTGCAGGATGAAGGCCTTCGGGTCGTAAACTGCGGTAGTAAGATAATAATGTAAATG
AATGTCTTACGGAAAGAGGTGGGTAACTACGTGCCAGCACCAGCGGTAATACGTAGACCT
CAAGCGTTATCCGGATTTATTGGGCGTAAAGGGCGCGTAGGTGGTTTCGTGCGTCTTCTG
TTAAAGCCCACCGCTCAACGGTGGAAGTGCAGGAGATACGACGGGACTAGAGGAGGTTAG
AGGTGCATAGAACACACGGTGTAGGGGTGAAATCCGTTGATATCGTGCGGAATACCAAAG
GCGAAGGCATTGCACTGGGACCTTCCTGACATTGATGCGCGAAAGCGTGGGGAGCAAAAA
GGATTAGATACCCCTGTAGTCC
>Otu4603
CCAGCCTATGGGGGGCAGCAGGCGCGAAACCTTTACAATGCACGAAAGTGTGATAGGGGA
ACTCAGCGTGCTCATGGAAACCATGGGCTTTTCTACAGGGTAAATACCTGTGGGAATAAA
GGATGGGCAAGACCGGTGCCAGCAGCCGCGGTAACACCGGCGTCCTAAGTGGGAATCACT
TATTAGGTCTAAAGCGCTCGTAGTTGGCTTTGTAAATTTCTTGTGAAATCGTGTCGCTTA
ACGGCACGGCGTGCAGGAAACACTGCAAGGCTTGGGACTGGGAGGCGTCAGAAGTATTTC
TTAGGGAGCGGTAAAATGTGATAATCTAAGAAGGACTACCTGTGGCGAAGGCGTCTGACG
AGAACAGGTTCGACAATCAGGAGCGAAGGCTAGGGGAGCAAACCGGATTAGATACCCCAG
TAGTCC
>Otu4605
CCAGCCTACGGGTCGCAGCAGTGGGGAATATTGGACAATGGGCGCAAGCCTGACCCAGCC
ATGCCGCGTGGACGACGAAGGCCCTCCGGGTCGTAAAGTCCTTTTATCTGGGGCTAAAAG
CCGTTTTTGAGCGGAATTGAAGGTACCAGAGGAATAAGCACCGGCAAACTACGTGCCAGC
AGCCGCGGTAATACGTAGGGTGCAAGCGTTGTCCGGATTTACTGGGTTTAAAGGGTGCGT
AGGCGGGAGGATAAGTCGGTGATGAAATGGGAGGGCTCAACCTTACCCGCTGTTACCGAT
ACTGCCCTTCTTGAGTTAAGCAGAGGTAGCTGGAATGAGCAGTGTAGCGGTGAAATGCGT
AGATATTACTCAGAACACCAATTGAGAAGTCAGGCTACTGGGCTTCAACTGACGCTGAGG
CACGAAAGCGTGGGGATCAAACAGGATTAGAAACCCTCGTAGTCC
>Otu4613
CCAGCCTATGGGATGCTGCAGTAACGAATATTCCGCAATGGGCGCAAGCCTGACGGAGCA
ATGCCGCGTGCAGGATGAAGCGGTTTCGCCGTGTAAACTGCTGTCAGACCCCAGAAACCA
TGATCGGGGTCAGAGGAAGGGCCGGCTAACCCTGTGCCAGCAGCCGCGGTAATACAGGGG
GCCCGAGCGTTAATCGGAATCACTGGGCTTAAAGGGTGCGTAGGCGGACTCGAAGGTGCA
TTGTGAAATCCCTTGGCTCAACCAAGGAATTGCAGTGCATACCACGAGTCTCGAGGCAGG
TAGGGGTAGCTGGAACGCTCGGTGGAGCGGTGAAATGCGTAGATATCGAGCGGAACGCCG
AAGGTGAAGACAAGCTACTGGGCCTGACCTGACGCTGAGGCACGAAAGCGTGGGGATCAA
ACAGGATTAGAAACCCCCGTAGTCC
>Otu4618
CCAGCCTATGGGAGGCAGCAGTGGGGAATATTGGACAATGGGCGCAAGCCTGATCCAGCC

ATGCCGCGTGAGTGATGACGGCCCTAGGGTTGTAAAGCTCTTTCACCCACGACGATAATG
ACGGTAGTCGGAGAAGAAGCCCCGGCTAACTTCGTGCCAGCAGCCGCGGTGATACGAAGG
GGGCTAGCGTTGTTCGGAATTACTGGGCGTAAAGCGCACGTAGGCGGATCGTTAAGTCGG
GGGTGAAAGCCCAGGGCTCAACCCTGGAATGGCCTTCGATACTGGCGATCTGGAGAATGG
GAGAGGTGAGTGGAATTCCTAGTGTAGAGGTGAAATTCGTAGATATTAGGAAGAACACCA
GTGGCGAAGGCGGCTCACTGGCCCATTTCTGACGCTGAGGTGCGAAAGCGTGGGGAGCAA
ACAGGATTAGATACCCTTGTAGTCC
>Otu4621
CCAGCCTATGGGTCGCACCAGTGGGGAATATTGGACAATGGGGGAAACCCTGATCTAGCA
ATGCCGCGTGCGTGATGAAGGCCTTCGGGTTGTAAAGCGCTTTTATAGGTGACGATAATG
ACGGTAACCTATGAATAAGCCCCGGCTAACTTTGTGCCAGCAGCCGCGGTAATACGAAGG
GGGCTAGCGTTGTTCGGAATGACTGGGCGTAAAGGGCGCGTAGGCGGTAAGATACGTTTG
GTGTGAAAGTCCTGGGCTTAACCTGGGGACTGCACTGAATACGGTTTTACTGGAGTGCGA
GAGGGGATAAGGGAATTGTGCGTGTAGAGGTGAAATTCGTAGATATGCACAAGAACACCG
GTGGCGAAGGCGCTTATCTGGCTCGCAACTGACGCTAAGGCGCGAAAGCGTGGGGAGCAA
ACAGGATTAGAGACCCCTGTAGTCC
>Otu4623
CCAGCCTATGGGTTGCTGCAGGCGCGCAAACTCTCCAATGTGCGAAAGCGTGAGAGGGGA
ATTCCTAGTGCTTACGGTTTATCCGTAGGCTTTTGCCATGTGTAAATAGCATGGCGAATA
AGTGGTGGGTAAGACTGGTGCCAGCCGCCGCGGTAACACCAGCGCCACAAGTGGCCATCG
CGATTATTGGGCCTAAAGCGTTCGTAGTCGGTTAAGTGCATCCTCTGTGAAATCGCAGCG
CCTAACGTTGCGGCATGCGGAGGACACGACTTAACTTGAGACCGGGAGGCGTCGAGGGTA
TTCCAAGGGGACCGGTAAAATGGTATAATCCTTGGAGGACCACCTGTGGCGAAGGCGCTC
GACGAGAACGGATCTGACGATGAGGAACGAAAGCCAGGGGAGCAAACCGGATTAGAGACC
CTCGTAGTCC
>Otu4626
CCAGCCTACGGGAGGCTGCAGTCGAGAATTTTTCTCAATGGGCGAAAGCCTGAAGGAGCG
ACGCCGCGTGGGGGATGAATGGCTTCGGCCCGTAAACCCCTGTCATTTGGGAACAATGTC
CCGACAATAACATTGTCGGGATTGATAGTACCGGAAGAGGAAGGGACGGCTAACTCTGTG
CCAGCAGCCGCGGTAATACAGAGGTCCCAAGCGTTGTTCGGATTTACTGGGCGTAAAGGG
TGCGTAGGTGGTCGGGTAAGTCTGATGTGAAATCTCGGAGCTTAACTCCGAAACTGCATT
GGATACTATTCGGCTTGAGGAATGGAGGGGAGACTGGAATACTTGGTGTAGCAGTGAAAT
GCGTAGATATCAAGTGGAACACCAGTGGCGAAGGCGAGTCTCTGGACATTTTCCTGACGC
TGAGGCACGAAAGCCAGGGGAGCAAACGGGATTAGAAACCCCGGTAGTCC
>Otu4630
CCAGCCTATGGGTGGCAGCAGTGGGGAATATTGGACAATGGGCGAAAGCCTGATCCAGCA
ATGCCGCGTGGATGAAGAAGGTCTTCGGATTGTAAAGTCCTTTCGACGGGGACGATGATG
ACGGTACCCGTAGAAGAAGCCCCGGCTAACTTCGTGCCAGCAGCCGCGGTAATACGAAGG
GGGCTAGCGTTGCTCGGAATGACTGGGCGTAAAGGGCGCGTAGGCGGTTTGGTTAGTTAG
ACGTGAAATTCCTGGGCTCAACCTGGGGGCTGCGTTTAATACAGCCAGACTTGAGTGTGA
AAGAGGGTCGTGGAATTCCCAGTGTAGAGGTGAAATTCGTAGATATTGGGAAGAATACCC
GTGGCGAAGGCGGCCAACTGGACCAACATTGACGCTGAGGCGCGAAAGCGTGGGTAGCAA
ACAGGATTAGATACCCCGGTAGTCC
>Otu4631
CCAGCCTATGGGTTGCTCCAGTAGGGAATATTGCAAAATGGGCGGAAGCCCGATGCAGCA
ACGCCGCGTGTGCGATGAAGGCCTTCGGGTCGTAAAGCACTTTTCAGAGGGATGAGGAAG
GACAGTACCTCTGGAATAAGTCTCGGCTAACTACGTGCCAGCAGCCGCGGTAAAACGTAG
GAGGCGAGCGTTATCCGGATTTACTGGGCGTAAAGCGCGTGCAGGCGGTTTGGAAAGTTG
GATGTGAAAGCTCCTGGCTCAACTAGGAGAGGTCGTTCAAGACTTTCAGACTAGAGGATG
GTAGAGGGAGGTGGAATTCCGGGTGTAGTGGTGAAATGCGTAGATATCCGGAGGAACACC
AGTGGCGAAAGCGGCCTTCTAGACCATAACTGACGCTCATACGCGAAAGCTAGGGTAGCA
AACGGGATTAGAAACCCCCGTAGTCC
>Otu4633
CCAGCCTATGGGAGGCACCAGTGGGGAATCTTTCACAATGGGGGCAACCCTGATGGAGCG
ACGCCGCGTGGAGGATAAGGTCTTCGGATTGTAAACTCCTGTCATGCGAGAGCAAGGCCT
GATGGTTAACACCCGTCATGCTTGATAGTATCGCAAGAGGAAGAGACGGCTAACTCTGTG
CCAGCAGCCGCGGTAATACAGAGGTCTCAAGCGTTGTTCGGAATCACTGGGCGTAAAGGG
TGTGCAGGCGGTGTGCTAAGTCAGATGTGAAAGCCCGGGGCTCAACCCCGGAACTGCATC

CGATACTGGCATGCTAGAGAATTGGAGAGGAGTCTAGAATTCACGGTGTAGCAGTGAAAT
GCGTGGATATCGTGAGGAATACCAAGTGCGAAGGCAGGACTCTGGACATTATCTGACGCT
GAGACACGAAGGCCAGGGGAGCAAACGGGATTAGATACCCGGGTAGTCC
>Otu4637
CCAGCCTATGGGTTGCAGCAGTGGGGAATCTTGGACAATGGGCGCAAGCCTGATCCAGCC
ATGCCGCGTGAGTGAAGGAGGTCTTCGGATTGTAAAGCTCTTTTGCCAGGGACGATAATG
ACGGTACCTGGAGAATAAGCCCCGGCAAACTTCGTGCCAGCAGCCGCGGTAATACGAAGG
GGGCTAGCGTTGTTCGGAATTACTGGGCGTAAAGCGCACGTAGGCGGGTCGTTAAGTCAG
GGGTGAAATCCCGGAGCTCAACTCCGGAACTGCCTTTGATACTGGCGATCTTGAGATCGG
AAGAGGTGAGTGGAATTCCCAGTGTAGAGGTGAAATTCGTAGATATTGGGAAGAACACCA
GTGGCGAAGGCGGCTCACTGGTCCGATACTGACGCTGAGGTGCGAAAGCGTGGGGAGCAA
ACAGGATTAGAAACCCCCGTAGTCC
>Otu4641
CCAGCCTACGGGTCGCAGCAGTGGGGAATCTTGCGCAATGGACGAAAGTCTGACGCAGCA
ACGCCGCGTGGGTGATGAAGGTCTTCGGATCGTAAAGCCCTGTCGGAGGGGAAGATGCTT
TCGCCGTCTAATAGATGGCGGAGGTGACGGTACCCTTAAAGGAAGCACCGGCTAACTCTG
TGCCAGCAGCCGCGGTAATACAGAGGGTGCAAGCGTTGTTCGGAATTATTGGGCGTAAAG
GGGGTGTAGGCGGCATGCATAGTCTTCTGTGAAATCTACGGGCTTACCCCGTAACGTGCA
GAGGATACTGGCAAGCTAGAGTGTCGGAGAGGATAGTGGAATTCCAGGTGTAGAGGTGAA
ATTCGTAGAGATCTGGAGGAACACCAGTGGCGAAGGCGACTATCTGGCTGACAACTGACG
CTGAGACTCGAAAGCATGGGGAGCAAACAGGATTAGATACCCGAGTAGTCC
>Otu4643
CCAGCCTACGGGTCGCAGCAGTAGGGAATATTGCGCAATGGGGGAAACCCTGACGCAGCG
ACGCCGCGTGAGTGATGAAGGCCTTAGGGTCGTAAAGCTCTGTTGCACGGGAAGAAAAAA
ATGACGGTACCGTGCGAGAAAGGATCGGCTAACTTCGTGCCAGCAGCCGCGGTAAGACGA
GGGATCCTAGCGTTGTTCGGAATCATTGGGCGTAAAGCGTATGTAGGCGGCCTTATAAGT
CAGGTGTGAAAGCCCCGGGCTCAACCCGGGAAGTGCATCTGATACTGTTTGGCTTGAGTA
CTGGAGAGGATAGTGGAATTCCTGGTGTAGTGGTGAAATACGTAGATATCAGGAGGAACA
CCGGTGGCGAAGGCGGCTATCTGGCCAAGTACTGACGCTGAGATACGAAAGCGTGGGGAG
CAAACAGGATTAGATACCCGTGTAGTCC
>Otu4650
CCAGCCTATGGGGGGCTGCAGTGGGGAATCTTCCGCAATGGACGAAAGTCTGACGGAGCA
ACGCCGCGTGAGTGAAGAAGGCCTTCGGGTTGTAAAGCTCTGTTATTGGGGACGAACGTT
CTTTATGTAAACAATGTGGAGAAGTGACGGTACCCAAGGAGGAAGCCACGGCTAACTACG
TGCCAGCAGCCGCGGTAATACGTAGGTGGCAAGCGTTGTCCGGAATTATTGGGCGTAAAG
CGCGCGTAGGTGGGATAGTAAGTCTGCTTTAAAAGTGCGGGGCTCAACCCCGTGATGGGG
TGGAAACTGCTATTCTTGAGTGCAGGAGAGGAAAGCGGAATTCCCAGTGTAGCGGTGAAA
TGCGTAGATATTGGGAGGAACACCAGTGGCGAAGGCGGCTTTCTGGACTGTGTCTGACAC
TGAGGCGCGAAAGCTAGGGGAGCGAACGGGATTAGAGACCCTAGTAGTCC
>Otu4660
CCAGCCTATGGGGGGCTGCAGTCGAGGATTTTTCTCAATGGGGGAAACCCTGAAGGAGCG
ACGCCGCGTGAGGGATGAAGGTCTTCGGATTGTAAACCTCTGTCATCTGGGATCAAAGTG
CTCTACCTAACACGTGGAGTATTGCTAGTACCAGAAGAGGAAGCAGTGGCTAACTCTGTG
CCAACAGCCGCGGTAATACAGAGACTGCAAGCGTTGTTCGGATTCATTGGGCGTAAAGGG
TGCGCAGGCGGTGTGCTAAGTCGGATGTGAAATCTCACAGCCTAACTGTGATAGGTCATT
CGAAACTGGCATGCTAGAGGATTGGAGAGGAGACTGGAATAGTTGGTGTAGCAGTGAAAT
GCGTAGAGATCAACTAGAACACCGGTGGCGAAGGCGAGTCTCTGGACAATTCCTGACGCT
CAGGCACGAAAGCCAGGGGAGCAAACGGGATTAGAAACCCGCGTAGTCC
>Otu4663
CCAGCCTACGGGGCGCTCCAGTAACGAATCTTCCGCAATGCACGAAAGTGTGACGGAGCG
ACGCCGCGTGTGGGAGAAGCCCTTCGGGGTGTAAACCGCTGTCAGGGGTTAGAAAGTTCT
GATCAACCCCAGAGGAAGGCACGGCTAACTCTGTGCCAGCAGCCGCGGTAAGACAGAGGT
GCCAAGCGTTAGGCGGAATCACTGGGCTTAAAGCGTGTGTAGGCGGGTGCCTAAGTACCT
TGTGAAATCCCATGGCTCAACCATGGAACTGCTTGGTATACTGAGCATCTTGAGCCACCT
AGGGGCTACTGGAACAAATGGTGGAGCGGTGAAATGCGTAGATATCATTTGGAACGCCAA
TGGTGAAAACAGGTAGCTGGGGGTGTGCTGACGCTGAGACACGAAAGCCAGGGGAGCAAA
CGGGATTAGAAACCCCAGTAGTCC
>Otu4664

CCAGCCTACGGGGGGCAGCAGTGGGGAATCTTGCACAATGGGGGAAACCCTGATGCAGCG
ACGCCGCGTGAGCGATGAAGCCCTTCGGGGTGTAAAGCTCTTTCGACGGGAACGATAATG
ACGGTACCCGGAGAAGAAGCTGCGGCTAACTACGTGCCAGCAGCCGCGGTAATACGTAGG
CAGCAAGCGTTGTTCGGAATTACTGGGCGTAAAGAGTATGTAGGCGGTTCGCCAAGTTTG
GTGTGAAATCTCCCGGCTTAACTGGGAGGGTGCGCCGAAAACTGGCGGGCTTGAGGGTGG
GAGAGGTAAGCGGAATTTCTGGTGTAGCGGTGAAATGCGTAGATATCAGAAGGAACACCT
GTGGTGTAGACAGCTTACTGGACCATTTCTGACGCTGAGATACGAAAGCGTGGGTAGCAA
ACAGGATTAGATACCCGTGTAGTCC
>Otu4665
CCAGCCTACGGGGCGCAGCAGTGGGGAATTTTACGCAATGGGCGAAAGCCTGACGTAGCG
ACACCGCGTGAGTGAAGAAGGACTTTGGTTCGTAAAACTCTGTCAACAGGGACGAAAAAA
ATGACGGTACCTGTGGAGGAAGCATCGGCTAACTACGTGCCAGCAGCCGCGGTAAGACGT
AGGATGCGAGCGTTGTCCGGATTTATTGGGCGTAAAGAGTTCGTAGGTGGTTTGTTAAGT
CTGATGTTAAAGACTGGGGCTCAACCTCGGAAATGCATTGGATACTGGCAGACTTGAGTG
TGGTAGAGGCAAGCGGAATTCCCAGTGTAGCGGTGAAATGCGTAGATATTGGGAAGAACA
CCGGTGGCGTAAGCGGCTTGCTGGGCCATAACTGACACTGAGGAACGAAAGCTAGGGTAG
CGAATGGGATTAGAAACCCCAGTAGTCC
>Otu4666
CCAGCCTATGGGGGGCAGCAGCAACGAATATTCCGCAATGGGCGAAAGCCTGACGGAGCG
ACGCCGCGTGAGGGATGAAGTCCCTTCGGGATGTAAACTTCAATAGTACGTCAGGAACCA
ATCCTGGCTAATACCTTGGAAAGATGACCAGCGTACGAAAGAGGCGGCTAACTCCGTGCC
AGCAGCCGCGGTAATACGGAGGCCTTGAGCGTTGTTCGGAATCACTGGGCTTAAAGCGCG
TGTAGGCGGGACGGTAAGTACCTTGTGAAATCCCCGGCTTCAATCCGGGAACTGCTTGGT
ATACTGCCGTTCTTGAGGCAGGTAGGGGTCACTGGAACCTTAGGTGGAGCGGTGAAATGC
GTAGATATCTAAGGGAACGCCTGTGGCGAAAGCGGGTGACTGGGCCTGACCTGACGCTGA
GACGCGAAAGCGTAGGGAGCAAACGGGATTAGATACCTTGTAGTCC
>Otu4668
CCAGCCTATGGGGGGCAGCAGTCGAGGATCTTCGTCAATGGACGAAAGTCTGAACGAGCG
ACGCCGCGTGTGCGATGAAGGCCTTCGGGTTGTAAAGCACAAAAGTGGGGATAAAAGCCG
AAAGGTCTGATTGATCCACAGTAAGCTCGGGCTAAGTTCGTGCCAGCAGCCGCGGTAAGA
CGAACCGAGCGAACGTTGTTCGGAATCACTGGGCTTAAAGGGCGCGTAGGCGGGCGATCA
AGTCGGTGGTGAAATCCTACAGCTTAACTGTAGAACTGCCTCCGATACTGGTTGTCTCGA
GGAAGGTAGGGGCGTACGGAACAGTTGGTGGAGCGGTGAAATGCGTTGATATCAACTGGA
ACTCCGGTGGCGAAGGCGGTACGCTGGACCTTTTCTGACGCTGAGGCGCGAAAGCCAGGG
GAGCAAACGGGATTAGAAACCCGCGTAGTCC
>Otu4669
CCAGCCTATGGGTGGCAGCAGTCGAGAATATTCCACAATGGGCGAAAGCCTGATGGAGCG
ACGCCGCGTGCAGGATGAAGGCCTTCGGGTCGTAAACTGCGGTAGTAAAGTAATAATGCA
AATGAATGCTTTATGGAAAGAGGTTGGTAACTACGTGCCAGCACCCGCGGTAAAACGTAG
ACCTCAAGCGTTATCCGGATTTATTGGGCGTAAAGAGCATGTAGGGGGTTTTGCGCGTCT
TCTGTTAAAGCTCGGGGCCTAACCCCGAAAGTGCAGGAGATACGGCAAGACTAGAGGATG
TTAGAGGTACCAGGAACACACGGTGTAGGGGTGAAATCCGTTGATATCGTGTGGAACACC
AAAGGCGAAGGCAGGGTACTGGGACACTCCTGACCCTGAGATGCGAAAGCGTGGGGAGCA
AAAAGGATTAGAAACCCTAGTAGTCC
>Otu4670
CCAGCCTATGGGTCGCACCAGCGACGAATCTTTCGCAATGGGCGCAAGCCTGACGGAGCA
ATGCCGCGTGTGGGATGAAGCACCTCCGGTGTGTAAACCACTGTCAGGGGACGGAACCAA
TGATTGTCCCCAAAGGAAGTGGCGACTAACTCTGTGCCAGCAGTCGCGGTAATACAGAGG
CCACGAGCGTTAGTCGGAATCACTGGGCTTAAAGGGTGCGTAGGCGGCTCAGCACGTGCT
TCGTGAAAGCCCCTGGCTCAACCAGGGAATTGCGGAGCATACGGCTGGGCTTGAGGCGTG
TAGGGGCTGTCAGAACAGTAGGTGGAGCGGTGAAATGCGTAGATATCTACTGGAATGCCG
ATGGTGAAGACGGGCAGCTGGGCACGACCTGACGCTGAGGCACGAAAGCGTGGGGAGCAA
ACAGGATTAGATACCCCCGTGTCC
>Otu4672
CCAGCCTATGGGTGGCACCAGTGAGGAATATTGGACAATGGGCGAAAGCCTGATCCAGCA
ATACCGCGTGAGTGAAGAAGGCCTTAGGGTTGTAAAGCTCTTTCAGCAGGGAAAATAATG
ATGGTACCTGCAAAAGAAGTCCCGGCTAACTCCGTGCCAGCAGCCGCGGTAATACGGAGG
GGACTAGCGTTGTTCGGATTTACTGGGCGTAAAGAGCGCGTAGGCGGGTTATTAAGTTAG

AAGTGAAAGCCCAGGGCTTAACCCTGGAACTGCTTCTAAAACTGGTAACCTAGAGTATGG
GAGGGGACAGTGGAATTCCTAGTGTAGAGGTGAAATTCTTAGATATTAGGAGGAACATCG
GTGGCGAAAGCGACTGTCTGGAACATTACTGACGCTGAGGTGCGAAAGCGCGGGGAGCAA
ACAGGATTAGAAACCCCAGTAGTCC
>Otu4675
CCAGCCTACGGGGCGCACCAGTCAAGAACCTTCCACAATGGACGAAAGTCTGATGGAGGG
ACGCCGCGTGATTGATGAAGTCCCTCTGGGACGTAAAGATCTTTTGTAGACTACTAATTT
TATTGAAGAGTCTAAGAATAAGGGGTTGCTAAACTCGTGCCAGCAGCAGCGGTAATACGA
GTGCCCCAAGCGTTATCCGGAATTACTGGGCGTAAAGGGTGTGTAGGTGGTCGTGTTAGT
CTCTCGTTAAATTCTTCGGCTTAACCGGGGGCATGCGGGGGAAACGGCACGACTTGAGGA
TGCGAGGGGCCTGTGGAACTCATGGAGTAGGGGTAATATCCGTTGATATCATGGGGAACA
CCGAAAGCGAAGGCAGCAGGCTGGCGCATTCCTGACACTGAAACACGAAAGCGTAGGTAG
CGAATGGGATTAGAAACCCTTGTAGTCC
>Otu4678
CCAGCCTACGGGGTGCACCAGTGAGGAATATTGCTCAATGGGCGAAAGCCTGAAGCAGCG
ACGCCGCGTGAGGGATGAAATCCGTTAGGATGTAAACCTCTTTTTCAGGGGATGAATGTA
CCCTTTTAGGGTATTGACCGTACCCTGAGAATAAGCACCGGCCAACTACGTGCCAGCAGC
CGCGGTAATACGTAGGGTGCGAGCGTTGTCCGGAATTACTGGGTGTAAAGGGTCCGCAGG
CGGGCAAGTAAGTCGGTGGTGAAATCTCACGGCCTAACCGTGAAACTGCCCTCGATACTA
CTTGTCTTGAGTTCGAGAGAGGATGATGGAATTCATGGTGTAGCGGTGAAATGCGTAGAG
ATCATGAAGAACACCAGTGGCGAAGGCGGTCATCTGGCTCGAAACTGACGCTCAGGGACG
AAAGCGTGGGTAGCAAACAGGATTAGAGACCCTAGTAGTCC
>Otu4684
CCAGCCTACGGGGGGCACCAGTCGGGAATTTTGCTCAATGGGCGCAAGCCTGAAGCAGCA
ACGCTGCGTGAGGGATGAAGGCCTTCGGGTTGTAAACCTCTTTTACCAGGGAAGACAATG
ACGGTACCTGGAGAATAAGTCACGGCTAACTACGTGCCAGCAGCCGCGGTAATACGTAGG
TGACGAGCGTTGTCCGGATTTACTGGGCGTAAAGAGCGCGCAGGCGGTCGTATAAGTCGA
GTGTGAAAGCCCCCGGCTCAACTGGGGAGGGTCGCTCGATACTATTCGACTCGAAGGTGG
GAGAGGGAAGCGGAATTCCCGGTGTAGTGGTGAAATGCGTAGATATCGGGAGGAACACCA
GTGGCGAAGGCGGCCTCCTGGCCCATTCTTGACGCTGAGGCGCGAAAGCTAGGGGAGCAA
ACGGGATTAGAAACCCGAGTAGTCC
>Otu4685
CCAGCCTATGGGGTGCTCCAGTGGGGAATATTGGACAATGGGCGAAAGCCTGATCCAGCA
ATGCCGCGTGTGTGAAGAAGGTCTTCGGATTGTAAAGCACTTTCGCACGCGACGATGATG
ACGGTAGCGTGAGAAGAAGCCCCGGCTAACTTCGTGCCAGCAGCCGCGGTAATACGAAGG
GGGCAAGCGTTGTTCGGAATTACTGGGCGTAAAGCGCACGCAGGCGGTTGTTCTAGTTAG
AAGTGAAAGCCCCGGGCTCAACCTGGGAACTGCTTTTAATACTGGACAGCTAGAATCATG
GAGAGGGTAACGGAATTCCGAGTGTAGAGGTGAAATTCGTAGATATTCGGAAGAACACCA
GCGGCGAAGGCGGTTACCTGGCCATGTATTGACGCTCATGTGCGAAAGCGTGGGGAGCAA
ACAGGATTAGATACCCTAGTAGTCC
>Otu4688
CCAGCCTATGGGTTGCTGCAGTGGGGAATTTTCCACAATGGGCGAAAGCCTGATGGAGCA
ACGCCGCGTGTGTGATGAAGGTCTTCGGATCGTAAAGCACTTTCGACCGGGACGAAGAAC
TGTTGGCTAATATCCAACAGCCTGACGGTACCGGGAGAAGAAGCACCGGCTAACTCTGTG
CCAGCAGCCGCGGTAATACAGAGGGTGCAAGCGTTGTTCGGAATTATTGGGCGTAAAGCG
CGTGTAGGCGGCTTATCAAGTCAGATGTGAAAGCCCTCGGCTCAACCGAGGAAGTGCGTC
TGAAACTGATGAGCTTGAGTACCGGAGAGGGTAGCGGAATTCCGCAAGTAGAGGTGAAAT
TCGTAGAGATGCGGAGGAACACCGGTGGCGAAGGCGGCTACCTGGACGGTCACTGACGCT
GAGACGCGAAAGCGTGGGGAGCAAACAGGATTAGAAACCCTTGTAGTCC
>Otu4690
CCAGCCTATGGGTTGCAGCAGTCGAGAGTCTTCGGCAATGGGCGAAAGCCTGACCGAGCG
ACGCCGCGTGGAGGATGAAGGCCTTCGGGTTGTAAACTCCCGTCACGGTGCAGGAAACGA
TCCAAGGTAATACTTAGGGAAACTGACCACACCGAGAGGAAGTCACGGCTAACTCTGTGC
CAGCAGCCGCGGTAAGACAGAGGTGGCAAGCGTTGTTCGGAATTACTGGGCATAAAGGGC
GCGTAGGCGGCGAGGATATGTCAGGGGTGAAAGCCCCCGGCTCAACCGGGGAACAGCCTC
TGAAACTACCTTGCTTGAGTGCAAGAGAGGAGAGTGGAACTCGAGGTGGAGCGGTGAAAT
GCGTAGATATCTCAGGGAACGCCGGTGGCGAAAGCGACTCTCTGGTTTGCAACTGACGCT
GAGGCGCGAAAGCCAGGGGAGCAAACGGGATTAGAAACCCCTGTAGTCC

>Otu4695
CCAGCCTACGGGTGGCAGCAGTAAGGGATATTGCGCAATGGGCGAAAGCCTGACCCAGCA
ACGCCGCGTGAGGGATGAAGGCCTTCGGGTCGTAAACCTCTTTTCTCAGGGAAGATAATG
ACGGTACCTGAGGAAGAAGCCACGGCTAACTACGTGCCAGCAGCCGCGGTAATACGTAGG
TGGCGAGCGTTGTCCGGATTTACTGGGCGTAAAGAGCGCGCAGGCGGTCGAATTAGTCGA
ATGTGAAAGCCCCCGGCTCAACTGGGGAGGGTCATTCGATACTGTTCGACTCGAAGGCAG
GAGAGGGAAGCGGAATTCCCGGTGTAGTGGTGAAATGCGTAGATATCGGGAGGAACACCA
GTGGCGAAGGCGGCTTCCTGGCCTGTTCTTGACGCTGAGGCGCGAAAGCTAGGGGAGCAA
ACGGGATTAGATACCCGTGTAGTCC
>Otu4699
CCAGCCTACGGGGCGCTGCAGTGGGGAATATTGCGCAATGGGGGAAACCCTGACGCAGCG
ACGCCGTGTGAGTGATGAAGACTTTCGGGTCGTAAAGCTCTGTCGGATGGGACGAATAAG
GGCTGTGAAAATATTGCAGTCCGATGACGGTACCGTCGAAGGAAGCACCGGCTAACTCCG
TGCCAGCAGCCGCGGTAATACGGGGGGTGCAAGCGTTGTTCGGAATTATTGGGCGTAAAG
AACGTGTAGGCGGGTCTGTATGTCAGATGTGAAAGCCCTTGGCTCAACTGGGGAAGTGCA
TCTGAAACTGCAGGTCTTGAGTACGAGAGAGGAGAGTGGAATTCCCAGTGTAGAGGTGAA
ATTCATAGATATTGGGAAGAACACCGGCGGCGAAAGCGGCTCTCTGGCTCGATACTGACG
CTGAGACGTGAAAGCGTGGGGAGCAAACAGGATTAGATACCCTCGTAGTCC
>Otu4705
CCAGCCTATGGGTCGCAGCAGTGGGGAATATTGGACAATGGGCGCAAGCCTGATCCAGCA
ATGCCGCGTGTGTGAAGAAGGTCTTCGGATTGTAAAGCACTTTCGACGGGGACGATGATG
ACGGTACCCGTAGAAGAAGCCCCGGCTAACTTCGTGCCAGCAGCCGCGGTAATACGAAGG
GGGCTAGCGTTGCTCGGAATGACTGGGCGTAAAGGGCGCGTAGGCGGATTGTACAGTCAG
ATGTGAAATTCCCGGGCTTAACCTGGGGGCTGCATTTGATACGTATGGTCTTGAGTGAGG
AAGAGGGTCGTGGAATTCCCAGTGTAGAGGTGAAATTCGTAGATATTCGCAAGAACACCA
GTGGCGAAGGCGGCTCACTGGCCCGGAACTGACGCTGAGACGCGAAAGCGTGGGGATCAA
ACAGGATTAGATACCCTTGTAGTCC
>Otu4708
CCAGCCTATGGGTTGCAGCAGGCGAGAAAATTTTGCAATGCGCGCAAGCGTGACAGAGCA
AGCCAGAGTGTTTTTCTTTATTTAAAGAACAACTTTTGCGGGATGTAAATAGTCTCGCGA
ATAAGGACTGGGCAAGACTGGTGCCAGCCGCCGCGGTAATCCCAGCAGTCCAAGTCGCAG
CCACTTTTATTGGGTCTAAAACATCCGTAGCTTGCTTAGTAAGTCTTTTGTCAAATCTGG
CATCTTAAGTGTCAGGCGAGCAGAAGATACTGCTTTGCTAGAGACCGGGAGACGTAAGGA
GTACAGTTTGGGTAGCGGTAAAATGCGTTGATCCTAACTGGACTAACAATAGCGAAGGCA
CCTTACGAGAACGGATCTGACAGTGAGAGATGAAGGCTAGGGGCGCAAATTGGATTAGAA
ACCCTCGTAGTCC
>Otu4717
CCAGCCTACGGGGGGCAGCAGTGGGGGATATTGCACAATGGGCGCAAGCCTGATGCAGCG
ACGCCGCGTGGAGGATGAAGGTTTTCGGATTGTAAACTCCTGTTACGTGGGAAAAAAATGC
TCTGTTTAAATAAAGCAGGGAGCTGATGGTACCACGAGAGAAAGCACCGGCAAACTTCGT
GCCAGCAGCCGCGGTAATACGAAGGGTGCAAGCGTTATTCGGAATAACTGGGTGTAAAGA
GCGTGCAGGCGGTTTTGTAAGTCATTTGTAAAAGAGCTCGGCTTAACTGAGCAACTGCAG
ATGAAACTGTAAGACTAGAGTGCAGGAGAGAAAGGTAGAATTCTCGGAGTAGCGGTAAAA
TGCGTAGATCTCGAGAGGAATACCGGTTGCGAAGGCGGCCTTTTGGCCTGTAACTGACGC
TCAAGCGCGAAAGCGTGGGGAGCAAACAGGATTAGAGACCCCCGAGTCC
>Otu4718
CCAGCCTATGGGAGGCTGCAGCTAAGAATATTCCGCAATGGACGAAAGTCTGACGGAGCG
ACGCCGCGTGGATGACGAAGGCCGAAAGGTTGTAAAGTCCTTTTGTTGGGGAAGAATAAC
CATGGGAGGGAATGCCCGTGGGATGACATGAACCGACGAATAAGCCCCGGCTAACTACGT
GCCAGCAGCCGCGGTAACACGTAGGGGGCGAGCGTTGTTCGGAATTATTGGGCGTAAAGG
GCGCGCAGGCGGTGCGGTAAGTCACCTGTGAAATCTCTGGGCTTAACTCAGAGCCTGCAG
GCGAAACTGCCGTGCTGGAGTGTGGGAGAGGTGCGTGGAATTCCCGGTGTAGCGGTGAAA
TGCGTAGATATCGGGAGGAACACCTGTGGCGAAAGCGGCGCACTGGACCACAACTGACGC
TGAGGCGCGAAAGCTAGGGGAGCAAACAGGATTAGAAACCCGGGTAGTCG
>Otu4726
CCAGCCTATGGGGTGCAGCAGTCGAGAATCATTTGCAATGGGCGAAAGCCTGACAATGCG
ACGCTGCGTGAGCGAAGAAGGCCTTCGGGTTGTAAAGCTCTTTCGCTTGGGAACAAGAGA
TGCCGGCGAACATCCGGCAAATTTGAGCGTACCAGGTAAAGAAGCACCGGCTAACTCCGT

GCCAGCAGCTGCGGTAATACGGAGGGTGCAAGCATTAATCGGATTTATTGGGCGTAAAGG
GCGCGTAGGCGGTTATGAAAGTCAGATGTGAAATCCCGGGGCTCACCCCCGGAACAGCAT
TTGAAACTCCATGACTAGAGGGTAGGCGGAGAAAACGGAATTCCACAAGTAGCGGTGAAA
TGCGTAGATATGTGGAAGAACACCAGTGGCGAAGGCGGTTTTCTAGCTTATTCCTGACGC
TGAGGCGCGAAAGCAAGGGGATCAAACAGGATTAGAAACCCTTGTAGTCC
>Otu4727
CCAGCCTATGGGTCGCAGCAGTAGGGAATATTGCGCAATGGGGGAAACCCTGACGCAGCG
ACGCCGCGTGGGCGACGAAGGCCTTCGGGTCGTAAAGCCCTTTTGGCAGGGACGAGTGAG
GACGGTACCTGCCGAATAAGTCTCGGCTAACTACGTGCCAGCAGCCGCGGTAAAACGTAG
GAGGCGAGCGTTATCCGGATTCACTGGGCGTAAAGCGAGCGTAGGCGGTTGGGTAAGTCG
GACGTGAAAGCCCCTGGCTCAACTGGGGGAGGCCGTTCGATACTGCCCGGCTTGAGGGTG
AGAGAGGGAAGTGGAATTCCCGGTGTAGCGGTGGAATGCGTAGAGATCGGGAGGAACACC
AGTGGCGAAAGCGGCTTCCTGGCTCACCCCTGACGCTGATAGCTCGAAAGCGTGGGGAGC
AAACGGGATTAGAAACCCTAGTAGTCC
>Otu4729
CCAGCCTACGGGGGGCACCAGTGAGGAATATTGGACAATGAGCGAAAGCTTGATCCAGCA
ATGTCACTTGGAGTGTGGAAGGCTATTTAGGTCGTAAAGCTCTTTTAATAGTTCTAATTG
TGATATCGGCTATTGAATAAGTCCCGGCTAACTTCGTGCCAGCAGCCGCGGTAAAACGGG
GGGGGCGAGCGTTATTCATAATGATTGGGCGTAAAGAGGGTGCGCAGGTGGCTTTACCAG
CTTTATATGAAAGAACAGGACTTAGTTCTGTGTTGGTATAAAGGACGTAATAAGCTAGGT
TTTTTGAGAAGAAGGCGGTACTCTTAGTGGTAGAGGTGAAATTCGGTAACAGTGTAGAGG
ACCAACAAGAGGCAAAGGCGGCTTTCTAGCAAAAATTGACACTCAGGCACGAAAAGCATG
GGTAGCAAATGGGATTAGATACCCGAGTAGTCC
>Otu4730
CCAGCCTACGGGTTGCACCAGTGAGGAATATTGGGCAATGCCCGTAAGGGTGACCCAGCA
ACGCCGCGTGGAGGATGAAGGTCGTAAGATTGTAAACTCCTGTTAGAGGGGACGAATAGC
TGTGATTCGTAAGGATCATAGTTTGACTGTACCCTCAGAGAAAGCCCCGGCTAACTACGT
GCCAGCAGCCGCGGTAATACGTAGGGGGCAAGCGTTGTCCGGATTTACTGGGTGTAAAGG
GCGCGCAGGCGGGCTTGTAAGTCGGTGGTGAAAGCCTGTGGCTTAACTACAGAACTGCCA
TTGATACTACAAGTCTTGAGTGCGGAAGAGAGAGACGGAATTCCAGGTGTAGTGGTGAAA
TACGTAGATATCTGGAAGAACACCAGTTGCGAAGGCGGTCTCTTGGTCCGTTACTGACGC
TCAGGCGCGAAAGCGTGGGTAGCAAACAGGATTAGAAACCCTGGTAGTCC
>Otu4731
CCAGCCTATGGGTGGCTCCAGTCGAGGATCATTTGCAATGGGCGAAAGCCTGACAGTGCG
ACGCTGCGTGGAGGATGAAGGCCTTCGGGTCGTAAACTCCTGTCATTCGGGAACAATAGT
CTGAGTGTTAATACCACTTGGAAATGATGGTACCGGAAGAGGAAGCCACGGCTAACTCTG
TGCCAGCAGCCGCGGTAATACAGAGGTGGCGAGCGTTGTTCGGATTTACTGGGCGTAAAG
GGTGCGTAGGTGGTTCCGTACGTCGGGTGTGAAATCCCACTGCTCAACGGTGGAAGGTCG
CCCGAAACTGCGGGGCTAGAGTGCAGGAGGGGAGAGCGGAATTCTTGGTGTAGCGGTGAA
ATGCGTTGATATCAAGAAGAACACCGGCGGCGAAGGCGGCTCTCTGGAATGCAACTGACA
CTGAGGCACGAAAGCTGGGGGAGCAAACAGGATTAGAGACCCCCGTAGTCC
>Otu4737
CCGCCTACGGGAGGCAGCAGGCGCGAAACCTTTGCCATGCGCGAAAGCGTGACAGGGGAA
TTCCGAGTGGTAGGATGGTTTTACCTTCCTATCTTTTGGCCAATCCAAACAATTGGCAGA
ATAAGATCTGGGTAAGACCAGTGCCAGCCGCCATGTGTGTGTAGTTGCACCCACATAGCA
CGGTACGCGGTAATACTGGCAGAGCGAGTGGTGTCCACGAATATTGGGTCTAAAGAGTCC
GTAGCTGGACTGTTAAGTCCACTGTGAAATCCGGCGGCTCAACCGCCGGGCGTGCAGTGG
ATACTGGCAGGCTTGGGAGCGGGGGAGGTCAGGAGTACTTGCGGGGTAAGAGTAAAATCT
GTTGATCCTGCAAGGACTACCAGTGGCGAAGGCGCCTGACCAAAACGCGTCCGACAGTGA
GGGACGAAGGCTAGGAGAACGAATCGGATTAGATACCCGAGTAGTCC
>Otu4740
CCGCCTATGGGTTGCAGCAGTGAGGAATATTGCGCAATGCCCGAAAGGGTGACGCAGCGA
CGCCGCGTGGAGGATGAAGGCCCTATGGGTCGTAAACTCCTTTTATGAGAGAATAATTTG
GCGCTATACGCGCCATTGAATGTATCTCAGGAAAAAGCATCGGCTAACTACGTGCCAGCA
GCCGCGGTAATACGTAGGATGCGAGCGTTGTCCGGATTCACTGGGTGTAAAGGGAGCGCA
GGCGGGTCGGTAAGTCATTGGTGAAATACTCGGGCTCAACCCGGGAACTGCCACTGATAC
TGCTGATCTTGAGTGCAGAAGAGGTTGATAGAATTCTAGGTGTAGCGGTGAAATGCGTAG
ATATCTAGAAGAATACCGATGGCGAAGGCAGTCAACTGGTCTGCTACTGACGCTCATGCT

CGAAAGCGTGGGGATCAAACAGGATTAGATACCCGCGTAGTCC
>Otu4745
CAGCCTATGGGATGCTGCAGCTAAGAATATTCCGCAATGGGAGAAATCCTGACGGAGCGA
CGCCGCGTGGAAGATGAAGGCCGAAAGGTTGTAAATTCCTTTTATACGGGAAGAATAAGG
TTAGGATGGGAATGCCTAATCGATGACGGTACCGTATGAATAAGCCCCGGCTAACTACGT
GCCAGCAGCCGCGGTAATACGTAGGGGGCAAGCGTTGTTCGGATTTACTGGGCGTAAAGG
GCATCTAGGCGGTTATGTAAGTGTGGATTTAAAGGCAGAGGCTTAACCTCTTGCATGGTC
TGCAAACTGCATAACTAGAGTGCAGAAGGGGAAACTGGAATTCCGCAAGTAGGGGTGAAA
TCTGTAGATATGCGGAAGAATACCGGTGGCGAAGGCGAGTTTCTGGTCTGTAACTGACGC
TGAAATGCGAAAGTGTGGGGAGCAAACAGGATTAGAAACCCCCGTAGTCC
>Otu4746
CCAGCCTACGGGGGGCAGCAGTGGGGAATTTTGCGCAATGGGGGAAACCCTGACGCAGCA
ACGCCGCGTGGAGGATGAAGCCCCTTGGGGCGTAAACTCCTTTCGACCGGGACGATACTG
ACGGTACCAGAGGAATAAGCATCGGCTAACTTCGTGCCAGCAGCCGCGGTAATACGAAGG
ATGCAAGCGTTATCCGGATTTATTGGGTTTAAAGGGTGCGTAGGCGGACTTATAAGTCAG
TGGTGAAATCTCATCGCTTAACGATGAACGTGCCATTGATACTGTAGGTCTTGAGTACAG
ATGCCGTTGGCGGAATGTGTCATGTAGCGGTGAAATGCATAGATATGACACAGAACACCG
ATTGCGAAGGCAGCTGACGAAACTGTAACTGACGCTGAGGCACGAAAGCGTGGGGATCAA
ACAGGATTAGATACCCTCGTAGTCC
>Otu4747
CCAGCCTACGGGATGCAGCAGTGGGGAATTTTGCGCAATGGGGGAAACCCTGACGCAGCA
ACGCCGCGTGGAGGATGAAGTACTTCGGTACGTAAACTCCTTTCGATCGGGACGATAATG
ACGGTACCGGAAGAAGAAGCCCCGGCTAACTTCGTGCCAGCAGCCGCGGTAATACAGAGG
TCCCAAGCGTTGTTCGGATTCACTGGGCGTAAAGGGTGCGTAGGTGGCGAAGTAAGTCGG
ATGTGAAATCTCCGAGCTCAACTCGGAAACTGCATTGGAAACTACTTTGCTCGAGGGTTG
GAGGGGGGACTGGAATACTTGGTGTAGCAGTGAAATGCGTAGATATCAAGTGGAACACCA
GTGGCGAAGGCGAGTCCCTGGACAACTCCTGACACTGAGGCACGAAAGCTAGGGGAGCAA
ACAGGATTAGAGACCCTAGTAGTCC
>Otu4752
CCAGCCTACGGGTTGCTGCAGTGGGGAATTTTGCGCAATGGGGGAAACCCTGACGCAGCA
ACGCCGCGTGAGTGATGAAGGCCTTCGGGTCGTAAAGCTCTGTCAGAGGGAAGGAAGTGT
AGGAGGGTTAATACCCCTTTTACTTGACGGTACCCTCAAAGGAAGCACCGGCTAACTCCG
TGCCAGCAGCCGCGGTAATACGGGGGGTGCTAGCGTTATTCGGAATTACTGGGCGTAAAG
CGCGTGCAGGCGGGCTAGCAAGTCTGATGTGAAAGCCCTGGGCTTAACCTGGGAAGTGCA
TTGGAAACTGCTGGTCTTGAGTGCTGGAGAGGAAGGGGGAATTCCCGGTGTAGAGGTGAA
ATTCGTAGAGATCGGGAGGAATACCAGTGGCGAAGGCGCCCTTCTGGACGGCAACTGACG
CTGAGACGCGAAAGCGTGGGGAGCAGACAGGATTAGATACCCTAGTAGTCC
>Otu4753
CCAGCCTACGGGGCGCACCAGTGGGGAATATTGGACAATGGGGGAAACCCTGATCCAGCA
ATGCCGCGTGGGTGAAGAAGGTCTTCGGATTGTAAAGCCCTTTCGGCGGGGACGATGATG
ACGGTACCCGCAGAAGAAGCCCCGGCTAACTTCGTGCCAGCAGCCGCGGTAATACGAAGG
GGGCTAGCGTTGCTCGGAATGACTGGGCGTAAAGGGCGCGTAGGCGGTTTGGACAGTCAG
ATGTGAAATTCCCGGGCTTAACCTGGGGGCTGCATTTGATACGTCCAGGCTTGAGTGAGG
AAGAGGGTTGTGGAATTCTCAGTGTAGAGGTGAAATTCGTAGATATTGGGAAGAACACCG
GTGGCGAAGGCGGCAACCTGGTCCTTTACTGACGCTCATACACGAAAGCTAGGGGAGCGA
ACGGGATTAGAAACCCCAGTAGTCC
>Otu4757
CCAGCCTACGGGTGGCAGCAGTGGGAATATTGGACAATGGGCGCAAGCCTGATCCAGCCA
TGCCGCGTGAGTGAAGAAGGCCTTAGGGTTGTAAAGCTCTTTTGGCGGGGACGATAATGA
CGGTACCCGCAGAATAAGCCCCGGCTAACTTCGTGCCAGCAGCCGCGGTAATACGAAGGG
GGCTAGCGTTGTTCGGAATCACTGGGCGTAAAGCGCACGTAGGCGGATCTTTAAGTCAGA
GGTGAAATGCCAGGGCTCAACCTTGGAACTGCCTTTGATACTGGAGATCTCGAGTCCGGG
AGAGGTGAGTGGAACTGCGAGTGTAGAGGTGAAATTCGTAGATATTCGCAAGAACACCAG
TGGCGAAGGCGGCTCACTGGCCCGGTACTGACGCTGAGGTGCGAAAGCGTGGGGGGCAAA
CAGGATTAGATACCCCAGTAGTCC
>Otu4758
CCAGCCTACGGGACGCAGCAGTGGGGAATTTTGGACAATGGGCGCAAGCCTGATCCAGCC
ATTCCGCGTGAGTGAAGAAGGCCTTCGGGTTGTAAAGCTCTTTCGGCAGGGAAGAAACGG

TATGGGTTAATACCCCGTGCTAATGACGGTACCTGAAGAAGAAGCAACGGCTAACTACGT
GCCAGCAGCCGCGGTAATACGTAGGGTGCGAGCGTTAATCGGAATTACTGGGCGTAAAGC
GTGCGCAGGCGGTTTTGTAAGACAGGTGTGAAATCCCCGGGCTTAACCTGGGAACTGCGC
TTGTGACTGCAAGACTCGAGTACGGCAGAGGGGGGTGGAATTCCACGTGTAGCAGTGAAA
TGCGTAGAGATGTGGAGGAACACCGATGGCGAAGGCAGCCCCCTGGGTCGATACTGACGC
TCATGCACGAAAGCGTGGGGAGCAAACAGGATTAGATACCCGTGTAGTCC
>Otu4760
CCAGCCTATGGGTGGCTGCAGTGGGGAATTTTGCGCAATGGGGGAAACCCTGACGCAGCA
ACGCCGCGTGGAGGATGAAGCCCCTTGGGGTGTAAACTCCTTTCGACTGGGAAGATTATG
ACGGTACCAGTGGAAGAAGCACCGGCTAACTCTGTGCCAGCAGCCGTGGTAATACGAGGG
GGGCAAGCGTTGTTCGGAATTATTGGGCGTAAAGGGTGCGTAGGCGGTTTGGTAAGTCTT
TTGTGAAATCTATGGGCTCAACTCATAGACTGCAAGGGAAACTGCCGGGCTTGAGTGTGG
GAGAGGTGCGTGGAATTCCCGGTGTAGCGGTGAAATGCGTAGATATCGGGAGGAACACCT
GTGGCGAAAGCGGCGCACTGGACCACTACTGACGCTGATGCGCGAAAGCTAGGGGAGCAA
ACAGGATTAGAAACCCGAGTAGTCC
>Otu4763
CCAGCCTACGGGGGGCTGCAGTCGAGAATCTTCCACAATGGACGAAAGTCTGATGGAGCG
ACGCCGCGTGATTGATGAAGTCCCTCTGGGACGTAAAGATCTTTTATGAGGGGAGAAGTT
TATTGACTGTACCTCATGAATAAGAGGCTCCTAATCTCGTGCCAGCAGGAGCGGTAATAC
GAGAGCCTCGAGCGTTATCCGGAATTATTGGGCGTAAAGGGTGCGTAGGTTGTTCTGTTA
GTCAATTGTTAAAACTCTGGGCTTAACCTAGAATCGGCGATTGAAACGGCAGAACTTGAA
AGTGCGAGAGGTTTACGGAACTCATGGTGTAGGGGTGAAATCCGTTGATATCATGGGGAA
CACCAAATGCGAAGGCAGTAAACTGGCGCATATTTGACACTGAAGCACGAAAGCGTGGGT
AGCGAATGGGATTAGATACCCCAGTAGTCC
>Otu4764
CCAGCCTACGGGGTGCTCCAGTCGAGAATCTTCCGCAATGGACGAAAGCCTGAAGGAGCG
ACGCCGCGTGGGGGATGAATGGCTTCGGCCCGTAAACCCCTGTCATTCGGGATCAATGCG
TCTGGGTGAACATCCCAGACGTTGATAGTACCGGAAGAGGAAGGGACGGCTAACTCTGTG
CCAGCAGCCGCGGTAATACAGAGGTCCCAAGCGTTGTTCGGATTTACTGGGCGTAAAGGG
TGCGTAGGTGGTTGGGTAAGTTTGATGTGAAATCTCCGGGCTCAACTCGGAGTCTGCAGG
AGAAACTGCCGTGCTTGAGTATGGGAGAGGTGAGTGGAATTCCTGGTGTAGCGGTGAAAT
GCGTAGATATCAGGAGGAACACCTGTGGCGAAAGCGGCTCACTGGACCATAACTGACGCT
GATGCACGAAAGCTAGGGGAGCAAACAGGATTAGATACCCCCGTAGTCC
>Otu4767
CCAGCCTACGGGGTGCAGCAGTAGGGAATTTTGGGCAATGGGCGAAAGCCTGACCCAGCA
ACGCCGCGTGAAGGATGAAGGCCTTCGGGTTGTAACCTTCTGTTGGATGGGAAAAAAATC
CCGACTTGTCGGGACTGATGGTACCATTCGAGTAAGCACCGGCTAACTACGTGCCAGCAG
CCGCGGTAATACGTAGGGTGCAAGCGTTGTTCGGAATTACTGGGCGTAAAGGGAGCGTAG
GCGGAAACCTAAGTTAGGAGTTTAATTCTCGGGCTTAACCCGAGGCTGGCTTTTAATACT
GGGTTTCTTGAGTATGGAAGAGGATGATGGAATTCCAGGTGTAGCGGTGGAATGCGTAGA
TATCTGGAAGAACACCAGTAGCGAAGGCGGTCATCTGGTCCAATACTGACGCTGAGGCTC
GAAAGCTAGGGGAGCAAACAGGATTAGAAACCCCCGTAGTCC
>Otu4768
CCAGCCTATGGGTTGCTGCAGTGGGGAATTTTGCGCAATGGGGGAAACCCTGACGCAGCA
ACGCCGCGTGAGTGATGAAGGCCTTCGGGTCGTAAAGCTCTGTCAGAGGGAAAGAAGTGT
AAGCTGGCTAATACCCGGTTTACTTGACGGTACCCTCAAAGGAAGCACCGGCTAACTCCG
TGCCAGCAGCCGCGGTAATACGGAGGGTGCGAGCGTTGTTCGGAATTATTGGGCGTAAAG
CGCGTGTAGGCGGTTTTTTAAGTCTGATGTGAAAGCCCTGGGCTCAACCCGGGAAGTGCA
TTGGAAACTGGGAGACTTGAATACGGGAGAGGGTAGTGGAATTCCTGGTGTAGGAGTGAA
ATCCGTAGATATCGGAAGGAACACCTGTGGCGAAAGCGGCTCACTGGACCACAACTGACG
CTGATGCACGAAAGCTAGGGGAGCAAACAGGATTAGAGACCCCAGTAGTCC
>Otu4772
CCAGCCTATGGGGGGCTGCAGTCGAGAATCTTCCGCAATGGGCGAAAGCCTGACGGAGCG
ACGTTACGTGAATGAAGAAGCCCTTCGGGGTGTTAAGTTCTTTTATATGGGAGGAAGTTA
TTGACAGTACCATATGAATAAGGGGATCCTAATTCTGTGCCAGCAGGAGCGGTAATACAG
AATCCCCAAGCGTTACCCGGATTTATTGGGCGTAAAGGGTCCGTAGGTGGTAAAGTAAGT
TGAAAGTTAAAGCTCATCGGCTTAACCTTTGAGATTCTTTCAATACTGCTATACTAGAGG
ATGTTAGGGGTGAACGGAACCGACGGAGTAGGGGTGAAATCCGTTGATATCGTCGGGAAC

ACCAAAAGCGAAGGCAGTTCACTAGGACAATCCTGACACTGAGGGACGAAAGCGTGGGGA
TCAAAAAGGATTAGAGACCCCTGTAGTCC
>Otu4773
CCAGCCTACGGGTTGCAGCAGTGGGGAATATTGGACAATGGGCGCAAGCCTGATCCAGCC
ATGCCGCGTGAGTGAAGAAGGCCTTAGGGTTGTAAAGCTCTTTTGGCGGGGACGATAATG
ACGGTACCCGCAGAATAAGCCCCGGCTAACTACGTGCCAGCAGCCGCGGTAACACGTAGG
GGGCGAGCGTTGTTCGGAATTACTGGGCGTAAAGGGCATGTAGGCGGCTTTTGTAAGCCC
GGCGTGGAAGCCCACGGCTCAACCGTGGGATTGCGTTGGGAACTGCGAGGCTTGAATCAT
GGAGAGGGAGCTAGAATTCCTGGTGTAGGGGTGAAATCTGTAGAGATCAGGAAGAATACC
AGTGGCGAAGGCGAGCTCCTGGCCAATGATTGACGCTGAGGTGCGAAAGTGTGGGGAGCA
AACCGGATTAGAGACCCGAGTAGTCC
>Otu4785
CCAGCCTATGGGGGGCAGCAGTTAAGAATCTTGGACAATGGACGAAAGTCTGATCCAGCG
ACGCCGCGTGAACGATGAAGGTCTTCGGATTGTAAAGTTCAATAAGATGGGGAGAAAAAG
CGCATGGTAATATCGTGCGTCTTGACGGTACCATCCTAAAGCCTCGGCTAAATACGTGCC
AGCAGCCGCGGTAATACGTATGAGGCAAGCGTTGTTCGGAATTATTGGGCGTAAAGGGCA
TCTAGGCGGGATGTTAAGTCAGGTGTGAAAGCTAAGGGCTCAACCCTTAAACTGCATTTG
ATACTGACATTCTTGAGTTCAGAAGAGGAAGGCGGAATTCCCGGTGTAGCGGTGAAATGC
GTAGATATCGGGAAGAACACCGGTGGCGAAGGCGGCCTTCTGGTCTTGAACTGACGCTGA
CATGCGAAAGTGTGGGGATCAAACGGGATTAGATACCCCCGTAGTCC
>Otu4791
CCAGCCTACGGGTTGCTGCAGTCGAGAATATTCCACAATGGGCGAAAGCCTGATGGAGCG
ACGCCGCGTGCGGGATGAAGGCCCTTGGGTTGTAAACCGCTTTTATAGAGGAAAAATGTA
ATGATGGTACTCTATGAATAAGAGGTTACTAACTCTGTGCCAGCAGTAGCGGTAATACAG
AGACCTCAAGCGTTATCCGGATTTATTGGGCGTAAAGAGCATGTAGGCGGTTTTGCTAGT
CAGATGTCAAATCTTTGAGCTCAACTCGAAAACTGCATTTGAAACGGCAAAACTAGAGAA
AGCGAGAGACCAGTGGAACATATGGTGTAGCAGTGAAATGCGTTGATATCATATGGAACA
CCAAAGGCGAAGGCATCTGGTTGGCGCTTTTCTGACGCTGAGATGCGAAAGCGTGGGTAG
CGAATGGGATTAGATACCCCGTAGTCC
>Otu4793
CCAGCCTATGGGTCGCTGCAGTGGGGAATCTTGCACAATGTGGGAAACCCTGATGCAGCG
ACGCCGCGTGGGGGGTGACGGCCTTCGGGGTGTAAACCCCTTTTCACGAGGAAGAAATAT
GACGGTACTCGTGGAATAAGTCTTGGCTAACTACGTGCCAGCAGCCGCGGTAATACGTAG
AAGGCGAACGTTATCCGGATTTACTGGGCGTAAAGCGGGTGTAGACGGTTTTTTAAGTCA
GTTGTGAAATCTCCTGACTCAATTGGGAGACTGCAATTGATACTGGAGGACTTGAGGGCA
TCAGAAGAAAGCGGAATTCCCGGTGTAGCGGTGAAATGCGTAGATATCGGGAGGAACACC
AGTGGCGAAGGCGGCTTTCTAGGATGCTCCTGACGTTGAGGCCCGAAAGTGTGGGGAGCG
AAACGGATTAGAAACCCGTGTAGTCC
>Otu4794
CCAGCCTACGGGTGGCTGCAGTCGGGAAACTTTTGCAATACACGAAAGTGTGACAAAGCA
AGCCAGAGTGCTTTTCATTTTTGAAAAGCTTTTGTGAGATGTAAAAAGTCTTACGAATAA
GGACTGGGCAAGACTGGTGCCAGCCGCCGCGGTAATCCCAGCAGTCCAAGTCGCAGCCAC
ATTTGTTGGGTCTAAAACATCCGTAGCTCGTCTTTCAAGTCTTTTGTGAAATCGGGACTC
TTAAGGTTCCGGCGGGCAAAAGATACTGTAAGGCTAGAGACCGGGAGGCGCAGAAAGTAC
ATATAGGGTAGCGGTAAAATGCTCTGATCCTATATGGACTAACAGTCGTGAAGGCGTTCT
GCGAGAACGGTTCTGACAGTGAGGGATGAAGGCTAGGGGCGCAAAAGGGATTAGAAACCC
TCGTAGTCC
>Otu4799
CCAGCCTACGGGGTGCAGCAGTGGGGAATTTTGCGCAATGGGGGAAACCCTGACGCAGCA
ACGCCGCGTGAGTGATGAAGGCCTTCGGGTCGTAAAGCTCTGTCAGAGGGAAAGAAGTGT
AGGAGGGTTAATACCCCTTTTACCTGACGGTACCCTCAAAGGAAGCACCGGCTAACTCCG
TGCCAGCAGCCGCGGTAATACGAAGGGGGCTAGCGTTGTTCGGAATCACTGGGCGTAAAG
CGCACGCAGGCGGATTGATAAGTCAGGGGTGAAATCCCGGGGCTCAACCTCGGAATTGCC
TTTGATACTGTCTATCTTCGAGTTCGGGAGAGGTTGGCGGAATTCCTAGTGTAGAGGTGA
AATTCGTAGATATTAGGAAGAACACCAGTGGCGAAGGCGGCCAACTGGCCCGATACTGAC
GCTCATGTGCGAAAGCGTGGGGAGCAAACAGGATTAGATACCCGAGTAGTCC
>Otu4801
CCAGCCTACGGGTGGCTGCAGTGAGGAATTTTGGACAATGGGGGAAACCCTGATCCAGCG

ACGCCGCGTGGAGGACGAAGGTCTTCGGATTGTAAACTCCTTTTGTCCGGGAATAATAAA
TCTGTAGAAATACAGAGATTGAATGTACCGGATGAATAAGCCACGGCTAACTTCGTGCCA
GCAGCCGCGGTAATACGAAGGTGGCAAGCGTTACTCGGAATTACTAGGCGTAAAGCGCAG
GTAGGTGGTTGTGTAAGTCTGCTGTGAAAGCTCCCGGCTTAACTGGGAGAGGTCAGCGGA
TACTGCATAGCTAGAGTGTGGGAGAGGGTACTGGAATTCCCGGTGTAGCGGTGAAATGCG
CAGAGATCGGGAGGAACACCAAAGGCGAAAGCAGGTACCTGGACCATTACTGACACTAAG
CTGCGAAAGCTAGGGGAGCAAACAGGATTAGAAACCCGTGTAGTCC
>Otu4802
CCAGCCTATGGGGTGCTGCAGTGGGGAATCTTGCGCAATGGGCGAAAGCCTGACGCAGCG
ACGCCGCGTGAGTGATGAAGGCCTTCGGGTTGTAAAGCTCTGTGGAGGGGGACGAATAAG
GCCGGGCGAACAATCCGGTCGATGACGGTACCTCTTTAGCAAGCACCGGCTAACTCTGTG
CCAACAGCCGCGGTAAGACAGAGGGTGCTAACGTTGTTCGGAATTACTGGGCGTAAAGCG
CGTGTAGGCGGCTTTGCAAGTCGGATGTGAAAGCCCTGGGCTCAACCTGGGAAGTGCATT
CGAAACTGCGGGGCTTGAGTTCTGGAGAGGATGGCGGAATTCTCGGTGTAGAGGTGAAAT
TCGTAGATATCGAGAGGAACACCGGCGGCGAAGGCGGCCATCTGGACAGCGACTGACGCT
GAGACGCGAAAGCGTGGGGAGCAAACAGGATTAGATACCCTCGTAGTCC
>Otu4810
CCAGCCTACGGGTTGCTGCAGTCGAGGATTTTTCTCAATGGGGGAAACCCTGAAGGAGCG
ACGCCGCGTGAGGGATGAAGGTCTTCGGATTGTAAACCTCTGTCATCCGGGATCAAAGCT
TACGACCTAACACGTCGTGAGTTGATAGTACCGGAAGAGGAAGCCGTGGCTAACTCTGTG
CCAGCAGCCGCGGTAATACAGAGACGGCAAGCGTTGCTCGGATTCATTGGGCGTAAAGGG
TGCGTAGGCGGTTCGTTAAGTCGGATGTGAAATCTCGCAGCCTAACTGTGATAGGTCATT
CGAAACTGACGAGCTTGAGGACTGGAGGGGAGACTGGAATCGCTGGTGTAGCGGTGAAAT
GCGTAGAGATCAGCGAGAACACCGGTGGCGAAGGCGGGTCTCTGGACAGTTCCTGACGCT
GAGGCACGAAAGCCAGGGGAGCAAACGGGATTAGATACCCCGGTAGTCC
>Otu4818
CCAGCCTATGGGACGCAGCAGACGAGAATATTCCGCAATGGGCGAAAGCCTGACGGAGCG
ACATCGCGTGATGGATGAAGTGCTTCGGTACGTAAACATCTTTTATCGGGGAAGAAACAA
ATGACGGTACCCGATGAATAAGGGGCTCCTAACTCTGTGCCAGCAGGAGCGGTAATACAG
AGGCCCCAAGCATTACCCGGAATCACTGGGCGTAAAGGGTGTCCAGGCGGCCCTATTAGT
CTCTCGTAAAATCCGTGAGCTCAACTTACGGCGCGCGAGGGAAACGGTAGGGCTCGAGGG
CGCGAGAGGTGCACGGAACTCATGGTGGAGGGGTGAAATCCGTTGATATCATGGGGAACA
CCAAAGGCGAAGGCAGTGCACTGGCGCGTTCCTGACGCTCACACACGAAAGCCAGGGTAG
CGAACGGGATTAGAAACCCTCGTAGTCC
>Otu4824
CCAGCCTATGGGAGGCAGCAGTGGGGAATATTGCACAATGGGCGAAAGCCTGATGCAGCG
ACGCCGCGTGGAGGATGAAGGTTTTCGGATTGTAAACTCCTGTTAAGTGGGAAGAAAGAC
TGATACCTAATACGTATTGGGGATGACGGTACCACTAGAGAAAGCACCGGCAAACTTCGT
GCCAGCAGCCGCGGTAATACGAAGGGTGCAAGTGTTATTCGGAATCACTGGGCGTAAAGA
GCGTGAAGGCGGCTTCTTAAGTCAACTGTTAAAGAGCTCGGCTTAACTGAGCAAGTGCGG
GTGATACTGGGAAGCTAGAGTGCAGGAGAGAAAGGTAGAATTCTCGGAGTAGCGGTAAAA
TGCGTAGATCTCGAGAGGAATACCGGTTGCGAAGGCGGCCTTTTGGCCTGCTACTGACGC
TAAAGCGCGAAAGCGTGGGGAGCAAACAGGATTAGATACCCTTGTAGTCC
>Otu4825
CCAGCCTATGGGAGGCTGCAGTGGGGAATATTGGACAATGGACGCAAGTCTGATCCAGCA
ATGCCGCGTGAGTGATGAAGGCCTTAGGGTTGTAAAGCTCTTTTACCAGGGATGATAATG
ACAGTACCTGGAGAATAAGCTCCGGCTAACTCCGTGCCAGCAGCCGCGGTAATACGGAGG
GAGCTAGCGTTGTTCGGAATTACTGGGCGTAAAGCGCACGTAGGCGGTTATTCAAGTCAG
AGGTGAAAGCCCGGAGCTCAACTTCGGAACTGCCTTTGAAACTAGATAACTAGAATCCTG
GAGAGGTGAGTGGAATTCCGAGTGTAGAGGTGAAATTCGTAGATATTAGGAAGAACACCA
GTGGCGAAGGCGGCCAACTGGCCCGATACTGACGCTCATGTGCGAAAGCGTGGGGAGCAA
ACAGGATTAGAAACCCCAGTAGTCC
>Otu4826
CCAGCCTACGGGTTGCAGCAGAGGAGGATCATTCGCAATGGGCGAAAGCCTGACGGTGCG
ACGCCGCGTGAGGGATGAAGGCCTTCGGGTTGTAAACCTCTGTCAGGAGTTAAGAACGAT
GGGGATGCTAATATCATCTCCATGTGACAAAGGCTCCAAAGGAAGCCACGGCTAACTACG
TGCCAGCAGCCGCGGTAATACGTAGGTGGGAAGCGTTACTCGGAATCACTGGGCATAAAG
GGCGTGTAGGCGGATGCGTAAGTCCGGTGTAAAAGCCCTCGGCTCAACCGGGGAAAGCCA

TCGGATACTGCGTGTCTCGAGTGCGGGAGGGGAGAGTGGAACTCTTGGTGGAGCGGTGAA
ATGCGTAGATATCAAGAGGAACGCCGGTGGCGAAAGCGGCTCTCTGGCCCGTTACTGACG
CTGAGGCGCGAAAGCTAGGGGAGCAAACGGGATTAGAAACCCTCGTAGTCC
>Otu4833
CCAGCCTATGGGGCGCTGCAGTCGAGAATCTTCGGCAATGGGCGAAAGCCTGACCGAGCG
ACGCCGCGTGTGGGATGAAGGCCTTCGGGTTGTAAACCACTGTCAGAGGGGAGGAAATAT
TGGGAGGATCACCTCTCAGTTTGACCTATCCTCAGAGGAAGGACGGCTAAGTTCGTGCC
AGCAGCCGCGGTAATACGAACCGTCCAAACGTTATTCGGAATCACTGGGCTTAAAGGGTG
CGTAGGCGGCGCGATAAGTTGGGTGTGAAATACCTCGGCTCAACCGAGGAATTGCGCCCA
AAACTGCCGTGCTCGAGGGAGACAGAGGTAAGCGGAACTTAGGGTGGAGCGGTGAAATGC
GTTGATATCCTAAGGAACACCGGTGGCGAAAGCGGCTTACTGGGTCTCTTCTGACGCTGA
GGCACGAAAGCTAGGGGAGCGAACAGGATTAGAGACCCTCGTAGTCC
>Otu4834
CCAGCCTACGGGTGGCTGCAGTGAGGAATCTTGCGCAATGGACGAAAGTCTGACGCAGCG
ACGCCGCGTGCGGGATGAAGGCCTTCGGGTTGTAAACCGCTTTCAGCAGGGACGAAATTG
ACGGTACCTGCAGAAGAAGCCCCGGCCAACTACGTGCCAGCAGCCGCGGTAATACGTAGG
GGGCAAGCGTTGTCCGGATTTATTGGGCGTAAAGAGCTCGTAGGTGGTTCGGTAAGTCGG
ATGTGAAATCTCCAGGCTCAACCTGGAGGGGTCATTCGATACTGCCGTGACTAGAGGTCG
GTAGGGGAGTGTGGAATTCCTGGTGGAGCGGTGAAATGCGCAGATATCAGGAGGAACACC
CGTAGCGAAGGCGGCACTCTGGGCCGATACTGACACTGAGGAGCGAAAGCGTGGGGAGCG
AACAGGATTAGAAACCCGCGTAGTCC
>Otu4836
CCAGCCTACGGGTGGCAGCAGTGGGGAATATTGGACAATGGGGGCAACCCTGATCCAGCC
ATGCCGCGTGAGTGAAGAAGGCCTTAGGGTTGTAAAGCTCTTTTGGCGGGGACGATAATG
ACGGTACCCGCAGAAGAAGCCCCGGCTAACTTCGTGCCAGCAGCCGCGGTAATACGAAGG
GGGCCAGCGTTGCTCGGAATCACTGGGCGTAAAGCGCACGTAGGCGGACTTTTAAGTCGG
AGGTGAAATCCTGGAGCTCAACTCCAGAACTGCCTTCGATACTGAGAGTCTTCGAGTTCG
GGAGAGGTGAGTGGAACTGCGAGTGTAGAGGTGAAATTCGTAGATATTCGCAAGAACACC
AGTGGCGAAGGCGGCTCACTGGCCCGATACTGACGCTGAGGTGCGAAAGCGTGGGGAGCA
AACAGGATTAGATACCCTTGTAGTCC
>Otu4842
CCAGCCTATGGGATGCTGCAGTGGGGAATATTGGACAATGGGGGAAACCCTGATCCAGCA
ATGCCGCGTGTATGATGAAGGTCTTCGGATTGTAAAGTACTTTTATTGGTGACGATGATG
ACGGTAACCAATGAATAAGCTCCGGCTAACTTCGTGCCAGCAGCCGCGGTAATACGAAGG
GAGCTAGCGTTGTTCGGAATAACTGGGCGTAAAGGGTGCGTAGGTGGTTATGTGCGTTAG
ATGTGAAATCCCCGGGCTTAACCTGGGAACTGCATTTAATACGGCATATCTAGAGTGCGA
GAGGGGAAAGCGGAATGGCGAGTGGAGCAGTGAAATGCATTGAGATTCGCTAGAACACCA
GAGGCGAAGGCGGCTTTCTGGCTCGCTACTGACACTAAGGCACGAAAGCGTGGGGAGCAA
ACAGGATTAGAAACCCGCGTAGTCC
>Otu4844
CCAGCCTACGGGAGGCAGCAGTGAGGAATATTGCGCAATGGGGGCAACCCTGACGCAGCA
ACGCCGCGTGAGTGAGGAAGGTTTTCGGATTGTAAAGCTCTGTCGAGCGGAAAGAAATGC
ATGGTGGTTAATATCCATTATGCTTGACGGTACCGCTAAAGGAAGCACCGGCTAACTCCG
TGCCAGCAGCCGCGGTAATACGGAGGGTGCGAGCGTTGTTCGGATTTATTGGGCGTAAAG
CGCGTGTAGGCGGTTTTTTAAGTCTGATGTGAAAGCCCTGGGCTCAACCCGGGAAGTGCA
TTGGAAACTGGGAGACTTGAATACGGGAGAGGGCAGTGGAATTCCTGGTGAAGGAGTGAA
ATCCGTAGATATCAGGAGGAACACCGGTGGCGAAGGCGGCTGCCTGGACCGATATTGACG
CTGAGACGCGAAAGCGTGGGGAGCAAACAGGATTAGAAACCCCCGTAGTCC
>Otu4845
CCAGCCTATGGGTGGCAGCAGTGAGGAATATTGCGCAATGGGGGCAACCCTGACGCAGCA
ACGCCGCGTGGGTGATGAAGGTCTTCGGATCGTAAAACCCTGTCGTCAGGGACGAAGGTC
GGGGTGTGAACAGCGCCTTGGCTTGACGGTACCTGGAGAGGAAGCCCCGGCTAACTCTGT
GCCAGCAGCCGCGGTAATACAGAGGGGGCAAGCGTTATTCGGAATTATTGGGCGTAAAGG
GCGCGTAGGCGGTTTTTTAAGTGGGATGTGCAATCCCCGAGCTTAACTTGGGAACTGCAT
CCCAGACTGGAAGGCTAGAGTGCTGGAGAGGGTGGTAGAATTCCACGTGTAGCGGTGAAA
TGCGTAGAGATGTGGAGGAATACCAGTGGCGAAGGCGGCCACCTGGACAGTAACTGACGC
TGAGGCGCGAAAGTGTGGGTAGCAAACAGGATTAGATACCCTAGTAGTCC
>Otu4850

CCAGCCTACGGGGGGCAGCAGTGGGGAATTTTGGACAATGGGCGCAAGCCTGATCCAGCA
ATGCCGCGTGAGCGATGAAGCCCTTCGGGGCGTAAAGCTCTTTCGACGGGAACGATAATG
ACGGTACCCGGAGAAGAAGCTGCGGCTAACTACGTGCCAGCAGCCGCGGTAATACGTAGG
CAGCAAGCGTTGTTCGGAGTTACTGGGCGTAAAGAGTGCGTAGGCGGTTCTGTAAGTCTG
GTGTGAAATCTCCCGGCTTAACTGGGAGGGTGCGCTAGAAACTGCGGGGCTGGAGTGTGG
GAAAGGTAGGCGGAATTCCTGGTGTAGCGGTGAAATGCGTAGATATCAGGAGGAACACCG
GCGGTGTAGACGGCTTACTAGACCATGACTGACGCTGAGGCACGAAAGCGTGGGTAGCAA
ACAGGATTAGATACCCCCGTAGTCC
>Otu4851
CCAGCCTATGGGTGGCAGCAGTAAAGAATCTTTCACAATGGGGGAAACCCTGATGAAGCA
ACACCGCGTGGAGGATGAAGGCGTTTTCGTTGTAAACTCCTTTTCTGAGGGAATAACTTT
GAAGGTACCTCAGGAATAAGCAGCGGCTAACTACGTGCCAGCAGCCGCGGTAATACGTAG
GCTGCAAGCGTTATCCGGAATTATTGGGCGTAAAGCGTACGCAGGCGGTTTGTTAAGTTG
GATGTCAAAGCTATGAGCTCAACTCATAACAGGTATTCAATACTGGCAAGCTGGAGATAG
GCAGAGGAAAGTGGAATTCTATATGTAGGGGTAATATCCGTAGATATATAGAGGAACACC
AAAAGCGAAGGCAGCTTTCTGGGCCTTTCCTGACGCTCATGTACGAAAGCGTGGGGAGCA
AACGGGATTAGAGACCCTAGTAGTCC
>Otu4858
CCAGCCTATGGGATGCACCAGTCGAGAATCTTCCGCAATGGGCGAAAGCCTGACGGAGCG
ACGTTACGTGAATGAAGAAGCTCTTCGGAGTGTAAAGTTCTTTTCTTCGGGAGGAAAGCC
GCAAGGCTAGACAGTACCGAAGGAATAAGGGGATCCTAATTCTGTGCCAGCAGGAGCGGT
AAGACAGAATCCCCAAGCGTTACCCGGATTTATTGGGCGTAAAGGGTCCGTAGGTTGTTT
GGTAAGTTAAAAGTTAAAGCTCATCGGCTTAACCTTTGAGATGCTTTTAATACTGCCAGA
CTTGAGGGCGTTAGGGGTTAACGGAACCGACGGAGTAGGGGTGAAATCCGTTGATATCGT
CGGGAACACCAAAAGCGAAGGCAGTTAACTAGAACGACCCTGACACTGAGGGACGAAAGC
GTGGGGAGCAAACAGGATTAGAGACCCCAGTAGTCC
>Otu4860
CCAGCCTATGGGTGGCAGCAGTGGGGAATCTTGCACAATGGGGGCAACTCTGATGCAGCG
ACGCCGCGTGAGCGATGAAGCCCTTCGGGGTGTAAAGCTCTTTCGGTAGGGACGATGATG
ACGGTACCTACAGAAGAAGCCCCGGCTAACTTCGTGCCAGCAGCCGCGGTAATACGAAGG
GGGCTAGCGTTGTTCGGAATTACTGGGCGTAAAGCGCACGCAGGCGGCTCGTTAAATTAG
AAGTGAAAGCCCTGGGCTCAACCTGGGAATTGCTTTTAAGACTGGCGAGCTTGAATTCGG
AAGAGGGCAGTGGAATTCCAAGTGTAGAGGTGAAATTCGTAGATATTTGGAAGAACACCA
GTGGCGAAGGCAACTGCCTGGTCCGACATTGACGCTCATGTGCGAAAGCGTGGGGAGCAA
ACAGGATTAGAAACCCCCGTAGTCC
>Otu4866
CCAGCCTATGGGGCGCAGCAGTTGGGAATCTTGCACAATGGGGGAAACCCTGATGCAGCG
ACGCCGCGTGGAGGATGAAGGATCTAGGTCTGTAAACTCCTTTTTCAGGGAAAGACTTAG
GACGGTACCCTGAGAATAAGGACCGGCTAACTACGTGCCAGCAGCCGCGGTAAGACGTAG
GGTCCAAGCGTTGTCCGGATTTACTGGGCGTAAAGAGCGCGTAGGCGGCTCGTTAAGTGT
GAAGCTAAAGCTCCGGGCTCAACTCGGAAACTATTTCGCATACTGGCAAGCTAGAGTGAT
GCAGAGGTTTGTGGAATTCCTGGTGTAGCGGTGAAATGCGTAGATATCAGGAGGAACACC
GGTGGTGTAGACGGCTTTCTGGACCGTAACTGACGCTGAGACACGAAAGCGTGGGTAGCA
AACAGGATTAGAAACCCTTGTAGTCC
>Otu4875
CCAGCCTACGGGAGGCAGCAGTGGGGAATTTTGCGCAATGGGGGAAACCCTGACGCAGCA
ACGCCGCGTGGAGGATGAAGCCCCTTGGGGCGTAAACTCCTTTCGACCGGGACGATACTG
ACGGTACCGGTGGAAGAAGCACCGGCTAACTCTGTGCCAGCAGCCGCGGTAATACAGGGG
GTGCAAGCGTTGTTCGGAATTATTGGGCGTAAAGGGCGTGTAGGCGGCTGGACAAGTCAG
ATGTGAAAGCCCTGGGCTTAACCTAGGAAGTGCATTTGGAACTGTCCGGCTTGAGTAAGG
GAGAGGAAAGTGGAATTCCTGGTGTAGAGGTGAAATTCGTAGATATCAGGAGGAACACCG
GTGGCGAAGGCGACTTTCTGGCCCTATACTGACGCTGAGACGCGAAAGCGTGGGTAGCAA
ACAGGATTAGATACCCGGGTAGTCC
>Otu4878
CCAGCCTACGGGGGGCTGCAGTGGGGAATTTTGGACAATGGGCGCAAGCCTGATCCAGCC
ATGCCGCGTGAGTGAAGAAGGCCTTCGGGTTGTAAAGCTCTTTTGTCTGGGAGCAAACCT
ACTGGGCTAATACCCCAGTGGCTGAGAGTACCAGAAGAATAAGCACCGGCTAACTACGTG
CCAGCAGCCGCGGTAATACGTAGGGTGCAAGCGTTAATCGGAATTACTGGGCGTAAAGCG

TGCGCAGGTGGTTATGTAAGCTTGATGTGAAATCCCCGGGCTCAACCTGGGAACTGCATT
GAGGACTGCATAGCTAGAGTGTGGCAGAGGGGGGTAGAATTCCACGTGTAGCAGTGAAAT
GCGTAGAGATGTGGAGGAATACCAATGGCGAAGGCAGCCCCCTGGGTTAACACTGACACT
CATGCACGAAAGCGTGGGGAGCAAACAGGATTAGAAACCCTCGTAGTCC
>Otu4881
CCAGCCTACGGGTTGCACCAGTGAGGAATATTGGTCAATGGGCGAAAGCCTGAACCAGCC
ATGCCGCGTGAAGGATGAAGGCCCTATGGGTTGTAAACTCCTTTTGTACGGGACGAACCA
TTTCTACGTGTAGAGATCTGACGGTACCGTATGAATAAGCAACGGCTAACTCCGTGCCAG
CAGCCGCGGTAATACGGAGGTTGCAAGCGTTATCCGGATTTATTGGGTTTAAAGGGTGCG
TAGGCGGGTCTGTAAGTCAGTGGTGAAAGCCTGTAGCTTAACTATAGAAATGCCATTGAT
ACTACAGAGCTTGAGTACAGTTGAGGTGGGCGGAATGTGTAATGTAGCGGTGAAATGCTT
AGATATTACACAGAACACCAATTGCGTAGGCAGCTCACTAAACTAATACTGACGCTGAGG
CACGAAAGCGTGGGGAGCAAACAGGATTAGAGACCCCTGTAGTCC
>Otu4886
CCAGCCTACGGGGTGCTGCAGTAGGGAATATTGGTAATCTGCGAAAGCGGGAACCAGCAA
CGCCGCGTGTGCGAAGAAGGCCTTAGGGTTGTAAAGCTCTTTTGGCGGGGACGATAATGA
CGGTACCCGCAGAATAAGCCCCGGCTAACTTCGTGCCAGCAGCCGCGGTAATACGAAGGG
GGCTAGCGTTGTTCGGAATCACTGGGCGTAAAGCGCACGCAGGCGGATTGATAAGTCAGG
GGTGAAATCCCGGGGCTCAACCTCGGAATTGCCTTTGATACTGTTAGTCTTCGAGTTCGG
GAGAGGTTGGCGGAATTCCTAGTGTAGAGGTGAAATTCGTAGATATTAGGAAGAACACCA
GTGGCGAAGGCGGCCAACTGGCCCGATACTGACGCTCATGTGCGAAAGCGTGGGGAGCAA
ACAGGATTAGATACCCTCGTAGTCC
>Otu4888
CCAGCCTACGGGACGCACCAGTTCGGAATATTCGGCAATGGGCGAAAGCCTGACCGAGCG
ACGCCGCGTGCGGGATGAAGGCCCTTTGGGTTGTAAACCGCTGTCAAAGGTGAGCAAATC
ACAGGACGATAACATCGTCTTGTGTTGAGTTAGGCTTGAGAGGAAGCCCCCGCTAATCAC
GTGCCAGCAGCGGCGGTAATACGTGAGGGGCGAACGTTGTTCGGTGTCACTGGGCTTAAA
GGGTGCGTAGGTGGCTGCGTAAGTAACGTGTGAAAGCCCTCGGCTTACCCGGGGAATTGC
GCGTTATACTGCGCGGCTTGAGGATCTCAGGGGAAGGGGGAACTCCAGGTGTAGCGGTGA
AATGCGCAGATATCTGGGGGAAGGCCGGTGGCGAAGGCGCCCTTCTGGGAGATTCCTGAC
ACTGAGGCACGAAAGCGTGGGGAGCAAACAGGATTAGATACCCTAGTAGTCC
>Otu4892
CCAGCCTATGGGTCGCACCAGTGGGGAATATTGCACAATGGGCGAAAGCCTGATGCAGCA
ACGCCGCGTGAGCGATGAAGGCCTTCGGGTCGTAAAGCTCTGTCCTCAAGGAAGATAATG
ACGGTACTTGAGGAGGAAGCCCCGGCTAACTACGTGCCAGCAGCCGCGGTAATACGTAGG
GGGCTAGCGTTATCCGGAATTACTGGGCGTAAAGGGTGCGTAGGTGGTTTTTTAAGTCAG
AAGTGAAAGGCTACGGCTCAACCGTAGTAAGCTTTTGAAACTAGAGAACTTGAGTGCAGG
AGAGGAGAGTAGAATTCCTAGTGTAGCGGTGAAATGCGTAGATATTAGGAGGAATACCAG
TAGCGAAGGCGGCTCTCTGGACTGTAACTGACACTGAGGCACGAAAGCGTGGGGAGCAAA
CAGGATTAGAAACCCTAGTAGTCC
>Otu4893
CCAGCCTATGGGTGGCACCAGTCGAGAATTTTTCACAATGGGCGCAAGCCTGATGGAGCG
ACGCCGCGTGGGGGATGAATGGCTTCGGCCCGTAAACCCCTGTCATTTGCGATCAAACGT
TATTATTTAAAAGATGATAACCTGATAGTAGCGAAAGAGGAAGGGACGGCTAGCTCTGTG
CCAGCAGCCGCGGTAATACAGAGGTCCCAAGCGTTGTTCGGATTCACTGGGCGTAAAGGG
TGCGTAGGTGGCGGGGTAAGTCGGATGTGAAATCTCCGAGCTCAACTCGGAAACTGCATT
GGAAACTACCTTGCTCGAGGGTTGGAGGGGGGACTGGAATACTTGGTGTAGCAGTGAAAT
GCGTAGATATCGAGAGGAACACCAGTGGCGAAGGCGAGTCCCTAGACAATTCCTGGCACT
GAGGCGCGAAAGCCAGGGGAGCAAACGGGATTAGATACCCCCGTAGTCC
>Otu4895
CCAGCCTATGGGGGGCTGCAGTAAGGAATATTGCGCAATGGGCGACAGCCTGACGCAGCA
ACGCCGCGTGGAGGATGAAGGCCTTCGGGTCGTAAACTCGTTTTGCCCGTGACGAGCAAG
GACGGTAGCGGGCGAAGAAGTCACGGCTAACTACGTGCCAGCAGCCGCGGTAAAACGTAG
GTGGCAAGCGTTATCCGGATTTACTGGGCGTAAAGGGTGTGCAGGCGGACGACCAAGTGG
TGTATGAAATCTCCGGGCTTAAGTCGGAGGGGTTAGGCCAGACTGGTTGTCTTGAGAGTG
AGAGAGGGACATGGAATTCTGGGTGTAGTGGTGAAATGCGTAGAGATCCGGAGGAACCTC
AGAGGCGAAGGCGGTGTCCTGGCTCATTTCTGACGCTCAGCCACGACAGCATGGGGAGCG
AGCGGGATTAGAAACCCCAGTAGCC

>Otu4896
CCAGCCTACGGGAGGCACCAGTCGAGAATCTTCGGCAATGGGCACAAGCCTGACCGAGCG
ACGCCGCGTGGAGGACGAAGGCCTTCGGGTTGTAAACTCCTGTCGAGGGGGAGGAAGGGG
TAACCTTGACCGATCCCTGGAGGAAGCACGGGCTAAGTTCGTGCCAGCAGCCGCGGTAAG
ACGAACCGTGCGAACGTTATTCGGAATCACTGGGCTTAAAGCGCGTGTAGGCGGGTGGGA
ACGTCGGCTTCTGAAAGCCCCCGGCTCAACCGGGGAAGTGGAACCGAAACGGCCCGCCTG
GAGGGACGTAGGGGGACCTGGAACTTCCGGTGGAGCGGTGAAATGCGTTGAGATCGGAAG
GAACGCCCGTGGCGAAAGCGAGGTCCTGGACGTCTGCTGACGCTGAGACGCGAAAGCTAG
GGGAGCGAACGGGATTAGATACCCCCGTAGTCC
>Otu4897
CCAGCCTATGGGGCGCTGCAGTCGAGAATTTTTCACAATGGGCGAAAGCCTGATGGAGCG
ACGCCGCGTGGGGGATGAATGGCTTCGGCCCGTAAACCCCTGTCATCTGGGATCAACCCC
AGCCGGTGAATAGCCGGCTGGCTGATAGTACCGGAAGAGGAAGGGACGGCTAGCTCTGTG
CCAGCAGCCGCGGTAAAACAGAGGTCCCAAGCGTTGTTCGGATTCACTGGGCGTAAAGGG
TGCGTAGGCGGTCGGGTAAGTCCGATGTGAAATCTCCAGGCTCAACCTGGAAACGGCATT
GGAAACTATCCGGCTGGAGGGTTGGAGGGGGGACTGGAATTCTCGGTGTAGCAGTGAAAT
GCGTAGATATCGAGAGGAACACCAGTGGCGAAGGCGAGTCCCTGGACAACTCCTGACGCT
GAGGCACGAAAGCTAGGGGAGCAAACAGGATTAGAAACCCCTGTAGTCC
>Otu4900
CAGCCTATGGGGTGCTGCAGTCGCGCAACCTTCGCAATGCGCGAAAGCGTGACGAGGGGA
TTCTGAGTGCCGACTTCACGTCGGCTTTTAGTCAGCTTGAAAAGCTGGCTGAATAAGGAG
CGGGTAATTGCTGTGCCAGCCGCCGCGGTAAAACAGCTGCTTCAAGTGGTGCCCACGAAT
ATTGAGCCTAAAACGAGTGTAGCGGGCTTGGCCAGTTCTCCGCGAAATCCACCAACTTAA
TTGGTGGGCGCGCGGGGAATACTACCAGGCTTGGGAATGGGGGAGGTCAGGGGTACTGCG
GGGGGAGGAGTAAAATCCTTTAATCCTCGCAGGACCATCGGTGGCGTAGGCGCCTGACCA
AAACATGTCCGACCGTGAGACTCGAAGGCCAGGGGAGCAAATCGGATTAGAAACCCCAGT
AGTCC
>Otu4909
CCAGCCTATGGGGGGCACCAGTGGGGAATTTTGCGCAATGGGGGAAACCCTGACGCAGCA
ACGCCGCGTGAGTGATGAAGGCCTTCGGGTCGTAAAGCTCTGTCAGAGGGAAAGAAGTGT
AGGAGGGTTAATACCCCTTTTATTTGACGGTACCGCAAGAATAAGCCCCGGCTAACTTCG
TGCCAGCAGCCGCGGTAATACGAAGGGGGCTAGCGTTGTTCGGAATTACTGGGCGTAAAG
CGCGTGTAGGCGGGTCTTTAAGTCAGGGGTGAAATGCCAAGGCTCAACCTTGGAACCGCC
TTTGATACTGGAGATCTTGAGTCCGGGAGAGGTGAGTGGAACTGCGAGTGTAGAGGTGAA
ATTCGTAGATATTCGCAAGAACACCAGTGGCGAAGGCGGCTCACTGGCCCGGAACTGACG
CTGAGACGCGAAAGCGTGGGGAGCAAACAGGATTAGAGACCCCTGTAGTCC
>Otu4915
CCAGCCTATGGGTTGCAGCAGTCAAGAATATTCCTCAATGGACGAAAGTCTGAAGGAGCG
ACGCCGCGTGATCGATGAAGTCCTTCGGGATGTAAAGATCTTTTATTAGGGAAGAATAAA
TGACGGTACCTAATGAATAAGAGGTTGCTAACTCTGTGCCAGCAGCAGCGGTAATACAGA
GACCTCGAGCGTTATCCGGATTGATTGGGCGTAAAGCGTGCGCAGACTGCCTTTTAAGTC
TGCGGTTAAATCCCGATGCTTAACATCGGGACTGCCGCGGAAACTGGATGGCTAGAGTGC
GGAAGAGGCTAGCGGAATTGCCAGTGTAACGGTAAAATGTGTTAATATTGGCAAGAACAC
CAGATGCGAAAGCGGCTAGCTGGTACGCAACTGACGTTGATGCACGAAAGCGTGGGTAGC
GAATGGGATTAGAGACCCCCGTAGTCC
>Otu4916
CCAGCCTACGGGTTGCAGCAGTGGGGAATCTTGCGCAATGGGCGAAAGCCTGACGCAGCA
ATGCCGCGTGAGGGACGAAGGCTTTCTGAGTTGTAAACCTCTTTCAGCAGGGACGATTGT
GACGGTACCTGCAGAAGAAGCACCGGCCAACTACGTGCCAGCAGCCGCGGTGATACGTAG
GGTGCAAGCGTTGTCCGGATTTATTGGGCGTAAGGGCTCGTAGGCGGTTCGATAAGTCGG
GTGTGAAATCCCCAGGCTCAACCTGGGGTCGCCACTCGATACTGTCGTGACTAGAATCAC
GTAGGGGACCACGGAATTCCAGGTGTAGCGGTGAAATGCGCAGATATCTGGAGGAACACC
AGCAGCGAAGGCGGTGGTCTGGGCGTGTATTGACGCTGAGGAGCGAAAGCGTGGGGAGCG
AACAGGATTAGAAACCCTAGTAGTCC
>Otu4918
CCAGCCTACGGGTGGCTGCAGTGAGGAATATTGGTCAATGGGGGAAACCCTGAACCAGCG
ACGCCGCGTGTAGGACGAAGGGGCTTTGCCCTGTAAACTACTGTCGGATGGGAAGAAAAG
CACCCTCGGGGTGCCGGGACTGTACCATCAAAGGAAGGATCGGCTAACTACGTACCAGCA

GCCGCGGTAATACGTAGGATCCAAGCGTTGTCCGGATTTACTGGGTGTAAAGGGTGCGTA
GGCGGACTTGTGCGTCAGAGGTTAAATGTCACGGCTTAACCGTGAACCTGCCTTTGATAC
GGCAAGTCTGGAGTGCGGGAGAGGAAGGTAGAATTCCTGGTGTAGCGGTGAAATGCGTAG
ATATCAGGAGGAATACCAGTGGCGAAGGCGGCCTTCTGGCCCGCAACTGACGCTGAGGCA
CGAAAGCGTGGGGAGCAAACAGGATTAGAGACCCTAGTAGTCC
>Otu4922
CCAGCCTACGGGGCGCTCCAGTGGGGAATTTTGCGCAATGGGGGAAACCCTGACGCAGCA
ACGCCGCGTGAGTGATGAAGGCCTTCGGGTCGTAAAGCTCTGTCAGAGGGAAAGAAGTGT
AGGAGGGTTAATACCCCTTTTACTTGACGGTACCCTCAAAGGAAGCACCGGCTAACTCCG
TGCCAGCAGCCGCGGTAATACGGAGGGTGCGAGCGTTGTTCGGAATTATTGGGCGTAAAG
CGCACGCAGGCGGCTCGTTAAATTAGAAGTGAAAGCCCTGGGCTCAACCCGGGAATTGCT
TTTAAGACTGGCGAGCTTGAATTCGGAAGAGGGCAGTGGAATTCCAAGTGTAGAGGTGAA
ATTCGTAGATATTTGGAAGAACACCAGTGGCGAAGGCGACTGCCTGGTCCGACATTGACG
CTCATGTGCGAAAGCGTGGGGAGCAAACAGGATTAGAGACCCTCGTAGTCC
>Otu4925
CCAGCCTACGGGAGGCTGCAGTGGGGAATTTTGCACAATGCCCGAAAGGGTGATGCAGCA
ACGCCGCGTGAGGGATGAAGGCCTTCGGGTCGTAAACCTCTTTCGACTGGGAAAAATGGG
CTGGTGAAAAGCCAGTTATGATGGTACCGGTGGAAGAAGCACCGGCTAACTCTGTGCCAG
CAGCCGCGGTAATACAGAGGGTGCGAGCGTTGTTCGGAATTATTGGGCGTAAAGGGCGCG
TAGGCGGTGTTGTAAGTCACCTGTGAAACCTCTAGGCTTAACCTAGAGCCTGCAGGCGAA
ACTGCAATGCTGGAGGGTGGGAGAGGTGCGTGGAATTCCCGGTGTAGCGGTGAAATGCGT
AGATATCGGGAGGAACACCTGTGGCGAAAGCGGCGCACTGGACCACTACTGACGCTGAGG
CGCGAAAGCCGGGGGAGCAAACAGGATTAGATACCCCCGTAGTCC
>Otu4938
CCAGCCTATGGGTGGCTCCAGTAGGGAATATTGGTAATGGGCGAAAGCCTGAACCAGCAA
CGCCGCGTGAGTGATGAAGGCCTTCGGGTTGTAAAACTCTTTTTATGGGGACGAGGAAGG
ACGGTACCCATAGAATAAGCTTCGGCTAACTACGTGCCAGCAGCCGCGGTAAAACGTAGG
AAGCGAGCGTTATCCGGATTTACTGGGCGTAAAGCGCGTGCAGGCGGTTTGGTAAGTTGG
ATGTGAAAGCTCCCGGCTTAACTGGGAGAGGTCGTTCAATACTGCCAGACTAGAGGATGG
TAGAGGGAGGTGGAATTCTGGGTGTAGTGGTGAAATGCGTAGATATCCAGAGGAACACCA
GTGGCGAAAGCGGCCTCCTGGACCATTTCTGACGCTCATACGCGAAAGCTAGGGTAGCAA
ACGGGATTAGAGACCCGCGTAGTCC
>Otu4939
CCAGCCTATGGGTGGCAGCAGGCGCGAAACCTTTACAATGCGCGAAAGCGTGATAGGGGG
ATCCTGAGTGCTTACGGTTTATCTGTAAGCTTTTGCCAAGAGTTCACATCTTGGAGAATA
AGTGGTGGGTAAGACCGGTGCCAGCAGCCGCGGTAACCCCGGCGCCACGAGTGGCAATCT
TATATTGGGCCTAAAGCATTCGTAGCAGGTCTGAAAAATCTTTTGTGAAATTGTCGAACT
CAATACGACAGCGTGCAGGAGACACTTTCAGACTAGAGACTGGGAGGAGTTGGGGGTATG
TCTAGGGGAGCGGTTAAATGTGATAATCCTAGACAGACCACCTGTGGCGAAGGCGCCCAA
CTAGAACAGTTCTGACTGTGAGGGATGAAAGCTGGGGGAGCAAACCGGATTAGATACCCG
CGTAGTCC
>Otu4943
CCAGCCTACGGGGTGCACCAGTGGGGAATTTTGGACAATGGGCGAAAGCCTGATCCAGCA
ACGCCGCGTGAAGGATGAAGGTCTTCGGATTGTAAACTTCTTTTAGAGGGAACGAATAAA
TTGACGGTACTCTCAGAAAAAGCCACGGCTAACTACGTGCCAGCAGCCGCGGTAATACGT
AGGTGGCAAGCGTTACTCGGATTTACTAGGCGTAAAGCGTGTGTAGGTGGTTGGATAAGT
CTGTGGTGAAATTTCCCGGCCCAACTGGGAAGGGTCCATAGAAACTATTCGGCTTGAGTG
TGGTAGGGGAAGACGGAATTCCTGGTGTAGCGGTGAAATGCGTAGATATCGGGAGGAACA
CCAATGGCGAAAGCAGTCAACTGGGCCAATACTGACACTGAGGAGCGAAAGCTAGGGGAG
CAAACAGGATTAGAAACCCGGGTAGTCC
>Otu4944
GGACTACGGGGGTATCTAATCCTGTTTGCTACCCACGCTTTCGTGTCTCAGCGTCAACAA
TGGTCCAGAAAGCCGTCTACACCGCAGGTGTTCCTCCTGATATCTACGCATTTCACCGCT
ACACCAGGAATTCCGCTTTCCTCTCCCACGTTCGAGCACCCCAGTTTCCGGCGCACCCTC
CCAGTTGAGCCGGGAGATTTCACACCGAACTTAAGGCACCGCCTACACACTCCTTACGCC
CAGTAATTCCGAACAACGCTCGCTGCCTACGTATTACCGCGGCTGCTGGCACGTAGTTAG
CCGCAGCTTCTTCTTCAGGTACCGTCATTATCTTCCCTGCCGACAGAGCTTTACACCCCG
AAGGGCTTCATCGCTCACGCGGCGTCGCTGCATCAGGGTTTCCCCCATTGTGCAAGATTC

CCCACTGCTGCCTCCCGTAGGCTGG
>Otu4947
CCAGCCTACGGGGCGCTCCAGTGGGGAATCTTGCGCAATGGGCGAAAGCCTGACGCAGCG
ACGCCGCGTGGGTGATGAAGGCCTTCGGGTTGTAAAGCCCTGTCAGGGGGAAAGAATGGA
GTGCCAGTATAATACGCTGGTGCTCTGACGGTACCCCAAAGGAAGCACCGGCTAACTCTG
TGCCAGCAGCCGCGGTAAGACAGAGGGTGCAAGCGTTGTTCGGAATTACTGGGCGTAAAG
GGCGCGTAGGCGGTTGGGAAAGTTGGATGTGAAATCCCTCGTTTCAAGCAAGGAACTGCA
TCCAAAACTACCCAGCTCGAGTCCCGGAGAGGAAAGTGGAATTCCCGGTGTAAAGGTGAA
ATTCGTAGAGATCGGGAGGAACACCAGAGGCGAAAGCGGCTTTCTGGACGGTGACTGACG
CTGAGGCGCGAAAGCGTGGGGAGCAAACAGGATTAGAAACCCTAGTAGTCC
>Otu4952
CCAGCCTATGGGACGCTCCAGTGGGGAATATTGGACAATGGGCGCAAGCCTGATCCAGCC
ATGCCGCGTGAGTGAAGAAGGCCTTAGGGTTGTAAAGCTCTGTCAGAGGGAAAGAAGTGT
AGGAGGGTTAATACCTCTTTTATTTGACGGTACCCTCAAAGGAAGCACCGGCTAACTCCG
TGCCAGCAGCCGCGGTAATACGGAGGGTGCGAGCGTTGTTCGGAATTATTGGGTGTAAAG
CGCGTGTAGGCGGTTTTTTAAGTCTGATGTGAAAGCCCTGGGCTCAACCCGGGAAGTGCA
TTGGAAACTGGGAGACTTGAATACGGGAGAGGGTAGTGGAATTCCTGGTGTAGGAGTGAA
ATCCGTAGATATCAGGAGGAACACCGGTGACGAAGGCGGCTGCCTGGACCGATATTGACG
CTGAGACGCGAAAGCGTGGGTAGCAAACCGGATTAGAAACCCGAGTAGTCC
>Otu4956
CCAGCCTATGGGAGGCAGCAGTCGAGGATCATTCGCAATGAGCGAAAGCTTGACGATGCG
ACGCTGTGTGAACGATGAAGGCCTTCGGGTCGTAAAGTTCTTTCGCTTGGGAACAAGAGA
TTGCGGTGAATAACCGTGAAATTTGAGGGTACCAGGTAAAGAAGCACCGGCTAACTCCGT
GCCAGCAGCTGCGGTAATACGGAGGGTGCAAGCATTAATCGGATTTATTGGGCGTAAAGG
GCGCGTAGGCGGTTATGTAAGTCAGATGTGAAATACCGAAGCTCAACCTCGGTGCTGCAT
TTGAAACTACAAGACTAGAGGGATGGCGGAGAAAACGGAATTCCACGTGTAGCGGTGAAA
TGCGTAGATATGTGGAAGAACACCTGTGGCGAAAGCGGTTTTCTAGCTGTTACCTGACGC
TGAGGCGCGAGAGCAAGGGGAGCAAACAGGATTAGATACCCCAGTAGTCC
>Otu4957
CCAGCCTACGGGTGGCAGCAGTTGGGAATCTTGCGCAATGGGGGCAACCCTGACGCAGCG
ACGCCGCGTGCGGGATGACGGTCTTCGGATTGTAAACCGCTTTTCTCGGGGACGATAATG
ACGGTACCCGAGGAATCAGCCCCGGCTAACTCCGTGCCAGCAGCCGCGGTAAGACGGAGG
GGGCAAGCGTTGTCCGGAATTACTGGGCGTAAAGCGCTCGTAGGCGGTCCTTTACGGCGG
TGGTGAAAGCTCCTGGCTTAACTGGGAGAGGCCCACCGCAACGGGAGGACTTGAGGGTGG
TAGAGGCAGATGGAATTCCTGGTGTAGCGGTGAAATGCGTAGAGATCAGGAGGAACACCT
ATGGCGAAGGCAGTCTGCTGGGCCACTCCTGACGCTGAGGAGCGAAAGCTAGGGGAGCAA
ACGGGATTAGAGACCCCTGTAGTCC
>Otu4961
CCAGCCTATGGGTGGCACCAGTCGAGAATTTTTCACAATGGGCGAAAGCCTGATGGAGCG
ACGCCGCGTGGGGGATGAATGGCTTCGGCCCGTAAACCCCTGTCATTCGGGATCAATGCG
TACTACTTAATACGTATTACGTTGATAGTACCGGAAGAGGAAGGGACGGCTAACTCTGTG
CCAGCAGCCGCGGTAATACAGAGGTCCCAAGCGTTGTTCGGATTTACTGGGCGTAAAGGG
TGCGTAGGTGGTCGGGTAAGTCTGACGTGAAATCTCCGAGCTTAACTCGGAAACTGCGTT
GGATACTATTCGGCTTGAGGGTTGGAGGGGGGACTGGAATTCTCGGTGTAGCAGTGAAAT
GCGTAGATATCGAGAGGAACACCAGTGGCGAAGGCGAGTCCCTGGACAACTCCTGACACT
AAGGCACGAAAGCTAGGGGAGCAAACAGGATTAGAAACCCGAGTAGTCC
>Otu4967
CCAGCCTACGGGGCGCAGCAGTGGGGAATTTTGCGCAATGGAGGAAACTCTGACGCAGCG
ACGCCGCGTGGGTGATGAAGGCCTTCGGGTTGTAAAGCCCTGTCGGCAGGGAAGAAAAGT
GCATTGGCTAATATCCAATGCGCCTTTGACGGTACCTGCAAAGGAAGCACCGGCTAACTC
CGTGCCAGCAGCCGCGGTAAGACGGAGGGTGCAAGCGTTGTTCGGATTGACTGGGCGTAA
AGGGCGTGTAGGCGGTTCGTTAAGTCGGGTGTGAAAGCCCGGGGCTCAGCTCCGGAAGTG
CGCCCGAAACTGGCGAGCTGGAGCGCGGTAGAGGAGGGTGGAATTCCTGGTGTAGCGGTG
AAATGCGTAGATATCAGGAGGAACACCGGCGGCGAAGGCGGCCCTCTGGACCGTTGCTGA
CGCTGAGACGCGAAAGCGTGGGGAGCAAACAGGATTAGATACCCCCGTAGTCC
>Otu4970
CCAGCCTATGGGTCGCTGCAGTCGAGAATTTTTCACAATGGGCGAAAGCCTGATGGAGCG
ACGCCGCATGGGGGATGAATGGCTTCGGCCCGTGAACCCCTGTCATTCGGGATCAATGTC

CCGACAATAACATTGTCGGGATTGATAGTACCGGAAGAGGAAGGGACGGCTAACTCTGTG
CCAGCAGCCGCGGTAATACAGAGGTCCCAAGCGTTGTTCGGATTTACTGGGCGTAAAGGG
TGCGTAGGTGGTTGGATAAGTCTGATGTGAAATCTCGGAGCTTAACTCCGAAACTGCATT
GGATACTATCTAGCTTGAGAGTTGGAGGGGGGACTGGAATTCTCGGTGTAGCAGTGAAAT
GCGTAGATATCGAGAGGAACACCAGTGGCGAAGGCGAGTCCCTGGACAACTTCTGACACT
GAGGCACGAAAGCCAGGGGAGCAAACGGGATTAGATACCCGTGTAGTCC
>Otu4975
CCAGCCTACGGGGGGCACCAGTGAGGAATATTGGTCAATGGACGCAAGTCTGAACCAGCC
ATGCCGCGTGAAGGATGAAGGCCTTCTGGGTTGTAAACTTCTTTTACCTGGGAAGAAACC
TCTGATTTCTATTAGAGTTGACGGTACCAGAGGAATAAGCACCGGCTAACTCCGTGCCAG
CAGCCGCGGTAATACGGAGGGTGCAAGCGTTATCCGGATTTATTGGGTTTAAAGGGTGCG
TAGGCGGACTTATAAGTCAGTGGTGAAATCTCATCGCTTAACGATGAACGTGCCATTGAT
ACTGTAGGTCTTGAGTACAGATGCCGTTGGCGGAATGTGTCATGTAGCGGTGAAATGCAT
AGATATGACACAGAACACCGATTGCGAAGGCAGCTGACGAAACTGTAACTGACGCTGAGG
CACGAAAGCGTGGGGATCAAACAGGATTAGAGACCCGAGTAGTCC
>Otu4978
CCAGCCTACGGGTCGCAGCAGTGGGGAATTTTAGACAATGGGCGAAAGCCTGATCTAGTA
ATATTTCGTGGATGATGAAGGCCTCAGCTGTAAAATTCTGTTTTCGATAATGACAATAAT
GATTACAATTATAAAAGAAGCCCCGGTAAATGTCTGTGCCAGCAGCCGCGGTAATACAGG
GGGGGCTAGTATTATTCTGCATGATTGGGCGTAAAGGATATCTAGGTGGTTTTTTTAATA
TTAAAAGAAAGACTATATTAAATGTATAGTAGGTTTTAGTATATTTTAAAACTTTGAGTT
TATAAAAGATAATAGAATGCTCAGAGTAACAGTGAAATGTATTGAAGCTGGGCGGAATTC
CAAATGGCGAAAGCAGTTATCTTGTTAAAACTGACACTAAAGTATGAAGGTATGGGGAGC
GAATAGGATTAGATACCCCCGTAGTCC
>Otu4981
CCAGCCTATGGGTCGCTGCAGTGGGGAATCTTGCACAATGGGGGAAACCCTGATGCAGCG
ACGCCGCGTGAGCGATGAAGCCCTTCGGGGTGTAAAGCTCTTTCGACGGGAACGATAATG
ACGGTACCCGGAGAAGAAGCTGCGGCTAACTACGTGCCAGCAGCCGCGGTAATACGTAGG
CAGCAAGCGTTGTTCGGAGTTACTGGGCGTAAAGAGTGCGTAGGCGGTGCCCTAAGTCTG
TTGTGAAATCTCCCGGCTTAACTGGGAGGGTGCGGTGGAAACTGGGGTGCTTGAGTGTGG
GAGAGGTAAGCGGAATTCCTGGTGTAGCGGTGAAATGCGTAGATATCAGGAGGAACGCCG
GTGGTGAAAGCGGCGAGCTGGATCACTACTGACGCTGAGGAACGAAAGCTAGGGGAGCAA
ACAGGATTAGAAACCCTTGTAGTCC
>Otu4987
CCAGCCTATGGGGGGCTGCAGTGGGGAATCTTGCACAATGGGGGAAACCCTGATGCAGCG
ACGCCGCGTGAGCGATGAAGCCCCTCGGGGTGTAAAGCTCTTTCGGTGGGGACGATTATG
ACGGTACCCGCAGAAGCAGCTGCGGCTAACTACGTGCCAGCAGCCGCGGTAATACGTAGG
CAGCGAGCGTTGTTCGGAGTTACTGGGCGTAAAGGGTGTGTAGGCGGTCCCCTAAGTTCG
GTGTGAAATCTCCCGGCTCAACTGGGAGGGTGCGCCGAAGACTGGAGGGCTTGGAGTGCG
GGATGGGAGAGTGGAATTCCCGGTGTAGCGGTGAAATGCGTAGATATCAGGAGGAACACC
GGTGGTGTAGACGGCTTTCTGGACCGTAACTGACGCTGAGACACGAAAGCGTGGGTAGCA
AACAGGATTAGAAACCCGTGTAGTCC
>Otu4988
CCAGCCTATGGGTGGCAGCAGTCGAGGATCTTCGGCAATGGGCGAAAGCCTGACCGAGCG
ACGCCGCGTGCGGGATGAAGGCCTTCGGGTTGTAAACCGCTTTCGTAGGGGAGGAAATTC
CGGTGGGATCACCCATCGGTTTGACCTAGCCTAGGAAGAAGGACGGGCTAAGTTCGTGCC
AGCAGCCGCGGTAATACGAACCGTCCAAACGTTATTCGGAATCACTGGGCTTAAAGGGTG
CGTAGGCGGCCCGGGAAGTTGGGTGTGAAATCCCTCGGCTCAACCGAGGAATTGCGCCCA
AAACTGCCGGGCTTGAGGGAGATAGAGGTGAGTGGAACTTAGGGTGGAGCGGTGAAATGC
GTTGATATCCTAAGGAACACCGGTGGCGAAAGCGACTCACTGGGTCTCTTCTGACGCTGA
GGCACGAAAGCTAGGGTAGCGAAGAGGATTAGAAACCCTCGTAGTCC
>Otu4990
CCAGCCTACGGGGTGCACCAGTGGGGAATATTGGACAATGGGGGAAACCCTGATCCAGCA
ATGCCGCGTGTGTGAAGAAGGCCTTCGGGTTGTAAAGCACTTTTGTTGAGGAGGAACGTT
GGTAGGTTAAGAGCTAACCAACTTGACTGTACTCAAAGAATAAGCACCGGCTAACTCTGT
GCCAGCAGCCGCGGTAATACAGAGGGTGCGAGCGTTAATCGGAATTACTGGGCGTAAAGG
GTGCGTAGGTGGAAAGATAAGTCGAACGTGAAAAGCCTGGGCTTAACCTAGGTCGTGCGT
GCGATACTGTTTTTCTAGAGTATGGCAGAGGAGAGTGGAATTTCAGGTGTAGCGGTGAAA

TGCGTAGATATCTGAAGGAACACCAGTGGCGAAGGCGACTCTCTGGGCTAATACTGACAC
TGAGGCACGAAAGCCAGGGGAGCAAACGGGATTAGATACCCCTGTAGTCC
>Otu4998
CCAGCCTATGGGGGGCTCCAGTCGAGAATATTCCACAATGGGCGAAAGCCTGATGGAGCG
ACGCCGCGTGCAGGATGAAGGTTTTAGGATCGTAAACTGCTTTTCTATGGGAGGAAGGTC
ACTTCGGTGATTTGACAGTACCGTAGGAATAAGGGGTTGCAAACTACGTGCCAGCAGCCG
CGGTAATACGTAGACCCCGAGCGTTATCCGGATTTATTGGGCGTAAAGAGTGTGTAGGGG
GCTAGGTGCGTCTCTTGTTAAATCTTGCAGCTTAACTGTGAGGCCGCAAGAGATACGACC
TAGCTAGAGAATACGAGAGGTTAGCGGAACGAACGGTGTAGGGGTGAAATCCGTTGATAT
CGTTCGGAACACCAAAAGCGAAGGCAGCTAACTGGCGTATTTCTGACCCTGAGACACGAA
AGCGTGGGGAGCAAAAAGGATTAGATACCCTCGTAGTCC
>Otu5000
CCAGCCTATGGGTTGCTGCAGTAGGGAATTTTGCGCAATGGACGAAAGTCTGACGCAGCA
ACGCCGCGTGAGTGATGAAGGTCTTCGGATTGTAAAGCTCTGTTGAGTGGGAAGAAAAGT
TTCAAGAATAATACCTTGAAATCTGACGGTACTACTTGAGGAAGCCCCGGCTAACTACGT
GCCAGCAGCCGCGGTAATACGTAGGGGGCAAGCGTTGTTCGGAATCACTGGGCGTAAAGG
GAGCGTAGGCGGAGAGGTAAGTTAGGAGTATAATATATGGGCTTAACCCGTAGTCTGCTT
CTAATACTGCTTTTCTTGAGTATGGGAGAGGGAGATGGAATTCCAGGTGTAGCGGTGGAA
TGCGTAGATATCTGGAAGAACACCAGCTGCGAAGGCGGTCTCCTGGCCCAATACTGACGC
TGAGGCTCGAAAGCTAGGGGAGCAAACAGGATTAGAAACCCTGGTAGTCC
>Otu5002
CCAGCCTACGGGTGGCAGCAGTTTGGAATATTCCACAATGGGCGAAAGCCTGATGGAGCG
ACACCGCGTGGAGGACGAAGGTCTTCGGATTGTAAACTCCTTTAGACCCGGATGAAGATT
CCAGGGTGAATAATCCTGGAATTTGACCGTACGGGTAAAATAAGCCACGGCTAACTCTGT
GCCAGCAGCCGCGGTAATACAGAGGTGGCAAACGTTGTCCGGAATTACTGGGTGTAAAGG
GTCCGCAGGCGGCCTTGTAAGTTGAAGGTGAAATGTCCCGGCTCAACCGGGTCACTGCCT
TCAAAACTGCAGGGCTTGAGTACGGTAGAGGAGAGTGGAATTCCCAGTGTAGCGGTGAAA
TGCGTAGATATTGGGAGGAACACCAGTGGCGAAGGCGGCTCTCTGGACCGATACTGACGC
TCAGGGACGAAAGCCAGGGGAGCAAACGGGATTAGATACCCTGGTAGTCC
>Otu5008
CCAGCCTATGGGTTGCTGCAGTCGAGAATCATTCGCAATGGGCGCAAGCCTGACGATGCG
ACGCCGTGTGAGCGATGAAGGCCTTAGGGTCGTAAAGCTCTTTCGCCTGGGAACAAGAGA
AGCTGACAAATAATCAGCCGATTTGAGGGTACCAGGTAAAGAAGCACCGGCTAACTCCGT
GCCAGCAGCTGCGGTAATACGGAGGGTGCAAGCATTAATCGGATTTATTGGGCGTAAAGG
GCGCGTAGGCGGAAAAGCAAGTCAGATGTGAAATCCCGGGGCTCACCCCCGGAACAGCAT
TTGAAACTGCATTCCTTGAGGGTAGGCGGAGAAAATGGAATTCCACAAGTAGCGGTGAAA
TGCGTAGATATGTGGAAGAACACCAGTGGCGAAGGCGATTTTCTAGCTTATACCTGACGC
TGAGGCGCGAAAGCAAGGGGATCAAACAGGATTAGAGACCCCAGTAGTCC
>Otu5011
CCAGCCTACGGGGGGCTGCAGTGGGGAATTTTGCGCAATGGGGGAAGCCCTGACGCAGCA
ATGCCGCGTGAGTGATGAAGGCCTTCGGGTCGTAAAGCTCTGTCAGAGGGAAAGAAGTGT
AGGAGGGTTAATACCCCTTTTATTTGACGGTACCCTCAAAGGAAGCACCGGCTAACTCCG
TGCCAGCAGCCGCGGTAATACGGAGGGTGCGAGCGTTGTTCGGAATTATTGGGCGTAAAG
CGCGTGTAGGCGGGTATTTAAGTCAGGGGTGAAATGCCAAGGCTCAACCTTGGAACTGCC
TTTGATACTGGATATCTTGAGTCCGGGAGAGGTGAGTGGAACTGCGAGTGTAGAGGTGAA
ATTCGTAGATATTCGCAAGAACACCAGTGGCGAAGGCGGCTCACTGGCCCGGTACTGACG
CTGAGGTGCGAAAGCGTGGGGAGCAAACAGGATTAGAAACCCCAGTAGTCC
>Otu5014
GGACTACGGGGGTATCTAATCCTGTTTGATCCCCACGCTTTCGAGCCTCAGCGTCAGTTA
CAGTTTTGTCAGCTGCCTTCGCAATCGGTGTTCTGTGTCATATCTAAGCATTTCACCGCT
ACATGACACATTCCGCCAACATCATCTGTACTCAAGTTCTACAGTATCAATGGCACGTTC
GTTGTTAAGCAACGAGATTTCACCACTGACTTATAAAACCGCCTACGCTCCCTTTAAACC
CAATAAATCCGGATAACGCTTGCATCCTTCGTATTACCGCGGCTGCTGGCACGAAGTTAG
CCGATGCTTATTCTTACGGTACTATCAGCCTCGCTCACGAGCGAGGGTTTTCTCCCGTAC
AAAAGCAGTTTACAACGCGTAGCGCCTTCTTCCTGCACGCGGGATGGCTGGTTCAGGCTT
GCGCCCATTGACCAATATTCCTCACTGCAGCGACCCGTAGGCTGG
>Otu5015
CCAGCCTATGGATGCAGCAGTGGGGAATATTGGGCAATGGGCGAAAGCCTGACCCAGCCA

CGCCGCGTGAGTGATGAAGGCCTTCGGGTCGTAAAGCTCTGTGGGGGGGGACGAACAAGC
AGGCACTAAATACGTGCAGGCCCTGACGGTACCTCCTTAGCAAGCACCGGCTAACCATGT
GCCAGCAGCCGCGGTAATACATGGGGTGCAAACGTTGCTCGGAATTATTGGGCGTAAAGC
GCGCGTAGGCGGTCGCTTAAGTCGGATGTGAAATCCCTTGGCTTAACTGAGGAAGTGCAT
CCGAGACTGAGCAGCTAGAGTACGAAAGAGGGTCGCGGAATTCCCGGTGTAGAGGTGAAA
TTCGTAGATATCGGGAGGAACACCAGTGGCGAAGGCGGTTTCCTGGCCTGATACTGACGC
TGAGGCGCGAAAGCGTGGGGAGCAAACAGGATTAGAAACCCCAGTAGTCC
>Otu5016
CCAGCCTACGGGGCGCACCAGTGGGGAATTTTGCGCAATGGGAGCAATCCTGACGCAGCG
ACGCCGCGTGGATGATGAAGGCCTTCGGGTTGTAAAGTCTAGTAAGCAGGGAAGAATAAG
CGTGGGGTAATATTCCATGTGATGACGGTACCTGCCTAAAGCCCCGGCTAGCTACGTGCC
AGCAGCCGCGGTAATACGTATGGGGCGAGCGTTGTTCGGAATTATTGGGCGTAAAGGGCG
CCTAGGCGGGGGACTAAGTTAGGTGTGAAAGCCATGGGCTCAACCCATGAACTGCATCTA
AGACTGGTTCTCTTGAGTTCGGGAGAGGAGAGCGGAATTCCAGGTGTAGCGGTGAAATGC
GTAGATATCTGGAAGAACACCAGTGGCGAAGGCGGCTCTCTGGCCTAGAACTGACGCTGA
AGCGCGAAAGCGTGGGGAGCGAACAGGATTAGATACCCTGGTAGTCC
>Otu5020
CCAGCCTACGGGTCGCAGCAGTCGAGAATTTTTCACAATGGGAGAAATCCTGACGGAGCG
ACGCCGCGTGGAAGATGAAGGCCGAAAGGTTGTAAATTCCTTTTATACGGGAAGAATAAG
TGTATTGAGAGAAACGGTGCATGATGACGGTACCGTATGAATAAGCCCCGGCTAACTACG
TGCCAGCAGCCGCGGTAATACGTAGGGGGCAAGCGTTGTTCGGATTCATTGGGCGTAAAG
GGCATGTAGGCTGTTATGTAAGTGTGGATTTAAAGGCGGAGGCTTAACCTCTAGTTTGGT
CTGCAAACTGCGTAGCTTGAGTGTAGAAGGGGAAACTGGAATTCCGCGTGTAGGGGTGAA
ATCTGTAGAGATCAGGAAGAATACCAGTGGTGAAGGCGAGCTCCTGGCCAATGATTGACG
CTGAGGTGCGAAAGTGTGGGGATCAAACAGGATTAGAGACCCCCGTAGTCC
>Otu5021
CCAGCCTATGGGAGGCAGCAGGAGGGAATTTTGCGCAATGGACGAAAGTCTGACGCAGCA
ACGCCGCGTGAGTGATGACGGTCTTCGGATTGTAAAGCTCTGTTGAGTGGGAAGAAAAGT
TTCGGTAATAATACTGCCGGAGCCTGACGGTACCGCTCGAGGAAGCCCCGGCTAACTACG
TGCCAGCAGCCGCGGTAATACGTAGGGGGCGAGCGTTGTTCGGAATCACTGGGCGTAAAG
GGAGCGTAGGCGGGATCGTAAGTTAGGAGTCTAATTCTCAGGCTTAACCTGAGGCCGGCT
CTTAATACTGCGGTTCTTGAGTAAGGAAGAGGGAGATGGAATTTCAGGTGTAGCGGTGGA
ATGCGTAGATATCTGAAAGAACACCAGCAGCGAAGGCGGTCTCCTGGTCCTATACTGACG
CTGAGGCTCGAAAGTTAGGGGAGCAAACAGGATTAGAAACCCGAGTAGTCC
>Otu5022
CCAGCCTACGGGGGGCAGCAGTGAGGAATATTGCGCAATGGGCGAAAGCCTGACGCAGCA
ACGCCGCATGAGGGACGAAGGCCTTCGGGTCGTAAACCTCTTTTCTGGGGGACGAGCAAG
GACGGTACCTCAGGAATAAGTCTCGGCTAACTACGTGCCAGCAGCCGCGGTAAAACGTAG
GAGGCGAGCGTTATCCGGATTTACTGGGCGTAAAGCGCACGCAGGCGGCTCAGCAAGTTG
GATGTGAAAGCTTCTGGCTTAACTGGGAGAGGTCATTCGAAACTGCTGGGCTTGAGGTTG
GGAGAGGGGTGTGGAATTCCCGGTGTAGCGGTGGAATGCGTAGATATCGGGAGGAACACC
TGTGGCGAAAGCGACACCCTGGCCCACACCTGACGCTGATGTGCGAAAGCGTGGGGAGCA
AACAGGATTAGAAACCCTTGTAGTCC
>Otu5025
CCAGCCTACGGGGGGCAGCAGTGAGGAGTATTGCTCAATGGGCGCAAGCCTGAAGCAGCG
ACGCCGCGTGAGGGATGAAATCCGTTAGGATGTAAACCTCTTTTGCTGGGGAAGAATGTT
CCAATTTATTGGAATTGACGGTACTCTGCGAATAAGCACCGGCAAACTACGTGCCAGCAG
CCGCGGTAATACGTAGGGTGCGAGCGTTGTCCGGAATTACTGGGTGTAAAGGGTTCGCAG
GCGGACAAGATAGTCAGTGGTGAAATTCTCCGGCTCAACCGGAGACGTGCTGTTGATACT
TCTTGTCTTGAGGTCGAGAAAGGATAATGGAATTCATGGTGTAGCGGTGAAATGCGTAGA
GATCATGAAGAACACCGGTAGCGAAGGCGGTTATCTGGCTCAAAACTGACGCTCAGGGAC
GAAAGCGTGGGTAGCAAACAGGATTAGAGACCCTAGTAGTCC
>Otu5026
CCAGCCTACGGGGGGCAGCAGTGGGGAATATTGCACAATGGGCGAAAGCCTGATGGAGCG
ACGCCGCGTGGGGGATGAATGGCTTCGGCCCGTAAACCCCTGTCATTCGCGATCAAACCT
TGCTATTTAAAGGATGGCAAGCTGATAGTACCGGAAGAGGAAGGGACGGCTAACTCTGTG
CCAGCAGCCGCGGTAATACAGAGGTCCCAAGCGTTGTTCGGATTCACTGGGCGTAAAGGG
TGCGTAGGTGGTTGGGTAAGTCTGATGTGAAATCTCGGAGCTTAACTCCGAAACTGCATT

GGATACTATCTAGCTTGAGAGTTGGAGGGGGGACTGGAATTCTCGGTGTAGCAGTGAAAT
GCGTAGATATCGAGAGGAACACCAGTGGCGAAGGCGAGTCCCTGGACAACTTCTGACACT
GAGGCACGAAAGCCAGGGGAGCAAACGGGATTAGATACCCTAGTAGTCC
>Otu5030
CCAGCCTACGGGATGCTCCAGTAGGGAATATTGGTAATCTGCGAAAGCGGGAACCAGCAA
CGCCGCGTGTGCGATGAAGGCCTTCGGGTCGTAAAGCACTTTTTGAGGAGACGAGGAAGG
ACGGTATCCTCAGAATAAGTCTCGGCTAACTACGTGCCAGCAGCCGCGGTAACACGTAGG
AGACGAGCGTTGTCCGGATTTATTGGGCGTAAAGCGCACGCAGGCGGTCGTATCAGTCCA
GGGTGACAGTTTCCGGCTTAACCGGAAAAGGACTCTGGAGACGGCACGACTTGAGGGCTT
CAGAGGGATGTGGAATTCCAGGTGGAGTGGTGAAATGCGTAGAGATCTGGAAGAACACCA
AAGGCGAAGGCAACATCCTGGGGAGTCACTGACGCTCGGGTGCGAAAGCTAGGGGAGCGA
ACGGGATTAGAAACCCCAGTAGTCC
>Otu5033
CCAGCCTACGGGTGGCTGCAGTAACGAATCTTCCGCAATGCACGCAAGTGTGACGGAGCG
ACGCCGCGTGTGGGACGAAGTCCTTCGGGATGTAAACCACTGTCAGGGGTAAGAAAGTTC
TGATCTACCCCAGAGGAAGGCACGGCTAACTCTGTGCCAGCAGCCGCGGTAAGACAGAGG
TGCCGAGCGTTAGGCGGAATCACTGGGCTTAAAGCGTGTGTAGGCGGGTCGTTAAGTACC
TTGTGAAATCCCATGGCTCAACCATGGAACGGCTGGGTATACTGGCGATCTTGAGCGGCC
TAGGGGCAGATGGAACAAGTGGTGGAGCGGTGAAATGCGTAGATATCACTTGGAACGCCA
AAGGTGAAAACAGTCTGCTGGGGGCCTGCTGACGCTGAGACACGAAAGCCAGGGGAGCGA
ACGGGATTAGAAACCCGAGTAGTCC
>Otu5034
CCAGCCTATGGGTGGCAGCAGTGGGGAATATTGGACAATGGGCGCAAGCCTGATCCAGCC
ATGCCGCGTGAGTGATGAAGGCCCTAGGGTTGTAAAGCTCTTTCGGCGGGGACGATGATG
ACGGTACCCGCAGAAGAAGCCCCGGCTAACTTCGTGCCAGCAGCCGCGGTAATACGAAGG
GGGCTAGCGTTGTTCGGAATTACTGGGCGTAAAGCGCACGCAGGCGGTCCGCATAGTCAG
GAGTGAAAGCCCTGGGCTCAACCCGGGAATTGCTTTTGATACTGGCGGACTAGACTCATG
GAGAGGGTAGCGGAATTCCGAGTGTAGAGGTGAAATTCGTAGATATTCGGAAGAACACCA
GTGGCGAAGGCGGCTACCTGGCCATCGAGTGACGCTCATGTGCGAAAGCGTGGGGAGCAA
ACAGGATTAGAAACCCGAGTAGTCC
>Otu5036
CCAGCCTACGGGTGGCAGCAGTAGGGAATATTGTGCAATGGGGGAAACCCTGACACAGCG
ACGCCGCGTGTGGGATGAAGGCCTTCGGGTTGTAAACCACTGTCAGAGGGGACGAATTTA
TCGTTTAATAAACGGTATTGACTTAACCCTCAGAGGAAGCAACCGCTAACTCCGTGCCAG
CAGCGGCGGTAATACGGAGGTTGCAAACGTTGTTCGGATTTACTGGGCGTAAAGAGTTCG
TAGGCGGTCGGGATTAATTAGACGTGAAAGCCTCCGGCTTACCCGGAGAATGGCATCTAA
TATTGCCCGACTTGAGTGTGAAAGAGGAAAGTGGAATTGTAGGTGGAGCGGTGAAATGCG
TAGATATCTACAGGAACACCGGATGCGAAGGCGACTTTCTAGTTCGCAACTGACGCTGAG
GAACGAAAGCTAGGGGATCAAACAGGATTAGATACCCTAGTAGTCC
>Otu5044
CCAGCCTACGGGTGGCTGCAGTCGAGAATCTTCGGCAATGGGCGCAAGCCTGACCGAGCG
ACGCCGCGTGGAGGATGAAGGCCTTCGGGTTGTAAACTCCTGTCGAGGGGGAACAAGGGG
CCGTGCAGAGCGGTCCTTGAGTGGTCCCTGGAGGAAGCACGGGCTAAGTTCGTGCCAGCA
GCCGCGGTAAGACGAACCGTGCGAACGTTATTCGGAATCACTGGGCTTAAAGCGCGTGTA
GGCGGTCCGGCACGTTCGTCGCTGAAATCCCCCGGCTCAACCGGGGAACGGGCGCGGATA
CGACCGGACTGGAGGGAGGTAGGGGGAGCCGGAACTTCCGGTGGAGCGGTGAAATGCGTT
GAGATCGGAAGGAACGCCCGTGGCGAAAGCGGGCTCCTAGACCTTACCTGACGCTGAGAC
GCGAAAGCCAGGGGAGCGAACGGGATTAGATACCCTGGTAGTCC
>Otu5045
CCAGCCTATGGGGGGCAGCAGTCGAGAATTTTTCTCAATGGGCGAAAGCCTGAAGGAGCG
ACGCCGCGTGGGGGATGAATGGCTTCGGCCCGTAAACCCCTGTCATTTGCGAACAAACCT
TTTCACCTAACACGTGAAGAGCTGATTGTAGCGGAAGAGGAAGAGACGGCTAACTCTGTG
CCAGCAGCCGCGGTAATACAGGGGTCTCAAGCGTTGTTCGGATTCACTGAGCGTAAAGGG
TGCGTAGGTGGAGGGGCAAGTCTGACGTGAAATCTCCGGGCCTAACCCGGAAACGGCGTT
GGAAACTGCCTTGCTCGAGGATTGGAGGGGAGACTGGAATACTTGGTGTAGCAGTGAAAT
GCGTAGATATCAAGTGGAACACCAGTGGCGAAGGCGAGTCTCTGGACAAATCCTGACACT
GAGGCACGAAAGCCAGGGGAGCAAACGGGATTAGATACCCTCGTAGTCC
>Otu5046

CAGCCTATGGGACGCTGCAGGCGCGAAAACTTTACAATGCGGGAAACCGTGATAAGGAAA
CCCCGAGTGCCAGCATAGGCTGGCTGTCCATCAGTGTAAATAACTGGTGAAGAAGGGGCC
GGGCAAGACCGGTGCCAGCCGCCGCGGTAATACCGGCGGCTCGAGTGGTGGCCGCTATTA
TTGGGCTTAAAGGGTCCGTAGCTGGATTCACAAGTCCCTTGAGAAATCCATCGGCTTAAC
TGCTGGGCGTTCAGGGGATACTGTGGTTCTAGGGACCGGGAGAGGTGAGAGGTACTGCCG
GGGTAGGAGTGAAATCCTGTAATCCTTGGGGGACCACCTGTGGCGAAGGCGTCTCACCAG
GACGGCTCCGACAGTGAGGAACGAAAGCTGGGGGAGCAAACCGGATTAGATACCCTGGTA
GTCC
>Otu5057
CCAGCCTACGGGGGGCACCAGGCTAAGCATTTTTCTCACAGCGATAGCGATAGCTTCCGG
GTTTTCTGGAGGAACGGTTTCTCCGCATCCGCTTTCCGCGACAATATCATTTTCGGCATC
GGTTGCGTGAATTACAGGCTTTCCAGCAAGCATGTAATCGAAGACTTTATTTGGAGAAAC
TCCAAATCTGTAAATATCCTTTCGTTGCCAGCCGATGAAGCATGCATCAAAATGAGCGAT
GATTGAGGGGACAAGCAACTTTGGAACAGCGGGGAGGAAGGAGACATTACTTAATGACAT
CTCACGGGCCAATTGTTCCAATCGTTGTTTTTCCGGACCCTGTCCAACAAGAACAACACT
AACATTGTCATCCCGTAAACGGTCTGCTGCCAGCAAGAGTGAATCAAGAGCATTAGATAC
CCTAGTAGTCC
>Otu5061
CCAGCCTATAGGCTGCAGTGGGGAATTTTGCGCAATGGGGGAAACCCTGACGCAGCAACG
CCGCGTGGAGGATGAAGGCCCTTGGGTCGTAAACTCCTTTCGACCGGGACGATTATGACG
GTACCGGTGGAAGAAGCACCGGCTAACTCTGTGCCAGCAGCCGCGGTAATACAGAGGGTG
CGAGCGTTGTTCGGAATTATTGGGCGTAAAGGGCGCGTAGGCTGTGCGGTAAGTCACCTG
TGAAATCCCCAGGCTTAACCTGGGGCCTGCAGGCGAAACTGCCGTGCTGGAGGGTGGGAG
AGGTGCGTGGAATTCCCGGTGTAGCGGTGAAATGCGTAGATATCGGGAGGAACACCGGTG
GTGTAGACGGCTTTCTGGACCGTAACTGACGCTGAAACACGAAAGCGTGGGTAGCAAACA
GGATTAGAGACCCTCGTAGTCC
>Otu5064
CCAGCCTATGGGTTGCTGCAGTGGGGAATATTGGACAATGGGGGAAACCCTGATCCAGCA
ATGCCGCGTGTGTGAAGAAGGTCTTCGGATTGTAAAGCACTTTTGGCAGGGACGATGATG
ACGGTACCTGCAGAATAAGCCCCGGCTAACTTCGTGCCAGCAGCCGCGGTAATACGAAGG
GGGCTAGCGTTGTTCGGAATCACTGGGCGTAAAGCGTGCGTAGGCGGTTTTCCAAGTCAG
GGGTGAAATCCTGGGGCTCAACCCCAGAATTGCCTTTGAAACTGGAAAGCTAGAGTACTG
GAGGGGATAGCGGAATTCCTAATGTAGAGGTGAAATTCGTAGATATTAGGAGGAACACCG
GTGGCGAAAGCGGCTATCTGGACAGTAACTGACGCTCTGGCACGAAAGTGTGGGGATCAA
ACAGGATTAGAGACCCTAGTAGTCC
>Otu5065
CCAGCCTACGGGTGGCTCCAGTGGGGAATTTTGCGCAATGGGGGAAACCCTGACGCAGCA
ACGCCGCGTGGAGGATGAAGTCCTTCGGGACGTAAACTCCTTTCGATCGGGACGATTATG
ACGGTACCGGAAGAAGAAGCCCCGGCTAACTTCGTGCCAGCAGCCGCGGTAATACGAGGG
GGGCGAGCGTTGTTCGGAATTATTGGGCGTAAAGGGTGCGTAGGCGGCTTGGTAAGTCTT
ATGTGAAATCTATGGGCTCAACTCATAGACTGCATGAGAAACTGCCGGGCTCGAGTGTGG
GAGAGGTGAGTGGAATTTCCGGTGTAGCGGTGAAATGCGTAGATATCGGAAGGAACTTCA
GTGGCGAAGGCGGCTTCCTGGTCCAGCACCGACGTTCAGGCACGAAAGCGTGGGGAGCAA
ACAGGATTAGAAACCCCCGTAGTCC
>Otu5069
CCAGCCTACGGGGTGCAGCAGTAGGGAATTTTCCACAATGGGCGAAAGCCTGATGGAGCA
ACGCCGCGTGCAGGATGAAGGCCCTCGGGTCGTAAACTGCTTTTATATGTGACGAATATG
ACGGTAGCATATGAATAAGGATCGGCTAACTCCGTGCCAGCGGCCGCGGTCATACGGAGG
ATCCAAGCGTTATCCGGAATTACTGGGCGTAAAGAGTTGCGTAGGTGGCATAGTAAGTAG
ATAGTGAAAGCCTGGGGCTCAACCCCTTACACATTATCTAAACTGCTAAGCTAGAGGATG
AGAGAGGTTATTGGAATTCTTAGTGTAGGAGTGAAATCCGTAGATATTAGGAGGAACACC
GATGGCGTAGGCAGATAACTGGCTCATTCCTGACACTAAGGCACGAAAGCGTGGGGAGCA
AACAGGATTAGAGACCCTAGTAGTCC
>Otu5072
CCAGCCTATGGGGTGCACCAGTGGGGAATATTGGACAATGGACGCAAGTCTGAGCCAGCC
ATGCCGCGTGGAGGATGAAGGTCCTCTGGATTGTAAACTTCTTTTATTTGGGAAGAAACC
CACGATTTCTATTGTGGTTGACGGTACCAGATGAATAAGCACCGGCTAACTCCGTGCCAG
CAGCCGCGGTAATACGGAGGGTGCAAGCGTTATCCGGATTCACTGGGTTTAAAGGGTGCG

TAGGCGGGCAGGTAAGTCAGTGGTGAAATCTCCGGGCTTAACCCGGAAACTGCCGTTGAT
ACTATTTGTCTTGAATGTCGTGGAGGTGAGCGGAATATGTCATGTAGCGGTGAAATGCTT
AGATATGACATAGAACACCCATTGCGAGGGCAGCTCACTACACGATGATTGACGCTGAGG
CACGAAAGCGTGGGGATCAAACAGGATTAGAAACCCCCGTAGTCC
>Otu5073
CCAGCCTACGGGTGGCTGCAGTGGGGAATATTGGACAATGGGCGAAAGCCTGATCCAGCA
ATGCCGCGTGAGTGACGAAGGCCTTAGGGTTGTAAAACTCTTTTGTCAGGGAAGATAATG
ACGGTACCTGCAGAAAAAGCTCCGGCTAACTCTGTGCCAGCAGCCGCGGTAATACAGAGG
GAGCTAGCGTTGTTCGGAATTACTGGGCGTAAAGCGCGCGTAGGCCGCTTGCCAAGTCAG
ATGTGAAATCCCGGGGCTTAACTCCGGAATTGCATTTGAAACTGGCGAGCTAGAGAATGG
TAGGGGATAGGGGAATTTCTAGTGTAGAGGTGAAATTCGTAGATATTAGAAGGAACACCA
GTGGCGAAGGCGCCTATCTGGGCCATTTCTGACGCTGAGGCGCGAAAGCGTGGGGAGCAA
ACAGGATTAGATACCCCAGTAGTCC
>Otu5074
CCAGCCTATGGGTGGCAGCAGTGGGGAATATTGGACAATGGGGGAAACCCTGATCCAGCA
ATGCCGCGTGGGTGAAGAAGGCCTGCGGGTTGTAAAGCCCTTTCGGTGAGGACAAAATGT
GTCGCATTAATACTGCGATGTTTTGATTTAACTCATTAAAGAAGCACCGGCTAACTCTGT
GCCAGCAGCCGCGGTAATACAGAGGGTGCGAGCGTTAATCGGAATTACTGGGCGTAAAGC
GCACGTAGGCGGTTAGGTAAGTTGGATGTGAAATCCCCGGGCTTAACTTGGGAATTGCAT
TCAAGACTGCCTGACTCGAGTGTGGGAGAGGGAAGCGGAATTCCAGGTGTAGCGGTGAAA
TGCGTAGATATCTGGAGGAACATCAGTGGCGAAGGCGGCTTTCTGGCCCAACACTGACGC
TGAGGTGCGAAAGCGTGGGGAGCAAACAGGATTAGATACCCTAGTAGTCC
>Otu5078
CCAGCCTATGGGGTGCAGCAGTGGGGAATTGTTCGCAATGGGCGCAAGCCTGACGACGCA
ACGCCGCGTGGAGGATGAAGACCTTCGGGTCGTAAACTCCTTTCGATCGAGACGAATGGC
CTCCGGGTGAACAATCCGGAGGAGTGACGGTACCGAGAGAAGAAGCCCCGGCTAACTCCG
TGCCAGCAGCCGCGGTAATACGGGGGGGGCAAGCGTTGTTCGGAATTACTGGGCGTAAAG
GGCTCGTAGGCGGCCAACTAAGTCAGACGTGAAATCCCTCGGCTTAACCGGGGAACTGCA
TCTGATACTGGATGGCTTGGGATCGGGAGAGGGATGCGGAATTCCAGGTGTAGTGGTGAA
ATGCGTAGATATCTGGAGGAACACCGGTGGCGAAGGCGGCATCCTGGACCGACACTGACG
CTGATGAGCGAAAGCCAGGGGAGCAAACGGGATTAGATACCCCTGTAGTCC
>Otu5082
CCAGCCTACGGGAGGCTGCAGTCGAGAATCTTCCGCAATGGACGAAAGTCTGACGGAGCG
ACGCCGCGTGGTTGATGAAGCGCTTCGGCGCGTAAAAACCTTTTATGAGCGACTAAGTTT
ATTGAAGAGCTCATGAATAAGAGGTTGCTAAACTCGTGCCAGCAGCAGCGGTAATACGAG
TGCCTCAAGCGTTATCCGGAATTATTGGGCGTAAAGGGTGTGTAGGTGGCTGTGTTAGTC
TCGCGTTAAATTCTTCGGCTCAACCGGGGACCTGCGCGGGAAACGGCACTGCTAGAGGAT
GCGAGGGGTCTTTGGAACTCATGGTGTAGCGGTGAAATGCGTTGATATCATGGGGAACAC
CGAAAGCGAAGGCAGAAGACTGGAGCACCCCTGACACTGAAACACGAAAGCGTGGGTAGC
GAATGGGATTAGATACCCTTGTAGTCC
>Otu5084
CCAGCCTATGGGGGGCAGCAGTAAGGAATATTGGTCAATGGACGCAAGTCTGAACCAGCC
ATGCCGCGTGAAGGATGAAGGCCTTAGGGTTGTAAAGCTCTTTTGGCAGGGACGATAATG
ACTGTACCTGCAGAATAAGCCCCGGCCAACTTCGTGCCAGCAGCCGCGGTAATACGAAGG
GGGCTAGCGTTGCTCGGAATCACTGGGCGTAAAGCGCACGTAGGCGGGTCGTTAAGTCGG
GGGTGAAATCCTGGAGCTCAACTCCAGAACTGCCTTCGATACTGGCGATCTTGGAGTCCG
GGAGAGGTGGGTGGAACTGCGAGTGTAGAGGTGAAATTCGTAGATATTCGCAAGAACACC
AGTGGCGAAGGCGGCTCACTGGCCCGGTACTGACGCTGAGGTGCGAAAGCGTGGGGAGCA
AACAGGATTAGAGACCCTAGTAGTCC
>Otu5085
CCAGCCTATGGGGTGCACCAGTGAGGAATATTCCACAATGGACGCAAGTCTGATGGAGCG
ACGCCGCGTGCAGGAAGAAGCCCTTCGGGGTGTAAACTGCTTTTTTTAGGGACGAACAAA
TGACGGTACCTAAAGAATAAGAGGCTGCTAACTTCGTGCCAGCAGCAGCGGTAATACGAA
GGCCTCAAGCGTTATCCGGAATTATTGGGCGTAAAGCGTCCGCAGGCGGTTTGGAAAGCG
TGTCGTTAAATTTCAGGGCTCAACCCCGAAACAGCGATGCGAACTTCCAAACTAGAGTGT
GGGAAAGGCATCTGGAATTGTCGGTGTAGTAGTAAAATGCGTTAATATCGACAGGAACAC
CAAAAGCGAAAGCATGATGCTGGAACACTACTGACGCTCAGGGACGAAAGCGTGGGGAGC
GAATCGGATTAGAAACCCCAGTAGTCC

>Otu5087
CCAGCCTACGGGAGGCACCAGTGGGGAATTTTGCGCAATGGGGGAAACCCTGACGCAGCA
ACGCCGCGTGGAGGATGAAGTACTTCGGTACGTAAACTCCTTTCGATCGGGACGATAATG
ACGGTACCGGAAGGAGAAGCCCCGGCTAACTTCGTGCCAGCAGCCGCGGTAATACGTAGG
CAGCAAGCGTTGTTCGGAATTACTGGGCGTAAAGAGTGCGTAGGCGGTGTTCTAAGTCTG
TTGTGAAATCTCCTGGCTCAACTGGGAGGGTGCGGCGGAGACTGGAATACTAGAGTATGG
GAGAGGTAAGCGGAATTCCTGGTGTAGCGGTGAAATGCGTAGATATCAGGAGGAACACCT
GTGGTGTAGACAGCTTACTGGACCATGACTGACGCTGAGGCACGAAAGCGTGGGTAGCAA
ACAGGATTAGATACCCCAGTAGTCC
>Otu5088
CCAGCCTATGGGTTGCTGCAGCTTGGAATATTGGGCAATGAGCGAAAGCTTGACCCAGCG
AGAATTCCTGCGTGAAGAAGGTTTTGTCGTAAAGCGCTTTTGTTAGAAAAGAAAAAAGAC
ATGATCTAATGTAAATAAGTCCCGGCTAATTCTGTGCCAGCAGCCGCGGCAATACAGAAG
GGGCGAGCGTTATTCATCTGTATTGGGTGTAAAGGGTATGTAGGTTGCTTTAAAAACCAT
TTGTTAAATTTTTGAGAAAAACTGAAGAAAAACAAATGCCAATTATAAAGCTTGAGTTAT
ACAGAAGTTGGGAGAATTTTATAATGAGAGATAAAATTCATAGATCTATAAAAGAAGACC
AAAGGCCGAGGCACCCTTCTAGGTATAACTGACACTGAGGTACGAAAGTATGGGTAGCAA
ACAGGATTAGAAACCCTCGTAGTCC
>Otu5090
CCAGCCTATGGGGGGCAGCAGTGGGGAATATTGGACAATGGGGGCAACCCTGATCCAGCG
ATGCCGCGTGGGTGAAGAAGGCCTTCGGGTTGTAAAGCCCTTTAGGCTGGGAAGAAGTAT
ATGTGGTGAATAGCCATGTATATTGACGGTACCAGCAGAATAAGCACCGGCAAACTCTGT
GCCAGCAGCCGCGGTAATACAGAGGGTGCGAGCGTTAATCGGATTTACTGGGCGTAAAGG
GCGCGTAGGCGGTTATATTAGTGTGATGTGAAAGCCCTGGGCTCAACCTAGGAAGTGCAT
CGCAAACGGTATAACTGGAGTATAAGAGAGGGCGGTGGAATTTCCGGTGTAGCGGTGAAA
TGCGTAGAGATCGGAAGGAACGTCAATGGCGAAGGCAGCCACCTGGCTTAATACTGACGC
TGAGGCGCGAAAGCGTGGGGAGCGAACAGGATTAGAGACCCGAGTAGTCC
>Otu5094
CCAGCCTACGGGGTGCTCCAGTAGGGAATATTGCGCAATGGACGAAAGTCTGACGCAGCC
ACGCCGCGTGAGTGAGGAAGGCCTTCGGGTTGTAAAGCTCTGTCATCCGGGAAAAACGGC
TATTGGGGGAAAGTTCCAGTAGCGTGATGGTACCGGAGAAGGAAGCACCGGCAAACTCTG
TGCCAGCAGCCGCGGTAATACAGAGGGTGCAAGCGTTGTTCGGAATTACTGGGCGTAAAG
CGTGCGTAATCGGTATTGAGAGTCGCGGGTGAAATCCCAAGGCTTAACCTTGGAACTGCC
TGTGAGACCTCAGTACTAGAGTGTGAGAGGGGATAGTGGAATACCCAGTGTAGCGGTGAA
ATGCGTAGAGATTGGGTGGAACACCGGTGGCGAAGGCGGCTATCTGGCTCACAACTGACG
ATCAGGCACGAAAGCGTGGGGAGCAAACAGGATTAGAAACCCCCGTAGTCC
>Otu5095
CCAGCCTATGGGAGGCAGCAGACGAGAATATTCGACAATGGGCGAAAGCCTGATCGAGGG
ATGCCGCGTGCGGGAAGAAGGGCTTCGGCCTGTAAACCGCTTTTATGTGGGAAGAACTCT
GACGGTACCACAAGAATAAGGGGTTGCTAACCTCGTGCCAGCAGCAGCGGTAATACGAGG
ACCCCAAGCGTTATCCGGAATTACTGGGCGTAAAGCGTGAGCAGCCGGTTTTGCTAGTCT
TATGTTAAATCTCACTGCTCAACGGTGAGACAGCGTGGGAAACGGCAATACTAGAGCGGG
TAAGAGGTAGACGGAACGTGCAGTGTAGGGGTGAAATCCATTGATATTGCACGGAACACC
AAAAGCGAAAGCAGTCTACTAGTGCTCTGCTGACGGTCAATCACTAAAGCGTGGGGATCA
AAAAGGATTAGATACCCGCGTAGTCC
>Otu5096
CCAGCCTACGGGGGGCTCCAGTAGGGAATATTGGACAATGGGCGGAAGCCTGATCCAGCC
ATGCCGCGTGCAGGAAGAAGGCGTTATGCGTTGTAAACTGCTTTTGATCGGGAATAAAAG
GACCCTGCGGGGTAAATTGAATGTACCGATAGAATAAGCCACGGCTAACTACGTGCCAGC
AGCCGCGGTAATACGTAGGTGGCGAGCGTTATCCGGATTTATTGGGTTTAAAGGGAGCGT
AGGCGGTTTTGTAAGTCAGTGGTGAAAGACGGCCGCTCAACGGTCGCATTGCCATTGATA
CTGCAAGGCTAGAGTACAGTAGAGGTAGGCGGAATTTAGGGTGTAGCGGTGAAATGCATA
GATACCCTAAAGAACACCGATTGCGAAGGCAGCTTACTGGACTGTAACTGACGCTGAGGC
ACGAAAGTGTGGGGATCAAACAGGATTAGAAACCCGCGTAGTCC
>Otu5098
CCAGCCTACGGGTGGCACCAGTGGGGAATATTGGACAATGGGGGGAACCCTGATCCAGCA
ATGCCGCGTGTGTGAAGAAGGCCTGCGGGTTGTAAAGCACTTTGGTTGGGGAGGAAAGGC
GGTTAGTGAATAACTAGCTGAGTTGACGTTACCCAAAGAACAAGCACCGGCTAACTCTGT

GCCAGCAGCCGCGGTAATACAGAGGGTGCAAGCGTTAATCGGAATTACTGGGCGTAAAGG
GCGCGTAGGCGGTTAAATAAGTTATGTGTGAAAGCTCTGGGCTTAACCTGGGAACTGCAC
ATAATACTGTTTAGCTAGAGTAGAGTAGAGGAAAGTGGAATTTCCGGTGTAGCGGTGAAA
TGCGTAGATATCGGAAGGAACACCAGTGGCGAAGGCGGCTTTCTGGACTCATACTGACGC
TGAGGCGCGAAAGCGTGGGGAGCAAACAGGATTAGAAACCCGCGTAGTCC
>Otu5100
CCAGCCTACGGGAGGCACCAGTGGGGAATATTGGACAATGGGGGAAACCCTGATCCAGCA
ATGCCGCGTGGGTGATGAAGGCCTTCGGGTTGTAAAGCCCTTTTGGCGGGGACGATGATG
ACGGTACCCGCAGAAGAAGCCCCGGCTAACTTCGTGCCAGCAGCCGCGGTAATACGAAAG
GGGCTAGCGTTGTTCGGAATTACTGGGCGTAAAGGGCGCGTAGGCGGCTTTGTTAGTTGG
GCGTGAAATGCCTGGGCTCAACCCAGGAATTGCGCTCAATACTTCGAGGCTAGAGTGTGG
GAGAGGATAGTGGAATTCCCAGTGTGGAGGTGAAATTCGTAGATATTGGGAAGAACACCG
GTGGCGAAGGCGGCTATCTGGACCACAACTGACGCTGAGGCGCGAAAGCGTGGGGAGCAA
ACAGGATTAGAAACCCCGGTAGTCC
>Otu5102
CCAGCCTATGGGGGGCACCAGCTAAGAATCTTCCGCAATGGACGAAAGTCTGACGGAGCG
ACGCCGCGTGGGTGATGAAGGCGGAAACGTTGTAAAACCCTTTTGTAGACGAGGAATAAG
CGTGGGAGGGAATGCCTGCGTGATGACGGTAGTCTGCGAATAAGCCCCGGCTAATTACGT
GCCAGCAGCCGCGGTAGCACGTATGGGGCGAGCGTTGTTCGGAATTATTGGGCGTAAAGG
GTGTGCAGGCGGTTATCTAAGTCTGGCGTGAAAGACCCGAGCTTAACTCGGGGAACGCGT
TGGAAACTGGGTGACTAGAGTTCTGGAGGGGTAGCTGGAATTCCAGGTGTAGGGGTGAAA
TCTGTAGATATCTGGAAGAACATCAGTGGCGAAGGCGAGCTACTAGCCAAGGACTGACGC
TGAGACACGAAAGCGTGGGGAGCAAACAGGATTAGATACCCCGTAGTCCC
>Otu5105
CCAGCCTATGGGAGGCAGCAGTGGGGAATATTGGACAATGGGGGCAACCCTGATCCAGCC
ATGCCGCGTGAGTGATGAAGGCCTTCGGGTTGTAAAGCTCTTTTGCAGGGGACGATGATG
ACGGTACCCTGAGAATAAGCTCCGGCTAACTTCGTGCCAGCAGCCGCGGTAATACGAAGG
GAGCTAGCGTTGTTCGGAATTACTGGGCGTAAAGGGCGTGTAGGCGGCTTGGCAAGTTGG
ATGTGAAAGCCCAGGGCTCAACCCTGGAATTGCATTCAAGACTGCCTTGCTTGAATTCGG
TAGAGGTTGGTGGAATTCCCAGTGTAGAGGTGAAATTCGTAGAGATTGGGAAGAACACCC
GTGGCGAAGGCGGCCAACTGGACCGACATTGACGCTGAGACGCGAAAGCGTGGGGATCAA
ACAGGATTAGATACCCTAGTAGTCC
>Otu5106
CCAGCCTACGGGTGGCAGCAGTGGGGAATCCTGCGCAATGGGCGAAAGCCTGACGCAGCG
ACGCCGTGTGTGCGATGAAGGTTTTCGGATCGTAAAGCACTGTCGCCCGGGAAAAAGAGC
GTGATGGTACCGGGAGAGGAAGCACCGGCTAACTCTGTGCCAGCAGCCGCGGTAATACAG
AGGGTGCGAGCGTTGTTCGGAATTATTGGGCGTAAAGCGCTGGTAGGTGGCCATTTAAGT
CGCGTGTGAAAGCTCCAGGCTCAACCTGGAAAGGGCGCGCGAAACTGGGAGGCTTGAGTA
CCGGAGAGGGTCGCGGAATTCCCGGTGTAGAGGTGAAATTCGTAGATATCGGGAGGAACA
CCAGAGGCGAAGGCGGCGACCTGGAAGGCAACTGACACTGAGCAGCGAAAGCGTGGGGAG
CAAACAGGATTAGAAACCCCGGTAGTCC
>Otu5108
CCAGCCTACGGGGTGCACCAGTGGGGAATTTTGCGCAATGGGGGAAACCCTGACGCAGCA
ACGCCGCGTGGAGGATGAAGTCCCTTGGGATGTAAACTCCTTTCGACCGGGAAGATGATG
ACGGTACCGGTAGAAGAAGCACCGGCTAACTCTGTGCCAGCAGCCGCGGTAATACAGAGG
GTGCGAGCGTTGTTCGGAATTATTGGGCGTAAAGGGCGCGTAGGCGGCGCGGTAAGTCAC
CTGTGAAATCTCCCGGCTCAACCGGGAGTCTGCAGGCGAAACTGCCGTGCTGGAGTATGG
GAGAGGTGCGTGGAATTCCCGGTGTAGCGGTGAAATGCGTAGATATCGGGAGGAACACCT
GTGGCGAAAGCGGCGCACTGGACCATAACTGACGCTGAGGCACGGAAGTGTGGGGATCAA
ACAGGATTAGATACCCCCGTAGTCC
>Otu5109
CCAGCCTATGGGTTGCAGCAGTAAGGAATATTGGACAATGGGCGCAAGCCTGATCCAGCC
ATGCCGCGTGCAGGATGAAGGTACTCTGCATTGTAAACTGCTTTTGTTTGGGAATAAACC
CTTCTACGTGTAGAGGGCTGAATGTACCAAAAGAATAAGGGACGGCTAACTTCGTGCCAG
CAGCCGCGGTAATACGAAGGTCCCGAGCGTTATCCGGATTTATTGGGTTTAAAGGGTGCG
TAGGCGGATTATTAAGTCAGTGGTGAAAGCCTACAGCTTAACTGTAGAATTGCCATTGAT
ACTGATGATCTTGAGTATGATTGAGGTGGGCGGAATGTGTCATGTAGCGGTGAAATGCTT
AGATATGACACAGAACACCGATTGCGAAGGCAGCTCACTAAGTCATAACTGACGCTGATG

CACGAAAGCGTGGGGAGCAAACAGGATTAGAGACCCCCGTAGTCC
>Otu5111
CCAGCCTATGGGACGCTGCAGTAGGGAATATTGGGCAATGGGCGCAAGCCTGACCCAGCC
ATGCCGCGTGCGGGAGGAAGGCCCTCTGGGTCGTAAACCGCTTTTGGGAGGGGACAAAAT
TCCCGTGCGCGGGCGATTGAGGGTACCTCCAGAATAAGCACCGGCTAACTCCGTGCCAGC
AGCCGCGGTAATACGGAGGGTGCGAGCGTTGTCCGGATTTATTGGGTTTAAAGGGTGCGT
AGGCGGCCCGATAAGTCAGCGGTGAAACCCACGGGCTCAACCCGTGGCGTGCCGTTGATA
CTGTTGGGCTTGGGTTGGCTCTGGGCGGGCGGAACGGAAGGTGTAGCGGTGAAATGCATA
GATATCTACCAGAACGCCGACAGCGAAGGCAGCTCGCTGGGGCCCGACCGACGCTGAGGC
ACGATAGCGTGGGGATCAAACAGGATTAGATACCCGAGTAGTCC
>Otu5112
CCAGCCTATGGGGTGCTCCAGTCGAGAATCTTCCGCAATGGGCGAAAGCCTGACGGAGCG
ACGCCGCGTGATTGATGAAGTCCTTCGGGACGTAAAGATCTTTTATGAGGGAGGAAGTCA
TTGACGTTACCTCATGAATAAGGGGCTCCTAACTCTGTGCCAGCAGGAGCGGTAATACAG
AGGCCCCAAGCATTATCCGGAATCACTGGGCGTAAAGGGTGTGTAGGCGGCGTAGTTAGT
CTTTCGTAAAAGATCTCTGGCTTAACTAGGGAAATGCGGGAGAAACGGCTATGCTCGAGG
ACGCGAGAGGTAAGGGGAACTCATGGTGTAGGGGTGAAATCCGTTGATATCATGGGGAAC
ACCAAATGCGAAGGCACCTTACTGGCGCGCTCCTGACGCTGAAACACGAAAGCGTGGGAA
TCGAACGGGATTAGATACCCCAGTAGTCC
>Otu5113
CCAGCCTATGGGGGGCAGCAGCTAAGAATCTTCCGCAATGGGCGAAAGCCTGACGGAGCG
ATGCCGCGTGATCGATGAAGGTCGAGAGATTGTAAAGATCTTTTACAGCTGAGGAATAAA
CCCAGGAGGGAATGCCTGGGAGATGACGTTAAGCTGTGAATAAGCCCCGGCTAATTACGT
GCCAGCAGCCGCGGTAACACGTAAGGGGCGAGCGTTGTTCGGAATTATTGGGCGTAAAGG
GCACGCAGGCGGCCTGACAAGTCTGGTGTGAAATCTCCGGGCTCAACTCGGGAAACGCGC
TGGAAACTGTCAAGCTAGAGTAGTAGAGGGGGAGTCGGAATTCCTAGTGTAGGGGTGAAA
TCTGTTGATATTAGGAAGAACACCGGTGGCGAAGGCGGACTCCTGGCTGGATACTGACGC
TGAGGTGCGAAAGCATAGGGAGCAAACAGGATTAGAGACCCGTGTAGTCC
>Otu5115
CCAGCCTACGGGGCGCAGCAGTAAGGAATATTGGACAATGCCCGCAAGGGTGATCCAGCC
ATGCCGCGTGCAGGAAGAAGGCCCTATGGGTCGTAAACTGCTTTTACACCGGAGAAAACC
CCCGATCGTGATCGGGGCTGATAGTATGGTGAGAATAAGCATCGGCTAACTTCGTGCCAG
CAGCCGCGGTAAGACGAAGGATGCAAGCGTTATCCGGATTCATTGGGTTTAAAGGGAGCG
TAGGTGGACTGATAAGTCAGTGGTGAAATCTTCGGGCTTAACCCGAAAATTGCCATTGAT
ACTGTTGGTCTTGAGTACAGTTGCTGTGGGCGGAATATGACATGTAGTGGTGAAATACAT
AGAGATGTCATAGAACACCGATTGCGAAGGCAGCTCACAAAGCTGTAACTGACACTGAGG
CTCGAAAGTGCGGGGATCAAACAGGATTAGAAACCCCAGTAGTCC
>Otu5117
CCAGCCTATGGGTCGCAGCAGTAAGGAATATTGGACAATGCCCGCCAGGGTGATCCAGCC
ATGCCGCGTGCAGGAAGAAGGCCCTATGGGTCGTAAACTGCTTTTACACCAGAGAAAACC
CTCCTTCGTGAAGGAGGCTGATAGTACGGTGAGAATAAGCATCGGCTAACTTCGTGCCAG
CAGCCGCGGTAAGACGAAGGATGCAAGCGTTATCCGGATTCATTGGGTTTAAAGGGAGCG
TAGGCGGCCTTATAAGTCAGTGGTGAAATCTCCTCGCTTAACGAGGAAATTGCCATTGAT
ACTGTAAGGCTTGAGTACAGTTGCCGTTGGCGGAATATGACATGTAGTGGTGAAATACAT
AGAGATGTCATAGAACACCGATTGCGAAGGCAGCTAACGAAACTGTAACTGACGCTGAGG
CTCGAAAGTGCGGGGATCAAACAGGATTAGAGACCCCCGTAGTCC
>Otu5118
CCAGCCTATGGGGTGCTCCAGAGAGCCCAACGCCGGCTTGCGCCATCGGGGCCGATCAAT
ATGACATCCATTTCTCTAACCAACTGAAAATATTCAATTAATCGCCGGACTCTACTGATT
CACCGACTATTTTGGTACAACGCGCGGTGCCCGCTTTTGCTCGAATTCCTGTCTTAGTCG
TACAGGCATAAGGCCAATAAGCGTGGTCGGCAGACCAGGGGGAGCTTCTGATTCAGCGCG
CGAACAGTGCCGACGGTCGGCTCGCAGAACGGCTCGCGGAAGATTACGGCGGCGACCTGG
TTCGATTTATCGCCCGGCGCCTTCGAACCAGAGCCGACGCCCAGGACTTGGCGCAGGAAG
TCTACTTACGGTTGCTGCGGCTGGAACGCAAAGACCTGATTAGAGACCCTTGTAGTCC
>Otu5119
CCACCTACGGGATGCAGCAGTAGGGAATATTGGGCAATGGGCGCAAGCCTGACCCAGCCA
TGCCGCGTGAAGGATGAAGGCCCTCTGGGTTGTAAACTTCTTTTATAGGGGACGAAAAAT
AGGTATTCTTACTTACTTGACGGTACCCTAGGAATAAGCACCGGCTAACTCCGTGCCAGC

AGCCGCGGTAATACGGAGGGTGCAAGCGTTATCCGGATTTACTGGGTTTAAAGGGTGCGT
AGGTGGGCAGGTAAGTCAGTGGTGAAATCTTCGAGCTTAACTCGGAAACTGCCATTGATA
CTATCTGTCTTGAATTATCTGGAGGTAAGCGGAATATGTCATGTAGCGGTGAAATGCTTA
GAGATGACATAGAACACCCATTGCGGAGGCAGCTTACTACGGCTCGATTGACACTGAGGC
ACGAAAGCGTGGGGATCAAACAGGATTAGATACCCCAGTAGTCC
>Otu5120
CCAGCCTATGGGGGGCAGCAGTCGAGGATCTTTCGCAATGGGCGCAAGCCTGACGAAGCG
ACGCTGCGTGAGCGATGAAGGCCTTCGGGTCGTAAAGCTCTTTCGCGCAAGAACAAGGAA
ATGATATGAATAATATCGTAACTTGAGGGTACTGCGTAAAGAAGCGCCGGCTAACTCCGT
GCCAGCAGCTGCGGTAATACGGAGAGCGCAAGCATTAATCGGAATTATTGGGCGTAAAGG
GCGCGTAGGCGGGACGGTAAGTCAGATGTGAAATTCCGAAGCTCAACTTCGGAGCTGCAT
TTGAAACTGCCATTCTAGAGGGTAGACGGAGAAAATGGAATTCCACGTGTAGCGGTGAAA
TGCGTAGATATGTGGAAGAACACCGGTGGCGAAAGCGGTTTTCTAGTTTATACCTGACGC
TGAGGCGCGAGAGCAAGGGGAGCAAACAGGATTAGATACCCCAGTAGTCC
>Otu5123
CCAGCCTATGGGGCGCAGCAGTCGAGAATCTTCCACAATGGACGAAAGTCTGATGGAGCG
ACGCCGCGTGATTGATGAAGTCCTTAGGGACGTAAAGATCTTTTATGAGGGAAGAAGTTT
ATTGACGGTACCTCATGAATAAGGGGCTCCTAATCTCGTGCCAGCAGGAGCGGTAATACG
AGAGCCCCGAGCGTTATCCGGAATTATTGGGCGTAAAGGGTGCGTAGGCGGTTTTATTAG
TCGAGCGTTAAATACCCGAGCTTAACTTGGGGACTGCGCTCGAAACGGTAAGACTTAGAA
TGAAGGAGAGGTGTGCGGAACTCATGGTGTAGGGGTGAAATCCGTTGATATCATGGGGAA
CACCGAAAGCGAAAGCAGCACACTGGCCTTTTATTGACGCTGATGCACGAAAGCGTGGGT
AGCGAATGGGATTAGAAACCCCCGTAGTCC
>Otu5124
CCAGCCTATGGGTGGCTGCAGTCGAGAATTTTTCACAATGGGGGAAACCCTGATGGAGCG
ACGCCGCGTGGGGGATGAATGGCTTCGGCCCGTAAACCCCTGTCATTTGCGAGCAAACCT
TTTCATTTAACAGACGAAAAGCTGATTGTAGTGGAAGAGGAAGGGACGGCTAACTCTGTG
CCAGCAGCCGCGGTAATACAGAGGTCCCAAGCGTTGTTCGGATTCACTGGGCGTAAAGGG
TGCGTAGGCGGTCGGGTAAGTCTGACGTGAAATCTCCGGGCCTAACCCGGAAACTGCGTC
GGATACTATCCGGCTAGAGGATTGGAAGGGAGACTGGAATACTTGGTGTAGCAGTGAAAT
GCGTAGATATCAAGTGGAACACCAGTGGCGAAGGCGAGTCTCTGGACAATTCCTGACGCT
GAGGCACGAAAGCCAGGGGAGCAAACGGGATTAGAGACCCTCGTAGTCC
>Otu5125
CCAGCCTATGGGTGGCTGCAGTAGGGAATATTGGACAATGGGCGAGAGCCTGATCCAGCC
ATGCTGCGTGCAGGAAGAAGGCCTTCTGGGTTGTAAACTGCTTTTGCCAGGGGATAAAAG
ACCCGTGCGCGGGGAATTGAAGGTACCTGGTGAATAAGCCACGGCTAACTACGTGCCAGC
AGCCGCGGTAATACGTAGGTGGCAAGCGTTGTCCGGATTTATTGGGTTTAAAGGGTGCGT
AGGCGGCCTGTTAAGTCAGGGGTGAAAGACGGTAGCTTAACTATCGCAGTGCCTTTGATA
CTGACGGGCTTGAATGTAGTTGAGGTAGGCGGAATGTGACAAGTAGCGGTGAAATGCATA
GATATGTCACAGAACTCCAATTGCGAAGGCAGCTTACTAAAGTATGATTGACGCTGAGGC
ACGAAAGCGTGGGGATCAAACAGGATTAGAAACCCTAGTAGTCC
>Otu5127
CCAGCCTACGGGGTGCTGCAGTCGAGAATCTTGGGCAATGCACGAAAGTGTGACCCAGCG
ACGCCGCGTGGAGGATGAAGGACCTCTGGTTCGTAAACTCCTTTTAGGGGAGAAGAAGCT
CTGACGGTATCCCCTGAATAAGCCACGGCTAACTACGTGCCAGCAGCCGCGGTAATACGT
AGGTGGCAAGCGTTGTCCGGATTTACTGGGCGTAAAGCGTATGCAGGCGGACCTTTAAGT
AGGAAGTGAAAGGTTGGAGCTCAACTCTAACACTGCTCCCTATACTGGAGGTCTTGAGTG
CCGGAGAGGAAGATGGAACTACACGTGTAGCGGTGAAATGCGTTGAGATGTGTAGGAACA
CCAATGGCGAGAGCAATCTTCTGGACGGATACTGACGCTGAGATACGAAAGCCAAGGTAG
CAAACAGGATTAGATACCCGTGTAGTCC
>Otu5129
CCAGCCTACGGGGGGCAGCAGTCAAGAACACTCGACAATGGGCGAAAGCCTGATCGAGCG
ACACCGCGTGCAGGAAGAAGGCCTTCGGGTCGTAAACTGCGGTAGTATGGTAACAATGTA
AATGAGTGCCATACGGAAAGAGGTGGGTAACTACGTGCCAGCACCAGCGGTAATACGTAG
ACCTCAAGCGTTATCCGGATTTATTGGGCGTAAAGAGCATGTAGGAGGTTTTGTGCGTCT
CTGGTTAAATCCCATCGCCTAACGGTGGACATGCCGGAGATACGACAAGACTAGAGGGGG
TTAGAGGTGCATGGAACGCACGGTGTAGGGGTGAAATCCGTTGATATCGTGCGGAACACC
AAAGGCGAGGGCAGTGCACTGGGACCTTCCTGACTCTGAGATGCGAAAGCGTGGGGAGCA

AAAAGGATTAGAAACCCTTGTAGTCC
>Otu5132
CCAGCCTACGGGATGCTGCAGTCGAGAATCTTCCACAATGGACGAAAGTCTGATGGAGCG
ACGCCGCGTGGTTGATGAAGTCCTTCGGGACGTAAAAACCTTTTATGAGGGAGGAAGTTA
TTGACGTTACCTCATGAATAAGGGGCTCCTAACTCTGTGCCAGCAGGAGCGGTAATACAG
AGGCCCCAAGCATTATCCGGAATCACTGGGCGTAAAGGGTGTGTAGGCGGTCGTATTAGT
CTTTTGTTAAAGTTCTCCGGCTTAACCGGAGAAATGCAGGAGAAACGGTACGACTCAGAG
GACGTAAGAGGTAAAGGGAACTCATGGTGTAGGGGTGAAATCCGTTGATATCATGGGGAA
CACCAAATGCGAAGGCACTTTACTGGTACGCTCCTGACGCTGAAACACGAAAGCGTGGGA
ATCGAACGGGATTAGATACCCGCGTAGTCC
>Otu5133
CCAGCCTATGGGGGGCACCAGTGGGGAATATTGGACAATGGGCGCAAGCCTGATCCAGCA
ATGCCGCGTGTGTGAAGAAGGTCTTCGGATTGTAAAGCACTTTTGGCAGGGATGATGATG
ACGGTACCTGCAGAATAAGCCCCGGCTAACTTCGTGCCAGCAGCCGCGGTAATACGAAGG
GGGCTAGCGTTGTCCGGATTTATTGGGTTTAAAGGGTGCGTAGGCGGCCCTGTAAGTCAG
TGGTGAAATACGGCAGCTCAACTGTCGAGGTGCCATTGATACTGCAGGGCTTGAGTACAG
ACGAGGTAGGCGGAATTGACGGTGTAGCGGTGAAATGCTTAGATATCGTCAAGAACACCG
ATAGCGAAGGCAGCTTACTAGGCTGTAACTGACGCTGAGGCACGAAAGTGCGGGGATCAA
ACAGGATTAGATACCCTCGTAGTCC
>Otu5136
CCAGCCTATGGGGGGCAGCAGTGGGGAATATTGGACAATGGGCGCAAGCCTGATCCAGCC
ATGCCGCGTAAGTGATGAAGGCCTTAGGGTTGTAAAGCTCTTTCGCTAGGGACGATAATG
ACGGTACCTAGACAAGAAGCCCCGGCTAACTTCGTGCCAGCAGCCGCGGTAATACGAAGG
GGGCTAGCGTTGTTCGGAATTACTGGGCGTAAAGCGCGTGTAGGCGGGCTTTTAAGTCAG
GGGTGAAATCCTGGAGCTCAACTCCAGAACTGCCCTTGATACTGAGAAGCTTGAGTCCAG
GAGAGGTGAGTGGAACTGCGAGTGTAGAGGTGAAATTCGTAGATATTCGCAAGAACACCA
GTGGCGAAGGCGGCTCACTGGCCCGGTACTGACGCTGAGGTGCGAAAGCGTGGGGAGCAA
ACAGGATTAGATACCCTAGTAGTCC
>Otu5139
CCAGCCTACGGGGCGCACCAGTAAGGAATATTGGACAATGGACGAAAGTCTGATCCAGCC
ATGCCGCGTGAAGGATTAAGGTCCTCTGGATTGTAAACTTCTTTTATCTGGGACGAAAAA
AGGGAATTCTTTCTCACTTGACGGTACCAGATGAATAAGCACCGGCTAACTCCGTGCCAG
CAGCCGCGGTAATACGGAGGGTGCAAGCGTTATCCGGATTCACTGGGTTTAAAGGGTGCG
TAGGCGGGTAGGTAAGTCAGTGGTGAAATACCGGAGCTTAACTCCGGAACTGCCATTGAT
ACTATCTATCTTGAATATTGTGGAGGTAAGCGGAATATGTCATGTAGCGGTGAAATGCTT
AGATATGACATAGAACACCCATTGCGAAGGCAGCTTACTACGCATATATTGACGCTGATG
CACGAAAGCGTGGGGATCAAACAGGATTAGAAACCCGAGTAGTCC
>Otu5140
CAGCCTACGGGTGGCTGCAGTGGGGAATCTTGCGCAATGGACGAAAGTCTGACGCAGCGA
CGCCGCGTGGGCGACGAAGGCCTTCGGGTTGTAAAGCCCTGTGGGGCGGGACGAACACGC
CCGGGTCGAATAGGCCCGGGTGCTGACGGTACCTGCTTAGCAAGCACCGGCTAACTCTGT
GCCAGCAGCCGCGGTAAGACAGAGGGTGCAAACGTTGCTCGGAATTACTGGGCGTAAAGC
GCGTGTAGGCGGTTTCGCAAGTCAGGTGTGAAAGCCCGGGGCTCAACCCCGGAAGTGCAC
TTGATACTACGGAGCTGGAGTACTGAAGAGGAGAGCGGAATTCCCGGTGTAGAGGTGAAA
TTCGTAGATATCGGGAGGAACACCAGTGGCGAAGGCGGCTCTCTGGCAGATACTGACGC
TGAGACGCGAAAGCGTGGGTAGCAAACAGGATTAGAAACCCGCGTAGTCC
>Otu5144
CCAGCCTACGGGGGGCAGCAGTCGAGAATCTTCCGCAATGGACGAAAGTCTGACGGAGCG
ACGCCGCGTGACTGATGAAGTCCTTAGGGACGTAAAGGTCTTTTATGAGGGAAGAAGTTT
ATTGACGGTACCTCAAGAATAAGGGGCTCCTAATCTCGTGCCAGCAGGAGCGGTAATACG
AGAGCCCCGAGCGTTATCCGGAATAACTGGGCGTAAAGGGTGTGTAGGTGGCATTATTAG
TCCGTTGTTAAAACCCCGAGCTCAACTTGGGAGATGCAATGGAAACGGTAAAGCTTAGAG
AGTGCGAGAGGTGAATGGAACTCATGGTGTAGGGGTGAAATCCGTTGATATCATGGGGAA
CACCGAAGGCGAAGGCAATTCACTGGCGCATTTCTGACACTGAAACACGAAAGCGTGGGT
AGCGAATGGGATTAGAAACCCTCGTAGTCC
>Otu5145
CCAGCCTACGGGATGCTGCAGTGGGGAATTTTGCGCAATGGGGGAAACCCTGACGCAGCA
ACGCCGCGTGGAGGATGAAGCCCCTTGGGGCGTAAACTCCTTTCGACCAAGACGATAATG

ACGGTACTGGTGGAAGAAGCACCGGCTAACTCTGTGCCAGCAGCCGCGGTAATACAGAGG
GTGCGAGCGTTGCTCGGAATTATTGGGCGTAAAGGGCGCGTAGGCGGTATGGTAAGTCAC
CTGTGAAACCTCTAAGCTTAACTTAGAGCCTGCAGGCGAAACTGCCATGCTGGAGGGTGG
GAGAGGTGCGTGGAATTCCCGGTGTAGCGGTGAAATGCGTAGATATCGGGAGGAACACCT
GTGGCGAAAGCGGCGCACTGGACCATAACTGACGCTGAGACGCGAAAGCCAGGGGAGCGA
ACGGGATTAGAAACCCTTGTAGTCC
>Otu5148
CCAGCCTACGGGATGCTCCAGTAGGGAATTTTGCGCAATGGACGAAAGTCTGACGCAGCA
ACGCCGCGTGCAGGAAGAAGGCCCTCGGGTCGTAAACTGCTTTTATGTGCGACGAATTTG
ACGGTAGCGTATGAATAAGGATCGGCTAACTCCGTGCCAGCAGCCGCGGTCATACGGAGG
ATCCAAGCGTTATCCGGAATTACTGGGCGTAGAGAGTTGCGTAGGTGGTTGATTAAGCAA
GGCATCAAATCTCACGGCTCAACCGTGAATCTATGTTTTGAACTAGTCGACTGGGGCATG
AGAGAGGTAACTGGAATTCCAGGTGTAGGAGTGAAATCCGTAGATATCTGGAGGAACACC
GATGGCGTAGGCAGGTTACTGGCTCATCGCCGACACTGAGGCACGAAAGCGTGGGGAGCG
AACGGGATTAGATACCCGCGTAGTCC
>Otu5149
CCAGCCTACGGGACGCAGCAGTGAGGAATTTTCGGCAATGGGGGAGACCCTGACCGAGCA
ATACTACGTGAAGGAAGAAGGTCTAAGGATTGTAAACTTCTGTTAGCGCGGAAGAAGGGC
TAAACGAAAGTTTAGTTTTGACGGTACGCGCAGAGAAAGCCACGACTAACTATGTGCCAG
CAGTCGCGGTAAGACATAGGTGGCAAACGTTATCCGGAATTATTGGGCGTAAAGGATGCG
TAGATGGTGTAATAAGTTATCAGTGGAAAATCGGGGCTCAACCTCGTGGAAGCTGGTAAT
ACTGTTATACTTGAGTTCAGGAGAGGTTAACGGAACTCCATGTGGAGCGGTAAAATGCGT
AGATATATGGAAGAACACCGATAAGGCGAAGGCAGTTAACTATTCTGTTACTGACATTGA
GGCATGAAAGCGTGGGGAGCAAAACGGATTAGAAACCCCCGTAGTCC
>Otu5150
CCAGCCTATGGGGGGCACCAGTGTGGAATTTTGGACAATGAACGAAAGTTTGATCCAGCG
AAATCGTGTGAGTGAAGAAGGTTCTTATCGTAAAACTTTATCGACATCGTATGAAAATGA
CAGAATGTTGTAGATGAAGTCCCGACTAACTTCGTGCCAGCAGTCGCGGTAAAACGGGGG
GGACTAGTGTTATTCATCATTACTGGGTGTAAAGCGCATGTAGACGATTTATTAAGTTTT
AATTATGACAATCCAAACTTAATTTGGAAGATAATTATTATACTAGTAAATTAGAGGTCA
AAGGAGGAAAAGCTATATTTCTGATGTAGGGATGAAATCCTAATAGTTTAGGGGGATAAC
CAGCAGGCGAAGGCGGCTTTCCATAATGATTCTGACGTTGAAGTGCTAAAGCGTGGGGCG
CAAATGGGATTAGATACCCGCGTAGTCC
>Otu5153
CCAGCCTATGGGTGGCTCCAGTGAGGAATCTTGCGCAATGGGGGAAACCCTGACGCAGCA
ACGCCGCGTGAGTGAGGAAGGCCTTCGGGTCGTAAAGCTCTGTCGGGTGGGAAGAAACGT
ATGAGGGTTAATACTCCTTGTACTTGACGGTACCGCCAAAGGAAGCACCGGCTAACTCCG
TGCCAGCAGCCGCGGTAATACGGGGGGTGCAAGCGTTGTTCGGAATTATTGGGCGTAAAG
AGCGTGTAGGCGGCGTGATAAGTCAGATGTGAAAGCCCTGGGCTTAACCCAGGAAGTGTA
TTTGAAACTGTCTTGCTTGAGTACGGGAGAGGGAAGAGGAATTCCTGGTGTAGAGGTGAA
ATTCGTAGATATCAGGAGGAACACCGGTGGCGAAGGCGACTTCCTGGCCCGATACTGACG
CTGAGACGCGAGAGCGTGGGGAGCAAACAGGATTAGATACCCTGGTAGTCC
>Otu5154
CCAGCCTACGGGGTGCTGCAGTCGAGAATCTTCCGCAATGGACGAAAGTCTGACGGAGCG
ACGCCGCGTGGAGGATGAAGTGCTTCGGCATGTAAACTCCTTTTGCCAGGGAAAAAGTTT
ATTGATTGTACCTGGAGAATAAGAGGTTGCTAAACTCGTGCCAGCAGCAGCGGTAATACG
AGTGCCTCGAGCGTTATCCGGAATCATTGGGCGTAAAGGGTGCGTAGGCGGTTCTGTTAG
TCCTGTGTTAAATCTACCGGCTCAACCGGTAGGCTGCATGGGAAACGGCAGAACTTGAGG
ACGGTAGGGGTCTCTGGAACTCATGGTGTAGCGGTGAAATGCGTTGATATCATGGGGAAC
ACCGAAAGCGAAGGCAAGAGACTGGACCGCTCCTGACGCTGAAGCACGAAAGCGTGGGTC
GCGAATGGGATTAGAAACCCGAGTAGTCC
>Otu5155
CCGCCTATGGGCGCAGCAGTGGGGAATTTTGGACAATGGGCGCAAGCCTGATCCAGCAAT
GCCGCGTGAGTGAAGAAGGCCCTCGGGTTGTAAAGCTCTTTTGTCAGGAACGAAATCCCT
TGGGCTAATACCTCGGGGGGATGACGGTACCTGAAGAATAAGCACCGGCTAACTACGTGC
CAGCAGCCGCGGTAATACGTAGGGTGCAAGCGTTAATCGGAATTACTGGGCGTAAAGCGT
GCGCAGGCGGTTTTGTAAGACAGGTGTGAGATCCCCGGGCTTAACCTGGGAACTGCATTT
GTGACTGCAAGGCTTGAGTGTGTCAGAGGGAGGTGGAATTCCGCGTGTAGCAGTGAAATG

CGTAGAGATGCGGAGGAACACCGATGGCGAAGGCAGCCTCCTGGGACAACACTGACGCTC
ATGCACGAAAGCGTGGGGAGCAAACAGGATTAGATACCCGAGTAGTCC
>Otu5156
CCAGCCTATGGGGGGCAGCAGTCGAGAATATTCGACAATGGACGAAAGTCTGATCGAGCG
ACACCGCGTGTAGGATGAAGGCCCTCGGGTCGTAAACTACGGTAGATAGTTAACAATGCA
AATGAGTGACTATTGGAAAGAGGTGGGTAACTACGTGCCAGCACCAGCGGTAATACGTAG
ACCTCAAGCGTTATCCGGATTTATTGGGCGTAAAGAGCATGTAGGAGGTTTTGTGCGTCT
TCTGTTAAAGCCCAGAGCCTAACTCTGGAAGTGCAGGAGATACGACAAGACTAGAGGGGG
TTAGAGGTGCATGGAACTCACGGTGTAGGGGTGAAATCCGTTGATATCGTGGGGAACACC
AAAGGCGAAGGCAGTGCACTGGGACCTTCCTGACTCTGAGTTGCGAAAGCGTGGGGAGCA
AAAAGGATTAGAAACCCCCGTAGTCC
>Otu5157
CCAGCCTATGGGGTGCAGCAGTGGGGAATCTTGCACAATGGGGGCAACCCTGATCCAGCC
ATGCCGCGTGAGTGAAGAAGGCCTTCGGGTTGTAAAGCTCTTTCAGACGGAAAGAAACGG
TTACGGCTAATACCCGTGACTAATGACGGTACCGTCAGAAGAAGCACCGGCTAACTACGT
GCCAGCAGCCGCGGTAATACGTAGGGTGCGAGCGTTAATCGGAATTACTGGGCGTAAAGC
GTGCGCAGGCGGTTTTGTAAGACAGGCGTGAAATCCCCGGGCTTAACCTGGGAACTGCGT
TTGTGACTGCAAGACTAGAGTGTGGCAGAGGGGGGGTAGAATTCCACGTGTAGCAGTGAA
ATGCGTAGATATGTGGAGGAATACCGATGGCGAAGGTAGCCCCCTGGGTCAACACTGACG
CTCATGCACGAAAGCGTGGGGAGCAAACAGGATTAGATACCCGAGTAGTCC
>Otu5158
CCAGCCTATGGGTGGCTGCAGTGGGGAATCTTGCACAATGGGGGAAACCCTGATGCAGCG
ACGCCGCGTGAGCGATGAAGCCCTTCGGGGTGTAAAGCTCTTTCGGCAGGGAAGATAGTG
ACGGTACCTGCAGAAGCAGCTGCGGCTAACTACGTGCCAGCAGCCGCGGTAATACGTAGG
CAGCGAGCGTTATTCGGATTCACTGGGCGTAAAGGGTGCGCAGGCGGCCAAGTGTGTTAG
GCGTGAAAGCCCGGGGCTTAACCCCGGAATTGCACCTAAAACTACATGGCTAGAGCATTG
GAGAGGGTAGCGGAATTCACGGTGTAGCAGTGAAATGCGTAGATATCGTGAGGAACACCA
GAGGCGAAGGCGGCTACCTGGACAATTGCTGACGCTCAGGCACGAAAGCGTGGGGAGCAA
AAGGGATTAGATACCCTCGTAGTCC
>Otu5159
CAGCCTACGGGTGGCTCCAGTGGGGAATATTGCGCAATGGGCGAAAGCCTGACGCAGCAA
CGCCGCGTGAAGGATGAAGGTCTTCGGATTGTAAACTTCTGTTAAGTGGGAAGAAAAATC
CGTTTCTAATACAGACGGTAATGACGGTACCATTAGAGAAAGCACCGGCTAACTTCGTGC
CAGCAGCCGCGGTAATACGAGGGGTGCAAGCGTTATTCGGAATTATTGGGCGTAAAGGGT
GCGTAGACGGCATATTAAGTCAACTGTTAAATTCCTCGGCCTAACTGGGGACCAGCGGTA
GATACTGATGTGCTAGAGGATGGAAGAGAGAAGTGGAATTCTCGGAGTAGCGGTAAAATG
CGTAGATCTCGAGAGGAACACCGATGGCGAAGGCAGCTTCTTGGTCCATTTCTGACGTTG
AGGCACGAAAGCGTGGGGAGCAAACAGGATTAGATACCCCAGTAGTCC
>Otu5161
CCAGCCTACGGGTCGCTGCAGCCGAGAATATTCGACAATGGGCGAAAGCCTGATCGAGCG
ATACCGCGTGGTGGATGAAGCGCTTCGGCGCGTAAACATCTTTTATGGAGGAGGAAGTTA
TTGACGTTACTCCATGAATAAGGGGCTCCTAACTCTGTGCCAGCAGGAGCGGTAATACAG
AGGCCCCGAGCGTTACCCGGAATTACTGGGCGTAAAGAGTGCGTAGGTGGTCGTGTTAGT
CGTCTGTCAAAACCCCCGGCTCAACCGGGGATCCGCGGGCGAAACGGCACGACTTGAGGG
CGTGAGAGGTACATGGAACTCATGGTGTAGGGGTGAAATCCGTTGATATCATGGGGAACA
CCGAAAGCGAAGGCAATGTACTGGCGCGTTCCTGACACTCAAGCACGAAAGCGTGGGTAG
CGAACGGGATTAGATACCCCCGTAGTCC
>Otu5163
CCAGCCTACGGGGTGCAGCAGACGAGAATATACCGCAATGGGCGAAAGCCTGACGGAGCG
ACGCCGCGTGATGGATGAAGTGCTTTGGTACGTAAACATCTTTTATCGGGGACGAAGTTA
TTGACGGTACCCGATGAATAAGGGGCTCCTAACACTGTGCCAGCAGGAGCGGTAATACAG
AGGCCCCAAGCATTACCCGGAATCACTGGGCGTAAAGGGTGTCAAGGCGGCCATATTAGT
CTTTCGTAAAATCCGTGGGCTCAACCTACGGCGCGCGAGAGAAACGGTATGGCTCGAGGG
CGTAAGAGGTGCATGGAACTCATGGTGGAGGGGTGAAATCCGTTGATATCATGGGGAACA
CCAAAGGCGAAGGCAGTGCACTGGTACGTTCCTGACGCTCACACACGAAAGCCAGGGTAG
CGAACGGGATTAGATACCCTAGTAGTCC
>Otu5167
CCAGCCTATGGGGGGCTGCAGTCGAGAATATTCGACAATGGACGAAAGTCTGATCGAGCG

ACGCCGCGTGCAGGATGAAGGCCTTCGGGTCGTAAACTGCGGTAGTAGCATAACAATGTA
AATGAGTGTGCTACGGAAAGAGGTGGGTAACTACGTGCCAGCACCAGCGGTAAAACGTAG
ACCTCGAGCGTTATCTGGATGTATTGGGCGTAAAGCGCATGTAGGAGGTTTTGTGCGTCT
TCTGTTAAAGCCCAGGGCCTAACCCTGGATCTGCAGGAGATACGACAGAACTAGAGGAGG
TTAGAGGTGCATGGAACTCACGGTGTAGGGGTGAAATCCGTTGATATCGTGGGGAACACC
AAAGGCGAAGGCAGTGCACTGGGACCTTCCTGACTCTGATATGCGAAAGCGTGGGGAGCA
AAAAGGATTAGATACCCGAGTAGTCC
>Otu5168
GCCAGCCTACGGGTGGCTCCAGTCAAGAACTTTCCACAATGGGCGAAAGCCTGATGGAGC
GACGCCGCGTGGTTGATGAAGTCCTTCGGGACGTAAAGACCTTTTATGGGGGAGGAAGTC
ATTGACGTTACCCCATGAATAAGGGGCTCCTAACTCTGTGCCAGTAGGAGCGGTAATACA
GAGGCCCCAAGCGTTATCCGGAATCACTGGGCGTAAAGGGTGTGTAGGCGGTCGTGTTAG
TCGTCTGTGAAAGACCTTGGGCTTAACCCAGGGGACGCACACGAAACGGCACGACTCAGA
GGTTGCGAGAGGTATACGGAACTCATAGTGTAGGGGTGAAATCCTTTGATATTATGGGGA
ACACCAAATGCGAAGGCAGTATACTGGCGCACACCTGACGCTGAGACACGAAAGCGTGGG
AATCGAACGGGATTAGAAACCCTAGTAGTCC
>Otu5175
CCAGCCTACGGGGGGCTGCAGTGGGGAATATTGGACAATGGGCGAAAGCCTGATCCAGCA
ATGCCGCGTGAGTGATGAAGGCCTTAGGGTTGTAAAGCTCTTTCGCCCGCGACGATAATG
ACGGTAGCGGGAGAAGAAGCCCCGGCTAACTTCGTGCCAGCAGCCGCGGTAATACGAAGG
GGGCAAGCGTTGTTCGGAATTACTGGGCGTAAAGGGCGCGCAGGCGGCCAAGGAAGTCAG
ATGTGAAAGCCCCGGGCTCAACCTGGGAACAGCATTTGGGACTCTTTGGCTAGAGTTCGG
GAGAGGTGGGTGGAATTCCCAGTGTAGAGGTGAAATTCGTAGATATTGGGAAGAACACCG
GTGGCGAAGGCGGCTCACTGGACCGATACTGACGCTGAGGCGCGAAAGCGTGGGGAGCAA
ACAGGATTAGATACCCTAGTAGTCC
>Otu5177
CCAGCCTATGGGTGGCACCAGTGAGGAATTTTCGTCAATGGGGGAAACCCTGAACGAGCA
ACGCCGCGTGCAGGAAGAAGGTTTTCGGATCGTAAACTGCAAATCTCTGGGAAGAGAGAG
GACGGTACCAGAGGAGAAGCCCCGGCTAACTACGTGCCAGCAGCCGCGGTAAAACGTAGG
GGGCCAGCGTTGCTCGGAATTACTGGGCGTAAAGGGTCCGTAGGCGGTGCGGCAAGTCGG
AAGTGAAATCTCTGGGCTTAACCCAGAGGCTGCTCCCGAAACTGCCGTGCTAGAGTGCGA
GAGAGGCGAGTGGAATTGCGGGTGTAGCGGTGAAATGCGTAGATATCCGCAGGAACATCC
GAGGCGAAAGCGGCTCGCTGGATCGCAACTGACGCTGAGGGACGAAAGCTAGGGGAGCAA
ACAGGATTAGAGACCCCAGTAGTCC
>Otu5178
CCAGCCTACGGGGGGCTGCAGTGGGGAATATTGGACAATGGGGGAAACCCTGATCCAGCA
ATGCCGCGTGTGTGAAGAAGGCCTTCGGGTTGTAAAGCACTTTCAGTGGGGAAGAACCAA
TTCAAGGCTAATACCCTTGAGTCTTGACGTTACCTACAGAAGAAGCACCGGCAAACTCTG
TGCCAGCAGCCGCGGTAATACAGAGGGTGCGAGCGTTAATCGGAATTACTGGGCGTAAAG
CGCACGTAGGTGGATATTTAAGTCGGAAGTGAAAGCCCTGGGCTTAACCTGGGAATTGCC
TCCGATACTGGCTATCTAGAGTATGGTAGAGGGTAGTGGAATTTCCGGTGTAGCGGTGAA
ATGCGTAGATATCGGAAGGAACACCAGTGGCGAAAGCGGCTACCTGGACCAATACTGACA
CTTAGGTGCGAAAGCGTGGGGAGCAAACAGGATTAGATACCCTTGTAGTCC
>Otu5182
CCAGCCTATGGGTCGCACCAGTGGGGAATTTTTCGCAATGGGGGAAACCCTGACGAAGCA
ACGCCGCGTGGAGGATGAAGGCCTTCGGGTCGTAAACTCCTGTCGACCGGGACGAAAGTA
GTCTGACCCAATACGTCGGGCTATCGACTGTACCGGTGGAGGAAGCCACGGCTAACTCTG
TGCCAGCAGCCGCGGTAATACAGAGGTGGCAAGCGTTGTTCGGAATTACTGGGCGTAAAG
GGCGCGTAGGCGGCCTTTCAAGTCCCGTGTGAAATCCCTCGGCTCAACTGAGGAATTGCA
CTGGAAACTGTTTGGCTTGAGTTCGGGAGAGGGAAGCGGAATTCCGGGTGTAGCGGTGAA
ATGCGTAGATATCCGGAGGAACACCGGTGGCGAAGGCGGCTTCCTGGACCGACACTGACG
CTGAGGCGCGAAAGCTAGGGGAGCAAACGGGATTAGAGACCCTGGTAGTCC
>Otu5186
CCAGCCTATGGGGTGCAGCAGTAACGAATCTTCCGCAATGCACGAAAGTGTGACGGAGCG
ACGCCGCGTGTAGGACGAAGCCCTTCGGGGTGTAAACTACTGTCAGGGGAAAGAAAGTTC
TGATCTACCCCAGAGGAAGAGACGGCTAACTCTGCGCCAGCAGCCGCGGTAAGACAGAGG
TCTCGAGCGTTAGGCGGAATCACTGGGCTTAAAGCGTGTGTAGGTGGATTTGTAAGTACC
TTGTGAAATCCCACGGCTTAACCGTGGAACTGCTTGGTATACTGCTTATCTTGGGCCATC

TAGGGGTTACCGGAACAAACGGTGGAGCGGTGAAATGCGTAGATAGCGTTTGGAACGCCA
ATGGTGAAAACAGGTAACTGGGGATGTGCCGACACTGAGACACGAAAGCCAGGGGAGCGA
ACGGGATTAGAAACCCGAGTAGTCC
>Otu5187
CCAGCCTACGGGTCGCTGCAGTAACGAATCTTCCGCAATGCACGAAAGTGTGACGGAGCG
ACGCCGCGTGTGGGACGAAGCCCTTCGGGGTGTAAACCACTGTCAGAGGATAGAAAGTTC
TGATCATCCTCAGAGGAAGGCACGGCTAACTCTGTGCCAGCAGCCGCGGTAAGACAGAGG
TGCCAAGCGTTAGGCGGAATCACTGGGCTTAAAGCGTGTGTAGGTGGATCCATAAGTACC
TTGTGAAATCCCACGGCTTAACCGTGGAACTGCTCGGTATACTGCGGATCTTGAGCCAAC
TAGGGGTTACCGGAACAAATGGTGGAGCGGTGAAATGCGTAGATATCATTTGGAACGCCA
ATGGTGAAAACAGGTAACTGGGGTTGTGCTGACACTGAGACACGAAAGCCAGGGGAGCAA
ACGGGATTAGATACCCGAGTAGTCC
>Otu5188
CCAGCCTATGGGTGGCTGCAGTCGAGGATCTTTGGCAATGGGCGCAAGCCTGACCAAGCG
ACGCCGCGTGTGCGATGAAGGCCTTCGGGTTGTAAAGCACTGTCGAGGGGGAGGAAGCCG
CAAGGTTGACCTATCCCTGGAGGAAGGACGGGCTAAGTTCGTGCCAGCAGCCGCGGTAAG
ACGAACCGTCCAAACGTTGTTCGGAATCACTGGGCTTAAAGGGCGCGTAGGCGGGCTACC
AAGTCCGGGGTGAAATCTTTCGGCTTAACCGGAAAACTGCCTTGGGTACTGGTAGTCTCG
AGGGAGGTAGGGGTCTGTGGAACTGATGGTGGAGCGGTGAAATGCGTTGATATCATCAGG
AACGCCGGTGGCGAAAGCGACGGACTGGACCTTCACTGACGCTGAGGCGCGAAAGCTAGG
AGAGCAAACGGGATTAGATACCCTCGTAGTCC
>Otu5191
CCAGCCTACGGGTCGCACCAGTGGGGAATATTGGACAATGGGCGCAAGCCTGATCCAGCA
ATGCCGCGTGTGTGAAGAAGGCCTTCGGGTTGTAAAGCACTTTTATCTGGAGCGAAACAC
GCAAGGTTAATACCTTTGCGGACTGACGGTACCAGAGGAATAAGCACCGGCTAACTTCGT
GCCAGCAGCCGCGGTAATACGAAGGGTGCAAGCGTTACTCGGAATTACTGGGCGCAAAGC
GTGAGTAGGTGGTTTCTTAAGTCTGCTGTGAAAGCCCCGGGCTCAACCTGGGAATTGCAG
TGGATACTGGGGAGCTAGAGCGCGGTAGAGGGTAGTGGAATTCCCGGTGTAGCGGTGAAA
TGCGTAGAGATCGGGAGGAACACCAGTGGCGAAGGCGGCTACCTGGACCAGCACTGACAC
TGAGTCACGAAAGCGTGGGGAGCAAACAGGATTAGAAACCCCTGTAGTCC
>Otu5192
CCAGCCTATGGGGGGCAGCAGTGGGGAATATTGGACAATGGGGGCAACCCTGATCCAGCA
ATGCCGCGTGGGTGACGAAGGCCTTCGGGTCGTAAAGCCCTGTCGGTAGGGAAGAAAAAC
GAGATGGCTAACATCCATTTTGCTTGACGGTACCTGCAAAGGAAGCACCGGCTAACTACG
TGCCAGCAGCCGCGGTAATACGTAGGGTGCTAGCGTTGTTCGGAATCATTGGGCGTAAAG
CGCGTGTAGGCGGCTATTTAAGTCGAGTGTGAAATCCCTAGGCTTAACCTAGGAAGTGCA
TTCGAAACTAACTAGCTGGAAGACGGTAGAGGAAGGTAGAATTCCTAGTGTAGAGGTGAA
ATTCGTAGATATTAGGAGGAACACCGGTGGCGAAGGCGGCCTTCTGGACCGTTCTTGACG
CTGAGACGCGAAAGCGTGGGTAGCAAACAGGATTAGATACCCCGGTAGTCC
>Otu5193
CCAGCCTACGGGTGGCACCAGTGGGGAATATTGGGCAATGGGCGCAAGCCTGACCCAGCC
ATGCCGCGTGAGTGATGAAGGCCTTCGGGTCGTAAAGCTCTGTGGGGAGGGAAGAACAAG
CTTGTGGCTAATATCCACGAGCCCTGACGGTACCTCCTTAGCAAGCACCGGCTAACTCCG
TGCCAGCAGCCGCGGTAATACGGAGGGTGCAAACGTTGCTCGGAATTATTGGGCGTAAAG
CGCACGTAGGCGGCCGACTAAGTCGGGTGTGAAAGCCCAGGGCTTAACCCTGGAAGTGCA
CTCGAAACTGGTCGGCTTGAATATGGAAGAGGGTCGCGGAATTCCCGGTGTAGAGGTGAG
ATTCGTAGATATCGGGAGGAACACCAGTGGCGAAGGCGGCGACCTGGGCCAATATTGACG
CTGAGGTGCGAAAGCGTGGGGAGCAAACAGGATTAGATACCCGAGTAGTCC
>Otu5195
CCAGCCTATGGGATGCACCAGTATCTCAAGAAATACTTTAAGCTCGTTATCCTTTCGAAC
GTGGACAACGAGACTTTTCTGGCGAGCAATCGCCGGCCGCAGGTCGAATTCGACGCGATC
TACACGGCGGAAGACATCGGATCGTACAAGCCTTCTTCACGCAATTTCGACTACATGATC
GAGAAGCTGGAAGGAAACGGAGTGGAGAAGGCAAAGATTCTGCACACCGCCGAGAGCATG
TTTCACGATCACAAGCCGGCAAACGATCACGGTCTTAAGTCGTGCTGGATCTATCGTCGC
CATGACAAACAGGGTTTCGGAGCAACCATGAATCCCGGCCAACTGCCGCATGTTGATTTT
AGGTTCAACAGCATGCACGAGCTCGTGAAGACGCATCAGGAGCAGTTGCGGGTTAAATGA
CAACATCGGCTACCCAATTAGAAACCCCTGTAGTCC
>Otu5196

CCAGCCTATGGGAGGCTCCAGTCGAGGATTTTTCTCAATGGGGGAAACCCTGAAGGAGCG
ACGCCGCGTGAGGGATGAAGGTCTTCGGATTGTAAACCTCTGTCATCTGGGAACAAAGCG
CTCCACCTAACACGTGGATTGTTGATAGTACCGGAAGAGGAAGCAGTGGCTAACTCTGTG
CCAGCAGCCGCGGTAATACAGAGACTGCAAGCGTTGTTCGGATTCATTGGGCGTAAAGGG
TGCGCAGGCGGTCCGGTAAGTTGGATGTGAAATACCATGGCTTAACCATGGAGGGTCATT
CAAAACTGCCGGGCTAGAGGACTGGAGAGGAGACTGGAATAGTCGGTGTAGCGGTGAAAT
GCGTAGAGATCGACTAGAACACCGGTGGCGAAGGCGAGTCTCTGGACAGTTCCTGACGCT
CAGGCACGAAAGCCAGGGGAGCAAACGGGATTAGATACCCCCGTAGTCC
>Otu5198
CCAGCCTACGGGACGCACCAGTAAGGGATATTGCGCAATGGGCGAAAGCCTGACGCAGCA
ACGCCGCGTGGAGGATGACGGTTTTCGGATTGTAAACTCCTTTTTGAAGGGACGAGGAAG
GACGGTACCTTCAGAATAAGTCACGGCTAACTACGTGCCAGCAGCCGCGGTAAAACGTAG
GTGGCGAGCGTTATCCGGATTTACTGGGTGTAAAGCGTGTGCAGGCGGTCTGTTAAGTGG
TGTATGAAATCGTTCGGCTCAACCGAGCGGGGTTATGCCAGACTGACAGACTAGAGGGGG
TGAGAGGGGCATGGAATTCCGGGTGTAGTGGTGAAATGCGTAGAGATCCGGAGGAACCCC
AGAGGCGAAGGCGGTGCCCTAGCACCCCCCTGACGCTCAGACACGATAGCATGGGGAGCG
AACGGGATTAGATACCCCCGTAGTCC
>Otu5204
CAGCCTATGGGGGGCTCCAGCAACGAATCTTCCCCAATGCCGGAAACGGTGAGGGAGCGA
CGCCGCGTGAAGGACGAAGTTCTTCGGAATGTAAACTTCTAAAGGGGTTATGAAAGATCA
GCGCCTAATACGCGCAAAGTTTGATCAGATCCTGATAAGGGGCGGCTAACTCTGTGCCAG
CAGCCGCGGTAATACAGAGGCCCCAAGCGTTACTGAGAATCACTGGGTTTAAAGGGTGCG
TAGGTGGCGCGTTAAGTCCGTTGTGAAATCCCTGGGCTCAACCCAGGAACTGCTTCGGAT
ACTGGCGCGCTTGAGGCCGGTAGGGGTCACTGGAACTGACGGTGGAGCGGTGAAATGCGT
AGATATCGTCAGGAACGCCGATGGTGAAGACAGGTGACTGGGCCGGAACTGACACTGAGG
CACGAAAGCGTGGGGAGCGAACGGGATTAGATACCCTAGTAGTCC
>Otu5205
CCAGCCTACGGGGTGCAGCAGTGAGGAATATTGCGCAATGGGCGAAAGCCTGACGCAGCG
ACGCCGCGTGGAGGATGAAGGTCTTCGGATTGTAAACTTCTGTTAAGTGGGAAGAAAATC
GGGGTTTTAATAGAGCCCTAAAATGACGGTACCACTAGAGAAAGCACCGGCTAACTTCGT
GCCAGCAGCCGCGGTAATACGAAGGGTGCGAGCGTTATTCGGATTTATTGGGCGTAAAGG
GTGCGCAGGCGGTGCGTTAAGTCAACTGTTAAATCTCCCGACCTAACCGGGAATCAGCGG
TAGATACTGGCGCGCTAGAGGGTGGAAGAGAGAAGTGGAATTCTTGGAGTAGCGGTGAAA
TGCGTAGATCTCAAGAGGAACACCGATGGCGAAGGCAGCTTCTTGGTCCACATCTGACGC
TCATGCACGAAAGCGTGGGGAGCAAACAGGATTAGATACCCGCGTAGTCA
>Otu5209
CCAGCCTATGGGACGCAGCAGTCGAGAATTTTTCTCAATGGGGGAAACCCTGAAGGAGCG
ACGCCGCGTGAAGGATGAAGGTCTTCGGATTGTAAACTTCTGTCATTAGAGAACAATTGT
CATCGAGTAACTATCGGTGGCTTGATAGTATCTGAAGAGGAAGAGACGGCTAACTCTGTG
CCAGCAGCCGCGGTAATACAGAGGTCTCAAGCGTTGTTCGGATTCATTGGGCGTAAAGGG
TGCGTAGGTGGCGGGGCAAGTTGGATGTGAAATCCCGGAACTTAACTCCGGAACTGCATT
CAATACTGCCTTGCTGGAGTACTGGAGAGGAGATTGGAATTTACGGTGTAGCAGTGAAAT
GCGTAGATATCGTAAGGAAGACCAGTGGCGAAGGCGGATCTCTGGACAGTTACTGACACT
GAGGCACGAAGGCCAGGGGAGCAAACGGGATTAGAAACCCCAGTAGTCC
>Otu5210
CCAGCCTACGGGGCGCACCAGTCGAGAATATTCCACAATGGGGGAAACCCTGATGGAGCG
ACACCGCGTGGTGGATGAAGGTCTTCGGATTGTAAACACCTTTTGCACGGGACGAAGCGT
AAGCTGACGGTACCGTGAGAATAAGAGGTTGCTAACTCTGTGCCAGCAGCAGCGGTAATA
CAGAGACCTCGAGCGTTATCCGGATTTACTGGGCGTAAAGCGTCCGTAGGCTGTTTGGTA
AGTCATCTGTCAAATCCCAGTGCTCAACATTGGGGCTGCGGGTGATACTGTCAAACTAGA
GGATGGAAGAGGCAAGGGGAACTGCCGGTGTAGTAGTAAAATGCGTTAATATCGGCAGGA
ACACCAAAGGCGAAGGCACCTTGCTAGGACATTCCTGACACTCAAGGACGAAAGCGTGGG
GAGCAAAACGGATTAGAACCCTAGTAGTCC
>Otu5212
CCAGCCTATGGGACGCTGCAGTTGGGAATCTTCTGCAATGCGCGCAAGCGTGACAGAGTG
AACCAAAGTGTTTCTTTTTTTAAGAAACTTTTGCCAGCTGTAAAAAGGCTGGCGAATAAG
GACTGGGCAAGATCGGTGCCAGCCGCCGCGGTAATCCCGACAGTCCGAGTCGCAGCCACA
ATTATTGGGTCTAAAATATCCGTAGCTTGCTAAAGAAGTCTCCTGTGAAATCCAGCATCT

TAAGTGTTGGGCGTGCAGGAGATACTCTTTGGCTAGAGACTGGAAGGCGTAAGAAGTACA
TTCGGGGTAGTGGTAAAATATTTTAATCCCGAGTGGACTGACAGTCGCGAAGGCGTCTTA
CGAGGACAGTTCTGACAGTGAGGGATGAAGGCTAGGGGCGCAAAAGGGATTAGATACCCC
CGTAGTCC
>Otu5213
CCAGCCTATGGGTGGCTGCAGTCGAGAATCTTCCGCAATGGACGAAAGTCTGACGGAGCG
ACGCCGCGTGGAGGATGAAGTTCTTCGGAATGTAAACTCCTTTTGCCAGGGAAAAAGTTA
TTGATTGTACCTGGAGAATAAGAAGTTGCTAAACTCGTGCCAGCAGCAGCGGTAATACGA
GTGCTTCAAGCGTTATCCGGAATCATTGGGCGTAAAGGGTGTGTAGGCGGTTCTGTTAGT
CTTGCGTAAAATCTCTCGGCTCAACCGAGAACTCGCGCAGGAAACGGCAGAACTTGAGGA
CGGAAGAGGTCTCTGGAACTCTAGGTGTAGCGGTGAAATGCGTTGATATCTAGGGGAACA
CCAAAAGCGAAGGCAAGAGACTAGTCCGCTCCTGACGCTGAAACACGAAAGCGTGGGTCG
CGAATGGGATTAGATACCCTCGTAGTCC
>Otu5214
CCAGCCTACGGGAGGCTCCAGTGGGGAATATTGGACAATGGGCGAAAGCCTGATCCAGCC
ATGCCGCGTGAGTGATGAAGGCCTTAGGGTTGTAAAGCTCTTTTGGCGGGGACGATAATG
ACGGTACCCGCAGAATAAGCCCCGGCTAACTACGTGCCAGCAGCCGCGGTAACACGTAGG
AGACGAGCGTTATCCGGATTTACTGGGCGTAAAGCGCGTGCAGGCGGTTTGGTAAGTTGG
ATGTGAAAGCTCCCGGCTTAACTGGGAGAGGTCGTTCAATACTGCCAGACTAGAGGATGG
TAGAGGGAGGTGGAATTCTGGGTGTAGTGGTGAAATGCGTAGATATCCAGAGGAACACCA
GTGGCGAAAGCGGCCTCCTGGACCATTTCTGACGCTCATACGCGAAAGCTAGGGTAGCAA
ACGGGATTAGAGACCCGAGTAGTCC
>Otu5215
CCAGCCTACGGGTTGCAGCAGTGAGGAATATTGCGCAATGGGGGCAACCCTGACGCAGCA
ACGCCGCGTGAGTGAGGAAGGTTTTCGGATTGTAAAGCTCTGTCGAGCGGAAAGAAATGC
ATTATGGCTAATATCCATAATGTTTGACGGTACCGCTAAAGGAAGCGCCGGCTAACTCCG
TGCCAGCAGCCGCGGTAATACGAAGGGGGCTAGCGTTGTTCGGAATCACTGGGCGTAAAG
CGTACGCAGGCGGATTGATAAGTCAGGGGTGAAATCCCGGAGCTCAACTTCGGAATTGCC
TTTGATACTGTCTATCTTCGAGTTCGGGAGAGGTTGGCGGAATTCCTAGTGTAGAGGTGA
AATTCGTAGATATTAGGAAGAACACCAGTGGCGAAGGCGGCCAACTGGCCCGATACTGAC
GCTCATGTACGAAAGCGTGGGGAGCAAACAGGATTAGAGACCCTAGTAGTCC
>Otu5217
CCAGCCTACGGGGGGCTGCAGTCGAGGATCTTTCTCAATGGGGGAAACCCTGAAGATGCG
ACGCCGCGTGAGGGATGAAGGCCTTCGGGTCGTAAACCTCTTTTAACAGGGAGGAATGCT
GTAGAGTGAATAACTCTGCAGATTGACTGTACCTGTGGAATAAGCCACGGCTAACTCTGT
GCCAGCAGCCGCGGTAATACAGAGGTGGCAAGCGTTACTCGGTTTGACTGGGTGTAAAGG
GTCCGCAGGCGGCTTTACAAGTAGAAAGTGAAATCCTTCAGCTTAACTGAAGAACTGCTT
TCTAAACTGTTTAGCTTGAGGATGGGAGAGGATAACGGAACTGCAGGTGTAAAGGTGAAA
TTTGTGGATATCTGCAGGAACGCCAGTGGAGAAGTCGGTTATCTGGTCCATTCCTGACGC
TCAGGGACGAAAGCTAGGGGAGCAAACAGAATTAGATACCCGAGTAGTCC
>Otu5218
CCAGCCTATGGGAGGCTCCAGTCGAGGATTTTTCTCAATGGGGGAAACCCTGAAGGAGCG
ACGCCGCGTGGGGGATGAAGGTCTTCGGATTGTAAACCCCTGTCATCTGAGAACAAAGTG
ATCTACCCAATACGTGGATTATTGATAGTACCGGAAGAGGAAGCCGTGGCTAACTCTGTG
CCAGCAGCCGCGGTAATACAGAGACGGCAAGCGTTGTTCGGATTCATTGGGCGTAAAGGG
TGCGCAGGCGGTCGGCCAAGTCTGATGTGAAAGCTCGCAGCCTAACTGCGAAATGGCGTC
GGAAACTAGCCGACTAGAGGACTGGAGAGGAGACTGGAATTGTCGGTGTAGCGGTGAAAT
GCGTAGAGATCGACAAGAACACCGGTGGCGAAGGCGGGTCTCTGGACAGTTCCTGACGCT
CAGGCACGAAAGCCAGGGGAGCAAACGGGATTAGATACCCGAGTAGTCC
>Otu5224
CCAGCCTACGGGGTGCAGCAGTTTGGAATTTTGGACAATGAGCGAAAGCCTGATCCAGCG
ACGCCGCGTGGACGATGAAGGCCTTCGGGTTGTAAAGTCCTTTTGCTCGGGAACAATTTT
TGAGCGTACCGAGAGAATAAGAGGTTGCTAACTCTGTGCCAGCAGCAGCGGTAATACAGA
GACCTCAAGCGTTATCCGGAATCATTGGGCGTAAAGCGTACCGATAGGTGGTTTAGTGTG
TCAGAAGTGAAATCTTCAGGCTTGACCTGGAGAATGTCTTTTGAAACTACTAAACTAGAG
GGGCAAAGAGGAAGCTGGAACGAACGGTGTAGTAGTGAAATGCGTTGATATCGTTCGGAA
CACCAATTAGCGTAGGCAGGCTTCTGGGTGCCACCTGACACTGCTAGGACGAAAGCGTGG
GGAGCGATAAGGATTAGATACCCTAGTAGTCC

>Otu5227
CCAGCCTACGGGATGCTGCAGCTAAGAATCTTCCGCAATGGGGGAAACCCTGACGGAGCG
ACGCCGCGTGGATGATGAAGGCCGTAAGGTTGTAAAATCCTTTTGTCGGTGAAGAATAAC
ACAAGGAGTGGAAAGCCTTGTGGATGACGTTAGCCGACGAATAAGCCCCGGCTAATTACG
TGCCAGCAGCCGCGGTAATACGTATGGGGCAAGCGGTGTTCGGAATTACTGGGCGTAAAG
GGCACGTAGGCGGCTTTGTAAGCCCGGCGTGGAAGCCCACGGCTCAACCGTGGGATTGCG
TTGGGAACTGCGAGGCTTGAATCATGGAGAGGGAGCTAGAATTCCTGGTGTAGGGGTGAA
ATCTGTAGAGATCAGGAAGAATACCAGTGGCGAAGGCGAGCTCCTGGCCAATGATTGACG
CTGAGGTGCGAAAGTGTGGGGATCAAACAGGATTAGAAACCCTAGTAGTCC
>Otu5230
CCAGCCTACGGGGCGCAGCAGGGAATTTTGCGCAATGGGCGAAAGCCTGACGCAGCAACA
CCGCGTGAGGGAAGACAGCCTTCGGGTCGTAAACCTCTTTTATCAGGGAAGAATAATGAC
GGTACCTGATGAATAAGTCACGGCTAACTACGTGCCAGCAGCTGCGGTAATACGTAGGTG
GCGAGCGTTATCCGGATTCATTGGGCGTAAAGAGCGCGTAGGTGGTCCTTCAAGTCAGAT
GTTAAATCTCTCGGCTTAACTGGGAGTTGTCATCTGATACTGCTGGACTTGAGGGCGGTA
GAGGGAGGTGGAATTCCCGGTGTAGTGGTGAAATGCGTAGATATCGGGAGGAACACCAGT
GGCGAAAGCGGCCTCCTAGGCCGTTCCTGACACTGAGGCGCGAAAGCGTGGGGAGCGAAC
AGGATTAGATACCCGGTAGTCC
>Otu5236
CCAGCCTACGGGGCGCTGCAGTGGGGAATTTTGCACAATGCCCGAAAGGGTGATGCAGCA
ACGCCGCGTGAGGGATGAAGGTCCTCGGATCGTAAACCTCTTTCGATCGGGAAAAATGGG
CGGGCGAACAACCCGTTCTGATGGTACCGGAAGAAGAAGCCCCGGCTAACTACGTGCCAG
CAGCCGCGGTAACACGTAGGGGGCGAGCGTTGTTCGGAATTACTGGGCGTAAAGGGCGTG
TAGGCGGTGCGGCAAGTCGGGCGTGAAATCCCCGGGCTTAACCCGGGGCGGTCGTTCGAA
ACTGCCGTGCTAGAGTGTGGGAGAGGGAAGTGGAATTCCCGGTGTAGCGGTGAAATGCGT
AGATATCGGGAGGAACACCTGCGGCGAAGGCGGCTTCCTGGACCATAACTGACGCTGAGG
CGCGAAAGCTAGGGGAGCAAACAGGATTAGAGACCCGAGTAGTCC
>Otu5240
CCAGCCTACGGGGCGCAGCAGTGGGGAATATTGGACAATGGGCGCAAGCCTGATCCAGCC
ATGCCGCGTGAGTGAAGAAGGCCTTCGGGGTGTAAAGCTCTTTCGGCAGGGACGATAATG
GCGGTACCTGCAGAAGAAGCTGCGGCTAACTACGTGCCAGCAGCCGCGGTAATACGTAGG
CAGCAAGCGTTGTTCGGAATTACTGGGCGTAAAGAGTGCGTAGGCGGTTTGGCAAGTTCG
GTGTGAAATCTCCCGGCTTAACTGGGAGGGTGCGCCGAAAACTGCCAGACTAGAGTGTGG
GAGAGGGAAGTGGAATTCCTGGTGTAGCGGTGAAATGCGTAGATATCAGGAGGAACACCT
GCGGTGTAGACGGCTTCCTGGACCACTACTGACGCTGAGGCACGAAAGCGTGGGGAGCAA
ACAGGATTAGATACCCTCGTAGTCC
>Otu5242
CCAGCCTACGGGATGCACCAGTCGAGAATCTTCGGCAATGGGCGCAAGCCTGACCGAGCG
ACGCCGCGTGCGGGATGGAGGCCTTCGGGTTGTAAACCGCTGTCAGAGGGGAGGAAATGC
CGGTGGGTTCTCTCATCGGTTTGACCTATCCTCAGAGGAAGGACGGCTAAGTTCGTGCC
AGCAGCCGCGGTAAGACGATCCGTCCAAACGTTATTCGGAATCACTGGGCTTAAAGGGTG
CGTAGGCGGTCCGGGAAGTTGGGTGTGAAAGCCCTCAGCTCAACTGAGGAATTGCGCCCA
AAACTGCCGGACTTGAGGGAGACATAGGTGAGCGGAACTTAGGGTGGAGCGGTGAAATGC
GTTGAGATCCTAAGGAACACCAGTAGCGAAAGCGGCTCACTGGGTCTCTTCTGACGCTGA
TGCACGAAAGCTAGGGGAGCGAACGGGATTAGATACCCTCGTAGTCC
>Otu5243
CCAGCCTACGGGTGGCAGCAGTAGGGAATATTGGGCAATGGGCGAAAGCCTGAAGGGGCG
ACGCCGCGTGGGGGATGAAGGGCTTCGGCCCGTAAACCCCTGTCATTCGCGAACAAACCC
TGCGATTTAATAGATCGCAGGCTGATTGTAGCGGAAGAGGAAGGGACGGCTAACTCTGTG
CCAGCAGCCGCGGTAATACGGAGGTCCCAAGCGTTGTTCGGATTCACTGGGCGTAAAGGG
TGCGTAGGTGGCAGGGTAAGTTTGATGTGAAATCTCCGGGCTTAACCCGGAAACGGCATT
GAATACTCTCCTGCTCGAGGGTTGGAAGGGGGACTGGAATACTTGGTGCAGCAGTGAAAT
GCGTAGATATCAAGTAGAACACCAGTGGCGAAGGCGAGTCCCTGGACAACTCCTGACACT
GAGGCACGAAAGCTATGGGAGCAAACAGGATTAGATACCCTTGTAGTCC
>Otu5245
CCAGCCTACGGGGGGCACCAGTGAGGAATTTTCCGCAATGGGCGAAAGCCTGACGGAGCG
ACGCCGTGTGGGTGATGAAGGCCTTCGGGTCTTAAAGCCCTGTCAGCGATGACGATATTG
ACGGTAATCGCAGAGGAAGCCCCGGCTAACTACGTGCCAGCAGCCGCGGTAATACGTAGG

GGGCGAACGTTGTCCGGAATTATTGGGCGTAAAGCGCATGTAGGCGGTTCTATAAGTCAG
GTGTAAAATCCCCCGGCTCAACCGGGGTTCGCACTTGATACTGTAGGGCTTGAGAGATAT
AGAGGAAAGCGGAACTTCAGGTGTAGCGGTGAAATGCGTAGATATCTGAAGGAACACCAG
TGGCGAAGGCGGCTTTCTGGATATTTTCTGACGCTGAGATGCGAAAGCAAGGGGAGCAAA
CGGGATTAGATACCCGAGTAGTCC
>Otu5247
CCAGCCTACGGGATGCTCCAGTAGGGAATATTGCTCAATGGGGGAAACCCTGAAGCAGCA
ACGCCGCGTGAGTGATGAAGGCCTTCGGGTCGTAAAGCTCTGTTGTACGGGGCGAATGCC
GTCATGGCTAATATCCATGGCGAGTGACAGTACCGTATAAGAAAGGACCGGCTAACTTCG
TGCCAGCAGCCGCGGTAAGACGGGGGGTCCAAGCGTTGTTCGGAATCATTGGGCGTAAAG
CGTGTGCAGGTGGTTTTGTAAGTTGGATGTGAAAGCCCCGGGCTTAACCCGGGAAGTGCA
TTCAAAACTGCAAAACTTGAGTATGGAAGAGGGTCGTGGAATTCCAGGCGTAGTGGTGAA
ATACGTAGATATCTGGAGGAACACCGGTGGCGAAGGCGGCGACCTGGTCCTATACTGACA
CTCATACACGAAAGCGTGGGGATCAAACAGGATTAGATACCCGCGTAGTCC
>Otu5250
CCAGCCACGGGGGGCAGCAGTGAGGAATATTGGTCAATGGACGCAAGTCTGAACCAGCCA
TGCCGCGTGAAGGATGAAGGCCCTATGGGTTGTAAACTTCTTTTATACGGGACGAATCTC
CCGCTCGTGAGCGGGATTGACGGTACTGTACGAATAAGCAACGGCTAACTCCGTGCCAGC
AGCCGCGGTAATACGGAGGTTGCAAGCGTTATCCGGATTTATTGGGTTTAAAGGGTGCGT
AGGCGGACTTATAAGTCAGTGGTGAAATCTCGTTGCTTAACAACGAACGTGCCATTGATA
CTGTAGGTCTTGAGTACAGATGCCGTTGGCGGAATGTGTCATGTAGCGGTGAAATGCATA
GATATGACACAGAACACCGATTGCGAAGGCAGCTGACGAAACTGTAACTGACGCTGAGGC
ACGAAAGCGTGGGGACCAAACAGGATTAGAAACCCCAGTAGTC
>Otu5257
CCAGCCTATGGGAGGCACCAGTGGGGAATCTTGGACAATGGGCGCAAGCCTGATCCAGCA
ATGCCGCGTGGGTGAAGACGGCCTGCGGGTTGTAAAGCTCTGTGGGGAGGGACGAATAAG
GGTTGGCTAATATCCAATCTGATGACGGTACCTCCTTAGCAGGCACCGGCTAACTCTGTG
CCAGCAGCCGCGGTAAGACAGAGGGTGCAAACGTTGTTCGGAATCACTGGGCGTAAAGCG
CGTGTAGGCGGCTACGTAAGTCGGACGTGAAAGCCCACGGCTCAACTGTGGAAGTGCGCT
CGAAACTGTGTAGCTTGAGTCTTGGAGAGGAAGGCGGAATACTTGGTGTAGAGGTGAAAT
TCGTAGATATCAGGTAGAACACCAGTGGCGAAGGCGGCCTTCTGGACAATGACTGACGCT
GAGACGCGAAAGCGTGGGGAGCAAACAGGATTAGATACCCCTGTAGTCC
>Otu5258
CCAGCCTACGGGTGGCACCAGTGGGGAATCTTGCACAATGGGGGAAACCCTGATGCAGCG
ACGCCGCGTGAGCGATGAAGCCCTTCGGGGTGTAAAGCTCTTTCGTCAGGGAAGATAGTG
ACGGTACCTGGAGAAGCAGCTGCGGCTAACTACGTGCCAGCAGCCGCGGTAATACGTAGG
CAGCGAGCGTTGTTCGGAGTTACTGGGCGTAAAGGGTGTGTAGGCGGTTGTTTAAGTTTG
GTGTGAAACCTCCCGGCTCAACTGGGAGGGTGCGCCGAGGACTGGAGAGCTTGGAGTGCG
GGATGGGAGAGTGGAATTCCCGGTGTAGCGGTGAAATGCGTAGATATCGGGAGGAACACC
TGTGGTGTAGACGGCTCTCTGGACCGTAACTGACGCTGAAACACGAAAGCGTGGGTAGCA
AACAGGATTAGATACCCCTGTAGTCC
>Otu5262
CCAGCCTACGGGGGGCAGCAGTGGGGAATTTTGGACAATGGGCGAAAGCCTGATCCAGCG
ACGCCGCGTGGAGGATGAAGGCCTTCGGGTTGTAAACTCCTTTTGGAGGGGACGAAATAA
ATGACGGTACCCTCAGAAAAAGCCACGGCTAACTACGTGCCAGCAGCCGCGGTAATACGT
AGGTGGCGAGCGTTACTCGGATTTACTAGGCGTAAAGCGCTTGCAGGTGGTTGGATAAGT
CCGTAGTGAAATTTCCCGGCTTAACTGGGAAGGGTCTACGGAAACTATTCGGCTTGAGTG
TGGGAGGGGTGGACGGAATTCCAGGTGTAGCGGTGAAATGCGTAGATATCTGGAGGAACA
CCGATGGCGAAAGCGGTCCACTGGCCCAATACTGACACTGAGGAGCGAAAGCTAGGGGAG
CAAACAGGATTAGATACCCCCGTAGTCC
>Otu5267
CCAGCCTACGGGTGGCAGCAGTGACGAAATCTGGACAATGCGCGAAAGCGTGATCTGGTA
ATACCTCTCAAGTGATGACGGTCAAGCGATTGTAAAACTTTGTATGTTAACTAAATAATG
ACACGGTTAATTAGGAAAAAGTCCTGGCCAACTCTGTGCCAGCAGCCGCGGTAATACAGA
GGGGGCAAGCATTATTCATCTTGACTGGGCGTAAAGGGTGTGTAGGTGGTCAACTTTATT
ATACGGTAAAGCCTGGGGTTATACTCAGAAGCTGTGTTAATATTAGTTAGACTTGAGTTT
ATGTGTGGTTATTAGAATTTTTGGTGGAGCAGTAAAATGCTTAGATACTACAAGGAATAC
CGAAGAGCGAAGGCGATTAACCAGCATTAAACTGACACTGAGGCACGAAGGCATGGGGAG

CAAACAGGATTAGATACCCTAGTAGTCC
>Otu5271
CCAGCCTACGGGTGGCACCAGTGAGGAATATTGGACAATGGGCAAACGCTTGATCCAGTA
ATAATTCATGAGTGAGGAAAGGAAAGCAGTAACGCTGTAAAACTCGAATAATAATAAAAA
TAATGATATTTTTTATAAAAAGCACTGGCTAATTCCGTGCCAGCAGCCGCGGTAAGACAG
GTAGTGCAGACGTTATTCATTTTGATTAGGTGTAAAGGATATGTAGATTGCTTTGTTTTG
AATATTTTCTAAAAATTCGAAGTGTAGTTCTTCGAAATGGAAAATTTACTCAAAAGCTAG
AGTTTGTGGTATGTTAAAAGAATTTTTTTTGTAAGGGTAGAATCTCTAAATAATTAAAAG
ACTACCAAGATAGTTGTGAAAACATTTTTCAGATCCAAACTGACATTGAGATATGAAAGT
ATGGGAATCAAACAGGATTAGATACCCCAGTAGTCC
>Otu5272
CCAGCCTATGGGGGGCTCCAGTCTAGAAAATTGGGCAATGGGCGCAAGCCTGACCCAGCG
ACGCCGCGTGGAGGATGAAGATCTTCGGGTTGTAAACTCCTGTCAGGCGGAACGATGCCT
TGTGCTCTAACTAAGCACAAGGTGACGGTACTGCCAAAGGAAGCCCCGGCTAACTACGTG
CCAGCAGCCGCGGTAAGACGTAGGGGGCGAGCGTTGTCCGGAATTACTGGGCGTAAAGGG
TGTGTAGGCGGTCTTGTAAGTCAGCAGTGAAAGCTCCGGGCTAAACTCGGAAACGGTTGC
TGATACTACCGGACTTGAGGGCAGGAGAGGAGGTTGGAACTTTCGGTGTAGCGGTAGAAT
GCGTAGATATCGAAAGGAACGCCGATAGCGAAGGCAAGCCTCTGGAATGTTCCTGACGCT
GAAACACGAAAGCTGGGGGAGCAAACAGGATTAGAAACCCCCGTAGTCC
>Otu5275
CCAGCCTATGGGGCGCAGCAGTGGGGAATTTTGCGCAATGGGGGAAACCCTGACGCAGCA
ACGCCGCGTGGAAGATGAAGCCCCTTGGGGCGTAAACTCCTTTCGACCGGGACGATTATG
ACGGTACCGGTGGAAGAAGCACCGGCTAACTCTGTGCCAGCAGCCGCGGTAATACAGAGG
GTGCGAGCGTTGTTCGGAATTATTGGGCGTAAAGCGCGTGTAGGCGGTTTGGTAAGTCTT
TTGTGAAATCTATGGGCTCAACTCATAGACTGCAAGGGAAACTGCCGGGCTTGAGTGTGG
GAGAGGTGAGTGGAATTTCCGGTGTAGCGGTGAAATGCGTAGATATCGGAAGGAACACCT
GTGGCGAAAGCGGCTCACTGGACCACAACTGACGCTGATGCACGAAAGCTAGGGGAGCAA
ACAGGATTAGAAACCCAGTAGTCC
>Otu5284
CCAGCCTATGGGGTGCTGCAGTGAGGAATTTTGGACAATGAACGAAAGTTTGATCCAGCA
ATATCGCGTGAGTGATAATTTAGTAAATTAGTTAATAGACTAATTTATAGAAGGTTAAAT
CCGTAAAACTCTTTCATTATTGATGAAAAAGACAGTAAATAAAAAAGAAGTCCCGACTAA
CTTCGTGCCAGCAGTCGCGGTAATACGGAGGGGGCAAGCGTTATTCACCTTGACTGGGCG
TAAAGGGTATGTAGATGGTTTTAAATGTTTAAAATAAGTAATCCAAGCTTAACTTTGAAG
TTATTTTAAAAACAATAAAACTAGAGGTTTTTGGAAGTAGGTTACATTTTTAATGCATAG
GTGAAATTATTTTATATTAAAGAGATATCCAGAAAGCGAAAGCGACCTACTAGATTTTTC
CTGACATTGAGGTACGAAAGCATGGGGATCAAACAGGATTAGAGACCCTTGTAGTCC
>Otu5286
CAGCCTATGGGAGGCTCCAGTGAGGAATCTTCCACAATGGGCGAAAGCCTGATGGAGCAA
CGCCGCGTGCAGGATGAAGGCCCTCGGGTCGTAAACTGCTTTTATAAGTGAAGAATATGA
CGGTAGCTTATGAATAAACACCGGCTAACTACGTGCCAGCAGCCGCGGTCATACGTAGGG
TGCAAGCATTATCCGGATTTACTGGGCGTAAAGCGCGTGCAGGCGGTTTGGTAAGTTGGA
TGTGAAAGCTCCTGGCTTAACTGGGAGAGGTCGTTCAGTACTGCCAGACTAGAGGATGGT
AGAGGGAGGTGGAATTCTGGGTGTAGTGGTGAAATGCGTAGATATCCAGAGGAACACCAG
TGGCGAAAGCGGCCTCCTGGACCATTTCTGACGCTCATACGCGAAAGCTAGGGTAGCAAA
CGGGATTAGAGACCCGAGTAGTCC
>Otu5291
CCAGCCTATGGGAGGCTGCAGTGGGGAATATTGGACAATGGGGGAAACCCTGATCCAGCA
ATGCCGCGTGAATGATGAAGGCCTTCGGGTTGTAAAGTTCTTTTACCCGAGACGATAATG
ACGGTATCGGGAGAATAAGCACCGGCTAACTTCGTGCCAGCAGCCGCGGTAATACGAAGG
GTGCAAGCGTTGTTCGGAATGACTGGGCGTAAAGGGCGCGTAGGCGGTTCGGTACGTTTG
ATGTGAAAGCCCCGGGCTCAACCTGGGAATTGCATCGAATACGGCCGAGCTTGAGTGCGA
GAGAGGAAAATGGAATAGTGCGTGTAGAGGTGAAATTCGTAGATATGCACTAGAACACCA
GAGGCGAAGGCGATTTTCTGGCTCGTAACTGACGCTGAGGCGCGAAAGCGTGGGGAGCAA
ACAGGATTAGAGACCCGAGTAGTCC
>Otu5294
CCAGCCTACGGGGTGCAGCAGCGGATTAACCTCAAGCAGATCGTCGGCACGGGGTCGAAG
TCCGATCAGACCGCTGATAATGAGATCGCAGTAGGTCGAGTGGTTGTAGTCTTTTCCTCG

TTCCCGAATTTGGAAACCCCGTTTCTGGAGCAAGCTGCGCGCGATCCAGTCCCCGTTCGT
CGGATTGATATCCTCGTCGATCCACGGCACCACTCGGCCATCTCCAAGTTGGAGATGCTG
AGATTTGGTATAGATCTTTAGGAGTTTAAAGTAGTCACTGGAGGAAATAATCTTTTGATC
GCTCCCGTTGAGCACGTTGGCCAAGCCACTTAACAGGATTGAAGTGGCGAACGGCCAGCT
TGGTCCGTTCCATTGACATTCGTGGCCCTCATTAGATACCCCAGTAGTCC
>Otu5295
CCAGCCTATGGGTGGCAGCAGTAGGGAATTTTGCGCAATGGACGAAAGTCTGACGCAGCA
ACGCCGCGTGAGTGATGAAGGCCTTCGGGTTGTAAAGCTCTGTTGAGTGGGGAGAAAAGC
TTTGGGGATAATCTACCAGAGCCTGACGGTACCATTCGAGGAAGCACCGGCTAACTACGT
GCCAGCAGCCGCGGTAATACGTAGGGTGCAAGCGTTGTTCGGAATCACTGGGCGTAAAGG
GAGCGTAGGTGGGGTTGTAAGTTAGGAGTTTAATACATGGGCTTAACCCATGGCTGGCTC
TTAAGACTGCGATACTTGAGTATTGGAGAGGGCGATGGAATTCCAGGTGTAGCGGTGGAA
TGCGTAGATATCTGGAAGAACACCAGAAGCGAAGGCGGTCGCCTGGCCAAATACTGACAC
TGAGGCTCGAAAGCTAGGGGAGCAAACAGGATTAGAAACCCTGGTAGTCC
>Otu5297
CCAGCCTACGGGTGGCAGCAGTCAAGAATATTCCTCAATGGACGAAAGTCTGAAGGAGCG
ACGCCGCGTGACTGATGAAGTCCTTCGGGATGTAAAGGTCTTTTCTTAAGGAAGAAACAA
TGACGGTACTTAAGGAATAAGAGGTTGCTAACTCTGTGCCAGCAGCAGCGGTAATACAGA
GACCTCAAGCGTTATCCGGCTTGATTGGGTGTAAAGCGTGCGCAGGCGGCTTGTTAAGCC
GACGGTTAAATCTTGTGGCTCAACTGCAAGATTGCCGCCGGAACTGGCAAACTAGAGCAT
GGAAGAGGCGAGCGGAATTGCCGGTGTAACGGTAAAATGTGTTAATATCGGCAAGAACAC
CAGAGGCGAAAGCGGCTCGCTGGTACATCGCTGACGCTGTGGCACGAAAGCGTGGGGAGC
AAATGGGATTAGAGACCCCCGTAGTCC
>Otu5298
CCAGCCTATGGGGGGCTCCAGCAGGGAATCTTGGTAATCTGCGAAAGCGGGAACCAGCAA
CGCCGCGTGTGCGATGAAGGCCTTCGGGTCGTAAAGCACTTTTCGAGGGGATGAGGAAGG
ACAGTACCCTCGGAATAAGTCTCGGCTAACTACGTGCCAGCAGCCGCGGTAAAACGTAGG
AGGCAAGCGTTATCCGGATTTACTGGGCGTAAAGCGTGTGCAGGCGGTTTGGTAAGTTGG
ATGTGAAAGCTCCTGGCTTAACTGGGAGGGGTCGTTCAATACTACCAGGCTAGAGGATGG
GAGAGGGAGGTGGAATTCCGGGTGTAGTGGTGAAACGCGTAGATATCCGGAGGAACACCA
GTGGCGAAAGCGGCCTCCTGGCCCATTTCTGACGCTCATACACGAAAGCTAGGGTAGCAA
ACGGGATTAGAGACCCTTGTAGTCC
>Otu5300
CCAGCCTATGGGAGGCTGCAGTCGAGAATTTTTCACAATGGGCGCAAGCCTGATGGAGCG
ACGCCGCGTGGGGGATGAATGGCTTCGGCCCGTAAACCCCTGTCATTTGCGATCAACCGT
TATTGTTTAAGAGATGATAACCTGATAGTAGCGAAAGAGGAAGGGACGGCTAACTCTGTG
CCAGCAGCCGCGGTAATACAGAGGTCCCAAGCGTTGTTCGGATTCACTGGGCGTAAAGGG
TGCGTAGGTGGCGAAGCAAGTCGGATGTGAAATCTCCGAGCTCAACTCGGAAACTGCATT
GGAAACTGCTTTGCTCGAGGATTGGAAGGGGGACTGGAATACTTGGTGTAGCAGTGAAAT
GCGTAGATATCAAGTGGAACACCAGTGGCGAAGGCGGCCTCCTGGACCATTTCTGACGCT
CAGACACGAAAGCTAGGGGAGCAAACGGGATTAGATACCCTAGTAGTCC
>Otu5305
CCAGCCTATGGGGGGCTGCAGCTAAGAATCTTCCGCAATGGGGGAAACCCTGACGGAGCG
ACGCCGCGTGGGGGATGACGGCCTTCGGGTTGTAAACCCCTTTTCTAGAGGAAGAAATAT
GACGGTACTCTAGGAATAAGTCTTGGCTAACTACGTGCCAGCAGCCGCGGTAATACGTAG
AAGGCGAACGGTATCCGGATTTACTGGGCGTAAAGCGGGTGTAGACGGTTTTTTAAGTCA
GTTGTGAAATCTCCTGACTCAATTGGGAGTCTGCAATTGATACTGGAGAACTTGAGGGCA
TCAGAAGAAAGCAGAATTCACGGTGTAGCGGTGAAATGCGTAGATATCGTGAGGAATACC
AGTGGCGCAAGCGGCTTTCTAGGATGCTCCTGACGTTGAGGCCCGAAAGTGTGGGGAGCG
AAACGGATTAGATACCCTCGTAGTCC
>Otu5306
CCAGCCTATGGGTCGCTCCAGTGGGGAATATTGGACAATGGGCGCAAGCCTGATCCAGCA
ATGCCGCGTGTGTGAAGAAGGCCTGCGGGTTGTAAAGCACTTTAAGCGGGGAAGAAGGCT
CTTGAGTGAATAGCTTGAGGGATTGACGTTACCTGCAGAATAAGCACCGGCTAACTCCGT
GCCAGCAGCCGCGGTAATACGGAGGGTGCGAGCGTTAATCGGAATGACTGGGCGTAAAGC
GCGCGTAGGCGGTTCGATCAGTCTGAGGTGAAAGCCCTGGGCTTAACCTGGGAACGGCCT
TGGAAACTGTCGAGCTAGAGTGTGGGAGAGGGGCGTGGAATTTCCGGTGTAGCAGTGAAA
TGCGTAGAGATCGGAAGGAACACCAGTGGCGAAGGCGGCGCCCTGGACCAACACTGACGC

TGAGGTGCGAAAGCGTGGGGAGCAAACAGGATTAGATACCCTCGTAGTCC
>Otu5313
CCAGCCTATGGGGGGCTGCAGTCGAGAATCTTTGGCAATGGGCGAAAGCCTGACCAAGCG
ACGCCGCGTGGAGGATGAAGGCCTTCGGGTTGTAAACTCCTGTTGGGGGGTATGATGCGG
GGAAGCGCTAATACCGTTTTCTCGTGACGTCAACCCCTGAGGAAGCCACGGCTAACTCTG
TGCCAGCAGCCGCGGTAAGACAGAGGTGGCAAGCGTTGTTCGGATTCACTGGGCTTAAAG
GGTGCGTAGGCGGCCGAGTATGTCTGGTGTGAAATCCCCCGGCCCAACCGGGGAACTGCG
CCGGAAACTGCTTGGCTCGAGGGCAGTAGAGGGGGGTGGAACTCCTGGTGTAGCGGTGAA
ATGCGTAGATATCAGGAGGAACGCCGGAGGCGAAAGCGACCCCCTGGTCTGTCCCTGACG
CTGTGGCACGAAAGCTAGGGGAGCAAACGGGATTAGAAACCCGGGTAGTCC
>Otu5315
CCAGCCTATGGGGGGCACCAGTCGAGGATCTTTGGCAATGAGCGCAAGCTTGACCAAGCG
ACGCCGCGTGTGCGATGAAGGCCTTCGGGTTGTAAAGCACTGTCGAGGGGGATCAAGGGT
AACTTGAGTGATCCCTGGAGGAAGCACGGGCTAAGTTCGTGCCAGCAGCCGCGGTAAGAC
GAACCGTGCGAACGTTGTTCGGAATCACCGGGCTTAAAGGGCGCGTAGGCGGCTCATCAA
GTCAGGGGTGAAATACTTCAGCTTAACTGGAGAAGTGCCTTTGATACTGGTGAGCTCGAG
GGAGGTAGGGGTGTGTGGAACTTCTGGTGGAGCGGTGAAATGCGTTGATATCAGAGGGAA
CGCCGGTGGCGAAAGCGACGCACTGGACCTCTTCTGACGCTGAGGCGCGAAAGCTAGGGG
AGCAAACGGGATTAGAAACCCTTGTAGTCC
>Otu5317
CCAGCCTATGGGGCGCAGCAGTGAGGAATATTGGACAATGGTGGCAACACTGATCCAGCC
ATGCCGCGTGCAGGATGAAGGCGCTACGCGTTGTAAACTGCTTTTGTACGGGAGAAAACC
TATCTACGTGTAGATAGCTGATAGTACCGTAAGAATAAGCATCGGCTAACTTCGTGCCAG
CAGCCGCGGTAATACGAAGGATGCAAGCGTTACCCGGATTTATTGGGCGTAAAGCGCGTG
TAGGCGGGTCTTTAAGTCAGGGGTGAAATGCCAAGGCTCAACCTTGGAACTGCCTTTGAT
ACTGGAGATCTTGAGTCCGGGAGAGGTGAGTGGAACTGCGAGTGTAGAGGTGAAATTCGT
AGATATTCGCAAGAACACCAGTGGCGAAGGCGGCTCACTGGCCCGGAACTGACGCTGAGA
CGCGAAAGCGTGGGGAGCAAACAGGATTAGAAACCCCCGTAGTCC
>Otu5318
CCAGCCTATGGGGTGCAGCAGTCGAGAATTTTTCTCAATGGGCGAAAGCCTGAAGGAGCG
ACGCCGCGTGGGGGATGAATGGCTTCGGCCCGTAAACCCCTGTCATTTGTGAACAAATTA
TTCCACCCAACACGTGGAGTATTGATAGTAACGGAAGAGGAAGGGACGGCTAACTCTGTG
CCAGCAGCCGCGGTAATACAGAGGTCCCAAGCGTTGTTCGGATTTACTGGGCGTAAAGGG
TACGTAGGCGGTTGGGTAAGTCTGACGTGAAATCTCCGGGCCTAACCCGGAAACTGCGTT
GGAAACTATCCAGCTAGAGGAATGGAGGGGAGACTGGAATACTTGGTGTAGCAGTGAAAT
GCGTAGATATCAAGTGGAACACCAGTGGCGAAGGCGAGCCTCTGGACAGCTCCTGACACT
CAGGGACGAAAGCTAGGGGAGCAAACGGGATTAGATACCCTGTAGTCC
>Otu5319
CCAGCCTACGGGGGGCAGCAGTCGAGAATTTTTCACAATGGGCGCAAGCCTGATGGAGCG
ACGCCGCGTGGGGGATGAATGGCTTCGGCCCGTAAACCCCTGTCATGTGCGATCAATGCT
GTATGGTGAATAATTGTACAGTTGATAGTAACGCAAGAGGAAGGGACGGCTAACTCTGTG
CCAGCAGCCGCGGTAATACAGAGGTCCCGAGCGTTGTTCGGATTCACTGGGCGTAAAGGG
TGCGTAGGCGGCTGGGTAAGTCTGATGTGAAATCTCCGGGCCCAACCCGGAAACGGCATT
GGATACTATCCGGCTCGAGGTTTGGAGGGGGGACTGGAATTCTCGGTGTAGCAGTGAAAT
GCGTAGATATCGAGAGGAACACCAGTGGCGAAGGCGGGTCCCTGGACAACACCTGACGCT
GAGGCACGAAAGTCGGGGGAGCAAACAGGATTAGAGACCCTAGTAGTCC
>Otu5325
CCAGCCTATGGGGCGCTCCAGTGGGGAATCTTGCACAATGGGGGAAACCCTGATGCAGCG
ACGCCGCGTGAGCGATGAAGCCCTTCGGGGTGTAAAGCTCTTTCGGCAGGGACGATAATG
ACGGTACCCGATGAATAAGCACCGGCTAACTACGTGCCAGCAGCCGCGGTAAGACGTAGG
GTGCGAGCGTTGTCCGGATTTACTGGGCGTAAAGGACGCGTAGGCGGTCTGTTTAGTGTG
AAGTGAAATCTCCAGAGCTCAACTCGGAAACTGCTTCGCATACTGGCAGACTAGAGGGAT
GCAAAGGTAGCTAGAATTCCTGGTGTAGCGGTGAAATGCGTTGATATCAGGAGGAATACC
CATGGCGAAGGCAGGCTACTGGGCATCATCTGACGCTGAGGCGTGAAAGCGTGGGTAGCA
AACAGGATTAGATACCCTGGTAGTCC
>Otu5326
CCAGCCTACGGGGCGCACCAGTCGAGAATTTTTCTCAATGGGCGAAAGCCTGAAGGAGCG
ACGCCGCGTGGGGGATGAATGGCTTCGGCCCGTAAACCCCTGTCATTCGGGATCAATGCT

TTCGACCTAATACGTCGAGAGTTGATAGTACCGAAAGAGGAAGAGACGGCTAACTCTGTG
CCAGCAGCCGCAGTAATACAGAGGTCTCAAGCGTTGTTCGGATTCACTGGGCGTAAAGGG
TGCGTAGGTGGCGGGGCAAGTCTGACGTGAAATCTCCGGGCCTAACCCGGAAACTGCGTT
GGAAACTGCCTTGCTAGAGGATTGGAGGGGAGACTGGAATACTTGGTGTAGCAGTGAAAT
GCGTAGATATCAAGTGGAACACCAGTGGCGAAGGCGAGTCTCTGGACAATTCCTGACACT
GAGGCACGAAAGCTAGGGGAGCAAACGGGATTAGATACCCTTGTAGTCC
>Otu5327
CCAGCCTATGGGGCGCAGCAGTTGGGAATCTTGGACAATGGGGGAAACCCTGATCCAGCC
ATGCCGCGTGAGTGATGAAGGCCTTAGGGTTGTAAAGCTCTTTTACCCGGGAAGATAATG
ACTGTACCGGGAGAATAAGCTCCGGCTAACTTCGTGCCAGCAGCCGCGGTAATACGAAGG
GGGCTAGCGTTGTTCGGAATTACTGGGCGTAAAGCGCGCGCAGGCGGTTCTCCAAGTCAG
TGGTGAAAGCCCAGAGCTCAACTCTGGAACTGCCATTGAAACTGTTGAACTTGAGGACGG
GAGAGGTGAGTGGAATTCCCAGTGTAGAGGTGAAATTCGTAGATATTAGGAAGAACACCA
GTGGCGAAGGCGGCCAACTGGCCCGATACTGACGCTCATGTGCGAAAGCGTGGGGAGCAA
ACAGGATTAGATACCCTTGTAGTCC
>Otu5330
CCAGCCTATGGGTCGCTCCAGTGGGGAATCTTGCACAATGGGGGAAACCCTGATGCAGCG
ACGCCGCGTGAGCGATGAAGCCCCTCGGGGTGTAAAGCTCTTTCGGCAGGGAAGACGATG
ACGGTACCTGCAGAAGCAGCTGCGGCTAACTACGTGCCAGCAGCCGCGGTAATACGTAGG
CAGCGAGCGTTGTTCGGAGTTACTGGGCGTAAAGGGTGTGTAGGCGGTTGTTTAAGTTTG
GTGTGAAATCTCCCGGCTCAACCGGGAGGGTGCGCCGAATACTGAGCGACTAGAGTGCGG
GAGAGGAGAGTGGAATTCCTGGTGTAGCGGTGAAATGCGTAGATATCGGGAGGAACACCT
GTGGCGAAAGCGGCGCACTGGACCACTTTTGACGCTGATGCGCGAAAGCTAGGGGAGCAA
ACAGGATTAGAGACCCCAGTAGTCC
>Otu5331
CCAGCCTACGGGGGGCAGCAGCTAGGAATATTGGGCAATGGACGAAAGTCTGACCCAGCG
ACACCGCGTGGAGGATGACAGCCCTCGGGTTGTAAACTCCTTTTAATAGGCCTTTCACGG
CTTATAGAATAAGCCCCTACTAACTACGTGCCAGAAGTCTCGGTAATACGTAGGGGGCAA
GCGTTATCCGGATTTATTGGGCGTAAAGTGTGCGTAGGCGCCTCGACAAGTCGTATCTTA
AAGACCAAGGCTTAACCTTGGGAGTGGATACGATACTGCCGAGGTTGAGGATTGTCAGGG
ATGTCGGAACAGTTAGTGTAGCAGTGAAATGCGTTGATATTAACTGGAACACCAAAGGCG
AAGGCAGACATCTGGGATTTTCCTGACGCTGAGGCACGAAAGCTAGGGTAGCGAAACAGA
TTAGAAACCCCTGTAGTCC
>Otu5333
CCCAGCCTACGGGGGGCAGCAGCGGGGAATTTTGGACAATGGGGGAAACCCTGATCCAGC
GATGCCGCGTGTGTGAAGAAGGCCTTCGGGTTGTAAAGCACTTTAGTTGGGGAAGAGAGG
TTATTTGCGAATAGTGGATGACTGAGACATTACCCAAAGAATAAGCACCGGCAAACTCTG
TGCCAGCAGCCGCGGTAATACAGAGGGTGCAAGCGTTAATCGGAGTGATTGGGCGTAAAG
GGCGCGTAGGCGGTGAGCTAAGTTAGTTGTGAAATACCAGAGCTTAACTTTGGGGAGTCA
ACTAAGACTGGCACACTAGAGTACAGTAGAGGGGAGTGGAATTTCCGGAGTAGCGGTGAA
ATGCGTAGATACCGGAAGGAACACCAGAGGCGAAGGCGACTCTCTGGGCTGTAACTGACG
CTGAGGCGCGAAAGCGTGGGTAGCAAACAGGATTAGAGACCCCTGTAGTCC
>Otu5335
CCAGCCTACGGGGGGCTCCAGTGGGGAATATTGGACAATGGGGGAAACCCTGATCCAGCA
ATGCCACGTGTGCGAAGAAGGCCTTAGGGTTGTAAAGCACTTTAGTAGGGGAGCAAGGTC
ATGACATTAATACCGTCATGATTTGAGAGTACCCTAAGAATAAGCACCGGCAAACTCTGT
GCCAGCAGCCGCGGTAATACAGAGGGTGCAAGCGTTAATCGGAATTACTGGGCGTAAAGG
GCTTGTAGGCGGTTAATTAAGTTAGATGTGAAATACCTGGGCTTAACCTGGGAATGGCAT
TTAATACTGGTTAGCTAGAGTATGGTAGAGGAAAGGGGAATTTCCGGTGTAGCAGTGAAA
TGCGTAGATATCGGAAGGAACATCAGTGGCGAAGGCGCCTTTCTGGACTAATACTGACGC
TGAGGAGCGAAGGCGTGGGGAGCAAACAGGATTAGATACCCGCGTAGTCC
>Otu5338
CCAGCCTATGGGGTGCAGCAGTAGGGAATTTTCCGCAATGGGCGAAAGCCTGACGGAGCA
ACGCCGCGTGAGGGATGAAGGGTTTCGGCTTGTAAACCTCTTTGGAGCGAGACGAACGGG
TATCCAGTTAACAATTGGGTACCGTGACTGTATCGCTAGAACAAGTGACGGCTAACTCTG
TGCCAGCAGCCGCGGTGATACAGAGGTCACGAACGTTGTCCGGAATTATTGGGCGTAAAG
GGCGCGTAGGTGGTTAGATAAGTCAGATGTGAAAGCTCTGGGCTCAACCCAGGAATTGCA
TTTGATACTGTTTGACTTGAGTACGGTAGAGGAGAGTGGAATTCCCAGTGTAGCGGTGAA

ATGCGTAGATATTGGGAGGAACACCGGTGGCGAAGGCGGCTCTCTGGACCGAAACTGACA
CTGAAGCGCGAAAGCCAGGGGAGCAAACGGGATTAGAAACCCCAGTAGTCC
>Otu5339
CCAGCCTACGGGTGGCAGCAGTGGGGAATTTTGCGCAATGGGGGAAACCCTGACGCAGCA
ACGCCGCGTGGAGGATGAAGTCCTTCGGGACGTAAACTCCTTTCGATCGGGACGATTATG
ACTGTACCGGGAGAATAAGCTCCGGCTAACTTCGTGCCAGCAGCCGCGGTAATACGAAGG
GGGCTAGCGTTGTTCGGAATTACTGGACGTAAAGCGCGCGCAGGCGGCCAAACAAGTCAG
GGGTGGAAGCCCAGAGCTCAACTCTGGAACTGCCTTTGAAACTTTTTGGCTAGAGGACGG
GAGAGGTGAGTGGAATTCCCAGTGTAGAGGTGAAATTCGTAGATATTGGGAAGAACACCG
GTGGCGAAGGCGGCTCACTGGCCCGTTTCTGACGCTCAGGCGCGACAGCGTGGGGATCAA
ACAGGATTAGATACCCTCGTAGTCC
>Otu5345
CCCAGCCTATGGGGCGCTGCAGTGGGGAATATTGGTAATCTGCGAAAGCGGGAACCAGCA
ACGCCGCGTGTGCGATGAAGGCCTTCGGGTCGTAAAGCACTTTTTGAGGAGACGAGGAAG
GACGGTATCCTCAGAATAAGTCTCGGCTAACTACGTGCCAGCAGCCGCGGTAATACGTAG
GGTGCAAGCGTTAATCGGAATTACTGGGCGTAAAGCGTGCGCAGGCGGTTTTGTAAGACA
GATGTGAAATGCCCGGGCTTAACCTGGGAACTGCATTTGTGACTGCAAGGCTAGAATCTG
GCAGAGGGGGGTAGAATTCCACGTGTAGCAGTGAAATGCGTAGAGATGTGGAGGAATACC
GATGGCGAAGGCAGCCCCCTGGGCCAAGATTGACGCTCATGCACGAAAGCGTGGGGAGCA
AACAGGATTAGAAACCCCCGTAGTCC
>Otu5347
CCAGCCTATGGGATGCTCCAGTCGAGAATCTTTCACAATAGGGGCAACCCTGATGGAGCG
ACGCCGCGTGGAGGATAAGGTCTTCGGATTGTAAACTCCTGTCATGTGGGAGCAAGGCAC
ACCAGTTAACACCTGGTTTGTTTGATAGTACCACAAGAGGAAGAGACGGCTAACTCTGTG
CCAGCAGCCGCGGTAATACAGAGGTCTCAAGCGTTGTTCGGAATCACTGGGCGTAAAGGG
TGTGCAGGCTGCGTGTTAAGTCAGATGTGAAAGCCCGGGGCTCAACCCCGGAACTGCATC
CGATACTGGCATGCTAGAGAATTGGAGAGGAGTCTAGAATTCACGGTGTAGCAGTGAAAT
GCGTAGATATCGTGAGGAATACCAAGTGCGAAGGCAGGACTCTGGACATTTTCTGACGCT
GAGACACGAAGGCCAGGGGAGCAAACGGGATTAGAACCCCAGTAGTCC
>Otu5353
CCAGCCTATGGGTGGCACCAGTAGGGAATTTTGCGCAATGGACGAAAGTCTGACGCAGCA
ACACCGTGTGAGGGATGAAGCATTTCGGTGTGTAAACCTCTGTCGATGATGAATAAGGTT
TCGGGGAGTGGAAAGCCTCGGGATTGAAGGTAGTCATGAAGGAAGCACCGGCTAACTCTG
TGCCAGCAGCCGCGGTAATACAGGGGGTGCAAGCGTTGTTCGGAATTACTGGGCGTAAAG
GGAGCGTAGGCGGAACTGCAAGACAGGGGTCAAATCTCCGGGCTCAACCCGGAACTTGCC
TTTGTGACTGCGGTTCTTGAGTATGGTAGAGGTCAGTGGAATTTCCGGTGTAGCGGTGGA
ATGCGTAGAGATCGGAAAGAACACCAGAGGCGAAGGCGACTGACTGGGCCAATACTGACG
CTGAGGCTCGAAAGCGTGGGGAGCAAACAGGATTAGATACCCTTGTAGTCC
>Otu5356
CCAGCCTATGGGTCGCACCAGTGGGGAATTTTGTGCAATGGGGGAAACCCTGACACAGCG
ACACCGCGTGAGCGAAGAAGCCCTTTGGGGTGTAAAGCTCTGTCAGCTGGAACGAAAAAA
ATGACGGTACCAGCAGAGGAAGCATCGGCTAACTACGTGCCAGCAGCCGCGGTAAGACGT
AGGATGCGAGCGTTGTCCGGATTTATTGGGCGTAAAGGGTTCGTAGGCGGTTCGTTAAGT
TTGGTGTTAAAGATCGGGGCTCAACCCCGGGAGTGCACTGAATACTGGCGGACTGGAGTG
TGGTAGAGGCTAGTGGAATTCCCAGTGTAGCGGTGAAATGCGTAGATATTGGGAAGAACA
CCGGTGGCGTAGGCGACTAGCTGGGCCATAACTGACGCTGAGGAACGAAAGCTAGGGGAG
CAAACGGGATTAGATACCCGCGTAGTCC
>Otu5361
CCAGCCTACGGGAGGCACCAGTCAAGCACGAGCCTAAGATCCGACTCGGCCTGTGCCGAA
AAGCCACGGATGGACGAGCCATCAAAAGTCAGGTTGTCTAAGCTCTTCAAGAGGAACTTC
TTGTCGTAATCCAGCATGTGGAGACGACCTTCGAGGTCGGTGAAGCAGACGGTAACGGCC
TTGATGGCCTTTTCGTCGGTCAGCTCTTCCGACCGTCGCTCGCGCAGCTGATCCGCGGGC
GTTCGGGCGAGTCGTTCTTCCTTTAGCTTGAGATTCTTTATCTCAAGCTGGTCATAGGGC
AGCTGGTAGAATTCGCGCAGGGGTGAGTCCATTAGAGTTTCTCCATTCCTATCGTAGAGG
ACGATTGACTGAAGTTGCGGTCTGGTGGCTCAAATTAGATACCCCCGTAGTCC
>Otu5362
CCAGCCTACGGGTCGCTCCAGTCGAGAATCATTCGCAATGGGCGAAAGCCTGACGATGCG
ACGCCGTGTGAGCGATGAAGGCCTTAGGGTCGTAAAGCTCTTTCGCTTGGGAACAAGAGA

AGCTGACGAATAATCAGCGGATTTGAGGGTACCAGGTAAAGAAGCACCGGCTAACTCCGT
GCCAGCAGCTGCGGTAATACGGAGGGTGCGAGCATTAATCGGATTTATTGGGCGTAAAGG
GCGCGTAGGCGGGAAGCTAAGTCAGATGTGAAATCCCGGGGCTCAACCCCGGAACAGCAT
ATGAAACTATTTTTCTTGAGGGTAGTCGGAGAGAATGGAATTCCACATGTAGCGGTGAAA
TGCGTAGATATGTGGAAGAACACCGGTGGCGAAGGCGATTCTCTAGGTTATTCCTGACGC
TAAAGCGCGAAAGCAAGGGGATCAAACAGGATTAGAGACCCGAGTAGTCC
>Otu5363
CCAGCCTATGGGTCGCTGCAGTGGGGAATCTTGCACAATGGAGGAAACTCTGATGCAGCG
ACGCCGCGTGAGCGATGAAGCCCTTCGGGGTGTAAAGCTCTTTCGACGGGAACGATAATG
ACGGTACCCGGAGAAGAAGCTGCGGCTAACTACGTGCCAGCAGCCGCGGTAATACGTAGG
CAGCAAGCGTTGTTCGGAATTACTGGGCGTAAAGAGTGTGTAGGCGGTTTTCTATGTTCG
GTGTGAAATCTCCCGACTTAACTGGGAGGGTGCGCCGAAAACTGGAAGGCTTGAGTATGG
GAGAGGAAAGCGGAATTCCTGGTGTAGCGGTGAAATGCGTAGATATCAGGAAGAACACCA
GTGGCGTAGGCGACTAACTGGGCCGTAACTGACGCTGAGGAACGAAAGCCAGGGGAGCGA
ATGGGATTAGAAACCCTTGTAGTCC
>Otu5364
CCAGCCTATGGGGGGCACCAGTGGGGAATATTGGACAATGGGCGCAAGCCTGATCCAGCA
ATGCCGCGTGAGTGAAGAAGGCCTTCGGGTTGTAAAGCTCTTTTGTCAGGGAAGAAACGG
TGAGGGCTAATATCTCTTGCTAATGACGGTACCTGAAGAATAAGCACCGGCTAACTACGT
GCCAGCAGCCGCGGTAATACGTAGGGTGCAAGCGTTAATCGGAATTACTGGGCGTAAAGC
GTGCGCAGGCGGTTTTGTAAGTCTGTCGTGAAATCCCCGGGCTCAACCTGGGAATGGCGA
TGGAGACTGCAAGGCTAGAATCTGGCAGAGGGGGGTAGAGTTCCACGTGTAGCAGTGAAA
TGCGTAGATATGTGGAGGAACACCGATGGCGAAGGCAGCCCCCTGGGCTAAGATTGACGC
TCATGCACGAAAGCGTGGGGAGCAAGCAGGATTAGATACCCGTGTAGTCC
>Otu5366
CCAGCCTATGGGTGGCAGCAGCAGTAGTTCCAGTCACAGCTTCTGCTGAAGGCTGTGTTC
CGTTTTCGTCGGTCATAGCGCCATCTGTCTCGCCGTCTCGGAAACGGTACTCTCTCCGAT
GGCATTGATCAACGCGCTCATGCACAGAGCCCGAGTTGCATGCCGGTCGATCGAATCCTT
AGTCTTGCTGTATGACTCGACGCCCGTCCAGCGAAAGCGGGATCTTTCTGAAGGGCGAGT
CTGTAGAAAGGGATGCCCGGAATTGCAAGTAACGCTCGACGCCGAGCAGCGCGAGTTGGT
CAATGAACGCGAAAAGCGCAGCTTTACGGAGAGTACGCTCACTGCTGCTCGCGGACGCTG
GCGGGACACAGCCTCTTTGGGGCGCCATCGGCCAATCCGAAAGGTAAGCCCTGCTGAAGC
CTGAGAACAGAACACTTGGCACGGACGTCTCGACATTAGAGACCCCTGTAGTCC
>Otu5372
CCAGCCTATGGGGTGCTGCAGTGGGGAATAGTGGACAATAAACGAAGGTTTAATCCTAAC
ATGTCGCGTGAGTGAAGAGCGATCTGTTAAGCGACATTAGGCTTGAATTGACGGTTAACT
CTGTAAAACTCTGTCGTCGGTAAAAAATAATGATGTTAACCGAAAATTATATTTTTAATT
GAAACCCTGACTAACTTCGTGCCAGCAGTCGCGGTGAAACGGGGAGGGTGGGTGTTCTTT
GTATTGACTGGGCGTAAAGCGTGCGTAGAAGGTTGGATAAGTTTAAATCTAAATTTTTAA
TTGGTATAATTAAAATTTTTTTAAAAACCTTCCGACTAGAGTTATATGGAAAATTATAGA
TTATTGAGTGTAGGGGTGAAATCCGATAATATTCAAGGGGCTAACAACGATGAAAATAAT
AATTTATTTATAACTGACTTTGAGGCACTAAAGCGTAGGTATCGAATAGGATTAGATACC
CTAGTAGTCC
>Otu5376
CCAGCCTACGGGTCGCAGCAGTGAGGAATCTTGCGCAATGGGCGAAAGCCTGACGCAGCG
ACGCCGCGTGAAGGATGAAGGCCTTAGGGTTGTAAACTTCTGTTAAGTGGGAAAAATTAC
CCGTTTTTAATAGAAACGGGGGATGATGGTACCACTAGAGAAAGCACCGGCTAATCTCGT
GCCAGCAGCCGCGGTAATACGAGAGGTGCGAGCGTTATTCGGAATCATTGGGCGTAAAGG
GTGCGTAGACGGTATGCTAAGTCAGTTGTTAAATTTCCCGGCCTAACCGGGAACCTGCGA
TTGAAACTAGCGTACTAGAGGGTGGAAGAGAGAAGTGGAATTCTCGGAGTAGCGGTAAAA
TGCGTAGATATCGAGAGGAACACCGATGGCGAAGGCAGCTTCTTGGGCCACACCTGACGT
TGAGGCACGAAAGCGTGGGGAGCAAACAGGATTAGAGACCCGTGTAGTCC
>Otu5377
CCAGCCTATGGGGGGCAGCAGTGGGGAATCTTGCGCAATGGACGAAAGTCTGACGCAGCA
ACGCCGCGTGGGGGATGAAGGTTTTCGGATTGTAAACTCCTGTCACTGCAGAACAAGGAT
GGACGGTTGAATAGACCGTCCATTTGATGGTATGCGGAGAGGAAGGGACGGCTAACTCTG
TGCCAGCAGCCGCGGTAAGACAGAGGTACCGAGCGTTGTTCGGATTCATTGGGCGTAAAG
GGTGTGTAGGAGGTCGGGTAAGTCAGGTGTGAAATCTCACAGCTTAACTGTGAAACTGCG

CTTGATACTGCCCGGCTAGAGGATCGGAGGGGGTAACGGAATTTATGGTGTAGCAGTGAA
ATGCGTAGATATCATAAGGAACACCGGTGGCGAAGGCGGTTACTTGGAAGATTCCTGACT
CTGAAACACGAAAGCCAGGGGAGCAAACGGGATTAGAGACCCTTGTAGTCC
>Otu5378
CCAGCCTACGGGAGGCTGCAGACGAGAATATTCGACAATGGACGAAAGTCTGATCGAGCG
ACGCCGCGTGGAGGATGAAGTGCTTCGGCATGTAAACTCCTTTTGCCAGGGAAGAAGTTA
TTGACGGTACCTGGAGAATAAGAGGTTGCTAAACTCGTGCCAGCAGCAGCGGTAATACGA
GTGCCTCGAGCGTTATCCGGAATTATTGGGCGTAAAGGGTGCGTAGGTGGTTGTGTTAGT
CTCATGTCAAATCTCCCGGCTCAACCGGGAACCTGCATGGGAAACGGTACGACTTAGAGG
TTGGAAGAGGTCTCGCGGAACTCATGGTGTAGCGGTGAAATGCGTTGATATCATGGGGAA
CACCAAAAGCGAAGGCAGCAGACTGGTCCATACCTGACACTGAAGCACGAAAGCGTGGGT
CGCGAATAGGATTAGAAACCCCTGTAGTCC
>Otu5383
CCAACCTATGGGAGGCAGCAGTGGGGAATATTGGACAATGGGCGAAAGCCTGATCCAGCC
ATGCCGCGTGAGTGATGAAGGCCTTAGGGTTGTAAAGCTCTTTTGTCAGGGAAGATAATG
ACGGTACCTGAAGAATAAGCACCGGCTAACTTCGTGCCAGCAGCCGCGGTAATACGAAGG
GTGCTAGCGTTGTTCGGAATTACTGGGCGTAAAGCGCGCGTAGGCGGGCTGTCAAGTCAG
GGGTGAAATCCCGGAGCTCAACTCCGGAACTGCCTTTGAAACTGTCAGCCTGGAGTGTGT
GAGGGGATAGCGGAATTCCAAATGTAGAGGTGAAATTCGTAGATATTTGGAGGAACACCG
GTGGCGAAGGCGGCTATCTGGCACACAACTGACGCTGAGGTGCGAAAGCGTGGGGATCAA
ACAGGATTAGATACCCGAGTAGTCC
>Otu5384
CCAGCCTACGGGGGGCAGCAGTGGGGAATATTGGACAATGGGGGCAACCCTGATCCAGCA
ATGCCGCGTGGATGAAGAAGGTCTTCGGATTGTAAAGTCCTTTCGACGGGGACGATGATG
ACGGTACCCGTAGAAGAAGCCCCGGCTAACTTCGTGCCAGCAGCCGCGGTAATACGAAGG
GGGCTAGCGTTGCTCGGAATGACTGGGCGTAAAGGGCGCGTAGGCGGCTTGAACAGTCAG
GCGTGAAATTCCTGGGCTTAACCTGGGGGCTGCGCTTGATACATTTGGGCTTGAGTGGGG
AAGAGGGTCGTGGAATTCCCAGTGTAGAGGTGAAATTCGTAGATATTGGGAAGAACACCG
GTGGCGAAGGCGGCGACCTGGTCCTTGACTGACGCTGAGGCGCGAAAGCGTGGGGAGCAA
TCAGGATTAGATACCCGCGTAGTCC
>Otu5388
CCAGCCTATGGGATGCAGCAGTAGGGAATATTGCGCAATGGAGGAAACTCTGACGCAGCG
ACGCCGCGTGGGTGACGAAGGCTTTCGGGTCGTAAAACCCTGTCGGAAGGAAAGAAAAGC
GAGCTGGCTAATATCCAGCTCGATCGACGGTACCTTTAAAGGAAGCACCGGCTAACTACG
TGCCAGCAGCCGCGGTAATACGTAGGGTGCAAGCGTTGTTCGGAATCATTGGGCGTAAAG
CGCGTGTAGGTGGTTGGCTAAGTCGATTGTAAAAGCCCTAGGCTCAACCAAGGAAGTGCA
TTCGAAACTAGCCAACTAGAGGATGGCAGAGGAAGGTAGAATTCCAAGTGTAGAGGTGAA
ATTCGTAGATATTTGGAGGAATACCGGCGGCGAAGGCGGCCTTCTGGACCATTCCTGACA
CTGAGACGCGAAAGCGTGGGGAGCAAACAGGATTAGAAACCCCCGTAGTCC
>Otu5391
CCAGCCTATGGGTGGCTCCAGTAACGAATCTTCCGCAATGCACGCAAGTGTGACGGAGCG
ACGCCGCGTGTGGGACGAAGCCCTTCGGGGTGTAAACCACTGTCAGGGATAGGAAAGTTC
TGATCCATCCCAGAGGAAGGCACGGCTAACTCTGTGCCAGCAGCCGCGGTAAGACAGAGG
TGCCAAGCGTTAGGCGGAATCACTGGGCTTAAAGCGTGTGTAGGCGGATCGTTAAGTACC
TTGTGAAATCCCCCGGCTCAACCGGGGAACTGCTGGGTATACTGGCGATCTTGAGCCACT
CAGGGGCAGATGGAACAGACGGTGGAGCGGTGAAATGCGTAGATATCGTCTGGAACGCCA
AAGGTGAAAACAGTCTGCTGGGGGTGTGCTGACGCTGAGACACGAAAGCCAGGGGAGCAA
ACGGGATTAGAAACCCGTGTAGTCC
>Otu5393
CCAGCCTACGGGACGCTCCAGTCGAGAATTTTTCACAATGGACGAAAGTCTGATGGAGCG
ACGCCGCGTGGAGGATGAAGGTTTTCGGATTGTAAACTCCTGTCACTGCAGAACAAGGCA
GTGCGTATGAATAGTACGTGCTGTTGATGGTATGCGGAGAGGAAGGGACGGCTAACTCTG
TGCCAGCAGCCGCGGTAATACAGAGGGTGCGAGCGTTAATCGGAATTACTGGGCGTAAAG
CGCGCGTAGACGGTTTTGTAAGTCAGATGTGAAATCCCTGGGCTCAACCTGGGAACTGCA
TTTGAGACTGCATGGCTAGAGTATCGAAGAGGGAAGTGGAATTTCCGGTGTAGCGGTGAA
ATGCGTAGATATCGGAAGGAACACCAGTGGCGAAAGCGACTTCCTGGTCGAATACTGACG
TTCATGTGCGAAAGCGTGGGGAGCAAACAGGATTAGAAACCCCAGTAGTCC
>Otu5403

CCAGCCTACGGGGTGCACCAGTGGGGAATATTGGACAATGGGGGAAACCCTGATCCAGCA
ATGCCGCGTGTGTGAAGAAGGCCTGAGGGTTGTAAGCACTTTCGTTGGGGAGGAAAGACT
CGATACTAATACTATTGAGGGATGACGCTACCCAAATAAGAAGCACCGGCTAACTCTGTG
CCAGCAGCCGCGGTAATACAGAGGGTGCGAGCGTTAATCGGAATTACTGGGCGTAAAGAG
CACGTAGGTTGGTAGATATGTTGGCTGTGAAATTCCTGAGCTTAACTTGGGAAGGTCAGC
CAAAACTGTCTATCTAGAGTATGGTAGAGGAAAGTGGAATTTCAGGTGTAGCGGTGAAAT
GCGTAGATATCTGAAGGAACATCAGTGGCGAAGGCGACTTTCTGGACTAATACTGACACT
GAGGTGCGAAAGCGTGGGGAGCAAACAGGATTAGAGACCCGAGTAGTCC
>Otu5404
CCGCCTACGGGGCGCACCAGTCGAGAATTTTTCACAATGGACGAAAGTCTGATGGAGCGA
CGCCGCGTGGAGGATGAAGGTTTTCGGATTATAAACTCCTGTCACTGCAGAACAAGGGTG
TGTGGTTGAACATTCCACACATTTGATGGTATGCGGAGAGGAAGGGACGGCTAACTCTGT
GCCAGCAGCCGCGGTAAGACAGAGGTCCCGAGCGTTGTTCGGATTCATTGGGCGTAAAGG
GTGTGTAGGAGGCTGGGTAAGTCGGGTGTGAAATCTCGCAGCTTAACTGCGAAACTGCGC
TTGATACTGCTCGGCTAGAGGATCGGAGGGGGTAACGGAATTTATGGTGTAGCAGTGAAA
TGCGTAGATATCATAAGGAACACCGGTGGCGAAGGCGGTTACCTGGAAGATTCCTGACTC
TGAAACACGAAAGCCAGGGGAGCAAACGGGATTAGAGACCCTTGTAGTCC
>Otu5406
CCAGCCTACGGGAGGCAGCAGTGGGGAATATTGGACAATGGGGGCAACCCTGATCCAGCG
ATGCCGCGTGAGTGAAGAAGGCCTTCGGGTCGTAAAGCTCTTTAGTCCGGGAAGAAGGTG
AAGTGGTGCATAGCCACGTTGCTTGACGGTACCGGAAGAATAAGCACCGGCAAACTCTGT
GCCAGCAGCCGCGGTAATACAGAGGGTGCGAGCGTTAATCGGAATTACTGGGTGTAAAGG
GCGCGTAGGCGGCAAGATGTGTGTGATGTGAAAGCCCCGGGCTTAACCTGGGAAGCGTAT
TGCAAACTGTCTGGCTGGAGTACATGAGAGGGTGGCGGAATTTCCGGTGTAGCGGTGAAA
TGCGTAGAGATCGGAAGGAACGTCAATGGCGAAGGCAGCCACCTGGCATGATACTGACGC
TGAGGCGCGAAAGCGTGGGGAGCGAACAGGATTAGATACCCTCGTAGTCC
>Otu5411
CCAGCCTACGGGGGGCTGCAGTAGGGAATATTGCGCAATGGAGGAAACTCTGACGCAGCG
ACGCCGCGTGGGTGACGAAGGCTTTCGGGTCGTAAAACCCTGTCGGAAGGGACGAATTGC
CATCTGATTAATACGCAGGTGGAGTGACGGTACCTTTAAAGGAAGCACCGGCTAACTACG
TGCCAGCAGCCGCGGTAATACGTAGGGTGCAAGCGTTGTTCGGAATTATTGGGCGTAAAG
CGCGTGTAGGTGGTTAATTAAGTCGATTGTAAAAGCCCTAGGCTCAACCAAGGAAGTGCA
TTCGAAACTAGTTGACTAGAGGACGGCAGAGGAAGGTGGAATTCCAAGTGTAGAGGTGAA
ATTCGTAGATATTTGGAGGAATACCGGCGGCGAAGGCGGCCTTCTGGACCGTTCCTGACA
CTGAGACGCGAAAGCGTGGGGAGCAAACAGGATTAGATACCCCCGTAGTCC
>Otu5413
CCAGCCTACGGGTTGCACCAGTGGGGAATCTTGCGCAATGGACGAAAGTCTGACGCAGCA
ACGCCGCGTGAGTGAAGAAGGCCTTCGGGTTGTAAAGCTCTGTCGAGCGGGACGAAAAGT
CTTTAAGTTAATAGCTTAGAGATGTGACGGTACCGCTAAAGGAAGCACCGGCTAACTCTG
TGCCAGCAGCCGCGGTAATACAGAGGGTGCTAGCGTTGCTCGGAATTATTGGGCGTAAAG
GGCAGGTAGGTGGTCTCGTTTGTCTGAGGTGAAAGCCCTGGGCTTAACCTAGGAAGTGCC
TCGGAAACGGCGAGACTAGAGTCTTGGAGAGGGTCGCGGAATACCCGGTGTAGCGGTGAA
ATGCGTAGAGATCGGGTGGAATACCAGAGGCGAAGGCGGCGACCTGGACAAGAGCTGACA
CTCAACTGCGAAAGCGTGGGGAGCAAACAGGATTAGAGACCCGCGTAGTCC
>Otu5416
CCAGCCTACGGGGGGCTGCAGTGGGGAATCTTGCGCAATAGGCGAAAGCCTGACGCAGCA
ACGCCGCGTGGGGGATGAAGCTTTTCGGAGTGTAAACCCCTTTCGACCCGGAAGAAGGCC
CGCAAGGGTCCGACGGTACGGGTATAAGAAGCCCCGGCTAACTACGTGCCAGCAGCCGCG
GTAAAACGTAGGGGGCCAGCGTTGCTCGGAATTACTGGGCGTAAAGAGTTGCGTAGGTGG
CATTGTAAGCAAGTAGTGAAAGCGTCCGGCTCAACCGGATAAACATTACTTGAACTGCAA
AGCTAGAGGATGAGAGAGGTTATTGGAATTCCCTGCGTAGGAGTGAAATCCGTAGATATA
GGGAGGAACACCGATGGCGTAGGCAGATAACTGGCTCATTCCTGACACTAAGGCACGAAA
GCGTGGGTAGCAAACGGGATTAGATACCCTTGTAGTCC
>Otu5418
CCAGCCTATGGGGGGCTGCAGTGGGGAATATTGGACAATGGGGGAAACCCTGATCCAGCC
ATGCCGCGTGAATGATGAAGGCCTTCGGGTTGTAAAGTTCTTTTGACAGGGAAGATGATG
ACGGTACCTGTAGAATAAGCTCCGGCTAACTTCGTGCCAGCAGCCGCGGTAATACGAAGG
GAGCTAGCGTTGTTCGGAATTACTGGGCGTAAAGGGCGCGTAGGCGGTTTAGCAAGTTGG

ATGTGAAAGCCCAGGGCTCAACCCTGGAATTGCATTCAAGACTACTTTACTTGAATTCGG
TAGAGGTTGGTAGAATTCCCAGTGTAGAGGTGAAATTCGTAGAGATTGGGAAGAATACCC
GTGGCGAAGGCGGCCAACTGGACCGACATTGACGCTGAGGCGCGAAAGCGTGGGGAGCAA
ACAGGATTAGAAACCCGGGTAGTCC
>Otu5422
CCAGCCTATGGGGTGCTGCAGTAGGGAATATTGCGCAATGGGCGAAAGCCTGACGCAGCG
ACGCCGCGTGAGTGATGAAGGCCTTAGGGTCGTAAAGCTCTGTTGTACGGGAAGAAAAAA
ATGACGGTACCGTATAAGAAAGGATCGGCTAACTTCGTGCCAGCAGCCGCGGTAATACGA
GGGATCCTAGCGTTGTTCGGAATTATTGGGCGTAAAGGGTGCGTAGGTGGCCTTGATAGT
CAGTTGTGAAATCCCCGGGCTCAACCCGGGAAGTGCTTCTGATACTACAAGGCTTGAGTA
CTGCAGAGGTGAGTGGAATTCCAGGTGTAGTGGTGAAATACGTAGATATCTGGAGGAACA
CCGGTGGCGAAGGCGGCTCACTGGGCATGTACTGACACTGAAGCACGAAAGCGTGGGGAG
CAAACAGGATTAGAAACCCCCGTAGTCC
>Otu5425
CCAGCCTATGGGGGGCTGCAGTTCGGAATATTCGGCAATGGGCGAAAGCCTGACCGAGCG
ACGCCGCGTGCGGGATGAAGGCCCTTTGGGTTGTAAACCGCTGTCACAGGTGAGCAAATC
ATTCTGGCTGAATAATCCAGGATGTTGAGTTAAGCTTGAGAGGAAGCCCCCGCTAATCAC
GTGCCAGCAGCGGCGGTAATACGTGAGGGGCGAACGTTGTTCGGTGTCACTGGGCTTAAA
GGGTGCGTAGGTGGCTGCGTAAGTAAGGTGTGAAAGCCCTCGGCTTACCCGGGGAATTGC
GCCTTATACTGCGCGGCTTGAGGATTTCAGGGGAAAGGGGAACTCCAGGTGTAGCGGTGA
AATGCGCAGATATCTGGGGGAAGGCCGGCGGCGAAGGCGCCTTTCTGGGAAATTCCTGAC
ACTGAGGCACGAAAGCGTGGGGAGCAAACAGGATTAGAGACCCTTGTAGTCC
>Otu5432
CGGGGGGCAGCAGCGAGGAATATTGGACAATGGGCGAAAGCTTGATCCAGCAATATCACA
TGAGAGAAGAAGACTTTTTGTTGTAAATCTATTGCGTTAAGGAGGATAATGACGAATACT
TAAAGAAGAAGTCCCGGCTAACTTCGTGCCAGCAGCCGCGGTAATACGGAGGGGACGAGT
GTTACTCATAATAACTGGGCGTAAAGGGTATGTAGGTGGTTTATTTAGTTAAATAAAAAA
AAATAAATTAATAGTTTATAAAATTATTTAAAACGTATAAACTAGAGTATAAGAGAGGAA
TATAGAATTTTTAGTGTAAGGGTGAAATCTAACGATAATAAAAAGACTATCAAATGCGAA
GGCAATATTCTAGATTATTACTGACACTGAGGTACTGAAGCATGGGGAGCAAATCGGATT
AGAAACCCCCGTAGTCC
>Otu5441
CCAGCCTATGGGAGGCTGCAGTAGGGAATCTTCCGCAATGGGCGAAAGCCTGACGGAGCA
ACGCCGCGTGAGTGAAGAAGGTCTTCGGATCGTAAAACTCTGTTATTAGGGAAGAACAAA
TGTGTAAGTAACTATGCACGTCTTGACGGTACCTAATCAGAGAGCCACGGCTAACTACGT
GCCAGCAGCCGCGGTAATACGTAGGTGGCAAGCGTTATCCGGAATTATTGGGCGTAAAGC
GCGCGTAGGCGGTTTTTTAAGTCTGATGTGAAAGCCCACGGCTCAACCGTGGAGGGTCAT
TGGAAACCGGAAAACTTGAGTGCAGAAGAGGAAAGTGGAATTCCATGTGTAGCGGTGAAA
TGCGCAGAGATATGGAGGAACACCAGTGGCGAAGGCGACTTTCTGGTCTGTAACTGACGC
TGATGTGCGAAAGCGTGGGGATCAAACAGGATTAGATACCCCCGTAGTCC
>Otu5443
CCAGCCTACGGGGCGCACCAGATGACCTGATCCTTGTCCAGGCGAATAATCCGGGCGGAG
CACTACCACAAGGAGTGAGTTCAAGCTGAGAGCCCCTTGAGAAGAGGCTGTACGAACTCG
TTGGCTCGGTGAATGTGTCGCGATACGTGAGCGAATCCCAATTAAAAGATGCGTGACCGC
GCGGGCAAGGGAGAGAGGTGGAATTTCGCTCATATGCCCCAAGGATTTCTCTCAGACGAC
TATTTTCTTGTCTAACGCGTTGCAATGGCGTCATCGTCACCATCTCTGGGTAGACGCAGT
GCTTGGGATCGCGGCATCGCTTGCAGGACGGCGTGGTCTTGTCGCATCTCTGGTAATTGA
ACTGTTGTCGATACGAAAAGAACGCCGATGGATTAGATACCCTGGTAGTCG
>Otu5455
CCAGCCTACGGGACGCTGCAGCCGAGAATCATTCGCAATGGGCGAAAGCCTGACGATGCG
ACGCCGTGTGAGCGATGAAGGCCTTCGGGTTGTAAAGCTCTTTCGCTTGGGAACAAGAGA
AGCCGGAGAATACCCGGCGGATTTGAGGGTACCAGGTAAAGAAGCACCGGCTAACTCCGT
GCCAGCAGCTGCGGTAATACGGAGGGTGCAAGCATTAATCGGATTTATTGGGCGTAAAGG
GCGCGTAGGCGGTGAAATAAGTCAGATGTGAAATCCCGGGGCTCAACCCCGGAACTGCAT
TTGAAACTGTTTTTCTAGAGGGTGGGCGGAGAAAACGGAATTCCACAAGTAGCGGTGAAA
TGCGTAGATATGTGGAAGAACACCGGTGGCGAAGGCGGTTTTCTAGCTTACACCTGACGC
TAATGCGCGAAAGCAAGGGGATCAAACAGGATTAGAAACCCGAGTAGTCC
>Otu5456

CCAGCCTACGGGTCGCAGCAGTCGAGAATTCTTCTCAATGGGCGAAAGCCTGAAGGAGCG
ACGCCGCGTGGGGGATGAATGGCTTCGGCCCGTAAACCCCTGTCATTTGCGAACAAATTG
ACTCATCTAACACGTGTGTCATTGATTGTAGTAGAAGAGGAAGGGACGGCTAACTCTGTG
CCAGCAGCCGCGGTAATACAGAGGTCCCAAGCGTTGTTCGGATTCACTGGGCGTAAAGGG
TGCGTAGGCGGTTAGGTAAGTCTGATGTGAAAGCTCGGAGCTTAACTCCGAAATGGCATC
GGATACTACTTGGCTAGAGGAATGGAAGGGAGACTGGAATACTTGGTGTAGCAGTGAAAT
GCGTAGATATCAAGTGGAACACCAGTGGCGAAGGCGAGTCTCTGGACATTTCCTGACGCT
GAGACGCGAAAGCGTGGGGAGCAAACAGGATTAGAAACCCTAGTAGTCC
>Otu5460
CCAGCCTACGGGTGGCTGCAGTAGGGAATATTGCGCAATGGGCGAAAGCCTGACGCAGCA
ATTCCGCGTGGAGGATGAAGGTTCTTGGATTGTAAACTCCTTTCGGTGGTGACGATAATG
ACGGTAGCCACAGAAGAAGCACCGGCTAACTACGTGCCAGCAGCCGCGGTAAGACGTAGG
GTGCAAGCGTTGTCCGGAATTATTGGGCGTAAAGAGTTCGTAGGCGGTTTGTCAAGTCTG
ATGTTAAATACCGAGGCTCAACCTCGACATGGCATCGGAAACTGACAGGCTAGAGTATGG
TAGAGGTAAAGGGAATTCCCGGTGTAGCGGTGAAATGCGTAGATATCGGGAGGAACACCA
GTGGCGAAAGCGCTTTACTGGACCATTACTGACGCTGAGGAACGAAAGCCAGGGTAGCGA
AAGGGATTAGAGACCCTAGTAGTCC
>Otu5470
CCAGCCTACGGGTCGCTGCAGTCGAGAATTTTTCTCAATGGGCGAAAGCCTTAAGGAGCG
ACGCCGCGTGGGGGATGAATGGCTTCGGCCCGTAAACCCCTGTCATTTGCGAACAAATTA
GTCCGCCCAACACGCGGACTATTGATTGTAGCGGAAGAGGAAGGGACGGCTAACTCTGTG
CCAGCAGCCGCGGTAATACAGAGGTCCCAAGCGTTGTTCGGATTCACTGGGCGTAAAGGG
TGCGTAGGCGGTTGGGTAAGTTTGACGTGAAATCTCCGGGCCTAACCCGGAAACTGCGTT
GAATACTACCCGGCTAGAGGATTGGAAGGGAGACTGGAATACTTGGTGTAGCAGTGAAAT
GCGTAGATATCAAGTGGAACACCAGTGGCGAAGGCGAGTCTCTGGACAATTCCTGGCGCT
GAGGCACGAAAGCTAGGGGAGCAAACAGGATTAGATACCCGAGTAGTCC
>Otu5472
CAGCCTATGGGAGGCTCCAGTGGGGAATTTTTTGCAATAAGTAAAAGCCTAACGAAGCAA
TGTCGCGTGGAAGTAGAAGGCCCACAGGTCGTAAATTCCTTTTCTCGGAGAAGAAGTAAT
GACGATATTTGAGGAATAAGCATCGACTAACTTTACGCCAACAGTTGTGGTAAGACAAAG
GATGTAAGCATTATTCAAAATGATTGGGCGTAAAGCATCTGTAGGTGGCTATAAATCCGT
TGTCAAATCCCAAGGCTCAACCCTAGATAGGCGATGGAAACTACTGAGTTAAAGTATGGT
AGGGGCAGAAGGAATTTCTAGTGGAATGGTGAAATGCATAAAGATTGGAAAAAATACCAA
CGACAAAAGCACTCTGCTAAGCCGACATTAACATTGAAAGACGAGAGCTAGGGGAGCAAA
TGGGATTAGAAACCCGGGTAGTCC
>Otu5473
CCAGCCTATGGGGGGCAGCAGTAGGGAATATTGGACAATGGAGGCAACTCTGATCCAGCC
ATGCCGCGTGAATGATGAAGGCCCTAGGGTTGTAAAGTTCTTTTAGCGGGGAAGATAATG
ACGGTACCCGCAGAAAAAGCCCCGGCTAACTCTGTGCCAGCAGCCGCGGTAATACAGAGG
GGGCTAGCGTTGTTCGGAATTACTGGGCGTAAAGGGCGCGTAGGCGGCTTTCTAAGTTAG
GAGTGAAATCCCAGGGCTTAACCCTGGAATTGCTCTTAATACTGGAAGGCTTGAGATCGG
TAGGGGATAGTGGAATTCCTAGTGTAGGAGTGAAATCCGTAGATATTAGGAGGAACACCA
GTGGCGAAGGCGGCTATCTGGACCGATTCTGACGCTGAGGCGCGAAAGTGTGCGGAGCAA
ACAGGATTAGAAACCCGGGTAGTCC
>Otu5474
CCAGCCTACGGGGGGCTCCAGTCGAGGATCTTTCGCAATGGGCGAAAGCCTGACGAAGCG
ACGCTGTGTGAGCGATGAAGGCCTTCGGGTTGTAAAGCTCTTTCGCATAAGAACAAGAGA
GATCGGTGAACAACCGGTCGATTTGAGGGTATTATGTAAAGAAGCACCGGCTAACTCCGT
GCCAGCAGCTGCGGTAATACGGAGGGTGCAAGCATTAATCGGATTTATTGGGCGTAAAGG
GCGCGTAGGCGGATCTGAAAGTCAGATGTGAAATTCCGAAGCTCAACTTCGGAGCTGCAT
TTGAAACTCCAGATCTCGAGGAATGGCGGAGAAAATGGAATTCCACGTGTAGCGGTGAAA
TGCGTAGATATGTGGAAGAACACCAGTGGCGAAAGCGGTTTTCTAGCTTTTTCCTGACGC
TGAGGCGCGAGAGCAAGGGGAGCAAACAGGATTAGATACCCGTGTAGTCC
>Otu5479
CCAGCCTATGGGTCGCAGCAGTCAAGAATATTCCTCAATGGCCGAAAGGCTGAAGGAGCG
ACGCCGCGTGCAGGATGAAGGTCTTCGGATTGTAAACTGCTTTTATAAGGGAAGAATTTG
TGACGGTACCTTATGAATAAGAGGTTGCTAACTCTGTGCCAGCAGCAGCGGTAATACAGA
GACCTCAAACGTTATCCGGATTTATTGGGCGTAAAGCGTCCGCAGGTGGTTTAGCAAGTC

AGAGGTTAAAACCTATCGCTTAACGATAGGGCCGCTTTTGATACTACTACGCTTGAGGTC
GGAAGAGGTGAGCGGAATTCTCGGTGTAGTCGTAATAAGCGTTGATATCGAGAAGAACAC
CAAATGCGAAGGCAGCTCACTGGTACGTACCTGACACTCATGGACGAAAGCGTGGGGAGC
AAACAGGATTAGAAACCCCAGTAGTCC
>Otu5482
CCAGCCTATGGGTGGCAGCAGTCAAGAAACTTCCACAATGGACGAAAGTCTGATGGAGCG
ACGCCGCGTGGTTGATGAAGTCCTTCGGGACGTAAAAACCTTTTATGAGGGAGGAAGTAA
TTGACGTTACCTCATGAATAAGGGGCTCCTAACTCTGTGCCAGCAGGAGCGGTAATACAG
AGGCCCCAAGCATTATCCGGAATTACTGGGCGTAAAGAGTGTGTAGGCGGTGCTCTAAGT
TTGGTGTGAAATCTCCCGGCTCAACTGGGAGGGTGCGCCGAAAACTGGGGTGCTCGAGCG
TGGGAGAGGAAAGCGGAATTCCTGGTGTAGCGGTGAAATGCGTAGATATCAGGAGGAACA
CCTGTGGTGTAGACGGCTTTCTGGACCATTGCTGACGCTGAGACACGAAAGCGTGGGTAG
CAAACAGGATTAGATACCCTAGTAGTCC
>Otu5487
CCAGCCTATGGGTTGCTGCAGTGAGGAATCTTGCGCAATGGGCGAAAGCCTGACGCAGCG
ACGCCGCGTGGAGGATGAAGGTTTTCGAATTGTAAACTCCTGTTAAGCGGGAAGAAAGGC
TCATCTTTAATACAGATGAGACATGACGGTACCGCTAGAGAAAGCACCGGCTAACTTCGT
GCCAGCAGCCGCGGTAATACGAGGGGTGCAAGTGTTATTCGGATTAACTGGGCGTAAAGG
GTGCGCAGACGGTTTGTTAAGTCACTTGTTAAATTCTTCGGCCTAACTGAAGGTATGCAA
GTGATACTGGCGATCTAGAGAGTGGAAGAGAGAAGTGGAATTCTCGGAGTAGCGGTAAAA
TGCGTAGATCTCGAGAGGAACACCAATGGCGAAGGCAGCTTCTTGGTCCATTTCTGACGT
TCATGCACGAAAGCGTGGGGAGCAAACAGGATTAGATACCCTAGTAGTCC
>Otu5488
CCAGCCTATGGGTGGCACCAGCCAGGAATATTGCGCAATGGACGAAAGTCTGACGCAGCG
ACGCCGCGTGGAGGAAGACGGCCTTAGGGTTGTAAACTCCTTTTAGAAAGCCGTTCACAG
CTTTCTGAATAAGCCTCTACTAACTACGTGCCAGAAGTCTCGGTAATACGTAGGAGGCGA
GCGTTATCCGGATTTACTGGGCGTAAAGTGTGCGTAGGCGCCCCATTAAGTCGTGCTTTA
AATACTAAGGCTTACCCTTAGAGCTGAGTACGATACTGTTGGGGTTGAGGATTGACAGGG
CAGCTGGAACAGTACGTGTAGCAGTGAAATGCGTTGATATGTACTGGAACACCAAGGGCG
AAGGCAGGCTGCTGGGTTTTTCCTGACGCTGAGGCACGAAAGCTAGGGTAGCGAAACAGA
TTAGAAACCCCAGTAGTCC
>Otu5494
CCAGCCTATGGGTTGCTGCAGTAGGGAATATTGCGCAATGGAGGAAACTCTGACGCAGCG
ACGCCGCGTGGGTGATGAAGGTCTTCGGATCGTAAAGCCCTGTCGGACGGGAAGAAAAAC
GTTATGGCTAACATCCATAGCGCTTGACGGTACCGTAAGAGGAAGCCCCGGCTAACTTCG
TGCCAGCAGCCGCGGTAATACGAAGGGGGCAAGCGTTGTTCGGAGTCATTGGGCGTAAAG
CGCGTGTAGGCGGTCTGTTAAGTCGATTGTGAAATCCCTGGGCTCAACCGAGGAAGTGCA
GTCGAAACTAACAAACTAGAAGATGGTAGAGGAAGGTGGAATTCCAGGTGTAGAGGTGAA
ATTCGTAGATATCTGGAGGAATACCGGTGGCGAAGGCGGCCTTCTGGGCCATTCTTGACG
CTGAGACGCGAAAGCGTGGGTAGCAAACAGGATTAGAAACCCCTGTAGTCC
>Otu5496
CCAGCCTACGGGGCGCACCAGTTGGGAATCTTGCACAATGGGGGCAACCCTGATGCAGCG
ACGCCGCGTGGAGGATGAAGGATCTAGGTCTGTAAACTCCTTTTGTTGGGAAAGACTTAG
GACGGTACCCAACGAATAAGGACCGGCCAACTACGTGCCAGCAGCCGCGGTAAGACGTAG
GGTCCGAGCGTTGTCCGGATTTACTGGGCGTAAAGAGCGCGTAGGCGGCTCGTTAAGTGT
GAAGCTAAATCTCCAGGGCTCAACCCGGAAACTATTTCGCATACTGACGAGCTTGAGGAA
TGCAGAGGTGAATGGAATTCCTGGTGTAGCGGTGAAATGCGTTGATATCAGGAGGAACAC
CCATGGCGAAGGCAGTTCACTGGGCATTATCTGACACTGAGGCGCGAAAGCGTGGGTAGC
AAACAGGATTAGAAACCCTCGTAGTCC
>Otu5500
CAGCCTATGGGATGCTGCAGTGTGGAATATTGGACAATGAACGACAGTTCGATCCAGCAA
TATTGCGTGACGGAAGAAGGTCCTTTTACCGTAAATGTCTTTTAGCAAAGTATGAAAATG
ACAAAATATTGAAGAAGAAGCCCCGACTAACTTCGTGCCAGCAGTCGCGGTAAAACGGGG
GGGGCTAGTGTTATTCATCATTACTGGGTGTAAAACGTGTGAAGATTGCTTTTTAAGTAT
TTATTAAGATAATCTGAGCATAACTTAGAAGTTAATAAATAGACTATTTAGCTAGAGGTT
GAAAGGGGTAAGCTATATTTTCGAGGAAGAGATAAAATTCTTAGATCTTGGGGAAATAAC
CAGCAGGCGAAAGCCGCTTGCCATGACAACACTGACGTTGTAGCACAAAAGCGTGGGGAT
CAAACGGGATTAGAAACCCGTGTAGTCC

>Otu5503
CCAGCCTATGGGTTGCAGCAGTCAAGAACTTTCCACAATGGGCGAAAGCCTGATGGAGCG
ACGCCGCGTGGTTGATGAAGTCCTTCGGGACGTAAAGACCTTTTATGGGGGAGGAAGTAA
TTGACGTTACCCCATGAATAAGGGGCTCCTAACTCTGTGCCAGCAGGAGCGGTAATACAG
AGGCCCCAAGCGTTATCCGGAATCACTGGGCGTAAAGGGTGTGTAGGCGGTTGTGTTAGT
CGTCTGTGAAAGACCTTGGGCTTAACCCAGGGGACGCAAACGAAACGGCATGACTTAGAG
GATGTGAGAGGTACAGGGAACTCATGGTGTAGGGGTGAAATCCGTTGATATCATGGGGAA
CACCAAATGCGAAGGCACTGTACTGGCACACTCCTGACGCTGAAACACGAAAGCGTGGGA
ATCGAACGGGATTAGAAACCCTCGTAGTCC
>Otu5506
CCAGCCTATGGGTCGCAGCAGTCGAGAATCTTCCACAATGGACGAAAGTCTGATGGAGCG
ACGCCGCGTGACTGATGAAGTCCTTCGGGACGTAAAGGTCTTTTGTGAGGTAGAAAGTTA
TTGATCGCCTCAAGAATAAGGGGTTGCTAAACTCGTGCCAGCAGCAGCGGTAATACGAGT
GCCCCAAGCGTTGTCCGGAATTATTGGGCGTAAAGGGTGTGTAGGCGGTTGTGTTAGTCT
TTTGTTAAAGCTCCCGGCTCAACCGGGAAGGTGCAAAAGAAACGGCACAACTAGAGGGTG
CGAGAGGTGTACAGAACTCATAGTGTAGGGGTGAAATCCGTTGATATTATGGGGAATACC
AAAAGCGAAGGCAGTACACTGGCGCATTCCTGACGCTGAGACACGAAAGCGTGGGTAGCG
AATGGGATTAGAAACCCGTGTAGTCC
>Otu5507
CCAGCCTACGGGGTGCTCCAGTAAGGAATATTGGTCAATGGGCGGAAGCCTGAACCAGCC
ATGCCGCGTGCAGGAAGACGGCCCTACGGGTTGTAAACTGCTTTTGCAGGGGAATAAACC
CCCGTATGTATACGGGGTTGAATGTACTCTGAGAATAAGGATCGGCTAACTCCGTGCCAG
CAGCCGCGGTAATACGGAGGATCCGAGCGTTATCCGGATTTATTGGGTTTAAAGGGTGCG
TAGGCGGCCTGTTAAGTCAGGGGTGAAAGACGGTAGCTTAACTATCGCAGTGCCTTTGAT
ACTGACGGGCTTGAATGTAGTTGAGGCAGGCGGAATGTGACAAGTAGCGGTGAAATGCAT
AGATATGTCACAGAACACCGATTGCGAAGGCAGCTGACGAAACTGTAACTGACGCTGAGG
CACGAAAGCGTGGGGATCAAACAGGATTAGAAACCCTGGTAGTCC
>Otu5508
CCAGCCTACGGGTCGCAGCAGTGAGGGATTTTGCGCAATGGAGGAAACTCTGACGCAGCG
ACGCCGCGTGCGGGACGAAGCCTTTCGGGGTGTAAACCGCTGTCAGTGGGGAAGAGAATG
GACGGTACCCACTGAGGAAGCCTCGGCTAACTACGTGCCAGCAGCCGCGGTAAGACGTAG
GGGGCGAGCGTTGTCCGGAATCACTGGGCGTAAAGAGCTCGTAGGCGGGGTTGCACGTCT
TGGAGTAAATCCATCTGCTCAACGGATGGGTCGTCTGAGATACGGCAGCTCTTGAGGACA
GAAGAGGAGAGCAGAATTCCGGGTGTAGCGGTGAAATGCGTAGATATCCGGAGGAATACC
AGTGGCGAAGGCGGCTTTCTGGTCTGATTCTGACGCTGAGGAGCGAAAGCCAGGGGAGCG
AACGGGATTAGAAACCCGCGTAGTCC
>Otu5510
CCAGCCTACGGGTCGCTCCAGTCGAGGATCTTCGGCAATGGGCGCAAGCCTGACCGAGCG
ACGCCGCGTGCGCGATGAAGGCCTTCGGGCTGTAAAGCGCTGTCGAGGGGAAGAAAGCCG
CAAGGTTGATCGACCCCTGGAGGAAGCACGGGCTAAGTTCGTGCCAGCAGCCGCGGTAAG
ACGAACCGTGCAAACGTTATTCGGAATCACTGGGCTTAAAGGGCGCGTAGGCGGACATTC
AAGTCCGGGGTGAAATCCTCCGGCCTAACCGGAGAATAGCCTTGGATACTGGATGTCTCG
AGGGAGGTAGGGGCGTGCGGAACTTCCGGTGGAGCGGTGAAACGCGTAGATATCGGAAGG
AACGCCGGTGGCGAAAGCGGCGCGCTGGACCTCTTCTGACGCCGAGGCGCGAAAGCCAGG
GGAGCGAACGGGATTAGATACCCGCGTAGTCC
>Otu5521
CCAGCCTACGGGTGGCACCAGTGCCAGCAGATCGTGGGTATGCCGCCTTCTTCGAGGTAC
CAGCGAGTGGCGCGATCATTGACGAAGGTCCACCTTTTTGGATCGATGTAGTCCAAGCCG
AGGATGGCGGGCTGTTTGCCGGTGGTGCGCTGCAGGTAGTCAAGCTCGGAGCGGGGTCCG
CTCCAGGCTGAGACCTGCTGGCCAGTGAGGGTCTTGTGGCCGTAGATGCTCCAAAGATAG
GCATAGAGCTTGCGGGCACGAGGGCTCGCATGGGGATTGCTGAGGTGCCTTTTGTGGTGG
AAGTGCCAGGGGAAGATGGAAGCCCGCGCGGAGAGCGACAGAACGGAAGCGGCACCGGCG
AGAAATGAGCGGCGAGTCGGTGCGGTTGGCAAAGGCATTCTCCGAGGATAGCGACGATAG
CAAGGTATTAGATACCCTCGTAGTCC
>Otu5525
CCAGCCTATGGGGGGCACCAGTGGGGAATATTGGACAATGGGGGAAACCCTGATCCAGCG
ACGCCGCGTGCGTGAAGAAGGCCTGCGGGTTGTAAAGCACTTTCAGTGGGGAGAAAGAAG
TTCAGGTTAATACCTTGGATGGATGATGTTACCCACAGAAGAAGCACCGGCTAACTCTGT

GCCAGCAGCCGCGGTAATACGGAGGGTGCGAGCGTTAATCGGAATTACTGGGCGTAAAGG
GTGCGTAGGTGGTTAAATAAGTTAGGTGTGAAATTCCTGAGCTCAACTTGGGGACTGCGC
CTGATACTGTTTGGCTAGAGTACTGTAGAGGGAAGTGGAATTTCCGGTGTAGCGGTGAAA
TGCGTAGATATCGGAAGGAACATCGGTGGCGAAGGCGACTCCCTGGACAGATACTGACAC
TGAGGCACGAAAGCGTGGGGAGCAAACAGGATTAGAAACCCCCGTAGTCC
>Otu5527
CCAGCCTACGGGGTGCAGCAGTGGGGAATATAGGACAATGGGCGCAAGCCTGATCCAGCC
ATGCCGCGTGTGTGAAGAAGGCCTTCGGGTTGTAAAGCACTTTTGTCCGGAAAGAAATCC
TGATGGCTAATATCCGTCGGGGATGACGGTACCGGAAGAATAAGCACCGGCTAACTACGT
GCCAGCAGCCGCGGTAATACGTAGGGTGCGAGCGTTAATCGGAATTACTGGGCGTAAAGC
GTGCGCAGGCGGTGATGTAAGACCGATGTGAAATCCCCGGGCTTAACCTGGGAACTGCAT
TGGTGACTGCATCGCTAGAGTATGGCAGAGGGGGGTAGAATTCCACGTGTAGCAGTGAAA
TGCGTAGAGATGTGGAGGAATACCGATGGCGAAGGCAGCCCCCTGGGCCAATACTGACGC
TCATGCACGAAAGCGTGGGGAGCAAACAGGATTAGATACCCCTGTAGTCC
>Otu5530
CCAGCCTACGGGGGGCAGCAGTGGGGAATATTGGACAATGGGGGCAACCCTGATCCAGCG
ATGCCGCGTGTGTGAAGAAGGCCTGCGGGTTGTAAAGCACTTTCAGTGATGAGGAAGGCT
GTTTATCGAATAGATAGACAGATTGACGTTAGTTACAGAAGAAGCACCGGCTAACCCCGT
GCCAGCAGCCGCGGTAATACGGAGGGTGCGAGCGTTAATCGGAATTACTGGGCGTAAAGG
GCGCGTAGGTGGTTGTATAAGTCGATTGTGAAATTCCCGGGCTTAACCTAGGCAGGTCAA
TCGATACTGTATAGCTAGAGTACTGTAGAGGGTAGTGGAATTTCCGGTGTAGCGGTGAAA
TGCGTAGATATCGGAAGGAACACCGGTGGCGAAGGCGGCTACCTGGACAGATACTGACAC
TGAGGCGCGAAAGCGTGGGTAGCGAACAGGATTAGAAACCCGAGTAGTCC
>Otu5532
CCAGCCTATGGGGGGCAGCAGTGGGGAATATTGGACAATGGGGGAAACCCTGATCCAGCC
ATGCCGCGTGAGTGATGAAGGCCTTAGGGTTGTAAAGCTCTTTTGTCAGGGAAGATGATG
ACGGTACCTGAAGAATAAGCACCGGCTAACTTCGTGCCAGCAGCCGCGGTAATACGAAGG
GTGCTAGCGTTGTTCGGAATCACTGGGCGTAAAGCGCGCGTAGACGGTTTTGTAAGTCAG
ATGTGAAATCCCCGGGCTCAACCTGGGAACTGCATTTGAGACTGCATGGCTAGAGTATCG
AAGAGGGAAGTGGAATTTCCGGTGTAGCGGTGAAATGCGTAGATATCGGAAGGAACACCA
GTGGCGAAAGCGACTTCCTGGTCGAATACTGACGTTCATGTGCGAAAGCGTGGGGAGCAA
ACAGGATTAGATACCCTGGTAGTCC
>Otu5541
CCAGCCTACGGGTTGCACCAGTGGGGAATTTTGGACAATGGGGGGAACCCTGACGCAGCA
ACGCCGCGTGAGTGAGGAAGGCCTTCGGGTTGTAAAGCTCTTTCGGCTGGGAAGAAGGGA
ACTGGAGTTAATAAGTTCAGTTTTTGATGGTACCAGAAGAAGAAGCACCGGCAAACTTCG
TGCCAGCAGCCGCGGTAATACGAAGGGTGCAAGCATTGTTCGGAATTATTGGGCGTAAAG
GGTTCGTAGGCGGGAGAACAAGTCAAGTGTGAAATATCTAGGCTAAACCTAGAAAGTGCA
TTTGAAACTGTTTTTCTTGAGTGTTAGAGAGGGTGGTGGAATTGCTGGTGTAGGAGTGAC
ATCCGTAGAGATCAGCAGGAACATCGGAGGCGAAGGCGGCCACCTGGCTAAATACTGACG
CTGAGGAACGAAAGCATGGGGAGCAAACAGGATTAGAAACCCGGGTAGTCC
>Otu5542
CCAGCCTATGGGAGGCTGCAGTGGGGAATATTGGACAATGAACGAAAGTTTGATCCAGCT
ATATTGCGTGAGTGACGAGTTATTAAAGATTAATAATCCGAAGGTCTCGAATTGTAAAAC
TCTTTTATAAGTGAAAATAATGATATTAACTTATGAATAAGTACCGGCTAACTTCGTGCC
AGCAGCCGCGGTAAAACGGAGGGTACAAGTGTTATTCGTGATAACTGGGCGTAAAGGATA
TGTAGGTGGTTTTACCCGTAATTTGTAAAAAACTGGAATTTAATTTCTTGAGTGCGAATT
AAACCGTAAAACTAGAGTATAAAAGAGGTTAGCAGTACTAATATTGTAAAGTTGGAATTT
GTTGATAATATTAGGACTATCAATTGGCGAAGGCGGCTAACTGGGTTATGACTGACACTG
AGATATGAAAGCATAGGTAGCAAAGAGGATTAGATACCCCCGTAGTCC
>Otu5545
CCAGCCTATGGGGGGCTGCAGTCGAGAATCTTTCGCAATGGGCGAAAGCCTGACGAAGCG
ACGCTGTGTGTGCGATGAAGGCCTTCGGGTTGTAAAGCACTTTCGCTTAGGAACAAGAGA
GGCCGGCTAATATCCGGCCGATTTGAGGGTACTAGGTAAAGAAGCACCGGCTAACTACGT
GCCAGCAGCTGCGGTAATACGTAGGGTGCAAGCATTAATCGGATTTATTGGGCGTAAAGG
GCGCGTAGGCGGGATCATAAGTCAGATGTTAAAGCCCGGGGCTCAACCCCGGAAAAGCAT
TTGAAACTGTAATCCTAGAGGGTAGGCGGAGAAAATGGAAATCCACAAGTAGCGGTGAAA
TGCGTAGATATGTGGAAGAACACCGGTGGCGAAGGCGGTTTTCTAGCTTATACCTGACGC

TGAAGCGCGAAAGCAAAGGGAGCAAACAGGATTAGAAACCCCGGTAGTCC
>Otu5565
CCAGCCTACGGGTCGCTCCAGTGGGGAATCTTGCGCAATGGGCGAAAGCCTGACGCAGCA
ACGCCGCGTGGGGGACGAAGGCTTTCTGAGTTGTAAACCCCTTTCAGCAGGAACGATAGT
GACGGTACCTGCAGAAGAAGCACCGGCCAACTACGTGCCAGCAGCCGCGGTGATACGTAG
GGTGCAAGCGTTGTCCGGATTTATTGGGCGTAAAGAGCTCGTAGGCGGTTTGATAAGTCG
GATGTGAAACCCCCAGGCTTAACCTGGGGCCGCCATTCGATACTGTCATGGCTAGAGTCC
GGTAGGGGACCATGGAATTCCTGGTGTAGCGGTGAAATGCGCAGATATCAGGAGGAACAC
CAGTGGCGAAGGCGGTGGTCTGGGCCGGAACTGACGCTGAGGAGCGAAAGCGTGGGGAGC
GAATAGGATTAGATACCCTCGTAGTCC
>Otu5579
CCAGCCTATGGGTCGCTGCAGTTTCGAATCATTCACAATGGGCGAAAGCCTGATGGTGCG
ACGCCGCGTGAGGGATGAAGGCCTTCGGGTTGTAAACCTCTGTCACCGGGGAAGAAACGC
TTCAAGTTAACAGCCTGAAGCCTGACTTAACCCGGAGAGGAAGCAGTGGCTAACTCTGTG
CCAGCAGCCGCGGTAATACAGAGACTGCGAGCGTTACTCGGATTCACTGGGCGTAAAGGG
TGCGCAGGCGGCCAGGTGTGTGAGGCGTGAAAGCCCGGGGCTTAACCCCGGAATTGCACC
TCAAACTACATGGCTAGAGTATTGGAGAGGGTAACGGAATTCACGGTGTAGCAGTGAAAT
GCGTAGATATCGTGAGGAACACCAGAGGCGAAGGCGGTTACCTGGACAATTACTGACGCT
CAGGCACGAAAGCGTGGGTAGCAAACAGGATTAGAGACCCTCGTAGTCC
>Otu5581
CCAGCCTATGGGGGGCTGCAGTAGGGAATATTGCGCAATGGAGGAAACTCTGACGCAGCG
ACGCCGCGTGGGTGACGAAGGCCTTCGGGTCGTAAAGCCCTGTCGGAAGGGACGAAAAAT
AGAATGGCTAATATCCATTTTACTTGACGGTACCTTTAAAGGAAGCACCGGCTAACTACG
TGCCAGCAGCCGCGGTAATACGTAGGGTGCGAGCGTTGTTCGGAATCATTGGGCGTAAAG
CGCGTGTAGGCGGCTAAATAAGTCGAGTGTGAAATCCCTGGGCTCAACCGAGGAAGTGCA
TTCGAAACTGTTTAGCTAGAGGACGGTAGAGGAAGGTGGAATTCCTAGTGTAGAGGTGAA
ATTCGTAGATATTAGGAGGAATACCGGTGGCGAAGGCGGCCTTCTGGGCCGTTCCTGACG
CTGAGACGCGAAAGCGTGGGGAGCAAACAGGATTAGATACCCCTGTAGTCC
>Otu5585
CCAGCCTATGGGAGGCAGCAGTGGGGAATATTGCGCAATGGGCGAAAGCCTGACGCAGCG
ACGCCGCGTGGAGGATGAAGGTTTTTGGATTGTAAACTCCTGTTAAGTGGGAAGAAAGTC
CTATTTCTAATAAATATAGGCTATGACGGTACCACTAGAGAAAGCACCGGCTAACTTCGT
GCCAGCAGCCGCGGTAATACGAGGGGTGCAAGCGTTATTCGGATTTATTGGGCGTAAAGG
GTGCGTAGACGGTTTGTTAAGTCTGTTGTTAAATTCTCCGACCTAATCGGAGGTAGGCGA
TGGAAACTGGCGGACTAGAGTGTGGGAGAGAGAAGTGGAATTCCCGGGGTAGCGGTAAAA
TGCACAGATATCGGGAGGAACACCAATGGCGAAGGCAGCTTCTTGGCCCATAACTGACGT
TGAGGCACGAAAGCGTGGGGAGCAAACAGGATTAGAGACCCGCGTAGTCC
>Otu5586
CCAGCCTACGGGATGCTGCAGTCGAGAATTTTTCTCAATGGGCGAAAGCCTGAAGGAGCG
ACGCCGCGTGTGCGATGAAGGCCTTCGGGTTGTAAAGCACTGTCGAGGGGGAGAAAGGTC
CGCAAGGGCTTGATCTATCCCTGGAGGAAGCACGGGCTAAGTTCGTGCCAGCAGCCGCGG
TAAGACGAACCGTGCGAACGTTGTTCGGAATCACTGGGCTTAAAGGGCGCGTAGGCGGGT
CTTCAAGTCAGGAGTGAAATCCTTCGGCTCAACCGGAGAAGCGCTTTTGATACTGGAGAC
CTCGAGGGGTGTAGGGGCATGTGGAACTTCCGGTGGAGCGGTGAAATGCGTAGATATCGG
AAGGAACGCCGGTGGCGAAAGCGACGTGCTGGACACCTACTGACGCTGAGGCGCGAAAGC
CAGGGGAGCAAACGGGATTAGATACCCCCGTAGTCC
>Otu5592
CCAGCCTATGGGGGGCAGCAGTGGGGAATATTGGACAATGGGGGCAACCCTGATCCAGCA
ATGCCGCGTGTGTGAAGAAGGCCTTAGGGTTGTAAAGCACTTTCAGTGGGGAGAAGGTTT
TAAGTGCTAATACCATTTAAAGTTGATGTTACCCACAGAAGAAGCACCGGCTAACTCTGT
GCCAGCAGCCGCGGTAATACAGAGGGTGCGAGCGTTAATCGGAATTACTGGGCGTAAAGG
GTGTGTAGGTGGTTAGGTAAGTTAGATGTGAAATCCCCGGGCTCAACCTGGGAATTGCGT
TTAAAACTGTTTAGCTAGAGTATTGTAGAGGATAGTGGAATTTCCAGTGTAGCGGTGAAA
TGCGTAGATATTGGAAGGAACACCAGTGGCGAAGGCGACTATCTGGGCAAATACTGACAC
TGAGACACGAGAGCATGGGGAGCAAACAGGATTAGAAACCCTAGTAGTCC
>Otu5597
CCAGCCTATGGGGGGCTGCAGTCGAGAATTTTTCTCAATGGGCGAAAGCCTGAAGGAGCG
ACGCCGCGTGGGGGATGAATGGCTTCGGCCCGTAAACCCCTGTCATTTGCGAACAAACCT

GTTCACCTAACACGTGAACAGCTGATTGTAGCGAAAGAGGAAGGGACGGCTAACTCTGTG
CCAGCAGCCGCGGTAATACAGAGGTCCCAAGCGTTGTTCGGATTCACTGGGCGTAAAGGG
TGCGTAGGTGGTGAGGTAAGTCTTATGTGAAAGCTCGGAGCTTAACTCCGAAATGGCATA
GGAAACGACCTTGCTGGAGGGTTGGAGGGGGGACTGGAATACTTGGTGGAGCAGTGAAAT
GCGTAGATATCAAGTGGAACACCAGTGGCGAAGGCGAGTCCCTGGACAACTCCTGACACT
GAGGCACGAAAGCTAGGGGAGCAAACGGGATTAGATACCCTCGTAGTCC
>Otu5599
CCAGCCTACGGGGGGCAGCAGTCGAGAATTTTTCTCAATGGGGGAAACCCTGAAGGAGCG
ACGCCGCGTGGAGGATGAAGGTCTTCGGATTGTAAACTCCTGTCATTTGGGAACAATTGT
TGGTCATTAACTGTGATCAGCTTGATAGTACCAGAAGAGGAAGAGACGGCTAACTCTGTG
CCAGCAGCCGCGGTAATACAGAGGTCTCAAGCGTTGTTTGGATTCATTGGGCGTAAAGGG
TGCGTAGGTGGCGAGGTTAGCCAGGTGTGAAATCTCGGGGCTCAACCCCGAAACTGCACT
TGATACTGCCTTGCTAGAGTACTGAAGAGGAGATTGGAATTTACGGTGTAGCAGTGAAAT
GCGTAGATATCGTAAGGAAGACCAGTGGCGAAGGCGAATCTCTGGACAGTTACTGACACT
GAGGCACGAAGGCCAGGGGAGCAAACGGGATTAGATACCCCCGTAGTCC
>Otu5604
CCAGCCTACGGGAGGCAGCAGTAGGGAATCTTCCACAATGGGCGAAAGCCTGATGGAGCA
ACGCCGCGTGCAGGACGAAAGCCTTCGGGTTGTAAACTGCTTTTATCTGTGACGAATATG
ACGGTAGCAGATGAATAAGGATCGGCTAACTCCGTGCCAGCAGCCGCGGTCATACGGAGG
ATCCAAGCGTTATCCGGAATTACTGGGCGTAAAGAGTTGCGTAGGTGGCATGGTAAGTTG
ATGGTGAAATCCTGGGGCTCAACCCCTTACCCATTATCAAAACTGCCAGGCTAGAGGATG
AGAGAGGTTATTGGAATTCCTAGTGTAGGAGTGAGATCCGTAGATATTAGGAGGGACACC
GATGGCGTAGGCAGGTTACTGGCTCGTTTCTGACACTAAGGCACGAAAGCGTGGGGAGCA
AACGGGATTAGAAACCCTTGTAGTCC
>Otu5608
CCAGCCTATGGGTTGCTCCAGTCGAGAATCTTTCGCAATGGGCGAAAGCCTGACGAAGCG
ACGCCGTGTGAGCGAAGAAGGCCTTCGGGTTGTAAAGCTCTTTCGCTAGGGAACAAGGAA
TATGGGATAACACCCCGTAAACTTGAGGGTACTTGGTAAAGAAGCACCGGCTAACTCCGT
GCCAGCAGCTGCGGTAATACGGAGGGTGCAAGCATTGATCGGAATTACTGGGCGTAAAGG
GCGCATAGGCGGCACAGAAAGTCAGATGTGAAATTCTCGGGGCTCAACCCCGGAACTGCA
TTTGAAACTCCTGAGCTAGAGGATGGTTAGGGAAAACGGAATTCCACGTGTAGCGGTGAA
ATGCGTAGATATGTGGAAGAACACCGGTGGTGAAGACGGTTTTCTGGGCCAAACCTGACG
CTGAGGCGCGAAAGCTAGGGGAGCAAACAGGATTAGAAACCCCAGTGGTCC
>Otu5610
CCAGCCTATGGGTCGCAGCAGTTTCGAATCATTCACAATGGACGAAAGTCTGATGGAGCG
ACGCCGCGTGGAGGATGAAGGTTTTCGGATTGTAAACTCCTGTCACTGCAGAACAAGGCT
GCGCGATTGAACATTTCGCGCAGTTGATGGTATGCGGAGAGGAAGGGACGGCTAACTCTG
TGCCAGCAGCCGCGGTAAGGCAGAGGTCCCGAGCGTTGTTCGGATTCATTGGGCGTAAAG
GGTGTGTAGGAGGCCAGGTAAGTCAGGTGTGAAATCTCAGAGCTTAACTCTGAAACTGCG
CTTGATACTGCTTGGCTCGAGGATCGGAGGGGGTATCGGAATTTATGGTGTAGCAGTGAA
ATGCGTAGATATCATAAGGAACACCGGTGGCGAAGGCGGATACCTGGAAGATTCCTGACT
CTGAAACACGAAAGCCAGGGGAGCAAACGGGATTAGATACCCTCGTAGTCC
>Otu5616
CCAGCCTACGGGGGGCAGCAGTCGAGGATCTTCGTCAATGGGCGCAAGCCTGAACGAGCG
ACGCCGCGTGTGCGATGAAGGCCTTCGGGTTGTAAAGCACTGTCGAGGGGGATAAAGGCC
GCAAGGCTTGATCGATCCCTGGAGGAAGCACGGGCTAAGTTCGTGCCAGCAGCCGCGGTA
AGACGAACCGTGCGAACGTTGTTCGGAATCACTGGGCTTAAAGGGCGCGTAGGCGGCAGG
CGAGGTCGGGGGTGAAATCCCACAGCTTAACTGTGGAAGAGCCTTCGATACCGGCTTGCT
GGAGGAAGGTAGGGGTGCGCGGAACTTCTGGTGGAGCGGTGAAATGCGTTGATATCAGAA
GGAACGCCGGTGGCGAAAGCGACGTGCTGGACCTCTTCTGACGCTGAGGCGCGAAAGCTA
GGGGAGCAAACGGGATTAGAGACCCGCGTAGTCC
>Otu5626
CCAGCCTACGGGGGGCTCCAGTGGGGAATATTGGGCAATGGGCGCAAGCCTGACCCAGCC
ATGCCGCGTGAGTGATGAAGGCCTTCGGGTCGTAAAGCTCTGTGGGGAGGGACGAAACAG
TTCGTGGCCAACACCCACGAACCTTGACGGTACCTCCTTAGCAAGCACCGGCTAACTCCG
TGCCAGCAGCCGCGGTAATACGGAGGGTGCAAACGTTGCTCGGAATCATTGGGCGTAAAG
CGCACGTAGGCGGCTCCTCAAGTCGGATGTGAAAGCCCTGGGCTCAACCCAGGAAGTGCA
TTCGAAACTGGCGAGCTTGAATATGGAAGAGGGTCGCGGAATTCCCGGTGTAGAGGTGAA

ATTCGTAGATATCGGGAGGAACACCAGTGGCGAAGGCGGCGACCTGGGCCAATATTGACG
CTGAGGTGCGAAAGCGTGGGGAGCAAACAGGATTAGATACCCTCGTAGTCC
>Otu5628
CCAGCCTATGGGGCGCAGCAGTGGGGAATATTGGACAATGAGCGCAAGCTTGATCCAGCA
ATATCGCGTGAATGACTACAGCCTGGATGGTTTTAAAGTTCTTTTGATGGATTATTATAA
TGATTGTATCCATAGAATAAGTCCCGGCTAACTTCGTGCCAGCAGCCGCGGTAAAACGGG
GGGGACTAGCGTTATTCGTCATTATTGGGCGTAAAGGGCAATTAGACGGTTAAAAAAGTT
TTGAGTTAAATTTAAGAGAAAGTATATCTCGGTTCAGCTTGAAATACTTTTTGGCTAGAG
TGCAATAGAGGATGAAAGTACTTTCAGTGTAAAAGTGAAATTTGACGATATTGAAGGGAC
TCCCTACAGGCGAAGGCGTTCATCTGGGTTGTTACTGACGTTCACAGGCGAAAGCGTGGG
GAGCAAACAGGATTAGAGACCCTAGTAGTCC
>Otu5639
CCAGCCTACGGGACGCAGCAGTAGGGAATATTGCGCAATGGAGGAAACTCTGACGCAGCG
ACGCCGCGTGGGTGATGAAGGCCTTCGGGTCGTAAAGCCCTGTCGGTAGGGAAGAAAAAC
GAGGTGGCTAACATCTATCTCGCTTGACGGTACCTGCAGAGGAAGCACCGGCTAACTACG
TGCCAGCAGCCGCGGTAATACGTAGGGTGCTAGCGTTGTTCGGAATCATTGGGCGTAAAG
CGCGTGTAGGTGGTTATTTAAGTCGAGTGTGAAATCCCTAGGCTTAACCTAGGAAGTGCA
TTCGAAACTAATTAGCTAGAAGACGGTAGAGGAAGGTAGAATTCCTAGTGTAGAGGTGAA
ATTCGTAGATATTAGGAGGAACACCGGTGGCGAAGGCGGCCTTCTGGACCGTTCTTGACA
CTGAGACGCGAAAGCGTGGGTAGCAAACAGGATTAGAAACCCGCGTAGTCC
>Otu5640
CCAGCCTACGGGGGGCAGCAGTCGAGAATCTTTCGCAATGGGCGCAAGCCTGACGGAGCG
ACGCTGTGTGAGTGATGAAGGCCTTCGGGTTGTAAAGCTCTTTCGCTGGGGAACAAGGGA
GGAAGAAGAATACTCTTCTAACTTGAGGGTACCCGGTAAAGAAGTACCGGCTAACTACGT
GCCAGCAGCTGCGGTAATACGTAGGGTACAAGCATTAATCGGATTTACTGGGCGTAAAGG
GTGCGTAGGCGGATAGACAAGTCAGGTGTGAAATTCCGGTACTCAATACCGGAGCTGCAC
TTGAAACTATCTTTCTAGAGGTCAGGCGGAGGAAACGGAATTCCATGTGTAGCGGTGAAA
TGCGTAGATATATGGAAGGACACCTGTGGCGAAAGCGGTTTCCTAGCTTGATTCTGACGC
TGATGCACGAGAGCGTGGGTAGCAAACAGGATTAGAAACCCTCGTAGTCC
>Otu5654
CCAGCCTATGGGATGCACCAGTTTGGAATTTTGGACAATGGGCGAAAGCCTGATCCAGCG
ACGCCGCGTGGACGATGAAGGTCTTCGGATCGTAAAGTCCTTTTAGTAGGGAACAATTTT
TGAGCGTACCTACAGAATAAGAGGTTGCTAACTCTGTGCCAGCAGCAGCGGTAATACAGA
GACCTCGAGCGTTATCCGGAATCATTGGGCGTAAAGCGTACCGATCGGTTGTTTTGAAAG
TCAGAGGTGAAATCACAAAGCTCAACTTTGTGACTGTCCTTTGAAACTTCAAAACTAGAG
GGGCAAAGAGGAAGCTGGAACAAACGGTGTAGTAGTGAAATGCGTTGATATCGTTTGGAA
CACCAATAGCGAAGGCAGGCTTCTGGGTGCCACCTGACACTGCTAGGACGAAAGCGTGGG
GAGCGAATGGGATTAGAAACCCTGGTAGTCC
>Otu5655
CCAGCCTACGGGAGGCACCAGTCGAGAATTTTTCTCAATGGGCGAAAGCCTGAAGGAGCG
ACGCCGCGTGGGGGATGAAGGGCTTCGGCCCGTAAACCCCTGTCATTTGCGAACAAACCT
TGCGTTTTAATAAAGCGCAAGCTGATTGTAGCGAAAGAGGAAGGGACGGCTAACTCTGTG
CCAGCAGCCGCGGTAATACAGAGGTCCCAAGCGTTGTTCGGATTCACTGGGCGTAAAGGG
TGCGTAGGTGGCTGGGTAAGTTTGATGTGAGATCTCCGGGCTTAACCCGGAAACGGCATT
GAATACTATCCAGCTGGAGGTTTGGAGGGGGGACTGGAATTCTCGGTGTAGCAGTGAAAT
GCGTAGATATCGAGAGGAACACCAGTGGCGAAGGCGAGTCCCTGGACAAAACCTGACACT
GAGGCACGAAAGCTAGGGGAGCAAACAGGATTAGAGACCCTTGTAGTCC
>Otu5656
CCAGCCTATGGGTTGCAGCAGTGGGGAATTTTGGACAATGGGGGCAACCCTGACCCAGCA
ATGCCGCGTGAGTGAAGAAGGCCTTCGGGTTGTAAAGCTCTTTTGTCAGGGAAGAAACGG
GTGTCTCTAATATAGATATCTAATGACGGTACCTGAAGAAGAAGCACCGGCTAACTCTGT
GCCAGCAGCCGCGGTAATACAGAGGGTGCGAGCGTTAATCGGAATTACTGGGCGTAAAGC
GCGCGTAGACGGTTTTGTAAGTCAGATGTGAAATCCCTGGGCTCAACCTGGGAACTGCAT
TTGAGACTGCATGGCTAGAGTATCGAAGAGGGAAGTGGAATTTCCAGTGTAGCGGTGAAA
TGCGTAGATATCGGAAGGAACACCAGTGGCGAAAGCGACTTCCTGGTCGAATACTGACGT
TCATGTGCGAAAGCGTGGGGAGCAAACAGGATTAGAAACCCTCGTAGTCC
>Otu5657
CCAGCCTATGGGGTGCTCCAGTCGAGAATCTTCCGCAATGGACGAAAGTCTGACGGAGCG

ACGCCGCGTGATTGATGAAGACCCTATGGGTCGTAAAGATCTTTTATGAGGGAAGAAGTT
TATTGACGGTACCTCATGAATAAGAGGCTCCTAATCTCGTGCCAGCAGGAGCGGTAATAC
GAGAGCCTCGAGCGTTATCCGGAATTATTGGGCGTAAAGGGTGCGTAGGTGGCGTTGTTA
GTCGAATGTTAAATCTCCGAGCTTAACTTGGAAACCGCATTCGAAACGGCAATGCTAGAG
AGGAGAAGAGGTATGCAGAACTCAAGGTGTAGGGGTGAAATCCGTTGATATCTTGGGGAA
TACCAAAAGCGAAGGCAGCATACTGGTCTCTTTCTGACACTGAAGCACGAAAGCGTGGGT
AGCGAATGGGATTAGAGACCCTTGTAGTCC
>Otu5663
CCAGCCTACGGGTCGCTGCAGTGGGGAATATTGGACAATGGGCGAAAGCCTGATCCAGCA
ATGCCGCGTGAATGATGAAGGCCTTAGGGTTGTAAAGTTCTTTTAGCAGGGAAGATAATG
ACGGTACCTGCAGAAAAAGCCCCGGCTAACTCCGTGCCAGCAGCCGCGGTAAGACGGAGG
GGGCTAGCGTTGTTCGGAATTACTGGGCGTAAAGAGCGCGTAGGCGGTTTGGTAAGTTGG
AAGTGAAATCCCGGGGCTTAACCTCGGAATTGCTTTCAAAACTGCCAGGCTAGAGTGTAG
TAGGGGATGATGGAATTCCTAGTGTAGAGGTGAAATTCTTAGATATTAGGAGGAACACCG
GTGGCGAAGGCGGTCATCTGGGCTACAACTGACGCTGATGCGCGAAAGCGTGGGGAGCAA
ACAGGATTAGAGACCCCAGTAGTCC
>Otu5673
CCAGCCTATGGGTGGCAGCAGCAACGAATATTCCGCAATGGGCGAAAGCCTGACGGAGCG
ACGCCGCGTGTGGGATGAAGTCCTTCGGGATGTAAACCACTGTCAGGGGCTACCAAGATC
TGAGGAGCCCCAGAGGAAGCCACGGCTAACTCTGTGCCAGCAGCCGCAGTAAGACAGAGG
TGGCTAGCGTTGTTCGGAATCATTGGGCTTAAAGCGCACGTAGGCGGCCCTTCAAGTGTC
TTGTGAAATCCCTCGGCTCAACCGAGGAATGGCTGGGCATACTGTCGGGCTTGAGGCAGG
TATGGGCGAGTGGAACTCTTGGTGGAGCGGTGGAATGCGTAGATATCAAGAGGAACGCCG
GTGGTGAAGACGACTCGCTGGGCCTGTCCTGACGCTGAGGTGCGAAAGCCAGGGGAGCAA
ACGGGATTAGAAACCCGAGTAGTCC
>Otu5674
CCAGCCTATGGGTGCACCAGTGGGGAATATTGGACAATGGGGGCGACCCTGATCCAGCCA
TGCCGCGTGGGTGAAGAAGGCCTTCGGGTTGTAAAGCCCTTTTGTACGGGAAGAAAAGCA
GCCGGTTAATAACTGGTTGTGCTGACGGTACCGTAAGAATAAGCACCGGCTAACTTCGTG
CCAGCAGCCGCGGTAATACGAAGGGTGCAAGCGTTACTCGGAATTACTGGGCGTAAAGCG
TGCGTAGGTGGTGGTTTAAGTCTGATGTGAAATCCCCGGGCTCAACCTGGGAATGGCATT
GGATACTGGGTCACTAGAGTGCGGTAGAGGGTGGTGGAATTCCCGGTGTAGCAGTGAAAT
GCGTAGAGATCGGGAGGAACATCTGTGGCGAAGGCGGCCACCTGGACCAGCACTGACACT
GAGGCACGAAAGCGTGGGGAGCAAACAGGATTAGAAACCCGCGTAGTCC
>Otu5678
CCAGCCTATGGGTGGCTCCAGTGGGGAATATTGGACAATGGGGGCAACCCTGATCCAGCC
ATGCCGCGTGAGTGATGAAGGCCTTCGGGTTGTAAAGCTCTTTTGGCGGGGACGATGATG
ACGGTACCCGCAGAATAAGCTCCGGCTAACTTCGTGCCGGCAGCCGCGGTAATACGAAGG
GAGCTAGCGTTGTTCGGAATTACTGGGCGTAAAGGGCGCGTAGGCGGCTTGGCAAGTTGG
GTGTGAAAGCCCAGGGCTCAACCCTGGAATAGCACTCAAGACTGCCTTGCTTGAATTCGG
TAGAGGTTGGTGGAATTCCCAGTGTAGAGGTGAAATTCGTAGATATTGGGAAGAACACCG
GTGGCGAAGGCGGCTCACTGGCCCGTAACTGACGCTCAGGCGCGACAGCGTGGGGATCAA
ACAGGATTAGAGACCCCGGTAGTCC
>Otu5681
CCAGCCTACGGGGCGCAGCAGTGGGGAATCTTGCACAATGGGGGCAACCCTGATGCAGCG
ACGCCGCGTGAGCGATGAAGCCCTTCGGGGTGTAAAGCTCTTTCGGCAGGGACGATAATG
ACGGTACCTGAAGAAGAAGCTGCGGCTAACTACGTGCCAGCAGCCGCGGTAATACGTAGG
GTGCAAGCGTTGTCCGGAATTATTGGGCGTAAAGAGCGTGTAGGCGGTCCGATCAGTCCG
CTGTGAAAGTCCAGGGCTCAACCCTGGGATGCCGGTGGATACTGTCGGACTAGAGTCCGG
AAGAGGCGAGTGGAATTCCTGGTGTAGCGGTGAAATGCGCAGATATCAGGAGGAACACCA
ATGGCGAAGGCAGCTCGCTGGGACGGTACTGACGCTGAGACGCGGAAGCGTGGGGAGCAA
ACAGGATTAGAAACCCCAGTAGTCC
>Otu5690
CCAGCCTACGGGACGCAGCAGTTCGGAATATTCGGCAATGGGCGAAACCCTGATGCAGCG
ACGCCGCGTGAGCGATGGAGCCCCTCGGGGTGTAAAGCTCTTTCGGCAGGGAAGATAATG
ACGGTACCTGCAGAAGCAGCTGCGGCTAACTACGTGCCAGCAGCCGCGGTAATACGTAGG
CAGCAAGCGTTGTTCGGAGTTACTGGGCGTAAAGGGTGTGTAGGCGGCTCTCTAAGTTTG
GTGTGAAATCTCCCGGCTCAACCGGGAGGGTGCGCCGAATACTGAAGGGCTAGAGTGCGG

GAGAGGAAAGTGGAATTCCTGGTGTAGAGGTGAAATGCGTAGATATCAGGAGGAACACCG
GTGGTGTAGACGGCTTTCTGGACCGTAACTGACGCTGAGACACGAAAGCGTGGGTAGCAA
ACAGGATTAGAAACCCTGGTAGTCC
>Otu5694
CCAGCCTATGGGTTGCTGCAGTCGAGAATCTTTCGCAATGGGCGAAAGCCTGACGAAGCG
ACGCCGTGTGAGCGAAGAAGGCCTTTGGGTTGTAAAGCTCTTTCGCCGGGGAACAAGGAA
CTAAAGTGAATAACTTTGGAACTTGAGGGTACTCGGTAAAGAAGCACCGGCTAACTCCGT
GCCAGCAGCTGCGGTAATACGGAGGGTGCAAGCATTAATCGGATTTATTGGGCGTAAAGG
GCGCGTAGGCGGGAAATTAAGTCAGGTGTGAAATCCCAAGGCTCAACCTTGGAACGGCAC
TTGATACTGCTTTTCTTGAGGGTAGACGGAGAAAGTGGAATTCCACAAGTAGCGGTGAAA
TGCGTAGATATGTGGAAGAACACCAGTGGCGTAGGCGACTTTCTAGTTTATACCTGACGC
TGAGGCGCGAAAGCTAGGGGAGCAAACAGGATTAGAAACCCCAGTAGTCC
>Otu5699
CCAGCCTATGGGTCGCTGCAGTGGGGAATATTGGACAATGGGCGCAAGCCTGATCCAGCA
ATGCCGCGTGAGTGAAGAAGGCCTGAGGGTTGTAAAGCACTTTCGGTGGGGAGGAGTTTA
TTTTGGTTAAGAGCTAGGATAAAGGACGTTACCCACATAAGAAGCACCGGCTAACTCTGT
GCCAGCAGCCGCGGTAATACGGAGGGTGCGAGCGTTAATCGGAATTACTGGGCGTAAAGG
GTGCGTAGGTGGTTTGATAAGTTAGCTGTGAAAGACCTGGGCTTAACCTGGGGGTGTCAG
CTAAGACTGTTGGACTCGAGTATGGGAGAGGGTAGTGGAATTTCCGGTGTAGCGGTGAAA
TGCGTAGAGATCGGAAGGAACACCAGTGGCGAAGGCGGCTACCTGGCCTAATACTGACAC
TGAGGCACGAAAGCTAGGGGAGCAAACAGGATTAGAAACCCTTGTAGTCC
>Otu5700
CCAGCCTATGGGTCGCTGCAGTGGGGAATATTGGACAATGGGCGCAAGCCTGATCCAGCA
ACGCCGCGTGAGTGATGAAGGCCCTCGGGTCGTAAAGCTCTTTCGGAAGGGACGAAACTG
GTCCGGGATAATAAACCGGGCTCCTGACGGTACCTTAAGAAGAAGCACCGGCTAACTCTG
TGCCAGCAGCCGCGGTAATACAGAGGGTGCAAGCGTTGTTCGGAATTATTGGGCGTAAAG
CGCGTGTAGGCGGCCAAGCATGTCGGGTGTGAAAGCCCTGGGCTTAACCCAGGAAGTGCG
CCCGAAACTACTTGGCTTGAGTGTCGGAGAGGATGGCGGAACTCCCGGTGTAGAGGTGAA
ATTCGTAGATATCGGGAAGAACACCAGTGGCGAAGGCGGCCATCTGGACGATAACTGACG
CTGAGACGCGAAAGCGTGGGGAGCAAACAGGATTAGAAACCCCGGTAGTCC
>Otu5707
CCAGCCTATGGGGCGCAGCAGTGAGGAATATTGCACAATGGGCGAAAGCCTGATGCAGCG
ACGCCGCGTGGAGGATGACGGTCTTCGGATTGTAAACTCCTGTTAAGTGGGAAAAAAGAA
TTGCTGTTAATAGCAGCGAGAGATGATGGTACCACTAGAGAAAGCGCCGGCTAACTTCGT
GCCAGCAGCCGCGGTAATACGAGGGGTGCAAACGTTATTCGGAATCACTGGGCGTAAAGG
GTGTGTAGACGGCGAAACAAGTCAGTTATAAAATCTCTTGGCTTAACCAAGAACCTGTAA
CCGAAACTGTTTTGCTTGAGGATGGAAGAGAGAAGCGGAATTCTCGGAGTAGCGGTAAAA
TGCGTAGATCTCGAGAGGAACACCGATGGCGAAGGCAGCTTCTTGGTCCATTTCTGACGT
TGAGACACGAAAGCGTGGGGAGCAAACAGGATTAGATACCCGTGTAGTCC
>Otu5714
CCAGCCTACGGGTGGCTGCAGTGGGGAATCTTGCGCAATGGGCGAAAGCCTGACGCAGCC
ATGCCGCGTGAGTGATGACGGCCTTAGGGTTGTAAAGCTCTTTCGCTAGGGACGATAATG
ACGGTACCTAGACAAGAAGCCCCGGCTAACTTCGTGCCAGCAGCCGCGGTAATACGAAGG
GGGCTAGCGTTGTTCGGAATTACTGGGCGTAAAGCGCGTGTAGGCGGGCGTTTAAGTCAG
GGGTGAAATGCCAAGGCTCAACCTTGGAACTGCCTTTGATACTGGACGCCTCGAGTCCGG
GAGAGGTGAATGGAACTGCGAGTGTAGAGGTGAAATTCGTAGATATTCGCAAGAACACCA
GTGGCGAAGGCGGCTCACTGGCCCGGAACTGACGCTGAGACGCGAAAGCGTGGGGAGCAA
ACAGGATTAGATACCCCAGTAGTCC
>Otu5727
CCAGCCTATGGGGCGCTGCAGTGGGGAATCTTGCGCAATGGGCGAAAGCCTGACGCAGCA
ACGCCGCGTGAGGGATGAAGGTCTTCGGGTTGTAAACCTCTTTCAAGCAGGGACGATAAT
GACGGTACCTGCAGAAGAAGCTCCGGCCAACTACGTGCCAGCAGCCGCGGTAATACGTAG
GGAGCGAGCGTTGTCCGGATTCATTGGGCGTAAAGAGCTCGTAGGCGACTTGGTAAGTCG
GGTGTGAAACCTCCAGGCTCAACCTGGAGATGCCACTCGATACTGCCATAGCTAGAGTCC
GGTAGGGGCCCACGGAATTCCTGGTGTAGCGGTGAAATGCGCAGATATCAGGAGGAACAC
CGGTGGCGAAGGCGGTGGGCTGGGCCGGCACTGACGCTGAGGAGCGAAAGCTAGGGGAGC
AAACAGGATTAGATACCCCCGTAGTCC
>Otu5728

CCAGCCTACGGGGGGCAGCAGTGAGGAATATTGGTCAATGGGTGCAAGCCTGAACCAGCC
ATCCCGCGTGAAGGACGACTGCCCTATGGGTTGTAAACTTCTTTTGTATAGGAATAAACC
TACCCTCGTGAGGGTAGCTGAAGGTACTATACGAATAAGCACCGGCTAACTCCGTGCCAG
CAGCCGCGGTAATACGGAGGGTGCAAGCGTTATCCGGATTTATTGGGTTTAAAGGGTCCG
TAGGCGGACTTATAAGTCAGTGGTGAAAGCCTGTCGCTTAACGATAGAACTGCCATTGAT
ACTGTAAGTCTTGAGTATATTTGAGGTAGCTGGAATAAGTAGTGTAGCGGTGAAATGCAT
AGATATTACTTAGAACACCAATTGCGAAGGCAGGTTACCAAGATATAACTGACGCTGGGG
GACGAAAGCGTGGGGAGCGAACAGGATTAGAGACCCCAGTAGTCC
>Otu5741
CCAGCCTACGGGGTGCACCAGTGGGGAATATTGGACAATGGGCGCAAGCCTGATCCAGCC
ATGCCGCGTGGATGATGAAGGCCTTAGGGTTGTAAAATCCTTTCGGCGGGGAAGATAATG
ACGGTACCCGCAGAAGAAGCCCCGGCTAACTTCGTGCCAGCAGCCGCGGTAATACGAAGG
GGGCTAGCGTTGTTCGGAATTACTGGGCGTAAAGCGCGCGCAGGCGGCTTTTCAAGTCAG
GGGTGAAAGCCCAGAGCTCAACTCTGGAACTGCCTTTGAAACTGTGAGGCTTGAGTACGG
GAGAGGTAAGTGGAATTCCCAGTGTAGAGGTGAAATTCGTAGATATTGGGAAGAACACCG
GTGGCGAAGGCGGCTCACTGGCCCGTTTCTGACGCTCATGCGCGACAGCGTGGGGATCAA
ACAGGATTAGATACCCTAGTAGTCC
>Otu5744
CCAGCCTACGGGGTGCTCCAGTGGGGAATATTGGACAATGGGGGCAACCCTGATCCAGCA
ATGCCACGTGTGTGAAGAAGGCCTGAGGGTTGTAAAGCACTTTAGCTGGGGAAGAAGGAA
TGCGTGCTAATACCACGTGTTATTGACATTACCCAAAGAATAAGCACCGGCTAACTCTGT
GCCAGCAGCCGCGGTAATACAGAGGGTGCAAGCGTTAATCGGAATTACTGGGCGTAAAGG
GCTCGTAGGTTGTTAAATAAGTCAGATGTGAAATCCCTGGGCTCAACCTAGGAATTGCAT
ATGATACTGTTTAGCTAGAGTATAGTAGAGGGAAGTGGAATTTCTGGTGTAGCGGTGAAA
TGCGTAGATATCAGAAGGAACATCAGTGGCGAAGGCGACTTCCTGGACTAATACTGACAC
TGAGGAGCGAAAGCGTGGGGATCAAACAGGATTAGAAACCCTAGTAGTCC
>Otu5746
CCAGCCTATGGGTTGCACCAGTAGGGAATCTTGCGCAATGGGCGAAAGCCTGACGATGCG
ACGCCGTGTGAGTGAAGAAGGCCTTAGGGTCGTAAAGCTCTTTCGCTTGGGAACAAGAAA
AGCTGGCTAACATCCAGCAGATTTGAGCGTACCAGGTAAAGAAGCACCGGCTAACTCCGT
GCCAGCAGCTGCGGTAATACGGAGGGTGCAAGTATTAATCGGATTTATTGGGCGTAAAGG
GCGCGTAGGCGGGCATGAAAGTCAGATGTGAAATCCCGGGGCTCAACCTCGGAACAGCAT
TTGAAACTCCATGTCTAGAGGGTAGGAGGAGAAAACGGAATTCCACAAGTAGCGGTGAAA
TGCGTAGATATGTGGAAGAACACCGGTGGCGAAGGCGGTTTTCTATCTTATTCTTGACGC
TGAGGCGCGAAAGCAAGGGGATCAAACAGGATTAGATACCCCTGTAGTCC
>Otu5752
CCAGCCTACGGGGGGCACCAGACGTCGGCTGCAACGCCGTATTTTTCCGCCAGAATACCC
TGCGCGCGCAACGCTTCGTTCAGAATGGGACCACTGCCGAAAAGCTGTACTCTTCCCCGA
CCTTCGGCGGCGGCCTTATACCGGTAGATACCTCTCAGAATGCCTTCCTGGGCGCCCTCC
GGCATCGGGGGCATTGGATAGTTCTCGTTATACAGTGTCAGGTAGTAGAACAGATCTTCC
TGCTCCTGATACATGCGGCGAATGCCATCCTGCACGATAACGGCGATTTCATACGCATAC
GCCGGATCGTAAGCGGCGCAGGTGGGCACGGTGCTGGCAAGCACGATGCTGTGGCCATCC
TGGTGTTGCAATCCCTCGCCCGATAGCGTGGTGCGTCCGGCTGTGCCGCCCATTAGAGAC
CCTTGTAGTCC
>Otu5754
CCAGCCTATGGGGTGCTGCAGTCAAGAACATTCGACAATGGGCGAAAGCCTGATCGAGCG
ACACCGCGTGCAGGATGAAGGCCTTCGGGTCGTAAACTGCGGTAATATAGTAACAATGCA
AATGAGTGCTATATGGAAAGAGGTGGGTAACTACGTGCCAGCACCAGCGGTAATGCGTAG
ACCTCAAGCGTTATCCGGATTTATTGGGCGTAAAGCGCGTGTAGGTGGTTTCGTGCGTCT
TCTGTTAAAGCCCACTGCCTAACGGTGGAAGTGCAGGAGATACGGCGAGACTAGAGGAGG
TTAGAGGTGCATAGAACGCACGGTGTAGGGGTGAAATCCGTTGATATCGTGCGGAATACC
AAAGGCGAAGGCATTGCACTGGGACCTTCCTGACATTGAGACGCGAAAGCGTGGGGAGCA
AAAAGGATTAGAAACCCGTGTAGTCC
>Otu5762
CCAGCCTATGGGAGGCACCAGTCGAGAATCTTTCGCAATGGGCGAAAGCCTGACGAAGCG
ACGCCGTGTGAGCGATGAAGGCCTTATGGTCGTAAAGCTCTTTCGCCTGTGAACAAGTGA
GTCTGACGAACAATCAGATAAATTGAGGGTAACAGGTAAAGAAGCACCGGCTAACTCCGT
GCCAGCAGCTGCGGTAATACGGAGGATGCAAGCATTAATCGGATTTATTGGGCGTAAAGG

GCGCGTAGGCCGGTCTTTAAGTCAGATGTGAAATCCCGAGGCTCAACCTCGGAACAGCAT
TTGAAACTGAAGACCTAGAGGGTAGAAGGAGAGAATGGAATTCCACAAGTAGCGGTGAAA
TGCGTAGATATGTGGAAGAACACCGGTGGCGAAGGCGATTCTCTATTTTATTCCTGACGC
TGAGGCGCGAAAGTAAGGGGATCAAACAGGATTAGAAACCCTCGTAGTCC
>Otu5763
CCAGCCTATGGGGGGCAGCAGTGGGGAATATTGGACAATGGGCGCAAGCCTGATCCAGCC
ATGCCGCGTGGAGGACGAAGGCCCTCTGGGTTGTAAACTCCTTTTATGGGGGACGAAAAA
GGGATTTTCTAATCCAACTGACGGTACCCCATGAATAAGCACCGGCTAACTCCGTGCCAG
CAGCCGCGGTAATACGGAGGGTGCAAGCGTTATCCGGATTCACTGGGTTTAAAGGGTGCG
TAGGCGGGCAGTTAAGTCAGTGGTGAAATCTTGGGGCTCAACCCCGAAACTGCCATTGAT
ACTATCTGCCTTGAATACCGTGGAGGTCAGCGGAATATGTCATGTAGCGGTGAAATGCTT
AGATATGACATAGAACACCGATTGCGAAGGCAGCTGGCTACACGGATATTGACGCTGAGG
CACGAAAGCGTGGGGATCAAACAGGATTAGAAACCCTTGTAGTCC
>Otu5766
CCAGCCTATGGGAGGCTCCAGTCGAGAATCTTTCGCAATGGGCGAAAGCCTGACGAAGCG
ACGCCGTGTGTGCGATGAAGGCCTTCGGGTTGTAAAGCACTTTCGCTTGGGAACAAGAGA
GATTGGCTAATATCCATTCAATTTGAGCGTACTAGGTAAAGAAGCACCGGCTAACTACGT
GCCAGCAGCTGCGGTAATACGTAGGGTGCAAGCATTAATCGGATTTATTGGGCGTAAAGG
GCGCGTAGGCGGGATGGTAAGTCAGATGTTAAAGCCCGGGGCTCAACCCCGGAAAAGCAT
TTGAAACTCCCATTCTAGAGGGTAGGCGGAGAAAATGGAATTCCACAAGTAGCGGTGAAA
TGCGTAGATATGTGGAAGAACACCAGTGGCGAAGGCGGTTTTCTAGCTTATACCTGACGC
TGAGGCGCGAAAGCAAGGGGATCAAACAGGATTAGAAACCCTAGTAGTCC
>Otu5767
CCAGCCTACGGGTTGCACCAGTGGGGAATCTTGCGCAATGGGCGAAAGCCTGACGCAGCA
ACGCCGCGTGAGGGACGAAGGCTTTCTGAGTTGTAAACCTCTTTCGACAGGAACGATTGT
GACGGTACCTGTAGAAGAAGCACCGGCCAACTATGTGCCAGCAGCCGCGGTGATACATAG
GGTGCAAGCGTTATTCGGATTTATTGGGCGTAAAGAGCTCGTAGGCGGTTCAGCAAGTCG
GGTGTTAAACCCCCAGGCTCAACCTGGGGCCGCCACCCGAAACTGTTGTGACTAGAGTTT
GGTAGGGGATCACGGAATTCCTGGTGTAGCGGTGGAATGCGCAGATATCAGGAGGAACAC
CTGCGGTGTAGACGGCTTTCTGGACCATTGCTGACGCTGAGACACGAAAGCGTGGGTAGC
AAACAGGATTAGAAACCCCCGTAGTCC
>Otu5769
CCAGCCTACGGGGGGCAGCAGTGAGGAATATTGGACAATGTGCGAAAGCATGATCCAGTA
ATGTCACTTTAGGGACGAAATCGTTTAGATGTAAACCTATGAAGTTGGTGAAGATAATGA
CATTAGCCAATGAATAGTCCTGGCTAACTTCGTGCCAGCAGCTGCGGTAAAACGGGGGGG
GCGAGTGTTACTCATATTGATTGGGCGTAAAGGGTGCGTAGATGGTTTTATTAAATTAAG
AAGTAAATATTAATGAAACGTGTTATAATAGTTTCTTAATACTATAAGACTTGAGTGCAT
CAGGGGAAAATGGTACTTTTGGTGAAGGGGTGAAATCCGTAGATATCTTAAGGACGACCG
AAGGCGAAAGCAATTTTCTAGGATGTGACTGACATTGAGGCACGAAAGCATGGGTAGCAA
ACAGGATTAGAGACCCTAGTAGTCC
>Otu5779
CCAGCCTATGGGGGGCAGCAGTCGAGAATCATTCGCAATGGGCGAAAGCCTGACGATGCG
ACGCCGTGTGTGTGATGAAGGCCTTCGGGTTGTAAAGCACTTTCGCCTGTGAACAAGAGA
AGCTAGCTAATATCTAGCGAATTTGAGGGTAGCAGGTAAAGAAGCACCGGCTAACTCCGT
GTCAGCAGCTGCGGTAATACGGAGGGTGCAAGCATTAATCGGATTTATTGGGCGTAAAGG
GCGCGTAGGCGGAAAGAAAAGTCAGATGTGAAAGACCGAGGCTTAACCTCGGAGCCGCAT
TTGAAACTCTCTTTCTAGAGGGTAGATGGAGAAAACGGAATTCCACGTGTAGCGGTGAAA
TGCGTAGATATGTGGAAGAACACCTGTGGCGAAGGCGGTTTTCTAATTTATTCCTGACGC
TGAGGCGCGAAAGTAAGGGGATCAAACAGGATTAGAAACCCCCGTAGTCC
>Otu5784
CCAGCCTACGGGATGCTGCAGTGGGGAATATTGGACAATGGGCGAAAGCCTGATCCAGCA
ATGCCGCGTGTGTGAAGAAGGTCTTCGGATTGTAAAGCACTTTTGGCAGGGACGATGATG
ACGGTACCTGCAGAATAAGCCCCGGCTAACTTCGTGCCAGCAGCCGCGGTAATACGAAGG
GGGCTAGCGTTGCTCGGAATGACTGGGCGTAAAGGGCGCGTAGGCGGCATACACAGTCAG
AAGTGAAATTCCTGGGCTCAACCTGGGGACTGCTTTTGATACGTGTGAGCTAGAGTGGAG
AAGAGGGTTGTGGAATTTCCAGTGTAGAGGTGAAATTCGTAGATATTGGGAAGAACACCG
GTGGCGAAGGCGGCTCACTGGCTCGTAACTGACGCTCAGGCGCGACAGCGTGGGGATCAA
ACAGGATTAGAAACCCTAGTAGTCC

>Otu5793
CCAGCCTATGGGTCGCACCAGTGGGGAATCTTTCACAATGGGGGAAACCCTGATGGAGCG
ACGCCGCGTGGAGGATGAAGGTTTTCGGATTGTAAACTCCTGTCACTGCAGAACAAGGGT
GCGTTTGTGAATAGCAGACGCATTTGATGGTATGCGGAGAGGAAGGGACGGCTAACTCTG
TGCCAGCAGCCGCGGTAAGACAGGGGTCCCGAGCGTTGTTCGGATTCATTGGGCGTAAAG
GGTGTGTAGGAGGTCGGGTAAGTCAGGTGTGAAATCTCAGAGCTTAACTCTGAAACTGCG
CTTGATACTGCCCGGCTAGAGGATCGGAGGGGGTATCGGAATTTATGGTGTAGCAGTGAA
ATGCGTAGATATCGTAAGGAAGACCAGTGGCGAAGGCGAATCTCTGGACAGTTACTGACG
CTGAGGCACGAAGGCCAGGGGAGCAAACGGGATTAGATACCCTAGTAGTCC
>Otu5796
CCAGCCTATGGGGCGCAGCAGTAAGGAATATTGGACAATGGTGGCAACACTGATCCAGCC
ATGCCGCGTGCAGGATGAAGGCGCTACGCGTTGTAAACTGCTTTTGTACCAGAGAAAACC
TATCTACGTGTAGATAGCTGATAGTATGGTAAGAATAAGCATCGGCTAACTTCGTGCCAG
CAGCCGCGGTAATACGAAGGATGCAAGCGTTATCCGGATTTATTGGGTTTAAAGGGTGCG
TAGGCGGATCAATAAGTCAGTGGTGAAAACCTTCAGCTTAACTGGAGACTTGCCATTGAT
ACTGTTAGTCTTGAGTACAGTCAAGGTAGGCGGAATGTGTAATGTAGCGGTGAAATGCTT
AGATATTACACAGAACACCGATTGCGAAGGCAGCTGACGAAACTGTAACTGACGCTGAGG
CACGAAAGCGTGGGGATCAAACAGGATTAGAAACCCTAGTAGTCC
>Otu5798
CCAGCCTATGGGATGCTGCAGTCGAGAATTTTTCACAATGGGCGCAAGCCTGATGGAGCG
ACGCCGCGTGGGGGATGAGTGGCTTCGGCCCGTAAACCCCTGTCATTTGCGATCAAACCT
CGTTATTTAAAAGATGACGAGCTGATAGTAGCGAAAGAGGAAGGGACGGCTAACTCTGTG
CCAGCAGCCGCGGTAATACAGAGGTCCCAAGCGTTGTTCGGATTCACTGGGCGTAAAGGG
TGCGTAGGCGGTTGGGTAAGTTTGACGTGAAATCTCCGGGCCTAACCCGGAAACTGCGTT
GAATACTATCTGACTAGAGGAATGGAGGGGAGACTGGAATACTTGGTGTAGCAGTGAAAT
GCGTAGATATCAAGTGGAACACCAGTGGCGAAGGCGAGTCTCTGGACATTTCCTGACGCT
GAGGCACGAAAGCCAGGGGAGCAAACGGGATTAGATACCCGTGTAGTCC
>Otu5804
CCAGCCTATGGGACGCAGCAGTGAGGAATATTGGTCAATGGGGGCAACCCTGAACCGGCC
ATGCCGCGTGAAGGATGAATGCCCTATGGGTTGTAAACTTCTTTTGTTAGGGACGAACCT
ATTATACGTGTATAGTACTGACGGTACCTAATGAATAAGCAACGGCTAACTCCGTGCCAG
CAGCCGCGGTAATACGGAGGTTGCAAGCGTTATCCGGATTTATTGGGTTTAAAGGGTGCG
TAGGCGGGATAATAAGTCAGTGGTGAAAACCTGCAGCTCAACTGTAGAACTGCCATTGAT
ACTGTTATTCTTGAGTTTGGTTGAGGTGGGCGGAATGTGTAGTGTAGCGGTGAAATGCTT
AGATATTACACAGAACACCGATTGCGTAGGCAGCTCACTAAACCAAAACTGACGCTGAGG
CACGAAAGCGTGGGGATCAAACAGGATTAGAAACCCCAGTAGTCC
>Otu5806
CCAGCCTACGGGGGGCTGCAGTCGAGAATATTCCTCAATGGACGAAAGTCTGAAGGAGCG
ACGCCGCGTGCAGGATGAAGGGCTTCGGCTCGTAAACTGCTTTTATTTGGGAAGAACTAA
TGACGGTACCAAATGAATAAGAGGTTGCTAACTCTGTGCCAGCAGCAGCGGTAATACAGA
GACCTCAAGCATTATCCGGATTTATTGGGCGTAAAGGGTCCGCAGGCGGTTACGCGCGTC
GATGGTTAAATCCTCGGGCTCAACTCGGGGACTGCTGTCGAAACGGCGCAACTAGAGGCC
GGGAGAGGTGAGCGGAATTGCCGGTGTAGTAGTAATATGCGTTGATATCGGCAAGAACAC
CAAATGCGAAGGCAGCTCACTAGAACGGTTCTGACGCTCAGGGACGAAAGCGTGGGGAGC
GAAAGGGATTAGATACCCTTGTAGTCC
>Otu5812
CCAGCCTACGGGGGGCAGCAGTGAGGAATTTTGCGCAATGGCCGCAAGGCTGACGCAGCA
ACGCCGCGTGGGTGAAGAAGGCCTTCGGGTCGTAAAGCCCTGTCAGGTGGGAAGAATGGC
CTGGGGATGAATAAGCCCCGGGAGTGACGGTACCACCAGAGGAAGCACCGGCTAACTCCG
TGCCAGCAGCCGCGGTAATACGGAGGGTGCAAGCGTTATTCGGAATTACTGGGCGTAAAG
CGCGTGCAGGCGGGCTAGCAAGTCTGATGTGAAAGCCCTGGGCTTAACCCGGGAAGTGCA
TTGGAAACTGCTAGTCTTGAGTGTTGGAGAGGAAGGGGGAATTCCCGGTGTAGAGGTGAA
ATTCGTAGAGATCGGGAGGAATACCGGTGGCGAAGGCGGCTACCTGGACCGATATTGACG
CTGAGACGCGAAAGCGTGGGGAGCAAACAGGATTAGAAACCCGTGTAGTCC
>Otu5813
CCAGCCTATGGGTTGCTCCAGTGGGGAATTTTGCGCAATGGGGGAAACCCTGACGCAGCA
ACGCCGCGTGAGTGATGAAGGCCTTCGGGTCGTAAAGCTCTGTCAGAGGGAAAGAAGTGT
AGGAGGGATAATACCCCTTTTACTTGACGGTACCCTCAAAGGAAGCACCGGCTAACTCTG

TGCCAGCAGCCGCGGTAATACAGAGGGGGCAAGCGTTGTTCGGAATTATTGGGCGTAAAG
GGTGCGTAGGCGGTGCGGTAAGTCTTTTGTGAAATCTCCGGGCTCAACTCGGAGTCTGCA
GGAGAAACTGCCGTGCTTGAGTATGGGAGAGGTGAGTGGAATTCCTGGTGTAGCGGTGAA
ATGCGTAGATATCAGGAGGAACACCTGTGGCGAAAGCGGCTCACTGGACCATAACTGACG
CTGAGGCACGAAAGCTAGGGGAGCAAACAGGATTAGATACCCGGGTAGTCC
>Otu5821
CCAGCCTATGGGTGGCAGCAGTGAGGAATCTTGCGCAATGGGGGCAACCCTGACGCAGCA
ACGCCGCGTGAGTGAGGAAGGTCTTCGGATCGTAAAGCTCTGTCGGGTGGGAAGAAATGT
ATGGAGGTGAATAATCTTCATGCTTGACGGTACCACCAAAGGAAGCACCGGCTAACTCCG
TGCCAGCAGCCGCGGTAATACGGGGGGTGCAAGCGTTGTTCGGAATCATTGGGCGTAAAG
GGCGTGTAGGCGGCTGGATAAGTCAGATGTGAAAGCCCTGGGCTTAACCCAGGAAGTGCA
TTTGAAACTGTCCGGCTTGAGTAGGGGAGAGGAAGGTGGAATTCCTGGTGTAGAGGTGAA
ATTCGTAGATATCAGGAGGAACACCGGTGGCGAAGGCGACCTTCTGGCCCTATACTGACG
CTGAGACGCGAAAGCGTGGGTAGCAAACAGGATTAGAGACCCTAGTAGTCC
>Otu5824
CCAGCCTACGGGGGGCAGCAGTTAGGAATCTTCTGCAATGCGCGCAAGCGTGACAGAGCG
AGCCAGAGTGTTTTTCATATGAAAAACTTTTGCCAGATGCAAAAAGTCTGGCGAATAAGG
ACTGGGAGAGACGGGTGCCAGCCGCCGCGGTAATACCCGCGGTCCAAGTCGCAGCCATCT
TTATTGGGTCTAAAACATCCGTAGCTTGCTTCGTAAGTCTCCTGTGAAATTCTATGTCTC
AAACATAGAGCGTGCAGGAGGTACTGCAAAGCTAGAGACTGGGAGACGTGAGAAGTATGT
TCAAAGTAGTAGTGAAATATTTTAATCTTGGACAGACTCACAATAGCGAAGGCATCTCAC
GAGAACAGTTCTGACAGTAAGGGATGAAGGCTAGGGTCGCGAAAGGGATTAGAGACCCCT
GTAGTCC
>Otu5834
CCAGCCTACGGGGTGCTGCAGTGGGGAATCTTGCGCAATGGACGAAAGTCTGACGCAGCA
ACGCCGCGTGGGTGATGAAGGTCTTCGGATCGTAAAGCCCTGTCGGAGGGGAAGATGCTT
TGCTTGGCTAATATCCAAGTGAGGTGACGGTACCTTTAAAGGAAGCACCGGCTAACTCTG
TGCCAGCAGCCGCGGTAATACAGAGGGTGCAAGCGTTGTTCGGAATTATTGGGCGTAAAG
GGAGTGTAGGCGGTTTTGATAGTCTTCTGTGAAATCTGCGGGCTCAGCCTGCAAAGTGCA
GAGGATACTGCAAGACTTGAGTGTCGGAGAGGATAGTGGAATTCCCGGTGTAGAGGTGAA
ATTCGTAGAGATCGGGAGGAACACCAGTGGCGAAGGCGACTGTCTGGCTGACAACTGACG
CTGAGACTCGAAAGCATGGGGAGCAAACAGGATTAGAGACCCTTGTAGTCC
>Otu5840
CCAGCCTACGGGGGGCTGCAGGGAATATTGCGCAATGGAGGAAACTCTGACGCAGCGACG
CCGCGTGGGTGACGAAGGCCTTCGGGTCGTAAAGCCCTGTCGGAAGGGACGAAAGACGTT
ATGGCTAACATCCATGACGAGTGACGGTACCTTCAGAGGAAGCACCGGCTAACTACGTGC
CAGCAGCCGCGGTAATACGTAGGGTGCTAGCGTTGTTCGGAATCATTGGGCGTAAAGCGC
GTGTAGGCGGTCAAATAAGTCGGGTGTGAAATCCCTGGGCTCAACCGAGGAAGTGCATCC
GAAACTATTTGGCTGGAAGATGGTAGAGGAAGGTGGAATTCCTAGTGTAGAGGTGAAATT
CGTAGATATTAGGAGGAATACCGGCGGCGAAGGCGGCCTTCTGGGCCATTCTTGACGCTG
AGACGCGAAAGCGTGGGGAGCAAACAGGATTAGATACCCCAGTAGTCC
>Otu5843
CCAGCCTACGGGTTGCAGCAGTGGGGAATATTGGGCAATGGGCGAAAGCCTGACCCAGCG
ACGCCATGTGGGTGATGAAGGCCTTCGGGTTGTAAAGCCCTGTTAGGTGGGAAGAAGGAT
CCAGAGGTAAATAATCTTTGGCTCTTGACGGTACCACCAGAGAAAGCACCGGCTAACTCC
GTGCCAGCAGCCGCGGTAATACGGAGGGTGCAAGCGTTGTTCGGAATTACTGGGCGTAAA
GGGCGCGTAGGTGGTTGCGTAAGTCGGATGTGAAATCCCCGGGCTTAACCCGGGAACTGC
ACCCGATACTGCGTGACTCGAGTGCAGCAGAGGGAAACGGAATTCCTGGTGTAGAGGTGA
AATTCGTAGATATCAGGAGGAACACCGGCGGCGAAGGCGGTTTCCTGGGCTGACACTGAC
ACTGAGGCGCGAAAGCGTGGGTAGCAAACAGGATTAGAAACCCCTGTAGTCC
>Otu5849
CCAGCCTATGGGGTGCAGCAGTGGGGAATATTGCACAATGGGCGAAAGCCTGATGCAGCG
ACGCCGCGTGAGGGATGACGGCCTTCGGGTTGTAAACCTCTTTCAGCAGGGAAGAAGCGT
GAGTGACGGTACCTGCAGAAGAAGCACCGGCTAACTACGTGCCAGCAGCCGCGGTAATAC
GTAGGGTGCGAGCGTTGTCCGGATTTATTGGGCGTAAAGAGCTCGTAGGCGGCTTGTCGC
GTCGGATGTGAAAGCCCGGGGCTCAACCCCGGGTTTGCATTCGATACGGGCAGGCTAGAG
TGTGGTAGGGGAGATCGGAATTCCTGGTGTAGCGGTGAAATGCGCAGATATCAGGAGGAA
CACCGGTGGCGAAGGCGGATCTCTGGGCCATTACTGACGCTGAGGAGCGAAAGCGTGGGG

AGCGAACAGGATTAGAGACCCCCGTAGTCC
>Otu5851
CCAGCTATGGGGTGCTGCAGTGGGGAATATTGGACAATGGGCGCAAGCCTGATCCAGCGA
TGCCGCGTGTGTGAAGAAGGTCTTAGGATTGTAAAGCACTTTCGGCGGGGATGATGATGA
CGGTACCCGCAGAAGAAGCCCCGGCTAACTTCGTGCCAGCAGCCGCGGTAATACGAAGGG
GGCTAGCGTTGCTCGGAATGACTGGGCGTAAAGGGCGCGTAGGCGGTTTGGACAGTCAGG
TGTGAAATTCCTGGGCTTAACCTGGGGGCTGCATTTGATACGTCCAGGCTTGAGTGAGGA
AGAGGGTCGTGGAATTCCCAGTGTAGAGGTGAAATTCGTAGATATTGGGAAGAACACCGG
TGGCGAAGGCGGCGACCTGGTCCTTAACTGACGCTGAGGCGCGAAAGCGTGGGGAGCAAA
CAGGATTAGATACCCGCGTAGTCC
>Otu5858
CCAGCCTACGGGGGGCTCCAGTCGAGAATCTTTCGCAATGGGCGAAAGCCTGACGAAGCG
ACGCCGTGTGAGCGAAGAAGGCCTTCAGGTTGTAAAGCTCTTTCGCTTGGGAACAAGAAA
GGGATACTAATACTGTCCTGATTTGAGGGTACTAGGTAAAGAAGCACCGGCTAACTCCGT
GCCAGCAGCTGCGGTAATACGGAGGGTGCAAGCATTGATCGGAATTACTGGGCGTAAAGG
GCGCGTAGGCGGACAGGAAAGTCAAATGTGAAATTCCGCGGCTCAACCGCGGAGCTGCAT
TTGAAACTTCCTGCCTGGAGCATAGTTAGGGAAAACGGAATTCCACGTGTAGCGGTGAAA
TGCGTAGATATGTGGAAGAACACCGGTGGTGAAGACGGTTTTCTGGGCTATTGCTGACGC
TGAGGCGCGAAAGCTAGGGGAGCAAACAGGATTAGAAACCCCAGTAGTCC
>Otu5865
CCAGCCTACGGGTCGCAGCAGTGAGGAATATTGGTCAATGGGCGAGAGCCTGAACCAGCC
ATCCCGCGTGCAGGAAGACGGCCCTATGGGTTGTAAACTGCTTTTTATGGGAAGAAAAA
GACCTACGTGTAGGTCACTGACGGTACCATATGAATAAGCATCGGCTAACTCCGTGCCAG
CAGCCGCGGTAATACGGAGGATGCGAGCGTTATCCGGATTTATTAGGTTTAAAGGGTGTG
TAGGCGGATAATTAAGTCAGTGGTGAAATCTCACAGCTTAACTGTGAAACTGCCATTGAT
ACTGGTAATCTTGAGTACGGTTGAAGTAGGCGGAATGTGTAATGTAGCGGTGAAATGCTT
AGATATTACACAGAACACCGATTGCGAAGGCAGCTTACTAAGCTGTTACTGACGCTGATG
CACGAAAGCGTGGGGAGCGAACAGGATTAGATACCCGCGTAGTCC
>Otu5869
CCAGCCTATGGGACGCAGCAGTGAGGAATATTGGGCAATGCCTGCAAAGGTGACCCAGCA
ACGCCGCGTGGAGGATGAAAACCGTAAGGTTGTAAACTCCTGTGAGAGGGGACGAATGGC
TAGTATCCGTAAGAATACTAGTATGACTGTACCCTCAGAGGAAGTCCCGGCTAACTACGT
GCCAGCAGCCGCGGTAATACGTAGGGGGCAAGCGTTGTCCGGATTTACTGGGTGTAAAGG
GCGCGTAGGCGGGTTTGTTAGTCAGAGGTGAAATCCTACAGCTCAACTGTAGAACTGCCT
TTGATACTGCAAATCTTGAGTTCAGAAGAGAGAAGCGGAATTCCAGGTGTAGTGGTGAAA
TACGTAGATATCTGGAAGAACACCAGTTGCGAAGGCGGCTTCTTGGTCTGTAACTGACGC
TCAGGCGCGAAAGCGTGGGTAGCAAACAGGATTAGATACCCCAGTAGTCA
>Otu5871
CCAGCCTATGGGGGGCAGCAGTCGAGAATCTTCCGCAATGGGGGAAACCCTGACGGAGCG
ACGCCGCGTGGAGGATGACATACTTCGGTGTGTAAACTCCTTTTGCCAGGGAAAAAGTTA
TTGATTGTACCTGGAGAATAAGAAGTTGCTAAACTCGTGCCAGCAGCAGCGGTAATACGA
GTGCTTCGAGCGTTATCCGGAATTATTGGGCGTACAGGGTGTGTAGGCGGCAGTGTTAGT
CTCTTGTAAAATTCCTCGGCTCAACCGAGGGCGCGCAAGAGAAACGGCACAGCTCGAGTA
TGGAAGGGGTCTTCGGAACTCATGGTGTAGCGGTGAAATGCGTTGATATCATGGGGAACA
CCAAAAGCGAAGGCAGAAGACTGGTCCATTACTGACGCTGAAACACGAAAGCGTGGGTCG
CGAATGGGATTAGAAACCCCCGTAGTCC
>Otu5874
CCAGCCTATGGGATGCTGCAGTAGGGAATATTCCACAATGGACGAAAGTCTGATGGAGCG
ACGCCGCGTGAGGGATGAAGGCCTTCGGGTTGTAAACCTCTTTCGACAGGGACTAAATTC
ACTGCAGTGAATAATTGCAGTGATTGACGGTACCTAGGGAAGAAGCTACGGCTAACTCTG
TGCCAGCAGCCGCGGTAATACAGAGGTAGCAAGCGTTGTCCGGATTCACTGGGCGTAAAG
GGCTTGTAGGTGGCTTTTTAAGTCAGGTGTGAAATCGTCCCGCTTAACGGGACAATTGCA
TTTGATACTGATAAGCTTGAGTGCGGGAAGGGAAACTGGAATTTCCGGTGTAGCGGTGAA
ATGCGTAGATATCGGAAAGAACACCAGTGGCGAAGGCGGGTTTCTGGCCTGCAACTGACA
CTGAGGAGCGAAAGCCAGGGGAGCAAACGGGATTAGATACCCTTGTAGTCC
>Otu5880
CCAGCCTATGGGGGGCTGCAGTCGAGAATCTTCGGCAATGGGCGCAAGCCTGACCGAGCG
ACGCCGCGTGCGGGATGAAGGCCTTCGGGTTGTAAACCGCTGTCAGTGTGGAGGAAATTC

CGTAGAGTTCTCTCTATGGATTGACCTATACGCAGAGGAAGGATGGGCTAAGTTCGTGCC
AGCAGCCGCGGTAAGACGAACCATCCAAACGTTATTCGGAATCACTGGGCTTAAAGGGTG
CGTAGGCGGCGCGTTAAGTCGGGTGTGAAATACCTCAGCCTAACTGAGGAATTGCGCCCG
AAACTAGCGTGCTTGAGGAAGACATAGGTGAGCGGAACTTAGGGTGGAGCGGTGAAATGC
GTTGATATCCTAAGGAACACCAGTAGCGAAAGCGGCTCACTGGGTCTTATCTGACGCTGA
GGCACGAAAGCCAGGGGAGCGAACGGGATTAGATACCCTAGTAGTCC
>Otu5884
CCAGCCTACGGGGCGCTCCAGTCGAGAATCTTTCGCAATGGGCGCAAGCCTGACGAAGCG
ACGCCGTGTGAGCGAAGAAGGCCTTCGGGTTGTAAAGCTCTTTCGCTAGGGAACAAGAGA
GGCGTGGTAATATAACGCCGATTTGATGGTACTTGGTAGAGAAGCACCGGCTAACTCCGT
GCCAGCAGCTGCGGTAATACGGAGGGTGCAAGCATTGATCGGAATTACTGGGCGTAAAGG
GCGCGTAGGCGGCTTTGAAAGTCAGATGTGAAATTCCGGGGCTCAACCCCGGAGCTGCAT
TTGAAACTTCAGAACTAGAGGTCAGTTAGGGAAAACGGAATTCCACGTGTAGCGGTGAAA
TGCGTAGATATGTGGAAGAACACCAGTGGTGAAGACGGTTTTCTGGGCTGATACTGACGC
TGAGGCGCGAAAGCAAGGGGAGCAAACAGGATTAGAGACCCTAGTAGTCC
>Otu5891
CCAGCCTACGGGGCGCACCAGTGGGGAATATTGGACAATGGGCGAAAGCCTGATCCAGCA
ATGCCGCGTGTGTGAAGAAGGCCTGCGGGTTGTAAAGCACTTTCAGTGGGGAAGAAAAGC
CCGGGACGAATAATCTCGGGTCTTGACGTTACCCACAGAAGAAGCACCGGCTAACTCCGT
GCCAGCAGCCGCGGTAATACGGAGGGTGCGAGCGTTAATCGGAATTACTGGGCGTAAAGC
GCGCGTAGGCGGTCGGGTAAGTCGGATGTGAAAGCCCTGGGCTTAACCTGGGAATTGCAT
TCGATACTGTCCGGCTAGAGTATGGGAGAGGGAAGTGGAATTTCCGGTGTAGCGGTGAAA
TGCGTAGATATCGGAAGGAACACCAATGGCGAAGGCAGCTTCCTGGCCCAATACTGACGC
TCAGGCGCGAAAGCGTGGGGAGCAAACAGGATTAGATACCCGGGTAGTCC
>Otu5893
CCAGCCTATGGGAGGCACCAGTAAGGAATATTGGTCAATGGGCGGAAGCCTGAACCAGCC
ATGCCGCGTGCAGGAAGACGGCCCTACGGGTTGTAAACTGCTTTTGCAGGGGAATAAACC
CCCGTACGTGTACGGGGCTGAATGTACTCTGAGAATAAGGATCGGCTAACTCCGTGCCAG
CAGCCGCGGTAATACGGAGGGTGCAAGCGTTATCCGGATTCACTGGGTTTAAAGGGTGCG
TAGGCGGGCAGGTAAGTCAGTGGTGAAATCTCCGGGCTTAACCCGGAAACTGCCGTTGAT
ACTATCTGTCTTGAATGTCGTGGAGGTGAGCGGAATATGTCATGTAGCGGTGAAATGCTT
AGATATGACATAGAACACCAATTGCGAAGGCAGCTCACTACACGATGATTGACGCTGAGG
CACGAAAGCGTGGGGATCAAACAGGATTAGAAACCCGCGTAGTCC
>Otu5895
CCAGCCTACGGGGGGCTCCAGTAGGGAATCTTGCGCAATGCGCGAAAGCGTGACGCAGCA
ACGCCGCGTGGGGGAAGAAGGCCTTCGGGTTGTAAACCCCTTTCAGTTGGGACGAAGCTT
CGTCGGTTAATAGCCGACTGGAGTGACGGTACCTTCACAAGAAGCACCGGCTAACTACGT
GCCAGCAGCCGCGGTAATACGTAGGGTGCAAGCGTTGTCCGGAATTATTGGGCGTAAAGA
GCTCGTAGGCGGCTTAGCAAGTCGGGTGTGAAACCTCCAGGCTCAACCTGGAGCCGCCAC
TCGATACTGCTATGGCTAGAGTCCGGTAGGGGACCACGGAATTCCTGGTGTAGCGGTGAA
ATGCGCAGATATCAGGAGGAACACCAGTGGCGAAGGCGGTGGTCTGGGCCGGAACTGACG
CTGAGGAGCGAAAGCGTGGGGAGCGAACAGGATTAGAAACCCTCGTAGTCC
>Otu5905
CCAGCCTATGGGGCGCTGCAGTGGGGAATCTTGCGCAATGGACGAAAGTCTGACGCAGCC
ACGCCGCGTGAGTGAAGAAGGCCTTCGGGTTGTAAAGCTCTGTCGAACGGGACGAAAACA
CTTGGGGTGAATAGCCTCAGGAATTGACGGTACCGTGAGAGGAGGCACCGGCTAACTCCG
TGCCAGCAGCCGCGGTAATACGGAGGGTGCAAGCGTTGCTCGGAATTATTGGGCGTAAAG
GGCAGGTAGGTGGTCTCATTTGTCTGAGGTGAAAGCCTTGAGCTTAACTCAAGAAGTGCC
TCAGAAACGGTGAGACTAGAGTCTTGGAGAGGGTCGCGGAATTCCCGGTGTAGCGGTGAA
ATGCGTAGAGATCGGGAGGAACACCAGAGGCGAAAGCGGCGGCCTAGACAAGTACTGACA
CTCAACTGCGAAAGCGTGGGGAGCAAACAGGATTAGAGACCCTTGTAGTCC
>Otu5911
CCAGCCTATGGGTCGCTGCAGTAGGGAATTTTCCACAATGGGCGAAAGCCTGATGGAGCA
ACGCCGCGTGCAGGATGAAGGCCTTAGGGTTGTAAACTGCTTTTCTCTGTGACGATTATG
ACGGTAACAGAGGAATAAGGACCTGCTAACTACGTGCCAGCAGCCGCGGTCATACGTAGG
GTCCAAGCGTTATCCGAAATTACTGGGCGTAAAGAGTTGCGTAGGTGGCATAGTAAGCGG
GTAGTGAAATCGCGTGGCTCAACCATACATCCATTACCTGAACTGCTAAGCTTGAGAACG
AGAGAGGTAGATGGAATTCCCAGTGTAGGAGTGAAATCCGTAGATATTGGGAGGAACACC

GATGGCGTAGGCAGTCTACTGGCTCGTTTCTGACACTAAGGCACGAAAGCGTGGGGAGCA
AACGGGATTAGAAACCCCAGTAGTCC
>Otu5928
CCAGCCTATGGGTGGCAGCAGTGGGGAATATTGCGCAATGGGGGAAACCCTGACGCAGCA
ACGCCGCGTGAGTGATGAAGGTCCTCGGATCGTAAAGCTCTGTCGGCAGGGAAGATGGTT
TGATTGGTTAATAACCAGTTAAGCTGACGGTACCTGCAAAGGAAGCACCGGCTAACTCTG
TGCCAGCAGCCGCGGTAATACAGAGGGTGCAAGCGTTGTTCGGAATTATTGGGCGTAAAG
GGCGCGTAGGCGGTCCCGAAAGTCGAATGTGAAAGCCCAGGGCTCAACCTTGGAAGTGCA
TCCGAAACTGCGGGACTAGAATCTCGGAAAGGGTGGTGGAATTCCCAGTGTAGAGGTGAA
ATTCGTAGATATTGGGAGGAACACCGGTGGCGAAGGCGACCACCTGGACGAAGATTGACG
CTGAGGCGCGAAAGCGTGGGGAGCAAACAGGATTAGAAACCCCCGTAGTCC
>Otu5936
CCAGCCTATGGGTCGCTGCAGTGAGGAACATTGGGCAATGGGCGAAAGCTTGACCTAGTA
ATGGTTCAAGAGTGAGAAATAGACGGCATTATCGCTGTAAAGCTCTTTCAAAAATGACGA
TAATGACTGTAATTAGAGAATAAGCACTGGCTAATTCTGTGCCAGCAGCCGCGGTAATAC
AGGGAGTGCAGACGTTATTCGGATGAATTTGGTGTAAAGTATATGTAGGCTGCTTTTTGA
ACTTTTTTAAAATCCTAGAATTAATCTTTTAGGAAAAATAAAGTAATCAAAAGCTAAGAG
TTTAAGAAAGGTTGAAAGAATTTCTTGTCTAAGGGTTGAATCTATAAAAATAAGGACGAC
TACCAATTTAGTAGCGAAAGCATTCTTCTGGCTTAATAACTGACGCTGAGATATGAAAGT
CTAGGGATCAAACAGGATTAGGGACCCTCGTAGTCC
>Otu5948
CCAGCCTACGGGGGGCAGCAGTGGGGAATATTGGACAATGGGCGAAAGCCTGATCCAGCA
ATGTCGCGTGAATGATGAAGGCCTTAGGGTTGTAAAGTTCTTTCGGTGGGGAAGATAATG
ACGGTACCCACATAAGAAGCTCCGGCTAACTCCGTGCCAGCAGCCGCGGTAATACGGAGG
GAGCTAGCGTTGTTCGGAATTACTGGGCGTAATGAGCGCGTAGGCGGCTAACTAAGTTGG
GAGTGAAATCCCAGGGCTTAACCCTGGAATGGCTTTCAAAACTGGTTAGCTAGAGGACGA
TAGAGGATGGTGGAATTCCTAGTGTAGAGGTGAAATTCTTAGATATTAGGAGGAACATCG
GAGGCGAAGGCGACCATCTGGGTCGTATCTGACGCTGAGGCGCGAAAGCGTGGGGAGCAA
ACAGGATTAGATACCCCCGTAGTCC
>Otu5952
CAGCCTATGGGTGGCAGCAGTGAGGAATATTGGTCAATGGACGCAAGTCTGAACCAGCCA
TGTCGCGTGCAGGAAGACGGCCTTATGGGTTGTAAACTGCTTTTCTATAGGGAGAAACCC
CCTTTCGTGAGAGGGGCTGACGGTACTATAGGAATAAACACCGGCTAACTCCGTGCCAGC
AGCCGCGGTAATACGGAGGGTGTAAGCGTTATCCGGAATCATTGGGTTTAAAGGGTCCGT
AGGCGGACTGATAAGTCAGAGGTGAAATCCCACAGCTTAACTGTGGAACTGCCTTTGATA
CTGTCAGTCTTGAGTTATATTGAAGTAGATGGAATGTGTAGTGTAGCGGTGAAATGCATA
GATATTACACAGAACACCGATTGCGAAGGCAGTCTACTAAGTATATACTGACGCTGAGGG
ACGAAAGCGTGGGGATCAAACAGGATTAGAAACCCTTGTAGTCC
>Otu5965
CAGCTACGGGGTGCTGCAGTGGGGAATATTGCGCAATGGACGAAAGTCTGACGCAGCAAC
GCCGCGTGAGTGAAGAAGGCCTTCGGGTTGTAAAGCTCTTTCGGCTGGGAAGAAGGGAGT
AAGGGCTAACAGTCCTTGCTTTTGATGGTACCAGAAGAAGAAGCACCGGCAAACTTCGTG
CCAGCAGCCGCGGTAATACGAAGGGTGCGAGCGTTGTTCGGAATTATTGGGCGTAAAGGG
TTCGTAGGCGGGATCGTAAGTCAAGTGTGAAATCCCCGAGCTTAACTCGGGACGTGCATT
TGAAACTGTGATTCTTGAGTCATTGAGAGGGTGGTGGAATTGTTGGTGTAGGAGTGACAT
CCGTAGAGATCAACAGGAACATCAGAGGCGAAAGCGGCCACCTGGCAATGTACTGACGCT
GAGGAACGAAAGCGTGGGGAGCAAACAGGATTAGATACCCTCGTAGTCC
>Otu5967
CCAGCCTACGGGTGGCAGCAGTAGGGAATCTTGCGCAATGCGCGAAAGCGTGACGCAGCA
ACGCCGCGTGGGGGAAGAAGGCTTTCGGGTTGTAAACCCCTTTCAGTTGGGACGAAGCTC
CGTCGGTTAATAGCCGGCTGGAGTGACGGTACCTTCACAAGAAGCACCGGCTAACTACGT
GCCAGCAGCCGCGGTAATACGTAGGGTGCAAGCGTTATTCGGATTTATTGGGCGTAAAGA
GCTCGTAGGCGGTTCAGCAAGTCGGGTGTTAAACCCCCAGGCTCAACCTGGGGCCGCCAC
CCGAAACTGTTGTGACTAGAGTTTGGTAGGGGATCACGGAATTCCTGGTGTAGCGGTGGA
ATGCGCAGATATCAGGAGGAACACCAGTAGCGAAGGCGGTGATCTGGGCCGGTACTGACG
CTGAGGAGCGAAAGCGTGGGGAGCGAACAGGATTAGAAACCCCGGTAGTCC
>Otu5968
CCAGCCTATGGGTCGCAGCAGTCGAGAATCATGCTCAATGCGCGAAAGCGTGAAGCTGTG

ATCCATCAGGAGTGAAGACGAGCAATCGTAAAGCTCATTAGAACGTTCACATTTTGAGTG
ACGTTTGAAAAGTAGTAGTTAATACATGTGCCAGCAGCTGCGGTCATACATGAGCTGCGG
GCATTAATTGGGAGGATTCGGTGTAAAGGGTACGTAGGCGGTAGCGATGAATCCACACGA
TGGACGAGATGCTCTCTCAGAGTTGTGTGGTGCCAGCTTCTTGAGATGGCTAGAGGAGAA
CGGAATGGTGAGGGGAGGAATGAAATCCTGAGATCTTCATTGGAACAACGAAGGCGAAGG
CAGTTCTCTGGGGCTTATCTGACGCTGAGGTACGAAAGGATGGGGAGCAAACGAGATTAG
AAACCCTAGTAGTCC
>Otu5970
CCAGCCTATGGGTCGCTGCAGTAAGGAATATTGGACAATGGGGGAAACCCTGATCCAGCA
ATGCCGCGTGAATGAAGAAGGCCTTCGGGTTGTAAAGTTCTTTCGCTCGCGAAGATAATG
ACGGTAGCGAGATAAGAAGGTCCGGCTAACTCTGTGCCAGCAGCCGCGGTGAGACAGAGG
GACCTAGCGTTGTTCGGAATGACTGGGCGTAAAGGGTGCGTAGGTGGTTATTTAAGTAAA
GTGTTAAAGCCCCAGGCTCAACCTGGGAATCGCATTTTATACTGGATAGCTAGAGTATGG
CAGAGGAAAATGGAATTCCTAGTGTAGAGGTGGAATTCGTAGATATTAGGAGGAACACCA
GAGGCGAAGGCGATTTTCTGGGCCATTACTGACACTGAGGCACGAAAGCGTGGGGAGCAA
ACAGGATTAGAGACCCTAGTAGTCC
>Otu5973
CCAGCCTACGGGGGGCAGCAGTCGAGAATCATTCGCAATGGGCGAAAGCCTGACGATGCG
ACGCCGTGTGAGCGATGAAGGCCTTCGGGTTGTAAAGCTCTTTCGCCTGGGAACAAGAGA
GATCGGCTAATATCCGGCCAATTTGAGGGTACCAGGTAAGGAAGCACCGGCTAACTCCGT
GCCAGCAGCTGCGGTAATACGGAGGGTGCAAGCATTAATCGGAATTATTGGGCGTAAAGG
GCGCGTAGGCGGGGAGATAAGTCAGATGTGAAATCCCGGGGCTCAACCTCGGAACAGCAT
TTGAAACTATCTTTCTTGAGGGTAGGAGGAGAAAACGGAATTCCACAAGTAGCGGTGAAA
TGCGTAGATATGTGGAAGAACACCGGTGGCGAAGGCGGTTTTCTATCTTATTCCTGACGC
TGAGGCGCGAAAGCAAGGGGATCAAACAGGATTAGAAACCCGCGTAGTCC
>Otu5974
CAAGCCTATGGGTCGCACCAGTGGGGAATATTGGACAATGGGCGCAAGCCTGATCCAGCA
ATGCCGCGTGAGTGAAGAAGGTCTTCGGATTGTAAAGCTCTTTCGACGGGGACGATGATG
ACGGTACCCGTAGAAGAAGCCCCGGCTAACTTCGTGCCAGCAGCCGCGGTAATACGAAGG
GGGCTAGCGTTGCTCGGAATGACTGGGCGTAAAGGGCGCGTAGGCGGCGATGATAGTCAG
GCGTGAAATTCCTGGGCTCAACCTGGGGACTGCGTTTGATACGTTATTGCTTGAGTTCGG
AAGAGGGTCGTGGAATTCCCAGTGTAGAGGTGAAATTCGTAGATATTCGCAAGAACACCA
GTGGCGAAGGCGGCTCACTGGCCCGGTACTGACGCTGAGGTGCGAAAGCGTGGGGAGCAA
ACGGGATTAGATACCCTCGTAGTCC
>Otu5981
CCAGCCTACGGGTTGCAGCAGTGGGGAATATTGCGCAATGGGCGAAAGCCTGACGCAGCG
ACGCCGCGTGGAGGATGAAGGCCTTCGGGTTGTAAACTTCTGTTAAGTGGGAAGAAATAA
GCGCTTCTAATACAGGCGCAAGATGACGGTACCATTAGAGAAAGCACCGGCTAAACTCGT
GCCAGCAGCCGCGGTAATACGAGTGGTGCAAGCGTTATTCGGAATCATTGGGCGTAAAGG
GTGTGTAGACGGTGTGCTAAGTCAACTGTTTAATTCTCCGGCCTAACCGGAGGCCAGCGG
TAGAAACTAGCATGCTAGAGGGTGGAAGAGAGAAGTGGAATTCTCGGAGTAGCGGTAAAA
TGCGTAGATCTCGAGAGGAACACCAATGGCGAAGGCAGCTTCTTGGTCCATTCCTGGCGT
TGAAACACGAAAGCGTGGGGAGCAAACAGGATTAGAAACCCTGGTAGTCC
>Otu6020
CCAGCCTATGGGTGGCAGCAGTGGGGAATATTGGACAATGGGCGCAAGCCTGATCCAGCA
ATGCCGCGTGAATGATGAAGGTCTTAGGGTTGTAAACTGCTTTTGCCGGGGGATAAAAGA
CCCCTGCGGGGGAAATTGAAGGTACCTGGTGAATAAGCCACGGCTAACTACGTGCCAGCA
GCCGCGGTAATACGTAGGTGGCAAGCGTTGTCCGGATTTATTGGGTTTAAAGGGTGCGTA
GGCGGTTCCTTAAGTCAGTGGTGAAATACAGCCGCTTAACGGTTGAGGTGCCAATGATAC
TGGGGGACTTGAGTACAGACGAGGTAGGCGGAATTGACGGTGTAGCGGTGAAATGCATAG
ATATCGTCAAGAACACCGATAGCGAAGGCAGCTTACTAGACTGTAACTGACGCTGATGCA
CGAAAGTGTGGGGATCAAACAGGATTAGAGACCCGTGTAGTCC
>Otu6024
CCAGCCTATGGGATGCACCAGTATACGGCGACTCAAGCCGCAAGTGCGCGCACACAGCGC
CGGCCGGCGCGCAATCAGATAGGAGGCAGTAGCCGCCTTCGGCGGACGCGATAGAGCAGG
GTCAGGAAGGTGACCGAGCCGCAGGTGATAAGGCTCGCGACCATGCCAAGGATCAGCGAG
AGGTCGTGCTGATAGACGCCGTGTACGATCGCGATCACCTGCAGCACGTTGAATCCCCCA
AAGGTGATCAGGGAGATTCCCTCGTCGCTCTTCTTGCGCCAGAGCGCCAGCACCTGCGGG

ACGAACAGCAGCGCGTTGCACAGGAGCGCCAGGCCAAAGATCACAGCGACAATCTGCTTC
ATGGGAACCTTCTCGTTGGAGGAGCTTCAAAAATTAGAGACCCTTGTAGTCC
>Otu6029
CCAGCCTATGGGGTGCACCAGTGGGGAATATTGGACAATGGGGGCAAGCCTGATCCAGCA
ATGCCGCGTGTGTGAAGAAGGCCTGCGGGTTGTAAAGCACTTTCAGTGGGGAGGAAGGCC
TTAGTGTTAATAGCACTGAGGATTGACGTTACCTACAGAAGAAGCACCGGCTAACTCTGT
GCCAGCAGCCGCGGTAATACAGAGGGTGCGAGCGTTAATCGGAATTACTGGGCGTAAAGG
GCGCGTAGGTGGTTAATTAAGTCAGATGTGAAATCCCTGGGCTTAACCTAGGAATTGCAT
ATGAAACTGATTAGCTAGAGTGCAAGAGAGGGAAGTGGAATTTCCGGTGTAGCGGTGAAA
TGCGTAGATATCGGAAGGAACACCAGTGGCGAAGGCGACTTCCTGGCTTGTTACTGACAC
TGAGGCGCGAAAGCGTGGGGAGCGAACAGGATTAGAAACCCCCGTAGTCC
>Otu6040
CCAGCCTATGGGGGGCTGCAGTGGGGAATATTGGACAATGGGCGCAAGCCTGATCCAGCC
ATGCCGCGTGGGTGATGAAGGCCCTAGGGTTGTAAAGCCCTTTCGGCGGGGAAGATAATG
ACGGTACCCGCAGAAGAAGCCCCGGCTAACTTCGTGCCAGCAGCCGCGGTAATACGAAGG
GGGCTAGCGTTGCTCGGAATGACTGGGCGTAAAGGGCGCGTAGGCGGCACATACAGTCAG
AAGTGAAATTCCTGGGCTCAACCTGGGGACTGCTTTTGATACGTGTGAGCTAGAGTGGAG
AAGAGGGTTGTGGAATTTCCAGTGTAGAGGTGAAATTCGTAGATATTGGGAAGAACACCG
GTGGCGAAGGCGGCGACCTGGTCCTTAACTGACGCTGAGGCGCGAAAGCGTGGGGAGCAG
ACAGGATTAGATACCCGAGTAGTCC
>Otu6044
CAGCCTATGGGGGGCAGCAGTGAGGAATCTTGCGCAATGGGCGAAAGCCTGACGCAGCGA
CGCCGCGTGAAGGATGAAGGTCTTCGGATTGTAAACTTCTGTTAAGCGGGAAGAAAGGTC
TATTTCTAATACAGATAGATGATGACGGTACCGCTAGAGAAAGCACCGGCTAACTTCGTG
CCAGCAGCCGCGGTAATACGAGGGGTGCAAGCGTTATTCGGATTTATTGGGCGTAAAGGG
TGTGCAGACGGTATATTAAGTCACTTGTTAAATTCTTCGGCCTAACTGAAGGTATGCAGG
TGATACTGGTAAACTAGAGGGTGGAAGAGAGAAGTGGAATTCTCGGAGTAGCGGTAAAAT
GCGTAGATCTCGAGAGGAACACCAATGGCGAAGGCAGCTTCTTGGTCCATTCCTGACGTT
CATACACGAAAGCGTGGGGAGCAAACAGGATTAGATACCCTAGTAGTCC
>Otu6048
CCAGCCTACGGGAGGCAGCGGGGAATTTTGGACAATGGACGCAAGTCTGATCCAGCCATG
CCGCGTGCGGGAAGAAGGCCTTCGGATTGTAAACCGCTTTTGTCAGGGAAGAAAAGTCTC
GAGTTAATACCTTGGGATGATGACGGTACCTGAAGAATAAGCACCGGCTAACTACGTGCC
AGCAGCCGCGGTAATACGTAGGGTGCAAGCGTTAATCGGAATTACTGGGCGTAAAGCGTG
CGCAGGCGGTCCGCTAAGACAGATGTGAAATCCCCGGGCTTAACCTGGGAACTGCATTTG
TGACTGGCGGGCTAGAGTATGGCAGAGGGGGGTAGAATTCCGCGTGTAGCAGTGAAATGC
GTAGAGATGTGGAGGAATACCGATGGCGAAGGCAGCCCCCTGGGCCAATACTGACGCTCA
TGCACGAAAGCGTGGGGAGCAAACAGGATTAGATACCCTTGTAGTCC
>Otu6053
CCAGCCTATGGGGCGCAGCAGTAGGGAATCTTCCACAATGGGCGAAAGCCTGATGGAGCA
ACGCCGCGTGCAGGATGAAGGCCTTCGGGTCGTAAACTGCTTTTATCTGTGACGATTATG
ACGGTAGCAGATGAATAAGGATCGGCTAACTCCGTGCCAGCAGCCGCGGTCATACGGAGG
ATCCAAGCGTTATCCGGAATTACTGGGCGTAAAGAGTTGCGTAGGTGGCAGAGTAAGTAG
GGCATGAAATCGTGTGGCTCAACCATACACACATGTTCTAAACTGCTCAGCTTGAGGACG
AGAGAGGTTATTGGAATTCCTAGTGTAGGAGTGAAATCCGTAGATATTAGGAGGAACACC
GATGGCGTAAGCAGATAACTGGCTCGTTCCTGACACTAAGGCACGAAAGCGTGGGGAGCA
AACGGGATTAGAAACCCTAGTAGTCC
>Otu6055
CCAGCCTATGGGGTGCACCAGTGGGGAATATTGGACAATGGGCGCAAGCCTGATCCAGCA
ATGCCGCGTGGATGAAGAAGGTCTTCGGATTGTAAAGTCCTTTCGGCGGGGACGATGATG
ACGGTACCCGCAGAAGAAGCCCCGGCTAACTTCGTGCCAGCAGCCGCGGTAATACGAAGG
GGGCTAGCGTTGCTCGGAATGACTGGGCGTAAAGGGCGCGTAGGCGGCTGATCTCGTCAG
GCGTGAAATTCCTGGGCTCAACCTGGGGGCTGCGTTTGATACAGATTGGCTGGAGTGGGG
AAGAGGGTCGTGGAATTCCCAGTGTAGAGGTGAAATTCGTAGATATTGGGAAGAACACCG
GTGGCGAAGGCGGCGACCTGGTCCTTGACTGACGCTGAGGCGCGAAAGCGTGGGGAGCAA
ACAGGATTAGATACCCCTGTAGTCC
>Otu6059
CCAGCCTATGGGGTGCAGCAGTGGGGAATATTGCACAATGGGCGGAAGCCTGATGCAGCG

ACGCCGCGTGAGGGATGACGGCCTTCGGGTTGTGAACCTCTTTCGACAGGGACGAAGCGT
GAGTGACGGTACCTGTAGAAGAAGCACCGGCCAACTACGTGCCAGCAGCCGCGGTAATAC
GTAGGGTGCGAGCGTTGTCCGGAATTACTGGGCGTAAAGAGCTTGTAGGCGGTCTGTCAC
GTCTTCTGTGAAAACTTGGGGCTCAACCTTAAGCTTGCAGGGGATACGGGCAGACTAGAG
TACTTCAGGGGAGACTGGAATTCCTGGTGTAGCGGTGAAATGCGCAGATATCAGGAGGAA
CACCGGTGGCGAAGGCGGGTCTCTGGGAAGTAACTGACGCTGAGAAGCGAAAGCGTGGGT
AGCGAACAGGATTAGAAACCCGCGTAGTCC
>Otu6060
CCAGCCTATGGGGGGCAGCAGTGGGGAATATTGGACAATGGGGGCAACCCTGATCCAGCG
ATGCCGCGTGGGTGAAGAAGGCCTTCGGGTTGTAAAGCCCTTTAGGTTGGGAAGAAGAGT
GTAGTGATACACTTTGACGGTACCAACAGAATAAGCACCGGCAAACTCTGTGCCAGCAGC
CGCGGTAATACAGAGGGTGCGAGCGTTAATCGGATTTACTGGGCGTAAAGGGCGCGTAGG
CGGTTTAGTGAGTGTGGTGTGAAAGCCCCGGGCTTAACCTGGGAAGTGCATCGCAAACGA
CTGAACTGGAGTATAAGAGAGGGTGGCGGAATTTCCGGTGTAGCGGTGAAATGCGTAGAG
ATCGGAAGGAACGTCAATGGCGAAGGCAGCCACCTGGCTTAATACTGACGCTGAGGCGCG
AAAGCGTGGGGATCAAACAGGATTAGATACCCTTGTAGTCC
>Otu6074
CCAGCCTACGGGTGGCAGCAGTCGAGAATCTTCCGCAATGGACGAAAGTCTGACGGAGCG
ACGCCGCGTGGTTGATGAAGTCCTTAGGGACGTAAAAACCTTTTATGTGGGAGAAAGTCA
TTGATAGTACCACATGAATAAGGGGTTGCTAAACTCGTGCCAGCAGCAGCGGTAATACGA
GTGCCCCAAGCGTTATCCGGAATTATTGGGCGTAAAGGGTGTGTAGGTGGTTGTGTTAGT
CTCTTGTTAAAGCTCTCGGCTCAACCGGGAACATGCAGGGGAAACGGCACAACTAGAGGA
TGTGAGAGGTCTGTGGAACTCATGGTGTAGGGGTGAAATCCGTTGATATCATGGGGAACA
CCAAAAGCGAAGGCAGCAGACTGGCGCATTCCTGACACTGAAACACGAAAGCGTGGGGAG
CAAACAGGATTAGAAACCCCAGTAGTCC
>Otu6076
CCAGCCTATGGGATGCACCAGTCGAGAATATTCGACAATGGGCGAAAGCCTGATCGAGCG
ACGCCGCGTGCAGGATGAAGGCCTTCGGGTCGTAAACTGCGGTAGTAGGTCAGCAATGCA
AATGAGCAACCTACGGAAAGAGGTGGGTAACTACGTGCCAGCACCAGCGGTAATACGTAG
ACCTCAAGCGTTATCCGGATTTATTGGGCGTAAAGCGTGTGTAGGAGGTTCGATGCGTCT
CATGTTAAAGCCCACCGCTCAACGGTGGATCTGCGTGGGATACGATCGAGCTCGAGGGGG
TTAGAGGTGCATGGAACTCACGGTGTAGGGGTGACATCCGTTGATATCGTGGGGAACACC
GAAGGCGAAGGCAGTGCACTGGGACCTTCCTGACTCTGAGACACGAAAGCGTGGGGAGCA
AAAAGGATTAGAAACCCGTGTAGTCC
>Otu6083
CCAGCCTATGGGGGGCTCCAGTGGGGAATATTGGACAATGGGCGCAAGCCTGATCCAGCA
ATACCGTGTGTGTGAAGAAGGCCTTTGGGTTGTAAAGCACTTTTGTGGGGGAAGAAGGGG
TATGGATGAATAGTTCATATTTTTGACGTTACCCCAAGAATAAGCACCGGCTAACTCTGT
GCCAGCAGCCGCGGTAATACAGAGGGTGCGAGCGTTAATCGGAATTATTGGGCGTAAAGG
GTGTGTAGGTGGTTTGATAAGTTAGGTGTGAAATACCTGGGCTCAACCTGGGTGCTGCAT
TTAATACTGTTGAGCTGGAGTATGGGAGAGGCAAGTGGAATTTCCGGTGTAGCGGTGAAA
TGCGTAGATATCGGAAGGAACATCGGTGGCGAAGGCGGCTTGCTGGACTGATACTGACAC
TGAGGCACGAGAGCATGGGGAGCAAACAGGATTAGAGACCCTAGTAGTCC
>Otu6092
CCAGCCTACGGGATGCAGCAGTGAAGAATCTTGGGCAATGGACTAACGTCTGACCCAGTG
AGAATACGTGCATGACGAATGCGCTCAGTTTTCTGTAAAGTGCTTTCAGCGGTGAAGAAG
AGGTCTGTAATCGCCAGAAGAAGCACCGGCTAATTCCGTGCCAGCAGCCGCGGTAATACG
GAAGGTGCGGGCGTTATTCGTTTTGATTGGGTGTAAAGGGTATGTAGGCGGCTGTTGTTG
TTGTTTAGGGCAAAAAAGCGGAGTTTTCCTATGCTCTGTCCATTTAGCGACACACAGCTT
GAGTTAAAGATAGGTCGGAATAATTTCCTGTGTAGGGGTAGAATCCATCCATACAGGAAC
GAGTGCCAACCGGCGAAGGCATCCCTCTCGCTTTAACTGACGCTAAGGTACGGAAGCCTA
GGGCGCAAACGGGATTAGAGACCCCCGTAGTCC
>Otu6099
CCAGCCTACGGGGCGCTGCAGTCGAGAGGCTTCGGCAATGGGGGAAACCCTGACCGAGCG
ACGCCGCGTGGGCGATGAAGGCCGTTAGGTTGTAAAGCCCTGTCGAGCGGGAAGAAATGC
AAGCGGGTAAACAATCCGCTTGTTTGACGGTACCGTTAAAGGAAGCTCCGGCCAACTCCG
TGCCAGCAGCCGCGGTAATACGGGGGGAGCAAACGTTGTTCGGAATTACTGGGCGTAAAG
GGCGTGCAGGCGGCCTTGTAAGTGGAATGTGAAATGCCCTGGCTCAACCAGGGAACTGCA

TCCCAGACTGCAAGGCTTGAGTATAGGAGAGGATGGGGGAATTCCCGGTGTAGCGGTGAA
ATGCATTGATATCGGGAGGAACACCAGTGGCGAAGGCGCCCATCTGGCCTAATACTGACG
CTCAGGCGCGAAAGCTAGGGGAGCAAACAGGATTAGAGACCCTGGTAGTCC
>Otu6100
CCAGCCTACGGGGTGCTGCAGGCGCGAAACCTTTACAATGCACGAAAGTGTGATAGGGGG
ACTCGAAGTGCTTATACAATAGTATAGGCTTTTGCCAACTCGAAATTGGTTGGAGAATAA
GTGGTGGGCAAGACTGGTGCCAGCCGCCGCGGTAACCCCAGCGCCACAAGTGGGAATCGG
TATTATTGGGTCTAAAGCGTCCGTAGCAGGTCTGCTAAATTGCTTGTGAAATCGCATCGC
TCAACGGTGCGGATTGCAGGTAAGACTGACAGACTAGAGACTGGGAGACGTTGGAGGTAT
TCAAAGGGGAGCGGTAAAATGCGAGAATCCTTTGAAGACCACCTGTTGCGAAGGCGTCCA
ACAATAACAGGTCTGACTGTGAGGGTCGAAAGCTAGGGGAGCGAACCGGATTAGAGACCC
TAGTAGTCC
>Otu6104
CCAGCCTACGGGGGGCACCAGTGGGGAATTTTCCACAATGGGCGCAAGCCTGATGGAGCA
ACGCCGCGTGTGTGATGAAGGTCTTCGGATCGTAAAGCACTTTCGACCGGGACGAAGAAC
CTCTGGCTAACATCCAGGGGCCTGACGGTACCGGGAGAAGAAGCACCGGCTAACTCTGTG
CCAGCAGCCGCGGTAATACAGAGGGTGCAAGCGTTGTTCGGAATTATTGGGCGTAAAGCG
CGTGTAGGCGGCTTTGCAAGTCAGATGTGAAAGCCCTCGGCTCAACCGAGGAAGTGCGTC
TGAAACTGCAGAGCTTGAGTACCGGAGAGGGTGGCGGAACTCTGCAAGTAGAGGTGAAAT
TTGTAGATATGCGGAAGAACACCGGTGGCGAAGGCGGCCACCTGGACGGTCACTGACGCT
GAGACGCGAAAGCGTGGGGAGCAAACAGGATTAGAAACCCCAGTAC
>Otu6105
CCAGCCTACGGGACGCAGCAGTGGGGAATATTGCACAATGGGCGAAAGCCTGATGCAGCG
ACGCCGCGTGGAGGATGACGGTCTTCGGATTGTAAACTCCTGTTAAGTGGGAAGAAAGAC
TGGCATCTAATACATGCTGGGGATGACGGTACCACTAGAGAAGGCACCGGCAAACTTCGT
GCCAGCAGCCGCGGTAATACGAAGGGTGCAAGCGTTATTCGGAATGACTGGGCGTAAAGA
GCGTGAAGGCGGTCCTTTAAGTCATTTGTTAAAGCGCTCGGCTTAACCGAGCATATGCGA
GTGAAACTGAAGGGCTAGAGTACAGGAGAGAAAGGTAGAATTCTCGGAGTAGCGGTAAAA
TGCGTAGATCTCGAGAGGAATACCGGTTGCGAAGGCGGCCTTTTGGCCTGTTACTGACGC
TTAAGCGCGAAAGCGTGGGGAGCAAACAGGATTAGAGACCCTAGTAGTCC
>Otu6106
CCAGCCTACGGGTTGCAGCAGTCGCGAAACCTTCGCAATGCGCGAAAGCGTGACGAGGGT
AATCTGAGTGCGAACTTTTAGTTCGCTTTTAGTCAGTTTGAATAACTGGCTGAATAAGGG
GTGGGTAAATCCTGTGCCAGCCGCCGCGGTAACACAGGTAGCCCGAGTAGTGCCCACGAA
TATTGAGTCTAAAACGCGCGTAGCCGGCACAGCCAGTTCCTTGTGAAATGCACGAGCCTA
ACTCGTGCGCGCGCAGGGAATACTACTGTGCTTGGGAGTGGAGGAGGCAGAGGGTACTTT
AGGGGGAGGAGTAAAATCCTGTAATCCTTAAAGGACCATCGGTGGCGAAAGCGCTCTGCC
AAGACACGTCCGACGGTGAGGCGCGAAGGCTAGGGGAGCAAATCGGATTAGAAACCCGCG
TAGTCC
>Otu6112
CCAGCCTATGGGTCGCTGCAGTGAGGAATATTGGTCAATGGGCGCAAGCCTGAGCCAGCC
ATGCCGCGTGAAGGATGAAGGCCTTCTGGGTTGTAAACTTCTTTTATGGGGGAAGAAAAT
TACGGTATCTACTGTAACCGACGGTACCCTAGGAATAAGCACCGGCTAACTCCGTGCCAG
CAGCCGCGGTAATACGGAGGGTGCAAGCGTTATCCGGATTTACTGGGTTTAAAGGGTGTG
TAGGCGGGCCCTTAAGTCAGTGGTGAAATCTCCGAGCTCAGCTTGGAAACTGCCATTGAT
ACTATAGGTCTTGAATTTTGTTGAGGTGGGCGGAATAAGTCATGTAGCGGTGAAATGCAT
AGATATGACTTAGAACCCCGATTGCGAAGGCAGCTCACTAAACAAACATTGACGCTGAGG
CACGAAAGCGTGGGGATCAAACAGGATTAGAAACCCTAGTAGTCC
>Otu6114
CCAGCCTATGGGTGGCAGCAGTGAGGAATATTGGTCAATGGACGCAAGTCTGAACCAGCC
ATGCCGCGTGAAGGATGAAGGCCTTCTGGGTTGTAAACTTCTTTTATCTGGGAAGAAACC
TCCGATTTCTATTGGAGCCGACGGTACCAGAGGAATAAGCACCGGCTAACTCCGTGCCAG
CAGCCGCGGTAATACGGAGGGTGCAAGCGTTATCCGGATTCACTGGGTTTAAAGGGTGTG
TAGGCGGGTATTTAAGTCAGTGGTGAAATCTCCGGGCTCAACCCGGAAACTGCCATTGAT
ACTATATATCTTGAATACTGTTGAGGTAGGCGGAATATGTCATGTAGCGGTGAAATGCAT
AGATATGACATAGAACACCGATTGCGAAGGCAGTTCACTAAGCTTGCATTGACACTGAGG
CACGAAAGCGTGGGGATCAAACAGGATTAGATACCCTAGTAGTCC
>Otu6118

CCAGCCTATGGGACGCAGCAGCGAGGAATATTGGTCAATGGACGAAAGTCTGAACCAGCC
ATGCCGCGTGAAGGATGACTGCCCTATGGGTTGTAAACTTCTTTTGTACGGGACGAATCT
TTCCTACGTGTAGGGAACTGACGGTACTGTACGAATAAGCATCGGCTAACTCCGTGCCAG
CAGCCGCGGTAATACGGAGGATGCAAGCGTTATCCGGATTTATTGGGTTTAAAGGGTGCG
TAGGCGGGACTGTAAGTCAGTGGTGAAATCTTGCAGCTTAACTGTAAAATTGCCATTGAT
ACTGCAGTTCTTGAGTCCAGTTGAAGTCGGCGGAATGTGTAGTGTAGCGGTGAAATGCTT
AGATATTACACAGAACACCAATTGCGAAGGCAGCTGACTAAACTGTCACTGACGCTGAGG
CACGAAAGCGTGGGGAGCAAACAGGATTAGATACCCTTGTAGTCC
>Otu6122
CCAGCCTATGGGAGGCTGCAGTCGAGAATATTCCGCAATGGACGAAAGTCTGACGGAGCG
ACGCCGCGTGGAGGATGAAGTATTTCGGTATGTAAACTCCTTTTGTAGACTAGTAATTTT
TGAACAGTCTAAGAATAAGAGGTTTCTAAACTCGTGCCAGCAGAAGCGGTAATACGAGTG
CCTCAAGCGTTATCCGGAATTATTGGGCGTAAAGGGTGTGTAGGTGGTTTTGTTAGTCTT
TTGTTAAATTTCTCGGCTTAACCGAGAACTTGCAAGAGAAACGGCAAGACTTAGAGTATG
CGAGGGGTCTGTGGAACTCATGGTGTAGCAGTGAAATGCGTTGATATCATGGGGAACACC
AAAAGCGAAGGTAGCAGACTGGAGCATTACTGACACTGAAACACGAAAGCGTGGGTAGCG
AATGGGATTAGATACCCCAGTAGTCC
>Otu6124
CTAGCCTACGGGAGGCTGCAGTCGAGAATCTTCGGCAATGGACGCAAGTCTGACCGAGCG
ACGCCGCGTGCGGGATGAAGGTCTTCGGATTGTAAACCGCTGTCGGAGGGGATGAAGTGC
AGGAGGGTACTCCCTCCTGTTTGACAGAGCCTCGGAGGAAGCACGGGCTAAGTTCGTGCC
AGCAGCCGCGGTAATACGAACTGTGCGAACGTTATTCGGAATCACTGGGCTTAAAGGGTG
CGTAGGCGGCGGCTCCAGTCAGGTGTGAAATCCCCCGGCTCAACCGGGGAACAGCGCTTG
AAACTAGGTCGCTCGAGTGAGGCAGGGGTGTATGGAACTTCCGGTGGAGCGGTGAAATGT
GTAGAGATCGGAAGGAACGCCGGCGGCGAAAGCGGTACACTGGGCCTTGTCTGACGCTGA
GGCACGAAAGCTAGGGGAGTGAACGGGATTAGAAACCCTCGTAGTCC
>Otu6127
CAGCCTACGGGACGCAGCAGTGGGGAATATTGGGCAATGGGCGCAAGCCTGACCCAGCCA
CGCCGCGTGAGTGATGAAGGCCTTCGGGTCGTAAAGCTCTGTGGGGAGGGACGAATCAGC
CTCGGGAACAATACCCCGGGGCCTTGACGGTACCTCCTTAGCAAGCACCGGCTAACTCCG
TGCCAGCAGCCGCGGTAATACGGAGGGTGCAAACGTTGCTCGGAATTATTGGGCGTAAAG
CGCACGTAGGCGGCTTGTTATGTCCGGTGTGAAATCCCTCGGCTTAACCGAGGAAGTGCA
TTGGAAACTGGCAAGCTTGAGTACGGAAGAGGGTCGCGGAATTCCCGGTGTAGAGGTGAA
ATTCGTAGATATCGGGAGGAACACCAGTGGCGAAGGCGGCGACCTGGGCCGATACTGACG
CTGAGGTGCGAAAGCGTGGGGAGCAAACAGGATTAGAGACCCTAGTAGTCC
>Otu6151
CCAGCCTATGGGGGGCACCAGTGGGGGATATTGGACAATGGGGCAACCCTGATCCAGCAA
TGCCGCGTGTGTGAAGAAGGCCTGAGGGTTGTAAAGCACTTTCAGTGGGGAGGAGGATTA
GTTGGTTAAGAGCTGATTAATTGGACGTTACCCACAGAAGAAGCACCGGCTAACTCCGTG
CCAGCAGCCGCGGTAATACGGAGGGTGCGAGCGTTAATCGGAATAACTGGGCGTAAAGGG
TGCGTAGGTGGTTTTATAAGTTATCTGTGAAATACCCGGGCTCAACCTGGGCAGGTCAGA
TAAGACTGTAAGACTCGAGTACAGGAGAGGGTAGTGGAATTTCCGGTGTAGCGGTGAAAT
GCGTAGAGATCGGAAGGAACACCAGTGGCGAAGGCGGCTACCTGGCCTGATACTGACACT
GAGGCACGAAAGCGTGGGGAGCAAACAGGATTAGAGACCCGCGTAGTCC
>Otu6152
CCAGCCTACGGGGTGCACCAGTGGGGAATTTTGGACAATGGGCGAAAGCCTGATCCAGCA
ACTCTGCGTGAGGGACGAAGCCCTTCGGGGCGTAAACCTCTTTTATTGGGGACGAATGCC
CGCAAGGGCTTGACGGTACCCAGTGAATAAGCCACGGCTAACTATGTGCCAGCAGCCGCG
GTAAGACATAGGTGGCGAGCGTTATTCGGAATTACTAGGCGTAAAGCGAGTGTAGGCGGG
CGCTTAAGTCTGTTGTGAAATCTCCCGGCTTAACTGGGAGGGGTCAATGGATACTGGGCG
TCTTGAGTGGGGTAGGGGGTACTGGAATTCCCGGTGTAGCGGTGAAATGCGTAGATATCG
GGAGGAACACCTATGGCGAAAGCAGGTACCTGGGCCCTTACTGACGCTAAGACTCGAAAG
CTAGGGGAGCAAACAGGATTAAAACCCCAGTAGTCC
>Otu6160
CCAGCCTATGGGTTGCACCAGTAAGGGATATTGCGCAATGGGCGAAAGCCTGACGCAGCA
ACGCCGCGTGGAGGATGACGGTTTTCGGATTGTAAACTCCTTTTTGAAGGGACGAGGAAG
GACGGTACCTTCAGAATAAGTCACGGCTAACTACGTGCCAGCAGCCGCGGTAAAACGTAG
GTGGCGAGCGTTATCCGGATTTACTGGGTGTAAAGCGTGTGCAGGCGGATGGTCAAGTGG

TGTATGAAATCGCCCGGCTCAACCGGGCGGGGTTATGCCAGACTGGTCATCTGGAGTGCG
AGAGAGGGGCGTGGAATTCCGGGTGTAGTGGTGAAATGCGTAGAGATCCGGAGGAACCCC
AGAGGCGAAGGCGGCGCCCTGGCTCGCAACTGACGCTCAGACACGACAGCATGGGGAGCG
AACGGGATTAGAAACCCGAGTAGTCC
>Otu6161
CCAGCCTACGGGGGGCAGCAGTCGAGAATTTTTCTCAATGGGCGAAAGCCTGAAGGAGCG
ACGCCGCGTGGGGGATGAATGGCTTCGGCCCGTAAACCCCTGTCATTTGCGAACAAATTG
ACTCACCTAACACGTGTGTCATTGATTGTAGCGGAAGAGGAAGGGACGGCTAACTCCGTG
CCAGCAGCCGCGGTAATACAGAGGTCCCAAGCGTTGTTCGGATTCACTGAGCGTAAAGGG
TGCGTAGGCGGTTTGACAAGTCTGATGTGAAATCCCGCAGCTTAACTGCGGAACTGCATT
GGAAACTGTTTGACTAGAGGAATGGAGGGGAGACTGGAATACTTGGTGTAGCAGTGAAAT
GCGTAGATATCAAGTGGAACACCAGTGGCGAAGGCGAGTCTCTGGACATTTCCTGACGCT
GAGGCACGAAAGCCAGGGGAGCAAACGGGATTAGAGACCCTCGTAGTCC
>Otu6163
CCAGCCTATGGGGTGCTGCAGTCGAGAATATTCCCCAATGGACGAAAGTCTGAGGGAGCG
ACGCCGCGTGCAGGATGAAGGGCTTCGGCTCGTAAACTGCTTTTACCAGGGAAGAATCAT
GACGGTACCTGGAGAATAAGAGGTTGCTAACTCTGTGCCAGCAGCAGCGGTAATACAGAG
ACCTCAAGCGTTATCCGGATTTATTGGGCGTAAAGGGTCCGCAGGTGGTCGTGTGCGTCG
GTGGTTAAATTTTCGGGCTCAACCCGGAAACTGCTGCCGATACGACTCGACTAGAGGCCG
GGAGAGGTAAGCGGAATTGCCGGTGTAGTAGTAATATGCGTTGATATCGGCAAGAACACC
AAATGCGTAGGCAGCTTACTGGAACGCGCCTGACACTCAGGGACGAAAGCGTGGGGAGCG
AAAGGGATTAGAAACCCTAGTAGTCC
>Otu6165
CCAGCCTACGGGGCGCAGCAGTGAGGAATTTTGGACAATGGGGGCAACCCTGATCCAGCG
ACGCCGCGTGGAGGACGAAGGTCTTCGGATTGTAAACTCCTTTTGCAGGGGACGAATGAG
TACCGTAACCCGATACTGTGACGGTACCCTGTGAATAAGCCACGGCTAACTTCGTGCCAG
CAGCCGCGGTAAGACGAAGGTGGCTAGCGTTACTCGGAATTACTAGGCGTAAAGGGCAGG
TAGGCGGCTCGGTGAGTCTATTGTGGAATCTTCCGGCTTAACTGGAAGGGGTCAATAGAT
ACTGCCGGGCTTGAGTGTGTGAGAGGGTGCTGGAATTCCCGGTGTAGCGGTGAAATGCGT
AGATATCGGGAGGAACACCTATGGCGAAAGCAGGCACCTGGCACATTACTGACGCTGAGC
TGCGAAAGCTAGGGGAGCAAACAGGATTAGATACCCGGGTAGTCC
>Otu6167
CCAGCCTATGGGTGGCTGCAGCAAGGAATAGTGGGCAATGGGCGAAAGCCTGACCCAGCA
ACGCCGCGTGGAGGATGAAGGCCTTCGGGTTGTAAACTCCTGTCAGAAGGGACGAAGGTG
TAATAGTGAATAACTGTTGCAGTTGACGGTACCTTCAAAGGAAGCCCCGGCTAATTACGT
GCCAGCAGCCGCGGTAATACGTATGGGGCAAGCGTTACTCGGATTTACTGGGCGTAAAGG
GCGCGTAGGTGGTGAAGAAAGTCAAATGTGAAAACCCATGGCTCAACTGTGGATCCGCAT
TTGAAACTTCTTTACTTGAGTTCAGGTGAGGAGAATGGAATTCCCGGTGTAGGGGTGAAA
TCTGTAGATATCGGGAAGAACACCAGTGGCGAAGGCGATTCTCTAACCTGATACTGACAC
TGAGGCGCGAAAGCGTGGGGAGCAAACAGGATTAGATACCCTAGTAGTCC
>Otu6169
CCAGCCTATGGGTTGCAGCAGTAAGGGATATTGCGCAATGGGCGAAAGCCTGACGCAGCA
ACGCCGCGTGGAGGAAGAAGGCCTTCGGGTCGTAAACTCCTTTTGTGAGGGACGAGGAAG
GACGGTACCTCACGAATAAGCCACGGCTAACTACGTGCCAGCAGCCGCGGTAAAACGTAG
GTGGCGAGCGTTATCCGGATTTACTGGGCGTAAAGCGTGTGCAGATGGGACAGCAAGTGG
TGTATGAAATCGCTCAGCTTAACTGGGCGGGGTTATGCCATACTGCTATTCTAGAGAACG
GGAGAGGGACGTGGAATTCCGGGTGTAGTGGTGAAGTGCGTAGAGATCCGGAGGAACCCC
TGAGGCGAAGGCGGCGTCCTGGCCCGATTCTGACATTCAGACACGACAGCATGGGGAGCA
AACGGGATTAGAAACCCGCGTAGTCC
>Otu6170
CCAGCCTATGGGGGGCAGCAGTGGGGAATTTTGCGCAATGGGGGAAACCCTGACGCAGCG
ATGCCGCGTGGAGGACGAAGGTCTTCGGATTGTAAACTCCTTTTACGGGGGACAAATGAC
GGCGTGAATAACGCCGGATGATGGTACTCCGCGAATAAGCCACGGCTAACTTCGTGCCAG
CAGCCGCGGTAAGACGAAGGTGGCGAGCGTTACTCGGAATTACTAGGCGTAAAGCGCGAG
CAGGCGGTTTGGTTAGTCCGTCGTGAAAGCCTTGGGCTCAACCCAGGGAGGTCGATGGAT
ACTGCCAGACTTGAATACGGGAGAGGTCGCTGGAATTCCTGGTGTAGCGGTGAAATGCGT
AGATATCAGGAGGAACACCAAAGGCGAAGGCAGGTGACTGGACCGTTATTGACGCTGAGT
CGCGAAAGCCGGGGGAGCAAACAGGATTAGATACCCTCGTAGTCC

>Otu6173
CCAGCCTATGGGTGGCTCCAGTGGGGAATATTGCGCAATGGACGAAAGTCTGACGCAGCG
ACGCCGCGTGTGGGATGAAGCCTTTCGGGGTGTAAACCACTGTTGCCCGGGACGAAACTC
TCCTTTCGAGGAGATTGACGGTACCGGGTGAGGAAGCACCGGCTAACTCTGTGCCAGCAG
CCGCGGTAATACAGAGGGTGCGAGCGTTGTCCGGAATCACTGGGCGTAAAGGGCGCGTAG
GTGGCGGGATAAGCGTGTGGTGAAAGCCCGGGGCTCAACCCCGTGTCTGCCATGCGGACT
GTCTTGCTCGAGCGTAGTAGAGGCAGGTGGAATTCCGGGTGTAGCGGTGGAATGCGTAGA
GATCCGGAAGAACACCGGTGGCGAAGGCGGCCTGCTGGGCTACTGCTGACACTGATGCGC
GACAGCGTGGGGAGCAAACAGGATTAGATACCCCTGTAGTCC
>Otu6186
CCAGCCTATGGGGCGCAGCAGTAAGGGATATTGCACAATGGGCGAAAGCCTGATGCAGCA
ACGCCGCGTGGAGGAAGAAGGCCTTCGGGTCGTAAACTCCTTTGGGTGGGGACGAGGAAG
GACGGTACCCATCAAACAAGTCACGGCTAACTACGTGCCAGCAGCCGCGGTAAAACGTAG
GTGGCAAGCGTTATCCGGAATTACTGGGCGTAAAGGGTGTGCAGGCGGGAACATAAGTGG
TGTATGAAATCTCCCAGCTTAACTGGGATAGGTTATGCCAGACTGTGTTTCTAGAGGACG
AGAGAGGGGGGTGGAATTCCGGGTGTAGTGGTGAAATGCGTAGAGATCCGGAGGAACCCC
AGGGGCGAAGGCGGCCCCCTGGCTCGTACCTGACGCTCAGCCACGAAAGCATGGGGAGCG
AACGGGATTAGATACCCCAGTAGTCC
>Otu6198
CCAGCCTACGGGGCGCAGCAGGCGCGAAACCTTCGCAATGCGCGTAAGCGTGACGAGGGG
ACTCTGAGTGGTATTAGCTCTTGCTAATGCCTTTTGCCCAGTGTAAATTCGAGTTGCACA
CCAACTCGTTATCAAGCTGGGAGAATAAGGGTGTGGGCAAGACTGGTGGCAGCCGCCGCG
GTAACACCAGCGCCTCGAGTGATACCCACGAATATTGGACCTAAAGCGTTCGTAGCCGGT
CCTGTAAGTCCGCTGTGAAATATTGGGGCTTGACCCTAATGCGTGCAGTGGATACTGCGG
GACTCGGGAGCGGGGGAGGGAAGAAGTACTTCTGGGGTAGCGGTAAAATGCGTTGAGCCT
AGAAGGACTTCCAGTGGCGAAGGCGTCTTCCTAAAACGCGTCCGACGGTGAGGAACGAAA
GCTGGGGGAGCAAATCGGATTAGATACCCGAGTAGTCC
>Otu6201
CCAGCCTACGGGTCGCAGCAGCTAAGAATCTTCCACAATGGGGGAAACCCTGACGGAGCG
ACGCCGCGTGGACGATGAAGGCCGTAAGGTTGTAAAGTCCTTTTCCTGGTGAAGAATAGG
CGAGGGAGTGGAAAGCCCTTGTGATGACGTTAACCAGGGAATAAGCCCCGGCTAATTACG
TGCCAGCAGCCGCGGTAATACGTATGGGGCGAGCGTTGTTCGGAGTTATTGGGCGTAAAG
GGCGTGTAGGCGGCCCGCCAAGTCTGGTGTGAAAGCGCAGGGCTCAACCCTGCGTAGGCG
CTGGAAACTGGCGGGCTTGAGTCTCGAGGGGGAAGCTGGAATTCCTGGTGTAGGGGTGAA
ATCTGTAGATATCAGGAAGAACACCGGTGGCGAAGGCGAGCTTCTACTCGTGGACTGACG
CTGAGGCGCGAAAGCGTGGGGAGCAAACAGGATTAGAAACCCGAGTAGTCC
>Otu6206
CCAGCCTGGGGGGCAGCAGTGAGGAATATTGCGCAATGGAGGAAACTCTGACGCAGCAAC
GCCGCGTGGGCGAAGAAGGCCTTCGGGTTGTAAAGCCCTGTCAGGTGGGACGAAACCACC
GATGGTTAATATCCTTCGGAACTGACTGTACCACCAAAGGAAGCACCGGCTAACTCCGTG
CCAGCAGCCGCGGTAAGACGGAGGGTGCAAGCGTTGTTCGGAATCACTGGGCGTAAAGCG
CGTGTAGGCGGTCCGAAAAGTCGAGTGTGAAAGCCCGAGGCTCAACCTCGGAAGTGCATT
CGAAACTCTCGGGCTTGAGTACTGGAGAGGGAAGCGGAATTCCTGGTGTAGAGGTGAAAT
TCGTAGATATCAGGAGGAACACCGGTGGCGAAGGCGGCTTCCTGGACAGATACTGACGCT
GAGACGCGAAAGCGTGGGGAGCAAACAGGATTAGAAACCCCCGTAGTCC
>Otu6210
CCAGCCTATGGGGGGCAGCAGTTAGGAATTTTCTGCAATGCGCGCAAGCGTGACAGAGCA
AGCCAGAGTGTTCCTCATAGAGGAACTTTTGCCAAATGCAAAAAGTTTGGCGAATAAGGA
CTGGGTAAGACAGGTGCCAGCCGCCGCGGTAATCCCTGCGGTCCAAGTCGCAGCCATCAT
TATTGGGTCTAAGACATCCGTAGCTTGTTTGGTAAGTTTTCTGTGAAATCCGGCATCTTA
AGTGTCGGGCGTGCAGAGAGTACTGCCAGGCTAGAGACTGGAAGACGTAAGAAGTATGTT
CAGAGTAGTAGTGAAATATTTTAATCCTGGACAGACTCACAACAGCGAAGGCATCTTACG
AGGACAGTTCTGACAGTAAGGGATGAAGGCTAGGGTCGCGAAAGGGATTAGATACCCCCG
TAGTCC
>Otu6216
CCAGCCTATGGGAGGCACCAGTAGGAAATATTGGGCAATGGGCGAAAGCTTGACCCAGTG
AGAACACATGCATGATGAATGCGCAAAGTTTTCTGTAAAGTGCTTTTCACGATTCGACGA
CAATGACGGTAGATCGAGAAGAAGTGCCGGCCAACTTCGTGCCAGCAGCTGCGGTAAGAC

GAAGGGCGCGGGCGTTATTCGTTATGATTGGGTGTAAAGGGTATGTAGGCGGTTTCCGGG
TTTTCTTGCCAAAAGAGCGGGGCGTTCCCTTGCGAGGGCAGTTTGAGTAGGAGGCTTGTG
TTGAGAAGAGGTTCGGTCAAATTTCTGTTCAGGGATAGAATTCTAGGAAATAGAAATGAA
AGCTAGCTAGCGAAAGCGCCCTTCTGGGCTCAACAGACGCTGAGGTACGGAAGCGTAGGT
AGCAAACAGGATTAGATACCCCAGTAGTCC
>Otu6218
CCAGCCTATGGGGTGCTCCAGTCGAGAATCTTCGGCAATGGGCGAAAGCCTGACCGAGCG
ACGCCGCGTGTGGGATGAAGGCCTTCGGGTTGTAAACCGCTGTCAGTGGGGAGGAAATGC
TGGTGGGTTCTCCCATCAGTTTGACCTATCCGCAGAGGAAGTACGGGCTAAGTTCGTGCC
AGCAGCCGCGGCAACACGAACCGTACGAACGTTATTCGGAATTACTGGGCTTAAAGGGTG
CGTAGGCGGCCCGGAAAGTTGGGTGTGAAAGCCCTCAGCTCAACTGAGGAATGGCGCCCA
AAACTGCCGGGCTTGAGGAAGACAGAGGTGAGTGGAACTTAGGGTGGAGCGGTGAAATGC
GTTGATATCCTAAGGAACACCAGTGGCGAAAGCGACTCACTGGGTCTTTTCTGACGCTGA
GGCACGAAAGCTAGGGGAGCGAACGGGATTAGATACCCGCGTAGTCC
>Otu6223
CCAGCCTATGGGTCGCTGCAGTAACGAATCTTCCCCAATGTACGAAAGTATGAGGGAGCG
ACGCTGCGTGGAGGATGAAGTCCTTCGGGATGTAAACTCCTGTCAGGGATTACCAAGTTC
TGAGGAATCCCAGAGGAAGAGGCGGCTAACTCTGTGCCAGCAGCCGCGGTAATACAGAGG
CCTCGAGCGTTAGGCGGAATCACTGGGCTTAAAGCGTGTGTAGGCGGATTTCTAAGTGCC
TTGTGAAATCCCATGGCTCAACCATGGAAATGCTCGGCATACTGGGAATCTTGAGCCATC
TAGGGGCAACTGGAACAAACGGTGGAGCGGTGAAATGCGTAGATATCGTTTGGAACGCCA
ATGGTGAAGACAGGTTGCTGGGGATGTGCTGACGCTGAGACACGAAAGCCAGGGGAGCAA
ACGGGATTAGAAACCCGAGTAGTCC
>Otu6224
CCAGCCTATGGGAGGCAGCAGTAACGAATATTGGGCAATGGGCGAAAGCCTGACCCAGCG
ACGCCGCGTGCGGGGGGAAGTCCCTCGGGATGTAAACCGCTGTCAGGGTGAAGAAAGACC
GCAAGGTTTGATCTAACCCAGAGGAAGCAACGGCTAACTTCGTGCCAGCAGCCGCGGTAA
TACGAAGGTTGCAAGCGTTGTTCGGAATCACTGGGCTTAAAGCGTATGTAGGCGGATGAG
TAAGCGTTTTGTGAAATCCCTCGGCTTAACCGAGGAATTGCTTGGCGAACTGCTCATCTT
GAGGCAAATAGGGGTGCATGGAACTCTTGGTGGAGCGGTGGAATGCGTAGATATCAAGAG
GAACGCCAGCGGCGAAAGTGGTGCACTGGATTTGTCCTGACGCTCAGATACGAAAGCGTG
GGGAGCAAACGGGATTAGAGACCCCCGTAGTCC
>Otu6226
CCAGCCTATGGTGCTGCAGTAGGGAATATTGCCCAATGGACGAAAGTCTGAGGCAGCAAC
GCCGCGTGCACGATGAAGGTCTTCGGATCGTAAAGTGCTTTTCTGAGAGATGAGAAAGGA
CAGTATCTCAGGAATAAGTCTCGGCTAACTACGTGCCAGCAGCCGCGGTAACACGTAGGA
GGCAAGCGTTATCCGGATTTACTGGGCGTAAAGCGTGTGCAGGCGGTTAGGTAAGTTGGA
TGTGAAAGCTCCCGGCTTAACTGGGAGAGGTCGTTCAAAACTACAAGACTTGAGAGTGGT
AGAGGAAGGTGGAATTCCGGGTGTAGTGGTGAAATGCGTAGATATCCGGAGGAACACCAG
TGGCGAAAGCGGCCTTCTGGACCATTTCTGACACTGATACGCGAAAGCTAAGGTAGCAAA
CGGGATTAGAAACCCGAGTAGTCC
>Otu6227
CCAGCCTACGGGGCGCTCCAGGCGCGAAAACTCTCCAATGCACGAAAGTGTGAGAGGGGA
ATCCCAAGTGCTTACGCATTACGCGTAGGCTTTTTCAATTGCAAAAAAATTGGAGAATA
AGTGGTGGGCAAGACCGGTGCCAGCAGCCGCGGTAACCCCGGCGCCACAAGTGGCAATCG
TGTATTTGGCCTAAAGCGTCCGTAGCCGGTTCTGTACATCCTTTGTGAAATTGTCCCGCT
TAACGGGGCAGCGTGCAGAGGACACGGCAGAGCTTGGAACTGGGAGGCGTCAGAGGTATT
CTTGGGGGAACGGTAAAATGTTATAATCCCAGGAAGACCACCGATGGCGAAGGCATCTGA
CGAGAACAGACTCGACGGTGAGGGACAAAAGCTAGGGGAGCAAACCGGATTAGATACCCC
CGTAGTCC
>Otu6230
CCAGCCTACGGGAGGCAGCAGTGGGGAATCTTGCGCAATGGACGAAAGCCTGACGCAGCG
ACGCCGCGTGAGTGAAGAAGGCCTTCGGGTTGTAAAGCTCTGTCAAGGGGGACGAAAACC
TGTCGGTGCATAGCCGGCAGACTGACGGTACCCTTGGAGGAAGCACCGGCTAACTCTGTG
CCAGCAGCCGCGGTAATACAGAGGGTGCAAGCGTTGCTCGGAATTATTGGGCGTAAAGGG
CAGGTAGGCGGTCTGATTTGTCAGGGGTGAAATCCCTGGGCTTAACCCAGGAAGTGCCCC
TGAAACGGTCAGACTGGAGTGCTGGAGAGGATCGTGGAATTCTCGGTGTAGCGGTGAAAT
GCGTAGAGATCGGGAGGAACACCAGAGGCGAAGGCGGCGATCTGGACAGCAACTGACGCT

CAACTGCGAAAGCGTGGGATCAAACAGGATTAGAAACCCGAGTAGTCC
>Otu6234
CCAGCCTATGGGGGGCAGCAGTGGGGAATTTTGGACAATGGGGGCAACCCTGATCCAGCC
ATGCCGCGTGAGTGAAGAAGGCCTTCGGGTTGTAAAGCTCTTTCGGACAGAAAGAAATCG
GTCGGGTAAATAGCTCGACTGGATGACGGTACTGTAAGAAGAAGCACCGGCTAACTACGT
GCCAGCAGCCGCGGTAATACGTAGGGTGCGAGCGTTAATCGGAATTACTGGGCGTAAAGC
GCGCGCAGGCGGTTTTGTAAGACAGCTGTGAAATCCCCGGGCTCAACCTGGGAACTGCGG
TTGTGACTGCAAGACTTGAGTACGGCAGAGGGGGGTGGAATTCCTGGTGTAGCAGTGAAA
TGCGTAGATATCAGGAGGAACACCGATGGCGAAGGCAGCCCCCTGGGCCTGTACTGACGC
TCATGCACTAAAGCGTGGGGAGCAAACAGGATTAGAGACCCCCGTAGTCC
>Otu6235
CCAGCCTATGGGGTGCAGCAGTCGCGAAAACTCCACAATGTGGGAAACCACGATGGGGGA
ACTCCAAGTGCACATACTTTGTATGTGCTTTTCTTCTGCCTAAAAAGCAGGAGGAATAAG
GGCCGGGTAAGACGGGTGCCAGCCGCCGCGGTAATACCCGCGGCCCGAGTGGTGCTCATT
ATTATTGAGCCTAAAGCGTCCGTAGCTGGTTCTGTAAATCTCTGGGTAAATCGGGCAGCT
TAACTGTCCGAATTCCGGAGAGACTGCAGGACTTGGGACTGGGAGAGGTTGAAGGTACTC
CAGGGGTAGGGGTAAAATCCTGTAATCCTTGGGGGACCACCAGTGGCGAAGGCGTTCAAC
TAGAACAGATCCGACAGTAAGGGACGAAGCCCTGGGGCGCAAACGGGATTAGAAACCCCC
GTAGTCC
>Otu6236
CCAGCCTACGGGGCGCTGCAGTGGGGAATTTTGGACAATGGGGGCAACCCTGATCCAGCC
ATTCCGCGTGAGTGAAGAAGGCCTTCGGGTTGTAAAGCTCTTTCGGCAGGAACGAAACGG
TGATCCCTAATACGGGTTACTAATGACGGTACCTGAAGAAGAAGCACCGGCTAACTACGT
GCCAGCAGCCGCGGTAATACGTAGGGTGCGAGCGTTAATCGGAATTACTGGGCGTAAAGC
GTGCGCAGGCGGTTTTGTAAGACAGCTGTGAAATCCCCGGGCTTAACCTGGGAACTGCGG
TTGTGACTGCAAGACTAGAGTCAGGCAGAGGGGGGTGGAATTCCACGTCTAGCAGTGAAA
TGCGTAGAGATGTGGAGGAACACCGATGGCGAAGGCAGCCCCCTGGGCTATGACTGACGC
TCATGCACGAAAGCGTGGGGAGCAAACAGGATTAGAGACCCCAGTAGTCC
>Otu6239
CCAGCCTACGGGAGGCAGCAGTGAGGAATATTGGTCAATGGACGCAAGTCCGGACCAGCC
ATGCCGCGTGAAGGATGAAGGCCTTCTGGGTTGTAAACTTCTTTTATCTGGGAAGAAACC
TCTGATTTCTATTAGAGCCGACGGTACCAGAGGAATAAGCACCGGCTAACTCCGTGCCAG
CAGCCGCGGTAATACGGAGGGTGCAAGCGTTATCCGGATTCACTGGGTTTAAAGGGTGTG
TAGGCGGGCAATTAAGTCAGCGGTGAAATCTCCGGGCTCAACCCGGAAACTGCCATTGAT
ACTATTTGTCTTGAATTTTGTTGAGGTAGGCGGAATAAGTCATGTAGCGGTGAAATGCAT
AGATATGACTTAGAACACCGATTGCGAAGGCAGCTTACTAAGCAAACGTTGACGCTGAGG
CACGAAAGCGTGGGGATCAAACAGGATTAGAAACCCCGGTAGTCG
>Otu6242
CCAGCCTATGGGAGGCAGCAGCGGGGAATATTGGACAATGGGCGCAAGCCTGATCCAGCA
ATGCCGCGTGGGTGAAGAAGGTCTTCGGATTGTAAAGCCCTTTCGGCGGGGACGATGATG
ACGGTACCCGCAGAAGAAGCCCCGGCTAACTTCGTGCCAGCAGCCGCGGTAATACGAAGG
GGGCTAGCGTTGCTCGGAATGACTGGTCGTAAAGGGCGCGTAGGCGGTTGACACAGTCAG
GCGTGAAATTCCCGGGCTTAACCTGGGGGCTGCGCTTGATACATGTTGACTTGAGTGTGG
AAGAGGGTCGTGGAATTCCCAGTGTAGAGGTGAAATTCGTAGATATTGGGAAGAACACCA
GTGGCGAAGGCGGCTATCTGGACGGTTACTGACGCTGAGGCGCGAAAGCGTGGGGAGCAA
ACAGGATTAGATACCCGCGTAGTCG
>Otu6244
CCAGCCTACGGGAGGCTGCAGTCGAGAATCTTCCGCAATGGGGGAAACCCTGACGGAGCG
ACGCCGCGTGGAGGATGAAGTACTTCGGTATGTAAACTCCTTTTGCCGGGGACGAAATTA
TTGACGGTACCCGGAGAATAAGAAGTTGCTAAACTCGTGCCAGCAGCAGCGGTAATACGA
GTGCTTCGAGCGTTATCCGGAATTATTGGGCGTAAAGGGTGTGTAGGCGGTTTCGTTAGT
CTCGCGTAAAATTCCTCGGCTCAACCGAGGGCGCGCGTGGGAAACGGCGAAACTTGAGGA
CGGAAGGGGTCTTCGGAACTCATGGTGTAGCGGTGAAATGCGTTGATATCATGGGGAACA
CCAAAAGCGAAGGCAGAAGACTGGTCCGCTCCTGACACTGAAACACGAAAGCGTGGGTCG
CGAATGGGATTAGAAACCCTGGTAGTCC
>Otu6249
CCAGCCTACGGGAGGCTGCAGTGGGGAATTTTGGACAATGGGCGCAACCCTGACGCAGCA
ACTCCGCGTGAGTGAGGAAGGTTTTCGGACCGTAAAGCTCTGTCGAGCGGGAAGAAATGC

CTGGAAGCCAATACCTTTCAGGCTTGACGGTACCGCTAAAGGAAGCACCGGCTAACTCCG
TGCCAGCAGCCGCGGTAATACGGGGGGTGCAAGCGTTGTTCGGAATCATTGGGCGTAAAG
AGCGTGTAGGCGGCTTAGTAAGTCAGATGTGAAAGCCCTGGGCTTAACCCAGGAAGTGCA
TTTGAAACTGCTGAGCTTGAGTAAGGGAGAGGAAAGTGGAATTCCTGGTGTAGAGGTGAA
ATTCGTAGATATCAAGAGGAACACCGGTGGCGAAGGCGACTTTCTGGCCCTATACTGACG
CTGAGACGCGAGAGCGTGGGTAGCAAACAGGATTAGATACCCTCGTAGTCC
>Otu6250
CCAGCCTACGGGTGGCTGCAGTGGGGAATATTGCGCAATGGGCGAAAGCCTGACGCAGCG
ACGCCGCGTGAGGGAAGAAGGCCTTCGGGTCGTAAACCTCTTTTCCTAGGGAAGAATAAT
GACGGTACCTGGGGAATAAGTCACGGCTAACTACGTGCCAGCAGCCGCGGTAATACGTAG
GCGGCGAGCATTATCCGGATTTATTGGGCGTAAAGCGAGTGTAGGTGGTTTTTCAAGTCG
GATGTAAAATTTCCCGGCTCAACTGGGAAGGGTCATTCGATACTGTTAAACTAGAGGGCA
GTAGAGGGAGGTGGAATTCCCGGTGTAGTGGTGAAATGCGTAGATATCGGGAGGAACACC
AGTGGCGAAAGCGGCCTCCTGGACTGTACCTGACACTGAGGCTCTAAAGCGTGGGTAGCA
AACAGGATTAGATACCCTGGTAGTCC
>Otu6259
CCAGCCTACGGGAGGCACCAGTAGGGAATATTGCACAATGGGCGGAAGCCTGATGCAGCA
ACGCCGCGTGCACGATGAAGGCCTTCGGGTCGTAAAGTGCTTTTCGGGGAGATGAGGAAG
GACAGTATCCCCGGAATAAGGATCGGCCAACTACGTGCCAGCAGCCGCGGTAAGACGTAG
GATCCGAGCGTTATCCGAATTCACTGGGCGTAAAGCGCGTGCAGGCGGCCTGCTAAGTTG
GATGTAAAACCTCCCGGCTCAACTGGGAGAGGACGTACAATACTAGCGGGCTAGAGGGCA
GTAGAGGGAGGTGGAATTCCCGGTGTAGTGGTGAAATGCGTAGATATCGGGAGGAACACC
AGTGGCGAAAGCGGCCTCCTGGGCTGCACCTGACGCTGAGATGCGAAAGCTAGGGGAGCA
AACGGGATTAGAGACCCGCGTAGTCC
>Otu6262
CCAGCCATGGGGGGCTGCAGTCGAGAATTTTTCACAATGGGCGAAAGCCTGACGCAGCAA
CGCCGCGTGTGTGATGAAGGTCTTCGGATCGTAAAGCACTGTCGCGAGGGACGAGAAGCC
ATCTGTGAACAGCGGATGGATAAGACGGTACCTCGAGAGGAAGCACCGGCTAACTCTGTG
CCAGCAGCCGCGGTAATACAGAGGGTGCGAGCGTTGTTCGGAATTATTGGGCGTAAAGCG
CTTGTAGGCGGTCTGTCAAGTCTATCGTGAAATCCCTTGGCTCAACCAAGGAAGTGCAGT
AGATACTAGCGGACTAGAGTGCCGGAGAGGGTCGCGGAATTCCCGGTGTAGAGGTGAAAT
TCGTAGATATCGGGAGGAACACCAGTGGCGAAGGCGGCGACCTGGACGGACACTGACGCT
GAGACGCGAAAGCGTGGGGAGCAAACAGGATTAGAAACCCTTGTAGTC
>Otu6266
CCAGCCTATGGGGCGCAGCAGTGGGGAATTTTTCGCAATGGGGGAAACCCTGACGAAGCA
ACGCCGCGTGGAGGATGAAGGCCTTCGGGTCGTAAACTCCTGTCGACTGGGAAGAAAGCA
CCCGACCTAATACGTCGAAGTGTTGACTGTACCGGTGGAGGAAGCCACGGCTAACTCTGT
GCCAGCAGCCGCGGTAATACAGAGGTGGCAAGCGTTGTTCGGAATTACTGGGCGTAAAGG
GCGCGTAGGCGGTTCGCCAAGTCCCGTGTGAAAGCCCCCGGCTCAACCGGGGAACGGCGC
GGGAAACTAGCAGGCTTGAGTTCGGGAGAGGGAAGCGGAATTCCGGGTGTAGCGGTGAAA
TGCGCAGATATCCGGAGGAACACCGGTGGCGAAGGCGGCTTCCTGGACCGACACTGACGC
TGAGGCGCGAAAGCGTGGGGAGCAAACAGGATTAGATACCCCAGTAGTCC
>Otu6267
CCAGCCTACGGGTCGCACCAGTCGAGAATTTTTCACAATGGGCGCAAGCCTGACCGAGCG
ACGCCGCGTGCGGGATGAAGGCCTTCGGGTTGTAAACCGCCGTCGGGATGGAGGAAATTC
CAAGGGGTAATCTCTTTGGATTGACCTATATTCAGAGGAAGGACGGGCTAAGTTCGTGCC
AGCAGCCGCGGTAAGACGAACCGTCCGAACGTTATTCGGATTCACTGGGCTTAAAGGGTG
CGTAGGCGGCTCAGAAAGTAGGGTGTGAAAGCCCTCGGCTCAACCGAGGAATTGCGCCCT
AAACTACTGGGCTTGAGGAACGCAGAGGTAAGCGGAACTTAGGGTGGAGCGGTGAAATGC
GTTGATATCCTAAGGAACACCAGGAGCGAAAGCGGCTTACTGGGCGTTTTCTGACGCTGA
TGCACGAAAGCTAGGGGAGCGAACGGGATTAGAAACCCGGGTAGTCC
>Otu6270
CCAGCCTATGGGTGGCAGCAGTGAGGAATATTGCTCAATGGGCGAAAGCCTGAAGCAGCG
ACGTCGCGTGAAGGATGAAATCCGTTAGGATGTAAACTTCTTTTGCAGGGGATGAATGTC
CCGACTTGTCGGGATTGACCGTACCCTGCGAATAAGCACCGGCCAACTACGTGCCAGCAG
CCGCGGTAATACGTAGGGTGCGAGCGTTGTCCGGAATTACTGGGTGTAAAGGGTCCGTAG
GCGGGCGAACAAGTCTGTGGTGAAAGTTCATGGCTCAACCGTGAAATTGCCTCCGATACT
GTTTGTCTTGAGTTCGAGAGAGGATGATGGAATTCATGGTGTAGCGGTGAAATGCGTAGA

GATCATGAAGAACACCAGTAGCGAAGGCGGTCATCTGGCTCGAAACTGACGCTCAGGGAC
GAAAGCGTGGGGAGCAAACAGGATTAGAAACCCTAGTAGTCC
>Otu6272
CCAGCCTATGGGACGCTCCAGTAGGGAATATTGGGCAATGGAGGCAACTCTGACCCAGCC
ATGCCGCGTGCAGGATGAAGGCCCTCTGGGTTGTAAACTGCTTTTATCAGGGAAGAAACG
CTTGGATTTATCCGAGCCTGACGGTACCTGAGGAATAAGCACCGGCTAACTCCGTGCCAG
CAGCCGCGGTAATACGGAGGGTGCAAGCGTTATCCGGATTTACTGGGTTTAAAGGGTGCG
TAGGCGGATTGTTAAGTCAGTGGTGAAAGATTCGGGCTCAACCTGGAAACTGCCATTGAT
ACTGATGATCTCGAGTACATTTGAGGTGGGCGGAATGTGCCATGTAGCGGTGAAATGCTT
AGATATGACACAGAACATCGATTGCGAAGGCAGCTCACTAAACTGTTACTGACGCTGAGG
CACGAAAGCGTGGGGATCAAACAGGATTAGAGACCCTAGTAGTCC
>Otu6277
CCAGCCTATGGGGGGCTCCAGGCGCGAAAACTCTACAATGCACGAAAGTGTGATAGGGGG
ACTCAAAGTGCTTATGTTTTACATAGGCTTTTGCCAACTCGAAATTGGTTGGCGAATAAG
TGGTGGGTAAGACTGGTGCCAGCCGCCGCGGTAACCCCAGCGCCACAAGTGGGAATCGAT
TTTATTGGGCTTAAAGCGTCCGTAGCAGGTCTTGTAAATTACTTGTGAAATCAAATAGCT
TAACTATTTGGAGTGCAGGTAACACTGCTAGACTAGAGACCGGGAGACGTTGGAGGTATT
CAGAGGGGAGCGGTAAAATGCGAGAATCCTCTGAGGACCACCTGTTGCGAAGGCGTCCAA
CAATAACGGGTCTGACTGTGAGGGTCGAAAGCTAGGGGAGCGAACCGGATTAGATACCCG
TGTAGTCC
>Otu6280
CCAGCCTATGGGTCGCACCAGCGGATCAGGCGGTGCTTTTCAAAACCGCAGTTAAGGAGA
TCGCCTACAAGCATGGAGTGCTGGCTACGTTTATGGCTAAGATTAGCGAGAATCTGCCTG
GATGCAGTGGACGCGTGCATCAATCTCTATGGGATAGCAAAAGCTCGAAGAACCTCTTCT
ATAGTGATAAGGACAAGTCAGGCATGAGTGAAACGATGAAAAGCTATATAGCGGGTCAAC
TCCACTGTCTGCCACATATACTGCCTATGGTAGCACCAACGATCAACTCCTACAAGAGAC
TGGTAGAAGGTGCGTGGGCACCGACCACAGTGACATGGGCAGTAGATAATCGTACGGTAG
CGCTTCGTGCCTTGCCGGCAGGTAGCAAATCTACCAGATTAGAAACCCTCGTAGTCC
>Otu6284
CCAGCCTACGGGACGCACCGGTGAGGAATATTGGTCAATGGGGGCAACCCTGAACCAGCC
ATGCCGCGTGGAGGAAGAAGGCCCTAAGGGTTGTAAACTTCTTTTGTACGGGGGTAAACT
CTCTTACGAGTAGGAGATTGAAAGTACTGTACGAATAAGCAACGGCTAACTCCGTGCCAG
CAGCCGCGGTAATACGGAGGTTGCAAGCGTTATCCGGATTTATTGGGTTTAAAGGGTGCG
TAGGCGGGTTTGTAAGTCAGTGGTGAAAGACTGTCGCTTAACGATAGCATTGCCATTGAT
ACTGCAAGTCTTGAGTATACTTGAGGTAGGCGGAATGTGTAGTGTAGCGGTGAAATGCTT
AGATATTACACAGAACACCAATTGCGAAGGCAGCTTACTAAATTATAACTGACGCTGAGG
CACGAAAGCGTGGGGATCAAACAGGATTAGAAACCCGGTATCC
>Otu6290
CCAGCCTATGGGTGGCTGCAGCTAGGAATCTTGCGCAATGGGGGAAACCCTGACGCAGCA
ACGCCGCGTGGGTGAAGAAGGCCTTCGGGTTGTAAAGCCCTTTTAGCTGGGACGAACGCA
ATGACGGTACCAGCAGAAAAAGCACCGGCTAACTCTGTGCCAGCAGCCGCGGTAATACAG
AGGGTGCAAGCGTTATTCGGAATTACTGGGCGTAAAGGGCGCGTAGGCGGCTTAGTAAGT
CGGATGTGAAATCCCTAGGGTCAACTTAGGAATTGCATTGGAAACTGCTTTGCTAGAGTG
CAGGAGAGGAGGGCGGAATTCCTGGTGTAGAGGTGAAATTCGTAGATATCAGGAGGAACA
CCGGTGGCGAAGGCGGCTCTCTGGACTGTTACTGACGCTGAGGCGCGAAAGCGTGGGGAG
CAAACAGGATTAGAAACCCCAGTAGTCC
>Otu6291
CCAGCCTACGGGTTGCACCAGTCGAGAATATTCCACAATGGGCGAAAGCCTGATGGAGCG
ACACCGCGTGCAGGATGAAGGTTTTAGGATCGTAAACTGCTTTTCTACAAGAGGAGGGTC
ACGCAAGTGATTTGACAGTACTGTAGGAATAAGGGGTTGCAAACTACGTGCCAGCAGCCG
CGGTAATACGTAGACCCCGAGCGTTATCCGGATTTATTGGGCGTAAAGGGTGTGTAGGGG
GCCAGGTGCGTCTTCTGTTAAATCTTGCAGCTTAACTGTGAGGCCGCAGGAGATACGACC
AGGCTAGAGAATGCGAGAGGTTAGCGGAACGAACGGTGTAGGGGTGAAATCCGTTGATAT
CGTTCGGAACACCAAAAGCGAAGGCAGCTAACTGGCGCATTTCTGACCCTGAAACACGAA
AGCGTGGGGAGCAAAAAGGATTAGATACCCGTGTAGTCC
>Otu6293
CCAGCCTACGGGAGGCACCAGTGGGGAATTTTGGACAATGGGGGCAACCCTGATCCAGCA
ATGCCGCGTGAGTGAAGAAGGCCCTCGGGTTGTAAAGCTCTTTTGTCAGGGAAGAAAAGG

TATCTCCTAATACGGGGTACTGATGACGGTACCTGAAGAATAAGCTCCGGCTAACTACGT
GCCAGCAGCCGCGGTAATACGTAGGGTGCAAGCGTTAATCGGAATTACTGGGCGTAAAGC
GTGCGCAGGCGGTTATGCAAGACAGAGGTGAAATCCCCGGGCTCAACCTGGGAACTGCCT
TTGTGACTGCATGGCTAGAGTGTGTCAGAGGGGGGTAGAATTCCACGTGTAGCAGTGAAA
TGCGTAGATATGTGGAGGAATACCGATGGCGAAGGCAGCCCCCTGGGATAACACTGACGC
TCATGCACGAAAGCGTGGGGAGCAAACAGGATTAGAAACCCCCGTAGTCC
>Otu6297
CCAGCCTATGGGGCGCACCAGTCGAGGATCTTCGGCAATGGGCGCAAGCCTGACCGAGCG
ACGCCGCGTGTGCGATGAAGGCCTTCGGGTTGTAAAGCACTGTCGAGGGGGAGGAAGCCG
CAAGGTTGACCTATCCCTGGAGGAAGCACGGGCTAAGTTCGTGCCAGCAGCCGCGGTAAG
GCGAACCGTGCTAACGTTATTCGGAATCACTGGGCTTAAAGGGCGCGTAGGCGGGTGACC
AAGTCAGGGGTGAAATCCTTCGGCTTAACCGGAGAACAGCTTCTGATACTGGTCGTCTGG
AGGGAGGTAGGGGCATGCGGAACTTCCGGTGGAGCGGTGAAATGCGTAGATATCGGAAGG
AACACCGGTGGCGAAGGCGGCGTGCTGGACCTCTTCTGACGCTGAGGCGCGAAAGCCAGG
GGAGCAAACGGGATTAGATACCCCAGTAGTCC
>Otu6299
CCAGCCTATGGGGGGCAGCAGTCGAGAATATTTGGCAATGGGCGAAAGCCTGACCTTGCG
ACGCCGCGTGCTGGACGAAGTCCTTCGGGACGTAAGCAGCTTTTGCGCGTGAGGAAGTAA
TTGACGTTAGCGCGCGAATAAGGGGCTCCTAACTCTGTGCCAGCAGGAGCGGTAATACAG
AGGCCCCGAGCATTATCCGGAATCACTGGGCGTAAAGGGTGTGTAGGCGGTCGCGTTAGT
CTTTTGTAAAAGGCCTCGGGCTTAACCCGGGGACTGCAGGGGAAACGGCGCGGCTTCGAG
GACGCGAGAGGTAGAGGGAACTCATGGTGTAGGGGTGAAATCCGTTGATACCATGGGGAA
CACCAAATGCGAAGGCACTCTACTGGCGCGCTCCTGACGCTGAAACACGAGAGCGTGGGT
AGCGAATGGGATTAGAAACCCCCGTAGTCC
>Otu6304
CCAGCCTATGGGTGGCACCAGTAACGAATCTTCCGCAATGCACGCAAGTGTGACGGAGCG
ACGCCGCGTGTGGGACGAAGTCCTTCGGGATGTAAACCACTGTCAGGGGAAAGAAAGTTC
TGATCTACCCCAGAGGAAGGCACGGCTAACTCTGTGCCAGCAGCCGCGGTAAGACAGAGG
TGCCGAGCGTTAGGCGGAATCACTGGGCTTAAAGCGTGTGTAGGCGGGTCGTTAAGTGTC
TTGTGAAATCCCCCGGCTCAACCGGGGAATGGCTGGGCATACTGGCGATCTTGAGCAGTC
TAGGGGCAGATGGAACAGACGGTGGAGCGGTGAAATGCGTAGATATCGTCTGGAACGCCA
AAGGTGAAAACAGTCTGCTGGGGACTTGCTGACGCTGAGACACGAAAGCCAGGGGAGCGA
ACGGGATTAGATACCCCTGTAGTCC
>Otu6311
CCAGCCTATGGGGCGCACCAGCGGGGAATCTTGCACAATGGGGGCAACCCTGATGCAGCG
ACGCCGCGTGAGCGATGAAGCCCTTCGGGGTGTAAAGCTCTTTCGGCAGGGAAGATGATG
ACGGTACCTGCAGAAGCAGCTGCGGCTAACTACGTGCCAGCAGCCGCGGTAATACGTAGG
CAGCAAGCGTTGTTCGGAGTTACTGGGCGTAAAGGGTGTGTAGGCGGCCTTCTAAGTTTG
GTGTGAAATCTCCCGGCTCAACCGGGAGGGTGCGCCGAAGACTGGGGGGCTAGAGTGCGG
GATGGGAGAGTAGAATTCCTGGTGTAGCGGTGAAATGCGTAGATATCAGGAGGAATACCT
GTGGTGTAGACGGCTCTCTGGACCGCAACTGACGCTGAGACACGAAAGCGTGGGTAGCAA
ACAGGATTAGAAACCCGGGTAGTCC
>Otu6315
CCAGCCTATGGGGTGCTGCAGTAAGGGATATTGCACAATGGGCGAAAGCCTGATGCAGCA
ACGCCGCGTGGAGGAAGAAGGCCTTCGGGTCGTAAACTCGTTTTGCGTCAGACGAGGAAG
GACGGTATGACGCGAATAAGTCACGGCTAACTACGTGCCAGCAGCCGCGGTAAAACGTAG
GTGGCAAGCGTTATCCGGATTTACTGGGCGTAAAGGGTGTGCAGGCGGGCAGACAAGTGG
TGTATGAAATGACCAGGCTCAACCTGGAACGGTTATGCCAGACTGTTTGTCTAGAGTGCG
AGAGAGGGGCGTGGAATTCCGGGTGTAGTGGTGAAATGCGTAGAGATCCGGAGGAACCCC
AGAGGCGAAGGCGGCGCCCTGGCTCGCAACTGACGCTCAGCCACGACAGCATGGGGAGCG
AACGGGATTAGATACCCTGGTAGTCC
>Otu6317
CCAGCCTACGGGTTGCACCAGTGGGGAATATTGGACAATGGGGGAAACCCTGATCCAGCA
ATGCCGCGTGAGTGATGAAGGCCTTCGGGTTGTAAAGCTCTTTTGTTAGGGACGATGATG
ACGGTACCTAACGAATAAGCTCCGGCTAACATCGTGCCAGCAGCCGCGGTAATACGAAGG
GAGCTAGCGTTGTTCGGAATTACTGGGCGTAAAGGTCGCGTAGGCGGCTTGATAAGTTTG
GTGTGAAAGCCCTGGGCTTAACCTAGGAATAGCATTGAATACTGTCAGGCTAGAGGCCGA
GAGAGGGAAATGGAATTGCGAGTGTAGAGGTGAAATTCGTAGATATTCGCAAGAACACCA

GTGGCGAAGGCGATTTCCTGGCTCGGTACTGACGCTAAGGCGCGAAAGCGTGGGGAGCAA
ACAGGATTAGAGACCCCCGTAGTCC
>Otu6338
CCAGCCTATGGGAGGCAGCAGTGGGGAATATTGGACAATGGGCGAAAGCCTGATCCAGCA
ATACCGCGTGTGTGAAGAAGGCCTTCGGGTTGTAAAGCACTTTCAGTAGGGAGGAAAGGC
TCAGAGCTAATATCTCTGAGAGTTGACGTTACCTACAGAAGAAGCACCGGCTAACTCTGT
GCCAGCAGCCGCGGTAATACAGAGGGTGCAAGCGTTAATCGGAATTACTGGGCGTAAAGC
GCTCGTAGGCGGATCTTTAAGTCGATTGTGAAATCCCCGGGCTCAACCTGGGAATGGCAC
TCGAAACTGGAGATCTAGAGTATGATAGAGGAAAGTAGAATTCCCGGTGTAGCGGTGAAA
TGCGTAGATATCGGGAGGAATACCAGTGGCGAAGGCGGCTTTCTGGATCAATACTGACGC
TGAGGGGCGAAAGCGTGGGGAGCAAACAGGATTAGATACCCCCGTAGTCC
>Otu6341
CCAGCCTACGGGACGCTGCAGTCGAGAATTTTTCTCAATGGGCGAAAGCCTGAGGGAGCG
ACGCCGCGTGGGGGATGAATGGCTTCGGCCCGTAAACCCCTGTCATTTGTGAACAAATTG
CTTCACCTAACACGTGAAGCATTGGTTGTAACGGAAGAGGAAGGGACGGCTAACTCTGTG
CCAGCAGCCGCGGTAATACAGAGGTCCCAAGCGTTGTTCGGATTCACTGGGCGTAAAGGG
TGCGTAGGCGGTTTGATAAGTCTGATGTGAAATCCCGCAGCTTAACTGCGGAACGGCATT
GGAAACTATTTGACTAGAGGAATGGAGGGGAGACTGGAATACTTGGTGTAGCAGTGAAAT
GCGTAGATATCAAGTGGAACACCAGTGGTGAAGGCGAGTCCCTGGACAACTTCTGACACT
GAGGCACGAAAGCCAGGGGAGCAAACGGGATTAGATACCCGTGTAGTCC
>Otu6343
CCAGCCTATGGGTTGCTGCAGTGGGGAATCTTGCGCAATGGCGAAAGCCTGACGCAGCGA
CGCCGCGTGGGGGATGAAGCTTCTCGGAGTGTAAACCCCTTTCGATCCGGACGAAACGCC
CGCAAGGGCTTGACGGTACGGGTATAAGAAGCCCCGGCTAACTACGTGCCAGCAGCCGCG
GTAAAACGTAGGGGGCCAGCGTTGCTCGGAATTACTGGGCGTAAAGGGTTCGTAGGCGGT
GCGGCAAGTCGGGAGTGAAATCTCTGGGCTTAACCCAGAGGCTGCTTCCGAAACTGCCAT
GCTAGAGTGTGAGAGAGGCGAGTGGAATTGCAGGTGTAGCGGTGAAATGCGTAGATATCT
GCAGGAACACCCGTGGCGAAGGCGGCTGGCTGGACCGCAACTGACGCTGAGGAACGAAAG
CTAGGGGAGCAAACAGGATTAGAAACCCCGGTAGTCC
>Otu6371
CCAGCCTACGGGACGCAGCAGTAGGGAATCTTCCGCAATGGACGAAAGTCTGACGGAGCA
ACGCCGCGTGAGTGATGAAGGTTCTCGGATCGTAAAGCTCTGTTGCCAAGGAAGAACAAC
CTAAAGAGTAACTGCTTTAGGGATGACGGTACTTGAGAAGAAAGCCCCGGCTAACTACGT
GCCAGCAGCCGCGGTAATACGTAGGGGGCGAGCGTTGTCCGGAATTATTGAGCGTAAAGC
GCGCGCAGGCGGTATCTTAAGTCTGATGTTTAATCCCAAGGCTCAACCTTGGTTCGCATT
GGAAACTGGGAAACTAGAGTGTAGGAGAGGAAAGTGGAATTCCACGTGTAGCGGTGAAAT
GCGTAGATATGTGGAGGAACACCAGTGGCGAAGGCGACTTTCTGGCCTATAACTGACGCT
GAGGCGCGAAAGCGTGGGGAGCAAACAGGATTAGAGACCCCCGTAGTCC
>Otu6375
CCAGCCTACGGGTCGCTGCAGTGAGGAATATTGCGCAATGGGCGAAAGCCTGACGCAGCG
ACGCCGCGTGGAGGATGAAGGTCTTCGGGTTGTAAACTCCTTTCAGTGGGAACGAAATTG
ACGGTACCCACAGAAGAAGCCCCGGCTAACTACGTGCCAGCAGCCGCGGTGATACGTAGG
GGGCGAGCGTTGTCCGGATTTACTGGGCGTAAAGAGCTCGTAGGCGGTTTGATAAGTCGG
GTGTGAAACCTCCAGGCTCAACCTGGAGTCGCCACTTGATACTGTCATGACTAGAGTCCG
GTAGGGGACCATGGAATTCCTGGTGTAGCGGTGAAATGCGCAGATATCAGGAGGAACACC
AGTAGCGAAGGCGGTGGTCTGGGCCGGTACTGACGCTGAGGAGCGAAAGCGTGGGGAGCG
AACAGGATTAGATACCCCGGTAGTCC
>Otu6379
CCAGCCTATGGGGTGCTCCAGTCAAGAATCTTCCCCAATGGCCGAAAGGCTGAGGGAGCG
ACGCCGCGTGTATGATGAAGCTCTTCGGAGTGTAAAATACTTTTCTCTGGGACGAATTTT
GACTTTACCAGAGGAATAAGGAGCCGCTAACCTCGTGCCAGCAGCGGCGGTAATACGAGG
GCTCCAAGCGTTATCCGGATTTATTGGGCGTAAAGGGTACGTAGGCGGTTTGACGCATCT
TTCGTTAAATCCTCAGGCTTAACCTGAGGGCCGCGGAAGAGATGATCAGACTAGAGACCG
GAAGAGGCAAACGGAATTGTTGGTGTAGGGGTTAAATCCGTTAATATCAACAAGAACACC
AAATGCGAAGGCAGTTTGCTAGGACGGTTCTGACGCTGAGGTACTAAAGCGTGGGTAGCG
AATAGGATTAGAAACCCTAGTAGTCC
>Otu6393
CCAGCCTATGGGACGCAGCAGTGGGGAATTTTGCGCAACGGAGGAAACCCTGACGCAGCA

ACGCCGCGTGGAGGATGAAGTCCCTTGGGACGTAAACTCCTTTCGACCGGGACGATTATG
ACGGTACCGGTGGAAGAAGCCCCGGCTAACTTCGTGCCAGCAGCCGCGGTAATACGAGGG
GGGCAAGCGTTGTTCGGAATTATTGGGCGTAAAGGGCGCGTAGGCGGCGCGGTAAGTCAC
CAGTGAAATCCCCGGGCTCAACTCGGGGCCTGCTGGCGAAACTGCCGTGCTGGAGTATGG
GAGGGATGCGTGGAATTCCCGGTGTAGCGGTGAAATGCGTAGATATCGGGAGGAACACCT
GTGGCGAAAGCGGCGCACTGGACCATAACTGACGCTGAGGCGCGAAAGCTAGGGGAGCAA
ACAGGATTAGAGACCCGTGTAGTCC
>Otu6435
CCAGCCTACGGGATGCTGCAGTCGAGAATATTCGACAATGGGCGAAAGCCTGATCGAGCG
ACACCGCGTGCAGGATGAAGGCCTTCGGGTCGTAAACTGCGGTAATAACATGACAATGTA
AATGAGTGTGTTATGGAAAGAGGTGGGTAACTACGTGCCAGCACCAGCGGTAAAACGTAG
ACCTCAAGCGTTATCCGGATTTATTGGGCGTAAAGAGCATGTAGGTGGCTTTGTGCGTCT
TCTGTTAAAGCCCGGGGCCTAACCCCGGAAGTGCAGGAGATACGGCAGAACTAGAGGAGG
TTAGAGGTGCATAGAACGCACGGTGTAGGGGTGAAATCCGTTGATATCGTGCGGAATACC
AAAGGCGAAGGCATTGCACTGGGACCTTCCTGACACTGAGATGCGAAAGCGTGGGGAGCA
AAAAGGATTAGAAACCCGAGTAGTCC
>Otu6440
CCAGCCTACGGGGGGCACCAGTCGAGAATCTTCCACAATGGACGAAAGTCTGATGGAGCG
ACGCCGCGTGATTGATGAAGACCCTCTGGGTCGTAAAAATCTTTTATGAGCTACTAAGTT
TATTGAAGAGCTCATGAATAAGGGGTTGCTAAACTCGTGCCAGCAGCAGCGGTAATACGA
GTGCCCCGAGCGTTATCCGGAATCATTGGGCGTAAAGGGTGTGTAGGTGGTCGTGTTAGT
CTTCCGTTAAAACCTGGGGCTTAACCTCAGATATGCGAAAGAGACGGCACGACTGGAGGG
TGCGAGGGGTCTGTGGAACTCATGGAGTAGGGGTGAAATCCGTTGATATCATGGGGAACA
CCAAAAGCGAAGGCAGCAGACTGGCGCATTCCTGACACTGAAACACGAAAGCGTGGGTAG
CGAATGGGATTAGAAACCCCAGTAGTCC
>Otu6445
CCAGCCTATGGGTTGCTGCAGTGGGGAATCTTGCGCAATGGGCGAAAGCCTGACGCAGCC
ACGCCGCGTGAGTGAAGAAGGCCTTCGGGTTGTAAAGCTCTGTCGGGCGGGACGAAAATG
GGATGCGTGAATAGCGCATCTTACTGACGGTACCGCCAAAGGAAGCACTGGCTAACTTCG
TGCCAGCAGCCGCGGTAATACGAGGGGTGCGAGCGTTGCTCGGAATTATTGGGCGTAAAG
GGTAGGTAGGTGGTCTCATTTGTCTGGGGTGAAAGCCTTGAGCTTAACTCAAGAAGTGCC
CCAGAAACGGTGAGACTCGAGTCTTGGAGAGGGTCGTGGAATTCCCGGTGTAGCGGTGAA
ATGCGTAGAGATCGGGAGGAACACCAGAGGCGAAGGCGGCGACCTGGACAAGTACTGACA
CTCAACTACGAAAGCGTGGGGAGCAAACAGGATTAGATACCCCAGTAGTCC
>Otu6455
CCAGCCTACGGGATGCAGCAGTGAGGAATATTGCTCAATGGACGAAAGTCTGAAGCAGCA
ACGCCGCGTGAGGGATGAAGGTGCTCTGCATCGTAAACCTCTGTAGTCGGGGACAAACGT
GGGGTTACTACCCCATTGATGGTACCCGAAAAGTAAGGATCGGCTAACTACGTGCCAGCA
GCCGCGGTAATACGTAGGATCCAAGCGTTGTCCGGATTTACTGGGTGTAAAGGGTGCGCA
GGCGGACTGGTAAGTCAGAAGTGAAATCTCGGCGCTCAACGCCGAAACGTCTTTTGATAC
TGCCAGTCTTGAATCGGGGAGAGATCCGTGGGATTCCGAGTGTAGCAGTGAAATGTGTAG
ATATTCGGAAGAACACCAGAGGCGAAGGCAGCGGATTGGCCCTTGATTGACGCTCAGGCA
CGAAAGCATGGGGATCAAACAGGATTAGATACCCCAGTAGTCC
>Otu6456
CCAGCCTACGGGGTGCACCAGTCGAGGATCTTCGGCAATGGGCGCAAGCCTGACCGAGCG
ACGCCGCGTGCGGGATGAAGGCCTTCGGGTTGTAAACCGCCGTCGGAGTGGAGGAAATTC
CAGGGGGTACTCCCTCTGGATTGACTAATACTCAGAGGAAGGACGGGCTAAGTTCGTGCC
AGCAGCCGCGGTAAGACGAACCGTCCAAACGTTATTCGGAATCACTGGGCTTAAAGGGTG
CGTAGGCGGCTCAGAAAGTAGGGTGTGAAAGCCCTCGGCTCAGCCGAGGAATTGCGCCCT
AAACTACTGGGCTTGAGGGAGACAGAGGTAAGCGGAACTTAGGGTGGAGCGGTGAAATGC
GTTGATATCCTAAGGAACACCAGGAGCGAAAGCGGCTTACTGGGTCTCTTCTGACGCTGA
GGCACGAAAGCGTGGGTAGCAAAAAGGATTAGAGACCCCAGTAGTCC
>Otu6460
CCAGCCTATGGGAGGCAGCAGTGGGGAATATTGGACAATGGGGGCAACCCTGATCCAGCC
ATGCCGCGTGAATGATGAAGGCCTTCGGGTTGTAAAGTTCTTTTGACGGGGACGATGATG
ACGGTACCCGTAGAATAAGCTCCGGCTAACTTCGTGCCAGCAGCCGCGGTAATACGAAGG
GAGCTAGCGTTGTTCGGATTTACTGGGCGTAAAGGGCGCGTAGGCGGTTTCGTAAGTTGG
AAGTGAAAGCCCTGGGCTCAACCCGGGAATTGCTTTCAAAACTACGTTACTCGAATTTGG

TAGAGGTTGGTAGAATTCCCAGTGTAGAGGTGAAATTCGTAGATATTGGGAAGAATACCC
GTGGCGAAGGCGGCCAACTGGACCAACATTGACGCTGAGGCGCGAAAGCGTGGGTAGCAA
ACGGGATTAGAGACCCTTGTAGTCC
>Otu6482
CCAGCCTATGGGTCGCAGCAGGCGCGAAACCTTCGCAATGCGCGAAAGCGTGACGAGGTT
AATCCGAGTGGTTCCCGCTGAGGGAATCTTTTGTCGGCTCTAAAAAGGCCGGCGAATAAG
GGGAGGGCAAGTCTGGTGTCAGCCGCCGCGGTAATACCAGCTCCTCGAGTGGTCGGGGCG
ATTATTGGGCCTAAAGCATCCGTAGCCGGTTCTGTAGGTCCTCTGTTAAATCCAACGGCT
TAACCGTTGGCCCGCAGAGGATACCGCAGGACTAGGAGGCGGGAGAGGCAGACGGTACTC
TACGTGTAGGGGTAAAATCCTTTGATCCGTAGAAGACCACCAGTGGCGAAGGCGGTCTGC
CAGAACGCGCTCGACGGTGAGGGATGAAAGCTGGGGGAGCGAACCGGATTAGAAACCCCG
GTAGTCC
>Otu6483
CCAGCCTACGGGACGCACCAGTAAGGAATCTTCCACAATGGACGAAAGTCTGATGGAGCA
ACGCCGCGTGCAGGATGACGGTCTTCGGATTGTAAACTGCTTTTATTTGGGAAGATTATG
ACGGTACCAAATGAATAAGGACCGGCTAACTACGTGCCAGCAGCCGCGGTAATACGTAGG
GTCCAAGCGTTATCCGGAATCACTGGGCGTAAAGCGTGCGTAGGTGGCGTATTAAGTGAG
ATGTGAAATCCCGTGGCTCAACCATGTGGACTGTGTCTCATACTGATATGCTCGAGGATG
GCAGAGGCAAGTGGAATTAGTAGTGTAGCAGTGAAATGCGTAGATATTACTAGGAACACC
AATGGCGAAGGCAGCTTGCTGGGCCACACCTGACACTAAGGCACGAAAGCGTGGGGAGCG
AACAGGATTAGATACCCTGGTAGTCC
>Otu6484
CCAGCCTACGGGTGGCAGCAGTGGGGAATATTGGACAATGGGGGAAACCCTGATCCAGCC
ATGCCGCGTGAGTGATGAAGGCCCTAGGGTTGTAAAGCTCTTTCACACGTGAAGATTATG
ACGGTAACGTGAGGAGAAGCCCCGGCTAACTTCGTGCCAGCAGCCGCGGTAATACGAAGG
GGGCTAGCGTTGTTCGGAATTACTGGGCGTAAAGCGCACGCAGGCGGCCTAGTTAGTTAG
GAGTGAAAGCCCTGGGCTTAACCTGGGAATTGCTCTTAATACGGCTAGGCTAGACTTATG
GAGAGGATAGCGGAATTCCGAGTGTAGAGGTGAAATTCGTAGATATTCGGAAGAACACCA
GTGGCGAAGGCGGCTATCTGGCCATTAAGTGACGCTCATGTGCGAAAGCGTGGGGAGCAA
ACAGGATTAGATACCCCCGTAGTCC
>Otu6489
CAGCCTATGGGTTGCTCCAGTGGGGAATCTTGCACAATGGGGGCAACCCTGATGCAGCGA
CGCCGCGTGAGCGATGAAGCCCTTCGGGGTGTAAAGCTCTTTCGACGGGGACGATGATGA
CGGTACCCGTAGAAGAAGCCCCGGCTAACTTCGTGCCAGCAGCCGCGGTAATACGAAGGG
GGCTAGCGTTGCTCGGAATGACTGGGCGTAAAGGGCGCGTAGGCGGATGATACAGTCAGA
TGTGAAATTCCTGGGCTCAACCTGGGGGCTGCATTTGATACGTGTTGTCTAGAGTGAGGA
AGAGGGTTGTGGAATTCCCAGTGTAGAGGTGAAATTCGTAGATATTGGGAAGAACACCGG
TGGCGAAGGCGGCAACCTGGTCCTTGACTGACGCTGAGGCGCGAAAGCGTGGGGAGCAAA
CAGGATTAGAGACCCCTGTAGTCC
>Otu6491
CCAGCCTACGGGATGCACCAGCTAAGAATATTCCGCAATGGACGAAAGTCTGACGGAGCG
ACGCCGCGTGGATGACGAAGGCCGAAAGGTTGTAAAGTCCTTTTGTTGGGGAAGAATAAC
CATGGGAGGGAATGCCCGTGGGATGACATGAACCGACGAATAAGCACCGGCTAACTCTGT
GCCAGCAGCCGCGGTAAGACGTAGGGGGCCAGCGTTGTTCGGAATTACTGGGTGTAGAGG
GTTCGTAGGCGGTGTGGCAAGTTGGGAGTGAAATCTCTGGGCTTAACCCAGAGGCTGCTT
CCAAAACTGCTGTGCTTGAGTGTGAGAGAGGCTCGTGGAATTGCAGGTGTAGCGGTGAAA
TGCGTAGATATCTGCAGGAACACCCGTGGCGAAAGCGGCGAGCTGGATCACAACTGACGC
TGAGGAACGAAAGCTAGGGGAGCAAACAGGATTAGATACCCCTGTAGTCC
>Otu6493
CTAGCCTATGGGGGGCAGCAGTGGGGAATTTTGGACAATGGGGGCAACCCCGATCCAGCC
ATGCCGCGTGAGTGAAGAAGGCCTTCGGGTTGTAAAGCTCTTTCAGCCGGAAAGAAAGCG
TACGGGTTAATAACCTGTGCGGATGACGGTACCGGAAGAAGAAGCACCGGCTAACTACGT
GCCAGCAGCCGCGGTAATACGTAGGGTGCGAGCGTTAATCGGAATTACTGGGCGTAAAGC
GTGCGCAGGCGGTTTTGTAAGACAGATGTGAAATCCCCGGGCTTAACCTGGGAACTGCAT
TTGTGACTGCAAGACTAGAGTACGGCAGAGGGGGGTAGAATTCCACGTGTAACAGTGAAA
TGCGTAGATATGTGGAGGAATACCGATGGCGAAGGCAGCCCCCTGGGTCGATACTGACGC
TCATGCACGAAAGCGTGGGGAGCAAACAGGATTAGAGACCCCCGTAGTCC
>Otu6494

CCAGCCTACGGGGTGCTCCAGTGGGGAATATTGCACAATGGGCGAAAGCCTGATGCAGCG
ACGCCGCGTGGAGGATGAAGGTTTTCGGATTGTAAACTCCTGTTAAGTGGGAAGAAAGAC
GCATACCTAATACGTATGCGGGATGACGGTACCACTAGAGAAAGCACCGGCAAACTTCGT
GCCAGCAGCCGCGGTAATACGAAGGGTGCGAGCGTTATTCGGAATGACTGGGTGTAAAGA
GCGTGGAGGCGGTTTGTTAAGTTAGTTGTTAAATCGCCCGGCTTAACCGGGCAATCGCGA
TTAAAACTGGCAAGCTAGAGTGTGGGAGAGAAAGGTAGAATTCTCGGAGTAGCGGCAAAA
TGCGTAGATCTCGAGAGGAATACCGGTTGCGAAGGCGGCCTTTTGGCCCACCACTGACGC
TGGAGCGCGAAAGCGTGGGGAGCAAACAGGATTAGATACCCGCGTAGTCC
>Otu6501
CCAGCCTACGGGGGGCTGCAGTGGGGAATTTTGCGCAATGGGGGAAACCCTGACGCAGCA
ACGCCGCGTGAGTGATGAAGGCCTTCGGGTCGTAAAGCTCTGTGGGGAGGGACGAATAAG
TGCTGGCTAATATCCAGCATGATGACGGTACCTCCTTAGCAAGCACCGGCTAACTCTGTG
CCAGCAGCCGCGGTAAGACAGAGGGTGCAAACGTTGTTCGGAATCACTGGGCGTAAAGCG
CGTGTAGGCGGCTACGTAAGTCGGACGTGAAAGCCCACGGCTCAACTGTGGAAGTGCGCT
CGAAACTGTGTAGCTTGAGTCTTGGAGAGGAGGGCGGAATACTTGGTGTAGAGGTGAAAT
TCGTAGATATCAGGTAGAACACCAGTGGCGAAGGCGGCCTTCTGGACAATGACTGACGCT
GAGACGCGAAAGCGTGGGGAGCAAACAGGATTAGAAACCCCCGTAGTCC
>Otu6509
CCAGCCTATGGGTGGCACCAGTCGAGAATCTTCCGCAATGGGCGAAAGCCTGACGGAGCG
ACGCCGCGTGGTTGATGAAGTCCTTCGGGACGTAAAAACCTTTTATGATTAAGAAAGTTT
ATTGATCTGATCATGAATAAGGGGCTCCAAACTCTGTGCCAGCAGGAGCGGTAATACAGA
GGCCCCAAGCGTTGTCCGGAATCACTGGGCGTAAAGGGTGTGTAGGTGGTCTTGTTAGTC
TCTCGTTAAAGCTCCCGACCTAATCGGGAAAATGCGAGAGATACGGCAAGCCTAGAGGAT
GCGAGAGGTATGTGGAATTCATGGAGTAGGGGTGAAATCCGTTGATATCGTGGGGAACAC
CAAAGGCGAAGGCAACATACTGGCGCATTCCTGACACTGAAACACGAAAGCGTGGGTAGC
GAATGGGATTAGATACCCGGGTAGTCC
>Otu6511
CCAGCCTACGGGGCGCAGCAGTGGGGAATATTGCGCAATGGGGGCAACCCTGACGCAGCG
ACGCCATGTGAAGGATGAAGGTCTTAGGATTGTAAACTTCTGTTAAGCGGGAATAAGGTC
TAGTTTACTAGACTTGACGGTACCGCTAGAGAAAGTACCGGCTAACTCCGTGCCAGCAGC
CGCGGTAATACGGAGGGTGCAAGCGTTATTCGGAATTACTAGGTGTAAAGGGTGCGTAGG
TGGTTTGTTAAGTCGAATGTTAAATCCACTAGCTTAACTAGTTGGCTGCATTCGAAACTG
ACGGACTTGAGTATAGAAGAGGAAAATGGAATTCCTAGTGTAGCGGTGAAATGCGTAGAT
ATTAGGAGGAACACCAATAGCGAAAGCAGTTTTCTGGTCTATAACTGACACTGAGGCACG
AAAGCTAGGGGATCAAACAGGATTAGAAACCCTAGTAGTCC
>Otu6516
CCAGCCTACGGGTCGCACCAGTGGGGAATTTTGGACAATGGGGGCAACCCTGATCCAGCC
ATGCCGCGTGAGTGAAGAAGGCCTTCGGGTTGTAAAGCTCTTTCGGTGGGAACGAAACGG
TTACCACTAACACTGGTGACTACTGACGGTACTCACGGAAGAAGCACCGGCTAACTACGT
GCCAGCAGCCGCGGTAATACGTAGGGTGCGAGCGTTAATCGGAATTACTGGGCGTAAAGC
GTGCGCAGGCGGTGGCTCAAGCCAGGCGTGAAATCCCCGGGCTTAACCTGGGAATGGCGT
TTGGGACTGGGCTGCTAGAGTGTGGCAGAGGGAGGTGGAATTCCACGTGTAGCAGTGAAA
TGCGTAGAGATGTGGAGGAACACCGATGGCGAAGGCAGCCTCCTGGGCCAGCACTGACGC
TCATGCACGAAAGCGTGGGGAGCAAACAGGATTAGATACCCCTGTAGTCC
>Otu6523
CCAGCCTATGGGTTGCAGCAGTCGAGAGGCTTTGTCAATGGGGGAAACCCTGAACAAGCG
ACGCCGCGTGAGGGATGAAGGCCTTTGGGTTGTAAACCTCTGTTGCACGGGAAGAAATAC
CGGTGGGTGAATAATCCGCCGGCTTGACGGTACCGTGTGAGGAAGCTCCGGCTAACTCCG
TGCCAGCAGCCGCGGTAATACGGGGGGAGCAAGCGTTGTTCGGAATCACTGGGCGTAAAG
GGCGCGTAGGCGGTTCGGTAAGTGAGATGTGAAAGACTCCGGCTCACCCGGAGAGATGCA
TCTCATACTGCTGAGCTGGAGTACAGGAGAGGAAACGAGAATTCCAGGTGTAGCGGTGAA
ATGCGTAGATATCTGGAGGAATACCGGTGGCGAAGGCGCGTTTCTGGCCTGTTACTGACG
CTGAGGCGCGATAGCCAGGGGAGCAAACGGGATTAGAGACCCCCGTAGTCC
>Otu6527
CAGCCTATGGGTCGCAGCAGTCGAGAGGCTTTGGCAATGGGGGAAACCCTGACCAAGCGA
CGCCGCGTGGAGGATGAAGGCCCTTGGGTTGTAAACTCCTTTTGCTGGGAAGAAGTGCG
ATCAGGTGAACAATCTGGCCGTTTGACGGTACCCAGCGAATAAGCTCCGGCTAACTCCGT
GCCAGCAGCCGCGGTAACACGGGGGGAGCAAGCGTTGTTCGGAATCACTGGGCGTAAAGG

GCGTGTAGGCGGTCAGGTAAGTGGGATGTGAAATGCCCCGGCTCACCCGGGGACCGGCAT
CCCAAACTGCCTGGCTTGAGTATGGGAGAGGATGGAGGAATTCCTGGTGTAGCGGTGAAA
TGCGTAGATATCAGGAGGAACACCGGTGGCGAAGGCGTCCATCTGGCCCAATACTGACGC
TGAGGCGCGAAAGCTAGGGGAGCGAACGGGATTAGAAACCCTCGTAGTCC
>Otu6531
CCAGCCTATGGGTTGCAGCAGGCGCGAAACCTCTGCAATGCGCGAAAGCGCGACAGGGCC
ACCCCGAGTGCTATCCGAAGAGGGTAGCTTTTCCTTAGTGTAGTAAGCTAGGGGAATAAG
CGGGGGGCAAGCCTGGTGTCAGCCGCCGCGGTAATACCAGCTCCGCGAGTGGTCGGGACG
ATTATTGGGCCTAAAGCGTTCGTAGCCGGCCTATCAAGTCTCTGGTTAAATTTCAAGGCT
TAACCTTGAGCATGCTGGAGATACTGTTAGGCTAGGGGGCGGGAGAGGTTGAGGGTACTT
CGCGGGTAGGGGCGAAATCCTATAATCCGCGAAGGACCACCAGTGGCGAAGGCGCTCAAC
TGGAACGCGCCCGACGGTGAGGGACGAAAGCCAGGGGAGCAAAGGGGATTAGAAACCCGA
GTAGTCC
>Otu6538
CCAGCCTATGGGATGCAGCAGTGGGGAATATTGGACAATGGGGGAAACCCTGATCCAGCA
ATGCCGCGTGTGCGATGAAGGCCTGCGGGTTGTAAAGCACTTTCGTTGGGGAAGAAATTC
TCAAATATAATACATTTGAGAGCTGACGTTACCCAAAGAAGAAGCACCGGCAAACTCTGT
GCCAGCAGCCGCGGTAATACAGAGGGTGCAAGCGTTAATCGGAGTTACTGGGCGTAAAGG
GCGCGTAGGCTGGTATTTAAGTCGATTGTGAAATCCCTGGGCTTAACCTAGGAATTGCAG
TCGATACTGAATATCTTGAGTATGGTAGAGGAAAGTGGAATTCTCGGTGTAGTGGTTAAA
TACGTAGATATCGAGAAGAACACCGGTGGCGAAGGCGACTTTCTGGACCAATACTGACGC
TGAGGCGCGAAAGCGTGGGGAGCAAACAGGATTAGAGACCCCTGTAGTCC
>Otu6539
CCAGCCTATGGGACGCAGCAGTAAGGGATATTGCGCAATGGGCGAAAGCCTGACGGAGCA
ACGCCGCGTGAGTGACGAAGCCCTTCGGGGTGTAAAACTCTTTTCTATGGGAAGAAGATC
TGACGGTACCATAGGAATAAGCATCGGCTAACTACGTGCCAGCAGCCGCGGTAATACGTA
GGATGCAAGCGTTGTCCGGATTTATTGGGCGTAAAGAGCGCGTAGGTGGTGTCTTAAGTT
AGGCGTGAAATCTCCCGGCTTAACTGGGAGGGGTCGTCTAAAACTGGGACGCTTGAGGGC
AGCAGAGGAAAGTGGAATTCCGGGTGTAGTGGTGAAATGCGTAGATATCCGGAGGAACAC
CAGTGGCGAAGGCGGCTTTCTGGGCTGTTCCTGACGCTGAGGCGCGAAGGCTAGGGTAGC
GAACGGGATTAGAAACCCCTGTAGTCC
>Otu6540
CCAGCCTACGGGTTGCTCCAGTTGGGAATTTTGCACAATGGGCGAAAGCCTGATGCAGCA
ACACTGCGTGAAGGATGAAGGTTTTCGGATCGTAAACTTCTTTTATCTGGGAATAATCTT
GAAGGTACCAGATGAATAAGCACCGGCTAACTTCGTGCCAGCAGCCGCGGTAACACGTAG
GAGGCAAGCGTTATCCGGATTTACTGGGCGTAAAGCGCATGCAGGTGGTTTGGTAAGTTG
GATGTGAAAGCTCCTGGCTCAACTGGGAGAGGTCGTTCAATACTACCAGACTTGAGAGCA
GTAGAGGGAGGTGGAATTCCCGGTGTAGTGGTGAAATGCGTAGATATCGGGAGGAACACC
AGTGGCGAAAGCGGCCTCCTGGACTGTTTCTGACACTCATATGCGAAAGCTAGGGTAGCA
AACGGGATTAGAAACCCCTGTAGTCC
>Otu6541
CCAGCCTATGGGGGGCAGCAGTCGCGAATCTTTTGCAATACACGAAAGTGTGACAAAGCG
AGCCAAAGTGTTTTCCGCACAGGAAAACTTTTGCCAGCTGTAAAAAGGCTGGCGAATAAG
GACTGGGCAAGACTGGTGCCAGCCGCCGCGGTAATCCCAGCAGTCCAAGTTGCAGCCAGC
TTTATTGGGTCTAAAACATCCGTAGCTTGTCTAATAAGTCTCTTGTGAAATCTGGCATCT
TAAGTGTCAGGCGCGCAAGAGATACTATTGGGCTTGAGACCGGTAGATGTAAGGAGTACA
TTTAAGGTAGTGGTAAAATACGTTAATCTTAGGTGGACTAACAATTGGCGAAGGCACCTT
ACAAGGACGGATCTGACAGTGAGGGATGAAGGCTAGGGGCGCAAAACGGATTAGATACCC
TAGTAGTCC
>Otu6544
CCAGCCTATGGGTTGCAGCAGTCGAGAATCTTTCGCAATGGGGGAAACCCTGACGAAGCG
ACGCCGCGTGAGCGATGAAGGTCTTCGGATTGTAAAGCTCTGTCACAAGGGACGAAATCT
TTTTTGGCTAACAGCCAAGAGAGTTGACGGTACCTTGAGAGGAAGCCCCGGCTAACTCTG
TGCCAGCAGCCGCGGTAATACAGAGGGGGCAAGCGTTGTTCGGAATTACTGGGTGTAAAG
GGTGCGTAGGTTGTTGGGTAAGTTAGATGTGAAATCTCAGGGCTTAACCCTGAAATTGCA
TTTAATACTGCTTGACTAGAATATGGGAGAGGGAAATGGAATTCTCGGTGTAAGAGTGAA
ATCTGCAGATATCGAGAGGAACACCAGTAGCGAAGGCGATTTCCTGGCCCAATATTGACA
CTGAGTGCACGAAAGCTAGGGGAGCAAACGGGATTAGAAACCCTGTAGTCC

>Otu6547
CCAGCCTATGGGATGCAGCAGTGGGGAATCTTGCACAATGGGGGAAACCCTGATGCAGCG
ACGCCGCGTGAGCGATGAAGCCCTTCGGGGTGTAAAGCTCTGTCTTTGGGGACGATAATG
ACGGTACCCAAGGAGGAAGCCACGGCTAACTACGTGCCAGCAGCCGCGGTAATACGTAGG
TGGCAAGCGTTGTCCGGATTTACTGGGCGTAAAGGATGCGTAGGTGGATATTTAAGTCAG
ATGTGAAATACCCGAGCTTAACTTGGGTGCTGCATTTGAAACTGGATATCTAGAGTGCAG
GAGAGGTAAGTGGAATTCCTAGTGTAGCGGCGAAATGCGTAGAGATTAGGAAGAACACCA
GTGGCGAAGGCGACTTACTGGACTGTAACTGACACTGAGGCATGAAAGCGTGGGGAGCAA
ACAGGATTAGAGACCCTCGTAGTCC
>Otu6549
CCAGCCTACGGGGTGCTGCAGCAAGGAATTTTCGGCAATGGGCGCAAGCCTGACCGAGCA
ACGCCGCGTGGTGGATGACGGCCTTCGGGTTGTAAACACCTTTTGGGGGGGAAGACATCG
ACGGTACCCCCCGAACAAGCCCCGGCTAACTCTGTGCCAGCAGCCGCGGTAAGACAGAGG
GGGCAAGCGTTGTCCGGAGTTACTGGGCGTAAAGCGCACGCAGGCGGCCCGGCACGTGTG
GGGTGAAAGCTCGGGGCTTAACCCAGAGTCGCCCCCGCATACGGCCGGGCTCGAGCCCGG
GAGAGGTCCCTCGAATTGCCGGTGTAGTGGTGAAATGCGTAGAGATCGGCAGGAAGACCA
AGGGGGAAGCCAGGGGACTGGCCCGGTGCTGACGCTCAGGTGCGATAGCGTGGGGAGCGA
ACCGGATTAGAGACCCTGGTAGTCC
>Otu6551
CCAGCCTACGGGGTGCAGCAGTAGGGAATATTGCGCAATGGACGAAAGTCTGACGGAGCG
ACGCCGCGTGGATGACGAAGGCCGAAAGGTTGTAAAGTCCTTTTGTTGGGGAAGAATAAC
CATGGGAGGGAATGCCCGTGGGATGACATGAACCGACGAATAAGCCCCGGCTAACTACGT
GCCAGCAGCCGCGGTAACACGTAGGGGGCGAGCGTTGTTCGGAATTACTGGGCGTAAAGG
GCATGTAGGCTGTTATGTAAGTGTGGATTTAAAGGCGGAGGCTTAACCTCTAGTTTGGTC
TGCAAACTGCGTAGCTTGAGTGTAGAAGGGGAAACTGGAATTCCGCGTGTAGGGGTGAAA
TCTGTAGATATGCGGAAGAATACCTGTGGCGAAGGCGAGTTTCTGGTCTATAACTGACGC
TGAAATGCGAAAGTGTGGGTAGCAAACAGGATTAGAAACCCTCGTAGTCC
>Otu6552
CCAGCCTACGGGTGGCTGCAGTCGAGGATCTTCGGCAATGGGCGCAAGCCTGACCGAGCG
ACGCCGCGTGTGCGATGAAGGCCTTCGGGTTGTAAAGCACTGTCGAGGGGGACGAAGCCG
CAAGGTTGACCGATCCCTGGAGGAAGCACGGGCTAAGTTCGTGCCAGCAGCCGCGGTAAG
ACGAACCGTGCAAACGTTGTTCGGAATCACTGGGCTTAAAGGGCGCGTAGGCGGCTTGCC
AAGTCAGGGGTGAAATACTTCAGCTTAACTGGAGAAGTGCCTTTGAAACTGGCGGGCTAG
AGGGAGGTAGGGGTGTGTGGAACTTCTGGTGGAGCGGTGAAATGCGTTGATATCAGAAGG
AACGCCGGTGGCGAAAGCGACGCACTGGACCTCTTCTGACGCTGAGGCGCGAAAGCTAGG
GGAGCAAACGGGATTAGATACCCTAGTAGTCC
>Otu6557
CCAGCCTACGGGTGGCAGCAGTAACGAATCTTCCGCAATGCACGAAAGTGTGACGGAGCG
ACGCCGCGTGTGGGACGAAGCCCTTCGGGGTGTAAACCACTGTCAGGGATAGGAAAGTTC
TGATCCATCCCAGAGGAAGGCACGGCTAACTCTGTGCCAGCAGCCGCGGTAAGACAGAGG
TGCCAAGCGTTAGGCGGAATCACTGGGCTTAAAGCGTGTGTAGGCGGATGCCTAAGTACC
TTGTGAAATCCCACGGCTTACCCGTGGAACTGCTGGGTATACTGGGTGTCTTGAGCCAAC
TAGGGGTCGCCGGAACAAGTGGTGGAGCGGTGAAATGCGTAGATATCACTTGGAACGCCA
ATGGTGAAAACAGGCGACTGGGGTTGTGCTGACGCTGAGACACGAAAGCCAGGGGAGCGA
ACGGGATTAGAAACCCCTGTAGTCC
>Otu6563
CCAGCCTATGGGGGGCTGCAGGAACGAATATTGGGCAATGGGCGAAAGCCTGACCCAGCG
ACGCCGCGTGTGGGAAGAAGTCCTTCGGGATGTAAACCACTGTCAGGGATCACCAAGCAA
CGGCGTACAATACGCGCCGAAGTTGAGGAGTCCCAAAGGAAGCCACGGCTAACTTCGTGC
CAGCAGCCGCGGTAAGACGAAGGTGGCAAGCGTTATTCGGAATCATTGGGCTTAAAGCGC
ACGTAGGCGGCCTGTTAAGTGTCTTGTGAAATCCCCCGGCTCAACCGGGGAATGGCTGGG
CATACTGACGGGCTTGAGGCAGGTAGGGGCAAGTGGAACTCTTGGTGGAGCGGTGGAATG
CGTAGATATCAAGAGGAACGCCGATGGTGAAGACAACTTGCTGGGCCTGTCCTGACGCTG
AGGTGCGAAAGCCAGGGGAGCAAACGGGATTAGAGACCCCAGTAGTCA
>Otu6564
CCAGCCTACGGGTTGCTGCAGTGAGGAATATTGGTCAATGGACGGAAGTCTGAACCAGCC
ATCCCGCGTGCAGGAAGAATGCCCTATGGGTTGTAAACTGTTTTTCCAGGGGAAGAAAAC
CTTCGACGAGTCGAAGCTTGACGGTACCTTGGGAATAAGCATCGGCTAACTCCGTGCCAG

CAGCCGCGGTAATACGGAGGATGCAAGCGTTATCCGGATTCATTGGGTTTAAAGGGTGCG
TAGGCGGACTGATAAGTCAGTGGTGAAAACCTGCAGCTTAACTGTAGACGTGCCATTGAT
ACTGTTAGACTTGAGTGTGGTCAAGGAAGGCGGAATGTGTAATGTAGCGGTGAAATGCTT
AGATATTACACAGAACACCTATTGCGAAGGCAGCTTTCTGGCCATTACTGACGCTGATG
CACGAAAGCGTGGGGAGCGAACAGGATTAGAAACCCGAGTAGTCC
>Otu6566
CCAGCCTACGGGGCGCACCAGTCGAGAATCTTCCGCAATGGACGAAAGTCTGACGGAGCG
ACGCCGCGTGGAGGATGAAGTTCTTCGGAATGTAAACTCCTTTTGCCAGGGAAAAAGTTA
TTGATTGTACCTGGAGAATAAGAAGTTGCTAAACTCGTGCCAGCAGCAGCGGTAATACGA
GTGCTTCGAGCGTTATCCGGAGTCATTGGGCGTAAAGGGTGCGTAGGCGGCGATGTTAGT
CTCATGTTAAATCTTTCGGCTTAACCGAGAGTCTGCATGGGAAACGGCATTGCTTAGAGG
ACGGAAGAGGTCACTGGGACTCATGGTGTAGCGGTGAAATGCGTTGATATCATGGGGAAC
ACCAAAAGCGAAGGCAAGTGACTGGTCCGCTCCTGACGCTGAAGCACGAAAGCGTGGGTC
GCGAATGGGATTAGATACCCCCGTAGTCC
>Otu6567
CCAGCCTACGGGGTGCTGCAGTCGAGAATATTCCACAATGGGCGAAAGCCTGATGGAGCG
ACGCCGCGTGCAGGAAGAAGGCCTTCGGGTTGTAAACTGCGGTAGCAGGGGAAAAACGCA
AGTGATGGTACCCTGCGGAAAGAGGTGGGTAACTACGTGCCAGCACCAGCGGTAATACGC
AGACCTCAAGCGTTATCCGGATTTATTGGGCGTAAAGCGCGTGTAGGGGGTCCTTTGCGT
CTTTGGTTAAAGCCCACCGCCTAACGGTGGAATCGCCAAAGATACGGAAGGACTTGAGGA
AGTTAGAGGCACATGGAACGCACGGTGTAGGGGTGAAATCCGTTGATATCGTGCGGAACA
CCAAAGGCGAAGGCAGTGTGCTGGGACTTTCCTGACCCTGAGACGCGAAAGCGTGGGGAG
CAAAAAGGATTAGAAACCCCTGTAGTCC
>Otu6574
CCAGCCTACGGGTGGCTGCAGGCGCGAAACCTTTGCCATGCGCGAAAGCGTGACAGGGGA
ACTCCGAGTGGTAGAAGGTTTTACTTTCTATCTTTTTGTCAATCCAAACAATTGGCAGAA
TAAGGTCTGGGTAAGGCCAGTGCCAGCCGCCATGTTGGGAGTGTTCACACCCCCGACACG
GTATGCGGTAGTACTGGCAGAACAAGTGGTACCCACGAATATTGGGTCTAAAGAGTCCGT
AGCGGGCCGACTAAGTCCACTGTGAAATCTGGCGGCTTAACCGTCAGGCGGGCAGTGGAT
ACTGATCGGCTTGGGAGCGGGGGAGGCCGGGAGTACTGGCGGGGTAACGGTAAAATGTTG
TAATCCTGCCAGGACTACCAGTGGCGAAGGCGCCCGGCCAAAACGCGTCCGACCGTGAGG
GACGAAGGCTAGGAGAACGAATCGGATTAGATACCCTAGTAGTCC
>Otu6575
CCAGCCTATGGGAGGCAGCAGTGGGGAATATTGGACAATGGGCGCAAGCCTGATCCAGCC
ATACCGCGTGAGTGATGAAGGCCTTAGGGTTGTAAAGCTCTTTCGCGCACGACGATAATG
ACGGTAGCGCGAGAAGAAGCCCCGGCTAACTTCGTGCCAGCAGCCGCGGTAATACGAAGG
GGGCTAGCGTTGTTCGGAATCACTGGGCGTAAAGCGCATGCAGGCGGATCGTTAAGTCGG
GGGTGAAATCCCGAGGCTCAACCTCGGAACTGCCTTCGATACTGGCGATCTTGAGTCCGG
GAGAGGTGAGTGGAACTGCGAGTGTAGAGGTGAAATTCGTAGATATTCGCAAGAACACCA
GTGGCGAAGGCGGCTCACTGGCCCGGTACTGACGCTCAGGTGCGAAAGCGTGGGGAGCAA
ACAGGATTAGAGACCCGCGTAGTCC
>Otu6581
CCAGCCTACGGGATGCTGCAGTCGAGGATTTTTCTCAATGGGGGAAACCCTGAAGGAGCG
ACGCCGCGTGGGGGATGAAGGTCTTCGGATTGTAAACCCCTGTCATCCGGGAACAATGTT
CCGCACCCAATACGTGCGGAATTGATAGTACTGGAAGAGGAAGCAGTGGCTAACTCTGTG
CCAGCAGCCGCGGTAATACAGAGACTGCAAGCGTTGCTCGGATTCATTGGGCGTAAAGGG
TGCGCAGGCGGTCGGGTAAGTCGGATGTAAAATCCCGCAGCTTAACTGCGGACGGTCATT
CGAAACTGCCCGGCTCGAGGACTGGAGAGGAGAATGGAATGGTCGGTGTAGCGGTGAAAT
GCGTAGAGATCGACCAGAACACCGGTGGCGAAGGCGGGTCTCTGGACAGTTCCTGACGCT
CAGGCACGAAAGCCAGGGGAGCAAACGGGATTAGATACCCCAGTAGTCC
>Otu6582
CCAGCCTATGGGGGGCTGCAGCCAGGAATATTGGGCAATGGACGCAAGTCTGACCCAGCG
ACGCCGCGTGGAGGAAGACGGCCTTAGGGTTGTAAACTCCTTTTGATAGACCCTTCACGG
TCTATAGAATAAGCCTCTACTAACTACGTGCCAGAAGTCTCGGTAATACGTAGGAGGCAA
GCGTTATCCGGATTCATTGGGCGTAAAGTGTGCGTAGGCGGTTTGATGTGTCTTCTCTTA
AAGACCGAAGCTTAACTTCGGGAGTGGGGAAGAAACTATCAAACTCGAGGATTATCAGGG
ATACTGGAACAGTTAGTGTAGCAGTGAAATGCGTTGATATTAACTGGAACACCAAGGGCG
AAGGCAAGTATCTGGGATTTTTCTGACGCTGAGGCACGACAGCTAGGGTAGCGAAACGGA

TTAGAGACCCTCGTAGTCC
>Otu6583
CCAGCCTATGGGTCGCAGCAGGCGCGAATACTTTACAATGCACGAAAGTGTGATAGGGGA
ACTCAAAGTGCTCATACACTGTATGGGCTTTTGCCAACTCAAAATTGGTTGGCGAATAAG
TGGTGGGAAAGACTGGTGCCAGCCGCCGCGGTAACACCAGCGCCACAAGTGGGAATCGCT
ATTACTGGGCCTAAAGCGTCCGTAGCAGGTTTTGTAAATTGCTTGTGAAATTGCATAGCT
CAACTATGCGAATTGCAAGCAACACTGCAAGACTAGAGACCGGGAGATGTTAGAGGTATT
CAGAGGGGAGCGGTAAAATGCGAGAATCCTCTGAGGACCACCTGTTGCGAAGGCGTCTAA
CAATAACGGGTCTGACTGTGAGGGTCCAAAGCTAGGGGAGCGAACCGGATTAGATACCCC
CGTAGTCG
>Otu6586
CTAGCCTATGGGTTGCTGCAGTGAGGAATCTTGCGCAATGGGGGAAACCCTGACGCAGCA
ACGCCGCGTGAGTGAGGAAGGTCTTCGGATTGTAAAGCTCTGTCAGGTGGAAAGAAGTGC
ATGGGAGTGAATAGCTACCATGTTTGACGGTACCACCAAAGGAAGCACCGGCTAACTCCG
TGCCAGCAGCCGCGGTAATACGGGGGGTGCAAGCGTTGTTCGGAATCATTGGGCGTAAAG
AGCGTGTAGGCGGCGTAATAAGTCAGATGTGAAATCCCCGGGCTCAACCCGGGAAGTGCA
TTTGAAACTGTTACGCTTGAGTAGGGGAGAGGGAAGTGGAATTCCTGGTGTAGAGGTGAA
ATTCGTAGATATCAGGAGGAACACCGGTGGCGAAGGCGACTTCCTGGCCCTATACTGACG
CTGAGACGCGAGAGCGTGGGTAGCAAACAGGATTAGATACCCTAGTAGTCC
>Otu6592
CCAGCCTACGGGTGGCACCAGTGGGGAATATTGCGCAATGGGCGAAAGCCTGATGGAGCG
ACGTCGCGTGACTGATGAAGTTCTTAGGGATGTAAAGGTCTTTTATGAGGGAGAAAGGCG
TCGCAAGACGTTTGATAGTACCTCATGAATAAGGGGTTGCTAAACTCGTGCCAGCAGCAG
CGGTAATACGAGTGCCCCGAGCGTTATCCGGAATTATTGGGCGTAAAGGGTATGTAGGTG
GTACTGTTAGTCTTTCGTTAAAGCTCCCGGCTTAACCGGGAAAAGGCGAAAGAAACGGCA
GCACTTGGAGAGTGCGAGAGGTGTATGGAACTCATGGTGTAGGGGTGAAATCCGTTAATA
TCATGGGGAACACCAAAGGCGAAGGCAATACACTGGCGCATTTCTGACACTGAAATACGA
AAGCGTGGGGAGCGAATGGGATTAGATACCCCTGTAGTCC
>Otu6596
CCAGCCTATGGGGGGCTGCAGTGGGGAATATTGGACAATGGGGGAAACCCTGATCCAGCG
ACGCCGCGTGTGTGAAGAAGGCCTGCGGGTTGTAAAGCACTTTTAGTGGGGATGAAACGC
TCAGACCTAACACGTCTGGGAATTGACCTAACCCAAAGAAAAAGCACCGGCTAACTCTGT
GCCAGCAGCCGCGGTAATACAGAGGGTGCAAGCGTTAATCGGAATTACTGGGCGTAAAGC
GTGCGTAGTCGGTTATTTAAGTCGGATGTGAAATCCCCGGGCTCAACCTGGGAATTGCAT
TCGATACTGGGTAGCTAGAGTTCGGCAGAGGGAAGTGGAATTTCCGGTGTAGCGGTGAAA
TGCGTAGATATCGGAAGGAACATCAGTGGCGGAAGCGACTTCCTGGACCAGAACTGACGA
TCAGGCACGAAAGCGTGGGGAGCAAACAGGATTAGATACCCGCGTAGTCC
>Otu6597
CCAGCCTACGGGATGCAGCAGTGGGGAATATTGGACAATGGGCGCAAGCCTGATCCAGCA
ATGCCGCGTGTGTGAAGAAGGCCTGCGGGTTGTAAAGCACTTTAAGCAGGAAAGAAGGGG
TGGGGGCGAATATCCTCCGCCATTGACGTTACCTGCAGAATAAGCACCGGCTAACTCCGT
GCCAGCAGCCGCGGTAATACGGAGGGTGCGAGCGTTAATCGGAATTACTGGGCGTAAAGC
GCGCGTAGGCGGTTCGATCAGTCTGAGGTGAAAGCCCTGGGCTCAACCTGGGAACGGCCT
TGGAAACTGTCGAGCTAGAGTGTGGGAGAGGGGCGTGGAATTTCCGGTGTAGCAGTGAAA
TGCGTAGAGATCGGAAGGAACACCAGTGGCGAAGGCGGCGCCCTGGCCCAACACTGACGC
TGAGGTGCGAAAGCGTGGGGAGCAAACAGGATTAGAAACCCGAGTAGTCC
>Otu6605
CCAGCCTACGGGACGCACCAGTCGGGAATTTTGGACAATGGGGGAACCCTGATCCAGCGA
CGCCGCGTGCAGGATGACATCCTTCGGGATGTAAACTGCTGTCGGGCGGGAAGAACGATC
CCGGTTCTAATACAATCGGGAAGTGACGGTACCGCCGAAGGAAGCACCGGCTAACTCTGT
GCCAGCAGCCGCGGTAAGACAGAGGGTGCAAGCGTTGTTCGGATTGACTGGGCGTAAAGG
GCGTGTGGGTGGTCAGGTAAGTTCGATGTGAAATCATCATCGCTTAACGAGATGTCCGCA
TCGAAAACTGTCTGACTTGAGTATCGGAGAGGATAACGGAATTCCTGGTGTAGCGGTGGA
ATGTGTAGAGATCAGGAGGAACACCTGTGGCGAAGGCGGTTATCTGGCCGAATACTGACA
CTGAGGCGCGAAAGCTAGGGGAGCAAACCGGATTAGATACCCCCGTAGTCC
>Otu6608
CCAGCCTATGGGGTGCTCCAGTCGAGAATCTTTCGCAATGGGCGAAAGCCTGACGAAGCG
ACGCCGTGTGAGCGAAGAAGGCCTTCGGGTTGTAAAGCTCTTTCGCTCGGGAACAAAGAA

GTTGACTGAATAAGTCATTACTTGAGTGTACCGGGTAAAGAAGCACCGGCTAACTCCGTG
CCAGCAGCTGCGGTAATACGGAGGGTGCAAGCATTAATCGGAATCATTGGGCGTACAGGG
TGCGTAGGTGGGTAATTAAGTCAGACGTGAAAGCCTGGGGCTCAACCCCAGAATGGCGTT
TGATACTGATTACCTTGAGGATAAGTGGGGAAAGTGGAATTCCGCAAGTAGCGGTGAAAT
GCGTAGATATGCGGAGGAACACCTGTGGTGAAGACGACTTTCTAACTTATTCCTGACACT
GAGGCACGAAAGCGAGGGGAGCAAACAGGATTAGAAACCCGAGTAGTCC
>Otu6609
CCAGACCTATGGGGGGCTCCAGTGAGGAATATTGGTCAATGGACGCAAGTCTGAACCAGC
CATGCCGCGTGCAGGAAGACGGCCCTATGGGTTGTAAACTGCTTTTGTAAGGGAATAACC
GCTCGTACGTGTACGAGTCTGAATGTACCTTACGAATAAGCATCGGCTAACTCCGTGCCA
GCAGCCGCGGTAATACGGAGGATGCAAGCGTTATCCGGATTTATTGGGTTTAAAGGGTCC
GCAGGCGGTATATTAAGTCAGTGGTGAAATCCGGCAGCTCAACTGTCGCACTGCCATTGA
TACTGGTATACTTGAGTGTAATTGAAGTAGGCGGAATAAGTCATGTAGCGGTGAAATGCA
TAGATATGACTTAGAACACCAATTGCGAAGGCAGCTTACTAAGTTACAACTGACGCTGAG
GGACGAAAGCGTGGGTAGCAAACAGGATTAGAAACCCCCGTAGTCCT
>Otu6610
CCAGCCTATGGGGTGCTGCAGTCGAGGATCTTTTGCAATGCCCGCAAGGGTGACAATGCG
ACGCCGCGTGAGCGATGAAGGCCTTCGGGTTGTAAAGCTCTGTCAGCATGGATGAAGTCT
TGAGAATTAATAACTCTCAAGATTGACGGTACATGCAGAGGAAGCCACGGCTAACTCTGT
GCCAGCAGCCGCGGTAATACAGAGGTGGCAAGCGTTGTTCGGATTTACTGGGTGTAAAGG
TCATGTAGGCGTTTTGTTAAGTCGGGCGTGAAATCCTATGGCTCAACCATAGAACTGCGC
TCGATACTGGCAGGATTGAGTACGGGAGAGGTAAGCGGAATTCCAGAAGTAAGGGTGAAA
TCTGTAGATATCTGGAAGAACACCATTAGCGAAGGCGGCTTACTGGTCCGATACTGACGC
TGAAATGAGAAAGCTAGGGGAGCAAACAGGATTAGATACCCTTGTAGTCC
>Otu6611
CCAGCCTATGGGTCGCTCCAGTCGAGAATTTTTCTCAATGGGCGAAAGCTTGAAGGAGCG
ACGCCGCGTGGGGGATGAAGGGCTTCGGCCTGTAAACCCCTGTCATTTGCGAACAAACCT
TTTCATTGAACAGGTGGAAAGTTGATAGTAGCGAAAGAGGAAGGGACGGCTAACTCTGTG
CCAGCAGCCGCGGTAATACAGAGGTCCCAAGCGTTGTTCGGATTCACTGGGCGTAAAGGG
TGCGTAGGTGGTCGGATAAGTTCGATGTGAAATCTCCGGGCTTAACTCGGAAATGGCATT
GAATACTATTCGGCTAGAGGGTCGGAGGGGAGACTGGAATTCTCGGTGTTGCAGTGAAAT
GCGTAGATATCGAGAGGAACACCAGTGGCGAAGGCGAGTCTCTGGACGACTCCTGACACT
GAGGCACGAAAGCTAGGGGAGCAAACAGGATTAGAAACCCGTGTAGTCC
>Otu6613
CCAGCCTATGGGTGGCACCAGTAACGAATATTGGGCAATGGGCGAAAGCCTGACCCAGCG
ACGCCGCGTGTGGGAGGAAGTCCTTCGGGACGTAAACCACTGTCAGGGGGAAGAAAGTCT
ATCGCAAGGTAGATTGATCTAACCCAGAGGAAGTCACGGCTAACTCTGTGCCAGCAGCCG
CGGTAAGACAGAGGTGGCTAGCGTTGTTCGGTGTCACTGGGCTTAAAGGGTGTGTAGGCG
GCTAGACAAGCGCGTTGTGAAATCCCTCGGCTTAACCGAGGAATTGCTTCGCGTACTATC
TGGCTTGAGGCAGGTATGGGTGCGGGGAACTGTTGGTGGAGCGGTGGAATGCGTAGATAT
CAACAGGAACGCCGGTGGTGAAGACGCTGCACTGGACCTGTCCTGACGCTGAGACACGAA
AGCGAGGGGACCGAACGGGATTAGAGACCCGCGTAGTCC
>Otu6615
CCAGCCTATGGGGGGCTGCAGTCGAGAATTTTTCACAATGGGCGCAACCCTGATCCAGCA
ATGCCGCGTGGGTGAAGAAGGCCTGCGGGTTGTAAAGCCCTTTAAGTGGGGAGGAACGGC
GGGATGCAAACAGCGTCCGGTTATGACGTTACCCACAGAATAAGCACCGGCTAACTCCGT
GCCAGCAGCCGCGGTAATACGGGGGGTGCAAGCGTTAATCGGAATTACTGGGCGTAAAGC
GTGCGTAGGCGGTTGGGTCAGTCAGCCGTGAAAGCCCTGGGCTTAACCTGGGAACGGCGG
TTGAGACGGCCCGACTGGAGTGGGCTAGAGGATTGTGGAATTCCCGGTGTAGCGGTGAAA
TGCGTAGAGATCGGGAGGAACACCGATGGCGAAGGCAGCAGTCTGGGGCCACACTGACGC
TGAGGCACGAAAGCGTGGGGAGCAAACAGGATTAGAAACCCTCGTAGTCC
>Otu6617
CCAGCCTACGGGATGCAGCAGTGAGGAATTTTGCGCAATGGCCGCAAGGCTGACGCAGCA
ACGCCGCGTGGGTGAAGAAGGCCTTCGGGTCGTAAAGCCCTGTCAGGTGGGAAGAATGAT
TCGGTTATGAATAATGGCCGAATGTGACGGTACCACCAGAGGAAGCACCGGCTAACTCCG
TGCCAGCAGCCGCGGTAATACGGAGGGTGCAAGCGTTATTCGGAATTACTGGGCGTAAAG
CGCGTGCAGGCGGGCGAGCAAGTCTGATGTGAAAGCCCCGGGCTTAACCTGGGAAGTGCA
TTGGAAACTGGGAGACTTGAATACGGGAGAGGGCAGTGGAATTCCTGGTGTAGGAGTGAA

ATCCGTAGATATCAGGAGGAACACCGGTGGCGAAGGCGGCTGCCTGGACCGATATTGACG
CTGAGACGCGAAAGCGTGGGGAGCAAACAGGATTAGAAACCCCCGTAGTCC
>Otu6620
CCAGCCTACGGGTCGCACCAGTGGGGAATTTTGCGCAATGGGCGAAAGCCTGACGCAGCA
ACGCCGCGTGGAGGATGAAGGTGCTCTGCATCGTAAACTCCTGTCGGCCGGGAAGAAAGG
GCAGCGCAGTAACTGGCGATGTCGTTGACTGTACCGGCAAAGGAAGCCCCGGCTAACTCC
GTGCCAGCAGCCGCGGTAATACGGAGGGGGCAAGCGTTACTCGGAATTATTGGGCGTAAA
GGGCGCGTAGGCGGTCGTGTGCGTCGGAGGTGAAATCCCCGGGCTTAACCCGGGAGCTGC
CTCCGATACGGCATGACTTGAGTCCGGGAGAGGGGAGCAGAATTCCCAGTGTAGCGGTGA
AATGCGTAGATATTGGGAGGAATACCAGTGGCGAAGGCGGCTCCCTGGACCGGTACTGAC
GCTGAGGCGCGAAAGCGTGGGTAGCAAACAGGATTAGATACCCCCGTAGTCC
>Otu6627
CCAGCCTATGGGAGGCTGCAGTCGAGGATCTTCGGCAATGGGGGCAACCCTGACCGAGCG
ACGCCGCGTGCGGGATGAAGGCCTTCGGGTTGTAAACCGCTGTCAGAGGGGAGGAAATGC
TGTGGGGTTCTCCCTGCAGTTTGACCTATCCTCAGAGGAAGTACGGGCTAAGTTCGTGCC
AGCAGCCGCGGTAAGACGAACCGTACGAACGTTATTCGGAATTACTGGGCTTAAAGGGTG
CGTAGGCGGCCCGGGAAGTTGGGTGTGAAATCCCTCGGCTCAACCGAGGAACTGCGCCCA
AAACTGCCGGGCTTGAGGGAGATAGAGGTGAACGGAACTTAGGGTGGAGCGGTGAAATGC
GTTGATATCCTAAGGAACACCAGTAGCGAAAGCGGTTCACTGGGTCTCTTCTGACGCTGA
GGCACGAAAGCTAGGGGAGCGAACGGGATTAGAAACCCCCGTAGTCC
>Otu6629
CCAGCCTATGGGGGGCACCAGTTAGGGAACTTTTACAATGCGCGCAAGCGTGATAAAGTA
AGCCAGAGTGTTATTCATAAAATTTGAATAACTTTTGTTCGTTGCAAAAAGACGGATGAA
TAAGGAGTGGGCAAGATCGGTGCCAGCCGCCGCGGTAATCCCGACGCTCCGAGTCGCAGC
CACAATTATTGGGTCTAAAACATTCGTAGCTTGTTTGTTAAGTTCTTTGTGAAATCTTAC
ATCTTAAGTGTAAGGCGTGCAAGGAATACTGGCAAACTTGAGACCGGAAGAAGCAAGAAG
TATGCTCGAGGTAGTGGTAAAATATGTTAATCTCGAGCAGACTAACAACAGCGAAGGCAT
CTTGCTAGTACGGATCTGACAGTGAGGAATGAAGGCTAGGGTCGCGAAGCGGATTAGATA
CCCGTGTAGTCC
>Otu6631
CCAGCCTATGGGAGGCTGCAGTCGAGAATTTTTCTCAATGGGCGAAAGCTTGAAGGAGCG
ACGCCGCGTGGGGGATGAAGGGCTTCGGCCTGTAAACCCCTGTCATTTGCGAACAAACCT
GCCCATTTAATACATGGGCGGCTGATAGTAGCGAAAGAGGAAGGGACGGCTAACTCTGTG
CCAGCAGCCGCGGTAATACAGAGGTCCCAAGCGTTGTTCGGATTCACTGGGCGTAAAGGG
TGCGTAGGTGGTGATGTAAGTTCGATGTGAAATCTCCGGGCTTAACCCGGAAAAGGCATA
GGAGACTATATCGCTGGAGGGTCGGAGGGGAGACTGGAATTCTCGGTGTAGCAGTGAAAT
GCGTAGATATCGAGAGGAACACCAGTGGCGAAAGCGAGTCTCTGGACGACTCCTGACACT
GAGGCACGAAAGCTAGGGGATCAAACAGGATTAGAAACCCTCGTAGTCC
>Otu6635
CCAGCCTATGGGTGGCTGCAGTCGAGAATATTCCCCAATGGCCGAAAGGCTGAGGGAGCG
ACGCCGCGTGTATGAAGACGCCCTTCGGGGTGTAAAATACTTTTAACCGGGACGAAACAA
TGACGGTACCGGTTGAATAAGGGGCTGCTAAACTCGTGCCAGCAGCAGCGGTAATACGAG
TGCCCCAAGCGTTATCCGGATTTATTGGGCGTAAAGCGTACGTAGGTGGTTTATCGCATC
TCCTGTTAAATCCCGAGGCTCAACCTCGGGGCCGCGGGAGAGATGGCTAAACTAGAGACA
GGGAGAGGCAAGTGGAATTGCTGGTGTAGGGGTTAAATCCGTTAATATCAGCAAGAACAC
CCAATGTGAAGACAGCTTGCTGGAACTGTTCTGACACTAAGGTACGAAAGCGTGGGGAGC
GAATGGGATTAGATACCCTCGTAGTCC
>Otu6636
CCAGCCTATGGGGGGCTCCAGCAACGAGTCTTCCCCAATGCCGGAAACGGTGAGGGAGCG
ACGCCGCGTGAAGGACGAAGTTCTTCGGAATGTAAACTTCTGTAAGGGACATGAAAGGTT
AGCGCCTAACACGCGCAAAATTTGATCTGTCCCCTAAAAGGGGCGGCTAACTCTGTGCCA
GCAGCCGCGGTAATACAGAGGCCCCAAGCGTTACTGAGAATCACTGGGTTTAAAGGGTGC
GTAGGTGGTCCGTTAAGTCCGTTGTGAAAGCCCTGGGCTCAACCCAGGAACTGCTTCGGA
TACTGGCGGACTTGAGGCCGGTAGGGGTCACTGGAACTGACGGTGGAGCGGTGAAATGCG
TAGATATCGTCAGGAACGCCAATGGTGAAGACAGGTGACTGGGCCGGTTCTGACACTGAG
GCACGAAAGCGTGGGGAGCAAACAGGATTAGATACCCGCGTAGTCC
>Otu6637
CCAGCCTACGGGACGCTCCAGGCGCGAAAACTTCACAATGCACGAAAGTGTGATGAGGGA

ATCCGAAGTGCTTACAGTTTACTGTAGGCTTTTGCCAAAAGTAAGGATTTTGGCGAATAA
GTGGTGGGCAAGACTGGTGCCAGCCGCCGCGGTAACACCAGCGCCACAAGTGGCAACCGT
ATTTATTTGGCCTAAAGCGTTCGTAGCCGGTTGGGTAAATCTTCTGTGAAATTGTTCAGC
TTAACTGCACAACGTGCAGAGGACACTGCTCAGCTAGGGGCCGGGAGGGGTATGGGGTAT
TCTTGAGGGAGCGGTAAAATGCTATAATCTCTTGAAGACCACCTGTGGCGAAGGCGCCAT
ACCAGCACGGATCCGACGGTGAGGGACGAAAGCCAGGGGAGCAATCCGGATTAGAGACCC
TAGTAGTCC
>Otu6638
CCAGCCTATGGGAGGCTGCAGTCGAGGATCTTTAGCAATGGACGAAAGTCTGACTATGCG
GCGCCGCGTGAGGGATGAAGGTTTTCGGATCGTAAACCTCTTTCGATAGGGAAGAAAGCC
TATGCTCTAACATAGTATAGGTGTGACGGTACCTAGAGAAGAAGCCACGGCCAACTCCGT
GCCAGCAGCCGCGGTAATACGGGGGTGGCGAGCGTTACTCGGATTTATTGGGTGTAAAGG
GCAGGTAGGCGTTCCTTCAAGTTAGAAGTGAAATCCTGTGGCTCAACCACAGAACTGCTT
CTAAAACTGTAGGAATTGAGACTGACAGAGGAGAGCGGAATTCCCGGTGTAAGGGTGAAA
TCTGTAGATATCGGGAGGAACACCAGTGGCGAAAGCGGCTCTCTGGGTTAGCTCTGACGC
TGAGCTGCGAAAGCTAGGGTAGCAAACAGGATTAGAAACCCGTGTAGTCC
>Otu6641
CCAGCCTATGGGGCGCAGCAGTGAGGAATGTTGCACAATGGGGGAAACCCTGATGCAGCA
ACGCCGCGTGGGTGATGAAGGCCTTCGGGTCGTAAAACCCTTTTCTGGGGGACGAGCAAG
GACGGTACCCCAGGAATAAGTCCCGGCTAACTACGTGCCAGCAGCCGCGGTAAAACGTAG
GGGGCGAGCGTTATCCGGAATTACTGGGCGTAAAGCGCGTGCAGGCGGCTTGATAGGTTG
GACGTGAAAGCTCCTGGCTCAACTGGGAGAGGCCGTTCAATACCGTCAGGCTTGAGGACG
GAAGAGGGGAGTGGAATTCCCGGTGTAGTGGTGGAATGCGTAGATATCGGGAGGAACACC
TGTGGCGAAAGCGGCTCCCTGGTCCGTATCTGACGCTCAGACGCGAAAGCGTGGGGAGCA
AACGGGATTAGAAACCCCGGTAGTCC
>Otu6642
CCAGCCTATGGGAGGCTCCAGGGACGAATCCTTCGCAATGCGCGCAAGCGTGACGAGGGG
ACTCCGAGTGATGGACCACGCGTTCATCTTTTCGCCAGACCAAACATCTGGCGGAATAAG
GGCTGGGCAATATCGGTGCCAGCCGCCGCGGTAACACCGAAAGCTCAAGTGGTGTCCACG
AATATTGGGCCTAAAGAGTCCGTAGCTGGCTTGTTAAGTTCTCTGTGAAATCTTCCAGCT
TAACTGGAAGGCGTGCAGGGGGTACTGACAGGCATGGGAGTGGGGGAAGTCAGGAGTACT
CTCGGGGTAGAGGTAAAATTCTGTAATCCTGTGAGGACTACCAGTGGCGAAGGCGCCTGA
CTAAAACATGTCCGGCAGTGAGGGACGAAGGCTAGGAGAACGAATCGGATTAGATACCCG
CGTAGTCC
>Otu6645
CCAGCCTATGGGTCGCAGCAGTCGAGAATCTTCCGCAATGGACGAAAGTCTGACGGAGCG
ACGCCGCGTGCAGGATGAAGTGCTTCGGCATGTAAACTGCTTTTGCCAGGGAAAAAGTTA
TTGATTGTACCTGGAGAATAAGAGGTTGCTAAACTCGTGCCAGCAGCAGCGGTAATACGA
GTGCCTCGAGCGTTATCCGGAATCATTGGGCGTAAAGGGTGTGTAGGCGGTTTCGTTAGT
CTTCCGTTAAATTCTTCGGCTCAACCGGGGGCATGCGGAGGAAACGGCGAAACTTAGAGG
ACGGAAGAGGTTTCTGGAACTCATGGTGTAGCGGTGAAATGCGTTGATATCATGGGGAAC
ACCGAAAGCGAAGGCAAGAAACTGGTCCGCTCCTGACGCTGAAACACGAAAGCGTGGGTC
GCGAATGGGATTAGAAACCCCCGTAGTCC
>Otu6650
CCAGCCTACGGGTGGCACCAGTAGGGAATATTCCGCAATGGGCGAAAGCCTGACGGAGCG
ACGCCGCGTGGAGGATGAAGGCCTTCGGGTCGTAAACTCCTGTCAGTGGTGAAGAATGCC
ACGCTTCGGCGTGGACTGACCTAAGCCGCAGAGGAAGCCCCGGCTAACTCCGTGCCAGCA
GCCGCGGTAAAACGGAGAGGGCGAGCGTTGCTCGGAATCACTGGGCGTAAAGCGCACGTA
GGCGGTTTGACAAGTTGGTTGTGAAATCTCATGGCTCACCCATGGAACGGCTTCCAATAC
TGTCGAACTGGAGTGCTGGAGGGGAGAGTGGAACTCTTGGTGGAGCGGTGAAATGCGTAG
ATATCAAGAGGAACGCCGGTGGCGAAGGCGACTCTCTGGACAGCAACTGACGCTGAGTGT
GCGAAAGCTAGGGGAGCAGACGGGATTAGAAACCCCAGTAGTCC
>Otu6658
CCAGCCTACGGGTGGCAGCAGCTAAGAATCTTCCGCAATGGGCGCAAGCCTGACGGAGCG
ACGCCGCGTGGATGAAGAAGGCCGCAAGGTTGTAAAATCCTTTTGTCAACGAAGAATAAG
GGTGCGAGGAAATGCGCATCTGATGACGTTAGTTGACGAATAAGCCCCGGCTAATTACGT
GCCAGCAGCCGCGGTAATACGTATGGGGCGAGCGTTGTTCGGAATTATTGGGCGTAAAGG
GCGTGTAGGCGGCTTGGCAAGCTTGGTGTGAAATCCCACGGCTTAACCGTGGATGTGCGC

TGAGAACTGCCAGACTTGAGTCCTCGGGAGGAAGATGGAATTCCTGGTGTAGGGGTGAAA
TCTGTAGATATCAGGAAGAACACCGATGGCGAAGGCAATCTTCTATCGAGAGACTGACGC
TGAGGCGCGAAAGCGTGGGGAGCAAACAGGATTAGAAACCCTTGTAGTCC
>Otu6660
CCAGCCTACGGGTTGCAGCAGGCGCGAAACCTTTGCCATGCGCGAAAGCGTGACAGGGGA
ACTCCGAGTGGTAAGAGGATTTATCTTCTTATCTTTTGGCCGATCCAAACAATCGGCAGA
ATAAGGTCTGGGTAAGACCAGTGCCAGCCGCCATGCTATGTTAACGCATCAGCATTGGCA
CGGTACGCGGTAATACTGGCAGAACAAGTGGTATCCACGAATATTGGGTCTAAAGAGTCC
GTAGCGGGCTGGTTAAGTCCACTGTGAAATCTAACGGCTCAACCGTTAGGCGTGCAGCGG
ATACTGATCGGCTTGGGAGTGGGGGAGGCCGGGAGTACTTGCGGGGTAGAGGTAAAATTC
TGTAATCCTGCAAGGACTACCAGTGGCGAAGGCGCCCGGCCAAAACACGTCCGACCGTGA
GGGACGAAGGCTAGGAGAACGAATCGGATTAGATACCCCCGTAGTCC
>Otu6661
CCAGCCTATGGGTCGCTGCAGTCGAGAATCTTCCACAATGGACGAAAGTCTGATGGAGCG
ACGCCGCGTGATTGATGAAGTCCCTTTGGGACGTAAAGATCTTTTATGAGGGGAGAAGTT
TATTGACTGTACCTCATGAATAAGAGGCTCCTAATCTCGTGCCAGCAGGAGCGGTAATAC
GAGAGCCTCGAGCGTTATCCGGAATTATTGGGCGTAAAGGGTGTGTAGGTTGTTCTGTTA
GTCTTTTGTCAAATCCCCGTGCTCAACGCGGGGTACGCTAGAGATACGGCAGGACTAAGA
AAGTGCGAAAGGTGTACGGAACTCATGGTGTAGGGGTGAAATCCGTTGATATCATGGGGA
ACACCAAAAGCGAAAGCAGTACACTGGCGCATATTTGACACTGAAACACGAAAGCGTGGG
GAGCAAACAGGATTAGAGACCCTAGTAGTCC
>Otu6665
CCAGCCTACGGGTCGCAGCAGTCGAGAATCATTCACAATGGGGGAAACCCTGATGGTGCA
ACGCCGCGTGGAGGATGAAGGTCCTTGGATTGTAAACTCCTGTCATCTGGGAGTAAGACC
TGACGGTGAATAGCCGGCAGGGTTGATAGTACCAGAAGAGGAAGGGACGGCTAACTTCGT
GCCAGCAGCCGCGGTAATACGAAGGTCCCAAGCGTTGTTCGGAATCACTGGGCGTAAAGG
GTGCGTAGGCGGCGTGGTAAGTCAGATGTGAAATCCCGGGGCTCAACCCCGGAACTGCAT
CCAATACTGCCATGCTTGAGGATTGGAGAGGAGACTGGAATTCTCGGTGTAGCAGTGAAA
TGCGTAGAGATCGAGAGGAACACTCGTGGCGAAGGCGAGTCTCTGGACAATTCCTGACGC
TGAGGCACGAAGGCCAGGGGAGCGAAAGGGATTAGAAACCCTTGTAGTCC
>Otu6675
CCAGCCTATGGGTTGCTGCAGGAGGGAGTATTGGTAATCTACGAAAGTGGGAACCAGCAA
CGCCGCGTGAGCGATGACGGCCTTCGGGTTGTAAAGCTCTTTTTGCCGGGACGAGGAAGG
ACGGTACCGGTAGAATAAGCCTCGGCTAACTACGTGCCAGCAGCCGCGGTAAAACGTAGG
AGGCGAGCGTTATCCGGATTTACTGGGTGTAAAGCGCATGCAGGCGGTTTACTAAGTTGG
GTGTGAAAGCTCCTGGCCCAACTGGGAGAGGTCGCTCAAGACTGGTAGACTAGAGAGTGG
TAAGGGAAGGTGGAATTCCGGGTGTAGTGGTGAAATGCGTAGATATCCGGAGGAACACCA
GTGGCGAAGGCGGCCTTCTGGACCACGACTGACGCTCAGATGCGAAAGCTAGGGTAGCAA
ACGGGATTAGATACCCCAGTAGTCC
>Otu6679
CCAGCCTACGGGTGGCACCAGTCGGGAAACTTCTGCAATGCGCGCAAGCGTGACAGAGCA
AACCAGAGTGCCTTTCCATATTGGAAATGCTTTTGTGAGATGTAAAAAGTCTTACGAATA
AGGGCCGGGCAAGACTGGTGCCAGCCGCCGCGGTAATCCCAGCGGTCCGAGTCGCAGCCA
CAATTATTGGGTCTAAAACATCCGTAGCTCGCTTTGTAAGTCTTTTGTGAAATCGGGACT
CTTAAGGTTCCGGCGAGCAAAAGATACTGCTTAGCTAGAGACCGGTAGACGTAACGAGTA
CGAACAGGGTAGCGGTAAAATGTGTTGATCCTATTCGGACTAACAACAGCGAAGGCACGT
TACGAGCACGGTTCTGACAGTGAGGGATGAAGGCTAGGGGCGCAAAGTGGATTAGAAACC
CGAGTAGTCC
>Otu6680
CCAGCCTATGGGTTGCTGCAGTGGGGAATTTTGGACAATGGGGGAAACCCTGATCCAGCA
ACGCCGCGTGAGCGATGAGGGTCTTCGGATCGTAAAGCTCTGTTAAGTGGAACGATGGGT
TCTGAGGCTAATATCCTTAGGATTTGACGGTACCACTGGAGGAAGTCACGGCTAACTACG
TGCCAGCAGCCGCGGTAATACGTAGGTGGCAAGCATTGTTCGGATTTACTAGGCGTAAAG
GGAGCGTAGGTGGCTATGTAAGTTGTAAGTGAAATTCCACGGCTTAACCGTGGAACGGCT
TGCAAAACTGCATGGCTTGAGTATAGGAGAGGGAAATGGAATTCCTAGTGTAGCGGTGAA
ATGCGTAGATATTAGGAAGAACACCTGTGGCGAAGGCGATTTCCTGGCCTAACACTGACG
CTGAGGCTCGAAAGCTAGGGGAGCAAACAGGATTAGAAACCCGCGTAGTCC
>Otu6685

CCAGCCTACGGGTTGCAGCAGTGAGGAATATTGGTCAATGGACGCAAGTCTGAACCAGCC
ATGCCGCGTGAAGGATGAAGGCCTTCTGGGTTGTAAACTTCTTTTACATGGGAAGAAAGG
CATTGTTTCTACGATGTCCGACGGTACCATGGGAATAAGCACCGGCTAACTCCGTGCCAG
CAGCCGCGGTAATACGGAGGGTGCAAGCGTTATCCGGATTTACTGGGTTTAAAGGGTGTG
TAGGCGGGCTGTTAAGTCAGTGGTGAAATCTCCGGGCTCAACCTGGAAACTGCCATTGAT
ACTATCAGTCTTGAATCTTGTTGAGGTTGGCGGAATATGTCATGTAGCGGTGAAATGCAT
AGATATGACATAGAACACCGATTGCGAAGGCAGCTAACTAAACAAGTATTGACGCTGAGG
CACGAAAGCGTGGGGATCAAACAGGATTAGATACCCCCGTAGTCC
>Otu6692
CCAGCCTACGGGAGGCACCAGTCGAGGATCTTCGGCAATGGGCGCAAGCCTGACCGAGCG
ACGCCGCGTGAGCGATGAAGGCCTTCGGGTTGTAAAGCTCTTTAGTGGGGGAGGAAGGGC
AACTTGACCGACCCCAAGAATAAGCACGGGCTAAGTTCGTGCCAGCAGCCGCGGTAAGAC
GAACCGTGCGAACGTTGTTCGGAATCACTGGGCTTAAAGGGCGCGTAGGCGGCCCGCCAA
GTCAGGGGTGAAAGCCCCCAGCTCAACTGGGGAAGTGCCTTTGATACTGGCGGGCTCGAG
CGAGGCAGGGGTCGTGGGAACTTCCGGTGGAGCGGTGAAATGCGTTGATATCGGAAGGAA
CGCCGGTGGCGAAAGCGCACGACTGGTCCTCTTCTGACGCTGAGGCGCGAAAGCCAGGGG
AGCGAACGGGATTAGAGACCCCTGTAGTCC
>Otu6693
CCAGCTACGGGTGGCAGCAGTGGGGAATTTTGGACAATGGGCGAAAGCCTGATCCAGCAA
CGCCGCGTGGAGGATGAAGGTCTTCGGATCGTAAACTCCTTTTGATGGGAACGAAAAAAA
TGACGGTACTCATAGAAAAAGCAACGGCTAACTACGTGCCAGCAGCCGCGGTAATACGTA
GGTTGCAAGCGTTACTCGGATTTACTAGGCGTAAAGCGTATGTAGGCGGTTATGTAAGTC
TGTGGTGAAATCTCCCGGCTCAACCGGGATAGGTCCACGGAAACTGCATGGCTTGAGTGT
GGGAGAGGGAGACGGAATTCCTGGTGTAGCGGTGAAATGCGTAGATATCAGGAGGAACAC
CAATGGCGAAAGCAGTCTCCTGGGCCAATACTGACGCTCAGATACGAAAGCTAGGGGAGC
AAACAGGATTAGAAACCCTAGTAGTCC
>Otu6696
CCAGCCTATGGGTCGCAGCAGTCGAGGAAATTTTTCAATGCCCGCAAGGGTGAACGAGTA
AGCCAGAGTGTTTTCTTTACGAAAACTTTTGCCTGATGTAAAAAGTCAGGCGAATAAGGA
CTGGGCAAGACCGGTGCCAGCCGCCGCGGTAATCCCGGCGGTCCAAGTCGCATCCACAAT
TATTGGGTCTAAAACATCCGTAGCTTGTTTTGTAAGTCTCTTGTGAAATCTCAAATCTCA
AGTTTGAGTCGTGCAAGAGATACTACATTGCTAGAGACTGGAAGACGCAAAGAGTACGTA
GGAGGTAGCGGTAAAATGTGTTAATCTTCTTCGGACTAACAATGGCGAAGGCACTTTGCG
AGTACAGATCTGACGGTGAGGGATGAAGGCTAGGGGCGCAAAAGGGATTAGATACCCGCG
TAGTCC
>Otu6697
CCAGCCTACGGGGCGCACCAGTGGGGAATTTTGCGCAATGGGGGAAACCCTGACGCAGCA
ACGCCGCGTGGGTGATGAAGGTCTTCGGATTGTAAAACCCTGTCGTCGGGGACGAAGGTG
GCTTGGCGAATAGCCGAGTTGCTTGACGGTACCCGGAGAGGAAGCCCCGGCTAACTCTGT
GCCAGCAGCCGCGGTAATACAGAGGGGGCAAGCGTTATTCGGAATTATTGGGCGTAAAGG
GCGCGTAGGCGGTGTTTTAAGTCGGATGTGTAATCCCCGGGCTCAACCTGGGAACTGCAT
CCGATACTGGGACGCTGGAGTGCTGGAGAGGGTGGTGGAATTCCTCGTGTAGCGGTGAAA
TGCGTAGAGATGAGGAGGAACACCAGTGGCGAAGGCGGCCACCTGGACAGTAACTGACGC
TGGGGCGCGAAAGTGTGGGGAGCAAACAGGATTAGATACCCTCGTAGTCC
>Otu6707
CCAGCCTATGGGTGGCTGCAGTCGAGAATTTTTCTCAATGGGGGAAACCCTGAAGGAGCG
ACGCCGCGTGGAGGATGAAGGTTTTCGGATTGTAAACTCCTGTCATTGGGGAACAATTTT
ACGGTTTTAACTGAGCCGTAATTGATAGTACCCGAAGAGGAAGAGACGGCTAACTCTGTG
CCAGCAGCCGCGGTAATACAGAGGTCTCAAGCGTTGTTCGGATTCATTGGGCGTAAAGGG
TGCGTAGGCTGTAAGGTATGTTGGATGTGAAATCCCGGGGCTTAACCCCGGAACTGCATT
CAATACTGCCCTACTAGAGTACTGGAGAGGAGATTGGAATTCACGGTGTAGCAGTGAAAT
GCGTAGATATCGTGAGGAAGACCAGTGGCGAAGGCGAATCTCTGGACAGTTACTGACGCT
GAGGCACGAAGGCCAGGGGAGCAAACGGGATTAGAAACCCTCGTAGTCC
>Otu6708
CCAGCCTATGGGGCGCAGCAGTGGGGAATATTGGACAATGGGGGCAACCCTGATCCAGCC
ATGCCGCGTGTGCGATGAAGGCCTTCGGGTTGTAAAGCACTTTCGCCGGTGAAGATGATG
ACGGTAGCCGGAGAAGGAGCCCCGGCTAACTCCGTGCCAGCAGCCGCGGTAATACGGAGG
GGGCAAGCGTTGTTCGGAATTACTGGGCGTAAAGGGCGCGTAGGCGGCTCGTTGCGTCAG

GTGTGAAAGCCCTGGGCTCAACCTGGGAACTGCACTTGATACGGGCGAGCTTGAATCCGG
GAGAGGATGGTGGAATTCCCAGTGTAGAGGTGAAATTCGTAGATATTGGGAAGAACACCG
ATGGCGAAGGCAGCCATCTGGCCCGGTTATTGACGCTGAGGCGCGAAAGCGTGGGGAGCA
AACAGGATTAGAAACCCTAGTAGTCC
>Otu6713
CCAGCCTATGGGGGGCTCCAGTGAGGAATATTGCGCAATGGCCGAAAGGCTGACGCAGCG
ACGCCGCGTAGGGGAAGACGGCCTTCGGGTTGTAAACCCTTGTCAGTGGGGAAGAAGAAA
GTGACGGTACCCACAGAGGAAGCCCCGGCTAACTACGTGCCAGCAGCCGCGGTAAGACGT
AGGGGGCGAGCGTTGTTCGGAATTACTGGGCGTAAAGAGCTCGTAGGCAGGAGATTAAGT
CTTGCAATAAAGTCCGTGGCTCAACTACGGGGAATTGCGAGATACTGATTTTCTTGAGAA
CAGGAGAGGGGAGTGGAATTCCTAGTGTAGCGGTGAAATGCGTAGATATTAGGAGGAACA
CCAGTGGCGAAGGCGGCTCTCTGGCCTGTTTCTGACGCTGAGGAGCGAAAGCTGGGGGAG
CGAACGGGATTAGAGACCCGTGTAGTCC
>Otu6721
CCAGCCTATGGGAGGCTCCAGTAAGGAATATTGGACAATGGTGGCAACACTGATCCAGCC
ATGCCGCGTGCAGGATGAAGGCGCTACGCGTTGTAAACTGCTTTTGTACGGGAGAAAACC
TATCTACGTGTAGATAGCTGATAGTACCGTAAGAATAAGCACCGGCTAACTCCGTGCCAG
CAGCCGCGGTAATGCGGAGGGTGCAAGCGTTATCCGGATTTATTGGGTTTAAAGGGTGCG
TAGGCGGTTAAATAAGACAGTGGTGAAAATCTGCAGCTCAACTGTAGGGGTGCCATTGAA
ACTGTTTTACTTGAGTAACCATGAGGTAGGCGGAATGTGTAGTGTAGCGGTGAAATGCTT
AGATATTACACAGAACACCGATTGCGTAGGCAGCTTACTAGCGGTTTACTGACGCTGATG
CACGAAAGCGTGGGGATCAAACAGGATTAGATACCCTCGTAGTCC
>Otu6723
CCAGGCCTACGGGGGGCTGCAGTGGGGAATTTTGGACAATGGGGGCAACCCTGATCCAGC
CATGCCGCGTGTCTGAAGAAGGCCTTCGGGTTGTAAAGGACTTTTGTCAGGGAGCAAATA
CTGCTTGTGAATAATGAGCGGGAATGAGAGTACCTGAAGAATAAGCTCCGGCTAACTACG
TGCCAGCAGCCGCGGTAATACGTAGGGTGCAAGCGTTAATCGGAATTACTGGGCGTAAAG
CGTGCGCAGGCGGTTTTGCAAGTCTGATGTGAAAGCCCCGGGCTTAACCTGGGAACGGCA
TTGGAGACTGCAAGGCTAGAGTACGTCAGAGGGGGGTAGAATTCCACGTGTAGCAGTGAA
ATGCGTAGAGATGTGGAGGAATACCGATGGCGAAGGCAGCCCCCTGGGATGATACTGACG
CTCATGCACGAAAGCGTGGGGAGCAAACAGGATTAGAAACCCCAGTAGTCC
>Otu6733
CCAGCCTATGGGGCGCAGCAGTCGAGAATATTCGACAATGGGCGAAAGCCTGATCGAGCG
ACGCCGCGTGCAGGATGAAGGTCTTAGGATTGTAAACTGCTTTTCCGCGGGAGGAAGTCG
AAGCACATAACTTGTTATGTGCTCGATTGACAGTACCGCGGGAATAAGGGGCTGCAAACT
TCGTGCCAGCAGCCGCGGTAATACGAAGGCCCCGAGCGTTATCCGGATTTATTGGGCGTA
AAGGGCGCGTAGGAGGTCCGATGCGTCTCTTGTAAAATTTCACCGCTCAACGGTGGAGCT
GCAAGGGATACGGTCGGACTTGAGGGCGTTAGAGGCAGATGGAACACACGGTGTAGGGGT
GAAATCCGTTGATATCGTGTGGAACACCAAAGGCGAAGGCATTCTGCTGGGACGTTCCTG
ACTCTGAGGCGCGAAAGCGTGGGGAGCAAAAAGGATTAGATACCCTTGTAGTCC
>Otu6734
CCAGCCTACGGGGGGCTGCAGCGAGGAATATTGGTCAATGGACGAGAGTCTGAACCAGCC
AAGTCGCGTGAAGGAAGAAGGTTCTATGGATTGTAAACTTCTTTTGTAAGGGAATAAAAC
AGGCCACGTGTGGCTTCTTGTATGTACTTTACGAATAAGGATCGGCTAACTCCGTGCCAG
CAGCCGCGGTAATACGGAGGATCCGAGCGTTATCCGGATTTATTGGGTTTAAAGGGTGCG
TAGGCGGACCTGTAAGTCAGTGGTGAAAGTTTGCGGCTCAACCGTAAAATTGCCATTGAA
ACTATAGGTCTTGAGTGTAAATGAGGTAGGCGGAATGTGTTGTGTAGCGGTGAAATGCTT
AGATATAACACAGAACACCAATTGCGAAGGCAGCTTACTAGAATACAACTGACGCTGAGG
CACGAAAGCGTGGGGATCAAACAGGATTAGATACCCCCGTAGTCC
>Otu6735
CCAGCCTACGGGGGGCTGCAGTGGGGAATATTGCGCAGTGGGCGAAAGCCTGACGCAGCG
ACGCCGCGTGGGTGATGAAGGCCTTCGGGTCGTAAAGCCCTGTCGGGAGGGAAGAAACCT
CGGCGACCGAATACGTCGTCGACCTGACGGTACCTCCGAAGGAAGCACCGGCTAACTCCG
TGCCAGCAGCCGCGGTAATACGGAGGGTGCGAGCGTTGTTCGGAATCACTGGGCGTAAAG
CGCGTGTAGGCGGCCTTCTAAGTCTGGTGCGAAAGCCCGGGGCTTACCCCCGGAACTGCG
CTGGAAACTGGGAGGCTCGAGTACCGGAGAGGAGGGTGGAATTCCTGGTGTAGCGGTGAA
ATGCGTAGATATCAGGAGGAACACCGGTGGCGAAGGCGGCCCTCTGGACGGATACTGACG
CTGAGACGCGAAAGCGTGGGGAGCAAACAGGATTAGATACCCGCGTAGTCC

>Otu6737
CCAGCCTATGGGGGGCTGCAGCAAGGAATCTTCCGCAATGGACGAAAGTCTGACGGAGCG
ACGCCGCGTGGAGGATGAAGTGCTTCGGCATGTAAACTCCTTTTGCCGGGGAAAAAGTTT
ATTGATTGTACCCGGAGAATAAGAAGTTGCTAAACTCGTGCCAGCAGCAGCGGTAATACG
AGTGCTTCGAGCGTTATCCGGAATTATTGGGCGTAAAGGGTGTGTAGGCGGCAGTGTTAG
TCTCTCGTAAAATTCCTCGGCTCAACCGAGGGCGCGCGAGAGAAACGGCACAGCTCGAGT
ATGGAAGGGGTCTTTGGAACTCATGGTGTAGCGGTGAAATGCGTTGATATCATGGGGAAC
ACCGAAAGCGAAGGCAGAAGACTGGTCCATTACTGACGCTGAAACACGAAAGCGTGGGTC
GCGAATGGGATTAGAAACCCGAGTAGTCC
>Otu6738
CCAGCCTACGGGACGCTCCAGGCGCGAAACCTTTACAATGCACGCAAGTGTGATAAGGGG
ACTCTAAGTGCATGCACAAAGTGCATGCTTTTGCCAAATGCAAATAGTTTGGCGAATAAG
TGGTGGGTAAGACGGGTGCCAGCAGCCGCGGTAACACCCGCGCCACAAGTGGCAACCGAT
TTTATTGGGCCTAAAGCGTCCGTAGCTGGTCTAGAAAATCTCTTGTGAAAATGTTGAGCT
TAACTTAACAAAGGGCAGGAGACACTACTAGACTCGGAACCGGTAGGGGTAAGAAGTATG
TTAGGGGTAGCGGTAAAATGTTATAATCCCTAATAGACTACCTGTGGCGAAGGCGTCTTA
CTAGGACGGATTCGACAGTGAGGGACGAAAGCCAGGGGAGCGATCCGGATTAGAGACCCG
AGTAGTCC
>Otu6739
CCAGCCTATGGGAGGCACCAGCAACGAATCTTCCCCAATGCCGGAAACGGTGAGGGAGCG
ACGCCGCGTGAAGGATGAAGTCTTTCGGGATGTAAACTTCTTTTAGGGATAAGAAAGCAG
AGGGACCTAATACGTCCTGAGTTGATCTATCCCAGAATAAGGGGCGGCTAACTCTGTGCC
AGCAGCCGCGGTAATACAGAGGCCCCAAGCGTTACTGAGAATCACTGGGTTTAAAGGGTG
CGTAGGTGGTCCGTTAAGTCAGTTGTGAAATCCCCGGGCTCAACCCGGGAACTGCTTCTG
ATACTGGCGGACTTGAGGCCAGTAGGGGTCACTAGAACTGACGGTGGAGCGGTGAAATGC
GTAGATATCGTCAGGAATGCCGGTGGTGAAGACGGGTGACTGGGCTGGTTCTGACACTGA
GGCACGAAAGCTAGGGGAGCGAACGGGATTAGAGACCCCTGTAGTCC
>Otu6744
CCAGCCTACGGGGGGCAGCAGTGGGGAATCTTCCACAATGGGCGAAAGCCTGATGGAGCA
ACGCCGCGTGAGTGATGAAGGCCTTCGGGTTGTAAAACTCTGTCTTGCAAGAATAAGGCT
GCCGGGTAAATAATCCGGCAGTTTGACTGTATTGCAGGAGGAAGACACGGCTAACTCTGT
GCCAGCAGCCGCGGTAATACAGAGGTGTCAAGTGTTGTCCGGAATTACTGGGCGTAAAGG
GCGTGTAGGCGGGTAGGCAAGTCAGATGTGAAATCACTGGGCTCAACCCAGTAACTGCAC
CTGAAACTGCCTGTCTTGAGTACAGAAGAGGAAAGCGGAATTCCCGGTGTAGCGGTGAAA
TGCGTAGATATCGGGAGGAACACCAGTGGCGAAGGCGGCTTTCTGGTCTGTTACTGACGC
TGAAACGCGAAAGCTAGGGGAGCAAACGGGATTAGAGACCCGAGTAGTCC
>Otu6750
CCAGCCTATGGGTGGCAGCAGTCGAGAATCATTTGCAATGGACGAAAGTCTGACAATGCG
ACGCCGCGTGCAGGAGGAAGGCCTTCGGGTTGTAAACTGCTGTCAGGGGTTATGAACGCC
GCGCGTGGACAATACGCGCGGTTTGACAAAGGCCCCAAAGGAAGCTCCGGCTAAATCCGT
GCCAGCAGCCGCGGTAATACGGATGGAGCAAGCGTTGTTCGGAATCACTGGGCATAAAGC
GCATGTAGGCGGCTCTGAAAGTCGGATGTGAAATCCCCCGGCCCACCCGGGGAACCGCAT
TCGAAACTCCAGGGCTTGAGTGAAGAAGGGGAGAGTGGAACTCCTGGTGGAGCGGTAAAA
TGCGTAGATATCAGGAGGAACGCCGGTGGAGAAGTCGACTCTCTGGTCTTTTACTGACGC
TGAGATGCGAAAGCTAGGGGAGCAAACAGGATTAGAAACCCCGGTAGTCC
>Otu6752
CCAGCCTACGGGTGGCAGCAGTGGGGAATATTGGACAATGGGCGCAAGCCTGATCCAGCC
ACGCCGCGTGAGTGATGAAGGTCTTCGGATTGTAAAGCTCTGTTGATTGGGAAGAAAAGT
TCCGGGAATAATACCTCGGGACGTGACGGTACCAGTCGAGGAAGCACCGGCTAACTACGT
GCCAGCAGCCGCGGTAATACGTAGGGTGCAAGCGTTGTTCGGAATCACTGGGCGTAAAGG
GAGAGTAGGCGGGAAAATAAGTTAGAAGTTTAATATATAGGCTTAACCTATAACCTGCTT
CTAATACTGTTTTTCTTGAGTATTGGAGAGGGAGATGGAATTCCAGGTGTAGCGGTGGAA
TGCGTAGATATCTGGAAGAACACCAGCTGCGAAGGCGGTCTCCTGGCCAAATACTGACGC
TGATGCTCGAAAGCTAGGGGAGCAAACAGGATTAGATACCCGAGTAGTCC
>Otu6756
CCAGCCTACGGGTTGCTGCAGTGGGGAATTTTGCGCAATGGGCGAAAGCCTGACGCAGCA
ACGCCGCGTGGGTGATGAAGGTCTTCGGATCGTAAAACCCTGTCGCCAGGGACGAAGGTC
GGTCGTTGAATAGGCGATTGGCTTGACGGTACCTGGAGAGGAAGCCCCGGCTAACTCTGT

GCCAGCAGCCGCGGTAATACAGAGGGGGCAAGCGTTATTCGGAATTATCGGGCATAAAGG
GCACATAAACGGTTTTTTAAGTAAGATGTGCAATCCCCGAGCTTAACTTGGGAACTGCAT
CTCAGACTGGAAGGCTAGAGTGCTGGAGAGGGTGGTAGAATTCCACGTGTAGCGGTGAAA
TGCGTAGAGATGTGGAGGAATACCAGTGGCGAAGGCGGCCACCTGGACAGTAACTGACGC
TGAGGCGCGAAAGTGTGGGTAGCGAACAGGATTAGAGACCCGAGTAGTCC
>Otu6761
CCGCCTACGGGTCGCAGCAGTCGAGAATCTTCGTCAATGCACGCAAGTGTGAACGAGCGA
CGCCGCGTGAGCGATGACGCCCTTCGGGGTGTAAAGCTCTTTTATTGGGGACAAACTTTG
ATGGTACCCAGTGAATAAGAGGTTGCTAACTCTGTGCCAGCAGCAGCGGTAATACAGAGA
CCTCAAGCGTTATCCGGATTTATTGGGCGTAAAGAGTCTGTAGGTTGAATAAATAGTCAG
ATGTCAAATTTTAGGGCTTAACCTTAAAACTGCATTTGAAACGTTTGTTCTAGAGGACAG
TAGGGGCAAGTAGAACTTATAGTGTAGCAGTAAAATGCGTTGATATTATAAGGAATACCA
AAAGCGAAGGCATCTTGCTGGACTGTTCCTGACACTGAGAGACGAAAGCGTGGGTAGCGA
ATGGGATTAGAGACCCTCGTAGTCC
>Otu6768
CCAGCCTATGGGTGGCAGCAGTGAGGAATATTGGTCAATGGACGGAAGTCTGAACCAGCC
ATCCCGCGTGAAGGAAGAAGGCGCTATGCGTTGTAAACTTCTTTTCCGAGAGAAGAAAAC
CCCCGACGAGTCGGGGCTTGGCGGTATCTTGGGAATAAGCATCGGCTAACTCCGTGCCAG
CAGCCGCGGTAATACGGAGGATGCAAGCGTTATCCGGATTTATTGGGTTTAAAGGGAGCG
TAGGCGGTTTTATAAGTCAGTGGTGAAATCTCGTTGCTTAACAACGAACGTGCCATTGAT
ACTGTAGAACTTGAGTACAGATGATGTTGGCGGAATGTGTCATGTAGCGGTGAAATGCTT
AGATATGACACAGAACACCGATTGCGAAGGCAGCTGACAAAACTGTAACTGACGCTGAGG
CTCGAAAGCGTGGGGATCAAACAGGATTAGAGACCCGAGTAGTCC
>Otu6774
CCAGCCTACGGGGGGCAGCAGTTTCGAATATTTCTCAATGGGGGAAACCCTGAAGGAGCG
ACGCCGCGTGGAGGATGAAGGGCTTCGGCTTGTAAACTCCTGTCATCTGGGAATAATGTG
CCGGCAGCTAATACCGGCCGGCATTGACTGTACCAGGAGAGGAAGGGACGGCTAACTCTG
TGCCAGCAGCCGCGGTAATACAGAGGTCCCGAGCGTTGTTCGGATTTATTGGGCGTAAAG
GGTGCGTAGGCGGTCATGTAAGTCGGATGTGAAATCCCGCAGCTCAACTGCGGAACGGCA
TCTGATACTGCTTGACTCGAAGACTGGAGAGGAGACTGGAATTTGCGGTGTAGCGGTGAA
ATGCGCAGATATCGCAAGGAACACCAGTGGCGAAGGCGGGTCTCTGGACAGTTCTTGACG
CTGAGGCACGAAAGCTAGGGGAGCAAACGGGATTAGATACCCCAGTAGTCC
>Otu6777
CCAGCCTACGGGTGGCAGCAGTCGAGGATCATTCGCAATGGGCGCAAGCCTGACGGTGCG
ACGCCGCGTGCAGGATGAAGGCCTTCGGGTTGTAAACTGCTGTCATGAGGGAGCAACGAC
TCTAGCTCTAATACCGCTAGGGAATGATAGTACCTCAGGAGGAAGCCACGGCTAACTCTG
TGCCAGCAGCCGCGGTAATACAGAGGCGGCAAGCGTTGTTCGGATTTACTGGGCGTAAAG
GGTGCGTAGGCGGTTCCGTGTGTCGGATGTGAAAGCCCGCAGCTCAACTGCGGAACGGCA
TTCGAGACTACGGTGCTGGAGTACGAGAGAGGAGAGCGGAATTCTTGGTGTAGCAGTGAA
ATGCGTAGATATCAAGAAGAACACCGGTGGCGAAGGCGGCTCTCTGGATCGATACTGACG
CTGAGGCACGAAAGCAGGGGGAGCAAACAGGATTAGAAACCCGCGTAGTCC
>Otu6779
CCAGCCTACGGGGCGCTGCAGTCGAGAGGCTTCGGCAATGGGGGAAACCCTGACCGAGCG
ACGCCGCGTGGGCGATGAAGGCCGTTAGGTTGTAAAGCCCTGTCGAGTAGGAAGAAACGC
AGGTGGGCGAACAGTCCATCTGCTTGACGGTACTGCTAAAGGAAGCTCCGGCCAACTCCG
TGCCAGCAGCCGCGGTAATACAGAGGTCCCAAGCGTTGTTCGGATTCACTGGGCGTAAAG
GGTGCGTAGGTGGTGAGGTAAGTCGGATGTGAAATCTCGGAGCTCAACTCCGAAATGGCA
TTGGATACTACCTTGCTCGAGGGTTGGAGGGGGGACTGGAATACTTGGTGTAGCAGTGAA
ATGCGTAGATATCAAGTGGAACACCAGTGGCGAAGGCGAGTCCCTGGATAACTCCTGACA
CTGAGGCACGAAAGCTAGGGGAGCAAACAGGATTAGATACCCTTGTAGTCC
>Otu6783
CCAGCCTACGGGGCGCTGCAGTGGGGAATTTTGGACAATGGGGGCAACCCTGATCCAGCC
ATTCCGCGTGAGTGAAGAAGGCCTTCGGGTTGTAAAGCTCTTTCGGCAGGAACGAAACGG
TGGAGGCTAATACCTTTCATCAATGACGGTACCGTAAGAATAAGCACCGGCTAACTACGT
GCCAGCAGCCGCGGTAATACGTAGGGTGCGAGCGTTAATCGGAATTACTGGGCGTAAAGC
GTGCGCAGGCGGTTATGTAAGACAGATGTGAAATCCCCGGGCTCAACCTGGGACCTGCAT
TTGTGACTGTATAGCTAGAGTACGGTAGAGGGGGATGGAATTCCGCGTGTAGCAGTGAAA
TGCGTAGATATGCGGAGGAACACCGATGGCGAAGGCAATCCCCTGGACCTGTACTGACGC

TCATGCACGAAAGCGTGGGGAGCAAACAGGATTAGATACCCGTGTAGTCC
>Otu6788
CCAGCCTATGGGGCGCAGCAGTAAGGAATATTGGTCAATGGACGAAAGTCTGAACCAGCC
ATGCCGCGTGAAGGATGAAGGCCCTCTGGGTTGTAAACTTCTTTTATCTGGGACGAAAAA
AGGGGATTCTTCCTCACCTGACGGTACCAGAGGAATAAGCACCGGCTAACTCCGTGCCAG
CAGCCGCGGTAATACGGAGGGTGCAAGCGTTATCCGGATTTACTGGGTTTAAAGGGTGCG
TAGGTGGCAGGTAAGTCAGTGGTGAAATCTTTGGGCTTAACCCGAAAACTGCCATTGAT
ACTATCTGTCTTGAATATCCTGGAGGTAAGCGGAATATGTCATGTAGCGGTGAAATGCTT
AGATATGACATAGAACACCCATTGCGAAGGCAGCTTACTACGGGATTATTGACACTGAGG
CACGAAAGCGTGGGTAGCGAATGGGATTAGAGACCCTCGTAGTCC
>Otu6789
CCAGCCTACGGGTCGCTGCAGTGGGGAATATTGGGCAATGGGCGAAAGCCTGACCCAGCG
ACGCCGTGTGGGCGATGAAGGCCTTCGGGTTGTAAAGCCCTGTTGGGTGGAAAGAAGGAA
CCAGAGACGAATAATCTTTGGCTCTTGACGGTACCACCAGAGAAAGCACCGGCTAACTCC
GTGCCAGCAGCCGCGGTAATACGGAGGGTGCAAGCGTTGTTCGGAATTACTGGGCGTAAA
GGGCGCGTAGGTGGTTGCGTAAGTCGGATGTGAAATCTCGGAGCTTAACTCCGAAATGGC
ATTGGAAACTACCTTGCTCGAGGGTTGGAGGGGGGACTGGAATACTTGGTGTAGCAGTGA
AATGCGTAGATATCAAGTGGAACACCAGTGGCGAAGGCGAGTCCCTGGACAACTCCTGAC
ACTGAGGCACGAAAGCTAGGGGAGCAAACAGGATTAGAGACCCGTGTAGTCC
>Otu6792
CCAGCCTACGGGAGGCTCCAGCAACGAATATTCCGCAATGGGCGCAAGCCTGACGGAGCG
ACGCCGCGTGAAGGATGAAGTCCTTCGGGATGTAAACTTCAATAGTTTGTCAGGAACCAA
ATTGGATTAATACGCTATGTTTGCTGACCAACAAACGAAAGGGGTGGCTAACTTCGTGCC
AGCAGCCGCGGTAAGACGGGGACCCCAAGCGTTGTTCGGAATCACTGGGCTTAAAGCGCG
TGTAGGCGGATCGGCAAGTACTTTGTGAAAGCCCCGGCTTCAACCCGGGAATCGCTGGGT
ATACTGCCGATCTTGAGGCAGGTAGGGGTTACTGGAACCTTAGGTGGAGCGGTGAAATGC
GTAGATATCTAAGGGAACGCCTGTGGCGAAAGCGGGTAACTGGGCCTGTCCTGACGCTGA
GACGCGAAAGCGTAGGGAGCAAACGGGATTAGATACCCGGGTAGTCC
>Otu6793
CCAGCCTATGGGAGGCTCCAGTGGGGAATTTTTCGCAATGGGGGCAACCCTGACGAAGCA
ACGCCGCGTGGAGGATGAAGGTCTTCGGATTGTAAACTCCTGTCGATCGGGACGAAAACG
GAGCGACCTAATACGTCGCATCGTTGACTGTACCGGTGGAGGAAGCCACGGCCAACTCTG
TGCCAGCAGCCGCGGTAATACAGAGGTGGCAAGCGTTGTTCGGAATTATTGGGCGTAAAG
GGCGCGTAGGTGGTCTAGCAAGTCCTGTGTGAAATCCCTCAGCTCAACTGAGGAACTGCA
CAGGAGACTGCCTGACTTGAGTTCGGGAGAGGGACGCGGAATTCCGGGTGTAGCGGTGAA
ATGCGTAGATATCCGGAGGAACACCAGAGGCGAAGGCGGCGTCCTGGACCGATACTGACA
CTGAGGCGCGAAAGCTAGGGGAGCAAACGGGATTAGAAACCCTAGTATCC
>Otu6794
CCAGCCTATGGGTCGCAGCAGTCGAGAATTTTTCTCAATGGGCGCAAGCTTGAAGGAGCG
ACGCCGCGTGGGGGATGAAGGGCTTCGGTCTGTAAACCCCTGTCATTTGCGAACAAACCT
TCGGATTGAATAGGTCCGGAGCTGATTGTAGTGAAAGAGGAAGGGACGGCTAACTCTGTG
CCAGCAGCCGCGGTAATACAGAGGTCCCAAGCGTCGTTCGGATTCACTGGGCGTAAAGGG
TGCGTAGGTGGTGAGGCAAGTTTGGTGTGAAATCTCCGAGCTCAACTCGGAAACGGCACT
AAAAACTGGTTTGCTGGAGGGTCGGAAGGGAGACTGGAATTCTCGGTGTAGCGGTGAAAT
GCGTAGATATCGAGAGGAACACCAGTGGCGAAAGCGAGTCTCTGGACGACACCTGACACT
GAGGCACGAAAGCTAGGGGAGCAAACAGGATTAGAAACCCTAGTAGTCC
>Otu6795
CCAGCCTATGGGGGGCTCCAGTGGGGAATATTGCACAATGGGCGCAAGCCTGATGCAGCG
ACGCCGCGTGGAGGATGAAGGTTTTCGGATTGTAAACTCCTGTTAAGTGGGAAGAAAGAC
CCATGGCTAATATCCATGGGGGATGACGGTACCGCTAGAGAAAGCACCGGCAAACTTCGT
GCCAGCAGCCGCGGTAATACGAAGGGTGCGAGCGTTATTCGGAATGACTGGGTGTAAAGA
GCGTGGAGGCGGCTTTGTAAGTCAGCTGTTAAATAGCCCGGCTTAACTGGGCAAATGCGG
TTGATACTATAAGGCTAGAGTACAAGAGAGAAGAGTGGAATTCTCGGAGTAGCGGTAAAA
TGCGTAGATCTCGAGAGGAACACCGGTTGCGAAGGCGGCTCTTTGGCTTGTAACTGACGC
TTAAGCGCGAAAGCGTGGGGAGCAAACAGGATTAGAAACCCCCGTAGTCC
>Otu6797
CCAGCCTATGGGGTGCTCCAGCTTAGGAAATTCTTCAATGCCCGCAAGGGTGAAAGAGTA
AACCAGAGTGTTTCCTTTTAGGAAACTTTTGTCTGCTGTAAAAAGGCAGATGAATAAGGA

CTGGGCAAGACCGGTGCCAGCCGCCGCGGTAATCCCGGCGGTCCGAGTCGCATCCACATT
TATTGGGTCTAAAACATCCGTAGCTTGTTTCATAAGTCTCTTGTGAAATCTCATTTCTCA
AACTTGAGGCGTGCAAGAGATACTATGTTGCTAGAGATCGGAAGACGTAAAGAGAACGAT
GGAGGTAGCGGTAAAATGTGTTAATCTTCGTCGGACTAACAATGGCGAAGGCACTTTACG
AGTACGAATCTGACAGTGAGGGATGAAGGCTAGGGGCGCAAAAGGGATTAGATACCCGCG
TAGTCC
>Otu6798
CCAGCCTATGGGAGGCTGCAGTGGGGAATCTTCCGCAATGGACGAAAGTCTGACGGAGCA
ACGCCGCGTGAGTGAAGAAGGCTTTCGGGTCGTAAAGCTCTGTTATTCGGGACGAACGGT
CAGAGTGTAAATAATGCTCTGGAGTGACGGTACCGAAGGAGGAAGCCACAGCTAACTACG
TGCCAGCAGCCGCGGTAATACGTAGGTGGCAAGCGTTGTCCGGAATTATTGGGCGTAAAG
CGCGCGCAGGTGGGATAGTAAGTCTTTTGTAAAAGTGCGGGGCTCAACCCCGTAAAGCGA
TGGATACTGCTATTCTTGAGTGCCGGAGAGGAAAGCGGAATTCCCAGTGTAGCGGTGAAA
TGCGTAGATATTGGGAGGAACACCAGTGGCGAAGGCGGCTTTCTGGACGGTGTCTGACAC
TGAGGCGCGAAAGCTAGGGGAGCGAACGGGATTAGATACCCAGTAGTCC
>Otu6801
CCAGCCTACGGGGGGCTGCAGTCGAGAATCTTTTCCAATGGACGAAAGTCTGAGAATGCG
ACGCCGCGTGAGGGACGAAGGTCTTCGGATTGTAAACCTCTTTTGTCGGGGAGGAAGTTG
CGCTTTTTAATAGAAGGCGTAATTGACTGTACCTGATGAATAAGCCACGGCTAACTCCGT
GCCAGCAGCCGCGGTAATACGGAGGTGGCAAGTGTTACTCGGTTTGACTGGGTGTAAAGG
GTCCGCAGGCGGTTTGATAAGTGAAAAGTGAAATCCTTTTGCTCAACGAAAGAAGTGCTT
TTCAAACTATCATACTTGAGGATGGGAGAGGAGAGTGGAACTGTCGGTGTAAAGGTGAAA
TTTGTGGAGATCGACAGGAATGCCCGTGGAGAAGTCGGCTCTCTGGTCCATACCTGACGC
TCAGGGACGAAAGCTAGGGGAGCAAACAGGATTAGATACCCGAGTAGTCC
>Otu6806
CCAGCCTATGGGGGGCAGCAGTCTCGAATCATCTGCAATGCGCGAAAGCGTGACAGTGCG
ACGCCGCGTGCGGGAGGAAGCCCTTCGGGGTGTAAACCGCTTTTCAGGGGCATGAAGTGC
CGGCGTTGAATAGGCGCCGGCATTGACCTAAACCCTGGAATAAGCCACGGCTAACTTCGT
GCCAGCAGCCGCGGTAAGACGAAGGTGGCGAGCGTTGCTCGGAATCACTGGGCTTAAAGC
GGGCGTAGGCGGCCCGGTCAGTCGGGTGTGAAAACCCTTGGCTCAACCAAGGAACGGCAC
CCGATACTACCGGGCTTGAGGCGATTAGGGGCGACTGGAACTCTCGGTGGAGCGGTGAAA
TGCGTAGATATCGAGAGGAACGCCAATGGTGAAGACAGGTCGCTAGGATCGTCCTGACGC
TGAGGCCCGAAAGCTAGGGGAGCAAACGGGATTAGAAACCCGCGTAGTCC
>Otu6809
CCAGCCTATGGGTCGCACCAGTTAAGAATCTTGCACAATGGGGGAAACCCTGAAGGAGCG
ACGCCGCGTGAAGGATGAAGGTCTTCGGATTGTAAACTTCTGTCATTAGAGAACAAGTGT
CGCCGGTTAACTGCCGGCGGCTTGATAGTATCTGAAGAGGAAGGGACGGCTAACTCTGTG
CCAGCAGCCGCGGTAATACAGAGGTCCCAAGCGTTGTTCGGATTCATTGGGCGTAAAGGG
TGCGTAGGTGGCGGGGTAAGTTGGATGTGAAATCCCGGGGCTCAACCCCGGAACTGCATT
CAATACTGCCCTGCTGGAGTACTGGAGAGGAGATTGGAATTTACGGTGTAGCAGTGAAAT
GCGTAGATATCGTAAGGAAGACCGGTGGCGAAGGCGGATCTCTGGACAGTTACTGACACT
GAGGCACGAAGGCCAGGGGAGCAAACGGGATTAGAAACCCTGGGTAGTCC
>Otu6811
CCAGCCTATGGGTCGCAGCAGTGGGGAATATTGCGCAATGGGCGAAAGCCTGACGCAGCG
ACGCAGCGTGAGGGACGACGGCCTTCGGGTTGTAAACCTCTTTCAGCGGGGACGAAGCGC
AAGTGACGGTACCCGCAGAAGAAGCACCGGCTAACTACGTGCCAGCAGCCGCGGTAATAC
GTAGGGTGCAAGCATTGTCCGGATTTATTGGGCGTAAAGAGCTCGTAGGCGGCTTGTCAC
GTCGGGTGTGAAAACTCGGGGCTTAACCCCGAGCCTGCACTCGATACGGGCTGGCTAGAG
TGTTGCAGGGGAGACTGGAATTCCTGGTGTAGCGGTGAAATGCGCAGATATCAGGAGGAA
CACCGGTGGCGAAGGCGGGTCTCTGGGCAACTACTGACGCTGAGGAGCGAAAGCGTGGGG
AGCGAACAGGATTAGAAACCCCCGTAGTCC
>Otu6815
CCAGCCTATGGGACGCTGCAGTAAGGAATATTGGACAATGGTGGCAACACTGATCCAGCC
ATGCCGCGTGCAGGATGAAGGCGCTACGCGTTGTAAACTGCTTTTGTACGGGAGAAAACC
TATCTACGTGTAGATAGCTGATAGTACCGTAAGAATAAGCATCGGCTAACTTCGTGCCAG
CAGCCGCGGTAATACGAAGGATGCAAGCGTTATCCGGATTCACTGGGTTTAAAGGGTGCG
TAGGTGGCTTTGTAAGTCAGTGGTGAAAGCCCGGAGCTCAACTCCGGAACTGCCATTGAT
ACTGCTTAGCTTGAATCACGTCGAGGTGGATGGAATAATACATGTAGCGGTGAAATGCTT

AGATATGTATTAGAACACCGATTGCGAAGGCAGTTCACTAGGCGTGTATTGACACTGAGG
CACGAAAGCGTGGGGATCAAACAGGATTAGATACCCCGGTAGTCC
>Otu6816
CCAGCCTACGGGGGGCTGCAGTGAGGAATTTTGCGCAATAGGGGAAACCCTGACGCAGCA
ACGCCGCGTGAGTGAAGAAGGCCTTCGGGTCGTAAAGCTCTGTCAAGTGGGAAAAAATTC
AACCGGTGAATAGCCGGTTGGCCTGATGGTACCACTAGAGGAAGCACCGGCTAACTCCGT
GCCAGCAGCCGCGGTAATACGGAGGGTGCAAGCGTTGTTCGGAATTACTGGGCGTAAAGC
GCGTGTAGGCGGTTTGGTAAGTCAGATGTGAAAGCCCTGGGCTCAACCCAGGAAGTGCAT
TTGAAACTGCCTTACTCGAGTATGGGAGAGGAGAGTGGAATTCCCAGTGTAGAGGTGAAA
TTCGTAGATATTGGGAGGAACACCCGTGGCGAAGGCGACTCTCTGGACCAATACTGACGC
TGAGACGCGAAAGCGTGGGGAGCAAACAGGATTAGATACCCGTGTAGTCC
>Otu6822
CCAGCCTATGGGAGGCAGCAGTGAGGAATCTTCCGCAATGGGCGAAAGCCTGACGGAGCG
ACACCGCGTGAAGGATGAAGCCCTTTCTGGGCGTAAACTTCTGTTCTGAGGGACGAAATT
TTTGACGGTACCTCAGGAGAAAGCATCGGCTAACCCTGTGCCAGCAGCCGCGGTAAGACA
GGGGATGCAAGCGTTACTCGGAATTACTGGGCGTAAAGCGTCTGTAGGCTTCTTTCCACG
TCTGGTGTTAAATGTCGGAGCCCAACTCCGTCACGTGCCGGAAACGAGAGAGATTGAGTC
AATCAGAGGCATCTGGAATGTCGTGTGTAGGGGTAAAATCCGTTGATCCACGATGGAACG
CCAAAAGCGAAGGCAGGATGCTGGGGTTGTACTGACGCTCAGAGACGAAAGCGTGGGGAG
CAAAGGGGATTAGATACCCTCGTAGTCC
>Otu6824
CCAGCCTATGGGGGGCTCCAGTGAGGAATATTCTGCAATGGACGAAAGTCTGACAGAGCA
ATACCGCGTGGGGGATATGAAGGCGTGATAGTTGTAAACCTCTTTACTCAAGGATGAACT
TTTTTGACACTACTTGTAGAAGTAAGTATCGGCTAAACCTGTGCCAGCAGCCGCGGTAAT
ACAGGAGATGCGAGTGTTATCCGGAATTATTGGGCGTAAAGTGTCTGTGGGCGGAAATCT
AAGTTCTATGTTAAATATTTAAGCTCAACTTAAAATAAAAGCATAGAATACTATTTTTCT
AGAGTATTTTAGAGGATCTGAGAATTTTCAGTGGAGCGGTAGAATGCATAGATATTGAAA
GGAATACCAAATGCGAAAGCACTTTTCTGGATTTTACTGACGCTAAGAGACGAAAGCTAG
GGGATCATACAGGATTAGAAACCCCAGTAGTCC
>Otu6827
CCAGCCTACGGGATGCTCCAGTCGAGAATTTTTCTCAATGGGCGAAAGCCTGATGGTGCG
ACGCCGCGTGGGGGATGAAGGTCTTCGGATTGTAAACCCCTGTCACCGGGGAAGAAACGC
TTCAAGTTAACAGCTTGAAGCCTGACTTAACCCGGAGAGGAAGCAGTGGCTAACTCTGTG
CCAGCAGCCGCGGTAATACAGAGACTGCAAGCGTTATTCGGATTCACTGGGCGTAAAGGG
TGCGCAGGCGGCCATGTGTGTTAGGCGTGAAAGCCCGAGGCTTAACCTCGGAATTGCACC
TAAAACTACATGGCTAGAGCATTGGAGAGGGTAGTGGAATTCATGGTGTAGCAGTGAAAT
GCGTAGATATCATGAGGAACACCAGTGGCGAAGGCGACTACCTGGACAATTGCTGACGCT
CAGGCACGAAAGCGTGGGGAGCAAAAGGGATTAGAAACCCTGGTAGTCC
>Otu6830
CAGCCTATGGGGGGCAGCAGTGAGGAATCTTGGACAATGGGCGCAAGCCTGATCCAGCAA
TGCCGCGTGGGTGATGAAGGCCTTCGGGTTGTAAAGCCCTTTCGGCGGGGAAGATGATGA
CGGTACCCGCAGAAGAAGCCCCGGCTAACTTCGTGCCAGCAGCCGCGGTAATACGAAGGG
GGCTAGCGTTGTTCGGAATTACTGGGCGTAAAGGGCGCGTAGGCTGTGCTGTAAGTCAGG
CGTGAAAGGCCCGGGCTCAGCCTGGGAACTGCGCTTGATACTGCGGCGCTGGAAGGCGGG
AGAGGATGGCGGAGTTCCCAGTGTAGAGGTGAAATCTGTAGATATCTGGAAGAACACCGG
TGGCGAAGGCGAGCTTCTATCAGAGGACTGACGCTGAGGCGCGAAAGCGTGGGGAGCAAA
CAGGATTAGAAACCCCCGTAGTCC
>Otu6835
CCAGCCTACGGGGGGCTGCAGTGAGGAATATTGGTCAATGGGGGCAACCCTGAACCAGCC
ATGCCGCGTGAAGGAAGACTGCCCTAAGGGTTGTAAACTTCTTTTGTACGGGGGTAATTT
TCACCTTGCGAGGTGAATTGAAAGTACTGTACGAATAAGCATCGGCTAACTCCGTGCCAG
CAGCCGCGGTAATACGGAGGATGCAAGCGTTATCCGGATTCATTGGGTTTAAAGGGTGCG
TAGGCGGGTTTGTAAGTCAGTGGTGAAAGACTGTCGCTTAACGATAGCATTGCCATTGAT
ACTGCAGGTCTTGAGTATACTTGAGGTAGGCGGAATGTGTAGTGTAGCGGTGAAATGCTT
AGATATTACACAGAACACCAATTGCGAAGGCAGCTTACTATGGTTTAATTGACGCTGAGG
CACGAAAGCGTGGGGATCAAACAGGATTAGATACCCCAGTAGTCC
>Otu6838
CCAGCCTACGGGGTGCAGCAGTTAGGAATCGTCTACAATGCGCGCAAGCGTGATAGCGCG

AGCCAGAGTGCCTCTCATTGAGAGGCTTTTGCCAAATCTAAAAAGTTTGGCGAATAAGGA
CTGGGTAAGACTAGTGCCAGCCGCCGCGGTAATCCTAGCGGTCCAAGTCGCAGCCAACAT
TATTGGGTCTAAAACATCCGTAGCTTGCTTGATAAGTTCCCTGTGAAATCCTGCGTCCTA
AATGCAGGGCGTGCAAGGAGTACTGTCAAGCTAGAGACTGGAAGACGTGGAGAGTACGTT
CGAAGTAGTGGTAAAATACGTTAATCTCGGACGGACTTACAATAGCGTAGGCACTCTACG
AGGACAGTTCTGACAGTGAGGGATGAAGGCTAGGGTCGCGAAAGGGATTAGATACCCGAG
TAGTCC
>Otu6841
CCAGCCTACGGGGCGCTGCAGTCGAGAATTTTTCACAATGGGCGCAAGCCTGATGGAGCG
ACGCCGCGTGGGGGATGAATGGCTTCGGCCCGTAAACCCCTGTCATTCGTGATCAACGCG
TTTGACCTAATACGTCAAACGTTGATAGTAACGGAAGAGGAAGGGACGGCTAACTCTGTG
CCAGCAGCCGCGGTAATACAGAGGTCCCAAGCGTTGTTCGGATTCACTGGGCGTAAAGGG
TGCGTAGGCGGTCGGGTAAGTCTGACGTGAAAGCCCGCAGCTCAACTGCGGAACGGCGTC
GGATACTATTCGGCTGGAGGGTGGGAAGGGGGACTGGAATTCTCGGTGTAGCAGTGAAAT
GCGTAGATATCGAGAGGAACACCAGTGGCGAAGGCGAGTCCCTGGACCACTCCTGACGCT
GAGGCACGAAAGCTAGGGGAGCAAACAGGATTAGAAACCCCTGTAGTCC
>Otu6843
CCAGCCTACGGGTTGCAGCAGCTAAGAATCTTCCGCAATGGGGGAAACCCTGACGGAGCG
ACGCCGCGTGGATGATGAAGGCCGTAAGGTTGTAAAATCCTTTTGTCGGTGAAGAATAAG
TGGAGGAGTGGAAAGCCTTCATGATGACGTTAGCCGACGAATAAGCCCCGGCTAATTACG
TGCCAGCAGCCGCGGTAATACGGAGGGTGCAAGCGTTATCCGGATTCACTGGGTTTAAAG
GGTGCGTAGGTGGGTCTGTAAGTCAGTGGTGAAATCCCCAAGCTTAACTTGGGAACTGCC
ATTGATACTATAGGTCTTGAATCATCTGGAGGTAAGCGGAATATGTCATGTAGCGGTGAA
ATGCTTAGATATGACATAGAACACCAATTGCGAAGGCAGCTTACTACGGGTGTATTGACA
CTGAGGCACGAAAGCGTGGGGATCAAACAGGATTAGATACCCTTGTAGTCC
>Otu6849
CCAGCCTACGGGAGGCAGCAGTGAGGAATATTGCGCAATGGGCGAAAGCCTGACGCAGCA
ACGCCGCGTGAGCGATGAAGGCCTTCGGGTCGTAAAGCTCTTTTCCAGGGGAAGAGGAAG
GACGGTACCCTGGGAATAAGCCCCGGCTAACTACGTGCCAGCAGCCGCGGTAACACGTAG
GGGGCAAGCGTTATTCGGATTTACTGGGCGTAAAGCGCATGCAGGCGGTCTTGCAAGTCG
GACGTTAAATCTCCCGGCTTAACTGGGAGGCGTCGTTCGAAACTGCGAGACTTGAGGTTG
GGAGAGGGAAGCGGAATTCCCGGTGTAGTGGTGGAATGCGTAGATATCGGGAGGAACACC
TGTGGCGAAAGCGGCTTCCTGGCCTACACCTGACGCTAAGATGCGAAAGCGTGGGGAGCG
AACAGGATTAGAAACCCTAGTAGTCC
>Otu6858
CCAGCCTATGGGATGCACCAGTCGAGAATATTCCACAATGGACGAAAGTCTGATGGAGCG
ACGCCGCGTGCAGGATGAAGGCCTTCGGGTCGTAAACTGCGGTAGTAAAGTAATAATGCA
AATGAATGCTTTATGGAAAGAGGTTGGTAACTACGTGCCAGCACCCGCGGTAAAACGTAG
ACCTCAAGCGTTATCCGGATTTACTGGGCGTAAAGAGCATGTAGGAGGTTTGGCGCGTCT
TCTGTTAAAGCCCACCGCCCAACGGTGGAAATGCAGGAGATACGGCGAGACTAGAGGATG
TTAGAGGTACCAGGAACACACGGTGTAGGGGTGAAATCCGTTGATATCGTGTGGAACACC
AAAGGCGAAGGCAAGGTACTGGGACGCTCCTGGCTCTGATATGCGAAAGCGTGGGGAGCA
AAAAGGATTAGATACCCGAGTAGTCC
>Otu6859
CCAGCCTATGGGAGGCACCAGTTAGGAATCTTTTGCAATGCGCGCAAGCGTGACAAAGCG
AGCCAGAGTGATTTTCAATTTGAAAATCTTTTGCCAAATGCAAAAAGTTTGGCGAATAAG
GACTGGGCAAGACTAGTGCCAGCCGCCGCGGTAATCCTAGCGGTCCAAGTCGCAGCCATC
ATTATTGGGTCTAAAACATCCGTAGCTTGCTGAATAAGTTTCCTGTGAAATCCGGCATCT
TAAGTGTCGGGCGCGCAAGAAATACTATTGAGCTAGAGACTGGAAGACGTAAAAAGTACG
TTTGAAGTAATGGTAAAATATGTTAATCTCAGACGGACTAACAACAGCGAAGGCATTTTA
CGAGGACAGTTCTGACAGTAAAGGATGAAGGCTAGGGGCGCAAAGTGGATTAGATACCCG
AGTAGTCC
>Otu6862
CCAGCCTATGGGAGGCAGCAGTGGGGAATATTGGACAATGGGGGAAACCCTGATCCAGCA
ATGCCGCGTGTGTGATGAAGGCCTGCGGGTTGTAAAGCACTTTCGGTGGGGAAGAAATAC
TCAGGGCTAATAACTCTGAGGGTTGACGTTACCCAAAGAAGAAGCACCGGCTAACTCTGT
GCCAGCAGCCGCGGTAATACAGAGGGTGCAAGCGTTAATCGGAGTTACTGGGCGTAAAGG
GCGCGTAGGCGGATATTTAAGTCGGATGTGAAAGCCCTGGGCTCAACCTAGGAATTGCAT

TCGATACTGGGTATCTAGAGTATGGTAGAGGAAAGTGGAATTCCCGGTGTAGCGGTGAAA
TGCGTAGAGATCGGGAAGAACACCAAGGCGAAGGCAGCTTTCTGGACCAATACTGACGCT
GAGGCGCGAAAGCGTGGGGAGCAAACAGGATTAGAAACCCGTGTAGTCC
>Otu6870
CCAGCCTACGGGTGGCAGCAGTGGGGAATATTGGACAATGGACGAAAGTTTGATCCAGTA
ATAGTTCATGAGAGAGAAAAGGAAAGCAATAACGCTGTAAATCTCAGAAAACAAAAAAAA
TAATGATTGTATTTGTATTAAGCACTGGCTAATTCTGTGCCAGCAGCCGCGGTAAGACAG
GGAGTGCTGATGTTATCCGTTTTAATTGGGTGTAAAGGATATGTAGATGGCTTTGTTATG
AAAATTTTATAAAAGTTCTAAAAAAAAAATTTAGAAAAGATAAGATTTATTCAAAAGCTTG
AGTGTGTCCGATGTTAAAAGAATTTTTTTTGTAGGGGTAGAATCCTTAAATATTCAAAAG
ACTATCAAAATAGTTGTGAAACCATTTTTCAAAGGCACACTGACATTGAGATATGAAAGT
ATAGGTATCGAACAGGATTAGAGACCCCAGTAGTCC
>Otu6871
CCAGCCTACGGGAGGCTCCAGTGGGGAATCTTGCGCAATGGGCGAAAGCCTGACGCAGCA
ACGCCGCGTGGAGGAAGAAGGCCTTCGGGTTGTAAACTCCTGTCAGGGGGAAAGAAGGCA
CCTGGGTTAATAGCCCAAGGGCTGACGGTACCCCCAGAGGAAGCCACGGCTAACTACGTG
CCAGCAGCCGCGGTAATACGTAGGTGGCGAGCATTGTCCGGAATTATTGGGCGTAAAGAG
CGCGTAGGCGGCTTGCCAGGTCCGATGTGAAATCTCAGGGCTCAACCCTGAACGTGCATC
GGAAACCGGCTTGCTAGAGTCTGGGAGAGGAGAGTGGAATTCCTGGTGTAGCGGTGAAAT
GCGCAGATATCAGGAGGAACACCAGTAGCGAAGGCGGCTCTCTGGAACAGTACTGACGCT
GAGGCGCGAAAGCTAGGGGAGCAAACAGGATTAGAAACCCGAGTAGTCC
>Otu6874
CCAGCCTATGGGGTGCTCCAGTAGGGAATATTGCTTAATGGGCGAAAGCCTGAAGCAGCA
ACGCCGCGTGTACGATGAAGGCCTTCGGGTTGTAAACCTCTTTTATCAGGGAAGAATAAT
GACGGTACCTGATGAATAAGTCACGGCTAACTACGTGCCAGCAGCTGCGGTAATACGTAG
GTGGCGAGCGTTATCCGGATTCATTGGGCGTAAAGAGCGCGTAGGTGGTCCTTCAAGTCA
GATGTGAAATCTTCCGGCTTAACTGGAAGAGGCCATCTGATACTGCTGGACTTGAGGGCG
GTAGAGGGAGGTGGAATTCCCGGTGTAGTGGTGAAATGCGTAGATATCGGGAGGAACACC
AGTGGCGAAAGCGGCCTCCTAGGCCGTTCCTGACACTGAGGCGCGAAAGCGTGGGGAGCG
AACAGGATTAGAAACCCCCGTAGTCC
>Otu6876
CCAGCCTACGGGGGGCAGCAGCGACGAATCTTCCGCAATGGGCGAAAGCCTGACGGAGCG
ATGCCGCGTGCGGGATGAAGCGGCTTTGCCGTGTAAACCGCTGTCAGGAGATACAAACAA
TGATGATCTCCAGAGGAAGGGCCGGCTAACCCTGTGCCAGCAGCCGCGGTAATACAGGGG
GCCCGAGCGTTAGTCGGAATCACTGGGCTTAAAGGGTGCGTAGGCGGACTTATAAGTGTC
GTGTGAAAGCCCACTGCTCAACGGTGGAACTGCACGGCAAACTGTAAGTCTCGAGGAAGT
TAGAGGTCGCTAGAACGTTTGGTGGAGCGGTGGAATGCGTAGATATCAAACGGAATGCCG
AAGGTGAAGACAGGCGACTAAGACTTTCCTGACGCTGAGGCACGAAAGCGTGGGGAGCAA
ACAGGATTAGAAACCCCTGTAGTCC
>Otu6880
CCAGCCTACGGGGGGCTCCAGTGACGAATATTCCGCAATGGGCGCAAGCCTGACGGAGCG
ATGCCGCGTGTGGGACGAAGCCCCTCGGGGTGTAAACCACTGTCAGGGGTTAGCAATAAA
TTGAGCAACCCCAAAGGAAGAGGCGACTAACTCTGTGCCAGCAGTCGCGGTAATACAGAG
GCCTCAAGCGTTAATCGGAATCACTGGGCTTAAAGGGTGCGTAGGCGGGCTTGCAAGTGC
CTTGTGAAATCCCACGGCTCAACCGTGGAATTGCTCGGTAGACTGCGAGTCTTGAGGTCG
GTAGGGGCCGCTGGAACTCTAGGTGGAGCGGTGAAATGCGTAGATATCTAGAGGAACGCC
CAAGGCGAAGGCAGGCGGCTGGGCCGAATCTGACGCTGAGGCACGAAAGCGTGGGGAGCG
AACGGGATTAGAGACCCCAGTAGTCC
>Otu6883
CCAGCCTACGGGAGGCAGCAGTTTGGAATATTCCACAATGGGCGAAAGCCTGATGGAGCG
ACACCGCGTGGAGGATGAAGGTCCTCGGATTGTAAACTCCTTTAGACCTAGATGAAGGCA
ACCGGGTGAATAATCCGGTTGTTTGACAGTATAGGTAGAATAAGCCACGGCTAACTCTGT
GCCAGCAGCCGCGGTAATACAGAGGTGGCGAACGTTGTCCGGATTTATTGGGTGTAAAGG
GCATGTAGGTGGTCTTGTAAGTCAAAGGTGAAATGGCCCGGCTTAACCAGGTCATTGCCT
TTGAAACTGCAGGGCTTGAGTGCGGAAGAGGAGAGCGGAATTCCCAGTGTAGCGGTGAAA
TGCGTAGATATTGGGAGGAACACCGGTGGCGAAGGCGGCTCTCTGGTCCGATACTGACAC
TGGGATGCGAAAGCCAGGGGAGCAAACGGGATTAGATACCCGCGTAGTCC
>Otu6885

CCAGCCTATGGGAGGCACCAGTAACGAATATTCCGCAATGGGCGAAAGCCTGACGGAGCG
ACGCCGCGTGTGGGAATAAGTCCTTCGGGATGTAAACCACTGTCAGGGGCTGCCAAGCGA
GCGGGGCCAATATCCCCGCAAGTTGAGGAGCCCCAGAGGAAGCCACGGCTAACTTCGTGC
CAGCAGCCGCGGTAATACGAAGGTGGCGAGCGTTGTTCGGAATCACTGGGCTTAAAGAGC
ATGTAGGCGGCTCATCAAGCGTCTTGTGAAATCCCTCGGCTCAACCGAGGAATGGCTGGG
CGAACTGATGGGCTTGAGGCAGGTAGGGGCGCGCGGAACTCTTGGTGGAGCGGTGGAATG
CGTAGATATCAAGAGGAACGCCGGTGGCGAAGGCGGCGCGCTGGGCTTGCCCTGACGCTG
AGATGCGAAAGCCAGGGGAGCAAACGGGATTAGATACCCTGGTAGTCC
>Otu6886
CCAGCCTACGGGGGGCAGCAGTGGGGAATCTTGCACAATGGGGGCAACCCTGATCCAGCG
ACGCCGCGTGAAGGATGAAGGTCTTCGGATTGTAAACTTCTTTTGGAGGGGATGAATGCC
CTGGTGAATAGCCAGGGGTGACAGTACCCTCTGAATAAGCCACGGCTAACTACGTGCCAG
CAGCCGCGGTAATACGTAGGTGGCAAGCGTTACTCGGAATCACTAGGCGTAAAGCGCAGG
TAGGCGGTTCAGTAAGTCTCTTGTGAAAGCTCCCGGCTCAACTGGGAGAGGCCAAGGGAA
ACTACTGGGCTAGAGTGTGGTAGAGGAAGCTGGAATTCCCGGTGTAGCGGTGAAATGCGT
AGAGATCGGGAGGAATACCTAAGGCGAAAGCAGGCTTCTGGGCCATCACTGACACTGATC
TGCGAAAGCTAGGGGAGCAAATAGGATTAGAAACCCCTGTAGTCC
>Otu6887
CCAGCCTACGGGACGCTCCAGTGGGGAATATTGGGCAATGGGCGAAAGCCTGACCCAGCC
ACGCCGCGTGAGTGATGAAGGCCTTCGGGTCGTAAAGCTCTGTGGGGAGGGACGAATAAG
CCGGCCAACAACCGGCCCTGACGGTACCTCCTTAGCAAGCACCGGCTAACCATGTGCCAG
CAGCCGCGGTAATACATGGGGTGCAAACGTTGCTCGGAATTATTGGGCGTAAAGCGCGCG
TAGGCGGTTACTTAAGTCGGATGTGAAATCCCTTGGCTTAACTGAGGAAGTGCATCCGAG
ACTGAGTAGCTAGAGTACGAAAGAGGGTCGCGGAATTCCCGGTGTAGAGGTGAAATTCGT
AGATATCGGGAGGAACACCGGCGGCGAAGGCGGCGACCTGGTTCGAGACTGACGCTGAGG
CGCGAAAGCGTGGGGAGCAAACAGGATTAGAAACCCGTGTAGTCC
>Otu6888
CCGCCTATGGGGGGCTGCAGTAGGGAATATTGGTAATGGGCGAAAGCCTGAACCAGCAAC
GCCGCGTGAGCGATGACGGCCTTCGGGTTGTAAAGCTCTTTTTAGAGGGAAGAGGAAGGA
CGGTACCTCTAGAATAAGCCTCGGCTAACTACGTGCCAGCAGCCGCGGTAAAACGTAGGA
GGCGAGCGTTATCCGGATTTACTGGGCGTAAAGCGCATGCAGGCGGTTCGGAAAGTTGGA
TGTGAAAGCTCTCGGCTTAACTGGGAGAGGTCGTTCAATACTTCCGGACTAGAGGATGTG
AGAGGAAAGTGGAATTCCGAGTGTAGTGGTGAAATGCGTAGATATTCGGAGGAACACCAG
TGGCGAAAGCGGCTTTCTGGCACATATCTGACGCTCATATGCGAAAGCTAGGGTAGCAAA
CGGGATTAGATACCCGCGTAGTCC
>Otu6889
CCAGCCTACGGGAGGCTCCAGTGGGGAATTTTGCGCAATGGGCGAAAGCCTGACGCAGCA
ATGCCGCGTGGGTGATGAAGGTCTTCGGATTGTAAAACCCTGTCGTCGGGGACGAAGGTT
GGGGGGTTAACAGCCCTTTAGCTTGACGGTACCCGGAGAGGAAGCCCCGGCTAACTCTGT
GCCAGCAGCCGCGGTAATACAGAGGGGGCAAGCGTTATTCGGAATTATTGGGCGTAAAGG
GCGCGTAGGCGGTGATTCAAGTCGGATGTGCAATCCCCGAGCTCAACTTGGGAACTGCAT
CCGAGACTGGATCGCTGGAGTGCTGGAGAGGGTGGTGGAATTCCTCGTGTAGCGGTGAAA
TGCGTAGAGATGAGGAGGAACACCAGTGGCGAAGGCGGCCACCTGGACAGTAACTGACGC
TGAGGCGCGAAAGTGTGGGTAGCAAACAGGATTAGAAACCCTTGAGTCC
>Otu6891
CCAGCCTACGGGTGGCTCCAGTCGAGGATCTTCGGCAATGGGCGCAAGCCTGACCGAGCG
ACGCCGCGTGTGTGACGAAGGCCTTCGGGTTGTAAAGCACTGTCGAGGGGGAGGAAAGCG
CAAGCTTGACCTATCCCTGGAGGAAGCACGGGCTAAGTTCGTGCCAGCAGCCGCGGTAAG
ACGAACCGTGCAAACGTTGTTCGGAATCACTGGGCTTAAAGGGCGCGTAGGCGGTTTGTC
AAGTCAGGGGTGAAATCTTTCGGCTCAACCGGAAAGGTGCCCTTGATACTGACAGACTAG
AGCGAGGTAGGGGCATGTGGAACTTCTGGTGGAGCGGTGAAATGCGTTGATATCAGAAGG
AACGCCGGTGGCGAAAGCGACGTGCTGGACCTCTTCTGACGCTGAGGCGCGAAAGCTAGG
GTAGCAAACGGGATTAGATACCCTTGTAGTCC
>Otu6893
CCGCCTATGGGGGGCAGCAGTAAGGGATATTGCGCAATGGGCGAAAGCCTGACGCAGCAA
CGCCGCGTGGAGGAAGAAGGCCTTCGGGTTGTAAACTTCTTTTAGCAGGGACGAGGAAGG
ACGGTACCTGCTGAATCAGTCACGGCTAACTACGTGCCAGCAGCCGCGGTAAAACGTAGG
TGGCAAGCGTTATCCGGATTTACTGGGCGTAAAGGGTGTGCAGGCGGGGATACAAGTGGT

GTATGAAATCTCCCAGCTTAACTGGGATAGGTTATGCCAGACTGTATTTCTAGAGGGCGA
GAGAGGGGGGTGGAATTCCGGGTGTAGTGGTGAAATGCGTAGAGATCCGGAGGAACCCCA
GAGGCGAAGGCGGCCCCCTGGCTCGTACCTGACGCTCAGCCACGAAAGCATGGGGAGCGA
ACGGGATTAGATACCCTAGTAGTCC
>Otu6897
CCAGCCTATGGGATGCACCAGTAGGGAATATTGGGCAATGGGCGAAAGCCTGACCCAGCA
ACGCCGCGTGCACGATGAAGGTCTTCGGATCGTAAAGTGCTTTTCTGAGAGATGAGAAAG
GACAGTATCTCAGGAATAAGTCTCGGCTAACTACGTGCCAGCAGCCGCGGTAACACGTAG
GAGGCGAGCGTTATCCGGATTTACTGGGCGTAAAGCGTATGCAGGCGGTTTGGTAAGTTG
GATGTGAAAGCTCCTGGCTTAACTGGGAGAGGTCGTTCAATACTACCAGGCTAGAGGATG
GGAGAGGGAGGTGGAATTCCGGGTGTAGTGGTGAAATGCGTAGATATCCGGAGGAACACC
AGTGGCGAAAGCGGCCTCCTGGCCCATTTCTGACACTGATACGCGAAAGCTAAGGTAGCA
AACGGGATTAGATACCCTCGTAGTCC
>Otu6899
CCAGCCTATGGGACGCAGCAGTGAGGAACATTGGTCAATAGGGGGAACCCTGAACCAGCC
ATGCCGCGTGAAGGAAGACTGCCCTAAGGGTTGTAAACTTCTTTTGTATGGGAGTAAAGT
CATTTACGAGTAAGTGATTGAAAGTACTGTACGAATAAGCGTCGGCTAACTCCGTGCCAG
CAGCCGCGGTAATACGGAGGATGCGAGCGTTATCCGGATTTATTGGGTTTAAAGGGTGCG
TAGGCGGGTTAGTAAGTCAGTGGTGAAAGACTGTCGCTTAACGATAGCATTGCCATAGAT
ACTGCTGATCTTGAGTACAGTTGAGGTAGGCGGAATGTGTAGTGTAGCGGTGAAATGCTT
AGATATTACACAGAACACCAATTGCGAAGGCAGCTTACCAGAGTGTAACTGACGCTGAGG
CACGAAAGCGTGGGGATCAAACAGGATTAGAGACCCCTGTAGTCC
>Otu6902
CCAGCCTATGGGGGGCTCCAGTCGAGAGGCTTTGTCAATGGGGGAAACCCTGAACAAGCG
ACGCCGCGTGCGCGATGAAGGCCTTCGGGTTGTAAAGCGCTGTCGAGGGGGAGGAAGACC
GCAAGGTTTGACCTATCCCTGGAGGAAGCACGGGCTAAGTTCGTGCCAGCAGCCGCGGTA
AGACGAACCGTGCAAACGTTGTTCGGAATCACTGGGCTTAAAGGGCGCGTAGGCGGGCCT
CCCAGTCCGGGGTGAAAGCTTTCGGCTTAACCGGAAAAGTGCCTTGGATACTGGAGGTCT
GGAGGGAGGTAGGGGCAGCTGGAACTTCCGGTGGAGCGGTGAAATGCGTTGATATCGGAA
GGAACGCCGGTGGCGAAAGCGAGCTGCTGGATCTCTACTGACGCTGAGGCGCGAAAGCCA
GGGGAGCGAACGGGATTAGAAACCCCAGTAGTCC
>Otu6904
CCAGCCTACGGGGTGCACCAGTGGGGAATATTCCGCAATGGGCGAAAGCCTGACGGAGCG
ACGCCGCGTGGATGATGAAGGTCTTCGGATCGTAAAGTCCTGTAGAGGGGGAAGATGGGT
GCAGCAATGCATCTGACAGTACTCCTAAAGTAAGCTACGGCAAACTCTGTGCCAGCAGCC
GCGGTTATACAGAGGTAGCAAGCGTTGTTCGGAATGACTGGGTGTAAAGCGCGTGTAGGT
GGTTTAGTAAGTTGAATGTGAAATCCCTTGGCTTAACCAAGGAACTGCATTCAAAACTAC
TTTACTTGAGTACGGGAGAGGAAAGTGGAATTTCTGGTGTAGCGGTAAAATGCGTAGATA
TCAGAAGGAACACCGGTGGCGAAGGCGACTTTCTGGTCCGTAACTGACACTGAGACGCGA
AAGCTAGGGGAGCAAACAGGATTAGAGACCCCCGTAGTCC
>Otu6907
CCAGCCTATGGGGGGCACCAGTCGAGAATTTTTCACAATGGGCGAAAGCCTGATGGAGCG
ACGCCGCGTGGGGGATGAATGGCTTCGGCCCGTAAACCCCTGTCATTTGCGAACAAATTG
GTTCTATTAACACCTGAACCATTGATAGTAGCGGAAGAGGAAGGGACGGCTAACTCTGTG
CCAGCAGCCGCGGTAATACAGAGGTCCCAAGCGTTGTTCGGATTCACTGGGCGTAAAGGG
TGCGTAGGTGGTCGGGTAAGTCTGACGTGAAATCTCCAAGCTCAACTTGGAAACTGCGTT
GGATACTACTCGGCTTGAGGAATGGAGGGGAGACTGGAATACTTGGTGTAGCAGTGAAAT
GCGTAGATATCAAGTGGAACACCAGTGGCGAAGGCGAGTCTCTGGACATTTCCTGACGCT
GAGGCACGAAAGCCAGGGGAGCAAACAGGATTAGATACCCTTGTAGTCC
>Otu6911
CCAGCCTATGGGGTGCTCCAGTAGGGAATTTTCCGCAATGGGCGAAAGCCTGACGGAGCG
ACGCCGCGTGGGTGAAGACGGCCTTTGGGTTGTAAAGCCCTGCTCATCGGGAAGAAGAAA
TGACGGTACTGGTGAAGAAAGCCCCGGCTAACTACGTGCCAGCAGCCGCGGTAATACGTA
GGGGGCGAGCATTGTCCGGGATGATTGGGCGTAAAGGGCGTGTAGGCGGATGCATAAGTC
GGGCGTGAAAAGCGCGGGCTCAACTCGTGTAAGCGTTCGAAACGGTGCATCTTGAGTGCA
GTAGAGGGAAGCGGAATTTCTGGTGTAGCGGTGGAATGCGTAGATATCAGAAGGAACACC
GGTGGCGAAGGCGGCTTCCTGGACTGCTACTGACGCTGAGGCGCGAAGGCGTGGGGAGCA
AACAGGATTAGATACCCCTGTAGTCC

>Otu6912
CCAGCCTATGGGAGGCTGCAGTAAGGAATATTGGACAATGGCCGCAAGGCTGATCCAGCC
ATGCCGCGTGCAGGAAGAAGGACCTATGGTTCGTAAACTGCTTTTGTAACAGAGAAAACC
CTCGTACGTGTACGGGGCTGATAGTATGTTAAGAATAAGCATCGGCTAACTTCGTGCCAG
CAGCCGCGGTGAGACGAAGGATGCAAGCGTTATCCGGATTCATTGGGTTTAAAGGGAGCG
TAGGCGGATAAATAAGTCAGTGGTGAAATCTCTGGGCTTAACCCAGAAACTGCCATTGAT
ACTGTTTATCTAGAGTACAGTTGCCGTTGGCGGAATATGACATGTAGTGGTGAAATACTT
AGATATGTCATAGAACACCGATTGCGAAGGCAGCTAACGAAACTGTAACTGACGCTGAGG
CTCGAAAGTGCGGGGATCAAACAGGATTAGATACCCTAGTAGTCC
>Otu6914
CCAGCCTACGGGTTGCAGCAGTAAGGAATATTGGACAATGCCCGGAAGGGTGATCCAGCC
ATGCCGCGTGCAGGAAGAAGGTCCTATGGATTGTAAACTGCTTTTACACCGGAGAAAACC
CCCCTTTGCGAAGGGGGCTGATAGTATGGTGAGAATAAGCATCGGCTAACTTCGTGCCAG
CAGCCGCGGTCAGACGAAGGATGCAAGCGTTATCCGGATTCATTGGGTTTAAAGGGAGCG
TAGGCGGCTTTATAAGTCAGTGGTGAAATCTCCTTGCTTAACGAGGATATTGCCATTGAT
ACTGTAGAGCTTGAGTACAGATGCCGTTGGCGGAATATGACATGTAGTGGTGAAATACAT
AGAGATGTCATAGAACACCGATTGCGAAGGCAGCTGACGAAACTGTAACTGACGCTGAGG
CTCGAAAGTGTGGGGATCAAACAGGATTAGATACCCGCGTAGTCC
>Otu6919
CCAGCCTACGGGAGGCACCAGTGGGGAATTTTGGACAATGGGAGCAACCCTGATCCAGCC
ATGCCGCGTGCGGGAAGAAGGCCTTCGGGTTGTAAACCGCTTTTATTTGGGAAGAAGGAG
TCGTGGTTAATACCTGCGATGACAGACGGTACCGGATGAATAAGCACCGGCTAACTACGT
GCCAGCAGCCGCGGTAATACGTAGGGTGCGAGCGTTAATCGGAATTACTGGGCGTAAAGC
GTGCGCAGGCGGTTCTGAAAGTCAGCTGTGAAAGCCCCGGGCTTAACCTGGGAATGGCGG
TTGAAACTACAGGACTAGAATCTGGCAGAGGGGGGTGGAATTCCACGTGTAGCAGTGAAA
TGCGTAGAGATGTGGAGGAACACCGATGGCGAAGGCAGCCCCCTGGGCTGAGATTGACGC
TCAGGCACGAAAGCGTGGGGAGCAAACAGGATTAGAGACCCTTGTAGTCC
>Otu6920
CCAGCCTACGGGGGGCTCCAGTGGGGAATTTTCCGCAATGGGCGAAAGCCTGACGGAGCA
ACGCCGCGTGGATGATGAAGTCTTTCGGGACGTAAAATCCTTTCAGCAGGGACGAAGCGA
GTGACGGTACCTGCAGAAGAAGCATCGGCTAACTACGTGCCAGCAGCCGCGGTAATACGT
AGGATGCGAGCGTTGTCCGGAATTATTGGGCGTAAAGAGAACGTAGGCGGTTTCTTAAGT
TTGGTCTTAAAGACCGGGGCTTAACTCCGGGAATGGATCGAAAACTGGGAGACTGGAGGA
CGTCAGAGGAAAGTGGAATTCCGAGTGTAGCGGTGAAATGCGTAGATATTCGGAGGAACA
CCAGTGGCGAAAGCGACTTTCTGGGACGTTCCTGACGCTGAGGTTCGAAAGCTCGGGGAG
CAAACAGGATTAGAGACCCCAGTAGTCC
>Otu6929
CCAGCCTATGGGGGGCAGCAGTAGGGAATCTTCCGCAATGGGCGAAAGCCTGACGGAGCA
ATGCCGCGTGAACGATGAAGGCCTTCGGGTCGTAAAGTTCTGTTGTGGGGGAAGAAATCC
TATACGGATAATACCCGTGTAGGTTTGACGGTACTCCACGAGGAAGCCCCGGCTAATTAC
GTGCCAGCAGCCGCGGTAATACGTAAGGGGCAAGCGTTGTCCGGAATCACTGGGCGTAAA
GCGCACGTAGGCGGTTTAGCGAGTTGGGGGTGTAAGCTCTAGGCTTAACCTAGAAATTGC
CTCCAAAACTACTTCACTTGAGTATTAGAGGGGAAGCTGGAATTCACGGTGTAGCGGTGA
AATGCGTAGATATCGTGGAGAACACCCGTGGCGAAGGCGGCTTCTGGCTAATGACTGAC
GCTGAGGTGCGAAAGCTAGGGGAGCGAACGGGATTAGAAACCCCCGTAGTCC
>Otu6930
CCAGCCTACGGGTCGCAGCAGTGGGGAATATTGGACAATGGGGGCAACCCTGATCCAGCG
ATGCCGCGTGAGTGAAGAAGGCCCTCGGGTCGTAAAGCTCTTTAGGCCGGGACGAAGTGC
GGAAGTTTAAGAGATGTCCGTGTTGACGGTACCGGCAGAATAAGCACCGGCAAACTCTGT
GCCAGCAGCCGCGGTAATACAGAGGGTGCGAGCGTTAATCGGAATTACTGGGCGTAAAGG
GCGCGTAGGCGGTTGTGCGAGTGTGGTGTGAAAGCCCCGGGCTTAACCTGGGAAGCGCAT
CGCAAACGGCATGACTAGAGTAGATGAGAGGGTGGCGGAATTTCCGGTGTAGCGGTGAAA
TGCGTAGAGATCGGAAGGAACGTCAATGGCGAAGGCAGCCACCTGGCATCATACTGACGC
TGAGGCGCGAAAGCGTGGGGAGCGAACAGGATTAGAAACCCGAGTAGTCC
>Otu6934
CCAGCCTATGGGGTGCTCCAGTGGGGAATATTGCACAATGGACGAAAGTCTGATGCAGCG
ACGCCGCGTGGAGGATGAAGGTTTTCGGATTGTAAACTCCTGTTGAGTGGGAAGAAAGGC
GACGACCTAATACGTCGTTGAGATGACGGTACCACTATAGAAAGCACCGGCAAACTTCGT

GCCAGCAGCCACGGTAATACGAAGGGTGCAAGCGTTATTCGGAGTTACTGGGCGTAAAGA
GCGCGTAGGCGGTTTTTTAAGTCATTTGTTAAATCTCCCGGCTTAACCGGGAACATGCAC
GTGAAACTGGAAGACTAGAGTGTAAGAGAGAGAAGTGGAATTCTCGGAGTAGCGGTAAAA
TGCGTAGATCTCGAGAGGAACACCGGAGGCGAAGGCGGCTTCTTGGCTTACAACTGGCGC
TCAAGTGCGAAAGCGTGGGGAGCAAACAGGATTAGATACCCTTGTAGTCC
>Otu6937
CCAGCCTATGGGGGGCACCAGTGGGGAATATTGGACAATGGGGGCAACCCTGATCCAGCG
ATGCCGCGTGTGTGAAGAAGGCCTGCGGGTTGTAAAGCACTTTCGGTGGTGAGGAGGGTT
GAGTGGATGAATAAGCCACTTGACTGACGCTAGCCACAAAAGAAGCACCGGCTAACTCTG
TGCCAGCAGCCGCGGTAATACAGAGGGTGCGAGCGTTAATCGGAATTATTGGGCGTAAAG
GGCGCGTAGGTGGATAAATAAGTTAGATGTGAAAGCCCTGGGCTTAACCTAGGAACGGCA
TCTGATACTGCTTGTCTGGAGTGGAGTAGAGGGTAGCGGAATTTCCGGTGTAGCGGTGAA
ATGCGTAGATATCGGAAAGAACACCAGTGGCGAAGGCGGCTACCTGGGCTCACACTGACA
CTGAGGCGCGAAAGCGTGGGGAGCGAACAGGATTAGAGACCCGCGTAGTCC
>Otu6938
CCAGCCTACGGGTGGCAGCAGTAGGGAATTTTCCACAATGGACGAAAGTCCGATGGAGCA
ACGCCGCGTGCAGGATGAAGGCCTTAGGGTTGTAAACTGCTTTTATTTGTGACGAATATG
ACGGTAGCAAATGAATAAGGATCGGCTAACTCCGTGCCAGCAGCCGCGGTCATACGGAGG
ATCCAAGCGTTATCCGGAATTACTGGGCGTAAAGAGTTGCGTAGGTGGCATTGTAAGTCC
CGATATAAAGACAACGGCTCAACCGTTGGAATCATCGGGAAACTGCAAAGCTAGAGGACG
AGAGAGGTTATTGGAATTCCTAGTGTAGGAGTGAAATCCGTAGATATTAGGAGGAACACC
GATGGCGTAGGCAGATAACTGGCTCGTTCCTGACACTGAGGCACGAAAGCGTGGGTAGCA
AACGGGATTAGAAACCCGTGTAGTCC
>Otu6939
CCAGCCTACGGGAGGCAGCAGCCGAGAATATTCGACAATGGGCGAAAGCCTGATCGAGCG
ACGCCGCGTGCAGGATGAAGTACTTCGGTACGTAAACTGCTTTTGCGGACTAAGAAGTTT
ATTGACTAGTCCGAGAATAAGAGGTTGCTAAACTCGTGCCAGCAGCAGCGGTAATACGAG
TGCCTCGAGCGTTATCCGGAATCATTGGGCGTAAAGGGTGTGTAGGCGGTCGTGTTAGTC
TCTCGTTAAATTCTTCGGCTCAACCGGGGGCTTGCGGGGGAAACGGCACGACTAGAGGGT
GCGAGAGGTGAATGGAACTCATGGTGTAGGGGTGAAATCCGTTGATATCATGGGGAACAC
CAAAAGCGAAGGCAATTCACTGGCGCATTCCTGACGCTCAAACACGAAAGCGTGGGTAGC
GAATGGGATTAGATACCCTGGTAGTCC
>Otu6945
CCAGCCTATGGGTCGCAGCAGTAGGGAATATTGGTCAATGGGCGCAAGCCTGAACCAGCC
ATGCCGCGTGCAGGATGAAGGCCCTACGGGTCGTAAACTGCTTTTGTACGGGAAGAAAAC
TCTCTACGTGTAGAGGGCTGACGGTACCGTGAGAATAAGGATCGGCTAACTTCGTGCCAG
CAGCCGCGGTAATACGAAGGATCCTAGCGTTGTCCGGATTTACTGGGTTTAAAGGGTGCG
TAGGCGGACTATTAAGTCAGTGGTGAAAGCCGGTAGCTTAACTATCGAATTGCCATTGAA
ACTGATAGTCTCGAGTATAGTTGAGGTAGTTGGAATGTATCATGTAGCGGTGAAATGCTT
AGATATGATACAGAACACCGATTGCGAAGGCAGATTACTAAACTATAACTGACGCTGAGG
CACGAAAGTGTGGGGATCAAACAGGATTAGAGACCCGCGTAGTCC
>Otu6947
CCAGCCTACGGGTCGCTGCAGTCGAGAATTTTTCACAATGGGGGAAACCCTGATGGAGCG
ACGCCGCGTGGGGGATGAATGGCTTCGGCCCGTAAACCCCTGTCATTTGCGAACAAACTT
TTTCACCTAACACGTGGAGAATTGATTGTAGCGGAAGAGGAAGGGACGGCTAACTCTGTG
CCAGCAGCCGCGGTAATACAGAGGTCCCAAGCGTTGTTCGGATTCACTGGGCGTAAAGGG
TGCGTAGGTGGCCGGGTAAGTCTAATGTGAAAGCTCGGAGCTCAACTCCGAAACGGCATT
GGATACTATCCGGCTGGAGGGTCGGAGGGGGGACTGGAATTCTCGGTGTAGCAGTGAAAT
GCGTAGATATCAAGTGGAACATCAGTGGCGAAGGCGAGTCTCTGGACAATTCCTGACACT
GAGGCACGAAAGCTAGGGGAGCAAACGGGATTAGAAACCCCAGTAGTCC
>Otu6949
CCAGCCTACGGGACGCTGCAGCCGAGAATATTCGACAATGGACGAAAGTCTGATCGAGCG
ACGCCGCGTGCAGGATGAAGTCCTTCGGGATGTAAACTGCTCTTGCGAGAGAGAAAGTTT
ATTGATCATCTCGCGAATAAGAGGTTGCTAAACTCGTGCCAGCAGCAGCGGTAATACGAG
TGCCTCAAGCGTTATCCGGGATTATTGGGCGTAAAGGGTGTGTAGGTGGTTATGTTAGTC
CTTCGTAAAATTCTCTGGCTCAACCAGAGTTCGCGCGGGATACGGCATGACTTGAGGGTG
TGAGAGGTCTGTGGAACTCATAGTGTAGCGGTGAAATGCGTTGATATTATGGGGAACACC
AAAAGCGAAGGCAACAGACTGGAACATTCCTGACACTGAGACACGAAAGCGTAGGTAGCG

AATGGGATTAGAAACCCGCGTAGTCC
>Otu6950
CCAGCCTACGGGACGCAGCAGTCGAGAATATTTCGCAATGGGCGCAAGCCTGACGAAGCG
ACGCCGTGTGAGTGAAGAAGGCCTTCGGGTCGTAAAGCTCTTTCGCCTGGGAACAAGAGA
AATCGGATAATATCCGACGTATTTGAGGGTACTAGGTAAAGAAGCACCGGCTAACTCCGT
GCCAGCAGCTGCGGTAATACGGAGGGTGCAAGCATTAATCGGATTTATTGGGCGTAAAGG
GGGCGTAGGCGGAAATGAAAGCCAGATGTGAAATCCCGGGGCTCAACCCAGGAACAGCAT
TTGAAACTCCTTTTCTAGAGGATAGGCGGAGAAAACGGAATTCCACATGTAGCGGTGAAA
TGCGTAGATATGTGGAAGAACACCGGTGGCGAAGGCGGTTTTCTAGCTTATTCCTGACGC
TGAGGCCCGAAAGCTAGGGGAGCAAACAGGATTAGAAACCCCTGTAGTCC
>Otu6954
CCAGCCTACGGGGTGCTGCAGTCGAGAATCATTCGCAATGGGCGAAAGCCTGACGATGCG
ACGCCGTGTGAGCGAAGAAGGCCTTAGGGTCGTAAAGCTCTTTCGCCTGGGAACAAGAGA
AGTTGACTAATAATCAGCTAATTTGAGAGTACTAGGTAAAGAAGCACCGGCTAACTCCGT
GCCAGCAGCTGCGGTAATACGGAGGGTGCAAGCATTAATCGGATTTATTGGGCGTAAAGG
GCGTGTAGGCGGGGATAATAGTCAGATGTGAAACCCTGGGGCTCAACCTCAGAACTGCAT
TTGAAACTGTATCTCTAGAGGATAGGCGGAGAAAACGGAATTCCACAAGTAGCGGTGAAA
TGCGTAGATATGTGGAAGAACACCGGTGGCGAAGGCGGTTTTCTAGCTTATTCCTGACGC
TGAGGCGCGAAAGCAAGGGGATTAAACAGGATTAGAACCCCCGTAGTCC
>Otu6955
CCAGCCTACGGGGCGCAGCAGTGGGGAATATTGCACAATGGGCGGAAGCCTGATGCAGCG
ACGCCGCGTGAGGGATGAAGGCCTTCGGGTTGTAAACCTCTTTCAGCACCGACGAATTTG
ACGGTAGGTGCAGAAGAAGCGCCGGCTAACTACGTGCCAGCAGCCGCGGTAATACGTAGG
GCGCAAGCGTTGTCCGGATTTATTGGGCGTAAAGAGCTCGTAGGCGGCTTGTCGCGTCGG
ACGTGAAAGCTCACAGCTTAACTGTGGGTCTGCGTCCGATACGGGCAGGCTAGAGGCAGG
TAGGGGAGAACGGAATTCCCGGTGTAGCGGTGAAATGCGCAGATATCGGGAGGAACACCG
GTGGCGAAGGCGGTTCTCTGGGCCTGTACTGACGCTGAGGAGCGAAAGCGTGGGGAGCGA
ACAGGATTAGATACCCCCGTAGTCC
>Otu6958
CCAGCCTACGGGTCGCAGCAGCCGAGAATATTCGACAATGGGCGAGAGCCTGATCGAGCG
ATACCGCGTGGTGGATGAAGCGCTTCGGCGCGTAAACATCTTTTATGAGGGAGGAAGTTA
TTGACGTTACCTCATGAATAAGGGGCTCCTAACTCTGTGCCAGCAGGAGCGGTAATACAG
AGGCCCCGAGCGTTACCCGGAATTACTGGGCGTAAAGAGTGCGTAGGTGGTCGTGTTAGT
CGTTTGTTAAAGCTCCCGGCTCAACCGGGAACATGTGGGCGAAACGGCACGACTTGAGGG
CGTGAGAGGTGCATAGAACTCTAGGTGTAGGGGTGAAATCCGTTGATATCTAGGGGAATA
CCGAAAGCGAGGGCAGTGCACTGGCGCGTTCCTGACACTCAAGCACGAAAGCGTGGGTAG
CGAACGGGATTAGATACCCTAGTAGTCC
>Otu6962
CCAGCCTACGGGGGGCTCCAGTCGAGGATCTTCGGCAATGGGCGCAAGCCTGACCGAGCG
ACGCCGCGTGTGCGATGAAGGCCTTCGGGTTGTAAAGCACTGTCGAGGGGGAGGAAAGCC
GCAAGGTCTGACCTATCCCTGGAGGAAGCACGGGCTAAGTTCGTGCCAGCAGCCGCGGTA
AGACGAACCGTGCGAACGTTGTTCGGAATCACTGGGCTTAAAGGGCGCGTAGGCGGTCGA
CCAAGTCTGGGGTGAAATCTTTCGGCTTAACCGGAAAAGTGCCTTGGATACTGGTCGGCT
GGAGGGAGGTAGGGGCAGCTGGAACTTCCAGTGGAGCGGCGAAATGCGTTGATATTGGAA
GGAACGCCGGAGGCGAAAGCGGGCTGCTGGACCTTCTCTGACGCTGAGGCGCGAAAGCTA
GGGTAGCAAAAAGGATTAGATACCCTGGTAGTCC
>Otu6963
CCAGCCTATGGGATGCTGCAGTGGGGAATATTGGGCAATGGGCGCAAGCCTGACCCAGCC
ACGCCGCGTGAGTGATGAAGGCCTTCGGGTCGTAAAGCTCTGTGGGGAGGGACGAATAAG
CACCGTGCTAACATCACGGGGCCCTGACGGTACCTCCTTAGCAAGCACCGGCTAACCATG
TGCCAGCAGCCGCGGTAATACATGGGGTGCAAACGTTGCTCGGAATTATTGGGCGTAAAG
CGCACGTAGGTGGTTTGTTATGTCGGATGTGAAAGCCCTCGGCTCAACTGAGGAAGTGCA
TCCGAGACTGGCAAGCTAGAGTATGTAAGAGGGTCGCGGAATTCCCGGTGTAGAGGTGAA
ATTCGTAGATATCGGGAGGAACACCGGTGGCGAAGGCGGCGACCTGGGACAACACTGACA
CTGAGGTGCGAAAGCGTGGGGAGCAAACAGGATTAGAAACCCGAGTAGTCC
>Otu6969
CCAGCCTATGGGGTGCAGCAGTCGAGAATATTCGACAATGGGCGAAAGCCTGATCGAGCG
ACACCGCGTGCAGGATGAAGGCCTTCGGGTCGTAAACTGCGGTAATAAAGTAACAATGCA

AATGAGTGCTTTATGGAAAGAGGCGGGTAACTACGTGCCAGCACCAGCGGTAATACGTAG
GCCTCAAGCGTTATCCGGATTTATTGGGCGTAAAGGGCATGTAGGAGGTTTTGCGCGTCT
TTTGTTAAAGCCCGGGGCCTAACCCCGGAGATGCAGGAGATACGGCATGACTAGAGGGGG
TTAGAGGTGCATGGAACTCACGGTGTAGGGGTGAAATCCGTTGATATCGTGGGGAACACC
AAAGGCGAAGGCAGTGCACTGGGGCCCTCCTGACTCTGATATGCGAAAGCGTGGGGAGCA
AAAAGGATTAGATACCCCCGTAGTCC
>Otu6970
CCAGCCTATGGGGGGCAGCAGTGGGGAATTTTGGACAATGGGGGCAACCCTGATCCAGCC
ATGCCGCGTGAGTGAAGAAGGCCTTCGGGTTGTAAAGCTCTTTCGGTAGGAACGAAACGG
TTACGGTTAATACCCGTGACTACTGACGGTACTTACAGAAGAAGCACCGGCTAACTACGT
GCCAGCAGCCGCGGTAATACGTAGGGTGCGAGCGTTAATCGGAATTACTGGGCGTAAAGC
GTGCGCAGGCGGTCTCGTAAGCTGGGTGTGAAAGCCCCGGGCTTAACCTGGGAATGGCAT
TCAGGACTGCGAGGCTCGAGTGTGGCAGAGGGAGGTGGAATTCCACGTGTAGCAGTGAAA
TGCGTAGAGATGTGGAGGAACACCGATGGCGAAGGCAGCCTCCTGGGCCAGCACTGACGC
TCATGCACGAAAGCGTGGGGAGCAAACAGGATTAGATACCCCGGTAGTCC
>Otu6971
CCAGCCTATGGGAGGCACCAGTAAGGAATATTGCTCAATGGGCGCAAGCCTGAAGCAGCA
ACGCCGCGTGCACGATGAAGGCCTTCGGGTCGTAAAGTGCTTTTACGGGAGATGAGGAAG
GACAGTATCCCTGGAATAAGGTACGGCTAACTACGTGCCAGCAGCCGCGGTAAAACGTAG
GTACCGAGCGTTATCCGGATTCACTGGGCGTAAAGCGCGTGCAGGTGGTTTGGTAAGTCG
GGCATGAAAACTCTTGGCTCAACTGAGAGACGCTGTCCGATACTGTCGAACTTGAGGGTG
TGAGAGGGAGGTGGAATTCCGCATGTAGTGGTGAAATGCGTAGATATGCGGAGGAACACC
AGTGGCGAAGGCGGCCTCCTGGCACACTCCTGACACTCATACGCGAAAGCTAGGGGAGCG
AACGGGATTAGAGACCCTCGTAGTCC
>Otu6972
CCAGCCTACGGGTTGCTGCAGTGGGGAATATTGGACAATGGGGGCAACCCTGATCCAGCC
ATGCCGCGTGAATGATGAAGGCCTTCGGGTTGTAAAGTTCTTTTGACGGGGACGATGATG
ACGGTACCCGCAGAATAAGCCCCGGCTAACTTCGTGCCAGCAGCCGCGGTAAGACGAAGG
GGGCTAGCGTTGTTCGGAATTACTGGGCGTAAAGCGCGTGTAGGCGGTTATCCAAGTCGG
GTGTGAAAGCCTTGAGCTTAACTCAAGAAATGCACTCGGTACTGGGTGACTAGAGGACCG
GAGAGGATAGTAGAATTCCCAGTGTAGTGGTGAAATACGTAGAGATTGGGAAGAACACCA
GTGGCGAAGGCGGCTATCTGGACGGTTTCTGACGCTAAGACGCGAAAGCGTGGGGAGCAA
ACAGGATTAGAAACCCTAGTAGTCC
>Otu6974
CCAGCCTATGGGGGGCTCCAGCCGAGAATATTCGACAATGGGCGAAAGCCTGATCGAGCG
ATACCGCGTGGTGGATGAAGTGCTTCGGCACGTAAACATCTTTTATGAGGGAGGAAGTTA
TTGACGTTACCTCATGAATAAGGGGCTCCTAACTCTGTGCCAGCAGGAGCGGTAATACAG
AGGCCCCGAGCGTTACCCGGAATTACTGGGCGTAAAGAGTGCGTAGGTGGTTGTATTAGT
CGTCTGTCAAATCTCCCGGCTCAACCGGGAAAACGCGGGCGAAACGGTACAACTCGAGGG
AGTGAGAGGTGAATGGAACTCATGGTGTAGGGGTGAAATCCGTTGATATCATGGGGAACA
CCGAAAGCGAAGGCAATTCACTGGCACTTTCCTGACACTCAAGTACGAAAGCGTGGGTAG
CGAACGGGATTAGAGACCCCCGTAGTCC
>Otu6976
CCAGCCTACGGGGGGCAGCAGTGGGGAATATTGGACAATGGGGGAAACCCTGATCCAGCA
ATGCCACGTGTGTGAAGAAGGTCTTCGGATTGTAAAGCTCTTTCACCCACGACGATAATG
ACGGTAGTGGGAGAAGAAGCCCCGGCTAACTTCGTGCCAGCAGCCGCGGTAATACGAAGG
GGGCTAGCGTTGTTCGGAATTACTGGGCGTAAAGCGCGTGTAGGCGGGTCCTTAAGTCAG
GGGTGAAATGCCAAGGCTCAACCTTGGAACTGCCTTTGATACTGGGGATCTTGAGTCCGG
GAGAGGTGAGTGGAACTGCGAGTGTAGAGGTGAAATTCGTAGATATTCGCAAGAACACCA
GTGGCGAAGGCGGCTCACTGGCCCGGAACTGACGCTGAGACGCGAAAGCGTGGGGAGCAA
ACAGGATTAGATACCCCTGTAGTCC
>Otu6984
CCAGCCTACGGGTCGCAGCAGTGGGGAATATTGGACAATGGGCGCAAGCCTGATCCAGCC
ATGCCGCGTGAGTGACGAAGGCCTTAGGGTTGTAAAGCTCTTTTGGCGGGGAAGATAATG
ACGGTACCCGCAGAATAAGCTCCGGCTAACTTCGTGCCAGCAGCCGCGGTAATACGAAGG
GAGCTAGCGTTGTTCGGAATCACTGGGCGTAGAGCGCACGTAGGCGGATGTGTCAGTCAG
GGGTGAAATCCCGGAGCTCAACTTCGGAACTGCCTTTGATACAGCACGTCTTGAGTCCGA
TAGAGGTGGGTGGAATTCCTAGTGTAGAGGTGAAATTCGTAGATATTAGGAAGAACACCG

GTGGCGAAGGCGGCCCACTGGATCGGTACTGACGCTGAGGTGCGAAAGCGTGGGGAGCAA
ACAGGATTAGAAACCCCTGTAGTCC
>Otu6988
CCAGCCTATGGGACGCTGCAGTGGGGAATCTTGCGCAATGGGCGAAAGCCTGACGCAGCA
ACGCCGCGTGAATGATGAAGGCCTTCGGGTTGTAAAGTTCTTTTGGCGGGGACGATGATG
ACGGTACCCGCAGAATAAGCTCCGGCTAACTTCGTGCCAGCAGCCGCGGTAATACGAAGG
GAGCTAGCGTTGTTCGGAATTACTGGGCGTAAAGGGCGCGTAGGCGGTTTGACAAGTTGG
ATGTGAAAGCCCAGGGCTCAACCCTGGAATTGCATTCAAGACTGTCTTACTTGAATTCGG
TAGAGGTTGGTGGAATTCCCAGTGTAGAGGTGAAATTCGTAGAGATTGGGAAGAACACCC
GTGGCGAAGGCGGCCAACTGGACCGACATTGACGCTGAGGCGCGAAAGCGTGGGTAGCAA
ACAGGATTAGAGACCCCAGTAGTCC
>Otu6989
CCAGCCTATGGGATGCAGCAGTCGAGAATTTTTCTCAATGGGCGAAAGCCTGAAGGAGCG
ACGCCGCGTGGGGGATGAAGGGCTTCGGCTCGTAAACCCCTGTCATTTGCGAACAAACCC
CGCTTTTTAATAGAGGGCGGGCTGATTGTAGCGAAAGAGGAAGGGACGGCTAACTCTGTG
CCAGCAGCCGCGGTAATACAGAGGTCCCAAGCGTTGTTCGGATTCACTGGGCGTAAAGGG
TGCGCAGGTGGCTGGGTAAGTTTGACGTGAAATCTCCGGGCTCAACCCGGAAAATGCGTT
GAATACTATCCGGCTGGAGGGTTGGAGGGGAGACTGGAATTCTCGGTGTAGCAGTGAAGT
GCGTAGATATCGAGAGGAACACCAGTGGCGAAGGCGAGTCTCTGGACAACTCCTGACACT
GAGGCACGAAGGCTAGGGGAGCAAACAGGATTAGATACCCCAGTAGTCC
>Otu6990
CCAGCCTATGGGGTGCTGCAGTGGGGAATCTTGCGCAATGGACGAAAGTCTGACGCAGCC
ACGCCGCGTGAGTGAAGAAGGCCTTCGGGTTGTAAAGCTCTGTCGGGCGGGACGAAAACT
TTTAGGGTTAACAGCCCTAGAAATTGACGGTACCGCCAAAGGAAGCACCGGCTAACTCTG
TGCCAGCAGCCGCGGTAATACAGAGGGTGCAAGCGTTGCTCGGAATCATTGGGCGTAAAG
GGCAAGTAGGTGGTCTCGTTTGTCTGAGGTGAAATCCCTAGGCTTAACCTGGGAACTGCC
TCAGAAACGGCGAGACTGGAGTACTGGAGAGGGTCGCGGAATTCCCGGTGTAGCGGTGAA
ATGCGTAGAGATCGGGAGGAATACCAGAGGCGAAAGCGGCGGCCTGGACAGTTACTGACA
CTCAACTGCGAAAGCGTGGGGAGCAAACAGGATTAGAAACCCCAGTAGTCC
>Otu6993
CCAGCCTACGGGTCGCTGCAGTAAGGAATATTGGTCAATGGGCGCAAGCCTGAACCAGCC
ATGCCGCGTGAAGGATGACTGCCCTATGGGTTGTAAACTTCTTTTGTACGGGAAAAAACC
CCTGCTCGTGTAGCGGGGCTGATGGTACCGTAAGAATAAGGATCGGCTAACTCCGTGCCA
GCAGCCGCGGTAATACGGAGGTTCCAAGCGTTATCCGGATTTATTGGGTTTAAAGGGTGC
GTAGGCGGATTATTAAGTCAGTGGTGAAAGCCTACAGCTTAACTGTAGAACTGCCATTGA
TACTGATGATCTTGAGTATAGTTGAGGTGGGCGGAATGTGTCATGTAGCGGTGAAATGCT
TAGATATGACACAGAACACCGATTGCGAAGGCAGCTCGCTAAGCTATAACTGACGCTGAG
GCACGAAAGCGTGGGGATCAAACAGGATTAGAAACCCTGTAGTCC
>Otu6994
CCAGCCTACGGGTGGCACCAGTGGGGAATCTTGCGCAATGGGCGAAAGCCTGACGCAGCA
ACGCCGCGTGAGGGACGAAGGTCTTCGGGTTGTAAACCTCTTTCAGCAGGGACGATTGTG
ACGGTACCTGCAGAAGAAGCCCCGGCCAACTACGTGCCAGCAGCCGCGGTAATACGTAGG
GGGCGAGCGTTGTCCGGATTCATTGGGCGTAAAGAGCTCGTAGGCGGCTTGGTTAGTTGG
GTGTGAAACCTCCAGGCTTAACCTGGAGACGCCACTCAATACTGCCATGGCTAGAGTCCG
GTAGGAGACCACGGAATCCCTGGTGTAGCGGTGAAATGCGCAGATATCAGGAGGAACACC
GGTGGCGAAGGCGGTGGTCTGGGCCGGCACTGACGCTGAGGAGCGAAGGCCAGGGGAGCA
AACGGGATTAGATACCCCCGTAGTCC
>Otu6998
CCAGCCTACGGGGTGCTGCAGTCGAGAATCTTCCGCAATGGGCGCAAGCCTGACGGAGCG
ACACCGCGTGCAGGATGAAGGCCTTCGGGTTGTAAACTGCTGTCAGGTTCTAGGAACCGC
TTGGTGTTAACAGCGCCAAGTCTGACCAAGACCAAAGGAAGCAACGGCTAACTCTGTGCC
AGCAGCCGCGGTAAGACAGAGGTTGCAAACGTTGTTCGGAATTACTGGGCATAAAGCGCA
CGTAGGCGGTCCGCTAAGTCAGGTGTGAAATCCCCCGGCTCAACTGGGGAATTGCGCCTG
ATACTGGCGGGCTTGAGATCGATAGGGGTGAGCGGAACTCCAGGTGGAGCGGTGAAATGC
GTAGATATCTGGAGGAACACCGGTGGCGAAAGCGGCTCACTGGATCGACACTGACGCTGA
GGTGCGAAAGCTAGGGTAGCAAACGGGATTAGAAACCCGAGTAGTCC
>Otu7002
CCAGCCTACGGGGGGCACCAGTAAGGAATATTGGTCAATGGACGCAAGTCTGAACCAGCC

ATGCCGCGTGAAGGATGAAGGCGCTATGCGTCGTAAACTTCTTTTATACGGGAGAAAACC
CCCCGAGTTCTCGGGGGCTGATAGTACTGTAAGAATAAGGGTCGGCTAACTTCGTGCCAG
CAGCCGCGGTAATACGAAGGACCCGAGCGTTATCCGGATTCACTGGGTTTAAAGGGTGCG
TAGGCGGGTAAGTAAGTCCGTGGTGAAATCTCTGAGCTTAACTCAGAAACTGCCGTGGAT
ACTATTTGCCTTGAATATTGTGGAGGTGAGCGGAATATGTCATGTAGCGGTGAAATGCTT
AGATATGACATAGAACACCAATTGCGAAGGCAGCTCACTACACAATCATTGACGCTGAGG
CACGAAAGCGTGGGGATCAAACAGGATTAGAAACCCGTGTAGTCC
>Otu7003
CCAGCCTATGGGTTGCTGCAGTCGAGAATGTTCCACAATGGACGAAAGTCTGATGGAGTG
ACGCCGCGTGATTGATGAAGTGGTTCGCCACGTAAAGATCTTTTATGAGGGAAGAAGTAT
ATTGACGGTACCTCATGAATAAGGGGCTCCTAACTCTGTGCCAGCAGGAGCGGTAATACA
GAGGCCCCAAGCGTTATCCGGAATTATTGGGCGTAAAGGGTGTGTAGGTGGCGTTGTTAG
TCGTTTGTTAAAGCCCGAGGCTCAACCTCGGAAGTGCGAACGAAACGGCAATGCTTGAGT
ATGTGAGAGGTAAGCGGAACTCATGGTGTAGGGGTGAAATCCGTTGATATCATGGGGAAC
ACCAAAAGCGAAGGCAGCTTACTGGCACATTACTGACACTGAAACACGAAAGCGTGGGTG
GCGAATGGGATTAGAAACCCCCGTAGTCC
>Otu7004
CCAGCCTATGGGGGGCTGCAGTCAAGAATATTCCTCAATGGCCGAAAGGCTGAAGGAGCG
ACGCCGCGTGTAGGATGAAGGTCTTCGGATTGTAAACTACTTTTATCAGGGAAGAATTTG
TGACGGTACCTGATGAATAAGAGGTTGCTAACTCTGTGCCAGCAGCAGCGGTAATACAGA
GACCTCAAGCGTTATCCGGATTTATTGGGCGTAAAGCGTCCGCAGGTGGTTTAGCCAGTC
AGGGGTCAAAACTTTCCGCTTAACGGAAAGGCTGCTTCTGATACTACTAAACTCGAGTAT
GGAAGAGGTTAGCGGAATTCTCGGTGTAGTCGTAATAAGCGTTGATATCGAGAAGAACAC
CAAATGCGAAGGCAGCTAACTGGTACATTACTGACACTCATGGACGAAAGCGTGGGGAGC
AAACAGGATTAGAAACCCGTGTAGTCC
>Otu7006
CCCAGCCTACGGGATGCTCCAGTGAGGAATATTGCTCAATGGGCGAAAGCCTGAAGCAGC
AACGCCGCGTGTGGGATGAAGGCCCTCTGGGTTGTAAACCACTGTAGAAAGGGACAAAGG
TCGCGCTCTGCGCGACTGGATGGTACCTTTAAAGTAAGCCTCGGCTAACTACGTGCCAGC
AGCCGCGGTAATACGTAGGAGGCAAGCGTTGTCCGGAATCACTGGGTGTAAAGGGTGCGC
TGGCGGGGAAGTAAGTCAATGGTGAAATCTCACGGCTTAACCGTGAAACTGCCACTGATA
CTGCTTTTCTTGAGTGCGGGAGAGGGTGGTGGAATTCTTGGTGTAGTGGTGAAATGCGTA
GATATCAAGAAGAACACCAGTGGCGAAGGCGGCCACCTGGCCCGTAACTGACGCTCATGC
ACGAAAGCGTGGGGATCAAACAGGATTAGAACCCCGGTAGTCC
>Otu7007
CCAGCCTATGGGGGGCTGCAGTGGGGAATCTTGCACAATGGGGGAAACCCTGATGCAGCG
ACGCCGCGTGAGTGATGAAGCCCCTCGGGGTGTAAAGCTCTTTCGACGGGAGAGATAATG
ACGGTACCCGAAGAAGAAGCTGCGGCTAACTACGTGCCAGCAGCCGCGGTAATACGTAGG
CAGCAAGCGTTGTTCGGAATTACTGGGCGTAAAGAGTGTGTAGGCGGTGCTCTAAGTCTG
TCGTGAAATCTCCCGGCTTAACTGGGAGGGTGCGGCGGAAACTAGTGTGCTAGAGGGTGG
GAGAGGTAAGCGGAACTCCTGGTGTAGCGGTGAAATGCGTAGATATCAGGAGGAACACCT
GTGGTGTAGACAGCTTACTGGACCACTTCTGACGCTGAGACACGAAAGCGTGGGTAGCAA
ACAGGATTAGAAACCCGAGTAGTCC
>Otu7009
CCAGCCTACGGGTGGCAGCAGTCGAGAATCATTCGCAATGGGCGCAAGCCTGACGATGCG
ACGCCGTGTGTGCGATGAAGGCCTTCGGGTTGTAAAGCACTTTCGCTTGGGAACAAGAGA
GGTTGGCTAATATCCAGCCGATTTGAGGGTACCAGGTAAAGAAGCACCGGCTAACTCCGC
GCCAGCAGCTGCGGTAATACGGAGGGTGCAAGCATTAATCGGATTTATTGGGCGTAAAGG
GCGCGTAGGCGGGCCTGTAAGTCAGATGTGAAATCCCGGAGCTCAACTCCGGAACAGCAT
TTGAAACTGTGGGTCTAGAGGATAGGCGGGGAAAATGGAATTCCACAAGTAGCGGTGAAA
TACGTAGATATGTGGAAGAACACCGGTGGCGAAGGCGATTTTCTAGCTTATACCTGACGC
TGAGGCGCGAAAGTAAGGGGATCAAACAGGATTAGATACCCTTGTAGTCC
>Otu7012
CCAGCCTATGGGTGGCACCAGTTTCGAATCATTCACAATGGGCGAAAGCCTGATGGTGCG
ACGCCGCGTGGGGGATGAAGGTCTTCGGATCGTAAACTCCTGTCATGAGGGAACAAACCC
GCGCGTTTTACCGATGCGCGGCTGATAGTACCTCAAGAGGAAGAGACGGCTAACTCTGTG
CCAGCAGCCGCGGTAATACAGAGGTCTCAAGCGTTGTTCGGATTCATTGGGCGTAAAGGG
TGCGTAGGCGGCGAAGCAAGTCAGATGTGAAATCCCGGGGCTCAACCCCGGAACTGCATT

TGATACTGCTTTGCTCGAGGACTGGAGAGGAGATCGGAATTCACGGTGTAGCAGTGAAAT
GCGTAGATATCGTGAGGAAGGCCAGTGGCGAAGGCGGATCTCTGGACAGTTCCTGACGCT
GAGGCACGAAGGCCAGGGGAGCAAACGGGATTAGAGACCCTAGTAGTCC
>Otu7018
CCAGCCTATGGGGTGCAGCAGTGGGGAATTTTGTGCAATGGGCGAAAGCCTGACACAGCG
ACACCGCGTGAGTGACGAAGCCCTTTGGGGTGTAAAACTCTGTCGGCTGGAACGAAAAAA
ATGACGGTACCAGCAAAGGAAGCATCGGCTGACTACGTGCCAGCAGCCGCGGTAAGACGT
AGGATGCGAGCGTTGTCCGGATTCATTGGGCGTAAAGGGTTCGTAGGCGGTTTGCCAAGT
CTGATGTTAAAGATCGGGGCTCAACCCCGGGAATGCATTGGAAACTGGCAGACTCGAGTA
CGGTAGAGGTAAGTGGAATTTCCAGTGTAGCGGTGAAATGCGTAGATATTGGAAAGAACA
CCAGTGGCGTAAGCGACTTACTGGGCCGTAACTGACGCTGAGGAACGAAAGCCAGGGTAG
CGAATGGGATTAGAAACCCTCGTAGTCC
>Otu7024
CCAGCCTACGGGAGGCACCAGTGGGGAATCTTGCGCAATGGGCGAAAGCCTGACGCAGCA
ACGCCGCGTGGGTGATGAAGGATTTCGGTCTGTAAAACCCTGTCGTCGGGGACGAAGGTG
GGCGTGTTAATAACGCGTTTACTTGACGGTACCCGGAGAGGAAGCCCCGGCTAACTCTGT
GCCAGCAGCCGCGGTAATACAGAGGGGGCAAGCGTTATTCGGAATTATTGGGCGTAAAGG
GCGCGTAGGCGGCTTTTTAAGTCGGATGTGTAATCCCCGAGCTTAACTTGGGAACTGCAT
CCGAGACTGGGAGGCTAGAGTGCTGGAGAGGATGGTAGAATTCCACGTGTAGCGGTGAAA
TGCGTAGAGATGTGGAGGAATACCGGTGGCGAAGGCGGCCATCTGGACAGCAACTGACGC
TGAGGCGCGAAAGTGTGGGTAGCAAACAGGATTAGAAACCCCTGTAGTCC
>Otu7025
CCAGCCTATGGGATGCACCAGTGGGGAATCTTGCGCAATGGGCGAAAGCCTGACGCAGCA
ACGCCGCGTGCGGGATGAAGGCCTTCGGGTTGTAAACCGCTTTCAGCAGGGACGAAAATG
ACGGTACCTGCAGAAGAAGCCCCGGCCAACTACGTGCCAGCAGCCGCGGTAACACGTAGG
GGGCAAGCGTTGTCCGGATTTATTGGGCGTAAAGAGCTCGTAGGCGGCTCGGTAAGTCGG
GTGTGAAACCTCCAGGCTCAACCTGGAGACGCCACCCGATACTGCTGTGGCTAGAGTCCG
GTAGGGGAGCGTGGAATTCCTGGTGTAGCGGTGAAATGCGCAGATATCAGGAGGAACACC
AGCGGCGAAGGCGGCGCTCTGGGCCGGAACTGACGCTGAGGAGCGAAAGCGTGGGGAGCA
AACAGGATTAGAAACCCCAGTAGTCC
>Otu7031
CCAGCCTACGGGGCGCAGCAGTCGAGAATCTTCCCCAATGGGCGAAAGCCTGAGGGAGCG
ACGCCGCGTGCGGGATGAAGGGCTTCGGCCTGTAAACCGCTTTTATTCGGGAAGAAGCGC
AAGCTGACGGTACCGAATGAATAAGAGGTTGCTAACTCTGTGCCAGCAGCAGCGGTAATA
CAGAGACCTCAAGCGTTATCCGGATTTATTGGGCGTAAAGCGTCCGCAGGTGGTTCGTTA
AGTCATTGGTCAAATCCCGGGGCTCAACCCCGGAACTGCCAATGATACTGGCGAACTTGA
GACCGGTAGAGGCAAGCGGAATTACAGGTGTAGCGGTAAAATGCGTTTATATCTGTAAGA
ACACCAAAGGCGAAAGCAGCTTGCTGGAACGGTTCTGACACTCATGGACGAAAGCGTGGG
GAGCGAAAAGGATTAGAAACCCGCGTAGTCC
>Otu7035
CCAGCCTATGGGGCGCAGCAGTGGGGAATATTGGACAATGGGCGAAAGCCTGATCCAGCA
ATGCCGCGTGTGTGAAGAAGGCCTTCGGGTTGTAAAGCACTTTAGGCAGGGAAGAAAAAA
TCGGCGTGAATATCGCCGGTCTTGACGGTACCTGAGGAATAAGCACCGGCTAACTCTGTG
CCAGCAGCCGCGGTAATACAGAGGGTGCAAGCGTTAATCGGATTTACTGGGCGTAAAGCG
TGTGTAGGTGGTCAAGAAAGTCGGTTGTGAAATCCCTGGGCTCAACCCGGGAACTGCTTC
CGATACTTCATGACTCGAATTCGGTAGAGGGAGGCGGAACTCCAGGTGTAGCGGTGAAAT
GCGTAGATATCTGGAAGAACACCAATGGCGAAGGCAACCTCCTGGGCCTGAATTGACACT
GAGACACGAAAGCGTGGGGATCAAACAGGATTAGAAACCCTCGTAGTCC
>Otu7036
CCAGCCTATGGGATGCTGCAGTGGGGAATATTGGACAATGAGCGAAAGCTTGATCCAACT
ATATCACATGAAAGAAGAAGGATTTCGGTCTGTAAATTTCGATAGCTAAAGAACATAATG
ATATTATTTAGCGAATTAGCCCTGGCTAACTTCGTGCCAGCAGCTGCGGTAATACGGAGG
GGGCGAGTGTTACTCATAATGATTGGGCGTAAAGGGTATGTAGGCGGTTTATTAAGTTCT
TGATTAAAACCCTTGAGGAATAAAGGAAATGATAGAATACTGATAAGCTTGAGTTTAGTG
GGGGAGAGCAGTACTGTAAGTGTAGGGGTAAAATCCGGTAAAATATTTATAGGACTATCA
ATGGCGAAAGCAGTAATCTGAATTAAACTGACGCTGAGGTACGAAAGCATAGGGATCAAA
AAGGATTAGAGACCCTCGTAGTCC
>Otu7040

CCAGCCTATGGGGCGCTGCAGTCGAGAATCTTCCACAATGGACGAAAGTCTGATGGAGCG
ACGTTACGTGAAGGATGAAGCCCTTCGGGGTGTAAACTTCTTTTATTTGGGAGGAAGTTA
TTGACAGTACCAAATGAATAAGGGGGTCCTAATTCTGTGCCAGCAGGAGCGGTAATACAG
AACCCCCGAGCGTTACCCGGATTTATTGGGCGTAAAGGGTCAGTAGGTGGCAAAGTAAGT
TTTGCGTTAAAGCTCATCGGCTCAACCTTTGAGATGCACAAAATACTGCTATGCTAGAGG
GCGTTAGAGGTGAATGGAACCGACGGAGTAGGGGTGAAATCCGTTGATCTCGTCGGGAAC
ACCAAAAGCGAAGGCAATTCACTGGGGCGACCCTGACACTGAATGACGAAAGCGTGGGGA
GCAAAAAGGATTAGATACCCTAGTAGTCC
>Otu7041
CCAGCCTATGGGTCGCAGCAGTCGAGAATTTTTCACAATGGGGGAAACCCTGATGGAGCG
ACGCCGCGTGGGGGATGAATGGCTTCGGCCCGTAAACCCCTGTCATTTGCGAACAAATTA
ATCTACCCAACACGTGGATTATTGATAGTAACGGAAGAGGAAGGGACGGCTAACTCTGTG
CCAGCAGCCGCGGTAATACAGAGGTCCCAAGCGTTGTTCGGATTCACTGGGCGTAAAGGG
TGCGTAGGCGGTCGGGTAAGTCTGACGTGAAATCTCCGGGCCTAACCCGGAAACTGCGTC
GGATACTATCCGGCTAGAGGATTGGAAGGGAGACTAGAATACTTGGTGTAGCAGTGAAAT
GCGTAGATATCAAGTGGAACACCAGTGGCGAAGGCGAGTCTCTGGACAATTCCTGACGCT
GAGGCACGAAAGCCAGGGGAGCAAACGGGATTAGAAACCCTAGTAGTCC
>Otu7052
CCAGCTATGGGTGGCTCCAGTGGGGAATCTTGCTCAATGGGCGAAAGCCTGAAGCAGCGA
CGCCGCGTGCGGGATGAAGGCCCTCGGGTTGTAAACCGCTTTCAGCAGGGACGAAATTGA
CGGTACCTGCAGAAGAAGGCCCGGCCAACTACGTGCCAGCAGCCGCGGTAATACGTAGGG
GCCTAGCGTTGTCCGGATTTATTGGGCGTAAAGAGCTCGTAGGCGGTTTGGTGAGTCGGG
TGTGAAATCTCCACGCTCAACGTGGAGGGGCCACCCGATACTGCCATGACTAGAGTCCGG
TAGGGGAGTGTGGAATTCCCGGTGTAGCGGTGAAATGCGCAGATATCGGGAGGAACACCA
GTAGCGAAGGCGACACTCTGGGCCGGTACTGACGCTGAGGAGCGAAAGCGTGGGGAGCAA
ACAGGATTAGATACCCTCGTAGTCCG
>Otu7056
CCAGCCTATGGGATGCAGCAGTGTGGAATATTGGGCAATGAACGCAAGTTCGATCCAGCA
ATATCGCGTGAGTGAGGAAGGTCCTTTACCGTAAAGCTCTTTCGACAATGTGAGAAAATG
ACAAAATATTGTAAAAGAAACCCCGACTAACTTCGTGCCAGCAGTCGCGGTAATACGGGG
GGGTTAGTGTTATTCATCATTACTGGGTGTAAAGCGCGCGTAGACTGTTTAAAAAGTTTT
AATTAAAACAATCTGAGCATAACTCAGAAATTAATTCTTATACTTTTAGACTTGAGGTTG
GAGGAAGAAAGCTATACTTCTCAGAGAAAAGTGAAATTTGTCCGAACTGAGGGGGATAAC
CAGCAGGCGAAAGCGGCTTTCTATGACAATGCTGACGTTTTGGCGCAAAAGCTTGGGGAT
CAAACGGGATTAGAAACCCTAGTAGTCC
>Otu7065
CCAGCCTACGGGGGGCAGCAGTCGAGAATCTTCCCCAATGGGCGAAAGCCTGAGGGAGCG
ACGCCGCGTGTGGGACGAAGTACTTCGGTATGTAAACCACTGTTAGGGTTATGAAAGCGA
CAGGACCTAATACGTCTTGAAGTTGATCTGACCCAGAGAAAGGGACGGCTAACTCTGTGC
CAGCAGCCGCGGTAATACAGAGGTCCCGAGCGTTACTGAGAATCACTGGGTTTAAAGGGT
GCGTAGGTGGTCCGTTAAGTCAGTTGTGAAATCCCCGGGCTCAACCCGGGAACTGCTTCT
GATACTGGCGGACTTGAGGCCAGTAGGGGTCACTAGAACTGACGGTGGAGCGGTGAAATG
CGTAGATATCGTCAGGAATGCCGGTGGTGAAGACGGGTGACTGGGCTGGTTCTGACACTG
AGGCACGAAAGCGTGGGGAGCGAACGGGATTAGATACCCTGGTAGTCC
>Otu7067
CCAGCCTACGGGTCGCACCAGTCGAGAATTTTTCTCAATGGGCGAAAGCCTGAAGGAGCG
ACGCCGCGTGGGGGATGAATGGCTTCGGCCCGTAAACCCCCGTCATTTGCGAACAAATTG
ACTCACCTAACACGTGTGTCATTGATTGTAGCGAAAGAGGAAGGGACGGCTAACTCTGTG
CCAGCAGCCGCGGTAATACAGAGGTCCCAAGCGTTGTTCGGATTCACTGGGCGTAAAGGG
TGCGTAGGTGGCAAGGTAAGTTTGATGTGAAATCTCCGGGCTTAACCCGGAAACTGCATT
GAATACTATCTCGCTCGAGGATTGGAGGGGAGACTGGAATACTTGGTGTAGCAGTGAAAT
GCGTAGATATCAAGTGGAACACCAGTGGCGAAGGCGAGTCTCTGGACAAATCCTGACACT
GAGGCACGAAAGCTAGGGGAGCAAACAGGATTAGATACCCCCGTAGTCC
>Otu7071
CCAGCCTATGGGTCGCAGCAGTGGGGAATCTTGCGCAATGGGCGAAAGCCTGACGCAGCG
ACGCCGCGTGGGTGATGAAGGCCTTCGGGTTGTAAAGCTCTGTGGGGAGGGACGAATAAG
TGTTGGCTAATATCCAGCATGATGACGGTACCTCTTTAGCAAGCACCGGCTAACTCTGTG
CCAGCAGCCGCGGTAAGACAGAGGGTGCAAACGTTGTTCGGAATTACTGGGCGTAAAGCG

TGTGTAGGCTGCCTCGTAAGTCGGATGTGAAAGCCCCGGGCTCAACCCGGGAAGTGCACT
CGATACTGCGGAGCTTGAGTGTCGGAGAGGTTGGTAGAATTCTCGGTGTAGAGGTGAAAT
TCGTAGATATCGAGGGGAATACCGGTGGCGAAGGCGGCCAACTGGACGAACACTGACGCT
GAGACACGAAAGCGTGGGTAGCAAACAGGATTAGATACCCGAGTAGTCC
>Otu7072
CCAGCCTACGGGGGGCAGCAGTGGGGAATATTGGGCAATGGGCGAAAGCCTGACCCAGCC
ACGCCGCGTGAGTGATGAAGGCCTTCGGGTCGTAAAGCTCTGTGGGGAGGGACGAACAAG
CACGGTGCTAACATCACCGGGCCCTGACGGTACCTCCTTAGCAAGCACCGGCTAACCCTG
TGCCAGCAGCCGCGGTAATACAGGGGGTGTAAACGTTGCTCGGAATCATTGGGCGTAAAG
CGCACGTAGGCGGTCTGTTATGTCGGATGTGAAAGCCCTTGGCTTAACCGAGGAAGTGCA
TCCGAAACTGGCAGGCTAGAGTACTAAAGAGGGTCGCGGAATTCCCGGTGTAGAGGTGAA
ATTCGTAGATATCGGGAGGAACACCGGTGGCGAAGGCGGCGACCTGGGTAGATACTGACG
CTGAGGTGCGAAAGCGTGGGGAGCAAACAGGATTAGATACCCCAGTAGTCC
>Otu7074
CCAGCCTACGGGTCGCAGCAGTTAGGAAATTTTGCCAATGGGGGAAACCCTGAGCGAGTA
ACGCCGCGTGGAGGACGAAGGCCTTCGGGTTGTAAACTCCTTTTCTGTGGGAAGATGATG
ACGGTACCACAGGAAGAAGCCCCGGCTAACTACGTGCCAGCAGCCGCGGTAATACGTAGG
GGGCGAGCGTTGTCCGGAATTACTGGGCGTAAAGAGCGCGTAGGCGGCACGATAAGTTCT
GTGTGAAAGCCCCCGGCTCAACTGGGGAGGGACGCGGAAAACTGTCGAGCTGGAGTGTCG
GAGAGGGAAGTGGAAGTCCCGGAGTAGGGGTGAAATCCGTAGAGACCGGGCCGAACACCA
GTGGCGAAGGCGACTTCCTGGCCGACGACTGACGCTGAGGCGCGAAAGCGTGGGGAGCGA
ACGGGATTAGATACCCGAGTAGTCC
>Otu7078
CCAGCCTACGGGTTGCAGCAGTAAGGAATATTGGACAATGGGCGCAAGCCTGATCCAGCC
ATGCCGCGTGCAGGATGAAAGTGCTCAGCACTGTAAACTGCTTTTGTTCGGGAAAAATTC
CCTGGTTTCTACCGGGGTTGATTGTACTGAAAGAATAAGGGACGGCTAACTTCGTGCCAG
CAGCCGCGGTAATACGAAGGTCCCAAGCGTTATCCGGATTTATTGGGTTTAAAGGGTGCG
TAGGCGGACTATTAAGTCAGTGGTGAAATCCTGCAGCCTAACTGTAGAATTGCCATTGAT
ACTGATGGTCTTAAGTACATTTGAAGTGGGCGGAATGTGTCATGTAGCGGTGAAATGCTT
AGATATGACACAGAACACCGATTGCGTAGGCAGCTCGCTAAACTGTAGCTGACGCTGAGG
CACGAAAGCGTGGGGAGCAAACAGGATTAGAAACCCTCGTAGTCC
>Otu7083
CCAGCCTACGGGATGCTCCAGTGGGGAATCTTGCACAATGGGGGCAACCCTGATGCAGCG
ACGCCGCGTGAGCGATGAAGCCCTTCGGGGTGTAAAGCTCTTTCGGCAGGGACGATAATG
ACGGTACCTGAAGAATAAGCACCGGCTAACTACGTGCCAGCAGCCGCGGTAATACGTAGG
GTGCAAGCGTTAATCGGAATTACTGGGCGTAAAGCGTGCGCAGGCGGTTCTATAAGACAG
ATGTGAAATCCCCGGGCTCAACCTGGGAACTGCATTTGTGACTGTAGAGCTGGAGTGCGG
CAGAGGGGGATGGAATTCCGCGTGTAGCAGTGAAATGCGTAGATATGCGGAGGAACACCG
ATGGCGAAGGCAATCCCCTGGGCCTGCACTGACGCTCATGCACGAAAGCGTGGGGAGCAA
ACAGGATTAGAGACCCTTGTAGTCC
>Otu7086
CCAGCCTATGGGGTGCAGCAGTCGAGAATCATTCGCAATGGGCGAAAGCCTGACGATGCG
ACGCCGTGTGAGCGAAGAAGGCCTTAGGGTCGTAAAGCTCTTTCGCTTGGGAACAAGAGG
AGTTGGTTAATACCCGATGACTTTGAGCGTACCAGGTAAAGAAGCACCGGCTAACTCCGT
GCCAGCAGCTGCGGTAATACGGAGGGTGCAAGCATTAATCGGATTTATTGGGCGTAAAGG
GCGCGTAGGCGGGGATAATAGTCAGATGTGAAATCCCGGGGCTCAACCTCGGAACTGCAT
TTGAAACTGTATCTCTAGAGGGTAGGCGGAGAAAACGGAATTCCACAAGTAGCGGTGAAA
TGCGTAGATATGTGGAAGAACACCGGTGGCGAAGGCGGTTTTCTAGCTTACTCCTGACGC
TGAGGCGCGAAAGCAAGGGGATCAAACAGGATTAGAGACCCGCGTAGTCC
>Otu7092
CCAGCCTATGGGTGGCAGCAGTGGGGAATATTGGACAATGAACAAACGTTCGATCCAGCA
ACATCGCGTGAGTGAAGTTTTTGTTATCTATTATTTATAAAATAAAAACATATAAAATGA
AACAGAATAGAAGGTCTAGAATTGTAAAGCTCTTTTATTAAGGAAGATAATGACGTTACT
TAATGAATAAGCCCCGGCTAACTTCGTGCCAGCAGCCGCGGTAAAACGGAGGGGGCTAGT
GTTATTCGTGTTGACTGGGCGTAAAGGGTATGTAGGCGGTTTGGCTAGTGCTTAGTAAAA
GACTGGAGCTTAACTTCTTGAATGCTTTGCAGACAGCTGAACTAGAGTACAGGAGAGGAT
AGTGGAACTATTAATGTAGAGGTAAAATTTGAAAATATTAATAGGAACATCAATAGGCGA
AGGCGACTATCTGGTCCGTAACTGACGCTGAGATACTAAAGCATGGGGAGCGAACAGGAT

TAGATACCCCGGTAGTCC
>Otu7099
CCAGCCTATGGGTCGCAGCAGTGGGGAATTTTGGACAATGGGGGAAACCCTGATCCAGCA
ACTCTGCGTGAGGGACGAAGCCTTTCGGGGCGTAAACCTCTTTTATCTGGGAAGAACACC
CGCAAGGGTCTGACGGTACCAGGTGAATAAGCCACGGCTAACTATGTGCCAGCAGCCGCG
GTAAGACATAGGTGGCGAGCGTTATTCGGAATTACTAGGCGTAAAGCGAGTGTAGGCGGG
CGCTTAAGTCTGTTGTGAAATCTCCCGGCTTAACTGGGAGGGGTCAATGGATACTGGGCG
TCTTGAGTGAGGTAGGGGGTACTGGAATTCCCGGTGTAGCGGTGAAATGCGTAGATATCG
GGAGGAACACCTATGGCGAAAGCGGCGCACTGGACCACATCTGACGCTGAGGCGCGAAAG
CTAGGGGAGCAAACAGGATTAGAGACCCCGGTAGTCC
>Otu7101
CCAGCCTATGGGGGGCAGCAGACGAGAATATTCCGCAATGGACGAAAGTCTGACGGAGCG
ACACTGCGTGATGGATGAAGTGCTTCGGTACGTAAACATCTTTTATCGGGGAAGAAGTCT
ATTGACGGTACCCGGTGAATAAGGGGCTCCTAACTCTGTGCCAGCAGGAGCGGTAATACA
GAGGCCCCGAGCGTTACCCGGAATCACTGGGCGTAAAGGGTGTCCAGGTGGCCATATTAG
TCTTTCGTAGAATCCGTGGGCTTAACCTACGGCGCGCGAGGGAAACGGTATGGCTCGAGG
GCGCGAGAGGTGCATGGAACTCATGGTGGAGGGGTGAAATCCGTTGATATCATGGGGAAC
ACCAAAGGCGAAGGCAGTGCACTGGCGCGTTCCTGACACTCACACACGAAAGCCAGGGGA
GCGAACGGGATTAGAGACCCTAGTAGTCC
>Otu7107
CCAGCCTATGGGTCGCAGCAGTGGGGAATTTTGGACAATGGGCGAAAGCCTGATCCAGCA
ATGCCGCGTGTGTGAAGAAGGCCTTCGGGTTGTAAAGCACTTTTGGCGGGAACGAAACGG
TTCTTTCTAATACAGAGGACTAATGACGGTACCCGCAGAATAAGCACCGGCTAACTACGT
GCCAGCAGCCGCGGTAATACGTAGGGTGCAAGCGTTAATCGGAATTACTGGGCGTAAAGC
GTGCGCAGGCGGTTATGTAAGACAGATGTGAAATGCCCGGGCTTAACCTGGGAACTGCAT
TTGTGACTGCATGGCTAGAATCTGGCAGAGGGGGGTAGAATTCCACGTGTAGCAGTGAAA
TGCGTAGATATGTGGAGGAACACCGATGGCGAAGGCAGCCCCCTGGGCTAAGATTGACGC
TCATGCACGAAAACGTGGGGAGCAAACAGGATTAGAAACCCCCGTAGTCC
>Otu7108
CCAGCCTATGGGAGGCAGCAGTGGGGAATATTGGACAATACGCAAAAGCGTGATCCATCG
ATATCGAGTGTGCGATGACGGCGGATTGTTGTAAAGTGCTTTTGCTAGTGAGGATTATGA
CGGTAACTAGAGAAATAAGCCCTGGCTAAATGACGTGCCAGCAGCCGCGGGAATACGTTA
GGGGCGAGCGTTATCCATATTTACTAGGCGTAAAGGGTGCGTAGATGGTTTGGGAACAAT
TTGGTAGAAAGAACAAGGTAACCCTTGTGTAGTGCGAAATTAATTCTTTTAACTTGAGTG
TCTAACAGGAATTTAGTATTTCTAAAGGGAGACGTTACAGTCTGAGAATTTAGGAGGATT
TCCATGTGCGAAGGCAAAATTCTGGTTGGATTCTGACGTTGAGGCACGAAGGCATGGGGA
GCAAAGCGGATTAGAAACCCCTGTAGTCC
>Otu7110
CCAGCCTACGGGGGGCACCAGCATGGAAGTCGTACAAACCAATATTCCTGATGAAGCTTA
CCGCAATATTTCTGAAGGCTGGGAAGAAGACTATTTCAATGGATTGAATGAGTTGTTCGA
GCCTTAGTATCCGCTAAAATTGTACGATAAGAATCGTCATTGCGAACAGAGTGAAGCAAT
CTGCTGATTAAAGATTGCTTCACTTCGTTCGCAATGACGTGACAAACTAACAATCCTACT
TGCCAAGCCCCTCGTTTCCGGTATCTGCAATAAATTTCCATTGCCCCTTTTCGTTTAATT
TCCAAACCGTCATATACGTTCCCTTGGTAGTATCCTGCTTAGCAATAAGCTTCCAGAAAC
CATAGGTGTAACCCAATTCACCACTTTCAGCAATAATTGCTTTGGCTGGATTCCAGATTA
GAAACCCCAGTAGTCC
>Otu7112
CCAGCCTACGGGGTGCTCCAGTCGAGGATCTTCGGCAATGGACGCAAGTCTGACCGAGCG
ACGCCGCGTGTGGGATGAAGGCCTTCGGGTTGTAAACCACTGTCAGTGGGGAGAAAATCG
GTGAGAGTCATCTCTCATCGTTGATCTATCCGCAGAGGAAGCAAGGGCTAAGTTCGTGCC
AGCAGCCGCGGTAAGACGAACCTTGCAAACGTTATTCGGAATCACTGGGCTTAAAGGGTG
CGTAGGCGGCGCGGTAAGTTGGGTGTGAAATACCTCGGCTCAACCGAGGAACTGCGCCCA
AAACTACCGTGCTTGAGGGAGATAGAGGTGAGCGGAACTTAGGGTGGAGCGGTGAAATGC
GTTGATATCCTAAGGAACACCCGTGGCGAAAGCGGCTCACTGGATCTCTTCTGACGCTGA
GGCACGAAAGCTAGGGGAGCGAACGGGATTAGAAACCCTAGTAGTCC
>Otu7115
CCAGCCTACGGGAGGCAGCAGTGGGGAATCTTGCACAATGGGCGCAAGCCTGATGCAGCA
ATGCCGCGTGAGTGATGAAGGCCTTCGGGTTGTAAATCTCTGTCGGCTGGGAAAAACGTC

TTTTCATCTAATAGGTGAAGAGAGTGATGGTACCAGCAAAGGAAGAGGCGGCTAACTCTG
TGCCAGCAGCCGCGGTAATACAGAGGCCTCGAGCGTTGTTCGGAATTACTGGGCGTAAAG
GGAATGTAGGTGGTGACGTAAGTCGGATGTGAAAGCCCTGGGCTTAACCTGGGAAGTGCA
TTTGATACTGCGTTGCTGGAGTGCGAGAGAGGAAAGCGGAATTCCCAGTGTGGGAGTGAA
ATCCGTAGATATTGGGAGGAATACCGATGGCGAAGGCAGCTTTCTGGCTCGTAACTGACA
CTGAGATTCGAAAGCGTGGGTAGCAAACAGGATTAGATACCCCCGTAGTCC
>Otu7118
CCAGCCTATGGGGCGCACCAGCGAGGAATATTGCACAATGGGCGCAAGCCTGATGCAGTA
ACGCCGCGTGAGGGATGAAGACCTTCGGGTTGTAAACCTCTTTTCAGCTTGACGAGTGAG
GACGGTAAAGTTGGAATAAGTCTCGGCTAACTACGTGCCAGCAGCCGCGGTAAGACGTAG
GAGGCGAGCGTTATCCGGATTCACTGGGCGTAAAGGGCATGTAGGCGGCTGGGCAAGTCC
TTCGTGAAAGCCCCTGGCTCAACTGGGGGAGGACGGAGGAGACTGTTCGGCTTGAGGGTA
GCAGAGGTCGGTGGAACTCCCGGTGTAGCGGTGGAATGCGTAGAGATCGGGAAGAACATC
AGTGGCGAAGGCGACCGACTGGGCTGCAACTGACGCTGAAATGCGAAAGTGTGGGGAGCA
AACGGGATTAGAAACCCTCGTAGTCC
>Otu7123
CCAGCCTATGGGGGGCAGCAGGCGCGCAAACTTCACAATGCACTTACGTGTGATGAGGGA
AGTCTGAGTGATTTCCTTCGGGAAATCTTTTGCCAACTTTAGTCAGGTTGGAGAATAGGT
GCTGGGTAAGACCGGTGGCAGCCGCCACGGTAATACCGGCGGCACAAGTGGTGTTCACTA
TTATTGGGCTTAAAGAGTCAGTAGCCGGCTGAGACAGTCTTGTGTGAAATATTGGTGCAT
AACATCAATACGTGCATAAGATACCTCTCAGCTAGGGAGCGGGGGAGGTTAGGAGTATTC
ATGGGGTAAGGGTAAAATCTTGTAATCCTATGAAGACTACCGGTGGCGAAGGCGCCTAAC
CAAAACGCATCCGACGGTGAGTGACGAAGGCCAGGAGAACGAATCGGATTAGAAACCCCC
GTAGTCC
>Otu7124
CCAGCCTACGGGTGGCTGCAGTGAGGAATATTGCTCAATGGACGAAAGTCTGAAGCAGCA
ACGCCGCGTGAGGGATGAAGGTGCTTTGCATTGTAAACCTCTGTAGTCGGAGACAAACGA
CGGGTTACTACCCGTTTGATGGTATCCGAAAAGTAAGGATCGGCTAACTACGTGCCAGCA
GCCGCGGTAATACGTAGGATCCAAGCGTTGTCCGGATTTACTGGGTGTAAAGGGTGCGCA
GGCGGGCTCATAAGTCAGGAGTGAAATCTCGGCGCTCAACGCCGAAACGTCTTTTGATAC
TATGAGCCTTGAATCAGCGAGAGATGTGCGGAATTCCGAGTGTAGCAGTGAAATGTGTAG
ATATTCGGAAGAACACCAGTGGCGAAGGCAGCACGTTGGCGCTCGATTGACGCTCAGGCA
CGAAAGCATGGGGATCAAACAGGATTAGATACCCTCGTAGTCC
>Otu7126
CCAGCCTATGGGTGGCAGCAGTAGGGAATCTTGCGCAATGGACGAAAGTCTGACGCAGCA
ACGCCATGTGTGGGATGACGCATTTCGGTGTGTAAACCACTGTCGGCAGGGAATAAAGTC
CGCTTTTTAGCGGAGGTGAATGTACCTGCAGAGGAAGCCCCGGCTAACTTCGTGCCAGCA
GCCGCGGTAATACGAGGGGGGCTAGTGTTGTTCGGAATTACTGGGCGTAAAGGGAGCGTA
GGCGGGTGCTTAAGTTGGATGTTTAAAACCGGGGCCCAACCCCGGCGTGGCATTCAAAAC
TGGGCATCTTGAATGGGACAGAGGCCAGTGGAATTGGAGGTGTAGCGGTGGAATGCGTAG
AGATCTCCAAGAACACCAGTTGCGAAGGCGGCTGGCTGGGTCCACATTGACGCTGAGGCT
CGAAAGCGTGGGGAGCAAACAGGATTAGATACCCAAGTAGTCC
>Otu7128
CCAGCCTATGGGAGGCTGCAGTAAGGAATATTGGACAATGGGGGCAACCCTGATCCAGCC
ATGCCGCGTGAGTGACGAAGGCCTTCGGGTTGTAAAGCTCTTTTGCCGGGGACGATAATG
ACGGTACCCGGAGAATAAGTCCCGGCTAACTTCGTGCCAGCAGCCGCGGTAATACGAAGG
GGGCTAGCGTTGTTCGGAATTACTGGGCGTAAAGCGCACGTAGGCCGAATGGTCAGTCAG
AGGTGAAATCCCGGAGCTCAACTTCGGAACTGCCTTTGATACAACCATTCTAGAGTCCGG
AAGAGGTGAGTGGAATTCCTAGTGTAGAGGTGAAATTCGTAGATATTAGGAAGAACACCA
GTGGCGAAGGCGGCTCACTGGTCCGGTACTGACGCTGAGGTGCGAAAGCGTGGGGAGCAA
ACAGGATTAGAAACCCGGGTAGTCC
>Otu7129
CCAGCCTATGGGAGGCAGCAGTAAGGAATATTGGACAATGGTCGCAAGACTGATCCAGCC
ATGCCGCGTGCAGGATGACGGCCCTATGGGTTGTAAACTGCTTTTATACGGGAAAAAACC
CCTCTACGTGTAGAGGGCTGATTGTACTGTAAGAATAAGGATCGGCTAGCTCCGTGCCAG
CAGCCGCGGTAATACGGAGGATCCAAGCGTTATCCGGATTCATTGGGTTTAAAGGGTGCG
TAGGCGGAATGATAAGTCAGTGGTGAAAGCCTGCAGCTTAACTGCAGAACTGCCATTGAT
ACTGTCAATCTTGAGTATATTTGATGTGGGCGGAATGTGTCATGTAGCGGTGAAATGCTT

AGATATGACACAGAACACCGATTGCGAAGGCAGCTCACAAAACTATAACTGACGCTGGGG
CACGAAAGCGTGGGTATCAAACAGGATTAGAAACCCGCGTAGTCC
>Otu7139
CCAGCCTACGGGAGGCACCAGTGGGGAATATTGGACAATGGGGGAAACCCTGATCCAGCG
ATGCCGCGTGAATGAAGAAGGCCTTAGGGTTGTAAAGTTCTTTCACCTGTGACGATAATG
ACGGTAACAGGAGAAGAAGCCCCGGCTAACTCCGTGCCAGCAGCCGCGGTAAGACGGAGG
GGGCTAGCGTTGTTCGGAATGACTGGGCGTAAAGGGCGCGTAGGCGGCCGATCAAGTCAG
GTGTGAAAGCCTCGGGCTTAACCCGAGAATTGCGCTTGATACTGGTTGGCTAGAAGATGG
AAGAGAAAAGCGGAATTCCTAGTGTAGAGGTGAAATTCGTAGATATTAGGAAGAACACCA
GAGGCGAAGGCGGCTTTTTGGTCCATTCTTGACGCTGAGGCGCGAAAGCGTGGGGAGCAA
ACAGGATTAGATACCCTCGTAGTCC
>Otu7140
CCAGCCTATGGGGGGCACCAGCACAGGTCTCGAAACTTCAAATCCGATGAAGTCATGATA
GCGATCGGAGGTGTAGCGATTCCTCGCGACCTGGGTCACGTCGAAACCAAATTCGGTGCG
AATGTGGGCTTTGGGATTATCCGCGTAGGTGACTTCGTCACCGAATTTCTTCCGCAGTTT
AGGAAACTCGACCGCTACTGCCCGATTCACGGTGGGATGGCCAACGTTGTCTGCCGAGTA
GTGCGCCAGGGCTCCGAGAGCGAAAGCGTATTCGTTGAGGTCCGTCGCCTCGTGGATGAG
ATTGACGACGAAATCTCCGCTGCGCACGTAATGAGTAAGGTCGCTAAAGTACTTGTTGCC
GAACGGGTAGTAGCCCATGTCTTGAATTAGAAACCCTAGTAGTCC
>Otu7142
CCAGCCTATGGGTCGCTCCAGTAGGGAATCTTCCGCAATGGGCGAAAGCCTGACGGAGCA
ACGCCGCGTGAGTGATGAAGGTTTTCGGATCGTAAAGCTCTGTTGCCAGGGAAGAATGCC
AGGGAGAGTAACTGCTCCCTGGGTGACGGTACCTGAGAAGAAAGCCCCGGCTAACTACGT
GCCAGCAGCCGCGGTAATACGTAGGGGGCAAGCGTTGTCCGGAATTATTGGGCGTAAAGC
GCGCGCAGGCGGCTAATTAAGTCTGGTGTCTAATCCCGGAGCTCAACTCCGGGTCGCACG
GGAAACTGGTTGGCTTGAGTACAGAAGAGGAAAGTGGAATTCCACGTGTAGCGGTGAAAT
GCGTAGAGATGTGGAGGAACACCAGTGGCGAAGGCGACTTTCTGGGCTGTAACTGACGCT
GAGGCGCGAAAGCGTGGGGAGCAAACAGGATTAGAAACCCTAGTAGTCC
>Otu7144
CCAGCCTACGGGTCGCTCCAGTGGGGAATTTTCCGCAATGGGCGCAAGCCTGACGGAGCA
ACGCCGCGTGGGTGACGAAGTCTTTCGGGATGTAAAACCCTTTCAGCTGGGAAGAACACA
ATGACGGTACCAGCAGAAGAAGCATCGGCTAACTACGTGCCAGCAGCCGCGGTAAGACGT
AGGATGCGAGCGTTGTCCGGAATTATTGGGCGTAAAGCGTACGTAGGCGGTTTCATAAGT
TAGAGGTTAAAGGCTGCGGCTCAACCGCAGAAATGCCTTTGATACTGTGAGACTGGAGGT
TGTCAGAGGAAGGCGGAATTCCCAGTGTAGCGGTGAAATGCGTAGAGATTGGGAGGAACA
CCAGAGGCGAAAGCGGCCTTCTGGGGCACACCTGACGCTGAGGTACGAAAGCCAGGGTAG
CAAACGGGATTAGAAACCCTAGTAGTCC
>Otu7150
CCAGCCTATGGGGCGCTGCAGTCGAGAATATTTGGCAATGGGCGAAAGTCTGACCATGCG
ACGCCGCGTGCTGGACGAAGTCCTTCGGGACGTAAACAGCTTTTGCGCGTGAGGAAGTAA
TTGACGTTAGCGCGCGAATAAGGGGCTCCTAACTCTGTGCCAGCAGGAGCGGTAATACAG
AGGCCCCGAGCATTATCCGGAATCACTGGGCGTAAAGGGTGTGTAGGCGGCTTTGTTAGT
CTTCCGTTAAAGTCTTTGGGCTCAACCCAGAGAATGCGGGAGAAACGGCAAGGCTTTGAG
GACGCGAGAGGTAAGGGGAACTCATGGTGTAGGGGTGAAATCCGTTGATATCATGGGGAA
CACCAAATGCGAAGGCACCTTACTGGCGCGCTCCTGACGCTGAAACACGAAAGCGTGGGT
AGCGAACGGGATTAGAAACCCTTGTAGTCC
>Otu7153
CCAGCCTACGGGTGGCAGCAGTCGAGAATCTTCCACAATGGACGAAAGTCTGATGGAGCG
ACGCCGCGTGATTGATGAAGTCCCTCTGGGACGTAAAGATCTTTTATGAGGGAAGAAGTT
TATTGACGGTACCTCATGAATAAGGGGCTCCTAACTCTGTGCCAGCAGGAGCGGTAATAC
AGAGGCCCCAAGCGTTATCCGGAATCACTGGGCGTAAAGGGTGTGTAGGCGGTCGTGTTA
GTCTTTCGTGAAAGATCTTGGGCCCAACCCAGGAGACGCGGGAGAAACGGCAGGACTCGA
GAGTGTGAGAGGTACAGGGAACTCATAGTGTAGGGGTGAAATCCGTTGATATTATGGGGG
ACACCAAATGCGAAGGCACTGTACTGGCACATTTCTGACGCTGAAACACGAAAGCGTGGG
CATCGAACGGGATTAGAAACCCCCGTAGTCC
>Otu7154
CCAGCCTACGGGGTGCTGCAGTCGAGAATCTTTCGCAATGGGCGAAAGCCTGACGAAGCG
ACGCCGTGTGAGCGAAGAAGGCCTTCGGGTTGTAAAGCTCTTTCGCTAGGGAACAAGAAA

GAATGGTTAATAACCATTCGATTTGATGGTACTTGGTAAAGAAGCACCGGCTAACTCCGT
GCCAGCAGCTGCGGTAATACGGAGGGTGCAAGCATTGATCGGAATTACTGGGCGTAAAGG
GCGCGTAGGCGGTCTGAAAAGTCGGATGTGAAATTCCGGGGCTCAACCCCGGAGCTGCAT
TTGAAACTTTCAGACTTGAGGTTGGCAAGGGAAAACGGAATTCCACGTGTAGCGGTGAAA
TGCGTAGATATGTGGAAGAACACCGGTGGTGAAGACGGTTTTCTGTGCTAAACCTGACGC
TGAGGCGCGAAAGCTAGGGGAGCAAACAGGATTAGATACCCCGGTAGTCC
>Otu7156
CCAGCCTATGGGGTGCAGCAGTAGGGAATTTTCCACAATGGGCGAAAGCCTGATGGAGCA
ACGCCGCGTGCAGGAGGAAGGCCCTCGGGTCGTAAACTGCTTTTATCTGTGATGATTTTG
ACAGTAACAGATGAATAAGGATCGGCTAACTCCGTGCCAGCAGCCGCGGTCATACGGAGG
ATCCAAGCGTTATCCGGAATTACTGGGCGTAAAGAGTTGCGTAGGTGGCATGGTAAGCAA
ATAGTGAAAGACTACGGCTCAACCGTAGATACATTGTTTGAACTGCCAAGCTAGAGCATA
GGAGAGGTAGCTGGAATTGCTAGTGTAGGAGTGAAATCCGTAGATATTAGCAGGAACACC
GATGGCGTAAGCAGGCTACTGGCCTATTGCTGACACTAAGGCACGAAAGCGTGGGGAGCG
AACGGGATTAGATACCCTTGTAGTCC
>Otu7161
CCAGCCTACGGGGCGCACCAGTCGAGAATCTTCCGCAATGGGCGAAAGCCTGACGGAGCG
ACGCCGCGTGGTTGATGAAGCACTTCGGTGCGTAAAAACCTTTTATGAGCGAGAAAGTCT
ATTGATCAGCTCATGAATAAGAGGTTGCTAAACTCGTGCCAGCAGCAGCGGTAATACGAG
TGCCTCAAGCGTTATCCGGAATCATTGGGCGTAAAGGGTGTGTAGGCGGTCCCGTTAGTC
TCCTGTTAAATTCTTCGGCTCAACCGGGGGTCTGCAGGGGAAACGGCGGGACTTGAGGAT
GCGAGGGGTCTCTGGAACTCATGGTGTAGCGGTGAAATGCGTTGATATCATGGGGAACAC
CGAAAGCGAAGGCAGGAGACTGGAGCATTCCTGACGCTGAAACACGAGAGCGTGGGTCGC
GAATGGGATTAGATACCCCAGTAGTCC
>Otu7162
CCAGCCTATGGGGGGCACCAGTCGAGAATCTTCCGCAATGGACGCAAGTCTGACGGAGCG
ACGCCGCGTGGTAGATGAAGTCCTTCGGGACGTAAATACCTTTTATGGGGGATGACATTT
CGACAGTACCTCATGAATAAGGGGCTCCTAACTCTGTGCCAGCAGGAGCGGTAATACAGA
GGCCCCAAGCATTATCCGGAATCACTGGGCGTAAAGGGTGCGTAGGTGGCGTGATTAGTC
CTCTTTCAAATCTTCCGGGCTCAACCCGGAAGCTCGAGGGGAAACGGTCATGCTCGAGGA
TGTACGAGGTATGTGGAACTCTAGGTGTAGGGGTGAAATCCGTTGATATCTAGGGGAACA
CCAAATGCGAAGGCAGCATACTGGGACATTCCTGACACTGAGGCACGAAAGCGTGGGTAG
CGAAAAGGATTAGATACCCGTGTAGTCC
>Otu7164
CCAGCCTATGGGGGGCACCAGTGAGGAATATTGCGCAATGGGCGAAAGCCTGACGCAGCG
ACGCCGCGTGGAGGATGAAGGCCTTCGGGTTGTAAACCCCTGTCAGTGGGGAAGAGAATG
GACGGTACCCACTGAGGAAGCTTCGGCTAACTACGTGCCAGCAGCCGCGGTAAGACGTAG
GGAGCAAGCGTTGTCCGGAATTACTGGGCGTAAAGAGCTCGTAGGCGGGATCGTAAGTCC
GAGAGGAAATCTCAAGGCTCAACTTTGGGGCTTTCTTGGATACTGCGATTCTTGAGTATA
TGAGAGGTAAGTAGAATTCCCGGTGTAGCGGTGAAATGCGTAGATATCGGGAGGAATACC
AGTGGCGAAGGCGACTTACTGGCATATTACTGACGCTGAGGAGCGAAAGCGTGGGGAGCA
AACGGGATTAGATACCCCCGTAGTCC
>Otu7165
CCAGCCTACGGGAGGCTGCAGTGAGGAATTTTGGACAATGAACGAAAGTTTGATCCAGCA
ATATCACGTGAATGATGAAGGTTTATTTATTTTTATCGTAAAGTTCTTTCAATGAGGAGG
ATAATGACGTTACTCATAGAAGAAGTCCTGACCAATCTCGTGCCAGCAGTCGCGGTAATA
CGGGGAGGGCAAGCGTTATTCTACGTTACTGGGTGTAAAAAGTATTAAGGCGGTTTAATA
AGTTTCTTTTGAAAGAATAGAATTCAATTCTAGAATTGACTGTAAATACTGTTAAACTTG
AGTTTATTAGGAGATAGTTGTATGTTTAGTGTAGGTCTAAAATCCTTCGATATTAAAAAG
ACATTCAATCGGTGAAGACTACTGTCTATAGTATAACTGACGTTGAAATACAAAAGCGTG
AGGAGCAAACAGGATTAGAAACCCGTGTAGTCC
>Otu7169
CCAGCCTACGGGTGGCAGCAGTAGGGAATATTGGTCAATGGGTGAGAGCCTGAACCAGCC
ATGCCGCGTGCAGGAAGAAGGCCTTCTGGGTTGTAAACTGCTTTTGCCAGGGGATAAAAG
ACCCTTGCGAGGGAAATTGAAGGTACCTGGTGAATAAGCCACGGCTAACTACGTGCCAGC
AGCCGCGGTAATACGTAGGTAGCAAGCGTTGTCCGGATTTATTGGGTTTAAAGGGTGCGT
AGGCGGTCCATTAAGTCAGTTGTGAAATACGGCAGCTTAACTGTCGAGGTGCAATTGATA
CTGGAGGACTTGAGTACAGACGAGGTAGGCGGAATTGACGGTGTAGCGGTGAAATGCATA

GATATGTCACAGAACACCAATTGCGAAGGCAGCTTGCTAAGTATGATTGACGCTGAGGCA
CGGAAGCGTGGGGATCAAACAGGATTAGAAACCCGTGTAGTCC
>Otu7170
CCAGCCTACGGGATGCACCAGTGGGGAATCTTGGACAATGGGGGAAACCCTGATGGAGCG
ACGCCGCGTGGGGGATGAATGGCTTCGGCCCGTAAACCCCTGTCATTTGCGAACAAACGG
TATTGTCTAAGAGATGATGCCTTGATAGTAGCGAAAGAGGAAGGGACGGCTAACTCTGTG
CCAGCAGCCGCGGTAATACAGAGGTCCCAAGCGTTGTTCGGATTCACTGGGCGTAAAGGG
TGCGTAGGTGGTCGGGTAAGTCTGATGTGAAAGCTCGAAGCTCAACTTCGAAATGGCATT
GGATACTATCCGGCTGGAGGGTCGGAGGGGGGACTGGAATTCTCGGTGTAGCAGTGAAAT
GCGTAGATATCGAGAGGAACACCAGTGGCGAAGGCGAGTCCCTGGACGACTCCTGACACT
GAGGCACGAAAGCTAGGGGAGCAAACAGGATTAGAAACCCTCGTAGTCC
>Otu7175
CCAGCCTATGGGTGGCAGCAGTGGGGAATATTAGACAATGGGCGAAAGCCTGATCTAGCA
ACATTTCGTGGAAGGAAGAAGGCCTCAGCTGTAATCTCCTATTTCGATAATAACAATAAT
GATTATAATTATAAAAGAAGCCCCGGTAAATATCTGTGCCAGCAGCCGCGGTAATGCAGG
GGGGGCAAGTATTATTCTACATGACTGGGCGTAAAGGATATGTAGGTGGTTTTTTAAATT
TTATTAATAAAGCTATACTACAAATATAGACATTTTTAAAATATTGTAAAACTTTGAGTT
TATACGAGATATTAGAACTTCTGGCGTTAGCAGTGAGATGTTTTCATAACAGAGGGACTT
CCTATTGGCGAAAGCAAATATCTAGTTAAAACTGACACTAAAATATGAAGGTTTGGGGAG
CAAACAGGATTAGAAACCCTAGTAGTCC
>Otu7176
CCAGCCTATGGGGTGCACCAGTCAGGAATATTGCGCAATGGAGGAAACTCTGACGCAGCG
ACGCCGCGTGGGTGATGAAGGCCTTCGGGTCGTAAAGCCCTGTCGGAGGGGAAGAAAAGC
TTGCTGGCTAATATCCAGCAGGCCCTTGACGGTACCCTCAAAGGAAGCACCGGCTAACTC
CGTGCCAGCAGCCGCGGTAAGACGGAGGGTGCAAGCGTTGTTCGGATTGACTGGGCGTAA
AGGGCGTGTAGGCGGTTGGTCAAGTCCGATGTGAAAGCCCGGGGCTTAGCCTCGGAAGTG
CATCGGAAACTAGCCAGCTGGAGCGCGATAGAGGAGGGTAGAATTCCTGGTGTAGCGGTG
AAATGCGTAGATATCAGGAGGAACACCTGTGGCGAAAGCGGCTCACTGGACCACAACTGA
CGCTGATGCACGAAAGCTAGGGGAGCAAACAGGATTAGATACCCTTGTAGTCC
>Otu7179
CCAGCCTATGGGGCGCTGCAGTGGGGAATATTGCGCAATGGGCGGAAGCCTGACGCAGCG
ACGCCGCGTGAGGGATGACGGCCTTCGGGTTGTAAACCTCTTTCAGCAGGGACGAAGAGT
GATTGACGGTACCTGCAGAAGAAGCGCCGGCCAACTACGTGCCAGCAGCCGCGGTAATAC
GTAGGGCGCAAGCGTTGTCCGGAATTATTGGGCGTAAAGAGCTCGTAGGCGGTTTGTCGC
GTCGGCTGTGAAATCCTGGGGCTTAACTCCGGGTCTGCAGTCGATACGGGCAGACTTGAG
TGTGGTAGGGGAGACTGGAATTCCTGGTGTAGCGGTGAAATGCGTAGATATCAGGAGGAA
CACCGGTGGCGAAGGCGGGTCTCTGGGCCATTACTGACGCTGAGGAGCGAAAGCGTGGGG
AGCGAACAGGATTAGAAACCCCAGTAGTCC
>Otu7181
CCAGCCTACGGGGGGCACCAGTGGGGAATATTGCGCAATGGGCGAAAGCCTGACGCAGCG
ACGCCGCGTGGAGGATGAAGGTCTTAGGATTGTAGACTTCTGTTAAGTGGGAAAAAAGAC
TCATTCATAATACGGACGGGGGATGATTGTACCACTAGAGAAAGCACCGGCTAAACTCGT
GCCAGCAGCCGCGGTAATACGAGTGGTGCAAGCGTTATTCGGAATCATTGGGCGTAAAGG
GTGCGTAGACGGTATGCTAAGTCTGCTGTTAAATCCCTCGGCTTAACCGAGGATATGCGG
CTGAAACTGGTGTACTTGAGGATGGAAGAGAGAAGTGGAATTCTCGGAGTAGCGGTAAAA
TGCGTAGATCTCGAGAGGAACACCAATGGCGAAGGCAGCTTCTTGGTCCATTCCTGACGT
TGAGGCACGAAAGCGTGGGGAGCAAACAGGATTAGAAACCCCAGTAGTCC
>Otu7186
CCAGCCTATGGGGGGCAGCAGTGAGGAATCTTCCGCAATGGGCGAAAGCCTGACGGAGCG
ACACCGCGTGGAGGATGACGCGTTTACTCGTGTAAACTCCTTTTCTGTGGGAAGATAATG
ACGGTACGACAGGAATAAGGGGCGGCAAACTACGTGCCAGCAGCCGCGGTAATACGTAGG
CCCCAAGCGTTATCCGGAATTACTGGGTGTAAAGCGTCTGTAGGCGGGATAACAAGTCTT
CCATGAAAGACCGGAGCTCAACTCCGCGTTTGTGGGAGATACTATTTTTCTAGAGTTAGG
GAGAGGGAAGCGGAATGGTATGAGTAGGGGTGCAATCCGTTGATACATATCAGAACACCA
AAAGCGAAGGCAGCTTCCTGGAACTATATTGACGCTGAGAGACGAAAGCGTGGGGAGCGA
AAAGGATTAGAAACCCCAGTAGTCC
>Otu7190
CCAGCCTACGGGTTGCACCAGTGGGGAATTTTGGACAATGGGGGAAACCCTGATCCAGCA

ACTCTGCGTGAGGGACGAAGCCTCTCGGGGCGTAAACCTCTTTTATCTGGGACGAAATCC
CGCAAGGGTCTGACGGTACCAGGTGAATAAGCCACGGCTAACTATGTGCCAGCAGCCGCG
GTAAGACATAGGTGGCGAGCGTTATTCGGAATTACTAGGCGTAAAGCGAGTGTAGGCGGG
CGCTTAAGTCCGTTGTGAAATCTCCCGGCTTAACTGGGAGGGGTCAACGGATACTGGGTG
TCTTGAGTGTGGTAGGGGGCAGTGGAATTCCCGGTGTAGCGGTGAAATGCGTAGATATCG
GGAGGAACACCTATGGCGAAAGCAGCTGCCTGGGCCATTACTGACGCTAAGACTCGAAAG
CTAGGGGAGCAAACAGGATTAGAAACCCGCGTAGTCC
>Otu7196
CCAGCCTATGGGAGGCAGCAGTTAGGAATCTTGGACAATGGACGCAAGTCTGATCCAGCC
ATGCCGCGTGAGTGATGAAGGCCTTCGGGTTGTAAAGCTCTTTAGCCGATGAATATAATG
ACTGTAGTCGGAGAATAAGCCCCGGCTAACTTCGTGCCAGCAGCCGCGGTAATACGAAGG
GGGCGAGCGTTGTTCGGAATCACTGGGCGTAAAGCGTGCGTAGGCGGCGATGAAAGTTAG
AAGTGAAAGCCCAGGGCTTAACCTTGGAATTGCTTTTAAAACTCCATTGCTCGAATCTCG
GAGAGGATAGCGGAATTGTCAGTGTAGCAGTGAAATGCGTAGATATTGACAGGAACACCA
GTGGCGTAAGCGGCTATCTGGACGAGTATTGACGCTGAGGCACGAAAGCGTGGGGATCAA
ACAGGATTAGATACCCTCGTAGTCC
>Otu7201
CCAGCCTATGGGATGCTGCAGTCGGGAATTTTCCACAATGGGCGAAAGCCTGATGGAGCA
ACGCCGCGTGCAGGATGAATGCCTTCGGGTTGTAAACTGCTTTTATATGTGAAGATTATG
ACGGTAGCATATGAATAAGGATCGGCTAACTCCGTGCCAGCAGCCGCGGTCATACGGAGG
ATCCAAGCGTTATCCGGAATTACTGGGCGTAAAGAGTTGCGTAGGTGGCATAGTAAGTAG
ACAGTGAAAGCGTGTGGCTCAACCATATACACATTGTCTAAACTGCTAAGCTAGAGTATG
AGAGAGGTAGATGGAATTCCCTGTGTAGGAGTGAAATCCGTAGATATAGGGAGGAACACC
GATGGCGTAGGCAGTCTACTGGCTCGTTACTGACACTCAGGCACGAAAGCGTGGGGAGCA
AACGGGATTAGATACCCGGGTAGTCC
>Otu7202
CCAGCCTACGGGTTGCTGCAGGCGAGAATATTCCGCAATGGACGCAAGTCTGACGGAGCG
ACGCCGCGTGATGGATGAAGTGCTTAGGTACGTAAACATCTTTTATCGGGGAAGAACTAA
TTGACGGTACCCGATGAATAAGGGGCTCCTAACTCTGTGCCAGCAGGAGCGGTAATACAG
AGGCCCCGAGCGTTACCCGGAATCACTGGGCGTAAAGGGTGTCCAGGCGGCCCTGTTAGT
CTCTTGTAAAATCTATGGGCTCAACCTATAGCGCGCAAGGGAAACGGCAGGGCTCGAGGG
CGCGAGAGGTACAGGGAACTCATGGTGGAGGGGTGAAATCCGTTGATATCATGGGGAACA
CCAAAGGCGAAGGCACTGTACTAGCGCGTTCCTGACGCTCACACACGAAAGCCAGGGGAG
CGAACGGGATTAGAAACCCCCGTAGTCC
>Otu7207
CCAGCCTACGGGGCGCTCCAGTAGGGAATATTGGACGATGGCCGCAAGGCTGATCCAGCC
ATGCCGCGTGCAGGAAGAAGGACCTATGGTTTGTAAACTGCTTTTGTAACAGAGAAAACC
CTCGTACGTGTACGGGGCTGATAGTATGGTAAGAATAAGCATCGGCTAACTTCGTGCCAG
CAGCCGCGGTAATACGAAGGATGCAAGCGTTATCCGGATTTATTGGGTTTAAAGGGTGCG
TAGGCGGACTTATAAGTCAGTGGTGAAATCTCGATGCTTAACATCGAACGTGCCATTGAT
ACTGTAGGTCTTGAGTACAGATGCCGTTGGCGGAATGTGTCATGTAGCGGTGAAATGCAT
AGATATGACACAGAACACCGATTGCGAAGGCAGCTGACGAAACTGTAACTGACGCTGAGG
CACGAAAGCGTGGGGATCAAACAGGATTAGAAACCCCCGTAGTCC
>Otu7213
CCAGCCTACGGGACGCTCCAGTGGGGAATCTTGGACAATGGGGGCAACCCTGATCCAGCG
ATGCCGCGTGAGTGAAGAAGGCCTTCGGGTCGTAAAGCTCTTTAGGTTGGGAGGAAGTCT
AGTATTTTAAGAGATAACTAGATTGACTGTACCGACAGAATAAGCACCGGCAAGCTCTGT
GCCAGCAGCCGCGGTAATACAGAGGGTGCGAGCGTTAATCGGATTTACTGGGCGTAAAGG
GCGCGTAGGCGGTTGTATGAGTGTGATGTGAAAGCCCCGGGCTTAACCTGGGAAGTGCAT
CGCAAACGATACGACTGGAGTAGGTGAGAGGGTGGCGGAATTTCCGGTGTAGCGGTGAAA
TGCGTAGAGATCGGAAGGAACGTCAATGGCGAAGGCAGCCACCTGGTACCATACTGACGC
TGAGGCGCGAAAGCGTGGGGATCGAACAGGATTAGATACCCCTGTAGTCC
>Otu7215
CCAGCCTATGGGTCGCACCAGTCGAGAATCTTTCACAATGGGGGCAACCCTGATGGAGCG
ACGCCGCGTGGAGGATAAGGTCTTCGGATTGTAAACTCCTGTCATGTGTGAACAAGGTTG
TCGTGTTAATAGCATGGCAAATTGATGGTAACACAAGAGGAAGAGACGGCTAACTCTGTG
CCAGCAGCCGCGGTAATACAGAGGTCTCAAGCGTTGTTCGGAATCACTGGGCGTAAAGGG
TGCGTAGGTGGCGTGGTAAGTCAGATGTGAAAGCCCGGGGCTCAACCTCGGAATTGCATC

CGATACTACCATGCTAGAGTACTGAAGAGGTGACTAGAATTCTCGGTGTAGCAGTGAAAT
GCGTAGATATCGAGAGGAATACCAACGGCGAAGGCAGGTCACTGGGCAGTTACTGGCACT
GAGGCACGAAGGCCAGGGGAGCAAACGGGATTAGAAACCCTTGTAGTCC
>Otu7217
CCAGCCTATGGGTGGCACCAGTGGGAATATTGGACAATGAGTGAAAGCTTGATCCAACAA
TACTGAATTTGTGATGACGGCATTTTTTGCTGTAAAGCAATTTTGTTGATGAGAATAATG
ATGGTAGTCAAAGAAATAAGTCCCGGCCAATGCCGTGCCAGCAGCCGCGGGAATACGGTA
GGGACAAACGTTATTCATATTTACTAGGCGTAAAGGGTACGTAGGTTGATTTATATCAAT
ATTAAATAAATAACAAAAATTACGTTTGTTTTAATAATTAAGAAGGTAAATCTTGAGTT
TTTATCGGGTTGGTAGTATTTTTTGAGTAGGTTTGAATATCGCAAAAAGTGAAAGGACTG
CTATACTAGCGAAGGCGACTTTCTGGATATTAACTGACGCTGAGGTACGGAGGTATAGGT
ATCGAACGGGATTAGAGACCCTTGTGGTCC
>Otu7218
CCAGCCTATGGGGGGCTGCAGTAGGGAATTTTGCGCAATGGGGGAAACCCTGACGCAGCA
ACGCCATGTGTGGGATGAAGCATTTCGGTGTGTAAACCACTGTCGGCAGGGAATAAAGCC
CGCATTTATGCGGGGGTGAATGTACCTGCAAAGGAAGCCCCGGCTAACTTCGTGCCAGCA
GCCGCGGTAATACGAGGGGGGCAAGTGTTGTTCGGAATTACTGGGCGTAAAGGGAGCGTA
GGCGGGCTCTTAAGTTGGATGTTTAAAACCGGGGCCTAACCCCGGCGTGGCATCCAAAAC
TGGGAGTCTTGAATGGGACAGAGGCCAGTGGAATTAGAGGTGTAGCGGTGGAATGCGTAG
ATATCTCTAAGAACACCAGTTGCGAAGGCGGCTGGCTGGGTCCACATTGACGCTGAGGCT
CGAAAGCGTGGGGAGCAAACAGGATTAGAAACCCCCGTAGTCC
>Otu7221
CCAGCCTATGGGGGGCACCAGTCGAGAATTTTTCACAATGGACGAAAGTCTGATGGAGCG
ACGCCGCGTGATTGATGAAGTCCTTCGGGACGTAAAGATCTTTTATGAGGGAAGAAGTTT
ATTGACGGTACCTCATGAATAAGGGGCTCCTAATCTCGTGCCAGCAGGAGCGGTAATACG
AGAGCCCCGAGCGTTATCCGGAATTATTGGGCGTAAAGGGTGTGTAGGTGGCATTGTTAG
TCGTTTGTTAAATCTCTGGGCTCAACCTAGAAATCGCGAACGAAACGGCAAAGCTTGAGA
ATAGAAGGGGTGCGCGGAGCTCATGGTGTAGGGGTGAAATCCGTTGATATCATGGGGAAC
ACCAAAAGCGAAGGCAGCGCACTGGTCTATATCTGACACTGAAACACGAAAGCGTGGGTA
GCGAATGGGATTAGAAACCCTCGTAGTCC
>Otu7224
CCAGCCTATGGGGCGCTCCAGTGTAGGATATTGGACAATGTACTAAAAGTATGATCCAGT
AATATTTCATAAATGATATTGACAGTATTAACACTGTAAAATTTTATTACTAAAGATAAT
TATGATGGTATTTCGTAAATTAGCACCGGCTAACCCTGTGCCAGCAGCCGCGGTAATACA
GGTGGTGCAAACATTATTCATTTTAATTTGGTGTAAAGAATACGTAGATTGCCATTTCAG
GTTAAAGAAAATTCTCATTAGGTTATGTGAATTAAAAAATTTATGTCTGGCTTTTGAGTT
CAATTGAGGTGGAAATAATTTCGTATGTAGTGGTAAAATTCTTATATATATGAAGGAATA
TTAAAATAGTTGTGCAAACATTTTTCTGAATTGAACTAACATTGAGGTATGAAGGTGTAG
GTATCAAATGGGATTAGAAACCCGCGTAGTCC
>Otu7225
CCAGCCTATGGGTTGCACCAGTCGAGAATATTCGACAATGGGCGCAAGCCTGATCGAGCG
ACGCCGCGTGCAGGAAGAAGGAGCTTTGCTCTGTAAACTGCTTTTGCGTGGGAAGAACTT
TGACGGTACCACGAGAATAAGGGGTTGCTAACCTCGTGCCAGCAGCAGCGGTAATACGAG
GACCCCGAGCGTTATCCGGAATTACTGGGCGTAAAGCGTGAGCATCCGGTCATGCTAGTC
TCATGTTAAATCTCATTGCTCAACGATGAGACCGCGTGGGAAACGGCACGACTAGAGCGG
GTAAGAGGCAGACGGAACGTGCAGTGTAGGGGTGAAATCCATTGATATTGCACGGAACAC
CAAAAGCGAAAGCAGTCTGCTAGTGCTCTGCTGACGGTCAATCACTAAAGCGTGGGGATC
AAAAAGGATTAGAAACCCCCGTAGTCC
>Otu7227
CCAGCCTACGGGGTGCTCCAGTCGAGAATTTTTCACAATGGGGGAAACCCTGATGGAGCG
ACGCCGCGTGGGGGATGAATGGCTTCGGCCCGTAAACCCCTGTCATTTGCGAACAAATTG
ATTCACTTAACACGTGAAGCATTGATAGTAGCGGAAGAGGAAGAGACGGCTAACTCTGTG
CCAGCAGCCGCGGTAATACAGAGGTCTCAAGCGTTGTTCGGATTCATTGGGCGTAAAGGG
TGCGCAGGCTGCGAGGTGAGTCGGACGTGAAATCCCGGGGCTTAACCCCGGAACTGCGTT
CGATACTGCCTTGCTTGAGGACTGGAGAGGAGACTGGAATTCCTGGTGTAGCAGTGAAAT
GCGTAGATATCAGGAGGAACGCCAATGGCGAAGGCAAGTCTCTGGACAGTTCCTGACGCT
CATGCACGAAGGCTAGGGGAGCAAACGGGATTAGAAACCCCAGTAGTCC
>Otu7235

CCAGCCTACGGGGGGCTGCAGTAAGGAATATTGGACAATGGGGGCAACCCTGATCCAGCC
ATGCCGCGTGAAGGATGAAGGCGCTATGCGTTGTAAACTTCTTTTGAACGGGAGAAAACC
CACCTACGTGTAGGTGGCTGATAGTACCGTTAGAATAAGGATCGGCTAACTCCGTGCCAG
CAGCCGCGGTAATACGGAGGATCCAAGCGTTATCCGGATTCATTGGGTTTAAAGGGTGCG
TAGGCGGACTGATAAGTCAGCGGTGAAATCTCTCAGCTTAACTGAGAAACTGCCGTTGAT
ACTGTTAGTCTTGAGTACAGATGATGTGGGCGGAATATGACATGTAGTGGTGAAATACTT
AGATATGTCATAGAACACCGATTGCGAAGGCAGCTCACAAAACTGTTACTGACGCTGAGG
CACGAAAGCGTGGGGATCAAACAGGATTAGAACCCGAGTAGTCC
>Otu7236
CCAGCCTATGGGACGCTGCAGTGGGGAATATTGCGCAATGGGCGAAAGCCTGACGCAGCG
ACGCCGCGTGGGGGATGACGCTTTTCGGAGTGTAAACCCCTTTCGATCGGGACGAATGCC
TCGCAAGAGGAGTGACGGTACCGGTAGAAGAAGCCCCGGCTAACTACGTGCCAGCAGCCA
CGGTAATACGTAGGGGGCCAGCGTTGCTCGGAATAACTGGGCGTAAAGGGTTCGTAGGCG
GTGCGGCAAGTTGGGAGTGAAATCTCTGGGCTTAACCCAGAGACTGCTTTCAAAACTGCC
GTGCTAGAGTGCGGGAGAGGCCAGTGGAATTGCAGGTGTAGCGGTGAAATGCGTAGATAT
CTGCAGGAACACCCGTGGCGAAGGCGGCTGGCTGGACCGCAACTGACGCTGAGGAACGAA
AGCTAGGGGAGCAAACAGGATTAGATACCCGAGTAGTCC
>Otu7238
CCAGCCTACGGGAGGCTCCAGTGGGGAATTGTTCGCAATGGGCGCAAGCCTGACGACGCA
ACGCCGCGTGGAGGATGAAGACCTTCGGGTCGTAAACTCCTTTCGATCGAGACGAATGTT
TTCTGGGTGAACAATCCGGAAAAGTGACGGTACCGAGAGAAGAAGCCCCGGCTAACTCTG
TGCCAGCAGCCGCGGTAATACAGGGGGGGGCAAGCGTTGTTCGGAATTACTGGGCGTAAA
GGGCTTGTAGGTGGCCAACTAAGTCAGACGTGAAATCCCTCAGCTTAACTGGGGAACTGC
GTCTGATACTGGATGGCTTGAGTGTGGGAGAGGGATGCGGAATTCCAGGTGTAGCGGTGA
AATGCGTAGATATCTGGAGGAACACCGGTGGCGAAGGCGGCATCCTGGACCAACACTGAC
ACTGAGGAGCGAAAGCCAGGGGAGCAAACGGGATTAGAGACCCTCGTAGTCC
>Otu7241
CCAGCCTTGGGATGCTCCAGTCGAGAATCTTCCGCAATGGACGAAAGTCTGACGGAGCGA
CGCCGCGTGGAGGATGAAGTGCTTCGGCATGTAAACTCCTTTTGCCAGGGAAAAAGTTTA
TTGATTGTACCTGGAGAATAAGAGGTTGCTAAACTCGTGCCAGCAGCAGCGGTAATACGA
GTGCCTCGAGCGTTATCCGGAATCATTGGGCGTAAAGGGTGCGTAGGCGGCGATGTTAGC
CTCCCGTAAAATCTCTGGGCTCAACCCAGAGTCCGCGGGAGATACGGCATTGCTAGAGGA
TGGTAGGGGTCTCTGGAACTCATGGTGTAGCGGTGAAATGCGTTGATATCATGGGGAACA
CCAAAAGCGAAGGCAAGAGACTGGACCACTCCTGACGCTGAAGCACGAAAGCGTGGGTCG
CGAATGGGATTAGAGACCCTCGTAGTCC
>Otu7242
CCAGCCTATGGGGTGCAGCAGTGGGGAATCTTGCGCAATGGACGAAAGTCTGACGCAGCG
ACGCCGCGTGGGTGATGAAGGCCTTCGGGTTGTAAAGCCCTGTGGGGAGGGACGAATAAG
TAGAAGCTAACACCTTCTATGATGACGGTACCTCCTTAGCAAGCACCGGCTAACTCTGTG
CCAGCAGCCGCGGTAAGACAGAGGGTGCAAACGTTGTTCGGAATTACTGGGCGTAAAGCG
TGTGTAGGCGGCTACGTAAGTCGGATGTGAAAGCCCTGGGCTCAACCCAGGAAGTGCACT
CGATACTGCGCAGCTTGAGTCTCGGAGAGGTTGGTGGAATTCTCGGTGTAGAGGTGAAAT
TCGTAGATATCGAGAGGAACACCGGTGGCGAAGGCGGCCAACTGGACGAAGACTGACGCT
GAGACACGAAAGCGTGGGGAGCAAACAGGATTAGATACCCGCGTAGTCC
>Otu7244
CCAGCCTACGGGGGGCTGCAGTCGAGAATATTCGACAATGGGCGAAAGCCTGATCGAGCG
ACGCCGCGTGCAGGAAGAAGGCCTTCGGGTCGTAAACTGCTTTTGCGTGGGACGAATTTT
GACGGTACCACGAGAATAAGGGGTTGCTAACTCTGTGCCAGCAGCAGCGGTAATACAGAG
ACCCCGAGCGTTATCCGGAATTACTGGGCGTAAAGCGTGAGCAGCCGGTTGTATTAGTCT
GGTGTCAAACCATGGGGCCCAACCCCATGCCTGCATTGGAAACGGTACAACTCGAGTGGG
TAAGAGGGAGGCGGAACGTATGGTGTAGGGGTGAAATCCACTGATATCATACGGAACACC
AAAAGCGAAGGCAGCCTCCTAGTGCTCTACTGACGGTCAATCACTAAAGCGTGGGGATCA
AAAAGGATTAGAAACCCGGGTAGTCC
>Otu7248
CCAGCCTACGGGTGGCAGCAGTAAGGAATATTGGTCAATGGGCGCAAGCCTGAACCAGCC
ATGCCGCGTGGAGGATGAAGGCCCTCTGGGTTGTAAACTTCTTTTATTTGGGAAGAAATC
CATTTTTTCTAAGATGGTTGACGGTACCAGATGAATAAGCACCGGCTAACTCCGTGCCAG
CAGCCGCGGTAATACGGAGGGTGCAAGCGTTATCCGGATTCACTGGGTTTAAAGGGTGCG

TAGGCGGGTTGGTAAGTCCGTGGTGAAATCTCCAAGCTTAACTTGGAAACTGCCGTGGAT
ACTCTCAATCTTGAATATAGTGGAGGTGAGCGGAATATGTCATGTAGCGGTGAAATGCTT
AGATATGACATAGAACACCCATTGCGAAGGCAGCTCGCTACACTATTATTGACGCTGAGG
CATGAAAGCGTGGGGATCAAACAGGATTAGAAACCCCAGTAGTCC
>Otu7252
CCAGCCTACGGGGTGCTGCAGTGGGGAATATTGCGCAATGGGCGAATGCCTGACGCAGCG
ACGCCGCGTGTGGGATGAAGGTCTTCGGATCGTAAACCACTGTCAGAGGGGACGAAGGCC
CGGAGCTAACTACTATCGGGATTGACGGTACCCTCGGAGGAAGCACCGGCTAACTCTGTG
CCAGCAGCCGCGGTAATACAGAGGGTGCGAGCGTTGTCCGGAATCACTGGGCGTAAAGGG
CGTGTAGGCGGCTGGGTAAGCCGCGTGTGAAATCCCGGGGCTCAACCCCGGAACGGCACG
CGGGACTGTCCGGCTAGAGAATGGTAGAGGCGAGTGGAATTCCTGGTGTAGCGGTGGAAT
GCGTAGAGATCGGGAAGAACACCGGTGGCGAAGGCGGCTCGCTGGGCCATTTCTGACGCT
GAGGCGCGAAAGCGTGGGGAGCAAACAGGATTAGAAACCCCTGTAGTCC
>Otu7259
CCAGCCTACGGGTTGCAGCAGTGGGGGATCTTGCGCAATGGGGGAAACCCTGACGCAGCG
ACGCCGCGTGGGTGAAGAAGGCCTTCGGGTTGTAAAGCCCTGTCGAACGGGACAAATGTC
TTCTGTGCTAATACCACAGAGGAGTGATGGTACCGTTAGAGGAAGCACCGGCTAACTCTG
TGCCAGCAGCCGCGGTAATACAGAGGGTGCAAGCGTTGTTCGGAATCATTGGGCGTAAAG
GGCAGGTAGGTGGTTTAGAAAGTCTACTGTGAAATCCCCGGGCTTAACCTGGGAACTGCG
GTGGAAACTACTTAAACTAGAGTGCTAGAGGGGTTCGTGGAATACTCGGTGTGGCGGTGA
AATGCGTAGATATCGAGTGGAATATCAGAGGCGAAGGCGACGAACTGGATAGTAACTGAC
ACTCAACTGCGAAAGCGTGGGGAGCAAACAGGATTAGAAACCCCAGTAGTCC
>Otu7262
CCAGCCTACGGGTGGCAGCAGTCGAGAATCTTTCGCAATGGGCGCAAGCCTGACGAAGCG
ACGCCGTGTGAGTGATGAAGGCCCTCGGGTCGTAAAGCTCTTTCATTTGGGAACAAAAGA
AGCCGACAAATAATCGGCTAATTTGAGTGTACTAGAAGAAGAAGCACCGGCTAACTCCGT
GCCAGCAGCTGCGGTAATACGGAGGGTGCAAGCATTAATCGGATTTATTGGGCGTAAAGG
GCGCGTAGGCGGGATTGTAAGTCAGATGTGAAATCCCGGGGCTCAACCCCGGAACGGCAT
TTGAAACTGCGATTCTTGAGGATAGGAGGAGAAAACGGAATTCCACATGTAGCGGTGAAA
TGCGTAGATATGTGGAAGAACACCAGTGGCGAAGGCGGTTTTCTATCTTATACCTGACGC
TGAGGCGCGAAAGCAAGGGGATCAAACAGGATTAGAAACCCGAGTAGTCC
>Otu7263
CCAGCCTACGGGGGGCAGCAGTCGAGAATCTTCCACAATGGACGAAAGTCTGATGGAGCG
ACGCCGCGTGCAGGATGAAGTTCTTCGGGATGTAAACTGCTTTTATGAGCGAGAAAGTTT
ATTGATCAGCTCATGAATAAGAGGTTGCTAAACTCGTGCCAGCAGCAGCGGTAATACGAG
TGCCTCGAGCGTTATCCGGAATTATTGGGCGTAAAGGGTGTGTAGGTGGTCGTGTTAGTC
CTTCGTAAAATTCTCTGGCTCAACCAGAGTACGCGCGGGATACGGCACGACTTGAGGGTG
CGAGGGGTCTGTGGAACTCATAGTGTAGCGGTGAAATGCGTTGATATTATGGGGAACACC
AAAAGCGAAGGCAGCAGACTGGAGCATTCCTGACACTGAAACACGAAAGCGTAGGTAGCG
AATGGGATTAGATACCCCCGTAGTCC
>Otu7264
CCAGCCTATGGGACGCTCCAGTGGGGAATATTGGACAATGGGGGAAACCCTGATCCAGCA
ATGCCGCGTGTGTGAAGAAGGTCTTCGGATTGTAAAGCACTTTTGGCAGGGACGATGATG
ACGGTACCTGCAGAATAAGCCCCGGCTAACTTCGTGCCAGCAGCCGCGGTAATACGTAGG
CAGCAAGCGTTGTTCGGAATTACTGGGCGTAAAGAGTGTGTAGGCGGTTCTCTATGTTCG
GTGTGAAATCTCCCGGCTTAACTGGGAGGGTGCGCCGAAAACTGGAGGGCTTGAGTATGG
GAGAGGAAAGCGGAATTCCTGGTGTAGCGGTGAAATGCGTAGATATCAGGAGGAACACCT
GCGGTGTAGACGGCTTTCTGGACCATCACTGACGCTGAGACACGAAAGCGTGGGTAGCAA
ACAGGATTAGAGACCCGTGTAGTCC
>Otu7265
CCAGCCTATGGGGTGCAGCAGTAGGGAATATTGGTCAATGGGCGAAAGCCTGAATCAGCA
ACGCCGCGTGAGTGACGAAGGCCTTCGGGTCGTAAAGTTCTGTCGTACGGGAAGAAAAAA
ATGACGGTACCGTACAAGAAAGGACCGGCCAACTTCGTGCCAGCAGCCGCGGTAAGACGA
GGGGTCCTAGCGTTGTTCGGAATTACTGGGCGTAAAGCGCATGTAGGTGGCTTAATAAGT
CAGATGTGAAAGCCTCGGGCTCAACCTGAGAAGTGCATTTGATACTGTTAAGCTTGAGTA
TGGGAGAGGGTAGTAGAATTCCTGGTGTAGTGGTGAAATACGTAGATATCAGGAGGAATA
CCGGTGGCGAAGGCGGCTACCTGGCCCAATACTGACACTGAGATGCGAAAGCGTGGGGAT
CAAACAGGATTAGATACCCGAGTAGTCC

>Otu7266
CCAGCCTATGGGGGGCAGCAGTCGAGAATTTTTCACAATGGACGAAAGTCTGATGGAGCG
ACGCCGCGTGGAGGATGAAGGTTTTCGGATTGTAAACTCCTGTCACTGCAGAACAAGGAT
GGGCGGTTGAACAGACCGCCCATTTGATGGTATGCGGAGAGGAAGGGACGGCTAACTCTG
TGCCAGCAGCCGCGGTAAGACAGAGGTCCCGAGCGTTGTTCGGATTCATTGGGCGTAAAG
GGTGTGTAGGAGGTCGGGTAAGTCAGGTGTGAAATCTCACAGCTTAACTGTGAAACTGCG
CTTGATACTGCCCGGCTAGAGGATCGGAGGGGGTAACGGAATTTATGGTGTAGCAGTGAA
ATGCGTAGATATCATAAGGAACACCGGTGGCGAAGGCGGCTACCTGGACAATTGCTGACG
CTCAGGCACGAAAGCGTGGGGAGCAAAAGGGATTAGAGACCCTAGTAGTCC
>Otu7269
CCAGCCTACGGGGGGCACCAGTCGAGGATCTTCGGCAATGGGCGCAAGCCTGACGACGCA
ACGCCGCGTGGAGGATGAAGACCTTCGGGTCGTAAACTCCTTTCGATCGAGACGAATGGC
CTTCGGGTTAATAACCTGAAGGAGTGACGGTACCGAGAGAAGAAGCCCCGGCTAACTCCG
TGCCAGCAGCCGCGGTAATACGGGGGGGGCAAGCGTTGTTCGGAATTACTGGGCGTAAAG
GGCACGTAGGTGGCCAACTAAGTCAGACGTGAAATCCCTCGGCTTAACCGGGGAACTGCG
TCTGATACTGGATGGCTTGAGTTTGGGAGAGGGATGCGGAATTCCAGGTGTAGCGGTGAA
ATGCGTAGATATCTGGAGGAACACCGGTGGCGAAGGCGGCATCCTGGACCAACACTGACA
CTGAGGAGCGAAAGCCAGGGGAGCAAACGGGATTAGAAACCCGCGTAGTCC
>Otu7275
CCAGCCTACGGGTTGCACCAGTGGGGAATATTGGACAATGGGGGCAACCCTGATCCAGCA
ATGCCGCGTGAGTGAAGAAGGCCTTCGGGTTGTAAAACTCTTTTGTTAGGGAAGATGATG
ACTGAACCTAACGAATAAGCCCCGGCTAACTTCGTGCCAGCAGCCGCGGTAATACGAAGG
GGGCTAGCGTTGTTCGGAATGACTGGGCGTAAAGGGTGTGTAGGCGGTTATACACGTCAG
ATGTGAAATCCCTGGGCTCAACCTGGGAATGGCATTTGATACGGTAGAGCTAGAGATTGG
TAGAGGGTAGAGGAATTGCGAGTGTAGAGGTGAAATTCGTAGAGATTCGCAGGAACACCG
GTGGCGAAGGCGGCTACCTGGGCCAATACTGACGCTGAGGCACGAGAGCGTGGGGAGCAA
ACAGGATTAGAAACCCCTGTAGTCC
>Otu7277
CCAGCCTACGGGGGGCTCCAGTGAGGAATATTGCCCAATGGGCGCAAGCCTGAGGCAGCG
ACGCCGCGTGGGTGAAGAAGGCCTTCGGGTCGTAAAGCCCTTTTCTGCGTGACGAGACAA
GGACGGTAGCGCAGGAAAAAGTCTCGGCTAACTACGTGCCAGCAGCCGCGGTAAAACGTA
GGAGGCAAGCGTTATCCGGAGTTACTGGGCGTAAAGCGTGCGTAGGCGGGAAGATAAGTG
GGTGGTGAAAGCGCCCGGCTAAACCGGGCGATGTCCATTCAGACTGTCTTTCTAGAGGCG
GATAGAGGAGTGCGGAATTCCGGGTGTAGCGGTGAAATGTGTAGAGATCCGGAGGAACAC
CAGTGGTGCAAACGGCGCTCTGGGTCCGTACTGACGCTGAAGCACGAAAGCGTGGGTAGC
AAACAGGATTAGATACCCTTGTAGTCC
>Otu7280
CCAGCCTATGGGAGGCAGCAGTTAGGAATATTGGACAATGGACGCAAGTCTGATCCAGCC
ATGCCGCGTGAGCGATGAAGGCCTTCGGGTTGTAAAGCTCTTTAGCCGACGAATATAATG
ACTGTAGTCGGAGAATAAGCCCCGGCTAACTTCGTGCCAGCAGCCGCGGTAATACGAAGG
GGGCGAGCGTTGTTCGGAATCACTGGGCGTAAAGAGTGCGTAGGCGGTTGTGAAAGTTAG
GAGTGAAAGCCCCAGGCTCAACTTGGGAATTGCTCTTAAAACTCCACAACTCGAATTCGG
GAGAGGATAGCGGAATTGTCAGTGTAGCAGTGAAATGCGTAGATATTGACAGGAACACCA
GTGGCGTAAGCGGCTATCTGGACCGACATTGACGCTGAGGCACGAAAGCGTGGGGATCAA
ACAGGATTAGAAACCCTCGTAGTCC
>Otu7282
CCAGCCTATGGGAGGCACCAGTCAAGAACATTCGACAATGGGCGAAAGCCTGATCGAGCG
ACGCCGCGTGTAGGATGAAGGCCTTCGGGTTGTAAACTACGGTAGATAGTTAATAATGTA
AATGAATGACTATCGGAAAGAGGTTGGTAACTACGTGCCAGCACCCGCGGTAATACGTAG
ACCTCAAGCGTTATCCGGATTTATTGGGCGTAAAGCGCGTGTAGGAGGTTTTGTGCGTCT
TTCGTTAAAGCCCGGGGCCTAACCCCGGAAATGCGGGAGATACGACAAGACTAGAGGAGG
TTAGAGGCGCATGGAACAAATGGTGTAGGGGTGAAATCCGTTGATATCATTTGGAACACC
AAAGGCGAAGGCAGTGCACTGGGACCTTCCTGACTCTGAGACGCGAAAGCGTGGGGAGCA
AAAAGGATTAGAAACCCTCGTAGTCC
>Otu7283
CCAGCCTACGGGTGGCACCAGTAGGGAATATTGGACAATGGGCGCAAGCCTGATCCAGCC
ACGCCGCGTGAGTGATGAAGGCCTTCGGGTCGTAAAGCTCTGTGGGGAGGGACGAACCGC
TGTTGGTTCATAACCAGCAGCATGACGGTACCTCCTTAGCAAGCACCGGCTAACTTCGTG

CCAGCAGCCGCGGTAATACGAAGGGTGCAAACGTTGCTCGGAATTATTGGGCGTAAAGCG
CACGTAGGCGGCTAGAAAAGTTGGATGTGAAATCCCTCGGCTTAACCAAGGAAGTGCATC
CAAAACTGCCTAGCTTGAGTACTTAAGAGGATCGCGGAATTCCCGGTGTAGAGGTGAAAT
TCGTAGATATCGGGAGGAACACCAGTGGCGAAGGCGGCGATCTGGGAAGATACTGACGCT
GAGGTGCGAAAGCGTGGGGAGCAAACAGGATTAGATACCCCCGTAGTCC
>Otu7286
CCAGCCTACGGGGTGCAGCAGTGGGGAATATTGGACAATGGGCGAAAGCTTGATCCAACG
ATATCATATTAGAGATGAAGGGTGTAAACTTGTAAATCTGGATAGCTAAAGAAAATTATG
ATATTATTTAGCGAATTTAGCCCTGGCTAACTTCGTGCCAGCAGCTGCGGTAATACGGAG
GGGGCGAGTGTTACTCGTATTGATTGGGCGTAAAGGGTATGTAGGCGGTTTAGTAAGTTC
TTGATAAAATATCTTTATAAAGTAAAGAAAATGATTGAATACTGCTAAACTGGAGTTTGA
TGGGAGTGATTAGTACTGTAAATGGAGGGGTAAAATCCAGCAAAATTTATAGGACTGGCA
GAAGGCGAAGGCAATGAACTATATTAAACTGACGCTGAGGTACGAAAGCATAGGGATCAA
AAAGGATTAGAGACCCTAGTAGTCC
>Otu7287
CCAGCCTATGGGGGGCAGCAGTAAGGAATATTGGTCAATGGGCGCAAGCCTGAACCAGCC
ATGCCGCGTGCAGGAAGACGGCCCTACGGGTTGTAAACTGCTTTTATTCGTGAATAAATC
TTTCTACGTGTAGGAAGCTGAAAGTAGCGAAAGAATAAGGATCGGCTAACTCCGTGCCAG
CAGCCGCGGTAATACGGAGGATCCAAGCGTTATCCGGATTTATTGGGTTTAAAGGGTGCG
TAGGCGGATTATTAAGTCAGAGGTGAAATTTTGCGGCTCAACCGTAAGCTTGCCTTTGAT
ACTGATGATCTTGAATTAACTTGAGGTAGGCGGAATGTGACAAGTAGCGGTGAAATGCAT
AGATATGTCACAGAACACCAATTGCGAAGGCAGCTTACTAAGGTTATATTGACGCTGAGG
CACGAAAGCGTGGGGATCAAACAGGATTAGATACCCCGTAGTCC
>Otu7289
CCAGCCTACGGGGCGCACCAGTCGAGAATTTTTCTCAATGGGCGAAAGCCTGAAGGAGCG
ACGCCGCGTGGGGGATGAATGGCTTCGGCCCGTAAACCCCTGTCATTTGCGAACAAACCT
TTTCATTTAATATGTGAAAAGCTGATTGTAGCGAAAGAGGAAGAGACGGCTAACTCTGTG
CCAGCAGCCGCGGTAATACAGAGGTCCCAAGCGTTGTTCGGATTCACTGGGCGTAAAGGG
TGCGTAGGCGGTGAGGTAAGTCTGACGTGAAATCTCCGGGCCTAACCCGGAAACTGCGTT
GGATACTATCTCGCTAGAGGAATGGAGGGGAGACTGGAATACTTGGTGTAGCAGTGAAAT
GCGTAGATATCAAGTGGAACACCAGTGGCGAAGGCGAGTCTCTGGACATCTCCTGACGCT
GAGGCACGAAAGCCAGGGGAGCAAACGGGATTAGAGACCCTCGTAGTCC
>Otu7290
CCAGCCTATGGGAGGCTGCAGTCGAGAATTTTTCACAATGGACGAAAGTCTGATGGAGCG
ACGCCGCGTGGAGGATGAAGGTTTTCGGATTGTAAACTCCTGTCACTGCAGAACAAGGAT
GTGCCAGTTAACAGCCGGCGCATTTGATGGTATGCGGAGAGGAAGGGACGGCTAACTCTG
TGCCAGCAGCCGCGGTAAGACAGAGGTCCCGAGCGTTGTTCGGATTCATTGGGCGTAAAG
GGTGTGTAGGAGGTTGGGTAAGTCAGGTGTGAAATCTCAGAGCTTAACTCTGAAACTGCG
CTTGATACTGCTCGGCTAGAGGATCGGAGGGGGTAACGGAATTCATGGTGTAGCAGTGAA
ATGCGTAGATATCATGAGGAACACCGGTGGCGAAGGCGGTTACCTGGAAGATTCCTGACT
CTGAAATACGAAAGCGTGGGGAGCAAAAAGGATTAGAAACCCGTGTAGTCC
>Otu7294
CCAGCCTACGGGGTGCTGCAGTGGGGAATCTTGGACAATGGGGGAAACCCTGATCCAGCC
ATGCCGCGTGAGTGATGAAGGCCTTAGGATTGTAAAGCTCTTTCGACGGGGACGATGATG
ACGGTACCCGTAGAAGAAGCCCCGGCTAACTTCGTGCCAGCAGCCGCGGTAATACGAAGG
GGGCTAGCGTTGCTCGGAATGACTGGGCGTAAAGGGCGCGTAGGCGGTTTGTACAGTCAG
ATGTGAAATTCCTGGGCTCAACCTGGGGGCTGCATTTGATACGTATGGACTGGAGTGAGG
AAGAGGGTCGTGGAATTCCCAGTGTAGAGGTGAAATTCGTAGATATTGGGAAGAACACCG
GTGGCGAAGGCGGCGACCTGGTCCTTTACTGACGCTGAGGCGCGAAAGCGTGGGGAGCAA
ACAGGATTAGAGACCCGCGTAGTCC
>Otu7304
CCAGCCTATGGGGGGCACCAGTCGAGGATCTTTGGCAATGAGCGCAAGCTTGACCAAGCG
ACGCCGCGTGTGCGATGAAGGCCTTCGGGTTGTAAAGCACTGTCGAGGGGAACGAAGCCG
AAAGGTTGACCGATCCCTGGAGGAAGCACGGGCTAAGTTCGTGCCAGCAGCCGCGGTAAG
ACGAACCGTGCGAACGTTGTTCGGAATCACTGGGCTTAAAGGGCGCGTAGGCGGCTCGTC
AAGTCGGGGGTGAAATACTCCAGCTCAACTGGAGAAGTGCCTTCGAAACTGACGAGCTCG
AGGGAGGTAGGGGTATGTGGAACTTCTGGTGGAGCGGTGAAATGCGTTGATATCAGAAGG
AACGCCGGTGGCGAAAGCGGCTCACTGGATCTCTTCTGACGCTGAGGCACGAAAGCTAGG

GTAGCGAACGGGATTAGAAACCCCCGTAGTCC
>Otu7305
CCAGCCTATGGGTCGCTGCAGCCGAGAATATTCGACAATGGGCGAAAGCCTGATCGAGCG
ACGCCGCGTGATTGATGAAGTCTTTCGGGATGTAAAGATCTTTTATAGGGGAGAAAGCCA
TGGTTGCCATGGTTGATGGTACTCTATGAATAAGGGGTTGCTAAACTCGTGCCAGCAGCA
GCGGTAATACGAGTGCCCCGAGCGTTATCCGGAATTATTGGGCGTAAAGGGTGTGTAGGT
GGTTGTGTTAGTCTTCTGTTAAATTCTTCGGCTTAACCGGGGACCTGCGGAAGAAACGGC
ACGACTAGAGAGTGCGAGAGGTGAGCGGAACTCATGGTGTAGGGGTGAAATCCGTTAATA
TGATGGGGAACACCGAAGGCGAAGGCAGCTCACTGGCGTACTTCTGACACTGAAACACGA
AAGCGTGGGTAGCGAATGGGATTAGAAACCCTAGTAGTCC
>Otu7306
CCAGCCTATGGGGGGCTGCAGTGGGGAATTTTGGACAATGGGGGCAACCCTGATCCAGCA
ATGCCGCGTGTGTGAAGAAGGCCTTCGGGTTGTAAAGCACTTTTGTCGGGAAAGAAATCA
TCCTGGCTAATATCCGGGGTGGATGACGGTACCGGAAGAATAAGCACCGGCTAACTCTGT
GCCAGTAGCCGCGGTAATACAGAGGGTGCGAGCGTTAATCGGAATTACTGGGCGTAAAGC
GTGCGTAGACGGTTTCGTAAGTCGGATGTGAAATCCCCGGGCTCAACCTGGGAATTGCAT
TCGAAACTGCAAAGCTAGGGTGCGGAAGAGGGAAGCGGAATTTCCGGTGTAGCGGTGAAA
TGCGTAGATATCGGAAGGAACATCAGTGGCGAAAGCGGCTTCCTGGTCCAGCACCGACGT
TCAGGCACGAAAGCGTGGGGAGCAAACAGGATTAGATACCCCAGTAGTCC
>Otu7308
CCAGCCTATGGGATGCTGCAGTCGAGAATCTTCCGCAATGGACGAGAGTCTGACGGAGCG
ACGCCGCGTGATTGATGAAGCACTTCGGTGCGTAAAGATCTTTTATGAGGGAAGAAGTTT
ATTGACTGTACCTCATGAATAAGGGGCTCCTAATCTCGTGCCAGCAGGAGCGGTAATACG
AGAGCCCCAAGCGTTATCCGGAATTATTGGGCGTAAAGGGTGTGTAGGTGGTTGTGTTAG
TCTTTCGTCAAATCTCCGAGCTTAACTTGGAGTCTGCGAGGGAAACGGCACGACTAGAAA
GTGCGAGAGGTGTACGGAACTCATGGTGTAGGGGTGAAATCTGTTGATATCATGGGGAAC
ACCAAAGGCGGAGGCAGTACACTGGCGCATATTTGACACTCAAACACGAAAGCGTGGGTA
GCGAATGGGATTAGAAACCCGAGTAGTCA
>Otu7310
CCAGCCTACGGGGGGCAGCAGCAAGGAATATTGGTCAATGGGCGAGAGCCTGAACCAGCC
ATGCCGCGTGCAGGAAGAAAGCCCTCCGGGTTGTAAACTGCTTTTGTACGGGAAGAAACC
CCGCCACGTGTGGCGGGTTGACGGTACTGTAAGAATAAGGATCGGCTAACTTCGTGCCAG
CAGCCGCGGTAATACGAAGGATCCAAGCGTTATCCGGATTTATTGGGTTTAAAGGGTGCG
TAGGCGGCTTATTAAGTCAGTGGTGAAAGCCCGATGCTTAACATCGGAACTGCCATTGAT
ACTGATGAGCTTGAATATACTTGAGGTAGGCGGAATGTGTCATGTAGCGGTGAAATGCTT
AGATATGACACAGAACACCGATTGCGAAGGCAGCTTGCTAAAGTATGATTGACGCTGAGG
CACTAAAGCGTGGGGATCAAACAGGATTAGAAACCCCCGTAGTCC
>Otu7313
CAGCCTATGGGACGCAGCAGTCAAGAACCTTCCACAATGGACGAAAGTCTGATGGAGCGA
CGCCGCGTGGTTGATGAAGTCCTTCGGGACGTAAAGACCTTTTATGAGGGAAGAAGTTTA
TTGACGGTACCTCATGAATAAGGGGCTCCTAATCTCGTGCCAGCAGGAGCGGTAATACGA
GAGCCCCGAGTGTTATCCGGAATTATTGGGCGTAAAGGGTGCGAAGGTGGTTGTGTTAGT
CATATGTTAAAACCCCAGGCTCAACTTGGGGACTGCATTTGAAACGGCACGACTAGAGGA
TGTGAGAGGTGTACAGAACTCATGGTGTAGGGGTGAAATCCGTTGATATCATGGGGAATA
CCAAAAGCGAAGGCAGTACACTGGTGCATACCTGACACTCCAGCACGAAAGCGTGGGTAG
CGAATGGGATTAGATACCCGAGTAGTCC
>Otu7314
CCAGCCTACGGGTCGCTGCAGTAGGGAATATTGGACAATGGGCGCAAGCCTGATCCAGCC
ATCCCGCGTGAAGGATTAAGGCCCTCTGGGTTGTAAACTTCTTTTTTTGGGGAATAAAAA
CTGGGATTTATCTCGGCTTGAAGGTACCCGAAGAATAAGCACCGGCTAACTCCGTGCCAG
CAGCCGCGGTAATACGGAGGGTGCAAGCGTTATCCGGATTCACTGGGTTTAAAGGGTGCG
TAGGTGGCTTTGTAAGTCAGTGGTGAAATCTCGGAGCTTAACTCCGAAACTGCCATTGAC
ACTGCTTAGCTTGAATCAAGTAGAGGTGGATGGAATATTACATGTAGCGGTGAAATGCTT
AGATATGTAATAGAACACCGATTGCGAAGGCAGTTCACTATGCTTGTATTGACACTGAGG
CACGAAAGCGTGGGGATCAAACAGGATTAGAAACCCCCGTAGTCC
>Otu7315
CCAGCCTATGGGTGGCTCCAGACGAGAATATTCGACAATGGGCGAAAGCCTGATCGAGCG
ACGCCGCGTGGAGGATGAAGTGCTTCGGCATGTAAACTCCTTTTGCCAGGGAAAAAGTTT

ATTGATTGTACCTGGAGAATAAGAAGTTGCTAAACTCGTGCCAGCAGCAGCGGTAATACG
AGTGCTTCGAGCGCTATCCGGAATCATTGGGCGTAAAGGGTGCGTAGGCGGCAGTGTTAG
TCTCGCGTAAAATCTTCCGGCTCAACCGGGAGTCTGCGCGGGAAACGGCACAGCTAGAGG
ACGGCAGAGGTCTCTGGAACTCATGGTGTAGCGGTGAAATGCGTTGATATCATGGGGAAC
ACCGAAAGCGAAGGCAAGAGACTGGGCCGCTCCTGACGCTGAAGCACGAAAGCGTGGGTC
GCGAATGGGATTAGAGACCCCCGTAGTCC
>Otu7321
CAGCCTACGGGAGGCTGCAGTAAGGAATATTGGACAATGGGGGCAACCCTGATCCAGCCA
TGCCGCGTGAAGGATGAAGGCCCTCTGGGTTGTAAACTTCTTTTATGCGGGAATAAACCC
CTCTACGTGTAGAGGGTTGAATGTACCGTAAGAATAAGGATCGGCTAACTTCGTGCCAGC
AGCCGCGGTAATACGAAGGATCCAAGCGTTATCCGGATTTATTGGGTTTAAAGGGTGCGT
AGGCGGGCTATTAAGTCAGTGGTGAAAACCCCAAGCTTAACTTGGGAACTGCCATTGATA
CTGATGGTCTTGAGTACACTTGAAGTGGGCGGAATGTGTCATGTAGCGGTGAAATGCTTA
GATATGACACAGAACACCGATTGCGAAGGCAGCTCACTAAATTGTAACTGACGCTGAGGC
ACGAAAGCGTGGGGATCAAACAGGATTAGAGACCCCCGTAGTCC
>Otu7332
CCAGCCTACGGGAGGCTGCAGTGGGGAATCTTGCGCAATGGGCGAAAGCCTGACGCAGCA
ACGCCGCGTGCGGGATGACGGCCTTCGGGTTGTAAACCGCTTTCAGGAGGGACGAAAATG
ACGGTACCTTCAGAAGAAGCCCCGGCTAACTACGTGCCAGCAGCCGCGGTAATACGTAGG
GGGCAAGCGTTGTCCGGATTTATTGGGCGTAAAGAGCTCGTAGGTGGTTCCGTAAGTCGC
GAAGTCAAATCTCTGGGCTCAACCCAGAATCGCTTCCGATACTGCGGTGACTGGAGTCCG
GTAGGGGAGCGTGGAATTCCTGGTGTAGCGGTGAAATGCGCAGATATCAGGAGGAACACC
AACAGCGAAGGCAGCGCTCTGGGCCGGTACTGACACTGAGGAGCGAAAGCGTGGGGAGCG
AACAGGATTAGATACCCCAGTAGTCC
>Otu7333
CCAGCCTACGGGTGGCAGCAGTAAGGGATATTGCACAATGGGCGCAAGCCTGATGCAGCA
ACGCCGCGTGGGGGAAGAAGGCCTTCGGGTCGTAAACTCGTTTTGCGTCTGAAGAGGAAG
GACGTTAGGACGCGAATAAGTCACGGCTAACTACGTGCCAGCAGCCGCGGTAAAACGTAG
GTGGCGAGCGTTATCCGGATTTACTGGGCGTAAAGGGTGTGCAGGCGGGAGTACAAGTGG
TGTATGAAAGGGCCGCGCTTAACGTGGCTAGGTTATGCCAGACTGTGCTTCTAGAGGGCG
AGAGAGGGGCGTGGAATTCCGGGTGTAGTGGTGAAATGCGTAGAGATCCGGAAGAACCCC
AGAGGCGAAGGCGGCGCCCTGGCTCGCACCTGACGCTCAGCCACGACAGCATGGGGAGCG
AACGGGATTAGAGACCCGCGTAGTCC
>Otu7336
CCAGCCTATGGGACGCAGCAGTCGAGAATTTTTCACAATGGGGGAAACCCTGATGGAGCG
ACGCCGCGTGGAGGATGAAGGTTTTCGGATTGTAAACTCCTGTCACTGCAGAACAAGGGT
GTGTTCGTTAATAGCGAATGCATTTGATGGTATGCGGAGAGGAAGGGACGGCTAACTCTG
TGCCAGCAGCCGCGGTAAGACAGAGGTCCCGAGCGTTGTTCGGATTCATTGGGCGTAAAG
GGTGTGTAGGAGGTCGGGTAAGTCAGGTGTGAAATCTCAGAGCTCAACTCTGAGACTGCG
CTCGATACTGCCCGGCTAGAGGATCGGAGGGGGTATCGGAATTTATGGTGTAGCAGTGAA
ATGCGTAGATATCATAAGGAACACCGGTGGCGAAGGCGGATACCTGGAAGATTCCTGACT
CTGAAACACGAAAGCCAGGGGAGCAAACGGATTAGAGACCCTCGTAGTCC
>Otu7337
CCAGCCTATGGGTTGCTGCAGTCGAGGATCTTCGGCAATGGGCGCAAGCCTGACCGAGCG
ACGCCGCGTGTGCGATGAAGGCCTTCGGGTTGTAAAGCACTGTCGAGGGGGAGGAAAGCC
GAAAGGTCTGACCTATCCCTGGAGGAAGCACGGGCTAAGTTCGTGCCAGCAGCCGCGGTA
AGACGAACCGTGCGAACGTTGTTCGGAATCACTGGGCTTAAAGGGCGCGTAGGCGGCTGG
CAAAGTCCGGGGTGAAAGCCCCCAGCTCAACTGGGGAAGGGCCTCGGATACTGGTCAGCT
CGAGGGAGGTAGGGGCGGATGGAACTTCCGGTGGAGCGGTGAAATGCGTTGATATCGGAA
GGAACGCCGGTGGCGAAAGCGATCCGCTGGCCCTCTACTGACGCTGAGGCGCGAAAGCCA
GGGGAGCAAACGGGATTAGAGACCCCGGTAGTCC
>Otu7346
CCAGCCTATGGGTGGCACCAGTAGGGAATTTTCCACAGTGGGCGAAAGCCTGATGGAGCA
ACGCCGCGTGCAGGATGAATGTCTTCGGATTGTAAACTGCTTTTATCTGTGACGAATATG
ACGGTAGCAGATGAATAAGGATCGGCTAACTCCGTGCCAGCAGCCGCGGTCATACGGAGG
ATCCAAGCGTTATCCGGAATTACTGGGCGTAAAGAGTTGCGTAGGTGGCAGAGTAAGCGA
ATAGTAAAATGATGTGGCTCAACCATATGTCTATTATTTGAACTGCTCAGCTAGAGGACG
AGAGAGGTTATTGGAATTCCTAGTGTAGGAGTGAAATCCGTAGATATTAGGAGGAACACC

GATGGCGTAGGCAGATAACTGGCTCGTTCCTGGCACTAAGGCACGAAAGCGTGGGTAGCA
AACGGGATTAGAAACCCGTGTAGTCC
>Otu7353
CCAGCCTATGGGGTGCTGCAGTCGAGGATCATTCGCAATGGGCGAAAGCCTGACGATGCG
ACGCTGTGTGAATGATGAAGGCCTTCGGGTTGTAAAGTTCTTTCGCTTAGGAACAAGAGA
GGCACGTGAACAACGAGCCGATTTGAGGGTACTAGGTAAAGAAGCACCGGCTAACTCCGT
GCCAGCAGCTGCGGTAATACGGAGGGTGCAAGCATTAATCGGATTTATTGGGCGTAAAGG
GCGCGTAGGCGGATTCGTAAGTCAGATGTGAAATTCCTAGGCTCAACCTAGGAGCTGCAT
TTGAAACTGTGAGTCTTGAGGATTGGCGGAGAAAATGGAATTCCACGTGTAGCGGTGAAA
TGCGTAGATATGTGGAAGAACATCGGTGGCGAAAGCGGTTTTCTAGCTTAGACCTGACGC
TGAGGCGCGAGAGCAAGGGGAGCAAACAGGATTAGATACCCCCGTAGTCC
>Otu7355
CCAGCCTACGGGGGGCTGCAGTGGGGAATATTGAACAATGCCCGCAAGGGTGATCCAGCC
ATGCCGCGTGCAGGAAGAAGGCCCTATGGGTTGTAAACTGCTTTTACACCGGAGAAAACC
CCCTTTCGTGAAAGGGGCTGATAGTATGGTGAGAATAAGCATCGGCTAACTTCGTGCCAG
CAGCCGCGGTAAGACGAAGGATGCAAGCGTTATCCGGATTTATTGGGTTTAAAGGGAGCG
TAGGTGGCCTTATAAGTCAGCGGTGAGAGACTAGAGCTTAACTCTGGAATTGCCATTGAT
ACTGTAGGGCTTGAGTATAGTTGCTGTGGGCGGAATATGACATGTAGTGGTGAAATACTT
AGAGATGTCATAGAACACCGATTGCGAAGGCAGCTCACAAAACTATAACTGACACTGAGG
CTCGAAAGTGCGGGGATCAAACAGGATTAGAAACCCCAGTAGTCC
>Otu7356
CCAGCCTACGGGATGCAGCAGTGAGGAATCTTCCGCAATGGGCGAAAGCCTGACGGAGCA
ACACCGCGTGCAGGATGACGGCTTTCGGGTTGTAAACTGCTTTTATTTGGGAAGATTATG
ACGGTACCAAATGAATAAGGACCGGCTAACTACGTGCCAGCAGCCGCGGTAATACGTAGG
GTCCAAGCGTTATCCGGAATCACTGGGCGTAAAGCGTACGTAGGCGGATGTTTAAGTGCA
ACATGAAATCCCGTGGCTCAACCATGTGGACTGTATTGCATACTGGACGTCTTGAGGACA
TGAGAGGCAAGTGGAATTACTGGTGTAGCAGTGAAATGCGTAGAGATCAGTAGGAACACC
AATGGCGAAGGCAGCTTGCTGGCATGTATCTGACGCTAAGGTACGAAAGCGTGGGGAGCG
AACAGGATTAGAGACCCGTGTAGTCC
>Otu7357
CCAGCCTATGGGGGGCACCAGTGGGGAATTTTGCGCAATGGGCGAAAGCCTGACGCAGCG
ACGCCGCGTGTGCGATGAAGGCCTTTGGGTCGTAAAGCACTGTCGATCGGGACGAAAGCT
CATCGGCTAATACCTGATGAGGTTGACGGTACCGGTAAAGGAAGCACCGGCCAACTCTGT
GCCAGCAGCCGCGGTAATACAGAGGGTGCAAACGTTGCTCGGAATGATTGGGCGTAAAGC
GCGTGTAGGCGGTCTAGTATGCCGGCTGTGAAAGCCCGTGGCTCAACCACGGAAGTGCAG
ACGGAACTGCTAGACTAGAGTGCCGAATAGGATGGCGGAATGCCCAGTGTAGAGGTGAAA
TTCGTAGATATTGGGTAGAACACCGGTGGCGAAGGCGGCCATCTGGGAGGTTACTGACGC
TGAGACGCGAAAGCGTGGGGAGCAAACAGGATTAGAAACCCTTGTAGTCC
>Otu7362
CCAGCCTATGGGGGGCTCCAGTCGAGAATATTTGGCAATGGGCGCAAGCCTGACCATGCG
ACGCCGCGTGGTTGACGAAGTCCTTCGGGACGTAAAAACCTTTTGCGCGTGAGGAAGTAA
TTGACGTTAGCGCGCGAATAAGGGGCTCCTAACTCTGTGCCAGCAGGAGCGGTAATACAG
AGGCCCCAAGCATTATCCGGAATCACTGGGCGTAAAGGGTGTGCAGGCGGCACTGCTAGT
CTTTCGTAAAAGCACCGGGGCTTAACCCCAGTACTGCGGAGGAAACGGCAGAGCTCGAGG
ACGCGAGAGGTAGAGGGAACTCATGGTGTAGGGGTGAAATCCGTTGATATCATGGGGAAC
ACCAAATGCGAAGGCACTCTACTGGCGCGCTCCTGACGCTGAGACACGAAAGCGTGGGAA
TCGAACGGGATTAGATACCCGAGTAGTCC
>Otu7364
CCAGCCTACGGGTCGCTGCAGTGGGGAATATTGCGCAATGGAGGAAACTCTGACGCAGCG
ACGCCGCGTGGGTGATGAAGGCCTTCGGGTCGTAAAGCCCTGTCGGAGGGGAAGAAAAGC
CGTCTGGCTAATATCCAGGCGGCATATGACGGTACCCTCAAAGGAAGCACCGGCTAACTC
CGTGCCAGCAGCCGCGGTAAGACGGAGGGTGCAAGCGTTGTTCAGATTGACTGGGCGTAA
AGGGCGTGTAGGCGGTTGGCCAAGTCCGATGTGAAAGCCCGGGGCTCAGCCTCGGAAGTG
CATCGGAAACTAGCCGGCTGGAGCGCGGTAGAGGATGGTAGAATTCCTGGTGTAGCGGTG
AAGTGCGTAGATATCAGGAGGAATACCGGCGGCGAAGGCGGCCCTCTGGATCGTTGCTGA
CGCTGAGACGCGAAAGCGTGGGGAGCAAACAGGATTAGAGACCCCGGTAGTCC
>Otu7370
CCAGCCTATGGGTGGCTGCAGTAGGGAATATTGCACAATGGGGGAAACCCTGATGCAGCA

ACGCCGCGTGAGTGACGAAGGCCTTCGGGTCGTAAAGCTCTGTTGTATGGGAAGAAAAAA
ATGACGGTACCTTGCGAGAAAGGACCGGCCAACTTCGTGCCAGCAGCCGCGGTAATACGA
GGGGTCCTAGCGTTGTTCGGAATCACTGGGCGTAAAGCGCATGTAGGCGGCTTCGTAAGT
CAGGTGTGAAAGCCCTGGGCTCAACCCAGGAAGTGCACTTGATACTGCAGAGCTTGAATA
TGTAAGAGGGTAGCGGAATTCCAGGTGTAGTGGTGAAATACGTAGATATCTGGAGGAACT
CCGGTGGCGAAGGCGGCTACCTGGTACAATATTGACGCTAAGATGCGAAAACGTGGGGAT
CAAACAGGATTAGAAACCCGAGTAGTCC
>Otu7372
CCAGCCTATGGGGGGCTGCAGTCGAGAATCTTCCACAATGGACGAAAGTCTGATGGAGCG
ACGCCGCGTGGTTGATGAAGTCCTTCGGGACGTAAAAACCTTTTATGAGGGAGGAAGTTA
TTGACGTTACCTCATGAATAAGGGGCTCCTAACTCTGTGCCAGCAGGAGCGGTAATACAG
AGGCCCCAAGCATTATCCGGAATCACTGGGCGTAAAGAGTGTGTAGGCGGTTGTGTTAGT
CTTTCGTGAAAGATCTCGGGCTTAACCCGGGAGACGCGGAGGAAACGGCACGGCTACGAG
GATGTGAGAGGTAAAGGGAACTCATGGTGTAGGGGTGAAATCCGTTGATATCATGGGGAA
CACCAAATGCGAAGGCACTTTACTGGCACACTCCTGACGCTGAAACACGAAAGCGTGGGA
ATCGAATGGGATTAGAGACCCGCGTAGTCC
>Otu7381
CCAGCCTATGGGGGGCAGCAGTCGAGAATCATTCGCAATGGGCGAAAGCCTGACGATGCG
ACGCCGTGTGAGCGATGAAGGCCTTCGGGTCGTAAAGCTCTTTCGCTTGGGAAAAAGAAA
GGTCGGCTAATATCCGGCCAATTTGATGGTACCAGGTAAAGAAGCACCGGCTAACTCCGT
GCCAGCAGCTGCGGTAATACGGGGGGTGCTAGCATTAATCGGATTTATTGGGCGTAAAGG
ACGCGTAGGCGGTAAGGTAAGTCGGGTGTGAAATCCCGGGGCTCAACCCCGGAACTGCAC
TTGAAACTGCCTTTCTTGAGGGTAGGCGGAGAAAACGGAATTCCACAAGTAGCGGTGAAA
TGCGTAGATATGTGGAAGAACACCTGTGGCGAAAGCGGTTTTCTAGCTTATTCCTGACGC
TGAGGCGTGAAAGCAAGGGGAGCAAACAGGATTAGATACCCCCGTAGTCC
>Otu7391
CCAGCCTATGGGGTGCAGCAGTAGGGAATTTTCCACAATGGGCGCAAGCCTGATGGAGCA
ACGCCGCGTGAGGGATGAAGGTCTTCGGATTGTAAACCTCTTTGGAGTGGGAAGAATGTC
TTCGGTGCTAACAGTACCGAGGAGTGACGGTACCACTAGAACAAGACACGGCTAACTCTG
TGCCAGCAGCCGCGGTAATACAGAGGTGTCAAACGTTGTCCGGAATTACTGGGCGTAAAG
GGCGCGTAGGCGGTTGGATAAGTCAGATGTGAAATCACTGGGCTCAACCCAGTAATTGCA
TTTGAAACTGTCCGACTCGAGTTGGGTAGAGGAGGGCGGAATTCCTGGTGTAGCGGTGAA
ATGCGTAGATATCAGGAGGAACACCTGTGGCGAAGGCGGCTCTCTGGTCCCATACTGACG
CTGAGGCGCGAAAGCTAGGGTAGCAAACAGGATTAGAAACCCCCGTAGTCC
>Otu7399
CCAGCCTACGGGTTGCACCAGTAACGAATCTTCCGCAATGCACGAAAGTGTGACGGAGCG
ACGCCGCGTGTGGGACGAAGCCCTTCGGGGTGTAAACCACTGTCAGAGGATAGAAAGTTC
TGATCATCCTCAGAGGAAGGCACGGCTAACTCTGTGCCAGCAGCCGCGGTAAGACAGAGG
TGCCAAGCGTTAGGCGGAATCACTGGGTTTAAAGCGTGTGTAGGTGGACCTGTAAGTACC
TTGTGAAATCCCACGGCTTAACCGTGGAACTGCTCGGTATACTGCGGGTCTGGAGGTACC
TAGGGGCGAGCGGAACAAATGGTGGAGCGGTGAAATGCGTAGATATCGGAAGGAACACCA
GTGGCGAAAGCGACTTCCTGGTCGAATACTGACGTTCATGTGCGAAAGCGTGGGGAGCAA
ACAGGATTAGAAACCCCCGTAGTCC
>Otu7404
CCAGCCTATGGGGCGCTGCAGTGGGGAATCTTGCACAATGGACGAAAGTCTGATGCAGCA
ACGCCGCGTGAGTGAAGAAGGCCCTTGGGTTGTAAAGCTCTTTCGGTCGGGAAGAAGGGG
AGTCGTGCTAATACCACGAGTTTCTGACGGTACCGGCATAAGAAGCACCGGCTAATCTCG
TGCCAGCAGCCGCGGTAATACGAGAGGTGCAAGCGTTGTTCGGAATGACTGGGCGTAAAG
CGCACGTAGGCGGGCAAGTAAGTCAGATGTGAAATCCCTGGGCTTAACCTGGGAACTGCA
TCTGAAACTGCATGTCTTGAATGTCTCAGAGGTTGGTGGAATTCCTGGTGTAGGAGTGAA
ATCCGTAGATATCAGGAGGAACATCGGAGGCGAAGGCGGCCAACTGGGAGCACATTGACG
CTGAGGTGCGAAAGCGTGGGGAGCAAACAGGATTAGAGACCCCAGTAGTCC
>Otu7409
CCAGCCTACGGGATGCTCCAGTCGAGGATCTTTCGCAATGGGCGCAAGCCTGACGAAGCG
ACGCTGTGTGAGTGATGAAGGCCTTCGGGTTGTAAAGCTCTTTCGCGCGGGAACAAGAGA
GGTTAGTGAATAACTAGCCGATGTGAGTGTACTGCGTAAAGAAGCACTGGCTAACTCCGT
GCCAGCAGCTGCGGTAATACGGAGGGTGCAAGCATTAATCGGAATTATTGGGCGTAAAGG
GCGCGTAGGCTGATGAACAAGTCAGATGTGAAATTTTGGAGCTCAGCTCCAAAGCTGCAT

TTGAAACTGTTTGTCTATAGAGGGATGGCGGAGAAAATGGAATTCCACATGTAGCGGTGA
AATGCGTAGATATGTGGAAGAACACCGGTGGCGAAGGCGGTTTTCTAGCTATTACCTGAC
GCTGAGGCGCGAGAGCATGGGGAGCAAACAGGATTAGATACCCCCGTAGTCC
>Otu7414
CCAGCCTACGGGTGGCAGCAGCGGGGAATATTGGACAATGGGGGCAACCCTGATCCAGCC
ATGCCGCGTGAGTGATGAAGGCCTTAGGGTTGTAAAGCTCTTTCGCAGGGGAAGATAATG
ACGGTACCCTGAGAAGAAGCCTCGGCTAACTCCGTGCCAGCAGCCACGGTAAGACGGAGG
AGGCTAGCGTTGTTCGGAATTACTGGGCGTAAAGCGTACGCAGGTGGCGGGACAAGTCGG
ATGTGAAAGCCCGGGGCTCAACCCCGGATGTGCATCCGTTACTGTTTTGCTAGAGTTCGG
GAGAGGAGAGCGGAATTCCCAGTGTAGAGGTGAAATTCGTAGATATTGGGAAGAACACCG
GTGGCGAAGGCGGCTCTCTGGACCGATACTGACACTCAAGTACGAAAGCGTGGGGAGCAA
ACAGGATTAGATACCCTGGTAGTCC
>Otu7415
CCAGCCTATGGGGCGCTGCAGTGGGGAATATTGGACAATGGGCGAAAGCCTGATCCAGCA
ATACCGCGTGTGTGAAGAAGGCCTTCGGGTTGTAAAGCACTTTAAGCAGGGAAGAAAAAA
CTCAAACTAATATTTTGAGTCTTGACGGTACCTGAAGAATAAGCACCGGCTAACTCTGTG
CCAGCAGCCGCGGTAATACAGAGGGTGCAAGCGTTAATCGGATTTACTGGGCGTAAAGCG
TGTGTAGGTGGTTGGGAAAGTCAATTGTGAAATCCCCGGGCTTAACTCGGAAACTGCTTT
TGATACTGTCTGACTAGAATTCGGTAGAGGGAGGCGGAACTCCAGGTGTAGCGGTGAAAT
GCGTAGATATCTGGAAGAACACCAATGGCGAAGGCAACCTCCTGGGCCTGAATTGACACT
GAGACACGAAAGCGTGGGGATCAAACAGGATTAGAGACCCTCGTAGTCC
>Otu7417
CCAGCCTATGGGGGGCTGCAGTGGGGAATATTGGACAATGGGGGCAACCCTGATCCAGCA
ATACCGCGTGTGTGAAGAAGGCCTTCGGGTTGTAAAGCACTTTAGGTTGGGAAGAAGGGG
CGAAGTTTAAGAGATGTCGCCTTTGACTGTACCGACAGAATAAGCACCGGCTAACTCTGT
GCCAGCAGCCGCGGTAATACAGAGGGTGCAAGCGTTAATCGGAGTTACTGGGCGTAAAGG
GCGCGTAGGCGGTTAGTTAAGTGAGATGTGAAAGCCCTGGGCTTAACCTAGGAATGGCAT
CTTAAACTGGTTAACTGGAGTACTGTAGAGGGTAGTGGAATTTCCGGTGTAGCGGTGAAA
TGCGTAGAGATCGGAAGGAACACCAGTGGCGAAGGCGGCTACCTGGACAGATACTGACGC
TGAGGCGCGAGAGCGTGGGGAGCAAACAGGATTAGAACCCGCGTAGTCC
>Otu7418
CCAGCCTATGGGTGGCAGCAGTCGAGAATCTTTCGCAATGAGCGCAGGCCTGACGAAGCG
ACGCCGTGTGAGCGAAGAAGGCCTTCGGGTTGTAAAGCTCTTTCGCTAGGGAACAAGAGA
GACGTGATAATACCACGTTGATTTGAGGGTACTTGGTAAAGAAGCACCGGCTAACTCCGT
GCCAGCAGCTGCGGTAATACGGAGGGTGCAAGCATTGATCGGAATTACTGGGCGTAAAGG
GTGCGTAGGCGGCCTGTTAAGTCAGATGTGAAAACCCACAGCTCAACTGTGGAACGGCAT
TTGAAACTGGCGGGCTCGAGGATAGTTAGGGAAAACGGAATTCCACATGTAGCGGTGAAA
TGCGTAGATATGTGGAAGAACACCGGTGGTGAAGACGGTTTTCTGGGCTATAACTGACGC
TGAGGCACGAAAGCTAGGGAAGCAAACAGGATTAGATACCCGTGTAGTCC
>Otu7419
CCAGCCTATGGGGCGCTGCAGTGGGGAATCTTGCGCAATGGGCGAAAGCCTGACGCAGCG
ACGCCGCGTGAGTGATGAAGGCCTTCGGGTTGTAAAGCTCTGTGGAGAGGGACGAATAAG
TGTTGGCTAACATCCAGCATGATGACGGTACCTCCTTAGCAAGCACCGGCTAACTCTGTG
CCAGCAGCCGCGGTAAGACAGAGGGTGCAAACGTTGTTCGGAATTACTGGGCGTAAAGCG
TGTGTAGGCGGCCATGTAAGTCGGATGTGAAAGCCCCGGGCTTAACCCGGGAAGTGCATT
CGATACTGCGTGGCTTGAGTATCGGAGAGGTTGGTGGAATTCTCGGTGTAGAGGTGGAAT
TCGTAGATATCGGGAGGAACACCGGTGGCGAAGGCGGCCAACTGGACGAATACTGACGCT
GAGACACGAAAGCGTGGGGAGCAAACAGGATTAGAAACCCTTGTAGTCC
>Otu7420
CCAGCCTACGGGGGGCTGCAGTGGGGAATTTTGTGCAATGGGCGAAAGCCTGACACAGCG
ACACCGCGTGAGCGAAGAAGCCCCTTGGGGTGTAAAGCTCTGTCGGCTGGAACGAAAAAA
ATGACGGTACCAGCAAAGGAAGCATCGGCTAACTACGTGCCAGCAGCCGCGGTAAGACGT
AGGATGCAAGCGTTGTCCGGATTTATTGGGCGTAAAGAGTTCGTAGGTGGTTCGTTAAGT
CCGATGTTAAATCCCGGGGCCCAACCCCGGAGCTGCATTGGATACTGGCGGACTTGAGTA
CAGTAGAGGCAAGCGGAATTCCCAGTGTAGCGGTGAAATGCGTAGATATTGGGAAGAACA
CCAGTGGCGTAAGCGGCTTGCTGGGCTGTAACTGACACTGAGGAACGAAAGCCAGGGGAG
CGAATGGGATTAGAAACCCGAGTAGTCC
>Otu7427

CCAGCCTATGGGAGGCTGCAGTAGGGAATCTTGCGCAATGGGCGAAAGCCTGACGCAGCA
ACACCGTGTGAAGGAAGAAGCCTCTTGGGGTGTAAACTTCTGTCGGCAGGGAAGATTATG
ACGGTACCTGCAAGGGAAGCATCGGCTAACTACGTGCCAGCAGCCGCGGTAAGACGTAGG
ATGCAAGCGTTGTCCGGAATTATTGGGCGTAAAGCGTTCGTAGGCGGTTGCCTAAGTCTG
GTGTTAAAGGCCTGAGCTTAACTCGGGTATGGCATTGGATACTGGGTGGCTAGAGTGCGG
TAGAGGTAACGGGAATTCCCGGTGTAGCGGTGAAATGCGTAGATATCGGGAGGAACACCA
GTGGCGAAAGCGCGTTACTGGACCGCAACTGACGCTGAGGAACGAAAGCCAGGGGAGCGA
AAGGGATTAGAAACCCTCGTAGTCC
>Otu7438
CCTACGGGGGGCAGCAGTGGGGAATTTTTCGCAATGGGGGAAACCCTGACGAAGCAACGC
CGCGTGGAGGATGAAGGCCTTCGGGTCGTAAACTCCTGTCGACTGGGACGAAAGCAGTCT
GGGCTAATATCCCGGGCTGTTGACTGTACCGGTGGAGGAAGCCACGGCTAACTCTGTGCC
AGCAGCCGCGGTAATACAGAGGTGGCAAGCGTTGTTCGGAATTACTGGGCGTAAAGGGCG
CGTAGGCGGCTCGACAAGTCCCGCGTGAAAGCCCCCGGCTCAACTGGGGAACTGCGCGGG
AAACTGTCGGACTTGAGTTCGGGAGAGGGAAGCGGAATTCCGGGTGTAGCGGTGAAATGC
GTAGATATCCGGAGGAACACCGGTGGCGAAGGCGGCTTCCTGGACCGACACTGACGCTGA
GGCGCGAAAGCTAGGGGAGCAAACGGGATTAGATACCCTAGTAGTCC
>Otu7439
CCAGCCTATGGGATGCTGCAGTCGAGAATCTTCCACAATGGACGAAAGTCTGATGGAGCG
ACATTTCGTGATGGATGAAGCCCTTCGGGGTGTAAACATCTTTTATCTGGGAGCAACTTA
ATGAGAGTACCAGATGAATAAGGGGATACTAATTCTGTGCCAGCAGTAGCGGTAATACAG
AATCCCTGAGCGTTACCCGGATTTATTGGGCGTAAAGGGTTCGTAGGTGGTAAAGTAAGT
TACAAATTAAACTTCATCGGCTTAACCTTTGAACTGTTTGTAATACTGCTATACTAAGAG
GGCGTTAGAGGCAAACGGAACCGACGGAGTAGGGGTGAAATCCGTTGATATCGTCGGGAA
CACCAAAAGCGAAGGCAGTTTGCTGGGACGACCCTGACACTGAGGAACGAAAGCGTGGGG
AGCAAAAAGGATTAGATACCCTAGTAGTCC
>Otu7443
CCAGCCTATGGGAGGCACCAGTAGGGAATTTTCCACAATGGACGAAAGTCTGATGGAGCA
ACGCCGCGTGCAGGATGAATGCCTTAGGGTTGTAAACTGCTTTTCTCTGTGACGAATATG
ACGGTAGCAGAGGAATAAGGACCTGCTAACTACGTGCCAGCAGCCGCGGTCATACGTAGG
GTCCAAGCATTATCCGGAATTACTGGGCGTAAAGAGTTGCGTAGGTGGCATAGTAAGCGG
GTAGTGAAGTCGTGTGGCTCAACCACATATACATTACCTGAACTGCTACGCTAGAGGACG
AGAGAGGTTATTGGAATTCCCAGTGTAGGAGTGAAATCCGTAGATATTGGGAGGAACACC
GATGGCGTAGGCAGATAACTGGCTCGTTCCTGACACTAAGGCACGAAAGCGTGGGTAGCA
AACGGGATTAGAAACCCTTGTAGTCC
>Otu7448
CAGCCTATGGGTGGCAGCAGTCGAGAATCATTCGCAATGGGCGCAAGCCTGACGATGCGA
CGCCGTGTGAGCGATGAAGGCCTTAGGGTCGTAAAGCTCTTTCGCTTGGGAACAAGAGAG
ATTGCCTAATAAGCAGTTAATTTGAGGGTACCAGGTAAAGAAGCACCGGCTAACTCCGTG
CCAGCAGCTGCGGTAATACGGAGGGTGCAAGCATTAATCGGATTTATTGGGCGTAAAGGG
CGCGTAGGCTGGACGATAAGTCAGATGTGAAATCCCGGGGCTCAACCCCGGAACAGCATT
TGAAACTGTCGTTCTTGAGGATAGAGGGAGAAAACGGAATTCCACAAGTAGCGGTGAAAT
GCGTAGATATGTGGAAGAACACCGGTGGCGAAGGCGGTTTTCTACTTTAGACCTGACGCT
GAGGCGCGAAAGCAAGGGGATCAAACAGGATTAGAGACCCTCGTAGTCC
>Otu7449
CCAGCCTACGGGTCGCTGCAGTGGGGAATATTGCGCAATGGGCGAAAGCCTGACGCAGCG
ACGCCGCGTGGAGGATGAAGGTCTTAGGATCGTAAACTTCTGTTAAGTGGGAAGAAATAC
CCGTTTCTAATACAAGCGGGGGATGACGGTACCATTAGAGAAAGCACCGGCTAACTTCGT
GCCAGCAGCCGCGGTAATACGAAGGGTGCGAGCGTTATTCGGAATCATTGGGCGTAAAGG
GTGCGTAGACGGTATATTAAGTCTGTTGTTAAATGCTTCGGCCTAACCGGAGACTTGCGG
TGGAAACTAATATGCTAGAGGGTGGAAGAGAGAAGTGGAATTCTCGGAGTAGCGGTAAAA
TGCGTAGATCTCGAGAGGAACACCAATGGCGAAGGCAGCTTCTTGGTCCATACCTGACGT
TGAGGCACGAAAGCGTGGGGAGCAAACAGGATTAGAGACCCTGGTAGTCC
>Otu7450
CCAGCCTATGGGTGGCAGCAGTGGGGAATCTTGCACAATGGGGGCAACCCTGATGCAGCG
ACGCCGCGTGAGCGATGAAGCCCTTCGGGGTGTAAAGCTCTTTCGGCAGGGACGATAATG
ACGGTACCTGCAGAAGCAGCTGCGGCTAACTACGTGCCAGCAGCCGCGGTAATACGTAGG
GGGCTAGCGTTGCTCGGAATCACTGGGCGTAAAGCGCACGTAGGCGGCTTTTTAAGTCAG

GGGTGAAATCCTGGAGCTCAACTCCAGAACTGCCTTTGATACTGAGAAGCTTGAGTCCGG
GAGAGGTGAGTGGAACTGCGAGTGTAGAGGTGAAATTCGTAGATATTCGCAAGAACACCA
GTGGCGAAGGCGGCTCACTGGCCCGGTACTGACGCTGAGATGCGAAAGCGTGGGGAGCAA
ACAGGATTAGATACCCTTGTAGTCC
>Otu7462
CCAGCCTACGGGGTGCTCCAGTAGGGAATCTTGCGCAATGGGCGAAAGCCTGACGCAGCA
ACGCCGCGTGGGGGATGAAGGCCTTCAGGTTGTAAACTCCTTTTAGTGGGAACGAAACTG
ACGGTACCCACAGAAAAAGCTCCGGCCAACCACGTGCCAGCAGCCGCGGTGATACGTAGG
GAGCAAGCGTTGTCCGGATTTATTGGGCGTAAAGAGCTCGTAGGCGGCTTGGCAAGTCGG
GTGTGAAACCTCCAGGCTCAACCTGGAGCCGCCACTCGATACTGCCATGGCTAGAGTCCG
GTAGGGGACCACGGAATTCCTGGTGTAGCGGTGAAATGCGCAGATATCAGGAGGAACACC
AGTGGCGAAGGCGGTGGTCTGGGCCGGAACTGACGCTGAGGAGCGAAAGCGTGGGGAGCG
AACAGGATTAGAAACCCTTGTAGTCC
>Otu7464
CCAGCCTACGGGGGGCTGCAGTAGGGAATATTGGACAATGGGCGAAAGCCTGATCCAGCC
ATGCCGCGTGAGTGATGAAGGCCTTAGGGTTGTAAAGCTCTTTTGGCGGGGAAGATAATG
ACGGTACCCGCAGAATAAGCACCGGCTGACTTCGTGCCAGCAGCCGCGGTAATACGAAGG
GTGCTAGCGTTGTTCGGAATTACTGGGCGTAAAGCGCGCGTAGGCGGCCATACTCGTCGG
GGGTGAAATCCCAGGGCTTAACCCTGGAACTGCCTTCGAAACTGTATGGCTGGAGTGTGT
GAGGGGATAGCGGAATTCCTAATGTAGAGGTGAAATTCGTAGATATTAGGAGGAACACCG
GTGGCGAAGGCGGCTATCTGGCACACAACTGACGCTGAGGCGCGAAAGCGTGGGGATCAA
ACAGGATTAGATACCCGCGTAGTCC
>Otu7470
CCAGCCTACGGGTCGCAGCAGTGGGGAATATTGGACAATGGAGGAAACTCTGATCCAGCC
ATGCCGCGTGAGTGATGAAGGCCTTCGGGTTGTAAAGCTCTTTTAGATGTGACGATGGTG
ACGGTAACATCAGAAAAAGCTCCGGCTAACTTCGTGCCAGCAGCCGCGGTAATACGAAGG
GAGCGAGCGTTGTTCGGAATCACTGGGCTTAAAGCGTGCGTAGGCGGGTTAGTAAGTCAG
AAGTGAAAGCCCAGGGCTCAACCCTGGAACTGCTTTTGAGACTGCTAATCTGGAATTCGG
TAGAGGTTCGTGGAACTCCTAGTGTAGAGGTGAAATTCGCAGATATTAGGAAGAACACCA
GTGGCGAAGGCGACGAACTGGGCCGATATTGACGCTGAGGCACGAAAGCGTGGGTAGCAA
ACAGGATTAGATACCCGGGTAGTCC
>Otu7471
CCAGCCTATGGGATGCTGCAGTGAGGAATTTTCCGCAATGGGCGAAAGCCTGACGGAGCG
ACGCCGCGTGTGGGAAGAAGGCCTTCGGGTCGTAAACCACTGTCGAAGGGGACGATATTG
ACGGTACCCTTGGAGGAAGCCCCGGCTAACTACGTGCCAGCAGCCGCGGTAAGACGTAGG
GGGCAAGCGTTGTCCGGAATTATTGGGCGTAAAGCGCCCGTAGGTGGATCAACAAGTCTG
CAAGCAAAGACCCGGGCTCAACTCGGGAATCCGTGTGGAAACTGTTGATCTTGAGGGAGT
CAGAGGATGATGGAATTCCCGGTGTAGCGGTGAAATGCGTAGATATCGGGAGGAACACCA
GTGGCGAAGGCGATCATCTGGGGCTAACCTGACACTGAGGAGCGAAAGCTAGGGGAGCAA
ACGGGATTAGAAACCCGAGTAGCC
>Otu7473
CCAGCCTATGGGTGGCACCAGTGGGGAATTTTGCGCAATGGACGAAAGTCTGACGCAGCA
ACGCCGCGTGAGGGATGAAGGCCTTCGGGTTGTAAACCTCTTTTCTCAGGGAAGATAGTG
ACGGTACCTGAGGAATAAGTCACGGCTAACTACGTGCCAGCAGCCGCGGTAATACGTAGG
TGGCTAGCGTTGTCCGGATTTACTGGGCGTAAAGAGCGCGCAGGCGGTCGTTCAAGTCGA
GTGTGAAAGCCCCCGGCTCAACTGGGGAGGGTCACTCGATACTGATCGACTCGAAGGCAG
GAGAGGGAAGCGGAATTCCCGGTGTAGTGGTGAAATGCGTAGATATCGGGAGGAACACCA
GTGGCGAAGGCGGCTTTCTGGCCTGTTCTTGACGCTGAGGCGCGAAAGCTAGGGTAGCAA
ACGGGATTAGATACCCGCGTAGTCC
>Otu7477
CCAGCCTACGGGTTGCTGCAGTAGGGAATTTTCCACAATGGACGGAAGTCTGATGGAGCA
ACGCCGCGTGCAGGATGAATGCCTTCGGGTTGTAAACTGCTTTTATGTGCGAAGATTATG
ACAGTAACACATGAATAAGGACCTGCTAACTACGTGCCAGCAGCCGCGGTCATACGTAGG
GTCCAAGCGTGATCCGGAATTACTGGGCGTAAGGAGTTGCGTAGGTGGCATGGTAAGTTG
GTAGTGAAAGCGTTCGGCTCAACCGAATATACATTATCAAAACTGCCAAGCTCGAGGACG
AGAGAGGCATCTGGAATTCCTAGTGTAGGAGTGAAATCCGTAGATATTAGGAGGAACACC
GATGGCGTAGGCAGGGTGCTGGCTCGTTTCTGACACTAAGGCACGAAAGCGTGGGGAGCG
AACGGGATTAGATACCCCAGTAGTCC

>Otu7480
CCAGCCTATGGGAGGCTGCAGTAGGGAATCTTGCGCAATGGGGCAACCCTGATCCAGCAA
TGCCGCGTGTGTGAAGAAGGCCTGCGGGTTGTAAAGCACTTTCGGTGGGGAGGAAAGCCT
TCGTGTTAATAGCGCGAGGGATTGACGTTACCCACATAAGAAGCACCGGCTAACTCTGTG
CCAGCAGCCGCGGTAATACAGAGGGTGCAAGCGTTAATCGGAATCACTGGGCGTAAAGAG
CGCGTAGGTGGTTATTTAAGTTGGATGTGAAAGCCCCGGGCTCAACCTGGGAAGGCCATC
CAAGACTGGGTAACTAGAGTACAGTAGAGGGAAGTGGAATTTCCGGTGTAGCGGTGAAAT
GCGTAGATATCGGAAGGAACACCAGTGGCGAAGGCGGCTTCCTGGACTGATACTGACACT
GAGGCGCGAAAGCGTGGGTAGCGAACAGGATTAGAAACCCTTGTAGTCC
>Otu7481
CCAGCCTACGGGGGGCTGCAGTAAGGGATATTGCGCAATGGGCGAAAGCCTGACGCAGCA
ACGCCGCGTGGAGGATGAAGGCCTTCGGGTCGTAAACTCCTTTTTTCAGGGACGAGCAAG
GACGGTACCTGGAGAATAAGTCACGGCTAACTACGTGCCAGCAGCCGCGGTAAAACGTAG
GTGGCGAGCGTTATCCGGATTTACTGGGTGTAAAGCGTGTGCAGGCGGATTACCAAGTGG
TGTATGAAAGGTTGGGGCTTAACTCCAACAGTGTATGCCAGACTGGTAGTCTGGAGTGCG
AGAGAGGGGCGTGGAATTCCGGGTGTAGTGGTGAAATGCGTAGAGATCCGGAGGAACCCC
AGAGGCGAAGGCGGCGCCCTGGCTCGCAACTGACGCTCAGACACGACAGCATGGGGAGCG
AACGGGATTAGATACCCCAGTAGTCC
>Otu7482
CCAGCCTACGGGGGGCAGCAGTAAGGAATATTGGTCAATGGACGCAAGTCTGAACCAGCC
ATGCCGCGTGAAGGAAGAAGGCCCTAGTGGTCGTAAACTTCTTTTATATGGGAAAAATTC
TTGGGTCGTGAATCCGAGTTGATGGTACTATATGAATAAGCAACGGCTAACTCCGTGCCA
GCAGCCGCGGTAATACGGAGGTTGCAAGCGTTATCCGGATTTATTGGGTTTAAAGGGTGC
GTAGGCGGACCTATAAGTCAGCGGTGAAATCCAACAGCTTAACTGTAGAATTGCCATTGA
TACTGTAGGTCTTGAGTATAGCTGAAGAGGGCGGAATGTGTTGTGTAGCGGTGAAATGCT
TAGATATAACACAGAACACCGATTGCGAAGGCAGCTCTCTAAACTATAACTGACGCTGAG
GCACGAAAGCGTGGGGATCAAACAGGATTAGATACCCTCGTAGTCC
>Otu7488
CCAGCCTATGGGATGCAGCAGCCGAGAATATTCGACAATGGGCGAAAGCCTGATCGAGCG
ACGCCGCGTGATTGATGAAGTTCCACTGGAATGTAAAAATCTTTTATAGAGGAGAAAGCC
GCATTTATGCGGTGTGATAGTACTCTAGGAATAAGGGGTTGCTAAACTCGTGCCAGCAGC
AGCGGTAATACGAGTGCCCCGAGCGTTATCCGGAATTATTGGGCGTAAAGGGTGTGTAGG
CGGTCTTGTTAGTCTTTTGTTAAAGCTCCTGGCTTAACCGGGAAAATGCAAAAGAAACGG
CAAGACTAGAGGGTGCAAGAGGCTTATGGAACTCATGGTGTAGGGGTGAAATCCGTTAAT
ATCATGGGGAACACCGAAGGCGAAGGCAGTAAGCTGGTGCACATCTGACGCTGAAACACG
AAAGCGTGGGTAGCGAATGGGATTAGAGACCCCAGTAGTCC
>Otu7490
CCAGCCTATGGGGGGCAGCAGTAGGGAATTTTCCACAATGGGCGAAAGCCTGATGGAGCA
ACGCCGCGTGAGGGATGACGCATCTCGGTGTGTAAACCTCTTTGGAGCGGGAAGAACGCC
GTTTGGGTGAATAATCCAGACGGGTGACGGTACCGCTAGAACAAGACACGGCTAACTTCG
TGCCAGCAGCCGCGGTAATACGAAGGTGTCGAACGTTGTCCGGAATTATTGGGCGTAAAG
GGCGCGTAGGCGGCCAGGTAAGTGGGATGTGAAAGCTCTGGGCTCAACCCAGGAATTGCA
TTCCATACTGCTTGGCTTGAGTTCGGTAGAGGAGGGCGGAATTCTAGGTGTAGCGGTGAA
ATGCGTAGATATCTAGAGGAACACCCGTGGCGAAGGCGGCTCTCTGGTCCGATACTGACG
CTGAGGCGCGAAAGGTAGGGGAGCAAACAGGATTAGATACCCTTGTAGTCC
>Otu7491
CCAGCCTACGGGAGGCTGCAGTGGGGAATATTGGACAATGAGGGCAACCTCGATCCAGCA
ATACTGAGTGGATGATGACGGTCGATTGATTGTAAAGTCCTTTTTATAAAGAGAACTATG
ATATTATTTATAGAATAAGTTCCGGCTAACTTCGTGCCGGCAGCCGCGGTAAAACGGGGG
GAACAAGCGTTATACATCATGATTGGGCGTAAAGGGCTTGAAGGTGGTTTTCTTAGCAAC
TGGTTGAGACGAGAAGACTAGAGTTCATGAGAGGATCGGGGTACTTTCAAAATAGAGGTG
AAATTTTTATATTGATAGGGACCACTGCCGGGCGAAAGCGCCGGTCTAACATGGCACTGA
CACTGATAAGCGGAAGCATGGGTATCAAACGGGATTAGAGACCCCCGTAGTCC
>Otu7494
CCAGCCTACGGGTCGCTGCAGTGGGGAATATTGGACAATGGGCGAAAGCCTGATCCAGCC
ATGCCGCGTGAGTGATGAAGGCTTTAGGGTTGTAAAGCTCTTTCGCCGATGAAGATAATG
ACGGTAGTCGGAGAAGAAGCCCCGGCTAACTTCGTGCCAGCAGCCGCGGTAATACGAAGG
GGGCTAGCGTTGTTCGGAATTACTGGGCGTAAAGCGCGCGTAGGCTGTGTCGCAAGTCAG

GGGTGAAATCCTGGGGCTTAACCCCAGAACTGCCTTTGAAACTGCGATGCTGGAATCTCA
GAGGGGATAGCGGAATTCCAAATGTAGGGGTGAAATCCGTAGATATTTGGAGGAACACCG
GTGGCGAAGGCGGCTATCTGGATGAGTATTGACGCTGAGGCGCGAAAGCGTGGGGATCAA
ACAGGATTAGAAACCCGCGTAGTCC
>Otu7495
CCAGCCTATGGGAGGCAGCAGTAGGGAATTTTCCACAATGGGCGAAAGCCTGATGGAGCA
ACGCCGCGTGCAGGATGAAGGCTTTCGGGTCGTAAACTGCTTTTCTTTGTGAGGATTATG
ACAGTAGCAGAGGAATAAGGATCGGCTAACTCCGTGCCAGCAGCCGCGGTCATACGGAGG
ATCCAAGCGTTATCCGGAATTACTGGGCGTAAAGAGTTGCGTAGGCGGCAGAGTAAGCAA
GATGTGAAAGCGTATGGCTCAACCATATATACATGTCCTGAACTGCTCAGCTAGAAGATG
AGAGAGGTGGCTGGAATTCCCAGTGTAGGAGTGAAATCCGTAGATATTGGGAGGAACACC
GATGGCGTAGGCAGGCCACTGGCTCATTCTTGACGCTAAGGCACGAAAGCGTGGGGAGCG
ACCGGGATTAGAGACCCTCGTAGTCC
>Otu7496
CCAGCCTATGGGGGGCACCAGTCGAGGATCTTCTGCAATGGGCGCAAGCCTGACAGAGCG
ACGCCGCGTGGGGGAGGAAGGCCTTCGGGTTGTAAACCCCTGTTGCCGGTTAAGAATGCT
GGGGCGCGAATAGCGCTTCCAGTTGACTAAGGCCGGTGAGGAAGCCACGGCTAACTACGT
GCCAGCAGCCGCGGTAAGACGTAGGTGGCGAGCGTTGTTCGGAATCACTGGGCATAAAGC
GCGCGTAGGCGGCCAAGTCAGTCGGGTGTGAAAGCCCTCGGCTCAACCGAGGAACTGCGC
CCGATACTGCTTGGCTTGAGGTCCGGAGGGGAGAGTGGAATTCCCGGTGGAGCGGTGAAA
TGCGTAGAGATCGGGAAGAACGCCGGCGGCGAAAGCGACTCTCTGGTCGGAATCTGACGC
TGAGGCGCGAAAGCTAGGGGAGCAAACGGGATTAGAGACCCGCGTAGTCC
>Otu7500
CCAGCCTATGGGAGGCAGCAGTGGGGAGTCTTGCGCAATGGGCGAAAGCCTGACGCAGCA
ACGCCGCGTGTGTGATGAAGGTCTTCGGATCGTAAAGCACTGTCGCGAGGGAAGATACGC
CTCTGGCTAATATTCAGAGGCTAAGACGGTACCTCGAGAGGAAGCACCGGCTAACTCTGT
GCCAGCAGCCGCGGTAATACAGAGGGTGCGAGTGTTGTTCGGAATTATTGGGCGTAAAGC
GCTTGTAGGCGGCTTATCAAGTCTCGTGTGAAATCCCCAGGCTTAACCTGGGAAGTGCGC
GGGAAACTGAAGAGCTTGAATGCCGGAGAGGGTCGCGGAATTCCCGGTGTAGAGGTGAAA
TTCGTAGATATCGGGAGGAACACCAGTGGCGAAGGCGGCGACCTGGACGGACATTGACGC
TGAGACGCGAAAGCGTGGGGAGCAAACAGGATTAGATACCCCAGTAGTCC
>Otu7502
CCAGCCTATGGGGGGCACCAGTGGGGAATTTTGGACAATGGGCGCAAGCCTGATCCAGCC
ATGCCGCGTGAGTGAAGAAGGCCTTCGGGTTGTAAAGCTCTTTCGGCGGGGACGAAATAG
CGCGGGCGAATATCCCGCGTTGATGACGGTACCCGTAGAAGAAGCACCGGCTAACTACGT
GCCAGCAGCCGCGGTAATACGTAGGGTGCGAGCGTTAATCGGAATTACTGGGCGTAAAGC
GTGCGCAGGCGGTCTCGCAAGTCCGATGTGAAATCCCCGGGCTCAACCTGGGAACTGCGT
TGGAAACTACGAGGCTGGAGTACGGCAGAGGGGGGTGGAATTCCACGTGTAGCAGTGAAA
TGCGTAGATATGTGGAGGAACACCGATGGCGAAGGCAGCCCCCTGGGCTAGTACTGACGC
TCATGCACGAAAGCGTGGGGAGCAAACAGGATTAGATACCCCGGTAGTCC
>Otu7506
CCAGCCTACGGGATGCACCAGTGGGGAATTTTGGACAATGGGGGCAACCCTGATCCAGCC
ATGCCGCGTGAGTGAAGAAGGCCTTCGGGTTGTAAAGCTCTTTCGGTGGGAACGAAACGG
TCGCGGTTAATAGCCGGGATCACTGACGGTACTCACAGAAGAAGCACCGGCTAACTACGT
GCCAGCAGCTGCGGTAATACGTAGGGTGCAGGCGTTAGTCGGAATTACTGGGCGTAAAGG
GTGCGCAGGCGGTTTTGTAAGTCAGGCGTGAAAGCCCCGGGCTTAACCTGGGAAATGCGT
TTGAGACTGCAAGGCTAGAGTGTGTCAGAGGGGGGTGGAATTCCACGTGTAGCAGTGAAA
TGCGTAGAGATGTGGAGGAACACCGATGGCGAAGGCAGCCCCCTGGGATAACACTGACGC
TCGGGCACGAAAGCGTGGGGAGCAAACAGGATTAGAACCCGAGTAGTCC
>Otu7508
CCAGCCTACGGGATGCAGCAGTAGGGAATCTTGCGCAATGGGCGAAAGCCTGACGCAGCA
ACGCCGCGTGGGGGATGAAGGCATTCGGGTTGTAAACTCCTTTCAGTGGGAACGAAATTG
ACGGTACCCACAAAAGAAGCCCCGGCCAACTACGTGCCAGCAGCCGCGGTGATGCGTAGG
GGGCGAGCGTTGTCCGGATTCATTGGGCGTAAAGAGCTCGTAGGCGGTTTGGTAAGTCGG
GTGTGAAACCTCCAGGCTCAACCTGGAGATGCCACTTGATACTGCCATGGCTAGAGTCCG
GTAGGGGACCATGGAATTCCTGGTGTAGCGGTGAAATGCGCAGATATCAGGAGGAACACC
AGTAGCGAAGGCGGTGGTCTGGGCCGGTACTGACGCTGAGGAGCGAAAGCGTGGGGAGCG
AACAGTATTAGAGACCCTAGTAGTCC

>Otu7509
CCAGCCTACGGGGGGCACCAGTAAGGAATATTGGACAATGGAGGCAACTCTGATCCAGCC
ATGCCGCGTGAAGGATGAAGGCGCTATGCGTCGTAAACTTCTTTTGTATGGGAAAAATAT
CCGTTTTTCTAAACGGATTGATGGTACCATAAGAATAAGGGTCGGCTAACTTCGTGCCAG
CAGCCGCGGTAATACGAAGGACCCGAGCGTTATCCGGATTCATTGGGTTTAAAGGGTGCG
TAGGTGGGCCTATAAGTCAGTGGTGAAAGCCCTTCGCTTAACGAAGGAATTGCCGTTGAT
ACTGTAGGTCTTGAGTACAGTTGAGGTGGGCGGAATGTGTCATGTAGCGGTGAAATGCTT
AGATATGACACAGAACACCGATTGCGAAGGCAGCTCGCTAAACTGTAACTGACACTGAGG
CACGAAAGCGTGGGTAGCAAACAGGATTAGAGACCCCCGTAGTCC
>Otu7510
CCAGCCTATGGGAGGCAGCAGTGGGGAATATTGGACAATGAGCGCAAGCTTGACCAAGCG
ACGCCGCGTGTGCGATGAAGGCCTTCGGGTTGTAAAGCACTGTCGAGGGGGACGAAGCCG
CAAGGTTGACCGATCCCTGGAGGAAGCTCGGGCTAAGTTCGTGCCAGCAGCCGCGGTAAG
ACGAACCGAGCGAACGTTGTTCGGAATCACTGGGCTTAAAGGGCGCGTAGGCGGCTCGCC
AAGTCGGGGGTGAAATACTTCAGCTTAACTGGAGAAGTGCCTTCGATACTGGTGGGCTAG
AGGGAGGTAGGGGCATGTGGAACTTCTGGTGGAGCGGTGAAATGCGTTGATATCAGAAGG
AACGCCGGTGGCGAAAGCGACGTGCTGGACCTTTTCTGACGCTGAGGCGCGAAAGCTAGG
GGAGCAAACGGGATTAGAGACCCCCGTAGTCC
>Otu7513
CCAGCCTATGGGAGGCTGCAGTGGGGAATCTTGCACAATGGGGGCAACCCTGATGCAGCG
ACGCCGCGTGAGCGATGAAGCCCTTCGGGGTGTAAAGCTCTTTCGGCAGGGACGATAATG
ACGGTACCTGAAGAAGAAGCTGCGGCTAACTACGTGCCAGCAGCCGCGGTGATACATAGG
GTGCGAGTGTGGTCCGGATTCATTGGGCGTAAAGAGCTCGTAGGCGGTTTGGTAAGTCGG
GTGTGAAACCTCCAGGCTCAACCTGGAGATGCCACTTGATACTGCCATGACTAGAGTCCG
GTAGGGGATCATGGAATTCCTGGTGTAGCGGTGAAATGCGCAGATATCAGGAGGAACACC
AGTAGCGAAGGCGGTGATCTGGGCCGGAACTGACGCTGAGGAGCGAAAGCGTGGGGAGCG
AACAGGATTAGAAACCCGAGTAGTCC
>Otu7516
CCAGCCTACGGGACGCAGCAGTGGGGAATTTTGCGCAATGGGCGAAAGCCTGACGCAGCG
ACGCCGCGTGTGCGATGAAGGCCTTTGGGTCGTAAAGCACTGTCGAGAGGGAAGAAAACT
TCCGGCGCTAATACCGCCGGGAATTGACGGTACCTTTAAAGGAAGCACCGGCTAACTCTG
TGCCAGCAGCCGCGGTAATACAGAGGGTGCAAACGTTGCTCGGAATTATTGGGCGTAAAG
CGCGTGTAGGCGGTTCATCAAGTCGGTTGTGAAAGCCCTTGGCTCAACCAAGGAAGTGCG
TCCGAAACTGGTGGGCTAGAGTTCTGAAGAGGATGGCGGAATGCGCAGTGTAGAGGTGAA
ATTCGTAGATATTGCGTAGAACACCGGTAGCGAAGGCGGCCATCTGGGAAGCGACTGACG
CTAAGACGCGAAAGCGTGGGGATCAAACAGGATTAGAAACCCGCGTAGTCC
>Otu7518
CCAGCCTATGGGATGCAGCAGTAGGGAATCTTGCGCAATGGGGGAAACCCTGACGCAGCG
ACGCCGCGTGCGGGATGAAGGGCTTCGGGTCGTAAGCCGCTTTCAGCAGGGACGAAATTG
ACGGTACCTGCAGAAGAAGGCCCGGCCAACTACGTGCCAGCAGCCGCGGTGATACGTAGG
GGTCTAGCGTTGTCCGGAATCATTGGGCGTAAAGAGCTCGTAGGCGGTTCGATTAGTCGG
GTGTGAAATCTCCACGCTCAACGTGGAGGGGCCACTCGATACTGTCGTGACTCGAGTCCG
GTAGGGGAGTTCGGAATTCCCGGTGTAGCGGTGAAATGCGCAGATATCGGGAGGAACACC
AGTGGCGAAGGCGGAACTCTGGGCCGGAACTGACGCTGAGGAGCGAAAGCGTGGGGAGCA
AACGGGATTAGATACCCGTGTAGTCC
>Otu7519
CCAGCCTATGGGTTGCTGCAGTCGAGAATCTTTCACAATGGGGGAAACCCTGATGGAGCG
ACGCCGCGTGGGGGATGAATGGCTTCGGCCCGTAAACCCCTATCATTTGCGAACAAACCT
TTTCATCTAACACATGAAAAGCTGATTGTAGCGGAAGAGGAAGGGACGGTTAACTCTGTG
CCAGCAGCCGCGGTAATACAGAGGTCCCAAGCGTTGTTCGGATTCACTGGGCGTAAAGGG
TGCGTAGGTGGCTGGGTAAGTCTGATGTGAAATCTCGGAGCTTAACTCCGAAACTGCATT
GGATACTACCCAACTTGAGGGTCGGAAGGGGGACTGGAATTCTCGGTGTAGCAGTGAAAT
GCGTAGATATCGAGAGGAACACCAGTGGCGAAGGCGAGTCCCTGGACGACTCCTGACACT
GAGGCACGAAAGCTAGGGGAGCAAACAGGATTAGAAACCCTAGTAGTCC
>Otu7522
CCAGCCTATGGGTGGCAGCAGTAGGGAATATTGGTCAATGGGGGCAACCCTGAACCAGCC
ATGCCGCGTGCAGGAAGAAGGCGTTCTGCGTTGTAAACTGCTTTTGATCGGGAACAAATG
ACTCTTGCGAGAGTAGCTGAGTGTACCGATAGAATAAGCCACGGCTAACTACGTGCCAGC

AGCCGCGGTAATACGTAGGTGGCAAGCGTTGTCCGGATTTATTGGGTTTAAAGGGTGCGT
AGGCGGCTGTTTAAGTCAGTGGTGAAAGCCGATCGCTTAACGATCGAACTGCCATTGATA
CTGAATAGCTTGAGTACAGATGAGGTAGGCGGAATTGACAGTGTAGCGGTGAAATGCATA
GATATTGTCAAGAACACCGATTGCGAAGGCAGCTTACTAAAGTGTCACTGACGCTGAGGC
ACGAAAGTGCGGGGATCAGACAGGATTAGAAACCCTAGTAGTCC
>Otu7523
CCAGCCTATGGGGTGCACCAGTGGGGAATATTGCGCAATGGGCGAAAGCCTGACGCAGCG
ATGCCGCGTGTGGGATGAAGGTCTTCGGATTGTAAACCACTGTCGGGAGGGACGAAGCTC
TGACGGTACCTCCAAAGGAAGCACCGGCTAACTCCGTGCCAGCAGCCGCGGTAATACGGA
GGGTGCAAGCGTTGTCCGGAATCACTGGGCGTAAAGGGCGCGTAGGCGGCTTGGTAAGTA
GGAGGTGAAATCTCGCGGCTCAACTGCGAGGCTGCCTTCTAGACTGCCAAGCTCGAGCAC
AGTAGAGGCAGGTGGAATTCCCGGTGTAGCGGTGGAATGCGTAGAGATCGGGAAGAACAT
CGGTGGCGAAGGCGGCCTGCTGGGCTGTTGCTGACGCTGATGCGCGACAGCGTGGGGAGC
AAACAGGATTAGAAACCCCAGTAGTCC
>Otu7524
CCAGCCTACGGGTTGCTGCAGTGAGGAATATTGCGCAATGGGCGAAAGCCTGACGCAGCG
ACGCCGCGTGGAGGATGAAGGTCTTTGGATCGTAAACTTCTGTTAAGTGGGAAGAAAAAG
TTGTTTCTAATACAGACAAAATATGACGGTACCTCTAAAGAAAGCACCGGCTAACTTCGT
GCCAGCAGCCGCGGTAATACGAAGGGTGCAAGCGTTATTCGGATTTATTGGGTGTAAAGG
GTGCGTAGACGGTACGTTAAGTCCGCTGTTAAATTTCCCAGCTCAACTGGGAGCCAGCGG
TGGATACTGGCGTGCTAGAGGATGGAAGAGAGAAGTGGAATTCCCGGAGTAGCGGTAAAA
TGCGTAGATCTCGGGAGGAACACCAAAGGCGAAGGCAGCTTCTTGGTCCATATCTGACGT
TGAGGCACGAAAGCGTGGGGAGCAAACGGGATTAGATACCCGAGTAGTCC
>Otu7527
CCAGCCTACGGGGGGCTCCAGTTAGGAATATTGGACAATGGGCGCAAGCCTGATCCAGCC
ATGCCGCGTGAGTGATGAAGGCCTTCGGGTTGTAAAGCTCTTTAGTCCACGAATATAATG
ACTGTAGTGGAAGAATAAGCCCCGGCTAACTTCGTGCCAGCAGCCGCGGTAATACGAGGG
GGGCGAGCGTTGTTCGGAATCACTGGGCGTAAAGAGTGCGTAGGCGGTCGTGAAAGTTAG
AAGTGAAAGCCCAAGGCTCAACCTTGGAATTGCTTTTAATACTACATGACTAGAATTCGG
GAGAGGATAGCGGAATTGTCAGTGTAGCAGTGAAATGCGTAGATATTGACAGGAACACCG
GTGGCGTAAGCGGCTATCTGGACCGACATTGACGCTGAGGTACGAAAGCGTGGGGATCAA
ACAGGATTAGATACCCTTGTAGTCC
>Otu7530
CCAGCCTACGGGTGGCACCAGTGGGGAATATTGGGCAATGGGCGAAAGCCTGACCCAGCC
ACGCCGCGTGAGTGATGAAGGCCTTCGGGTCGTAAAGCTCTGTGGGGAGGGACGAATAAG
CCGGGTGCTAATATCACCAGGCCCTGACGGTACCTCCTTAGCAGGCACCGGCTAACCATG
TGCCAGCAGCCGCGGTAATACATGGGGTGCAAACGTTGCTCGGAATTATTGGGCGTAAAG
CGCACGTAGGCGGTTCACTAAGTCGGATGTGAAAGCCCTCGGCTCAACCGAGGAAGTGCA
TCCGAAACTGGCGAGCTTGAGTACGAAAGAGGGTCGCGGAATTCCCGGTGTAGAGGTGAA
ATTCGTAGATATCGGGAGGAACACCAGTGGCGAAGGCGGCGACCTGGGTCGATACTGACG
CTGAGGTGCGAAAGCGTGGGGAGCAAACAGGATTAGAAACCCTAGTAGTCC
>Otu7533
CCAGCCTATGGGGGGCTGCAGTGGGGAATATTGGACAATGGGCGAAAGCCTGATCCAGCA
ATGCCGCGTGTGTGAAGAAGGCCTGAGGGTTGTAAAGCACTTTCAGTGGGGAGAAAGAGG
CCATTGTTAATACCAATGGTGGATGATGTTACCCACAGAAGAAGCACCGGCTAACTCTGT
GCCAGCAACCGCGGTAATACAGAGGGTGCAAGCGTTAATCGGAATTACTGGGCGTAAAGG
GTGCGTAGGTGGTTTTGTAAGTCAGATGTGAAATTCCTGGGCTCAACCTGGGGACTGCGT
CTGATACTGTGAAGCTAGAGTACTGTAGAGGAAAGTGGAATTTCCGGTGTAGCAGTGAAA
TGCGTAGATATCGGAAGGAACATCAGTGGCGAAGGCGACTTTCTGGACAGATACTGACAC
TGAGGCACGAAAGCGTGGGGAGCAAACAGGATTAGAACCCCAGTAGTCC
>Otu7536
CCAGCCTACGGGAGGCAGCAGTGGGGAATCTTGCGCAATGGGCGAAAGCCTGACGCAGCG
ACGCCGCGTGGAGGATGAAGGCCTTCGGGTTGTAAACTTCTGTTAAGTGGGAAGAAATAA
CTGTCCCTAATACGGACGGGAGATGACTGTACCATTAGAGAAAGCACCGGCTAAACTCGT
GCCAGCAGCCGCGGTAATACGAGTGGTGCAAGCGTTATTCGGAATCATTGGGCGTAAAGG
GTGTGTAGACGGCATATTAAGTCAATTGTTTAATTCTCCGGCCTAACCGGAGTCCAGCGG
TAGATACTGGTATGCTAGAGGGTGGAAGAGAGAAGTGGAATTCTCGGAGTAGCGGTAAAA
TGCGTAGATCTCGAGAGGAACACCAATGGCGAAGGCAGCTTCTTGGTCCACACCTGACGT

TGAGACACGAAAGCGTGGGGATCAAACAGGATTAGATACCCCCGTAGTCC
>Otu7538
CCAGCCTACGGGTTGCACCAGTAGGGAATATTGGACAATGGAGGAAACTCTGATCCAGCC
ATGCCGCGTGAATGATGAAGGCCTTCGGGTTGTAAAGTTCTTTAGTCCGGGAAGATAATG
ACGGTACCGGAAGAATAAGCTCCGGCTAACTCTGTGCCAGCAGCCGCGGTAATACAGAGG
GAGCTAGCGTTGTTCGGAATTACTGGGCGTAAAGCGCGCGCAGGCGGCCATTTAAGTCAG
ATGTGAAAGCCCGGGGCTTAACCTCGGAACTGCATTTGAAACTGAGTGGCTAGAGTATGG
TAGGGGAGAGGGGAATTCCTAGTGTAGCGGTGAAATGCGTAGAGATTAGGAGGAACACCA
GTGGCGAAGGCGCCTCTCTGGGCCATTACTGACGCTGAGGCGCGAAAGCGTGGGGAGCAA
ACAGGATTAGATACCCTAGTAGTCC
>Otu7542
CCAGCCTATGGGAGGCTCCAGTCGAGAATTTTTCTCAATGGGCGAAAGCCTGAAGGAGCG
ACGCCGCGTGGGGGATGAATGGCTTCGGCCCGTAAACCCCTGTCATTTGCGAACAAACCT
TTTCATTTAACAGACGAAAAGCTGATAGTAGCGGAAGAGGAAGGGACGGCTAACTCTGTG
CCAGCAGCCGCGGTAATACAGAGGTCCCAAGCGTTGTTCGGATTCACTGGGCGTAAAGGG
TGCGTAGGTGGTCAGATAAGTCTGATGTGAAATCTCGGAGCTTAACTCCGAAACTGCATT
GGATACTATTTGGCTTGAGGGTCGGAGGGGGGACTGGAATTCTCGGTGTAGCAGTGAAAT
GCGTAGATATCAAGTGGAACACCAGTGGCGAAGGCGAGTCCCTAGACAACTCCTGACACT
GAGGCACGAAAGCTAGGGGAGCAAACAGGATTAGATACCCGTGTAGTCC
>Otu7543
CCAGCCTACGGGGGGCTGCAGTGGGGAATCTTGCGCAATGGGCGAAAGCCTGACGCAGCA
ACGCCGCGTGGAGGATGAAGGCCTTCGGGTCGTAAACTTCTTTTAGCAGGGACGAGGAAG
GACGGTACCTGCGGAATCAGTCACGGCTAACTACGTCCCAGCAGCCGCGGTAAAACGTAG
GTGGCAAGCGTTATCCGGATTTACTGGGCGTAAAGGGTGTGCAGGCGGGAGTGCAAGTGG
TGTATGAAATATCCCAGCTTAACTGGGAGGGGTTATGCCAGACTGCACTTCTAGAGGACG
AGAGAGGGGGGTGGAATTCCGGGTGTAGTGGTGAAATGCGTAGAGATCCGGAGGAACCCC
AGAGGCGAAGGCGGCCCCCTGGCTCGTATCTGACGCTCAGCCACGAAAGCATGGGGAGCG
AACGGGATTAGATACCCCCGTAGTCC
>Otu7550
CCAGCCTATGGGTTGCAGCAGTGGGGAATATTGGGCAACGGAGTAATTTCTGACCCAGCG
AGAATGCCTGCATGACGAAGGCGTGAAGTTTTCTGTAAAGTGCTGTCGGTGATAACGATA
ATACCAGTAATCATAAATAAGAAGTGCCGGCTAACTCCGTGCCAGCAGCTGCGGTAAGAC
GGAGGGCGCGGGCGTTATCCGTTGTGATTGGGTGTAAAGGGTACGTAGGCTGCTGTTGAT
AATTTAAAACTTAAAATGCGGGGCAATCCTACGCATAAAGTTTTTTAAAGGATTCAGCTT
GTGTTGAATGCAGGTTAGTTTAATTTTTTGGGTAATAGGGGTAGAATCCCACGAGCCAAG
AAAGAATACCAACGAGCGGAGGCATCTTTCTAGTTTCAACAGACGCTAAGGTACGGAAGC
TGCGAAATCAAACAGGATTAGATACCCGCGTAGTCC
>Otu7556
CCAGCCTACGGGTGGCAGCAGTGGGGAATTTTGGACAATGGGCGAAAGCCTGATCCAGCG
ACGCCGCGTGGAGGATGAAGGCCTTCGGGTTGTAAACTCCTTTTAGAGGGAACGAATAAA
TTGACGGTACCCTCAGAAAAAGCCACGGCTAACTACGTGCCAGCAGCCGCGGTAATACGT
AGGTGGCGAGCGTTACTCGGATTTACTAGGCGTAAAGCGCTTGTAGGTGGTTGGATAAGT
CTGTAGTGAAATTTCACGGCTTAACTGTGAACTGTCTATAGAAACTATCCGGCTTGAGTG
TGGAAGGGGGCAACGGAATTCCCGGTGTAGCGGTGAAATGCGTAGATATCGGGAGGAACA
CCAATGGCGAAAGCAGTTGCCTGGTCCAATACTGACACTGAGGAGCGAAAGCTAGGGGAG
CAAACAGGATTAGATACCCTCGTAGTCC
>Otu7559
CCAGCCTACGGGGGGCAGCAGTAGGGAATTTTCCACAATGGGCGAAAGCCTGATGGAGCA
ACGCCGCGTGCAGGATGAAGGCTCTAGGGTCGTAAACTGCTTTTATCTGTGACGATTATG
ACGGTAGCAGATGAATAAGGATCGGCTAACTCCGTGCCAGCAGCCGCGGTCACACGGAGG
ATCCAAGCGTTATCCGGAATTACTGGGCGTAAAGAGTTGCGTAGGTGGCAGAGTAAGTTG
GTAGTGAAAGCGTGTGGCTCAACCACATAAACATTACTAAAACTGTTCAGCTAGAGGACG
AGAGAGGTTATTGGAATTTCTAGTGTAGGAGTGAAATCCGTAGATATTAGAAGGAACACC
GATGGCGTAGGCAGATAACTGGCTCGCTCCTGACACTAAGGCACGAAAGCGTGGGTAGCA
AACGGGATTAGATACCCCAGTAGTCC
>Otu7564
CCAGCCTATGGGTGGCAGCAGTGAGGGATATTGCGCAATGGGCGAAAGCCTGACGCAGCG
ACACCGCGTGAAGGGTGAAGGCTTTCGGGTCGTAAACTTCTGTTAAGTGGGAAGAAAGGC

CTGGTTCTAATACAGCCAGGAAATGACGGTACCACTAGAGAAAGCACCGGCTAACTTCGT
GCCAGCAGCCGCGGTAAGACGAGGGGTGCGAACGTTATTCGGAATGATTGGGTGTAAAGG
GTGCGTAGGCGGCATGTTAAGTCAATCGTTAAATTTTTCAGCTTAACTGAAAGTAGGCGG
TAGAAACTGGCGTGCTTGAGGGTGGAAGAGAGAAGTAGAATTCTCGGAGTAGCGGTAAAA
TGCGTAGATCTCGAGAGGAATACCAATGGCGAAGGCAGCTTCTTGGTCCATTTCTGACGC
TGAGGCACGAAAGCGTGGGGAGCAAACAGGATTAGAGACCCGTGTAGTCC
>Otu7566
CCAGCCTATGGGATGCAGCAGTTTCGAATCATTCACAATGGGCGAAAGCCTGATGGTGCG
ACGCCGCGTGAGGGATGAAGGTCTTCGGATTGTAAACCTCTGTCACCGGGGAAGAAACGC
TTCATTTTAATAGAATGAAGCCTGACTTAACCCGGAGAGGAAGCAGTGGCTAACTCTGTG
CCAGCAGCCGCGGTAATACAGAGACTGCAAGCGTTATTCGGATTCACTGGGCGTAAAGGG
TGCGCAGGTGGCTGGGTGTGTCAGATGTGAAATCCCGAGGCTCAACCTCGGAACTGCGTC
TGAAACTACTCGGCTAGAGTATTGGAGAGGGTAACGGAATTCACGGTGTAGCAGTGAAAC
GCGTAGATATCGTGAGGAACACCAGAGGCGAAGGCGGTTACCTGGACAATTACTGACACT
CAGGCACGAAAGCGTGGGGAGCAAAAGGGATTAGATACCCGCGTAGTCC
>Otu7568
CCAGCCTATGGGGGCTGCAGTGGGGAATATTGGACAATGGGGGAAACCCTGATCCAGCAA
TGCCGCGTGTGTGAAGAAGGCCTTCGGGTTGTAAAGCACTTTCGGTGGGGAAGAAAGACC
TAAGGTTAATATCCTTAGGAGGTGACGTTACCCACAGAAGAAGCACTGGCTAACTCTGTG
CCAGCAGCCGCGGTAATACAGAGAGTGCAAGCGTTAATCGGAATTACTGGGCGTAAAGCG
CATGTAGGTGGATATTTAAGTCGGATGTGAAAGCCCTAGGCTCAACCTGGGAATTGCATC
TGATACTGGGTATCTAGAGTATGGTAGAGGGAAGTGGAATTTCCGGTGTAGCGGTGAAAT
GCGTAGATATCGGAAAGAACACCAGTGGCGAAGGCGGTTTCCTGGACCAATACTGACACT
CAGGTGCGAAAGCGTGGGGAGCAAACAGGATTAGAAACCCCCGTAGTCC
>Otu7571
CCAGCCTATGGGTGGCTGCAGTGGGGAATTTTGGACAATGGGGGCAACTCTGATCCAGCG
ACGCCGCGTGAAGGATGAAGGTCTTCGGATTGTAAACTTCTTTTGGAGGGGATGAATGCC
TGAGTGAAAAATTCAGGGTGACAGTACTCTCTGAATAAGCCACGGCTAACTACGTGCCAG
CAGCCGCGGTAATACGTAGGTGGCAAGCGTTACTCGGAGTCACTAGGCGTAAAGCGCAGG
TAGGCGGTTCGGTAAGTCTGTTGTGAAAGCTCCCGGCTCAACTGGGAGAGGCCAACGGAA
ACTACCGGGCTAGAGTGTGGTAGAGGTAGCTGGAATTCCCGGTGTAGCGGTGAAATGCGT
AGATATCGGGAGGAATACCTATGGCGAAAGCAGGCTACTGGACCATCACTGACGCTGATC
TGCGAAAGCTAGGGGAGCAAACAGGATTAGAAACCCTAGTAGTCC
>Otu7572
CCAGCCTACGGGGGGCAGCAGTAAGGAATATTGGTCAATGGGCGAAAGCCTGAACCAGCC
ATGCCGCGTGCAGGATGACGGCCCTATGGGTTGTAAACTGCTTTTATCAGGGAAGAAACC
CCTTTACGTGTAGAGGGTTGACGGTACCTGAAGAATAAGGATCGGCTAACTCCGTGCCAG
CAGCCGCGGTAATACGGAGGATCCAAGCGTTATCCGGATTTATTGGGTTTAAAGGGTGCG
TAGGCGGCCTATTAAGTCAGAGGTGAAAGCCTGGAGCCTAACTCCAGAACTGCCTTTGAT
GCTGATGGGCTTGAATATAGTTGAGGTGGGCGGAATGTGTCATGTAGCTGTGAAATGCTT
AGATATGACGCAGAACACCAATTGCGAAGGCAGCTCGCTAAACTATTATTGACGCTGAGG
CACTAAAGCGTGGGGATCAAACAGGATTAGAGACCCTCGTAGTCC
>Otu7574
CCAGCCTACGGGGGGCAGCAGTGGGGAATCTTAGACAATGGGCGAAAGCTTGATCTAGCA
ATATTGCGTGAATGAACATTGACAGCGGAGATGCTGTAAAATTCTATCGATCTAGGGATT
ATTATGGCTGTACCTAGCGACGAAGTCCTGGCTTACTTCGTGCCAGCAGCCGCGGTAATA
CGAGGGGGACGAGCGTTATCCGGATGGATTGGGCGTAAAACGTTTGTAGGTGGTTTTTTA
AAATCTTTATGAAATTCTAGAGCCTAACTTTAGAGCACATATTGATGAAGTTAATCCTTG
AGTTTATTTCAGGAGTTTTGAATTTTATAAGTAAGAGTGAAATCTAACGATCTATAAAAG
AAAATCGATAGCGAAGGCTATTCTCTTGAATATTCTGACATTGAGAAACGAAAGCGTAGG
GAGCCAATAGGATTAGATACCCTGGTAGTCC
>Otu7576
CCAGCCTACGGGAGGCTCCAGTGGGGAATATTGGACAATGGGCGCAAGCCTGATCCAGCC
ATGCCGCGTGGGTGATGAAGGCCCTAGGGTTGTAAAGCCCTTTCGGCGGGGACGATGATG
ACGGTACCCGCAGAAGAAGCCCCGGCTAACTTCGTGCCAGCAGCCGCGGTAATACGAAGG
GGGCTAGCGTTGCTCGGAATGACTGGGCGTAAAGGGCGCGTAGGCGGTCTGGACAGTCAG
GCGTGAAATTCCCGGGCTCAACCTGGGGGCTGCGCTTGATACATCTCGACTCGAGTGAGG
AAGAGGGTCGTGGAATTCCCAGTGTAGAGGTGAAATTCGTAGATATTGGGAAGAACACCG

GTGGCGAAGGCGGCGACCTGGTCCTTGACTGACGCTGAGGCGCGAAAGCGTGGGGAGCAA
ACAGGATTAGAAACCCTTGTAGTCC
>Otu7577
CAGCCTATGGGGCGCTGCAGTGAGGAATATTGCTCAATGGACGAAAGTCTGAAGCAGCAA
CGCCGCGTGAGGGATGAAGGTGCTCTGCATTGTAAACCTCTGTAGTCGGGGAAAAACGGC
GGGTTATTACCCGTTTGATGGTACCCGAAAAGTAAGGATCGGCTAACTACGTGCCAGCAG
CCGCGGTAATACGTAGGATCCAAGCGTTGTCCGGATTTACTGGGTGTAAAGGGTGCGCAG
GCGGACTTGTAAGTTAGAGGTGAAATCTCACGGCTCAACCGTGAAACTGCCTTTAATACT
GCAAGTCTTGAATTGGGGAGAGAGTCGCGGAATTCCGAGTGTAGCAGTGAAATGTGTAGA
TATTCGGAAGAACACCAGTGGCGAAGGCAGCGACTTGGCCCTATATTGACGCTCAGGCAC
GAAAGCATGGGGATCAAACAGGATTAGAGACCCTCGTAGTCC
>Otu7579
CCAGCCTATGGGAGGCTGCAGTCGAAAATATTCGACAATGGGCGAAAGCCTGATCGAGCG
ATACCGCGTGGTGGATGAAGCGATTCGTCGCGTAAACATCTTTTATGAGGGAGGAAGTTA
TTGACGTTACCTCATGAATAAGGGGCTCCTAACTCTGTGCCAGCAGGAGCGGTAATACAG
AGGCCCCGAGCGTTACCCGGAATTACTGGGCGTAAAGAGTGCGTAGGTGGTCGTGTTAGT
CGTCTGTTAAACCCCGGAGCTCAACTCCGGATCTGCAGACGAAACGGCACGACTTGAGGG
CGTGAGAGGTACATGGAACTCATGGTGTAGGGGTGAAATCCGTTGATATCATGGGGAACA
CCGAAAGCGAAGGCAATGTACTGGCGCGTTCCTGACACTCAAGCACGAAAGCGTGGGTAG
CGAACGGGATTAGAGACCCCCGTAGTCC
>Otu7595
CCAGCCTATGGGATGCAGCAGTAGGGAATATTGGACAATGGGCGCAAGCCTGATCCAGCC
ATCCCGCGTGAAGGATTAAGGCCCTATGGGTTGTAAACTTCTTTTCTCTGGGAATAAAAA
GTGGAGTTCACTCCGCCCTGAAGGTACCAGAGGAATAAGCACCGGCTAACTCCGTGCCAG
CAGCCGCGGTAATACGTAGGTGGCAAGCGTTGTCCGGATTTATTGGGTTTAAAGGGTGCG
TAGGCGGCCCTGTAAGTCAGTGGTGAAATACGGCAGCTCAACTGTCGAGGTGCCATTGAT
ACTGCAGGGCTTGAGTACAGACGAGGTAGGCGGAATTGACGGTGTAGCGGTGAAATGCTT
AGATATCGTCAAGAACACCGATAGCGAAGGCAGCTTACTAGGCTGTAACTGACGCTGAGG
CACGAAAGTGTGGGGATCAAACAGGATTAGAGACCCTCGTAGTCC
>Otu7597
CCAGCCTACGGGAGGCTCCAGTGGGGAATCTTGCGCAATGGGCGAAAGCCTGACGCAGCG
ACGCCGCGTGGGTGATGAAGGCCTTCGGGTTGTAAAGCCCTGTGGGGAGGGACGAATAAG
GCTGGGTGAACAATCCAGTTGATGACGGTACCTCCTTAGCAAGCACCGGCTAACTCTGTG
CCAGCAGCCGCGGTAATACAGAGGGTGCAAACGTTGTTCGGATTTACTGGGCGTAAAGCG
CGTGTAGGCGGCCGGCCAAGTCGGATGTGAAAGCCCGGGGCTCAACCTCGGAAGTGCATT
CGATACTGGTCGGCTGGAGTCTCGGAGAGGCGGGTGGAACTCTCGGTGTAGAGGTGAAAT
TCGTAGATATCGAGAAGAACACCGGTGGCGAAGGCGGCCCGCTGGACGAAGACTGACGCT
GAGACGCGAAAGCGTGGGGAGCAAACAGGATTAGAAACCCTCGTAGTCC
>Otu7598
CCAGCCTATGGGGCGCTGCAGTGGGGAATATTGGACAATGGGCGCAAGCCTGATCCAGCG
ATGCCGCGTGTGTGAAGAAGGCCTTTGGGTTGTAAAGCACTTTTTTTGGGGAAGAACGTT
ATGGATTCGAATAGGGTCCATAAGTGACGGTACCTAACGAATAAGCACCGGCTAACTCTG
TGCCAGCAGCCGCGGTAATACAGAGGGTGCGAGCGTTGTTCGGAATTATTGGGCGTAAAG
GGTGCGTAGGCGGTGCCACAAGTCACTTGTGAAATCCCCAGGCTTAACCTGGGGCCTGCA
GGCGAAACTGTGGTGCTGGAGTATGGGAGAGGTGCGTGGAATTCCCGGTGTAGCGGTGAA
ATGCGTAGATATCGGGAGGAACACCTGTGGCGAAAGCGGCGCACTGGACCATAACTGACG
CTGATGCACGAAAGCTAGGGGAGCAAACAGGATTAGAAACCCGAGTAGTCC
>Otu7599
CCAGCCTATGGGTCGCAGCAGTGGGGAATTTTCCACAATGGGCGAAAGCCTGATGGAGCA
ACGCCGCGTGCAGGATGAATGCCTTCGGGTCGTAAACTGCTTTTTTTTGTGAGGATTATG
ACAGTAGCAGAAGAATAAGGATCGGCTAACTCCGTGCCAGCAGCCGCGGTCATACGGAGG
ATCCAAGCGTTATCCGGAATTACTGGGCGTAAAGAGTTGCGTAGGCGGCATAGTAAGCAG
AGTGTGAAATCGTGTGGCTCAACCATACATCCATACTTTGAACTGCTAAGCTAGAGAATG
AGAGAGGTGGCTGGAATTCCCAGTGTAGGAGTGAAGTCCGTAGATATTGGGAGGAACACC
GATGGCGTAGGCAGGCCACTGGCTCATTTCTGACGCTCAGGCACGAAAGCGTGGGGAGCG
ACTGGGATTAGAAACCCGAGTAGTCC
>Otu7601
CCAGCCTACGGGAGGCAGCAGTCGAGGATCTTTCGCAATGGGCGCAAGCCTGACGAAGCG

ACGCCGTGTGAGCGAAGAAGGCCTTCGGGTTGTAAAGCTCTTTCGCCTGGGAACAAGAGA
CTCTAGCGAATAACTAGGGGATTTGAGGGTACTAGGTAAAGAAGCACCGGCTAACTCCGT
GCCAGCAGCTGCGGTAATACGGAGGGTGCAAGCATTAATCGGATTTATTGGGCGTAAAGA
GGGCGTAGGCGGACATGTAAGTCAGATGTGAAAGCCCGGGGCTCAACCCCGGAACGGCAT
TTGAAACTGCATATCTAGAGGATAGGCGGAGAAAACGGAATTCCACATGTAGCGGTGAAA
TGCGTAGATATGTGGAAGAACACCGGTGGCGAAGGCGGTTTTCTAGCTTAGCCCTGACGC
TGAGGCCCGAAAGCAAGGGGAGCAAACAGGATTAGAAACCCGCGTAGTCC
>Otu7604
CCAGCCTATGGGGTGCAGCAGTGGGGAATCTTGCGCAATGGGAGAAATCCTGACGCAGCA
ACGCCGCGTGGGTGATGAAGGTCTTCGGATCGTAAAGCCCTGTCGGAGGGGAAGATGCTT
GTTTGTGCTAATAGTACAGACAGGTGACGGTACCCTCAAAGGAAGCACCGGCTAACTCTG
TGCCAGCAGCCGCGGTAATACAGAGGGTGCAAGCGTTGTTCGGAATTATTGGGCGTAAAG
GGAGTGTAGGCGGTTTTCTAAGTCTTCTGTGAAATCTACGGGCTTAGCTCGTAACGTGCA
GAGGAAACTGGAAGACTTGAGTGTCGGAGAGGGTAGCGGAATTCCCGGTGTAGAGGTGAA
ATTCGTAGAGATCGGGAGGAACACCAGTGGCGAAGGCGGCTACCTGGCTGACAACTGACG
CTGAGACTCGAAAGCATGGGGAGCAAACAGGATTAGATACCCGGGTAGTCC
>Otu7606
CCAGCCTATGGGACGCAGCAGTGAGGAATCTTGCGCAATGGGCGAAAGCCTGACGCAGCG
ACGCCGCGTGGAGGATGAAGGTCTTCGGATTGTAAACTTCTGTTAAGTGGGGCGAAATAC
CCATCTCTAATAAAGATGGGGGATGACAGTACCACTAGAGAAAGCACCGGCTAAACTCGT
GCCAGCAGCCGCGGTAATACGAGTGGTGCAAGCGTTATTCGGATTTATTGGGCGTAAAGG
GTGTGCAGACGGCTTATTAAGTCACTTGTTAAATTCTCCGGCCTAACTGGAGGTATGCGG
GTGAAAATGATGAGCTTGAGGATGGAAGAGAGAAGCGGAATTCTCGGAGTAGCGGTAAAA
TGCGTAGATCTCGAGAGGAACACCAATGGCGAAGGCAGCTTCTTGGTCCATTCCTGACGT
TCATACACGAAAGCGTGGGGAGCAAACAGGATTAGAGACCCTCGTAGTCC
>Otu7607
CCAGCCTATGGGAGGCACCAGTGAGGGATATTGCACAATGGGCGCAAGCCTGATGCAGCG
ACGCCGCGTGGGTGAAGAAGGTCTTCGGATCGTAAAGCCCTTTTCTGCGTGACGAGGAAC
AGACGGTAGCGCAGGAAGAAGTCTCGGCTAACTACGTGCCAGCAGCCGCGGTAAAACGTA
GGAGGCGAGCGTTATCCGGAGTTACTGGGCGTAAAGCGCGTGTAGGTGGTCTGGTAAGCT
GGATGTGAAAGATCCCGGCTCAACCGGGAAAAGCCATAGAGGACTGCCAGACTCGAGGGG
GGTAGAGGGGCGTAGAATTCCGGGTGTAGCGGTGAAATGTGTAGAGATCCGGAGGAATAC
CAGAGGTGAAGACGGCGCCCTGGGCCCGACCTGACACTGAGACGCGAAAGCGTGGGGAGC
GAACGGGATTAGAAACCCCCGTAGTCC
>Otu7614
CCAGCCTATGGGAGGCTCCAGTGGGGAATATTGCACAATGGGCGAAAGCCTGATGCAGCG
ACGCCGCGTGGAGGATGAAGGTTTTCGGATTGTAAACTCCTGTTAAGTGGGAAGAGAGAA
CCTGTGTTAATACCACAGGGAGATGACGGTACCACTAGAGAAAGCACCGGCTAACTTCGT
GCCAGCAGCCGCGGTAATACGAGGGGTGCAAGCGTTATTCGGAATAACTGGGCGTAAAGA
GCGCGTAGGCGGCTTGTTAAGTCTGTTGTTAAATTGCCCGGCTTAACCGGGAAACGGCAA
TAGAAACTGGCGAGCTAGAGGACAAGAGAGAGAAGTGGAATTCTCGGAGTAGCGGTAAAA
TGCGTAGATCTCGAGAGGAACACCGATGGCGAAGGCAGCTTCTTGGCTTGTATCTGACGC
TCAAGTGCGAAAGCGTGGGGAGCAAACAGGATTAGATACCCTCGTAGTCC
>Otu7617
CCAGCCTACGGGTTGCTGCAGTAGGGAATATTGCACAATGGAGGAAACTCTGATGCAGCA
ACGCCGCGTGAGTGACGAAGGCCTTCGGGTCGTAAAGCTCTGTTGTACGGGAAGAAAAAA
ATGACGGTACCGTATAAGAAAGGACCGGCCAACTTCGTGCCAGCAGCCGCGGTAATACGA
AGGGTCCAAGCGTTGTTCGGAATCACTGGGCGTAAAGCGCATGTAGGAGGCTTAATAAGT
CGAATGTGAAATCCCCGGGCTCAACCCGGGACGTGCATTCGATACTGTTGAGCTTGAGTA
TGAGAGAGGGTAGTAGAATTCCAGGTGTAGTGGTGAAATACGTAGATATCTGGAGGAATA
CCGGTGGCGAAGGCGGCTACCTGGCTCAATACTGACTCTTAGGTGCGAAAGCGTGGGGAT
CAAACAGGATTAGAGACCCGGGTAGTCC
>Otu7619
CCAGCCTATGGGGGGCAGCAGTGGGGAATATTGGACAATGGGGGCAACCCTGATCCAGCA
ATGCCGCGTGAGTGATGAAGGCCTTCGGGTTGTAAAGCTCTTTTGTCAGGGACGATAATG
ACGGTACCTGACGAATAAGCTCCGGCTAACTTCGTGCCAGCAGCCGCGGTAATACGAAGG
GAGCTAGCGTTGTTCGGAATTACTGGGCGTAAAGGGCGCGTAGGCGGCTTTATAAGTCAG
GTGTGAAATCCCTGGGCTTAACCTAGGAATGGCATTTGAAACTGTAAGGCTAGAGATCGA

GAGAGGATAGTGGAATTGCGAGTGTAGAGGTGAAATTCGTAGATATTCGCAAGAACACCG
GTGGCGAAGGCGGCTATCTGGCTCGATACTGACGCTGAGACGCGAAAGCGTGGGGAGCAA
ACAGGATTAGAAACCCTTGTAGTCC
>Otu7622
CCAGCCTATGGGGTGCTGCAGTGGGGAATATTGCACAATGGGCGAAAGCCTGATGCAGCG
ACGCCGCGTGAGGGATGACGGCCTTCGGGTTGTAAACCTCTTTCGACAGGGACGAAGGGA
AACTGACGGTACCTGTAGAAGAAGCACCGGCTAACTACGTGCCAGCAGCCGCGGTAATAC
GTAGGGTGCGAGCGTTGTCCGGAATTACTGGGCGTAAAGAGCTTGTAGGCGGTCTGTCAC
GTCGATTGTGAAGACTCACAGCTCAACTGTGGGCCTGCAGTCGATACGGGCAGACTAGAG
TACTTCAGGGGAGACTGGAATTCCTGGTGTAGCGGTGAAATGCGCAGATATCAGGAGGAA
CACCGGTGGCGAAGGCGGGTCTCTGGGAAGTAACTGACGCTGAGAAGCGAAAGCGTGGGT
AGCGAACAGGATTAGAAACCCCTGTAGTCC
>Otu7624
CCAGCCTATGGGGGGCACCAGTGGGGAATATTGGACAATGGGGGAAACCCTGATCCAGCA
ATGCCGCGTGTGTGAAGAAGGCCTTCGGGTTGTAAAGCACTTTCAGTAGGGAAGAAAGAC
TTAAGACTAATAATCTTAGGAGTTGACGTTACCTAAAGAAGAAGCACTGGCTAACTCTGT
GCCAGCAGCCGCGGTAATACAGAGAGTGCAAGCGTTAATCGGAATCATTGGGCGTAAAGC
GCACGTAGGTGGATACTTAAGTCGAACGTGAAATCCCCGGGCTTAACCTGGGAACTGCGT
CCGATACTGAGCATCTAGAGTAGGGTAGAGGGAAGTGGAATTTCCGGTGTAGCGGTGAAA
TGCGTAGATATCGGAAAGAACACCAGTGGCGAAGGCGGCTTCCTGGACCAATACTGACGC
TGAGGAGCGAAAGCGTGGGGAGCGAACAGGATTAGATACCCTCGTAGTCC
>Otu7625
CCAGCCTATGGGTGGCAGCAGTAAGGAATATTGGACAATGGGCGCAAGCCTGATCCAGCC
ATGCCGCGTGAAGGATGAAGGCGCTATGCGTCGTAAACTTCTTTTGTATGGGAAAAATTC
CCCGCTTTCTAGCGGGGTTGATGGTACCATAAGAATAAGGGTCGGCTAACTTCGTGCCAG
CAGCCGCGGTAATACGAAGGACCCGAGCGTTATCCGGATTCATTGGGTTTAAAGGGTGCG
TAGGCGGACCTGTAAGTCAGTGGTGAAATCCCTTCGCTTAACGAAGGAATTGCCATTGAT
ACTGCAGGTCTTGAGTACAGTTGAGGTTGGCGGAATGTGTCATGTAGCGGTGAAATGCTC
AGATATGACACAGAACACCGATTGCGAAGGCAGCTAGCTAAACTGTAACTGACGCTGAGG
CACGAAAGCGTGGGTAGCAAACAGGATTAGAAACCCGAGTAGTCC
>Otu7627
CCAGCCTATGGGGGGCAGCAGTTTGGAATATTGGTCAATGGGGGAAACCCTGAACCAGCG
ACGCCGCGTGGAGGATGAAGGATCTAGGTCTGTAAACTCCTTTTGTCGGGAAAGACTTAG
GACGGTACCCGACGAATAAGCTCCGGCTAACTACGTGCCAGCAGCCGCGGTAAGACGTAG
GGAGCGAGCGTTGTCCGGATTTACTGGGCGTAAAGGGCGCGTAGGCGGCTCGTTAAGTGT
GAAGTGAAATCTCCAGGGCTCAACCCGGAAACTGCTTTACATACTGGCGAGCTAGAGGGA
TGGAGAGGTAAGTGGAATTCCTGGTGTAGCGGTGAAATGCATAGATATCAGGAGGAACAC
CCATGGCGAAGGCAGCTTACTGGCCATCTCCTGACGCTGAGGCGCGAAAGCGTGGGTAGC
AAACAGGATTAGAGACCCCAGTAGTCC
>Otu7629
CCAGCCTATGGGGGGCTGCAGTGGGGAATTTTGGACAATGGGGGCAACCCTGATCCAGCG
ACGCCGCGTGAAGGATGAAGGTCTTCGGATTGTAAACTTCTTTTAGAGGGGATGAATGTC
TTGGTGAATAGCCAAGCGTGACAGTACTCTTTGAATAAGCCACGGCTAACTACGTGCCAG
CAGCCGCGGTAATACGTAGGTGGCAAGCGTTACTCGGAATCACTAGGCGTAAAGCGCAGG
TAGGCGGTTGGGTAAGTCTTTTGTGAAAGCTCCCGGCTCAACTGGGAGAGGTCAAAGGAA
ACTACTCGGCTAGAGTTTGGTAGGGGATACTGGAATTCCTGGTGTAGCGGTGAAATGCGT
AGATATCAGGAGGAATACCTATGGCGAAAGCAGGTATCTGGGCCACAACTGACGCTGAGC
TGCGAAAGCTAGGGGAGCAAACAGGATTAGATACCCCCGTAGTCA
>Otu7631
CCAGCCTACGGGGGGCACCAGTGGGGAATCTTGCGCAATGGGCGAAAGCCTGACGCAGCA
ACGCCGCGTGTGTGATGAAGGTCTTCGGATCGTAAAGCACTGTCGCGAGGGAAGACGAGA
CGGGTTCTAACAGGACCTGTTTGAGACGGTACCTCGAGAGGAAGCACCGGCTAACTCTGT
GCCAGCAGCCGCGGTAATACAGAGGGTGCGAGCGTTGTTCGGAATTATTGGGCGTAAAGC
GCATGTAGGCGGCCTGTTAAGTTCCGTGTGAAATCCCTAGGCTTAACCTAGGAAGTGCGC
GGAAAACTGGTAGGCTTGAGTGCTGGAGAGGGTCGCGGAATTCCCGGTGTAGAGGTGAAA
TTCGTAGATATCGGGAGGAACACCAGTGGCGAAGGCGGCGACCTGGACAGACACTGACGC
TGAGACGCGAAAGCGTGGGGCGCAAACAGGATTAGATACCCGCGTAGTCC
>Otu7632

CCAGCCTATGGGACGCACCAGTAGGGAATATTGCACAATGGAGGAAACTCTGATGCAGCG
ACGCCGCGTGAGTGACGAAGGCCTTCGGGTCGCAATGCTCTGTTCTCAGGGAAAAAGAAA
GTGATTGTACCTGAGAAGAAAGGACCGGCTAACTTCGTGCCAGCAGCCGCGGTAAGACGA
GGGGTCCAAGCGTTGTCCGGAATTATTGGGCGTAAAGCGGGTGTAGGTGGCTCTATAAGT
CAGAAGTGAAAGCCCCGAGCTTAACTTGGGAAGTGCTTTTGATACTGCAGAGCTTGAATG
TGGGAGAGGATCGTGGAGTTCCAGGTGTAGTGGTGAAATACGTAGATATCTGGAGGAACA
CCGGTGGCGAAGGCGGCGATCTGGCCCAACATCGACACTGAGACCCGAAAGCGTGGGGAT
CAAACAGGATTAGAAACCCGAGTAGTCC
>Otu7637
CCAGCCTATGGGTGGCAGCAGTCGAGGATCTTTCGCAATGGGCGAAAGCCTGACGAAGCG
ACGCCGTGTGAATGATGAAGGCCTTCGGGTTGTAAAGTTCTTTCGCCTGGGAACAAGAGA
GGCTAGTGAATAACTTAGCCGATTTGAGGGTACCAGGTAAAGAAGCACCGGCTAACTCCG
TGCCAGCAGCTGCGGTAATACGGAGGGTGCGAGCATTAATCGGAATTATTGGGCGTAAAG
GGCGCGTAGGCGGGCAAACAAGTCAGATGTGAAATTCCGGAGCTCAACTTCGGAGCTGCA
TTTGAAACTGTTAGTCTAGAGGCAAGACGGAGAAAATGGAATTCCACGTGTAGCGGTGAA
ATGCGTAGATATGTGGAAGAACACCAGTGGCGAAAGCGGTTTTCTGGTTTATGCCAGACG
CTGAGGCGCGAGAGCAAGGGGAGCAAACAGGATTAGAGACCCTTGTAGTCC
>Otu7638
CCAGCCTATGGGGGCAGCAGTGGGGAATATTGGACAATGGGGGAAACCCTGATCCAGCGA
CGCCGCGTGTGTGAAGAAGGCCTTCGGGTTGTAAAGCACTTTAGTCCGGGAAGAAGCGTA
GTTGTTTAAGAGATGGCTATGTTGACGGTACCGGAAGAATAAGCACCGGCTAACTCTGTG
CCAGCAGCCGCGGTAATACAGAGGGTGCAAGCGCTAATCGGAGTTACTGGGCGTAAAGGG
CGCGTAGGCGGCCATGTAAGTTAGGTGTGAAATCCCTGGGCTTAACCTAGGAACTGCACC
TAAGACTGTGTGACTGGAGTACAGTAGAGGGCAGTGGAATTTCCGGTGTAGCGGTGAGAT
GCGTAGAGATCGGAAGGAACACCAGTGGCGAAGGCGGCTGCCTGGACTGATACTGACGCT
GAGGCGCGAAAGCGTGGGGAGCAAACAGGATTAGATACCCTTGTAGTCC
>Otu7641
CCAGCCTATGGGGTGCAGCAGTCGAGAATTTTTCTCAATGGGCGAAAGCCTGACGCAGCA
ACGCCGCGTGGAGGATGAAGGCCTTCGGGTTGTAAACTTCTTTGGGCAGGGACGAGGAAG
GACGGTACCTGCCAAACAAGTCACGGCTAACTACGTGCCAGCAGCCGCGGTAAAACGTAG
GTGGCAAGCGTTATCCGGAATTACTGGGCGTAAAGGGTGTGCAGGCGGGGCTGTAAGTGG
TGTATGAAATCTCCCAGCTTAACTGGGATAGGTTATGCCAGACTGCAGTTCTAGAGGACG
AGAGAGGGGGGTGGAATTCCGGGTGTAGTGGTGAAATGCGTAGAGATACGGAGGAACCCC
AGAGGCGAAGGCGGCCCCCTGGCTCGTACCTGACGCTCAGCCACGAAAGCATGGGGAGCG
AACGGGATTAGAAACCCCGGTAGTCC
>Otu7642
CCAGCCTATGGGTGGCTCCAGTCAAGAAACTTTGGCAATGCACGAAAGTGTGACCAAGCG
ACGCCGCGTGATTGATGAGGCCCTTCGGGGTGTAAAGATCTTTTATGCGTGAAAAAATTA
TTGATATTAGCGCATGAATAAGGGGCTCCTAACTCTGTGCCAGCAGGAGCGGTAATACAG
AGGCCCCGAGCGTTATCCGGAATTATTGGGCGTAAAGGGTGCGTAGGCGGCAATGTTAGT
CGAATGTCAAAAATCAAGGCTCAACCTTGGGGAGGCATTCGAAACGGCATAGCTCGAGTG
TGTGAGAGGTGAATGGAACTCATGGTGTAGGGGTGAAATCCGTTGATATCATGGGGAACT
CCTAATGCGAAGGCAATTCACTGGCACATTACTGACGCTGAAGCACGAAAGCGTGGGTAG
CGAATGGGATTAGAAACCCCAGTAGTCC
>Otu7645
CCAGCCTATGGGACGCTGCAGTGGGGAATCTTGCGCAATGGGCGAAAGCCTGACGCAGCA
ACGCCGCGTGAGGGATGAAGGTCTTCGGGTTGTAAACCTCTTTCGAGCAGGGACGATTGT
GACGGTACCTGCAGAAGAAGCTCCGGCCAACTACGTGCCAGCAGCCGCGGTGATACGTAG
GGAGCGAGCGTTGTCCGGATTTATTGGGCGTAAAGAGCTCGTAGGCGGCTCAGTAAGTCA
GGTGTGAAATCCTCAGGCTCAACCTGGGGCTGCCATCTGATACTGCTGTGGCTAGAGCCC
GGTAGGGGTCCACGGAATTCCTGGTGTAGCGGTGAAATGCGCAGATATCAGGAGGAACAC
CTGCGGTGTAGACGGCTTTCTGGACCATTGCTGACGCTGAGACACGAAAGCGTGGGTAGC
AAACAGGATTAGAAACCCCCGTAGTCC
>Otu7653
CCAGCCTACGGGGGGCAGCAGTAACGAATCTTCCGCAATGCGCGCAAGCGTGACGGAGCG
ACGCCGCGTGTGGGACGAGGCCCTTCGGGGTGTAAACCACTGTCAGAGGATAGAAAGTTC
TGATCGTCCTCAGAGGAAGGCACGGCTAACTCTGTGCCAGCAGCCGCGGTAAGACAGAGG
TGCCGAGCGTTAGGCGGAATCACTGGGCTTAAAGCGTGTGTAGGCGGACCGTTAAGTGCC

TTGTGAAATCCCCCGGCTCAACCGGGGAACGGCTGGGCATACTGGCGGTCTTGAGTCACT
CAGGGGTCAGCGGAACAAACGGTGGAGCGGTGAAATGCGTAGATATCGTTTGGAACGCCG
ACGGCGAAAGCAGCTGACTGGGAGTGATCTGACGCTGAGACACGAAAGCCAGGGGAGCGA
ACGGGATTAGAAACCCCTGTAGTCC
>Otu7655
CCAGCCTATGGGTGGCAGCAGTGGGGAATCTTGCACAATGGAGGAAACTCTGATGCTGCG
ACGCCGCGTGAGCGATGAAGCCCTTCGGGGTGCAAAGCTCTTTCGACGGGAACGATAATG
ACGGTACCTGAAGAAGCAGCTGCGGCTAACTCTGTGCCAGCAGCCGCGGTAATACAGAGG
TCCCAAGCGTTGTTCGGATTCACTGGGCGTAAAGGGTGCGTAGGCGGTCGGGTAAGTCTG
ACGTGAAATCTCCGGGCCTAACCCGGAAACTGCGTCGGATACTATCCGGCTAGAGGATTG
GAAGGGAGACTGGAATACTTGGTGTAGCAGTGAAATGCGTAGATATCAAGTGGAACACCA
GTGGCGAAGGCGAGTCTCTGGACAATTCCTGACGCTGAGGCACGAAAGCCAGGGGAGCAA
ACGGGATTAGATACCCTTGTAGTCC
>Otu7660
CCAGCCTACGGGATGCACCAGTCGAGAATCTTTTGCAATGGGGGAAACCCTGACAAAGCG
ACGCCGTGTGAGCGATGAAGGCCTTCGGGTTGTAAAGCTCTTTCGCCAGGGAACAAGAGA
GGAGAGTTAATAGCTCTCTGATTTGAGGGTACCTGGTAAAGAAGTACCGGCTAACTCCGT
GCCAGCAGCTGCGGTAATACGGAGGGTACAAGCATTAATCGGATTTACTGGGCGTAAAGG
GTGCGTAGGCGGATAGGAAAGTTAGATGTGAAATTCCAGTACTCAATACTGGAGCTGCAT
TTAAAACTCCCTTTCTCGAGGATAGACGGAGAGAACGGAATTCCATGTGTAGCGGTGAAA
TGCGTAGATATATGGAAGAACACCTGTGGCGTAAGCGGTTCTCTAGTTTATTTCTGACGC
TGAGGCACGAGAGTGTGGGGAGCAAACAGGATTAGAAACCCTTGTAGTCC
>Otu7662
CCAGCCTACGGGGTGCAGCAGTGGGGAATATTGCACAATGGGCGCAAGCCTGATGCAGCG
ACGCCGCGTGGAGGATGAAGGTTTTCGGATTGTAAACTCCTGTTAAGTGGGAAAAAAGAC
GCATACCTAATACGTATGCGGGATGATGGTACCACTAGAGAAAGCACCGGCAAACTTCGT
GCCAGCAGCCGCGGTAATACGAAGGGTGCGAGCGTTATTCGGAATAACTGGGTGTAAAGA
GCGTGTCGGCGGTTTCTTAAGTCATTCGTTAAAGGGCTCAGCTTAACTGAGTATCATGCG
GGCGAAACTGGGAAACTAGAGTGCGGGAGAGAAGAGTGGAATTCTCGGAGTAGCGGTAAA
ATGCGTAGATCTCGAGAGGAACACCGGTTGCGAAGGCGGCTCTTTGGTCCGTAACTGACG
CTTAAGCGCGAAAGCGTGGGGAGCAAACAGGATTAGAAACCCCCGTAGTCC
>Otu7664
CCAGCCTATGGGGGGCTGCAGTGGGGAATCTTGCACAATGGGCGAAAGCCTGATGCAGCG
ACGCCGCGTGGGGGGTGAAGCATTTCGGTGTGTAAACCCCTTTCGACCGGGACGAATGCC
CGCAAGGGAGTGACGGTACCGGTATAAGAAGCCCCGGCTAACTACGTGCCAGCAGCCGCG
GTAAGACGTAGGGGGCCAGCGTTGTTCGGAATTACTGGGTGTAAAGGGTTCGTAGGCGGT
GCGGCAAGTTGGGAGTGAAATCTCTGGGCTCAACCCAGAGGCTGCTTCCAAAACTGCTGT
GCTTGAGTGTGAGAGAGGCTCGTGGAATTGCAGGTGTAGCGGTGAAATGCGTAGATATCG
GAAGGAACACCTGTGGCGAAAGCGGCTCACTGGACCACAACTGACGCTGATGCACGAAAG
CTAGGGGAGCAAACAGGATTAGATACCCGTGTAGTCC
>Otu7670
CCAGCCTATGGGTGGCAGCAGTGGGGAATATTGGACAATGGGCGCAAGCCTGATCCAGCA
ATGCCGCGTGTGTGAAGAAGGCCTGCGGGTTGTAAAGCACTTTCGGTGGGGAGGAGAAGC
TCTGAGTTAATACCTTAGAGTGGAGACGTTACCCACAGAAGAAGCACCGGCTAACTCTGT
GCCAGCAGCCGCGGTAATACAGAGGGTGCAAGCGTTAATCGGAATTACTGGGCGTAAAGG
GTGCGTAGGTGGTTTTTTAAGTTAGGTGTGAAAGTCCTGGGCTTAACCTGGGGATTGCGC
CTAATACTGTGAGACTAGAGTACTGTAGAGGAAGGTGGAATTTCCGGTGTAGCGGTGAAA
TGCGTAGATATCGGAAGGAACATCAGTGGCGAAGGCGACTTTCTGGACAGATACTGACAC
TGAGGCACGAAAGCGTGGGGAGCAAACAGGATTAGAAACCCGAGTAGTCC
>Otu7671
CCAGCCTACGGGTCGCAGCAGTCGAGAATCTTCGGCAATGAGCGCAAGCTTGACCGAGCG
ACGCCGCGTGTGCGATGAAGGCCTTCGGGTTGTAAAGCACTGTCGAGGGGGAGAAAGGCG
AAAGCTTGATCTATCCCTGGAGGAAGCACGGGCTAAGTTCGTGCCAGCAGCCGCGGTAAG
ACGAACCGTGCAAACGTTATTCGGAATCACTGGGCTTAAAGGGCGCGTAGGCGGCCACC
AAGTCCGGGGTGAAATCCTCCAGCTTAACTGGAGAACTGCCTCGGATACTGGAGGTCTCG
AGGATGATAGGGGCATGCGGAACTGTAGGTGGAGCGGTGAAATGCGTTGATATCTACAGG
AACTCCGGTGGCGAAGGCGGCATGCTGGATCATTTCTGACGCTGAGGCGCGAAAGCCAGG
GGAGCGAACGGGATTAGATACCCCCGTAGTCC

>Otu7674
CCAGCCTATGGGGTGCTCCAGTAAAGAATCTTCCACAATGGACGAAAGTCTGATGGAGCG
ACGCCGCGTGCAGGATGAAGCCCCTCGGGGTGTAAACTGCTTTTGTGTGGGAATACATTT
TTGAAGGTACCACACGAATAAGCACCGGCCAATCTCGTGCCAGCAGCCGCGGTAATACGA
GAGGTGCAAGCGTTATCCGGAATCACTGGGCGTAAAGCGTCCGCAGTCGGCAAGTTAAGT
TCGAGGTTAAATCCCATCGCTCAACGATGGATTCGTCTCGAAAACTGGCTCGCTAGAGGA
ATGCAGGGGCAGGCGGAATTCGTGGTGTAGGGGTAAAATCCGTTGATACCACGAGGAACA
CCAAAAGCGAAGGCAGCCTGCTGGGCATTTCCTGACGATCATGGACGAAAGCGTGGGGAG
CAAACAGGATTAGATACCCGTGTAGTCC
>Otu7677
CCAGCCTACGGGGCGCTCCAGTGCAGAATCTTGGACAATAAGCGCAAGCTTGATCCAGCA
ATACTACGTGAGTGAAGAAGGTCTTTGAAATGGATTGTAAAGCTCTTTTACTGAGGATGA
TGATGACAGTACTCAGGGAATAAGTCCCGACTAACTTCGTGCCAGCAGTCGCGGTAATAC
GGGGGGGACAAGTATTACTCAGCATAATTGGGCGTAAAGGGTGCGTAGATGGTTTTTTTT
GTTAGAAGTTAAATACAAGAGCTTAACTTTTGAAAGACTTCTAATACGATTAGACTTGAG
TATGATAAAAGATAGTAGTATTCTTAGTGGAAAGGTGGAATTTGTAGATACTAAGAGGAC
TTTTAATAGGCGAAAGCGACTATCTAGATCAGAACTGACGTTGAGTGCACGAAAGCATGG
GGAGCAAACAGGATTAGATACCCGAGTAGTCC
>Otu7683
CCAGCCTATGGGTGGCTCCAGTAAGGAATATTGGGCAATGGACGAAAGTCTGACCCAGCC
ATGCCGCGTGGAGGATGAAGGTCCTCTGGATTGTAAACTTCTTTTATTTGGGACGAAAAA
AGGGAATTCTTTCTCACTTGACGGTACCAGATGAATAAGCACCGGCTAACTCCGTGCCAG
CAGCCGCGGTAATACGGAGGGTGCAAGCGTTATCCGGATTCACTGGGTTTAAAGGGTGCG
TAGGCTGGCAGGTAAGTCAGTGGTGAAATCTCCGGGCTTAACCCGGAAACTGCCGTTGAT
ACTATCTGTCTTGAATGCCGTGGAGGTTAGCGGAATATGTCATGTAGCGGTGAAATGCTT
AGATATGACATAGAACACCTATTGCGAAGGCAGCTAACTACGCGGTGATTGACGCTGAGG
CACGAAAGCGTGGGGATCAAACAGGATTAGAAACCCTCGTAGTCC
>Otu7687
CCAGCCTATGGGTTGCTGCAGTCGAGAATATTCGACAATGGGCGAAAGCCTGATCGAGCG
ACACCGCGTGCAGGATGAAGGCCTTCGGGTTGTAAACTGCGGTAGTATGCCAAGAATGCA
AATGACTAGCATATGGAAAGAGGTGGGTAACTTCGTGCCAGCACCAGCGGTAATACGTAG
ACCTCAAGCGTTATCCGGATTTATTGGGCGTAAAGAGCATGTAGGAGGTTTTGTGCGTCT
TCTGTTAAAGCCCAGAGCCTAACTCTGGAGATGCAGGAGATACGACAAGACTAGAGGGGG
TTAGAGGTGCATGGAACTCACGGTGTAGGGGTGAAATCCGTTGATATCGTGGGGAACACC
AAAGGCGAAGGCAGTGCACTGGGACCTTCCTGACTCTGAGATGCGAAAGCGTGGGGAGCA
AAAAGGATTAGAAACCCTAGTAGCC
>Otu7689
CCAGCCTATGGGTGGCACCAGTGGGGAATTTTGCACAATGGGCGCAAGCCTGATGCAGCA
ACGCCGCGTGTGTGATGAAGGTCTTCGGATCGTAAAGCACTTTCGACCGGGACGAACAAG
CTACTCCTAACACGAGTGGCCCTGACGGTACCGGGAGAAGAAGCACCGGCTAACTCTGTG
CCAGCAGCCGCGGTAATACAGAGGGTGCAAGCGTTGTTCGGAATTATTGGGCGTAAAGCG
CGGGTAGGCGGCTTGAAAAGTCGGGTGTGAAAGCCCTCAGCTCAACTGAGGAAGTGCGCC
CGAAACTCTCGAGCTTGAGTGCCGGAGAGGATGGCGGAATGCCCCAAGTAGAGGTGAAAT
TCGTAGATATGGGGCGGAACACCGGTGGCGAAGGCGGCCATCTGGACGGTAACTGACGCT
GAGCCGCGAAAGCGTGGGTAGCAAACAGGATTAGAAACCCCTGTAGTCC
>Otu7690
CCAGCCTATGGGTGGCAGCAGTCGAGAATCTTCCGCAATGGACGAAAGTCTGACGGAGCG
ACGCCGCGTGATTGATGAAGTCCTTCGGGACGTAAAGATCTTTTATGGGGGAGGAAGTAA
TTGACGTTACCCCATGAATAAGAGGCTCCTAACTCTGTGCCAGCAGGAGCGGTAATACAG
AGGCCTCAAGCGTTACCCGGAATCACTGGGCGTAAAGGGTGTGTAGGTGGCAGTGTTAGT
CTTTCGCCAAAGCTCTCGGGCTCAACCCGGGAAATGCGGAGGAAACGGCACAGCTTTGAG
GGTGTGAGAGGTATATGGAACTCATGGTGTAGGGGTGAAATCCGTTGATATCATGGGGAA
CACCAAATGCGAAGGCAATATACTGGCACATTTCTGACACTGAAACACGAAAGCGTAGGT
AGCGAATGGGATTAGAGACCCCTGTAGTCC
>Otu7693
CCAGCCTACGGGTTGCAGCAGTGGGGAATCTTGCGCAATGGGCGAAAGCCTGACGCAGCG
ACGCCGCGTGAGTGATGAAGGCCTTCAGGTTGTAAAGCTCTGTGGGGAGGAACGAATAAG
TCATGGCTAATACTCATGATGATGACGGTACCTCCTTAGCAAGCACCGGCTAACTCTGTG

CCAGCAGCCGCGGTAAGACAGAGGGTGCAAACGTTGTTCGGAATCACTGGGCGTAAAGCG
TGTGTAGGTGGTCCTATAAGTCGGATGTGAAAGCCCCGGGCTCAACCCGGGAAGTGCACC
CGATACTGTAGGACTTGAGTACCGGAGAGGTTGGTGGAATTCTCGGTGTAGAGGTGAAAT
TCGTAGATATCGAGAGGAACATCGGTGGCGAAGGCGGCCAACTGGACGGATACTGACACT
GAGACACGAAAGCGTGGGGAGCAAACAGGATTAGATACCCGAGTAGTCC
>Otu7698
CCAGCCTATGGGTCGCTGCAGTGGGGAATCTTGAGCAATGGACTAACGTCTGACTCAGTG
AGAACACATGCATGACGAATGCGCCCAGTTTTCTGTAAAGTGCTTTCAAGGGAAAAAGAC
AATGACTTCACCCCAAGATGAAGGGCCGACTAACTTCGTGCCAGCAGTCGCGGTAATACG
AAGGGCCCGGGTGTTACTCGTTTTGATTGGGTGTAAAGGGTAAGTAGGCGGTCGATGGGA
TTTTTAGGCTAAAGCGCGGAGTGTTCCTATGCGAAAGCCCAATCTGTAGCATCTGACTAG
TGTTGAGGAGAGGTTTAGGCAAATTCGTAGGGAAGGGTAAAATCTTGCGAGCTACGAATG
AACGCCGGCTGGCAAAGGCGCTTTTCCAGCCTCAACAGACGCTGAGGTACGGAAGCGTAG
GGAGCAAACGGGATTAGAAACCCGGGTAGTCC
>Otu7705
CCAGCCTATGGGAGGCAGCAGCCGAGAATATTCGACAATGGACGAAAGTCTGATCGAGCG
ACGCCGCGTGATGGATGAAGACCTTCGGGTCGTAAACATCTTTTATGAGGGAGAAAGTTT
ATTGATAGTACCTCACGAATAAGGGGTTGCTAAACTCGTGCCAGCAGCAGCGGTAATACG
AGTGCCCCGAGCGTTATCCGGAATCATTGGGCGTAAAGGGTGTGTAGGTGGCAGTGTTAG
TCGATTGTTAAAACCTGGGGCTCAACCCCAGATGTGCGATCGAAACGGCACAGCTAGAGG
ATGGCAGGGATGTATGGAACTCATGGAGTAGGGGTGAAATCCGTTGATATCATGGGGAAC
ACCGAAAGCGAAGGCAGTACATTGGGCCACTCCTGACACTGAAACACGAAAGCGTGGGTA
GCGAATGGGATTAGATACCCCGGTAGTCC
>Otu7708
CCAGCCTACGGGTGGCACCAGTGGGGAATATTGGACAATGGGCGCAAGCCTGATCCAGCC
ATGCCGCGTGAGTGATGAAGGCCCTAGGGTTGTAAAGCTCTTTTGTGTGGGAAGATAATG
ACGGTACCGCAAGAATAAGCCCCGGCTAACTTCGTGCCAGCAGCCGCGGTAATACGTAGG
TGGCAAGCGTTGTCCGGATTTATTGGGTTTAAAGGGTGCGTAGGCGGTTCTTTAAGTCAG
TGGTGAAATACTTCGGCTCAACCGGAGAGGTGCCAATGATACTGGGGGACTTGAGTACAG
ACGAGGTAGGCGGAATTGACGGTGTAGCGGTGAAATGCATAGATATCGTCAAGAACACCG
ATAGCGAAGGCAGCTTACTAGACTGTAACTGACGCTGATGCACGAAAGTGTGGGGATCAA
ACAGGATTAGAGACCCTGGTAGTCC
>Otu7710
CCAGCCTATGGGTTGCTGCAGTGGGGAATTTTGGACACTGGGCGTAAGCTTGATCCAGCA
ATACTGAGTGAATGAGGACGGCTATTTGGTTGTAAAATTCTTTTGTTATAGAAAACATAG
CTTGTATGTAAAGAAAAAGCTCCGGCTAACTTCGTGCCAGCAGCCGCGGTAAAACGGGGG
GAGCTAGCGTTATTCAACTTGACTGGGCGTAAAGGGCGAGTAGGTTGTTAATTTTGTGCT
TTGTCAGACATATTAACTAGAGTATATGAAAGGATTAGGGTACCTTTAAAGTAGAGGTGA
TATTCGACTATAGTACAGGGACCACCGACGGGCGAAAGCGCTGATCTAGCATATTACTGA
CGCTCAGGAGCGAAAGTATGGGTAGCGAATGGGATTAGATACCCCCGTAGTCC
>Otu7715
CCAGCCTATGGGTGGCTCCAGTGGGGAATCTTGGACAATGGGGGCAACCCTGATCCAGCC
ATGCCGCGTGAGTGAAGAAGGCCTTCGGGTTGTAAGGCTCTTTCGGACGGGACGAAATGG
TTACAGTGAATAACTGTGATTGATGACGGTACCGTAATAAGAAGCACCGGCTAACTACGT
GCCAGCAGCCGCGGTAATACGTAGGGTGCGAGCGTTAATCGGAATTACTGGGCGTAAAGC
GTGCGCAGGCGGTTCGCCAAGTCAGGTGTGAAATCCCCGGGCTCAACCTGGGAATTGCGC
TTGAAACTAGCGAGCTTGAGTGTGGCAGAGGGGGGTGGAATTCCACGTGTAGCAGTGAAA
TGCGTAGAGATGTGGAGGAACACCGATGGCGAAGGCAGCCCCCTGGGTCAACACTGACGC
TCATGCACGAAAGCGTGGGGAGCAAACAGGATTAGAAACCCCGGTAGTCC
>Otu7716
CCAGCCTACGGGTTGCACCAGTCGAGAATATTCCACAATGGACGAAAGTCTGATGGAGCG
ACGCCGCGTGCAGGATGAAGGCCTTCGGGTCGTAAACTGCGGTAGACAAATAATAATGCA
AATGAATGTTTGTCGGAAAGAGGTGGGTAACTACGTGCCAGCACCAGCGGTAAAACGTAG
ACCTCAAGCGTTATTCGGATTTATTGGGCGTAAAGCGCGTGTAGGGGGGTTTCGTGCGTCT
TCTGTTAAAGCCCACCGCCTAACGGTGGAACTGCAGGAGATACGGCGAGACTAGAGGATG
TTAGAGGTACCAGGAACACACGGTGTAGGGGTGAAATCCGTTGATATCGTGTGGAACACC
AAAGGCGAAGGCAAGGTACTGGGACACTCCTGACCCTGAGACGCGAAAGCGTGGGGAGCA
AAAAGGATTAGAGACCCGTGTAGTCC

>Otu7721
CCAGCCTATGGGGGGCAGCAGCCGAGAATATTCGACAATGGGCGAAAGCCTGATCGAGCG
ATGCCGCGTGATGGATGAAGCGCTTCGGCGCGTAAACATCTTTTATGGAGGAGGAAGTTA
TTGACGTTACTCCATGAATAAGGGGCTCCTAACTCTGTGCCAGCAGGAGCGGTAATACAG
AGGCCCCAAGCGTTACCCGGAATTACTGGGCGTAAAGGGTGCGTAGGTGGTCATATTAGT
CGTTCGTTAAACACTCGGGCTCAACCCGAGGACTGCGGGCGAAACGGTATGACTTGAGAG
CGTGAGAGGTGCATGGAACTCATGGTGTAGGGGTGAAATCCGTTGATATCATGGGGAACA
CCGAAAGCGAAGGCAATGCACTGGCGCGTTTCTGACACTCAAGCACGAAAGCGTGGGTAG
CGAACGGGATTAGAAACCCGAGTAGTCC
>Otu7723
CCAGCCTATGGGATGCAGCAGTAAGGAATATTGGACAATGGGCGCAAGCCTGATCCAGCC
ATGCCGCGTGCAGGATGAAGGCTCTATGGGTCGTAAACTGCTTTTGTACGGAAGAAACCC
TCCTATGTATAGGGGGCTGACGGTACCGTAAGAATAAGGATCGGCTAACTCCGTGCCAGC
AGCCGCGGTAATACGGAGGATCCAAGCGTTATCCGGATTTATTGGGTTTAAAGGGTGCGT
AGGCGGACTTGTAAGTCAGTGGTGAAAGCCTGCGGCTTAACTGCAGAACTGCCATTGATA
CTGCTGGTCTTGAGTATAGTTGAGGTGGGCGGAATGTGTCATGTAGCGGTGAAATGCTTA
GATATGACACAGAACACCGATTGCGAAGGCAGCTCGCTAAACTATAACTGACGCTGAGGC
ACGAAAGCGTGGGTAGCAAACAGGATTAGATACCCCAGTAGTCC
>Otu7728
CCAGCCTATGGGACGCACCAGTAAGGGATATTGCGCAATGGGCGAAAGCCTGACGCAGCA
ACGCCGCGTGAAGGATGAAGGCCTTCGGGTCGTAAACTTCTTTTCTCACTGACGAGGAAG
GACGGTAGGTGAGGAATCAGTCACGGCTAACTACGTGCCAGCAGCCGCGGTAAAACGTAG
GTGGCGAGCGTTATCCGGATTTACTGGGCGTAAAGCGTGTGCAGGCGGAAGAACAAGTGG
TGTATGAAATCGCTCGGCTTAACCGGGTGCTGTTATGCCAGACTGTTCTTCTAGAGTGCG
AGAGAGGGGCGTGGAATTCCGGGTGTAGTGGTGAAATGCGTAGAGATCCGGAGGAACCCC
AGAGGCGAAGGCGGCGCCCTGGCTCGCAACTGACGCTCAGACACGACAGCATGGGGAGCG
AACGGGATTAGATACCCCTGTAGTCC
>Otu7730
CCAGCCTATGGGGGGCACCAGTAGGGAATATTGGACAATGGGCGCAAGCCTGATCCAGCA
ATGCCGCGTGAGTGAAGAAGGCCTTCGGGTTGTAAAGCTCTTTCACCCGTGACGATGATG
ACGGTAACGGGAGAAGAAGCCCCGGCTAACTCCGTGCCAGCAGCCGCGGTAATACGGAGG
GGGCAAGCGTTGTTCGGAATTACTGGGCGTAAAGGGCGCGTAGGCGGCTTGGCAAGTTAG
GCGTGAAAGCCCTGGGCTTAACCCAGGAATTGCGCTTGAGACTGCTAGGCTTGAGTGCGG
AAGAGGTGAGTGGAATTCCCAGTGTAGAGGTGAAATTCGTAGATATTGGGAAGAACACCA
GTGGCGAAGGCGGCTCACTGGTCCGCAACTGACGCTGAGGCGCGAAAGCGTGGGGAGCAA
ACAGGATTAGAAACCCGAGTAGTCC
>Otu7735
CCGCCTACGGGTTGCAGCAGTGAAGAATCTTGGGCAATGGACCGATGTCTGACCCAGTGA
GAATACATGCATGACGAAGGCGCTCAGTTTTCTGTAAAGTGCTTTTAACAACGAAAAGAC
TGTCTGAAGTTGTCAAAGAAGCACCGGCCAACTCCGTGCCAGCAGCCGCGGTAAGACGGA
GGGTGCGGGCGTTATTCGTTTTGATTGGGTGTAAAGGGTATGTAGGTGGCATCGAAAAT
TTTAGACAAAATGGCGAAGTAGTTCTATGCACTGTCTTTTTTTATAAACTGGTGTCTAGA
GTTGAAGATAAGTCGGAGTAATTTCCTAAGTAGGGGTAAAATCCGTCTATATAGGAACGA
ACGCCAACAGGCGAAGGCATCCCTCTCGCTTCAACCAACGCTAATGTACGGAAGCCTAGG
GCGCAAACGGGATTAGAAACCCTCGTAGTCC
>Otu7744
CCAGCCTACGGGTGGCACCAGTGGGGAATATTGGGCAATGGGCGCAAGCCTGACCCAGCC
ATGCCGCGTGAGTGATGAAGGCCTTCGGGTCGTAAAGCTCTGTGGGGAGGGACGAACAGT
CTTGGTGTTAATACCACCAGGGCCTGACGGTACTTCCTTAGCAAGCACCGGCTAACTCCG
TGCCAGCAGCCGCGGTAATACGGAGGGTGCAAACGTTGCTCGGAATCATTGGGCGTAAAG
CGCACGTAGGCGGTCTTTTAAGTCGGGTGTGAAAGCCCTGGGCTCAACCCAGGAAGTGCA
CTCGAAACTGAAAGGCTAGAGTACGGAAGAGGGTCTCGGAATTCCCGGTGTAGAGGTGAA
ATTCGTAGATATCGGGAGGAACACCAGTGGCGAAGGCGGAGACCTGGGCCGATACTGACG
CTGAGGTGCGAAAGCGTGGGGAGCAAACAGGATTAGAGACCCCGGTAGTCC
>Otu7746
CCAGCCTACGGGGCGCTGCAGTGGGGAATATTGCACAATGGGCGCAAGCCTGATGCAGCG
ACGCCGCGTGGAGGATGAAGGTTTTCGGATTGTAAACTCCTGTTAAGTGGGAAGAAAGAA
CCGGTATTAATACCATCGGGAGATGACGGTACCACTAGAGAAAGCACCGGCTAACTTCGT

GCCAGCAGCCGCGGTAATACGAGGGGTGCAAGCGTTATTCGGAATAACTGGGCGTAAAGA
GCGCGTAGGCGGCTTGTTAAGTTTGTTGTTAAATTGTTCGGCCTAACCGAGCATCGGCAG
TAAATACTGGCAAGCTAGAGGACAAGAGAGGGGTGGAATTCTCGGAGTAGCGGTAAAA
TGCGTAGATCTCGAGAGGAACACCGATGGCGAAGGCAGCCCCTTGGCTTGTATCTGACGC
TCAAGTGCGAAAGCGTGGGGAGCAAACAGGATTAGATACCCGAGTAGTCC
>Otu7749
CCAGCCTACGGGTGGCACCAGTGGGGAATTTTGGACAATGGGGGCAACCCTGATCCAGCC
ATGCCGCGTGTGTGAAGAAGGCCTTCGGGTTGTAAAGCACTTTCGGACGGAACGAAATCG
CACGGGTGAATATCCCGTGTGGATGACGGTACTGTAAGAAGAAGCACCGGCTAACTACGT
GCCAGCAGCCGCGGTAATACGTAGGGTGCGAGCGTTAATCGGAATTACTGGGCGTAAAGC
GTGCGCAGGCGGCTTCGCAAGTCAGACGTGAAATCCCCGGGCTTAACCTGGGAATGGCGT
TTGAAACTACGGAGCTGGAGTGTGGCAGAGGGAGGTAGAATTCCACGTGTAGCGGTGAAA
TGCGTAGATATGTGGAGGAATACCGATGGCGAAGGCAGCCTCCTGGGCCAACACTGACGC
TCATGCACGAAAGCGTGGGGAGCAAACAGGATTAGATACCCCAGTAGTCC
>Otu7750
CCAGCCTATGGGGCGCTGCAGTGGGGAATATTGGACAATGGGGGCAACCCTGATCCAGCG
ATGCCGCGTGGGTGAAGAAGGCCTTCGGGTTGTAAAGCCCTTTAGGTTGGGACGAAGAGT
GGTAGGTTAATACCCTATTATTTTGACGGTACCGACAGAATAAGCACCGGCAAACTCTGT
GCCAGCAGCCGCGGTAATACAGAGGGTGCGAGCGTTAATCGGAATCACTGGGCGTAAAGG
GCGCGTAGGCGGTTAGATGTGTGTGATGTGAAAGCCCTGGGCTTAACCTAGGAAGTGCAT
CGCAAACTATATAACTGGAGTAGATGAGAGGGTGGCGGAATTTCCGGTGTAGCGGTGAAA
TGCGTAGAGATCGGAAGGAACGTCAATGGCGAAGGCAGCCACCTGGCATCATACTGACGC
TGAGGCGCGAAAGCGTGGGGATCGAACAGGATTAGATACCCTCGTAGTCC
>Otu7753
CCAGCCTACGGGGGGCAGCAGTGGGGAATATTGGACAATGGGCGCAAGCCTGATCCAGCC
ATGCCGCGTGTGTGATGAAGGCCTTAGGGTTGTAAAGCACTTTCACCGGAGAAGATAATG
ACGGTATCCGGAGAAGAAGCCCCGGCTAACTTCGTGCCAGCAGCCGCGGTAATACGAAGG
GGGCTAGCGTTGTTCGGAATTACTGGGCGTAAAGCGCACGTAGGCGGATATTTAAGTCAG
GGGTGAAATCCCAGAGCTCAACTCTGGAGCTGCCTTTGATACTGGGTATCTTGAGTATGG
AAGAGGTAAGTGGAATTGCGAGTGTAGAGGTGAAATTCGTAGATATTCGCAGGAACACCA
GTGGCGAAGGCGGCTTACTGGTCCATTACTGACGCTGAGGTGCGAAAGCGTGGGGAGCAA
ACAGGATTAGAAACCCCTGTAGTCC
>Otu7754
CCAGCCTATGGGGTGCAGCAGTGGGGAATATTGGACAATGGGGGAAACCCTGATCCAGCA
ATGCCGCGTGTGTGAAGAAGGCCTTCGGGTTGTAAAGCACTTTTGTTGGGGAGGAAGGCG
TGGAAACACGATTGACTTAACCCAAAGAAACAGCACCGGCTAACTCTGTGCCAGCAGCCG
CGGTAATACAGAGGGTGCAAGCGTTAATCGGAATTATTGGGCGTAAAGCGCACGTAGGCG
GTTTGATAAGTTAGATGTGAAATACCTGGGCTTAACTTGGGGATGTCATCTAATACTGTC
AGACTAGAGTAGAGTAGAGGAAGGCGGAATTCCCGGTGTAGCGGTGAAATGCGTAGATAT
CGGGAGGAACATCAGTGGCGAAGGCGGCCTTCTGGACAAATACTGACGCTGAGGAGCGAA
AGCGTGGGGAGCAAACAGGATTAGAAACCCGCGTAGTCC
>Otu7757
CCAGCCTACGGGGGGCTGCAGTCGAGGATCTTCGTCAATGGGCGAAAGCCTGAACGAGCG
ACGCCGCGTGCATGATGAAGGCCTTCGGGTTGTAAAGTGCGAAAGTGGGGACAAAGCCGC
AAGGTTGATTGATCCACAGTAAGGACGGGCTAAGTTCGTGCCAGCAGCCGCGGTAAGACG
AACTGTCCTAACGTTGTTCGGAATCACTGGGCTTAAAGGGCGCGTAGGCGGTCGACCAAG
TCAGGGGTGAAATCCCGCAGCTTAACTGCGGAAGTGCCTTTGATACTGGTTGTCTCGAGG
GAGGTAGGGGTGTGTGGAACTCACAGTGGAGCGGTGAAATGCGTTGATATTGTGAGGAAC
GCCGGAGGCGAAAGCGACGCACTGGACCTCTTCTGACGCTGAGGCGCGAAAGCTAGGGGA
GCAAACGGGATTAGATACCCGTGTAGTCC
>Otu7760
CCAGCCTATGGGTGGCACCAGTGAGGAATCTTCCACAATGGGCGAAAGCCTGATGGAGCG
ACGCCGCGTGCAGGATGAAGGCCTTCGGGTTGTAAACTGCTTTTATAAGTGAAGAATATG
ACGGTAACTTATGAATAAGCACCGGCTAACTACGTGCCAGCAGCCGCGGTCATACGTAGG
GTGCAAGCATTATCCGGAGTGACTGGGCGTAAAGAGTTGCGTAGGTGGACAAGTAAGCGA
ATAGTGAAATTTGACGGCTCAACCGTACAAACTATTATTCGAACTGCTTGTCTCGAGAAT
GGTAGAGGTAACCGGAATTTCTTGTGTAGGAGTGAAATCCGTAGATATAAGAAGGAACAC
CAATGGCGTAGGCAGGTTACTGGACCATTTCTGACACTAAGGCACGAAAGCGTGGGGAGC

GAACCGGATTAGAAACCCCCGTAGTCC
>Otu7763
CCAGCCTATGGGATGCTCCAGTGGGGAATCTTGCGCAATGGGCGAAAGCCTGATGCAGCA
ACGCCGCGTGGAGGATGAAGGCTTTCGGGTCGTAAACTCCTTTTTGACCTGACGAGGAAG
GACGGTAGGGTCAGAATAAGTCACGGCTAACTACGTGCCAGCAGCCGCGGTAAAACGTAG
GTGGCGAGCGTTATCCGGATTTACTGGGCGTAAAGCGTGTGCAGGCGGGCTGTCAAGTGG
TGTATGAAAGGTCTGGGCTAAACCCAGATAGGTTATGCCAGACTGGCAGTCTTGAGTACG
AGAGAGGGACGTGGAATTCCGGGTGTAGTGGTGAAATGCGTAGAGATCCGGAGGAACCCC
AGAGGCGAAGGCGGCGTCCTGGCTCGATACTGACGCTCAGACACGACAGCATGGGGAGCG
AACGGGATTAGAGACCCGTGTAGTCC
>Otu7765
CCAGCCTACGGGGGGCAGCAGTCGAGAATCTTTCACAATGGGGGCAACCCTGATGGAGCG
ACGCCGCGTGGAGGATAAGGTCTTCGGATTGTAAACTCCTGTCATGTGAGAACAAAGCCC
TTTGGGTCTGATAGTATCACAAGAGGAAGAGACGGCTAACTCTGTGCCAGCAGCCGCGGT
AATACAGAGGTCTCAAGCGTTGTTCGGAATCACTGGGCGTAAAGGGTGTGTAGGCGGCGT
GGAAAGTCAGATGTGAAATCCGGGGGCTCAACCCCCGAATAGCATCCGATACTGCCATGC
TCGAGGTCTGTAGAGGAGTCTGGAATTTTCGGTGTAGCAGTGAAATGCGTAGATATCGAA
AGGAACACTTATAGCGAAGGCAAGACTCTGGGCAGAATCTGACGCTGAAACACGAAGGCC
AGGGTAGCGAACGGGATTAGATACCCTTGTAGTCC
>Otu7776
CCAGCCTACGGGTGGCACCAGTCGAGAATCTTCGGCAATGTGCGCAAGCCTGACCGAGCG
ACGCCGCGTGGAGGACGAAGGCCTTCGGGTTGTAAACTCCTGTCGAGGGGAAGGAAGAGA
CGGCGCAGAGCCGTTTTTGACCGCTCCCTGGAGGAAGCACGGGCTAAGTTCGTGCCAGCA
GCCGCGGTAAGACGAACCGTGCGAACGTTATTCGGAGTCACTGGGCTTAAAGCGCGTGTA
GGCGGACTGCCACGTCGGGCGCTGAAATCCCCCGGCTCAACCGGGGAATGGGCACCGATA
CGAGCAGTCTCGAGGGGAGTAGGGGGACCTGGAACTTCCGGTGGAGCGGTGAAATGCGTT
GAGATCGGAAGGAACGCCCGTGGCGAAAGCGAGGTCCTGGGCTTCTACTGACGCTGAGAC
GCGAAAGCTAGGGGAGCAAACGGGATTAGAAACCCCAGTAGTCC
>Otu7781
CCAGCCTATGGGGGGCAGCAGTTAGGAATCTTGCACAATGGGGGAAACCCTGATGCAGCG
ACGCCGCGTGCGGGACGAAGGCCCTCGGGTCGTAAACCGCTTTCAGCAGGAGCGAAATTG
ACGGTACCTGCAGAAGAAGCCCCGGCCAACTACGTGCCAGCAGCCGCGGTAATACGTAGG
GGGCAAGCGTTGTCCGGATTCATTGGGCGTAAAGAGCTCGTAGGCGGTTCGGTAAGTCAG
GTGTGAAATCTCTGCGTTCAACGCAGAGCGGCCACCTGATACTGCCGTGACTCGAGTCCG
GTAGGGGAGTGTGGAATTCCCGGTGTAGCGGTGAAATGCGCAGATATCGGGAGGAACACC
AGTAGCGAAGGCGGCACTCTGGGCCGTGACTGACGCTGAGGAGCGAAAGCGTGGGTAGCA
AACAGGATTAGATACCCCCGTAGTCC
>Otu7782
CCAGCCTATGGGTGGCTGCAGTAGGGAATATTGGACAATGGGCGCAAGCCTGATCCAGCC
ATCCCGCGTGAAGGATTAAGGTCCTATGGATTGTAAACTTCTTTTCTCTGGGAATAAAAA
CCCCGACTTGTCGGGGCTTGAAGGTACCAGAGGAATAAGCACCGGCTAACTCCGTGCCAG
CAGCTGCGGTAATACGGAGGGTGCAAGCGTTATCCGGATTCACTGGGTTTAAAGGGTGCG
TAGGTGGCTTTGTAAGTCAGTGGTGAAAGCCCGGAGCTCAACTCCGGAACTGCCATTGAT
ACTGCTTAGCTTGAATCAGGTAGAGGTGGATGGAATATTACATGTAGCGGTGAAATGCTT
AGAGATGTAATAGAACACCAATTGCGAAGGCAGTTCACTATGCCTGCATTGACACTGAGG
CACGAAAGCGTGGGGATCAAACAGGATTAGAAACCCGAGTAGTCC
>Otu7785
CCAGCCTACGGGTTGCACCAGCCGAGAATATTCGACAGTGGGCGCAAGCCTGATCGAGCG
ATACCGCGTGGTGGATGAAGCGCTTCGGCGCGTAAACATCTTTTATGGAGGAGGAAGTAA
TTGACGTTACTCCATGAATAAGGGGCTCCTAACTCTGTGCCAGCAGGAGCGGTAATACAG
AGGCCCCGAGCGTTACCCGGAATTACTGGGCGTAAAGAGTGTGTAGGTGGTCATGTTAGT
CGTTCGTTGAATCCCGGGGCTCAACCCCGGAATCGCGGGCGAAACGGCATGACTTGAGGG
CGTGAGAGGTATGTGGAACTCAAGGTGTAGGGGTGAAATCCGTTGATATCTTGAGGAACA
CCGAAAGCGAAGGCAGCATACTGGCGCGTTCCTGACACTCAAACACGAAAGCGTGGGTAG
CGAACAGGATTAGAAACCCGCGTAGTCC
>Otu7789
CCAGCCTACGGGTGGCAGCAGTCGAGAATCTTCGGCAATGAGCGCAAGCTTGACCGAGCG
ACGCCGCGTGAGCGATGAAGGCCTTCGGGTTGTAAAGCTCTTTAGTGGGGAGAAAAGCC

GAAAGGTCTGATCTATCCCTGGAGGAAGCACGGGCTAAGTTCGTGCCAGCAGCCGCGGTA
AGACGAACCGTGCGAACGTTGTTCGGAATCACTGGGCTTAAAGGGCGCGTAGGCGGGCCG
CAAAGTCAGGGGTGAAATCCTCCAGCTTAACTGGAGAACTGCCTTTGATACTGGCGGTCT
GGAGTAAGGTAGGGGCATGCGGAACTGATGGTGGAGCGGTGAAATGTGTTGATATCATCA
GGAACTCCGGTGGCGAAAGCGGCGTGCTGGACCTTTACTGACGCTGATGCGCGAAAGCCA
GGGGAGCAAACGGGATTAGAAACCCGAGTAGTCC
>Otu7791
CCAGCCTATGGGAGGCAGCAGTCGAGAATTTTTCTCAATGGGGGAAACCCTGAAGGAGCG
ACGCCGCGTGGAGGATGAAGGTCTTCGGATTGTAAACTCCTGTCATTAGAGAACAAAACC
CACTTAGTAACTGATGTTGGGTTTGATAGTATCTGAAGAGGAAGAGACGGCTAACTCTGT
GCCAGCAGCCGCGGTAATACAGAGGTCTCAAGCGTTGTTCGGATTCATTGGGCGTAAAGG
GTGCGCAGGCTGCGAGGTGAGTCGGACGTGAAATCCCGGGGCTTAACCCCGGAACTGCGT
TCGATACTGCCTTGCTTGAGGACTGGAGAGGAGACTGGAATTCCTGGTGTAGCAGTGAAA
TGCGTAGATATCAGGAGGAACACCAGTGGCGAAGGCGAGTCCCTGGACGACTCCTGACAC
TGAGGCACGAAAGCTAGGGGAGCAAACAGGATTAGAAACCCCAGTAGTCC
>Otu7792
CCAGCCTACGGGGGGCAGCAGTTAGGAATTTTGCGCAATGGCCGAAAGGCTGACGCAGCA
ACGCCGCGTGAGGGATGACGGTCTTCGGATTGTAAACCTCTTTTAGTTAGGAAAAAACAA
ATGATGGTACTGACAGAAAAAGCACCGGCTAACTACGTGCCAGCAGCCGCGGTAATACGT
AGGGTGCAAGCGTTATTCGGAATTATTGGGCGTAAAGCGCATGTAGGTGGCTTCGTAAGT
CTGATGTGAAATCCCTGGACTCAATTCAGGAACTGCATTGGATACTGCGAGGCTAGAGTA
GAGGAGGGGTAGGCGGAATTCCAGGTGTAGAGGTGAAATTCGTAGATATCTGGAGGAACA
ACAGTGGCGAAGGCGGCTTACTGGACTCTAACTGACACTGAGATGCGAAAGTGTGGGGAG
CAAACAGGATTAGATACCCTCGTAGTCC
>Otu7796
CCAGCCTATGGGGCGCTGCAGTGGGGAATATTGGACAATGGGGGAAACCCTGATCCAGCG
ACGCCGCGTGTGTGAAGAAGGCCTGCGGGTTGTAAAGCACTTTTAGTGGGGATGAAAAGT
TCAGGGCTAATACCCTTGGATGTTGACCTAACCCACAGAAAAAGCACCGGCTAACTCTGT
GCCAGCAGCCGCGGTAATACAGAGGGTGCAAGCGTTAATCGGAATTACTGGGCGTAAAGC
GTGCGTAGGCGGTTTCGTAAGTCGGGTGTGAAAGCCCCGGGCTCAACCTGGGAATTGCAT
TCGAGACTGCGAAGCTAGGGTGCGGAAGAGGGAAGCGGAATTTCTGGTGTAGCGGTGAAA
TGCGTAGATATCGGAAGGAACACCAGTGGCGAAAGCGACTTCCTGGTCGAATACTGACGT
TCATGTGCGAAAGCGTGGGGAGCAAACAGGATTAGATACCCCAGTAGTCC
>Otu7801
CCAGCCTATGGGACGCAGCAGTAGGGAATATTGGGCAATGGGCGCAAGCCTGACCCAGCC
ATCCCGCGTGAAGGATGAAGGCCCTCTGGGTCGTAAACTTCTTTTGTTGGGGAAGAAAAA
TTGGGATTCATCCCGACTTGACGGTACCCAACGAATAAGCACCGGCTAACTCCGTGCCAG
CAGCCGCGGTAATACGGAGGGTGCAAGCGTTATCCGGATTCACTGGGTTTAAAGGGTGCG
TAGGCGGCTATGAAAGTCAGTGGTGAAAGCCCGCAGCTTAACTGCGGAACTGCCATTGAA
ACTTCATAGCTGGAGTATTATTGAAGTGGGCGGAATACATCATGTAGCGGTGAAATGCTT
AGATATGATGTAGAACTCCGATTGCGAAGGCAGCTCACTAAATAATAACTGACGCTGAAG
CACGAAAGCGTGGGTAGCAAACAGGATTAGAAACCCGTGTAGTCC
>Otu7809
CCAGCCTACGGGTCGCACCAGTTTCGAATCATTCACAATGGGCGAAAGCCTGATGGTGCG
ACGCCGCGTGAGGGATGAAGGTCTTCGGATTGTAAACCTCTGTCACTAGGGAAGAAACGC
TTCAAGTTAACAGCTTGAAGCCTGACTTAACCTGGAGAGGAAGCAGTGGCTAACTCTGTG
CCAGCAGCCGCGGTAATACAGAGACTGCAAGCGTTATTCGGATTCACTGGGCGTAAAGGG
TGCGTAGGCGGTTGGGTAAGTCTGACGTGAAATCTCCGGGCCTAACCCGGAAACTGCGTT
GGATACTATCCGGCTAGAGGATTGGAAGGGAGACTGGAATACTTGGTGTAGCAGTGAAAT
GCGTAGATATCAAGTGGAACACCAGTGGCGAAGGCGAGTCTCTGGACAATTCCTGACGCT
GAGGCACGAAAGCCAGGGGAGCAAACGGGATTAGAAACCCGGGTAGTCC
>Otu7812
CCAGCCTATGGGTCGCAGCAGTCGAGAATTTTTCTCAATGGCGGAAGCCTGAACCAGCCA
TGCCGCGTGCAGGAAGACGGCCCTACGGGTTGTAAACTGCTTTTGCAGGGGAATAAACCT
CCCGACGAGTCGGGAGTTGAATGTACTCTGAGAATAAGGATCGGCTAACTCCGTGCCAGC
AGCCGCGGTAATACGGAGGATCCGAGCGTTATCCGGATTTATTGGGTTTAAAGGGTGCGT
AGGCGGCTTTTTAAGTCAGGGGTGAAAGACGGTAGCTTAACTATCGCAGTGCCCTTGATA
CTGAAGAGCTTGAATGTACTAGAGGTAGGCGGAATGTGACAAGTAGCGGTGAAATGCATA

GATATGTCACAGAACACCAATTGCGAAGGCAGCTTACTATGGTATGATTGACGCTGAGGC
ACGAAAGCGTGGGGATCAAACAGGATTAGAAACCCTCGTAGTCC
>Otu7813
CCAGCCTATGGGTGGCAGCAGTGAGGAATATTGGACAATGGGGGAAACCCTGATCCAGCC
ATGCCGCGTGAGTGATGAAGGCCTTCGGGTTGTAAAACTCTTTTAGATGTGACGATGATG
ACGGTAACATCAGAAAAAGCTCCGGCTAACTTCGTGCCAGCAGCCGCGGTAATACGAAGG
GAGCGAGCGTTGTTCGGAATCACTGGGCTTAAAGCGTGCGTAGGCGGAAATGTAAGTCAG
AAGTGAAAGCCCAGGGCTCAACCCTGGAACTGCTTTTGAGACTGCAATTCTAGAATTCGG
TAGAGGCTAGTGGAATGTCCAGTGTAGAGGTGAAATTCGCAGATATTGGACGGAACACCA
GTGGCGAAAGCGACTAGCTGGGCCGATATTGACGCTGAGGTACGAAAGCGTGGGTAGCAA
ACAGGATTAGAAACCCCTGTAGTCC
>Otu7815
CCAGCCTATGGGACGCTGCAGTGGAGAATCTTGGACAATGGGCGAAAGCCTGATCCAGTC
ACGTTGCGTGAGATCTCTCTTTTTTCAATGAAGATCATGACCGTAATTGAAGAATAAGTC
CTGGCTAACCTCGTGCCAGCAGCTGCGGTAAGACGAGGAGGACAAGCGTTCTTCGATATC
ACTAGGCGTAAAGTCCAGTAAGGTGGTACTAAGTAAAAGAACTTCATCTGAAAGTCAAAA
TTCAGCAAGTTCTCAAACTTCTACTAGAGCATGAAGAAAGAAAAAGGAATTTCCAAAGGA
GTGGTAAAATACGTTGAGATTGGAGGGAAGACCAAAGGCGAAGGCATTTTTCTATTTCAT
ACTGACACTGATCTGGGAAAGTCTGGGGAGCAAATGGGATTAGAGACCCCAGTAGTCC
>Otu7825
CCAGCCTATGGGGGGCAGCAGTGGGGAATCTTGCGCAATGGGCGAAAGCCTGACGCAGCG
ACGCCGCGTGTGCGATGAAGGTCTTCGGATCGTAAAGCACTGTCGCCCGGGAAGAAACCT
GACGGTACCGGGAGAGGAAGCACCGGCTAACTCTGTGCCAGCAGCCGCGGTAATACAGAG
GGTGCGAGCGTTGTTCGGAATTATTGGGCGTAAAGCGCTGGTAGGTGGCAAGGCCAGTCG
CGTGTGAAATCTCCCGGCTCAACCGGGAAAGTGCGCGCGAAACTACCTTGCTTGAGTGAC
GGAGAGGGTCGCGGAATTCCCGGTGTAGAGGTGAAATTCGTAGATATCGGGAGGAACACC
AGAGGCGAAGGCGGCGACCTGGAAGTCCACTGACACTGAGCAGCGAAAGCGTGGGGAGCA
AACAGGATTAGAGACCCTCGTAGTCC
>Otu7826
CCAGCCTATGGGGTGCAGCAGTAGGGAATATTGCGCAATGGACGAAAGTCTGACGCAGCG
ACGCCGCGTGAGTGATGAAGGCCTTAGGGTCGTAAAGCTCTGTTGAATGGGAAGAAAAAA
ATGACGGTACCATTCGAGAAAGGATCGGCTAACTTCGTGCCAGCAGCCGCGGTAATACGA
GGGATCCTAGCGTTGTTCGGAATTATTGGGCGTAAAGGGTGTGTAGGCTGCCATACAAGT
CAGTTGTGAAAGCCCCGGGCTCAACCCGGGAAGTGCTTCTGATACTGTTTGGCTTGAATA
CTGGAGAGGACAGTGGAATTCCTGGTGTAGTGGTGAAATACGTAGATATCAGGAGGAACA
CCGGCGGCGAAGGCGGCTGTCTGGCCATGTATTGACGCTGAAACACGAAAGCGTGGGGAG
CAAACAGGATTAGATACCCCAGTAGTCC
>Otu7828
CCAGCCTACGGGGGGCAGCAGTAGGGAATATTGGACAATGAGCGAAAGCTTGATCCAGCA
ATACCGTATGAGCGATGAAGGCCAAACTGGTTGTAAAGCTATTTCGTTGAGGAAGATAAT
GACACGAATCAAAGAAAGAAGTCCCGGCCAATGCCGTGCCAGCAGCCGCGGAAATACGGT
AGGGGCAAGCGTTATTCACATTATATGGGCGTAAAGGGTGCGTAGGTGGTATTAATAACT
TTTTTATAAAAAACAAAGCATAACTTTGTAAAATAATTAATAATGTTAATTCTAGAGTGA
GGTTGAGGTTAATGGAATTTCTAGTGGAGAGGTAGAATTCTGCGATATTAGAAGGAACAC
CAAAGGCGAAGGCAATTTTCTGGGTCTACACTGACATTGAGGCACGAAGGCATAGGGATC
AAATGGGATTAGAAACCCCCGTAGTCC
>Otu7838
CCAGCCTATGGGTCGCTGCAGTGGGGAATTTTGGACAATGGGGGCAACCCTGATCCAGCA
ATGCCGCGTGAGTGAAGAAGGCCTTCGGGTTGTAAAGCTCTTTTGTCCGGGAAGAGACGG
TTTGGGCTAATACCCTGGGCTAATGACGGTACCTGAAGAATAAGCACCGGCTAACTACGT
GCCAGCAGCCGCGGTAATACGTAGGGTGCAAGCGTTAATCGGAATTACTGGGCGTAAAGC
GTGCGCAGGCGGTTGTGTAAGACAGGTGTGAAATCCCCGGGCTTAACCTGGGAATGGCAT
TTGTGACTGCACGGCTAGAGTGTGTCAGAGGGGGGTAGAATTCCACGTGTAGCAGTGAAA
TGCGTAGAGATGTGGAGGAATACCGATGGCGAAGGCAGCCCCCTGGGGTAACACTGACGC
TCATGCACGAAAGCGTGGGGAGCAAACAGGATTAGATACCCCAGTAGTCC
>Otu7844
CCAGCCTACGGGGTGCAGCAGTGGGGAATATTGGACAATGGGGGAAACCCTGATCCAGCA
ATGCCGCGTGAGTGATGAAGGCCCTAGGGTTGTAAAGCTCTTTTATTGGGGAAGATTATG

ACGGTACCCAAGGAATAAGCACCGGCTAACTTCGTGCCAGCAGCCGCGGTAATACGAAGG
GTGCAAGCGTTGTTCGGAATGACTGGGCGTAAAGGGAGCGTAGGCGGTTTTGTACGTTTG
ATGTGAAAGTCCTGGGCTCAACCTGGGGACGGCATGAAATACGGCAAGACTTGAGGCCTT
GAGAGGAAGATGGAATTGTGTGTGTAGAGGTGAAATTCGTAGATATACACAAGAACACCA
GTGGCGAAGGCGATTTTCTGGCAAGGACCTGACGCTAAGGCTCGAAAGCGTGGGGAGCAA
ACAGGATTAGATACCCCCGTAGTCC
>Otu7848
CCAGCCTACGGGGCGCTGCAGTGGGGAATATTGGACAATGGGCGCAAGCCTGATCCAGCA
ATGCCGCGTGAGTGATGAAGGCCTTAGGGTTGTAAAGCTCTTTCGGCGGGGACGATGATG
ACGGTACCCGCAGAAGAAGCCCCGGCTAACTTCGTGCCAGCAGCCGCGGTAATACGAAGG
GGGCAAGCGTTGTTCGGAATTACTGGGCGTAAAGAGTGTGTAGGCGGTGCTCTAAGTTTG
GTGTGAAATCTCCCGGCTCAACTGGGAGGGTGCGCCGAAGACTGGAGTGCTCGAGCGTGG
GAGAGGAAAGCGGAATTCCTGGTGTAGCGGTGAAATGCGTAGATATCAGGAGGAACACCT
GTGGTGTAGACGGCTTTCTGGACCATTGCTGACGCTGAGACACGAAAGCGTGGGTAGCAA
ACAGGATTAGAAACCCTAGTAGTCC
>Otu7849
CCAGCCTATGGGGGGCAGCAGTGGGGAATCTTGCACAATGGGGGAAACCCTGATGCAGCG
ACGCCGCGTGAGCGATGAAGCCCCTCGGGGTGTAAAGCTCTTTCGACGGGAACGATTGTG
ACGGTACCTGTAGAAGAAGCCCCGGCTAACTTCGTGCCAGCAGCCGCGGTAATACGAAGG
GGGCTAGCGTTGCTCGGAATTACTGGGCGTAAAGGGCGCGTAGGCGGGCGTTTAAGTCAG
AGGTCAAAGCCCAGGGCTCAACCCTGGAACTGCCTTTGATACTGGGCGCCTAGAGTGCGG
GAGAGGTGAGTGGAACTCCGAGTGTAGAGGTGAAATTCGTAGATATTCGGAAGAACACCA
GTGGCGAAGGCGACTCACTGGCCCGTTACTGACGCTGAGGCGCGAAAGCGTGGGGAGCAA
ACAGGATTAGAAACCCCCGTAGTCC
>Otu7851
CCAGCCTATGGGGGGCACCAGTGGGGAATTTTACACAATGGGCGAAAGCCTGATGTAGCG
ACACCGCGTGAGCGAAGAAGCCCTTTGGGGTGTAAAGCTCTGTCAACAGGGACGAACACA
ATGACGGTACCAGTGGAGGAAGCATCGGCTAACTACGTGCCAGCAGCCGCGGTAAGACGT
AGGATGCAAGCGTTGTCCGGATTTATTGGGCGTAAAGAGTTCGTAGGCGGTTTGTTAAGT
CTGGTGTTAAAGATCGGGGCTCAACCTCGGGAGTGCATTGGATACTGGCAGACTTGAGTA
CAGTAGAGGTAAGTGGAATTCCCAGTGTAGCGGTGAAATGCGTAGATATTGGGAAGAACA
CCAGTGGCGTAGGCGACTTACTGGGCTGTTACTGACGCTGAGGAACGAAAGCCAGGGGAG
CGAATGGGATTAGAAACCCCAGTAGTCC
>Otu7852
CCAGCCTATGGGAGGCTCCAGTAGGGAATTTTCCACAATGGGCGAAAGCCTGATGGAGCA
ACGCCGCGTGCAGGATGAAGGCCTTAGGGTTGTAAACTGCTTTTCTCTGTGACGATTATG
ACGCTAACAGAGGAATAAGGATCTGCTAACTCCGTGCCAGCAGCCGCGGTCATACGGGGG
ATCCAAGCGTTATCCGGAATTACTGGGCGTAAAGAGTTGCGTAGGTGGCATGGTAAGTTG
GTATTGAAAGCGTGTGGCTCAACCACATAAACAATACTAAAACTGCCAAGCTAGAGGGCG
AGTGAGGTACCTGGAATTCTCAGTGTAGGAGTGAAATCCGTAGATATTGGGAGGAACACC
AATGGCGTAAGCAGGGTACTGGCTCGTTCCTGACACTCAGGCACGAAAGCGTGGGGAGCA
AACGGGATTAGATACCCTCGTAGTCC
>Otu7857
CCAGCCTATGGGGGGCTGCAGTGGGGAATCTTGCACAATGGACGAAAGTCTGATGCAGCA
ACGCCGCGTGGGGGATGAAGCTTTTCGGAGTGTAAACCCCTTTCGACCCGGAAGAATGCC
CGCAAGGGCTTGACGGTACCGGAAGAAGAAGCCCCGGCTAACTTCGTGCCAGCAGCCGCG
GTAATACGAGGGGGGCGAGCGTTGTTCGGAAGTATTGGGCGTAAAGGGTGCGTAGGCGGT
TTGGTAAGTCTTATGTGAAATCTATGGGCTCAACTCATAGACTGCATGAGAAACTGCAGG
GCTTGAGTGTGGGAGAGGTGAGTGGAATTTCCGGTGTAGCGGTGAAATGCGTAGATATCG
GAAGGAACACCTGTGGCGAAAGCGGCTCACTGGACCATAACTGACGCTGAGGCACGAAAG
CTAGGGGAGCAAACAGGATTAGAAACCCCAGTAGTCC
>Otu7865
CCAGCCTACGGGGGGCAGCAGTGGGGAATATTGGACAATGGGGGAAACCCTGATCCAGCA
ATGCCGCGTGCAAGATGAAGGCCTTAGGGTTGTAAACTGCTTTTATGTGTGACGAATATG
ACGGTAGCATATGAATAAGGATCGGCTAACTCCGTGCCAGCAGCCGCGGTCATACGGAGG
ATCCAAGCGTTATCCGGAATTACTGGGCGTAAAGAGTTGCGTAGGTGGCAGAGTAAGCAG
GTAGTGAAAGAGTACGGCTCAACCGTATAGACATTATCTGAACTGCTCAGCTAGAGGACG
AGAGAGGTAACTGGAATTTCTAGTGTAGGAGTGAAATCCGTAGATATTAGAAGGAACACC

GATGGCGTAGGCAGGTTACTGGCTCGTTTCTGACACTGAGGCACGAAAGCGTGGGGAGCA
AACAGGATTAGATACCCGCGTAGTCC
>Otu7876
CCAGCCTACGGGAGGCACCAGTGGGGAATATTGGACAATGGAGGAAACTCTGACGCAGCG
ACGCCGCGTGGGTGATGAAGGCCTTCGGGTCGTAAAGCCCTGTCGGAAGGGACGAAAAAC
AATTTGGCTAACATCCAAGTTGCTTGACGGTACCTTCAGAGGAAGCACCGGCTAACTACG
TGCCAGTAGCCGCGGTAATACGTAGGGTGCTAGCGTTGTTCGGAACCATTGGGCGTAAAG
CGCGTGTAGGTGGTTAGGTAAGTCGGGTGTGAAATCCCTGGGCTCAACCGAGGAAGTGCA
TCCGAAACTGCCTAGCTAGAGGACGGTAGAGGAAGGTGGAATTCCTAGTGTAGAGGTGAA
ATTCGTAGATATTAGGAGGAATACCGGTGGCGAAGGCGGCCTTCTGGGCCGTTCCTGACA
CTGAGACGCGAAAGCGTGGGGAGCAAACAGGATTAGAAACCCGAGTAGTCC
>Otu7878
CCAGCCTATGGGGCGCTGCAGCCGAGAATATTCGACAATGGGCGAAAGCCTGATCGAGCG
ATACCGCGTGGTCGATGAAGTGCTTCGGCACGTAAAGACCTTTTATGGAGGAGGAAGTAA
TTGACGTTACTCCATGAATAAGGGGCTCCTAACTCTGTGCCAGCAGGAGCGGTAATACAG
AGGCCCCGAGCATTACCCGGAATTATTGGGCGTAAAGAGTGCGTAGGTGGTCATGTTAGT
CGTTTGTTAAAACCCCGAGCTCAACTCGGGATCTGCGAGCGAAACGGCATGACTTGAGGG
CGTGAGAGGTACATGGAACTCATGGTGTAGGGGTGAAATCCGTTGATATCATGGGGAACA
CCGAAAGCGAAGGCAATGTACTGGCGCGTTCCTGACACTCAAGCACGAAAGCGTGGGTAG
CGAACGGGATTAGAAACCCGAGTAGTCC
>Otu7882
CCAGCCTATGGGTCGCAGCAGTAGGGAATTTTGCACAATGGGCGCAAGCCTGATGCAGCA
ATGCCGCGTGAGTGAAGAAGGCTCTTGGGTTGTAAAGCTCTTTCGGCTGGAAAGAAGGGG
TAGCCTGCGAACAGTTGGCTATCTTGACGGTACCAGAAGAAGAAGCACCTGCTAACTTCG
TGCCAGCAGCAGCGGTAATACGAAGGGTGCAAGCGTTGTTCGGAATTACTGGGCGTAAAG
CGCGCGTAGGCGGGTCGGTAAGTCGATTGTGAAAGCCCAGGGCTTAACCTTGGAATCGCA
GTCGAAACTGCCGATCTTGAATGTCTGAGAGGATGGCGGAATTCCCGGTGTAGTAGTGAA
ATACGTAGATATCGGGAAGAACACCTGAGGCGAAGGCGGCTATCTGGCGGTACATTGACG
CTGAGGCGCGAAAGCGTGGGGAGCAAACAGGATTAGATACCCCTGTAGTCC
>Otu7889
CCAGCCTATGGGTCGCAGCAGTCGAGAATCTTCGGCAACGCACGAAAGTGTGACCGAGCG
ACGCCGCGTGATTGATGAAGCCCTTCGGGGTGTAAAGATCTTTTATGCATGAAGAAATTA
TTGACATTAGTGCATGAATAAGGGGCTCCTAACTCTGTGCCAGCAGGAGCGGTAATACAG
AGGCCCCGAGCGTTATCCGGAATTATTGGGCGTAAAGGGTGCGTAGGTGGTTTTATTAGT
CGACTGTGAAAGATCCGGGCTCAACCCGGGGAACGCAATCGAAACGGTAAGACTTGAGTG
TGTGAGAGGTGAACGGAACTCATGGAGTAGGGATGAAATCCGTTGATATCATGGGGAACA
CCAAATGCGAAGGCAGTTCACTGGCACATTACTGACACTGAAGCACGAAAGCGTGGGTAG
CGAATGGGATTAGATACCCTGGTAGTCC
>Otu7895
CCAGCCTACGGGAGGCTGCAGTTGGGAATCTTGCACAATGGGGGGAACCCTGATGCAGCG
ACGCCGCGTGGAGGACGAAGGGTCTAGGCCTGTAAACTCCTTTTATCGGGAAAGACTTAG
GACGGTACCCGATGAATAAGCACCGGCTAACTACGTGCCAGCAGCCGCGGTAAGACGTAG
GGTGCAAGCGTTGTCCGGATTTACTGGGCGTAAAGAGCGCGTAGGCGGTCTGTTAAGTGT
AGAGTGAAATCTCCAGGGCTCAACCCGGAAACTGCTCTGCATACTGGCAGACTGGAGGAT
TGCAGAGGTATGTGGAATTCCCGGTGTAGCGGTGAAATGCGTTGATATCGGGAGGAACAC
TCATGGCGAAGGCAGCATACTGGGCAATATCTGACGCTGAGGCGCGAAAGCGTGGGTAGC
AAACAGGATTAGAGACCCCCGTAGTCC
>Otu7896
CTAGCCTACGGGTGGCTGCAGTGGGGAATATTGGACAATGGGCGCAAGCCTGATCCAGCC
ATGCCGCGTGGGTGATGAAGGCCCTAGGGTTGTAAAGCCCTTTCGACGGGGACGATAATG
ACGGTACCTATAGAAGAAGCCCCGGCTAACTTCGTGCCAGCAGCCGCGGTAATACGAGGG
GGGCAAGCGTTGTTCGGAATTATTGGGCGTAAAGGGTGCGTAGGCGGTTTGACAAGTCTT
ATGTGAAATCTATGGGCTCAACCCATAGTCTGCATGGGAAACTGTCGGGCTTGAGTATGG
GAGAGGTGAGTGGAATTTCCGGTGTAGCGGTGAAATGCGTAGATATCGGAAGGAACACCT
GTGGCGAAAGCGGCTCACTGGACCATAACTGACGCTGATGCACGAAAGCTAGGGGAGCAA
ACAGGATTAGAAACCCTAGTAGTCC
>Otu7902
CCAGCCTATGGGGTGCAGCAGTAGGGAATCTTGCGCAATGCGCGAAAGCGTGACGCAGCA

ACGCCGCGTGGGGGAAGAAGGCTTTCGGGTTGTAAACCCCTTTCAGTTGGGACGAAGCTC
CGTCGGTTAATAGCCGGCTGGAGTGACGGTACCTTCACAAGAAGCACCGGCTAACTACGT
GCCAGCAGCTGCGGTAATACGTAGGGTGCAAGCGTTGTCCGGAATCATTGGGCGTAAAGA
GCGTGTAGGCGGTTCGATCAGTCCGCTGTAAAAGTCCAGGGCTCAACCCTGGGAGGCCGG
TGGATACTGTCGGGCTAGAGTCCGGAAGAGGCGAGTGGAATTCCTGGTGTAGCGGTGAAA
TGCGCAGATATCAGGAGGAACACCAGCAGCGAAGGCGGTGGTCTGGGCCGACATTGACGC
TGAGGAGCGAAAGCGTGGGGAGCGAACAGGATTAGATACCCTTGTAGTCC
>Otu7908
CCAGCCTATGGGTCGCTGCAGTTGGGAATCTTGGGCAATGGGGGAAACCCTGACCCAGCC
ATGCCGCGTGAATGATGAAGCCCTTAGGGTTGTAAAGTTCTTTTAGCAGGGACGATGATG
ACGGTACCTGCAGAATAAGCTCCGGCTAACTTCGTGCCAGCAGCCGCGGTAATACGAAGG
GAGCTAGCGTTGTTCGGAATCACTGGGCGTAAAGCGTGCGCAGGCGGCTATTTAAGTCAG
AGGTGAAATGCCAAGGCTCAACCTTGGAACTGCCTTTGAAACTGGGTAGCTAGAGTACTG
GAGGGGTTAGGGGAATTTCTAATGTAGCGGTGAAATGCGTAGATATTAGAAGGAACACCA
GCGGCGAAGGCGCCTAACTGGACAGTCACTGACGCTCATGCACGAAAGCGTGGGGATCAA
ACAGGATTAGAAACCCTAGTAGTC
>Otu7913
CCAGCCTACGGGGCGCACCAGTGGGGAATCTTGCGCAATGCGCGAAAGCGTGACGCAGCA
ACGCCGCGTGGGGGAAGACGGCCTTCGGGTTGTAAACCCCTTTCAGTTGGGACGAAGCTT
CGCCGGTGAATAGCCGGTTGGAGTGACGGTACCTTCAGAAGAAGCCCCGGCTAACTACGT
GCCAGCAGCCGCGGTAATACGTAGGGAGCGAGCGTTGTCCGGAATCATTGGGCGTAAAGG
GTTCGTAGGCGGGAATGCAAGTCAAGTGTGAAATCCCCAGGCTCAACCTGGGACGTGCAT
TTGAGACTGTGTTTCTTGAGTTTCGGAGAGGGTGGTGGAATTGCTGGTGTAGGAGTGACA
TCCGTAGAGATCAGCAGGAACACCGGAGGCGAAGGCGACCACCTGGCCGAATACTGACGC
TGAGGAACGAAAGCGTGGGGAGCAAACAGGATTAGAAACCCGAGTAGTCC
>Otu7915
CCAGCCTACGGGTTGCTGCAGTCGAGAATTTTTCTCAATGGGCGAAAGCCTGAAGGAGCG
ACGCCGCGTGGGGGATGAATGGCTTCGGCCCGTAAACCCCTGTCATTTGTGAACAAATTA
ATCTACCCAACACGTGGATTATTGATAGTAACGGAAGAGGAAGGGACGGCTAACTCTGTG
CCAGCAGCCGCGGTAATACAGAGGTCCCAAGCGTTGTTCGGATTCACTGGGCGTAAAGGG
TTCGTAGGCGGGAATGCAAGTCAAGTGTGAAATCCCCAGGCTCAACCTGGGACGTGCATT
TGAGACTGTGTTTCTTGAGTTTCGGAGAGGGTGGTGGAATTGCTGGTGTAGGAGTGACAT
CCGTAGAGATCAGCAGGAACACCGGAGGCGAAGGCGACCACCTGGCCGAATACTGACGCT
GAGGAGCGAAAGCCAGGGGAGCGAACGGGATTAGAAACCCCAGTAGTCC
>Otu7916
CCAGCCTACGGGGTGCTGCAGTCGAGAATTTTTCACAGTGGACGAAAGTCTGATGGAGCG
ACGCCGCGTGGAGGATGAAGTGCTTCGGCATGTAAACTCCTTTTGCCAGGGAAAAAGTTT
ATTGATTGTACCTGGAGAATAAGAAGTTGCTAAACTCGTGCCAGCAGCAGCGGTAATACG
AGTGCTTCGAGCGTTATCCGGAATCATTGGGCGTAAAGGGTGTGTAGGCGGCGATGTTAG
TCTCCCGTAAAATTCTCCGGCTCAACCGGAGGCGCGCGGGAGAAACGGCATCGCTCGAGG
GCAGGAGAGGTCTCTGGAACTCATGGTGTAGCGGTGAAATGCGTTGATATCATGGGGAAC
ACCAAAAGCGAAGGCAAGAGACTGGCCTGTTTCTGACGCTGAAACACGAAAGCGTGGGTC
GCGAATGGGATTAGATACCCCGGTAGTCC
>Otu7919
CCAGCCTACGGGAGGCAGCAGTGGGGAATATTGCGCAATGGGGGAAACCCTGACGCAGCA
ACGCCGCGTGAGTGATGAAGGTCCTCGGATCGTAAAGCTCTGTCGTTAGGAAAGATGGTT
TATGTAGTTAATACCTATGTAAGCTGACGGTACCTAAAAAGGAAGCACCGGCTAACTCTG
TGCCAGCAGCCGCGGTAATACAGAGGGTGCAAGCGTTGTTCGGAATCATTGGGCGTAAAG
GGCGCGTAGGCGGTCCAGCAAGTCGAATGTGAAAGCCCAGGGCTCAACCTTGGAAGTGCA
TCCGAAACTGCCGGACTCGAATCTCGGAGAGGGTGGTGGAATTCCCAGTGTAGAGGTGAA
ATTCGTAGATATTGGGAGGAACACCGGTGGCGAAGGCGACCGCCTGGACGAAGATTGACG
CTGAGGCGCGAGAGCGTGGGGAGCAAACAGGATTAGAAACCCGGGTAGTCC
>Otu7920
CCAGCCTATGGGGTGCAGCAGTGGGGAATATTGGACAATGGGGGAAACCCTGATCCAGCG
ACGCCGCGTGGGTGAAGAAGGTCTTCGGATTGTAAAGCCCTTTTATTGGGGAAGATAATG
ACGGTACCCAATGAATAAGCACCGGCTAACTTCGTGCCAGCAGCCGCGGTAATACGAAGG
GTGCTAGCGTTGTTCGGAATGACTGGGCGTAAAGGGCGCGTAGGTGGTTTGATGCGTCTG
ATGTGAAATACCTGGGCTTAACCTGGGAACTGCATCAGATACGGTCAGGCTTGAGTATGA

GAGGGGAAGATGGAATTGCAGGTGGAGAGGTGAAATTCGTAGATATCTGCAGGAACACCG
GTGGCGAAGGCGATCTTCTGGCTCATTACTGACACTGAGGCGCGAAAGCGTGGGGAGCAA
ACAGGATTAGATACCCCCGTAGTCC
>Otu7924
CCAGCCTATGGGTGGCTGCAGTGGGGAATATTGGACAATGGGCGAAAGCCTGATCCAGCA
ATGCCGCGTGAGTGATGAAGGCCTTAGGGTTGTAAAGCTCTTTTGCCAGGGATGATAATG
ACAGTACCTGGAGAATAAGCTCCGGCTAACTCCGTGCCAGCAGCCGCGGTAATACGGAGG
GAGCTAGCGTTGTTCGGAATTACTGGGCGTAAAGCGCGCGTAGGCGGTTTTTCAAGTCAG
AGGTGAAAGCCCGGAGCTTAACTCCGGAACTGCCTTTGAAACTAGAGAACTAGAAGATGG
GAGAGGTGAGTGGAATTCCGAGTGTAGAGGTGAAATTCGTAGATATTCGGAAGAACACCA
GTGGCGAAGGCGGCTCACTGGACCATTCTTGACGCTGAGGTGCGAAAGCGTGGGGAGCAA
ACAGGATTAGAAACCCTGGTAGTCC
>Otu7933
CCAGCCTATGGGGTGCTGCAGTGGGGAATATTGGACAATGGGCGCAAGCCTGATCCAGCG
ATGCCGCGTGTGTGAAGAAGGCCTGCGGGTTGTAAAGCACTTTCAGTGGGGAGAACGAGG
CGTAGTTTAATAGACTATGCGGAAGATGTTACCCACAGAAGAAGCACCGGCTAACTCTGT
GCCAGCAGCCGCGGTAATACAGAGGGTGCAAGCGTTAATCGAAATTACTGGGCGTAAAGG
GTGCGTAGGTGGTTTGATAAGTTAGATGTGAAAGTCCTGGGCTCAACCTGGGGATTGCGT
CTGATACTGTTAAGCTAGAGTTCTGTAGAGGAAGGTGGAATTTCCGGAGTAGCGGTGAAA
TGCGTAGATATCGGAAGGAACATCGGTGGCGAAGGCGGCCTTCTGGACAGGAACTGACAC
TGAGGCACGAAAGCGTGGGGAGCAAACAGGATTAGAAACCCGGGTAGTCC
>Otu7934
CCAGCCTACGGGTGGCAGCAGTAGGGAATATTGCTCAATGGAGGAAGCTCTGATGCAGCG
ACGCCGCGTGAGTGATGAAGGACTTCGGTTCGTAAAGCTCCGTTGCAAGGGAAAAATAAA
TTGATGGTACTTTGCGAGAAAGGGTCGGCTAACTTCGTGCCAGCAGCCGCGGTAAGACGA
GGGACCCTAGCGTTGTTCGGAATCATTGGGCGTAAAGCGGGTGTAGGTGGCCTTGTAAGT
CAGGTGTGAAAGCCCAGGGCTTAACCTTGGAAGTGCATTTGATACTGCGAAGCTTGAGTG
CTGGAGAGGTTACTAGAATTCCTGGTGTAGTGGTGAAATACGTAGATATCAGGAGGAATA
CCGGAGGCGAAGGCGGGTAACTGGCCAGACACTGACACTCAGACCCGAAAGTGTGGGGAT
CAAACAGGATTAGGTACCCCGGTAGTCC
>Otu7936
CCAGCCTATGGGTCGCAGCAGTTGGGAATCTTGCACAATGGGGGAAACTCTGATGCAGCG
ACGCCGCGTGAGCGATGAAGCCCCTCGGGGTGTAAAGCTCTTTCGGCAGGAACGATAATG
ACGGTACCTGAAGAAGAAGCTGCGGCTAACTACGTGCCAGCAGCCGCGGTAATACGTAGG
CAGCGAGCGTTGTTCGGAATTACTGGGCGTAAAGAGTGTGTAGGCGGTGCTCTAAGTTTG
GTGTGAAATCTCCCGGCTCAACTGGGAGGGTGCGCCGAAAACTGGGGTGCTCGAGCGTGG
GAGAGGAAAGCGGAATTCCTGGTGTAGCGGTGAAATGCGCAGATATCAGGAGGAACACCA
ATGGCGAAGGCAGCCCGCTGGGACGGTACTGACGCTGAGACGCGAAAGCGTGGGGAGCAA
ACAGGATTAGAGACCCTAGTAGTCC
>Otu7942
CCAGCCTACGGGTTGCAGCAGTCAGGAATATTGCGCAATGGAGGAAACTCTGACGCAGCG
ACGCCGCGTGGGTGATGAAGGCCTTCGGGTCGTAAAGCCCTGTCGGAAGGGACGAAAAGC
GGTATGGCTAATATCTTTACCGTTTGACGGTACCTTTAAAGGAAGCACCGGCTAACTACG
TGCCAGCAGCCGCGGTAATACGTAGGGTGCAAGCGTTGTTCGGAATCATTGGGCGTAAAG
CGCGTGTAGGTGGTTATGTAAGTCGAGTGTGAAATCCCTGGGCTCAACCGAGGAAGTGCA
TCCGAAACTACATAGCCAGAGGACGGTAGAGGAAGGTGGAATTCCTAGTGTAGAGGTGAA
ATTCGTAGATATTAGGAGGAATACCGGTGGCGAAGGCGGCCTTCTGGGCCGGACCTGACA
CTGAGACGCGAAAGCGTGGGTAGCAAACAGGATTAGAAACCCTCGTAGTCC
>Otu7948
CCAGCCTATGGGTCGCAGCAGTGGGGAATATTGCGCAATGGGCGAAAGCCTGACGCAGCA
ACGCCGCGTGAGTGAAGAAGGCTTTAGGGTTGTAAAACTCTTTCGGTTGGGAAGAAGGAG
TGTAGTATGAATAGTATTATACTTTGATGGTACCAGCAGAAGAAGCACCGGCAAACTTCG
TGCCAGCAGCCGCGGTAATACGAAGGGTGCAAGCGTTGTTCGGAATTATTGGGCGTAAAG
GGTTCGTAGGCGGTATCTTAAGTCAAGTGTAAAATATCTAAGCTTAACTTGGAGGAGTCA
TTTGAAACTGAGGTACTTGAGTTTCGGAGAGGGTGGTGGAATTGCTGGTGTAGGAGTGAT
ATCCGTAGAGATCAGCAGGAACACCGGAGGCGAAAGCGGCCACCTGGCCGAAGACTGACG
CTGAGGAACGAAAGCGTGGGTAGCAAACAGGATTAGAAACCCTGGTAGTCC
>Otu7949

CCAGCCTATGGGATGCTGCAGTGGGGAATATTGGACAATGGGCGCAAGCCTGACCCAGCC
ATGCCGCGTGAATGATGAAGGCCTTCGGGTTGTAAAGTTCTTTTAGTGGAGAAAATAATG
ATGGTATCCACAGAAAAAGCCCCGGCTAACTTCGTGCCAGCAGCCGCGGTAATACGAAGG
GGGCTAGCGTTGTTCGGAATTACTGGGCGTAAAGCGTTTGTAGGCGGTAGAACAAGTCAG
TGGTGAAAGCCCAGGGCTTAACCCTGGAATTGCCATTGAAACTGTTCTGCTAGAGAGTGT
TAGAGGAAAGTGGAATTCCTAGTGTAGGAGTGAAATCCGTAGAGATTAGGAGGAATATCG
ATGGCGAAGGCAGCTTTCTGGACATTTCTGACGCTGAGAAACGAAAGCGTGGGTAGCAA
ACAGGATTAGAGACCCCAGTAGTCC
>Otu7955
CCAGCCTACGGCACCAGTCGAGAATTTTTCACAATGGACGAAAGTCTGATGGAGCGACGC
CGCGTGGAGGATGAAGGTTCTTGGATTGCAAACTCCTGTCACTGCAGAACAAGGGTGCGC
ATGTTAATAGCAAGCGCATTTGATGGTATGCGGAGAGGAAGGGACGGCTAACTCTGTGCC
AGCAGCCGCGGTAAGACAGAGGTCCCGAGCGTTGTTCGGATTCATTGGGCGTAAAGGGTG
TGTAGGAGGTCGGGTCAGTCAGGTGTGAAATCTCCCAGCTTAACTGGGAAACTGCGCTTG
ATACTGCCCGGCTAGAGGATCGGAGGGGGTATCGGAATTTATGGTGTAGCAGTGAAATGC
GTAGATATCATAAGGAACACCGGTGGCGAAGGCGGATACCTGGAAGATTCCTGACTCTGA
AACACGAAAGCCAGGGGAGCAAACGGGATTAGAAACCCTAGTAGTCC
>Otu7958
CCAGCCTATGGGGCGCAGCAGTGGGGAATTTTGGACAATGGGGGCAACCCTGATCCAGCC
ATGCCGCGTGAGTGAAGAAGGCCTTCGGGTTGTAAAGCTCTTTTGTCCGGGAAGAAAAGG
CATCTCTTAATACGGGGTGTTCTTGACGGTACTGGAAGAATAAGCACCGGCTAACTACGT
GCCAGCAGCCGCGGTAATACGTAGGGTGCGAGCGTTAATCGGAATTACTGGGCGTAAAGC
GTGCGCAGGCGGTTCGTTAAGTCAGATGTGAAAGCCCCGGGCTCAACCTGGGAACGGCAT
TTGAGACTGGCGAGCTGGAGTGTGGCAGAGGGGGGTGGAATTCCACGTGTAGCAGTGAAA
TGCGTAGAGATGTGGAGGAACACCGATGGCGAAGGCAGCCCCCTGGGCTGACACTGACGC
TCAGGCACGAAAGCGTGGGGAGCAAACAGGATTAGATACCCCCGTAGTCC
>Otu7961
CAGCCTATGGGGGGCAGCAGTAACGAATATTCCGCAATGGGCGAAAGCCTGACGGAGCAA
TGCCGCGTGCAGGATGAAGCAGCTTCGCTGTGTAAACTGCTGTCAGGGGTTACTAACACT
GAAGAACTCCAGAGGAAGGGCCGGCTAAACTCGTGCCAGCAGCCGCGGTAAGACGAGTGG
CCCAAGCGTTAGTCGGAATCACTGGGCTTAAAGGGTGCGTAGGCGGATCGGTAAGTGTCT
TGTGAAAGCCCACGGCTCAACCGTGGAACCGCAGGGCATACTGCCGATCTTGAGGTAGGT
AGAGGCCGGTAGAACGATCGGTGGAGCGGTGGAATGCGTAGATATCGATCGGAACGCCAA
AGGTGAAGACAGCCGGCTGGGCCTTACCTGACGCTGAGGCACGAAAGCATGGGGATCAAA
CAGGATTAGAAACCCCCGTAGTCC
>Otu7963
CCAGCCTATGGGTTGCAGCAGTAACGAATCTTCCGCAATGCTCGAAAGAGTGACGGAGCG
ACGCCGCGTGTGGGACGAAGTCCTTCGGGATGTAAACCACTGTCAGGGGCTACCAAGTTC
TGAGGAGCCCCAGAGGAAGGCACGGCTAACTCTGTGCCAGCAGCCGCGGTAATACAGAGG
TGCCAAGCGTTAGGCGGAATCACTGGGCTTAAAGCGTGTGTAGGCGGACGGGTAAGTACT
TTGTGAAATCCCACGGCTCAACCGTGGAACTGCTGGGTATACTGTCCGTCTTGAGCGACC
TAGGGGCTATCGGAACAAACGGTGGAGCGGTGAAATGCGTAGATATCGTTTGGAACGCCA
ATGGTGAAAACAGATAGCTGGGGGTCTGCTGACGCTGAGACACGAAAGCCAGGGGAGCAA
ACGGGATTAGAAACCCTTGTAGTCC
>Otu7964
CCAGCCTACGGGGGGCTGCAGTGAGGAATTTTCTCAATGGGGGAAACCCTGAAGGAGCGA
CGCCGCGTGGAGGATGAAGGTCTTCGGATTGTAAACTCCTGTCATTTGGGAACAAGTTGC
ATTGATTAACTGTCGATGCATTGATAGTACCAGAAGAGGAAGAGACGGCTAACTCTGTGC
CAGCAGCCGCGGTAATACAGAGGTCTCAAGCGTTGTTCGGATTCATTGGGCGTAAAGGGT
GCGTAGGTGGCGGGGTAAGTCAGGTGTGAAATCTCGAGGCTCAACCTCGAAACTACACTT
GATACTGCCTTGCTCGAGTACTGGAGAGGAGATTGGAATTTACGGTGTAGCAGTGAAATG
CGTAGATATCGTGAGGAAGACCAGTGGCGAAGGCGGATCTCTGGACAGTTCCTGACACTG
AGGCACGAAGGCCAGGGGAGCAAACGGGATTAGATACCCCAGTAGTCC
>Otu7967
CCAGCCTATGGGATGCACCAGTCGAGAATTTTTCACAATGGGGGAAACCCTGATGGAGCG
ACGCCGCGTGGAGGATGAAGGCCTTCGGGTCGTAAACTCCTGTCACTGCAGAACAAGGTT
CAAGTAATTAACATGTTCTCGAGAGTGCTAGTATGCGGAGAGGAAGGGACGGCTAACTCT
GTGCCAGCAGCCGCGGTAATACAGAGGTCCCAAGCGTTGTTCGGATTCACTGGGCGTAAA

GGGTGTGTAGGAGGTTGTCTAAGTCGGGCGTGAAATCCCACCGCTTAACGGTGGAACTGC
GTTCGATACTGGATAGCTGGAGGATCGGAGAGGAGAGCGGAATTCTCGGTGTAGCGGTGA
AATGCGTAGATATCGAGAGGAACGCCAGCGGCGAAAGCGGCTCTCTGGACGATTCCTGAC
TCTGAAACACGAAAGCCAGGGGAGCGAACGGGATTAGATACCCCTGTAGTCC
>Otu7983
CCAGCCTATGGGGGGCAGCAGCTAAGAATATTCCGCAATGGACGAAAGCCTGATCCAGCC
ATGCCGCGTGAGTGAAGAAGGCCTTCGGGTTGTAAAGCTCTTTCGGACAGAAAGAAATCG
CACTGGTAAATAATCAGTGTGGATGACGGTACTGTAAGAAGAAGCACCGGCTAACTACGT
GCCAGCAGCCGCGGTAATACGTAGGGTGCGAGCGTTAATCGGAATTACTGGGCGTAAAGC
GTGCGCAGGCGGTTACATAAGACAGATGTGAAATCCCCGGGCTTAACCTGGGAACTGCGT
TTGTGACTGTGTAACCGGAGTACGGCAGAGGGGGGTGGAATTCCTGGTGTAGCAGTGAAA
TGCGTAGATATCAGGAGGAACACCGATGGCGAAGGCAGCCCCCTGGGCCTGTACTGACGC
TCATGCACGAAAGCGTGGGGAGCAAACAGGATTAGATACCCCCGTAGTCC
>Otu7989
CCAGCCTATGGGACGCAGCAGTGGGGAATATTGGACAATGGGCGCAAGCCTGATCCAGCC
ATGCCGCGTGAGTGAAGAAGGCCTTAGGGTTGTAAAGCTCTTTTGGCGGGGACGATAATG
ACGGTACCCGCAGAATAAGCCCCGGCTAACTTCGTGCCAGCAGCCGCGGTAATACGAAGG
GGGCTAGCGTTGTTCGGAATCACTGGGCGTAAAGCGCACGCAGGCGGATTGATAAGTCAG
GGGTGAAATCCCGGGGCTCAACCTCGGAATTGCCTTTGATACTGTCTGTCTTTGAGTTCG
GAAGAGGTTGGCGGAATTCCTAGTGTAGAGGTGAAATTCGTAGATATTAGGAAGAACACC
AGTGGCGAAGGCGAGTCTCTGGACATTTCCTGACGCTGAGGCACGAAAGCCAGGGGAGCA
AACAGGATTAGATACCCCGGTAGTCC
>Otu7991
CCAGCCTATGGGTGGCAGCAGTGGGGAATATTGGACAATGGGCGCAAGCCTGATCCAGCC
ATGCCGCGTGAGTGATGAAGGCCTTAGGGTTGTAAGCTCTTTTACCTGGGAAGATAATGA
CGGTACCAGGAGAATAAGCCCCGGCTAACTTCGTGCCAGCAGCCGCGGTAATACGAAGGG
GGCTAGCGTTGTTCGGATTTACTGGGCGTAAAGCGCACGTAGGCGGATCTTTAAGTCAGG
GGTGAAATCCCAAGGCTCAACCTTGGAACTGCCTTTGATACTGGGGATCTCGAGTCCGGA
AGAGGTTGGTGGAACTCCGAGTGTAGAGGTGAAATTCGTAGATATTCGCAAGAACACCAG
TGGCGAAGGCGGCTCACTGGCCCGATACTGACGCTGAGGTGCGAAAGCGTGGGGAGCAAA
CAGGATTAGATACCCGCGTAGTCC
>Otu8003
CCAGCCTATGGGGCGCACCAGTCGAGAATCTTCCGCAATGGACGAAAGTCTGACGGAGCG
ACGTTTCGTGAATGATGAAGCCCTTCGGGGTGTAAAGTTCTTTTACTTGGGAGGAAGCCG
CAAGGCAGACAGTACCAGGTGAATAAGAGGATCCTAATTCTGTGCCAGCAGGAGCGGTAA
TACAGAATCCTCGAGCGTTACCCGGATTTATTGGGCGTAAAGGGTCCGTAGGTGGTCAAG
TAAGTTGAAAGTTAAAACTCATCGGCTTAACCTTTGAGGTGCTTTCAATACTGCTAGACT
AGAGGATGTTAGGGGTTAACGGAACCGACGGAGTAGGGGTGAAATCCGTTGATATCGTCG
GGAACACCAAAAGCGAAGGCAGTTAACTAGGACAATCCTGACACTGAGGGACGAAAGCGT
GGGGAGCAAAAAGGATTAGAAACCCTCGTAGTCC
>Otu8014
CCAGCCTATGGGATGCTGCAGTAAGGAATATTGGACAATGGGGGAAACCCTGACGCAGCA
ACGCCGCGTGGAGGATGAAGTCCCTTGGGACGTAAACTCCTTTCGACCGGGACGATGATG
ACGGTACCGGTGGAAGAAGCCCCGGCTAACTTCGTGCCAGCAGCCGCGGTAATACGAGGG
GGGCGAGCGTTGTTCGGAATTATTGGGCGTAAAGGGCGCGTAGGTGGCGCGGTAAGTCAC
CTGTGAAACCTCCGGGCTCAACTCGGAGCCTGCAGGCGAAACTGCCGTGCTGGAGTATGG
GAGAGGTGCGTGGAATTCCCGGTGTAGCGGTGAAATGCGTAGATATCGGGAGGAACACCT
GTGGCGAAAGCGGCGCACTGGACCATAACTGACGCTGAGGCGCGGAAGCTAGGGGAGCAA
ACAGGATTAGAAACCCCAGTAGTCC
>Otu8024
CCAGCCTATGGGGGGCACCAGTGGGGAATATTGGACAATGGGCGCAAGCCTGATCCAGCC
ATGCCGCGTGAGTGATGACGGCCTTAGGATTGTAAAGCCCTTTCGACAGGGACGATGATG
ACGGTACCTGTAGAAGAAGCCCCGGCTAACTTCGTGCCAGCAGCCGCGGTAATACGAAGG
GGGCTAGCGTTGCTCGGAATTACTGGGCGTAAAGGGCGCGTAGGCGGTTCTGTTAGTCAG
GCGTGAAATTCCTGGGCTCAACCTGGGGGCTGCGCTTGATACGGCGGGACTAGAGGATGG
AAGAGGCTCGTGGAATTCCCAGTGTAGAGGTGAAATTCGTAGATATTGGGAAGAACACCG
GTGGCGAAGGCGGCGAGCTGGTCCATTACTGACGCTGAGGCGCGACAGCGTGGGGAGCAA
ACAGGATTAGATACCCTGGTAGTCC

>Otu8025
TCAGCCTATGGGAGGCACCAGTGGGGAATCTTGCACAATGGGGGAAACCCTGATGCAGCG
ACGCCGCGTGAGCGATGAAGCCCTTCGGGGTGTAAAGCTCTTTCGTCAGGGAAGATAGTG
ACGGTACCCGCAGAATAAGCTCCGGCTAACTTCGTGCCAGCAGCCGCGGTAATACGAAGG
GAGCTAGCGTTGTTCGGAATTACTGGGCGTAAAGGGCGCGTAGGCGGTTTGACAAGTTGG
ATGTGAAAGCCCAGGGCTCAACCCTGGAATTGCATTCAAGACTGTCTTACTTGAATTCGG
TAGAGGTGAGTGGAATTCCCAGTGTAGAGGTGAAATTCGTAGATATTGGGAAGAACACCG
GTGGCGAAGGCGGCTCACTGGACCGACATTGACGCTGAGGCGCGAAAGCGTGGGGAGCAA
ACAGGATTAGAAACCCCAGTAGTCC
>Otu8035
CCAGCCTATGGGTCGCAGCAGTAGGGAATTTTCCACAATGGGCGAAAGCCTGATGGAGCA
ACGCCGCGTGAGGGATGAAGGGTTTCGGCTTGTAAACCTCTTTGGAGTGAGACGAACGAG
CTTTCGGTTAACAATCGGAGGCAGTGACGGTATCACTAGAACAAGTCACGGCTAACTCTG
TGCCAGCAGCCGCGGTAATACAGAGGTGACTAACGTTGTCCGGAATTATTGGGCGTAAAG
GGCGCGTAGGCGGTTGGGTAAGTCAAATGTGAAATACCCGGGCTCAACCCGGGGGTTGCA
TCTGATACTGCTCAGCTTGAGTACGGTAGAGGAAGGCGGAATTCCCAGTGTAGCGGTGAA
ATGCGTAGATATTGGGAGGAACACCGGTGGCGAAGGCGGCCTTCTGGACCGAAACTGACG
CTGAAGCGCGAAAGCTAGGGGAGCAAACGGGATTAGAAACCCGCGTAGTCC
>Otu8039
CCAGCCTACGGGGGGCTCCAGTCGAGAATTTTCCACAATGGGCGAAAGCCTGATGGAGCG
ACGCCGCGTGGGGGATGAAGGGCTTCGGCTCGTAAACCCCTGTCAGCTGTGAACAAACCC
TGTGGGTGAATAGCTTACAGGTTGATAGTAGCAGCAGAGGAAGGGACGGCTAACTCTGTG
CCAGCAGCCGCGGTAATACAGAGGTCCCAAGCGTTGTTCGGATTCACTGGGCGTAAAGGG
TGCGTAGGTGGCCAGGTAAGTCTAGCGTGAAATCTCCAAGCTCAACTTGGAAAGTGCGCT
GGATACTATCTGGCTAGAGTTCCGGAGGGGAGATTGGAATTCTCGGTGTAGCAGTGAAAT
GCGTAGATATCGAGAGGAACATCAGCGGCGAAGGCGAGTCTCTGGACGGATACTGACACT
GAGGCACGAAAGCTAGGGGATCAAACGGGATTAGATACCCGAGTAGTCC
>Otu8047
CCAGCCTATGGGTGCTCAGTCGAGAATCTTCGGCAATGGGCGCAAGCCTGACCGAGCGAC
GCCGCGTGCGGGATGAAGGCCCTCGGGTTGTAAACCGCTGTCAGAGGGGAGGAAATTCCA
TGGGGTTCACCCCGTGGTTTGACCTATCTTCAGAGGAAGTACGGGCTAAGTTCGTGCCAG
CAGCCGCGGTAAGACGAACCGTACGAACGTTACTCGGAATCACTGGGCTTAAAGGGTGCG
TAGGCGGTCCGGTAAGTCGGGTGTGAAATCCCTCAGCTTAACTGAGGAATAGCGCCCGAA
ACTGTCGGACTTGAGGGAGACAGAGGTGGGCGGAACTTAGGGTGGAGCGGTGAAATGCGT
TGATATCCTAAGGCACACCAGTGGCGAAAGCGGCTCACTGGGTCTCTTCTGACGCTGAGG
CACGAAAGCTAGGGGAGCAAACGGGATTAGATACCCGAGTAGTCC
>Otu8050
CCAGCCTACGGGTGGCACCAGTGGGGAATTTTGCGCAATGGGGGAAACCCTGACGCAGCA
ACGCCGCGTGAGTGAGGAAGGATTTCGGTCTGTAAAGCTCTGTCGAGGTGGAAAAATGAG
CCATGTCTAATAGGCATGGATAGTGATGGTACACCTGAAGGAAGCACCGGCAAACTTCGT
GCCAGCAGCCGCGGTAATACGAAGGGTGCGAGCGTTGTTCGGATTTATTGGGCGTAAAGC
GCGCGCAGGCGGGATGGCAAGTCAGATGTGAAATGTCGGGGCTCAACCTCAAATCTGCGT
CTGAAACTACCGTTCTAGAATACTGGAGAGGGTAAGGGAATATCGCATGTAGGGGTAAAA
TCCGTAGATATGCGATGGAACATCGGAGGCGAAGGCGCTTACCTGGACAGTTATTGACGC
TGAGGCGCGAAAGCGTGGGGAGCAAACAGGATTAGAAACCCGCGTAGTCC
>Otu8055
CCAGCCTATGGGTGGCTGCAGTGGGGAATTTTGGACAATGGGGGCAACCCTGATCCAGCC
ATGCCGCGTGAGTGAAGAAGGCCTTCGGGTTGTAAAGCTCTTTTGTTCGGGAAGAAGGAG
TGGAGGTTAATACCCTTCGTGACAGACGGTACCGGAAGAATAAGCACCGGCTAACTACGT
GCCAGCAGCCGCGGTAATACGTAGGGTGCGAGCGTTAATCGGAATTACTGGGCGTAAAGC
GTGCGCAGGCGGTTCGTTAAGACAGCTGTGAAAGCCCCGGGCTTAACCTGGGAATGGCGG
TTGTGACTGGCGAGCTAGAGTGTAGCAGAGGGGGGTGGAATTCCACGTGTAGCAGTGAAA
TGCGTAGAGATGTGGAGGAACACCAATGGCGAAGGCAGCCCCCTGGGCTAACACTGACGC
TCAGGCACGAAAGCGTGGGGAGCAAACAGGATTAGAAACCCCTGTAGTCC
>Otu8057
CCAGCCTACGGGGTGCACCAGTTGGGAATATTGCGCAATGGGCGAAAGCCTGACGCAGCA
ACGCCGCGTGAGCGATGAAGGTCTTCGGATCGTGAAGCTCTTTCGGCCGGGACGAAAACC
CATCACTTCATACGTGGTGGCTTGACGGTACCGGAAGAAGAAGCACCGGCTAACTCTGTG

CCAGCAGCCGCGGTAATACAGAGGGTGCAAGCGTTGTTCGGATTTATTGGGCGTAAAGCG
CGTGTAGGCGGTCTGTTATGTCGGGTGTGAAAGCCCTCAGCTTAACTGAGGAAGTGCGCC
CGAAACTGGCAGACTTGAGTACCGGAGAGGAGAGCGGAACACCCGGTGTAGAGGTGAAAT
TCGTAGATATCGGGTAGAACACCAGTGGCGAAGGCGGCTCTCTGGACGGATACTGACGCT
GAGACGCGAAAGCGTGGGGAGCAAACAGGATTAGAGACCCCAGTAGTCC
>Otu8058
CCAGCCTATGGGGCGCTGCAGTGGGGAATATTGCGCAATGGGCGAAAGCCTGACGCAGCA
ACGCCGCGTGCGGGATGAAGGCCTTCGGGTTGTAAACCGCTTTCAGTGGGGACGAAATTG
ACGGTACCCACAGAAGAAGCCCCGGCCAACTACGTGCCAGCAGCCGCGGTAACACGTAGG
AGGCGAGCGTTGTCCGGATTTATTGGGCGTAAAGAGCTCGTAGGCGGCTCAGTAAGTCAG
GTGTGAAAACTCCAGGCTCAACCTGGAGACGCCACCTGATACTGCTGTGGCTAGAGTCCG
GTAGGGGAGCATGGAATTTGTGGTGTAGCGGTGAAATGCGCAGATATCACAAGGAACACC
AGTGGCGAAGGCGGTGCTCTGGGCCGGAACTGACGCTGAGGAGCGAAAGCGTGGGGAGCA
AACAGGATTAGAGACCCTAGTAGTCC
>Otu8060
CCAGCCTATGGGGTGCAGCAGCAAGGAATTTTCCGCAATGGGAGCAACCCTGACGGAGCA
ACGCCGCGTGGGCGAGGACGCTTTTCGGAGTGTAAAGCCCTTTTCAGACGGAAGAGAATG
ACGGTACGTCTGGAAGAAGCATCGGCTAACTACGTGCCAGCAGCCGCGGTAATACGTAGG
ATGCAAGCGTTGTCCGGATTTACTGGGCGTAAAGCGTGTGCAGGCGGAATGTTAGGTAGA
TTGTGAAAGCCCCCGGCTTAACTGGGGAAGGTCAGTCTAAACCGGCAATCTGGAAGCAAG
CAGAGGGGTGTGGAATTCCCGGTGTAGTGGTGAAATGCGTAGAGATCGGGAGGAACACCA
GAGGCGAAGGCGACACCCTGGGCTTGGCTTGACGCTCAGACACGAAGGCGTGGGGAGCGA
ACAGGATTAGAGACCCTCGTAGTCC
>Otu8062
CCAGCCTATGGGTCGCACCAGTGGGGAATTTTACGCAATGGGGGAAACCCTGACGTAGCG
ACACCGCGTGAGCGAAGAAGCCCTTTGGGGTGTAAAGCTCTGTCAGCTGGAACGAAAATA
AATATGACGGTACCAGCAGAGGAAGCATCGGCTAACTACGTGCCAGCAGCCGCGGTAAGA
CGTAGGATGCGAGCGTTGTCCGGATTCATTGGGCGTAAAGAGTTCGTAGGTGGTTTGTTA
AGTCTGGTGTTAAAGAATGGGGCCTAACCCCAGAGATGCATTGGATACTGGCAAACTTGA
GTGCGTTAGAGGTGAGTGGAATTCCCAGTGTAGCGGTGAAATGCATAGATATTGGGAAGA
ACACCGGTGGCGTAAGCGACTCACTGGGACGTAACTGACACTGAGGAACGAAAGCCGGGG
GAGCGAATGGGATTAGAAACCCGGGTAGTCC
>Otu8065
CCAGCCTACGGGTTGCAGCAGTGGGGAATCTTGCGCAATGGGCGAAAGCCTGACGCAGCA
ACGCCGCGTGCGGGATGACGGCCTTCGTGGTTGTAAACCGCTTTCAGCAGGGACGAAAAT
GACGGTACCTGCAGAAGAAGCCCCGGCTAACTACGTGCCAGCAGCCGCGGTAATACGTAG
GGGGCAAGCGTTGTCCGGATTCATTGGGCGTAAAGAGCTCGTAGGCGGTTCAGTAAGTCG
GGTGTGAAAGCTCCGAGCTCAACTCGGAGAGGCCACCCGATACTGCTGTGACTTGAGTCC
AGTAGAGGGGCGTGGAACTCCTGGTGTAGCGGTGAAATGCGCAGATATCAGGAAGAACAC
CTGCAGCGAAGGCGGCGCTCTGGGCTGGAACTGACGCTGAGGAGCGAAAGCGTGGGTAGC
AAACAGGATTAGAAACCCTAGTAGTCC
>Otu8071
CCAGCCTATGGGACGCTCCAGTCAAGAACCTTCCACAATGGACGAAAGTCTGATGGAGCG
ACGCCGCGTGATGGATGAAGTGCTTCGGCATGTAAACATCTTTTGTACGCAAGAAAGTAA
TTGATCAGCGTAAGAATAAGGAGTTGCTAAACTCGTGCCAGCAGCAGCGGTAATACGAGT
GCTCCAAGCGTTATCCGGAATCATTGGGCGTAAAGGGTGTGTAGGCGGTTGTGTTAGTCT
TCTGTTAAATTCTTCGGCTTAACCGGGGGCATGCAGGGGAAACGGCGCAACTAGAGGATG
CGAGAGGTGAAAGGAACTCATGGAGTAGGGGTGAAATCCGTTGATATCATGGGGAACACC
AAATGCGAAGGCAATTCACTGGCGCACTACTGACGCTGAGACGCGAAAGCGTGGGGAGCA
AACAGGATTAGATACCCCAGTAGTCC
>Otu8084
CCAGCCTATGGGGTGCTGCAGTGGGGAATATTGGACAATGGGGGAAACCCTGATCCAGCA
ATGCCGCGTGGGTGAAGAAGGTCTTCGGATTGTAAAGCCCTTTCGGCGGGGACGATGATG
ACGGTACCCGCAGAAGAAGCCCCGGCTAACTTCGTGCCAGCAGCCGCGGTAATACGAAGG
GGGCGAGCGTTGCTCGGAATGACTGGGCGTAAAGGGCGCGTAGGTGGCGTGCACAGTCAG
GCGTGAAATTCCTGGGCTCAACCTGGGGGCTGCGCTTGATACGTGCGTGCTAGAGTTCGG
AAGAGGGTCGTGGAATTCCCAGTGTAGAGGTGAAATTCGTAGATATTGGGAAGAACACCG
GTGGCGAAGGCGGCGACCTGGTCCGGGACTGACGCTGAGGCGCGAAAGCGTGGGGAGCAA

ACAGGATTAGAGACCCTTGTAGTCC
>Otu8088
CCAGCCTATGGGGGGCAGCAGTGGGGAATATTGGACAATGGACGAAAGTCTGACGCAGCG
ACGCCGCGTGGGTGATGAAGGCCTTCGGGTTGTAAAGCCCTGTGGGGCGGGACGAATTAG
TAGAAGCTAATACCTTCTATGATGACGGTACCGCCTTAGCAAGCACCGGCTAACTCTGTG
CCAGCAGCCGCGGTAAGACAGAGGGTGCAAACGTTGTTCGGGATTACTGGGCGTAAAGCG
TGTGTAGGCGGCCAAGCAAGTCGGATGTGAAAGCCCCAGGCTTAACCTGGGAAGTGCACC
CGATACTGCCTGGCTTGAGTATCGGAGAGGTTGGTGGAATTCTCGGTGTAGAGGTGAAAT
TCGTAGATATCGAGAGGAACACCGGTGGCGAAGGCGGCCAACTGGACGAATACTGACGCT
GAGACACGAAAGCGTGGGGAGCAAACAGGATTAGATACCCCAGTAGTCC
>Otu8089
CCAGCCTACGGGGGGCTGCAGTCGAGGATCTTCGGCAATGGGCGCAAGCCTGACCGAGCG
ACGCCGCGTGTGCGATGAAGGCCTTCGGGTTGTAAAGCACTGTCGAGGGGGAGGAAAGCC
CGTAAGGGTTTGACCTATCCCTGGAGGAAGCACGGGCTAAGTTCGTGCCAGCAGCCGCGG
TAAGACGAACCGTGCGAACGTTGTTCGGAATCACTGGGCTTAAAGGGCGCGTAGGCGGGC
AAGCAAGTCCGGGGTGAAAGCTTTCGGCTCAACCGGAAAAGTGCCTTGGATACTGCTTGT
CTTGAGGGAGGTAGGGGCATGTGGAACTTCCGGTGGAGCGGTGAAATGCGTTGATATCGG
AAGGAACGCCGGTGGCGAAAGCGACGTGCTGGCCCTCTTCTGACGCTGAGGCGCGAAAGC
CAGGGGTGCGAACGGGATTAGATACCCGTGTAGTCG
>Otu8090
CCAGCCTACGGGGTGCTCCAGTGGGGAATATTGCACAATGGACGAAAGTCTGATGCAGCC
ATGCCGCGTGAGTGATGAAGGCCTTCGGGTTGTAAAGCTCTTTTGGCGGGGACGATAATG
ACGGTACCCGCGGAATAAGCCCCGGCTAACTTCGTGCCAGCAGCCGCGGTAATACGAAGG
GGGCAAGCATTGTTCGGAATTACTGGGCGTAAAGGGTGTGTAGGCGGATCGATCAGTCAG
ATGTGAAAGCCCAAGGCTCAACCTTGGAATTGCATTTGATACTGTCAGTCTGGAATTGTG
GAGAGGAACGTGGAGTTCTCAGTGTAGAGGTGAAATTCGTAGATACTGAGAGGAACACCA
GTGGCGTAGGCGACGTTCTGGCCAACAATTGACGCTGAGGCACGAAAGTGTGGGGAGCAA
ACAGGATTAGAGACCCGTGTAGTCC
>Otu8093
CCAGCCTATGGGAGGCTGCAGTGGGGAATCTTGGACAATGGGGGCAACCCTGATCCAGCG
ATGCCGCGTGGGTGAAGAAGGCCTTCGGGTTGTAAAGCCCTTTAGGTTGGGACGAAGTGT
GGGAGTTTAAGAGATACTCACATTGACGGTACCGACAGAATAAGCACCGGCAAACTCTGT
GCCAGCAGCCGCGGTAATACAGAGGGTGCGAGCGTTAATCGGAGTTACTGGGCGTAAAGG
GCGCGTAGGCGGTTGTGTGAGTGTGATGTGAAAGCCCTGGGCTCAACCTAGGAAGTGCAT
CGCAAACGACATGACTGGAGTATATGAGAGGGTGGCGGAATTTCCGGTGTAGCGGTGAAA
TGCGTAGAGATCGGAAGGAACGTCGATGGCGAAGGCAGCCACCTGGCATAATACTGACGC
TGAGGCGCGAAAGCGTGGGGATCAAACAGGATTAGAAACCCGCGTAGTCC
>Otu8100
CCAGCTATGGGGGGCTGCAGTGGGGAATATTGGACAATGGGGGAAACCCTGATCCAGCGA
CGCCGCGTGTGTGAAGAAGGCCTGCGGGTTGTAAAGCACTTTTAGTGGGGATGAAATGTG
CAGGGTTAATACCTCTGCATTTTGACCTAACCCACAGAAAAAGCACCGGCTAACTCTGTG
CCAGCAGCCGCGGTAATACAGAGGGTGCAAGCGTTAATCGGAATTACTGGGCGTAAAGCG
TGCGCAGATGGTTCGATAAGTCGGGTGTGAAAGCCCCGGGCTCAACCTGGGAATTGCATT
CGAGACTGTCGAGCTAGGGTGCGGAAGAGGGAAGCGGAATTTCCGGTGTAGCGGTGAAAT
GCGTAGATATCGGAAGGAACACCAGTGGCGAAAGCGGCTTCCTGGTCCAGCACCGACATT
CAGGCACGAAAGCGTGGGGAGCAAACAGGATTAGAGACCCTAGTAGTCC
>Otu8120
CCAGCCTACGGGACGCACCAGTCGAGGATCTTTCGCAATGGGCGCAAGCCTGACGAAGCG
ACGCTGCGTGAGCGATGAAGGCCTTCGGGTTGTAAAGCTCTTTCGCGCAAGAACAAGGAA
AAGGTGTGAATAGCACTTTAACTTGAGGGTACTGCGTAAAGAAGCACCGGCTAACTCCGT
GCCAGCAGCTGCGGTAATACGGAGGGTGCAAGCATTAATCGGAATAATTGGGCGTAAAGG
GCGCGTAGGCGGGAAGGCAAGTCAGATGTGAAATTCCGGAGCTCAACTTCGGAGCTGCAT
TTGAAACTATCTTTCTAGAGGGAGGACGGAGAAAACGGAATTCCACGTGTAGCGGTGAAA
TGCGTAGATATGTGGAAGAACACCTGTGGCGAAAGCGGTTTTCTAGTTTATATCCTGACG
CTGAGGCGCGAGAGCAAGGGGAGCAAACAGGATTAGAAACCCCGGTAGTCC
>Otu8127
CCAGCCTACGGGTTGCAGCAGTAAGGAATATTGGTCAATGGACGCAAGTCTGAACCAGCC
ATGCCGCGTGAAGGATGAAGGCCCTCTGGGTTGTAAACTTCTTTTATAGGGGACGAAAAA

AGGTCATTCTTGATCACTTGACGGTACCCTAGGAATAAGCACCGGCTAACTCCGTGCCAG
CAGCCGCGGTAATACGGAGGGTGCAAGCGTTATCCGGATTTACTGGGTTTAAAGGGTGCG
TAGGTGGGCAGGTAAGTCAGTGGTGAAATCTTCGAGCTTAACTCGGAAACTGCCATTGAT
ACTATCTGTCTCGAATTATCTGGGGGTGAGCGGAATATGTCATGTAGCGGTGAAATGCTT
AGAGATGACATAGAACACCCATTGCGAAGGCAGCTCACTACCGATATATTGACACTGAGG
CACGAAAGCGTGGGGATCAAACAGGATTAGAAACCCGAGTAGTCC
>Otu8132
CCAGCCTACGGGGTGCTCCAGTGGGGAATATTGCGCAATGGGGGAAACCCTGACGCAGCG
ACGCCGCGTGGAGGATGAAGGTCTTAGGATTGTAAACTTCTGTTAAGTGGGAAGAAATGT
TGTGACCTAATACGTCACAAAGCTGACGGTACCACTAGAGAAAGCACCGGCTAACTTCGT
GCCAGCAGCCGCGGTAATACGAAGGGTGCTAACGTTATTCGGAGTTATTGGGCGTAAAGA
GCGTGTAGGCGGTCTGTTAAGTTGACTGTTAAATCTCCCGGCCTAACCGGGAAAATGCAG
TCAAAACTGGCAAGACTAGAGGGCAGGAGAGAGAAGTGGAATTCTCGGAGTAGCGGTAAA
ATGCGTAGATCTCGAGAGGAACACCGATTGCGAAGGCAGCTTCTTGGCCTGTACCTGACG
CTGAGACGCGAAAGCGTGGGGAGCAAACAGGATTAGAAACCCTCGTAGTCC
>Otu8137
CCAGCCTATGGGTTGCAGCAGTAAGGAATATTGGACAATGGGCGCAAGCCTGATCCAGCC
ATGCCGCGTGCAGGATGACAGCGTTATGCGTTGTAAACTGCTTTTATACGGGAAAAAAAT
CTTATACGTGATATAGGACTGATGGTACCGTAAGAATAAGGACCGGCTAACTTCGTGCCA
GCAGCCGCGGTAATACGAAGGGTCCGAGCGTTATCCGGATTTATTGGGTTTAAAGGGTGC
GTAGGCGGCTTATTAAGTCAGTGGTGAAAGCCTGCAGCTTAACTGCAGAACTGCCATTGA
TACTGATGAGCTTGAGTACATTTGAGGCGGGCGGAATGTGTCATGTAGCGGTGAAATGCT
TAGATATGACACAGAACACCGATTGCGAAGGCAGCTCGCTAAACTGTAACCGACGCTGAG
GCACTAAAGCGTGGGGAGCAAACAGGATTAGATACCCGTGTAGTCC
>Otu8143
CCAGCCTATGGGTGGCAGCAGTCGAGAATTTTTCTCAATGGGCGAAAGCCTGAAGGAGCG
ACGCCGCGTGAGTGAAGAAGGCCTTCGGGTTGTAAAGCTCTTTTGTCAGGGAAGAAACGG
GTGGCTCTAATACAGCTATCTAATGACGGTACCTGAAGAATAAGCACCGGCTAACTACGT
GCCAGCAGCCGCGGTAATACGTAGGGTGCAAGCGTTAATCGGAATTACTGGGCGTAAAGC
GTGCGCAGGCGGTTTGCTAAGTCAGCTGTGAAATCCCCGGGCTTAACCTGGGAATGGCAG
TTGAGACTGGCAGGCTAGAGTGTGTCAGAGGGGGGTAGAATTCCACGTGTAGCAGTGAAA
TGCGTAGAGATGTGGAGGAATACCGATGGCGAAGGCAGCCCCCTGGGATAACACTGACGC
TCATGCACGAAAGCGTGGGGAGCAAACAGGATTAGATACCCGGGTAGTCC
>Otu8155
CCAGCCTATGGGTGGCTCCAGTCGAGAATTTTTCACAATGGGGGAAACCCTGATGGAGCG
ACGCCGCGTGGGGGATGAATGGCTTCGGCCCGTAAACCCCTGTCATTTGTGAACAAATTG
CTTCACCCAACACGTGAAGCATTGATTGTAACGGAAGAGGAAGGGACGGCTAACTCTGTG
CCAGCAGCCGCGGTAATACAGAGGTCCCAAGCGTTGTTCGGATTCACTGGGCGTAAAGGG
TGCGTAGGCGGTTTGACAAGTCTGATGTGAAATCCCGCAGCTTAACTGCGGAACGGCATT
GGAAACTGTCAGACTAGAGGAATGGAGGGGAGACTGGAATACTTGGTGTAGCAGTGAAAT
GCGTAGATATCAAGTGGAACACCAGTGGCGAAGGCGAGTCTCTGGACATTTCCTGACGCT
GAGGCACGAAAGCCAGGGGAGCAAACGGGATTAGAAACCCTTGTAGTCC
>Otu8166
CCAGCCTACGGGACGCACCAGTCGAGAATCATTCGCAATGGGCGCAAGCCTGACGATGCG
ACGCCGTGTGAGCGAAGAAGGCCTTCGGGTCGTAAAGCTCTTTCGCTTGGGAACAAGAGA
GACCAACTAATAATTGGTTAATTTGAGTGTACCAGGTAAAGAAGCACCGGCTAACTCCGT
GCCAGCAGCTGCGGTAATACGGAGGGTGCAAGCATTAATCGGATTTATTGGGCGTAAAGG
GCGCGTAGGCGGAAAGGAAAGTCAGATGTGAAATCCCGGGGCTCAACCCCGGAACAGCAT
TTGAAACTCCCTATCTAGAGGATAGGCGGAGAAAACGGAATTCCACAAGTAGCGGTGAAA
TGCGTAGATATGTGGAAGAACACCGGTGGCGAAGGCGGTTTTCTAGCTTATTCCTGACGC
TGATGCGCGAAAGCAAGGGGATCAAACAGGATTAGAGACCCCAGTAGTCC
>Otu8171
CCAGCCTACGGGAGGCAGCAGTGGGGAATATTGGACAATGGGGGCAACCCTGATCCAGCA
ATGCCGCGTGAGTGATGAAGGCCTTCGGGTTGTAAAGCTCTTTCGCCCACGACGATGATG
ACGGTAGTGGGAGAAGAAGCCCCGGCTAACTTCGTGCCAGCAGCCGCGGTAATACGAAGG
GGGCTAGCGTTGTTCGGAGTTACTGGGCGTAAAGGGCGCGTAGGCGGCTGTCCAAGTCGG
GTGTGAAAGCCCAGGGCTCAACCCTGGAATAGCACTCGAGACTGGGCGGCTCGAGGACGG
GAGAGGATGGTGGAATTCCTAGTGTAGAGGTGAAATTCGTAGATATTAGGAAGAACACCG

GTGGCGAAGGCGGCCATCTGGCTCGGTACTGACACTCAAGCGCGAAAGCGTGGGGAGCAA
ACAGGATTAGAAACCCCCGTAGTCC
>Otu8181
CCAGCCTATGGGTGGCACCAGTGAGGAATATTGGTCAATGGACGCAAGTCTGAACCAGCC
ATGCCGCGTGAAGGATGAAGGCCTTATGGGTTGTAAACTTCTTTTACGAGGGAAGAAACC
CCGGATTTCGATTCGGGTTGACGGTACCTTGGGAATAAGCACCGGCTAACTCCGTGCCAG
CAGCCGCGGTAATACGGAGGGTGCAAGCGTTATCCGGATTTACTGGGTTTAAAGGGTGTG
TAGGCGGGTCATTAAGTCAGTGGTGAAATCTCCGGGCTCAACCCGGAAACTGCCATTGAT
ACTATTGGCCTTGAATGCTGTTGAGGGGGGCGGAATATGTCATGTAGCGGTGAAATGCAT
AGGTATGACATAGAACACCGATAGCGAAGGCAGCCCTCCAAGCAGTTATTGACGCTGAGG
CACGAAAGCGTGGGGATCAAACAGGATTAGATACCCGTGTAGTCC
>Otu8182
CCAGCCTACGGGGGGCAGCAGTCGAGAATTTTTCACAATGGGGGAAACCCTGATGGAGCG
ACGCCGCGTGGAGGATGAAGGTTCTTGGATTGTAAACTCCTGTCACTGCAGAACAAGGGT
AGCCGGTTGAATATTCCGGCTATTTGATGGTATGCGGAGAGGAAGGGGCGGCTAACTCTG
TGCCAGCAGCCGCGGTAAGACAGAGGTCCCGAGCGTTGTTCGGATTCATTGGGCGTAAAG
GGTGTGTAGGAGGTCGGGTTAGTCAGGTGTGAAATCTCCCAGCTTAACTGGGAAACTGCG
CTTGATACTGCCCGGCTAGAGGATCGGAGGGGGTATCGGAATTTATGGTGTAGCAGTGAA
ATGCGTAGATATCATAAGGAACACCGGTGGCGAAGGCGGATACCTGGAAGATTCCTGACT
CTGAAACACGAAAGCCAGGGGAGCAAACGGGATTAGAAACCCCAGTAGTCC
>Otu8186
CCAGCCTATGGGTTGCTGCAGTGGGGAATCTTGCGCAATGGGCGAAAGCCTGACGCAGCG
ACGCCGCGTGAGTGACGAAGGCCTTCGGGTTGTAAAGCTCTGTGGGGAGGGAAGAATAAG
TCGACGTTAATAGCGTCGATGATGACGGTACCTCCTTAGCAAGCACCGGCTAACTCCGTG
CCAGCAGCCGCGGTAAGACAGAGGGTGCGAACGTTGTTCGGAATTACTGGGCGTAAAGCG
CGTGTAGGCTGCTTGGCAAGTCGGATGTGAAAGCCCAGGGCTCAACCCTGGAAGTGCACT
CGATACTGCCGAGCTGGAGTCCCGGAGAGGAAGGCGGAATTCTCGGTGTAGAGGTGAAAT
TCGTAGATATCGAGAGGAACACCTGTGGCGAAGGCGGCCTTCTGGACGGTGACTGACGCT
GAGACGCGAAAGCGTGGGGAGCAAACAGGATTAGATACCCGTGTAGTCC
>Otu8187
CCAGCTATGGGTGGCAGCAGTGGGGAATTTTGGACAATGGGGGCAACCCTGATCCAGCAA
TGCCGCGTGTGTGAAGAAGGCCTTCGGGTTGTAAAGCACTTTTGTCAGGAACGAAACGGT
TCGGGCTAATACCCTGGGCTAATGACGGTACCTGAAGAATAAGCACCGGCTAACTACGTG
CCAGCAGCCGCGGTAATACGTAGGGTGCGAGCGTTAATCGGAATTACTGGGCGTAAAGCG
TGCGCAGGCGGTTGTGTAAGACAGATGTGCAATGCCCGGGCTTAACCTGGGAACTGCATT
TGTGACTGCACGGCTAGAGTTTGGCAGAGGGGGGTAGAATTCCACGTGTAGCAGTGAAAT
GCGTAGATATGTGGAGGAATACCGATGGCGAAGGCAGCCCCCTGGGTCAAAACTGACGCT
CATGCACGAAAGCGTGGGGAGCAAACAGGATTAGATACCCCGGTAGTCC
>Otu8198
CCAGCCTATGGGGGGCACCAGTAAGGAATTTTGGGCAATGAGGTAACCCTTGACCCGGCG
AGAGCGCGTGCATGATGAATGCGGTCAGTTTTCTGTAAAGTGCTTTTAAGGAATCAAGAT
ACTGACTGTAGTTCCTATAAGAAGCACCGGCTAACTCCGTGCCAGCAGCTGCGGTAAGAC
GGAGGGTGCGGGCCTTATTCATTATGATTGGGTGTAAAGGGTGGGTAGGTGGCTACTAAC
GGCGTTGATCGAAACTGCAGAGAGTTCCTGTGCTATTTTCAGTGGACACGAAGGTACGCT
AGAGTTGGGTATTGATCGCGGCAAAATCTCATGGAGAGGTTGAATCTTAGGAGATGAGAC
TGAACATCGGCAAGCGAAGGCGCGCGTTTTGTCTCAACTGACGCTGAGTCACGGAAGCCA
GGGGAGCAAACTGGATTAGATACCCCTGTAGTCC
>Otu8203
CCAGCCTATGGGGTGCACCAGTGGGGAATATTGGACAATGGGCGAAAGCCTGATCCAGCA
ATGCCGCGTGAGTGAGGAAGGCCTTAGGGTTGTAAAGCTCTTTCGGCGGGGACGATGATG
ACGGTACCCGCAGAAGAAGCCCCGGCTAACTTCGCGCCAGCAGCCGCGGTAATACGAAGG
GGGCTAGCGTTGCTCGGAATGACTGGGCGTAAAGGGCGCGTAGGCGGTTTGTACAGTCAG
ATGTGAAATTCCCGGGCTCAACCTGGGGACTGCATTTGATACGTGCAGGCTTGAGTTCGG
AAGAGGGTCGTGGAATTCCCAGTGTAGAGGTGAAATTCGTAGATATTGGGAAGAACACCG
GTGGCGAAGGCGGCGACCTGGTCCGAGACTGACGCTGAGGCGCGAAAGCGTGGGGAGCAA
ACAGGATTAGATACCCTCGTAGTCC
>Otu8207
CCAGCCTACGGGTCGCTGCAGTCGAGGATCTTCGGCAATGAGCGCAAGCTTGACCGAGCG

ACGCCGCGTGTGCGACGAAGGCCTTCGGGTTGTAAACTCCTGTCGAGGGGGAGGAAGGGG
TGACCTTGACCGATCCCTGGAGGAAGCACGGGCTAAGTTCGTGCCAGCAGCCGCGGTAAG
ACGAACCGTGCTAACGTTATTCGGAATCACTGGGCTTAAAGCGCGTGTAGGCGGATGGGA
ACGTCGGTTGTTGAAAGCCCCCGGCTTAACCGGGGAACTGGCGCCGAAACGCCCCGTCTG
GAGGGACGTAGGGGGACCTGGAACTTCCGGTGGAGCGGTGAAATGCGTTGAGATCGGAAG
GAACGCCCGTGGCGAAAGCGAGGTCCTGGACGTTTTCTGACGCTGAGACGCGAAAGCTAG
GGGAGCGAACGGGATTAGATACCCCCGTAGTCC
>Otu8212
CCAGCCTATGGGATGCTGCAGTGGGGAATATTGGACAATGGGCGAAAGCCTGATCCAGCC
ATGCCGCGTGAGTGATGAAGGCCTTAGGGTTGTAAAGCTCTTTTGCCCGGGAAGATAATG
ACGGTAACGTGAGAAGAAGCCCCGGCTAACTTCGTGCCAGCAGCCGCGGTAATACGAAGG
GGGCAAGCGTTGTTCGGAATTACTGGGCGTAAAGGGCGCGTAGGCGGTGCACTAAGTCAG
GCGTGAAAGCCCTGGGCTCAACCCGGGAATTGCGCTTGATACTGGTGCGCTCGAATCCAG
GAGAGGTTGGTGGAATTCCCAGTGTAGAGGTGAAATTCGTAGAGATTGGGAAGAACACCG
GTGGCGAAGGCGGCCAACTGGACTGGTATTGACGCTGAGGTGCGAAAGCGTGGGGAGCAA
ACAGGATTAGAGACCCCTGTAGTCC
>Otu8226
CCAGCCTACGGGTTGCTCCAGTCGAGAATCTTCGGCAATGGGCGCAAGCCTGACCGAGCG
ACGCCGCGTGGAGGATGAAGGCCTTCGGGTTGTAAACTCCTGTCGAGGGGGAGGAAGGTC
GGGTAACCGGCTTGACCGATCCCTGGAGGAAGCACGGGCTAAGTTCGTGCCAGCAGCCGC
GGTAAGACGAACCGTGCGAACGTTATTCGGAATCACTGGGCTTAAAGCGCGTGTAGGCGG
ATAGGGACGTCGGTCGCTGAAATCCCCCGGCTCAACCGGGGAACGGGCACCGAAACGCCC
TGTCTGGAGGGACGTAGGGGGACCTGGAACATCCGGTGGAGCGGTGAAATGCGTTGAGAT
CGGATGAAACGCCCGTGGCGAAAGCGAGGTCCTGGACGTCTTCTGACGCTGAGACGCGAA
AGCTAGGGTAGCGAACGGGATTAGAAACCCTCGTAGTCC
>Otu8232
CAGCCTACGGGTGGCTGCAGTCGAGAATTTTTCTCAATGGGCGAAAGCCTGAAGGAGCGA
CGCCGCGTGGGGGATGAATGGCTTCGGCCCGTAAACCCCTGTCATTTGTGAACAAATTGC
TTCACCCAACACGTGAAGCATTGATAGTAACGGAAGAGGAAGGGACGGCTAACTCTGTGC
CAGCAGCCGCGGTAATACAGAGGTCCCAAGCGTTGTTCGGATTCACTGGGCGTAAAGGGT
GCGTAGGCGGTCGGGTAAGTCTGACGTGAAATCTCCGGGCCTAACCCGGAAACTGCGTCG
GATACTATCCGGCTAGAGGATTGGAAGGGAGACTGGAATACTTGGTGTAGCGGTGAAATG
CGCAGATATCAGGAGGAACACCAATGGCGAAGGCAGCTCGCTGGGACGTGACTGACGCTG
AGACGCGAAAGCGTGGGGAGCAAACAGGATTAGAAACCCCAGTAGTCC
>Otu8233
CCAGCCTACGGGGCGCTGCAGTAAGGAATATTGGTCAATGGGCGAAAGCCTGAACCAGCC
ATGCCGCGTGGAGGATGAAGGCCCTCTGGGTTGTAAACTTCTTTTATTTGGGAAGAAATC
CATTTTTTCTAAGGTGGTTGACGGTACCAGATGAATAAGCACCGGCTAACTCCGTGCCAG
CAGCCGCGGTAATACGGAGGGTGCAAGCGTTATCCGGATTCACTGGGTTTAAAGGATGCG
TAGGCGGGCAGGTAAGTCAGTGGTGAAATCTCCGGGCTTAACCCGGAAACTGCCGTTGAT
ACTATCTGTCTTGAATGTCGTGGAGGTGAGCGGAATATGTCATGTAGCGGTGAAATGCTT
AGATATGACATAGAGCACCAATTGCGAAGGCAGCTCACTACACGATGATTGACGCTGAGG
GACGAAAGCTAGGGGAGCAAACAGGATTAGAGACCCGAGTAGTCC
>Otu8238
CCAGCCTACGGGACGCAGCAGTGGGGAATATTGGACAATGGGGGAAACCCTGATCCAGCG
ACGCCGCGTGTGTGAAGAAGGCCTGCGGGTTGTAAAGCACTTTTAGTGGGGACAAAAAGC
TACGGATTAATACTCTGTGGTCTTGATTTAACCCAAAGAAAAAGCACCGGCTAACTCTGT
GCCAGCAGCCGCGGTAATACAGAGGGTGCGAGCGTTAATCGGAATTACTGGGCGTAAAGC
GCGCGTAGACGGTTTTGCAAGTCTGATGTGAAAGCCCCGGGCTCAACCTGGGAACGGCAT
TGGAGACTGCAAGACTAGAGTGCGTCAGAGGGGGGTAGAATTCCGCGTGTAGCAGTGAAA
TGCGTAGAGATGCGGAGGAATGCCGATGGCGAAGGCAGCCCCCTGGGATGACACTGACGC
TCATGCACGAAAGCGTGGGGAGCAAACAGGATTAGATACCCGTGTAGTCC
>Otu8249
CCAGCCTACGGGGCGCTCCAGTGGGGAATATTGGACAATGGGCGCAAGCCTGATCCAGCC
ATGCCGCGTGAGTGATGAAGGCCTTAGAGTTGTAAAGCTCTTTTGGCGGGGAAGATAATG
ACGGTACCCGCAGAATAAGCCCCGGCTAACTTCGTGCCAGCAGCCGCGGTAATACGAAGG
GGGCAAGCATTGTTCGGAATTACTGGGCGTAAAGGGTGCGTAGGCGGATTGATCAGTCAG
GTGTGAAAGCCCAAGGCTCAACCTTGGAATTGCATTTGATACTATCAGTCTGGAATATTG

GAGAGGAACGTGGAATTCTCAGTGTAGAGGTGAAATTCGTAGATATTGAGAGGAACACCA
GTGGCGTAGGCGACGTTCTGGCCCATTACTGACGCTGAGGCGCGAAAGCGTGGGGAGCAA
ACAGGATTAGAAACCCCCGTAGTCC
>Otu8263
GGACTACTGGGGTATCTAATCCTGTTTGCTCCCCTAGCTTTCGTGCCTCAGCGTCAGTTA
TGGTCCAGTGAGCCGCTTTCGCCACAGGTGTTCCTTCCGATATCTACGCATTTCACCGCT
ACACCGGAAATTCCACTCACCTCTCCCATACTCAAGCCCGGCAGTTTCTCATGCAGACTA
TGGGTTGAGCCCATAGATTTCACATAAGACTTACAAAACCGCCTACGCACCCTTTACGCC
CAATAATTCCGAACAACGCTCGCCCCCCTCGTATTACCGCGGCTGCTGGCACGAAGTTAG
CCGGGGCTTCTTCTTCCGGTACCGTCATTATCGTCCCGATCGAAAGGAGTTTACGTCCCG
AAGGACTTCATCCTCCACGCGGCGTTGCTGCGTCAGGGTTTCCCCCATTGCGCAAAATTC
CCCACTGCTGCCTCCCATAGGCTGG
>Otu8265
CCAGCCTATGGGAGGCTCCAGGTTCGTCGCAAACTGGACCGTATAGGCAACGCCGTTTTG
CCCGGCGAACTCGAACTGAAAACCGTTGCTGTTTACCATCGGAGATTGCAGTTGCGGCAT
GTTGATCGGCGAAACGAAAACATTGGTGCCCACCGAGACCCCGGCGGGATTGTCATCGTT
ATAAACAGTGTAAGTGACCGTGTATTCACCAGGAGTGAAATAGAAGTGATCGAGGACGAC
AGTGTTTGAGGCTGACTCGCCGTCACCGAAACTCCAGTTGATCGCGGAGGCGCGGCCGGT
GAAGGAGCCGGTTAAAAAGGCCGAGCGATTGACCGGAATATTGGTTTGGTTGCAAACGAG
GCTAACCGTGAGCGGCCCCACGAGATTAGATACCCTAGTAGTCC
>Otu8275
CCAGCCTACGGGTGGCACCAGTGGGGAATCTTGCGCAATGGACGAAAGTCTGACGCAGCC
ACGCCGCGTGAGTGAAGAAGGCCTTCGGGTTGTAAAGCTCTGTCGCGCGGGACGAAAAGT
CATATGGCTCATACCCATGTGACCTGACGGTACCGCGAAAGGAAGCACCGGCTAACTCTG
TGCCAGCAGCCGCGGTAATACAGAGGGTGCAAGCGTTGCTCGGAATTATTGGGCGTAAAG
GGTAGGTAGGTGGTCTCATTTGTCGGGGGTGAAAGCCTTGGGCTTAACCCAAGAAGTGCC
TTCGAAACGGTGAGACTAGAGTTCTGGAGAGGGTCGTGGAATTCCCGGTGTAGCGGTGAA
ATGCGTAGAGATCGGGAGGAACACCAGAGGCGAAGGCGGCGACCTGGACAGATACTGACA
CTCAACTACGAAAGCGTGGGGAGCAAACAGGATTAGAAACCCGTGTAGTCC
>Otu8277
AGCCTATGGGTGGCAGCAGTCGAGAATCATTCGCAATGGGCGAAAGCCTGACGATGCGAC
GCCGTGTGAGCGAAGAAGGCCTTCGGGTCGTAAAGCTCTTTCGCTTGGGAACAAGAAAAG
CTGACTAATAATCAGCAGATTTGAGCGTACCAGGTAAAGAAGCACCGGCTAACTCCGTGC
CAGCAGCTGCGGTAATACGGAGGGTGCAGGCATTAATCGGATTTATTGGGCGTAAAGGGC
GCGTAGGCGGGATATAAGTCAGACGTGAAATCCCGAGGCTCAACCTCGGAACTGCATTT
GAAACTATATTTCTAGAGGGTAGGAGGAGAAAACGGAATTCCACAAGTAGCGGTGAAATG
CGTAGATATGTGGAAGAACACCGGTGGCGAAGGCGGTTTTCTATCTTACTCCTGACGCTG
ATGCGCGAAAGCAAGGGGATCAAACAGGATTAGATACCCCTGTAGTCC
>Otu8279
CCAGCCTACGGGAGGCAGCAGTGGGGAATTCTTGGCAATGCGCGAAAGCGTGACCGGGCG
AGAATTCATACGTGAAGAAGACGTATTGTCGTAAAGCGTAGTTTATGCAAGCGAGGTATA
ACAAAGGACATACTCGGTAGTGTAAAGTACTGGTTAATTCTGTGCCAGCAGCCGCGGTAA
GACAGAAAGTGCAGGCGTTATTCGGATTTACTGGGCGTTAAGGGTATGTAGGCAGCTAGA
TGTAACTAAGAAGGAAAGGCTCAGGAATCCGCTGAGAAGTATTCTTAAAGATTATTAAGC
TAGAGTTTATGCGGAGTTGGGAGAATTAACGAAGTAATGGAGTTAAATCCTGCGATATTG
TTAGGAAGGCCAAAGGCGGAAGCACCTTTCTGGCATAAACTGACGCTGAGGTACGAAAGA
TTGGGGATCAAAGCGGATTAGAGACCCGAGTAGTCC
>Otu8286
CCAGCCTATGGGTGGCTGCAGTGGGGAATATTGGACAATGGGGGCAACCCTGATCCAGCA
ATGCCGCGTGTGTGAAGACGGCCTTCGGGTTGTAAAGCACTTTAGTTGGGGAGGAAGGTT
AGTAGTTTAAGAGATGACTAATTTGACTGTACCCAAAGAATAAGCACCGGCTAACTCTGT
GCCAGCAGCCGCGGTAATACAGAGGGTGCGAGCGTTAATCGGAATTACTGGGCGTAAAGG
GCGCGTAGGCGGTATATTAAGTTGGATGTGAAATCCCTGGGCTTAACCTAGGAACTGCAT
CTAATACTGGTATGCTAGAGTGCTGTAGAGGATAGCGGAATTTCCGGTGTAGCGGTGAAA
TGCGTAGATATCGGAAGGAACACCAGTGGCGAAGGCGGCTATCTGGACAGACACTGACGC
TGAGGCGCGAGAGCGTGGGGAGCAAACAGGATTAGAAACCCTGGTAGTCC
>Otu8289
CCAGCCTACGGGAGGCACCAGTGGGGAATCTTGCGCAATGGACGAAAGTCTGACGCAGCC

ACGCCGCGTGAGTGAAGAAGGCCTTCGGGTTGTAAAGCTCTGTCGGGCGGGACGAAAATA
CTTCGGGCTAATACCTCGAGGAGTTGACGGTACCGCCAAAGGAAGCACCGGCTAACTTCG
TGCCAGCAGCCGCGGTAATACGAGGGGTGCAAGCGTTGCTCGGAATTATTGGGCGTAAAG
GGCAGGTAGGTGGTCTGATTTGTCTAGGGTGAAAGCCTTGAGCTCAACTCAAGAAGTGCC
CTAGAAACGGTCAGACTGGAGTCTCGGAGAGGGTCGTGGAATTCCCGGTGTAGCGGTGAA
ATGCGTAGAGATCGGGAGGAACACCAGCGGCGAAGGCGGCGACCTGGACGAGTACTGACA
CTCAACTGCGAAAGCGTGGGGAGCAAACAGGATTAGAAACCCCCGTAGTCC
>Otu8310
CCAGCCTATGGGGGGCTGCAGTGGGGAATATTGGACAATGGGGGAAACCCTGATCCAGCC
ATGCCGCGTGAATGATGAAGGCCTTCGGGTTGTAAAGTTCTTTTGACAGGGAAGATGATG
ACTGTACCTGTAGAATAAGCTCCGGCTAACTTCGTGCCAGCAGCCGCGGTAATACGAAGG
GAGCTAGCGTTGTTCGGAATTACTGGGCGTAAAGGGCGCGTAGGCGGTTTAGTAAGTTGG
AAGTGAAATCCCTGGGCTCAACCCGGGAATTGCTTTCAAGACTATTAGACTTGAATTCGG
TAGAGGTTGGTAGAATTTCCAGTGTAGAGGTGAAATTCGTAGATATTGGAAAGAATACCG
GTGGCGAAGGCGGCAAACTGGGCATCATCTGACGCTGAGGCGCGAAAGCGTGGGTAGCAA
ACAGGATTAGAGACCCGCGTAGTCC
>Otu8319
CCAGCCTATGGGAGGCTGCAGTGGGAATATTGCACAATGGGGGAAACCCTGATCCAGCAA
TGCCGCGTGTGTGAAGAAGGCCTTCGGGTTGTAAAGCACTTTTGTCAGGAACGAAACGGT
CGGGGCTAATATCCCTGGCTAATGACGGTACCTGAAGAATAAGCACCGGCTAACTACGTG
CCAGCAGCCGCGGTAATACGTAGGGTGCAAGCGTTAATCGGAATTACTGGGCGTAAAGCG
TGCGCAGGCGGTTATACAAGACAGATGTGAAATCCCCGGGCTCAACCTGGGAACTGCATT
TGTGACTGTATGGCTAGAATCTGGCAGAGGGGGGTAGAATTCCACGTGTAGCAGTGAAAT
GCGTAGATATGTGGAGGAATACCGATGGCGAAGGCAGCCCCCTGGGCTAAGATTGACGCT
CATGCACGAAAGCGTGGGGAGCAAACAGGATTAGATACCCTAGTAGTCC
>Otu8337
CCAGCCTACGGGTTGCAGCAGTCGAGAATATTCGACAATGGGCGAAAGCCTAATCGAGCG
ACACCGCGTGTAGGATGAAGGCCCTCGGGTCGTGAACTACGGTAGCTAGGGAGGAATGCA
AATGACTGTACCTAGCGGAAAGAGGTGGGTAACTACGTGCCAGCACCAGCGGTAATACGT
AGACCTCAAGCGTTATCCGGATTTATTGGGCGTAAAGAGCATGTAGGAGGTTTCGCGCGT
CTTTTGTTAAAGCCCACCGCCCAACGGTGGAAGTGCAGGAGATACGGCGGGACTAGAGGA
GGTTAGAGGTGCATGGAACTCACGGTGTAGGGGTGAAATCCGTTGATATCGTGGGGAGCA
CCAAAGGCGAAGGCAGTGCACTGGGACCTTCCTGACTCTGAGATGCGAAAGCGTGGGGAG
CAAAAAGGATTAGATACCCGAGTAGTCC
>Otu8344
CCAGCCTACGGGGGGCAGCAGTGGGAAATTTTGGACAATGGGGGCAACCCTGATCCAGCA
ATGCCGCGTGTGTGAAGAAGGCCTTCGGGTTGTAAAGCACTTTTGTCAGGAAAGAAATTG
TCCGGGCTAATATCCTGGATAGATGACGGTACCTGAAGAATAAGCACCGGCTAACTACGT
GCCAGCAGCCGCGGTAATACGTAGGGTGCAAGCGTTAATCGGAATTACTGGGCGTAAAGC
GTGCGCAGGCGGTTTTGTAAGTCTGATGTGAAATCCCCGGGCTCAACCTGGGAATGGCAT
TGGAGACTGCAAGGCTAGAATCTGGCAGAGGGGGGTAGAATTCCACGTGTAGCAGTGAAA
TGCGTAGAGATGTGGAGGAATACCGATGGCGAAGGCAGCCCCCTGGGCTAAGATTGACGC
TCATGCACGAAAGCGTGGGGAGCAAACAGGATTAGAAACCCTCGTAGTCC
>Otu8350
GGACTACGAGGGTATCTAATCCTGTTTGCTCCCCACGCTTTCGCGTCTCAGCGTCAGTAC
CGTCCCAGCGAGCTGCCTTCGCCATTGGTGTTCCTCCTGATATCTGCGCATTTCACCGCT
ACACCAGGAATTCCACTCGCCTCTTCCGGACTCTAGTCCGACAGTATCCACCGGCATCCC
AGGGTTGAGCCCTGGACTTTCACAGCGGACTGATCGGACCGCCTACACGCTCTTTACGCC
CAATAATTCCGGACAACGCTTGCACCCTACGTATTACCGCGGCTGCTGGCACGAAGTTAG
CCGGGGCTTCTTCTTCCGGTACCGTCATTATCGTCCCGATCGAAAGGAGTTTACGTACCG
AAGTACTTCATCCTCCACGCGGCGTTGCTGCGTCAGGGTTTCCCCCATTGCGCAAAATTC
CCCACTGCTGCCCCCCATAGGCTGG
>Otu8358
CCAGCCTACGGGATGCTGCAGTGAGGAATATTGCACAATGGGCGAAAGCCTGATGCAGCG
ACGCCGCGTGAAGGATGAAGGTCTTCGGATTGTAAACTTCTGTTAAATGGGAAAAAAGAG
ACCTTCTGAATAGGAAGGGAAGATGATGGTACCATTAGAGAAAGCACCGGCTAACTTCGT
GCCAGCAGCCGCGGTAATACGAGGGGTGCAAGCGTTATTCGGATTTATTGGGCGTAAAGG
GTTTGTAGGCTGTTCGTTAAGTCATCTATTAAATCTCCTAGCTTAACTAGGAAAATGTGG

GTGAAACTGGCGGACTAGAGGATGGAAGAGAGAAGTGGAATTCTCGGAGTAGCGGTAAAA
TGCGTAGATCTCGAGAGGAACACCGATGGCGAAGGCAGCTTCTTGGTCCATATCTGACGC
TGAAAAACGAAAGCGTGGGGAGCAAACAGGATTAGAAACCCGCGTAGTCC
>Otu8359
CCAGCCTACGGGGTGCTGCAGTGGGGAATATTGCGCAATGGGGGAAACCCTGACGCAGCA
ACGCCGCGTGAGTGAGGAAGGCCTTCGGGTTGTAAAGCTCTTTCGGTTGGGAAGAAGGGG
ATTTTTGCTAATAACAGAAGTTCTTGATGGTACCAAAAGAAGAAGCACCGGCTAACTTCG
TGCCAGCAGCCGCGGTAATACGAAGGGTGCAAGCGTTATTCGGAATTATTGGGCGTAAAG
GGTTCGTAGGCGGAGAACAAGTCAAGTGTGAAATGCCCGGGCTTAACCTGGAGAGTGCA
TTTGAAACTGTTTTTCTTGAGTATTGGAGAGGGTGGCGGAATTGCTGGTGTAGGAGTGAC
ATCCGTAGAGATCAGCAGGAACATCGGAGGCGAAGGCGGCCACCTGGACAATTACTGACG
CTGAGGAACGAAAGCATGGGGAGCAAACAGGATTAGATACCCCCGTAGTCC
>Otu8370
CCAGCCTATGGGGCGCTGCAGTGGGGAATATTGGACAATGGGCGCAAGCCTGATCCAGCC
ATGCCGCGTGAGTGATGACGGCCTTAGGGTTGTAAAGCTCTTTCGGCAGGGACGATAATG
ACGGTACCTGCAGAAGCAGCACCGGCTAACTACGTGCCAGCAGCCGCGGTAATACGTAGG
GTGCAAGCGTTAATCGGAATTACTGGGCGTAAAGCGTGCGCAGGCGGTTTATTAAGTCAG
CTGTGAAATCCCCGGGCTTAACCTGGGAATGGCAGTTGAGACTGGTAAGCTGGAGTGTGT
CAGAGGGGGGTAGAATTCCACGTGTAGCAGTGAAATGCGTAGAGATGTGGAGGAATACCG
ATGGCGAAGGCAGCCCCCTGGGATAACACTGACGCTCATGCACGAAAGCGTGGGGAGCAA
ACAGGATTAGATACCCGAGTAGTCC
>Otu8375
CCAGCCTACGGGGGGCACCAGTGGGGAATATTGGACAATGGGGGAAACCCTGATCCAGCG
ATGCCGCGTGTGTGAAGAAGGCCTGAGGGTTGTAAAGCACTTTCAGTGGGGAAGAATCGT
CTTAGGTTAAGAGCTAAAGGCGTTGACGTTACCCAAAGAAGAAGCACCGGCTAACTCCGT
GCCAGCAGCCGCGGTAATACGGAGGGTGCAAGCGTTAATCGGAATTACTGGGCGTAAAGC
GTGCGTAGGTGGTTAGATAAGTTGTCTGTGAAAGCCCTGGGCTTAACCCGGGAAGGTCAG
GCAAAACTGTTTAGCTTTGAGTACAAGAGAGGGTAGTGGAATTTCCGGTGTAGCGGTGAA
ATGCGTAGATATCGGAAGGAACACCAGTGGCGAAGGCGGCTACCTGGCTTGATACTGACA
CTGAGGCACGAAAGCGTGGGGAGCAAACAGGATTAGAAACCCCCGTAGTCC
>Otu8406
CCAGCCTATGGGTCGCAGCAGTAAGGAATATTGGTCAATGGGCGCAAGCCTGAACCAGCC
ATGCCGCGTGGAGGATGAAGGCCCTCTGGGTTGTAAACTTCTTTTACCTGGGACGAAAAA
CGGGGATTCCTCCCCGCCTGACGGTACCAGGTGAATAAGCACCGGCTAACTCCGTGCCAG
CAGCCGCGGTAATACGGAGGGTGCGAGCGTTATCCGGATTCACTGGGTTTAAAGGGTGCG
TAGGCGGGTTTTTAAGTCAGTGGTGAAATCTTTGGGCTTAACCCGGAAACTGCCATTGAT
ACTGTTTATCTTGAATATTGTGGAGGCCTGCGGAATATGTCATGTAGCGGTGAAATGCTT
AGATATGACATAGAACACCAATTGCGAAGGCAGCAGGCTACGCATATATTGACGCTGAGG
CACGAAAGCGTGGGGATCAAACAGGATTAGAAACCCTAGTAGTCC
>Otu8407
CCAGCCTACGGGGCGCTGCAGTGGAGAATATTAGTCAATGAATGAAAGTTTGAACTAGCG
TTTTGCGGAGATGATAGATTCATAGAATCGTTAAGACTAGGATAGTTGTAAAGTTTGTGC
GCAAGTTAAAAAAATGATATTATACTTGAAGAGAACCCCGGGCCAACAACGTGCCAGCAG
CTGCGGTAAGACGTTGACGGGGAAGCGTTACTAATCGTTACTAGGTGTAAAGAGTGACTA
GATGGTTTAAGAATTTATAAATAAAATCATAGAATAGGCGAAGATTTTTATAAAACCTTA
ATACTTGAAAATAAAAGAGTTTAGTGATAAGCACAATCTAGAGATAAAATTCTGATAGAT
TGTAAGGATCTTCAGCGGCCGCGGCGACTTCTCATTTTAATTTAACATCGAATCATTGAA
GTACGAGTGTCAATCAGGATTAGAGACCCGCGTAGTCC
>Otu8413
CCAGCCTATGGGATGCACCAGTGGGGAATATTGGACAATGGGCGCAAGCCTGATCCAGCA
ATGCCGCGTGTGTGAAGAAGGCCTGCGGGTTGTAAAGCACTTTCAGTGGGGAAGAAGGCC
TTGGTGTTAATATCACTGAGGATTGACATTACCCACAGAAGAAGCACCGGCTAACTCTGT
GCCAGCAGCCGCGGTAATACAGAGGGTGCAAGCGTTAATCGGAATTACTGGGCGTAAAGG
GTGCGTAGGTGGTTAATTAAGTCAGATGTGAAATCCCCAGGCTTAACCTGGGAATTGCAT
TTGAAACTGGTAGACTAGAGTGCGGTAGAGGGGAGTGGAATTTCCGGTGTAGCGGTGAAA
TGCGTAGATATCGGAAGGAACACCAGTGGCGAAGGCGACTTCCTGGACCGTTACTGACAC
TGAGGCACGAAAGCGTGGGGAGCAAACAGGATTAGAGACCTCGTAGTTC
>Otu8414

CCAGCCTACGGGTTGCTCCAGTGGGGAATATTGGACAATGGGCGCAAGCCTGATCCAGCA
ATGCCGCGTGGATGAAGAAGGTTTTCGGATTGTAAAGTCCTTTCGGCGGGGACGATGATG
ACGGTACCCGCAGGAGAAGCCCCGGCTAACTTCGTGCCAGCAGCCGCGGTAATACGAAGG
GGGCTAGCGTTGCTCGGAATGACTGGGCGTAAAGGGCGCGTAGGCGGCTTGGCTTGTTAG
ACGTGAAATTCCTGGGCTCAACCTGGGGACTGCGTTTAAGACGGCTGGGCTGGAGTTCGG
AAGAGGGTCGTGGAATTCCCAGTGTAGAGGTGAAATTCGTAGATATTGGGAAGAACACCG
GTGGCGAAGGCGGCGACCTGGTCCGATACTGACGCTGAGGCGCGAAAGCGTGGGGAGCAA
ACAGGATTAGATACCCTCGTAGTCC
>Otu8417
CCAGCCTACGGGGGGCTCCAGTCAAGAGTCGTAGGCAATGAGTTAACGCTAGACCACGCT
ACCTCTAAGGCAGAAGAAGACTTAGGTTGTAAAGGCCTAATTGTAGAACAGAAGAATGAA
AGCTATCAAGGATAGAGCCGATTAATCATAGTGCCAGCAATAGCGGTCACGCTAGAGGCT
CAAACGTTCGCCTCATGAACTGGGTCTGAAGCATAGGTAGGAGGCTGAAGGAACCGCGGT
AAAGGAAGCCTAGAAGGAAGAACTAGACGAATCCGTGTAACGGCAAGCTAGAGTTGTAGA
GAAGCTATTAGAACGAAGGTTGTACTAGTAAAATGGGAAGAAACCCTTTAGGATGCCAGT
AGGCGTAGGCGAATAGCTAGATACTAACTGACTCTGAGTTATGATACCAGAGAGAACAAA
GAGGATTAGAACCCCGGTAGTCC
>Otu8420
CCAGCCTATGGGGGGCTCCAGTGGGGAATTTTACACAATGGGGGAAACCCTGATGTAGCG
ACACCGCGTGAGCGAAGAAGCCCTTTGGGGTGTAAAGCTCTGTCAGCAGGGACGAAAAAA
ATGACGGTACCTGCAGAGGAAGCATCGGCTAACTACGTGCCAGCAGCCGCGGTAAGACGT
AGGATGCGAGCGTTGTCCGGATTTATTGGGCGTAAAGAGTTCGTAGGTGGTTTCATAAGT
CTGGTGTTAAAGACCGGGGCTCAACCTCGGGATCGCACTGGATACTGTGAGACTGGAGTT
AGGTAGAGGCAATCGGAATTCCCAGTGTAGCGGTGAAATGCGTAGATATTGGGAAGAACA
CCGGTGGCGTAAGCGGATTGCTGGGCCTAAACTGACACTGAGGAACGAAAGCCGGGGGAG
CAAATGGGATTAGAAACCCTCGTAGTCC
>Otu8427
CCAGCCTATGGGGGGCACCAGTGGGGAATATTGCGCAATGGGCGAAAGCCTGACGCAGCA
ATTCCGCGTGGAGGATGAGGTTTCTTGGAATGTAAACTCCTTTCAGTGGTGACGATAATG
ACGGTAGCCACAGAAGAAGCACCGGCTAACTACGTGCCAGCAGCCGCGGTAAGACGTAGG
GTGCAAGCGTTGTCCGGAATTATTGGGCGTAAAGAGTTCGTAGGCGGTTTACCAAGTCTG
GTGTTAAATACTGAGGCTCAACCTCGGCATGGCATCGGAAACTGGTAGGCTAGAGTATGG
TAGAGGCAAGGGGAATTCCCGGTGTAGCGGTGAAATGCGTAGATATCGGGAGGAACACCA
GTGGCGAAAGCGCCTTGCTGGACCATTACTGACGCTGAGGAACGAAAGCCAGGGTAGCGA
AAGGGATTAGAAACCCGTGTAGTCC
>Otu8430
CCAGCCTACGGGATGCTCCAGTGGGGAATATTGCGCAATGGGCGAAAGCCTGACGCAGCA
ATTCCGCGTGGAGGATGAAGGTTCTTGGATCGTAAACTCCTTTCAGTGGTGACGATTATG
ACGGTAGCCACAGAAGAGGCACCGGCTAACTACGTGCCAGCAGCCGCGGTAAGACGTAGG
GTGCAAGCGTTGTCCGGAATTATTGGGCGTAAAGAGTTCGTAGGCGGTTTGCCAAGTCTG
ATGTTAAAGCCCGAAGCTCAACTTCGGTATGGCATCGGAAACTGGCAGGCTAGAGTGTGG
TAGAGGCAAGGGGAATTCCCGGTGTAGCGGTGAAATGCGTAGATATCGGGAGGAACACCA
GTGGCGAAAGCGCCTTGCTGGGCCATTACTGACGCTGAGGAACGAAAGCCAGGGTAGCGA
AAGGGATTAGATACCCCAGTAGTCC
>Otu8442
CCAGCCTATGGGTGGCAGCAGTCGGGAAAACTTCGCAATGCACGTAAGTGTGACGAGGGA
ATTCTCAGTGCTCAGGTTTAACCTGGGCTTTTGCCAAGAGCAAGAATCTTGGCGAATAAG
TGGTGGGCAAGACTGGTGCCAGCCGCCGCGGTAACCCCAGCGCCACGAGTGGGAATCGCT
TTTATCGGGTCTAAAGCATCCGTAGCCGGTCCGTTAAATGTTCTGTGAAATCGTGGCGCT
CAACGTCACGACGCGCAGAGCACACTGACAGACTTGGGACCAGGAAAGGTATTCGGTATT
CCAGGGGGAGCGGTAAAATGTGATAATCCTTGGAGGACCACCAATGGCGAAGGCAGAATA
CTAGAATGGATCCGACGGTGAGGGATGAAAGCTAGGGGAGCGATCCGGATTAGAAACCCG
TGTAGTCC
>Otu8444
CCAGCCTATGGGGTGCTGCAGTGGGGAATTTTGCGCAATGGGCGAAAGCCTGACGCAGCG
ACGCCGCGTGAGTGATGAAGGCCTTCGGGTTGTAAAGCTCTGTGGGCGGGGACGAATAAG
GGTTGGCTAATATCCAACCTGATGACGGTACCCGTTTAGCAAGCACCGGCTAACTCTGTG
CCAGCAGCCGCGGTAAGACAGAGGGTGCAAACGTTGTCCGGAATTACTGGGCGTAAAGCG

CGTGTAGGCTGCTCCGCAGGTCGGATGTGAAAGCCCTGGGCTCAACCTAGGAAGTGCATT
CGATACTGCAGAGCTTGAGTCCTGGAGAGGAAGGCGGAATTCTCGGTGTAGAGGTGAAAT
TCGTAGATATCGAGAGGAACACCGGTGGCGAAGGCGGCCTTCTGGACAGTGACTGACGCT
GAGACGCGAAAGCGTGGGGAGCAAACAGGATTAGAAACCCTCGTAGTCC
>Otu8445
CCAGCCTACGGGGGGCAGCAGCTAAGAATATTCCACAATGGACGCAAGTCTGATGGAGCG
ACGCCGCGTGGACGATGAAGGCCTTCGGGTTGTAAAGTCCTTTCGTGCGGGAAGATTTTT
CCGCCGGTGAACAATCGGTGGGATCGACTGTACCGCAGGAAGAAGCCCCGGCTAATTACG
TACCGGCAGCCGCGGTAATACGTATGGGGCGAGCGTTATTCGGATTCATTGGGCGTAAAG
GGTATGCAGGGGGCTTGTTAAGTCGGAAGTGAAATTTCCGGGCTCAACCCGGGACTTGCT
TTCGATACTGGCAAGCTAGAGTTTGGCAGAGGAGAATGGAATTCCAGGTGTAGGGGTGAA
ATCTGTAGATATCTGGAAGAACACCAGTGGTGAAGACGATTCTCTGGGCCAAAACTGACC
CTCAGATACGAAAGCCAGGGGAGCAAACGGGATTAGAGACCCTAGTAGTCC
>Otu8448
CCAGCCTACGGGGCGCAGCAGTGGGGAATATTGGACAATGGGCGCAAGCCTGATCCAGCC
ATGCCGCGTGAGTGATGACGGCCCTAGGGTTGTAAAGCTCTTTCACCCACGACGATAATG
ACGGTAGTGGGAGAAGAAGCCCCGGCTAACTTCGTACCAGCAGCCGCGGTAATACGAAGG
GGGCTAGCGTTGCTCGGAATGACTGGGCGTAAAGGGCGCGTAGGCGGATGGCACAGTCAG
ATGTGAAATTCCCGGGCTTAACCTGGGGGCTGCATTTGATACGTGTTGTCTGGAGTGAGG
AAGAGGGTCGTGGAATTCCCAGTGTAGAGGTGAAATTCGTAGATATTGGGAAGAACACCG
GTGGCGAAGGCGGCGACCTGGTCCTTGACTGACGCTGAGGCGCGAAAGCGTGGGGAGCAA
ACAGGATTAGATACCCTTGTAGTCC
>Otu8450
CCAGCCTACGGGTTGCTGCAGTAACGAATATTCCGCAATGCGCGAAAGCGTGACGGAGCA
ATGCCGCGTGCAGGATGAAGCAGCTTCGCTGTGTAAACTGCTGTCAGGGGTTACTAACAC
TGAAGAACTCCAGAGGAAGGACCGGCTAAACTCGTGCCAGCAGCCGCGGTAATACGAGTG
GTCCAAGCGTTAGTCGGAATCACTGGGCTTAAAGGGTGCGTAGGCGGACCAGCAAGTATC
TTGTGAAACCCCACGGCTCAACCGTGGAACTGCAGGGTAAACTGCTGGTCTTGAGGAAGC
TAGAGGTCGACAGAACGATCGGTGGAGCGGTGGAATGCGTAGATATCGATCGGAATGCCA
AAGGTGAAGACAGTCGACTGGGGCTTTCCTGACGCTCAGGCACGAAAGCGTGGGGATCAA
ACAGGATTAGAAACCCCTGTAGTCC
>Otu8451
CCAGCCTATGGGGCGCAGCAGTCGAGAATTTTTCTCAATGGGCGAAAGCCTGAAGGAGCG
ACGCCGCGTGGGGGATGAATGGCTTCGGCCCGTAAACCCCTGTCATTTGCGAACAAATTG
GTTCACCTAACACGTGAACCATTGATAGTAGCGGAAGAGGAAGGGACGGCTAACTCTGTG
CCAGCAGCCGCGGTAATACAGAGGTCCCAAGCGTTGTTCGGATTCACTGGGCGTAAAGGG
TGCGTAGGTGGCGAAGTAAGTCGGATGTGAAATCCCGGAGCTTAACTCCGGAACTGCATT
CGATACTGCGGTGCTTGAGGACTGGAGAGGAGATCGGAATTCACGGTGTAGCAGTGAAAT
GCGTAGATATCGTGAGGAAGACCAGTGGCGAAGGCGGATCTCTGGACAGTTCCTGACACT
GAGGCACGAAGGCCAGGGGAGCAAACGGGATTAGATACCCCTGTAGTCC
>Otu8452
CCAGCCTATGGGGTGCTGCAGCTAAGAATATTCCGCAATGGACGAAAGTCTGACGGAGCG
ACGCCGCGTGGATGATGAAGGCTTTCGGGTTGTAAAATCCTTTCGTGTGGGAAGATTTTC
CAGTTTGTGAATAACAAACAGGATTGACTGTACCGCAGGAAGAAGCCCCGGCTAATTACG
TGCCAGCAGCCGCGGTAATACGTATGGGGCGAGCGTTATTCGGATTTATTGGGCGTAAAG
CGTAAGCAGGTGGTCTGTTAAGTTGAGTGTGAAATTTTCAGGCTTAACCTGAAACTTGCA
TTCAAAACTGGCAGACTCGAGTTTGGAAGAGGATAATGGAATTCCAGGTGTAGGGGTGAA
ATCTGTAGATATCTGGAAGAACACCAGTGGCGAAGGCGATTATCTGGTCCAAAACTGACA
CTCATTTACGAAAGCCAGGGGAGCAAACGGGATTAGATACCCGCGTAGTCC
>Otu8453
CCAGCCTATGGGAGGCTGCAGTGAGGAATATTGGTCAATGGGCGCAAGCCTGAACCAGCC
ATGCCGCGTGAAGGATGAAGGCCTTCTGGGTTGTAAACTTCTTTTATGGGGGAAGAAAAT
TATGGTTTCTACTATAACCGACGGTACCCTAGGAATAAGCACCGGCTAACTCCGTGCCAG
CAGCCGCGGTAATACGGAGGATGCGAGCGTTATCCGGATTTATTGGGTTTAAAGGGAGCG
TAGGCGGTTTTATAAGTCAGTGGTGAAATCTCGTTGCTTAACAACGAACGTGCCATTGAT
ACTGTAGAACTTGAGTACAGATGATGTTGGCGGAATGTGTCATGTAGCGGTGAAATGCTT
AGATATGACACAGAACACCGATTGCGAAGGCAGCTGACAAAACTGTAACTGACGCAGAGG
CTCGAAAGCGTGGGGATCAAACAGGATTAGATACCCGGGTAGTCC

>Otu8456
CCAGCCTACGGGTGGCTCCAGACGAGAATATTCGACAATGGGCGAAAGCCTGATCGAGCG
ACGCCGCGTGGAGGATGAAGTTCTTCGGGACGTAAACTCCTTTTGCCAGGGAAAAAGTTA
TTGATTGTACCTGGAGAATAAGAAGTTGCTAAACTCGTGCCAGCAGCAGCGGTAATACGA
GTGCTTCGAGCGTTATCCGGAATTATTGGGCGTAAAGGGTGTGTAGGCGGCTATATTAGT
CTCGCGTTAAATTCTCCGGCTCAACCGGGGAGCTGCGTGGGAAACGGTATGGCTTGAGGA
CGGAAGAGGTCTCTGGAACTCATGGTGTAGCGGTGAAATGCGTTGATATCATGGGGAACA
CCAAAAGCGAAGGCAAGAGACTGGTCCGCTCCTGTCGCTGAAACACGAAAGCGTGGGTCG
CGAATGGGATTAGATACCCCAGTAGTCC
>Otu8460
CCAGCCTACGGGGCGCACCAGCAGGGAATCGTACGCAATGGACGAAAGTCTGACGTCGCA
ATGCCGCGTGGAGGATGAAGGCCTTCGGGTCGTAAACTCCTTTTACGGTAGCAGAGATGC
TACCGAGAATAAGCACCTGCTAACTCTGTGCCAGCAGCCGCGGTAATACAGAGGGTGCAA
GCGTTATCCGGATTTATTGGGCGTAAAGCGTTTCGTAGGCGATTTGGCAAGTTATTTTTC
AAATACCACGGCTTAACCGGGGGAAGGGAGATAATACTACCAGATTTGATTTTTGGTGGG
GCATCTGGAACTGATGGTGTAGTAGTGAAATACGTTGATATCATCAGGAACTCCAAGGGC
GAAGGCAGGATGCTAACCAATTAATGACGCTGAGGAACGACAGCTAGGGGAGCGAAAGGG
ATTAGATACCCCTGTAGTCC
>Otu8471
CCAGCCTATGGGTGGCACCAGTGAGGAATTTTGCTCAATGGGCGCAAGCCTGAAGCAGCG
ACGCCGCGTGAATGATGAAGTTCTTCGGAATGTAAAGTTCTTTTGGCAGGGAAAATAATG
ATGGTACCTGCAGAATAAGCACCGGCAAACTATGTGCCAGCAGCCGCGGTAATACATAGG
GTGCAAGCGTTGTCCGGATTTACTGGGCGTAAAGAGCGTGTAGGTGGAAATTTAAGTCAA
ACTTGAAATACTAGAGCTTAACTCTAGTCTGGGTTTGATACTGATTTTCTAGAGATTGGG
AGAGGAAAGTGGAACTCACAGTGTAGCGGTGAAATGCGTAGATATTGTGAGGAACACCCG
TGGCGAAGGCGGCTTTCTGGTCCAATTCTGACACTGAGACGCGAAAGCGTGGGGATCAAA
CAGGATTAGAAACCCCAGTAGTCC
>Otu8474
CCAGCCTATGGGGGGCTGCAGTGGGGAATTTTGCGCAATGGACGAAAGTCTGACGCAGCG
ACGCCGCGTGGGTGATGAAGGCCTTCGGGTTGTAAAGCCCTGTGGGGAGGGAAGAATAAG
GTGTGGCTAATAACCGCACTGATGACGGTACCTCCTTAGCAAGCACCGGCTAACTCTGTG
CCAGCAGCCGCGGTAAGACAGAGGGTGCGAACGTTGTTCGGAATTACTGGGCGTAAAGCG
CGTGTAGGCGGCCGCGCAAGTCGGGTGTGAAAGCCCTCGGCTCAACCGAGGAAGTGCACT
CGAAACTGCGCGGCTTGAGTCCCGGAGAGGATAGTGGAATTCTCGGTGTAGAGGTGAAAT
TCGTAGATATCGAGAGGAACACCGGTGGCGAAGGCGGCTATCTGGACGGTGACTGACGCT
GAGACGCGAAAGCATGGGGAGCAAACAGGATTAGAGACCCGAGTAGTCTC
>Otu8478
CCAGCCTACGGGTTGCAGCAGTAGGGAATATTGCGCAATGGAGGAAACTCTGACGCAGCG
ACGCCGCGTGAGTGATGAAGGCCTTCGGGTCGTAAAGCTCTGTCGTATGGGAATAATAAA
TGAATGTACCATGCAAGAAAGGATCGGCTAACTTCGTGCCAGCAGCCGCGGTAAGACGAG
GGATCCTAGCGTTGTTCGGAATTATTGGGCGTAAAGCGGGCGCAGGCTGCTTTGTAAGTC
AGGTGTGAAATCCCAGGGCTCAACCCTGGACGTGCATTTGATACTGCAAAGCTTGAGTGT
AGGAGAGGTTACTGGAATTCCTGGTGTAGTGGTGAAATACGTAGATATCAGGAGGAACTC
CGGAGGCGAAGGCGGGTAACTGGCCTAACACTGACGCTCATGCCCGAAAGCGTGGGGATC
AAACAGGATTAGAGACCCTCGTAGTCC
>Otu8481
CCAGCCTACGGGGGGCAGCAGTAACGAATATTGGGCAATGGGCGAAAGCCTGACCCAGCG
ACGCCGCGTGCAGGAGGAAGTCCTTCGGGATGTAAACTGCTGTCAGGGGTTAGCAAGCAA
AGGGACCTAACAAGTTCCGGCGTTGAGCAGCCCCAAAGGAAGCCTCGACTAACTCTGTGC
CAGCAGTCGCGGTAAGACAGAGGAGGCAAGCGTTGTTCGGAATCACTGGGCTTAAAGCGC
GTGTAGGCGGCTAGGTAAGTGTCGTGTGAAATCCAACGGCTCAACCGTTGAACGGCACGG
CAAACTTCCTGGCTTGAGGTCGGTAGAGGTGCCTGGAACTCTCGGTGGAGCGGTGAAATG
CGTAGATATCGAGAGGAACGCCAGAGGCGAAAGCGGGGCACTGGGCCGATTCTGACGCTG
AGACGCGAAAGCGTGGGGAGCAAACAGGATTAGAAACCCGCGTAGTCC
>Otu8482
CCAGCCTACGGGTGGCACCAGCTAAGAATATTCCGCAATGGGCGCAAGCCTGAACCAGCA
ACGCCGCGTGAGTGATGAAGGCCTTCGGGTTGTAAAACTCTTTTTATGGGGACGAGGAAG
GACGGTACCCATAGAATAAGCTTCGGCTAACTACGTGCCAGCAGCCGCGGTAAAACGTAG

GAAGCGAGCGTTATCCGGATTTACTGGGCGTAAAGCGCATGCAGGCGGTTTGGAAAGTTG
GATGTGAAAGCTCCTGGCTTAACTGGGAGAGGTCGTTCAAAACTACCAGACTAGAGGATG
TGAGAGGAAAGTGGAATTCCGAGTGTAGTGGTGAAATGCGTAGATATTCGGAGGAACACC
AGTGGCGAAAGCGGCTTTCTGGCACATAACTGACGCTCATATGCGAAAGCTAGGGTAGCA
AACGGGATTAGAGACCCGCGTAGTCC
>Otu8484
CCAGCCTATGGGTCGCAGCAGTGGGGAATACTGCTCAATGGGCGAAAGCCTGAAGCAGCG
ACGCCGCGTGAACGAAGAAGTCCTTCGGGATGTAAAGTCCTTTCGACAGGGAAAATAATG
ATGGTACCTGGAGAAGAAGGCCCGGCTAACTATGTGCCAGCAGCCGCGGTAATACATAGG
GGCCAAGCGTTATCCGGATTTATTGGGCGTAAAGCGCGTGTAGGTGGCTGACTAAGTCAG
GCGTTAAATCTAGAAGCTCAACTTCTAATCGCGTCTGAAACTGGTCGGCTTCGAGGTCGG
GAGAGGATAGTGGAACTCACAGTGTAGCGGTGAAATGCGTAGATATTGTGAGGAACACCA
GTGGCGAAGGCGACTATCTGGACCGAATCTGACGCTGAGACGCGAAAGCGTGGGGAGCAA
ACAGGATTAGAGACCCCAGTAGTCC
>Otu8487
CCAGCCTACGGGGGGCTGCAGTGGGGAATCTTGCACAATGGGCGAAAGCCTGATGCAGCA
ACGCCGCGTGAGTGAAGAAGGCCCTTGGGTCGTAAAGCTCTTTAGGCTGGGAAGAGGGGA
AACGTAGCTAATACCTACGATTTTTGACGGTACCAGCAGAATAAGCACTGGCTAATTCCG
TGCCAGCAGCCGCGGTAATACGGAAGGTGCAAGCGTTGTTCGGAGTGACTGGGCGTAAAG
CGCACGTAGGCGGGTTTGTATGTCGATTGTGAAAGCCCCAGGCTTAACCTGGGAATGGCA
GTCGAAACTGCAGATCTTGAATGCCCGAGAGGTTAGTGGAATTCCAGGTGTAGGAGTGAA
ATCCGTAGATATCTGGAGGAACACCAGAGGCGAAGGCGGCTAACTGGTGGTGCATTGACG
CTGAGGTGCGAAAGCGTGGGGAGCAAACAGGATTAGATACCCTAGTAGTCC
>Otu8491
CCAGCCTACGGGATGCTGCAGCAACGAATCTTCCGCAATGGGCGAAAGCCTGACGGAGCA
ATGCCGCGTGTGGGATGAAGTATCTATGGTATGTAAACCACTGTCAGAAAGCAGAAACAC
TGATTGCTTTCAAAGGAAGTGGCGACTAACTCTGTGCCAGCAGTCGCGGTAATACAGAGG
CCACGAGCGTTAGTCGGAATCACTAGGCTTAAAGGGTGCGTAGGCGGATTTCCAAGCGTC
CTGTGAAAGCCTCCCGCTCAACGGGAGAACAGCAGGGCGAACTGGAAGTCTCGAGGTTGG
TAGGGGCTCACAGAACAGTCGGTGGACCGGTGAAATGGGTAGAGATCGACTGGAATGCCG
ATGGCGAAGGCGGTGAGCTGGGCCAATTCTGACGCTGAGGCACGAAAGCGTGGGGAGCAA
ACAGGATTAGATACCCTTGTAGTCC
>Otu8497
CCAGCCTACGGGGCGCTCCAGTGGGGAATTTTGCGCAATGGGCGAAAGCCTGACGCAGCA
ACGCCGCGTGGGTGATGAAGGCCTTCGGGTTGTAAAGCTCTGTCAGGAGGGACGAAGCCA
CTCGGGTTAATAGCCCAGAGGGTGACGGTACCTCCAAAGGAAGCCCCGGCTAACTACGTG
CCAGCAGCCGCGGTAATACGTAGGGGGCAAGCGTTGTCCGGAATTATTGGGCGTAAAGAG
CGTGTAGGCGGCTCGTCAAGTCCGGTGTGAAATCTCGGGGCTCAACCCCGAAACTGCATT
GGAAACTGGCGAGCTAGAGTCCGGAAGGGGAGATTGGAATTCCTGGTGTAGCGGTGAAAT
GCGCAGATATCAGGAAGAACACCCGTAGCGAAGGCGGATCTCTGGGACGGTACTGACGCT
GAGACGCGAAAGCGTGGGGAGCAAACAGGATTAGAAACCCCCGTAGTCC
>Otu8502
CCAGCCTACGGGGCGCAGCAGTCGAGGATCTTCCGCAATGGGCGAAAGCCTGACGGAGCG
ACGCCGCGTGTGGGATGAAGGCCTTCGGGTTGTAAACCACTGTCACTCGTTATGAAAGGA
TCCGGTGTTAACAGCATCGGGAACTGACAAAGGCGAGAGAGGGAGCCACGGCTAACTTCG
TGCCAGCAGCCGCGGTAAGACGAAGGTGGCGAGCGTTGTTCGGAATCACTGGGCATAAAG
CGCACGTAGGCGGCGATGTAAGTCGGATGTGAAATCCCTCGGCCCAACCGAGGAACTGCG
TCCGAAACTGCATTGCTTGAGGATCAAAGGGGTGCTCGGAATTTCCGGTGGAGCGGTGAA
ATGCGTTGATATCGGAAGGAACGTCGGCGGCGAAAGCGGAGCACTGGTTGATTATTGACG
CTGAGGTGCGAAAGCCAGGGGAGCAAACGGGATTAGAAACCCGAGTAGTCC
>Otu8508
CCAGCCTATGGGTGGCTGCAGTGAGGAATCTTCGGCAATGGGCGCAAGCCTGACCGAGCA
ACGCCGCGTGCGGGACGGAGGCCTTCGGGTCGTAAACCGCTTTTCCCAGGGGCGAGAACG
GACGGTACCTGGGGAAGAAGCCTCGGCTAACTACGTGCCAGCAGCCGCGGTAATACGTAG
GAGGCGAGCGTTGTCCGGAGTTACTGGGCGTAAAGGGTGCGCAGGCGGCCTGCCGCGTGG
GGGGTGAAATCGCCCGGCTTAACCGGGCGGGGGCTTTCCAGACGGGCAGGCTTGAGGGGC
GGAGAGGGGCGCGGAATTCCGGGTGGAGCGGTGAAATGCGTAGAGATCCGGAAGAACACC
GACGGCGAAGGCAGCGCCCTGGACGCCAACTGACGCTCGGGCACGAAAGCGCGGGGAGCG

AACAGGATTAGAAACCCGAGTAGTCC
>Otu8517
CCAGCCTATGGGGCGCAGCAGTCGAGAATCTTCCACAATGGACGAAAGTCTGATGGAGCG
ACGCCGCGTGTTGGATGAAGTGCTTAGGTACGTAAACAACTTTTATGAGGGACGAAATTA
TTGACGGTACCTCATGAATAAGGGGCTCCTAACTCTGTGCCAGCAGGAGCGGTAATACAG
AGGCCCCAAGCATTACCCGGAATCACTGGGCGTAAAGGGTGTCGAGGCGGTCGTATTAGT
CGTTCGTAAAATCCGCGCGCTTAACGTGCGGTCCGCGAACGAAACGGTACGACTCGAGGG
TGGGAGAGGTGCGTGGAACTCACGGTGTAGGGGTGAAATCCGTTGATATCGTGGGGAACA
CCAAAGGCGAAGGCAGCGCACTGGCCATTCCTGACGCTCACACACGAAAGCCAGGGTAGC
GAACGGGATTAGAAACCCGCGTAGTCC
>Otu8518
CCAGCCTACGGGGGGCTCCAGTCGAGAATCATTCGCAATGGGCGCAAGCCTGACGATGCG
ACGCCGTGTGTATGATGGAGGCCTTCGGGTCGTAAAGTACTTTCGCCCGGGAACAAGAGA
TGTTGGCTAATATCCAGCAAATTTGAGTGTACCGGGTAAAGAAGCGCCGGCTAACTACGT
GCCAGCAGCTGCGGTAATACGTAGGGCGCAAGCGTTAATCGGAATTATTGGGCGTATAGG
GCGCGTAGGCGGACCAATTAGTCGGATGTGAAATCCCAGGGCTCAACCCTGGAACAGCAT
CCGAAACTGTTGTGTCTAGAGGGTAGAAGGCGGAAACGGAATTCCACAAGTAGCGGTGAA
ATGCGTAGATATGTGGAAGAACACCTGTGGCGAAGGCGGTTTCCGATTTTACTCCTGACG
CTGAGGCGCGAAAGCAAGGGGATCAAACAGGATTAGATACCCGGGTAGTCC
>Otu8519
CCAGCCTACGGGTCGCTCCAGTGGGGAATTTTGCGCAATGGGGGAAACCCTGACGCAGCA
ACGCCGCGTGGAGGATGAAATATCTTGGTATGTAAACTCCTTTCGATGGGGAAGATTATG
ACGGTACCCATAGAAGAAGCCCCGGCTAACTTCGTGCCAGCAGCCGCGGTAATACGAGGG
GGGCAAGCGTTGTTCGGATTTATTAGGCGTAAAGGGTGCGTAGGCGGTTTGACAAGTCTT
GTGTGAAATCTATGGGCTCAACCCATAGTCTGCACAGGAAACTGTCGGGCTTGAGTATGG
GAGAGGTGAGTGGAATTTCCGGTGTAGCGGTGAAACGCGTAGATATCGGAAGGAACACCA
GTGGCGAAAGCGACTTCCTGGTCCAATACTGACGTTCATGTGCGAAAGCGTGGGGAGCAA
ACAGGATTAGAAACCCTCGTAGTCC
>Otu8532
CCAGCCTATGGGGGGCAGCAGTCGAGAATTTTTCTCAATGGGGGAAACCCTGATGCAGCG
ACGCCGCGTGAGCGATGAAGCCCTTCGGGGTGTAAAGCTCTTTCGGCAGGGACGATTATG
ACGGTACCTGCAGAAGCAGCTGCGGCTAACTACGTGCCAGCAGCCGCGGTAATACGTAGG
CAGCAAGCGTTGTTCGGAGTTACTGGGCGTAAAGGGTGCGTAGGCGGTCCTATAAGTATG
GTGTGAAATCTCCCGGCTTAACTGGGAGGGTGCGCTATAGACTGTGGGACTAGAGTGTGG
GAGAGGAAAGTGGAATTCCTGGTGTAGCGGTGAAATGCGTAGATATCAAGTGGAACACCA
GTGGCGAAGGCGAGTCTCTGGACAATTCCTGACGCTGAGGCACGAAAGCCAGGGGAGCAA
ACGGGATTAGAAACCCCAGTAGTCC
>Otu8533
CAGCCTACGGTGGCAGCAGTAAGGAATATTGGTCAATGGGCGGAAGCCTGAACCAGCCAT
GCCGCGTGCAGGAAGACGGCCCTACGGGTTGTAAACTGCTTTTGCAGGGGAATAAACCCC
CGGTCGTGATCGGGGCTGAATGTACTCTGAGAATAAGGATCGGCTAACTCCGTGCCAGCA
GCCGCGGTAATACGGAGGATCCGAGCGTTATCCGGATTTATTGGGTTTAAAGGGTGCGTA
GGCGGCCCTGTAAGTCAGGGGTGAAAGACGGTAGCTTAACTATCGCAGTGCCTTTGATAC
TGCAGGGCTTGAGTACAGACGAGGTAGGCGGAATTGACGGTGTAGCGGTGAAATGCTTAG
ATATCGTCAAGAACACCGATAGCGAAGGCAGCTTACTAGGCTGTAACTGACGCTGAGGCA
CGAAAGTGTGGGGATCAAACAGGATTAGATACCCCCGTAGTCC
>Otu8535
CAGCCTACGGGGCGCTCCAGTGGGGAATCTTGCACAATGGGCGAAAGCCTGATGCAGCAA
CGCCGCGTGGGGGATGAAGCTTTTCGGAGTGTAAACCCCTTTCGACCCGGAAGAAAGCCC
GCAAGGGTTTGACGGTACGGGTATAAGAAGCCCCGGCTAACTACGTGCCAGCAGCCGCGG
TAATACGTAGGGGGCCAGCGTTGCTCGGAATTACTGGGCGTAAAGGGTCTGTAGGCGGTG
TAGCAAGTCGAGAGTGAAATCCCTGGGCTTAACCCAGGGGCTGCTAACGAAACTGCTATG
CTAGAGTGTGAGAGAGGCAAGTGGAATTACGGGTGTAGCGGTGAAATGCGTAGATATCGG
AAGGAACACCTGTGGCGAAAGCGGCTCACTGGACAATAACTGACGCTGATGCACGAAAGC
TAGGGGAGCAAACAGGATTAGAGACCCGCGTAGTCC
>Otu8539
CCAGCCTATGGGTGGCTGCAGTGAGGAATTTTCCGCAATGGGCGAAAGCCTGACGGAGCG
ACGCCGCGTGCAGGAGGAAGGCCCTCGGGTCGTAAACTGCTTTTATTGGTGACGATTTTG

ACGGTAGCCAATGAATAAGGGCCTGCTAACTACGTGCCAGCAGCCGCGGTCATACGTAGG
ACCCAAGCGTTATCCGGAATTACTGGGCGTAAAGAGTTGCGTAGGTGGCATAGTAAGCAA
AAAGTGAAATGACTCGGCTCAACCGAGTATACATTTTTTGAACTGCTAAGCTTGAGTATG
AGAGAGGTAGATGGAATTTCTAGTGTAGGAGTGAAATCCGTAGATATTAGAAGGAACACC
GATGGCGTAAGCAGTCTACTAGCTCATTACTGACACTAAGGCACGAAAGCGTGGGGAGCG
AACGGGATTAGATACCCCTGTAGTCC
>Otu8550
CCAGCCTACGGGAGGCACCAGTGGGGAATATTGCGCAATGGGCGGAAGCCTGACGCAGCG
ACGCCGCGTGGGTGATGAAGGTTTTCGGATCGTAAAGCCCTGTTGGAGAGGGAAGAACAA
CTTGGAGGAGTAATCCCCTTCGAGCTTGACGGTACCTCTTCAGAAAGCACCGGCTAACTC
CGTGCCAGCAGCCGCGGTAATACGGAGGGTGCTAACGTTGTTCGGAATTACTGGGCGTAA
AGTGCGTGTAGGCGGTTTGGTGTGTCAGATGTGAAATCCCTTGGCTCAACTGAGGACGTG
CATTTGAAACTGCCAGACTGGAGTACGGGAGAGGGTAGTAGAATTCCCGGTGTAGAGGTG
AAATTCGTAGATATCGGGAGGAATACCCGTGGCGAAGGCGGCTACCTGGACCGATACTGA
CGCTGATACGCGAAAGCGTGGGGAGCAAACAGGATTAGATACCCCTGTAGTCC
>Otu8551
CCAGCCTACGGGATGCTCCAGGGACGAATATTCCGCAATGGGCGAAAGCCTGACGGAGCG
ACGCCGCGTGTGGGAGGAAGTCCTTCGGGATGTAAACCACTGTCAGGGGCTACCAAGTGA
ACCGCCATAGCATGGCGGGGAATTGAGGCGCCCCAGAGGAAGCCACGGCTAACTTCGTGC
CAGCAGCCGCGGTAAGACGAAGGTGGCTAGCGTTGTTCGGAATCACTGGGCTTAAAGAGC
ACGCAGGCGGCCTGTTAAGCACCTTGTGAAATCCCTCGGCTCAACCGGGGAATTGCTTGG
TGTACTGGCAGGCTTGAGGCAAGTAAGGGTGCGTGGAACTCTTGGTGGAGCGGTGAAATG
CGTAGATATCAAGAGGAACGCCGGAGGCGAAAGCGATGCACTGGGCTTGTCCTGACGCTG
AGGTGCGAAAGCCAGGGGAGCAAACGGGATTAGAAACCCTCGTAGTTCC
>Otu8552
CCAGCCTATGGGATGCTCCAGTGGGGAATTTTGCGCAATGGGGGAAACCCTGACGCAGCA
ACGCCGCGTGGAGGATGAAGCCCTTTGGGGCGTAAACTCCTTTCGACTGGGAAGATAATG
ACGGTACCAGTGGAAGAAGCACCGGCTAACTCCGTGCCAGCAGCCGCGGTAATACGGAGG
GTGCGAGCGTTGTTCGGAATTATTGGGCGTAAAGCGCGTGTAGGCGGTTTTTTAAGTCTG
ATGTGAAAGCCCTGGGCTCAACCCGGGAAGTGCATTGGAAACTGGGAGACTTGAATGCGG
GAGAGGGTAGTGGAATTCCTGGTGTAGGAGTGAAATCCGTAGATATCAGGAGGAACACCG
GTGGCGAAGGCGGCTACCTGGACCGATATTGACGCTGAGACGCGAAAGCGTGGGGAGCAA
ACAGGATTAGATACCCTAGTAGTCC
>Otu8557
CCAGCCTACGGGAGGCAGCAGTGGGGAATCTTGCGCAATGGACGAAAGTCCGACGCAGCC
ACGCCGCGTGAGTGAAGAAGGCCTTCGGGTTGTAAAGCTCTGTCGGAGGGGACAAAAACC
GAGTCAGTTAATAGCTGATTCGCTTGATGGTACCCTCAAAGGAAGCACCGGCTAACTTCG
TGCCAGCAGCCGCGGTAATACGAAGGGTGCAAGCGTTGCTCGGAATTATTGGGCGTAAAG
GGTAGGTAGGTGGTTACGTATGTCTGGGGTGAAATCCCTGAGCTCAACTCAGGACGTGCC
TTGGAAACGGCGTAACTAGAGTACTAGAGAGGTTCGTGGAATTCTTGGTGTAGCGGTGAA
ATGCGTAGAGATCAAGAGGAACATCAGCGGCGAAGGCGGCGAACTGGATAGTAACTGACA
CTGAAGTGCGAAAGTGTGGGGATCAAACAGGATTAGATACCCCTGTAGTCC
>Otu8564
CCAGCCTACGGGTGGCAGCAGTAAGGAATATTGCACAATGGGCGCAAGCCTGATGCAGCA
ACGCCGCGTGGAGGAAGAAGGCCTTCGGGTCGTAAACTCGTTTTGTGTCTGACGAGATAG
GACGGTAGGACACGAATAAGTCACGGCTAACTACGTGCCAGCAGCCGCGGTAAAACGTAG
GTGGCAAGCGTTATCCGGATTTACTGGGCGTAAAGGGTGTGCAGGCGGGCCACCAAGTGG
TGTATCAAAGTGCGGCGCTTAACGTCGCAAGGTTATGCCAGACTGGTGGTCTAGAGTGCG
AGAGAGGGGCGTGGAATTCCGGGTGTAGTGGTGAAATGCGTAGAGATCCGGAGGAACCCC
AGAGGCGAAGGCGGCGCCCTGGCTCGCAACTGACGCTCAGCCACGACGGCATGGGGAGCG
AACGGGATTAGAAACCCGAGTAGTCC
>Otu8565
CCAGCCTATGGGGTGCTCCAGTAACGAATCTTCCGCAATGGGCGCAAGCCTGACGGAGCA
ATGCCGCGTGCCAGAAGAAGCTCCTCGGAGTGTAAATGGCTTTCAGGGTTAGGTAATCTC
GAACCGACTCAGAAGAAGGGGCGACTAACTTCGTGCCAGCAGTCGCGGTAAGACGAAGGC
CCCGAGCGTTACTCGGATTCACTGGGCTTAAAGGGTGCGTAGGCGGATCGGAATGTGCTT
GGTGAAATCCCACGGCTCAACCGTGGAACTGCCAGGTAGACAACCGGTCTGGAGATGGCT
AGGGGCAACTGGAACTCTAGGTGGAGCGGTGAAATGCGTAGATATCTAGGGGAACGCCGA

TGGTGAAAACAGGTTGCTGGGGCCATTCTGACGCTGAGGCACGAAAGCGTGGGGAGCAAA
CGGGATTAGAAACCCCCGTAGTCC
>Otu8566
CAGCCTATGGGGGCACCAGGCGCGAAACCTTTGCAATGCACGTAAGTGTGACAAGGGAA
CCCAGAGTGCTCATACACAGTATGGGCTTTTGCCAAGAGCAAATATCTTGGAGAATAAGT
GGTGGGAAAGACTGGTGCCAGCCGCCGCGGTAACCCCAGCGCCACAAGTGGGGATCGCGT
TTATTGGGCCTAAAGCATCCGTAGCCGGTTTTGTAAATCTTTTGTGAAATTGTTGGGCTC
AACCTAACAGCGTGCAGGAGACACTGCAAAACTAGGGACCGGGAGGGGTTAGGGGTATGC
AATGGGGAGCGGTAAAATGCTATAATCCATTGTAGACCACCTGTGGCGAAGGCGCCTAAC
CAGAACGGGTCCGACGGTGAGGGATGAAAGCCAGGGGAGCAAACCGGATTAGATACCCGA
GTAGTCC
>Otu8567
CAGCCTATGGGGGCTGCAGTAGGGAATATTGGGCAATGGTCGGAAGACTGACCCAGCCAT
GCCGCGTGCGGGAAGAAGGCGCTATGCGTCGTAAACCGCTTTTATCGGGGAAGAAGAGAG
GGATGCGTCCCGAATTGACGGTACCCGAGGAATAAGCATCGGCTAACTCCGTGCCAGCAG
CCGCGGTAATACGGAGGATGCGAGCATTGTCCGGATTTATTGGGTTTAAAGGGTGCGTAG
GCGGCCCGTTAAGTCATCGGTTAAATGCCCCGGCTCAACCGGGGGAATGCCGGAGATACT
GACGGGCTTGAGTGTCGTATAAGCGGGCGGAATCGGCGGAGTAGCGGTGAAATGCATAGA
TACCGCCGGGAACACCGATAGCGAAGGCAGCTCGCGGGTCGACCACTGACGCTGAGGCAC
GAAAGCGTGGGGATCAAACAGGATTAGAGACCCCAGTAGTCC
>Otu8569
CCAGCCTATGGGGGGCAGCAGTAAGGGATATTGCGCAATGGGCGAAAGCCTGACGCAGCG
ACGCCGCGTGCGGGAGGAAGGCCTTCGGGTTGTAAACCGCTTTCAGGGGGGACGAAGCCA
CTCGGGTTAATAGCCCAGAGGGTGACGGTACCTTCAGAAGAAGCCCCGGCTAACTACGTG
CCAGCAGCCGCGGTAATACGTAGGGGGCAAGCGTTGTCCGGATTCATTGGGCGTAAAGAG
CGCGTAGGCGGTCAGGCAGGTCCGTCGTGAAAACTCGAGGCTCAACCTCGAGGTGTCGGT
GGAAACCTTCTGGCTAGAGTCCGGAAGAGGAGAGTGGAATTCCTGGTGTAGCGGTGGAAT
GCGCAGATATCAGGAAGAACACCCGTGGCGAAGGCGGCTCTCTGGGACGGTACTGACGCT
GAGGCGCGAAAGCGTGGGGAGCAAACAGGATTAGATACCCCCGTAGTCC
>Otu8573
CCAGCCTATGGGTTGCAGCAGTGGGGAATCTTGCGCAATGGACGAAAGTCTGACGCAGCC
ACGCCGCGTGAGTGAAGAAGGCCTTCGGGTTGTAAAGCTCTGTCGCCCGGGACGAAAATG
CTTGAGGTTAACAGCCTCAGGTACTGACGGTACCGGGAGAGGAAGCACCGGCTAACTTCG
TGCCAGCAGCCGCGGTAATACGAGGGGTGCAAGCGTTGCTCGGAATTATTGGGCGTAAAG
GGTAGGTAGGTGGTCTTATTTGTCTGGGGTGAAAGCCTTGAGCTCAACTCAAGAAGTGCC
CCAGAAACGGTAAGACTAGAGTCCTGGAGAGGGTCGTGGAATTCCCGGTGTAGCGGTGAA
ATGCGTAGAGATCGGGAGGAACACCAGAGGCGAAGGCGGCGACCTGGACAGGCACTGACA
CTCAACTACGAAAGCGTGGGGAGCAAACAGGATTAGAAACCCTCGTAGTCC
>Otu8576
CCAGCCTACGGGTCGCAGCAGTAACGAATCTTCCGCAATGCACGAAAGTGTGACGGAGCG
ACGCCGCGTGTGGGATGAAGTTCTTCGGAATGTAAACCACTGTCAGGGGAAAGAAAGTAC
TGATCTACCCCAGAGGAAGGGACGGCTAACTCTGTGCCAGCAGCCGCGGTAAGACAGAGG
TCCCGAGCGTTAGGCGGAATCACTGGGCTTAAAGCGTGTGTAGGCGGATCGTTAAGTGCC
TTGTGAAATCCCACGGCTTAACCGTGGAATGGCTGGGCAGACTGGCGATCTTGAGCCATC
TAGGGGCGACCGGAACAAACGGTGGAGCGGTGAAATGCGTAGATATCGTTTGGAACGCCA
ACGGTGAAAACAGGTCGCTGGGGATGTGCTGACGCTGAGACACGAAAGCCAGGGGAGCAA
ACGGGATTAGAAACCCTTGTAGTCC
>Otu8583
CCAGCCTATGGGTGGCAGCAGTGAGGAATATTGGTCAATGGGCGCAAGCCTGAACCAGCC
ATGCCACGTGCAGGATGACGGCCTTTTAGGTTGTAAACTGCTTTTGTATGGGGTGAATCT
CTCGAACGTGTTCGAGATTGACGGTACCGTACGAATAAGGATCGGCTAACTCCGTGCCAG
CAGCCGCGGTAATACGGAGGATCCAAGCGTTATCCGGATTCATTGGGTTTAAAGGGTGAG
TAGGCGGGATAATAAGTCAGTGGTGAAATCTTGCAGCTTAACTGTAAAATTGCCATTGAT
ACTGTTATTCTTGAATACAGTTGAGGTAGGCGGAATGTGTAATGTAGCGGTGAAATGCGT
AGATATTACACAGAACACCGATTGCGAAGGCAGCTTACTAAACTGTTATTGACGCTGAGG
CACGAAAGCGTGGGGATCAAACAGGATTAGATACCCGTGTAGTCC
>Otu8584
CCAGCCTACGGGACGCTGCAGTGAGGAATTTTCGTCAATGGGGGCAACCCTGAACGAGCA

ACGCCGCGTGCAGGATGACGGTTTTCGGATCGTAAACTGCTTTTCTCAGGGACGAGCAAG
GACGGTACCTGAGGAATCAGCCCCGGCTAACTACGTGCCAGCAGCCGCGGTAATACGTAG
GGGGCAAGCGTTGTCCGGATTTATTGGGCGTAAAGCGCACGCAGGCGGTCTTTTAAGTTC
GGAGTGACAGTCGTTGGCTTAACCGACGAAGGCCTTGGGAAACTGGAAGACTTGAGGGCT
CAAGAGGGACACGGAATTCCGGGTGTAGTGGTGAAATGCGTAGATATCCGGAGGAACACC
AATGGCGTAGGCAGTGTCCTGGGGAGTACCTGACGCTCAGGTGCGAAAGCCAGGGGAGCG
AACGGGATTAGATACCCGGGTAGTCC
>Otu8586
CCAGCCTATGGGTGGCAGCAGCAAGGAATTTTCGGCAATGGAGGAAACTCTGACCGAGCA
ACGCCGCGTGGGCGAAGAAGTCTTTCGGGATGTAAAGCCCTTTTGTGTGAGAAGAAGGAA
AGACGGTATCACACGAATAAGCATCGGCTAACTACGTGCCAGCAGCCGCGGTAATACGTA
GGATGCAAGCGTTGTCCGGAATTATTGGGCGTAAAGGGCGTGTAGGCGGTAAACCAAGTC
AGATGTGAAATATCCCGGCTTAACTGGGAGGGGCCATTTGAAACTGGTTAACTAGAGTGC
AGGAGAGGTCAGTAGAATTCCCGGTGTAGTGGTGAAATGCGTAGATATCGGGAGGAATAC
CAGTGGCGAAGGCGGCTGACTGGCCTGTAACTGACGCTGAAACGCGAAGGCTAGGGGAGC
AAACAGGATTAGAAACCCCCGTAGTCC
>Otu8587
CCAGCCTATGGGTCGCACCAGTAAGGAATATTGGGCAATGGGCGCAAGCCTGACCCAGCC
ATGCCGCGTGAAGGATGAAGGCCCTATGGGTTGTAAACTTCTTTTGTACGGGAAAAAACC
TCCGGTCGTGAACCGGAGCTGATGGTACCGTAAGAATAAGCATCGGCTAACTCCGTGCCA
GCAGCCGCGGTAATACGGAGGATGCAAGCGTTATCCGGATTCATTGGGTTTAAAGGGTGC
GCAGGCGGAGTGATAAGTCAGTGGTGAAATCCTACGGCTCAACTGTAGAACTGCCATTGA
TACTGTCATTCTTGAGTACTGTTGAGGTGGGCGGAATGTGTCATGTAGCGGTGAAATGCT
TAGATATGACACAGAACACCGATCGCGAAGGCAGCTCACCAAGCAGAAACTGACGCTCAT
GCACGAAAGCGTGGGGATCAAACAGGATTAGAAACCCCCGTAGTCG
>Otu8588
CCAGCCTATGGGTTGCTGCAGTCGAGAATTTTTCACAATGGGCGAAAGCCTGATGGAGCG
ACGCCGCGTGGGGGATGAATGGCTTCGGCTCGTAAACCCCTGTCATTTGCGAACAAACCC
TTGTATTGAACAGATGCAAGGCTGATAGTAGTGAAAGAGGAAGGGACGGCCAACTCTGTG
CCAGCAGCCGCGGTAATACAGAGGTCCCAAGCGTTGTTCGGATTCACTGGGCGTAAAGGG
TGCGTAGGTGGCGGGATAAGTTTGATGTGAAATCTCCGGGCTTAACCTGGAAAATGCATT
AAAAACTGTTCTGCTAGAGGGTTGGAGGGGGGACTGGAATTCTCGGTGTAGCAGTGAAAT
GCGTAGATATCGAGAGGAACACCAGTGGCGAAAGCGAGTCCCTGGACAGCTCCTGACACT
GAGGCACGAAAGCTAGGGGAGCAAACAGGATTAGATACCCTAGTAGTCC
>Otu8592
CCAGCCTATGGGACGCACCAGTCGGGAAACTTTTGCAATACACGAAAGTGTGACAAAGCA
AGCCAAAGTGCTCTTCTTTTTAGAAGAGCTTTTGTGAGATGTAAAAAGTCTTACGAATAA
GGACTGGGCAAGACTGGTGCCAGCCGCCGCGGTAATCCCAGCAGTCCAAGTCGCAGCCAC
ATTTGTTGGGTCTAAAATATCCGTAGCTTGCTTTTCAAGTCTCTTGTGAAATCGGGACTC
TTAAGGTTCCGGCGGGCAAGAGATACTGTTTAGCTAGAGACCGGGAGGCGCAAGAAGTAC
GATTAGGGTAGCGGTAAAATGCGTTGATCCTAATTGGACTAACAGTCGCGAAGGCGTCTT
GCGAGAACGGTTCTGACAGTGAGGGATGAAGGCTAGGGGCGCAAAGTGGATTAGAGACCC
CCGTAGTCC
>Otu8593
CCAGCCTACGGGTGGCACCAGTGGGGAATTTTGCGCAATGGGGGAAACCCTGACGCAGCA
ACGCCGCGTGGAGGATGAAGTTTTTCGGAATGTAAACTCCTACCGCGAGGGAAGAAACGC
CGATTGGTCGGCCTGACGGTACCTCGTGAGAAAGCACCGGCTAACTACGTGCCAGCAGCC
GCGGTAATACGTAGGGTGCAAGTGTTGTTCGGATTTACTGGGTGTAAAGGGAAGTGTAGG
CGGATATATAAGTCAAAGGTGAAAACCCCGCGCTTAACGCGGGATCTGCCTTTGATACTG
TGTATCTTGAGTGTTGGAGAGGGCAACAGAATTCCAGGTGTAGCGGTGGAATGCGTAGAT
ATCTGGAAGAATACCAGTTGCGAAGGCGGTTACCTGGCCACTCACTGACGCTGAGGCTCG
AAAGCTGGGGGAGCAAACAGGATTAGAGACCCCGGTAGTCC
>Otu8595
GCCAGCCTACGGGTGGCTCCAGTCGAGAATCTTCCACAATGGACGAAAGTCTGATGGAGC
GACGCCGCGTGGTTGACGAAGTCCTTCGGGACGTAAAAACCTTTTATGGGGGAGGAAGTA
ATTGACGTTACCCCATGAATAAGAGGCTCCTAACTCTGTGCCAGCAGGAGCGGTAATACA
GAGGCCTCAAGCGTTACCCGGATTCACTGGGCGTAAAGGGTGTGTAGGTGGCGATGTTAG
TCTCTCGTGAAAGCTTTTGGGCTTAACCCAGAAAACGCGGGGGAAACGGCATTGCTTCGA

GGGTGTGAGAGGTGCACGGAACTCATGGTGTAGGGGTGAAATCCGTTGATATCATGGGGA
ACACCAAATGCGAAGGCAGTGCACTGGCACATTTCTGACACTGAAACACGAAAGCGTGGG
TAGCGAGCGGGATTAGATACCCCAGTAGTCC
>Otu8598
CCAGCCTACGGGGTGCACCAGTAAGGAATATTGGACAATGGTGGCAACACTGATCCAGCC
ATGCCGCGTGCAGGATGAAGGCGCTACGCGTTGTAAACTGCTTTTGTACGGGAGAAAACC
TATCTACGTGTAGATAGCTGATAGTACCGTAAGAATAAGCATCGGCTAACTACGTGCCAG
CAGCCGCGGTAATACGTAGGATGCTAGCGTTGTCCGGATTTACTGGGTGTAAAGGGAGCG
CAGGCGGGTTGGTAAGTCAGTGGTGAAATTCTCAGGCTTAACCTGAGGACTGCCACCGAT
ACTGCCAATCTTGAGTGCAGAAGAGGTTGATGGAATTCTAGGTGTAGCGGTGAAATGCGT
AGATATCTAGAAGAACACCGATGGCGAAGGCAGTCAACTGGTCTGCTACTGACGCTCATG
CTCGAAAGCGTGGGGATCAAACAGGATTAGAAACCCCATAGTCC
>Otu8603
CCAGCCTATGGGTTGCACCAGTAACGAATATTCCGCAATGGGCGAAAGCCTGACGGAGCG
ACGCCGCGTGTAGGAATAAGCTCTTCGGAGTGTAAACTACTGTCAGGGGTTACCAAGCAA
AGAGGGCTAATACCCTTCTGAATTGAGGAGCCCCAGAGGAAGCCACGGCTAACTTCGTGC
CAGCAGCCGCGGTAAGACGAAGGTGGCAAGCGTTGTTCGGAATCACTGGGCTTAAAGAGC
ACGTAGGCGGCTCATCAAGTGTCTTGTGAAATCCCCCGGCTCAACCGGGGAATCGCTGGA
TAGACTGACGAGCTTGAGGCAGGTAGGGGCGAGTGGAACTCTTGGTGGAGCGGTGGAATG
CGTAGATATCAAGAGGAACGCCGGAGGTGAAGACGACTCGCTGGGCCTGTCCTGACGCTG
AGGTGCGAAAGCCAGGGGAGCAAACGGGATTAGAAACCCTGGCAGTCC
>Otu8604
CCAGCCTACGGGGCGCAGCAGGGAATATTGGTAATCTGCGAAAGCGGGAACCAGCAACGC
CGCGTGTGCGATGAAGGCCTTCGGGTCGTAAAGCACTTTTTGAGGGGACGAGGAAGGACG
GTACCCTCAGAATAAGTCTCGGCTAACTACGTGCCAGCAGCCGCGGTAACACGTAGGAGA
CGAGCGTTATCCGGATTTACTGGGCGTAAAGGGTGCGTAGGCGGTTTGGTAAGTTGGATG
TGAAAGCTCCTGGCTTAACTGGGAGAGGTCGTTCAATACTACCGGACTTGAGAGCAGTAG
AGGAAAGTGGAATTCCCGGTGTAGTGGTGAAATGCGTAGATATCGGGAGGAACACCAGTG
GCGAAAGCGGCTTTCTGGACTGTTTCTGACACTCATATGCGAAAGCTAGGGTAGCAAACG
GGATTAGATACCCCAGTAGTCC
>Otu8613
CCAGCCTACGGGTCGCACCAGTGGGGAATATTGGACAATGGGCGCAAGCCTGATCCAGCC
ATGCCGCGTGAGTGATGACGGCCTTAGGGTTGTAAAGCTCTTTCGCCAGGGACGATAATG
ACGGTACCTGGATAAGAAGCCCCGGCTAACTTCGTGCCAGCAGCCGCGGTAATACGAAGG
GGGCTAGCGTTGTTCGGAATTACTGGGCGTAAAGCGCGCGCAGGCGGCCAATCAAGTCAG
GGGTGAAAGCCCAGAGCTCAACTCTGGAACTGCCTTTGAAACTGTTTGGCTTGAGTACGA
GAGAGGTGAGTGGAATTCCCAGTGTAGAGGTGAAATTCGTAGATATTGGGAAGAACACCG
GTGGCGAAGGCGGCTCACTGGCTCGTTACTGACGCTCAGGCGCGACAGCGTGGGGATCAA
ACAGGATTAGATACCCCTGTAGTCC
>Otu8614
CCAGCCTATGGGACGCTCCAGTCGAGAATTTTTCTCAATGGGGGAAACCCTGAAGGAGCG
ACGCCGCGTGGGGGATGAAGGACTTCGGTTCGTAAACCCCTGTCACTTGGGAACAAACGT
CCCAACCTAACACGTTGGGATTTGATAGTACCAGGAGAGGAAGGCACGGCTAACTCTGTG
CCAGCAGCCGCGGTAATACAGAGGTGCCAAGCGTTGTTCGGATTTACTGGGCGTAAAGGG
TGCGTAGGCGGTCATGTAAGTCGGGTGTGAAATCCCGGAGCCTAACTCCGGAATGGCATT
CGAAACTGCATGGCTCGAGATCTGGAGAGGTGATTGGAATTCTCGGTGTAGCAGTGAAAT
GCGTAGATATCGAGAGGAACACCAGAGGCGAAGGCGAATCACTGGACAGTATCTGACGCT
GAGGCACGAAAGCCAGGGGAGCAAACGGGATTAGAGACCCTTGTAGTCC
>Otu8619
CCAGCCTATGGAAGGCAGCAGTGGGGAATATTGCGCAATGGCCGAAAGGCTGACGCAGCG
ACGCCGCGTGGGGGATGAAGCATTTCGGTGTGTAAACCCCTGTTGCTCGGGACGAACAAG
CGGCTTTAGCCGCTCTGACGGTACCGGGTGAGGAAGCACCGGCTAACTCCGTGCCAGCAG
CCGCGGTAATACGGAGGGTGCGAGCGTTGTCCGGAATCACTGGGCGTAAAGGGCGCGTAG
GCGGTCTGCTAAGCGTGTGGTGAAAGCTCGGGGCTCAACCCCGAGTCGGCCATGCGAACT
GGTGGACTAGAGCACTGTAGAGGCAGGTGGAATTCCGGGTGTAGCGGTGGAATGCGTAGA
GATCCGGAAGAACACCGGTGGCGAAGGCGGCCTGCTGGGCAGTAGCTGACGCTGAGGCGC
GACAGCGTGGGGAGCAAACAGGATTAGAAACCCCAGTAGTCC
>Otu8627

CCAGCCTACGGGTCGCTGCAGTCGAGGATCTTCGGCAATGGGCGCAAGCCTGACCGAGCG
ACGCCGCGTGTGCGATGAAGGCCTTCGGGTTGTAAAGCACTGTCGAGGGGAAGAAAAGCC
CGCAAGGGTGTGATCGACCCCTGGAGGAAGCACGGGCTAAGTTCGTGCCAGCAGCCGCGG
TAAGACGAACCGTGCGAACGTTGTCCGGAATCACTGGGCTTAAAGGGCGCGTAGGCGGGC
GAGCGAGTCAGGGGTGAAAGCCTCCAGCTTAACTGGAGAAGTGCCTTTGATACTGCGAGT
CTCGAGGGAGGTAGGGGCGTGCGGAACTTCCGGTGGAGCGGTGAAATGCGTAGATATCGG
AAGGAACATCGGTGGCGAAAGCGGCGCGCTGGACCTCTACTGACGCTGAGGCGCGAAAGC
CAGGGGAGCAAACGGGATTAGAAACCCTAGTAGTCC
>Otu8639
CCAGCCTATGGGTTGCAGCAGTGGAGAATATTAGTCAATGAATGAAAATTTGAACTAGCG
TTTTGCGGAAATGAAAGATTCATAGAATCGTTAAGACTAAGATAGTTGTAAAGTTTGTGC
GCAAGTTAAAAAACGATATTATACTTGAAGAGATCCCCGGGCCAACAACGTGCCAGCAGC
TGCGGTAAGACGTTGACGGGGAAGCGTTACTAATCGTTACTAGGCGTAAAGAATGACTAG
ATAGTTTAAGAATTTATAAATAAAATCATAGAATAGACGAAGATTTTTATAAAACCTTAG
TACTTGAAAATAAAAGAGTTTGGTGATAAGCACAATCTAGAGATAAAATTCTAATAGATT
GTAAGGATCTTCACTAGCCGCGGCAACCTCTCATTTTAATTTAACGTCGAATCATTGAAG
TGCGAGTATCAATCAGGATTAGATACCCGGGTAGTCC
>Otu8642
CCAGCCTACGGGATGCTCCAGTCGAGAATCTTTCGCAATGGGCGCTAAGCCTGACGAAGC
GACGCCGTGTGTGTGATGAAGGCCCTCGGGTTGTAAAGCACTTTCGCCTAAGAACAAGAA
AACGCCGCTAATATCGGCGCGCTTTGAGAGTACTAGGTAAAGAAGCACCGGCTAACTACG
TGCCAGCAGCTGCGGTAATACGTAGGGTGCAAGCATTAATCGGATTTATTGGGCGTAAAG
GGCGCGTAGGCGGGAGAGTAAGTCAGATGTGAAAGCCCGGAGCTCAACTCCGGAACAGCA
TTTGAAACTGCTCATCTAGAGGGTAGGCGGAGAAAACGGAATTCCACAAGTAGCGGTGAA
ATGCGTAGATATGTGGAAGAACACCAGTGGCGAAGGCGGTTTTCTAGCTTACACCTGACG
CTGAGGCGCGAAAGCAAGGGGAGCAAACAGGATTAGAAACCCTAGTAGTC
>Otu8649
CCAGCCTATGGGAGGCTGCAGTGGGGAATCTTGCGCAATGGGCGAAAGCCTGACGCAGCA
ACGCCGCGTGAGGGAAGAAGGTCTTAGGATTGTAAACCTCTTTCAGTAGGGACGAAGCTT
CCAAGGTTAATAGCCCTGTGGAGTGACGGTACCTACAGAAGAAGCCCCGGCTAACTACGT
GCCAGCAGCCGCGGTAATACGTAGGGGGCAAGCGTTGTCCGGAATCATTGGGCGTAAAGC
GCGTGTAGGCGGCTCGGAAAGTCTGGTGTGAAAACTTGAGGCTCAACCTCAAGGGTGTCG
CTGGATACTATCGAGCTCGAGTCCGGAAGAGGAGAGTGGAATTCCTGGTGTAGCGGTGGA
ATGCGCAGATATCAGGAGGAACACCAATGGCGAAGGCAGCTCTCTGGTACGTGACTGACG
CTGAGACGCGAAAGCGTGGGGAGCGAACAGGATTAGATACCCCTGTAGTCC
>Otu8658
CCAGCCTACGGGGCGCTGCAGTGGGGAATTTTGCGCAATGGGGGAAACCCTGACGCAGCC
ACGCCGCGTGAGTGAAGAAGGCCTTCGGGTCGTAAAGCTCTGTCGGAGGGGACGAAAAGC
TTCTGGGTTAACAGCCCAAGAGTCTGACGGTACCCTCAAAGGAAGCACCGGCTAACTTCG
TGCCAGCAGCCGCGGTAATACGAGGGGTGCGAGCGTTGCTCGGAATTATTGGGCGTAAAG
GGTAGGTAGGTGGTCTCATTTGTCTGGGGTGAAAGCCTTGAGCTTAACTCAAGAAGTGCC
CTAGAAACGGTGAGACTAGAATCTGTGAGAGGGTCGTGGAATTCCCGGTGTAGCGGTGAA
ATGCGTAGAGATCGGGAGGAACACCAGAGGCGAAGGCGGCGACCTGGAGCAGTATTGACA
CTCAACTACGAAAGCGTGGGTAGCAAACAGGATTAGAAACCCTAGTAGTCC
>Otu8659
CCAGCCTACGGGGGGCTCCAGTGGGGAATTTTGCGCAATGGGGGAAACCCTGACGCAGCA
ACGCCGCGTGGAGGATGAAGTATCTCGGTACGTAAACTCCTTTCGATAGGGAAGATAATG
ACGGTACCTATAGAAGAAGCCCTGGCTAACTTCGTGCCAGCAGCCGCGGTAATACGAGGG
GGGCAAGCGTTGTTCGGATTTATTGGGCGTAAAGCGTGCGCAGGCGGTTATGTAAGACAG
ATGTGAAATGCCCGGGCTTAACCTGGGAACTGCATTTGTGACTGCATGGCTAGAATCTGG
CAGAGGGGGGTAGAATTCCACGTGTAGCAGTGAAATGCGTAGATATGTGGAGGAATACCG
ATGGCGAAGGCAGCCCCCTGGGCTAAGATTGACGCTCATGCACGAAAGCGTGGGGAGCAA
ACAGGATTAGAAACCCCAGTAGTCC
>Otu8662
CCAGCCTATGGGGCGCTGCAGTCGAGGATCTTTCGCAATGGGCGAAAGCCTGACGAAGCG
ACGCCGTGTGAGCGATGAAGGCCTTCGGGTTGTAAAGCTCTTTCGCCTGTGAACAAGAGA
GAGGTGCGAATAACACCTTAATTTGAGCGTAGCAGGTAAAGAAGCACCGGCTAACTACGT
GCCGGCAGCTGCGGTAATACGTAGGGTGCAAGCATTAATCGGAATTATTGGGCGTAAAGG

GCGTGTAGGCGGTTTAATAAGTCAGATGTGAAATTCCGGGCTCAACCTCGGAGCTGCAT
TTGAAACTATTAGACTAGAGGGTTGACGGAGAAAACGGAATTCCACGTGTAGCGGTGAAA
TGCGTAGATATGTGGAAGAACACCTGTGGCGAAAGCGGTTTTCTAGTTAACAACTGACGC
TGAGGCGCGAGAGCAAGGGGAGCAAACAGGATTAGAAACCCGAGTAGTCC
>Otu8665
CAGCCTACGGGGGGCTGCAGTAGGGAATCTTGCGCAATGGGCGAAAGCCTGACGCAGCCA
TGCCGCGTGAATGATGAAGGTCTTAGGATTGTAAAATTCTTTCACCGGGGAAGATAATGA
CGGTACCCGGAGAAGAAGCCCCGGCTAACTTCGTGCCAGCAGCCGCGGTAATACGAAGGG
GGCTAGCGTTGCTCGGAATTACTGGGCGTAAAGGGCGCGTAGGCGGATATTTAAGTTAGA
GGTGAAAGCCCAGGGCCCAACCCTGGAACAGCCTTTAATACTGGATATCTTGAGTTCGGG
AGAGGTGAGTGGAACTCCGAGTGTAGAGGTGAAATTCGTAGATATTCGGAAGAACACCAG
TGGCGAAGGCGACTCACTGGCCCGATACTGACGCTGAGGCGCGAAAGCGTGGGGAGCAAA
CAGGATTAGAAACCCGCGTAGTCC
>Otu8669
CCAGCCTACGGGGGGCAGCAGTGGGGAATCTTGCGCAATGGGCGAAAGCCTGACGCAGCG
ACGCCGTGTGTGCGATGAAGGTTTTCGGATCGTAAAGCACTGTCGCCCGGGAAAAAGAAA
TGATGGTACCGGGAGAGGAAGCACCGGCTAACTCTGTGCCAGCAGCCGCGGTAATACAGA
GGGTGCAAGCGTTAATCGGAATTACTGGGCGTAAAGCGTGCGCAGGCGGTTCGCTAAGAC
AGATGTGAAATCCCCGGGCTTAACCTGGGAACTGCATTTGTGACTGGCGGGCTAGAGTAT
GGCAGAGGGGGGTAGAATTCCACGTGTAGCAGTGAAATGCGTAGAGATGTGGAGGAATAC
CGATGGCGAAGGCAGCCCCCTGGGCCAATACTGACGCTCATGCACGAAAGCGTGGGGAGC
AAACAGGATTAGATACCCCAGTAGTCC
>Otu8685
CCAGCCTACGGGTGGCTGCAGTGGGGTATCTTGCGCAATGGACGAAAGTCTGACGCAGCA
ACGCCGCGTGAGTGATGAAGGTCTTCGGATCGTAAAGCTCTGTCGAGGGGGAAGATGCTT
GGTGCATCGAATAGATACATCAGGTGACGGTACCCTTAAAGGAAGCACCGGCTAACTCTG
TGCCAGCAGCCGCGGTAATACAGAGGGTGCAAGCGTTGCTCGGAATTATTGGGCGTAAAG
GGAGTGTAGGCGGCTAGGTAAGTCTTCTGTGAAATCTACGAGCTTAACTCGTTAAAGTGC
AGAAGATACTGCTTAGCTTGAGTGTCGGAGAGGATAGTGGAATTCCCGGTGTAGAGGTGA
AATTCGTAGAGATCGGGAGGAACACCAGTGGCGAAGGCGACTGTCTGGCTGACAACTGAC
GCTGAGACTCGAAAGCATGGGGAGCAAACAGGATTAGAAACCCTAGTAGTCC
>Otu8686
CCAGCCTACGGGTGGCTCCAGGTTTCAGCAGAAAGCCAACGATCTTTTACTTTTGGAACT
GCAAAAGGTGTCCGATTCTGTTGAGGGATTAAATTTGGGATGGGAACCCTAGGGGTTACG
ATAGTATTGTGGAGAACAGCCGAATTGCGTAGGTCAGGAGTAGAGATTGAGTTTTTGAGT
TTCGGGGTTCTTGAGCTGGCAAGGCGAGTGAATGAAGAATTAGGAGGGTCGATACTGTCA
GCATTGTGTGATGACGGAGTGAATGTAGATCGTGGCAGTACCGGCGGGGTTTTGGCATTC
CGGGGTATTTCACGGGCGTAGGGGTGAACCGGCCTTGTAGAAAATGAATTTTGGCGGTCT
AGGGTGGACTGGTCGGACGGAGGTTGCCGGGAACGATGCAAGTCCTCAGAGTGAGACGGA
GAAAGGCGGCGACGTTGGCGGTGATTAGATACCCCTGTAGTCC
>Otu8688
CCAGCCTATGGGGTGCAGCAGTGAAGAATCTTGGGCAATGGACAAAAGTCTGACCCAGTG
AGAATTACTCCATGACGAATGCGCCCAGTCTTCTGTAAAGTGGTTTCGCGAGATGAGGAA
AATGACTGTAACTCGGGATGAAGCGCCGACCAACTTCGTGCCAGCAGTCGCGGTAAGACG
AAGGGCGCGGGTGTTATTCGTTTTGATTGGGTGTAAAGGGTAGGTAGGCGGGCTCGAGTT
TCTTTGGACAAAATCGCGGAGAGTTCCTACGCGAATTCCTTTTATTAAACGGGTCTTGTG
TTGAGAGGGGGTTTAGGCAAATTCGCTTTAGTAGGGGTAGAATCCATCAATATGCGAATG
AACGTCAGCTGGCGCAGGCGCTTTTCCGATTTCAACAGACGCTGAGCTACGGAAGCTTAG
GGAGCAAACGGGATTAGATACCCGAGTAGTCC
>Otu8692
CCAGCCTATGGGTTGCAGCAGTGGGGAATTTTGGACAATGGGGGGAACCCTGATCCAGCA
ACGCCGCGTGAATGAAGAAGGCCTTCGGGTTGTAAAGTTCTTTTATTTGGGAGCAAAGGA
TGTGGGTTAATAGCTTACATATATGAGAGTACCAAAGGAATAAGCACCGGCTAACTACGT
GCCAGCAGCCGCGGTAATACGTAGGGTGCGAGCGTTAATCGGAATTACTGGGCGTAAAGG
GTGCGTAGGCGGATATATAAGTTAGATGTGAAAAGCCCGGGCTCAACCTTGGTCGTGCAT
TTAAGACTGTATGTCTAGAGTTTGTAAGAGGGGGGTAGAATTCAGGTGTAGCAGTGAAAT
GCGTAGAGATCTGGAGGAATACCGATGGCGAAGGCAGCCCCCTGGGATAGAACTGACGCT
GAGGCACGAAAGCGTGGGTAGCAAACAGGATTAGAGACCCGAGTAGTCC

>Otu8701
CCAGCCTATGGGAGGCAGCAGTGGGGAATATTGCGCAATGGGCGAAAGCCTGACGCAGCA
ACGCCGCGTGAGTGAAGAAGGCCTTCGGGTTGCAAAGCTCTTTCGGCTGGGAAGAAGGGT
GTGCGGATTAATACACCGTACATTTGATGGTACCAGAATAAGAAGCACCGGCTAACTTCG
TGCCAGCAGCCGCGGTAATACGAAGGGTGCAAGCGTTGTTCGGAATTATTGGGCGTAAAG
GGATCGTAGGTGGAAAGATAAGTCAAGTGTGAAATCCCCGAGCTTAACTTGGGACGTGCA
CCTGAAACTATTTTTCTTGAGTATCGGAGAGGGTAGTGGAATTGCTGGTGTAGGAGTGAC
ATCCGTAGAGATCAGCAGGAACACCGGAGGCGAAAGCGACTGCCTGGCCGAAAACTGACA
CTGAGGAACGAAAGCGTGGGGAGCAAACAGGATTAGATACCCGGGTAGTCC
>Otu8702
CCAGCCTATGGGAGGCAGCAGTGGGGAATATTGGGCAATGGGCGCAAGCCTGACCCAGCC
ATGCCGCGTGAGTGATGAAGGCCTTCGGGTCGTAAAGCTCTGTGGGGAGGGACGAATAGT
CCTGTGGCTAACATCCATGGGATTTGACGGTACTTCCTTAGCAAGCACCGGCTAACTCCG
TGCCAGCAGCCGCGGTAATACGGAGGGTGCAAACGTTGCTCGGAATTATTGGGCGTAAAG
CGCACGTAGGCGGTTTGTTAAGTCGGGTGTGAAAGCCCTGGGCTCAACCCAGGAAGTGCA
TTCGAAACTGGCAAGCTTGAGTATGGAAGAGGGTCTCGGAATTCCCGGTGTAGAGGTGAA
ATTCGTAGATATCGGGAGGAACACCAGTGGCGAAGGCGGAGACCTGGGCCAATACTGACG
CTGAGGTGCGAAAGCGTGGGGAGCAAACAGGATTAGAAACCCGAGTAGTCC
>Otu8705
CCAGCCTACGGGAGGCTGCAGTCGAGAATCTTCCGCAATGGGCGAAAGCCTGACGGAGCG
ACGCCGCGTGATTGATGAAGGCCTTCGGGTTGTAAAACTCTTTCGCGCACGACGATAATG
ACGGTAGTGCGAGAAGAAGCTCCGGCTAACTTCGTGCCAGCAGCCGCGGTAATACGAAGG
GGGCTAGCGTTGTTCGGAATTACTGGGCGTAAAGCGCGCGCAGGCGGTCTTCCAAGTCAG
TGGTGAAAGCCCGGAGCTCAACTCCGGAACTGCCATTGAAACTGTTAGACTTGAGGACGA
GAGAGGTGAGTGGAATTCCCAGTGTAGAGGTGAAATTCGTAGATATTGGGAAGAACACCG
GTGGCGAAGGCGGCTCACTGGCTCGTTTCTGACGCTCAGGCGCGACAGCGTGGAGATCAA
ACAGGATTAGAAACCCTAGTAGTCC
>Otu8712
CCAGCCTACGGGACGCTGCAGTAGGGAATCTTCCGCAATGGACGAAAGTCTGACGGAGCA
ACGCCGCGTGAGTGATGAAGGTTTTCGGATCGTAAAGCTCTGTTGCCAGGGAAGAACGTC
CGGTAGAGTAACTGCTACCGGAGTGACGGTACCTGAGAAGAAAGCCCCGGCTAACTACGT
GCCAGCAGCCGCGGTAATACGTAGGGGGCAAGCGTTGTCCGGAATTATTGGGCGTAAAGC
GCGCGCAGGCGGTCATTTAAGTCTGGTGTTTAAACCTTGGGCTCAACCTAAGGTCGCACT
GGAAACTGGGTGACTTGAGTACAGAAGAGGAAAGTGGAATTCCACGTGTAGCGGTGAAAT
GCGTAGATATGTGGAGGAACACCAGTGGCGAAGGCGACTCTCTGGGCTGTAACTGACGCT
GAGGCGCGAAAGCGGGGGGAGCAAACAGGATTAGATACCCTCGTAGTCC
>Otu8715
CCAGCCTACGGGGGGCTGCAGTGGGGAATATTGGACAATGGGCGCAAGCCTGATCCAGCC
ATGCCGCGTGAGTGATGACGGCCTTAGGGTTGTAAAGCTCTGTCAGGGGGAAAGAAAAAG
TCGGTTAACAGCCGATGTTGACGGTACCCCCAAAGGAAGCACCGGCTAACTCTGTGCCAG
CAGCCGCGGTAATACAGAGGGTGCAAGCGTTGTTCGGAATTACTGGGCGTAAAGCGAGTG
TAGGCGGCCTATTAAGTCAGATGTGAAATCCCTTGGCTCAACCGAGGAACTGCATCTGAT
ACTGGTAGGCTTGAGTGTCGGAGGGGATGGTAGAATTCCTGGTGTAGAGGTGAAATTCGT
AGATATCAGGAGGAATACCTGTAGCGAAGGCGACCATCTGGAAGACAACTGACGCTGAGA
CTCGAAAGCGTGGGTAGCAAACAGGATTAGAAACCCCCGTAGTCC
>Otu8718
CAGCCTATGGGGCGCTGCAGTGGGGAATATTGCACAATGGGCGAAAGCCTGATGCAGCGA
CGCCGCGTGAGGGATGACGGCCTTCGGGTTGTAAACCTCTTTCAGCAGGGACGAAGCGCA
AGTGACGGTACCTGCAGAAGAAGCACCGGCTAACTACGTGCCAGCAGCCGCGGTAATACG
TAGGGTGCAAGCGTTGTCCGGAATTACTGGGCGTAAAGAGTTCGTAGGCGGTTTGTCGCG
TCGTTTGTGAAAACCAGCAGCTCAACTGCTGGCTTGCAGGCGATACGGGCAGACTTGAGT
ACTGCAGGGGAGACTGGAATTCCTGGTGTAGCGGTGAAATGCGCAGATATCAGGAGGAAC
ACCGGTGGCGAAGGCGGGTCTCTGGGCAGTAACTGACGCTGAGGAACGAAGGCGTGGGTA
GCGAACAGGATTAGAAACCCTAGTAGTCC
>Otu8720
CCAGCCTATGGGTCACCAGTAGGGAATATTGCGCAATGGAGGAAACTCTGACGCAGCGAC
GCCGCGTGGGTGATGAAGGTCTTCGGATCGTAAAGCCCTGTCGGAAGGGACGAAAAATGA
GATGGCTAACATCCATTTCACTTGACGGTACCTTCAAAGGAAGCACCGGCTAACTACGTG

CCAGCAGCCGCGGTAATACGTAGGGTGCTAGCGTTGTTCGGAATCATTGGGCGTAAAGCG
CGTGTAGGTGGTTAGTTAAGTCGGGTGTGAAATCCCTGGGCTCAACCGAGGAAGTGCATT
CGAAACTAACTAACTCGAGAGTGGTAGAGGAAGGTGGAATTCCGTGTGTAGAGGTGAAAT
TCGTAGATATACGGAGGAATACCGGTGGCGAAGGCGGCCTTCTGGACCATTTCTGACACT
GAGACGCGAAAGCGTGGGGAGCAAACAGGATTAGAAACCCGAGTAGTCC
>Otu8728
CCAGCCTATGGGATGCTCCAGTGGGGAATCTTGCACAATGGACGAAAGTCTGATGCAGCA
ACGCCGCGTGGGGGATGAAGCTTTTCGGAGTGTAAACCCCTTTCGACCCGGAAGAAAGCC
CGCAAGGGTTTGACGGTACGGGTATAAGAAGCCCCGGCTAACTACGTGCCAGCAGCCGCG
GTAATACGTAGGGGGCCAGCGTTGTTCGGAATTATTGGGCGTAAAGGGTGCGTAGACGGT
GCCACAAGTCACTTGTGAAATCCCCAGGCTTAACCTGGGGCCTGCAGGCGAAACTGTGGT
GCTGGAGTATGGGAGAGGTGCGTGGAATTCCCGGTGTAGCGGTGAAATGCGTAGATATCG
GGAGGAACACCTGTGGCGAAAGCGGCGCACTGGACCATAACTGACGCTGATGCACGAAAG
CTAGGGGAGCAAACAGGATTAGAAACCCTAGTAGTCC
>Otu8739
CCAGCCTATGGGACGCTGCAGTGGGGAATCTTGCACAATGGGCGAAAGCCTGAACGAGCG
ACGCCGCGTGCGCGATGAAGGCCTTCGGGTTGTAAAGCGCGAAAGATGGGACGAAAGCCG
AAAGGTTTGACCGATCATCAGTAAGCTCGGGCTAAGTTCGTGCCAGCAGCCGCGGTAAGA
CGAACCGAGCAAACGTTATTCGGAATCACTGGGCTTAAAGGGCGCGTAGGCGGGCATTCA
AGTCTATGGTGAAATCCAACAGCTCAACTGTTGAACTGCCGCGGATACTGGGTGTCTCGA
GGGAGGTAGGGGCGTGCGGAACTATTGGTGGAGCGGTGAAATGCGTTGATATCAATAGGA
ACTCCGGTGGCGAAGGCGGCACGCTGGACCTCTTCTGACGCTGAGGCGCGAAAGCCAGGG
GAGCAAACGGGATTAGAGACCCTTGTAGTCC
>Otu8748
CCAGCCTATGGGGGGCACCAGTAGGGAATATTGCGCAATGGGGGAAACCCTGACGCAGCG
ACGCCGCGTGAGTGATGAAGGCCTTCGGGTCGTAAAGCTCTGTTGCAAGGGAATAAAAAA
ATGAATGTACCTTGTGAGGAAGGATCGGCTAACTTCGTGCCAGCAGCCGCGGTAAGACGA
GGGATCCTAGCGTTGCTCGGAATCATTGGGCGTAAAGCGGGTGTAGGTGGCTTTGTAAGT
CAGGTGTGAAAGCCCAGGGCTCAACCCTGGAAGTGCATTTGATACTGCGAAGCTTAAGTG
TTGGAGAGGTCATCGGAATACCTGGTGTAGTGGTGAAATACGTAGATATCAGGTGGAACA
CCGGAGGCGAAGGCGGGTGACTGGCCAAACACTGACACTGAGACCCGAAAGCGTGGGGAT
CAAACAGGATTAGATACCCTCGTAGTCC
>Otu8753
CAGCCTACGGGGTGCAGCAGTCGAGAATCTTCCGCAATGGACGAAAGTCTGACGGAGCGA
CGTTACGTGAAGGATGAAGCCCTTCGGGGTGTAAACTTCTTTTATATGGGAGCAAATTAT
TGAGAGTACCATATGAATAAGGGGATCCTAATTCTGTGCCAGCAGGAGCGGTAATACAGA
ATCCCCGAATGTTACCCGGATTTATTGGGCGTAAAGGGTGTGTAGGTGGTTTGGTAAGTT
CAAAATTAAACCTCATCGGCTTAACCTTTGAGCTGTTTTGAATACTGCCAGACTAAGAGG
GCGTTAGAGGTGAACGGAACCGATGGAGTAGGGGTGAAATCCGTTGATATCATCGGGAAC
ACCAAAAGCGAAGGCAGTTCACTGGGGCGACCCTGACACTGAAACACGAAAGCGTGGGGA
GCAAAAAGGATTAGAAACCCCGGTAGTCC
>Otu8754
CCAGCCTACGGGGCGCTCCAGTGGGGAATTTTGCGCAATGGGGGAAACCCTGACGCAGCA
ACGCCGCGTGGAGGATGAAGTATCTTGGTACGTAAACTCCTTTCGATGGGGAAGATAATG
ACGGTACCCATAGAAGAAGCTCCGGCTAACTTCGTGCCAGCAGCCGCGGTAATACGAAGG
GGGCTAGCGTTGTTCGGAATTACTGGGCGTAAAGCGCGCGCAGGCGGTTCTCCAAGTCAG
TGGTGAAAGCCCGGAGCTCAACTCCGGAACTGCCATTGAAACTGTTGAACTTGAGGACGA
GAGAGGTGAGTGGAATTCCCAGTGTAGAGGTGAAATTCGTAGATATTGGGAAGAACACCG
GTGGCGAAGGCGGCTCACTGGCTCGTTTCTGACGCTCAGGCGCGACAGCGTGGGGATCAA
ACAGGATTAGATACCCTAGTAGTCC
>Otu8755
CCAGCCTATGGGTGGCACCAGTGAGGAATCTTGCACAATGGGCGAAAGCCTGATGCAGCG
ACGCCGCGTGGAGGATGACGGTCTTCGGATTGTAAACTCCTGTTAAACGGGAAGAAAGAC
CTGTTTCTAATACAGACAGGTTATGGCGGTACCGTTAGAGAAAGCACCGGCTAACTTCGT
GCCAGCAGCCGCGGTAATACGAGGGGTGCAAGCGTTATTCGGATTGATTGGGTGTAAAGG
GTGTGCAGACGGCTTACTAAGTCACTTGTTAAATTCCTCGGCCTAACTGAGGATCTGCAG
GTGAGACTGGAAAGCTAGAGGGTGGAAGAGAGAAGTGGAATTCTCGGAGTAGCGGTAAAA
TGCGTAGATCTCGAGAGGAACACCAATGGCGAAGGCAGCTTCTTGGTCCATTCCTGACGT

TCATACACGAAAGCGTGGGGAGCAAACAGGATTAGAGACCCCCGTAGTCC
>Otu8756
CCAGCCTACGGGGTGCTCCAGGGAATATTGCACAATGGGCGCAAGCCTGGTGCAGCAATG
CCGCGTGAAGGATGAAGGTCTTCGGATTGTAAACTTCTTTTGTTGGGGATGAAGAAATCC
TGGTGAATATCCAGGAGATTTGACCGTACCCAAAGAATAACCACCGGCTAACTCCGTGCC
AGCAGCTGCGGTAATACGGAGGGTGGAAGCGTTGACCGGATTGACTGGGCGTAAAGGGAG
CGTAGGCGGCTATATAAGTCGATGGTGAAATTTCTGGGCTTAACCCAGGAGCTGCTGTCG
AAACTGTATAACTAGAGGATATTAAGGGAAAGCGGAATACCACATGTAGCGGTGAAATGC
GTAGATATGTGGTAGAACACCGGTGGTGAAGACGGCTCTCTGTGATATACCTGACGCTGA
GGCTCGAAAGTATGGGGATCAAACAGGATTAGAAACCCTGGTAGTCG
>Otu8770
CCAGCCTATGGGATGCAGCAGTTAGGAATCTTCCGCAATGGGCGAAAGCCTGACGGAGCG
ACACCGCGTGAAGGATGACACCCTTTACTGGGCGTAAACTTCTGTTGTGAGGGACGAAAT
TTTTGACGGTACCTCACGAGAAAGCACCGGCTAATTCTGTGCCAGCAGCCGCGGTAAGAC
AGAAGGTGCAAACGTTACTCGGAATTACTGGGCGTAAAGCGTCTGCAGGTGTCCTGCCAC
GTCCGGCGTCAAAACGCGAGGCTCAACTTCGCGTCCGTGCCGGAAACGAGCGGGATCGAG
CCATTCAGAGGCATCTGGAATGTCGTGTGTAGGGGTAAAATCCGTAGATCCACGATGGAA
CGCCAAAAGCGAAGGCAGGATGCTGGGGATGCGCTGACACTCAGAGACGAAAGCGTGGGG
AGCAAAGGGGATTAGAGACCCTTGTAGTCC
>Otu8771
CCAGCCTACGGGGGGCACCAGTCGAGAATTTTTCTCAATGGGCGAAAGCCTGAAGGAGCG
ACGCCGCGTGGGGGATGAATGGCTTCGGCCCGTAAACCCCTGTCATTTGTGAACAAATTG
CTTCACCCAACACGTGAAGCATTGATAGTAACGGAAGAGGAAGGGACGGCTAACTCTGTG
CCAGCAGCCGCGGTAATACAGAGGTCCCAAGCGTTGTTCGGATTCACTGGGCGTAAAGGG
TGCGTAGGCGGCGGGGTAAGTCGGGTGTGAAATCTCAGGGCTCAACCCTGAAACTGCACT
CGATACTGCTCTGCTTGAGGACTGGAGAGGAGACTGGAACTCACGGTGTAGCAGTGAAAT
GCGTAGAGATCGTGAGGAAGACCAGTGGCGAAGGCGGGTCTCTGGACAGTTCCTGACGCT
GAGGCACGAAGGCCAGGGGAGCAAACGGGATTAGAAACCCTTGTAGTCG
>Otu8785
CCAGCCTACGGGGGGCACCAGTGGGGAATAATGGACAATGGGGGAAACCCTGATCCAGCG
ACGCCGCGTGTGTGAAGAAGGCCTGCGGGTTGTAAAGCACTTTTAGTGGGGACAAAAAGC
TACGGATTAATACTCTGTGGTCTTGATTTAACCCAAAGAAAAAGCACCGGCTAACTCTGT
GCCAGCAGCCGCGGTAATACAGAGGGTGCGAGCGTTAATCGGAATTACTGGGCGTAAAGC
GCGCGTAGACGGTTTTGTAAGTCAGATGTGAAATCCCCGGGCTTAACCTGGGAACTGCAT
CTGTGACTGCAAGGCTAGAATATAGCAGAGGGGGGTAGAATTCCACGTGTAGCAGTGAAA
TGCGTAGATATGTGGAGGAATACCGATGGCGAAGGCAGCCCCCTGGGCTAATATTGACGC
TCATGCACGAAAGCGTGGGGAGCAAACAGGATTAGATACCCGAGTAGTCC
>Otu8788
CCAGCCTATGGGAGGCAGCAGTCGAGGATTTTTCTCAATGGGGGAAACCCTGAAGGAGCG
ACGCCGCGTGGGGGATGAAGGTCTTCGGATTGTAAACCCCTGTCATCTGGGAACAATGCT
AGTCACCTAACACGTGCCTAGTTGATAGTACCGGAAGAGGAAGCAGTGGCTAACTCTGTG
CCAGCAGCCGCGGTAATACAGAGACTGCAAGCGTTGTTCGGATTCATTGGGCGTAAAGGG
TCCGCAGGCGGTCTGTTAAGTCGGGTGTGAAATCTCACAGCCTAACTGTGATAGGTCATT
CGAAACTAGCAGGCTCGAGGAATGGAGAGGAAACTGGAATAGCTGGTGTAGCGGTGAAAT
GCGTAGAGATCAGCTAGAACACCGGTGGCGAAGGCGGGTTTCTGGACATTTCCTGACGCT
CAGGGACGAAAGCCAGGGGAGCAAACAGGATTAGATACCCTAGTAGTCC
>Otu8798
CAGCCTATGGGTCGCTGCAGTGGGGAATTTTGCACAATGGGGGAAACCCTGATGCAGCAA
CGCCGCGTGAGGGAAGAAGTCTTTCGGGATGTAAACCTCTTTTCGATGGGAAGACAAGCA
AACGGACGGTACCATCGGAATAAGCATCGGCTAACTACGTGCCAGCAGCCGCGGTAATAC
GTAGGATGCAAGCGTTGTCCGGAATTATTGGGCGTAAAGGGCGTGCAGGCGGCTTGTCAA
GTCAGACGTGAAATTCTTCGGCTTAACCGGAGCGGGTCATCTGAGACTGGCGGGCTAGAG
TGCAGGAGAGGAAAGCGGAATTCCCGGTGTAGTGGTGAAATGCGTAGATATCGGGAGGAA
CACCAGAGGCGAAAGCGGCTTTCTGGCCTGTAACTGACGCTGAGGCGCGAAGGCTAGGGT
AGCGAACAGGATTAGATACCCTTGTAGTCC
>Otu8823
CCAGCCTATGGGTGGCAGCAGTGAGGAATATTGGTCAATGGGCGCAAGCCTGAACCAGCC
ATGCCGCGTGAAGGATGAAGGCCTTCTGGGTTGTAAACTTCTTTTATGAGGGAAGAAAAC

TGGGGTTTCTACTCCAGCCGACGGTACCTTAGGAATAAGCACCGGCTAACTCCGTGCCAG
CAGCCGCGGTAATACGGAGGGTGCAAGCGTTATCCGGATTTACTGGGTTTAAAGGGTGTG
TAGGCGGGTCAGTAAGTCAGTGGTGAAATCTCCGGGCTCAACCCGGAAACTGCCATTGAT
ACTATTGGTCTTGAATTCTGTTGAGGAGGGCGGAATATGTCATGTAGCGGTGAAATGCAT
AGATATGACATAGAACACCGATAGCGAAGGCAGCTCTCAAAGCAGATATTGACGCTGAGG
CACGAAAGCGTGGGGATCAAACAGGATTAGAAACCCCCGTAGTCC
>Otu8826
CCAGCCTACGGGGGGCTCCAGTGGGGAATTTTGCGCAATGGGGGAAACCCTGACGCAGCA
ACGCCGCGTGGAGGATGAAGTACCTTGGTACGTAAACTCCTTTCGATCGGGACGATAATG
ACGGTACCGAGAGAAGAAGCCCCGGCTAACTTCGTGCCAGCAGCCGCGGTAATACGAGGG
GGGCGAGCGTTGTTCGGAATTATTGGGCGTAAAGGGCGCGTAGGCGGTGTTGTAAGTCAC
CTGTGAAACCTCTGGGCTTAACTCAGAGCCTGCAGGCGAAACTGCAATGCTGGAGGGTGG
GAGAGGTGCGTGGAATTCCCGGTGTAGCGGTGAAATGCGTAGATATCGGGAGGAACACCT
GTGGCGAAAGCGGCGCACTAGACCACTACTGACGCTGAGGCGCGAAAGCTAGGGGAGCAA
ACAGGATTAGATACCCCAGTAGTCC
>Otu8827
CCAGCCTACGGGGGGCAGCAGTCGAGAATCTTCGGCAATGAGCGCAAGCTTGACCGAGCG
ACGCCGCGTGCGGGATGAAGGCCTTCGGGTTGTAAACCGCTTTGGTCCGAAAGAAAGGGT
AAAACTTGATCGATCGGAGAAACAAGGACGGGCTAAGTTCGTGCCAGCAGCCGCGGTAAG
ACGAACCGTCCAAACGTTATTCGGAATCACTGGGCTTAAAGGGCGCGTAGGCGGCCGAGC
GGGTCACGGGTGAAATCCTCCAGCTTAACTGGAGAACTGCCCTTGATACCACTGCGGCTC
GAGGAAGGAAGGGGCAATCGGAACTGTCGGTGGAGCGGTGAAATGCGTTGATATCGACAG
GAACGCCGGTGGCGAAAGCGGATTGCTGGTCCTTTTCTGGCGCTGAGGCGCGAAAGCCAG
GGGAGCAAACGGGATTAGAAACCCTCGTAGTCC
>Otu8840
CCAGCCTACGGGGGCAGCAGTCGAGAATTTTTCACAATGGGCGAAAGCCTGATGGAGCGA
CGCCGCGTGGGGGATGAATGGCTTCGGCCCGTAAACCCCTGTCATTCGGGATCAATGCGT
TTGGGTGAATATCCCAAACGTTGATAGTACCGGAAGAGGAAGGGACGGCTAACTCTGTGC
CAGCAGCCGCGGTAATACAGAGGTCCCAAGCGTTGTTCGGATTCACTGGGCGTAAAGGGT
GCGTAGGTGGTCGGATAAGTTTGATGTGAAATCTCCGGGCTTAACCCGGAAATGGCATTG
AATACTATTCGGCTTGAGGATTGGAGGGGGGACTGGAATACTTGGTGTAGCAGTGAAATG
CGTAGATATCAAGTGGAACACCAGTGGCGAAGGCGAGTCCCTGGACAAATCCTGACACTA
AGGCACGAAAGCTAGGGGAGCAAACAGGATTAGAAACCCTGTAGTCC
>Otu8845
CCAGCCTATGGGAGGCACCAGTGGGGAATCTTGCACAATGGGGGAAACCCTGATGCAGCG
ACGCCGCGTGAGCGATGAAGCCCTTCGGGGTGTAAAGCTCTTTCGACGGGAACGATAATG
ACGGTACCCGGAGAAGAAGCTGCGGCTAACTACGTGCCAGCAGCCGCGGTAATACGTAGG
CAGCAAGCGTTGTTCGGAATTATTGGGCGTAAAGGGCGCGTAGGCGGTGCGGTAAGTCAT
CTGTGAAATCTTCCGGCTCAACCGGGAGACTGCAGGCGAAACTGCCGTGCTGGAGTGTGG
GAGAGGTGAGTGGAATTCCCGGTGTAGCGGTGAAATGCGTAGATATCGGGAGGAACACCT
GTGGCGAAAGCGGCGCACTGGACCATTACTGACGCTGAGGCGCGAAAGCTAGGGGAGCAA
ACAGGATTAGAAACCCTTGTAGTCC
>Otu8855
CCAGCCTATGGGGGGCTGCAGTCGAGAATATTCGACAATGGGCGAAAGCCTGATCGAGCG
ACACCGCGTGCAGGATGAAGGCCTTCGGGTCGTAAACTGCGGTAGCTAGGGAAGAATGCA
AATGACTGTACCTAGCGGAAAGAGGTGGGTAACTACGTGCCAGCACCAGCGGTAATACGT
AGACCTCAAGCGTTATCCGGATTTATTGGGCGTAAAGCGTATGTAGGAGGTTTTGCGCGT
CTTTGGTTAAAGCCCACTGCTCAACGGTGGAAGTGCCAAGGATACGGCAGAACTAGAGGG
AGTTAGAGGTGAATGGAACTCACGGTGTAGGGGTGAAATCCGTTGATATCGTGGGGAACA
CCAAAGGCGAAGGCAGTTCACTGGGACTTTCCTGACTCTGAGTTGCGAAAGCGTGGGGAG
CAAAAAGGATTAGATACCCGAGTAGTCC
>Otu8857
CCAGCCTACGGGAGGCTGCAGCAACGAATCTTCCCCAATGGGCGAAAGCCTGAGGGAGCG
ACGCCGCGTGTGGGTTAAGTCCTTCGGGATGTCAACCACTGTCAGGGCTACGAAAGCGTA
AGGACCTAATACGTCCTGAGTTGATCTAGTCCAAAGGAAGGGACGGCTAACTCTGTGCCA
GCAGCCGCGGTAATACAGAGGTCCCAAGCGTTACTGAGAATCACTGGGTTTAAAGGGTGC
GTAGGTGGTCCGTTAAGTTCGTTGTGAAATCCCTGGGCTCAACCCAGGAACTGCTTCGAA
TACTGGCGGACTTGAGGCCAGTAGAGGTCACTGGAACTGACGGTGGAGCGGTGAAATGCG

TAGATATCGTCAGGAACGCCGGTGGTGAAGACGGGTGACTGGGCTGGTTCTGACACTGAG
GCACGAAAGCGTGGGGAGCGAACGGGATTAGAAACCCTAGTAGTCC
>Otu8876
CCAGCCTACGGGAGGCAGCAGTGAGGAATTTTGGACAATGGGCGCAAGCCTGATCCAGCA
ATATTTATCGTGTGAAAGAGACGGCTAGCTTCGTAAAACACTTTTGTAAATAGCAATAAT
GATACATATTTAAGTAATTTAGTCCCGGCTAATTTCGTGCCAGCAGCCGCGGTAATACGG
GAGGGGCAAGCGTTATTCATCATAAGCGGGCGTAGAGGGTGAGTAGACGATGTAAACTAG
TTAATGTTATAAAAAACAAAGAACTTTTTGTTATTTGTTAACTAAAAATAAATTAGCGAC
AAAAAAGTGTGGATGGAATTACTAATGTAAAGGTAAAATATAATGATATTAGTACGTACA
CCAGCGGCGAAGGCAGCTCACATTTTAGTCGGACGTTGAGGTGCGAAAGCATGGGAATCA
AACGGGATTAGATACCCGAGTAGTC
>Otu8888
CCAGCCTACGGGAGGCACCAGTGGGGAATCTTGGACAATGGGCGCAAGCCTGATCCAGCC
ATGCCGCGTGAGTGAAGAAGGCCTTAGGGTTGTAAAGCTCTTTCGCCGACGAAGATAATG
ACGGTAGTCGGAGAAGAAGCCCCGGCTAACTTCGTGCCAGCAGCCGCGGTGATACGAAGG
GGGCTAGCGTTGTTCGGAATTACTGGGCGTAAAGCGCACGTAGGCGGACTGTTAAGTCGG
GGGTGAAAGCCCAGGGCTCAACCCTGGAATGGCCTTCGATACTGGCAGTCTGGAGAACGG
GAGAGGTGAGTGGAATTCCTAGTGTAGAGGTGAAATTCGTAGATATTAGGAAGAACATCG
GTGGCGAAGGCGGCGACCTGGTCCATTACTGACGCTGAGGCGCGAAAGCGTGGGGGGCAA
ACAGGATTAGAAACCCTGGTAGTCC
>Otu8895
CCAGCCTACGGGATGCTGCAGTCGAGAATTTTTCACAATGGGCGCAAGCCTGATGGAGCG
ACGCCGCGTGGGGGATGAATGGCTTCGGCCCGTAAACCCCTGTCATTCGCGATCAACCGT
TGTTGTTTAAGAGATGACAATCTGATAGTAGCGGAAGAGGAAGGGACGGCTAACTCTGTG
CCAGCAGCCGCGGTAATACAGAGGTCCCAAGCGTTGTTCGGATTCACTGGGCGTAAAGGG
TGCGTAGGTGGTGAGGTAAGTCGGATGTGAAAGCTCGGAGCTCAACTCCGAAATGGCATT
GGAAACTACCTGACTGGAGGGTCGGAGGGGGGACTGGAATTCTCGGTGTAGCAGTGAAAT
GCGTAGATATCGTGAGGAAGACCAGTGGCGAAGGCGAATCTCTGGACAGTTACTGACGCT
GAGGCACGAAGGCCAGGGGAGCAAACAGGATTAGATACCCTCGTAGTCC
>Otu8897
CCAGCCTACGGGGGGCAGCAGTAAGGGATATTGCACAATGGGCGAAAGCCTGATGCAGCA
ACGCCGCGTGGAGGATGAAGGCCTTCGGGTCGTAAACTCGTTTTGACGCTGACGAGCAAG
GACGGTAGGCGTCGAATAAGTCACGGCTAACTACGTGCCAGCAGCCGCGGTAAAACGTAG
GAGGCGAGCGTTATCCGGATTTACTGGGCGTAAAGCGCGTGCAGGTGGTTTGGTAAGTTG
GATGTGAAAGCTCCCGGCTCAACTGGGAGAGGTCGTTCAATACTACCAGACTTGAGAGCA
GTAGAGGGAGGTGGAATTCCCGGTGTAGTGGTGAAATGCGTAGATATCGGGAGGAACACC
AGTGGCGGAAGCGGCCTCCTGGACTGTTTCTGACACTCAGACGCGAAAGCTAGGGTAGCA
AACGGGATTAGAAACCCTAGTAGTCC
>Otu8899
CCAGCCTACGGGACGCAGCAGTCGAGAGGCTTCGGCAATGGGGGAAACCCTGACCGAGCG
ACGCCGCGTGGAGGATGAAGGCCCTTGGGTTGTAAACTCCTTTTGTCAGCGAAGAAGTGC
GATCAGGTGAATAATCTGGTCGTTTGACGGTACCTGACGAATAAGCTCCGGCTAACTCCG
TGCCAGCAGCCGCGGTAACACGGGGGGAGCAAGCGTTGTTCGGAATCACTGGGCGTAAAG
GGCGTCTAGGCGGTCAGGTAAGTGGGACGTGAAATACCTCGGCTCACCCGAGGGACTGCG
TTCCAAACTGCTTGGCTTGAGTATGGGAGAGGATGTGGGAATTCCTGGTGTAGCGGTGAA
ATGCGTAGATATCAGGAGGAACACCGGTGGCGAAGGCGCACATCTGGCCCAATACTGACG
CTGAAGCGCGAAAGCTAGGGGAGCAAACGGGATTAGATACCCTAGTAGTCC
>Otu8904
CAGCCTATGGGGGGCAGCAGTGGGGAATATTGGACAATGGGGGAAACCCTGATCCAGCCA
TGCCGCGTGAGCGATGAAGGCCTTCGGGTTGTAAAGCTCTTTTGGCGGGGACGATGATGA
CGGTACCCGCAGAATAAGCTCCGGCTAACTTCGTGCCAGCAGCCGCGGTAATACGAAGGG
GGCTAGCGTTGTTCGGAATTACTGGGCGTAAAGGGCGCGTAGGCGGTTTGGCAAGTTGGA
TGTGAAAGCCCAAAGCTCAACTTTGGAATTGCATTCAAGACTGTCAGACTTGAATTCGGT
AGAGGTAAGTGGAATTCCCAGTGTAGAGGTGAAATTCCTAGATATTGGGAAGAACACCCG
TGGCGAAGGCGGCTTACTGGACCGACATTGACGCTGAGGCGCGAAAGCGTGGGGATCAAA
CAGGATTAGATACCCCTGTAGTCC
>Otu8912
CCAGCCTACGGGTCGCTGCAGTCGAGAATTTTTCACAATGGGCGAAAGCCTGATGGAGCG

ACGCCGCGTGGGGGATGAAGGGCTTCGGCTCGTAAACCCCTGCCATTCGTGAACAAGGCA
GGCAGGTGAACAATCTGTTTGTTGATAGTAGCGGAAGAGGAAGGGACGGCTAACTCTGTG
CCAGCAGCCGCGGTAATACAGAGGTCCCGAGCGTTGTTCGGATTTACTGGGCGTAAAGGG
TGCGTAGGCGGTGAGGTAAGTTTGGTGTGAAATCTCTGGGCCTAACCCAGAAAATGCATT
GAATACTATCTGACTAGAGTCTTGGAGGGGAGACCGGAATTCTCGGTGTAGCAGTGAAAT
GCGTAGATATCGAGAGGAACATCAGTGGCGAAGGCGGGTCTCTGGACAAGTACTGACGCT
GAGGCACGAAAGCCAGGGGAGCAAACGGGATTAGAAACCCTTGTAGTCC
>Otu8913
CCAGCCTATGGGAGGCTGCAGACGAGAATATTCGACAATGGACGAAAGTCTGATCGAGCG
ACGCCGCGTGGAAGATGAAGTGCTTCGGCATGTAAATTCCTTTTGCCAGGGAAGAAGTTT
ATTGATTGTACCTGGAGAATAAGAGGTTGCTAAACTCGTGCCAGCAGCAGCGGTAATACG
AGTGCCTCGAGCGTTATCCGGAATCATTGGGCGTAAAGGGTGCGTAGGCGGCTATGTTAG
TCTCGTGTAAAATCTTTCGGCTCAACCGAGAGTCTGCATGGAAACGGCATAGCTTGAGG
GCGGGAGAGGTCTCTGGAACTCATGGTGTAGCGGTGAAATGCGTTGATATCATGGGGAAC
ACCAAAAGCGAAGGCAAGAGACTGGCCCGCTCCTGACGCTGAAGCACGAAAGCGTGGGTC
GCGAATGGGATTAGAGACCCCTGTAGTCC
>Otu8919
CCAGCCTATGGGGCGCTGCAGTCGAGAATTTTTCTCAATGGGCGAAAGCCTGAAGGAGCG
ACGCCGCGTGGGGGATGAATGGCTTCGGCCCGTAAACCCCTGTCATTTGCGAACAAATTG
ACTCGCCTAACACGTGTGTCATTGATTGTAGCGGAAGAGGAAGGGACGGCTAACTCTGTG
CCAGCAGCCGCGGTAATACAGAGGTCCCAAGCGTTGTTCGGATTCATTGGGCGTAAAGGG
TGCGCAGGCTGTGGGGTAAGTCGGATGTGAAATTTAGGGGCTCAACCCCTAAACTGCATT
CGATACTGCTCCGCTAGAGGTATGTAGAGGAGATTGGAATTCACGGTGTAGCAGTGAAAT
GCGTAGATATCGTGAGGAAGACCAGTGGCGAAGGCGAATCTCTGGGCATTACCTGACGCT
CATGCACGAAGGCCAGGGGAGCAAACGGGATTAGATACCCCTGTAGTCC
>Otu8924
CCAGCCTATGGGGGGCTGCAGGTTCGAGTTCTCCACACTGCATGAAAATGCGATGGGGGA
ATCCCACGTGCTTATGTCTAACATAGGCTTTTGCCAAGACCAGAAACCTTGGCGAATAAG
GGGTGGGCAAGACCGGTGCCAGCCGCCGCGGTAACACCGGCGCCCCAAGTGGCACTCGCG
ATTATTGGGCCTAAAGCGTCCGTAGCCGGATTGGTAAATCCTCTGTGAAATCGTGCTACT
CAACGGTACGGCGCGCAGGGGACACTGCTAGTCTCGGGACCGGGAGGGGTCAGAGGTATG
CCGTGGGGACCGGTAAAATGGGATAATCCACGGTAGACCACCAATGGCGAAGGCATCTGA
CCAGAACGGATCCGACGGTGAGGGACGAAAGCCAGGGGAGCGAACCGGATTAGAGACCCG
AGTAGTCC
>Otu8927
CCAGCCTATGGGGTGCTGCAGACGAGAATATTCGACAATGGGCGAAAGCCTGATCGAGCG
ACGCCGCGTGGAGGATGAAGTTCTTCGGAATGTAAACTCCTTTTGCCGGGGAAAAAGTTA
TTGATTGTACCCGGAGAATAAGAAGTTGCTAAACTCGTGCCAGCAGCAGCGGTAATACGA
GTGCTTCAAGCGTTATCCGGAATCATTGGGCGTAAAGGGTGTGTAGGCGGCGATGTTAGT
CTCGCGTTAAATCTTTCGGCTCAACCGAGAGTCCGCGCGGGAAACGGCATTGCTTGAGGA
TGGAAGGGGTCTGCGGAACTCATGGTGTAGCGGTGAAATGCGTTGATATCATGGGGAACA
CCGAAAGCGTAGGCAGCAGACTGGTCCACTCCTGACGCTGAAACACGAAAGCGTGGGTCG
CGAATGGGATTAGAAACCCCCGTAGTCC
>Otu8928
CCAGCCTATGGGGTGCTGCAGTCGAGGATCTTCGGCAATGGGCGCAAGCCTGACCGAGCG
ACGCCGCGTGGACGATGAAGGCCTTCGGGTTGTAAAGTCCTGTCGAGGGGGAGGAAGCCG
CAAGGTTGACCTATCCCTGGAGGAAGCACGGGCTAAGTTCGTGCCTGCAGCCGCGGTAAG
ACGAACCGTGCGAACGTTATTCGGAATCACTGGGCTTAAAGGGCGCGTAGGCGGGTCGCC
AAGTCAGGGGTGAAATCTTCCGGCTTAACCGGAAAACGGCCCCCGATACTGGCGGTCTTG
AGGGGGGTAGGGGCACGCGGAACATCCAGTGGAGCGGTGAAATGCGTTGATATCGGATGG
AACGCCGGTGGCGAAAGCGGCGTGCTGGACCTCATCTGACGCTGAGGCGCGAAAGCCAGG
GTAGCGAACGGGATTAGAGACCCCTGTAGTCC
>Otu8929
CCAGCCTATGGGGTGCTGCAGTCGAGAATCTTCGGCAATGGGGGAAACCCTGACCGAGCG
ACGCCGCGTGTGAGATGAAGGCCTTCGGGTTGTAAATCACTGTCGTAGAGGAGGAAATGT
AGGTGGGTTCTCCCACTTATTTGACCTATTCTAGGAGGAAGGACGGGCTAAGTTCGTGCC
AGCAGCCGCGGTAAGACGAACCGTCCAAACGTTATTCGGAATTACTGGGCTTAAAGGGTG
CGTGGGCGGTCTGACAAGTTGGGTGTGAAAGCCCTCAGCTCAACTGAGGAACTGCGCCCA

AAACTGTTGAACTCGAGGGAGACATAGGTGAGCGGAACTTAGGGTGGAGCGGTGAAATGC
GTTGAGATCCTAAGGAACACCAGTAGCGTAAGCGGCTCACTGGGTCTCTTCTGACGCTGA
TGCACGAAAGCTAGGGGAGCGAAGAGGATTAGATACCCCAGTAGTCC
>Otu8934
CCAGCCTACGGGGGGCTGCAGTGAGGAATATTGCGCAATGGACGGAAGTCTGACGCAGCA
ATGCCGCGTGCGGGATGAAGGCGCTATGCGTCGTAAACCGCTGTAGCGTGGGGCAAAAAG
CACCCTTGGGGTGTTAGATGGTACCACGAAAGTAAGCATCGGCTAACTACGTGCCAGCAG
CCGCGGTAATACGTAGGATGCAAGCGTTGTCCGGATTTACTGGGTGTAAAGGGTGCGCAG
GCGGGCTTGTAAGTCAGAGGTGAAAGCCCGGAGCTTAACTCCGGAAATGCCTTTGATACT
GCAGGTCTTGAATCTGATAGAGGCTCATGGAATATGTGGTGTAGCGGTGAAATGCGTAGA
TATCACATAGAACACCAGTGGCGAAGGCGGTGAGCTGGGTCAGTATTGACGCTCATGCAC
GAAAGTGTGGGTAGCAAACAGGATTAGAAACCCTCGTAGTCC
>Otu8936
CCAGCCTATGGGGGGCTCCAGCGGGGAATTTTGCACAATGGGGGAAACCCTGATGCAGCA
ACGCCGCGTGGAGGATGAAGTCCCTTGGGATGTAAACTCCTTTCGACCGGGACGATAATG
ACGGTACCGGTGGAAGAAGCCCCGGCTAACTCTGTGCCAGCAGCCGCGGTAATACAGAGG
TCCCAAGCGTTGTTCGGATTCACTGGGCGTAAAGGGTGCGTAGGTGGCGGGGTTAGTCGG
ATGTGAAATCTCCGAGCTCAACTCGGAAATGGCATTGGAAACTGCCTTGCTCGAGGGTTG
GAGGGGGGACTGGAATACTTGGTGTAGCAGTGAAATGCGTAGATATCAAGTGGAACACCA
GTGGCGAAGGCGAGTCCCTGACAACTCCTGACACTGAGGCACGAAAGCTAGGGGAGCAAA
CAGGATTAGAAACCTGAGTAGTCC
>Otu8939
CCAGCCTATGGGGGGCTCCAGTGGGGAATATTGGACAATGGGCGAAACCCTGATCCAGCG
ACGCCGCGTGTGTGAAGAAGGCCTGCGGGTTGTAAAGCACTTTTAGTGGGGACAAAAAGC
CGAGGGCTAATACCTTTCGGTCTTGATTTAACCCAAAGAAAAAGCACCGGCTAACTCTGT
GCCAGCAGCCGCGGTAATACAGAGGGTGCAAGCGTTAATCGGAATTACTGGGCGTAAAGC
GAGCGTAGTCGGTTATTTAAGTCGGGTGTGAAAGCCCCGGGCTCAACCTGGGAATTGCAT
TCGATACTGGATAGCTAGAGTTCGGCAGAGGGAAGCGGAATTTCCGGTGTAGCGGTGAAA
TGCGTAGATATCGGAAGGAACATCAGTGGCGAAAGCGGCTTCCTGGACCAGAACTAACGA
TCAGGCTCGAAAGCGTGGGGAGCAAACAGGATTAGATACCCGCGTAGTCC
>Otu8943
CCAGCCTATGGGTTGCAGCAGTGGGGAATTTTGGACAATGGGGGCAACCCTGATCCAGCG
ATGCCGCGTGTGTGAAGAAGGCCTTCGGGTTGTAAAGCACTTTTAGTGGGGAAGAAAAGC
GTAAGGTTAATACCCTTGCGTCTTGACGTTACCTACAGAAAAAGCACCGGCTAACTCTGT
GCCAGCAGCCGCGGTAATACAGAGGGTGCAAGCGTTAATCGGAATTATTGGGCGTAAAGC
GCACGTAGGTGGATATTTAAGTCGGGTGTGAAAGCCCCGGGCTCAACCTGGGAATCGCAT
TCGATACTGAATACCTAGAGTATGGTAGAGGGAGGTAGAACTTCCGGTGTAGCGGTGAAA
TGCGTAGATATCGGAAAGAATACCAGTGGCGAAGGCGGCCTCCTGGACTAATACTGACAC
TGAGGTGCGAAAGCGTGGGGAGCAAACAGGATTAGAGACCCTAGTAGTCC
>Otu8944
CCAGCCTATGGGGCGCTGCAGCAACGAATCTTCCCCAATGGACGAAAGCCTGATGGAGCG
ACGCCGCGTGGGGGATGAATGGCTTCGGCCCGTAAACCCCTGTCATTTGCGATCAACCGT
TATTATTTAAAAGATGATGACCTGATAGTAGCGAAAGAGGAAGGGACGGCTAACTCTGTG
CCAGCAGCCACGGTAATACAGAGGTCCCAAGCGTTGTTCGGATTCACTGGGCGTAAAGGG
TGCGTAGGTGGCGAGGTAAGTCGGATGTGAAATCTCCAAGCTCAACTTGGAAAATGCATT
GGAAACTACCTTGCTCGAGGGTTGGAGGGGGGACTGGAATACTTGGTGTAGCAGTGAAAT
GCGTAGATATCAAGTGGAACACCAGTGGCGAAAGCGAGTCCCTGGACAACTCCTGACACT
GAGGCACGAAAGCTAGGGGAGCAAACAGGATTAGATACCCTCGTAGTCC
>Otu8949
CCAGCCTATGGGTGGCAGCAGTGGGGAATCTTCCGCAATGGACGAAAGTCTGACGGAGCG
ACGCCGCGTGAGTGACGAAGGCCCTCGGGTCGTAAAGCTCTGTCATTGGGGACGAACGGC
CAGGTCAGGAAATGGGCCTGGAGTGACGGTACCCGAGAAGGAAGCCACGGCTAACTACGT
GCCAGCAGCCGCGGTAATACGTAGGTGGCGAGCGTTGTCCGGAATTATTGGGCGTAAAGC
GCGCGCAGGTGGGATCTTAAGTCTGTTTTCAAAGTGCGGAGCTTAACTCCGTGATGGGAT
GGAAACTGGGATTCTTGAGTACCGGAGAGGAAAGCGGAATTCCCCGTGTAGCGGTGAAAT
GCGTAGATATGGGGAGGAACACCAGTGGCGAAGGCGGCTTTCTGGACGGTGTCTGACACT
GAGGCGCGAAAGCCAGGGGAGCGAACGGGATTAGAAACCCTAGTAGTCC
>Otu8955

CCAGCCTACGGGTGGCAGCAGTCGAGAATCTTTCACAATGGGGGAAACCCTGATGGAGCG
ACGCCGCGTGGAGGATAAGGTCTTCGGATTGTAAACTCCTGTCATGCGAGAACAAGAAAG
TGATAGTATCGCAAGAGGAAGAGACGGCTAACTCTGTGCCAGCAGCCGCGGTAATACAGA
GGTCTCAAGCGTTGTTCGGAATCACTGGGCGTAAAGGGTGCGTAGGCGGCGCGGAAAGTC
AGATGTGAAATCCGGAGGCTCAACCTCCGAATAGCATCCGATACTACCGCGCTAGAGAGC
TGTAGGGAAGTCTGGAATTCTCGGTGTAGCAGTGAAATGCGTAGATATCGAGAGGAACGC
TCGTGGCGAAGGCGAGACTTTGGACAGCAACTGACGCTGAGGCACGAAGGCTAGGGGAGC
AAACGGGATTAGAGACCCGCGTAGTCC
>Otu8956
CCAGCCTATGGGGGGCTGCAGCTAAGAATCTTCCGCAATGGGGGAAACCCTGACGGAGCG
ACGCCGCGTGGGGGATGAATGGCTTCGGCCCGTAAACCCCTGTCATTTGCGATCAACCGT
TGTTATTTAAAAGATGACAGCCTGATAGTAGCGAAAGAGGAAGGGACGGCTAACTCTGTG
CCAGCGGCCGCGGTAATACAGAGGTCCCAAGCGTTGTTCGGATTCACTGGGCGTAAAGGG
TGCGTAGGTGGCGGGGTAAGTCGGATGTGAAATCTCGGAGCTCAACTCCGAAACTGCATT
GGAAACTACCTTGCTTGAGGGTTGGAGGGGGGACTGGAATACTTGGTGTAGCAGTGAAAT
GCGTAGATATCAAGTGGAACACCAGTGGCGAAGGCGAGTCCCTGGACAACTCCTGACACT
GAGGCACGAAAGCTAGGGGAGCAAACAGGATTAGAAACCCTCGTAGTCC
>Otu8967
CCAGGCCTACGGGGGGCAGCAGTGGGGAATATTGGACAATGGGGGAAACCCTGATCCAGC
AATGCCGCGTGTGTGAAGAAGGCCTGAGGGTTGTAAAGCACTTTCAGTGGGGAGGAGGAT
TATCAGGTTAAGAGCTGGATAATTGGACGTTACCCACAGAAGAAGCACCGGCTAACTCCG
TGCCAGCAGCCGCGGTAATACGGAGGGTGCGAGCGTTAATCGGAATTACTGGGCGTAAAG
GGTGCGTAGGTGGTTGGATAAGTTATCTGTGAAAGCCCCGGGCTCAACCTGGGAAGGTCA
GATAAGACTGTTCGACTCGAGTATAGGAGAGGGTAGTGGAATTTCCGGTGTAGCGGTGAA
ATGCGTAGAGATCGGAAGGAACACCAGTGGCGAAGGCGGCTACCTGGCCTGATACTGACA
CTGAAGCACGAAAGCGTGGGGAGCAAACAGGATTAGAAACCCGAGTAGTCC
>Otu8969
CCAGCCTAGGGTTGCAGCAGTAGGGAATATTGGACAATGGGCGCAAGCCTGATCCAGCCA
TGCCGCGTGCAGGAAGAAGGCCTTCTGGGTTGTAAACTGCTTTTGACAGAGAACAAAAAG
ACCCTGCGGGGTAAATTGAGTGTATCTGTAGAATAAGCCACGGCTAACTACGTGCCAGCA
GCCGCGGTAATACGTAGGTGGCAAGCGTTATCCGGATTTATTGGGTTTAAAGGGTGCGTA
GGCGGTTATGTAAGTCAGTGGTGAAAGACTGCCGCTCAACGGTAGCATTGCCATTGATAC
TGCCTAGCTAGAGTACAGTAGAGGTAGGCGGAATTTATGGTGTAGCGGTGAAATGCATAG
ATACCCTAAAGAACACCGATTGCGAAGGCAGCTTACTGGACTGTAACTGACGCTGAGGCA
CGAAAGTATGGGAATCAAACAGGATTAGAGACCCTGGTAGTCC
>Otu8970
CCAGCCTACGGGGACGCAGCAGTGGGGAATTTTCCGCAATGGGCGAAAGCCTGACGGAGC
AATACCGCGTGAGGGAGGAAGGCTCTTGGGTTGTAAACCTCTTTTCTCAGGGAAGAAAAA
AATGACGGTACCTGAGGAATCAGCATCGGCTAACTCCGTGCCAGCAGCCGCGGTAATACG
GAGGATGCAAGCGTTATCCGGAATGATTGGGCGTAAAGCGTCCGCAGGTGGTAGTTCAAG
TCTGCTGTTAAAAAGTTTGGCTCAACCAAATAAGGGCGGTGGAAACTGAATAACTAGAGT
GTGATAGGGGTAGAGGGAATTCCTGGTGTAGCGGTGAAATGCGTAGAGATCAGGAAGAAC
ACCGGTGGCGAAAGCGCTCTGCTAGGTCACGACTGACACTGAGGGACGAAAGCTAGGGGA
GCGAATGGGATTAGATACCCGCGTAGTCC
>Otu8971
CCAGCCTACGGGAGGCTCCAGTGGGGAATCTTGGACAATGGGCGCAAGCCTGATCCAGCC
ATGCCGCGTGAGTGATGAAGGCCTTAGGGTTGTAAAACTCTTTCGCCGAGGACGATAATG
ACGGTACTCGGAGAAGAAGCTCCGGCTAACTTCGTGCCAGCAGCCGCGGTAATACGAAGG
GGGCTAGCGTTGTTCGGATTTACTGGGCGTAAAGCGCGTGTAGGCGGATTTGTAAGTTGG
GGGTGAAATCCCGAGGCTCAACCTCGGAACTGCCTCCAAAACTACAAGTCTGGAGCAAGA
TAGAGGCAAGTGGAATTGCGAGTGTAGAGGTGAAATTCGTAGATATTCGCAGGAACACCA
GTGGCGAAGGCGACTTGCTGGATCTTTGCTGACGCTGAGACGCGAAAGCGTGGGGAGCAA
ACAGGATTAGAAACCCGCGTAGTCC
>Otu8977
CCAGCCTATGGGATGCAGCAGCCAAGAATCTTCCGCAATGGGGGAAACCCTGACGGAGCG
ACGCCGCGTGAAGGATGAAGGTCTTCGGATTGTAAACTTCTTTTATCTGGGAAGAATAAG
TCCGGTAGGAAATGACCGGATGATGACGGTACCAGAGGAATAAGCCCCGGCTAATTACGT
GCCAGCAGCCGCGGTAATACGTAAGGGGCGAGCGTTGCTCGGAATTACTGGGCGTAAAGG

GCTTGTAGGCGGCCTTGTAAGTCGAATGTGAAAAACCGTGGCTCAACCCCGGAAACGCGT
TCGAAACTGCGAGGCTTGAGTTTGGTGGAGGGCAATGGAACACCCGGTGTAGCGGTGAAA
TGCGTAGATATCGGGTAGAACACCGAAGGCTAAAGCAGTTGTCCATGCCAAAACTGACGC
TGAGGAGCGAAAGTATGGGGATCAAACAGGATTAGATACCCGTGTAGTCC
>Otu8991
CCAGCCTACGGGTTGCAGCAGACGAGAATATTCCGCAATGGGCGAAAGCCTGACGGAGCG
ACGCCGCGTGATTGATGAAGTGCTTCGGTACGTAAAGATCTTTTATCGGGGACGAAGTTT
ATTGACGGTACCCGATGAATAAGGGGCTCCTAACTCTGTGCCAGCAGGAGCGGTAATACA
GAGGCCCCAAGCATTACCCGGAATCACTGGGCGTAAAGGGTGTCTAGGCGGCCATATTAG
TCGTTCGTTAAATCTGCGGGCTCAACCTGCAGGCTGCGAGCGAAGCGGTATGGCTCGAGG
GCGTAAGAGGTGCACGGAACTCATGGTGGAGGGGTGAAATCCGTTGATATCATGGGGAAC
ACCAAAGGCGAAGGCAGTGCACTGGTACGTTCCTGACGCTCAAACACGAAAGCCAGGGTA
GCGAACGGGATTAGATACCCCTGTAGTCC
>Otu8992
CCAGTTCGCGATGCCCGAAAGGGTGACGAAGCGACGCCGCGTGTGGAAGAAGGCCTTCGG
GTTGTAAACCACTGTCGGGAGTTAAGAAGTGTAAGGGGGTGAATAGTCCTCTTGCTTGAC
GTTAGCTCCGGAGGAAGCCACGGCTAACTCTGTGCCAGCAGCCGCGGTAATACAGAGGTG
GCAAGCGTTGTTCGGAATTATTGCGCGTAAAGAGCACGTAGGTGGCCCTACAAGTCAGCT
GTGAAATCCTTCTGCTCAACGGAAGAACGGCAGTTGATACTATAGGGTTCGAGTGCGGGA
GGGGAGAGTGGAACTTCTGGTGGAGCGGTGAAATGCGTAGATATCAGAAGGAACATCGGC
GGCGAAAGCGACTCTCTGGCCCGTAACTGACGCTGAGGTGCGAAAGCTAGGGGAGCAAAC
GGGATTAAAAACCCCTCTACTTAGTGCC
>Otu8994
CCAGCCTATGGGGGGCTGCAGTCGAGGATCTTTTGCAATGGGCGAAAGCCTGACAAAGCG
ACGCTGCGTGGAGGATGAAGGCCTTCGGGTTGTAAACTCCTGTCATTCGGAAACAATTGC
CGATCGGCTAATATCCGATCGGTTTGATGGTACCGGAAGAGGAAGCCACGGCTAACTCTG
TGCCAGCAGCCGCGGTAATACAGAGGTGGCGAGCGTTGTTCGGATTTACTGGGCGTAAAG
GGTGCGTAGGTGGTCTTGTGTGTCGGATGTGAAATCCCACTGCTCAACGGTGGAACGGCA
TCGGAAACTGCAAGACTCGAGTGCAGGAGGGGAGAGCGGAATACTTGGTGTAGCGGTGAA
ATGCGTTGATATCAAGTAGAACACCGGTGGCGTAGGCGGCTCTCTGGAATGCAACTGACA
CTGAGGCACGAAAGCTAGGGAAGCAAACAGGATTAGATACCCTAGTAGTCC
>Otu9006
CCAGCCTACGGGTCGCTGCAGTGGGGAATTTTGGACAATGGGGGCAACCCTGATCCAGCC
ATGCCGCGTGAGTGAAGAAGGCCTTCGGGTTGTAAAGCTCTTTCGCGAGGGAAGAAAAGG
TTCTGGCTAATATCCAGAGCTCATGACGGTACCTTGATAAGAAGCACCGGCTAACTACGT
GCCAGCAGCCGCGGTAATACGTAGGGTGCGAGCGTTAATCGGAATTACTGGGCGTAAAGC
GTGCGCAGGCGGTTTTGTAAGACAGACGTGAAATCCCCGGGCTCAACCTGGGAACTGCGT
TTGTGACTGCAAGACTAGAGTGTGGCAGAGGGGGGTAGAATTCCACGTGTAGCAGTGAAA
TGCGTAGAGATGTGGAGGAATACCGATGGCGAAGGCAGCCCCCTGGGCTAACACTGACGC
TCATGCACGAAAGCGTGGGGAGCAAACAGGATTAGATACCCGAGTAGTCC
>Otu9008
CCAGCCTATGGGTCGCACCAGTGGGGAATTTTGCACAATGGGGGAAACCCTGATGCAGCA
ATGCCGCGTGGAGGATGAAGTTCTTTGGAACGTAAACTCCTTTCGATCGGGACGATTATG
ACGGTACCGGAAGAAGAAGCCCCGGCTAACTCTGTGCCAGCAGCCGCGGTAATACAGAGG
GGGCAAGCGTTGTTCGGAATTATTGGGCGTAAAGGGTGCGTAGGCGGTGCGGTAAGTCTT
TTGTGAAATCTCCGGGCTCAACTCGGAGCCTGCAAAGGATACTGTCGTGCTTGAGTATGG
GAGAGGTGAGTGGAATTCCTGGTGTAGCGGTGAAATGCGTAGATATCAGGAGGAACACCT
GTGGCGAAGGCGGCTCACTGGACCATTACTGACGCTGATGCACGAAAGCTAGGGGAGCAA
ACAGGATTAGATACCCTTGTAGTCC
>Otu9014
CCAGCCTATGGGACGCAGCAGTGGGGAATATTCCGCAATGGGCGAAAGCCTGACGGAGCG
ACGCCGCGTGATGGATGAAGTGCTTTGGTACGTAAACATCTTTTATCGGGGACGAAGTTT
ATTGACGGTACCCGATGAATAAGGGGCTCCTAACTCTGTGCCAGCAGGAGCGGTAATACA
GAGGCCCCAAGCATTACCCGGAATTACTGGGCGTAAAGAGTGTCGAGGCGGCCATATTAG
TCTCTCGTAAAATCCGTGGGCTCAACCTACGGCGCGCGGGGGAAACGGTATGGCTCGAGG
GCGCGAGAGGTGCACGGAACTCATGGTGGAGGGGTGAAATCCGTTGATATCATGGGGAAC
ACCAAAGGCGAAGGCAGTGCACTAGTGCGTTCCTGACGCTCAAACACGAAAGCCAGGGTA
GCGAATGGGATTAGAACCCTAGTAGTCC

>Otu9016
CCAGCCTACGGGGCGCTGCAGTAGGGAATTTTGCGCAATGGGCGAAAGCCTGACGCAGCA
ACGCCGCGTGAGTGATGAAGCCCTTCGGGATGTAAAGCTCTGTTGAGTGGGAAGAAAAAT
TATCAGTCTAATAAACTGAGAGTCTGACGGTACCATTCGAGTAAGCACCGGCTAACTACG
TGCCAGCAGCCGCGGTAATACGTAGGGTGCAAGCGTTGTTCGGAATCACTGGGTGTAAAG
GGAGCGTAGGCGGGTTTATAAGTTGGAAGTTTAATACACAGGCTTAACCTGTGAACGGCT
TTCAAGACTGTATATCTTGAGTATGGGAGAGGGTGATGGAATTCCAGGTGTAGCGGTGGA
ATGCGTAGATATCTGGAAGAACACCAGCTGCGAAGGCGGTCGCCTGGCCCAATACTGACG
CTGAGGCTCGAAAGCTAGGGGAGCAAACAGGATTAGAGACCCCTGTAGTCC
>Otu9029
CCAGCCTATGGGGTGCTGCAGTCGAGAATTTTTCACAATGGGGGAAACCCTGATGGAGCG
ACGCCGCGTGGGGGATGAATGGCTTCGGCCCGTAAACCCCTGTCATTTGCGAACAAACTT
TACGTGTTAATAGCGCGTGAATTGATTGTAGCGGAAGAGGAAGGGACGGCTAACTCTGTG
CCAGCAGCCGCGGTAATACAGAGGTCCCAAGCGTTGTTCGGATTCACTGGGCGTAAAGGG
TGCGTAGGTGGTCGGGTAAGTCTGATGTGAAATCTCGCAGCTCAACTGCGAAACGGCATT
GGATACTATCCGGCTAGAGGATCGGAGGGGGGACTGGAATCCTCGGTGTAGCAGTGAAAT
GCGTAGATATCGAGAGGAACACCAGTGGCGAAGGCGAGTCCCTGGACGACTCCTGACACT
GAGGCACGAAAGCTAGGGGAGCAAACAGGATTAGAAACCCTGGTAGTCC
>Otu9035
CCAGCCTATGGGGGGCTGCAGTAGGGAATATTGCGCAATGGAGGAAACTCTGACGCAGCG
ACGCCGCGTGAGTGACGAAGGACTTCGGTTCGTAAAGCTCTGTTGCAGGGGAATAAGAAA
GTGAATGTACCCTGTGAGGAAGGGTCGGCTAACTTCGTGCCAGCAGCCGCGGTAAGACGA
GGGACCCTAGCGTTGTTCGGAATCATTGGGCGTAAAGCGGATGTAGGTGGCTTGGTAAGC
CAGGTGTGAAAGCCCGGGGCTTAACCCTGGAAGTGCATTTGATACTGCTGAGCTTGAGTG
CTGGAGAGGCTGCTAGAATTCCTGGTGTGGTGGTGAAATACGTAGATATCAGGTGGAATA
CCGGAGGCGAAGGCGGGTAACTAGCTAGACACTGACACTGAGATCCGAAAGTGTGGGGAT
CAAACAGGATTAGAAACCCTAGTAGTCC
>Otu9047
CAGCCTATGGGTCGCTCCAGTGGGGAATTTTGCGCAATGGGGGAAACCCTGACGCAGCAA
CGCCGCGTGGAGGATGAAGTACCTTGGTACGTAAACTCCTTTCGATCGGGACGATAATGA
CGGTACCGAGAGAAGAAGCCCCGGCTAACTTCGTGCCAGCAGCCGCGGTAATACGAGGGG
GGCGAGCGTTGTTCGGAATTATTGGGCGTAAAGGGTGCGTAGGCGGTTTTGTAAGTCGGA
TGTGAAATCCCCAGGCTCAACTTGGGAACCGCATCTGATACTGCATGACTAGAGTACTGC
AGAGGGAAACGGAATTCCTGGTGTAGAGGTGAAATTCGTAGATATCAGGAGGAACACCGG
TGGCGAAGGCGGTTTCCTGGGCAGATACTGACACTGAGGCGCGAAAGCGTGGGTAGCAAA
CAGGATTAGAAACCCCTGTAGTCC
>Otu9050
CCAGCCTACGGGAGGCTGCAGGGAATCGTACACAATGGACGAAAGTCTGATGTCGCAATG
CCGCGTGGAGGATGAAGGCCTTCGGGTTGTAAACTCCTTTTACAGTAATAGAGATGTTAT
TGAGAATAAGCACCTGCTAACTCTGTGCCAGCAGCCGCGGTAATACAGAGGGTGCAAGCG
TTATCCGGATTTATTGGGCGTAAAGCGTTTCGTAGGTGACCAGGTAAGTTATCTTTCAAA
GACCACGGCTCAACCGGGGGAAGGGAGGTAATACTGCCGGGTTTGATTTATGGTGGGGCT
TCTGGAACTGATGGTGTAGTAGTGAAATACGTTGATATCATCAGGAACTCCAAGGGCGAA
GGCAGGAAGCTAACCATGTAATGTCACTGAGGAACGACAGCTAGGGGAGCGAAAGGGATT
AGAAACCCGCGTAGTCC
>Otu9058
CCAGCCTATGGGGGGCTCCAGTGGGGAATATTGGGCAATGGGCGAAAGCCTGACCCAGCA
ATGCCGCGTGAGTGATGAAGGTCGAAAGATTGTAAAGCTCTTTTGCCGGGGACGATTATG
ACGGTACCCGGAGAATAAGGACTGACTAACTACGTGCCAGCAGTCGCGGTAATACGTAGA
GTCCAAGCGTTACTCGGAATTACTGGGCGTAAAGTATGCGTAGGCGGTTTTGTAAGTCAG
TAGTGAAATACCGGGGCTCAACTTCGGTGCTGCTATTGATACTGCATTACTAGAGTCTGG
TAGAGGGAAACGGAATTCTTAGTGTAGGGGTAAAATCCATAGATATTAAGAGGAACGCCG
GAGGCGAAAGCGGTTTCCTGGGCCATGACTGACGCTGAGGCATGACAGCGTGGGGAGCAA
ACAGGATTAGAAACCCCAGTAGTCC
>Otu9065
CCAGCCTACGGGATGCAGCAGTGGGGAATATTGGACAATGGGCGCAAGCCTGGTCCAGCC
ATGCCGCGTGAGTGATGAAGGCCTTCGGGTTGTAAAGCTCTTTCGACGGGGACGATGATG
ACGGTACCCGTAGAAGAAGCCCCGGCTAACTTCGTGCCAGCAGCCGCGGTAATACGAAGG

GGGCAAGCGTTGTTCGGAATTACTGGGCGTAAAGGGCGCGTAGGCGGCTTCGTCAGTCAG
GTGTGAAAGCCCTGGGCTCAACCCGGGAACTGCATTTGATACTGCGAAGCTCGAGTCTCG
GAGAGGTTGGTGGAATTCCCAGTGTAGAGGTGAAATTCGTAGAGATTGGGAAGAACACCG
GTGGCGAAGGCGGCCAACTGGACGAGCACTGACGCTGAGGCGCGAAAGCGTGGGGAGCAA
ACAGGATTAGATACCCCTGTAGTCC
>Otu9067
CCGCCTACGGGGGGCTGCAGTGGGGAATTTTGGACAATGGGCGCAAGCCTGATCCAGCCA
TGCCGCGTGAGTGAAGAAGGCCTTCGGGTTGTAAAGCTCTTTCGCGAGGGAAGAAAAGGC
TACAGATAATACCTGTGGCCCATGACGGTACCTTGATAAGAAGCACCGGCTAACTACGTG
CCAGCAGCCGCGGTAATACGTAGGGTGCGAGCGTTAATCGGAATTACTGGGCGTAAAGCG
AGCGCAGGCGGTTCTAAAAGCCAGATGTGAAATCCCCGAGCTCAACTTGGGAACTGCGTT
TGGAACTCTAGGACTAGAGTGTGTCAGAGGGAGGTAGAATTCCACGTGTAGCAGTGAAAT
GCGTAGAGATGTGGAGGAACACCGATGGCGAAGGCAGCCTCCTGGGATAACACTGACGCT
CATGCTCGAAAGCGTGGGGAGCAAACAGGATTAGAGACCCCAGTAGTCC
>Otu9071
CCAGCCTACGGGAGGCACCAGTAGGGAATATTGCACAATGGAGGAAACTCTGATGCAGCG
ACGCCGCGTGAGTGATGAAGGACTTCGGTTCGTAAAGCTCTGTCGCAGGGGAATAACACA
ATGAAGGTACTCTGCGAGAAAGGGTTGGCTAACTTCGTGCCAGCAGCCGCGGTAAGACGA
GGGACCCTAGCGTTGTTCGGAATCATTGGGCGTAAAGCGGGTGTAGGTGGCTTTGTGAGT
CAGATGTGAAAGCCCAGGGCTCAACCCTGGAAGTGCATTTGGTACTGCGAAGCTTGAGTG
CTGGAGAGGTTACTGGAATTCCTGGTGTAGTGGTGAAATACGTAGATATCAGGAGGAATA
CCGGAGGCGAAGGCGGGTAACTGGACAGACACTGACACTCAAACCCGAAAGCGTGGGGAT
CAAACAGGATTAGATACCCTTGTAGTCC
>Otu9072
CCAGCCTACGGGTTGCAGCAGCTAAGAATATTCCACAATGGACGAAAGTCTGATGGAGCG
ACGCCGCGTGGACGATGAAGGCCTTCGGGTTGTAAAGTCCTTTCGTGCGGGAAGATCTTT
CTGCCGGTTAATAATCGGCGGAATCGACTGTACCGCAGGAAGAAGCCCCGGCTAATTACG
TGCCAGCAGCCGCGGTAATACGTATGGGGCGAGCGTTATTCGGATTTATTGGGCGTAAAG
GGTATGCAGGGGGCCTGTTAAGTCGGGAGTGAAATTTCTAAGCTCAACTTAGAAATTGCT
TTCGATACTGGCAGGCTAGAGTTTGGCAGAGGAGAATGGAATTCCAGGTGTAGGGGTGAA
ATCTGTAGATATCTGGAAGAACACCAGTGGTGAAGACGATTCTCTGGGCCAAAACTGACC
CTCAGATACGAAAGCCAGGGGAGCAAACGGGATTAGATACCCCAGTAGTCC
>Otu9075
CCAGCCTATGGGGTGCACCAGTCGAGAATTTTTCTCAATGGGCGAAAGCCTGAAGGAGCG
ACGCCGCGTGGGGGATGAATGGCTTCGGCCCGTAAACCCCTGTCATTTGCGAACAAATTG
ACTCGCTTAACACGTGTGTCATTGATTGTAGCGAAAGAGGAAGGGACGGCTAACTCTGTG
CCAGCAGCCGCGGTAATACAGAGGTCCCAAGCGTTGTTCGGATTCACTGGGCGTAAAGGG
TGCGTAGGTGGCGAAGTAAGTCGGATGTGAAATCTCCGAGCTCAACTCGGAAACTGCATT
GGAAACTACTTTGCTTGAGGGTTGGAGGGGGGACTGGAATACTTGGTGTAGCAGTGAAAT
GCGTAGATATCAAGTGGAACACCAGTGGCGAAGGCGAGTCCCTGGACAACTCCTGACACT
GAGGCACGAAAGCTAGGGGAGCAAACAGGATTAGAGACCCCGGTAGTCC
>Otu9083
CCAGCCTACGGGTGGCTGCAGTGGGGAATATTGGACAATGGGGGAAACCCTGATCCAGCC
ATGCCGCGTGAGTGATGAAGGCTTTAGGGTTGTAAAGCCCTTTCACCGATGAAGATAATG
ACGGTAGTCGGAGAAGAAGCCCCGGCTAACTTCGTGCCAGCAGCCGCGGTAATACGAAGG
GGGCTAGCGTTGTTCGGAATTACTGGGCGTAAAGCGAGCGTAGGTTGCTCGACAAGTCAG
TGGTGAAATCCCAGAGCTTAACTCTGGAACTGCCATTGAAACTGTCGAGCTGGAATCTCA
GAGGGGGTAGCGGAATTCCAAATGTAGGGGTGAAATCCGTAGATATTTGGAGGAACACCG
GTGGCGAGGGCGGCTACCTGGATGAGTATTGACACTGAGGCTCGAAAGCGTGGGGATCAA
ACAGGATTAGAAACCCGGGTAGTCC
>Otu9086
CCAGCCTATGGGTGGCTGCAGCAACGAATCTTCCCCAATGGCGAAAGCCTGAGGGAGCGA
CGCCGCGTGGAGGACGAAGTACTTCGGTATGTAAACTCCTGTTAGGGTTACGAAAGCGTA
AGGGGCTAATACCCTCTGAGTTGATCTAATCCAGAGAAAGGGACGGCTAACTCTGTGCCA
GCAGCCGCGGTAATACAGAGGTCCCAAGCGTTACTGAGAATCACTGGGTTTAAAGGGTGC
GTAGGTGGTCTGTTAAGTTCCTTGTGAAATCCCCGGGCTCAACCCGGGAACGGCTGGGAA
TACTGGCAGACTTGAGGCCAGTATGGGTCACTGGAACTGACGGTGGAGCGGTGAAATGCG
TAGATATCGTCAGGAACGCCGGTGGTGAAGACGGGTGACTGGGCCGGTTCTGACACTGAG

GCACGAAAGCGTGGGGAGCGAACGGGATTAGAAACCCTTGTAGTCC
>Otu9089
CCAGCCTCGGGGTGCTGCAGTAACGAATCTTCCGCAATGGGCGAAAGCCTGACGGAGCGA
CGCCGCGTGCAGGACGAAGTCCTTCGGGATGTAAACTGCTGTCAGGGGTTAGAAAGTTCT
GATCAACCCCAGAGGAAGGCATGGCTAACTCTGTGCCAGCAGCCGCGGTAAGACAGAGGT
GCCAAGCGTTAGGCGGAATCACTGGGCTTAAAGCGTGTGTAGGCGGATGCCTAAGTACCT
TGTGAAATCCCACGGCTCAACCGTGGAACTGCTGGGTATACTGGGTGTCTTGAGCCATTC
AGGGGCGACTGGAACAAACGGTGAGCGGTGAAATGCGTAGATATCGTTTTGAACGCCAAA
GGTGAAAACAGGTCGCTGGGGATGTGCTGACGCTGAGACACGAAAGCCAGGGGAGCAAAC
GGGATTAGATACCCCTGTAGTCC
>Otu9095
CCAGCCTATGGGGGGCTGCAGTCGGGAATTTTTCACAATGGGCGCAAGCCTGATGGAGCG
ACGCCGCGTGGGGGATGAATGGCTTCGGCCCGTAAACCCCTGTCATTTGCGAACAAGTTG
GTTCAACTAATACCTGAACCATTGATAGTAGCGAAAGAGGAAGGGACGGCTAACTCTGTG
CCAGCAGCCGCGGTAATACAGAGGTCCCAAGCGTTGTTCGGATTCACTGGGCGTAAAGGG
TGCGTAGGTGGTGAGGTAAGTCGGATGTGAAATCTCCGAGCTCAACTCGGAAACTGCATT
GGAAACTACCTTGCTCGAGGGTTGGAGGGGGGACTGGAATGCTTGGTGTAGCAGTGAAAT
GCGTAGATATCAAGCGGAACACCAGTGGCGAAGGCGAGTCCCTGGACAACTCCTGACACT
GAGGCACGAAAGCTAGGGGAGCAAACAGGATTAGAAACCCCAGTAGTCC
>Otu9097
CCAGCCTATGGGTGGCTCCAGTGGGGAATCTTGCGCAATGGACGAAAGTCTGACGCAGCC
ACGCCGCGTGAGTGAAGAAGGCCTTCGGGTTGTAAAGCTCTGTCGGAAGGGACAAAAACT
ATGTGGGTTAATAGCCCACGTACTTGATGGTACCTTTAAAGGAAGCACCGGCTAACTTCG
TGCCAGCAGCCGCGGTAATACGAAGGGTGCAAGCGTTGCTCGGAATTATTGGGCGTAAAG
GGTAGGTAGGTGGTTACGTATGTCTGAGGTGAAATCCCTGAGCTTAACTCAGGAAGTGCC
TTGGAAACGGCGTAACTAGAGTATTGGAGAGGTTCGTGGAATTCCCAGTGTAGCGGTGAA
ATGCGTAGAGATTGGGAGGAACACCAGAGGCGAAGGCGGCGAACTGGACAATTACTGACA
CTGAACTACGAAAGCGTGGGGAGCAAACAGGATTAGATACCCGTGTAGTCC
>Otu9107
CCAGCCTACGGGTCGCTGCAGTGGGGAATACTGGACAATGGGCGAAAGCCTGATGGTGCG
ACGCCGCGTGAGGGGCGAAGGCCTTCGGGTTGTAAACCTCTGTCACCGGGGAAGAAACGC
TACATTTTAATAGAATGTAGCCTGACTTAACCCGGAGAAGAAGCAGTGGCTAACTCTGTG
CCAGCAGCCGCGGTAATACAGAGACTGCAAGCGTTATTCGGATTCACTGGGCGTAAAGGG
TGCGCAGGCGGCCAGGTGTGTCAGGTGTGAAAGCCCGGGGCTTAACCCCGGAATTGCGCC
TGAAACTACTTGGCTAGAGCATTGGAGAGGGTAGCAGAATTCACGGTGTAGCAGTGAAAT
GCGTAGATATCGTGAGGAATACCAGAGGCGAAGGCGGCTACCTGGACAATTGCTGACGCT
CAGGCACGAAAGCGTGGGGAGCAAAAGGGATTAGAAACCCGAGTAGTCC
>Otu9108
CCAGCCTATGGGAGGCTGCAGCTGAGAATCTTCCACAATGGACGAAAGTCTGATGGAGCG
ACATCGCGTGAAGGATGAAGGCCTTCGGGTTGTAAACTTCTTTTGACGCTGACGAATAAC
TGACGGTAAGCGTCGAATAAGCCTCGGCTAACTACGTGCCAGCAGCCGCGGTAATACGTA
GGAGGCAAACGTTGTTCGGATTCACTGGGCGTAAAGGGTGAGTAGGCGGAACTGTAAGTC
ATTGGTGAAAGACTGGGGCTTAACCCCAGGAACGCCTTTGATACTACAGTTCTCGAGTGC
GATAGAGGATGGTGGAATTCCTGGTGTAAGGGTGGAATCTGTAGATATCAGGAGGAACAT
CGATGGCGAAGGCAGCCATCTGGGTCGCAACTGACGCTGAATCACGAGAGCTGGAGGAGC
AAACGGGATTAGAAACCCCTGTAGTCC
>Otu9110
CCAGCCTATGGGGTGCTGCAGCAACGAATATTGGACAATGGACGCAAGTCTGATCCAGCG
ACGCCGCGTGTGGGATGAAGTCCTTCGGGATGTAAACCACTGTCAGGGGTAACCAATCAA
TTGAGGTATCCCAGAGGAAGCCACGGCTAACTCCGTGCCAGCAGCCGCGGTAAGACGGAG
GTGGCGAGCGTTACTCGGATTCACTGGGCTTAGAGCGGGTGCAGGCGGCCATATAAGCCT
GGAGTGAATTCCCTCGGCTCAACCGAGGAATTGTTTCGGATACTGTATGGCTTGAGGCAA
GCAGGGGTCAGTGGAACGTTTGGTGTAGCGGTGAAATGCGTAGATATCAAACGGAACGCC
GGTGGTGAAGACGGCTGACTGGGCTTGACCTGACGCTCAGACCCGAAAGCGTGGGGAGTG
AACCGGATTAGAAACCCCTGTAGTCC
>Otu9113
CCAGCCTACGGGATGCAGCAGTGGGGAATTTTGGACAATGGGCGAAAGCCTGATCCAGCA
ACGCCGCGTGGAGGATGAAGGTCTTCGGATTGTAAACTCCTTTTGAGGGGAACGAATAAA

TTGACGGTACCCCTAGAAAAAGCCACGGCTAACTACGTGCCAGCAGCCGCGGTAATACGT
AGGTGGCGAGCGTTACTCGGAATTACTAGGCGTAAAGAGCATGTAGGTGGTTAGGTAAGT
CTGTGGTGAAATTCTCCGGCTTAACTGGAGGGGGTTCACAGATACTACCTGGCTTGAGTG
TGGGAAGGGATGGCGGAATTCCTGGTGTAGCGGTGAAATGCGTAGATATCAGGAGGAACA
CCGATGGCGAAGGCGGCTATCTGGCCCAATACTGACACTGAGATGCGAAAGCTAGGGGAG
CAAACAGGATTAGAAACCCCAGTAGTCC
>Otu9124
CCAGCCTACGGGTGGCTGCAGTGAGGAATATTGGTCAATGGACGGAAGTCTGAACCAGCC
ATCCCGCGTGCAGGATGAAGGCGCTACGCGTTGTAAACTGCTTTTGTACCAGAGAAAACC
TATCTACGTGTAGATAGCTGATAGTATGGTAAGAATAAGCATCGGCTAACTTCGTGCCAG
CAGCCGCGGTAATACGGAGGATGCAAGCGTTATCCGGATTCATTGGGTTTAAAGGGAGCG
TAGGCGGTTTTATAAGTCAGTGGTGAAATCTCGTTGCTTAACAACGAACGTGCCATTGAT
ACTGTAGAACTTGAGTACAGATGATGTTGGCGGAATGTGTCATGTAGCGGTGAAATGCTT
AGATATGACACAGAACACCGATTGCGAAGGCAGCTGACAAAACTGTAACTGACGCTGAGG
CACGAAAGCGTGGGGATCAAACAGGATTAGAAACCCCCGTAGTCC
>Otu9127
CCAGCCTATGGGTGGCACCAGGCGCGAAACCTCTACAATGCACGAAAGTGTGATAGGGGA
ATCCCAAGTGCTTACACAAAGTGTAGGCCTTTGCCAAGAGTAAATATCTTGGCGAATAAG
TGGTGGGAAAGACTGGTGCCAGCAGCCGCGGTAACCCCAGCGCCACAAGTGGCATACACA
TTTATTGGGCCTAAAGCGTCCGTAGCCGGTCGGTTAAATCTCTTGTGAAATCATGGTGCT
TAACTCCATGGCGTGCAGGAGACACTGACTGACTTGGAACCGGGAGGAGTCAGAGGTATT
CTAGGGGGAGCGGTAAAATGTTATAATCCCTAGAGGACCACCTGTGGCGAAGGCGTCTGA
CTAGAACGGATTCGACGGTGAGGGACGAAGGCCAGGGGAGCGAACCGGATTAGAGACCCC
CGTAGTCC
>Otu9146
CCAGCCTACGGGTCGCAGCAGTGGGGAATCTTGGACAATGGGCGCAAGCCTGATCCAGTA
ATATTTATTGTGCGAAAGAGAAGGCTAGCTTCGTAAAGCACTTTTGTAAACGAAAATAAT
GATAAAAGTTTAAGTAATTTAGTCCCGGCTAATTTCGTGCCAGCAGCCGCGGTAATACGG
GAGGGGCAAGCGTTATTCATCATAAGCGGGCGTAAAGGGTGAGTAGGCAATGGTAGATAA
TTAACAAGAAATAACAAAGTACACTTTGTTGCTTGTGTATTAGAAACTAGTTAATAGAGA
CGACGAAATGGGGTTAGAATTACTAATGGAAAGGTAAAATATATTGATATTAGTACGAAT
GCCGAAGGCGAAGGCAAATTTCATTCTAGTCTAACGCTGAGGTGCGAAAGCTTGGGAATC
AAACGGGATTAGATACCCCAGTAGTCC
>Otu9149
CCAGCCTATGGGGCGCTCCAGGTGCGAAACCTTTACAATGCGCGAAAGCGTGATAAGGGA
ACTCTGAGTGGCAGCCGGCTTTTGCCAACTGTCTTTTGGCGAACTTAAAACGTTCGCAGA
ATAAGTTCTGGGTAAGACCAGTGCCAGCCGCCATGTGCACTTGCACAATGCGCACGGTAC
GCGGTAATACTGGCAGAACGAGTGGTGCCCACGAATATTGGGTCTAAAGAGACCGTAGCT
GGCCCGTTAAGTCCACTGTGAAATCTTGGGGCTCAACCTCAAGGCGTGCAGCGGATACTG
GCGGGCTTGAGAGCGGGGGAGGTCAGGGGTACTTACGGGGTAAGAGTAAAATCTGTTGAT
CCTGTAAGGACCACCAGTGGCGAAGGCGCCTGACCGAAACGCGTCTGACAGTGAGGGTCG
AAGGCTAGGAGAACGAATCGGATTAGATACCCCAGTAGTCC
>Otu9160
CCAGCCTATGGGGTGCTGCAGTGAGGAATCTTGGGCAATGAACTAAAGTTTGACCCAGCA
ATATTTCATGGATGATGACAGCTGCTATAGCCGTAAAGTTCGTTTATAAAAGAAGATAAT
GACTGTATTTTATTTAAAGTCCCGGCTAATATTTGTGCCAGCAGCCGCGGTAAGACAAAT
GGGGCAAGCGTTATTCATAATTATTGGGCGTAAAGGGCACGTAGGCTGCTAAATCAATCA
AAGATAAAATACTAGAATAAAGTTTTAGAAACATTATTGATATTGTTTAGCTCAGAGTTA
AGAACAGGTTGAAAGAATTTTTAATGGAGGGGTAAAATCCATAGATATTAAAAAGAATAC
CAACAGTGAAGACATTTGGCTAGTTTTAACTGACGCTGAGGTGCGAAAGCTTAGGTATCA
AACAGGATTAGATACCCCTGTAGTCC
>Otu9162
CCGCCTATGGGGCGCACCAGCTAGGAATATTGCGCAATGGGCGAAAGCCTGACGCAGCAA
CGCCGCGTGCGGGATGACGGCCTTCGGGTTGTAAACCGCTTTTAGTAAGAACGATTATGA
CGGTACTTACAGAAAAAGGTCCGGCTAACTACGTGCCAGCAGCCGCGGTAATACGTAGGG
ACCGAGCGTTGTCCGGATTTATTGGGCGTAAAGAGCTCGTAGGCGGCACGGTAAGTCAGA
AGTGAAAATGCAAGGCTTAACCTTGCATTGCTTTTGATACTGCCGAGCTTGAGTATGGCA
GGGGAAAAGGGAATTCCACGTGTAGCGGTGAAATGCGCAGATATGTGGAGGAACACCAGT

GGCGAAGGCGCTTTTCTGGCCATTACTGACGCTGAGGAGCGAAAGCGTGGGGAGCGAAC
AGGATTAGATACCCTCGTAGTCC
>Otu9164
CCAGCCTACGGGGTGCAGCAGACGAGAATATTCGACAATGGGCGAAAGCCTGATCGAGCG
ACGCCGCGTGGAGGATGAAGTTCTTCGGAATGTAAACTCCTTTTGCCAGGGAAAAAGTTA
TTGATTGTACCTGGAGAATAAGAGGTTGCTAAACTCGTGCCAGCAGCAGCGGTAATACGA
GTGCCTCAAGCGTTATCCGAAATCATTGGGCGTAAAGGGTTTGTAGGTGGCCGTGTTAGT
CTTGTGTTAAATTTACCGGCTCAACCGGTAAGCTGCATGGGAAACGGCATAGCTTCGAGG
ACGGTAGAGGTCTCTGGTACTCATAGTGTAGCGGTGAAATGCGTTGATATTATGGGGAAC
ACCAAAAGCGAAGGCAAGAGACTGGACCGCTCCTGACACTGAAAAACGAAAGCGTGGGTC
GCGAATGGGATTAGAGACCCGAGTAGTCC
>Otu9165
CCAGCCTACGGGTTGCACCAGTGGGGAATATTGGACAATGGGCGCAAGCCTGATCCAGCA
ATGCCGCGTGTGTGAAGAAGGCCTGCGGGTTGTAAAGCACTTTCAGTGGGGAGGAAGGCC
TAATGGTAAATAGCCATTAGGATTGACGTTACCCACAGAAGAAGCACCGGCTAACTCTGT
GCCAGCAGCCGCGGTAATACAGAGGGTGCGAGCGTTAATCGGAATTACTGGGCGTAAAGG
GTGCGTAGGTGGTCCCTTAAGTTAGATGTGAAATCCCTGGGCTTAACCTGGGAATGGCAT
TTAAGACTGAGGGGCTAGAGTTTGGTAGAGGGAAGTGGAATTTCCGGTGTAGCGGTGAAA
TGCGTAGATATCGGAAGGAACATCAGTGGCGAAGGCGACTTCCTGGACCGATATTGACAC
TGAGGCACGAAAGCGTGGGGAGCGAACAGGATTAGATACCCGAGTAGTCC
>Otu9166
CCAGCCTATGGGGCGCAGCAGTTTCGAATCATTCACAATGGGGGAAACCCTGATCCAGCG
ACGCCGCGTGTGTGAAGAAGGCCTGCGGGTTGTAAAGCACTTTTAGTGGGGACAAAAAGC
TACGGATTAATACTTCGTGGTCTTGATTTAACCCAAAGAAAAAGCACCGGCTAACTCTGT
GCCAGCAGCCGCGGTAATACAGAGGGTGCGAGCGTTAATCGGAATTACTGGGCGTAAAGC
GTGCGTAGACGGTTTTTTAAGTCGGATGTGAAATCCCCGGGCTCAACCTGGGAATTGCAT
TCGAGACTGGAAGGCTAGGGTGCGGAAGAGGGAAGCGGAATTTCTGGTGTAGCGGTGAAA
TGCGTAGATATCAGAAGGAACATCAGTGGCGAAAGCGGCTTCCTGGTCCAGCACCGACGT
TCAGGCACGAAAGCGTGGGGAGCAAACAGGATTAGAAACCCTTGTAGTCC
>Otu9169
CCAGCCTATGGGTCGCTGCAGTGGGGAATTTTGCGCAATGGGCGAAAGCCTGACGCAGCG
ACACCGCGTGAACGAAGAAGCCATTTGGGGTGTAAAGTTCTGTCGGCTGGGAAGAAAAAA
ATGACGGTACCAGCAAAGGAAGCATCGGCTAACTACGTGCCAGCAGCCGCGGTAAGACGT
AGGATGCAAGCGTTGTCCGGATTTATTGGGCGTAAAGAGTTCGTAGGCGGTTTGTTAAGT
CTGACGTTAAAGATCAGGGCTCAACCCTGGGATTGCTTCGGATACTGGCAGACTGGAGTG
TGGTAGAGGTAAGTGGAATTTCTGGTGTAGCGGTGAAATGCGTAGATATCAGGAAGAACA
CCGGTGGCGTAGGCGGCTGACTGGATCGCAACTGACGCTGAGGAACGAAAGCTAGGGGAG
CAAACAGGATTAGAACCCCAGTAGTCC
>Otu9171
CCAGCCTATGGGTTGCAGCAGTGGGGGATATTGGACAATGGGCGAAAGCCTGATCCAGCA
ATACCGCGTGTGTGAAGAAGGCCTGCGGGTTGTAAAGCACTTTAGTGGGGGAAGAAGTTC
ACGCAAGTGGATTGACGGTACCCCAAGAATAAGCACCGGCCAACTCTGTGCCAGCAGCCG
CGGTAATACAGAGGGTGCGAGCGTTAATCGGAATGACTGGGCGTAAAGGGCGCGTAGGCG
GATAAGTGTGTTGGGTGTGAAATCCCCGAGCTCAACTCGGGAATTGCGTCCAAAACTGTT
TATCTAGAGTGAAGTAGAGGTGAGTGGAATTTCCGGAGTAGCGGTGAAATGCGTAGATAC
CGGAAGGAACACCAGTGGCGAAGGCGGCTCACTGGACTTTTACTGACGCTGAGGCGCGAA
AGCGTGGGGAGCAAACAGGATTAGAAACCCCCGTAGTCC
>Otu9172
CCAGCCTATGGGTCGCAGCAGTCGAGAATTTTTCTCAATGGGCGAAAGCCTGATCGAGCG
ACGCCGCGTGATGGATGAAGTGCTTCGGCATGTAAACATCTTTTGTAGGCCAGAAAGCTA
TTGATCAGCCTAAGAATAAGGAGTTGCTAAACTCGTGCCAGCAGCAGCGGTAATACGAGT
GCTCCAAGCGTTATCCGGAATCATTGGGCGTAAAGGGTGTGTAGGTGGTTGTGTTAGTCT
TCTGTTAAATTCTTCGGCTTAACCGGGGGCATGCAGGGGAAACGGCACAACTAGAGGATG
CGAGAGGTATATGGAACTCATGGAGTAGGGGTGAAATCCGTTGATATCATGGGGAACACC
AAATGCGAAGGCAATATACTGGCGCACTCCTGACACTGAAACACGAAAGCGTAGGTAGCG
AATGGGATTAGATACCCGCGTAGTCC
>Otu9182
CCAGCCTACGGGTCGCAGCAGTGGGGAATCTTGCGCAATGGGCGAAAGCCTGACGCAGCA

ACGCCGCGTGGAGGACGAAGGCTTTCTGAGTTGTAAACTCCTTTCAGCAGGGACGATAGT
GACGGTACCTGCAGAAGAAGCACCGGCCAACTACGTGCCAGCAGCCGCGGTGATACGTAG
GGTGCAAGCGTTGTCCGGATTTATTGGGCGTAAAGAGCTCGTAGGCGGTTCGACAAGTCG
GGTGTTAAACCCCCAGGCTCAACCTGGGGCCGCCACCCGAAACTGTCGTGACTAGAGTTT
GGTAGGGGATCACGGAATTCCTGGTGTAGCAGTGGAATGCGCAGATATCAGGAGGAACAC
CAGTAGCGAAGGCGGTGATCTGGGCCAATACTGACGCTGAGGAGCGAAAGCGTGGGGAGC
GAACAGGATTAGAAACCCTGGTAGTCC
>Otu9188
CCAGCCTATGGGGGGCAGCAGTGGGGAATCTTGGACAATGGGGGCAACCCTGATCCAGCC
ATGCCGCGTGAGTGAAGAAGGCCTTCGGGTTGTAAAGCTCTTTCGGCAGGAACGAAATGG
TTCGTGCTAATACCACGGACTGATGACGGTACCTGAAGAAGAAGCACCGGCTAACTACGT
GCCAGCAGCCGCGGTAATACGTAGGGTGCAGGCGTTAATCGGAATTACTGGGCGTAAAGC
GTGCGCAGGCGGTTTCTTAAGTCAGATGTGAAAGCCCAGGGCTTAACCTGGGAACTGCGT
TTGAAACTGGGAGACTTGAGTGTGGCAGAGGGGGGTGGAATTCCACGTGTAGCAGTGAAA
TGCGTAGATATGTGGAGGAACACCGATGGCGAAGGCAGCCCCCTGGGCTAACACTGACGC
TCATGCACGAAAGCTAGGGGAGCAAACAGGATTAGATACCCGAGTAGTCC
>Otu9195
CCAGCCTATGGGAGGCTGCAGTTGGGAATCTTGGACAATGGGGGAAACCCTGATCCAGCC
ATGCCGCGTGAGTGATGAAGGCCTTAGGGTTGTAAAGCTCTTTCGCCGGGGACGATAATG
ACGGTACCCGGATAAGAAGCTCCGGCTAACTTCGTGCCAGCAGCCGCGGTAATACGAAGG
GGGCTAGCGTTGTTCGGAATTACTGGGCGTAAAGCGCGCGCAGGCGGTTCTCCAAGTCAG
TGGTGAAAGCCCGGAGCTCAACTTCGGAATTGCCATTGAAACTGTTGAACTTGAGTACGA
GAGAGGTGAGTGGAATTCCCAGTGTAGAGGTAAAATTCGTAGATATTCGCAAGAACACCA
GTGGCGAAGGCGGCTCACTGGCCCGGAACTGACGCTGAGACGCGAAAGCGTGGGGAGCAA
ACAGGATTAGAAACCCCCGTAGTCC
>Otu9196
CCAGCCTATGGGAGGCAGCAGCTAGGAATCTTGGGAATGCGCGAAAGCGTGACCCAGCAA
CGCCGCGTGGGCGATGAAGGCCTTCGGGTCGTAAAGCCCTTTTGCAGGGGACGATAGTGA
CGGTACCTTGCGAATAAGCCACGGCTAACTACGTGCCAGCAGCCGCGGTAATACGTAGGT
GGCAAGCGTTGTCCGGATTTACTGGGCGTAAAGGGCGTGCAGGCGGTCTGTTAAGTTCAG
GGTGAAAGCTCCCGGCTCAACTGGGAGAGGTCCTTGGATACTGGCAGACTTGAGGGAGGT
AGAGGAGAGTGGAATTCCCGGTGTAGTGGTGATATGCGTAGATATCGGGAGGAACACCAG
TGGCGAAGGCGGCTCTCTGGGCCTTTCCTGACGCTGAGACGCGAAAGCGTGGGGAGCGAA
CCGGATTAGAGACCCTGGTAGTCC
>Otu9197
CCAGCCTATGGGGGGCACCAGTGGGGAATTGTTCGCAATGGGCGCAAGCCTGACGACGCA
ACGCCGCGTGGAGGATGAAGACTTTCGGGTCGTAAACTCCTGTCGAATGGGACGAAAAAG
ACGCGGGTTAACAACCTGTGTTTCTGACGGTACCGTTAAAGGAAGCCCCGGCTAACTCCG
TGCCAGCAGCCGCGGTAATACGGGGGGGGCAAGCGTTGTTCGGAATTACTGGGCGTAAAG
GGCTCGTAGGCGGCCAACTAAGTCAGACGTGAAATCCCTCAGCTTAACTGAGGAACTGCG
TCTGATACTGGATGGCTTGGATTCGGGAGAGGGATGCGGAATTCCAGGTGTAGCGGTGAA
ATGCGTAGATATCTGGAGGAACACCGGTGGCGAAGGCGGCATCCTGGACCGATATTGACG
CTGAGGAGCGAAAGCCAGGGGAGCAAACGGGATTAGAAACCCCCGTAGTCC
>Otu9198
CCAGCCTACGGGTCGCTCCAGTCGAGAATATTCGACAATGGGCGAAAGCCTGATCGAGCG
ACACCGCGTGCAGGATGAAGGCCTTCGGGTCGTAAACTGCGGTAGTAGCATAACAATGCA
AATGAGTGTGCTACGGAAAGAGGTGGGTAACTACGTGCCAGCACCAGCGGTAATACGTAG
ACCTCAAGCGTTATCCGGATTTATTGGGCGTAAAGAGCATGTAGGATGTTTTGCGCGTCT
TTTGTTAAAGCCCAGGGCCTAACCCTGGAGATGCAAGAGATACGGCAGGACTAGAGGAGG
TTAGAGGTGCATGGAACTCACGGTGTAGGGGTGAAATCCGTTGATATCGTGGGGAACACC
AAGGGCGAAGGCAGTGCACTGGGACCTTCCTGACTCTGAGATGCGAAAGCGTGGGGAGCA
AAAAAGATTAGAGACCCCTGTAGTCC
>Otu9200
CCAGCCTATGGGGGGCAGCAGTGGGGAATATTGGACAATGGGGGCAACCCTGATCCAGCA
ATGCCGCGTGAGTGATGAAGGCCTTAGGGTTGTAAAGCTCTTTTACCCACGACGATGATG
ACGGTAGTGGGAGAATAAGCCCCGGCTAACTTCGTGCCAGCAGCCGCGGTAATACGAAGG
GGGCTAGCGTTGTTCGGAGTTACTGGGCGTAAAGGGCGCGTAGGCGGCGCACCAAGTCAG
GCGTGAAAGCCCTGGGCTCAACCCGGGAATTGCGCTTGAGACTGGTGTGCTTGAGTACGG

GAGAGGATAGTGGAATTCCCAGTGTAGAGGTGAAATTCGTAGATATTGGGAAGAACACCG
GTGGCGAAGGCGGCTATCTGGACCGTAACTGACGCTGAGGCGCGAAGGCTAGGGGAGCAA
ACAGGATTAGATACCCCCGTAGTCC
>Otu9202
CCAGCCTATGGGTCGCTGCAGTGGGGAATCTTGGACAATGGGGGAAACCCTGATCCAGCC
ATGCCGCGTGAGTGATGAAGGCCTTAGGGTTGTAAAACTCTTTCGGCGGGGACGATAATG
ACGGTACCCGGAGAAGAAGCTGCGGCTAACTACGTACCAGCAGCCGCGGTAATACGTAGG
CAGCAAGCGTTGTTCGGAATTACTGGGCGTAAAGAGTGTGTAGGCGGTTTTCTATGTTCG
GTGTGAAATCTCCCGGCTTAACTGGGAGGGTGCGCCGAAAACTGGAAGGCTTGAGTATGG
GAGAGGAGAGCGGAATTCCTGGTGTAGCGGTGAAATGCGTAGATATCAGGAGGAACACCT
GCGGTGTAGACGGCTTTCTGGACCATCACTGACGCTGAGACACGAAAGCGTGGGTAGCAA
ACAGGATTAGAAACCCGGGTAGTCC
>Otu9203
CCAGCCTATGGGGGGCTGCAGTGAGGAATATTGCTCAATGGGCGAAAGCCTGAAGCAGCG
ACGCCGCGTGAGGGATGAAATCCGTTAGGATGTAAACCTCTTTTCTAAGGGAAGAATATC
CTGATCAATCAGGAAGTGACTGTACTTTAGGAATAAGGATCGGCCAACTACGTGCCAGCA
GCCGCGGTAATACGTAGGATCCGAGCGTTGTCCGGAATTACTGGGTGTAAAGGGTCCGCA
GGTGGGCAAGCAAGTCGGTGGTGAAATCTCTCCGCTCAACGGAGAAACTGCCCTCGATAC
TGCTTGTCTTGAGTTCGAGAGAGGGTAACGGAATTCATGGTGTAGCGGTGAAATGCGTAG
ATATCATGAAGAACACCGGTCGCGTAGGCGGTTACCTGGCTCGCAACTGACACTCAGGGA
CGAAAGCATGGGTAGCAAACAGGATTAGAAACCCGGGTAGTCC
>Otu9209
CCAGCCTACGGGAGGCAGCAGTCGAGGATCTTCGGCAATGGGCGCAAGCCTGACCGAGCG
ACGCCGCGTGTGCGATGAAGGCCTTCGGGTTGTAAAGCACTGTCGAGGGGGAGAAAAGCC
CGCAAGGGTCTGATCTATCCCTGGAGGAAGCACGGGCTAAGTTCGTGCCAGCAGCCGCGG
TAAGACGAACCGTGCGAACGTTGTTCGGAATCACTGGGCTTAAAGGGCGCGTAGGCGGGC
CATCAAGTCCGTGGTGAAATACTTCGGCTTAACCGGAGAAGTGCTGCGGATACTGGTGGT
CTCGAGGGGGGTAGGGGCATGTGGAACTTCCGGTGGAGCGGTGAAATGCGTAGATATCGG
AAGGAACGCCGGTGGCGAAAGCGACGTGCTGGACCCCATCTGACGCTGAGGCGCGAAAGC
CAGGGGAGCAAACGGGATTAGAAACCCGCGTAGTCC
>Otu9211
CCAGCCTATGGGACGCAGCAGTGGGGAATATTGGACAATGGACGAAAGTCTGACGCAGCC
ACGCCGCGTGAGTGAAGAAGGCCTTCGGGTTGTAAAGCTCTGTCGGAGGGGACGAAAAGC
ATTAGGGTTAACAGCCCTAGTGCCTGACGGTACCCTTAAAGGAAGCACCGGCTAACTCTG
TGCCAGCAGCCGCGGTAATACAGAGGGTGCAAGCGTTGCTCGGAATCATTGGGCGTAAAG
GGCAAGTAGGTGGTCTCATTTGTCTAGGGTGAAATCCTTGAGCTTAACTCAAGAAGTGCC
CTAGAAACGGTGAGACTCGAGTTCTGGAGAGGGTCGTGGAATTCCCGGTGTAGCGGTGAA
ATGCGTAGAGATCGGGAGGAACACCAGAGGCGAAGGCGGCGACCTGGACAGATACTGACA
CTCAACTGCGAAAGCGTGGGGAGCAAACAGGATTAGATACCCTAGTAGTCC
>Otu9215
CCAGCCTATGGGTCGCAGCAGTGAGGAATATTGCACAATGGGCGAAAGCCTGATGCAGCG
ACGCCGCGTGGAGGATGAAGGTCTTCGGATTGTAAACTCCTGTTAAGTGGGAAAAAAGAA
TTGCTCTGAATAGGAGCAAGAGATGATTGTACCACTAGAGAAAGCACCGGCTAACTTCGT
GCCAGCAGCCGCGGTAATACGAGGGGTGCAAACGTTATTCGGAATCATTGGGCGTAAAGG
GTGCGTAGACGGCAAATCAAGTCAGTTATTAAATCTTCTAGCTCAACTAGAAATCTGTGG
CTGAAACTAGCTTGCTTGAGTATGGAAGAGAGAAGCGGAATTCTCGGAGTAGCGGTAAAA
TGCGTAGATCTCGAGAGGAACACCGATGGCGAAGGCAGCTTCTTGGTCCATTTCTGACGT
TGAGGCACGAAAGCGTGGGGAGCAAACAGGATTAGATACCCTAGTAGTCC
>Otu9217
CCAGCCTACGGGACGCACCAGTGGGGAATATTGGGCAATGGGCGAAAGCCTGACCCAGCC
ACGCTGCGTGAGTGATGAAGGCCTTCGGGTCGTAAAGCTCTGTGGGGAGGGACGAACAAG
CCTGGTGCTAATATCACCGGGCCCTGACGGTACCTCCTTAGCAAGCACCGGCTAACCCTG
TGCCAGCAGCCGCGGTAATACAGGGGGTGCAAACGCTGCTCGGAATCATTGGGCGTAAAG
CGCACGTAGGCGGTTCGTTATGTCGGATGTGAAAGCCCTCGGCTTAACTGAGGAAGTGCA
TCCGAAACTGGCGAGCTAGAGTACTAAAGAGGGTCGCGGAATTCCCGGTGTAGAGGTGAA
ATTCGTAGATATTGGGAAGAACACCGGTGGCGAAGGCGGCTCACTGGCTCATATCTGACG
CTCAGGCGCGACAGCGTGGGGATCAAACAGGATTAGATACCCCAGTAGTCC
>Otu9221

CCAGCCTATGGGTGGCACCAGTGGGGAATCTTGCGCAATGGGCGAAAGCCTGACGCAGCA
ACGCCGCGTGGAGGACGAAGGCTTTCTGAGTTGTAAACTCCTTTCAGCAGGGACGATTGT
GACGGTACCTGCAGAAGAAGCACCGGCCAACTACGTGCCAGCAGCCGCGGTGATACGTAG
GGTGCAAGCGTTGTCCGGATTTATTGGGCGTAAAGGGTGCGTAGGCGGCTTTTTAAGTCA
GAGGTGAAATCCTGGAGCTCAACTCCAGAACTGCCTTTGATACTGAAGAGCTTGAGTCCG
GGAGAGGTGAGTGGAACTGCGAGTGTAGAGGTGAAATTCGTAGATATTCGCAAGAACACC
AGTGGCGAAGGCGGCTCACTGGCCCGGTACTGACGCTGAGGTGCGAAAGCGTGGGGAGCA
AACAGGATTAGATACCCTCGTAGTCC
>Otu9222
CCAGCCTATGGGGGGCACCAGTGGGGAATCTTGGACAATGGGGGCAACCCTGATCCAGCG
ATGCCGCGTGGGTGAAGAAGGCCTTCGGGTTGTAAAGCCCTTTAGGCTGGGAAGAAGAAT
GCGGGATGAATAATCCTGTATTTTGACGGTACCAGCAGAATAAGCACCGGCAAACTCTGT
GCCAGCAGCCGCGGTAATACAGAGGGTGCGAGCGTTAATCGGATTTACTGGGCGTAAAGG
GCGCGTAGGCGGTCAAATGAGTGTGATGTGAAAGCCCCGGGCTTAACCTGGGAAGTGCAT
CGCAAACGATTTGACTGGAGTAGATGAGAGGGTGGTGGAATTTCCGGTGTAGCGGTGAAA
TGCGTAGAGATCGGAAGGAACGTCAATGGCGAAGGCAGCCACCTGGCATCATACTGACGC
TGAGGCGCGAAAGCGTGGGGAGCGAACAGGATTAGAGACCCTAGTAGTCC
>Otu9225
CCAGCCTATGGGTCGCTGCAGTAAGGAATATTGGTCAATGGACGCAAGTCTGAACCAGCC
ATGCCGCGTGGAGGATGAAGGTCCTCTGGATTGTAAACTTCTTTTATTTGGGAAGAAATG
CATTTTTTCTAAAGTGCTTGACGGTACCAGATGAATAAGCACCGGCTAACTCCGTGCCAG
CAGCCGCGGTAATACGGAGGGTGCAAGCGTTATCCGGATTTACTGGGTTTAAAGGGTGCG
CAGGCGGGTACGTAAGTCAGTGGTGAAATCTCTAGGCTTAACCTGGGAATTGCCATTGAT
ACTATATATCTTGAATGCTGTGGAGGTAAGCGGAATATGTCATGTAGCGGTGAAATGCTT
AGAGATGACATAGAACACCTATTGCGAAGGCAGCTTACTACGCAGAGATTGACGCTCATG
CACGAAAGCGTGGGGATCAAACAGGATTAGATACCCCTGTAGTCC
>Otu9227
CCAGCCTACGGGGTGCACCAGTCGAGAATTTTTCTCAATGGGCGAAAGCCTGAAGGAGCG
ACGCCGCGTGGGGGATGAAGGGCTTCGGCTCGTAAACCCCTGTCATTTGCGAACAAACCC
TGTCATTTAACAGACGACAGGCTGATTGTAGCAGAAGAGGAAGGGACGGCTAACTCTGTG
CCAGCAGCCGCGGTAATACAGAGGTCCCAAGCGTTGTTCGGATTCACTGGGCGTAAAGGG
TGCGCAGGTGGCAAGGTAAGTTTGATGTGAAATCTCCGGGCTTAACCCGGAAACTGCATT
GAATACTCTCTTGCTAGAGGATTGGAGGGGGGACTGGAATACTTGGTGTAGCAGTGAAAT
GCGTAGATATCAAGTGGAACACCAGTGGCGAAGGCGAGTCTCTGGACATTTCCTGACGCT
GAGGCACGAAAGCCAGGGGAACGAACGGGATTAGAAACCCTTGTAGTCC
>Otu9230
CCAGCCTATGGGATGCTGCAGTCGAGAATTTTTCACAATGGGCGAAAGCCTGATGGAGCG
ACACCGCGTGGGGGATGAAGGGCTTCGGCTCGTAAACCCCTGTCATGCATGGGCAATGCT
GTTGACCTAACACGTCAGCAGTTGATAGTAATGCAAGAGGAAGGGACGGCTAACTCTGTG
CCAGCAGCCGCGGTAATACAGAGGTCCCAAGCGTTGTTCGGATTCACTGGGCGTAAAGGG
TGCGTAGGTGGCAAGGTAAGTTCGATGTGAAATCTCCGAGCTCAACTCGGAAATGGCATT
GAATACTCTCTTGCTAGAGGGTCGGAGGGGAGACTGGAATTCTCGGTGTAGCAGTGAAAT
GCGTAGATATCGAGAGGAACACCAGTGGCGAAGGCGAGTCTCTGGACGACAACTGACACT
GAAGCACGAAAGCTAGGGGAGCAAACAGGATTAGATACCCGGGTAGTCC
>Otu9238
CCAGCCTATGGGGCGCTGCAGTGGGGAATATTGCGCAATGGGCGAAAGCCTGACGCAGCG
ACGCCGCGTGAAGGATGAAGGCCTACGGGTTGTAAACTTCTGTTAAGCGGGAAGAAAACT
CTATCTCTAATACAGATAGATATGACGGTACCGCTAGAGAAAGCACTGGCTAAACTCGTG
CCAGCAGCCGCGGTAATACGAGTGGTGCAAGCATTATTCGGAATCATTGGGCGTAAAGGG
TGCGTAGGTTGTATACTAAGTCTGTTGTTAAATTTCTCGGCCTAACCGGGAACCTGCAGT
GGAAACTAGTGTACTAGAGGATGGAAGAGAGAAGTGGAATTCTCGGAGTAGCGGTAAAAT
GCGTAGATCTCGAGAGGAACACCGATGGCGAAAGCAGCTCCTTGGTCCAATCCTGACACT
GATGCACGAAAGCGTGGGGAGCAAACAGGATTAGAAACCCCGGTAGTCC
>Otu9240
CCAGCCTACGGGGGGCACCAGCCAAGAACATTCGACAATGGGCGAAAGCCTGATCGAGCG
ATACCGCGTGGTGGATGAAGCGCTTCGGCGCGTAAACACCTTTTATGGAGGAGGAAGTTA
TTGACGTTACTCCATGAATAAGGGGCTCCTAACTCTGTGCCAGCAGGAGCGGTAATACAG
AGGCCCCGAGCGTTACCCGGAATTACTGGGCGTAAAGGGTGTGTAGGTGGTCGTGTTAGT

CGTCCGTCAAAACCTGGGGCTCAACCCCAGATCCGCGGGCGAAACGGCACGACTCGAGGG
CGTGAGAGGTACATGGAACTCATGGTGTAGGGGTGAAATCCGTTGATATCGTGGGGAACA
CCGAAAGCGAAGGCAATGTACTGGCGCGTTCCTGACACTGAAACACGAAAGCGTGGGTAG
CGAACGGGATTAGAGACCCTAGTAGTCC
>Otu9254
CCAGCCTATGGGGCGCACCAGTCGAGAATTTTTCACAATGGGGGAAACCCTGATGGAGCG
ACGCCGCGTGGAGGATGAAGGTCTTCGGATTGTAAACCTCTGTCACTGGGGAAGAAACGC
TTCAAGTAAACATCTTGAAGCCTGACTTAACCCGGAGAGGAAGCAGTGGCTAACTCTGTG
CCAGCAGCCGCGGTAATACAGAGACTGCAAGCGTTATTCGGATTCACTGGGCGTAAAGGG
TGCGCAGGCGGCCGTGTGTGTGAGGCGTGAAAGCCCGGAGCTCAACTCCGGAATTGCACC
TCAAACTACACGGCTAGAGCATTGGAGAGGGTAGCAGAATTCACGGTGTAGCAGTGAAAT
GCGTAGATATCGTGAGGAATACCAGAGGCGAAGGCGGCTACCTGGACAATTGCTGACGCT
CAGGCACGAAAGCGTGGGGAGCAAAAGGGATTAGAAACCCGCGTAGTCC
>Otu9271
CCAGCCTATGGGGGGCTCCAGTGGGGAATATTGCGCAATGGGCGAAAGCCTGACGCAGCA
ACGCCGCGTGAGTGAAGAAGGCCTTCGGGTTGTAAAGCTCTGTCAGAGGGAAAGAATATG
GCTGGCAACACCAGCCTTTGACGGTACCCTCGAAGGAAGCACTGGCTAACTCCGTGCCAG
CAGCCGCGGTAATACGGAGAGTGCAAGCGTTGTTCGGAATTATTGGGCGTAAAGCGCGTG
TAGGTGGCTTGACAAGTCTGATGTGAAAGCCCAGGGCTCAACCCTGGAAGTGCACCGGAT
ACTGTCGAGCTTGAGTACTGGAGGGGGAAGTGGAATTCTCGAGTGTAGAGGTGAAATTCG
TAGATATTCGGAAGAACATCAGTGGCGAAGGCGACTTCCTGGACAGATACTGACACTGAG
ACGCGAAAGCGTGGGTAGCAATCAGGATTAGATACCCGAGTAGTCC
>Otu9274
CCAGCCTACGGGGGGCAGCAGTGGGGAATCTTGGACAATGGAGGAAACTCTGCTCCAGCC
ATGCCGCGTGAGTGGTGAAGGCCTTCGGGTTGTAAAACTCTTTTACCCGGGAAGATAATG
ACTGTACCGGGAGAATAAGCTCCGGCTAACTTCGTGCCAGCAGCCGCGGTAATACGAAGG
GGGCTAGCGTTGTTCGGAATTACTGGGCGTAAAGCGCGCGCAGGCGGTTCTTCAAGTCAG
GGGTGAAAGCCCAGAGCTCAACTCTGGAATTGCCTTTGAAACTGTAGAACTTGAGTACGA
GAGAGGTGAGTGGAATTCCCAGTGTAGAGGTGAAATTCGTAGATATTGGGAAGAACACCG
GTGGCGAAGGCGGCTCACTGGCTCGTTACTGACGCTCAGGCGCGACAGCGTGGGGATCAA
ACAGGATTAGAGACCCGCGTAGTCC
>Otu9277
CCAGCCTACGGGGGGCTGCAGTCGAGAATCTTCCGCAATGGACGAAAGTCTGACGGAGCG
ACGCCGCGTGATTGATGAAGTCCCTTTGGGACGTAAAGATCTTTTATGAGGGAAGAAGTT
TATTGACTGTACCTCATGAATAAGAGGCTCCTAATCTCGTGCCAGCAGGAGCGGTAATAC
GAGAGCCTCGAGCGTTATCCGGAATTATTGGGCGTAAAGGGTGCGTAGGTTGTTCTGTTA
GTCTTCCGTCAAATTCTTCGGCTTAACCGGGGGCATGCGGGGGAAACGGCAGGACTTGAA
AGTGCGAGGGGTGTACGGAACTCATGGTGTAGGGGTGAAATCCGTTGATATCATGGGGAA
CACCGAAAGCGAAAGCAGTACACTGGCGCATATTTGACACTGAAGCACGAAAGCGTGGGT
AGCGAATGGGATTAGAAACCCTCGTAGTCC
>Otu9278
CCAGCCTATGGGTGGCTCCAGTGGGGAATATTGGACAATGGGGGAAACCCTGATCCAGCA
ATGCCGCGTGTGTGAAGAAGGCCTGCGGGTTGTAAAGCACTTTTGTTGGGGAAGAAGGAT
ACGAAAGTATTTTGACGGTACCCAAAGAATAAGCACCGGCTAACTCTGTGCCAGCAGCCG
CGGTAATACAGAGGGTGCGAGCGTTAATCGGAATTACTGGGCGTAAAGCGCGCGTAGGTG
GTTTTGTGTGTTGGGTGTGAAATCCCTGGGCTTAACCTAGGAATTGCATTCAAAACTGCA
AGACTTGAGTGGAGTAGAGGTGAGTGGAATTTCCGGAGTAGCGGTGAAATGCGTAGATAC
CGGAAGGAACACCAGTGGCGAAGGCGACTCACTGGACTCTAACTGACACTGAGGTGCGAA
AGCGTGGGGAGCGAACAGGATTAGAAACCCCAGTAGTCC
>Otu9281
CCAGCCTACGGGACGCTGCAGTGGGGAATCTTGCGCAATGGGCGAAAGCCTGACGCAGCG
ACGCCGCGTGGAGGATGAAGGTCTTCGGATTGTAAACTTCTGTTAAGTGGGAAGAAATGC
CTATCAATAATACTGATAGGAGCTGACTGTACCATTAGAGAAAGCACCGGCTAAACTCGT
GCCAGCAGCCGCGGTAATACGAGTGGTGCAAGCGTTATTCGGAATCATTGGGCGTAAAGG
GTGTGTAGACGGCATATCAAGTCAACTGTTTAATTCTCCGGCCTAACCGGAGGCATGCGG
TAGAAACTAATGTGCTCGAGGTTGGAAGAGAGAAGTGGAATTCTCGGAGTAGCGGTAAAA
TGCGTAGATCTCGGGAGGAACACCAATGGCGAAGGCAGCTTCTTGGTCCATACCTGACGT
TGAGACACGAAAGCGTGGGGAGCAAACAGGATTAGATACCCCGGTAGTCC

>Otu9285
CCAGCCTACGGGTCGCTCCAGTGGGGAATCTTGCGCAATGGACGAAAGTCTGACGCAGCC
ACGCCGCGTGAGTGAAGAAGGCCTTCGGGTTGTAAAGCTCTGTCGGGAGGGACGAAAAAC
CTTGAGGTTAACAGCCTCAGGACTTGACGGTACCTCCAAAGGAAGCACCGGCTAACTTCG
TGCCAGCAGCCGCGGTAATACGAGGGGTGCAAGCGTTGCTCGGAATTATTGGGCGTAAAG
GGTAGGTAGGTGGTCTCATTTGTCTGGGGTGAAAGCCTTGAGCTTAACTCAAGAAGTGCC
CTAGAAACGGTGAGACTAGAGTCTTGGAGAGGGTCGTGGAATTCCCGGTGTAGCGGTGAA
ATGCGTAGAGATCGGGAGGAACACCAGAGGCGAAGGCGGCGACCTGGACAAGTACTGACA
CTCAACTACGAAAGCGTGGGTAGCAAACAGGATTAGAAATCCGTGTAGTCC
>Otu9289
CCAGCCTATGGGAGGCAGCAGTCGAGAATTTTTCACAATGGGGGAAACCCTGATGGAGCG
ACGCCGCGTGGAGGATGAAGGTCTTCGGATCGTAAACTCCTGTCACTACAGAACAAGGGT
AGTAGCATTAATAAAGCTACTACTTGATGGTATGTGGAGAGGAAGGAACGGCTAACTCTG
TGCCAGCAGCCGCGGTGATACAGAGGTTCCAAGCGTTGTTCGGATTCACTGGGCGTAAAG
GGTGCGTAGGAGGTCAGATAAGTAGGATGTGAAATCCTGTCGCCTAACGACAGAACTGCA
TTCTAAACTGCCTGACTAGAGGATCGGAGAGGAGAGCGGAATTCTTGGTGTAGCGGTGAA
ATGCGTAGATATCAAGAGGAACGCCAACAGCGGAGGCAGCTCTCTGGAAGACTCCTGACT
CTGAGGCACGAAGGCCAGGGTAGCAAACGGGATTAGAAACCCCTGTAGTCC
>Otu9294
CCAGCCTATGGGTTGCACCAGTAAGGAATATTGGTCAATGGACGCAAGTCTGAACCAGCC
ATGCCGCGTGGAGGATGAAGGTCCTCTGGATTGTAAACTTCTTTTATTTGGGAAGAAACC
CATTTTTTCTAAAGTGGTTGACGGTACCAGATGAATAAGCACCGGCTAACTCCGTGCCAG
CAGCCGCGGTAATACGGAGGGTGCAAGCGTTATCCGGATTCACTGGGTTTAAAGGGTGCG
TAGGTGGGCAGGTAAGTCAGTGGTGAAATCTCCGGGCTTAACCCGGAAACTGCCGTTGAT
ACTATCTGTCTTGAATATCGTGGAGGTGAGCGGAATATGTCATGTAGCGGTGAAATGCAT
AGATATGACACAGAACACCGATTGCGAAGGCAGCTGACGAAACTGTAACTGACGCTGAGG
CACGAAAGCGTGGGGATCAAACAGGATTAGAAACCCGCGTAGTCC
>Otu9295
CCAGCCTACGGGGGGCAGCAGTAGGGAATCTTGCGCAATGGGCGAAAGCCTGACGCAGCG
ACGCCGCGTGCGGGATGAAGCCCTTCGGGGTGTAAAGCTCTTTCGGCGGGGACGATGATG
ACGGTACCCGCAGAAGAAGCCCCGGCTAACTTCGTGCCAGCAGCCGCGGTAATACGAAGG
GGGCTAGCGTTGCTCGGAATGACTGGGCGTAAAGGGCGCGTAGGCGGTTCGTACAGTCAG
ACGTGAAATTCCCGGGCTCAACCCGGGGGCTGCGTTTGATACGTGCGGGCTTGAGTTCGG
AAGAGGGTCGTGGAATTCCCAGTGTAGAGGCGAAATTCGTAGATATTGGGAAGAACACCG
GTGGCGAAGGCGGCGACCTGGTCCGATACTGACGCTGAGGCGCGAAAGCGTGGGGAGCAA
ACAGGATTAGAGACCCTCGTAGTCC
>Otu9296
CCAGCCTATGGGGTGCTCCAGTGGGGAATCTTGCACAATGGGCGAAAGCCTGATGCAGCA
ACGCCGCGTGAGTGAAGAAGGCCCTTGGGTTGTAAAGCTCTTTCGGTTGAGAAGAAGGGG
AAGTTAGCTAATACCTTACTTCTTTGACGGTACCAGCAGAAGAAGCACCGGCTAATTCCG
TGCCAGCAGCCGCGGTAATACGGGAGGTGCAAGCGTTGTTCGGAGTGACTGGGCGTAAAG
CGCACGTAGGCGGGTTTGTAAGTCGGATGTGAAATCCCTAGGCTTAACCTGGGAACTGCA
TCCGAAACTATAAGTCTTGAATACCTCAGAGGTTGGTAGAATTCCTGGTGTAGGAGTGAA
ATCCGTAGATATCAGGAGGAATACCGGAGGCGAAGGCAGCCAACTGGGAGAGTATTGACG
CTGAGGTGCGAAAGCGTGGGGAGCAAACAGGATTAGAGACCCCTGTAGTCC
>Otu9307
CCAGCCTACGGGTCGCTGCAGTCGAGAATTTTTCACAATGGACGAAAGTCTGATGGAGCA
ACGCCGCGTGGAGGATGAAGGTTTTCGGATTGTAAACTCCTGTCACTGCAGAACAAGGTA
ACGTCAGTTAACAGCCGGCGTTATTGATGGTATGCGGAGAGGAAGGGACGGCTAACTCTG
TGCCAGCAGCCGCGGTAAGACAGAGGTCCCGAGCGTTGTTCGGATTCATTGGGCGTAAAG
GGTGTGTAGGAGGTCGGGTCAGTCAGGTGTGAAATCTCAGGGCTTAACCCTGAAACTGCG
CTTGATACTGCCCGGCTAGAGGATCGGAGGGGGTATCGGAATTTATGGTGTAGCAGTGAA
ATGCGTAGATATCATAAGGAACACCGGTGGCGAAGGCGGATACCTGGAAGATTCCTGACT
CTGAAACACGAAAGCCAGGGGAGCAAACGGGATTAGAAACCCGGGTAGTCC
>Otu9315
CCAGCCTATGGGACGCTGCAGCCGAGAATCTTCCACAATGGACGAAAGTCTGATGGAGCG
ACGCCGCGTGATTGATGAAGTCCCTCTGGGACGTAAAGATCTTTTATGAGGGAAGAAGTT
TATTGACGGTACCTCATGAATGAGGGGCTCCTAATCTCGTGCCAGCAGGAGCGGTAATAC

GAGAGCCCCGAGCGTTATCCGGAATTATTGGGCGTAAAGGGTGTGTAGGTGGCATTGTTA
GTCTTTTGTTAAATCCCAGAGCTTAACTTTGGAATCGCAAGAGAAACGGCAAAGCTAGAG
AGTGTGAGAGGTCTATGGAACTCATGGTGTAGGGGTGAAATCCGTTGATATCATGGGGAA
CACCAAAAGCGAAGGCAATAGACTGGCGCATTTCTGACACTGAAACACGAAAGCGTGGGT
AGCGAATGGGATTAGAAACCCGCGTAGTCC
>Otu9316
CCAGCCTACGGGGTGCACCAGTGGGGAATTTTGGACAATGGGGGCAACCCTGATCCAGCC
ATGCCGCGTGAGTGAAGAAGGCCTTCGGGTTGTAAAGCTCTTTCGGCGGGGACAAAACGG
CGACTGCGAATAACGGTCGTTAATGATGGTACCGGAAGAATAAGCACCGGCTAACTACGT
GCCAGCAGCCGCGGTAATACGTAGGGTGCAAGCGTTAATCGGAATTACTGGGCGTAAAGC
GTGCGCAGGCGGTTCGCTAAGACAGATGTGAAATCCCCGGGCTTAACCTGGGAACTGCAT
TTGTGACTGGCGGGCTAGAGTATGACAGAGGGGGGTAGAATTCCACGTGTAGCAGTGAAA
TGCGTAGAGATGTGGAGGAATACCGATGGCGAAGGCAGCCCCCTGGGCCAATACTGACGC
TCATGCACGAAAGCGTGGGGAGCAAACAGGATTAGATACCCCTGTAGTCC
>Otu9319
CCAGCCTATGGGTGGCACCAGTGAGGAATCTTGGACAATGAACGAAAGTTTGATCCAGCA
AAGTCACGTGAATGATGAAAATAATAATTGTAACATTCTTTTTTCAGTGAAGATCATGAC
ATTAATTGAAGAATAAGCTCCGACCAACTTCGTGCCAGCAGTCGCGGTAATACGGGGGGA
GCTAGTGTTATTCGCAGTGACTGGGCGTAAAGGGCATCTAGGTGGCATTTGTAGTTTTTA
ATGAAACATTGAGGCCTAACCTCATTCTTATTACTAACACTGTTTGCTAAGGGTTTGAAA
AAAGAAAACAGAATCTTTAGTTGAGAGGTGAAATTCTACGACATTAAAGAGGCTATCAAA
AGCGAAGGCAGTTTTCTAGTTTAAACTGACACTGAAAGGCAAAAGCATGTGTAGCAAACT
GGATTAGAGACCCGGGTAGTCC
>Otu9320
CCAGCCTATGGGTGGCTGCAGTGGGGAATCCTGGGCAATGGACTAACGTCTGACCTGGTG
AGAACACGTGCACGACGAAGGCGCTAGTTTTTTGTAAAGTGCTTTTACCGACGAGAAAAA
GGTCAGTACTCGGCGAAAAAAGCGTCGGCCAACTTCGTGCCAGCAGCTGCGGTAAGACGA
AGGACGCGGGTGTTATTCGTTTTGATTGGGTGTAAAGGGTATGTAGGTGGTTTCTTAGTT
TTTGGCTAAAAGAGCAACGAGTATCGATGCGTCTGGCCAATTCGCTAGAATCTTGAGCTG
GTTAAAGGTATAAACTATTCCTTACGTAAAGGTTCAATTTTTGGAGGTACGGAAGACCGT
CGACAGGCGAAAGCATTTTTCCAGTTCCAGCTGACGCTGAGGTACGGAAGCTTAGGGAGC
GAACGGGATTAGATACCCCCGTAGTCC
>Otu9328
CCAGCCTATGGGTGGCACCAGTGGGGAATCTTGCACAATGGAGGCAACTCTGATGCAGCG
ACGCCGCGTGAGCGATGAAGCCCCTCGGGGTGTAAAGCTCTTTCGGCAGGGACGATAATG
ACGGTACCTGAAGAAGAAGCTGCGGCTAACTACGTGCCAGCAGCCGCGGTAATACGTAGG
CAGCGAGCGTTGTTCGGAATTACTGGGCGTAAAGAGCGTGTAGGCGGTGCTCTAAGTTCG
GTGTGAAATCTCCTGGCTTAACTGGGAGGGTGCGCCGAAAACTGGAGTGCTCGAGCGTGG
GAGAGGAAAGCGGAATTCCTGGTGTAGCGGTGAAATGCGCAGATATCAGGAGGAACACCA
GTAGCGAAGGCGGTGATCTGGGCCAATACTGACGCTGAGGAGCGAAAGCGTGGGGAGCGA
ACAGGATTAGAACCCCCTGTAGTCC
>Otu9333
CCAGCCTACGGGTCGCAGCAGTCGAGAATCTTTCGCAATGGGCGCAAGCCTGACGAAGCG
ACGCCGTGTGAGCGAAGAAGGCCTTCGGGTTGTAAAGCTCTTTCGCTAGGGAACAAGAAA
GGCTGGATAATACCCAGCCAATTTGATGGTACTTGGTAAAGAAGCACCGGCTAACTCCGT
GCCAGCAGCTGCGGTAATACGGAGGGTGCAAGCATTGATCGGAATTACTGGGCGTAAAGG
GCGCGTAGGCGGCCTGGAAAGTCAGATGTGAAATTCCTGGGCTCAACCCAGGAGCGGCAT
TTGAAACTTCCAGACTAGAGTGCAATTAGGGAAAACGGAATTCCACGTGTAGCGGTGAAA
TGCGTAGATATGTGGAAGAACACCGGTGGTGAAGACGGTTTTCTGGGTTGTTACTGACGC
TGAGGCGCGAAAGCTTGGGGAGCAAACAGGATTAGAGACCCTTGTAGTCC
>Otu9336
CCAGCCTACGGGGGGCACCAGTGGGGAATCTTGCGCAATGGACGAAAGTCTGACGCAGCC
ACGCCGCGTGAGTGAAGAAGGCCTTCGGGTTGTAAAGCTCTGTCGGAGGGGACGAAAAGC
TTTGGAGTGAATAGCTTCAGAGCCTGACGGTACCCTTAAAGGAAGCACCGGCTAACTCTG
TGCCAGCAGCCGCGGTAATACAGAGGGTGCAAGCGTTGCTCGGAATCATTGGGCGTAAAG
GGCAAGTAGGTGGTCTCATTTGTCTCGGGTGAAAGCCTTGGGCTTAACCCAAGAAGTGCC
CGAGAAACGGTGAGACTCGAGTTTCGGAGAGGGTCGTGGAATTCCCGGTGTAGCGGTGAA
ATGCGTAGAGATCGGGAGGAACACCAGCGGCGAAGGCGGCGACCTGGTCCTTTACTGACG

CTGAGGCGCGAAAGCGTGGGGAGCAAACAGGATTAGAAACCCTGGTAGTCC
>Otu9337
CCAGCCTATGGGAGGCAGCAGTGGGGAATCTTGCGCAATGGGGGAAACCCTGACGCAGCG
ACGCCGCGTGGGCGAAGAAGGCCTTCGGGTTGTAAAGCCCTGTGGGGCGGGACGAATACC
TCACGGTAAACAATCGTGAGGAGTGACGGTACCGCCTTAGCAAGCACCGGCTAACTCTGT
GCCAGCAGCCGCGGTAAGACAGAGGGTGCAAACGTTGCTCGGAATTACTGGGCGTAAAGC
GCGTGTAGGCGGCTTTGTAAGTCCGATGTGAAAGCCCTTCGCTCAACGAGGGAAGCGCAT
TGGATACTGCAGAGCTCGAGTCCTGGAGGGGAGAGCGGAATTCTCGGTGTAGAGGTGAAA
TTCGTAGATATCGGGAGGAACACCAGTGGCGAAGGCGGCTCTCTGGACAGGTACTGACGC
TGAGACGCGAAAGCATGGGGAGCGAACAGGATTAGATACCCCGGTAGTCC
>Otu9344
CCAGCCTATGGGAGGCAGCAGTCGAGGATCTTCGGCAATGGGCGCAAGCCTGACCGAGCG
ACGCCGCGTGCGGGATGAAGGCCTTCGGGTTGTAAACCGCTGTCAGTGGGGAGGAAATGC
GTAGGGGTTCTCCCCTACGTTTGACCTATCCGCAGAGGAAGTACGGGCTAAGTTCGTGCC
AGCAGCCGCGGTAAGACGAACCGTACGAACGTTATTCGGAATCACTGGGCTTAAAGGGTG
CGTAGGCTGCGCAGCAAGTTGGGTGTGAAATCCCTCGGCTCAACCGAGGAACTGCGCCCA
AAACTGCTGTGCTCGAGGGAGACAGAGGTGAGCGGAACTTAGGGTGGAGCGGTGAAATGC
GTTGATATCCTAAGGAACACCCGTGGCGAAAGCGGCTCACTGGGTCTCTTCTGACGCTGA
GGCACGAAAGCTAGGGTAGCAAACAGGATTAGAGACCCCTGTAGTCC
>Otu9346
CCAGCCTATGGGTCGCAGCAGTCGAGAATCTTCCACAATGGACGAAAGTCTGATGGAGCG
ACGCCGCGTGGTTGATGAAGTCCTTCGGGACGTAAAAACCTTTTGTGAGGGAGGAAGTTA
TTGACGTTACCTCATGAATAAGGGGCTCCTAACTCTGTGCCAGCAGGAGCGGTAATATAG
AGGCCCCAAGCATTATCCGGAATCACTGGGCGTAAAGGGTGTGTAGGCGGTCGTGTTAGT
CTTTCGTGAAAGATCTTCGGCTTAACCGAGGAGACGCGGGAGAAACGGCACGGCTTCGAG
GACGCGAGAGGTATATGGAACTCATGGTGTAGGGGTGAAATCCGTTGATATCATGGGGAA
CACCAAATGCGAAGGCAATATACTGGCGCGCTCCTGACGCTGAAACACGAAAGCGTGGGA
ATCGAACGGGATTAGATACCCGTGTAGTCC
>Otu9349
CCAGCCTATGGGACGCTGCAGTGGGGAATTTTGGACAATGGGCGCAAGCCTGATCCAGCA
ATGCCGCGTGAGTGAAGAAGGCCTTCGGGTTGTAAAGCCCTTTTGTCAGGGAAGAAACGG
GTTGGTCTAATACACTGATCTAATGACGGTACCTGAAGAATAAGCACCGGCTAACTACGT
GCCAGCAGCCGCGGTAATACGCAGGGTGCAAGCGTTAATCGGAATTACTGGGCGTAAAGC
GTGCGCAGGCGGTTATGTAAGACAGATGTGAAATGCCCGGGCTTAACCTGGGAACTGCAT
TTGTGACTGCATGGCTAGAGTTTGGCAGAGGGGGGTAGAATTCCACGTGTAGCAGTGAAA
TGCGTAGATATGTGGAGGAACACCGATGGCGAAGGCAGCCCCCTGGGTCAAGACTGACGC
TCATGCACGAAAGCGTGGGGAGCAAACAGGATTAGATACCCGTGTAGTCC
>Otu9350
CCAGCCTATGGGTCGCTGCAGTGGGGAATTTTTCTCAATGGGGGAAACCCTGAAGGAGCG
ACGCCGCGTGGAGGATGAAGGTCTTCGGATTGTAAACTCCTGTCATCAGGGAACAATTTG
CATGCGTTAACTGTGTATGCATTGATAGTACCTGAAGAGGAAGAGACGGCTAACTCTGTG
CCAGCAGCCGCGGTAATACAGAGGTCTCAAGCGTTGTTCGGATTCATTGGGCGTAAAGGG
TGCGTAGGTGGTGTCGTAAGTCGGGTGTGAAATCCCGGGGCTCAACCCCGGAACTGCATT
CGATGCTGCGATGCTCGAGGGCTGGAGAGGAGATTGGAATTCACGGTGTAGCAGTGAAAT
GCGTAGATATCGTGAGGAAGACCAGTGGCGAAGGCGAATCTCTGGACAGTTCCTGACACT
GAGGCACGAAGGCCAGGGGAGCAAACGGGATTAGAGACCCTTGTAGTCC
>Otu9354
CCAGCCTACGGGTGGCACCAGTCGAGAATCTTCCGCAATGGACGAAAGTCTGACGGAGCG
ACGCCGCGTGGAGGATGAAGTGCTTCGGCATGTAAACTTCTTTTGCCAGGGAAAAAGTTA
TTGATTGTACCTGGAGAATAAGAAGTTGCTAAACTCGTGCCAGCAGCAGCGGTAATACGA
GTGCTTCGAGCGTTATCCGGAATCATTGGGCGTAAAGGGTGTGTAGGTGGTAGTGTTAGT
CTCGTGTTAAATCTTTCGGCTCAACCGAGAGGCTGCATGGGAAACGGCACAACTAAGAGG
ACGGAAGAGGTCTCTGGAACTCATGGTGTAGCGGTGAAATGCGTTGATATCATAGGGAAC
ACCAAAAGCGAAGGCAAGAGACTGGTCCGCTCCTGACACTGAAACACGAAAGCGTGGGTC
GCGAATGGGATTAGAAACCCTCGTAGTCC
>Otu9357
CCAGCCTACGGGGCGCTCCAGTGTGGAATATTGGACAATAGGCTACCGCCTGATCCAGCA
ATACTTATTGGATGAAGTGACGGTGAAAATTGTAAAGTCCTCTCGTTGTTAAAGATGATG

ACAAATAGCAAAGCAAGAAGTCCTGACAAAACTCTGTGCCAGCAGCCGCGGTAAGACAGA
GGGGGCAAGCGTTATTCCTCGTTATTCGGCGTAAAGGGTACGTAGATGAACAACTAATAA
TATTATGTTAAATTCTAGAAGATTTTTTAGACTAATGTTTTAAATTGTTTTGTACAAAGA
ATTAAACAAGATTTATAGGAACTTTATAAGTAGGGGTAAAATCCGACGATATGTAAAGGA
ACACCAATAGCTAATGCATATATTTAGGTTTAATTGTCATTGAGGTACGAAGGTATAGGT
AGCAAACAGGATTAGAAACCCGAGTAGTCC
>Otu9359
CCAGCCTATGGGTGGCACCAGTCAAGAAACTTCCACAATGGACGAAAGTCTGATGGAGCG
ACGCCGCGTGGTTGATGAAGTCCTTCGGGACGTAAAAACCTTTTATGAGGGAGGAAGTAA
TTGACGTTACCTCATGAATAAGGGGCTCCTAACTCTGTGCCAGCAGGAGCGGTAATACAG
AGGCCCCAAGCATTATCCGGAATCACTGGGCGTAAAGGGTGTGTAGGCGGCCATATTAGT
CTGTCGTGAAAGGCCTTTGGCTCAACCAAGGGACCGCGGGAGAAACGGTATGGCTTAGAG
GATGTGAGAGGTAAAGGGAACTCATGGTGTAGGGGTGAAATCCGTTGATATCATGGGGAA
CACCAAATGCGAAGGCACTTTACTGGCACACTCCTGACGCTGAAACACGAAAGCGTGGGA
ATCGAATGGGATTAGAAACCCCCGTAGTCC
>Otu9363
CCAGCCTACGGGTGGCTCCAGTCGAGGATCTTTCGCAATGGGCGAAAGCCTGACGAAGCG
ACGCTGTGTGAACGATGAAGGCCTTCGGGTCGTAAAGTTCTTTCGCCTAAGAACAAGAGA
GAGACGTGAATAACGTTTTGATTTGAGGGTACTAGGTAAAGAAGCACCGGCTAACTCCGT
GCCAGCAGCTGCGGTAATACGGAGGGTGCAAGCATTAATCGGATTTATTGGGCGTAAAGG
GCGCGTAGGCGGAAGAGCAAGTCAGGTGTGAAATTCCGAAGCTCAACTTCGGAGCTGCAC
TTGAAACTGCTCATCTAGAGGATTGGCGGAGAAAACGGAATTCCACGAGTAGCGGTGAAA
TGCGTAGATATGTGGAAGAACACCGGTGGCGAAAGCGGTTTTCTAGCTTATTCCTGACGC
TGAGGCGCGAGAGCAAGGGGAGCAAACAGGATTAGAAACCCCCGTAGTCC
>Otu9372
CCAGCCTACGGGGTGCTCCAGTCGAGAATCTTCCGCAATGGACGAAAGTCTGACGGAGCG
ACGCCGCGTGATTGATGAAGTCCTTCGGGACGTAAAGATCTTTTATGGAGGAGGAAGTAA
TTGACGTTACTCCATGAATAAGGGGCTCCTAACTCTGTGCCAGCAGGAGCGGTAATACAG
AGGCCCCAAGCATTATCCGGAATCACTGGGCGTAAAGGGTGTGTAGGCGGTCGTGTTAGT
CTTTTGTTAAAGTTCTTCGGCTTAACCGAGGAAATGCAGAGGAAACGGCACGACTTAGAG
GTTGCGAGAGGTACAGGGAACTCATAGTGTAGGGGTGAAATCCGTTGATATTATGGGGAA
CACCAAATGCGAAGGCACTGTACTGGCGCACACCTGACGCTGAGACACGAAAGCGTGGGA
ATCGAACGGGATTAGAGACCCCCGTAGTCC
>Otu9374
CCAGCCTATGGGTCGCTGCAGTCGAGAATCATTCGCAATGGGCGAAAGCCTGACGATGCG
ACGCCGCGTGTGTGATGAAGGCCTTCGGGTCGTAAAGCACTTTCGCCTGGGAACAAGAGA
GGTAGGCTAATAATCTGCCGATTTGAGGGTACCAGGTAAAGAAGCACCGGCTAACTACGT
GCCAGCAGCTGCGGTAATACGTAGGGTGCTAGCGTTAATCGGAATTATTGGGCGTATAGG
GCGCGTAGGCGGATTGATAAGTTGGATGTGAAATCCCAGAGCTCAACTCTGGAACAGCAT
CCAAAACTGTCGATCTAGAGGGTAGATGGGGAAAATGGAATTCCACAAGTAGCGGTGAAA
TGCGTAGATATGTGGAAGAACACCTGTGGCGAAAGCGGTTTTCCAATTTACACCTGACGC
TGAGGCGCGAAAGCAAGGGGATCAAACAGGATTAGATACCCCAGTAGTCC
>Otu9382
CCAGCCTACGGGAGGCAGCAGTAAGGGATATTGCGCAATGGGCGAAAGCCTGACGCAGCA
ACGCCGCGTGGAGGACGACGGCCTTCGGGTTGTAAACTCCTTTTGGTCGGGACGAGGAAG
GACGGTACCGACCGAATAAGCTTCGGCTAACTACGCGCCAGCAGCCGCGGTAAGACGTAG
GAAGCGAGCGTTATCCGGATTTACTGGGCTTAAAGGGTGTGTAGGCGGTTAGCCAAGTTG
TGCATGAAAGCTGTCCGCTCAACGGGCAGAGGCTGTGCAATACTGGCTGACTAGAGGCTG
TGAGAGGGACGCGGAATTCCGGGTGTAGCGGTGAAATGCGTAGAGATCCGGAAGAACACC
AGAGGCGAAGGCGGCGTCCTGGCACAGCACTGACGCTGAGACACGACAGCATGGGGAGCG
AACGGGATTAGAAACCCCCGTAGTCC
>Otu9389
CCAGCCTATGGGAGGCAGCAGTGGGGAATTTTGCGCAATGGGGGAAACCCTGACGCAGCA
ACGCCGCGTGGAGGATGAAGGCCCTCGGGTCGTAAACTCCTGTCAAACGGGAACAAGAAA
GTGATAGTACCGTTAGAGGAAGAGACGGCTAACTCTGTGCCAGCAGCCGCGGTAATACAG
AGGTCTCAAGCGTTGTTCGGATTCATTGGGCGTAAAGGGCGCGCAGGCTGTGGGGTAAGT
CGGGTGTGAAATTTAGAGGCTCAACCTCTAAACTGCATTCGATACTGCTCTGCTAGAGGA
CTGTAGAGGAGATTGGAATTCACGGTGTAGCAGTGAAATGCGTAGATATCGTGAGGAAGA

CCAGTGGCGAAGGCGAATCTCTGGGCAGTTCCTGACGCTCATGCACGAAGGCCAGGGGAG
CAAACGGGATTAGAAACCCCTGTAGTCC
>Otu9392
CCAGCCTACGGGTGGCAGCAGTGGGGAATATTGCACAATGGGGGAAACCCTGATGCAGCG
ACGCCGCGTGAGTGAAGAAGTATTTCGGTATGTAAAGCTCTATCAGCAGGGACGAAGCGG
AGTTCGTGAGAACTCCATTGACGGTACCTGACTAAGAAGCCCCGGCTAACTACGTGCCAG
CAGCCGCGGTAATACGTAGGGGGCAAGCGTTATCCGGATTTACTGGGTGTAAAGGGAGCG
TAGACGGTAATGTAAGTCAGATGTGAAAGCCCGGGGCTCAACCCCGGGACTGCATTTGAA
ACTATGATACTAGAGTGCAGGAGAGGTAAGTGGAATTCCTAGTGTAGCGGTGAAATGCGT
AGATATTAGGAGGAACACCAGTGGCGAAGGCGGCTTACTGGACTGTAACTGACGTTGAGG
CTCGAAAGCGTGGGGAGCAAACAGGATTAGAGACCCCCGTAGTCC
>Otu9403
CCAGCCTATGGGATGCTGCAGTCTAGAGGCTTCGGCAATGGGCGAAAGCCTGACCGAGCG
ACGCCGCGTGCGGGATGAAGCCCCTTGGGGTGTAAACCGCTTTCGATCGGGAAAAAGGCC
CGGGTGGCGAACCCGGGATTGATGGTACCGGAAGAAGAAGCACCGGCTAACTCCGTGCCA
GCAGCCGCGGTAATACGGAGGGTGCGAGCGTTATTCGGATTCATTGGGCGTAAAGGGCGC
GTAGGTGGTTGGGTAAGTCCGGTGTGAAAACGCTATCGCTCACCGAAGCGAATGCGCTGG
ATACTGCTTAACTTGAGTTCGAGAGAGGTTGGCGGAATACCTGGTGTAGCGGTGGAATGC
GTAGATATCAGGTAGAACATCTGTGGCGAAGGCGGCCAACTGGCTCGACACTGACACTGA
GGCGCGAAAGCGTGGGGAGCAAACGGGATTAGAAACCCTAGTAGTCC
>Otu9404
CCAGCCTACGGGTTGCTGCAGTGGGGAATCTTGCGCAATGCGCGAAAGCGTGACGCAGCA
ACGCCGCGTGGGGGAAGACGGCCTTAGGGTTGTAAACCCCTTTCAGGAGGGACGAAGGTT
CACCGGTGAATAGCCGTGTGGATTGACGGTACCTCCACAAGAAGCACCGGCTAACTACGT
GCCAGCAGCCGCGGTAATACGTAGGGTGCAAGCGTTGTCCGGAATCATTGGGCGTAAAGC
GCGCGTAGGTGGCCATTTAAGTCCGCTGTGAAAGTCAAAGGCTCAACCTTTGAAAGCCGG
TGGATACTGGATGGCTAGAGTACGGAAGAGGCGAGTGGAATTCCTGGTGTAGCGGTGGAA
TGCGCAGATATCAGGAGGAACACCAATAGCGAAGGCAGCTCGCTGGGACGTTACTGACAC
TGAGGCGCGAAAGCGTGGGGAACGAACAGGATTAGAAACCCTCGTAGTCC
>Otu9405
CCAGCCTACGGGGGCTGCAGTGAAGAATCTTGGGCAATGGACTAACGTCTGACCCAGTGA
GAACACATGCATGACGAATGCGCCCAGTTTTCTGTAAAGTGCTTTCGCGAGATGAAGATA
TTGACTTTAGCTCGGGATGAAGCGCCGGCCAACTCCGTGCCAGCAGCCGCGGTAAGACGG
AGGGCGCGAGTGTTATTCGTTTTGATTGGGTGTAAAGGGTATGTAGGCGGCACCGGATTT
TTTGGCTTAAAGCGCGGAGTATTCCTATGCGTAAGCCTTCTTCTGAAGGAGCTAGAGTTG
GAAATGGGTTTAGTCAAGTTCGCTAGTAGGGGTAAAATCCAGCGATATGCGAACGAACGT
CAGCTGGCGCAGGCGCTTCTCCGGTTCCAACTGACGCTGAGGTACGGAAGCTTAGGGAGC
AAACGGGATTAGAAACCCTGGTAGTCC
>Otu9416
CCGCCTACGGGGTGCTGCAGTGGGGAATATTGCGCAATGGGGGAAACCCTGACGCAGCAA
CGCCGCGTGGGTGAAGAAGGCCTTCGGGTTGTAAAGCCCTGTCAGGAGGAAAGAAAGCGC
AGCCAATCAATACCTGGTTGCGTTGACGGTACCTCCAGAGGAAGCACCGGCTAACTCCGT
GCCAGCAGCCGCGGTAATACGGAGGGTGCGAGCGTTGTTCGGAATCATTGGGCGTAAAGC
GCGTGCAGGCGGTCCGGCAAGTCGGGTGTGAAAGCCCTCGGCTCAACCGAGGAATTGCAT
TCGAAACTGCCGGACTTGAGTGCGGGAGAGGGAAGTGGAACTCATAGTGTAGAGGTGAAA
TTCGTAGATATTATGAGGAACACCTGTGGCGAAGGCGGCTTCCTGGACCGACACTGACGC
TGAGACGCGAAAGCGTGGGGAGCAAACAGGATTAGAGACCCTTGTAGTCC
>Otu9431
GGACTACGAGGGTATCTAATCCTGTTTGCTACCCACGCTTTCGTGCATCAGCGTCAGTTA
TCGTCCAGTAAGCCGTTTACACCACCGGTGTTCCTCCTGATATCTACGCATTTCACCGCT
ACACCAGGAATTCCACTTACCTCTCCGACACTCTAGCCTCTCAGTATTCGGCGCACCCTC
CCGGTTGAGCCGGGAGATTTCACACCAAACTTAAAAAGCCGCCTACGCACCCTTTACGCC
CAGTAACTCCGAACAACGCTTGCTGCCTACGTATTACCGCGGCTGCTGGCACGTAGTTAG
CCGCAGCTGCTTCTGCAGGTACCGTCATAACCTTCCCTGCCGAAAGAGCTTTACACCCCG
AAGGGCTTCATCGCTCACGCGGCGTCGCTGCATCAGAGTTTCCTCCATTGTGCTAGATTC
CCCACTGCTGCATCCCATAGGCTGG
>Otu9433
CCAGCCTACGGGGTGCTGCAGTGGGGAATCTTGCGCAATGGGCGAAAGCCTGACGCAGCG

ACGCCGCGTGAAGGATGAAGGTCTTCGGATTGTAAACTTCTGTTAAGTGGGAAGAAAAGC
CCATCCCTAATACGGATGTGGTTATGACGGTACCATTAAAGAAAGCACCGGCTAATCTCG
TGCCAGCAGCCGCGGTAATACGAGAGGTGCAAGCGTTATTCGGAATTACTGGGCGTAAAG
GGTGCGTAGACGGTATGCTAAGTCTGTTGTTAAATCCTCCGGCCTAACCGGAGATCTGCA
GTGGAAACTGGCGTACTAGAGGGTGAAAGAGAGAAGTGGAATTCCCGGAGTAGCGGTAAA
ATGCGTAGATCTCGGGAGGAACACCAATGGCGAAGGCAGCTTCTTGGCTCATTCCTGACG
TTGAGGCACGAAAGCGTGGGGAGCAAATAGGATTAGAAACCCCCGTAGTCC
>Otu9442
CCAGCCTACGGGTGGCTGCAGTGGGGAATCTTGCGCAATGGACGAAAGTCTGACGCAGCC
ACGCCGCGTGAGTGAAGAAGGCCTTCGGGCTGTAAAGCTCTGTCGGAGGGGACGAAAATG
TTCTGGGTTAACAGCCCAGGGTACTGACGGTACCCTCAAAGGAAGCACCGGCTAACTTCG
TGCCAGCAGCCGCGGTAATACGAGGGGTGCAAGCGTTGCTCGGAATTATTGGGCGTAAAG
GGTAGGTAGGTGGTCTCATTTGTCTGATGTGAAAGCCTTGGGCTTAACCCAAGAAGTGCG
TCAGAAACGGTGAGACTAGAGTCTTGGAGAGGGTCGTGGAATTCCCGGTGTAGCGGTGAA
ATGCGTAGAGATCGGGAGGAACACCAGAGGCGAAGGCGGCGACCTGGACAGGTACTGACA
CTCAACTACGAAAGCGTGGGGAGCAAACAGGATTAGAAACCCGGGGTAGTCC
>Otu9450
CCAGCCTATGGGTGGCTCCAGTGGGGAATCTTGCGCAATGGGCGAAAGCCTGACGCAGCG
ACGCCGCGTGAGTGATGAAGGCCTTCGGGTTGTAAAGCTCTGTGGAGAGGGACGAATAAG
GGCTGGCTAATATCCATCCTGATGACGGTACCTCTTTAGCAAGCACCGGCTAACTCTGTG
CCAGCAGCCGCGGTAAGACAGGGGGTGCAAACGTTGTTCGGAATTACTGGGCGTAAAGCG
CGTGTAGGCTGCTCCAAAAGTCGGGTGTGAAAGCCCTGGGCTCAACCTAGGAAGTGCACT
CGAAACTGCGGAGCTGGAGTCCTGGAGAGGAAGGCGGAATTCTCGGTGTAGAGGTGAAAT
TCGTAGATATCGGGAGGAACACCGGTGGCGAAGGCGGCCTTCTGGACAGTGACTGACGCT
GAGACGCGAAAGCGTGGGGAGCAAACAGGATTAGAAACCCCCTAGTCC
>Otu9454
CCAGCCTACGGGGTGCAGCAGTGGGGAATTTTGGACAATGAGGGAAACCCTGATCCAGCC
ATGCCGCGTGAGTGAAGAAGGCCTTCGGGTTGTAAAGCTCTTTTGGTCGGAAAGAAGGGG
TTCGGGTGAATAACCTGGATTTTTGACGGTACCGGCAGAATAAGCACCGGCTAACTACGT
GCCAGCAGCCGCGGTAATACGTAGGGTGCGAGCGTTAATCGGAATTACTGGGCGTAAAGA
GTGCGTAGGCGGTCTTTCAAGTCCGTTGTGAAAGCCCCGGGCTCAACCTGGGAATGGCAG
TGGAAACTAGAAGACTTGAATCTGGCAGAGGGGGGTGGAATTCCACGTGTAGCAGTGAAA
TGCGTAGAGATGTGGAGGAACACCGATGGCGAAGGCAGCCCCCTGGGCTGAGATTGACGC
TCAGGCACGAAAGCGTGGGGAGCAAACAGGATTAGAAACCCCTGTAGTCC
>Otu9465
CCAGCCTACGGGTGGCTGCAGTCGAGAATCTTCGGCAATGGACGCAAGTCTGACCGAGCG
ACGCCGCGTGTGGGATGAAGGCCTTCGGGTTGTAAACCACTGTCAGAGAGGATGAAATGC
TGGTGGGTACTCCCATCAGTTTGACATATTCTCAGAGGAAGGACGGGCTAAGTTCGTGCC
AGCAGCCGCGGTAAGACGAACCGTCCAAACGTTATTCGGAATCACTGGGCTTAAAGGGTG
CGTAGGCGGCGCGGAAAGTAGGGTGTGAAATCCCTCGGCTTAACCGAGGAACTGCGCTCT
AAACTACCGTGCTCGAGGGAGATAGAGGTGAGCGGAACTTAGGGTGGAGCGGTGAAATGC
GTTGATATCCTGAGGAACACCGGTGGCGAAAGCGACGTACTGGACCTCTTCTGACGCTGA
GGCGCGAAAGCTAGGGGGGCAAACGGGATTAGAAACCCGAGTAGTCC
>Otu9467
CCAGCCTATGGGTGGCACCAGTAGGGAATTTTCCACAATGGGCGAAAGCCTGATGGAGCA
ACGCCGCGTGCAGGATGAATGCCTTAGGGTTGTAAACTGCTTTTATATGTGAAGAATATG
ACGGTAGCATGTGAATAAGGATCGGCTAACTCCGTGCCAGCAGCCGCGGTCATACGGAGG
ATCCAAGCGTTATCCGGAATTACTGGGCGTAAAGAGTTGCGTAGGTGGCATAGTAAGTTG
ATAGTGAAAGCGTGCGGCTCAACCGCATACACATTACCAAAACTGCTAAGCTAGAGGACG
AGAGAGGTAGATGGAATTTCCAGTGTAGGAGTGAAATCCGTAGATATTGGAAGGAACACC
GATGGCGTAGGCAGTCTACTGGCTTGTTCCTGACACTAAGGCACGAAAGCGTGGGGAGCG
AACGGGATTAGATACCCTTGTAGTCC
>Otu9468
CCAGCCTACGGGTGGCTGCAGTGGGGAATATTGGACAATGAGCGAAAGCTTGATCCAACA
ATATCACATAAGAGAAGAAGGGCTTTGTGCTTGTAAATCTTGGTAGTTGATGAAGATAAT
GACATTAGTCAACGAAAAAATGCCCTGGCTAACTTCGTGCCAGCAGCTGCGGTAATACGG
AGGGGGCAAGTGTTACTCATAATGATTGGGCGTAAAGTGTATGTAGATGGTGTATTAAGT
TCTTGATAAAAATCCCTTCTATGCAGTAGGGAAAGATTGAATACTAATATACTAGAGTCT

AATAGGAGAGAGCGGTACTGTAAGTGCAGGGGTAGAATCCAGTAAAAGATTTATAGGACA
AACAGCTAGCGAAGGCAGCAATCTTGGTTAAACTGACATTGAGGTACGAAAGCACAGGGA
TCAAAAAGGATTAGATACCCGCGTAGTCC
>Otu9474
CCAGCCTACGGGGGGCAGCAGCCGAGAATATTCGACAATGGGCGAAAGCCTGATCGAGCG
ATACCGCGTGGTGGATGAAGTGCTTCGGCACGTAAACATCTTTTATGAGGGAGGAAATTA
TTGACGTTACCTCATGAATAAGGGGCTCCTAACTCTGTGCCAGCAGGAGCGGTAATACAG
AGGCCCCGAGCGTTACCCGGAATCACTGGGCGTAAAGCGTGTGTAGGAGGTCGTATTAGT
CGTCCGTTAAATCCCGGGGCTTAACCCCGGACTCGCGGGCGAAACGGTATGACTAGAGGG
TGTGAGAGGTGAATGGAACTCATGGTGTAGGGGTGAAATCCGTTGATATCATGGGGAACA
CCGAAAGCGAAGGCAATTCACTGGCGCATTCCTGACTCTGAAACACGAAAGCGTGGGTAG
CGAACGGGATTAGAACCCTCGTAGTCC
>Otu9481
CCAGCCTATGGGTTGCTCCAGTCGAGGATCTTTGGCAATGGGCGAAAGCCTGACCGAGCG
ACGCCGCGTGTGGGATGAAGGCCCTTGGGTTGTAAACCACTGTCAGAGGGGATGAAGTGC
AGGTGAGCTATCTCATCTGTTTGACATAGCCTCAGAGGAAGCACGGGCTAAGTTCGTGCC
AGCAGCAGCGGTAATACGAACTGTGCAAACGTTATTCGGAATCACTGGGCTTAAAGGGTG
CGTAGGCGGTCGTTTAAGTAGGGTGTGAAAGCCCTCGGCTCAACCGAGGAATTGCGCCCT
AAACTGGATGGCTGGAGTGAGATAGGGGTGTGTGGAACTTCCGGTGGAGCGGTGAAATGT
GTTGATATCGGAAGGAACGCCGGTGGCGAAAGCGACACACTGGGTCTTAACTGACGCTGA
GGCACGAAAGCCAGGGGAGCAAACGGGATTAGAAACCCTTGTAGTCC
>Otu9486
CCAGCCTATGGGGGGCTCCAGTCGAGAATCATTCGCAATGGGCGAAAGCCTGACGATGCG
ACGCTGCGTGAGCGATGAAGGCCTTCGGGTCGTAAAGCTCTTTCGCCGGGGAACAAGGGA
GGATGGCTAATATCCATCCGACTTGAGGGTACTCGGTAAAGAAGCACCGGCTAACTCCGT
GCCAGCAGCTGCGGTAATACGGAGGGTGCAAGCGTTAATCGGATTTATTGGGCGTAAAGG
GGGCGTAGGCGGGAAGGCAAGTCAGATGTGAAATTCCAGGGCTTAACCTTGGAGCTGCAT
TTGAAACTACCTTTCTTGAGGAAAGGCGGAGAAAACGGAATTCCACAAGTAGCGGTGAAA
TGCGTAGATATGTGGAAGAACACCTGTGGCGAAAGCGGTTTTCTAGCTTATTCCTGACGC
TGAGGCCCGAAAGCTAGGGGAGCAAACAGGATTAGAAACCCTAGTAGTCC
>Otu9489
CCAGCCTACGGGAGGCAGCAGTGGGGAATCTTGCACAATGGAGGAAACTCTGATGCAGCG
ACGCCGCGTGAGCGATGAAGCCCTTCGGGGTGTAAAGCTCTTTCGACGGGAACGATAATG
ACGGTACCCGAAGAAGAAGCTGCGGCTAACTACGTGCCAGCAGCCGCGGTGATACGTAGG
GTGCAAGCGTTAATCGGAATTACTGGGCGTAAAGCGCACGTAGGCGGCTTTGCAAGTCGG
ATGTGAAATCCCTCGGCTTAACCAAGGAAGTGCATCCGAAACTGCAGAGCTTGAGTACTT
AAGAGGATCGCGGAATTCCCGGTGTAGAGGTGAAATTCGTAGATACCGGGAGGAACACCA
GTGGCGAAGGCGGCGATCTGGGAAGATACTGACGCTGAGGTGCGAAAGCGTGGGGAGCAA
ACAGGATTAGAGACCCGAGTAGTCC
>Otu9497
CCAGCCTATGGGTGGCAGCAGTAGGGAATATTGCTCAATGGGCGCAAGCCTGAAGCAGCA
ATGCCGCGTGAAGGATGAAGGCCTTCGGGTTGTAAACTTCTTTTGTCAGGGATGAAGAAA
TTTGGATTAATAATCCGAAGATTTGACCGTACCTGAAGAATAACCACCGGCTAACTCCGT
GCCAGCAGCTGCGGTAATACGGAGGGTGGAAGCGTTGATCGGATTGACTGGGCGTAAAGG
GAGCGTAGGCGGCTTGATAAGTCGATGGTGAAATTTCTGGGCTTAACCCAGAAGCTGCTG
TTGAAACTATCAAACTAGAGGATATTAAGGGAAAGCGGAATACCACATGTAGCGGTGAAA
TGCGTAGATATGTGGTAGAACACCGGTGGTGAAGACGGCTTTCTGTGATATACCTGACGC
TGAGGCTCGAAAGTATGGGGATCAAACAGGATTAGAAACCCCCGTGGTCC
>Otu9500
CCAGCCTATGGGGGGCACCAGTCGAGAATTTTTCTCAATGGGCGAAAGCCTGATGGTGCG
ACGCCGCGTGAGGGATGAAGGTCTTCGGATTGTAAACCTCTGTCACCGGGGAAGAAACGC
TACGGGTTAACAGCCCGTAGCCTGACTTAACCCGGAGAGGAAGCAGTGGCTAACTCTGTG
CCAGCAGCCGCGGTAATACAGAGACTGCGAGCGTTATTCGGATTCACTGGGCGTAAAGGG
TGCGCAGGTGGCCGGGTGTGTGAGGCGTGAAAGCCCGGAGCTTAACTCCGGAATTGCACC
TCAAACTACACGGCTAGAGCATTGGAGAGGGGAGCAGAATTCACGGTGTAGCAGTGAAAT
GCGTAGATATCGTGAGGAATACCAGAGGCGAAGGCGGCTCCCTGGACAATTGCTGACACT
CAGGCACGAAAGCGTGGGGAGCAAAAGGGATTAGAAACCCTCGTAGTCC
>Otu9501

CCAGCCTATGGGGGGCAGCAGTCGAGAATTTTTCTCAATGGGCGAAAGCCTGAAGGAGCG
ACGCCGCGTGGGGGATGAAGGGTTTCGATCCGTAAACCCCTGTCATTTGCGAACAAACCC
TGGAGTTTAAGAGATGCCAGGCTGATTGTAGCGAAAGAGGAAGGGACGGCTAACTCTGTG
CCAGCAGCCGCGGTAATACAGAGGTCCCAAGCGTTGTTCGGATTCACTGGGCGTAAAGGG
TGCGTAGGTGGCATGGTAAGTTTGATGTGAAATCTCCGGGCTTAACCCGGAAATGGCATT
GAATACTATTGTGCTGGAGGTTTGGAGGGGGGACTGGAATTCTCGGTGTAGCAGTGAAAT
GCGTAGATATCGAGAGGAACACCAGTGGCGAAGGCGAGTCCCTGGACAACACCTGACACT
GAGGCACGAAAGCTAGGGGAGCAAACAGGATTAGAAACCCCAGTAGTCC
>Otu9505
CCAGCCTACGGGGGGCTCCAGTGGGGAATCTTGCACAATGGGCGAAAGCCTGATGCAGCC
ATGCCGCGTGTGTGAAGAAGGCCTTCGGGTTGTAAAGCACTTTAAGAGGGGAGAAAAGGG
TAGTCGTTAATAGCGGCTATCTGTGATGTTACCCTCAGAATAAGCACCGGCAAACTTCGT
GCCAGCAGCCGCGGTAATACGAAGGGTGCGAGCGTTAATCGGAATTACTGGGCGTAAAGA
GCACGTAGGCGGTCTGATAAGCGGAATGTGAAATCCCCGGGCTTAACCTGGGAACTGCAT
TCCGAACTGTCAGGCTAGAGTATGATAGAGGTGAGCGGAATTTCCTGTGTAGCGGTGAAA
TGCGTAGATATAGGAAGGAACATCAGTTGCGAAGGCGGCTCACTGGATCAATACTGACGC
TGAGGTGCGAAAGCGTGGGTAGCGAACAGGATTAGAACCCCTGTAGTCC
>Otu9506
CCAGCCTATGGGGGGCTCCAGTAGGGAATATTGGGCAATGGGCGCAAGCCTGACCCAGCC
ATGCCGCGTGAAGGATGAAGGTCCACAGGATTGTAAACTTCTTTTATCGGGGAATAAGGC
AGATGTAACTCATCTGATTGAAGGTACCCGAGGAATAAGCACCGGCTAACTACGTGCCAG
CAGCCGCGGTAATACGTAGGGTGCAAGCGTTGTCCGGATTTACTGGGTTTAAAGGGTGCG
TAGGCGGTTTCATAAGTCAGTGATGAAATGTCGGGGCTCAACCCCGTACCTGTTACTGAT
ACTGCGAAACTTGAGAATGGATGAGGTAGCTGGAATGTGTGGTGTAGCGGTGAAATGCGT
AGAGATCACACAGAACACCAATTGCGAAGGCAGGTTACTGGGCCATTTCTGACGCTGAGG
CACGAAAGCGTGGGGAGCAAACAGGATTAGAAACCCTGGTAGTCC
>Otu9524
CCAGCCTACGGGTTGCTCCAGTGGGGAATCTTTCGCAATGGGCGAAAGCCTGACGAAGCG
ACGCCGTGTGTGTGATGAAGGCCTTCGGGTTGTAAAGCACTTTCGCCTGGGAACAAGAAA
CTTTAGCTAATAACTAAGGAATTTGAGAGTATCAGGTAAAGAAGTACCGGCTAACTACGT
GCCAGCAGCTGCGGTAATACGTAGGGTACAAGCATTAATCGGATTTATTGGGCGTAAAGG
GCGCGTAGGCGGAGATGCAAGTCAGATGTGAAATCCCGAAGCTCAACTTCGGAACAGCAT
TTGAAACTACATCCCTAGAGGATAGACGGAGAAAACGGAATTCCACAAGTAGCGGTGAAA
TGCGTAGATATGTGGAAGAACACCAGTGGCGAAGGCGGTTTTCTAGTTTATACCTGACGC
TGAGGCGCGAAAGCAAGGGGAGCAAACAGGATTAGAAACCCTAGTAGTCC
>Otu9533
CCAGCCTATGGGAGGCAGCAGTGTGGAATCTTGGGCAATGAGGGAAACCTTGACCCAGCG
TTATCGCCAGGGTGATTAAAAGCTGTAGAAATAACCAGGCTCTAAATCTCTCAGGAGAGG
TCTTCAAACCGAGGGGATCTTTCTGTATTCCCGGACAATCTCGTGCCAGCAGTCGCGGTA
ATACGGGAGGGAAGAGTGCTGCTCGTCGCGACTGGGAGTACGAGTTCGTAGGCGGCCTAT
GCTTCAGGCCTCGAGTGGTTACGGAGTAGCCGTGAATGGGGGGTTGTCGGAGAAGGCTAG
AGTGCTTCGAGGGGTCGATGGTATTGCTGGATTAACGTTGGAATGTGACTGATGCCTGCA
AGAGGACCGGAGGCGAAGGCGATCGACCGGAAGTTACTGACGCTCAAGAACGAAATCGGA
GGGAGCGAACGGGATTAGAGACCCTCGTAGTCC
>Otu9538
CCAGCCTATGGGGGGCACCAGTGAGGAATTTTCCGCAATGGGCGAAAGCCTGAAGGAGCG
ACGCCGCGTGGGGGATGAAGGGCTTCGGCTCGTAAACCCCTGTCATTTGCGAACAAACCT
TACGATTGAATAGATCGTGAGCTGATTGTAGCGAAAGAGGAAGGGACGGCTAACTCTGTG
CCAGCAGCCGCGGTAATACAGAGGTCCCAAGCGTTGTTCGGATTCACTGGGCGTAAAGGG
TGCGTAGGTGGCAGGGTAAGTCTGACGTGAAATCTCCGGGCCTAACCCGGAAACTGCGTC
GGATACTATCCGGCTAGAGGATTGGAAGGGAGACTGGAATACTTGGTGTAGCAGTGAAAT
GCGTAGATATCAAGTGGAACACCAGTGGCGAAGGCGAGTCTCTGGACAATTCCTGACGCT
GAGGCACGAAAGCCAGGGGAGCAAACGGGATTAGAGACCCCGGTAGTCC
>Otu9544
CCAGCCTATGGGGTGCTGCAGTCGAGAATCTTCCACAATGGACGAAAGTCTGATGGAGCG
ACGCCGCGTGGTTGATGAAGTCCTTCGGGACGTAAAAACCTTTTATGGAGGAGGAAATTT
TGACGTTACTCCATGAATAAGGGGCTCCTAACTCTGTGCCAGCAGGAGCGGTAATACAGA
GGCCCCAAGCATTATCCGGAATCACTGGGCGTAAAGGGTGTGTAGGCGGTTATGTTAGTC

TTTCGTGAAAGATCTTCGGCTTAACCGAGGGGACGCGGGAGAAACGGCACAACTTAGAGG
TTGCGAGAGGTAAAGGGAACTCATAGTGTAGGGGTGAAATCCGTTGATATTATGGGGAAC
ACCAAATGCGAAGGCACTTTACTGGCGCACACCTGACGCTGAAACACGAAAGCGTGGGAA
TCGAACGGGATTAGAGACCCCTGTAGTCC
>Otu9546
CCAGCCTACGGGGGGCTGCAGTGGGGAATATTGGACAATGGGGGAAACCCTGATCCAGCG
ACGCCGCGTGTGTGAAGAAGGCCTGCGGGTTGTAAAGCACTTTTAGTGGGGACAAAAAGC
CATCGCCTAACACGCTGTGGTCTTGATTTAACCCAAAGAAAAAGCACCGGCTAACTCTGT
GCCAGCAGCCGCGGTAATACAGAGGGTGCGAGCGTTAATCGGAATTACTGGGCGTAAAGC
GCGCGTAGACGGTTTTGTAAGTCAGATGTGAAATCCCTGGGCTCAACCTGGGAACTGCAT
TTGAGACTGCATGGCTTGAGTGTGGGAGAGGTGAGTGGAATTTCCGGTGTAGCGGTGAAA
TGCGTAGATATCGGAAGGAACACCTGTGGCGAAAGCGGCTCACTGGACCACAACTGACGC
TGATGCACGAAAGCTAGGGGAGCAAACAGGATTAGAGACCCCTGTAGTCC
>Otu9551
CCAGCCTACGGGTGGCTGCAGTGGGGAATATTGCGCAATGGCCGAAAGGCTGACGCAGCG
ACGCCGCGTGTAGGATGAGGCCCTTCGGGGTGTAAACTACTGTTGCCCGGGACGAACGGC
AGACTTTGGTCTGCGTGACGGTACCGGGTGAGGAAGCACCGGCTAACTCCGTGCCAGCAG
CCGCGGTAATACGGAGGGTGCGAGCGTTGTCCGGAATCACTGGGCGTAAAGGGCGCGTAG
GTGGCTTGGTAAGGGGGCGGTGAAAGCCCGGGGCTCAACCCCGGGTCTGCCGGACCGACT
GCTAGGCTAGAGCACACTAGAGGCAAGTGGAATTCCGGGTGTAGCGGTGGAATGCGTAGA
TATCCGGAAGAACACCGGTGGCGAAGGCGGCTTGCTGGGGTGTGGCTGACACTGAGGCGC
GACAGCGTGGGGAGCAAACAGGATTAGATACCCGCGTAGTCC
>Otu9552
CCAGCCTATGGGGCGCTGCAGTCGAGGATCTTCGGCAATGGGCGCAAGCCTGACCGAGCG
ACGCCGCGTGGGCGATGAAGGCCTTCGGGTTGTAAAGCCCTGTCGAGGGGGAGAAAGGGA
AACTTGATCTATCCCTGGAGGAAGCACGGGCTAAGTTCGTGCCAGCAGCCGCGGTAAGAC
GAACCGTGCGAACGTTGTTCGGAATCACTGGGCTTAAAGGGCGCGTAGGCGGCCGCCAA
GTCTGTGGTGAAATCCCCCGGCTCAACCGGGGAACTGCCGTGGATACTGGAGGTCTCGAG
GGAGGTAGGGGCATGCGGAGCTACGGGTGGAGCGGTGAAATGCGTTGATATCCGTAGGAA
CTCCGGTGGCGAAGGCGGCGTGCTGGACCTCTTCTGACGCTGAGGCGCGAAAGCCAGGGG
AGCGAACGGGATTAGATACCCTCGTAGTCC
>Otu9553
CCAGCCTATGGGAGGCTGCAGTGGGGAATCTTGCGCAATGGGCGAAAGCCTGACGCAGCA
ACGCCGCGTGGAGGACGAAGGCCTTCTCGGTTGTAAACTCCTTTCGGCAGGGACGAAAAC
ACCGACGGTACCTGCAGAAGAAGCCCCGGCCAACTACGTGCCAGCAGCCGCGGTAATACG
TAGGGGGCGAGCGTTGTCCGGATTTATTGGGCGTAAAGAGCTCGTAGGCGGCTTGGTTAG
TTGGGTGTGAAATCTCCAGGCTCAACCTGGAGGGGCCACCCAATACTGCCATGGCTAGAG
TCCGGTAGGGGGCCACGGAATTCCTGGTGTAGCGGTGAAATGCGCAGAGATCAGGAGGAA
CACCGGTGGCGAAGGCGGTGGCCTGGGCCGGAACTGACGCTGAGGAGCGAAAGCGTGGGG
AGCGAACAGGATTAGATACCCCAGTAGTCC
>Otu9555
CCAGCCTACGGGGGGCTGCAGTGGGGAATATTGCGCAATGGGCGAAAGCCTGACGCAGCG
ACGCCGCGTGGAGGATGAAGGTCTTCGGATTGTAAACTTCTGTTAAGTGGGAAGAAAAGT
CTATTCCTAATACGGATAGATGTTGACGGTACCACTAGAGAAAGCACTGGCTAATCTCGT
GCCAGCAGCCGCGGTAATACGAGAGGTGCGAGCGTTATTCGGAATTATTGGGCGTAAAGG
GTGTGTAGACGGCATGTTAAGTCAACTGTTAAATTCTCCGGCCTAACCGGAGTTCTGCGG
TAGATACTGGCATACTAGAGGGTGGAAGAGAGAAGTGGAATTCTCGGAGTAGCGGTAAAA
TGCGTGGATCTCGAGAGGAACACCAATGGCGAAGGCAGCTTCTTGGTCCACTCCTGACGT
TGAGACACGAAAGCGTGGGGAGCAAACAGGATTAGATACCCTAGTAGTCC
>Otu9557
CCAGCCTACGGGGGGCTCCAGTCGAGAATCTTTCGCAATGGCGCAAGCCTGACGAAGCGA
CGCCGTGTGAGCGAAGAAGGCCTTCGGGTTGTAAAGCTCTTTCGCTAGGGAACAAGAGAG
GCTGGATAATAACCAGTTAATTTGATGGTACTTGGTAAAGAAGCACCGGCTAACTCCGTG
CCAGCAGCTGCGGTAATACGGAGGGTGCAGGCATTGATCGGAATTACTGGGCGTAAAGGG
CGCGTAGGCGGCAAGATAAGTCAGATGTGAAATTCCGGGGCTCAACCCCGGAGCTGCATT
TGAAACTGTCTAGCTAGAGGTCAGTTAGGGAAAACGGAATTCCACGTGTAGCGGTGAAAT
GCGTAGATATGTGGAAGAACACCAGTGGTGAAGACGGTTTTCTGGGCTGATACTGACGCT
GAGGCGCGAAAGCAAGGGGAGCGAACAGGATTAGATACCCTCGTAGTCC

>Otu9566
CCAGCCTACGGGTTGCTGCAGTAGGGAATCTTGCGCAATGGACGAAAGTCTGACACAGCA
ACGCCGCGTGAGGGACGAAGGCTTTCTGAGTTGTAAACCTCTTTCGGCAGGAACGATTGT
AACGGTACCTGCAGAAGAAGCACCGGCCAACTACGTGCCAGCAGCCGCGGTGATACGTAG
GGTGCAAGCGTTATTCGGATTTATTGGGCGTAAAGAGCTCAGGCGGTTCGATAAGTTGGG
TGTGAAACCCCCAGGCTTAACCTGGGGCAGCCACCCAAAACTGTCGTGACTAGAGTTTGG
TAGGGGATCACGGAATTCCTGGTGTAGCGGTAGAATGCGCAGATATCAGGAGGAACACCA
GTAGCGAAGGCGGTGATCTGGGCCAATACTGACGCTGAGGAGCGAAAGCGTGGGGAGCGA
ACAGGATTAGATACCCTTGTAGTCC
>Otu9567
CCAGCCTATGGGGCGCAGCAGTGGGGAATCTTGCGCAATGGACGAAAGTCTGACGCAGCC
ACGCCGCGTGAGTGAAGAAGGCCTTCGGGTTGTAAAGCTCTGTCGGGCGGGACGAAAATC
TTAGACGTGAATAGCGTCTAGGACTGACGGTACCGCCAAAGGAAGCACCGGCTAACTTCG
TGCCAGCAGCCGCGGTAATACGAGGGGTGCAAGCGTTGCTCGGAATTATTGGGCGTAAAG
GGTAGGTAGGTGGTCTCATTTGTCTGGGGTGAAAGCCTTGAGCTTAACTCAAGAAGTGCC
CCAGAAACGGTGAGACTGGAGTCTCGGAGAGGGTCGTGGAATTCCCGGTGTAGCGGTGAA
ATGCGTAGAGATCGGGAGGAACACCAGAGGCGAAGGCGGCGACCTGGAAGATTCCTGACT
CTGAAACACGAAAGCCAGGGGAGCAAACGGGATTAGATACCCGTGTAGTCC
>Otu9579
CCAGCCTATGGGAGGCAGCAGTGGGGAATCTTGCACAATGGACGAAAGTCTGATGCAGCA
ACGCCGCGTGGGGGATGAAGCTTTTCGGAGTGTAAACCCCTTTCGACCCGGAAGAAAGCC
CGCAAGGGTTTGACGGTACGGGTATAAGAAGCCCCGGCTAACTACGTGCCAGCAGCCGCG
GTAAAACGTAGGGGGCCAGCGTTGCTCGGAATTACTGGGCGTAAAGGGTCCGTAGGCGGT
GTGGCAAGTCGGTAGTGAAATCTCAGAGCTTAACTCTGAAACTGCGCTTGATACTGCTCG
GCTAGAGGATCGGAGGGGGTAACGGAATTTATGGTGTAGCAGTGAAATGCGTAGATATCA
TAAGGAACACCGGTGGCGAAGGCGGTTACCTGGAAGATTCCTGACTCTGAAACACGAAAG
CCAGGGGAGCAAACGGGATTAGATACCCTGGTAGTCC
>Otu9588
CCAGCCTATGGGGGGCTGCAGTGAGGAATATTGCACAATGGGCGAAAGCCTGATGCAGCG
ACGCCGCGTGGAGGATGACGGTCTTCGGATTGTAAACTCCTGTTAAGTGGGAAAAAAGAC
TTGTTCTTAATAGGAACGAGGGATGATGGTACCACTAAAGAAAGCACCGGCTAACTTCGT
GCCAGCAGCCGCGGTAACACGAGGGGTGCAAACGTTATTCGGAATCATTGGGCGTAAAGG
GTACGTAGACGGCGAAATAAGTCAGTCATTAAATCTTCCAGCTTAACTGGAAACTTGTGG
CTGAAACTGATTTGCTTGAGGATGGAAGAGAGAAGCGGAATTCTCGGGGTAGCGGTAAAA
TGCGTAGATCTCGAGAGGAACACCGATGGCGAAGGCAGCTTCTTGGTCCATTTCTGACGT
TGAGGTACGAAAGCGTGGGGAGCAAACAGGATTAGAAACCCCCGTAGTCC
>Otu9595
CCAGCCTATGGGGGGCAGCAGTGGGGAATATTGGACAATGGGCGAAAGCCTGATCCAGCA
ATGCCGCGTGTGTGAAGAAGGCCTTCGGGTTGTAAAGCACTTTAGTTGGGGAGGAAGTTG
TGTAGTTTAAGAGATAGCACAATTGACTGTACCCAAAGAATAAGCACCGGCTAACTCTGT
GCCAGCAGCCGCGGTAATACAGAGGGTGCGAGCGTTAATCGGAATTACTGGGCGTAAAGG
GCGCGTAGGCGGTTTATTAAGTTAGATGTGAAATCCCTGGGCTTAACCTAGGAACTGCAT
ATAATACTGATAAGCTGGAGTGCGGTAGAGGATAGTGGAATTTCCGGTGTAGCGGTGAAA
TGCGTAGATATCGGAAGGAACACCAGTGGCGAAGGCGGCTATCTGGACTGACACTGACGC
TGAGGCGCGAAAGCGTGGGGAGCAAACAGGATTAGAGACCCTAGTAGTCC
>Otu9596
CCAGCCTATGGGTCGCAGCAGTGGGGAATTTTGGACAATGGGGGCAACCCTGATCCAGCC
ATTCCGCGTGAGTGAAGAAGGCCTTCGGGTTGTAAAGCTCTTTCGGCAGGAACGAAACGG
GTTACGTAAATAGCGTAGCTTACTGACGGTACCTGAAGAAGAAGCACCGGCTAACTACGT
GCCAGCAGCCGCGGTAATACGTAGGGTGCGAGCGTTAATCGGAATTACTGGGCGTAAAGC
GTGCGCAGGCGGTTTGTTAAGACAGGTGTGAAATCCCCGGGCTTAACCTGGGAATGGCGT
TTGTTACTGGCAAGCTAGAGTGTGTCAGAGGGAGGTAGAATTCCACGTGTGGCAGTGAAA
TGCGTAGAGATGTGGAGGAATACCGATGGCGAAGGCAGCCTCCTGGGATAACACTGACGC
TCATGCACGAAAGCGTGGGGAGCAAACAGGATTAGATACCCCAGTAGTCC
>Otu9599
CCAGCCTATGGGTGGCTGCAGTCGAGAATATTTGACAATGGAGGAAACCCTGATGCAGCG
ACGCCGCGTGAGCGATGAAGCCCTTCGGGGTGTAAAGCTCTTTCGGCAGGGACGATTATG
ACGGTACCTGCAGAAGCAGCTGCGGCTAACTACGTGCCAGCAGCCGCGGTAATACGTAGG

CAGCAAGCGTTGTTCGGAGTTACTGGGCGTAAAGGGTGTGTAGGCGGTTCTCTAAGTTTG
GTGTGAAATCTCCCGGCTCAACTGGGAGGGTGCGCCGAAAACTGGGGGGCTAGAGTGCGG
GATGGGAGAGTGGAATTCCTGGTGTAGCGGTGAAATGCGTAGATATCAGGAGGAACACCT
GTGGTGTAGACGGCTCTCTGGACCGCAACTGACGCTGAGACACGAAAGCGTGGGGAGCAA
AAAGGATTAGATACCCGTGTAGTCC
>Otu9607
CCAGCCTACGGGGGGCAGCAGTGGGGAATATTGGACAATGGGCGGAAGCCTGATCCAGCA
ATGCCGCGTGTGTGAAGAAGGCCTGCGGGTTGTAAAGCACTTTCAGTGGGGAGGAACGAC
TTTAAACTAATACTTTGAGGAGATGACGTTACCCACAGAAGAAGCACCGGCTAACTCTGT
GCCAGCAGCCGCGGTAATACAGAGGGTGCGAGCGTTAATCGGAATTACTGGGCGTAAAGG
GTGCGTAGGTGGTTTGATAAGTTAGGTGTGAAAGTCCTGGGCTTAACCTGGGGATTGCGC
CTGATACTGTTAGACTAGAGTACTGTAGAGGAAAGTGGAATTTCCGGTGTAGCGGTGAAA
TGCGTAGATATCGGAAGGAACACCAGTGGCGAAGGCGACTTTCTGGACAGATACTGACAC
TGAGGCACGAAAGCATGGGGAGCGAACAGGATTAGAGACCCTAGTAGTCC
>Otu9621
CCAGCCTATGGGTCGCTGCAGTGGGGAATATTGGACAATGGGCGCAAGCCTGATCCAGCC
ATGCCGCGTGAGTGATGAAGGCCTTAGGGTTGTAAAGCTCTTTTAGCAGGGAAGATAATG
ACGGTACCTGCAGAAAAAGCCCCGGCTAACTTCGTGCCAGCAGCCGCGGTAATACGAAGG
GGGCTAGCGTTGCTCGGAATTACTGGGCGTAAAGCGCACGTAGGCGGCTTCTTAAGTCGG
TGGTGAAATCCTGGAGCTCAACTCCAGAACTGCCTTCGATACTGGGAAGCTCGAGTCCGG
GAGAGGTGAGTGGAACTGCGAGTGTAGAGGTGAAATGCGTAGAGATGTGGAGGAACACCT
GTGGCGAAAGCGGCGCACTGGACCATAACTGACGCTGATGCACGAAAGCTAGGGGAGCAA
ACAGGATTAGAAACCCCCGTAGTCC
>Otu9624
CCAGCCTATGGGGGGCAGCAGTGGGGAATCTTGCACAATGGAGGAAACTCTGATGCAGCG
ACGCCGCGTGAGCGATGACGCCCTTCGGGGTGTAAAGCTCTTTCGGCAGGGAAGATAATG
ACGGTACCTGCAGAAGCAGCTGCGGCTAACTACGTGCCAGCAGCCGCGGTAATACGTAGG
CAGCAAGCGTTGTTCGGAGTTACTGGGCGTAAAGGGTGCGTAGGCGGCTTTTTAAGTTTG
GTGTGAAATCTCCCGGCTCAACTGGGAGGGTGCGCCGAATACTGAGAGGCTAGAGTGTCG
GAGAGGTAAGTGGAATTTCCGGTGTAGCGGTGAAATGCGTAGATATCGGAAGGAACACCT
GTGGCGAAAGCGGCTCACTGGACCACAACTGACGCTGATGCACGAAAGCTAGGGGAGCAA
ACAGGATTAGATACCCCAGTAGTCC
>Otu9630
CCAGCCTACGGGTGGCAGCAGTGGGGAATTTTGCGCAATGGGGGAAACCCTGACGCAGCA
ACGCCGCGTGGAGGATGAAGTACTTCGGTACGTAAACTCTTTCGACGGGGACGATAATGA
CGGTACCCGTAGAAGAAGCTCCGGCTAACTTCGTGCCAGCAGCCGCGGTAATACGAAGGG
GGCTAGCGTTGTTCGGAATTACTGGGCGTAAAGCGCGCGCAGGCGGCTTCTCAAGTCAGG
GGTGAAAGCCCAGAGCTCAACTCTGGAACTGCCCTTGAAACTGTGGAGCTTGAGTACGGG
AGAGGTGAGTGGAATTCCCAGTGTAGAGGTGAAATTCGTAGATATTGGGAAGAACACCGG
TGGCGAAGGCGGCTCACTGGCCCGTTACTGACGCTCAGGCGCGACAGCGTGGGGATCAAA
CAGGATTAGATACCCGGGTAGTCC
>Otu9632
CCAGCCTATGGGGGGCTCCAGTGAGGAATATTGCTCAATGGACGAAAGTCTGAAGCAGCA
ACGCCGCGTGAGGGATGAAGGTGCTCTGCATTGTAAACCTCTGTAGTCGGGGAAAAATGG
CGGGTTACTACCCGTTTGATGGTACCCGAAAAGTAAGGATCGGCTAACTACGTGCCAGCA
GCCGCGGTAATACGTAGGATCCAAGCGTTGTCCGGATTTACTGGGTGTAAAGGGTGCGCA
GGCGGACTGGTTAGTCAGAGGTGAAATCCCATCGCTCAACGATGGACCTGCCTTTGATAC
TGCCAGTCTTGAATAAGCGAGAGATTCGTGGAATTCCGAGTGTAGCAGTGAAATGTGTAG
ATATTCGGAAGAACACCAGAGGCGAAGGCAGCGAATTGGCGCTTTATTGACGCTCAGGCA
CGAAAGCATGGGGATCAAACAGGATTAGATACCCTCGTAGTCC
>Otu9636
CCAGCCTATGGGAGGCTCCAGTGGGGAATATTGGACAATGGGTGAGAGCCTGATCCAGCC
ATGCCGCGTGCAGGAAGAAGGCCTTCTGGGTTGTAAACTGCTTTTGCCAGGGGATAAAAG
ACCCTTGCGAGGGAAATTGAAGGTACCTGGTGAATAAGCCACGGCTAACTACGTGCCAGC
AGCCGCGGTAATACGTAGGTGGCAAGCGTTGTCTGGATTTATTGGGTTTAAAGGGTGCGT
AGGCGGCCCCGTAAGTCAGTGGTGAAATACGGCAGCTCAACTGTCGAGGTGCCATTGATA
CTGCGGGGCTTGAGTATGGACGAGGTGGGCGGAATTGACGGTGTAGCGGTGAAATGCTTA
GATATCGTCAAGAACACCGATAGCGAAGGCAGCTCACTAGGCCATAACTGACGCTGAGGC

ACGAAAGTGCGGGGATCAAACAGGATTAGAAACCCCAGTAGTCC
>Otu9644
CCGGCCTACGGGTGGCAGCAGTAAGGAATATTGGACAATGGTCACAAGACTGATCCAGCC
ATGCCGCGTGCAGGATGACAGCCCTATGGGTCGTAAACTGCTTTTGTGCGAGAAAAAACC
ACTCTACGTGTAGAGTGCTGATTGTATCGTAAGGATAAGGATCGGCTAACTCCGTGCCAG
CAGCCGCGGTAATACGGAGGATCCAAGCGTTATCCGGATTCATTGGGTTTAAAGGGTGCG
TAGGCGGAATGATAAGTCAGTGGTGAAAGCCTGTTGCTTAACAACAGAACTGCCATTGAT
ACTGTCAATCTTGAGTACATTTGATGTGGGCGGAATGTGTCATGTAGCGGTGAAATGCTT
AGATATGACACAGAACACCGATTGCGAAGGCAGCTCACAAAACTGTAACTGATGCTGAGG
CACGAAAGCGTGGGTATCAAACAGGATTAGAGACCCGTGTAGTCC
>Otu9646
CCAGCCTATGGGGGGCAGCAGTGGGGAATTTTGGACAATGGGGGAAACCCTGATCCAGCA
ACTCTGCGTGAGGGACGAAGGGCTTCGGCCCGTAAACCTCTTTTGCACGGGGCGAACACC
CGCAAGGGTTTGACGGTACCGTGCGAATAAGCCACGGCTAACTATGTGCCAGCAGCCGCG
GTAAGACATAGGTGGCGAGCGTTATTCGGAATTACTAGGCGTAAAGCGAGTGTAGGCGGA
TGCTTAAGTCCGACGTTAAATCTCCAGGCTTAACTTGGAGCTGTCGTCGGATACTGGGCA
CCTCGAATGGGGTAGGGGGCAGCGGAATTCCCGGTGTAGCGGTGAAATGCGTAGATATCG
GGAGGAACACCTATGGCGAAAGCAGCTGCCTGGGCCTCTATTGACGCTAAGACTCGAAAG
CTGGGGGAGCAAACAGGATTAGATACCCTAGTAGTCC
>Otu9651
CCAGCCTATGGGTTGCTGCAGTCAAGAACATTCGACAATGGGCGAAAGCCTGATCGAGCG
ACGCCGCGTGCGGGAAGAAGCAGCTTTGCTGTGTAAACCGCTTTTGCGTGGGAAGAACTT
TGACGGTACCACGAGAATAAGGGGTTGCTAACCTCGTGCCAGCAGCAGCGGTAATACGAG
GACCCCGAGCGTTATCCGGAATCACTGGGCGTAAAGCGTGAGCATCTGGTTGTGTTAGTC
CACGGTTAAATCCTACTGCTCAACGGTGGGACTGCGGTGGAAACGGCACGACTTGAACGG
GTGAGAGGCAAGCGGAACGTGCAGTGTAGGGGTGAAATCCATTGATATTGCACGGAACAC
CAAAAGCGAAAGCAGTCTACTAGTGCTCTGCTGACGGTCAATCACTAAAGCGTGGGGATC
AAAAAGGATTAGAGACCCCCGTAGTCC
>Otu9652
CAGCCTACGGGAGGCAGCAGTGGGGAATCTTGCGCAATGGACGAAAGTCTGACGCAGCCA
CGCCGCGTGAGTGAAGAAGGCCTTCGGGTTGTAAAGCTCTGTCGTGCGGGACGAACATCG
AAACAGTTAAGAGCTGGTTCGACTGACGGTACCGCAAAAGGAAGCACCGGCTAACTTCGT
GCCAGCAGCCGCGGTAATACGAGGGGTGCAAGCGTTGCTCGGAATTATTGGGCGTAAAGG
GTAGGTAGGTGGTCTCATTTGTCTGGGGTGAAAGCCTTGGGCTTAACTCAAGAAGTGCCC
CGGAAACGGTGAGACTAGAGTGCTGGAGAGGGTCGTGGAATTCCCGGTGTAGCGGCGAAA
TGCGTAGAGATCGGGAGGAACACCAGAGGCGAAGGCGGCGACCTGGACAGCTACTGACAC
TCAACTACGAAAGCGTGGGGAGCAAACAGGATTAGAACCCGAGTAGTCC
>Otu9662
CCAGCCTACGGGATGCTCCAGTGGGGAATTTTGGACAATGGGCGAAAGCCTGATCCAGCA
ACCCTGCGTGAGGGACGAAGGGCTTCGGCCCGTAAACCTCTTTTACATGGGACGAAACCC
CGCAAGGGTGTGACGGTACCATGCGAATAAGCCACGGCTAACTATGTGCCAGCAGCCGCG
GTAAGACATAGGTGGCGAGCGTTATTCGGAATTACTAGGCGTAAAGCGAGTGTAGGCGGA
CTCTTAAGTCTGACGTTAAATCTCCGGGCTTAACTCGGAGCTGTCGTCGGATACTGAGAG
CCTCGAATGGAGTAGGGGGCAGGGGAATTCCCGGTGTAGCGGTGAAATGCGTAGATATCG
GGAGGAACACCTATGGCGAAAGCACCTGCCTGGGCTTCTATTGACGCTAAGACTCGAAAG
CTGGGGGAGCAAACAGGATTAGATACCCGCGTAGTCC
>Otu9666
CCAGCCTATGGGGGGCACCAGTGGGGAATTTTGGACAATGGGCGCAAGCCTGATCCAGCA
ATGCCGCGTGTGTGAAGAAGGCCTTCGGGTTGTAAAGCTCTTTTGTCGGGGACGAAACGG
TTTTGGCGAATAACCAAGACTACTGACGGTACCCGAAGAATAAGCACCGGCTAACTACGT
GCCAGCAGCCGCGGTAATACGTAGGGTGCGAGCGTTAATCGGAATTACTGGGCGCAAAGC
GTGCGCAGGCGGTTGTATAAGCCAGATGTGAAATCCCCGGGCTCAACCTGGGAATGGCAT
TTGGGACTGTACAGCTGGAGTGTGGCAGAGGAGACTAGAATTCCTGGTGTAGCAGTGAAA
TGCGTAGATATCAGGAGGAATACCGATGGCGAAGGCAGGTCTCTGGGCTGACACTGACGC
TCATGCACGAAAGCGTGGGGAGCAAACAGGATTAGAAACCCCAGTAGTCC
>Otu9669
CAGCCTACGGGAGGCTGCAGCGAGGAATTTTGCACAATCGACGCAAGTCGGATGCAGCAA
CGCCGCGTGGGGGAAGAAGGCTTTCGAGTTGTAAACCCCTTTTGTGAGGGAAGAAGATCT

GACGGTACCTCACGAATAAGTCACGGCTAACTACGTGCCAGCAGCCGCGGTAATACGTAG
GTGGCGAACGTTGTCCGGATTTACTGGGCGTAAAGCGCGCGCAGGCGGACCGATAAGTCA
GAGGTGAAATCTCCCGGCTCAACTGGGAGGGGCCCTTTGATACTGTCGGTCTTGAGATGC
CTAGAGGAGAGTGGAATTCCCGGTGTAGTGGTGGAATGCGTAGATATCGGGAGGAACACC
AGTGGCGAAAGCGGCTCTCTGGGGGCGATCTGACGCTGAGGCGCGAAAGCGTGGGGAGCA
AACCGGATTAGAGACCCCAGTAGTCC
>Otu9673
CCAGCCTACGGGTCGCTGCAGTCGAGAATCTTTCACAATGGGGGCAACCCTGATGGAGCG
ACGCCGCGTGGAGGATAAGGTCTTCGGATTGTAAACTCCTGTCATGCGAGAGCAAGACAT
GGCAACATGTTTGATAGTATCGCAAGAGGAAGAGACGGCTAACTCTGTGCCAGCAGCCGC
GGTAATACAGAGGTCTCAAGCGTTGTTCGGAATAACTGGGCGTAAAGGGTGCGTAGGCGG
CGGGGAAAGTCGGGTGTGAAATTTGAGGGCTCAACCCTCACAAAGCACCTGATACTACTC
TGCTCGAGACCTGAAGAGGAGCTTGGAATTCTCAGTGTAGCAGTGAAATGCGTAGATTTT
GAGAAGAACGCTCGCAGCGAAAGCGAAGCTCTGGTCAGCGCCTGACGCTGAGGCACGAAG
GCTAGGGGAGCAAACAGGATTAGAAACCCTAGTAGTCC
>Otu9679
CCAGCCTATGGGGGGCTGCAGTGGGGAATATTGGACAATGGGCGAAAGCCTGATCCAGCA
ATGCCGCGTGAGTGATGAAGGCCTTCGGGTTGTAAAGCTCTGTCGGGAGGGACGAAAAAA
CGGATTAACAAACCGTATTGACGGTACCTCCAAAGGAAGCACCGGCTAACTCTGTGCCAG
CAGCCGCGGTAATACAGAGGGTGCAAGCGTTGCTCGGAATTACTGGGCGTAAAGCGCGTG
TAGGCGGCGACATAAGTCGGGTGTGAAATCCCTCGGCTCAACCGAGGAAGTGCACTCGAT
ACTGCGTTGCTTGAATGTCGGAGGGGATGGCGGAATTCCCGGTGTAGAGGTGAAGTTCGT
AGATATCGGGAGGAACACCTGTGGCGAAGGCGGCCATCTGGACGATTATTGACGCTGAGA
CGCGAAAGCGTGGGTAGCAAACAGGATTAGAAACCCCGGTAGTCC
>Otu9682
CCAGCCTACGGGGGGCTCCAGCTAAGAATCTTCCGCAATGGGCGAAAGCCTGACGGAGCG
ATGCCGCGTGAAGGATGAAGGTCTTCGGATTGTAAACTTCTGTCATTAGAGAACAAGTGC
CGCCGAGTAACTACCGCCGGCTTGATAGTATCTGAAGAGGAAGGGACGGCTAACTCTGTG
CCAGCAGCCGCGGTAATACAGAGGTCCCAAGCGTTGTTCGGATTCATTGGGCGTAAAGGG
TGCGTAGGTGGCGGGGTAAGTTGGATGTGAAATCCTGGGGCTCAACCCTAGAACTGCATT
CAATACTGCCTTGCTAGAGTACTGGAGAGGAGATTGGAATTTACGGTGTAGCAGTGAAAT
GCGTAGATATCGTAAGGAAGACCGGTGGCGAAGGCGGATCTCTGGACAGTTACTGACACT
GAGGCACGAAGGCCAGGGGAGCAAACGGGATTAGATACCCCCGTAGTCC
>Otu9686
CCAGCCTATGGGTTGCAGCAGTAAGGAATATTGGTCAATGGACGCAAGTCTGAACCAGCC
ATGCCGCGTGAAGGATGAAGGCGCTATGCGTCGTAAACTTCTTTTATACGGGAGAAAACC
CCCCGAGTTCTCGGGGGCTGATAGTACTGTAAGAATAAGGGTCGGCTAACTTCGTGCCAG
CAGCCGCGGTCATACGAAGGACCCGAGCGTTATCCGGATTCATTGGGTTTAAAGGGTGCG
TAGGCGTCCTTGTAAGTTTTCGGTGAAATCCTCAAGCTTAACTTGAGAACTGCCGAGAAT
ACTACTAGGATTGAGTACAGTTGAAGCGGGCGGAATGTGTCATGTAGCGGTGAAATGCTT
AGATATGACACAGAACACCAATTGCGAAGGCAGCTCGCTAAGCTGTAACTGACGCTGAGG
CACGAAAGCGTGGGTAGCAAACAGGATTAGATACCCCCGTAGTCC
>Otu9687
CCAGCCTACGGGAGGCAGCAGTGGGGAATATTGCGCAATGGGCGAAAGCCTGACGCAGCA
GCGCCGCGTGAGTGAGGAAGGCCTTCGGGTTGTAAAGCTCTTTCGGTTGGGAAGAAGGGA
ATAACTATTAATAAGAGTTATTTTTGATGGTACCAAAAGAAGAAGCACCGGCAAACTTCG
TGCCAGCAGCCGCGGTAATGCGAAGGGTGCGAGCGTTATTCGGATTTATTGGGCGTAAAG
GGTTCGTAGGCGGGAATGCAAGTCAAGTGTGAAATGTCCGGGCTTAACCCGGAGAGTGCA
TTTGAAACTGTTTTTCTTGAGTGTTGAAGAGGGTGGTGGAATTGCTGGTGTAGGAGTGAC
ATCCGTAGAGATCAGCAGGAACATCGGAGGCGAAGGCGGCCGACTGGTCAATCACTGACG
CTGAGGAACGAAAGCATGGGGAGCAAACAGGATTAGAAACCCCAGTAGTCC
>Otu9688
CCAGCCTATGGGGCGCTGCAGTTTCGAATCATTCACAATGGGCGAAAGCCTGATGGTGCG
ACGCCGCGTGAGGGATGAAGGTCTTCGGATTGTAAACCTCTGTCACCGGGGAAGAAACGC
TTCAAGTTAATAGCTTGAAGCCTGACTTAACCCGGAGAGGAAGCAGTGGCTAACTCTGTG
CCAGCAGCCGCGGTAATACAGAGACTGCGAGCGTTATTCGGATTCACTGGGCGTAAAGGG
TGCGCAGGCGGCTAAGTGTGTTAGGCGTGAAAGCCCGAGGCTTAACCTCGGAATTGCACC
TAAAACTATTTGGCTAGAGTACTGGAGAGGGAAACGGAATTCACGGTGTAGCAGTGAAAT

GCGTAGATATCGTGAGGAACACCAGAGGCGAAGGCGGTTTCCTGGACAGTTACTGACGCT
CAGGCACGAAAGCGTGGGGAGCAAAAGGGATTAGATACCCCAGTAGTCC
>Otu9692
CCAGCCTACGGGGTGCAGCAGTGGGGAATCTTGGACAATGGGGGCAACCCTGATCCAGCC
ATGCCGCGTGAGTGATGAAGGCCTTCGGGTTGTAAAACTCTTTCGACGGGGACGATAATG
ACGGTACCCGTAGAAGAAGCTCCGGCTAACTTCGTGCCAGCAGCCGCGGTAATAAGAAGG
GGGCTAGCGTTGTTCGGAGTTACTGGGCGTAAAGGGCGCGTAGGCGGCTGTCCAAGTCGG
GTGTGAAAGCCCAGGGCTCAACCCTGGAATAGCACTCGAGACTGGGCGGCTCGAGGACGG
GAGAGGATGGTGGAATTCCTAGTGTAGAGGTGAAATTCGTAGATATTGGGAAGAACACCG
GTGGCGAAGGCGGCCATCTGGACCGTTTCTGACGCTGAGGCGCGAAAGCGTGGGGAGCAA
ACAGGATTAGAAACCCCTGTAGTCC
>Otu9693
CCAGCCTATGGGGGGCAGCAGTGACGAAATCTGGACAATGCGCGACAGCGTGATCTGGTA
ATACTTCTTAAGTGATGACGGTCAAGCGGTTGTAAAGCTTTTTATAATGACTAAATAATG
ACAAGGTTAATTAGGAAAAAGTCCTGGCCAACTCTGTGCCAGCAGCCGCGGTAATACAGA
GGGGGCGAGCATTATTCATCTTGACTGGGCGTAAAGGGTGTGTAGGTGGCCAACTCAATT
ATGCGGTAAAGCCTGGGGTAGTACTCAGAAACTGTGTTAATATTAATTCGGCTTGAGTTA
GTGTGGGGTTATTAGAATTTTTGGTGGAGCAGTAAAATGCTTAGATACTACAAGGAATAC
CAAAGAGTGGAAGCAATTAACTATTGCTAACTGACGCTGAGGCACGAAGGCATGGGGAGC
AAACAGGATTAGAAACCCCCGTAGTCC
>Otu9695
CCAGCCTATGGGGGGCTGCAGTCGAGGATCTTCGGCAATGGGCGCAAGCCTGACCGAGCG
ACGCCGCGTGTGCGATGAAGGCCTTCGGGTTGTAAAGCACTGTCGAGGGGGAGAAAAGGG
AAACCTTGATCTATCCCCGGAGGAAGCACGGGCTAAGTTCGTGCCAGCAGCCGCGGTAAG
ACGAACCGTGCGAACGTTGTTCGGAATCACTGGGCTTAAAGGGCGCGTAGGCGGGCTGCC
AAGTCTGTCGTGAAATCCCGCGGCTCAACCGCGGAACTGCGATGGATACTGGCGGTCTAG
AGGGGGGTAGGGGCATGCGGAACTGTGGGTGGAGCGGTGAAATGCGTTGATATCCACAGG
AACTCCGGTGGCGAAGGCGGCGTGCTGGACCTCTTCTGACGCTGAGGCGCGAAAGCTAGG
GGAGCAAACAGGATTAGATACCCCTGTAGTCC
>Otu9698
CCAGCCTATGGGTGGCTCCAGTCGAGAATTTTTCTCAATGGGCGAAAGCCTGAAGGAGCG
ACGCCGCGTGGGGGATGAATGGCTTCGGCCCGTAAACCCCTGTCATTCGGGATCAATGCT
TGTTTCCCAACACGAGACAAGTTGATAGTACCGGAAGAGGAAGGGACGGCTAACTCTGTG
CCAGCAGCCGCGGTAATACAGAGGTCCCAGGCGTTGTTCGGATTTACTGGGCGTAAAGGG
TGCGTAGGTGGTCGGGTAAGTCTGATGTGAAATCTCCGGGCTCAACCCGGAAATGGCATT
GGATACTATTCGACTAGAGGGTTGGAGGGGGGACTGGAATACTTGGTGTAGCAGTGAAAT
GTGTAGATATCAAGTGGAACACCAGTGGCGAAGGCGAGTCCCTGGACAACTCCTGACACT
GAGGCACGAAAGCTAGGGGAGCAAACAGGATTAGATACCCTCGTAGTCC
>Otu9702
CCAGCCTATGGGTGGCTCCAGGGCCTGAACATCTTTGGGGAAGCATGAACCACCGTAGCC
GCAACCTGCATACAGGAAGCTGTGTCCTATGCGCGGGTCCGAGCCTATGCCATGCCTTAC
GGCTTCGATATCGACACCAACATGATCTGCCAGATTGGCCAGTTCATTCATGAAGGAGAT
GCGCGTAGCCAGCATGGCGTTGGCTGCATATTTGGTAAATTCTGCGGAGCGTACGTCCAT
CCAGTAAGTACGTTCGTGATTGCGGTTAAACGGCGCGTACAAGGCTTTCATATGATGATG
TGCGGTACTGCCTTCTGGCGTGTCATCACAGCCGATCACGATGCGATCAGGGCGCATGAA
ATCTTCTACCGCCGCGCCTTCTTTCAGAAACTCTGGATTAGATACCCTTGTAGTCC
>Otu9706
GGACTACCGGGGTATCTAATCCTGTTTGCTACCCACGCTTTCGTGCCTCAGCGTCAGTCA
TGGTCCAGTAAGCTGTCTACACCACAGGTGTTCCTCCTGATATCTACGCATTTCACCGCT
ACACCAGGAATTCCGCTTACCTCTCCCATACTCTAGCATTCCAGTCTCCGCCGCACCCTC
CCAGTTGAGCCAGGAGATTTCACAACAGACTTAGAACACCGCCTACGCACTCTTTACGCC
CAGTAATTCCGAACAACGCTTGCTGCCTACGTATTACCGCGGCTGCTGGCACGTAGTTAG
CCGCAGCTTCTTCTTCGGGTACCGTCATTATCGTTCCCGTCGAAAGAGCTTTACACCCCG
AAGGGCTTCATCGCTCACGCGGCGTCGCTGCATCAGGGTTTCCCCCATTGTGCAAGATTC
CCCACTGCTGCGCCCCGTAGGCTGG
>Otu9710
CCAGCCTATGGGGCGCTCCAGTCAAGAACATTTGGCAATGGGCGAAAGCCTGACCATGCG
ACGCCGCGTGCTGGATGAAGTCCTTCGGGACGTAAACAGCTTTTATGCGTGAAGAAGTAA

TTGACATTAGCGCATGAATAAGGGGCTCCTAACTCTGTGCCAGCAGGAGCGGTAATACAG
AGGCCCCAAGCATTATCCGGAATCACTGGGCGTAAAGGGTGTGTAGGCGACGATATTAGT
CTTTTGTTAAAGTTCTCCGGCTTAACCGGGGAAATGCAGGAGAAACGGTATCGCTTTGAG
GGCGCGAGAGGTACAGGGAACTCATGGTGTAGGGGTGAAATCCGTTGATATCATGGGGAA
CACCAAATGCGAAGGCACTGTACTGGCGCGTTTCTGACGCTGAAACACGAAAGCGTGGGT
AGCGAACGGGATTAGATACCCGAGTAGTCC
>Otu9729
CCAGCCTACGGGGTGCAGCAGTAGGGAATTTTCCACAATGGACGAAAGTCTGATGGAGCA
ACGCCGCGTGCAGGATGAATGCCTTAGGGTTGTAAACTGCTTTTATGATCGACGATTATG
ACGGTAGATCATGAATAAGGATCGGCTAACTCCGTGCCAGCAGCCGCGGTCATACGGAGG
ATCCAAGCGTTATCCGGAATTACTGGGCGTAAAGAGTTGCGTAGGTGGCATGGTAAGTTG
GTAGTGAAAGCGTTCGGCTCAACCGAATATACATTATCAAAACTGCCAAGCTTGAGAGCG
AGAGAGGTAACTGGAATTCCTAGTGTAGGAGTGAAATCCGTAGATATTAGGAGGAACACC
AATGGCGTAGGCAGGTTACTGGCTCGTCTCTGACACTAAGGCACGAAAGCGTGGGGAGCA
AACGGGATTAGATACCCCAGTAGTCC
>Otu9754
CCAGCCTATGGGTGGCAGCAGTAAGGAATCTTTCGCAATGGGGGAAACCCTGACGAAGCG
ACACCGCGTGAACGATGAAGGTCGGAAGATCGTAAAGTTCTTTTCTGAGGGAGAAATTCT
GATAGTACCTCAGGAATAAGCACCGGCTAACTCCGTGCCAGCAGCCGCGGTAAGACGGAG
GGTGCAAGCGTTATCCGGAATTATTGGGTGTAAAGTGTTCTCAGGTGGTCCTGTAAGTCA
GATGTCAAAGCCTGAAGCTTAACTTCAGTCTGGTATCTGATACTGCAAGACTCGAGATCT
CGAGAGGCAAGTGGAATTTTGCGTGTAGGGGTAAAATCCGTTGATACGCAAAGGAACACC
AAAAGCGAAAGCAGCTTGCTGGCGAGTTTCTGACACTTATGAACGAAAGCGTGGGGAGCG
AAAAGGATTAGAGACCCTAGTAGTCC
>Otu9755
CCAGCCTATGGGGCGCACCAGTGGGGAATATTGCGCAATGGGCGAAAGCCTGACGCAGCG
ACGCCGCGTGAAGGATGAAGGCCTACGGGTTGTAAACCTCTGTTAAGCGGGAAGAAAATT
CTATTTCTAATAAAAATAGACATGACGGTACCGCTAGAGAAAGCACTGGCTAAACTCGTG
CCAGCAGCCGCGGTAATACGAGTGGTGCAAGCATTATTCGGAATCATTGGGCGTAAAGGG
TGCGTAGGCGGCGCATTAAGTCTACTGTTAAATATCTCGGCCTAACCGAGAAGCTGCAGT
GGATACTGGTGTGCTAGAGGATGGGAGAGAGAAGTGGAATTCTCGGAGTAGCGGTAAAAT
GCGTAGATCTCGAGAGGAACACCGATGGCGAAAGCAGCTTCTTGGACCAATCCTGACGCT
GATGCACGAAAGCGTGGGGAGCAAACAGGATTAGAAACCCTAGTAGTCC
>Otu9756
CCAGCCTATGGGGCGCAGCAGTGGGGAATATTGGACAATGGGCGAAAGCCTGATCCAGCC
ATGCCGCGTGAGTGATGAAGGCCTTAGGGTTGTAAAGCACTTTTAATGGGGAGGAACTCC
TGGCGGCGAACACCCGGCAGACTGACATTACCCATAGAAAAAGCACCGGCTAACTCCGTG
CCAGCAGCCGCGGTAATACGGAGGGTGCAAGCGTTAATCGGAATTACTGGGCGTAAAGCG
TGCGTAGGCGGCCCGTTAAGTCAGATGTGAAAGCCCCGGGCTCAACCTGGGAACGGCATT
TGAAACTGGCGGGCTAGAGTTAGGTAGAGGGGAGTGGAATTTCAGGTGTAGCGGTGAAAT
GCGTAGAGACCTGAAGGAACACCAGTGGCGAAGGCGACTCCCTGGACCCAAACTGACGCT
GAGGTACGAAAGCGTGGGTAGCAAACAGGATTAGATACCCGAGTAGTCC
>Otu9768
CCAGCCTATGGGGCGCAGCAGTGAGGAATATTGGTCAATGGACGCAAGTCTGAACCAGCC
ATGCCGCGTGAAGGATGAAGGCCTTCTGGGTTGTAAACTTCTTTTACCTGGGAAGAAACC
ACTATTTTCTAATAGTGTTGACGGTACCAGAGGAATAAGCACCGGCTAACTCCGTGCCAG
CAGCCGCGGTAATACGGAGGGTGCAAGCGTTATCCGGATTTACTGGGTTTAAAGGGTGTG
TAGGCGGGCTGTTAAGTCAGTGGTTAAAGCCTGCAGCTTAACTGTAGAATTGCCGTTGAT
ACTGTCGTTCTTGAGTATATTTGATGTGGGCGGAATGTGTCATGTAGCGGTGAAATGCTT
AGATATGACACAGAACACCGATTGCGAAGGCAGCTCACAAAACTATAACTGACGCTGAGG
CACGAAAGCGTGGGGATCAAACAGGATTAGAAACCCTCGTAGTCC
>Otu9775
CCAGCCTACGGGTGGCTCCAGGTTCGCATAGCCCCGCATACAAGCTTCGTGCAGCGGACC
TCGCCCATCTAAAGGATGTACATACGTCGCGTCCGCATGCCGCCGCAGCAACGCCTCCAC
CGCCAACGTCTGCCCGTGTAGCACCGCGCTATCGAGTGGCGTATTGCCATTCGCATCCAG
CGCCTGCACATCCGCCTTCGCCCCAATCAGCAAATCGATAATCGGCGCAAACCCCCGCGC
CGCCGCCACGTGCAACAGGCTCTGCCCGTCGCGATACTCGCCGCCTATTTGTGCACCTGC
GTTTAACAAGAGTTTGGCCATGCTCGCCCGTCCGGTGAGCACCGCATACTGCAATGGGGT

CGACCCCGCCTCGCCATGCTGCGCATTCACATCTGCCCCATGCGCAATTAGAAACCCGAG
TAGTCC
>Otu9782
CCAGCCTATGGGAGGCTCCGGCCAATGCCTTACTGCGTATGTTGCTATTGCGTTATCTCT
GTTATATCCTCTAATTTTCTCATTTATTTTACTATCTATTTCTTTTAGTGCTTCTGTGTT
AAACGTTAAAGTTACGAAGTAACCCTTGCCGTTATTTTTGTTTTTTAATTCTTCATTCAG
TCTTATTCTCCACTGTGATGCTCTTTTTTTATTGCATTCTTTGCAGTTGTTGCATCCGAT
CGGCACATACAAAAGCCGTCGGTCGGACGGCTGTGTATTTGTTTTCTTGTATTTCGGATT
TTTTATCAGTTTCGGATATAAACACATTTCTTTGAGTTATTTTCATTTATTTAATCGTAG
CACCAGCACCCCAATATCCTTTGAATTACCTCGCCGAAGGCATTTTTTTCTATCTTCAGG
TAATTATTATTTACTTCCTTTCATTAGAGACCCCAGTAGTCC
>Otu9783
CCAGCCTATGGGTGGCAGCAGTGGGGAATCTTGCACAATGGGCGAAAGCCTGATGCAGCA
ACGCCGCGTGGGTGAAGAAGGTCTTTGGATTGTAAAGCCCTTTCGGCTAGGAAGAAGGGC
CTACGGATTAATACTCCGTAGGTTTGACGGTACTAGAAGAAGAAGCACCGGCTAATTCCG
TGCCAGCAGCCGCGGTAATACGGAAGGTGCAAGCGTTATTCGAAATTACTGGGCGTAAAG
AGCACGTAGGTGGGTATGTGTGTCGGATGTGAAATCCCTGGGCTCAACCTGGGAATTGCA
TTCGAAACTGCATATCTTGAATACTTGAGGGGTTGGAGAAATTCCAGGTGTAGGAGTGAA
ATCCGTAGAGATCTGGAGGAATACCGGAGGCGAAGGCGTCCAACTGGCAATGTATTGACA
CTGAGGTGCGAAAGTGCGGGGAGCAAACAGGATTAGATACCCGGGTAGTCC
>Otu9792
CCAGTCTACGGGTGGCAGCAGTGGGGAATATTGGACAATGGGGGCAACCCTGATCCAGCG
ACGCCGCGTGTGTGAAGAAGGCCTGCGGGTTGTAAAGCACTTTTAGTGGGGACAAAAAGC
GACGGACTAATACTCTGTGGTCTTGATTTAACCCAAAGAAAAAGCACCGGCTAACTCCGT
GCCAGCAGCCGCGGTAATACGGAGGGTGCAAGCGTTATCCGGATTCACTGGGTTTAAAGG
GTGCGTAGGCGGGTAAGTAAGTCCGTGGTGAAATCTCTGAGCTTAACTCAGAAACTGCCG
TGGATACTATTTGCCTTGAATATTGTGGAGGTGAGCGGAATATGTCATGTAGCGGTGAAA
TGCTTAGATATGACATAGAACACCAATTGCGAAGGCAGCTCACTACACAATCATTGACGC
TGAGGCACGAAAGCGTGGGGATCAAACAGGATTAGAGACCCGGGTAGTCC
>Otu9795
CCAGCCTATGGGGTGCACCAGTGGGGAATATTGGACAATGGGCGCAAGCCTGATCCAGCC
ATGCCGCGTGAGTGATGAAGGCCCTAGGGTTGTAAAGCTCTTTTGTGCGGGAAGATAATG
ACGGTACCGCAAGAATAAGCCCCGGCTAACTTCGTGCCAGCAGCCGCGGTAATACGAAGG
GGGCTAGCGTTGCTCGGAATGACTGGGCGTAAAGGGCGCGTAGGCGGATGGTACAGTCAG
ATGTGAAATTCCCGGGCTTAACCTGGGGGCTGCATTTGATACGTGCTGTCTAGAGTGCGG
AAGAGGGTCGTGGAATTCCCAGTGTAGAGGTGAAATTCGTAGATATTGGGAAGAACACCG
GTGGCGAAGGCGGCGACCTGGTCCGTAACTGACGCTGAGGCGCGAAAGCGTGGGGAGCAA
ACAGGATTAGATACCCTGGTAGTCC
>Otu9801
CCAGCCTATGGGTTGCACCAGTGGGGAATTTTGGACAATGGGGGAAACCCTGATCCAGCC
ATGCCGCGTGCGGGAAGAAGGCCTTCGGGTTGTAAACCGCTTTTAGCCGGAAAGAAGGGG
TTTGGGTGAATAACCTAGGTCTTTGACGGTACCGGCGGAATAAGCACCGGCTAACTACGT
GCCAGCAGCCGCGGTAATACGTAGGGTGCGAGCGTTAATCGGAATTACTGGGCGTAAAGA
GTGCGTAGGCGGTTGCTGAAGTCTGCTGTGAAAGCCCTGGGCTCAACCTAGGAATGGCAG
TGGAAACTAGGCGACTTGAATCTGGCAGAGGGGGGTGGAATGCCACGTGTAGCAGTGAAA
TGCGTAGAGATGTGGCGGAACACCGATGGCGAAGGCAGCCCCCTGGGCTGAGATTGACGC
TCAGGCACGAAAGCGTGGGGAGCAAACAGGATTAGAAACCCTCGTAGTCC
>Otu9806
CCAGCCTATGGGAGGCAGCAGTGGGGAATATTGGACAATGGGCGAAAGCCTGAGGGAGCG
ACGCCGCGTGGGGGATGAATGGCTTCGGCCCGTAAACCCCTGTCATTTGCGAACAAACCT
TTTCATTTAACAGACGAAAAGCTGATAGTAGCGGAAGAGGAAGGGACGGCTAACTCTGTG
CCAGCAGCCGCGGTAATACAGAGGTCCCAAGCGTTGTTCGGATTCACTGGGCGTAAAGGG
TGCGTAGGCGGTTTGACAAGTCTGATGTGAAATCCCGCAGCTTAACTGCGGAACTGCATT
GGAAACTGTCTGGCTAGAGGAATGGAGGGGAGACTGGAATGCTTGGTGTAGCAGTGAAAT
GCGTAGATATCAAGCGGAACACCAGTGGCGAAGGCGAGTCTCTGGACATTTCCTGACGCT
GAGGCACGAAAGCCAGGGGAGCAAACGGGATTAGATACCCGGGTAGTCC
>Otu9808
CCAGCCTATGGGAGGCAGCAGTGAGGGATTTTGCGCAATGGAGGAAACTCTGACGCAGCG

ACGCCGCGTGCGGGACGAAGCCTTTCGGGGTGTAAACCGCTGTCAGTGGAGAAGAGAATG
GACGGTACCCACTGAGGAAGCCTCGGCTAACTACGTGCCAGCAGCCGCGGTAAGACGTAG
GGGGCGAGCGTTGTTCGGAATCACTGGGCGTAAAGAGCTCGTAGGCGGGGCTGCGCGTCG
CGGAGTAAATCCATCTGCTTAACGGATGGTTCATCTGCGATACGGCAGTTCTTGAGGACA
GGAGAGGGAAGCGGAATTCCGAGTGTAGCGGTAAAATGCGTAGAGATTCGGAGGAACACC
GGTGGCGAAGGCGGCTTCCTGGCCTGATTCTGACGCTGAGGAGCGAAAGCCAGGGGAGCG
AACGGGATTAGAAACCCGCGTAGTCC
>Otu9815
CCCAGCCTACGGGGGGCTCCAGTAAGGAATATTGGACAATGGAGGCAACTCTGATCCAGC
CATGCCGCGTGAAGGATGAAGGTCCTATGGATTATAAACTTCTTTTGAACGGGAGAAAAC
CCTAGGTCGTGTACCTAGGCTGATAGTACCGTTAGAATAAGGGTCGGCTAACTTCGTGCC
AGCAGCCGCGGTAATACGAAGGACCCGAGCGTTATCCGGATTCATTGGGTTTAAAGGGTG
CGTAGGCGGAACGATAAGTCAGTGGTGAAAGCCTGCAGCTTAACTGCAGAATTGCCATTG
ATACTGTCGATCTTGAGTACATTTGATGTGGGCGGAATGTGCCATGTAGCGGTGAAATGC
TTAGATATGGCACAGAACACCGATCGCGAAGGCAGCTCACAAAACTGTAACTGACGCTGA
GGCACGAAAGCGTGGGGATCAAACAGGATTAGATACCCCAGTAGTCC
>Otu9816
CCAGCCTACGGGGGGCAGCAGTGGGGAATTTTACGCAATGGGCGAAAGCCTGACGTAGCG
ACACCGCGTGAGCGACGAAGCCCTTTGGGGTGTAAAGCTCTGTCGGCTGGAACGAAAAAA
ATGACGGTACCAGCAGAGGAAGCATCGGCTAACTACGTGCCAGCAGCCGCGGTAAGACGT
AGGATGCGAGCGTTGTCCGGATTTATTGGGCGTAAAGAGTTCGTAGGTGGTTTGTTAAGT
CTGATGTTAAATACCGAGGCTCAACTTCGGAGGTGCATTGGATACTGGCAGACTTGGGTA
CAGTAGAGGCAAGCGGAATTCCCAGTGTAGCGGTGAAATGCGTAGATATTGGGAAGAACA
CCAGTGGCGTAGGCGGCTTGCTGGACTGTAACTGACACTGAGGAACGAAAGCCAGGGGAG
CGAATGGGATTAGATACCCTTGTAGTCC
>Otu9820
CCAGCCTATGGGTGGCTGCAGTGGGGAATATTGGACAATGGGCGCAAGCCTGATCCAGCC
ATGCCGCGTGAGTGATGACGGCCCTAGGGTTGTAAAGCTCTTTCGCAAGGGAAGAAAACT
TAGTCTCTAACATAGGCTGAGGTTGACGGTACCTTGATAAGAAGCACCGGCTAACTACGT
GCCAGCAGCCGCGGTAATACGTAGGGTGCGAGCGTTAATCGGAATTACTGGGCGTAAAGC
GTGCGCAGGCGGTTTTGTAAGTCAGATGTGAAATCCCCGAGCTCAACTTGGGAACTGCGT
TTGAAACTACAAGACTAGAATATGTCAGAGAGGGGTAGAATTCCACGTGTAGCAGTGAAA
TGCGTAGAGATGTGGAGGAATACCAATGGCGAAGGCAGCCCCCTGGGATAATATTGACGC
TCATGCACGAAAGCGTGGGGAGCAAACAGGATTAGAGACCCTAGTAGTCC
>Otu9829
CCAGCCTACGGGGTGCTGCAGTAGGGAATCTTGCGCAATGGACGAAAGTCTGACGCAGCA
ACGCCATGTGTGGGATGACGCATTTCGGTGTGTAAACCACTGTCGGCAGGGAATAAAGCC
TCGTCTTTGGCGAGGGATGAATGTACCTGCAGAGGAAGCCCCGGCTAACTTCGTGCCAGC
AGCCGCGGTAATACGAGGGGGGCTAGTGTTGTTCGGAATCACTGGGCGTAAAGGGAGCGT
AGGCGGGTGCTTAAGTTGGATGTTTAAAACCGGGGCCCAACCCCGGCGTGGCATTCAAAA
CTGGGCATCTTGAATGGGACAGAGGCTGGTGGAATTGGGGGTGTGGCGGTGGAATGCGTA
GAGATTCCCAAGAACACCTGTTGCGAAGGCGGCCAGCTGGGTCCACATTGACGCTGAGGC
TCGAAAGCGTGGGGAGCAAACAGGATTAGATACCCTTGTAGTCC
>Otu9838
CCAGCCTATGGGGTGCACCAGCCAAGAACCTTCCACAATGGACGAAAGTCTGATGGAGCG
ACGCCGCGTGGTTGATGAAGTGCTTCGGCACGTAAAGACCTTTTATGAGGGAAGAAGTTT
ATTGACGGTACCTCATGAATAAGGGGCTCCTAACTCTGTGCCAGCAGGAGCGGTAATACA
GAGGCCCCGAGCGTTATCCGGAATTATTGGGCGTAAAGGGTGTGTAGGTGGCGTAGTTAG
TCGATTGTTAAAGACCCGGGCTTAACCTGGGGACTGGGATCGAAACGGCTATGCTTGAGT
ATGTGAGAGGTAAGCGGGACTCAAGGTGTAGGGGTGAAATCCGTTGATATCTTGGGGAAC
ACCAAAAGCGAAGGCAGCTTACTAGCACATTACTGACACTGAAACACGAAAGCGTGGGTA
GCGAATGGGATTAGAAACCCGCGTAGTCC
>Otu9842
CCAGCCTACGGGGCGCACCAGTGGGGAATCTTGCACAATGGGGGAAACCCTGATGCAGCG
ACGCCGCGTGAGCGATGAAGCCCCTCGGGGTGTAAAGCTCTTTCGGCAGGGAAGATTATG
ACGGTACCTGCAAAAGCAGCTGCGGCGAACTACGTGCCAGCAGCCGCGGTAATACGTAGG
CAGCAAGCGTTGTTCGGAGTTACTGGGCGCAAAGGGTGTGTAGGCGGCTTTTTAAGTTTG
GTGTGAAATCTCCCGGCTCAACTGGGAGGGTGCGCCGAATACTGAGAGGCTAGAGTGTGG

GAGAGGAAAGTGGAATTCCTGGTGCAGCGGTGAAATGCGTAGATATCAGGAGGAACACCT
GCGGTGTAGACGGCTTTCTGGACCATCACTGACGCTGAGACACGAAAGCGTGGGGAGCAA
ACAGGATTAGAGACCCTCGTAGTCC
>Otu9843
CCAGCCTATGGGTCGCTGCAGTCGGGAATTTTGCGCAATGGACGAAAGTCTGACGCAGCA
ACGCCGCGTGCAGGAGGAAGGCCTTCGGGTTGTAAACTGCTTTTATCTGTGACGATTTTG
ACGGTAACAGAAGAATAAGGATCGGCTAACTCCGTGCCAGCAGCCGCGGTCATACGGAGG
ATCCAAGCGTTATCCGGAATTACTGGGCGTAAAGAGTTGCGTAGGTGGCAAAGTAAGCAA
ATAGTGAAATGATGCGGCTCAACCGCATGTCCATTATTTGAACTGCTTAGCTAGAACATA
GGAGAGGTACCTGGAATTCCCAGTGTAGGAGTGAAATCCGTAGATATTGGGAGGAACACC
GATGGCGTAGGCAGGGTACTAGCCTATTGTTGACACTAAGGCACGAAAGCGTGGGGAGCG
AACGGGATTAGAAACCCTTGTAGTCC
>Otu9846
CCAGCCTACGGGGCGCAGCAGTGGGGAATCTTGCGCAATGGGCGAAAGCCTGACGCAGCG
ACGCCGCGTGGGCGACGAAGGCCTTCGGGTTGTAAAGCCCTGTGGGGCGGGACGAATAAG
CGTTGGTCAAATAGGCCAGCGCCCTGCCGGTACCGCCTTAGCAAGCACCGGCTAACTCTG
TGCCAGCAGCCGCGGTAAGACAGAGGGTGCGAACGTTGCTCGGAATTACTGGGCGTAAAG
CGCGTGCAGGTGGTTCTCCACGTCCGGTGTGAAAGCCCGGGGCTCAACCCCGGAAGCGCG
CTGGATACGAGGGAACTGGAGGGCTGGAGAGGCAGGCGGAATTCCCGGTGTAGAGGTGAA
ATTCGTAGATATCGGGAGGAACACCGGTGGCGAAGGCGGCCTGCTGGACAGCACCTGACA
CTGAGACGCGAAAGCGTGGGTAGCAAACAGGATTAGATACCCCAGTAGTCC
>Otu9852
CCAGCCTTCGGGATGCAGCAGTAACGAATCTTCCGCAATGCACGAAAGTGTGACGGAGCG
ACGCCGCGTGTGGGATGAAGTTCTTCGGAATGTAAACCACTGTCAGGGGTTAGAAAGTTC
TGATCAACCCCAGAGGAAGGCACGGCTAACTCTGTGCCAGCAGCCGCGGTAAGACAGAGG
TGCCGAGCGTTAGGCGGAATCACTGGGCTTAAAGCGTGTGTAGGCGGGTCGATAAGTACC
TTGTGAAATCCCACGGCTCAACCGTGGAACTGCTGGGTATACTGTCGATCTTGAGCTACC
TAGGGGCAGTCGGAACAAGTGGTGGAGCGGTGAAATGCGTAGATATCACTTGGAACGCCA
ATGGTGAAAACAGACTGCTGGGGGTATGCTGACGCTGAGACACGAAAGCCAGGGGAGCAA
ACGGGATTAGAGACCCTTGTAGTCC
>Otu9861
CAGCCTATGGGTGGCAGCAGTGGGGAATATTGGACAATGGGCGAAAGCCTGATCCAGCAA
TACCGCGTGTGTGAAGAAGGCCTGAGGGTTGTAAAGCACTTTCAATGGGAAGGAATACCT
ATGGGCGAATACCCTGTAGACTGACATTACCCATAGAAGAAGCACCGGCTAACTCCGTGC
CAGCAGCCGCGGTAATACGGAGGGTGCGAGCGTTAATCGGAATTACTGGGCGTAAAGAGT
GCGTAGGCGGTTCGTTAAGTCAGATGTGAAAGCCCTGGGCTCAACCTGGGAACTGCATTT
GATACTGGCGAACTCGAGTTGAGTAGAGGAGAGTGGAATTTCAGGTGTAGCGGTGAAATG
CGTAGAGATCTGAAGGAACACCAGTGGCGAAGGCGGCTCTCTGGACTCAAACTGACGCTG
AGGTACGAAAGCGTGGGTAGCAAACAGGATTAGATACCCGAGTAGTCG
>Otu9872
CCAGCCTACGGGTCGCTGCAGTAGGGAATTTTGCGCAATGGGCGAAAGCCTGACGCAGCA
ACACCGCGTGAGGGATGAAGCTTCTTGGAGTGTAAACCTCTGTCAGTGGGGACGAAGCCT
GACGGTACCCACAGAGGAAGCATCGGCTAACTACGTGCCAGCAGCCGCGGTAAGACGTAG
GATGCAGGCGTTGTCCGGGTTTATTGGGCGTAAAGAGTTCGTAGGTGGCATATCAAGTCT
GGTGTTAAATCCCGAAGCTCAACTTCGGACCGGCACTGGATACTGATAAGCTGGAGTACG
GTAGAGGCAAGGGGAATTCCCAGTGTAGCGGTGAAATGCGTAGATATTGGGAGGAACACC
GGTGGCGTAAGCGCCTTGCTGGGCCGTTACTGACGCTGAGGAACGAAAGCCAGGGGAGCA
AATGGGATTAGATACCCCGGTAGTCC
>Otu9875
CCAGCCTACGGGGCGCTGCAGTCGAGAATTTTTCACAATGGGCGCAAGCCTGATGGAGCG
ACGCCGCGTGGGGGATGAATGGCTTCGGCCCGTAAACCCCTGTCATTTGCGATCAACCGT
TATTGTTTAAGAGATGATAACCTGATAGTAGCGAAAGAGGAAGGGACGGCTAACTCTGTG
CCAGCAGCCGCGGTAATACAGAGGTCCCAAGCGTTGTTCGGATTCACTGGGCGTAAAGGG
TGCGTAGGTGGCGAAGTAAGTCACCTGTGAAATCTCTGGGCTTAACTCAGAGCCTGCAGG
CGAAACTGCCGTGCTGGAGTGTGGGAGAGGTGCGTGGAATTCCCGGTGTAGCGGTGAAAT
GCGTAGATATCGGGAGGAACACCTGTGGCGAAAGCGGCGCACTGGACCACAACTGACGCT
GAGGCGCGAAAGCTAGGGGAGCAAACAGGATTAGAGACCCCCGTAGTCC
>Otu9877

CCAGCCTACGGGGCGCAGCAGTGGGGAATTTTCCGCAATGGGCGCAAGCCTGATCCAGCC
ATGCCGCGTGAGTGATGAAGGTCTTCGGATTGTAAACTTCTTTAGACCTAGATGAAGATT
GCAAGGTAAATAATCTTGCAATTTGACAGTATAGGTAAAATAGGCCACGGCTAACTCTGT
GCCAGCAGCCGCGGTAATACAGAGGTGGCAAACGTTGTCCGGATTTATTGGGTGTAAAGG
GCATGTAGGTGGTCTTGTAAGTCAAAGGTGAAATGGCCCGGCTCAACCAGGTCATTGCCT
TTGAAACTGCAGGGCTTGAGTACGGAAGAGGAGAGTGGAATTCCCAGTGTAGCGGTGAAA
TGCGTAGATATTGGGAGGAACACCGGTGGCGAAGGCGGCTCTCTGGTCCGAAACTGACAC
TGAGATGCGAAAGCCAGGGGAGCAAACGGGATTAGAAACCCCAGTAGTCC
>Otu9879
CTACGGGAGGCTGCAGTGGGGAATTTTGCGCAATGGGGGAAACCCTGACGCAGCAACGCC
GCGTGGAGGATGAAGCCCCTTGGGGCGTAAACTCCTTTCGACCGGGACGATTATGACGGT
ACCGGTGGAAGAAGCACCGGCTAACTCTGTGCCAGCAGCCGCGGTAATACGTAGGGGGCA
AGCGTTGTTCGGAATCACTGGGCGTAAAGGGAGCGTAGGCGGGGAAATAAGTTAGAAGTT
TAATGTCCGGGCTTAACCCGGAATCTGCTTCTAATACTGTTTTTCTTGAGTATTGGAGAG
GGAGATGGAATTCCAGGTGTAGCGGTGGAATGCGTAGATATCTGGAAGAACACCAGCTGC
GAAGGCGGTCTCCTGGCCAAATACTGACGCTGATGCTCGAAGGCTAGGGGAGCAAACAGG
ATTAGATACCCCCGTAGTCC
>Otu9887
CCAGCCTATGGGTTGCTCCAGTCGAGAATCTTCCACAATGGACGAAAGTCTGATGGAGCG
ACGCCGCGTGATTGATGAAGTCCCTTTGGGACGTAAAGATCTTTTGTCAGGGATGAAGTT
TATTGACCGTACCTGAAGAATAAGAGGCTCCTAATCTCGTGCCAGCAGGAGCGGTAATAC
GAGAGCCTCGAGCGTTATCCGGAATTATTGGGCGTAAAGGGTGCGTAGGTTGTTTTGTTA
GTCATTTGTTAAAACCCCGGGCTTAACCTGGGGCAGGCAAGTGAAACGGCAAGACTTGAA
AGTGTGAGAGGAGTACGGAACTTATGGTGTAGGGGTGAAATCCGTTGATATCATAGGGAA
CACCAAATGCGAAGGCAGTACTCTGGCGCATATTTGACACTGAAGCACGAAAGCGTGGGT
AGCGAATGGGATTAGAAACCCTAGTAGTCC
>Otu9890
CCAGCCTACGGGGGGCAGCAGTTAGGAATTTTGCGCAATGGACGAAAGTCTGACGCAGCG
ACATCGCGTGAAGGATGAAGGCCCTCGGGTCGTAAACTTCTTTTTTCCGGCCATGAAGGC
CGGTAGAATCAGTGACTACTAACTACGTGCCAGCAGTCGCGGTAATACGTAGGTCACAAG
CGTTACCCGGTTTTATTGGGCGTAAAGAGTCTGTAGGTGGTTTTTTAAGTCTTGTTTCAA
AGACCTCGGCTCAACCGAGGACAGGGGCAGGATACTGGAAGACTAGAGTGATATCGGGGT
TACTGGAATTTGTAGTGTAGGGGTTAAATCCGTGGATACTACAAAGAACACCAAGCGCGA
AGGCGGGTAACCAGGTATATACTGACACTCAGAGACGAAAGCTAGGGTAGCCAATCAGAT
TAGATACCCGAGTAGTCC
>Otu9902
CCAGCCTATGGGTCGCTGCAGTGGGGAATCTTGCACAATGGGGGAAACCCTGATGCAGCG
ACGCCGCGTGAGCGATGAAGCCCTTCGGGGTGTAAGGCTCTTTCGGCAGGGAAGATTATG
ACGGTACCTGCAAAAGCAGCTGCGGCTAACTACGTGCCAGCAGCCGCGGTAATACGTAGG
CAGCGAGCGTTGTTCGGAGTTACTGGGCGTAAAGGGTGTGTAGGCGGTTGTTTAAGTTGA
ATGTGAAATCCCCGGGCTCAACCTGGGAACTGCATCCAAAACTGGCGAGCTAGAGTATGG
TAGAGGGTGGTGGAATTGCCTGTGTAGCGGTGAAATGCGTAGATATAGGAAGGAACACCA
GTGGCGAAGGCGACCACCTGGACTGATACTGACACTGAGGTGCGAAAGCGTGGGGAGCAA
ACAGGATTAGAAACCCGTGTAGTCC
>Otu9910
CCAGCCTATGGGGTGCAGCAGTCGAGAATTTTTCACAATGGGCGAAAGCCTGATGGAGCG
ACGCCGCGTGGGGGACGAATGGCTTCGGCCCGTAAACCCCTGTCATTCGCGATCAAACCT
TGGTGTTTAAGAGATGCCAAGCTGATAGTAGCGGAAGAGGAATGGACGGCTAACTCTGTG
CCAGCAGCCGCGGTAATACAGAGGTCCCGAGCGTTGTTCGGATTCACTGGGCGTAAAGGG
TGCGTAGGTGGTGGAGCAAGTCGGATGTGAAATCTCGGAGCTTAACTCCGAAACGGCATT
GGAAACTGCTCTGCTCGAGGGTCGGAAGGGGGACTGGAATTCTCGGTGTAGCAGTGAAAT
GCGTAGATATCGAGAGGAACACCGGTGGCGAAGGCGAGTCCCTGGACGACTCCTGACACT
GAGGCACGAAAGCTAGGGGAGCAAACAGGATTAGATACCCCCGTAGTCC
>Otu9911
CAGCCTACGGGGTGCAGCAGTGGGGAATATTGCGCAATGGGGGAAACCCTGACGCAGCGA
CGCCGCGTGGGTGAAGAAGGCCTTCGGGTTGTAAAGCCCTTTCGGTAGGGGAGAAGGGTA
TTGTGGTGAATAGCCATAGTATTTGATGGTACCTAAAGAAGAAGCACCGGCTAACTTCGT
GCCAGCAGCCGCGGTAATACGAAGGGTGCGAGCGTTGTTCGGAATTATTGGGCGTAAAGG

GTTCGTAGGCGGGAAGATAAGTAAAGTGTGAAATCCCAGGGCTAAACCTTGGAGAGTCAT
TTTAGACTGTTTTTCTTGAGTATCTGAGAGGGTGGTGGAATTGCTGGTGTAGGAGTGACA
TCCGTAGAGATCAGCAGGAACATCGGAGGCGAAGGCGACCACCTGGCAGAGTACTGACGC
TGAGGAACGAAAGCGTGGGGAGCAAACAGGATTAGAAACCCCAGTAGTCC
>Otu9913
CCAGCCTATGGGTGGCACCAGTGGGGAATTTTGCGCAATGGGGGAAACCCTGACGCAGCA
ACGCCGCGTGAGTGATGAAGGCCTTCGGGTCGTAAAGCTCTGTCAGAGGGAAAGAAGTGT
AGGAGGGTTAATACCCCTTTTATTTGACGGTACCGCTAAAGGAAGCACCGGCTAACTCCG
TGCCAGCAGCCGCGGTAATACGGGGGGTGCTAGCGTTGTTCGGAATTATTGGGCGTAAAG
AGCGTGTAGGCGGCTGGATAAGTCAGATGTGAAAGCCCTGGGCTTAACCCAGGAAGTGCA
TTTGAAACTGTCCGGCTTGAGTAAGGGAGAGGAAAGTGGAATTCCTGGTGTAGAGGTGAA
ATTCGTAGATATCAGGAGGAACACCGGTGGCGAAGGCGACTTTCTGGCCCTATACTGACG
CTGAGACGCGAGAGCGTGGGTAGCAAACAGGATTAGATACCCCTGTAGTCC
>Otu9924
CCTACGGGACGCAGCAGTCGAGGATCTTCGGCAATGGGCGCAAGCCTGACCGAGCGACGC
CGCGTGCGGGATGAAGGCCTTCGGGTTGTAAACCGCCGTCGGAGGGGAGGAAATGCCATG
GGGTTCTCCCTGTGGTTTGACCTATCCTCAGAGGAAGGACGGGCTAAGTTCGTGCCAGCA
GCCGCGGTAAGACGAACCGTCCAAACGTTATTCGGAATCACTGGGCTTAAAGGGTGCGTA
GGCGGCCCGGAAAGTTGGGTGTGAAATCCCTCGGCTTAACCGAGGAATTGCGCCCAAAAC
TACCGGGCTCGAGGGAGATAGAGGTGAGCGGAACTTAGGGTGGAGCGGTGAAATGCGTTG
ATATCCTAAGGAACACCAGGAGCGAAAGCGGCTCACTGGGTCTCTTCTGACGCTGAGGCA
CGAAAGCTAGGGGAGCGAACGGGATTAGATACCCCAGTAGTCC
>Otu9938
CCAGCCTATGGGTCGCTGCAGTGGGGAATTTTGCGCAATGGGGGAAACCCTGACGCAGCA
ACGCCGCGTGAGTGATGAAGGCCTTCGGGTCGTAAAGCTCTGTCAGAGGGAAAGAAGTGT
AGGAGGGTTAATACCCCTTTTACTTGACGGTACCCTCAAAGGAAGCACCGGCTAACTCCG
TGCCAGCAGCCGCGGTAATACGGAGGGTGCGAGCGTTGTTCGGATTTATTGGGCGTAAAG
CGCGTGTAGGCGGTTTTTTAAGTCTGATGTGAAATCTCCCGGCTCAACTGGGAGGGTGCG
CCGAATACTGAAGGGCTAGAGTGCGGGAGAGGAGAGTGGAATTCCTGGTGTAGCGGTGAA
ATGCGTAGATATCAGGAGGAACACCGGTGGTGTAGACGGCTCTCTGGACCGTAACTGACG
CTGAGACACGAAAGCGTGGGTAGCAAACAGGATTAGATACCCGCGTAGTCC
>Otu9939
CCAGCCTACGGGTCGCACCAGTGGGGAATATTGGACAATGGGCGCAAGCCTGATCCAGCA
ATGCCGCGTGTGTGAAGAAGGCCTGCGGGTTGTAAAGCACTTTGAGCGGGAAAGAAGGCG
CGGGGCTTAACACGTTCCGCGATTGACGTTACCCGCAGAACAAGCACCGGCTAACTCCGT
GCCAACAGCCGCGGTAATACGGAGGGTGCGAGCGTTAATCGGAATTACTGGGCGTAAAGC
GTGCGTAGGCGGCCCGGCCAGTCCGACGTGAAAGCCCCGGGCTCAACCTGGGAACTGCGT
CGGATACTGCCGGGCTGGAGTGCGGGAGAGGGCGGTGGAATTTCCGGTGTAGCAGTGAAA
TGCGTAGAGATCGGAAGGAACACCAGTGGCGAAGGCGGCCGCCTGGACCGACACTGACGC
TGAGGCACGAAAGCGTGGGGAGCAAACAGGATTAGATACCCCAGTAGTCC
>Otu9943
CCAGCCTACGGGATGCTCCAGTGGGGAATTTTGCGCAATGGGGGAAACCCTGACGCAGCA
ACGCCGCGTGGAGGATGAAGTACTTCGGTACGTAAACTCCTTTCGATCGGGACGATAATG
ACGGTACCGGAAGAAGAAGCCCCGGCTAACTTCGTGCCAGCAGCCGCGGTAATACGAGGG
GGGCGAGCGTTGTTCGGAATTATTGGGCGTAAAGGGTGCGTAGGCGGTTTGGTAAGTCTT
ATGTGAAATCTCCGGGCCTAACCCGGAAACTGCGTTGGATACTATCCGGCTAGAGGAATG
GAGGGGAGACTGGAATACTTGGTGTAGCAGTGAGATGCGTAGATATCAAGTGGAACACCA
GTGGCGAAGGCGAGTCTCTGGACATTTCCTGACGCTGAGGCACGAAAGCCAGGGGAGCAA
ACGGGATTAGAACCCGAGTAGTCC
>Otu9944
CCAGCCTATGGGGTGCTGCAGTCGAGAATTTTTCACAATGGGGGAAACCCTGATGGAGCG
ACGCCGCGTGGGGGATGAATGGCTTCGGCTCGTAAACCCCTGTCATTCGGGATCAATGCC
TGCTATTTAAAAGATGGTAGGTTGATAGTACCGAAAGAGGAAGGGACGGCTAACTCTGTG
CCAGCAGCCGCGGTAATACAGAGGTCCCAAGCGTTGTTCGGATTCACTGGGCGTAAAGGG
TGCGTAGGCGGTTGGGTAAGTCTGACGTGAAATCTCCGGGCCTAACCCGGAAACTGCGTT
GGAAACTATCTGACTAGAGGAATGGAGGGGAGACTGGAATACTTGGTGTAGCAGTGAAAT
GCGTAGATATCAAGTGGAACACCAGTGGCGAAGGCGAGTCTCTGGACATTTCCTGACGCT
GAGGCACGAAAGCCAGGGGAGCAAACGGGATTAGAGACCCCAGTAGTCC

>Otu9947
CCAGCCTATGGGTTGCAGCAGTGAGGAATATTGGGCAATGCCCGCAAGGGTGACCCAGCA
ACGCCGCGTGAAGGATGAAAGCCGTAAGGTTGTAAACTTCTGTTAGAGGGGACGAATAGC
TTCGATCAAATCGAAGTTTGACGGTACCCGCAGAATAAGCCCCGGCTAACTTCGTGCCAG
CAGCCGCGGTAATACGAAGGGGGCTAGCGTTGTTCGGAATCACTGGGCGTAAAGCGTACG
CAGGCGGATTGATAAGTCAGGGGTGAAATCCCGGGGCTCAACCTCGGAATTGCCTTTGAT
ACTGTCTATCTTCGAGTTCGGGAGAGGTTGGCGGAATTCCTAGTGTAGAGGTGAAATTCG
TAGATATTAGGAAGAACACCAGTGGCGAAGGCGGCCAACTGGCCCGATACTGACGCTCAT
GTGCGAAAGCGTGGGGAGCAAACAGGATTAGAGACCCTCGTAGTCC
>Otu9953
CCAGCCTATGGGGCGCAGCAGTCGAGGATCTTCGGCAATGGGCGCAAGCCTGACCGAGCG
ACGCCGCGTGGGCGATGAAGGCCTTCGGGTTGTAAAGCCCTGTCGGGGAGGAGAAAAGGG
TAAAACCTTGATCTATTCCCGGAGGAAGCACGGGCTAAGTTCGTGCCAGCAGCCGCGGTA
AGACGAACCGTGCGAACGTTGTTCGGAATCACTGGGCTTAAAGGGCGCGTAGGCGGGCCT
CCAAGTCCGGGGTGAAATCCTCCAGCCTAACTGGAGAAGAGCCTCGGAGACTGGGGGCCT
CGAGAAGGGTAGGGGCAAGCGGAACTATGGGTGGAGCGGTGAAATGCATTGATATCCATA
GGAACTCCGGTGGCGAAGGCGGCTTGCTGGACCCTTTCTGACGCTGAGGCGCGAAAGCCA
GGGGAGCAAACGGGATTAGAAACCCTCGTAGTCC
>Otu9956
CCAGCCTATGGGGGGCACCAGTGGGGAATCTTGCGCAATGGGCGAAAGCCTGACGCAGCC
ACGCCGCGTGAGTGAAGAAGGCCTTCGGGTTGTAAAGCTCTGTCGGAGGGGACGAAAACT
TCTATGGTTAATAGCCATAGGAATTGACGGTACCCTCAAAGGAAGCACCGGCTAACTTCG
TGCCAGCAGCCGCGGTAATACGAGGGGTGCAAGCGTTGCTCGGAATTATTGGGCGTAAAG
CGCGTGTAGGCGGTTTTTTAAGTCTGATGTGAAAGCCCTGGGCTCAACCCGGGAAGTGCA
TTGGAAACTGGGAGACTTGAATACGGGAGAGGGTAGTGGAATTCCTGGTGTAGGAGTGAA
ATCCGTAGATATCAGGAGGAACACCGGTGGCGAAGGCGGCTACCTGGACCGATATTGACG
CTGAGACGCGAAAGCGTGGGGAGCAAACAGGATTAGAGACCCGAGTAGTCC
>Otu9960
CCAGCCTATGGGGGGCACCAGTGGGGAATCTTGCGCAATGGACGAAAGTCTGACGCAGCC
ACGCCGCGTGAGTGAAGAAGGCCTTCGGGTTGTAAAGCTCTGTCGGGAGGGACGAACATC
CATTGGGTTAACAGCCCAGTGGACTGACGGTACCTTCAAAGGAAGCACCGGCTAACTCTG
TGCCAGCAGCCGCGGTAATACAGAGGGTGCAAGCGTTGCTCGGAATCATTGGGCGTAAAG
GGTAGGTAGGTGGTCTCATTTGTCTGGGGTGAAAGCCCAGGGCTTAACCCTGGAAGTGCC
CCGGAAACGGTGAGACTGGAGTCTTGGAGAGGGTCGTGGAATTCCCGGTGTAGCGGTGAA
ATGCGTAGAGATCGGGAGGAACACCAGAGGCGAAGGCGGCGACCTGGACAAGTACTGACA
CTCAACTACGAAAGTGTGGGGAGCAAACAGGATTAGATACCCAGTAGTCC
>Otu9962
CCAGCCTACGGGAGGCAGCAGCAACGAATATTGGGCAATGGGCGAAAGCCTGACCCAGCG
ACGCCGCGTGCGGGATGAAGCCCTTCGGGGTGTAAACCGCTGTCAGGGTTAGAGAATCTG
TCGGTGTGAATAATGCCGGCAGTTGACTCATCCCAGAGGAAGCCACGGCTAACTCCGTGC
CAGCAGCCGCGGTAAGACGGAGGTGGCGAGCGTTACTCGGATTCACTGGGCTTAGAGCGG
GTGCAGGCGGTCCCGCAAGTCCGGAGTGAAATCCCTCGGCTCAACCGAGGAATTGCTTCG
GATACTGCGGGACTTGAGGCAGGTAGGGGCAAGTGGAACGTTTGGTGTAGCGGTGAAATG
CGTAGATATCAAACGGAACGCCGGTGGTGAAGACGGCTTGCTGGACCTGTCCTGACGCTC
AGACCCGAAAGCGTGGGGAGTGAACCGGATTAGATACCCGAGTAGTCC
>Otu9966
CCAGCCTACGGGATGCTGCAGTCGAGGATCTTCGGCAATGGGCGCAAGCCTGACCGAGCG
ACGCCGCGTGTGCGATGAATGGCTTCGGCCCGTAAACCCCTGTCATTTGCGAACAAATTG
CTTCACCCAACACGTGAAGCATTGATAGTAGCGGAAGAGGAAGGGACGGCTAACTCTGTG
CCAGCAGCCGCGGTAATACAGAGGTCCCAAGCGTTGTTCGGATTCACCGGGCGTAAAGGG
TGCGTAGGCGGTTGGGTAAGTCTGACGTGAAATCTCCGGGCCTAACCCGGAAACTGCGTT
GGATACTATCCGGCTAGAGGAATGGAGGGGAGACTGGAATACTTGGTGTAGCAGTGAAAT
GCGTAGATATCAAGTGGAACACCAGTGGCGAAGGCGAGTCTCTGGACATTTCCTGACGCT
GAGGCACGAAAGCCAGGGGAGCAAACGGGATTAGAAACCCGAGTAGTCC
>Otu9967
CCAGCCTACGGGTCGCAGCAGTCGAGAATCTTCCGCAATGGGCGAAAGCCTGACGGAGCG
ACGCCGCGTGGTTGATGAAGTACTTCGGTACGTAAAAACCTTTTATGAGCGACTAAGTTT
ATTGAAGAGCTCATGAATAAGAGGTTGCTAAACTCGTGCCAGCAGCAGCGGTAATACGAG

TGCCTCAAGCGTTATCCGGAATTATTGGGCGTAAAGGGTGTGTAGGCGGCTGTGTTAGTC
TCGCGTTAAATTCTTCGGCTCAACCGGGGACCTGCGCGGGAAACGGCACAGCTAGAAGAT
GCGAGGGGTCTCTGGAACTCATGGTGTAGCGGTGAAATGCGTTGATATCATGGGAACAC
CAAAAGCGAAGGCAGGAGACTGGAGCATTCTTGACGCTGAAACACGAAAGCGTGGGGAGC
AAACAGGATTAGATACCCTAGTAGTCC
>Otu9968
CCAGCCTATGGGAGGCACCAGTAGGGAATATTGGACAATGGACGCAAGTCTGATCCAGCC
ATCCCGCGTGAAGGATTAAGGCCCTCTGGGTTGTAAACTTCTTTTCTCTGGGAATAAAAA
GCGGTATTCTTATCGCCTTGAAGGTACCAGAGGAATAAGCACCGGCTAACTCCGTGCCAG
CAGCCGCGGTAATACGGAGGGTGCAAGCGTTATCCGGATTCACTGGGTTTAAAGGGTGCG
TAGGTGGTCTGTTAAGTCAGCGGTGAAAGCCTGGAGCTCAACTCCAGAATTGCCATTGAT
ACTGACGGACTTGAATCAAGTTGAGGTGGATGGAATATTACATGTAGCGGTGAATGCTTA
GATATTACACAGAACACCGATTGCGAAGGCAGCTTACTGGGCCATTACTGACGCTGATGC
ACGAAAGCGTGGGGAGCGAACAGGATTAGATACCCGTGTAGTCC
>Otu9972
CCAGCCTATGGGAGGCTCCAGTGGGGAATCTTGCACAATGGGCGAAAGCCTGATGCAGCG
ACGCCGCGTGAAGGATGAAGGCCTTCGGGTTGTAAACTTCTTTTGCAGGGGAAGATAATG
ACGGTACCCTGCGAATAAGCCACGGCTAACTCTGTGCCAGCAGCCGCGGTAAGACAGAGG
TGGCGAGCGTTGTTCGGAATTACTGGGCTTAAAGGGCGCGTAGGCGGTGATACAAGTCTG
GGGTGGAAGCCCATAGCTTAACTATGGGACTGCCCTGGAAACTGTATTGCTTGAGTCGGA
CAGGGGAAGGCGGAATTCCAGGTGTAACGGTGAAATGCGTAGATATCTGGAGGAAGGCCT
GTGGTGAAGACGGCCTTCTGGGTCTTGACTGACGCTCATACGCGAAAGCTAGGGTAGCAA
ACCGGATTAGATACCCGCGTAGTCC
>Otu9973
CCAGCCTACGGGACGCAGCAGTGAGGAATATTGGTCAATGGGGGCAACCCTGAACCAGCC
ATGCCGCGTGAAGGAAGACGGTCCTAAGGATTGTAAACTTCTTTTGTACGGGGGTAACCT
CGGGTACGTGTACCCGACTGAAAGTACTGTACGAATAAGCAACGGCTAACTCCGTGTCAG
CAGCCGCGGTAATACGGAGGTTGCAAGCGTTATCCGGATTTATTGGGTTTAAAGGGTGCG
TAGGCGGGTATATAAGTCAGCGGTGAAAGACTGTCGCTTAACGATAGCATTGCCATTGAT
ACTGTATGTCTTGAGTACATTTGAGGTGGGCGGAATGAGTAGTGTAGCGGTGAAATGCTT
AGATATTACTCAGAACACCAATTGCGAAGGCAGCTCACTAAACTGTCACTGACGCTGAGG
CACGAAAGCGTGGGGATCAAACAGGATTAGAAACCCCGAGTAGTCC
>Otu10002
GGACTACTGGGGTCTCTAATCCTGTTTGCTCCCCACGCTTTCGCGCCTCAGCGTCAGTCC
TCTGATAGAAGCTCGCCTTCGCCACCGGTGTTCTTCCAGATATCTACAGATTTCACCCCT
ACACCTGGAATTCCAGCTCCCCCTCAAGAACTCAAGCCCACCAGTTTCCAGCGCATATGC
AGGGTTAAGCCCTGCGATTTCACACCAGACTTGATGAGCCGCCTACACGCCCTTTACGCC
CAATAAATCCGAACAACGCTTGCCCCATACGTATTACCGCGGCTGCTGGCACGTAATTAG
CCGGGGCTTATTCGTCGGCTAACGTCATCAGGTGGGCTTTCCACTCCCACCCTTATTCTT
CACCAACAAAAGGATTTTACAACCTTACGGCCTTCATCATCCACGCGGCGTCGCTCCGTC
AGGGTTTCCCCCATTGCGGAAGATTCTTAGCTGCAGCGACCCATAGGCTGG
>Otu10012
CCAGCCTATGGGAGGCTCCAGGCGCGAAAACTTTACAGTGCCGGCAACGGCGATAAGGGG
ACCTCGAGTGCCAGGATACAATCCTGGCTGTCGTAATGCCTAAAAAGCACTATATAGCAA
GGGCCGGGCAAGACCGGTGCCAGCCGCCGCGGTAACACCGGCGGCTCGAGTGGTAACCGC
TATTATTGGGTCTAAAGGGTCTGTAGCCGGCCTGGTAAGTCTTTTGGGAAATCCGGCAGC
TCAACTGTCGGGCTTTCAGAGGATACTGCTAGGCTCGAGACCGGGAGAGGTGAGAGGTAC
TTCATGGGTAGGGGTGAAATCTTGTAATCCTTGAAGGACCACCAGTGGCGAAGGCGTCTC
ACCAGAACGGATCTGACGGCAAGGACGAAAGCTAGGGGCACGAACCGGATTAGAAACCC
GAGTAGTCC
>Otu10022
CCAGCCTATGGGTCGCAGCAGTGGGGAATATTGGACAATGGGGGAAACCCTGATCCAGCC
ATGCCGCGTGAGTGAAGAAGGCCTTAGGGTTGTAAAGCTCTTTTGGCGGGGACGATAATG
ACGGTACCCGCAGAATAAGCCCCGGCTAACTTCGTGCCAGCAGCCGCGGTAATACGAAGG
GGGCTAGCGTTGTTCGGAATCACTGGGCGTAAAGCGTACGCAGGCGGATTGATAAGTCTG
ATGTGAAAGCCCTGGGCTCAACCCGGGAAGTGCATTGGAAACTGGGAGACTTGAATACGG
GAGAGGGTAGTGGAATTCCTGGTGTAGGAGTGAAATCCGTAGATATCAGGAGGAACACCG
GTGGCGAAGGCGGCTACCTGGACCGATATTGACGCTGAGACGCGAAAGCGTGGGGAGCAA

ACAGGATTAGAGACCCCCGTAGTCC
>Otu10041
CCAGCCTATGGGTCGCAGCAGTAGGGAATATTGGTCAATGGGCGCAAGCCTGAACCAGCC
ATGCCGCGTGCAGGAAGAAGGCGTTACGCGTTGTAAACTGCTTTTGAACGGGAATAAAAA
GGCCATGCGTGGCAAATTGAATGTACCGTTAGAATAAGCCACGGCTAACTACGTGCCAGC
AGCCGCGGTAATACGTAGGTGGCAAGCGTTATCCGGATTTATTGGGTTTAAAGGGGGCGT
AGGCGGTCCTGTAAGTCAGTGGTGAAAGACTCCGGCTCAACCGGAGCATTGCCATTGATA
CTGCAGGGCTAGAGTACGGTAGAGGTGGGCGGAATTTACGGTGTAGCGGTGAAATGCATA
GATACCGTAAAGAACACCGATTGCGAAGGCAGCTCACTGGACCGTAACTGACGCTGAGGC
CCGAAAGTGCGGGTATCAAACAGGATTAGAAACCCCCGTAGTCC
>Otu10045
CCAGCCTATGGGGGGCTGCAGTGGGGAATTTTGCGCAATGGGGGAAACCCTGACGCAGCA
ACGCCGCGTGGAGGAAGAAGTATTTCGGTACGTAAACTCCTTTCGATCGGGACGATAATG
ACGGTACCGAGAGAAGAAGCCCCGGCTAACTTCGTGCCAGCAGCCGCGGTAATACGAGGG
GGGCAAGCGTTATCCGGATTCATTGGGTTTAAAGGGTGCGTAGGCGGACTTATAAGTCAG
TGGTGAAATCTCATCGCTTAACGATGAACGTGCCATTGATACTGTAGGTCTTGAGTACAG
ATGCCGTTGGCGGAATGTGTCATGTAGCGGTGAAATGCATAGATATGACACAGAACACCG
ATTGCGAAGGCAGCTGACGAAACTGTAACTGACGCTGAGGCACGAAAGCGTGGGGATCAA
ACAGGATTAGATACCCTTGTAGTCC
>Otu10051
CCAGCCTATGGGAGGCACCAGTAAGGAATATTGGACAATGGTGGCAACACTGATCCAGCC
ATGCCGCGTGCAGGATGAAGGCGCTATGCGTTGTAAACTGCTTTTCCAGGGGAAGAAAAC
CCCCGACGTGTCGGGGCTTGCCGGTACCCTGGGAATAAGCATCGGCTAACTCCGTGCCAG
CAGCCGCGGTAATACGGAGGATGCGAGCGTTATCCGGATTTATTGGGTTTAAAGGGTGCG
TAGGCGGATAAATAAGTCAGTGGTGAAAACCTGCAGCTTAACTGTAGAATTGCCATTGAT
ACTGTTAGTCTTGAGTACGGTCAAGGTAGGCGGAATGTGTAATGTAGCGGTGAAATGCTT
AGATATTACACAGAACACCGATTGCGAAGGCAGCTTACTGGCCATTACTGACGCTGATG
CACGAAAGCGTGGGGAGCGAACAGGATTAGAAACCCCTGTAGTCC
>Otu10052
CCAGCCTACGGGTTGCAGCAGTGGGGAATATTGGACAATGGGCGAAAGCCTGATCCAGCC
ATGCCGCGTGAGTGATGAAGGCCTTCGGGTTGTAAAACTCTTTTCTGAGGGAAGAACAAC
CGGGCGAGGAAATGCGCCCGGCCTGACGGTACCTCAGGAATAAGCAACGGCTAACTCCGT
GCCAGCAGCCGCGGTAATACGGAGGTTGCGAGCGTTGTTCGGAATAACTGGGCGTAAAGG
GAGCGCAGGCGGTTTGGTAAGTCTGAAGTGAAAGCCCGGGGCTTAACCCCGGAAGTGCTT
TGGAAACTGTCAAACTAGAGTAAGTGAGAGGAGAGCGGAATTCAGGGTGTAGCGGTGAAA
TGCGTAGATATCCTGAGGAACACCGGTGGCGAAAGCGGCTCTCTGGTACGAAACTGACGC
TGAGGCTCGAAAGCGTGGGGATCAAACGGGATTAGATACCCCAGTAGTCC
>Otu10054
CCAGCCTACGGGTGGCAGCAGTGAGGAATATTGGTCAATGGGCGCAAGTCTGACCCAGCC
ATGCCGCGTGAAGGAAGAAGGCCCTAGTGGTCGTAAACTTCTTTTATATGGGAAAAATTC
TCGGATCGTGAATCCGGGTTGATGGTACTATATGAATAAGCATCGGCTAACTCCGTGCCA
GCAGCCGCGGTAATACGGAGGATGCAAGCGTTATCCGGATTTATTGGGTTTAAAGGGTGC
GTAGGCGGGACGATAAGTCAGTGGTGAAATCCTACGGCTTAACTGTAGAATTGCCATTGA
TACTGTCGTTCTTGAGTGCAGCTGAAGAGGGCGGAATGTGTTGTGTAGCGGTGAAATGCT
TAGATATAACACAGAACACCGATTGCGAAGGCAGCTCTCTAAACTGCAACTGACGCTGAG
GCACGAAAGCGTGGGGATCAAACAGGATTAGAAACCCCAGTAGTCC
>Otu10055
CCAGCCTATGGGTGGCTGCAGTCGAGAATCTCCGGCAATGGGCGCAAGCCTGACCGAGCG
ACGCCGCGTGGAGGATGAAGGCCTTCGGGTTGTAAACTCCTGTCGAGGGGGAGCAAGGTG
CCGTGAAGAACGGTGCTCGAGCGATCCCTGGAGGAAGCACGGGCTAAGTTCGTGCCAGCA
GCCGCGGTAAGACGAACCGTGCGAACGTTATTCGGAATCACTGGGCTTAAAGCGCGTGCA
GGCGGTCCGGCACGTCCGTCGCTGAAATCCCCCGGCTCAACCGGGGAACCGGCGCGGATA
CGACCGGGCTGGAGGAGGGTAGGGGGATCGGGAACTTCCGGTGGAGCGGTGAAATGCGTT
GAGATCGGAAGGAACGCCCGTGGCGAAAGCGCGGTCCTGGACCCTTCCTGACGCTGAGAC
GCGAAAGCCAGGGGAGCGAACGGGATTAGAGACCCGCGTAGTCC
>Otu10058
CTAGCCTATGGGTCGCAGCAGTCGAGAATTTTTCACAATGGGGGAAACCCTGATGGAGCG
ACGCCGCGTGGGGGATGAATGGCTTCGGCCCGTAAACCCCTGTCATTTGTGAACAAACCT

TTTCACTGAACAAGTGGAAAGTTGATAGTAACGGAAGAGGAAGGGACGGCTAACTCTGTG
CCAGCAGCCGCGGTAATACAGAGGTCCCAAGCGTTGTTCGGATTCACTGGGCGTAAAGGG
TGCGTAGGTGGCGGGGTAAGTCGGATGTGAAATCTCCGAGCTCAACTCGGAAATGGCATG
GGAAACTGCTCTGCTCGAGGGTTGGAGGGGGGACTGGAATACTTGGTGTAGCAGTGAAAT
GCGTAGATATCAAGTGGAACACCAGTGGCGAAGGCGAGTCCCTGGACAACTCCTGACACT
GAGGCACGAAAGCTAGGGGAGCAAACAGGATTAGAAACCCGCGTAGTCC
>Otu10070
CCAGCCTATGGGTGGCTGCAGTGGGGAATATTGGGCAATAGGCGCAAGCCTGACCCAGCA
ATGCCGCGTGTGTGAAGAAGGCCTGCGGGTTGTAAAGCACTTTGGGCGGGGAAGAAGCGC
GCGCCGCGAACACCGGCGCGCGTTGGCGCTACCCGCGGAACAAGCACCGGCTAACTCCGT
GCCAGCAGCCGCGGTAATACGGAGGGTGCGAGCGTTAATCGGAATTACTGGGCGTAAAGC
GTGCGTAGGCGGCCCGGTCAGTCTCGCGTGAAAGCCCCGGGCTCAACCTGGGAACTGCGC
GGGAAACTGCCGGGCTGGAGTGCGGGAGAGGGCGGTGGAATTTCCGGTGTAGCAGTGAAA
TGCGTAGAGATCGGAAGGAACACCAGTGGCGAAGGCGGCCGCCTGGACCGGCACTGACGC
TGAGGCACGAAAGCGTGGGGAGCAAACAGGATTAGATACCCGCGTAGTCC
>Otu10075
CCAGCCTATGGGGCGCAGCAGTGGGGAATTTTGCGCAATGGGGGAAACCCTGACGCAGCA
ACGCCGCGTGGAGGATGAAGTCCCTTGGGACGTAAACTCCTTTCGACCGGGACGATAATG
ACGGTACCGGTGGAAGAAGCCCCGGCTAACTCCGTGCCAGCAGCCGCGGTAATACGGGGG
GGGCAAGCGTTGTTCGGAATTATTGGGCGTAAAGGGCGCGTAGGCGGTGCGGTAAGTCAT
TTGTGAAAGCTCCCGGCTCAACTGGGAGACTGCAGGCGAAACTGCCGTGCTGGAGGGTGG
GAGAGGTGAGTGGAATTCCCGGTGTAGCGGTGGAATGCGTAGATATCTGGAAGAACACCA
GCTGCGAAGGCGGTCTCCTGGCCAAATACTGACGCTGATGCTCGAAAGCTAGGGGAGCAA
ACAGGATTAGATACCCCAGTAGTCC
>Otu10079
CCAGCCTACGGGTGGCAGCAGTGGGGAATTTTGCGCAATGGGGAAACCCTGACGCAGCAA
CGCCGCGTGAGTGATGAAGGCCTTCGGGTCGTAAAGCTCTGTCAGAGGGAAAGAAGTGTA
GGAGGGTTAATACCCCTTTTACTTGACGGTACCCTCAAAGGAAGCACCGGCTAACTCCGT
GCCAGCAGCCGCGGTAATACGGAGGGTGCGAGCGTTGTTCGGATTTATTGGGCGTAAAGC
GCGTGTAGGCGGTTTTTTAAGTCTGATGTGAAAGCCCTGGGCTCAACCCGGGAAGTGCAT
TGGAAACTGAGAGACTTGAATACGGGAGAGGGTAGTGGAATTCCTGGTGTAGTGGTGAAA
TGCGTAGATATCCAGAGGAACACCAGTGGCGAAAGCGGCCTCCTGGACCATTTCTGACGC
TCATACGCGAAAGCTAGGGTAGCAAACGGGATTAGAAACCCCAGTAGTCC
>Otu10080
CCAGCCTACGGGGTGCAGCAGTCGAGAATTTTTCACAATGGGGGAAACCCTGATGGAGCG
ACGCCGCGTGGGGGATGAATGGCTTCGGCCCGTAAACCCCTGTCATTTGTGAACAAACCT
TACCGCTGAACAAGCGGGAAGCTGATTGTAACGGAAGAGGAAGGGACGACTAACTCTGTG
CCAGCAGCCGCGGTAATACAGAGGTCCCAAGCGTTGTTCGGATTCACTGGGCGTAAAGGG
TGCGTAGGTGGCCGGGTAAGTCTGATGTGAAATCTCCGGGCCTAACCCGGAAACTGCGTT
GGATACTATCCGGCTAGAGGAATGGAGGGGAGACTGGAATACTTGGTGTAGCAGTGAAAT
GCGTAGATATCAAGTGGAACACCAGTGGCGAAGGCGAGTCTCTGGACATTTCCTGACGCT
GAGGCACGAAAGCCAGGGGAGCAAACGGGATTAGAAACCCCTGTAGTCC
>Otu10088
CCAGCCTATGGGTCGCTGCAGTGAGGAATATTGGTCAATGGGTGCAAACCTGAACCAGCC
ATGCCGCGTGAAGGATGAAGGCTCTACGAGTCGTAAACTTCTTTTATACGGGAATAAACC
TCTCTACGTGTAGAGAGTTGAAGGTACTGTAAGAATAAGCATCGGCTAACTCCGTGCCAG
CAGCCGCGGTAATACGGAGGATGCAAGCGTTATCCGGATTTATTAGGTTTAAAGGGTGTG
TAGGCGGACTGGTAAGTCAGCGGTGAAATCTCTCAGCTTAACTGAGAAACTGCCATTGAT
ACTGTCAGTCTTGAATGCAGATGAGGTAAGCGGAATATGTAATGTAGCGGTGAAATGCTT
AGATATTACATAGAACACCAATTGCGAAGGCAGCTTACTAAATTGTGATTGACGCTGAGA
CACGAAAGCGTGGGGAGCGAACAGGATTAGAGACCCCGGTAGTCC
>Otu10092
CCAGCCTACGGGGCGCTGCAGTGGGGAATTTTGCGCAATGGACGAAAGTCTGACGCAGCG
ACGCCGCGTGGGTGATGAAGGCCTTCGGGTTGTAAAGCCCTGTGGGGCGGGAAGAATAAG
TGGTGACTAATACTTGCCATGATGACGGTACCGCCTTAGCAAGCACCGGCTAACTCTGTG
CCAGCAGCCGCGGTAAGACAGAGGGTGCAAACGTTGTTCGGAATTACTGGGCGTAAAGCG
CGTGTAGGCGGCTTAGCAAGTCGGCTGTGAAAGCCCTTGGCTCAACCAAGGAAGTGCACT
CGAAACTGCTAAGCTTGAGTCTCGGAGAGGATGGTGGAATTCTCGGTGTAGAGGTGAAAT

TCGTAGATATCGAGAGGAACACCAGTGGCGAAGGCGGCCATCTGGACGATGACTGACGCT
GAGACGCGAAAGCATGGGGAGCAAACAGGATTAGAGACCCCAGTAGTCC
>Otu10103
CCAGCCTATGGGAGGCAGCAGCCGAGGATTTTTCTCAATGGGCGAAAGCCTGAAGGAGCG
ACGCCGCGTGGGGGATGAAGGTCTTCGGATTGTAAACCCCTGTCATCTGGGAACAATGGT
TACGACCCAATACGTCGTGACTTGATAGTACCGGAAGAGGAAGCCGTGGCTAACTCTGTG
CCAGCAGCCGCGGTAATACAGAGACGGCAAGCGTTGTTCGGATTCATTGGGCGTAAAGGG
TCCGTAGGCGGTCGGGCAAGTCGGATGTGAAATCCCGCAGCCTAACTGCGGTGGGTCATT
CGATACTGCTCGGCTCGAGGGCTGGAGAGGAGACTGGAATTGTCGGTGTAGCGGTGAAAT
GCGTAGAGATCGACAAGAACACCGGTGGCGAAGGCGGGTCTCTGGACAGTTCCTGACGCT
GAGGGACGAAAGCTAGGGGAGCAAACGGGATTAGATACCCGGGTAGTCC
>Otu10112
CCAGCCTACGGGAGGCAGCAGTCGAGAATTTTTCTCAATGGGGGAAACCCTGAAGGAGCG
ACGCCGCGTGAAGGATGAAGGTCTTCGGATTGTAAACTTCTGTCATTAGGGAACAAATCT
TCCGCGTAACTGCCGGAAGTCTGATAGTACCTGAAGAGGAAGAGACGGCTAACTCTGTGC
CAGCAGCCGCGGTAATACAGAGGTCTCAACCGTTGTTCGGATTCATTGGGCGTAAAGGGT
GCGTAGGCTGCGGAGTAAGTCTGGTGTGAAATCTCGGGGCTCAACCCCGAAACTGCACTG
GATACTGCTTCGCTCGAGTATTGGAGAGGAGATTGGAATTTACGGTGTAGCAGTGAAATG
CGTAGATATCGTAAGGAAGACCAGTGGCGAAGGCGAATCTCTGGACAATTACTGACGCTG
AGGCACGAAGGCCAGGGGAGCAAACGGGATTAGATACCCTCGTAGTCC
>Otu10122
CCAGCCTACGGGAGGCACCAGGGTTTTCAGTCAACGATCCTAGCGCCGCTCCCAGTCGCG
ACTCAAGCATCCCTCGCACAACCTTCGGTCGGACCAGAATCTCAGCATCGACGTGTGCAT
TCTCCTCCGGCACGCCCCCGCTAGCCCCTAGTCCCTCTTCCATACTCCCTACTCCCTGCT
CCTCCACCACTGCCGCAATCGCCATTGGCTGCGCGAAGAGCGAACTCGATCCGGATTGCT
CGTTCTGAATCCGCATTCCCAGCGTCGAAAACCCAGCCGGCCCCCGCAAGCGTCCATTTT
CAAAGAGGAAGACCGCCACCTCATCTACATTCGCCGACGCCTGCAAAATGACGGCTCTCA
ACTGGCTCAGCGGGCGCACCAGCTCGCTTGCCAACGCGCGCACGGCCTCAACGCGCTGCA
CCTGCGCATGGAGCGCAGCGGCGGATTCGAATTAGATACCCCAGTAGTCC
>Otu10126
CAGCCTATGGGTTGCAGCAGTCGAGAATCTTCCGCAATGGACGAAAGTCTGACGGAGCGA
CGCCGCGTGATGGATGAAGACCCTCTGGGTCGTAAACATCTTTTGTGAGGGAGAAAGTTT
ATTGATAGTACCTCAAGAATAAGGGGTTGCTAAACTCGTGCCAGCAGCAGCGGTAATACG
AGTGCCCCGAGCGTTATCCGGAATTATTGGGCGTAAAGGGTGTGTAGGTGGTTTTGTTAG
TCGATCGTTAAAACCCGGAGCTTAACTCCGGATCTGCGTTCGAAACGGCAAGACTAGAGG
ACGGCAGGGATGAGTGGAACTCATGGAGTAGGGGTGAAATCCGTTGATATCATGGGGAAC
ACCGGAAGCGAAGGCAGCTCATTGGGCCGCTCCTGACACTGAAACACGAAAGCGTGGGTA
GCGAAAGGGATTAGAAACCCTCGTAGTCC
>Otu10130
CCAGCCTATGGGGCGCAGCAGTAGGGAATATTGGACAATGGACTAAAGTCTGATTCAGTT
ACAATCATAGATATTTAAATTATGCTTTTATATTTTAAAGTCCTGACTAATTATGTGCCA
GCAGTCGCGGTAATACATAAAGGGCGAAGGTTCATCAAAATGACTGGGTATAAAGCGTTT
GAAGAGTCTCAATTAACATTTGTAAAAATTTATCAAGACCATTATTAGTTGGTTTTTACG
CTTTTATGTTTTTGTGTTTGAGTTTTTTTTAGGAATAGAGAATTTTATAGGGAGCAATGA
AATGCTATTATCTCTAAAGGAATTTCAATAGTTAATACACTATTCTAAAATTTAACTGAC
CTTTAAAAACGAAAGTCAAGGGAGTAAATAGGATTAGATACCCTCGTAGTCC
>Otu10142
CCAGCCTATGGGACGCAGCAGCCAAGAACATTCGACAATGGGCGAAAGCCTGATCGAGCG
ACGCCGCGTGGAGGATGAAGTCCTTCGGGATGTAAACTCCTTTTGCCAGGGAGGAAGTCT
ATTGACGTTACCTGGAGAATAAGAGGTTGCTAAACTCGTGCCAGCAGCAGCGGTAATACG
AGTGCCTCAAGCGTTATCCGGAATTATTGGGCGTAAAGGGTGTGTAGGTGGTCATGTTAG
TCTTTCGTTAAAGCTCCTGGCTCAACCGGGAAAATGCGGAGGAAACGGCATGACTATGAG
GGTGTGAGAGGTTTGCGGAACTCATGGTGTAGGGGTAAAATCCGTTGATATCATGGGGAA
CACCAAAAGCGAAGGCAGCAAACTGGCGCATTCCTGACACTGAAACACGAAAGCGTGGGG
AGCAAATGGGATTAGATACCCCCGTAGTCC
>Otu10144
CCAGCCTATGGGGGGCTGCAGTGGGGAATATTGGACAATGGGCGAAAGCCTGATCCAGCA
ATGCCGCGTGAGTGATGAAGGCCTTAGGGTTGTAAAGCTCTTTTAGTAGGGAAGATAATG

ACGGTACCTACAGAAAAAGCCCCGGCAAACTTCGTGCCAGCAGCCGCGGTAATACGAAGG
GGGCTAGCGTTGTTCGGAATCACTGGGCGTAAAGCGCGCGCAGGCGGCTTGTCAAGTTGG
GAGTGAAAGCCCGGAGCTTAACTTCGGAATAGCTTTCAAAACTGACAGGCTAGAGATGGA
TAGAGGATAGTGGAATTCCTAGTGTAGAGGTGAAATTCTTAGATATTAGGAGGAACACCG
GAGGCGAAGGCGGCTATCTGGGTCCATACTGACGCTGAGGCGCGAGAGCGTGGGGAGCAA
ACAGGATTAGATACCCCCGTAGTCC
>Otu10145
CCAGCCTATGGGAGGCAGCAGTGGGGAATATTGGACAATGGGGGAAACCCTGATCCAGCA
ATGCCGCGTGAGTGATGAAGGCCTTAGGGTTGTAAAGCTCTTTTACCCGGGATGATAATG
ACAGTACCGGGAGAATAAGCTCCGGCTAACTCCGTGCCAGCAGCCGCGGTAATACGGAGG
GAGCGAGCGTTGTTCGGAATTACTGGGCGTAAAGGGCACGTAGGCGGCTTTGTAAGCCCG
GCGTGGAAGCCCACGGCTCAACCGTGGGATTGCGTTGGGAACTGCGAGGCTTGAATCATG
GAGAGGGAGCTAGAATTCCTGGTGTAGGGGTGAAATCTGTAGAGATCAGGAAGAATACCA
GTGGCGAAGGCGAGCTCCTGGCCAATGATTGACGCTGAGGTGCGAAAGTGTGGGGGGCAA
ACAGGATTAGAAACCCCTGTAGTCC
>Otu10148
CCAGCCTACGGGAGGCAGCAGTTAGGAATTTTGCACAATGGGGGAAACCCTGATGCAGCA
ACGCCGCGTGGATGATGAAGGCCTTCGGGTCGTAAAATCCTTTTCAACGGGAGGATTCGG
ACGGTACCGTTGGAATAAGCATCGGCTAACTACGTGCCAGCAGCCGCGGTAATACGTAGG
ATGCGAGCGTTATCCGGATTTACTGGGCGTAAAGAGCGCGTAGGTGGTTCCGTAAGTTGG
GCGTGAAAGCCCCCGGCTTAACTGGGGAGGGTCGTTCAATACTGCGGGACTTGAGGGTGG
ATGAGGAGACTGGAATTCCCGGTGTAGTGGTGAAATGCGTAGATATCGGGAGGAACACCT
GTGGCGAAGGCGAGTCTCTAGGCCACTCCTGACACTGAGGCGCGAAAGCGTGGGGAGCGA
ACGGAATTAGAGACCCGGGTAGTCC
>Otu10163
CCAGCCTATGGGTTGCTGCAGGGAATATTGCATAATGGGCGAAAGCCTGATGCAGCAACG
CCGCGTGTGCGATGAAGGCCTTCGGGTTGTAAAGCACTTTTCGAGGGGACGAGGAAGGAC
AGTACCCTCGGAATAAGCCTCGGCTAACTACGTGCCAGCAGCCGCGGTAACACGTAGGAG
ACGAGCGTTATCCGGATTTACTGGGCGTAAAGCGCGTGCAGGCGGTTTGGTAAGTTGGAT
GTGAAAGCTCCTGGCTTAACTGGGAGAGGTCGTTCAATACTGCCAGACTAGAGGATGGTA
GAGGGAGGTGGAATTCTGGGTGTAGTGGTGAAATGCGTAGATACCCAGAGGAACACCAGT
GGCGAAAGCGGCCTCCTGGACCATTTCTGACGCTCATACGCGAAAGCTAGGGTAGCAAAC
GGGATTAGAAACCCTCGTAGTCC
>Otu10171
CCAGCCTACGGGTGGCACCAGTGGAGAATCATCGTCGATGGGCGAAAGCCTGAGCGTGCG
ATGCCACAACATGAAGACGGCCTCTGCTGTAAAGTGTCCAGGGGACGATACAGAAGGAGT
GCGTCCTACGGTAGCCCTGTACAACTTCGGTGCCAGCATACGCGGTCATACCGAGAGGGC
GAGACATGCTTGTGAGGATTAGGTGTAGAGCGTCCGTAGACGGCTTGGCTGATGTCATCT
GTGCATGGCGGGCATAACCTGCCTTGTGGATGAGTGGATTGGCCAGCTTGGATGGTTAAG
AAGAGCACGGGAGACATCAGGGACCGGTGAAATGGGTTGATCTGATGGGGACTTCGTAGG
CGGAAGCAGTGCTCTTGTGGCCAGTGACGTTGAGGGACGAGAGCACGGGGAGCAAAGTGG
ATTAGAACCCTTGAAGTCC
>Otu10173
CCAGCCTATGGGGTGCTGCAGCCGAGAATATTCGACAATGGGCGAAAGCCTGATCGAGCG
ATACCGCGTGGTGGATGAAGTGCTTCGGCACGTAAACATCTTTTATGGAGGAGGAAGTCA
TTGACGTTACTCCATGAATAAGGGGCTCCTAACTCTGTGCCAGCAGGAGCGGTAATACAG
AGGCCCCGAGCGTTACCCGGAATTACTGGGCGTAAAGAGTGCGTAGGCGGTTGCGTTAGT
CGTTCGTTAAATCCCGGGGCTCAACTCCGGACCAGCGAGCGAAACGGCGCGACTTGAGGG
CGCGAGAGGTACAGGGAACTCATGGTGTAGGGGTGAAATCCGTTGATATCATGGGGTACA
CCGAAAGCGAAGGCACTGTACTGGCGCGTTCCTGACGCTGAGGCACGAAAGCGTGGGTAG
CGAACGGGATTAGAAACCCCAGTAGTCC
>Otu10174
CCAGCCTACGGGAGGCAGCAGTGGGGAATTTTGGACAATGGGCGCAAGCCTGATCCAGCC
ATGCCGCGTGTCTGAAGAAGGCCTTCGGGTTGTAAAGGACTTTTGTCTGGGAGCAAAGCC
TAGAGATGAAGAATTACTAGGGCTGAGAGTACCGGAAGAATAAGCACCGGCTAACTACGT
GCCAGCAGCCGCGGTAATACGTAGGGTGCAAGCGTTAATCGGAATGACTGGGCGTAAAGA
GTGCGTAGGCGGATTCGCGAGCTTGATGTGAAATCCCCGGGCTTAACCTGGGAATGGCAT
GGAGGACAGTGAGTCTAGAGTCTGTCAGAGGTGGGTAGAATTCCCCGTGTAGCAGTGAAA

TGCGTAGAGATGGGGAGGAATACCGATGGCGAAGGCAGCCCACTGGGATAAGACTGACGC
TGAGGTACGAAAGCGTGGGGAGCAAACAGGATTAGAAACCCGCGTAGTCC
>Otu10178
CCAGCCTATGGGAGGCACCAGTGAGGAATTTTGGTCAATGGGGGAAACCCTGAACCAGCG
ACGCCGCGTGCAGGATGAAGGAGCTTTGCTCTGTAAACTGCTGTCGGATGGGAAAAAAAG
CGGGCTCTGTCCGCTGGGATTGTACCATCAAAGGAAGGATCGGCTAACTACGTGCCAGCA
GCCGCGGTAATACGTAGGATCCAAGCGTTGTCCGGATTTACTGGGTGTAAAGGGTGCGTA
GGCGGATTCGTGCGTCAGAGGTTAAATCTCCCGGCTTAACCGGGAACCTGCCTTTGATAC
GGCGAGTCTTGAGTGCGAGAGAGGACGGTAGAATTCCTGGTGTAGCAGTGAAATGCGTAG
ATATCAGGAGGAATACCAGTGGCGAAGGCGGCCGTCTGGCTCGCAACTGACGCTCAGGCA
CGAAAGCGTGGGGAGCAAACAGGATTAGAAACCCTCGTAGTCC
>Otu10183
CCAGCCTACGGGATGCTCCAGTAGGGAATATTGGTAATCTGCGAAAGCGGGAACCAGCAA
CGCCGCGTGTGCGATGAAGGCCTTCGGGTCGTAAAGCACTTTTTGAGGGGACGAGGAAGG
ACGGTACCCTCAGAATAAGTCTCGGCTAACTACGTGCCAGCAGCCGCGGTAACACGTAGG
AGACGAGCGTTATCCGGATTTACTGGGCGTAAAGCGCGTGCAGGCGGTTTGGTAAGTTGG
ATGTGAAAGCTCCCGGCTTAACTGGGAGAGGTCGTTCAATACTGCCAGACTTGAGAGCAG
TAGAGGAAGATGGAATTCCCGGTGTAGTGGTGAAATGCGTAGATATCGGGAGGAACACCA
GTGGCGAAGGCGATCTTCTGGACTGTTTCTGACGCTCATATGCGAAAGCTAGGGTAGTAA
ACAGGATTAGATACCCTCGTAGTCC
>Otu10197
CCAGCCTATGGGTGGCTGCAGTCGAGGATCTTCGGCAATGGGCGCAAGCCTGACCGAGCG
ACGCCGCGTGCGGGATGAAGGCCTTCGGGTTGTAAACCGCCGTCGGAGGGGAGGAAATGC
CAGGGGGTTCTCCCCCTGGTTTGACCTATCCTCAGAGGAAGTACGGGCTAAGTTCGTGCC
AGCAGCCGCGGTAAGACGAACCGTACGAACGTTATTCGGAATCACTGGGCTTAAAGGGTG
CGTAGGCGGCCCGGAAAGTTGGGTGTGAAATCCCTCGGCTCAACCGAGGAATTGCGCCCA
AAACTACCGGGCTTGAGGGACACAGAGGTGAGCGGAACTTAGGGTGGAGCGGTGAAATGC
GTTGATATCCTAAGGAACACCAGGAGCGAAAGCGGCTCACTGGACCATAACTGACGCTGA
TGCACGAAAGCTAGGGGAGCAAACAGGATTAGATACCCCTGTAGTCC
>Otu10198
CCAGCCTATGGGTTGCTGCAGCCAAGAATCTTCCGCAATGGGGGAAACCCTGACGGAGCG
ACGCCGCGTGAGGGAAGAAGGTCTTCGGATTGTAAACCTCTTTTCTTGGGGAAGAATAAG
ACTACCAGGAAATGGGTAGTTGATGACGGTACCCGAGGAATAAGCCCCGGCTAATTACGT
GCCAGCAGCCGCGGTAATACGTAAGGGGCGAGCGTTGCTCGGAATTACTGGGCGTAAAGG
GCGTGTAGGCGGCTTGATAAGTCGAATGTAAAAGACCGCGGCTTAACCGCGGGAATGCGT
TCGAAACTGTCAAGCTTGAGTTTGGTGGAGGGTGATGGAATTCCCGGTGTAGCGGTGAAA
TGCGTAGATATCGGGAAGAACACCGAAGGCGAAAGCAGTCATCTATGCCAAAACTGACGC
TGAGGCGCGAAAGTATGGGGATCAAACAGGATTAGAAACCCTAGTAGTCC
>Otu10203
CCAGCCTATGGGTCGCAGCAGTCGAGGATCTTTGGCAATGGGCGCAAGCCTGACCAAGCG
ACGCCGCGTGTGCGATGAAGGCCTTCGGGTTGTAAAGCACTGTCAGGGGGGAACAAATCA
ATGAGTGATCCCCAGAGGAAGCACGGGCTAAGTTCGTGCCAGCAGCCGCGGTAAGACGAA
CCGTGCGAACGTTGTTCGGAATCACTGGGCTTAAAGGGCGCGTAGGTGGTTTGCCAAGTC
GGGGTTGAAATCTTTCGGCTTAACCGGAAAAGTGGCTTCGATACTGGCAAGCTGGAGGGA
GGTAGGGGCGGATGGAACTTCCGGTGGAGCGGTGAAATGTGTTGATATCGGAAGGAACGC
CGGTGGCGAAAGCGATCCGCTGGACCTCTTCTGACACTGAGGCGCGAAAGCCAGGGGAGC
AAACGGGATTAGATACCCCTGTAGTCC
>Otu10208
CCAGCCTACGGGAGGCAGCAGTCGAGAATATTCCACAATGGGCGAAAGCCTGATGGAGCG
ACGCCGCGTGCAGGATGAAGCCCTTCGGGGTGTAAACTGCTTTTATTACAGAAGAAGTTA
TTGACTGTATGTAGTGAATAAGGGGCTGCAAACTTCGTGCCAGCAGCCGCGGTAATACGA
AGGCCCCAAGCGTTATCCGGATTTATTGGGCGTAAAGCGTGCGTAGGAGGTTCGGTAAGT
TCATTGTTAAATTTCGCTGCTTAACGGCGGAGCCGCAATGAATACTGCCAAACTAGAGTG
TGCGAGAGGCTAATAGAACTCACGGTGTAGGGGTGAAATCCGTTGATATCGTGGGGAATA
CCAAAGGCGAAGGCATTTAGCTGGCGCATTACTGACTCTAAGGCACGAAAGCGTGGGGAG
CAAAGAGGATTAGAGACCCTCGTAGTCC
>Otu10210
CCAGCCTACGGGGGGCTGCAGTGGGGAATTTTGCGCAATGGGGGCAACCCTGAACCAGCC

ATGCCGCGTGAAGGATGACAGCCCTACGGGTTGTAAACTTCTTTTGTACGGGAAAAAACC
CCTGGTCGTGAACCAGGGCTGATGGTACCGTAAGAATAAGCATCGGCTAACTCCGTGCCA
GCAGCCGCGGTAGTACGGAGGATGCAAGCGTTATCCGGATTCATTGGGTTTAAAGGGTGC
GCAGGCGGAATGATAAGTCAGTGGTGAAATCCTACAGCTCAACTGTAGAACTGCCATTGA
TACTATCGTTCTTGAGTACATTTGAAGTGGGCGGAATGTGTCATGTAGCGGTGAAATGCT
TAGATATGACACAGAACACCGATCGCGAAGGCAGCTCACTAAGTTGTAACTGACGCTCAT
GCACGAAAGCGTGGGGATCAAACAGGATTAGATACCCGAGTAGTCC
>Otu10211
CCAGCCTATGGGGGGCAGCAGTAGGGAATCTTGCGCAATGGGGGCAACCCTGACGCAGCG
ATGCCGCGTGAGTGAGGAAGGCCTTCGGGTTGTAAAACTCTGTTCTGCGGGAAAAACGGA
CTTCCGGTAAATAATCGGAGGTAGTGATGGTACCGCAGGAGAAAGCACCGGCTAACTTCG
TGCCAGCAGCCGCGGTAATACGAAGGGTGCAAGCGTTGTTCGGAATTATTGGGCGTAAAG
GGCGAGTAGGCGGATGGAGAAGTCAGATGTGAAATCTCGGGGCCTAACCCCGAAACTGCG
TCTGAAACTTTCCATCTAGAATCTCGAAGAGGGAAGGGGAATTTCGCATGTAGGAGTAAA
ATCCGTAGAGATGCGAAGGAACACCAGAGGCGAAGGCGCCTTCCTGGGAGAGTATTGACG
CTGAGGCGCGAAAGCGTGGGGAGCAAACAGGATTAGATACCCCGGTAGTCC
>Otu10212
CCGCCTACGGGAGGCAGCAGTGAGGAATATTGGTCAATGGGCGCAAGCCTGAACCAGCCA
TCCCGCGTGCAGGAAGAAGGCGCTATGCGTCGTAAACTGCTTTTCCGGAGGAAGAAAACC
CGCTACGAGTAGCGGTTTGCCGGTACTCTGGGAATAAGCATCGGCTAACTCCGTGCCAGC
AGCCGCGGTAATACGGAGGATGCAAGCGTTATCCGGATTCATTGGGTTTAAAGGGTGCGC
AGGCGGAATGATAAGTCAGTGGTGAAATCCTACAGCTTAACTGTAGAACTGCCATTGATA
CTGTCGTTCTTGAGTACAGTTGGAGTGGGCGGAATGTGTCATGTAGCGGTGAAATGCTTA
GATATGACACAGAACACCGATTGCGAAGGCAGCTGACGAAACTGTAACTGACGCTGAGGC
ACGAAAGCGTGGGGATCAAACAGGATTAGATACCCTCGTAGTCC
>Otu10218
CCAGCCTATGGGGGGCACCAGTCGAGAATTTTTCACAATGGGCGAAAGCCCGATGGAGCG
ACGCCGCGTGGGGGATGAATGGCTTCGGCCCGTAAACCCCTGTCATTCGGGAGCAATGCG
TCTGGGTGAATAGCTCAGACGTTGATAGTACCGGAAGAGGAAGGGACGGCTAACTCTGTG
CCAGCAGCCGCGGTAATACAGAGGTCCCAAGCGTTGTTCGGATTTACTGGGCGTAAAGGG
TGCGTAGGTGGTTGGGTAAGTCTGATGTGAAATCTCCGAGCTCAACTCGGAAATGGCATT
GGAAACTGCTCTGCTCGAGGGTTGGAGGGGGGACTGGAATACTTGGTGTAGCAGTGAAAT
GCGTAGATATCAAGTGGAACACCAGTGGCGAAGGCGAGTCCCTGGACAACTCCTGACACT
GAGGCACGAAAGCTAGGGGAGCAAACAGGATTAGATACCCCTGTAGTCC
>Otu10229
TCAGCCTATGGGTGGCACCAGTAGGGAATATTGGTAATCTGCGAAAGCGGGAACCAGCAA
CGCCGCGTGTGCGATGAAGGCCTTCGGGTCGTAAAGCACTTTTTAGAGGGATGAGGAAGG
ACAGTACCTTTAGAATAAGCATCGGCTAACTTCGTGCCAGCAGCCGCGGTAATACGAAGG
ATGCAAGCGTTATCCGGATTTATTGGGTTTAAAGGGTGCGTAGGCGGACTTATAAGTCAG
TGGTGAAATCTCGTTGCTTAACAACGAACGTGCCATTGATACTGTAGGTCTTGAGTACAG
ATGCCGTTGGCGGAATGTGTCATGTAGCGGTGAAATGCATAGATATGACACAGAACACCG
ATTGCGAAGGCAGCTGACGAAACTGTAACTGACGCTGAGGCACGAAAGCGTGGGGATCAA
ACAGGATTAGAGACCCCAGTAGTCC
>Otu10236
CCAGCCTACGGGAGGCACCAGCTAAGAATATTCCGCAATGGACGAAAGTCTGACGGAGCG
ACGCCGCGTGGATGACGAAGGCCGAAAGGTTGTAAAGTCCTTTTGTTGGGGAAGAATAAC
CATGGGAGGGAATGCCCGTGGGATGACATGAACCGACGAATAAGCCCCGGCTAACTACGT
GCCAGCAGCCGCGGTAACACGTGGGGGGCGAGCGTTGTTCGGAATTATTGGGCGTAAAGC
GCGTGTAGGCGGCTTAGCAAGTCAGGTGTGAAAGCCCTCGGCTTAACCGAGGAAGTGCGC
CTGAAACTATTGAGCTTGAGTACCGGAGAGGGCGGCGGAATTCCCGGTGTAGAGGTGAAA
TTCGTAGATATCGGGAGGAACACCAGCGGCGAAGGCGGCCGCCTGGACGGTCACTGACGC
TGAGACGCGAAAGCGTGGGTAGCAAACAGGATTAGAAACCCCAGTAGTCC
>Otu10252
CCAGCCTACGGGAGGCTCCAGTGGGGAATATTGGACAATGGGCGAAAGCCTGATCCAGCG
ACGCCGCGTGAGTGATGAAGGCCTTCGGGTCGTAAAGCTCTGTCGGCGGGAACGAACAAG
GGTGACCTAACACGTTATCCCCTGACGGTACTCGCAAAGGAAGCACCGGCTAACTCTGTG
CCAGCAGCCGCGGTAAGACAGAGGGTGCAAGCGTTGTTCGGAATTACTGGGCGTAAAGGG
AGTGTAGGCGGCGGAGTGTGTCCGGCGTGAAAGACCTCGGCTCAACCGGGGAAGTGCGTT

GGATCCTACTTTGCTAGAGTGTGAGAGAGGGTGGCGGAATTGTCAGTGTAGAGGTGAAAT
TCGTAGATACCGGCAGGAACACCGGAGGCGAAGGCGGCCACCTGGATCACAACTGACGCT
GAAACTCGAAAGCGTGGGTAGCAAACAGGATTAGATACCCTCGTAGTCC
>Otu10254
CCAGCCTACGGGGCGCTCCAGTGAGGAATATTGCGCAATGGGCGAAAGCCTGATGGAGCG
ACGCCGCGTGGGGGATGAATGGCTTCGGCCCGTAAACCCCTGTCATTCGGGATCAATGCC
TGTTTCCTAACACGAAGCAGGTTGATAGTACCGGAAGAGGAAGGGACGGCTAACTCTGTG
CCAGCAGCCGCGGTAATACAGAGGTCCCAAGCGTTGTTCGGATTTACTGGGCGTAAAGGG
TGCGTAGGCGGTTGGGTAAGTCTGATGTGAAATCTCTGGGCTCAACCCAGAAATGGCATT
GGATACTATCCGGCTTGAGAGTTGGAGGGGGGACTGGAATACTTGGTGTAGCAGTGAAAT
GCGTAGATATCAAGTGGAACACCAGTGGCGAAGGCGAGTCCCTGGACAACTTCTGACGCT
GAGGCACGAAAGCTAGGGGAGCAAACAGGATTAGAAACCCTGGTAGTCC
>Otu10255
CCAGCCTTGGGATGCTCCAGTGGGGAATCTTGCACAATGGGGGCAACCCTGATGCAGCGA
CGCCGCGTGAACGATGAAGCCCTTCGGGGTGTAAAGTTCTTTCGGCAGGGAAGATAGTGA
CGGTACCTGCAGAAGCAGCTGCGGCTAACTACGTGCCAGCAGCCGCGGTAATACGTAGGC
AGCGAGCGTTGTTCGGAGTTACTGGGCGTAAAGGGTGTGTAGGCGGTGCTTTAAGTTTGG
TGTGAAATCTCCCGGCTCAACTGGGAGGGTGCGCCGAATACTGAAGTGCTAGAGTATGGG
AGAGGGAAGTGGAATTCCTGGTGTAGCGGTGAAATGCGTAGATATCAGGAGGAACACCTG
TGGTGTAGACGGCTTCCTAGACCATAACTGACGCTGAGACACGAAAGCGTGGGTAGCAAA
CAGGATTAGAAACCCCCGTAGTCC
>Otu10260
CCAGCCTATGGGGGGCTGCAGTAAGGAATATTGGACAATGGTGGCAACACTGATCCAGCC
ATGCCGCGTGCAGGATGAAGGCGCTACGCGTTGTAAACTGCTTTTGTACGAGAGAAAACC
TATCTACGTGTAGATAGCTGATAGTATGGTAAGAATAAGCATCGGCTAACTTCGTGCCAG
CAGCCGCGGTAATACGAAGGATGCAAGCGTTATCCGGATTTATTGGGCGTAAAGCGCGTG
TAGGCGGTTTGGTAAGTTGGATGTGAAAGCTCCTGGCTTAACTGGGAGAGGTCGTTCAAT
ACTGCCAGACTAGAGGATGGTAGAGGGAGGTGGAATTCTGGGTGTAGTGGTGAAATGCGT
AGATATTCGGAGGAACACCAGTGGCGAAAGCGGCCTCCTGGACCATTTCTGACGCTCATA
CACGAAAGCTAGGGGAGCAAACGGGATTAGAAACCCGTGTAGTCC
>Otu10263
CCAGCCTACGGGTCGCAGCAGTAAGGAATATTGGACAATGGGGGCAACCCTGATCCAGCC
ATGCCGCGTGAAGGATGAAGGCGCTATGCGTCGTAAACTTCTTTTAACCAGGAAAAATTC
TTATGTTTCTACATGAGCTGATTGTACCGGGAGAATAAGGATCGGCTAACTTCGTGCCAG
CAGCCGCGGTAATACGAAGGATCCAAGCGTTATCCGGATTTATTGGGTTTAAAGGGTGCG
TAGGCGGGTTGTTAAGTCAGTGGTGAAATCTTGCAGCTTAACTGTAAAATTGCCATTGAT
ACTGACGATCTTGAGTACGTTTGACGTGGGCGGAATATGTCATGTAGCGGTGAAATGCTT
AGAGATGACATAGAACACCGATTGCGAAGGCAGCTCACGAAACCGTAACTGACGCTGAGG
CACGAAAGCGTGGGGATCAAACAGGATTAGAAACCCTGGTAGTCC
>Otu10264
CCAGCCTATGGGTGGCAGCAGTGAGGAATTTTCGTCAATGGGGGCAACCCTGAACGAGCA
ACGCCGCGTGCAGGAAGAAGGTTTTCGGATCGTAAACTGCTTTTCTCTGGGACGAGAACG
GACGGTACCAGAGGAATCAGCCTCGGCTAACTACGTGCCAGCAGCCGCGGTAATACGTAG
GAGGCAAGCGTTGTCCGGATTTATTGGGCGTAAAGCGCACGTAGGCGGTCGTATCAGTCC
AGGGTGACAGTTTCCGGCTTAACCGGAAAAGGACTCTGGAGACGGCACGACTTGAGGGCT
TCAGAGGGATGTGGAATTCCAGGTGGAGTGGTGAAATGCGTAGAGATCTGGAAGAACACC
AAAGGCGAAGGCAACATCCTGGGGAGTCACTGACGCTCGGGTGCGAAAGCTAGGGGAGCG
AACGGGATTAGATACCCCCGTAGTCC
>Otu10272
CCAGCCTATGGGTCGCTCCAGCAGGGAATATTGGTAATCTGCGAAAGCGGGAACCAGCAA
CGCCGCGTGTGCGATGAAGGTCTTCGGATCGTAAAGTGCTTTTCTGAGAGATGAGAAAGG
ACAGTATCTCAGGAATAAGTCTCGGCTAACTACGTGCCAGCAGCCGCGGTAACACGTAGG
AGGCAAGCGTTATCCGGATTTACTGGGCGTAAAGCGCATGCAGGTGGTTCGGTAAGTTGG
ATGTGAAAGCTCCTGGCTCAACTGGGAGAGGTCGTTCAATACTACCGGACTTGAGAGCAG
TAGAGGGAGGTGGAGTTCCCGGTGTAGTGGTGAAATGCGTAGATATCGGGAGGAACACCA
GTGGCGAAAGCGGCCTCCTGGACTGTTTCTGACACTCATATGCGAAAGCTAGGGTAGCAA
ACGGGATTAGATACCCCTGTGTCC
>Otu10274

CCAGCCTATGGGGGGCACCAGTAAGGAATATTGGACAATGGTGGCAACACTGATCCAGCC
ATGCCGCGTGCAGGATGAAGGCGCTACGCGTTGTAAACTGCTTTTGTACGGGAGAAAACC
TATCTACGTGTAGATAGCTGATAGTACCGTAAGAATAAGCATCGGCTAACTTCGTGCCAG
CAGCCGCGGTAATACGAAGGATGCAAGCGTTATCCGGATTTATTGGGTTTAAAGGGTGCG
TAGGCGGACTTATAAGTCAGTGGCGAAATCTCGTTGCTTAACAACGAACGTGCCATTGAT
ACTGTAGGTCTTGAGTACAGATGCCGTTGGCGGAATGTGTCATGTAGCGGTGAAATGCAT
AGATATCGGAAGGAACACCTGTGGCGAAAGCGGCTCACTGGACCATAACTGACGCTGATG
CACGAAAGCTAGGGGGGCAAACAGGATTAGAAACCCCAGTAGTCC
>Otu10275
CCAGCCTATGGGATGCAGCAGTGGGGAATTTTGGACAATGGGCGAAAGCCTGATCCAGCG
ACGCCGCGTGGAGGATGAAGGCCTTCGGGTTGTAAACTCCTTTTAGAGGGAACGAAAAAA
ATGACGGTACCCTCAGAAAAAGCCACGGCTAACTACGTGCCAGCAGCCGCGGTAATACGT
AGGTGGCGAGCGTTACTCGGATTTACTAGGCGTAAAGCGCTTGTAGGTGGTCATATAAGT
CTGTAGTGAAATCTCCCGGCTCAACTGGGAGGGGTCTACAGAAACTATATGGCTTGAGTG
TGGCAGGGGGAAACGGAATTCCCGGTGTAGCGGTGAAATGCGTAGATATCGGGAGGAACA
CCAATGGCGAAAGCAGTTTCCTGGGCCAATACTGACACTGAGGAGCGAAAGCTAGGGGAG
CAAACAGGATTAGAGACCCTTGTAGTCC
>Otu10283
CCAGCCTATGGGGGGCACCAGTGGGGAATATTGGACAATGGGCGCAAGCCTGATCCAGCC
ATGCCGCGTGAGTGAAGAAGGCCTTAGGGTTGTAAAGCTCTTTTGGCGGGGACGATAATG
ACGGTACCCGCAGAATAAGCCCCGGCTAACTTCGTGCCAGCAGCCGCGGTAATACGAAGG
GGGCTAGCGTTGTTCGGAATCACTGGGCGTAAAGCGTACGCAGGCGGTTGGGTAAGTCTG
ACGTGAAATCTCCGGGCCTAACCCGGAAACTGCGTTGGATACTATCCGGCTAGAGGAATG
GAGGGGAGACTGGAATACTTGGTGTAGCAGTGAAATGCGTAGATATCAAGTGGAACACCA
GTGGCGAAGGCGAGTCTCTGGACATTTCCTGACGCTGAGGCACGAAAGCCAGGGGAGCAA
ACAGGATTAGAGACCCCTGTAGTCC
>Otu10299
CCAGCCTATGGGATGCTGCAGTGGGGAATATTGGACAATGGGGGCAACCCTGATCCAGCA
ATGCCGCGTGTGTGAAGAAGGCCTGAGGGTTGTAAAGCACTTTCAGTGGGGAGGAGGGTT
GTTTGGTTAAGAGCTGAATAATTGGACGTTACCCACAGAAGAAGCACCGGCTAACTCCGT
GCCAGCAGCCGCGGTAATACGGAGGGTGCGAGCGTTAATCGGAATTACTGGGCGTAAAGG
GTGCGTAGGTGGTTGGATAAGTTATCTGTGAAATCCCCGGGCTCAACCTGGGAAGGTCAG
ATAAGACTGTTTGACTCGAGTATAGGAGAGGGTAGTGGAATTTCCGGTGTAGCGGTGAAA
TGCGTAGAGATCGGAAGGAACACCGGTGGCGAAGGCGGCTTCCTGGCATGTAACTGACGC
TCATGTTCGAAAGCGTGGGTAGCAAACAGGATTAGAAACCCCTGTAGTCC
>Otu10326
CCAGCCTACGGGGTGCTGCAGTGAGGAATCTTCCACAATGGGCGAAAGCCTGATGGAGCA
ACGCCGCGTGCAGGACGAAGGCCCTCGGGTCGTAAACTGCTTTTATAAGTGAAGAATATG
ACGGTAGCTTATGAATAAGCACCGGCTAACTACGTGCCAGCAGCCGCGGTCATACGTAGG
GTGCAAGCATTATCCGGAGTGACTGGGCGTAAAGAGTTGCGTAGGTGGTTTGTTAAGTGA
ATGGTGAAATCTGGTGGCTCAACCACACAGACTATTATTCAAACTGGCAAACTCGAGAGT
AGCAGAGGTAACTGGAATTTCTAGTGTAGGAGTGAAATCCGTAGATATTAGAAGGAACAC
CAATGGCGTAGGCAGGTTACTAGGCTATTTCTGACACTGAGGCACGAAAGCGTGGGGAGC
GAACCGGATTAGAAACCCCTGTAGTCC
>Otu10334
CCAGCCTATGGGGCGCACCAGTGGGGAATATTGGACAATGGGGGAAACCCTGATCCAGCC
ATGCCGCGTGAGTGAAGAAGGCCTTAGGGTTGTAAAGCACTTTCGACGGGGACGATGATG
ACGGTACCCGTAGAAGAAGCCCCGGCTAACTTCGTGTCAGCAGCCGCGGTAATACGAAGG
GGGCTAGCGTTGCTCGGAATGACTGGGCGTAAGGGGCGCGTAGGCGGTTTGGTGAGTTAG
GCGTGAAATTCCTGGGCTTAACCTGGGGACTGCGTTTAATACCGCCAGACTTGAGTGAGG
AAGAGGGTTGTGGAATTCCCAGTGTAGAGGTGAAATTCGTAGATATTGGGAAGAACACCG
GTGGCGAAGGCGGCAACCTGGTCCTTGACTGACGCTGAGGCGCGAAAGCGTGGGGAGCAA
ACAGGATTAGAAACCCGCGTAGTCC
>Otu10336
CCAGCCTACGGGGGGCACCAGTGAGGAATTTTGGGCAATGGGCGAAAGCCTGACCCAGCG
ACGCCGCGTGGGTGATGAAGGCCTTCGGGTCGTAAAACCCTGTGAGATGGGAAGAAGCCG
TTTCGTGCTAATACCACGGCGCGGTGACAGTACCATCAGAGGAAGCCCCGGCTAACTACG
TGCCAGCAGCCGCGGTAAAACGTAGGGGGCTAGCGTTGTTCGGAATTACTGGGCGTAAAG

CGCGTGTAGGCGGCTTGGCAAGTCGGAAGTGAAAGCCCCAGGCTCAACTTGGGAATTGCT
TTCGAAACTGCCTAGCTTGAGTGCGGGAGGGGAGAGCGGAATTCCAGGTGTAGCGGTGAA
ATGCGTAGATATCTGGAGGAACACCGGTGGCGAAGGCGGCTCTCTGGACCGTAACTGACG
CTGAGACGCGAAAGCGTGGGGAGCAAACAGGATTAGAAACCCTTGTAGTCC
>Otu10350
CCAGCCTACGGGGTGCACCAGTGGGGAATCTTGCACAATGGGGGAAACCCTGATGCAGCG
ACGCCGCGTGAGCGATGGAGCCCTTCGGGGTGTAAAGCTCTTTCGGCAGGGAAGATTATG
ACGGTACCAGTGGAAGAAGCACCGGCTAACTCTGTGCCAGCAGCCGCGGTAATACAGAGG
GTGCGAGCGTTGTTCGGAATTATTGGGCGTAAAGGGCGCGTAGGCGGTGTTGTAAGTCAC
CTGTGAAACCTCTGGGCTTAACTCAGAGCCTGCAGGTGAAACTGCAATGCTGGAGGGTGG
GAGAGGTGCGTGGAATTCCCGGTGTAGCGGTGAAATGCGTAGATATCGGGAGGAACACCT
GTGGCGAAAGCGGCGCACTGGACCACTACTGACGCTGAGGCGCGAAAGCCGGGGGAGCAA
ACAGGATTAGATACCCGTGTAGTCC
>Otu10355
CCAGCCTACGGGTCGCACCAGTCGAGAATCATTCGCAATGGGCGAAAGCCTGACGATGCG
ACGCCGTGTGAGCGATGAAGGCCTTAGGGTTGTAAAGCTCTCTCGCCTGGGAACAAGAGG
ATAGGCATAAAACGCTTAGAATTTGAGGGTACCAGGTAAAGAAGCACCGGCTAACTCCGT
GCCAGCAGCTGCGGTAATACGGAGGGTGCAAGCATTAATCGGATTTATTGGGCGTAAAGG
GCGTGTAGGCGGGGATAAAAGTCAGATGTGAAATCCCGGGGCTCAACCTCGGAACAGCAT
TTGAAACTCTATTTCTTGAGGGTAGGAGGAGAAAACGGAACTCCACATGTAGCGGTGAAA
TGCGTAGATATGCGGAAGAACACCGGTGGCGAAGGCGGTTTTCTATCTTACTCCTGACGC
TGAGGCGCGAAAGCAAGGGGAGCAAACAGGATTAGATACCCTTGTAGTCC
>Otu10357
CCAGCCTACGGGTCGCAGCAGCTAGGAATATTCCGCAATGGACGAAAGTCTGACGGAGCG
ACGCCGCGTGGACGGTGAAGGCCTTCGGGTTGTAAAGTCCTTTCGTGCGGGAAGATTTTT
TCCGGTGTGAACAATGCCGGAGATCGACTGTACCGCAGGAAGAAGCCCCGGCTAATTACG
TGCCTGCAGCCGCGGTATTACGTATGGGGCGAGCGTTATTCGGATTCATTGGGCGTAAAG
CGTATGTCGGGGGTCTGAAAAGTCGGAGGTGAAATTTTCCTGCTTAACATGAAAATTGCT
TCCGAAACTGCCAGACTGGAGTTTTGGAGAGGAGATTGGAATTCCAGGTGTAGGGGTGAA
ATCTGCAGATATCTGGAAGAACACCAGTGGCGAAGGCGAATCTCTGGCCAAAAACTGACC
CTTAGATACGAAAGCCAGGGGAGCAAACGGGATTAGATACCCCGGTAGTCC
>Otu10359
GGACTACGGGGGTTTCTAATCCTGTTTGCTCCCCTAGCTTTCGTTCCTCAGCGTCAGTAG
TGATCCAGCTCGCCGCTTTCGCCACGGGTGTTCCTGCAGATATCTACGCATTTCACCGCT
ACACCTGCAATTCCACGAGCCTCTCTCACACTCAAGCACAGCAGTTTTGGAAGCAGCCTC
TGGGTTAAGCCCAGAGATTTCACTCCCAACTTGCCGCACCGCCTACGAACCCTTTACACC
CAGTAATTCCGAACAACGCTGGCCCCCTACGTCTTACCGCGGCTGCTGGCACGTAGTTAG
CCGGGGCTTCTTATACTGGTACCGTCAATCCCTTGCGGGTTTTCGTCCCAGTCGAAAGGG
GTTTACACTCCGAAAAGCTTCATCCCCCACGCGGCGTCGCTGCATCAGGCTTTCGCCCAT
TGTGCAAGATTCCCCACTGCTGCCCCATAGGCTGG
>Otu10361
CCAGCCTATGGGTTGCTGCAGTTCGGAATATTCGGCAATGGGCGCAAGCCTGACCGAGCG
ACGCCGCGTGCGGGATGAAGGCCCTCTGGGTTGTAAACCGCTGTCACAGGTGAGCAAATC
AGCCGGTTCCAACAAAGCCGGCTGTTGAGTTAAGCTTGAGAGGAAGCCCCCGCTAATCAC
GTGCCAGCAGCGGCGGTAATACGTGAGGGGCGAACGTTGTTCGGTGTCACTGGGCTTAAA
GGGCACGTAGGTGGCTGCGTAAGTAAGGTGTGAAAGCCCTCGGCTTACCCGGGGAATCGC
GCCTTATACTGCGTGGCTTGAGGGTCACAGGGGAAGGGGGAACTCCAGGTGTAGCGGTGA
AATGCGCAGATATCTGGGGGAAGGCCGGCGGCGAAGGCGACCTTCTGGGTGATTCCTGAC
ACTGAGGCGCGAAAGCGTGGGGAGCAAACAGGATTAGATACCCGTGTAGTCC
>Otu10368
CCAGCCTATGGGGGGCAGCAGGCATTGAGCCGGAGGAAGCAAGAGAAATGTATAGCCGGT
TTTTAATAACAAGGATTGAAAATGCTGATATATTTTTAAACGAAGCAAAACATGCCAGGA
AATCACTTATTTGAGTATGCCGTTATACGTGTAATGCCAAAAGTGGAATGTGAAGAGTTT
CTCAATGTGGGTGTGATCCTGTATTGTAAGGATAAAAAGTTTTTGCAGGCTGTTTTTAAA
CTGAATGAGGAACGGCTCCGTAATTTTTCACAACAGATCGATATTGAAGAGTTAAAAGAA
CACCTGTGTTCTTTTAAACAGATATGCCGTGGGGGTGCCGACGCAGGACCTATCGGTAAA
CTCGATATTGCTTCCCGTTTCCGATGGCTCACAGGTACCCGTAGTACCATTGTTCAAACA
TCGAAAGTGCACCCTGGTTTGTGCAACGATCCATTAGATACCCCTGTAGTCC

>Otu10371
CCAGCCTACGGGATGCAGCAGTGGGGAATATTGCACAATGGGCGAAAGCCTGATGCAGCG
ACGCCGCGTGGAGGATGAAGGTTTTCGGATTGTAAACTCCTGTTAAGTGGGAAGAAAGAC
CGTTTTCTAATAAAAAACGGGGATGACGGTACCACTAGAGAAAGCACCGGCAAACTTCGT
GCCAGCAGCCGCGGTAATACGAAGGGTGCGAGCGTTATTCGGAATAACTGGGCGTAGAGA
GTTTGAAGGCGGCTTTTTAAGTCAGCTGTTAAATCCCCCGGCTTAACCGGGGATATGCAG
TTGATACTGGAGAGCTAGAGTTTGGGAGAGAAGGGTAGAATTCTCGGAGTAGCGGTAAAA
TGCGTAGATCTCGAGAGGAATACCGGTTGCGAAGGCGGCCCTTTAGCCCAATACTGACGC
TCAAGAACGAAAGCGTGGGGAGCAAACAGGATTAGATACCCTCGTAGTCC
>Otu10376
CCAGCCTATGGGATGCACCAGTTAGGAATCTTGCGCAATGGGCGAAAGCCTGACGCAGCA
ACGCCGCGTGAGGGATGAATGCTTTCGGGTTGTAAACCTCTTTCAGCAGGAACGAAAATG
ACGGTACCTGCAGAAGAAGCTCCGGCCAACTACGTGCCAGCAGCCGCGGTAATACGTAGG
GAGCAAGCGTTGTCCGGATTTATTGGGCGTAAAGAGCTCGTAGGCGGTTTAGAAAGTCAG
GTGTGAAACCTCCAGGCTCAACCTGGAGACGCCACTTGATACTTCTATGACTTGAGTTCG
GTAGGGGACCATGGAATTCCTGGTGTAGCGGTGAAATGCGCAGATATCAGGAGGAACACC
GATGGCGAAGGCAGTGGTCTGGGCCGATACTGACGCTGAGGAGCGAAAGCGTGGGGAGCG
AACAGGATTAGAAACCCCAGTAGTCC
>Otu10378
CCAGCCTACGGGGCGCAGCAGTGGGGAATATTGGACAATGGGGGGAACCCTGATCCAGCA
ATGCCGCGTGTGTGAAGAAGGCCTGAGGGTTGTAAAGCACTTTCAGTGGGGAGGAGGCGT
GACAGGTTAAGAGCTAGTTAAGCGGACGTTACCCACAGAAGAAGCACCGGCTAACTCCGT
GCCAGCAGCCGCGGTAATACGGAGGGTGCGAGCGTTAATCGGAATTACTGGGCGTAAAGG
GTGCGTAGGTGGTTTATTAAGTTATCTGTGAAAGCCCCGGGCTTAACCTGGGAAAGTCAG
ATAAGACTGATAGACTTGAGTATGGGAGAGGGTAGTGGAATTTCCGGTGTAGCGGTGAAA
TGCGTAGAGATCGGAAGGAACACCAGTGGCGAAGGCGGCTACCTGGCCTAATACTGACAC
TGAGGCACGAAAGCGTGGGGAGCAAACAGGATTAGATACCCTAGTAGTCC
>Otu10382
CCAGCCTACGGGTTGCTCCAGTAGGGAATCTTCCGCAATGGACGAAAGTCTGACGGAGCA
ACGCCGCGTGAGTGAGGAAGGCCTTCGGGTCGTAAAGCTCTGTTGCCAGGGAAGAACGGG
TGGAAGAGTAACTGCTTCCGCCATGACGGTACCTGAGAAGAAAGCCCCGGCTAACTACGT
GCCAGCAGCCGCGGTAATACGTAGGGGGCAAGCGTTGTCCGGAATTATTGGGCGTAAAGC
GCGCGCAGGCGGCTTTGTAAGTCCGGTGTTTAATCTTGGGGCTCAACCCCAAGTCGCACG
GGAAACTGCAAGGCTTGAGTGCAGAAGAGGAAAGTGGAATTCCACGTGTAGCGGTGAAAT
GCGTAGAGATGTGGAGGAACACCAGTGGCGAAGGCGACTTTCTGGGCTGTAACTGACGCT
GAGGCGCGAAAGCGTGGGGAGCAAACAGGATTAGATACCCTAGTAGTCC
>Otu10386
CCAGCCTATGGGGGGCTGCAGTCGAGAATCTTTCGCAATGGGCGAAAGCCTGACGAAGCG
ACGCCGTGTGAGTGATGAAGGCCTTCGGGTCGTAAAGCTCTTTCGCTTGGGAACAAGAGA
GGCCGGCTAATATCCGGCCAATTTGAGGGTACTAGGTAAAGAAGCACCGGCTAACTCCGT
GCCAGCAGCTGCGGTAATACGGAGGGTGCGAGCATTAATCGGATTTATTGGGCGTAAAGG
GCGCGTAGGCGGGAATGTAAGTCAGATGTGAAATCCCGGGGCTCAACCCCGGAACAGCAT
TTGAAACTGCATTTCTCAGAGGGTAGACGGAGAAAATGGAATTCCACAAGTAGCGGTGAA
ATGCGTAGATATGTGGAAGAACACCAGTGGCGAAGGCGATTTTCTAGTTTATACCTGACG
CTAAAGCGCGAAAGCTAGGGGAGCAAACAGGATTAGAAACCCCCGTAGTCC
>Otu10395
CCAGCCTACGGGGTGCTCCAGTTAGGGAACTTTCACACTGGGCGCAAGCCTGATGGAGTA
ACGCCACGTGGGCGATGAAGGTCTTCGGATCGTAAAGCCCTTTTCTGGGGGACGAGGAGA
AGACGGTACCCCAGGAAGAAGTCTCGGCTAACTACGTGCCAGCAGCCGCGGTAATACGTA
GGAGGCGAGCGTTGTCCGGAATTACTGGGCGTAAAGAGCGTGTAGGCGGTCCGTCATGTC
GCCCTTGAAAGCCCTCTGCTCAACGGGGGGAGGGAGGGCGAAACGGGCGGACTCGATGGA
GTCTGGTAGGGGGGAGGCGGAACGGCCGGTGGAGCGGTGAAATGCGTAGAGATCGGCCAGA
ACACCAGTGGCGAAGGCGGCCTCCTGGGCCAGAACTGACGCTGAGACGCGACAGCGTGGG
GAGCGAACGGAATTAGAGACCCGCGTAGTCC
>Otu10398
CCAGCCTACGGGAGGCAGCAGTCGAGAATTTTTCTTAATGGGGGCAACCCTGAAGGAGCG
ACGCCGCGTGGAGGATGAAGGTCTTCGGATTGTAAACTCCTGTCATTGGTGAACAAGGCC
TTCCGGTTAACTGCCGGTGAGGTTCGATAGTAGCCGAAGAGGAAGGGACGGCTAACTCTG

TGCCAGCAGCCGCGGTAATACAGAGGTCCCAAGCGTTGTTCGGATTCATTGGGCGTAAAG
GGTGCGTAGGCGGCGGGGTCAGTCTGATGTGAAAGGCCGGGGCTCAACCCCGGAACGGCA
TTGGATACTGCCTTGCTAGAGAACTGGAGAGGAGACTGGAATTTACGGTGTAGCAGTGAA
ATGCGTAGAGATCGTAAGGAAGACCAGTGGCGAAGGCGAGTCTCTGGACAGATTCTGACG
CTGAGGCACGAAGGCCAGGGGAGCAAACGGGATTAGATACCCCTGTAGTCCC
>Otu10408
CCAGCCTACGGGTTGCAGCAGTGGGGAGTATTGCGCAATGGGCGAAAGCCTAACGCAGCG
ACGCCGCGTGAAGGATGAAGGTCTTCGGATTGTAAACTTCTGTTAAGTGGGAAAAAAGCA
TCATGCTTAATACGTATGGTGGATGATGGTACCACTAGAGAAAGCACCGGCTAATCTCGT
GCCAGCAGCCGCGGTAATACGAGAGGTGCAAGCGTTATTCGGAATTATTGGGCGTAAAGG
GTGCGTAGACGGTGTGCTTAGTCTGTTGTTAAATCTTTCGGCTTAACCGAAAATCAGCAA
TGGATACTGGCATGCTAGAGTGTGAAAGAGAGAAGTGGAATTCTTGGAGTAGCGGTAAAA
TGCGTAGATCTCAAGAGGAACACCGATGGCGAAGGCAGCTTCTTGGTTCACTACTGACGT
TGAGGCACGAAAGCGTGGGGAGCAAACAGGATTAGAGACCCCAGTAGTCC
>Otu10414
CCAGCCTATGGGTGGCAGCAGTGGGGAATATTGGACAATGGGGGGAACCCTGATCCAGCA
ATGCCGCGTGTGTGAAGAAGGCCTGGGGGTTGTAAAGCACTTTCAGTGGGGAGGAGGATT
GGCAGGTTAAGAGCTGGCTGATTGGACGTTTCCCACAGAAGAAGCACCGGCTAACTCCGT
GCCAGCAGCCGCGGTAATACGGAGGGTGCAAGCGTTAATCGGAATTACTGGGCGTAAAGG
GTGCGCAGGTGGTTTGATAAGATATCTGTGAAATCCCCGGGCTCAACCTGGGAAGGTCAG
ATGTGACTGTCAGACTCGAGTATGGGAGAGGGTAGTGGAATTTCCGGTGTAGCGGTGAAA
TGCGTAGAGATCGGAAGGAACACCAGTGGCGAAGGCGGCTACCTGGCCTGATACTGACAC
TGAGGCACGAAAGCGTGGGGAGCAAACAGGATTAGATACCCTCGTAGTCC
>Otu10421
CCAGCCTATGGGAGGCACCAGTCAAGAACATTCGACAATGGGCGAAAGCCTGATCGAGCG
ACACCGCGTGCAGGATGAAGGCCTTCGGGTCGTAAACTGCGGTAGTATGGTAACAATGCA
AATGAGTGCCATACGGAAAGAGGTGGGTAACTACGTGCCAGCACCAGCGGTAATACGTAG
ACCTCAAGCGTTATCCGGATTTATTGGGCGTAAAGAGCATGTAGGAGGTTTTGCACGTCT
GTGGTTAAAGCCCATCGCCTAACGATGGAATCGCCACGGATACGGCAAGACTAGAGGGGG
TTAGAGGTGCATAGAACGCACGGTGTAGGGGTGAAATCCGTTGATATCGTGCGGAATACC
AAAGGCGAAGGCAGTGCACTGGGACCTTCCTGACTCTGAGATGCGAAAGCGTGGGGAGCA
AAAAGGATTAGAAACCCGCGTAGTCC
>Otu10443
CCAGCCTATGGGGCGCTGCAGTGGGGAATCTTGCACAATGGGCGAAAGCCTGATGCAGCA
ATGCCGCGTGAGTGATGAAGGCCTTCGGGTTGTAAATCTCTGTCGGCCGGGACAAATGTG
TTCTTATTTAATAAATAAGGGCAGCGATGGTACCGGTAAAGGAAGAGGCGGCTAATTCTG
TGCCAGCAGCCGCGGTAATACAGAAGCCTCAAGCGTTGTTCGGAATTACTGGGCGTAAAG
GGAATGTAGGCGGTAGCGTAAGTCAGATGTGAAAGCCCGGGGCTTAACCCTGGAAGTGCA
TTTGATACTGCGTTGCTAGAGTGCGAAAGAGGAAAGTGGAATTCCATGTGTAGGGGTGAA
ATCCGTAGATATGTGGAGGAATACCAATGGCGAAAGCAGCTTTCTGGTTCGTAACTGACG
CTGAGATTCGAAAGCGTGGGTAGCAAACAGGATTAGAAACCCTAGTAGTCC
>Otu10445
CCAGCCTATGGGAGGCACCAGTGAGGAATATTGGTCAATGGGCGCAAGCCTGAACCAGCC
ATCCCGCGTGCAGGATGAAGGCCCTATGGGTCGTAAACTGCTTTTATACACCAAGAAAAA
CATCCACGTGTGGGTGCCCGACGGTAGTGTATGAATAAGCATCGACTAACTCCGTGCCAG
CAGCCGCGGTAATACGGAGGATGCAAGCGTTATCCGGATTCATTGGGTTTAAAGGGTGCG
TAGGCGGCTTTTTAAGTCAGTGGTGAAATCTTGCCGCTTAACGGTAAAATTGCCATTGAT
ACTGAAGAGCTTGAGTACAGTTGAGGTAAGCGGAATGTGTAGTGTAGCGGTGAAATGCTT
AGATATTACACAGAACACCGATTGCGAAGGCAGCTTACTAAGCTGTTACTGACGCTGATG
CACGAAAGCGTGGGGAGCGAACAGGATTAGATACCCCAGTAGTCC
>Otu10447
CCAGCCTATGGGGGGCACCAGTGGGGAATCTTGCACAATGGACGAAACCCTGATCCAGCC
ATGCCGCGTGGATGATGAAGGCCCTAGGGTTGTAAAGTCCTTTCGACGGGGAAGATAATG
ACGGTACCCGTAGAAGAAGCCCCGGCTAACTTCGTGCCAGCAGCCGCGGTAATACGAAGG
GGGCTAGCGTTGCTCGGAATCACTGGGCGTAAAGCGCACGTAGGCGGACTCTTAAGTCGG
TGGTGAAATCCTGGAGCTCAACTCCAGAACTGCCTTCGATACTGAGAGTCTTGAGTCCGA
GAGAGGTGAGTGGAACTGCGAGTGTAGAGGTGAAATTCGTAGATATTCGCAAGAACACCA
GTGGCGAAGGCGGCTCACTGGCTCGGTACTGACGCTGAGGTGCGAAAGCGTGGGGAGCAA

ACAGGATTAGAAACCTCGTAGTCC
>Otu10450
CCAGCCTACGGGTGGCACCAGTTGGGAATCTTGGACAATGGGGGAAACCCTGATCCAGCC
ATGCCGCGTGAGTGATGAAGGCCTTCGGGTTGTAAAGCTCTTTTACCCGGGAAGATAATG
ACGGTACCGGAAGAAGAAGCCCCGGCTAACTTCGTGCCAGCAGCCGCGGTAATACGAGGG
GGGCAAGCGTTGTTCGGAATTATTGGGCGTAAAGGGTGCGTAGGCGGTTTGGTAAGTCTT
TTGTGAAATCTATGGGCTCAACTCATAGACTGCAAGGGAAACTGCCGGGCTTGAGTGTGG
GAGAGGTGAGTGGAATTTCCGGTGTAGCGGTGAAATGCGTAGATATCGGAAGGAACACCT
GTGGCGAAAGCGGCTCACTGGACCACAACTGACGCTGATGCACGAAAGCTAGGGGAGCAA
ACAGGATTAGATACCCCAGTAGTCC
>Otu10465
CCAGCCTACGGGTCGCACCAGTAGGGAATTTTCCACAATGGGCGAAAGCCTGATGGAGCA
ACGCCGCGTGCAGGATGAATGCCTTCGGGTTGTAAACTGCTTTTGTTAGTGACGATTATG
ACGGTAGCTAACGAATAAGGGTCTGCTAACTACGTGCCAGCAGCCGCGGTCATACGTAGG
ACCCAAGCGTTATCCGGAATTACTGGGCGTAAAGAGTTGCGTAGGTGGCAAAGTAAGCGA
ATAGTGAAAGCGTGTGGCTCAACCATACATCCATTATTCGGACTGCTTAGCTAGAGAACG
AGAGAGGTAGATGGAATTTCTAATGTAGGGGTAATATCCGTAGATATTAGAAGGAACACC
GATGGCGTAGGCAGTCTACCGGCTCGTTTCTGACACTAATGCACGAAAGCATGGGGAGCA
AACAGGATTAGAAACCCGGGTAGTCC
>Otu10467
CCAGCCTATGGGTTGCTCCAGTGGGGAATCTTGGACAATGGGGGAGACCCTGATCCAGCC
ATGCCGCGTGAGTGATGAAGGCCTTCGGGTTGTAAAACTCTTTCGACGGGGACGATAATG
ACGGTACCCGCAGAAGAAGCCCCGGCTAACTTCGTGCCAGCAGCCGCGGTAATACGAAGG
GGGCTAGCGTTGCTCGGAATGACTGGGCGTAAAGGGCGCGTAGGCGGATGGCACAGTCAG
ATGTGAAATTCCCGGGCTTAACCTGGGGGCTGCATTTGATACGTGGTGTCTGGAGTGAGG
AAGAGGGTTGTGGAATTCCCAGTGTAGAGGTGAAATTCGTAGATATTGGGAAGAACACCG
GTGGCGAAGGCGGCAACCTGGTCCTTGACTGACGCTGAGGCGCGAAAGCGTGGGGAGCAA
ACAGGATTAGAAACCCGCGTAGTCC
>Otu10472
CCAGCCTACGGGGGGCACCAGTGGGGAATCTTGCGCAATGGACGAAAGTCTGACGCAGCA
ACGCCGCGTGAGTGAAGAAGGCCTTCGGGTTGTAAAGCTCTGTCGAGCGAGACGAAAAAT
TGGCTGGTGAATAGCCAGCCAATCTGACGGTACCGCTAAAGGAAGCACCGGCTAACTCCG
TGCCAGCAGCCGCGGTAATACGGAGGGTGCAAGCGTTGCTCGGAATTATTGGGCGTAAAG
GGTAGGTAGGTGGTCTCGTTTGTCTGGGGTGAAATCCTTGAGCTTAACTCAAGAAGTGCC
TCAGAAACGGCGAGACTAGAGTTCTGGAGAGGGTCGTGGAATTCCCGGTGTAGCGGTGAA
ATGCGTAGATATCGGGAGGAACACCAGAGGCGAAGGCGGCGACCTGGACAGATACTGACA
CTAAACTACGAAAGCGTGGGGAGCAAACAGGATTAGATACCCTAGTAGTCC
>Otu10478
CCAGCCTATGGGTTGCAGCAGTGGGGAATTTTACACAATGGGGGAAACCCTGATGTAGCG
ACACCGCGTGAGCGAAGAAGCCCTTTGGGGTGTAAAGCTCTGTCGTGAGGGACGAAAAAA
ATGACGGTACCTCAAGAGGAAGCATCGGCTAACTACGTGCCAGCAGCCGCGGTAAGACGT
AGGATGCGAGCGTTGTCCGGATTTATTGGGCGTAAAGAGTTCGTAGGTGGTTCACTAAGT
CTGGTGTAAAAGGCACGGGCTCAACTCGTGTATCGCGTTGGATACTGGTGGACTGGAGTG
GAGTAGAGGCAAGCGGAATTCCCAGTGTAGCGGTGAAATGCGTAGATATTGGGAAGAACA
TCGGTGGCGCAAGCGGCTTGCTGGGCTTTAACTGACACTGAGGAACGAAAGCTAGGGGAG
CAAATGGGATTAGAGACCCGCGTAGTCC
>Otu10483
CCAGCCTATGGGTTGCTCCAGTGGGGAATCCTGGACAATGGGGGAAACCCTGATCCAGCC
ATGCCGCGTGAGTGATGAAGGCCTTAGGGTTGTAAAACTCTTTCGACGGGGACGATAATG
ACGGTACCCGTAGAAGAAGCTCCGGCTAACTTCGTGCCAGCAGCCGCGGTAATACGAAGG
GGGCTAGCGTTGTTCGGAATTACTGGGTGTAAAGCGCGCGCAGGCGGCCTTCCAAGTCAG
GGGTGAAAGCCCAGAGCTCAACTCTGGAATTGCCTTTGAAACTGTTAGGCTTGAGTACGG
GAGAGGTGAGTGGAATTCCCAGTGTAGAGGTGAAATTCGTAGATATTGGGAAGAACACCG
GTGGCGAAGGCGGCTCACTGGACCACAACTGACGCTGATGCACGAAAGCTAGGGGAGCAA
ACAGGATTAGAAACCCGAGTAGTCC
>Otu10484
CCAGCCTATGGGGTGCTGCAGTAGGGAATTTTCCGCAATGGGCGAAAGCCTGACGGAGCA
ACGCCGCGTGAGTGAAGACGGTTTTCGGATTGTAAAGCTCTGTTCTGTGGGAAGAAAAAA

ATGACGGTACCATAGGAGAAAGCCCCGGCTAACTACGTGCCAGCAGCCGCGGTAATACGT
AGGGGGCAAGCGTTGTCCGGAATTATTGGGCGTAAAGTGCGCGCAGGCGGTTAATTAAGT
CAGGTGTGAAAGGCTACGGCTCAACCGTAGAGTTGCATTTGAAACTGGTTAACTTGAGTG
CAGGAGAGGTAAGTGGAATTCCCGGTGTAGCGGTGGAATGCGTAGAGATCGGGAGGAACA
CCAGTGGCGAAGGCGGCTTACTGGCCTGCAACTGACGCTGAGGCGCGAAAGCGTGGGGAG
CAAACAGGATTAGATACCCGAGTAGTCC
>Otu10485
CCAGCCTACGGGGGGCTCCAGTGAGGAATCTTGGACAATGGGGGCAACCCTGATCCAGCG
ATGCCGCGTGAGTGAAGAAGGCCTTCGGGTCGTAAAGCTCTTTAGGTTGGGACGAAGGTT
ATGTGGGGATAATCCATGTAGCTTGACGGTACTAACAGAATAAGCACCGGCAAACTCTGT
GCCAGCAGCCGCGGTAATACAGAGGGTGCAAGCGTTAATCGGAGTTACTGGGCGTAAGGG
GCGCGTAGGCGGTGAATTGGGTGTGGTGTGAAAGCCCCGGGCTTAACCTGGGAAGTGCAT
CGCAAACTAATTGACTGGAGTAGATGAGAGGGTGGCGGAATTTCCGGTGTAGCGGTGAAA
TGCGTAGAGATCGGAAGGAACGTCAATGGCGAAGGCAGCCACCTGGCATCATACTGACGC
TGAGGCGCGAGAGCGTGGGGAGCAAACAGGATTAGAAACCCCTGTAGTCC
>Otu10486
CCAGCCTACGGGGGGCTGCAGTAACGAATCTTCCGCAATGGGCGCAAGCCTGACGGAGCG
ACGCCGCGTGTGGGACGAAGTCCTTCGGGATGTAAACCACTGTCAGGGGTTAGAAAGTTC
TGATCAACCCCAGAGGAAGGCACGGCTAACTCTGTGCCAGCAGCCGCGCTAAGACAGAGG
TGCCAAGCGTTAGGCGGAATCACTGGGCTTAAAGCGTGTGTAGGTGGATGCCTAAGTGCC
TTGTGAAATCCCACGGCTCAACCGTGGAATTGCTGGGCATACTGGGTGTCTTGAGCCATC
CAGGGGTCGCTGGAACAAACGGTGGAGCGGTGGAATGCGTAGATATCGTTTGGAACGCCA
ATGGTGAAAACAGGCGACTGGGGATGAGCTGACACTGAGACACGAAAGCCAGGGGAGCAA
ACGGGATTAGAAACCCTTGTAGTCC
>Otu10488
CCAGCCTACGGGGGGCAGCAGCCGAGGATCATTCGCAATGGGCGAAAGCCTGACGATGCG
ACGCCGTGTGAGCGATGAAGGCCTTCGGGTTGTAAAGCTCTTTCGCTTAGGAACAAGGGA
ATATAGTGAATAACTATGTGACTTGAGGGTACTAGGTAAAGAAGCACCGGCTAACTCCGT
GCCAGCAGCTGCGGTAATACGGAGGGTGCAAGCATTAATCGGAATTATTGGGCGTAAAGG
GCGCGTAGGCGGATTTGTAAGTCAGATGTGAAATTCCGAAGCTCAACTTCGGAGCTGCAT
TTGAAACTGTGAGTCTAGAGGGATGGCGGAGAAAATGGAATTCCACGTGTAGCGGTGAAA
TGCGTAGATATGTGGAAGAACACCGGTGGCGAAGGCGATTTTCTAGCTTTTTCCTGACGC
TGAGGCGCGAGAGCAAGGGGAGCAAACAGGATTAGAAACCCCAGTAGTCC
>Otu10489
CCAGCCTATGGGAGGCTCCAGTGGGGAATATTGGTCAATGGGCGAAAGCCTGAACCAGCA
ATGCCGCGTGAGTGATGAAGGCCTTCGGGTTGTAAAGCTCTGTCGGTGGGAACGAATAAG
TCGGTTAATACTCGATGATGACGGTACCCACAAAGGAAGCACCGGCTAACTCTGTGCCAG
CAGCCGCGGTAATACAGAGGGTGCAAGCGTTGTTCGGAATTACTGGGCGTAAAGCGCGTG
TAGGCGGTCTGATAAGTCGGTCGTGAAAGCCCTTGGCTTAACCAAGGAACTGCGATCGAA
ACTGCCGGACTAGAGTATCGGAGGGGATGGTGGAATTCCCGGTGTAGAGGTGAAATTCGT
AGATATCGGGAGGAACACCTGTGGCGAAGGCGACCATCTGGAAGATTACTGACGCTGAGA
CGCGAAAGCGTGGGGAGCAAACAGGATTAGATACCCCAGTAGTCC
>Otu10494
CCAGCCTACGGGGGGCAGCAGTGGGGAATTTTGGACAATGGGCGCAAGCCTGATCCAGCA
ATGCCGCGTGTGTGAAGAAGGCCTTCGGGTTGTAAAGCACTTTTGTCCGGAAAGAAATCC
TTTGAGTTAATACCTCGGGGGGATGACGGTACCGGAAGAATAAGCACCGGCTAACTCTGT
GCCAGCAGCCGCGGTAATACAGAGGGTGCGAGCGTTAATCGGAATTACTGGGCGTAAAGC
GCGCGTAGACGGTTTGGTAAGTCAGATGTGAAATCCCCGGGCTCAACCTGGGAACTGCAT
TTGATACTGCCTGGCTAGAGTATCGAAGAGGGAAGTGGAATTTCCGGTGTAGCGGTGAAA
TGCGTAGATATCGGAAGGAACACCAGTGGCGAAAGCGACTTCCTGGTCGAATACTGACGT
TCATGTGCGAAAGCGTGGGGAGCAAACAGGATTAGAAACCCCAGTAGTCC
>Otu10497
CCAGCCTATGGGTCGCAGCAGTGGGGAATCTTGCGCAATGGACGAAAGTCTGACGCAGCG
ACGCCGCGTGGGCGACGAAGGCCTTCGGGTTGTAAAGCCCTGTGGGGAGAGACGAACACG
CTAAGCTCAAATAGGGCGGAGCGCTGACGGTATCTCCTTAGCAAGCACCGGCTAACTCTG
TGCCAGCAGCCGCGGTAAGACAGAGGGTGCAAACGTTGCTCGGAATTACTGGGCGTAAAG
CGCGTGTAGGCGGGCCTGCCAGTCAGGTGTGAAAGCCCGGGGCTCAACCCCGGAAGTGCA
TTTGATACTACAGGTCTTGAGTACTGACGAGGAGAGCGGAATTCCCGGTGTAGAGGTGAA

ATTCGTAGATATCGGGAGGAACACCAGTGGCGAAGGCGGCTCTCTGGGCAGATACTGACG
CTGAGACGCGAAAGCATGGGGAGCAAACAGGATTAGATACCCTTGTAGTCC
>Otu10500
CCAGCCTACGGGGGGCTGCAGTGGGGAATTTTGGGCAATGGCCGAAAGGCTGACCCAGCA
ACGCCGCGTGAAGGATGAAATCCTTCGGGATGTAAACTTCGCAAATAAGGGAAGAATAAA
GGTAAGCTAATACCCTATCATATGACGGTACCTTATGTAAGCCCCGGCTAACTCCGTGCC
AGCAGCCGCGGTAATACGGGGGGGGCAAGCGTTGTTCGGAACTACTGGGCGTAAAGGGCG
CGTAGGCGGTCAGACCAAGTCGAATGTGAAATACCAGGGCTTAACTCTGGCAGGTCGTTC
GATACTGGTCGACTAGAGTGTGGAAGAGGATGTCGGAATTCCCGGTGTAGCGGTGAAATG
CGTAGATATCGGGAGGAACACCAGCGGCGAAGGCGGGCATCTGGGCCAACACTGACGCTG
ATGCGCGAAAGCTAGGGGAGCAAACAGGATTAGATACCCTAGTAGTCC
>Otu10503
CCAGCCTATGGGGGGCACCAGTGGGGAATTTTGCGCAATGGGGGAAACCCTGACGCAGCA
ACGCCGCGTGGAGGATGAAGCCCCTTGGGGCGTAAACTCCTTTCGACCAAGACGATGATG
ACGGTACTGGTGGAAGAAGCACCGGCTAACTCTGTGCCAGCAGCCGCGGTAATACAAAGG
GTGCGAGCGTTGTTCGGAATTATTGGGCGTAAAGGGCGCGTAGGCGGTGCCACAAGTCAC
TTGTGAAATCCCCGGGCTTAACCCGGGGCCTGCAGGCGAAACTGTGGTGCTGGAGTATGG
GAGAGGTAAGCGGAATTCCTGGTGTAGCGGTGAAATGCGTAGATATCAGGAGGAACACCT
GTGGTGTAGACAGCTTACTGGACCATCACTGACACTGAGACACGAAAGCGTGGGTAGCAA
ACAGGATTAGAACCCCCGTAGTCC
>Otu10505
CCAGCTATGGGTTGCAGCAGTCGAGAATATTCGACAATGGGCGAAAGCCTGATCGAGCGA
CACCGCGTGCGGGAAGAAGCTCTTCGGAGCGTAAACCGCTTTTGTCAGGGAAGAATTTTG
ACGGTACCTGAAGAATAAGGGGTTGCTAACCTCGTGCCAGCAGCAGCGGTAATACGAGGA
CCCCGAGCGTTATCCGGAATTACTGGGCGTAAAGCGTGAGCATCCGGTTGTGTTAGTCTC
ATGTTAAATCTCACTGCTCAACGGTGAGACCGCGTGGGAAACGGCACGACTAGAGCGGGT
AAGAGGCAAGCGGAACGTGCAGTGTAGGGGTGAAATCCATTGATATTGCACGGAACACCA
AAAGCGAAAGCAGCTTGCTAGTGCTCTGCTGACGGTCAATCACGAAAGCGTGGGGATCAA
AAAGGATTAGAGACCCCAGTAGTCC
>Otu10509
CCAGCCTATGGGGGGCAGCAGTGGGGAATATTGCGCAATGGGCGAAAGCCTGACGCAGCG
ACACCGCGTGGAGGATGAAGGCCTTCGGGTTGTAAACTTCTGTTAAGTGGGAAGAAAGAA
CCATTTTTAATAAAAATGGGTGATGACGGTACCATTAGAGAAAGCACCGGCTAAACTCGT
GCCAGCAGCCGCGGTAATACGAGTGGTGCAAGCGTTATTCGGAATAATTGGGCGTAAAGG
GTGTGTAGACGGTATATTAAGTCAACTGTTAAATTCTTCGGCCTAACCGGAGGCAAGCGG
TAGAAACTGATATGCTAGAGGGTGGGAGAGAGAAGTGGAATTCTCGGAGTAGCGGTAAAA
TGCGTAGATCTCGAGAGGAACACCGATGGCGAAGGCAGCTTCTTAGTCCATTCCTGACGT
TGAGACACGAAAGCGTGGGGAGCAAACAGGATTAGATACCCCTGTAGTCC
>Otu10537
CCAGCCTACGGGGGGCTGCAGTCGAGGATCTTTAGCAATGCCCGAAAGGGTGACTATGCG
ACGCCGCGTGGAGGATGAAGGCCTTCGGGTTGTAAACTCCTGTCAGGGGGGAAGAAACTT
TGCAGGTTAATAGCTTGCAAACTGACATCACCCCCAAAGGAAGCCACGGCTAACTCTGTG
CCAGCAGCCGCGGTAAGACAGAGGTGGCAAGCGTTGTTCGGATTCATTGGGTGTAAAGGG
CAGGTAGGCGGTTTTATAAGTCCGGCGTGAAATCCCAGAGCTCAACTCTGGAACTGCGTT
GGAAACTATATGACTTGAGTATCGGAGAGGTGAGGGGAATTCTCGGTGTAAGGGTGAAAT
CTGTAGATATCGAGAGGAACACCAGTGGCGAAGGCGCCTCACTGGCCGGTTACTGACGCT
GAGCTGCGAAAGCAAGGGGAGCAAACAGGATTAGAGACCCCCGTAGTCC
>Otu10550
CCAGCCTACGGGTCGCTCCAGCGACGAATCTTCCGCAATGGGCGCAAGCCTGACGGAGCG
ACGCCGCGTGTGGGATGAAGCGCCTTCGGTGTGTAAACCACTGTCAGGGGATAGAACCAA
TGATCATCCCCAAAGGAAGAGACGGCTAACCCTGTGCCAGCAGCCGCGGTAAGACAGGGG
TCTCGAGCGTTAATCGGAATTACTGGGCTTAAAGGGTGCGTAGGCCGGCCCGCAAGTGTC
TTGTGAAATCCCCTGGCTCAACCAGGGAACAGCTCGACATACTGCGGGTCTTGAGGAAGG
TAGGGGTTGCCAGAACTGTAGGTGGAGCGGTGAAATGCGTAGATATCTACAGGAATGCCG
ATGGTGAAGACGGGCAACTGGGCCTTTCCTGACGCTGAGGCACGAAAGCGTGGGGAGCAA
ACAGGATTAGAGACCCTCGTAGTCC
>Otu10551
CAGCCTATGGGGGGCTGCAGTGGGGAATATTGGACAATGGGCGCAAGCCTGATCCAGCAA

TGCCGCATGTGTGAAGAAGGCCTTCGGGTTGTAAAGCACTTTAGGTTGGGAAGAAAAAAT
TCGGGCGAATATCCCGAGTCTTGACGGTACCGACAGAATAAGCACCGGCTAACTCTGTGC
CAGCAGCCGCGGTAATACAGAGGGTGCTAGCGTTAATCGGATTTACTGGGCGTAAAGCGT
GCGTAGGCGGTTTGTTAAGTCGGTTGTGAAAGCCCTGGGCTCAACCTGGGAATTGCAGTC
GATACTGGCGGACTAGAGATCGGTAGAGGGGGGTGGAACTCCAGGTGTAGCGGTGAAATG
CGTAGATATCTGGAAGAACACCGATGGCGAAGGCAGCCCCCTGGGCCTGATCTGACGCTG
AGGCACGAAAGCGTGGGTAGCAAACAGGATTAGAAACCCTCGTAGTCC
>Otu10554
CCAGCCTACGGGTGGCTGCAGTCGAGAATTTTTCTCAATGGGGGAAAGCCTGACCATGCG
ACGCCGCGTGCTGGATGAAGTCCTTCGGGACGTAAACAGCTTTTACGCGTGAAGAAGTAA
TTGACATTAGCGCGAGAATAAGGGGCTCCTAACTCTGTGCCAGCAGGAGCGGTAATACAG
AGGCCCCGAGCATTACCCGGAATCACTGGGCGTAAAGGGTGTGTAGGCGGTGGTATTAGT
CTTTCGTAAAAGCACTGGGGCTTAACCCCGGTAATGCGATAGAAACGGTACCGCTACGAG
GATGCGAGAGGTAAAGGGAACTCATGGTGTAGGGGTGAAATCCGTTGATATCATGGGGAA
CACCAAATGCGAAGGCACTTTACTGGCGCACTCCTGACGCTGAAACACGAAAGCGTGGGA
ATCGAACGGGATTAGATACCCTAGTAGTCC
>Otu10556
CCAGCCTATGGGGTGCTGCAGCAACGAATATTCCGCAATGGGCGAAAGCCTGACGGAGCG
ACGCCGCGTGAAGGATGAAGTCCTTCGGGATGTAAACTTCATTAGTTTGTCAGAAAAGAA
CCCGACCTAACACGTCGGGGAGTTGATCAGCAAACGAAAGGGACGGCTAACTCCGTGCCA
GCAGCCGCGGTAAGACGGAGGTCCCGAGCGTTGTTCGGAATTATTGGGCTTAAAGCGCGT
GTAGGCGGAGCCGTAAGTACCTTGTGAAATCCCCGGACTCAATCCGGGAACTGCTCGGTA
TACTGCGGTTCTCGAGGCAGGTAGAGGTCACTGGAACCTTAGGTGGAGCGGTGAAATGCG
TAGATATCTAAGGGAACGCCTGTGGCGAAAGCGGGTGACTGGGCCTGTCCTGACGCTGAG
ACGCGAAAGCGTAGGGAGCAAACGGGATTAGATACCCGAGTAGTCC
>Otu10561
CCAGCCTATGGGAGCTCCAGTCGAGAATTTTTCTCAATGGGCGAAAGCCTGAAGGAGCAA
CGCCGCGTGGAGGATGAAATCCCTTGGGATGTAAACTCCTTTCGATCGGGAAGATAATGA
CGGTACCGGAAGAAGAAGCCCCGGCTAACTTCGTGCCAGCAGCCGCGGTAATACGAGGGG
GGCGAGCGTTGTTCGGAATTATTGGGCGTAAAGGGCGCGTAGGCGGTTTTACAAGTCTTG
TGTGAAATCTTCGGGCTCAACTCGAAGTCTGCACGAGAAACTGTAAGGCTAGAGTGTGGG
AGGGGTGCGTGGAATTTCCGGTGTAGCGGTGAAATGCGTAGATATCGGAAGGAACACCTG
TGGCGAAAGCGGCGCACTGGACCACAACTGACGCTGAGGCGCGAAAGCTAGGGGAGCAAA
CAGGATTAGAAACCCTTGTAGTCC
>Otu10566
CCAGCCTATGGGTGGCAGCAGTTGGGAATATTGCACAATGGGCGAAAGCCTGATGCAGCG
ACGCCGCGTGGAGGATGAAGGTTTTCGGATTGTAAACTCCTGTTAAGTGGGAAGAAAGAC
GCGTACCTAATACGTACGTGGGATGACGGTACCACTAGAGAAAGCACCGGCAAACTTCGT
GCCAGCAGCCGCGGTAATACGAAGGGTGCGAGCGTTATTCGGAATAACTGGGTGTAAAGA
GCGTGGAGGCGGTTGATTAAGTTAGTTGTTAAATCGCTCGGCTTAACCGGGCACATGCGA
TTAAAACTGATCAGCTAGAGTGCGAGAGAGAAGGGTAGAATTCTCGGAGTAGCGGTAAAA
TGCGTAGATCTCGAGAGGAATACCGGTTGCGAAGGCGGCCTTTTGGCTCGTTACTGACGC
TGGAGCGCGAAAGCGTGGGGAGCAAACAGGATTAGATACCCTAGTAGTCC
>Otu10571
CCAGCCTACGGGCGCACCATTGAGGAATTTTGGACAATGGGGGAAACCCTGATCCAGCG
ATGTCGCGTGTATGATGAAGGCCTTTTGGTTGTAAAGTACTTTCGTCGGTGAAGATAATG
ACGGTAACCGAAAAAGAAGCTCCGGCTAACTTCGTGCCAGCAGCCGCGGTAAGACGAAGG
GAGCGAGCGTTGCTCGGAATAATTGGGCGTAAAGGGTGCGTAGGTGGTTTTTTAAGTGAG
ATGTTAAAGACCTGGACTCAATCTAGGGAATGCATTTCATACTGAAAAACTTGAGTGTGG
GAGAGGAAACAGGAATTCCTCATGTAGGGGTGAAATTCGTAGATATGAGGAGGAACACCA
AAGGCGAAGGCAAGTTTCTGGAACACAACTGACACTGAGGCACGAAAGCGTGGGGAGCAA
ACAGGATTAGATACCCCGGTAGTCC
>Otu10574
CCGCCTACGGGATGCAGCAGTGGGGAATATTGGACAATGGGCGCAAGCCTGATCCAGCCA
CGCCGCGTGAGTGATGAAGGCCTTCGGGTCGTAAAGCTCTGTGGGGAGGGACGAACCGCT
GCACGTTCATATCGTGCAGCATGACGGTACCTTCTTAGCAAGCACCGGCTAACTTCGTGC
CAGCAGCCGCGGTAATACGAAGGGTGCAAACGTTGCTCGGAATTATTGGGCGTAAAGCGC
ACGTAGGCGGCTTAGCAAGTCGGATGTGAAATCCCTCGGCTCAACCAAGGAAGTGCATCC

GAAACTGCTGAGCTTGAGTACTTAAGAGGATCGCGGAATTCCCGGTGTAGAGGTGAAATT
CGTAGATATCGGGAGGAACACCAGTGGCGAAGGCGGCGATCTGGGAAGATACTGACGCTG
AGGTGCGAAAGCGTGGGGAGCAAACAGGATTAGAGACCCTAGTAGTCC
>Otu10575
CCAGCCTACGGGTCGCTCCAGTCGAGAATTTTTCTCAATGGGGGAAACCCTGAAGGAGCG
TCGCCGCGTGGAGGATGAAGGTCTTCGGATTGTAAACTCCTGTCATTAGGGAACAATTGT
CCGCGATTAACTGTCGCGGGCTTGATAGTACCTGAAGAGGAAGAGACGGCTAACTCTGTG
CCAGCAGCCGCGGTAATACAGAGGTCTCAAGCGTTGTTCGGATTCATTGGGCGTAAAGGG
TGCGTAGGCGGCGGGGCAAGTCAAGTGTGAAATCTCGGGGCTCAACCCCGAAACTGCACT
TGATACTGCCTTGCTAGAGTGCTGGAGAGGAGATTGGAATTTACGGTGTAGCAGTGAAAT
GCGTAGATATCGGAAGGAACATCAGTGGCGAAAGCGGCTTCCTGGTCCAGCACCGACGTT
CAGGCACGAAAGCGTGGGGAGCAAACAGGATTAGATACCCCCGTAGTCC
>Otu10578
CCAGCCTATGGGATGCTCCAGTGGGGAATATTGGACAATGGGCGCAAGCCTGAACGAGCG
ACGCCGCGTGCGCGATGAAGGCCTTCGGGTTGTAAAGCGCGAAAGTGGGGACGAAAGCTG
AAAGGTTTGACCGATCCACAGTAAGCTCGGGCTAAGTTCGTGCCAGCAGCCGCGGTAAGA
CGAACCGAGCGAACGTTGTCCGGAATCACTGGGCTTAAAGGGCGCGTAGGCGGGCATTCA
AGTCTGCGGTGAAATACTCCAGCTTAACTGGAGAACTGCCGTGGATACTGGGTGTCTCGA
GGGAGGTAGGGGCGTGCGGAACTATTGGTGGAGCGGTGAAATGCGTTGATATCGATAGGA
ACTCCGGTGGCGAAGGCGGCACGCTGGACCTCTTCTGACGCTGAGGCGCGAAAGCCAGGG
GAGCAAACGGGATTAGAAACCCTCGTAGTCC
>Otu10584
CCAGCCTACGGGTCGCAGCAGTCGAGAATCTTTCGCAATGGGCGAAAGCCTGACGAAGCG
ACGCCGTGTGAGCGATGAAGGCCTTAGGGTTGTAAAGCTCTTTCGCTTGTGAACAAGAGA
GGTTAGCTAATATCTAGCCAATTTGAGGGTAGCAGGTAAAGAAGCACCGGCTAACTCTGT
GCCAGCAGCTGCGGTAATACAGAGGGTGCAAGCATTAATCGGATTTATTGGGCGTAAAGA
GCGCGTAGGCGGGTTTTTAAGTCAGATGTGAAATCCCGGGGCTCAACCCCGGAACAGCAT
TTGAAACTGAAAATCTTGAGGGTAGTCGGAGAAAATGGAATTCCACGTGTAGCGGTGAAA
TGCGTAGATATGTGGAAGAACACCGGTGGCGAAGGCGATTTTCTAGATTATTCCTGACGC
TGAGGCGCGAAAGCAAGGGGATCAAACAGGATTAGAAACCCGAGTAGTCC
>Otu10589
CCAGCCTATGGGGCGCAGCAGTCGAGAATCTTTCGCAATGGGCGAAAGCCTGACGAAGCG
ACGCCGTGTGTGCGATGAAGGCCTTCGGGTTGTAAAGCACTTTCGCTTGGGAACAAGAGA
AGCTGGCTAACATCCAGCAGATTTGAGCGTACCAGGTAAAGAAGCACCGGCTAACTCCGT
GCCAGCAGCTGCGGTAATACGGAGGGTGCAAGCATTAATCGGATTTATTGGGCGTAAAGG
GCGCGTAGGCGGGGATAAAAGTCAGATGTGAAATCCCGGGGCTCAACCCCGGAACAGCAT
TTGAAACTCTATCTCTAGAGGGTAGGAGGAGAAAACGGAATTCCACAAGTAGCGGTGAAA
TGCGTAGATATGTGGAAGAACACCGGTGGCGAAGGCGGTTTTCTATCTTATTCCTGACGC
TGAGGCGCGAAAGCAAGGGGATCAAACAGGATTAGAGACCCGCGTAGTCC
>Otu10593
CCAGCCTACGGGTCGCAGCAGTCGAGAATCTTCCGCAATGGACGAAAGTCTGACGGAGCG
ACGCCGCGTGAAGGATGAAGCGCTTCGGCGTGTAAACTTCTTTTATAGGGGAGAAAGTTT
ATTGATAGTACCCTACGAATAAGAGGCTACAAACTCTGTGCCAGCAGTAGCGGTAATACA
GAGGCCTCAAGCGTTATCCGGAATTACTGGGCGTAAAGGGTGTGTAGGTGGTCTAGTTAG
TCTTGTGTTAAATTCTTCGGCTTAACCGGGGGCATGCATAGGAAACGGCTAGACTTGAGG
GTGCGAGAGGCGCATGGAACTCATGGAGTAGGGGTGAAATCCGCTGATATCATGAGGAAC
ACCAAAAGCGAAGGCAGTGCACTGGCGCATTTCTGACACTCAAACACGAAAGCGTGGGTA
GCGAATGGGATTAGAAACCCTCGTAGTCC
>Otu10597
CCAGCCTACGGGTGGCTGCAGTAAGGAATATTGGTCAATGGGCGAAAGCCTGAACCAGCC
ATGCCGCGTGCAGGAAGACGGCCCTATGGGTTGTAAACTGCTTTTATTCGGGAATAAATC
TTCTTACGTGTAAGAAGCTGAATGTACTGAAAGAATAAGGATCGGCTAACTCCGTGCCAG
CAGCCGCGGCAATACGGAGGATCCAAGCGTTATCCGGATTTATTGGGTTTAAAGGGTGCG
TAGGCGGCCTATTAAGTCAGAGGTGAAATTTTGCAGCTTAACTGTAACATTGCCTTTGAT
ACTGATTGGCTTGAATTAACTTGAGGTAGGCGGAATGTGACAAGTAGCGGTGAAATGCAT
AGATATGTCACAGAACACCAATTGCGAAGGCAGCTTACTAAGGTTTTATTGACGCTGAGG
CACGAAAGCGTGGGGATCAAACAGGATTAGAGACCCCTGTAGTCC
>Otu10601

CCAGCCTATGGGGGGCACCAGTGGGGAATATTGGACAATGGGCGCAAGCCTGATCCAGCA
ATGCCGCGTGTGTGAAGAAGGTCTTCGGATTGTAAAGCACTTTTGGCAGGGACGATGATG
ACGGTACCTGCAGAATAAGCCCCGGCTAACTTCGTGCCAGCAGCCGCGGTAATACGAAGG
GGGCTAGCGTTGCTCGGAATGACTGGGCGTAAAGGGCGCGTAGGCGGCTGTCACAGTCAG
ATGTGAAATTCCTGGGCTCAACCTGGGGACTGCATTTGATACGTGGCGGCTTGAGTGGAG
AAGAGGGTTGTGGAATTTCCAGTGTAGCGGTGAAATGCGCAGATATCGGAAGGAACACCT
GTGGCGAAAGCGGCTCACTGGACCATAACTGACGCTGATGCACGAAAGCTAGGGGAGCAA
ACAGGATTAGATACCCCCGTAGTCC
>Otu10602
CCAGCCTACGGGAGGCAGCAGTGGGGAATCTTGGACAATGGGGGAAACCCTGATCCAGCC
ATGCCGCGTGAGTGATGAAGGCCCTAGGGTTGTAAAACTCTTTCGCGCACGACGATAATG
ACGGTAGTGCGAGAAGAAGCTCCGGCTAACTTCGTGCCAGCAGCCGCGGTAATACGAAGG
GGGCTAGCGTTGTTCGGAATTACTGGGCGTAAAGCGCGCGCAGGCGGTCCTTCCAGTCAG
AGGTGAAAGCCCAGAGCTCAACTCTGGAACTGCCTTTGAAACTAGAGGACTTGGGTACGG
GAGAGGTGAGTGGAATTCCGAGTGTAGAGGTGAAATTCGTAGATATTCGGAAGAACACCA
GTGGCGAAGGCGGCTCACTGGCCCGTTACCGACGCTCAGGCGCGACGGCGTGGGGAGCAA
ACAGGATTAGATACCCCCGTAGTCC
>Otu10605
CCAGCCTACGGGACGCTGCAGTGGGGAATTTTGCGCAATGGGGGAAACCCTGACGCAGCA
ACGCCGCGTGGAGGATGAAGTCCTTCGGGACGTAAACTCCTTTCGATCGGGACGATGATG
ACGGTACCACCAGAAGAAGCCCCGGCTAACTTCGTGCCAGCAGCCGCGGTAATACGAAGG
GGGCTAGCGTTGTTCGGAATTACTGGGCGTAAAGCGCACGCAGGCGGCTCGTTAAATTAG
AAGTGAAAGCCCTGGGCTCAACCCGGGAATTGCTTTTAAGACTGGCGAGCTAGAATTCGG
AAGAGGGCAGTGGAATTCCAAGTGTAGAGGTGAAATTCGTAGATATTTGGAAGAACACCA
GTGGCGAAGGCGACTGCCTGGTCCGATATTGACGCTCATGTGCGAAAGCGTGGGGAGCAA
ACAGGATTAGAAACCCGTGTAGTCC
>Otu10608
CCAGCCTATGGGGGGCAGCAGTAGGGAATTTTCCACAATGGGCGAAAGCCTGATGGAGCA
ACGCCGCGTGCAGGATGAAGGCTTTCGGGTCGTAAACTGCTTTTATCTGTGATAATTATG
ACAGTAACAGAAGAATAAGGGTCTGCTAACTACGTGCCAGCAGCCGCGGTCATACGTAGG
ACCCAAGCGTTATCCGGAATTACTGGGCGTAAGGAGTTGCGTAGGTGGCAAAGTAAGCAA
ATAGTGAAAACGTATGGCTCAACGATATACACATTGTTTGAACTGCTTAGCTAGAGCACA
AGAGAGGTAGCTGGAATTTCTAGTGAAGGAGTGAAATCCGTAGATATTAGAAGGAACACC
GATGGCGTAAGCAGGCTACTGGCTTGTTGCTGACACTCAGGCACGAAAGCGTGGGGAGCG
AACGGGATTAGAAACCCTAGTAGTCC
>Otu10617
CCAGCCTATGGGTCGCTCCAGTGGGGAATTTTGGACAATGGGGGCAACCCTGATCCAGCG
ACGCCGCGTGAAGGATGACGGTCTTCGGATTGTAAACTTCTTTTGGAGGGGATGAATGCC
TGAGTGAATAACTCAGGGTGACAGTACTCTCTGAATAAGCCACGGCTAACTACGTGCCAG
CAGCCGCGGTAATACGTAGGTGGCAAGCGATACTCGGAATCACTAGGCGTAAAGCGCAGG
TAGGTGGTTCGGTAAGTCTTTTGTGAAAGCTCCTGGCTCAACTGGGAGAGGTCAAGGGAA
ACTGCCGGACTAGAGTTTGGTAGGGGATACTGGAAATCCTGGCGCAGCGGTGAAATGCGT
AGATATCAGGAGGAATACCTATGGCGAAAGCAGGTATCTGGGCCACAACTGACACTGAGC
TGCGAAAGCTAGGGGAGCAAACAGGATTAGATACCCTCGTAGCC
>Otu10620
CCAGCCTACGGGTGGCTCCAGTAGGGAATCTTCCACAATGGGCGAAAGCCTGATGGGGCA
ACGCCGCGTGCAGGATGAAGGCCTTCGGGTTGTAAACTGCTTTTCTTTGTGAGGACTATG
ACAGTAGCAGAGGAATAAGGATCGGCTAACTCCGTGCCAGCAGCCGCGGTCATACGGAGG
ATCCAAGCGTTATCCGGAATCACTGGGCGTAAAGAGTTGCGTAGGCGGCAGAGTTAACAG
AGTGTGTAATAGTATGGCTCAACCATATAAACATGCTCTGCATTGCTCAGCTAGAGGGCG
AAAGAGGTAGCTGGAATTCCTAGTGTAGGAGTGAAATCCGTAGATATTAGGAGGAACACC
GATGGCGTAGGCAGGCTACTGGTTCGACCCTGACGCTAAGGCACGAAAGCGTGGGGAGCG
ACCGGGATTAGAAACCCGCGTAGTCC
>Otu10622
CCAGCCTATGGGAGGCACCAGTCGAGAATCTTCCACAATGGACGAAAGTCTGATGGAGCG
ACGCCGCGTGGTTGATGAAGTCCTTCGGGACGTAAAAACCTTTTATGAGGGAGGAAGTAA
TTGACGTTACCTCATGAATAAGGGGCTCCTAACTCTGTGCCAGCAGGAGCGGTAATACAG
AGGCCCCAAGCATTATCCGGAATCACTGGGCGTAAAGGGTGTGTAGGCGGTTGTATTAGT

CTTTTGTTAAAGTTCTGGGGCTTACCCTCGGAAATGCAAGAGAAACGGTACAACTACGAG
GATGCGAGAGGTATATGGAACTCATGGTGTAGGGGTGAAATCCGTTGATATCATGGGGAA
CACCAAATGCGAAGGCAATATACTGGCGCACTCCTGACGCTGAAACACGAAAGCGTGGGA
ATCGAACGGGATTAGATACCCTAGTAGTCC
>Otu10627
CCAGCCTATGGGGCGCAGCAGTCGAGAATCATTCGCAATGGGCGAAAGCCTGACGATGCG
ACGCCGTGTGTGCGATGAAGGCCTTCGGGTTGTAAAGCACTTTCGCCTGGGAACAAGAGA
AGCCGGCTAATATCCGGCAGATTTGAGGGTACCAGGTAAAGAAGCACCGGCTAACTACGT
GCCAGCAGCTGCGGTAATACGTAGGGTGCGAGCATTAATCGGATTTATTGGGCGTAAAGG
GCGCGTAGGCGGGAATGTAAGTCGGTCGTGAAATCCCGGGGCTCAACCCCGGAACAGCGT
TCGAAACTACATTTCTGGAGGGTAGGCGGAGAAAATGGAATTCCACATGTAGCGGTGAAA
TGCGTAGATATGTGGAAGAACACCGGTGGCGAAGGCGGTTTTCTAGCTTATACCTGACGC
TGAGGCGCGAAAGCAAGGGGATCAAACAGGATTAGAAACCCCAGTAGTCC
>Otu10631
CCAGCCTACGGGGTGCACCAGTGGGGAATCTTGCACAATGGGGGAAACCCTGATGGAGCG
ACGCCGCGTGGGGGATGACTGGCTTCGGCCCGTAAACCCCTGTCATTTGCGAACAAATTG
ATTCACCTAACACGTGAAGCATTGATAGTAGCGGAAGAGGAAGGGACGGCTAACTCTGTG
CCAGCAGCCGCGGTAATACAGAGGTCCCAAGCGTTGTTCGGATTCACTGGGCGTAAAGGG
TGCGTAGGTGGCCGGGTAAGTCTGATGTGAAATCTCGCAGCTTAACTGCGAAACTGCATT
GGATACTATCCGACTAGAGGAATGGAGGGGAGACTGGAATACTTGGTGTAGCAGTGAAAT
GCGTAGATATCAAGTGGAACACCAGTGGCGAAGGCGAGTCTCTGGACATTTCCTGACGCT
GAGGCACGAAAGCCAGGGGAGCAAACGGGATTAGAGACCCGAGTAGTCC
>Otu10632
CCAGCCTATGGGAGGCTGCAGTCGAGGATCTTCGGCAATGGACGCAAGTCTGACCGAGCG
ACGCCGCGTGGATGATGAAGGCCTTCGGGTTGTAAAATCCTGTCGAGGGGGACGAAGCCG
CAAGGTTGACCGATCCCTGGAGGAAGGGCGGGCTAAGTTCGTGCCAGCAGCCGCGGTAAA
ACGAACTGCCCAAACGTTGTTCGGAATCACTGGGCTTAAAGGGCGCGTAGGCGGCGAATC
AAGTCAGGGGTGAAATCCGGCAGCTTAACTGTCGAAGTGCCTTTGATACTGGTTTGCTCG
AGGAAGGTAGGGGTGTGTGGAACTTCTGGTGGAGCGGTGAAATGCGTTGATATCAGAAGG
AACGCCGGTGGCGAAAGCGACGCACTGGACCTTTCCTGACGCTGAGGCGCGAAAGCTAGG
GGAGCAAACGGGATTAGATACCCTAGTAGTCC
>Otu10633
CCAGCCTACGGGTCGCTGCAGCCGAGAATATTCGACAATGGGCGAAAGCCTGATCGAGCG
ACGCCGCGTGCTGGATGAAGTGCTTCGGTACGTAAACAGCTTTTATTGGGGACGAAGTAA
TTGACGGTACCCAATGAATAAGGGGCTCCTAACTCTGTGCCAGCAGGAGCGGTAATACAG
AGGCCCCGAGCATTATCCGGAATCACTGGGCGTAAAGGGTGTCCAGGTGGTCGTATTAGT
CGTTCGTAAAATCCGCAAGCTCAACTTGCGGCCCGCGAGCGAAACGGTACGACTAGAGGG
CGCGAGAGGTGCACGGAACTCATGGTGGAGGGGTGAAATCCGTTGATATCATGGGGAACA
CCAAAGGCGAAGGCAGTGCACTGGCGCGTTCCTGACACTCACACACGAAAGCCAGGGGAG
CGAACGGGATTAGAGACCCGAGTAGTCC
>Otu10634
CCAGCCTATGGGGTGCACCAGTCGAGAATTTTTCTCAATGGGCGGAAGCCTGAAGGAGCG
ACGCCGCGTGGGGGATGAATGGCTTCGGCCCGTAAACCCCTGTCATTTGCGAACAAATTG
CTTCACCCAACACGTGTGTCATTGATTGTAGCGGAAGAGGAAGGGACGGCTAACTCTGTG
CCAGCAGCCGCGGTAATACAGAGGTCCCAAGCGTTGTTCGGACTCACTGGGCGTAAAGGG
TGCGTAGGCGGTTGGGTAAGTCTGACGTGAAATCTCTGGGCTTAACTCAGAGGCTGCTGC
CGAAACTGCTGTGCTAGAGTGCGAGAGAGGCGAGTGGAATTGCGGGTGTAGCGGTGAAAT
GCGTAGATATCCGCAGGAACATCCGAGGCGAAAGCGGCTCGCTGGATCGCAACTGACGCT
GAGGGACGAAAGCTAGGGGAGCAAACAGGATTAGATACCCTGGTAGTCC
>Otu10638
CCAGCCTATGGGGTGCAGCAGTCGAGAATATTTGGCAATGGGCGAAAGCCTGACCATGCG
ACGCCGCGTGCTGGACGAAGTCCTTCGGGACGTAAACAGCTTTTATGGAGGAAGAAGTAA
TTGACATTACTCCATGAATAAGGGGCTCCTAACTCTGTGCCAGCAGGAGCGGTAATACAG
AGGCCCCGAGCATTATCCGGAATCACTGGGCGTAAAGGGTGTGTAGGCGGTCGTGTTAGT
CCTTTGTAAAAGGTCTTCGGCTTAACCGAGGAACTGCAGGGGAAACGGCACGACTTCGAG
GACGCGAGAGGTATGGGGAACTCATGGTGTAGGGGTGAAATCCGTTGATATCATGGGGAA
CACCAAATGCGAAGGCACTTTACTGGTACGCTCCTGACGCTGAAACACGAAAGCGTGGGA
ATCGAACGGGATTAGATACCCCGGTAGTCC

>Otu10659
CCAGCCTACGGGATGCAGCAGCCGAGAATCATTCGCAATGGGCGAAAGCCTGACGATGCG
ACGCCGTGTGAGCGATGAAGGCCTTAGGGTTGTAAAGCTCTTTCGCTTGGGAACAAGAGA
GGTCAGCTAACATCTGACTAATTTGAGGGTACCAGGTAAAGAAGCACCGGCTAACTCCGT
GCCAGCAGCTGCGGTAATACGGAGGGTGCAAGCATTAATCGGATTTATTGGGCGTAAAGG
GCGCGTAGGCGGAGTGAAAAGTCAGATGTGAAATCCCGGGGCTCAACCTCGGAACAGCAT
TTGAAACTCTCACCCTAGAGGGTAGGCGGAGAAAACGGAATTCCACAAGTAGCGGTGAAA
TGCGTAGATATGTGGAAGAACACCGGTGGCGAAGGCGGTTTTCTAGCTTATTCCTGACGC
TGAGGCGCGAGAGCAAGGGGATCAAACAGGATTAGAAACCCCTGTAGTCC
>Otu10664
CCAGCCTACGGGTCGCTCCAGGTCCATGTATCCGATGACGTTCGGTCTCGCTTGTTGTGC
GGTGGAAATGATCCATGCAGGTTGCTCGCGTTATGACCTGGATCGCTTTGGGGTCGTGTT
TCGCGCGTCGCCGCGTCAATCCGATGTCATGATTGTTGCCGGTACTCTGTGCAACAAGAT
GGCGCCGGCCTTGCGCAAGGTGTACGACCAGATGGCCGAGCCGCGCTGGGTGATCTCGAT
GGGGTCGTGTGCCAACGGTGGCGGCTATTACCATTATTCGTATTCCGTCGTGCGCGGCTG
TGATCGCATCGTACCGGTTGATGTCTATGTTCCGGGTTGTCCTCCGACTGCTGAGGCATT
GTTGTACGGCATCATGCAATTGCAAAACAAGATCAAGCGCACCAATACCATCGCGCGCTA
AGCGGCAAGCGCACACTATGACGACGAAATTAGAGACCCCCGTAGTCC
>Otu10668
CCAGCCTACGGGAGGCAGCAGTGGGGAATATTGGACAATGGGGGCAACCCTGATCCAGCC
ATGCCGCGTGAATGATGAAGGCCTTCTGGGTTGTAAACTGCTTTTGCCTGGGGATAAAAG
ACCCTTGCGAGGGAAATTGAAGGTACCGGGTGAATAAGCCACGGCTAACTACGTGCCAGC
AGCCGCGGTAATACGTAGGTGGCAAGCGTTGTCCGGATTTATTGGGTTTAAAGGGTGCGT
AGGCGGTTCATTAAGTCAGTTGTGAAATACGTCAGCTTAACTGACGAGGTGCAATTGATA
CTGGAGAACTTGAGTACAGACGAGGTAGGCGGAATTGACGGTGTAGCGGTGAGATGCATA
GATATCGTCAAGAACACCGATAGCGAAGGCAGCTTACTAGACTGTAACTGACGCTGATGC
ACGAAAGTGTGGGGATCAAACAGGATTAGAAACCCCCGTAGTCC
>Otu10669
CCCAGCCTACGGGAGGCACCAGTGGGGAATCTTGCACAATGGGCGAAAGCCTGATGCAGC
GACGCCGCGTGAGGGAAGAAGGCCTTCGGGTTGTAAACCTCTTTCAGGAGGGACGAAGCC
ACTCGGGTTAATAGCCCAGAGGGTGACGGTACCTCCAGAAGAAGCCCCGGCTAACTACGT
GCCAGCAGCCGCGGTAATACGTAGGGGGCAAGCGTTGTCCGGACTTATTGGGCGTAAAGA
GCGTGTAGGCGGCCATACAGGTCAGCTGTGAAAACTCGAGGCTCAACCTCGAGACGTCGG
TTGAAACCGTACGACTAGAGTCCGGAAGAGGAGAGTGGAATTCCCGGTGTAGCGGTGAAA
TGCGCAGATATCGGGAAGAACACCTATGGCGAAGGCAGCTCTCTGGGACGGTACTGACGC
TGAGACGCGAAAGCGTGGGGAGCGAACAGGATTAGATACCCTAGTAGTCC
>Otu10672
CCAGCCTATGGGTGGCACCAGTGGAGAATATTGGTCAATGAACGAAAGTTTGAACTAGTG
GATTGCGGAAATGAAAGATTCATCGAATCGTGAAGGCTATGACAGTTGTAAAGTTTGTGC
GCAGATTAAAATCTTGTTTTTACATCTGAAGAGAACCCCGGGCCAACAACGTGCCAGCAG
CTGCGGTAAGACGTTGACGGGGAAGCGTTACTGGTCATTACTAGACGTAAAGAGTGGCTA
GATTGTTTAAGTTTTTTTGTTGAAAATCAAAGAATTGACAAAGACAAACAAAAAACCTTA
ATACTTGAGGAATAAGGAGATTTGTGATAAGTTTAATCTAGAGATAGAATTCTAAAAGAT
TAAAAGGATCTTCAAATGTCACGACAGCAATTTACTTATTATCCTGACATCAAACCATTA
AAGTATAGGTATCAATCAGGATTAGAAACCCTGGTAGTCC
>Otu10673
CCAGCCTATGGGTCGCAGCAGTGGGGAATATTGGACAATGGGGGCAACCCTGATCCAGCA
ATGCCGCGTGTGTGAAGAAGGCCTGAGGTTTGTAAAGCACTTTCAGTAGGGAGGAGGACG
TAAAGGTTAAGAGCTATTATGTTGGACGTTACCTACAGAAGAAGCACCGGCTAACTCCGT
GCCAGCAGCCGCGGTAATACGGAGGGTGCGAGCGTTAATCGGAATTACTGGGCGTAAAGG
GTGTGTAGGTGGTTTGATAAGTTATCTGTGAAAGTCCTGGGCTTAACCTGGGAAGGTCAG
ATAAGACTGTTAGACTTGAGTATAGGAGAGGGTAGTGGAATTTCCGGTGTAGCGGTGAAA
TGCGTAGAGATCGGAAGGAACACCAGTGGCGAAGGCGGCTACCTGGCCTAATACTGACAC
TGAGGCACGAAAGCGTGGGGAGCAAACAGGATTAGAAACCCCGGTAGTCC
>Otu10684
CCAGCCTACGGGTGGCTCCAGTGGGGAATATTGCGCAATGGGCGAAAGCCTGACGCAGCG
ACGCCGCGTGGAGGACGAAGGCCTTAGGGTCGTAAGCTCCTGTCAGATGGAAAGAAAAGC
TTGCTCTCTAGTAAAGACAAGTATGACGGTACCATCAGAGGAAGCACCGGCTAACTTCGT

GCCAGCAGCCGCGGTAATACGAAGGGTGCGAGCGTTATTCGAAATTACTGGGCGTAAAGG
GTGCGTAGACGGTGTGTTAAGTTTGTTGTTAAAGACTCCGGCCTAACTGGAGGGTTGCAA
TGAAAACTGGCATGCTAGAGGGTGAGAGAGAGAAGTGGAATTCCCGGAGTAGCGGTAAAA
TGCGTGGATCTCGGGAGGAACACCAATGGCGAAGGCAGCTTCTTGGCTCATTTCTGACGT
TGAGGCACGAAAGCGTGGGGAGCAAACAGGATTAGAAACCCCAGTAGTCC
>Otu10687
CCAGCCCGGGGCGCTGCAGTCGAGAATATTCGACAATGGGCGAAAGCCTGATCGAGCGAC
ACCGCGTGCAGGATGAAGGCCCTCGGGTCGTAAACTGCGGTAGTATGATAAGAATGCAAA
TGACTGTCATACGGAAAGAGGTGGGTAACTACGTGCCAGCACCAGCGGTAATACGTAGAC
CTCAAGCGTTATTCGGATTTATTGGGCGTAAAGCGCACGTAGGAGGTTTTGCGCGTCTTT
TGTTAAAGCCCGGGGCCTAACCCCGGAAGTGCGGGAGATACGGCAGAACTAAAGGAGGTT
AGAGGTGCATGGAACTCACGGTGTAGGGGTGAAATCCGTTGATATCGTGCGGAATACCAA
AGGCGAAGGCATTGCACTGGGACCTTCCTGACACTGAGACGCGAAAGCGTGGGGAGCAAA
AAGGATTAGATACCCTAGTAGTCC
>Otu10688
CCAGCCTACGGGGCGCTCCAGTGGGGAATATTGGACAATGGGCGGGAGCCTGATCCAGCC
ATGCCGCGTGCAGGAAGAAGGCCTTCTGGGTTGTAAACTACTTTTGCCAGGGGATAAAAG
ACCCGTGCGCGGGGAATTGAAGGTACCTGGTGAATAAGCCACGGCTAACTACGTGCCAGC
AGCCGCGGTAATACGTAGGTGGCAAGCGTTGTCCGGATTTATTGGGTTTAAAGGGTGTGT
AGGCGGGCATGTAAGTCAGTGGTGAAATCTCCGGGCTCAACCCGGAAACTGCCATTGATA
CTATATGCCTTGAATTCTGTTGAGGTTGGCGGAATAAGTCATGTAGCGGTGAAATGCATA
GATATGACTTAGAACACCGATTGCGAAGGCAGCTAACTAAGCAGATATTGACGCTGAGGC
ACGAAAGCGTGGGGATCAAACAGGATTAGAGACCCGAGTAGTCC
>Otu10691
CCAGCCTACGGGTGGCAGCAGACGAGAATATTCCGCAATGGACGAAAGTCTGACGGAGCG
ACATCGCGTGATGGATGAAGTGCTTAGGTACGTAAACATCTTTTATCGGGGACGAAGTAA
TTGACGTTACTCCATGAATAAGGGGCTCCTAACTCTGTGCCAGCAGGAGCGGTAATACAG
AGGCCCCGAGCATTACCCGGAATCATTGGGCGTAAAGAGTGCGTAGGTGGTCATGTTAGT
CGGGCGTTAAACCCCGGGGCTCAACCCCGGAATCGCACCCGAAACGGCATGACTCGAGGG
CGTGAGAGGTACATGGAACTCATGGTGTAGGGGTGAAATCCGTTGATATCATGGGGAACA
CCGAAAGCGAAGGCAATGTACTGGCGCGTTCCTGACACTCAAGCACGAAAGCGTGGGTAG
CGAACGGGATTAGATACCCGCGTAGTCC
>Otu10707
CCAGCCTATGGGAGGCAGCAGTCGAGAATTCTTCACAATGGGCGAAAGCCTGATGGAGCG
ACGCCGCGTGGGGGATGAATGGCTTCGGCCCGTAAACCCCTGTCATTTGCGAACAAACCT
CGCCATTTAACAGACGGCGAGCTGATTGTAGCGAAAGAGGAAGGGACGGCTAACTCTGTG
CCAGCAGCCGCGGTAATACAGAGGTCCCAAGCGTTGTTCGGATTCACTGGGCGTAAAGGG
TGCGTAGGCGGTCGGGTAAGTCTGACGTGAAATCTCGCGGCTTAACCGCGAAAAGGCGTC
GGATACTATCCGGCTTGAGGAGTGGAGGGGAGACTGGAATTCTCGGTGTAGCAGTGAAAT
GCGTAGATATCGAGAGGAACACCAGTGGCGAAGGCGAGTCTCTGGACACTTCCTGACGCT
GAGGCACGAAAGCCAGGGGAGCAAACGGGATTAGAAACCCCTGTAGTCC
>Otu10730
CCAGCCTATGGGATGCTGCAGCAGGGAATCGTACGCAATGGACGAAAGTCTGACGTCGCA
ATGCCGCGTGGAGGATGAAGGCCTTCGGGTCGTAAACTCCTTTTACGGTAATAGAGATAT
TATCGAGAATAAGCACCTGCTAACTCTGTGCCAGCAGCCGCGGTAATACAGAGGGTGCAA
GCGTTATCCGGATTTATTGGGCGTAAAGCGTTTCGTAGGTGACACGGCAAGTTATTTTTC
AAATACCACGGCTTAACCGGGGGAAGGGAGATAATACTACCGAGTTTGACTTCTGGTGGG
CATCTGGAACTGATGGTGTAGTAGTGAAATACGTTGATATCATCAGGAACTCCAAGGGCG
AAGGCAGGATGCTAACCAGTTAGTGACACTGAGGAACGACAGCTAGGGGAGCGAAAGGGA
TTAGAAACCCTAGTAGTCC
>Otu10739
CCAGCCTATGGGAGGCTCCAGTCGAGAATTTTTCTCAATGGGCGAAAGCCTGAAGGAGCG
ACGCCGCGTGGGGGATGAATGGCTTCGGCCCGTAAACCCCTGTCATTTGTGAACAAATTG
CTTCACCCAACACGTGAAGCATTGATTGTAACGGAAGAGGAAGGGACGGCTAACTCTGTG
CCAGCAGTCGCGGTAATACGTAGGCAGCAAGCGTTGTTCGGAGTTACTGGGCGTAAAGGG
TGCGTAGGCGGCTTTTTAAGTTTGGTGTGAAATCTCCCGGCTCAACTGGGAGGGTGCGCC
GAATACTGAGAGGCTAGAGTGTGGGAGAGGAAAGTGGAATTCCTGGTGTAGCGGTGAAAT
GCGTAGATATCAGGAGGAACACCGGTGGTGTAAACGGCTTTCTGGACCATAACTGACGCT

GAGGCACGAAAGCGTGGGTAGCAAACAGGATTAGATACCCGTGTAGTCC
>Otu10744
CCAGCCTATGGGTTGCAGCAGTGGGGAATATTGGACAATGGGCGAAAGCCTGATCCAGCA
ATACCGCGTGTGTGAAGAAGGCCCTCGGGTTGTAAAGCACTTTCAGTAGGGAGGAAAGAA
TTAGGCTAATACCCCTGAGAGAAGACGTTACCTACAGAAGAAGCACCGGCTAACTCTGTG
CCAGCAGCCGCGGTAATACAGAGGGTGCAAGCGTTAATCGGAATTACTGGGCGTAAAGCG
CTCGTAGGCGGGTGTTTAAGTCGATCGTGAAAGCCCTGGGCTTAACCTGGGAATGGCGGT
CGATACTGGGCACCTAGAGTATGGTAGAGGATAGTGGAATTCCCGGTGTAGCAGTGAAAT
GCGTAGATATCGGGAGGAACACCAGTGGCGAAGGCGACTATCTGGACCAATACTGACGCT
GAGGAGCGAAAGCGTGGGGAGCAAACAGGATTAGAAACCCGTGTAGTCC
>Otu10749
CCAGCCTACGGGAGGCTCCAGCTAAGAATATTCCGCAATGGACGAAAGTCTGATGGAGCG
ACGCCGCGTGGGGGATGAATGGCTTCGGCCCGTAAACCCCTGTCATTTGCGATCAACCGT
TATTGTTTAAGAGATGATAACCTGATAGTAGCGAAAGAGGAAGGGACGGCTAACTCTGTG
CCAGCAGCCGCGGTAATACAGAGGTCCCAAGCGTTGTTCGGATTCACTGGGCGTAAGGGG
TGCGTAGGTGGCGAAGTAAGTCGGATGTGAAATCTCCGAGCTCAACTCGGAAACTGCATT
GGAAACTACTTTGCTCGAGGATTGGAGGGGGGACTGGAATACTTGGTGTAGCAGTGAAAT
GCGTAGATATCAAGTGGAACACCAGTGGCGAAGGCGAGTCCCTGGACAATTCCTGACACT
GAGGCACGAAAGCTAGGGGAGCAAACAGGATTAGAGACCCCAGTAGTCC
>Otu10766
CCAGCCTATGGGAGGCTGCAGCCGAGAATATTCGACAATGGGCGAAAGCCTGATCGAGCG
ATACCGCGTGGTGGATGAAGTGCTTCGGCACGTAAATACCTCTTATGGAGGAGGAAGTTA
TTGACGTTACTTCATGAATAAGGGGCTCCTAACTCTGTGCCAGCAGGAGCGGTAATACAG
AGGCCCCGAGCATTACCCGGAATTATTGGGCGTAAAGAGTGCGTAGGTGGTCATATTAGT
CGGTCGTTAAAACCCGGGGCTCAACCCCGGAGGTGCGACCGAAACGGTATGACTCGAGGG
CGTGAGAGGTACATGGAACTCATGGTGTAGGGGTGAAATCCGTTGATATCATGGGGAACA
CCGAAAGCGAAGGCAATGTACTGGCGCGTTTCTGACACTCAAGCACGAAAGCGTGGGTAG
CGAACGGGATTAGATACCCCGGTAGTCC
>Otu10810
CCAGCCTACGGGGGGCACCAGTGGGGAATATTGGACAATGGGCGAAAGCCTGATCCAGCA
ATGCCGCGTGTGTGAAGAAGGCCTTCGGGTTGTAAAGCACTTTAGTGGGGGAAGAAAGCT
ACGCAAGTAGTTTGACGGTACCCCAAGAATAAGCAACGGCTAACTCTGTGCCAGCAGCCG
CGGTAATACAGAGGTTGCGAGCGTTAATCGGAATGACTGGGCGTAAAGGGCGCGTAGGTG
GATGTGTGTGTTGGATGTGAAAGCCCCGGGCTTAACCTGGGAATTGCATACAAAACTGCA
CATCTAGAGTAGAGCAGAGGTAAGTGGAATTTCCGGAGTAGCGGTGAAATGCGTAGATAC
CGGAAGGAACACCAGTGGCGAAGGCGACTTACTGGGCTCAAACTGACACTGAGGCGCGAA
AGCGTGGGGAGCAAACAGGATTAGAAACCCCCGTAGTCC
>Otu10839
CCAGCCTACGGGGGGCAGCAGCCGAGAATATTCGACAATGGGCGAAAGCCTGATCGAGCG
ATACCGCGTGGTGGATGAAGCGCTTCGGCGCGTAAACATCTTTTATGGAGGAGGAAGTTA
TTGACGTTACTCCATGAATAAGGGGCTCCTAACTCTGTGCCAGCAGGAGCGGTAATACAG
AGGCCCCGAGCGTTACCCGGAATTACTGGGCGTAAAGAGTGCGTAGGTGGTCGTGTTAGT
CGCGCGTCAAAACCCGGAGCTCAACTCCGGATCCGCGCGCGAAACGGCACGACTCGAGGG
CGTGAGAGGTGCATAGAACTCTAGGTGTAGGGGTGAAATCCGTTGATATCTAGGGGAATA
CCGAAAGCGAAGGCAGTGCACTGGCGCGTTCCTGACACTCAAGCACGAAAGCGTGGGGAG
CAAAAAGGATTAGATACCCCTGTAGTCC
>Otu10849
CCAGCCTATGGGTGGCTCCAGTCGAGGATCTTTCGCAATGGGCGCAAGCCTGACGAAGCG
ACGCTGCGTGAACGATGAAGGCCTTCGGGTCGTAAAGTTCTTTCGCGCAAGAACAAGGAA
ATGATGTGAATAACATCGTAACTTGAGGGTACTGCGTAAAGAAGCACCGGCTAACTCCGT
GCCAGCAGCTGCGGTAATACGGAGAGTGCAAGCATTAATCGGAATTATTGGGCGTAAAGG
GCGCGTAGGCGGAAGTGTAAGTCAGATGTGAAATTCCGAAGCTCAACTTCGGAGCTGCAT
TTGAAACTACACATCTAGAGGGTTGACGGAGAAAACGGAATTCCACGTGTAGCGGTGAAA
TGCGTAGATATGTGGAAGAACACCGGTGGCGAAAGCGGTTTTCTGGGCTGAGACTGACGC
TGAGGCGCGAAAGCAAGGGGAGCAAACAGGATTAGAAACCCGAGTAGTCC
>Otu10857
CCAGCCTACGGGTTGCTCCAGTGGGGAATTTTGGACAATGGGGGAAACCCTGATCCAGCA
ATGCCGCGTGTGTGAAGAAGGCCTTCGGGTTGTAAAGCACTTTTGTCCGGAAAGAAATCC

TGTGGGCTAATACCCTGTGGGGATGACGGTACCGGAAGAATAAGCACCGGCTAACTACGT
GCCAGCAGCCGCGGTAATACGTAGGGTGCAAGCGTTAATCGGAATTACTGGGCGTAAAGC
GTGCGCAGGCGGTTCGCTAAGACAGATGTGAAATCCCCGGGCTTAACCTGGGAACTGCAT
TTGAGACTGCATGGCTAGAGTATCGAAGAGGGAAGTGGAATTTCCGGTGTAGCGGTGAAA
TGCGTAGATATCGGAAGGAACACCAGTGGCGAAAGCGACTTCCTGGTCGAATACTGACGT
TCATGTGCGAAGGCGTGGGGAGCAAACAGGATTAGAAACCCCAGTAGTCC
>Otu10860
CCAGCCTACGGGGGGCTGCAGTCGAGAATATTCGACAATGGACGAAAGTCTGATCGAGCG
ACACCGCGTGTAGGATGAAGGCCCTCGGGTCGTAAACTACGGTAGTATGATAAGAATGCA
AATGACTGTCATACGGAAAGAGGTGGGTAACTACGTGCCAGCACCAGCGGTAATACGTAG
ACCTCAAGCGTTATCCGGATTTATTGGGCGTAAAGAGCATGTAGGAGGTTTCGCGCGTCT
TTTGTTAAAGCCCAGGGCCTAACCCTGGAAGTGCAGGAGATACGGCGGAACTAGAGGAGG
TTAGAGGTGCATGGAACTCATGGTGTAGGGGTGAAATCCGTTGATATCATGGGGAACACC
AAATGCGAAGGCAGTACACTGGCGCATATTTGACACTGAAGCACGAAAGCGTGGGTAGCG
AATGGGATTAGATACCCGCTAGTCC
>Otu10862
CCAGCCTACGGGAGGCTGCAGTCGAGGATCTTCGGCAATGGGCGAAAGCCTGACCGAGCG
ACGCCGCGTGCGCGATGAAGGCCTTCGGGTTGTAAAGCGCTGTCGAGGGGGAGGAAGCCG
AAAGGTTGACCTATCCCTGGAGGAAGCACGGGCTAAGTTCGTGCCAGCAGCCGCGGTAAG
ACGAACCGTGCGAACGTTGTTCGGAATCACTGGGCTTAAAGGGCGCGTAGGCGGGCGATC
AAGTCAGGGGTGAAATCTTTCGGCTTAACCGGAAAATAGCCTTTGATACTGGTTGTCTTG
AGGGAGGTAGGGGCATGTGGAACTTCCGGTGGAGCGGTGAAATGTGTTGATATCGGAAGG
AACGCCGGTGGCGAAAGCGACGTGCTGGATCTCTTCTGACGCTGAGGCGCGAAAGCTAGG
GTAGCGAACGGGATTAGAACCCTTGTAGTCC
>Otu10865
CCAGCCTACGGGGGGCAGCAGTGGGGAATCTTGCGCAATGGGCGAAAGCCTGACGCAGCG
ACGCCGCGTGAGTGATGAAGGCCTTCGGGTTGTAAAGCTCTGTGGGGAGAGACGAATAAG
TCTTGGTGAATATCCACGATGATGACGGTATCTCCTTAGCAAGCACCGGCTAACTCTGTG
CCAGCAGCCGCGGTAAGACAGAGGGTGCAAACGTTGTTCGGAATTACTGGGCGTAAAGCG
TGTGTAGGCGGTCTAACAAGTCGGATGTGAAAGCCCCGGGCTCAACCCGGGAAGTGCACT
CGAAACTGGTAGACTGGAGTGTCGGAGAGGTGGGTAGAATTCTCGGTGTAGAGGTGAAAT
TCGTAGATATCGAGAGGAATACCGGCGGCGAAGGCGGCCCACTGGACGAACACTGACGCT
GAGACACGAAAGCGTGGGGAGCAAACAGGATTAGATACCCCAGTAGTCC
>Otu10871
CCAGCCTATGGGTGGCTCCAGTCGAGAATCTTCCGCAATGGACGAAAGTCTGACGGAGCG
ACGCCGCGTGGAGGATGAAGTTCTTCGGAATGTAAACTCCTTTTGCCAGGGAAAAAGTTA
TTGATTGTACCTGGAGAATAAGAAGTTGCTAAACTCGTGCCAGCAGCAGCGGTAATACGA
GTGCTTCGAGCATTATCCGGAATCATTGGGCGTAAAGGGTGCGTAGGTGGTAGTGTCAGT
CTCGTGTAAAATCTTCCGGCTCAACCGGGAACTCGCACGAGAAACGGCACAACTAAGAGG
ACGAGAGAGGTTTCTGGAACTCATGGTGTAGCGGTGAAATGCGTTGATATCATGGGGAAC
ACCAAAAGCGAAGGCAAGAAACTGGCTCGCTCCCGACACTGAAGCACGAAAGCGTGGGTC
GCGAATGGGATTAGATACCCGCGTAGTCC
>Otu10876
CCAGCCTACGGGGTGCAGCAGTCGAGAATCATTCGCAATGGGCGAAAGCCTGACGATGCG
ACGCTGCGTGAGTGATGAAGGCCTTCGGGTCGTAAAGCTCTTTCGCCTGGGAACAAGAGA
GGTCTGCTAATATCAGGCCAATTTGAGGGTATCAGGTAAAGAAGCACCGGCTAACTCCGT
GCCAGCAGCTGCGGTAATACGGAGGGTGCAAGCATTAATCGGATTTATTGGGCGTAAAGG
GCGCGTAGGCGGGGAGGAAAGTCAGATGTGAAACGCCGGGGCTCAACCCCGGAACTGCAT
TTGAAACTTCCTTTCTTGAGGGTAGGCGGAGATAATGGAATTCCACAAGTAGCGGTGAAA
TGCGTAGATATGTGGAAGAACACCGGTGGCGAAGGCGATTATCTAGCTTATTCCTGACGC
TGAGGCGCGAAAGCTAGGGGAGCAAACAGGATTAGATACCCCAGTAGTCC
>Otu10878
CCAGCCTATGGGGTGCAGCAGTGGGGAATCTTGCGCAATGGGCGAAAGCCTGACGCAGCA
ACGCCGCGTGAGGGACGAAGGCTTTCTGAGTTGTAAACCTCTTTCAGCAGGGACGATTGT
GACGGTACCTGCAGAAGAAGCACCGGCCAACTACGTGCCAGCAGCCGCGGTGATACGTAG
GGTGCAAGCGTTGTCCGGATTTATTGGGCGTAAAGAGCTCGTAGGCGGTTTGATAAGTCG
GATGTGAAACCCCCAGGCTTAACCTGGGGCCGCCATTCGATACTGTCATGACTTGAGTCC
GGTAGGGGACCACGGAATTCCTGGTGTAGCGGTGAAATGCGCAGATATCAGGAGGAACAC

CAGCGGCGAAGGCGGTGGTCTGGGCCGGCACTGACGCTGAGGCACGAAAGTGTGGGGATC
AAACAGGATTAGAGACCCCTGTAGTCC
>Otu10880
CCAGCCTATGGGTGGCACCAGTCGAGGATCTTCGGCAATGTGCGCAAGCACGACCGAGCG
ACGCCGCGTGGGCGACGAAGGCCTTCGGGTTGTAAAGCACTGTCGAGGGGGAGAAAAGCC
CGCAAGGGTCTGATCTATCCCTGGAGGAAGCACGGGCTAAGTTCGTGCCAGCAGCCGCGG
TAAGACGAACCGTGCGAACGTTGTTCGGAATCACTGGGCTTAAAGGGCGCGTAGGCGGGT
CGTCAAGTCAGGGGTGAAATACTTCGGCTCAACCGGAGAAGTGCTTCTGATACTGGCGAC
CTCGAGGGGTGCAGGGGCATGTGGAACTTCCGGTGGAGCGGTGAAATGCGTAGATATCGG
AAGGAACGCCGGTGGCGAAAGCGACGTGCTGGACACCTTCTGACGCTGAGGCGCGAAAGC
CAGGGGAGCAAACGGGATTAGAGACCCGTGTAGTCC
>Otu10881
CCAGCCATGGGGGGCTGCAGTCAAGAACATTTGGCAATGGGCGCAAGCCTGACCATGCGA
CGCCGCGTGGTTGACGAAGTCCTTCGGGACGTAAAGACCTTTTACGCGTGAAGAAGTAAT
TGACATTAGCGCGTGAATAAGGGGCTCCTAACTCTGTGCCAGCAGGAGCGGTAATACAGA
GGCCCCAAGCATTATCCGGAATCACTGGGCGTAAAGGGTGTGTAGGCGGTCATATTAGTC
CTTCGTAAAAGCACTGGGGCTTAACCCCGGTAATGCGGTGGAAACGGTATGACTCGAGGA
CGCGAGAGGTAAGGGGAACTCATGGTGTAGGGGTGAAATCCGTTGATATCATGGGGAACA
CCAAATGCGAAGGCACCTTACTGGCGCGCTCCTGACGCTGAAACACGAAAGCGTGGGAAT
CGAACGGGATTAGAAACCCCCGTAGTCC
>Otu10886
CCAGCCTACGGGTGGCAGCAGTGGGGAATATTGGACAATGGGCGCAAGCCTGATCCAGCC
ATGCCGCGTGAGTGATGAAGGCCTTCGGGTTGTAAAGCTCTTTAGCCGATGATTATAATG
ACTGTAGTCGGAAAATAAGCCCCGGCTAACTTCGTGCCAGCAGCCGCGGTAATACGAAGG
GGGCGAGCGTTATTCGGAATCACTGGGCGTAAAGCGTGCGTAGGCTGTTTCACAAGTTGG
AAGTGAAAGCCCAGGGCTCAACCCTGGAATTGCTTTCAAAACTGTGAGACTCGAATCTCG
GAGAGGATAGCGGAATTTCCAGTGTAGTAGTGAAATACGTAGATATTGGAAGGAACACCA
GTGGCGCAAGCGGCTATCTGGACGAGTATTGACGCTGAGGCACGAAAGCGTGGGGATCAA
ACAGGATTAGAAACCCTAGTAGTCC
>Otu10892
GGACTACCGGGGTTTCTAATCCTGTTTGCTACCCACGCTTTCGTGTCTCAGCGTCAGAAG
TGGTCCAGTAAGCTGTCTACACCACAGGTGTTCCTCCTGATATCTACGCATTTCACCGCT
ACACCAGGAATTCCGCTTACCTCTCTCACCCTCTAGCATTCTAGTTTCCGCTGCACCCTC
CCAGCTAAGCCGGGAGATTTCACAACAGACTGGGAACACCGCCTACACACTCTTTACGCC
CAGTAATTCCGAACAACGCTCGCTGCCTACGTATTACCGCGGCTGCTGGCACGTAGTTAG
CCGCAGCTTCTTCTCCGGGTACCGTCATTATCGTTCCCGTCGAAAGAGCTTTACACCCCG
AAGGGCTTCATCGCTCACGCGGCGTCGCTGCATCAGGCTTTCGCCCATTGTGCAAGATTC
CCCACTGGTGCCTCCCATAGGCTGG
>Otu10900
GGACTACGCGGGTATCTAATCCTGTTTGATCCCCACGCTTTCGTGCCTCAGCGTCAATCA
TACTTTAGTAAGCTGCCTTCGCAATTGGTGTTCTGTGACATATCTATGCATTTCACCGCT
ACTTGTCACATTCCGCCTACCTCAAGTACATTCAAGCCCATCAGTATCAAAGGCACTGCG
ATAGTTGAGCTACCGTCTTTCACCCCTGACTTAATAGGCCGCCTACGCACCCTTTAAACC
CAATAAATCCGGATAACGCTTGGATCCTCCGTATTACCGCGGCTGCTGGCACGGAGTTAG
CCGATCCTTATTCTCAGAGTACATTCAACCCCGTATACATACGGGGGTTTATTCCCCTGC
AAAAGCAGTTTACAACCCGTAGGGCCGTCTTCCTGCACGCGGCATGGCTGGTTCAGGCTT
GCGCCCATTGACCAATATTCCTTACTGCTGCCACCCGTAGGCTGG
>Otu10903
CCAGCCTACGGGTGGCTGCAGTAGGGAATTTTCCACAATGGGCGAAAGCCTGATGGAGCA
ACGCCGCGTGCAGGATGAAGGCTTTCGGGTCGTAAACTGCTTTTCTGATCGACGATTATG
ACGGTAGGTCAGGAATAAGGGTCTGCTAACTACGTGCCAGCAGCCGCGGTCATACGTGGG
ACCCAAGCGTTATCCGGAATTACTGGGCGTAAAGAGTTGCGTAGGTGGCAGAGTAAGCAA
GATATGAAAGCGTGTGGCTCAACCATACATGGCATATTTTGAACTGCTCAGCTAGAGTAT
GGGAGAGGTAGATGGAATTTCTAGTGTAGGAGTGAAATCCGTAGATATTAGAAGGAACAC
CGATGGCGTAGGCAGTCTACCGGCCCATTACTGACACTAAGTCACGAAAGCGTGGGGAGC
AAACAGGATTAGAGACCCTCGTAGTCC
>Otu10914
CCAGCCTACGGGACGCAGCAGTAGGGAATATTGCACAATGGAGGAAACTCTGATGCAGCG

ACGCCGCGTGAGTGATGAAGGACTTCGGTTTGTAAAGCTCTGTTCTCAGGGAATAAAAAA
ATGAATGTACTTGAGGAGAAAGGGACGGCTAACTTCGTGCCAGCAGCCGCGGTAATACGA
AGGTCCCAAGCGTTGCTCGGAATCATTGGGCGTAAAGCGAGCGCAGGTGGTTTTGTAAGT
CAGGAGTGAAAGCCCAAGGCTTAACCTTGGAAGTGCTTTTGATACTGCAAAACTTGAGTG
TGGGAGAGGGTGCTAGAATTCCTGGTGTAGTGGTGAAATACGTAGAGATCAGGAGGAACA
CCGGTGGCGAAGGCGGGCACCTGGCCCAACACTGACACTTAGGCTCGAAAGCGTGGGGAT
CAAACAGGATTAGAAACCCCAGTAGTCC
>Otu10920
CCAGCCTACGGGTCGCAGCAGTGAGGGATATTGCACAATGGGCGAAAGCCTGATGCAGCG
ACGCCGCGTGGGTGATGAAGGTCTTCGGATCGTAAAGCCCTTTTCTGTGTGACGAAAACA
GACGGTAGCACAGGAATAAGTCTCGGCTAACTACGTGCCAGCAGCCGCGGTAAAACGTAG
GAGGCAAGCGTTATCCGGAGTTACTGGGCGTAAAGCGTGTGTAGGTGGTTATTCAAGTTC
GGTGTGAAAGCGCCCGGCTCAACTGGGCGAGGACATCGAAGACTGAATGGCTAGAGGATG
GTAGAGGGGCGTAGAATTCCGGGTGTAGCGGTGAAATGTGTAGAGATCCGGAGGAATACC
AGTGGTGCAGACGGCGCCCCGGGCCATACCTGACACTGAGACACGAAAGCGTGGGGAGCG
AACGGGATTAGAAACCCTGGTAGTCC
>Otu10921
CCAGCCTATGGGTTGCTGCAGTGAGGAATATTGCGCAATGGGCGAAAGCCTGACGCAGCG
ACGCCGCGTGGAGGATGAAGGTCTTAGGATTGTAAACTTCTGTTAAGTGGGAAGAAAATC
TCTTTTCTAATAAAAAAGAGCTATGACGGTACCATTAGAGAAAGCACCGGCTAACTTCGT
GCCAGCAGCCGCGGTAATACGAAGGGTGCAAGCGTTATTCGGAATAATTGGGCGTAAAGG
GTGCGCAGGCTGTATGTTAAGTCAACTGTTAAATTCTTCAGCCTAACTGAGGGTCTGCGG
TAGATACTGGCAAACTAGAGTATGGAAGAGAGAAGTGGAATTCTCGGAGTAGCGGTAAAA
TGCGTAGATCTCGAGGGGAACACCGATGGCGAAGGCAGCTTCTTGGTCCATTACTGACGC
TCAAGCACGAAAGCGTGGGGAGCAAACAGGATTAGATACCCTAGTAGTCC
>Otu10926
CCAGCCTATGGGAGGCAGCAGTCAAGAACCTTCCACAATGGACGAAAGTCTGATGGAGCG
ATGCCGCGTGATTGATGAAGTGCTTCGGCACGTAAAGGTCTTTTATGAGGGAAGAAATTA
TTGACGGTACCTCATGAATAAGGGGCTCCTAACTCTGTGCCAGCAGGAGCGGTAATACAG
AGGCCCCGAGCGTTATCCGGAATTATTGGGCGTAAAGGGTGTGTAGGTGGCGTTGTTAGT
CGTTTGTGAAAGCCCGGGGCTCAACCTCGGAGATGCGAACGAAACGGCAATGCTTGAGTA
TGTGAGAGGTAAGCGGAACTCATGGTGTAGGGGTGAAATCCGTTGATATCATGGGGAACA
CCAAATGCGAAGGCAATATACTGGCGCACTCCTGACACTGAAACACGAAAGCGTAGGTAG
CGAATGGGATTAGATACCCCAGTAGTCC
>Otu10933
CCAGCCTATGGGTCGCACCAGTGGGGAATTGTTCGCAATGGGCGAAAGCCTGACGACGCA
ACGCCGCGTGGAGGATGAAGACCTTCGGGTCGTAAACTCCTTTCGACCGAGATGAATATT
CATCGGCTTAATACACCGATGGAGTGACAGTATCGAGGGAAGAAGCCCCGGCTAACTCTG
TGCCAGCAGCCGCGGTAATACAGGGGGGGCAAGCGTTGTTCGGAATTACTGGGCGTAAAG
GGTTCGTAGGTGGCTTGTTGAGTCAGACGTGAAATCCCTCAGCTTAACTGGGGAACTGCG
TCTGATACTGACGAGTTTGAGTGCAGGAGAGGAACGCGGAATTCCAGGTGTAGCGGTGAA
ATGCGTAGATATCTGGAGGAACACCGGTGGCGAAGGCGGCGTTCTGGACTGCAACTGACA
CTGAGGAACGAAAGCCAGGGGAGCAAACGGGATTAGAGACCCCCGTAGTCC
>Otu10945
CCAGCCTACGGGAGGCAGCAGTAGGGAATTTTCCACAATGGGCGAAAGCCTGATGGAGCA
ACGCCGCGTGAGGGATGAAGGTTTTCGGATCGTAAACCTCTTTGGAGCGGGAAGAATAGC
GCTTGGGTGAATAATCCAGGCGCCTGACGGTACCGCTAGAACAAGACACGGCTAACTCTG
TGCCAGCAGCCGCGGTAATACAGAGGTGTCAAACGTTGTCCGGAATTATTGGGCGTAAAG
GGCGCGTAGGCGGCCGGGTAAGTGAGATGTGAAAGCTCTGGGCTCAACCCAGGAATTGCA
TTTCATACTGCTCGGCTAGAGATTGGTAGAGGATGGCGGAATTCCAGGTGTAGCGGTGAA
ATGCGTAGATATCTGGAGGAACACCTGTGGCGAAGGCGGCTATCTGGTCCAATTCTGACG
CTGAGGCGCGAAAGGTAGGGGAGCAAACAGGATTAGATACCCGGGTAGTCC
>Otu10954
CCAGCCTACGGGGTGCTCCAGTGGGGAATTTTGCGCAATGGGGGAAACCCTGACGCAGCA
ACGCCGCGTGGAGGATGAAGTCCCTTGGGACGTAAACTCCTTTCGACCAAGACGATAATG
ACGGTACTGGTGGAAGAAGCACCGGCCAACTCTGTGCCAGCAGCCGCGGTAATACAGAGG
GTGCAAGCGTTGTTCGGAATTATTGGGCGTAAAGGGTTCGTAGGCGGGAATGCAAGTCAA
GTGTGAAATCCCCAGGCTCAACCTGGGACGTGCATTTGAGACTGTGTTTCTTGAGTTTCG

GAGAGGGTGGTGGAATTGCTGGTGTAGGAGTGACATCCGTAGAGATCAGCAGGAACACCG
GAGGCGAAGGCGACCACCTGGCCGAATACTGACGCTGAGGAACGAAAGCGTGGGGAGCAA
ACAGGATTAGAAACCCTGGTAGTCC
>Otu10956
CCAGCCTATGGGTTGCAGCAGTAGGGAATTTTGCACAATGGGCGCAAGCCTGATGCAGCA
ATGCCGCGTGAGTGAAGAAGGCTCTTGGGTTGTAAAGCTCTTTCGGCTGGGAAGAAGGGG
TAGCACTCGAACAGAAGGCTATTCTGACGGTACCAGAAGAAGAAGCACCTGCTAACTTCG
TGCCAGCAGCAGCGGTAATACTAAGGGTGCAAGCGTTGTTCGGAATTACTGGGCGTAAAG
CGCGCGTAGGCGGTTAGTAAGTCGATTGTGAAATCCCTGGGCTTAACCTGGGAACTGCA
GTCGAAACTACTAATCTTGAATGTCTGAGAGGATGGCGGAATAACCGGTGTAGTAGTGAA
ATACGTAGATATCGGTTAGAACATCTGAGGCGAAGGCGGCTATCTGGCGGTACATTGACG
CTGATGCGCGAAAGCGTGGGGAGCAAACAGGATTAGATACCCCCGTAGTCC
>Otu10963
CCAGCCTACGGGTGGCAGCAGTGGGGAATTTTGCGCAATGGGCGAAAGCCTGACGCAGCG
ACGCCGCGTGCGGGATGAAGGCCTTCGGGTCGTAAACCGCTTTCAGCAGGGACGAAATTG
ACGGTACCTGCAAAAGAAGGCCCGGCCAACTACGTGCCAGCAGCCGCGGTGATACGTAGG
GGTCTAGCGTTGTCCGGAATCATTGGGCGTAAAGAGCTCGTAGACGGTTCGGCAAGTCGG
ATGTGAAATCTCCACGCTCAACGTGGAGGGGCCATTCGAAACTGCTGTGACTCGAGTCCG
GTAGGGGAGTTCGGAATTCCCGGTGTAGCGGTGAAATGCGCAGATATCGGGAGGAACACC
AGTGGCGAAGGCGGAACTCTGGGCCGGAACTGACGCTGAGGAGCGAAAGCGTGGGGAGCG
AACAGGATTAGAAACCCTCGTAGTCC
>Otu10966
CCAGCCTACGGGGGGCAGCAGTGGGGAATATTGCGCAATGGGCGAAAGTCTGACCGAGCG
ACGCCGCGTGCGGGATGAAGGCCCTTTGGGTTGTAAACCGCTGTCACAGGTGAGCAAATC
ACGTTCAGTGAACAACTGGACGTGTTGAGTTAAGCTTGAGAGGAAGCCCCCGCTAATCAC
GTGCCAGCAGCGGCGGTAATACGTGAGGGGCGAACGTTGTTCGGTGTCACTGGGCTTAAA
GGGTGCGTAGGTGGCTGCGTAAGTAACGTGTGAAAGCCCTCGGCTTACCCGGGGAATTGC
GCGTTATACTGCGCGGCTTGAGGATTTCAGGGGAAAGGGGAACTCCGAGTGTAGCGGTGA
AATGCGCAGATATTCGGGGGAAGGCCGGCGGCGAAGGCGCCTTTCTGGGAAATTCCTGAC
ACTGAGGCACGAAAGCGTGGGGAGCAAACAGGATTAGAAACCCTGGTAGTCC
>Otu10968
CTAGCCTACGGGTGGCTGCAGTCAAGAATTTTCCCCAATGGACGAAAGTCTGAGGGAGCG
ACGCCGCGTGCAGGAAGAAACTCTTCGGGGTGTAAACTGCTTTTGCATGGGAATAAGATC
TGAATGTACCATGCGAATAAGGACCTGCTAAACTCGTGCCAGCAGCAGCGGTAATACGAG
TGGTCCAAGCGTTATCCGGTTTTATTGGGCGTAAAGGGTCCGCAGGTGGTTCGATAAGTC
TGGTGTTAAATTCTGGCGCTCAACGTCAGAGCCGCATTGGAAACTATCGAGCTAGAGGCT
GGGAGAGGTGCATGGAATTGTCGGTGTAGTAGTAAAATGCGTTAATATCGACAAGAACAC
CAAAGGCGAAGGCATTGCACTAGAACAGTTCTGACACTCAGGGACGAAAGCGTGGGGAGC
GAATGGGATTAGAGACCCGCGTAGTCC
>Otu10969
CCAGCCTATGGGGGGCACCAGTGGGGAATCTTGCGCAATGGGCGAAAGCCTGACGCAGCC
ACGCCGCGTGAGTGAAGAAGGCCTTCGGGTTGTAAAGCTCTGTCGCCCGGGACGAAAAGC
CCTTGGACTAATACTCCAGGGGCCTGACGGTACCGGGAAAGGAAGCACCGGCTAACTCTG
TGCCAGCAGCCGCGGTAATACAGAGGGTGCAAGCGTTGCTCGGAATTATTGGGCGTAAAG
GGTAGGTAGGTGGTCTCATTTGTCTCGTGTGAAAGCCTTGGGCTTAACTCAAGAAGTGCG
CGAGAAACGGTGAGACTGGAGTCCTGGAGAGGGTCGTGGAATTCCCGGTGTAGCGGTGAA
ATGCGTAGAGATCGGGAGGAACACCAGAGGCGAAGGCGGCGACCTGGACAGGTACTGACA
CTCAACTACGAAAGCGTGGGGAGCAAACAGGATTAGATACCCGCGTAGTCC
>Otu10977
CCAGCCTATGGGGTGCTCCAGTGGGGAATATTGGACAATGGGCGCAAGCCTGATCCAGCC
ATGCCGCGTGGATGATGAAGGCCCTAGGGTTGTAAAGTCCTTTCGGCGGGGAAGATAATG
ACGGTACCCGCAGAAGAAGCCCCGGCTAACTTCGTGCCAGCAGCCGCGGTAATACGAAGG
GGGCTAGCGTTGCTCGGAATCACTGGGCGTAAAGCGCACGTAGGCGGCTTTTTAAGTCAG
GGGTGAAATCCTGGAGCTCAACTCCAGAACTGCCTTTGATACTGAGAAGCTTGAGTCCGG
GAGAGGTGAGTGGAACTGCGAGTGTAGAGGTGAAATTCATAGATATCCGCAGGAACATCC
GAGGCGAAAGCGGCTCGCTGGATCGCAACTGACGCTGAGGGACGAAAGCTAGGGGAGCAA
ACAGGATTAGAGACCCTCGTAGTCC
>Otu10978

CCAGCCTATGGGGTGCTGCAGTCGAGGATCTTTCGCAATGGGCGCAAGCCTGACGAAGCG
ACGCCGTGTGAGCGATGAAGGCCTTAGGGTCGTAAAGCTCTTTCGCTAGGGAGCAAGAGA
GGCTGTCTAATAAGCAGTTAATTTGAGAGTACCTGGTAAAGAAGCACCGGCTAACTCCGT
GCCAGCAGCTGCGGTAATACGGAGGGTGCAAGCATTAATCGGAATTATTGGGCGTAAAGG
GCGCGTAGGCGGTTTTTTAAGTCAGATGTGAAATTCTGGGGCTCAACCCCAGAGCTGCAT
TTGAAACTGGGAGACTGGAGGGTAGGCGGAGAAAGCGGAATTCCACGTGTAGCGGTGAAA
TGCGTAGATATGTGGAAGAACACCGGTGGCGAAGGCGGCTTTCTAGCTTATTCCTGACGC
TGAGGCGCGAAAGCAAGGGGAGCAAACAGGATTAGATACCCTAGTAGTCC
>Otu10982
CCAGCCTATGGGTGGCACCAGTAGGGAATTGTCGGCAATGGGCGAAAGCCTGACCGCGCG
ACGCCGCGTGGAGGAAGAAGGCCTTCGGGTTGTAAACTTCTTTTGTGGGGGACGATTATG
ACGGTACCCCAAGAATAAGGGCCTGCTAACTACGTGCCAGCAGCAGCGGTAATACGTAGG
GCCCGAGCATTATCCGGAATTATTGGGCGTAAAGAGCGTGTAGGTGCTTTATTAAGTCGG
TCGTTAAATCTCACTGCTCAACAGTGAGGCCGCGAGCGATACTAATGAAATTGAGGTTTG
CAGGGGTGAGCAGAACTCTTGGAGTAGGGGTGAAATCCGTTGATACCAAGAAGAATACCA
ATGGCGAAGGCAGCTCACTGGGCAACACCTGACACTGAGACGCGAAAGCGTGGGGAGCAA
ACAGGATTAGAGACCCTAGTAGTCC
>Otu10985
CCAGCCTACGGGATGCTGCAGTAAGGAATATTGGACAATGGAGGCAACCCTGATCCAGCA
ATGCCGCGTGAGTGATGAAGGCCTTCGGGTTGTAAAGCTCTTTTGTCAGGGACGATGATG
ACGGTACCTGACGAATAAGCTCCGGCTAACTTCGTGCCAGCAGCCGCGGTAATACGAGGG
GAGCTAGCGTTGTTCGGAATTACTGGGCGTAAAGGGCGCGTAGGCGGTTCTATAAGTCAG
ATGTGAAAGCCCCGGGCTTAACCTGGGAGGTGCATTTGAAACTGTAGGGCTTGAGATCGA
GAGAGGAAAGTGGAATTACGAGTGTAGAGGTGAAATTCGTAGATATTCGTAAGAACACCA
GTGGCGAAGGCGACTTTCTGGCTCGATACTGACGCTAAGGCGCGAAAGCGTGGGGAGCAA
ACAGGATTAGAAACCCTTGTAGTCC
>Otu11000
CCAGCCTATGGGTTGCTGCAGTCGAGGATTTTTCTCAATGGAGGAAACCCTGAAGGAGCG
ACGCCGCGTGAGGGATGAAGGTCTTCGGATTGTAAACCTCTGTCATCTGGGAACAATGCT
GTTCACCTAACACGTGAACAGTTGATAGTACCGGAAGAGGAAGCAGTGGCTAACTCTGTG
CCAGCAGCCGCGGTAATACAGAGACTGCAAGCGTTGTTCGGATTCATTGGGCGTAAAGGG
TCCGCAGGCGGTCGGTTAAGTCGGATGTGAAATCTCACAGCCTAACTGTGATAGGTCATT
CGAAACTAGCCGGCTTGGGGAGTGGAGAGGAGATTGGAATTGCTGGTGTAGCAGTGAAAT
GCGTAGATATCAGCAAGAACACCGGTGGCGAAGGCGAATCTCTGGACACTTCCTGACACT
CAGGGACGAAAGCCAGGGGAGCAAACGGGATTAGATACCCGGGTAGTCC
>Otu11006
CCAGCCTATGGGTGGCTGCAGTAGGGAATATTGGACAATGGGCGCAAGCCTGATCCAGCC
ATCCCGCGTGAAGGATTAAGGTCCTATGGATTGTAAACTTCTTTTCTCTGGGAATAAAAA
GTGGAGTTCACTCCGCCTTGAAGGTACCAGAGGAATAAGCACCGGCTAACTCCGTGCCAG
CAGCCGCGGTAATACGGAGGGTGCAAGCGTTATCCGGATTCACTGGGTTTAAAGGGTGCG
TAGGTGGCTTTGTAAGTCAGTGGTGAAAGCCCGGAGCTCAACTCCGGAACTGCCATTGAT
ACTGCTTAGCTTGAATCAAGTCGAGGTGGATGGAATAATACATGTAGCGGTGAAATGCTT
AGATATGTATTAGAACACCGATTGCGAAGGCAGTTCACTAGGCGTGTATTGACACTGAGG
CACGAAAGCGTGGGGATCAAACAGGATTAGAAACCCCCGTAGTCC
>Otu11021
CCAGCCTACGGGATGCAGTAGGGAATATTGGGCAATGGGCGAAAGCCTGACCCAGCAACG
CCGCGTGCACGATGAAGGTCTTCGGATCGTAAAGTGCTTTTCTGAGGGATGAGAAAGGAC
AGTATCTCAGGAATAAGTCTCGGCTAACTCTGTGCCAGCAGCCGCGGTAATACAGAGGGG
GCAAGCGTTATTCGGAATTATTGGGCGTAAAGGGCGCGTAGGCGGTGTTATAAGTCAGAT
GTGTAATCCCCGAGCTCAACTTGGGAACTGCATCTGAGACTGTAGTACTAGAGTGCTGGA
GAGGGTGGTAGAATTCCACGTGTAGCGGTGAAATGCGTAGAGATGTGGAGGAATACCAGT
GGCGAAGGCGGCCACCTGGACAGTAACTGACGCTGAGGCGCGAAAGTGTGGGTAGCAAAC
AGGATTAGAAACCCGAGTAGTCC
>Otu11022
CCAGCCTACGGGGGGCAGCAGCTAAGAATCTTCCGCAATGGGGGAAACCCTGACGGAGCG
ACGCCGCGTGGATGATGAAGGCCGTAAGGTTGTAAAATCCTTTTGTCGGTGAAGAATAAA
TCAAGGAGTGGAAAGCCTTGGTGATGACGTTAGCCGACGAATAAGCCCCGGCTAATTACG
TGCCAGCAGCCGCGGTAATACGAAGGGGGCTAGCGTTGTTCGGAATCACTGGGCGTAAAG

CGTACGCAGGCGGATTGATAAGTCAGGGGTGAAATCCCGGAGCTCAACTTCGGAATTGCC
TTTGATACTGTCTATCTTCGAGTTCGGGAGAGGTTGGCGGAATTCCTAGTGTAGAGGTGA
AATTCGTAGATATTAGGAAGAACACCAGTGGCGAAGGCGGCCAACTGGCCCGATACTGAC
GCTCATGTACGAAAGCGTGGGGAGCAAACAGGATTAGATACCCCAGTAGTCC
>Otu11024
CCAGCCTACGGGTGGCACCAGTTTAGAAATTTGCGCAATGGGCGAAAGCCTGACGCAGCG
ACGCCGCGTGAGGGATGAAGGTCTTCGGATTGTAAACCTCTGTCGAGCAGAACGAAACCC
TATCGGTTAATCATCGGTAGGTTTGACGGTACTGTTAGAGGAAGCTATGGCTAACTCTGT
GCCAGCAGCCGCGGTAAAACAGAGGTAGCGAGCGTTGTCCGGAATTACTGGGCGTAAAGG
GCGTGTAGGTGGCCTTGTAAGTCCAGCGTTAAATTTCCCAGCTTAACTGGGATCAGTCGC
TGGATACTGCTTGGCTTGAGGACAGTAGAGGAGAGTGGAATTCCCGGTGTAGCGGTGAAA
TGCGTAGATATCGGGAGGAACACCTGTGGCGAAGGCGGCTCTCTGGTCTGTCCCTGACAC
TAAGGCGCGAAAGCTAGGGGAGCAAACGGGATTAGAAACCCCCGTAGTCC
>Otu11025
CCAGCCTACGGGATGCAGCAGTGGGGAATATTGGACAATGGGGGCAACCCTGATCCAGCA
ATGCCGCGTGTGTGAAGAAGGCCTGAGGGTTGTAAAGCACTTTCAGTGGGGAGGAGAGAT
GACTGTTTAAGAGATGGTTATAAGGACGTTACCCACAGAAGAAGCACCGGCTAACTCCGT
GCCAGCAGCCGCGGTAATACGGAGGGTGCGAGCGTTAATCGGAATTACTGGGCGTAAAGA
GTGCGTAGGTGGTTGATTAAGTTATATGTGAAATCCCTGGGCTTAACCTGGGCAGGTCAT
ATAATACTGATTGACTCGAGTATGGGAGAGGGTAGTGGAATTTCCGGTGTAGCGGTGAAA
TGCGTAGATATAGGAAGGAACACCAGTGGCGAAGGCGACCACCTGGACTGATACTGACAC
TGAGGTGCGAAAGCGTGGGGAGCAAACAGGATTAGAGACCCGAGTAGTCC
>Otu11026
CCAGCCTACGGGATGCACCAGTTTCGAATTTTTCGCAATGGGCGAAAGCCTGACGGAGCG
ACGCCGCGTGGGGGATGAAGGTCTTCGGATTGTAAACCCCTGTCAGCTGGGAACAAGGTC
TCGTGGTGAATAACCATGAGATTTGATAGTACCGGCAGAGGAAGCCGTGGCTAACTCTGT
GCCAGCAGCCGCGGTAATTCAGAGACGGCAAGCGTTGTTCGGATTCACTGGGCGTAAAGG
GTCCGCAGGTGGCGAAGCAAGTCTGACGTGAAATCCCGCGGCCTACCGCGGAACTGCGTC
GGATACTGCCTTGCTAGAGGACGGCAGAGGAGACTGGAATTCATGGTGTAGCGGTGAAAT
GCGTAGATATCATGAGGAACACCGGTGGCGAAGGCGAGTCTCTGGACCGTTCCTGACACT
CAGGGACGAAAGCGTGGGGAGCAAACGGGATTAGATACCCTCGTAGTCC
>Otu11033
CCAGCCTATGGGGGGCTGCAGTCGAGAATTTTTCTCAATGGGGGAAACCCTGAAGGAGCG
ACGCCGCGTGGAGGATGAAGGTTTTCGGATTGTAAACTCCTGTCATTGGGGAACAATTGC
ATGGGTTTAACTGACCTGTGTTTGATAGTACCCGAAGAGGAAGAGACGGCTAACTCTGTG
CCAGCAGCCGCGGTAATACAGAGGTCTCAAGCGTTGTTCGGATTCATTGGGCGTAAAGGG
TGCGTAGGCGGTGGTGCAAGTTGGATGTGAAATCCCGGGGCTTAACCCCGGAACTGCATT
CAATACTGCTCTGCTAGAGTACTGGAGAGGAGATTGGAATTCACGGTGTAGCAGTGAAAT
GCGTAGATATCGTGAGGAAGACCAGTGGCGAAGGCGAATCTCTGGACAGTTACTGACACT
CATATGCGAAAGCTAGGGTAGCAAACGGGATTAGAAACCCTCGTAGTCC
>Otu11043
CCAGCCTACGGGGCGCAGCAGTTTGGAATATTCCACAATGGGCGAAAGCCTGATGGAGCG
ACGCCGCGTGCAGGATGAAGGCCTTCGGGTCGTAAACTGCGGTAGTAAGCAAAGAATGCA
AATGACTAGCTTACGGAAAGAGGTGGGTAACTACGTGCCAGCACCAGCGGTAAAACGTAG
ACCTCAAGCGTTATCCGGATTTATTGGGCGTAAAGAGCATGTAGGAGGTTTTGCGCGTCT
TTTGTTAAAGCCCACCGCCTAACGGTGGGAGTGCACGAGATACGGCAAGACTAGAGGATG
TTAGAGGTACCAGGAACTCACGGTGTAGGGGTGAAATCCGTTGATATCGTGGGGAACACC
AAAGGCGAAGGCAAGGTACTGGGACACTCCTGACTCTGAGATGCGAAAGCGTGGGGAGCA
AAAAGGATTAGAAACCCGCGTAGTCC
>Otu11045
CCAGCCTATGGGGGGCTCCAGTGGGGAATTTTGCGCAATGGGGGAAACCCTGACGCAGCA
ACGCCGCGTGGAGGATGAAGTACTTCGGTACGTAAACTCCTTTCGATCGGGACGATAATG
ACGGTACCGGAAGAAGAAGCCCCGGCTAACTTCGTGCCAGCAGCCGCGGTAATACGAGGG
GGGCGAGCGTTGTTCGGAATTATTGGGCGTAAAGGGTGCGTAGGCGGTTTTGTAAGTCTT
ATGTGAAATCTATGGGCTCAACTCATAGACTGCATGAGAAACTGCAGGGCTTGAGTGTGG
GAGAGGTGAGTGGAATTCCCGATGTAGCGGTGAAATGCGTAGATATCAGGAGGAACACCG
GTGGCGAAGGCGACTTTCTGGCCCTATACTGACGCTGAGACGCGAAAGCGTGGGTAGCAA
ACAGGATTAGAGACCCTTGTAGTCC

>Otu11058
CCAGCCTACGGGTGGCAGCAGTGGGGAATATTGCACAATGGGCGAAAGCTTGATGCAGCG
ACGCCGCGTGGAGGATGAAGGTTTTCGGATTGTAAACTCCTGTTAAGTGGGATGAAAGAC
TGATACCTAATACGTATTGGGGATGACAGTACCACTAGAGAAAGCACCGGCAAACTTCGT
GCCAGCAGCCGCGGTAATACGAAGGGTGCGAGCGTTATTCGGAATCACTGGGCGTAAGGA
GCGTGAAGGCGGTTTTTTAAGTCATTTGTTAAAGTACCCGGCTTAACTGGGTAAATGCGA
GTGATACTGGAAGGCTAGAGTGCAGGAGAGAAAGGTAGAATTCTCGGAGTAGCGGTAAAA
TGCGTAGATCTCGAGAGGAACACCGGTTGCGAAGGCGGCCTTTTGGCCTGTTACTGACGC
TAAAGCGCGAAAGCGTGGGGAGCAAACAGGATTAGATACCCTAGTAGTCC
>Otu11064
CCAGCCTATGGGTCGCTGCAGTGGGGAATTTTGGACAATGGGGGCAACCCTGATCCAGCC
ATGCCGCGTGCGGGAAGAAGGCCTTCGGGTTGTAAACCGCTTTTGGTCGGGAAGAAAGGG
TTCGTCCTAACAGGGCGGACTGTTGACGGTACCGGCAGAATAAGCACCGGCTAACTACGT
GCCAGCAGCCGCGGTAATACGTAGGGCGCGAGCGTTAATCGGAATTACTGGGCGTAAAGC
GTGCGCAGGCGGTAAGTTAAGTCTGATGTGAAAGCCCCGGGCTCAACCTGGGAATGGCAT
TGGAAACTGGCTTGCTGGAGTGTGGCAGAGGGGGGTGGAATTCCGCGTGTAGCAGTGAAA
TGCGTAGAGATGCGGAGGAACACCGATGGCGAAGGCAGCCCCCTGGGCCAACACTGACGC
TCAGGCACGAAAGCGTGGGGAGCAAACAGGATTAGATACCCTTGTAGTCC
>Otu11080
CCAGCCTACGGGTGGCTGCAGTGGGGAATTTTGGACAATGGACGAAAGTCTGATCCAGCG
ACGCCGCGTGAGGGACGAAGGTTTTCGGATCGTAAACTTCTTTTGCAGGGGACGAAAGCG
CGCAAGCGTTTGACAGTACTCTGCGAATAAGCCACGGCTAACTACGTGCCAGCAGCCGCG
GTAATACGTAGGTGGCGAGCGTTACTCGGAATTACTAGGCGTAAAGCGTGTGTAGGCGGG
AGCTTAAGTCTGCTGTTAAATCTCATAGCTCAACTATGAAATGCCGGCAGATACTGGGCT
TCTTGAATTCGGTAGAGGAAACTGGAATTCCAGGTGTAGCGGTGAAATGCGCAGATATCT
GGAAGAACGCCAAAGGCGAAGGCAGGTTTCTGGGCCGCAATTGACGCTGAGACACGAAAG
CTAGGGGAGCAAACAGGATTAGATACCCGAGTAGTCC
>Otu11088
CCAGCCTACGGGGGGCTGCAGTGGGGAATTTTGCGCAATGGGCGAAAGCCTGACGCAGCA
ACGCCGCGTGGGTGATGAAGGTCTTCGGATCGTAAAACCCTGTCGTCAGGGACGAAGGTC
ATCGGTTGAACATTCCGGTGGCTTGACGGTACCTGGAGAGGAAGCCCCGGCTAACTCTGT
GCCAGCAGCCGCGGTAATACGAAGGGGGCTAGCGTTGTTCGGAATCACTGGGCGTAAAGC
GCACGCAGGCGGATTGATTAGTCAGGGGTGAAATCCCGGGGCTCAACCTCGGAATTGCCT
TTGATACTGTCAGTCTTGAGTTCGGGAGTGGTTGGCGGAATTCCTAGTGTAGAGGTGAAA
TTCGTAGATATTAGGAAGAACACCAGTGGCGAAGGCGGCCAACTGGCCCGATACTGACGC
TCATGTGCGAAAGCGTGGGGAGCAAACAGGATTAGATACCCTAGTAGTCC
>Otu11089
CCAGCCTACGGGGGGCAGCAGTCGAGAATTTTTCACAATGGGGGAAACCCTGATGGAGCG
ACGCCGCGTGGGGGATGAATGGCTTCGGCCCGTAAACCCCTGTCATTCGGGATCAATGCC
TGCTATTTAAAAGATGGTAGGTTGATAGTACCGGAAGAGGAAGGGACGGCTAACTCTGTG
CCAGCAGCCGCGGTAATACAGAGGTCCCAAGCGTTGTTCGGATTCACTGGGCGTAAAGGG
TGCGTAGGTGGCGGGGTAAGTCGGATGTGAAATCTCCGAGCTCAACTCGGAAATGGCATT
GGAAACTACTCTGCTCGAGGGTTGGAGGGGGGACTGGAATACTTGGTGTAGCAGTGAAAT
GCGTAGATATCAAGTGGAACACCAGTGGCGAAGGCGAGTCCCTGGACAACTCCTGACACT
GAGGCACGAAAGCTAGGGGAGCAAACAGGATTAGAAACCCCCGTAGTCC
>Otu11090
CCAGCCTATGGGACGCAGCAGTCGAGAATCTTCGGCAATGGGGGAAACCCTGACCGAGCG
ACGCCGCGTGTGGGATGAAGGCCTTCGGGTTGTAAACCGCTGTCAGAGGGGAGGAAATGT
CGGTGGGTTCTCCCATCGATTTGACCTATCCTCAGAGGAAGGACGGGCTAAGTTCGTGCC
AGCAGCCGCGGTAAGACGAACCGTCCAAACGTTATTCGGAATCACTGGGCTTAAAGGGTG
CGTAGGCGGCCCGGGAAGTTGGGTGTGAAATCCCTCGGCTCAACCGAGGAACTGCGCCCA
AAACTGCAGGGCTGGAGGGAGACATAGGTGAGCGGAACTTAGGGTGGAGCGGTGAAATGC
GTTGAGATCCTAAGGAACACCAGTAGCGAAGGCGGCTCACTGGGTCTCTTCTGACGCTGA
TGCACGAAAGCTAGGGGAGCGAAGAGGATTAGATACCCTAGTAGTCC
>Otu11091
CCAGCCTATGGGGGGCTGCAGGCGAGAATATTCCGCAATGGGCGAAAGCCTGACGGAGCG
ACGCCGCGTGATGGATGAAGTGCTTCGGTACGTAAACATCTTTTATCGAGGAAGAACTAA
ATGACGGTACTCGATGAATAAGGGGCTCCTAACTCTGTGCCAGCAGGAGCGGTAATACAG

AGGCCCCAAGCATTACCCGGAATCACTGGGCGTAAAGGGTGTCTAGGCGGCTATGTTAGT
CTCTCGTAAAATCTGCGGGCTCAACCTGCAGCGCGCGGGGGAAACGGCATAGCTCGAGAG
CGCGAGAGGTAGAGGGAACTCACGGTGGAGGGGTGAAATCCGTTGATATCGTGGGGAACA
CCAAAGGCGAAGGCACTCTACTAGCGCGTTTCTGACGCTCAAACACGAAAGCCAGGGGAG
CGAACGGGATTAGAGACCCGTGTAGTCC
>Otu11119
CCAGCCTACGGGTCGCTGCAGCTAAGAATATTCCGCAATGGGAGAAATCCTGACGCAGCG
ACGCCGCGTGAGCGATGAAGGCCTTCGGGTTGTAAAGCTCTGTGGGGAGGGACGAATAAG
GGTTGGCTAATATCCAATCTGATGACGGTACCTCCTTAGCAAGCACCGGCTAACTCTGTG
CCAGCAGCCGCGGTAAGACAGAGGGTGCAAACGTTGTTCGGAATTACTGGGCGTAAAGCG
CGTGTAGGCGGCTACGTAAGTCGGACGTGAAAGCCCACGGCTCAACTGTGGAAGTGCGCT
CGAAACTGCGTAGCTTGAGTTTTGGAGAGGAAGGCGGAATACTTGGTGTAGAGGTGAAAT
TCGTAGATATCAGGTAGAACACCAGTGGCGAAGGCGGCCTTCTGGACAATGACTGACGCT
GAAACGCGAAAGCGTGGGGAGCAAACAGGATTAGATACCCGCGTAGTCC
>Otu11130
CCAGCCTATGGGGTGCAGCAGTGGGGAATTTTGCGCAATGGGGGAAACCCTGACGCAGCA
ACGCCGCGTGAGTGATGAAGGCCTTCGGGTCGTAAAGCTCTGTCAGAGGGAAAGAAGTGT
AGGAGGGCTAATACCCCTTTTACTTGACGGTACCCTCAAAGGAAGCACCGGCTAACTCCG
TGCCAGCAGCCGCGGTAATACGGAGGGTGCGAGCGTTGTTCGGAATTATTGGGCGTAAAG
CGCGTGCAGGCGGTTTGGTAAGTTGGATGTGAAAGCTCCTGGCTTAACTGGGAGAGGTCG
TTCAATACTGCCAGACTAGAGGATGGTAGAGGGAGGTGGAATTCTGGGTGTAGTGGTGAA
ATGCGTAGATATCCAGAGGAACACCAGTGGCGAAAGCGGCCTCCTGGACCATTTCTGACG
CTCATACGCGAAAGCTAGGGTAGCAAACGGGATTAGAAACCCTAGTAGTCC
>Otu11143
CCAGCCTATGGGTGGCTGCAGTAGGGAATATTGGTAATGTGCGAAAGCGCGAACCAGCAA
CGCCGCGTGCACGATGAAGGCCTTCGGGTTGTAAAGTGCTTTTAGAGAGGATGAGGAAGG
ACAGTACTCTCTGAATAAGCCTCGACTAACTACGTGCCAGCAGCCGCGGTAAAACGTAGG
AGGCAAGCGTTATCCGGATTCACTGGGCGTAAAGCGCATGCAGGCGGTTCGGTAAGTTGG
GCGTGAAATCTCCCGGCTCAACTGGGAGAGGTCGTTCAATACTACCGGGCTAGAGAGCAG
TAGAGGAAGATGGAATTCCCGGTGTAGTGGTGAAATGCGTAGATATCGGGAGGAACACCA
GTGGCGAAGGCGATCTTCTGGGCTGTTTCTGACGCTCATATGCGAAAGCTAGGGTAGTAA
ACAGGATTAGAGACCCCCGTAGTCC
>Otu11147
CCAGCCTATGGGGGGCTCCAGTGAGGAATATTGCGCAATGGCCGAAAGGCTGACGCAGCG
ACGCCGCGTGAAGGATGAAGTCCGTTAGGATGTAAACTTCTTTTGCAGGGGATGAATGTG
GCGCCTTCGGGTGCCATTGACCGTACCCTGCGAATAAGTCTCGGCTAACTACGTGCCAGC
AGCCGCGGTAAAACGTAGGAGGCAAGCGTTATCCGGATTCACTGGGCGTAAAGCGCGTGC
AGGCGGTTCGGTAAGTTGGGCGTGAAATCTCCCGGCTCAACTGGGAGAGGTCGTTCAATA
CTACCGGGCTAGAGAGCAGTAGAGGAAGATGGAATTCCCGGTGTAGTGGTGAAATGCGTA
GATATCGGGAGGAACACCAGTGGCGAAAGCGGCCTTCTGGACTGTTTCTGACACTCATAT
GCGAAAGCTAGGGTAGCAAACGGGATTAGAAACCCCCGTAGTCC
>Otu11151
CCAGCCTACGGGGTGCTCCAGTGGGGAATATTGGACAATGGGCGCAAGCCTGATCCAGCC
ATGCCGCGTGTGTGATGAAGGCCTTAGGGTTGTAAAGCACTTTCGCCGGTGAAGATAATG
ACGGTAACCGGAGAAGAAGCCCCGGCTAACTTCGTGCCAGCAGCCGCGGTAATACGAAGG
GGGCTAGCGTTGTTCGGAATTACTGGGCGTAAAGCGCACGTAGGCGGACTTTTAAGTCAG
GGGTGAAATCCCGGGGCTCAACCCCGGAACTGCCCTTGATACTGGAAGTCTTGAGTTCGA
GAGAGGTGAGTGGAATTCCGAGTGTAGAGGTGAAATTCGTAGATATTCGGAAGAACACCA
GTGGCGAAGGCGGCTCACTGGCTCGATACTGACGCTGAGGTGCGAAAGCGTGGGGAGCAA
ACAGGATTAGATACCCTAGTAGTCC
>Otu11152
CCAGCCTACGGGGGGCAGCAGTAGGGAATTTTCCACAATGGGCGAAAGCCTGATGGAGCA
ACGCCGCGTGCAGGATGAAGGCCTTCGGGTTGTAAACTGCTTTTATCTGTGACGAATATG
ACGGTAGCAGATGAATAAGGATCGGCTAACTCCGTGCCAGCAGCCGCGGTCATACGGAGG
ATCCAAGCGTTATCCGGAATTACTGGGCGTAAAGAGTTGCGTAGGTGGCAGAGTAAGTCA
GTAGTGAAAGCGTGCGGCTCAACCGCATATCCATTACTGAAACTGCTCAGCTAGAGGACG
AGAGAGGTACCTGGAATTCCTAGTGTAGGAGTGAAATCCGTAGATATTAGGAGGAACACC
GATGGCGTAGGCAGGGTGCTGGCTCGTTCCTGACACTAAGGCACGAAAGCGTGGGTAGCG

AACGGGATTAGATACCCGAGTAGTCC
>Otu11155
CAGCCTATGGGTTGCTGCAGTCGGGAATCTTCCGCAATGGGCGAAAGCCTGACGGAGCGA
CGCTGCGTGCGGGAAGAAGGCCTTCGGGTTGTAAACCGCTGTCAGGGGTTATGAAATCCA
TCGGCAGCAATGCCGGTGGTCGACAAAGGCCCCAGAGGAAGCCATGGCTAACTCCGTGCC
AGCAGCCGCGGTAATACGGAGATGGCAAGCGTTGTTCGGAATCACTGGGCGTAAAGCGCA
TGTAGGCGGTCAGGCAAGTTGGGTGTGGAAGCGCTCGGCTCAACCGGGCAATTGCGCCCA
AAACTGCCGGACTGGAGTGCGGTAGGGGAGAGTGGAACTCCTGGTGGAGCGGTGAAATGC
GTAGATATCAGGAGGAACGCCGGCGGTGAAGACGACTCTCTGGGCCGTCACTGACGCTGA
GGTGCGAAAGCCAGGGGAGCAAACGGGATTAGAGACCCTCGTAGTCC
>Otu11156
CCAGCCTACGGGTTGCAGCAGTGGGGAATCTTGGACAATGGGCGAAAGCCCGATCCAGCA
ATATGGCGTGAGTGCAGAAGGGCAATGCCGCTCGTAAAGCTCTTTCGTCGAGTGCGCGAT
CATGACAGGACTCGAGGAAGAAGCCCCGGCTAACTCCGTGCCAGCAGCCGCGGTAATACG
TAGGGGGCGAGCGTTGTTCGGAATTACTGGGCGTAAAGGGCGTGTAGGCGGTGTGGTAAG
TTGGGCGTGAAATCCCCGGGCTTAACCCGGGGGGGCCGTTCAAGACTGCCATGCTAGGGT
GCGGGAGAGGGCAGTGGAATTCCCGGTGTAGCGGTGAAATGCGTAGATATCGGGAGGAAC
ACCTGCGGCGAAGGCGGCTGCCTGGACCGTAACCGACGCTGAGGCGCGAAAGCTAGGGGA
GCAAACAGGATTAGAAACCCCTGTAGTCC
>Otu11157
CCAGCCTATGGGTGGCAGCAGTCGAGAATCTTCCGCAATGGGGGAAACCCTGACGGAGCG
ACGCCGCGTGGAGGATGAAGTTCTTCGGAATGTAAACTCCTTTTGCCAGGGACGAAGTCT
ATTGACGGTACCTGGAGAATAAGAAGTTGCTAAACTCGTGCCAGCAGCAGCGGTAATACG
AGTGCTTCAAGCGTTATCCGGAATCATTGGGCGTAAAGGGTGTGTCGGCGGCGATGTTAG
TCTTCCGTAAAATTCTTCGGCTCAACCGGGGGCTCGCGGTAGAAACGGCATTGCTAGAGG
ACGGGAAAGGTTTCTGGAACTCATGGTGTAGCGGTGAAATGCGTTGATATCATGGGGAAC
ACCAAAAGCGAAGGCAAGAAACTGGCCCGCTCCTGACGCTCTAACACGAAAGCGTGGGTC
GCGAATGGGATTAGATACCCTCGTAGTCC
>Otu11162
CCAGCCTACGGGGGGCAGCAGTAGGGAATATTGGGCAATGGGCGCAAGCCTGACCCAGCA
ACGCCGCGTGGAGGATGAAGTCCTTCGGGACATAAACTCCTTTTGGGGGGGACGAATAAA
ATGACGGTACCCCTTGAATAAGCCTCGGCCAACTCCGTGCCAGCAGCCGCGGTAATACGG
GGGAGGCAAGCGTTGTTCGGAATTACTGGGTGTAAAGGGCATGTAGGCGGGTCACTAAGT
CATTCGTGTAATCTCTGGGCTTAACCCAGAGGCTGCGATTGATACTGGCGATCTTGAGTG
GGGGAGAGGAAATCGGAATACCCGGTGTAGCGGTGGAATGCGTAGATATCGGGTAGAACA
CCAGTAGCGAAGGCGGATTTCTGGACCCTAACTGACGCTGAGGTGCGAAAGCGTGGGTAG
CAAACAGGATTAGAGACCCGAGTAGTCC
>Otu11177
CCAGCCTACGGGTGGCACCAGTCGAGAATCTTTCGCAATGGGCGCAAGCCTGACGAAGCG
ACGCCGTGTGAGCGAAGAAGGCCTTCGGGTTGTAAAGCTCTTTCGCTAGGGAACAAGAAA
GGTGTATGAACAATACATCGATTTGATGGTACTTGGTAAAGAAGCACCGGCTAACTCCGT
GCCAGCAGCTGCGGTAATACGGAGGGTGCAAGCATTGATCGGAATTACTGGGCGTAAAGG
GTGCGTAGGCGGATTGTTAAGTCAGGTGTGAAATTCCGCGGCTCAACTGCGGAGCTGCAT
TTGAAACTGGCAATCTAGAGCATAGATAGGGAAAACGGAATTCCACGTGTAGCGGTGAAA
TGCGTAGATATGTGGAAGAACACCAGTGGTGAAGACGGTTTTCTGGGCTATGGCTGACGC
TGAGGCACGAAAGCTAGGGGAGCAAACAGGATTAGAAACCCCAGTAGTCC
>Otu11181
CCAGCCTATGGGTCGCACCAGGCCGGCAGCTCGTTTTCCAGCAGTCTGCCTATGACGTTG
GGCGGCGGGGCGACCTTCAATACGGCCGGCTACGCCGTGACTCTCTCCGGTTCCCTTTCC
GGTCCCGGCAGCCTTACCAAGATCGGCAGCGGCTCGTTGACCTTGGCAGCTACGGACACG
TTCAGCGGCACTACGCTGATCGGCGGCGGCACGCTCGCGCTGGGCAGTCCGCTGGCCTTG
CAGAATAGCACGCTGGACGGCAGCGGCAGCGGGGTTTTGAGTTTCGGATCGCTGACCGCG
GCCACCTTCGGCGGCCTGACCGGCCCCGGCACGCTCAGCCTGAGCAATACCGCGTCCGCC
GCCGTGGCCCTCAGTGTGGGCAACAACAACGCCAACACCACGTTCTCCGGCATGATTAGA
AACCCTTGTAGTCC
>Otu11183
CCAGCCTATGGGTCGCTGCAGTAACGAATATTCCGCAATGGACGAAAGTCTGACGGAGCG
ACGCCGCGTGTGGGATGAAGTCCTTCGGGATGTAAACCACTGTCAGGGGCTACCAAGTGA

GCGCGCCTAATATGCGCGCAAGCTGAGGAGCCCCAGAGGAAGCCACGGCTAACTTCGTGC
CAGCAGCCGCGGTAAGACGAAGGTGGCAAGCGTTGTTCGGAATCACTGGGCTTAAAGAGC
ACGTAGGCGGCTTACCAAGTACTTTGTGAAATCCCTCGGCTCAACCGAGGAATTGCTTGG
TAAACTGGTAGGCTTGAGGCAGGTAAGGGCGCGCGGAACTCTTGGTGGAGCGGTGAAATG
CGTAGATATCAAGAGGAACGCCGGCGGTGAAGACGGCGCGCTAGGCCTGTCCTGACGCTG
AGGTGCGAAAGCCAGGGGAGCAAACGGGATTAGAAACCCCAGTAGTCC
>Otu11205
CCAGCCTACGGGGTGCACCAGTGGGGAATTTTCCTCAATGAGCGAAAGCCTGAAGGAGCG
ACGCCGCGTGGGGGATGAAGGGCTTCGGCTCGTAAACCCCTGTCATTTGCGAACAAACCT
TACGATCTAACACATCGTGAGCTGATTGTAGCGAAAGAGGAAGGGACGGCTAACTCTGTG
CCAGCAGCCGCGGTAATACAGAGGTCCCAAGCGTTGTTCGGATTCACTGGGCGTAAAGGG
TGCGTAGGTGGCCGGGTAAGTTTGATGTGAAATCTCCGAGCTTAACTCGGAAACTGCATT
GAATACTATCCGGCTCGAGGGTTGGAGGGGGACTGGAATTCTCGGTGTAGCAGTGAAATG
CGTAGATATCGAGAGGAACACCAGTGGCGAAGGCGAGTCCCTGGACAACTCCTGACACTG
AGGCACGAAAGCTAGGGGAGCAAACAGGATTAGAAACCCGTGTAGTCC
>Otu11222
CCAGCCTATGGGGCGCAGCAGTGGGGAATTTTGGACAATGGGGGCAACCCTGATCCAGCC
ATGCCGCGTGAATGAAGAAGGCCTTAGGGTTGTAAAGTTCTTTTGTACGGGAGCAAATTG
CGTGGAGTAATATTCTATGTAGATGAGAGTACCGTAAGAATAAGCACCGGCTAACTACGT
GCCAGCAGCCGCGGTAATACGTAGGGTGCGAGCGTTAATCGGAATTACTGGGCGTAAAGG
GTGCGCAGGCGGATATACAAGTTAGGTGTGAAATTCCTGGGCTTAACCTAGGAATGGCAT
TTAAGACTGTATGTCTAGAGTTTATGAGAGGGTGGTAGAATTCCGAGTGTAGCAGTGAAA
TGCGTAGAGATTCGGAGGAATACCTGTGGCGAAGGCGGCCACCTGGGATAGAACTGACGC
TGAGGCACGAAAGCGTGGGGAGCAAACAGGATTAGAAACCCGCGTAGTCC
>Otu11227
CCAACCTATGGGGGGCAGCAGTGGGGAATATTGGGCAATGGGCGCAAGCCTGACCCAGCC
ATGCCGCGTGAGTGATGAAGGCCTTCGGGTCGTAAAGCTCTGTGGGGAGGGACGAACAAG
CACCTGGCTAACATCCAGGGGCCCTGACGGTACCTCCTTAGCAAGCACCGGCTAACTCTG
TGCCAGCAGCCGCGGTAATACAGAGGGTGCAAACGTTGCTCGGAATCATTGGGCGTAAAG
CGCACGTAGGCGGTCCGATAAGTCGGGTGTGAAAGCCCTCGGCTCAACCGAGGAAGTGCA
TTCGATACTGTCAGACTTGAGTATGGAAGAGGGTCGCGGAATTCCCGGTGTAGAGGTGAA
ATTCGTAGATATCGGGAGGAACACCAGTGGCGAAGGCGGCGACCTGGGCCAATACTGACG
CTGAGGTGCGAAAGCGTGGGGAGCAAACAGGATTAGAAACCCGAGTAGTCC
>Otu11229
CCAGCCTATGGGTGGCACCAGACGAGAATATTCCGCAATGGACGAAAGTCTGACGGAGCG
ACGCCGCGTGATGGATGAAGTGCCTTGGTACGTAAACATCTTTTATCGGGGACGAAGTTT
ATTGACGGTACCCGATGAATAAGGGGCTCCTAACTCTGTGCCAGCAGGAGCGGTAATACA
GAGGCCCCAAGCATTACCCGGAATCACTGGGCGTAAAGGGTGTCTAGGCGGCCATATTAG
TCTCTCGTTAAATCCGTGGGCTCAACCTGCGGTCTGCGAGGGAAACGGTATGGCTCGAGG
GCGTAAGAGGTGCACGGAACTCATGGTGGAGGGGTGAAATCCGTTGATATCATGGGGAAC
ACCAAAGGCGAAGGCACTCTATTGGCGCGTTCCTGACGCTCACACACGAAAGCCAGGGTA
GCGAACAGGATTAGAGACCCTAGTAGTCC
>Otu11230
CCAGCCTATGGGTGGCTGCAGTTCGGAATATTCGGCAATGGGCGAAAGCCTGACCGAGCG
ACGCCGCGTGGAGGAAGGGCCCTCTGGGTCGTATACTCCTGTCAAAGGTTACCAAGTCAT
TCTGGATGAACAATCCGGGATGTTGAGTAAGGCTTGAGAGGAAGCCCCCGCTAATCACGT
GCCAGCAGCGGCGGTAATACGTGAGGGGCAAACGTTGTTCGGTGTCACTGGGCTTAAAGG
GCGCGTAGGTGGCTATGCAAGTAAGGTGTGAAAGCCCTCGGCTTATCCGAGGAACCGCGC
CTTAAACTGCATAGCTTGAGGACCAGAGAGGTGGGAAGAACCCTAGGTGTAGCGGTGAAA
TGCGCAGATATCTAGGGGAAGGCCGGAGGCGAAGGCGTCCCACTGGCTGGTTTCTGACAC
TGAGGCGCGAAAGCGTGGGGAGCAAACAGGATTAGAAACCCTTGTAGTCC
>Otu11232
CCAGCCTACGGGTCGCTGCAGTGAGGAATTTTCCACAATGGGCGAAAGCCTGATGGAGCA
ACGCCGCGTGCAGGATGAAGGCCTTCGGGTCGTAAACTGCTTTTATGTATGACGATTATG
ACGGTAGTACATGAATAAGGATCGGCTAACTCCGTGCCAGCAGCCGCGGTCATACGGAGG
ATCCAAGCGTTATCCGGAATTACTGGGCGTAAAGAGTTGCGTAGGTGGTATAGTAAGCAG
GGCATGAAATCGTGTGGCTCAACCATACAGACATGTTCTGAACTGCTAAGCTTGAGGACG
AGAGAGGTAACTGGAATTCCTAGTGTAGGAGTGAAACCCGTAGATATTAGGAGGAACACC

GATGGCGTAGGCAGGTTACTAGCTCGTTCCTGACACTCAGGCACGAAAGCGTAGGGAGCA
AACGGGATTAGATACCCGTGTAGTCA
>Otu11234
CCAGCCTATGGGTGCTCCAGTGGGGAATATTGGACAATGGGCGAAAGCCTGATCCAGCAA
TGCCGCGTGTGTGAAGAAGGCCTTCGGGTTGTAAAGCACTTTCAGCGGGGAGGAAAGCCT
TAGGGTTAATAACCCTGAGGGATGACGTTACCCGCAGAAGAAGCACCGGCTAACTCTGTG
CCAGCAGCCGCGGTAATACAGAGGGTGCAAGCGTTAATCGGAATTACTGGGCGTAAAGCG
CTCGTAGGCGGGTGCCTAAGTCGATTGTGAAAGCCCTGGGCTTAACCTGGGAACTGCAGT
CGATACTGGGTATCTAGAGTATGGTAGAGGATAGTGGAATTCCCGGTGTAGCAGTGAAAT
GCGTAGATATCGGGAGGAACACCAGTGGCGAAGGCGACTTTCTGGACCAATACTGACGCT
GAGGAGCGAAAGCGTGGGGAGCAAACAGGATTAGATACCCCCGTAGTCC
>Otu11236
CCAGCCTACGGGGCGCACCAGTGGGGAATATTGGACAATGGGGGCAACCCTGATCCAGCA
ATGCCGCGTATGTGAAGAAGGCCTTCGGGTTGTAAAGCACTTTAGGCTGGGAAGAAGGTA
TCTAGTTTAAGAGATAAGATATTTGACTGTACCGGCAGAATAAGCACCGGCTAACTCTGT
GCCAGCAGCCGCGGTAATACAGAGGGTGCGAGCGTTAATCGGAATTACTGGGCGTAAAGG
GCGCGTAGGTGGTTATGTAAGATTGATGTGAAATCCCTGGGCTTAACCTAGGAACTGCGT
CGATGACTGCATAGCTGGAGTACTGTAGAGGGTAGTAGAATTTCCGGTGTAGCGGTGAAA
TGCGTAGAGATCGGAAGGAATACCAGTGGCGAAGGCGGCTACCTGGACAGATACTGACAC
TGAGGCGCGAAAGCGTGGGGAGCAAACAGGATTAGAAACCCTTGTAGTCC
>Otu11239
CCAGCCTACGGGATGCAGCAGTTAGGAATCTTGGACAATGGGCGAAAGCCTGATCCAGCC
ATGCCGCGTGAGTGATGAAGGCCTTCGGGTTGTAAAGCTCTTTAGCCGACGAATATAATG
ACTGTAGTCGGAGAATAAGCCCCGGCTAACTTCGTGCCAGCAGCCGCGGTAATACGAAGG
GGGCGAGCGTTGTTCGGAATCACTGGGCGTAAAGCGTGCGTAGGCGGCGATAAAAGTTAG
AAGTGAAAGCCCAGGGCTCAACCTTGGAATTGCTTTTAAAACTAGATTGCTAGAATTCGG
GAGAGGATAGCGGAATTGTCAGTGTAGCAGTGAAATGCGTAGATATTGACAGGAACACCA
GTGGCGTAAGCGGCTATCTGGACCGACATTGACGCTGAGGCACGAAAGCTAGGGGAGCAA
ACAGGATTAGAAACCCGCGTAGTCC
>Otu11249
CCAGCCTACGGGTGGCAGCAGTGGGGAATCTTGCACAATGGGCGAAAGCCTGATGCAGCA
ACGCCGCGTGGGGGATGAAGCTTTTCGGAGTGTAAACCCCTTTCGACCCGGAAGAAAGCC
CGCAAGGGTTTGACGGTACCTGCAGAAGAAGCCCCGGCCAACTACGTGCCAGCAGCCGCG
GTAATACGTAGGGGGCAAGCGTTGTCCGGACTTATTGGGCGTAAAGAGCTCGTAGGCGGC
TTGGCAAGTCGGATGTGAAACCTCCAGGCTTAACCTGGAGTCGCCATTCGATACTGCCAT
GGCTAGAGTTTGGTAGGGGACCACGGAATTCCTGGTGTAGCGGTGAAATGCGCAGATATC
AGGAGGAACACCAGCGGCGAAGGCGGTGGTCTGGGCCAATACTGACGCTGAGGAGCGAAA
GCGTGGGGAGCGAACAGGATTAGAAACCCCCGTAGTCC
>Otu11254
CCAGCCTATGGGGGGCTGCAGTCGAGAATCTTCCACAATGGACGAAAGTCTGATGGAGCG
ACGCCGCGTGATTGATGAAGTCCCTCTGGGACGTAAAAATCTTTTATGAGCTACTAAGTT
TATTGAAGAGCTCATGAATAGGGGGTTGCTAAACTCGTGCCAACAGCAGCGGTAATACGA
GTGCCCCAAGCGTTATCCGGAATTATTGGGCGTAAAGGGTGTGTAGGTGGTTGTGTTAGT
CTTCCGTCAAAACCTCGGGCTCAACCCGAGATCTGCGAGAGAAACGGCACAACTAGAGGA
TGCGAGGGGTGAATGGAACTCATGGAGTAGGGGTGAAATCCGTTGATATCATGGGGAACA
CCAAAAGCGAAGGCAGTTCACTGGCGCATTCCTGACACTGAAACACGAAAGCGTGGGTAG
CGAATGGGATTAGATACCCCTGTAGTCC
>Otu11265
CCAGCCTATGGGAGGCAGCAGTCGAGGATCTTCGGCAATGAGCGCAAGCTTGACCGAGCG
ACGCCGCGTGCGGGATGAAGGCTTTCGGGTTGTAAACCGCCGTCGGAGTGGAGGAAATTC
CATAGGGTACTCTTTATGGATTGACCTATACTCAGAGGAAGGACGGGCTAAGTACGTGCC
AGCAGCCGCGGTAATACGTACCGTCCAAACGTTATTCGGATTTACTGGGCTTAAAGGGTG
CGTAGGCGGCTCAGAAAGTAGGGTGTGAAAGCCCTCGGCTTAACCGAGGAATTGCGCCCT
AAACTACTAAGCTTGAGGAACGCAGAGGTAAGCGGAACTTACGGTGGAGCGGTGAAATGC
GTTGATATCGTAAGGAACACCAGGAGCGAAAGCGGCTTACTGGGCGTTTTCTGACGCTGA
TGCACGAAAGCTAGGGGAGCGAACGGGATTAGATACCCTTGTAGTCC
>Otu11274
CCAGCCTACGGGGGGCACCAGTGGGGAATTTTGGACAATGGGCGCAAGCCTGATCCAGCC

ATGCCGCGTGAGTGAAGAAGGCCTTTGGGTTGTAAAGCTCTTTCGGTTGGGAAGAAGGGA
GTAGTTGCTAATACCAACTATTTTTGACGGTACCAGCATAAGAAGCACCGGCTAATTCCG
TGCCAGCAGCCGCGGTAATACGGAGGTGCAAGCGTTGTTCGGAGTGACTGGGCGTAAAG
CGCACGTAGGTGGCTTGTAAGTCGAATGTGAAATCCCTGGCTTAACCTGGGAACTGCA
TCCGAAACTACAAGTCTTGAATGCTTCAGAGGTTGGTAGAATTCCAGGTGTAGGAGTGAA
ATCCGTAGATATCTGGAGGAATACCGGAGGCGAAGGCGGCCAACTGGGAATGCATTGACA
CTGAGGTGCGAAAGCGTGGGGAGCAAAAAGGATTAGATACCCCCGTAGTCC
>Otu11277
CCAGCCTACGGGTGGCAGCAGTGGGGAATCTTGCACAATGGGCGAAAGCCTGATGCAGCA
ATGCCGCGTGAGTAATGAAGGCCTTCGGGTTGTAAATCTCTGTCGGCTGGGACAAACGAG
CGCATATCTAATAGGTATGCGAAGTGATGGTACCAGCAAAGGAAGAGGCGGCTAACTCTG
TGCCAGCAGCCGCGGTAATACAGAGGCCTCGAGCGTTGTTCGGAATTACTGGGCGTAAAG
GGAATGTAGGTGGTGATGTAAGTCAGATGTGAAAGCCCTGGGCTTAACCCGGGAAGTGCA
TTTGATACTGCATCGCTAGAGTGCGAGAGAGGAAAGTGGAATTTCCAGTGTAGGAGTGAA
ATCCGTAGATATTGGAAGGAATACCGATGGCGAAGGCAACTTTCTGGCTCGTAACTGACA
CTGAGATTCGAAAGCGTGGGTAGCAAACAGGATTAGATACCCGCGTAGTCC
>Otu11279
CCAGCCTACGGGTGGCAGCAGTGGGGAATCTTGCACAATGGGGGAAACCCTGATGCAGCG
ACGCCGCGTGAGCGATGAAGCCCTTCGGGGTGTAAAGCTCTTTCGGCAGGGACGATAATG
ACGGTACCTGCAGAAGCAGCTGCGGCTAACTACGTGCCAGCAGCCGCGGTAATACGTAGG
CAGCAAGCGTTGTTCGGAGTTACTGGGCGTAAAGGGTGCGTAGGCGGCTCGATAAGTATG
GTGTGAAATCTCCCGGCTTAACTGGGAGGGTGCGCTATAGACTGCCGGGCTAGAGGGTGG
GAGAGGAAAGTGGAATTCCTGGTGTAGCGGTGAAATGCGTAGATATCCGTAGGAACATCC
GAGGCGAAAGCGGCGCACTGGACCATAACTGACGCTGAGGCGCGAAAGCTAGGGGAGCAA
ACAGGATTAGAGACCCGAGTAGTCC
>Otu11281
CCAGCCTATGGGGGGCTGCAGTGGGGAATCTTGCACAATGGACGAAAGTCTGATGCAGCA
ACGCCGCGTGGGGGATGAAGCTTCTCGGAGTGTAAACCCCTTTCGACCCGGACGAATGCC
CGCAAGGGTTTGACGGTACGGGTATAAGAAGCCCCGGCTAACTACGTGCCAGCAGCCGCG
GTAATACGTAGGGGGCCAGCGTTGCTCGGAATTACTGGGCGTAAAGGGTCTGTAGGCGGT
GCGGCAAGTCGGATGTGAAATCTCTGGGCTTAACCCAGAGGCTGCTTCCGAAACTGCCGC
GCTAGAGGATGAGAGAGGCGAGTGGAATTGCGGGTGTAGCGGTGAAATGCGTAGATATCT
GCAGGAACACCCGTGGCGAAAGCGGCGCACTGGACCACAACTGACGCTGAGGAACGAAAG
CTAGGGGAGCAAACAGGATTAGAGACCCCGGTAGTCC
>Otu11291
CCAGCCTACGGGTGCTCCAGGTAATCTTTCAGTAATGAATGAACGACATTGACCCAATGC
CTATGGTTTTGAAATGAGCTAGAGAACTACTCTGTATTCATTGGTTCTCACTTGTTCGTG
GCTGAAGACCCTTTTAATGTCATGAAGCCGCACACCGCGTTTGACCAAGAGGCCGTGCTC
AATTTTGAGCGTAGTCTCTCCGCAGACAAAGCAAGAAGCGCTTCTTAAGAGATCATATGT
TTCAGTGACTGCCCCAAATATTGAATTTTCGTGAGGCGCTAGTCCTAGTGGAATATTTTT
TGATAGAATAGCGGAGACATCTTGGCTTACTTCAGTGTACCATAATTGAAAGATATAAAA
CACTGATAACCTGATATCGAAGGCTGTTGCTCGTATATCGCGCCAGAACCAAAGACATTC
TGAGCTGCCTGTGCAGGAACAATATTTCGATTAGAAACCCTCGTAGTCC
>Otu11293
CCAGCCTATGGGTGGCTCCAGTGGGGAATATTGCGCAATGGACGAAAGTCTGACGCAGTA
ACGCCGCGTGAGGGAGGAAGACCTTCGGGTTGTAAACCTCTTTTGGGTAGGACGAGTGAG
GACGGTACTATCAGAATAAGTCTCGGCTAACTACGTGCCAGCAGCCGCGGTAAGACGTAG
GAGGCGAGCGTTATCCGGATTCACTGGGCGTAAAGGGCATGTAGGCGGTTAGATAAGTCG
TGTGTTAAAGCCCCCGGCTCAACTGGGGGAGGTCATGCGAGACTATTTGACTTGAGGGCA
GCAGAGGAGCATGGAACTCCCGGTGTAGTGGTGGAATGCGTAGATATCGGGAAGAACACC
AGTGGCGAAGGCGGTGCCCTGGGCTGCAACTGACGCTGAAATGCTAAAGTGTGGGGAGCA
AACGGGATTAGAGACCCTAGTAGTCC
>Otu11310
CAGCCTACGGGGCGCAGCAGTGGGGAATATTGGACAATGGGCGCAAGCCTGATCCAGCCA
TGCCGCGTGAGTGATGAAGGCCTTAGGGTTGTAAAGCTCTTTCGCTAGGGACGATGATGA
CGGTACCCGCAGAATAAGCCCCGGCTAACTTCGTGCCAGCAGCCGCGGTAAGACGAAGGG
GGCTAGCGTTGTTCGGAATTACTGGGCGTAAAGCGTGTGTAGGTGGTTGTCATAGTCAGG
TGTGAAAGCCTTGAGCTCAACTCAAGAAATGCACTTGATACTGGGCGACTAGAGGACCGG

AGAGGATAGTGGAATTCCCAGTGTAGTGGTGAAATACGTAGAGATTGGGAAGAACACCAG
TGGCGAAGGCGGCTATCTGGACGGTTTCTGACACTAAGACACGAAAGCGTGGGGAGCAAA
CAGGATTAGATACCCGAGTAGTCC
>Otu11320
CCAGCCTACGGGGTGCACCAGTCGAGAATCTTCCACAATGGACGAAAGTCTGATGGAGCG
ACGCCGCGTGATTGATGAAGCTTTTCGGAGTGTAAAGATCTTTTATGAGGGAAGAAGTTT
ATTGACGGTACCTCATGAATAAGGGGCTCCTAATCTCGTGCCAGCAGGAGCGGTAATACG
AGAGCCCCGAGCGTTATCCGGAATTATTGGGCGTAAAGGGTGCGTAGACGGTTGTGCTAG
TCGATTGTTAAAACCTGGGGCTTAACCCCAGATCTGCGATCGAAACGGCACGACTAGAGA
GTGTGAGAGGTATAGGGAACTCATGGTGTAGGGGTGAAATCCGTTGATATCATGGGGAAC
ACCAAAAGCGAAGGCACTATACTGGCACACATCTGACGTTCAAGCACGAAAGCGTGGGTA
GCGAATGGGATTAGAGACCCTTGTAGTCC
>Otu11321
CCAGCCTACGGGTGGCAGCAGTGGGGAATATTGGACAATGGGCGCAAGCCTGATCCAGCC
ATGCCGCGTGGATGATGAAGGCCTTAGGGTTGTAAAATCCTTTCGACGGGGAAGATAATG
ACGGTACCTGCAGAATAAGCCCCGGCTAACTTCGTGCCAGCAGCCGCGGTAATACGAAGG
GGGCTAGCGTTGCTCGGAATGACTGGGCGTAAAGGGCGCGTAGGCGGCCCACACAGTCAG
ATGTGAAATTCCTGGGCTCAACCTGGGGACTGCATTTGATACGTGTGAGCTTGAGTGGAG
AAGAGGGTTGTGGAATTTCCAGTGTAGAGGTGAAATTCGTAGATATTGGAAAGAACACCG
GTGGCGAAGGCGGCAACCTGGTCTTCAACTGACGCTGAGGCGCGAAAGCGTGGGGAGCAA
ACAGGATTAGATACCCTTGTAGTCC
>Otu11324
CCAGCCTACGGGTTGCAGCAGTGCGGAATATTGGACAATGTGCGAAAGCGTGATCTAGTA
ATATCTCGTCTGGGAAAGAAGGTCCATGAGACTGTAAACCAGTGTTGAGCAATTAAGGAT
AATGACAATAATATCTGCTTGAAAGAAGTCCTGGCTAAACCCTGTGCCAGCCGCCGCGGT
AATACAGGGAGGGCGAGCGTTATTCATCGTTACTGGGCGTAAAGGGTACGTAGGTGGCTA
GTTAAATTAAATGTGAAATGCTAGAGCTAAGCTTTAGAAGGGCATATTAATATTGACTTG
AGCTAAGAGTTAAATTGAGTTTTAAGTAATACTAGAAGGAAAGGTAAAATTTTACGATCT
CTAGTAGAACACCGGTGGTGAAAACCTTATTTCAAATTTAACTGACACTGAGGTACGAAG
GAATAGGGAGCAAACAGGATTAGAAACCCGAGTAGTCC
>Otu11326
CCAGCCTATGGGTTGCTGCAGTGAGGGATATTGCGCAATGGGCGAAAGCCTGACGCAGCG
ACACCGCGTGAAGGATGACGGCCTTAGGGTTGTAAACTTCTGTTAAGTGGGAAGAAAGTG
ACGGTCTTAATACGACCGTAAGATGACGGTACCACTAGAGAAAGCACCGGCTAACTTCGT
GCCAGCAGCCGCGGTAAGACGAGGGGTGCGAACGTCATTCGGAATGATTGGGCGTAAAGG
GTGCGTAGGCGGCTTGTTAAGTCAACTGTTAAATTTCTCAGCCTAACTGGGAGTATGCGG
TAGAAACTGGCGAGCTTGAGGATGGAAGAGAGAAGTGGAATTCTCGGAGTAGCGGTAAAA
TGCGTAGATCTCGAGAGGAACACCGATGGCGAAGGCAGCTTCTTAGTCCATATCTGACGC
TGAGGCACGAAAGCGTGGGGAGCAAACAGGATTAGAAACCCTTGTAGTCC
>Otu11341
CCAGCCTATGGGAGGCTCCAGTGGGGAATATTGCACAATGGGCGCAAGCCTGATGCAGCG
ACGCCGCGTGGAGGATGAAGGTTTTCGGATTGTAAACTCCTGTTACGTGGGAAGAAAGAC
GCTTACCTAATACGTAAGTGGGATGACGGTACCATGAGAGAAAGCACCGGCAAACTTCGT
GCCAGCAGCCGCGGTAATACGAAGGGTGCGAGCGTTATTCGGAATGACTGGGCGTAAAGA
GCGAGGAGGCGGTCTTTTAAGTCGCTTGTGAAAGTCCCTGGCTTACCCAGGGGAATGCGG
GTGAAACTGGAAGACTAGAGTACAAGAGAGAAGAGTGGAATTCTTGGAGTAGCGGTAAAA
TGCGTAGATCTCAAGAGGAACACCGATTGCGAAGGCAGCTCTTTGGCTTGTAACTGACGC
TGGAGCGCGAAAGTGTGGGGAGCAAACAGGATTAGAAACCCCGGTAGTCC
>Otu11345
CCAGCCTATGGGATGCAGCAGAGGAGAATATTGGTCAATGAACGAAAGTTTGAACCAGCG
TTTTGCGGAGATGATAGATTCAAAGAATCATGAAGGCTAAGGCAGCTGTAAAGTCTGTGC
ATAGATGAAAATGATGCTTTTATGTCTGAGGAGAATCCCGGGCCAACAACGTGCCAGCAG
CTGCGGTAAGACGTTGACGGGGGAGCGTTACTGGCCGTTACTAGGCGTAAAGTATGGCTA
AATTGTTTAAGAAATTATACCTCAAATCAAAGAAAAGACGAAGGCGTGATATAGTACCTT
AATACTGGAGAAACAAGGAGGTTTGCGATGAGCATGGTCTAGAGATAAAATTCAGATAGA
CCATAAGGATCTCCGGCGGCCGCGGCGGCTTCCCACTTGTTTTCTGACGTTGAACCATTA
AAGTACGAGTATCAATCAGGATTAGATACCCGCGTAGTCC
>Otu11350

CCAGCCTATGGGTGGCACCAGCTAAGAATCTTCCGCAATGGGCGAAAGCCTGACGGAGCG
ATGCCGCGTGATCGATGAAGGTCGAGAGATTGTAAAGATCTTTTCCACATGAGGAATAAG
GTTGGGAGGGAATGCCCAATCGATGACGTTAGTGTGGGAATAAGCCCCGGCTAATTACGT
GCCAGCAGCCGCGGTAACACGTAAGGGGCGAGCGTTGTTCGGAATTATTGGGCGTAAAGG
GCACGCAGGCGGTTATACAAGTCCGATGTGAAAGACCTGGGCTTAACTCAGGGGCCGCGT
TGGAAACTGTATGACTAGAATCTTGGAGGGGGAGTCGGAATTCCTAGTGTAGGGGTGAAA
TCTGTTGATATTAGGAAGAACACCGGTGGCGAAGGCGGACTCCTGGCCGAAGATTGACGC
TGAGGTGCGAAAGCATAGGGAGCAAACAGGATTAGATACCCTCGTAGTCC
>Otu11361
CCAGCCTATGGGTTGCAGCAGTGGGGAGTATTGCGCAATGGGCGAAAGCCTGACGCAGCA
ACGCCGCGTGAGTGAAGAAGGCCTTCGGGTTGTAAAGCTCTTTCGATGGGGGTGAAGGGC
AGTATTACAAATAATAATACTGTCTGACGGTACCTGAAGAAGAAGCACCTGCTAACTCCG
TGCCAGCAGCAGCGGTAATACGGAGGGTGCAAGCGTTGTTCGGAATTACTGGGCGTAAAG
CGCACGTAGGCGGATTTGTAAGTCGAGTGTTAAATCCCTGGGCTTAACCCAGGATCTGCA
TTCGAAACCGCAAACCTTGAATGCCAGAGAGGGTGGGAGAATTCCTGGTGTAGAAGTGAA
ATTCGTAGATATCAGGAGGAATACCTGAGGCGAAGGCGCCTGCCTGGCTGTGCATTGACG
CTAAGAGTGCGAAAGCGTGGGGAGTAAACAGGATTAGAGACCCCTGTAGTCC
>Otu11375
CCAGCCTATGGGGGGCTCCAGTAAGGAATATTGGACAATGGGCGAAAGCCTGGTCCAGCC
ATGCCGCGTGAAGGATTAAGGTCCTCTGGATTGTAAACTTCTTTTATTTGGGACGAAAAA
AGGAAATTCTTTTCCACTTGACGGTACCAGATGAATAAGCACCGGCTAACTCCGTGCCAG
CAGCCGCGGTAATACGGAGGGTGCAAGCGTTATCCGGATTTACTGGGTTTAAAGGGTGCG
TAGGTGGGTAGGTAAGTCAGTGGTGAAATCTCTGAGCTTAACTCGGAAACTGCCATTGAT
ACTATTTATCTTGAATATTGTGGAGGTAAGCGGAATATGTCATGTAGCGGTGAAATGCTT
AGATATGACATAGAACACCAATTGCGAAGGCAGCTTACTACACATATATTGACACTGAGG
CACGAAAGCGTGGGGATCAAACGGGATTAGAAACCCCTGTAGTCC
>Otu11377
CCAGCCTATGGGTGGCTCCAGTGGGGAATATTGCACAATGGGGGAAACCCTGATGCAGCG
ACGCCGCGTGAGCGATGAAGCCCTTTGGGGTGTAAAGCTCTTTCGGCAGGGAAGATTATG
ACGGTACCTGCAGAAGCAGCTGCGGCTAACTACGTGCCAGCAGCCGCGGTAATACGTAGG
CAGCAAGCGTTGTTCGGAATTACTGGGCGTAAAGGGTGTGTAGGCGGTTGCTTAAGTTTG
GTGTGAAATCTCCCGGCTCAACTGGGAGGGTGCGCCGAATACTGAGTAACTAGAGTGCGG
GAGAGGAAAGTGGAATTCCTGGTGTAGCGGTGAAATGCGTAGATATTAGGAAGAACACCA
GTGGCGAAGGCGGCCAACTGGCCCGATACTGACGCTCATGTGCGAAAGCGTGGGGAGCAA
ACAGGATTAGATACCCCAGTAGTCC
>Otu11378
CCAGCCTATGGGGCGCAGCAGTTGGGAATTTTGCACAATGGGCGCAAGCCTGATGCAGCA
ACACTGCGTGAAGGATGAAGGTTTTCGGATCGTAAACTTCTTTTATCTGGGAATAATCTT
GAAGGTACCAGATGAATAAGCACCGGCTAACTACGTGCCAGCAGCCGCGGTAATACGTAG
GGTGCAAACGTTATCCGGATTTATTGGGCGTAAAGAGCTCGTAGGTGGACTTATAAGTTG
GATATTAAATACTTTGGCTCAACCAAAGAAATGTATCCAAAACTATTTGTCTTGAGGTAT
GTAGGGGAGGATGGAACTTACGGTGTAGCAGTGAAATGCGTAGATATCGTAAGGAACGCC
GATGGTGAAGACAGTCCTCTGGACATAACCTGACACTGAGGAGCGAAAGCGTGGGGAGCG
AACGGGATTAGAGACCCCCGTAGTCC
>Otu11381
CCAGCCTACGGGGGGCTGCAGTGGGGAATTTTGCACAATGAGCGAAAGCTTGATGCAGCG
ACGCCGCGTGGAGGATGACGGTCTTCGGATTGTAAACTCCTGTTAAGTGGGAAAAAATGT
CGATACCTAATACGTATCGAAGCTGATAGTACCACTAGAGAAAGCACCGGCAAACTTCGT
GCCAGCAGCCGCGGTAATACGAAGGGTGCTAGCGTTATTCGGAATAACTGGGCGTAAAGA
GCGTGAAGACGGCTTTTTAAGTCATCTGTTAAATAGCCCGGCTTAACTGGGCATCTGCGG
GTGAAACTGAAGAGCTAGAGTGCAGGAGAGAAAGGTAGAATTCTCGGAGTAGCGGTAAAA
TGCGTAGATCTCGAGAGGAATACCGGTTGCGAAGGCGGCCTTTTGGCCTGTTACTGACGT
TCAAGCGCGAAGGCGTGGGGAGCAAACAGGATTAGAAACCCGAGTAGTCC
>Otu11383
CCAGCCTACGGGACGCTGCAGGGAAGCTCTGGGGCTGCTTCAGGTGAGATGTGGGGACGC
TTTGGGTGAAGTCACCGGGGGGTCATCGCCACTCGTGACCCCATTCCCGAAGCACGCCTT
CCCCGGTCCCGTCACCGCAGAATCGAATGAGACGCGCGAGACGAGAGCGATTCAACGCCT
TGATCGTCTCGTGCAATCGCCGCTTGGGGCACTGTTCGTCGCGCGGTGGCAATGGATCGA

AAATTCGAGCAGGCCAACCTTCTTCCTCGAACGCTGCAAGCACGATTTCCTGATTGGCGG
CCGGCAGCCGAAACCGCTTGATGATTTGCCCTTCGACCACCAATTCCCGCCGCAGCGAAT
CCCACCGCGGCACGAGTGGGGCCTCGGCCGTCGATGAATCGCGGTGTCCGTTTGAACCAT
TAGAGACCCCAGTAGTCC
>Otu11384
CCAGCCTATGGGGGGCAGCAGTCGAGAATTTTTCACAATGGGCGAAAGCCTGATGGAGCG
ACGCCGCGTGGGGGATGAATGGCTTCGGCCCGTAAACCCCTGTCATTTGCGATCAACCGT
TGTTGTTTAAGAGATGACAACCTGATAGTAGCGAAAGAGGAAGGGACGGCTAACTCTGTG
CCAGCAGCCGCGGTAATACAGAGGTCCCAAGCGTTGTTCGGATTCACTGGGCGTAAAGGG
TGCGTAGGTGGCGAAGTAAGTCGGATGTGAAATCTCCGAGCTCAACTCGGAAACTGCATT
GGAAACTACTTTGCTCGAGGGTTGGAAGGGGGACTGGAATACTTGGTGTAGCAGTGAAAT
GCGTAGATATCGGGAGGAACACCTGTGGCGAAAGCGGCGCACTGGACCACTACTGACGCT
GAGGCGCGAAAGCCGGGGGAGCAAACAGGATTAGATACCCCTGTAGTCC
>Otu11392
CCAGCCTATGGGATGCTGCAGCTAAGAATCTTCCGCAATGGGGGAAACCCTGACGGAGCG
ACGCCGCGTGGATGATGAAGGCCGTAAGGTTGTAAAATCCTTTTGTCGGTGAAGAATAAA
TCAAGGAGTGGAAAGCCTTGGTGATGACGTTAGCCGACGAATAAGCCCCGGCTAATTACG
TGCCAGCAGCCGCGGTAATACGTATGGGGCAAGCGTTGTTCGGATTTATTGGGCGTAAAG
GGCGTGTAGGCGGCTCATCAAGTCTGGTGTGGAAATGCAGGGCCTAACTCTGCAAATGCG
CTGGAAACTGGTGGGCTTGAGTTCTTGAGGGGAAGCTGGAATTCCAGGTGTAGGGGTGAA
ATCTGTAGATATGCGGAAGAATACCTGTGGCGAAGGCGAGTTTCTGGTCTATAACTGACG
CTGAAATGCGAAAGTGTGGGTAGCAAACAGGATTAGAAACCCCAGTAGTCC
>Otu11395
CCAGCCTACGGGTGGCTCCAGTCGAGAATCTTCCGCAATGGGCGAAAGCCTGACGGAGCG
ACGCCGCGTGATCGATGAAGTGCTTCGGCATGTAAAGATCTTTTATGAGGGACGAAGTTT
ATTGACGGTACCTCATGAATAAAGGGTTGCTAAACTCGTGCCAGCAGCAGCGGTAATACG
AGTGCCCTAAGCGTTATCCGGAATCATTGGGCGTAAAGGGTGTGTAGGTGGTCGCGTTAG
TCTTCTGTTAAATTCTTCGGCTCAACCGGGGGCATGCAGGGGAAACGGCGCGACTAGAGG
ATGCGAGGGGCAAATGGAACTCATGGAGTAGGGGTGAAATCCGTTGATATCATGGGGAAC
ACCAAAAGCGAAGGCAGTTTGCTGGCGCATTCCTGACACTGAAACACGAAAGCGTGGGTA
GCGAATGGGATTAGAAACCCCCGTAGTCC
>Otu11399
GCCAGCCTACGGGGGGCACCAGTGAGGAATATTGGTCAATAGGGGGAACCCTGAACCAGC
CATGCCGCGTGAAGGAAGAATGCCCTAAGGGTTGTAAACTTCTTTTGTACGGGAGTAATG
ACAGGTACGTGTACCTGAGTGAAAGTACCGTACGAGTAAGCATCGGCTAACTCCGTGCCA
GCAGCCGCGGTAATACGGAGGGTGCAAGCGTTATCCGGATTTACTGGGTTTAAAGGGTGT
GTAGGCGGGCTTTTAAGTCAGTGGTGAAATCTCCGGGCTCAACTTGGAAACTGCCATTGA
TACTATTAGTCTCGAATTCTGTTGAGGTAGGCGGAATAAGTCATGTAGCGGTGAAATGCA
TAGATATGACTTAGAACACCAATTGCGAAGGCAGCTTACTAAGCAGATATTGACGCTGAG
GCACGAAAGCGTGGGGATCAAACAGGATTAGATACCCGAGTAGTCC
>Otu11403
CCAGCCTACGGGTCGCACCAGTCGAGAATCTTTCACAATGGGGGCAACCCTGATGGAGCG
ACGCCGCGTGGAGGATAAGGTCTTCGGATTGTAAACTCCTGTCATGCGAGAGCAAGGCAC
ATCAGTTAACACCTGGTTTGCTTGATAGTATCGCAAGAGGGAGAGACGGCTAACTCTGTG
CCAGCAGCCGCGGTAATACAGAGGTCTCAAGCGTTGTTCGGAATCACTGGGCGTAAAGGG
TGTGTAGGCGGTGCGTTAAGTCAGATGTGAAAGCCCAGGGCTCAACCTTGGAACTGCATC
CGATACTGGCGCGCTAGAGAATTGGAGAGGAGTCTAGAATTCACGGTGTAGCAGTGAAAT
GCGTGGATATCGTGAGGAATACCAAGTGCGAAGGCAGGACTCTGGACATTTTCTGACGCT
GAGACACGAAGGCTAGGGGAGCAAACGGGATTAGAAACCCGCGTAGTCC
>Otu11409
CCAGCCTTATGGGTGGCTCCAGTCGAGAATTTTTCTCAATGGGCGAAAGCCTGAAGGAGC
GACGCCGCGTGGGGGATGAATGGCTTCGGCCCGTAAACCCCTGTCATTTGTGAACAAATT
AATCTACCCAACACGTGGATTATTGATAGTAACGGAAGAGGAAGGGACGGCTAACTCTGT
GCCAGCAGCCGCGGTAATACAGAGGTCCCAAGCGTTGTTCGGATTCACTGGGCGTAAAGG
GTGCGTAGGTGGTTGGATAAGTTTGATGTGAAATCTCGGAGCTCAACTCCGAAACTGCAT
TGAATACTATCTAGCTTGAGGATTGGAGGGGGGACTGGAATTCTCGGTGTAGCAGTGAAA
TGCGTAGATATCGAGAGGAACACCAGTGGCGAAGGCGAGTCCCTGGACAATACCTGACAC
TGAGGCACGAAAGCTAGGGGAGCAAACAGGATTAGATACCCCTGTAGTCC

>Otu11418
CCAGCCTACGGGAGGCTGCAGTCGAGGATCTTCGGCAATGGGCGAAAGCCTGACCGAGCG
ACGCCGCGTGCGGGATGAAGGCCTTCGGGTTGTAAACCGCTGTCAGTGAGGAGGAAGGCT
CCGTGAAGAGCGGGGTTTGACCTATTCGCAGAGGAAGTGTGGGCTAAGCTCGTGCCAGCA
GCCGCGGTAAGACGAGCCACACAAACGTTATTCGGAATTACTGGGCTTAAAGGGTGCGTA
GGCGGTGCGGAAAGTAGGGTGTGAAATCCCTCGGCTCAACCGGGGAACAGCGCTCTAAAC
TACCGTGCTTGAGGGAGACAGAGGTAAGCGGAACTTCGGGTGGAGCGGTGAAATGCGTTG
ATATCCGAAGGAACACCGGTGGCGAAAGCGGCTTACTGGGTCTTCTCTGACGCTGAGGCA
CGAAAGCTAGGGTAGCAAACGGGATTAGAAACCCTGGTAGTCC
>Otu11419
CCAGCCTATGGGAGGCTGCAGTGAGGAATTTTCCGCAATGGGCGCAAGCCTGACGGAGCG
ACGCCGCGTGGAGGACGAAGTCTTTCGGGATGTAAACTCCTTTTGCCCGGGAAGAACACA
ATGACGGTACCGGGTGAATAAGCCTCGGCTAACTACGTGCCAGCAGCCGCGGTAAGACGT
AGGAGGCAAGCGTTGTTCGGAATTACTGGGCGTAAAGCGTCCGTAGGCGGGTCGGTAAGT
GGCTAGTTAAAACCCGGGGCTCAACCCCGAGGGTGCTAGCCAAACTGCCAATCTGGAGGT
CGGCAGAGGGTGATGGAATTCCCGGTGTAGCGGTGAAATGCGTAGATATCGGGAGGAACA
CCAGTGGCGCAAGCGATCACCTGGGCTAGACCTGACGCTGAGGGACGAAAGCGTGGGGAG
CAAACGGGATTAGAAACCCTAGTAGTCC
>Otu11433
CCAGCCTATGGGTTGCTGCAGTGTGGAATCTTGGACAATGGGCGTAAACAGCCTGATCCA
GTTACATCTCTCAAGTGATTGACTACATTTGTCGTAAAACTGTCTGGTTAGCTGCCTCAA
TCATGCTTATTTGGAGGCGGTTAATATAAATAGCTCTGGGCAACTTCTGTGCCAGCAGCC
GCGGGAATACAGAGAGAGTAAGCATTATCCATCCTAACTGGGTGTAAAAGGTGCGTAGAT
GAATATTACTTAGAAGACTAGATGATAATTGCTGTGTTGGCAGGCATTTCTAGTACCAAG
TATTTTGAGACACACAGAAGTTGTTAGAACACATAGTGTTCAATTGTTCAGGAAACTGTT
GATCTTATGGAGACTACTCAGAGCGAAGGCATTCAACTTGTTGTGATCTGACGTTGAGGC
ACGAAAGCGTGGGGAGCAAAAGGGATTAGATACCCCAGTTAGTCC
>Otu11449
CCAGCCTACGGGGGGCAGCAGACGAGAATATTCCGCAATGGACGCAAGTCTGGCGGAGCG
ACGCCGCGTGATGGATGAAGTGCTTTGGTATGTAAACATCTTTTATCGGGGACGAGTTTA
TTGACGGTACCCGATGAATAAGGGGCTCCTAACTCTGTGCCAGCAGGAGCGGTAATACAG
AGGCCCCAAGCATTACCCGGAATTACTGGGCGTAAAGGGTGTGTAGGCGGTCATATTAGT
CGTTCGTTAAATCCGTGGGCTCAACCTACGGCATGCGAGCGAAACGGTATGACTGGAGGG
CGCGAGAGGTGCACGGAACTCATGGTGGAGGGGTGAAATCCGTTGATATCATGGGGAACA
CCAAGGGCGAAGGCAGTGCACTAGCGCGTTTCTGACGCTGAAACACGAAAGCCAGGGGTG
CGAACGGGATTAGATACCCCGGTAGTCC
>Otu11455
CCAGCCTATGGGTGGCTGCAGTAGGGAATTTTGCGCAATGGGCGAAAGCCTGACGCAGCA
ACACCGCGTGAGTGATGAAGCATCTTGGTGTGTAAAACTCTGTCAGAGGGGACGAATTTT
GACGGTACCCTCAAAGGAAGCATCGGCTAACTACGTGCCAGCAGCCGCGGTAAGACGTAG
GATGCGAGCGTTGTCCGGAATTATTGGGCGCAAAGAGTTCGTAGGCGGCATGTAAAGTCT
GGTGTTAAATCCCGGAGCTCAACTCCGGTTCGGCATTGGATACTTACAAGCTTGAGTACA
GAAGAGGCAAAGGGAATTCCTAGTGTAGCGGTGAAATGCGTAGATATTAGGAGGAACACC
GGTGGCGTAAGCGCTTTGCTGGTCTGTTACTGACGCTGAGGAACGAAAGCCAGGGGAGCA
AATGGGATTAGAAACCCGCGTAGTCC
>Otu11463
CCAGCCTACGGGATGCTCCAGTGGGGAATATTGGACAATGGAGGAAACTCTGACGCAGCG
ACGCCGCGTGGGTGACGAAGGTCTTCGGATCGTAAAGCCCTGTCGGATGGGAAGAAAAAC
GGAATGGCTAACATCCATACCGCTTGACGGTGCCATAAGAGGAAGCCCCGGCTAACTTCG
TGCCAGCAGCCGCGGTAATACGAAGGGGGCAAGCGTTGTTCGGAATCATTGGGCGTAAAG
CGCGTGTAGGCTGTCTATTAAGTCGGGTGTGAAATCCCTGGGCTCAACCGAGGAAGTGCA
TTCGAAACTGATAGACTAGAAGATGGTAGAGGAAGGTGGAATTCCAAGTGTAGAGGTGAA
ATTCGTAGATATTTGGAGGAATACCGGTGGCGAAGGCGGCCTTCTGGGCCATTCTTGACG
CTGAGACGCGAAAGCGTGGGGAGCAAACAGGATTAGAGACCCGAGTAGTCC
>Otu11464
CCAGCCTATGGGTGGCACCAGTGGGGAATATTGCGCAATGGGGGAAACCCTGACGCAGCG
ACGCCGCGTGCGGGATGAAAGCCTTCGGGTTGTAAACCGCTTTCACCAGGGAAGAATCCA
GACGGTACCTGGGGAAGAAGCGCCGGCTAACTACGTGCCAGCAGCCGCGGTAATACGTAG

GGCGCAAGCGTTGTCCGGATTTATTGGGCGTAAAGAGCTCGTAGGCGGCCTGTCGCGTCT
GCTGTGAAATCCCGGGGCTTAACCCCGGGCTTGCAGTGGATACGGGCTGGCTGGAGGCAG
GCAGGGGAGAACGGAATTCTCGGTGTAGCGGTGAAATGCGCAGATATCGGGAGGAACACC
GGTGGCGAAGGCGGTTCTCTGGGCCTGTTCTGACGCTGAGGGACGAAAGCTAGGGGGGCA
AACAGGATTAGATACCCTCGTAGTCC
>Otu11466
CCAGCCTACGGGATGCTGCAGTGAGGAATATTTGGCGATGAAAAAAATTTTGACATAGTA
ATAATTCTAGAGTGAATAATAGACAGCAATATCGCTGTAAAACTCTTTTGATAAAATGAA
TAATGACTACATTTAAAGAATAAGCACTGGCTAATTCTGTGCCAGCAGCCGCGGTAATAC
AGGGAGTGCAGACGTTATTCATATGCATTGGGTGTAAAGGATATGTAGGTTGCTTTGAAT
GGACATTTTTTAAAAAACTAGAATAAATCTTTTAGAATTAAAATGTAACCAAAAGCTATG
AGTTTATACGAAGTTGAAAGAATTTTTTAGGCAGGGGTTGAATCCATAGAGCTAAGAACG
ACTACCAAAATCGTAGTGAAAACATTCTTCTAGTATAACACTAACACTGAGATATGAAAG
TTTAGGAATCAAACAGGATTAGAGACCCCAGTAGTCA
>Otu11480
CCAGCCTACGGGTTGCACCAGTGGGGAATATTGGACAATGGGCGCAAGCCTGATCCAGCC
ATGCCGCGTGAGTGATGAAGGCCCTAGGGTTGTAAAGCTCTTTTGTGCGGGAAGATAATG
ACGGTACCGCAAGAATAAGCCCCGGCTAACTTCGTGCCAGCAGCCGCGGTAATACGAAGG
GGGCTAGCGTTGCTCGGAATCACTGGGCGTAAAGGGTGCGTAGGCGGGTCTTTAAGTCAG
GGGTGAAATGCCAAGGCTCAACCTTGGAACTGCCTTTGATACTGGAAGTCTTGAGTCCGG
GAGAGGTGAGTGGAACTGCGAGTGTAGAGGTGAAATTCGTAGATATTCGGAAGAACACCA
GTGGCGAAGGCGACACACTGGCCCGTTACTGACGCTGAGGCGCGAAAGCGTGGGGAGCAA
ACAGGATTAGAGACCCGAGTAGTCC
>Otu11485
CCAGCCTACGGGTCGCACCAGGCACTTGATGACGACCTTTTTACCATCGAACTTTCGCTG
CGCTCGGTACACCACCTTGCTGGTGCTCTTGTAGAGCATCTCCAGGTTCTCATAGGCCTG
AAGTTGATAGCATCAGCGGCAAGCAAAAGATGAGAACTACCAACATTAAGCCGCTCCGGT
CGGTCCGGCAGCGTGTCCACCTCGGCCGTAAAACCCCCGGCCTTGCGCTTTGCGGGGCGG
TGCCCTGACGTCGTTGGCAGCAGCACCGCTTATTATCGTTTGCAAGTCGGGGCGTTTGCG
TCGTCTAAATCCTAATTCTACGTAATTTGCCCTCGGCCTGTGAGGGCAAATTCGACTTTG
CCCTCGGATTGGGGGTGCTGATTAGAGACCCCTGTAGTCC
>Otu11487
CCAGCCTACGGGGTGCACCAGTCGAGGATCTTCGGCAATGGGCGCAAGCCTGACCGAGCG
ATGCCGCGTGGGCGATGAAGGCCTTCGGGTTGTAAAGCCCTGTCGAGAGGGAGAAAGGGG
AAACCTTGATCTATCTCTGGAGGAAGGACGGGCTAAGTTCGTGCCAGCAGCCGCGGTAAG
ACGAACCGTCCAAACGTTGTTCGGAATCACTGGGCTTAAAGGGAGCGTAGGCGGGCTGCC
AAGTCCGGGGTGAAATCCTCCAGCTTAACTGGAGAACTGCCTCGGATACTGGAAGTCTCG
AGGAGGGTAGGGGCAAGTGGAACCGTGGGTGGAGCGGTGAAATGCGTTGATATCCATGGG
AACTCCGGTGGCGAAGGCGACTTGCTGGACCTTTTCTGACGCTGAGGCTCGAAAGCCAGG
GGAGCAAACGGGATTAGATACCCTAGTAGTCC
>Otu11488
CCAGCCTATGGGTGGCTCCAGTAGGGAATATTGCGCAATGGGGGAAACTCTGACGCAGCG
ACGCCGCGTGGGTGATGAAGGCTTTCGGGTCGTAAAGCCCTGTCGGAAGGGAAGAAAAAT
ATTATGGCTAACATCCATAGTACTTGACGGTACCTTCAAAGGAAGCACCGGCTAACTACG
TGCCAGCAGCCGCGGTAATACGTAGGGTGCAAGCGTTGTTCGGAATCATTGGGCGTAAAG
CGCGTGTAGGTGGTTAAGTAAGTCGGGTGTGAAAGCCCTCGGCTCAACCGGGGAAGTGCA
TCCGAAACTGCTTAGCTAGAGGACGGTAGAGGAAGGTGGAATTCCTAGTGTAGAGGTGAA
ATTCGTAGATATTAGGAGGAATACCGGCGGCGAAGGCGGCCTTCTGGGCCGTTCCTGACA
CTGAGACGCGAAAGCGTGGGGAGCAAACAGGATTAGATACCCCAGTAGTCC
>Otu11490
CCAGCCTACGGGTGGCAGCAGTAAGGAATATTGGTCAATGGGCGGAAGCCTGACCGAGCG
ACGCCGCGTGGAGGACGAAGGCCTTCGGGTTGTAAACTCCTGTCGAGGGGGAGGAAGAGG
TGGTGCAGAGCCATCTTTGACCGATCCCTGGAGGAAGCACGGGCTAAGTTCGTGCCAGCA
GCCGCGGTAAGACGAACCGTGCGAACGTTATTCGGAATCACTGGGCTTAAAGCGCGTGTA
GGCGGTCCGGCACGTCGAGCGTTGAAATCCCCTGGCTCAACCAGGGAAGTGGCATCGATA
CGACCGGACTGGAGGGGGGTAGGGGGCGCCGGAACTTCCGGTGGAGCGGTGAAATGCGTT
GAGATCGGAAGGAACGCCCGTGGCGAAAGCGGGCGCCTGGACCCCACCTGACGCTGAGAC
GCGAAAGCCAGGGGAGCGAACGGGATTAGATACCCCGGTAGTCC

>Otu11500
CCAGCCTATGGGGCGCTCCAGTAAGGAATATTGGTCAATGGACGCAAGTCTGAACCAGCC
ATGCCGCGTGGAGGATGAAGGCCCTCTGGGTTGTAAACTTCTTTTATTTGGGAAGAAACC
CATCCTTTCTAGGGTGGCTGACGGTACCAGATGAATAAGCACCGGCTAACTCCGTGCCAG
CAGCCGCGGTAATACGGAGGGTGCAAGCGTTATCCGGATTCACTGGGTTTAAAGGGTGCG
TAGGTGGGCAGGTAAGTCAGTGGTGAAATCTCTAAGCTTAACTTAGAAACTGCCGTTGAT
ACTATCTGTCTTGAATATCGTGGAGGTGAGCGGAATATGTCATGTAGCGGTGAAATGCTT
AGATATGACATAGAACACCAATTGCGAAGGCAGCTCGCTACACGGTTATTGACACTGAGG
CACGAAAGCGTGGGGATCAAACAGGATTAGAGACCCGAGTAGTCC
>Otu11503
CCAGCCTATGGGATGCTCCAGTAGGGAATATTGCGCAATGGAGGAAACTCTGACGCAGCG
ACGCCGCGTGGGTGACGAAGGCCTTCGGGTTGTAAAGCCCTGTCGGAAGGGACGAAAAAT
GGTATGGCTAATATCCATATCACTTGACGGTACCTTTAAAGGAAGCACCGGCTAACTACG
TGCCAGCAGCCGCGGTAATACGTAGGGTGCAAGCGTTGTTCGGAATCATTGGGCGTAAAG
CGCGTGTAGGTGGTGGAGCAAGTCGGACGTGAAATCCCTGGGCTTAACCGAGGAAGTGCA
TTCGATACTGCTTAGCTAGAGGACGGTAGAGGAAGGTAGAATTCCTAGTGTAGAGGTGAA
ATTCGTAGATATTAGGAGGAATACCGGTGGCGAAGGCGGCCTTCTGGGCCGTTCCTGACA
CTGAGACGCGAAAGCGTGGGGAGCAAACAGGATTAGATACCCGAGTAGTCC
>Otu11507
CCAGCCTATGGGTGGCTGCAGTGGGGAATATTGGACAATGGGCGCAAGCCTGATCCAGCC
ATGCCGCGTGAGTGATGAAGGCCCTAGGGTTGTAAAGCTCTTTTGTGCGGGAAGATAATG
ACGGTACCCACAGAAGAAGCCCCGGCTAACTTCGTGCCAGCAGCCGCGGTAATACGAAGG
GGGCTAGCGTTGTTCGGATTTACTGGGCGTAAAGCGCACGTAGGCGGATTGTTAAGTGAG
GGGTGAAATCCCAGGGCTCAACCCTGGAACTGCCTTTCATACTGGCAATCTCGAGTCCGG
AAGAGGTGAGTGGAACTCCTAGTGTAGAGGTGGAATTCGTAGATATTAGGAAGAACACCA
GTGGCGAAGGCGGCTCACTGGTCCGGTACTGACGCTGAGGTGCGAAAGCGTGGGGAGCAA
ACAGGATTAGAAACCCTAGTAGTCC
>Otu11528
CCAGCCTATGGGGTGCAGCAGTGGGGAATATTGGGCAATGGGCGAAAGCCTGACCCAGCG
ACGCCGTGTGGGCGATGAAGGCCTTCGGGTTGTAAAGCCCTGTGAGGTGGAAAGAAGGGC
CTAGAGGTTAATACCCTTTAGGCTTTGACGGTACCACCAGAGGAAGCACCGGCTAACTCC
GTGCCAGCAGCCGCGGTAATACGGAGGGTGCAAGCGTTGTTCGGAATTACTGGGCGTAAA
GGGCGCGTAGGTGGTTGTGTAAGTCAGATGTGAAATCCCGAGGCTTAACCTCGGAACTGC
ATCTGATACTGCATGACTCGAGTACAGCAGAGGGAAACGGAATTCCTGGTGTAGAGGTGA
AATTCGTAGATATCAGGAGGAACACCGGTGGCGAAGGCGGTTTCCTGGGCTGATACTGAC
ACTGAGGCGCGAAAGCGTGGGGAGCAAACAGGATTAGATACCCCTGTAGTCC
>Otu11535
CCAGCCTACGGGGCGCTCCAGTCGAGGATCTTCGGCAATGGGCGCAAGCCTGACCGAGCG
ACGCCGCGTGCGGGATGAAGGCCTTCGGGTTGTAAACCGCCGTCAGAGGGGAGGAAATGC
CGGGAAGTAATAAATAATCGACCCGGTTTGACCTATCCTCAGAGGAAGTACGGGCTAAGT
TCGTGCCAGCAGCCGCGGTAAGACGAACCGTACGAACGTTATTCGGAATCACTGGGCTTA
AAGGGTGCGTAGGCGGCCCGGAAAGTTGGGTGTGAAAGCCCTCGGCTCAACCGAGGAATT
GCGCCCAAAACTACCGGGCTCGAGGGAGACAGAGGTGAGCGGAACTTAGGGTGGAGCGGT
GAAATGCGTTGATATCCTAAGGAACACCAGGAGCGAAAGCGGCTCACTGGGTCTCTTCTG
ACGCTGAGGCACGAAAGCTAGGGGAGCGAACGGGATTAGAAACCCCTGTAGTCC
>Otu11537
CCAGCCTACGGGGGGCAGCAGTAGGGAATATTGGGCAATGGGCGCAAGCCTGACCCAGCG
ACGCCGCGTGGGTGATGAAGATCTTTGGATCGTAAAACCCTGTCAGTGGGGAAGAACCGC
ACCAGAAGTTACTGTCTGGTGCCTGACGGTACCCACAGAGGAAGCTCCGGCTAACTCCGT
GCCAGCAGCCGCGGTAATACGGGGGGAGCGAGCGTTGTTCGGATTCACTGGGCGTAAAGG
GCGTGTAGGTGGGTCTTCAAGTCATATGTGAAAACCGACAGCTTAACTGTCGGCCTGCAT
CTGAAACTGGGGACCTTGAGTACGGGAGAGGAAAGCGGAATTCCTAGTGTAGCGGTGAAA
TGCGTAGATATCAGGAGGAACACCGGTGGCGAAGGCGGCTTTCTGGCCCGATACTGACAC
TGAGGCGCGAAGGCCGGGGGAGCAAACAGGATTAGAGACCCAGTAGTCC
>Otu11541
CCAGCCTATGGGGGGCAGCAGCAAGGAATTTTGCGCAATGGGCGAAAGCCTGACGCAGCA
ACGCCGCGTGAAGGATGAAGGCCTTCGGGTTGTAAACTTCTTTTCTCAGGGACGAATAAT
GACGGTACCTGGGGAATAAGCTTCGGCTAACTACGTGCCAGCAGCCGCGGTAATACGTAG

GAAGCAAGCGTTATCCGGATTTACTGGGCGTAAAGTGGGCGTAGGTGGCCTCACAAGTCG
GATGTGAAATCTCCTGGCTTAACTGGGAGGAGTCATCCGATACTGTGGGGCTAGAGGACA
GCAGGGGAAGGTGGAATTCCAGGTGTAGTGGTGAAATGCGTAGATATCTGGAGGAACACC
AGTGGCGAAGGCGGCCTTCCAGGCTGTTTCTGACACTGAGGCCCGAAAGTGTGGGGAGCG
AACAGGATTAGAAACCCCGGTAGTCC
>Otu11544
CCAGCCTATGGGACGCTCCAGTCGAGAATCTTCCGCAATGGACGAAAGTCTGACGGAGCG
ACGCCGCGTGATGGATGAAGTGGTTCGCCATGTAAACATCTTTTATGAGCTACTAAATTT
TGAAGAGCTCATGAATAAGGAGTTGCTAAACTCGTGCCAGCAGCAGCGGTAATACGAGTG
CTCCAAGCGTTATCCGGAATTATTGGGCGTAAAGGGTGTGTAGGTTGTCCTGTTAGTCAG
ATGTTAAAACTCTCCGCTTAACGGAGAAAGGGCATTTGAAACGGCAGAACTAGAGGATGC
GAGAGGTGAACAGAACTCATGGTGTAGGGGTGAAATCCGTTGATATCATGGGGAATACCA
AATGCGAAGGCAGTTCACTGGCGCACTCCTGACACTGAAACACGAAAGCGTGGGTAGCGA
ACTGGATTAGAAACCCGTGTAGTCC
>Otu11548
CCAGCCTATGGGTTGCAGCAGTGGGGAATATTGGACAATGGGCGAAAGCCTGATCCAGCG
ACGCCGCGTGTGTGAAGAAGGCCTGCGGGTTGTAAAGCACTTTTAGTGGGGACAAAAAGC
CATGGGCTAATACCCTGTGGTCTTGATTTAACCCAAAGAAAAAGCACCGGCTAACTCTGT
GCCAGCAGCCGCGGTAATACAGAGGGTGCAAGCGTTAATCGGAATTACTGGGCGTAAAGC
GTGCGTAGACGGTTATCTAAGTCGGATGTGAAATCCCCGGGCTCAACCTGGGAATTGCAT
TCGAGACTGAATAGCTAGGGTGCGGAAGAGGGAAGCGGAATTTCCGGTGTAGCAGTGAAA
TGCGTAGGTATCGGAAGGAACATCAGTGGCGAAAGCGGCTTCCTGGTCCAGCACCGACGT
TCAGGCACGAAAGCGTGGGGAGCAAACAGGATTAGAGACCCCGGTAGTCC
>Otu11550
CCAGCCTATGGGAGGCTCCAGTGGGGAATATTGGACAATGGGGGCAACCCTGATCCAGCA
ATGCCGCGTGTGTGAAGAAGGCCTTCGGGTTGTAAAGCACTTTTATCCGGAACGAAACGC
GCAGGGTGAATATCCTTGCGAACTGACGGTACCGGAGGAATAAGCACCGGCTAACTTCGT
GCCAGCAGCCGGGGTAATACGTAGGCAGCAAGCGTTGTTCGGAATTACTGGGCGTAAAGG
GTGCGTAGGCGGGTCTTTAAGTCAGGGGTGAAATCCTGGAGCTCAACTCCAGAACTGCCT
TTGATACTGAGGATCTTGAGTTCGGGAGAGGTGAGTGGAACTGCGAGTGTAGAGGTGAAA
TTCGTAGATATTCGCAAGAACACCAGTGGCGAAGGCGGCTCACTGGCCCGATACTGACGC
TGAGGCACGAAAGCGTGGGGAGCAAACAGGATTAGAGACCCCCGTAGTCC
>Otu11562
GGACTACTGGGGTATCTAATCCTGTTTGCTACCCACACTTTCGCATTTCAGCGTCAGTTA
TAGACCAGAAACTCGCCTTCGCCACAGGTATTCTTCCGCATATCTACAGATTTCACCCCT
ACACGCGGAATTCCAGTTTCCCCTTCTACACTCAAGCTACGCAGTTTGCAGACCAAACTA
GAGGTTAAGCCTCCGCCTTTAAATCCACACTTACATAACAGCCTACATGCCCTTTACGCC
CAATGAATCCGAACAACGCTTGCCCCCTACGTATTACCGCGGCTGCTGGCACGAAGTTAG
CCGGGGCTTATTCTGCGGGTACCGTCATTATCGTCCCCGCCAAAAGAGCTTTACAACCCT
AAGGCCTTCTTCACTCACGCGGCATGGCTGGATCAGGCTTGCGCCCATTGTCCAATATTC
CCCACTGGTGCGACCCGTAGGCTGG
>Otu11563
CCAGCCTACGGGTGGCAGCAGTCGAGGATTTTTCTCAATGGGGGAAACCCTGAAGGAGCG
ACGCCGCGTGAGGGATGAAGGTCTTCGGATTGTAAACCTCTGTCATCTGGGAACGATGTG
ATCTACCTAACACGTGGATTATTGATAGTACCGGAAGAGGAAGCAGTGGCTAACTCTGTG
CCAGCAGCCACGGTAATACAGAGACTGCAAGCGTTGTTCGGATTCATTGGGCGTAAAGGG
TGCGCAGGCGGTCTGTTAAGTCGGATGTGAAATCTCACAGCCTAACTGTGATAGGTCATT
CGAAACTGGCGGACTAGAGGACTGGAGAGGAGACTGGAATAGTCGGTGTAGCGGTGAAAT
GCGTAGAGATCGACTAGAACACCGGTGGCGAAGGCGAGTCTCTGGACAGTTCCTGACGCT
CAGGTGCGAAAGTGTGGGGATCAAACAGGATTAGAAACCCCTGTAGTCC
>Otu11568
CCAGCCTATGGGGGGCTCCAGTGAGGAATCTTGCGCAATGGGGGAAACCCTGACGCAGCA
ACGCCGCGTGAGTGATGAAGGCCTTCGGGTCGTAAAGCTCTGCCAGATGGGAAGAATGTC
CCGGCGGTGAATAATCGTTGGGATCGACGGTACCGTCAGAGGAAGCACCGGCTAACTCCG
TGCCAGCAGCCGCGGTAATACGGAGGGTGCAAGCGTTGTTCGGAATTATTGGGCGTAAAG
AGCGTGTAGGCGGCCTTGTAAGTCAGATGTGAAATACCTTGGCTCGACCAAGGAAGTGCA
TTTGAAACTGCTTGGCTTGAGTACGGGAGGGGGAAGCGGAATTCCCGGTGTAGAGGTGAA
ATTCGTAGATATCGGGAGGAACACCAGTGGCGAAGGCGGTTTCCTGGCCTGATACTGACG

CTGAGGCGCGAAAGCGTGGGGAGCAAACAGGATTAGATACCCTCGTAGTCC
>Otu11579
CCAGCCTACGGGTTGCTGCAGTCGAGAATCTTGGTCAATGGGCGTAAGCCTGAACCAGCG
AAGCTACGTGGGTGATGACGGCTATTTGGTCGTAAAGTCCCGGAACCTAGACTAGTGTTT
AACGAAGTTTAGGTGATGAAGTTCTGACTAACTGCCGTGCCAGCAGTCGCGGTAAATCGG
CGAGAGCGAGTGTTGGTGTAAAATGGGCGTAAAGGATCCGTAGGCGGCTTGGCATTGGCC
TAATGTGAAGCTCGACTGGCTAGTGGTTAATTCCATTAGCAAAGTTGTAGCGTTAGGTAC
CACTAAGCTAGAGTTGGGTAGAGGAAAGTGGAATACTCAAGCGACAGCTAAAATTGGTAG
ACCTTGAGTGGAACACCAGAGGCGAAGGCGACTTTCTGGACCCATACTGACGCTGAGGGA
TGAAGGCTGGGGTAGCGAAACGGATTAGAGACCCCGGTAGTCC
>Otu11584
CCAGCCTACGGGTTGCAGCAGCTGGAGGGAGACCTTTAGATCAACCAGAACAAAGAACAG
ATTTCCCGCTATTGTTACCGATGCTGCCATAATTCCTAGAAACAAGGACAGGTACATAAT
TCCAGCACCTAAATTGTTTGGCTTGAAGTAAACTACTGTCAGAGTGACAGGAATCACGTA
TCCAAGGATTGTCAATATCACGAACATGACCAATGCAAACCATACACATTCAGCATAGTA
CTGAAGATCATTTCTTTGCAAATGCAAGTTGTTCAACCTCTTTGTCGTATAGAGAAAGCT
TGGTATCAGAATGCAGGATTGAAGTAATAGAACAATACCAAATGCCAACCAAACTACATG
CACCTTGTGGCTACATGTGATGTAGATCATTAGAAACCCCCGTATCC
>Otu11585
CCAGCCTATGGGTTGCACCAGTGGGGAATATTGGACAATGGGCGCAAGCCTGATCCAGCC
ACGCCGCGTGAGTGATGAAGGCCTTCGGGTCGTAAAGCTCTGTGGGGAGGGACGAACCGC
TGATGGTTAATACCCAGCAGCATGACGGTACCTCCTTAGCAAGCACCGGCTAACTTCGTG
CCAGCAGCCGCGGTAATACGAAGGGTGCAAACGTTGCTCGGAATTATTGGGCGTAAAGCG
CACGTAGGCGGCTCTACAAGTCGGATGTGAAAGCCTTCGGCTTAACCGAAGAAGTGCATC
CGAAACTGCAGAGCTTGAGTACCAAAGAGGATCGCGGAATTCCCGGTGTAGAGGTGAAAT
TCGTAGATATTCGCAAGAACACCAGTGGCGAAGGCGGCTCACTGGCCCGATACTGACGCT
GAGGCACGAAAGCGTGGGGAGCAAACAGGATTAGATACCCCCGTAGTCC
>Otu11593
CCAGCCTATGGGGTGCAGCAGTGGGGAATCTTGAACAATGGGCGAAAGCTTGATTCAGCG
ATACTACGTGAATGAGAGAAGGCCAAATGGTCGTAAAGTTCTTTATATCAATAAAAGTGT
GATTTTAATTGGTAGGAAATAGCCCCGGCTAATATCTGTGCCAGCAGCCGCGGTAATACA
GGGGGGGCAAGCGTTACTCGTCTTGATTGGGCGCAAAGGGCATGTAGGTGGTTTATTGTG
AGTGTTGGTAAAATTCTAGAGTTTTTAAGCTTTAGTTCTGCCGGCACTATCGATACAACT
TGAGTTAGTAAATCGTTAATAGAATTCTTAGTGTAGCGCTAAAATGTTACGATATTAGGA
GGAATACTGTAAATGAAAATATTTTTCGGTTATTAACTGACGCTGAGGTGCGAAGGCATG
GGGAGCAAACAGGATTAGAAACCCGAGTAGTCC
>Otu11595
CCAGCCTATGGGTGGCACCAGGCATATTTACACGCTCAAAGAAGTCGCCGTCATGATCGG
AGAGAACATTGAACTGCTCGAGGAAATCATCGCCAACCCAGACAATATTGCCGAAGGCGA
AATGGTCTATGTCCGCGACGGTAGTGAAGATGGCACGACGGGTCTGACCGAGAACGGCGT
TAACGACCTTCAGGAGCTTCTCGCGGACATACGCACATGGGATGGTGGAATCCGAGAGTC
CCTCGTGGGAGAACAATGCGATCCGGAGATTATCGAGCGCATCCTAGCGGACGAGCTAAA
ACGCCAGCCCTAAAATGCCTCTCGATGGCCGCAAAGGCGTTCCCCGGACGCTTACCTTCC
AGCAGCCGCCCGGTGCCGTGCGGATGAAGGGCAAAAAGCGCGAGCGGTCCGGCCCGTTGA
CGGGATCAAAGGGTCCACCAGGATCCGCGAATTAGAAACCCTCGTAGTCC
>Otu11596
CCAGCCTATGGGGGGCACCAGTGGGGAATATTGGACAATGGGGGCAACCCTGATCCAGCG
ATGCCGCGTGTGTGAAGAAGGCCTGAGGGTTGTAAAGCACTTTCGGTGGGGAGGAGGGTT
TATTGGTTAAGAGCTGATGAACTGGACGTTACCTGCAGAAGAAGCACCGGCTAACTCCGT
GCCAGCAGCCGCGGTAATACGGAGGGTGCGAGCGTTAATCGGAATTACTGGGCGTAAAGG
GTGCGTAGGTGGTTTGATAAGTCACATGTAGAAGCCCCGGGCTCAACCTGGGAATGTCGT
GTGAAACTGTTAGACTTGAGTACGGGAGAGGGCAGTGGAATTTCCGGTGTAGCGGTGAAA
TGCGTAGATATCGGAAGGAACACCGGTGGCGAAGGCGGCTGCCTGGCCTGATACTGACAC
TGAGGCACGAAAGCGTGGGGAGCAAACAGGATTAGATACCCGGGTAGTCC
>Otu11599
CCAGCCTATGGGGTGCTGCAGTGAGGAATCTTGCGCAATGGGCGAAAGCCTGACGCAGCG
ACGCCGCGTGGAGGATGAAGGTTTTCGGATCGTAAACTTCTGTTAAGTGGGAAGAAACAC
CTGTTGTTAATACCAGCAGGAACTGACGGTACCATTAGAGAAAGCACCGGCTAACTACGT

GCCAGCAGCCGCGGTAATACGTAGGGTGCTAGCGTTATTCGGAATTACTGGGCGTACAGG
GTGCGTAGACGGCTCATTAAGTCGATTGTTAAAGATCCCGGCCTAACCGGGAAAAAGCGG
TCGATACTGTTGAGCTAGAGGTTAGAAGAGAGAAGTGGAATTCTCGGAGTAGCGGTAAAA
TGCGTAGATATCGAGAGGAACACCAATGGCGAAGGCAGCTTCTTGGTCTATACCTGACGT
TGAGGCACGAAAGCGTGGGGAGCAAACAGGATTAGATACCCGCGTAGTCC
>Otu11603
CCAGCCTATGGGTCGCAGCAGTGGGAAATCTTGCGCAATGGACGAAAGACTGACGCAGCA
ACGCCGCGTGAGGGACGAAGGCTTTCTGAGTTGTAAACCTCTTTCGGTAGGAACGATTGT
GACGGTACCTACAGAAGAAGCACCGGCCAACTATGTGTCAGCAGCCGCGGTGATACATAG
GGTGCAAGCGTTGTTCGGATTTATTGGGCGTAAAGAGCTCGTAGGCGGTTCGATAAGTTG
GGTGTTAAATCTCCAGGCTTAACCTGGAGCAGCCATCCGAAACTGTCGTGACTAGAGTTT
GGTAGGGGATCACGGAATTCCTGGTGTAGCGGTGGAATGCGCAGATATCAGGAGGAACAC
CAGTAGCGAAGGCGGTGATCTGGGCCAATACTGACGCTGAGGAGCGAAAGCGTGGGGAGC
GAACAGGATTAGAAACCCTAGTAGTCC
>Otu11604
CCAGCCTATGGGGCGCTGCAGTCGAGAATCATTCGCAATGGGCGAAAGCCTGACGGTGCG
ACGCCGTGTGAGTGAAGAAGGCCTTCGGGTCGTAAAGCTCTTTCGCCTGGGAACAAGGGA
GGCTGGCAAATAACCAGCCAACTTGAGGGTACCAGGAGAAGAAGCACCGGCTAACTACGT
GCCAGCAGCTGCGGTAATACGTAGGGTGCAAGCATTAATCGGATTTATTGGGCGTAAAGG
GCGCGTAGGCGGGAAGGTAAGTCAGATGTGAAATCCCGGAGCTTAACTCCGGAACGGCAT
TTGAAACTACCTATCTAGAGGGTAGGCGGAGATAACGGAATTCCACAAGTAGCGGTGAAA
TGCGTAGATATGTGGAAGAACACCTGTGGCGAAAGCGGTTTTCTAGCTTACTCCTGACGC
TGAAGCGCGAAAGCTAGGGGAGCAAACAGGATTAGAAACCCTCGTAGTCC
>Otu11610
CCAGCCTATGGGTGGCTCCAGTGGGGAATCTTGCACAATGGGCGAAAGCCTGATGGAGCG
ACGCCGCGTGGAGGATGAAGGTTTTCGGATTGTAAACTCCTGTCACTGCAGAACAAGGCT
ATGCGGTTTAATAGACCATGTAGTTGATGGTATGCGGAGAGGAAGGGACGGCTAACTCTG
TGCCAGCAGCCGCGGTAAGACAGAGGTCCCGAGCGTTGTTCGGATTCATTGGGCGTAAAG
GGTGTGTAGGAGGTCGGGTCAGTCAGGTGTGAAATCTCAGGGCTTAACCCTGAAACTGCG
CTTGATACTGCCCGGCTAGAGGATCGGAGGGGGTATCGGAATTTATGGTGTAGCAGTGAA
ATGCGTAGATATCATAAGGAACACCGGTGGCGAAGGCGGATACCTGGAAGATTCCTGACT
CTGAAACACGAAAGCCAGGGGAGCAAACGGGATTAGAAACCCCAGTAGTCC
>Otu11620
CCAGCCTATGGGTGGCAGCAGTAACGAATCTTCCGCAATGCACGCAAGTGTGACGGAGCA
ACGCCGCGTGTGGGATGAAGTCCTTCGGGATGTAAACCACTGTCAGGGGTAGGAAAGTTC
TGATCCACCCCAGAGGAAGGCACGGCTAACTCTGTGCCAGCAGCCGCGGTAAGACAGAGG
TGCCGAGCGTTAGGCGGAATCACTGGGCTTAAAGCGTGTGTAGGCGGATCGTTAAGTACC
TTGTGAAATCCCACGGCTCAACCGTGGAACTGCTGGGTATACTGGCGGTCTTGAGTCACC
TAGGGGCTACCGGAACAAATGGTGGAGCGGTGAAATGCGTAGATATCATTTGGAACGCCA
ATGGCGAAAGCAGGTAGCTGGGGGTGAACTGACGCTGAGACACGAAAGCCAGGGGAGCAA
ACGGGATTAGATACCCGAGTAGTCC
>Otu11630
CCAGCCTACGGGTGGCAGCAGTAAGGAATATTGGTCAATGGGCGGAAGCCTGAACCAGCC
ATGCCGCGTGCAGGAAGACGGCCCTACGGGTTGTAAACTGCTTTTGCAGGGGAATAAACC
TTCGTATGTATACGAAGCTGAATGTACTCTGAGAATAAGGATCGGCTAACTCCGTGCCAG
CAGCCGCGGTAATACGGAGGATCCAAGCGTTATCCGGATTTATTGGGTTTAAAGGGTGCG
TAGGTGGCTTATTAAGTCAGGGGTGAAAGACGGTAGCTTAACTATCGCGGTGCCTTTGAT
ACTGATGAGCTTGAATGAACTAGAGGTAGGCGGAATGTGACAAGTAGCAGTGAAATGCAT
AGATATGTCACAGAACACCAATTGCGAAGGCAGCTTACTATGGTTTTATTGACACTGAGG
CACGAAAGCGTGGGGATCAAACAGGATTAGATACCCCCGTAGTCC
>Otu11631
CCAGCCTATGGGGTGCACCAGTAAGGAATATTGGTCAATGGGCGGAAGCCTGAACCAGCC
ATGCCGCGTGCAGGAAGACGGCCCTACGGGTTGTAAACTGCTTTTGCAGGGGAATAAACC
CCGTTACGTGTAACGGGTTGAATGTACTCTGAGAATAAGGATCGGCTAACTCCGTGCCAG
CAGCCGCGGTAATACGGAGGATCCGAGCGTTATCCGGATTTATTGGGTTTAAAGGGTGCG
TAGGCGGCCTGTTAAGTCAGGGGTGAAAGACGGTAGCTTAACTATCGCAGTGCCTTTGAT
ACTGACGGGCTTGAATGTAGTTGAGGTAGGCGGAATGTGACAAGTAGCAGTGAAATGCGT
AGATATCAAGTGGAACACCAGTGGCGAAGGCGAGTCTCTGGACAACTTCTGACACTGAGG

CACGAAAGCTAGGGGAGCAAACAGGATTAGATACCCTAGTAGTCC
>Otu11632
CAGCCTATGGGTGGCAGCAGTAAGGAATATTGGTCAATGGGCGGAAGCCTGAACCAGCCA
TGCCGCGTGCAGGAAGACGGCCCTACGGGTTGTAAACTGCTTTTGCAGGGGAATAAACCC
CCATATGTATATGGGGCTGAATGTACTCTGAGAATAAGGATCGGCTAACTCTGTGCCAGC
AGCCGCGGTAATACAGAGGGTGCGAGCGTTAATCGGAATTACTGGGCGTAAAGCGCGCGT
AGACGGTTTTGTAAGTCAGATGTGAAATCCCTGGGCTCAACCTGGGAGCTGCATTTGAGA
CTGCATGGCTAGAGTATCGAAGAGGGAAGTGGAATTTCCGGTGTAGCGGTGAAATGCGTA
GATATCGGAAGGAACACCAGTGGCGAAAGCGACTTCCTGGTCGAATACTGACGTTCATGT
GCGAAAGCGTGGGGAGCAAACAGGATTAGAAACCCGCGTAGTCC
>Otu11639
CCAGCCTATGGGGGGCAGCAGTGGGGAATATTGGACAATGGGCGAAAGCCTGATCCAGCA
ATACCGCGTGTGCGATGAAGGTCTTCGGATTGTAAAGCACTTTTAATTGGGAAGAAGGTT
AGCGTGATAACACCATGACTAATTGACGCTACCGATAGAAAAAGCACCGGCTAACTCTGT
GCCAGCAGCCGCGGTAATACAGAGGGTGCAAGCGTTAATCGAAATAACTGGGCGTAAAGA
GTACGTAGGCGGAATTATAAGTCGTGTGTGAAAAACTGGGGCTCAACCTCGGGACTGCAC
ACGATACTGTAAATCTAGAGTTTGGTAGAGGTAAGTGGAATTTCCGGTGTAGCGGTGAAA
TGCATAGATATCGGAAGGAACACTAGTGGCGAAGGCGACTTACTGGGCCAATACTGACGC
TGAGGTACGAAAGCGTGGGGAGCAAACAGGATTAGAAACCCTGGTAGTCC
>Otu11641
CCAGCCTATGGGTGGCAGCAGCTAAGAATATTCCGCAATGGGAGAAATCCTGACGGAGCG
ACGCCGCGTGGGTGATGAAGGTCTTCGGACTGTAAAACCCTGTCGTCAGGGACGAAGGTG
GCGGAGTTAACAACTCCGTTACTTGACGGTACCTGGAGAGGAAGCCCCGGCTAACTCTGT
GCCAGCAGCCGCGGTAATACAGAGGGGGCAAGCGTTATTCGGAATTATTGGGCGTAAAGG
GCGCGTAGGCGGTTTTTTAAGTCAGATGTGTAATCCCCGAGCTTAACTTGGGAACTGCAT
CTGAGACTGGGAGGCTAGAGTACTGGAGAGGGTGGTGGAATTCCTCGTGTAGCGGTGAAA
TGCGTAGAGATGAGGAGGAACACCAGTGGCGAAGGCGGCCACCTGGACAGTAACTGACGC
TGAGGCGCGAAAGTGTGGGTAGCAAACAGGATTAGATACCCGCGTAGTCC
>Otu11644
CCAGCCTACGGGGTGCTCCAGTGGGGAATATTGGGCAATGGGCGCAAGCCTGACCCAGCA
ACGCCGCGTGAGGGAAGAAGGTCTTCGGATTGTAAACCTCTGTCCTTAGGGACGAAAAAA
ATGACGGTACCTAAGGAGGAAGCTCCGGCTAACTACGTGCCAGCAGCCGCGGTAATACGT
AGGGAGCAAGCGTTGTCCGGAATTACTGGGCGTAAAGGGTGCGTAGGCGGCGCTGCAAGT
CAGATGTGAAATCCCCGGGCTTAACCCGGGACCTGCATTTGAAACTGCAGTGCTTGAGTG
CAGGAGAGGAAAGCGGAATTCCTAGTGTAGCGGTGAAATGCGTAGATATTAGGAAGAACA
CCAGTGGCGAAGGCGGCTTTCTGGACTGTAACTGACGCTGAGGCACGAAAGCGTGGGGAG
CAAACAGGATTAGAAACCCGCGTAGTCC
>Otu11645
CCAGCCTATGGGATGCAGCAGTAGGGAATTTTGCGCAATGGGCGAAAGCCTGACGCAGCA
ACGCCGCGTGAGTGATGAAGGTCTTCGGATTGTAAAGCTCTGTTGAGTGGGAAGAAAAGT
TCCGGCGTTAATATTCCGGGATATGACGGTACCATTCGAGTAAGCCCCGGCTAACTACGT
GCCAGCAGCCGCGGTAATACGTAGGGGGCAAGCGTTGTTCGGAATTACTGGGCGTAAAGG
GAGCGTAGGCGGAAACGTAAGTTAGAAGTTTAACATATGGGCTTAACCCATAATCTGCTT
TTAATACTGCGTTTCTTGAGTATGGAAGAGGGAGATGGAATTCCAGGTGTAGCGGTGGAA
TGCGTAGATATCTGGAAGAACACCAGTTGCGAAGGCGGTCTCCTGGTCCAATACTGACGC
TGAGGCTCGAAAGCTAGGGGAGCAAACAGGATTAGATACCCCCGTAGTCC
>Otu11648
CCAGCCTATGGGTGGCTGCAGTGGGGAATTTTGCGCAATGGGGGAAACCCTGACGCAGCA
ACGCCGCGTGGAGGATGAAGCCCCTTGGGGTGTAAACTCCTTTCGACTGGGAAGATTATG
ACGGTACCAGTGGAAGAAGCACCGGCTAACTCTGTGCCAGCAGCCGCGGTAATACAGAGG
GTGCGAGCGTTGTTCGGAATTATTGGGCGTAAAGGGCGCGTAGGCGGTGTTGTAAGTCAC
CTGTGAAACCTCTGGGCTTAACTCAGAGCCTGCAGGCGAAACTGCAATGCTGGAGGGTGG
GAGAGGTGCGCGGAATTCCCGGTGTAGCGGTGAAATGCGTAGATATCGGGAGGAACACCA
GTGGCGAAGGCGGCTTTCTGGTCTGCAACTGACGCTGAAACGCGAAAGCTAGGGGAGCAA
ACGGGATTAGATACCCCCGTAGTCC
>Otu11649
CCAGCCTACGGGTGGCACCAGTGAGGAATATTGGTCAATGGGCGCAAGCCTGAACCAGCC
ATCCCGCGTGCAGGAAGAAGGCGCTATGCGTTGTAAACTGCTTTTCCGAGGGAAGAAAAC

CCCCGACGTGTCGGGGCTTGTCGGTACTTTGGGAATAAGCATCGGCTAACTCCGTGCCAG
CAGCCGCGGTAATACGGAGGATGCGAGCGTTATCCGGATTCATTGGGTTTAAAGGGTGCG
TAGGCGGACTGATAAGTCAGTGGTGAAAACCTGCAGCTTAACTGTAGAACTGCCATTGAT
ACTGTCGGACTTGAGTATGGTCAAGGTAGGCGGAATGTGTAATGTAGCGGTGAAATGCAT
AGATATTACACGGAACACCGATTGCGAAGGCAGCTGACAAAACTGTAACTGACGCTGAGG
CTCGAAAGCGTGGGGATCAAACAGGATTAGAAACCCCTGTAGTCC
>Otu11652
CCAGCCTATGGGATGCTGCAGTCGAGAATCTTCCACAATGGACGAAAGTCTGATGGAGCG
ACGCCGCGTGGTTGACGAAGTCCTTCGGGACGTAAAAACCTTTTATGGGGGAGGAAGTAA
TTGACGTTACCCCATGAATAAGGGGCTCCTAACTCTGTGCCAGCAGGAGCGGTAATACAG
AGGCCCCGAGCATTATCCGGAATCACTGGGCGTAAAGGGTGTGTAGGCGGTCATATTAGT
CGACTGTGAAAGATCTCGGGCTTAACCCGGGAGACGCAATGGAAACGGTATGACTTAGAG
GATGTGAGAGGTACAGGGAACTCATGGTGTAGGGGTGAAGTCCGTTGATATCATGGGGAA
CACCAAATGCGAAGGCACTGTACTGGCACATTCCTGACGCTGAGACACGAAAGCGTGGGA
ATCGAACGGGATTAGAGACCCTTGTAGTCC
>Otu11655
CCTACGGGTGGCACCAGGTCAGTCGGAACAGTGTACTGAACCAGGCCGCCTGCTTTCTGA
CTGCTCTTCTCTTCGGAAAAAAAGAAGAACAGTCTGTCCCGGTTCCGGTTGAACTTTCCA
GGGTAATAGATCGGTCCTCCAACGGTGACCCCGAATGTGTTGTAACGGTAGATCGCGCGC
GGCACGGAATTGAGGTTGTTGAAGTAGGTGTTCGCGTTCAATGACTCGTTGCGAATATAT
TCATATGCGCTGCCGTGGAACGTACGTGTCCCGGACTTCGTCACGCCAACGATAACGGCG
CTGCCGTTTCTGCCATACTCGGCCCGGAAATTGTTGACGAGAACCGTCACCTCGCTGATG
GCGTCAATGTTGGAAGTGAACGGGTACTGCGGATTAGATACCCGTGTAGTCC
>Otu11657
CCAGCCTATGGGGTGCTGCAGTGAAGAATCTTGGGCAATGGACGAAAGTCTGACCCAGTG
AGAATACATGCATGACGAATGCGCATAGTTTTCTGTAAAGTGCTATTCGCGGATTGAAGA
AAATGACTAAACCGAGATGAAGCGCCGGCCAACTCCGTGCCAGCAGCCGCGGCAAGACGG
AGGGCGCGAGCGTTATTCGTTTTGATTGGGTGTAAAGGGTACGTAGGCTGCCCCTAGTTT
TTTGACCAAAAGAGCGGAGTGTTCCTACGCGACGGTCTTTTAAAAAGGGGGCTTGTGTTA
GAGATAGGTCGGGTCAAATTCTTAAGTAGGGGTAAGATCCAACGAGATGAGAATGAACGT
CAGCTGGCGAAGGCGCCTCTCCCGCTCTAACAGGCGCTAAGGTACGGAAGCTTAGGTAGC
AAACAGAATTAGATACCCGCGTAGTCC
>Otu11662
CCAGCCTATGGGGGGCACCAGTAGGGAATATTGGGCAATGACCGAAAGGTTGACCCAGCG
ACGCCGCGTGGAGGATGAAGGCTTTCGGGTTGTAAACTCCTGTCAGAGGGGAAGAAACTC
TTGTTTAACAAACGGGATTGACTTAACCCTCAGAGGAAGCACCGGCTAACTCCGTGCCAG
CAGCTGCGGTAATACGGAGAGTGCAAGCGTTGTTCGGATTTACTGGGTGTAAAGGACCTG
TAGGCGTCCAGATCAATCAGACGTGTAATACCCCGGCTTACCCGGGGAACGGCATCTGAT
ATTGTCTGGATCGAGGATGGGATAGGAGAGCGGAACTGTAGGTGTAGCGGTGAAATGTGT
AGATATCTACAGGAACACCAGAGGCGCAGGCGGCTCTCTGGCCCATATCTGACGCTGAGG
GGTGAAAGCTAGGGGAGCAAACAGGATTAGAAACCCGGGTAGTCC
>Otu11663
CCAGCCTACGGGGGGCTGCAGCAACGAATATTGGGCAATGGGCGAAAGCCTGACCGAGCG
ACGCCGCGTGCAGGATGAAGGCCCTCGGGTTGTAAACTGCTTTTCTTCAGGATGAAGTAT
GGTCCGCAAGGGTCATATTGACGGTACTGAGGGAATAAGTGGCTGCAAACTTCGTGCCAG
CAGCCGCGGTAATACGAAGGCCACAAGCGTTATCCGGATTTATTGGGCGTAAAGGGTGCG
TAGGAGGACCGGTGCGTCTCTTGTTAAATTTCACCGCTCAACGGTGAAGCCGCAAGGGAT
ACGATCGGACTAGAGGGCGTTAGAGGCAGATGGAACACACGGTGTAGGGGTGAAATCCGT
TGATATCGTGTGGAACACCAAAGGCGAAGGCATTCTGCTGGGACGTTCCTGACTCTGAGG
CACGAAAGCGTGGGGAGCAAAAAGGATTAGAAACCCTCGTAGTCC
>Otu11665
CCAGCCTACGGGTGGCTGCAGTGGGGAATCTTGCGCAATGGGCGAAAGCCTGACGCAGCA
ACGCCGTGTGAGTGATGAAGGCTTTCGGGTCGTAAAGCTCTGTCAGATGGGAAGAATTAG
TCTGCTGAAAATATCTGCAGATGATGACGGTACCATCGAAGGAAGCACCGGCTAACTCCG
TGCCAGCAGCCGCGGTAATACGGAGGGTGCAAGCGTTGTTCGGAATTATTGGGCGTAAAG
CGCGTGTAGGCGGATAGATAAGTCAGGTGTGAAATCCCTTGACTCAATCAAGGAACTGCA
CTTGAAACTATTTATCTCGAGTACGATAGGGGAGGGCGGAATTCCCAGTGTAGAGGTGAA
ATTCATAGATATTGGGAGGAACACCAGTGGCGAAGGCGACCCTCTGGATCGATACTGACG

CTGAGACGCGAAAGCGTGGGTAGCAAACAGGATTAGAAACCCGGGTAGTCC
>Otu11668
CCAGCCTACGGGTCGCTGCAGTGGGGAATATTGGACAATGGGCGCAAGCCTGATCCAGCC
ATGCCGCGTGAGTGATGAAGGCCCTAGGGTTGTAAAGCTCTTTCACCGGAGAAGATAATG
ACGGTATCCGGAGGAGAAGCCCCGGCTAACTTCGTGCCAGCAGCCGCGGTAATACGAAGG
GGGCTAGCGTTGTTCGGATTTACTGGGCGTAAAGCGCACGTAGGCGGATCGATCAGTCAG
GGGTGAAATCCCAGAGCTCAACTCTGGAACTGCCTTTGATACTGTCGATCTGGAGTATGG
AAGAGGTGAGTGGAATTCCGAGTGTAGAGGTGAAATTCGTAGATATTCGGAAGAACACCA
GTGGCGAAGGCGGCTCACTGGACAGGTATTGACGCTGAGGTGCGAAAGCGTGGGGAGCAA
ACAGGATTAGAAACCCCAGTAGTCA
>Otu11675
CCAGCCTATGGGTTGCAGCAGTCGAGGATCTTCGGCAATGGGCGCAAGCCTGACCGAGCG
ACGCCGCGTGCGGGATGAAGGCCTTCGGGTTGTAAACCGCTGTCAGTGGGGAGGAAGGCT
CTGTGAAGAGCGGAGTTTGACCTATCCGCAGAGGAAGTACGGGCTAAGTTCGTGCCAGCA
GCCGCGGTAAGACGAACCGTACGAACGTTATTCGGAATCACTGGGCTTAAAGGGTGCGTA
GGCGGTCCGGTAAGTCGGGTGTGAAATACCTCAGCTTAACTGAGGAATTGCGCCCGAAAC
TGTCGGACTTGAGGGAGACAGAGGTGAGCGGAACTTAGGGTGGAGCGGTGAAATGCGTTG
ATATCCTAAGGAACACCAGGGGCGAAAGCGGCTCACTGGGTCTCTTCTGACGCTGAGGCA
CGAAAGCTAGGGTAGCGAAGAGGATTAGAAACCCTTGTAGTCC
>Otu11677
CCAGCCTACGGGGCGCTGCAGTCGAGAATCTTCCACAATGGACGAAAGTCTGATGGAGCG
ACGCCGTGTGATTGATGAAGTCCCTCTGGGACGTAAAGATCTTTTATGAGGGAAGAAGTT
TATTGACTGTACCTCATGAATAAGAGGCTCCTAATCTCGTGCCAGCAGGAGCGGTAATAC
GAGAGCCTCGAGCGTTATCCGGAATTATTGGGCGTAAAGGGTGCGTAGGTTGTTCTGTTA
GTCTTTTGTCAAACTCGCTGGCTCAACTGGCGGCATGCGGAAGAAACGGCAGAACTTGAA
AGTGCGAGAGGTATACAGAACTCATGGTGTAGGGGTGAAATCCGTTGATATCATGGGGAA
TACCAAATGCGAAGGCAGTATACTGGCGCATATTTGACACTGAAGCACGAAAGCGTGGGT
AGCGAATGGGATTAGAAACCCTAGTAGTCC
>Otu11688
CCAGCCTACGGGTTGCTGCAGTGAAGAATCTTGGGCAATGGAGGAAACTCTGACCCAGTG
AGAGTACATGCATGATGGAGGCGCGTAGTTTTCTGTAAAGTGCTTTCAGTGACGAAGATA
ATGTCCGTAGTCACAGTAAGAAGCGCCGGCCAACTCCGTGCCAGCAGCCGCGGTAAGACG
GAGGGCGCGGGCGTTATTCGTTTTGATTGGGTGTAAAGGGTACGTAGGCTGCTGAAAAAA
ATTCTTGGTTCAAATCGCGGAGTGTTCCTACGCGTGGTCCTCTTATTGATTCAGCTTGTG
TTGATGATGGGTCGGGGGAATTTCCTGTGTAGGGGTAAGATCCAGCCAGATAGGAACGAA
CGCCAGCTAGCGAAGGCGCCCTTCTAGTTTCAACAGACGCTAAGGTACGGAAGCCTGGGG
AGCAAACAGAATTAGAAACCCTGGTAGTCC
>Otu11690
GGACTACTAGGGTTTCTAATCCTGTTTGCTCCCCACGCTTTCGCGTCTCAGCGTCAATAT
CGGTCCAGGTAGCCGCCTTCGCCACCGGTGTTCCTCCTGATATCTACGGATTTCACTCCT
ACACCAGGAATTCCACTACCCTCTCCCGTATTCAAGTCTCCCAGTTTCCAATGCACTTCC
CGGGTTGAGCCCAGGGCTTTCACATCAGACTTAAAAAACCGCCTACACGCGCTTTACGCC
CAATAATTCCGAACAACGCTCGCACCCTCCGTATTACCGCGGCTGCTGGCACGGAGTTAG
CCGGTGCTTCCTTTGAGGGTACCGTCAAGTAAAAGGGGTATTAACCCTCTCACACTTCTT
TCCCTCTGACAGAGCTTTACGACCCGAAGGCCTTCATCACTCACGCGGCGTTGCTGCGTC
AGGGTTTCCCCCATTGCGCAAAATTCCCCACTGCTGCCCCCCATAGGCTGG
>Otu11695
CCAGCCTACGGGGGGCAGCAGTCGCGAATCATTGGCCAATGTGCGAAAGCATGACCATGC
GACGCCGCGTGGGGGAGGAAGGTCTTCGGATCGTAAACCCCTGTCACCGGGGAATAATGA
CCGGCCTTTAATAGGGGCCGGGCGAGAACCAACCCGGAGAGGAAGTAGTGGCTAACTCCG
TGCCAGCAGCCGCGGTAATACGGAGACTACGAGCGTTACTCGGATTCACTGGGCGTAAAG
GGTGCGTAGGCGGACAGGTGTGTCAGGTGTGAAATCCCGGGGCTTAACTCCGGAACGGCG
CTTGAAACTACCTGTCTAGAGACTTGGAGAGGGCAGTGGAATTCTCGGTGTAGCGGTGAA
ATGCGTAGATATCGAGAGGAACACCAACGGCGAAGGCAGCTGCTTGGACAAGTACTGACG
CTGAAGCACGAAAGCGTGGGGAGCAAAAGGATTAGATACCCGAGTAGTCC
>Otu11700
CCAGCCTACGGGGGCTCCAGTGAGGAATTTTGGGCAATGGGCGAAAGCCTGACCCAGCGA
CGCCGCGTGGGTGATGAAGGCCTTCGGGTCGTAAAACCCTGTGAGCCGAGAAGAAGCCGC

GCCGTGCTAATACCACGGTGCGGTGACAGTATCGGCAGAGGAAGCCCCGGCCAACTACGT
GCCAGCAGCCGCGGTAATACGTAGGGGGCAAGCGTTGTTCGGAATTACTGGGCGTAAAGC
GCGTGTAGGCGGTCTGATTAGTCGGAAGTGAAATCCCTCGGCTCAACCGGGGAACTGCTT
TCGATACTGTCAGACTTGAGTGCGGGAGGGGAGAGCAGAATTCCCGGTGTAGCGGTGAAA
TGCGTAGATATCGGGAGGAATACCGGTGGCGAAGGCGGCTCTCTGGACCGCGACTGACGC
TGAGACGCGAAAGCGTGGGGATCAAACAGGATTAGAAACCCGAGTAGCC
>Otu11703
CCAGCCTACGGGACGCTGCAGTAAGGGATATTGCGCAATGGGCGAAAGGCTGAGGGAGCG
ACGCCGCGTGTATGATGAAGCTCTTCGGAGTGTAAAATACTTTTCTCTGGGACGAATTTT
GACTTTACCAGAGGAATAAGGAGCCGCTAACCTCGTGCCAGCAGCGGCGGTAATACGAGG
GCTCCAAGCGTTATCCGGATTTATTGGGCGTAAAGGGTACGTAGGTGGTTTGATGCATCT
TTCGTTAAATCCTCAGGCTTAACCTGAGGGCCGCGGAAGAGATGGTCAGACTAGAGACCG
GAAGAGGCAAGCGGAATTGTTGGTGTAGGGGTTAAATCCGTTAATATCAACAAGAACACC
AAAAGCGAAGGCAGCTTGCTAGGACGGTTCTGACACTGAGGTACTAAAGCGTGGGGAGCG
AACAGGATTAGAGACCCCCGTAGTCC
>Otu11711
CCAGCCTACGGGAGGCTGCAGTCGAGAATCTTTCGCAATGGGGGAAACCCTGACGACGCG
ACGCCGCGTGGAGGATGAAGGCCTTCGGGTTGTAAACTCCTGTCAGCTGGGAGGAATAAG
CGGTTCATTAACAATGGATCGCCTTGACAGTACCAGCAGAGGAAGCCACGGCTAACTCTG
TGCCAGCAGCCGCGGTAATACAGAGGTGGCAAGCGTTGTTCGGATTTATTGGGCGTAAAG
GGTCCGTAGGCGGCCTAGAAAGTCGGATGTGAAATCCCACAGCTTAACTGTGGAACTGCA
TTCGAAACTTCTAAGCTCGAGTGTGAGAGGGGCAAAGGGAATTCCCGGTGTAAGAGTGAA
ATCTGTAGAGATCGGGAGGAACACCAGTCGCGTAGGCGCTTTGCTGGCTCACTACTGACG
CTGAGGGGCGAAAGCCAGGGGAGCAAACGGGATTAGAGACCCGCGTAGTCC
>Otu11714
CCAGCCTATGGGGGGCAGCAGTCGAGAATATTCCACAATGGGCGAAAGCCTGATGGAGCG
ACGCCGCGTGCAGGAAGAAGCCCTTCGGGGTGTAAACTGCTTTTATTAAAGAGGAAGTTA
ATGACTATTTAGTGAATAAGGGGCTGCAAACTTCGTGCCAGCAGCCGCGGTAATACGAAG
GCCCCGAGCGTTATCCGGATTTATTGGGCGTAAAGCGTGCGTAGGAGGTTTAGTGAGTCT
GTTGTTAAATTTCGCTGCTTAACGGCGGAGCCGCAACAGATACTACTAGACTAGAGTGTG
TGAGAGGCTAATAGAACTCACGGTGTAGGGGTGAAATCCGTTGATATCGTGGGGAATACC
AAAGGCGAAGGCATTTAGCTAGCGCATTACTGACTCTAAGGCACGAAAGCGTGGGGAGCA
AAAAGGATTAGAGACCCCGGTAGTCC
>Otu11717
CCAGCCTACGGGACGCAGCAGTAAGGAATATTGGGCAATGGGCGAAAGCCTGACCCAGCA
ACGCCGCGTGGAGGATGAAGGCCTTCGGGTCGTAAACTCCTTTTGTCGGGGACGAGGAAG
GACGGTACCCGGCGAATCAGTCACGGCTAACTACGTGCCAGCAGCCGCGGTAAAACGTAG
GTGGCAAGCGTTATCCGGATTTACTGGGCGTAAAGCGTGTGCAGGCGGGACTCCAAGTGG
TGTATGAAAGCACCCGGCTCAACCGGGTGAGGTTATGCCAGACTGGAGTTCTGGAGGCTG
AGAGAGGGACATGGAATTCCGGGTGTAGTGGTGAAATGCGTAGAGATCCGGAGGAACTCC
AGAGGCGAAGGCGGTGTCCTGGCTCAGACCTGACGCTCAGACACGAAAGCATGGGTAGCG
AACGGGATTAGAAACCCTAGTAGTCC
>Otu11718
CCAGCCTACGGGGGGCTCCAGCTAAGAATATTCCGCAATGGACGCAGGTCTGACGGAGCG
ACGCCGCGTGATGGATGAAGTGCTTTGGTATGTAAACATCTTTTATCGGGGACGAAGTTT
ATTGACGGTACCCGATGAATAAGGGGCTCCTAACTCTGTGCCAGCAGGAGCGGTAATACA
GAGGCCCCAAGCATTACCCGGAATAACTGGGCGTAAAGGGTGTGCAGGCGGTCATATTAG
TCCTTCGTAAAATCCGTGGGCTCAACCTACGGTCCGCGAAGGAAACGGTATGACTCGAGG
GCGCGAGAGGTGCACAGAACTCATGGTGGAGGGGTGAAATCCGTTGATATCATGGGGAAC
ACCAAAGGCGAAGGCAGTGCACTGGTGCGTTCCTGACGCTCAAACACGAAAGCCAGGGTA
GCGAACGGGATTAGAAACCCCCGTAGTCC
>Otu11719
CCAGCCTATGGGTCGCAGCAGTGAGGAATTTTCCGCAATGGGCGAAAGCCTGACGGAGCG
ACGCCGCGTGGGTGAAGAAGGTTTTCGGATCGTAAAGCCCTGTCAGGGGGAAAGAATCCC
CTCCGTGTAAATAATGCGGAGGGCTGACGGTACCCCCAAAGGAAGCTCCGGCCAACTACG
TGCCAGCAGCCGCGGTAATACGTAGGGAGCGAGCGTTATCCGGAATCACTGGGCGTAAAG
AGCTCGTAGGCGGTTCGTTAAGTCGCATGTTAAATCCCCGGGCTCAACTCGGGAAATGCG
TGCGAAACTGACAAACTAGAGGACGGGAGAGGTAAGTGGAATTCATGGTGTAGCGGTGAA

ATGCATAGATATCATGAAGAACACCAGTGGCGAAGGCGGCTTACTGGACCGTTCCTGACG
CTGAGGAGCGAAAGCCAGGGGAGCAAACGGGATTAGAAACCCGCGTAGTCC
>Otu11726
CCAGCCTACGGGGTGCTGCAGTCGAGAATTTTTCTCAATGGGCGAAAGTCTGACGGAGCG
ACGCCGCGTGGAGGATGAAGTTCTTCGGAATGTAAACTCCTTTTGCCAGGGAAAAAGTTA
TTGATTGTACCTGGAGAATAAGAAGTTGCTAAACTCGTGCCAGCAGCAGCGGTAATACGA
GTGCTTCAAGCGTTATCCGGAATCATTGGGCGTAAAGGGTGTGTAGGCGGCGATGTTAGT
CTCGCGTTAAATCTTTCGGCTCAACCGAGAGTCCGCGTGGGAAACGGCATTGCTAAGAGG
ATGGCAGAGGTCTCTGGAACTCATGGTGTAGCGGTGAAATGCGTTGATATCATGGGGAAC
ACCAAAAGCGAAGGCAAGAGACTGGGCCACTCCTGACGCTGAAACACGAAAGCGTGGGTC
GCGAATGGGATTAGAAACCCGAGTAGTCC
>Otu11735
CCAGCCTATGGGTGGCACCAGTAGGGAATTTTGCGCAATGGGCGAAAGCCTGACGCAGCA
ACGCCGCGTGAGGGATGAAGGTCTTCGGATTGTAAACCTCTGTTGAGTGGGAAGAAACGT
ACCGAGGCGAATACCCTCGGTGCCTGACGGTACCATTCGAGGAAGCCCCGGCTAACTACG
TGCCAGCAGCCGCGGTAATACGTAGGGGGCTAGCGTTGTTCGGAATCACTGGGCGTAAAG
GGAGTGTAGGCGGGGATGTAAGTCAGGAGTGTAATCTACGGGCTCAACCCGTAATTTGCT
CTTGAGACTGCGTTTCTTGAGTAGTGGAGAGGGAGACGGAATTCCAGGTGTAGCGGTGGA
ATGCGTAGATATCTGGAAGAACACCAGTAGCGAAGGCGGTCTCCTGGCCACATACTGACG
CTGAGGCTCGAAAGTGTGGGGAGCGAAACGGATTAGAAACCCGAGTAGTCC
>Otu11741
CCAGCCTACGGGGGGCAGCAGCTAAGAATCTTCCGCAATGGGCGAAAGCCTGACGGAGCG
ATGCCGCGTGATCGATGAAGGTCGAGAGATTGTAAAGATCTTTTCCACATGAGGAATAAC
CATCGGAGGGAATGCCGGTGGGATGACGTCAGTGTGGGAATAAGCCCCGGCTAATTACGT
GCCAGCAGCCGCGGTAACACGTAAGGGGCGAGCGTTGTTCGGAATTATTGGGCGTAAAGG
GCACGCAGGCGGCCCGGCAAGTCTGATGTGAAAGACCTGGGCTCAACTCAGGGACCGCGT
TGGAAACTGCCGAGCTGGAATCTTGGAGGGGGAGTCGGAATTCCTAGTGTAGGGGTGAAA
TCTGTTGATATTAGGAAGAACACCGGTGGCGAAGGCGGACTCCTGGCCGAAGATTGACGC
TGAGGTGCGAAAGCATAGGGAGCAAACAGGATTAGAAACCCCAGTAGTCC
>Otu11751
CCAGCCTACGGGGTGCTCCAGTGAGGGATATTGCACAATGGGGGAAACCCTGATGCAGCG
ACGCCGCGTGGGTGATGAAGGACTTCGGATCGTAAAGCCCTTTTCTGCGTGACGAGGAAG
GACGGTAGCGCAGGAAGAAGTCTCGGCTAACTACGTGCCAGCAGCCGCGGTAAAACGTAG
GAGGCAAGCGTTATCCGGAGTTACTGGGCGTAAAGCGCGTGCAGGTGGTTGGACAAGTTC
GGTGTGAAAGCGCCCGGCTCAACTGGGCGAGGACATTGAAGACTGTTCGACTGGAGGCGG
GTAGAGGGGCGTAGAATTCCGGGTGTAGCGGTGAAATGTGTAGAGATCCGGAGGAATACC
AGTGGTGAAGACGGCGCCCTGGCCCGACCTGACACTGAGACGCGAAAGCGTGGGGAGCG
AACGGGATTAGAAACCCTAGTAGTCC
>Otu11753
CCAGCCTATGGGGGGCACCAGTGAGGAATATTGGGCAATGGACGAAAGTCTGACCCAGCA
ACGCCGCGTGGAGGATGAAGGTCGTAAGATCGTAAACTCCTTTTGAGAGGGATGAATATA
CAGATTTCGTAAGGGATTTGTACTGACCGTACCTCTAGAAGAAGCCCCGGCTAACTACGT
GCCAGCAGCCGCGGTAATACGTAGGGGGCAAGCGTTGTCCGGATTTACTGGGTGTAAAGG
GCGCGTAGGCGGATTTGTAAGTCAGAGGTGAAAGCCCGCAGCTTAACTGCGGAACTGCCT
TTGATACTGCATATCTTGAATTCGGAAGAGAGAAGCGGAATTCCAGGTGTAGTAGTGAAA
TACGTAGATATCTGGAAGAACACCAGTGGCGAAGGCGGCTTCTTGGTCCGTAATTGACGC
TGAGGCGCGAAAGCGTGGGGAGCAAACAGGATTAGAGACCCTCGTAGTCC
>Otu11755
CCAGCCTATGGGAGGCTGCAGTTAGGGAACTTTTCCAATGCGCGCAAGCGTGAGAAAGTA
AGCCAGAGTGTTATTCATAAATTTTGAATAACTTTTGTTTGTTGTAAAAAGACAGACGAA
TAAGGAGTGGGCAAGATCGGTGCCAGCCGCCGCGGTAATCCCGACGCTCCAAGTCGCAGC
CACAATTATTGGGTCTAAAACATTCGTAGCTTGCCTTGCAAGTCTTTTGTGAAATCTTAA
CTCTTAAAGTTGAGGCGTGCAAGAGATACTGCTTGGCTTGGGACCGGAAGAAGCAAGAAG
TATGTTCGAGGTAGTGGTAAAATATGTTAATCTTGAACAGACTAACAACAGCGAAGGCAT
CTTGCTAGTACGGATCTGACAGTGAGGAATGAAGGCTAGGGTCGCGAAACGGATTAGATA
CCCTAGTAGTCC
>Otu11758
CCAGCCTACGGGTCGCTCCAGTGGGGAATTTTGCGCAATGGACGAAAGTCTGACGCAGCA

ACGCCGCGTGGGTGATGAAGGTCTTCGGATTGTAAAACCCTGTCGTCAGGGACGAAGGTT
GTGTTTTGAATATGGATGCAACTTGACGGTACCTGGAGAGGAAGCCCCGGCTAACTCTGT
GCCAGCAGCCGCGGTAATACAGAGGGGGCAAGCGTTATTCGGAATTATTGGGCGTAAAGG
GCGCGTAGGCGGTTTCGTAAGTAGGATGTGTAATCCCCGGGCTTAACCTGGGAACTGCAT
CCTAGACTGCGAGGCTAGAGTGCTGGAGAGGGTGGTAGAATTCCACGTGTAGCGGCGAAA
TGCGTAGAGATGTGGAGGAATACCAGTGGCGAAGGCGGCCACCTGGTCAGTAACTGACGC
TGATGCGCGAAAGTGTGGGGAGCAAACAGGATTAGATACCCGAGTAGTCC
>Otu11765
CCTATGGGAGGCAGCAGGCGCGAAACCTTTGCAATGCGCGTAAGCGTGACAGGGGAACTC
TGAGTGGTAGGGAACTAAGTTCCCTATCTTTTGACAACCCTAAATCGGTTGTAGAGTAAG
GTCTGGGCAAGACCAGTGCCAGCCGCCATCGGTATATGCTAAATGCACCATACCAACGGT
AAGCGGTAATACTGGCAGAACGAGTGGTATCCACGAATATTGGGTCTAAAGGGTCCGTAG
CTGGCCTGTTAAGTTCTCTGTGAAATCTTGGAGCTCAACTTCAAGGCGTGCAGGGAATAC
TGGCGGGCTTGAGAGTGGGGGAGGTTAGGGGTACTCACGGGGTAAGAGTAAAATCTTGTA
ATCCTGTGAGGACTTCCAGTGGCGTAGGCGCCTAACCAAAACACGTCTGACAGTGAGGGA
CGAAGGCTAGGAGAACGAATCGGATTAGAAACCCGTGTAGTCC
>Otu11769
CCAGCCTACGGGGTGCTGCAGTGAGGAGTATTGCTCAATGGGCGAAAGCCTGAAGCAGCG
ACGCCGCGTGAGGGATGAAATCCGTTAGGATGTAAACCTCTTTTGCAGGGGAAGAATGTT
CCGAATTCGTCCGGAATTGACGGTACTCTGCGAATAAGCACCGGCAAACTACGTGCCAGC
AGCCGCGGTAATACGTAGGGTGCGAGCGTTGTCCGGAATTACTGGGTGTAAAGGGTCCGC
AGGCGGACATGTAAGTCGATGGTGAAATCTCCTGGCTCAACCAGGAACTTGCTGTCGATA
CTATGGGTCTTGAGTTCGAGAGAGGATAATGGAATTCATGGTGTAGCGGTGAAATGCGTA
GAGATCATGAAGAACACCGGTAGCGAAGGCGGTTATCTGGCTCGATACTGACGCTCAGGG
ACGAAAGCGTGGGTAGCAAACAGGATTAGATACCCCTGTAGTCC
>Otu11774
CCAGCCTATGGGGGGCTGCAGTGAGGAATATTGCGCAATGGACGAAAGTCTGACGCAGCG
ACGCCGCGTGAGTGACGAAGCCCTTAGGGGTGTAAAGCTCTTTTGTTGGGGACGAATGCC
TACTACGGTAGGAGCGACGGTACCCAACGAATAAGGACCGGCTAACTCCGTGCCAGCAGC
CGCGGTGATACGGGGGGTCCAAGCGTTGTCCGGATTTACTGGGCGTAAAGGGCGCGTAGG
CGGGATGGTAAGTCGGTGGTGAAATGTGCGGGCTTAACCTGCAGATTGCCTTCGATACTG
CCGTTCTTGAGTGAAGAAGGGGAAAGCGGAATACCTGGTGTAGCGGTGGAATGCGCAGAT
ATCAGGTAGAACACCGGTGGCGAAGGCGGCTTTCTGGTCTTACACTGACGCTGAGGCGCG
AAAGCGTGGGGAGCAAACAGGATTAGAACCTCGTAGTCC
>Otu11776
CCAGCCTATGGGTGGCTCCAGTGGGGAATCTTGCGCAATGGGGGAAAGCCTGATGGAGCG
ACGCCGCGTGGGGGATGAATGGCTTCGGCCCGTAAACCCCTGTCATTTGCGATCAAACCT
CGTTATTTAAAAGATGACGAGCTGATAGTAGCGAAAGAGGAAGGGACGGCTAACTCTGTG
CCAGCAGCCGCGGTAATACAGAGGTCCCAAGCGTTGTTCGGATTCACTGGGCGTAAAGGG
CGCGTAGGTGGTGAGGTAAGTCGGATGTGAAATCTCGGAGCTTAACTCCGAAACTGCATT
GGAAACTACCTTGCTCGAGGATTGGAGGGGGGACTGGAATACTTGGTGTAGCAGTGAAAT
GCGTAGATATCAAGTGGAACACCAGTGGCGAAGGCGAGTCCCTGGACAATTCCTGACACT
GAGGCACGAAAGCTAGGGGAGCAAACAGGATTAGATACCCCAGTAGTCC
>Otu11780
CCAGCCTATGGGTTGCTGCAGTGGGGAATATTGGGCAATGGGCGAAAGCCTGACCCAGCG
ACGCCGCGTGGGTGATGAAGGCCTTCGGGTCGTAAAACCCTGTCATGCGGAAAGAACAGC
CTCGCGACTAATACTCGCGGGGTCTGACGGTACCGCAGGAGGAAGCACCGACTAACTCCG
TGCCAGCAGCCGCGGTAATACGGAGGGTGCAAGCGTTGTTCGGAATTATTGGGCGTAAAG
GGCGCGTAGGCGGTTGCGTTAGTCAGATGTGAAATACTTCGGCTCAACTGGAGAATTGCA
TTTGATACTGCGTAACTAGAGTACAGGAGAGGGAAACGGAATTCCAGGTGTAGAGGTGAA
ATTCGTAGATATCTGGAGGAACACCGGTGGCGAAGGCGGTTTCCTGGCCTGATACTGACA
CTGAGGCGCGAAAGCGTGGGGAGCAAACAGGATTAGAAACCCCTGTAGTCC
>Otu11783
CAGCCTACGGGGCGCAGCAGTCGAGAATTTTTCACAATGGGCGCAAGCCTGATGGAGCGA
CGCCGCGTGGGGGATGAATGGCTTCGGCCCGTAAACCCCTGTCATTTGGGATCAATGCGT
CCAGGTGAATAGCCTGGATGTTGATAGTACCAGAAGAGGAAGGGACGGCTAACTCTGTGC
CAGCAGCCGCGGTAATACAGAGGTCCCAAGCGTTGTTCGGATTTACTGGGCGTAAAGGGT
GCGTAGGTGGTCGGGTAAGTCTGATGTGAAATTTCGGAGCCTAACTCCGAAACTGCATTG

GATACTATTCGGCTAGAGGTTGGGAGGGGAGACTGGAATTCTCGGTGTAGCAGTGAAATG
CGTAGATATCGAGAGGAACACCAGTGGCGAAGGCGAGTCTCTGGACCAATCCTGACGCTG
AGGCACGAAAGCCAGGGGAGCAAACGGGATTAGATACCCGAGTAGTCC
>Otu11785
CCAGCCTATGGGGTGCTCCAGCAAGGAATTTTGTACAATGGGCGAAAGCCTGATACAGCG
ACGCCGCGTGAAGGATGAAGACCTTAGGGTTGTAAACTTCTTTTCCCAGGGAAGAATAAT
GACGGTACTTGGGGAATAAGCTTCGGCTAACTACGTGCCAGCAGCCGCGGTAATACGTAG
GAAGCGAGCGTTATCCGGATTTATTGGGCGTAAAGAGTGCGCAGGCGGTCCTTCAAGTCG
AATGTTAAAACTCCCGGCTTAACTGGGAGAGGCCATTCGATACTGTTGGACTCGAGGGCT
GTAGGGGGAGATGGAATTCCCGGTGTAGCGGTGAAATGCGTAGATATCGGGAGGAACACC
AGTGGCGAAAGCGGTCTCCTTGGCAGTTTCTGACGCTTAAGCACGAAAGCGTGGGGAGCA
AACAGGATTAGAAACCCGCGTAGTCC
>Otu11787
CCAGCCTATGGGATGCACCAGTGGGGAATATTAGACAATGGGCGAAAGCTTGATCTAGCT
ATACTTCGTGGGGGACGAAAGCCTCAGTTGTAAACTCCTATATCGATAATGAAAATAATG
ATTGTAATTATAAATGAAGCCCCGGTAAATATCTGTGCCAGCAGCCGCGGTGATACAGGG
GGGGCGAGTATTATTCTACATGACTGGGCGTAAAGGATATGTAGGCGGTTTTTTAAATAT
TGAAAGAAAAGCTATAAAAAAATTATGGATAGTTTCAATATATTGTAAAACTTTGAGTTT
ATAGGAGATATTAGAACCCGGTAGAGTAAATGTGAAATGTTTTGAGACTAGGGGGAATTC
CGAATAGCGAAAGCATTTATCTTGTTAAAACTGACGCTGAAATATGAAGGTGTGGGTAGC
GAATGGGATTAGAGACCCTAGTAGTCC
>Otu11795
CCAGCCTACGGGTCGCTGCAGTAACGAATCTTCCGCAATGCACGAAAGTGTGACGGAGCG
ACGCCGCGTGTGGGACGAAGTTCTTCGGAATGTAAACCACTGTCAGGGGTTAGAAAGTTC
TGATCAACCCCAGAGGAAGGCACGGCTAACTCTGTGCCAGCAGCCGCGGTAATACAGAGA
CTGCAAGCGTTATTCGGATTCACTGGGCGTAAAGGGTGCGCAGGTGGCCAAGTGTGTGAG
GCGTGAAAGCCCGGAGCTCAACTCCGGAATTGCACCTCAAACTACATGGCTAGAGCATTG
GAGAGGGGAGCAGAATTCACGGTGTAGCGGTGAAATGCGTAGATATCGTGAGGAGTACCA
GAGGCGAAGGCGGCTCCCTGGACAATAGCTGACACTCAGGCACGAAAGCGTGGGGAGCAA
AAGGGATTAGAGACCCTTGTAGTCC
>Otu11797
GGACTACAGGGGTATCTAATCCCTTTTGCTCCCCATACTTTCGAGCCTGAGTGTCAGGAT
TTGTCCAGCGGGTTGCCTTCGCCGTCGGTGTTCCTCCAGATATCTACGCATTTCACCGCT
ACACCTGGAATTCCGCCCGCCTCTCCAATCCTCCAGACAAGTAGTTTCGGGTGCAGTTCC
GGAGTTAAGCCCCGGGATTTCACACCCGACACACCTGCCCACCTGCGCTCCCTTTACGCC
CAGTGAATCCGAGTAACGCTTGCAGTCTCTGTATTACCGCGGCTGCTGGCACAGAGTTAG
CCACTGCTTCCTCTCCTGGTTGAATCAGCCCGGCTCTTCGCCGGGGTTTTGTCCCAGGTG
ACAGGGGTTTACAATCCGAAGACCTTCATCCCCCACGCGGCGTCGCACCATCAGGGTTGC
CCCCATTGTGAATGATTCGAAACTGCTGCACCCCGTAGGCTGG
>Otu11803
CCAGCCTATGGGGGGCAGCAGGTACAGCCGGCTGTCTCCCACATGCCCGATGTGCACATC
CCGGCCCTTGAAGATGCAGACGTTCATCGTCGTCGCCATCCGCGTCGCCTTCCGGTCATT
CTCGTGGGAGTCGTAAAGCGACATGTTGGCCGCAAAGAAAATCTCCCGCAGGAGGGCCTT
TGCGCCGGTGTCAGGGCTGCACTCCTGGAACTTCCTGATCGCAGTCTCAACCGCCAGACG
GCTCGCCACATCCCCGCTCCCATGCCCGCCCACGCCGTCGGCAATAATCGCGATCGACCC
CCGCGACTGCCGCTCGTCAGGCTCCTCGGGTTCCCAAAAGCCGGCATAATCCTCGTTTTT
CGGCCGGACCGGCCCCTTCGAGGAGAGGGTGAAGACGTTTAGTTCCATGGAGAACCAGAG
GAAATAAGGGAATCACGCGTCATAAGGCAATCGTCGATTAGATACCCCCGTAGTCC
>Otu11806
CCAGCCTATGGGGTGCAGCAGTGAGGAATTTTCCACAATGGACGAAAGTCTGATGGAGCA
ACGCCGCGTGCAGGATGACGGCCTTCGGGTTGTAAACTGCTTTTTCTAGTGAAGATTATG
ACGGTAGCTAGAGAATAAGGATCGGCTAACTACGTGCCAGCAGCCGCGGTAATACGTAGG
ATCCAAGCGTTATCCGGAATTACTGGGCGTAAAGCGTACGTAGGTGGAGACTTAAGTGAG
ATGCGAAATCTGGCGGCTCAACCGTCTAGACTGTATCTCATACTGGGTTTCTTGAGGTTG
GCAGAGGCAAGTGGAATTTCCGGTGTAGCAGTGAAATGCGTAGATATCGGAAGGAACACC
AATGGCGAAGGCAGCTTGCTGGGCCACACCTGACACTGAGGTACGAAAGCGTGGGGAGCG
AACAGGATTAGATACCCGCGTAGTCC
>Otu11812

CCAGCCTATGGGTCGCAGCAGTAAGGAATATTGGTCAATGGACGAAAGTCTGAACCAGCC
ACGCCGCGTGAGGGATGAAGGCCTTCGGGTTGTAAACCTCTGTCACCGGGGAAGAAACGC
TTCAAGTTAACAGCTTGAAGCCTGACTTAACCCGGAGAGGAAGCAGTGGCTAACTCTGTG
CCAGCAGCCGCGGTAATACAGAGACTGCAAGCGTTATTCGGATTCACTGGGCGTAAAGGG
TGCGCAGGCGGCCAAGTGTGTTAGGCGTGAAAGCCCGGGGCTTAACCCCGGAATTGCACC
TAAAACTACATGGCTAGAGCATTGGAGAGGGTAGCGGAATTCACGGTGTAGCAGTGAAAT
GCGTAGATATCGTGAGGAACACCAGAGGCGAAGGCGGCTACCTGGACAATTGCTGACGCT
CAGGCACGAAAGCGTGGGGAGCAAAAGGGATTAGAAACCCGCGTAGTCC
>Otu11814
CCAGCCTACGGGTCGCTGCAGTAGGGAATTTTCCACAATGGGCGAAAGCCTGATGGAGCA
ACGCCGCGTGCAGGATGAATGCCTTCGGGTTGTAAACTGCTTTTATATGTGACGATTATG
ACGGTAGCATATGAATAAGGATCGGCTAACTCCGTGCCAGCAGCCGCGGTCATACGGAGG
ATCCAAGCGTTATCCGGAATTACTGGGCGTAAAGAGTTGCGTAGGCGGCATCGTAAGCGA
GTAGTGAAAGCGTGTGGCTCAACCACATATACATTACTCGAACTGCGAAGCTAGAGGACA
AGAGAGGTACCTGGAATTCCTAGTGTAGGGGTGAAATCCGTAGATATTAGGAAGAACACC
GATGGCGTAGGCAGGGTACTGGCTTGTTCCTGACGCTCAGGCACGAAAGCGTGGGGAGCG
AACGGGATTAGAGACCCGAGTAGTCC
>Otu11817
CCAGCCTATGGGGGGCACCAGTCGAGGATCTTCCGCAATGAGCGCAAGCTTGACGGAGCG
ACGCCGCGTGTGGGATGAAGGCCTTCGGGTTGTAAACCACTGTCGCCGGTTATGAAAGGG
GCTGGTGTTAACAGCATCAGCAACTGACAAAGGCCGGATAGGAAGCCACGGCTAACTTCG
TGCCAGCAGCCGCGGTAAGACGAAGGTGGCGAGCGTTGTTCGGAATCACTGGGCATAAAG
CGCACGTAGGCGGCGGCGTAAGTCGGATGTGAAATCCCTCGGCCCAACCGAGGAACTGCG
TCCGATACTGCGTTGCTTGAGGACCAAAGGGGTGCATGGAATTCCCGGTGGAGCGGTGAA
ATGCGTTGATATCGGGAGGAACGCCGGCGGCGAAAGCGGTGCACTGGTTGGTTTCTGACG
CTGAGGTGCGAAAGCCAGGGGAGCAAACGGGATTAGAAACCCCAGTAGTCC
>Otu11818
CCAGCTACGGGTCGCACCAGTGAAGAATCTTGGGCAATGGACTAACGTCTGACCCAGCGA
GAATCCATACGTGAAAGAAAGGCACAAGTTGTAAAACGCGAAATTACCAGCACAGATAAT
AACTTAGCTGATGAATGAAGTACCTGCCAACCTCGTGCCAGCAGCAGCGGTAATACGGGG
GGTGCGTGCGTTATTCGTTTTGATTGGGTGTATAGGGTACGTAGGCTAGTATCTATTAAC
ATAATGTTAAATCTTGGGTATCTTACACAAGGAAACCTTATGAAAAATTTACTATGTGTT
TAGAGCGGGTTGGTTAAATTACTCAAGTAATGTGGGGTAAAATCCCTAGAAATGAGTAGG
AATTCCGTGGGCGAAAGCATCTTACTTGCCTAAACATGCGCTGAGAGTACGGAAGCGTAG
GTATCAAACAGGATTAGAAACCCCAGTAGTCC
>Otu11821
CCAGCCTACGGGAGGCACCAGTGGGGAATCTTGCACAATGGGGGCAACCCTGATGCAGCG
ACGCCGCGTGAGCGATGAAGCCCTTCGGGGTGTAAAGCTCTTTCGGCAGGGAAGACAATG
ACGGTACCTGCAGAAGCAGCTGCGGCTAACTACGTGCCAGCAGCCGCGGTAATACGTAGG
CAGCAAGCGTTGTTCGGAGTTACTGGGCGTAAGGGGTGTGTAGGCGGCTTTCAAAGTTTG
TTGTGAAATCTCCCGGCTCAACCGGGAGGGTGCGCCGAATACTGAAGGGCTAGAGTGTGG
GAGAGGAAAGTGGAATTCCTGGTGTAGCGGTGAAATGCGTAGATATCAGGAGGAACACCG
GTGGTGTAGACGGCTTTCTGGACCATAACTGACGCTGAGACACGAAAGCGTGGGTAGCAA
ACAGGATTAGAGACCCGCGTAGTCG
>Otu11829
CCAGCCTACGGGGGGCTCCAGTCGAGGATCTTCGGCAATGGGCGCAAGCCTGACCGAGCG
ACGCCGCGTGTGCGATGAAGGCCTTCGGGTTGTAAAGCACTGTCGAGGGGGAGGAAGCTC
CGCAAGGAGTTGACCTATCCCTGGAGGAAGCACGGGCTAAGTTCGTGCCAGCAGCCGCGG
TAAGACGAACCGTGCGAACGTTGTTCGGAATCACTGGGCTTAAAGGGCGCGTAGGCGGGC
AACCAAGTCTGGGGTGAAATCTTTCGGCTTAACCGGAAAATAGCCCTGGATACTGGCTGT
CTGGAGGGAGGTAGGGGCATGTGGAACTTCCGGTGGAGCGGTGAAATGCGTAGATATCGG
AAGGAACGCCGGTGGCGAAAGCGACGTGCTGGACCTCTACTGACGCTGAGGCGCGAAAGC
CAGGGGAGCAAACGGGATTAGATACCCGTGTAGTCC
>Otu11834
CCAGCCTACGGGAGGCTCCAGTAAGGAATATTGGTCAATGGGCGGAAGCCTGAACCAGCC
ATGCCGCGTGCAGGAAGACGGCCCTACGGGTTGTAAACTGCTTTTGTACGGGAATAAACC
CCTCTACGTGTAGGGGGCTGAATGTACTGTAAGAATAAGGATCGGCTAACTCCGTGCCAG
CAGCCGCGGTAATACGGAGGATCCAAGCGTTATCCGGATTTATTGGGTTTAAAGGGTGCG

TAGGCGGTTTATTAAGTCAGGGGTGAAAGACGGCAGCTTAACTGTCGCAGTGCCTTTGAT
ACTGATGAACTTGAATGTACTAGAAGTAGGCGGAATGTGACAAGTAGCGGTGAAATGCAT
AGATATGTCACAGAACACCGATTGCGAAGGCAGCTTACTATGGTATTATTGACGCTGAGG
CACGAAAGCGTGGGGATCAAACAGGATTAGAAACCCTCGTAGTCC
>Otu11836
CCAGCCTACGGGTCGCTGCAGTCGAGGATCTTCGGCAATGGGCGCAAGCCTGACCGAGCG
ACGCCGCGTGTGCGATGAAGGCCTTCGGGTTGTAAAGCACTGTCGAGGGGGAGGAAAGCC
GCAAGGTCTGACCTATCCCTGGAGGAAGCACGGGCTAAGTTCGTGCCAGCAGCCGCGGTA
AGACGAACCGTGCGAACGTTGTTCGGAATCACTGGGCTTAAAGGGCGCGTAGGCGGTTTA
TCAAGCCCAGGGTGAAATCTTTCGGCTTAACCGGAAAAGTGCCTTGGATACTGGTGAACT
GGAGGGAGGTAGGGGCAGATGGAACTTCCGGTGGAGCGGTGAAATGCGTTGATATCGGAA
GGAACGCCGGTGGCGAAAGCGATCTGCTGGCCCTCTTCTGACGCTGATGCGCGAAAGCTA
GGGGAGCAAACGGGATTAGATACCCCTGTAGTCC
>Otu11837
CCAGCCTATGGGTCGCACCAGTAGGGAATCTTGCGCAATGGGCGAAAGCCTGACGCAGCC
ATGCCGCGTGAATGATGAAGGTCTTAGGATTGTAAAATTCTTTCAGCAGGGAAGATAATG
ACTGTACCTGCAGAAGAAGCCCCGGCTAACTTCGTGCCAGCAGCCGCGGTAATACGAAGG
GGGCTAGCGTTGCTCGGAATTACTGGGCGTAAAGGGCGCGTAGGCGGGTATTTAAGTCAG
AGGTTAAATCCCAGGGCTCAACCCTGGAACTGCCTTTGATACTGGATATCTAGAGTTCGG
GAGAGGTGTGTGGAACTCCGAGTGTAGAGGTGAAATTCGTAGATATTCGCAAGAACACCA
GTGGCGAAGGCGGCTCACTGGCCCGATACTGACGCTGAGGCACGAAAGCGTGGGGAGCAA
ACAGGATTAGAGACCCTCGTAGTCC
>Otu11838
CCAGCCTATGGGGGGCTCCAGGCATAAACGGCGATGACCAGCGCGCAAACCGCGCCTAGA
ACAGCGACACGCCTCCATGGGGCAAGGGGGGCGGTCACGGCAACTGGCGTGGAAACTCCA
CGGCCCGCCACCTCCTCCTTCTGGGATCGTTCGGCCATACGGTCTGCCTGACGAGAATGA
ACCCTTTCTGCCTGCATTGAAGCAGTTTTTTCTTTTCGGTTGGCCCGGGCGATCATTGCC
GCACGCCGAGGCCTTGTTGGACGACAACTTGCTTGGATGCTCCGGGCAAAGGTGGCCGTG
GATTTTCACCGTGACCGCCCAGTCACTACGCCGTAGAAAGAACCATGACCTCGGACCCAG
CCAATCCGACGCCGCGCTACGTCCCCTACAAGCCCCTGTACATCACGCGGTTTTTTCACC
TCGGCTCCAAGCTCTTCCGGATCCGGGAAGCTCTCATCGGGCTATTTGGAGAGGATTAGA
GACCCCAGTAGTCC
>Otu11842
CCAGCCTACGGGTGGCAGCAGTGGGGAATCTTGGACAATGGGGGCAACCCTGATCCAGCG
ATGCCGCGTGTGTGAAGAAGGCCTGCGGGTTGTAAAGCACTTTTGGTTGGGAAGAAGGGT
AGAGAGTGAATAGCTTTCTGCTTTGACGGTACCGACAGAATAAGCACCGGCTAACTCTGT
GCCAGCAGCCGCGGTAATACAGAGGGTGCAAGCGTTAATCGGAATGACTGGGCGTAAAGG
GCGAGTAGGCGATTGTATAGGTGTGGTGTGAAAGACACGGGCTCAACCTGTGAATGTCGC
CGCAAACGGTATGACTAGAGAGCAGTAGAGGGGAGTGGAATTTCCGGAGGAGCGGTGAAA
TGCGTAGATCTCGGAAGGAACACCAGTGGCGAAGGCGACTCCCTGGACTGCAACTGACGC
TGAGGTGCGAAAGCGTGGGGAGCAAACAGGATTAGATACCCTCGTAGTCC
>Otu11849
CCAGCCTATGGGATGCAGCAGTGGGGAATTTTGGACAATGGGGGAAACCCTGATCCAGCA
ATGCCGCGTGAGTGAAGAAGGTCTTCGGATTGTAAAGCTCTTTCGACGGGGACGATGATG
ACGGTACCCGTAGAAGAAGCCCCGGCTAACTTCGTGCCAGCAGCCGCGGTAATACGAGGG
GGGCAAGCGTTGTTCGGAATTATTGGGCGTAAAGGGTGCGTAGGCGGTCTGATAAGTCTG
ATGTGAAATCGATGGGCTCAACCCATCGTCTGCATTAGAAACTGTCGGGCTTGAGTGTGG
GAGAGGTGAGTGGAATTTCCGGTGTAGCGGTGAAATGCGTAGATATCGGAAGGAACACCT
GTGGCGAAAGCGGCTCACTGGACCATTACTGACGCTGATGCACGAAAGCTAGGGGAGCAA
ACAGGATTAGAGACCCTTGTAGTCC
>Otu11855
CCAGCCTACGGGGTGCTGCAGTGGGGAATATTGGACAATGGGGGCAACCCTGATCCAGCG
ATGCCGCGTGTGTGAAGAAGGCCTGCGGGTTGTAAAGCACTTTCGGTGGGGAGGAGGACT
ATGAGGTTAAGAGCTATATAGTTGGACGTTACCCACATAAGAAGCACCGGCTAACTCCGT
GCCAGCAGCCGCGGTAATACGGAGGGTGCAAGCGTTAATCGGAATTACTGGGCGTAAAGG
GTGTGTAGGCGGTTTAATAAGTTAAGTGTGAAATTCCCGGGCTCAACCTGGGGTAGTCAT
TTAAGACTGTTAGACTAGAGTATGGGAGAGGGTAGTGGAATTTCCGGTGTAGCGGTGAAA
TGCGTAGAGATCGGAAGGAACACCAGTGGCGAAGGCGGCTACCTGGCCTAATACTGACGC

TGAGGCACGAAAGCGTGGGGAGCAAACAGGATTAGATACCCTTGTAGTCC
>Otu11864
CCAGCCTATGGGTGGCAGCAGTCGAGAATCTTTCGCAATGGGCGCAAGCCTGACGAAGCG
ACGCCGTGTGAGTGAAGAAGGCCTTCGGGTTGTAAAGCTCTTTCGCTAGGGAACAAGAGA
TGTGGCCTAATACTCCACAAATTTGATGGTACTTGGTAAAGAAGCACCGGCTAACTCCGT
GCCAGCAGCTGCGGTAATACGGAGGGTGCAAGCATTGATCGGAATTACTGGGCGTAAAGG
GCGCGTAGGCGGTCAGATAAGTCAGATGTGAAATTCCGGGGCTCAACCCCGGAGCTGCAT
TTGAAACTATCTGACTCGAGGACAGTTAGGGTAAACGGAATTCCACGTGTAGCGGTGAAA
TGCGTAGATATGTGGAAGAACACCGGTGGTGAAGACGGCTTACTGGGCTGTAACTGACGC
TGAGGCGCGAAAGCTAGGGGAGCAAACAGGATTAGAGACCCGTGTAGTCC
>Otu11866
CCAGCCTACGGGGTGCACCAGTGGGGAATATTGGACAATGGGCGCAAGCCTGACCGAGCG
ACGCCGCGTGGAGGATGAAGGCCTTCGGGTTGTAAACTCCTGTCGAGGGGGAGGAAGTCC
CGAGCGATCGGGATTGACCGATCCCTGGAGGAAGCACGGGCTAAGTTCGTGCCAGCAGCC
GCGGTAAGACGAACCGTGCGAACGTTATTCGGAATCACTGGGCTTAAAGCGCGTGTAGGC
GGATGGGAACGTCGGTTGCTGAAAGCCCCCGGCTCAACCGGGGAACTGGCGCCGAAACGC
CCCGTCTGGAGGGACGTAGGGGGACCTGGAACTTCCGGTGGAGCGGTGAAATGCGTTGAG
ATCGGAAGGAACGCCCGTGGCGAAAGCGAGGTCCTGGACGTCTACTGACGCTGAGACGCG
AAAGCTAGGGGAGCGAACGGGATTAGAGACCCCCTAGTCC
>Otu11872
CCAGCCTATGGGGTGCAGCAGTCGAGAATCATTCGCAATGGGCGAAAGCCTGACGATGCG
ACGCCGTGTGCGTGATGAGGGCCTTCGGGTTGTAAAGCGCTTTCGCCCGGGAACAAGAGA
AGCAGGCTAATATCCTGCGGATTTGAGGGTACCGGGTAAAGAAGCGCCGGCTAACTCCGT
GCCAGCAGCTGCGGTAATACGGAGGGCGCGAGCATTAATCGGATTTATTGGGCGTAAAGG
GCGCGTAGGCGGAGATGTAAGTCAGATGTGAAATCCCGGGGCTCAACCCCGGAACAGCAT
TTGATACTGCTTCTCTCGAGGGTAGGCGGAGAAAATGGAATTCCACAAGTAGCGGTGAAA
TGCGTAGATATGTGGAAGAACACCGGTGGCGAAGGCGATTTTCTAGCTTATACCTGACGC
TGATGCGCGAAAGCAAGGGGATCAAACAGGATTAGATACCCGTGTAGTCC
>Otu11876
GGACTACAGGGGTTTCTAATCCCGTTTGCTCCCCTGGCTTTCGCGTCTCAGCGTCAGGAC
ATCCCCAGCGAGCTGTTTTCACCATTGGCGTTCCAAACGATATCTACGCATTTCACCGCT
CCACCGTTTGTTCCGCTCGCCCCTAGATGCCTCAAGAAGACCAGTATACCCAGCAGTTCC
CGGGTTGAGCCCGGGGATTTCACAAGGTACTTAGCCCTCCGCCTACACGCGCTTTAAGCC
CAGTGATTCCGCCTAACGCTCGGGGCCTCTGTCTTACCGCGACTGCTGGCACAGAGTTAG
TCGCCCCTTCCTCTGGGGATCCTCAGAACTTGGTATCCCCTGACAGAAGTTTACATTCCG
AAGAACTTCGTCCTCCACGCGGCGTCGCTCCGTCACACTTTCGTGCATTGCGGAAGATTC
GTTACTGGTGCCCCCCATAGGCTGG
>Otu11879
CCAGCCTATGGGTCGCTGCAGTCGAGAATTTTTCTCAATGGGGGAAACCCTGAAGGAGCG
ACGCCGCGTGGAGGATGACGGTCTTCGGATTGTAAACTCCTGTCATGAGGGAACAAACTT
ACGCCAGTAACTACGGCGTGATTGATAGTACCTCAAGAGGAAGAGACGGCTAACTCTGTG
CCAGCAGCCGCGGTAATACAGAGGTCTCAAGCGTTGTTCGGATTCATTGGGCGTAAAGGG
TGCGTAGGCGGCGAGGCAAGTCAGGTGTGAAATCCCGGAGCTTAACTCCGGAACTGCACT
TGATACTGCCTTGCTCGAGGACTGGAGAGGAGATCGGAATTCACGGTGTAGCAGTGAAAT
GCGTAGATATCGTGAGGAAGGCCAGTGGCGAAGGCGGATCTCTGGACAGTTCCTGACGCT
GAGGCACGAAGGCCAGGGGAGCAAACGGGATTAGATACCCCCGTAGTCC
>Otu11885
CCAGCCTATGGGATGCAGCAGTAAGGAATATTGGTCAATGGGCGGAAGCCTGAACCAGCC
ATGCCGCGTGCAGGAAGACGGCCCTACGGGTTGTAAACTGCTTTTGTACCGGAATAAACC
TCAATTCGTGAATTGAGCTGAATGTACGGTAAGAATAAGGATCGGCTAACTCCGTGCCAG
CAGCCGCGGTAATACGGAGGATCCAAGCGTTATCCGGATTTATTGGGTTTAAAGGGTGCG
TAGGCGGCCTTGTAAGTCAGGGGTGAAAGACGGTAGCTCAACTATCGCAGTGCCTTTGAT
ACTGCAGGGCTTGAATGTACTTGAGGTAGGCGGAATGTGACAAGTAGCGGTGAAATGCAT
AGATATGTCACAGAACACCAATTGCGAAGGCAGCTTACTATGGTTTGATTGACACTGAGG
CACGAAAGCGTGGGGATCAAACAGGATTAGAGACCCCTGTAGTCC
>Otu11888
CCAGCCTATGGGAGGCAGCAGTAGGGAATATTGCGCAATGGAGGAAGCTCTGACGCAGCG
ACGCCGCGTGGGTGATGAAGGCCTTCGGGTCGTAAAGCCCTGTCGGAGGGGAAGAAAAAC

ATACTCGATAATAATAAGTATGCTTGACGGTACCCTTAAAGGAAGCACCGGCTAACTACG
TGCCAGCAGCCGCGGTAATACGTAGGGTGCAAGCGTTGTTCGGAATCATTGGGCGTAAAG
CGCGCGTAGGCTGTTAATTAAGTCGGATGTGAAATCCCTGGGCTCAACCGAGGAAGTGCA
TTCGAAACTGATTAGCTCGAAGACGGTAGAGGAAGGTGGAATTCCTTGTGTAGAGGTGAA
ATTCGTAGATATTAGGAGGAATACCAGTGGCGAAGGCGGCCTTCTGGACCGTTCTTGACG
CTGAGGCGCGAAAGCGTGGGTAGCAAACAGGATTAGATACCCTTGTAGTCC
>Otu11893
CCAGCCTACGGGTTGCTGCAGCTCTCGTTGCGCTTGGAGTAGTCGGCGATGACCAGGAAG
CTCACATCGTCGGACGGGGTGAGCAGGTACTGGCCGCGTACCGTGTAGACATTGCGATTG
TTGGAATCGCCGTCGGTGTTCGGACCGAGACCGGTATCGACGGGGGAGAAGCCGTCGCGT
TTTTGGTAGCCCGCGTACAGGCGGAAGGCGGATATGCCGCCAACGGCTCCGGTGATGGAC
CCGGAGATTTCGCGGTCGTTGTAATTCCCGAAGGTGATTTCACCCGTCACCCCGAACGTG
GTCGAGGGCCGCTTGGTCACGATGTTAATGACGCCCGCATCGTTGTTCTTGCCGAACAAC
TCTCCCTGCGGGCCCTCGAGAATTTCGATGCGATCGATTTCTCCGAGGTTGCCGAAGCCC
ACCCCGTTGCGGGGGCGGTAGACGCCGTCGATGACGATGCCCACGGAGGATTAGATACCC
TTGTAGTCC
>Otu11904
CCAGCCTACGGGTCGCTGCAGTTCAGAATATTCGGCAATGGACGAAAGTCTGACCGAGCG
ACGCCGCGTGAGGGATGAAGGCCCTATGGGTTGTAAACCTCTTTCGCGATTTAACAAGTC
ATAGTCATTTAACAAATGGTTATGTTGAGCAAGGATCGTAAAGAAGCCCCCGCTAATCAC
GTGCCAGCAGCGGCGGTAATACGTGAGGGGCGAACGTTGTTCGGTGTCACTGGGCTTAAA
GGGTGCGTAGGTGGTTGCGTAAGTAACGTGTGAAATCCCTCGGCTTACCCGGGGAATTGC
ATGTTATACTGCGTGACTTGAGGTCTAAAGGGGAAAAGGGAACTCCAGGTGTAGCGGTGA
AATGCGCAGATATCTGGGGGAAGGCCGGCGGCGAAGGCGCTTTTCTGGTTAGATCCTGAC
ACTGAGGCACGAAAGCGTGGGGAGCAAACAGGATTAGATACCCCGGTAGTCC
>Otu11905
CCAGCCTATGGGAGGCTCCAGTGGGGAATATTGGACAATGGGGGCAACCCTGATCCAGCA
ATGCCGCGTGTGTGAAGAGGGTCTTCGGATTGTAAAGCACTTTTGGCAGGGACGATAATG
ACGGTACCCGCAGAAGAAGCTCCGGCTAACTTCGTGCCAGCAGCCGCGGTAATACGAAGG
GGGCTAGCGTTGTTCGGAATTACTGGGCGTAAAGCGCGCGCAGGCGGCCTTTCAAGTCAG
GGGTGAAAGCTCGGAGCTCAACTCCGGAATTGCCTTTGAAACTGCAAGGCTTGAGTACGG
GAGAGGTGAGTGGAATTCCCAGTGTAGAGGTGAAATTCGTAGATATTGGGAAGAACACCG
GTGGCGAAGGCGGCTCACTGGCCCGTGACTGACGCTCAAGCGCGACAGCGTGGGGAGCAA
ACAGGATTAGAAACCCCCGTAGTCG
>Otu11912
CCAGCCTATGGGTCGCAGCAGTGGGGAATATTGGACAATGGGGGCAACCCTGATCCAGCG
ATGCCGCGTGTGTGAAGAAGGCCTGAGGGTTGTAAAGCACTTTCGGTTGGGAAGAGGGCA
TTTTTGCTTAATACGCAAGAGTGTTGACGTTACTAACAGAAGAAGCACCGGCAAACCCTG
TGCCAGCAGCCGCGGTAATACAGGGGGTGCAAGCGTTAATCGGAATGACTGGGCGTAAAG
GGCACGTAGGCGGCCATGTAAGTTGGGTGTTAAAGACCAGGGCTTAACCTTGGGAGTGCA
TTCAAGACTGTGTGGCTAGAATATAGAAGAGGTTGATGGAATTTCCGGTGTAGCGGTGAA
ATGCGTAGAGATCGGAAGGAACACCAGTGGCGAAGGCGATTGGCTGGTCTAATATTGACG
CTGAGGTGCGAAAGCGTGGGGAGCAAACAGGATTAGAAACCCGTGTAGTCC
>Otu11917
CGAGCCTACGGGTGGCTGCAGTAGGGAATATTGCACAATGGAGGAAACTCTGATGCAGCG
ACTCCGCGTGAGTGATGAAGGACTTCGGTTCGTAAAGCTCTGTTGCAAGGGAAAAAGAAA
GTGATTGTACTTTGCGAGAAAGGGTCGGCTAACTTCGTGCCAGCAGCCGCGGTAAGACGA
GGGACCCTAGCGTTGTTCGGAATCATTGGGCGTAAAGCGGGTGTAGGTGGCTTTGTAAGT
CAGGTGTGAAAGCCCGGGGCTCAACCTCGGAAGGGCATTTGATACTGCGAAGCTTGAGTG
CTGGAGAGGCTACTAGAATTCCTGGTGTAGTGGTGAAATACGTAGATATCAGGAGGAATA
CCAGAGGCGAAGGCGGGTAGCTGGCCAGACACTGACACTTAGACCCGAAAGCGTGGGGAT
CAAACAGGATTAGGACCCGCGAGTCC
>Otu11925
CCAGCCTATGGGGGGCACCAGTTTCGAATCATTCACAATGGGCGAAAGCCTGATGGTGCA
ACGCCGCGTGGGGGACGAAGGTCTTCGGATTGTAAACCCCTGTCATTGAGGAACAAAACC
CGGACTCATACTCCGGCTTGAGTTAACTCAAAGAGGAAGTAGTGGCAAACTCCGTGCCAG
CAGCCGCGGTAATACGGAGACTACGAGCGTTACTCGGATTCACTGGGCGTAAAGGGTGCG
CAGGCGGCCGAATGTGTTGGGTGTGAAATCCTGGGGCTTAACTCCAGGGCTGCGCTCAAA

ACTGTTCGGCTAGAGTCTCGGAGAGGTAAGCGGAATTCCTAGTGTAGCAGTGAAATGCGT
AGATATTAGGAGGAACACCAAAGGCGAAGGCAGCTTACTGGACGAGTACTGACGCTCATG
CACGAAAGCGTGGGGAGCAAAAAGGATTAGATACCCGCGTAGTCC
>Otu11936
CCAGCCTACGGGGGGCTCCAGTGGGGAATATTGGACAATGGGCGCAAGCCTGATCCAGCA
ATGCCGCGTGAGTGAAGAAGGTCTTCGGATTGTAAAGCTCTTTCGACGGGGACGATGTTG
ACGGTACCCGTAGAAGAAGCCCCGGCTAACTTCGTGCCAGCAGCCGCGGTAATACGAAGG
GGGCTAGCGTTGCTCGGAATGACTGGGCGTAAAGGGCGCGTAGGCGGCGATGGTAGTCAG
GCGTGAAATTCCTGGGCTCAACCTGGGAACTGCATTTGAGACTGCATGGCTAGAGTATCG
AAGAGGGAAGTGGAATTTCCGGTGTAGCGGTGAAATGCGTAGATATCGGAAGGAACACCA
GTGGCGAAAGCGACTTCCTGGTCGAATACTGACGTTCATGTGCGAAAGCGTGGGGAGCAA
ACAGGATTAGAAACCCGAGTAGTCC
>Otu11942
CCAGCCTACGGGGTGCAGCAGTCGAGAATTTTTCACAATGGACGAAAGTCTGATGGAGCG
ACGCCGCGTGGAGGATGAAGGTTTTCGGATTGTAAACTCCTGTCACTGCAGAACAAGGTA
ACGCCAGTTAACAGCCGGCGTTATTGATGGTATGCGAAGAGGAAGGGACGGCTAACTCTG
TGCCAGCAGCCGCGGTAATACAGAGGTCCCAAGCGTTGTTCGGATTCACTGGGCGTAAAG
GGTGCGTAGGCGGTAGGGTAAGTCTGTTGTGAAATCTCCGAGCTTAACTCGGAAACTGCA
ATGGAAACTATCCGACTAGAGGAATGGAGGGGAGACTGGAATACTTGGTGTAGCAGTGAA
ATGCGTAGATATCAAGTGGAACACCAGTGGCGAAGGCGAGTCTCTGGACATTTCCTGACG
CTGAGGCACGAAAGCGTGGGGAGCAAACAGGATTAGATACCCTAGTAGTCC
>Otu11954
CCAGCCTACGGGGTGCAGCAGTCGAGAATTTTTCTCAATGGGGGAAACCCTGAAGGAGCG
ACGCCGCGTGGAGGATGAAGGTCTTCGGATTGTAAACTCCTGTCATTAGGGAACAATGGC
GAGCGATTAACTGTCGCTTGTTTGATAGTACCTGAAGAGGAAGAGACGGCTGACTCTGTG
CCAGCAGCCGCGGTAATACAGAGGTCTCAAGCGTTGTTCGGATTCATTGGGCGTAAAGGG
TGCGTAGGCGGCGGGGCAAGTCAGGTGTGAAATCTCGGGGCTCAACTCCGAAACTGCACT
TGATACTGCCTTGCTAGAGTACTGGAGAGGAGATTGGAATTTCCGGTGTAGCGGTGAAAT
GCGTAGATATCGGAAGGAACACCTGTGGCGAAAGCGGCTCACTGGACCATAACTGACGCT
GATGCACGAAAGCTAGGGGAGCAAACAGGATTAGAAACCCCCGTAGTCC
>Otu11961
CCAGCCTACGGGACGCACCAGTGGGGAATATTGGACAATGGGCGAAAGCCTGATCCAGCA
ATGCCGCGTGAGTGATGAAGGCCTTAGGGTTGTAAAGCTCTTTCGCACGCGACGATGATG
ACGGTAGCGTGAGAAGAAGCCCCGGCTAACTTCGTGCCAGCAGCCGCGGTAATACGAAGG
GGGCTAGCGTTGTTCGGAATTACTGGGCGTAAAGGGCGCGTAGGCGGCGACGATAGTCAG
AAGTGAAAGCCCCGGGCTCAACCTGGGAATTGCTTTTGATACTGTGTTGCTTGAGTTCGG
GAGAGGTGAGTGGAATTCCCAGTGTAGAGGTGAAATTCGTAGATATTGGGAAGAACACCG
GTGGCGAAGGCGGCGACCTGGTCCGATACTGACGCTGAGGCGCGAAAGCGTGGGGAGCAA
ACAGGATTAGAGACCCTCGTAGTCC
>Otu11970
CCAGCCTACGGGGGGCAGCAGTGGGGAATATTGGACAATGGGCGCAAGCCTGATCCAGCA
ATGCCGCGTGAGTGATGAAGGCCTTAGGGTTGTAAAGCTCTTTCGCACGCGACGATGATG
ACGGTAGCGTGAGAAGAAGCCCCGGCTAACTTCGTGCCAGCAGCCGCGGTAATACGAAGG
GGGCTAGCGTTGCTCGGAATGACTGGGCGTAAAGGGCGCGTAGGCGGTTTGAACAGTTGG
GCGTGAAATTCCAGGGCTTAACCTTGGGACTGCGTTCAATACGTTTAGACTTGAGTTCGG
AAGAGGGTCGTGGAATTCCCAGTGTAGAGGTGAAATTCGTAGATATTGGGAAGAACACCG
GTGGCGAAGGCGGCGACCTGGTCCGAAACTGACGCTGAGGCGCGAAAGCGTGGGGAGCAA
GCAGGATTAGATACCCGCGTAGTCC
>Otu11978
CCAGCCTACGGGTGGCTGCAGTGGGGAATTTTGGACAATGGGCGCAAGCCTGATCCAGCA
ATGCCGCGTGAGTGAAGAAGGCCTTCGGGTTGTAAAGCTCTTTTGTCAGGGAAGAAACGG
GTCTTTCTAATACAGAGGCCTAATGACGGTACCGGAAGAATAAGCACCGGCTAACTACGT
GCCAGCAGCCGCGGTAATACGTAGGGTGCAAGCGTTAATCGGAATTACTGGGCGTAAAGC
GTGCGCAGGCGGTCCGCTAAGACAGATGTGAAATCCCCGGGCTTAACCTGGGAACTGCAT
TTGTGACTGGCGGGCTAGAGTATGGCAGAGGGGGGTAGAATTCCACGTGTAGCAGTGAAA
TGCGTAGAGATGTGGAGGAATACCGATGGCGAAGGCAGCCCCCTGGGCCAATACTGACGC
TCATGCACGAAAGCGTGGGGAGCAAACAGGATTAGAAACCCCAGTAGTCC
>Otu11982

CCAGCCTATGGGGGGCAGCGGTGGGGAATATTGGACAATGAGCGAAAGCTTGATCCAGCA
ATACCAAGTTATTGAAGAAGGCCAAACCGGCTGTAAATTTATTTCGTTGAGGAAGATAAT
GACATGAATCAAAGAAAGAAGTCCCGGCCAATGCCGTGCCAGCAGCCGCGGAAATACGGT
AGGGGCAAGCGTTATTCACATTGTATGGGCGTAAAGGGTGCGTAGGTAGTAATTTGAACT
TTTTTGTAAAGAACAAAAGTGTACTTTTGTAAAGCAAAAAATAATCTTATTTCTAGAGTT
GGAAAGAGGTTGACGGAATTCCTAGTGGAGAGGTAGAATTCAACAATATTAGGAGGAACA
TCAAAGGCGAAGGCAGTTTTCTGGGTCCAGACTGACGCTGAGGCACGAAGGCATAGGGAT
CAAATGGGATTAGAAACCCGTGTAGTCC
>Otu11986
CCAGCCTATGGGTCGCAGCAGTTGGGAATCTTGGACAATGGGGGAAACCCTGATCCAGCC
ATGCCGCGTGAGTGATGAAGGCCTTCGGGTTGTAAAACTCTTTCGACGGGGACGATAATG
ACGGTACCCGTAGAAGAAGCTCCGGCTAACTTCGTGCCAGCAGCCGCGGTAATACGAAGG
GGGCTAGCGTTGTTCGGAATTACTGGGCGTAAAGCGCGCGCAGGCGGCTATCCAAGTCAG
TGGTGAAAGCCCGGAGCTCAACTCCGGAACTGCCACTGAAACTGTTTAGCTTGAGGACGA
GAGAGGTGAGTGGAATTCCCAGTGTAGAGGTGAAATTCGTAGATATTGGGAGGAACACCC
GTGGCGAAGGCGGCCAACTGGACCGACATGGACGCTGAGGCGCGAAAGCGTGGGGATCAA
ACAGGATTAGAAACCCGGGTAGTCC
>Otu12000
CCAGCCTATGGGTGGCTGCAGTGGGGAATATTGGACAATGGGCGCAAGCCTGAACCAGCT
ATGCCGCGTGTGTGAAGAAGGCCTTCGGGTTGTAAAGCACTTTTGTCCGGAAAGAAATCC
TCTTCGATAATACCGAGGGGGGATGACGGTACCTGAAGAATAAGCACCGGCTAACTACGT
GCCAGCAGCCGCGGTAATACGTAGGGTGCAAGCGTTAATCGGAATTACTGGGCGTAAAGC
GTGCGCAGGCGGTTATGTAAGACAGATGTGAAATGCCCGGGCTTAACCTGGGAACTGCAT
TTGTGACTGCATGGCTAGAATCTGGCAGAGGGGGGTAGAATTCCACGTGTAGCAGTGAAA
TGCGTAGATATGTGGAGGAATACCGATGGCGAAGGCAGCCCCCTGGGCTAAGATTGACGC
TCATGCACGAAAGCGTGGGGAGCAAACAGGATTAGATACCCGCGTAGTCC
>Otu12011
CAGCCTATGGGTGGCAGCAGCCGAGAATTTTTCACAATGGGGGAAACCCTGATGGAGCGA
CGCCGCGTGGAGGATGAAGGTTTTCGGATTGTAAACTCCTGTCACTGCAGAACAAGGGTG
TGCCAGTTAACAGCCGGCGCATTTGATGGTATGCGGAGAGGAAGGGACGGCTAACTCCGT
GCCAGCAGCCGCGGTAAGACAGAGGTCCCAAGCGTTGTTCGGATTCATTGGGCGTAAAGG
GTGTGTAGGAGGTAGGGTAAGTCAGGTGTGAAATCCCAGAGCTTAACTCTGAAACTACGC
TTGATACTGCTCTGCTAGAGGATCGGAGGGGGTATCGGAATTTATGGTGTAGCAGTGAAA
TGCGTAGATATCATAAGGAACACCGGTGGCGAAGGCGGATACCTGGAAGATTCCTGACTC
TGAAACACGAAAGCCAGGGGAGCAAACGGGATTAGATACCCCCGTAGTCC
>Otu12012
CCAGCCTACGGGGGGCAGCAGTGGGGAATATTGGGCAATGTGCGACAGCGTGACCTGATA
ATAATTCCAGAGTGAGAAAATGGCAGCAACAACGCTGTAAACCTCTTTTGTTAAAAAAAA
TAATGACTGTATTTAAAGAATTAACACTGGCTAATTCTGTGCCAGCAGCCGCGGTAACAC
AGGGAGTGTAGACGTTATTCATCATTACTGGGTGTAAAGATACGTAGGCTGTCTTGGATT
GGATATTTTGTAAAAAGCTTGGAGTATTCTCGAAGAATGACAAAAGTTACCCAAAGACTA
AAGAGTTTAAATCAAGTTAAGAGAATTTCTTGTGTAAGGGTAGAATCTATATATATTGGG
AGGACTACCAAAACAGTTGCGAAAGCACTTTTCAAGATAGAACTGACGCTAAGGTATGAA
AGAATAGGTATCAAACAGGATTAGATACCCTAGTAGTCC
>Otu12017
CCTATGGGGGGCAGCAGTAAGGAATATTGGTCAATGGGCGCAAGCCTGAACCAGCCATGC
CGCGTGCAGGAAGACGGCCCTACGGGTTGTAAACTGCTTTTGTACTGGAATAAACCTTGG
TATGTATACTAAGCTGAATGTACAGTAAGAATAAGGATCGGCTAACTCCGTGCCAGCAGC
CGCGGTAATACGGAGGATCCGAGCGTTATCCGGATTTATTGGGTTTAAGGGGTGCGTAGG
CGGCCTTGTAAGTCAGGGGTGAAAGACGGTAGCTCAACTATCGCAGTGCCTTTGATACTG
CAGGGCTTGAATGTACTTGAGGTAGGCGGAATGTGACAAGTAGCGGTGAAATGCATAGAT
ATGTCACAGAACACCAATTGCGAAGGCAGCTTACTAAAGTATGATTGACGCTGAGGCACG
AAAGCGTGGGGATCAAACAGGATTAGATACCCTCGTAGTCC
>Otu12034
CCAGCCTATGGGGGGCAGCAGTGGGGAATATTGGACAATGGGGGAAACCCTGATCCAGCA
ATGCCGCGTGAGTGATGAAGGCCTTCGGGTTGTAAAGCTCTTTCGCCTGTGAAGATAATG
ACGGTAACAGGATAAGAAGTCCCGGCTAACTCCGTGCCAGCAGCCGCGGTAAGACGGAGG
GGACTAGCGTTGTTCGGAATGACTGGGCGTAAAGGGTGCGTAGGCGGTCCCTTAAGTAAG

GCGTGAAAGCCCTGAGCTCAACTCGGGAATTGCGCTTTAAACTGAGGGGCTTGAGGGTGA
AAGAGGAAAGTGGAATTCCTAGTGTAGGGGTGAAATCCGTAGATATTAGGAAGAACACCA
GAGGCGAAGGCGACTTTCTGGTTCATCACTGACGCTGAGGCACGATAGCGTGGGGAGCGA
AAAGGATTAGATACCCCAGTAGTCC
>Otu12039
CCAGCCTACGGGGGGCTGCAGTGGGGAATTTTGCGCAATGGGGGAAACCCTGACGCAGCA
ACGCCGCGTGGAGGATGAAGCCCCTTGGGGTGTAAACTCCTTTCGACCGGGAAAATAATG
ATGGTACCGGTGGAAGAAGCACCGGCTAACTCTGTGCCAGCAGCCGCGGTAATACAGAGG
GTGCGAGCGTTGTTCGGAATTATTGGGCGTAAAGGGCGCGTAGGCGGTATGGTAAGTCAC
CTGTGAAACCTCTAAGCTTAACTTAGAGCCTGCAGGCGAAACTGCCATGCTGGAGGGTGG
GAGAGGTGCGTGGAATTCCTGGTGTAGCGGTGAAATGCGTAGATATCAGGAGGAACACCG
GTGGTGTAAACGGCTTTCTGGACCATAACTGACGCTGAGACACGAAAGCGTGGGTAGCAA
ACAGGATTAGAAACCCCAGTAGTCC
>Otu12041
CCAGCCTATGGGTGGCACCAGTGGGGAATATTGGACAATGGGCGCAAGCCTGATCCAGCA
ATGCCGCGTGAGTGATGAAGGCCTTAGGGTTGTAAAGCTCTTTCGCACGCGACGATGATG
ACGGTAGCGTGAGAAGAAGCCCCGGCTAACTTCGTGCCAGCAGCCGCGGTAATACGAAGG
GGGCTAGCGTTGTTCGGAATTACTGGGCGTAAAGGGCGCGTAGGCGGCGCCGGAAGTCAG
ATGTGAAAGCCCCGGGCTCAACCTGGGAATTGCATTTGATACTCTGGTGCTTGAGTTCGG
GAGAGGTGAGTGGAATTCCCAGTGTAGAGGTGAAATTCGTAGATATTGGGAAGAACACCG
GTGGCGAAGGCGGCTCACTGGCTTGTAACTGACGCTCAGGCGCGACAGCGTGGGGAGCAA
ACAGGATTAGATACCCTGTAGTCC
>Otu12042
CCAGCCTATGGGTGGCAGCAGTAGGGAATATTGCTCAATGGGGGAAACCCTGAAGCAGCA
ACGCCGCGTGAGTGATGAAGGCCTTCGGGTCGTAAAGCTCTGTTGTACGGGAAGAATGCC
CAAGTAGCTAACATCTACTTGGGGTGACGGTACCGTATAAGAAAGGACCGGCTAACTTCG
TGCGAGCAGCCGCGGTAAGACGGGGGGTCCAAGCGTTGTTCGGAATCATTGGGCGTAAAG
CGTGTGCAGGTGGTTTTGAAAGTTAGATGTGAAAGCCTCGGGCTCAACCCGAGAAGTGCA
TTTAAAACTCCAAAACTTGAGTAAGGAAGAGGGTCGTGGAATTCCAGGTGTAGTGGTGAA
ATACGTAGATATCTGGAGGAACACCGGTGGCGAAGGCGGCGACCTGGTCCTATACTGACA
CTCATACACGAAAGCGTGGGGATCAAACAGGATTAGAGACCCGTGTAGTCC
>Otu12044
CCAGCCTACGGGGGCACCAGTGGGGAATTTTGCGCAATGGGGGAAACCCTGACGCAGCAA
CGCCGCGTGGAGGATGAAATATCTTGGTATGTAAACTCCTTTCGATGGGGAAGATAATGA
CGGTACCCATAGAAGAAGCCCCGGCTAACTTCGTGCCAGCAGCCGCGGTAATACGAAGGG
GGCTAGCGTTGCTCGGAATCACTGGGCGTAAAGCGCACGTAGGCGGGTCCTTAAGTCAGA
GGTGAAATCCTGGAGCTCAACTCCAGAACTGCCTTTGATACTGAGGATCTCGAGTTCGGG
AGAGGTGAGTGGAACTGCGAGTGTAGAGGTGAAATTCGTAGATATTCGCAAGAACACCAG
TGGCGAAGGCGGCTCACTGGCCCGATACTGACGCTGAGGTGCGAAAGCGTGGGGAGCAAA
CAGGATTAGATACCCTCGTAGTCC
>Otu12051
CCAGCCTACGGGGTGCTGCAGTGGGGAATATTGGACAATGGGGGAAACCCTGATCCAGCC
ATGCCGCGTGAACGATGAAGGCCTTCGGGTTGTAAAGTTCTTTTGGTGGGGACGATGATG
ACGGTACCCACAGAAAAAGCACCGGCTAACTCTGTGCCAGCAGCCGCGGTAATACAGAGG
GTGCGAGCGTTAATCGGAATTACTGGGCGTAAAGCGCGCGTAGACGGTTTGGTAAGTCAG
ATGTGAAATCCCTGGGCTCAACCTGGGAACTGCATTTGATACTGCCTGGCTAGAGTATCG
AAGAGGGAAGTGGAATTTCCGGTGTAGCGGTGAAATGCGTAGATATCGGAAGGAACACCA
GTGGCGAAAGCGACTTCCTGGTCGAATACTGACGTTCATGTGCGAAAGCGTGGGGAGCAA
ACAGGATTAGAAACCCTCGTAGTCC
>Otu12053
CCAGCCTACGGGGGGCAGCAGTCGAGGATCTTTCTCAATGGGCGAAAGCCTGAAGGAGCG
ACGCCGCGTGGGGGATGAAGGTCTTCGGATTGTAAACCCCTGTCATCTGGGAACAATGCT
CCGCACCCAACACGTGCGGAGTTGATAGTACCGGAAGAGGAAGCCGTGGCTAACTCTGTG
CCAGCAGCCGCGGTAATACAGAGACGGCAAGCGTTGCTCGGATTCATTAGGCGTAAAGGG
TCCGCAGGCGGTCGGCTAAGTCGGATGTGAAATCCCGAGGCTTAACCTCGGAAAGTCATT
CGAAACTAGTCGACTAGAGGACTGGAGAGGAGACCGGAATTGTCGGTGTAGCGGTGAAAT
GCGTAGATATCGACAAGAACACCAGTGGCGAAGGCGGGTCTCTGGCCAATTCCTGACGCT
CAGGGACGAAAGCGTGGGGAGCAAACGGGATTAGATACCCTTGTAGTCC

>Otu12059
CCAGCCTATGGGATGCTGCAGTCGCGAATCATTGGCTAATGCACGCAAGTGTGACCATGC
GACGCCGCGTGGGGGAGGAAGGTCTTCGGATCGTAAACCCCTGTCACCGGGGAATAATGA
CCGGCCCTGAATAGGGGCCGGGCGAGAACCAACCCGGAGAGGAAGTAGTGGCTAACTCCG
TGCCAGCAGCCGCGGTAATACGGAGACTACGAGCGTTACTCGGATTCACTGGGCGTAAAG
GGTGCGTAGGCGGTCAGGTGTGTCAGGTGTGAAATCCCGGGGCTTAACCCCGGAACGGCG
CTTGAAACTACCTGGCTAGAGGCTTGGAGAGGGCAGTGGAATTCTCAGTGTAGCGGTGAA
ATGCGTAGATATTGAGAGGAACACCAACGGCGAAGGCAGCTGCCTGGACAAGATCTGACG
CTGAAGCACGAAAGCGTGGGTAGCAAAAGGGATTAGAAACCCCTGTAGTCC
>Otu12066
CCAGCCTACGGGGCGCTGCAGGAACGAATATTGGGCAATGGGCGAAAGCCTGACCGAGCG
ACGCCGCGTGCGGGATGAAGGCCTTCGGGTTGTAAACCGCTGTCAGTGGGGAGGAAATCT
GGGAGGGATCACCCTCTCAGTTGACCTATCCGCAGAGGAAGGACGGGCTAAGTTCGTGCC
AGCAGCCGCGGTAATACGAACCGTCCTAACGTCATTCGGAATCACTGGGCTTAAAGGGTG
CGTAGGCGGTCCGGTAAGTTGGGTGTGAAATCCCTCGGCTTATCCGAGGAATTGCGCTCA
AAACTGTCGGACTTGAGGGAGACAGAGGTGAGTGGAACTTAGGGTGGAGCGGTGAAATGC
GTTGATATCCTAAGGAACACCGGTGGCGAAAGCGACTCACTGGGTCTCTTCTGACGCTGA
GGCACGAAAGCTAGGGTAGCGAACGGGATTAGAAACCCGCGTAGTCC
>Otu12070
CCAGCCTATGGGAGGCTCCAGTGGGGAATATTGGACAATGGGCGCAAGCCTGATCCAGCC
ATGCCGCGTGAGTGATGACGGCCCTAGGGTTGTAAAGCTCTTTCACCCACGACGATAATG
ACGGTAGTGGGAGAAGAAGCCCCGGCTAACTTCGTGCCAGCAGCCGCGGTAATACGAAGG
GGGCTAGCGTTGTTCGGATTTACTGGGCGTAAAGCACACGTAGGCGGATCTTTAAGTCAG
GGGTGAGATGCCGAGGCTCAACTTCGGAACTGCCTTTGATACTGGAGATCTTGAGTTCGG
GAGAGGTGAGTGGAACTGCGAGTGTAGAGGTGAAATTCGTAGATATCTGCAGGAACACCC
GTGGCGAAAGCGGCGAGCTGGATCACTACTGACGCTGAGGAACGAAAGCTAGGGGAGCAA
GCAGGATTAGAAACCCCTGTAGTCC
>Otu12077
CCAGCCTACGGGTGGCACCAGCAACGAATATTCCGCAATGGGCGCAAGCCTGACGGAGCG
ACGCCGCGTGGGGGATGAAGTCCTTCGGGATGTAAACTCTAATAGTATGTTAGGAAGCAA
GTCGACCTAACACGTCGGCAAGTTGACCGACATACGAAAGGGGCGGCTAACTCCGTGCCA
GCAGCCGCGGTAAGACGGAGGCCCCGAGCGTTGTTCGGAATCACTGGGCTTAAAGCGCGT
GTAGGCGGGACGGTAAGTACCTTGTGAAATCCCCGGCTTCAACCCGGGAACTGCTCGGTA
TACTGCCGTTCTTGAGGCAGGTAGGGGTCGCTGGAACCTTAGGTGGAGCGGTGAAATGCG
TAGATATCTAAGGGAACGCCTGTGGCGAAAGCGGGCGACTGGGCCTGTCCTGACGCTGAG
ACGCGAAAGCGTAGGGAGCAAACGGGATTAGATACCCCGGTAGTCC
>Otu12080
CCAGCCTATGGGTGGCTGCAGTTAGGAATTTTGCGCAATGGACGAAAGTCTGACGCAGCG
ACACCGCGTGAGGGATGAAGGTCTTCGGATCGTAAACCTCTTTTATTGGAGCATGAAGCT
CTTTTGAATAAGGAACTACTAACTACGTGCCAGCAGTCGCGGTAATACGTAGGTTCCGAG
CGTTACCCGGTTTTATTGGGCGTAAAGAGTCTGTAGGTGGCTTTCTCAGTCTTGTTTCAA
AGACTCCGACTCAATCGGAGGCAGGGGCAGGATACTAGGAGGCTCGAGTTATATCGGGGT
TACTGGAATTTGCAGTGTAGGGGTAAAATCCGTGGATACTGCAAAGAACACCAAGCGCGA
AGGCGGGTAACTAGGTATTTACTGACACTCAGAGACGAAAGCTAGGGTAGCCAATCAGAT
TAGAGACCCCAGTAGTCC
>Otu12084
CCAGCCTACGGGGGGCTGCAGTCAAGAATATTCCTCAATGACCGAAAGGCTGAAGGAGCG
ACGCCGCGTGTATGAAGAAGCCCTCCGGGGCGTAAAGTACTTTTAACTGGGACGAAACTA
TGACGGTACCAGTTGAATAAGGGGCTGCTAACCTCGTGCCAGCAGCAGCGGTAATACGAG
GGCCCCAAGCATTATCCGGATTCATTGGGCGTAAAGCGTCCGCAGACGGTTTGTCGCATC
TCCTGTTAAATCACAAGGCTCAACCTTGTGGCCGCGGGAGAGATGGATAAGCTAGAGACT
GGAAGAGGCAAGCGGAATTGCTGGTGTAGGGGTTAAATCCGTTAATATCAGCAGGAACAC
TTAATGCGAAGGCAGCTTGCTAGGACAGTTCTGACGTTCAGGGACGAAAGCGTGGGGAGC
GAATGGGATTAGATACCCCTGTAGTCC
>Otu12088
CCAGCCTATGGGAGGCAGCAGTGGGGAATATTGCACAATGGGGGAAACCCTGATGCAGCA
ACACCGCGTGAGCGACGAAGGCCTTCGGATCGTAAAACCCTGTCGTCGGGGACGAAGGTT
TGGGGTTGAACAGGCCCTGAGCTTGACGGTACCCGGAGAGGAAGCCCCGGCTAACTCTGT

GCCAGCAGCCGCGGTAATACAGAGGGGGCAAGCGTTATTCGGAATTATTGGGCGTAAAGG
GCGCGTAGGCGGTGTTTTAAGTGAGATGTGCAATCCCCGGGTTTAACCTGGGAACTGCAA
CTCAGACTGGAACGCTAGAGTACTGGAGAGGGTGGTAGAATTCCACGTGTAGCGGTGAAA
TGCGTAGAGATGTGGAGGAATACCAGTGGCGAAGGCGGCCACCTGGACAGTAACTGACGC
TGAGGCGCGAAAGTGTGGGTAGCAAACAGGATTAGATACCCGTGTAGTCC
>Otu12094
CCAGCCTACGGGGGGCTGCAGTCGAGAATTTTTCACAATAGGCGAAAGCCTGATGGAGCG
ACGCCGCGTGGGGGATGAAGGGCTTCGGCTTGTAAACCCCTGTCATTCGTGAACAAAGCT
GTCGGGTGAATAGCTTGGCAGTTGATAGTAGCGGAAGAGGAAGGGACGGCTAACTCTGTG
CCAGCAGCCGCGGTAATACAGAGGTCCCGAGCGTTGTTCGGATTTACTGGGCGTAAAGGG
TGCGTAGGCGGTGAGGTAAGTCTGGTGTGAAATCTCCGGGCCTAACCCGGAAAATGCATT
GGAAACTATCTGACTAGAGACTTGGAGGGGAGATCGGAATTCTCGGTGTAGCAGTGAAAT
GCGTAGATATCGAGAGGAACATCAGTGGCGAAGGCGGATCTCTGGACAAGTACTGACGCT
GAGGCACGAAAGCCAGGGGAGCAAACGGGATTAGAAACCCGCGTAGTCC
>Otu12095
CCAGCCTATGGGAGGCTGCAGTCGAGAATTCTTCACAATGGGCGAAAGCCTGATGGAGCG
ACGCCGCGTGGGGGATGAATGGCTTCGGCCCGTAAACCCCTGTCATTCGCGATCAATGCG
TCCGGCCTAACACGCTGGACGTTGATAGTAGCGGAAGAGGAAGGGACGGCTAACTCTGTG
CCAGCAGCCGCGGTAATACAGAGGTCCCAAGCGTTGTTCGGATTCACTGGGCGTAAAGGG
TGCGTAGGCGGCCGGGTCAGTCTGATGTGAAATCTCGGAACTTAACTCCGAAACTGCATT
GGATACTATTCGGCTAGAGGGTTGGAGGGGGGACTGGAATTCTCGGTGTAGCAGTGAAAT
GCGTAGATATCGAGAGGAACACCAGTGGCGAAGGCGAGTCCCTGGACAACTCCTGACGCT
GAGGCACGAAAGCGTGGGGAGCAAACAGGATTAGAGACCCCTGTAGTCC
>Otu12103
CCAGCCTATGGGGCGCTCCAGTGGGGAACCTTGCGCAATGGGCGAAAGCCTGACGCAGCG
ACGCCGCGTGAGTGATGAAGGCCTTCGGGTTGTAAAGCTCTGTGGGGAGAGAAGAATTAG
TGCGGGCTAATACTCCGCATGATGACGGTATCTCCTTAGCAAGCACCGGCTAACTCTGTG
CCAGCAGCCGCGGTAAGACAGAGGGTGCAAACGTTGTTCGGAATTACTGGGCGTAAAGCG
TGTGTAGGCGGCCATGTAAGTTGGATGTGAAAGCCCCGGGCTCAACCCGGGAAGTGCATT
CAAAACTGCGTGGCTTGAGTACTGGAGAGGTTGGTAGAATTCTCGGTGTAGAGGTGAAAT
TCGTAGATATCGAGAGGAATACCGGTGGCGAAGGCGGCCAACTGGACAGATACTGACGCT
GAGACACGAAAGCGTGGGGAGCAAACAGGATTAGATACCCTAGTAGTCC
>Otu12104
CCAGCCTACGGGGTGCTCCAGCTAAGGAACTTCTTCAATGCGCGTAAGCGTGAAAGAGTA
AGCCGGAGTGTTGTTCATAATTTTGAACAACTTTTATTTGTTGCAAAAAGACAGATGAAT
AAGGACTGGGCAAGATCGGTGCCAGCCGCCGCGGTAATCCCGACGGTCCAAGTCGCAGCC
ACAATTATTGGGTCTAAAACATTCGTAGCTTGTTTATTAAGTTCTTTGTGAAATCTCAAA
TCTTAAATTTGAGGCGTGCAAAGAATACTGGTAAACTGGAGACCGGAAGAAGCAAGGAGT
ATGTTTAAGGTAGCGGTAAAATGTGTTAGTCTTAAGCAGACTAACAAGAGCGAAGGCACC
TTGCTAGTACGGATCTGACAGTGAGGAATGAAGGCTAGGGGCGCGAAACGGATTAGAAAC
CCTCGTAGTCC
>Otu12107
CCAGCCTACGGGGGGCAGCAGTGGGGAATATTGCACAATGGGCGCAAGCCTGATGCAGCG
ACGCCGCGTGGAGGATGAAGGTTTTCGGATTGTAAACTCCTGTTAAGTGGGAAAAAAGAC
TGATATTTAAAAGATGTCGGGGATGATGGTACCACTAGAGAAAGCGCCGGCAAACTTCGT
GCCAGCAGCCGCGGTAATACGAAGGGTGCAAGCGTTATTCGGAATCACTGGGCGTAAAGA
GTGCGGAGACGGCATTGTAAGTCAACTGTTAAAGAGCCCGGCTTAACCGGGCAAAGGCGG
GTGATACTGCAAAGCTAGAGTGCAGGAGAGAAAGGTAGAATTCTCGGAGTAGCGGTAAAA
TGCGTAGATCTCGAGAGGAATACCGGTTGCGAAGGCGGCCTTTTGGCCTGCAACAGACGT
TTAAGCACGAAAGCGTGGGGAGCAAACAGGATTAGAAACCCCAGTAGTCC
>Otu12108
CCAGCCTATGGGTGGCTCCAGTCGAGAATATTTCGCAATGGGCGAAAGCCTGACGAAGCG
ACGCCGTGTGGATGAAGAAGGCCTTCGGGTTGTAAAATCCTGTCACTTGGGAGCAAATAG
TTGGTGTGAATAACACTAGTTAGTGAGAGTACCAAGGGAGGAAGCACTGGCTAACTCCGT
GCCAGCAGCTGCGGTAATACGGAGGGTGCAAGCGTTAGTCGGAATAACTGGGCGTAAAGG
GTGCGTAGGCGGGCATATAAGTTAGGTGTGAAATTACCGGGCTCAACTTGGTAACGGCAC
TTAATACTGTATGTCTAGAGGAATGGTGGGGAAAACGGAATTCCATGTGTAGCGGTGAAA
TGCGTAGATATATGGAAGAACACCTGTGGCGAAAGCGGTTTTCTAACCTACTCCAGACGC

TGAGGCACGAAAGCAAGGGGATCAAACAGGATTAGAAACCCCCGTAGTCC
>Otu12113
CCAGCCTACGGGACGCTCCAGTCGAGAATCTTCCCCAATGGCCGAAAGGCTGAGGGAGCG
ACGCCGCGTGCAGGAAGAAGCCCTTCGGGGTGTAAACTGCTTTTCTTCAAGAACAACTCT
GAGTGTATTGAAGGAATAAGGGGCTGCTAACTTCGTGCCAGCAGCAGCGGTAATACGAAG
GCCCCGAGCGTTATCCGGATTTATTGGGCGTAAAGCGTTCAACGGTGGCGTAGTGTGTTC
TTGGTTAAATCTCACGGCTCAACCGTGGGGCTGCCGGGAAAACTGCTATGCTTGAGGGCG
GAAGAGGTGTGCGGAACTACCGGTGTAGCGGTAAAATGCGTTCATATCGGTAGGAACACC
GAAGGCGAAAGCAGCACACTAGGACGACCCTGACACTCAACGAACGAAAGCGTGGGTAGC
GAATGGGATTAGAAACCCATGTAGTCC
>Otu12118
CCAGCCTATGGGTGGCTCCAGGCGCGAAAACTTTACAATGCGCGAAAGCGTGATAAGGGG
ATCCTGAGTGCTCATACACAGTATGGGCTTTTTTCAAGAGTAAAAATCTTGAAGAATAAG
TGGTGGGCAAGACTGGTGCCAGCCGCCGCGGTAACACCAGCGCCACGAGTGGCATTCGTG
TATTTGGCCTAAAGCGTCCGTAGCTGGTTCTGTAAATCTTTCGTGAAATTGTTATTGTCA
ACGTAACAGCGTGCGAAAGACACTGCAGGACTTGAGACCGGGAGGAGTCAGAGGTATTCT
TGGGGGAGCGGTAAAATGTTATAATCCCAAGAAGACCACCAGTGGCGAAGGCGTCTGACT
AGAACGGATCTGACAGTGAGGGACGAAAGCTAGGGGAGCGATCCGGATTAGAAACCCGAG
TAGTCC
>Otu12123
CCAGTCTATGGGGGGCTGCAGTCGAGGATCTTTAGCAATGGGCGAAAGCCTGACTATGCG
ACGCCGCGTGGACGATGAAGGCCTTCGGGTCGTAAAGTCCTGTCAGACGGGATGAATATT
TTTAACCTAATACGTTGAAAAGGTGACAGTACCGTCAGAGGAAGCCACGGCTAACTCCGT
GCCAGCAGCCGCGGTAATACGGAGGTGGCGAGCGTTACTCGGATTTACTGGGTGTAAAGG
GTATGTAGGCGGGCTTGTAAGTCATGCGTGAAAGCCCTTGGCTCAACCAAGGAAATGCGC
GTGAAACTGCTTGTCTTGAGTGCGGGAGAGGGGAACGGAATTCCCGGTGTAAGGGTGAAA
TCTGTAGAGATCGGGAGGAACACCAGTGGCGAAAGCGGTTCCCTGGTCCGTAACTGACGC
TGAAATACGAAAGCTTGGGGAGCAAACAGGATTAGATACCCGAGTAGTCC
>Otu12131
CCAGCCTACGGGGGGCTGCAGTGGGGAATTTTGCGCAATGGGCGAAAGCCTGACGCAGCA
ACGCCGCGTGGAGGATGAAGGCCTTCGGGTCGTAAACTCCTGTCAGTGGGGACGAATGCT
TCGCAAGAAGTTTGACTGTACCCGTTGAGGAAGCCACGGCTAACTCCGTGCCAGCAGCCG
CGGTAATACGGAGGTGGCAAGCGTTGTTCGGAATTACTGGGCGTAAAGGGCGCGTAGGCG
GCTTTGTAAGTCAGATGTGAAAGCCCCGGGCTTAACCCGGGAATTGCATCTGAAACTGCT
TTGCTTGAGTTCTGGAGGGGGTAGTGGAATTCCCAGTGTAGCGGTGAAATGCGTAGATAT
TGGGAGGAACACCGGTGGCGAAGGCGACTTTCTGGCCCTATACTGACGCTGAGACGCGAG
AGCGTGGGGAGCAAACAGGATTAGATACCCTCGTAGTCC
>Otu12138
CCAGCCTATGGGACGCTGCAGCAGGGAATTTTGCGCAATGGGCGAAAGCCTGACGCAGCA
ACGCCGCGTGAGGGATGAAGGCCTTCGGGTTGTAAACCTCTTTTTTCAGGGAAGAATAAT
GACGGTACCTGAAGAATAAGTCACGGCTAACTACGTGCCAGCAGCCGCGGTAATACGTAG
GTGGCAAGCGTTATCCGGATTCACTGGGCGTAAAGAGCGCGTAGGTGGTTCTTCAAGTCA
GATGTTAAATCTCCCGGCTTAACTGGGAGCCATCATCTGATACTGATGGACTTGAGGGTG
GTAGAGGGAGGTGGAATTCCCGGTGTAGTGGTGAAATGCATAGATATCGGGAGGAACACC
AGTGGCGAAAGCGGCCTCCTAGGCCATTCCTGACACTGAGGCGCGAAAGCGTGGGGAGCG
AACAGGATTAGAGACCCGGGTAGTCC
>Otu12142
CCAGCCTATGGGTGGCAGCAGTGGGGAATCTTGCGCAATGGGCGAAAGCCCGACGCAGCC
ATGCCGCGTGAATGATGAAGGTCTTAGGATTGTAAAATTCTTTCACCGGGGACGATAATG
ACGGTACCCGGAGAAGAAGCCCCGGCTAACTTCGTGCCAGCAGCCGCGGTAATACGAAGG
GGGCTAGCGTTGCTCGGAATTACTGGGCGTAAAGGGAGCGTAGGCGGACTGTTAAGTTAG
AGGTGAAAGCCCAGGGCTCAACCTTGGAATTGCCTTTGATACTGGCAGTCTTGAGTACGG
AAGAGGTATGTGGAACTCCGAGTGTAGAGGTGAAATTCGTAGATATTCGGAAGAACACCA
GTGGCGAAGGCGACATACTGGTCCGTTACTGACGCTGAGGCTCGAAAGCGTGGGGAGCAA
ACAGGATTAGAAACCCCGGTAGTCC
>Otu12146
CCAGCCTATGGGGGGCAGCAGTCGCGAATCTTTTGCAATACACGAAAGTGTGACAAAGCG
AGCCAAAGTGATTCCCGCACAGGGAATCTTTTGCCAGCTGTAAAAAGGCTGGCGAATAAG

GACTGGGCAAGACTGGTGCCAGCCGCCGCGGTAATCCCAGCAGTCCAAGTTGCAGCCAGC
ATTATTGGGTCTAAAACATCCGTAGCTTGTTTAACAAGTTCTTTGTGAAATCTGGCATCT
TAAGTGTCGGGCGTGCAAAGAATACTGTTAGGCTAGAGACCGGTAGATGTAAGGAGTACA
CCTAAGGTAGTGGTAAAATACGTTAATCTTAGGTGGACTAACAATTGGCGAAGGCACCTT
ACAAGGACGGATCTGACAGTGAGGGATGAAGGCTAGGGGCGCAAAACGGATTAGATACCC
CTGTAGTCC
>Otu12148
CCAGCCTATGGGGGGCACCAGTAACGGATATTGGGCAATGGGCGAAAGCCTGACCCAGCG
ACGCCGCGTGCAGGATGAAGGTCTTCGGATTGTAAACTGCTGTCAGGGGCTACCAAGTGA
CCGTGGCCAACATCCACGGAAGCTGAGGCGCCCCAGAGGAAGCCACGGCTAACTTCGTGC
CAGCAGCCGCGGTAAGACGAAGGTGGCAAGCGTTGTTCGGAATCACTGGGCTTAAAGAGC
ACGTAGGCGGGCCGTTAAGCACCTTGTGAAAGCCCCCGGCTCAACCGGGGAACTGCTTGG
TGTACTGGCGGTCTTGAGGCAGGTAGGGGCGTGGAGAACTCTTGGTGGAGCGGTGAAATG
CGTAGATATCAAGAGGAATGCCGGAGGTGAAGACGCCGCGCTGGGCCTGTCCTGACGCTG
AGGTGCGAAAGCCAGGGGAGCAAACGGGATTAGATACCCTGGTAGTCC
>Otu12153
CCAGCCTATGGGTGGCAGCAGTAAGGAATTTTCCACAATGCCCGAAAGGGTGATGGAGCG
ACGCTGCGTGGGTGATGAAGGCCTTCGGGTTGTAAAACCCTTTTGTAAAGGATGAACTTA
CAGTACTTTACGAATAAGCTTCGGCCAACTCTGTGCCAGCAGCCGCGGTAAGACAGAGGA
AGCAAGCATTATCCGGAATGATTGGGTGTAAAATGTGTGTAGGTCGTTATATAAGTCAAA
TGTGAAATCCTGATGCTTAACATCAGAACGGCATTTGATACTGTTTAACTTGAGTTCGAG
CGAGGAGGGCGGAATTCCCGGTGGAGCGGTGAAATGCGTTGATATCGGGGGGAACACCAA
TAGCGAAGGCAGCTTTCTAGCTCGAAACTGACACTGAAACACGAAAGCTAGGGGAGCAAA
CAGGATTAGATACCCTAGTAGTCC
>Otu12155
CCAGCCTATGGGAGGCAGCAGTCGAGAATATTCCTCAATGGCCGAAAGGCTGAAGGAGCG
ACGCCGCGTGATCGAAGAAGCCCTTCGGGGTGTAAAGATCTTTTTTATGGAACGAACGAT
GACGGTACCATAAGAATAAGGGGTTACTAACTCTGTGCCAGCAGTAGCGGTAATACAGAG
ACCCCAAACATTATCCGGATTTATTGGGCGTAAAGCGTCCGCAGGTGGCTTGGCAAGTTT
CGTTTCAAATCTCAGCGCTCAACGTTGGGACCGGACGAAAAACTGCTAAGCTTGAGGTTG
GGAGAAGTCAGTGGAATGGCCGGTGTAGGGGTAAAATCCGTTAATATCGGCTAGAACACC
AGTGGCGAAAGCGACTGACTAGAACACACCTGACACTCATGGACGAAAGCGTGGGTAGCG
AACAGGATTAGAGACCCCCGTAGTC
>Otu12157
CCAGCCTACGGGTTGCTGCAGTCGAGAATCTTTCCCAATGGACGAAAGTCTGAGGATGCG
ACGCCGCGTGAGGGATGAAGGTCTTCGGATCGTAAACCTCTTTTGTCGGGGAGGAAACTC
CGTCGGTGAACAACCGCCGGAACTGACTGTACCTGGCGAATAAGCCACGGCTAACTCCGT
GCCAGCAGCCGCGGTAATACGGAGGTGGCAAGCGTTACTCGGTATGACTAGGTGTAAAGG
GTTCGTAGGCGGCCTTGTAAGTGGAAGGTGAAATCTTTTAGCTCAACTAAAAAACTGCCT
TTCAAACTGCTAGGCTAGAGGCTGGAAGAGGAGAGCGGAACTGCTAGTGTAAAGGTGAAA
TTTGTGGATATTAGCAGGAACGCCTGTGGAGAAGTCGGCTCTCTGGTCCCGTCCTGACGC
TGAGGAACGAAAGCTAGGGGAGCAAACAGGATTAGATACCCCCGTAGTCC
>Otu12158
CCAGCCTACGGGATGCTCCAGCAAGGAATTTTCGGCAATGGGCGCAAGCCTGACCGAGCA
ACGCCGCGTGCGGGATGACGGCCTTCGGGTTGTAAACCGCTTTTGCGGGGGACGATCATG
ACGGTACCCCGCGAATAAGGCTCGGCTAACTCTGTGCCAGCAGCCGCGGTAAGACAGAGG
AGCCAAGCGTTGTCCGGATTAACTGGGCGTAAAGCGCACGCAGGCGGTCACGCGCGTGGG
GTGTGAAAACGAGCCGCTTAACGGCTCGAGGCCACTTCATACGGCGCGACTGGAGCCTGG
CAGAGGTCCGTGGAATTGCCGGTGTAGTGGTGAAATGCGTAGAGATCGGCAGGAACACCC
AGGGGGAAACCAGCGGACTGGGCCAGTGCTGACGCTCAGGTGCGAAAGCGTGGGGAGCGA
ACCGGATTAGATACCCTGGTAGTCC
>Otu12159
CCAGCCTACGGGATGCACCAGTGGGGAATATTCCGCAATGGACGAAAGTCTGACGGAGCG
ACGCCGCGTGGATGACGAAGGCCGAAAGGTTGTAAAGTCCTTTTGCTGGGGAAGAATAAC
CATGGGAGGGAATGCCCGTGGGATGACATGAACCGGCGAATAAGCCCCGGCTAACTACGT
GCCAGCAGCCGCGGTAACACGTAGGGGGCGAGCGTTGTTCGGAATTACTGGGCGTAAAGG
GCATGTAGGCGGCCCTATAAGCCTGGCGTGAAAGTCACCGGCTCAACCGGTGGATTGCGC
TGGGAACTGCAGGGCTTGAATCATGGAGAGGGAGCTAGAATTCCTGGTGTAGGGGTGAAA

TCTGTAGAGATCAGGAAGAATACCAGTGGCGAAGGCGAGCTCCTGGCCAATGATTGACGC
TGAGGTGCGAAAGTGTGGGGATCAAACAGGATTAGAAACCCTCGTAGTCC
>Otu12165
CCAGCCTATGGGGGGCAGCAGCAGGGAATCTTGCGCAATGGGCGAAAGCCTGACGCAGCA
ACACCGCGTGGGGGATGAAGGCTCATGGGTCGTAAACCCCTTTTCTCAGGGAAGAAAAAA
ATGACGGTACCTGAGGAATAAGCATCGGCCAACTACGTGCCAGCAGCCGCGGTAATACGT
AGGATGCAAGCGTTATCCGGATTCACTGGGCGTAAAGAGCGCGTAGGCGGTCCTTTAAGT
TAGGTGTGAAAACTCTCAGCTCAACTGAGAGACGCCATCTAATACTGGGGGGCTTGAGGG
CAGCAGAGGAAGATGGAATTCCCGGTGTAGTGGTGAAATGCGTAGATATCGGGAGGAACA
CCAGTGGCGAAGGTGGTCTTCTGGGCTGTACCTGACGCTGAGGCGCGAAAGCGTGGGTAG
CAAACAGGATTAGATACCCTTGTAGTCC
>Otu12166
CCAGCCTATGGGATGCAGCAGGTGCGAAACCTCTGCAATGCGCGAAAGCGTGACAGGGGG
ACTCTGAGCGCTACACCACGCGTGTAGCTTTTTGCAAGCCTAGACAGCTTGTAGAATAAG
GGCTGGGTAAGACCAGTGCCAGCCGCCGCGGTAATACTGGCGGCCCGAGTGGTGTCCACG
AATATTGGGCCTAAAGAGTCCGTAGCTGGCCTGTTAAGTTTCCTGTGAAATCTCCGGGCT
TAACTCGGAGGCGTGCAGGGAATACTGACGGGCTTGAGAGCGGGGGAGGCCAAGAGTACT
TATGGGGTAGGGGTAAAATCCTGTAATCCTATAAGGACTACCAGTGGCGAAGGCGCTTGG
CCAAAACGCGTCTGACAGTGAGGGACGAAGGCTAGGAGAACGAATCGGATTAGATACCCC
CGTAGTCC
>Otu12167
CCAGCCTACGGGTTGCTGCAGTAAGGAATATTGGGCAATGGGCGGGAGCCTGACCCAGCC
ATGCCGCGTGCAGGAAGACGGCCTTATGGGTTGTAAACTGCTTTTGTACGGGAATAAATC
TCTCTACGTGTAGAGAGTTGAAGGTACCGTAAGAATAAGCATCGGCTAACTCCGTGCCAG
CAGCCGCGGTAATACGGAGGATGCAAGCGTTATCCGGATTCATTGGGTTTAAAGGGTGCG
CAGGCGGACTAATAAGTCAGCGGTGAAATTCTACGGCTCAACTGTAGAACTGCCGTTGAT
ACTGTTAGACTAGAGTATAATTGGAGTGGGCGGAATGTGTCATGTAGCGGTGAAATGCAT
AGATATGACACAGAACACCGATCGCGAAGGCAGCTCACTAAGTTACAACTGACGCTCATG
CACGAAAGCGTGGGGATCAAACAGGATTAGAAACCCTGGTAGTCC
>Otu12175
CCAGCCTACGGGGGGCAGCAGTTAAGAATCTTGCGCAATGGGGGAAACCCTGACGCAGCG
ACGCCGCGTGGACGATGAAGGCCTTCGGGTTGTAAAGTCCAATAAGCAGGGAAGAATAAG
CAGCGGGAAATATTCGCTGTGATGACGGTACCTGCCTAAAGCCCCGGCTAACTACGTGCC
AGCAGCCGCGGTAATACGTATGGGGCTAGCGTTGTTCGGAATTATTGGGCGTAAAGGGCG
TCTAGGCGGGGGATTAAGTTAGGTGTGAAATCCCTGGGCTCAACCCAGGAACTGCATCTA
AAACTGGTTCTCTTGAGTTCGGGAGAGGAGAGCGGAATTCCCGGTGTAGCGGTGAAATGC
GTAGATATCGGGAAGAACACCAGTGGCGAAGGCGGCTCTCTGGCCTAGAACTGACGCTGA
AGCGCGAAAGCGTGGGGGGCGAACGGGATTAGATACCCGCGTAGTCC
>Otu12191
CCAGCCTACGGGTGGCAGCAGCAAGGAATATTGCGCAATGGGCGAAAGCCTGACGCAGCG
ACGCCGCGTGAGGGAAGAAGGTCTTCGGATTGTAAACCTCTTTTCTTAGGGAAGAATAAT
GACGGTACCTGAGGAATAAGTCACGGCTAACTACGTGCCAGCAGCCGCGGTAATACGTAG
GTGGCTAGCGTTATCCGGATTTACTGGGCGTAAAGAGGGTGCAGGCGGCTCTTCAAGTCG
GTTATAAAATCTCTTGGCTTAACTAAGAGGGACTGACCGATACTGTTGGGCTGGAGAGTA
GCAGAGGGAGATGGAATTCCCGGTGTAGTGGTGAAATGCGTAGATATCGGGAGGAACATC
AGTGGCGAAAGCGGTCTCCTAGGCTATTTCTGACGCTCAGGCCCTAAAGCGTGGGGAGCA
AACAGGATTAGAAACCCCGGTAGTCC
>Otu12195
CCAGCCTACGGGTGGCTGCAGTGGGGAATATTGCGCAATGGGCGGAAGCCTGACGCAGCA
ACGCCGCGTGAGGGATGGAGGCCTTCGGGTTGTAAACCTCTTTCAGCAGGGAAGAAGCGA
GAGTGACGGTACCTGCAGAAGAAGCACCGGCTAACTACGTGCCAGCAGCCGCGGTGATAC
GTAGGGTGCAAGCGTTGTCCGGAATTATTGGGCGTAAAGAGCTCGTAGGCGGTCTGTCGC
GTCTGCTGTGAAAACCTGGGGCTTAACCCCGGGCCTGCAGTGGGTACGGGCAGACTAGAG
TGCGGTAGGGGAGACTGGAATTCCTGGTGTAGCGGTGAAATGCGCAGATATCAGGAGGAA
CACCGATGGCGAAGGCAGGTCTCTGGGCCGTAACTGACGCTGAGGAGCGAAAGCGTGGGG
AGCGAACAGGATTAGAGACCCTAGTAGTCC
>Otu12200
CCAGCCTACGGGAGGCTGCAGTCGAGGATCTTCGGCAATGGGCGCAAGCCTGACCGAGCG

ACGCCGCGTGCGCGATGAAGGCCTTCGGGTTGTAAAGCGCTGTCGAGGGGGAGGAAAACC
CGCAAGGGTCTGACCTATCCCTGGAGGAAGCACGGGCTAAGTTCGTGCCAGCAGCCGCGG
TAAGACGAACCGTGCAAACGTTGTTCGGAATCACTGGGCTTAAAGGGCGCGTAGGCGGCT
TGTCAAGTCCGGGGTGAAATCTTTCGGCTCAACCGGACAAGGGCCTTGGATACTGACAAG
CTGGAGGAAGGTAGGGGCATGTGGAACTTCCGGTGGAGCGATGAAATGCGTTGATATCGG
AAGGAACGCCGGTGGCGAAAGCGACGTGCTGGACCTTTTCTGACGCTGAGGCGCGAAAGC
TAGGGGAGCAAACGGGATTAGAGACCCTAGTAGTCC
>Otu12201
CCAGCCTACGGGTGGCTGCAGTGGGGAATTTTGCTCAATGGGGGAAACCCTGAAGCAGCG
ACGCCGCGTGGAGGATGAAGGTTCTCTGAATCGTAAACTCCTTTTACAGGGGACAAAGGC
CCGCAAGGGTTTGATGGTACTCTGTGAATAAGCATCGGCCAACTACGTGCCAGCAGCCGC
GGTAATACGTAGGATGCGAGCGTTGTTCGGAATTACTAGGTGTAAAGCGTCTGTAGGTGT
CCCGAAAAGTTTCGTGTGAAAACCCCCGGCTTAACCGGGGAATTGCGCGAAAAACTATCG
GGATTGAGTGCTAGAGGGGAAACTGGAATTCCCGGTGTAGCGGTGAAATGCGTAGATATC
GGGAGGAACACCAACGGCGAAGGCAGGTTTCTGGCTAGTTACTGACACTGAGAGACGAAA
GCGTGGGGAGCAAACAGGATTAGAGACCCGAGTAGTCC
>Otu12208
CCAGCCTATGGGTTGCTCCAGCTAAGAATATTCCGCAATGGGCGCAAGCCTGACGGAGCG
ACGCCGCGTGGACGATGAAGGCTTTCGGGTTGTAAAGTCCTTTCGTGCAAGAAGATTCTA
CACCGGGTAAATAATCCGGTGTTTTGACTGTATTGCAGGAAGAAGCCCCGGCTAATTACG
TGCCAGCAGCCGCGGTAATACGTATGGGGCGAGCGTTATTCGGATTTATTGGGCGTAAAG
CGTATGTAGGTGGTTTGATAAGTTGAATGTGAAATTTCCGGGCTTAACCCGGAACCTGCA
TTCAAAACTGCCAGACTTGAGTTCAGAGGAGGATAATGGAATTCCAGGTGTAGGGGTGAA
ATCTGTAGATATCTGGAAGAACACCAGTGGCGAAGGCGATTATCTGGTCTGATACTGACA
CTTAGATACGAAAGCCAGGGGAGCAAACGGGATTAGAAACCCCTGTAGTCC
>Otu12210
CCAGCCTATGGGGTGCAGCAGTGGGGAATATTGGGCAATGGGCGAAAGCCTGACCCAGCC
ACGCCGCGTGAGTGATGAAGGCCTTCGGGTCGTAAAGCTCTGTGGGGAGGGACGAATAAG
CGCGTATTAAATAAATACGCGCCCTGACGGTACCTCCTTAGCAAGCACCGGCTAACCATG
TGCCAGCAGCCGCGGTAATACATGGGGTGCAAACGTTGCTCGGAATCATTGGGCGTAAAG
CGCGCGTAGGCGGTCGCTTAAGTCGGATGTGAAATCCCTCGGCTTAACTGAGGAAGTGCA
TCCGAAACTGAATGGCTTGAGTACGAAAGAGGGTCGCGGAATTCCCGGTGTAGAGGTGAA
ATTCGTAGATATCGGGAGGAACACCTGTGGCGAAAGCGGCTCACTGGACCACAACTGACG
CTGATGCACGAAAGCTAGGGGAGCAAACAGGATTAGATACCCTAGTAGTCC
>Otu12214
CCAGCCTACGGGGTGCACCAGTGGGGAATCCTGCACAATGGGGGAAACCCTGATGCAGCG
ACGCCGCGTGAGCGATGAAGCCCTTCGGGGCGTAAAGCTCTTTCGACGGGAACGATAATG
ACGGTACCCGGAGAAGAAGCTGCGGCTAACTACGTGCCAGCAGCCGCGGTAATACGTAGG
CAGCAAGCGTTGTTCGGAGTTACTGGGCGTAAAGAGTGCGTAGGCGGTTCTGTAAGTCTG
GTGTGAAAGCCCTGGGCTCAACCCGGGAAGTGCATTGGAAACTGGGAGACTTGAATACGG
GAGAGGGTAGTGGAATTCCTGGTGTAGGAGTGAAATCCGTAGATATCAGGAGGAACACCG
GTGGCGAAGGCGGCTACCTGGACCGATATTGACGCTGAGACGCGAAAGCGTGGGGAGCAA
ACAGGATTAGAAACCCGGGTAGTCC
>Otu12216
CCAGCCTACGGGTGGCAGCAGTCGAGAATCTTCGGCAATGGACGCAAGTCTGACCGAGCG
ACGCCGCGTGGAGGATGAAGGCCTTCGGGTTGTAAACTCCTGTCACGGGACAAGAATAAG
CGGACGTTAATACCGTCCGCGATGACTATCCCGAGAGGAAGCCACGGCTAACTCTGTGCC
AGCAGCCGCGGTAATTCAGAGGCGGCAAGCGTTGTTCGGAATCACTGGGCATAAAGGGCG
CGTAGGTGGTCAAGTAGGTCAAGGGTGAAAGCCCCCGGCTCAACCGGGGTCCCGCCTTTG
AAACCACTTGGCTGGAGCGGAACAGGGGAGAGTGGAACTCGTGGTGGAGCGGTGAAATGC
GTAGATATCACGAGGAACGCCGGCGGTGAAGACGACTCTCTGGGTTCTAGCTGACACTGA
GGCGCGAAAGCTGGGGGAGCAAACGGGATTAGAGACCCTTGTAGTCC
>Otu12219
CCAGCCTATGGGTTGCAGCAGTGGGGAATATTGGACAATGGGCGCAAGCCTGATCCAGCC
ATGCCGCGTGAGTGATGACGGCCTTAGGGTTGTAAAGCTCTTTCGCCAGGGACGATAATG
ACGGTACCCGCAGAATAAGCCCCGGCTAACTTCGTGCCAGCAGCCGCGGTAATACGAAGG
GGGCTAGCGTTGTTCGGAATCACTGGGCGTAAAGCGCACGCAGGCGGATTGATAAGTCAG
TGGTGAAATCTCGTTGCTTAACAACGAACGTGCCATTGATACTGTAGGTCTTGAGTACAG

ATGCCGTTGGCGGAATGTGTCATGTAGCGGTGAAATGCATAGATATGACACAGAACACCG
ATTGCGAAGGCAGCTGACGAAACTGTAACTGACGCTGAGGCACGAAAGCGTGGGGATCAA
ACAGGATTAGAAACCCCAGTAGTCC
>Otu12221
CCAGCCTACGGGACGCTGCAGTGAGGAATTTCGCGCAATGGCCGCAAGGCTGACGCAGCA
ACGCCGCGTGGGTGAAGAAGGCCTTCGGGTCGTAAAGCCCTGTCAGGTGGGAAGAACGGC
CAAGGGGTTAATAGCCTCTTGGAGTGACGGTACCACCAGAGGAAGCACCGGCTAACTCCG
TGCCAGCAGCCGCGGTAATACGGAGGGTGCAAGCGTTATTCGGAATTACTGGGCGTAAAG
CGCGTGCAGGCGGCTAGCAAGTCTGATGTGAAAGCCCTGGGCTTAACCTGGGGAGTGCA
TTGGAAACTGCTAGTCTTGAGTGTTGGAGAGGAAGGGGGAATTCCCGGTGTAGAGGTGAA
ATTCGTAGAGATCGGGAGGAATACCAGTGGCGAAGGCGCCCTTCTGGACGACAACTGACG
CTGAGACGCGAAAGCGTGGGGAGCGAACAGGATTAGATACCCGAGTAGTCC
>Otu12223
CCAGCCTATGGGGGGCTGCAGTGAGGAATATTGCGCAATGGACGAAAGTCTGACGCAGCG
ACGCCGCGTGAAGGATGAAGGCCCTTTGGGTCGTAAACTTCTGTAGAGAGGGAAGAATAT
CCGCCTTGGGCGGACTGACGGTACCTCTAAAGTAAGGATCGGCTAACTACGTGCCAGCAG
CCGCGGTAATACGTAGGATCCGAGCGTTGTCCGGATTTATTGGGTGTAAAGGGAGCGCAG
GCGGGTCTATAGTCGAGGGTGAGATCTTACAGCTTAACTGTAAAACTGCCTTCGATACTG
TAGATCTTGAGTGTAGGAAAGGGCGATGGAATTCATGGTGTAGCGGTGAAATGCGTAGAT
ATCATGAAGAACACCAGTTGCGAAGGCGGTCGCCTAGCCTATCACTGACGCTCATGCTCG
AAAGTGTGGGGAGCAAACAGGATTAGATACCCCAGTAGTCC
>Otu12224
CCAGCCTATGGGAGGCTGCAGTGGGGAATATTGGACAATGGGGGAAACCCTGATCCAGCA
ATGCCGCGTGAGTGATGAAGGCCTTCGGGTCGTAGAGCTCTTTTACCAGGGATGATAATG
ACAGTACCTGGAGAATAAGCTCCGGCTAACTCCGTGCCAGCAGCCGCGGTAATACGAAGG
GGGCTAGCGTTGTTCGGAATCACTGGGCGTAAAGCGCACGCAGGCGGATTGATAAGTCAG
GGGTGAAATCCCGGGGCTCAACCTCGGAATTGCCTCTGATACTGTCTGTCTTGAGTTCGG
GAGAGGTTGGCGGAATTCCTAGTGTAGAGGTGAAATTCGTAGATATTAGGAAGAACACCA
GTGGCGAAGGCGGCCAACTGGCCCGATGCTGACGCTCATGTGCGAAAGCGTGGGGAGCAA
ACAGGATTAGAAACCCTTGTAGTCC
>Otu12230
CCAGCCTATGGGATGCTGCAGTGGGGAATATTGGACAATGGGCGCAAGCCTGATCCAGCC
ATGCCGCGTGAGTGAAGAAGGCCTTAGGGTTGTAAAGCTCTTTTGGCGGGGACGATAATG
ACGGTACCCGCAGAATAAGCCCCGGCTAACTTCGTGCCAGCAGCCGCGGTAATACGTAGG
GTGCGAGCGTTAATCGGAATTACTGGGCGTAAAGCGTGCGCAGGCGGTTATGTAAGACAG
ATGTGAAATCCCCGGGCTCAACCCGGGACCTGCATTTGTGACTGCATAGCTAGAGTACGG
TAGAGGGGGATGGAATTCCGCGTGTAGCAGTGAAATGCGTAGATATGCGGAGGAACACCG
ATGGCGAAGGCAATCCCCTGGACCTGTACTGACGCTCATGCACGAAAGCGTGGGGAGCAA
ACAGGATTAGAAACCCTCGTAGTCC
>Otu12244
CCAGCCTATGGGTCGCTGCAGCAACGAATCTTCCCCAATGCCGGAAACGGTGAGGGAGCG
ACGCCGCGTGTGGGATGAAGTACTTCGGTATGTAAACCACTGTTAGGGCTACGAAAGCAA
TGCGCGCTAATACCGCGCAGAGTTGATCTAGTCCAGAGAAAGGGACGGCTAACTCTGTGC
CAGCAGCCGCGGTAATACAGAGGTCCCAAGCGTTACTGAGAATCACTGGGTTTAAAGGGT
GCGTAGGTGGTCCGTTAAGTCGGTTGTGAAATCCCCGGGCTCAACCCGGGAACTGCTTCC
GATACTGGCGGACTTGAGGCCTGTAGGGGTCACTGGAACTGACGGTGGAGCGGTGAAATG
CGTAGATATCGTCAGGAACGCCTGTGGTGAAGACGGGTGACTGGGCAGGTTCTGACACTG
AGGCACGAAAGCGTGGGGAGCGAACGGGATTAGAGACCCGTGTAGTCC
>Otu12261
CCAGCCTACGGGTTGCAGCAGTCGAGAATCTTCCACAATGGACGAAAGTCTGATGGAGCG
ACGCCGCGTGATTGATGAAGTCCTTAATCGGACGTAAAGATCTTTTATGAGGGAAGAAGT
TTATTGACGGTACCTCATGAATAAGAGGCTCCTAATCTCGTGCCAGCAGGAGCGGTAATA
CGAGAGCCTCGAGCGTTATCCGGAATTATTGGGCGTAAAGGGTGCGTAGGTTGTTTTGTT
AGTCTTTTGTCAAACTCGCTGGCTCAACTGGCGGAACGCAAAAGAAACGGCAAAACTTGA
AAGTGCGAGAGGCGTACGGAACTTATGGTGTAGGGGTAAAATCCGTTGATATCGTAGGGA
ACACCAAATGCGAAGGCAGTACGCTGGCGCATGTTTGACACTGAAGCACGAAAGCGTGGG
TCGCGAATGGGATTAGATACCCCTGTAGTCC
>Otu12265

CCAGCCTATGGGTTGCAGCAGTAGGGAATTGTCGGCAATGGGCGAAAGCCTGACCGCGCG
ACGCCGCGTGGAGGAAGAAGGCCTTCGGGTTGTAAACTCCTTTTATGAGGGACGATTATG
ACGGTACCTCATGAATAAGGGCCTGCTAACTACGTGCCAGCAGCAGCGGTAATACGTAGG
GCCCGAGCGTTACCCGGAATTACTGGGCGTAAAGAGTACGTAGGTGTTCCAACAAGTCGG
TCGTTAAATCTCACGGCTTAACCGCGAGACCGCGAGCGATACTGTTGGAATTGAGTTTTG
CAGGGGCAAGTGGAATTCCCGGAGTAGGGGTGAAATCCGTTGATACCGGGAGGAACACCA
ATGGCGAAGGCAACTTGCTAGGCAATGACTGACACTGAGGTACGAAAGCGTGGGGAGCAA
ACAGGATTAGAAACCCTGGTAGTCC
>Otu12267
CCAGCCTACGGGATGCTGCAGTAGGGAATATTGGGCAATGGGCGAAAGCCTGACCCAGCA
ACGCCGCGTGAGTGAGGAAGGTTTTCGGATCGTAAAGCTCTGTCAGGTGGGAAGAAATGT
ATAGAAGCAAATAGCCTCTATACTTGACGGTACCACCAGAGGAAGCACCGGCTAACTCCG
TGCCAGCAGCCGCGGTAATACGGGGGGTGCAAGCGTTGTTCGGAATTATTGGGCGTAAAG
AGCGTGTAGGCGGCTAGATAAGTCGGATGTGAAAGCCCTGGGCTTAACCCGGGAAGTGCA
TTTGAAACTGTCTTGCTAGAGTAAGGGAGAGGGAAGTGGAATTCCTGGTGTAGAGGTGAA
ATTCGTAGATATCAGGAGGAACACCGGTGGCGAAGGCGACTTTCTGGCCCTATACTGACG
CTGAGACGCGAGAGCGTGGGGAGCAAACAGGATTAGATACCCCTGTAGTCC
>Otu12271
CCAGCCTATGGGGGGCTCCAGTCGAGAATCTTCCGCAATGGGCGAAAGCCTGACGGAGCG
ATGCCGCGTGAGGGATGAAGGCCTTCGGGTTGTAAACCTCTGTCGCCCGGGACGAAACCA
TGACGGTACCGGGAGAGGAAGCTACGGCTAACTACGTGCCAGCAGCCGCGGTAATACGTA
GGTAGCGAGCGTTATTCGGATTCACTGGGCGTAAAGGGTCCGCAGGAGGCCTGTCAAGCC
AGACGTGAAAGCCCAAGGCTCAACCTTGGAATGGCGTCTGGGACTGCCGGGCTTGAGTGC
ACGAGGGGAAAGCAGAATTCCTGGTGTAGCGGTGGAATGCGTAGATATCAGGAGGAATAC
CGATGGCGAAGGCGGCTTTCTGGCGTGCAACTGACTCTCAGGGACGAAAGTGTGGGTAGC
AAACAGGATTAGATACCCCCGTAGTCC
>Otu12297
CCAGCCTATGGGGTGCTGCAGTGGGGAATCTTGGACAATGAGTGAAAACTTGATCCAGCG
ATATCACATGAGTGAAGAAGGCCATTTGGTTGTAAAGCTCTTTCGTTAATGAAGATAATG
ACAGTAATTAAAGTAAGAAGTCCTGGCAAATCCTGTGCCAGCAGCCGCGGTAATACAGGA
GGGGCAAGCGTTATTCGGATTTAATGGGCGTAAAGGGTGCGTAGGCTATAAATGCGATTA
AATATGAAAGACCAGAGCTTAACTCTGGAACTGTATTTAAAATCAATTTATTTGAGTTAG
ATAGGGGTTTGTGGAATTCCTAAAGTAAAGGTAATATTTTTCGAGATTAGGCGGAATATC
CGAAGCGAAGGCGGCATTCTGGATCTACACTGACGCTGAGGCACGAAAGCGTGGGTAGCA
AACAGGATTAGAAACCCCTGTAGTCC
>Otu12307
CCAGCCTACGGGGGGCAGCAGCTAAGAATCTTCCGCAATGGGGGAAACCCTGACGGAGCG
ACGCCGCGTGGATGATGAAGGCCGTAAGGTTGTAAAATCCTTTTGTCGGTGAAGAATAAG
GGAGGGAGTGGAAAGCCCTCCTGATGACGTTAGCCGGCGAATAAGCCTCGGCTAATTACG
TGCCAGCAGCCGCGGTAATACGTATGGGGCAAGCGTTGTTCGGATTTACTGGGCGTAAAG
CGCATGCAGGTGGTTTGGTAAGTTGGATGTGAAAGCTCCTGGCTCAACTGGGAGAGGTCG
TTCAATACTACCAGACTTGAGAGCAGTAGAGGGAGGTGGAATTCCCGGTGTAGTGGTGAA
ATGCGTAGATATCGGGAGGAACACCAGTGGCGAAAGCGGCCTCCTGGACTGTTTCTGACA
CTCATATGCGAAAGCTAGGGTAGCAAACGGGATTAGAGACCCTCGTAGTCC
>Otu12311
CCAGCCTACGGGACGCTGCAGTGGGGAATATTGGGCAATGGGCGAAAGCCTGACCCAGCG
ACGCCGCGTGGGTGATGAAGGCCTTCGGGTTGTAAAGCCCTTTCGTGCGGAAAGAACAGT
CCCGTATCTAATACATGCGGGATTTGACGGTACCGCAGGAAGAAGCACCGGCTAACTCCG
TGCCAGCAGCCGCGGTAATACGGAGGGTGCAAGCGTTGTTCGGAATTACTGGGCGTAAAG
GGCGCGTAGGCGGTAATGTAAGTCAGATGTGAAATCCTTTGGCTCAACTGAAGAACTGCA
TCTGAAACTGCATAACTCGAGTACAGAAGAGGGAAACGGAATTCCCGGTGTAGAGGTGAA
ATTCGTAGATATCGGGAGGAACACCAGTGGCGAAGGCGGTTTCCTGGTCTGATACTGACG
CTGTGGCGCGAAAGCGTGGGGAGCAAACAGGATTAGAAACCCTTGTAGTCC
>Otu12328
CCAGCCTATGGGGGGCAGCAGAGGAGGATCATCGTCAATGGGCGAAAGCCTGAACGTGCG
ACGCCGCGTGGGGGAAGAAGGCCTTCGGGTCGTAAACCCCTGTCAGAGGTCTCTAAGTTA
TGCCGCGCAAATAGCGCGGTATATTGACAAGCCTCAGAGGAAGGGACGGCTAACTTCGTG
CCAGCAGCAGCGGTAATACGGAGGTCCCGAGCGTTGTTCGGAATCACTGGGCGTAAAGGG

CGTCTAGGCGGGATGGCAAGTCGGGTATGAAATCCTTTGGCTCAACCAGAGAATTGTGCC
CGAAACTGCCGTCCTTGAGTATGGCAGAGGACAGTGGGACTCCTGGTGGAGCGGTGAAAT
GCGTAGATATCAGGAGGAACGCCGGAGGCGAAAGCGATTGTCTGGGCCATTACTGACGCT
GAAGCGCGAAAGCTAGGGGAGCAAACGGGATTAGATACCCCCGTAGTCC
>Otu12329
CCAGCCTACGGGTGGCAGCAGCTAAGAATATTCCGCAATGGGAGAAATCCTGACGGAGCG
ACGCCGCGTGAATGAAGAGGGCAGAAATGTTGTAAAATTCTTTTATACGGGAAGAATAAG
GTGAGTAGTGGAAAGACTCATCGATGACGGTACCGTATGAATAAGCCCCGGCTAACTACG
TGCCAACAGCCGCGGTAATACGTAGGGGGCTAGCGTTGTTCGGATTTATTGGGCGTAAAG
GGCATGTAGGCGGTTATGCAAGTGTGGGTTTAAAGGCGCAGGCTCAACCTGTAGCATGGT
CTGCAAACTACATAACTAGAGTTCGGGATGGGAGACTGGAATTCCGCGTGTAGGGGTGAA
ATCTGTAGAGATGCGGAAGAATACCGGCGGCGAAGGCGAGTCTCTGGCCTGAAACTGACG
CTGAGATGCGAAAGTGTGGGGAGCAAACAGGATTAGAAACCCTTGTAGTCC
>Otu12330
CCAGCCTATGGGACGCTGCAGTCGAGGATCATTCGCAATGGGCGCAAGCCTGACGATGCA
ACGCTGCGTGGAGGATGAAGGCCTTCGGGTCGTAAACTCCTGTCATGCGGGAGCAATGGT
CCCGTCGCGAATAGCGGCGGGCAGGGATAGTACCGCAAGAGGAAGCCACGGCTAACTCTG
TGCCAGCAGCCGCGGTAATACAGAGGTGGCGAGCGTTGTTCGGATTTACTGGGCGTAAAG
GGCGCGTAGGCGGTCTCGTGTGTCGGATGTGAAATCCCGTCGCTCAACGACGGAACGCCA
TTCGAAACTGCGGGACTCGAGTGCAGGAGAGGAGAGCGGAACTCTTGGTGTAGCGGTGAA
ATGCGTTGATATCAAGAAGAACACCAGTGGCGAAGGCGGCTCTCTGGAATGCAACTGACG
CTGAGGCACGAAAGCGTGGGGAGCAAACAGGATTAGAGACCCCAGTAGTCC
>Otu12341
CCAGCCTATGGGGCGCTGCAGTAAGGAATATTGGGCAATGGACGCAAGTCTGACCCAGCC
ATGCCGCGTGAAGGATGAATGTCCTATGGATTGTAAACTTCTTTTATGCAGGAAAAACAT
CCCGGTCGTGAACCGGGACTGATGGTACTGTATGAATAAGCATCGGCTAACTCCGTGCCA
GCAGCCGCGGTAATACGGAGGATGCAAGCGTTATCCGGATTCATTGGGTTTAAAGGGTGC
GCAGGCGGAATAATAAGTCAGTGGTGAAATCTCCCGGCTCAACCGGGAAACTGCCATTGA
TACTGTTGTTCTTGAGTACAGTTGAAGTGGGCGGAATGTGTCATGTAGCGGTGAAATGCT
TAGATATGACACAGAACACCGATAGCGAAGGCAGCTCACTAAGCTGTAACTGACGCTCAT
GCACGAAAGCGTGGGGATCAAACAGGATTAGAAACCCCAGTAGTCC
>Otu12348
CCAGCCTATGGGGTGCAGCAGTCGAGAATATTCGTCAATGCGCGAAAGCGTGAACGAGCG
ATGCCGCGTGCGGGATGAAGGCCTTCGGGTTGTAAACCGCTTTTTTCAGGGAGCAATTAT
TGAGCGTACCTGACGAATAAGAGGTTACTAACTCTGTGCCAGCAGTAGCGGTAATACAGA
GACCTCAAGCGTTATCCGGATTTATTGGGCGTAAAGCGCGGGTAGGTGGACATATTAGTC
AGATGTCAAATCCTCCGACTTAATCGGAGAACTGCATTTGAAACGGTATGTCTAGAGAAA
GCGAGAGACCAGTGGAACTCATGGTGTAGTGGTGATATGCGTTGATATCATGAGGAACAC
CAAAGGCGAAAGCATCTGGTTGGCACTTTTCTGACACTGATCCGCGAAAGCGTGGGTAGC
GAATGGGATTAGATACCCTTGTAGTCC
>Otu12349
CCAGCCTACGGGTTGCAGCAGTTGGGAATCTTGGACAATGGGGGAAACCCTGATCCAGCC
ATGCCGCGTGAGTGATGAAGGCCTTAGGGTTGTAAAGCTCTTTTACCCGGGAAGATAATG
ACTATACCGGGAGAATAAGCTCCGGCTAACTTCGTGCCAGCAGCCGCGGTAATACGAAGG
GGGCTAGCGTTGTTCGGAATTACTGGGCGTAAAGCGTGCGCAGGCGGTCTCTCAAGTCAG
GGGTGAAAGCCCAGAGCTCAACTCTGGAATTGCCTTTGAAACTGTGAGGCTCGAGTGCGG
GAGAGGTGAGTGGAATTCCCAGTGTAGAGGTGAAATTCGTAGATATTGGGAAGAACACCG
GTGGCGAAGGCGGCTTCCTGGCCCTATACTGACGCTGAGACGCGAAAGCGTGGGTAGCAA
ACAGGATTAGATACCCCAGTAGTCC
>Otu12350
CCAGCCTACGGGGCGCTGCAGTGGGGAATATAGGACAATGGGGGCAACCCTGATCCAGCA
ATGCCGCGTGAGTGATGAAGGCCTTCGGGTTGTAAAGCTCTTTTGCCAGGGACGATAATG
ACGGTACCTGGAGAATAAGCCCCGGCTAACTTCGTGCCAGCAGCCGCGGTAATACGAAGG
GGGCTAGCGTTGTTCGGAATTACTGGGCGTAAAGGGCGCGTAGGCGGTCTGATAAGTTAG
GTGTGAAATTCCCGGGCTTAACCTGGGCGCTGCACTTAATACTGTCAGGCTTGAATCCGG
GAGAGGATAGCGGAATTCCCAGTGTAGAGGTGAAATTCGTAGATATTGGGAAGAACACCA
GTGGCGAAGGCGGCTATCTGGACCGGCATTGACGCTGAGGCGCGAAAGCGTGGGGAGCAA
ACAGGATTAGAGACCCGAGTAGTCC

>Otu12352
CCAGCCTACGGGTCGCAGCAGTGGGGAGTATTGGACAATGGGGGCAACCCTGATCCAGCA
ATGCCGCGTGAGTGATGAAGGCCTTAGGGTTGTAAAGCTCTTTCGACGGGGAAGATGATG
ACGGTACCCGTAGAAGAAGCCCCGGCTAACTTCGTGCCAGCAGCCGCGGTAATACGAAGG
GGGCTAGCGTTGTTCGGATTTACTGGGCGTAAAGGGCGCGTAGGCGGTGCAATAAGTCAG
GCGTGAAAGCCCCGGGCTCAACCCGGGAATTGCGCTTGATACTGTTGCGCTTGAATCCAG
GAGAGGATGGCGGAATTCCCAGTGTAGAGGTGAAATTCGTAGATATTGGGAAGAACACCA
GTGGCGAAGGCGGCCATCTGGCCTGGAATTGACGCTGAGGCGCGAAAGCGTGGGGAGCAA
ACAGGATTAGAAACCCTCGTAGTCC
>Otu12356
CCAGCCTACGGGATGCAGCAGGCGCGAAAACTTTACAATGCGGGAAACCGTGATAGGGGA
ACTCTGAGTGCCCGTTAAATCGGGCTGTCCATCAGTTTAAATAACTGGTGAAGAAAGGGC
CGGGCAAGACCGGTGCCAGCCGCCGCGGTAATACCGGCGGCTCGAGTGGTGGCCACTATT
ACTGGGCTTAAAGCGTTCGTAGCTGGTTTGTTAAGTCTCTGGGGAAATCTTCCGGCTTAA
CCGGAAGGCGTCTCAGGGATACTGGCAGACTAGGGACCGGGAGAGGTGAGAGGTACCTCA
GGGGTAGGGGTGAAATCTTGTAATCCTTGAGGGACCACCAGTGGCGAAGGCGTCTCACCA
GAACGGATCTGACGGCAAGGGACGAAAGCTAGGGGCACGAACCGGATTAGATACCCGAGT
AGTCC
>Otu12357
CCAGCCTACGGGTCGCAGCAGTAGGGAATTTTCCGCAATGGGCGCAAGCCTGACGGAGCA
ACGCCGCGTGAGGGATGAAGGTCTTCGGATCGTAAACCTCTTTGGAGCGAGACGAATGGG
TTTTCGGTGAACAACCGGGAACCGTGACTGTATCGCTAGAACAAGTCACGGCTAACTCTG
TGCCAGCAGCCGCGGTAATACAGAGGTGACTAACGTTGTCCGGAATTATTGGGCGTAAAG
GGCGCGTAGGTGGTTGGATAAGTCAGATGTGAAAGCTCTGGGCTTAACCCAGGAATTGCA
TTTGATACTGTTTGACTTGAGTACGGTAGAGGAGAGTGGAATTCCCAGTGTAGCGGTGAA
ATGCGTAGATATTGGGAAGAACACCGGTGGCGAAGGCGGCTCTCTGGACCGAAACTGACA
CTGAAGCGCGAAAGCTAGGGGAGCAAACGGGATTAGAAACCCTGGTAGTCT
>Otu12365
CCAGCCTATGGGTTGCTGCAGTCGGGAAAATTTTGCAATGCACGAAAGTGTGACAAAGCA
AGCCAAAGTGCTGTTCTTTTTTTGAACAGCTTTCGTGAGATGCAAAAAGTCTCACGAATA
AGGACTGGGTAAGACTGGTGCCAGCCGCCGCGGTAATCCCAGCAGTCCAAGTCGCAGCCA
CAATTATTGGGTCTAAAACATCCGTAGCTTGCTTTGTAAGTCTTTTGTAAAATCGGGAAT
CTTAAGTTTCCGGCGCGCAAAAGATACTGCTTAGCTAGAGACCGGGAGACGTAAGAAGTA
CGGGTAGAGTAACGGTAAAATGTGTTGATCCTACCTGGACTAACAATCGCGAAGGCATCT
TACGAGAACGGATCTGACAGTGAGGGATGAAGGCTAGGGGCGCAAAACGGATTAGAAACC
CTAGTAGTCC
>Otu12369
CCAGCCTACGGGGGGCACCAGTGGGGAATATTGGTCAATGGGCGCAAGCCTGAACCAGCC
ATGCCGCGTGTAGGAAGACGGCCCTATGGGTTGTAAACTACTTTTGCAGGGGAATAAACC
CCGGTACGTGTACCGGGTTGAATGTACTCTGAGAATAAGGATCGGCTAACTCCGTGCCAG
CAGCCGCGGTAATACGGAGGATCCGAGCGTTATCCGGATTTATTGGGTTTAAAGGGTGCG
TAGGCGGCCTATTAAGTCAGGGGTGAAAGACGGTAGCTTAACTATCGCAGTGCCTTTGAT
ACTGATGGGCTTGAATACACTAGAGGTAGGCGGAATGTGACAAGTAGCGGTGAAATGCTT
AGATATGTCACAGAACACCAATTGCGAAGGCAGCTTACTATGGTGTTATTGACGCTGAGG
CACGAAAGCGTGGGGATCAAACAGGATTAGAGACCCGCGTAGTCC
>Otu12371
CCAGCCTATGGGATGCAGCAGAGGGGAATATTGGGCAATGGGCGAAAGCCTGACCCAGCC
ACGCCGCGTGAGTGATGAAGGCCTTTGGGTTGTAAACCCCTGTCAGGGGGGACGAAAGCC
TTCCGGTGAATAGCCGGGAGGGTTGACGGTACCCCCAAAGGAAGCCCCGGCTAACTCCGT
GCCAGCAGCCGCGGTAATACGGAGGGGGCAAGCGTTATTCGGAATTACTGGGCGTAAAGC
GCGCGCAGGTGGTGCGGTAAGTGGCAGGTGAAAGCCCTTGGCTTAACCAAGGAACTGCCT
GCCAAACTGTTGCGCTGGAGGCCGGGAGGGGAGAGCGGAATTCCTGGTGTAGCGGTGAAA
TGCGTAGATACCAGGAGGAACACCGGTGGCGAGGGCGGCTCTCTGGACCGGTTCTGACAC
TCATGCGCGAAAGCGTGGGGAGCAAACAGGATTAGATACCCGTGTAGTCC
>Otu12376
CCAGCCTACGGGTTGCAGCAGTGGGGAATCTTGCACAATGGGGGAAACCCTGATGCAGCG
ACGCCGCGTGAGCGATGAAGCCCTTCGGGGTGTAAAGCTCTTTCGGCGGGAACGAAACCT
GACGGTACCCGCAGAAGAAGCTGCGGCTAACTACGTGCTAGCAGCCGCGGTAATACGTAG

GCAGCGAGCGTTGTTCGGAGTTACTGGGCGTAAAGAGTATGTAGGCGGTGCTGCAAGTTT
GGTGTGAAATCTCCCGGCTTAACTGGGAGGGAGCGCCGAAAACTGCAGGGCTAGAGTGCG
GGAGAGGAAAGCGGAATTCCTGGTGTAGCGGTGAAATGCGTAGATATCAGGAGGAACACC
TGCGGTGTAGACGGCTTTCTGGACCACTACTGACGCTGAGGCACGAAAGCGTGGGGAGCA
AACAGGATTAGAAACCCGAGTAGTCC
>Otu12379
CCAGCCTATGGGGCGCTCCAGTCAAGAATATTCCCCAATGGACGAAAGTCTGAGGGAGCG
ACGCCGCGTGCAGGATGAAGGGCTTCGGCTTGTAAACTGCTTTTCTTTGGGACGAAGCTT
CGGCTGACGGTACCAAGGGAATAAGAGGTTGCTAACTCTGTGCCAGCAGCAGCGGTAATA
CAGAGACCTCAAGCGTTATCCGGATTTATTGGGCGTAAAGCGTCCGCAGGCGGCACGTTA
AGTCTCTCGTCAAATCTATGGGCTCAACTCATAGGCTGCGGGGGAAACTGGCGAGCTAGA
GGTTGGGAGAGGTGAGCGGAATTACCGGTGTAGTAGTAAAATGCGTTAATATCGGTAAGA
ACACCAAAGGCGAAGGCAGCTCACTGGAACACACCTGACGCTAAGGGACGAAAGCACGGG
GAGCGAAGCGGATTAGAAACCCTAGTAGTCC
>Otu12384
CCAGCCTATGGGTGGCTCCAGACGAGAATATTCGACAATGGACGAAAGTCTGATCGAGCG
ACGCCGCGTGGAGGATGAAGTGCTTCGGCATGTAAACTCCTTTTGCCAGGGAAAAAGTCT
ATTGATTGTACCTGGAGAATAAGAGGTTGCTAAACTCGTGCCAGCAGCAGCGGTAATACG
AGTGCCTCGAGCGTTATCCGGAATTATTGGGCGTAAAGGGTGCGTAGGCGGTTCCGTTAG
TCCCATGTTAAATCTCCCGGCTCAACCGGGAACTCGCACGGGAAACGGCGGAACTATGAG
GGTGGAAGGGGTCTGTGGAACTCATGGTGTAGCGGTGAAATGCGTTGATATCATGGGGAA
CACCAAAAGCGAAGGCAGCAGACTGGTCCACTTCTGACGCTGAAGCACGAAAGCGTGGGT
CGCGAATGGGATTAGAGACCCCCGTAGTCC
>Otu12389
CCAGCCTACGGGGGGCTGCAGTCGAGAATATTCCCCAATGGGCGAAAGCCTGAGGGAGCG
ACGCCGCGTGCAGGATGAAGGACTTCGGTTCGTAAACTGCTTTTGCCGAGGACGAATACT
GACGGTACTCGGAGAATAAGAGGTTGCTAACTCTGTGCCAGCAGCAGCGGTAATACAGAG
ACCTCAAACATTGCCCGGATTTATTGGGCGTAAAGGGTCCGCAGGCGGCCTGGTGTGTCC
CGCGTTAAATGTCAAGGCTCAACTTTGACACCGCGCTGGATACGACCAAGCTTGAGAGTG
GGAGAGGCGAGCGGAATTACCGGTGTAGTAGTAAAATGCGTTAATATCGGTAAGAACACC
AAATGCGAAGGCAGCTCGCTAGAACACTTCTGACGCTCAGGGACGAAAGCGTGGGGAGCG
AATGGGATTAGATACCCCTGTAGTCC
>Otu12392
CCAGCCTACGGGGGGCACCAGTGGGGGATATTGCACAATGGAGGAAACTCTGATGCAGCG
ACGCCGCGTGAGGGAAGAAGGTCTTCGGATTGTAAACCTTTGTCTTCAGGGAAGAACGGT
GACGAAAGTCACTCTGACGGTACCTGAGGAGGAAGCCACGGCTAACTACGTGCCAGCAGC
CGCGGTAATACGTAGGTGGCAAGCGTTGTCCGGAATTACTGGGTGTAAAGGGTGCGCAGG
CGGGACAATAAGTTGGATGTGAAATACCCGGGCTCAACTCGGGTGCTGCATTCAAAACTA
TTGTTCTTGAGTGAAGTAGAGGTAGGCGGAATTCCCGGTGTAGCGGTGAAATGCGTAGAT
ATCGGGAGGAACACCAGTGGCGAAGGCGGCCTACTGGGCTCTTACTGACGCTGAGGCACG
AAAGCATGGGTAGCAAACAGGATTAGATACCCGCGTAGTCC
>Otu12395
CCAGCCTATGGGTCGCTCCAGTTAAGAATCTTGGACAATGGACGCAAGTCTGATCCAGCG
ACGCCGCGTGAACGATGAAGGTCTTCGGATTGTAAAGTTCGGTAAGATGGGACGAAGAAG
CTGAAGATAATACCTTCAGTGGTGACGGTACCATCCTAAAGCCTCGGCTAAATACGTGCC
AGCAGCCGCGGTAATACGTATGAGGCAAGCGTTGTTCGGAATTATTGGGCGTAAAGGGCA
TCTAGGCGGGATGTTAAGTCAGGTGTGAAAGCCAAGGGCTCAACCCTTGAACTGCATTTG
ATACTGACGTTCTTGAGTTCGGAAGAGGAAGGCGGAATTCCCGGTGTAGCGGTGAAATGC
GTAGATATCGGGAAGAACACCGGTGGCGAAGGCGGCCTTCTGGTCTAGAACTGACGCTGA
CATGCGAAAGTGTGGGGATCAAACGGGATTAGAACCCCTGTAGTCC
>Otu12402
CCAGCCTATGGGACGCACCAGTGGGGAATATTGGGCAATGGGCGAAAGCCTGACCCAGCG
ACGCCGTGTGGGCGATGAAGGCCTTCGGGTTGTAAAGCCCTGTTGGGTGGAAAGAAGGGT
TCGGAGACGAATAATCTCTGAACCTTGACGGTACCACCAGAGAAAGCACCGGCTAACTCC
GTGCCAGCAGCCGCGGTAATACGGAGGGTGCAAGCGTTGTTCGGAATTACTGGGCGTAAA
GGGCGCGTAGGTGGTTGCGTAAGTCGGATGTGAAATCCCCGGGCTTAACCCGGGAACTGC
ACCCGATACTGCGTGACTCGAGTGCAGCAGAGGGAAACGGAATTCCTGGTGTAGAGGTGA
AATTCGTAGATATCAGGAGGAACACCGGTGGTGTAGACGGCTTTCTGGACCGTAACTGAC

GCTGAGACACGAAAGCGTGGGTAGCAAACAGGATTAGATACCCCTGTAGTCC
>Otu12407
CCAGCCTTGGGTCGCAGCAGTGGGGAATATTGGACAATGGGCGCAAGCCTGATCCAGCCA
TGCCGCGTGAGTGAAGAAGGCCTTAGGGTTGTAAAGCTCTTTTGGCGGGGACGATAATGA
CGGTACCCGCAGAATAAGCCCCGGCTAACTTCGTGCCAGCAGCCGCGGTAATACGAAGGG
GGCTAGCGTTGCTCGGAATCACTGGGCGTAAAGGGTGCGTAGGCGGGTCTTTAAGTCAGG
GGTGAAATGCCGAGGCTCAACTTCGGAACTGCCTTTGATACTGGAGATCTTGAGTTCGGG
AGAGGTGAGTGGAACTGCGAGTGTAGAGGTGGAATTCGTAGACATTCGCAAGAACACCAG
TGGCGAAGGCGGCTCACTGGCCCGATACTGACGCTGAGGTGCGGAAGCGTGGGGAGCAAA
CAGGATTAGAAACCCCTGTAGTCC
>Otu12420
CCAGCCTATGGGGGGCAGCAGTAAGGGGTATTGCGCAATGGGCGAAAGCCTGACGCAGCA
ACGCCGCGTGGAGGATGAAGTCCCTCGGGATGTAAACTCCAATAGTTTGTGAGGAAGTAA
CCCGGCCTAATACGTCGGGAAGTTGACCAGCAGATGAAAGGGGCGGCTAACTCCGTGCCA
GCAGCCGCGGTAAGACGGAGGCCCCGAGCGTTGTTCGGAATTACTGGGCTTAAAGCGCGT
GTAGGCGGGTCGGTAAGTGCTTTGTGAAATCCCCGGGCTCAACCCGGGAATAGCTTGGCA
TACTGCCGGTCTTGAGGCAGGTATGGGTCATTGGAACCTTAGGTGTAGCGGTGAAATGCG
TAGATATCTAAGGGAACGCCGGTGGCGAGAGCGGGTGACTGGGCCTGTCCTGACGCTGAG
ACGCGAAAGCGTAGGGAGCAAACGGGATTAGATACCCGCGTAGTCC
>Otu12424
CCAGCCTACGGGGGGCTGCAGTGGGGAATATTGGTCAATGGGCGAAAGCCTGAACCAGCC
ACGCCGCGTGAGTGATGAAGGCCTTCGGGTCGTAAAGCTCTGTGGGGAGGGACGAAAGCC
TGAACATAAACAATGTTCAGGGTTGACGGTACCTCCTTAGCAAGCACCGGCTGACCCTGT
GCCAGCAGCCGCGGTAATACAGGGGGTGCAAACGTTGCTCGGAATTATTGGGCGTAAAGC
GCACGTAGGCGGTCTCGCAAGTTGGATGTGAAAGCCCTCGGCTTAACCGAGGAAGTGCAT
CCAAAACTACGAGGCTTGAATATGGAAGAGGGTCGCGGAATTCCCGGTGTAGAGGTGAAA
TTCGTAGATATCGGGAGGAACACCAGTGGCGAAGGCGGCGACCTGGTCCAATATTGACGC
TGAGGTGCGAAAGCGTGGGGAGCAAACAGGATTAGAGACCCCCGTAGTCC
>Otu12429
CCAGCCTATGGGTGGCAGCAGTAGGGAATATTGCACAATGGACGAAAGTCTGATGCAGCA
ACGCCGCGTGCACGATGAAGGCCTTAGGGTTGTAAAGCTCTTTTACCCGGGAAGATAATG
ACTGTACCGGGAGAATAAGCTCCGGCTAACTTCGTGCCAGCAGCCGCGGTAATACGAAGG
GGGCTAGCGTTGTTCGGAATTACTGGGCGTAAAGCGCGCGCAGGCGGCCTGACAAGTCAG
GGGTGAAAGCCCAGAGCTCAACTCTGGGACTGCCCTTGAAACTTTCAGGCTAGAGGACGG
GAGAGGTGAGTGGAATTCCCAGTGTAGAGGTGAAATTCGTAGATATTGGGAAGAACACCG
GTGGCGAAGGCGGCTCACTGGCCCGTTTCTGACGCTCAGGCGCGACAGCGTGGGGATCAA
ACAGGATTAGAAACCCGAGTAGTCC
>Otu12436
CCAGCCTACGGGGTGCACCAGGCGCGAAAACTTTACAATGCACGAAAGTGTGATAGGGGG
ACTCAAAGTGCTTATACTTTTAGTATAGGCTTTTGCCAAGTTAAAATTGCTTGGCGAATA
AGTGATGGGCAAGACTGGTGCCAGCCGCCGCGGTAACCCCAGCGTCACGAGTGGGAATCA
GTATTATTGGGCCTAAGGCGTCCGTAGCAGGTCTTCTAAATTATTTGTGAAATCGCATAG
CTTAACTATGCGGATTGCAGATAGGACTGGAAGACTTGGGATCGGGAGAAGTCGAAGGTA
CTCAGAGGGGAGCGGTAAAATGCGAGAATCCTCTGAAGACCATCTGTTGCGAAGGCGTTC
GACAATAACGAGTCTGACTGTGAGGGACGAAAGCTAGGGGAGCGAACCGGATTAGAAACC
CTAGTAGTCC
>Otu12443
CCAGCCTACGGGTCGCACCAGTAGGGAATATTGGACAATGGGGGAAACCCTGATCCAGCA
ACGCCGCGTGGATGATGAAGCTTCTTGGAGTGTAAAATCCTGTCAGTGGGGACGAAACGT
CCGTGGAGGAAATGCCACGGGCCTGACTGTACCCGCAGAGGAAGTTCCGGCCAACTCCGT
GCCAGCAGCCGCGGTAATACGGGGGGAACGAGCGTTGTTCGGATTTACTGGGCGTAAAGG
GCACGCAGGTGGGAAAGAAAGTCAGGTGTGAAATCCCGAGGCTTAACTTCGGGACTGCAT
TTGATACTTCTTTTCTTGAGTACGAGAGAGGATGGCGGAATTCTTGGTGTAGCGGTGAAA
TGCGTAGATATCAAGAAGAACACCGGTGGCGAAGGCGGCCATCTGGCTCGATACTGACGC
TCATGTGCGAAGGCCGGGGGAGCAAACAGGATTAGAGACCCGAGTAGTCC
>Otu12448
CCAGCCTATGGGTTGCAGCAGTCGAGAGGCTTCGTCAATGGGGGAAACCCTGAACGAGCG
ACGCCGCGTGAGGGATGAAGGCCTTCGGGTTGTAAACCCCTGTCGCGCGGGGAGAAGGAT

TCGGCGGCGAACAGTCGCAGGATCTCGACGGTACCGCAAAAGGAAGCTCCGGCTAACTCC
GTGCCAGCAGCCGCGGTAATACGGGGGGAGCGAACGTTGTTCGGAATTACTGGGCGTAAA
GGGCGCGCAGGCGGCCTCATAAGTGGGATGTGAAAGCCCCTGGCTCAACCAGGGAAATGC
GTCCCATACTGTGGGGCTTGAGTGCAGAGGAGGATGGGGGAATTCCCGGTGTAGCGGTGA
AATGCATTGATATCGGGAGGAACACCAGTGGCGAAGGCGCCCATCTGGCCTGCTACTGAC
GCTCAGGCGCGAGAGCCGGGGGAGCAAACAGGATTAGAGACCCCTGTAGTCC
>Otu12451
CCAGCCTATGGGTTGCTCCAGTAGGGGATATTGGTAATCTACGGAAGTGGGAACCAGCAA
CGCCGCGTGTGCGATGACGGCCTTCGGGTTGTAAAGCACTTTTCATGGGGACGAGGAAGG
ACGGTACCCGTGGAATAAGCCTCAGCTAACTACGTGCCAGCAGCCGCGGTAAAACGTAGG
AGGCGAGCGTTATCCGGATTTACTGGGTGTAAAGCGCATGCAGGCGGTTCGCTAAGTTGG
GTGTGAAAGCTCCCGGCTCAACTGGGAGAGGTCGCTCAAGACTGGCGGACTGGAGCGTGG
TAAGGGAAGGCGGAATTCCGGGTGTAGTGGTGAAATGCGTAGATATCCGGAGGAACACCA
GTGGCGAAGGCGGCCTTCTGGGCCACGACTGACGCTCAGATGCGACAGCTAGGGGAGCAA
ACGGGATTAGATACCCGAGTAGTCC
>Otu12453
CCAGCCTATGGGTTGCTCCAGGGACGAATATTCGTCAATGGACGAAAGTCTGAACGAGCG
ACGCCGCGTGTGGGAGGAAGTCCTTCGGGATGTAAACCACTGTCAGGGGCTACCAAGTGA
CCGTTCCTAACAGGGACGGAAGTTGAGGGGCCCCAGAGGAAGCCACGGCTAACTTCGTGC
CAGCAGCCGCGGTAAGACGAAGGTGGCAAGCGTTGTTCGGAATCACTGGGCTTAAAGGGC
GCGTAGGCGGCCTGTTAAGCGCCTTGTGAAATCCCCCGGCTCAACCGGGGAATTGCTCGG
CGAACTAGCAGGCTTGAGGCAGGTAAGGGCGTGAGGAACTCTTGGTGGAGCGGTGAAATG
CGTAGATATCAAGAGGAACGCCGGTGGTGAAGACGTCACGCTGGGCCTGTCCTGACGCTG
AGGCGCGAAAGCCAGGGGAGCAAACGGGATTAGATACCCCAGTAGTCC
>Otu12454
CCAGCCTATGGGGTGCTCCAGTCGGGAAATTTCTGCAATGCGCGCAAGCGTGACAGAGCA
AGCCAGAGTGCTTTTCTATTATTGAAAAGCTTTTGTGAAATGTAAAAATTTTTACGAATA
AGGACCGGGCAAGACTGGTGCCAGCCGCCGCGGTAATCCCAGCGGTCCGAGTCGCAGCCA
CAATTATTGGGTCTAAAACATCCGTAGCTTGTCTTATAAGTCTCTTGTGAAATCTGGCTT
CTTAAAAGTCGGGCGAGCAAGAGATACTGTTTGACTAGAGACTGGGAGACGTAAGAAGTA
CGTATAGGGTAACGGTAAAATGTGTTGATCCTATATGGACTAACAAGAGCGAAGGCATCT
TACGAGCACAGATCTGACAGTGAGGGATGAAGGCTAGGGGCGCAAATTGGATTAGAAACC
CCTGTAGTCC
>Otu12459
CCAGCCTACGGGGTGCACCAGTAGGGAATATTGGTTAATCTACGAAAGTGGGAACCAGCA
ACGCCGCGTGCACGATGAAGGCCTTCGGGTTGTAAAGTGCTTTTTGAGAGGATGAGGAAG
GACAGTACTCTTAGAATAAGCTTCGGCTAACTACGTGCCAGCAGCCGCGGTAAAACGTAG
GAAGCGAGCGTTATCCGGATTTACTGGGCGTAAAGCGTGTGTAGGCGGTTTGATAAGTTG
GATGTGAAAGCTCCTGGCTTAACTGGGAGAGGTCGTTCAAAACTATCAGACTCGAGAGTG
GTAGAGGGAGGTGGAATTCCGGGTGTAGTGGTGAAATGCGTAGATATCCGGAGGAACACC
AGTGGCGAAAGCGGCCTCCTGGCCCATTTCTGACGCTCAGACACGAAAGCTAAGGTAGCA
AACGGGATTAGATACCCGCGTAGTCC
>Otu12463
CCAGCCTATGGGGGGCTGCAGTGGGGAATATTCCGCAATGGGCGAAAGCCTGACGGAGCG
ACGCCGCGTGGGTGATGAAGGTCTTCGGATCGTAAAGCCCTGTGGAGCGGGAAGAAGGGT
GCCGTAAGGCATTTGACAGTACCGCTAAAGCAAGCTACGGCAAACTCTGTGCCAGCAGCC
GCGGTAATACAGAGGTAGCGAGCGTTGTTCGGAATGACTGGGTGTAAAGCGCGTGTAGGC
GGTCTCGTAAGTTGAATGTGAAATCCCTTGGCTTAACCAAGGAACGGCATTCAAAACTGC
GTTACTTGAGTGCGGAAGAGGAAAGTGGAATTTCTGGTGTAGCGGTAAAATGCGTAGATA
TCAGAAGGAACACCGGTGGCGAAGGCGACTTTCTGGTCCGCAACTGACGCTGAGACGCGA
AAGCTAGGGGAGCAAACAGGATTAGATACCCGAGTAGTCC
>Otu12465
CCAGCCTACGGGAGGCTCCAGGCGCGAAACCTCTGCAACGCGCAAGCGTGACAGGGGAAG
TCTGAGTGGCGGAAACTTAGTTTCCGTCTTTTGCCAAGTGCAAAAAGCTTGGAGAATAAG
GGTGTGGGCAAACACACCTTATTTATTGGTGACAAACAGTCAATAGGGAGTGGCCTATGA
CTGGTGGCAGCCGCCGCGGTAACACCAGCGCCTCGAGTGGTACCCACGAATATTGGGCCT
AAAGTGTCCGTAGCCGGACTGTTAAGTCCGCTGTGAAATGTTGGGGCTCAACCCTAACGC
GTGCAGTGGATACTGTCAAGTCTTGAGAACGGGGGAGGGTCGGAGTACTTCCAGGGTAGG

GGTAAAATCCGTTGAGCCTGGAAGGACTACCAGTGGCGAAGGCGCCGATCCAAAACGTGT
CCGACGGTGAGGGACGAAAGCTGGGGGAGCGAACGGGATTAGAAACCCGGGTAGTCC
>Otu12468
CCAGCCTACGGGTTGCACCAGTGAGGAATATTGGTCAATGGACGCAAGTCTGAACCAGCC
ATGCCGCGTGAAGGATGAGGGCCTTCTGGGTTGTAAACTTCTTTTATCTGGGAAGAAAAC
TACGATTTCTATTGTAGCCGACGGTACCAGAGGAATAAGCACCGGCTAACTCCGTGCCAG
CAGCCGCGGTAATACGGAGGGTGCAAGCGTTATCCGGATTTACTGGGTTTAAAGGGTGTG
TAGGCGGGTCTTTAAGTCAGTGGTGAAATCTCCGAGCTCAACTTGGAAACTGCCATTGAT
ACTATTGATCTTGAATTTTGTTGAGGTAGGCGGAATATGTCATGTAGCGGTGAAATGCAT
AGATATGACACAGAACACCGATTGCGAAGGCAGCTGACGAAACTGTAACTGACGCTGAGG
CACGAAAGCGTGGGGATCAAACAGGATTAGAACCCCGGTAGTCC
>Otu12474
CCAGCCTACGGGTTGCTCCAGTGAGGAATATTGGACAATGGGAGAAATCCTGATCCAGCA
ATGTTGCTAGGGTGAGAAAGGCTGTAAAGATAAAGGGCCGTAAACACTCTGTTGCCAAAG
AAGATAAAGACGGTATTTGGAAAAGTAGTCCCGGCTAACCTCGTGCCAGCAGCCGCGGTA
ATACGAGGGGGACAAGTGTTATTCGTTCATACTGGGCGTAAAGCGTTTTTAGGTGGTTCT
TTGCGAGTTAGTAACTAAAGTTCAGAGGGCGACCCTGGAATGTTTACTAAGACCATTTTT
CTAGAGTTTTGGAGGGGTGAGTGGTATTTCTTCAATAAAGTTAAAATTTTATGATAGAAG
AAAGAGGTCCACGGGTGAAGACAACTCACTAGTCAAAACTGACACCAAAAGACTAAAGCA
TGGGGAGCGATCGGGATTAGAGACCCGCGTAGTCC
>Otu12477
CCAGCCTACGGGGCGCACCAGTGGGGAATTTTGCGCAATGGGCGAAAGCCTGACGCAGCA
ACGCCGCGTGAGCGAAGAAGGCCTTCGGGTTGTAAAGCTCTGTCAGGGGGAAAGAAGGCA
CCTGGGTTAATAGCCCAAGGGCTGACGGTACCCCCAAAGGAAGCCACGGCTAACTACGTG
CCAGCAGCCGCGGTAATACGTAGGTGGCGAGCGTTGTCCGGAATCATTGGGCGTAAAGAG
CGCGTAGGTGGTTTGTCAGGTCTGATGTGAAATCCTGGGGCTCAACTCCAGAACTGCATC
GGAAACCGGCTTACTAGAGTCTGGGAGAGGAGAGTGGAATTCCTGGTGTAGCGGTGAAAT
GCGTAGATATCGGGAGGAACACCGGTAGCGAAGGCGGCTCTCTGGAACAGTACTGACGCT
GAGACGCGAAAGCGTGGGTAGCAAACAGGATTAGAGACCCCCGTAGTCC
>Otu12479
CCAGCCTACGGGGGGCAGCAGTGAGGAATTTTGCACAATGGGCGAAAGCCTGATGCAGCG
ACGCCGCGTGGACGAAGAAGGCCTTCGGGTTGTAAAGTCCTGTCAGTGGGAACGATAATG
ACGGTACCCGCTGAGGAAGCTCCGGCTGACTCCGTGCCAGCAGCCGCGGTAAGACGGAGG
GAGCAAGCGTTGTCCGGAATCACTGGGCGTAAGGAGCTCGTAGGCGGGGGCTTAAGTCTG
GGAGTAAATCCAACGGCTCAACCGTTGGGTCATCCCGGATACTGGGTCTCTTGAGGACAG
AAGGGGAAAGGGGAATTCCAGGTGTAGCGGTGAAATGCGTAGATATCTGGAGGAACACCG
GTGGCGAAGGCGCCTTTCTGGTCTGATTCTGACGCTGAGGAGCGAAAGCCAGGGGAGCGA
ACGGGATTAGATACCCGTGTAGTCC
>Otu12481
CCAGCCTATGGGAGGCAGCAGTCGAGAATTCTTCACAATGGGCGAAAGCCTGATGGAGCG
ACGCCGCGTGGGGGATGAATGGCTTCGGCCCGTAAACCCCTGTCGTTCGGGATCAATGCG
CTTGACCTAATACGTCAGGCGTTGATAGTACCGGAAGAGGAAGGGACGGCTAACTCTGTG
CCAGCAGCCGCGGTAATACAGAGGTCCCAAGCGTTGTTCGGATTCACTGGGCGTAAAGGG
TGCGTAGGCGGCCAGGTAAGTCTGGTGTGAAATCTCGGAGCTCAACTCCGAAACGGCACT
GGAAACTATCTGGCTCGAGGTTGGGAGGGGGGACTGGAATTCTCGGTGTAGCAGTGAAAT
GCGTAGATATCGAGAGGAACACCAGTGGCGAAGGCGAGTCCCTGGACGACACCTGACACT
GAGGCACGAAAGCTAGGGGAGCAAACAGGATTAGAAACCCTAGTAGTCC
>Otu12491
CCAGCCTATGGGTGGCTGCAGTCGAGAATTTTTCTCAATGGGCGAAAGCCTGAAGGAGCG
ACGCCGCGTGGGGGATGAAGGGCTTCGGCCCGTAAATCCCTGTCATTTGCGAACAAAGCT
TGCGATCTAATAAGTCGCAAGTTGGTAGTAGCGAAAGAGGAAGGGACGGCTAACTCTGTG
CCAGCAGCCGCGGTAATACAGAGGTCCCAAGCGTTGTTCGGATTCACTGGGCGTAAAGGG
TGCGTAGGTGGCGAGGTAAGTTTGATGTGAAATCTCCGGGCTTAACCCGGAAACTGCATT
GAATACTATCTCGCTCGAGGGTTGGAAGGGGGACTGGAATACTTGGTGTAGCAGTGAAAT
GCGTAGATATCAAGTGGAACACCAGTGGCGAAGGCGAGTCCCTGGACAACTCCTGATACT
GAGGCACGAAAGCTAGGGGAGCAGACAGGATTAGAAACCCTTGTAGTCC
>Otu12495
CCAGCCTATGGGTCGCTGCAGACGAGAATATTCGACAATGGGCGAAAGCCTGATCGAGCG

ACGCCGCGTGATGGATGAAGTCCTTCGGGACGTAAACATCTTTTATGGGGGAGGAAGTTA
TTGACGTTACCCCATGAATAAGAGGCTCCTAACTCTGTGCCAGCAGGAGCGGTAATACAG
AGGCCTCAAGCATTATCCGGAATCACTGGGCGTAAAGGGTGTGTAGGCGGTCGTGTTAGT
CTTTCGTGAAAGACCGTGGGCTTAACCCATGGGACGCGGGGGAAACGGCACGACTTCGAC
GACGCGAGAGGTAGAGGGAACTCATGGTGTAGGGGTGAAATCCGTTGATATCATGGGGAA
CACCAAATGCGAAGGCACTCTACTGGCGCGCTCCTGACGCTGAAACACGAAAGCGTGGGA
ATCGAACGGGATTAGAAACCCGTGTAGTCC
>Otu12496
CCAGCCTATGGGATGCAGCAGTCGAGGATCATTCGCAATGGGCGCAAGCCTGACGATGCG
ACGCTGCGTGGAGGATGAAGGCCTTCGGGTTGTAAACTCCTGTCATTCGGGAACAACGAG
CCTGCGGTGAATAGCCGCGGGAAATGATAGTACCGGAAGAGGAAGCCACGGCTAACTCTG
TGCCAGCAGCCGCGGTAATACAGAGGTGGCGAGCGTTGTTCGGATTTACTGGGCGTAAAG
GGTGCGTAGGTGGCTTCGTGTGTCGGATGTGAAAGCTCACTGCTTAACGGTGAAACGGCA
TTCGAAACTGCGGAGCTTGAGTGCAGGAGAGGAGAGCGGAATTCTTGGTGTAGCGGTGGA
ATGCGTTGATATCAAGAAGAACACCGGTGGCGTAGGCGGCTCTCTAGAATGACACTGACA
CTGAGGCACGAAAGTTGGGGGAGCAAACAGGATTAGAGACCCGCGTAGTCC
>Otu12498
CCAGCCTACGGGGGGCAGCAGTCGGGAATTTTGCTCAATGGACGAAAGTCTGAAGCAGCA
ACGCCGCGTGAGGGATGAAGGCCTTCGGGTTGTAAACCTCTTTTATCAGGGACGATAATG
ACGGTACCTGATGAATAAGCCACGGCTAACTACGTGCCAGCAGCCGCGGTAATACGTAGG
TGGCAAGCGTTGTCCGGATTTACTGGGCGTAAAGAGCGCGCAGGCGGTCGTTTAAGTCGA
ATGTGAAAGCCCCCGGCTCAACTGGGGAGGGTCATTCGATACTGATCGACTTGAAGGCAG
GAGAGGGTAGTGGAATTCCCGGTGTAGTGGTGAAATGCGTAGATATCGGGAGGATCACCA
GTGGCGAAGGCGACTACCTGGCCTGTTCTTGACGCTGAGGCGCGAAAGCTGGGGGAGCAA
ACGGGATTAGATACCCCTGTAGTCC
>Otu12500
CCAGCCTATGGGATGCTGCAGTAGGGAATATTGGGCAATGGGCGAAAGCCTGACCCAGCA
ACGCCGCGTGCACGATGAAGGTCTTCGGATCGTAAAGTGCTTTTCTGAGAGATGAGAAAG
GACAGTATCTCAGGAATAAGTCTCGGCTAACTACGTGCCAGCAGCCGCGGTAAAACGTAG
GAGGCAAGCGTTATCCGGATTTACTGGGCGTAAAGCGTGTGCAGGCGGTTTGGTAAGTTG
GATGTGAAAGCTCCTGGCTCAACTGGGAGAGGTCGTTCAATACTACCAGACTTGAGAGTG
GTAGAGGGAGGTGGAATTCCCGGTGTAGTGGTGAAATGCGTAGATATCGGGAGGAACACC
TGTGGCGAAAGCGGCTCACTGGACCACAACTGACGCTGATGCGCGAAAGCTAGGGGAGCA
AACAGGATTAGAGACCCCCGTAGTCC
>Otu12519
CCAGCCTATGGGAGGCACCAGTGGGGAATATTGGACAATGGGCGCAAGCCTGATCCAGCC
ATGCCGCGTGAGTGAAGAAGGCCTTAGGGTTGTAAAGCTCTTTTGGCGGGGACGATAATG
ACGGTACCCACAGAATAAGCTCCGGCTAACTTCGTGCCAGCAGCCGCGGTAATACGAAGG
GAGCTAGCGTTGTTCGGAATTACTGGGCGTAAAGGGCGCGTAGGCGGCTTGATAAGTTGG
GTGTGAAAGCCCAGGGCTCAACCCTGGAACTGCACTCAAGACTATCTTGCTTGAATTCGG
TAGAGGTTGGTGGAATTCCCAGTGTAGAGGTGAAATTCGTAGATATTGGGAAGAATATCC
GTGGCGAAGGCGGCTCACTGGACCGACACTGACGCTGAGGCGCGAAAGCGTGGGGAGCAA
ACAGGATTAGAAACCCTCGTAGTCC
>Otu12521
CCGCCTATGGGGGGCAGCAGTGAGGAATATTGCACAATGCCCGAAAGGGTGATGCAGCGA
CGCCGCGTGAAGGATGAAGGCCCTATGGGTCGTAAACTTCTTTTGAGGGGGAAGAACGTA
TCGCTACAGGCGATATTGACTGTACCCCTAGAAAAAGCATCGGCTAACTACGTGCCAGCA
GCCGCGGTAATACGTAGGATGCGAGCGTTGTCCGGAATCACTGGGTGTAAAGGGAGCGCA
GGCGGTTTAGCAAGTCATTGGTGAAATTCTCAGGCTCAACCTGGGGGCTGCCAATGATAC
TGCTGATCTTGAGTGTGGAAGAGGCGGATGGAATTCCTGGTGTAGCGGTGAAATGCGTAG
ATATCAGGAAGAACACCGATGGCGAAGGCAGTCCACTGGTCCATTACTGACGCTCATGCT
CGAAAGCGTGGGGATCAAACAGGATTAGATACCCCTGTAGTCC
>Otu12527
CCAGCCTATGGGTCGCAGCAGTCGAGAATTTTTCTCAATGGGCGAAAGCCTGACCAAGCG
ACGCCGCGTGGACGATGAAGGCCTTCGGGTTGTAAAGTCCTGTCGAGGGGGATCAAGGGG
CAACCTTGAGTGATCCCTGGAGGAAGCTCGGGCTAAGTTCGTGCCAGCAGCCGCGGTAAG
ACGAACCGAGCGAACGTTGTTCGGAATCACTGGGCTTAAAGGGCGCGTAGGCGGCTTACC
AAGTCAGGGGTGAAATTTTGCGGCTTAACCGTAACAGTGCCTTTGATACCGGTAGGCTCG

AGGGAGATAGGGGTCTGCGGAACTTCTGGTGGAGCGGTGAAATGCGTTGATATCGGAAGG
AACGCCGGTGGCGAAAGCGGCGGACTGGATCTCTTCTGACGCTGAGGCGCGAAAGCTAGG
GGAGCAAACGGGATTAGAAACCCGAGTAGTCC
>Otu12531
CCAGCCTACGGGGGGCTGCAGCTAAGAATCTTCCGCAATGGGCGAAAGCCTGACGGAGCG
ACGCCGCGTGGACGAAGAAGGCCGAAAGGTTGTAAAGTCCTTTTCTCTGTGAAGAATAAG
GGTGGAAGGAAATGTTCACCTGATGACGTTAGCAGAGGAATAAGCCCCGGCCAATTACGT
GCCAGCAGCCGCGGTAATACGTAAGGGGCGAGCGTTGTTCGGAATTATTGGGCGTAAAGG
GCGCGTAGGCGGTCTGGTAAGTCTGGTGTTTAATCCCGCGGCTTAACCGTGGAAGAGCAC
TGGAAACTGTCGGGCTTGAGTTCAGGAGGGGGTACTGGAATTCCAGGTGTAGGGGTGAAA
TCTGTAGATATCTGGAAGAACACCGGTGGCGAAGGCGAGTACCTGGCCATGGACTGACGC
TGAGGCGCGAAAGCGTGGGGAGCAAACAGGATTAGATACCCGAGTAGTCC
>Otu12533
CCAGCCTATGGGAGGCAGCAGTCGAGAATAATTGTCAATGCTCGAAAGAGTGAACATGCG
ACGCCGCGTGCGGGATGAAGGCCCTTGGGTTGTAAACCGCTTTTATAAGGGAAAAATGTA
ATGATGGTACCTTATGAATAAGAGGTTACTAACTCTGTGCCAGCAGTAGCGGTAATACAG
AGACCTCAAGCGTTATCCGGATTTATTGGGCGTAAAGAGCATGTAGGCGGATATATTAGT
CAGACGTCAAATCTTCGAGCTTAACTCGAAAACTGCGTTTGAAACGGTATATCTAGAGGA
AGTGAGAGACCAGTGGAACATATGGTGTAGCAGTGAAATGCGTTGATATCATATGGAACA
CCAAAGGCGAAGGCATCTGGTTGGCACTTTCCTGACGCTGAGATGCGAAAGCGTGGGTAG
CGAATGGGATTAGATACCCGCGTAGTCC
>Otu12538
CCAGCCTACGGGATGCTGCAGTGGGGAATTTTGGACAATGGACGCAAGTCTGATCCAGCC
ATGCCGCGTGAGTGAAGAAGGCCTTCGGGTTGTAAAGCTCTTTTGTCCGGGAGCAAATCC
TGGTTCCTAATACGAACCGGGGATGAGAGTACCGGAAGAATAAGGACCGGCTAACTACGT
GCCAGCAGCCGCGGTAATACGTAGGGTCCAAGCGTTAATCGGAATTACTGGGCGTAAAGG
GTGCGCAGGTGGTTACGCAAGTCTGATGTGAAATCCCCGGGCTCAACCTGGGAACTGCAT
TGGATACTGTGTAACTAGAGTGTGGCAGAGGGGGGTGGAATTCCGCGTGTAGCAGTGAAA
TGCGTAGAGATGCGGAGGAACACCAATGGCGAAGGCAGCCCCCTGGGTTAACACTGACAC
TCATGCACGAAAGCGTGGGGAGCAAACAGGATTAGAAACCCCAGTAGTCC
>Otu12539
CCAGCCTACGGGTGGCAGCAGTGGGGAATCTTGCGCAATGGGCGAAAGCCTGACGCAGCA
ACGCCGCGTGTGTGATGAAGGTCTTCGGATCGTAAAGCACTGTCGGGAGGGACGAATAAG
GGCCGGGTGAACAATCCAGCATGATGACGGTACCTCCAAAGGAAGCACCGGCTAACTCTG
TGCCAGCAGCCGCGGTAATACAGAGGGTGCAAGCGTTGTTCGGAATTATTGGGCGTAAAG
CGCGTGTAGGCGGTCTCGCAAGTCGGATGTGAAAGCCCTCGGCTTAACCGAGGAAGTGCG
TTCGAAACTACGAGACTTGAGTACCGGAGAGGGTGGCGGAATTCCCGGTGTAGAGGTGAA
ATTCGTAGATATCGGGAGGAACACCAGTGGCGAAGGCGGCCACCTGGACGGCTACTGACG
CTGAGACGCGAAAGCGTGGGGAGCAAACAGGATTAGAAACCCCCGTAGTCC
>Otu12546
CCAGCCTATGGGAGGCAGCAGTCGAGAATATTCCACAATGGGGGAAACCCTGACGCAGCA
ACGCCGCGTGGGTGAAGAAGGCCTTCGGGTCGTAAAGCCCTGTCAGGTGGGAAGAAAGGT
TTCAAGGAGAAACACTTGGGATTTGACGGTACCACCAGAGGAAGCACCGGCTAACTCCGT
GCCAGCAGCCGCGGTAATACGGAGGGTGCAAGCGTTATTCGGAATTACTGGGCGTAAAGC
GCGTGTAGGCGGGGATGCAAGTCTGATGTGAAAGCCCTGGGCTTAACCTGGGAAGTGCAT
TGGAAACGGCATCTCTTGAGTACTGGAGAGGAAGGGGGAATTCCCGGTGTAGAGGTGAAA
TTCGTAGAGATCGGGAGGAATACCAGTGGCGTAGGCGCCTTTCTGGACGGTTACTGACGC
TGAGACGCGAAAGCGTGGGGAGCAAACAGGATTAGATACCCTCGTAGTCC
>Otu12564
CCAGCCTATGGGGGGCACCAGTGAGGAATCTTCCGCAATGGGCGAAAGCCTGACGGAGCG
ACACCGCGTGAAGGATGAAGCCCTTTCCGGGCGTAAACTTCTGTTCTGAGGGACGAAATT
TTTGACGGTACCTCAGGAGAAAGCATCGGCTAACCCTGTGCCAGCAGCCGCGGTAAGACA
GGGGATGCAAGCGTTACTCGGAATTACTGGGCGTAAAGCGTCTGCAGGCGTTCTCCCACG
TCTGGCGTCAAATGACGGAGCTTAACTCCGTCAACGTGCCGGAAACGAGGAGAATTGAGT
CATTCAGAGGCATCTGGAATGTCGTGTGTAGGGGTAAAATCCGTTGATCCACGATGGAAC
GCCAAAAGCGAAGGCAGGATGCTGGGGATGTACTGACGCTCAGAGACGAAAGCGTGGGGA
GCAAAGGGGATTAGAAACCCGTGTAGTCC
>Otu12574

CCAGCCTACGGGGTGCACCAGTCGAGAATTTTTCACAATGGGCGCAAGTCTGATGGAGCG
ACGCCGCGTGGACGATGAAGGCCTTCGGGTTGTAAAGTCCTTTCGTGCGGGAAGATTTTT
CCGCCGGTGAACAATCGGCGGGACCGACTGTACCGCAGGAAGAAGCCCCGGCTAATTACG
TGCCAGCAGCCGCGGTAACACGTATGGGGCGAGCGTTATTCGGATTCATTGGGCGTAAAG
GGTATGCAGGGGGCTTGCTAAGTCGGGAGTGAAATTTCCAGGCTCAACCTGGAACTTGCT
TTCGATAATGGCAGGCTAGAGTTTGGAAGAGGAGAATGGAATTCCAGGTGTAGGGGTGAA
ATCTGTAGATATCTGGAAGAACACCAGTGGTGAAGACGATTCTCTGGTCCAAAACTGACC
CTCAGATACGAAAGCCAGGGGAGCAAACGGGATTAGAAACCCTTGTAGTCC
>Otu12582
CCAGCCTACGGGGTGCACCAGTGGGGAATTTTGCGCAATGGGGGAAACCCTGACGCAGCA
ACGCCGCGTGAGTGATGAAGGCCTTCGGGTCGTAAAGCTCTGTCAGTGGGAAAGAAGTGT
ATTGCGGCTAATACCTGCGATACTTGACGGTACCCACAAAGGAAGCACCGGCTAACTCCG
TGCCAGCAGCCGCGGTAATACGGAGGGTGCGAGCGTTGTTCGGATTTATTGGGCGTAAAG
CGCGTGTAGGCGGCTTCTTAAGTCAGATGTGAAAGCCCCGGGCTCAACCTGGGAAGTGCA
TTGGAAACTGGGGAACTTGAATACGGGAGAGGGTAGTGGAATTCCTGGTGTAGGAGTGAA
ATCCGTAGATATCAGGAGGAACACCGGTGGCGAAGGCGGCTACCTGGACCGATATTGACG
CTGAGACGCGAAAGCGTGGGGAGCAAACAGGATTAGAGACCCTAGTAGTCC
>Otu12583
CCAGCCTATGGGTCGCAGCAGTCGAGAATAGTCTACAATGGACGAAAGTCTGATAGTGCG
ACGCCGCGTGAGCGAAGAAACCTTTCGGGGTGTAAAGTTCTTTTCTGTGCGAGCAGTGGT
GGCTAGAGTAATATGCTGGTCATTTTGATATTAGTACAGGAATAAGCCACGGCTAACTCC
GTGCCAGCAGCCGCGGTAACACGGGGGTGGCAAGCGTTGTCCGGAATTACTAGGCGTAAA
GGGCAGGTAGGCGGATTTGTAAGTCGGACGTTAAATCCTCAGTCTCAAGCTGAGACATGC
GTTCGATACTGTGAATCTTGAATATGGTGGGGGAAGACGGAATTCCAGGTGTAGCGGTGG
AATGCGCAGATATCTGGAGGAACACCGAAGGCGAAGGCAGTCTTCTATACCAAAATTGAC
GCTAAACTGCGAAGGTGTGGGGATCAAACAGGATTAGAAACCCCCGTAGTCC
>Otu12585
CCAGCCTACGGGGGGCTCCAGTTAGGAATATTGCACAATGGGCGAAAGCTTGATGCAGCG
ACGCCGCGTGGGGGATGACGTCTTTCGGGATGTAAACCCCTTTTGTCGGGAAAGAACTAA
GACGGTACCCGGCGAATAAGCCCCGGCTAACTACGTGCCAGCAGCCGCGGTAATACGTGG
GGGGCGAGCGTTGTCCGGAATTACTGGGCGTAAAGAGCGTGTAGGCGGTCGGTCAAGTCG
GATGTGAAATCTCCAGGGCTCAACCCGGAAACTGCATTTGATACTGACTGACTTGAGAGA
TGAAGGGGCAAGTGGAATTCCCGGTGTAGCGGTGAAATGCATTGATATCGGGAGGAACAC
CCGTAGTGAAGGCGGCTTGCTGGGCATCTTCTGACGCTGAGACGCGAAAGCAGGGGGAGC
AAACGGGATTAGATACCCGCGTAGTCC
>Otu12600
CCAGCCTACGGGGTGCACCAGTCGAGAATCTTCCGCAATGGGCGAAAGCCTGACGGAGCG
ACGTTACGTGAATGATGAAGCCCTTAGGGGTGTAAAGTTCTTTTATATGGGAGGAAGCCG
CAAGGCAGACAGTACCATATGAATAAGGGGATCCTAATTCTGTGCCAGCAGGAGCGGTAA
TACAGAATCCCCAAGCGTTACCCGGATTTATTGGGCGTAAAGGGTTCGTAGGTGGTATAG
TAAGTTGAAAGTTAAAACTCATCGGCTTAACCTTTGAGGTGCTTTCAATACTGCTACACT
TGAGGGCGTTAGGGGTTAGCGGAACCGACAGAGTAGGGGTGAAATCCGTTGATATTGTCG
GGAACACCAAAAGCGAAGGCAGCTAACTAGGACGACCCTGACACTGAGGAACGAAAGCGT
GGGGAGCAAACAGGATTAGAAACCCCCGTAGTCC
>Otu12602
CCAGCCTATGGGGGGCTGCAGTGGGGAATATTGGACAATGGGGGCAACCCTGATCCAGCC
ATGCCGCGTGAGTGATGAAGGCCTTAGGGTTGTAAAGCTCTTTCGGCGGGGACGATAATG
ACGGTACCCGCAGAAGAAGCCCCGGCTAACTTCGTGCCAGCAGCCGCGGTAATACGAAGG
GGGCTAGCGTTGCTCGGAATCACTGGGCGTAAAGCGCACGTAGGCGGATCTTTAAGTCAG
GGGTGAAATCCCAAGGCTCAACCTTGGAACTGCCTTTGATACTGGGGATCTCGAGTCCGG
AAGAGGTTGGTGGAACTCCGAGTGTAGAGGTGAAATTCGTAGATATTAGGAAGAACACCA
GTGGCGAAGGCGGCCAACTGGCCCGATACTGACGCTCATGTACGAAAGCGTGGGGAGCAA
ACAGGATTAGATACCCTCGTAGTCC
>Otu12623
CCAGCATGGGTCGCTCCAGTGGGGAATATTGGACAATGGGCGCAAGCCTGATCCAGCCAT
GCCGCGTGAGTGATGACGGCCCTAGGGTTGTAAAGCTCTTTCACCCACGACGATAATGAC
GGTAGTGGGAGAAGAAGCCCCGGCTAACTTCGTGCCAGCAGCCGCGGTAATACGAAGGGG
GCTAGCGTTGTTCGGATCTACTGGGCGTAAAGCGCACGTAGGCGGATCTTTAAGTCAGGG

GTGAAATGCCGAGGCTCAACTTCGGAATTGCCTTTGATACTGTCTATCTTCGAGTTCGGG
AGAGGTTGGCGGAATTCCTAGTGTAGAGGTGAAATTCGTAGATATTAGGAAGAACACCAG
TGGCGAAGGCGGCCAACTGGCCCGATACTGACGCTCATGTACGAAAGCGTGGGGAGCAAA
CAGGATTAGAGACCCCTGTAGTCC
>Otu12629
CCAGCCTATGGGGGGCAGCAGTGGGGAATATTGGACAATGGGCGCAAGCCTGATCCAGCC
ATGCCGCGCGAGTAAGAAGGCCTTAGGGTTGTAAAGCCCTTTTGGCGGGGAAGAAGGCCC
TGAGTTGAACAGGCTTGGGGGGTGACGTTACCCGCTGAAGAAGCACCGGCCAACTCCGTG
CCAGCAGCCGCGGTAATACGGAGGGTGCGAGCGTTAATCGGAATTACTGGGCGTAAAGCG
CACGTAGGCGGTGGGTTAAGTCAGTTGTGAAAGCCCCGGGCTCAACCTGGGAATGGCAAT
TGGTACTGACCGACTGGAGTGTAACAGAGGACCGTGGAATTCCCGGTGTAGCGGTGAAAT
GCGTAGAGATCGGGAAGAACACCGATGGCGAAGGCAGCGGTCTGGGTTAACACTGACGCT
GAAGAGCGAAAGCGTGGGGAGCAACCAGGATTAGATACCCTAGTAGTCC
>Otu12631
CCAGCCTATGGGATGCTGCAGTCGGGAAACTTCTGCAATGCGCGCAAGCGTGACAGAGCA
AACCAGAGTGCGTTTCCATATTGGAAACGCTTCTGTGAGATGCAAAAAGTCTTACGAATA
AGGACCGGGCAAGACTGGTGCCAGCCGCCGCGGTAATCCCAGCAGTCCGAGTCGCAGCCA
CATTTGTTGGGTCTAAAACATCAGTAGCTCGCCATCTAAGTCTCTTGTCAAATCGGGCCT
CTTAAGGGTCCGGCGAGCAAGAGATACTGTTTGGCTAGAGACCGGGAGACGTAAGGAGTA
CTATGGGAGTAGCGGTAAAATGCGTTGATCCTCATAGGACTAACAATAGCGAAGGCACCT
TACGATAACGGTTCTGACAGTGAGGGATGAAGGCTAGGGGCGCAAAGTGGATTAGATACC
CCAGTAGTCC
>Otu12638
CCAGCCTATGGGGGGCAGCAGTGAGGAATATTGGACAATGAACGACAGTTTGATCCAGCA
ATACTAAATGGGTGATGAACGCTCAAATGGCCGTAAAACCCTTTCGACGAGGAAGATCAT
GACATTAATCGAGGAAAGAAGTCCTGGCCAATGCCGTGCCAGCAGCCGCGGAAATACGGT
AAGGGCAAGCGTTATTCATAATTAATAGGCGTAAAGGGTGCGTAGGTGGTTTTAAAAAAA
TTTTTGTATAAAAAAACAAAGCGGAACTTTGTAAATTGCAAAAAAATGTTAATTCATGAGT
TAGACTCGCGTTAATAGAATATCTAAAGTAATGATAAAATGTTGCGAGATTAGATGGAAT
ACCGAAAGCGAAGGCAGTTTTCTAGAGCTAACTGACATTGAGGCACGAAGGCATAGGGAT
CAAATGGGATTAGAGACCCTTGTAGTCC
>Otu12646
CCAGCCTACGGGAGGCAGCAGTAAGGAATATTGGTCAATGGACGCAAGTCTGAACCAGCC
ATGCCGCGTGAAGGATGAAGGCCTTCTGGGTTGTAAACTTCTTTTATCTGGGAAGAAAAC
TACGGTTTCTACTGTAGCCGACGGTACCAGAGGAATAAGCACCGGCTAACTCCGTGCCAG
CAGCCGCGGTAATACGGAGGGTGCAAGCGTTATCCGGATTTACTGGGTTTAAAGGGTGTG
TAGGCGGGTCTTTAAGTCAGTGGTGAAATCTCCGAGCTCAACTTGGAAACTGCCATTGAT
ACTATTGATCTTGAATTCTGTTGAGGTGGGCAGAATATGTCATGTAGCGGTGAAATGCTT
AGATATGACATAGAACCCCAATTGCGAAGGCAGCTCACTAACCAGACATTGACGCTGAGG
CACGAAAGCGTGGGGATCAAACAGGATTAGAAACCCTCGTAGTCC
>Otu12647
CCAGCCTACGGGGCGCTGCAGTCAAGAACATTCGACAATGGGCGAAAGCCTGATCGAGCG
ACGCCGCGTGCAGGATGAAGGTCTTCGGATCGTAAACTGCTTTTATGTTTTAGAAAGTTA
TTGATCGAGACATGAATAAGGAGCTGCAAACTTCGTGCCAGCAGCCGCGGTAATACGAAG
GCTCCAAGCGTTATCCGGATTTATTGGGCGTAAAGGGCGCGTAGGGGGTTTTGTGTGTTT
CCGGTTAAAGACCGAGGCTTAACTTCGGTATCGCCGGGAAAACTACATAACTAGAGGGTG
TTAGAGGTCTGTAGAACGCACGGTGTAGGGGTGAAATCCGTTGATATCGTGCGGAATACC
AAAGGCGAAGGCAGCAGACTGGGACATTCCTGACCCTGAGGCGCGAAAGCGTGGGGAGCA
AAAAGGATTAGATACCCTCGTAGTCC
>Otu12663
CCAGCCTATGGGATGCAGCAGTCGGGGATCTTTTGCAATGCGCGCAAGCGTGACAAAGCG
AGCCAAAGTGTTTTTCTTTTTAGAAAAACTTTTGCCAGCTGTAAAAAGGCTGGCGAATAA
GGACTGGGCAAGACCGGTGCCAGCCGCCGCGGTAATCCCGGCAGTCCAAGTCGCAGCCAC
ATTTATTGGGTCTAAAATATCCGTAGCTTGCTTAAAAAGTTTCTTGTGAAATTCTGTCTC
TTAAGGTCAGAGCGCGCAAGAAATACTCTTAAGCTAGAGACCGGTAGGCGTAAGAAGTAC
AGTCAGGGTAGTGGTAAAATATTTTAATCCTGATTGGACTCACAGTCGCGAAGGCGTCTT
ACGAGGACGGTTCTGACAGTGAGGGATGAAGGCTAGGGGCGCAAAATGGATTAGAAACCC
TCGTAGTCC

>Otu12673
CCAGCCTATGGGACGCAGCAGTGAGGAATATTGGTCAATGGACGGAAGTCTGAACCAGCC
ATGCCGCGTGCAGGAAGAAGGCCCTATGGGTCGTAAACTGCTTTTGTACCAGAGAAAACC
CGAGTACGTGTACTCGGTTGATAGTATGGTAAGAATAAGCATCGGCTAACTTCGCGCCAG
CAGCCGCGGTAAGACGAAGGATGCAAGCGTTATCCGGATTCATTGGGTTTAAAGGGTGCG
TAGGCGGACTTATAAGTCAGTGGTGAAATCTCGTCGCTTAACGACGAACGTGCCATTGAT
ACTGTAGGTCTTGAGTACAGATGCCGTTGGCGGAATGTGTCATGTAGCGGTGAAATGCAT
AGATATGACACAGAACACCGATTGCGAAGGCAGCTGACGAAACTGTAACTGACGCTGAGG
CACGAAAGCTGGGGGAGCAAACCGGATTAGAAACCCGTGTAGTCC
>Otu12674
CCAGCCTATGGGGGGCACCAGTCGAGAATTTTTCACAATGGGGGAAACCCTGATGGAGCG
ACGCCGCGTGGGGGATGAATGGCTTCGGCCCGTAAACCCCTGTCATTTGCGAACAAATTT
TGCGCATGAACAATGCGCAAATTGATTGTAGCGGAAGAGGAAGGGACGGCTAACTCTGTG
CCAGCAGCCGCGGTAATACAGAGGTCCCAAGCGTTGTTCGGATTCACTGGGCGTAAAGGG
TGCGTAGGTGGCAAGGTAAGTCTGATGTGAAATCTCGCGGCTTAACCGCGAAACGGCATC
GGATACTATTTAGCTAGAGGGTCGGAGGGGGGACTGGAATTCTCGGTGTAGCAGTGAAAT
GCGTAGATATCGAGAGGAACACCAGTGGCGAAGGCGAGTCCCTGGACGACTCCTGACACT
GAGGCACGAAAGCTAGGGGAGCAAACAGGATTAGATACCCTAGTAGTCC
>Otu12679
CCAGCCTATGGGTTGCTGCAGTGGGGAATTTTGGACAATGGACGCAAGTCTGATCCAGCC
ATTCCGCGTGCAGGATGAAGGCCTTCGGGTTGTAAACTGCTTTTGTACGGAACGAAAAGG
CTCTGATTAATACTCGGGGCTCATGACGGTACCGTAAGAATAAGCACCGGCTAACTACGT
GCCAGCAGCCGCGGTAATACGTAGGGTGCAAGCGTTAATCGGAATTACTGGGCGTAAAGC
GTGCGCAGGCGGTTATATAAGACAGATGTGAAATCCCCGGGCTCAACCTGGGAACTGCAT
TTGTGACTGTATAGCTAGAGTACGGTAGAGGGGGATGGAATTCCGCGTGTAGCAGTGAAA
TGCGTAGATATGCGGAGGAACACCGATGGCGAAGGCAATCCCCTGGACCTGTACTGACGC
TCATGCACGAAAGCGTGGGGAGCAAACAGGATTAGAGACCCTCGTAGTCC
>Otu12680
CCAGCCTATGGGACGCAGCAGTCGAGAATTTTTCACAATGGGGGCAACCCTGATGGAGCG
ACGCCGCGTGGAGGATGAAGGCCTTCGGGTCGTAAACTCCTGTCACTACAGAACAAGAGT
ATAACAGCTAACATCTGTTATGCTTGATAGTATGTGGAGAGGAAGGGACGGCTAACTCTG
TGCCAGCAGCCGCGGTGATACAGAGGTCCCAAGCGTTGTTCGGATTTACTGGGCGTAAAG
GGTGCGTAGGAGGCTGGGAAAGTCGGATGTGAAAGCTCACTGCTTAACGGTGAAACTGCA
TTCGAAACTTCCTGGCTAGAGGATCAGAAAGGAAAGCGGAATTCTTGGTGTAGCGGTGAA
ATGCGTAGATATCAAGAGGAACGCCGATGGCGAAAGCAGCTTTCTGGAGGATTCCTGACT
CTGAGGCACGAAGGCTAGGGTAGCAAACAGGATTAGATACCCCCGTAGTCC
>Otu12686
CCAGCCTATGGGACGCTGCAGTGAGGAATATTGCGCAATGAGCGAAAGCCTGACGCAGCA
ACGCCGCGTGAAGGATGAAGGTGCTCTGCATTGTAAACTTCTGTAGGGGGGGACGAATAT
CCCGGTATTGCCGGGAATGACGGTACCCCCAAAGTAAGCACCGGCTAACTCCGTGCCAGC
AGCCGCGGTAATACGGAGGGTGCAAGCGTTGTCCGGATTTATTGGGTGTAAAGGGCGTGT
AGGCGGAGTGATGTGTCGAGAGTGAAATCGTGCGGCTTAACCGTATCAATTGCTCTCGAA
ACTGTCACCCTTGAGTACGAGAGGGGCAGATGGAATTCCGGGTGTAGCGGTGGAATGCGT
AGATATCTGGAAGAACACCGGTGGCGAAGGCGGTCTGCTGGCTCGATACTGACGCTGAGG
CACGAAAGCCAGGGGAGCAAACGGGATTAGAAACCCCAGTAGTCC
>Otu12687
CCAGCCTACGGGATGCTGCAGTGAGGAATTTTCCGCAATGGGCGAAAGCCTGACGGAGCG
ACGCCGTGTGGGTGATGAAGGTTTTCGGATCTTAAAGCCCTGTCAGCGAGAACGATATTG
ACGGTACTCGCAGAGGAAGCCCCGGCTAACTACGTGCCAGCAGCCGCGGTAATACGTAGG
GGGCGAACGTTGTCCGGAATTATTGGGCGTAAAGCGCATGTAGGCGGTCAGGTAAGTCAG
GTGTCAAATCCTTTCGCTCAACGAAGGGCCGCACTTGATACTGCTTGGCTAGAGAGATGT
AGAGGAAAGCGGAACTTCCGGTGTAGCGGTGAAATGCGTAGATATCGGAAGGAACACCAG
TGGCGAAGGCGGCTTTCTGGTCATTTTCTGACGCTGAGATGCGAAAGCAAGGGGAGCAAA
CGGGATTAGAGACCCGGGTAGTCC
>Otu12700
CCAGCCTACGGGTGGCAGCAGTCAAGAATTTTCCTCAATGGCCGAAAGGCTGAAGGAGCG
ACGCCGCGTGTAGGATGAAGGTCTTCGGATTGTAAACTACTTTTATAGGGGACGAACTAG
TGACGGTACCCTAAGAATAAGAGGTTGCTAACTCTGTGCCAGCAGCAGCGGTAATACAGA

GACCTCAAGCGTTATCCGGATTTATTGGGCGTAAAGCGTCCGCAGATGGTTTGGCGGGTG
AGGAGTTAAAATCCATAGCTTAACTATGGAACTGCTTCTCAAACTACCAAACTCGAGGGT
GGGAGAGGTAAGCGGAATTCTCGGTGTAGTCGTAATAAGCGCTGATATCGAGAAGAACAC
CAAATGCGAAGGCAGCTTACTGGAACACTCCTGACATTCAGGGACGAAAGCGTGGGGAGC
AAACAGGATTAGAGACCCTTGTAGTCC
>Otu12701
CCAGCCTACGGGGCGCTCCAGTGGGGAATATTGGACAATGGGGGAAACCCTGATCCAGCG
ACGCCGCGTGTGTGAAGAAGGCCTTCGGGTTGTAAAGCACTTTTGTCCGGAAAGAAATCC
CTGGTCCTAATATGGCCGGGGGATGACGGTACCGGAAGAATAAGCACCGGCTAACTACGT
GCCAGCAGCCGCGGTAATACGTAGGGTGCAAGCGTTAATCGGAATTACTGGGCGTAAAGC
GTGCGCAGGCGGTGATGTAAGACCGATGTGAAATCCCCGGGCTCAACCTGGGAACTGCAT
TGGTGACTGCATCGCTTGAGTATGGCAGAGGGGGGTAGAATTCCACGTGTAGCAGTGAAA
TGCGTAGAGATGTGGAGGAATACCGATGGCGAAGGCAGCCCCCTGGGTCAATACTGACGC
TCATGCACGAAAGCGTGGGGAGCAAACAGGATTAGAAACCCCCGTAGTCC
>Otu12702
CCAGCCTATGGGAGGCACCAGTCGAGAATTTTTCACAATGGGGGAAACCCTGATGGAGCG
ACGCCGCGTGGGGGATGACTGGCTTCGGCCCGTAAACCCCTGTCATTTGTGAACAAACCT
ATCCACCTAACACGTGGAGAGTTGATAGTAACGGAAGAGGAAGGGACGGCTAACTCTGTG
CCAGCAGCCGCGGTAATACAGAGGTCCCAAGCGTTGTTCGGATTCACTGGGCGTAAAGGG
TGCGTAGGTGGCCAGGTAAGTTTGATGTGAAATCTCGAAGCTTAACTTCGAAACTGCATT
GAATACTATTTGGCTGGAGGGTCGGAGGGGGGACTGGAATTCTCGGTGTAGCAGTGAAAT
GCGTAGATATCGAGAGGAACACCAGTGGCGAAGGCGAGTCCCTGGACGACTCCTGACACT
GAGGCACGAAAGCTAGGGGAGCAAACAGGATTAGAGACCCTCGTAGTCC
>Otu12708
CCAGCCTACGGGGGGCAGCAGTGGGGAATTTTGCGCAATGGGGGAAACCCTGACGCAGCA
ACGCCGCGTGGAGGATGAAGCCCCTTGGGGTGTAAACTCCTTTCGATCGGGACGATAATG
ACGGTACCGGAAGAAGAAGCCCCGGCTAACTTCGTGCCAGCAGCCGCGGTAATACGAGGG
GGGCAAGCGTTGTTCGGAATTATTGGGCGTAAAGAGCACGTAGGAGGTTTCGCGCGTCTT
TTGTTAAAGCCCAGGGCCTAACCCTGGAAGTGCAGGAGATACGGCGGAACTAGAGGAGGT
TAGAGGTGCATGGAACTCACGGTGTAGGGGTGAAATCCGTTGATATCGTGGGGAACACCA
AAGGCGAAGGCAGTGCACTGGGACCTTCCTGACTCTGAGATGCGAAAGCGTGGGGAGCAA
AAAGGATTAGAAACCCCCGTAGTCC
>Otu12709
CCAGCCTATGGGTGGCAGCAGGCGAGAATATTCCGCAATGGACGCAAGTCTGACGGAGCG
ACGCCGCGTGATGGATGGAGTGCTTCGGTACGTAAACATCTTTTATCGGGGAAGAACTAA
TTGACGGTACCCGATGAATAAGGACCTCCTAACTCTGTGCCAGCAGGAGCGGTAATACAG
AGGGACCGAGCGTTACCCGGAATCACTGGGCGTAAAGGGTGTCCAGGCGGCCTTGTTAGT
CTCGCGTAAAATCTGTGGGCTCAACCTACAGCGCGCGCGGGAAACGGCAAGGCTCGAGGG
CGCGAGAGATAGAGGGAACTCATGGTGGAGGGGTGAAATCCGTTGATATCATGGGGAACA
CCAAAGGCGAAGGCACTCTATTGGCGCGTTCCTGACGCTCACACACGAAAGCCAGGGTAG
CGAACGGGATTAGAGACCCTCGTAGTCC
>Otu12715
CCAGCCTACGGGGGGCTCCAGTGGGGAATTTTGGACAATGGGGGAAACCCTGATCCAGCA
ACTCTGCGTGAGGGACGAAGCCCTTCGGGGTGTAAACCTCTTTTATCTGGGACGAACGCC
AGCAATGGTTTGACGGTACCAGGTGAATAAGCAACGGCTAACTACGTGCCAGCAGCCGCG
GTAAGACGTAGGTTGCAAGCGTTATTCGGAATTACTAGGCGTAAAGCGAGTGTAGGCGGG
TGCTTAAGTCCGTCGTGAAATCTCCTGGCTCAACTGGGAGGGGTCGATGGATACTGGGCG
TCTTGAGTGGAGTAGGGGGCAGTGGAATTCCCGGTGTAGCGGTGAAATGCGTAGATATCG
GGAGGAACACCTATGGCGAAAGCAGCTGCCTGGGCTCTTACTGACGCTGAGGCTCGAAAG
CTAGGGGAGCAAACAGGATTAGATACCCCCGTAGTCC
>Otu12718
CCAGCCTATGGGGTGCAGCAGTAACGAATCTTCCGCAATGCACGAAAGTGTGACGGAGCG
ACGCCGCGTGTGGGACGAAGTTCTTCGGAATGTAAACCACTGTCAGGGGTTAGAAAGTTC
TGATCAACCCCAGAGGAAGGCACGGCTAACTCTGTGCCAGCAGCCGCGGTAATACAGAGG
TGCCAAGCGTTAGGCGGAATCACTGGGCTTAAAGCGTGTGTAGGCGGTCCTTTTAGTACC
TTGTGAAATCCCATGGCTCAACCATGGAACTGCTTGGTATACTGGGAGACTTGAGCCACT
TAGGGGTCAGCGGAACAAATGGTGGAGCGGTGAAATGCGTAGATATCATTTGGAACGCCG
ATGGTGAAAACAGCTGACTGGGAGTGTGCTGACGCTGAGACACGAAAGCCAGGGTAGCGA

ACGGGATTAGAAACCCTCGTAGTCC
>Otu12731
CCAGCCTATGGGGGGCAGCAGTAGGGAATTTTCCACAATGGACGAAAGTCTGATGGAGCA
ACGCCGCGTGCAGGATGAAGGCCTTAGGGTTGTAAACTGCTTTTCTCTGTGACGATTATG
ACGGTAGCAGAGGAATAAGGATCGGCTAACTCCGTGCCAGCAGCCGCGGTCATACGGAGG
ATCCAAGCGTTATCCGGAATTACTGGGCGTAAAGAGTTGCGTAGGTGGCATAGTAAGCAA
GTAATGAAATCGTGTGGCTCAACCATGCTCACATTATTTGAACTGCTAAGCTAGAGAACG
GGAGAGGTAGATGGAATTCCCAGTGTAGGAGTGAAATCCGTAGATATTGGGAGGAACACC
GATGGCGTAAGCAGTCTACTGGCCTGTTTCTGACACTAAGGCACGAAAGCGTGGGGAGCA
AACGGGATTAGAAACCCGGGTAGTCC
>Otu12738
CCAGCCTACGGGGCGCAGCAGTGGGGAATCTTGCGCAATGGGCGAAAGCCTGACGCAGCA
ACGCCGCGTGAGGGATGAAGGCCTTCGGGTTGTAAACCTCTTTCAAGCAGGGACGATTGT
GACGGTACCTGCAGAAGAAGCTCCGGCCAACTACGTGCCAGCAGCCGCGGTAATACGTAG
GGAGCGAGCGTTGTCCGGAATCATTGGGCGTAAAGAGCTCGTAGGCGGCTCGGTAAGTCG
GATGTGAAACCTCCAGGCTCAACCTGGAGTCGCCATCCGATACTGCCGTGGCTAGAGTCC
GGTAGGGGCCCACGGAACTCCTGGTGTAGCGGTGAAATGCGCAGATATCAGGAAGAACAC
CGGTGGCGAAGGCGGCCAACTGGACCGACATTGACGCTGAGGCGCGAAAGCGTGGGGAGC
AAACAGGATTAGATACCCTCGTAGTCC
>Otu12750
CCAGCCTATGGGGTGCACCAGTGGGGAATCTTGCGCAATGGACGAAAGTCTGACGCAGCC
ACGCCGCGTGAGTGAAGAAGGCCTTCGGGTTGTAAAGCTCTGTCGGAGGGGACAAAAACC
TCTTAGGTTAATAGCCTAGGAGCTTGATGGTACCCTTAAAGGAAGCACCGGCTAACTTCG
TGCCAGCAGCCGCGGTAATACGAAGGGTGCAAGCGTTGCTCGGAATTATTGGGCGTAAAG
GGTAGGTAGGTGGTGACAAAAGTCTAGGGTGAAATCCCTGAGCTCAACTCAGGACGTGCC
TTGGAAACTTTGTCACTAGAGTGCTAGAGAGGTTCGCAGGATTCCCGGTGTAGCGGTGAA
ATGCGTAGAGATCGGGAGGAATACCAGAGGCGAAGGCGGCGAACTGGATAGCAACTGACA
CTAAACTACGAAAGCGTGGGGAGCAAACAGGATTAGAAACCCCCGTAGTCC
>Otu12763
CCAGCCTATGGGGGGCTCCAGTGGGGAATTTTCCACAATGGGCGAAAGCCTGATGGAGCA
ACGCCGCGTGTGTGATGAAGGTCTTCGGATCGTAAAGCACTTTCGACCGGGACAAAAACG
CCATGGCTAACATCCATGGAACTGACGGTACCGGGAGAAGAAGCACCGGCTAACTCTGTG
CCAGCAGCCGCGGTAATACAGAGGGTGCAAGCGTTGTTCGGAATTATTGGGCGTAAAGCG
CGTGTAGGCTGCTTGATAAGTCACATGTGAAATCCCTCGGCTTAACCGAGGAAGTGCGTG
TGAAACTGTCGAGCTTGAGTACCGGAGAGGATGGCGGAATTCTGCAAGTAGAGGTGAAAT
TCGTAGATATGCAGAGGAACACCGGTGGCGAAGGCGGCCATCTGGACGGTAACTGACGCT
GAGACGCGAAAGCGTGGGGAGCAAACAGGATTAGAGACCCCGGTAGTCC
>Otu12765
CCAGCCTACGGGTGGCTCCAGTCGAGAATCTTCCACAATGGGCGCAAGCCTGATGGAGCG
ACGCCGCGTGGTTGATGAAGTCCTTCGGGACGTAAAAACCTTTTATGAGGGAGGAAGTTA
TTGACGTTACCTCATGAATAAGGGGCTCCTAACTCTGTGCCAGCAGGAGCGGTAATACAG
AGGCCCCAAGCATTATCCGGAATCACTGGGCGTAAAGGGTGTGTAGGCGGTCGCGTTAGT
CGTTTGTGAAAGACCTTGGGCCCAACCCAGGGGACGCAACCGAAACGGCGCGACTTCGAG
GACGCGAGAGGTCTGGGGAACTCATGGTGTAGGGGTGAAATCCGTTGATATCATGGGGAA
CACCAAATGCGAAGGCACCAGACTGGCGCGCTCCTGACGCTGAAACACGAAAGCGTGGGA
ATCGAACGGGATTAGATACCCGCGTAGTCC
>Otu12770
CCAGCCTACGGGGGGCAGCAGCCGAGAATATTCGACAATGGACGAAAGTCTGATCGAGCG
ACGCCGCGTGCGGGATGACGTGCTTCGGCATGTAAACCGCTTTTGCCGGGGAAAAAGTTT
ATTGATGGTACCCGGCGAATAAGGAGTTGCTAAACTCGTGCCAGCAGCAGCGGTAATACG
AGTGCTCCAAGCGTTATCCGGAATTATTGGGCGTAAAGGGTGTGTAGGCGGTGACGTTAG
TCCTCTGTTAAATTCTTCGGCTTAACCGGGGGTCTGCGGAGGAGACGGCATCACTTGAGG
ATGCGAGAGGTATGCGGAACTCATGGTGTAGGGGTGAAATCCGTTGATATCATGGGGAAC
ACCAAAAGCGAAGGCAGCATACTGGCGCACTCCTGACGCTGAAACACGAAAGCGTGGGTA
GCGAATGGGATTAGAAACCCGCGTAGTCC
>Otu12779
CCAGCCTATGGGAGGCAGCAGTGGGGAATTTTGCGCAATGGGCGAAAGCCTGACGCAGCG
ACGCCGCGTGGAGGATGAAGGTTCTAGGATTGTAAACTCCTGTTAAGTGGGAAGAAAAAC

CTGTTCCTAATATGAACAGGGCATGACTGTACCATTAGAGAAAGCACCGGCTAACTTCGT
GCCAGCAGCCGCGGTAATACGAGGGGTGCAAGCGTTATTCGGAATAATTGGGCGTAAAGG
GTGTGTAGACGGCTTATTAAGTCAGTTGTTAAATCTTCCGGCCTAACTGGGAGCTCGCGA
TTGAAACTGGTATTGCTAGAGGGTGGAAGAGAGAAGTGGAATTCTCGGAGTAGCGGTAAA
ATGCGTAGATCTCGAGAGGAACACCGATGGCGAAGGCAGCTTCTTGGTCCATTTCTGACG
TTGAAACACGAAAGCGTGGGGAGCAAACAGGATTAGAAACCCCTGTAGTCC
>Otu12782
CCAGCCTATGGGGGGCAGCAGTGGGGAATCTTGCACAATGGAGGAAACTCTGATGCAGCG
ACGCCGCGTGAGCGATAAAGCCCCTCGGGGTGTAAAGCTCTTTCGGCAGGGAAGATAATG
ACGGTACCTGCAGAAGCAGCTGCGGCTAACTACGTGCCGGCAGCCGCGGTAATACGTAGG
CAGCAAGCGTTGTTCGGAGTTACTGGGCGTAAAGGGTGCGTAGGCGGCTTTTTAAGTTTG
GTGTGAAATCTCCCGGCTCAACTGGGAGGGTGCGCCGAATACTGAGAGGCTAGAGTGTGG
GAGAGGAAAGTGGAATTCCTGGTGTAGCGGTGAAATGCGTAGATATCAGGAAGAACACTA
GTGGCGTAGGCGACTTACTGGGCCATAACTGACGCTGAGGAACGAAAGCCAGGGGAGCAA
ATGGGATTAGAGACCCCTGTAGTCC
>Otu12784
CCAGCCTATGGGGGGCACCAGTGGGGAATATTGCGCAATGGCCGAAAGGCTGACGCAGCG
ACGCCGCGTGTGGGGTGACGCCCTTCGGGGTGTAAACCACTGTTGCCCGGGACGAACGGG
CGGATTATTCCGCCGTGACGGTACCGGGTGAGGAAGCACCGGCTAACTCCGTGCCAGCAG
CCGCGGTAATACGGAGGGTGCGAGCGTTGTCCGGAATCACTGGGCGTAAAGGGCGCGTAG
GCGGCTTGGTAAGGGGGCGGTGAAAGCCCGGGGCTCAACCCCGGGTCTGCCGGACCGACT
GCCGAGCTGGAGCACACTAGAGGCGAGTGGAATTCCGGGTGTAGCGGTGGAATGCGTAGA
GATCCGGAAGAACACCGGTGGCGAAGGCGGCTCGCTGGGGTGTGGCTGACGCTGAGGCGC
GACAGCGTGGGGAGCAAACAGGATTAGAAACCCTTGTAGTCC
>Otu12795
CCAGCCTACGGGTTGCAGCAGTCGAGGATCTTCGGCAATGGGCGCAAGCCTGACCGAGCG
ACGCCGCGTGTGGGATGAAGGCCCTTGGGTTGTAAACCACTGTCAGAGGGGATGAAGTGC
CGGGAGGCTATCCTTCCGGCTTGACATAGCCTCAGAGGAAGCACGGGCTAAGTTCGTGCC
AGCAGCCGCGGTAATACGAACTGTGCAAACGTTATTCGGAATCACTGGGCTTAAAGGGTG
CGTAGGCGGCTGTCTAAGTAGGGTGTGAAAGCCCCCGGCTCAACCGGGGAATTGCGCCCT
AAACTGGATGGCTGGAGTGAGGCAGGGGTGTGTGGAACTTCCGGTGGAGCGGTGAAATGT
GTTGATATCGGAAGGAACGCCGGTGGCGAAAGCGACACACTGGGTCTCAACTGACGCTGA
GGCACGAAAGCCAGGGGAGCAAACGGGATTAGAAACCCGAGTAGTCC
>Otu12803
CCAGCCTACGGGAGGCACCAGTCGAGAATCTTCCGCAATGGGCGAAAGCCTGATCGAGCG
ACACCGCGTGCAGGATGAAGGCCTTCGGGTCGTAAACTGCGGTAGATAAGTGACAATGCA
AATGAGTGCTTATCGGAAATAGGTGGGTAACTACGTGCCAGCACCAGCGGTAAAACGTAG
ACCTCAAGCGTTATCCGGATTTATTGGGCGTAAAGCGCATGTGGGTGGTTTCGCGCGTCT
TCTGCTAAAGCCCACCGCCCAACGGTGGAAGTGCAGGAGATACGGCGAGACTAGAGGAGG
TTAGAGGTGCATAGAACGCACGGTGTAGGGGTGAAATCCGTTGATATCGTGCGGAATACC
AAAGGCGAAGGCATTGCACTGGGACCTTCCTGACATTGAGATGCGAAAGCGTGGGGAGCA
AAAAGGATTAGATACCCGCGTAGTCC
>Otu12806
CCAGCCTACGGGAGGCACCAGTGGGGAATATTGGACAATGGGGGAAACCCTGATCCAGCG
ATGCCGCGTGTGTGAAGAAGGCCTGAGGGTTGTAAAGCACTTTCAGTGGGGAGGATAGTT
TGCAGGTTAAGAGCTGGCAAGCGGGACGTTACCCACAGAAGAAGCACCGGCTAACTCCGT
GCCAGCAGCCGCGGTAATACGGAGGGCGCGAGCGTTAATCGGAATTACTGGGCGTAAAGG
GTGCGCAGGTGGTTTGGCAAGATATCTGTGAAATCCCCGGGCTTAACCTGGGAGCGTCAG
ATAAGACTGCGAGACTCGAGTACAGGAGAGGGCAGTGGAATTTCCGGTGTAGCGGTGAAA
TGCGTAAAGATCGGAAGGAACACCGGTGGCGAAGGCGGCTGCCTGGCCTGATACTGACAC
TGAGGCACGAAAGCGTGGGGAGCAAACAGGATTAGAGACCCGAGTAGTCC
>Otu12807
CCAGCCTATGGGGTGCTGCAGTGGGGAATCTTGCGCAATGGGCGAAAGCCTGACGCAGCG
ACGCCGCGTGGGGGATGAAGCTTCTCGGAGTGTAAACCCCTTTCGACCCGGACGAAACGC
CCGCAAGGGCTTGACGGTACGGGTATAAGAAGCCCCGGCTAACTACGTGCCAGCAGCCGC
GGTAATACGTAGGGGGCCAGCGTTGCTCGGAATTACTGGGCGTAAAGGGTTCGTAGGCGG
TGCGGCAAGTCGGGAGTGAAATCTCTGGGCTCAACTCAGAGGCTGCTTCCGAAACTGCCG
CGCTAGAGTGCGGGAGGGGCCAGTGGAATTGCGGGTGTAGCGGTGAAATGCGTAGATATC

CGTAGGAACATCCGAGGCGAAAGCGGCTCGCTGGATCACAACTGACGCTGAGGGACGAAA
GCTAGGGGAGCAAACAGGATTAGATACCCTAGTAGTCC
>Otu12815
CCAGCCTATGGGGCGCACCAGTCGAGAATCTTCCGCAATGGACGAAAGTCTGACGGAGCG
ACGCCGCGTGATTGATGAAGCTTTTCGGAGTGTAAAGATCTTTTATGAGGGAAGAAGTTT
ATTGACGGTACCTCATGAATAAGGGGCTCCTAATCTCGTGCCAGCAGGAGCGGTAATACG
AGAGCCCCGAGCGTTATCCGGAATTATTGGGCGTAAAGGGTGCGTAGGTGGCACTGTTAG
TCGTTTGTCAAATCTTCCCGCTTAACGAGAAATCCGCGGACGAAACGGCAGAGCTAGAGA
GTGTGAGGGGTGAATGGAACTCATGGTGTAGGGGTGAAATCCGTTGATATCATGGGGAAC
ACCAAATGCGAAGGCAGTACACTGGCGCATATTTGACACTGAAGCACGAAAGCGTGGGTA
GCGAATGGGATTAGATACCCCAGTAGTCC
>Otu12820
CCAGCCTACGGGGTGCACCAGTCGAGAATCTTCCGCAATGGGCGAAAGCCTGACGGAGCG
ACGCCGCGTGACTGATGAAGTCCTTCGGGACGTAAAGGTCTTTTGTGAGGTAGAAAGTAA
TTGATCGCCTCAAGAATAAGGGGTTGCTAAACTCGTGCCAGCAGCAGCGGTAATACGAGT
GCCCCAAGCGTTATCCGGAATTATTGGGCGTAAAGGGTGCGTAGGCGGTTATATTAGTCT
TTTGTTAAAGCTTCCGGCTCAACCGGAAAAATGCAATTGATACGGTATAACTAGAGGGTG
TGAGAGGTGTACAGAACTCATAGTGTAGGGGTGAAATCCGTTGATATTATGGGGAATACC
AAAAGCGAAGGCAGTACACTGGCACATTCCTGACGTTGAGGCACGAAAGCGTGGGTAGCG
AATGGGATTAGATACCCTAGTAGTCC
>Otu12821
CTAGCCTATGGGTGGCTGCAGTGGGGAATATTGGACAATGAGCGAAAGCTTGATCCAGCA
ATACCTCATGGAGGACGAAGGTTTATTGATTGTAAACTCCTTTTAGTAAAGAAAATTGTG
ATTATATTTACAGAATAAGCCCCGGCTAATTCCGTGCCAGCAGCCGCGGTAATACGGGAG
GGGCTAGTGTTATTCGGAATGACTGGGCGTAAAGGGCATCTAGGTGGATTTTTACGTTTT
TTAAGAAATCTCATGGCTTAACTAATGAAACTTAAAAAAAACGATTAATCTTGAGTATGG
CAGAGGGTAACAGAATTCTTAGTGTAACAGCAATATGTGTGAATATTAAGAGGACTGCCA
AAGCGAAGGCGGTTATCTGGACCATTACTGACGCTGAAATGCGAAAGCATGGGGATCAAA
TCGGATTAGATACCCGCGTAGTCC
>Otu12824
CCAGCCTACGGGATGCTGCAGTCGAGAATCTTTCGCAATGGGCGAAAGCCTGACGAAGCG
ACGCTGTGTGGACGATGAAGGCCTTCGGGTTGTAAAGTCCTTTCGCGTAAGAACAAGAGA
GACCTTCTAACACAAGGTTGATTTGAGGGTACTACGTAAAGAAGCACCGGCTAACTCCGT
GCCAGCAGCTGCGGTAATACGGAGGGTGCAAGCATTAATCGGATTTACTGGGCGTAAAGG
GCGCGTAGGCGGGTCTACAAGTCAAATGTGAAATTCCGGAGCTCAACTTCGGAGCGGCAT
TTGAAACTGTAGGTCTATAGAGGGATGGCGGAGAAAACGGAATTCCACGTGTAGCGGTGA
AATGCGTAGATATGTGGAAGAACACCTGTGGCGAAAGCGGTTTTCTAGCTATTTCCTGAC
GCTGAGGCGCGAGAGCATGGGGAGCAAACAGGATTAGATACCCCGGTAGTCC
>Otu12828
CCAGCCTATGGGATGCACCAGACGAGAATATTCGACAATGGGCGAAAGCCTGATCGAGCG
ACGCTGCGTGGAGGATGAAGTTCTTCGGGACGTAAACTCCTTTTGCCAGGGAAAAAGTTA
TTGATTGTACCTGGAGAATAAGAAGTTGCTAAACTCGTGCAGCAGCAGCGGTAATACGAG
TGCTTCGAGCGTTATCCGGAATCATTGGGCGTAAAGGGTGTGTAGGCGGTTTTGTTAGTC
TCGTGTAAAATCTCCCGGCTCAACCGGGAACTCGCACGGGAAACGGCAAGACTTAGAGGA
CGGAAGAGGTCTCTGGAACTCATGGTGTAGCGGCGAAATGCGTTGATATCATGGGGAACA
CCGAAAGCGAAGGCAAGAGACTGGTCCGCTCCTGACGCTGAAACACGAAAGCGTGGGTCG
CGAATGGGATTAGAAACCCGTGTAGTCC
>Otu12839
CCAGCCTACGGGGTGCTGCAGTACGATCTCAACATAGTCTTTTTTCGTGTTGCTGACCAC
ACCAACTGTAACTGCGGTGCCGACCGAGAGCATCAGCGGCTCCGAGTGCTTCAATGGCTC
AATAATGAGCTCACTTGTTGCTCCGACCACCCGTTCCATAAGGGTAACACTGCACCGGAT
CTTATCCCAGACCGGGGGAAGTTTCCCCTCATGACCAAGTACCTGGCCGGCGAGTGCATC
ACTCTTGGTAAGGGCCGGGTCCAGTTTTGTCCCTACACCCAGCAATCCCCCGGGGGTTGC
CTCGTCAACCTGCTTGGACCCGGCATTAATTGAGGTTATCGTCGTGATAATGGGGATCCA
TTTGATCCTGTTCTCAATCTGTGTCTGCCTGCCGGGACGGATCTCAATTTTTTCGTCTGC
TCGGAGGATGCCCCGGATTAGATACCCCCGTAGTCC
>Otu12848
CCAGCCTATGGGACGCAGCAGTCGAGAATTTTTCTCAATGGGCGAAAGCCTGAAGGAGCG

ACGCCGCGTGGGGGATGAAGGCCTTCGGGTTGTAAACTCCTTTTGTAGGGGAAGATAATG
ACGGTACCCTGCGAATAAGCCACGGCTAACTCTGTGCCAGCAGCCGCGGTAAGACAGAGG
AGGCGAGCGTTGTTCGGAATTACTGGGCTTAAAGGGCGCGTAGGCGGTGATATAAGTCTG
GGGTGGAAGCCCATAGCTTAACTATGGGACTGCCCTGGAAACTGTATTGCTTGAGTCGGA
CAGGGGAAGGCGGAATTCCAGGTGTAGCGGTGAAATGCGTAGATATCTGGAGGAAGGCCT
GTGGTGAAGACGGCCTTCTGGGTCTTGACTGACGCTGAGGCGCGAAAGCGTGGGTAGCAA
ACAGGATTAGATACCCCAGTAGTCC
>Otu12855
CCAGCCTACGGGAGGCTGCAGTGGGGAATATTGCACAATGGGGGAAACCCTGACGCAGCA
ACGCCGCGTGAGTGAGGAAGGTTTTCGGATCGTAAAGCTCTGTCGAGCGGGAAGAAATGT
GTGGTGGCTAATATCCATCATGCTTGACGGTACCGCTAAAGGAAGCACCGGCCAACTCCG
TGCCAGCAGCCGCGGTAATACGGAGGGTGCGAGCGTTGTTCGGAATTATTGGGCGTAAAG
CGCGTGTAGGCGGTTTTTTAAGTCTGATGTGAAAGCCCTGGGCTCAACCCGGGAAGTGCA
TTGGAAACTGGGAGACTTGAATACGGGAGAGGGTAGTGGAATTCCTGGTGTAGGAGTGAA
ATCCGTAGATATCAGGAGGAACACCGGTGGCGAAGGCGGCTACCTGGACCGATATTGACG
CTGAGACGCGAAAGCGTGGGGAGCAAACAGGATTAGATACCCCAGTAGTCC
>Otu12857
CCAGCCTATGGGACGCACCAGTCAGGAATATTCCTCAATGGCCGAAAGGCTGAAGGAGCG
ACGCCGCGTGACTGATGAAGCTCTTCGGAGCGTAAAGGTCTTTTATTAGGGACGAATTTC
GACGGTACCTAATGAATAAGGGGCTGCTAACCTCGTGCCAGCAGCAGCGGTAATACGAGG
GCCCCAAGCATTATCCGGATTTATTGGGCGTAAAGCGTCCGCAGACGGTTTGTCGCATCT
CCTGTTAAATCCTCGGGCTTAACCCGGGGGCCGCGGGAGAGATGGATAAACTAGAGACTG
GAAGAGGTAAGCGGAATTGCTGGTGTAGGGGTTAAATCCGTTAATATCAGCAAGAACATT
AAATGCGTAGGCAGCTTACTAGGACAGTTCTGACGTTCAGGGACGAAAGCGTGGGTAGCG
AATGGGATTAGAGACCCCCGTAGTCC
>Otu12862
CCAGCCTATGGGACGCAGCAGTGGGGAATTTTGCGCAATGGGGGAAACCCTGACGCAGCA
ACGCCGCGTGGAGGATGAAGTACTTCGGTACGTAAACTCCTTTCGATCGGGACGATAATG
ACGGTACCCGGAGAAGAAGCCCCGGCTAACTTCGTGCCAGCAGCTGCGGTAATACGAAGG
GGGCTAGCGTTGCTCGGAATTACTGGGCGTAAAGGGCGCGTAGGCGGGTCGTTAAGTTGG
GGGTGAAAGCCCGGGGCTCAACCTCGGAATTGCCTTCAATACTGGCGACCTTGAGTATGG
GAGAGGTGAGTGGAACTCCGAGTGTAGAGGTGAAATTCGTAGATATTCGGAAGAACACCA
GTGGCGAAGGCGACTCACTGGCCCATTACTGACGCTGAGGCGCGAAAGCGTGGGGAGCAA
ACAGGATTAGATACCCGTGTAGTCC
>Otu12871
CCAGCCTATGGGTGGCAGCAGCAGGTAGACCGGAGCGAGATAGATCATTGCCAAAAGTCC
GTTCACCACGAGGCCGCCTCGCCCAGAGGAGCCAGCGCTACTCAGTTTAAACAGTGAAAA
CGCGAAAATAGCGATGAACGGAAAGAGCCAAAAAAGGCTGTCGAAATGCGCGAGAATCGG
GTAGACGGGAAGATAAATTACGGTGACTGCTGCCTGCGCTATCATATGACGCAGTTCCCC
CACGGACAGAAGAACGGGCAGCAGCAGCAAAACCAAGTCGTGCGGGTAGACGTGATAGCC
CGCCAGGATCAAGCATACCAGTGCAAAAGAAAACGCTCGCTCCAGCGAGCGAGCGCTTTT
CCACTGCCTCCAAGCGAGCAGGATTAGATACCCTTGTAGTCC
>Otu12878
CCAGCCTATGGGAGGCAGCAGTCGAGAATCTTCCACAATGGACGAAAGTCTGATGGAGCG
ACGCCGCGTGACTGATGAAATCCTTCGGGATGTAAAGGTCTTTTGTGAGGGACGAAGTTT
ATTGACGGTACCTCAAGAATAAAGGGTTGCTAAACTCGTGCCAGCAGCAGCGGTAATACG
AGTGCCCAAAACGTTATCCGGAATTATTGGGCGTAAAGGGTGTGTAGGTGGTTGCGTTAG
TCTTCTGTTAAATTCTTCGGCTTAACCGGGGGCATGCGGAGGAAACGGCGCGACTAGAGG
ATGCGAGAGGCACAGGGAACTCATGGAGTAGGGGTGAAATCCGTTGATATCATGGGGAAC
ACCGAAAGCGAAGGCACTGTGCTGGCGCATTCCTGACGCTGAGACACGAAAGCGTAGGTA
GCGAATGGGATTAGAAACCCGTGTAGTCC
>Otu12885
CCAGCCTATGGGGCGCAGCAGTGGGGAATATTGGACAATGGGCGAAAGCCTGATCCAGCA
ATGCCGCGTGTGTGATGAAGGTCTTCGGATCGTAAAGCACTGTCGCGAGGGACGAATAAG
GGTCGGGCTAACATCCCGGTCCGATGACGGTACCTCGAGAGGAAGCACCGGCTAACTCTG
TGCCAGCAGCCGCGGTAATACAGAGGGTGCAAGCGTTGTTCGGAATTATTGGGCGTAAAG
CGCGTGTAGGCGGCTTAGCAAGTCAGGTGTGAAAGCCCTCGGCTTAACCGAGGAAGTGCG
CCTGAAACTATTGAGCTTGAGTACCGGAGAGGGCGGCGGAATTCCCGGTGTAGAGGTGAA

ATTCGTAGATATCGGGAGGAACACCAGCGGCGAAGGCGGCCGCCTGGACGGTCACTGACG
CTGAGACGCGAAAGCGTGGGTAGCAAACAGGATTAGATACCCGAGTAGTCC
>Otu12892
CCAGCCTACGGGGCGCACCAGTCGAGAATCTTCCGCAATGGACGAAAGTCTGACGGAGCG
ACGCCGCGTGGAGGATGAAGTTCTTCGGAATGTAAACTCCTTTTGCCAGGGAAAAAGTTA
TTGATTGTACCTGGAGAATAAGAAGTTGCTAAACTCGTGCCAGCAGCAGCGGTAATACGA
GTGCTTCAAGCGTTATCCGGAATCATTGGGCGTAAAGGGTGTGTAGGTGGTTTTGTTAGT
CTCACGTAAAATCTCTCGGCTTAACCGAGAATCTGCGCGGGAAACGGCAAGACTCGAGGA
CGGAAGAGGTTTCTGGAACTCATGGTGTAGCGGTGAAATGCGTTGATATCATGGGGAACA
CCGAAAGCGAAGGCAAGAAACTGGTCCGCTCCTGACACTGAAACACGAAAGCGTGGGTCG
CGAATGGGATTAGATACCCGTGTAGTCC
>Otu12902
CCAGCCTATGGGGGGCTCCAGTGAGGAATATTGGACAATGGGCGCAAGCCTGATCCAGCC
ATGCCGCGTGAGTGATGAAGGCCTTAGGGTTGTAAAACTCTTTTAGTGGGGAAAATAATG
ATGGTACCCACAGAAAAAGCTCCGGCTAACTTCGTGCCAGCAGCCGCGGTAATACGAAGG
GAGCTAGCGTTACTCGGAATTACTGGGCGTAAAGCGCATGTAGGCGGAATTTCAAGTCAG
GGGTGAAATCCCGGGGCTCAACCCCGGAATTGCCATTGAAACTGTAATTCTTGAGATTGA
CAGAGGATGGTGGAATTTCCAGTGTAGAGGTGAAATTCGTAGATATTGGAAAGAACACCG
GTGGCGAAGGCGACCATCTGGGTCAATACTGACGCTGAGATGCGAAAGCGTGGGGAGCAA
ACGGGATTAGAGACCCCCGTAGTCC
>Otu12912
CCAGCCTATGGGATGCACCAGTAAGGAATATTGGACAATGGTGGCAACACTGATCCAGCC
ATGCCGCGTGCAGGATGAAGGCGCTACGCGTTGTAAACTGCTTTTGTACCAGAGAAAACC
TATCTACGTGTAGATAGCTGATAGTATGGTAAGAATAAGCATCGGCTAACTTCGTGCCAG
CAGCCGCGGTAATACGAAGGATGCAAGCGTTATCCGGATTTATTGGGTTTAAAGGGTGCG
TAGGCGGCCCTGTAAGTCAGTGGTGAAATCTTTGGGCTTAACCCAAAAATTGCCATTGAT
ACTGCAGGGCTTGAGTACAGTTGCTGTGGGCGGAATATGACATGTAGTGGTGAAATACAT
AGAGATGTCATAGAACACCGATTGCGAAGGCAGCTCACAAAACTGTAACTGACGCTGAGG
CTCGAAAGTGCGGGGATCAAACAGGATTAGATACCCGTGTAGTCC
>Otu12914
CCAGCCTATGGGACGCTCCAGTGGGGAATATTGCGCAATGGGCGAAAGCCTGACGCAGCG
ACGCCGCGTGGGGGATGAAGGCCTTCGGGTTGTAAACCCCTTTCACCAGGAGCGAAATCA
GGCGGTACCTGGTGAATAAGCCACGGCTAACTACGTGCCAGCAGCCGCGGTAATACGTAG
GTGGCAAGCGTTGTCCGGATTTATTGGGTTTAAAGGGTGCGTAGGCGGCCCTGTAAGTCA
GTGGTGAAATACGGCAGCTCAACTGTCGAGGTGCCATTGATACTGCAGGGCTTGAGTACA
GACGAGGTAGGCGGAATTGACGGTGTAGCGGTGAAATGCTTAGATATCGTCAAGAACACC
GATAGCGAAGGCAGCTTACTAGGCTGTAACTGACGCTGAGGCACGAGAGTGTGGGGATCA
AACAGGATTAGAAACCCTGGTAGTCC
>Otu12923
CCAGCCTACGGGGGGCACCAGTGAGGAATATTGGACAATGAACGAAAGTTTGATCCAGCA
ATGTCGCGTGAGTGATACTAATAAAATTAAAAATACAAATAGTATTTTTAAGGCAGGTTA
GATCCGTAAAACTCTTTCAACATTGATGAAAAAGACCGTATTTGTAGAAGAAGTCCCGAC
TAACTTCGTGCCAGCAGTCGCGGTAAAACGGAGGGGGCAAGCGTTATTCACCTTGACTGG
GCGTAAAGGGTATGTAGGTTGTTTTTATTTATATAATAAGTAATCCAAGCATAACTTTT
GAAGTTATTTTTTATAAATTTAGACTAGAGGTAATAAGAAGTAAGTTACATTTTTAAAGT
AGGGGTGAAATCCTTAAATATTAAAAAGATAGCCGAATGGCGAAAGCTACTTACTATGAT
TATCCTGACACTGAAATACGAAAGCATGGGGATCAAACAGGATTAGAAACCCGAGTAGTC
C
>Otu12924
CCAGCCTACGGGTGGCTGCAGTCGAGAATTTTTCACAATGGGGGAAACCCTGATGGAGCG
ACGCCGCGTGGGGGATGAATGGCTTCGGCCCGTAAACCCCTGTCATTTGCGAACAAACGG
CATTATTTAAAAGATGATGTTTTGATAGTAGCGAAAGAGGAAGGGACGGCTAACTCTGTG
CCAGCAGCCGCGGTAATACAGAGGTCCCAAGCGTTGTTCGGATTCACTGGGCGTAAAGGG
TGCGTAGGTGGTCGGGTAAGTCTGATGTGAAATCTCGCAGCTCAACTGCGAAACTGCATT
GGATACTATCCGGCTGGAGGGTCGGAGGGGGGACTGGAATTCTCGGTGTAGCAGTGAAAT
GCGTAGATATCGAGAGGAGCACCAGTGGCGAAGGCGAGTCCCTGGACGACTCCTGACGCT
GAGGCACGAAAGCCAGGGGAGCAAACGGGATTAGAAACCCTAGTAGTCC
>Otu12925

CCAGCCTATGGGTGGCAGCAGTAAGGGATTTTCGGCAATGGGCGAAAGCCTGACCGAGCA
ACGCCGCGTGTGTGAAGACGGCCTTCGGGTTGTAAAGCACTTTTCATGGGGACGAGAACG
GACGGTACCTGTGGAAGAAGCCCCGGCTAACTACGTGCCAGCAGCCGCGGTAATACGTAG
GGGGCGAGCGTTGTCCGGAGTTATTGGGCGTAAAGCGCGTGCAGGCGGCCTGGAAGGTGC
GGTGTGAAAGCTCCCGGCTCAACTGGGAGAGTGCGTCGCAGACCGCCAGGCTCGAGGGCT
GGAGAGGAAGATGGAATTCCCGGTGTAGCGGTGGAATGCGTAGAGATCGGGAGGAACACC
AGTGGCGAAGGCGGTCTTCTGGACAGCCCCTGACGCTCAAGCGCGAAAGCGAGGGGAGCG
AACGGGATTAGAAACCCTCGTAGTCC
>Otu12954
CCAGCCTACGGGTGGCAGCAGTCGAGAATTTTTCACAATGGGCGAAAGCCTGATGGAGCG
ACGCCGCGTGGGGGATGAATGCCTTCGGGTCGTAAACTGCGGTAGATAGGTAACAATGTA
AATGAGTGCCTATCGGAAAGAGGTGGGTAACTACGTGCCAGCACCAGCGGTAAAACGTAG
ACCTCAAGCGTTATCCGGATTTATTGGGCGTAAAGCGCGTGTAGGTGGTTCGGTGCGTTT
CTGGTTAAATCCCATTGCCTAACAATGGACCCGCCAGGAATACGGCCGGACTAGAGGAGG
TTAGAGGTGCATAGAACGCACGGTGTAGGGGTGAAATCCGTTGATATCGTGCGGAATACC
AAAGGCGAAGGCATTGCACTGGGGCCTTCCTGACACTGAGACGCGAAAGCGTGGGGAGCA
AAAAGGATTAGAAACCCGCGTAGTCC
>Otu12962
CCAGCCTACGGGGGGCTGCAGTCGAGGATCTTCGGCAATGGGCGCAAGCCTGACCGAGCG
ACGCCGCGTGTGCGATGAAGGCCTTCGGGTTGTAAAGCACTGTCGAGGGGGAGAAAAGCC
CGCAAGGGTTTGATCTATCCCTGGAGGAAGCACGGGCTAAGTTCGTGCCAGCAGCCGCGG
TAAGACGAACCGTGCGAACGTTATTCGGAATCACTGGGCTTAAAGGGCGCGTAGGCGGGT
TCTCAAGTCAGGGGTGAAATCCTCCAGCTTAACTGGAGAAGTGCTTTTGATACTGGGAAT
CTCGAGTAAGGTAGGGGCATGTGGAACTTCCGGTGGAGCGGTGAAATGCGTAGATATCGG
AAGGAACGCCGGTGGCGAAAGCGACGTGCTGGACCTTTTCTGACGCTGAGAGACGAAAGC
TAGGGGAGCAAACAGGATTAGATACCCTAGTAGTCC
>Otu12963
CCAGCCTACGGGTTGCTGCAGTGGGGAATTTTGGACAATGGGGGCAACCCTGATCCAGCC
ATGCCGCGTGAGTGAAGAAGGCCTTCGGGTTGTAAAGCTCTTTCGGCCGGGACGAAATCG
CATGGGTGAATATCCTGTGTGGATGACGGTACCGGAAGAAGAAGCACCGGCTAACTACGT
GCCAGCAGCCGCGGTAACACGTAGGGTGCGAGCGTTAATCGGAATTACTGGGCGTAAAGC
GTGCGCAGGCGGTTTTGTAAGCCAGATGTGAAATCCCCGGGCTCAACCTGGGAACTGCAT
TTGGGACTGCGAGGCTGGAGTACGGCAGAGGGGGGTAGAATTCCTGGTGTAGCAGTGAAA
TGCGTAGATATCAGGAGGAATACCGATGGCGAAGGCAGCCCCCTGGGTCGATACTGACGC
TCATGCACGAAAGCGTGGGGAGCAAACAGGATTAGAAACCCGAGTAGTCC
>Otu12966
CCAGCCTATGGGTCGCACCAGTGGGGAATATTGGACAATGGGCGCAAGCCTGATCCAGCA
ATGCCGCGTGAATGATGAAGGCCTTAGGGTTGTAAAGTTCTTTTAGTGGAGAAGAAAATG
ACGGTACCCACAGAAAAAGCTCCGGCTAACTCCGTGCCAGCAGCCGCGGTAATACGGAGG
GAGCTAGCGTTTTTCGGAATTACTGGGCGTAAAGAGCGCGTAGGCGGCTAGCTAAGTTGA
AAGTGAAAGCCCAAGGCTTAACCTTGGAACTGCTTTCAAAACTAGTTAGCTTGAGATCGG
TAGGGGACAGTAGAATTCCTAGTGTAGAGGTGAAATTCTTAGATATTAGGAGGAATACCG
GTGGCGAAAGCGACTGTCTGGACCGTTTCTGACGCTAAGGCGCGAAAGCGTGGGGAGCAA
ACAGGATTAGAGACCCCAGTAGTCC
>Otu12969
CCAGCCTACGGGGGGCACCAGCAACGAATCTTCCCCAATGGGCGAAAGCCTGAGGGAGCG
ACGCCGCGTGTGGGATGAAGTACTTCGGTATGTAAACCACTGTTAGGGTTAGGAAAGCGA
TGCGCACTAATAGTGCGCAAAGTTGATCTAACCCAGAGAAAGGGACGGCTAACTCTGTGC
CAGCAGCCGCGGTAATACAGAGGTCCCAAGCGTTACTGAGATTCACTGGGTTTAAAGGGT
GCGTAGGTGGTCCGTTAAGTCAGTTGTGAAATCCCCGGGCTCAACCTGGAACTGCTTCT
GATACTGGCGGACTTGAGGCCTGTAGGGGTCACTGGAACTGACGGTGGAGCGGTGAAATG
CGTAGATATCGTCAGGAACGCCTGTGGTGAAGACGGGTGACTGGGCAGGCTCTGACACTG
AGGCACGAAAGCGTGGGGAGCGAACGGGATTAGATACCCCCGTAGTCC
>Otu12975
CCAGCCTACGGGTTGCAGCAGTGGGGAATATTGCGCAATGGCCGAAAGGCTGACGCAGCG
ACGCCGCGTGTGGGATGACGGCCTTCGGGTTGTAAACCACTGTCGGGAGGAACGAATACC
GGGCGTCGAATAGGCGACCCGGGGCGACGGTACCTCCAAAGGAAGCACCGGCTAACTCCG
TGCCAGCAGCCGCGGTAATACGGAGGGTGCGAGCGTTGTCCGGAATCACTGGGCGTAAAG

GGTGCGTAGGTGGCTCGTTAAGTGGCTGGTGAAATCCCGGGGCTCAACTCCGGGGCTGCC
GGTCAGACTGGCGGGCTCGAGCACGGTAGAGGCAGATGGAATTCCCGGTGTAGCGGTGGA
ATGCGTAGATATCGGGAAGAATACCGGTGGCGAAGGCGTTCTGCTGGACCGTTGCTGACA
CTGAGGCACGACAGCGTGGGGAGCAGACAGGATTAGATACCCTGGTAGTCC
>Otu12976
CCAGCCTATGGGACGCTGCAGTGGGGAATTTTGCACAATGGACGAAAGTCTGATGCAGCG
ACGCTGCGTGAAGGATGAAGGCCCTAGGGTCGTAAACTTCTTTTAGCGGGGAGTAATCAT
GAACGTACCCGCAGAATAAGCACCGGCTAACTACGTGCCAGCAGCCGCGGTAATACGTAG
GGTGCGAGCGTTATTCGGATTTACTGGGCGTAAAGAGCGTGTAGGTTGTTTGATGCGTCT
TCGATTAAAGACCCAAGCTCAACTTGGGGAGTGTTGAGGATACGATCAGACTAGAGGTTT
GCAGGGGCAGATGGAATTCCTGGTGTAGCAGTGAAATGCGTAGATATCAGGACGAACACC
AATGGCGAAAGCAGTCTGCTGGGCTTACTCTGACACTGAGATGCGAAAGCGTTGGGAGCG
AACGGGATTAGAACCCGTGTAGTCC
>Otu12977
CCAGCCTACGGGGGGCAGCAGTGAGGAATTTTCCGCAATGGGCGAAAGCCTGACGGAGCG
ACGCCGCGTGTGGGATGAAGGCCTTCGGGTCGTAAACCACTGTCGAAGGGGACGATATTG
ACGGTACCCTTGGAGGAAGCCCCGGCTAACTACGTGCCAGCAGCCGCGGTAAGACGTAGG
GGGCAAGCGTCGTCCGGAATTATTGGGCGTAAAGCGCTCGTAGGTGGATCGGCAAGTCCG
CAAGCAAAGCCCTGAGCTCAACTCAGGAATCCGTGTGGAAACTGCCGACCTTGAGGTAAT
CAGAGGATGATGGAATTCCCGGTGTAGCGGTGAAATGCGTAGATATCGGGAGGAACACCA
GTGGCGAAGGCGATCATCTGGGGTTAACCTGACACTGAGGAGCGAAAGCTAGGGGAGCAA
ACGGGATTAGAAACCCTCGTAGTCC
>Otu12980
CCAGCCTATGGGTGGCTCCAGTCGAGGATCTTCGTCAATGGGCGCAAGCCTGAACGAGCG
ACGCCGCGTGCGCGATGAAGGCCTTCGGGTTGTAAAGCGCAAAAGAGAAGAGAAAGCCGA
AAGGTTGATCAATTCTCAGTAAGGACGGGCTAAGTTCGTGCCAGCAGCCGCGGTAAGACG
AACTGTCCTAACGTTGTGCGGAATCACTGGGCTTAAAGGGCGCGTAGGCGGTCTGCCAAG
TCGGGGGTGAAACCCTGCAGCTTAACTGTAGTAGTGCCTTCGATACTGGCAGTCTGGAGG
GAGATAGGGGTGTGCGGAACTTCCAGTGGAGCGGTGAAATGCGTTGATATTGGAAGGAAC
GCCGGAGGCGAAAGCGGCGCACTGGATCTCTTCTGACGCTGAGGCGCGAAAGCTAGGGGA
GCAAACGGGATTAGAGACCCCTGTAGTCC
>Otu12988
CCGCCTACGGGGGGCTCCAGTAGGGAATCTTGCGCAATGGGCGAAAGCCTGACGCAGCCA
TGCCGCGTGAATGATGAAGGTCTTAGGATTGTAAAATTCTTTCAGCGGGGTCGATAATGA
CGGTACCCGCAGAAGAAGCCCCGGCTAACTTCGTGCCAGCAGCCGCGGTAATACGAAGGG
GGCTAGCGTTGCTCGGAATTACTGGGCGTAAAGGGCGCGTAGGCGGCTGTCATAGTCAGA
TGTGAAATTCCTGGGCTTAACCTGGGGGCTGCATTTGATACGTGGCGGCTAGAGTGCGGA
AGAGGGTCGTGGAATTCCCAGTGTAGAGGTGAAATTCGTAGATATTGGGAAGAACACCGG
TGGCGAAGGCGGCGACCTGGTCCGATACTGACGCTGAGGCGCGAAAGCGTGGGGAGCAAA
CAGGATTAGATACCCGGGTAGTCC
>Otu12992
CCAGCCTACGGGGCGCACCAGTGGGGAAAACTTTACAATGCGCGAAAGCGTGATAAGGGA
ATCCTTCGTGCTCTAGTCTAACTAGGGCTTTTGCCAAGGGTAAAATCCTTGGCGAATAAG
TGGTGGGCAAGACTGGTGCCAGCCGCCGCGGTAACCCCAGCGCCACGAGTGGCATCCAGC
TTTATTTGGTCTAAAGCGTCCGTAGCTGGCGCGTTACATTCTCTGTGAAATCGTGGCGCT
CAACGTCACGACGTGCAGAGAAGACGGACACGCTAGGGACCAGGAGAGGTCAGCAGTATT
CTGTGGGGAGCGGTAAAATGCTATAATCCACAGAGGACTACCAATGGCGAAGGCAGCTGG
CCAGAATGGATCCGACAGTGAGGGACGAAAGCTAGGGGAGCGATCCGGATTAGAAACCCC
AGTAGTCCC
>Otu13000
CCAGCCTATGGGGGGCACCAGTCGAGAATCTTCCGCAATGGACGAAAGTCTGACGGAGCG
ACGCCGCGTGGTCGATGAAGCGCTTCGGCGCGTAAAAACCTTTTATGAGCGACTAAGTTT
ATTGAAGAGCTCATGAATAAGTGGTTGCTAAACTCGTGCCAGCAGCAGCGGTAATACGAG
TGCCCCAAGCGCTATCCGGAATTATTGGGCGTAAAGGGTGTGTAGGCGGTCGTGTTAGTC
TCGCGTTAAATTCTTCGGCTTAACCGGGGGCATGCGCGGGAAACGGCACGACTAGAAGAT
GCGAGGGGTCTCTGGAACTCATGGTGTAGCGGTGAAATGCGTTGATATCATGGGGAACAC
CGAAAGCGAAGGCAGGAGACTAGAGCATTCTTGACGCTGAAACACGAAAGCGTGGGTAGC
GAATGGGATTAGAAACCCTTGTAGTCC

>Otu13005
CCAGCCTACGGGAGGCTCCAGTCGAGAATCATTCGCAATGGGCGCAAGCCTGACGATGCG
ACGCCGTGTGGACGATGAAGGCCTTCGGGTTGTAAAGTCCTTTCGCCTGGGAACAAGAAA
TGCAAACTAATACTTTGCAAATTTGAGGGTACCAGGTAAAGATGCACCGGCTAACTCCGT
GCCAGCAGCTGCGGTAATACGGAGGGTGCAAGCATTAATCGGATTTATTGGGCGTAAAGG
GGGCGTAGGCGGAGATAAAAGTCAGGCGTGAAATTACGGGGCTCAACCCCGTAGCCGCAC
CTGAAACTCTATTTCTAGAGGATAGACGGAGAAAACGGAATTCCACAAGTAGCGGTGAAA
TGCGTAGATATGTGGAAGAACATCTGTGGCGAAGGCGGTTTTCTAGTTTAAACCTGACGC
TAAGGCCCGAAAGCGAGGGGAGCAAACAGGATTAGAGACCCCTGTAGTCC
>Otu13011
CCAGCCTACGGGTGGCACCAGTTGGGAATATTGCGCAATGGGCGAAAGCCTGACGCAGCA
ACGCCGCGTGTGCGATGAAGGTCTTCGGATCGTAAAGCACTTTCGGCCGGGAAGAAAACC
TGCCCATTCATAATGGGTAGCTTGACTGTACCGGAAGAAGAAGCACCGGCTAACTCTGTG
CCAGCAGCCGCGGTAATACAGAGGGTGCAAGCGTTGTTCGGAATTATTGGGCGTAAAGCG
CGTGTAGGCGGCTCGTTATGTCGGGTGTGAAAGCCCTCGGCTCAACCGAGGAAGTGCGCC
CGAAACTGGCGAGCTTGAGTGCCGGAGAGGACAGTGGAACACCCGGTGTAGAGGTGAAAT
TCGTAGATATCGGGTAGAACACCAGTGGCGAAGGCGACTGTCTGGACGGACACTGACGCT
GAGACGCGAAAGCGTGGGGAGCAAACAGGATTAGATACCCGCGTAGTCC
>Otu13016
CCAGCCTACGGGAGGCACCAGTTAGGAATCTCCCACAATGGGCGAAAGCCTGATGGAGCG
ACGCCGCGTGAAGGATGAAGCCCTTACCGGGTGTAAACTTCTGTTGTGAGGGACGAAATT
TTTGACGGTACCTCACGAGAAAGCACCGGCTAATTCTGTGCCAGCAGCCGCGGTAAGACA
GAAGGTGCAAGCGTTACTCGGAATTACTGGGCGTAAAGCGTCTGTAGGTGTCCTGTCACG
TCTGAGGACAAATCGCGGGGCCCAACCCCGCGCTCTTCTTGGATACGAGCAGGATGGAGC
CACTCAGAGGCATCTGGAATGTCGTGTGTAGGGGTAAAATCCGTAGATACACGATGGAAC
GCCAAAAGCGAAAGCAGGATGCTGGGGGTGTGCTGACACTCAGAGACGAAAGCGTGGGGA
GCAAAGGAGATTAGAAACCCCAGTAGTCC
>Otu13020
CCAGCCTACGGGATGCAGCAGGGAATATTGGACAATGGGCGAGAGCCTGATCCAGCCATG
CCGCGTGCAGGAAGAAGGCCTTCTGGGTTGTAAACTGCTTTTGCCAGGGGATAAAAGACC
CGTGCGCGGGGAATTGAAGGTACCTGGTGAATAAGCCACGGCTAACTACGTGCCAGCAGC
CGCGGTAATACGTAGGTGGCAAGCGTTGTCCGGATTTATTGGGTTTAAAGGGTGCGTAGG
CGGACTTATAAGTCAGTGGTGAAATCTCGATGCTTAACATCGAACGTGCCATTGATACTG
TAGGTCTTGAGTACAGATGCCGTTGGCGGAATGTGTCATGTAGCGGTGAAATGCATAGAT
ATGACACAGAACACCGATTGCGAAGGCAGCTGACGAAACTGTAACTGACGCTGAGGCACG
AAAGCGTGGGGATCAAACAGGATTAGATACCCGCGTAGTCC
>Otu13031
CCAGCCTACGGGAGGCAGCAGTGGGGAATATTGGACAATGGGGGCAACCCTGATCCAGCG
ACGCCGCGTGTGTGAAGAAGGCCTGCGGGTTGTAAAGCACTTTTAGTGGGGATGAAAAGC
TCAGGGCTAATACCCCCGGGTCTTGACCTAACCTACAGAAAAAGCACCGGCTAACTCTGT
GCCAGCAGCCGCGGTAATACAGAGGGTGCGAGCGTTGTTCGGAATTATTGGGCGTAAAGG
GTGCGTAGGCGGTGCCACAAGTCACTTGTGAAATCCCCAGGCTTAACCTGGGGCCTGCAG
GCGAAACTGTGGTGCTGGAATATGGGAGAGGTGCGTGGAATTCCCGGTGTAGCGGTGAAA
TGCGTAGATATCGGGAGGAACACCTGTGGCGAAAGCGGCGCACTGGACCATAACTGACGC
TGATGCACGAAAGCTAGGGGAGCAAACAGGATTAGAAACCCCTGTAGTCC
>Otu13032
CCAGCCTATGGGTCGCAGCAGTAAGAAATATTGGTCAATGGACGCAAGTCTGAACCAGCC
ATGCCGCGTGGAGGATGAATGCCCTCTGGGTTGTAAACTCCTTTTATCTTGGAAGAAACC
CGAGAATTCTTTTTCGGTTGACGGTACCAGAGGAATAAGCACCGGCTAACTCCGTGCCAG
CAGCCGCGGTAATACGGAGGGTGCAAGCGTTATCCGGATTCACTGGGTTTAAAGGGTGCG
TAGGTGGCAGGTAAGTCAGTGGTGAAATCTTTGGGCTTAACCCGAAAACTGCCATTGAT
ACTATCTGTCTTGAATGTTGTGGAGGTGAGCGGAATATGTCATGTAGCGGTGAAATGCTT
AGATATGACATAGAACATCGATTGCGAAGGCAGCTCGCTACGCAAATATCGACACTGAGG
CACGAAAGCGTGGGGATCAAACAGGATTAGAGACCCCCGTAGTCC
>Otu13048
CCAGCCTATGGGGCGCACCAGTGGGGAATATTGGACAATGGGCGAAAGCCTGATCCAGCA
ATGCCGCGTGAGTGATGAAGGCCTTAGGGTTGTAAAGCTCTTTCGGGTGGGACGATGATG
ACGGTACCACCAGAAGAAGCCCCGGCTAACTTCGTGCCAGCAGCCGCGGTAATACGAAGG

GGGCTAGCGTTGTTCGGAATTACTGGGCGTAAAGCGCGCGCAGGCGGCTTCTCAAGTCAG
GGGTGAAAGCCCAGAGCTCAACTCTGGAACTGCCCTTGAAACTGTGGAGCTTGAGTACGG
GAGAGGTGAGTGGAATTCCCAGTGTAGAGGTGAAATTCGTAGATATTGGGAAGAACACCG
GTGGCGAAGGCGGCTCACTGGCCCGTTACTGACGCTCAGGCGCGACAGCGTGGGGATCAA
ACAGGATTAGAAACCCGTGTAGTCC
>Otu13061
CCAGCCTATGGGTCGCAGCAGTCGAGAATCTTTCGCAATGGGCGAAAGCCTGACGAAGCG
ACGCCGTGTGAGCGAAGAAGGCCTTCGGGTTGTAAAGCTCTTTCGCCGGGGAACAAGAGA
CTAGGAATAACACTCCTAGAATTTGAGGGTACTCGGTAAAGAAGCACCGGCTAACTCCGT
GCCAGCAGCTGCGGTAATACGGAGGGTGCAAGCATTAATCGGATTTATTGGGCGTAAAGG
GCGCGTAGGCGGGAAGGTCAGTCAGATGTGAAATCCCTAGGCTTAACCTTGGAACTGCAT
TTGATACTGCCTTTCTTGAGGGTAGAAGGAGAAAGTGGAATTCCACAAGTAGCGGTGAAA
TGCGTAGATATGTGGAAGAACACCAGTGGCGAAGGCGACTTTCTATTTTACTCCTGACGC
TGAGGCGCGAAAGCTAGGGGAGCAAACAGGATTAGAAACCCGCGTAGTCC
>Otu13067
CCAGCCTACGGGGGGCTGCAGTTGGGAATCTTGGACAATGGGGGAAACCCTGATTCAGCC
ATGCCGCGTGAGTGATGAAGGCCTTCGGGTTGTAAAACTCTTTCGACGGGGACGATAATG
ACTGTACCGGGAGAATAAGCTCCGGCTAACTTCGTGCCAGCAGCCGCGGTAATACGAAGG
GGGCCAGCGTTGCTCGGAATTACTGGGCGTAAAGGGTCCGTAGGCGGTGTGGCAAGTCGG
TAGTGAAATCTCTGGGCTTAACTCAGAGGCTGCTGCCGAAACTGCTGTGCTAGAGTGCGA
GAGAGGCGAGTGGAATTGCGGGTGTAGCGGTGAAATGCGTAGATATCCGCAGGAACATCC
GAGGCGAAAGCGGCTCGCTGGATCGCAACTGACGCTGAGGGACGAAAGCTAGGGGAGCAA
ACAGGATTAGATACCCTCGTAGTCC
>Otu13075
CCAGCCTATGGGGTGCTGCAGTGGGGAATTGTTCGCAATGGGCGCAAGCCTGACGACGCA
ACGCCGCGTGGAGGATGAAGCCCCTTGGGGCGTAAACTCCTTTCGACTGGGAAGATAATG
ACGGTACCAGTGGAAGAAGCACCGGCTAACTCTGTGCCAGCAGCCGCGGTAATACAGAGG
GTGCGAGCGTTGTTCGGAATTATTGGGCGTAAAGGGCGCGTAGGCGGTATTGTAAGTCAC
CTGTGAAACCTCTAGGCTTAACCTAGAGCCTGCAGGCGAAACTGCAATGCTGGAGGGTGG
GAGAGGTGCGTGGAATTCCCGGTGTAGCGGTGAAATGCGTAGATATCGGGAGGAACACCT
GTGGCGAAAGCGGCGCACTGGACCACTACTGACGCTGAGGCGCGAAAGCCGGGGGAGCAA
ACAGGATTAGAAACCCCAGTAGTCC
>Otu13081
CCAGCCTACGGGTTGCTCCAGACGAGAATATTCCGCAATGGACGAAAGTCTGACGGAGCG
ACGCCGCGTGATGGATGAAGTGCCTTGGTACGTAAACATCTTTTATCGGGGACGAAGTTT
ATTGACGGTACCCGATGAATAAGGGGCTCCTAACTCTGTGCCAGCAGGAGCGGTAATACA
GAGGCCCCAAGCATTACCCGGAATCACTGGGCGTAAAGGGTGTCTAGGCGGTCATATTAG
TCTCTCGTTAAATGTCCGGGCTCAACCTGGTCACCGCGAGGGAAACGGTATGACTCGAGG
GCGTAAGAGGTGCACGGAACTCATGGTGGAGGGGTGAAATCCGTTGATATCATGGGGAGC
ACCAAATGCGAAGGCAGTACACTGGCGCATATTTGACACTGAAGCACGAAAGCGTGGGTA
GCGAATGGGATTAGATACCCTTGTAGTCC
>Otu13092
CCAGCCTATGGGGTGCACCAGTCGAGAATCTTCCGCAATGGGCGAAAGCCTGACGGAGCG
ACGCCGCGTGATTGATGAAGTCCTTCGGGACGTAAAAATCTTTTGGAAGGGAGAAAGTTA
TTGATAGTATCTTCAGAATAAGGGGTTGCTAAACTCGTGCCAGCAGCAGCGGTAATACGA
GTGCCCCAAGCGTTATCCGGAATTATTGGGCGTAAAGGGTGTGTAGGCGGTCTTGTTAGT
CTTCTGTCAAATTCTTCGGCTTAACCGGGGGTCCGCAGAGGAAACGGCAAGACTTGAGGA
TGCGAGAGGTACATGGAACTCATAGTGTAGGGGTGAAATCCGTTGATATTATGGGGAACA
CCGAAAGCGAAGGCAATGTACTGGCGCACTCCTGACGCTGAAACACGAAAGCGTGGGTAG
CGAATGGGATTAGATACCCTTGTAGTCC
>Otu13099
CCAGCCTATGGGTTGCAGCAGTCGAGAATCTTCCGCAATGGACGAAAGTCTGACGGAGCG
ACGCCGCGTGGAGGATGAAGTCCTTAGGGACGTAAACTCCTTTTATAGGTTAGAAAGTTA
TTGATCAACCTAGGAATAAGAGGTTGCTAAACTCGTGCCAGCAGCAGCGGTATACGAGTG
CCTCAAGCGTTATCCGGAATTACTGGGCGTAAAGGGTGTGTAGGCGGTTCTATTAGTCTT
GTGTAAAATCTTCGCGCTTAACGCGAAATCTGCATAGGAAACGGTAGAACTAGAGGATGC
AAGGGGCACATAGAACTCATGGTGTAGGGGTGAAATCCGTTGATATCATGGGGAATACCA
AGAGCGAAGGCAGTGTGCTGGTGCATTCCTGACGTTGAAACACGAAAGCGTGGGTAGCGA

ACGGGATTAGAAACCCCAGTAGTCC
>Otu13105
CCAGCCTATGGGGGGCTGCAGTGGGGAATATTGGACAATGGGCGAAAGCCTGATCCAGCA
ATGCCGCGTGAGTGACGAAGGCCTTAGGGTTGTAAAGCTCTTTCGCAGGGGAAGATAATG
ACGGTACCCTGATAAGAAGCACCGGCTAACTCCGTGCCAGCAGCCGCGGTAATACGGAGG
GTGCTAGCGTTGTTCGGAATTACTGGGCGTAAAGCGCGCGTAGGTGGTGCAGCAAGTTGG
GAGTGAAATCCCAGAGCTTAACTCTGGAATTGCTTTCAAGACTGCTGCGCTAGAGTATGG
CAGGGGATAGTGGAATTCCTAGTGTAGGGGTGAAATCCGTAGATATTAGGAGGAACACCA
GAGGCGAAGGCGACTATCTGGGCCATTACTGACGCTGATGCGCGAAAGCGTGGGGAGTAA
ACAGGATTAGATACCCTAGTAGTCC
>Otu13115
CCAGCCTATGGGAGGCAGCAGTGGGGAATATTGGACAATGGGGGCAACCCTGATCCAGCA
ATGCCGCGTGAGTGATGAAGGCCTTAGGGTTGTAAAGCTCTTTCGGCGGGGACGATGATG
ACGGTACCCGCAGAAGAAGCCCCGGCTAACTTCGTGCCAGCAGCCGCGGTAATACGAAGG
GGGCAAGCGTTGTTCGGAATTACTGGGCGTAAAGGGCGCGCAGGCGGCCATCCTAGTCAG
GCGTGAAAGCCCCGGGCTTAACCTGGGAATTGCGTTTGATACTTGGTGGCTGGAGTTCGG
GAGAGGTTGGTGGAATTCCCAGTGTAGAGGTGAAATTCGTAGATATTGGGAAGAACACCG
GTGGCGAAGGCGGCCAACTGGACCGAAACTGACGCTGAGGCGCGAAAGCGTGGGGAGCAA
ACAGGATTAGAAACCCCTGTAGTCC
>Otu13116
GGACTACTGGGGTATCTAATCCTGTTTGATCCCCACGCTTTCGTGCATCAGCGTCAATAT
ATGCGTAGTGAGCTGCCTTCGCAATGGGTATTCTATGTCATATCTAAGCATTTCACCGCT
ACATGACATATTCTGCTCACCTCCACAATATTCAAGACAGATAGTATCAATGGCAGTTTT
CGGGTTAAGCCCAAAGATTTCACCACTGACTTACCTGCCCGCCTACGCACCCTTTAAACC
CAGTGAATCCGGATAACGCTTGCACCCTCCGTATTACCGCGGCTGCTGGCACGGAGTTAG
CCGGTGCTTATTCATCTGGTACCGTCAAGTGAGAAAGAATTCCCTTTTTTCGTCCCAGAT
AAAAGAAGTTTACAATCCAGAGGACCTTCATCCTCCACGCGGCATGGCTGGTTCAGGCTT
TCGCCCATTGACCAATATTCCTTACTGGAGCACCCCGTAGGCTGG
>Otu13126
CCAGCCTATGGGACGCACCAGTGGGGAATTTTGGACAATGGGGGCAACCCTGATCCAGCC
ATGCCGCGTGAGTGAAGAAGGCCTTCGGGTTGTAAAGCTCTTTCGGCGGGGACGATGATG
ACGGTACCCGTAGAATAAGCACCGGCTAACTACGTGCCAGCAGCCGCGGTAATACGTAGG
GTGCAAGCGTTAATCGGAATTACTGGGCGTAAAGCGTGCGCAGGCGGTTCGCTAAGACAG
ATGTGAAATCCCCGGGCTTAACCTGGGAACTGCATTTGTGACTGGCGGGCTAGGGTATGG
CAGAGGGGGGTAGAATTCCACGTGTAGCAGTGAAATGCGTAGAGATGTGGAGGAATACCG
ATGGCGAAGGCAGCCCCCTGGGCCAATACTGACGCTCATGCACGAAAGCGTGGGGAGCAA
ACAGGATTAGATACCCCTGTAGTCC
>Otu13131
CCAGCCTACGGGACGCTCCAGTGGGGAATATTGGACAATGGGGGAAACCCTGATCCAGCG
ACGCCGCGTGTGTGAAGAAGGCCCGCGGGTTGTAAAGCACTTTTAGTGGGGACAAAAAGC
TACGGATTAATACTTCGTGGTCTTGATTTAACCCAAAGAAAAAGCACCGGCTAACTCTGT
GCCAGCAGCCGCGGTAATACAGAGGGTGCGAGCGTTAATCGGAATTACTGGGCGTAAAGC
GCGCGTAGACGGTTCGCTAAGACAGATGTGAAAGCCCCGGGCTCAACCTGGGAATGGCAT
TTGAGACTGCGAAGCTAGAGTACGGCAGAGGGAGGTGGAATTCCACGTGTAGCAGTGAAA
TGCGTAGATATGTGGAGGAACACCGATGGCGAAGGCAGCCTCCTGGGCCAGTACTGACGC
TCATGCACGAAAGCGTGGGGAGCAAACAGGATTAGAAACCCCAGTAGTCC
>Otu13151
CCAGCCTACGGGTCGCACCAGTCGAGAATCTTTCGCAATGGGCGCAAGCCTGACGAAGCG
ACGCCGTGTGAGCGAAGAAGGCCTTCGGGTTGTAAAGCTCTTTCGCTAGGGAACAAGAAA
GACAGACTAATAACCTGTCGATTTGATGGTACTTGGTAAAGAAGCACCGGCTAACTCCGT
GCCAGCAGCTGCGGTAATACGGAGGGTGCAAGCATTGATCGGAATTACTGGGCGTAAAGG
GCGCGTAGGCGGTCAGGAAAGTCAGACGTGAAATCCCGGGGCTCAACCCCGGGGCTGCAT
TTGAAACTTCCTATCTAGAGTTCAGTTAGGGAAAACGGAATTCCACGTGTAGCGGTGAAA
TGCGTAGATATGTGGGAGAACACCAGTGGTGAAGACGGTTTTCTGGGCTGATACAGACGC
TGAGGCGCGAAAGCAAGGGGAGCAAACAGGATTAGATACCCCCGTAGTCC
>Otu13152
CCAGCCTATGGGGCGCAGCAGTAGGGAATCTTGGACAATGGGCGAAAGCCTGATCCAGCC
ATGCCGCGTGTGTGATGAAGGCCTTAGGGTTGTAAAGCACTTTCGCCAGGGAAGATAATG

ACGGTACCTGGATAAGAAGCCCCGGCTAACTTCGTGCCAGCAGCCGCGGTAATACGAAGG
GGGCTAGCGTTGTTCGGAATTACTGGGCGTAAAGCGCACGTAGGCGGTTTTTCAAGTTGG
AGGTGAAATCCCCGGACTTAATCTGGGAACTGCCTTCAAGACTGGGAGACTTGAGTCCGA
GAGAGGTGAGTGGAATTTCCAGTGTAGAGGTGAAATTCGTAGATATTGGAAAGAACACCA
GTGGCGAAGGCGGCTCACTGGCTCGGTACTGACGCTGAGGAGCGACAGCGTGGGGAGCAA
ACAGGATTAGAACCCTTGTAGTCC
>Otu13159
CCAGCCTATGGGACGCTGCAGTAAGGAATATTGGACAATGCTCGCAAGAGTGATCCAGCC
ATGCCGCGTGCAGGAAGACGGCCCTATGGGTTGTAAACTGCTTTTGAACTAGAGAAAACC
CCACTACGTGTAGTGGGCTGATAGTATAGTTAGAATAAGCATCGGCTAACTTCGTGCCAG
CAGCCGCGGTAAGACGAAGGATGCAAGCGTTATCCGGATTCATTGGGTTTAAAGGGAGCG
TAGGTGGACTTATAAGTCAGTGGTGAAATACGGCAGCTCAACTGTCGAGGTGCCATTGAT
ACTGCAGGGCTTGAGTACAGACGAGGTAGGCGGAATTGACGGTGTAGCGGTGAAATGCTT
AGATATCGTCAAGAACACCGATAGCGAAGGCAGCTTACTAGGCTGTAACTGACGCTGAGG
CACGAAAGTGTGGGGATCAAACAGGATTAGATACCCGCGTAGTCC
>Otu13161
CCAGCCTACGGGGGGCAGCAGTAGGGAATTTTCCACAATGGGCGCAAGCCTGATGGAGCA
ACGCCGCGTGCAGGATGAATGTCTTCGGATTGTAAACTGCTTTTCTCTGTGACGATTTTG
ACGGTAGCAGAGGAATAAGGACCTGCTAACTACGTGCCAGCAGCCGCGGTCATACGTAGG
GTCCAAGCGTTATCCGGAATTACTGGGCGTAAAGAGTTGCGTAGGTGGCAGAGTAAGTCT
GTAGTGAAATCGTGTGGCTCAACCACATATACATTATGGAAACTGCTCAGCTAGAGGACG
AGAGAGGTTATTGGAATTCCCAGTGTAGGAGTGAAATCCGTAGATATTGGGAGGAACACC
GATGGCGTAGGCAGATAACTGGCTCGTTCCTGACACTAAGGCACGAAAGCGTGGGTAGCA
AACGGGATTAGATACCCGGTCC
>Otu13173
CCAGCCTATGGGGCGCTCCAGTGGGGAATATTGGACAATGGGGGCAACCCTGATCCAGCA
ATGCCGCGTGTGTGAAGAAGGCCTTCGGGTTGTAAAGCACTTTAGTTGGGGAAGAAGGTA
ATTAGTTTAAGAGATAAATTATTTGACGGTACCCAAAGAATAAGCACCGGCTAACTCTGT
GCCAGCAGCCGCGGTAATACAGAGGGTGCAAGCGTTAATCGGAGTTACTGGGCGTAAAGG
GCGCGTAGGCGGTTATTTAAGTTGGATGTGAAATCCCTGGGCTTAACCTAGGAACTGCAT
TCAAAACTGGGTAACTAGAGTACTGTAGAGGGTAGTGGAATTTCCGGTGTAGCGGTGAAA
TGCGTAGAGATCGGAAGGAACACCAGTGGCGAAGGCGGCTACCTGGACAGATACTGACGC
TGAGGCGCGAGAGCGTGGGGAGCAAACAGGATTAGAAACCCCTGTAGTCC
>Otu13176
CCAGCCTACGGGTGGCAGCAGTAAGGGATATTGCGCAATGGGCGAAAGCCTGACGCAGCA
ACGCCGCGTGGAGGATGAAGGCCTTCGGGTCGTAAACTTCTTTTAGCAGGGACGAGGAAG
GACGGTACCTGCGGAATCAGTCACGGCTAACTACGTGCCAGCAGCCGCGGTAAAACGTAG
GTGGCAAGCGTTATCCGGATTTACTGGGCGTAAAGGGTGTGCAGGCGGGACTGCAAGTGG
TGTATGAAATCTCCAGGCTCAACCTGGAGGGGTTATGCCAGACTGCAGTTCTAGAGGGCG
AGAGAGGGGGGTGGAATTCCGGGTGTAGTGGTGAAATGCGTAGAGATCCGGAGGAACCCC
AGAGGCGAAGGCGGCCCCCTGGCTCGTACCTGACGCTCAGCCACGAAAGCTAGGGGAGCA
AACAGGATTAGATACCCTTGTAGTCA
>Otu13181
CCAGCCTATGGGGTGCAGCAGTAGGGAATATTGGACAATGGACGAAAGTCTGATCCAGCA
ATGCCGCGTGAATGATGAAGGCCTTAGGGTTGTAAAGTTCTTTAAGTGGGGAAGATAATG
ACGGTACCCACAGAATAAGCACCGGCTAACTCCGTGCCAGCAGCCGCGGTAATACGGAGG
GTGCAAGCGTTGTTCGGAATTACTGGGCGTAAAGCGCGCGTAGGCGGCTTTGTAAGTTGG
GAGTGAAATCCCGGGGCTTAACTCCGGAATTGCTTTCAAAACTACAAAGCTAGAGTATGG
CAGGGGACAGTGGAATTCCTAGTGTAGAGGTGAAATTCGTAGATATTAGGAGGAACACCA
GTGGCGAAAGCGACTGTCTGGACCATTACTGACGCTGAGGCGCGAAAGCGTGGGGAGCAA
ACAGGATTAGATACCCCGGTAGTCC
>Otu13186
CCAGCCTATGGGTTGCACCAGTAGGGAATTTTCCACAATGGGCGCAAGCCTGATGGAGCA
ACGCCGCGTGCAGGATGAATGTCTTCGGATTGTAAACTGCTTTTCTCTGTGACGAATATG
ACGGTAGCAGAAGAATAAGGACCTGCTAACTACGTGCCAGCAGCCGCGGTCATACGTAGG
GTCCAAGCGTTATCCGGAATTACTGGGCGTAAAGAGTTGCGTAGGTGGCATTGTAAGCGG
GTAGTGAAATGACGAGGCTCAACCTCGTGTCCATTACCTGAACTGCAAAGCTAGAGGATG
AGAGAGGTTATTGGAATTCCCTGTGTAGGAGTGAAATCCGTAGATATAGGGAGGAACACC

GATGGCGTAGGCAGATAACTGGCTCATTCCTGACACTAAGGCACGAAAGCGTGGGGAGCA
AACAGGATTAGAAACCCCTGTAGTCC
>Otu13191
CCAGCCTACGGGTTGCTGCAGTCGAGGATCTTTCGCAATGGGCTAAAGCCTGACGAAGCG
ACGCTGTGTGAGTGATGAAGGCCTTCGGGTTGTAAAGCTCTTTCGCTTGGGAACAAGAAA
TGGTAATGAATAATTGCCAAATTTGAGGGTACCAGGTAAAGAAGCACCGGCTAACTACGT
GCCAGCAGCTGCGGTAATACGTAGGGTGCAAGCATTAATCGGAATTATTGGGCGTAAAGG
GCGCGTAGGCGGAAACGCAAGTCAGATGTGAAATTCCAAAGCTCAACTTTGGAGCTGCAT
TTGAAACTGCGATTCTTTGAGGTATGGCGGAGAAAGCGGAATTCCACGTGTAGCGGTGAA
ATGCGTAGATATGTGGAAGAACACCAGTGGCGAAAGCGGCTTTCTAGTTTTTACCTGACG
CTGAGGCGCGAGAGCAAGGGGAGCAAACAGGATTAGAAACCCTCGTAGTCC
>Otu13195
CCAGCCTACGGGGGGCACCAGTCGAGAATCTTCGGCAATGGGCGCAAGCCTGACCGAGCG
ACGCCGCGTGTGGGATGAAGGCCCTTGGGTTGTAAACCACTGTCAGAGGGGATGAAGTGC
CGGAGAGCTATCTCTCCGGTTTGACATAGCCTCAGTGGAAGCACGGGCTAAGTTCGTGCC
AGCAGCCGCGGTAATACGAACTGTGCAAACGTTATTCGGAATCACTGGGCTTAAAGAGTG
CGTAGGCGGCTGTTTAAGTAGGGTGTGAAAGCCCCCGGCTCAACCGGGGAATGGCGCCCT
AAACTAGACAGCTGGAGTGGGACAGGGGTGTACGGAACTTCCGGTGGAGCGGTGAAATGT
GTTGATATCGGAAGGAACGCCGGTGGCGAAAGCGGTACACTGGGTCTCAACTGACGCTGA
GGCACGAAAGCCAGGGGAGCAAACGGGATTAGAAACCCGTGTAGTCC
>Otu13196
CCAGCCTATGGGTGGCAGCAGTAGGGAATTTTCCACAATGGACGCAAGTCTGATGGAGCA
ACGCCGCGTGCAGGAGGAAGGCCTTCGGGTTGTAAACTGCTTTTATTTGTGAAGATTTTG
ACTGTAACAAATGAATAAGGACCTGCTAACTACGTGCCAGCAGCCGCGGTCATACGTAGG
GTCCAAGCGTTATCCGGAATTACTGGGCGTAAAGAGTTGCGTAGGTGGCATAGTAAGCAA
ATAGTGAAAGCGTGAGGCTCAACCTCATATCCATTGTTTGAACTGCTAAGCTTGAGCATG
AGAGAGGTAGATGGAATTTCTAGTGTAGGAGTGAAATCCGTAGATATTAGAAGGAACACC
GATGGCGTAGGCAGTCTACTGGCTCATTGCTGACGCTGAGACATGAAAGCGTGGGAATCG
AACGGGATTAGAGACCCTCGTTAGTCC
>Otu13198
CCAGCCTACGGGGGGCTGCAGTGGGGAATCTTGGACAATGGGGGCAACCCTGATCCAGCC
ATGCCGCGTGAGTGATGAAGGCCTTCGGGTTGTAAAACTGTTTCGACGGGGACGATGATG
ACGGTACCCGTAGAAGAAGCCCCGGCTAACTTCGTGCCAGCAGCCGCGGTAATACGAAGG
GGGCTAGCGTTGCTCGGAATGACTGGGCGTAAAGGGCGCGTAGGCGGTTCGTACAGTCAG
GCGTGGAATTCCCGGGCTCAACCTGGGGGCTGCGTTTGATACGTACGGGCTTGAGTTCGG
AAGAGGGTCGTGGAATTCCCAGTGTAGAGGTGAAATTCGTAGATATTGGGAAGAACACCG
GTGGCGAAGGCGGCGATCTGGGCCAATACTGACGCTGAGGAGCGAAAGCGTGGGGAGCGA
ACAGGATTAGAAACCCGTGTAGTCC
>Otu13202
CCAGCCTACGGGGGGCTGCAGTAGGGAATATTGGACAATGGGCGAGAGCCTGATCCAGCC
ATGCCGCGTGCAGGAAGAAGGCCTTCTGGGTTGTAAAGCACTTTTAGTGGGGATGAAAAG
TTCAGGGCTAATACCCTTGGATCTTGACCTAACCTACAGAAAAAGCACCGGCTAACTCTG
TGCCAGCAGCCGCGGTAATACAGAGGGTGCGAGCGTTAATCGGAATTACTGGGCGTAAAG
CGTGCGTAGACGGTTCTGTAAGTCGGATGTGAAATCCCCGGGCTCAACCTGGGAATTGCA
TTCGAGACTGCAAAGCTAGGGTGCGGAAGAGGGAAGCGGAATTTCCGGTGTAGCGGTGAA
ATGCGTAGATATCGGAAGGAACATCAGTGGCGAAGGCGGCTTCCTGGTCCAGCACCGACG
TTCAGGCACGAAAGCGTGGGGAGCAAACAGGATTAGAGACCCTTGTAGTCC
>Otu13211
CCAGCCTACGGGACGCTGCAGTGGGGAATCTTGCACAATGGAGGAAATTCTGATGCAGCG
ACGCCGCGTGAGCGATGAAGCCCTTCGGGGTGTAAAGCTCTTTCGACGGGAACGATCATG
ACGGTACCTGAGGAAGAAGCTGCGGCTAACTACGTGCCAGCAGCCGCGGTAAGACGTAGG
ATGCAAGCGTTGTCCGGATTTATTGGGCGTAAAGAGTTCGTAGGCGGTTTGTTAAGTCTG
ACGTTAAAGATCAGGGCCCAACCCTGGGAATGCTTTGGATACTGACAGACTGGAGTGTGG
TAGAGGTAAGTGGAATTTCTGGTGTAGCGGTGAAATGCGTAGATATCAGAAAGAACACCA
GTGGCGTAGGCGACTTACTGGGCCATAACTGACGCTGAGGAACGAAAGCCAGGGGAGCGA
ATGGGATTAGAAACCCCTGTAGTCC
>Otu13225
CCAGCCTATGGGTCGCACCAGTGAGGAATATTGCACAATGGGCGAAAGCCTGATGCAGCG

ACGCCGCGTGGAGGATGAGGGTCTTCGGATTGTAAACTCCTGTTAAATGGGAAAAAAGAG
TCCTTCTGAATAGGGAGGAAAGATGATGGTACCATTAAAGAAAGCACCGGCTAACTTCGT
GCCAGCAGCCGCGGTAATACGAGGGGTGCAAGCGTTATTCGGATTTATTGGGCGTAAAGG
GTGTGTAGGCGGTTTTTTAAGTCATTTATTAAATGCCTTGGCTTAACCAAGGAAATGTGG
GTGAAACTAGAAGACTAGAGTGTGGAAGAGAGAAGTGGAATTCTCGGAGTAGCGGTAAAA
TGCGTAGATCTCGAGAGGAACACCGATGGCGAAGGCAGCTTCTTGGTCCATTACTGACGC
TCAAACACGAAAGCGTGGGGAGCAAACAGGATTAGATACCCGGGTAGTCC
>Otu13230
CCAGCCTATGGGTGGCAGCAGTGAGGAATTTTGGGCAATGAGCTAACGCTTGACCCATTG
ACGGTTTATGAGTGAGAAAAGGACAGCAATAACGCTGTAAAGCTCTTTTGTTGAGGAAGA
TAAATGACTGTACTCAAAGAATAAGCACTGGCTAATTCTGTGCCAGCAGCCGCGGTAACA
CAGGGAGCGCGAACGTTATCCGGAATTATTGGGCGTAAAGGATATGTAGGCAGTTTTGGT
TGGATATTTTGTGAAATCCTCGAGAAAATCCTTGCGGAGGACAAAAGTTACCCAAAAACT
TTGAGTTTTGAGAAAGGTTAAAAGAATTTCTTGTGTAAGGGTAGAATCTTAACATACTAG
AGAGACTGCCGAAACAGTAGCGAAGGCATTTTTCTAGCTCTAACTGACGCTGAGATATGA
AAGGATAGGGAGCAAACGGGATTAGAAACCCCGGTAGTCC
>Otu13242
CCACCCTATGGGAGGCAGCAGCTTGGAATATTGCACAATGGGGGAAACCCTGATGCAGCG
ACGCCGCGTGGAGGATGAAGGATCTAGGTCTGTAAACTCCTTTTATCGGGAAAGACTTAG
GACGGTACCCGATGAATAAGCACCGGCTAACTACGTGCCAGCAGCCGCGGTAAGACGTAG
GGTGCAAGCGTTGTCCGGATTTACTGGGCGTAAAGGACGCGTAGGCGGTCCGTTAAGTGT
GAAGTGAAATCTCCAGGGCTCAACCCGGAAACTGCTTCGCATACTGTCGGACTAGAGGGA
TGCAGAGGTAGCTAGAATTCCTGGTGTAGCGGTGAAATGCGTTGATATCAGGAGGAATAC
CCATGGCGAAGGCAGGCTACTGGGCATCATCTGACGCTGAGGCGTGAAAGCGTGGGTAGC
AAACAGGATTAGAGACCCCCGTAGTCC
>Otu13265
CCAGCTATGGGGTGCAGCAGTGGGGAATCTTGCACAGTGGGCGAAAGCCTGATGCAGCGA
CGCCGCGTGGGGGATGAAGCTTTTCGGAGTGTAAACCCCTTTCGACTGGGACGAATGCCC
GCAAGGGAGTGACGGTACCAGTATAAGAAGCCCCGGCTAACAACGTGCCAGCAGCCGCGG
TAAGACGTAGGGGGCCAGCGTTGTTCGGAATTACTGGGTGTAAAGGGTTCGTAGGCGGTG
CGGCAAGTTGGGAGTGAAATCTCTGGGCTCAACCCAGAGGCTGCTTCCAAAACTGCTGTG
CTTGAGTGTGAGAGAGGCTCGTGGAATTGCAGGTGTAGCGGTGAAATGCGTAGATATCAG
GAGGAACACCGGTGGTGTAGACGGCTTTCTGGACCGTAACTGACGCTGAAACACGAAAGC
GTGGGTAGCAAACAGGATTAGATACCCCCGTAGTCC
>Otu13266
CCAGCCTATGGGGGGCTGCAGTAACGAGTATTGGGCAATGGGCGAAAGCCTGACCCAGCG
ACGCCGCGTGCAGGATGAAGTCCTTCGGGATGTAAACTGCTGTCAGGGGCTGCCAAGCAA
CCGTAATTAACATCTACGGAAGTTGAGGAGCCCCAGAGGAAGCCACGGCTAACTTCGTGC
CAGCAGCCGCGGTAAGACGAAGGTGGCAAGCGTTGTTCGGAATCATTGGGCTTAAAGCGC
ACGTAGGCGGCTCTTCAAGTGTCTTGTGAAATCCCTCGGCTCAACCGAGGAATTGCTGGG
CATACTGTCGAGCTTGAGGTAGGTATGGACGAGTGGAACTCTTGGTGGAGCGGTGGAATG
CATAGATATCAAGAGGAACGCCGGTGGTGAAGACGACTCGCTGGGCCTATCCTGACGCTG
AGGTGCGAAAGCCAGGGGAGCAAACGGGATTAGAAACCCCTGTAGTCC
>Otu13271
CCAGCCTACGGGGGGCAGCAGTGAGGAATATTGGACAATGGTGGCAACACTGATCCAGCC
ATGCCGCGTGCAGGATGAAGGCGCTACGCGTTGTAAACTGCTTTTGTACCAGAGAAAACC
TATCTACGTGTAGATAGCTGATAGTATGGTAAGAATAAGCATCGGCTAACTTCGTGCCAG
CAGCCGCGGTAATACGGAGGGTGCAAGCGTTATCCGGATTTACTGGGTTTAAAGGGTGCG
TAGGCGGTGCGGTAAGTCTTCTGTGAAATCTCCGGGCTCAACTCGGAGTCTGCAGGAGAA
ACTGCCGTGCTTGAGTATGGGAGAGGTGAGTGGAATTCCTGGTGTAGCGGTGAAATGCGT
AGATATCAGGAGGAACACCTGTGGCGAAAGCGGCTCACTGGACCATAACTGACGCTGATG
CACGAAAGCTAGGGGAGCAAACAGGATTAGAAACCCGCGTAGTCC
>Otu13278
CAGCCTATGGGGGGCAGCAGTGGGGAATATTGCACAATGGGCGCAAGCCTGATGCAGCGA
CGCCGCGTGGAGGAAGAAGGTTTTCGGATTGTAAACTCCTGTTAAGTGGGAAAAAATCCT
GACGGATAATACCCGATTGGGGATGATGGTACCACTAGAGAAAGTACCGGCAAACTTCGT
GCCAGCAGCCGCGGTAATACGAAGGGTACAAGCGTTATTCGGAGTTACTGGGCGTAAAGA
GCGTGTAGGCGGCTTGTTAAGTTGGATGTTAAATTCCCCGGCCTAACCGGGGATGTGCAT

CCGAAACTGGCGAGCTAGAGTGCAAGAGAGAGAAGTAGAATTCCCGGAGTAGCGGTAAAA
TGCGTAGATCTCGGGAGGAATACCGATGGCGAAGGCAGCTTCTTGGCTTGTAACTGACGC
TAAAGCGCGAAAGCGTGGGGAGCAAACAGGATTAGATACCCCAGTAGTCC
>Otu13308
CCAGCTACGGGTGGCTGCAGTGAGGAATTTTCCGCAATGGGCGAAAGCCTGACGGAGCGA
CGCCGCGTGTGGGATGAAGGCCTTCGGGTCGTAAACCACTGTCGAAGGGGACTATACTGA
AGGTACCCTTGGAGGAAGCCCCGGCTAACTACGTGCCAGCAGCCGCGGTAAGACGTAGGG
GGCAAGCGTTATTCGGAATTATTGGGCGTAAAGCGCTCGTAGGCGGATGAGCAAGTTCGT
GAAGAAAGACCCGGGCTCAGCTCGGGGAACGGCACGAATACTGCTCGTCTTGAGGCAATC
AGAGGGTGATGGAATTCCCGGTGTAGCGGTGAAATGCGTAGATATCGGGAGGAACACCAG
TGGCGAAGGCGATCACCTGGGGTTGTTCTGACGCTGAGGAGCGAAAGCTGGGGGAGCAAA
CGGGATTAGAAACCCGCGTAGTCC
>Otu13310
CCAGCCTATGGGTGGCAGCAGTCGAGAATATTTCGCAATGGGCGCAAGCCTGACGAAGCG
ACGCCGTGTGAGCGATGAAGGCCTTAGGGTCGTAAAGCTCTTTCGCCTAAGAACAAGAGA
AGATAGTGAATAGCTGTCGGATTTGAGCGTACTAGGTAAAGAAGCACCGGCTAACTCCGT
GCCAGCAGCTGCGGTAATACGGAGGGTGCAAGCATTAATCGGATTTATTGGGCGTAAAGG
GGGCGTAGGCGGATCAGCAAGCCAGATGTGAAATCCCGAGGCTCAACCTCGGAACAGCAT
TTGGTACTACTAATCTAGAGGGTAGACGGAGAAAACGGAATTCCACATGTAGCGGTGAAA
TGCGTAGATATGTGGAAGAACACCGGTGGCGAAAGCGGTTTTCTAGTTTATTCCTGACGC
TGAGGCCCGAAAGCTAGGGGAGCAAACAGGATTAGAAACCCCCGTAGTCC
>Otu13321
CCAGCCTATGGGTCGCAGCAGGGAATATTGGTAATGTGCGAAAGCGCGAACCAGCAACGC
CGCGTGCACGATGAAGGCCTTCGGGTTGTAAAGTGCTTTTAGAGAGGATGAGGAAGGACA
GTACTCTCTGAATAAGCCTCGGCTAACTACGTGCCAGCAGCCGCGGTAAAACGTAGGAGG
CAAGCGTTATCCGGATTTACTGGGCGTAAAGCGCGTGTAGGTGGTTTTGTAAGCCCGGCG
TGGAAGCCCACGGCTCAACCGTGGGATTGCGTTGGGAACTGCGAGGCTTGAATCATGGAG
AGGGAGCTAGAATTCCTGGTGTAGGGGTGAAATCTGTAGAGATCAGGAAGAATACCAGTG
GCGAAGGCGAGCTCCTGGCCAATGATTGACGCTGAGGCGCGAAAGCGTGGGGAGCAAACA
GGATTAGAAACCCGAGTAGTCC
>Otu13324
CCAGCCTATGGGGGGCTGCAGTCAAGAACATTTGGCAATGGGCGAAAGCCTGACCATGCG
ACGCCGCGTGCTGGACGAAGTCCTTCGGGACGTAAACAGCTTTTATGGGGGAAGAAGTAA
TTGACATTACCCCATGAATAAGGGGCTCCTAACTCTGTGCCAGCAGGAGCGGTAATACAG
AGGCCCCGAGCATTATCCGGAATCACTGGGCGTAAAGGGTGTGTAGGCGGTCGTGTTAGT
CTTTTGTAAAAGATCTCGGGCTTAACCCGGGAGATGCGGAGGAAACGGCACGACTCAGAG
GACGCGAGAGGTAGAGGGAACTCATGGTGTAGGGGTGAAATCCGTTGATATCATGGGGAA
CACCAAATGCGAAGGCACTCTACTGGCGCGCTCCTGACGCTGAAACACGAAAGCGTGGGT
AGCGAATGGGATTAGAGACCCGTGTAGTCC
>Otu13336
GGACTACAAGGGTATCTAATCCGGTTCGCTCCCCCAGCTTTCATCCCTCACCGTCGGGCG
CGTTCTAGCAGACAGCCTTCGCCACTGGTGGTCTTCCCGGGATTATAGGATTTCACCCCT
ACCCCGGAAATACCGTCTGCCTCTCTCGTCCCCTAGCCAAGCAGTATCCCCCGCAATCCA
ACGATTAATTCGGTGGATTTAACAGGGGACTAATCCAGCCAGCTACGGATGCTTTAGGCC
CAATAAACATCCACACCACTCGGAGAGCTGGTATTACCGCGGCGGCTGACACCAGACTTG
CCCTCTCCTTATTCCCCCAGCGTTCTAGACTGGGTAAAAGCCTTCCTCAGCGGATGGCAC
TCGGGATAGCCCTGTCAAGCTTTCGCTTATTGCAAAGTTTTCGCGACTGCAGCCCCCCAT
AGGCTGG
>Otu13343
CCAGCCTATGGGTGGCACCAGCTAAGAATATTCCGCAATGGACGAAAGTCTGACGGAGCG
ACGCCGCGTGGATGACGAAGGCCGAAAGGTTGTAAAGTCCTTTTGTTGGGGAAGAATAAG
CATGGGAGGGAATGCCCGTGTGATGACATGAACCGACGAATAAGCCCCGGCTAACTACGT
GCCAGCAGCCGCGGTAACACGTAGGGGGCGAGCGTTGTTCGGAATTACTGGGCGTAAAGG
GCATGTAGGCGGCTTTGTAAGTCCGGCGTGGAAGCCCACGGCTCAACCGTGGGATTGCGC
TGGAAACTGCGGAGCTTGAATCATGGAGAGGGAGCTAGAATTCCTGGTGTAGGGGTGAAA
TCTGTAGATATGCGGAAGAATACCTGTGGCGAAGGCGAGTTTCTGGTCTATAACTGACGC
TGAAATGCGAAAGTGTGGGTAGCAAACAGGATTAGAAACCCCAGTAGTCC
>Otu13345

GGACTACAAGGGTTTCTAATCCTGTTTGCTACCCACGCTTTCGTGTCTCAGCGTCAGTCG
CGGTCCAGAGAGCCGTCTACACCGCCGGTGTTCCTCCTGATATCTACGCATTTCACCGCT
ACACCAGGAATTCCACTCTCCTCTCCCGCACTCTAGTCGCTCAGTATTCGGCGCACCCTC
CCGGTTGAGCCGGGAGATTTCACACCAAACTTAAACAACCGCCTACACACCCTTTACGCC
CAGTAACTCCGAACAACGCTCGCTGCCTACGTATTACCGCGGCTGCTGGCACGTAGTTAG
CCGCAGCTGCTTCTGCAGGTACCGTCATCGTCTTCCCTGCCGAAAGAGCTTTACACCCCG
AGGGGCTTCATCGCTCACGCGGCGTCGCTGCATCAGGGTTTCCCCCATTGTGCAAGATTC
CCCACTGCAGCGACCCGTAGGCTGG
>Otu13351
CCCTATGGGAGGCAGCAGCTAAGAATCTTCCGCAATGGGCGAAAGCCTGACGGAGCGATG
CCGCGTGATCGATGAAGGTCGAGAGATTGTAAAGATCTTTTCCACATGAGGAATAACCAT
CGGAGGGAATGCCGGTGGGATGACGTTAGTGTGGGAATAAGCCCCGGCTAATTACGTGCC
AGCAGCCGCGGTAACACGTAAGGGGCGAGCGTTGTTCGGAATTATTGGGCGTAAAGGGCA
CGCAGGCGGTTCTGCAAGTCTGATGTGAAAACTCCGGGCTCAACTCGGAATGCGCGTTGG
AAACTGTAGGACTAGAGTATTAGAGGGGGAGTCGGAATTCCTAGTGTAGGGGTGAAATCT
GTTGATATTAGGAAGAACACCGGTGGCGAAGGCGGACTCCTGGCTGGATACTGACGCTGA
GGTGCGAAAGCATAGGGAGCGAACAGGATTAGAGACCCTCGTAGTCC
>Otu13367
CCAGCCTACGGGTGGCAGCAGTCGAGAATCATTCGCAATGGGCGCAAGCCTGACGATGCG
ACGCCGTGTGGACGATGAAGGCCTTCGGGTTGTAAAGTCCTTTCGCCTGGGAACAAGAGA
TACGTAGTAATACTGCGTAAATTTGAGGGTACCAGGTAAAGAAGCACCGGCTAACTCCGT
GCCAGCAGCTGCGGTAATACGGAGGGTGCAAGCGTTAATCGGATTTATTGGGCGTAAAGG
GGGCGTAGGCTGGGACGAAAGTCAGACGTGAAATTCCGGGGCTCAACTCCGGACCTGCAT
CTGAAACTTCATCTCTAGAGGTTAGACGGAGAAAACGGAATTCCACAAGTAGCGGTGAAA
TGCGTAGATATGTGGAAGAACACCGGTGGCGAAGGCGGTTTTCTAGTTTATACCTGACGC
TGAGGCCCGAAAGCGAGGGGAGCAAACAGGATTAGATACCCGGGTAGTCC
>Otu13403
CCAGCCTATGGGATGCTGCAGTCGAGAATCATTCGCAATGGGCGCAAGCCTGACGATGCG
ACGCCGCGTGAGCGATGAAGGCCTTCGGGTTGTAAAGCTCTTTCGCCTGGGAACAAGGAA
AGCTGGCGAATACCCAGTGACTTGAGGGTACCAGGTAAAGAAGCACCGGCTAACTCCGTG
CCAGCAGCTGCGGTAATACGGAGGGTGCAAGCATTAATCGGATTTATTGGGCGTAAAGGG
CGCGTAGGCGGGAAGGTAAGTCAAATGTGAAAGCCCGGGGCTCAACCTCGGAGCCGCATG
TGAAACTGCCTTTCTTGAGGGTAGGCGGAGAAAACGGAATTCCACAAGTAGCGGTGAAAT
GCGTAGATATGTGGAAGAACACCGGTGGCGAAAGCGGTTTTCTAGCTTATTCCTGACGCT
GAGGCGCGAAAGCAAGGGGAGCAAACAGGATTAGAAACCCTGGTAGTCC
>Otu13412
CCAGCCTATGGGGGGCTGCAGTGGGGAATCTTGCACAATGGAGGAAACTCTGATGCAGCG
ACGCCGCGTGAGCGATGAAGCCCTTCGGGGTGTAAAGCTCTTTCGGCAGGGAAGATTATG
ACGGTACCTGCAGAAGCAGCTGCGGCTAACTACGTGCCAGCAGCCGCGGTAATACGTAGG
CAGCAAGCGTTGTTCGGAGTTACTGGGCGTAAAGGGTATGTAGGCGGTTGCCTAAGTTTG
GTGTGAAATCTCCCGGCTTAACCGGGAGGGTGCGCCGAAAACTGGGTGACTAGAGTATGG
GATGGGTAAGTGGAATTCCTGGTGTAGCGGTGAAATGCGTAGATATCAGGAGGAACATCC
GAGGCGAAAGCGGCTCGCTGGATCGCAACTGACGCTGAGGGACGAAAGCTAGGGGAGCAA
ACAGAATTAGAGACCCCAGTAGTCC
>Otu13421
CCAGCCTACGGGTCGCTCCAGTGGGGAATATTGCACAATGGACGAAAGTCTGATGCAGCG
ACGCCGCGTGGAGGATGAAGGTTTTCGGATTGTAAACTCCTGTTGAGTGGGAAGAAAGAC
CTCTACCTAATACGTAGAGGGGATGACGGTACCACTAGAGAAAGCACCGGCAAACTTCGT
GCCAGCAGCCGCGGTAATACGAAGGGTGCAAGCGTTATTCGGAGTTACTGGGCGTAAAGA
GCGCGTAGGCGGTTTTTTAAGTCATGTGTTAAATCTCCCGGCTTAACCGGGAACATGCAT
GTGATACTGGGAAACTAGAGTATAAGAGAGAGAAGCGGAATTCTCGGAGTAGCGGTAAAA
TGCGTAGATCTCGAGAGGAACACCGGAAGCGAAGGCGGCTTCTTGGCTTATAACTGACGC
TGATGCACGAAAGCTAGGGGAGCAAACAGGATTAGAAACCCCAGTAGTCC
>Otu13431
CCAGCCTACGGGTTGCAGCAGTAGGGAATATTGGGCAATGGGCGAAAGCCTGACCCAGCC
ATGCCGCGTGAAGGATGAAGGTCCACAGGATTGTAAACTTCTTTTATCAGGGAATAAGGC
AGATGTAACTCATCTGATTGAAGGTACCTGGGGAATAAGCACCGGCTAACTTCGTGCCAG
CAGCCGCGGTAATACAGAGGTCTCAAGCGTTGTTCGGATTCATTGGGCGTAAAGGGTGCG

TAGGCGGCGAAGCAAGTCAGATGTGAAATCCCGGGGCTCAACCCCGGAACTGCATTTGAT
ACTGCTTTGCTCGAGGACTGGAGAGGAGATCGGAATTCACGGTGTAGCAGTGAAATGCGT
AGATATCGTGAGGAAGGCCAGTGGCGAAGGCGGATCTCTGGACAGTTCCTGACGCTGAGG
CACGAAGGCCAGGGGAGCAAACGGGATTAGAAACCCTTGTAGTCC
>Otu13440
CCAGCCTATGGGAGGCTGCAGTAAGGAATATTGGTCAACGGGCGGAAGCCTGAACCAGCC
ATGCCGCGTGGAGGATGAAGGCCCTCTGGGTTGTAAACTTCTTTTATTGGGGAAGAAACC
GGTTTTTTCTAAAGCCGTTGACGGTACCCATTGAATAAGCACCGGCTAACTCCGTGCCAG
CAGCCGCGGTAATACGGAGGGTGCAAGCGTTATCCGGATTCACTGGGTTTAAAGGGTGCG
TAGGTGGACAGGTAAGTCAGTGGTGAAATCTCTAAGCTTAACTTAGAAACTGCCGTTGAT
ACTATCTGCCTTGAATTTAGTGGAGGTGAGCGGAATATGTCATGTAGCGGTGAAATGCTT
AGATATGACATAGAACACCCATTGCGAAGGCAGCTCGCTACACTACGATTGACACTGAGG
CACGAAAGCGTGGGGATCAAACAGGATTAGAAACCCTCGTAGTCC
>Otu13457
CCAGCCTACGGGAGGCAGCAGTAGGGAATCTAGTGCAATGGGCGAAAGCCTGACGCAGCC
ATGCCGCGTGAATGATGAAGGTCTTAGGATTGTAAAATTCTTTCAGCGGGGACGATAATG
ACGGTACCCGCAGAAGAAGCCCCGGCTAACTTCGTGCCAGCAGCCGCGGTAATACGAAGG
GGGCTAGCGTTGCTCGGAATTACTGGGCGTAAAGCGCGCGTAGACGGTTTTGTAAGTCAG
ATGTGAAATCCCCGGGCTCAACCTGGGAACTGCATTTGAGACTGCATGGCTAGAGTATCG
AAGAGGGAAGTGGAATTTCCGGTGTAGCGGTGAAATGCGTAGATATCGGAAGGTACACCA
GTGGCGAAAGCGACTTCCTGGTCGAATACTGACGTTCATGTGCGAAAGCGTGGGGAGCAA
ACAGGATTAGAAACCCGAGTAGTCC
>Otu13458
CCAGCCTACGGGATGCAGCAGTGGGGAATCTTGCACAATGGGGGCAACCCTGATGCAGCG
ACGCCGCGTGAACGATGAAGCCCTTCGGGGCGTAAAGCTCTTTCGGAAGGGACGAAACCG
GTCCGGGATAATAAACCGGGCTCTTGACGGTACCTTAGGAAGAAGCACCGGCTAACTCTG
TGCCAGCAGCCGCGGTAATACAGAGGGTGCAAGCGTTGTTCGGAATTATTGGGCGTAAAG
CGCGTGTAGGCGGCAAGGCATGTCGGGTGTGAAAGCCCTCGGCTTAACCGAGGAAGTGCG
CCCGAAACTATCTTGCTTGAGTTTCGGAGGGGATGGCGGAACTCCCGGTGTAGAGGTGAA
ATTCGTAGATATCGGGAAGAACACCAGTGGCGAAGGCGGCCATCTGGACGAAAACTGACG
CTGAGACGCGAAAGCGTGGGGAGCAAACAGGATTAGAAACCCTTGTAGTCC
>Otu13474
CCAGCCTATGGGGGGCTGCAGTTGGGAATCTTGCACAATGGGGGAAACCCTGATGCAGCG
ACGCCGCGTGGAGGACGAAGGGTCTAGGCCTGTAAACTCCTTTTTTCGGGAAAGACTTAG
GACGGTACCCGAAGAATAAGGACCGGCTAACTACGTGCCAGCAGCCGCGGTAAGACGTAG
GGTCCAAGCGTTGTCCGGATTTACTGGGCGTAAAGAGCGCGTAGGCGGTCTGTTAAGTGT
AGAGTGAAATCTCTGGGCTCACCCCAGAAACTGCTCTGCATACTGGCAGACTTGAGGGAT
GCAGAGGTTTATGGAATTCCTGGTGTAGCGGTGAAATGCGTTGATATCAGGAGGAACACC
CATGGCGAAGGCAGCAAACTGGGCATCATCTGACGCTGAGGCGCGAAAGCGTGGGTAGCA
AACAGGATTAGATACCCGCGTAGTCC
>Otu13475
CCAGCCTATGGGTGGCAGCAGTGACAAAGGCTACTAAATGTTATGCTCTCTGGCCGGAAA
AACAGGAAGCAATAAAAATTAAAAATAAAGAAATTAAAATTTTATTAAAAAACAAAAAAA
CAAAAAAACACACAAGGGATTGTAGAAAGACAAAAATTACTGTTTTTTTAGAGATTTTTT
CAAACAATTTTTTTAATGCGCTTAGGGGCCAAGTGCATGATTACATGACCAAATGCATGA
TTACATGACTAAGTGCAAAATTAGCGAATTGGGTATTATGTGAAGAATTGATGAGGTTTG
ATTGAATAATTGTAATATAGACGTTTAAGGGTTAACCCCATCGATGTCCAACTAAAGGGT
TCCCTATGTATACTACATCAACCATAATAGTTATGTAGTGCAGTTGACCTTGGATATTAG
AGACCCGAGTAGTCC
>Otu13477
CCAGCCTATGGGATGCTGCAGTAGGGAATCTTCCACAATGGGCGAAAGCCTGATGGAGCA
ACGCCGCGTGCAGGATGAAGGTCTTCGGATTGTAAACTGCTTTTATATGCGACGATTATG
ACGGTAGCATATGAATAAGGATCGGCTAACTCCGTGCCAGCAGCCGCGGTCATACGGAGG
ATCCAAGCGTTATCCGGAATTACTGGGCGTAAAGAGTTGCGTAGGTGGCAGCGTAAGCAA
GTAGTGAAATTCTGGGGCTCAACCCCTTGTCCATTACTTGAACTGCGCAGCTAGAGGATG
AGAGAGGTTATTGGAATTCCTAGTGTAGGAGTGAAATCCGTAGATATTAGGAGGAACACC
GATGGCGTAGGCAGATAACTGGCTCATTCCTGACACTAAGGCACGAAAGCATGGGTAACA
AACGGGATTAGATACCCCAGTAGTCC

>Otu13482
CCAGCCTACGGGACGCACCAGTGGGGAATCTTGCACAATGGGGGCAACCCTGATGCAGCG
ACGCCGCGTGAGCGATGAAGCCCTTCGGGGTGTAAAGCTCTTTCGGCAGGGAAGATAATG
ACGGTACCTGCAGAAGCAGCTGCGGCTAACTACGTGCCAGCAGCCGCGGTAATACGTAGG
CAGCAAGCGTTGTTCGGAGTTACTGGGCGTAAAGGGTGTGTAGGCGGCTTTCTAAGTTTG
TTGTGAAATCTCCCGGCTTAACCGGGAGGGTGCGGCGAAGACTGGAAGGCTAGAGTATGG
GATGGGAGAGTGGAATTCCTGGTGTAGCGGTGAAATGCGTAGATATCAGGAGGAACACCA
ATGGCGAAGGCAGCTCGCTGGACGTGACTGACGCTGAGACGCGAAAGCGTGGGGAGCAA
ACAGGATTAGATACCCTGGTAGTCC
>Otu13489
CCAGCCTACGGGATGCACCAGTCGAGAATTTTTCACAATGGGCGCAAGCCTGATGGAGCG
ACGCCGCGTGGGGGATGAAGGGCTTCGGCTCGTAAACCCCTGTCATTCGTGAACAAAGCC
TGGGGGTAAACAGCCTTCAGGGTGATAGTAACGGAAGAGGAAGGGACGGCTAACTCTGTG
CCAGCAGCCGCGGTAATACAGAGGTCCCGAGCGTTGTTCGGATTTACTGGGCGTAAAGGG
TGCGTAGGCGGCCGGGTAAGTCCAGCGTGAAATCTCCAAGCTTAACTTGGAAAGGGCGCT
GGAAACTATCAGGCTCGAGTGCCGGAGAGGAGACTGGAATTCTCGGTGTAGCAGTGAAAT
GCGTAGATATCGAGAGGAACACCAGTGGCGAAGGCGGGTCTCTGGACGGATACTGACGCT
GAGGCACGAAAGCCAGGGGAGCAAACGGGATTAGATACCCGAGTAGTCC
>Otu13507
CCAGCCTACGGGTCGCAGCAGTCGAGAATCTTCGGCAATGGGCGCAAGCCTGACCGAGCG
ACGCCGCGTGGAGGATGAAGGCCTTCGGGTTGTAAACTCCTGTCGCAGGGGAGCAAGGGC
CGGCGAAGAGCCGGTCTTGACCGATCCTGGGAGGAAGCACGGGCTAAGTTCGTGCCAGCA
GCCGCGGTAAGACGAACCGTGCGAACGTTATTCGGAATCACTGGGCTTAAAGCGCGTGTA
GGCGGGTCGACGCATCGGTCGTTGAAATCCCCCGGCTCAACCGGGGAACTGGCGCCGAGA
TGGTCGATCTGGAGGGGAGCAGGGGGACCTGGAACTTCCGGTGGAGCGGTGAAATGCGTT
GAGATCGGAAGGAACGCCCGTGGCGAAAGCGAGGTCCTGGACTTTTTCTGACGCTGAGAC
GCGAAAGCTAGGGGAGCAAACGGGATTAGAGACCCGCGTAGTCC
>Otu13514
CCAGCCTACGGGTGGCAGCAGTGAGGAATCTTGCGCAATGGGCGAAAGCCTGACGCAGCG
ACGCCGCGTGGAGGATGACGGTCTTCGGATTGTAAACTCCTGTTAAGTGGGAAGAAAAGC
CTGTTTTTAATAGAAACAGGTAATGACGGTACCATTAGAGAAAGCACCGGCTAACTTCGT
GCCAGCAGCCGCGGTAATACGAGGGGTGCTAGCGTTATTCGGAATTATTGGGCGTAAAGG
GTGCGTAGGTGGCGCGCTAAGTCAACTGTTAAATTCCTCGGCCTAACCGAGGATCTGCGG
TAGATACTGGCGAGCTAGAGGGTGGAAGAGAGAAGTGGAATTCTCGGAGTAGCGGTAAAA
TGCGTAGATCTCGAGAGGAACACCAATGGCGAAGGCAGCTTCTTGGTCCATACCTGACAC
TGAGGCACGAAAGCTAGGGGAGCAAACAGGATTAGATACCCCTGTAGTCC
>Otu13534
CCAGCCTATGGGGGGCTGCAGTAAGGAATATTGGACAATGGTGGCAACACTGATCCAGCC
ATGCCGCGTGCAGGATGAAGGCGCTACGCGTTGTAAACTGCTTTTGTACCAGAGAAAACC
TATCTACGTGTAGATAGCTGATAGTATGGTAAGAATAAGCATCGGCTAACTTCGTGCCAG
CAGCCGCGGTAATACGAAGGATGCAAGCGTTATCCGGATTTATTGGGTTTAAAGGGTGCG
TAGGCGGGTTTGTAAGTCAGCGGTGAAAGACTGTCGCTTAACGATAGCATTGCCATTGAT
ACTGCAGATCTTGAGTACAGTTGAGGTAGGCGGAATGTGTAGTGTAGCGGTGAAATGCTT
AGATATGACACAGAACACCGATTGCGAAGGCAGCTGACGAAACTGTAACTGACGCTGAGG
CACGAAAGCGTGGGGATCAAACAGGATTAGATACCCCGGTAGTCC
>Otu13537
CCAGCCTATGGGTGGCAGCAGTAAGGAATATTGGTCAATGGACGCAAGTCTGAACCAGCC
ATGCCGCGTGAAGGATGACAGCCCTACGGGTTGTAAACTTCTTTTGTACGGGAAAAAACC
CCTGGTCGTGAACCAGGGCTGATGGTACCGTAAGAATAAGCATCGGCTAACTCCGTGCCA
GCAGCCGCGGTAATACGGAGGATGCAAGCGTTATCCGGATTCATTGGGTTTAAAGGGTGC
GCAGGCGGAATGATAAGTCAGTGGTGAAATCCTACAGCTCAACTGTAGAACTGCCATTGA
TACTATCGTTCTTGAGTACATTTGAAGTGGGCGGAATGTGTCATGTAGCGGTGAAATGCT
TAGATATGACACAGGACACCGATTGCGAAGGCAGCTTACTGGGCCATTACTGACGCTGAT
GCACGAAAGCGTGGGGAGCGAACAGGATTAGAAACCCTAGTAGTCC
>Otu13541
CCAGCCTATGGGGTGCTGCAGCTAAGAATATTCCGCAATGGACGAAACTCTGATGCAGCG
ACGCCGCGTGAGTGACGAAGGCCTTCGGGTTGTGAAGCTCTGTTCTCAGGGAAAAAGAAA
GTGATTGTACCTGAGGAGAAAGGACCGGCTAACTTCGTGCCAGCAGCCGCGGTAAGACGA

GGGGTCCAAGCGTTGTTCGGAATCATTGGGCGTAAAGCGGGTGTAGGTGGCTCTGTAAGT
CAGGAGTGAAATCCCCAAGCTTAACTTGGGAAGTGCTTTTGAAACTGCAGAGCTTGAGTG
TGGGAGAGGATCGTGGAATTCCAGGTGTAGTGGTGAAATACGTAGATATCTGGAGGAACA
CCGGTGGCGAAGGCGGCGATCTGGCCCAACACTGACACTGAGACCCGAAAGCGTGGGGAT
CAAACAGGATTAGAGACCCGTGTAGTCC
>Otu13542
CAGCCTACGGGGTGCACCAGGCGCGAAAACTTTACAATGCGGGAAACCGTGATAGGGGAA
CTCTGAGTGCCCGTTAAATCGGGCTGTCCATCAGTTTAAATAACTGGTGAAGAAAGGGCC
GGGCAAGACCGGTGCCAGCCGCCGCGGTAATACCGGCGGCTCGAGTGGTGGCCGCTATTA
TTGGGCTTAAAGGGTCCGTAGCTGGATTCACAAGTCCCTTGAGAAATCCATCGGCTTAAC
TGCTGGGCGTTCAGGGGATACTGTGGTTCTAGGGACCGGGAGAGGTGAGAGGTACTGCCG
GGGTAGGAGTGAAATCCTGTAATCCCGGTGGGACCACCTATGGCGAAGGCATCTCACCAG
AACGGCTCCGACAGTGAGGGGCGAAAGCTGGGGGAGCAAACCGGATTAGATACCCTCGTA
GTCC
>Otu13549
CCAGCCTACGGGTCGCACCAGTGGGGAATCTTGCGCAATGGGCGAAAGCCTGACGCAGCG
ACGCCGCATGGGGGATGAAGCTTCTCGGAGTGTAAACCCCTTTCGACTGGGACGAATGCC
TCGCAAGAGGAGTGACGGTACCAGTAGAAGAAGCCCCGGCTAACTACGTGCCAGCAGCCG
CGGTAATACGAAGGGGGCTAGCGTTGTTCGGAATTACTGGGCGTAAAGCGCGTGTAGGCG
GGTCTTTAAGTCAGGGGTGAAATGCCAAGGCTCAACCTTGGAACTGCCTTTGATACTGGA
GATCTTGAGTCCGGGAGAGGTGAGTGGAACTGCGAGTGTAGAGGTGAAATTCGTAGATAT
TCGCAAGAACACCAGTGGCGAAGGCGGCTCACTGGCCCAGAACTGACGCTGAGACGCGAA
AGCGTGGGGAGCAAACAGGATTAGATACCCTCGTAGTCC
>Otu13550
CAGCCTACGGGTGGCTGCAGTCGAGAATTTTTCACAATGGGGGAAACCCTGATGGAGCGA
CGCCGCGTGGGGGATGAATGGCTTCGGCCCGTAAACCCCTGTCATTTGCGAACAAAGTTG
GCGCATGAACAATGCGTTAATTGATTGTAGCGAAAGAGGAAGGGACGGCTAACTCTGTGC
CAGCAGCCGCGGTAATACAGAGGTCCCAAGCGTTGTTCGGATTCACTGGGCGTAAAGGGT
GCGTAGGTGGCCGGGTAAGTCTGATGTGAAATCTCGGAGCTCAACTCCGAAACGGCATTG
GATACTCTCCGGCTGGAGGGTCGGAGGGGGGACTGGAATTCTCGGTGTAGCAGTGAAATG
CGTAGATATCGAGAGGAACACCAGTGGCGAAGGCGAGTCCCTAGACAATTCCTGACACTG
AGGCACGAAAGCCAGGGGAGCAAACGGGATTAGATACCCGAGTAGTCC
>Otu13553
CCAGCCTACGGGTTGCTCCAGTCAAGAATCATGGTCAATGCGCGACAGCGTGAACCTGCG
ACCTGTCAGAAGTGAAGACGGCATTGGTCTTAAATCTTTGTAGGTTGGTTCCATCATGAG
GGGCCAGCCGTACAGTCGTGATTAATCCTTGTGCCAGCAATAGCGGTCATACAAGAGCGG
CGAATGTTGCTCGATGGAATTCGGTGTAAAACGTGGGTAGGCGGTCGAACCCAACAAGAC
TTTGAAGCAAGAGGGACACTCCGGAACGGGTCTTGGTTGCTCGACTCGAGATGAGACGGG
GAAAACGGAATGATGGGAGAACGGTTAGAATCGGTCGATCCCCATTGGAACGTCAGAGGC
GAAGGCGGTTTTCTGGGCTCAATCTGACGCTGATGTCACGAAAGCTGGGGGAGCAAATCG
GATTAGATACCCGCGTAGTCC
>Otu13554
CCAGCCTACGGGGGGCACCAGTCGCGAATCTTTTGCAATGCGCGCAAGCGTGACAAAGCG
AGCCAAAGTGCTTTTCATTTTAGAAAAGCTTTTTTTCATTTTAAAAAGATGGAAGAATAA
GGACTGGGCAAGACCGGTGCCAGCCGCCGCGGTAATCCCGGCAGTCCAAGTTGCAGCCAA
CTTTATTGGGTCTAAAACATCCGTAGCTTGTCTGTTAAGTTCCTTGTGAAATCCCTCATC
TTAAGTGTGGGGCTTGCGGGGAATACTGGCAGACTCGGGACCGGTAGATGTGGGGAGTAC
ACTGGAGGTAGTGGTAAAATACGTTAATCTTCAGTGGACTAACAATGAGCGAAGGCACCC
TACAAGGACGGATCTGACAGTGAGGGATGAAGGCCAGGGGCGCAAAACGGATTAGATACC
CCAGTAGTCC
>Otu13563
CCAGCCTATGGGAGGCAGCAGTCGAGAATTTTTCTCAATGGGGGAAACCCTGAAGGAGCG
ACGCCGCGTGAAGGATGAAGGTCTTCGGATCGTAAACTTCTGTCATTAGAGAACAATTCG
CCGGGGAGTAACTGCCTCGGCGTTGATAGTATCTGAAGAGGAAGGGACGGCTAACTCTGT
GCCAGCAGCCGCGGTAATACAGAGGTCCCAAGCGTTGTTCGGATTCATTGGGCGTAAAGG
GTGCGTAGGTGGTGCCGTAAGTCGGATGTGAAATCCCGGAGCTTAACTCCGGAACTGCAT
TCGATACTGCGGTGCTGGAGGACTGGAGAGGAGATCGGAATTCACGGTGTAGCAGTGAAA
TGCGTAGATATCGTGAGGAAGACCAGTGGCGAAGGCGGATCTCTGGACAGTTCCTGACAC

TGAGGCACGAAGGCCAGGGGAGCAAACGGGATTAGATACCCCCGTAGTCC
>Otu13564
CCAGCCTACGGGAGGCTGCAGTGGGGAATCTTGCGCAATGGGCGAAAGCCTGACGCAGCG
ACGCCGCGTGAGTGATGAAGGCCTTCGGGTTGTAAAGCTCTGTGGGGAGAGAAGAATTAG
TGCAGGCTAATCACTGCACGATGACGGTATCTCCTTAGCAAGCACCGGCTAACTCTGTGC
CAGCAGCCGCGGTAAGACAGAGGGTGCAAACGTTGTTCGGAATTACTGGGCGTAAAGCGT
GTGTAGGCTGCTATGCAAGTCGGGTGTGAAATCCCTGGGCTCAACCCAGGAAGTGCACTC
GAAACTGCATGGCTCGAGTACCGGAGAGGTTGGTAGAATTCTCGGTGTAGAGGTGAAATT
CGTAGATAGCGAGAGGAATACCTGTGGCGAAGGCGGCCAACTGGACGAGTACTGACGCTG
AGACACGAGAGCGTGGGGAGCAAACAGGATTAGAAACCCTTGTAGTCT
>Otu13568
CCAGCCTATGGGTTGCAGCAGTTTAGAAATTTGCGCAATGGGCGAAAGTCTGACGATGCG
ACGCCGCGTGAACGATGAAGGCCTTCGGGTTGTAACGTTCTGTTGTCCTGGAACAAGAGT
GGTTGACGAATAGTTGATCACGATGAGGGTACAGGAAGAGGAAGCACCGGCTAATTCCGT
GCCAGCAGCTGCGGTAATACGGAAGGTGCAAGCGTTAGTCGGAATTACTGGGCGTAAAGG
GTGCGTAGGCGGAGATGTACGTCGGATGTGAAATCCCGGGGCTTAACCCCGGAACTGCAT
ACGAAACTGCATCCCTTGGGGAATGGCGGAGAAAATGGAATTCCATATGTAGCGGTGAAA
TGCGTAGATATATGGAAGAACACCTGTGGCGAAAGCGGTTTTCTAGCCTCCTCCCGACGC
TGAGGCACGAAAGCAAGGGGAGCAAACAGGATTAGAGACCCCAGTAGTCC
>Otu13575
CCAGCCTACGGGTGGCAGCAGTAAGGAATATTGGACAATGGTGGCAACACTGATCCAGCC
ATGCCGCGTGCAGGATGAAGGCGCTACGCGTTGTAAACTGCTTTTGTACGGGAGAAAACC
TATCTACGTGTAGATAGCTGATAGTACCGTAAGAATAAGCATCGGCTAACTTCGTGCCGG
CAGCCGCGGTAATACGAAGGATGCGAGCGTTGTCCGGATTCACTGGGTGTAAAGGGAGCG
CAGGCGGGTCGGTAAGTCATTGGTGAAATTTTCGGGCTCAACCCGAAGACTGCCACTGAT
ACTGCCGATCTTGAGTGTGGAAGAGGTTGGTGGAATTCATGGTGTAGCGGTGAAATGCGT
AGATATCATGAAGAACACCGATGGCGAAGGCAGCCAACTGGTCCATTACTGACGCTCATG
CTCGAAAGCGTGGGGATCAAACAGGATTAGATACCCCGGTAGTCC
>Otu13583
CCAGCCTACGGGTTGCTCCAGGCGCGAAAACTTTACAATGCGGGAAACCGTGATAAGGGG
ACACCGAGTGCCAGCATCATATGCTGGCTGTCCGGGCGTGTAAAATACACCTGTTAGCAA
GGGCCGGGCAAGACCGGTGCCAGCCGCCGCGGTAACACCGGCGGCCCGAGTGGTGATCGT
GATTATTGGGTCTAAAGGGTCCGTAGCCGGTTTGGTCAGTCCTCCGGGAAATCTGACGGC
TTAACCGTTAGGCTTTCGGGGGATACTGCCAGACTTGGAACCGGGAGAGGTGAGAGGTAC
TCCAGGGGTAGGAGTGAAATCCTGTAATCCTTGGGGGACCACCTGTGGCGAAGGCGTCTC
ACCAGAACGGCTCCGACAGTGAGGGACGAAAGCTGGGGGAGCAAACCGGATTAGATACCC
TAGTAGTCC
>Otu13595
CCAGCCTACGGGGTGCACCAGTGGGGAATTTTGCGCAATGGGGGAAACCCTGACGCAGCA
ACGCCGCGTGAGTGATGAAGGCCTCCGGGTCGTAAAGCTCTGTCAGAGGGAAAGAAGTGT
AGGAGGGTTAATACCCCTTTTACTTGACGGTACCCTCAAAGGAAGCACCGGCTAACTCCG
TGCCAGCAGCCGCGGTAATACGGAGGGTGCGAGCGTTGTTCGGATTTATTGGGCGTAAAG
CGCGTGTAGGCGGTTTTTTAAGTCTGATGTGAAAGCCCTGGGCTCAACCCGGGAAGTGCA
TTGGAAACTGGGAGACTTGAATACGGGAGAGGGTAGTGGAATTCCTGGTGTAGCAGTGAA
ATGCGTAGATATCAAGTGGAACACCAGTGGCGAAAGCGAGTCCCTGGACAACTCCTGACA
CTGAGGCACGAAAGCTAGGGGAGCAAACAGGATTAGAAACCCCCGTAGTCC
>Otu13608
CCAGCCTACGGGGTGCTGCAGTCGAGAATTTTTCTCAATGGGCGAAAGCCTGAAGGAGCG
ACGCCGCGTGGGGGATGAATGGCTTCGGCCCGTAAACCCCTGTCATTTGCGAACAAATTG
TTTCACCTAACACGTGAATCATTGATTGTAGCGGAAGAGGAAGGGACGGCTAACTCTGTG
CCAGCAGCCGCGGTAATACAGAGGTCCCAAGCGTTGTTCGGATTCACTGGGCGTAAAGGG
TGCGTAGGCGGTCGGGTAAGTCTGATGTGAAATCTCGGAGCTCAACTCCGGAACGGCATT
GGATACTATCCGGCTGGAGGGTCGGAAGGGGGACTGGAATTCTCGGCGTAGCAGTGAAAT
GCGTAGATATCGAGAGGAACACCAGCGGCGAAGGCGAGTCCCTGGACGACTCCTGACACT
GAGGCACGAAAGCTAGGGGAGCAAACAGGATTAGAAACCCCAGTAGTCC
>Otu13619
GGACTACCGGGGTCTCTAATCCCGTTTGCTCCCCTGGCTTTCGTGCCTCAGTGTCAGGAA
TTGTCTAGGGACTCGCCTTCGCCACTGGTGTTCCGCTCGATATCTACGCATTTCACTGCT

ACACCGAGCATTCCAGTCCCCCCTCCAATCCTCAAGCTGGATAGTATTCAATGCATTTTC
CGGGTTAAGCCCGGAGATTTCACATCAAACTTACCCAACCACCTACGCACCCTTTACGCC
CAGTAAATCCGAACAACGCTTGGGACCTCTGTATTACCGCGGCTGCTGGCACAGAGTTAG
CCGTCCCTTCCTCTGCCGGTACTATCAATCCCGACAATGTTATTGCCGGGACATTGATCC
CGAATGACAGGGGTTTACGGGCCGAAGCCATTCATCCCCCACGCGGCGTCGCTCCTTCAG
GCTTTCGCCCATTGAGAAAAATTCTCGACTGCTGCACCCCGTAGGCTGG
>Otu13629
CCAGCCTATGGGGTGCTGCAGTCGAGAATTTTTCACAATGGGCGGAAGCCTGATGGAGCG
ACGCCGCGTGGGGGATGAATGGCTTCGGCCCGTAAACCCCTGTCATTTGCGATCAACCCT
TACGACCTAACACGTCGTGAGCTGATAGTAGCGAAAGAGGAAGGGACGGCTAACTCTGTG
CCAGCAGCCGCGGTAATACAGAGGTCCCAAGCGTTGTTCGGATTCACTGGGCGTAAAGGG
TGCGTAGGCGGTCGGGTAAGTCTGATGTGAAAGCTCGCGGCTCAACCGCGAAATGGCATC
GGATACTATCCGGCTGGAGGAATGGAAGGGAGACTGGAATACTTGGTGTAGCAGTGAAAT
GCGTAGATATCAAGTGGAACACCAGTGGCGAAGGCGAGTCTCTGGACATTTCCTGACGCT
GAGGCACGAAAGCCAGGGGAGCAAACGGGATTAGATACCCTAGTAGTCC
>Otu13639
CCAGCCTACGGGTGGCACCAGTGGGGAATATTGGACAATGGGCGCAAGCCTGATCCAGCC
ATGCCGCGTGAGTGAAGAAGGCCTTAGGGTTGTAAAGCTCTTTTGGCGGGGGCGATAATG
ACGGTACCCGCAGAATAAGCCCCGGCTAACTTCGTGCCAGCAGCCGCGGTAATACGAAGG
GGGCTAGCGTTGCTCGGAATGACTGGGCGTAAAGGGCGCGTAGGCGGATGACACAGTCAG
GCGTGAAATTCCTGGGCTCAACCTGGGGGCTGCGCTTGATACATGTTGTCTAGAGTGTGA
AAGAGGGTCGTGGAATTCCCAGTGTAGAGGTGAAATTCGTAGATATTGGGAAGAACACCG
GTGGCGAAGGCGGCGACCTGGTTCATTACTGACGCTGAGGCGCGAAAGCGTGGGGAGCAA
ACAGGATTAGAGACCCTAGTAGTCC
>Otu13643
CCAGCCTACGGGATGCTCCAGTGAGGAATATTGGTCAATGGACGCAAGTCTGAACCAGCC
ATGCCGCGTGAAGGATGAAGGCCTTCTGGGTTGTAAACTTCTTTTACCTGGGAAGAAACC
CCGATTTTCTAATCGGGTTGACGGTACCAGAGGAATAAGCACCGGCTAACTCCGTGCCAG
CAGCCGCGGTAATACGGAGGGTGCAAGCGTTATCCGGATTTATTGGGTTTAAAGGGTGCG
TAGGCGGACTTATAAGTCAGTGGTGAAAACCTTCAGCTTAACTGGAGACTTGCCATTGAT
ACTGTTAGTCTTGAGTACGGTCAAGGTAGGCGGAATGTGTAATGTAGCGGTGAAATGCTT
AGATATTACACAGAACACCGATTGCGAAGGCAGCTTACTGGGCCATTACTGACGCTGATG
CACGAAAGCGTGGGGAGCGAACAGGATTAGAAACCCCAGTAGTCC
>Otu13646
GGACTACTAGGGTTTCTAATCCTGTTTGCTCCCCACGCTTTCGTGCCTCAGCGTCAGTAT
CGGGCCAGTGAGCCGCCTTCGCCACTGGTGTTCTTGCGAATATCTACGAATTTCACCTCT
ACACTCGCAGTTCCACTCACCTCTCCCGAACTCAAGATCCTCAGTATCAAAGGCAGTTCT
GGAGTTGAGCTCCAGGATTTCACCCCTGACTTAAAGACCCGCCTACGCACCCTTTACGCC
CAGTGATTCCGAGCAACGCTAGCCCCCTTCGTATTACCGCGGCTGCTGGCACGAAGTTAG
CCGGGGCTTATTCTTGCGGTACCGTCATTATCTTCCCGCACAAAAGAGCTTTACAACCCT
AGGGCCTTCATCACTCACGCGGCATGGCTGGATCAGGCTTGCGCCCATTGTCCAATATTC
CCCACTGCAGCCCCCCGTAGGCTGG
>Otu13652
CCAGCCTACGGGGGGCTGCAGTCGAGAATCTTCTGCAATGGACGAAAGTCTGACAGAACG
ACGCCGCGTGGAGGAAGAGGGCCTTCGGGTTGTAAACTCCTGTCACAGGTTATGAAGGGT
GTTCTGCATAACATGCGGGACTCTCGGACAAAGGCCTGAGAGGAAGCCACGGCTAACTCT
GTGCCAGCAGCCGCGGTAAGACAGAGGTGGCAAGCGTTGTTCGGAACCACTGGGCGTAAA
GGGCGCGTAGGCGGCGATACAAGTCGGGTGTGAAATCCCTCGGCTCAGCCGAGGCATTGC
TTCCGATACTGTATTGCTAGAGTGCGGGAGGGGAGAGTGGAACTTCTGGTGGAGCGGTGA
AATGCGTAGATATCAGAAGGAACGCCGGAGGCGAAGGCGACTCTCTGGACCGTTACTGAC
GCTGAGGCGCGAAAGCTAGGGGAGCAAATGGGATTAGATACCCTAGTAGTCC
>Otu13660
CCAGCCTATGGGATGCAGCAGTGGGGAATTTTGCGCAATGGGCGAAAGCCTGACGCAGCA
ACGCCGCGTGGGTGATGAAGGTCTTCGGATCGTAAAACCCTGTCGTCAGGGACGAAGGGC
TTGTGCCTAATACGTGCAAGTCTTGACGGTACCTGGAGAGGAAGCCCCGGCTAACTCTGT
GCCAGCAGCCGCGGTAATACAGAGGGGGCAAGCGTTATTCGGAATTATTGGGCGTAAAGG
GCGCGTAGGCGGTGTTTTAAGTGAGATGTGTAATCCCCGAGCTTAACTTGGGAACTGCGT
CTCAGACTGGGACGCTAGAGTGCTGGAGAGGGTGGTAGAATTCCACGTGTAGCGGTGAAA

TGCGTAGAGATGTGGAGGAATACCAGTGGCGAAGGCGGCCACCTGGACAGTAACTGGCGC
TGAGGCGCGAAAGTGTGGGTAGCAAACAGGATTAGATACCCTAGTAGTCC
>Otu13665
CCAGCCTACGGGGGGCACCAGTGGGGAATTTTGCGCAATGGGGGAAACCCTGACGCAGCA
ACGCCGCGTGGAGGATGAAGTACTTCGGTACGTAAACTCCTTTCGATCGGGAAGATAATG
ACGGTACCGCAAGAATAAGCCCCGGCTAACTTCGTGCCAGCAGCCACGGTAATACGAAGG
GGGCTAGCGTTGCTCGGAATCACTGGGCGTAAAGGGTGCGTAGGCGGGTCTTTAAGTCAG
AGGTGAAATCCTGGAGCTCAACTCCAGAACTGCCTTTGATACTGAGGGTCTTGAGTTCGG
GAGAGGTGAGTGGAACTGCGAGTGTAGAGGTGAAATTCGTAGATATTCGCAAGAACACCA
GTGGCGAAGGCGGCTCACTGGCTCGATACTGACGCTGAGGTGCGAAAGCGTGGGGAGCAA
ACAGGATTAGATACCCCAGTAGTCC
>Otu13668
CCAGCCTATGGGACGCTGCAGTGGGGAATCTTGCGCAATGGGCGAAAGCCTGACGCAGCA
ACGCCGCGTGAGTGATAAAGGTCTTCGGATCGTAAAGCTCTGTCGACAGGAAAGACGGTT
TGATTGGCCAACATCCAATTGAGCTGACGGTACCTGTAAAGGAAGCACCGGCTAACTCTG
TGCCAGCAGCCGCGGTAAGACAGAGGGTGCAAGCGTTGTTCGGAATTATTGGGCGTAAAG
GGCGCGTAGGCGGCGTCGTAAGTCACGTGTGAAAGCCCAGGGCTTAACCCTGGAAGTGCG
CGTGAAACTGCGATGCTTGAGTCTCGGAGAGGGTGGTGGAATTCCCAGTGTAGAGGTGAA
ATTCGTAGATATTGGGAGGAATACCGGTGGCGAAGGCGACCACCTGGACGAAGACTGACG
CTGAGGCGCGAAAGCGTGGGGAGCAAACAGGATTAGATACCCGGGTAGTCC
>Otu13669
CCAGCCTATGGGGGGCAGCAGTAAGGAATATTGCTCAATGGCCGAAAGGCTGAAGCAGCA
ACGCCGCGTGAAGGATGAAGGGATTTTTCTTGTAAACTTCTGTAACGAGGGAAAAAACTT
CCCCTCTGGGGAAATTGATTGTACCTCGAAAGTAAGCCTCGGCTAACTACGTGCCAGCAG
CCGCGGTAATACGTAGGAGGCGAGCGTTGTCCGGATTTACTGGGTGTAAAGGGTGCTCAG
GTGGTTGTGCAAGTCAGGGGTGAAATCCCGGAGCTTAACTCCGGAACTGCCTTTGATACT
GCTCAGCTTGAATATGGAAGAGGCTGATGGAATTTATGGTGTAGCAGTGAAATGCGTAGA
TATCATAAAGAACACCAGTGGCGAAGGCGGTCAGCTGGTCCATTATTGACACTAAAGCAC
GAAAGCGTGGGGAGCAAACAGGATTAGATACCCCTGTAGTCC
>Otu13676
CCAGCCTATGGGGGGCTCCAGCTGGGAATCTTCTGCAATACACGCAAGTGTGACAGAGCG
AGCCAGAGTGCTCCTCATTGAGGAGCTTTTGCTAAATCTAAAAAGTTTGGCGAATAAGGA
CTGGGCAAGACCGGTGCCAGCCGCCGCGGTAATACCGGCGGTCCAAGTCGCAGCCACTTT
TATTGGGTCTAAAACATCCGTAGCTTGTTCGGTGAGTTCCTTGTGAAATCCTGTCTCTTA
AGGTCAGGGCGAGCAAGGAGTACTACCGGGCTAGAGACTGGAAGACGTAAGAAGTACGTT
TGAAGTAGCGGTTAAATGCTTTAATCTTAGACGGACTCACAACAGCGAAGGCATCTTACG
AGGACAGTTCTGACAGTGAGGGATGAAGGCCAGGGGCGCAAAATGGATTAGATACCCTAG
TAGTCC
>Otu13680
CCAGCCTACGGGGTGCACCAGTCGAGGATTTTTCTCAATGGGGGAAACCCTGAAGGAGCG
ACGCCGCGTGGGGGATGAAGGTCTTCGGATTGTAAACCCCTGTCATCTGGGAACAAAGCG
CTCCACCTAACACGTGGATTGTTGATAGTACCGGAAGAGGAAGCCGTGGCTAACTCTGTG
CCAGCAGCCGCGGTAATACAGAGACGGCAAGCGTTGTTCGGATTCATTGGGCGTAAAGGG
TGCGCAGGCGGCTCGTTAAGTCGGATGTGAAATCTCACAGCCTAACTGTGATAGGTCATT
CGAAACTGGCGGGCTCGAGGACTGGAGAGGAGACTGGAATGGTCGGTGTAGCGGTGAAAT
GCGTAGAGATCGACCAGAACACCGGTGGCGAAGGCGAGTCTCTGGACAGTAACTGACACT
GAGGCACGAAGGCCAGGGGAGCAAACGGGATTAGATACCCCGGTAGTCC
>Otu13697
CCAGCCTACGGGGCGCTGCAGTGAGGAATATTGGGCAATGGACGCAAGTCTGACCCAGCC
AAGCCGCGTGAAGGATGAAGGCTCTACGAGTCGTAAACTTCTTTTATAGAGGAAGAAAAT
GCTCTACGTGTAGAGAACTGACTGTACTTTATGAATAAGCATCGGCTAACTCCGTGCCAG
CAGCCGCGGTAATACGGAGGATGCAGGCGTTATCCGGATTCATTGGGTTTAAAGGGTGCG
TAGGCGGAATAATAAGTCAGTGGTGAAATACTGCAGCTTAACTGTAGAACTGCCATTGAT
ACTGTTATTCTTGAATGCAGTTGAGGTAGGCGGAATGTGTAGTGTAGCGGTGAAATGCTT
AGATATTACACAGAACACCGATTGCGAAGGCAGCTTACTAAACTGTGATTGACGCTGAGG
CACGAAAGCGTGGGGATCAAACAGGATTAGAAACCCGGGTAGTCC
>Otu13702
CCAGCCTATGGGATGCAGCAGTCGAGAATTTTTCTCAATGGGCGAAAGCCTGAAGGAGCG

ACGCCGCGTGGGGGATGAATGGCTTCGGCCCGTAAACCCCTGTCATTTGTGACCAAACCT
TTTCATTTAATATGTGAAAAGCTGATGGTAGCGGAAGAGGAAGGGACGGCTAACTCTGTG
CCAGCAGCCGCGGTAATACAGAGGTCCCAAGCGTTGTTCGGATTCACTGGGCGTAAAGGG
TGCGTAGGTGGCGGGATAAGTCTGATGTGAAATCTCGAAGCTTAACTTCGAAACTGCATT
GGATACTATTCTGCTGGAGGGTTGGAGGGGGGACTGGAATTCTCGGTGTAGCAGTGAAAT
GCGTAGATATCGAGAGGAACACCAGTGGCGAAGGCGAGTCCCTGGGCAACTCCTGACACT
GAGGCACGAAAGCTAGGGGAGCAAACAGGATTAGATACCCCAGTAGTCC
>Otu13703
CCAGCCTACGGGTGGCTGCAGTGGGGAATCTTGCACAATGGGGGAAACCCTGATGCAGCG
ACGCCGCGTGAGCGATGAAGCCCTTCGGGGTGTAAAGCTCTTTCGGCAGGGAAGATAATG
ACGGTACCACCAGAAGAAGCCCCGGCTAACTTCGTGCCAGCAGCCGCGGTAATACGGAGG
GTGCGAGCGTTGTTCGGAATTATTGGGCGTAAAGCGCGTGTAGGCGGTTTTTTAAGTCTG
ATGTGAAAGCCCTGGGCTCAACCCGGGAAGTGCATTGGAAACTGGGAGACTTGAATACGG
GAGAGGGTAGTGGAATTCCTGGTGTAGGAGTGAAATCCGTAGATATCAGGAGGAACACCG
GTGGCGAAGGCGGCTACCTGGACCGATATTGACGCTGAGACGCGAAAGCGTGGGGAGCAA
ACAGGATTAGAAACCCCCGTAGTCC
>Otu13707
CCAGCCTATGGGGGGCAGCAGTGGGGAATATTGGGCAATGGGCGAAAGCCTGACCCAGCG
ACGCCGCGTGGACGATGAAGGCCTTCGGGTTGTAAAGTCCTGTCATGCGGAAAGAATTGC
CTTATGGTTAATACCCATGAGGTTTGACGGTACCGCAGGAGGAAGCACCGGCTAACTCCG
TGCCAGCAGCCGCGGTAATACGGAGGGTGCAGGCGTTGTTCGGAATTATTGGGCGTAAAG
GACGCGTAGGCGGTAATGAAAGTCAGATGTGAAATACTTCGGCTCAACTGGAGAACTGCA
TCTGAAACTTCATAACTCGAGTACAGAAGAGGGAAACGGAATTCCCGGTGTAGAGGTGAA
ACTCGTAGATATCGGGAGGAACACCAGTGGCGAAGGCGGTTTCCTGGTCTGATACTGACG
CTGAGGCGTGAAAGTGTGGGGAGCAAACAGGATTAGAAACCCCCGTAGTCC
>Otu13717
CCAGCCTACGGGGTGCTGCAGTCAAGAATATTCCCCAATGGCCGCAAGGCTGAGGGAGCG
ACGCCGCGTGCAGGAAGAAGCCCTTCGGGGTGTAAACTGCTTTTCTTCGAGAACAACTCT
GAGTGTACCGAAGGAATAAGGGGCTGCTAACTTCGTGCCAGCAGCAGCGGTAATACGAAG
GCCCCGAGCGTTATCCGGATTTATTGGGCGTAAAGCGTTCAACGGTGGCATGGTGTGTTC
TTGGTTAAATCCCACAGCTCAACTGTGGGGCTGCCGGGAAAACTGCCAAGCTTGAGGACG
GAAGAGGCGCGCGGAACTACCGGTGTAGCGGTAAAATGCGTTCATATCGGTAGGAACACC
GAAGGCGAAAGCAGCGCGCTGGGACGTCTCTGACACTAAAGAACGAAAGCGTGGGTAGCG
AATGGGATTAGAGACCCCCGTAGTCC
>Otu13720
CCAGCCTATGGGGGGCTCCAGGCGCGAAACCTTTACAATGCGCGAAAGCGTGATAGGGGA
ATTCTGAGTGATGTTGGAACTACGTTCCTCATCTTTTGCCAAGTCCAAACAGCTTGGCGA
ATAAGTGCTGGGTAAGACCAGTGCCAGCCGCCGCGGTAATACTGGCAGCACGAGTGGTAT
CCACGAATATTGGGTCTAAAGAGTCCGTAGCCGGTCCTTTAAGTCTTCTGTGAAATCTTG
AGGCTTAACTTCAAGGCGTGCAGAAGATACTGCTGGACCTGAGAGTGGGGGAGGTCAGGA
GTACTTATGGGGTAGTGGTAAAATACTGTAATCCTATAAGGACTCTCAGTGGCGAAGGCG
CCTGACCAAAACACGTCTGACGGTGAGGGACGAAGGCTAGGAGAACGAATCGGATTAGAG
ACCCGAGTAGTCC
>Otu13721
CCAGCCTACGGGAGGCAGCAGTAGGGAATATTGCGCAATGGGGGAAACCCTGACGCAGCG
ACGCCGTGTGAGGGAAGAAGGCTTTCGGGTCGTAAACCTCTGTCAGAAAGGAAGAACCTC
CCATATCCGAACAGGGTGTGGGACTGACGGTACTTTCAAAGGAAGCGCCGGCTAACTCCG
TGCCAGCAGCCGCGGTAATACGGAGGGCGCAAGCGTTAATCGGAATCACTGGGCGTAAAG
CGCGCGTAGGCGGCTTTCTAAGTCGGGCGTGAAAGCCCTCGGCTCAACCGGGGAACTGCG
TTCGAAACTGGGAGGCTTGAGTCCTGGAGAGGGTGGCGGAATTCCGGGTGTAGGAGTGAA
ATCCGTAGATATCCGGAGGAACACCGGTGGCGAAGGCGGCCACCTGGACAGGTACTGACG
CTGAGGCGCGAAAGCGTGGGGAGCAAACAGGATTAGATACCCCTGTAGTCC
>Otu13725
CCAGCCTATGGGTCGCTGCAGTCGGGAAATTTTTGCAATGCGCGAAAGCGTGACAGAGCA
AGTCAGAGTGCTTTTCCATATTGGAAAAGCTTTTGTGTGATGTAAACATTCACACGAATA
AGGAGTGGGAGAGACTGGTGCCAGCCGCCGCGGTAATCCCAGCACTCCAAGTCGCAGCCA
CAATTATTGGGTCTAAAACATCCGTAGCTTGCTTTGTAAGTCTTTTGTCAAATCGGGACT
CTTAAGGTTCCGGCGAGCAAAAGATACTGTTTAGCTAGGGACCGGGAGATGTGAAGAGTA

CGAATTAGGTAGCGGTAAAATGCGTTGATCTTTTTCGGACTAACAACAGCGAAGGCACTT
CACAAGAACGGATCCGACAGTGAGGGATGAAGGCTAGGGGCGCAAATTGGATTAGATACC
CGTGTAGTCC
>Otu13726
CCAGCCTACGGGGCGCTGCAGTCGAGAATTTTTCACAATGGGCGAAAGCCTGATGGAGCG
ACGCCGCGTGGGGGATGAATGGCTTCGGCCCGTAAACCCCTGTCATTCGCGAACAATGCC
TGCGACTTAATACGTCGCAGGTTGATAGTAGCGGAAGAGGAAGGGACGGCTAACTCTGTG
CCAGCAGCCGCGGTAATACAGAGGTCCCAAGCGTTGTTCGGATTCATTGGGCGTAAAGGG
TGCGTAGGTGGTGCCGTAAGTCGGATGTGAAATCCCGGAGCTTAACTCCGGAACTGCATT
CGATACTGCGGTGCTTGAGGACTGGAGAGGAGATCGGAATTCACGGTGTAGCAGTGAAAT
GCGTAGATATCGTGAGGAAGACCGGTGGCGAAGGCGGATCTCTGGACAGTTCCTGACACT
GAGGCACGAAGGCCAGGGGAGCAAACGGGATTAGAGACCCGCGTAGTCC
>Otu13728
CCAGCCTACGGGAGGCAGCAGGCGCGAAACCTTTGCAATACACGAAAGTGTGACAAGGGG
ATTCCAAGTGTGCATATACCATATGCACTTTTTCGAAGCGTAAAAAACTTTGGGAATAAG
GGGTGGGCAAGACTAGTGCCAGCCGCCGCGGTAAAACTAGCATCTCGAGTGGCAGCCACA
TTTACTGGGCTTAAAGCGTCCGTAGCTCGTTCAGAAAGTCCCCGGTGAAATCCTACGGCT
TAACCGTAGGGCTTGCCAGGGAAACTCCTGGACTAGAAGGCAGGAGAGGTCAGGGGTACG
TCTGGGGTAGGGGTGAAATCCTATAATCCTAGACGGACCAACAGTGGCGAAGGCGCCTGA
CGTGAATGCGCTTGACAGTGAGGGACGAGAGCCAGGGTCGCGAAACGGATTAGATACCTA
GTCC
>Otu13734
CCAGTCTATGGGTGGCAGCAGTGAGGAATCTTGGACAATGGGGGCAACCCTGATCCAGCG
ACGCCGCGTGAAGGATGAAGGTCTTCGGATTGTAAACTTCTTTTGTTCGGGAATAACGAA
TCTGTGGAAACACGGAGACTGAAGGTACCGGATGAATAAGCCACGGCTAACTTCGTGCCA
GCAGCCGCGGTAATACGAAGGTGGCAAGCGTTACTCGGAATTACTAGGCGTAAAGGGCAG
GTAGGTGGTTCGGTAAGTCTGTTGTGTAAGCTCCCGGCTTAACTGGGAGAGGTCAACGGA
AACTACCGGACTTGAGTATAGGAGAGGTTACTGGAATTCCCGGTGTAGCGGTGAAATGCG
CAGAGATCGGGAGGAACACCAATGGCGAAAGCAGGTAACTGGACTATTACTGACACTCAG
CTGCGAAAGCTAGGGGAGCAAACAGGATTAGAAACCCCCGTAGTCC
>Otu13735
CCAGCCTATGGGAGGCTGCAGTGGGGAATATTGGGCAATGGGCGAAAGCCTGACCCAGCG
ACGCCGTGTGGGCGATGAAGGCCTTCGGGTTGTAAAGCCCTGTTGGGTGGAAAGAAGGGC
TTTTGGACGAATAATCCAGAAGCTTTGACGGTACCACCAGAGGAAGCACCGGCTAACTCC
GTGCCAGCAGCCGCGGTAATACGGAGGGTGCAAGCGTTGTTCGGAATTACTGGGCGTAAA
GGGCGCGTAGGTGGTTGTGTAAGTCAGATGTGAAATCCCGAGGCTCAACTTCGGAACTGC
ATCTGATACTGCATGACTCGAGTGCAGCAGAGGGAAACGGAATTCCTGGTGTAGAGGTGA
AATTCGTAGATATCAGGAGGAACACCGGTGGCGAAGGCGGTTTCCTGGGCTGACACTGAC
ACTGAGGCGCGAAAGCGTGGGTAGCAAACAGGATTAGAAACCCTGGTAGTCC
>Otu13743
CCAGCCTATGGGGTGCTCCAGTGGGGAATATTGGACAATGGGCGCAAGCCTGATCCAGCC
ATGCCGCGTGAGTGATGAAGGCCTTCGGGTTGTAAAGCTCTTTCGCACGTGACGATGATG
ACGGTAACGTGAGAAGAAGCCCCGGCTAACTTCGTGCCAGCAGCCGCGGTAATACGGAGG
GTGCAAGCGTTGTTCGGAATTACTGGGCGTAAAGGGCGCGTAGGTGGTTGCGTAAGTCGG
ATGTGAAATCCCCGGGCTTAACCCGGGAACTGCACCCGATACTGCGTGACTCGAGTGCAG
CAGAGGGAAACGGAATTCCTGGTGTAGAGGTGAAATTCGTAGATATCAGGAGGAACACCG
GTGGCGAAGGCGGTTTCCTGGGCTGACACTGACACTGAGGCGCGAAAGCGTGGGTAGCAA
ACAGGATTAGAGACCCTCGTAGTCC
>Otu13747
CCAGCCTATGGGTTGCACCAGTAGGGAATATTGGTCAATGGGCGCAAGCCTGAACCAGCC
ATGCCGCGTGAGTGATGAAGGCCTTCGGGTCGTAAAACTCTTTTCTGAGGGAAGAACATC
CTGCGTAGGAAATGACGTGGGCTTGACGGTACCTCAGGAATAAGCAACGGCTAACTCCGT
GCCAGCAGCCGCGGTAATACGGAGGTTGCGAGTGTTGTTCGGAATAACTGGGCGTAAAGG
GAGCGCAGGCGGTTGGGTAAGTCTAAAGTGAAAGCCCGGGGCTCAACTCCGGAAGTGCTT
TGGATACTACCCCACTCGAGTTCGTGAGAGGAAAGCGGAATTCCAGGTGTAGCGGTGAAA
TGCGTAGATATCTGGAGGAACACCGGTGGCGAAAGCGGCTTTCTGGCACGATACTGACGC
TGAGGCTCGAAAGCGTGGGGAGCAAACAGGATTAGAGACCCGCGTAGTCC
>Otu13752

CCAGCCTATGGGGTGCTCCAGGCGCGAAACCTTTACAATGCGCGAAAGCGTGATAAGGGA
ACTCTGAGTGGCCGTCGGCTTTTGCCGACTGTCTTTTGGCAAACTCAAAACGTTTGCAGA
ATAAGTTCTGGGTAAGACCAGTGCCAGCCGCCATGTGCTATGCACATTGCACACGGTACG
CGGTAATACTGGCAGAACGAGTGGTGTCCACGAATATTGGGTCTAAAGAGACCGTAGCTG
GCCTGTTAAGTTCACTGTGAAATCTTGGGGCTCAACCTCAAGGCGTGCAGTGAATACTGG
CAGGCTCGAGAGCGGGGGAGGTTAGGGGTACTTACGGGGTAAGAGTAAAATCTGTTGATC
CTGTAAGGACCACCAGTGGCGAAGGCGCCTAACCAAAACGCGTCTGACAGTGAGGGTCGA
AGGCTAGGAGAACGAATCGGATTAGAAACCCTGGTAGTCC
>Otu13768
CCAGCCTACGGGGGGCTGCAGTGGGGAATTTTGGACAATGGGCGGAAGCCCGATCCAGCC
ATTCCGCGTGAGTGATGAAGGCCTTCGGGTCGTAAAGCTCTGTCAAAGGGAAAGAAGTGT
ATGGTGGTTAATACCCATTATACTTGACGGTACCCTCAAAGGAAGCACCGGCTAACTCCG
TGCCAGCAGCCGCGGTAATACGGAGGGTGCGAGCGTTGTTCGGAATTATTGGGCGTAAAG
CGCGTGTAGGCGGTTTTTTAAGTCTGATGTGAGAGCCCTGGGCTCAACCCGGGAAGTGCA
TTGGAAACTGGGAGACTTGAATACGTGAGAGGGTAGTGGAATTCCTGGTGTAGGAGTGAA
ATCCGTAGATATCAGGAGGAACACCGGTGGCGAAGGCGGCTACCTGGACCGATATTGACG
CTGAGACGCGAAAGCGTGGGGAGCAAACAGGATTAGATACCCGCGTAGTCC
>Otu13775
CCAGCCTACGGGAGGCAGCAGGCGAGGAAATTTTCCAATGTCCGCAAGGATGAGAAAGGA
ATCCGAAGTGCTTTTCTTTTTAGAAAAGCTTTTATCCACTGCAAAAAGGTGGAGGAATAA
GGACTGGGCAAGACCGGTGCCAGCCGCCGCGGTAATCCCAGCAGTCCGAGTCGTGGCCAC
ATTTATTAGGTCTAAAACATTCGTAGCTTGCTTTGTAAGTTCCTTGTGAAATTCTGTCGC
TTAACGGCAGAGCGTGCAAGGGATACTGCATTGCTAGAGACTGGTAGACGTAAGGAGTAC
GGTTGAGGTAGCGGTAAAATGTTTTAATCTCAATCGGACTAACAATGGCGAAGGCACCTT
ACGAGGACAGATCTGACAGTGAGGAATGAAGGCCAGGGGCGCAAAATGGATTAGAAACCC
TCGTAGTCC
>Otu13786
CCAGCCTATGGGGGGCTCCAGGGCAATGAAGATCCCGGCCCGGAAACGGACATCTATCTC
CCGGTGCGTTTGGAATCCTTCAGGATCGAGGGCTTTGCCGGCTCCCGGACATGGAGCCAT
ATCACCCGGAGGCCTAGTGGTGACTCCGATCCGGATACCCTGGTGTTGGACCTTCACCTT
TACGACGATACATGGCGATTGGTCGGGGAAGTTCTCGGCTTACATTTCAAGTCAGTAGAC
CGCCGGGTGTTGTTGCGCGCTCGCCAGGGTCGTTTGGCCGAGCGCCTCCACGAGATGAAT
TGGCTACCCCGGCAACGTGCTGGTTTTGATCCAGCGGGTTTGCAGCCGGACTACTTTCCA
TCCCCTGATCGAATCGTGGATGCGGTCGTTCCTGCACTCGGCCGATTAGATACCCTCGTA
GTCC
>Otu13789
CCAGCCTACGGGTGGCTCCAGTGGGGAATCTTGCACAATGGGGGAAACCCTGATGCAGCG
ACGCCGCGTGAGCGATGAAGCCCTTCGGGGTGTAAAGCTCTTTCGGCAGGGACGATTATG
ACGGTACCTGCAGAAGAAGCTGCGGCTAACTACGTGCCAGCAGCCGCGGTAATACGTAGG
CAGCAAGCGTTGTTCGGAATTACTGGGCTTAAAGAGTGCGTAGGCGGTTGGGCAAGTCTG
TTGTGAAATCTCCCGGCTTAACTGGGAGGGTGCGGTGGAAACTGCCCGGCTAGAGTGTGG
GAGAGGGAAGTGGAATTCCTGGTGTAGCGGTGAAATGCGAAGACATCAGGAGGAACACCG
GTGGTGTAGACGGCTTTCTGGACCGTAACTGACGCTGAGACACGAAAGCGTGGGTAGCAA
ACAGGATTAGAGACCCGTGTAGTCC
>Otu13790
CCAGCCTATGGGAGGCTGCAGTCGGGAAACTTTTGCAATACACGTAAGTGTGACAAAGCA
AGCCAGAGTGTTTTTCTTTTTAGAAAAACTTTTGTGAGATGTAAAAAGTCTCACGAATAA
GGACCGGGCAAGACTGGTGCCAGCCGCCGCGGTAATCCCAGCAGTCCAAGTCGCAGCCAC
ATTTGTTGGGTCTAAAACATCCGTAGCTTGCCTTTCAAGTCTTTTGTGAAATCGGGACTC
TTAAGGTTCCGGCGGGCAAAAGATACTGTAAGGCTAGAGACCGGGAGGCGTAAGAAGTAC
GGACAGGGTAACGGTAAAATGTGTTGATCCTGTTTGGACTAACAGTCGCGAAGGCGTCTT
ACGAGAACGGTTCTGACAGTGAGGGATGAAGGCTAGGGGCGCAAAGTGGATTAGATACCC
CAGTAGTCC
>Otu13806
CCAGCCTACGGGACGCACCAGCTAAGAATATTCCGCAATGGGAGATATCCTGACGGAGCG
ACGCCGCGTGTATGAAGAAGGCAGAAATGTTGTAAAGTACTTTTATACGGGAAGAATAAG
GCAGGTAGTGGAAAAATCTGTTGATGACGGTACCGTATGAATAAGCCACGGCTAACTACG
TGCCAGCAGCTGCGGTAATACGTAGGTGGCAAGCGTTGTTCGGATTTATTGGGCGTAAAG

GGCATGTAGGCGGTTATATAAGTGTGAATTGAAATACCGGAGCTTAACTCCGCGTTTGGT
TTGCAAACTGTATGACTAGAGTGTAGAAGGGGAAACTGGAATTCCGCGTGTAGGGGTGAA
ATCTGTAGATATGCGGAAGAATACTTGTGGCGAAGGCGAGTTTCTGGTCTATAACTGACG
CTGAAATGCGAAAGTGTGGGGAGCAAACAGGATTAGAGACCCCCGTAGTCC
>Otu13816
CCAGCCTACGGGGGGCTCCAGTGGGGAATATTGGACAATGGGCGCAAGCCTGATCCAGCC
ATGCCGCGTGAGTGACGAAGGCCTTAGGGTTGTAAAGCTCTTTTGCCGGGGAAGATAATG
ACGGTACCCGGAGAATAAGTCCCGGCTAACTTCGTGCCAGCAGCCGCGGTAATACGAAGG
GGACTAGCGTTGTTCGGAATCACTGGGCGTAAAGCGCACGTAGGCGGACTTTCAAGTCGG
GGGTGAAATCCCAGGGCTCAACCCTGGAACTGCCTTCGATACTGTGAGTCTCGAGTCCGG
AAGAGGTGGGTGGAATTCCTAGTGTAGAGGTGAAATTCGTAGATATTAGGAAGAACACCG
GTGGCGAAGGCGGCCCACTGGTCCGGTACTGACGCTGAGGTGCGAAAGCGTGGGGAGCAA
ACAGGATTAGATACCCTAGTAGTCC
>Otu13820
CCAGCCTATGGGAGGCTGCAGTAACGAATATTCCGCAATGGGCGCAAGCCTGACGGAGCG
ATGCCGCGTGCAGGACGAAGCCCTTCGGGGTGTAAACTGCTGTCAGGGTTTAGGAATCAA
TTGACCAAACCCAAAGGAAGAGGCGACTAACTCTGTGCCAGCAGTCGCGGTAATACAGAG
GCCTCGAGCGTTAATCGGAATCACTGGGCTTAAAGGGTGCGTAGGCGGGACCGCAAGTGC
CTTGTGAAATCCCACGGCTTAACCGTGGAACTGCTTGGTAGACTTCGGTCCTTGAGGTCG
GTAGGGGTTGCTGGAACTCTAGGTGGAGCGGTGAAATGCGTAGATATCTAGAGGAACGCC
AATGGTGAAGACAGGCAACTGGGCCGATTCTGACGCTGAGGCACGAAAGCGTGGGGAGCG
AACGGGATTAGAGACCCGCGTAGTCC
>Otu13837
CCAGCCTATGGGGTGCAGCAGTGGGGAATATTGCACAATGGGCGCAAGCCTGATGCAGCG
ACGCCGCGTGGGGGACGAAGGCCTTCGGGTTGTAAACCCCTGTCGGCAGGGACGAATGGG
TGGGTAGCTAACATCTGCCTATCTTGACGGTACCTGTAAAGGAAGCACCGGCTAACTCTG
TGCCAGCAGCCGCGGTAAGACAGAGGGTGCAAGCGTTGTTCGGAATTACTGGGCGTAAAG
GGCGCGTAGGCGGTTCTGCAAGTCGGATGTGAAATCCCTCGTTTCAAGCGAGGAACTGCA
TTCGAAACTGCAAGACTAGAGTCCTGGAGAGGGAAGTGGAATTCCTGGTGTAGAGGTGAA
ATTCGTAGAGATCAGGAGGAACACCCGAGGCGAAGGCGGCTTCCTGGACAGTGACTGACG
CTGAGGCGCGAAAGCGTGGGTAGCAAACAGGATTAGAAACCCCAGTAGTCC
>Otu13838
CCAGCCTACGGGTCGCTCCAGTGACGAATATTCCGCAATGGGCGAAAGCCTGACGGAGTG
ACGCCGCGTGTGGGACGAGGCCCTTCGGGGTGTAAACCACTGTCAGGGAACAAGAACGCA
ATGACTATTCCCAGAGGAAGGGGCGACTAACTCTGTGCCAGCAGTCGCGGTAATTCAGAG
GCCCCAAGCGTTAATCGGAGTCACTGGGCTTAAAGGGTGCGTAGGCGGACTTGTAAGTGC
CTTGTGAAATCCCACGGCTCAACCGTGGAACCGCTCGGCATACTGCTCGTCTTGAGGTCG
GTAGAGGTCGCTGGAACTCTAGGTGTAGCGGTGAAATGCGTAGATATCTAGAGGAACGCC
AATGGTGAAGACAGGCGACTGGGCCGATTCTGACGCTGAGGCACGAAAGCGTGGGGAGCA
AACGGGATTAGAAACCCCAGTAGTCC
>Otu13839
CCAGCCTATGGGGGGCAGCAGTCAAGAATCTTCCACAATGGCCGAAAGGCTGATGGAGCG
ACGCCGCGTGTATGAAGACGCCCCTCGGGGTGTAAAGTACTTTTTTTAGGGATGAAACAA
TGACAGTACCTAAAGAATAAGGGGCTGCTAACCTCGTGCCAGCAGCAGCGGTAATACGAG
GGCCCCAAGCTGTATCCGGATTTATTGGGCGTAAAGCGTGTGTAGGTGGTTTGTAACATC
TTTTGTTAAATCTGCGGGCTTAACCCGTAGGCCGCGGAAGATATGGACAGACTAGAGACA
GGGAGAGGTAAGCGGAATTGCTGGTGTAGGGGTTAAATCCGTTAATATCAGCAGGAACAC
CAAATGCGTAGGCAGCTTACTAGAACTGTTCTGACACTGAGACACGAAAGCGTGGGGAGC
GAATGGGATTAGAAACCCCCGTAGTCC
>Otu13848
CCAGCCTATGGGTTGCTGCAGTAAGGAATATTGGTCAATGGAGGCAACTCTGAACCAGCC
ATGCCGCGTGCAGGAAGACGGCCCTATGGGTTGTAAACTGCTTTTGAATGGGAGAAAACC
CCGGTACGTGTACCGGGTTGATAGTACCATTAGAATAAGCATCGGCTAACTTCGTGCCAG
CAGCCGCGGTAAGACGAAGGATGCAAGCGTTATCCGGATTCATTGGGTTTAAAGGGTGCG
TAGGCGGACTTGTAAGTCAGTGGTGAAATCTCTCAGCTTAACTGAGAAACTGCCCTTGAT
ACTGCAGGTCTAGAGTATAGATGACGTTGGCGGAATATGACATGTAGTGGTGAAATACTT
AGATATGTCATAGAACACCGATTGCGAAGGCAGCTAACGAAACTATAACTGACGCTGAGG
CACGAAAGTGCGGGGAGCAAACCGGATTAGAAACCCCTGTAGTCC

>Otu13850
GGACTACAGGGGTTTCTAATCCTGTTTGCTCCCCTAGCTTTCGCGCCTGAGCGTCAGTAT
TAGGCCAGATGGGCGCCTTCGCCACTGGTGTTCCTCCCGATATCAATGCATTTCACCGCT
ACACCGGGAATTCCCCCATCCTCTCCTATACTCAAGCCTTGCAGTCTGAGATGCATTTCC
CTGGTTGAGCCAGGGCATTTCACATCCCACTTACAAGGCCGCCTGCACGCCCTTTACGCC
CAGTAATTCCGAACAACGTTCGCTCCCCCCGTATTACCGCGGCTGCTGGCACGGAGTTGG
CCGGAGCTTCCTTTAGCAGTACCGTCAAGCAGATGGACTGTTCGCCCACCTGCGTTTCTT
CCTACTCGACAGGGCTTTACAACCTAACGGCCTTCATCGCCCACGCGGCGTCGCTCGGTC
AGGGTTTCCCCCATTGCCGAAGCCTCTCGACTGCTGCCACCCATAGGCTGG
>Otu13862
CCAGCCTACGGGACGCTCCAGTGAGGAATATTGCGCAATGGACGAAAGTCTGACGCAGCG
ACGCCGCGTGAGCGATGAAGGCCTTCGGGTCGTAAAGCTCTTTTAGCAGGGACGAGTGAG
GACGGTACCTGTTGAATAAGTCTCGGCTAACTACGTGCCAGCAGCCGCGGTAAAACGTAG
GAGGCGAGCGTTATCCGGATTCACTGGGCGTAAAGCGTGCGTAGGCGGTTGGGTAAGTCG
GACGTTAAAGCCCCTGGCTCAACTAGGGGAGGTCGTTCGATACTGCTTGGCTTGAGGATG
ATAGAGGAAAGCGGAATTCCCGGTGTAGCGGTGGAATGCGTAGAGATCGGGAGGAACACC
AGTGGCGAAAGCGGCTTTCTGGCTCATTTCTGACGCTGAGTGCACGAAAGCGTGGGGAGC
AAACGGGATTAGAAACCCCAGTAGTCC
>Otu13870
GGACTACGCGGGTATCTAATCCTGTTTGCTCCCCACGCTTTCGTACATCAGCGTCAGTAA
TGGCCCAGTAAGCTGCCTTCGCAATTGGTGTTCTGTGTAATATCTAAGCATTTCACCGCT
ACATTACACATTCCGCCTACCTTGACCGTACTCAAGATTAACAGTATCAATGGCAAGTCT
CCAGTTAAGCTGAAGGTTTTCACCACTGACTTATTATTCCGCCTACGCACCCTTTAAACC
CAATAAATCCGGATAACGCTCGCATCCTCCGTATTACCGCGGCTGCTGGCACGGAGTTAG
CCGATGCTTATTCCCAGGGTACCGGCAAGCCACGACACGTCGTAGGTTTTCTTCCCCTGG
AAAAGCAGTTTACAACGCATAGCGCCGTCTTCCTGCACGCGGGATGGCTGGTTCAGGCTT
GCGCCCATTGACCAATATTCCTCACTGGTGCCCCCCGTAGGCTGG
>Otu13871
CCAGCCTATGGGTTGCTGCAGTCGAGAATTTTTCTCAATGGGCGAAAGCCTGAAGGAGCG
ACGCCGCGTGCGAGATGAAGGCCTTCGGGTTGTAAATCGCTGTCAGAGGGGAGGAAATGC
TAGTGGGTTCTCCCATTAGTTTGACCTATCCTCAGAGGAAGGACGGGCTAAGTTCGTGCC
AGCAGCCGCGGTAAGACGAACCGTCCAAACGTTATTCGGAATCACTGGGCTTAAAGGGTG
CGTAGGCGGTCCGGGAAGTTGGGTGTGAAAGCCCTCGGCTCAACCGAGGAACTGCGTCCA
AAACTGCCGGACTTGAGGGAGACATAGGTGAGCGGAACTTAGGGTGGAGCGGTGAGATGC
GTTGAGATCCTAAGGAACACCAGTAGCGAAAGCGGCTCACTGGGTCTCTTCTGACGCTGA
TGCACGAAAGCTAGGGGAGCGAAGAGGATTAGAAACCCTCGTAGTCC
>Otu13878
CCAGCCTACGGGATGCTGCAGTCACGAATCTTCCTCAATGGCCGAAAGGCTGAAGGAGCG
ACGCCGCGTGTATGAAGACGCCCTTCGGGGTGTAAAGTACTTTTATCTGGGAAGAATCCA
TGACGGTACCAGATGAATAAGGAGCTGCTAACCTCGTGCCAGCAGCAGCGGTAATACGAG
GGCTCCAAGCGTTATCCGGATTTATTGGGCGTAAAGGGTCCGTAGGCGGTTTTTCGCATC
TCCTGTTAAATCCCGAGGCTCAACCTCGGATCAGCGGGAGAGATGGACAAACTAGAGGCC
AAGAGAGGCAAGCGGAATTGCTGGTGTAGGGGTTAAATCCGTTAATATCAGCAGGAACAC
CGAATGCGAAAGCAGCTTGCTAGAATGGTCCTGACGCTAAGGGACGAAAGCGTGGGTAGC
GAATGGGATTAGAAACCCTCGTAGTCC
>Otu13879
CCAGCCTACGGGGGGCAGCAGTCGAGAATCATTCGCAATGGGCGCAAGCCTGACGATGCG
ACGCCGCGTGCGGGATGGAGGCCTTCGGGTTGTAAACCGCTGTCAAAGGGGAAGAAACGC
GCGAACGCTAACATCGTTCGCGTTTGACGTTACTCTTAAAGGAAGCCACGGCTAACTCCG
TGCCAGCAGCCGCGGTAATACGGAGGTGGCGAGCGTTGTTCGGAATTACTGGGCGTAAAG
GGTGTGTAGGCGGTCGGACGTGTTCGGTGTGAAAGCCCCCGGCTCACTCGGGGAACTGCA
TCGAAAACTGTCCGGCTTGAGTGCAGGAGAGGAGAATGGAATTTCCGGTGTAGCGGTGAA
ATGCGTAGATATCGGAAGGAACGCCAATGGCGAAAGCAGTTCTCTGGCCTGTTACTGACG
CTGAGGCACGAAAGCCAAGGGAGCAAACGGGATTAGATACCCCCGTAGTCC
>Otu13889
CCAGCCTATGGGGGGCTCCAGTTGGGAATCTTGCACAATGGGGGAAACCCTGATGCAGCG
ACGCCGCGTGGAGTATGAAGGATCTAGGTCTGTAAACTCCTTTTGTCAGGAAAGACTTAG
GACGGTACCTGACGAATAAGCCCCGGCTAACTACGTGCCAGCAGCCGCGGTAAGACGTAG

GGGGCGAGCGTTGTCCGGATTTACTGGGCGTAAAGGGCGCGTAGGCGGCTAGTTAAGTGA
GAAGTGAAATCTCCAGGGCTCAACCCGGAAACTGCTTTTCATACTGGCTAGCTAGAGGAA
CGCAGAGGTGTGTGGAATTCCTGGTGTAGCGGTGAAATGCATAGATATCAGGAGGAACAC
CCATGGCGAAGGCAGCACACTGGGCGTTTCCTGACGCTGAGGCGCGAAAGCGTGGGTAGC
AAACAGGATTAGAAACCCTTGTAGTCC
>Otu13895
CCAGCCTATGGGTTGCAGCAGTCGAGAATTTTTCACAATGGGCGCAAGCCTGATGGAGCG
ACGCCGCGTGGGGGATGAATGGCTTCGGCCCGTAAACCCCTGGCATTCGGGATCAATGCG
TCTGACCTAACACGTCAGACGTTGATAGTACCGGAAGAGGAAGGGACGGCTAACTCTGTG
CCAGCAGCCGCGGTAATACAGAGGTCCCAAGCGTTGTTCGGATTTACTGGGCGTAAAGGG
TGCGTAGGCGGTCGGATAAGTTTGATGTGAAAGCTCGGAGCCTAACTCCGAAACGGCATT
GAATACTATCTGGCTAGAGGTTGGGATGGGGGACTGGAATTCTCGGTGTAGCAGTGAAAT
GCGTAGATATCGAGAGGAACACCAGTGGCGAAGGCGAGTCCCTGGACCAATCCTGACGCT
GAGGCACGAAAGCCAGGGGAGCAAACGGGATTAGATACCCTCGTAGTCC
>Otu13901
CCAGCCTACGGGGGGCACCAGTGGGGAATTTTGGACAATGGGCGCAAGCCTGATCCAGCC
ATGCCGCGTGTCTGAAGAAGGCCTTCGGGTTGTAAAGGACTTTTGTCCGGGAGCAAATGC
CCTGTGCGAATAACACAGGGAGCTGAGAGTACCGGAAGAATAAGCACCGGCTAACTACGT
GCCAGCAGCCGCGGTAATACGTAGGGTGCAAGCGTTAATCGGAATTACTGGGCGTAAAGC
GTGCGCAGGCGGTTTTGTAAGCCAGATGTGAAAGCCCCGGGCTCAACCTGGGAACGGCGT
TTGGAACTACAAGACTAGAGTGTGTCAGAGGGGGGTGGAATTCCGCGTGTAGCAGTGAAA
TGCGTAGAGATGCGGAGGAACACCGATGGCGAAGGCAGCCCCCTGGGATAACACTGACGC
TCATGCACGAAAGCGTGGGGAGCAAACAGGATTAGAGACCCCAGTAGTCC
>Otu13904
CCAGCCTATGGGTGGCAGCAGTAAGGAATATTGGGCAATGGACGCAAGTCTGACCCAGCC
ATGCCGCGTGGAGGATGAAGGTCCTCTGGATTGTAAACTTCTTTTCTCTGGGGCGAAAAA
TAGGTATTCTTACTTACTTGACAGTACCAGAGGAATAAGCACCGGCTAACTCCGTGCCAG
CAGCCGCGGTAATACGGAGGGTGCAAGCGTTATCCGGATTCACTGGGTTTAAAGGGTGCG
TAGGTGGGCAGGTAAGTCAGCGGTGAAATCCCCGAGCTTAACTTGGGAACTGCCATTGAT
ACTATCTGTCTTGAATACCTTGGAGGTGAGCGGAATATGTCATGTAGCGGTGAAATGCTT
AGATATGACATAGAACACCAATTGCGAAGGCAGCTCACTACACGGTCATTGACACTGAGG
CACGAAAGCGTGGGGATCAAACAGGATTAGAAACCCCCGTAGTCC
>Otu13909
CCAGCCTACGGGTCGCAGCAGTCGAGGATATTTAGCAATGGACGAAAGTCTGACTATGTG
ACGCCGCGTGAGGGATGAAGGTCTTCGGATCGTAAACCTCTTTCGATAGGGAAGAAAACC
TGTTCTTAACGGAACAGACTGACGGTACCTAGAGAAGAAGCCACGGCCAACTCTGTGCCA
GCAGCCGCGGTAATACAGAGGTGGCGAGCGTTACTCGGATTTATTGGGTGTAAAGGGCAA
GTAGGCGTCTTAACAAGTTAGGAGTGAAATCCTGCAGCTTAACTGCAGAACTGCTTTTAA
AACTGTTGAGATTGAGGCTGGGAGAGGAGAGCGGAATTCTCGGTGTAAGAGTGAAATCTG
TAGATATCGAGAGGAACACCAGTGGCGAAGGCGGCTCTCTGGTCCAGTACTGACGCTGAG
TTGCGAAAGCTAGGGGAGCAAACAGGATTAGATACCCCTGTAGTCC
>Otu13911
CCAGCCTATGGGTGGCTGCAGTCAAGAATATTCCTCAATGGCCGAAAGGCTGAAGGAGCG
ACGCCGCGTGAAGGATGAAGTCCCTCGGGATGTAAACTTCTTTTTTTCGGGAAGAAACAA
TGACGGTACCGAAAGAATAAGGGGCTGCTAAACTCGTGCCAGCAGCAGCGGTAATACGAG
TGCCCCGAGCGTTATCCGGATTTATTGGGCGTAAAGCGCGCGTAGACGGTTTGGAACATC
TTTTGTTAAATTCCAGGGCTCAACCCTGGGGCCGCAGGAGATATGGCCAGACTACGAGGC
TGGAAGAGGCAAACGGAATTGTTGGTGTAGGGGTTAAATCCGTTAATATCAACAAGAACA
CCAAAAGCGAAAGCAGTTTGCTGGGACAGTTCTGACGTTAAGGCGCGAAAGCGTGGGGAG
CGAATGGGATTAGAAACCCCCGTAGTCC
>Otu13914
CCAGCCTACGGGTGGCTGCAGTGGGGAATATTGGACAATGGGGGCAACCCTGATCCAGCA
ATGCCGCGTGTGTGAAGAAGGCCTGAGGGTTGTAAAGCACTTTCAGTGGGGAGGAGGTCT
TGTAGGTTAAGAGCTGGCAAGATGGACGTTACCCACAGAAGAAGCACCGGCTAACTCCGT
GCCAGCAGCCGCGGTAATACGGAGGGTGCGAGCGTTAATCGGAATTACTGGGCGTAAAGG
GTGCGTAGGTGGTAGATTAAGTTAGCATTGAAATTCCTGGGCTTAACCTGGGGTTGTGTG
CTAAAACTGGTTTACTTGAGTATAGGAGAGGGTAGTGGAATTTCCGGTGTAGCGGTGAAA
TGCGTAGAGATCGGAAGGAACACCAGTGGCGAAGGCGGCTACCTGGCCAGATACTGACAC

TGAGGCACGAAAGCGTGGGGAGCAAACAGGATTAGAAACCCCAGTAGTCC
>Otu13925
CCAGCCTATGGGAGGCTGCAGTCGAGAATTTTTCACAATGGGCGAAAGCCTGATGGAGCG
ACGCCGCGTGGGGGATGAATGGCTTCGGCCCGTAAACCCCTGTCATTCGGGATCAATGCC
TGCTATTTAAAAGATGGCAGGTTGATAGTACCGGAAGAGGAAGGGACGGCTAACTCTGTG
CCAGCAGCCGCGGTAATACAGAGGTCCCAAGCGTTGTTCGGATTCACTGGGCGTAAAGGG
TGCGTAGGTGGTCGGGTAAGTTTGATGTGAAATCTCGGAGCTTAACTCCGAAACGGCATT
GAATACTATTCGGCTGGAGGGTTGGAGGGGAGACTGGAATTCTCGGTGTAGCAGTGAAAT
GCGTAGATATCGAGAGGAACACCAGTGGCGAAGGCGAGTCTCTGGACAACTCCTGACACT
AAGGCACGAAAGCTAGGGGAGCAAACAGGATTAGAAACCCGTGTAGTCC
>Otu13927
CCAGCCTATGGGTCGCACCAGTGGGGAATATTGGACAATGGGCGCAAGCCTGATCCAGCC
ATGCCGCGTGAGTGATGACGGCCTTAGGGTTGTAAAGCTCTTTCACCCACGACGATAATG
ACGGTACCTGGATAAGAAGCCCCGGCTAACTTCGTGCCAGCAGCCGCGGTAATACGAAGG
GGGCTAGCGTTGTTCGGATTTACTGGGCGTAAAGCGCACGCAGGCGGATTGATAAGTCAG
GGGTGAAATCCCGGGGCTCAACCTCGGAATTGCCTTTGATACTGTCTGTCTTGAGTTCGG
GAGAGGTTGGCGGAATTCCTAGTGTAGAGGTGAAATTCGTAGATATTAGGAAGAACACCA
GTGGCGAAGGCGGCCAACTGGCCCGACACTGACGCTCATGTGCGAAAGCGTGGGGAGCAA
ACAGGATTAGAAACCCTTGTAGTCC
>Otu13929
CCAGCCTATGGGGGGCTGCAGTCGAGAATTTTTCACAATGGGCGAAAGCCTGATGGAGCG
ACGCCGCGTGGGGGATGAAGGCCTTCGGGTCGTAAACCCCTGTCAGTTGCGAACAAAGGT
TATGGGTGAACATCCCATAATTTGATGGTAGTGACAGAGGAAGGGACGGCTAACTCTGTG
CCAGCAGCCGCGGTAATACAGAGGTCCCAAGCGTTGTTCGGATTTACTGGGCGTAAAGGG
TGCGTAGGCGGCTGGGTAAGTCGAGTGTGAAAGCCCCGGGCTCAACCCGGGAACGGCACT
GGAAACTGCCTGGCTAGAGGGTTGGAGGGGGGACTGGAATTCCCGGTGTAGCAGTGAAAT
GCGTAGATATCGGGAGGAACGCCAGTGGCGAAGGCGAGTCCCTGGACAACCCCTGACGCT
GAGGCACGAAAGCTAGGGGAGCAAACAGGATTAGAAACCCCTGTAGTCC
>Otu13930
CAGCCTATGGGTGGCAGCAGTGGGGAATTTTGGACAATGGGGGCAACCCTGATCCAGCGA
CGCCGCGTGGAGGACGAAGGTCTTCGGATTGTAAACTCCTTTTAGAGGGGATGAATGCCT
GCGTGAATAGCGCAGGGTGACAGTACCCTTTGAATAAGCCACGGCTAACTACGTGCCAGC
AGCCGCGGTAATACGTAGGTGGCAAGCGTTACTCGGAATCACTAGGCGTAAAGCGCAGGT
AGGCGGTTCGGTAAGTCTTTTGTGAAAGCTCCTGGCTCAACTGGGAGAGGTCAAAGGAAA
CTGCCGGACTAGAGTGTGGTAGGGGATACTGGAATTCCCGGTGTAGCGGTGAAATGCGTA
GATATCGGGAGGAATACCTATGGCGAAAGCAGGTATCTGGGCCATCACTGACGCTGAGCT
GCGAAAGCTAGGGGAGCAAACAGGATTAGAAACCCCTGTAGTCC
>Otu13931
CCAGCCTATGGGACGCAGCAGTAAGGAATATTGGTCAATGGGCGCAAGCCTGAACCAGCC
ATGCCGCGTGAAGGATGACGGCCCTATGGGTTGTAAACTTCTTTTATACGGGAAAAAACC
CCCCGATTCCTCGGGGGCTGATGGTACCGTAAGAATAAGGATCGGCTAACTCCGTGCCAG
CAGCCGCGGTAATACGGAGGATCCAAGCGTTATCCGGATTTATTGGGTTTAAAGGGTGCG
TAGGCGGAATGATAAGTCAGTGGTGAAATCCTACGGCTCAACTGTAGAACTGCCATTGAT
ACTGTCAATCTTGAGTACACTTGAAGTGGGCGGAATGTGTCATGTAGCGGTGAAATGCTT
AGAGATGACACAGAACACCGATAGCGAAGGCAGCTCACTAAATTGTAACTGACGCTGAGG
CACGAAAGCGTGGGGATCAAACAGGATTAGAAACCCGAGTAGTCC
>Otu13934
CCAGCCTACGGGACGCTGCAGGCGAGAAAACTTTACAATGCGCGCAAGCGTGATAGGGGA
ACTTCGAGTGCTAGCTGTGCTAGCTTTTGCCAAGTCTAGAAAGCTTGGCGAATAAGTGGG
GGGTAATATACGTGCCAGCCGCCGCGGTAATACGTAATCCACAAGTGGTATCCACTTATA
TTGGGCCTAAAACCTTTGTAGCCGGTTGTGCGCGTCGTTGGTGAAATCCAAGAGCTTAAC
TTTTGGAATGGCTGACGATACGGCACGGCTGTGAGACCAAGAGATGTCAAGGGAATTCTA
GGGGTACCGGTAAAATGGTGTGATCCTTAGAGGACCTCCAGTAGCGAAGGCGCTTGACAA
GAATGGTTCTGACGGTGAGAGAGGAAAGCTGGGGGAGCGACCCGGATTAGATACCCCAGT
AGTCC
>Otu13936
CCAGACCTATGGGGGGCTGCAGTGGGGAATATTGGACAATGGGCGCAAGCCTGATCCAGC
CATCCCGCGTGCAGGAATAAGGCCCTATGGGTTGTAAACTGCTTTTCTTAGGGAAGAAAA

ACTCCGATGTATCGGAGCTTGACGGTACCTAAGGAATAAGCACCGGCTAACTCCGTGCCA
GCAGCCGCGGTAATACGGAGGGTGCAAGCGTTATCCGGATTTACTGGGTTTAAAGGGTGC
GAAGGCGGTAAAGTAAGTCAGTGGTGAAAGCTTCCAGCTCAACTGGAAAATTGCCATTGA
TACTGCTTTACTTGAGTTTGGTTGAGGTAGGCGGAATACATCAAGTAGCGGTGAAATGCA
TAGATATGATGTAGAACACCAATTGCGAAGGCAGCTTACTAAACCAATACTGACGCTCAT
GCACGAAAGCGTGGGGAGCAAACAGGATTAGAAACCCTCGTAGTCCT
>Otu13955
CCAGCCTATGGGGTGCACCAGCCGAGAATATTGGACAATGGGGGAAACCCTGATCCAGCG
ACGTTGCGTGGAGGAAGAAGATCTTCGGATTGTAAACTTCTTTTCTAAGGGAAGAAGTAA
[truncated: 766,166 more chars]
